# Phytochemical Characterization and Biological Evaluation of *Camellia hakodae* Ninh Flowers

**DOI:** 10.3390/molecules31071088

**Published:** 2026-03-26

**Authors:** Nguyen Hoang Thao My, Nguyen Huu Lac Thuy, Vo Thi Kim Khuyen, Nguyen Duc Tuan

**Affiliations:** 1 School of Pharmacy, University of Medicine and Pharmacy at Ho Chi Minh City, 41-43 Đinh Tien Hoang, Saigon Ward, Ho Chi Minh City 700000, Vietnam; nhtmy@ntt.edu.vn (N.H.T.M.); vtkkhuyen@ump.edu.vn (V.T.K.K.); 2 Faculty of Medical Laboratory Technology, Nguyen Tat Thanh University, 300A Nguyen Tat Thanh, Xom Chieu Ward, Ho Chi Minh City 700000, Vietnam

**Keywords:** *Camellia hakodae* Ninh, phytochemistry, mass spectrometry, extraction, polyphenols, pharmacological activities

## Abstract

*Camellia hakodae* Ninh flowers are an endemic Vietnamese species with limited phytochemical and biological characterization. This study aimed to characterize the phytochemical profile and evaluate antioxidant and anti-inflammatory activities of the total flower extract. Ultrasonic-assisted extraction (UAE) and maceration with methanol and ethanol at different concentrations were carried out to evaluate the efficiency of extracting total phenolic content (TPC) and total flavonoid content (TFC), quantified by colorimetric assays, along with the antioxidant and anti-inflammatory activities of the resulting extracts. The highest TPC (94.9 ± 4.5 mg GAE/g) and TFC (3.1 ± 0.2 mg QE/g) were obtained using UAE with 70% methanol, while maceration with 70% ethanol showed comparable TPC values. The optimized extract exhibited strong antioxidant activity with an IC_50_ of 29.06 µg/mL, close to that of ascorbic acid (28.16 µg/mL) and significant anti-inflammatory activity in the proteinase inhibition assay (IC_50_ = 2.72 mg/mL) compared to acetylsalicylic acid (IC_50_ = 3.16 mg/mL). GC-MS and LC-QTOF-MS/MS analyses revealed diverse metabolites, including phenolic acids, flavonoids, fatty acids, terpenoids, and nitrogen-containing compounds, with representative constituents, such as quinic acid, catechins, flavonol glycosides, and loliolide, providing strong chemical evidence for the observed bioactivities. This integrated study demonstrates that *C. hakodae* flower is a rich source of multifunctional bioactive compounds and highlights its strong potential for applications in nutraceuticals, functional foods, and cosmeceuticals.

## 1. Introduction

Natural products derived from medicinal plants continue to attract significant scientific interest due to their remarkable chemical diversity and broad spectrum of biological activities. Among them, phenolic compounds and flavonoids represent two of the most extensively studied classes of plant secondary metabolites because they play crucial roles in protecting plants against oxidative stress and environmental challenges, offering substantial health benefits when incorporated into the human diet or formulated as nutraceuticals and cosmeceuticals [1,2]. In recent years, the global demand for natural antioxidants and anti-inflammatory agents has steadily increased, driven by growing concerns over the long-term safety of synthetic additives and pharmaceuticals.

*Camellia* is a large genus of the family Theaceae with more than 200 species distributed mainly in East and Southeast Asia, many of which have been traditionally used as beverages, medicinal plants, or ornamental species [3]. The most famous representative, *Camellia sinensis* (green tea), is well known for its rich polyphenolic profile, particularly catechins [4], which are responsible for its potent antioxidant and health-promoting effects [4,5]. Beyond *C. sinensis*, increasing attention has been paid to other *Camellia* species such as *C. japonica* (ornamental flowers), *C. oleifera* (edible oil), and *C. nitidissima*, which have been reported to contain diverse phenolic acids, flavonoids, saponins, and fatty acids with promising biological activities, including anti-inflammatory, cardioprotective, and anti-cancer effects [6]. *Camellia hakodae* Ninh, a less-studied species endemic to Vietnam, represents a particularly valuable yet underexplored botanical resource. Traditionally, the flowers of this species have been appreciated not only for their ornamental value but also for their potential medicinal properties in folk medicine. Compared with the leaves, which have been the focus of phytochemical studies in the genus *Camellia*, flowers remain relatively under-investigated, despite evidence suggesting that floral tissues often accumulate unique flavonoid glycosides, phenolic acids, and aroma-related compounds that differ markedly from those in vegetative parts [7]. Phytochemical investigations of endemic *Camellia* species remain limited, particularly with respect to flower-derived extracts, which are often overlooked despite their potential chemical diversity and biological relevance. The exploration of the chemical composition and biological potential of *C. hakodae* flowers, therefore, holds great promise for uncovering novel bioactive compounds and expanding the economic and therapeutic value of this endemic species.

From a phytochemical perspective, the efficiency of extracting polyphenols and flavonoids from plant matrices is strongly influenced by several factors, including extraction technique, solvent composition, temperature, and extraction time [8]. Polar organic solvents such as methanol and ethanol are commonly used due to their strong solvation capacity for hydroxylated compounds, while aqueous mixtures often provide superior performance by balancing polarity and mass transfer efficiency [9]. In parallel, the development of advanced extraction techniques, such as ultrasonic-assisted extraction [10], microwave-assisted extraction [11], and pressurized liquid extraction, has significantly improved the recovery of thermolabile and structurally sensitive phytochemicals. UAE has gained widespread popularity because acoustic cavitation can disrupt plant cell walls, enhance solvent penetration, and accelerate mass transfer. This allows for the reduction in extraction time and solvent consumption, and even an increase in the extraction yields, which aligns with the trends in Green Chemistry [12,13]. However, despite the advantages of UAE, its performance may vary depending on solvent composition and the physicochemical nature of target compounds. In some cases, prolonged or intense ultrasonic treatment may lead to partial degradation or re-association of labile phenolics, highlighting the need for systematic optimization of extraction conditions rather than assuming a universally superior technique. Consequently, comparative investigations of conventional methods such as maceration or maceration and modern techniques like UAE are essential for establishing robust, scalable extraction protocols, particularly when the goal is industrial or pilot-scale application.

A comprehensive chemical characterization is essential for understanding the functional potential of complex plant extracts. Plant-derived extracts typically consist of diverse classes of compounds with a wide polarity range, from volatile to non-volatile constituents. The complementary use of gas chromatography–mass spectrometry (GC-MS) and liquid chromatography–quadrupole time-of-flight tandem mass spectrometry (LC-QTOF-MS/MS) has therefore become an indispensable strategy in natural product research. While GC-MS enables effective profiling of volatile and semi-volatile compounds such as fatty acids, terpenoids, and sterols, LC-QTOF-MS/MS provides high-resolution detection of non-volatile phenolic acids, flavonoids, glycosides, and nitrogen-containing metabolites. These not only allow the tentative identification of secondary metabolites but also provide valuable insights into the chemical diversity underlying biological activity [14]. Linking phytochemical composition to biological activity is critical for substantiating the functional value of plant extracts. Antioxidant activity, commonly evaluated by free radical scavenging assays such as DPPH and ABTS, serves as a primary indicator of the health-promoting potential of polyphenol-rich extracts. Numerous studies have demonstrated strong correlations between total phenolic content (TPC), total flavonoid content (TFC), and antioxidant capacity across a wide range of medicinal plants [1,15,16,17]. In addition, increasing evidence suggests that many phenolic acids and flavonoids also exert anti-inflammatory effects by modulating key molecular targets involved in inflammation, including cyclooxygenases (COX), lipoxygenases, and pro-inflammatory cytokines regulated by the NF-κB and MAPK signaling pathways [18,19,20,21,22].

Despite the growing interest in *Camellia* species, integrated studies that simultaneously address extraction optimization, biological activity assessment, and advanced metabolite profiling remain scarce, particularly for *C. hakodae* flowers. Most existing investigations focus either on leaf extracts or on isolated biological endpoints, without sufficiently exploring the interrelationship between extraction conditions, chemical composition, and multifunctional bioactivities. This knowledge gap limits both scientific understanding and the rational development of value-added products from this promising botanical resource. Therefore, this study aims to provide a comprehensive investigation of the total flower extract of *C. hakodae* Ninh by integrating comparative evaluation via ultrasonic-assisted extraction and conventional maceration methods, quantitative determination of total phenolic and flavonoid contents, along with in-depth phytochemical profiling, and their typical biological activities. By correlating chemical composition with biological performance, this work seeks to elucidate the functional significance of *C. hakodae* flowers and to highlight their potential as a rich source of multifunctional natural products for applications in nutraceuticals, functional foods, and cosmeceuticals.

## 2. Results and Discussion

### 2.1. Extraction Investigation of Total Phenolic and Flavonoid Contents

**Figure 1:**
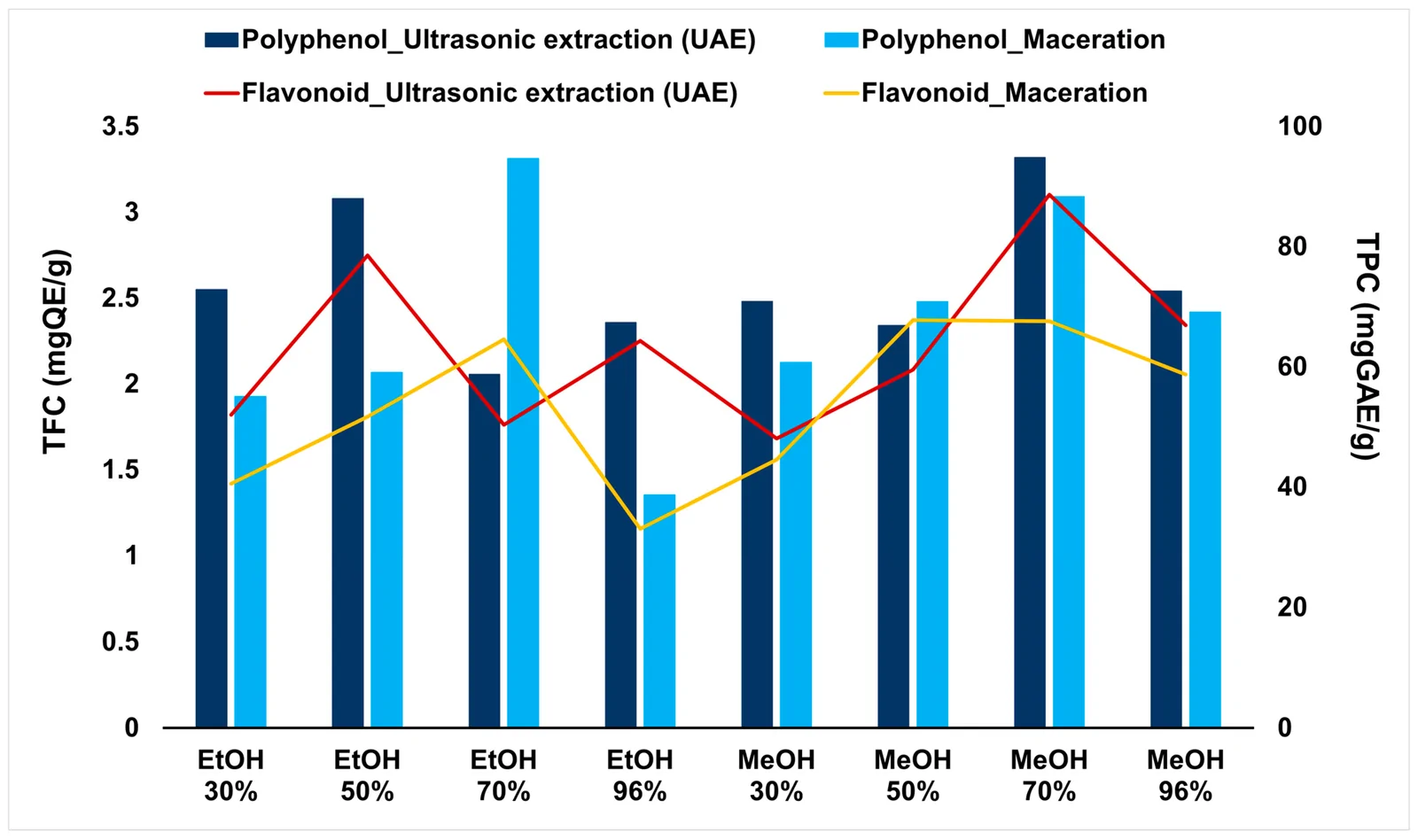
Effects of extraction conditions on total phenolic and flavonoid contents.

Ultrasonic-assisted extraction, especially with higher solvent concentrations, generally yielded 58.7 ± 2.5 to 94.9 ± 4.5 mgGAE/g of dry material, higher than maceration (38.7 ± 1.9 to 94.7 ± 4.5 mgGAE/g). This can be attributed to the mechanical effects of ultrasonic waves that disrupt plant cell walls and facilitate the release of intracellular phenolic compounds into the extraction medium. However, it is important to note that UAE is not always effective, since efficiency also depends on the type and concentration of the solvent. For the case of ethanol, at solvent concentrations of 50% and 70%, maceration occasionally resulted in comparable or even higher phenolic yields (Figure 1). UAE with 70% methanol and maceration in 70% ethanol gave the highest TPC values, reaching 94.9 ± 4.5 mgGAE/g and 94.7 ± 4.5 mgGAE/g, respectively. The results reveal that *C. hakodae* flowers are a rich phenolic source, with the TPC comparable to or higher than several reported values in commonly studied *Camellia* species, such as *C. sinensis*, containing 43.21 to 139.02 mgGAE/g in leaves [23], and *C. japonica* [24], depending on plant part and extraction conditions. This evidence supports the potential of flower-derived extracts as valuable alternatives to leaf-based materials, which have traditionally dominated phytochemical investigations within the genus *Camellia*.

The TFC ranged from 1.7 ± 0.1 to 3.1 ± 0.2 mgQE/g for ultrasonic-assisted extraction, and from 1.2 ± 0.1 to 2.4 ± 0.1 mgQE/g for maceration. The flavonoid levels are comparable to those reported for other *Camellia* species; for instance, C. japonica flowers have shown values around 0.566–1.081 mg QE/g dry weight [24]. In both extraction methods, methanol could extract flavonoids better than ethanol, especially at high concentrations from 50%. Particularly, 70% methanol yielded the highest TFC from ultrasonic-assisted extraction (3.1 ± 0.2 mgQE/g) (Figure 1), followed by 50% methanol via the maceration method. This suggests that increasing solvent polarity within an optimal range enhances the solubilization of flavonoid compounds and promotes their transfer into the extraction medium [10,25,26]. Moreover, hydroalcoholic mixtures often enhance the recovery of phenolic and flavonoid compounds due to a synergistic effect between water and organic solvents. The presence of water improves the swelling of plant cell walls and increases matrix permeability, thereby facilitating solvent penetration into intracellular structures and mass transfer. This enhanced diffusion allows for intracellular phytochemicals to be released more efficiently into the extraction medium. In contrast, absolute solvents, such as 96% methanol or ethanol, may reduce matrix hydration and limit the diffusion of polar compounds, resulting in lower extraction efficiency [9,26,27,28,29].

By systematically comparing a modern extraction and a conventional maceration across different solvent compositions, this work highlights the critical influence of extraction parameters on the recovery of phenolic compounds and flavonoids, which are widely regarded as key contributors to antioxidant and anti-inflammatory activities. The results demonstrated that UAE, particularly in methanol-based systems, could maximize the extraction efficiency of total phenolic and flavonoid contents compared with maceration. While prolonged solvent–matrix contact can enhance phenolic solubilization, ultrasonic treatment under certain solvent conditions may induce partial degradation or re-association of labile phenolic compounds [27]. Therefore, the application of ultrasound markedly reduces extraction time and solvent consumption, which ultimately makes this technique advantageous for scale-up in pilot and industrial applications. Although 70% methanol provided the highest efficiency in ultrasonic botanical extraction, its practical application in large-scale processes will be limited due to the toxicity of methanol. In contrast, ethanol represents a safer and more suitable alternative because it is widely accepted as a food-grade solvent and is commonly used in the extraction of bioactive compounds for food, nutraceutical, and cosmetic applications. In our present study, maceration with 70% ethanol yielded the highest polyphenol and flavonoid contents, while in ultrasonic extraction, 50% ethanol produced TPC and TFC values comparable to those obtained with 70% methanol. These findings demonstrate that ethanol–water mixtures can effectively replace methanol in achieving comparable extraction efficiency while offering a safer and more industry-compatible alternative for the recovery of bioactive compounds from *C. hakodae* flowers.

### 2.2. Phytochemical Profile of Camellia hakodae Ninh Flowers

The combined GC-MS and LC-QTOF-MS/MS analyses provided complementary information for the comprehensive profiling of metabolites present in *C. hakodae* flowers. GC-MS detects volatile and semi-volatile constituents such as fatty acids, esters, terpenoids, and sterols, whereas LC-QTOF-MS/MS enables the identification of more polar and non-volatile metabolites, including polyphenols, catechins, and flavonoid glycosides. The coexistence of phenolic compounds, terpenoid lactones, unsaturated fatty acids, and phytosterols suggests that the biological effects of *C. hakodae* flower extracts can collectively contribute to the observed biological activities through complementary effects [30].

GC-MS analysis revealed a complex chemical profile, which is dominated by phenolic derivatives, fatty acids and their esters, terpenoid lactones, and phytosterols (Table 1). Early eluting phenolic compounds, such as 4-vinylphenol (4.47 min), and furan derivatives are well known for their radical-scavenging and antimicrobial activities, which are largely attributed to the presence of phenolic hydroxyl groups that confer strong electron-donating capacity [1,15]. Notably, loliolide was detected as one of the prominent constituents. This monoterpene-derived lactone has been widely reported to exert antioxidant, anti-inflammatory, and immunomodulatory effects in both plant and marine sources [31,32], suggesting that it may play a contributory role in the biological activities observed for *C. hakodae* flower extracts. A substantial proportion of the GC-MS profile consisted of long-chain fatty acids, including α-linolenic acid (11.02 min), stearic acid (11.16 min), together with their corresponding esters. Unsaturated fatty acids are known to modulate oxidative stress and inflammatory signaling pathways, partly through the regulation of eicosanoid synthesis and membrane fluidity [33]. Their presence, therefore, provides a rationale for the strong antioxidant and proteinase-inhibitory activities recorded in this study. Finally, phytosterols such as chondrillasterol (20.16 min) and stigmast-7-en-3-ol (20.73 min) were identified. Plant sterols have been reported to exhibit anti-inflammatory and immunomodulatory effects by downregulating pro-inflammatory mediators and inhibiting key enzymes involved in inflammatory cascades [34,35]. The occurrence of these compounds in *C. hakodae* flowers supports their anti-inflammatory results in the proteinase inhibition assay.

Complementarily, LC-QTOF-MS/MS analysis of the total extract of *C. hakodae* flowers in both negative and positive ionization modes (Table 2) revealed a chemically diverse metabolite profile dominated by precursors of phenolic acids, flavonoids, amino acid derivatives, organic acids, and vitamin-related metabolites, which underlies the strong antioxidant and anti-inflammatory properties observed experimentally, reinforcing the potential of this endemic Vietnamese species as a valuable source of multifunctional natural products for nutraceutical, cosmeceutical, and functional food applications. In the negative ion mode, several highly polar metabolites related to phenolic metabolism were detected at early retention times. Notably, quinic acid (0.759 min) is an important precursor of hydroxycinnamic and chlorogenic acids, which are widely recognized for their strong antioxidant properties through hydrogen-donating and metal-chelating mechanisms [15,36]. Its presence, therefore, offers a convincing explanation for the pronounced radical-scavenging activity observed in the DPPH assay. In the positive ion mode, a similar pattern of polar bioactive metabolites was observed. Powerful antioxidant polyphenols, such as procyanidin B2 (3.320 min) and catechin 7-β-D-xylopyranoside (3.982 min), and common flavonoids, such as isoquercitrin (7.942 min), quercetin 3-O-glucoside (9.5 min), quercetin (11.256 min), myricitrin (10.419 min), kaempferol 7-galactoside (10.872 min), and rutin (10.436 min), were identified. These compounds are well known for their roles in cellular redox regulation and metabolic homeostasis, further supporting the biological relevance of the extract. Beyond classical phenolics, the occurrence of amino acid derivatives such as D-pipecolic acid (1.035 min) suggests an additional layer of antioxidant protection. They have been reported to participate in redox homeostasis and stress-response pathways, thereby complementing the activity of polyphenols [1]. Moreover, vitamin-related compounds such as nicotinic acid (niacin) (RT 1.174 min) regulate NAD^+^/NADH redox balance and are involved in cellular defense against oxidative stress. Phenolic acid-related compounds and nitrogen-containing metabolites are known to modulate inflammatory responses through multiple mechanisms, including the inhibition of pro-inflammatory enzymes and suppression of key signaling pathways such as NF-κB and MAPK [18].

### 2.3. Assessment of Biological Activities

**Figure 2:**
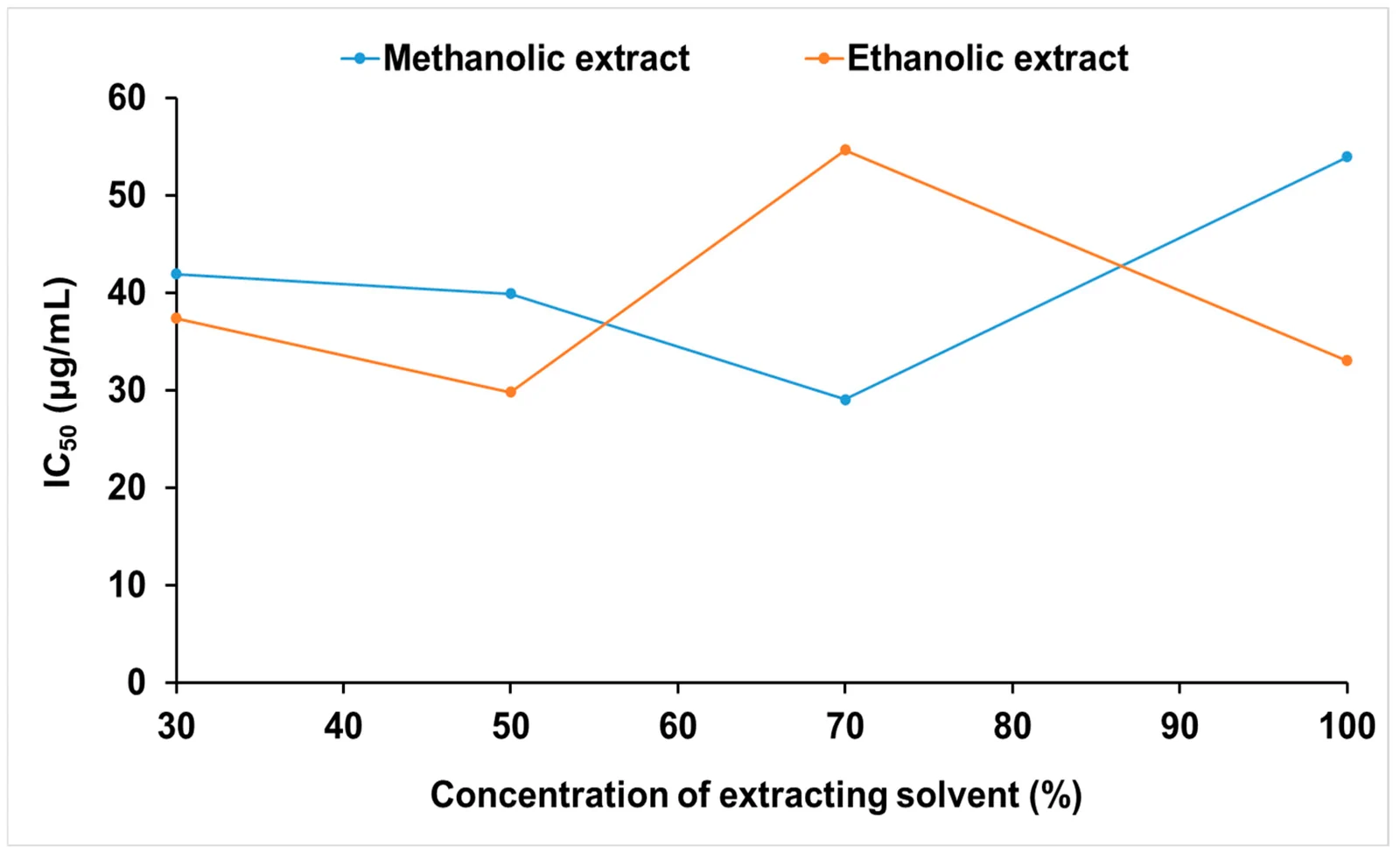
Antioxidant activities of the total extract from *Camellia hakodae* Ninh flowers.

The results demonstrated a clear dependence of antioxidant capacity on solvent type and its concentration (Figure 2). For methanolic extracts, antioxidant activity increased as the methanol concentration increased from 30% to 70%, reaching the lowest IC_50_ value at 70% methanol (29.06 µg/mL). However, a reduction in antioxidant activity was observed at methanol, suggesting that excessively high solvent strength may diminish extraction efficiency or affect the stability of compounds, as pure organic solvents often yield lower antioxidant activity than aqueous solvent mixtures in plant extracts [9,26,27,28,29]. The decline in antioxidant activity observed at methanol suggests that excessively high solvent strength may compromise extraction efficiency or affect the stability of certain compounds, corroborating previous findings that aqueous–organic solvent mixtures often outperform absolute organic solvents in recovering antioxidant constituents from plant matrices [9]. In contrast to the methanolic extract, the lowest activity was observed at 70% ethanol. The extract obtained with 50% ethanol exhibited the strongest activity (IC_50_ = 29.81 µg/mL). Both methanol and ethanol proved to be suitable solvents for extracting polyphenols and flavonoids from *C. hakodae* flowers. The IC_50_ values of the extract at the optimal solvent concentration are comparable to those of ascorbic acid (28.16 µg/mL), demonstrating the strong antioxidant capacity of *C. hakodae* flowers and confirming their potential as a rich natural source of polyphenols and flavonoids with potential applications in functional foods, cosmetics, and health-promoting products.

The antioxidant power also appears to be related to the contents of polyphenol as well as other phytochemical compositions of the extract. Extracts obtained under conditions that produced higher TPC and TFC generally exhibited stronger radical-scavenging capacity, as reflected by lower IC_50_ values in the DPPH assay. This observation is consistent with numerous studies [8,15,17,26]. However, this relationship is not always strictly proportional. Although the total phenolic contents of the maceration extracts obtained with 70% methanol (88.3 mgGAE/g, the second highest TPC) and 50% ethanol (59.1 mgGAE/g, the second highest TPC) differed, their antioxidant power was found to be comparable. This observation indicates that antioxidant capacity is not solely determined by the total amount of phenolic compounds but also by the qualitative composition of phytochemicals and their interactions within the extract matrix. LC-QTOF-MS/MS analysis revealed the presence of several antioxidant-related metabolites, including phenolic acid derivatives such as quinic and hydroxycinnamic acids, as well as flavonoid-related compounds such as flavonol glycosides and catechin derivatives. These compounds are known to exhibit significant radical-scavenging capacity due to their hydroxylated aromatic structures and conjugated systems [9,22]. In addition, GC-MS analysis detected other classes of bioactive compounds, including fatty acids, terpenoid derivatives such as loliolide, and phytosterols, which have also been reported to contribute to antioxidant and anti-inflammatory activities through complementary mechanisms. The coexistence of these diverse metabolite classes may lead to cooperative or synergistic interactions among phytochemicals, thereby enhancing the overall antioxidant performance of the extracts. Such synergistic effects among phenolics and other secondary metabolites have been widely documented in plant extracts and may explain why samples with different total phenolic levels can exhibit similar antioxidant capacities [30]. Therefore, the comparable antioxidant activities observed in this study are likely associated with differences in phytochemical composition and the combined biological effects of multiple metabolite classes identified by LC-MS and GC-MS analyses. The strong antioxidant activity observed for extracts obtained under optimized conditions further supports the functional relevance of the phenolic-rich profile of *C. hakodae* flowers. The IC_50_ values of DPPH radical scavenging, comparable to ascorbic acid, indicate a high radical-quenching capacity, which can be mechanistically linked to the abundance of hydroxylated phenolic structures capable of donating hydrogen atoms and stabilizing reactive oxygen species.

The ethanolic extract from *C. hakodae* flowers exhibited a concentration-dependent inhibitory effect on proteinase (trypsin) activity. At lower concentrations (0.625–1.25 mg/mL), the inhibition rates were only about 13–24%, but an increasing trend was obviously observed. When the concentration increased to 2.5 mg/mL, the inhibition exceeded 50% (51.96%), and at 10 mg/mL, the inhibitory effect reached 86.9%, demonstrating a strong anti-inflammatory activity. The extract showed a strong inhibitory potency with an IC_50_ value of 2.72 mg/mL, which was lower than that of acetylsalicylic acid, indicating superior enzyme inhibition. The lower IC_50_ value relative to acetylsalicylic acid highlights the potent enzyme-inhibitory activity of the extract and indicates a complementary contribution of multiple phytochemical classes, including polyphenols, flavonoids, and saponins, that can interact directly with proteolytic enzymes [30].

The phytochemical composition identified in *Camellia hakodae* flowers is consistent with reports on other species within the genus *Camellia*. The most extensively studied species, *Camellia sinensis*, has been shown to contain abundant phenolic compounds, including catechins, flavonol glycosides, and phenolic acid derivatives [23], which are largely responsible for its well-documented antioxidant and anti-inflammatory activities [4,5]. Similarly, phytochemical investigations of *Camellia japonica* flowers have revealed the presence of flavonoids and phenolic constituents associated with significant antioxidant potential [24]. The LC-QTOF-MS/MS analysis in the present study revealed comparable classes of metabolites in *C. hakodae* flowers, including phenolic acid derivatives and flavonoid-related compounds, suggesting that this species shares common phytochemical features with other members of the genus. Extracts of *C. nitidissima* have been reported to inhibit nitric oxide production and downregulate iNOS expression in LPS-stimulated RAW264.7 macrophages, with IC_50_ values ranging from 15 to 25 µg/mL, demonstrating strong anti-inflammatory activity primarily attributed to flavonoids and polyphenols [37]. Similarly, saponins isolated from *C. oleifera* have shown potent inhibitory effects on inflammatory enzymes and cytokines through suppression of NF-κB and MAPK signaling pathways, highlighting the important role of saponin-type compounds in anti-inflammatory responses [38]. In comparison, the 96% ethanolic extract of *C. hakodae* flowers exhibited even stronger proteinase inhibition than acetylsalicylic acid, suggesting that flavonoids and other phenolic compounds may play an important role in the observed anti-inflammatory activity. Although saponins have been reported in other *Camellia* species with anti-inflammatory properties, their presence in *C. hakodae* flowers was not specifically investigated in the present study. Though *C. japonica* seed oil has been shown to reduce nitric oxide production and suppress iNOS and COX-2 expression via modulation of NF-κB and AP-1 pathways, its anti-inflammatory efficacy is generally lower than that of flavonoid-rich extracts [39]. Collectively, these findings indicate that *C. hakodae* represents a particularly promising medicinal resource within the genus, underscoring its potential for development into natural anti-inflammatory formulations derived from Vietnamese plants.

Taken together, these findings demonstrate that the biological activities of *C. hakodae* flower extracts cannot be attributed to a single compound class but rather arise from the concerted action of multiple metabolite groups, including phenolics, flavonoids, fatty acids, terpenoids, and sterols. This multifactorial nature of activity not only reinforces the relevance of whole-extract approaches in functional product development but also underscores the importance of advanced chromatographic and mass spectrometric techniques in elucidating complex phytochemical–bioactivity relationships. From an applied perspective, the identification of optimal extraction conditions, combined with the demonstration of strong antioxidant and anti-inflammatory activities and a rich, chemically diverse metabolite profile, positions *C. hakodae* flowers as a promising candidate for further development in nutraceuticals, functional foods, and cosmeceuticals. Moreover, as an endemic species, the valorization of *C. hakodae* also carries implications for sustainable utilization of Vietnamese plant resources and the creation of region-specific high-value natural products. Future studies on the isolation of key bioactive constituents, in vivo validation of biological effects, and scale-up will be essential to fully translate these findings into practical applications.

## 3. Materials and Methods

### 3.1. Chemicals and Reagents

All solvents used for chromatographic analysis were of LC-MS or GC-MS grade from Merck (Darmstadt, Germany). If not otherwise stated, all reagents were of analysis grade and were used directly without further purification.

### 3.2. Plant Materials

**Figure 3:**
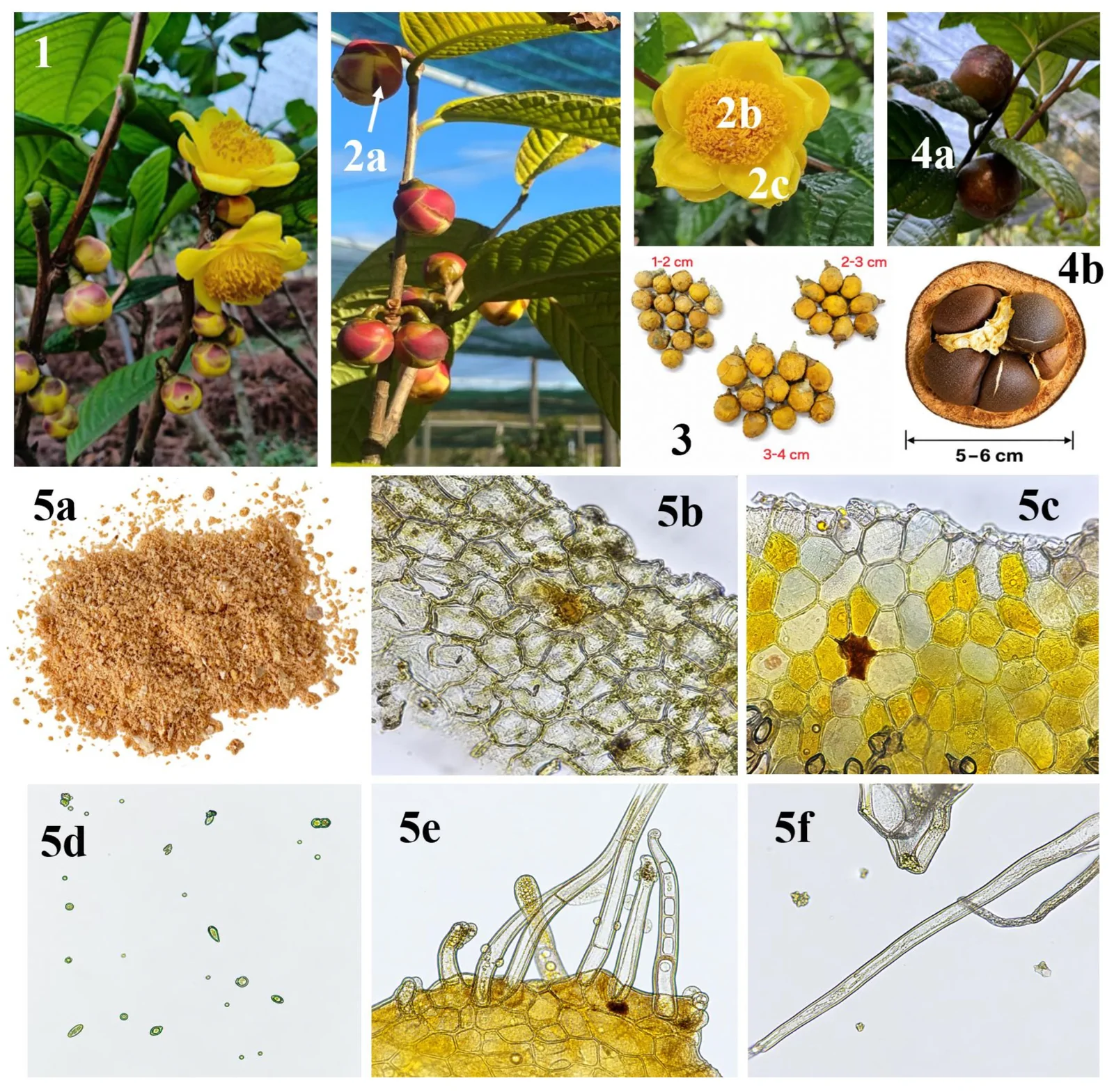
Morphological characteristics of *Camellia hakodae* Ninh flowers: (**1**) whole plant; (**2**) flower—(**2a**) sepal, (**2b**) petal, and (**2c**) stamen; (**3**) dried flowers of different sizes; (**4**) fruit—(**4a**) fruit and (**4b**) transverse section of mature fruit showing a four-locular ovary; and (**5**) microscopic characteristics of powdered flowers—(**5a**) flower powder, (**5b**) fragment of sepal epidermis, (**5c**) fragment of petal epidermis, (**5d**) pollen grain, (**5e**) fragment of petal with covering trichomes, and (**5f**) fragment of filament.

Fresh flowers of *C. hakodae* Ninh were harvested in Vinh Phuc province, Vietnam and thoroughly cleaned to remove dust and pest-damaged parts. The plant material was taxonomically identified based on morphological characteristics according to the botanical description of the species (Figure 3), and further confirmed by DNA barcoding analysis conducted by Phu Sa Genomics Co., Ltd. (Can Tho City, Vietnam). Only the intact flowers were selected and carefully separated from other plant parts. The flowers were then dried under controlled conditions at 40 °C to preserve the bioactive compounds and natural aroma of the material, reaching a final moisture of 8.02 ± 0.11%. The dried flowers were subsequently ground into fine powder and stored in airtight containers at 4 °C, protected from light and moisture, until further extraction.

### 3.3. Extraction Procedure

Flower powder of *C. hakodae* Ninh (2.0 g) was extracted by maceration with methanol (MeOH) and ethanol (EtOH), respectively at different concentrations including 96%, 30%, 50%, and 70% *v*/*v* prepared by dilution of analytical-grade solvent (96%) with distilled water for 24 h to evaluate the effects of solvent composition and extraction method on the recovery of bioactive compounds. A solvent-to-material ratio of 25 mL/g was applied. After extraction, the mixtures were filtered, and the extracts were adjusted to a final volume of 50 mL. All experiments were conducted at room temperature (25 ± 2 °C).

In addition, ultrasonic-assisted extraction was performed, using the same solvent composition and solvent-to-material ratio as in maceration described above, to enable a direct comparison between extraction techniques. Samples were sonicated at 40 kHz for three consecutive cycles of 15 min each, for a total of 45 min [13,25,27,28]. After extraction, all samples were filtered and diluted to a final volume of 50 mL.

The resulting extracts were used for the determination of total polyphenol and flavonoid contents to optimize the extraction procedure. All the steps were carried out under light-protected conditions to avoid degradation of compounds.

### 3.4. Determination of Total Phenolic Content (TPC) and Flavonoid Content (TFC)

Total polyphenol content (TPC) was determined using the Folin–Ciocalteu colorimetric method with gallic acid as the reference standard [40]. The crude extract was diluted 50-fold with MeOH. Then, 30 µL of the diluted extract or gallic acid standards (10–60 µg/mL) were respectively mixed with 150 µL of 10% (*v*/*v*) Folin–Ciocalteu reagent in a 96-well microplate. The reaction mixtures were thoroughly mixed to ensure homogenization before incubation. After 5 min, 120 µL of 7.5% (*w*/*v*) sodium carbonate solution was added, and the reaction mixtures were incubated in the dark at room temperature for 60 min prior to absorbance measurement at 765 nm.

Total flavonoid content (TFC) was assessed by an aluminum chloride colorimetric assay using quercetin as the reference standard [41]. The crude extract was diluted 2-fold with MeOH. In brief, 50 µL of the diluted extract or quercetin standards (3.2–50 µg/mL) were respectively mixed with 10 µL of 1 M sodium acetate, 10 µL of 10% (*w*/*v*) AlCl_3_ solution, and 150 µL of methanol in a 96-well microplate. The mixtures were thoroughly mixed, and the microplate was covered with a plate lid to minimize solvent evaporation during incubation. The mixtures were allowed to react at room temperature in the dark for 40 min prior to measurement at 415 nm.

All measurements were performed in triplicate, together with a blank sample containing only reagents for TPC and TFC. Linear regression analysis was applied to calculate the slope, intercept, and coefficient of determination (R^2^), with accepted values determined to be above 0.990. The TPC (expressed as mg GAE/g of dried sample) and TFC (expressed as mgQE/g) in samples were quantified from the calibration curve y = 0.0121x + 0.0703 (R^2^ = 0.9952), and y = 0.0171x − 0.0132 (R^2^ = 0.9956), respectively.

### 3.5. Determination of Biological Activities

Antioxidant activity was evaluated using the DPPH radical scavenging assay following Khorasani et al. (2015) [42]. Briefly, 225 µL of diluted sample solutions of methanolic and ethanolic extracts obtained by maceration, respectively, were mixed with 75 µL of 0.3 mM DPPH solution in a 96-well microplate and incubated in the dark at 37 °C for 30 min. Absorbance was measured at 517 nm. Radical scavenging activity was calculated as (A_0_ − Aₜ)/A_0_ × 100, where A_0_ represents the absorbance of the control solution containing all reagents except the test sample, and Aₜ represents the absorbance of the sample or extract. Ascorbic acid was used as a positive control. Also, the concentration required to inhibit 50% of DPPH radicals (IC_50_) was determined from the dose–response curve. The extracts were evaluated at 10, 25, 32, 40, 50, 100, 200, and 400 µg/mL. Ascorbic acid, used as the positive control, was tested at 5, 10, 20, 30, 40, and 50 µg/mL. This range was selected to cover the reported IC_50_ value of ascorbic acid (28.16 µg/mL) and to ensure reliable interpolation of the concentration–response relationship. All experiments were performed in triplicate, and results were expressed as mean values.

Anti-inflammatory activity was assessed based on proteinase inhibition according to Oyedepo et al. (1995) [43] with minor modifications. The reaction mixture contained 200 µL of trypsin and 200 µL of solution from ethanolic extracts at 6 concentrations (0.625, 1.25, 2.5, 5, 10 and 20 mg/mL). Tris–HCl buffer (20 mM, pH 7.4) and acetylsalicylic acid (2, 2.75, 3.25, 3.5, 3.75 and 4 mg/mL) were used as negative and positive controls, respectively. After incubation at 37 °C for 5 min, 300 µL of 0.65% casein was added, and the mixture was further incubated for 30 min. The reaction was terminated with 400 µL of 10% TCA, followed by centrifugation at 14,000 rpm for 5 min. A defined volume of the extract solution (50 µL) was mixed with 625 µL of 500 mM sodium carbonate and 125 µL of 0.5 N Folin reagent, and absorbance was recorded at 660 nm. Proteinase inhibition was calculated as (C − D)/C × 100, where C and D are the absorbance of the negative control and sample or positive control, respectively. The IC_50_ of acetylsalicylic acid was 3.16 mg/mL.

The absorbance for the assays of TPC, TFC, antioxidant and anti-inflammatory activities was recorded on a Multiskan SkyHigh Microplate Spectrophotometer (Thermo Scientific, Singapore).

### 3.6. GC-MS and LC-QTOF-MS/MS Analysis

The dried material was extracted with n-hexane for GC-MS and methanol for LC-MS. The extracts were filtered through a 0.22 µm membrane filter to remove particulate matter and transferred into amber glass vials and stored at 4 °C prior to analysis.

GC–MS analysis was carried out on an Agilent 8890 GC (Santa Clara, CA, USA) coupled with a triple quadrupole mass spectrometer (Agilent 7010B) using an HP-5MS capillary column (30 m × 0.25 mm, 0.25 µm film thickness). Samples (1 µL) were injected in splitless mode with helium as the carrier gas at a constant flow rate of 1.0 mL/min. The injector temperature was set at 280 °C. Electron impact ionization was set at 70 eV, with the ion source and quadrupole temperatures of 280 °C and 150 °C, respectively. The temperature gradient is detailed in Table 3 for the total run time of 30 min. Mass spectra were acquired over an *m*/*z* range of 30–600, and interpretation was based on comparison with the NIST 2020 mass spectral library and MSD ChemStation software (https://agilent-msd-chemstation-classic-data-ana.software.informer.com/, accessed on 18 December 2024), with the accepted match index ≥ 80%.

LC-QTOF-MS/MS analysis was performed on an Agilent 1290 Infinity II UHPLC with Agilent 6546 Quadrupole Time-of-Flight (Q-TOF), equipped with an electrospray ionization (ESI) source. Chromatographic separation was carried out on an Agilent ZORBAX Eclipse XDB-C18 (2.1 × 100 mm, 1.8 μm), maintained at 35 °C. The binary mobile phase consisted of 5 mM ammonium formate and 0.1% formic acid in water (A) and 5 mM ammonium formate and 0.1% formic acid in methanol (B) adjusted to a constant flow rate of 0.3 mL/min according to the gradient elution program specified in Table 3. Mass spectra were acquired in both positive and negative ionization modes to improve metabolite detection and characterization.

## 4. Conclusions

This study provides a comprehensive insight into the phytochemical composition and biological potential of *Camellia hakodae* Ninh flowers, highlighting this endemic Vietnamese species as a valuable yet underexplored source of multifunctional natural products. The study shows that hydroalcoholic solvent systems significantly influence the recovery of phenolic and flavonoid compounds. Ultrasonic-assisted extraction with 70% methanol yielded the highest TPC and TFC, while ethanol-based extraction gave comparable extraction performance. Notably, extracts obtained with 70% methanol and 50% ethanol exhibited similar antioxidant activities, indicating that ethanol–water mixtures may represent a practical and safer alternative for the extraction of bioactive constituents. Comprehensive GC–MS and LC–QTOF–MS profiling revealed a chemically diverse metabolite composition, including phenolic acids, flavonoids, fatty acids, terpenoids, and phytosterols. These metabolites classes may collectively contribute to the antioxidant and anti-inflammatory activities observed in the extracts. Overall, the present findings contribute to the current knowledge of the phytochemistry and biological potential of this endemic Vietnamese species, and support their potential application as a natural source of bioactive compounds. Future investigations focusing on the isolation of key bioactive constituents, in vivo validation of biological effects, and process scale-up will be essential to facilitate the development of value-added products derived from this plant resource.

## Figures and Tables

**Table 1:** GC-MS analysis results of *Camellia hakodae* Ninh flowers.

No	Proposed Compounds	Retention Time (min)	Molecular Formula	Peak Area	Match Index (%)
1	4-Vinylphenol	4.4752	C_8_H_8_O	1,886,792,917.7	80.3
2	5-Hydroxymethylfurfural	4.5341	C_6_H_6_O_3_	3,716,254,587.5	82.4
3	Loliolide	8.1019	C_11_H_16_O_3_	962,805,901.3	90.3
4	Hexadecanoic acid, methyl ester	9.1252	C_17_H_34_O_2_	410,747,455.0	88.0
5	n-Hexadecanoic acid	9.4693	C_16_H_32_O_2_	11,325,485,523.5	93.1
6	Hexadecanoic acid, ethyl ester	9.7047	C_18_H_36_O_2_	1,388,637,728.4	93.1
7	9,12-Octadecadienoic acid, methyl ester	10.6012	C_19_H_34_O_2_	430,280,469.0	91.6
8	9,12,15-Octadecatrienoic acid, methyl ester	10.6601	C_19_H_32_O_2_	474,549,552.8	90.0
9	9,12,15-Octadecatrienoic acid	11.0268	C_18_H_30_O_2_	18,690,994,969.8	89.4
10	Octadecanoic acid	11.1626	C_18_H_36_O_2_	3,131,142,734.4	88.1
11	Hexadecanoic acid, 2-hydroxy-1- (hydroxymethyl)ethyl ester	14.0061	C_19_H_38_O_4_	2,039,430,851.1	92.3
12	Bis(2-ethylhexyl) phthalate	14.3185	C_24_H_38_O_4_	1,390,480,245.2	90.0
13	1,2-cyclohexanedicarboxylic acid, bis(2-ethylhexyl) ester	14.7260	C_24_H_44_O_4_	4,212,231,008.3	85.1
14	E,E,Z-1,3,12-nonadecatriene -5,14-diol	15.3644	C_19_H_34_O_2_	1,365,589,744.8	84.3
15	Chondrillasterol	20.1637	C_29_H_48_O	3,307,195,243.4	93.5
16	Stigmast-7-en-3-ol	20.7342	C_29_H_50_O	461,651,156.8	86.3

*The full list of compouds detected by GC-MS is available in Supplementary Materials.*

**Table 2:** Representative polyphenols and flavonoids in *Camellia hakodae* flowers identified by LC-QTOF-MS/MS.

No	Proposed Compounds	Retention Time (min)	Molecular Formula	Ionization ESI (+/−)	Mass (*m*/*z*)	Mass Error (ppm)	Match Index (%)
1	Proanthocyanidin A1	14.452	C_30_H_24_O_12_	−	576.1268	−0.64	93.88
2	Pyrocatechol (catechol)	1.579	C_6_H_6_O_2_	−	110.0367	−0.71	99.59
3	Catechin	3.041	C_15_H_14_O_6_	+	290.0795	1.73	96.73
4	Procyanidin B2	3.320	C_30_H_26_O_12_	+	578.1434	1.61	98.59
5	Isoquercitrin	7.942	C_21_H_20_O_12_	+	464.0958	0.73	98.80
6	Quercetin 3-O-glucoside (isoquercetin)	9.500	C_21_H_20_O_12_	+	464.0968	2.90	94.76
7	3-O-Methylquercetin	9.791	C_16_H_12_O_7_	+	316.0586	1.09	98.34
8	Myricitrin	10.419	C_21_H_20_O_12_	+	464.0958	0.68	98.92
9	Rutin	10.436	C_27_H_30_O_16_	+	610.1534	0.07	98.52
10	Naringin	11.047	C_27_H_32_O_14_	+	580.1795	3.71	85.28
11	Quercetin	11.256	C_15_H_10_O_7_	+	302.0435	2.93	95.08
12	Kaempferol 7-galactoside	10.872	C_21_H_20_O_11_	+	448.1008	0.44	98.28
13	Luteolin 7-galactoside	12.460	C_21_H_20_O_11_	+	448.1013	1.57	98.59
14	Isohamnetin 3-glucoside	13.157	C_22_H_22_O_12_	+	478.1115	0.69	98.11
15	Isovitexin	16.876	C_21_H_20_O_10_	−	432.1055	−0.26	99.46
16	Kaempferol	21.446	C_15_H_10_O_6_	−	286.0476	−0.63	99.47

*The full list of compouds detected by LC-QTOF-MS/MS is available in Supplementary Materials.*

**Table 3:** Summary of GC temperature gradient and LC gradient conditions.

Technique	Program
GC-MS	60 °C (hold 2 min) → 170 °C at 40 °C/min → 310 °C at 10 °C/min (hold 10 min)
LC-QTOF-MS/MS	98% A (0–0.5 min) → 50% A (1 min) → 35% A (4 min) → 5% A (10 min) → 0% A (12–16 min) → 98% A (18 min)

## Data Availability

Data is contained within the article or Supplementary Materials.

## Supplementary Materials

The following supporting information can be downloaded at: https://www.mdpi.com/article/10.3390/molecules31071088/s1.

## Abbreviations

The following abbreviations are used in this manuscript:

UAE	Ultrasonic-assisted extraction
GC–MS	Gas chromatography–mass spectrometry
LC-QTOF-MS/MS	Liquid chromatography-quadrupole time-of-flight tandem mass spectrometry
DPPH	2,2-diphenyl-1-picrylhydrazyl
ABTS	2,2′-azino-bis(3-ethylbenzothiazoline-6-sulfonic acid
TPC	Total phenolic content
TFC	Total flavonoid content
NF-Κb	Nuclear factor-κB
MAPK	Mitogen-activated protein kinase
COX	Cyclooxygenases
MeOH	Methanol
EtOH	Ethanol
NADH	Nicotinamide adenine dinucleotide + hydrogen
RT	Retention time
IC_50_	Half maximal inhibitory concentration
