# Correction: My et al. Phytochemical Characterization and Biological Evaluation of *Camellia hakodae* Ninh Flowers. *Molecules* 2026, *31*, 1088

**DOI:** 10.3390/molecules31213913

**Published:** 2026-10-30

**Authors:** Nguyen Hoang Thao My, Nguyen Huu Lac Thuy, Vo Thi Kim Khuyen, Nguyen Duc Tuan

**Affiliations:** 1 School of Pharmacy, University of Medicine and Pharmacy at Ho Chi Minh City, 41-43 Đinh Tien Hoang, Saigon Ward, Ho Chi Minh City 700000, Vietnam; nhtmy@ntt.edu.vn (N.H.T.M.); vtkkhuyen@ump.edu.vn (V.T.K.K.); 2 Faculty of Medical Laboratory Technology, Nguyen Tat Thanh University, 300A Nguyen Tat Thanh, Xom Chieu Ward, Ho Chi Minh City 700000, Vietnam

In the original publication [1], Figure 3 included images of different parts of the plant, whereas the figure title, research scope and results presented in this manuscript focus exclusively on the flowers. Therefore, the authors have replaced the original images with new images showing only the flowers and powdered flower material, ensuring consistency with the actual research subject of this study. The corrected Figure 3 appears below. The authors state that the scientific conclusions are unaffected. This correction was approved by the Academic Editor. The original publication has also been updated.

## Figures and Tables

**Figure 3:**
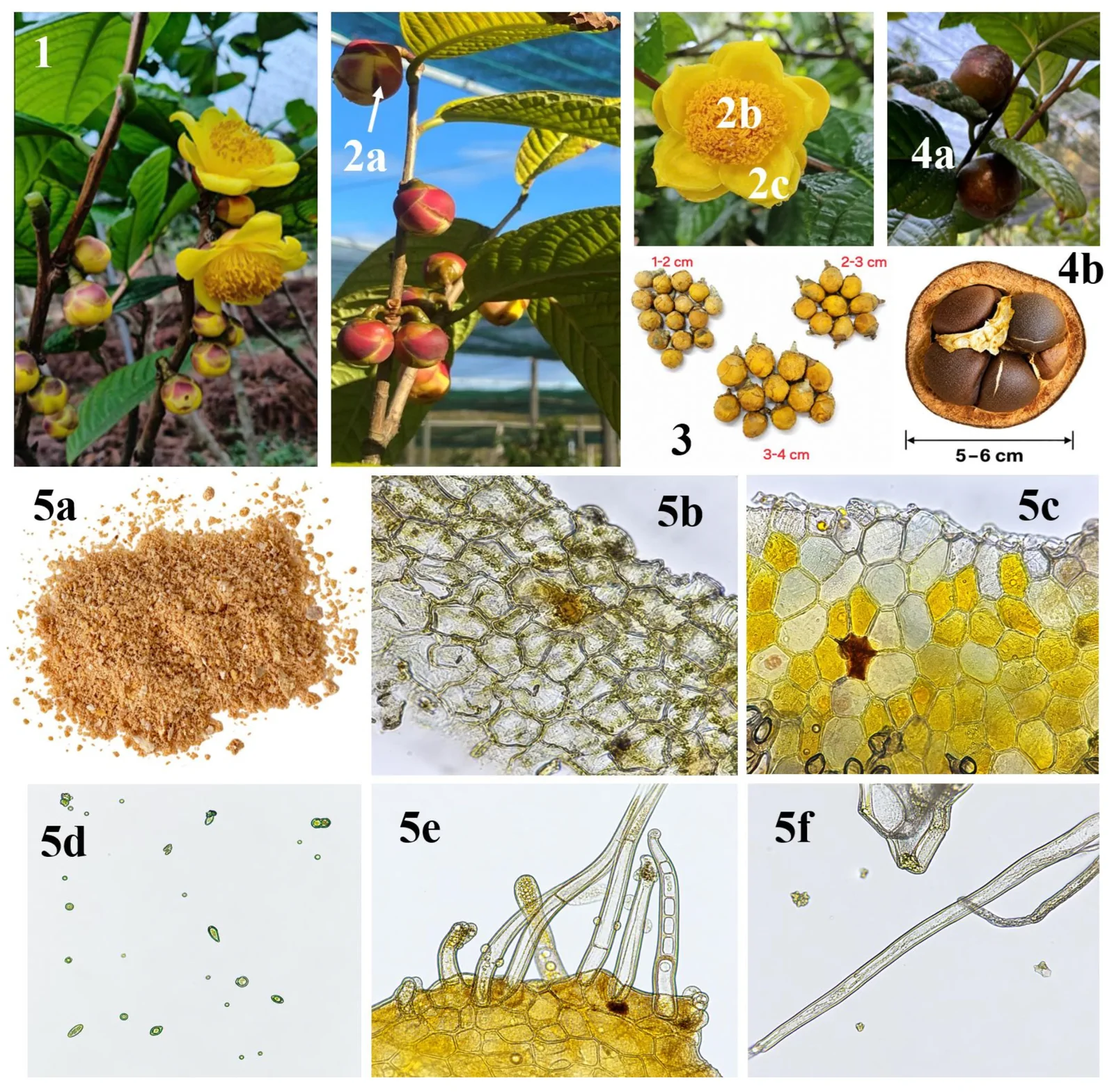
Morphological characteristics of *Camellia hakodae* Ninh flowers: (**1**) whole plant; (**2**) flower—(**2a**) sepal, (**2b**) petal, and (**2c**) stamen; (**3**) dried flowers of different sizes; (**4**) fruit—(**4a**) fruit and (**4b**) transverse section of mature fruit showing a four-locular ovary; and (**5**) microscopic characteristics of powdered flowers—(**5a**) flower powder, (**5b**) fragment of sepal epidermis, (**5c**) fragment of petal epidermis, (**5d**) pollen grain, (**5e**) fragment of petal with covering trichomes, and (**5f**) fragment of filament.
