# Supplementary material for: Phytochemical Characterization and Biological Evaluation of Camellia hakodae Ninh Flowers
Source: Molecules. 2026 Mar 26;31(7):1088. doi: 10.3390/molecules31071088 (PMC13074563; doi:10.3390/molecules31071088)

Library Search Results - NonTarget Hits with Details

**Batch Path**	D:\COE\Demo mau CHI MY\dATA		
**Analysis File Name**	Mau Chi My.uaf		
**Analyst Name**	DEMO	**Analysis Time**	12/18/2024 10:36:48 AM
**File Name**	Cao TP gcms re-2.D	**Path Name**	D:\COE\Demo mau CHI MY\dATA
**Sample Name**	Cao TP gcms	**Sample Type**	Sample
**Method Path Name**		**Method Version**	
**Acq. Method File**	fULLSCAN 2024	**Acq. Method Path**	D:\COE\Demo mau CHI MY\
**Acq. Date-Time**	11/15/2024 8:40:19 PM	**Acq. Operator**	
**Instrument Name**	GCTQ 7010B ALS	**Dil.**	1

Component RT	Compound Name	CAS#	Formula	Component Area	Match Factor	Estimated Conc.
3.8730	1,2,4-Cyclopentanetrione, 3-methyl-	4505-54-8	C6H6O3	330300837.9	72.4	
4.1809	4H-Pyran-4-one, 2,3-dihydro-3,5-dihydroxy-6-methyl-	28564-83-2	C6H8O4	1096529072.7	76.6	
4.4752	4-Vinylphenol	2628-17-3	C8H8O	1886792917.7	80.3	
4.5341	5-Hydroxymethylfurfural	67-47-0	C6H6O3	3716254587.5	82.4	
4.8646	1,3-Benzenediol, 2-methyl-	608-25-3	C7H8O2	1469604077.0	72.6	
4.9732	2-Methoxy-4-vinylphenol	7786-61-0	C9H10O2	1583931250.0	70.0	
7.1465	.epsilon.-N-Formyl-L-lysine	1190-48-3	C7H14N2O3	6790335298.4	70.3	
7.7442	Tetradecanoic acid	544-63-8	C14H28O2	1117071763.7	71.0	
8.1019	Loliolide	5989-02-6	C11H16O3	962805901.3	90.3	
8.5320	Acetamide, N-(4-ethoxy-3-hydroxyphenyl)-	16060-48-3	C10H13NO3	814099700.5	70.9	
9.1252	Hexadecanoic acid, methyl ester	112-39-0	C17H34O2	410747455.0	88.0	
9.4693	n-Hexadecanoic acid	57-10-3	C16H32O2	11325485523.5	93.1	
9.7047	Hexadecanoic acid, ethyl ester	628-97-7	C18H36O2	1388637728.4	93.1	
10.6012	9,12-Octadecadienoic acid (Z,Z)-, methyl ester	112-63-0	C19H34O2	430280469.0	91.6	
10.6601	9,12,15-Octadecatrienoic acid, methyl ester, (Z,Z,Z)-	301-00-8	C19H32O2	474549552.8	90.0	
11.0268	9,12,15-Octadecatrienoic acid, (Z,Z,Z)-	463-40-1	C18H30O2	18690994969.8	89.4	
11.1626	Octadecanoic acid	57-11-4	C18H36O2	3131142734.4	88.1	
14.0061	Hexadecanoic acid, 2-hydroxy-1-(hydroxymethyl)ethyl ester	23470-00-0	C19H38O4	2039430851.1	92.3	
14.3185	Bis(2-ethylhexyl) phthalate	117-81-7	C24H38O4	1390480245.2	90.0	
14.7260	1,2-Cyclohexanedicarboxylic acid, bis(2-ethylhexyl) ester	84-71-9	C24H44O4	4212231008.3	85.1	
15.2422	1,2-Cyclohexanedicarboxylic acid, bis(2-ethylhexyl) ester	84-71-9	C24H44O4	2646825304.6	77.1	
15.3644	E,E,Z-1,3,12-Nonadecatriene-5,14-diol	1000131-11-4	C19H34O2	1365589744.8	84.3	
15.4505	4,11-Dimethyl-8-(propan-2-yl)-5,12-dioxatricyclo[9.1.0.04,6]dodecan-7-ol	1000493-61-5	C15H26O3	730090951.4	74.1	
20.1637	Chondrillasterol	481-17-4	C29H48O	3307195243.4	93.5	
20.7342	Stigmast-7-en-3-ol, (3.beta.,5.alpha.)-	521-03-9	C29H50O	461651156.8	86.3	

Sample Chromatogram

+EI TIC Scan Cao TP gcms re-2.D (Cao TP gcms)

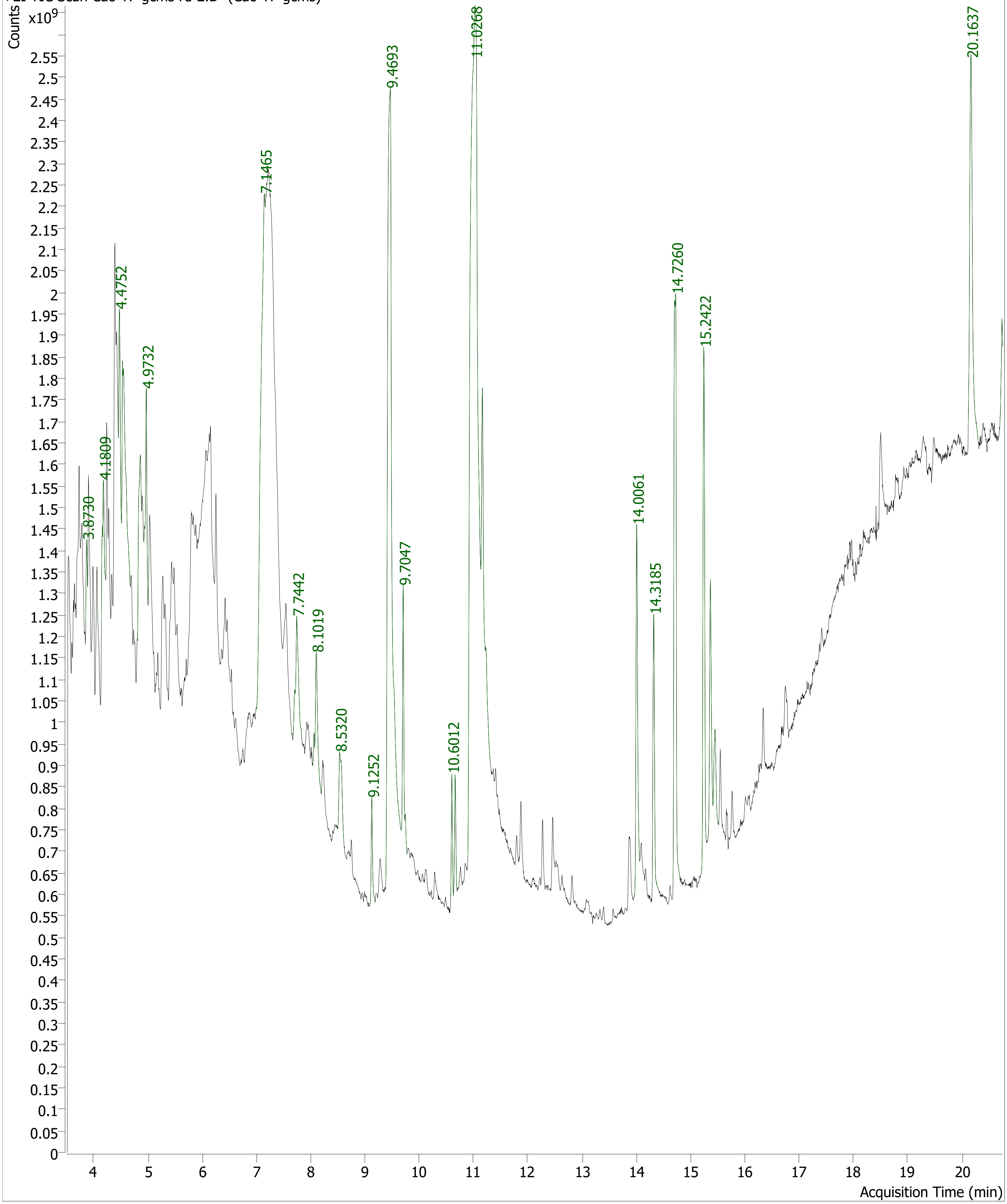

Library Search Results - NonTarget Hits with Details

Component RT	Compound Name	Component Area	Match Factor	CAS#	Formula	Estimated Conc.
3.8730	1,2,4-Cyclopentanetrione, 3-methyl-	330300837.9	72.4	4505-54-8	C6H6O3	

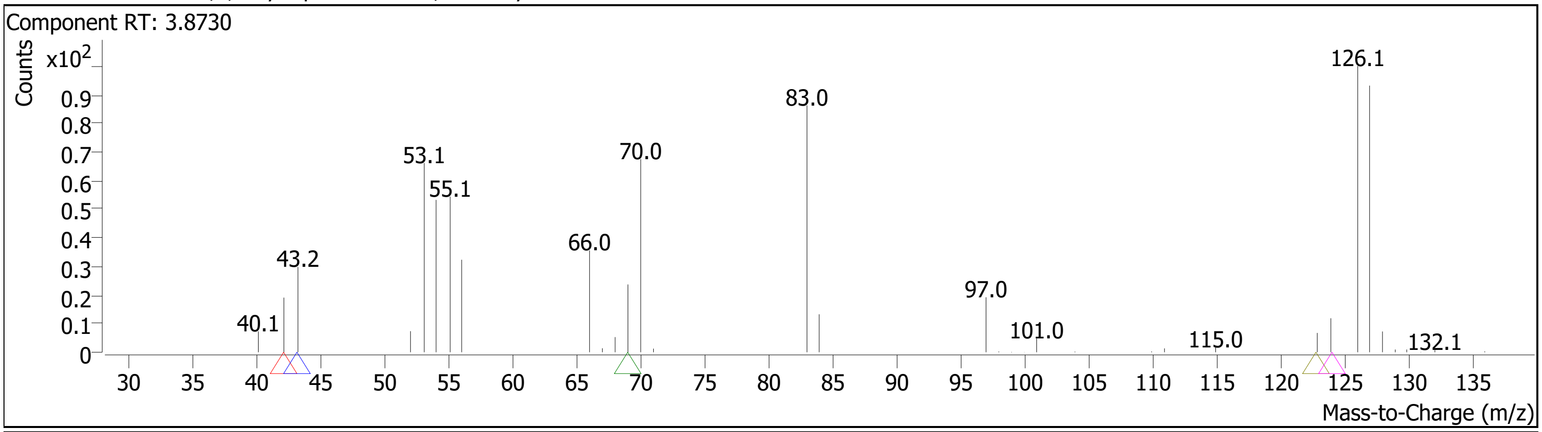

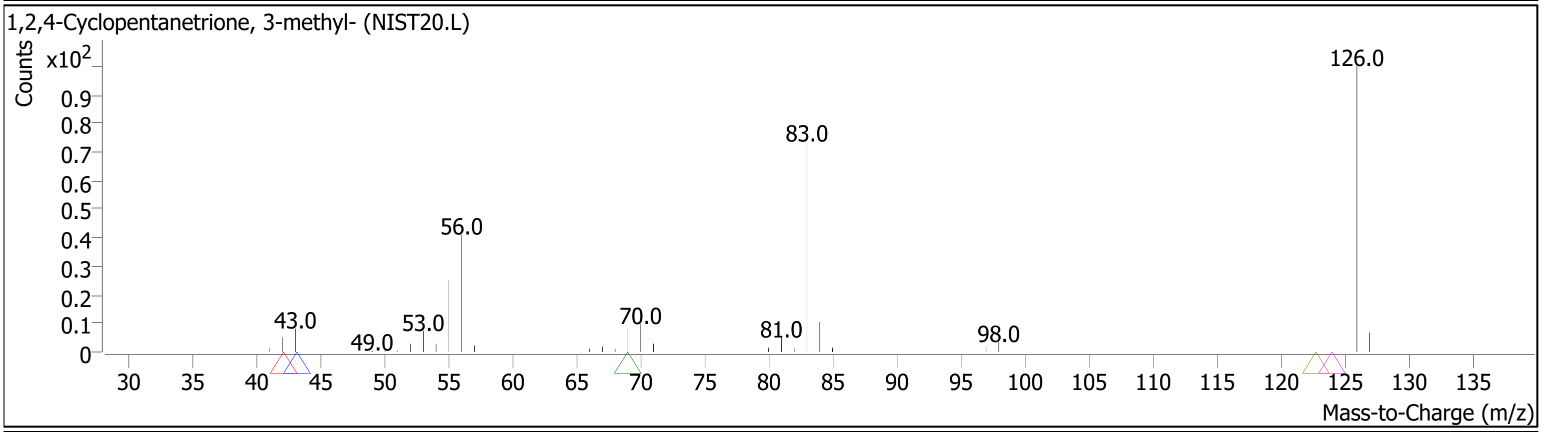

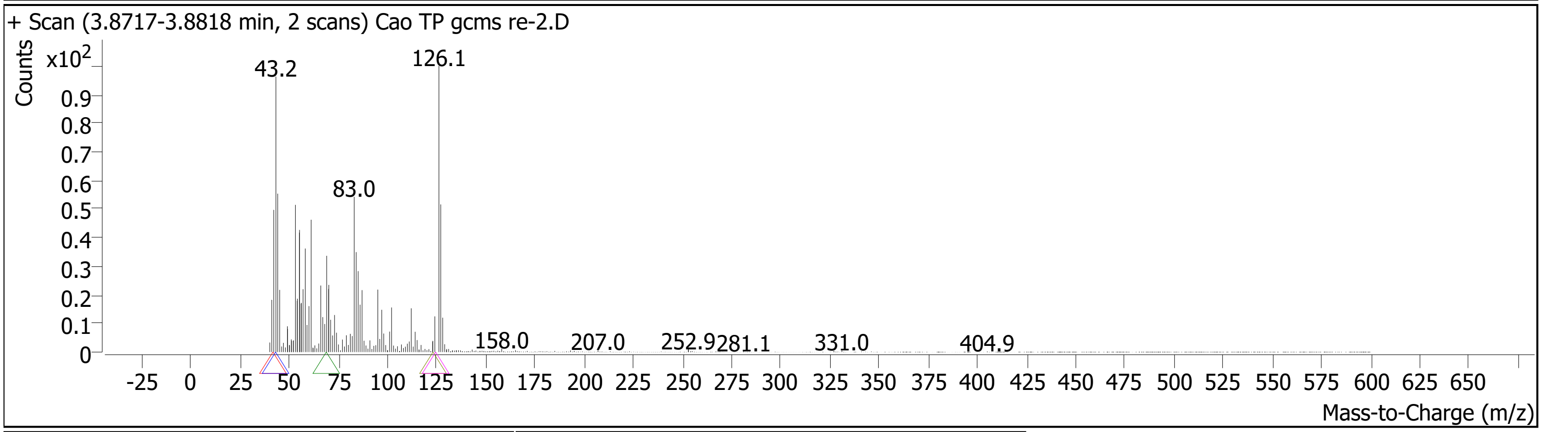

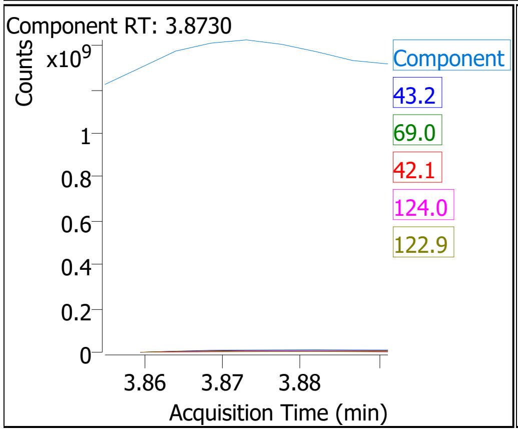

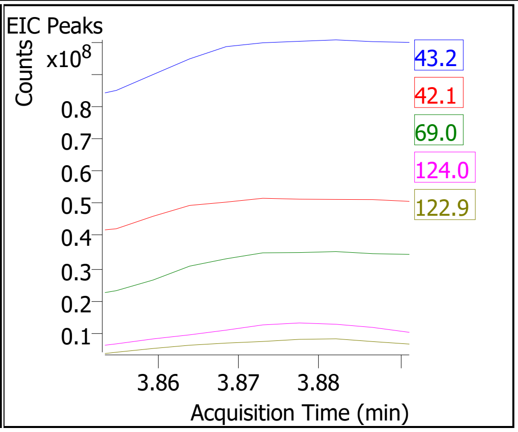

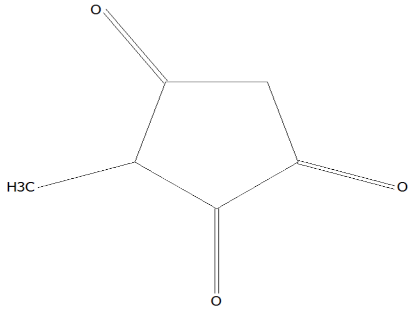

Library Search Results - NonTarget Hits with Details

Component RT	Compound Name	Component Area	Match Factor	CAS#	Formula	Estimated Conc.
4.1809	4H-Pyran-4-one, 2,3-dihydro-3,5-dihydroxy-6-methyl-	1096529072.7	76.6	28564-83-2	C6H8O4	

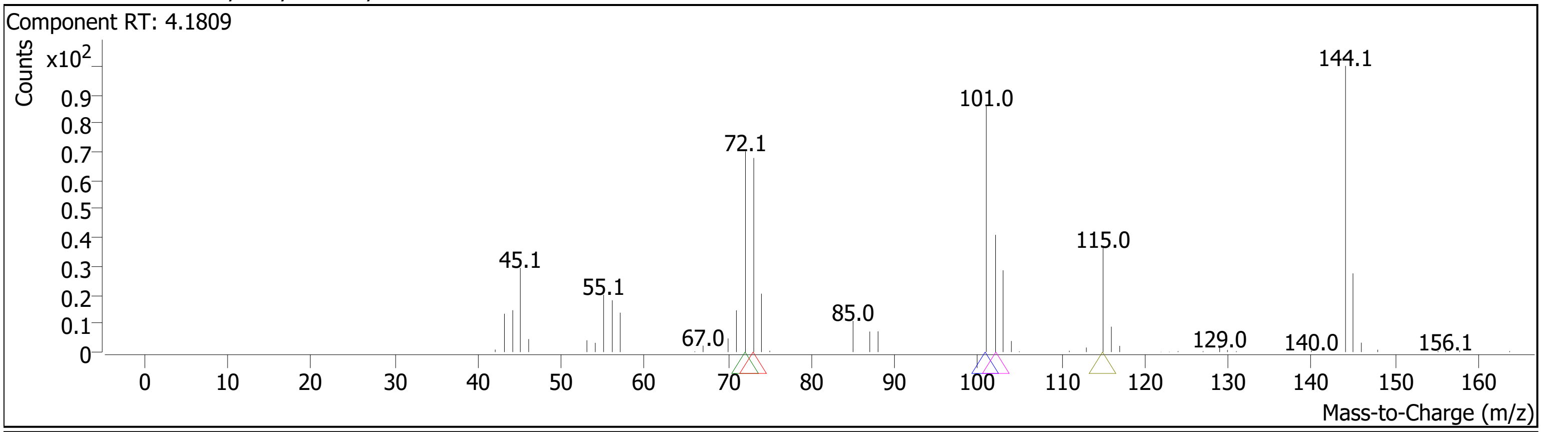

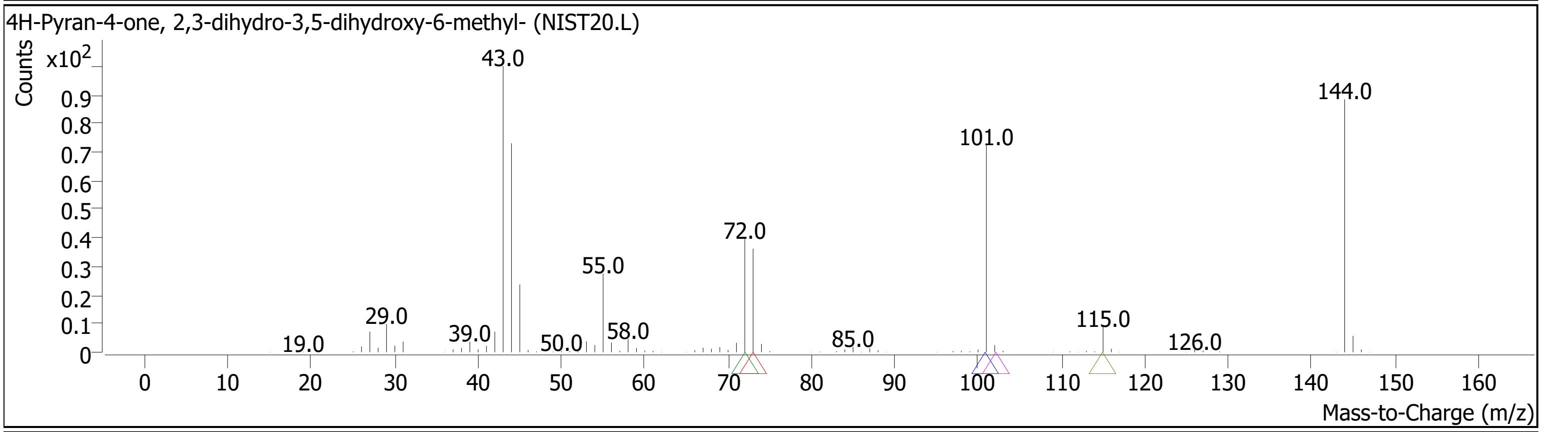

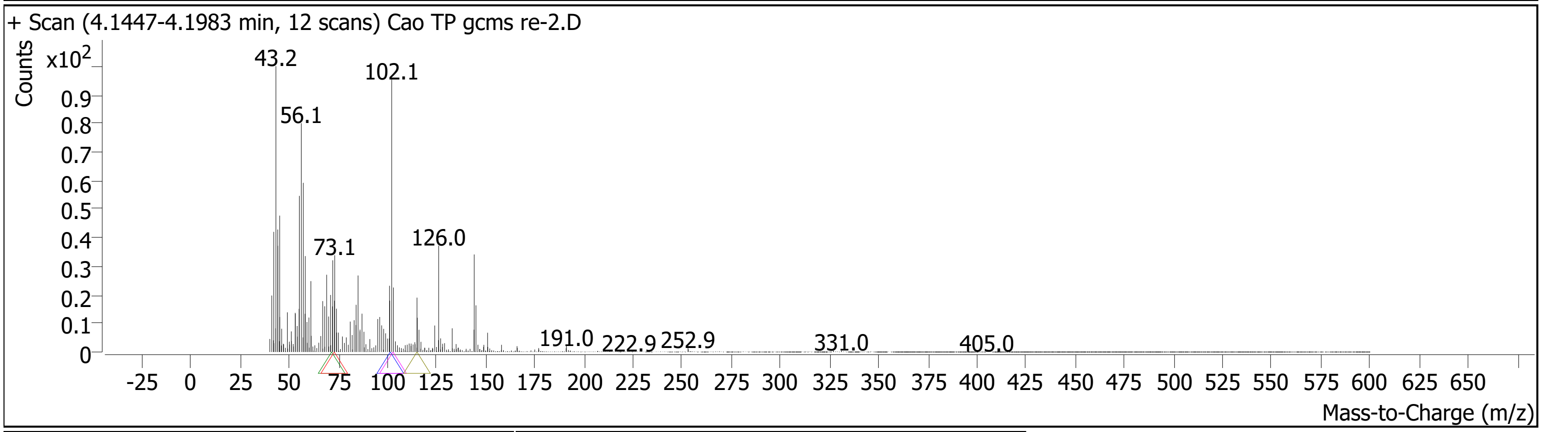

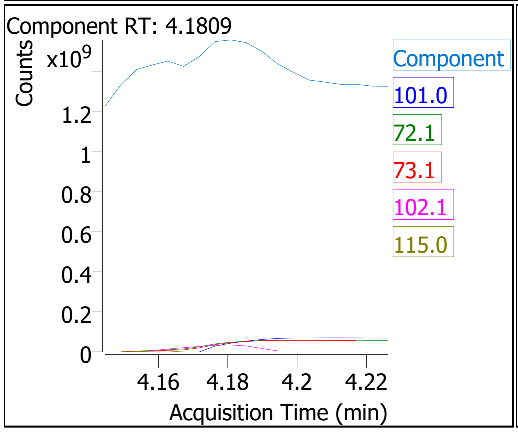

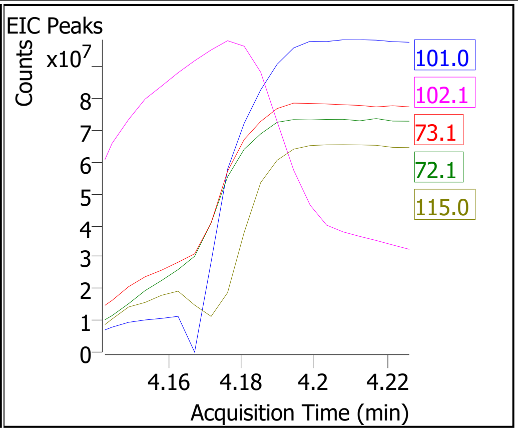

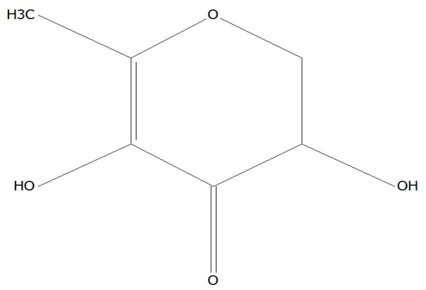

Library Search Results - NonTarget Hits with Details

Component RT	Compound Name	Component Area	Match Factor	CAS#	Formula	Estimated Conc.
4.4752	4-Vinylphenol	1886792917.7	80.3	2628-17-3	C8H8O	

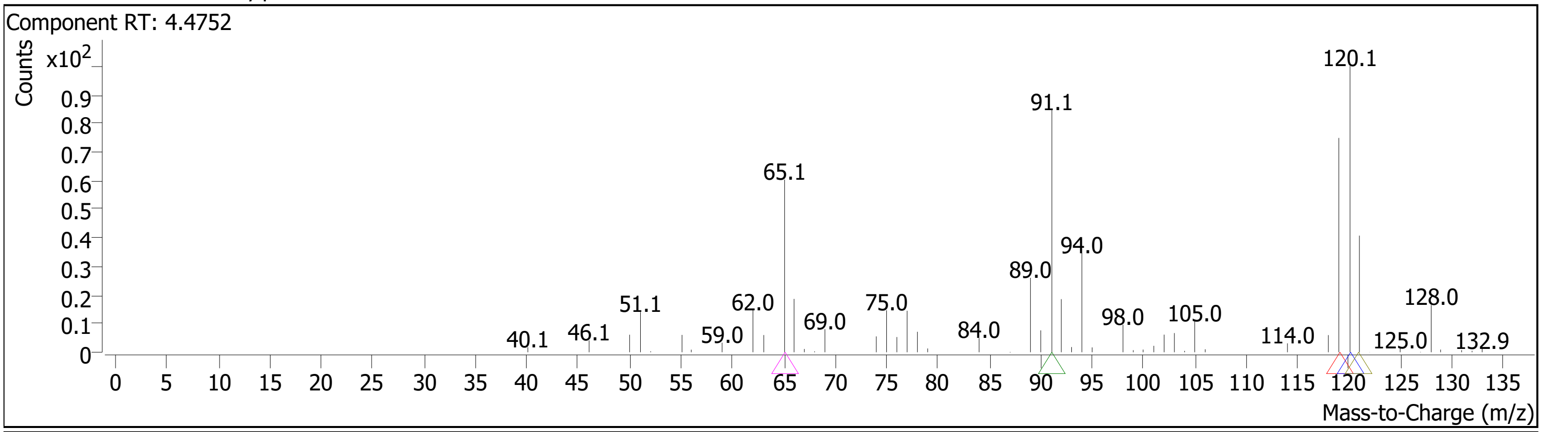

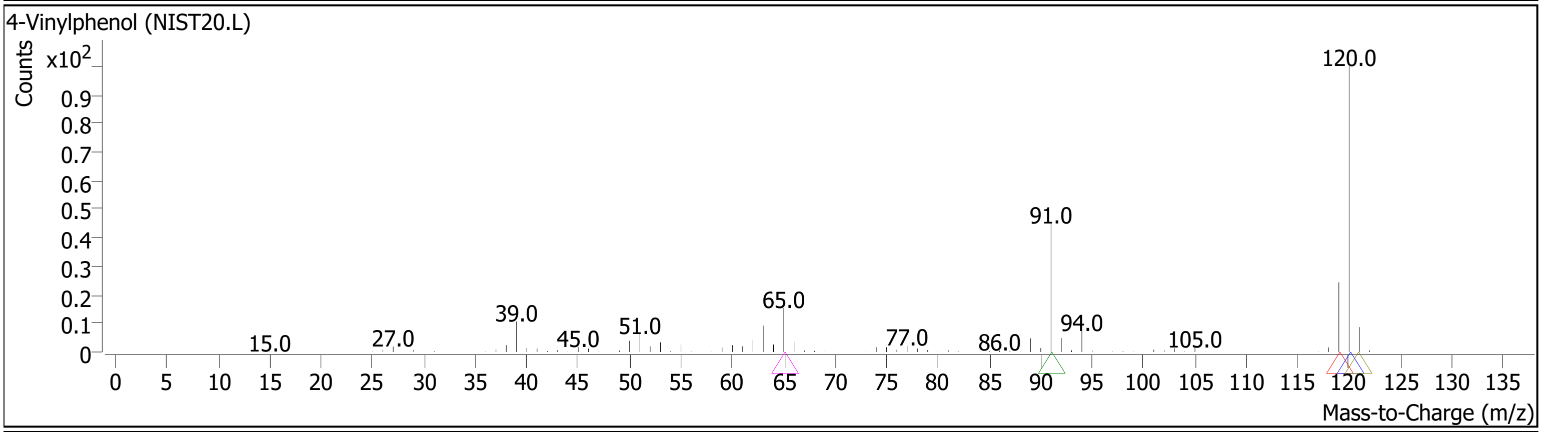

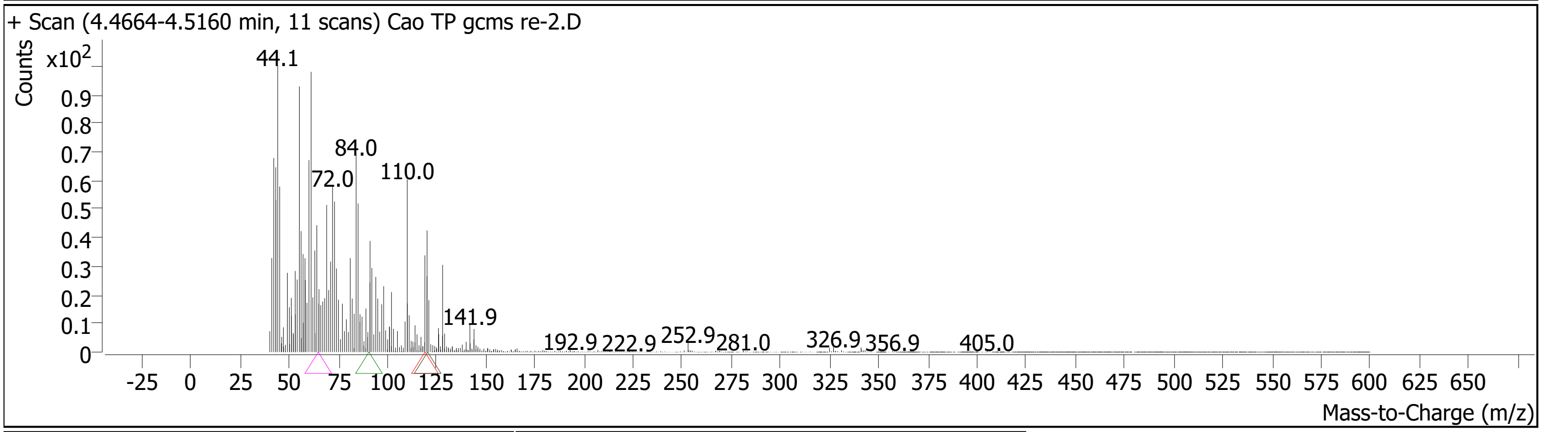

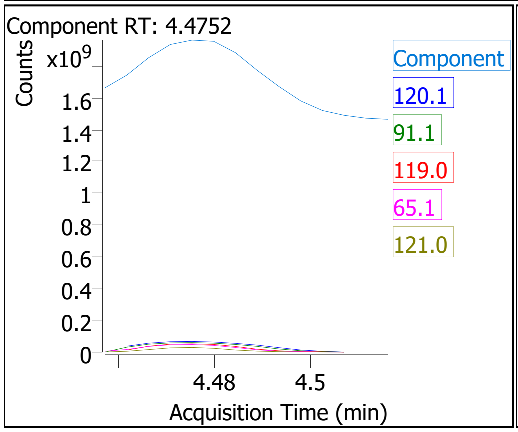

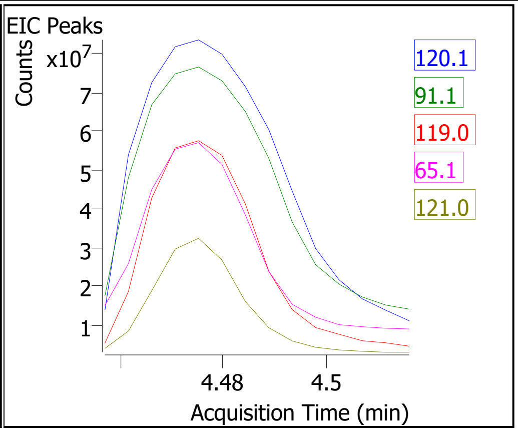

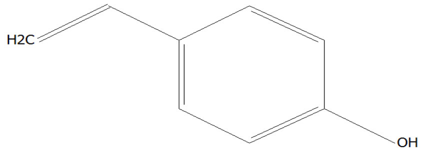

Component RT	Compound Name	Component Area	Match Factor	CAS#	Formula	Estimated Conc.
4.5341	5-Hydroxymethylfurfural	3716254587.5	82.4	67-47-0	C6H6O3	

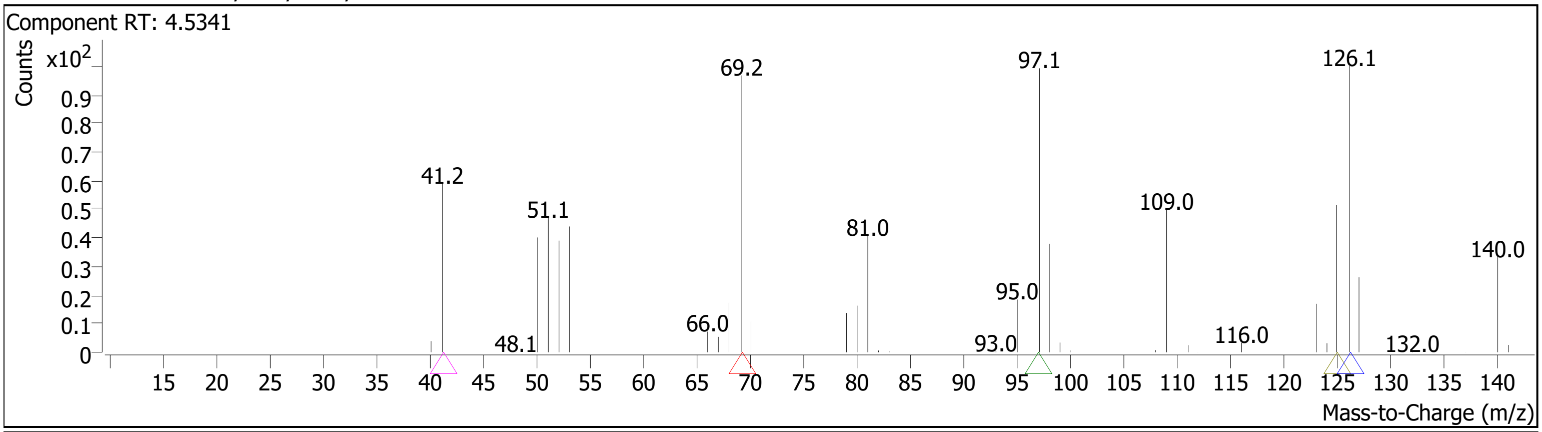

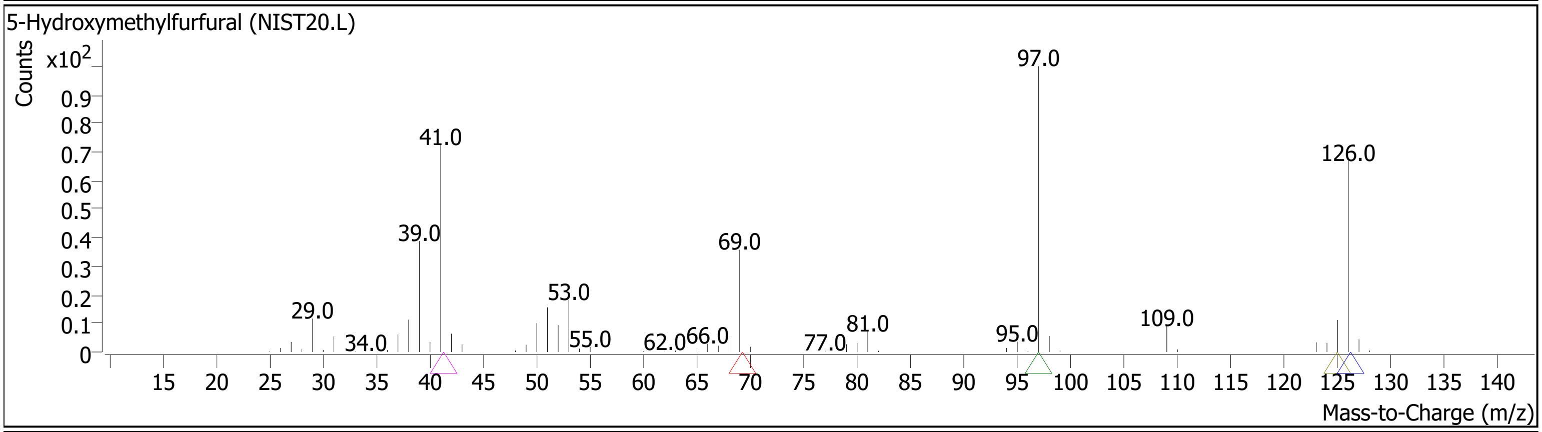

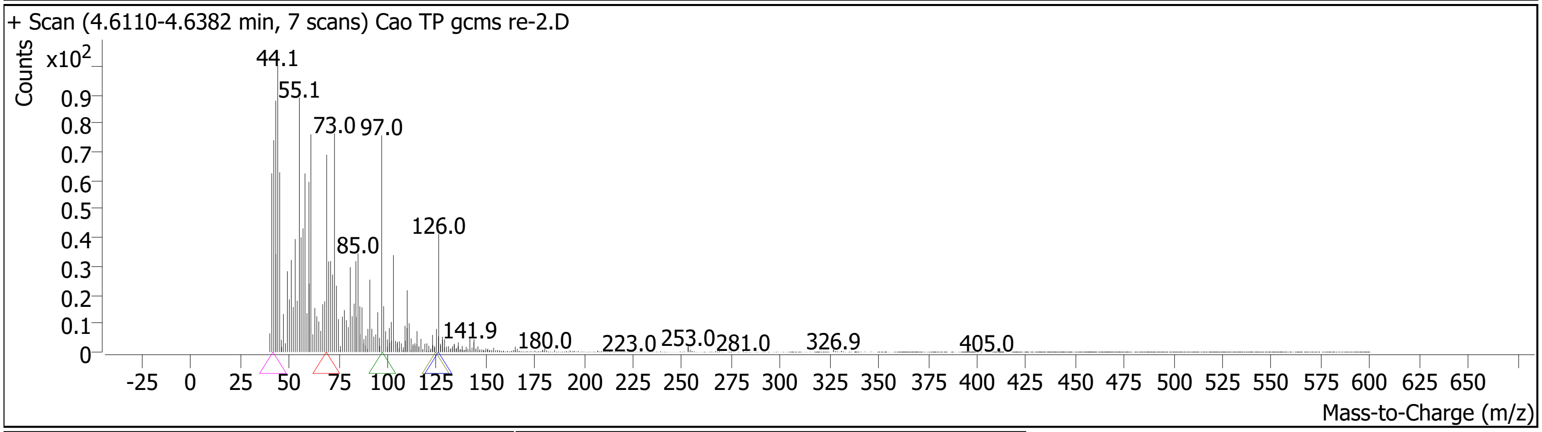

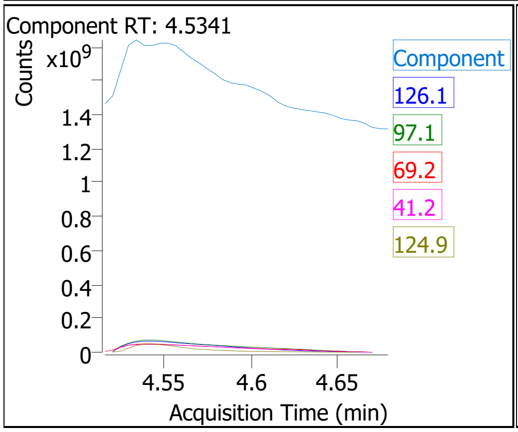

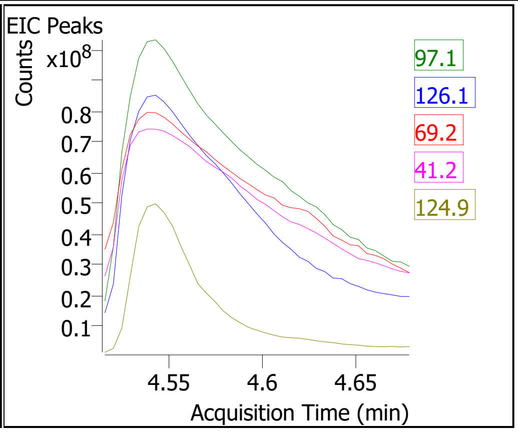

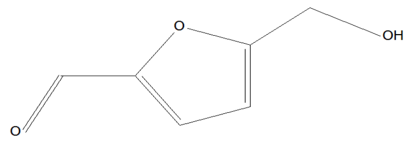

Library Search Results - NonTarget Hits with Details

Component RT	Compound Name	Component Area	Match Factor	CAS#	Formula	Estimated Conc.
4.8646	1,3-Benzenediol, 2-methyl-	1469604077.0	72.6	608-25-3	C7H8O2	

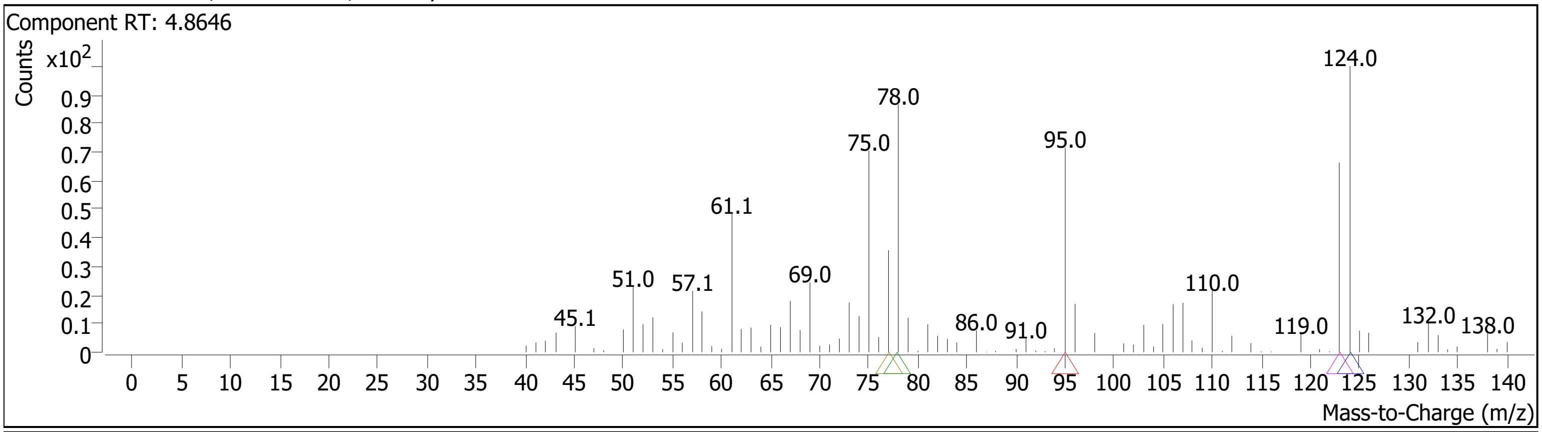

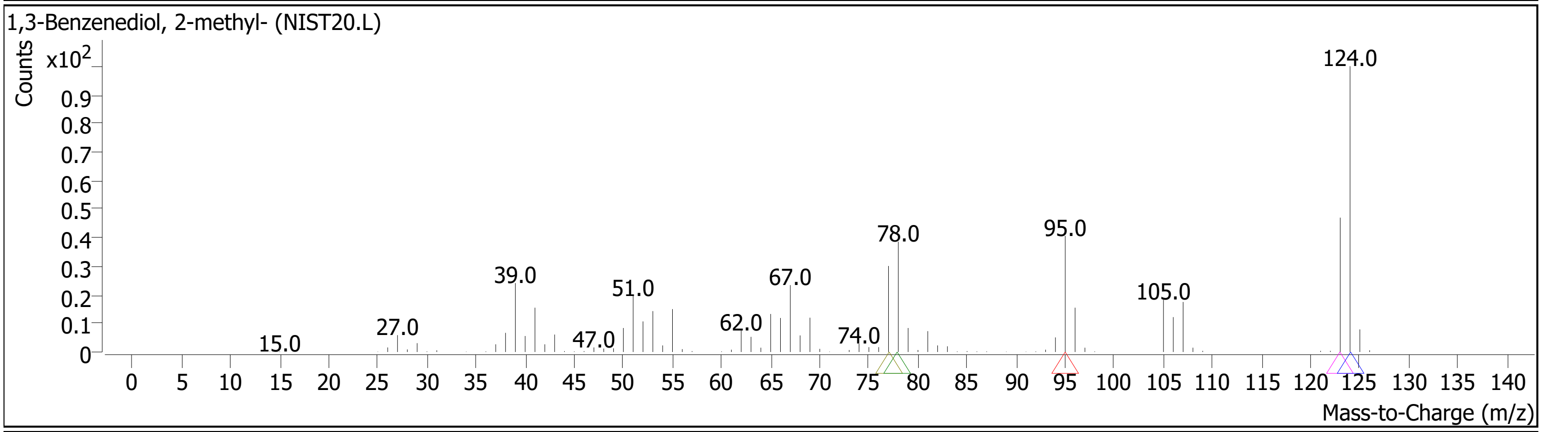

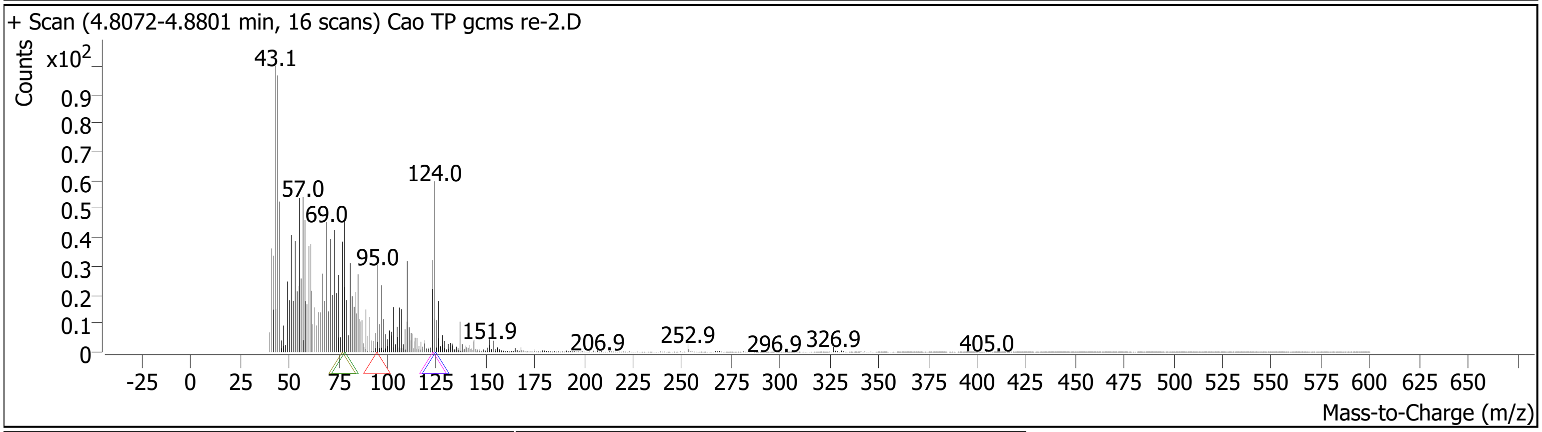

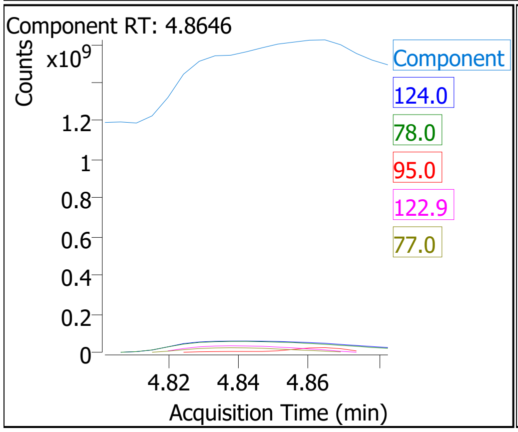

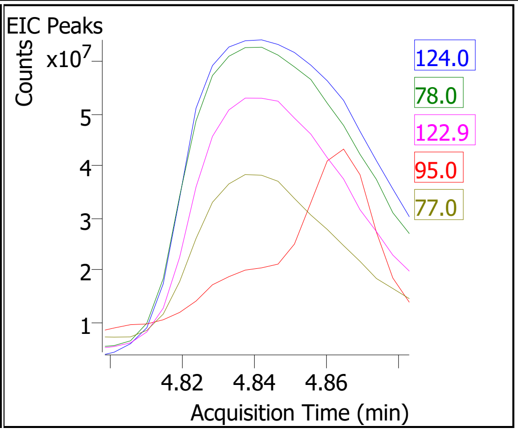

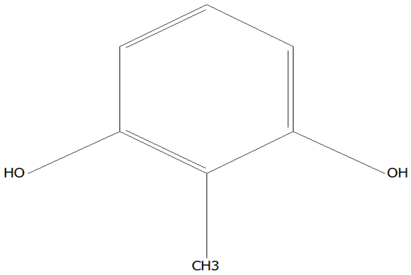

Library Search Results - NonTarget Hits with Details

Component RT	Compound Name	Component Area	Match Factor	CAS#	Formula	Estimated Conc.
4.9732	2-Methoxy-4-vinylphenol	1583931250.0	70.0	7786-61-0	C9H10O2	

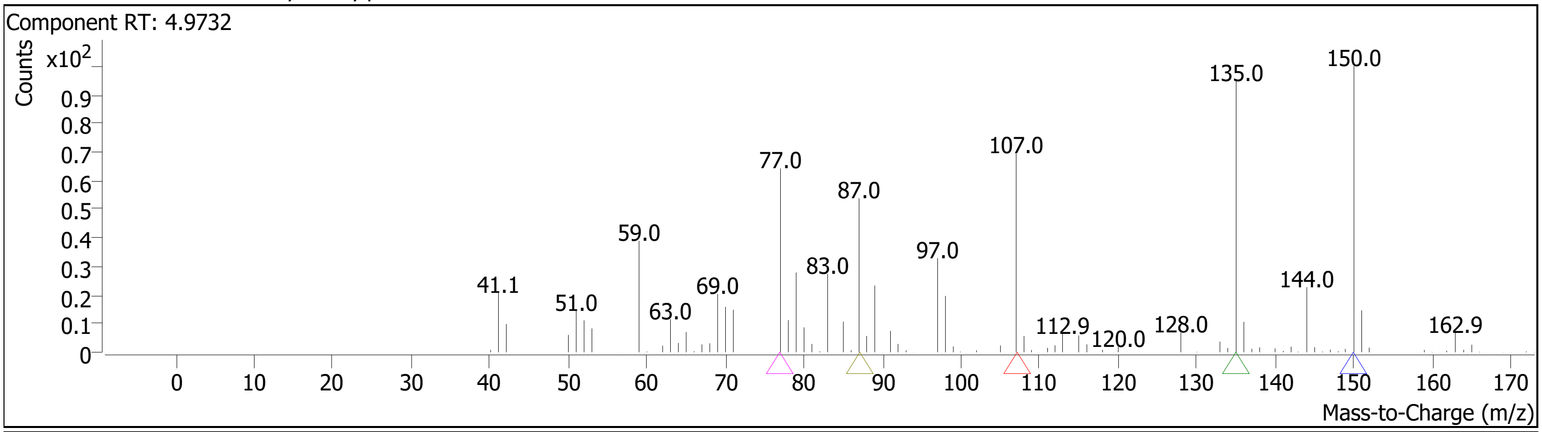

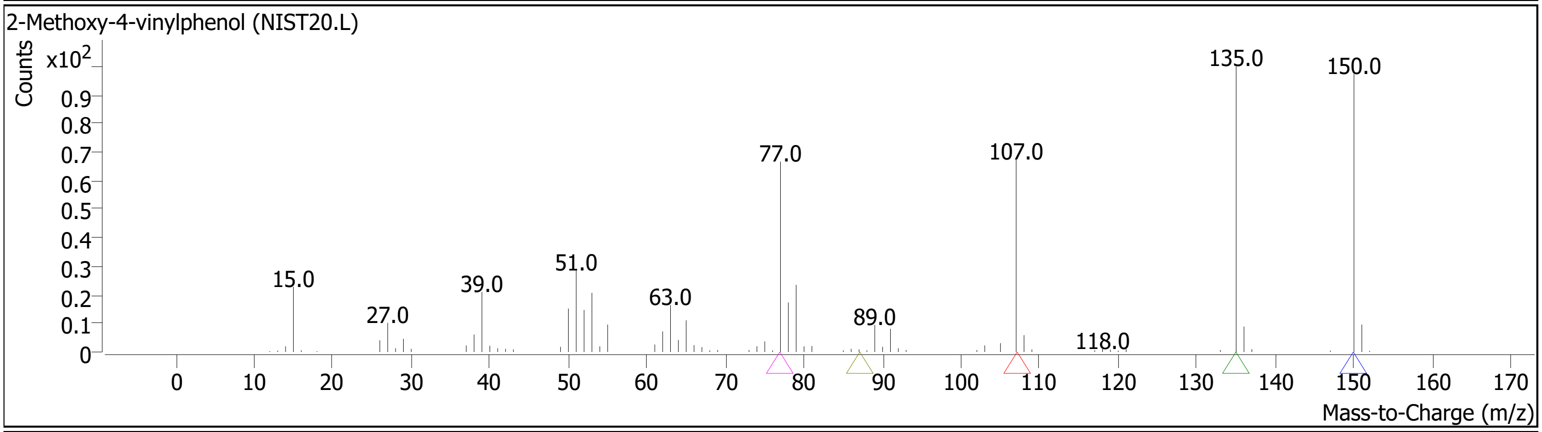

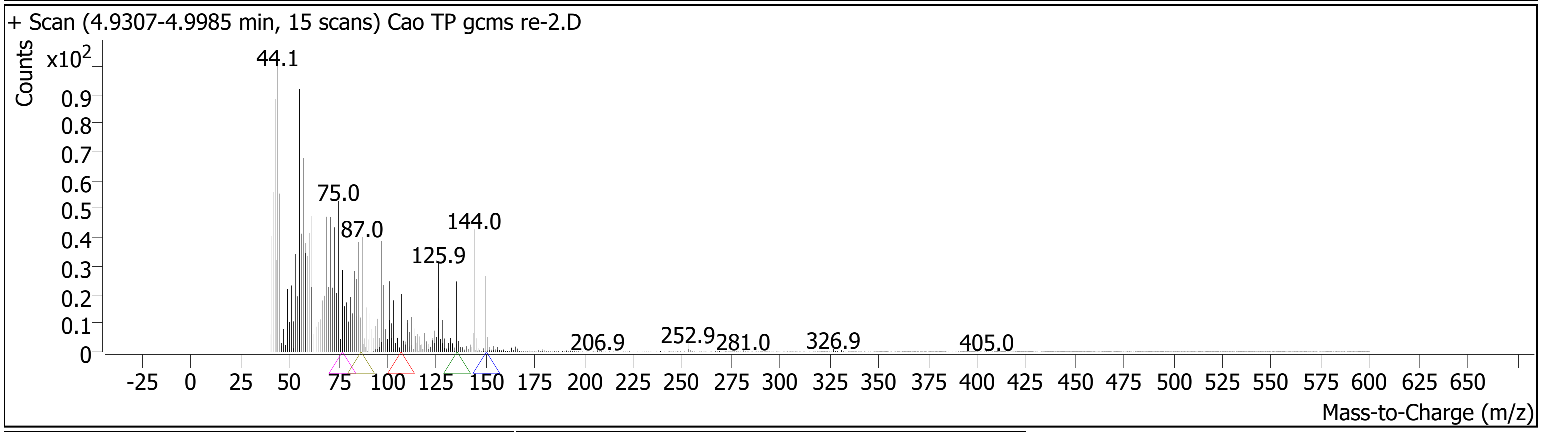

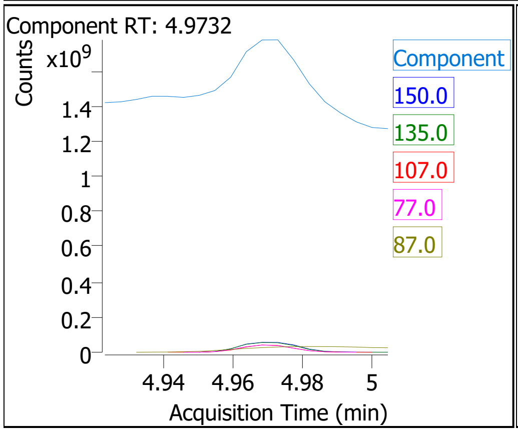

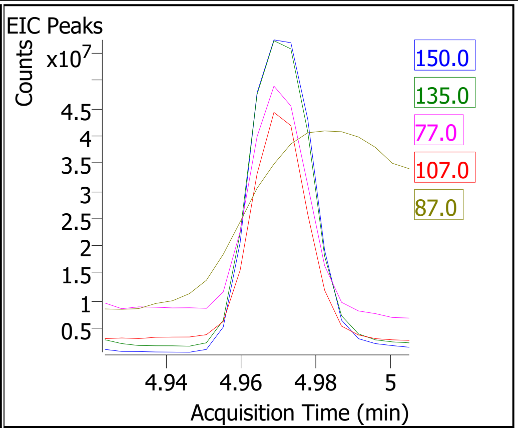

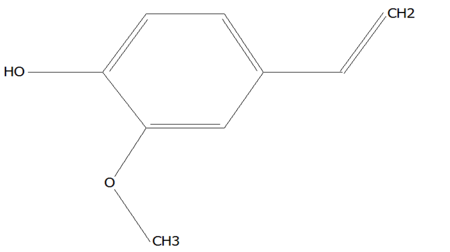

Library Search Results - NonTarget Hits with Details

Component RT	Compound Name	Component Area	Match Factor	CAS#	Formula	Estimated Conc.
7.1465	.epsilon.-N-Formyl-L-lysine	6790335298.4	70.3	1190-48-3	C7H14N2O3	

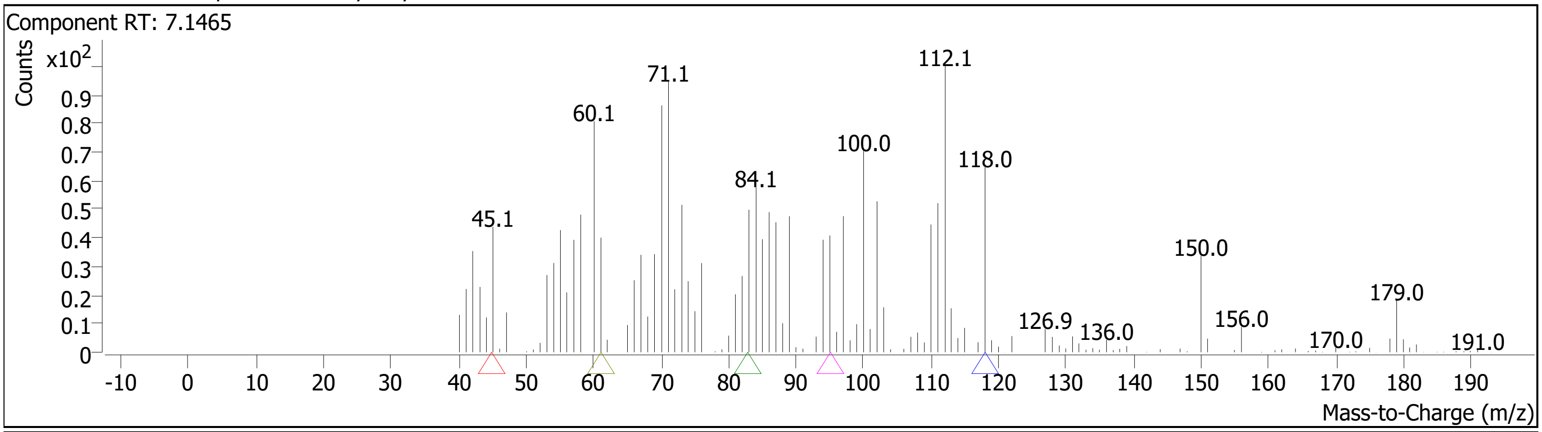

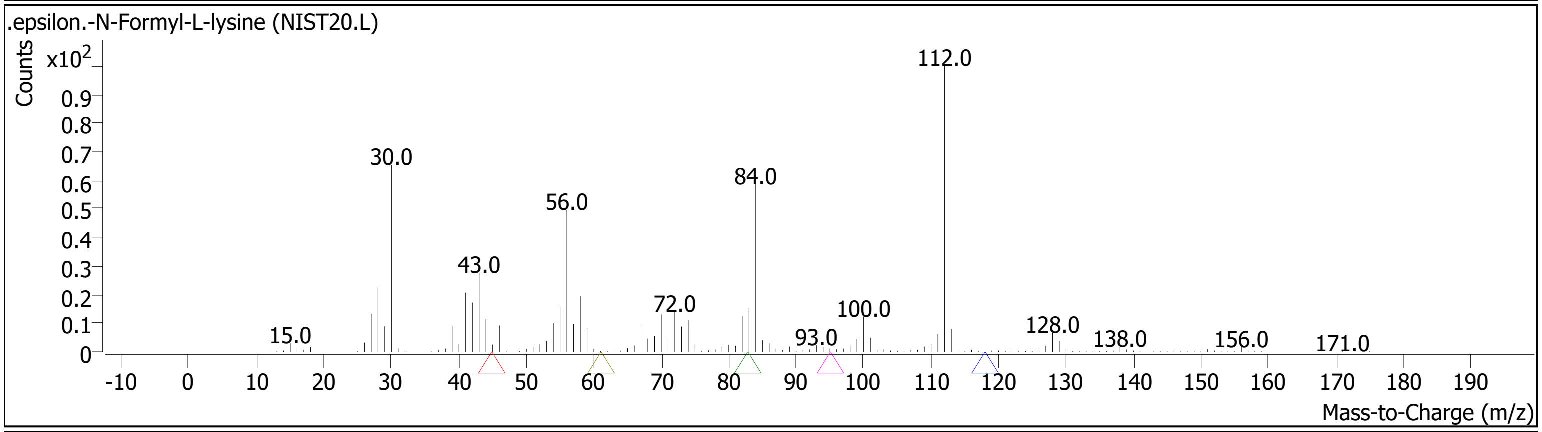

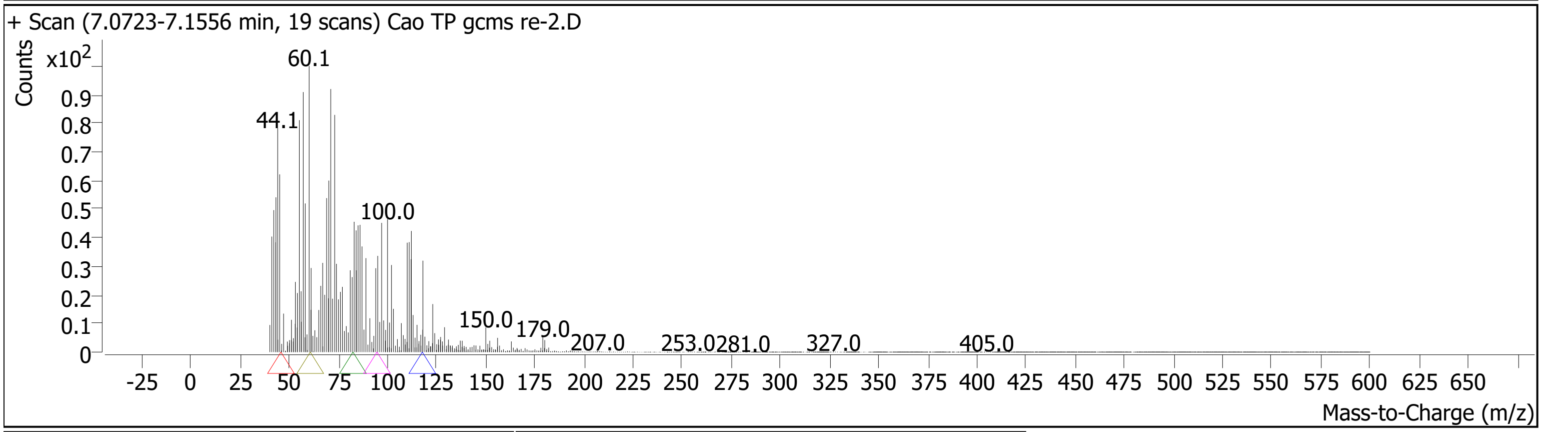

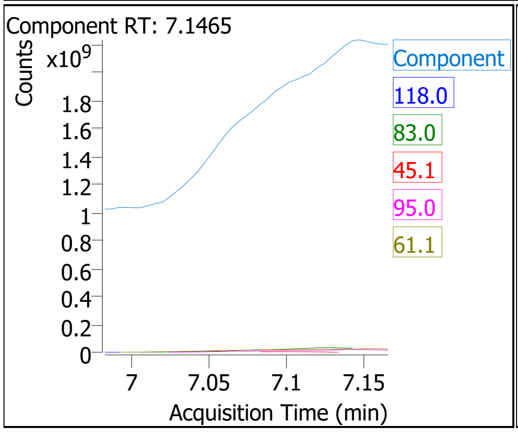

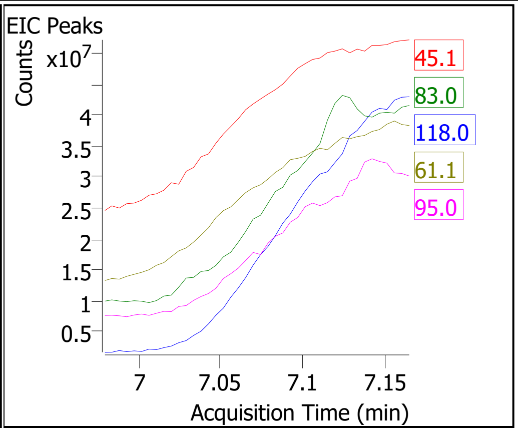

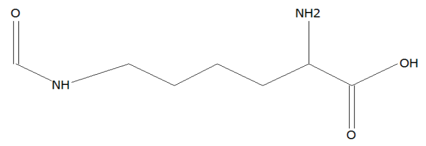

Library Search Results - NonTarget Hits with Details

Component RT	Compound Name	Component Area	Match Factor	CAS#	Formula	Estimated Conc.
7.7442	Tetradecanoic acid	1117071763.7	71.0	544-63-8	C14H28O2	

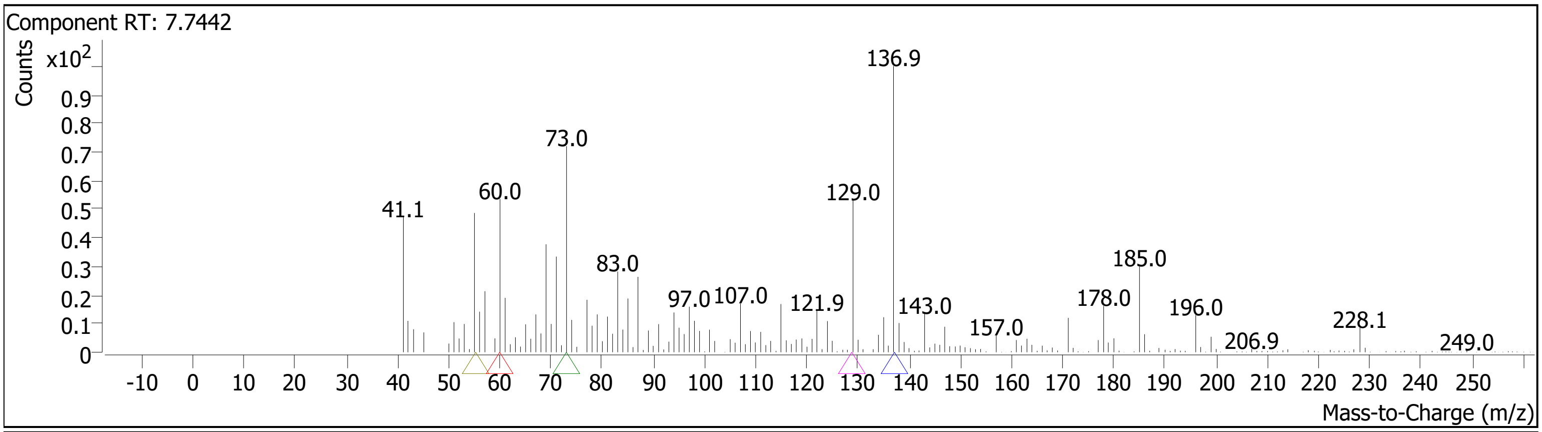

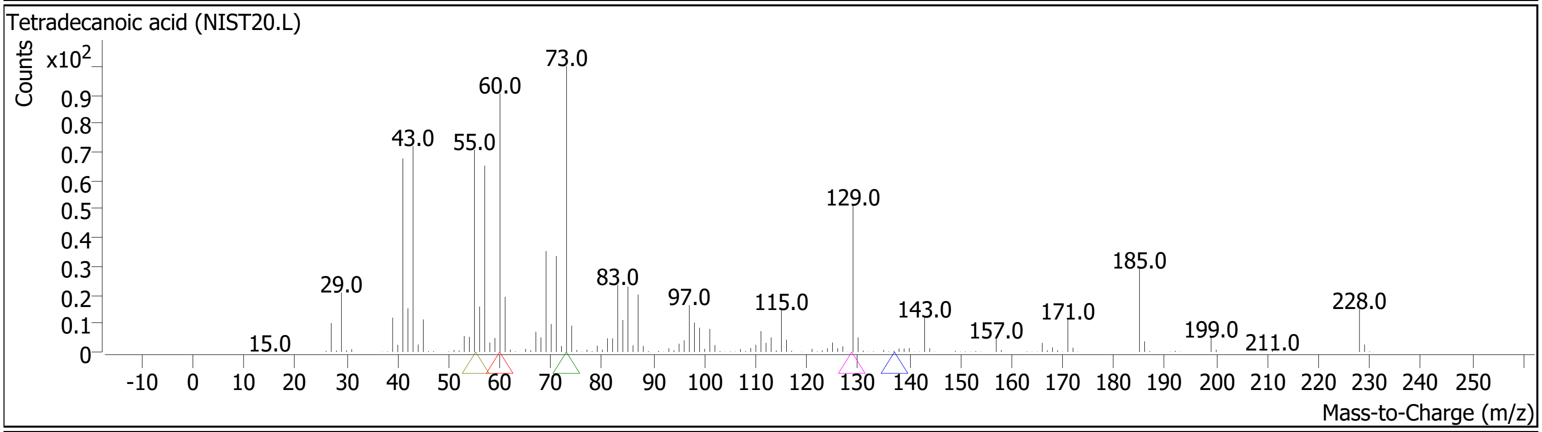

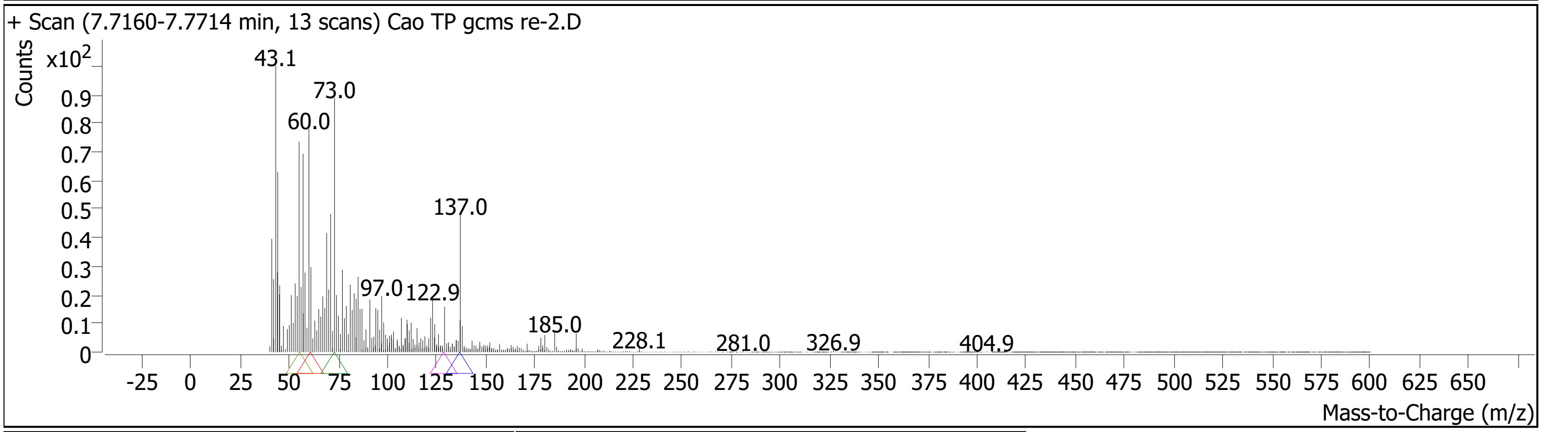

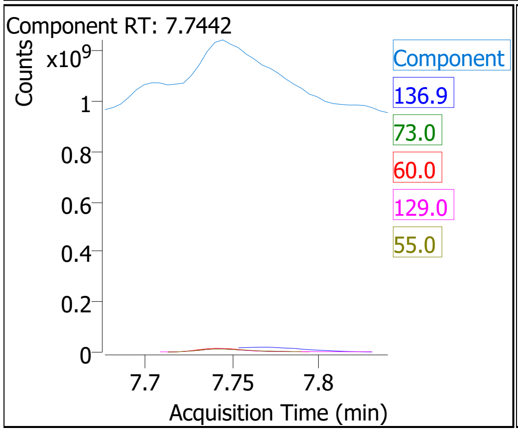

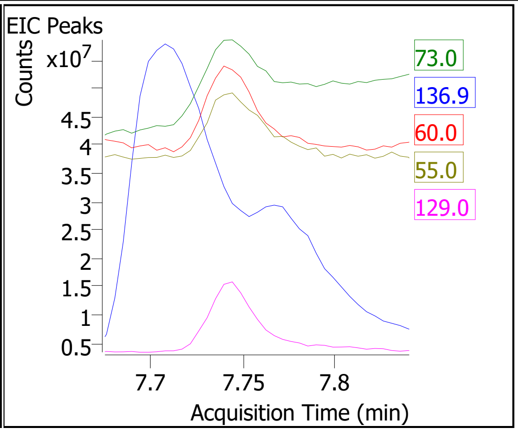

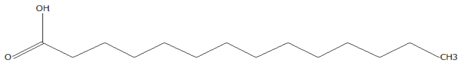

Generated at 10:40 AM on 12/18/2024

Library Search Results - NonTarget Hits with Details

Component RT	Compound Name	Component Area	Match Factor	CAS#	Formula	Estimated Conc.
8.1019	Loliolide	962805901.3	90.3	5989-02-6	C11H16O3	

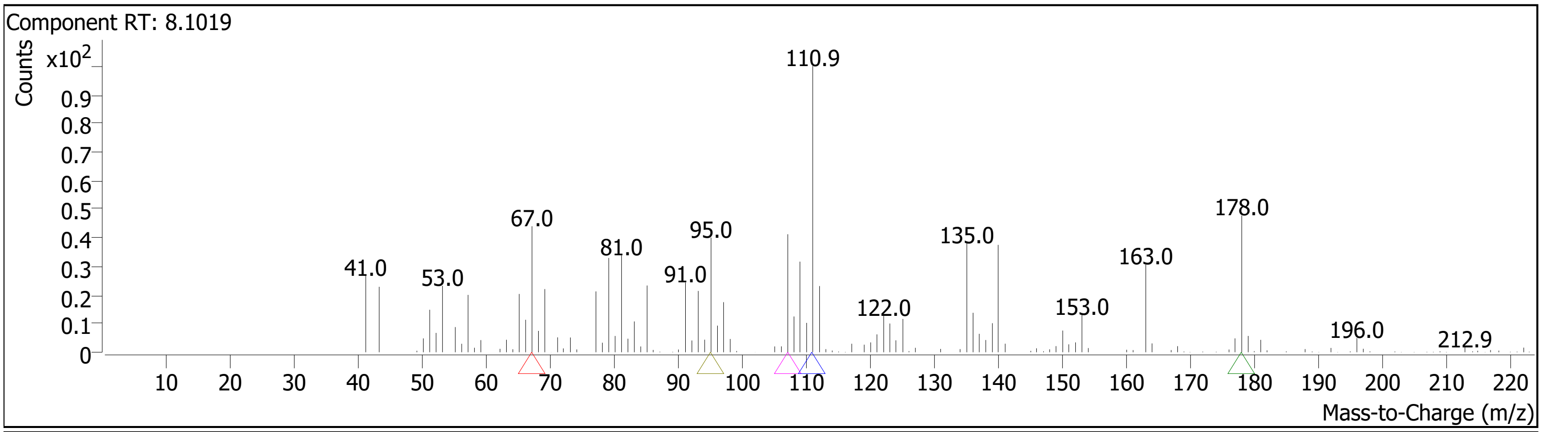

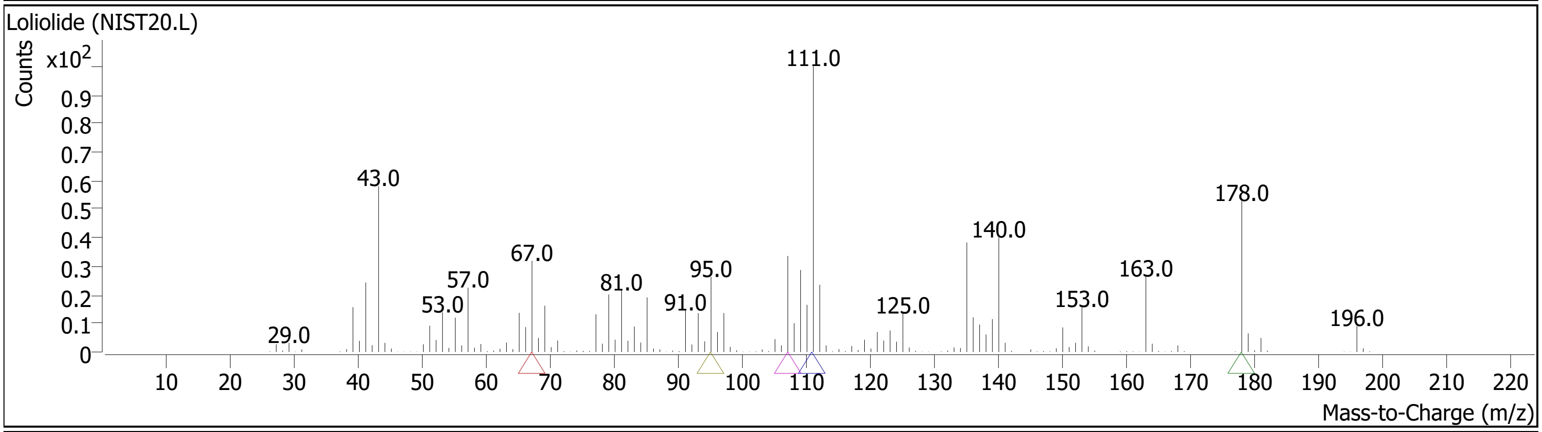

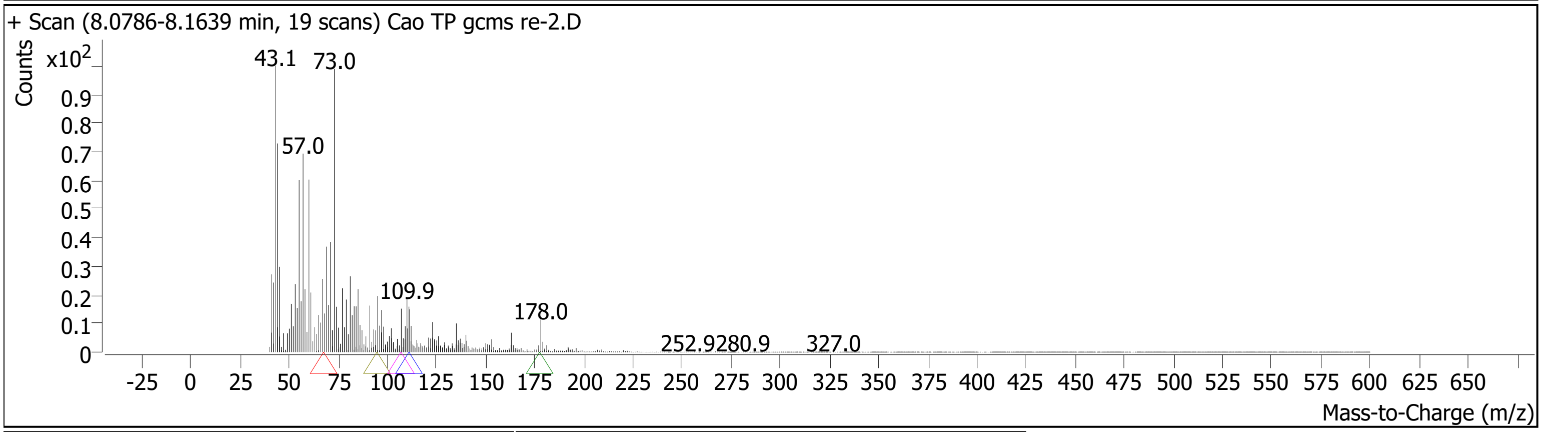

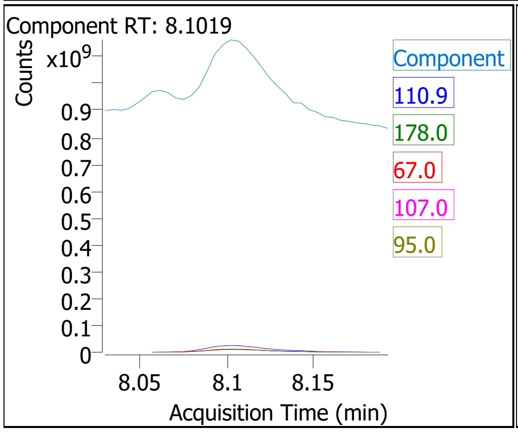

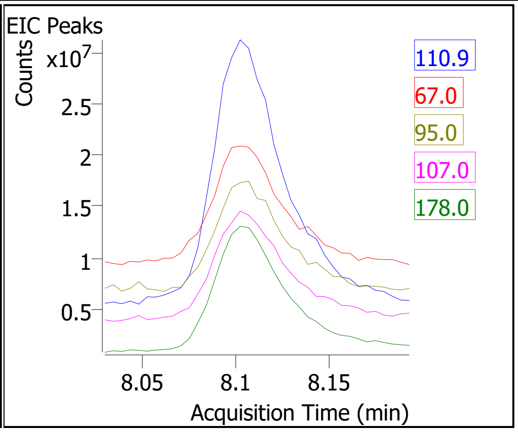

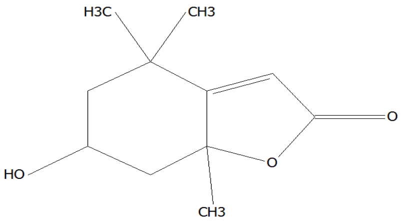

 Generated at 10:40 AM on 12/18/2024

Library Search Results - NonTarget Hits with Details

Component RT	Compound Name	Component Area	Match Factor	CAS#	Formula	Estimated Conc.
8.5320	Acetamide, N-(4-ethoxy-3-hydroxyphenyl)-	814099700.5	70.9	16060-48-3	C10H13NO3	

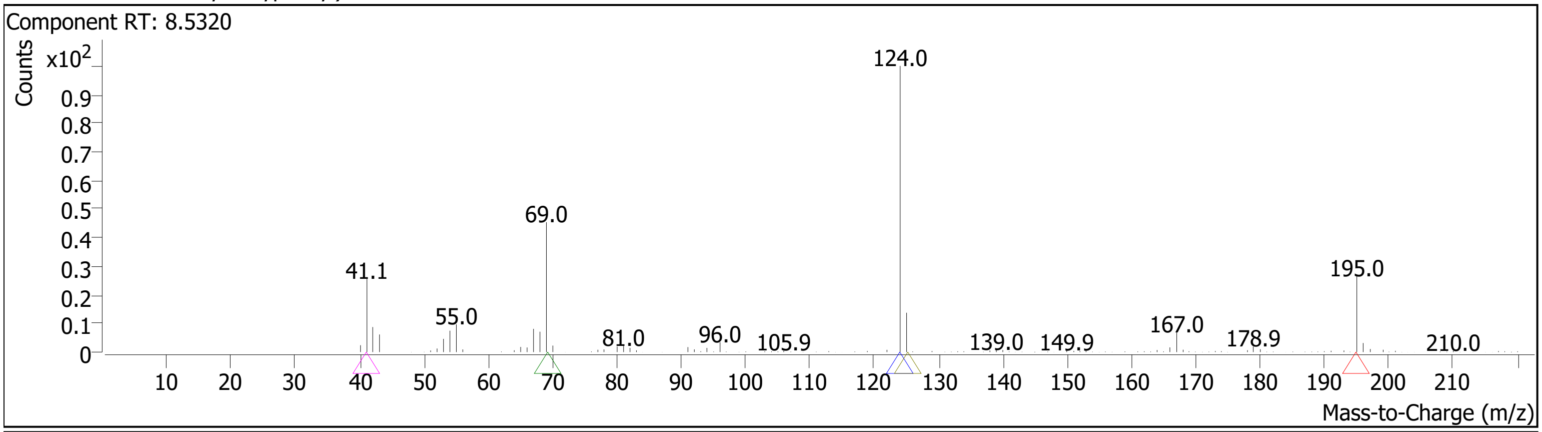

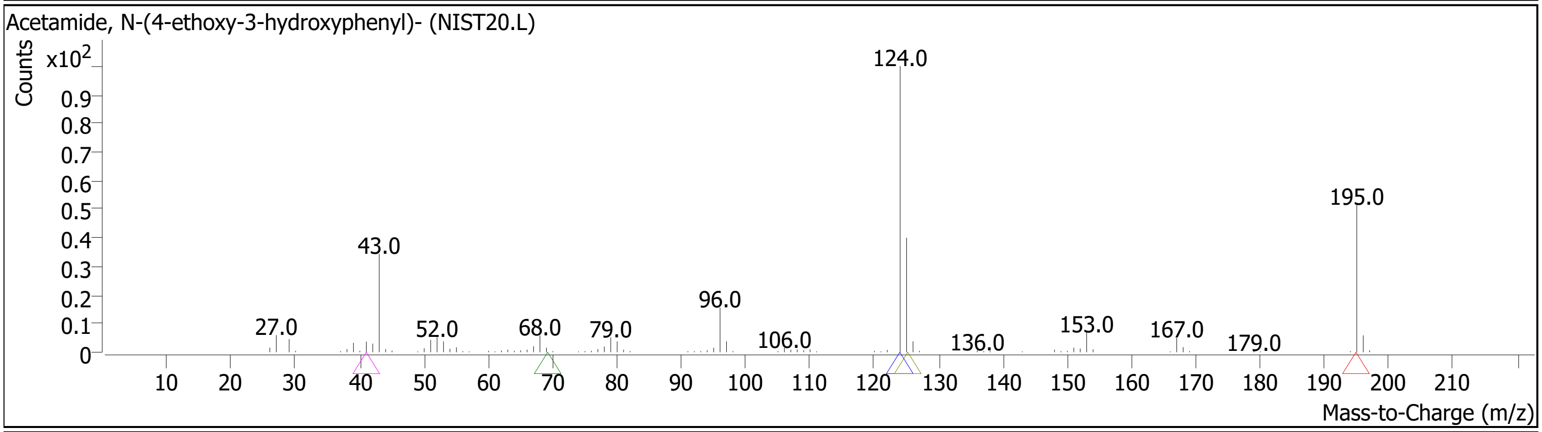

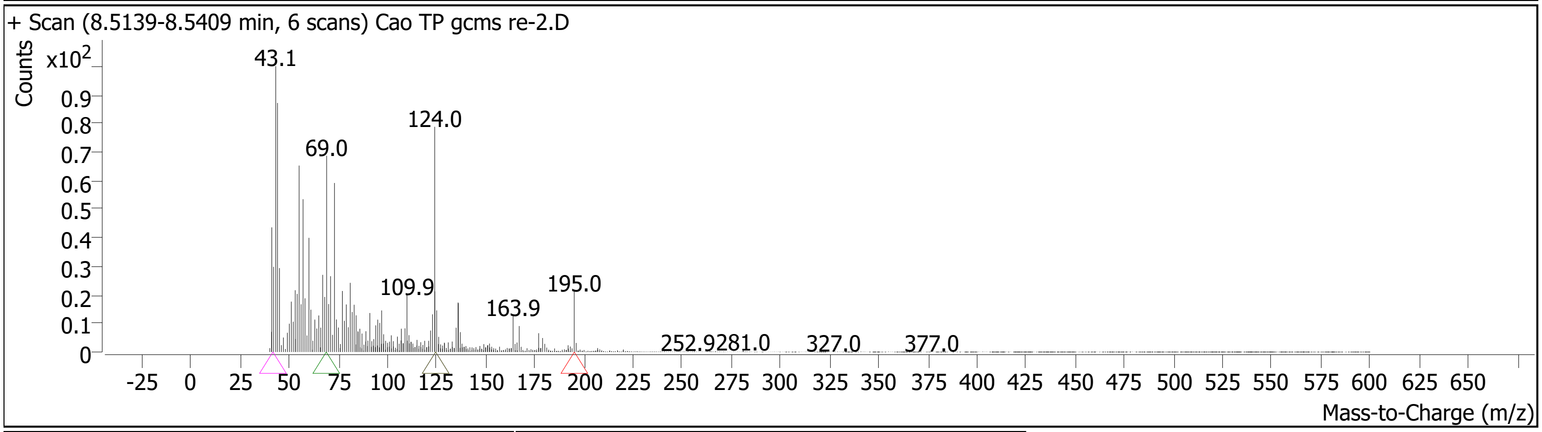

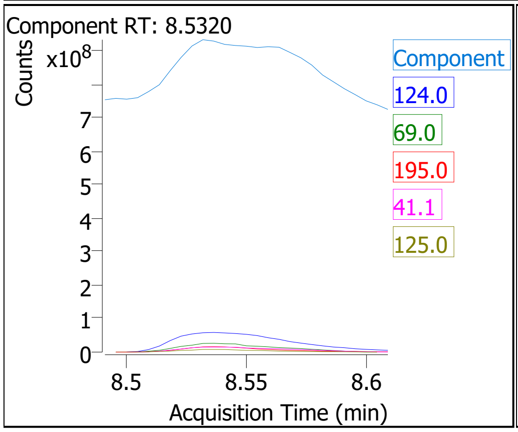

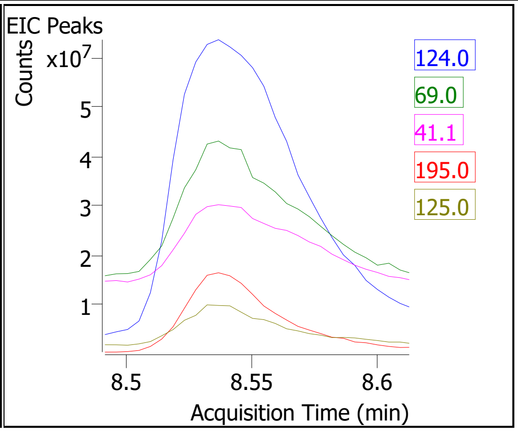

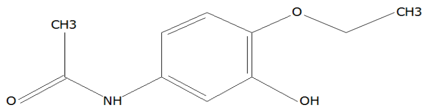

Generated at 10:40 AM on 12/18/2024

Library Search Results - NonTarget Hits with Details

Component RT	Compound Name	Component Area	Match Factor	CAS#	Formula	Estimated Conc.
9.1252	Hexadecanoic acid, methyl ester	410747455.0	88.0	112-39-0	C17H34O2	

Component RT: 9.1252

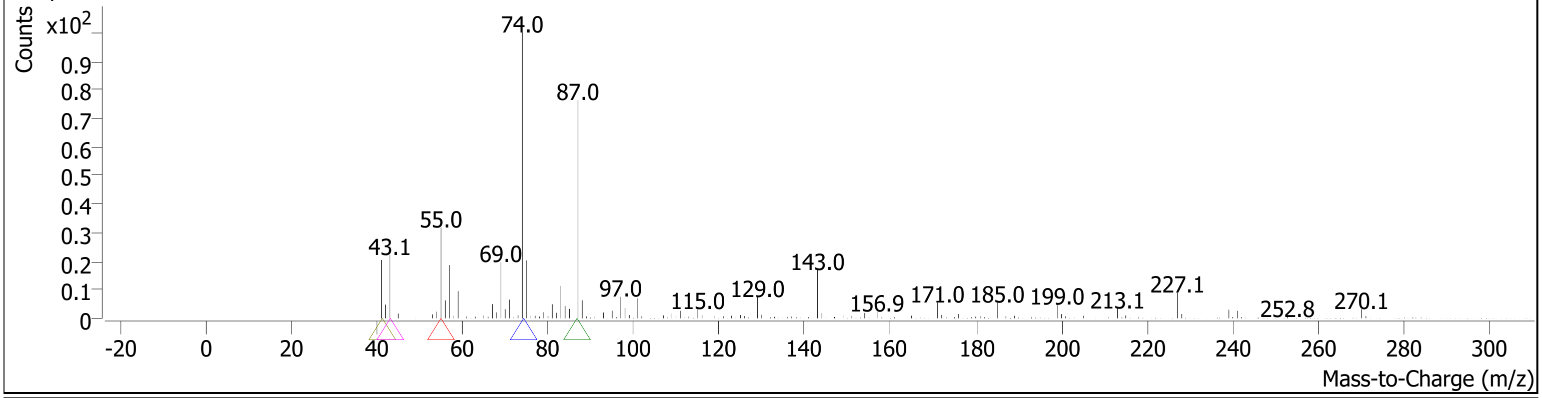

Hexadecanoic acid, methyl ester (NIST20.L)

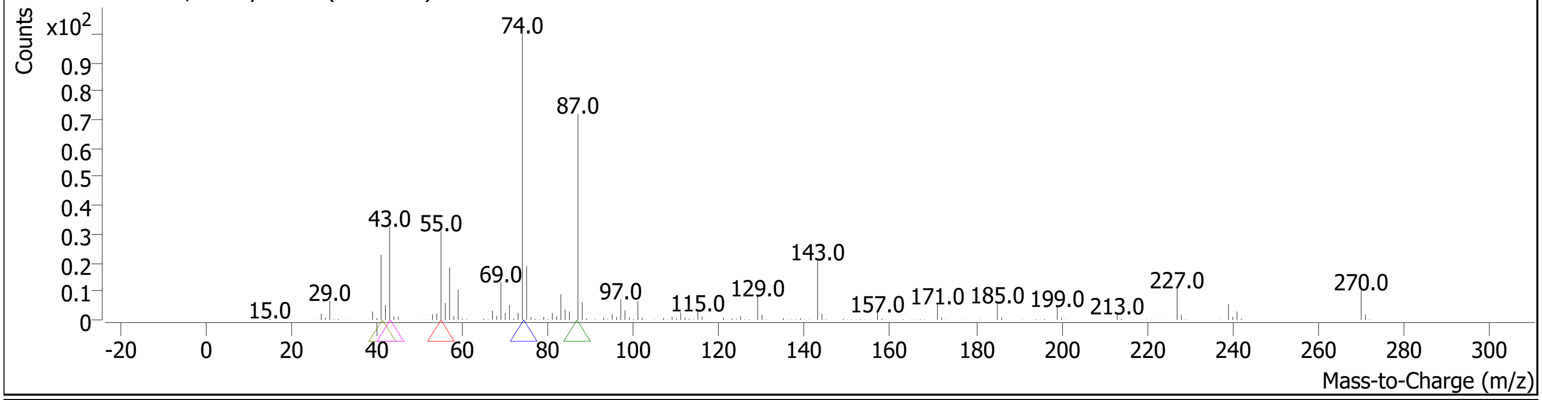

+ Scan (9.1070-9.1566 min, 11 scans) Cao TP gcms re-2.D

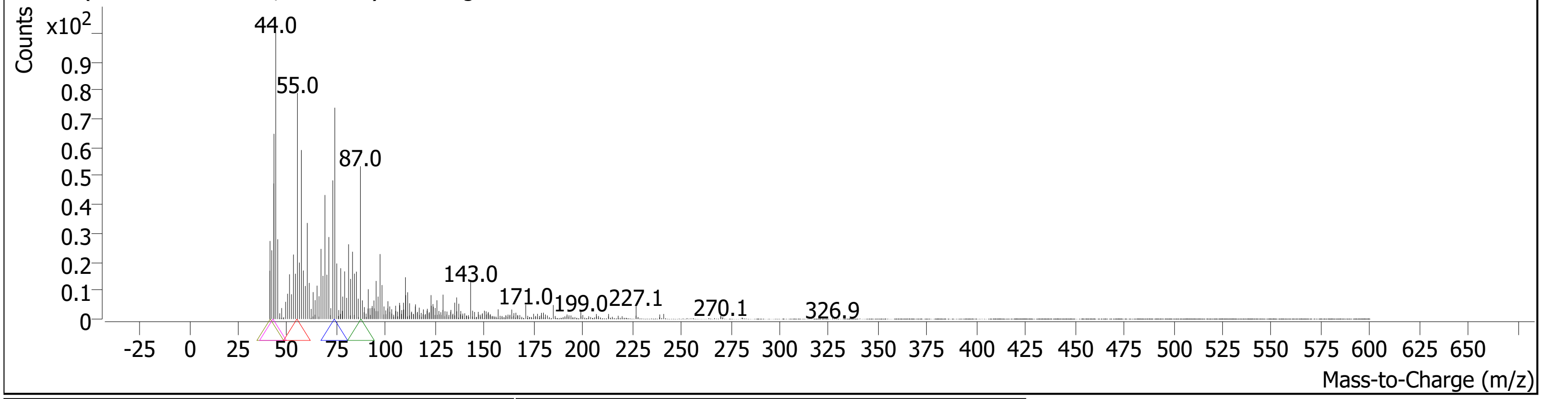

Component RT: 9.1252

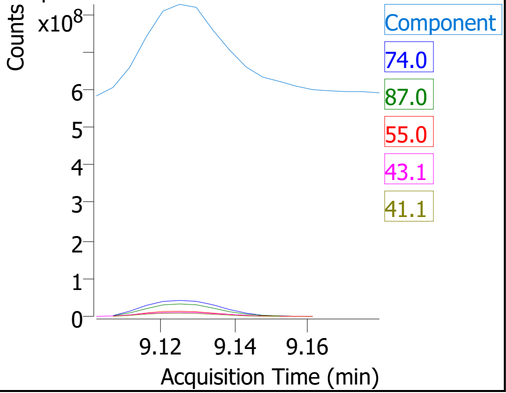

EIC Peaks

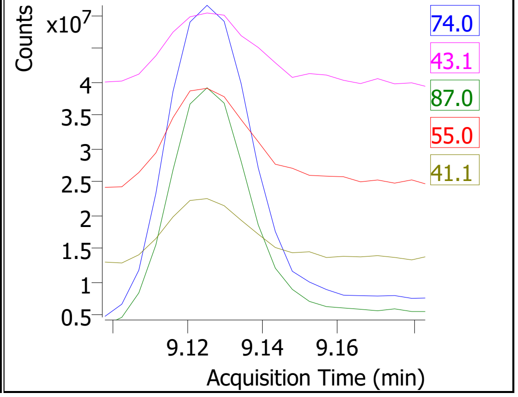

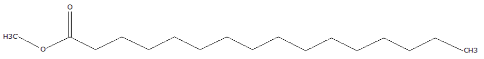

Library Search Results - NonTarget Hits with Details

Component RT	Compound Name	Component Area	Match Factor	CAS#	Formula	Estimated Conc.
9.4693	n-Hexadecanoic acid	11325485523.5	93.1	57-10-3	C16H32O2	

Component RT: 9.4693

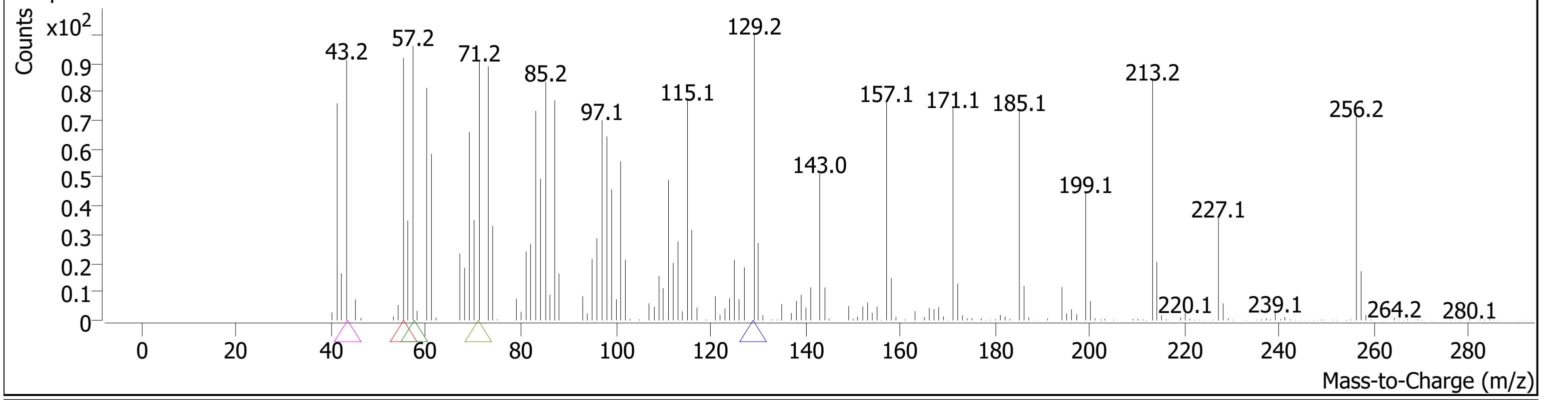

n-Hexadecanoic acid (NIST20.L)

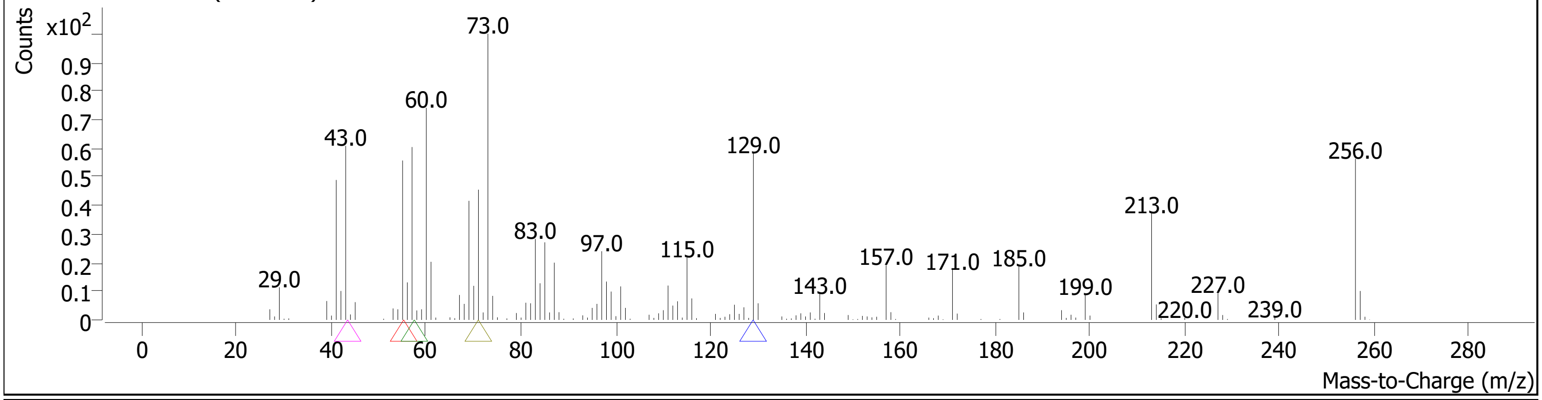

+ Scan (9.3926-9.5779 min, 41 scans) Cao TP gcms re-2.D

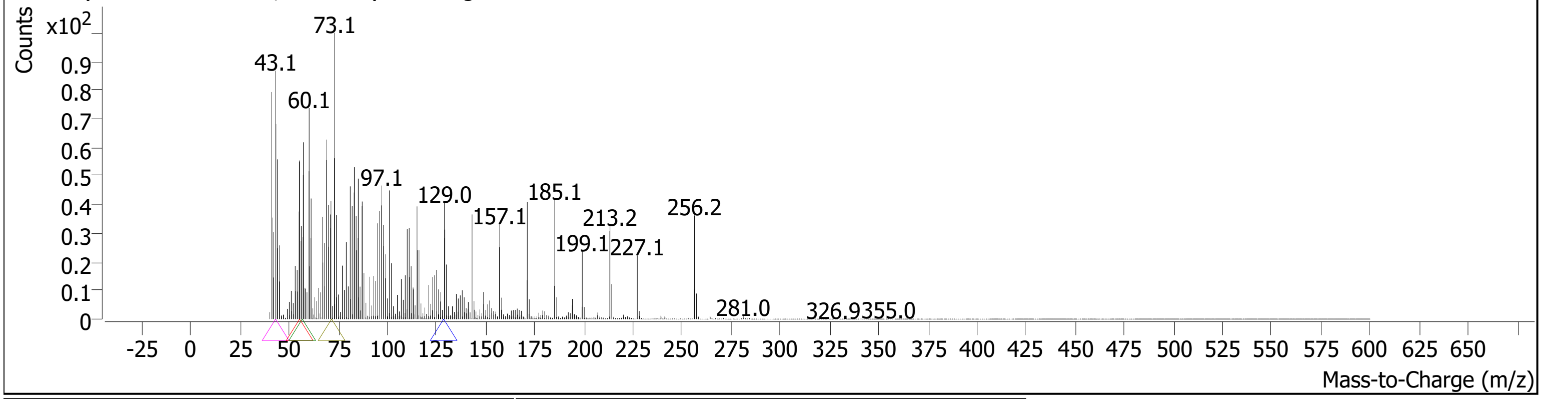

Component RT: 9.4693

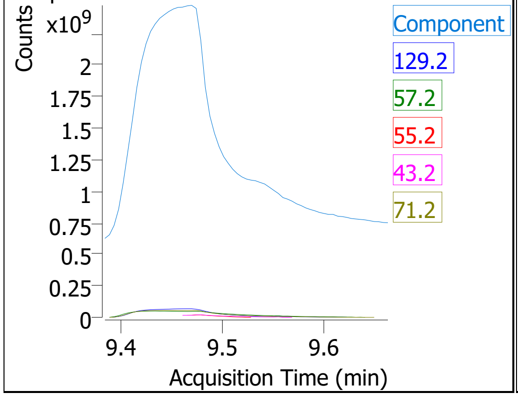

EIC Peaks

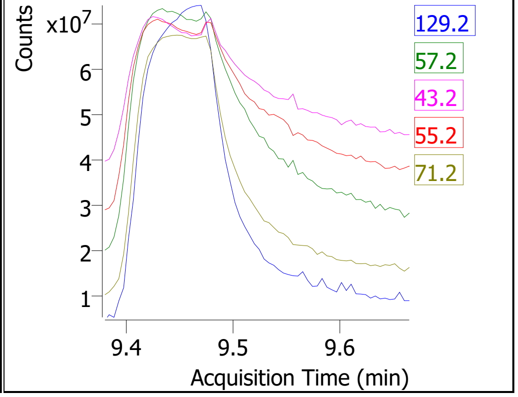

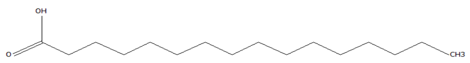

Library Search Results - NonTarget Hits with Details

Component RT	Compound Name	Component Area	Match Factor	CAS#	Formula	Estimated Conc.
9.7047	Hexadecanoic acid, ethyl ester	1388637728.4	93.1	628-97-7	C18H36O2	

Component RT: 9.7047

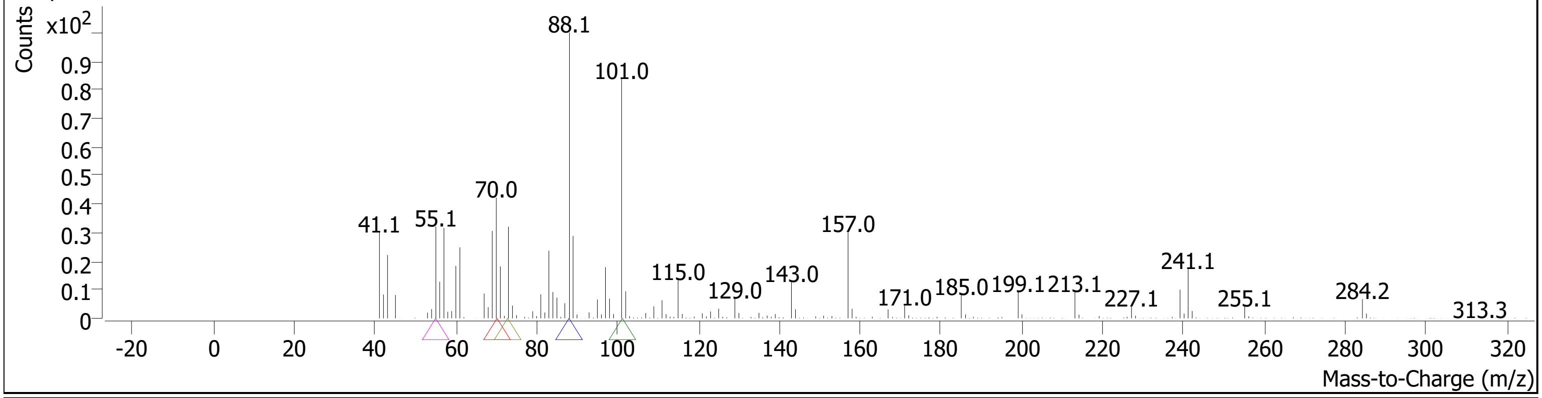

Hexadecanoic acid, ethyl ester (NIST20.L)

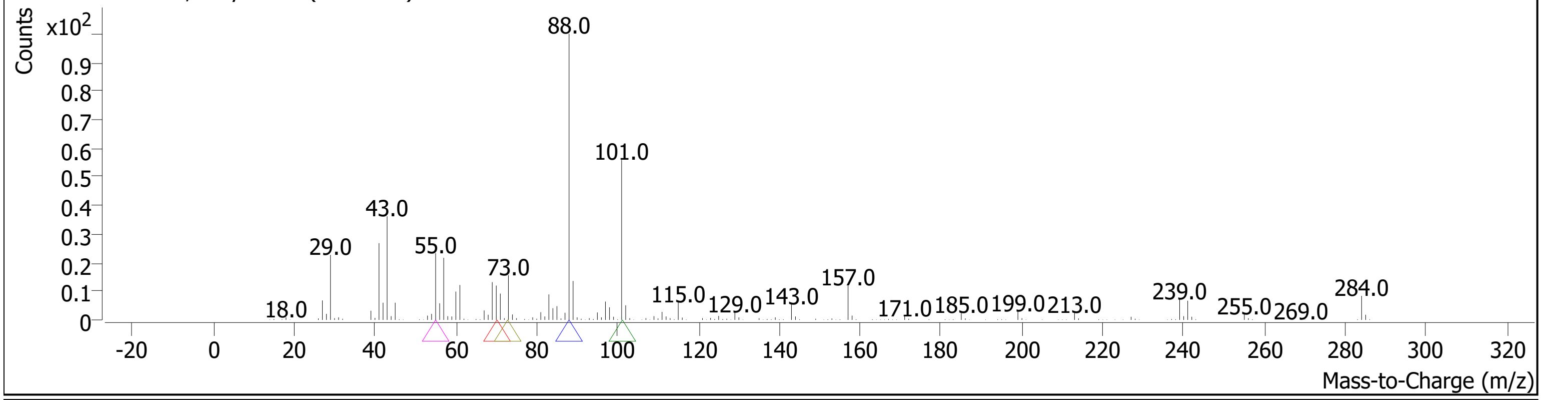

+ Scan (9.6866-9.7636 min, 18 scans) Cao TP gcms re-2.D

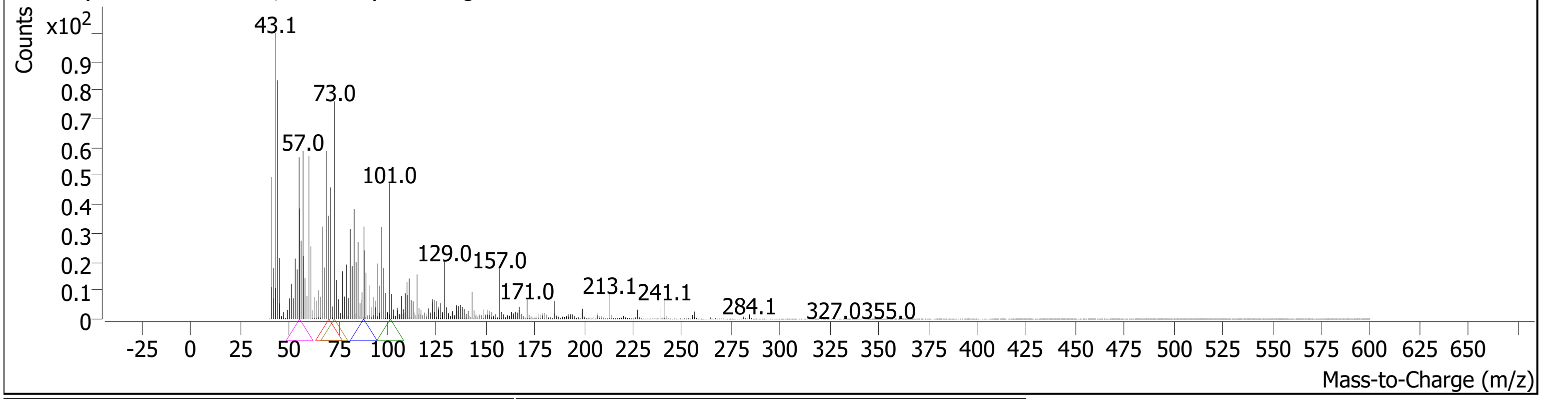

Component RT: 9.7047

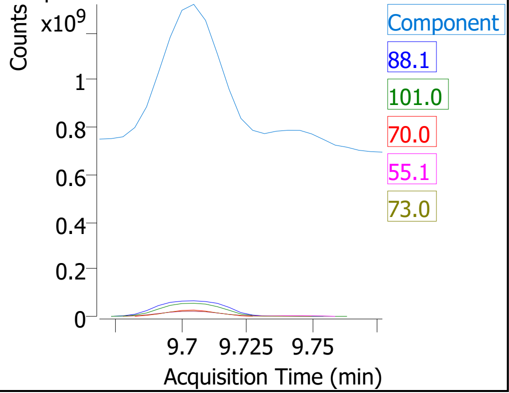

EIC Peaks

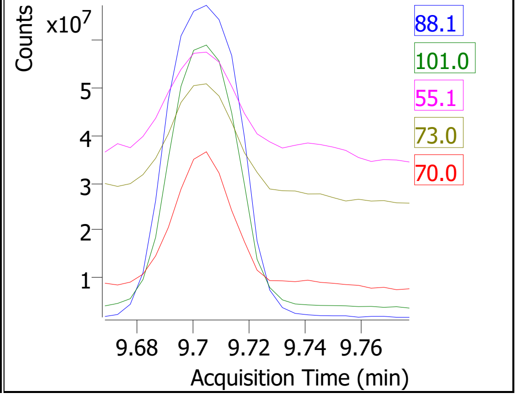

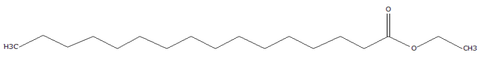

Library Search Results - NonTarget Hits with Details

Component RT	Compound Name	Component Area	Match Factor	CAS#	Formula	Estimated Conc.
10.6012	9,12-Octadecadienoic acid (Z,Z)-, methyl ester	430280469.0	91.6	112-63-0	C19H34O2	

Component RT: 10.6012

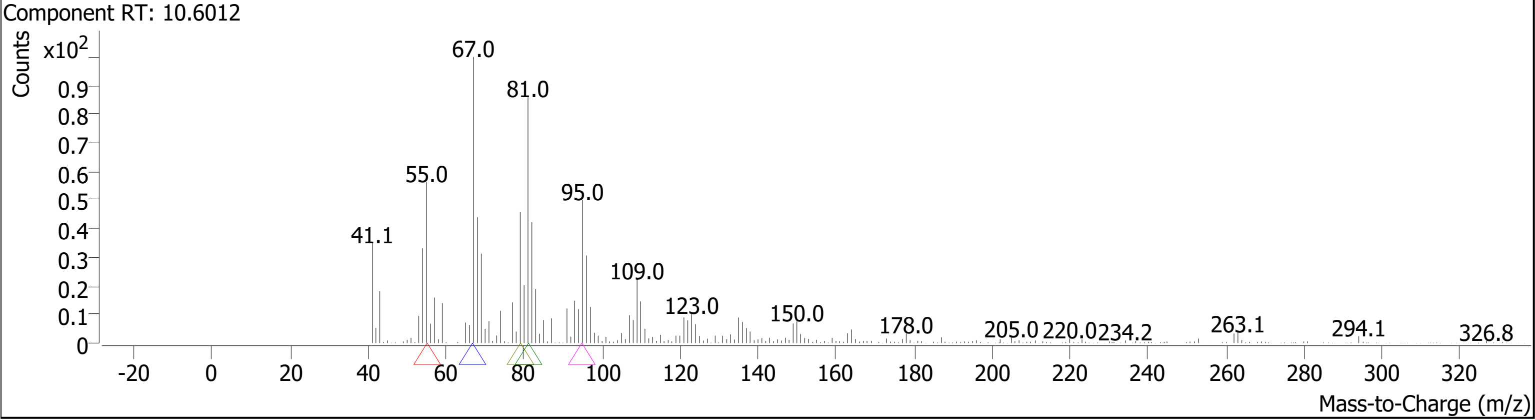

9,12-Octadecadienoic acid (Z,Z)-, methyl ester (NIST20.L)

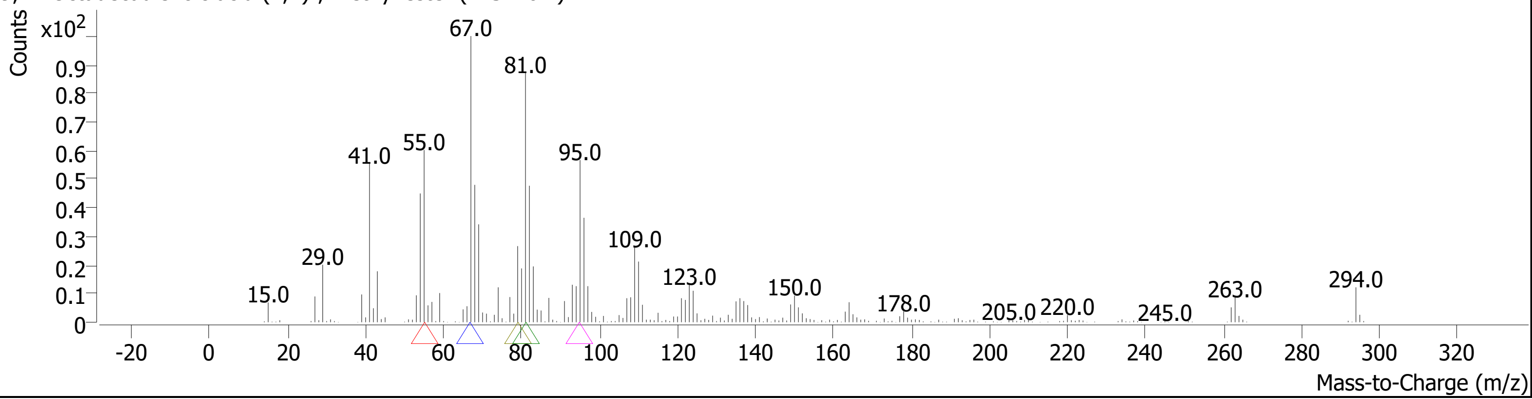

+ Scan (10.5818-10.6174 min, 8 scans) Cao TP gcms re-2.D

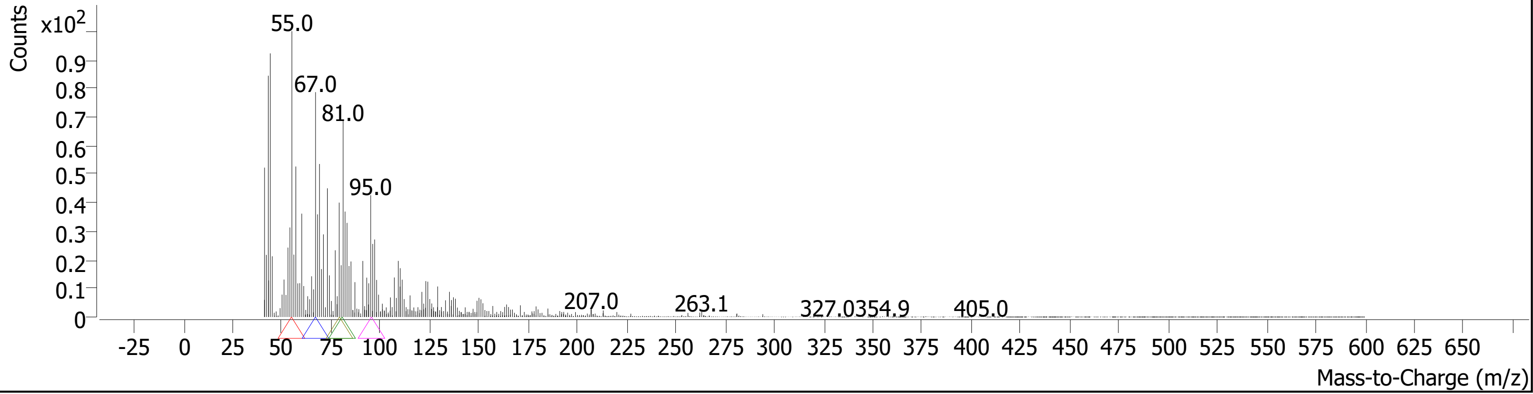

Component RT: 10.6012

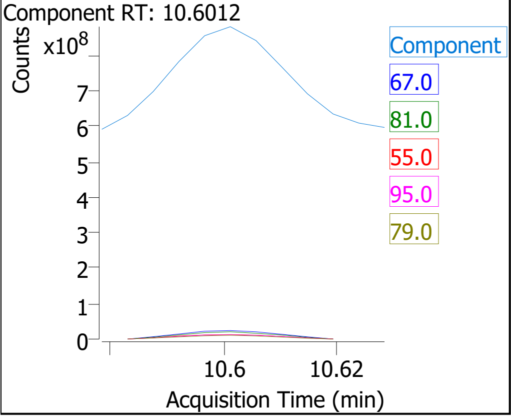

EIC Peaks

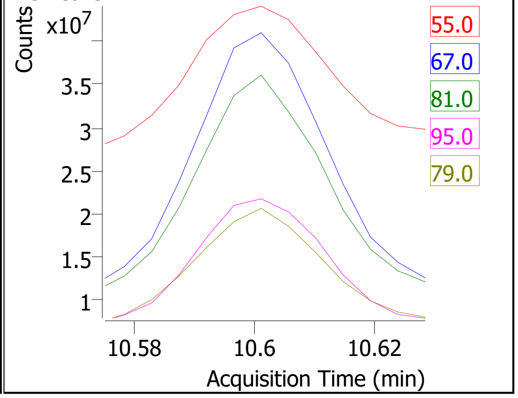

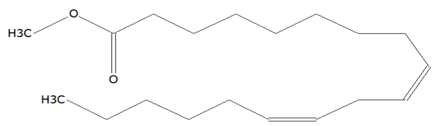

Library Search Results - NonTarget Hits with Details

Component RT	Compound Name	Component Area	Match Factor	CAS#	Formula	Estimated Conc.
10.6601	9,12,15-Octadecatrienoic acid, methyl ester, (Z,Z,Z)-	474549552.8	90.0	301-00-8	C19H32O2	

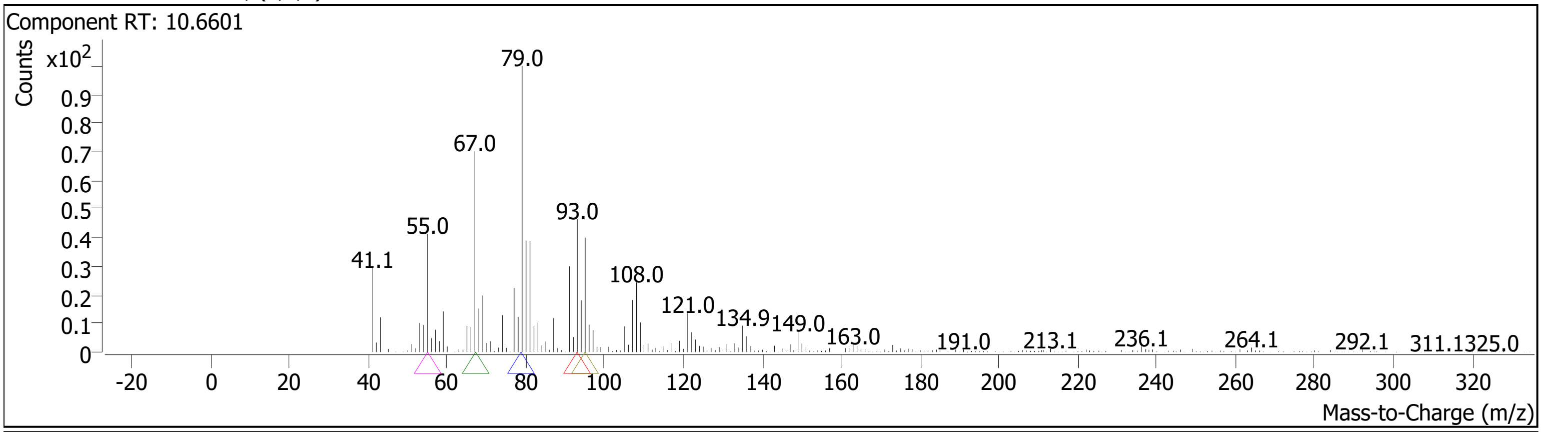

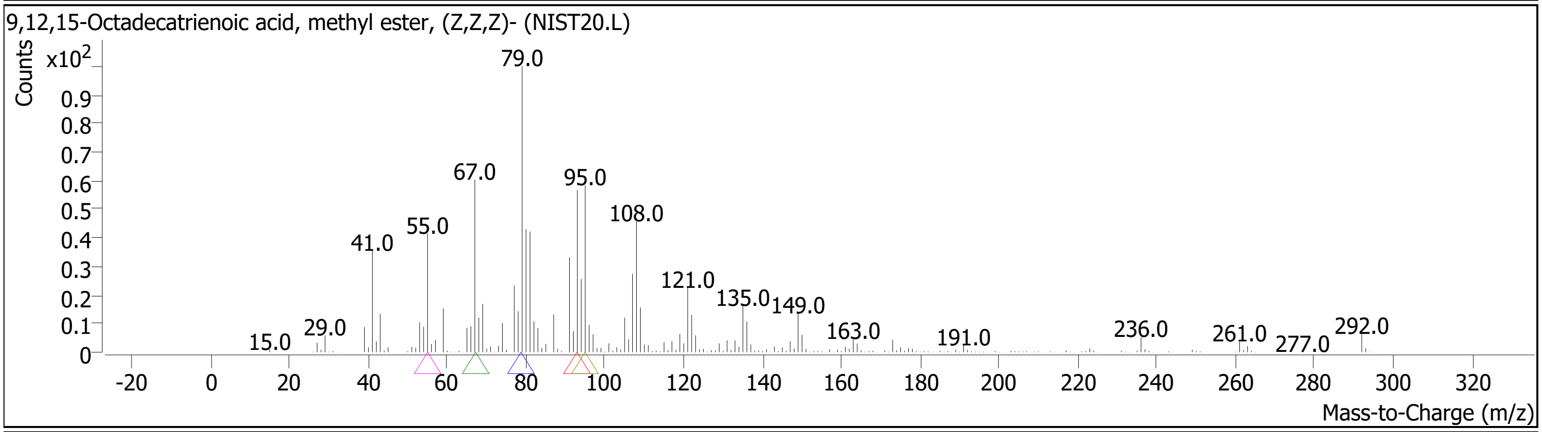

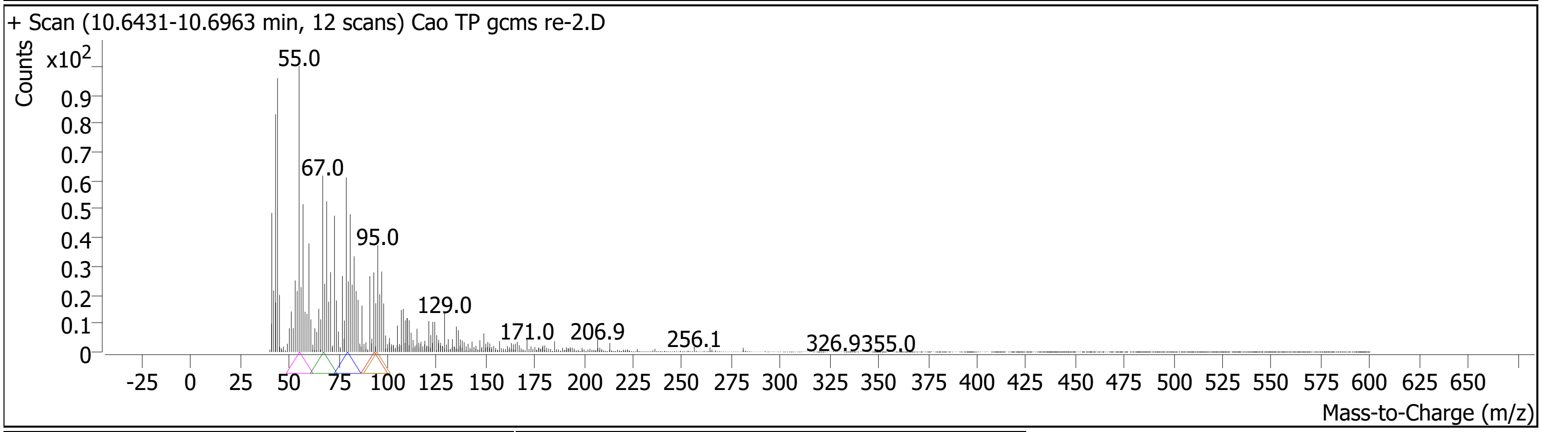

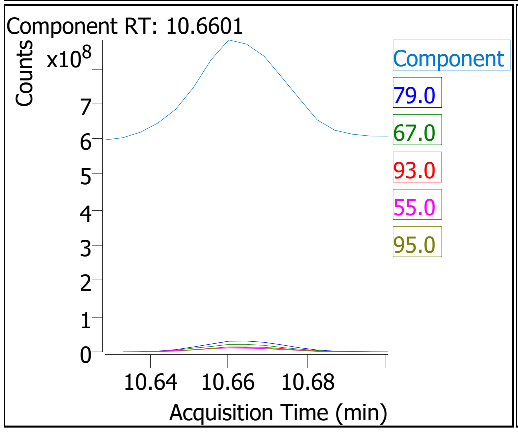

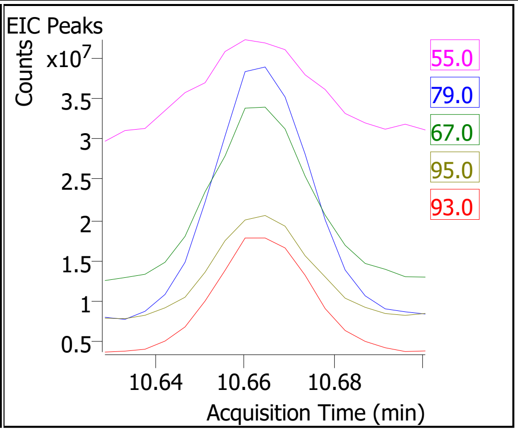

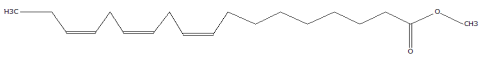

Library Search Results - NonTarget Hits with Details

Component RT	Compound Name	Component Area	Match Factor	CAS#	Formula	Estimated Conc.
11.0268	9,12,15-Octadecatrienoic acid, (Z,Z,Z)-	18690994969.8	89.4	463-40-1	C18H30O2	

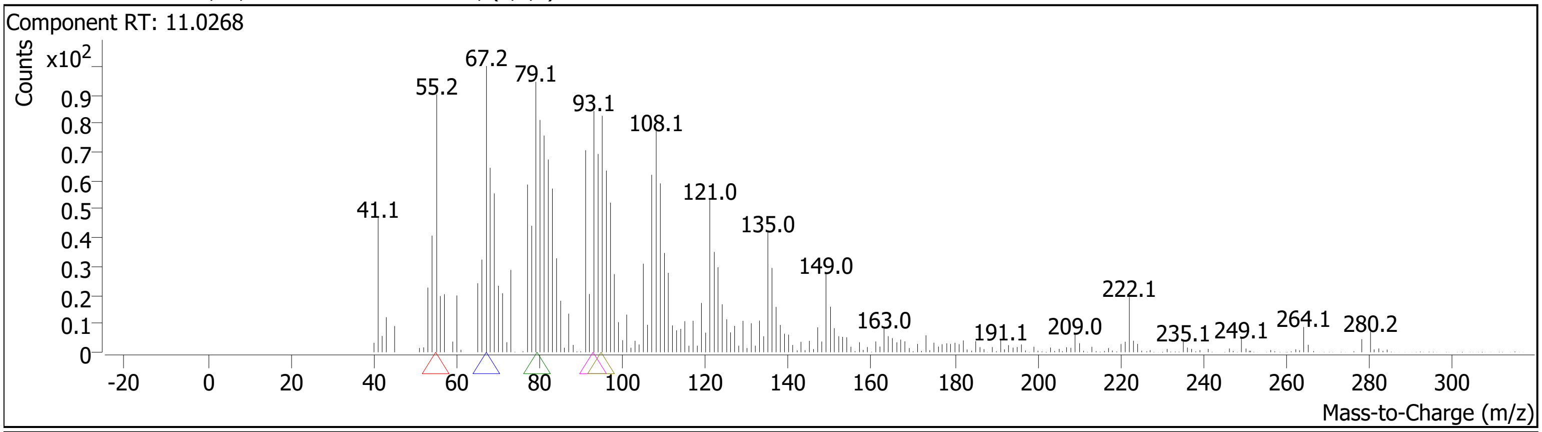

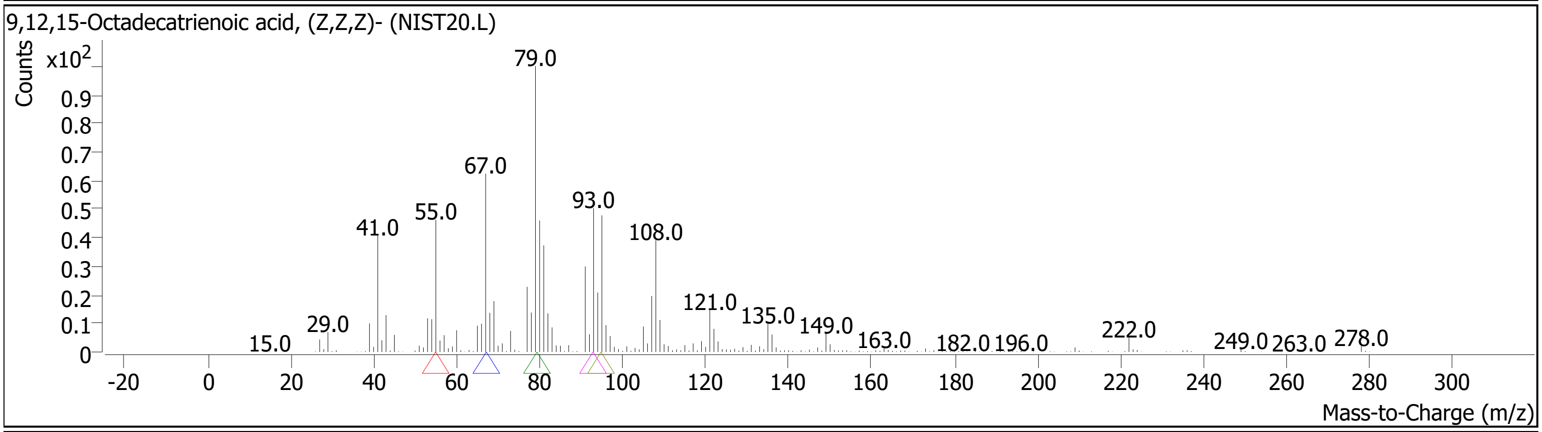

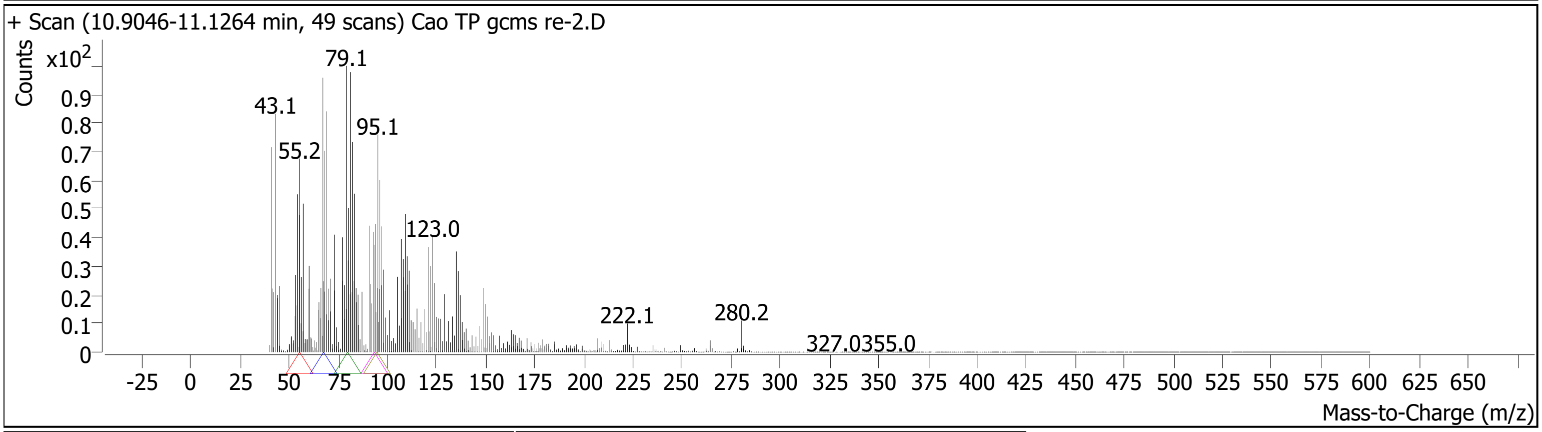

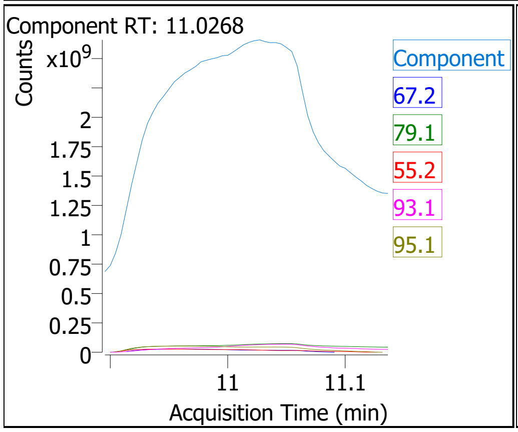

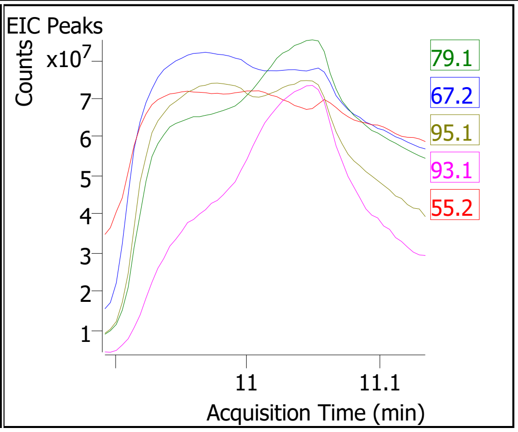

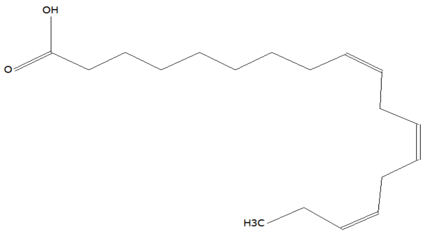

Generated at 10:40 AM on 12/18/2024

Library Search Results - NonTarget Hits with Details

Component RT	Compound Name	Component Area	Match Factor	CAS#	Formula	Estimated Conc.
11.1626	Octadecanoic acid	3131142734.4	88.1	57-11-4	C18H36O2	

Component RT: 11.1626

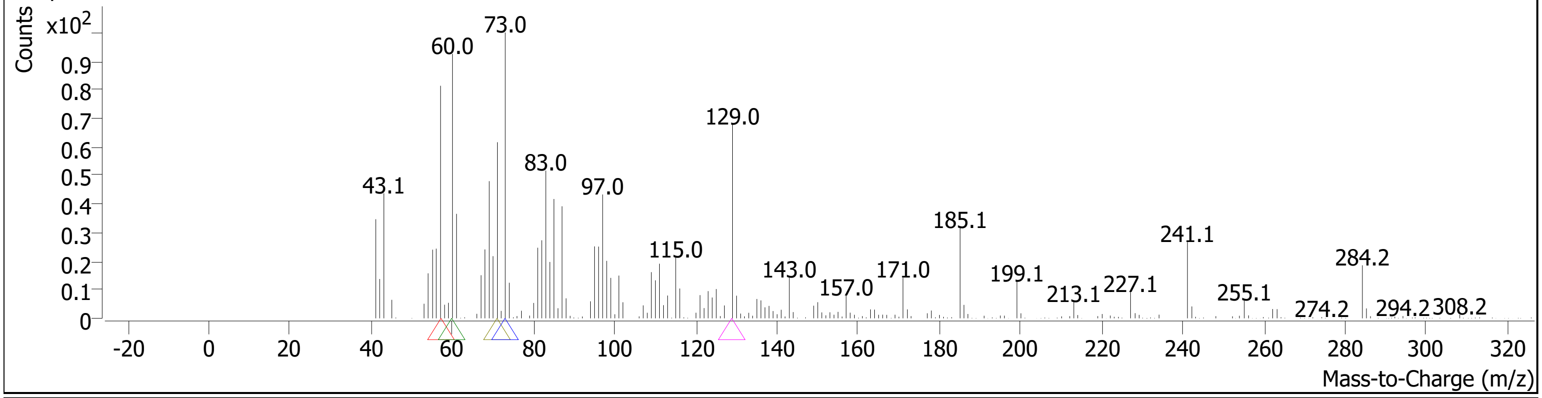

Octadecanoic acid (NIST20.L)

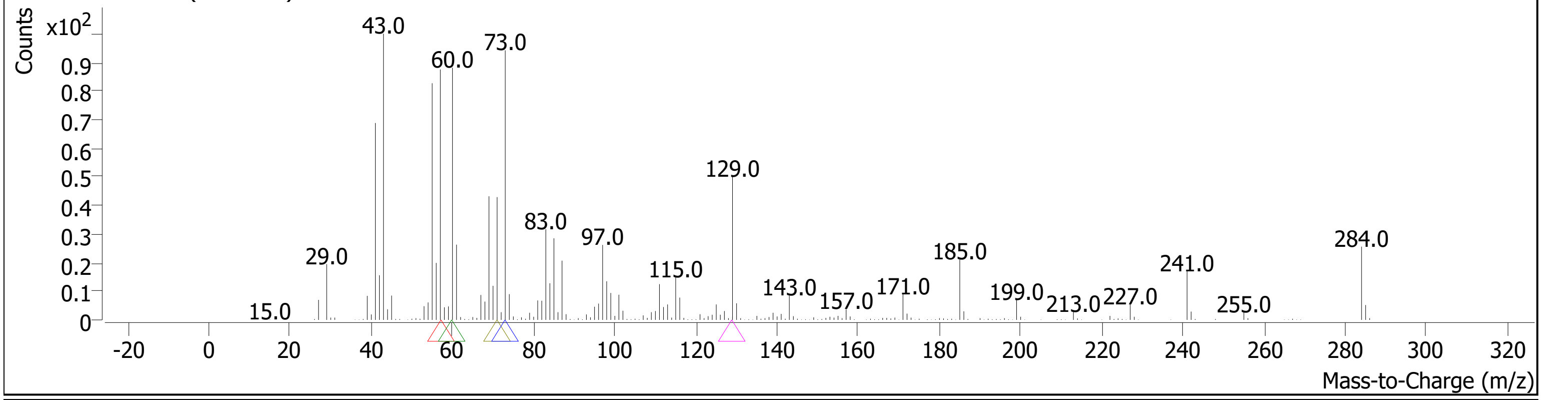

+ Scan (11.1491-11.1898 min, 10 scans) Cao TP gcms re-2.D

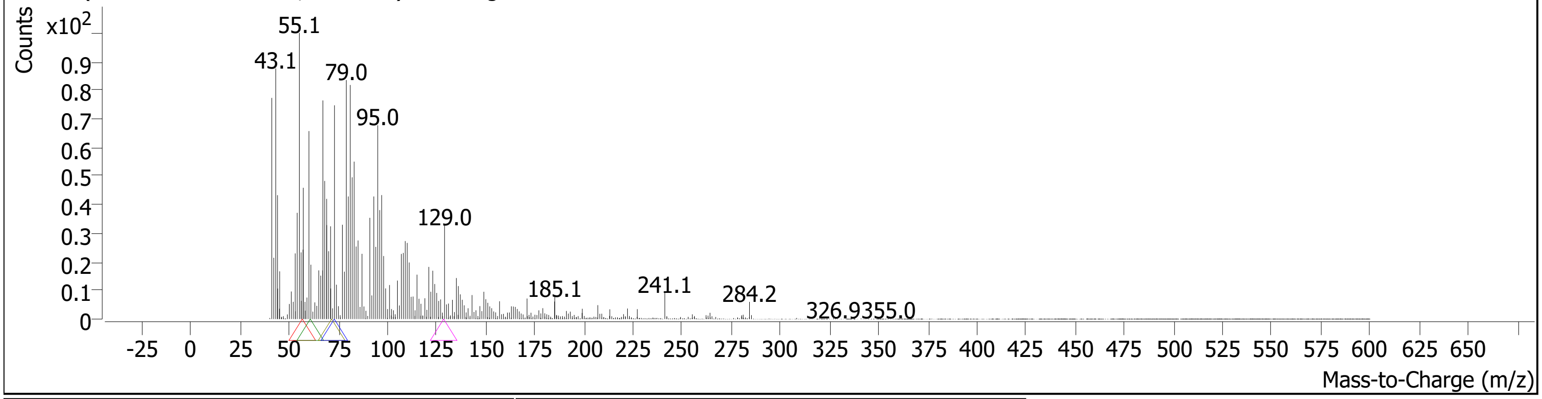

Component RT: 11.1626

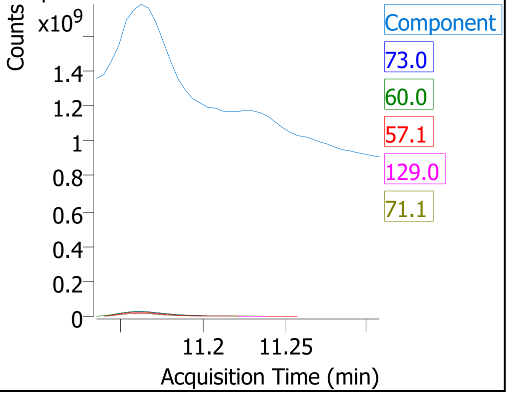

EIC Peaks

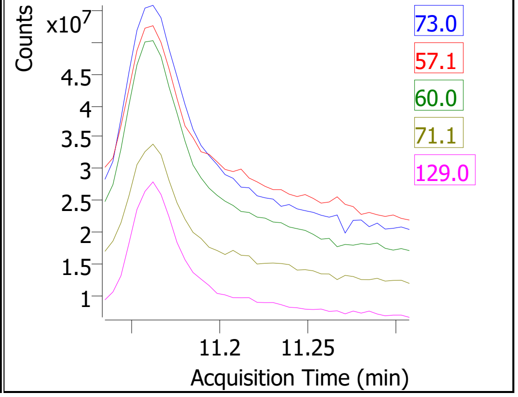

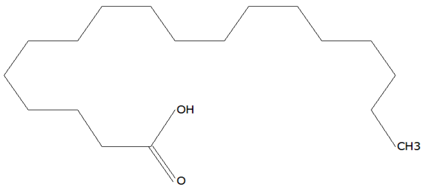

Library Search Results - NonTarget Hits with Details

Component RT	Compound Name	Component Area	Match Factor	CAS#	Formula	Estimated Conc.
14.0061	Hexadecanoic acid, 2-hydroxy-1-(hydroxymethyl)ethyl ester	2039430851.1	92.3	23470-00-0	C19H38O4	

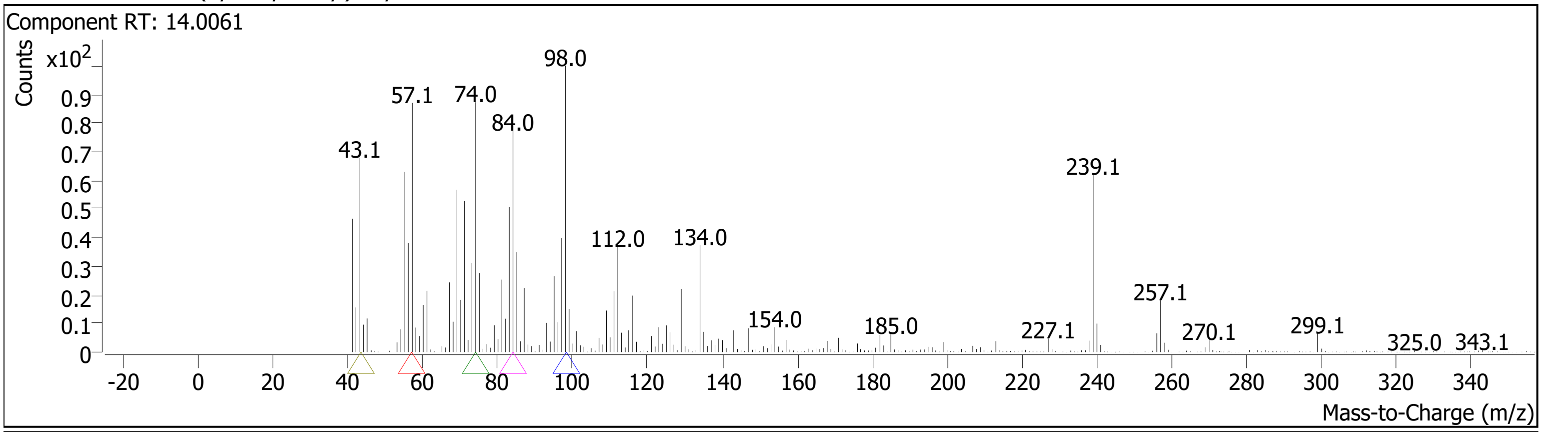

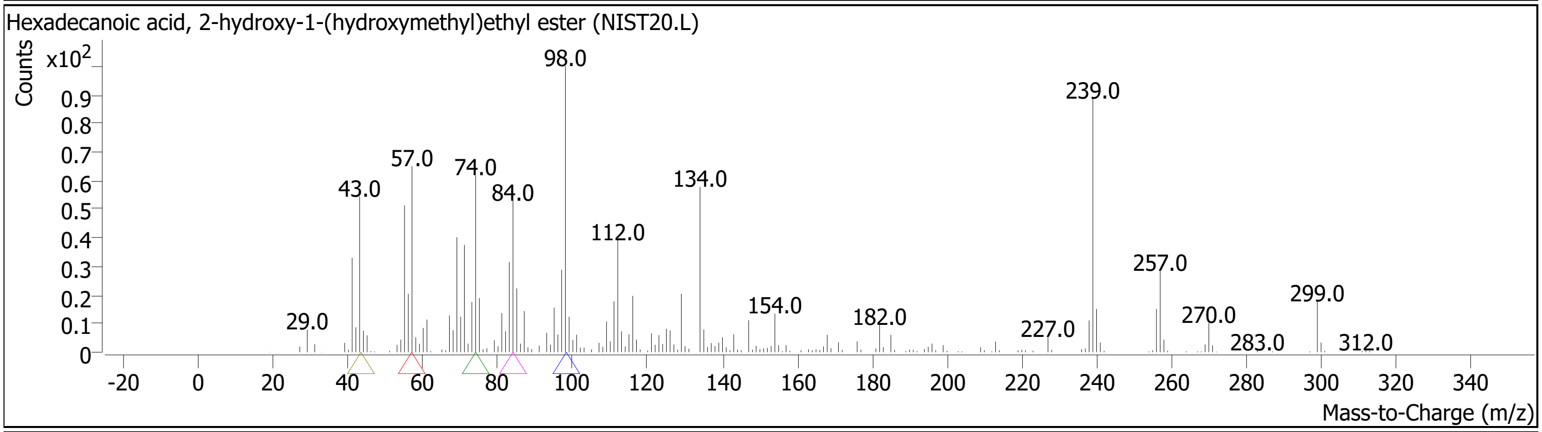

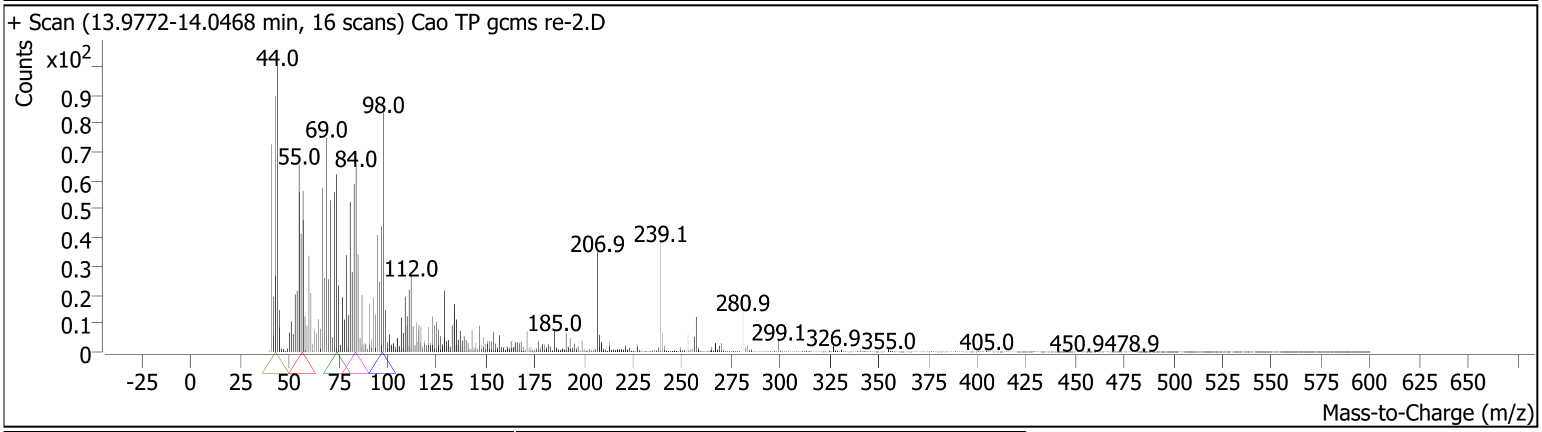

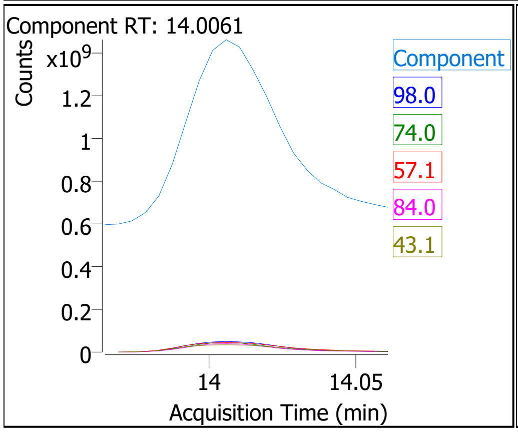

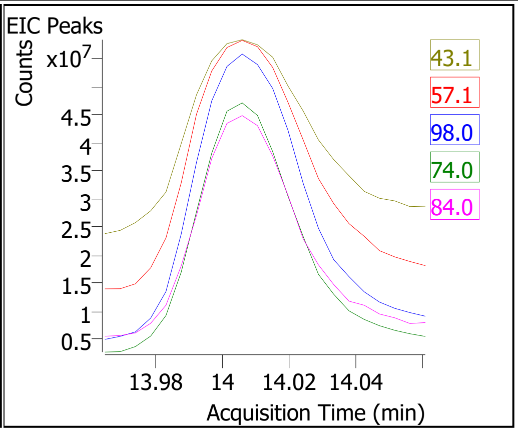

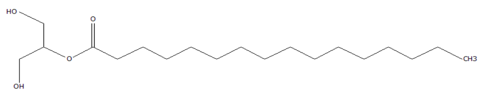

Generated at 10:40 AM on 12/18/2024

Library Search Results - NonTarget Hits with Details

Component RT	Compound Name	Component Area	Match Factor	CAS#	Formula	Estimated Conc.
14.3185	Bis(2-ethylhexyl) phthalate	1390480245.2	90.0	117-81-7	C24H38O4	

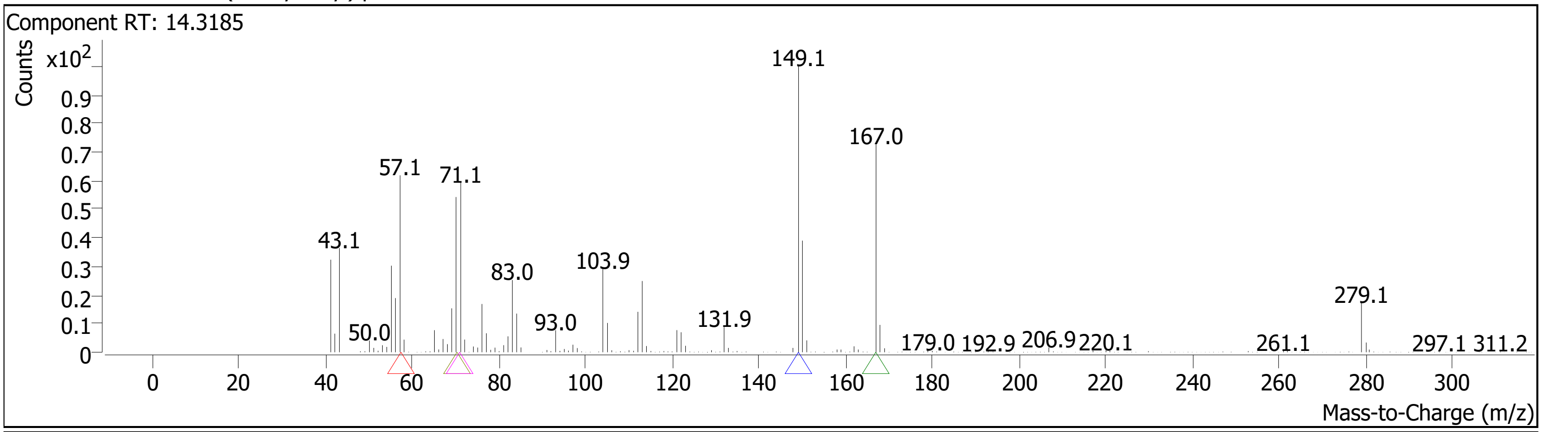

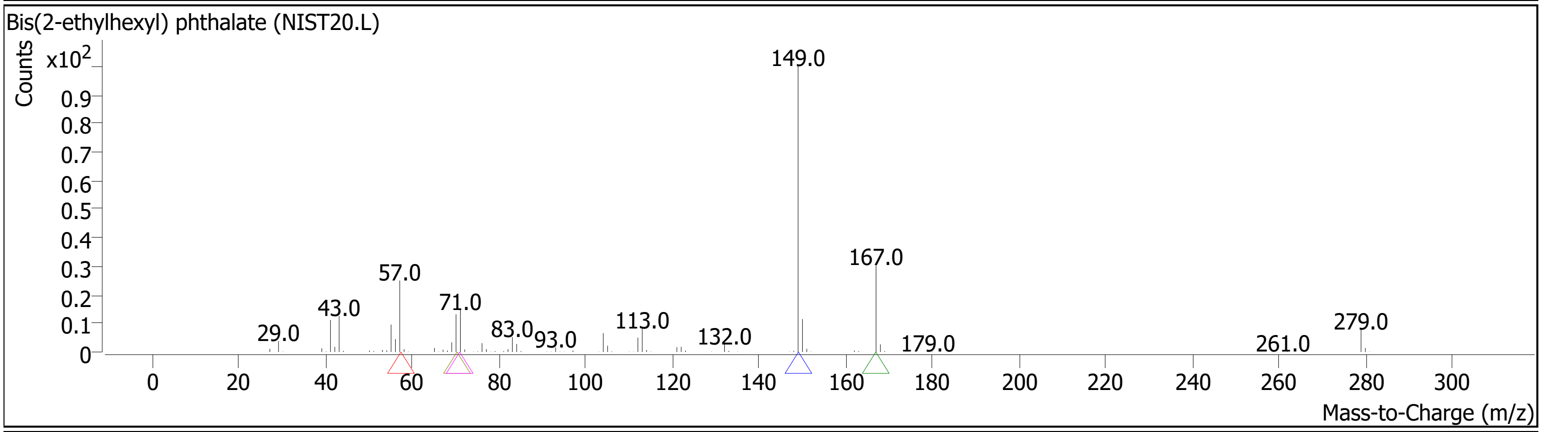

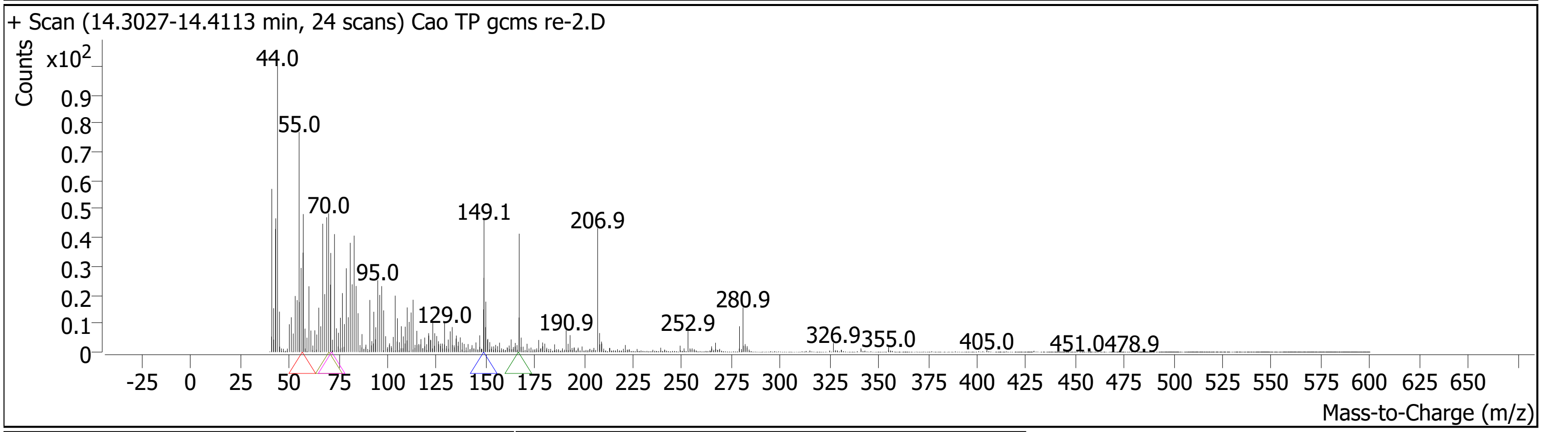

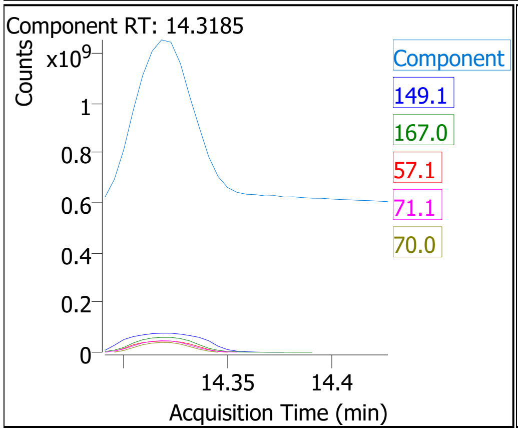

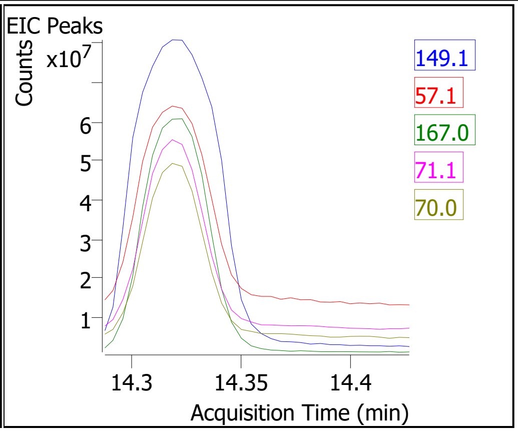

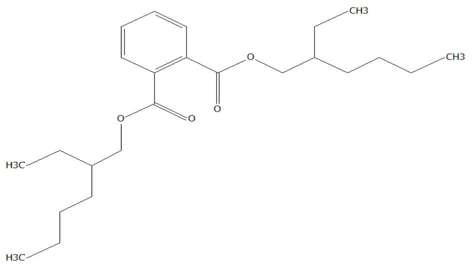

Library Search Results - NonTarget Hits with Details

Component RT	Compound Name	Component Area	Match Factor	CAS#	Formula	Estimated Conc.
14.7260	1,2-Cyclohexanedicarboxylic acid, bis(2-ethylhexyl) ester	4212231008.3	85.1	84-71-9	C24H44O4	

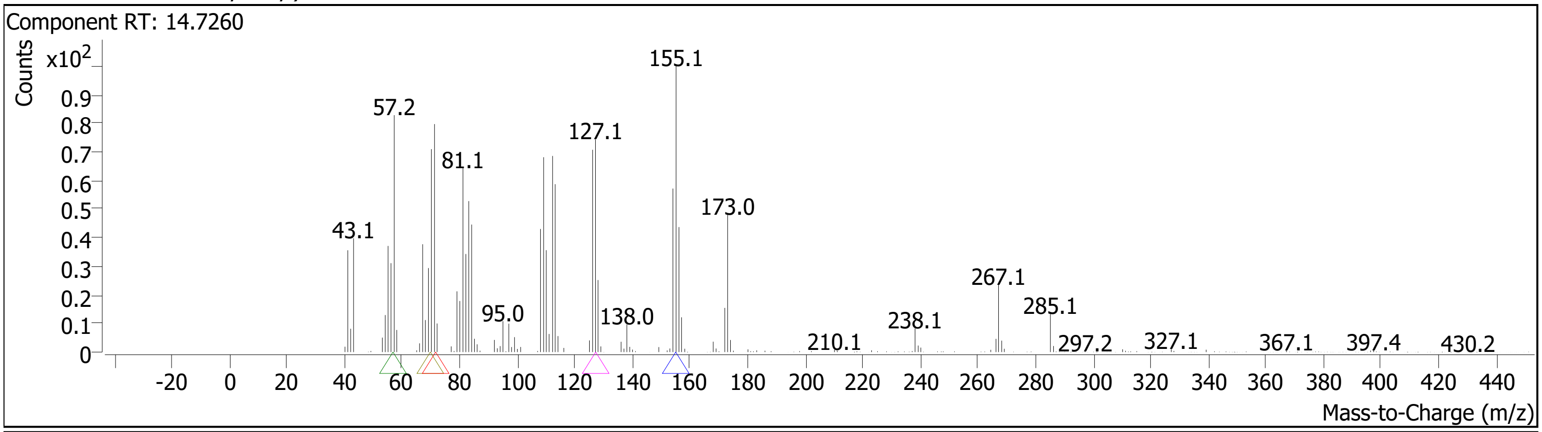

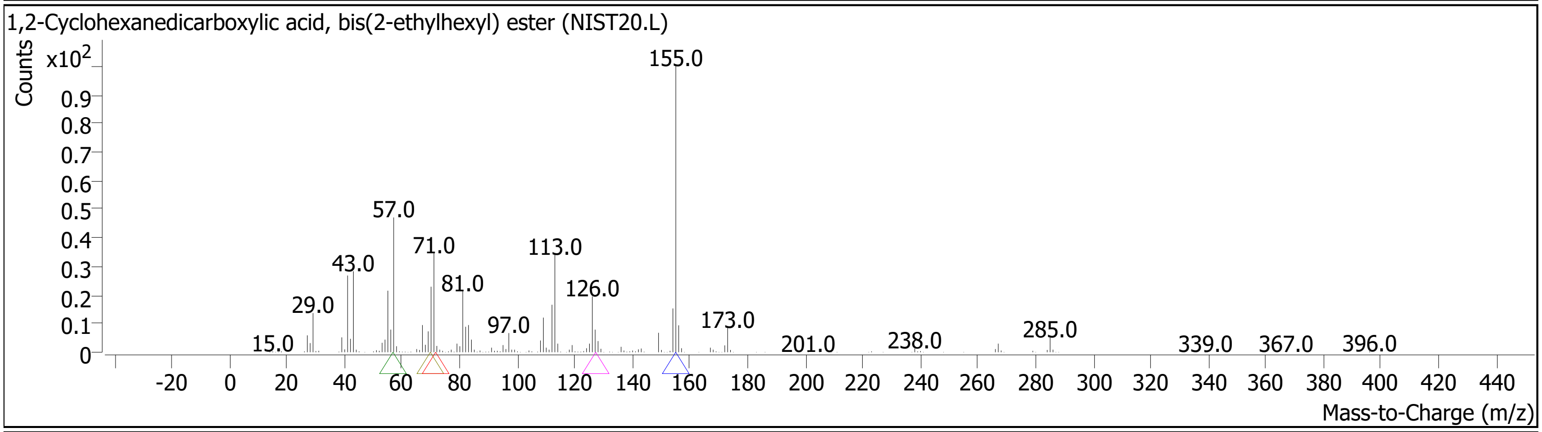

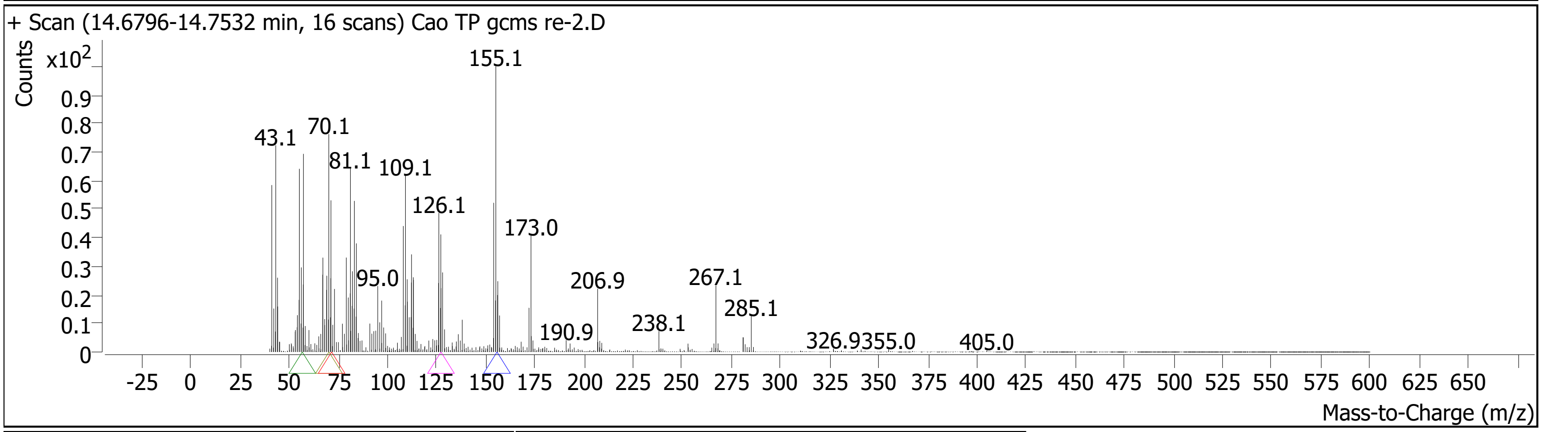

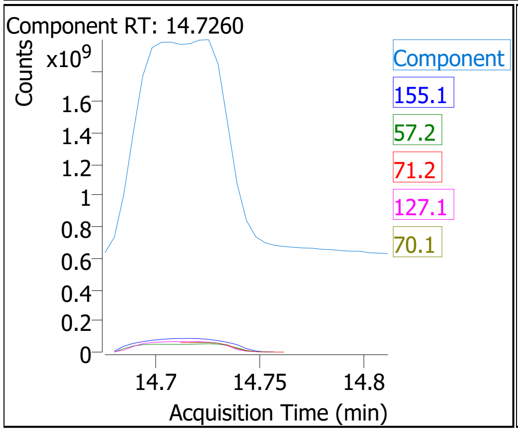

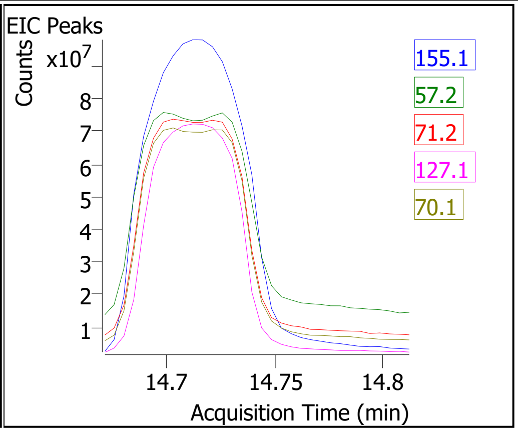

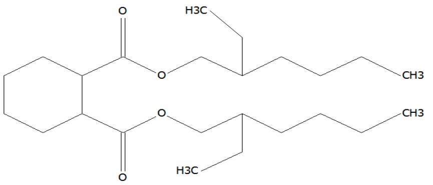

 Generated at 10:40 AM on 12/18/2024

Component RT	Compound Name	Component Area	Match Factor	CAS#	Formula	Estimated Conc.
15.2422	1,2-Cyclohexanedicarboxylic acid, bis(2-ethylhexyl) ester	2646825304.6	77.1	84-71-9	C24H44O4	

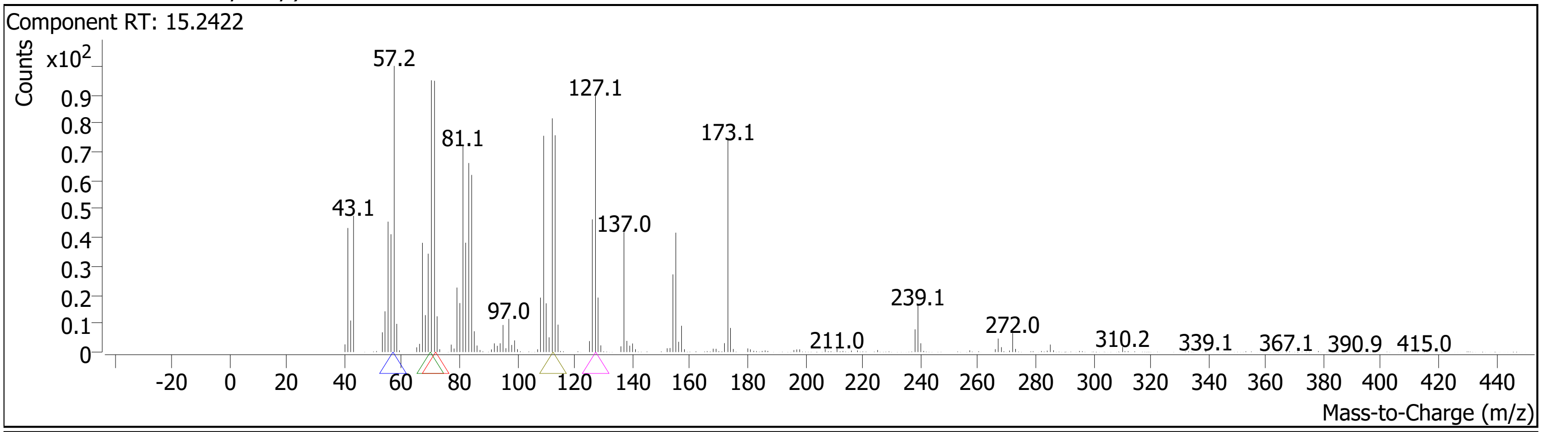

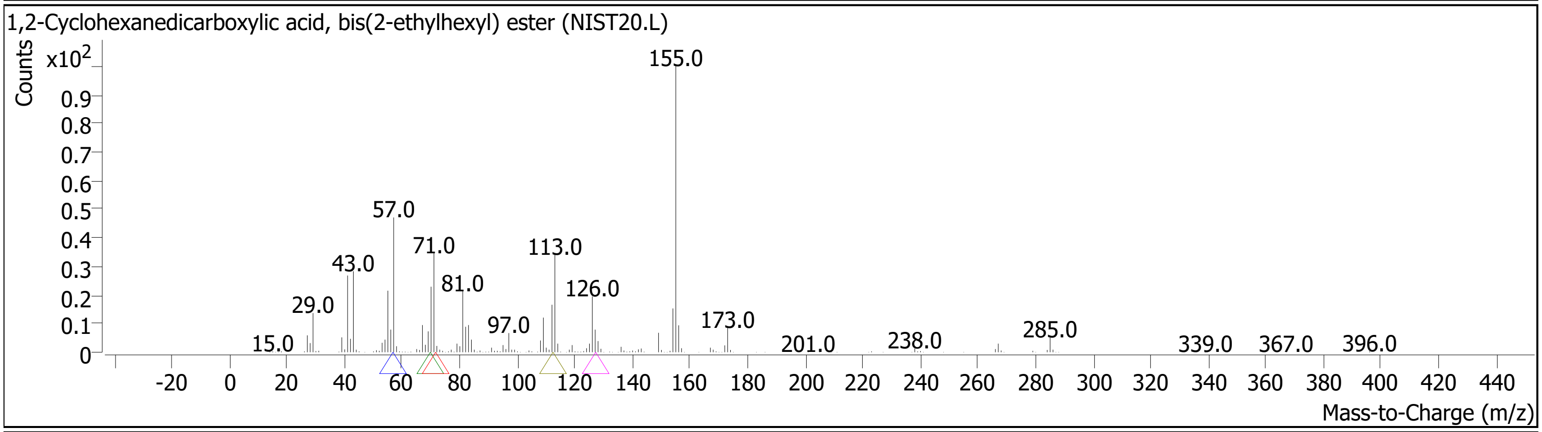

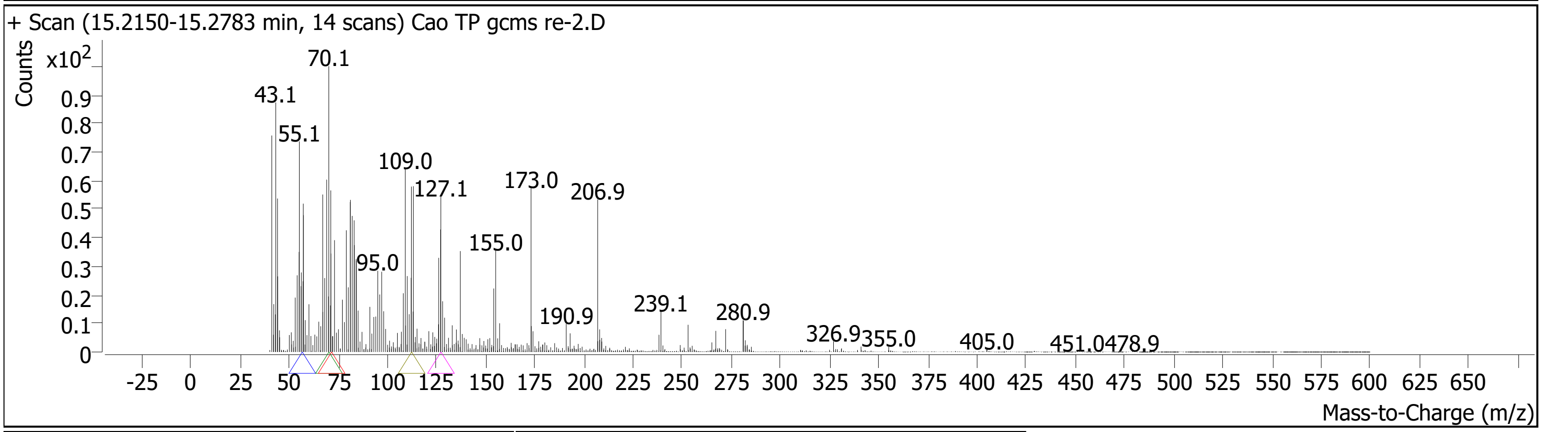

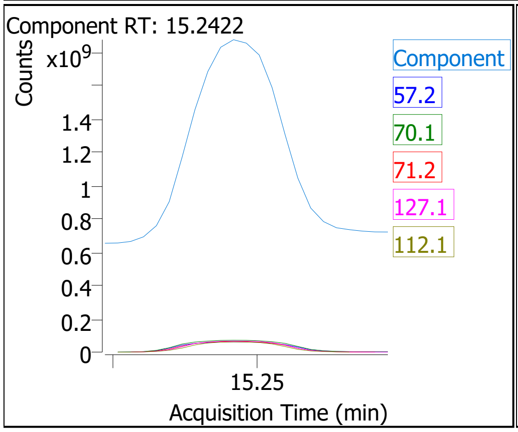

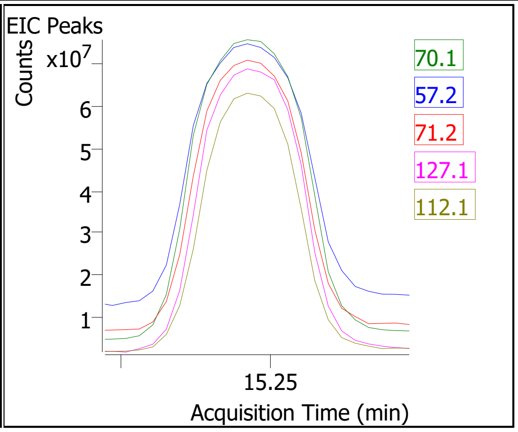

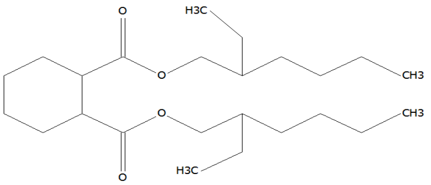

Library Search Results - NonTarget Hits with Details

Component RT	Compound Name	Component Area	Match Factor	CAS#	Formula	Estimated Conc.
15.3644	E,E,Z-1,3,12-Nonadecatriene-5,14-diol	1365589744.8	84.3	1000131-11-4	C19H34O2	

Component RT: 15.3644

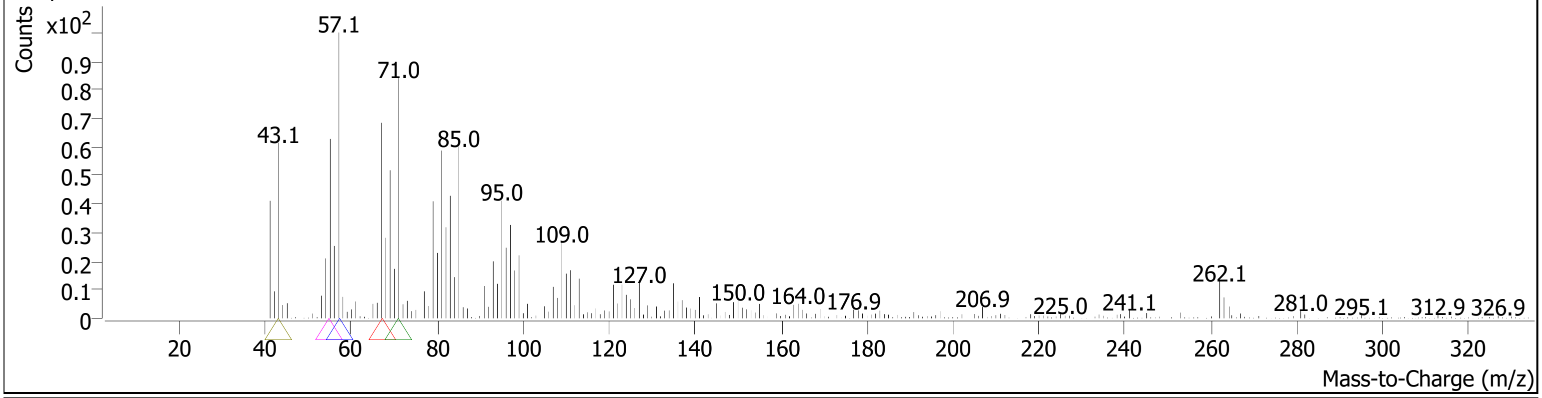

E,E,Z-1,3,12-Nonadecatriene-5,14-diol (NIST20.L)

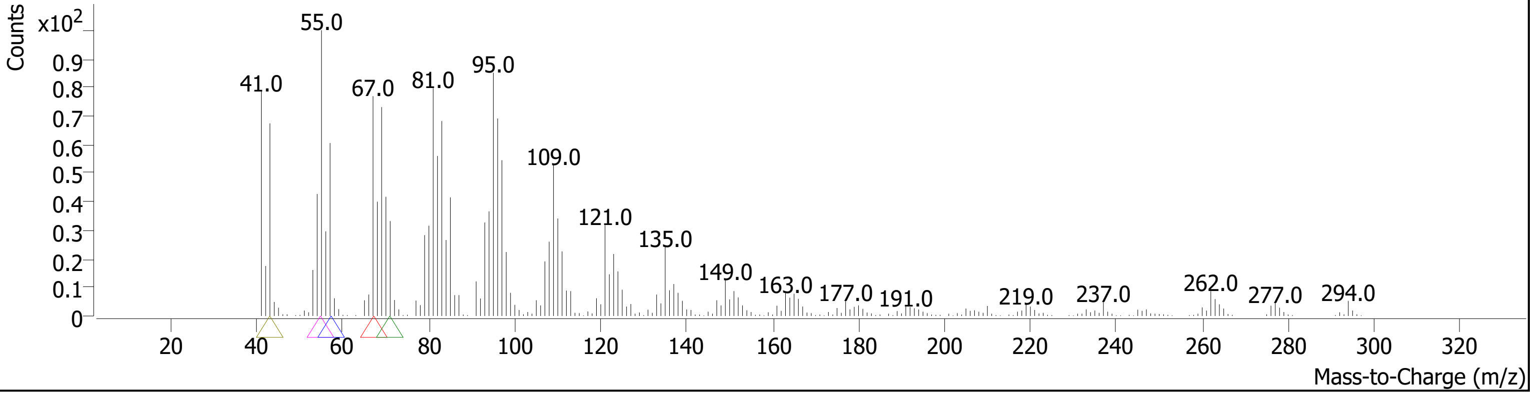

+ Scan (15.3396-15.4097 min, 16 scans) Cao TP gcms re-2.D

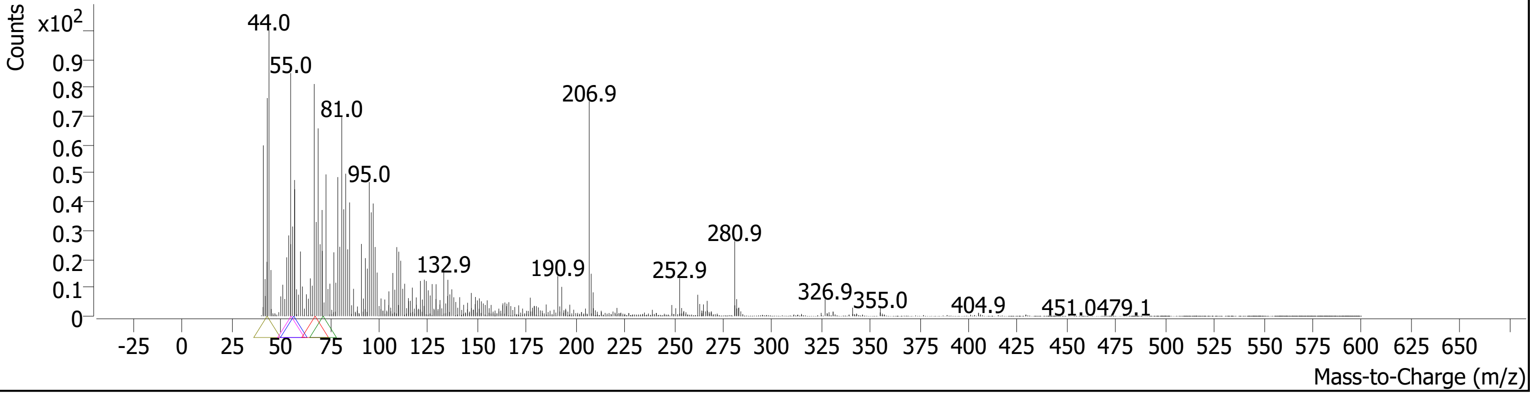

Component RT: 15.3644

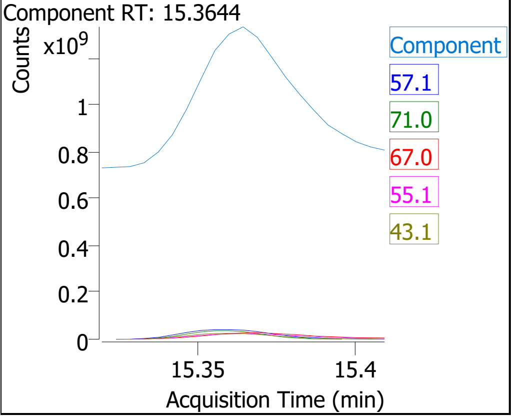

EIC Peaks

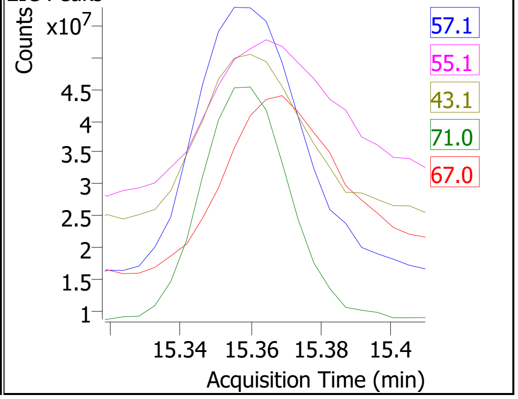

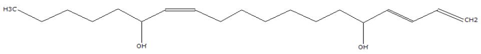

Library Search Results - NonTarget Hits with Details

Component RT	Compound Name	Component Area	Match Factor	CAS#	Formula	Estimated Conc.
15.4505	4,11-Dimethyl-8-(propan-2-yl)-5,12-dioxatricyclo[9.1.0.04,6]dodecan-7-ol	730090951.4	74.1	1000493-61-5	C15H26O3	

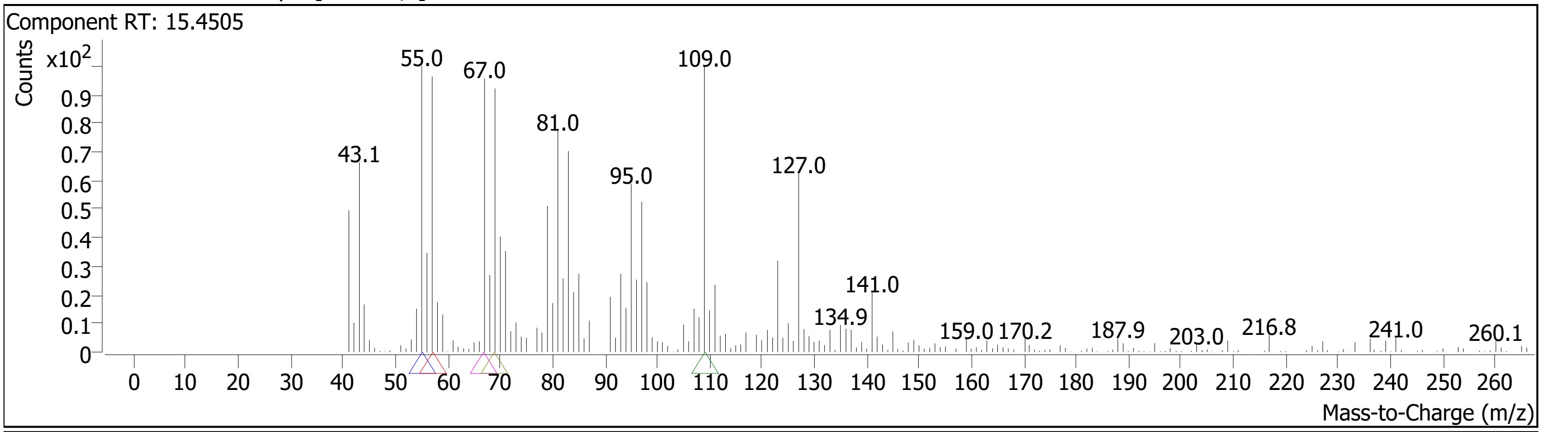

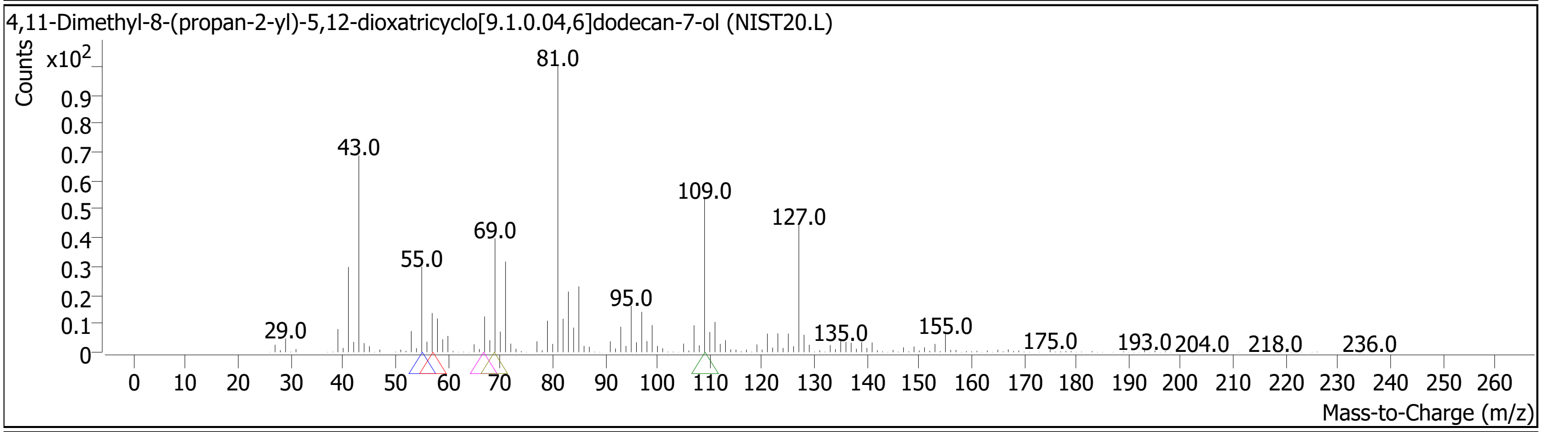

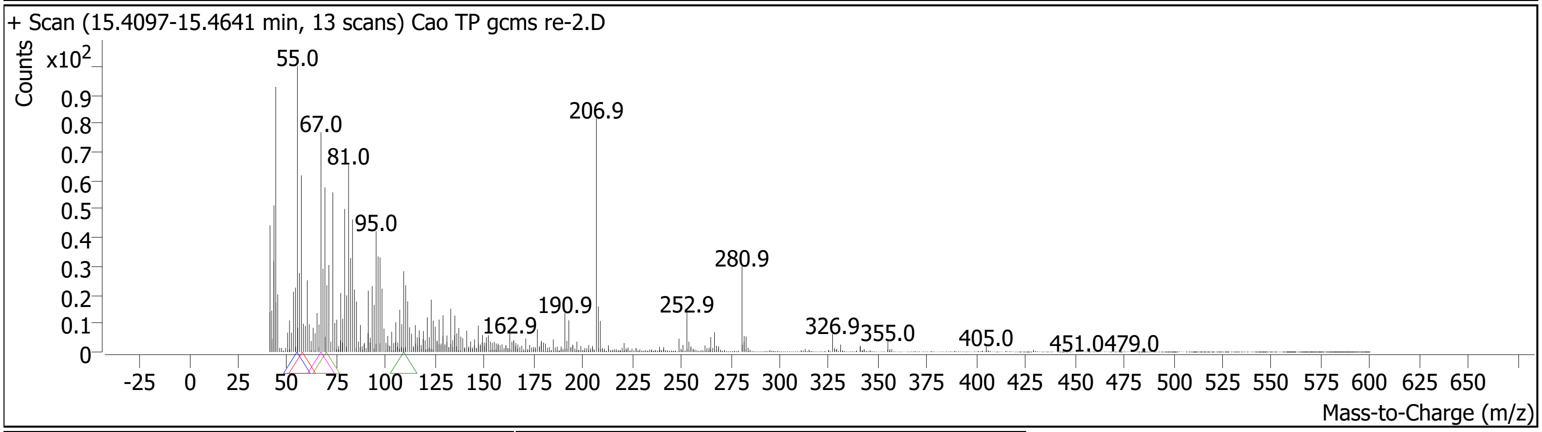

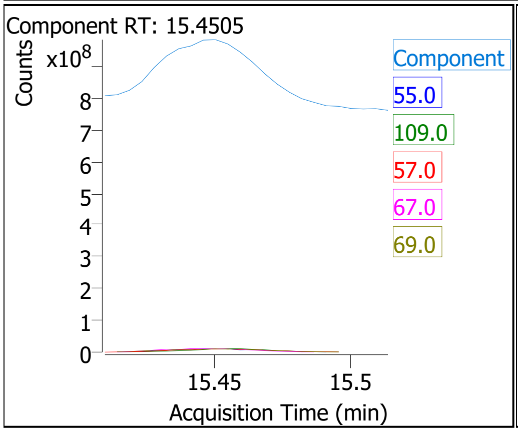

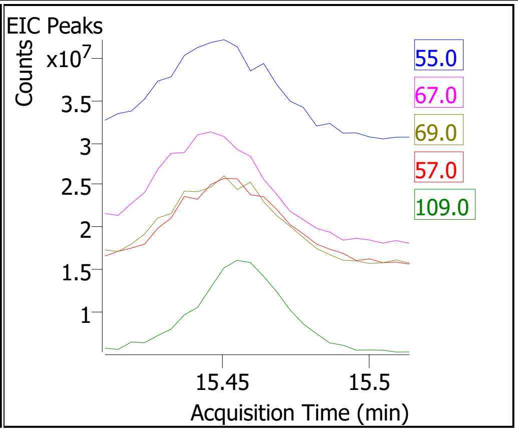

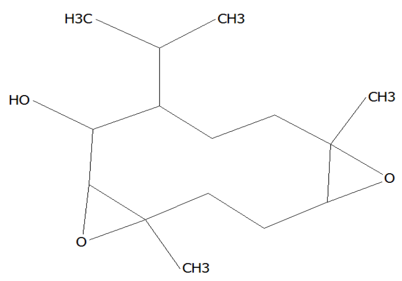

Generated at 10:40 AM on 12/18/2024

Component RT	Compound Name	Component Area	Match Factor	CAS#	Formula	Estimated Conc.
20.1637	Chondrillasterol	3307195243.4	93.5	481-17-4	C29H48O	

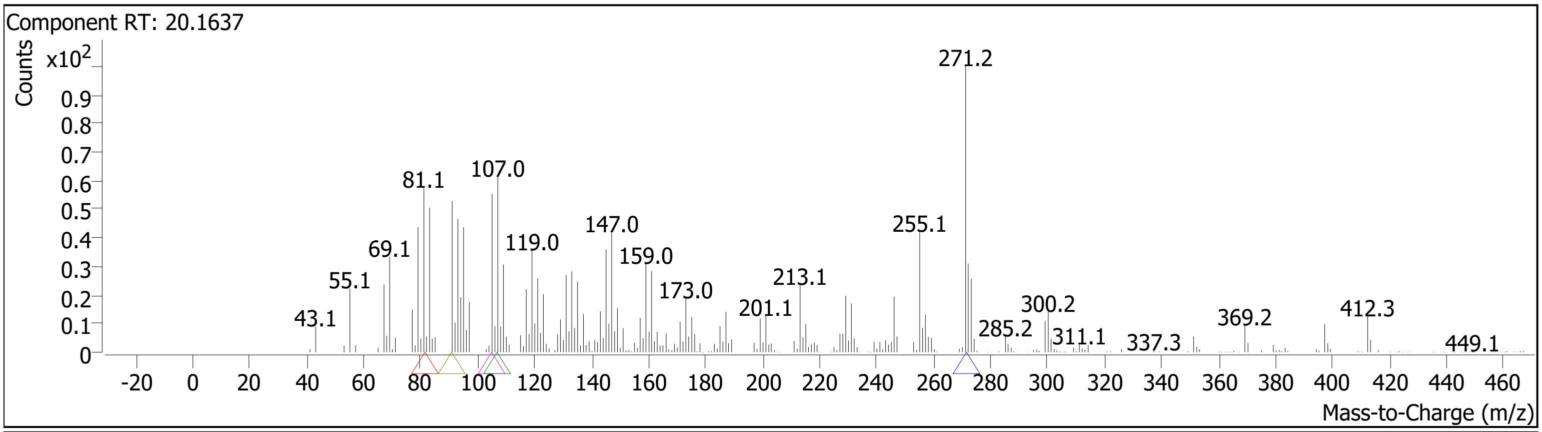

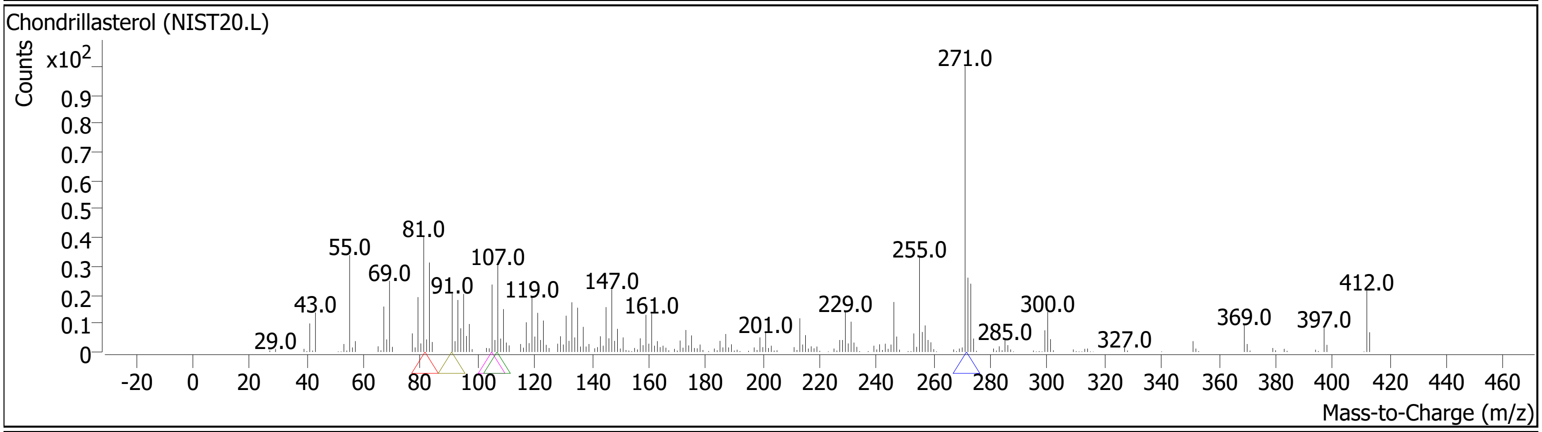

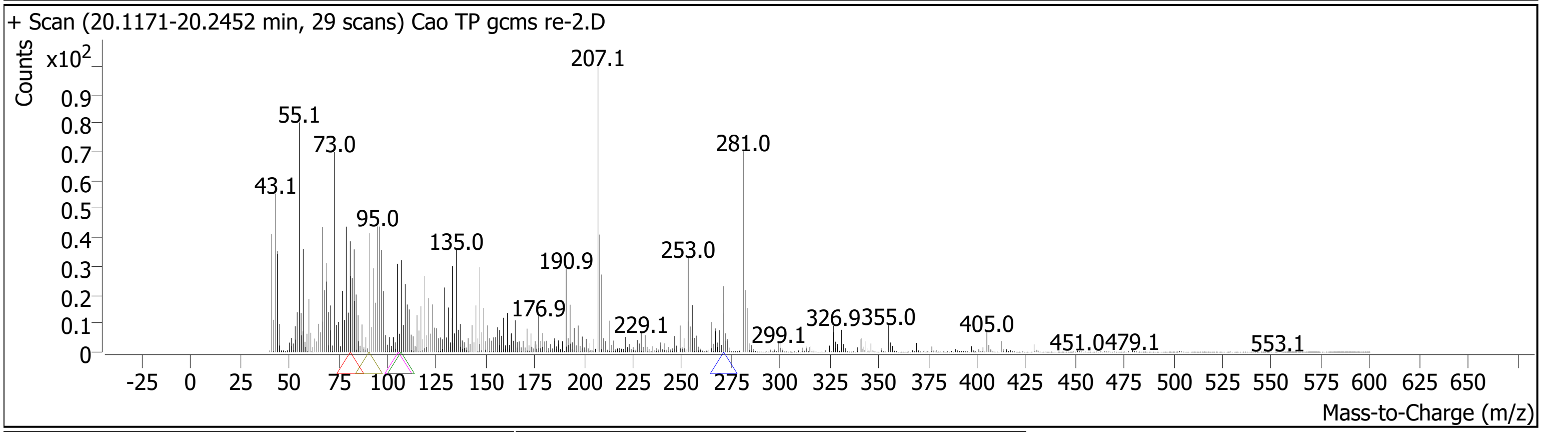

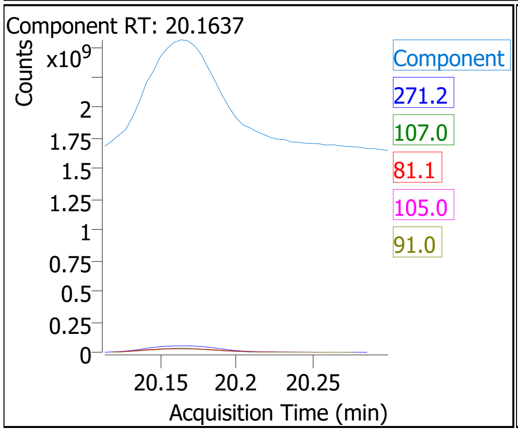

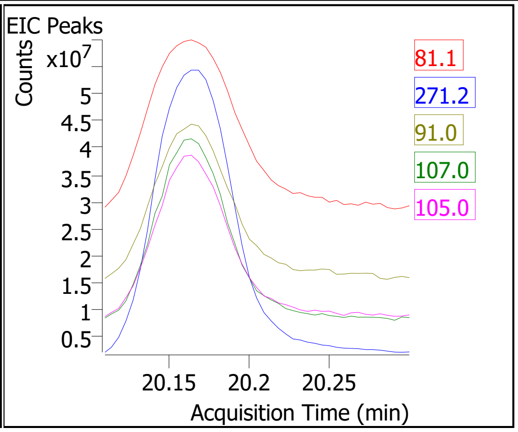

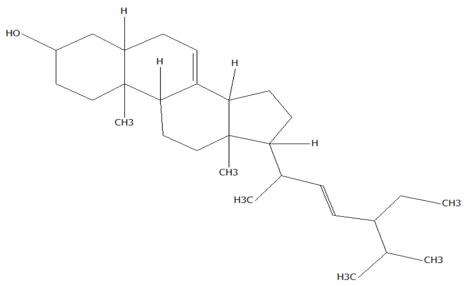

Component RT	Compound Name	Component Area	Match Factor	CAS#	Formula	Estimated Conc.
20.7342	Stigmast-7-en-3-ol, (3.beta.,5.alpha.)-	461651156.8	86.3	521-03-9	C29H50O	

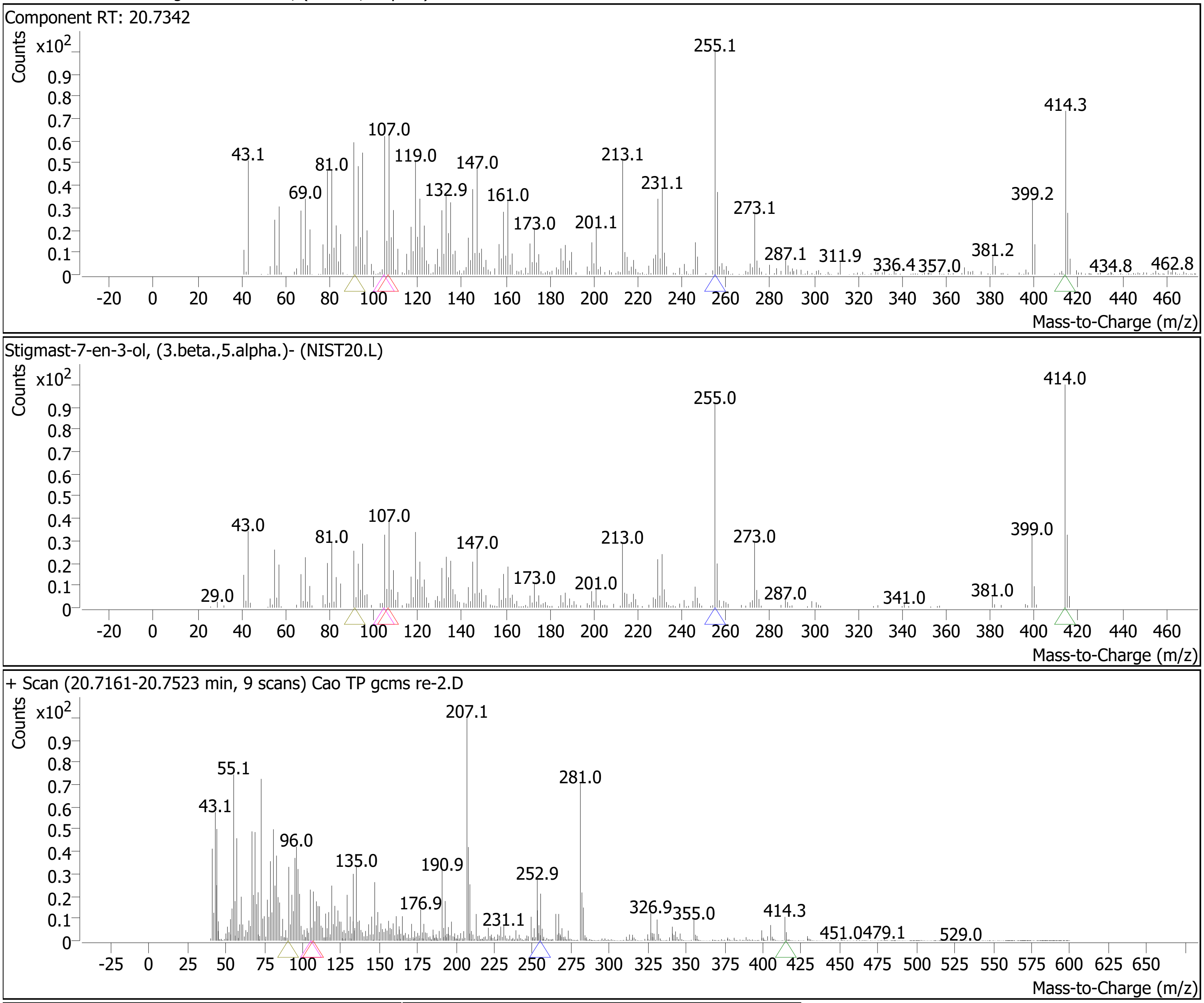

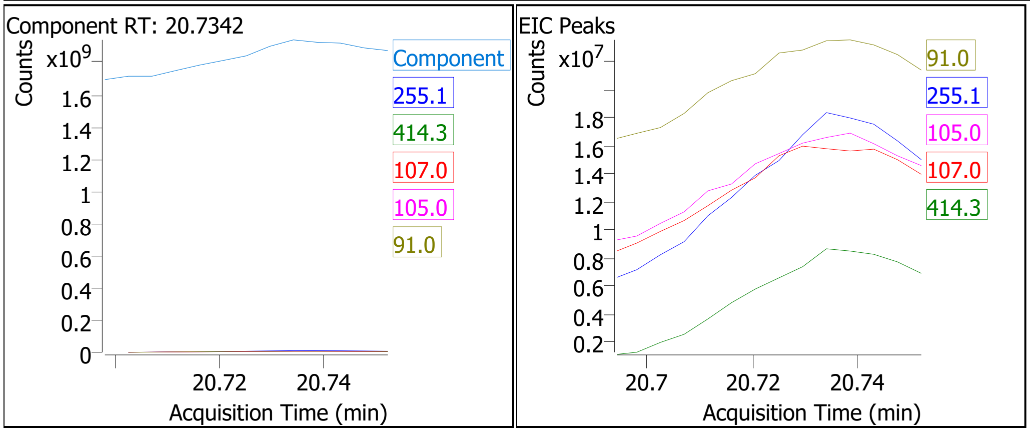

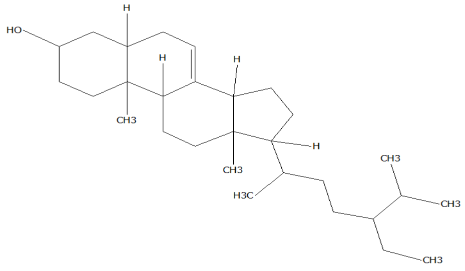

Supplement: Supplementary file 1 [file molecules-31-01088-s001.zip › GC-MS data.pdf]
