# Supplementary material for: Phytochemical Characterization and Biological Evaluation of Camellia hakodae Ninh Flowers
Source: Molecules. 2026 Mar 26;31(7):1088. doi: 10.3390/molecules31071088 (PMC13074563; doi:10.3390/molecules31071088)
Supplement: Supplementary file 1 [file molecules-31-01088-s001.zip › LC-QTOF-MS-MS_negative mode.pdf]

Compound Screening Report

Sample Information

**Sample Name**	Cao TP	**Data File Path**	D:\Trung Pham\Chi My NTT\data\neg\cao TP.d
**Sample ID**		**Acq Time (Local)**	11/18/2024 1:16:02 AM (UTC+07:00)
**Instrument**	LCQTOF	**Acq Method Path**	D:\Trung Pham\Chi My NTT\Method\Negative 150V Long MSMS.m
**MS Type**	QTOF	**Acq SW Version**	6200 series TOF/6500 series Q-TOF 10.1 (48.0)
**Inj Vol (ul)**	5	**IRM Status**	All ions missed
**Sample Position**	P2-F3	**DA Method Path**	D:\Trung Pham\VIGH\Method processing data\Method screening.m
**Plate Position**		**Target Source Path**	D:\MassHunter\PCDL\METLIN PCDL B.08.00\Metlin_AMRT_PCDL All compound.cdb
**Acq Operator**		**Result Summary**	225 qualified (110923 targets)

RT	Name	Formula	CAS	Mass	Mass (Tgt)	Diff (Tgt, ppm)	Score	Algorithm
0.759	<Quinic acid>	C7 H12 O6	77-95-2	192.0633	192.0634	-0.58	99.76	FBF
1.126	<Valiolone>	C7 H12 O6		192.0634	192.0634	-0.14	99.91	FBF
1.579	<Pyrocatechol>	C6 H6 O2	120-80-9	110.0367	110.0368	-0.71	99.59	FBF
1.597	3,4-Dihydroxybenzoic acid	C7 H6 O4	99-50-3	154.0265	154.0266	-0.68	99.58	FBF
3.742	4,4'-Dinitrodiphenylurea	C13 H10 N4 O5	587-90-6	302.0636	302.0651	-4.91	88.36	FBF
4.039	4,4'-Dinitrodiphenylurea	C13 H10 N4 O5	587-90-6	302.0638	302.0651	-4.51	90.03	FBF
5.469	3-O-p-Coumaroylquinic acid	C16 H18 O8		338.0999	338.1002	-0.69	99.60	FBF
5.922	1-Caffeoyl-4-deoxyquinic acid	C16 H18 O8	153444-59-8	338.1000	338.1002	-0.49	99.74	FBF
8.975	alpha-Hydrojuglone 4-O-b-D-glucoside	C16 H18 O8	39015-63-9	338.1000	338.1002	-0.39	99.56	FBF
9.079	Cinnamtannin A1	C45 H38 O18		866.2054	866.2058	-0.53	99.06	FBF
9.358	8,8'-Methylenebiscatechin	C31 H28 O12	81555-08-0	592.1576	592.1581	-0.76	99.40	FBF
9.899	Hyperoside	C21 H20 O12	482-36-0	464.0954	464.0955	-0.25	99.42	FBF
10.405	Depressonol A	C32 H38 O20		742.1956	742.1956	-0.04	99.64	FBF
10.667	6-Hydroxykaempferol 3-rutinoside-6-glucoside	C33 H40 O21		772.2062	772.2062	0.03	99.69	FBF
10.911	Cinnamtannin A2	C60 H50 O24		1154.2679	1154.2692	-1.12	98.35	FBF
11.260	<Arthromerin B>	C21 H24 O10		436.1368	436.1369	-0.33	99.06	FBF
11.295	C.I. Pigment Red 149	C40 H26 N2 O4	4948-15-6	598.1899	598.1893	1.01	82.43	FBF
11.295	Geniposide pentaacetate	C27 H34 O15	49776-64-9	598.1899	598.1898	0.16	99.73	FBF
11.295	7-Hydroxytrifluoperazine glucuronide	C27 H32 F3 N3 O7 S		599.1930	599.1913	2.86	87.76	FBF
11.451	<Hyperin>	C21 H20 O12		464.0954	464.0955	-0.13	99.67	FBF
11.591	<2''-O-beta-L-galactopyranosylorientin>	C27 H30 O16		610.1533	610.1534	-0.06	99.90	FBF
12.097	6-Hydroxyluteolin 6-xyloside	C20 H18 O11		434.0849	434.0849	-0.09	99.82	FBF
12.516	<Troxerutin>	C33 H42 O19	7085-55-4	742.2319	742.2320	-0.18	99.13	FBF
13.091	Camelliaside B	C32 H38 O19		726.2006	726.2007	-0.13	99.75	FBF
13.527	N(omega)-(ADP-D-ribosyl)-L-arginine	C21 H35 N9 O15 P2		715.1748	715.1728	2.82	83.17	FBF
13.649	<Streptonigrin>	C25 H22 N4 O8	3930-19-6	506.1423	506.1438	-2.96	95.66	FBF
13.719	(Indol-3-yl)glycolaldehyde	C10 H9 N O2		175.0633	175.0633	-0.36	99.77	FBF
13.893	Malvidin 3-glucoside-5-(6-acetylglucoside)	C30 H35 O19		699.1796	699.1773	3.39	92.74	FBF
13.911	Carnocin CP 5	C23 H19 N3 O5 S	149983-81-3	449.1038	449.1045	-1.73	86.71	FBF
13.911	<Kaempferol 3-alpha-D-galactoside>	C21 H20 O11		448.1006	448.1006	0.01	99.74	FBF
14.452	Proanthocyanidin A1	C30 H24 O12	103883-03-0	576.1264	576.1268	-0.64	93.88	FBF
14.539	Indole-3-carboxylic acid	C9 H7 N O2	771-50-6	161.0476	161.0477	-0.73	99.43	FBF
14.957	Foramsulfuron	C17 H20 N6 O7 S	173159-57-4	452.1111	452.1114	-0.71	84.62	FBF
15.027	Camelliaside A	C33 H40 O20		756.2112	756.2113	-0.14	99.00	FBF
15.550	6''-Caffeoylhyperin	C30 H26 O15	84575-22-4	626.1272	626.1272	0.08	99.74	FBF
15.742	Sennoside E	C44 H38 O23	11137-63-6	934.1830	934.1804	2.76	94.26	FBF
15.847	Hyperoside	C21 H20 O12	482-36-0	464.0955	464.0955	0.16	99.88	FBF
15.934	Apigenin 4'-[feruloyl-(->2)-glucuronyl-(1->2)-glucuronide]	C37 H34 O20		798.1640	798.1643	-0.49	99.64	FBF
15.969	(2S,3S)-3,5,7-trihydroxy-6-methyl-2-(3,4,5-trihydroxyphenyl)-2,3-dihydrochromen-4-one	C16 H14 O8	1212351-36-4	334.0687	334.0689	-0.53	99.77	FBF
16.876	<Isovitexin>	C21 H20 O10	38953-85-4	432.1055	432.1056	-0.26	99.46	FBF
17.155	N-Caffeoyltryptophan	C20 H18 N2 O5	109163-69-1	366.1215	366.1216	-0.21	99.90	FBF
17.312	Quercetin 3-(3''-sulfatoglucoside)	C21 H20 O15 S		544.0521	544.0523	-0.31	98.25	FBF
17.801	Quercetin	C15 H10 O7	117-39-5	302.0427	302.0427	0.15	99.52	FBF
17.910	3,5,7,3',4'-Pentahydroxy-6,8-dimethoxyflavone 3-alpha-L-arabinopyranoside	C22 H22 O13		494.1061	494.1060	0.03	99.58	FBF
19.859	dTDP-β-L-rhodinose	C16 H26 N2 O13 P2		516.0932	516.0910	4.32	82.72	FBF
19.998	4''-O-Acetylmyricitrin	C23 H22 O13		506.1059	506.1060	-0.29	99.70	FBF
19.998	6-Hydroxyluteolin 7-(6''-malonylglucoside)	C24 H22 O15		550.0956	550.0959	-0.40	99.57	FBF
20.051	dTDP-β-L-rhodinose	C16 H26 N2 O13 P2		516.0935	516.0910	4.80	80.18	FBF
20.312	Patuletin 3-(6''-p-coumaroylglucoside)	C31 H28 O15		640.1427	640.1428	-0.19	99.68	FBF
20.417	2-Hydroxybenzaldehyde O-[xylosyl-(1->6)-glucoside]	C18 H24 O11	14907-56-3	416.1316	416.1319	-0.68	99.41	FBF
20.644	Na-p-Hydroxycoumaroyltryptophan	C20 H18 N2 O4		350.1264	350.1267	-0.63	99.70	FBF
20.644	N-dodecanoyl-L-Homoserine lactone-3-hydrazone-fluorescein	C37 H40 N4 O8 S		700.2534	700.2567	-4.71	83.48	FBF
20.748	6''-O-Caffeoylastragalin	C30 H26 O14	190328-43-9	610.1324	610.1323	0.19	99.35	FBF
21.115	2''-O-Feruloylorientin	C31 H28 O14		624.1478	624.1479	-0.20	99.01	FBF
21.446	<Kaempferol>	C15 H10 O6	520-18-3	286.0476	286.0477	-0.63	99.47	FBF
21.760	PRE	C44 H44 O24	160564-02-3	956.2225	956.2223	0.21	98.08	FBF
21.760	1,3,5,8-Tetrahydroxy-6-methoxy-2-methylanthraquinone 8-O-b-D-glucoside	C22 H22 O12	101508-15-0	478.1111	478.1111	0.04	99.86	FBF
22.403	N1,N5,N10-Tricoumaroyl spermidine	C34 H37 N3 O6		583.2680	583.2682	-0.40	99.68	FBF
22.806	6'''-Deamino-6'''-dehydro-6'''-oxoneomycin C	C23 H43 N5 O14		613.2785	613.2807	-3.55	80.34	FBF
22.946	Tiliroside	C30 H26 O13	20316-62-5	594.1371	594.1373	-0.43	99.07	FBF
23.365	N1,N5,N10-Triferuloyl spermidine	C37 H43 N3 O9		673.2996	673.2999	-0.54	99.71	FBF

RT	Name	Formula	CAS	Mass	Mass (Tgt)	Diff (Tgt, ppm)	Score	Algorithm
24.516	Eucaglobulin	C23 H30 O12	241130-84-7	498.1735	498.1737	-0.37	99.81	FBF
28.336	(+)-9,10,18-trihydroxy-12Z-octadecenoic acid	C18 H34 O5		330.2405	330.2406	-0.32	99.65	FBF
28.545	Phloionolic acid	C18 H36 O5		332.2562	332.2563	-0.16	99.53	FBF
28.841	(+)-9,10,18-trihydroxy-12Z-octadecenoic acid	C18 H34 O5		330.2404	330.2406	-0.71	99.21	FBF
28.894	10-hydroxy-hexadecan-1,16-dioic acid	C16 H30 O5		302.2092	302.2093	-0.54	99.62	FBF
28.894	Emedastine	C17 H26 N4 O		302.2092	302.2107	-4.72	88.50	FBF
28.964	6-Hydroxyluteolin 6-sulfate	C15 H10 O10 S		381.9993	381.9995	-0.53	97.02	FBF
29.121	(+)-9,10,18-trihydroxy-12Z-octadecenoic acid	C18 H34 O5		330.2404	330.2406	-0.71	98.58	FBF
29.347	PE-Cer(d15:2(4E,6E)/18:0(2OH))	C35 H69 N2 O7 P		660.4810	660.4842	-4.91	85.76	FBF
29.347	(+)-9,10,18-trihydroxy-12Z-octadecenoic acid	C18 H34 O5		330.2405	330.2406	-0.50	99.75	FBF
29.661	10,16-dihydroxy-palmitic acid	C16 H32 O4		288.2303	288.2301	0.85	98.96	FBF
32.330	<Polyscioside D>	C54 H86 O24	202585-62-4	1118.5501	1118.5509	-0.70	99.35	FBF
36.743	Camelliasaponin A1	C58 H92 O25	183020-18-0	1188.5922	1188.5928	-0.47	97.47	FBF
36.987	Congmunoside XIV	C57 H92 O23	329969-14-4	1144.6024	1144.6029	-0.47	99.74	FBF
37.301	Camelliasaponin A2	C58 H92 O25	183183-15-5	1188.5921	1188.5928	-0.56	99.45	FBF
38.208	5-Oxoavermectin "1b" aglycone	C33 H44 O8		568.3030	568.3036	-1.12	99.09	FBF
38.208	Ceanothine E	C34 H40 N4 O4	23926-98-9	568.3031	568.3050	-3.35	93.71	FBF
38.365	<9(S)-HOTrE>	C18 H30 O3	89886-42-0	294.2192	294.2195	-0.89	98.95	FBF
40.214	Soyasaponin bg	C54 H84 O21	143519-54-4	1068.5504	1068.5505	-0.07	99.04	FBF
40.371	<9(R)-HODE>	C18 H32 O3	10075-11-3	296.2351	296.2351	-0.20	99.80	FBF
42.481	MGDG(18:5(3Z,6Z,9Z,12Z,15Z)/18:5(3Z,6Z,9Z,12Z,15Z))	C45 H66 O10		766.4684	766.4656	3.60	92.53	FBF
42.534	SQDG(22:5(5Z,8Z,11Z,14Z,17Z)/16:1(13Z))	C45 H76 O12 S		840.5056	840.5057	-0.19	99.10	FBF
42.970	Mycalamide A	C24 H41 N O10	115185-92-7	503.2727	503.2730	-0.72	99.08	FBF
43.685	DGDG(18:5(3Z,6Z,9Z,12Z,15Z)/18:4(6Z,9Z,12Z,15Z))	C51 H78 O15		930.5364	930.5341	2.54	94.58	FBF
44.348	Chukrasin methyl ether	C43 H58 O16	1045017-87-5	830.3751	830.3725	3.17	93.47	FBF
44.348	2-Dodecylbenzenesulfonic acid	C18 H30 O3 S		326.1917	326.1916	0.46	91.55	FBF
44.819	MGDG(18:5(3Z,6Z,9Z,12Z,15Z)/18:4(6Z,9Z,12Z,15Z))	C45 H68 O10		768.4842	768.4812	3.78	91.34	FBF
46.074	LysoPC(24:1(15Z))	C32 H65 N O7 P		606.4492	606.4499	-1.10	97.21	FBF
46.249	LysoPC(24:1(15Z))	C32 H65 N O7 P		606.4493	606.4499	-0.99	97.03	FBF
46.284	<α-Linolenic Acid>	C18 H30 O2	463-40-1	278.2252	278.2246	2.29	96.48	FBF
46.284	PC(O-5:0/0:0)[R]	C13 H31 N O6 P		328.1878	328.1889	-3.46	81.11	FBF
46.301	PA(O-16:0/12:0)	C31 H63 O7 P		578.4308	578.4311	-0.60	96.22	FBF
46.301	2-Hexaprenyl-3-methyl-5-hydroxy-6-methoxy-1,4-benzoquinol	C38 H58 O4		578.4308	578.4335	-4.73	90.13	FBF
46.790	Tetradecyl sulfate	C14 H30 O4 S	139-88-8	294.1863	294.1865	-0.73	96.65	FBF
47.191	<Δ2-cis-Hexadecenoic Acid>	C16 H30 O2	2825-68-5	254.2244	254.2246	-0.88	99.05	FBF
47.888	16-Hydroxy hexadecanoic acid	C16 H32 O3	506-13-8	272.2353	272.2351	0.44	99.35	FBF
47.941	<Linoelaidic Acid>	C18 H32 O2	506-21-8	280.2409	280.2402	2.50	96.06	FBF
47.941	2-methylbacteriohopane-32,33,34,35-tetrol	C36 H64 O4		560.4819	560.4805	2.54	99.62	FBF
48.290	1α-hydroxy-23-[3-(1-hydroxy-1-methylethyl)phenyl]-22,22,23,23-tetradehydro-24,25,26,27-tetranorvitamin D3 / 1α-hydroxy-23-[3-(1-hydroxy-1-methylethyl)phenyl]-22,22,23,23-tetradehydro-24,25,26,27-tetranorcholecalciferol	C32 H42 O3		474.3130	474.3134	-0.78	99.09	FBF
48.830	MGDG(18:3(9Z,12Z,15Z)/18:4(6Z,9Z,12Z,15Z))	C45 H72 O10		772.5149	772.5125	3.09	94.07	FBF
49.092	Asparagoside A	C33 H54 O8	14835-43-9	578.3814	578.3819	-0.74	99.12	FBF
49.179	<Myristic Acid ethyl ester>	C16 H32 O2	124-06-1	256.2408	256.2402	2.37	95.73	FBF
49.685	12-OAHSA	C36 H68 O4	101901-73-9	564.5124	564.5118	1.14	98.81	FBF
49.685	Erythrinasinate A	C38 H66 O4	102607-46-5	586.4933	586.4961	-4.81	88.33	FBF
51.586	decyl octanoate	C18 H36 O2		284.2715	284.2715	-0.19	99.33	FBF
51.918	DG(13:0/20:5(5Z,8Z,11Z,14Z,17Z)/0:0)[iso2]	C36 H60 O5		572.4439	572.4441	-0.38	99.75	FBF
52.894	DG(13:0/18:2(9Z,12Z)/0:0)[iso2]	C34 H62 O5		550.4592	550.4597	-1.00	99.22	FBF
53.104	1,2-di-(9Z,12Z,15Z-octadecatrienoyl)-3-(8-(2E,4Z-decadienoyloxy)-5,6-octadienoyl)-sn-glycerol	C57 H88 O8		900.6502	900.6479	2.56	95.57	FBF
53.104	DG(13:0/20:4(5Z,8Z,11Z,14Z)/0:0)[iso2]	C36 H62 O5		574.4593	574.4597	-0.73	99.72	FBF
53.296	DG(13:0/20:4(5Z,8Z,11Z,14Z)/0:0)[iso2]	C36 H62 O5		574.4593	574.4597	-0.80	99.02	FBF
54.185	Fasciculic acid A	C36 H60 O8	126906-00-1	620.4284	620.4288	-0.65	99.21	FBF
54.342	<Arachidic acid>	C20 H40 O2	506-30-9	312.3026	312.3028	-0.80	99.60	FBF
54.429	DG(14:1(9Z)/17:1(9Z)/0:0)[iso2]	C34 H62 O5		550.4595	550.4597	-0.41	99.06	FBF
54.673	DG(15:1(9Z)/16:1(9Z)/0:0)[iso2]	C34 H62 O5		550.4597	550.4597	-0.12	97.40	FBF
55.162	(20R)-Ginsenoside Rh2	C36 H62 O8	78214-33-2	622.4441	622.4445	-0.52	99.50	FBF
58.581	<16-Methylheptadecyl isobutyrate>	C22 H44 O2		340.3339	340.3341	-0.66	99.48	FBF
59.226	Lauryl hydrogen sulfate	C12 H26 O4 S	151-21-3	266.1549	266.1552	-1.02	96.63	FBF
61.424	PS(17:1(9Z)/22:2(13Z,16Z))	C45 H82 N O10 P		827.5670	827.5676	-0.74	99.59	FBF

Compound Details

Cpd 90: <Quinic acid>

Name	Formula	RT	RI	Mass	Diff (Tgt, ppm)	CAS	ID Source	Score	Algorithm
<Quinic acid>	C7 H12 O6	0.759		192.0633	-0.58	77-95-2	M-FBF	99.76	FBF

Species	m/z	Score (Tgt)	Score (Lib)	Score (DB)	Score (MFG)	Score (RT)
(M-H)-	191.0560	99.76				

Compound Screening Report

Compound Chromatograms (overlaid)

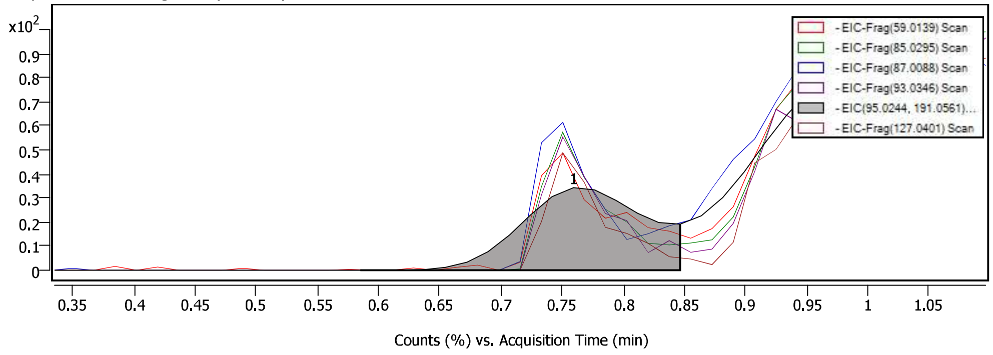

Structure

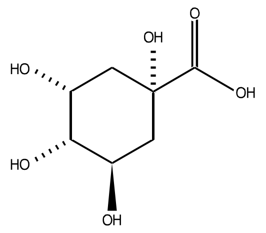

Coelution Plot

Compound Spectra (overlaid)

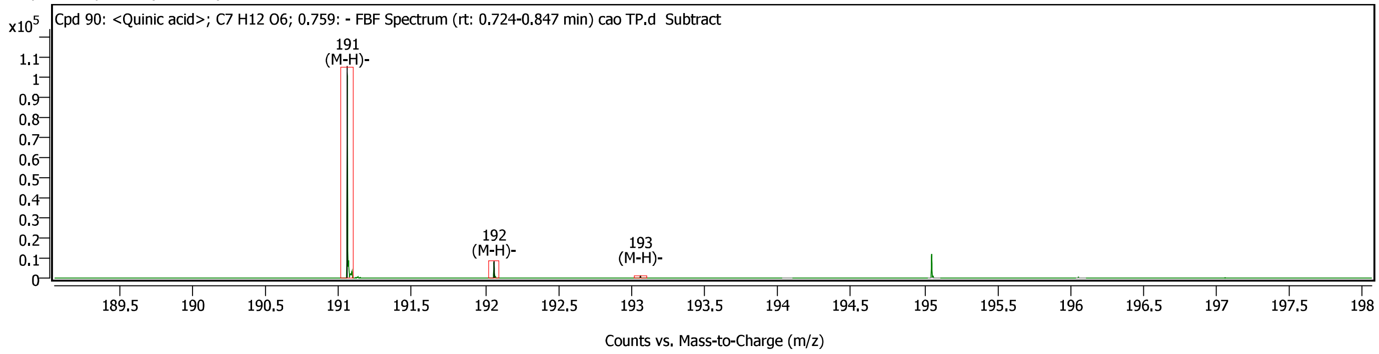

Fragment Spectrum (raw)

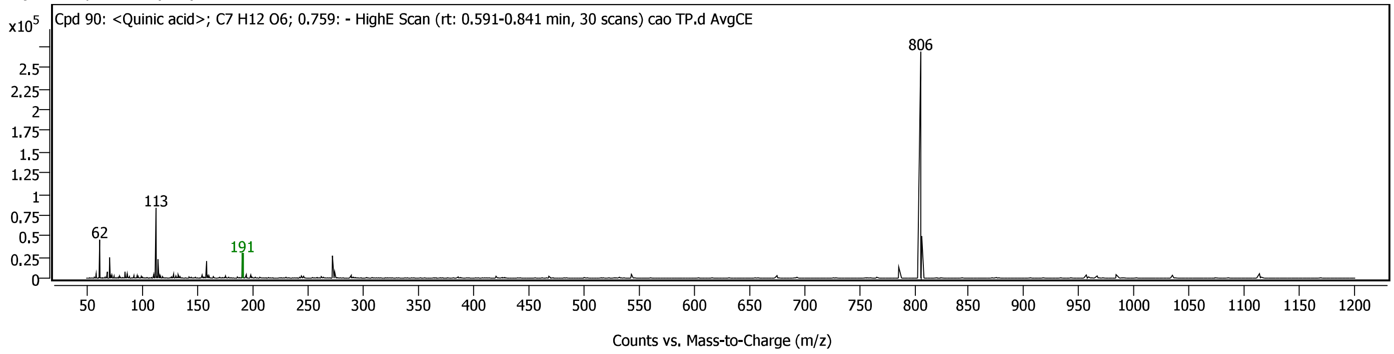

Compound ID Table

Name	Formula	Species	RT	RT Diff	Mass	CAS	ID Source	Score	Score (Lib)	Score (Tgt)
<Quinic acid>	C7 H12 O6	(M-H)-	0.759		192.0633	77-95-2	FBF	99.76		99.76
<2D-5-O-Methyl-2,3,5/4,6-pentahydroxycyclohexanone>	C7 H12 O6	(M-H)-	0.759		192.0633		FBF	99.76		99.76
<Valiolone>	C7 H12 O6	(M-H)-	0.759		192.0633		FBF	99.76		99.76

Cpd 91: <Valiolone>

Name	Formula	RT	RI	Mass	Diff (Tgt, ppm)	CAS	ID Source	Score	Algorithm
<Valiolone>	C7 H12 O6	1.126		192.0634	-0.14		M-FBF	99.91	FBF

Species	m/z	Score (Tgt)	Score (Lib)	Score (DB)	Score (MFG)	Score (RT)
(M-H)-	191	99.91				

Compound Chromatograms (overlaid)

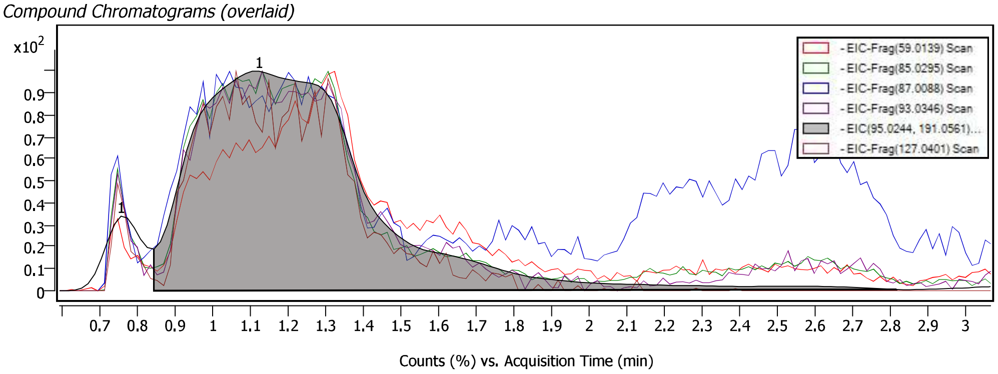

Structure

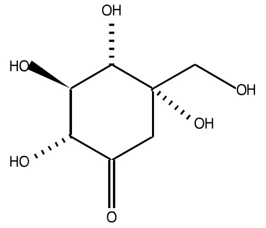

Coelution Plot

Compound Spectra (overlaid)

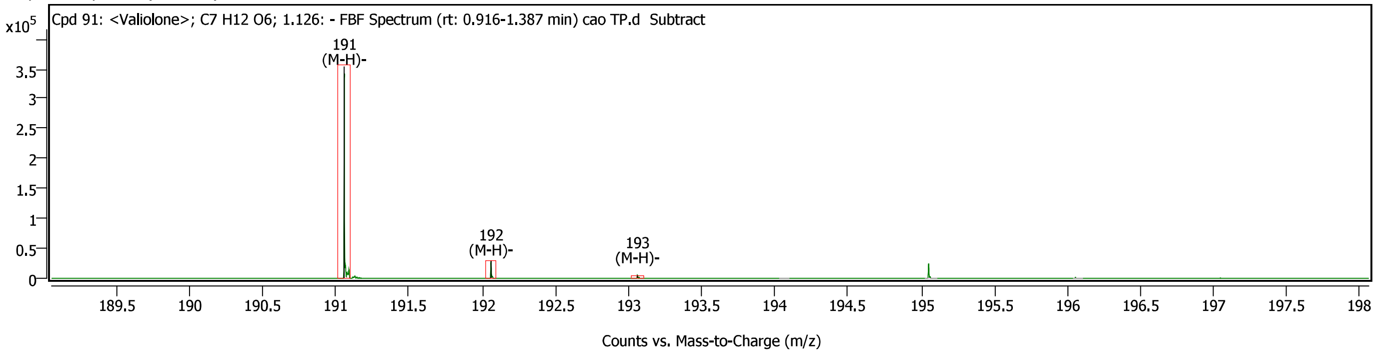

Fragment Spectrum (raw)

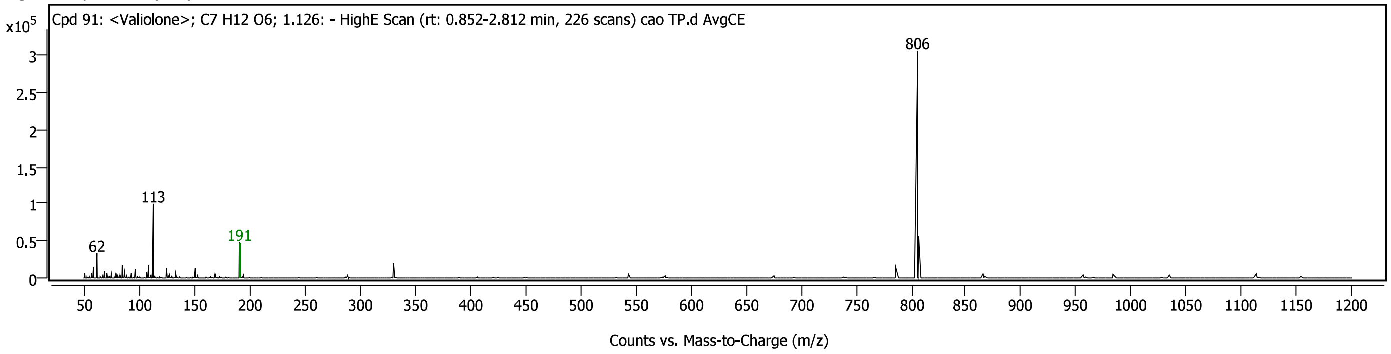

Compound ID Table

Name	Formula	Species	RT	RT Diff	Mass	CAS	ID Source	Score	Score (Lib)	Score (Tgt)
<Valiolone>	C7 H12 O6	(M-H)-	1.126		192.0634		FBF	99.91		99.91
<2D-5-O-Methyl-2,3,5/4,6-pentahydroxycyclohexanone>	C7 H12 O6	(M-H)-	1.126		192.0634		FBF	99.91		99.91
<Quinic acid>	C7 H12 O6	(M-H)-	1.126		192.0634	77-95-2	FBF	99.91		99.91

Cpd 222: <Pyrocatechol>

Name	Formula	RT	RI	Mass	Diff (Tgt, ppm)	CAS	ID Source	Score	Algorithm
<Pyrocatechol>	C6 H6 O2	1.579		110.0367	-0.71	120-80-9	M-FBF	99.59	FBF

Species	m/z	Score (Tgt)	Score (Lib)	Score (DB)	Score (MFG)	Score (RT)
(M-H)-	109	99.59				

Compound Chromatograms (overlaid)

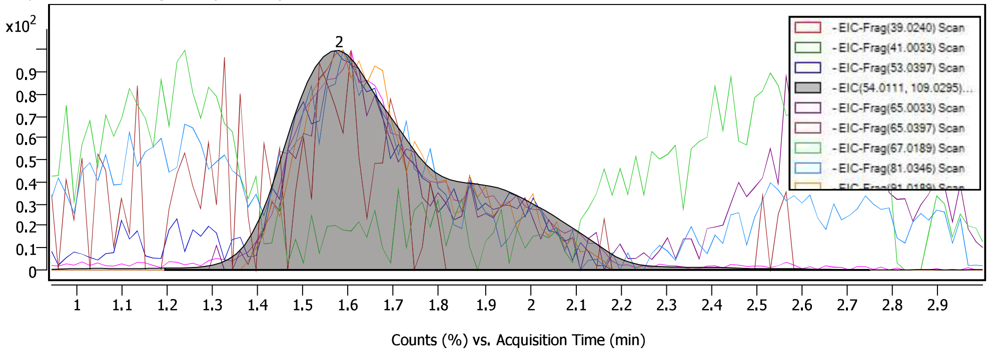

Structure

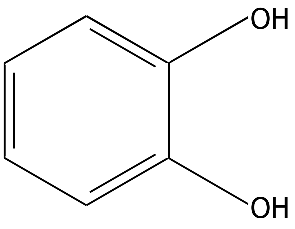

Coelution Plot

Compound Screening Report

Compound Spectra (overlaid)

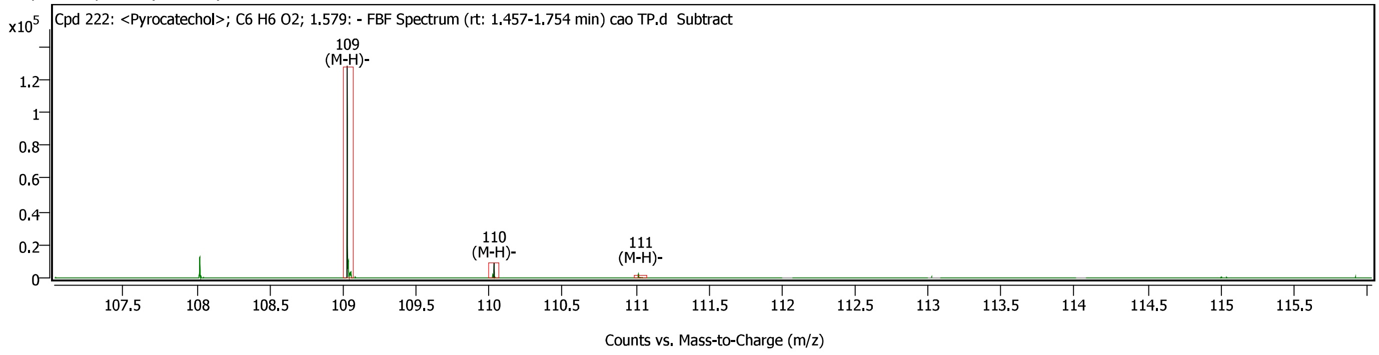

Fragment Spectrum (raw)

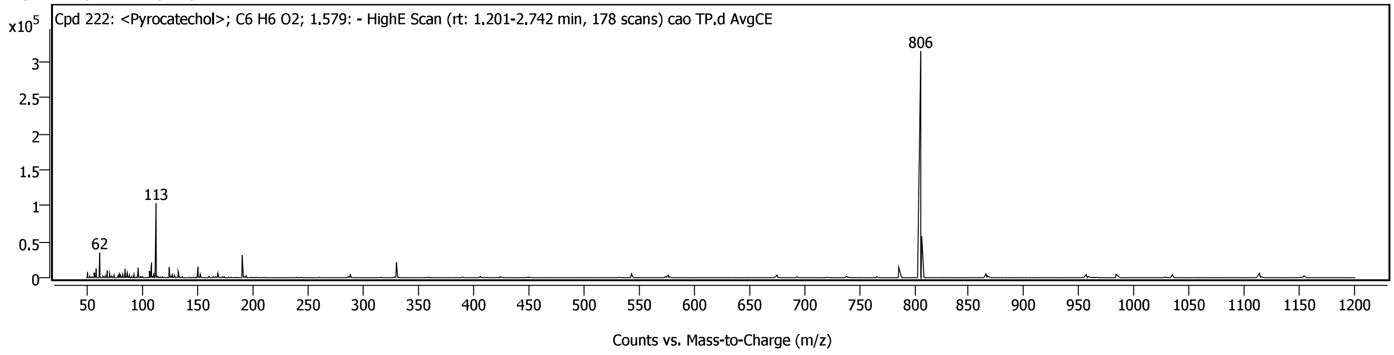

Compound ID Table

Name	Formula	Species	RT	RT Diff	Mass	CAS	ID Source	Score	Score (Lib)	Score (Tgt)
<Pyrocatechol>	C6 H6 O2	(M-H)-	1.579		110.0367	120-80-9	FBF	99.59		99.59
<Hydroquinone>	C6 H6 O2	(M-H)-	1.579		110.0367	123-31-9	FBF	99.59		99.59
<2-Acetylfuran>	C6 H6 O2	(M-H)-	1.579		110.0367	1192-62-7	FBF	99.59		99.59
<5-Methyl-2-furaldehyde>	C6 H6 O2	(M-H)-	1.579		110.0367	620-02-0	FBF	99.59		99.59
<Muconic dialdehyde>	C6 H6 O2	(M-H)-	1.579		110.0367		FBF	99.59		99.59
<Resorcinol>	C6 H6 O2	(M-H)-	1.579		110.0367	108-46-3	FBF	99.59		99.59

Cpd 238: 3,4-Dihydroxybenzoic acid

Name	Formula	RT	RI	Mass	Diff (Tgt, ppm)	CAS	ID Source	Score	Algorithm
3,4-Dihydroxybenzoic acid	C7 H6 O4	1.597		154.0265	-0.68	99-50-3	M-FBF-FragConfirm	99.58	FBF

Species	m/z	Score (Tgt)	Score (Lib)	Score (DB)	Score (MFG)	Score (RT)
(M-H)-	153	99.58				

Compound Chromatograms (overlaid)

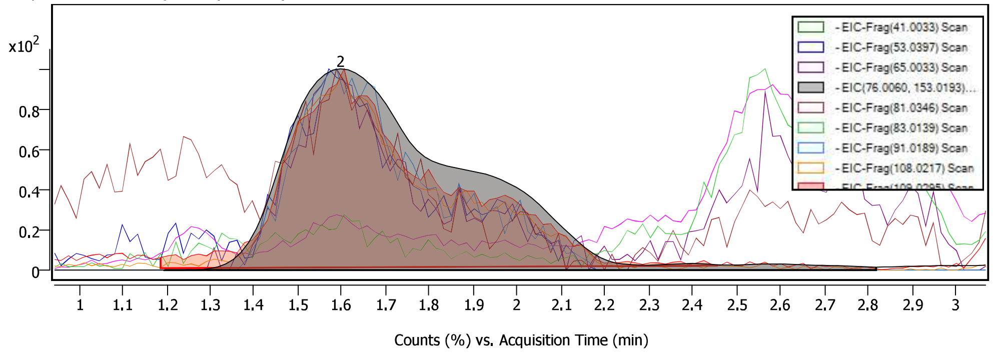

Structure

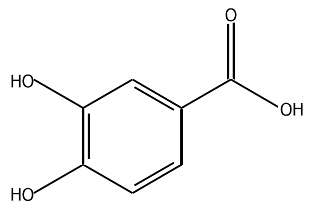

Compound Screening Report

Coelution Plot

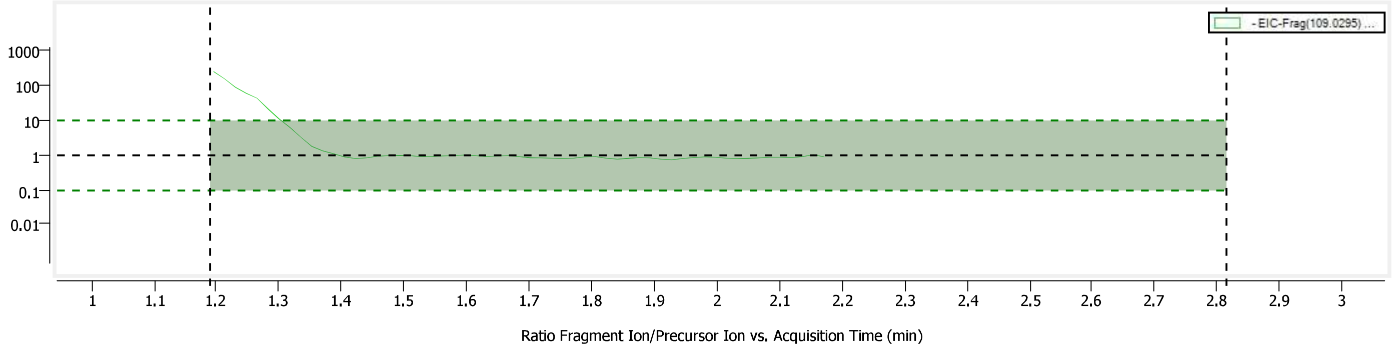

Compound Spectra (overlaid)

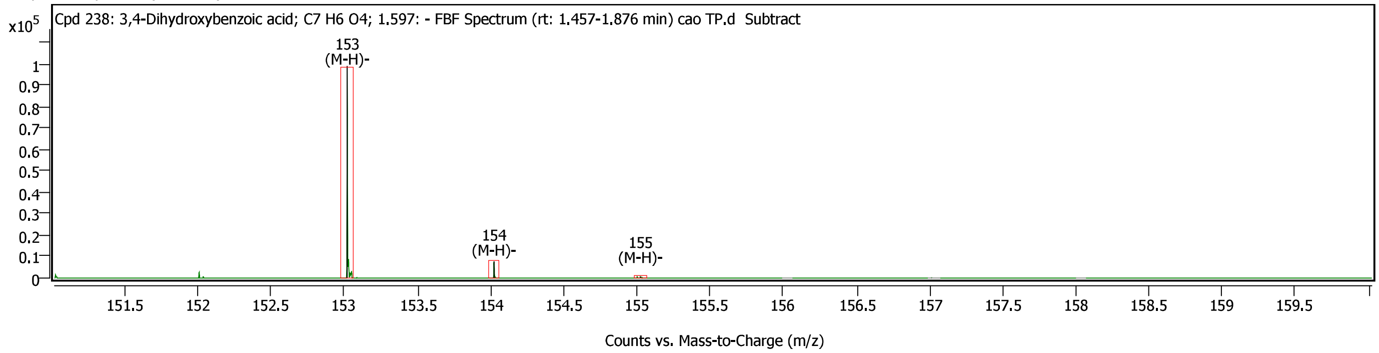

Fragment Spectrum (clean)

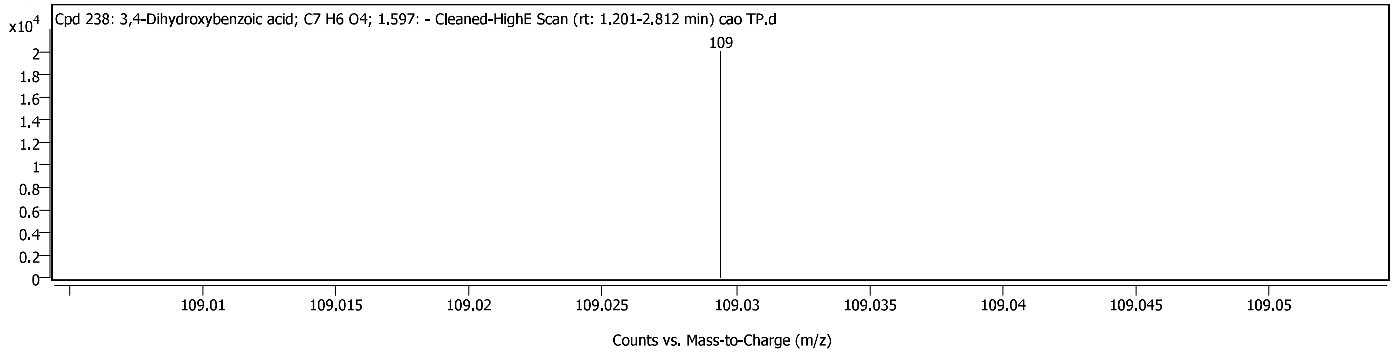

Fragment Spectrum (raw)

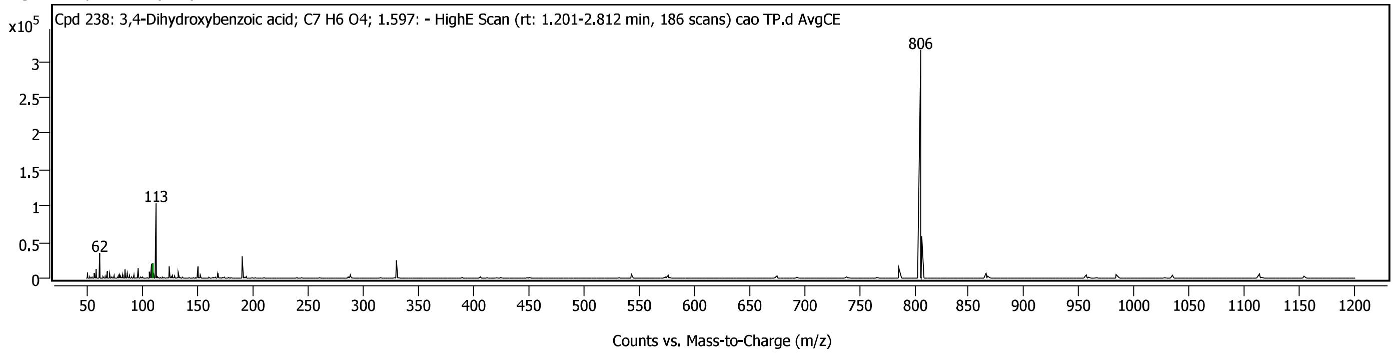

Compound ID Table

Name	Formula	Species	RT	RT Diff	Mass	CAS	ID Source	Score	Score (Lib)	Score (Tgt)
3,4-Dihydroxybenzoic acid	C7 H6 O4	(M-H)-	1.597		154.0265	99-50-3	FBF-FragConfirm	99.58		99.58
Patulin	C7 H6 O4	(M-H)-	1.597		154.0265	149-29-1	FBF-FragConfirm	99.58		99.58
2,6-dihydroxybenzoic acid	C7 H6 O4	(M-H)-	1.597		154.0265	303-07-1	FBF-FragConfirm	99.58		99.58
2,3-Dihydroxybenzoic acid	C7 H6 O4	(M-H)-	1.597		154.0265	303-38-8	FBF-FragConfirm	99.58		99.58
2,4-Dihydroxybenzoic acid	C7 H6 O4	(M-H)-	1.597		154.0265	89-86-1	FBF-FragConfirm	99.58		99.58
Gentisic acid	C7 H6 O4	(M-H)-	1.597		154.0265	490-79-9	FBF-FragConfirm	99.58		99.58
3,5-dihydroxybenzoic acid	C7 H6 O4	(M-H)-	1.597		154.0265	99-10-5	FBF-FragConfirm	99.58		99.58

Cpd 78: 4,4'-Dinitrodiphenylurea

Name	Formula	RT	RI	Mass	Diff (Tgt, ppm)	CAS	ID Source	Score	Algorithm
4,4'-Dinitrodiphenylurea	C13 H10 N4 O5	3.742		302.0636	-4.91	587-90-6	FBF	88.36	FBF

Species	m/z	Score (Tgt)	Score (Lib)	Score (DB)	Score (MFG)	Score (RT)
(M-H)-	301	88.36				

Compound Chromatograms (overlaid)

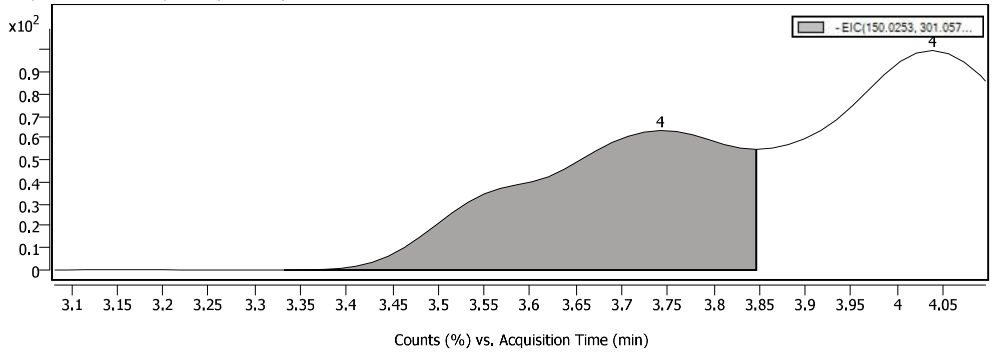

Structure

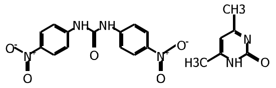

Compound Spectra (overlaid)

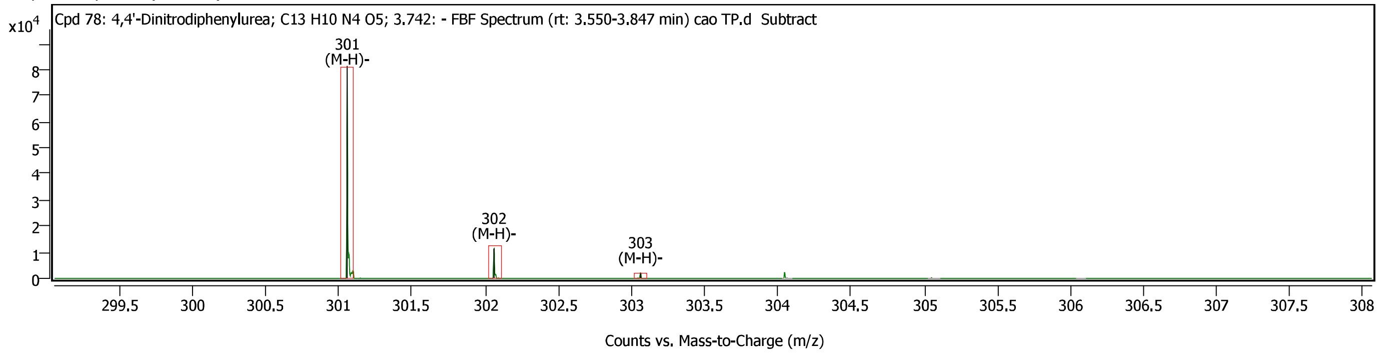

Compound ID Table

Name	Formula	Species	RT	RT Diff	Mass	CAS	ID Source	Score	Score (Lib)	Score (Tgt)
4,4'-Dinitrodiphenylurea	C13 H10 N4 O5	(M-H)-	3.742		302.0636	587-90-6	FBF	88.36		88.36

Cpd 79: 4,4'-Dinitrodiphenylurea

Name	Formula	RT	RI	Mass	Diff (Tgt, ppm)	CAS	ID Source	Score	Algorithm
4,4'-Dinitrodiphenylurea	C13 H10 N4 O5	4.039		302.0638	-4.51	587-90-6	FBF	90.03	FBF

Species	m/z	Score (Tgt)	Score (Lib)	Score (DB)	Score (MFG)	Score (RT)
(M-H)-	301	90.03				

Compound Chromatograms (overlaid)

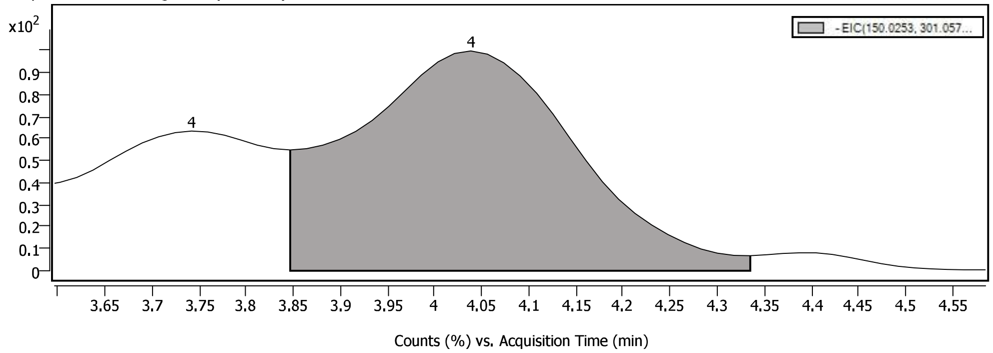

Structure

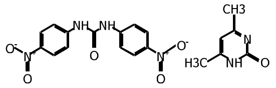

Compound Spectra (overlaid)

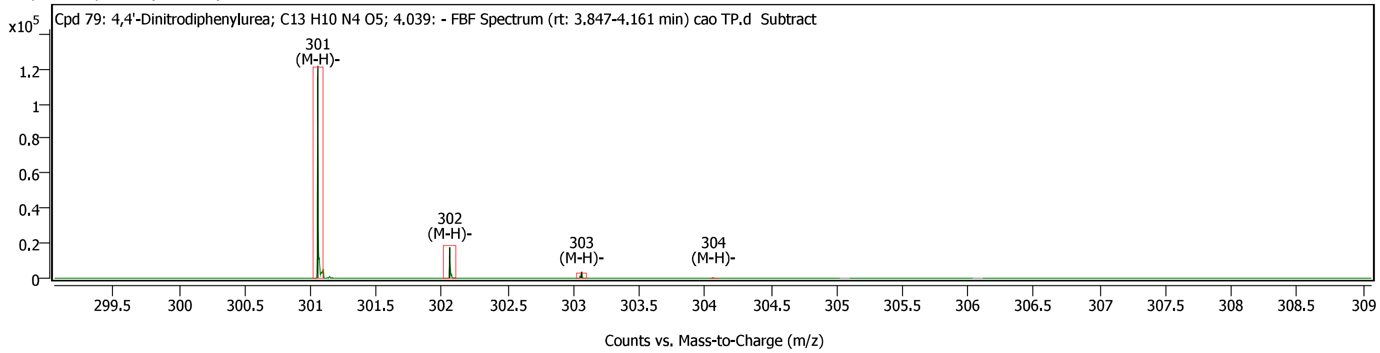

Compound Screening Report

Compound ID Table

Name	Formula	Species	RT	RT Diff	Mass	CAS	ID Source	Score	Score (Lib)	Score (Tgt)
4,4'-Dinitrodiphenylurea	C13 H10 N4 O5	(M-H)-	4.039		302.0638	587-90-6	FBF	90.03		90.03

Cpd 208: 3-O-p-Coumaroylquinic acid

Name	Formula	RT	RI	Mass	Diff (Tgt, ppm)	CAS	ID Source	Score	Algorithm
3-O-p-Coumaroylquinic acid	C16 H18 O8	5.469		338.0999	-0.69		M-FBF	99.60	FBF

Species	m/z	Score (Tgt)	Score (Lib)	Score (DB)	Score (MFG)	Score (RT)
(M-H)-	337	99.60				

Compound Chromatograms (overlaid)

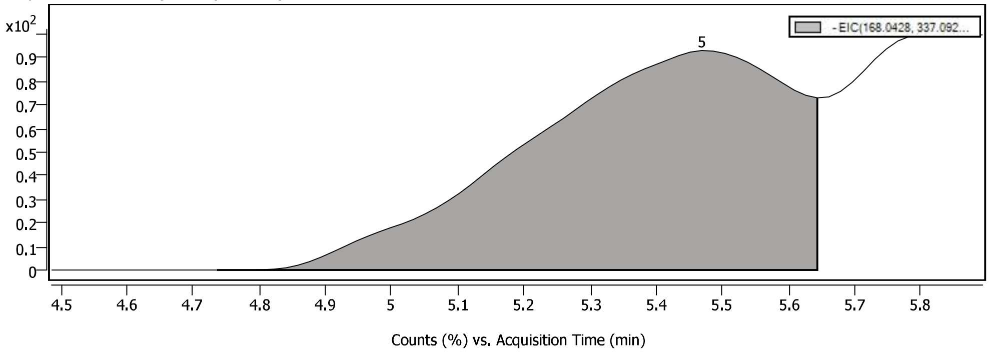

Structure

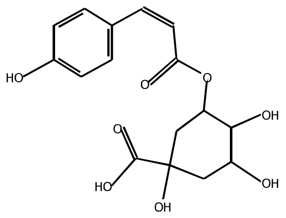

Compound Spectra (overlaid)

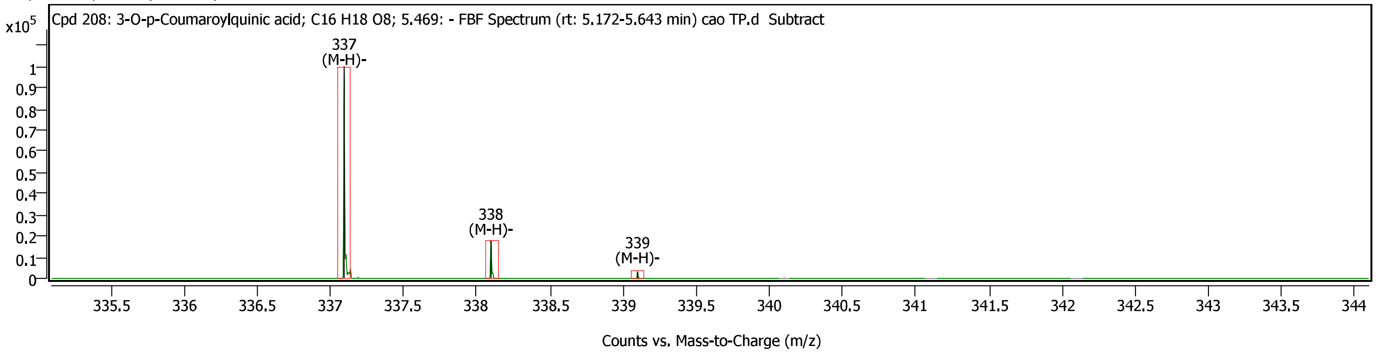

Compound ID Table

Name	Formula	Species	RT	RT Diff	Mass	CAS	ID Source	Score	Score (Lib)	Score (Tgt)
3-O-p-Coumaroylquinic acid	C16 H18 O8	(M-H)-	5.469		338.0999		FBF	99.60		99.60
p-Coumaroyl quinic acid	C16 H18 O8	(M-H)-	5.469		338.0999		FBF	99.60		99.60
Hydrojuglone glucoside	C16 H18 O8	(M-H)-	5.469		338.0999	22427-33-4	FBF	99.60		99.60
alpha-Hydrojuglone 4-O-b-D-glucoside	C16 H18 O8	(M-H)-	5.469		338.0999	39015-63-9	FBF	99.60		99.60
4-p-Coumaroylquinic acid	C16 H18 O8	(M-H)-	5.469		338.0999	93451-44-6	FBF	99.60		99.60
1-Caffeoyl-4-deoxyquinic acid	C16 H18 O8	(M-H)-	5.469		338.0999	153444-59-8	FBF	99.60		99.60

Cpd 209: 1-Caffeoyl-4-deoxyquinic acid

Name	Formula	RT	RI	Mass	Diff (Tgt, ppm)	CAS	ID Source	Score	Algorithm
1-Caffeoyl-4-deoxyquinic acid	C16 H18 O8	5.922		338.1000	-0.49	153444-59-8	M-FBF	99.74	FBF

Species	m/z	Score (Tgt)	Score (Lib)	Score (DB)	Score (MFG)	Score (RT)
(M-H)-	337	99.74				

Compound Chromatograms (overlaid)

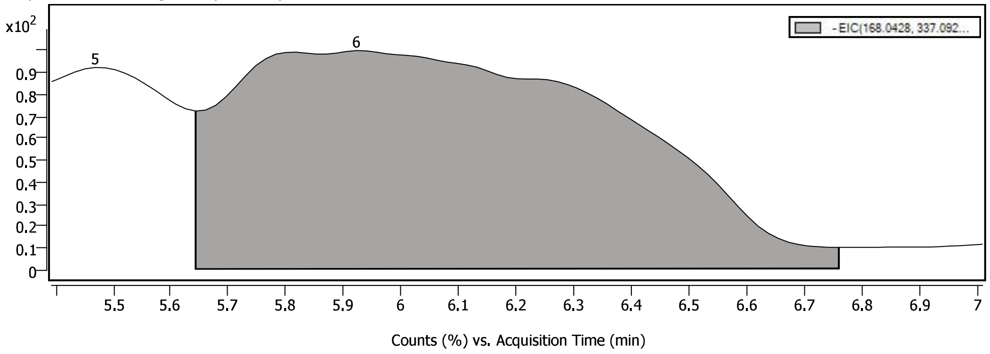

Structure

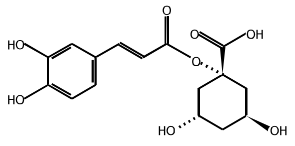

Compound Spectra (overlaid)

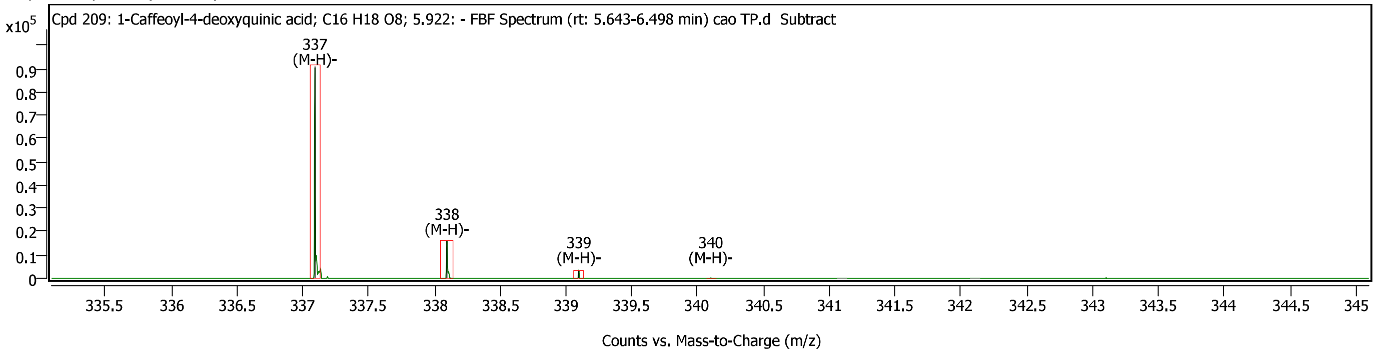

Compound ID Table

Name	Formula	Species	RT	RT Diff	Mass	CAS	ID Source	Score	Score (Lib)	Score (Tgt)
1-Caffeoyl-4-deoxyquinic acid	C16 H18 O8	(M-H)-	5.922		338.1000	153444-59-8	FBF	99.74		99.74
p-Coumaroyl quinic acid	C16 H18 O8	(M-H)-	5.922		338.1000		FBF	99.74		99.74
Hydrojuglone glucoside	C16 H18 O8	(M-H)-	5.922		338.1000	22427-33-4	FBF	99.74		99.74
alpha-Hydrojuglone 4-O-b-D-glucoside	C16 H18 O8	(M-H)-	5.922		338.1000	39015-63-9	FBF	99.74		99.74
4-p-Coumaroylquinic acid	C16 H18 O8	(M-H)-	5.922		338.1000	93451-44-6	FBF	99.74		99.74
3-O-p-Coumaroylquinic acid	C16 H18 O8	(M-H)-	5.922		338.1000		FBF	99.74		99.74

Cpd 210: alpha-Hydrojuglone 4-O-b-D-glucoside

Name	Formula	RT	RI	Mass	Diff (Tgt, ppm)	CAS	ID Source	Score	Algorithm
alpha-Hydrojuglone 4-O-b-D-glucoside	C16 H18 O8	8.975		338.1000	-0.39	39015-63-9	M-FBF	99.56	FBF

Species	m/z	Score (Tgt)	Score (Lib)	Score (DB)	Score (MFG)	Score (RT)
(M-H)-	337	99.56				

Compound Chromatograms (overlaid)

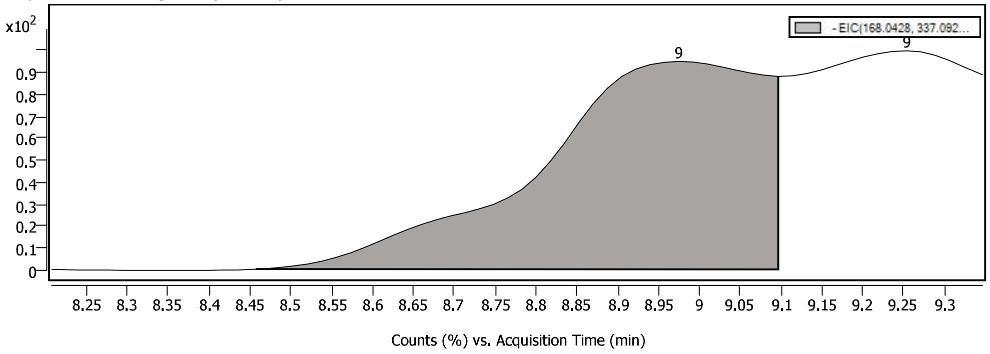

Structure

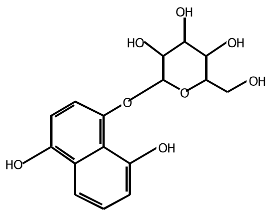

Compound Spectra (overlaid)

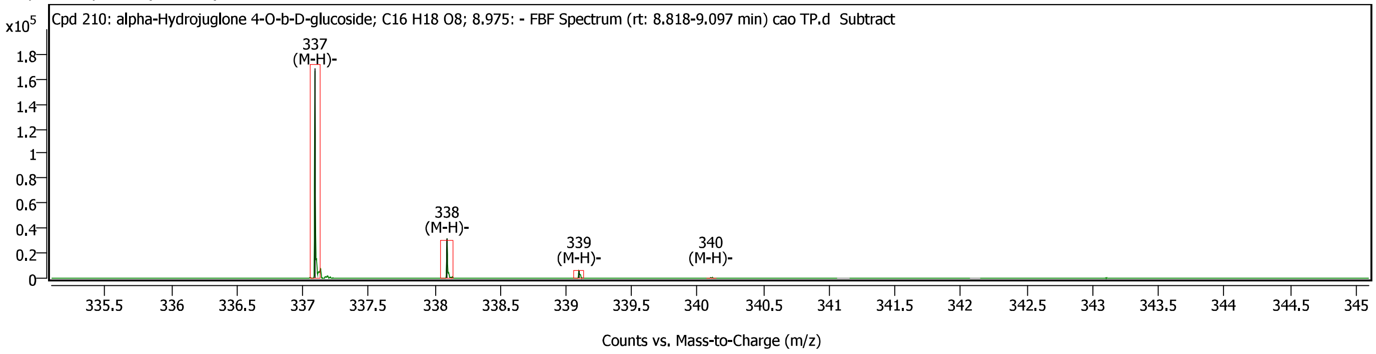

Compound ID Table

Name	Formula	Species	RT	RT Diff	Mass	CAS	ID Source	Score	Score (Lib)	Score (Tgt)
alpha-Hydrojuglone 4-O-b-D-glucoside	C16 H18 O8	(M-H)-	8.975		338.1000	39015-63-9	FBF	99.56		99.56
p-Coumaroyl quinic acid	C16 H18 O8	(M-H)-	8.975		338.1000		FBF	99.56		99.56
Hydrojuglone glucoside	C16 H18 O8	(M-H)-	8.975		338.1000	22427-33-4	FBF	99.56		99.56
4-p-Coumaroylquinic acid	C16 H18 O8	(M-H)-	8.975		338.1000	93451-44-6	FBF	99.56		99.56
3-O-p-Coumaroylquinic acid	C16 H18 O8	(M-H)-	8.975		338.1000		FBF	99.56		99.56
1-Caffeoyl-4-deoxyquinic acid	C16 H18 O8	(M-H)-	8.975		338.1000	153444-59-8	FBF	99.56		99.56

Cpd 213: Cinnamtannin A1

Name	Formula	RT	RI	Mass	Diff (Tgt, ppm)	CAS	ID Source	Score	Algorithm
Cinnamtannin A1	C45 H38 O18	9.079		866.2054	-0.53		M-FBF	99.06	FBF

Species	m/z	Score (Tgt)	Score (Lib)	Score (DB)	Score (MFG)	Score (RT)
(M-2H)-2 (M-H)-	432 865	99.06				

Compound Chromatograms (overlaid)

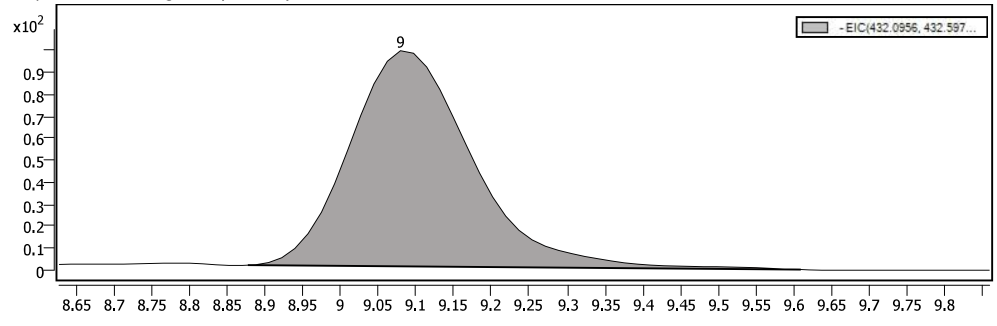

Structure

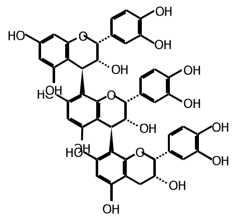

Compound Spectra (overlaid)

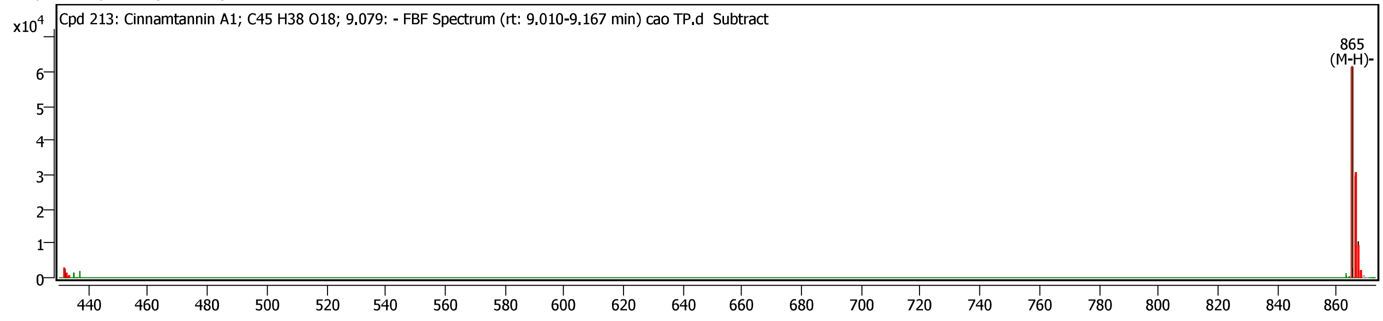

Compound ID Table

Name	Formula	Species	RT	RT Diff	Mass	CAS	ID Source	Score	Score (Lib)	Score (Tgt)
Cinnamtannin A1	C45 H38 O18	(M-2H)-2 (M-H)-	9.079		866.2054		FBF	99.06		99.06
Robinetinidol-(4alpha->8)-catechin-(6->4alpha)-robinetinidol	C45 H38 O18	(M-2H)-2 (M-H)-	9.079		866.2054		FBF	99.06		99.06

Cpd 18: 8,8'-Methylenebiscatechin

Name	Formula	RT	RI	Mass	Diff (Tgt, ppm)	CAS	ID Source	Score	Algorithm
8,8'-Methylenebiscatechin	C31 H28 O12	9.358		592.1576	-0.76	81555-08-0	M-FBF	99.40	FBF

Species	m/z	Score (Tgt)	Score (Lib)	Score (DB)	Score (MFG)	Score (RT)
(M-H)-	591	99.40				

Compound Chromatograms (overlaid)

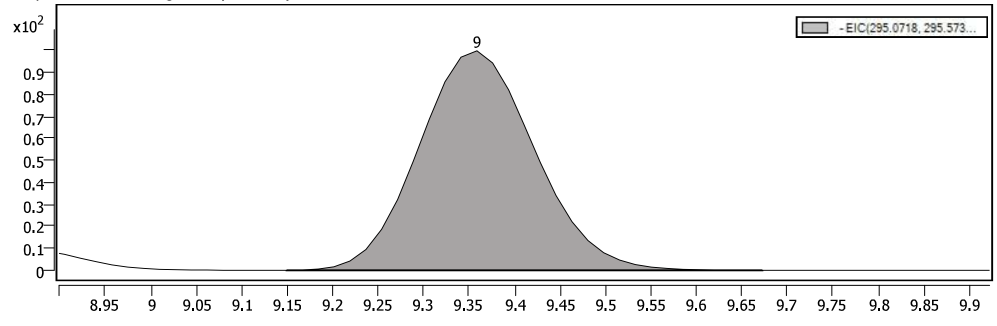

Structure

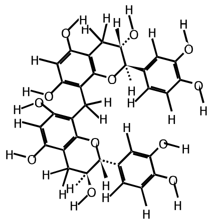

Compound Spectra (overlaid)

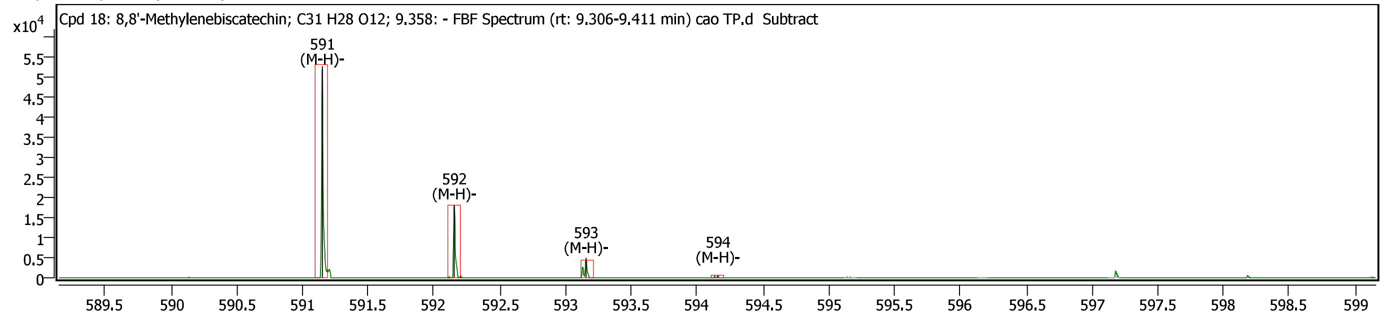

Compound ID Table

Name	Formula	Species	RT	RT Diff	Mass	CAS	ID Source	Score	Score (Lib)	Score (Tgt)
8,8'-Methylenebiscatechin	C31 H28 O12	(M-H)-	9.358		592.1576	81555-08-0	FBF	99.40		99.40
Aurasperone C	C31 H28 O12	(M-H)-	9.358		592.1576	41689-66-1	FBF	99.40		99.40

Cpd 201: Hyperoside

Name	Formula	RT	RI	Mass	Diff (Tgt, ppm)	CAS	ID Source	Score	Algorithm
Hyperoside	C21 H20 O12	9.899		464.0954	-0.25	482-36-0	FBF-FragConfirm	99.42	FBF

Species	m/z	Score (Tgt)	Score (Lib)	Score (DB)	Score (MFG)	Score (RT)
(M-H)-	463	99.42				

Compound Chromatograms (overlaid)

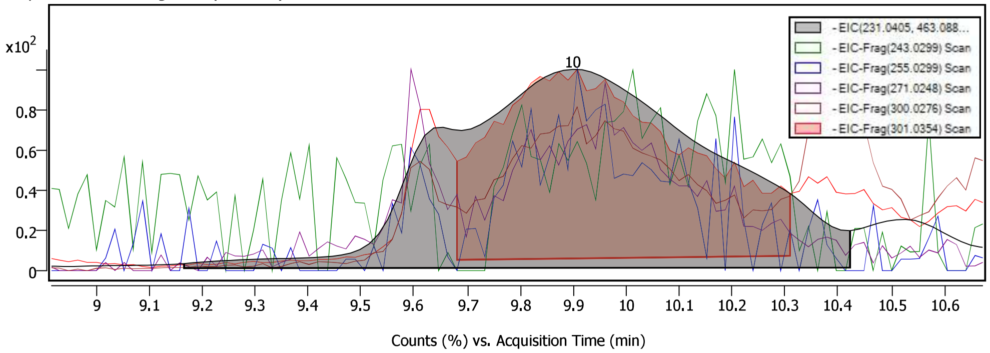

Structure

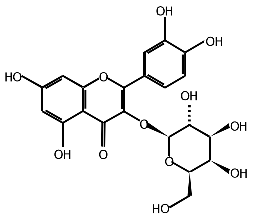

Coelution Plot

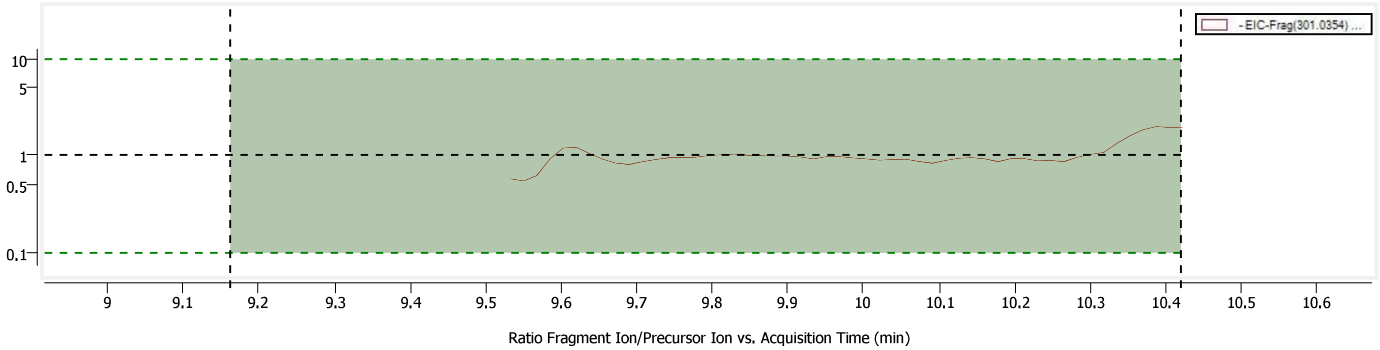

Compound Spectra (overlaid)

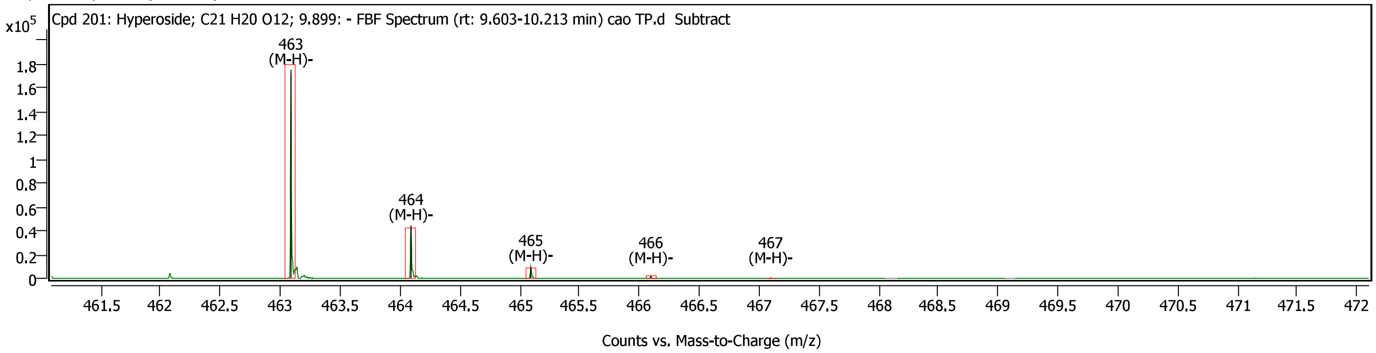

Fragment Spectrum (clean)

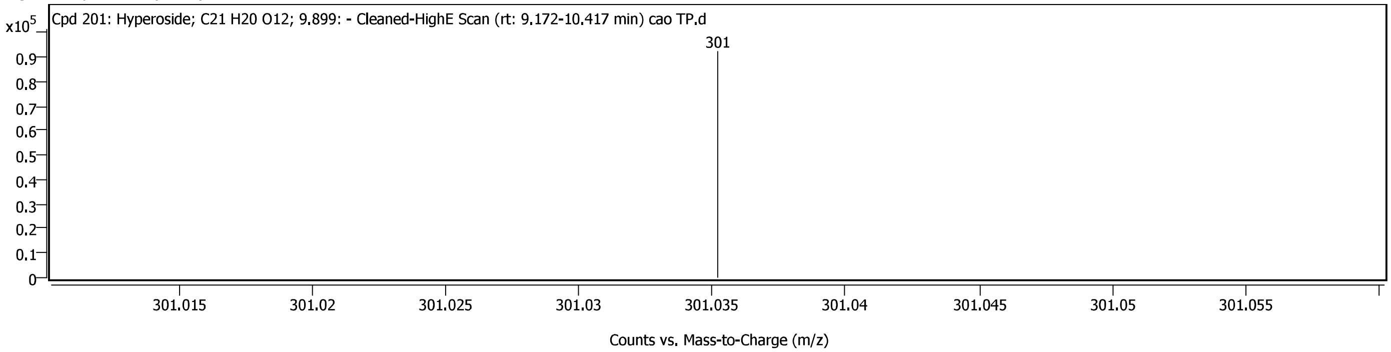

Fragment Spectrum (raw)

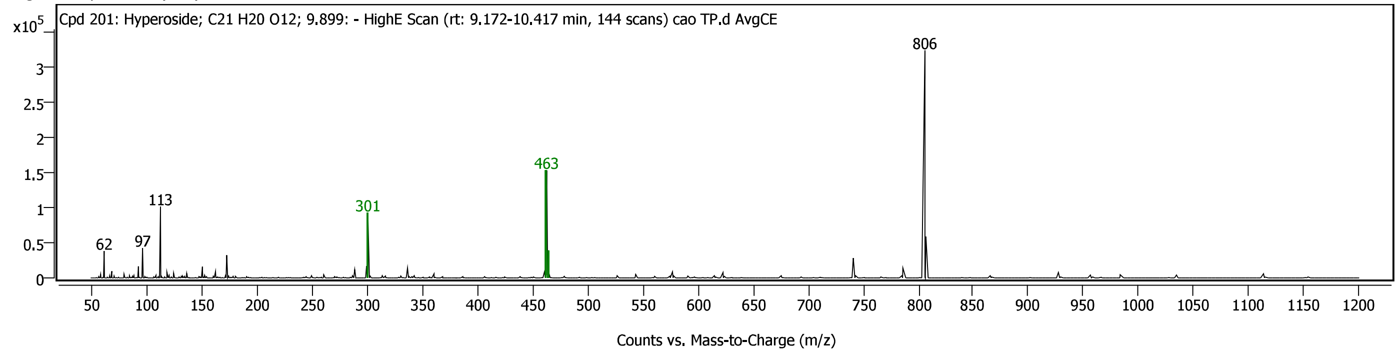

Compound ID Table

Name	Formula	Species	RT	RT Diff	Mass	CAS	ID Source	Score	Score (Lib)	Score (Tgt)
Hyperoside	C21 H20 O12	(M-H)-	9.899		464.0954	482-36-0	FBF-FragConfirm	99.42		99.42
Bracteatin 6-O-glucoside	C21 H20 O12	(M-H)-	9.899		464.0954		FBF-FragConfirm	99.42		99.42
Quercetagetin 3-rhamnoside	C21 H20 O12	(M-H)-	9.899		464.0954		FBF-FragConfirm	99.42		99.42
8-Hydroxyluteolin 8-glucoside	C21 H20 O12	(M-H)-	9.899		464.0954		FBF-FragConfirm	99.42		99.42
Annulatin 3'-xyloside	C21 H20 O12	(M-H)-	9.899		464.0954		FBF-FragConfirm	99.42		99.42
Bracteatin 6-glucoside	C21 H20 O12	(M-H)-	9.899		464.0954		FBF-FragConfirm	99.42		99.42
Gossypetin 7-rhamnoside	C21 H20 O12	(M-H)-	9.899		464.0954		FBF-FragConfirm	99.42		99.42
Bractein	C21 H20 O12	(M-H)-	9.899		464.0954		FBF-FragConfirm	99.42		99.42
Corniculatusin 3-alpha-L-arabinofuranoside	C21 H20 O12	(M-H)-	9.899		464.0954		FBF-FragConfirm	99.42		99.42
Eriodictyol 7-glucuronide	C21 H20 O12	(M-H)-	9.899		464.0954		FBF-FragConfirm	99.42		99.42
6-Hydroxyluteolin 7-glucoside	C21 H20 O12	(M-H)-	9.899		464.0954		FBF-FragConfirm	99.42		99.42
Gossypetin 8-rhamnoside	C21 H20 O12	(M-H)-	9.899		464.0954		FBF-FragConfirm	99.42		99.42
6-Hydroxytricetin 5-rhamnoside	C21 H20 O12	(M-H)-	9.899		464.0954		FBF-FragConfirm	99.42		99.42
6-Hydroxykaempferol 3-glucoside	C21 H20 O12	(M-H)-	9.899		464.0954		FBF-FragConfirm	99.42		99.42
6-Hydroxyluteolin 7-galactoside	C21 H20 O12	(M-H)-	9.899		464.0954		FBF-FragConfirm	99.42		99.42
6-Hydroxyluteolin 6-glucoside	C21 H20 O12	(M-H)-	9.899		464.0954		FBF-FragConfirm	99.42		99.42
6-Hydroxyluteolin 5-glucoside	C21 H20 O12	(M-H)-	9.899		464.0954		FBF-FragConfirm	99.42		99.42
6-Hydroxykaempferol 7-glucoside	C21 H20 O12	(M-H)-	9.899		464.0954		FBF-FragConfirm	99.42		99.42
6-C-Glucosylquercetin	C21 H20 O12	(M-H)-	9.899		464.0954		FBF-FragConfirm	99.42		99.42
6-C-beta-D-Glucopyranosyl-5,7,2',4',5'-pentahydroxyflavone	C21 H20 O12	(M-H)-	9.899		464.0954		FBF-FragConfirm	99.42		99.42
5,7,3',4',5'-Pentahydroxyflavone 8-C-glucopyranoside	C21 H20 O12	(M-H)-	9.899		464.0954		FBF-FragConfirm	99.42		99.42
5,6,7,3',4'-Pentahydroxy-8-methoxyflavone 7-apioside	C21 H20 O12	(M-H)-	9.899		464.0954		FBF-FragConfirm	99.42		99.42
3,5,7,2',6'-Pentahydroxyflavone 2'-glucoside	C21 H20 O12	(M-H)-	9.899		464.0954		FBF-FragConfirm	99.42		99.42
2'-Hydroxyisoorientin	C21 H20 O12	(M-H)-	9.899		464.0954		FBF-FragConfirm	99.42		99.42
(2S)-5,7,3',4'-Tetrahydroxyflavanone 7-glucuronide	C21 H20 O12	(M-H)-	9.899		464.0954		FBF-FragConfirm	99.42		99.42
Herbacetin 3-beta-D-glucofuranoside	C21 H20 O12	(M-H)-	9.899		464.0954		FBF-FragConfirm	99.42		99.42
8-Hydroxyluteolin 7-glucoside	C21 H20 O12	(M-H)-	9.899		464.0954		FBF-FragConfirm	99.42		99.42
Herbacetin 3-glucoside	C21 H20 O12	(M-H)-	9.899		464.0954		FBF-FragConfirm	99.42		99.42
Tricetin 7-glucoside	C21 H20 O12	(M-H)-	9.899		464.0954		FBF-FragConfirm	99.42		99.42
Quercetin 3-alloside	C21 H20 O12	(M-H)-	9.899		464.0954		FBF-FragConfirm	99.42		99.42
Herbacetin 7-glucoside	C21 H20 O12	(M-H)-	9.899		464.0954		FBF-FragConfirm	99.42		99.42
Tricetin 3'-glucoside	C21 H20 O12	(M-H)-	9.899		464.0954		FBF-FragConfirm	99.42		99.42
Spiraeoside	C21 H20 O12	(M-H)-	9.899		464.0954		FBF-FragConfirm	99.42		99.42
Robinetin 7-glucoside	C21 H20 O12	(M-H)-	9.899		464.0954		FBF-FragConfirm	99.42		99.42
Quercimeritrin	C21 H20 O12	(M-H)-	9.899		464.0954		FBF-FragConfirm	99.42		99.42
Quercetin 5-glucoside	C21 H20 O12	(M-H)-	9.899		464.0954		FBF-FragConfirm	99.42		99.42
Quercetin 4'-glucoside	C21 H20 O12	(M-H)-	9.899		464.0954	20229-56-5	FBF-FragConfirm	99.42		99.42
Quercetin 3-β-D-glucoside	C21 H20 O12	(M-H)-	9.899		464.0954	482-35-9	FBF-FragConfirm	99.42		99.42
Quercetin 3-O-glucoside	C21 H20 O12	(M-H)-	9.899		464.0954	21637-25-2	FBF-FragConfirm	99.42		99.42
Quercetin 3'-glucoside	C21 H20 O12	(M-H)-	9.899		464.0954		FBF-FragConfirm	99.42		99.42
Quercetin 7-galactoside	C21 H20 O12	(M-H)-	9.899		464.0954		FBF-FragConfirm	99.42		99.42
Myricetin 3'-rhamnoside	C21 H20 O12	(M-H)-	9.899		464.0954		FBF-FragConfirm	99.42		99.42
Patuletin 3-xyloside	C21 H20 O12	(M-H)-	9.899		464.0954		FBF-FragConfirm	99.42		99.42
Isoetin 5'-glucoside	C21 H20 O12	(M-H)-	9.899		464.0954		FBF-FragConfirm	99.42		99.42
Myricitrin	C21 H20 O12	(M-H)-	9.899		464.0954	17912-87-7	FBF-FragConfirm	99.42		99.42
Herbacetin 8-glucoside	C21 H20 O12	(M-H)-	9.899		464.0954		FBF-FragConfirm	99.42		99.42
Hyperin	C21 H20 O12	(M-H)-	9.899		464.0954		FBF-FragConfirm	99.42		99.42
Isoaffinetin	C21 H20 O12	(M-H)-	9.899		464.0954		FBF-FragConfirm	99.42		99.42
Isoetin 7-glucoside	C21 H20 O12	(M-H)-	9.899		464.0954		FBF-FragConfirm	99.42		99.42
Isoquercitrin	C21 H20 O12	(M-H)-	9.899		464.0954		FBF-FragConfirm	99.42		99.42
Larycitrin 3-alpha-L-arabinofuranoside	C21 H20 O12	(M-H)-	9.899		464.0954		FBF-FragConfirm	99.42		99.42
Herbacetin 4'-glucoside	C21 H20 O12	(M-H)-	9.899		464.0954		FBF-FragConfirm	99.42		99.42
Myricetin 7-rhamnoside	C21 H20 O12	(M-H)-	9.899		464.0954	184533-14-0	FBF-FragConfirm	99.42		99.42

Cpd 134: Depressonol A

Name	Formula	RT	RI	Mass	Diff (Tgt, ppm)	CAS	ID Source	Score	Algorithm
Depressonol A	C32 H38 O20	10.405		742.1956	-0.04		M-FBF	99.64	FBF

Species	m/z	Score (Tgt)	Score (Lib)	Score (DB)	Score (MFG)	Score (RT)
(M-H)-	741	99.64				

Compound Chromatograms (overlaid)

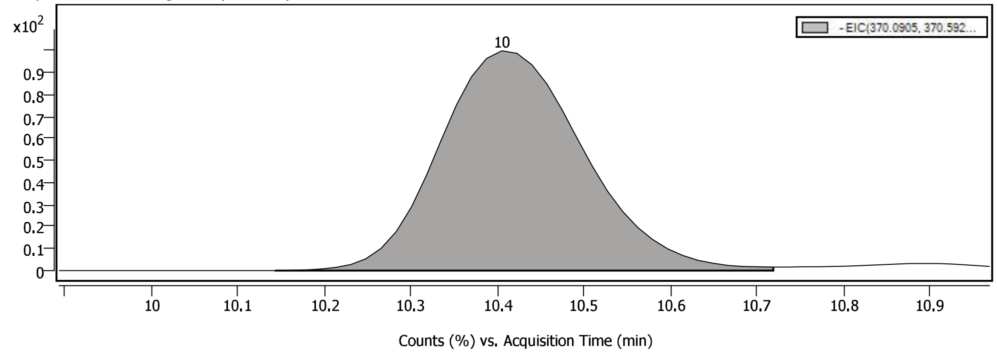

Structure

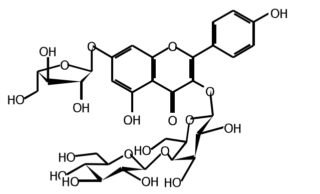

Compound Spectra (overlaid)

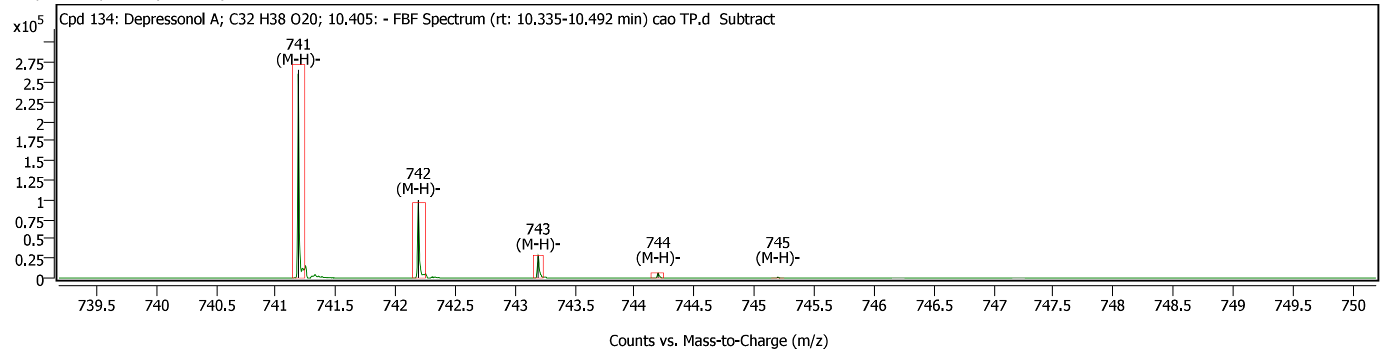

Compound ID Table

Name	Formula	Species	RT	RT Diff	Mass	CAS	ID Source	Score	Score (Lib)	Score (Tgt)
Depressonol A	C32 H38 O20	(M-H)-	10.405		742.1956		FBF	99.64		99.64
Kaempferol 3-apiosyl-(1->2)-glucoside-4'-glucoside	C32 H38 O20	(M-H)-	10.405		742.1956		FBF	99.64		99.64
Kaempferol 3-sambubioside-7-glucoside	C32 H38 O20	(M-H)-	10.405		742.1956		FBF	99.64		99.64
Kaempferol 3-sophoroside-7-alpha-L-arabinofuranoside	C32 H38 O20	(M-H)-	10.405		742.1956		FBF	99.64		99.64
Quercetin 3-(2Gal-apiosylrobinobioside)	C32 H38 O20	(M-H)-	10.405		742.1956		FBF	99.64		99.64
Quercetin 3-(2G-apiosylrutinoside)	C32 H38 O20	(M-H)-	10.405		742.1956		FBF	99.64		99.64
Quercetin 3-(2G-xylosylrutinoside)	C32 H38 O20	(M-H)-	10.405		742.1956		FBF	99.64		99.64
Quercetin 3-(2R-apiosylrutinoside)	C32 H38 O20	(M-H)-	10.405		742.1956		FBF	99.64		99.64
Quercetin 3-glucosyl-(1->4)-xylosyl-(1->4)-rhamnoside	C32 H38 O20	(M-H)-	10.405		742.1956		FBF	99.64		99.64
Quercetin 3-xylosyl-(1->2)-rhamnosyl-(1->6)-glucoside	C32 H38 O20	(M-H)-	10.405		742.1956		FBF	99.64		99.64
Quercetin 7-(2G-xylosylrutinoside)	C32 H38 O20	(M-H)-	10.405		742.1956		FBF	99.64		99.64
Quercetin 3-rhamnosyl-(1->2)-alpha-L-arabinopyranoside-7-glucoside	C32 H38 O20	(M-H)-	10.405		742.1956		FBF	99.64		99.64
Quercetin 3-sambubioside-7-rhamnoside	C32 H38 O20	(M-H)-	10.405		742.1956		FBF	99.64		99.64
Quercetin 3-rutinoside-3'-apioside	C32 H38 O20	(M-H)-	10.405		742.1956		FBF	99.64		99.64

Cpd 135: 6-Hydroxykaempferol 3-rutinoside-6-glucoside

Name	Formula	RT	RI	Mass	Diff (Tgt, ppm)	CAS	ID Source	Score	Algorithm
6-Hydroxykaempferol 3-rutinoside-6-glucoside	C33 H40 O21	10.667		772.2062	0.03		M-FBF	99.69	FBF

Species	m/z	Score (Tgt)	Score (Lib)	Score (DB)	Score (MFG)	Score (RT)
(M-H)-	771	99.69				

Compound Chromatograms (overlaid)

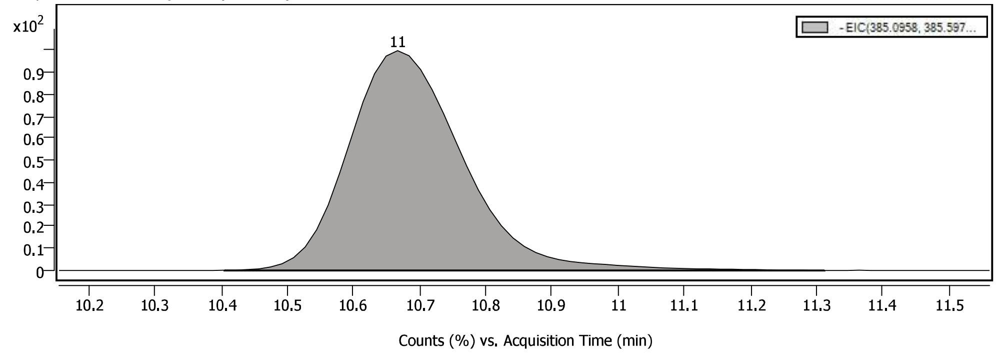

Structure

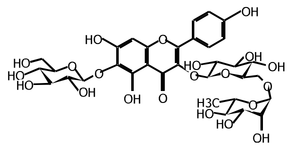

Compound Spectra (overlaid)

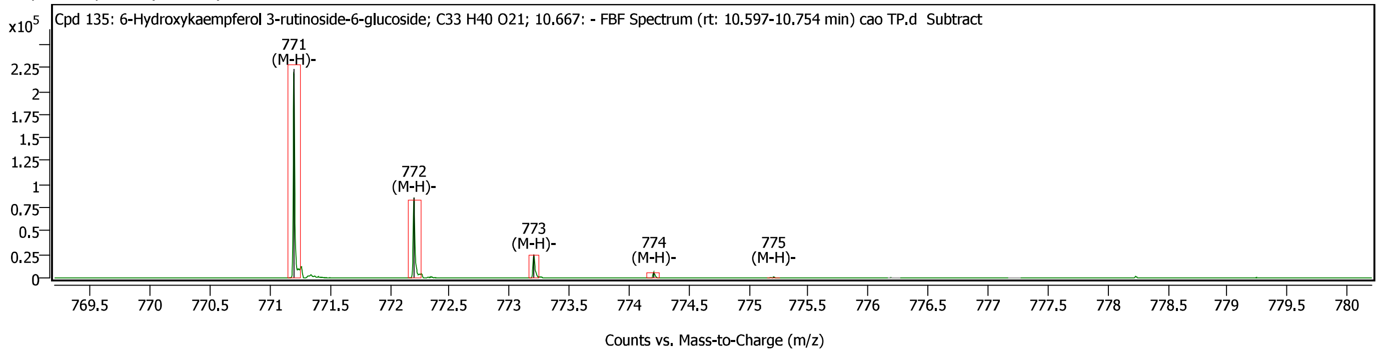

Compound ID Table

Name	Formula	Species	RT	RT Diff	Mass	CAS	ID Source	Score	Score (Lib)	Score (Tgt)
6-Hydroxykaempferol 3-rutinoside-6-glucoside	C33 H40 O21	(M-H)-	10.667		772.2062		FBF	99.69		99.69
Kaempferol 7-(3G-glucosylgentiobioside)	C33 H40 O21	(M-H)-	10.667		772.2062		FBF	99.69		99.69
Kaempferol 3-glucoside-7-gentiobioside	C33 H40 O21	(M-H)-	10.667		772.2062		FBF	99.69		99.69
Kaempferol 3-sophoroside-4'-glucoside	C33 H40 O21	(M-H)-	10.667		772.2062		FBF	99.69		99.69
Kaempferol 3-sophoroside-7-glucoside	C33 H40 O21	(M-H)-	10.667		772.2062		FBF	99.69		99.69
Kaempferol 3-sophorotrioside	C33 H40 O21	(M-H)-	10.667		772.2062		FBF	99.69		99.69
Myricetin 3-(2G-rhamnosylrutinoside)	C33 H40 O21	(M-H)-	10.667		772.2062		FBF	99.69		99.69
Lucenin-2,3'-O-glucoside	C33 H40 O21	(M-H)-	10.667		772.2062		FBF	99.69		99.69
Luteolin 7-gentiobioside-4'-glucoside	C33 H40 O21	(M-H)-	10.667		772.2062		FBF	99.69		99.69
Kaempferol 3-glucosyl-(1->2)-gentiobioside	C33 H40 O21	(M-H)-	10.667		772.2062		FBF	99.69		99.69
Myricetin 3-robinobioside-7-rhamnoside	C33 H40 O21	(M-H)-	10.667		772.2062		FBF	99.69		99.69
Kaempferol 3-O-β-D-glucosyl-(1->2)-β-D-glucosyl-(1->2)-β-D-glucoside	C33 H40 O21	(M-H)-	10.667		772.2062	80714-53-0	FBF	99.69		99.69
Myricetin 3-rutinoside-7-rhamnoside	C33 H40 O21	(M-H)-	10.667		772.2062		FBF	99.69		99.69
Kaempferol 3-glucosyl-(1->2)-galactoside-7-glucoside	C33 H40 O21	(M-H)-	10.667		772.2062		FBF	99.69		99.69
Quercetin 3-rhamnosyl-(1->2)[glucosyl-(1->6)-galactoside]	C33 H40 O21	(M-H)-	10.667		772.2062		FBF	99.69		99.69
Kaempferol 3-glucoside-7-sophoroside	C33 H40 O21	(M-H)-	10.667		772.2062		FBF	99.69		99.69
Quercetin 3-(2G-glucosylrutinoside)	C33 H40 O21	(M-H)-	10.667		772.2062		FBF	99.69		99.69
Kaempferol 3-gentiobioside-7-glucoside	C33 H40 O21	(M-H)-	10.667		772.2062		FBF	99.69		99.69
Kaempferol 3-gentiobioside-4'-glucoside	C33 H40 O21	(M-H)-	10.667		772.2062		FBF	99.69		99.69
Kaempferol 3,7,4'-triglucoside	C33 H40 O21	(M-H)-	10.667		772.2062		FBF	99.69		99.69
Isoorientin 4',2''-di-O-glucoside	C33 H40 O21	(M-H)-	10.667		772.2062		FBF	99.69		99.69
Isoorientin 3'-O-sophoroside	C33 H40 O21	(M-H)-	10.667		772.2062		FBF	99.69		99.69
Hypolaetin 8-glucoside-3'-rutinoside	C33 H40 O21	(M-H)-	10.667		772.2062		FBF	99.69		99.69
Hyperin 6''-[glucosyl-(1->3)-rhamnoside]	C33 H40 O21	(M-H)-	10.667		772.2062	134953-93-8	FBF	99.69		99.69
Kaempferol 3-glucosyl-(1->2)-galactosyl-(1->2)-glucoside	C33 H40 O21	(M-H)-	10.667		772.2062		FBF	99.69		99.69
Kaempferol 3-sophoroside 7-glucoside	C33 H40 O21	(M-H)-	10.667		772.2062		FBF	99.69		99.69
Luteolin 7-sophorotrioside	C33 H40 O21	(M-H)-	10.667		772.2062		FBF	99.69		99.69
Quercetin 3-glucosyl-(1->3)-rhamnosyl-(1->6)-galactoside	C33 H40 O21	(M-H)-	10.667		772.2062		FBF	99.69		99.69
Quercetin 3-rhamnosyl-(1->2)-galactoside-7-glucoside	C33 H40 O21	(M-H)-	10.667		772.2062		FBF	99.69		99.69
Sorboside	C33 H40 O21	(M-H)-	10.667		772.2062		FBF	99.69		99.69
Quercetin 7-methyl ether 3-alpha-L-arabinopyranosyl-(1->3)-[galactosyl-(1->6)-galactoside]	C33 H40 O21	(M-H)-	10.667		772.2062		FBF	99.69		99.69
Quercetin 3-sophoroside-7-rhamnoside	C33 H40 O21	(M-H)-	10.667		772.2062		FBF	99.69		99.69
Quercetin 3-(3R-glucosylrutinoside)	C33 H40 O21	(M-H)-	10.667		772.2062		FBF	99.69		99.69
Quercetin 3-rutinoside-7-galactoside	C33 H40 O21	(M-H)-	10.667		772.2062		FBF	99.69		99.69
Quercetin 3-rutinoside-4'-glucoside	C33 H40 O21	(M-H)-	10.667		772.2062		FBF	99.69		99.69
Quercetin 3-rutinoside 7-galactoside	C33 H40 O21	(M-H)-	10.667		772.2062	50867-29-3	FBF	99.69		99.69
Quercetin 3-robinobioside-7-glucoside	C33 H40 O21	(M-H)-	10.667		772.2062		FBF	99.69		99.69
Quercetin 3-rhamnosyl-(1->6)-glucosyl-(1->6)-galactoside	C33 H40 O21	(M-H)-	10.667		772.2062		FBF	99.69		99.69
Quercetin 3-(2G-rhamnosylgentiobioside)	C33 H40 O21	(M-H)-	10.667		772.2062		FBF	99.69		99.69
Quercetin 3-rhamnosyl-(1->2)-glucosyl-(1->6)-galactoside	C33 H40 O21	(M-H)-	10.667		772.2062		FBF	99.69		99.69
Quercetin 3-rutinoside-7-glucoside	C33 H40 O21	(M-H)-	10.667		772.2062		FBF	99.69		99.69
Quercetin 3-O-glucosyl-rutinoside	C33 H40 O21	(M-H)-	10.667		772.2062		FBF	99.69		99.69
Quercetin 3-glucoside-7-rutinoside	C33 H40 O21	(M-H)-	10.667		772.2062		FBF	99.69		99.69
Quercetin 3-neohesperidoside-7-glucoside	C33 H40 O21	(M-H)-	10.667		772.2062		FBF	99.69		99.69
Quercetin 3-galactoside-7-glucosyl-(1->4)-rhamnoside	C33 H40 O21	(M-H)-	10.667		772.2062		FBF	99.69		99.69
Quercetin 3-glucoside-7-glucosyl-(1->4)-rhamnoside	C33 H40 O21	(M-H)-	10.667		772.2062		FBF	99.69		99.69
Quercetin 3-glucoside-7-neohesperidoside	C33 H40 O21	(M-H)-	10.667		772.2062		FBF	99.69		99.69
Quercetin 3-galactoside-7-neohesperidoside	C33 H40 O21	(M-H)-	10.667		772.2062		FBF	99.69		99.69
Quercetin 3-glucosyl-(1->2)-[rhamnosyl-(1->6)-galactoside]	C33 H40 O21	(M-H)-	10.667		772.2062		FBF	99.69		99.69
Quercetin 3-glucosyl-(1->2)-rhamnoside-7-glucoside	C33 H40 O21	(M-H)-	10.667		772.2062		FBF	99.69		99.69
Quercetin 3-glucosyl-(1->6)-glucosyl-(1->4)-rhamnoside	C33 H40 O21	(M-H)-	10.667		772.2062		FBF	99.69		99.69

Cpd 81: Cinnamtannin A2

Name	Formula	RT	RI	Mass	Diff (Tgt, ppm)	CAS	ID Source	Score	Algorithm
Cinnamtannin A2	C60 H50 O24	10.911		1154.2679	-1.12		FBF	98.35	FBF

Species	m/z	Score (Tgt)	Score (Lib)	Score (DB)	Score (MFG)	Score (RT)
(M-2H)-2 (M-H)-	576 1153	98.35				

Compound Screening Report

Compound Chromatograms (overlaid)

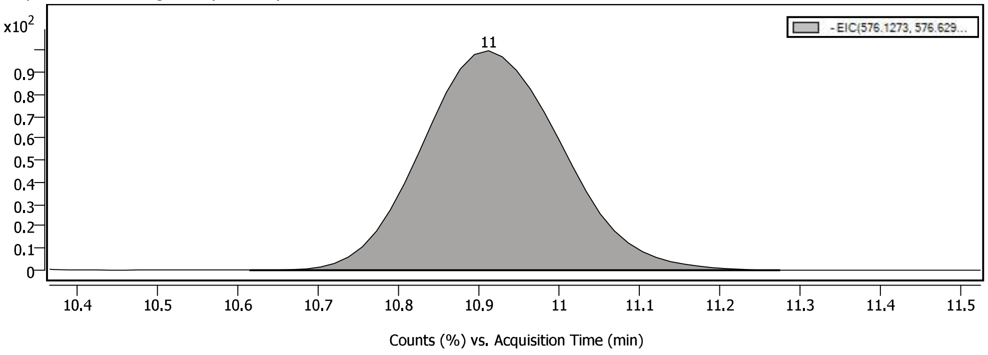

Structure

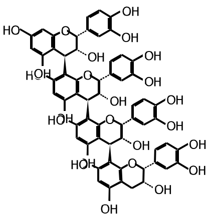

Compound Spectra (overlaid)

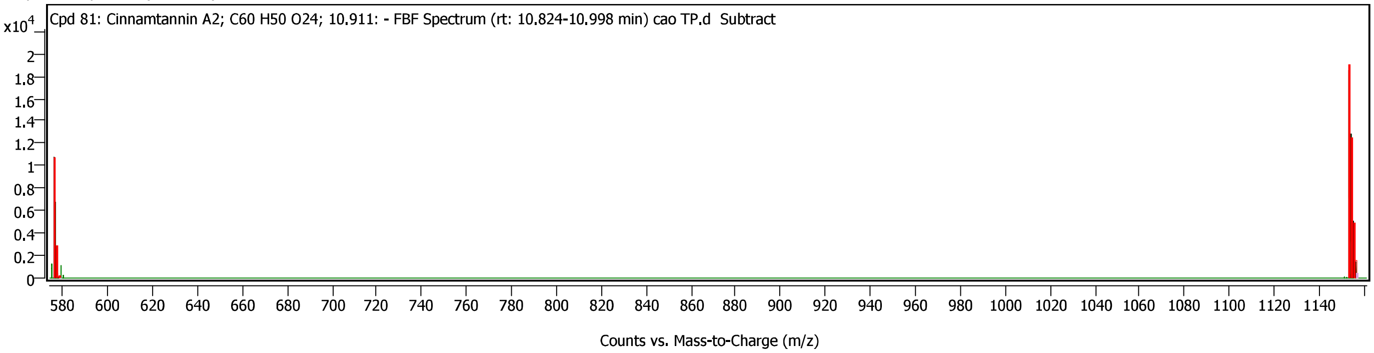

Compound ID Table

Name	Formula	Species	RT	RT Diff	Mass	CAS	ID Source	Score	Score (Lib)	Score (Tgt)
Cinnamtannin A2	C60 H50 O24	(M-2H)-2 (M-H)-	10.911		1154.2679		FBF	98.35		98.35

Cpd 152: <Arthromerin B>

Name	Formula	RT	RI	Mass	Diff (Tgt, ppm)	CAS	ID Source	Score	Algorithm
<Arthromerin B>	C21 H24 O10	11.260		436.1368	-0.33		M-FBF	99.06	FBF

Species	m/z	Score (Tgt)	Score (Lib)	Score (DB)	Score (MFG)	Score (RT)
(M-H)-	435	99.06				

Compound Chromatograms (overlaid)

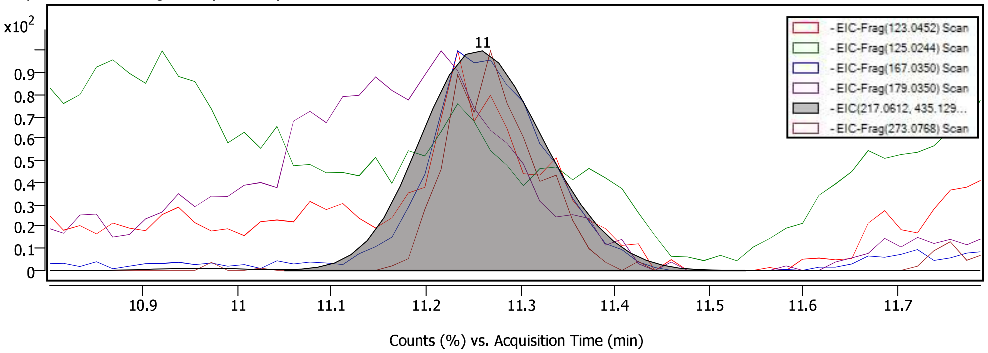

Structure

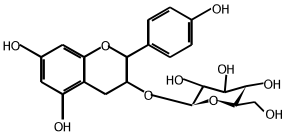

Coelution Plot

Compound Spectra (overlaid)

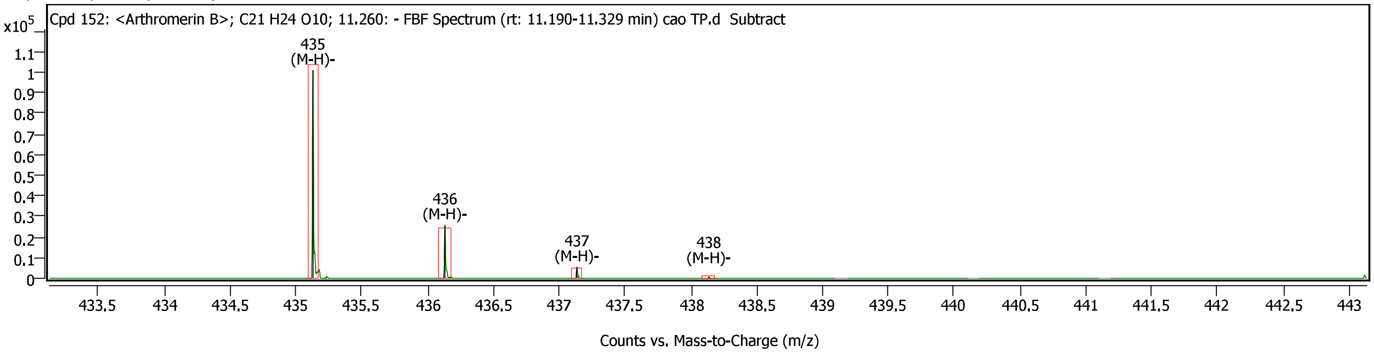

Fragment Spectrum (raw)

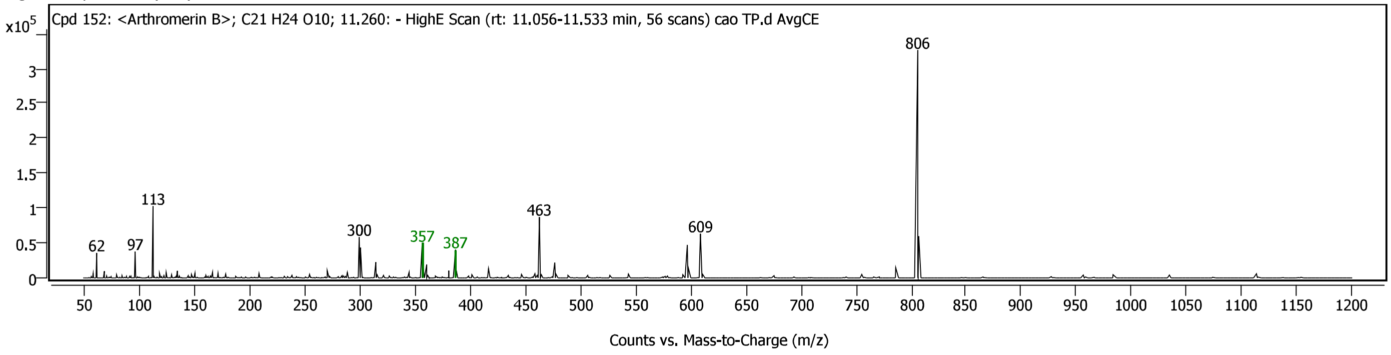

Compound ID Table

Name	Formula	Species	RT	RT Diff	Mass	CAS	ID Source	Score	Score (Lib)	Score (Tgt)
<Arthromerin B>	C21 H24 O10	(M-H)-	11.260		436.1368		FBF	99.06		99.06
<Ent-afzelechin-7-O-beta-D-glucopyranoside>	C21 H24 O10	(M-H)-	11.260		436.1368		FBF	99.06		99.06
<Catechin 7-O-alpha-L-rhamnopyranoside>	C21 H24 O10	(M-H)-	11.260		436.1368		FBF	99.06		99.06
<Coatline A>	C21 H24 O10	(M-H)-	11.260		436.1368		FBF	99.06		99.06
<Epiafzelechin 3-O-beta-D-glucopyranoside>	C21 H24 O10	(M-H)-	11.260		436.1368		FBF	99.06		99.06
<Catechin 3-O-alpha-L-rhamnoside>	C21 H24 O10	(M-H)-	11.260		436.1368		FBF	99.06		99.06
<Afzelechin 4'-O-beta-D-glucopyranosdie>	C21 H24 O10	(M-H)-	11.260		436.1368		FBF	99.06		99.06
<Epiafzelechin 3-O-beta-D-allopyranoside>	C21 H24 O10	(M-H)-	11.260		436.1368		FBF	99.06		99.06
<Nothofagin>	C21 H24 O10	(M-H)-	11.260		436.1368		FBF	99.06		99.06
<Epiafzelechin 5-O-beta-D-glucopyranoside>	C21 H24 O10	(M-H)-	11.260		436.1368		FBF	99.06		99.06
<Trilobatin>	C21 H24 O10	(M-H)-	11.260		436.1368		FBF	99.06		99.06
<Phenethyl 6-galloylglucoside>	C21 H24 O10	(M-H)-	11.260		436.1368		FBF	99.06		99.06
<Phlorhizin>	C21 H24 O10	(M-H)-	11.260		436.1368	60-81-1	FBF	99.06		99.06
<Pterosupin>	C21 H24 O10	(M-H)-	11.260		436.1368		FBF	99.06		99.06
<Rocymosin B>	C21 H24 O10	(M-H)-	11.260		436.1368		FBF	99.06		99.06
<Licoagroside F>	C21 H24 O10	(M-H)-	11.260		436.1368		FBF	99.06		99.06

Cpd 30: C.I. Pigment Red 149

Name	Formula	RT	RI	Mass	Diff (Tgt, ppm)	CAS	ID Source	Score	Algorithm
C.I. Pigment Red 149	C40 H26 N2 O4	11.295		598.1899	1.01	4948-15-6	FBF	82.43	FBF

Species	m/z	Score (Tgt)	Score (Lib)	Score (DB)	Score (MFG)	Score (RT)
(M-H)-	597	82.43				

Compound Chromatograms (overlaid)

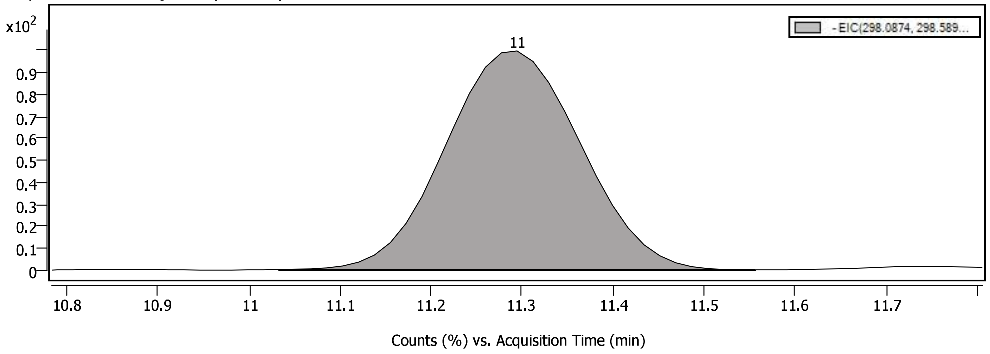

Structure

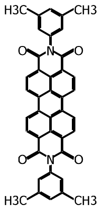

Compound Spectra (overlaid)

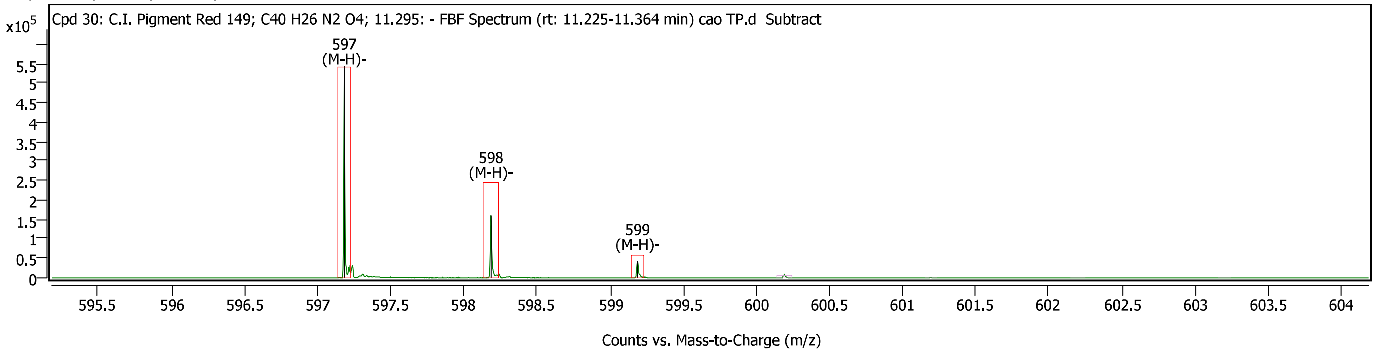

Compound ID Table

Name	Formula	Species	RT	RT Diff	Mass	CAS	ID Source	Score	Score (Lib)	Score (Tgt)
C.I. Pigment Red 149	C40 H26 N2 O4	(M-H)-	11.295		598.1899	4948-15-6	FBF	82.43		82.43

Cpd 108: Geniposide pentaacetate

Name	Formula	RT	RI	Mass	Diff (Tgt, ppm)	CAS	ID Source	Score	Algorithm
Geniposide pentaacetate	C27 H34 O15	11.295		598.1899	0.16	49776-64-9	M-FBF	99.73	FBF

Species	m/z	Score (Tgt)	Score (Lib)	Score (DB)	Score (MFG)	Score (RT)
(M-H)-	597	99.73				

Compound Chromatograms (overlaid)

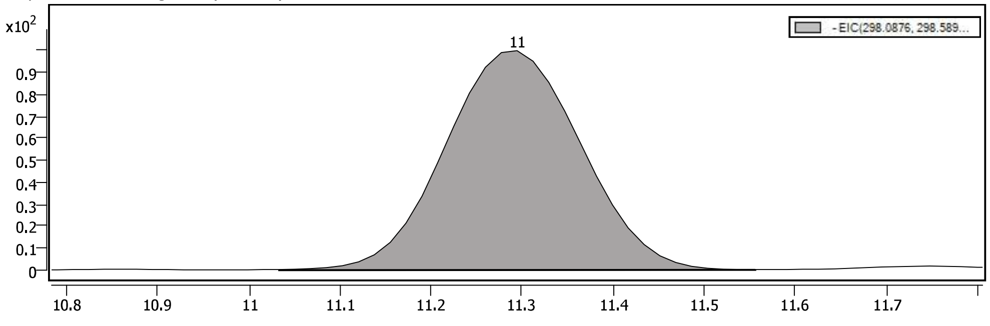

Structure

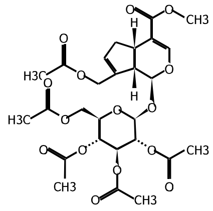

Compound Spectra (overlaid)

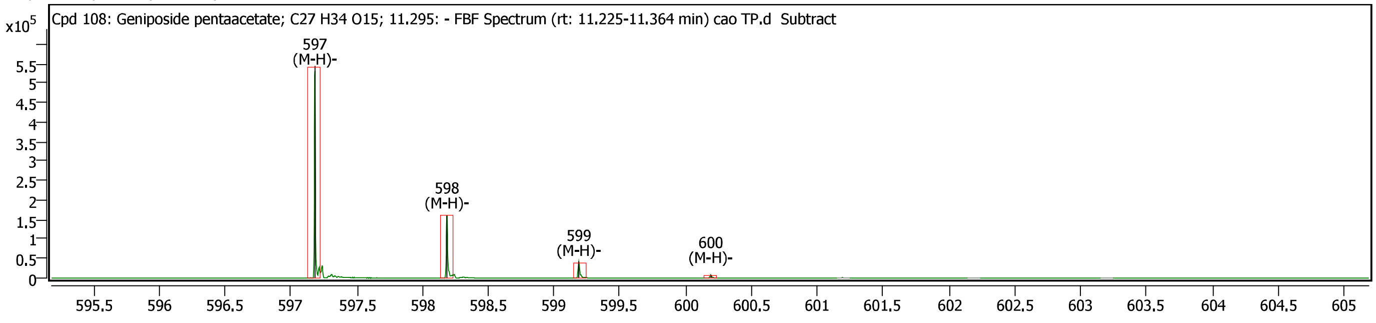

Compound ID Table

Name	Formula	Species	RT	RT Diff	Mass	CAS	ID Source	Score	Score (Lib)	Score (Tgt)
Geniposide pentaacetate	C27 H34 O15	(M-H)-	11.295		598.1899	49776-64-9	FBF	99.73		99.73
Phloretin 3',5'-Di-C-glucoside	C27 H34 O15	(M-H)-	11.295		598.1899		FBF	99.73		99.73
Catechin 3-O-rutinoside	C27 H34 O15	(M-H)-	11.295		598.1899		FBF	99.73		99.73
10-Acetoxyoleuropein	C27 H34 O15	(M-H)-	11.295		598.1899		FBF	99.73		99.73

Cpd 187: 7-Hydroxytrifluoperazine glucuronide

Name	Formula	RT	RI	Mass	Diff (Tgt, ppm)	CAS	ID Source	Score	Algorithm
7-Hydroxytrifluoperazine glucuronide	C27 H32 F3 N3 O7 S	11.295		599.1930	2.86		FBF	87.76	FBF

Species	m/z	Score (Tgt)	Score (Lib)	Score (DB)	Score (MFG)	Score (RT)
(M-H)-	598	87.76				

Compound Chromatograms (overlaid)

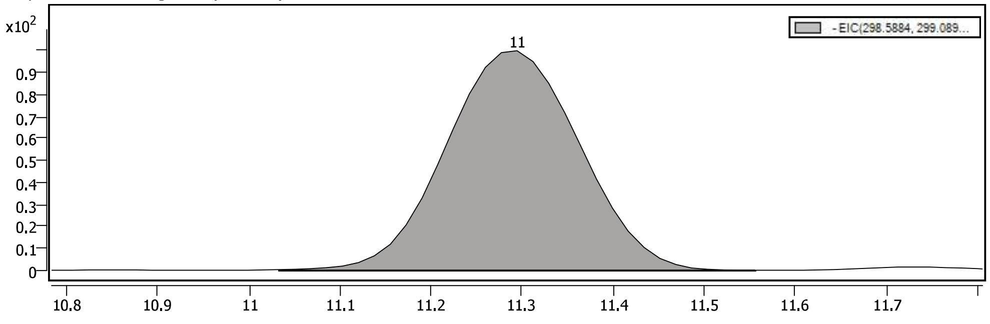

Structure

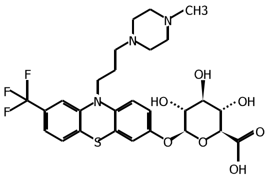

Compound Screening Report

Compound Spectra (overlaid)

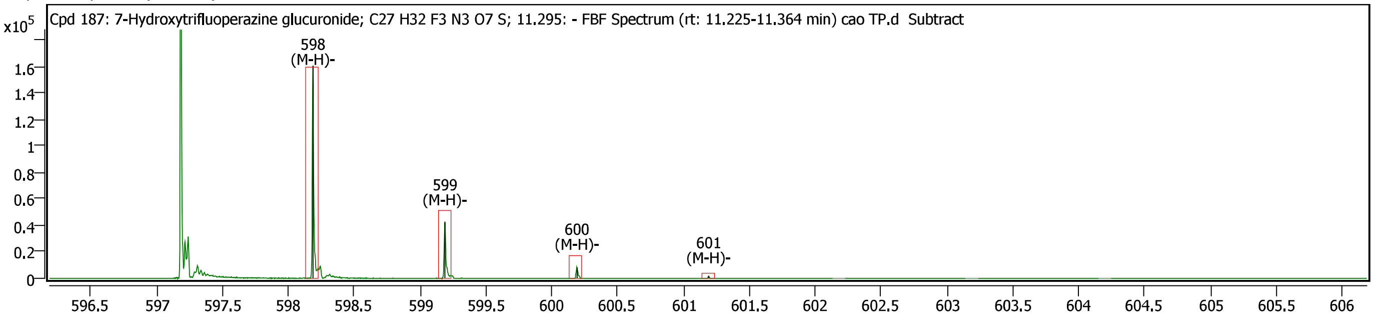

Counts vs. Mass-to-Charge (m/z)

Compound ID Table

Name	Formula	Species	RT	RT Diff	Mass	CAS	ID Source	Score	Score (Lib)	Score (Tgt)
7-Hydroxytrifluoperazine glucuronide	C27 H32 F3 N3 O7 S	(M-H)-	11.295		599.1930		FBF	87.76		87.76

Cpd 202: <Hyperin>

Name	Formula	RT	RI	Mass	Diff (Tgt, ppm)	CAS	ID Source	Score	Algorithm
<Hyperin>	C21 H20 O12	11.451		464.0954	-0.13		M-FBF	99.67	FBF

Species	m/z	Score (Tgt)	Score (Lib)	Score (DB)	Score (MFG)	Score (RT)
(M-H)-	463	99.67				

Compound Chromatograms (overlaid)

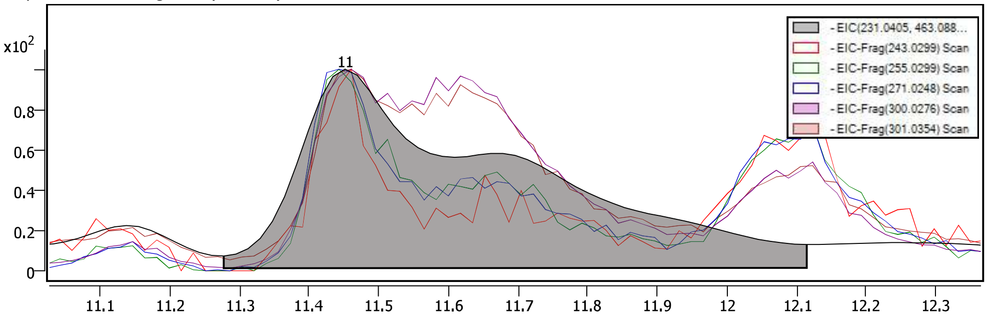

Counts (%) vs. Acquisition Time (min)

Structure

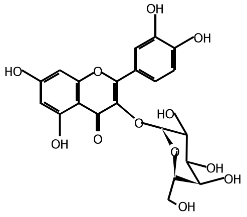

Coelution Plot

Compound Spectra (overlaid)

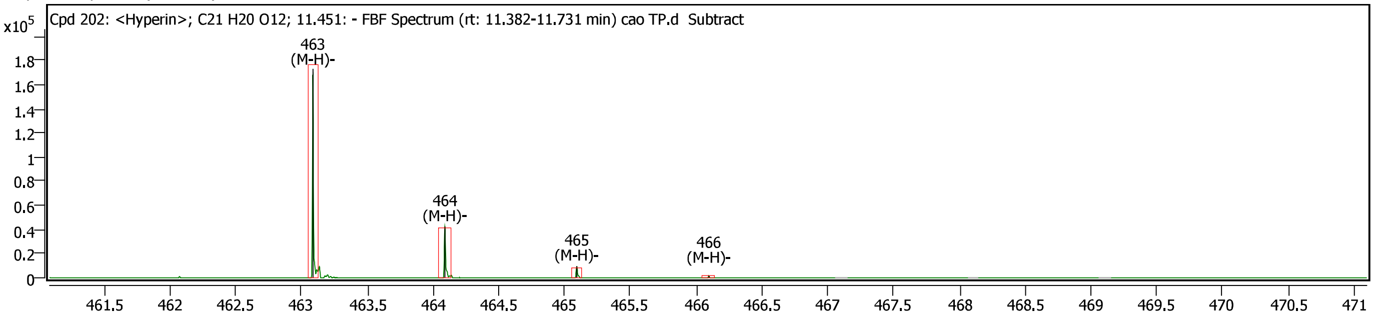

Counts vs. Mass-to-Charge (m/z)

Fragment Spectrum (raw)

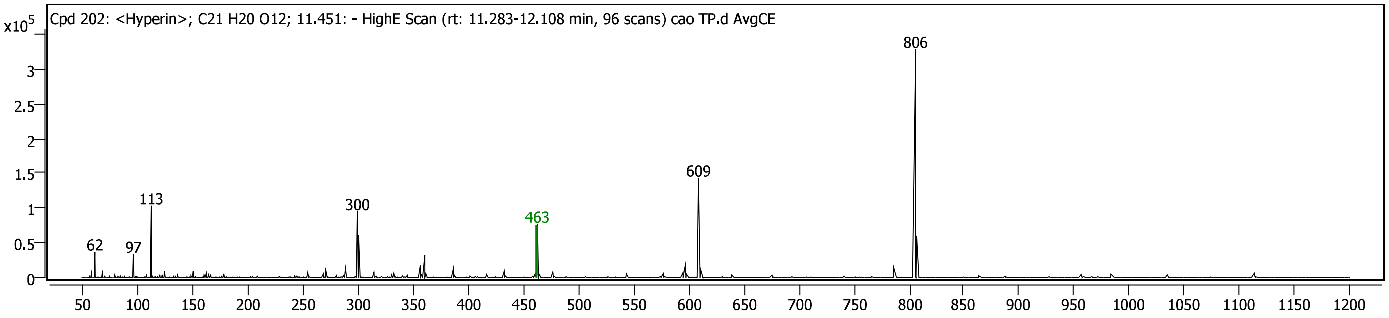

Counts vs. Mass-to-Charge (m/z)

Compound Screening Report

Compound ID Table

Name	Formula	Species	RT	RT Diff	Mass	CAS	ID Source	Score	Score (Lib)	Score (Tgt)
<Hyperin>	C21 H20 O12	(M-H)-	11.451		464.0954		FBF	99.67		99.67
<Bracteatin 6-O-glucoside>	C21 H20 O12	(M-H)-	11.451		464.0954		FBF	99.67		99.67
<Quercetagetin 3-rhamnoside>	C21 H20 O12	(M-H)-	11.451		464.0954		FBF	99.67		99.67
<8-Hydroxyluteolin 8-glucoside>	C21 H20 O12	(M-H)-	11.451		464.0954		FBF	99.67		99.67
<Annulatin 3'-xyloside>	C21 H20 O12	(M-H)-	11.451		464.0954		FBF	99.67		99.67
<Bracteatin 6-glucoside>	C21 H20 O12	(M-H)-	11.451		464.0954		FBF	99.67		99.67
<Gossypetin 7-rhamnoside>	C21 H20 O12	(M-H)-	11.451		464.0954		FBF	99.67		99.67
<Bractein>	C21 H20 O12	(M-H)-	11.451		464.0954		FBF	99.67		99.67
<Corniculatusin 3-alpha-L-arabinofuranoside>	C21 H20 O12	(M-H)-	11.451		464.0954		FBF	99.67		99.67
<Eriodictyol 7-glucuronide>	C21 H20 O12	(M-H)-	11.451		464.0954		FBF	99.67		99.67
<6-Hydroxyluteolin 7-glucoside>	C21 H20 O12	(M-H)-	11.451		464.0954		FBF	99.67		99.67
<Gossypetin 8-rhamnoside>	C21 H20 O12	(M-H)-	11.451		464.0954		FBF	99.67		99.67
<6-Hydroxytricetin 5-rhamnoside>	C21 H20 O12	(M-H)-	11.451		464.0954		FBF	99.67		99.67
<6-Hydroxykaempferol 3-glucoside>	C21 H20 O12	(M-H)-	11.451		464.0954		FBF	99.67		99.67
<6-Hydroxyluteolin 7-galactoside>	C21 H20 O12	(M-H)-	11.451		464.0954		FBF	99.67		99.67
<6-Hydroxyluteolin 6-glucoside>	C21 H20 O12	(M-H)-	11.451		464.0954		FBF	99.67		99.67
<6-Hydroxyluteolin 5-glucoside>	C21 H20 O12	(M-H)-	11.451		464.0954		FBF	99.67		99.67
<6-Hydroxykaempferol 7-glucoside>	C21 H20 O12	(M-H)-	11.451		464.0954		FBF	99.67		99.67
<6-C-Glucosylquercetin>	C21 H20 O12	(M-H)-	11.451		464.0954		FBF	99.67		99.67
<6-C-beta-D-Glucopyranosyl-5,7,2',4',5'-pentahydroxyflavone>	C21 H20 O12	(M-H)-	11.451		464.0954		FBF	99.67		99.67
<5,7,3',4',5'-Pentahydroxyflavone 8-C-glucopyranoside>	C21 H20 O12	(M-H)-	11.451		464.0954		FBF	99.67		99.67
<5,6,7,3',4'-Pentahydroxy-8-methoxyflavone 7-apioside>	C21 H20 O12	(M-H)-	11.451		464.0954		FBF	99.67		99.67
<3,5,7,2',6'-Pentahydroxyflavone 2'-glucoside>	C21 H20 O12	(M-H)-	11.451		464.0954		FBF	99.67		99.67
<2'-Hydroxyisoorientin>	C21 H20 O12	(M-H)-	11.451		464.0954		FBF	99.67		99.67
<(2S)-5,7,3',4'-Tetrahydroxyflavanone 7-glucuronide>	C21 H20 O12	(M-H)-	11.451		464.0954		FBF	99.67		99.67
<Herbacetin 3-beta-D-glucofuranoside>	C21 H20 O12	(M-H)-	11.451		464.0954		FBF	99.67		99.67
<8-Hydroxyluteolin 7-glucoside>	C21 H20 O12	(M-H)-	11.451		464.0954		FBF	99.67		99.67
<Herbacetin 3-glucoside>	C21 H20 O12	(M-H)-	11.451		464.0954		FBF	99.67		99.67
<Tricetin 7-glucoside>	C21 H20 O12	(M-H)-	11.451		464.0954		FBF	99.67		99.67
<Quercetin 3-alloside>	C21 H20 O12	(M-H)-	11.451		464.0954		FBF	99.67		99.67
<Herbacetin 7-glucoside>	C21 H20 O12	(M-H)-	11.451		464.0954		FBF	99.67		99.67
<Tricetin 3'-glucoside>	C21 H20 O12	(M-H)-	11.451		464.0954		FBF	99.67		99.67
<Spiraeoside>	C21 H20 O12	(M-H)-	11.451		464.0954		FBF	99.67		99.67
<Robinetin 7-glucoside>	C21 H20 O12	(M-H)-	11.451		464.0954		FBF	99.67		99.67
<Quercimeritrin>	C21 H20 O12	(M-H)-	11.451		464.0954		FBF	99.67		99.67
<Quercetin 5-glucoside>	C21 H20 O12	(M-H)-	11.451		464.0954		FBF	99.67		99.67
<Quercetin 4'-glucoside>	C21 H20 O12	(M-H)-	11.451		464.0954	20229-56-5	FBF	99.67		99.67
<Quercetin 3-β-D-glucoside>	C21 H20 O12	(M-H)-	11.451		464.0954	482-35-9	FBF	99.67		99.67
<Quercetin 3-O-glucoside>	C21 H20 O12	(M-H)-	11.451		464.0954	21637-25-2	FBF	99.67		99.67
<Quercetin 3'-glucoside>	C21 H20 O12	(M-H)-	11.451		464.0954		FBF	99.67		99.67
<Quercetin 7-galactoside>	C21 H20 O12	(M-H)-	11.451		464.0954		FBF	99.67		99.67
<Myricetin 3'-rhamnoside>	C21 H20 O12	(M-H)-	11.451		464.0954		FBF	99.67		99.67
<Patuletin 3-xyloside>	C21 H20 O12	(M-H)-	11.451		464.0954		FBF	99.67		99.67
<Isoetin 5'-glucoside>	C21 H20 O12	(M-H)-	11.451		464.0954		FBF	99.67		99.67
<Myricitrin>	C21 H20 O12	(M-H)-	11.451		464.0954	17912-87-7	FBF	99.67		99.67
<Herbacetin 8-glucoside>	C21 H20 O12	(M-H)-	11.451		464.0954		FBF	99.67		99.67
<Hyperoside>	C21 H20 O12	(M-H)-	11.451		464.0954	482-36-0	FBF	99.67		99.67
<Isoaffinetin>	C21 H20 O12	(M-H)-	11.451		464.0954		FBF	99.67		99.67
<Isoetin 7-glucoside>	C21 H20 O12	(M-H)-	11.451		464.0954		FBF	99.67		99.67
<Isoquercitrin>	C21 H20 O12	(M-H)-	11.451		464.0954		FBF	99.67		99.67
<Larycitrin 3-alpha-L-arabinofuranoside>	C21 H20 O12	(M-H)-	11.451		464.0954		FBF	99.67		99.67
<Herbacetin 4'-glucoside>	C21 H20 O12	(M-H)-	11.451		464.0954		FBF	99.67		99.67
<Myricetin 7-rhamnoside>	C21 H20 O12	(M-H)-	11.451		464.0954	184533-14-0	FBF	99.67		99.67

Cpd 124: <2''-O-beta-L-galactopyranosylorientin>

Name	Formula	RT	RI	Mass	Diff (Tgt, ppm)	CAS	ID Source	Score	Algorithm
<2''-O-beta-L-galactopyranosylorientin>	C27 H30 O16	11.591		610.1533	-0.06		M-FBF	99.90	FBF

Species	m/z	Score (Tgt)	Score (Lib)	Score (DB)	Score (MFG)	Score (RT)
(M-H)-	609	99.90				

Compound Chromatograms (overlaid)

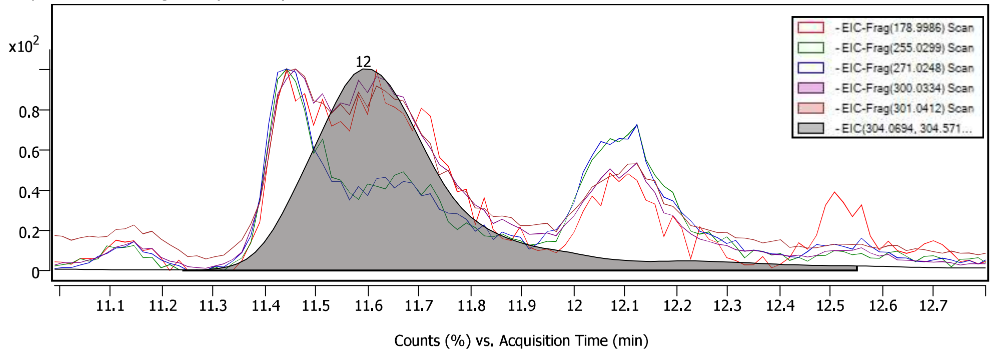

Structure

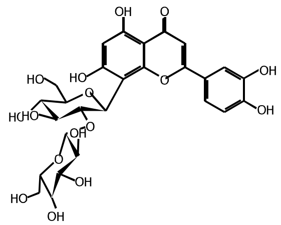

Coelution Plot

Compound Spectra (overlaid)

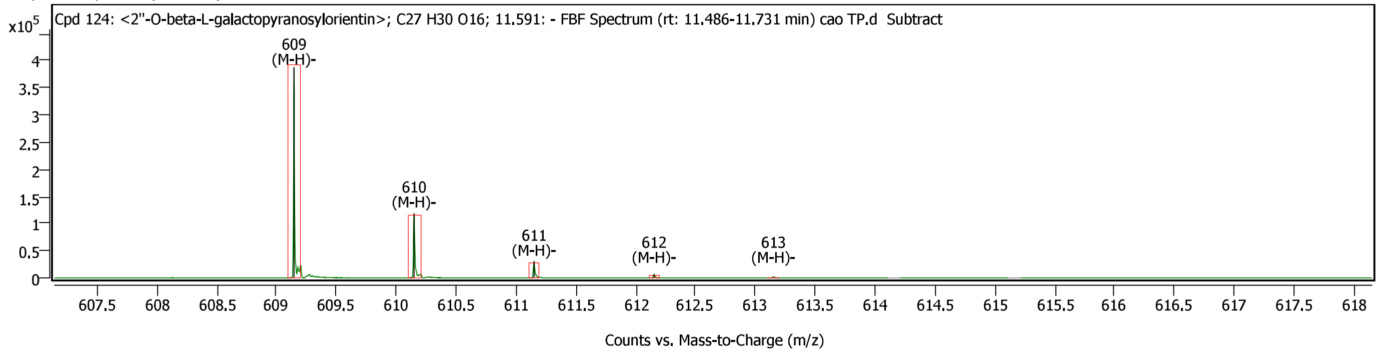

Fragment Spectrum (raw)

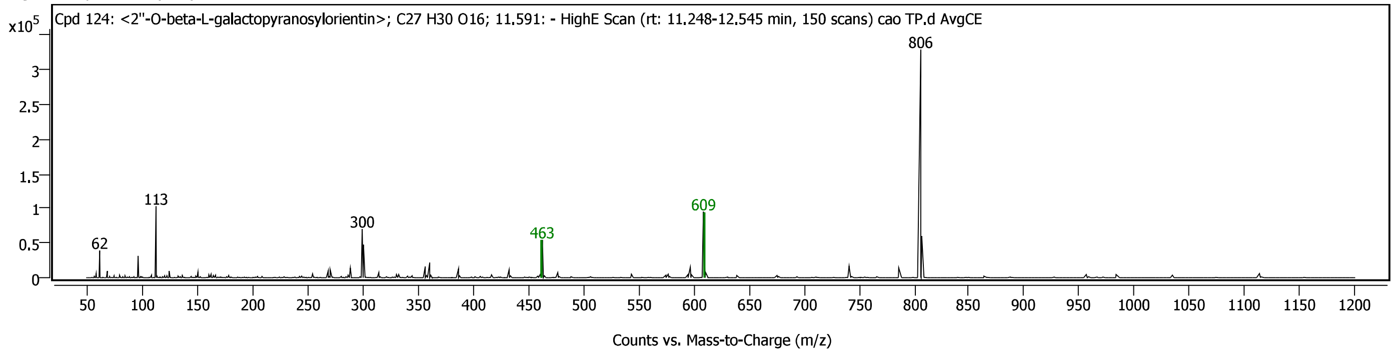

Compound ID Table

Name	Formula	Species	RT	RT Diff	Mass	CAS	ID Source	Score	Score (Lib)	Score (Tgt)
<2''-O-beta-L-galactopyranosylorientin>	C27 H30 O16	(M-H)-	11.591		610.1533		FBF	99.90		99.90
<Isorhamnetin 3-apiosyl-(1->2)-galactoside>	C27 H30 O16	(M-H)-	11.591		610.1533		FBF	99.90		99.90
<Kaempferol 3-galactoside-4'-glucoside>	C27 H30 O16	(M-H)-	11.591		610.1533		FBF	99.90		99.90
<Isorhamnetin 3-L-arabinopyranosyl-(1->6)-galactoside>	C27 H30 O16	(M-H)-	11.591		610.1533		FBF	99.90		99.90
<Isorhamnetin 3-glucoside-7-xyloside>	C27 H30 O16	(M-H)-	11.591		610.1533		FBF	99.90		99.90
<Isorhamnetin 3-glucoside-7-alpha-L-arabinopyranoside>	C27 H30 O16	(M-H)-	11.591		610.1533		FBF	99.90		99.90
<Isorhamnetin 3-apiosyl-(1->2)-glucoside>	C27 H30 O16	(M-H)-	11.591		610.1533		FBF	99.90		99.90
<Isoorientin 4'-O-glucoside>	C27 H30 O16	(M-H)-	11.591		610.1533		FBF	99.90		99.90
<Isoquercitrin 4''-rhamnoside>	C27 H30 O16	(M-H)-	11.591		610.1533	123160-33-8	FBF	99.90		99.90
<Isoorientin 7-O-galactoside>	C27 H30 O16	(M-H)-	11.591		610.1533		FBF	99.90		99.90
<Isoorientin 7-glucoside>	C27 H30 O16	(M-H)-	11.591		610.1533	35450-86-3	FBF	99.90		99.90
<Isorhamnetin 3-sambubioside>	C27 H30 O16	(M-H)-	11.591		610.1533		FBF	99.90		99.90
<Isoorientin 2''-O-alpha-D-mannoside>	C27 H30 O16	(M-H)-	11.591		610.1533		FBF	99.90		99.90
<Kaempferol 3-gentiobioside>	C27 H30 O16	(M-H)-	11.591		610.1533		FBF	99.90		99.90
<Isorhamnetin 3-vicianoside>	C27 H30 O16	(M-H)-	11.591		610.1533		FBF	99.90		99.90
<Isorhamnetin 3-xylosyl-(1->2)-galactoside>	C27 H30 O16	(M-H)-	11.591		610.1533		FBF	99.90		99.90
<Isorhamnetin 3-xylosyl-(1->6)-glucoside>	C27 H30 O16	(M-H)-	11.591		610.1533		FBF	99.90		99.90
<Isoscutellarein 7-allosyl-(1->2)-glucoside>	C27 H30 O16	(M-H)-	11.591		610.1533		FBF	99.90		99.90
<Kaempferol 3,4'-diglucoside>	C27 H30 O16	(M-H)-	11.591		610.1533		FBF	99.90		99.90
<Kaempferol 3,5-digalactoside>	C27 H30 O16	(M-H)-	11.591		610.1533		FBF	99.90		99.90
<Kaempferol 3,5-diglucoside>	C27 H30 O16	(M-H)-	11.591		610.1533		FBF	99.90		99.90
<Kaempferol 3,7-digalactoside>	C27 H30 O16	(M-H)-	11.591		610.1533		FBF	99.90		99.90
<Kaempferol 3,7-diglucoside>	C27 H30 O16	(M-H)-	11.591		610.1533		FBF	99.90		99.90
<Isorhamnetin 3-O-[b-D-xylopyranosyl-(1->6)-b-D-glucopyranoside]>	C27 H30 O16	(M-H)-	11.591		610.1533	142905-19-9	FBF	99.90		99.90
<Isoorientin 3'-O-glucoside>	C27 H30 O16	(M-H)-	11.591		610.1533		FBF	99.90		99.90
<Rheinoside A>	C27 H30 O16	(M-H)-	11.591		610.1533	111545-28-9	FBF	99.90		99.90
<Allivicin>	C27 H30 O16	(M-H)-	11.591		610.1533	71939-16-7	FBF	99.90		99.90
<8-C-Glucosylquercetin 2''-O-rhamnoside>	C27 H30 O16	(M-H)-	11.591		610.1533		FBF	99.90		99.90
<8-Hydroxyapigenin 8-sophoroside>	C27 H30 O16	(M-H)-	11.591		610.1533		FBF	99.90		99.90
<3,7,2',3',4'-Pentahydroxyflavone 3-neohesperidoside>	C27 H30 O16	(M-H)-	11.591		610.1533		FBF	99.90		99.90
<3-[(2S,3R,4S,5S,6R)-4,5-Dihydroxy-6-(hydroxymethyl)-3-[(2S,3R,4S,5R)-3,4,5-trihydroxyoxan-2-yl]oxyoxan-2-yl]oxy-2-(3,4-dihydroxyphenyl)-5-hydroxy-7-methoxychromen-4-one>	C27 H30 O16	(M-H)-	11.591		610.1533		FBF	99.90		99.90
<6-C-Glucosylkaempferol 3-O-glucoside>	C27 H30 O16	(M-H)-	11.591		610.1533		FBF	99.90		99.90
<6-Hydroxykaempferol 3-rutinoside>	C27 H30 O16	(M-H)-	11.591		610.1533		FBF	99.90		99.90
<6-Hydroxykaempferol 7-rutinoside>	C27 H30 O16	(M-H)-	11.591		610.1533		FBF	99.90		99.90
<6-Hydroxyluteolin 6-glucoside-3'-rhamnoside>	C27 H30 O16	(M-H)-	11.591		610.1533		FBF	99.90		99.90
<6-Hydroxyluteolin 7-rutinoside>	C27 H30 O16	(M-H)-	11.591		610.1533		FBF	99.90		99.90
<Aureusidin 4,6-diglucoside>	C27 H30 O16	(M-H)-	11.591		610.1533		FBF	99.90		99.90
<Herbacetin 8-rutinoside>	C27 H30 O16	(M-H)-	11.591		610.1533		FBF	99.90		99.90
<Herbacetin 7-rhamnoside-8-glucoside>	C27 H30 O16	(M-H)-	11.591		610.1533		FBF	99.90		99.90
<Annulatin 7-rhamnoside-3'-xyloside>	C27 H30 O16	(M-H)-	11.591		610.1533		FBF	99.90		99.90
<Kaempferol 3-glucoside-7-galactoside>	C27 H30 O16	(M-H)-	11.591		610.1533		FBF	99.90		99.90
<Calendoflavobioside>	C27 H30 O16	(M-H)-	11.591		610.1533	32453-36-4	FBF	99.90		99.90
<Camelliaside C>	C27 H30 O16	(M-H)-	11.591		610.1533		FBF	99.90		99.90
<Fisetin 3,7-diglucoside>	C27 H30 O16	(M-H)-	11.591		610.1533		FBF	99.90		99.90
<Flavocannabiside>	C27 H30 O16	(M-H)-	11.591		610.1533		FBF	99.90		99.90
<Gossypetin 8-methyl ether 3-xylosyl-(1->2)-rhamnoside>	C27 H30 O16	(M-H)-	11.591		610.1533		FBF	99.90		99.90
<Herbacetin 3-rhamnoside-8-glucoside>	C27 H30 O16	(M-H)-	11.591		610.1533		FBF	99.90		99.90
<Herbacetin 7-glucosyl-(1->3)-rhamnoside>	C27 H30 O16	(M-H)-	11.591		610.1533		FBF	99.90		99.90
<Isoorientin 2''-O-glucopyranoside>	C27 H30 O16	(M-H)-	11.591		610.1533		FBF	99.90		99.90
<Luteolin 7-gentiobioside>	C27 H30 O16	(M-H)-	11.591		610.1533		FBF	99.90		99.90
<Quercetin 3-glucosyl-(1->2)-rhamnoside>	C27 H30 O16	(M-H)-	11.591		610.1533		FBF	99.90		99.90
<Quercetin 3-glucosyl-(1->4)-rhamnoside>	C27 H30 O16	(M-H)-	11.591		610.1533		FBF	99.90		99.90
<Orobol 6,8-di-C-glucoside>	C27 H30 O16	(M-H)-	11.591		610.1533		FBF	99.90		99.90
<Panasenoside>	C27 H30 O16	(M-H)-	11.591		610.1533		FBF	99.90		99.90
<Quercetin 3-(2-glucosylrhamnoside)>	C27 H30 O16	(M-H)-	11.591		610.1533	143016-74-4	FBF	99.90		99.90
<Quercetin 3-galactoside 7-rhamnoside>	C27 H30 O16	(M-H)-	11.591		610.1533	38784-81-5	FBF	99.90		99.90
<Quercetin 3-galactoside-7-rhamnoside>	C27 H30 O16	(M-H)-	11.591		610.1533		FBF	99.90		99.90
<Quercetin 3-galactosyl-(1->2)-rhamnoside>	C27 H30 O16	(M-H)-	11.591		610.1533		FBF	99.90		99.90
<Quercetin 3-galactosyl-(1->4)-rhamnoside>	C27 H30 O16	(M-H)-	11.591		610.1533		FBF	99.90		99.90
<Quercetin 3-glucoside-7-rhamnoside>	C27 H30 O16	(M-H)-	11.591		610.1533		FBF	99.90		99.90

Compound Screening Report

Compound ID Table

Name	Formula	Species	RT	RT Diff	Mass	CAS	ID Source	Score	Score (Lib)	Score (Tgt)
<Quercetin 3-methyl ether 7-alpha-L-arabinofuranosyl-(1->6)-glucoside>	C27 H30 O16	(M-H)-	11.591		610.1533		FBF	99.90		99.90
<Luteolin 7,4'-diglucoside>	C27 H30 O16	(M-H)-	11.591		610.1533		FBF	99.90		99.90
<Robinetin 3-rutinoside>	C27 H30 O16	(M-H)-	11.591		610.1533		FBF	99.90		99.90
<Quercetin 3-rhamnoside-3'-glucoside>	C27 H30 O16	(M-H)-	11.591		610.1533		FBF	99.90		99.90
<Quercetin 3-rhamnoside-7-glucoside>	C27 H30 O16	(M-H)-	11.591		610.1533		FBF	99.90		99.90
<Quercetin 3-rhamnosyl-(1->2)-galactoside>	C27 H30 O16	(M-H)-	11.591		610.1533		FBF	99.90		99.90
<Quercetin 3-robinobioside>	C27 H30 O16	(M-H)-	11.591		610.1533		FBF	99.90		99.90
<Quercetin 7-(rhamnosylglucoside)>	C27 H30 O16	(M-H)-	11.591		610.1533	73432-00-5	FBF	99.90		99.90
<Quercetin 7-methyl ether 3-alpha-L-arabinopyranosyl-(1->3)-galactoside>	C27 H30 O16	(M-H)-	11.591		610.1533		FBF	99.90		99.90
<Quercetin 7-rutinoside>	C27 H30 O16	(M-H)-	11.591		610.1533		FBF	99.90		99.90
<Quercetin 8-C-(2"-rhamnosylglucoside)>	C27 H30 O16	(M-H)-	11.591		610.1533	182062-20-0	FBF	99.90		99.90
<Kaempferol 3-glucosyl-(1->6)-galactoside>	C27 H30 O16	(M-H)-	11.591		610.1533		FBF	99.90		99.90
<Kaempferol 3-O-β-D-glucosyl-(1->2)-β-D-glucoside>	C27 H30 O16	(M-H)-	11.591		610.1533	19895-95-5	FBF	99.90		99.90
<Quercetin 3-neohesperidoside>	C27 H30 O16	(M-H)-	11.591		610.1533		FBF	99.90		99.90
<Myricetin 3-rhamnosyl-(1->2)-rhamnoside>	C27 H30 O16	(M-H)-	11.591		610.1533		FBF	99.90		99.90
<Rutin>	C27 H30 O16	(M-H)-	11.591		610.1533	153-18-4	FBF	99.90		99.90
<Luteolin 7-galactosyl-(1->6)-galactoside>	C27 H30 O16	(M-H)-	11.591		610.1533		FBF	99.90		99.90
<Kaempferol 3-O-β-D-glucosylgalactoside>	C27 H30 O16	(M-H)-	11.591		610.1533		FBF	99.90		99.90
<Orientin 7-glucoside>	C27 H30 O16	(M-H)-	11.591		610.1533		FBF	99.90		99.90
<Kaempferol 7,4'-diglucoside>	C27 H30 O16	(M-H)-	11.591		610.1533		FBF	99.90		99.90
<Kaempferol 7-sophoroside>	C27 H30 O16	(M-H)-	11.591		610.1533		FBF	99.90		99.90
<Lucenin 2>	C27 H30 O16	(M-H)-	11.591		610.1533		FBF	99.90		99.90
<Luteolin 3',4'-diglucoside>	C27 H30 O16	(M-H)-	11.591		610.1533		FBF	99.90		99.90
<Luteolin 7,3'-diglucoside>	C27 H30 O16	(M-H)-	11.591		610.1533		FBF	99.90		99.90
<Luteolin 7-allosyl-(1->2)-glucoside>	C27 H30 O16	(M-H)-	11.591		610.1533		FBF	99.90		99.90
<Orobol 7-O-sophoroside>	C27 H30 O16	(M-H)-	11.591		610.1533		FBF	99.90		99.90
<Luteolin 7-galactoside-4'-glucoside>	C27 H30 O16	(M-H)-	11.591		610.1533		FBF	99.90		99.90
<Luteolin 6-C-glucoside 8-C-arabinoside>	C27 H30 O16	(M-H)-	11.591		610.1533		FBF	99.90		99.90
<Orientin 3'-O-glucoside>	C27 H30 O16	(M-H)-	11.591		610.1533		FBF	99.90		99.90
<Luteolin 7-laminaribioside>	C27 H30 O16	(M-H)-	11.591		610.1533		FBF	99.90		99.90
<Lutonarin>	C27 H30 O16	(M-H)-	11.591		610.1533		FBF	99.90		99.90
<Meloside L>	C27 H30 O16	(M-H)-	11.591		610.1533	55196-48-0	FBF	99.90		99.90
<Multinoside A>	C27 H30 O16	(M-H)-	11.591		610.1533	59262-54-3	FBF	99.90		99.90
<Myricetin 3,4'-di-O-alpha-L-rhamnopyranoside>	C27 H30 O16	(M-H)-	11.591		610.1533		FBF	99.90		99.90
<Orientin 4'-glucoside>	C27 H30 O16	(M-H)-	11.591		610.1533		FBF	99.90		99.90

Cpd 237: 6-Hydroxyluteolin 6-xyloside

Name	Formula	RT	RI	Mass	Diff (Tgt, ppm)	CAS	ID Source	Score	Algorithm
6-Hydroxyluteolin 6-xyloside	C20 H18 O11	12.097		434.0849	-0.09		M-FBF	99.82	FBF

Species	m/z	Score (Tgt)	Score (Lib)	Score (DB)	Score (MFG)	Score (RT)
(M-H)-	433	99.82				

Compound Chromatograms (overlaid)

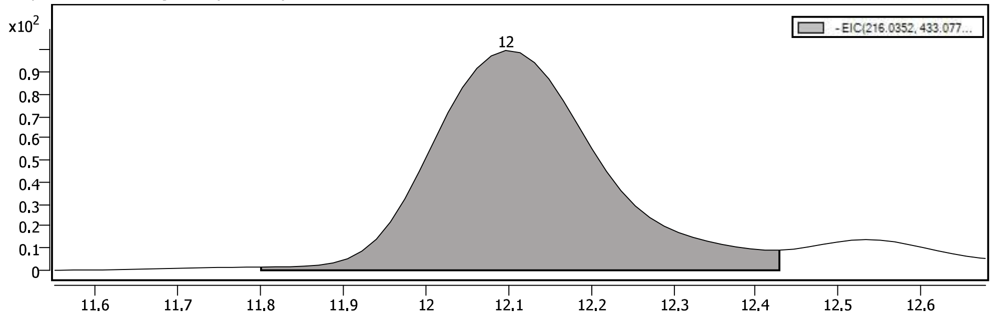

Structure

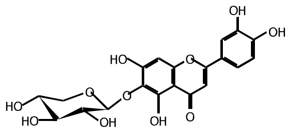

Compound Screening Report

Compound Spectra (overlaid)

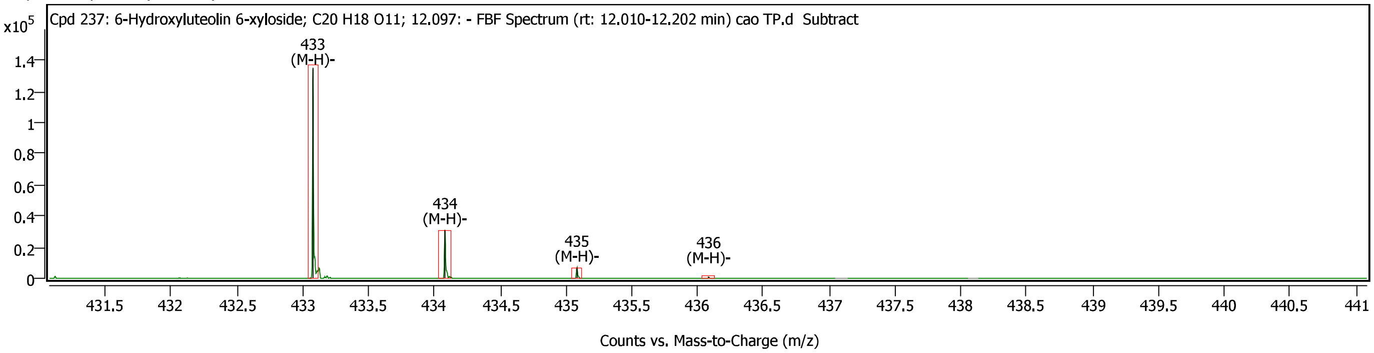

Compound ID Table

Name	Formula	Species	RT	RT Diff	Mass	CAS	ID Source	Score	Score (Lib)	Score (Tgt)
6-Hydroxyluteolin 6-xyloside	C20 H18 O11	(M-H)-	12.097		434.0849		FBF	99.82		99.82
Guajavarin	C20 H18 O11	(M-H)-	12.097		434.0849	22255-13-6	FBF	99.82		99.82
Quercetin 3-O-alpha-D-arabinopyranoside	C20 H18 O11	(M-H)-	12.097		434.0849		FBF	99.82		99.82
Quercetin 3-beta-L-arabinopyranoside	C20 H18 O11	(M-H)-	12.097		434.0849		FBF	99.82		99.82
Isoetin 2'-xyloside	C20 H18 O11	(M-H)-	12.097		434.0849		FBF	99.82		99.82
Herbacetin 8-xyloside	C20 H18 O11	(M-H)-	12.097		434.0849		FBF	99.82		99.82
Herbacetin 8-alpha-L-arabinopyranoside	C20 H18 O11	(M-H)-	12.097		434.0849		FBF	99.82		99.82
Herbacetin 7-beta-L-arabinopyranoside	C20 H18 O11	(M-H)-	12.097		434.0849		FBF	99.82		99.82
Quercetin 3'-xyloside	C20 H18 O11	(M-H)-	12.097		434.0849		FBF	99.82		99.82
Tricetin 3'-xyloside	C20 H18 O11	(M-H)-	12.097		434.0849		FBF	99.82		99.82
Avicularin	C20 H18 O11	(M-H)-	12.097		434.0849	572-30-5	FBF	99.82		99.82
8-Hydroxyluteolin 7-xyloside	C20 H18 O11	(M-H)-	12.097		434.0849		FBF	99.82		99.82
6-Hydroxyluteolin 7-xyloside	C20 H18 O11	(M-H)-	12.097		434.0849		FBF	99.82		99.82
6-Hydroxyluteolin 7-arabinopyranoside	C20 H18 O11	(M-H)-	12.097		434.0849		FBF	99.82		99.82
6-Hydroxyluteolin 7-apioside	C20 H18 O11	(M-H)-	12.097		434.0849		FBF	99.82		99.82
Fukinolic acid	C20 H18 O11	(M-H)-	12.097		434.0849	50982-40-6	FBF	99.82		99.82
Quercetin 7-xyloside	C20 H18 O11	(M-H)-	12.097		434.0849		FBF	99.82		99.82

Cpd 223: <Troxerutin>

Name	Formula	RT	RI	Mass	Diff (Tgt, ppm)	CAS	ID Source	Score	Algorithm
<Troxerutin>	C33 H42 O19	12.516		742.2319	-0.18	7085-55-4	M-FBF	99.13	FBF

Species	m/z	Score (Tgt)	Score (Lib)	Score (DB)	Score (MFG)	Score (RT)
(M-2H)-2 (M-H)-	370 741	99.13				

Compound Chromatograms (overlaid)

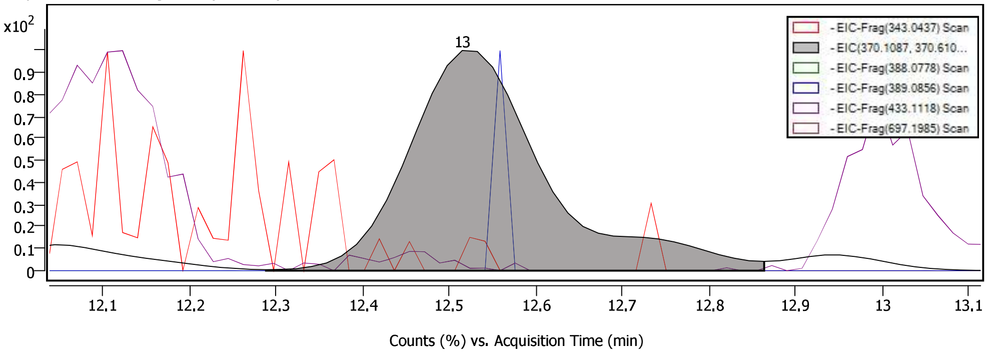

Structure

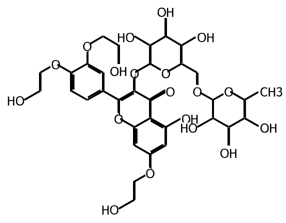

Coelution Plot

Compound Spectra (overlaid)

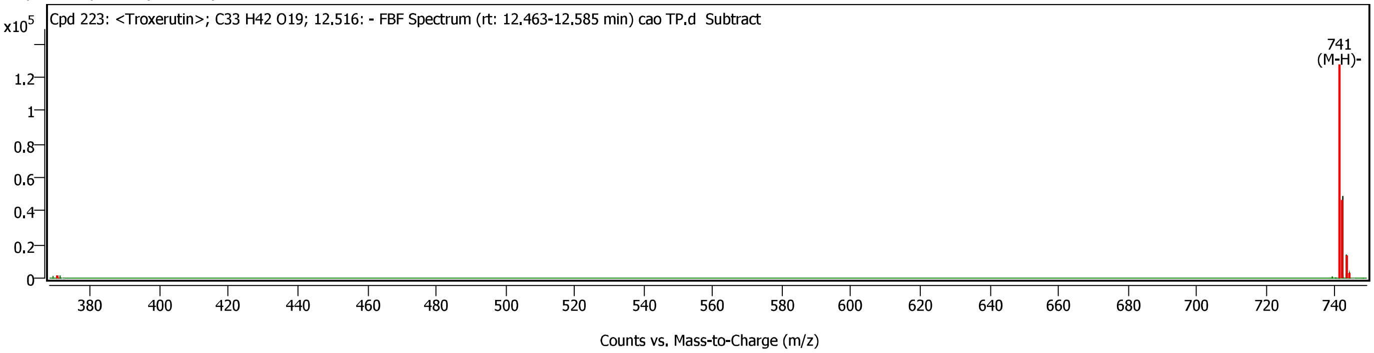

Fragment Spectrum (raw)

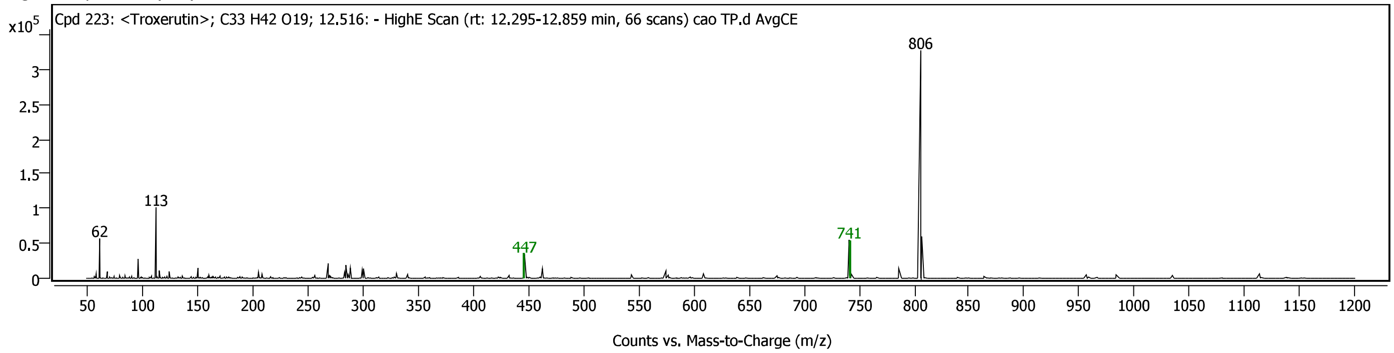

Compound ID Table

Name	Formula	Species	RT	RT Diff	Mass	CAS	ID Source	Score	Score (Lib)	Score (Tgt)
<Troxerutin>	C33 H42 O19	(M-2H)-2 (M-H)-	12.516		742.2319	7085-55-4	FBF	99.13		99.13
<Narirutin 4'-glucoside>	C33 H42 O19	(M-2H)-2 (M-H)-	12.516		742.2319		FBF	99.13		99.13
<Naringin 4'-glucoside>	C33 H42 O19	(M-2H)-2 (M-H)-	12.516		742.2319		FBF	99.13		99.13

Cpd 133: Camelliaside B

Name	Formula	RT	RI	Mass	Diff (Tgt, ppm)	CAS	ID Source	Score	Algorithm
Camelliaside B	C32 H38 O19	13.091		726.2006	-0.13		M-FBF	99.75	FBF

Species	m/z	Score (Tgt)	Score (Lib)	Score (DB)	Score (MFG)	Score (RT)
(M-H)-	725	99.75				

Compound Chromatograms (overlaid)

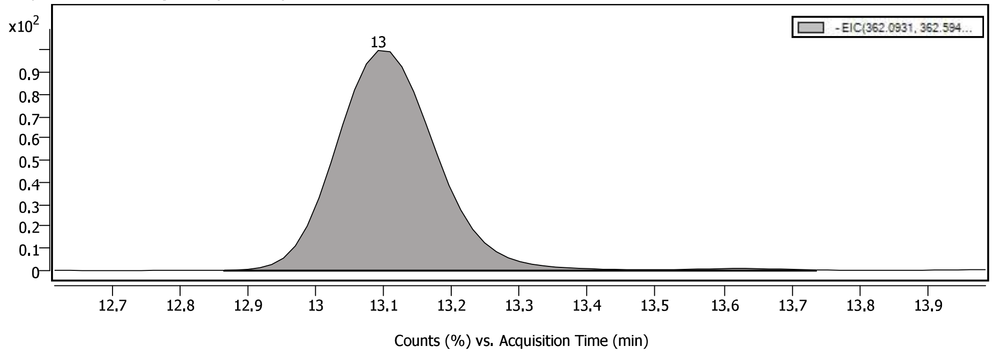

Structure

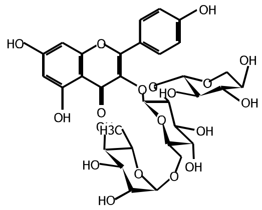

Compound Spectra (overlaid)

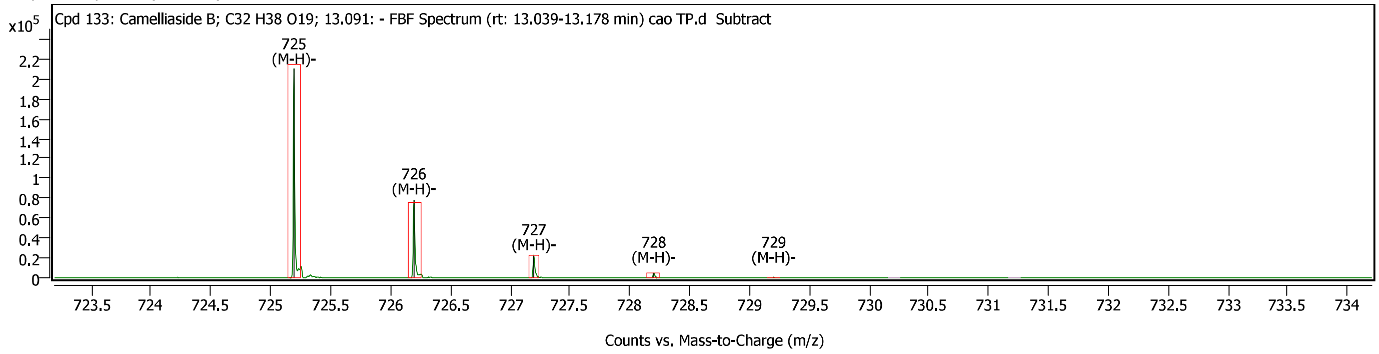

Compound Screening Report

Compound ID Table

Name	Formula	Species	RT	RT Diff	Mass	CAS	ID Source	Score	Score (Lib)	Score (Tgt)
Camelliaside B	C32 H38 O19	(M-H)-	13.091		726.2006		FBF	99.75		99.75
Kaempferol 3-apioside-7-rhamnosyl-(1->6)-galactoside	C32 H38 O19	(M-H)-	13.091		726.2006		FBF	99.75		99.75
Kaempferol 3-(2G-apiosylrobinobioside)	C32 H38 O19	(M-H)-	13.091		726.2006	132185-73-0	FBF	99.75		99.75
Kaempferol 3-xylosyl-(1->3)-rhamnosyl-(1->6)-galactoside	C32 H38 O19	(M-H)-	13.091		726.2006		FBF	99.75		99.75
Isomollupentin 7,2''-di-O-glucoside	C32 H38 O19	(M-H)-	13.091		726.2006		FBF	99.75		99.75
Isoschaftoside 4'-glucoside	C32 H38 O19	(M-H)-	13.091		726.2006	151922-19-9	FBF	99.75		99.75
Isovitexin 4'-O-glucoside 2''-O-arabinoside	C32 H38 O19	(M-H)-	13.091		726.2006		FBF	99.75		99.75
Isovitexin 7-O-glucoside 2''-O-arabinoside	C32 H38 O19	(M-H)-	13.091		726.2006		FBF	99.75		99.75
Isovitexin 7-O-xyloside-2''-O-glucoside	C32 H38 O19	(M-H)-	13.091		726.2006		FBF	99.75		99.75
Kaempferol 3-(2Gal-apiosylrobinobioside)	C32 H38 O19	(M-H)-	13.091		726.2006		FBF	99.75		99.75
Isoschaftoside 4'-O-glucoside	C32 H38 O19	(M-H)-	13.091		726.2006		FBF	99.75		99.75
Kaempferol 7-methyl ether 3-apiosyl-(1->5)-apioside-4'-glucoside	C32 H38 O19	(M-H)-	13.091		726.2006		FBF	99.75		99.75
Kaempferol 3-lathyroside-7-rhamnoside	C32 H38 O19	(M-H)-	13.091		726.2006		FBF	99.75		99.75
Quercetin 3-xylosyl-(1->2)-rhamnoside-4'-rhamnoside	C32 H38 O19	(M-H)-	13.091		726.2006		FBF	99.75		99.75
Kaempferol 3-robinobioside-7-alpha-L-arabinofuranoside	C32 H38 O19	(M-H)-	13.091		726.2006		FBF	99.75		99.75
Schaftoside 6''-O-glucoside	C32 H38 O19	(M-H)-	13.091		726.2006		FBF	99.75		99.75
Schaftoside 4'-O-glucoside	C32 H38 O19	(M-H)-	13.091		726.2006		FBF	99.75		99.75
Schaftoside 4'-glucoside	C32 H38 O19	(M-H)-	13.091		726.2006	151922-20-2	FBF	99.75		99.75
Isovitexin 7-O-arabinoside 2''-O-glucoside	C32 H38 O19	(M-H)-	13.091		726.2006		FBF	99.75		99.75
Kaempferol 3-xylosyl-(1->6)-glucosyl-(1->2)-rhamnoside	C32 H38 O19	(M-H)-	13.091		726.2006		FBF	99.75		99.75

Cpd 92: N(omega)-(ADP-D-ribosyl)-L-arginine

Name	Formula	RT	RI	Mass	Diff (Tgt, ppm)	CAS	ID Source	Score	Algorithm
N(omega)-(ADP-D-ribosyl)-L-arginine	C21 H35 N9 O15 P2	13.527		715.1748	2.82		M-FBF	83.17	FBF

Species	m/z	Score (Tgt)	Score (Lib)	Score (DB)	Score (MFG)	Score (RT)
(M-H)-	714	83.17				

Compound Chromatograms (overlaid)

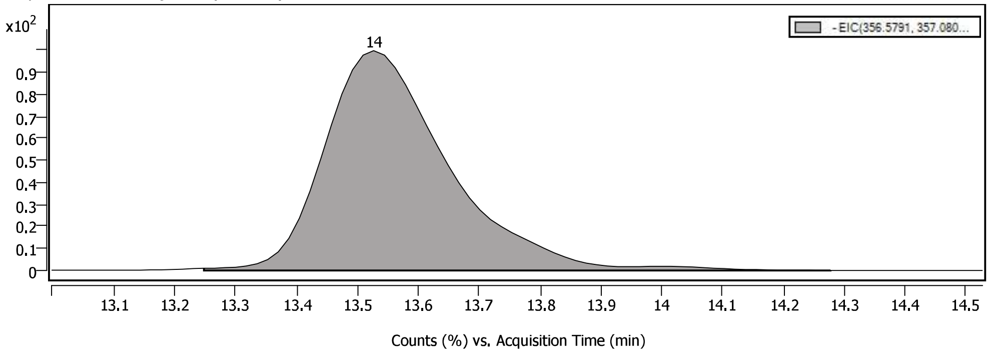

Structure

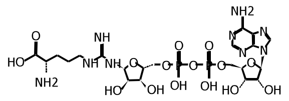

Compound Spectra (overlaid)

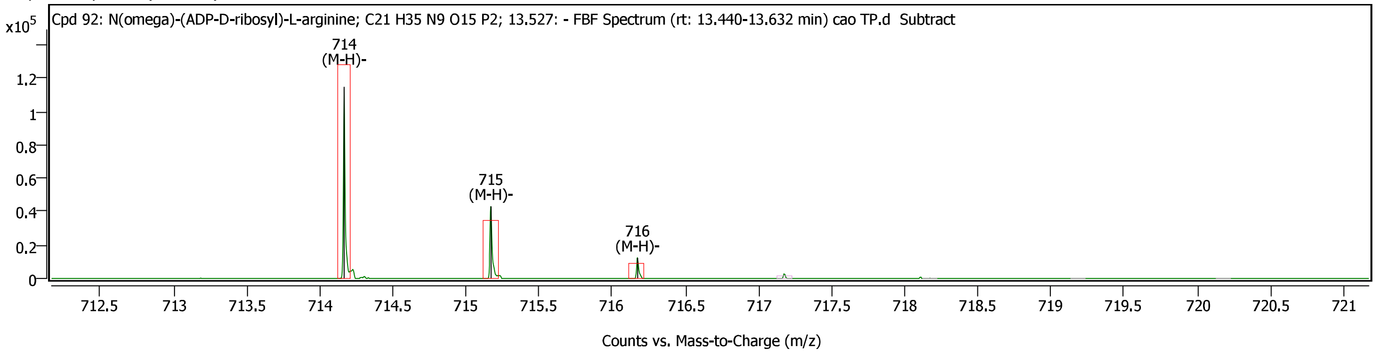

Compound ID Table

Name	Formula	Species	RT	RT Diff	Mass	CAS	ID Source	Score	Score (Lib)	Score (Tgt)
N(omega)-(ADP-D-ribosyl)-L-arginine	C21 H35 N9 O15 P2	(M-H)-	13.527		715.1748		FBF	83.17		83.17
N2-(ADP-D-Ribosyl)-L-arginine	C21 H35 N9 O15 P2	(M-H)-	13.527		715.1748	103960-56-1	FBF	83.17		83.17

Cpd 220: <Streptonigrin>

Name	Formula	RT	RI	Mass	Diff (Tgt, ppm)	CAS	ID Source	Score	Algorithm
<Streptonigrin>	C25 H22 N4 O8	13.649		506.1423	-2.96	3930-19-6	FBF	95.66	FBF

Species	m/z	Score (Tgt)	Score (Lib)	Score (DB)	Score (MFG)	Score (RT)
(M-H)-	505	95.66				

Compound Screening Report

Compound Chromatograms (overlaid)

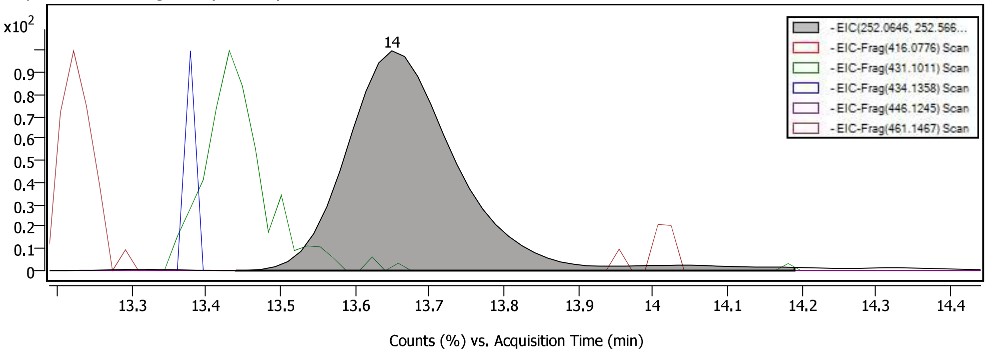

Structure

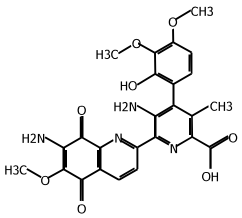

Coelution Plot

Compound Spectra (overlaid)

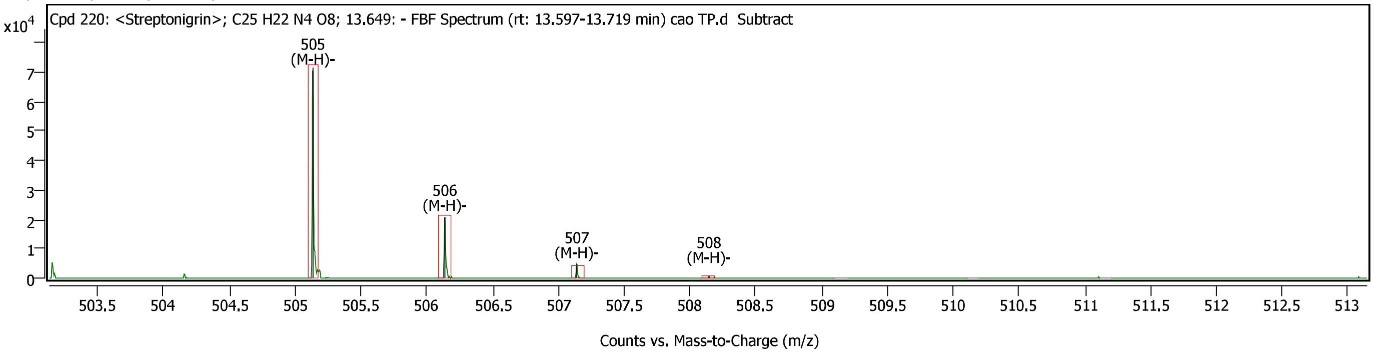

Fragment Spectrum (raw)

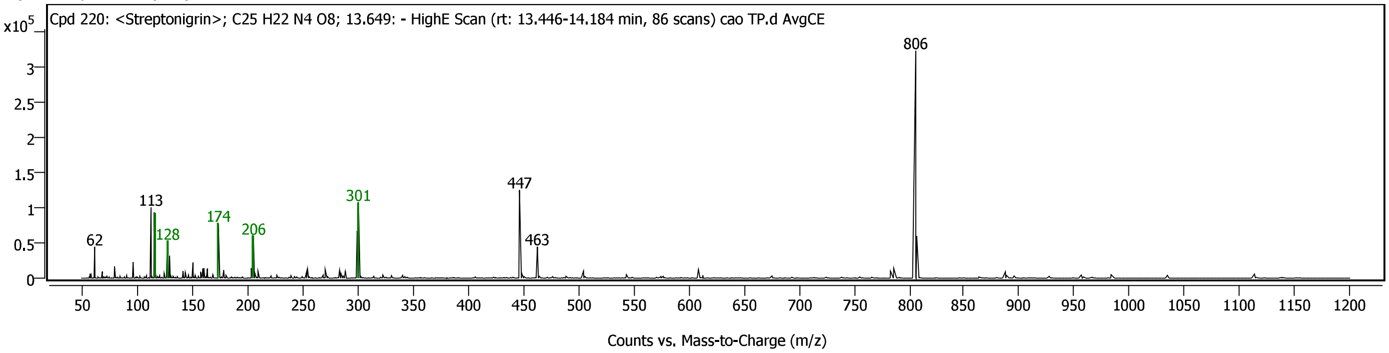

Compound ID Table

Name	Formula	Species	RT	RT Diff	Mass	CAS	ID Source	Score	Score (Lib)	Score (Tgt)
<Streptonigrin>	C25 H22 N4 O8	(M-H)-	13.649		506.1423	3930-19-6	FBF	95.66		95.66

Cpd 89: (Indol-3-yl)glycolaldehyde

Name	Formula	RT	RI	Mass	Diff (Tgt, ppm)	CAS	ID Source	Score	Algorithm
(Indol-3-yl)glycolaldehyde	C10 H9 N O2	13.719		175.0633	-0.36		M-FBF	99.77	FBF

Species	m/z	Score (Tgt)	Score (Lib)	Score (DB)	Score (MFG)	Score (RT)
(M-H)-	174	99.77				

Compound Chromatograms (overlaid)

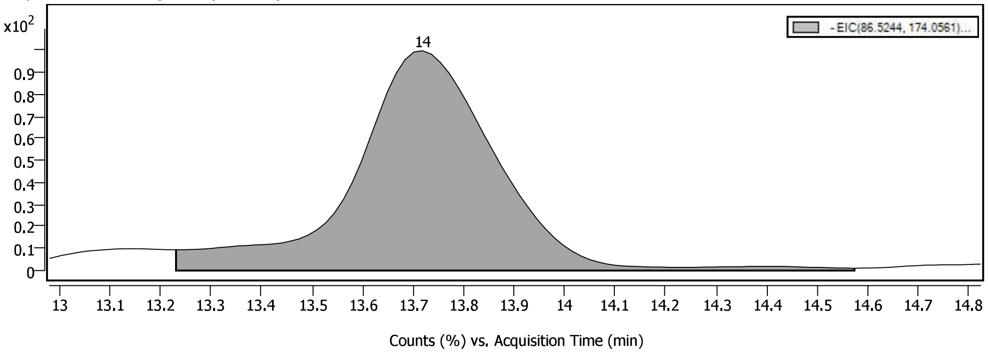

Structure

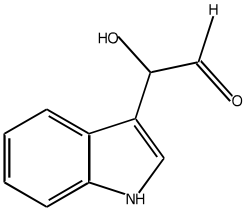

Compound Screening Report

Compound Spectra (overlaid)

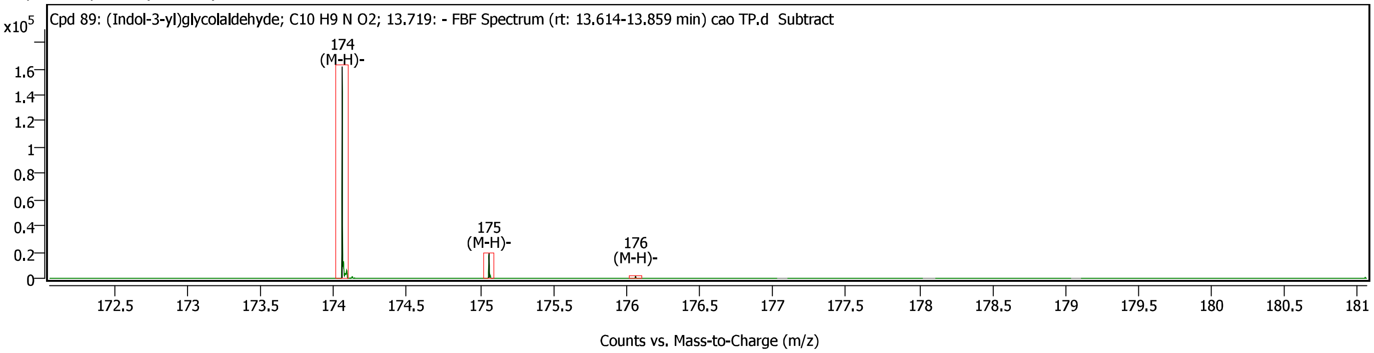

Compound ID Table

Name	Formula	Species	RT	RT Diff	Mass	CAS	ID Source	Score	Score (Lib)	Score (Tgt)
(Indol-3-yl)glycolaldehyde	C10 H9 N O2	(M-H)-	13.719		175.0633		FBF	99.77		99.77
2-Methylquinoline-3,4-diol	C10 H9 N O2	(M-H)-	13.719		175.0633		FBF	99.77		99.77
3-Methyl-quinolin-2,8-diol	C10 H9 N O2	(M-H)-	13.719		175.0633		FBF	99.77		99.77
1-Methoxy-1H-indole-3-carboxaldehyde	C10 H9 N O2	(M-H)-	13.719		175.0633	67282-55-7	FBF	99.77		99.77
3-Hydroxy-2-methyl-1H-quinolin-4-one	C10 H9 N O2	(M-H)-	13.719		175.0633		FBF	99.77		99.77
IAA / 3-Indoleacetic acid	C10 H9 N O2	(M-H)-	13.719		175.0633	87-51-4	FBF	99.77		99.77
5-Hydroxyindoleacetaldehyde	C10 H9 N O2	(M-H)-	13.719		175.0633	1892-21-3	FBF	99.77		99.77
N-Acetylindoxyl	C10 H9 N O2	(M-H)-	13.719		175.0633		FBF	99.77		99.77
NH2Mec	C10 H9 N O2	(M-H)-	13.719		175.0633	26093-31-2	FBF	99.77		99.77
Gentianine	C10 H9 N O2	(M-H)-	13.719		175.0633	439-89-4	FBF	99.77		99.77
Hydroxymethyl indol-3-yl ketone	C10 H9 N O2	(M-H)-	13.719		175.0633	2400-51-3	FBF	99.77		99.77

Cpd 154: Malvidin 3-glucoside-5-(6-acetylglucoside)

Name	Formula	RT	RI	Mass	Diff (Tgt, ppm)	CAS	ID Source	Score	Algorithm
Malvidin 3-glucoside-5-(6-acetylglucoside)	C30 H35 O19	13.893		699.1796	3.39		FBF	92.74	FBF

Species	m/z	Score (Tgt)	Score (Lib)	Score (DB)	Score (MFG)	Score (RT)
(M-H)-	698	92.74				

Compound Chromatograms (overlaid)

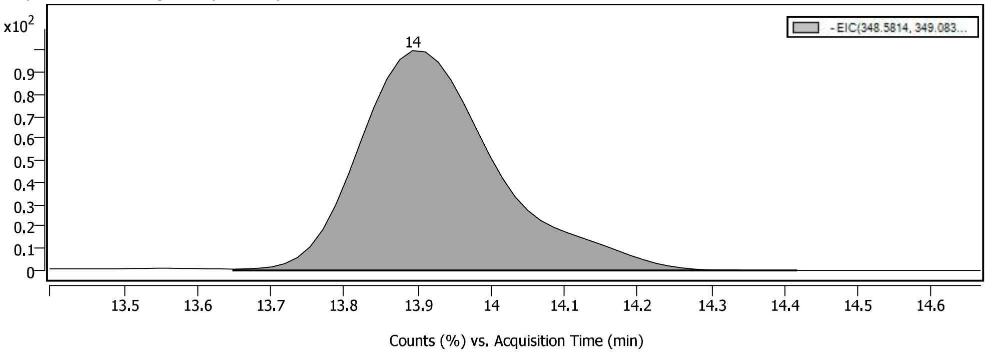

Structure

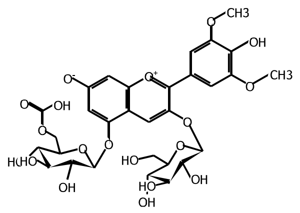

Compound Spectra (overlaid)

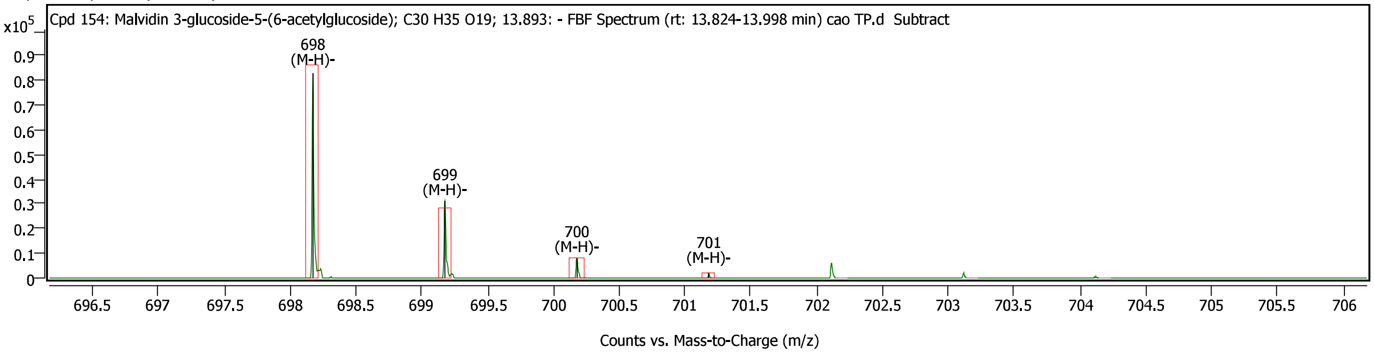

Compound ID Table

Name	Formula	Species	RT	RT Diff	Mass	CAS	ID Source	Score	Score (Lib)	Score (Tgt)
Malvidin 3-glucoside-5-(6-acetylglucoside)	C30 H35 O19	(M-H)-	13.893		699.1796		FBF	92.74		92.74

Cpd 24: Carnocin CP 5

Name	Formula	RT	RI	Mass	Diff (Tgt, ppm)	CAS	ID Source	Score	Algorithm
Carnocin CP 5	C23 H19 N3 O5 S	13.911		449.1038	-1.73	149983-81-3	FBF	86.71	FBF

Species	m/z	Score (Tgt)	Score (Lib)	Score (DB)	Score (MFG)	Score (RT)
(M-H)-	448	86.71				

Compound Chromatograms (overlaid)

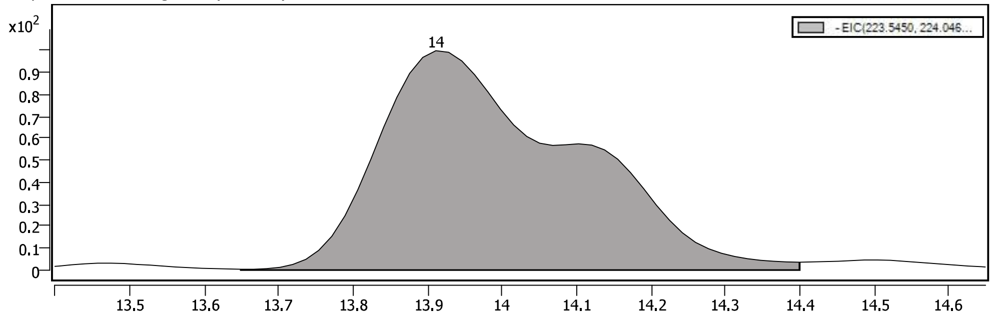

Structure

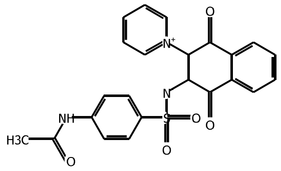

Compound Spectra (overlaid)

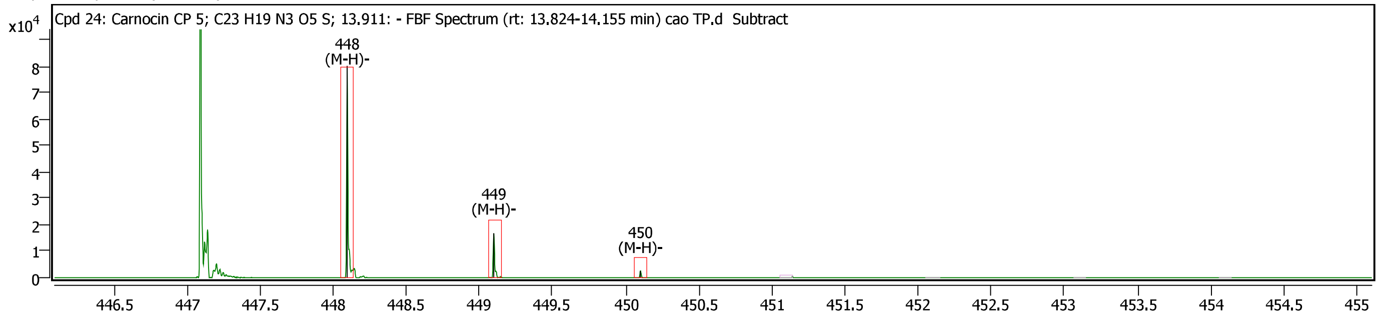

Compound ID Table

Name	Formula	Species	RT	RT Diff	Mass	CAS	ID Source	Score	Score (Lib)	Score (Tgt)
Carnocin CP 5	C23 H19 N3 O5 S	(M-H)-	13.911		449.1038	149983-81-3	FBF	86.71		86.71

Cpd 110: <Kaempferol 3-alpha-D-galactoside>

Name	Formula	RT	RI	Mass	Diff (Tgt, ppm)	CAS	ID Source	Score	Algorithm
<Kaempferol 3-alpha-D-galactoside>	C21 H20 O11	13.911		448.1006	0.01		M-FBF	99.74	FBF

Species	m/z	Score (Tgt)	Score (Lib)	Score (DB)	Score (MFG)	Score (RT)
(M-H)-	447	99.74				

Compound Chromatograms (overlaid)

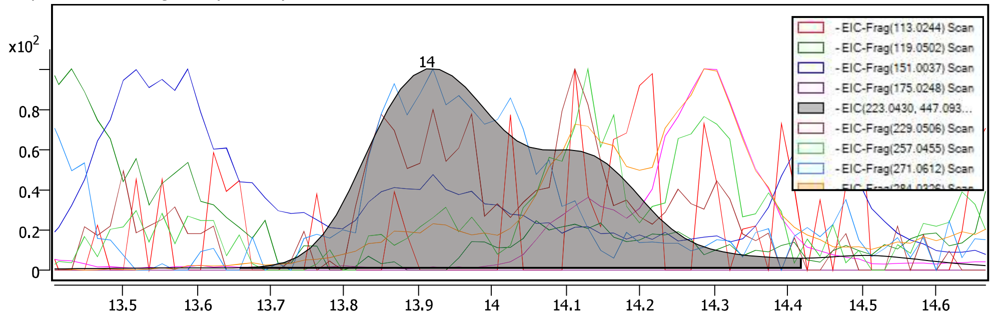

Structure

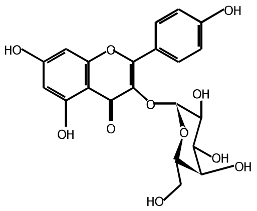

Coelution Plot

Compound Spectra (overlaid)

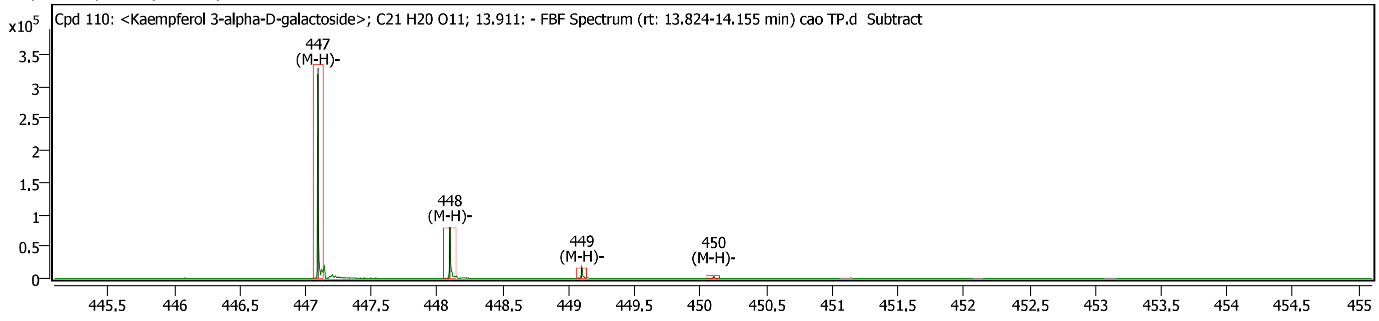

Compound Screening Report

Fragment Spectrum (raw)

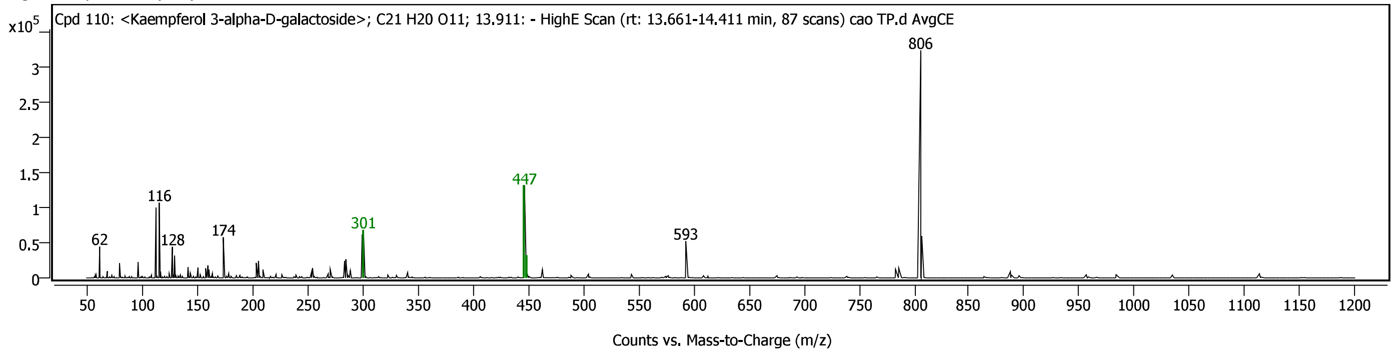

Compound ID Table

Name	Formula	Species	RT	RT Diff	Mass	CAS	ID Source	Score	Score (Lib)	Score (Tgt)
<Kaempferol 3-alpha-D-galactoside>	C21 H20 O11	(M-H)-	13.911		448.1006		FBF	99.74		99.74
<Carthamone>	C21 H20 O11	(M-H)-	13.911		448.1006		FBF	99.74		99.74
<Fisetin 3-glucoside>	C21 H20 O11	(M-H)-	13.911		448.1006		FBF	99.74		99.74
<Azaleatin 3-arabinoside>	C21 H20 O11	(M-H)-	13.911		448.1006	63742-73-4	FBF	99.74		99.74
<Aureusin>	C21 H20 O11	(M-H)-	13.911		448.1006		FBF	99.74		99.74
<Herbacetin 8-rhamnoside>	C21 H20 O11	(M-H)-	13.911		448.1006		FBF	99.74		99.74
<Fisetin 8-C-glucoside>	C21 H20 O11	(M-H)-	13.911		448.1006		FBF	99.74		99.74
<Fisetin 7-glucoside>	C21 H20 O11	(M-H)-	13.911		448.1006		FBF	99.74		99.74
<Fisetin 4'-glucoside>	C21 H20 O11	(M-H)-	13.911		448.1006		FBF	99.74		99.74
<evolvuside B>	C21 H20 O11	(M-H)-	13.911		448.1006		FBF	99.74		99.74
<Petunidin-3-O-arabinoside>	C21 H20 O11	(M-H)-	13.911		448.1006		FBF	99.74		99.74
<Herbacetin 7-rhamnoside>	C21 H20 O11	(M-H)-	13.911		448.1006		FBF	99.74		99.74
<Dihydronorwogonin 7-O-glucuronide>	C21 H20 O11	(M-H)-	13.911		448.1006		FBF	99.74		99.74
<Dihydrobaicalein 7-O-glucuronide>	C21 H20 O11	(M-H)-	13.911		448.1006		FBF	99.74		99.74
<Datiscanin>	C21 H20 O11	(M-H)-	13.911		448.1006		FBF	99.74		99.74
<Cynaroside>	C21 H20 O11	(M-H)-	13.911		448.1006	5373-11-5	FBF	99.74		99.74
<Cernuoside>	C21 H20 O11	(M-H)-	13.911		448.1006		FBF	99.74		99.74
<6-C-Galactosylisoscutellarein>	C21 H20 O11	(M-H)-	13.911		448.1006		FBF	99.74		99.74
<Aureusidin 6-O-glucoside>	C21 H20 O11	(M-H)-	13.911		448.1006	633-15-8	FBF	99.74		99.74
<8-C-Glucosylorobol>	C21 H20 O11	(M-H)-	13.911		448.1006		FBF	99.74		99.74
<8-C-Galactosylluteolin>	C21 H20 O11	(M-H)-	13.911		448.1006		FBF	99.74		99.74
<8-C-beta-D-Glucopyranosylkaempferol>	C21 H20 O11	(M-H)-	13.911		448.1006		FBF	99.74		99.74
<6-Hydroxyluteolin 7-rhamnoside>	C21 H20 O11	(M-H)-	13.911		448.1006		FBF	99.74		99.74
<6-Hydroxyluteolin 6-rhamnoside>	C21 H20 O11	(M-H)-	13.911		448.1006		FBF	99.74		99.74
<Astragalin>	C21 H20 O11	(M-H)-	13.911		448.1006	480-10-4	FBF	99.74		99.74
<Isoorientin>	C21 H20 O11	(M-H)-	13.911		448.1006		FBF	99.74		99.74
<6-Hydroxyluteolin 5-rhamnoside>	C21 H20 O11	(M-H)-	13.911		448.1006		FBF	99.74		99.74
<6-C-Galactosylluteolin>	C21 H20 O11	(M-H)-	13.911		448.1006		FBF	99.74		99.74
<Isorhamnetin 3-alpha-L-arabinofuranoside>	C21 H20 O11	(M-H)-	13.911		448.1006		FBF	99.74		99.74
<5,7,3',4'-Tetrahydroxy-4-phenylcoumarin 5-O-glucoside>	C21 H20 O11	(M-H)-	13.911		448.1006		FBF	99.74		99.74
<5,7,2',6'-Tetrahydroxyflavone 2'-O-glucoside>	C21 H20 O11	(M-H)-	13.911		448.1006		FBF	99.74		99.74
<1,2,6,8-Tetrahydroxy-3-methylanthraquinone 2-O-b-D-glucoside>	C21 H20 O11	(M-H)-	13.911		448.1006		FBF	99.74		99.74
<6-C-Glucosylorobol>	C21 H20 O11	(M-H)-	13.911		448.1006		FBF	99.74		99.74
<6-C-Glucopyranosylkaempferol>	C21 H20 O11	(M-H)-	13.911		448.1006		FBF	99.74		99.74
<Distichin>	C21 H20 O11	(M-H)-	13.911		448.1006		FBF	99.74		99.74
<8-C-Methylquercetin 3-xyloside>	C21 H20 O11	(M-H)-	13.911		448.1006		FBF	99.74		99.74
<Hypolaetin 8-rhamnoside>	C21 H20 O11	(M-H)-	13.911		448.1006		FBF	99.74		99.74
<Naringenin 5-O-glucuronide>	C21 H20 O11	(M-H)-	13.911		448.1006		FBF	99.74		99.74
<Naringenin-4'-O-glucuronide>	C21 H20 O11	(M-H)-	13.911		448.1006		FBF	99.74		99.74
<Naringenin-4'-O-β-D-Glucuronide>	C21 H20 O11	(M-H)-	13.911		448.1006	158196-35-1	FBF	99.74		99.74
<Naringenin-7-O-glucuronide>	C21 H20 O11	(M-H)-	13.911		448.1006		FBF	99.74		99.74
<Naringenin-7-O-β-D-Glucuronide>	C21 H20 O11	(M-H)-	13.911		448.1006		FBF	99.74		99.74
<Orientin>	C21 H20 O11	(M-H)-	13.911		448.1006		FBF	99.74		99.74
<Orobol 7-O-glucoside>	C21 H20 O11	(M-H)-	13.911		448.1006		FBF	99.74		99.74
<Quercetin 3-methyl ether 3'-xyloside>	C21 H20 O11	(M-H)-	13.911		448.1006		FBF	99.74		99.74
<Naringenin 4'-O-glucuronide>	C21 H20 O11	(M-H)-	13.911		448.1006		FBF	99.74		99.74
<Quercetin 7-rhamnoside>	C21 H20 O11	(M-H)-	13.911		448.1006		FBF	99.74		99.74
<Quercitrin>	C21 H20 O11	(M-H)-	13.911		448.1006	522-12-3	FBF	99.74		99.74
<Rhamnetin 3-alpha-L-arabinofuranoside>	C21 H20 O11	(M-H)-	13.911		448.1006		FBF	99.74		99.74
<Scutellarein 6-glucoside>	C21 H20 O11	(M-H)-	13.911		448.1006		FBF	99.74		99.74
<Scutellarein 7-glucoside>	C21 H20 O11	(M-H)-	13.911		448.1006		FBF	99.74		99.74
<Trifolin>	C21 H20 O11	(M-H)-	13.911		448.1006		FBF	99.74		99.74
<Rhamnetin 3-alpha-L-arabinopyranoside>	C21 H20 O11	(M-H)-	13.911		448.1006		FBF	99.74		99.74
<Luteolin 7-galactoside>	C21 H20 O11	(M-H)-	13.911		448.1006		FBF	99.74		99.74
<Luteolin 5-glucoside>	C21 H20 O11	(M-H)-	13.911		448.1006		FBF	99.74		99.74
<Luteolin 4'-O-glucoside>	C21 H20 O11	(M-H)-	13.911		448.1006	6920-38-3	FBF	99.74		99.74
<Luteolin 3'-glucoside>	C21 H20 O11	(M-H)-	13.911		448.1006		FBF	99.74		99.74
<Luteolin 7-glucoside>	C21 H20 O11	(M-H)-	13.911		448.1006		FBF	99.74		99.74
<Kaempferol 7-galactoside>	C21 H20 O11	(M-H)-	13.911		448.1006		FBF	99.74		99.74
<Isorhamnetin 3-xyloside>	C21 H20 O11	(M-H)-	13.911		448.1006		FBF	99.74		99.74
<Kaempferol 5-glucoside>	C21 H20 O11	(M-H)-	13.911		448.1006		FBF	99.74		99.74
<Kaempferol 3-O-β-D-galactoside>	C21 H20 O11	(M-H)-	13.911		448.1006		FBF	99.74		99.74
<Kaempferol 3-alpha-D-glucoside>	C21 H20 O11	(M-H)-	13.911		448.1006		FBF	99.74		99.74
<Isoscutellarein 7-glucoside>	C21 H20 O11	(M-H)-	13.911		448.1006		FBF	99.74		99.74
<Maritimein>	C21 H20 O11	(M-H)-	13.911		448.1006		FBF	99.74		99.74
<Kaempferol 7-alloside>	C21 H20 O11	(M-H)-	13.911		448.1006	765949-92-6	FBF	99.74		99.74
<Maritimetin 7-glucoside>	C21 H20 O11	(M-H)-	13.911		448.1006		FBF	99.74		99.74
<Kaempferol 4'-glucoside>	C21 H20 O11	(M-H)-	13.911		448.1006		FBF	99.74		99.74
<Kaempferol 7-O-glucoside>	C21 H20 O11	(M-H)-	13.911		448.1006	16290-07-6	FBF	99.74		99.74

Cpd 248: Proanthocyanidin A1

Name	Formula	RT	RI	Mass	Diff (Tgt, ppm)	CAS	ID Source	Score	Algorithm
Proanthocyanidin A1	C30 H24 O12	14.452		576.1264	-0.64	103883-03-0	M-FBF	93.88	FBF

Species	m/z	Score (Tgt)	Score (Lib)	Score (DB)	Score (MFG)	Score (RT)
(M-2H)-2 (M-H)-	287 575	93.88				

Compound Chromatograms (overlaid)

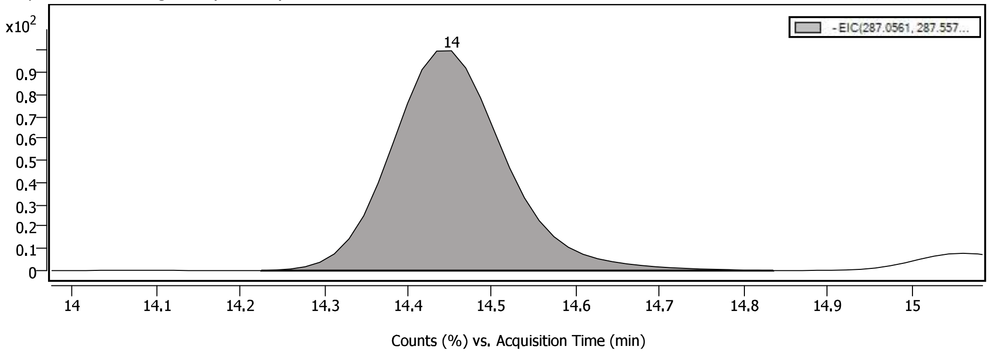

Structure

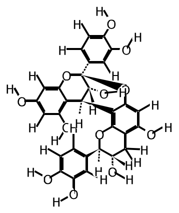

Compound Spectra (overlaid)

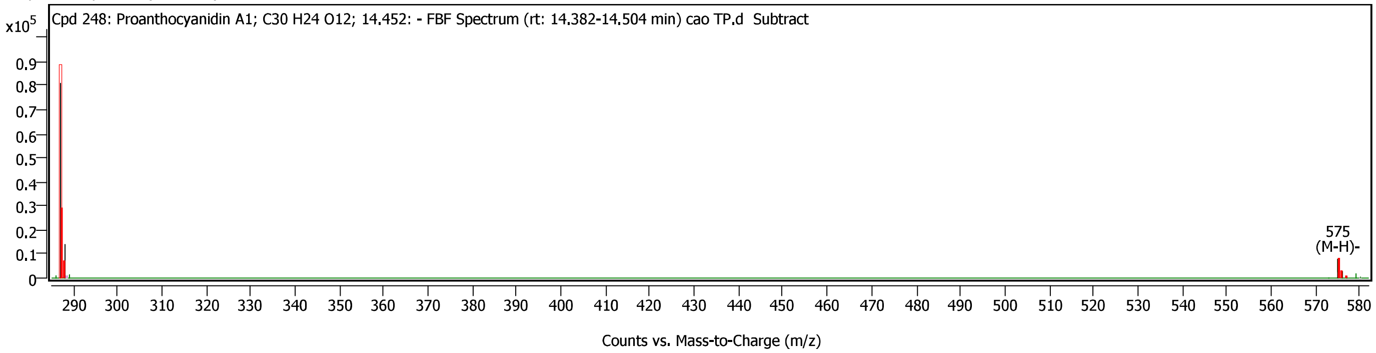

Compound ID Table

Name	Formula	Species	RT	RT Diff	Mass	CAS	ID Source	Score	Score (Lib)	Score (Tgt)
Proanthocyanidin A1	C30 H24 O12	(M-2H)-2 (M-H)-	14.452		576.1264	103883-03-0	FBF	93.88		93.88
Proanthocyanidin A5'	C30 H24 O12	(M-2H)-2 (M-H)-	14.452		576.1264	111466-30-9	FBF	93.88		93.88
Proanthocyanidin A2	C30 H24 O12	(M-2H)-2 (M-H)-	14.452		576.1264	41743-41-3	FBF	93.88		93.88
Pavetannin A2	C30 H24 O12	(M-2H)-2 (M-H)-	14.452		576.1264	130853-74-6	FBF	93.88		93.88
Epicatechin-(2beta->7,4beta->6)-catechin	C30 H24 O12	(M-2H)-2 (M-H)-	14.452		576.1264		FBF	93.88		93.88
Epicatechin-(2beta->5,4beta->6)-ent-epicatechin	C30 H24 O12	(M-2H)-2 (M-H)-	14.452		576.1264	135095-75-9	FBF	93.88		93.88

Cpd 87: Indole-3-carboxylic acid

Name	Formula	RT	RI	Mass	Diff (Tgt, ppm)	CAS	ID Source	Score	Algorithm
Indole-3-carboxylic acid	C9 H7 N O2	14.539		161.0476	-0.73	771-50-6	M-FBF-FragConfirm	99.43	FBF

Species	m/z	Score (Tgt)	Score (Lib)	Score (DB)	Score (MFG)	Score (RT)
(M-H)-	160	99.43				

Compound Chromatograms (overlaid)

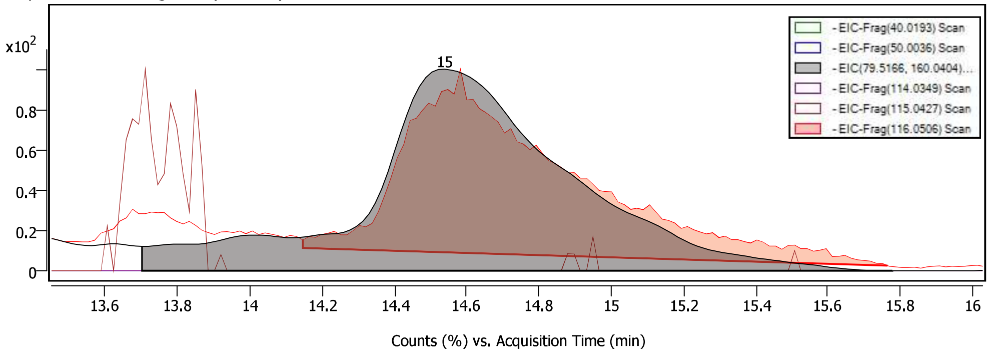

Structure

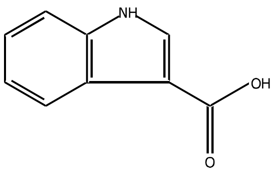

Coelution Plot

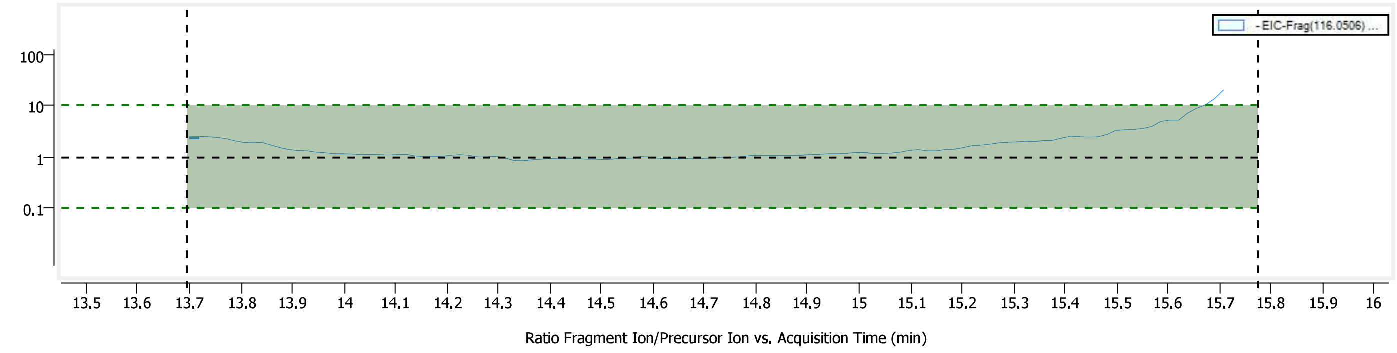

Compound Spectra (overlaid)

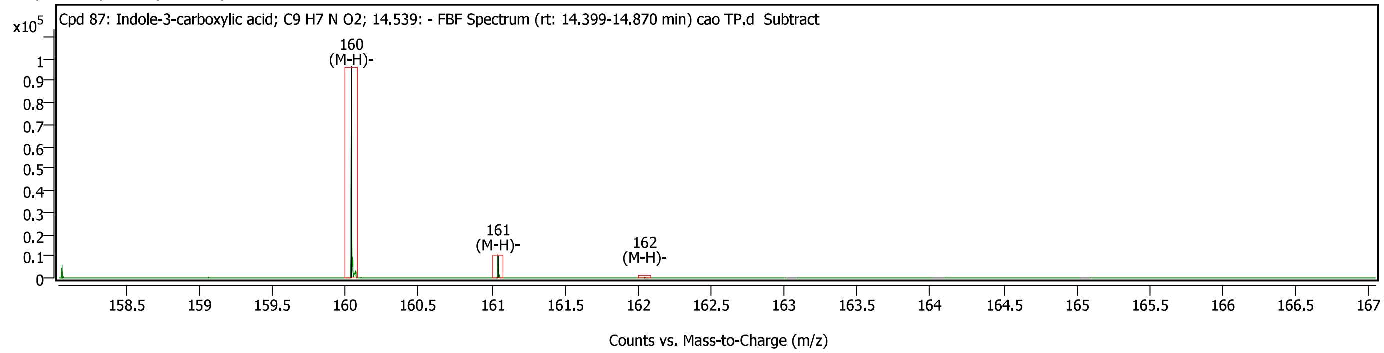

Fragment Spectrum (clean)

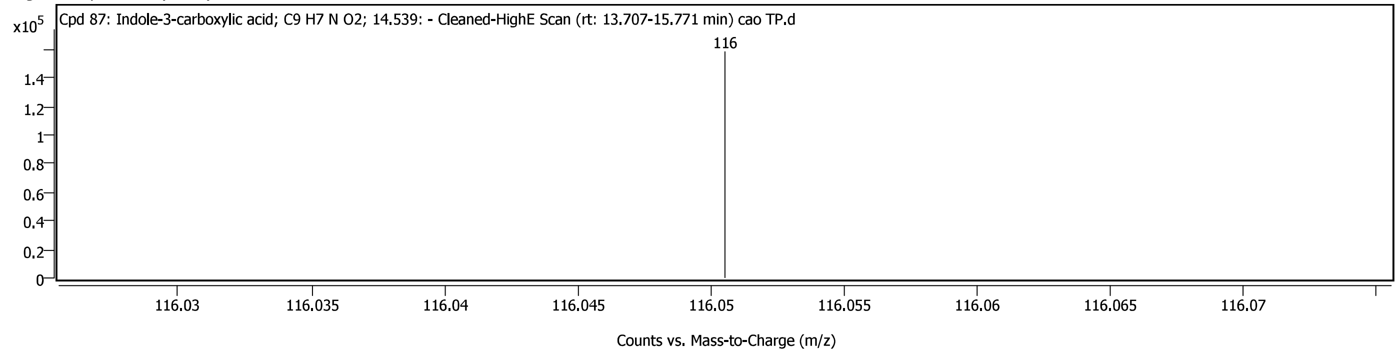

Fragment Spectrum (raw)

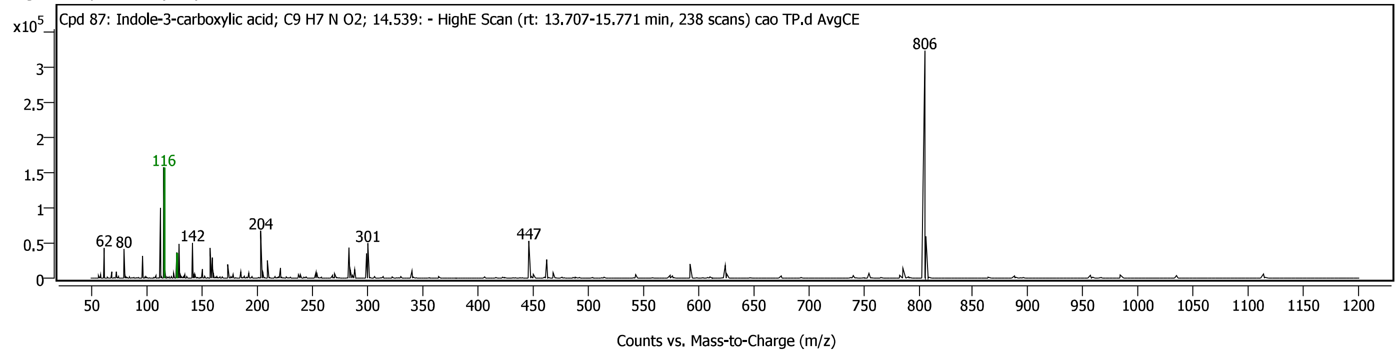

Compound ID Table

Name	Formula	Species	RT	RT Diff	Mass	CAS	ID Source	Score	Score (Lib)	Score (Tgt)
Indole-3-carboxylic acid	C9 H7 N O2	(M-H)-	14.539		161.0476	771-50-6	FBF-FragConfirm	99.43		99.43
3-Hydroxy-1H-quinolin-4-one	C9 H7 N O2	(M-H)-	14.539		161.0476		FBF-FragConfirm	99.43		99.43
2-4-Quinolinediol	C9 H7 N O2	(M-H)-	14.539		161.0476	86-95-3	FBF-FragConfirm	99.43		99.43
2-Indolecarboxylic acid	C9 H7 N O2	(M-H)-	14.539		161.0476	1477-50-5	FBF-FragConfirm	99.43		99.43
3-Formyl-6-hydroxyindole	C9 H7 N O2	(M-H)-	14.539		161.0476	192184-71-7	FBF-FragConfirm	99.43		99.43
4,8-Dihydroxyquinoline	C9 H7 N O2	(M-H)-	14.539		161.0476		FBF-FragConfirm	99.43		99.43
4,6-Dihydroxyquinoline	C9 H7 N O2	(M-H)-	14.539		161.0476	3517-61-1	FBF-FragConfirm	99.43		99.43
Quinoline-3,4-diol	C9 H7 N O2	(M-H)-	14.539		161.0476		FBF-FragConfirm	99.43		99.43
Quinolin-2,8-diol	C9 H7 N O2	(M-H)-	14.539		161.0476		FBF-FragConfirm	99.43		99.43

Cpd 2: Foramsulfuron

Name	Formula	RT	RI	Mass	Diff (Tgt, ppm)	CAS	ID Source	Score	Algorithm
Foramsulfuron	C17 H20 N6 O7 S	14.957		452.1111	-0.71	173159-57-4	FBF	84.62	FBF

Species	m/z	Score (Tgt)	Score (Lib)	Score (DB)	Score (MFG)	Score (RT)
(M-H)-	451	84.62				

Compound Chromatograms (overlaid)

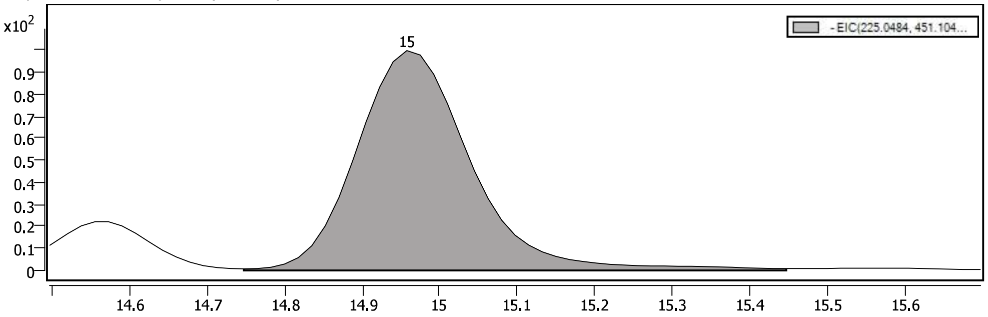

Structure

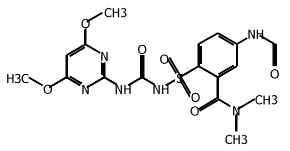

Compound Spectra (overlaid)

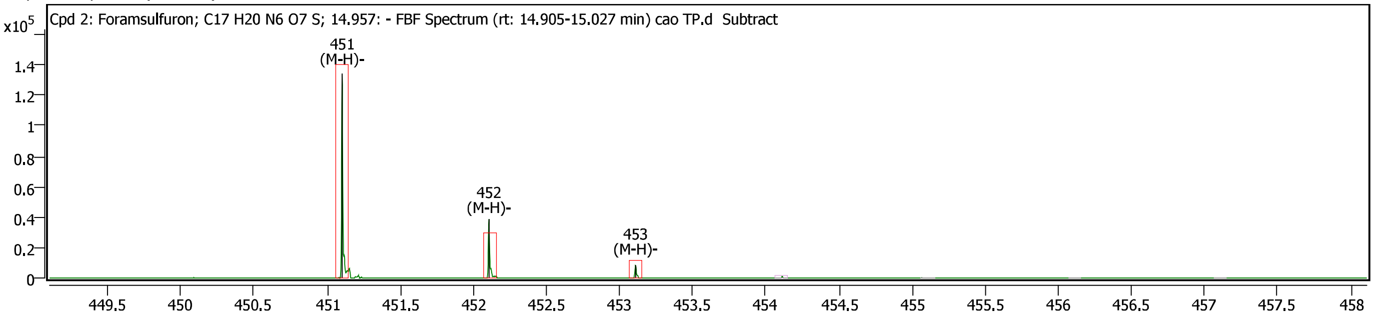

Compound ID Table

Name	Formula	Species	RT	RT Diff	Mass	CAS	ID Source	Score	Score (Lib)	Score (Tgt)
Foramsulfuron	C17 H20 N6 O7 S	(M-H)-	14.957		452.1111	173159-57-4	FBF	84.62		84.62

Cpd 137: Camelliaside A

Name	Formula	RT	RI	Mass	Diff (Tgt, ppm)	CAS	ID Source	Score	Algorithm
Camelliaside A	C33 H40 O20	15.027		756.2112	-0.14		M-FBF	99.00	FBF

Species	m/z	Score (Tgt)	Score (Lib)	Score (DB)	Score (MFG)	Score (RT)
(M-H)-	755	99.00				

Compound Chromatograms (overlaid)

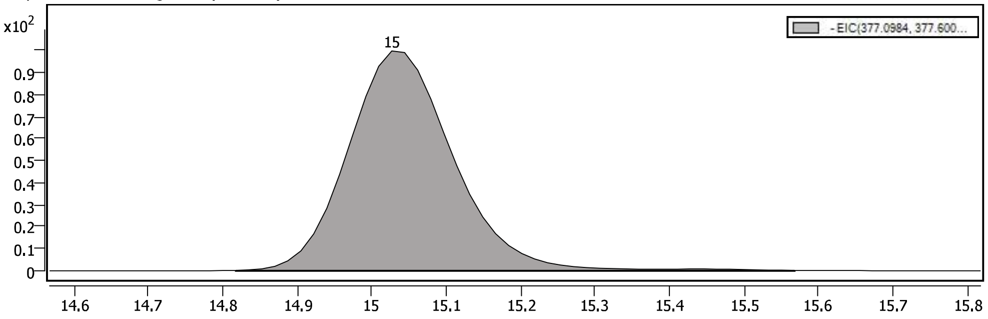

Structure

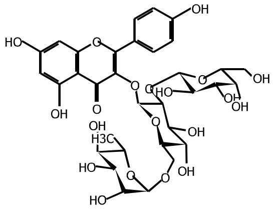

Compound Spectra (overlaid)

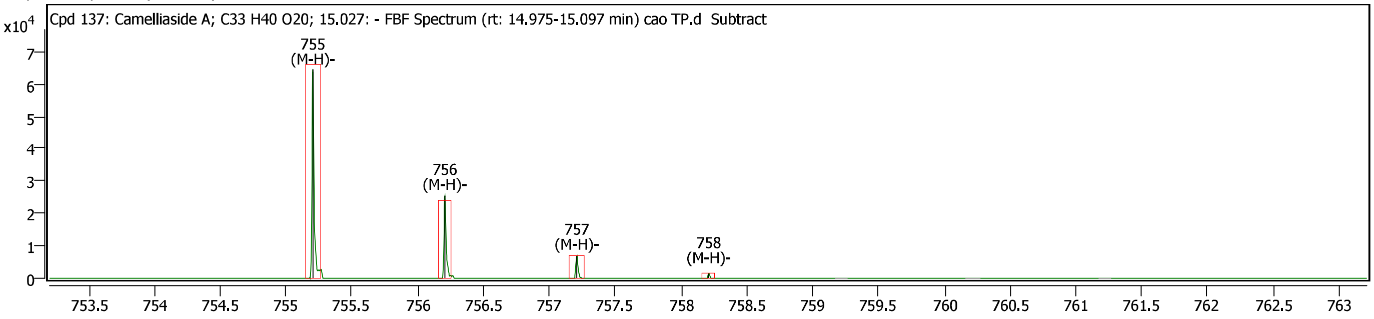

Compound ID Table

Name	Formula	Species	RT	RT Diff	Mass	CAS	ID Source	Score	Score (Lib)	Score (Tgt)
Camelliaside A	C33 H40 O20	(M-H)-	15.027		756.2112		FBF	99.00		99.00
Kaempferol 3-gentiobioside-7-rhamnoside	C33 H40 O20	(M-H)-	15.027		756.2112		FBF	99.00		99.00
Kaempferol 3-[glucosyl-(1->3)-rhamnosyl-(1->6)-galactoside]	C33 H40 O20	(M-H)-	15.027		756.2112	136449-09-7	FBF	99.00		99.00
Kaempferol 3-gentiobioside 7-rhamnoside	C33 H40 O20	(M-H)-	15.027		756.2112	173740-43-7	FBF	99.00		99.00
Kaempferol 3-(2G-rhamnosylgentiobioside)	C33 H40 O20	(M-H)-	15.027		756.2112		FBF	99.00		99.00
Isovitexin 7-O-galactoside-2''-O-glucoside	C33 H40 O20	(M-H)-	15.027		756.2112		FBF	99.00		99.00
Kaempferol 3-(3G-glucosylneohesperidoside)	C33 H40 O20	(M-H)-	15.027		756.2112		FBF	99.00		99.00
Kaempferol 3-(3Rha-glucosylrutinoside)	C33 H40 O20	(M-H)-	15.027		756.2112		FBF	99.00		99.00
Kaempferol 3-(6''-rhamnosylsophoroside)	C33 H40 O20	(M-H)-	15.027		756.2112	55696-58-7	FBF	99.00		99.00
Kaempferol 3-glucosyl-(1->4)-rhamnosyl-(1->2)-glucoside	C33 H40 O20	(M-H)-	15.027		756.2112		FBF	99.00		99.00
Kaempferol 3-rhamnosyl-(1->2)-[glucosyl-(1->4)-glucoside]	C33 H40 O20	(M-H)-	15.027		756.2112		FBF	99.00		99.00
Kaempferol 3-neohesperidoside-7-glucoside	C33 H40 O20	(M-H)-	15.027		756.2112		FBF	99.00		99.00
Kaempferol 3-glucosyl-(1->2)-[glucosyl-(1->3)-rhamnoside]	C33 H40 O20	(M-H)-	15.027		756.2112		FBF	99.00		99.00
Kaempferol 3-glucosyl-(1->2)[rhamnosyl-(1->6)-galactoside]	C33 H40 O20	(M-H)-	15.027		756.2112		FBF	99.00		99.00
Kaempferol 3-glucosyl-(1->2)-rhamnoside-7-glucoside	C33 H40 O20	(M-H)-	15.027		756.2112		FBF	99.00		99.00
Kaempferol 3-glucosyl-(1->3)-rhamnosyl-(1->6)-galactoside	C33 H40 O20	(M-H)-	15.027		756.2112		FBF	99.00		99.00
Kaempferol 3-laminaribioside-7-rhamnoside	C33 H40 O20	(M-H)-	15.027		756.2112		FBF	99.00		99.00
Kaempferol 3-(2G-glucosylrutinoside)	C33 H40 O20	(M-H)-	15.027		756.2112		FBF	99.00		99.00
Isovitexin 7,2''-di-O-glucoside	C33 H40 O20	(M-H)-	15.027		756.2112		FBF	99.00		99.00
Isovitexin 4',7-diglucoside	C33 H40 O20	(M-H)-	15.027		756.2112		FBF	99.00		99.00
Cynarotrioside	C33 H40 O20	(M-H)-	15.027		756.2112	20056-21-7	FBF	99.00		99.00
Marginatoside	C33 H40 O20	(M-H)-	15.027		756.2112		FBF	99.00		99.00
Isovitexin 7,2''-Di-O-galactoside	C33 H40 O20	(M-H)-	15.027		756.2112		FBF	99.00		99.00
3-[6-[[(2R,3R,4R,5S,6S)-3,5-Dihydroxy-6-methyl-4-[(2S,3R,4R,5R,6S)-3,4,5-trihydroxy-6-methyloxan-2-yl]oxyoxan-2-yl]oxymethyl]-3,4,5-trihydroxyoxan-2-yl]oxy-2-(3,4-dihydroxyphenyl)-5,7-dihydroxychromen-4-one	C33 H40 O20	(M-H)-	15.027		756.2112		FBF	99.00		99.00
Apigenin 7-cellobioside-4'-glucoside	C33 H40 O20	(M-H)-	15.027		756.2112		FBF	99.00		99.00
Apigenin 7-sophorotrioside	C33 H40 O20	(M-H)-	15.027		756.2112		FBF	99.00		99.00
Clovin	C33 H40 O20	(M-H)-	15.027		756.2112	81970-00-5	FBF	99.00		99.00
Isoorientin 3'-O-neohesperidoside	C33 H40 O20	(M-H)-	15.027		756.2112		FBF	99.00		99.00
Isovitexin 4',2''-di-O-glucoside	C33 H40 O20	(M-H)-	15.027		756.2112		FBF	99.00		99.00
Isorhamnetin 3-(2G-apiosylrutinoside)	C33 H40 O20	(M-H)-	15.027		756.2112		FBF	99.00		99.00
Isorhamnetin 3-apiosyl-(1->6)-glucoside-7-rhamnoside	C33 H40 O20	(M-H)-	15.027		756.2112		FBF	99.00		99.00
Isorhamnetin 3-O-[b-D-xylopyranosyl-(1->2)-[a-L-rhamnopyranosyl-(1->6)]-b-D-glucopyranoside]	C33 H40 O20	(M-H)-	15.027		756.2112		FBF	99.00		99.00
Isorhamnetin 3-xylosyl-(1->2)-glucoside-7-rhamnoside	C33 H40 O20	(M-H)-	15.027		756.2112		FBF	99.00		99.00
Isorhamnetin 3-xylosyl-(1->3)-rhamnosyl-(1->6)-glucoside	C33 H40 O20	(M-H)-	15.027		756.2112		FBF	99.00		99.00
Isorhamnetin 3-xylosylrhamnosyl-(1->6)-galactoside	C33 H40 O20	(M-H)-	15.027		756.2112		FBF	99.00		99.00
Kaempferol 3-neohesperidoside-4'-glucoside	C33 H40 O20	(M-H)-	15.027		756.2112		FBF	99.00		99.00
Kaempferol 3-rhamnoside-7,4'-digalactoside	C33 H40 O20	(M-H)-	15.027		756.2112		FBF	99.00		99.00
Vicenin-2,6''-O-glucoside	C33 H40 O20	(M-H)-	15.027		756.2112		FBF	99.00		99.00
Quercetin 3-rhamninoside	C33 H40 O20	(M-H)-	15.027		756.2112		FBF	99.00		99.00
Quercetin 3-rhamnosyl-(1->2)-rhamnosyl-(1->6)-glucoside	C33 H40 O20	(M-H)-	15.027		756.2112		FBF	99.00		99.00
Quercetin 3-(2Gal-rhamnosyl-robinobioside)	C33 H40 O20	(M-H)-	15.027		756.2112		FBF	99.00		99.00
Quercetin 3-glucosyl-(1->2)-rhamnoside-7-rhamnoside	C33 H40 O20	(M-H)-	15.027		756.2112		FBF	99.00		99.00
Quercetin 3-isorhamninoside	C33 H40 O20	(M-H)-	15.027		756.2112		FBF	99.00		99.00
Quercetin 3-neohesperidoside-7-rhamnoside	C33 H40 O20	(M-H)-	15.027		756.2112		FBF	99.00		99.00
Quercetin 3-O-[6''-O-rhamnosyl)glucoside] 7-O-rhamnoside	C33 H40 O20	(M-H)-	15.027		756.2112		FBF	99.00		99.00
Quercetin 3-rhamnosyl-(1->4)-rhamnoside-7-galactoside	C33 H40 O20	(M-H)-	15.027		756.2112		FBF	99.00		99.00
Kaempferol 3-rhamnosyl-(1->2)-glucosyl-(1->6)-galactoside	C33 H40 O20	(M-H)-	15.027		756.2112		FBF	99.00		99.00
Wyomin	C33 H40 O20	(M-H)-	15.027		756.2112		FBF	99.00		99.00
Quercetin 3-rhamnosyl-(1->4)-rhamnosyl-(1->6)-glucoside	C33 H40 O20	(M-H)-	15.027		756.2112		FBF	99.00		99.00
Quercetin 3-robinobioside-7-rhamnoside	C33 H40 O20	(M-H)-	15.027		756.2112		FBF	99.00		99.00
Quercetin 3-rutinoside-7-rhamnoside	C33 H40 O20	(M-H)-	15.027		756.2112		FBF	99.00		99.00
Retamatrioside	C33 H40 O20	(M-H)-	15.027		756.2112		FBF	99.00		99.00
Rhamnocitrin 3-apiosyl-(1->2)-glucoside-4'-glucoside	C33 H40 O20	(M-H)-	15.027		756.2112		FBF	99.00		99.00
Kaempferol 3-rhamnosyl-(1->2)-galactoside-7-glucoside	C33 H40 O20	(M-H)-	15.027		756.2112		FBF	99.00		99.00
Manghaslin	C33 H40 O20	(M-H)-	15.027		756.2112	55696-57-6	FBF	99.00		99.00
Luteolin 7-rutinoside-4'-glucoside	C33 H40 O20	(M-H)-	15.027		756.2112		FBF	99.00		99.00

Compound ID Table

Name	Formula	Species	RT	RT Diff	Mass	CAS	ID Source	Score	Score (Lib)	Score (Tgt)
Kaempferol 3-rutinoside-7-glucoside	C33 H40 O20	(M-H)-	15.027		756.2112		FBF	99.00		99.00
Kaempferol 3-sophoroside 7-rhamnoside	C33 H40 O20	(M-H)-	15.027		756.2112	93098-79-4	FBF	99.00		99.00
Polygonatiin	C33 H40 O20	(M-H)-	15.027		756.2112		FBF	99.00		99.00
Kaempferol 7-galactoside 3-rutinoside	C33 H40 O20	(M-H)-	15.027		756.2112	111137-46-3	FBF	99.00		99.00
Kaempferol 3-rhamnosyl-(1->6)-glucosyl-(1->6)-galactoside	C33 H40 O20	(M-H)-	15.027		756.2112		FBF	99.00		99.00
Kaempferol 3-robinobioside-7-glucoside	C33 H40 O20	(M-H)-	15.027		756.2112		FBF	99.00		99.00
Kaempferol 3-rutinoside-7-galactoside	C33 H40 O20	(M-H)-	15.027		756.2112		FBF	99.00		99.00
Kaempferol 3-sophoroside-7-rhamnoside	C33 H40 O20	(M-H)-	15.027		756.2112		FBF	99.00		99.00
Orientin 4'-O-glucoside-2''-O-rhamnoside	C33 H40 O20	(M-H)-	15.027		756.2112		FBF	99.00		99.00
Quercetin 3-[rhamnosyl-(1->2)-rhamnosyl-(1->6)-glucoside]	C33 H40 O20	(M-H)-	15.027		756.2112	32453-37-5	FBF	99.00		99.00
Kaempferol 3-rutinoside-4'-glucoside	C33 H40 O20	(M-H)-	15.027		756.2112	89439-58-7	FBF	99.00		99.00
Lucenin 2,7-O-rhamnoside	C33 H40 O20	(M-H)-	15.027		756.2112	29432-27-7	FBF	99.00		99.00
Luteolin 7-glucoside-4'-neohesperidoside	C33 H40 O20	(M-H)-	15.027		756.2112		FBF	99.00		99.00
Luteolin 7-neohesperidoside-4'-glucoside	C33 H40 O20	(M-H)-	15.027		756.2112		FBF	99.00		99.00
Luteolin 7-rutinoside-3'-glucoside	C33 H40 O20	(M-H)-	15.027		756.2112		FBF	99.00		99.00
Lucenin-2,7-O-rhamnoside	C33 H40 O20	(M-H)-	15.027		756.2112		FBF	99.00		99.00

Cpd 119: 6''-Caffeoylhyperin

Name	Formula	RT	RI	Mass	Diff (Tgt, ppm)	CAS	ID Source	Score	Algorithm
6''-Caffeoylhyperin	C30 H26 O15	15.550		626.1272	0.08	84575-22-4	M-FBF	99.74	FBF

Species	m/z	Score (Tgt)	Score (Lib)	Score (DB)	Score (MFG)	Score (RT)
(M-H)-	625	99.74				

Compound Chromatograms (overlaid)

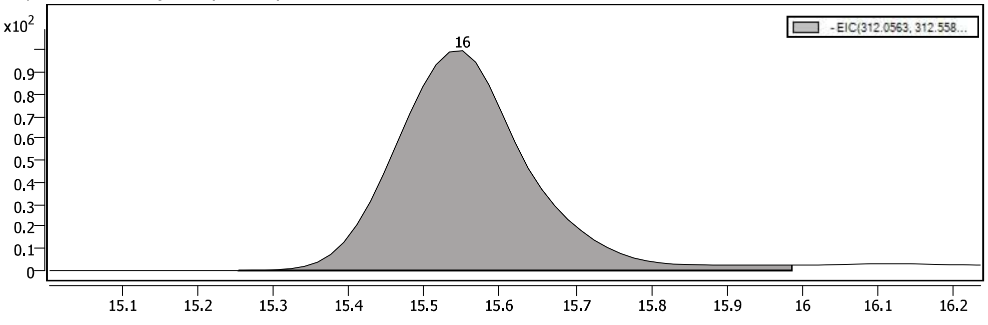

Structure

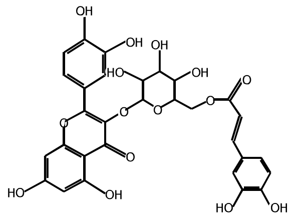

Compound Spectra (overlaid)

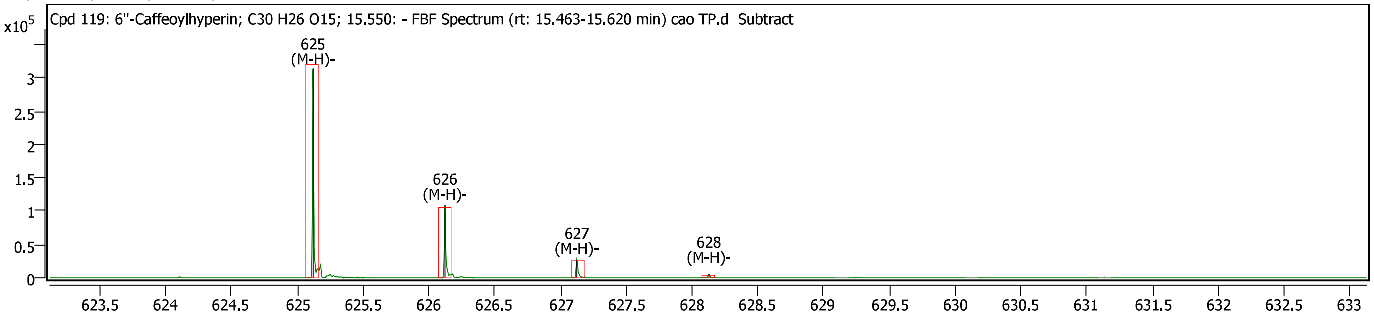

Compound ID Table

Name	Formula	Species	RT	RT Diff	Mass	CAS	ID Source	Score	Score (Lib)	Score (Tgt)
6''-Caffeoylhyperin	C30 H26 O15	(M-H)-	15.550		626.1272	84575-22-4	FBF	99.74		99.74
Myricetin 3'-O-(6''-p-coumaroyl)glucoside	C30 H26 O15	(M-H)-	15.550		626.1272		FBF	99.74		99.74
6-Hydroxykaempferol 7-(6''-(E)-caffeylglucoside)	C30 H26 O15	(M-H)-	15.550		626.1272		FBF	99.74		99.74
Myricetin 3-(6''-p-coumaroylglucoside)	C30 H26 O15	(M-H)-	15.550		626.1272		FBF	99.74		99.74
Nympholide A	C30 H26 O15	(M-H)-	15.550		626.1272		FBF	99.74		99.74
Nympholide B	C30 H26 O15	(M-H)-	15.550		626.1272		FBF	99.74		99.74
Quercetin 3-(6''-caffeylgalactoside)	C30 H26 O15	(M-H)-	15.550		626.1272		FBF	99.74		99.74
Spicoside A	C30 H26 O15	(M-H)-	15.550		626.1272		FBF	99.74		99.74

Cpd 239: Sennoside E

Name	Formula	RT	RI	Mass	Diff (Tgt, ppm)	CAS	ID Source	Score	Algorithm
Sennoside E	C44 H38 O23	15.742		934.1830	2.76	11137-63-6	M-FBF	94.26	FBF

Species	m/z	Score (Tgt)	Score (Lib)	Score (DB)	Score (MFG)	Score (RT)
(M-H)-	933	94.26				

Compound Screening Report

Compound Chromatograms (overlaid)

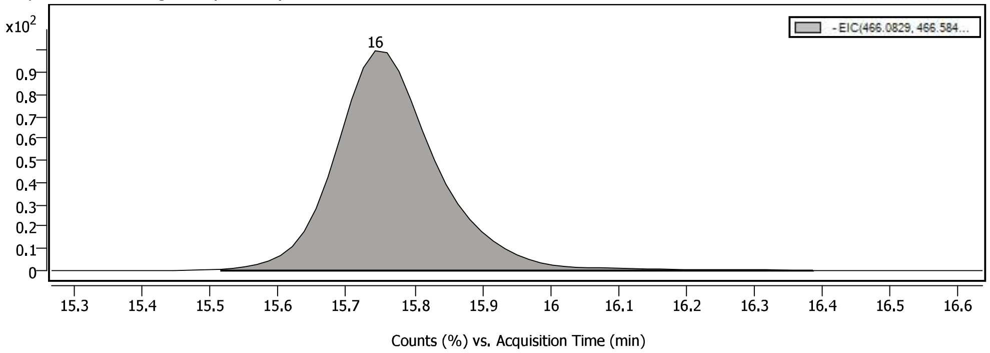

Structure

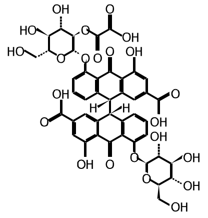

Compound Spectra (overlaid)

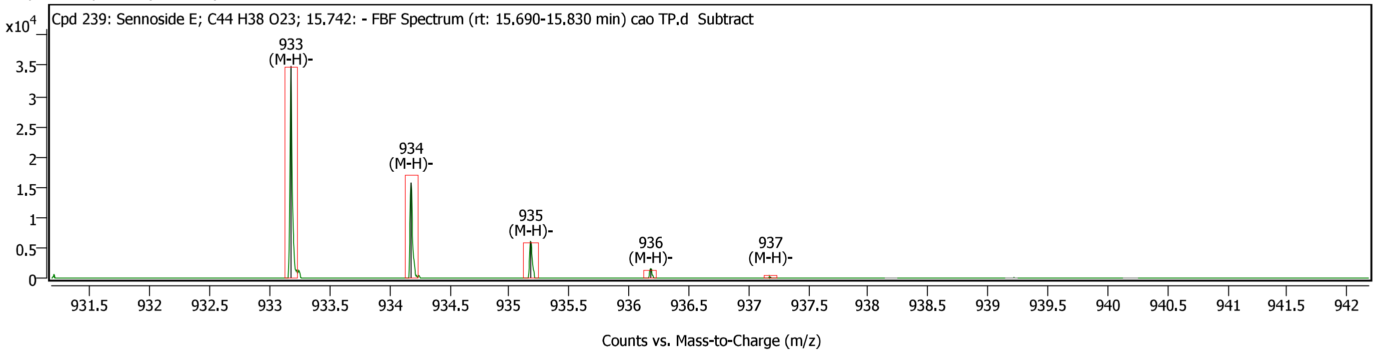

Compound ID Table

Name	Formula	Species	RT	RT Diff	Mass	CAS	ID Source	Score	Score (Lib)	Score (Tgt)
Sennoside E	C44 H38 O23	(M-H)-	15.742		934.1830	11137-63-6	FBF	94.26		94.26
Sennoside F	C44 H38 O23	(M-H)-	15.742		934.1830	52842-23-6	FBF	94.26		94.26

Cpd 204: Hyperoside

Name	Formula	RT	RI	Mass	Diff (Tgt, ppm)	CAS	ID Source	Score	Algorithm
Hyperoside	C21 H20 O12	15.847		464.0955	0.16	482-36-0	FBF-FragConfirm	99.88	FBF

Species	m/z	Score (Tgt)	Score (Lib)	Score (DB)	Score (MFG)	Score (RT)
(M-H)-	463	99.88				

Compound Chromatograms (overlaid)

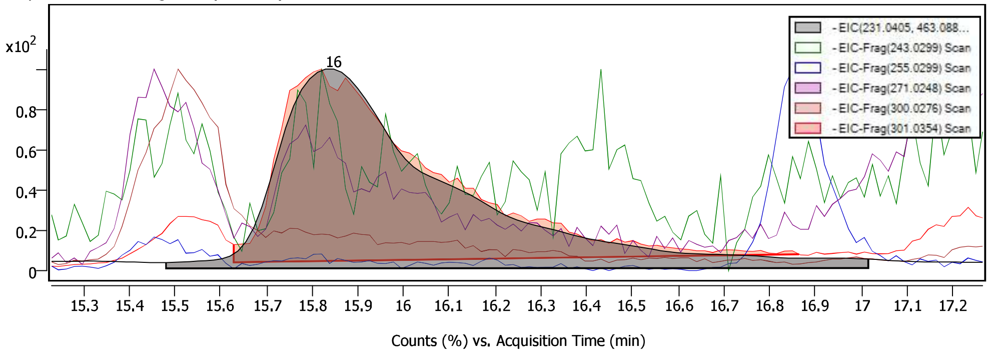

Structure

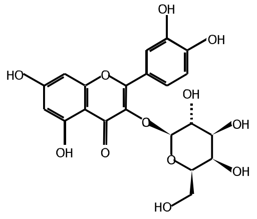

Coelution Plot

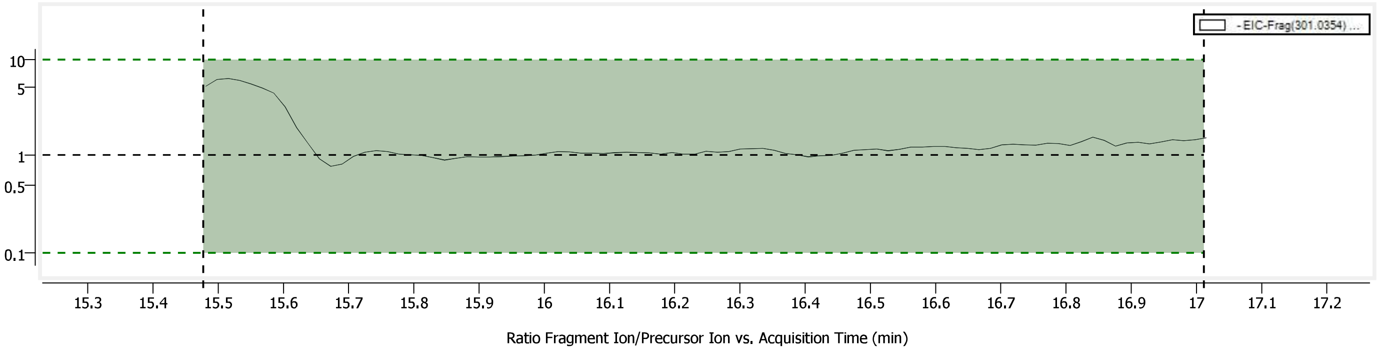

Compound Spectra (overlaid)

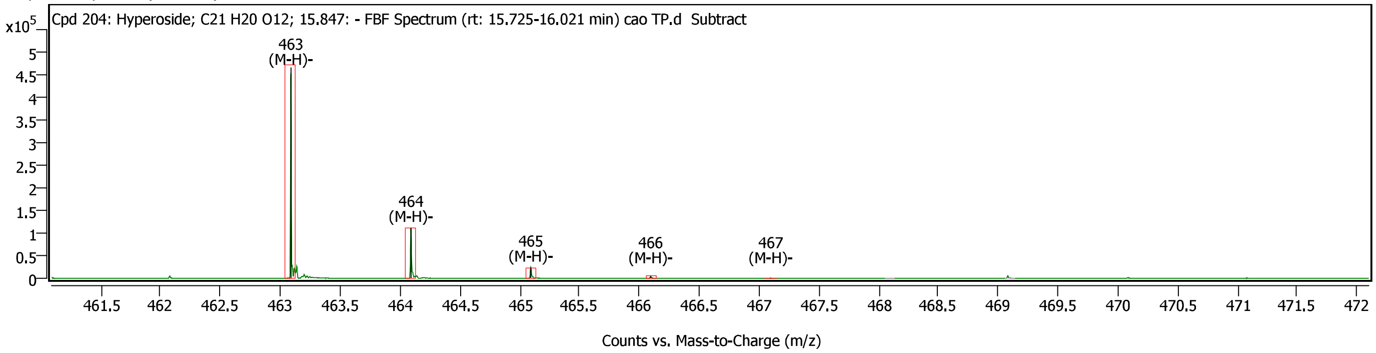

Fragment Spectrum (clean)

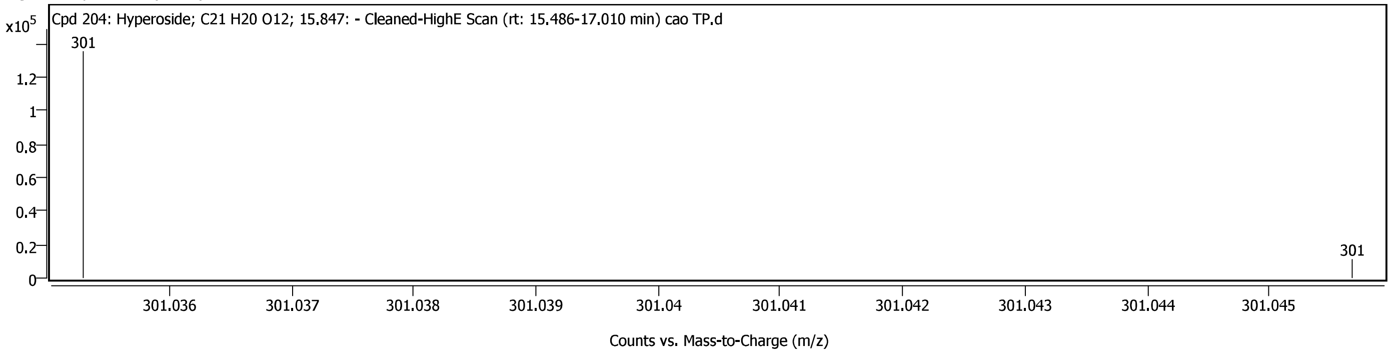

Fragment Spectrum (raw)

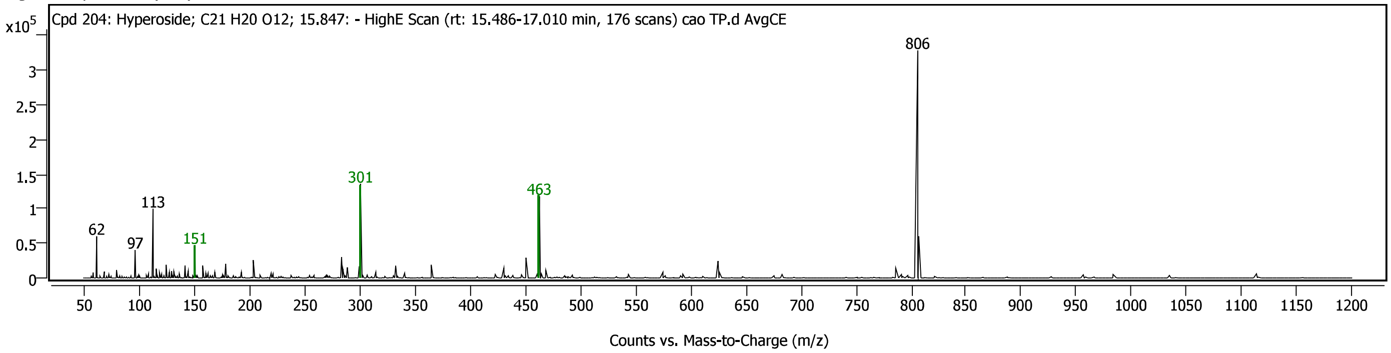

Compound ID Table

Name	Formula	Species	RT	RT Diff	Mass	CAS	ID Source	Score	Score (Lib)	Score (Tgt)
Hyperoside	C21 H20 O12	(M-H)-	15.847		464.0955	482-36-0	FBF-FragConfirm	99.88		99.88
Bracteatin 6-O-glucoside	C21 H20 O12	(M-H)-	15.847		464.0955		FBF-FragConfirm	99.88		99.88
Quercetagetin 3-rhamnoside	C21 H20 O12	(M-H)-	15.847		464.0955		FBF-FragConfirm	99.88		99.88
8-Hydroxyluteolin 8-glucoside	C21 H20 O12	(M-H)-	15.847		464.0955		FBF-FragConfirm	99.88		99.88
Annulatin 3'-xyloside	C21 H20 O12	(M-H)-	15.847		464.0955		FBF-FragConfirm	99.88		99.88
Bracteatin 6-glucoside	C21 H20 O12	(M-H)-	15.847		464.0955		FBF-FragConfirm	99.88		99.88
Gossypetin 7-rhamnoside	C21 H20 O12	(M-H)-	15.847		464.0955		FBF-FragConfirm	99.88		99.88
Bractein	C21 H20 O12	(M-H)-	15.847		464.0955		FBF-FragConfirm	99.88		99.88
Corniculatusin 3-alpha-L-arabinofuranoside	C21 H20 O12	(M-H)-	15.847		464.0955		FBF-FragConfirm	99.88		99.88
Eriodictyol 7-glucuronide	C21 H20 O12	(M-H)-	15.847		464.0955		FBF-FragConfirm	99.88		99.88
6-Hydroxyluteolin 7-glucoside	C21 H20 O12	(M-H)-	15.847		464.0955		FBF-FragConfirm	99.88		99.88
Gossypetin 8-rhamnoside	C21 H20 O12	(M-H)-	15.847		464.0955		FBF-FragConfirm	99.88		99.88
6-Hydroxytricetin 5-rhamnoside	C21 H20 O12	(M-H)-	15.847		464.0955		FBF-FragConfirm	99.88		99.88
6-Hydroxykaempferol 3-glucoside	C21 H20 O12	(M-H)-	15.847		464.0955		FBF-FragConfirm	99.88		99.88
6-Hydroxyluteolin 7-galactoside	C21 H20 O12	(M-H)-	15.847		464.0955		FBF-FragConfirm	99.88		99.88
6-Hydroxyluteolin 6-glucoside	C21 H20 O12	(M-H)-	15.847		464.0955		FBF-FragConfirm	99.88		99.88
6-Hydroxyluteolin 5-glucoside	C21 H20 O12	(M-H)-	15.847		464.0955		FBF-FragConfirm	99.88		99.88
6-Hydroxykaempferol 7-glucoside	C21 H20 O12	(M-H)-	15.847		464.0955		FBF-FragConfirm	99.88		99.88
6-C-Glucosylquercetin	C21 H20 O12	(M-H)-	15.847		464.0955		FBF-FragConfirm	99.88		99.88
6-C-beta-D-Glucopyranosyl-5,7,2',4',5'-pentahydroxyflavone	C21 H20 O12	(M-H)-	15.847		464.0955		FBF-FragConfirm	99.88		99.88
5,7,3',4',5'-Pentahydroxyflavone 8-C-glucopyranoside	C21 H20 O12	(M-H)-	15.847		464.0955		FBF-FragConfirm	99.88		99.88
5,6,7,3',4'-Pentahydroxy-8-methoxyflavone 7-apioside	C21 H20 O12	(M-H)-	15.847		464.0955		FBF-FragConfirm	99.88		99.88
3,5,7,2',6'-Pentahydroxyflavone 2'-glucoside	C21 H20 O12	(M-H)-	15.847		464.0955		FBF-FragConfirm	99.88		99.88
2'-Hydroxyisoorientin	C21 H20 O12	(M-H)-	15.847		464.0955		FBF-FragConfirm	99.88		99.88
(2S)-5,7,3',4'-Tetrahydroxyflavanone 7-glucuronide	C21 H20 O12	(M-H)-	15.847		464.0955		FBF-FragConfirm	99.88		99.88
Herbacetin 3-beta-D-glucofuranoside	C21 H20 O12	(M-H)-	15.847		464.0955		FBF-FragConfirm	99.88		99.88
8-Hydroxyluteolin 7-glucoside	C21 H20 O12	(M-H)-	15.847		464.0955		FBF-FragConfirm	99.88		99.88
Herbacetin 3-glucoside	C21 H20 O12	(M-H)-	15.847		464.0955		FBF-FragConfirm	99.88		99.88
Tricetin 7-glucoside	C21 H20 O12	(M-H)-	15.847		464.0955		FBF-FragConfirm	99.88		99.88
Quercetin 3-alloside	C21 H20 O12	(M-H)-	15.847		464.0955		FBF-FragConfirm	99.88		99.88
Herbacetin 7-glucoside	C21 H20 O12	(M-H)-	15.847		464.0955		FBF-FragConfirm	99.88		99.88
Tricetin 3'-glucoside	C21 H20 O12	(M-H)-	15.847		464.0955		FBF-FragConfirm	99.88		99.88
Spiraeoside	C21 H20 O12	(M-H)-	15.847		464.0955		FBF-FragConfirm	99.88		99.88
Robinetin 7-glucoside	C21 H20 O12	(M-H)-	15.847		464.0955		FBF-FragConfirm	99.88		99.88
Quercimeritrin	C21 H20 O12	(M-H)-	15.847		464.0955		FBF-FragConfirm	99.88		99.88
Quercetin 5-glucoside	C21 H20 O12	(M-H)-	15.847		464.0955		FBF-FragConfirm	99.88		99.88
Quercetin 4'-glucoside	C21 H20 O12	(M-H)-	15.847		464.0955	20229-56-5	FBF-FragConfirm	99.88		99.88
Quercetin 3-β-D-glucoside	C21 H20 O12	(M-H)-	15.847		464.0955	482-35-9	FBF-FragConfirm	99.88		99.88
Quercetin 3-O-glucoside	C21 H20 O12	(M-H)-	15.847		464.0955	21637-25-2	FBF-FragConfirm	99.88		99.88
Quercetin 3'-glucoside	C21 H20 O12	(M-H)-	15.847		464.0955		FBF-FragConfirm	99.88		99.88
Quercetin 7-galactoside	C21 H20 O12	(M-H)-	15.847		464.0955		FBF-FragConfirm	99.88		99.88
Myricetin 3'-rhamnoside	C21 H20 O12	(M-H)-	15.847		464.0955		FBF-FragConfirm	99.88		99.88
Patuletin 3-xyloside	C21 H20 O12	(M-H)-	15.847		464.0955		FBF-FragConfirm	99.88		99.88
Isoetin 5'-glucoside	C21 H20 O12	(M-H)-	15.847		464.0955		FBF-FragConfirm	99.88		99.88
Myricitrin	C21 H20 O12	(M-H)-	15.847		464.0955	17912-87-7	FBF-FragConfirm	99.88		99.88
Herbacetin 8-glucoside	C21 H20 O12	(M-H)-	15.847		464.0955		FBF-FragConfirm	99.88		99.88
Hyperin	C21 H20 O12	(M-H)-	15.847		464.0955		FBF-FragConfirm	99.88		99.88
Isoaffinetin	C21 H20 O12	(M-H)-	15.847		464.0955		FBF-FragConfirm	99.88		99.88
Isoetin 7-glucoside	C21 H20 O12	(M-H)-	15.847		464.0955		FBF-FragConfirm	99.88		99.88
Isoquercitrin	C21 H20 O12	(M-H)-	15.847		464.0955		FBF-FragConfirm	99.88		99.88
Larycitrin 3-alpha-L-arabinofuranoside	C21 H20 O12	(M-H)-	15.847		464.0955		FBF-FragConfirm	99.88		99.88
Herbacetin 4'-glucoside	C21 H20 O12	(M-H)-	15.847		464.0955		FBF-FragConfirm	99.88		99.88
Myricetin 7-rhamnoside	C21 H20 O12	(M-H)-	15.847		464.0955	184533-14-0	FBF-FragConfirm	99.88		99.88

Cpd 23: Apigenin 4'-[feruloyl-(->2)-glucuronyl-(1->2)-glucuronide]

Name	Formula	RT	RI	Mass	Diff (Tgt, ppm)	CAS	ID Source	Score	Algorithm
Apigenin 4'-[feruloyl-(->2)-glucuronyl-(1->2)-glucuronide]	C37 H34 O20	15.934		798.1640	-0.49		M-FBF	99.64	FBF

Species	m/z	Score (Tgt)	Score (Lib)	Score (DB)	Score (MFG)	Score (RT)
(M-H)-	797	99.64				

Compound Chromatograms (overlaid)

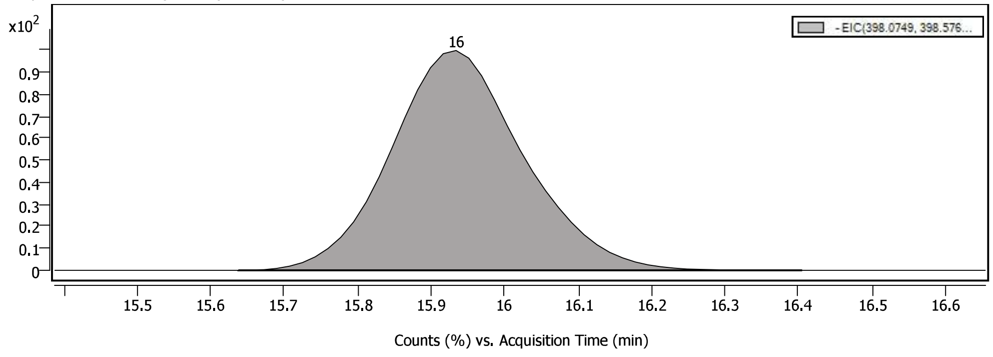

Structure

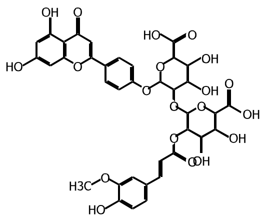

Compound Spectra (overlaid)

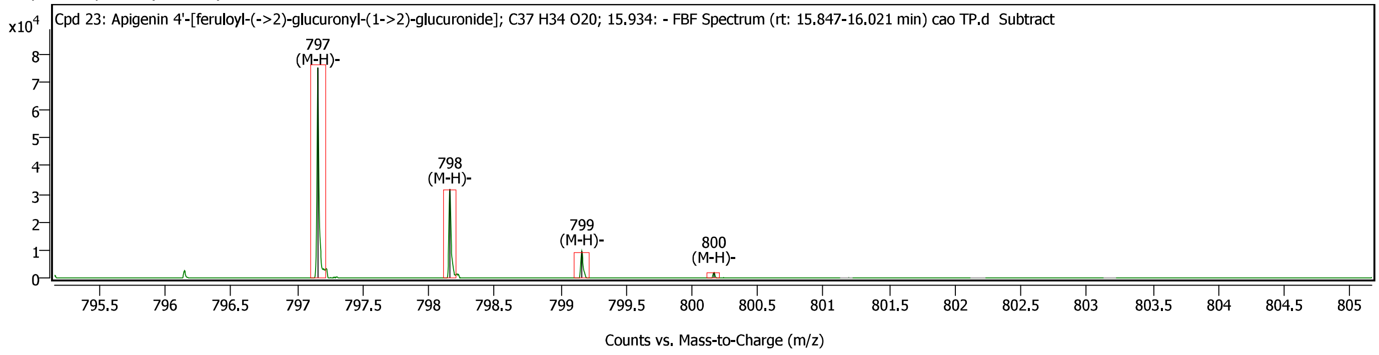

Compound ID Table

Name	Formula	Species	RT	RT Diff	Mass	CAS	ID Source	Score	Score (Lib)	Score (Tgt)
Apigenin 4'-[feruloyl-(->2)-glucuronyl-(1->2)-glucuronide]	C37 H34 O20	(M-H)-	15.934		798.1640		FBF	99.64		99.64
Apigenin 4'-(2''-feruloylglucuronosyl)-(1->2)-glucuronide	C37 H34 O20	(M-H)-	15.934		798.1640		FBF	99.64		99.64

Cpd 88: (2S,3S)-3,5,7-trihydroxy-6-methyl-2-(3,4,5-trihydroxyphenyl)-2,3-dihydrochromen-4-one

Name	Formula	RT	RI	Mass	Diff (Tgt, ppm)	CAS	ID Source	Score	Algorithm
(2S,3S)-3,5,7-trihydroxy-6-methyl-2-(3,4,5-trihydroxyphenyl)-2,3-dihydrochromen-4-one	C16 H14 O8	15.969		334.0687	-0.53	1212351-36-4	M-FBF	99.77	FBF

Species	m/z	Score (Tgt)	Score (Lib)	Score (DB)	Score (MFG)	Score (RT)
(M-H)-	333	99.77				

Compound Chromatograms (overlaid)

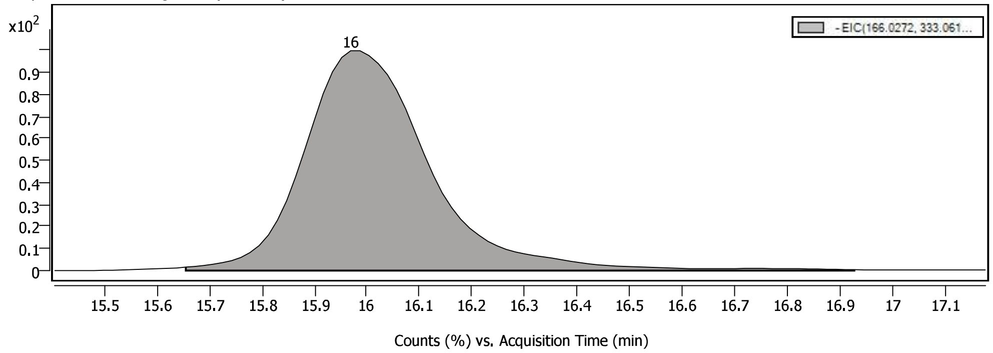

Structure

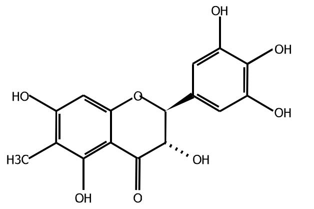

Compound Spectra (overlaid)

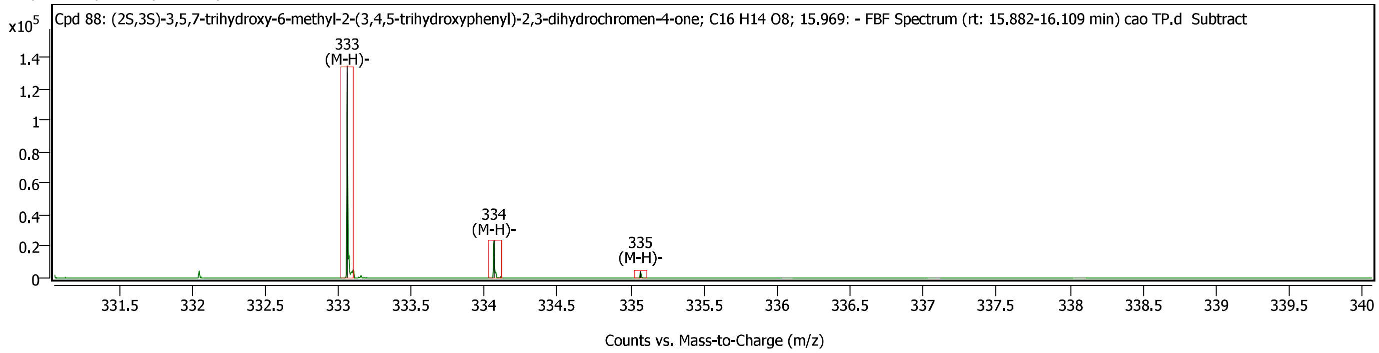

Compound ID Table

Name	Formula	Species	RT	RT Diff	Mass	CAS	ID Source	Score	Score (Lib)	Score (Tgt)
(2S,3S)-3,5,7-trihydroxy-6-methyl-2-(3,4,5-trihydroxyphenyl)-2,3-dihydrochromen-4-one	C16 H14 O8	(M-H)-	15.969		334.0687	1212351-36-4	FBF	99.77		99.77
6-Methoxytaxifolin	C16 H14 O8	(M-H)-	15.969		334.0687	31076-39-8	FBF	99.77		99.77
5,5'-Dehydrodivanillate	C16 H14 O8	(M-H)-	15.969		334.0687	2134-90-9	FBF	99.77		99.77
Amaronol B	C16 H14 O8	(M-H)-	15.969		334.0687		FBF	99.77		99.77
Hovenitin I	C16 H14 O8	(M-H)-	15.969		334.0687	71106-82-6	FBF	99.77		99.77
Xanthotoxol arabinoside	C16 H14 O8	(M-H)-	15.969		334.0687	160845-06-7	FBF	99.77		99.77

Cpd 141: <Isovitexin>

Name	Formula	RT	RI	Mass	Diff (Tgt, ppm)	CAS	ID Source	Score	Algorithm
<Isovitexin>	C21 H20 O10	16.876		432.1055	-0.26	38953-85-4	M-FBF	99.46	FBF

Species	m/z	Score (Tgt)	Score (Lib)	Score (DB)	Score (MFG)	Score (RT)
(M-H)-	431	99.46				

Compound Screening Report

Compound Chromatograms (overlaid)

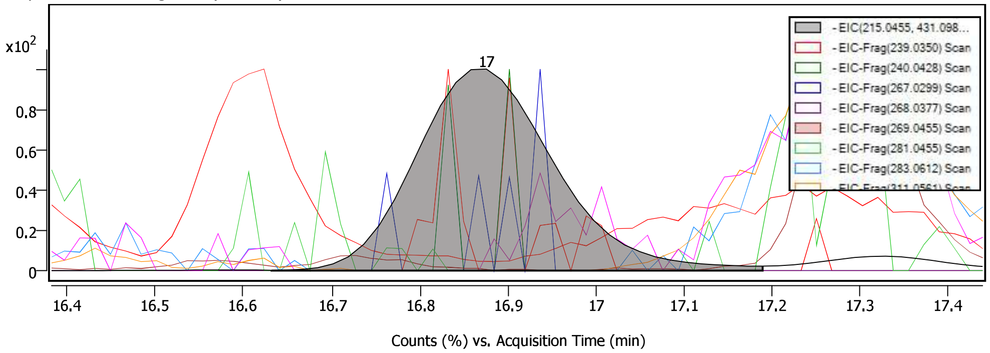

Structure

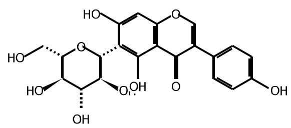

Coelution Plot

Compound Spectra (overlaid)

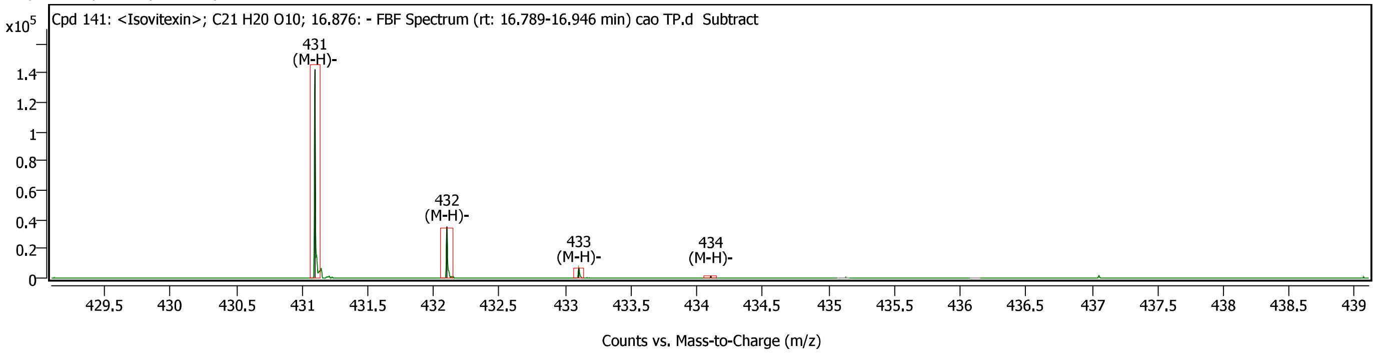

Fragment Spectrum (raw)

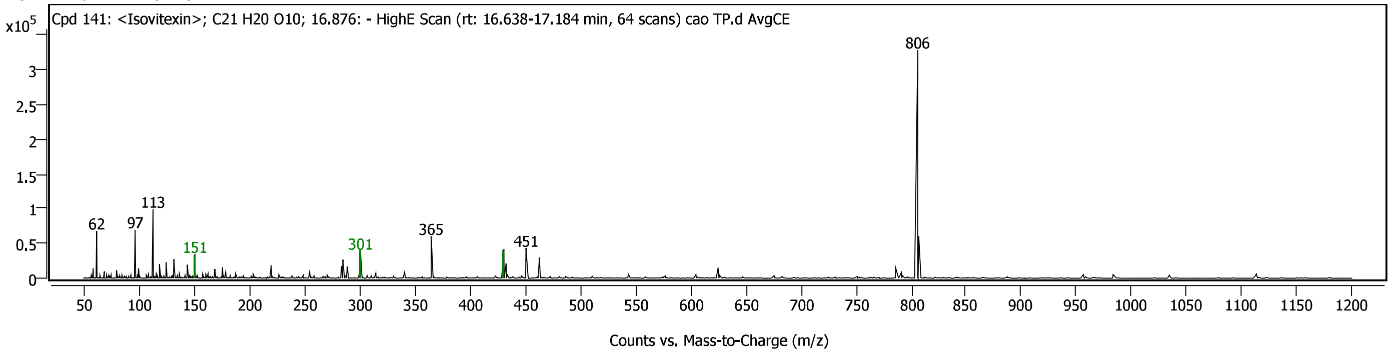

Compound ID Table

Name	Formula	Species	RT	RT Diff	Mass	CAS	ID Source	Score	Score (Lib)	Score (Tgt)
<Isovitexin>	C21 H20 O10	(M-H)-	16.876		432.1055	38953-85-4	FBF	99.46		99.46
<8-C-Rhamnopyranosylluteolin>	C21 H20 O10	(M-H)-	16.876		432.1055		FBF	99.46		99.46
<7,3',4'-Trihydroxyflavone 7-glucoside>	C21 H20 O10	(M-H)-	16.876		432.1055		FBF	99.46		99.46
<Apigenin 4'-glucoside>	C21 H20 O10	(M-H)-	16.876		432.1055		FBF	99.46		99.46
<Apigenin 4'-O-glucoside>	C21 H20 O10	(M-H)-	16.876		432.1055	20486-34-4	FBF	99.46		99.46
<Apigenin 5-galactoside>	C21 H20 O10	(M-H)-	16.876		432.1055		FBF	99.46		99.46
<Apigenin 7-glucoside>	C21 H20 O10	(M-H)-	16.876		432.1055	578-74-5	FBF	99.46		99.46
<Apigenin 5-glucoside>	C21 H20 O10	(M-H)-	16.876		432.1055		FBF	99.46		99.46
<Apigenin 7-galactoside>	C21 H20 O10	(M-H)-	16.876		432.1055		FBF	99.46		99.46
<Aureusidin 6-rhamnoside>	C21 H20 O10	(M-H)-	16.876		432.1055		FBF	99.46		99.46
<Baicalein 6-glucoside>	C21 H20 O10	(M-H)-	16.876		432.1055		FBF	99.46		99.46
<8-C-beta-D-Galactopyranosylapigenin>	C21 H20 O10	(M-H)-	16.876		432.1055		FBF	99.46		99.46
<Demethyltexasin 4'-O-glucoside>	C21 H20 O10	(M-H)-	16.876		432.1055		FBF	99.46		99.46
<8-C-Glucosyl-5-deoxykaempferol>	C21 H20 O10	(M-H)-	16.876		432.1055		FBF	99.46		99.46
<6-C-beta-D-Galactosylapigenin>	C21 H20 O10	(M-H)-	16.876		432.1055		FBF	99.46		99.46
<7,3',4'-Trihydroxyflavone 7-galactoside>	C21 H20 O10	(M-H)-	16.876		432.1055		FBF	99.46		99.46
<Afzelin>	C21 H20 O10	(M-H)-	16.876		432.1055	482-39-3	FBF	99.46		99.46
<7,3',4',5'-Tetrahydroxyflavone 7-rhamnoside>	C21 H20 O10	(M-H)-	16.876		432.1055		FBF	99.46		99.46
<6-Hydroxydaidzein 4'-glucoside>	C21 H20 O10	(M-H)-	16.876		432.1055		FBF	99.46		99.46
<6-C-Fucosylluteolin>	C21 H20 O10	(M-H)-	16.876		432.1055		FBF	99.46		99.46
<6-C-Fucopyranosylluteolin>	C21 H20 O10	(M-H)-	16.876		432.1055		FBF	99.46		99.46
<6-C-Chinovopyranosylluteolin>	C21 H20 O10	(M-H)-	16.876		432.1055		FBF	99.46		99.46
<5,7,8-Trihydroxyflavone 7-galactoside>	C21 H20 O10	(M-H)-	16.876		432.1055		FBF	99.46		99.46
<5,7,8-Trihydroxyflavone 5-glucoside>	C21 H20 O10	(M-H)-	16.876		432.1055		FBF	99.46		99.46
<5,7,2'-Trihydroxy 7-glucoside>	C21 H20 O10	(M-H)-	16.876		432.1055		FBF	99.46		99.46
<3'-Hydroxy-3,5,8,4',5'-pentamethoxy-6,7-methylenedioxyflavone>	C21 H20 O10	(M-H)-	16.876		432.1055		FBF	99.46		99.46
<1-O-beta-D-Glucopyranosylaloeemodin>	C21 H20 O10	(M-H)-	16.876		432.1055		FBF	99.46		99.46
<Emodin 8-glucoside>	C21 H20 O10	(M-H)-	16.876		432.1055	23313-21-5	FBF	99.46		99.46
<Genistein 5-glucoside>	C21 H20 O10	(M-H)-	16.876		432.1055	128508-06-5	FBF	99.46		99.46
<alpha-Rhamnorobin>	C21 H20 O10	(M-H)-	16.876		432.1055	5041-74-7	FBF	99.46		99.46
<Dihydrodaidzein 7-O-glucuronide>	C21 H20 O10	(M-H)-	16.876		432.1055		FBF	99.46		99.46
<w-O-beta-D-Glucopyranosylaloeemodin>	C21 H20 O10	(M-H)-	16.876		432.1055	50488-89-6	FBF	99.46		99.46
<Luteolin 3'-methyl ether 7-xyloside>	C21 H20 O10	(M-H)-	16.876		432.1055		FBF	99.46		99.46
<Vitexin>	C21 H20 O10	(M-H)-	16.876		432.1055	3681-93-4	FBF	99.46		99.46
<Sulfurein>	C21 H20 O10	(M-H)-	16.876		432.1055		FBF	99.46		99.46
<Scutellarein 7-rhamnoside>	C21 H20 O10	(M-H)-	16.876		432.1055		FBF	99.46		99.46
<Resokaempferol 7-glucoside>	C21 H20 O10	(M-H)-	16.876		432.1055		FBF	99.46		99.46
<Resokaempferol 4'-glucoside>	C21 H20 O10	(M-H)-	16.876		432.1055		FBF	99.46		99.46
<Galanginin>	C21 H20 O10	(M-H)-	16.876		432.1055		FBF	99.46		99.46
<Pueraria glycoside 1>	C21 H20 O10	(M-H)-	16.876		432.1055		FBF	99.46		99.46
<Peonidin pentose>	C21 H20 O10	(M-H)-	16.876		432.1055	741197-64-8	FBF	99.46		99.46
<Neovitexin>	C21 H20 O10	(M-H)-	16.876		432.1055		FBF	99.46		99.46
<Luteolin 7-rhamnoside>	C21 H20 O10	(M-H)-	16.876		432.1055		FBF	99.46		99.46
<Luteolin 3'-rhamnoside>	C21 H20 O10	(M-H)-	16.876		432.1055		FBF	99.46		99.46
<Kaempferol 7-rhamnoside>	C21 H20 O10	(M-H)-	16.876		432.1055		FBF	99.46		99.46
<Kaempferol 5-rhamnoside>	C21 H20 O10	(M-H)-	16.876		432.1055		FBF	99.46		99.46
<1,8-Dihydroxy-3-hydroxymethylanthraquinone 8-O-b-D-glucoside>	C21 H20 O10	(M-H)-	16.876		432.1055	33037-46-6	FBF	99.46		99.46
<Kaempferol 4'-rhamnoside>	C21 H20 O10	(M-H)-	16.876		432.1055		FBF	99.46		99.46
<Kaempferol 3-O-alpha-L-rhamnofuranoside>	C21 H20 O10	(M-H)-	16.876		432.1055	5041-73-6	FBF	99.46		99.46
<Kaempferide 3-alpha-L-arabinopyranoside>	C21 H20 O10	(M-H)-	16.876		432.1055		FBF	99.46		99.46
<Isogenistein 7-O-glucoside>	C21 H20 O10	(M-H)-	16.876		432.1055		FBF	99.46		99.46
<Isogenistein 7-glucoside>	C21 H20 O10	(M-H)-	16.876		432.1055	70943-69-0	FBF	99.46		99.46
<Glucoemodin>	C21 H20 O10	(M-H)-	16.876		432.1055	34298-85-6	FBF	99.46		99.46
<Genistin>	C21 H20 O10	(M-H)-	16.876		432.1055	529-59-9	FBF	99.46		99.46
<Genistein 8-C-glucoside>	C21 H20 O10	(M-H)-	16.876		432.1055		FBF	99.46		99.46
<Genistein 7-O-glucoside>	C21 H20 O10	(M-H)-	16.876		432.1055		FBF	99.46		99.46
<Genistein 5-O-glucoside>	C21 H20 O10	(M-H)-	16.876		432.1055		FBF	99.46		99.46
<Resokaempferol 3-glucoside>	C21 H20 O10	(M-H)-	16.876		432.1055		FBF	99.46		99.46
<Galangin 7-glucoside>	C21 H20 O10	(M-H)-	16.876		432.1055		FBF	99.46		99.46
<Genistein 4'-O-glucoside>	C21 H20 O10	(M-H)-	16.876		432.1055	152-95-4	FBF	99.46		99.46

Cpd 230: N-Caffeoyltryptophan

Name	Formula	RT	RI	Mass	Diff (Tgt, ppm)	CAS	ID Source	Score	Algorithm
N-Caffeoyltryptophan	C20 H18 N2 O5	17.155		366.1215	-0.21	109163-69-1	FBF	99.90	FBF

Species	m/z	Score (Tgt)	Score (Lib)	Score (DB)	Score (MFG)	Score (RT)
(M-H)-	365	99.90				

Compound Chromatograms (overlaid)

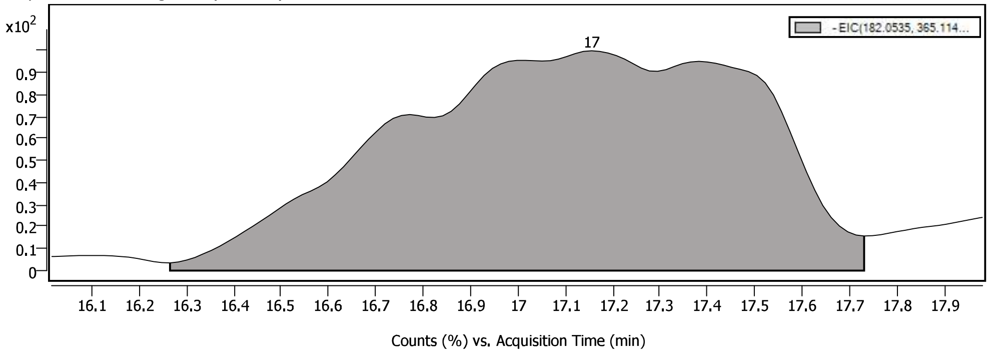

Structure

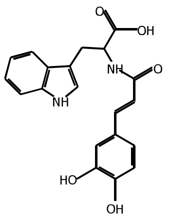

Compound Spectra (overlaid)

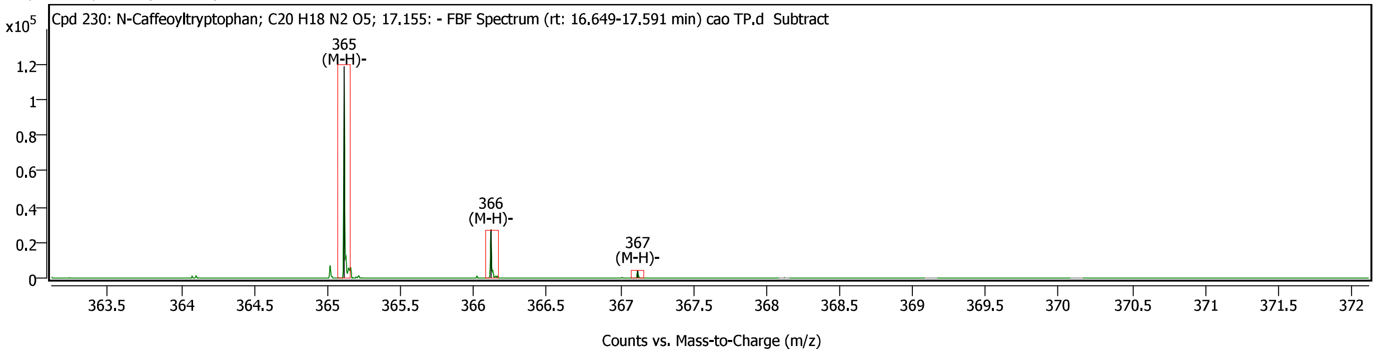

Compound ID Table

Name	Formula	Species	RT	RT Diff	Mass	CAS	ID Source	Score	Score (Lib)	Score (Tgt)
N-Caffeoyltryptophan	C20 H18 N2 O5	(M-H)-	17.155		366.1215	109163-69-1	FBF	99.90		99.90

Cpd 125: Quercetin 3-(3''-sulfatoglucoside)

Name	Formula	RT	RI	Mass	Diff (Tgt, ppm)	CAS	ID Source	Score	Algorithm
Quercetin 3-(3''-sulfatoglucoside)	C21 H20 O15 S	17.312		544.0521	-0.31		M-FBF	98.25	FBF

Species	m/z	Score (Tgt)	Score (Lib)	Score (DB)	Score (MFG)	Score (RT)
(M-H)-	543	98.25				

Compound Chromatograms (overlaid)

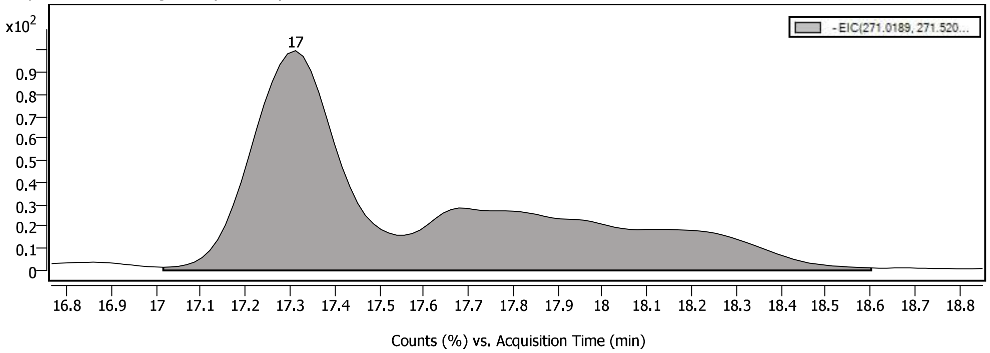

Structure

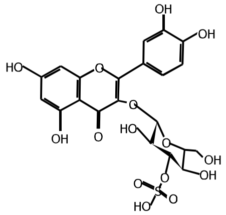

Compound Spectra (overlaid)

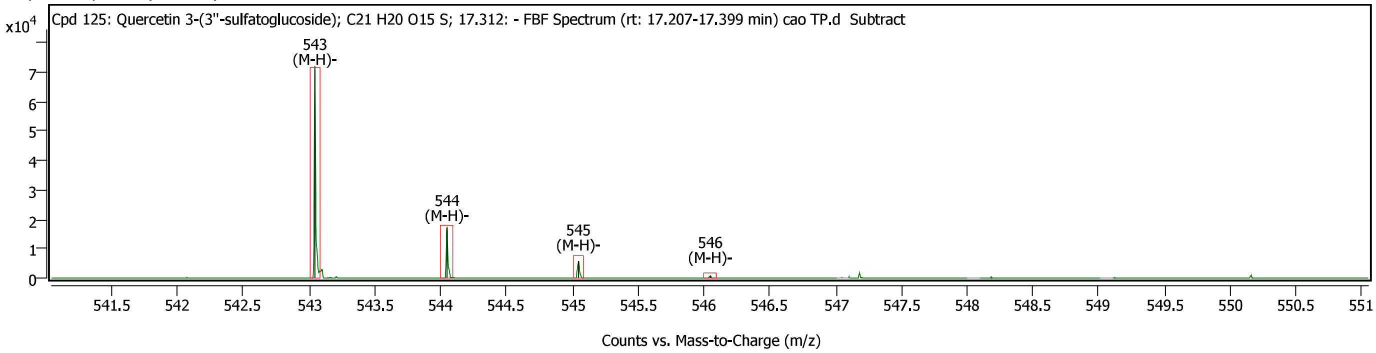

Compound Screening Report

Compound ID Table

Name	Formula	Species	RT	RT Diff	Mass	CAS	ID Source	Score	Score (Lib)	Score (Tgt)
Quercetin 3-(3''-sulfatoglucoside)	C21 H20 O15 S	(M-H)-	17.312		544.0521		FBF	98.25		98.25
Quercetin 3-glucoside-3'-sulfate	C21 H20 O15 S	(M-H)-	17.312		544.0521		FBF	98.25		98.25
Hypolaetin 7-sulfate-8-glucoside	C21 H20 O15 S	(M-H)-	17.312		544.0521		FBF	98.25		98.25
8-Hydroxyluteolin 8-glucoside-3'-sulfate	C21 H20 O15 S	(M-H)-	17.312		544.0521		FBF	98.25		98.25
8-Hydroxyluteolin 8-glucoside 3'-sulfate	C21 H20 O15 S	(M-H)-	17.312		544.0521	63109-34-2	FBF	98.25		98.25

Cpd 215: Quercetin

Name	Formula	RT	RI	Mass	Diff (Tgt, ppm)	CAS	ID Source	Score	Algorithm
Quercetin	C15 H10 O7	17.801		302.0427	0.15	117-39-5	M-FBF-FragConfirm	99.52	FBF

Species	m/z	Score (Tgt)	Score (Lib)	Score (DB)	Score (MFG)	Score (RT)
(M-H)-	301	99.52				

Compound Chromatograms (overlaid)

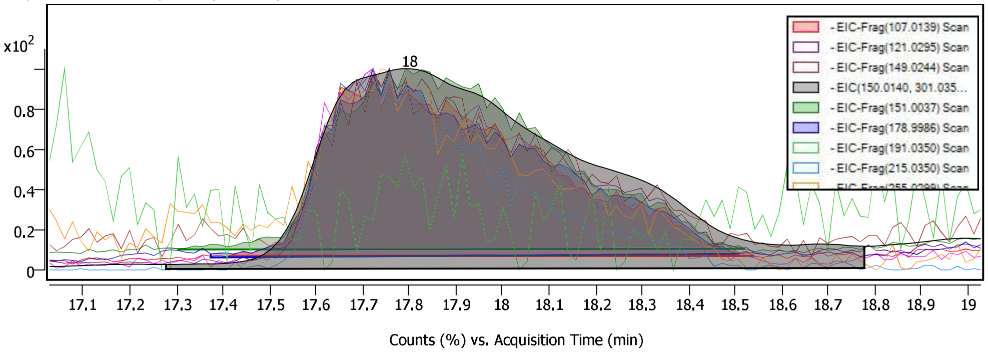

Structure

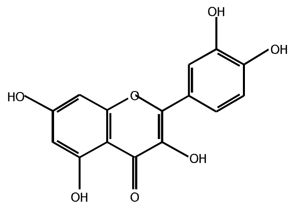

Coelution Plot

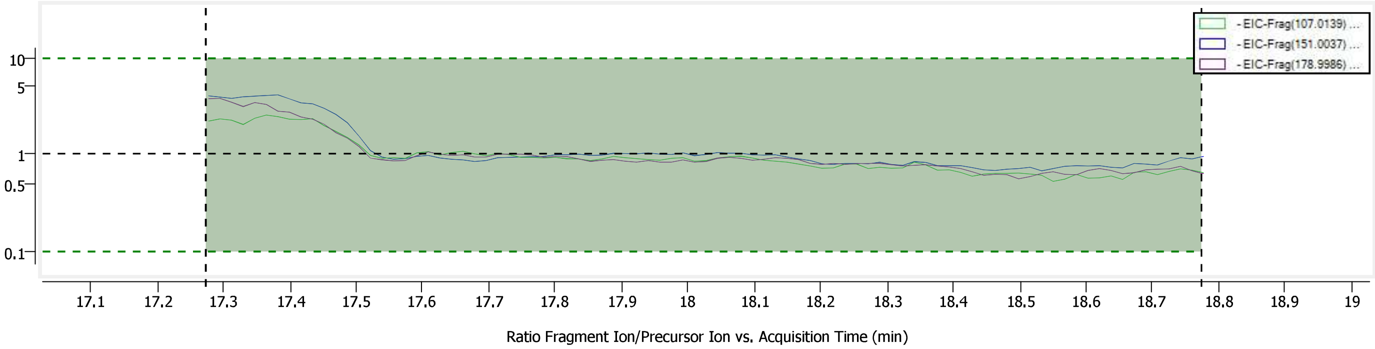

Compound Spectra (overlaid)

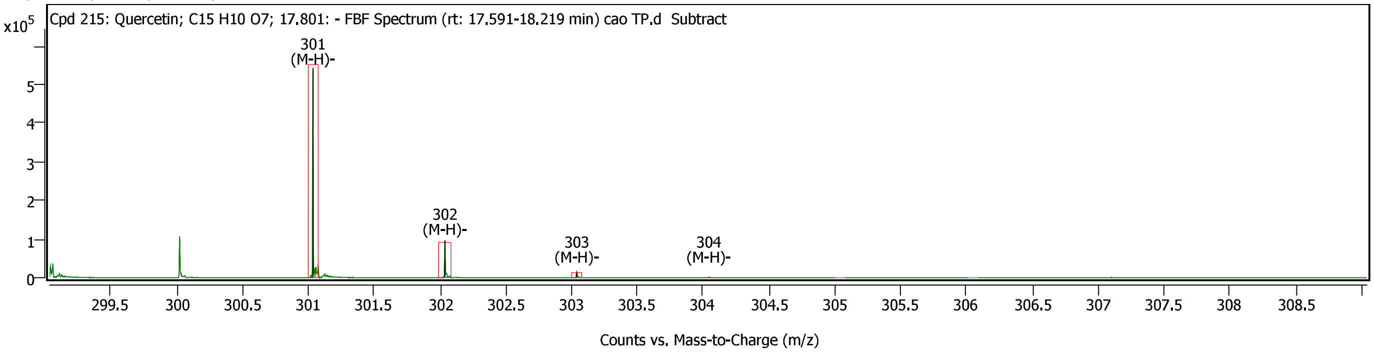

Fragment Spectrum (clean)

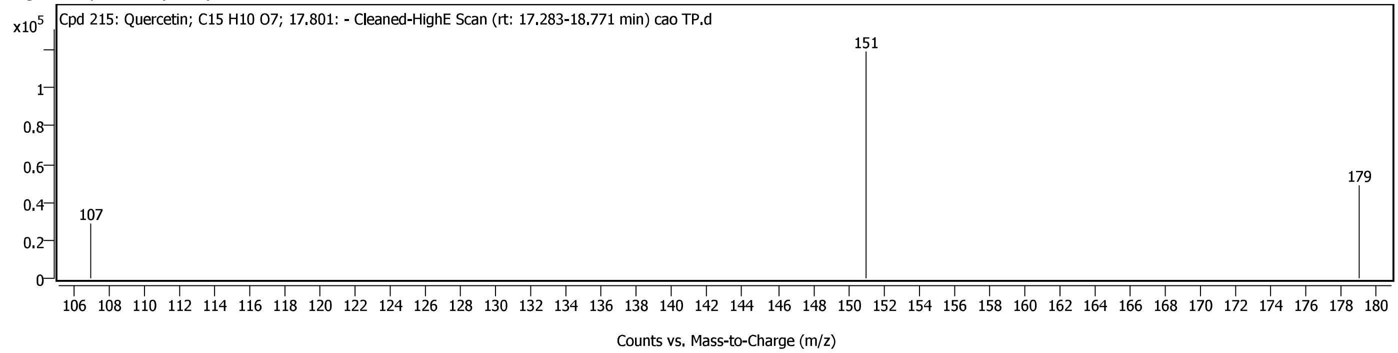

Fragment Spectrum (raw)

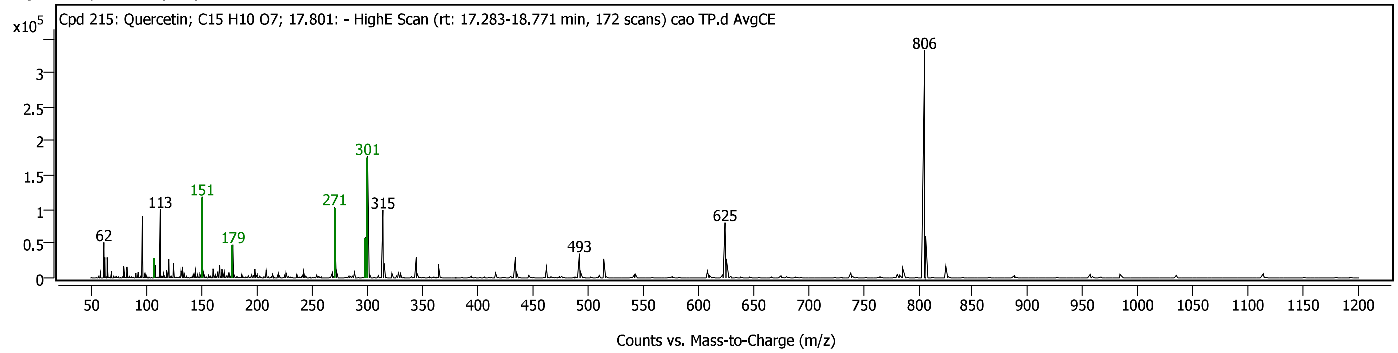

Compound ID Table

Name	Formula	Species	RT	RT Diff	Mass	CAS	ID Source	Score	Score (Lib)	Score (Tgt)
Quercetin	C15 H10 O7	(M-H)-	17.801		302.0427	117-39-5	FBF-FragConfirm	99.52		99.52
6-Hydroxyluteolin	C15 H10 O7	(M-H)-	17.801		302.0427		FBF-FragConfirm	99.52		99.52
5,7,8,3',4'-Pentahydroxyisoflavone	C15 H10 O7	(M-H)-	17.801		302.0427		FBF-FragConfirm	99.52		99.52
Herbacetin	C15 H10 O7	(M-H)-	17.801		302.0427		FBF-FragConfirm	99.52		99.52
2-(4-Hydroxyphenyl)-5,6,7,8-tetrahydroxy-4H-1-benzopyran-4-one	C15 H10 O7	(M-H)-	17.801		302.0427	577-26-4	FBF-FragConfirm	99.52		99.52
2',3',4',5,7-Pentahydroxyflavone	C15 H10 O7	(M-H)-	17.801		302.0427	144707-17-5	FBF-FragConfirm	99.52		99.52
2'-Hydroxypseudobaptigenin	C15 H10 O7	(M-H)-	17.801		302.0427		FBF-FragConfirm	99.52		99.52
3,5,7,2',5'-Pentahydroxyflavone	C15 H10 O7	(M-H)-	17.801		302.0427		FBF-FragConfirm	99.52		99.52
5,6,7,3',4'-Pentahydroxyisoflavone	C15 H10 O7	(M-H)-	17.801		302.0427		FBF-FragConfirm	99.52		99.52
5,7,8,2',4'-Pentahydroxyisoflavone	C15 H10 O7	(M-H)-	17.801		302.0427		FBF-FragConfirm	99.52		99.52
6-Hydroxykaempferol	C15 H10 O7	(M-H)-	17.801		302.0427		FBF-FragConfirm	99.52		99.52
Tricetin	C15 H10 O7	(M-H)-	17.801		302.0427	520-31-0	FBF-FragConfirm	99.52		99.52
Bracteatin	C15 H10 O7	(M-H)-	17.801		302.0427		FBF-FragConfirm	99.52		99.52
Hypolaetin	C15 H10 O7	(M-H)-	17.801		302.0427		FBF-FragConfirm	99.52		99.52
Isoetin	C15 H10 O7	(M-H)-	17.801		302.0427		FBF-FragConfirm	99.52		99.52
Melanoxetin	C15 H10 O7	(M-H)-	17.801		302.0427		FBF-FragConfirm	99.52		99.52
Morin	C15 H10 O7	(M-H)-	17.801		302.0427	480-16-0	FBF-FragConfirm	99.52		99.52
Rhynchosin	C15 H10 O7	(M-H)-	17.801		302.0427		FBF-FragConfirm	99.52		99.52
Robinetin	C15 H10 O7	(M-H)-	17.801		302.0427		FBF-FragConfirm	99.52		99.52
Viscidulin I	C15 H10 O7	(M-H)-	17.801		302.0427	92519-95-4	FBF-FragConfirm	99.52		99.52

Cpd 117: 3,5,7,3',4'-Pentahydroxy-6,8-dimethoxyflavone 3-alpha-L-arabinopyranoside

Name	Formula	RT	RI	Mass	Diff (Tgt, ppm)	CAS	ID Source	Score	Algorithm
3,5,7,3',4'-Pentahydroxy-6,8-dimethoxyflavone 3-alpha-L-arabinopyranoside	C22 H22 O13	17.910		494.1061	0.03		M-FBF	99.58	FBF

Species	m/z	Score (Tgt)	Score (Lib)	Score (DB)	Score (MFG)	Score (RT)
(M-H)-	493	99.58				

Compound Chromatograms (overlaid)

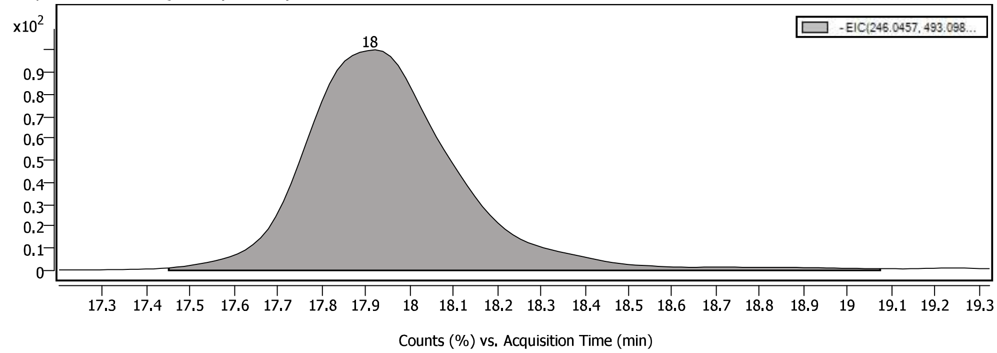

Structure

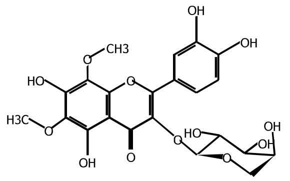

Compound Spectra (overlaid)

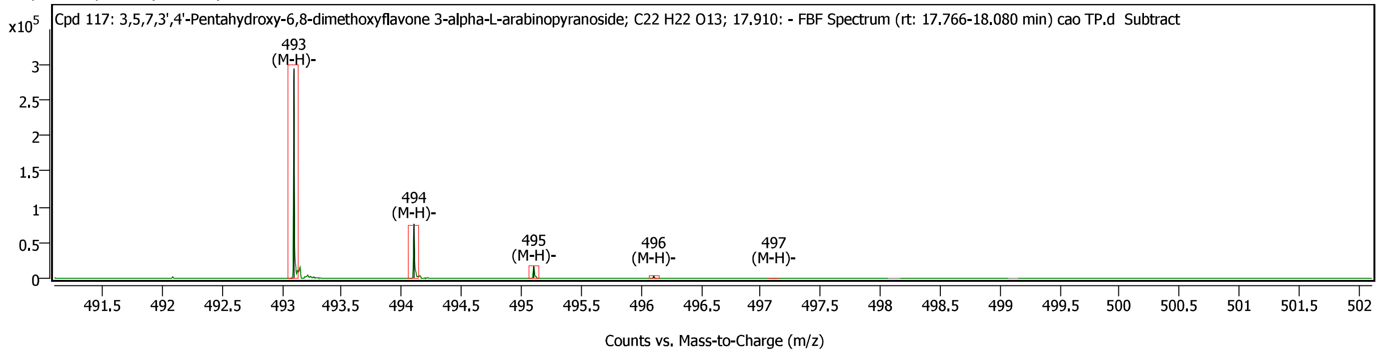

Compound ID Table

Name	Formula	Species	RT	RT Diff	Mass	CAS	ID Source	Score	Score (Lib)	Score (Tgt)
3,5,7,3',4'-Pentahydroxy-6,8-dimethoxyflavone 3-alpha-L-arabinopyranoside	C22 H22 O13	(M-H)-	17.910		494.1061		FBF	99.58		99.58
Laricitrin 3-galactoside	C22 H22 O13	(M-H)-	17.910		494.1061		FBF	99.58		99.58
Myricetin 5-methyl ether 7-glucoside	C22 H22 O13	(M-H)-	17.910		494.1061		FBF	99.58		99.58
Patuletin 7-galactoside	C22 H22 O13	(M-H)-	17.910		494.1061		FBF	99.58		99.58
Myricetin 5-methyl ether 3-glucoside	C22 H22 O13	(M-H)-	17.910		494.1061		FBF	99.58		99.58
Myricetin 5-methyl ether 3-galactoside	C22 H22 O13	(M-H)-	17.910		494.1061		FBF	99.58		99.58
Myricetin 4'-methyl ether 3-O-beta-D-galactopyranoside	C22 H22 O13	(M-H)-	17.910		494.1061		FBF	99.58		99.58
Laricitrin 7-glucoside	C22 H22 O13	(M-H)-	17.910		494.1061		FBF	99.58		99.58
Laricitrin 5'-glucoside	C22 H22 O13	(M-H)-	17.910		494.1061		FBF	99.58		99.58
Laricitrin 3-glucoside	C22 H22 O13	(M-H)-	17.910		494.1061		FBF	99.58		99.58
Patuletin 3-galactoside	C22 H22 O13	(M-H)-	17.910		494.1061		FBF	99.58		99.58
Haploside B	C22 H22 O13	(M-H)-	17.910		494.1061		FBF	99.58		99.58
Europetin 3-galactoside	C22 H22 O13	(M-H)-	17.910		494.1061		FBF	99.58		99.58
Corniculatusin 3-glucoside	C22 H22 O13	(M-H)-	17.910		494.1061		FBF	99.58		99.58
Corniculatusin 3-galactoside	C22 H22 O13	(M-H)-	17.910		494.1061		FBF	99.58		99.58
Annulatin 3'-glucoside	C22 H22 O13	(M-H)-	17.910		494.1061		FBF	99.58		99.58
Hibiscetin 8-methyl ether 3-rhamnoside	C22 H22 O13	(M-H)-	17.910		494.1061		FBF	99.58		99.58
Patuletin 7-glucoside	C22 H22 O13	(M-H)-	17.910		494.1061		FBF	99.58		99.58
Patuletin 3-glucoside	C22 H22 O13	(M-H)-	17.910		494.1061		FBF	99.58		99.58
Quercetagetin 3-methyl ether 7-glucoside	C22 H22 O13	(M-H)-	17.910		494.1061		FBF	99.58		99.58
Patuletin 5-glucoside	C22 H22 O13	(M-H)-	17.910		494.1061		FBF	99.58		99.58
Ranupenin 8-glucoside	C22 H22 O13	(M-H)-	17.910		494.1061		FBF	99.58		99.58
Quercetagetin 7-methyl ether 6-glucoside	C22 H22 O13	(M-H)-	17.910		494.1061		FBF	99.58		99.58
Quercetagetin 7-methyl ether 4'-glucoside	C22 H22 O13	(M-H)-	17.910		494.1061		FBF	99.58		99.58
Quercetagetin 7-methyl ether 3-glucoside	C22 H22 O13	(M-H)-	17.910		494.1061		FBF	99.58		99.58
Quercetagetin 3'-methylether 7-glucoside	C22 H22 O13	(M-H)-	17.910		494.1061	29741-08-0	FBF	99.58		99.58
Ranupenin 3-galactoside	C22 H22 O13	(M-H)-	17.910		494.1061		FBF	99.58		99.58
Quercetagetin 3'-methyl ether 7-glucoside	C22 H22 O13	(M-H)-	17.910		494.1061		FBF	99.58		99.58
Quercetagetin 3'-methyl ether 3-glucoside	C22 H22 O13	(M-H)-	17.910		494.1061		FBF	99.58		99.58
Quercetagetin 3'-methyl ether 3-galactoside	C22 H22 O13	(M-H)-	17.910		494.1061		FBF	99.58		99.58
Pleurostimin 7-glucoside	C22 H22 O13	(M-H)-	17.910		494.1061		FBF	99.58		99.58
Quercetagetin 3-methyl ether 6-glucoside	C22 H22 O13	(M-H)-	17.910		494.1061		FBF	99.58		99.58

Cpd 206: dTDP-β-L-rhodinose

Name	Formula	RT	RI	Mass	Diff (Tgt, ppm)	CAS	ID Source	Score	Algorithm
dTDP-β-L-rhodinose	C16 H26 N2 O13 P2	19.859		516.0932	4.32		FBF	82.72	FBF

Species	m/z	Score (Tgt)	Score (Lib)	Score (DB)	Score (MFG)	Score (RT)
(M-H)-	515	82.72				

Compound Chromatograms (overlaid)

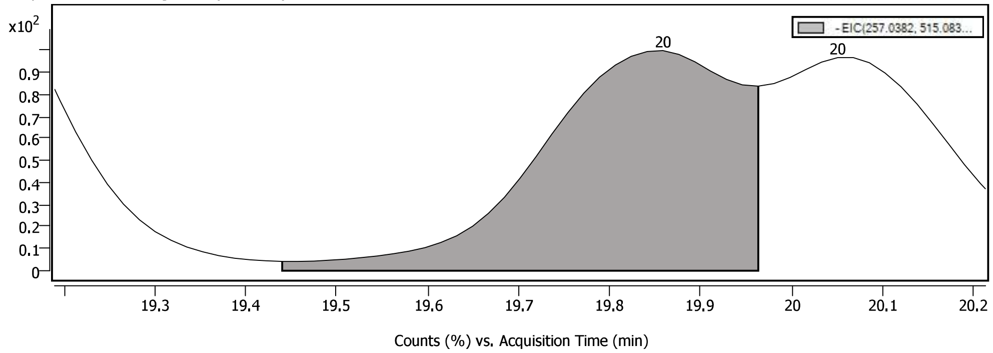

Structure

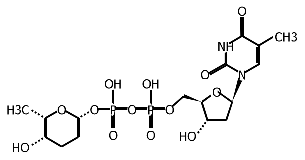

Compound Spectra (overlaid)

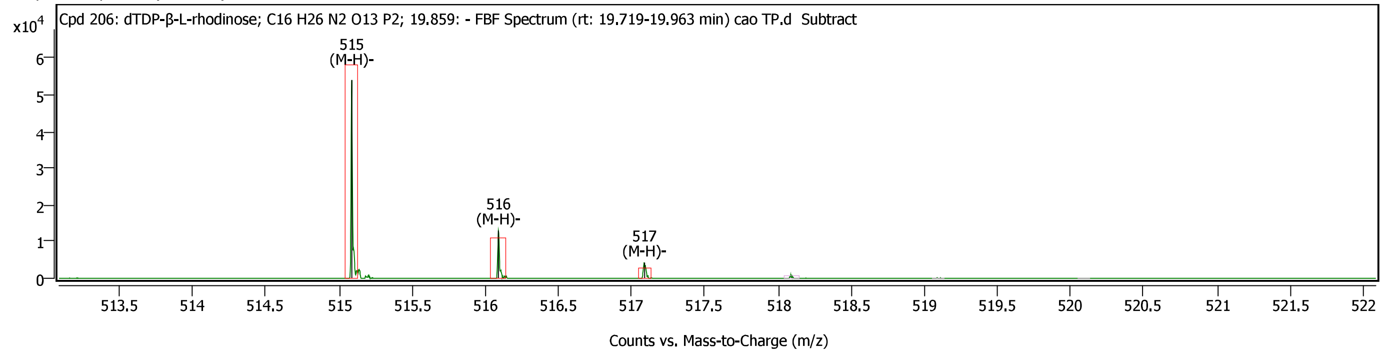

Compound ID Table

Name	Formula	Species	RT	RT Diff	Mass	CAS	ID Source	Score	Score (Lib)	Score (Tgt)
dTDP-β-L-rhodinose	C16 H26 N2 O13 P2	(M-H)-	19.859		516.0932		FBF	82.72		82.72

Cpd 22: 4''-O-Acetylmyricitrin

Name	Formula	RT	RI	Mass	Diff (Tgt, ppm)	CAS	ID Source	Score	Algorithm
4''-O-Acetylmyricitrin	C23 H22 O13	19.998		506.1059	-0.29		M-FBF	99.70	FBF

Species	m/z	Score (Tgt)	Score (Lib)	Score (DB)	Score (MFG)	Score (RT)
(M-H)-	505	99.70				

Compound Chromatograms (overlaid)

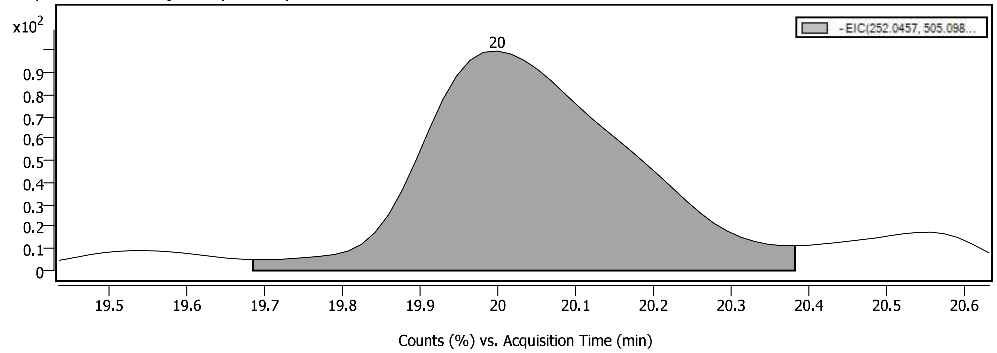

Structure

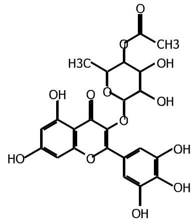

Compound Spectra (overlaid)

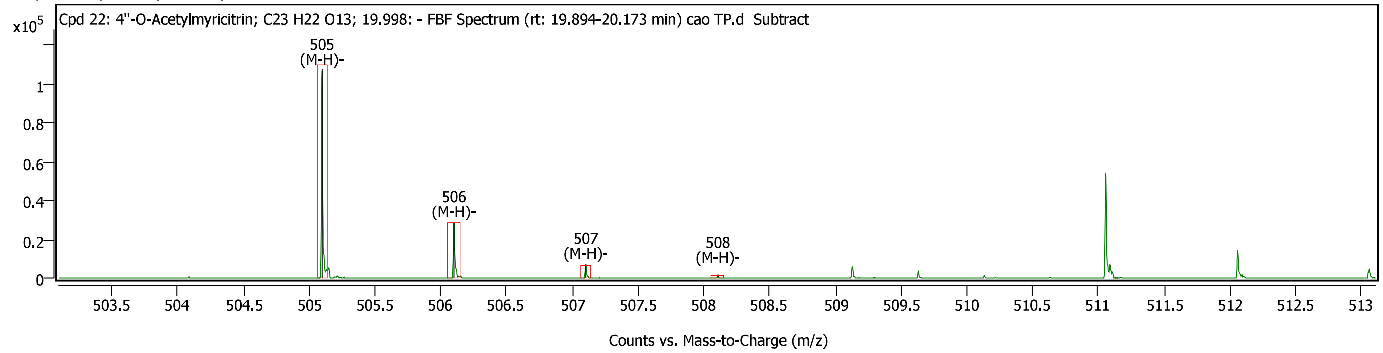

Compound ID Table

Name	Formula	Species	RT	RT Diff	Mass	CAS	ID Source	Score	Score (Lib)	Score (Tgt)
4''-O-Acetylmyricitrin	C23 H22 O13	(M-H)-	19.998		506.1059		FBF	99.70		99.70
Glyphoside	C23 H22 O13	(M-H)-	19.998		506.1059	11029-60-0	FBF	99.70		99.70
Quercetin 3-(2''-acetylgalactoside)	C23 H22 O13	(M-H)-	19.998		506.1059		FBF	99.70		99.70
Tricin 7-glucuronoside	C23 H22 O13	(M-H)-	19.998		506.1059	32769-02-1	FBF	99.70		99.70
Myricetin 3-acetylrhamnoside	C23 H22 O13	(M-H)-	19.998		506.1059		FBF	99.70		99.70
Myricetin 3-(2''-acetylrhamnoside)	C23 H22 O13	(M-H)-	19.998		506.1059		FBF	99.70		99.70
Isobiflorin 6''-gallate	C23 H22 O13	(M-H)-	19.998		506.1059	152041-17-3	FBF	99.70		99.70
6-Methoxyluteolin 7-glucuronide methyl ester	C23 H22 O13	(M-H)-	19.998		506.1059		FBF	99.70		99.70
5,2',6'-Trihydroxy-7,8-dimethoxyflavone 2'-glucuronide	C23 H22 O13	(M-H)-	19.998		506.1059		FBF	99.70		99.70
Quercetin 3- (3''-acetylgalactoside)	C23 H22 O13	(M-H)-	19.998		506.1059		FBF	99.70		99.70
Tricin 7-glucuronide	C23 H22 O13	(M-H)-	19.998		506.1059		FBF	99.70		99.70
Quercetin 3-(6''-ethylglucuronide)	C23 H22 O13	(M-H)-	19.998		506.1059		FBF	99.70		99.70
Quercetin 3-(6''-acetylgalactoside)	C23 H22 O13	(M-H)-	19.998		506.1059		FBF	99.70		99.70
Quercetin 3'-glucoside-7-acetate	C23 H22 O13	(M-H)-	19.998		506.1059		FBF	99.70		99.70
Quercetin 3-O-(6''-acetyl-glucoside)	C23 H22 O13	(M-H)-	19.998		506.1059		FBF	99.70		99.70
Quercetin 7-(6''-acetylglucoside)	C23 H22 O13	(M-H)-	19.998		506.1059		FBF	99.70		99.70
Tricetin 3',4'-dimethyl ether 7-glucuronide	C23 H22 O13	(M-H)-	19.998		506.1059		FBF	99.70		99.70
Quercetin 3-(6''-acetylglucoside)	C23 H22 O13	(M-H)-	19.998		506.1059		FBF	99.70		99.70

Compound Screening Report

Cpd 118: 6-Hydroxyluteolin 7-(6''-malonylglucoside)

Name	Formula	RT	RI	Mass	Diff (Tgt, ppm)	CAS	ID Source	Score	Algorithm
6-Hydroxyluteolin 7-(6''-malonylglucoside)	C24 H22 O15	19.998		550.0956	-0.40		M-FBF	99.57	FBF

Species	m/z	Score (Tgt)	Score (Lib)	Score (DB)	Score (MFG)	Score (RT)
(M-H)-	549	99.57				

Compound Chromatograms (overlaid)

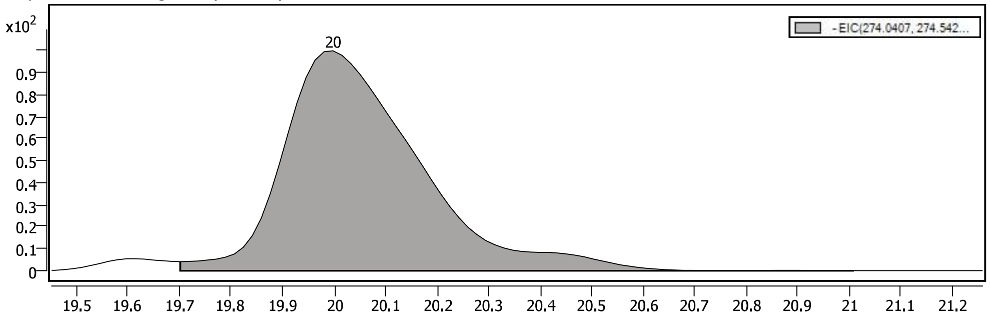

Counts (%) vs. Acquisition Time (min)

Structure

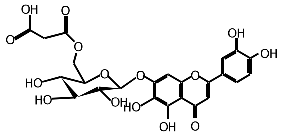

Compound Spectra (overlaid)

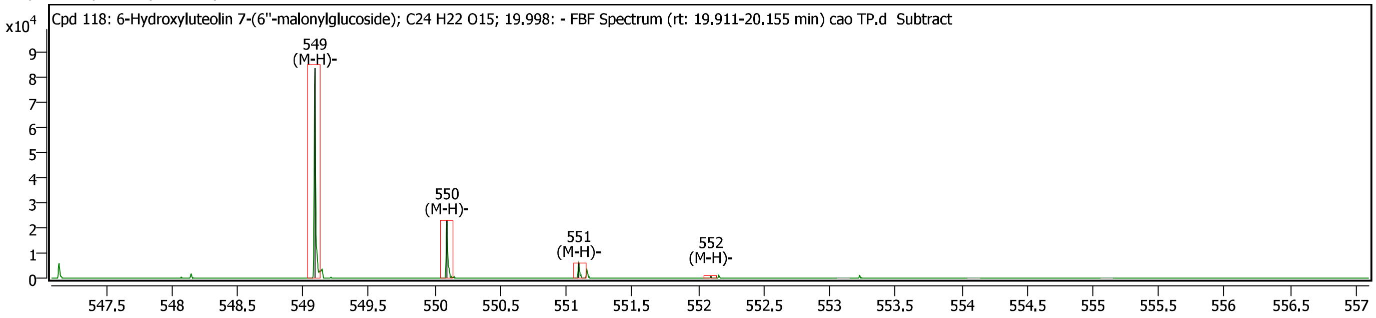

Counts vs. Mass-to-Charge (m/z)

Compound ID Table

Name	Formula	Species	RT	RT Diff	Mass	CAS	ID Source	Score	Score (Lib)	Score (Tgt)
6-Hydroxyluteolin 7-(6''-malonylglucoside)	C24 H22 O15	(M-H)-	19.998		550.0956		FBF	99.57		99.57
Myricetin 3-(4''-malonylrhamnoside)	C24 H22 O15	(M-H)-	19.998		550.0956		FBF	99.57		99.57
Quercetin 3-O-malonylglucoside	C24 H22 O15	(M-H)-	19.998		550.0956	96862-01-0	FBF	99.57		99.57
Quercetin 3-(6''-malonylgalactoside)	C24 H22 O15	(M-H)-	19.998		550.0956		FBF	99.57		99.57
Quercetin 3-(6''-malonylglucoside)	C24 H22 O15	(M-H)-	19.998		550.0956		FBF	99.57		99.57

Cpd 207: dTDP-β-L-rhodinose

Name	Formula	RT	RI	Mass	Diff (Tgt, ppm)	CAS	ID Source	Score	Algorithm
dTDP-β-L-rhodinose	C16 H26 N2 O13 P2	20.051		516.0935	4.80		FBF	80.18	FBF

Species	m/z	Score (Tgt)	Score (Lib)	Score (DB)	Score (MFG)	Score (RT)
(M-H)-	515	80.18				

Compound Chromatograms (overlaid)

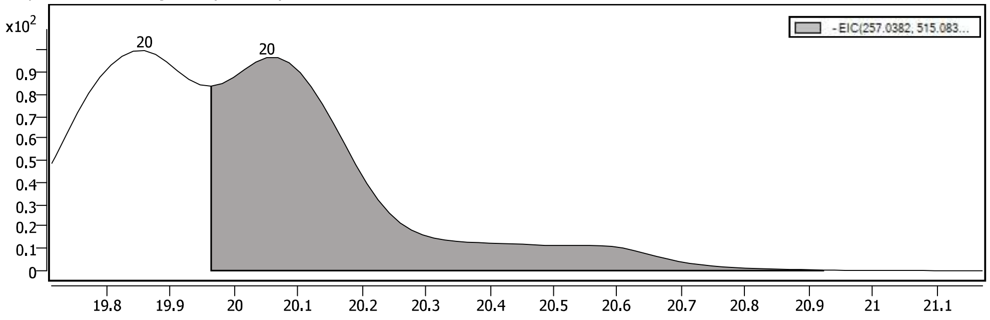

Counts (%) vs. Acquisition Time (min)

Structure

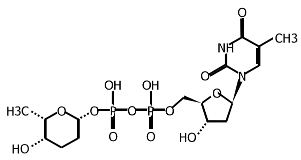

Compound Spectra (overlaid)

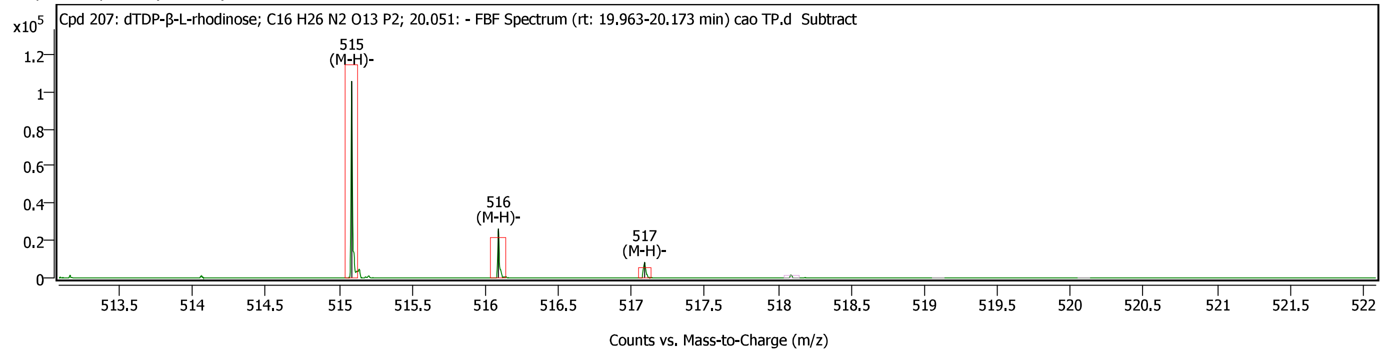

Compound ID Table

Name	Formula	Species	RT	RT Diff	Mass	CAS	ID Source	Score	Score (Lib)	Score (Tgt)
dTDP-β-L-rhodinose	C16 H26 N2 O13 P2	(M-H)-	20.051		516.0935		FBF	80.18		80.18

Cpd 127: Patuletin 3-(6''-p-coumaroylglucoside)

Name	Formula	RT	RI	Mass	Diff (Tgt, ppm)	CAS	ID Source	Score	Algorithm
Patuletin 3-(6''-p-coumaroylglucoside)	C31 H28 O15	20.312		640.1427	-0.19		M-FBF	99.68	FBF

Species	m/z	Score (Tgt)	Score (Lib)	Score (DB)	Score (MFG)	Score (RT)
(M-H)-	639	99.68				

Compound Chromatograms (overlaid)

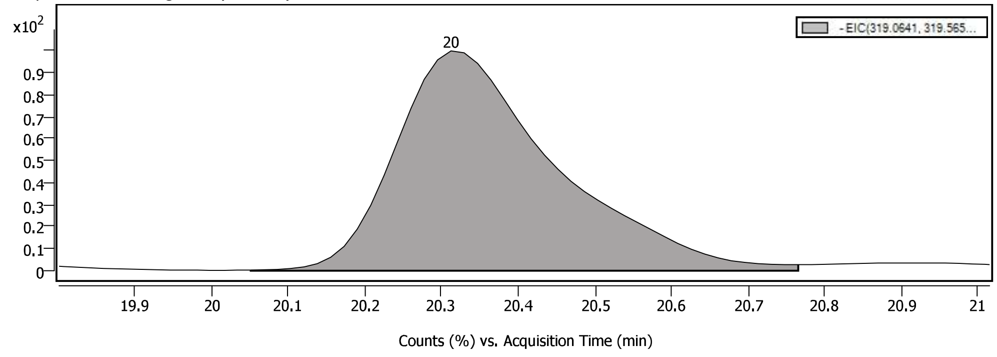

Structure

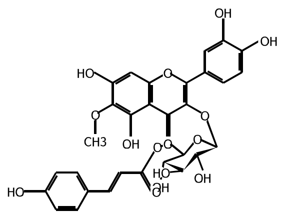

Compound Spectra (overlaid)

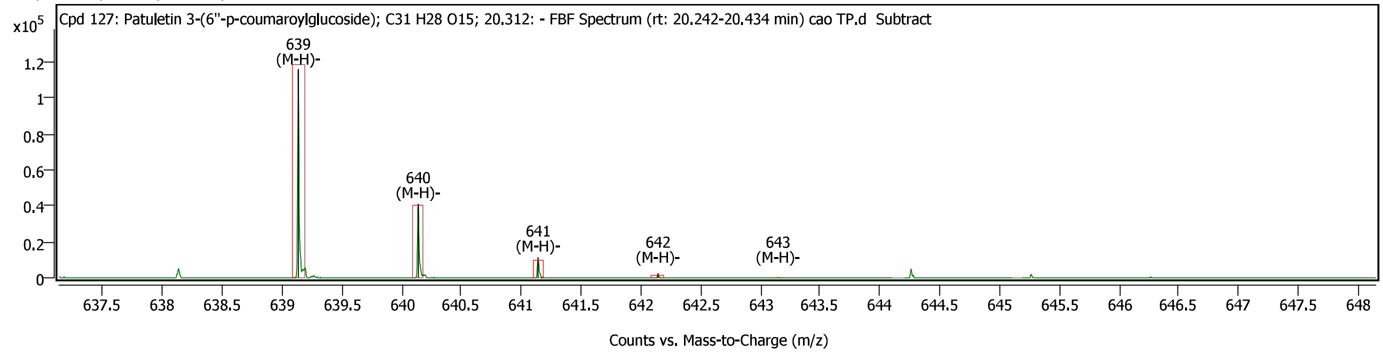

Compound ID Table

Name	Formula	Species	RT	RT Diff	Mass	CAS	ID Source	Score	Score (Lib)	Score (Tgt)
Patuletin 3-(6''-p-coumaroylglucoside)	C31 H28 O15	(M-H)-	20.312		640.1427		FBF	99.68		99.68
Quercetin 3-(6''-ferulylglucoside)	C31 H28 O15	(M-H)-	20.312		640.1427		FBF	99.68		99.68
Quercetin 3-(6''-feruloylgalactoside)	C31 H28 O15	(M-H)-	20.312		640.1427		FBF	99.68		99.68

Cpd 26: 2-Hydroxybenzaldehyde O-[xylosyl-(1->6)-glucoside]

Name	Formula	RT	RI	Mass	Diff (Tgt, ppm)	CAS	ID Source	Score	Algorithm
2-Hydroxybenzaldehyde O-[xylosyl-(1->6)-glucoside]	C18 H24 O11	20.417		416.1316	-0.68	14907-56-3	FBF	99.41	FBF

Species	m/z	Score (Tgt)	Score (Lib)	Score (DB)	Score (MFG)	Score (RT)
(M-H)-	415	99.41				

Compound Screening Report

Compound Chromatograms (overlaid)

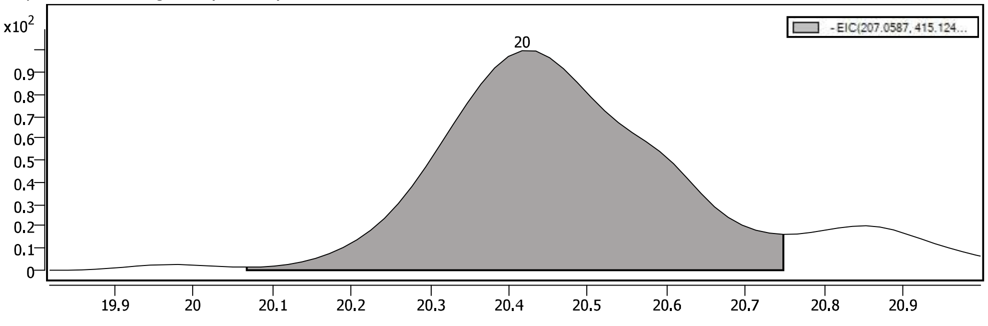

Structure

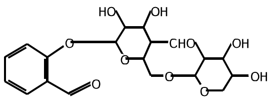

Compound Spectra (overlaid)

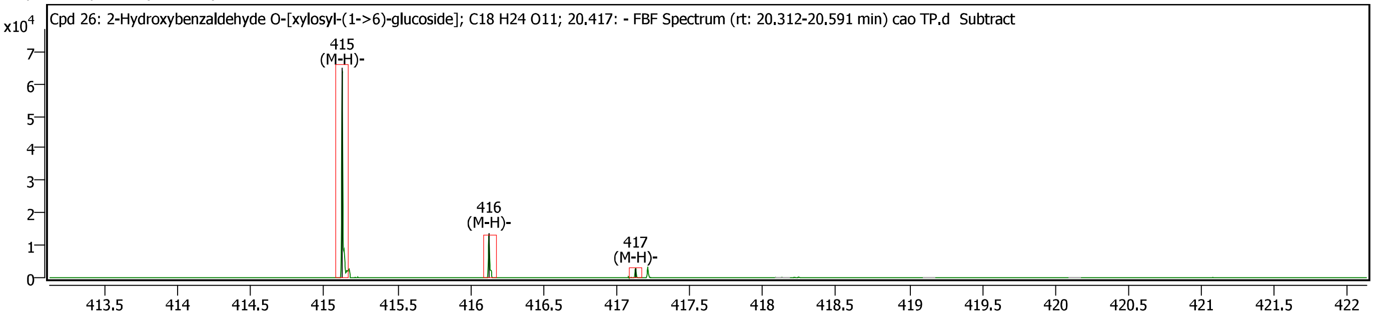

Compound ID Table

Name	Formula	Species	RT	RT Diff	Mass	CAS	ID Source	Score	Score (Lib)	Score (Tgt)
2-Hydroxybenzaldehyde O-[xylosyl-(1->6)-glucoside]	C18 H24 O11	(M-H)-	20.417		416.1316	14907-56-3	FBF	99.41		99.41

Cpd 29: Na-p-Hydroxycoumaroyltryptophan

Name	Formula	RT	RI	Mass	Diff (Tgt, ppm)	CAS	ID Source	Score	Algorithm
Na-p-Hydroxycoumaroyltryptophan	C20 H18 N2 O4	20.644		350.1264	-0.63		M-FBF	99.70	FBF

Species	m/z	Score (Tgt)	Score (Lib)	Score (DB)	Score (MFG)	Score (RT)
(M-H)-	349	99.70				

Compound Chromatograms (overlaid)

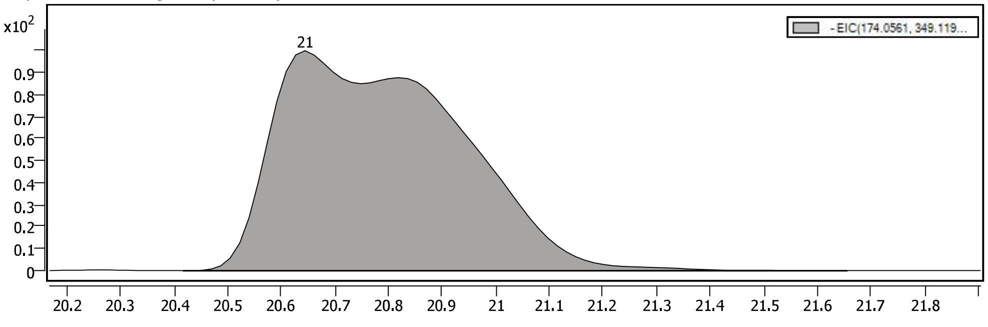

Structure

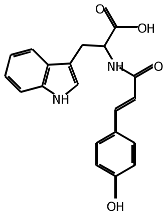

Compound Spectra (overlaid)

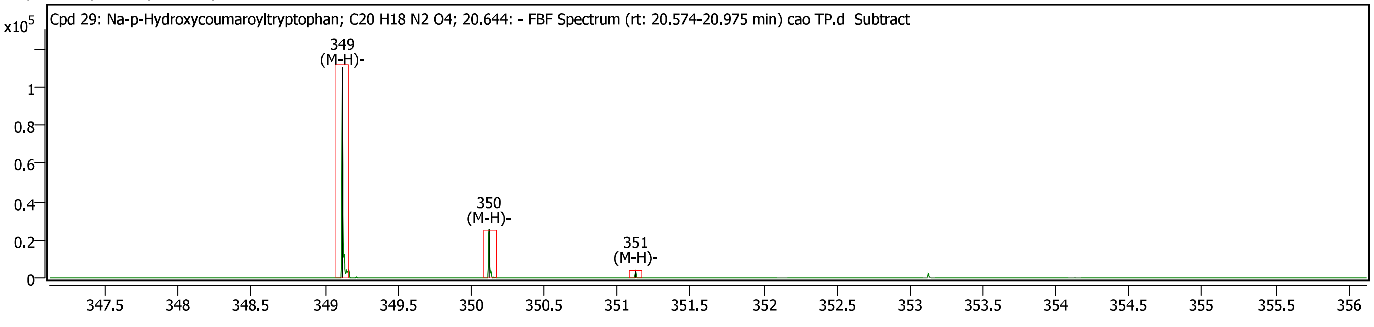

Compound ID Table

Name	Formula	Species	RT	RT Diff	Mass	CAS	ID Source	Score	Score (Lib)	Score (Tgt)
Na-p-Hydroxycoumaroyltryptophan	C20 H18 N2 O4	(M-H)-	20.644		350.1264		FBF	99.70		99.70
Moschamindole	C20 H18 N2 O4	(M-H)-	20.644		350.1264	99615-94-8	FBF	99.70		99.70

Cpd 94: N-dodecanoyl-L-Homoserine lactone-3-hydrazone-fluorescein

Name	Formula	RT	RI	Mass	Diff (Tgt, ppm)	CAS	ID Source	Score	Algorithm
N-dodecanoyl-L-Homoserine lactone-3-hydrazone-fluorescein	C37 H40 N4 O8 S	20.644		700.2534	-4.71		FBF	83.48	FBF

Species	m/z	Score (Tgt)	Score (Lib)	Score (DB)	Score (MFG)	Score (RT)
(M-2H)-2 (M-H)-	349 699	83.48				

Compound Chromatograms (overlaid)

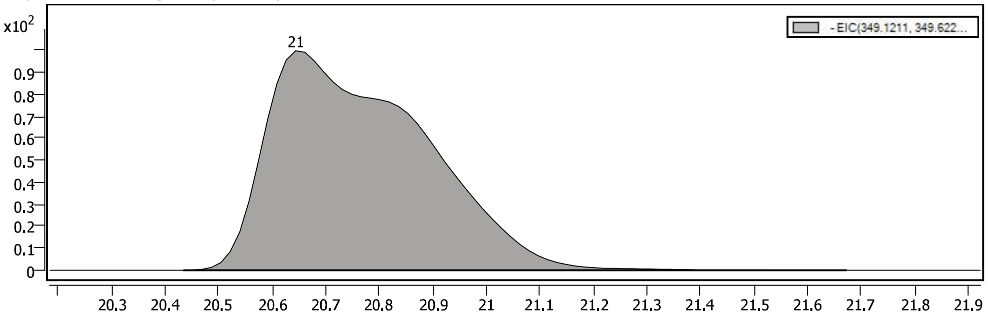

Structure

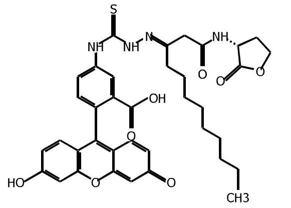

Compound Spectra (overlaid)

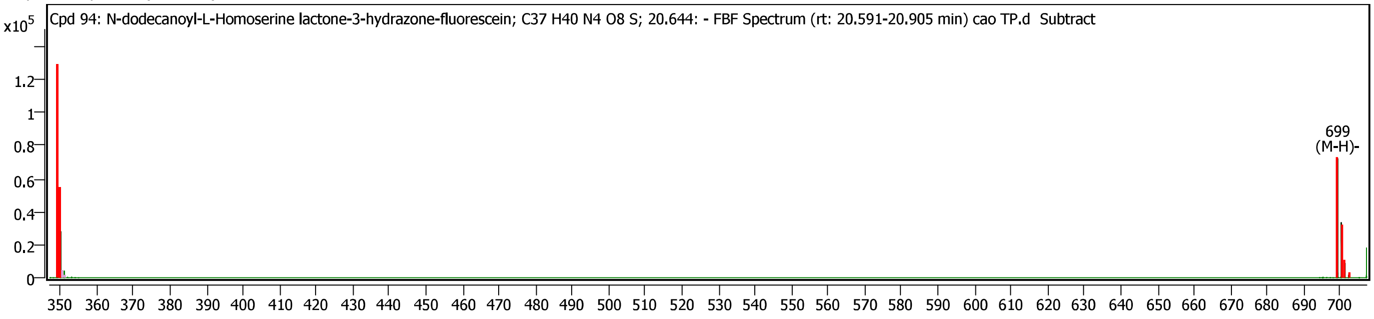

Compound ID Table

Name	Formula	Species	RT	RT Diff	Mass	CAS	ID Source	Score	Score (Lib)	Score (Tgt)
N-dodecanoyl-L-Homoserine lactone-3-hydrazone-fluorescein	C37 H40 N4 O8 S	(M-2H)-2 (M-H)-	20.644		700.2534		FBF	83.48		83.48

Cpd 131: 6''-O-Caffeoylastragalin

Name	Formula	RT	RI	Mass	Diff (Tgt, ppm)	CAS	ID Source	Score	Algorithm
6''-O-Caffeoylastragalin	C30 H26 O14	20.748		610.1324	0.19	190328-43-9	M-FBF	99.35	FBF

Species	m/z	Score (Tgt)	Score (Lib)	Score (DB)	Score (MFG)	Score (RT)
(M-2H)-2 (M-H)-	304 609	99.35				

Compound Chromatograms (overlaid)

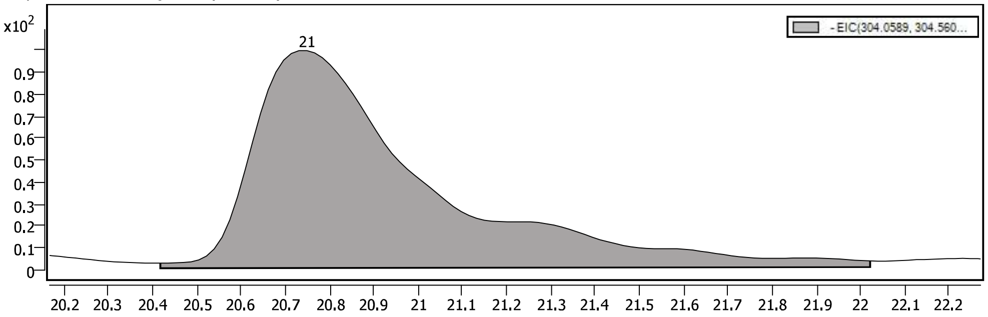

Structure

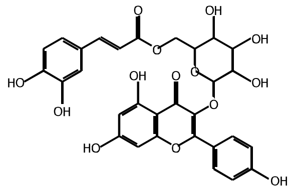

Compound Spectra (overlaid)

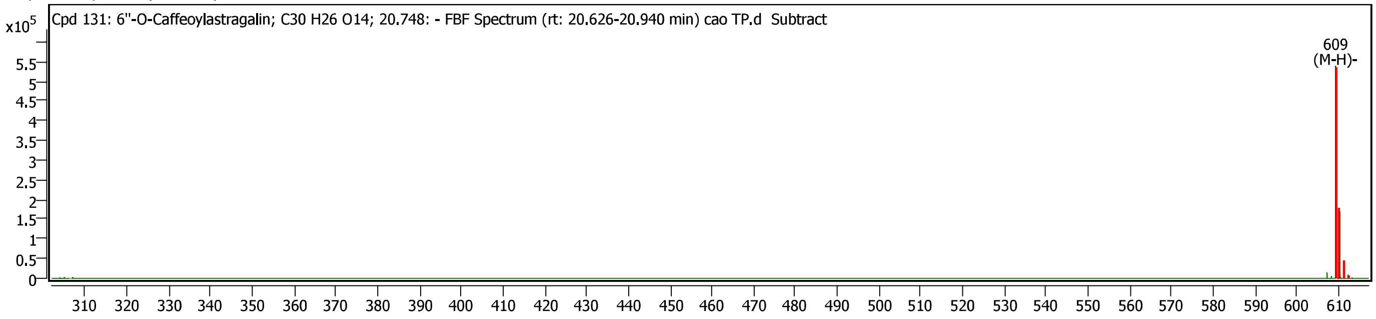

Counts vs. Mass-to-Charge (m/z)

Compound ID Table

Name	Formula	Species	RT	RT Diff	Mass	CAS	ID Source	Score	Score (Lib)	Score (Tgt)
6''-O-Caffeoylastragalin	C30 H26 O14	(M-2H)-2 (M-H)-	20.748		610.1324	190328-43-9	FBF	99.35		99.35
Kaempferol 3-(6''-caffeoylglucoside)	C30 H26 O14	(M-2H)-2 (M-H)-	20.748		610.1324		FBF	99.35		99.35
6''-Caffeoylisoorientin	C30 H26 O14	(M-2H)-2 (M-H)-	20.748		610.1324		FBF	99.35		99.35
Gallocatechin-(4alpha->8)-epigallocatechin	C30 H26 O14	(M-2H)-2 (M-H)-	20.748		610.1324		FBF	99.35		99.35
Helichrysoside	C30 H26 O14	(M-2H)-2 (M-H)-	20.748		610.1324		FBF	99.35		99.35
Isoorientin 2''-O-(E)-caffeate	C30 H26 O14	(M-2H)-2 (M-H)-	20.748		610.1324		FBF	99.35		99.35
Isoorientin 6''-O-caffeate	C30 H26 O14	(M-2H)-2 (M-H)-	20.748		610.1324		FBF	99.35		99.35
Prodelphinidin B	C30 H26 O14	(M-2H)-2 (M-H)-	20.748		610.1324	86631-36-9	FBF	99.35		99.35
Orientin 2''-O-caffeate	C30 H26 O14	(M-2H)-2 (M-H)-	20.748		610.1324		FBF	99.35		99.35
Quercetin 3- (3''-p-coumarylglucoside)	C30 H26 O14	(M-2H)-2 (M-H)-	20.748		610.1324		FBF	99.35		99.35
Quercetin 3-(2''-p-coumarylglucoside)	C30 H26 O14	(M-2H)-2 (M-H)-	20.748		610.1324		FBF	99.35		99.35
Quercetin 3-(3-p-coumaroylglucoside)	C30 H26 O14	(M-2H)-2 (M-H)-	20.748		610.1324	76211-70-6	FBF	99.35		99.35
Theasinensin C	C30 H26 O14	(M-2H)-2 (M-H)-	20.748		610.1324	89013-69-4	FBF	99.35		99.35
Orientin 7-O-caffeate	C30 H26 O14	(M-2H)-2 (M-H)-	20.748		610.1324		FBF	99.35		99.35

Cpd 140: 2''-O-Feruloylorientin

Name	Formula	RT	RI	Mass	Diff (Tgt, ppm)	CAS	ID Source	Score	Algorithm
2''-O-Feruloylorientin	C31 H28 O14	21.115		624.1478	-0.20		M-FBF	99.01	FBF

Species	m/z	Score (Tgt)	Score (Lib)	Score (DB)	Score (MFG)	Score (RT)
(M-H)-	623	99.01				

Compound Chromatograms (overlaid)

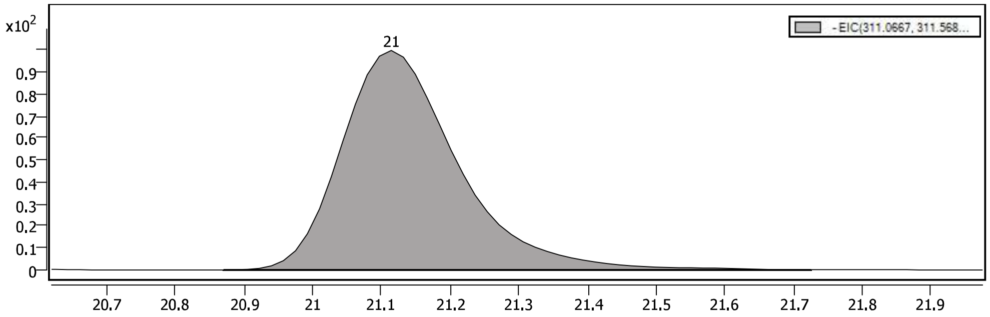

Counts (%) vs. Acquisition Time (min)

Structure

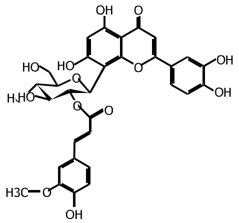

Compound Spectra (overlaid)

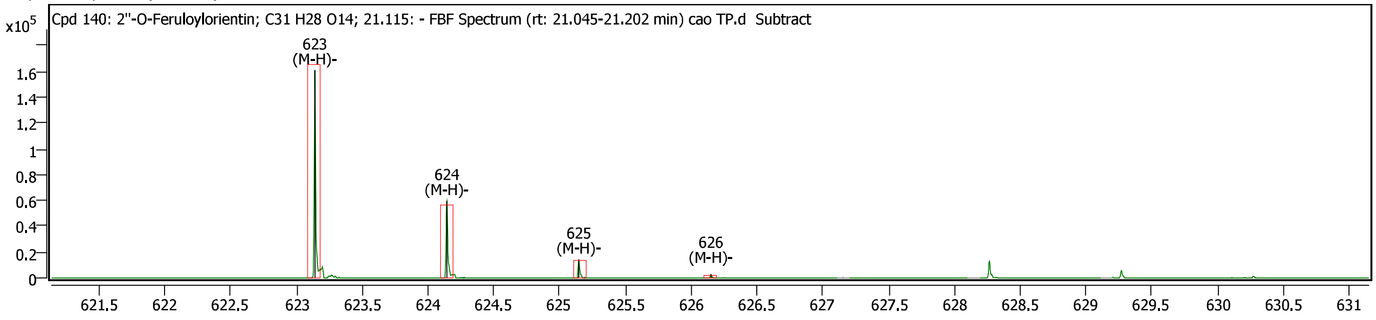

Counts vs. Mass-to-Charge (m/z)

Compound Screening Report

Compound ID Table

Name	Formula	Species	RT	RT Diff	Mass	CAS	ID Source	Score	Score (Lib)	Score (Tgt)
2''-O-Feruloylorientin	C31 H28 O14	(M-H)-	21.115		624.1478		FBF	99.01		99.01
4''-Methyl-6''-(3,4-dihydroxy-E-cinnamoyl)isoorientin	C31 H28 O14	(M-H)-	21.115		624.1478		FBF	99.01		99.01
Isoorientin 2''-O-(E)-ferulate	C31 H28 O14	(M-H)-	21.115		624.1478		FBF	99.01		99.01
6-Hydroxykaempferol 6-methyl ether 3-(6''-p-coumaroylglucoside)	C31 H28 O14	(M-H)-	21.115		624.1478		FBF	99.01		99.01
Isorhamnetin 3-(6''-p-coumarylglucoside)	C31 H28 O14	(M-H)-	21.115		624.1478		FBF	99.01		99.01
Isorhamnetin 7-(6''-p-coumarylglucoside)	C31 H28 O14	(M-H)-	21.115		624.1478		FBF	99.01		99.01
Kaempferol 3-(6''-ferulylglucoside)	C31 H28 O14	(M-H)-	21.115		624.1478		FBF	99.01		99.01
Luteolin 7-(6''-ferulylglucoside)	C31 H28 O14	(M-H)-	21.115		624.1478		FBF	99.01		99.01

Cpd 138: <Kaempferol>

Name	Formula	RT	RI	Mass	Diff (Tgt, ppm)	CAS	ID Source	Score	Algorithm
<Kaempferol>	C15 H10 O6	21.446		286.0476	-0.63	520-18-3	M-FBF	99.47	FBF

Species	m/z	Score (Tgt)	Score (Lib)	Score (DB)	Score (MFG)	Score (RT)
(M-H)-	285	99.47				

Compound Chromatograms (overlaid)

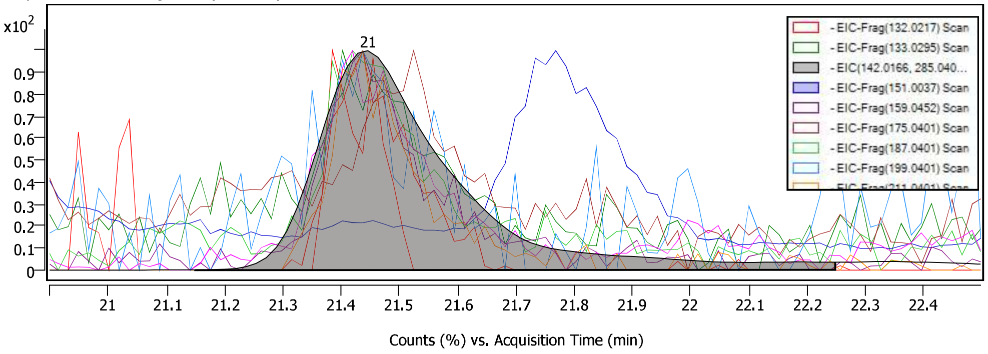

Structure

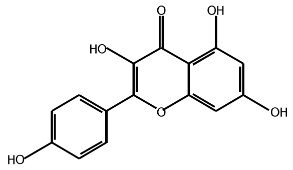

Coelution Plot

Compound Spectra (overlaid)

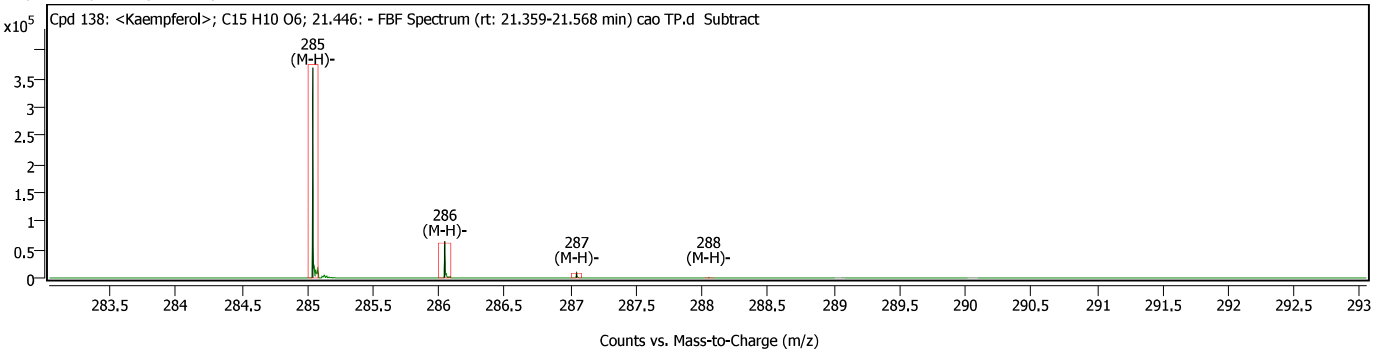

Fragment Spectrum (raw)

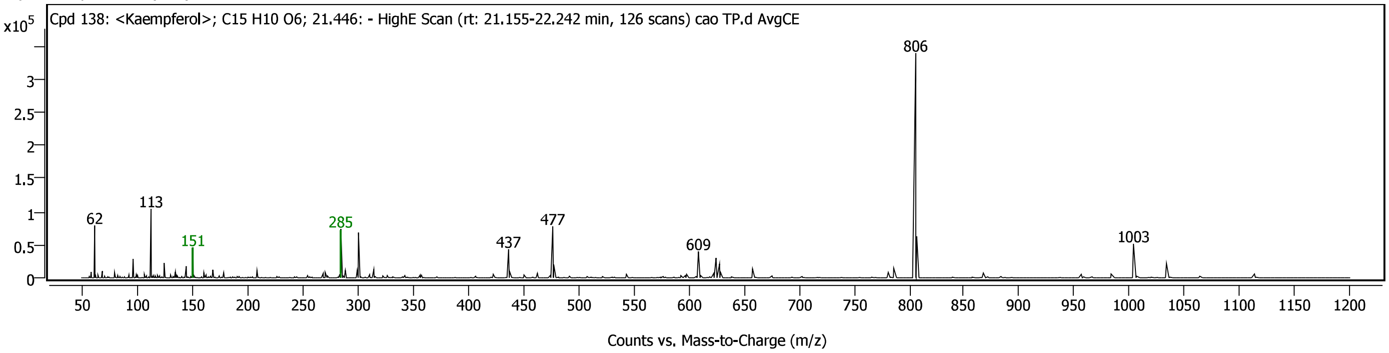

Compound Screening Report

Compound ID Table

Name	Formula	Species	RT	RT Diff	Mass	CAS	ID Source	Score	Score (Lib)	Score (Tgt)
<Kaempferol>	C15 H10 O6	(M-H)-	21.446		286.0476	520-18-3	FBF	99.47		99.47
<3,7,8,4'-Tetrahydroxyflavone>	C15 H10 O6	(M-H)-	21.446		286.0476		FBF	99.47		99.47
<Datiscetin>	C15 H10 O6	(M-H)-	21.446		286.0476		FBF	99.47		99.47
<8-Hydroxygalangin>	C15 H10 O6	(M-H)-	21.446		286.0476		FBF	99.47		99.47
<6-Demethoxycapillarisin>	C15 H10 O6	(M-H)-	21.446		286.0476	61854-36-2	FBF	99.47		99.47
<7,8,2',4'-Tetrahydroxyisoflavone>	C15 H10 O6	(M-H)-	21.446		286.0476		FBF	99.47		99.47
<7,3',4',5'-Tetrahydroxyflavone>	C15 H10 O6	(M-H)-	21.446		286.0476		FBF	99.47		99.47
<6-Hydroxygenistein>	C15 H10 O6	(M-H)-	21.446		286.0476		FBF	99.47		99.47
<6-Hydroxygalangin>	C15 H10 O6	(M-H)-	21.446		286.0476		FBF	99.47		99.47
<2'-Hydroxygenistein>	C15 H10 O6	(M-H)-	21.446		286.0476		FBF	99.47		99.47
<5,7,2',6'-Tetrahydroxyflavone>	C15 H10 O6	(M-H)-	21.446		286.0476		FBF	99.47		99.47
<5,7,2',5'-Tetrahydroxyflavone>	C15 H10 O6	(M-H)-	21.446		286.0476		FBF	99.47		99.47
<5,7,2',3'-Tetrahydroxyflavone>	C15 H10 O6	(M-H)-	21.446		286.0476		FBF	99.47		99.47
<Aureusidin>	C15 H10 O6	(M-H)-	21.446		286.0476		FBF	99.47		99.47
<7,8,3',4'-Tetrahydroxyisoflavone>	C15 H10 O6	(M-H)-	21.446		286.0476		FBF	99.47		99.47
<Citreorosein>	C15 H10 O6	(M-H)-	21.446		286.0476	481-73-2	FBF	99.47		99.47
<Baptigenin>	C15 H10 O6	(M-H)-	21.446		286.0476		FBF	99.47		99.47
<Orobol>	C15 H10 O6	(M-H)-	21.446		286.0476		FBF	99.47		99.47
<Norartocarpetin>	C15 H10 O6	(M-H)-	21.446		286.0476		FBF	99.47		99.47
<Maritimetin>	C15 H10 O6	(M-H)-	21.446		286.0476		FBF	99.47		99.47
<Luteolin>	C15 H10 O6	(M-H)-	21.446		286.0476	491-70-3	FBF	99.47		99.47
<Scutellarein>	C15 H10 O6	(M-H)-	21.446		286.0476		FBF	99.47		99.47
<Isoscutellarein>	C15 H10 O6	(M-H)-	21.446		286.0476		FBF	99.47		99.47
<Fisetin>	C15 H10 O6	(M-H)-	21.446		286.0476	528-48-3	FBF	99.47		99.47
<Helmon>	C15 H10 O6	(M-H)-	21.446		286.0476		FBF	99.47		99.47

Cpd 16: PRE

Name	Formula	RT	RI	Mass	Diff (Tgt, ppm)	CAS	ID Source	Score	Algorithm
PRE	C44 H44 O24	21.760		956.2225	0.21	160564-02-3	FBF	98.08	FBF

Species	m/z	Score (Tgt)	Score (Lib)	Score (DB)	Score (MFG)	Score (RT)
(M-2H)-2 (M-H)-	477 955	98.08				

Compound Chromatograms (overlaid)

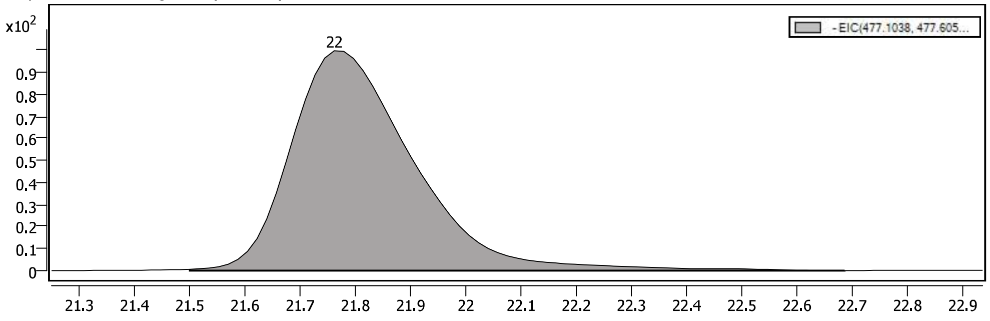

Structure

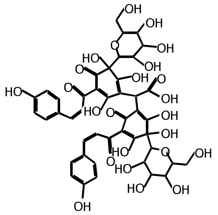

Compound Spectra (overlaid)

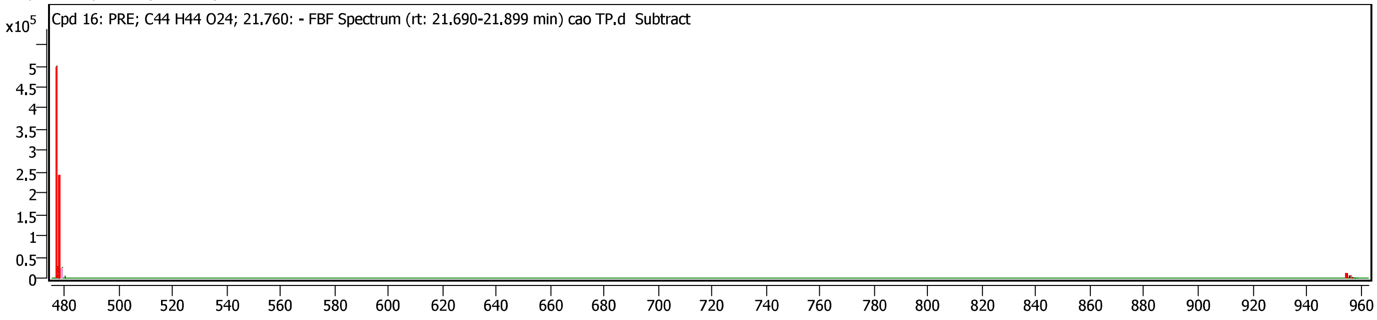

Compound ID Table

Name	Formula	Species	RT	RT Diff	Mass	CAS	ID Source	Score	Score (Lib)	Score (Tgt)
PRE	C44 H44 O24	(M-2H)-2 (M-H)-	21.760		956.2225	160564-02-3	FBF	98.08		98.08

Cpd 116: 1,3,5,8-Tetrahydroxy-6-methoxy-2-methylanthraquinone 8-O-b-D-glucoside

Name	Formula	RT	RI	Mass	Diff (Tgt, ppm)	CAS	ID Source	Score	Algorithm
1,3,5,8-Tetrahydroxy-6-methoxy-2-methylanthraquinone 8-O-b-D-glucoside	C22 H22 O12	21.760		478.1111	0.04	101508-15-0	M-FBF	99.86	FBF

Species	m/z	Score (Tgt)	Score (Lib)	Score (DB)	Score (MFG)	Score (RT)
(M-H)-	477	99.86				

Compound Chromatograms (overlaid)

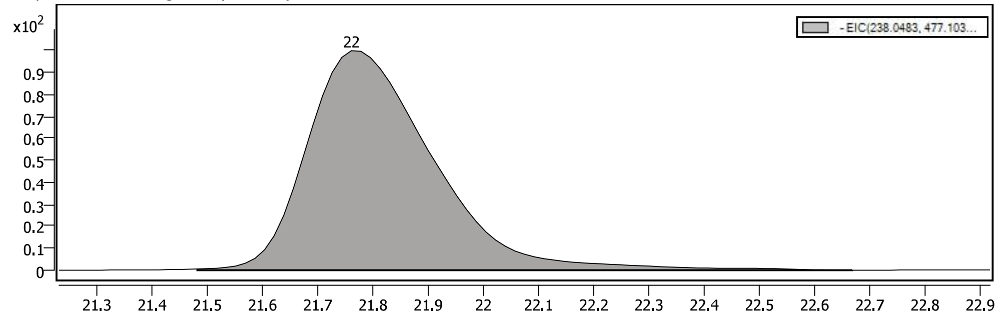

Structure

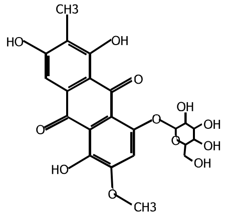

Compound Spectra (overlaid)

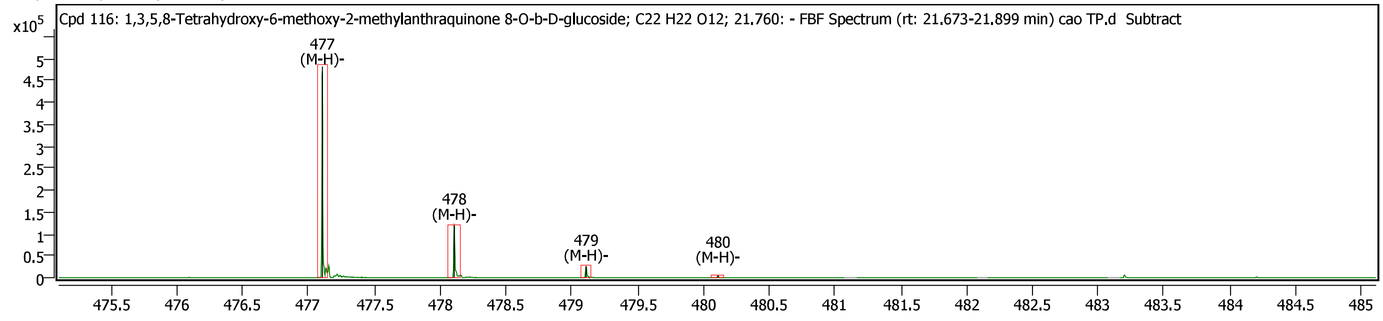

Compound ID Table

Name	Formula	Species	RT	RT Diff	Mass	CAS	ID Source	Score	Score (Lib)	Score (Tgt)
1,3,5,8-Tetrahydroxy-6-methoxy-2-methylanthraquinone 8-O-b-D-glucoside	C22 H22 O12	(M-H)-	21.760		478.1111	101508-15-0	FBF	99.86		99.86
Azaleatin 3-glucoside	C22 H22 O12	(M-H)-	21.760		478.1111		FBF	99.86		99.86
6-Hydroxykaempferol 3-methyl ether 7-glucoside	C22 H22 O12	(M-H)-	21.760		478.1111		FBF	99.86		99.86
8-Hydroxyluteolin 3'-methyl ether 7-glucoside	C22 H22 O12	(M-H)-	21.760		478.1111		FBF	99.86		99.86
8-Hydroxyluteolin 4'-methyl ether 8-glucoside	C22 H22 O12	(M-H)-	21.760		478.1111		FBF	99.86		99.86
Alliumoside A	C22 H22 O12	(M-H)-	21.760		478.1111	58902-89-9	FBF	99.86		99.86
Azaleatin 3-galactoside	C22 H22 O12	(M-H)-	21.760		478.1111		FBF	99.86		99.86
Eupatolitin 3-apioside	C22 H22 O12	(M-H)-	21.760		478.1111		FBF	99.86		99.86
Estragonoside	C22 H22 O12	(M-H)-	21.760		478.1111	181020-33-7	FBF	99.86		99.86
Eupafolin 4'-glucoside	C22 H22 O12	(M-H)-	21.760		478.1111	112208-83-0	FBF	99.86		99.86
Europetin 3-rhamnoside	C22 H22 O12	(M-H)-	21.760		478.1111		FBF	99.86		99.86
Herbacetin 7-methyl ether 3-glucoside	C22 H22 O12	(M-H)-	21.760		478.1111		FBF	99.86		99.86
6-Methoxyluteolin 7-glucoside	C22 H22 O12	(M-H)-	21.760		478.1111		FBF	99.86		99.86
7-O-Methylgossypetin 3-rhamnoside	C22 H22 O12	(M-H)-	21.760		478.1111	56768-33-3	FBF	99.86		99.86
6-Methoxyluteolin 3'-glucoside	C22 H22 O12	(M-H)-	21.760		478.1111	112208-84-1	FBF	99.86		99.86
Isorhamnetin 3-galactoside	C22 H22 O12	(M-H)-	21.760		478.1111	5041-82-7	FBF	99.86		99.86
6-Methoxykaempferol 7-glucoside	C22 H22 O12	(M-H)-	21.760		478.1111		FBF	99.86		99.86
6-Methoxykaempferol 3-glucoside	C22 H22 O12	(M-H)-	21.760		478.1111		FBF	99.86		99.86
6-Methoxykaempferol 3-galactoside	C22 H22 O12	(M-H)-	21.760		478.1111		FBF	99.86		99.86
6-Hydroxyluteolin 7-methyl ether 6-glucoside	C22 H22 O12	(M-H)-	21.760		478.1111		FBF	99.86		99.86
6-Hydroxyluteolin 7-methyl ether 6-galactoside	C22 H22 O12	(M-H)-	21.760		478.1111		FBF	99.86		99.86
6-Hydroxykaempferol 4'-methyl ether 7-glucoside	C22 H22 O12	(M-H)-	21.760		478.1111		FBF	99.86		99.86
Isorhamnetin 3-glucoside	C22 H22 O12	(M-H)-	21.760		478.1111		FBF	99.86		99.86
6-Hydroxykaempferol 3-methyl ether 6-glucoside	C22 H22 O12	(M-H)-	21.760		478.1111		FBF	99.86		99.86
5,2',4',5'-Tetrahydroxy-7-methoxy-4-phenylcoumarin 5-O-glucoside	C22 H22 O12	(M-H)-	21.760		478.1111		FBF	99.86		99.86
2-O-(4-Hydroxycinnamoyl)-1-O-galloyl-beta-D-glucopyranoside	C22 H22 O12	(M-H)-	21.760		478.1111	94356-18-0	FBF	99.86		99.86
Pedaliin	C22 H22 O12	(M-H)-	21.760		478.1111	22860-72-6	FBF	99.86		99.86
8-C-Rhamnosyleuropetin	C22 H22 O12	(M-H)-	21.760		478.1111		FBF	99.86		99.86
Annulatin 7-rhamnoside	C22 H22 O12	(M-H)-	21.760		478.1111		FBF	99.86		99.86
Tricetin 3'-methyl ether 7-glucoside	C22 H22 O12	(M-H)-	21.760		478.1111		FBF	99.86		99.86
Quercetin 3-methyl ether 7-glucoside	C22 H22 O12	(M-H)-	21.760		478.1111		FBF	99.86		99.86
Isorhamnetin 7-glucoside	C22 H22 O12	(M-H)-	21.760		478.1111		FBF	99.86		99.86
Tamarixin	C22 H22 O12	(M-H)-	21.760		478.1111		FBF	99.86		99.86
Tamarixetin 7-glucoside	C22 H22 O12	(M-H)-	21.760		478.1111		FBF	99.86		99.86
Tamarixetin 3-galactoside	C22 H22 O12	(M-H)-	21.760		478.1111		FBF	99.86		99.86
Syringetin 3-xyloside	C22 H22 O12	(M-H)-	21.760		478.1111		FBF	99.86		99.86
Sexangularetin 3-glucoside	C22 H22 O12	(M-H)-	21.760		478.1111		FBF	99.86		99.86
Rhamnetin 5-glucoside	C22 H22 O12	(M-H)-	21.760		478.1111		FBF	99.86		99.86
Rhamnetin 3-glucoside	C22 H22 O12	(M-H)-	21.760		478.1111		FBF	99.86		99.86
Rhamnetin 3-galactoside	C22 H22 O12	(M-H)-	21.760		478.1111		FBF	99.86		99.86
Ranupenin 3-rhamnoside	C22 H22 O12	(M-H)-	21.760		478.1111		FBF	99.86		99.86
Quercetin 3-methyl ether 5-glucoside	C22 H22 O12	(M-H)-	21.760		478.1111		FBF	99.86		99.86
Sexangularetin 3-galactoside	C22 H22 O12	(M-H)-	21.760		478.1111		FBF	99.86		99.86
Isorhamnetin 4'-glucoside	C22 H22 O12	(M-H)-	21.760		478.1111		FBF	99.86		99.86
Quercetin 3-methyl ether 4'-glucoside	C22 H22 O12	(M-H)-	21.760		478.1111		FBF	99.86		99.86
Nepetin 4'-glucoside	C22 H22 O12	(M-H)-	21.760		478.1111		FBF	99.86		99.86
Laricitrin 3-rhamnoside	C22 H22 O12	(M-H)-	21.760		478.1111		FBF	99.86		99.86
Quercetin 3-methyl ether 3'-glucoside	C22 H22 O12	(M-H)-	21.760		478.1111		FBF	99.86		99.86
Keyakinin B	C22 H22 O12	(M-H)-	21.760		478.1111		FBF	99.86		99.86
Mearnsitrin	C22 H22 O12	(M-H)-	21.760		478.1111	30484-88-9	FBF	99.86		99.86
Myricetin 3,4'-dimethyl ether 3'-xyloside	C22 H22 O12	(M-H)-	21.760		478.1111		FBF	99.86		99.86
Myricetin 5-methyl ether 3-rhamnoside	C22 H22 O12	(M-H)-	21.760		478.1111		FBF	99.86		99.86
Nepitrin	C22 H22 O12	(M-H)-	21.760		478.1111	569-90-4	FBF	99.86		99.86
Patuletin 3-rhamnoside	C22 H22 O12	(M-H)-	21.760		478.1111		FBF	99.86		99.86
Quercetin 3-methyl ether 7-galactoside	C22 H22 O12	(M-H)-	21.760		478.1111		FBF	99.86		99.86
Pollenin B	C22 H22 O12	(M-H)-	21.760		478.1111	30484-94-7	FBF	99.86		99.86

Cpd 198: N1,N5,N10-Tricoumaroyl spermidine

Name	Formula	RT	RI	Mass	Diff (Tgt, ppm)	CAS	ID Source	Score	Algorithm
N1,N5,N10-Tricoumaroyl spermidine	C34 H37 N3 O6	22.403		583.2680	-0.40		FBF	99.68	FBF

Species	m/z	Score (Tgt)	Score (Lib)	Score (DB)	Score (MFG)	Score (RT)
(M-H)-	582	99.68				

Compound Chromatograms (overlaid)

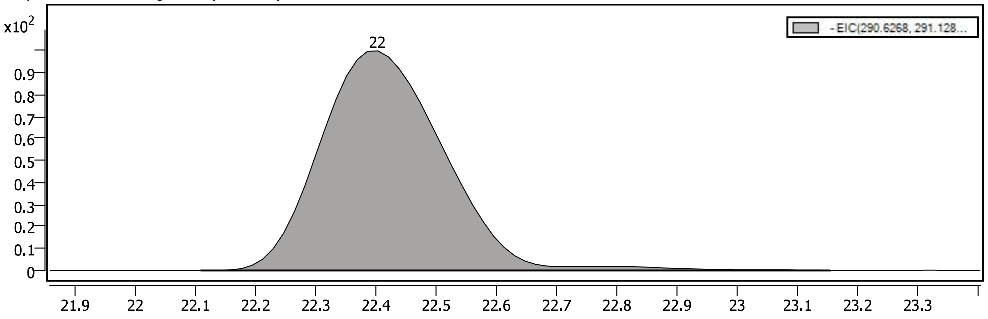

Structure

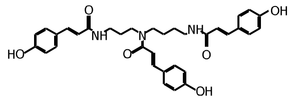

Compound Spectra (overlaid)

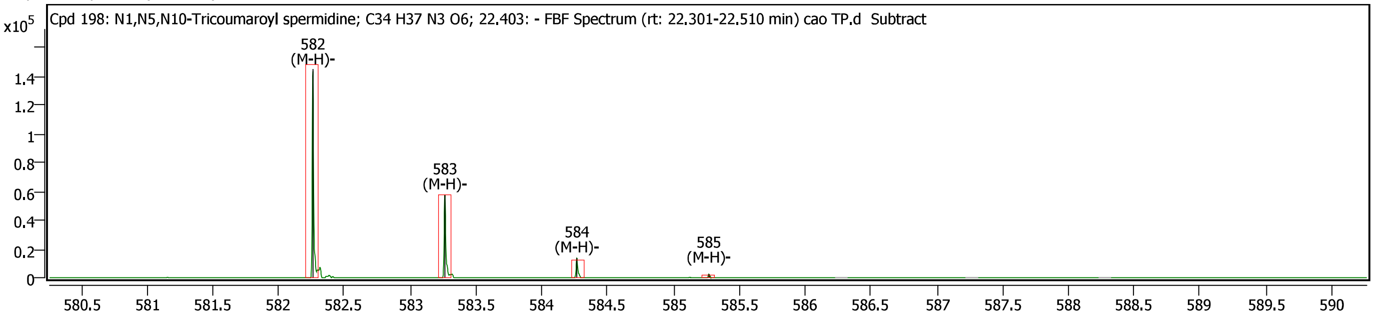

Compound ID Table

Name	Formula	Species	RT	RT Diff	Mass	CAS	ID Source	Score	Score (Lib)	Score (Tgt)
N1,N5,N10-Tricoumaroyl spermidine	C34 H37 N3 O6	(M-H)-	22.403		583.2680		FBF	99.68		99.68

Cpd 199: 6'''-Deamino-6'''-dehydro-6'''-oxoneomycin C

Name	Formula	RT	RI	Mass	Diff (Tgt, ppm)	CAS	ID Source	Score	Algorithm
6'''-Deamino-6'''-dehydro-6'''-oxoneomycin C	C23 H43 N5 O14	22.806		613.2785	-3.55		FBF	80.34	FBF

Species	m/z	Score (Tgt)	Score (Lib)	Score (DB)	Score (MFG)	Score (RT)
(M-H)-	612	80.34				

Compound Chromatograms (overlaid)

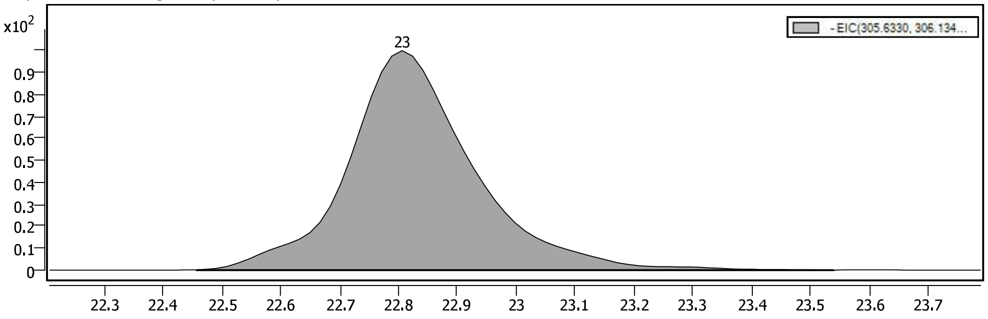

Structure

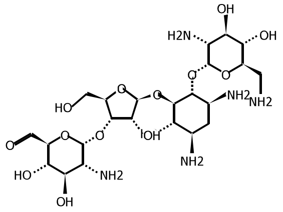

Compound Spectra (overlaid)

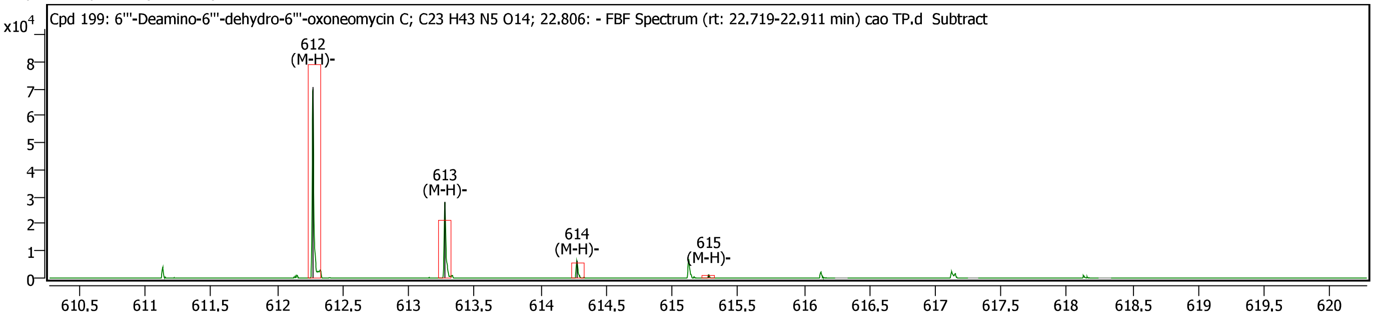

Compound ID Table

Name	Formula	Species	RT	RT Diff	Mass	CAS	ID Source	Score	Score (Lib)	Score (Tgt)
6'''-Deamino-6'''-dehydro-6'''-oxoneomycin C	C23 H43 N5 O14	(M-H)-	22.806		613.2785		FBF	80.34		80.34

Cpd 143: Tiliroside

Name	Formula	RT	RI	Mass	Diff (Tgt, ppm)	CAS	ID Source	Score	Algorithm
Tiliroside	C30 H26 O13	22.946		594.1371	-0.43	20316-62-5	M-FBF	99.07	FBF

Species	m/z	Score (Tgt)	Score (Lib)	Score (DB)	Score (MFG)	Score (RT)
(M-H)-	593	99.07				

Compound Chromatograms (overlaid)

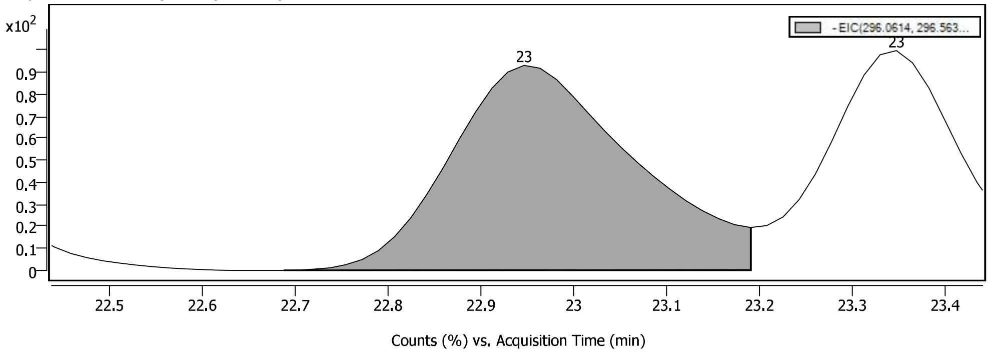

Structure

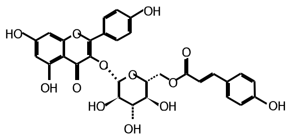

Compound Spectra (overlaid)

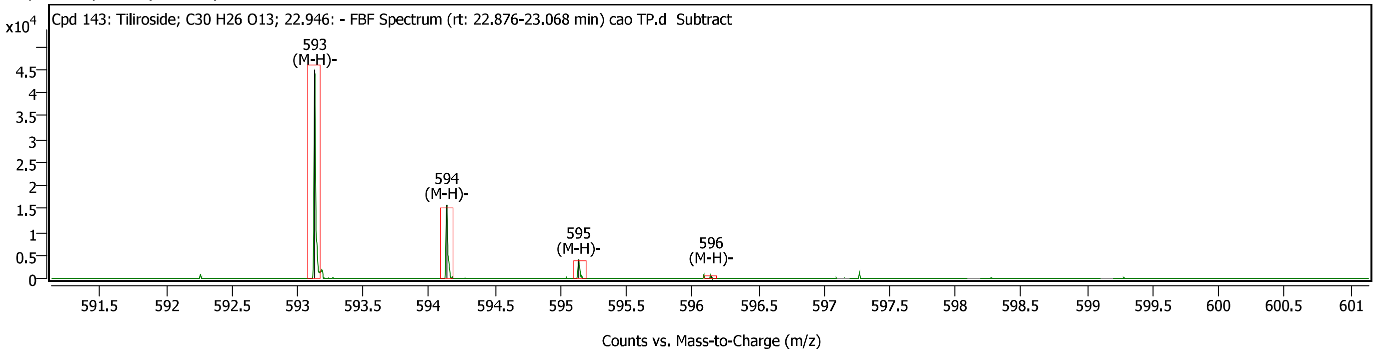

Compound ID Table

Name	Formula	Species	RT	RT Diff	Mass	CAS	ID Source	Score	Score (Lib)	Score (Tgt)
Tiliroside	C30 H26 O13	(M-H)-	22.946		594.1371	20316-62-5	FBF	99.07		99.07
Apigenin 7-(6''-E-Caffeoylglucoside)	C30 H26 O13	(M-H)-	22.946		594.1371		FBF	99.07		99.07
2''-O-trans-p-Coumaroylastragalin	C30 H26 O13	(M-H)-	22.946		594.1371	137018-32-7	FBF	99.07		99.07
Piperitoside	C30 H26 O13	(M-H)-	22.946		594.1371	20196-92-3	FBF	99.07		99.07
Epigallocatechin-(4beta->8)-catechin	C30 H26 O13	(M-H)-	22.946		594.1371	77983-30-3	FBF	99.07		99.07
Epicatechin-(4beta->8)-gallocatechin	C30 H26 O13	(M-H)-	22.946		594.1371		FBF	99.07		99.07
Buddlenoid A	C30 H26 O13	(M-H)-	22.946		594.1371	142750-32-1	FBF	99.07		99.07
Apigenin 7-glucoside-4'-trans-caffeate	C30 H26 O13	(M-H)-	22.946		594.1371		FBF	99.07		99.07
6''-O-p-Coumaroyltrifolin	C30 H26 O13	(M-H)-	22.946		594.1371	68170-52-5	FBF	99.07		99.07
8-Hydroxyapigenin 8-(6''-E-p-coumaroylglucoside)	C30 H26 O13	(M-H)-	22.946		594.1371		FBF	99.07		99.07
7-O-(4-Hydroxycinnamoyl) astragalin	C30 H26 O13	(M-H)-	22.946		594.1371	51795-36-9	FBF	99.07		99.07
3''-O-Caffeoylcosmosiin	C30 H26 O13	(M-H)-	22.946		594.1371	79366-62-4	FBF	99.07		99.07
Isoorientin 2''-O-(E)-p-coumarate	C30 H26 O13	(M-H)-	22.946		594.1371		FBF	99.07		99.07
Maritimetin 6-(6''-p-coumarylglucoside)	C30 H26 O13	(M-H)-	22.946		594.1371		FBF	99.07		99.07
Gallocatechin-(4alpha->8)-epicatechin	C30 H26 O13	(M-H)-	22.946		594.1371	79199-56-7	FBF	99.07		99.07
Luteolin 7-(6''-p-coumarylglucoside)	C30 H26 O13	(M-H)-	22.946		594.1371		FBF	99.07		99.07
Kaempferol 3- (6''-p-coumarylgalactoside)	C30 H26 O13	(M-H)-	22.946		594.1371		FBF	99.07		99.07
Kaempferol 3-(2''-p-coumarylglucoside)	C30 H26 O13	(M-H)-	22.946		594.1371		FBF	99.07		99.07
Kaempferol 3-(3''-p-coumarylglucoside)	C30 H26 O13	(M-H)-	22.946		594.1371		FBF	99.07		99.07
Kaempferol 3-(4''-p-coumaroylglucoside)	C30 H26 O13	(M-H)-	22.946		594.1371		FBF	99.07		99.07
Kaempferol 3-(5''-feruloylapioside)	C30 H26 O13	(M-H)-	22.946		594.1371		FBF	99.07		99.07
Kaempferol 7-(6''-p-coumarylglucoside)	C30 H26 O13	(M-H)-	22.946		594.1371		FBF	99.07		99.07
Luteolin 7-(2''-p-coumaroylglucoside)	C30 H26 O13	(M-H)-	22.946		594.1371		FBF	99.07		99.07
Kaempferol 3-(2''-(Z)-p-coumaroylglucoside)	C30 H26 O13	(M-H)-	22.946		594.1371		FBF	99.07		99.07

Cpd 95: N1,N5,N10-Triferuloyl spermidine

Name	Formula	RT	RI	Mass	Diff (Tgt, ppm)	CAS	ID Source	Score	Algorithm
N1,N5,N10-Triferuloyl spermidine	C37 H43 N3 O9	23.365		673.2996	-0.54		FBF	99.71	FBF

Species	m/z	Score (Tgt)	Score (Lib)	Score (DB)	Score (MFG)	Score (RT)
(M-H)-	672	99.71				

Compound Chromatograms (overlaid)

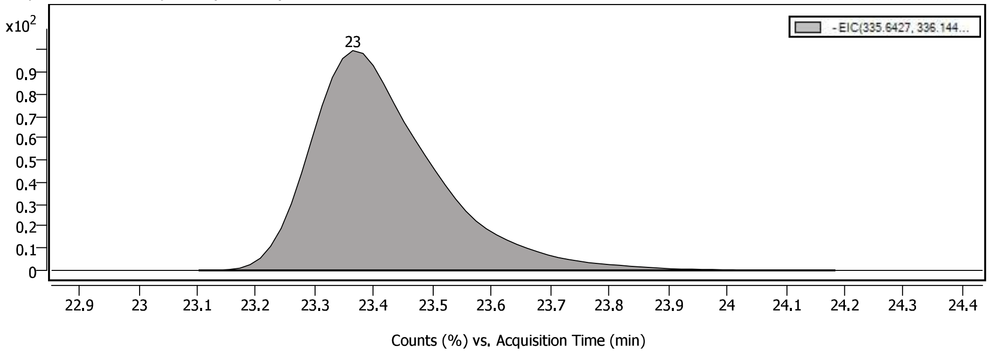

Structure

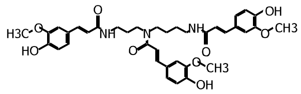

Compound Spectra (overlaid)

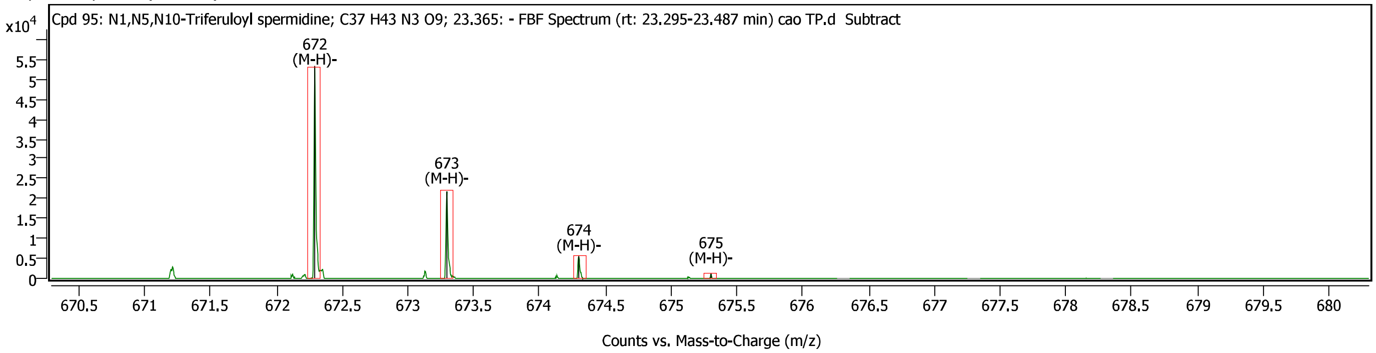

Compound ID Table

Name	Formula	Species	RT	RT Diff	Mass	CAS	ID Source	Score	Score (Lib)	Score (Tgt)
N1,N5,N10-Triferuloyl spermidine	C37 H43 N3 O9	(M-H)-	23.365		673.2996		FBF	99.71		99.71

Cpd 25: Eucaglobulin

Name	Formula	RT	RI	Mass	Diff (Tgt, ppm)	CAS	ID Source	Score	Algorithm
Eucaglobulin	C23 H30 O12	24.516		498.1735	-0.37	241130-84-7	M-FBF	99.81	FBF

Species	m/z	Score (Tgt)	Score (Lib)	Score (DB)	Score (MFG)	Score (RT)
(M-H)-	497	99.81				

Compound Chromatograms (overlaid)

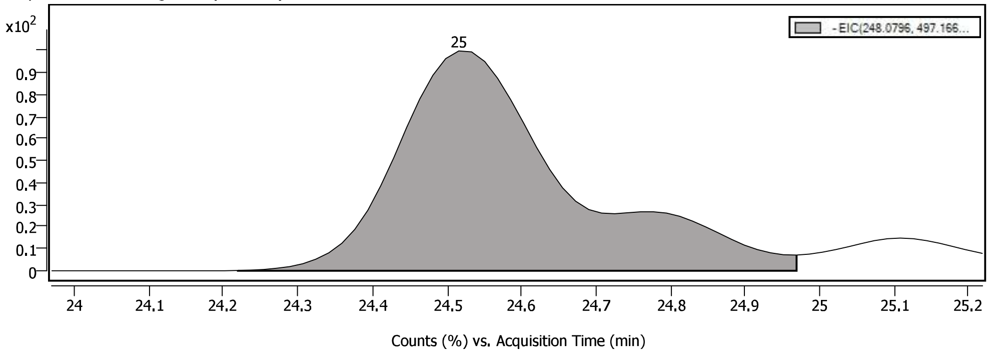

Structure

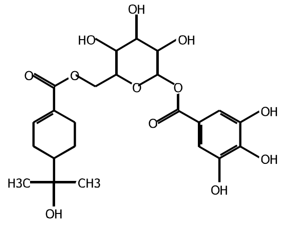

Compound Spectra (overlaid)

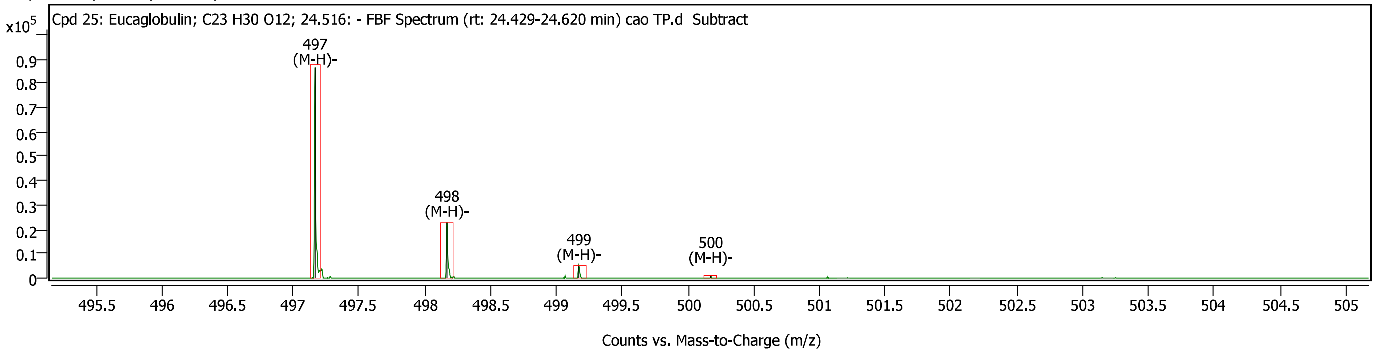

Compound ID Table

Name	Formula	Species	RT	RT Diff	Mass	CAS	ID Source	Score	Score (Lib)	Score (Tgt)
Eucaglobulin	C23 H30 O12	(M-H)-	24.516		498.1735	241130-84-7	FBF	99.81		99.81
Musabalbisiane B	C23 H30 O12	(M-H)-	24.516		498.1735	143199-58-0	FBF	99.81		99.81

Cpd 64: (+)-9,10,18-trihydroxy-12Z-octadecenoic acid

Name	Formula	RT	RI	Mass	Diff (Tgt, ppm)	CAS	ID Source	Score	Algorithm
(+)-9,10,18-trihydroxy-12Z-octadecenoic acid	C18 H34 O5	28.336		330.2405	-0.32		M-FBF	99.65	FBF

Species	m/z	Score (Tgt)	Score (Lib)	Score (DB)	Score (MFG)	Score (RT)
(M-H)-	329	99.65				

Compound Chromatograms (overlaid)

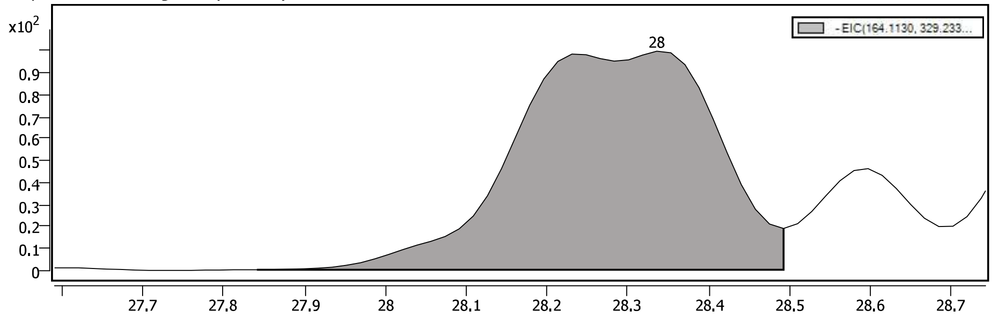

Structure

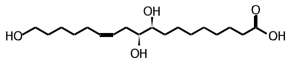

Compound Spectra (overlaid)

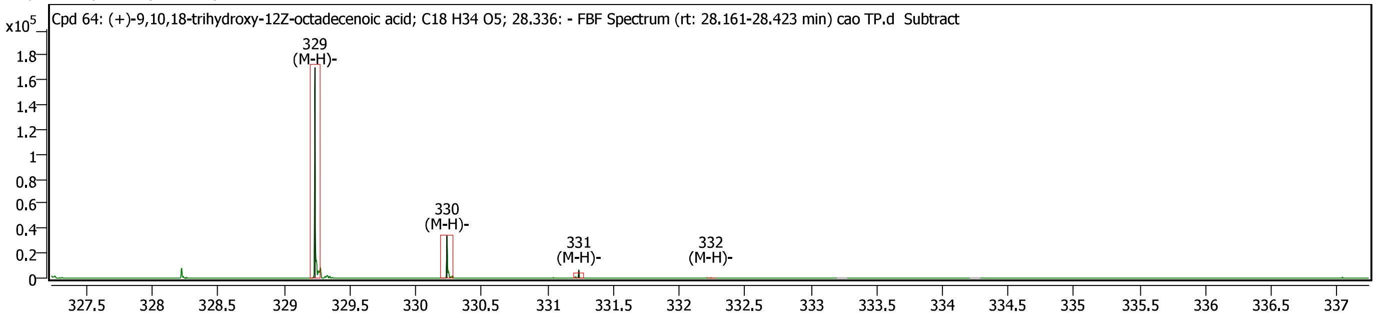

Compound ID Table

Name	Formula	Species	RT	RT Diff	Mass	CAS	ID Source	Score	Score (Lib)	Score (Tgt)
(+)-9,10,18-trihydroxy-12Z-octadecenoic acid	C18 H34 O5	(M-H)-	28.336		330.2405		FBF	99.65		99.65
9,10,18-TriHOME(12)	C18 H34 O5	(M-H)-	28.336		330.2405		FBF	99.65		99.65
11,12,13-TriHOME	C18 H34 O5	(M-H)-	28.336		330.2405		FBF	99.65		99.65
11,12,13-trihydroxy-9-octadecenoic acid	C18 H34 O5	(M-H)-	28.336		330.2405		FBF	99.65		99.65
5,8,12-TriHOME(9)	C18 H34 O5	(M-H)-	28.336		330.2405		FBF	99.65		99.65
5,8,12-trihydroxy-9-octadecenoic acid	C18 H34 O5	(M-H)-	28.336		330.2405		FBF	99.65		99.65
9,10,13-trihydroxy-11-octadecenoic acid	C18 H34 O5	(M-H)-	28.336		330.2405		FBF	99.65		99.65
9,10,13-TriHOME(11)	C18 H34 O5	(M-H)-	28.336		330.2405		FBF	99.65		99.65
9,10,18-TriHOME(12Z)	C18 H34 O5	(M-H)-	28.336		330.2405		FBF	99.65		99.65
9,10-Dihydroxy-12,13-epoxyoctadecanoate	C18 H34 O5	(M-H)-	28.336		330.2405		FBF	99.65		99.65
9,12,13-TriHOME(10)	C18 H34 O5	(M-H)-	28.336		330.2405		FBF	99.65		99.65
9,12,13-trihydroxy-10-octadecenoic acid	C18 H34 O5	(M-H)-	28.336		330.2405		FBF	99.65		99.65
9S,10S,11R-trihydroxy-12Z-octadecenoic acid	C18 H34 O5	(M-H)-	28.336		330.2405		FBF	99.65		99.65
9S,12S,13S-trihydroxy-10E-octadecenoic acid	C18 H34 O5	(M-H)-	28.336		330.2405		FBF	99.65		99.65
9,10,18-trihydroxy-12-octadecenoic acid	C18 H34 O5	(M-H)-	28.336		330.2405		FBF	99.65		99.65

Cpd 231: Phloionolic acid

Name	Formula	RT	RI	Mass	Diff (Tgt, ppm)	CAS	ID Source	Score	Algorithm
Phloionolic acid	C18 H36 O5	28.545		332.2562	-0.16		M-FBF	99.53	FBF

Species	m/z	Score (Tgt)	Score (Lib)	Score (DB)	Score (MFG)	Score (RT)
(M-H)-	331	99.53				

Compound Chromatograms (overlaid)

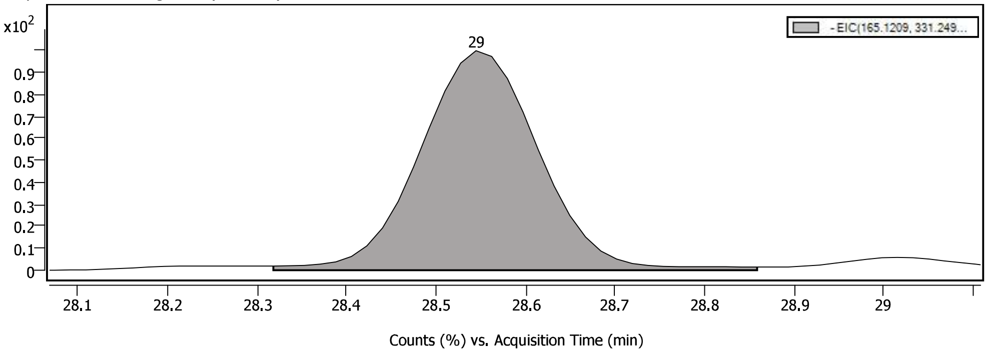

Structure

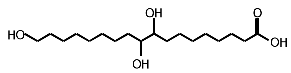

Compound Spectra (overlaid)

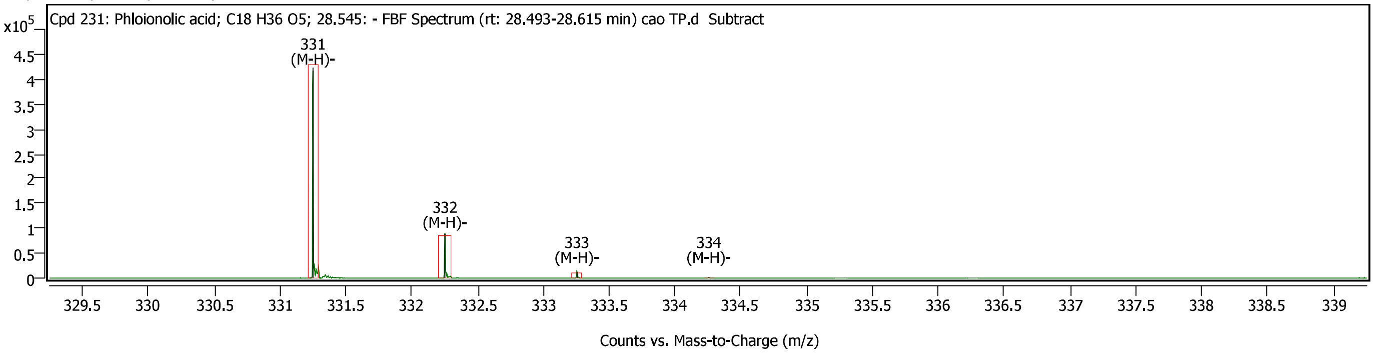

Compound ID Table

Name	Formula	Species	RT	RT Diff	Mass	CAS	ID Source	Score	Score (Lib)	Score (Tgt)
Phloionolic acid	C18 H36 O5	(M-H)-	28.545		332.2562		FBF	99.53		99.53
9,10,13-Trihydroxystearic acid	C18 H36 O5	(M-H)-	28.545		332.2562	50439-74-2	FBF	99.53		99.53
18-hydroxy-9S,10R-dihydroxy-stearic acid	C18 H36 O5	(M-H)-	28.545		332.2562		FBF	99.53		99.53
9R,10S,18-trihydroxy-stearic acid	C18 H36 O5	(M-H)-	28.545		332.2562		FBF	99.53		99.53

Cpd 65: (+)-9,10,18-trihydroxy-12Z-octadecenoic acid

Name	Formula	RT	RI	Mass	Diff (Tgt, ppm)	CAS	ID Source	Score	Algorithm
(+)-9,10,18-trihydroxy-12Z-octadecenoic acid	C18 H34 O5	28.841		330.2404	-0.71		M-FBF	99.21	FBF

Species	m/z	Score (Tgt)	Score (Lib)	Score (DB)	Score (MFG)	Score (RT)
(M-H)-	329	99.21				

Compound Chromatograms (overlaid)

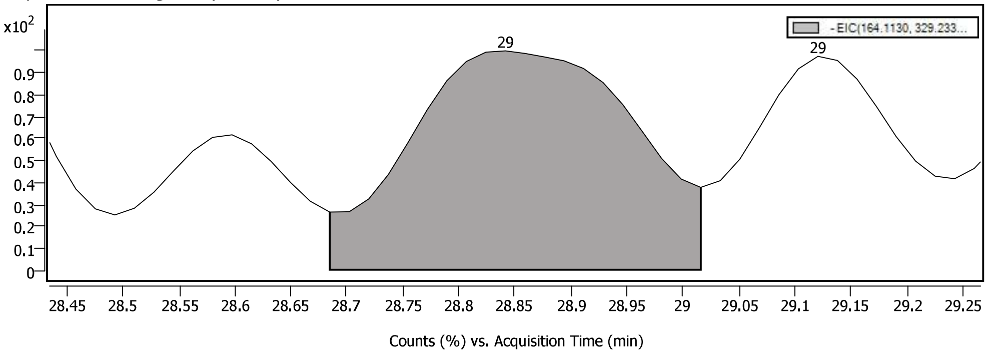

Structure

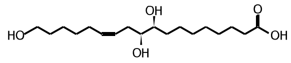

Compound Spectra (overlaid)

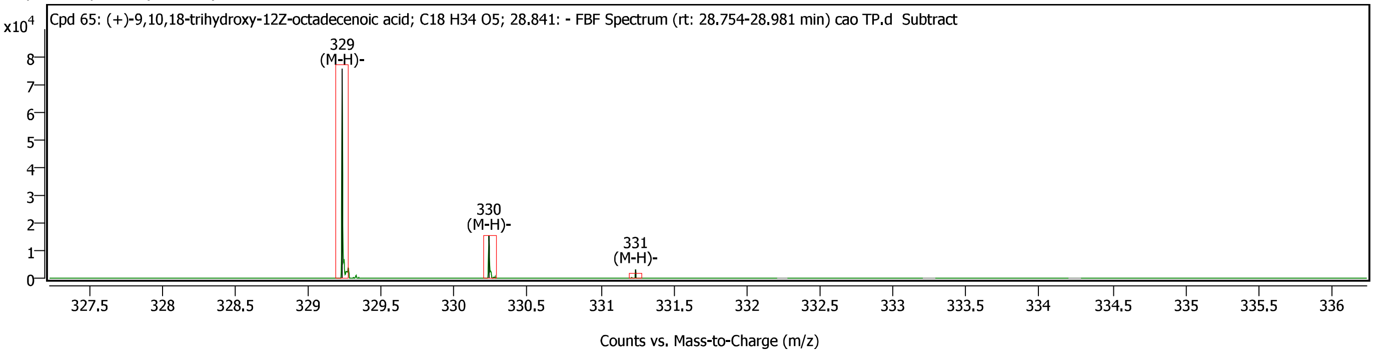

Compound ID Table

Name	Formula	Species	RT	RT Diff	Mass	CAS	ID Source	Score	Score (Lib)	Score (Tgt)
(+)-9,10,18-trihydroxy-12Z-octadecenoic acid	C18 H34 O5	(M-H)-	28.841		330.2404		FBF	99.21		99.21
9,10,18-TriHOME(12)	C18 H34 O5	(M-H)-	28.841		330.2404		FBF	99.21		99.21
11,12,13-TriHOME	C18 H34 O5	(M-H)-	28.841		330.2404		FBF	99.21		99.21
11,12,13-trihydroxy-9-octadecenoic acid	C18 H34 O5	(M-H)-	28.841		330.2404		FBF	99.21		99.21
5,8,12-TriHOME(9)	C18 H34 O5	(M-H)-	28.841		330.2404		FBF	99.21		99.21
5,8,12-trihydroxy-9-octadecenoic acid	C18 H34 O5	(M-H)-	28.841		330.2404		FBF	99.21		99.21
9,10,13-trihydroxy-11-octadecenoic acid	C18 H34 O5	(M-H)-	28.841		330.2404		FBF	99.21		99.21
9,10,13-TriHOME(11)	C18 H34 O5	(M-H)-	28.841		330.2404		FBF	99.21		99.21
9,10,18-TriHOME(12Z)	C18 H34 O5	(M-H)-	28.841		330.2404		FBF	99.21		99.21
9,10-Dihydroxy-12,13-epoxyoctadecanoate	C18 H34 O5	(M-H)-	28.841		330.2404		FBF	99.21		99.21
9,12,13-TriHOME(10)	C18 H34 O5	(M-H)-	28.841		330.2404		FBF	99.21		99.21
9,12,13-trihydroxy-10-octadecenoic acid	C18 H34 O5	(M-H)-	28.841		330.2404		FBF	99.21		99.21
9S,10S,11R-trihydroxy-12Z-octadecenoic acid	C18 H34 O5	(M-H)-	28.841		330.2404		FBF	99.21		99.21
9S,12S,13S-trihydroxy-10E-octadecenoic acid	C18 H34 O5	(M-H)-	28.841		330.2404		FBF	99.21		99.21
9,10,18-trihydroxy-12-octadecenoic acid	C18 H34 O5	(M-H)-	28.841		330.2404		FBF	99.21		99.21

Cpd 63: 10-hydroxy-hexadecan-1,16-dioic acid

Name	Formula	RT	RI	Mass	Diff (Tgt, ppm)	CAS	ID Source	Score	Algorithm
10-hydroxy-hexadecan-1,16-dioic acid	C16 H30 O5	28.894		302.2092	-0.54		M-FBF	99.62	FBF

Species	m/z	Score (Tgt)	Score (Lib)	Score (DB)	Score (MFG)	Score (RT)
(M-H)-	301	99.62				

Compound Chromatograms (overlaid)

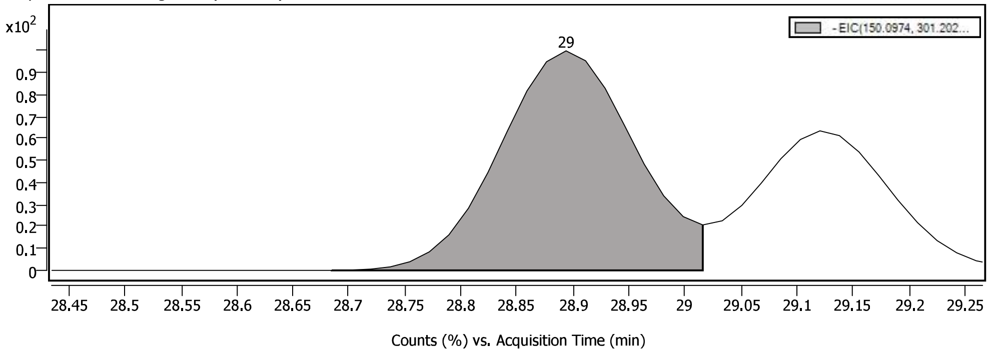

Structure

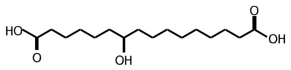

Compound Spectra (overlaid)

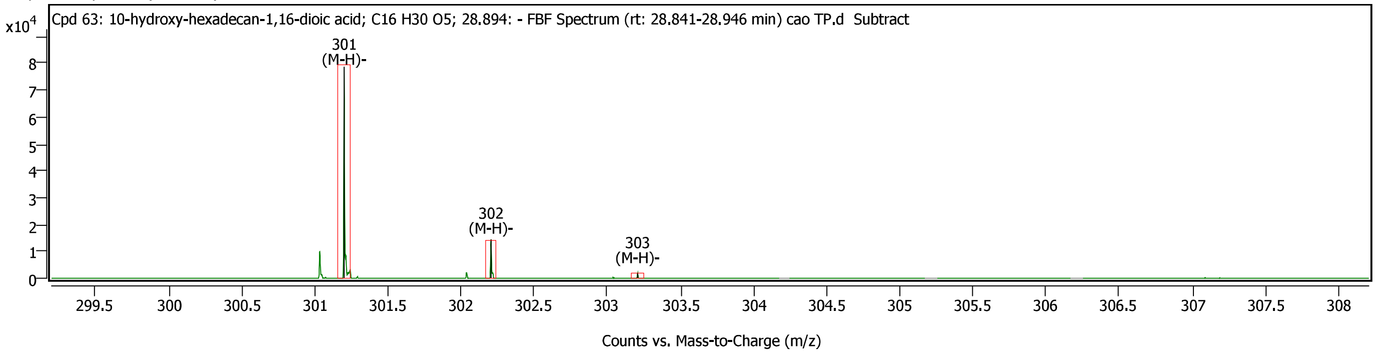

Compound ID Table

Name	Formula	Species	RT	RT Diff	Mass	CAS	ID Source	Score	Score (Lib)	Score (Tgt)
10-hydroxy-hexadecan-1,16-dioic acid	C16 H30 O5	(M-H)-	28.894		302.2092		FBF	99.62		99.62
9-hydroxy-hexadecan-1,16-dioic acid	C16 H30 O5	(M-H)-	28.894		302.2092		FBF	99.62		99.62

Cpd 228: Emedastine

Name	Formula	RT	RI	Mass	Diff (Tgt, ppm)	CAS	ID Source	Score	Algorithm
Emedastine	C17 H26 N4 O	28.894		302.2092	-4.72		FBF	88.50	FBF

Species	m/z	Score (Tgt)	Score (Lib)	Score (DB)	Score (MFG)	Score (RT)
(M-H)-	301	88.50				

Compound Chromatograms (overlaid)

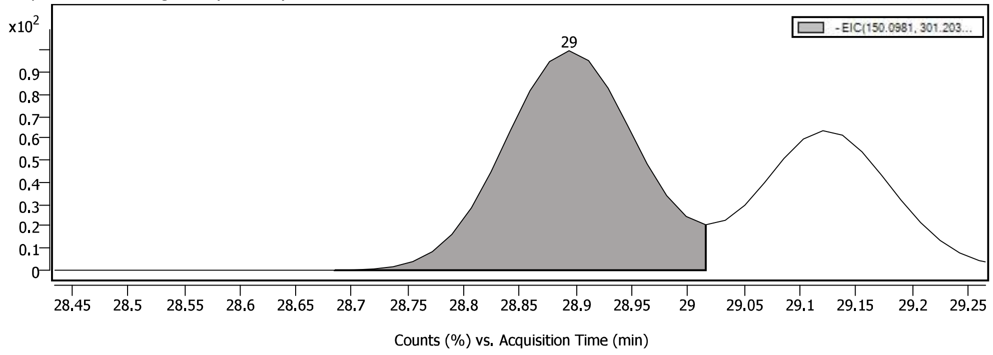

Structure

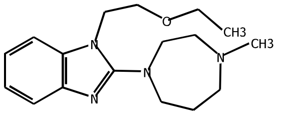

Compound Spectra (overlaid)

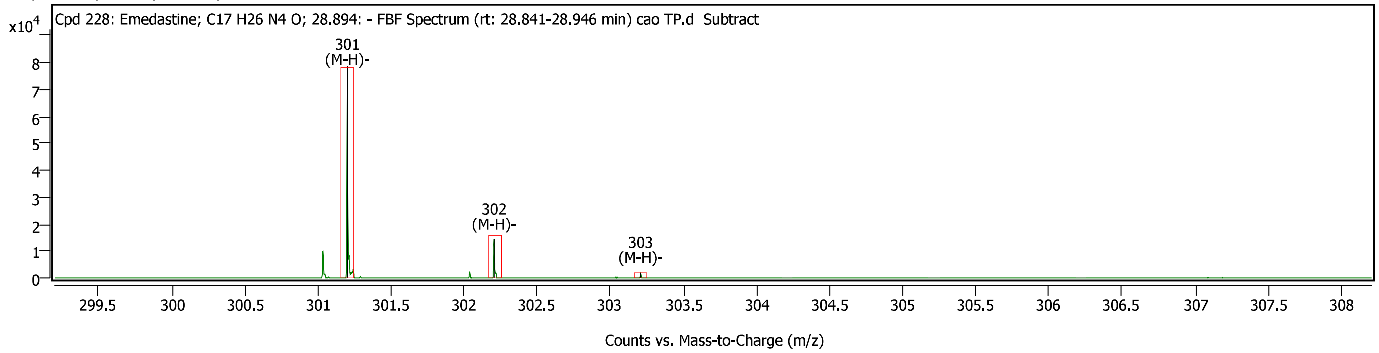

Compound ID Table

Name	Formula	Species	RT	RT Diff	Mass	CAS	ID Source	Score	Score (Lib)	Score (Tgt)
Emedastine	C17 H26 N4 O	(M-H)-	28.894		302.2092		FBF	88.50		88.50

Cpd 247: 6-Hydroxyluteolin 6-sulfate

Name	Formula	RT	RI	Mass	Diff (Tgt, ppm)	CAS	ID Source	Score	Algorithm
6-Hydroxyluteolin 6-sulfate	C15 H10 O10 S	28.964		381.9993	-0.53		M-FBF	97.02	FBF

Species	m/z	Score (Tgt)	Score (Lib)	Score (DB)	Score (MFG)	Score (RT)
(M-H)-	381	97.02				

Compound Chromatograms (overlaid)

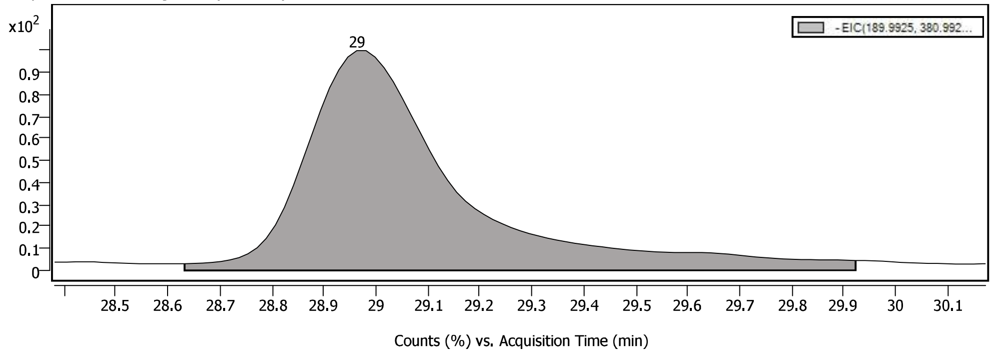

Structure

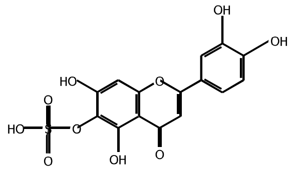

Compound Spectra (overlaid)

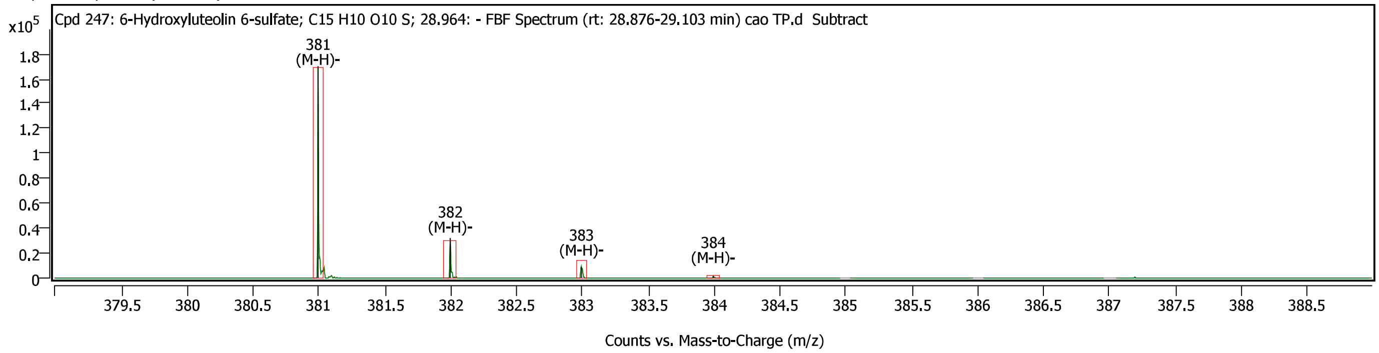

Compound ID Table

Name	Formula	Species	RT	RT Diff	Mass	CAS	ID Source	Score	Score (Lib)	Score (Tgt)
6-Hydroxyluteolin 6-sulfate	C15 H10 O10 S	(M-H)-	28.964		381.9993		FBF	97.02		97.02
8-Hydroxyluteolin 8-sulfate	C15 H10 O10 S	(M-H)-	28.964		381.9993		FBF	97.02		97.02
6-Hydroxyluteolin 7-sulfate	C15 H10 O10 S	(M-H)-	28.964		381.9993		FBF	97.02		97.02
8-Hydroxyluteolin 7-sulfate	C15 H10 O10 S	(M-H)-	28.964		381.9993		FBF	97.02		97.02
Quercetin 3-O-sulfate	C15 H10 O10 S	(M-H)-	28.964		381.9993		FBF	97.02		97.02
Quercetin 3'-O-sulfate	C15 H10 O10 S	(M-H)-	28.964		381.9993		FBF	97.02		97.02
Tricetin 3'-sulfate	C15 H10 O10 S	(M-H)-	28.964		381.9993		FBF	97.02		97.02
Quercetin 7-O-sulfate	C15 H10 O10 S	(M-H)-	28.964		381.9993		FBF	97.02		97.02

Cpd 66: (+)-9,10,18-trihydroxy-12Z-octadecenoic acid

Name	Formula	RT	RI	Mass	Diff (Tgt, ppm)	CAS	ID Source	Score	Algorithm
(+)-9,10,18-trihydroxy-12Z-octadecenoic acid	C18 H34 O5	29.121		330.2404	-0.71		M-FBF	98.58	FBF

Species	m/z	Score (Tgt)	Score (Lib)	Score (DB)	Score (MFG)	Score (RT)
(M-H)-	329	98.58				

Compound Chromatograms (overlaid)

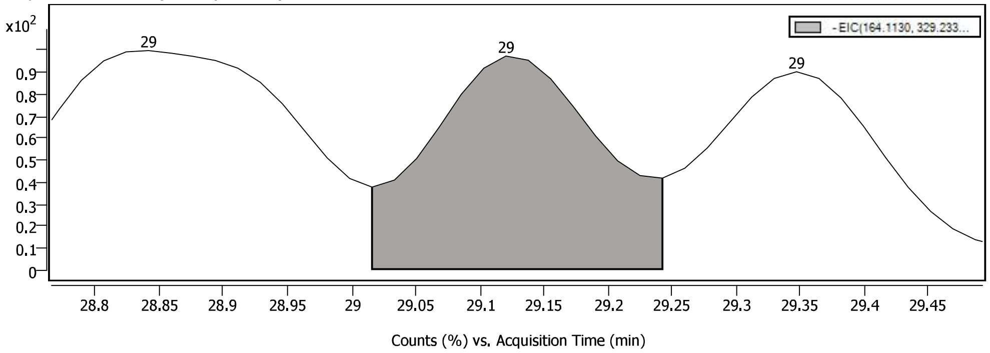

Structure

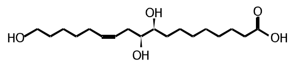

Compound Spectra (overlaid)

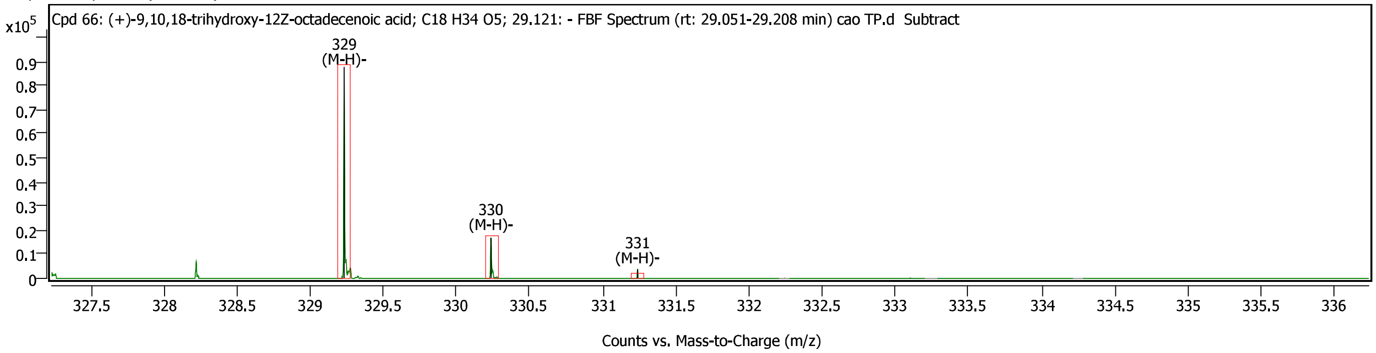

Compound ID Table

Name	Formula	Species	RT	RT Diff	Mass	CAS	ID Source	Score	Score (Lib)	Score (Tgt)
(+)-9,10,18-trihydroxy-12Z-octadecenoic acid	C18 H34 O5	(M-H)-	29.121		330.2404		FBF	98.58		98.58
9,10,18-TriHOME(12)	C18 H34 O5	(M-H)-	29.121		330.2404		FBF	98.58		98.58
11,12,13-TriHOME	C18 H34 O5	(M-H)-	29.121		330.2404		FBF	98.58		98.58
11,12,13-trihydroxy-9-octadecenoic acid	C18 H34 O5	(M-H)-	29.121		330.2404		FBF	98.58		98.58
5,8,12-TriHOME(9)	C18 H34 O5	(M-H)-	29.121		330.2404		FBF	98.58		98.58
5,8,12-trihydroxy-9-octadecenoic acid	C18 H34 O5	(M-H)-	29.121		330.2404		FBF	98.58		98.58
9,10,13-trihydroxy-11-octadecenoic acid	C18 H34 O5	(M-H)-	29.121		330.2404		FBF	98.58		98.58
9,10,13-TriHOME(11)	C18 H34 O5	(M-H)-	29.121		330.2404		FBF	98.58		98.58
9,10,18-TriHOME(12Z)	C18 H34 O5	(M-H)-	29.121		330.2404		FBF	98.58		98.58
9,10-Dihydroxy-12,13-epoxyoctadecanoate	C18 H34 O5	(M-H)-	29.121		330.2404		FBF	98.58		98.58
9,12,13-TriHOME(10)	C18 H34 O5	(M-H)-	29.121		330.2404		FBF	98.58		98.58
9,12,13-trihydroxy-10-octadecenoic acid	C18 H34 O5	(M-H)-	29.121		330.2404		FBF	98.58		98.58
9S,10S,11R-trihydroxy-12Z-octadecenoic acid	C18 H34 O5	(M-H)-	29.121		330.2404		FBF	98.58		98.58
9S,12S,13S-trihydroxy-10E-octadecenoic acid	C18 H34 O5	(M-H)-	29.121		330.2404		FBF	98.58		98.58
9,10,18-trihydroxy-12-octadecenoic acid	C18 H34 O5	(M-H)-	29.121		330.2404		FBF	98.58		98.58

Cpd 3: PE-Cer(d15:2(4E,6E)/18:0(2OH))

Name	Formula	RT	RI	Mass	Diff (Tgt, ppm)	CAS	ID Source	Score	Algorithm
PE-Cer(d15:2(4E,6E)/18:0(2OH))	C35 H69 N2 O7 P	29.347		660.4810	-4.91		FBF	85.76	FBF

Species	m/z	Score (Tgt)	Score (Lib)	Score (DB)	Score (MFG)	Score (RT)
(M-2H)-2 (M-H)-	329 659	85.76				

Compound Chromatograms (overlaid)

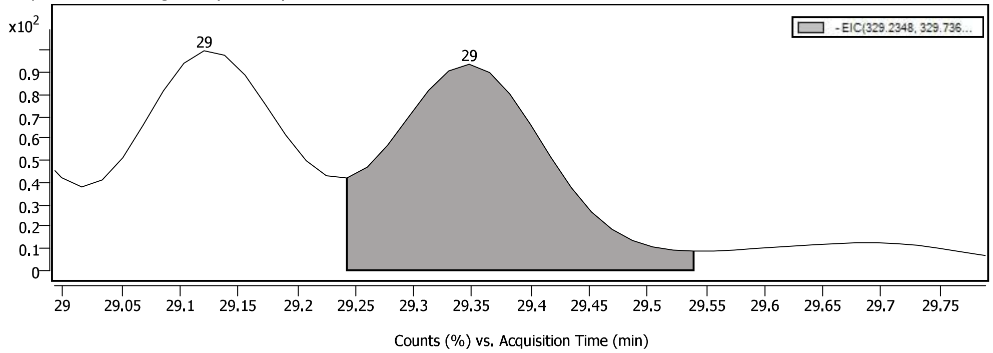

Structure

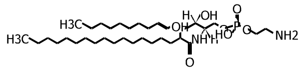

Compound Spectra (overlaid)

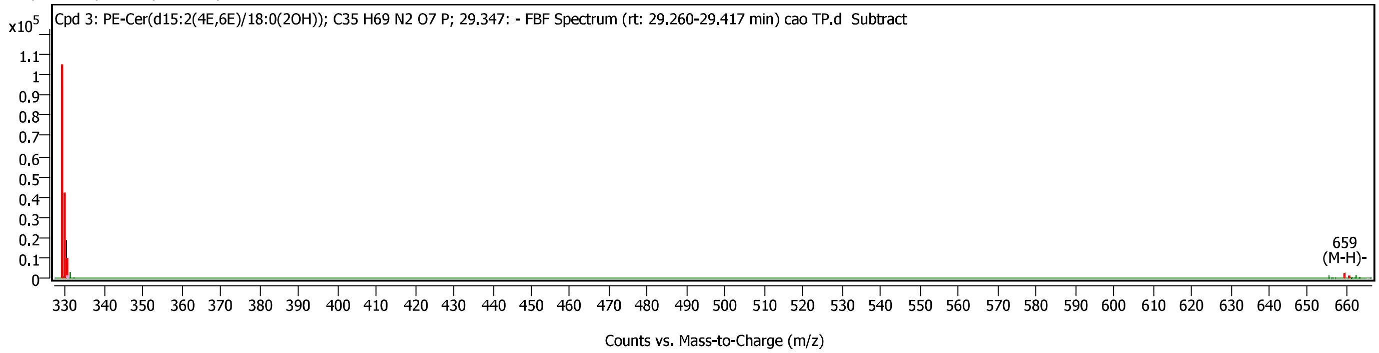

Compound ID Table

Name	Formula	Species	RT	RT Diff	Mass	CAS	ID Source	Score	Score (Lib)	Score (Tgt)
PE-Cer(d15:2(4E,6E)/18:0(2OH))	C35 H69 N2 O7 P	(M-2H)-2 (M-H)-	29.347		660.4810		FBF	85.76		85.76

Cpd 67: (+)-9,10,18-trihydroxy-12Z-octadecenoic acid

Name	Formula	RT	RI	Mass	Diff (Tgt, ppm)	CAS	ID Source	Score	Algorithm
(+)-9,10,18-trihydroxy-12Z-octadecenoic acid	C18 H34 O5	29.347		330.2405	-0.50		M-FBF	99.75	FBF

Species	m/z	Score (Tgt)	Score (Lib)	Score (DB)	Score (MFG)	Score (RT)
(M-H)-	329	99.75				

Compound Chromatograms (overlaid)

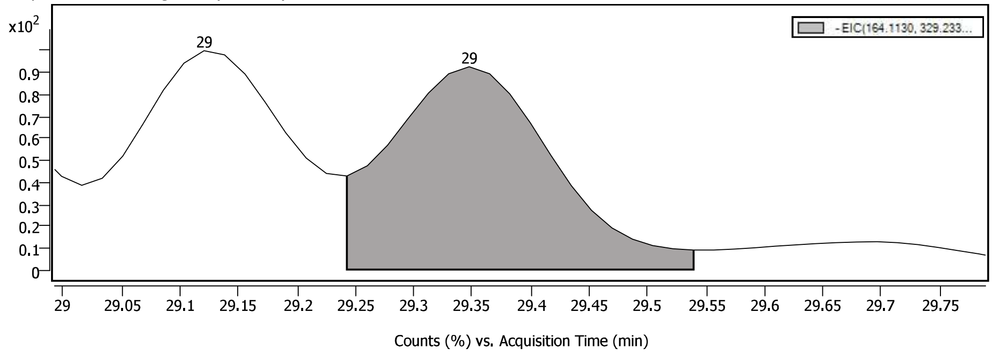

Structure

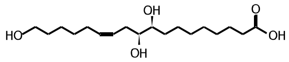

Compound Spectra (overlaid)

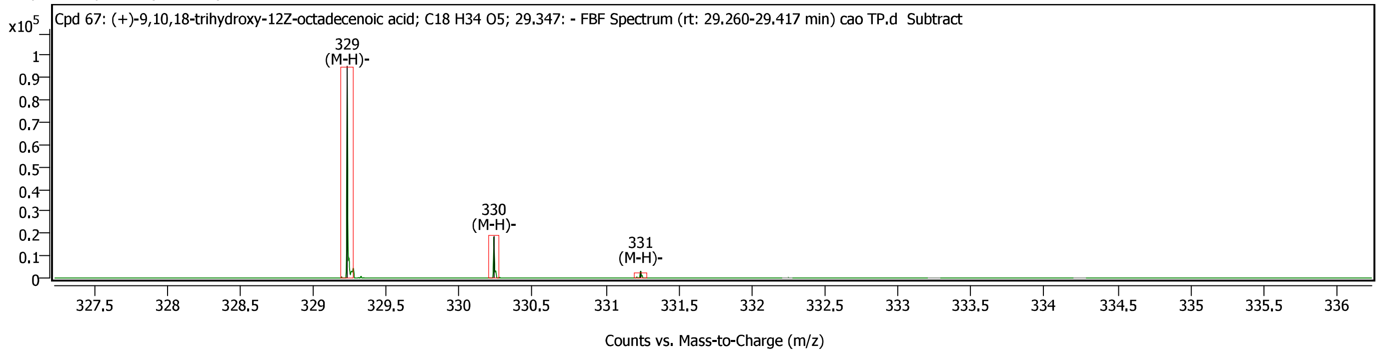

Compound Screening Report

Compound ID Table

Name	Formula	Species	RT	RT Diff	Mass	CAS	ID Source	Score	Score (Lib)	Score (Tgt)
(+)-9,10,18-trihydroxy-12Z-octadecenoic acid	C18 H34 O5	(M-H)-	29.347		330.2405		FBF	99.75		99.75
9,10,18-TriHOME(12)	C18 H34 O5	(M-H)-	29.347		330.2405		FBF	99.75		99.75
11,12,13-TriHOME	C18 H34 O5	(M-H)-	29.347		330.2405		FBF	99.75		99.75
11,12,13-trihydroxy-9-octadecenoic acid	C18 H34 O5	(M-H)-	29.347		330.2405		FBF	99.75		99.75
5,8,12-TriHOME(9)	C18 H34 O5	(M-H)-	29.347		330.2405		FBF	99.75		99.75
5,8,12-trihydroxy-9-octadecenoic acid	C18 H34 O5	(M-H)-	29.347		330.2405		FBF	99.75		99.75
9,10,13-trihydroxy-11-octadecenoic acid	C18 H34 O5	(M-H)-	29.347		330.2405		FBF	99.75		99.75
9,10,13-TriHOME(11)	C18 H34 O5	(M-H)-	29.347		330.2405		FBF	99.75		99.75
9,10,18-TriHOME(12Z)	C18 H34 O5	(M-H)-	29.347		330.2405		FBF	99.75		99.75
9,10-Dihydroxy-12,13-epoxyoctadecanoate	C18 H34 O5	(M-H)-	29.347		330.2405		FBF	99.75		99.75
9,12,13-TriHOME(10)	C18 H34 O5	(M-H)-	29.347		330.2405		FBF	99.75		99.75
9,12,13-trihydroxy-10-octadecenoic acid	C18 H34 O5	(M-H)-	29.347		330.2405		FBF	99.75		99.75
9S,10S,11R-trihydroxy-12Z-octadecenoic acid	C18 H34 O5	(M-H)-	29.347		330.2405		FBF	99.75		99.75
9S,12S,13S-trihydroxy-10E-octadecenoic acid	C18 H34 O5	(M-H)-	29.347		330.2405		FBF	99.75		99.75
9,10,18-trihydroxy-12-octadecenoic acid	C18 H34 O5	(M-H)-	29.347		330.2405		FBF	99.75		99.75

Cpd 176: 10,16-dihydroxy-palmitic acid

Name	Formula	RT	RI	Mass	Diff (Tgt, ppm)	CAS	ID Source	Score	Algorithm
10,16-dihydroxy-palmitic acid	C16 H32 O4	29.661		288.2303	0.85		M-FBF	98.96	FBF

Species	m/z	Score (Tgt)	Score (Lib)	Score (DB)	Score (MFG)	Score (RT)
(M-H)-	287	98.96				

Compound Chromatograms (overlaid)

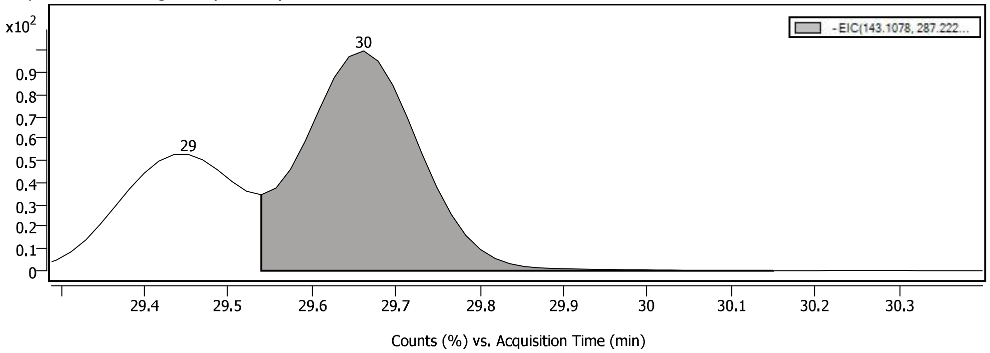

Structure

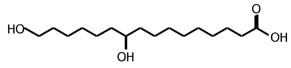

Compound Spectra (overlaid)

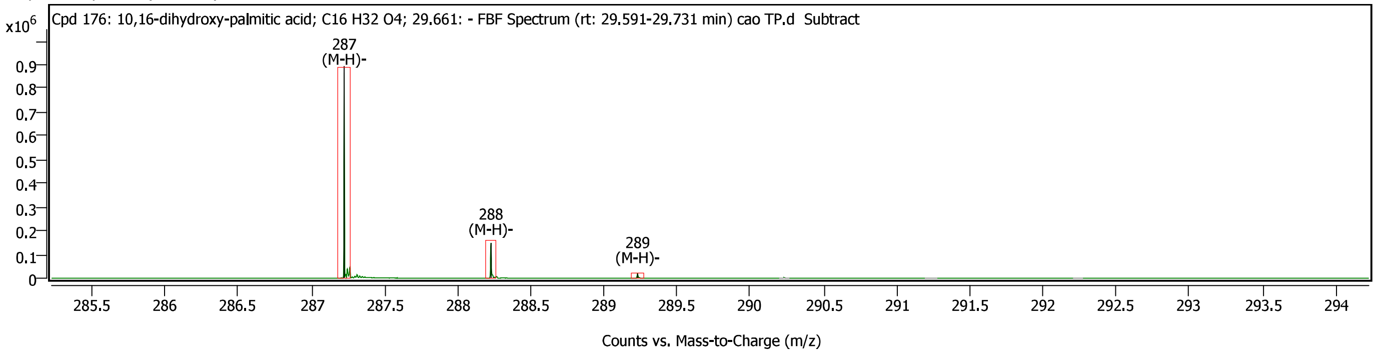

Compound ID Table

Name	Formula	Species	RT	RT Diff	Mass	CAS	ID Source	Score	Score (Lib)	Score (Tgt)
10,16-dihydroxy-palmitic acid	C16 H32 O4	(M-H)-	29.661		288.2303		FBF	98.96		98.96
Ustilic acid A	C16 H32 O4	(M-H)-	29.661		288.2303		FBF	98.96		98.96
9,16-dihydroxy-palmitic acid	C16 H32 O4	(M-H)-	29.661		288.2303		FBF	98.96		98.96
9,10-dihydroxy-hexadecanoic acid	C16 H32 O4	(M-H)-	29.661		288.2303		FBF	98.96		98.96
4,12-dihydroxy-hexadecanoic acid	C16 H32 O4	(M-H)-	29.661		288.2303		FBF	98.96		98.96
3,12-dihydroxy palmitic acid	C16 H32 O4	(M-H)-	29.661		288.2303		FBF	98.96		98.96

Cpd 185: <Polyscioside D>

Name	Formula	RT	RI	Mass	Diff (Tgt, ppm)	CAS	ID Source	Score	Algorithm
<Polyscioside D>	C54 H86 O24	32.330		1118.5501	-0.70	202585-62-4	M-FBF	99.35	FBF

Species	m/z	Score (Tgt)	Score (Lib)	Score (DB)	Score (MFG)	Score (RT)
(M-H)-	1118	99.35				

Compound Screening Report

Compound Chromatograms (overlaid)

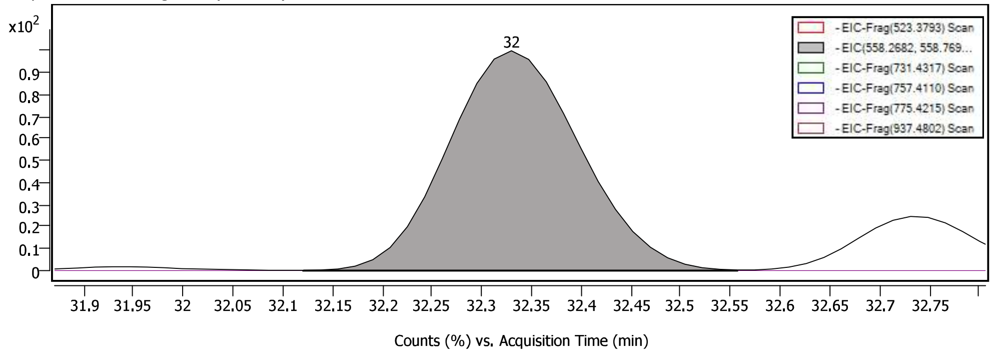

Structure

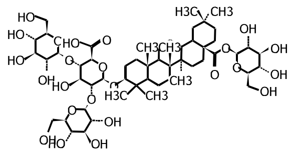

Coelution Plot

Compound Spectra (overlaid)

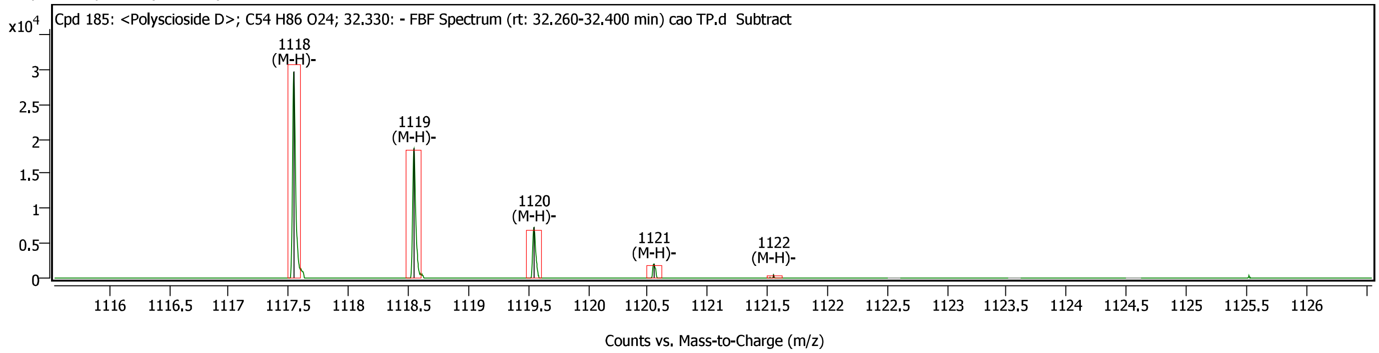

Fragment Spectrum (raw)

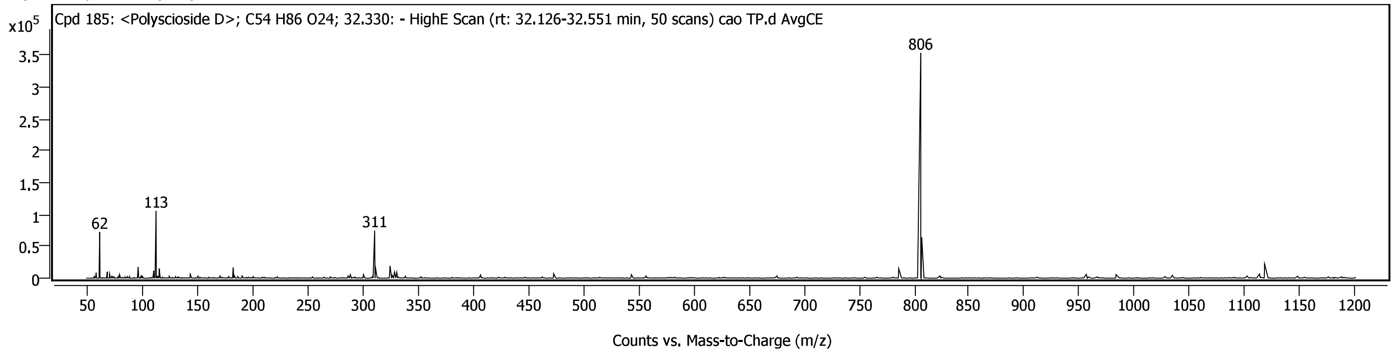

Compound ID Table

Name	Formula	Species	RT	RT Diff	Mass	CAS	ID Source	Score	Score (Lib)	Score (Tgt)
<Polyscioside D>	C54 H86 O24	(M-H)-	32.330		1118.5501	202585-62-4	FBF	99.35		99.35
<Elatoside D>	C54 H86 O24	(M-H)-	32.330		1118.5501	156856-40-5	FBF	99.35		99.35
<Calendulaglycoside A>	C54 H86 O24	(M-H)-	32.330		1118.5501	29660-94-4	FBF	99.35		99.35

Cpd 31: Camelliasaponin A1

Name	Formula	RT	RI	Mass	Diff (Tgt, ppm)	CAS	ID Source	Score	Algorithm
Camelliasaponin A1	C58 H92 O25	36.743		1188.5922	-0.47	183020-18-0	M-FBF	97.47	FBF

Species	m/z	Score (Tgt)	Score (Lib)	Score (DB)	Score (MFG)	Score (RT)
(M-H)-	1188	97.47				

Compound Chromatograms (overlaid)

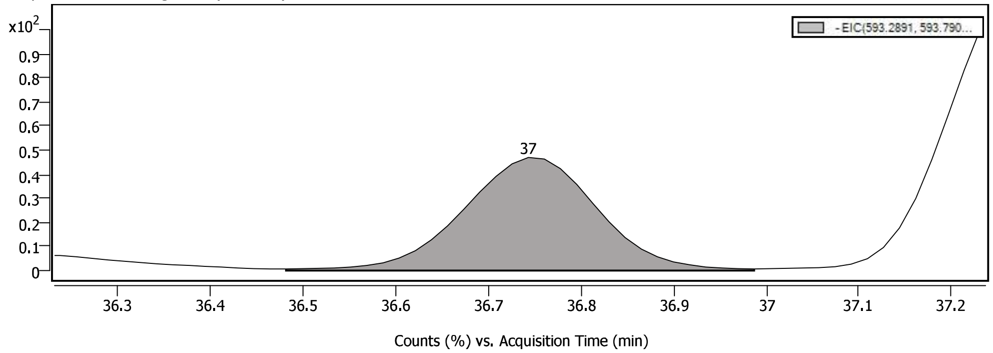

Structure

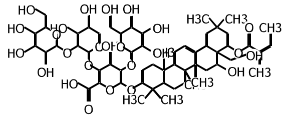

Compound Screening Report

Compound Spectra (overlaid)

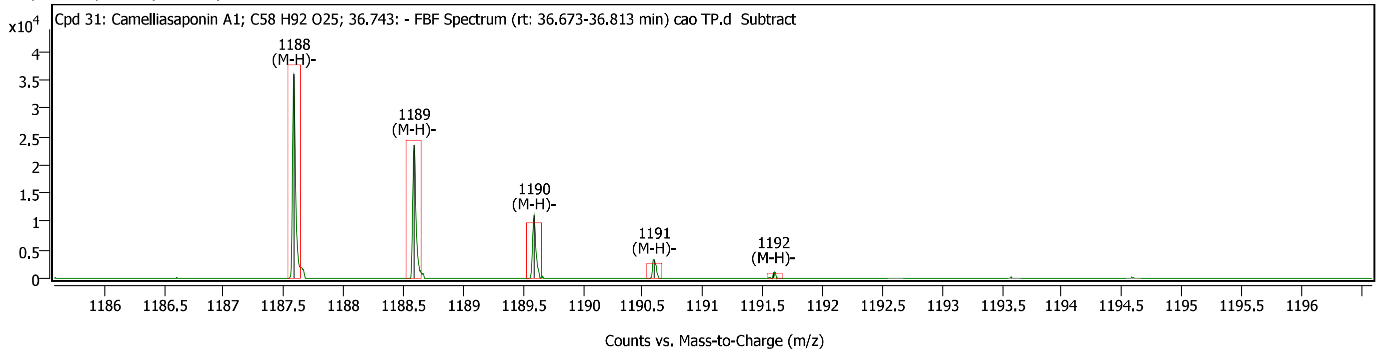

Compound ID Table

Name	Formula	Species	RT	RT Diff	Mass	CAS	ID Source	Score	Score (Lib)	Score (Tgt)
Camelliasaponin A1	C58 H92 O25	(M-H)-	36.743		1188.5922	183020-18-0	FBF	97.47		97.47
Camelliasaponin A2	C58 H92 O25	(M-H)-	36.743		1188.5922	183183-15-5	FBF	97.47		97.47

Cpd 254: Congmunoside XIV

Name	Formula	RT	RI	Mass	Diff (Tgt, ppm)	CAS	ID Source	Score	Algorithm
Congmunoside XIV	C57 H92 O23	36.987		1144.6024	-0.47	329969-14-4	FBF	99.74	FBF

Species	m/z	Score (Tgt)	Score (Lib)	Score (DB)	Score (MFG)	Score (RT)
(M-H)-	1144	99.74				

Compound Chromatograms (overlaid)

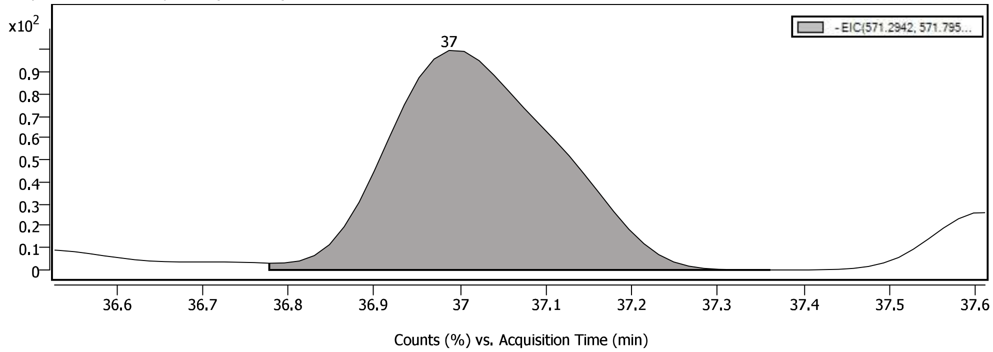

Structure

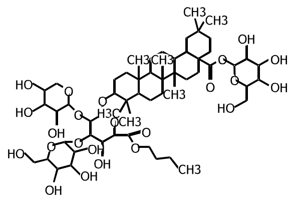

Compound Spectra (overlaid)

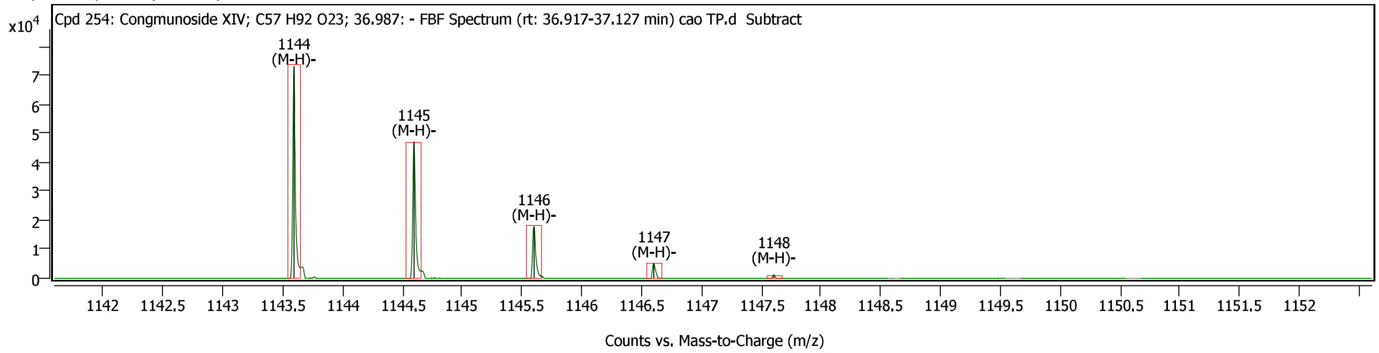

Compound ID Table

Name	Formula	Species	RT	RT Diff	Mass	CAS	ID Source	Score	Score (Lib)	Score (Tgt)
Congmunoside XIV	C57 H92 O23	(M-H)-	36.987		1144.6024	329969-14-4	FBF	99.74		99.74

Cpd 32: Camelliasaponin A2

Name	Formula	RT	RI	Mass	Diff (Tgt, ppm)	CAS	ID Source	Score	Algorithm
Camelliasaponin A2	C58 H92 O25	37.301		1188.5921	-0.56	183183-15-5	M-FBF	99.45	FBF

Species	m/z	Score (Tgt)	Score (Lib)	Score (DB)	Score (MFG)	Score (RT)
(M-H)-	1188	99.45				

Compound Chromatograms (overlaid)

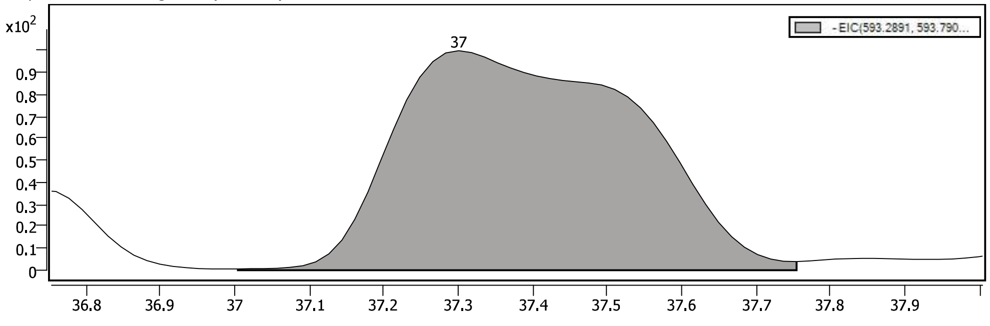

Structure

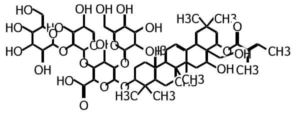

Compound Spectra (overlaid)

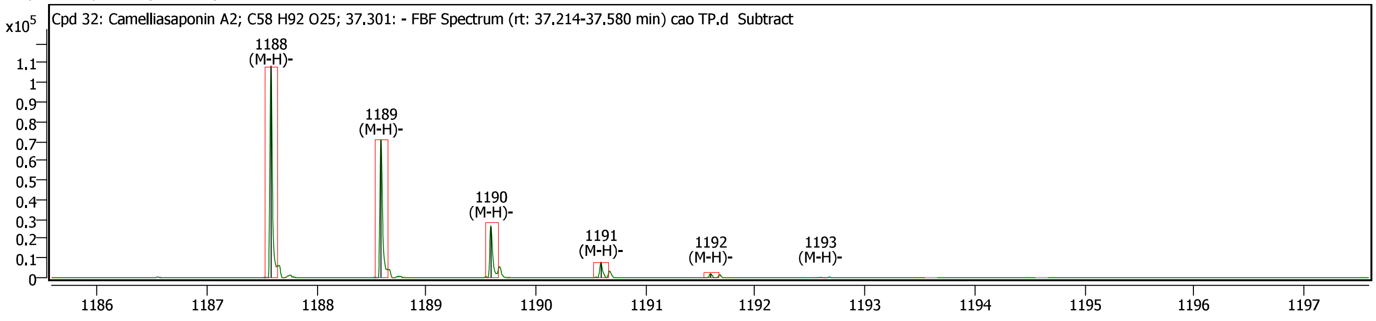

Compound ID Table

Name	Formula	Species	RT	RT Diff	Mass	CAS	ID Source	Score	Score (Lib)	Score (Tgt)
Camelliasaponin A2	C58 H92 O25	(M-H)-	37.301		1188.5921	183183-15-5	FBF	99.45		99.45
Camelliasaponin A1	C58 H92 O25	(M-H)-	37.301		1188.5921	183020-18-0	FBF	99.45		99.45

Cpd 96: 5-Oxoavermectin "1b" aglycone

Name	Formula	RT	RI	Mass	Diff (Tgt, ppm)	CAS	ID Source	Score	Algorithm
5-Oxoavermectin "1b" aglycone	C33 H44 O8	38.208		568.3030	-1.12		FBF	99.09	FBF

Species	m/z	Score (Tgt)	Score (Lib)	Score (DB)	Score (MFG)	Score (RT)
(M-H)-	567	99.09				

Compound Chromatograms (overlaid)

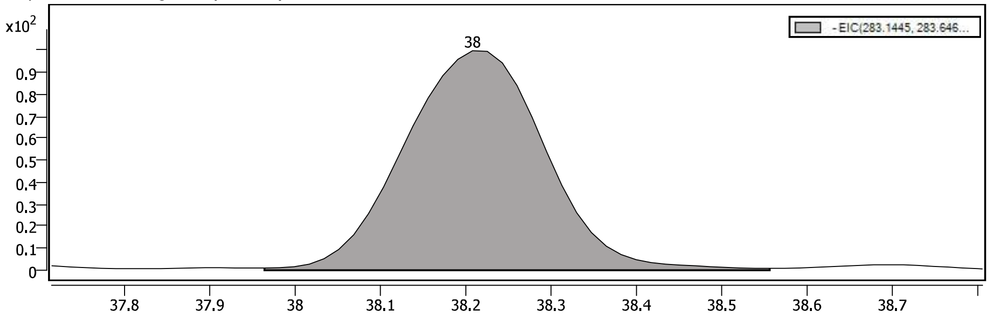

Structure

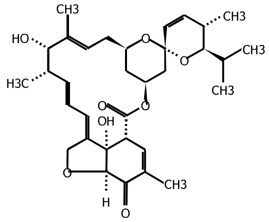

Compound Spectra (overlaid)

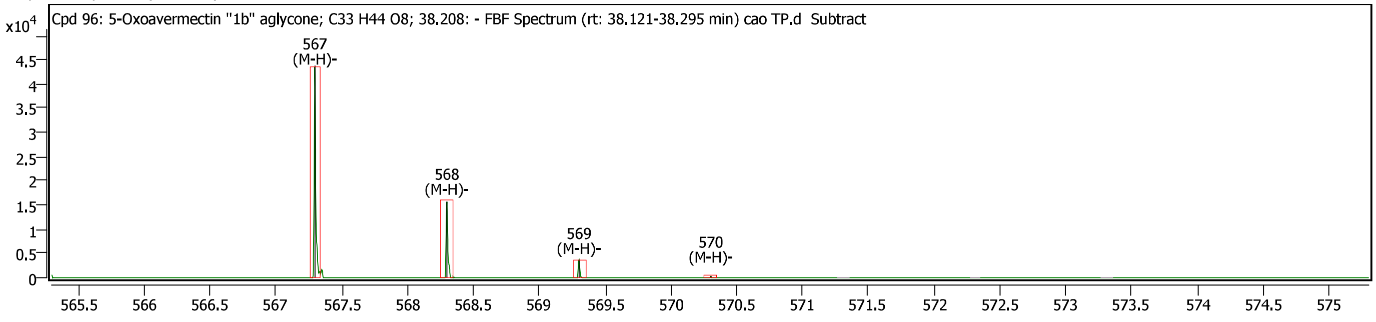

Compound ID Table

Name	Formula	Species	RT	RT Diff	Mass	CAS	ID Source	Score	Score (Lib)	Score (Tgt)
5-Oxoavermectin "1b" aglycone	C33 H44 O8	(M-H)-	38.208		568.3030		FBF	99.09		99.09

Cpd 181: Ceanothine E

Name	Formula	RT	RI	Mass	Diff (Tgt, ppm)	CAS	ID Source	Score	Algorithm
Ceanothine E	C34 H40 N4 O4	38.208		568.3031	-3.35	23926-98-9	M-FBF	93.71	FBF

Species	m/z	Score (Tgt)	Score (Lib)	Score (DB)	Score (MFG)	Score (RT)
(M-H)-	567	93.71				

Compound Chromatograms (overlaid)

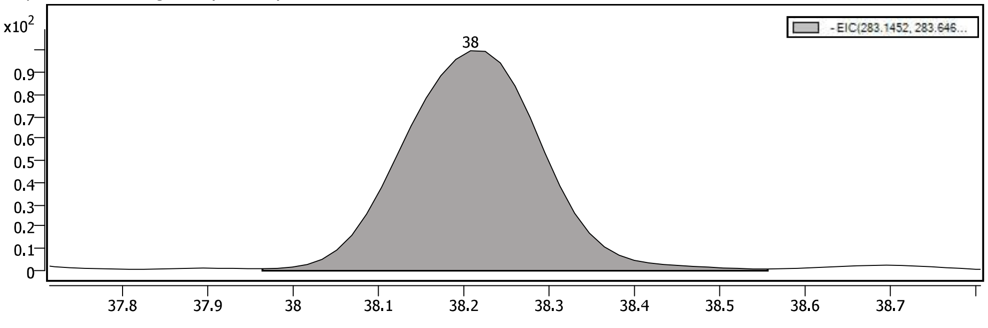

Structure

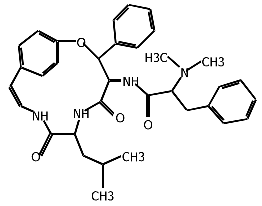

Compound Spectra (overlaid)

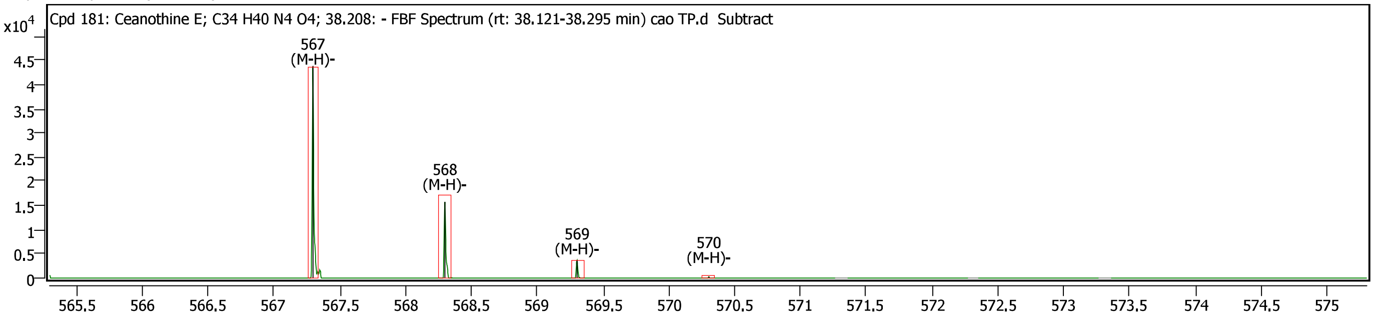

Compound ID Table

Name	Formula	Species	RT	RT Diff	Mass	CAS	ID Source	Score	Score (Lib)	Score (Tgt)
Ceanothine E	C34 H40 N4 O4	(M-H)-	38.208		568.3031	23926-98-9	FBF	93.71		93.71
Protoporphyrinogen IX	C34 H40 N4 O4	(M-H)-	38.208		568.3031	7412-77-3	FBF	93.71		93.71
Crenatine A	C34 H40 N4 O4	(M-H)-	38.208		568.3031	52801-20-4	FBF	93.71		93.71
Adouetine Y	C34 H40 N4 O4	(M-H)-	38.208		568.3031	19542-38-2	FBF	93.71		93.71

Cpd 173: <9(S)-HOTrE>

Name	Formula	RT	RI	Mass	Diff (Tgt, ppm)	CAS	ID Source	Score	Algorithm
<9(S)-HOTrE>	C18 H30 O3	38.365		294.2192	-0.89	89886-42-0	M-FBF	98.95	FBF

Species	m/z	Score (Tgt)	Score (Lib)	Score (DB)	Score (MFG)	Score (RT)
(M-H)-	293	98.95				

Compound Chromatograms (overlaid)

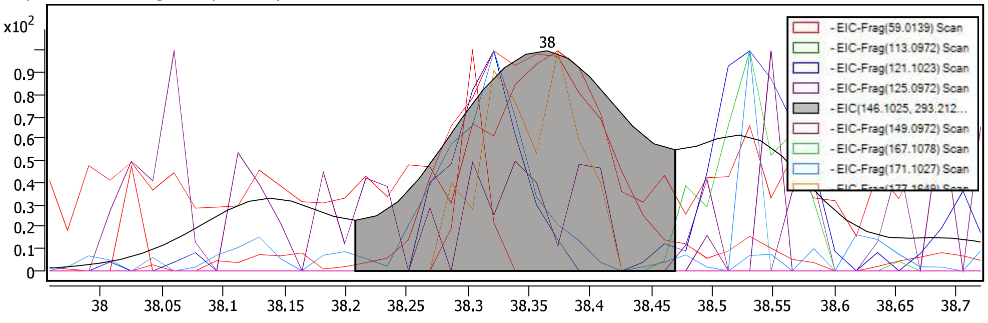

Structure

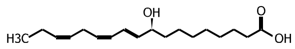

Coelution Plot

Compound Spectra (overlaid)

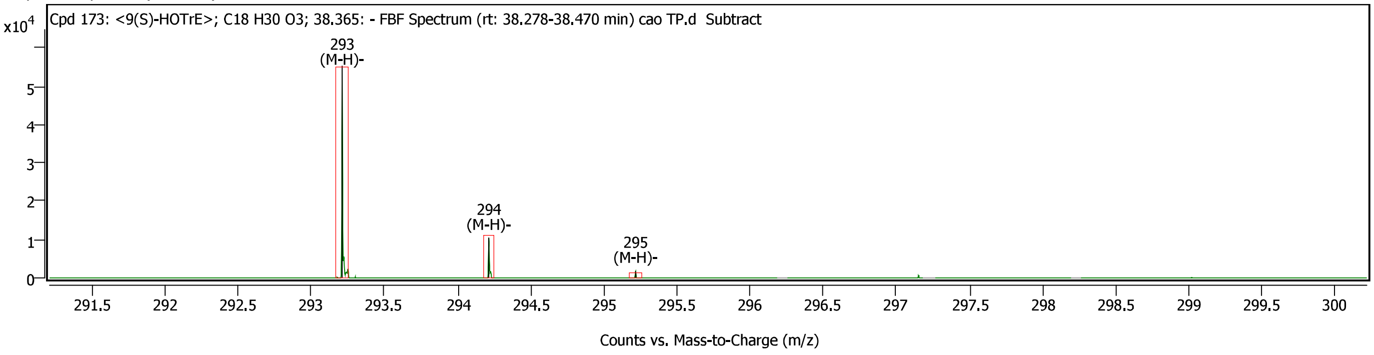

Fragment Spectrum (raw)

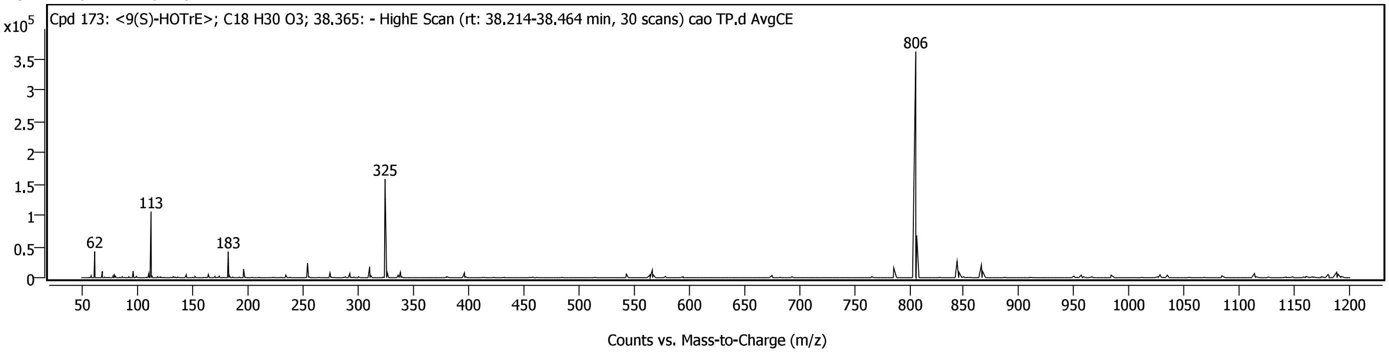

Compound ID Table

Name	Formula	Species	RT	RT Diff	Mass	CAS	ID Source	Score	Score (Lib)	Score (Tgt)
<9(S)-HOTrE>	C18 H30 O3	(M-H)-	38.365		294.2192	89886-42-0	FBF	98.95		98.95
<γ- 9(10)-EpODE>	C18 H30 O3	(M-H)-	38.365		294.2192		FBF	98.95		98.95
<12,13-Epoxy-9,15-octadecadienoic acid>	C18 H30 O3	(M-H)-	38.365		294.2192	88159-18-6	FBF	98.95		98.95
<17-hydroxy-linolenic acid>	C18 H30 O3	(M-H)-	38.365		294.2192		FBF	98.95		98.95
<15,16-Epoxy-9,12-octadecadienoic acid>	C18 H30 O3	(M-H)-	38.365		294.2192		FBF	98.95		98.95
<13S-HOTrE(gamma)>	C18 H30 O3	(M-H)-	38.365		294.2192	74784-20-6	FBF	98.95		98.95
<13-OxoODE>	C18 H30 O3	(M-H)-	38.365		294.2192	54739-30-9	FBF	98.95		98.95
<13-HOTE>	C18 H30 O3	(M-H)-	38.365		294.2192		FBF	98.95		98.95
<13(S)-HOTrE>	C18 H30 O3	(M-H)-	38.365		294.2192	87984-82-5	FBF	98.95		98.95
<12-oxo-9-octadecynoic acid>	C18 H30 O3	(M-H)-	38.365		294.2192		FBF	98.95		98.95
<12,13S-EpODE>	C18 H30 O3	(M-H)-	38.365		294.2192		FBF	98.95		98.95
<10-Oxo-11-octadecen-13-olide>	C18 H30 O3	(M-H)-	38.365		294.2192		FBF	98.95		98.95
<2-hydroxy-9Z,12Z,15Z-octadecatrienoic acid>	C18 H30 O3	(M-H)-	38.365		294.2192		FBF	98.95		98.95
<(9Z,12Z,14E)-16-Hydroxy-9,12,14-octadecatrienoic acid>	C18 H30 O3	(M-H)-	38.365		294.2192	81325-65-7	FBF	98.95		98.95
<(9S,13S)-15,16-dihydro-12-oxo-10-phytoenoic acid>	C18 H30 O3	(M-H)-	38.365		294.2192		FBF	98.95		98.95
<γ- 6(7)-EpODE>	C18 H30 O3	(M-H)-	38.365		294.2192		FBF	98.95		98.95
<(9S,13S)-10,11-dihydro-12-oxo-15-phytoenoic acid>	C18 H30 O3	(M-H)-	38.365		294.2192		FBF	98.95		98.95
<(9R,13R)-15,16-dihydro-12-oxo-10-phytoenoic acid>	C18 H30 O3	(M-H)-	38.365		294.2192		FBF	98.95		98.95
<(9R,13R)-10-oxo-11-phytoenoic acid>	C18 H30 O3	(M-H)-	38.365		294.2192		FBF	98.95		98.95
<(9R,13R)-10,11-dihydro-12-oxo-15-phytoenoic acid>	C18 H30 O3	(M-H)-	38.365		294.2192		FBF	98.95		98.95
<9-OxoODE>	C18 H30 O3	(M-H)-	38.365		294.2192	54232-59-6	FBF	98.95		98.95
<17-Hydroxylinolenic acid>	C18 H30 O3	(M-H)-	38.365		294.2192		FBF	98.95		98.95
<(9E,11E)-13-oxooctadeca-9,11-dienoic acid>	C18 H30 O3	(M-H)-	38.365		294.2192		FBF	98.95		98.95
<alpha-kamlolenic acid>	C18 H30 O3	(M-H)-	38.365		294.2192		FBF	98.95		98.95
<Colneleic acid>	C18 H30 O3	(M-H)-	38.365		294.2192		FBF	98.95		98.95
<2R-hydroxy-9Z,12Z,15Z-octadecatrienoic acid>	C18 H30 O3	(M-H)-	38.365		294.2192		FBF	98.95		98.95
<γ- 12(13)-EpODE>	C18 H30 O3	(M-H)-	38.365		294.2192		FBF	98.95		98.95
<α-9(10)-EpODE>	C18 H30 O3	(M-H)-	38.365		294.2192		FBF	98.95		98.95
<α-12(13)-EpODE>	C18 H30 O3	(M-H)-	38.365		294.2192		FBF	98.95		98.95
<Sterebin D>	C18 H30 O3	(M-H)-	38.365		294.2192		FBF	98.95		98.95
<Squamostanal A>	C18 H30 O3	(M-H)-	38.365		294.2192	156764-90-8	FBF	98.95		98.95
<Juvenile hormone I>	C18 H30 O3	(M-H)-	38.365		294.2192	13804-51-8	FBF	98.95		98.95
<2-Hydroxylinolenic acid>	C18 H30 O3	(M-H)-	38.365		294.2192		FBF	98.95		98.95
<α-15(16)-EpODE>	C18 H30 O3	(M-H)-	38.365		294.2192		FBF	98.95		98.95
<2-HoTrE>	C18 H30 O3	(M-H)-	38.365		294.2192		FBF	98.95		98.95
<9S,10-epoxy-10,12Z-octadecadienoic acid>	C18 H30 O3	(M-H)-	38.365		294.2192		FBF	98.95		98.95
<9-oxo-10,12-octadecadienoic acid>	C18 H30 O3	(M-H)-	38.365		294.2192		FBF	98.95		98.95
<9-hydroxy-10E-octadecen-12-ynoic acid>	C18 H30 O3	(M-H)-	38.365		294.2192		FBF	98.95		98.95
<9-HOTE>	C18 H30 O3	(M-H)-	38.365		294.2192		FBF	98.95		98.95
<8-hydroxy-11Z-octadecen-9-ynoic acid>	C18 H30 O3	(M-H)-	38.365		294.2192		FBF	98.95		98.95
<8-(5-hexyl-furan-2-yl)-octanoic acid>	C18 H30 O3	(M-H)-	38.365		294.2192		FBF	98.95		98.95
<beta-kamlolenic acid>	C18 H30 O3	(M-H)-	38.365		294.2192		FBF	98.95		98.95
<(9S,13S)-10-oxo-11-phytoenoic acid>	C18 H30 O3	(M-H)-	38.365		294.2192		FBF	98.95		98.95
<3,4-Dimethyl-5-pentyl-2-furanheptanoic acid>	C18 H30 O3	(M-H)-	38.365		294.2192	92745-17-0	FBF	98.95		98.95

Cpd 20: Soyasaponin bg

Name	Formula	RT	RI	Mass	Diff (Tgt, ppm)	CAS	ID Source	Score	Algorithm
Soyasaponin bg	C54 H84 O21	40.214		1068.5504	-0.07	143519-54-4	FBF	99.04	FBF

Species	m/z	Score (Tgt)	Score (Lib)	Score (DB)	Score (MFG)	Score (RT)
(M-H)-	1068	99.04				

Compound Chromatograms (overlaid)

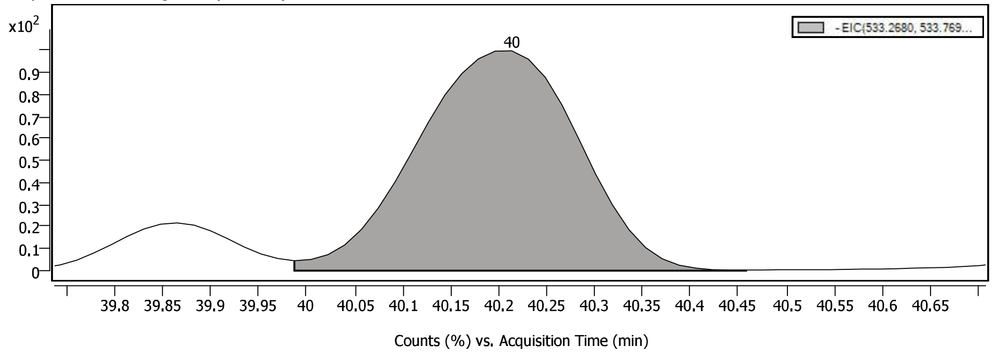

Structure

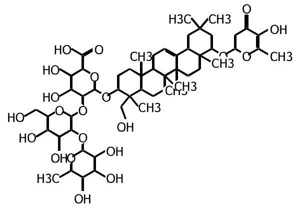

Compound Spectra (overlaid)

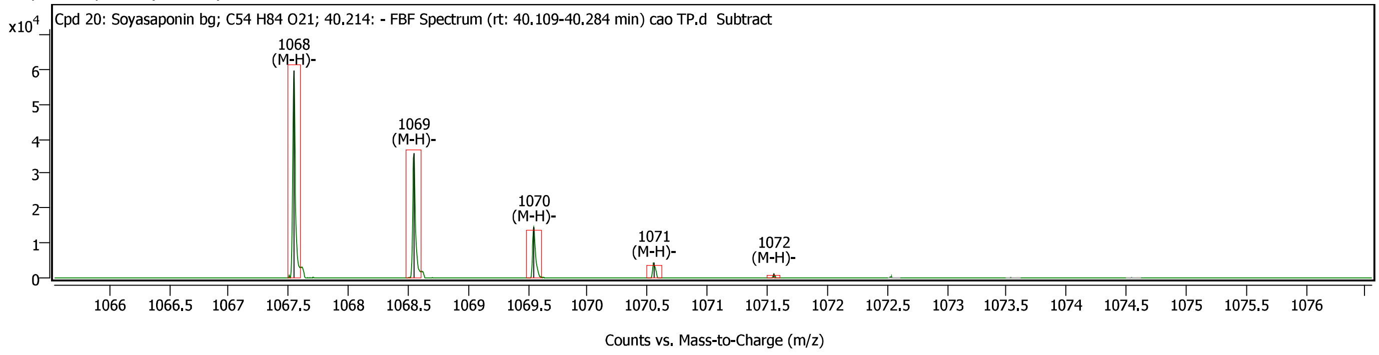

Compound ID Table

Name	Formula	Species	RT	RT Diff	Mass	CAS	ID Source	Score	Score (Lib)	Score (Tgt)
Soyasaponin bg	C54 H84 O21	(M-H)-	40.214		1068.5504	143519-54-4	FBF	99.04		99.04

Cpd 174: <9(R)-HODE>

Name	Formula	RT	RI	Mass	Diff (Tgt, ppm)	CAS	ID Source	Score	Algorithm
<9(R)-HODE>	C18 H32 O3	40.371		296.2351	-0.20	10075-11-3	M-FBF	99.80	FBF

Species	m/z	Score (Tgt)	Score (Lib)	Score (DB)	Score (MFG)	Score (RT)
(M-H)-	295	99.80				

Compound Chromatograms (overlaid)

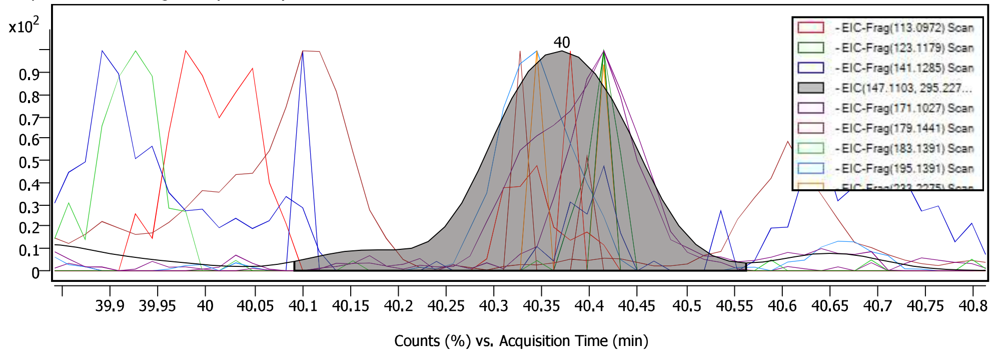

Structure

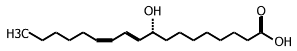

Coelution Plot

Compound Spectra (overlaid)

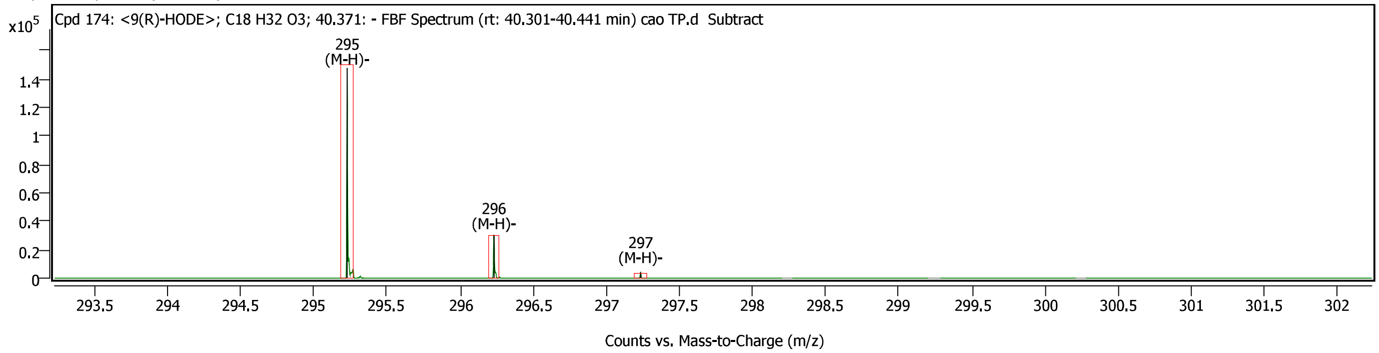

Fragment Spectrum (raw)

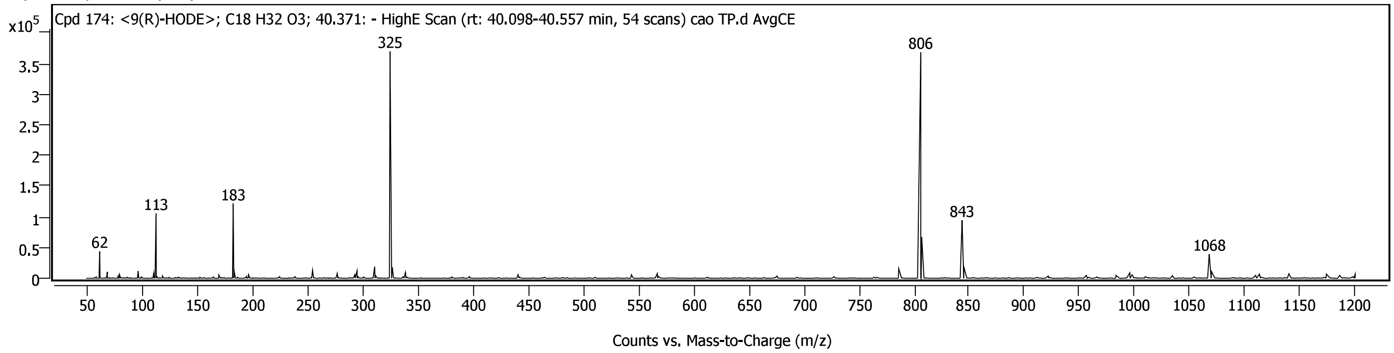

Compound Screening Report

Compound ID Table

Name	Formula	Species	RT	RT Diff	Mass	CAS	ID Source	Score	Score (Lib)	Score (Tgt)
<9(R)-HODE>	C18 H32 O3	(M-H)-	40.371		296.2351	10075-11-3	FBF	99.80		99.80
<12-oxo-9Z-octadecenoic acid>	C18 H32 O3	(M-H)-	40.371		296.2351		FBF	99.80		99.80
<12,13-epoxy-9-octadecenoic acid>	C18 H32 O3	(M-H)-	40.371		296.2351		FBF	99.80		99.80
<12-oxo-10Z-octadecenoic acid>	C18 H32 O3	(M-H)-	40.371		296.2351		FBF	99.80		99.80
<12-oxo-9E-octadecenoic acid>	C18 H32 O3	(M-H)-	40.371		296.2351		FBF	99.80		99.80
<12R,13S-epoxy-9Z-octadecenoic acid>	C18 H32 O3	(M-H)-	40.371		296.2351		FBF	99.80		99.80
<12-OxoOME(10E)>	C18 H32 O3	(M-H)-	40.371		296.2351		FBF	99.80		99.80
<12-OxoOME(10Z)>	C18 H32 O3	(M-H)-	40.371		296.2351		FBF	99.80		99.80
<12-OxoOME(9E)>	C18 H32 O3	(M-H)-	40.371		296.2351		FBF	99.80		99.80
<12-OxoOME(9Z)>	C18 H32 O3	(M-H)-	40.371		296.2351		FBF	99.80		99.80
<12-hydroxy-10-octadecynoic acid>	C18 H32 O3	(M-H)-	40.371		296.2351		FBF	99.80		99.80
<12-Hydroxy-8,10-octadecadienoic acid>	C18 H32 O3	(M-H)-	40.371		296.2351	170171-31-0	FBF	99.80		99.80
<10R-HODE>	C18 H32 O3	(M-H)-	40.371		296.2351		FBF	99.80		99.80
<12,13-EpOME(9)>	C18 H32 O3	(M-H)-	40.371		296.2351		FBF	99.80		99.80
<12-hydroxy-9-octadecynoic acid>	C18 H32 O3	(M-H)-	40.371		296.2351		FBF	99.80		99.80
<12(13)-EpOME>	C18 H32 O3	(M-H)-	40.371		296.2351		FBF	99.80		99.80
<10S-HODE>	C18 H32 O3	(M-H)-	40.371		296.2351		FBF	99.80		99.80
<10-keto-12Z-octadecenoic acid>	C18 H32 O3	(M-H)-	40.371		296.2351		FBF	99.80		99.80
<(Z)-13-Oxo-9-octadecenoic acid>	C18 H32 O3	(M-H)-	40.371		296.2351	38205-11-7	FBF	99.80		99.80
<(1S,2S)-3-oxo-2-pentyl-cyclopentaneoctanoic acid>	C18 H32 O3	(M-H)-	40.371		296.2351		FBF	99.80		99.80
<(1R,2R)-3-oxo-2-pentyl-cyclopentaneoctanoic acid>	C18 H32 O3	(M-H)-	40.371		296.2351		FBF	99.80		99.80
<(±)9-HODE>	C18 H32 O3	(M-H)-	40.371		296.2351	98524-19-7	FBF	99.80		99.80
<(±)13-HODE>	C18 H32 O3	(M-H)-	40.371		296.2351	73804-64-5	FBF	99.80		99.80
<12R-hydroxy-9Z,15Z-octadecadienoic acid>	C18 H32 O3	(M-H)-	40.371		296.2351		FBF	99.80		99.80
<6-hydroxy-9Z,12Z-octadecadienoic acid>	C18 H32 O3	(M-H)-	40.371		296.2351		FBF	99.80		99.80
<12-oxo-10E-octadecenoic acid>	C18 H32 O3	(M-H)-	40.371		296.2351		FBF	99.80		99.80
<12R-HODE>	C18 H32 O3	(M-H)-	40.371		296.2351		FBF	99.80		99.80
<12S-hydroxy-9-octadecynoic acid>	C18 H32 O3	(M-H)-	40.371		296.2351		FBF	99.80		99.80
<8S-HODE>	C18 H32 O3	(M-H)-	40.371		296.2351		FBF	99.80		99.80
<Dimorphecolic acid>	C18 H32 O3	(M-H)-	40.371		296.2351		FBF	99.80		99.80
<Densipolic acid>	C18 H32 O3	(M-H)-	40.371		296.2351		FBF	99.80		99.80
<beta-Dimorphecolic acid>	C18 H32 O3	(M-H)-	40.371		296.2351		FBF	99.80		99.80
<Avenoleic acid>	C18 H32 O3	(M-H)-	40.371		296.2351		FBF	99.80		99.80
<9S-hydroxy-10E,12E-octadecadienoic acid>	C18 H32 O3	(M-H)-	40.371		296.2351		FBF	99.80		99.80
<9R,10S-EpOME>	C18 H32 O3	(M-H)-	40.371		296.2351		FBF	99.80		99.80
<9-HODE>	C18 H32 O3	(M-H)-	40.371		296.2351		FBF	99.80		99.80
<9,10-epoxy-12-octadecenoic acid>	C18 H32 O3	(M-H)-	40.371		296.2351		FBF	99.80		99.80
<9,10-EpOME(12)>	C18 H32 O3	(M-H)-	40.371		296.2351		FBF	99.80		99.80
<9(S)-HODE>	C18 H32 O3	(M-H)-	40.371		296.2351	73543-67-6	FBF	99.80		99.80
<13S-hydroxy-9E,11Z-octadecadienoic acid>	C18 H32 O3	(M-H)-	40.371		296.2351		FBF	99.80		99.80
<(±)12(13)-EpOME>	C18 H32 O3	(M-H)-	40.371		296.2351		FBF	99.80		99.80
<13(R)-HODE>	C18 H32 O3	(M-H)-	40.371		296.2351	10219-69-9	FBF	99.80		99.80
<8R-HODE>	C18 H32 O3	(M-H)-	40.371		296.2351		FBF	99.80		99.80
<13(S)-HODE>	C18 H32 O3	(M-H)-	40.371		296.2351	29623-28-7	FBF	99.80		99.80
<13R-HODE>	C18 H32 O3	(M-H)-	40.371		296.2351		FBF	99.80		99.80
<13R-hydroxy-9E,11Z-octadecadienoic acid>	C18 H32 O3	(M-H)-	40.371		296.2351		FBF	99.80		99.80
<18-Oxooleate>	C18 H32 O3	(M-H)-	40.371		296.2351		FBF	99.80		99.80
<2R-hydroxy-linoleic acid>	C18 H32 O3	(M-H)-	40.371		296.2351		FBF	99.80		99.80
<6-HODE>	C18 H32 O3	(M-H)-	40.371		296.2351		FBF	99.80		99.80
<alpha-artemisic acid>	C18 H32 O3	(M-H)-	40.371		296.2351		FBF	99.80		99.80
<12S,13R-EpOME>	C18 H32 O3	(M-H)-	40.371		296.2351	503-07-1	FBF	99.80		99.80
<7-Methoxy-9-methyl-hexadeca-4E,8E-dienoic acid>	C18 H32 O3	(M-H)-	40.371		296.2351		FBF	99.80		99.80

Cpd 157: MGDG(18:5(3Z,6Z,9Z,12Z,15Z)/18:5(3Z,6Z,9Z,12Z,15Z))

Name	Formula	RT	RI	Mass	Diff (Tgt, ppm)	CAS	ID Source	Score	Algorithm
MGDG(18:5(3Z,6Z,9Z,12Z,15Z)/18:5(3Z,6Z,9Z,12Z,15Z))	C45 H66 O10	42.481		766.4684	3.60		FBF	92.53	FBF

Species	m/z	Score (Tgt)	Score (Lib)	Score (DB)	Score (MFG)	Score (RT)
(M-H)-	765	92.53				

Compound Chromatograms (overlaid)

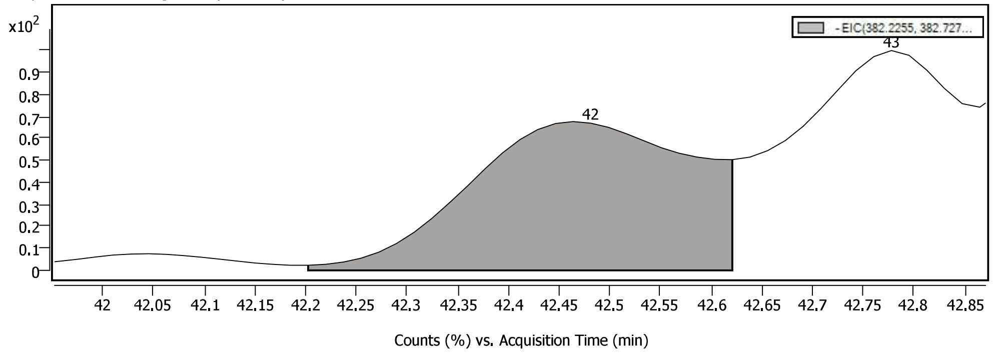

Structure

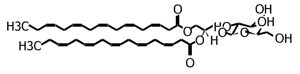

Compound Spectra (overlaid)

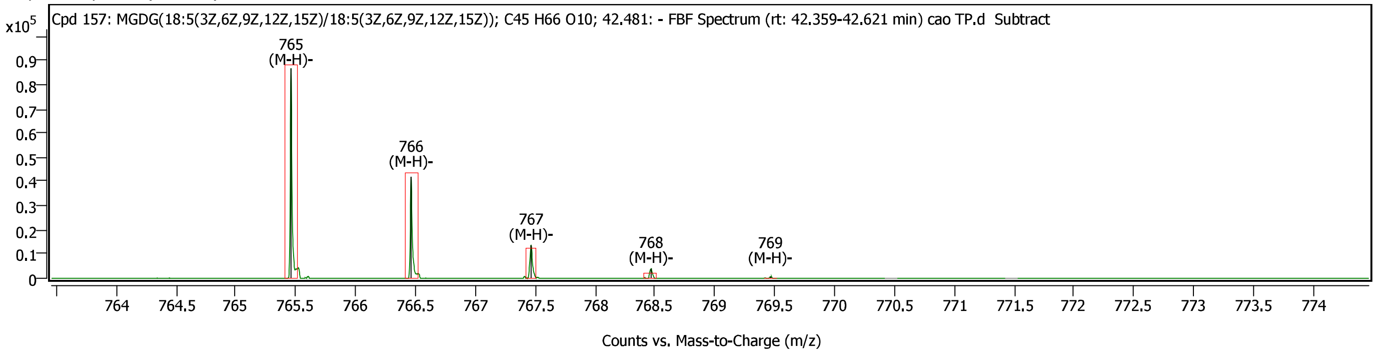

Compound ID Table

Name	Formula	Species	RT	RT Diff	Mass	CAS	ID Source	Score	Score (Lib)	Score (Tgt)
MGDG(18:5(3Z,6Z,9Z,12Z,15Z)/18:5(3Z,6Z,9Z,12Z,15Z))	C45 H66 O10	(M-H)-	42.481		766.4684		FBF	92.53		92.53

Cpd 160: SQDG(22:5(5Z,8Z,11Z,14Z,17Z)/16:1(13Z))

Name	Formula	RT	RI	Mass	Diff (Tgt, ppm)	CAS	ID Source	Score	Algorithm
SQDG(22:5(5Z,8Z,11Z,14Z,17Z)/16:1(13Z))	C45 H76 O12 S	42.534		840.5056	-0.19		FBF	99.10	FBF

Species	m/z	Score (Tgt)	Score (Lib)	Score (DB)	Score (MFG)	Score (RT)
(M-H)-	839	99.10				

Compound Chromatograms (overlaid)

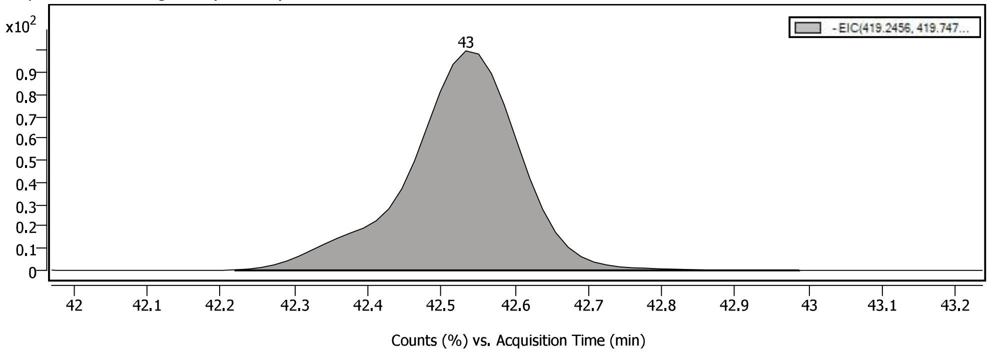

Structure

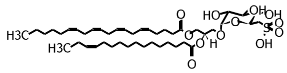

Compound Spectra (overlaid)

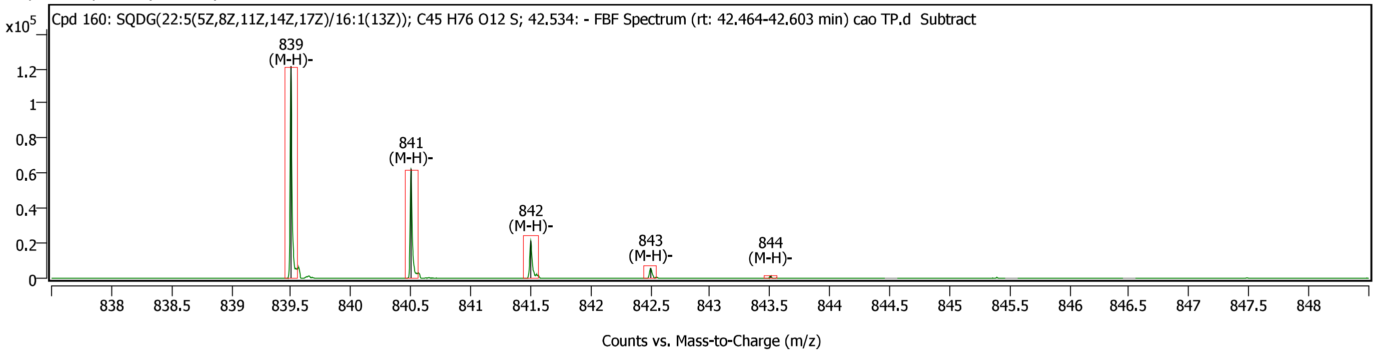

Compound ID Table

Name	Formula	Species	RT	RT Diff	Mass	CAS	ID Source	Score	Score (Lib)	Score (Tgt)
SQDG(22:5(5Z,8Z,11Z,14Z,17Z)/16:1(13Z))	C45 H76 O12 S	(M-H)-	42.534		840.5056		FBF	99.10		99.10

Cpd 200: Mycalamide A

Name	Formula	RT	RI	Mass	Diff (Tgt, ppm)	CAS	ID Source	Score	Algorithm
Mycalamide A	C24 H41 N O10	42.970		503.2727	-0.72	115185-92-7	FBF	99.08	FBF

Species	m/z	Score (Tgt)	Score (Lib)	Score (DB)	Score (MFG)	Score (RT)
(M-H)-	502	99.08				

Compound Screening Report

Compound Chromatograms (overlaid)

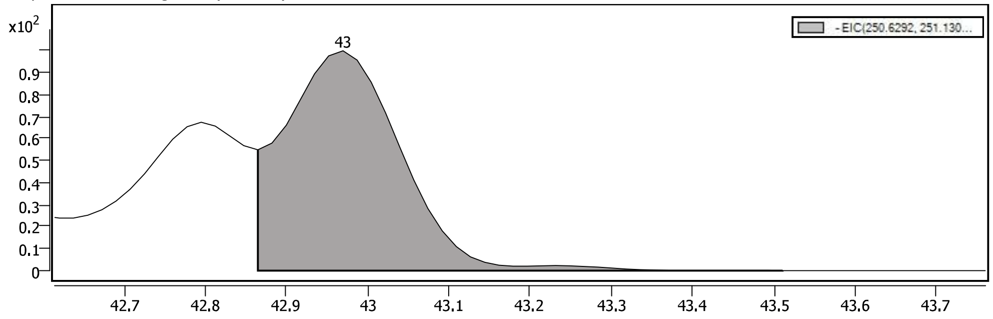

Structure

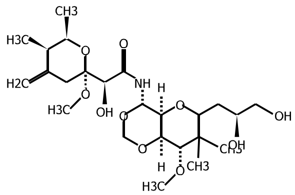

Compound Spectra (overlaid)

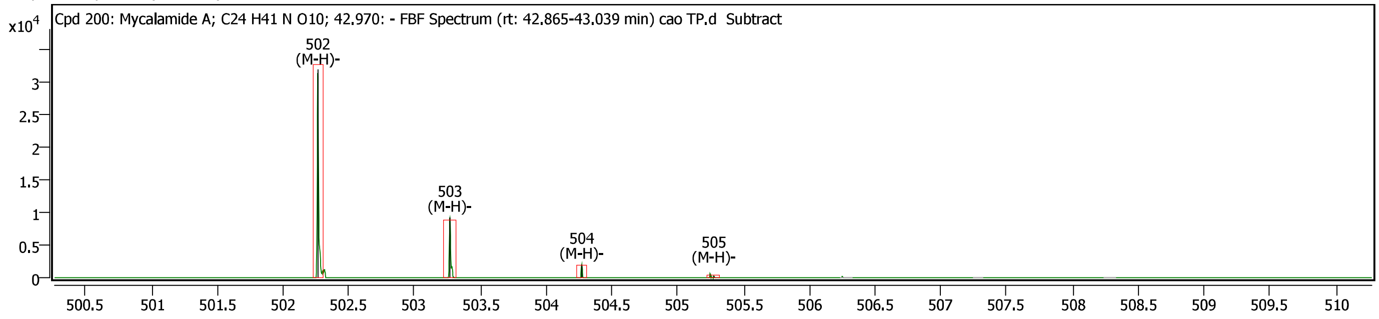

Compound ID Table

Name	Formula	Species	RT	RT Diff	Mass	CAS	ID Source	Score	Score (Lib)	Score (Tgt)
Mycalamide A	C24 H41 N O10	(M-H)-	42.970		503.2727	115185-92-7	FBF	99.08		99.08

Cpd 159: DGDG(18:5(3Z,6Z,9Z,12Z,15Z)/18:4(6Z,9Z,12Z,15Z))

Name	Formula	RT	RI	Mass	Diff (Tgt, ppm)	CAS	ID Source	Score	Algorithm
DGDG(18:5(3Z,6Z,9Z,12Z,15Z)/18 :4(6Z,9Z,12Z,15Z))	C51 H78 O15	43.685		930.5364	2.54		FBF	94.58	FBF

Species	m/z	Score (Tgt)	Score (Lib)	Score (DB)	Score (MFG)	Score (RT)
(M-H)-	930	94.58				

Compound Chromatograms (overlaid)

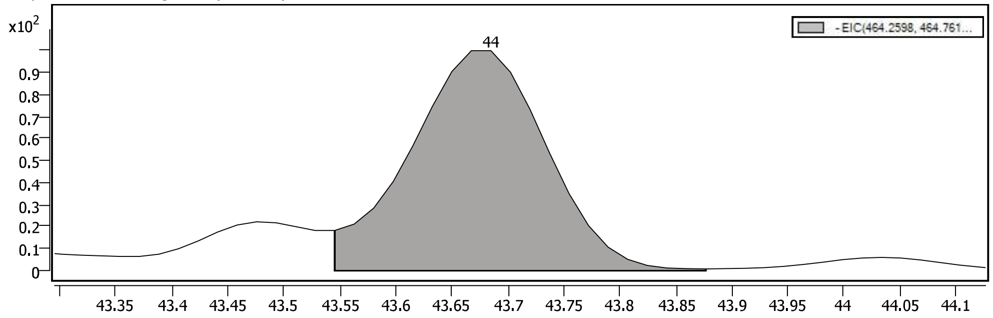

Structure

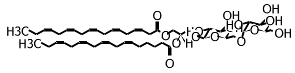

Compound Spectra (overlaid)

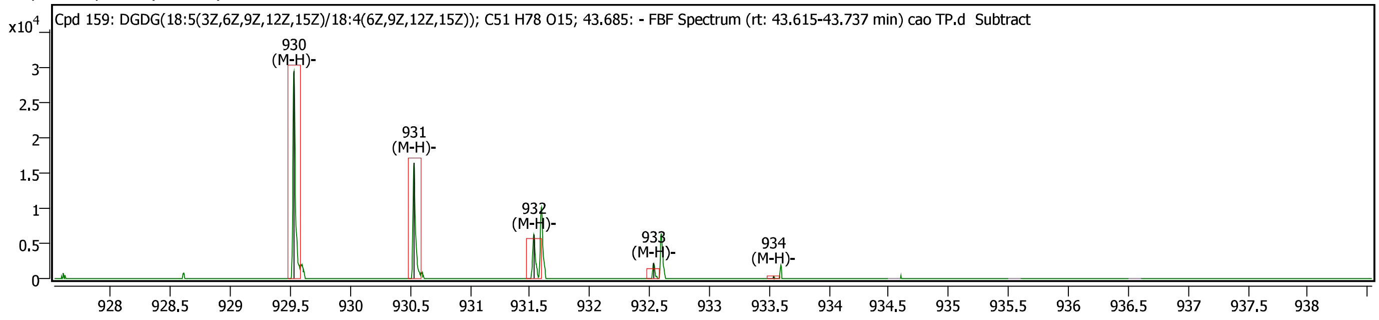

Compound ID Table

Name	Formula	Species	RT	RT Diff	Mass	CAS	ID Source	Score	Score (Lib)	Score (Tgt)
DGDG(18:5(3Z,6Z,9Z,12Z,15Z)/18:4(6Z,9Z,12Z,15Z))	C51 H78 O15	(M-H)-	43.685		930.5364		FBF	94.58		94.58

Cpd 166: Chukrasin methyl ether

Name	Formula	RT	RI	Mass	Diff (Tgt, ppm)	CAS	ID Source	Score	Algorithm
Chukrasin methyl ether	C43 H58 O16	44.348		830.3751	3.17	1045017-87-5	FBF	93.47	FBF

Species	m/z	Score (Tgt)	Score (Lib)	Score (DB)	Score (MFG)	Score (RT)
(M-H)-	829	93.47				

Compound Chromatograms (overlaid)

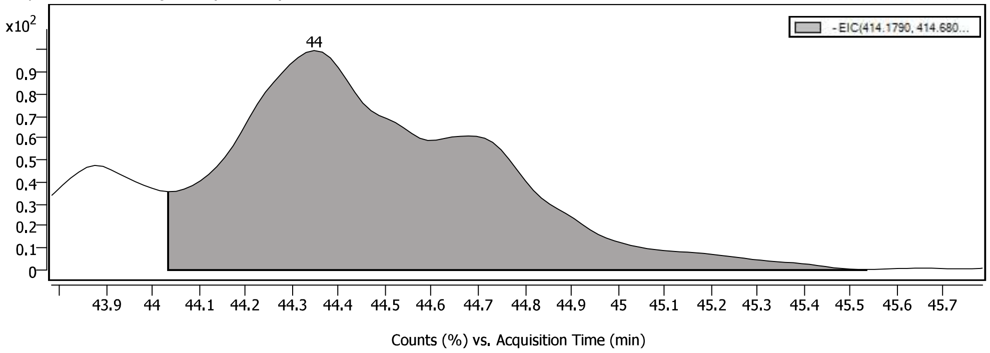

Structure

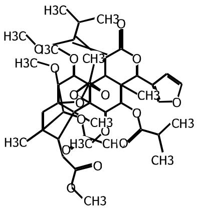

Compound Spectra (overlaid)

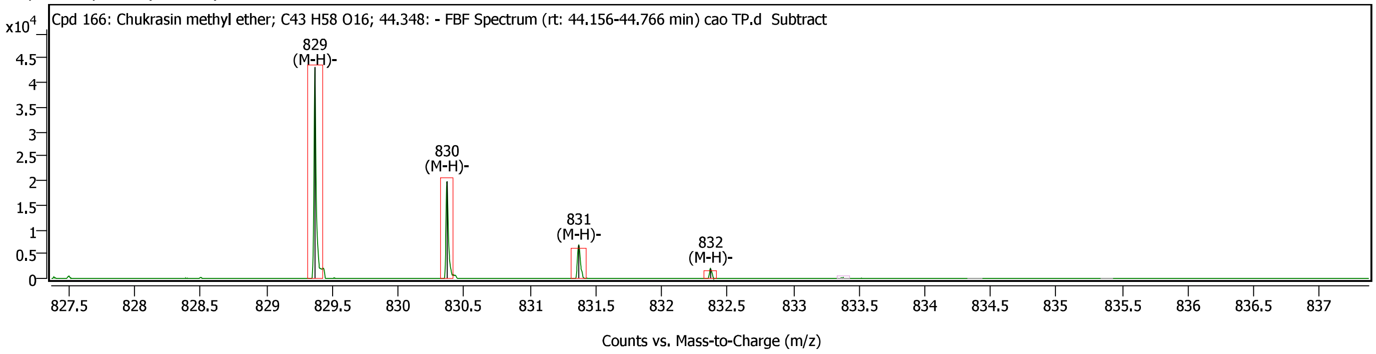

Compound ID Table

Name	Formula	Species	RT	RT Diff	Mass	CAS	ID Source	Score	Score (Lib)	Score (Tgt)
Chukrasin methyl ether	C43 H58 O16	(M-H)-	44.348		830.3751	1045017-87-5	FBF	93.47		93.47

Cpd 232: 2-Dodecylbenzenesulfonic acid

Name	Formula	RT	RI	Mass	Diff (Tgt, ppm)	CAS	ID Source	Score	Algorithm
2-Dodecylbenzenesulfonic acid	C18 H30 O3 S	44.348		326.1917	0.46		M-FBF	91.55	FBF

Species	m/z	Score (Tgt)	Score (Lib)	Score (DB)	Score (MFG)	Score (RT)
(M-H)-	325	91.55				

Compound Chromatograms (overlaid)

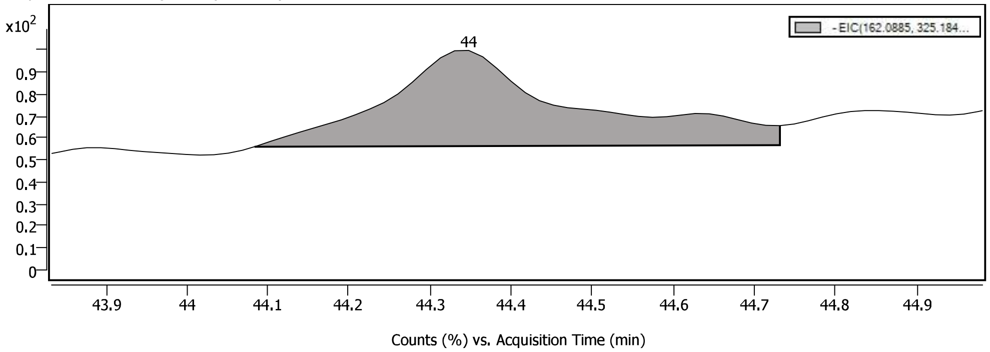

Structure

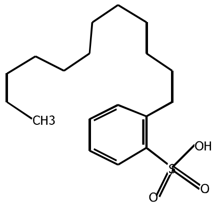

Compound Screening Report

Compound Spectra (overlaid)

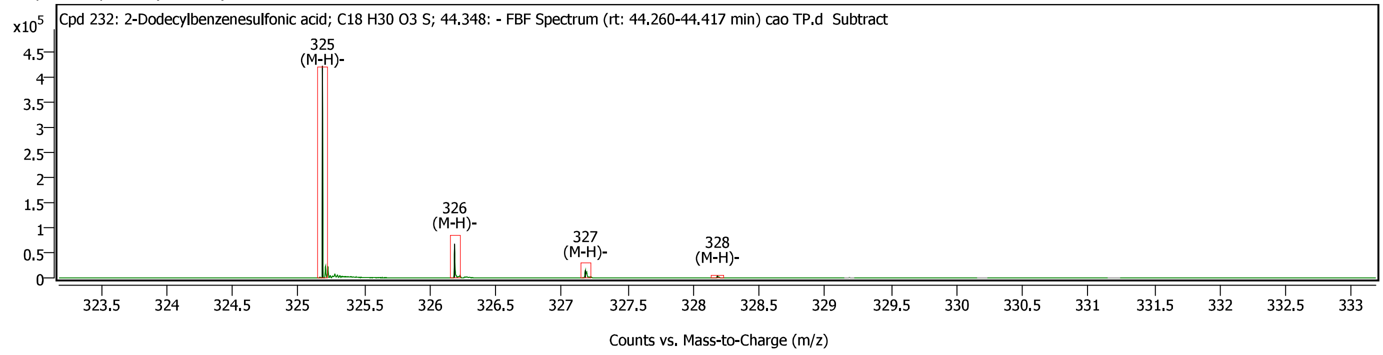

Compound ID Table

Name	Formula	Species	RT	RT Diff	Mass	CAS	ID Source	Score	Score (Lib)	Score (Tgt)
2-Dodecylbenzenesulfonic acid	C18 H30 O3 S	(M-H)-	44.348		326.1917		FBF	91.55		91.55
4-Dodecylbenzenesulfonic acid	C18 H30 O3 S	(M-H)-	44.348		326.1917	121-65-3	FBF	91.55		91.55

Cpd 158: MGDG(18:5(3Z,6Z,9Z,12Z,15Z)/18:4(6Z,9Z,12Z,15Z))

Name	Formula	RT	RI	Mass	Diff (Tgt, ppm)	CAS	ID Source	Score	Algorithm
MGDG(18:5(3Z,6Z,9Z,12Z,15Z)/18:4(6Z,9Z,12Z,15Z))	C45 H68 O10	44.819		768.4842	3.78		FBF	91.34	FBF

Species	m/z	Score (Tgt)	Score (Lib)	Score (DB)	Score (MFG)	Score (RT)
(M-H)-	767	91.34				

Compound Chromatograms (overlaid)

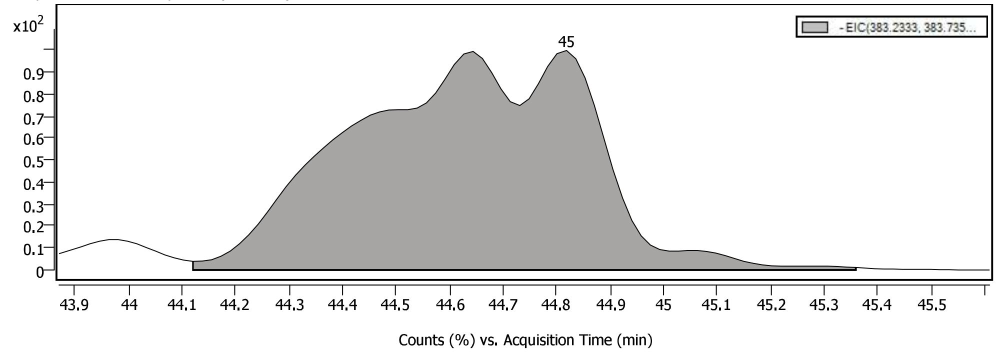

Structure

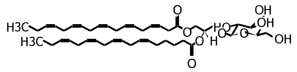

Compound Spectra (overlaid)

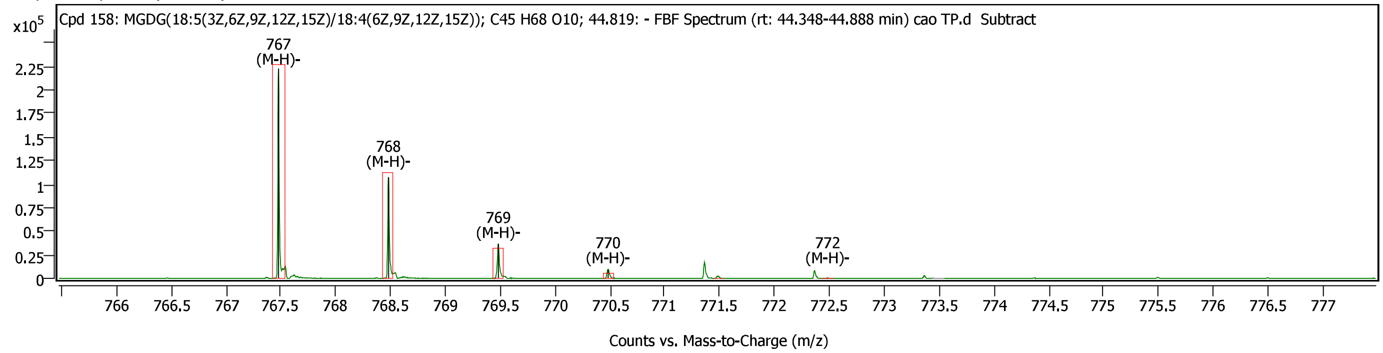

Compound ID Table

Name	Formula	Species	RT	RT Diff	Mass	CAS	ID Source	Score	Score (Lib)	Score (Tgt)
MGDG(18:5(3Z,6Z,9Z,12Z,15Z)/18:4(6Z,9Z,12Z,15Z))	C45 H68 O10	(M-H)-	44.819		768.4842		FBF	91.34		91.34

Cpd 225: LysoPC(24:1(15Z))

Name	Formula	RT	RI	Mass	Diff (Tgt, ppm)	CAS	ID Source	Score	Algorithm
LysoPC(24:1(15Z))	C32 H65 N O7 P	46.074		606.4492	-1.10		FBF	97.21	FBF

Species	m/z	Score (Tgt)	Score (Lib)	Score (DB)	Score (MFG)	Score (RT)
(M-H)-	605	97.21				

Compound Chromatograms (overlaid)

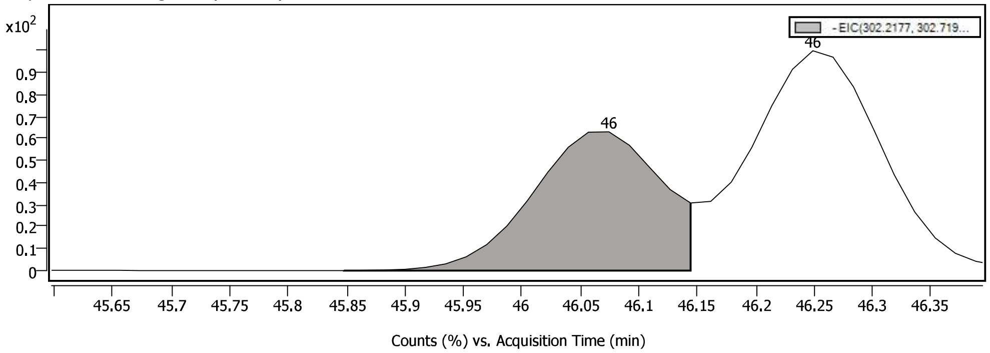

Structure

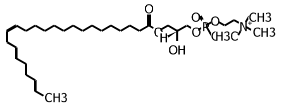

Compound Spectra (overlaid)

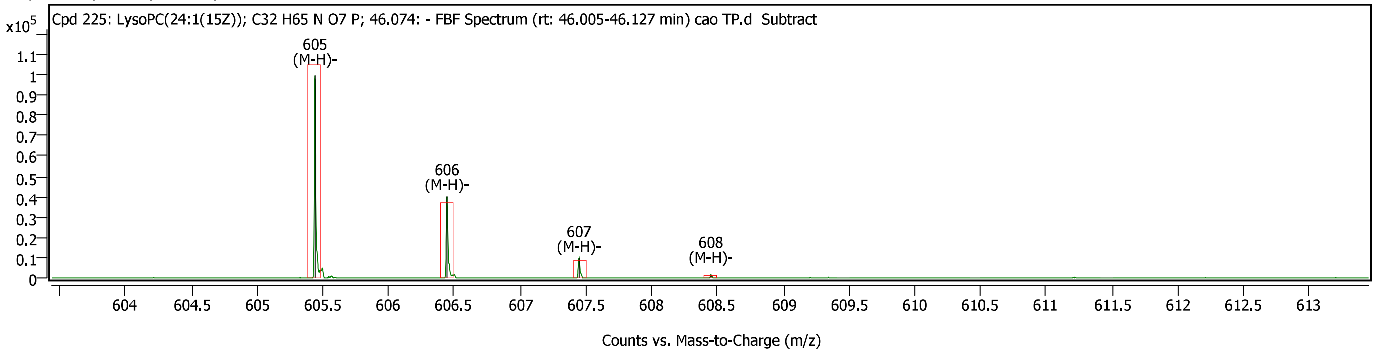

Compound ID Table

Name	Formula	Species	RT	RT Diff	Mass	CAS	ID Source	Score	Score (Lib)	Score (Tgt)
LysoPC(24:1(15Z))	C32 H65 N O7 P	(M-H)-	46.074		606.4492		FBF	97.21		97.21

Cpd 226: LysoPC(24:1(15Z))

Name	Formula	RT	RI	Mass	Diff (Tgt, ppm)	CAS	ID Source	Score	Algorithm
LysoPC(24:1(15Z))	C32 H65 N O7 P	46.249		606.4493	-0.99		FBF	97.03	FBF

Species	m/z	Score (Tgt)	Score (Lib)	Score (DB)	Score (MFG)	Score (RT)
(M-H)-	605	97.03				

Compound Chromatograms (overlaid)

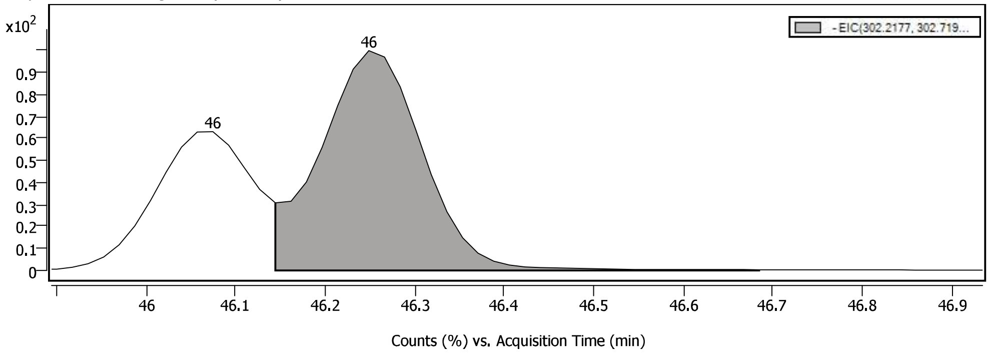

Structure

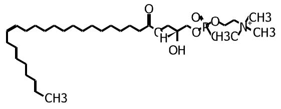

Compound Spectra (overlaid)

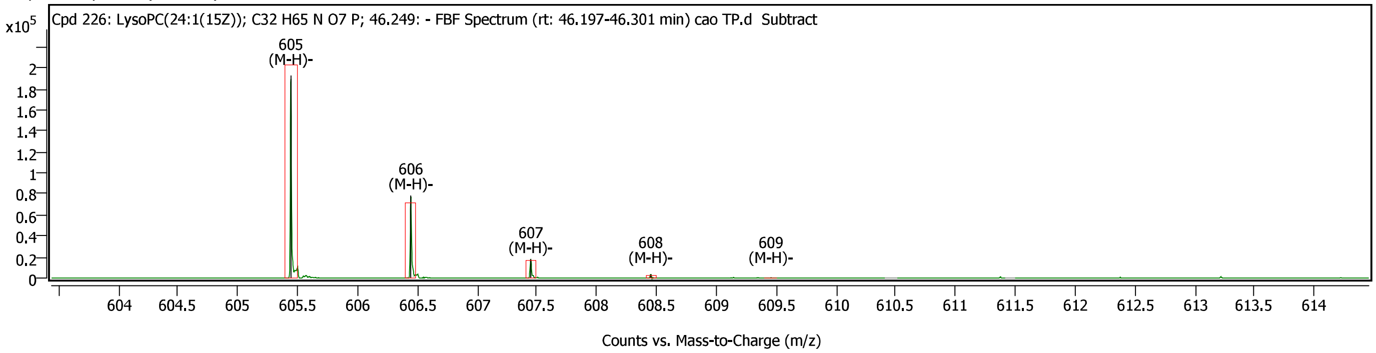

Compound ID Table

Name	Formula	Species	RT	RT Diff	Mass	CAS	ID Source	Score	Score (Lib)	Score (Tgt)
LysoPC(24:1(15Z))	C32 H65 N O7 P	(M-H)-	46.249		606.4493		FBF	97.03		97.03

Cpd 74: <α-Linolenic Acid>

Compound Screening Report

Name	Formula	RT	RI	Mass	Diff (Tgt, ppm)	CAS	ID Source	Score	Algorithm
<α-Linolenic Acid>	C18 H30 O2	46.284		278.2252	2.29	463-40-1	M-FBF	96.48	FBF

Species	m/z	Score (Tgt)	Score (Lib)	Score (DB)	Score (MFG)	Score (RT)
(M-H)-	277	96.48				

Compound Chromatograms (overlaid)

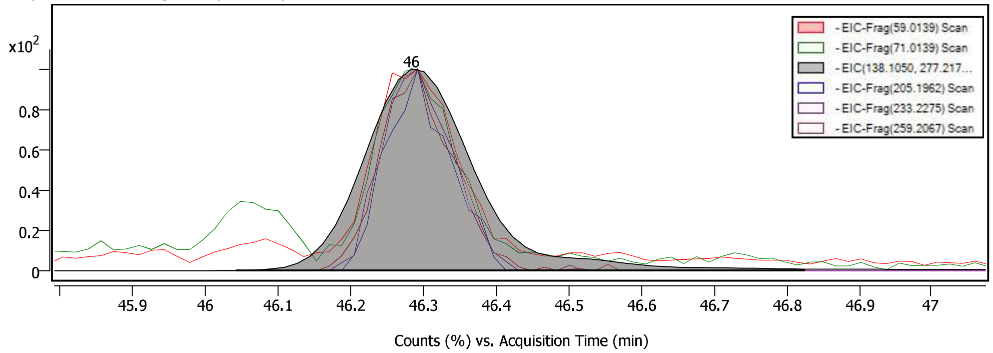

Structure

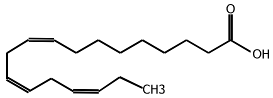

Coelution Plot

Compound Spectra (overlaid)

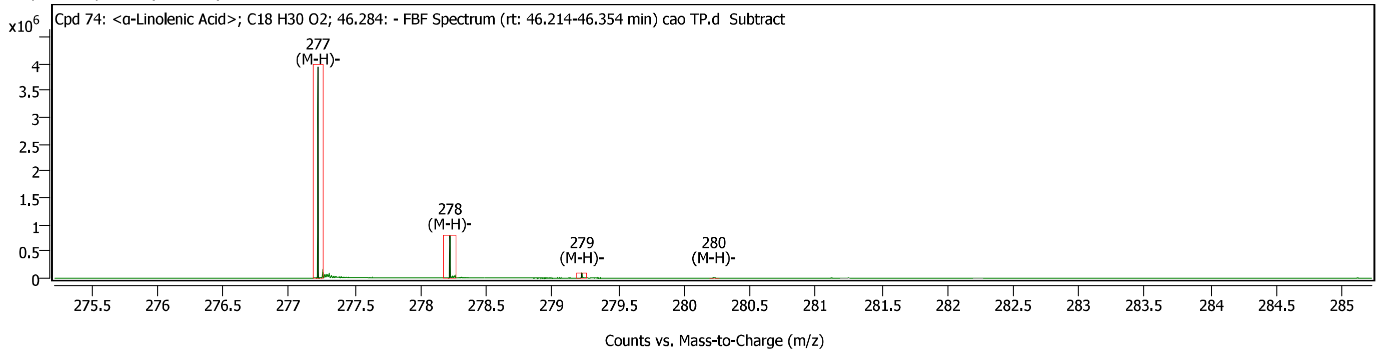

Fragment Spectrum (raw)

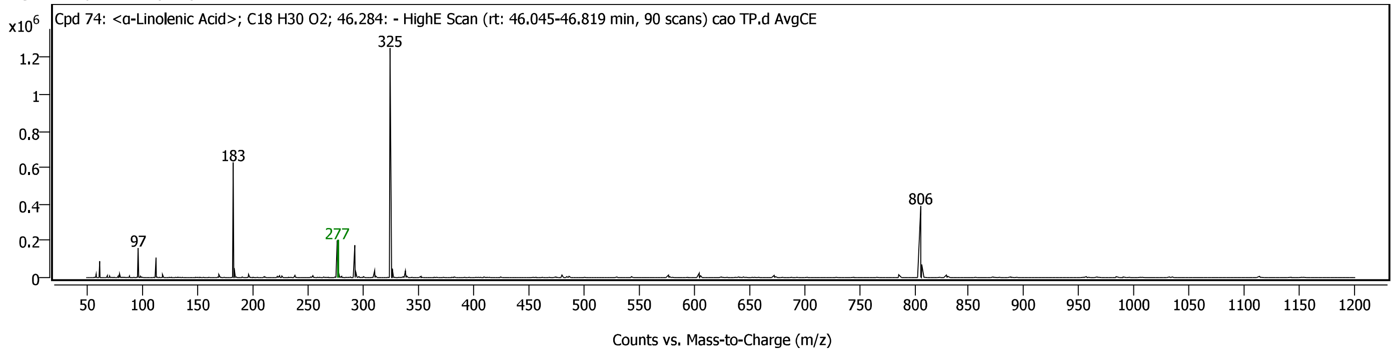

Compound Screening Report

Agilent

Compound ID Table

Name	Formula	Species	RT	RT Diff	Mass	CAS	ID Source	Score	Score (Lib)	Score (Tgt)
<α-Linolenic Acid>	C18 H30 O2	(M-H)-	46.284		278.2252	463-40-1	FBF	96.48		96.48
<7Z,9Z,12Z-Octadecatrienoic acid>	C18 H30 O2	(M-H)-	46.284		278.2252		FBF	96.48		96.48
<3E,9Z,12Z-Octadecatrienoic acid>	C18 H30 O2	(M-H)-	46.284		278.2252	2277-04-5	FBF	96.48		96.48
<5,9,12-Octadecatrienoic acid>	C18 H30 O2	(M-H)-	46.284		278.2252		FBF	96.48		96.48
<5Z,9Z,12E-Octadecatrienoic acid>	C18 H30 O2	(M-H)-	46.284		278.2252		FBF	96.48		96.48
<6,10,14-Octadecatrienoic acid>	C18 H30 O2	(M-H)-	46.284		278.2252	873392-34-8	FBF	96.48		96.48
<7E,9Z,12Z-Octadecatrienoic acid>	C18 H30 O2	(M-H)-	46.284		278.2252		FBF	96.48		96.48
<9Z,12E,15E-Octadecatrienoic acid>	C18 H30 O2	(M-H)-	46.284		278.2252		FBF	96.48		96.48
<8-Hydroxy-15,16-bisnor-11-labden-13-one>	C18 H30 O2	(M-H)-	46.284		278.2252	16736-49-5	FBF	96.48		96.48
<8Z,10E,12Z-Octadecatrienoic acid>	C18 H30 O2	(M-H)-	46.284		278.2252	28872-28-8	FBF	96.48		96.48
<9,12,14-Octadecatrienoic acid>	C18 H30 O2	(M-H)-	46.284		278.2252	374621-93-9	FBF	96.48		96.48
<9E,12E,15Z-Octadecatrienoic acid>	C18 H30 O2	(M-H)-	46.284		278.2252	21661-13-2	FBF	96.48		96.48
<9E,12Z,15E-Octadecatrienoic acid>	C18 H30 O2	(M-H)-	46.284		278.2252		FBF	96.48		96.48
<9E,12Z,15Z-Octadecatrienoic acid>	C18 H30 O2	(M-H)-	46.284		278.2252	21661-10-9	FBF	96.48		96.48
<4E,6E,10Z-Hexadecatrienyl acetate>	C18 H30 O2	(M-H)-	46.284		278.2252		FBF	96.48		96.48
<4E,6E,11Z-Hexadecatrienyl acetate>	C18 H30 O2	(M-H)-	46.284		278.2252		FBF	96.48		96.48
<10E,12E,14E-Hexadecatrienyl acetate>	C18 H30 O2	(M-H)-	46.284		278.2252		FBF	96.48		96.48
<(E)-octadec-9-en-12-ynoic acid>	C18 H30 O2	(M-H)-	46.284		278.2252		FBF	96.48		96.48
<4E,6Z,10Z-Hexadecatrienyl acetate>	C18 H30 O2	(M-H)-	46.284		278.2252		FBF	96.48		96.48
<(E,E)-3,7,11-Trimethyl-2,6,10-dodecatrienyl propionate>	C18 H30 O2	(M-H)-	46.284		278.2252		FBF	96.48		96.48
<(R)-lamenallenic acid>	C18 H30 O2	(M-H)-	46.284		278.2252		FBF	96.48		96.48
<(S)-lamenallenic acid>	C18 H30 O2	(M-H)-	46.284		278.2252		FBF	96.48		96.48
<10,12,14-octadecatrienoic acid>	C18 H30 O2	(M-H)-	46.284		278.2252		FBF	96.48		96.48
<10,12,15-Octadecatrienoic acid>	C18 H30 O2	(M-H)-	46.284		278.2252	374621-94-0	FBF	96.48		96.48
<Columbinic acid>	C18 H30 O2	(M-H)-	46.284		278.2252		FBF	96.48		96.48
<10E,12E,14Z-Hexadecatrienyl acetate>	C18 H30 O2	(M-H)-	46.284		278.2252		FBF	96.48		96.48
<11Z,13E,15-Hexadecatrienyl acetate>	C18 H30 O2	(M-H)-	46.284		278.2252		FBF	96.48		96.48
<11Z-octadecen-9-ynoic acid>	C18 H30 O2	(M-H)-	46.284		278.2252		FBF	96.48		96.48
<13Z-Hexadecen-11-ynyl acetate>	C18 H30 O2	(M-H)-	46.284		278.2252		FBF	96.48		96.48
<16-methyl-6Z,9Z,12Z-heptadecatrienoic acid>	C18 H30 O2	(M-H)-	46.284		278.2252		FBF	96.48		96.48
<17-octadecen-9-ynoic acid>	C18 H30 O2	(M-H)-	46.284		278.2252		FBF	96.48		96.48
<2E,9Z,12Z-octadecatrienoic acid>	C18 H30 O2	(M-H)-	46.284		278.2252		FBF	96.48		96.48
<11E-octadecen-9-ynoic acid>	C18 H30 O2	(M-H)-	46.284		278.2252		FBF	96.48		96.48
<5,8,11-octadecatrienoic acid>	C18 H30 O2	(M-H)-	46.284		278.2252		FBF	96.48		96.48
<9E-Octadecen-12-ynoic acid>	C18 H30 O2	(M-H)-	46.284		278.2252		FBF	96.48		96.48
<Gorlic acid>	C18 H30 O2	(M-H)-	46.284		278.2252		FBF	96.48		96.48
<Elaidolinoleic acid>	C18 H30 O2	(M-H)-	46.284		278.2252		FBF	96.48		96.48
<octadeca-5S,6,16E-trienoic acid>	C18 H30 O2	(M-H)-	46.284		278.2252		FBF	96.48		96.48
<octadeca-9Z,11E,14Z-trienoic acid>	C18 H30 O2	(M-H)-	46.284		278.2252		FBF	96.48		96.48
<octadeca-9Z,11E,15Z-trienoic acid>	C18 H30 O2	(M-H)-	46.284		278.2252		FBF	96.48		96.48
<Pinolenic Acid>	C18 H30 O2	(M-H)-	46.284		278.2252	16833-54-8	FBF	96.48		96.48
<Pseudoeleostearic acid>	C18 H30 O2	(M-H)-	46.284		278.2252		FBF	96.48		96.48
<trans-3, cis-9, cis-12-octadecatrienoic acid; C18:3n-6,9,15>	C18 H30 O2	(M-H)-	46.284		278.2252		FBF	96.48		96.48
<estrane-3alpha,17alpha-diol>	C18 H30 O2	(M-H)-	46.284		278.2252		FBF	96.48		96.48
<α-Calendic acid>	C18 H30 O2	(M-H)-	46.284		278.2252	5204-87-5	FBF	96.48		96.48
<α-ESA>	C18 H30 O2	(M-H)-	46.284		278.2252	506-23-0	FBF	96.48		96.48
<β-Calendic acid>	C18 H30 O2	(M-H)-	46.284		278.2252	822-19-5	FBF	96.48		96.48
<β-Eleostearic acid>	C18 H30 O2	(M-H)-	46.284		278.2252	544-73-0	FBF	96.48		96.48
<γ-Linolenic Acid>	C18 H30 O2	(M-H)-	46.284		278.2252	506-26-3	FBF	96.48		96.48
<Punicic acid>	C18 H30 O2	(M-H)-	46.284		278.2252		FBF	96.48		96.48
<estrane-3α,17α-diol>	C18 H30 O2	(M-H)-	46.284		278.2252		FBF	96.48		96.48
<C18:3n-4,8,12>	C18 H30 O2	(M-H)-	46.284		278.2252		FBF	96.48		96.48
<9E,11Z,13Z-octadecatrienoic acid>	C18 H30 O2	(M-H)-	46.284		278.2252		FBF	96.48		96.48
<9Z-Octadecen-12-ynoic acid>	C18 H30 O2	(M-H)-	46.284		278.2252		FBF	96.48		96.48
<Acetylenic acids; 11-Octadecen-9-ynoic acid, (E)-; Ximenynic acid; Santalbic acid; trans-11-Octadecen-9-ynoic acid; Ximeninic acid>	C18 H30 O2	(M-H)-	46.284		278.2252		FBF	96.48		96.48
<Acetylenic acids; 11-Octadecen-9-ynoic acid, (Z)-; cis-11-Octadecen-9-ynoic acid>	C18 H30 O2	(M-H)-	46.284		278.2252		FBF	96.48		96.48
<Acetylenic acids; 17-Octadecen-9-ynoic acid>	C18 H30 O2	(M-H)-	46.284		278.2252		FBF	96.48		96.48
<Crepenynic acid>	C18 H30 O2	(M-H)-	46.284		278.2252		FBF	96.48		96.48
<C18:3n-4,6,9>	C18 H30 O2	(M-H)-	46.284		278.2252		FBF	96.48		96.48
<C18:3n-3,6,8>	C18 H30 O2	(M-H)-	46.284		278.2252		FBF	96.48		96.48
<C18:3n-5,7,9>	C18 H30 O2	(M-H)-	46.284		278.2252		FBF	96.48		96.48
<C18:3n-6,9,16>	C18 H30 O2	(M-H)-	46.284		278.2252		FBF	96.48		96.48
<C18:3n-7,10,13>	C18 H30 O2	(M-H)-	46.284		278.2252		FBF	96.48		96.48
<Catalpic acid>	C18 H30 O2	(M-H)-	46.284		278.2252		FBF	96.48		96.48
<cis-8, trans-10, cis-12-octadecatrienoic acid; C18:3n-6,8,10>	C18 H30 O2	(M-H)-	46.284		278.2252		FBF	96.48		96.48
<octadeca-11E,13E,15Z-trienoic acid>	C18 H30 O2	(M-H)-	46.284		278.2252		FBF	96.48		96.48

Compound Screening Report

Compound ID Table

Name	Formula	Species	RT	RT Diff	Mass	CAS	ID Source	Score	Score (Lib)	Score (Tgt)
<9Z,12E,15Z-Octadecatrienoic acid>	C18 H30 O2	(M-H)-	46.284		278.2252		FBF	96.48		96.48
<9Z,12Z,15E-Octadecatrienoic acid>	C18 H30 O2	(M-H)-	46.284		278.2252	21661-08-5	FBF	96.48		96.48

Cpd 170: PC(O-5:0/0:0)[R]

Name	Formula	RT	RI	Mass	Diff (Tgt, ppm)	CAS	ID Source	Score	Algorithm
PC(O-5:0/0:0)[R]	C13 H31 N O6 P	46.284		328.1878	-3.46		FBF	81.11	FBF

Species	m/z	Score (Tgt)	Score (Lib)	Score (DB)	Score (MFG)	Score (RT)
(M-H)-	327	81.11				

Compound Chromatograms (overlaid)

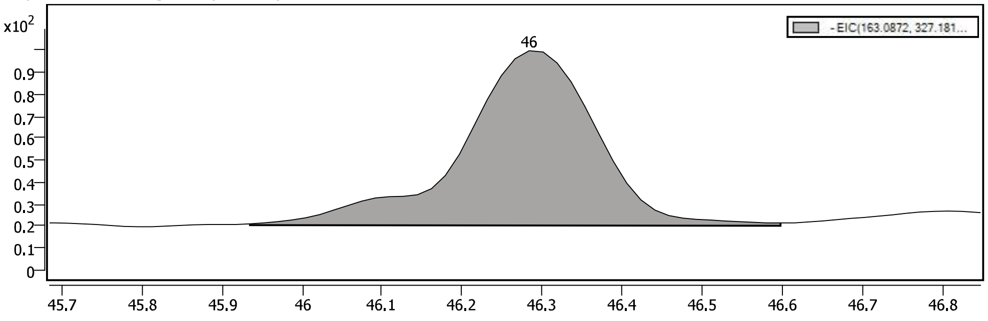

Structure

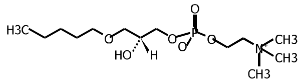

Compound Spectra (overlaid)

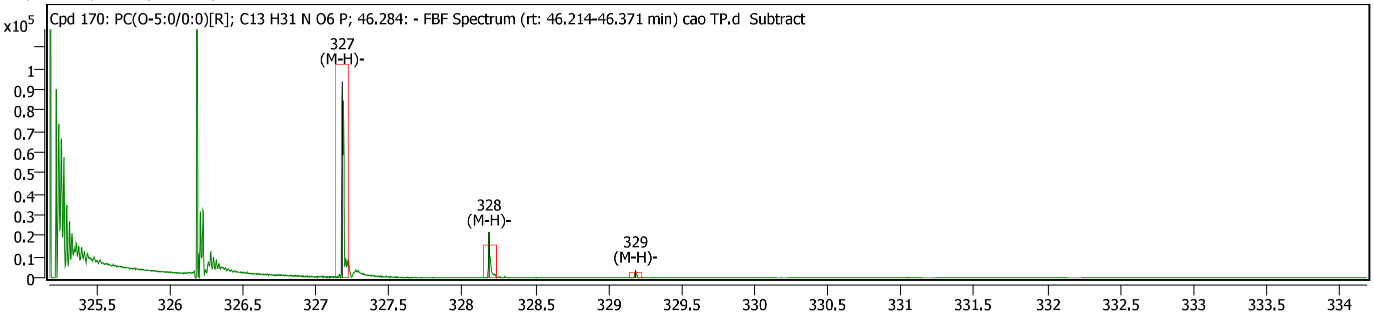

Compound ID Table

Name	Formula	Species	RT	RT Diff	Mass	CAS	ID Source	Score	Score (Lib)	Score (Tgt)
PC(O-5:0/0:0)[R]	C13 H31 N O6 P	(M-H)-	46.284		328.1878		FBF	81.11		81.11

Cpd 38: PA(O-16:0/12:0)

Name	Formula	RT	RI	Mass	Diff (Tgt, ppm)	CAS	ID Source	Score	Algorithm
PA(O-16:0/12:0)	C31 H63 O7 P	46.301		578.4308	-0.60		FBF	96.22	FBF

Species	m/z	Score (Tgt)	Score (Lib)	Score (DB)	Score (MFG)	Score (RT)
(M-H)-	577	96.22				

Compound Chromatograms (overlaid)

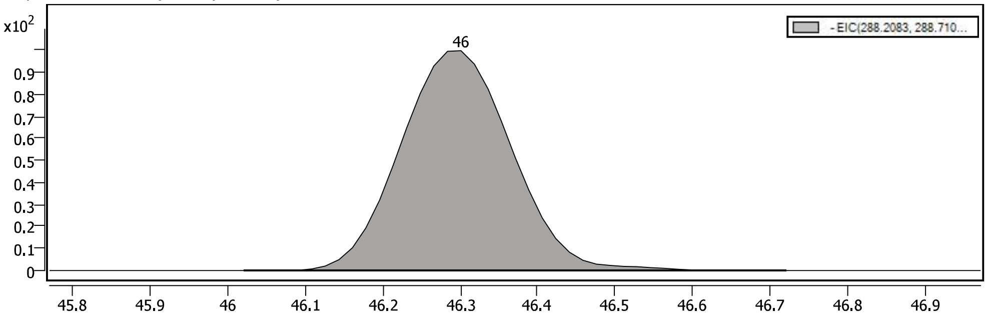

Structure

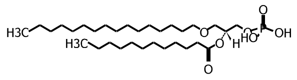

Compound Spectra (overlaid)

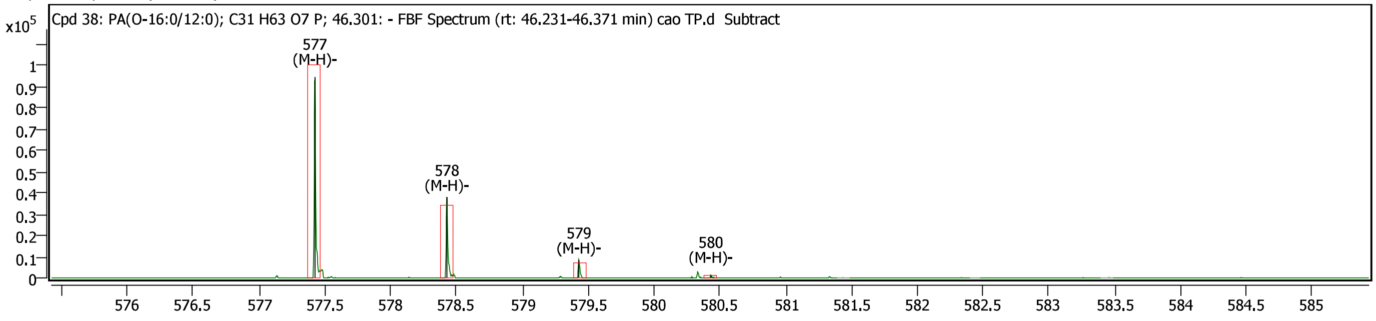

Compound ID Table

Name	Formula	Species	RT	RT Diff	Mass	CAS	ID Source	Score	Score (Lib)	Score (Tgt)
PA(O-16:0/12:0)	C31 H63 O7 P	(M-H)-	46.301		578.4308		FBF	96.22		96.22

Cpd 227: 2-Hexaprenyl-3-methyl-5-hydroxy-6-methoxy-1,4-benzoquinol

Name	Formula	RT	RI	Mass	Diff (Tgt, ppm)	CAS	ID Source	Score	Algorithm
2-Hexaprenyl-3-methyl-5-hydroxy-6-methoxy-1,4-benzoquinol	C38 H58 O4	46.301		578.4308	-4.73		FBF	90.13	FBF

Species	m/z	Score (Tgt)	Score (Lib)	Score (DB)	Score (MFG)	Score (RT)
(M-H)-	577	90.13				

Compound Chromatograms (overlaid)

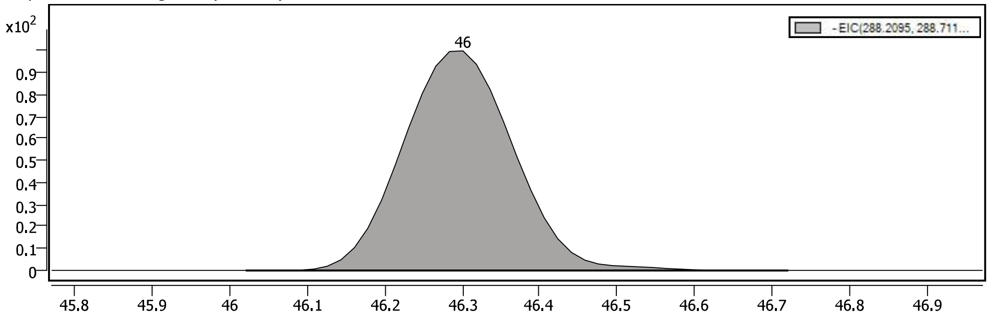

Structure

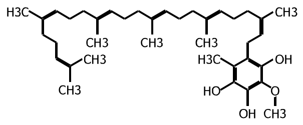

Compound Spectra (overlaid)

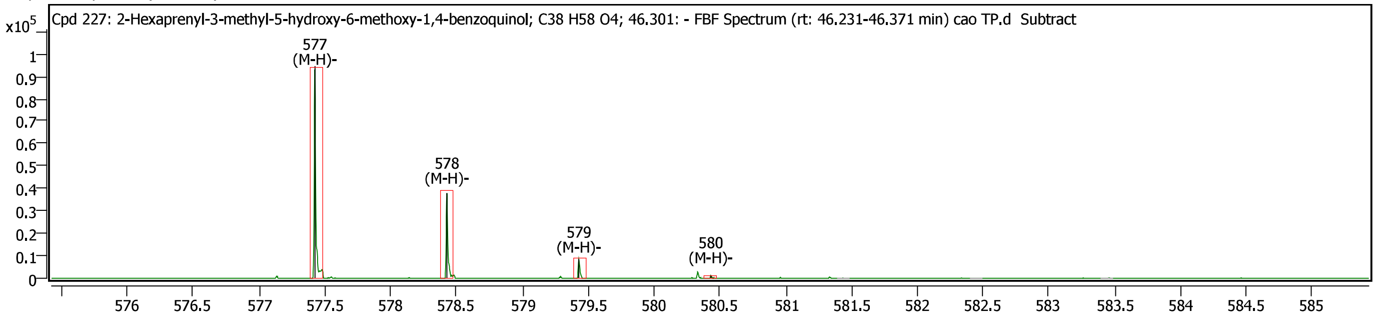

Compound ID Table

Name	Formula	Species	RT	RT Diff	Mass	CAS	ID Source	Score	Score (Lib)	Score (Tgt)
2-Hexaprenyl-3-methyl-5-hydroxy-6-methoxy-1,4-benzoquinol	C38 H58 O4	(M-H)-	46.301		578.4308		FBF	90.13		90.13

Cpd 193: Tetradecyl sulfate

Name	Formula	RT	RI	Mass	Diff (Tgt, ppm)	CAS	ID Source	Score	Algorithm
Tetradecyl sulfate	C14 H30 O4 S	46.790		294.1863	-0.73	139-88-8	M-FBF	96.65	FBF

Species	m/z	Score (Tgt)	Score (Lib)	Score (DB)	Score (MFG)	Score (RT)
(M-H)-	293	96.65				

Compound Chromatograms (overlaid)

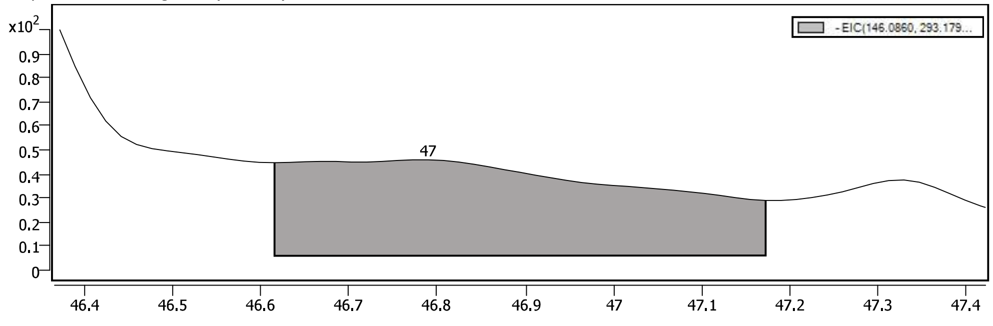

Structure

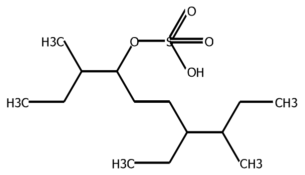

Compound Spectra (overlaid)

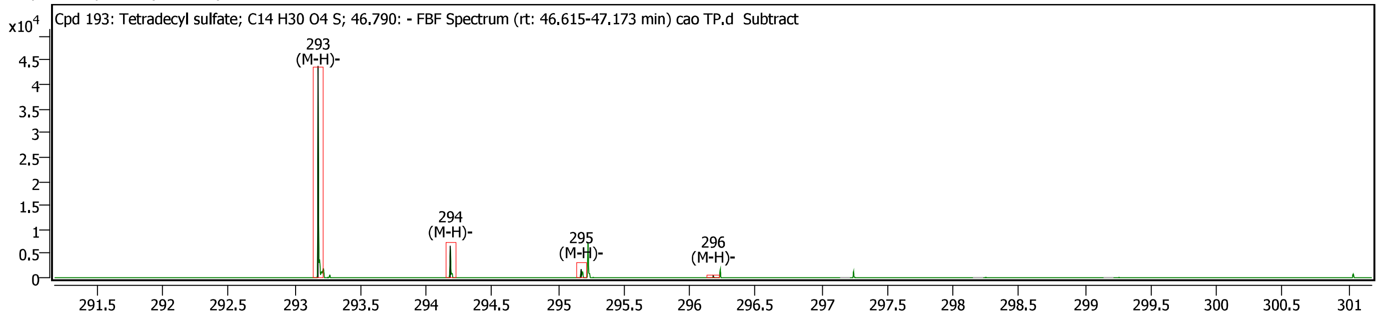

Compound ID Table

Name	Formula	Species	RT	RT Diff	Mass	CAS	ID Source	Score	Score (Lib)	Score (Tgt)
Tetradecyl sulfate	C14 H30 O4 S	(M-H)-	46.790		294.1863	139-88-8	FBF	96.65		96.65
Sodium Tetradecyl Sulfate	C14 H30 O4 S	(M-H)-	46.790		294.1863	1191-50-0	FBF	96.65		96.65

Cpd 9: <Δ2-cis-Hexadecenoic Acid>

Name	Formula	RT	RI	Mass	Diff (Tgt, ppm)	CAS	ID Source	Score	Algorithm
<Δ2-cis-Hexadecenoic Acid>	C16 H30 O2	47.191		254.2244	-0.88	2825-68-5	M-FBF	99.05	FBF

Species	m/z	Score (Tgt)	Score (Lib)	Score (DB)	Score (MFG)	Score (RT)
(M-H)-	253	99.05				

Compound Chromatograms (overlaid)

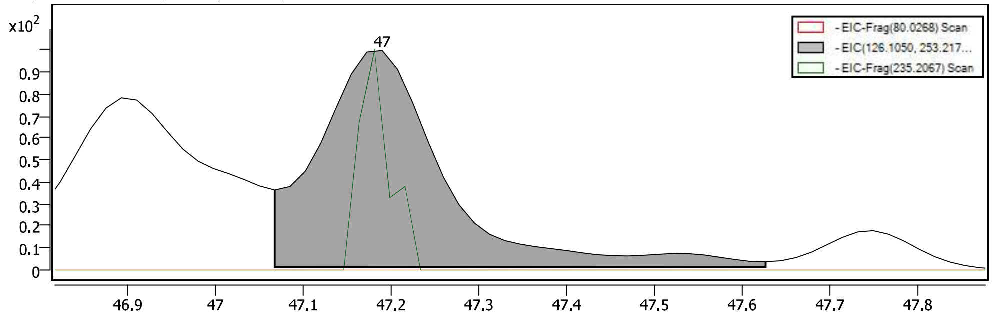

Structure

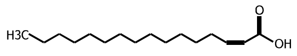

Coelution Plot

Compound Spectra (overlaid)

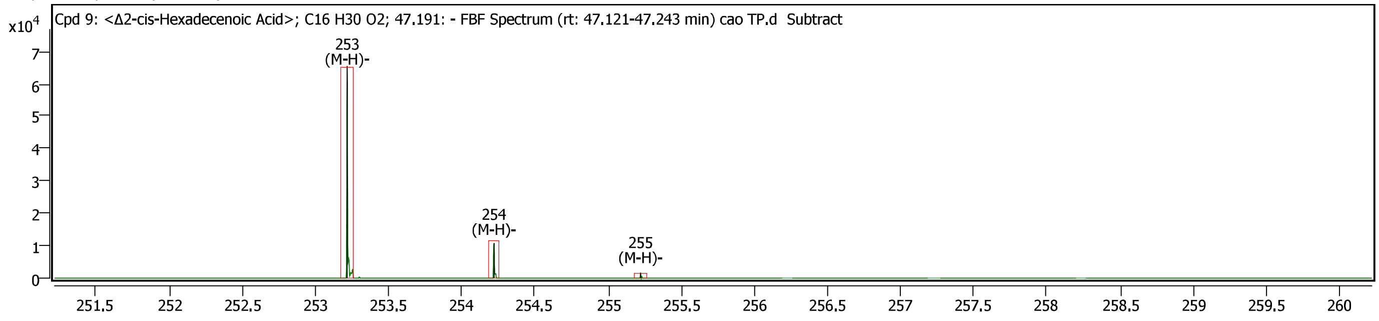

Fragment Spectrum (raw)

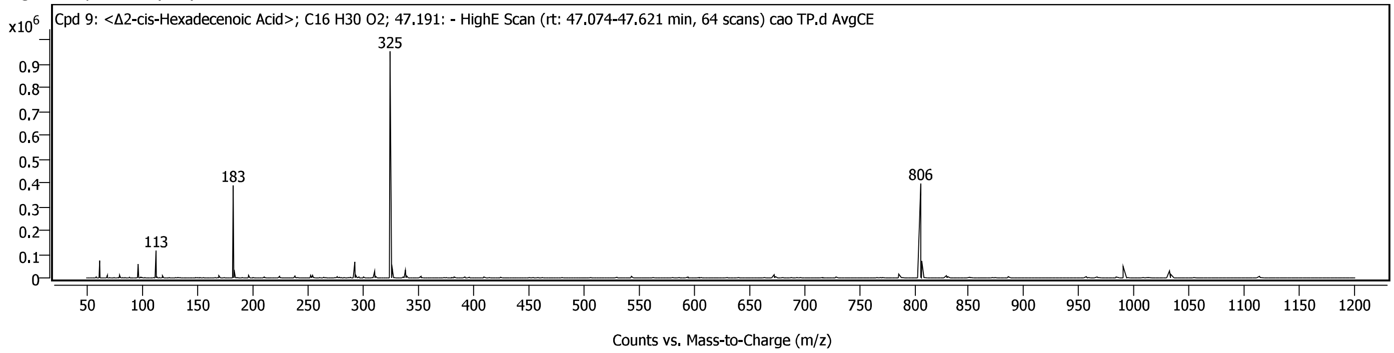

Compound ID Table

Name	Formula	Species	RT	RT Diff	Mass	CAS	ID Source	Score	Score (Lib)	Score (Tgt)
<Δ2-cis-Hexadecenoic Acid>	C16 H30 O2	(M-H)-	47.191		254.2244	2825-68-5	FBF	99.05		99.05
<16-Hexadecanolide>	C16 H30 O2	(M-H)-	47.191		254.2244		FBF	99.05		99.05
<15:1(4)(13Me)>	C16 H30 O2	(M-H)-	47.191		254.2244		FBF	99.05		99.05
<15R-Hexadecanolide>	C16 H30 O2	(M-H)-	47.191		254.2244		FBF	99.05		99.05
<16:1(4)>	C16 H30 O2	(M-H)-	47.191		254.2244		FBF	99.05		99.05
<16:1(5Z)>	C16 H30 O2	(M-H)-	47.191		254.2244		FBF	99.05		99.05
<5-Dodecyldihydro-2(3H)-furanone>	C16 H30 O2	(M-H)-	47.191		254.2244	730-46-1	FBF	99.05		99.05
<2,4-dimethyl-2E-tetradecenoic acid>	C16 H30 O2	(M-H)-	47.191		254.2244		FBF	99.05		99.05
<2-hexyl-2-decenoic acid>	C16 H30 O2	(M-H)-	47.191		254.2244		FBF	99.05		99.05
<3E-Hexadecenoic acid>	C16 H30 O2	(M-H)-	47.191		254.2244	2457-70-7	FBF	99.05		99.05
<3E-Tetradecenyl acetate>	C16 H30 O2	(M-H)-	47.191		254.2244		FBF	99.05		99.05
<3Z-Tetradecenyl acetate>	C16 H30 O2	(M-H)-	47.191		254.2244		FBF	99.05		99.05
<13Z-Hexadecenoic acid>	C16 H30 O2	(M-H)-	47.191		254.2244	144462-52-2	FBF	99.05		99.05
<14-methyl-4-pentadecenoic acid>	C16 H30 O2	(M-H)-	47.191		254.2244		FBF	99.05		99.05
<C16:1n-6>	C16 H30 O2	(M-H)-	47.191		254.2244		FBF	99.05		99.05
<11Z-Hexadecenoic acid>	C16 H30 O2	(M-H)-	47.191		254.2244	2416-20-8	FBF	99.05		99.05
<12Z-Tetradecenyl acetate>	C16 H30 O2	(M-H)-	47.191		254.2244		FBF	99.05		99.05
<12E-Tetradecenyl acetate>	C16 H30 O2	(M-H)-	47.191		254.2244		FBF	99.05		99.05
<11Z-Tetradecenyl acetate>	C16 H30 O2	(M-H)-	47.191		254.2244		FBF	99.05		99.05
<6E-Tetradecenyl acetate>	C16 H30 O2	(M-H)-	47.191		254.2244		FBF	99.05		99.05
<11-Hexadecenoic acid>	C16 H30 O2	(M-H)-	47.191		254.2244	2271-34-3	FBF	99.05		99.05
<11E-Tetradecenyl acetate>	C16 H30 O2	(M-H)-	47.191		254.2244		FBF	99.05		99.05
<10Z-Tetradecenyl acetate>	C16 H30 O2	(M-H)-	47.191		254.2244		FBF	99.05		99.05
<10-Hexadecenoic acid>	C16 H30 O2	(M-H)-	47.191		254.2244	25976-13-0	FBF	99.05		99.05
<10E-Tetradecenyl acetate>	C16 H30 O2	(M-H)-	47.191		254.2244		FBF	99.05		99.05
<(Z)-7-Dodecenyl butyrate>	C16 H30 O2	(M-H)-	47.191		254.2244		FBF	99.05		99.05
<(Z)-5-Hexadecenoic acid>	C16 H30 O2	(M-H)-	47.191		254.2244	7056-90-8	FBF	99.05		99.05
<(Z)-14-Methyl-6-pentadecenoic acid>	C16 H30 O2	(M-H)-	47.191		254.2244	123739-73-1	FBF	99.05		99.05
<(E)-3-Hexadecenoic acid>	C16 H30 O2	(M-H)-	47.191		254.2244	1686-10-8	FBF	99.05		99.05
<13-Hexadecenoic acid>	C16 H30 O2	(M-H)-	47.191		254.2244	14134-46-4	FBF	99.05		99.05
<15-Hexadecanolide>	C16 H30 O2	(M-H)-	47.191		254.2244	69297-56-9	FBF	99.05		99.05
<5Z-Tetradecenyl acetate>	C16 H30 O2	(M-H)-	47.191		254.2244		FBF	99.05		99.05
<Δ2-trans-Hexadecenoic Acid>	C16 H30 O2	(M-H)-	47.191		254.2244	929-79-3	FBF	99.05		99.05
<5E-Tetradecenyl acetate>	C16 H30 O2	(M-H)-	47.191		254.2244		FBF	99.05		99.05
<Vittatalactone>	C16 H30 O2	(M-H)-	47.191		254.2244		FBF	99.05		99.05
<trans-9-Palmitoleic acid>	C16 H30 O2	(M-H)-	47.191		254.2244	10030-73-6	FBF	99.05		99.05
<Sapienic acid>	C16 H30 O2	(M-H)-	47.191		254.2244		FBF	99.05		99.05
<Hypogeic acid>	C16 H30 O2	(M-H)-	47.191		254.2244		FBF	99.05		99.05
<hexadec-7Z-enoic acid>	C16 H30 O2	(M-H)-	47.191		254.2244		FBF	99.05		99.05
<6Z-Hexadecenoic acid>	C16 H30 O2	(M-H)-	47.191		254.2244	17004-51-2	FBF	99.05		99.05
<ethyl 9Z-tetradecenoate>	C16 H30 O2	(M-H)-	47.191		254.2244		FBF	99.05		99.05
<ethyl 9E-tetradecenoate>	C16 H30 O2	(M-H)-	47.191		254.2244		FBF	99.05		99.05
<ethyl 7E-tetradecenoate>	C16 H30 O2	(M-H)-	47.191		254.2244		FBF	99.05		99.05
<dodecyl 2E-butenoate>	C16 H30 O2	(M-H)-	47.191		254.2244		FBF	99.05		99.05
<delta-hexadecalactone>	C16 H30 O2	(M-H)-	47.191		254.2244		FBF	99.05		99.05
<Citronellyl hexanoate>	C16 H30 O2	(M-H)-	47.191		254.2244	10580-25-3	FBF	99.05		99.05
<Gaidic acid>	C16 H30 O2	(M-H)-	47.191		254.2244		FBF	99.05		99.05
<cis-9-Palmitoleic acid>	C16 H30 O2	(M-H)-	47.191		254.2244	373-49-9	FBF	99.05		99.05
<8Z-Tetradecenyl acetate>	C16 H30 O2	(M-H)-	47.191		254.2244		FBF	99.05		99.05
<cis-7-Hexadecenoic Acid>	C16 H30 O2	(M-H)-	47.191		254.2244	2416-19-5	FBF	99.05		99.05
<6Z-Tetradecenyl acetate>	C16 H30 O2	(M-H)-	47.191		254.2244		FBF	99.05		99.05
<7E-Tetradecenyl acetate>	C16 H30 O2	(M-H)-	47.191		254.2244		FBF	99.05		99.05
<7Z-Tetradecenyl acetate>	C16 H30 O2	(M-H)-	47.191		254.2244		FBF	99.05		99.05
<8E-Tetradecenyl acetate>	C16 H30 O2	(M-H)-	47.191		254.2244		FBF	99.05		99.05
<7-Palmitoleic acid>	C16 H30 O2	(M-H)-	47.191		254.2244	2197-46-8	FBF	99.05		99.05
<9E-Tetradecenyl acetate>	C16 H30 O2	(M-H)-	47.191		254.2244		FBF	99.05		99.05
<9Z-Tetradecenyl acetate>	C16 H30 O2	(M-H)-	47.191		254.2244		FBF	99.05		99.05
<C16:1n-14>	C16 H30 O2	(M-H)-	47.191		254.2244		FBF	99.05		99.05
<C16:1n-5>	C16 H30 O2	(M-H)-	47.191		254.2244		FBF	99.05		99.05
<cis-Palmitvaccenic acid>	C16 H30 O2	(M-H)-	47.191		254.2244		FBF	99.05		99.05
<6-isopentyl-9-methyl-5-decenoic acid>	C16 H30 O2	(M-H)-	47.191		254.2244		FBF	99.05		99.05
<cis-10-Palmitoleic acid>	C16 H30 O2	(M-H)-	47.191		254.2244	2511-97-9	FBF	99.05		99.05

Cpd 71: 16-Hydroxy hexadecanoic acid

Name	Formula	RT	RI	Mass	Diff (Tgt, ppm)	CAS	ID Source	Score	Algorithm
16-Hydroxy hexadecanoic acid	C16 H32 O3	47.888		272.2353	0.44	506-13-8	FBF-FragConfirm	99.35	FBF

Species	m/z	Score (Tgt)	Score (Lib)	Score (DB)	Score (MFG)	Score (RT)
(M-H)-	271	99.35				

Compound Screening Report

Compound Chromatograms (overlaid)

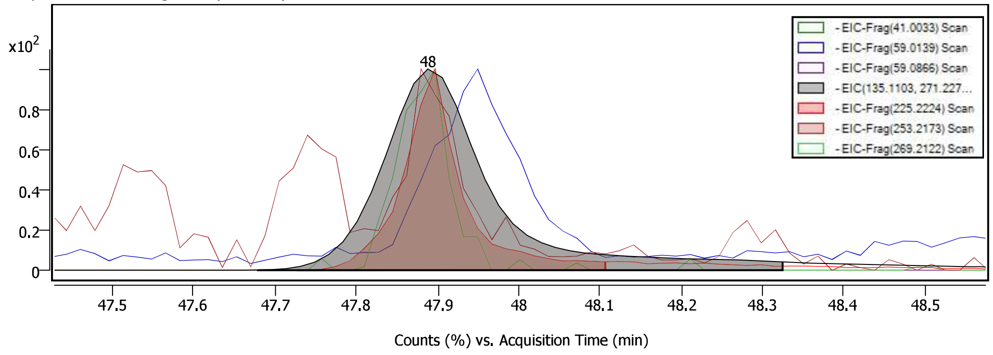

Structure

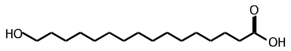

Coelution Plot

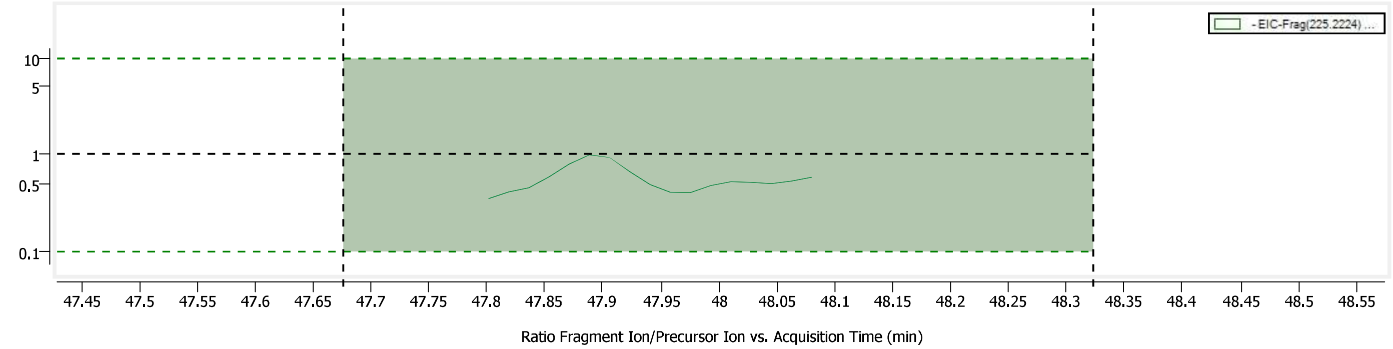

Compound Spectra (overlaid)

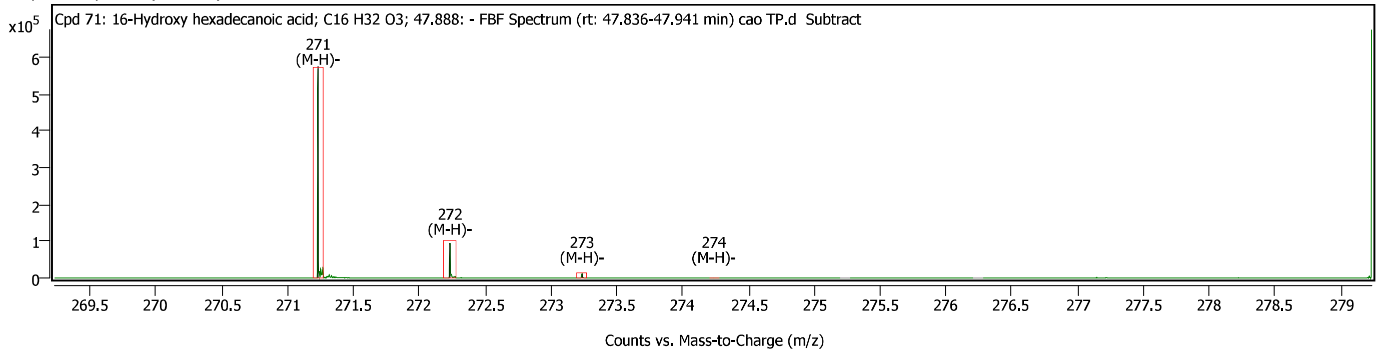

Fragment Spectrum (clean)

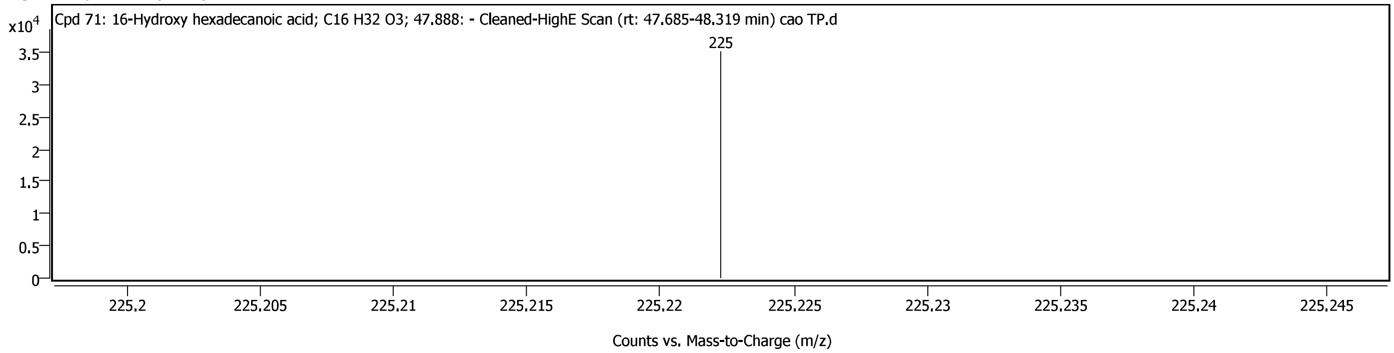

Fragment Spectrum (raw)

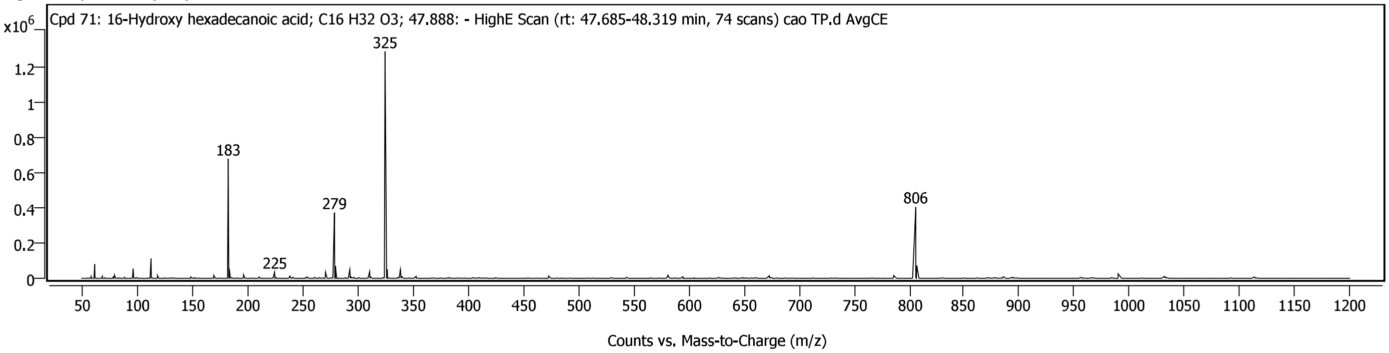

Compound ID Table

Name	Formula	Species	RT	RT Diff	Mass	CAS	ID Source	Score	Score (Lib)	Score (Tgt)
16-Hydroxy hexadecanoic acid	C16 H32 O3	(M-H)-	47.888		272.2353	506-13-8	FBF-FragConfirm	99.35		99.35
15-hydroxy-hexadecanoic acid	C16 H32 O3	(M-H)-	47.888		272.2353		FBF-FragConfirm	99.35		99.35
7R-hydroxy-hexadecanoic acid	C16 H32 O3	(M-H)-	47.888		272.2353		FBF-FragConfirm	99.35		99.35
3R-hydroxypalmitic acid	C16 H32 O3	(M-H)-	47.888		272.2353		FBF-FragConfirm	99.35		99.35
3-hydroxy-hexadecanoic acid	C16 H32 O3	(M-H)-	47.888		272.2353	928-17-6	FBF-FragConfirm	99.35		99.35
2-Hydroxyhexadecanoic acid	C16 H32 O3	(M-H)-	47.888		272.2353	764-67-0	FBF-FragConfirm	99.35		99.35
2-hydroxy palmitic acid	C16 H32 O3	(M-H)-	47.888		272.2353		FBF-FragConfirm	99.35		99.35
(+)-15S-hydroxy-hexadecanoic acid	C16 H32 O3	(M-H)-	47.888		272.2353		FBF-FragConfirm	99.35		99.35
14S-hydroxy-hexadecanoic acid	C16 H32 O3	(M-H)-	47.888		272.2353		FBF-FragConfirm	99.35		99.35
14-hydroxy palmitic acid	C16 H32 O3	(M-H)-	47.888		272.2353		FBF-FragConfirm	99.35		99.35
11S-hydroxy-hexadecanoic acid	C16 H32 O3	(M-H)-	47.888		272.2353		FBF-FragConfirm	99.35		99.35
3S-hydroxypalmitic acid	C16 H32 O3	(M-H)-	47.888		272.2353		FBF-FragConfirm	99.35		99.35
11-hydroxy palmitic acid	C16 H32 O3	(M-H)-	47.888		272.2353		FBF-FragConfirm	99.35		99.35
Hexadecanoic acid, 5-hydroxy-, (R)-	C16 H32 O3	(M-H)-	47.888		272.2353		FBF-FragConfirm	99.35		99.35
5S-hydroxy-hexadecanoic acid	C16 H32 O3	(M-H)-	47.888		272.2353		FBF-FragConfirm	99.35		99.35
4-hydroxy palmitic acid	C16 H32 O3	(M-H)-	47.888		272.2353		FBF-FragConfirm	99.35		99.35
Hexadecanoic acid, 11-hydroxy-, (S)-; (+)-Jalapinolic acid; (11S)-Jalapinolic acid; Jalapinolic acid	C16 H32 O3	(M-H)-	47.888		272.2353		FBF-FragConfirm	99.35		99.35
5-hydroxy-hexadecanoic acid	C16 H32 O3	(M-H)-	47.888		272.2353		FBF-FragConfirm	99.35		99.35
Hexadecanoic acid, 8-hydroxy-, (S)-; Hexadecanoic acid, 8-hydroxy-, (+)-	C16 H32 O3	(M-H)-	47.888		272.2353		FBF-FragConfirm	99.35		99.35
Hexadecanoic acid, 5-hydroxy-, (S)-	C16 H32 O3	(M-H)-	47.888		272.2353		FBF-FragConfirm	99.35		99.35
Hexadecanoic acid, 14-hydroxy-, (S)-; Hexadecanoic acid, 14-hydroxy-, L-	C16 H32 O3	(M-H)-	47.888		272.2353		FBF-FragConfirm	99.35		99.35
Hexadecanoic acid, 15-hydroxy-, (S)-(+)-; Hexadecanoic acid, 15-hydroxy-, L-	C16 H32 O3	(M-H)-	47.888		272.2353		FBF-FragConfirm	99.35		99.35
9-methoxy-pentadecanoic acid	C16 H32 O3	(M-H)-	47.888		272.2353		FBF-FragConfirm	99.35		99.35
8S-hydroxy-hexadecanoic acid	C16 H32 O3	(M-H)-	47.888		272.2353		FBF-FragConfirm	99.35		99.35
5R-hydroxy-hexadecanoic acid	C16 H32 O3	(M-H)-	47.888		272.2353		FBF-FragConfirm	99.35		99.35

Cpd 73: <Linoelaidic Acid>

Name	Formula	RT	RI	Mass	Diff (Tgt, ppm)	CAS	ID Source	Score	Algorithm
<Linoelaidic Acid>	C18 H32 O2	47.941		280.2409	2.50	506-21-8	M-FBF	96.06	FBF

Species	m/z	Score (Tgt)	Score (Lib)	Score (DB)	Score (MFG)	Score (RT)
(M-H)-	279	96.06				

Compound Chromatograms (overlaid)

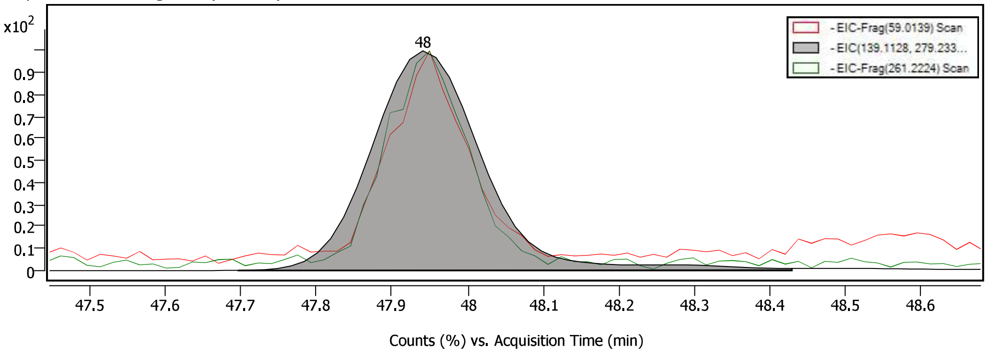

Structure

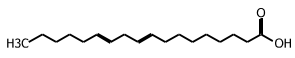

Coelution Plot

Compound Spectra (overlaid)

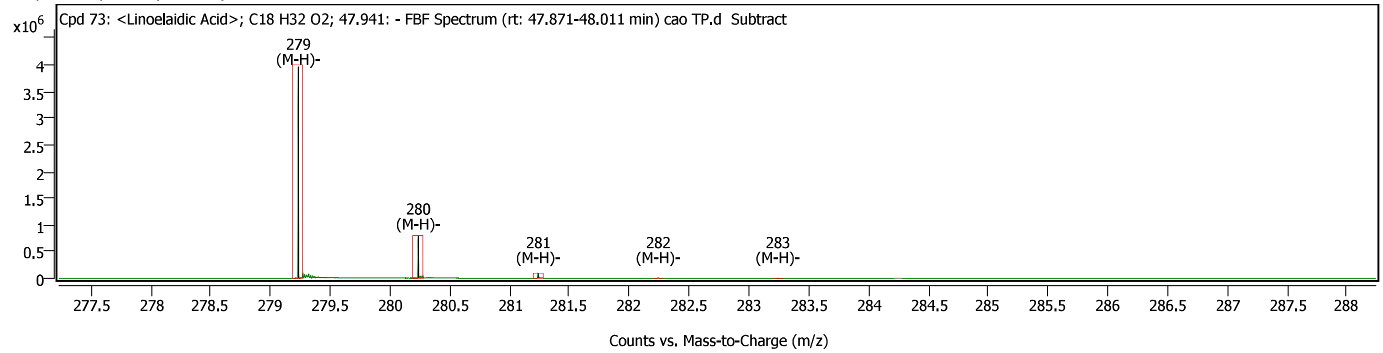

Fragment Spectrum (raw)

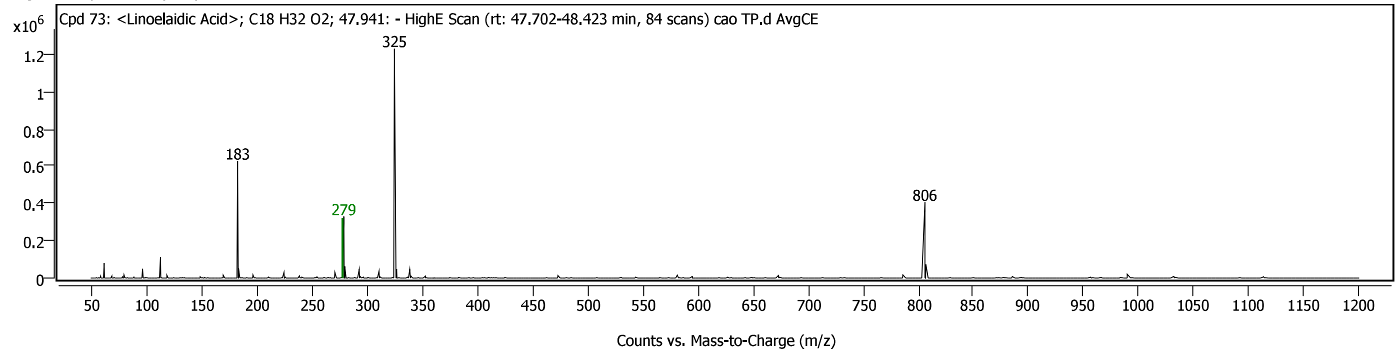

Compound Screening Report

Compound ID Table

Name	Formula	Species	RT	RT Diff	Mass	CAS	ID Source	Score	Score (Lib)	Score (Tgt)
<Linoelaidic Acid>	C18 H32 O2	(M-H)-	47.941		280.2409	506-21-8	FBF	96.06		96.06
<4-Octadecynoic acid>	C18 H32 O2	(M-H)-	47.941		280.2409		FBF	96.06		96.06
<3Z,12Z-octadecadienoic acid>	C18 H32 O2	(M-H)-	47.941		280.2409		FBF	96.06		96.06
<6E,9E-octadecadienoic acid>	C18 H32 O2	(M-H)-	47.941		280.2409		FBF	96.06		96.06
<4,9-octadecadienoic acid>	C18 H32 O2	(M-H)-	47.941		280.2409		FBF	96.06		96.06
<3Z,7Z-Octadecadienoic acid>	C18 H32 O2	(M-H)-	47.941		280.2409		FBF	96.06		96.06
<3Z,6Z-octadecadienoic acid>	C18 H32 O2	(M-H)-	47.941		280.2409		FBF	96.06		96.06
<2E,6E-Octadecadienoic acid>	C18 H32 O2	(M-H)-	47.941		280.2409		FBF	96.06		96.06
<3-Octadecynoic acid>	C18 H32 O2	(M-H)-	47.941		280.2409		FBF	96.06		96.06
<3E,7E-Octadecadienoic acid>	C18 H32 O2	(M-H)-	47.941		280.2409		FBF	96.06		96.06
<2Z,6Z-Octadecadienoic acid>	C18 H32 O2	(M-H)-	47.941		280.2409		FBF	96.06		96.06
<2Z,5Z-octadecadienoic acid>	C18 H32 O2	(M-H)-	47.941		280.2409		FBF	96.06		96.06
<2-Octadecynoic acid>	C18 H32 O2	(M-H)-	47.941		280.2409		FBF	96.06		96.06
<2,4-octadecadienoic acid>	C18 H32 O2	(M-H)-	47.941		280.2409		FBF	96.06		96.06
<17-Octadecynoic Acid>	C18 H32 O2	(M-H)-	47.941		280.2409	34450-18-5	FBF	96.06		96.06
<4Z,8Z-Octadecadienoic acid>	C18 H32 O2	(M-H)-	47.941		280.2409		FBF	96.06		96.06
<5Z,9Z-octadecadienoic acid>	C18 H32 O2	(M-H)-	47.941		280.2409		FBF	96.06		96.06
<16-Octadecynoic acid>	C18 H32 O2	(M-H)-	47.941		280.2409		FBF	96.06		96.06
<4E,8E-Octadecadienoic acid>	C18 H32 O2	(M-H)-	47.941		280.2409		FBF	96.06		96.06
<4Z,7Z-octadecadienoic acid>	C18 H32 O2	(M-H)-	47.941		280.2409		FBF	96.06		96.06
<16-methyl-6Z,9Z-heptadecadienoic acid>	C18 H32 O2	(M-H)-	47.941		280.2409		FBF	96.06		96.06
<5,10-octadecadienoic acid>	C18 H32 O2	(M-H)-	47.941		280.2409		FBF	96.06		96.06
<6E,10E-octadecadienoic acid>	C18 H32 O2	(M-H)-	47.941		280.2409		FBF	96.06		96.06
<6,11-Octadecadienoic acid>	C18 H32 O2	(M-H)-	47.941		280.2409		FBF	96.06		96.06
<6, 8-Octadecadienoic acid>	C18 H32 O2	(M-H)-	47.941		280.2409	335606-45-6	FBF	96.06		96.06
<16-methyl-9Z,12Z-heptadecadienoic acid>	C18 H32 O2	(M-H)-	47.941		280.2409		FBF	96.06		96.06
<5Z,9E-Octadecadienoic acid>	C18 H32 O2	(M-H)-	47.941		280.2409		FBF	96.06		96.06
<5Z,12Z-Otadecadienoic acid>	C18 H32 O2	(M-H)-	47.941		280.2409	50499-21-3	FBF	96.06		96.06
<5,11-Octadecadienoic acid>	C18 H32 O2	(M-H)-	47.941		280.2409		FBF	96.06		96.06
<5Z,12E-Otadecadienoic acid>	C18 H32 O2	(M-H)-	47.941		280.2409		FBF	96.06		96.06
<5Z,11Z-Octadecadienoic acid>	C18 H32 O2	(M-H)-	47.941		280.2409		FBF	96.06		96.06
<5-Octadecynoic acid>	C18 H32 O2	(M-H)-	47.941		280.2409		FBF	96.06		96.06
<5E,9Z-Octadecadienoic acid>	C18 H32 O2	(M-H)-	47.941		280.2409		FBF	96.06		96.06
<5E,12Z-Otadecadienoic acid>	C18 H32 O2	(M-H)-	47.941		280.2409		FBF	96.06		96.06
<5E,12E-Otadecadienoic acid>	C18 H32 O2	(M-H)-	47.941		280.2409		FBF	96.06		96.06
<5Z,8Z-octadecadienoic acid>	C18 H32 O2	(M-H)-	47.941		280.2409		FBF	96.06		96.06
<5,6-octadecadienoic acid>	C18 H32 O2	(M-H)-	47.941		280.2409		FBF	96.06		96.06
<12E,16E-octadecadienoic acid>	C18 H32 O2	(M-H)-	47.941		280.2409		FBF	96.06		96.06
<16:2(2E,4E)(4Me,6Me[S])>	C18 H32 O2	(M-H)-	47.941		280.2409		FBF	96.06		96.06
<6E,11Z-Octadecadienoic acid>	C18 H32 O2	(M-H)-	47.941		280.2409		FBF	96.06		96.06
<11E,14Z-Octadecadienoic acid>	C18 H32 O2	(M-H)-	47.941		280.2409	17027-26-8	FBF	96.06		96.06
<10Z,14Z-Octadecadienoic acid>	C18 H32 O2	(M-H)-	47.941		280.2409		FBF	96.06		96.06
<10Z,13Z-Octadecadienoic acid>	C18 H32 O2	(M-H)-	47.941		280.2409	5027-60-1	FBF	96.06		96.06
<10Z,12Z-Octadecadienoic acid>	C18 H32 O2	(M-H)-	47.941		280.2409	7307-45-1	FBF	96.06		96.06
<10Z,12E-Hexadecadienyl acetate>	C18 H32 O2	(M-H)-	47.941		280.2409		FBF	96.06		96.06
<10-Octadecynoic acid>	C18 H32 O2	(M-H)-	47.941		280.2409		FBF	96.06		96.06
<9-octadecen-4-olide>	C18 H32 O2	(M-H)-	47.941		280.2409		FBF	96.06		96.06
<10E,14E-Octadecadienoic acid>	C18 H32 O2	(M-H)-	47.941		280.2409		FBF	96.06		96.06
<10E,12Z-Hexadecadienyl acetate>	C18 H32 O2	(M-H)-	47.941		280.2409		FBF	96.06		96.06
<10E,12E-Octadecadienoic acid>	C18 H32 O2	(M-H)-	47.941		280.2409	1072-36-2	FBF	96.06		96.06
<10E,12E-Hexadecadienyl acetate>	C18 H32 O2	(M-H)-	47.941		280.2409		FBF	96.06		96.06
<(Z)-3,7-Dimethyl-2,6-octadienyl octanoate>	C18 H32 O2	(M-H)-	47.941		280.2409		FBF	96.06		96.06
<(S)-laballenic acid>	C18 H32 O2	(M-H)-	47.941		280.2409		FBF	96.06		96.06
<(R)-laballenic acid>	C18 H32 O2	(M-H)-	47.941		280.2409		FBF	96.06		96.06
<(E)-3,7-Dimethyl-2,6-octadienyl octanoate>	C18 H32 O2	(M-H)-	47.941		280.2409		FBF	96.06		96.06
<10E,12Z-Octadecadienoic acid>	C18 H32 O2	(M-H)-	47.941		280.2409	2420-56-6	FBF	96.06		96.06
<11E,13E-Hexadecadienyl acetate>	C18 H32 O2	(M-H)-	47.941		280.2409		FBF	96.06		96.06
<11E,13Z-Hexadecadienyl acetate>	C18 H32 O2	(M-H)-	47.941		280.2409		FBF	96.06		96.06
<15-Octadecynoic acid>	C18 H32 O2	(M-H)-	47.941		280.2409		FBF	96.06		96.06
<11-Hexadecynyl acetate>	C18 H32 O2	(M-H)-	47.941		280.2409		FBF	96.06		96.06
<14Z,17-octadecadienoic acid>	C18 H32 O2	(M-H)-	47.941		280.2409		FBF	96.06		96.06
<14-Octadecynoic acid>	C18 H32 O2	(M-H)-	47.941		280.2409		FBF	96.06		96.06
<13Z,16Z-octadecadienoic acid>	C18 H32 O2	(M-H)-	47.941		280.2409		FBF	96.06		96.06
<13-Octadecynoic acid>	C18 H32 O2	(M-H)-	47.941		280.2409		FBF	96.06		96.06
<13E,17-octadecadienoic acid>	C18 H32 O2	(M-H)-	47.941		280.2409		FBF	96.06		96.06
<12Z,15Z-octadecadienoic acid>	C18 H32 O2	(M-H)-	47.941		280.2409		FBF	96.06		96.06
<12-Octadecynoic acid; 12-Stearolic acid>	C18 H32 O2	(M-H)-	47.941		280.2409		FBF	96.06		96.06
<12-octadecynoic acid>	C18 H32 O2	(M-H)-	47.941		280.2409		FBF	96.06		96.06
<11Z,15Z-octadecadienoic acid>	C18 H32 O2	(M-H)-	47.941		280.2409		FBF	96.06		96.06
<11Z,14Z-octadecadienoic acid>	C18 H32 O2	(M-H)-	47.941		280.2409		FBF	96.06		96.06
<11Z,14E-Hexadecadienyl acetate>	C18 H32 O2	(M-H)-	47.941		280.2409		FBF	96.06		96.06
<11Z,13Z-Hexadecadienyl acetate>	C18 H32 O2	(M-H)-	47.941		280.2409		FBF	96.06		96.06
<11Z,13E-Hexadecadienyl acetate>	C18 H32 O2	(M-H)-	47.941		280.2409		FBF	96.06		96.06
<11-Octadecynoic acid>	C18 H32 O2	(M-H)-	47.941		280.2409		FBF	96.06		96.06
<16:2(2E,4E)(5Me,7Me[S])>	C18 H32 O2	(M-H)-	47.941		280.2409		FBF	96.06		96.06
<6E,11Z-Hexadecadienyl acetate>	C18 H32 O2	(M-H)-	47.941		280.2409		FBF	96.06		96.06
<6E,12E-octadecadienoic acid>	C18 H32 O2	(M-H)-	47.941		280.2409		FBF	96.06		96.06
<Linalyl caprylate>	C18 H32 O2	(M-H)-	47.941		280.2409	10024-64-3	FBF	96.06		96.06
<cis-9, cis-11-octadecadienoic acid; C18:2n-7,9>	C18 H32 O2	(M-H)-	47.941		280.2409		FBF	96.06		96.06
<cis-5, trans-12-octadecadienoic acid; C18:2n-6,13>	C18 H32 O2	(M-H)-	47.941		280.2409		FBF	96.06		96.06

Compound ID Table

Name	Formula	Species	RT	RT Diff	Mass	CAS	ID Source	Score	Score (Lib)	Score (Tgt)
<cis-5, cis-12-octadecadienoic acid; C18:2n-6,13>	C18 H32 O2	(M-H)-	47.941		280.2409		FBF	96.06		96.06
<cis-10, cis-13-octadecadienoic acid; C18:2n-5,8>	C18 H32 O2	(M-H)-	47.941		280.2409		FBF	96.06		96.06
<cis-10, cis-12-octadecadienoic acid; C18:2n-6,8>	C18 H32 O2	(M-H)-	47.941		280.2409		FBF	96.06		96.06
<cis,cis-6,9-octadecadienoic acid; C18:2n-9,12>	C18 H32 O2	(M-H)-	47.941		280.2409		FBF	96.06		96.06
<C18:2n-9,14>	C18 H32 O2	(M-H)-	47.941		280.2409		FBF	96.06		96.06
<C18:2n-6,10>	C18 H32 O2	(M-H)-	47.941		280.2409		FBF	96.06		96.06
<C18:2n-9,12>	C18 H32 O2	(M-H)-	47.941		280.2409		FBF	96.06		96.06
<C18:2n-8,13>	C18 H32 O2	(M-H)-	47.941		280.2409		FBF	96.06		96.06
<C18:2n-8,11>	C18 H32 O2	(M-H)-	47.941		280.2409		FBF	96.06		96.06
<C18:2n-7,11>	C18 H32 O2	(M-H)-	47.941		280.2409		FBF	96.06		96.06
<C18:2n-7,10>	C18 H32 O2	(M-H)-	47.941		280.2409		FBF	96.06		96.06
<C18:2n-6,15>	C18 H32 O2	(M-H)-	47.941		280.2409		FBF	96.06		96.06
<cis-9, trans-12-octadecadienoic acid; C18:2n-6,9>	C18 H32 O2	(M-H)-	47.941		280.2409		FBF	96.06		96.06
<Chaulmoogric acid>	C18 H32 O2	(M-H)-	47.941		280.2409	29106-32-9	FBF	96.06		96.06
<cis-8, cis-11-octadecadienoic acid; C18:2n-7,10>	C18 H32 O2	(M-H)-	47.941		280.2409		FBF	96.06		96.06
<C18:2n-6,12>	C18 H32 O2	(M-H)-	47.941		280.2409		FBF	96.06		96.06
<C18:2n-5,9>	C18 H32 O2	(M-H)-	47.941		280.2409		FBF	96.06		96.06
<Ethyl (Z,Z)-11,13-hexadecadienoate>	C18 H32 O2	(M-H)-	47.941		280.2409		FBF	96.06		96.06
<6-Octadecynoic acid; Tariric acid; 6,7-Stearolic acid; 6-Stearolic acid>	C18 H32 O2	(M-H)-	47.941		280.2409		FBF	96.06		96.06
<trans-9, trans-11-octadecadienoic acid; C18:2n-7,9>	C18 H32 O2	(M-H)-	47.941		280.2409		FBF	96.06		96.06
<trans-9, cis-12-octadecadienoic acid; C18:2n-6,9>	C18 H32 O2	(M-H)-	47.941		280.2409		FBF	96.06		96.06
<trans-8, trans-10-octadecadienoic acid; C18:2n-8,10>	C18 H32 O2	(M-H)-	47.941		280.2409		FBF	96.06		96.06
<trans-5, trans-12-octadecadienoic acid; C18:2n-6,13>	C18 H32 O2	(M-H)-	47.941		280.2409		FBF	96.06		96.06
<trans-5, cis12-octadecadienoic acid; C18:2n-6,13>	C18 H32 O2	(M-H)-	47.941		280.2409		FBF	96.06		96.06
<trans-10, trans-12-octadecadienoic acid; C18:2n-6,8>	C18 H32 O2	(M-H)-	47.941		280.2409		FBF	96.06		96.06
<trans-10, cis-12-octadecadienoic acid; C18:2n-6,8>	C18 H32 O2	(M-H)-	47.941		280.2409		FBF	96.06		96.06
<Taxoleic acid>	C18 H32 O2	(M-H)-	47.941		280.2409		FBF	96.06		96.06
<Stearolic acid>	C18 H32 O2	(M-H)-	47.941		280.2409	506-24-1	FBF	96.06		96.06
<Sebaleic acid>	C18 H32 O2	(M-H)-	47.941		280.2409		FBF	96.06		96.06
<Mangiferic acid>	C18 H32 O2	(M-H)-	47.941		280.2409	18402-92-1	FBF	96.06		96.06
<Malvalic acid>	C18 H32 O2	(M-H)-	47.941		280.2409		FBF	96.06		96.06
<Linoleic acid>	C18 H32 O2	(M-H)-	47.941		280.2409	60-33-3	FBF	96.06		96.06
<Ethyl 2E,4Z-hexadecadienoate>	C18 H32 O2	(M-H)-	47.941		280.2409		FBF	96.06		96.06
<C18:2n-6,11>	C18 H32 O2	(M-H)-	47.941		280.2409		FBF	96.06		96.06
<9E,11Z-Hexadecadienyl acetate>	C18 H32 O2	(M-H)-	47.941		280.2409		FBF	96.06		96.06
<4E,6Z-Hexadecadienyl acetate>	C18 H32 O2	(M-H)-	47.941		280.2409		FBF	96.06		96.06
<7Z,11Z-octadecadienoic acid>	C18 H32 O2	(M-H)-	47.941		280.2409		FBF	96.06		96.06
<8Z,11Z-Octadecadienoic acid>	C18 H32 O2	(M-H)-	47.941		280.2409	4906-90-5	FBF	96.06		96.06
<8Z,10Z-Hexadecadienyl acetate>	C18 H32 O2	(M-H)-	47.941		280.2409		FBF	96.06		96.06
<8-Octadecynoic acid>	C18 H32 O2	(M-H)-	47.941		280.2409		FBF	96.06		96.06
<8E,10E-Octadecadienoic acid>	C18 H32 O2	(M-H)-	47.941		280.2409	115863-92-8	FBF	96.06		96.06
<C18:2n-4,7>	C18 H32 O2	(M-H)-	47.941		280.2409		FBF	96.06		96.06
<8,11-octadecadienoic acid>	C18 H32 O2	(M-H)-	47.941		280.2409		FBF	96.06		96.06
<7Z,11Z-Hexadecadienyl acetate>	C18 H32 O2	(M-H)-	47.941		280.2409		FBF	96.06		96.06
<9,13-octadecadienoic acid>	C18 H32 O2	(M-H)-	47.941		280.2409		FBF	96.06		96.06
<7Z,11E-Hexadecadienyl acetate>	C18 H32 O2	(M-H)-	47.941		280.2409		FBF	96.06		96.06
<7Z,10Z-octadecadienoic acid>	C18 H32 O2	(M-H)-	47.941		280.2409		FBF	96.06		96.06
<7-trans,9-cis-octadecadienoic acid>	C18 H32 O2	(M-H)-	47.941		280.2409		FBF	96.06		96.06
<7-octadecynoic acid>	C18 H32 O2	(M-H)-	47.941		280.2409		FBF	96.06		96.06
<7E,12E-octadecadienoic acid>	C18 H32 O2	(M-H)-	47.941		280.2409		FBF	96.06		96.06
<6Z,11Z-Octadecadienoic acid>	C18 H32 O2	(M-H)-	47.941		280.2409		FBF	96.06		96.06
<6-octadecynoic acid>	C18 H32 O2	(M-H)-	47.941		280.2409		FBF	96.06		96.06
<9(E),11(E)-Conjugated Linoleic Acid>	C18 H32 O2	(M-H)-	47.941		280.2409	544-71-8	FBF	96.06		96.06
<6Z,9Z-octadecadienoic acid>	C18 H32 O2	(M-H)-	47.941		280.2409		FBF	96.06		96.06
<C18:2n-12>	C18 H32 O2	(M-H)-	47.941		280.2409		FBF	96.06		96.06
<9(Z),11(E)-Conjugated Linoleic Acid>	C18 H32 O2	(M-H)-	47.941		280.2409	2540-56-9	FBF	96.06		96.06
<C18:2n-2,6>	C18 H32 O2	(M-H)-	47.941		280.2409		FBF	96.06		96.06
<C18:2n-2,5>	C18 H32 O2	(M-H)-	47.941		280.2409		FBF	96.06		96.06
<C18:2n-14,16>	C18 H32 O2	(M-H)-	47.941		280.2409		FBF	96.06		96.06
<C18:2n-3,7>	C18 H32 O2	(M-H)-	47.941		280.2409		FBF	96.06		96.06
<C18:2n-13,16>	C18 H32 O2	(M-H)-	47.941		280.2409		FBF	96.06		96.06
<C18:2n-12,15>	C18 H32 O2	(M-H)-	47.941		280.2409		FBF	96.06		96.06
<C18:2n-11,14>	C18 H32 O2	(M-H)-	47.941		280.2409		FBF	96.06		96.06
<9E,12Z-Octadecadienoic acid>	C18 H32 O2	(M-H)-	47.941		280.2409	2420-42-0	FBF	96.06		96.06
<C18:2n-1,5>	C18 H32 O2	(M-H)-	47.941		280.2409		FBF	96.06		96.06
<C18:2n-1,4>	C18 H32 O2	(M-H)-	47.941		280.2409		FBF	96.06		96.06
<9Z-octadecadien-4R-olide>	C18 H32 O2	(M-H)-	47.941		280.2409		FBF	96.06		96.06
<9Z,12E-Octadecadienoic acid>	C18 H32 O2	(M-H)-	47.941		280.2409	2420-55-5	FBF	96.06		96.06
<9Z,11Z-Octadecadienoic acid>	C18 H32 O2	(M-H)-	47.941		280.2409	544-70-7	FBF	96.06		96.06
<8,12-octadecadienoic acid>	C18 H32 O2	(M-H)-	47.941		280.2409		FBF	96.06		96.06
<C18:2n-8,12>	C18 H32 O2	(M-H)-	47.941		280.2409		FBF	96.06		96.06
<C18:2n-3,6>	C18 H32 O2	(M-H)-	47.941		280.2409		FBF	96.06		96.06

Cpd 105: 2-methylbacteriohopane-32,33,34,35-tetrol

Name	Formula	RT	RI	Mass	Diff (Tgt, ppm)	CAS	ID Source	Score	Algorithm
2-methylbacteriohopane-32,33,34,35-tetrol	C36 H64 O4	47.941		560.4819	2.54		M-FBF	99.62	FBF

Species	m/z	Score (Tgt)	Score (Lib)	Score (DB)	Score (MFG)	Score (RT)
(M-2H)-2 (M-H)-	279 559	99.62				

Compound Chromatograms (overlaid)

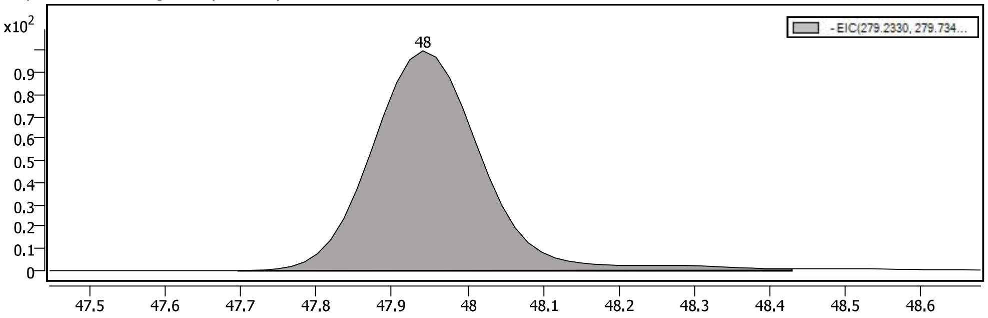

Counts (%) vs. Acquisition Time (min)

Structure

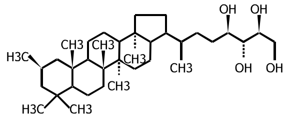

Compound Spectra (overlaid)

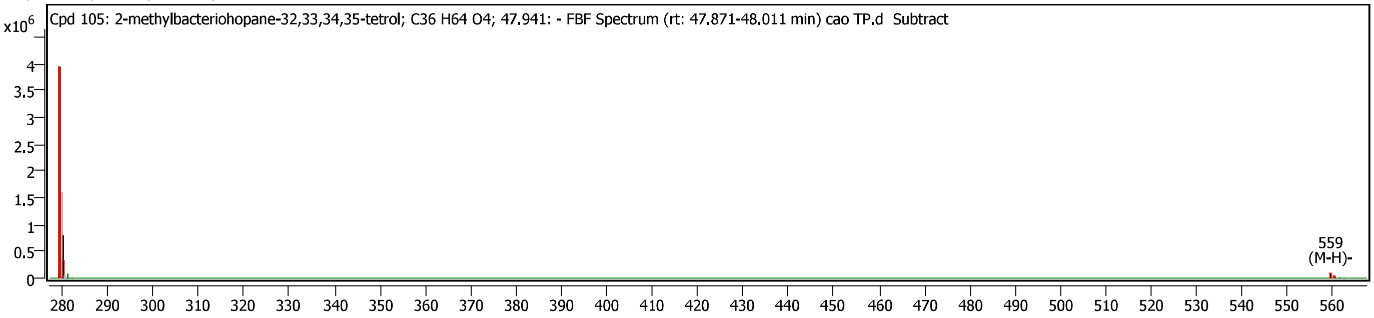

Counts vs. Mass-to-Charge (m/z)

Compound ID Table

Name	Formula	Species	RT	RT Diff	Mass	CAS	ID Source	Score	Score (Lib)	Score (Tgt)
2-methylbacteriohopane-32,33,34,35-tetrol	C36 H64 O4	(M-2H)-2 (M-H)-	47.941		560.4819		FBF	99.62		99.62
Mayolene-18	C36 H64 O4	(M-2H)-2 (M-H)-	47.941		560.4819		FBF	99.62		99.62

Cpd 169: 1α-hydroxy-23-[3-(1-hydroxy-1-methylethyl)phenyl]-22,22,23,23-tetradehydro-24,25,26,27-tetranorvitamin D3 / 1α-hydroxy-23-[3-(1-hydroxy-1-methylethyl)phenyl]-22,22,23,23-tetradehydro-24,25,26,27-tetranorcholecalciferol

Name	Formula	RT	RI	Mass	Diff (Tgt, ppm)	CAS	ID Source	Score	Algorithm
1α-hydroxy-23-[3-(1-hydroxy-1-methylethyl)phenyl]-22,22,23,23-tetradehydro-24,25,26,27-tetranorvitamin D3 / 1α-hydroxy-23-[3-(1-hydroxy-1-methylethyl)phenyl]-22,22,23,23-tetradehydro-24,25,26,27-tetranorcholecalciferol	C32 H42 O3	48.290		474.3130	-0.78		FBF	99.09	FBF

Species	m/z	Score (Tgt)	Score (Lib)	Score (DB)	Score (MFG)	Score (RT)
(M-H)-	473	99.09				

Compound Chromatograms (overlaid)

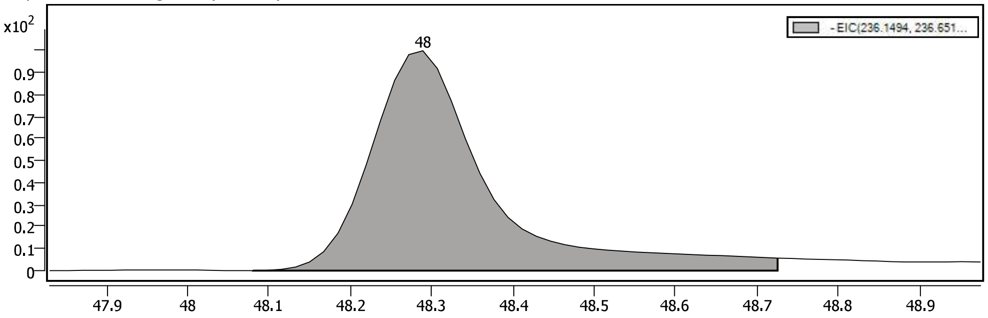

Counts (%) vs. Acquisition Time (min)

Structure

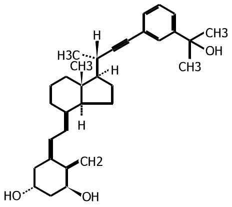

Compound Spectra (overlaid)

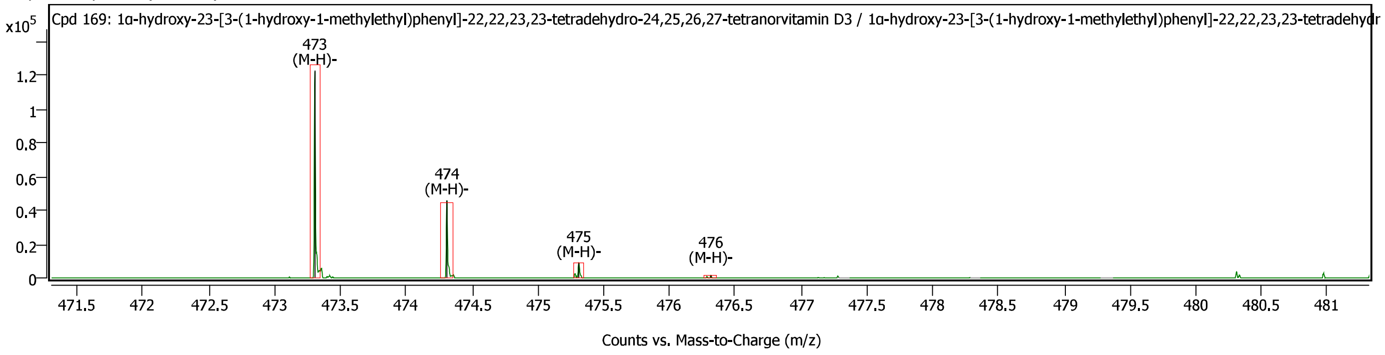

Compound ID Table

Name	Formula	Species	RT	RT Diff	Mass	CAS	ID Source	Score	Score (Lib)	Score (Tgt)
1α-hydroxy-23-[3-(1-hydroxy-1-methylethyl)phenyl]-22,22,23,23-tetradehydro-24,25,26,27-tetranorvitamin D3 / 1α-hydroxy-23-[3-(1-hydroxy-1-methylethyl)phenyl]-22,22,23,23-tetradehydro-24,25,26,27-tetranorcholecalciferol	C32 H42 O3	(M-H)-	48.290		474.3130		FBF	99.09		99.09

Cpd 156: MGDG(18:3(9Z,12Z,15Z)/18:4(6Z,9Z,12Z,15Z))

Name	Formula	RT	RI	Mass	Diff (Tgt, ppm)	CAS	ID Source	Score	Algorithm
MGDG(18:3(9Z,12Z,15Z)/18:4(6Z, 9Z,12Z,15Z))	C45 H72 O10	48.830		772.5149	3.09		FBF	94.07	FBF

Species	m/z	Score (Tgt)	Score (Lib)	Score (DB)	Score (MFG)	Score (RT)
(M-H)-	772	94.07				

Compound Chromatograms (overlaid)

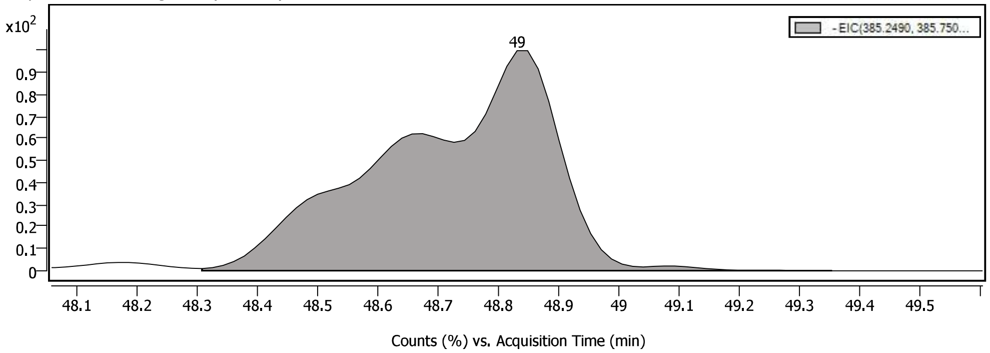

Structure

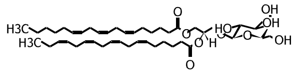

Compound Spectra (overlaid)

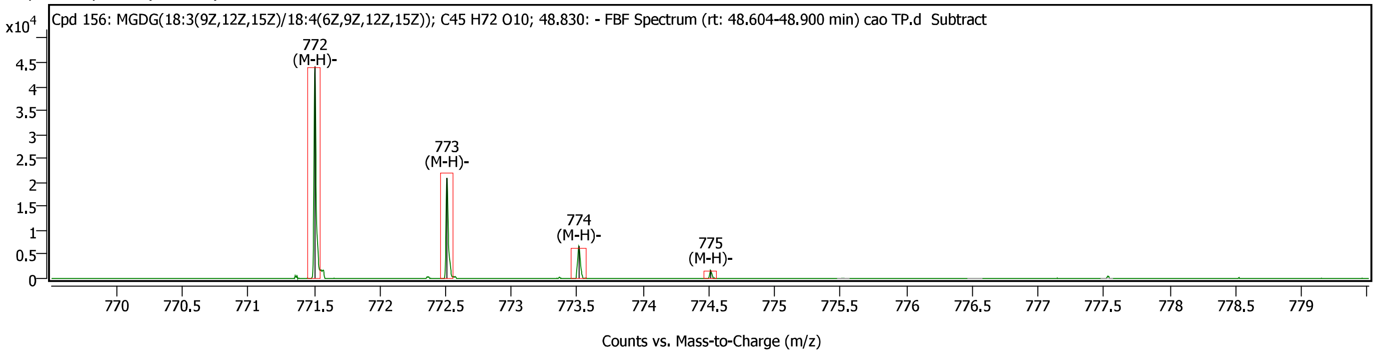

Compound ID Table

Name	Formula	Species	RT	RT Diff	Mass	CAS	ID Source	Score	Score (Lib)	Score (Tgt)
MGDG(18:3(9Z,12Z,15Z)/18:4(6Z, 9Z,12Z,15Z))	C45 H72 O10	(M-H)-	48.830		772.5149		FBF	94.07		94.07

Cpd 168: Asparagoside A

Name	Formula	RT	RI	Mass	Diff (Tgt, ppm)	CAS	ID Source	Score	Algorithm
Asparagoside A	C33 H54 O8	49.092		578.3814	-0.74	14835-43-9	M-FBF	99.12	FBF

Species	m/z	Score (Tgt)	Score (Lib)	Score (DB)	Score (MFG)	Score (RT)
(M-H)-	577	99.12				

Compound Chromatograms (overlaid)

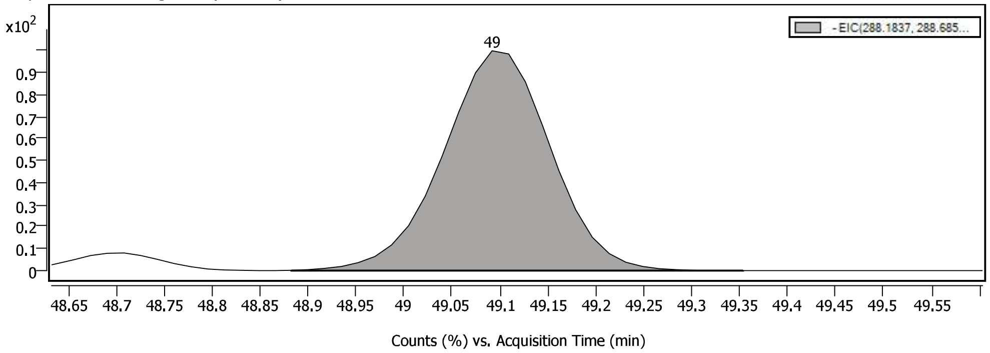

Structure

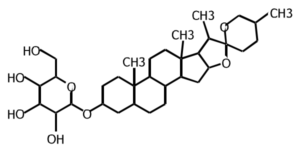

Compound Spectra (overlaid)

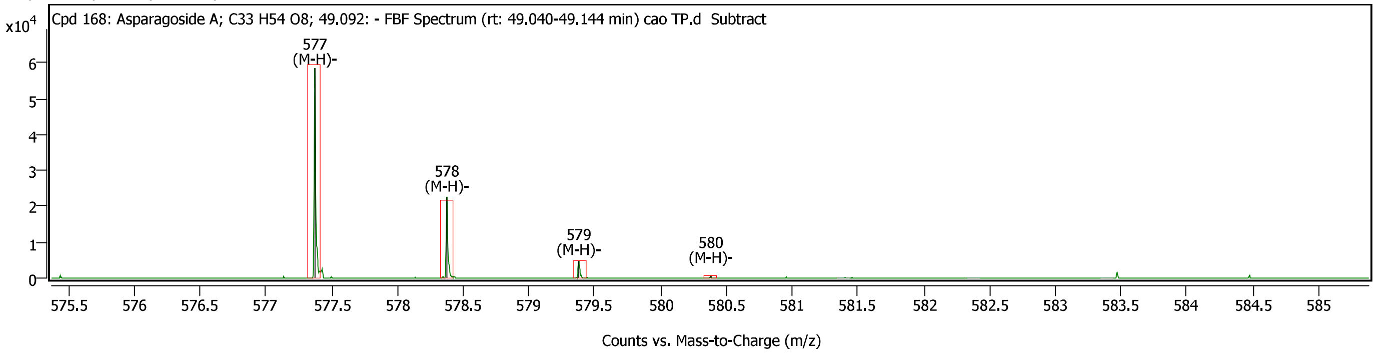

Compound ID Table

Name	Formula	Species	RT	RT Diff	Mass	CAS	ID Source	Score	Score (Lib)	Score (Tgt)
Asparagoside A	C33 H54 O8	(M-H)-	49.092		578.3814	14835-43-9	FBF	99.12		99.12
1,25-Dihydroxyvitamin D3 3-glycoside	C33 H54 O8	(M-H)-	49.092		578.3814		FBF	99.12		99.12
(25S)-5beta-spirostan-3beta-yl beta-D-glucoside	C33 H54 O8	(M-H)-	49.092		578.3814		FBF	99.12		99.12
Pandaroside D	C33 H54 O8	(M-H)-	49.092		578.3814		FBF	99.12		99.12

Cpd 75: <Myristic Acid ethyl ester>

Name	Formula	RT	RI	Mass	Diff (Tgt, ppm)	CAS	ID Source	Score	Algorithm
<Myristic Acid ethyl ester>	C16 H32 O2	49.179		256.2408	2.37	124-06-1	M-FBF	95.73	FBF

Species	m/z	Score (Tgt)	Score (Lib)	Score (DB)	Score (MFG)	Score (RT)
(M-H)-	255	95.73				

Compound Chromatograms (overlaid)

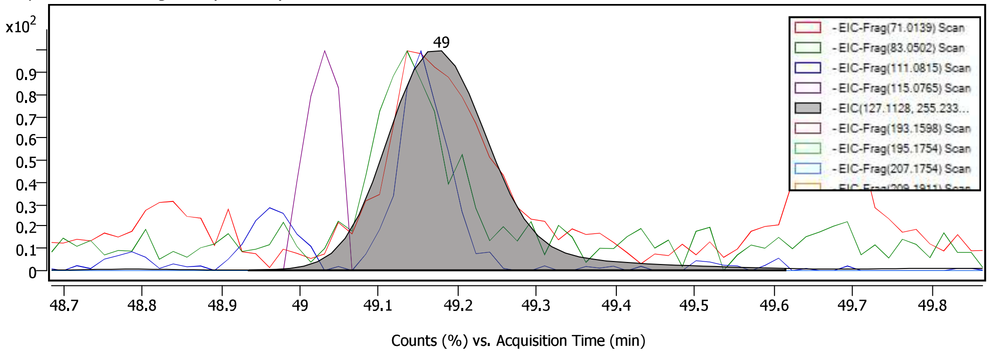

Structure

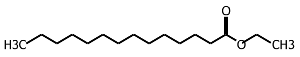

Coelution Plot

Compound Screening Report

Compound Spectra (overlaid)

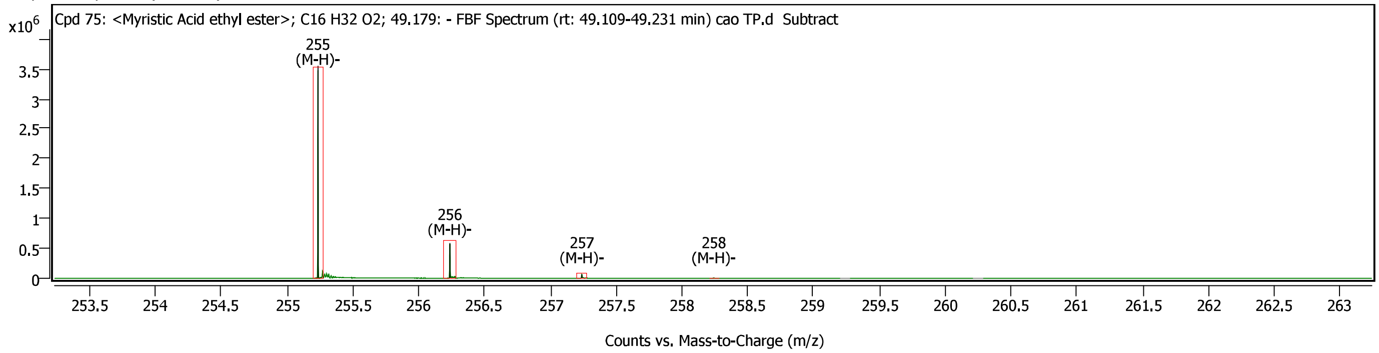

Fragment Spectrum (raw)

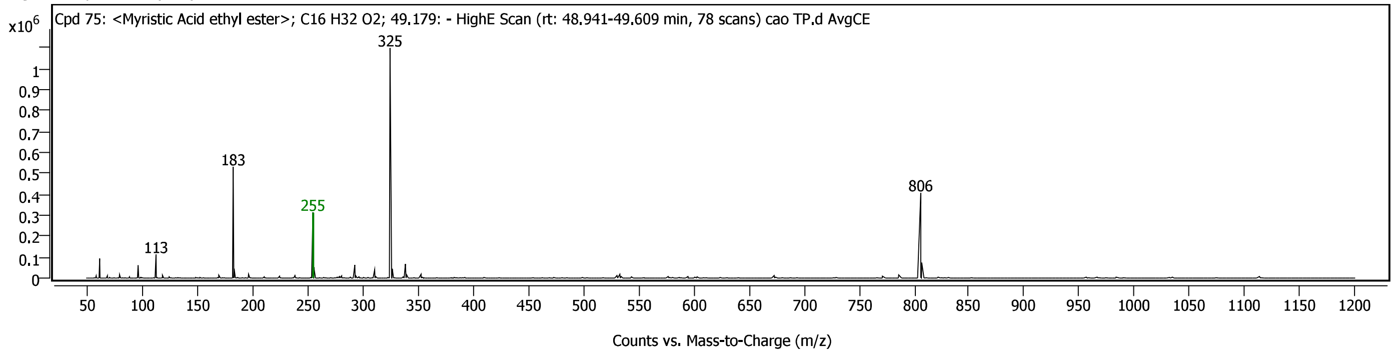

Compound ID Table

Name	Formula	Species	RT	RT Diff	Mass	CAS	ID Source	Score	Score (Lib)	Score (Tgt)
<Myristic Acid ethyl ester>	C16 H32 O2	(M-H)-	49.179		256.2408	124-06-1	FBF	95.73		95.73
<2-heptyl-nonanoic acid>	C16 H32 O2	(M-H)-	49.179		256.2408		FBF	95.73		95.73
<Butyl dodecanoate>	C16 H32 O2	(M-H)-	49.179		256.2408	106-18-3	FBF	95.73		95.73
<3-methyl-pentadecanoic acid>	C16 H32 O2	(M-H)-	49.179		256.2408		FBF	95.73		95.73
<3-ethyl-3-methyl-tridecanoic acid>	C16 H32 O2	(M-H)-	49.179		256.2408		FBF	95.73		95.73
<3,5-dimethyl-tetradecanoic acid>	C16 H32 O2	(M-H)-	49.179		256.2408		FBF	95.73		95.73
<2-propyl-tridecanoic acid>	C16 H32 O2	(M-H)-	49.179		256.2408		FBF	95.73		95.73
<2-hydroxyhexadecanal>	C16 H32 O2	(M-H)-	49.179		256.2408		FBF	95.73		95.73
<2-Hexyldecanoic acid>	C16 H32 O2	(M-H)-	49.179		256.2408	25354-97-6	FBF	95.73		95.73
<4,8,12-Trimethyl-tridecanoic acid>	C16 H32 O2	(M-H)-	49.179		256.2408	10339-73-8	FBF	95.73		95.73
<13-methyl-pentadecanoic acid>	C16 H32 O2	(M-H)-	49.179		256.2408		FBF	95.73		95.73
<2-ethyl-2-butyl-decanoic acid>	C16 H32 O2	(M-H)-	49.179		256.2408		FBF	95.73		95.73
<2,8-Dimethyl-tetradecanoic acid>	C16 H32 O2	(M-H)-	49.179		256.2408		FBF	95.73		95.73
<2,6-Dimethyl-tetradecanoic acid>	C16 H32 O2	(M-H)-	49.179		256.2408		FBF	95.73		95.73
<2,4-dimethyl-tetradecanoic acid>	C16 H32 O2	(M-H)-	49.179		256.2408		FBF	95.73		95.73
<14:0(10Me,13Me)>	C16 H32 O2	(M-H)-	49.179		256.2408		FBF	95.73		95.73
<13,13-dimethyl-tetradecanoic acid>	C16 H32 O2	(M-H)-	49.179		256.2408		FBF	95.73		95.73
<2-hexyl-decanoic acid>	C16 H32 O2	(M-H)-	49.179		256.2408		FBF	95.73		95.73
<7-Methyloctyl 5-methylhexanoate>	C16 H32 O2	(M-H)-	49.179		256.2408		FBF	95.73		95.73
<4-hexyl-decanoic acid>	C16 H32 O2	(M-H)-	49.179		256.2408		FBF	95.73		95.73
<Hexyl decanoate>	C16 H32 O2	(M-H)-	49.179		256.2408	10448-26-7	FBF	95.73		95.73
<Tridecanoic acid, 4,8,12-trimethyl-; 4,8,12-Trimethyltridecanoic acid>	C16 H32 O2	(M-H)-	49.179		256.2408		FBF	95.73		95.73
<Tetradecyl acetate>	C16 H32 O2	(M-H)-	49.179		256.2408		FBF	95.73		95.73
<Palmitic Acid>	C16 H32 O2	(M-H)-	49.179		256.2408	57-10-3	FBF	95.73		95.73
<Octyl octanoate>	C16 H32 O2	(M-H)-	49.179		256.2408	2306-88-9	FBF	95.73		95.73
<Isopalmitic acid>	C16 H32 O2	(M-H)-	49.179		256.2408	4669-02-7	FBF	95.73		95.73
<6-ethyl-tetradecanoic acid>	C16 H32 O2	(M-H)-	49.179		256.2408		FBF	95.73		95.73
<formyl 2,6,10-trimethyl-dodecanoate>	C16 H32 O2	(M-H)-	49.179		256.2408		FBF	95.73		95.73
<Dodecyl 2-methylpropanoate>	C16 H32 O2	(M-H)-	49.179		256.2408	6624-71-1	FBF	95.73		95.73
<decyl hexanoate>	C16 H32 O2	(M-H)-	49.179		256.2408		FBF	95.73		95.73
<Dodecyl butyrate>	C16 H32 O2	(M-H)-	49.179		256.2408	3724-61-6	FBF	95.73		95.73

Cpd 1: 12-OAHSA

Name	Formula	RT	RI	Mass	Diff (Tgt, ppm)	CAS	ID Source	Score	Algorithm
12-OAHSA	C36 H68 O4	49.685		564.5124	1.14	101901-73-9	M-FBF	98.81	FBF

Species	m/z	Score (Tgt)	Score (Lib)	Score (DB)	Score (MFG)	Score (RT)
(M-2H)-2 (M-H)-	281 564	98.81				

Compound Chromatograms (overlaid)

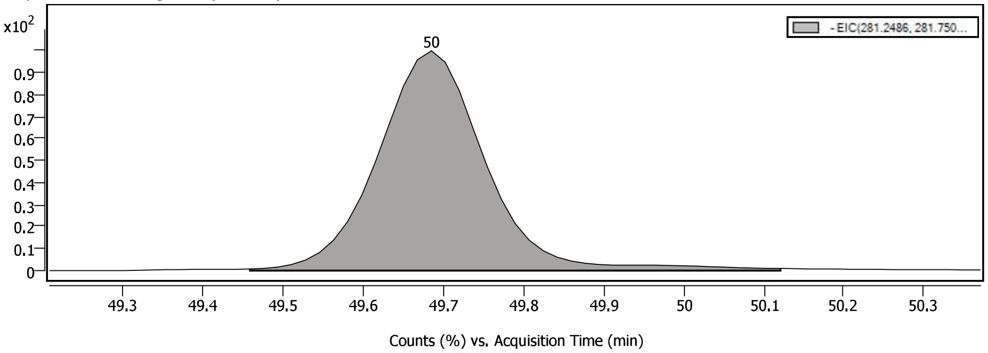

Structure

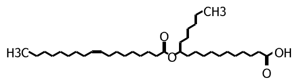

Compound Spectra (overlaid)

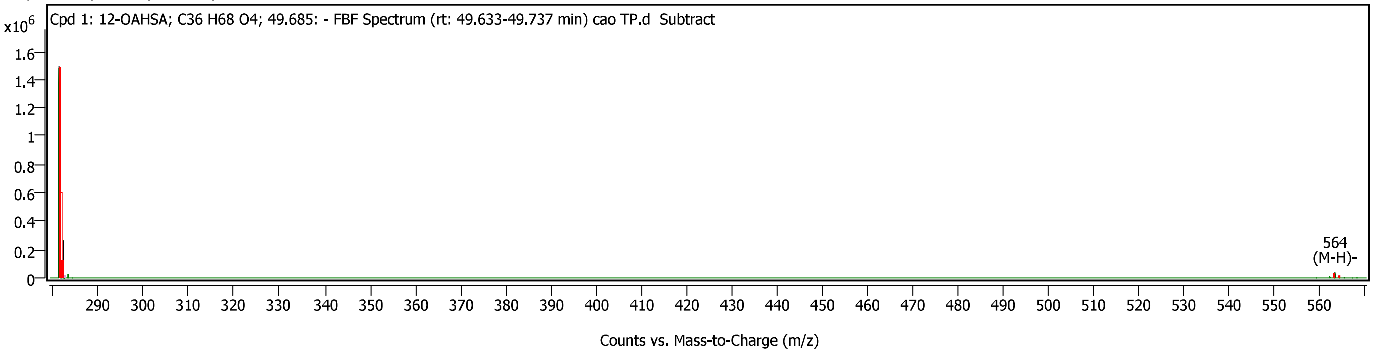

Compound ID Table

Name	Formula	Species	RT	RT Diff	Mass	CAS	ID Source	Score	Score (Lib)	Score (Tgt)
12-OAHSA	C36 H68 O4	(M-2H)-2 (M-H)-	49.685		564.5124	101901-73-9	FBF	98.81		98.81
9-OAHSA	C36 H68 O4	(M-2H)-2 (M-H)-	49.685		564.5124	154086-90-5	FBF	98.81		98.81

Cpd 21: Erythrinasinate A

Name	Formula	RT	RI	Mass	Diff (Tgt, ppm)	CAS	ID Source	Score	Algorithm
Erythrinasinate A	C38 H66 O4	49.685		586.4933	-4.81	102607-46-5	FBF	88.33	FBF

Species	m/z	Score (Tgt)	Score (Lib)	Score (DB)	Score (MFG)	Score (RT)
(M-H)-	585	88.33				

Compound Chromatograms (overlaid)

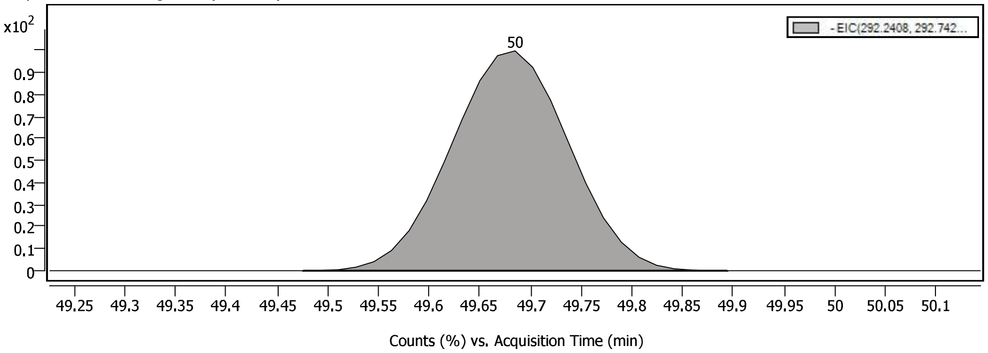

Structure

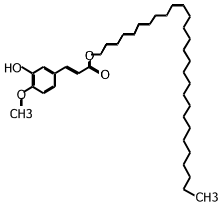

Compound Spectra (overlaid)

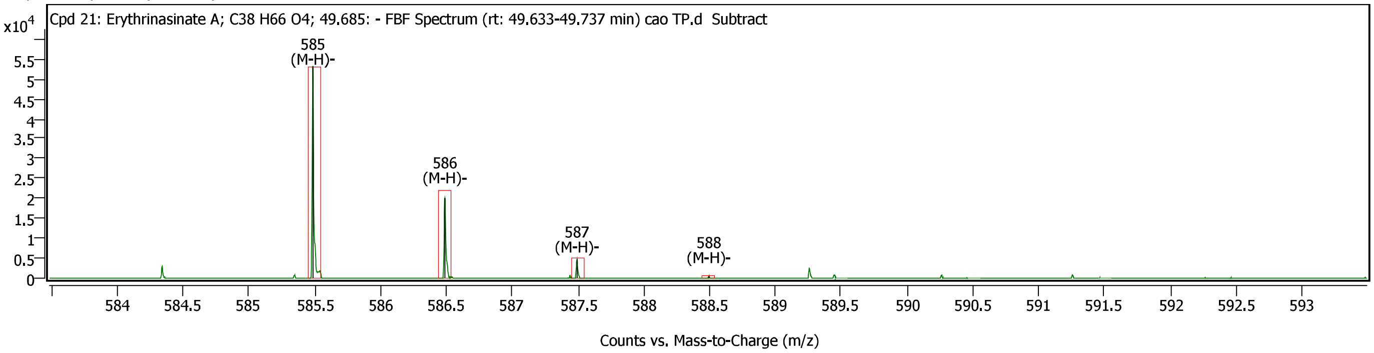

Compound ID Table

Name	Formula	Species	RT	RT Diff	Mass	CAS	ID Source	Score	Score (Lib)	Score (Tgt)
Erythrinasinate A	C38 H66 O4	(M-H)-	49.685		586.4933	102607-46-5	FBF	88.33		88.33

Cpd 11: decyl octanoate

Name	Formula	RT	RI	Mass	Diff (Tgt, ppm)	CAS	ID Source	Score	Algorithm
decyl octanoate	C18 H36 O2	51.586		284.2715	-0.19		M-FBF	99.33	FBF

Species	m/z	Score (Tgt)	Score (Lib)	Score (DB)	Score (MFG)	Score (RT)
(M-H)-	283	99.33				

Compound Chromatograms (overlaid)

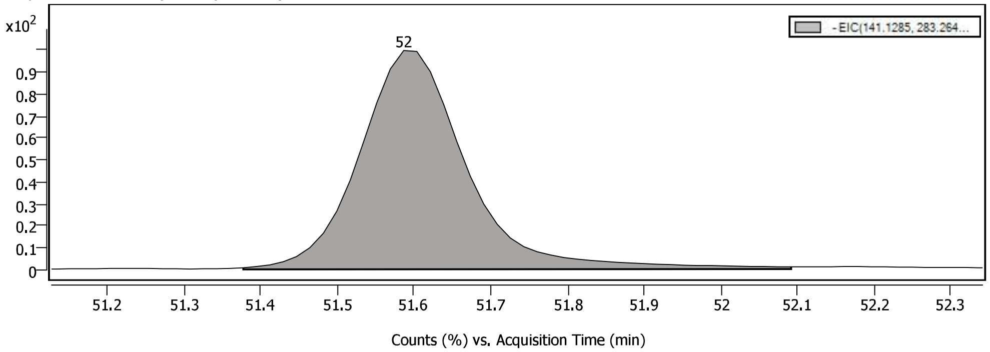

Structure

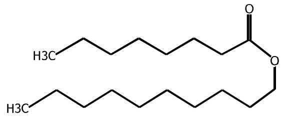

Compound Spectra (overlaid)

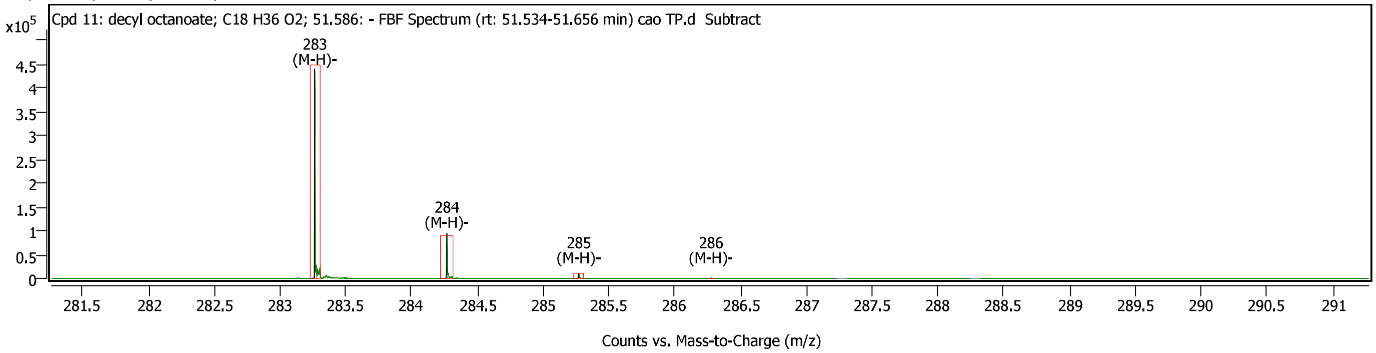

Compound ID Table

Name	Formula	Species	RT	RT Diff	Mass	CAS	ID Source	Score	Score (Lib)	Score (Tgt)
decyl octanoate	C18 H36 O2	(M-H)-	51.586		284.2715		FBF	99.33		99.33
7-Methyloctyl 7-methyloctanoate	C18 H36 O2	(M-H)-	51.586		284.2715		FBF	99.33		99.33
hexyl dodecanoate	C18 H36 O2	(M-H)-	51.586		284.2715		FBF	99.33		99.33
6,14-Dimethyl-hexadecanoic acid	C18 H36 O2	(M-H)-	51.586		284.2715		FBF	99.33		99.33
(+)-Isostearic acid	C18 H36 O2	(M-H)-	51.586		284.2715	2724-58-5	FBF	99.33		99.33
10-Methyl-heptadecanoic acid	C18 H36 O2	(M-H)-	51.586		284.2715	26429-10-7	FBF	99.33		99.33
11,15-dimethyl-hexadecanoic acid	C18 H36 O2	(M-H)-	51.586		284.2715		FBF	99.33		99.33
14-Methylheptadecanoic acid	C18 H36 O2	(M-H)-	51.586		284.2715		FBF	99.33		99.33
15-methyl-heptadecanoic acid	C18 H36 O2	(M-H)-	51.586		284.2715		FBF	99.33		99.33
2,14-Dimethyl-hexadecanoic acid	C18 H36 O2	(M-H)-	51.586		284.2715	133488-85-4	FBF	99.33		99.33
2,6-Dimethyl-5-heptenyl nonanoate	C18 H36 O2	(M-H)-	51.586		284.2715		FBF	99.33		99.33
2,6-Dimethyl-hexadecanoic acid	C18 H36 O2	(M-H)-	51.586		284.2715		FBF	99.33		99.33
4,14-Dimethyl-hexadecanoic acid	C18 H36 O2	(M-H)-	51.586		284.2715		FBF	99.33		99.33
4,8-Dimethyl-hexadecanoic acid	C18 H36 O2	(M-H)-	51.586		284.2715		FBF	99.33		99.33
lambda Isostearic acid	C18 H36 O2	(M-H)-	51.586		284.2715		FBF	99.33		99.33
Hexadecyl acetate	C18 H36 O2	(M-H)-	51.586		284.2715		FBF	99.33		99.33
Neostearic acid	C18 H36 O2	(M-H)-	51.586		284.2715		FBF	99.33		99.33
Tetradecyl isobutyrate	C18 H36 O2	(M-H)-	51.586		284.2715		FBF	99.33		99.33
tetradecyl butyrate	C18 H36 O2	(M-H)-	51.586		284.2715		FBF	99.33		99.33
Stearic acid	C18 H36 O2	(M-H)-	51.586		284.2715	57-11-4	FBF	99.33		99.33
Palmitic Acid ethyl ester	C18 H36 O2	(M-H)-	51.586		284.2715	628-97-7	FBF	99.33		99.33
dodecyl hexanoate	C18 H36 O2	(M-H)-	51.586		284.2715		FBF	99.33		99.33
octyl decanoate	C18 H36 O2	(M-H)-	51.586		284.2715		FBF	99.33		99.33
Methyl 14-methyl-8-hexadecenoate	C18 H36 O2	(M-H)-	51.586		284.2715		FBF	99.33		99.33
formyl heptadecanoate	C18 H36 O2	(M-H)-	51.586		284.2715		FBF	99.33		99.33

Cpd 7: DG(13:0/20:5(5Z,8Z,11Z,14Z,17Z)/0:0)[iso2]

Name	Formula	RT	RI	Mass	Diff (Tgt, ppm)	CAS	ID Source	Score	Algorithm
DG(13:0/20:5(5Z,8Z,11Z,14Z,17Z)/0:0)[iso2]	C36 H60 O5	51.918		572.4439	-0.38		FBF	99.75	FBF

Species	m/z	Score (Tgt)	Score (Lib)	Score (DB)	Score (MFG)	Score (RT)
(M-H)-	571	99.75				

Compound Chromatograms (overlaid)

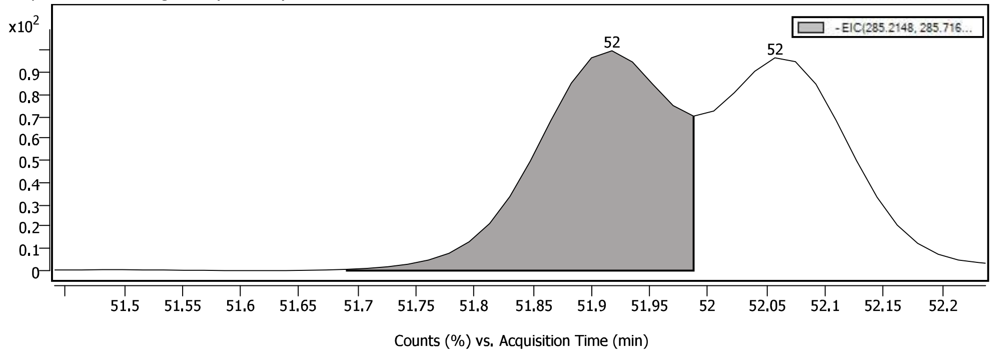

Structure

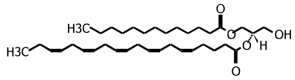

Compound Spectra (overlaid)

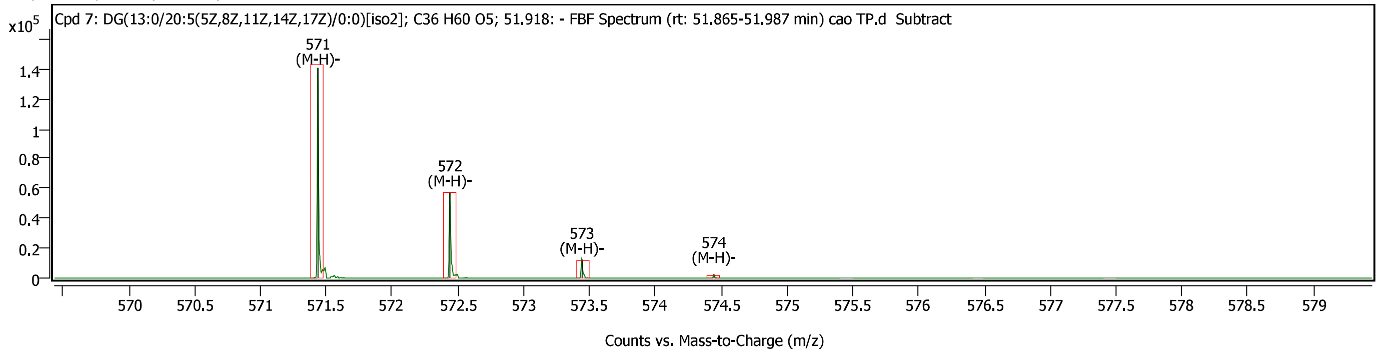

Compound ID Table

Name	Formula	Species	RT	RT Diff	Mass	CAS	ID Source	Score	Score (Lib)	Score (Tgt)
DG(13:0/20:5(5Z,8Z,11Z,14Z,17Z)/0:0)[iso2]	C36 H60 O5	(M-H)-	51.918		572.4439		FBF	99.75		99.75

Cpd 4: DG(13:0/18:2(9Z,12Z)/0:0)[iso2]

Name	Formula	RT	RI	Mass	Diff (Tgt, ppm)	CAS	ID Source	Score	Algorithm
DG(13:0/18:2(9Z,12Z)/0:0)[iso2]	C34 H62 O5	52.894		550.4592	-1.00		M-FBF	99.22	FBF

Species	m/z	Score (Tgt)	Score (Lib)	Score (DB)	Score (MFG)	Score (RT)
(M-H)-	549	99.22				

Compound Chromatograms (overlaid)

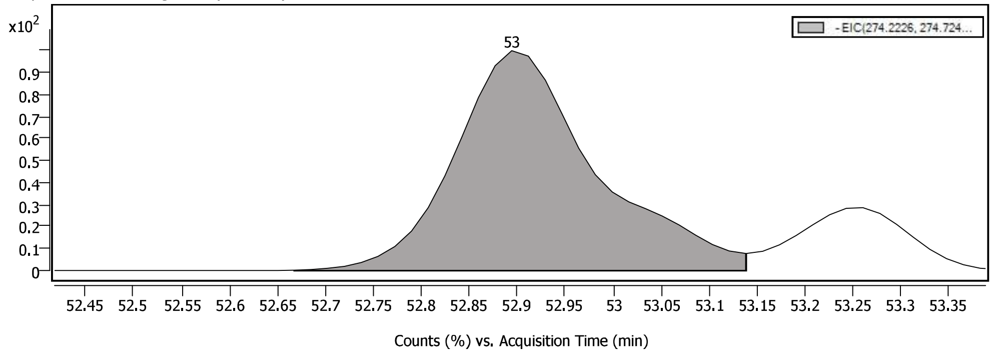

Structure

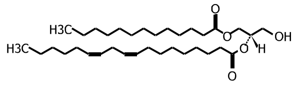

Compound Spectra (overlaid)

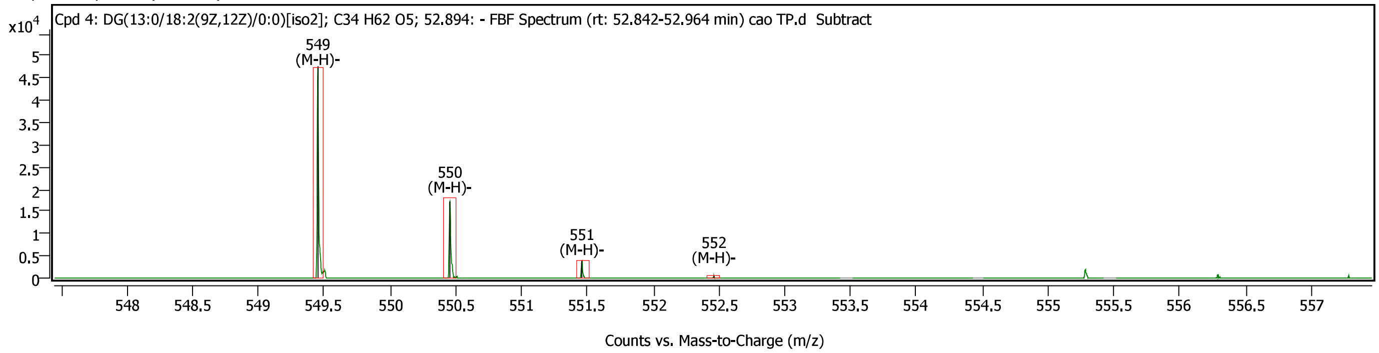

Compound ID Table

Name	Formula	Species	RT	RT Diff	Mass	CAS	ID Source	Score	Score (Lib)	Score (Tgt)
DG(13:0/18:2(9Z,12Z)/0:0)[iso2]	C34 H62 O5	(M-H)-	52.894		550.4592		FBF	99.22		99.22
DG(15:1(9Z)/16:1(9Z)/0:0)[iso2]	C34 H62 O5	(M-H)-	52.894		550.4592		FBF	99.22		99.22
DG(14:1(9Z)/17:1(9Z)/0:0)[iso2]	C34 H62 O5	(M-H)-	52.894		550.4592		FBF	99.22		99.22
DG(14:0/17:2(9Z,12Z)/0:0)[iso2]	C34 H62 O5	(M-H)-	52.894		550.4592		FBF	99.22		99.22

Cpd 61: 1,2-di-(9Z,12Z,15Z-octadecatrienoyl)-3-(8-(2E,4Z-decadienoyloxy)-5,6-octadienoyl)-sn-glycerol

Name	Formula	RT	RI	Mass	Diff (Tgt, ppm)	CAS	ID Source	Score	Algorithm
1,2-di-(9Z,12Z,15Z-octadecatrienoyl)-3-(8-(2E,4Z-decadienoyloxy)-5,6-octadienoyl)-sn-glycerol	C57 H88 O8	53.104		900.6502	2.56		FBF	95.57	FBF

Species	m/z	Score (Tgt)	Score (Lib)	Score (DB)	Score (MFG)	Score (RT)
(M-H)-	900	95.57				

Compound Chromatograms (overlaid)

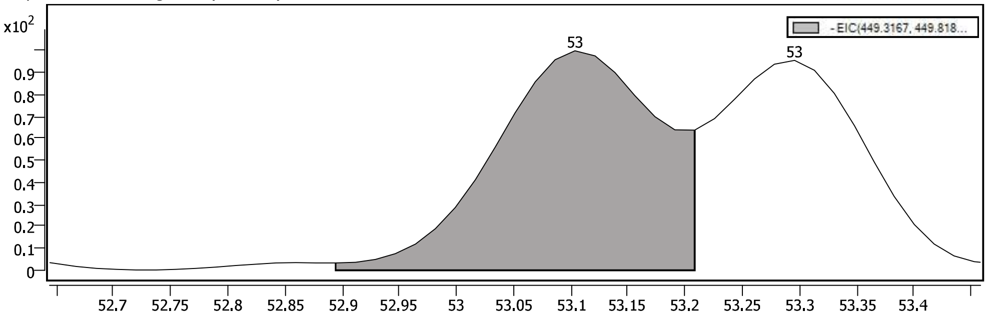

Structure

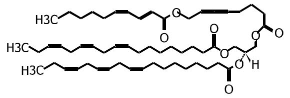

Compound Spectra (overlaid)

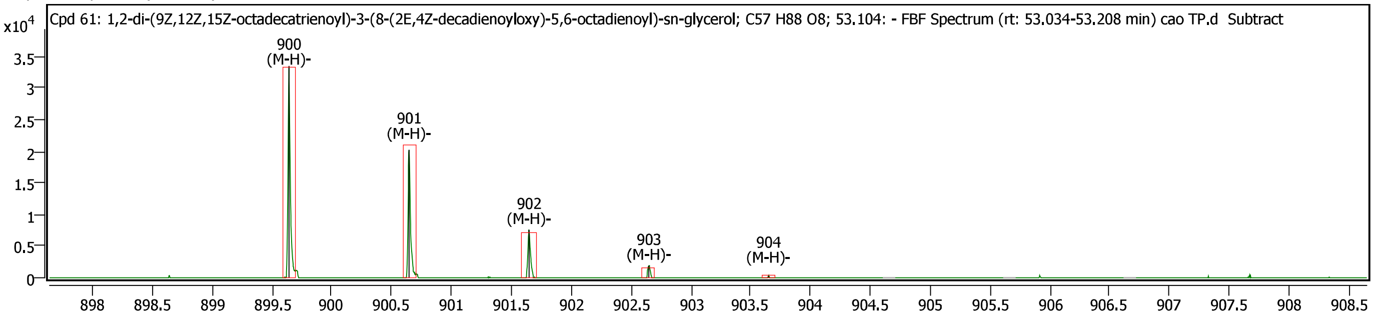

Compound ID Table

Name	Formula	Species	RT	RT Diff	Mass	CAS	ID Source	Score	Score (Lib)	Score (Tgt)
1,2-di-(9Z,12Z,15Z-octadecatrienoyl)-3-(8-(2E,4Z-decadienoyloxy)-5,6-octadienoyl)-sn-glycerol	C57 H88 O8	(M-H)-	53.104		900.6502		FBF	95.57		95.57

Cpd 99: DG(13:0/20:4(5Z,8Z,11Z,14Z)/0:0)[iso2]

Name	Formula	RT	RI	Mass	Diff (Tgt, ppm)	CAS	ID Source	Score	Algorithm
DG(13:0/20:4(5Z,8Z,11Z,14Z)/0:0)[iso2]	C36 H62 O5	53.104		574.4593	-0.73		M-FBF	99.72	FBF

Species	m/z	Score (Tgt)	Score (Lib)	Score (DB)	Score (MFG)	Score (RT)
(M-H)-	573	99.72				

Compound Chromatograms (overlaid)

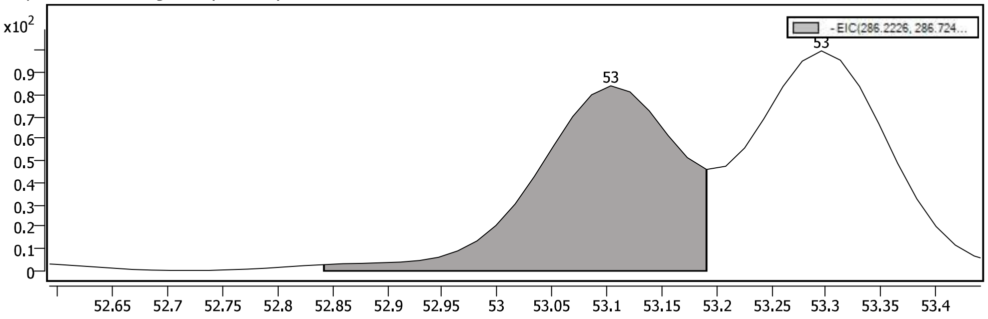

Structure

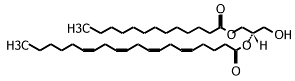

Compound Spectra (overlaid)

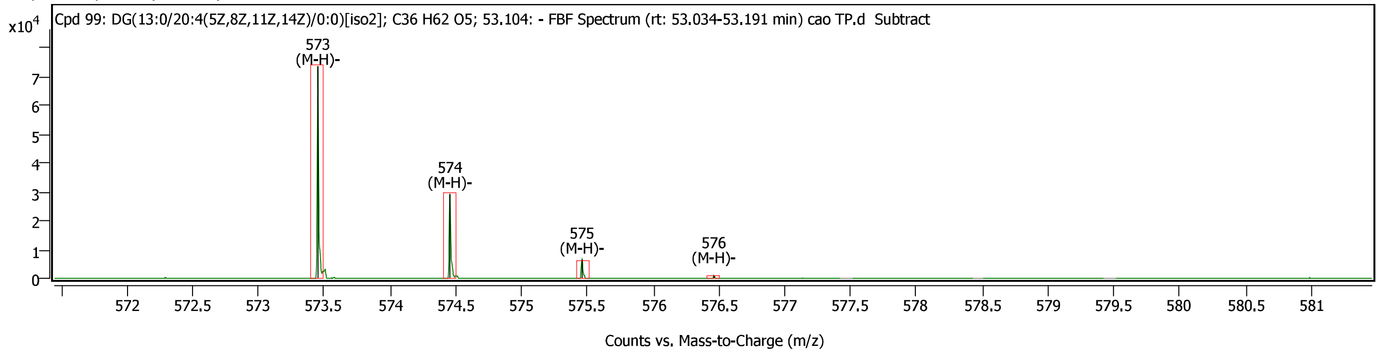

Compound ID Table

Name	Formula	Species	RT	RT Diff	Mass	CAS	ID Source	Score	Score (Lib)	Score (Tgt)
DG(13:0/20:4(5Z,8Z,11Z,14Z)/0:0)[iso2]	C36 H62 O5	(M-H)-	53.104		574.4593		FBF	99.72		99.72
DG(18:4(6Z,9Z,12Z,15Z)/15:0/0:0)	C36 H62 O5	(M-H)-	53.104		574.4593		FBF	99.72		99.72
DG(15:1(9Z)/18:3(9Z,12Z,15Z)/0:0)[iso2]	C36 H62 O5	(M-H)-	53.104		574.4593		FBF	99.72		99.72
DG(15:0/18:4(6Z,9Z,12Z,15Z)/0:0)	C36 H62 O5	(M-H)-	53.104		574.4593		FBF	99.72		99.72

Cpd 100: DG(13:0/20:4(5Z,8Z,11Z,14Z)/0:0)[iso2]

Name	Formula	RT	RI	Mass	Diff (Tgt, ppm)	CAS	ID Source	Score	Algorithm
DG(13:0/20:4(5Z,8Z,11Z,14Z)/0:0)[iso2]	C36 H62 O5	53.296		574.4593	-0.80		M-FBF	99.02	FBF

Species	m/z	Score (Tgt)	Score (Lib)	Score (DB)	Score (MFG)	Score (RT)
(M-H)-	573	99.02				

Compound Chromatograms (overlaid)

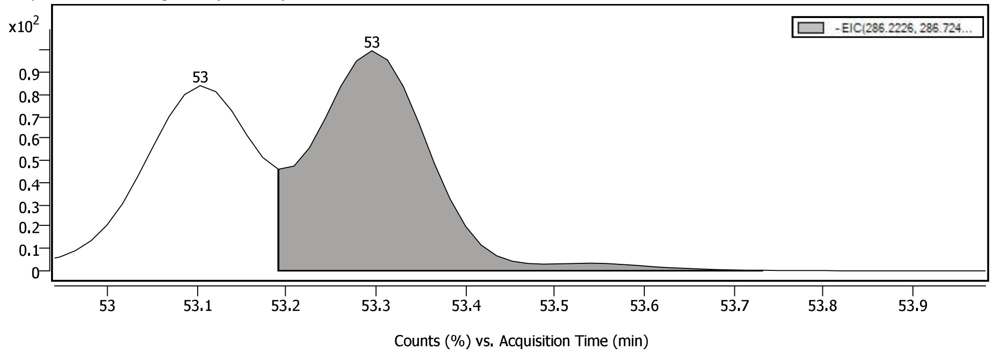

Structure

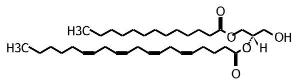

Compound Spectra (overlaid)

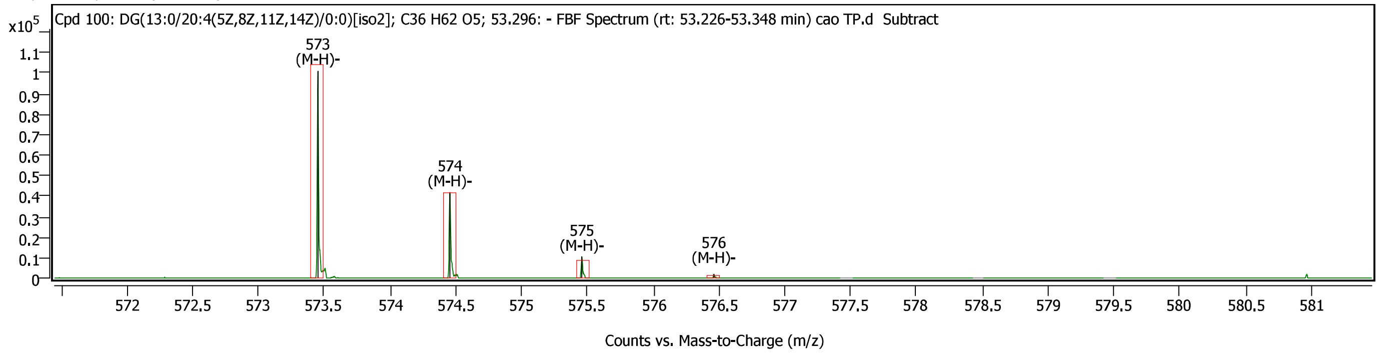

Compound ID Table

Name	Formula	Species	RT	RT Diff	Mass	CAS	ID Source	Score	Score (Lib)	Score (Tgt)
DG(13:0/20:4(5Z,8Z,11Z,14Z)/0:0)[iso2]	C36 H62 O5	(M-H)-	53.296		574.4593		FBF	99.02		99.02
DG(18:4(6Z,9Z,12Z,15Z)/15:0/0:0)	C36 H62 O5	(M-H)-	53.296		574.4593		FBF	99.02		99.02
DG(15:1(9Z)/18:3(9Z,12Z,15Z)/0:0)[iso2]	C36 H62 O5	(M-H)-	53.296		574.4593		FBF	99.02		99.02
DG(15:0/18:4(6Z,9Z,12Z,15Z)/0:0)	C36 H62 O5	(M-H)-	53.296		574.4593		FBF	99.02		99.02

Cpd 244: Fasciculic acid A

Name	Formula	RT	RI	Mass	Diff (Tgt, ppm)	CAS	ID Source	Score	Algorithm
Fasciculic acid A	C36 H60 O8	54.185		620.4284	-0.65	126906-00-1	M-FBF	99.21	FBF

Species	m/z	Score (Tgt)	Score (Lib)	Score (DB)	Score (MFG)	Score (RT)
(M-H)-	619	99.21				

Compound Chromatograms (overlaid)

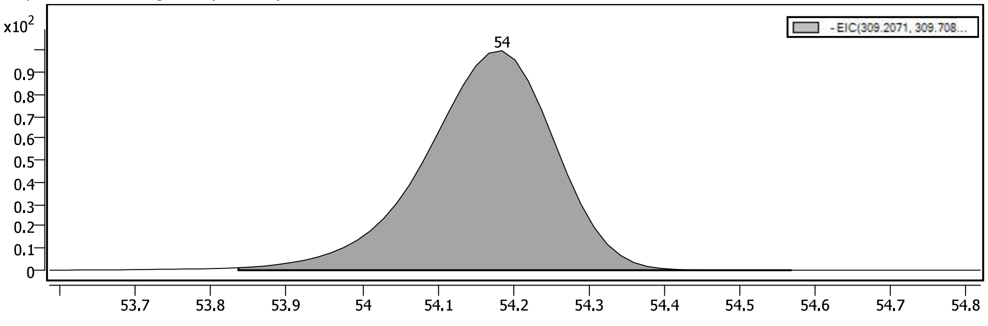

Structure

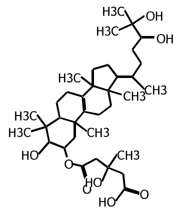

Compound Spectra (overlaid)

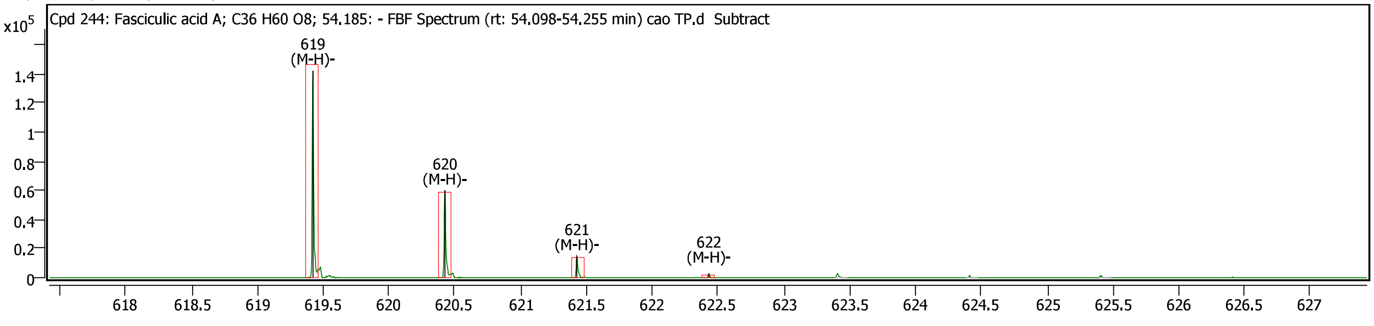

Compound ID Table

Name	Formula	Species	RT	RT Diff	Mass	CAS	ID Source	Score	Score (Lib)	Score (Tgt)
Fasciculic acid A	C36 H60 O8	(M-H)-	54.185		620.4284	126906-00-1	FBF	99.21		99.21
(3b,7b,22x)-Cucurbita-5,24-diene-3,7,23-triol 7-glucoside	C36 H60 O8	(M-H)-	54.185		620.4284		FBF	99.21		99.21
Soyasapogenol B 24-O-b-D-glucoside	C36 H60 O8	(M-H)-	54.185		620.4284	115334-08-2	FBF	99.21		99.21
Ginsenoside Rh4	C36 H60 O8	(M-H)-	54.185		620.4284	174721-08-5	FBF	99.21		99.21

Cpd 10: <Arachidic acid>

Name	Formula	RT	RI	Mass	Diff (Tgt, ppm)	CAS	ID Source	Score	Algorithm
<Arachidic acid>	C20 H40 O2	54.342		312.3026	-0.80	506-30-9	M-FBF	99.60	FBF

Species	m/z	Score (Tgt)	Score (Lib)	Score (DB)	Score (MFG)	Score (RT)
(M-H)-	311	99.60				

Compound Chromatograms (overlaid)

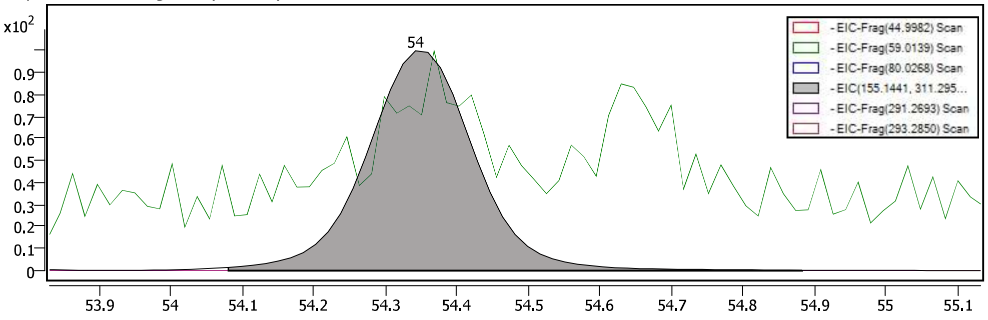

Structure

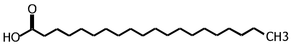

Coelution Plot

Compound Spectra (overlaid)

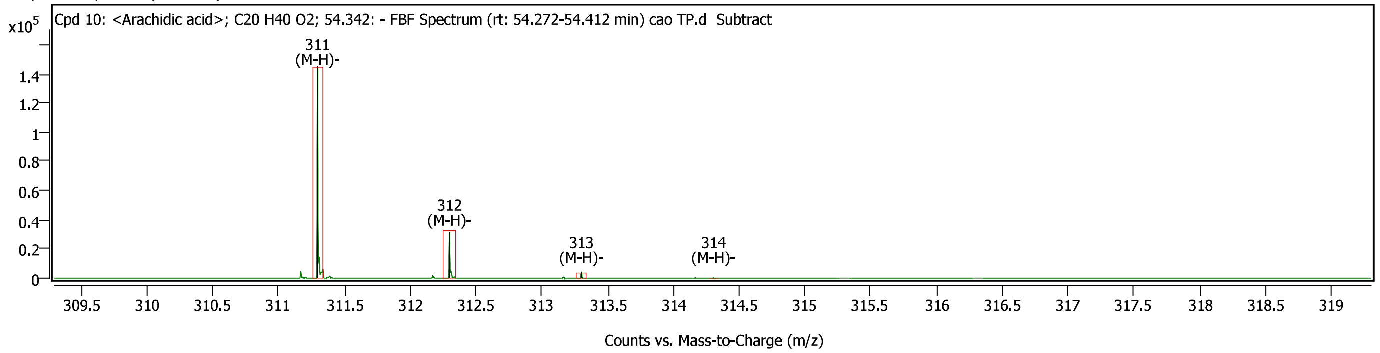

Fragment Spectrum (raw)

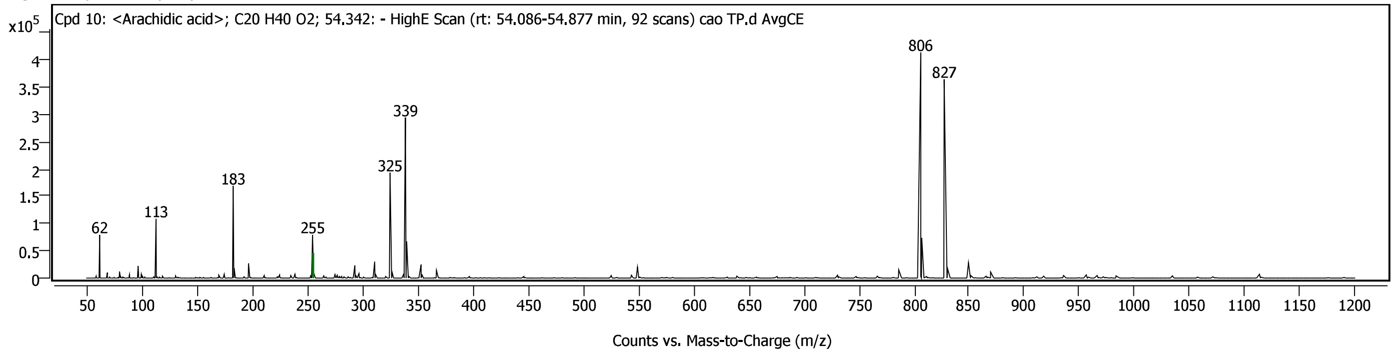

Compound ID Table

Name	Formula	Species	RT	RT Diff	Mass	CAS	ID Source	Score	Score (Lib)	Score (Tgt)
<Arachidic acid>	C20 H40 O2	(M-H)-	54.342		312.3026	506-30-9	FBF	99.60		99.60
<14-Methylpentadecyl isobutyrate>	C20 H40 O2	(M-H)-	54.342		312.3026		FBF	99.60		99.60
<4-Hydroxy-6-eicosanone>	C20 H40 O2	(M-H)-	54.342		312.3026		FBF	99.60		99.60
<dodecyl octanoate>	C20 H40 O2	(M-H)-	54.342		312.3026		FBF	99.60		99.60
<17-methyl-nonadecanoic acid>	C20 H40 O2	(M-H)-	54.342		312.3026		FBF	99.60		99.60
<19:0(11Me)>	C20 H40 O2	(M-H)-	54.342		312.3026		FBF	99.60		99.60
<6,14-Dimethyl-octadecanoic acid>	C20 H40 O2	(M-H)-	54.342		312.3026		FBF	99.60		99.60
<3,7-Dimethyl-6-octenyl 3,7-dimethyl-6-octenoate>	C20 H40 O2	(M-H)-	54.342		312.3026		FBF	99.60		99.60
<3,7-Dimethyl-6-octenyl decanoate>	C20 H40 O2	(M-H)-	54.342		312.3026		FBF	99.60		99.60
<3L,7D,11D-phytanic acid>	C20 H40 O2	(M-H)-	54.342		312.3026	31653-05-1	FBF	99.60		99.60
<3-Methylbutyl pentadecanoate>	C20 H40 O2	(M-H)-	54.342		312.3026		FBF	99.60		99.60
<4,14-Dimethyl-octadecanoic acid>	C20 H40 O2	(M-H)-	54.342		312.3026		FBF	99.60		99.60
<4,16-Dimethyl-octadecanoic acid>	C20 H40 O2	(M-H)-	54.342		312.3026		FBF	99.60		99.60
<5-Hydroxy-7-eicosanone>	C20 H40 O2	(M-H)-	54.342		312.3026		FBF	99.60		99.60
<hexadecyl butyrate>	C20 H40 O2	(M-H)-	54.342		312.3026		FBF	99.60		99.60
<2,14-Dimethyl-octadecanoic acid>	C20 H40 O2	(M-H)-	54.342		312.3026		FBF	99.60		99.60
<6,16-Dimethyl-octadecanoic acid>	C20 H40 O2	(M-H)-	54.342		312.3026		FBF	99.60		99.60
<Isoarachidic acid>	C20 H40 O2	(M-H)-	54.342		312.3026	6250-72-2	FBF	99.60		99.60
<7-Methyloctyl 9-methyldecanoate>	C20 H40 O2	(M-H)-	54.342		312.3026		FBF	99.60		99.60
<tetradecyl hexanoate>	C20 H40 O2	(M-H)-	54.342		312.3026		FBF	99.60		99.60
<Stearic Acid ethyl ester>	C20 H40 O2	(M-H)-	54.342		312.3026	111-61-5	FBF	99.60		99.60
<Phytanic Acid>	C20 H40 O2	(M-H)-	54.342		312.3026	14721-66-5	FBF	99.60		99.60
<Octadecyl acetate>	C20 H40 O2	(M-H)-	54.342		312.3026		FBF	99.60		99.60
<2,2,6,10,14-Pentamethylpentadecanoic acid>	C20 H40 O2	(M-H)-	54.342		312.3026	122706-67-6	FBF	99.60		99.60
<hexyl tetradecanoate>	C20 H40 O2	(M-H)-	54.342		312.3026		FBF	99.60		99.60
<decyl decanoate>	C20 H40 O2	(M-H)-	54.342		312.3026		FBF	99.60		99.60

Cpd 5: DG(14:1(9Z)/17:1(9Z)/0:0)[iso2]

Name	Formula	RT	RI	Mass	Diff (Tgt, ppm)	CAS	ID Source	Score	Algorithm
DG(14:1(9Z)/17:1(9Z)/0:0)[iso2]	C34 H62 O5	54.429		550.4595	-0.41		M-FBF	99.06	FBF

Species	m/z	Score (Tgt)	Score (Lib)	Score (DB)	Score (MFG)	Score (RT)
(M-H)-	549	99.06				

Compound Chromatograms (overlaid)

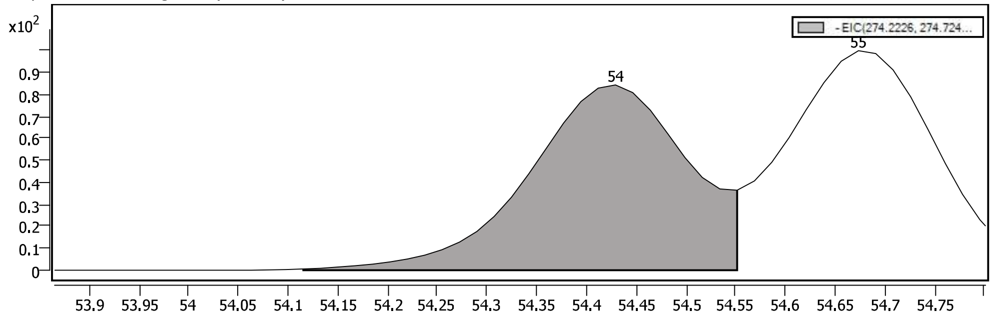

Structure

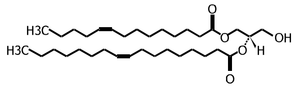

Compound Spectra (overlaid)

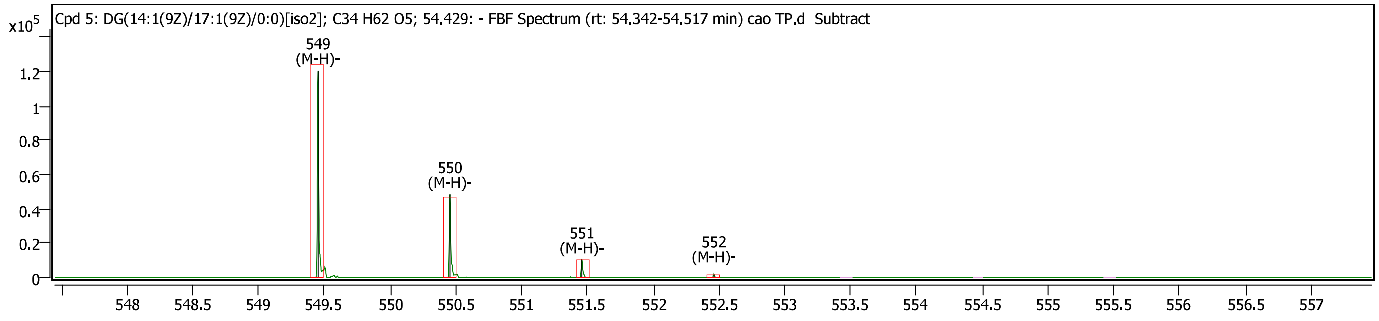

Compound ID Table

Name	Formula	Species	RT	RT Diff	Mass	CAS	ID Source	Score	Score (Lib)	Score (Tgt)
DG(14:1(9Z)/17:1(9Z)/0:0)[iso2]	C34 H62 O5	(M-H)-	54.429		550.4595		FBF	99.06		99.06
DG(15:1(9Z)/16:1(9Z)/0:0)[iso2]	C34 H62 O5	(M-H)-	54.429		550.4595		FBF	99.06		99.06
DG(14:0/17:2(9Z,12Z)/0:0)[iso2]	C34 H62 O5	(M-H)-	54.429		550.4595		FBF	99.06		99.06
DG(13:0/18:2(9Z,12Z)/0:0)[iso2]	C34 H62 O5	(M-H)-	54.429		550.4595		FBF	99.06		99.06

Cpd 6: DG(15:1(9Z)/16:1(9Z)/0:0)[iso2]

Name	Formula	RT	RI	Mass	Diff (Tgt, ppm)	CAS	ID Source	Score	Algorithm
DG(15:1(9Z)/16:1(9Z)/0:0)[iso2]	C34 H62 O5	54.673		550.4597	-0.12		M-FBF	97.40	FBF

Species	m/z	Score (Tgt)	Score (Lib)	Score (DB)	Score (MFG)	Score (RT)
(M-H)-	549	97.40				

Compound Chromatograms (overlaid)

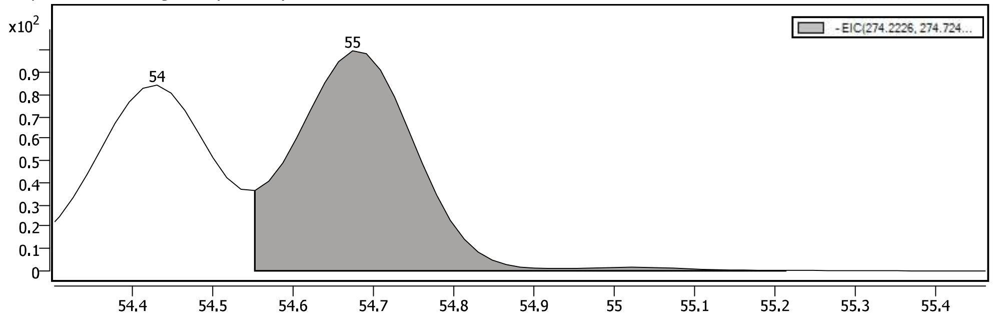

Structure

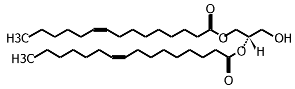

Compound Spectra (overlaid)

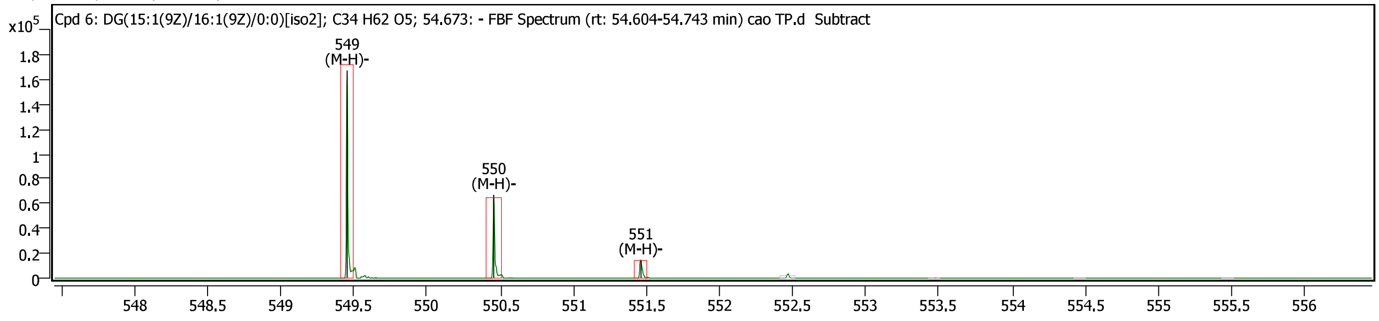

Compound Screening Report

Compound ID Table

Name	Formula	Species	RT	RT Diff	Mass	CAS	ID Source	Score	Score (Lib)	Score (Tgt)
DG(15:1(9Z)/16:1(9Z)/0:0)[iso2]	C34 H62 O5	(M-H)-	54.673		550.4597		FBF	97.40		97.40
DG(14:1(9Z)/17:1(9Z)/0:0)[iso2]	C34 H62 O5	(M-H)-	54.673		550.4597		FBF	97.40		97.40
DG(14:0/17:2(9Z,12Z)/0:0)[iso2]	C34 H62 O5	(M-H)-	54.673		550.4597		FBF	97.40		97.40
DG(13:0/18:2(9Z,12Z)/0:0)[iso2]	C34 H62 O5	(M-H)-	54.673		550.4597		FBF	97.40		97.40

Cpd 15: (20R)-Ginsenoside Rh2

Name	Formula	RT	RI	Mass	Diff (Tgt, ppm)	CAS	ID Source	Score	Algorithm
(20R)-Ginsenoside Rh2	C36 H62 O8	55.162		622.4441	-0.52	78214-33-2	M-FBF	99.50	FBF

Species	m/z	Score (Tgt)	Score (Lib)	Score (DB)	Score (MFG)	Score (RT)
(M-H)-	621	99.50				

Compound Chromatograms (overlaid)

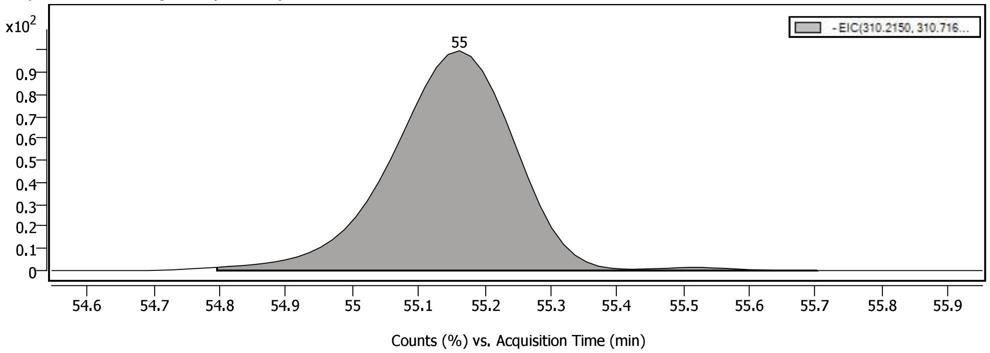

Structure

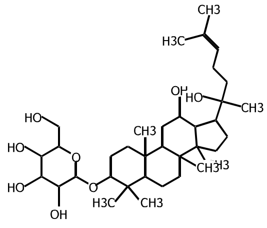

Compound Spectra (overlaid)

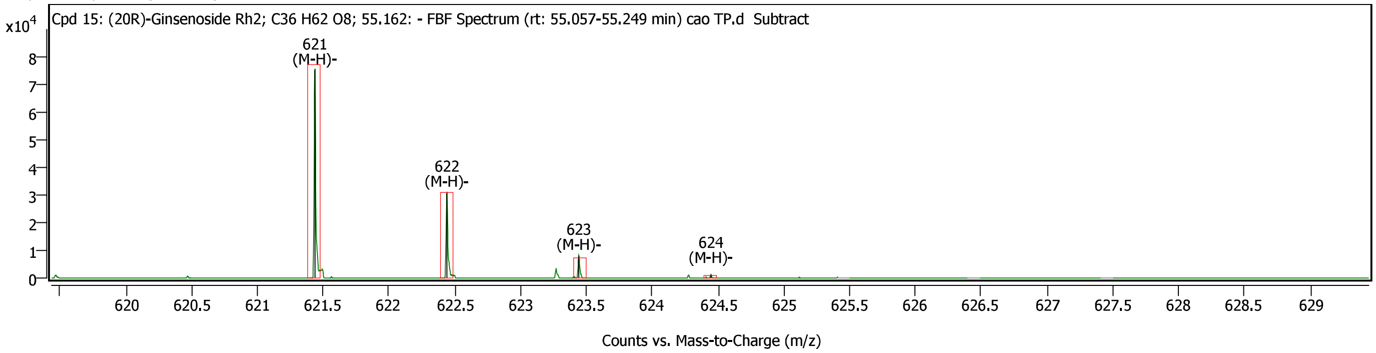

Compound ID Table

Name	Formula	Species	RT	RT Diff	Mass	CAS	ID Source	Score	Score (Lib)	Score (Tgt)
(20R)-Ginsenoside Rh2	C36 H62 O8	(M-H)-	55.162		622.4441	78214-33-2	FBF	99.50		99.50
Squamosinin A	C36 H62 O8	(M-H)-	55.162		622.4441	163597-71-5	FBF	99.50		99.50

Cpd 17: <16-Methylheptadecyl isobutyrate>

Name	Formula	RT	RI	Mass	Diff (Tgt, ppm)	CAS	ID Source	Score	Algorithm
<16-Methylheptadecyl isobutyrate>	C22 H44 O2	58.581		340.3339	-0.66		M-FBF	99.48	FBF

Species	m/z	Score (Tgt)	Score (Lib)	Score (DB)	Score (MFG)	Score (RT)
(M-H)-	339	99.48				

Compound Chromatograms (overlaid)

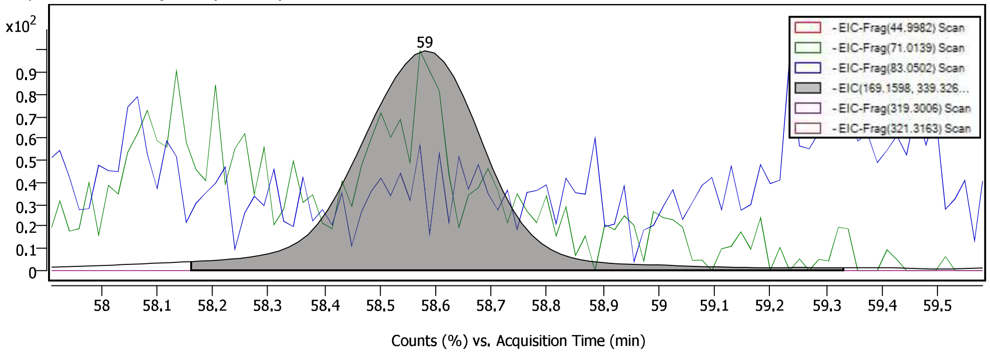

Structure

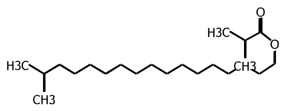

Coelution Plot

Compound Screening Report

Compound Spectra (overlaid)

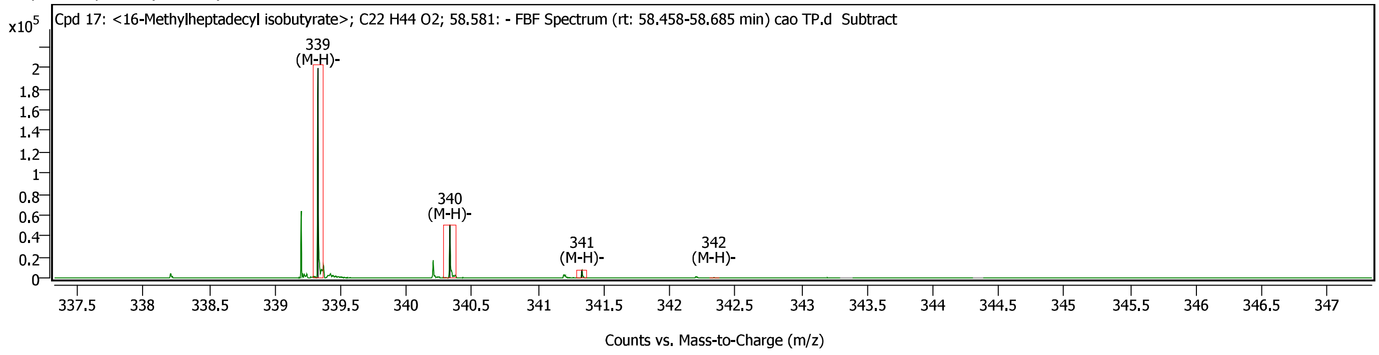

Fragment Spectrum (raw)

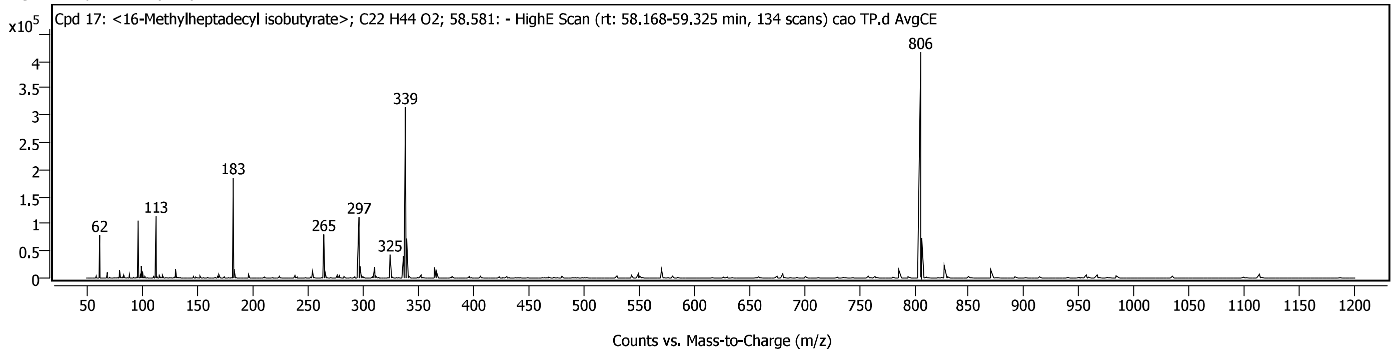

Compound ID Table

Name	Formula	Species	RT	RT Diff	Mass	CAS	ID Source	Score	Score (Lib)	Score (Tgt)
<16-Methylheptadecyl isobutyrate>	C22 H44 O2	(M-H)-	58.581		340.3339		FBF	99.48		99.48
<Butyl octadecanoate>	C22 H44 O2	(M-H)-	58.581		340.3339	123-95-5	FBF	99.48		99.48
<6-Hydroxy-8-docosanone>	C22 H44 O2	(M-H)-	58.581		340.3339		FBF	99.48		99.48
<tetradecyl octanoate>	C22 H44 O2	(M-H)-	58.581		340.3339		FBF	99.48		99.48
<19-methyl-heneicosanoic acid>	C22 H44 O2	(M-H)-	58.581		340.3339		FBF	99.48		99.48
<3-methyl-heneicosanoic acid>	C22 H44 O2	(M-H)-	58.581		340.3339		FBF	99.48		99.48
<4-Hydroxy-6-docosanone>	C22 H44 O2	(M-H)-	58.581		340.3339		FBF	99.48		99.48
<5-Hydroxy-7-docosanone>	C22 H44 O2	(M-H)-	58.581		340.3339		FBF	99.48		99.48
<6,10,13-Trimethyltetradecyl 3-methylbutanoate>	C22 H44 O2	(M-H)-	58.581		340.3339		FBF	99.48		99.48
<8-Hydroxy-6-docosanone>	C22 H44 O2	(M-H)-	58.581		340.3339		FBF	99.48		99.48
<Octadecyl isobutyrate>	C22 H44 O2	(M-H)-	58.581		340.3339		FBF	99.48		99.48
<dodecyl decanoate>	C22 H44 O2	(M-H)-	58.581		340.3339		FBF	99.48		99.48
<Docosanoic acid>	C22 H44 O2	(M-H)-	58.581		340.3339	112-85-6	FBF	99.48		99.48
<Eicosyl acetate>	C22 H44 O2	(M-H)-	58.581		340.3339		FBF	99.48		99.48
<hexadecyl hexanoate>	C22 H44 O2	(M-H)-	58.581		340.3339		FBF	99.48		99.48
<hexyl hexadecanoate>	C22 H44 O2	(M-H)-	58.581		340.3339		FBF	99.48		99.48
<octadecyl butyrate>	C22 H44 O2	(M-H)-	58.581		340.3339		FBF	99.48		99.48
<Isobehenic acid>	C22 H44 O2	(M-H)-	58.581		340.3339		FBF	99.48		99.48

Cpd 102: Lauryl hydrogen sulfate

Name	Formula	RT	RI	Mass	Diff (Tgt, ppm)	CAS	ID Source	Score	Algorithm
Lauryl hydrogen sulfate	C12 H26 O4 S	59.226		266.1549	-1.02	151-21-3	FBF-FragConfirm	96.63	FBF

Species	m/z	Score (Tgt)	Score (Lib)	Score (DB)	Score (MFG)	Score (RT)
(M-H)-	265	96.63				

Compound Chromatograms (overlaid)

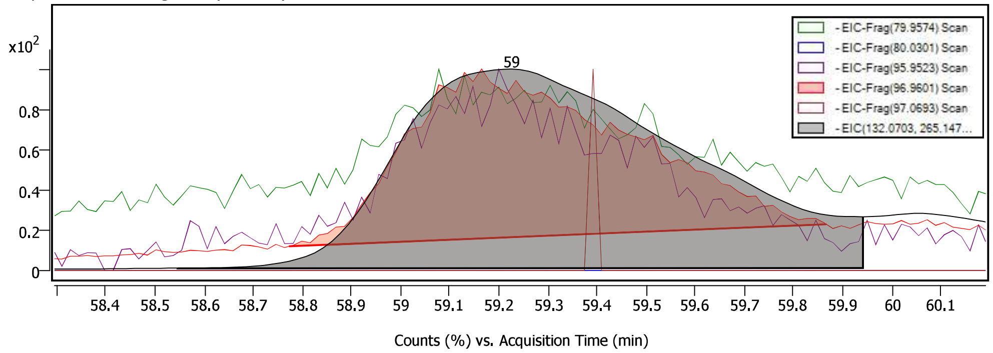

Structure

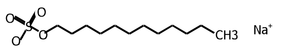

Compound Screening Report

Coelution Plot

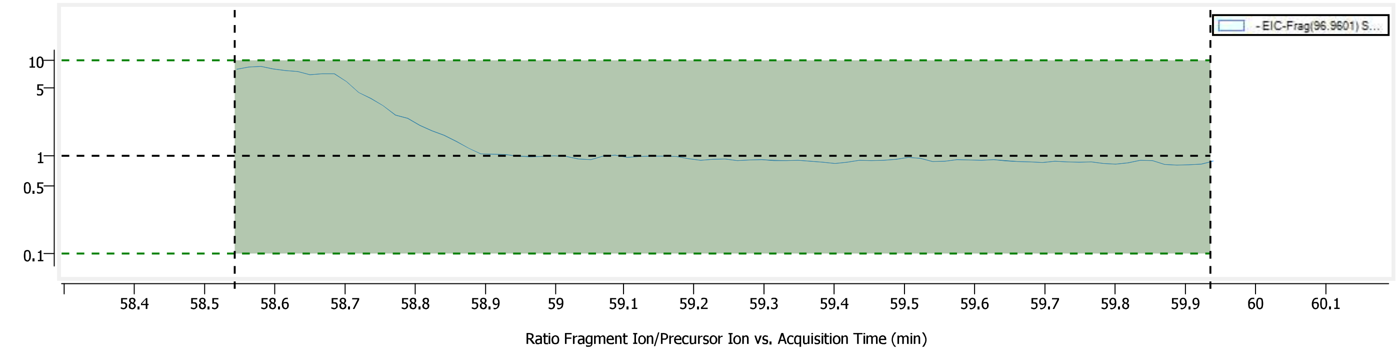

Compound Spectra (overlaid)

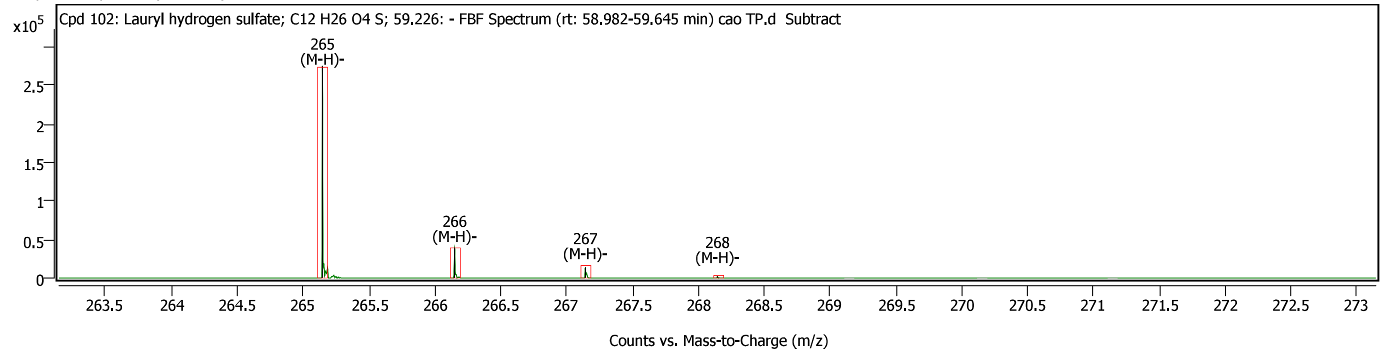

Fragment Spectrum (clean)

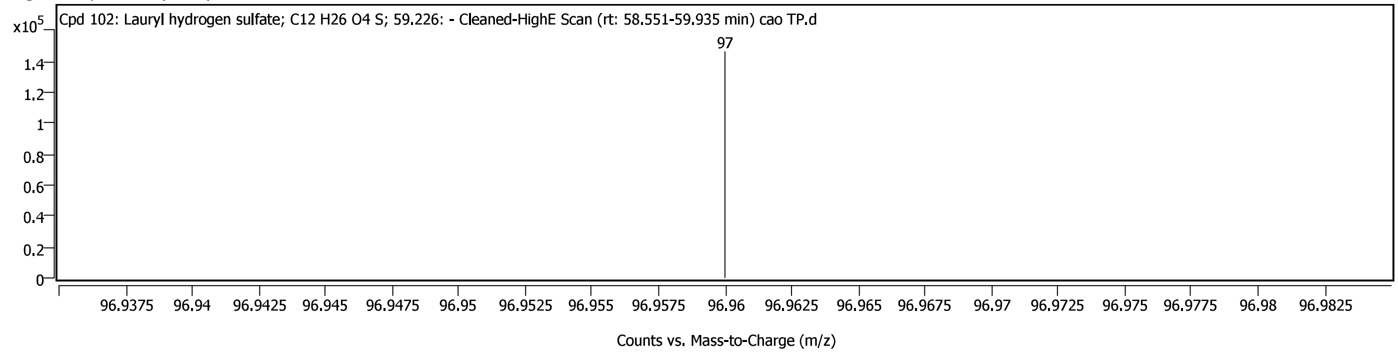

Fragment Spectrum (raw)

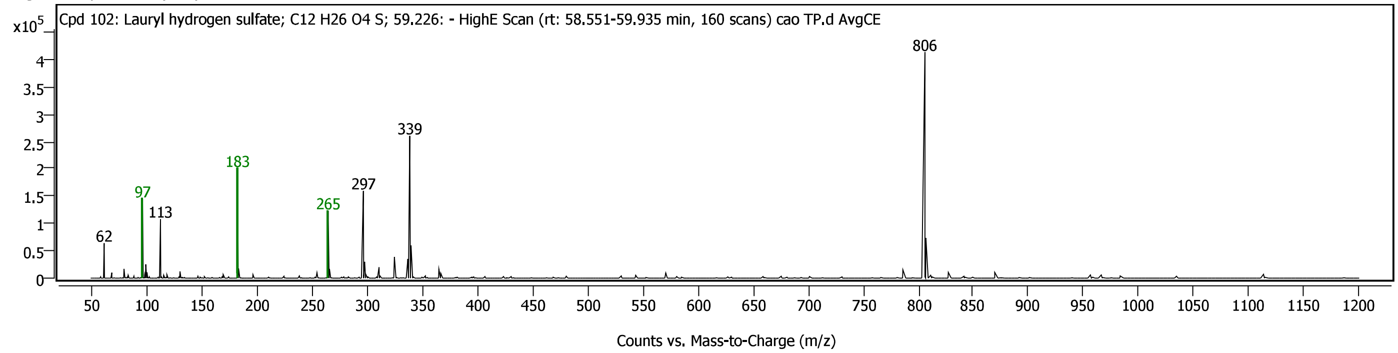

Compound ID Table

Name	Formula	Species	RT	RT Diff	Mass	CAS	ID Source	Score	Score (Lib)	Score (Tgt)
Lauryl hydrogen sulfate	C12 H26 O4 S	(M-H)-	59.226		266.1549	151-21-3	FBF-FragConfirm	96.63		96.63

Cpd 58: PS(17:1(9Z)/22:2(13Z,16Z))

Name	Formula	RT	RI	Mass	Diff (Tgt, ppm)	CAS	ID Source	Score	Algorithm
PS(17:1(9Z)/22:2(13Z,16Z))	C45 H82 N O10 P	61.424		827.5670	-0.74		M-FBF	99.59	FBF

Species	m/z	Score (Tgt)	Score (Lib)	Score (DB)	Score (MFG)	Score (RT)
(M-H)-	827	99.59				

Compound Screening Report

Compound Chromatograms (overlaid)

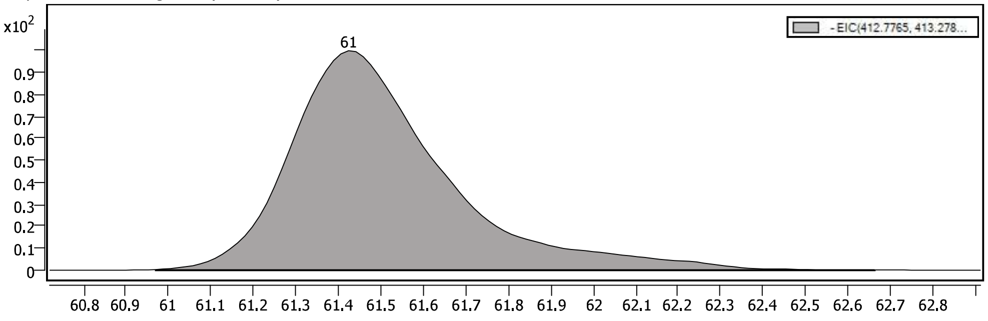

Structure

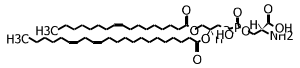

Compound Spectra (overlaid)

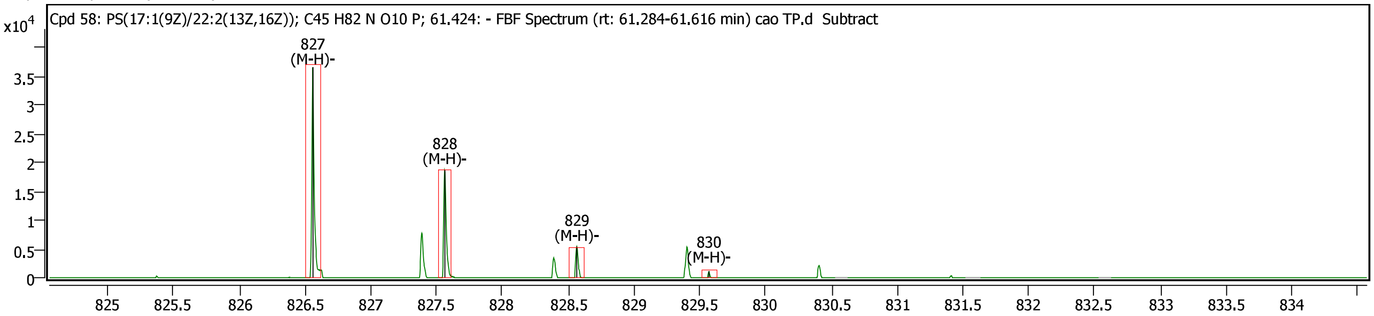

Compound ID Table

Name	Formula	Species	RT	RT Diff	Mass	CAS	ID Source	Score	Score (Lib)	Score (Tgt)
PS(17:1(9Z)/22:2(13Z,16Z))	C45 H82 N O10 P	(M-H)-	61.424		827.5670		FBF	99.59		99.59
PS(19:1(9Z)/20:2(11Z,14Z))	C45 H82 N O10 P	(M-H)-	61.424		827.5670		FBF	99.59		99.59
PS(17:2(9Z,12Z)/22:1(11Z))	C45 H82 N O10 P	(M-H)-	61.424		827.5670		FBF	99.59		99.59
PS(18:3(6Z,9Z,12Z)/21:0)	C45 H82 N O10 P	(M-H)-	61.424		827.5670		FBF	99.59		99.59
PS(18:3(9Z,12Z,15Z)/21:0)	C45 H82 N O10 P	(M-H)-	61.424		827.5670		FBF	99.59		99.59
PS(19:0/20:3(8Z,11Z,14Z))	C45 H82 N O10 P	(M-H)-	61.424		827.5670		FBF	99.59		99.59
PS(20:2(11Z,14Z)/19:1(9Z))	C45 H82 N O10 P	(M-H)-	61.424		827.5670		FBF	99.59		99.59
PS(21:0/18:3(6Z,9Z,12Z))	C45 H82 N O10 P	(M-H)-	61.424		827.5670		FBF	99.59		99.59
PS(21:0/18:3(9Z,12Z,15Z))	C45 H82 N O10 P	(M-H)-	61.424		827.5670		FBF	99.59		99.59
PS(22:1(11Z)/17:2(9Z,12Z))	C45 H82 N O10 P	(M-H)-	61.424		827.5670		FBF	99.59		99.59
PS(22:2(13Z,16Z)/17:1(9Z))	C45 H82 N O10 P	(M-H)-	61.424		827.5670		FBF	99.59		99.59
PS(20:3(8Z,11Z,14Z)/19:0)	C45 H82 N O10 P	(M-H)-	61.424		827.5670		FBF	99.59		99.59

MassHunter Qual 12.0
(End of Report)
