# Supplementary material for: Phytochemical Characterization and Biological Evaluation of Camellia hakodae Ninh Flowers
Source: Molecules. 2026 Mar 26;31(7):1088. doi: 10.3390/molecules31071088 (PMC13074563; doi:10.3390/molecules31071088)
Supplement: Supplementary file 1 [file molecules-31-01088-s001.zip › LC-QTOF-MS-MS_positive mode.pdf]

Compound Screening Report

Agilent

Sample Information

**Sample Name**	Cao TP	**Data File Path**	D:\Trung Pham\Chi My NTT\data\Positive\Cao TP re.d
**Sample ID**		**Acq Time (Local)**	11/16/2024 11:21:59 PM (UTC+07:00)
**Instrument**	LCQTOF	**Acq Method Path**	D:\Trung Pham\Chi My NTT\Method\Positive 150VAll MSMS long .m
**MS Type**	QTOF	**Acq SW Version**	6200 series TOF/6500 series Q-TOF 10.1 (48.0)
**Inj Vol (ul)**	5	**IRM Status**	All ions missed
**Sample Position**	P2-F3	**DA Method Path**	D:\Trung Pham\VIGH\Method processing data\Method screening.m
**Plate Position**		**Target Source Path**	D:\MassHunter\PCDL\METLIN PCDL B.08.00\Metlin_AMRT_PCDL All compound.cdb
**Acq Operator**		**Result Summary**	1312 qualified (110923 targets)

RT	Name	Formula	CAS	Mass	Mass (Tgt)	Diff (Tgt, ppm)	Score	Algorithm
1.035	DL-pipecolic acid	C6 H11 N O2	535-75-1	129.0791	129.0790	0.72	99.59	FBF
1.174	<Nicotinic acid>	C6 H5 N O2	59-67-6	123.0322	123.0320	1.25	98.22	FBF
1.174	<1-Aminocyclobutane carboxylic acid>	C5 H9 N O2	22264-50-2	115.0635	115.0633	1.06	98.72	FBF
1.244	<Pirbuterol>	C12 H20 N2 O3	38677-81-5	240.1479	240.1474	2.18	97.67	FBF
1.314	<Nitisinone>	C14 H10 F3 N O5	104206-65-7	329.0518	329.0511	2.20	83.20	FBF
1.314	<Mecarbam>	C10 H20 N O5 P S2	2595-54-2	329.0520	329.0521	-0.10	96.47	FBF
1.366	<Ribothymidine>	C10 H14 N2 O6	1463-10-1	258.0857	258.0852	1.89	85.49	FBF
1.366	Pyroglutamic acid	C5 H7 N O3	98-79-3	129.0428	129.0426	1.22	86.25	FBF
1.471	<Pirbuterol>	C12 H20 N2 O3	38677-81-5	240.1480	240.1474	2.68	96.57	FBF
1.488	(S)-5'-Deoxy-5'-(methylsulfinyl)adenosine	C11 H15 N5 O4 S	897-42-7	313.0851	313.0845	2.11	97.17	FBF
1.540	Piperidine	C5 H11 N	110-89-4	85.0891	85.0891	-0.52	95.99	FBF
1.575	<N-benzyl-1-methyl-1H-pyrazolo[3,4-d]pyrimidin-4-amine>	C13 H13 N5		239.1162	239.1171	-3.90	92.19	FBF
1.575	Methyldopate	C12 H17 N O4		239.1161	239.1158	1.36	96.53	FBF
1.575	<Pro Ile>	C11 H20 N2 O3		228.1484	228.1474	4.34	90.72	FBF
1.575	Adenine	C5 H5 N5	73-24-5	135.0548	135.0545	2.07	80.64	FBF
1.575	Adenosine	C10 H13 N5 O4	58-61-7	267.0975	267.0968	2.84	95.94	FBF
1.593	<D-Cathinone>	C9 H11 N O	71031-15-7	149.0841	149.0841	0.51	99.69	FBF
1.680	Tiapamil	C26 H37 N O8 S2	57010-31-8	555.1955	555.1961	-1.05	83.82	FBF
1.680	<2-Amino-3-methyl-1-butanol>	C5 H13 N O	473-75-6	103.0998	103.0997	0.63	99.19	FBF
1.680	N-(1-Deoxy-1-fructosyl)isoleucine	C12 H23 N O7	87304-79-8	293.1483	293.1475	2.94	95.59	FBF
2.011	L-Phenylalanine	C9 H11 N O2	63-91-2	165.0794	165.0790	2.75	96.56	FBF
2.099	N-(1-Deoxy-1-fructosyl)phenylalanine	C15 H21 N O7	87251-83-0	327.1331	327.1318	3.88	91.24	FBF
2.099	Tranylcypromine glucuronide	C15 H19 N O6		309.1223	309.1212	3.46	93.03	FBF
2.256	<trans-Zeatin>	C10 H13 N5 O	1637-39-4	219.1112	219.1120	-3.57	91.90	FBF
2.256	D-Pantothenic acid	C9 H17 N O5	79-83-4	219.1112	219.1107	2.20	97.58	FBF
2.325	Tiapamil	C26 H37 N O8 S2	57010-31-8	555.1958	555.1961	-0.41	85.91	FBF
2.430	Succinoadenosine	C14 H17 N5 O8	4542-23-8	383.1078	383.1077	0.33	98.83	FBF
2.604	Octylamine	C8 H19 N	111-86-4	129.1519	129.1517	0.89	86.91	FBF
2.622	Semilepidinoside B	C17 H22 N2 O7		366.1437	366.1427	2.71	96.26	FBF
2.674	DL-Tryptophan	C11 H12 N2 O2	54-12-6	204.0901	204.0899	1.26	99.18	FBF
2.674	4-Formyl Indole	C9 H7 N O	1074-86-8	145.0528	145.0528	0.12	98.29	FBF
2.674	3-Amino-2-naphthoic acid	C11 H9 N O2	5959-52-4	187.0638	187.0633	2.42	97.13	FBF
2.901	Cinnamtannin A2	C60 H50 O24		1154.2684	1154.2692	-0.70	99.46	FBF
2.971	1,8-Diazacyclotetradecane-2,9-dione	C12 H22 N2 O2		226.1685	226.1681	1.80	97.95	FBF
2.971	<ε-Caprolactam>	C6 H11 N O	105-60-2	113.0842	113.0841	1.36	98.45	FBF
3.041	<(-)-Catechin>	C15 H14 O6	18829-70-4	290.0795	290.0790	1.73	96.73	FBF
3.163	(2S,4S)-Monatin	C14 H16 N2 O5	146142-94-1	292.1060	292.1059	0.12	98.34	FBF
3.180	2,4,6-Octatriyn-1-ol	C8 H6 O	13033-21-1	118.0416	118.0419	-2.15	97.04	FBF
3.215	Indol-3-ylacetyl-myo-inositol L-arabinoside	C21 H27 N O11		469.1586	469.1584	0.45	99.74	FBF
3.320	Procyanidin B2	C30 H26 O12		578.1434	578.1424	1.61	98.59	FBF
3.477	Indol-3-ylacetyl-myo-inositol L-arabinoside	C21 H27 N O11		469.1586	469.1584	0.42	98.09	FBF
3.634	De-O-methylsimmondsin	C15 H23 N O9	135105-75-8	361.1376	361.1373	0.87	98.26	FBF
3.982	<Catechin 7-β-D-xylopyranoside>	C20 H22 O10	42830-48-8	422.1217	422.1213	0.85	98.25	FBF
4.052	<Val Val>	C10 H20 N2 O3		216.1476	216.1474	0.98	99.20	FBF
4.523	1,2-O-Diacetylzephyranthine	C20 H23 N O6		373.1529	373.1525	0.91	99.44	FBF
4.715	Gambiriin A2	C30 H28 O12		580.1606	580.1581	4.27	90.36	FBF
4.715	2,5-Dihydroxybenzaldehyde	C7 H6 O3	1194-98-5	138.0319	138.0317	1.52	98.77	FBF
4.715	(-)-Catechin	C15 H14 O6	18829-70-4	290.0802	290.0790	4.04	90.41	FBF
4.855	<a-Asarone>	C12 H16 O3	2883-98-9	208.1102	208.1099	1.09	98.91	FBF
4.907	ent-Fisetinidol-(4beta->8)-catechin-(6->4beta)-ent-fisetinidol	C45 H38 O17		850.2112	850.2109	0.30	99.28	FBF
5.011	<Asn Asn Asn>	C12 H20 N6 O7		360.1406	360.1393	3.46	94.48	FBF
5.413	<Pyridine-2-azo-p-dimethylaniline>	C13 H14 N4		226.1210	226.1218	-3.54	93.61	FBF
5.413	Allixin	C12 H18 O4	125263-70-9	226.1210	226.1205	2.03	97.26	FBF
5.517	ent-Fisetinidol-(4beta->8)-catechin-(6->4beta)-ent-fisetinidol	C45 H38 O17		850.2112	850.2109	0.31	99.46	FBF
5.796	4-Dedimethyl-6-dehydro-anhydrotetracycline	C20 H18 N2 O7		398.1116	398.1114	0.62	99.27	FBF
5.796	S-Adenosyl-4-methylthio-2-oxobutanoate	C15 H20 N5 O6 S	22365-11-3	398.1120	398.1134	-3.67	81.56	FBF
5.866	Stilbamidine	C16 H16 N4	122-06-5	264.1365	264.1375	-3.88	92.54	FBF
5.866	<γ-CEHC>	C15 H20 O4	178167-75-4	264.1364	264.1362	0.89	98.61	FBF
5.971	1-(alpha-Methyl-4-(2-methylpropyl)benzeneacetate)-beta-D-Glucopyranuronic acid	C19 H26 O8	115075-59-7	382.1613	382.1628	-3.86	92.80	FBF
6.145	Riboflavin	C17 H20 N4 O6	83-88-5	376.1389	376.1383	1.53	98.79	FBF
6.198	4-Dedimethyl-6-dehydro-anhydrotetracycline	C20 H18 N2 O7		398.1117	398.1114	0.80	99.14	FBF
6.198	S-Adenosyl-4-methylthio-2-oxobutanoate	C15 H20 N5 O6 S	22365-11-3	398.1120	398.1134	-3.52	82.16	FBF
6.355	<Cuminaldehyde>	C10 H12 O	122-03-2	148.0888	148.0888	0.07	99.65	FBF
6.494	Butopyronoxyl	C12 H18 O4	532-34-3	226.1206	226.1205	0.52	99.02	FBF
6.721	Cinnamoylglycine	C11 H11 N O3	16534-24-0	205.0743	205.0739	2.15	97.57	FBF

RT	Name	Formula	CAS	Mass	Mass (Tgt)	Diff (Tgt, ppm)	Score	Algorithm
6.808	Istamycin C1	C19 H37 N5 O6		431.2736	431.2744	-1.83	97.20	FBF
7.087	N1-Caffeoyl-N10-feruloylspermidine	C26 H33 N3 O6	114916-07-3	483.2374	483.2369	0.88	99.49	FBF
7.436	5,7,3'-Trihydroxy-6,4',5'-trimethoxyflavanone	C18 H18 O8		362.1004	362.1002	0.73	81.27	FBF
7.471	Myricetin 3-galactoside	C21 H20 O13	15648-86-9	480.0906	480.0904	0.48	99.50	FBF
7.698	1,2-O-Diacetylzephyranthine	C20 H23 N O6		373.1529	373.1525	0.94	99.29	FBF
7.942	Isoquercitrin	C21 H20 O12		464.0958	464.0955	0.73	98.80	FBF
8.081	Netilmicin	C21 H41 N5 O7		475.2996	475.3006	-2.19	97.46	FBF
8.116	PAC-1	C23 H28 N4 O2	315183-21-2	392.2218	392.2212	1.35	96.79	FBF
8.116	Dihydrofukinolide	C22 H32 O6	41059-95-4	392.2217	392.2199	4.72	98.62	FBF
8.116	<Trospium>	C25 H30 N O3	10405-02-4	392.2217	392.2226	-2.17	84.73	FBF
8.134	<Sophoraflavanone B>	C20 H20 O5		340.1315	340.1311	1.14	98.33	FBF
8.169	N-trans-Feruloyl-4-O-methyldopamine	C19 H21 N O5	78510-20-0	343.1426	343.1420	1.87	97.51	FBF
8.273	<Lunarine>	C25 H31 N3 O4	24185-51-1	437.2319	437.2315	1.04	98.10	FBF
8.343	Dianthramine	C14 H11 N O6	136945-65-8	289.0589	289.0586	0.82	98.88	FBF
8.430	N1-Caffeoyl-N10-feruloylspermidine	C26 H33 N3 O6	114916-07-3	483.2368	483.2369	-0.27	99.12	FBF
8.552	Pyranodelphinin A	C30 H33 O16		649.1793	649.1769	3.81	88.35	FBF
8.639	Matairesinol	C20 H22 O6	580-72-3	358.1419	358.1416	0.80	98.06	FBF
8.639	Dide-O-methyl-4-O-alpha-D-glucopyranosylsimmondsin	C20 H31 N O14	370068-00-1	509.1720	509.1745	-4.92	82.62	FBF
9.128	8,8'-Methylenebiscatechin	C31 H28 O12	81555-08-0	592.1581	592.1581	0.08	99.24	FBF
9.180	Lunarine	C25 H31 N3 O4	24185-51-1	437.2322	437.2315	1.72	98.40	FBF
9.267	N1,N10-Diferuloylspermidine	C27 H35 N3 O6	70185-61-4	497.2528	497.2526	0.49	99.54	FBF
9.390	Rhamnetin	C16 H12 O7	90-19-7	316.0586	316.0583	0.95	98.42	FBF
9.494	N-Adenylylanthranilate	C17 H19 N6 O8 P		466.1013	466.1002	2.41	93.16	FBF
9.500	Quercetin 3-O-glucoside	C21 H20 O12	21637-25-2	464.0968	464.0955	2.90	94.76	FBF
9.512	<Herbacetin>	C15 H10 O7		302.0429	302.0427	0.95	99.23	FBF
9.651	N1,N10-Diferuloylspermidine	C27 H35 N3 O6	70185-61-4	497.2529	497.2526	0.61	99.33	FBF
9.791	3-O-Methylquercetin	C16 H12 O7	1486-70-0	316.0586	316.0583	1.09	98.34	FBF
9.791	7-O-Methylgossypetin 3-rhamnoside	C22 H22 O12	56768-33-3	478.1114	478.1111	0.67	98.78	FBF
9.791	5,7-Dihydroxy-2-(4-hydroxy-3-methoxyphenyl)-3-[3,4,5-trihydroxy-6-[[(2R,3R,4R,5R,6S)-3,4,5-trihydroxy-6-methyloxan-2-yl]oxymethyl]oxan-2-yl]oxychromen-4-one	C28 H32 O16	1393485-18-1	624.1694	624.1690	0.65	99.65	FBF
9.948	Ikarisoside F	C31 H36 O14		632.2085	632.2105	-3.20	81.89	FBF
10.419	Myricitrin	C21 H20 O12	17912-87-7	464.0958	464.0955	0.68	98.92	FBF
10.436	Rutin	C27 H30 O16	153-18-4	610.1534	610.1534	0.07	98.52	FBF
10.663	Cinnamtannin A2	C60 H50 O24		1154.2695	1154.2692	0.29	99.15	FBF
10.750	(±)-Naringenin	C15 H12 O5	67604-48-2	272.0688	272.0685	1.31	97.45	FBF
10.750	Helichrysin A	C21 H22 O10	529-41-9	434.1215	434.1213	0.38	98.55	FBF
10.837	Citbismine F	C36 H34 N2 O10		654.2225	654.2213	1.69	97.54	FBF
10.872	Kaempferol 7-galactoside	C21 H20 O11		448.1008	448.1006	0.44	98.28	FBF
10.942	SB 243213	C22 H19 F3 N4 O2		428.1451	428.1460	-2.23	97.21	FBF
11.047	10-Acetoxyoleuropein	C27 H34 O15		598.1906	598.1898	1.34	98.52	FBF
11.047	7-Hydroxytrifluoperazine glucuronide	C27 H32 F3 N3 O7 S		599.1935	599.1913	3.71	85.28	FBF
11.047	Naringin	C27 H32 O14	10236-47-2	580.1795	580.1792	0.50	98.83	FBF
11.239	N-Adenylylanthranilate	C17 H19 N6 O8 P		466.1014	466.1002	2.50	92.39	FBF
11.256	Quercetin	C15 H10 O7	117-39-5	302.0435	302.0427	2.93	95.08	FBF
11.343	Rutin	C27 H30 O16	153-18-4	610.1543	610.1534	1.55	98.26	FBF
11.762	N-Caffeoyltryptophan	C20 H18 N2 O5	109163-69-1	366.1223	366.1216	2.06	97.65	FBF
11.989	epi-4'-hydroxyjasmonic acid	C12 H18 O4		226.1208	226.1205	1.35	97.71	FBF
12.163	Polyethylene, oxidized	C12 H20 O5	68441-17-8	244.1315	244.1311	1.83	97.59	FBF
12.268	<Troxerutin>	C33 H42 O19	7085-55-4	742.2323	742.2320	0.43	98.72	FBF
12.285	Prunin 6''-O-gallate	C28 H26 O14		586.1323	586.1323	0.13	99.31	FBF
12.442	15-Acetoxyscirpene-3,4-diol 4-O-a-D-glucopyranoside	C23 H34 O11	99124-45-5	486.2079	486.2101	-4.55	90.63	FBF
12.442	Glucosylgalactosyl hydroxylysine	C18 H34 N2 O13	32448-35-4	486.2080	486.2061	3.85	90.91	FBF
12.460	N,N'-(((4-methyl-1,3-phenylene)bis(azanediyl))bis(carbonothioyl))dibenzamide	C23 H20 N4 O2 S2		448.1016	448.1028	-2.59	80.73	FBF
12.460	Luteolin 7-galactoside	C21 H20 O11		448.1013	448.1006	1.57	98.59	FBF
12.786	Coumarin	C9 H6 O2	91-64-5	146.0368	146.0368	0.14	99.65	FBF
13.157	Isorhamnetin 3-glucoside	C22 H22 O12		478.1115	478.1111	0.69	98.11	FBF
13.210	Genistein 4'-O-glucoside	C21 H20 O10	152-95-4	432.1056	432.1056	-0.15	97.60	FBF
13.210	1-Methyl-4-nitro-5-(S-Gluctathionyl) Imidazole	C14 H20 N6 O8 S	36892-55-4	432.1060	432.1063	-0.79	90.30	FBF
13.297	<Quercetin 3-O-glucoside>	C21 H20 O12	21637-25-2	464.0960	464.0955	1.20	98.83	FBF
13.401	C.I. Pigment Red 149	C40 H26 N2 O4	4948-15-6	598.1897	598.1893	0.74	81.58	FBF
13.628	Carnocin CP 5	C23 H19 N3 O5 S	149983-81-3	449.1040	449.1045	-1.12	89.07	FBF
13.820	Carnocin CP 5	C23 H19 N3 O5 S	149983-81-3	449.1040	449.1045	-1.29	92.78	FBF
13.890	Coumarin	C9 H6 O2	91-64-5	146.0369	146.0368	0.63	99.38	FBF
14.047	<7-[(2S,3R,4S,5S,6R)-6-[[(2S,3R,4R)-3,4-Dihydroxy-4-(hydroxymethyl)oxolan-2-yl]oxymethyl]-3,4,5-trihydroxyoxan-2-yl]oxy-5-hydroxy-3-(4-hydroxyphenyl)-6-methoxychromen-4-one>	C27 H30 O15	365544-05-4	594.1590	594.1585	0.88	99.25	FBF
14.064	3,5-Dicaffeoyl-4-succinoylquinic acid	C29 H28 O15	179761-31-0	616.1405	616.1428	-3.78	92.17	FBF
14.675	<Foramsulfuron>	C17 H20 N6 O7 S	173159-57-4	452.1112	452.1114	-0.41	86.40	FBF
14.675	<5,7-Dihydroxy-2-(4-hydroxy-3-methoxyphenyl)-3-[3,4,5-trihydroxy-6-[[(2R,3R,4R,5R,6S)-3,4,5-trihydroxy-6-methyloxan-2-yl]oxymethyl]oxan-2-yl]oxychromen-4-one>	C28 H32 O16	1393485-18-1	624.1691	624.1690	0.09	99.75	FBF
14.745	<Morin>	C15 H10 O7	480-16-0	302.0430	302.0427	0.99	99.10	FBF
14.762	5'-Butyrylphosphoinosine	C14 H19 N4 O9 P		418.0905	418.0890	3.56	84.77	FBF
14.867	<n-Propyl cinnamate>	C12 H14 O2	7778-83-8	190.0996	190.0994	1.02	99.04	FBF
15.006	<Tolmetin>	C15 H15 N O3	26171-23-3	257.1055	257.1052	1.11	98.90	FBF
15.111	Liatrin	C22 H26 O8	34175-79-6	418.1629	418.1628	0.28	99.75	FBF

RT	Name	Formula	CAS	Mass	Mass (Tgt)	Diff (Tgt, ppm)	Score	Algorithm
15.111	<Fluridone>	C19 H14 F3 N O	59756-60-4	329.1028	329.1027	0.29	98.35	FBF
15.216	<12-Hydroxy-4,4-bisnor-4,8,11,13-podocarpatetraen-3-one>	C15 H16 O2	108123-97-3	228.1154	228.1150	1.74	98.18	FBF
15.320	Neobavaisoflavone	C20 H18 O4	41060-15-5	322.1208	322.1205	0.81	99.40	FBF
15.407	Rhizocticin A	C11 H22 N5 O6 P	114301-25-6	351.1303	351.1308	-1.45	98.44	FBF
15.425	Na-p-Hydroxycoumaroyltryptophan	C20 H18 N2 O4		350.1272	350.1267	1.42	98.31	FBF
15.564	N-Caffeoyltryptophan	C20 H18 N2 O5	109163-69-1	366.1220	366.1216	1.15	98.94	FBF
15.582	Hyperoside	C21 H20 O12	482-36-0	464.0963	464.0955	1.86	97.50	FBF
15.687	Morfamquat	C26 H34 N4 O4	4636-83-3	466.2568	466.2580	-2.48	96.15	FBF
16.001	Aurasperone C	C31 H28 O12	41689-66-1	592.1577	592.1581	-0.66	80.23	FBF
16.088	<10-Hydroxycamtothecin>	C20 H16 N2 O5	19685-09-7	364.1061	364.1059	0.61	99.34	FBF
16.384	Piperenol A triacetate	C27 H26 O10	134476-92-9	510.1503	510.1526	-4.56	90.55	FBF
16.576	Petasitenine	C19 H27 N O7	60102-37-6	381.1772	381.1788	-4.20	85.95	FBF
16.576	Vomicine	C22 H24 N2 O4	125-15-5	380.1742	380.1736	1.68	98.56	FBF
16.925	10-Fluoro-capric acid	C10 H19 F O2		190.1360	190.1369	-4.85	84.22	FBF
16.995	<10-Hydroxycamtothecin>	C20 H16 N2 O5	19685-09-7	364.1066	364.1059	1.90	98.16	FBF
17.222	<Quinaprilat>	C23 H26 N2 O5	82768-85-2	410.1848	410.1842	1.45	98.55	FBF
17.396	Kaempferol 7-methyl ether 3-xylosyl-(1->2)-[rhamnosyl-(1->6)-glucoside]	C33 H40 O19		740.2163	740.2164	-0.14	99.93	FBF
17.448	Quinalizarin	C14 H8 O6	81-61-8	272.0324	272.0321	1.00	98.27	FBF
17.489	Morin	C15 H10 O7	480-16-0	302.0437	302.0427	3.46	92.87	FBF
17.571	Nympholide A	C30 H26 O15		626.1275	626.1272	0.51	98.10	FBF
17.728	<(±)-Jasmonic Acid>	C12 H18 O3	77026-92-7	210.1259	210.1256	1.33	98.20	FBF
17.762	Oxprenolol glucuronide	C21 H31 N O9	53564-66-2	441.1982	441.1999	-3.76	85.97	FBF
17.762	Diferuloylputrescine	C24 H28 N2 O6	42369-86-8	440.1953	440.1947	1.18	99.04	FBF
17.850	N-dodecanoyl-L-Homoserine lactone-3-hydrazone-fluorescein	C37 H40 N4 O8 S		700.2558	700.2567	-1.27	87.20	FBF
17.850	Rhizocticin A	C11 H22 N5 O6 P	114301-25-6	351.1304	351.1308	-0.94	99.18	FBF
18.006	N1,N5,N10-Tricaffeoyl spermidine	C34 H37 N3 O9		631.2532	631.2530	0.38	99.16	FBF
18.303	Isopimpinellin	C13 H10 O5	482-27-9	246.0531	246.0528	1.14	97.88	FBF
18.774	6alpha,9-Difluoro-11beta-hydroxypregn-4-ene-3,20-dione	C21 H28 F2 O3		366.2022	366.2007	4.27	90.51	FBF
20.240	Kolaflavanone	C31 H24 O12		588.1266	588.1268	-0.22	99.52	FBF
20.711	N1,N5,N10-Tricoumaroyl spermidine	C34 H37 N3 O6		583.2685	583.2682	0.53	98.49	FBF
20.798	Luteolin 7-(6''-ferulylglucoside)	C31 H28 O14		624.1485	624.1479	1.00	99.02	FBF
20.798	Allamandin	C15 H16 O7	51820-82-7	308.0900	308.0896	1.29	98.47	FBF
21.042	Loxtidine	C19 H29 N5 O2	76956-02-0	359.2312	359.2321	-2.64	96.51	FBF
21.042	Kolaflavanone	C31 H24 O12		588.1269	588.1268	0.20	99.38	FBF
21.042	6alpha,9-Difluoro-11beta-hydroxypregn-4-ene-3,20-dione	C21 H28 F2 O3		366.2020	366.2007	3.71	90.43	FBF
21.182	Dihydromorelloflavone	C30 H22 O11		558.1162	558.1162	-0.06	99.29	FBF
21.484	N1,N5,N10-Triferuloyl spermidine	C37 H43 N3 O9		673.3000	673.2999	0.09	99.15	FBF
21.828	<KOdiA-PC>	C32 H59 N O11 P	439904-33-3	664.3821	664.3826	-0.66	97.25	FBF
21.828	Caloxanthin sulfate	C40 H56 O6 S		664.3824	664.3798	3.96	88.69	FBF
21.828	<18α-Glycyrrhetinic acid>	C30 H46 O4	1449-05-4	470.3395	470.3396	-0.14	98.89	FBF
22.089	Cer(t18:0/18:0)	C36 H73 N O4		583.5561	583.5540	3.64	84.13	FBF
22.089	N1,N5,N10-Tricoumaroyl spermidine	C34 H37 N3 O6		583.2699	583.2682	2.89	95.49	FBF
22.334	Helichrysin A	C21 H22 O10	529-41-9	434.1215	434.1213	0.51	99.38	FBF
22.477	6'''-Deamino-6'''-dehydro-6'''-oxoneomycin C	C23 H43 N5 O14		613.2805	613.2807	-0.24	84.50	FBF
22.506	GW 6471	C35 H36 F3 N3 O5	436159-64-7	635.2609	635.2607	0.28	99.38	FBF
23.032	N1,N5,N10-Triferuloyl spermidine	C37 H43 N3 O9		673.3016	673.2999	2.53	96.32	FBF
23.642	Louisfieserone A	C22 H24 O5		368.1637	368.1624	3.66	92.03	FBF
23.642	Asp His Val	C15 H23 N5 O6		369.1664	369.1648	4.26	90.63	FBF
23.642	Prasterone sulfate	C19 H28 O5 S	651-48-9	368.1639	368.1657	-4.96	82.01	FBF
23.642	(E)-3-(2-Methylpropylidene)-1(3H)-isobenzofuranone	C12 H12 O2	56014-69-8	188.0839	188.0837	1.16	98.80	FBF
24.619	<13-epi-12-oxo Phytodienoic Acid>	C18 H28 O3	71606-07-0	292.2042	292.2038	1.28	98.96	FBF
24.706	Kaempferol 3-apioside-7-rhamnosyl-(1->6)-(2''-(E)-caffeoylglactoside)	C41 H44 O22		888.2300	888.2324	-2.69	96.36	FBF
25.055	L-Olivosyl-oleandolide	C26 H44 O10		516.2913	516.2934	-4.11	92.20	FBF
25.160	(8S,Z)-6-((S)-3-Hydroxy-2-methylpropylidene)-8-methyloctahydroindolizin-8-ol	C13 H23 N O2		225.1731	225.1729	0.83	98.86	FBF
25.404	<9-OxoOTrE>	C18 H28 O3	125559-74-2	292.2043	292.2038	1.45	98.79	FBF
25.701	4,4-Difluoropregn-5-ene-3,20-dione	C21 H28 F2 O2		350.2070	350.2057	3.72	92.77	FBF
26.660	<Confertifoline>	C15 H22 O2	1811-23-0	234.1624	234.1620	1.85	97.65	FBF
26.748	Tragopogonsaponin K	C50 H72 O15		912.4871	912.4871	-0.01	99.81	FBF
26.765	Ephedrannin A	C30 H20 O11	82001-39-6	556.1005	556.1006	-0.13	99.44	FBF
27.254	C16 Sphinganine	C16 H35 N O2		273.2674	273.2668	2.26	96.92	FBF
27.585	Kurilensoside G	C32 H54 O10		598.3718	598.3717	0.21	98.99	FBF
27.672	Chikusetsusaponin Ia	C41 H70 O12		754.4845	754.4867	-2.92	95.15	FBF
27.777	<thio-Miltefosine>	C21 H47 N O3 P S	943022-11-5	424.3021	424.3014	1.56	86.61	FBF
27.864	4,4-Difluoropregn-5-ene-3,20-dione	C21 H28 F2 O2		350.2070	350.2057	3.70	93.59	FBF
27.917	Botrydial	C17 H26 O5		310.1782	310.1780	0.43	98.68	FBF
27.986	3,3-Difluoro-5alpha-androstan-17beta-yl acetate	C21 H32 F2 O2	1827-75-4	354.2387	354.2370	4.63	90.42	FBF
28.004	12,13-DiHOME	C18 H34 O4		314.2461	314.2457	1.39	98.09	FBF
28.091	PI(10:0/16:0)	C35 H67 O13 P		726.4317	726.4319	-0.29	91.29	FBF
28.283	Notoginsenoside R10	C30 H50 O9	335157-20-5	554.3455	554.3455	0.08	99.73	FBF
28.423	Evasterioside D	C33 H58 O10		614.4025	614.4030	-0.74	99.61	FBF
28.440	Corrinoid	C19 H22 N4	262-76-0	306.1834	306.1844	-3.28	94.39	FBF
28.510	Notoginsenoside T2	C37 H62 O10	343962-54-9	666.4320	666.4343	-3.47	92.63	FBF
28.510	<PAz-PC>	C33 H65 N O10 P	117746-89-1	666.4320	666.4346	-3.90	91.88	FBF
28.632	Corrinoid	C19 H22 N4	262-76-0	306.1833	306.1844	-3.70	93.49	FBF
28.894	(+)-Vulgraon B	C16 H24		216.1881	216.1878	1.27	99.01	FBF

RT	Name	Formula	CAS	Mass	Mass (Tgt)	Diff (Tgt, ppm)	Score	Algorithm
29.016	endo-1,4-beta-Xylanase	C34 H50 N4 O2	9025-57-4	546.3922	546.3934	-2.17	97.57	FBF
29.120	5-Ethyl-7-methyl-3E,5E,7E-undecatriene	C14 H24		192.1878	192.1878	0.03	86.72	FBF
29.435	28-Homobrassinolide	C29 H50 O6	80483-89-2	494.3609	494.3607	0.40	99.75	FBF
29.435	PC(P-17:0/0:0)	C25 H53 N O6 P		494.3610	494.3610	-0.17	96.20	FBF
29.609	PC(P-19:1(12Z)/0:0)	C27 H55 N O6 P		520.3765	520.3767	-0.34	97.90	FBF
29.609	Cyclopassifloic acid B	C31 H52 O6	292167-35-2	520.3765	520.3764	0.20	99.83	FBF
29.801	Cyclopassifloic acid E	C31 H52 O8	301540-74-9	552.3665	552.3662	0.57	82.72	FBF
29.818	<Linoleoyl glycine>	C20 H35 N O3	2764-03-6	337.2621	337.2617	1.17	98.62	FBF
30.115	trans-EKODE-(E)-Ib	C18 H30 O4	478931-82-7	310.2148	310.2144	1.33	98.01	FBF
30.150	Cyclopassifloic acid A	C31 H52 O7	292167-34-1	536.3712	536.3713	-0.17	99.64	FBF
30.150	1-Hydroxyvitamin D3 3-D-glucopyranoside	C33 H52 O8		576.3639	576.3662	-4.00	92.76	FBF
30.150	<ponasterone A>	C27 H44 O6		464.3130	464.3138	-1.67	97.74	FBF
30.307	9,11alpha-epoxy-6alpha-acetoxy-cholest-7-en-3beta,5alpha,19-triol	C29 H46 O6		490.3272	490.3294	-4.48	89.28	FBF
31.040	(25R)-5alpha-spirostan-3beta,6alpha,23S-triol 6-O-beta-D-glucopyranoside	C34 H58 O10		626.4029	626.4030	-0.17	99.84	FBF
31.528	<15-methyl-15(S)-PGE1>	C21 H36 O5	35700-26-6	368.2566	368.2563	0.85	99.38	FBF
31.546	<Eplerenone>	C24 H30 O6	107724-20-9	414.2046	414.2042	0.77	98.21	FBF
32.331	Asebotoxin II	C23 H36 O6	23984-18-1	408.2492	408.2512	-4.95	88.50	FBF
32.331	<3-methoxy Prostaglandin F1α>	C21 H38 O6	54432-43-8	386.2672	386.2668	0.89	99.05	FBF
32.401	<Dehydro (11,12)ursolic acid lactone>	C30 H46 O3	35959-05-8	454.3449	454.3447	0.51	99.14	FBF
32.575	delta5-Demissine	C50 H81 N O20	195433-57-9	1015.5355	1015.5352	0.32	99.58	FBF
33.290	delta5-Demissine	C50 H81 N O20	195433-57-9	1015.5354	1015.5352	0.16	99.64	FBF
34.006	9,11-Octadecadiynoic acid	C18 H28 O2		276.2096	276.2089	2.36	96.95	FBF
34.058	Delcorine	C26 H41 N O7	52358-55-1	479.2883	479.2883	0.03	99.77	FBF
34.180	PI(12:0/12:0)	C33 H63 O13 P		698.4031	698.4006	3.51	89.87	FBF
34.302	Stearidonic Acid	C18 H28 O2	20290-75-9	276.2102	276.2089	4.63	86.97	FBF
34.459	PI(O-20:0/0:0)	C29 H59 O11 P		614.3821	614.3795	4.31	85.38	FBF
34.459	Synaptolepis factor K1	C36 H54 O8	66268-94-8	614.3821	614.3819	0.43	99.47	FBF
34.669	Brevetoxin B	C50 H70 O14	79580-28-2	894.4769	894.4766	0.35	90.78	FBF
34.721	Dequalinium	C30 H40 N4	522-51-0	456.3242	456.3253	-2.35	97.18	FBF
34.948	Zizyphine A	C33 H49 N5 O6	51059-42-8	611.3674	611.3683	-1.49	97.67	FBF
35.715	Terminaline	C23 H41 N O2	15112-49-9	363.3140	363.3137	0.68	99.06	FBF
35.977	12(S),20-DiHETE	C20 H32 O4	89614-44-8	336.2286	336.2301	-4.32	91.57	FBF
36.117	Lauroyl diethanolamide	C16 H33 N O3	120-40-1	287.2464	287.2460	1.14	98.39	FBF
36.256	Phytosphingosine	C18 H39 N O3	554-62-1	317.2933	317.2930	0.94	98.42	FBF
36.623	Hypromellose	C32 H60 O19		748.3723	748.3729	-0.84	96.10	FBF
37.164	Soyasaponin bg	C54 H84 O21	143519-54-4	1068.5506	1068.5505	0.11	94.23	FBF
37.443	<Dodecylphosphocholine>	C17 H39 N O4 P	29557-51-5	352.2615	352.2617	-0.47	97.35	FBF
37.879	<Etiocholanolone>	C19 H30 O2	53-42-9	290.2248	290.2246	0.83	99.35	FBF
38.647	TR-Saponin B	C52 H80 O20	288153-02-6	1024.5244	1024.5243	0.12	98.77	FBF
38.839	5-Oxoavermectin "1b" aglycone	C33 H44 O8		568.3037	568.3036	0.19	99.04	FBF
39.118	19-Norandrosterone	C18 H28 O2	1225-01-0	276.2097	276.2089	2.71	94.81	FBF
39.484	<19-Norandrosterone>	C18 H28 O2	1225-01-0	276.2098	276.2089	3.22	92.00	FBF
39.519	N-Oleoyl-L-Serine	C21 H39 N O4		369.2881	369.2879	0.56	99.62	FBF
39.763	<γ-Linolenic Acid>	C18 H30 O2	506-26-3	278.2256	278.2246	3.63	91.02	FBF
40.252	Acetyl tributyl citrate	C20 H34 O8	77-90-7	402.2258	402.2254	0.96	98.17	FBF
40.496	<Oxybutynin>	C22 H31 N O3	5633-20-5	357.2309	357.2304	1.54	97.50	FBF
40.618	Corchorusoside A	C35 H54 O14	210637-15-3	698.3495	698.3514	-2.66	95.45	FBF
41.072	Hericenone E	C37 H54 O6	137592-05-3	594.3896	594.3920	-4.12	92.43	FBF
41.124	PE-Cer(d15:1(4E)/18:0)	C35 H71 N2 O6 P		646.5018	646.5050	-4.90	89.80	FBF
41.211	Pipecuronium	C35 H62 N4 O4	68399-58-6	602.4758	602.4771	-2.18	97.59	FBF
41.333	Corchorosol A	C29 H44 O9	23838-13-3	536.2963	536.2985	-4.17	91.92	FBF
41.473	Drotaverine	C24 H31 N O4	985-12-6	397.2255	397.2253	0.58	98.72	FBF
41.473	<AT-56>	C25 H27 N5	162640-98-4	397.2256	397.2266	-2.59	96.77	FBF
41.578	Dicyclohexyl phthalate	C20 H26 O4	84-61-7	330.1834	330.1831	0.95	98.01	FBF
41.735	Tacrolimus	C44 H69 N O12	104987-11-3	803.4818	803.4820	-0.26	98.80	FBF
41.787	Pipecuronium	C35 H62 N4 O4	68399-58-6	602.4760	602.4771	-1.84	98.07	FBF
41.874	Chalcomycin	C35 H56 O14	20283-48-1	700.3648	700.3670	-3.19	95.25	FBF
41.874	Bergamottin	C21 H22 O4	7380-40-7	338.1522	338.1518	1.14	98.95	FBF
42.031	Dequalinium	C30 H40 N4	522-51-0	456.3235	456.3253	-3.85	86.99	FBF
42.066	Tacrolimus	C44 H69 N O12	104987-11-3	803.4819	803.4820	-0.10	99.75	FBF
42.066	rhodexin A	C29 H44 O9		536.2968	536.2985	-3.18	94.41	FBF
42.363	α-Linolenoyl Ethanolamide	C20 H35 N O2	57086-93-8	321.2677	321.2668	2.95	94.08	FBF
42.450	Ganoderic acid Mg	C35 H54 O8	110042-11-0	602.3820	602.3819	0.26	99.63	FBF
42.572	4-Ketonostoxanthin 3-sulfate	C40 H53 Na O8 S		716.3384	716.3359	3.51	89.78	FBF
42.572	Chalcomycin	C35 H56 O14	20283-48-1	700.3652	700.3670	-2.62	95.86	FBF
42.642	5,7-Dihydroxy-4-methylcoumarin	C10 H8 O4	2107-76-8	192.0424	192.0423	0.75	99.36	FBF
42.677	Actinonin	C19 H35 N3 O5	13434-13-4	385.2594	385.2577	4.55	89.89	FBF
42.781	DG(12:0/17:2(9Z,12Z)/0:0)[iso2]	C32 H58 O5		522.4285	522.4284	0.18	99.89	FBF
42.781	Epomusenin A	C37 H66 O3	178330-55-7	558.5027	558.5012	2.75	94.71	FBF
42.781	PC(O-18:0/4:0)	C30 H63 N O7 P		580.4320	580.4342	-3.78	93.24	FBF
42.973	Ganoderol A	C30 H46 O2	104700-97-2	438.3500	438.3498	0.40	97.98	FBF
43.148	8-Azaadenosine	C9 H12 N6 O4	10299-44-2	268.0932	268.0920	4.29	83.42	FBF
43.165	(S)-Nerolidol 3-O-[a-L-Rhamnopyranosyl-(1->4)-a-L-rhamnopyranosyl-(1->2)-b-D-glucopyranoside]	C33 H56 O14		676.3652	676.3670	-2.69	94.87	FBF
43.200	<Doxapram>	C24 H30 N2 O2	309-29-5	378.2312	378.2307	1.33	98.00	FBF
43.270	PA(21:0/0:0)	C24 H49 O7 P		480.3218	480.3216	0.50	89.52	FBF
43.270	EB 1213	C31 H44 O4		480.3219	480.3240	-4.28	90.90	FBF
43.567	8-Azaadenosine	C9 H12 N6 O4	10299-44-2	268.0928	268.0920	2.88	82.46	FBF
43.881	Gingerglycolipid A	C33 H56 O14	145937-22-0	676.3650	676.3670	-2.91	95.53	FBF
43.916	PE(19:0/0:0)	C24 H50 N O7 P		495.3331	495.3325	1.17	99.14	FBF
44.212	PE-Cer(d15:2(4E,6E)/18:0)	C35 H69 N2 O6 P		644.4905	644.4893	1.75	80.92	FBF
44.317	Cholesteryl linolenate	C45 H74 O2		646.5670	646.5689	-2.95	81.65	FBF
44.317	Linoleoyl Ethanolamide	C20 H37 N O2	68171-52-8	323.2835	323.2824	3.37	92.98	FBF
44.614	Progesterone 3-biotin	C38 H59 N5 O5 S		697.4253	697.4237	2.31	91.21	FBF
44.788	Sambutoxin	C28 H39 N O4	160047-56-3	453.2859	453.2879	-4.54	85.99	FBF

RT	Name	Formula	CAS	Mass	Mass (Tgt)	Diff (Tgt, ppm)	Score	Algorithm
44.823	Hovenidulcioside A2	C38 H58 O12	171499-80-2	706.3904	706.3928	-3.44	93.76	FBF
44.823	4-Hydroxy-3-methoxycinnamaldehyde	C10 H10 O3	458-36-6	178.0633	178.0630	1.64	98.11	FBF
44.945	Hydroxysintaxanthin 5,6-epoxide	C31 H42 O3		462.3114	462.3134	-4.42	91.23	FBF
44.945	Pfaffic acid	C29 H44 O3	86432-14-6	440.3299	440.3290	2.03	97.12	FBF
45.276	Homodolichosterone	C29 H48 O5	85797-14-4	476.3503	476.3502	0.34	98.63	FBF
45.521	Palmitoyl Ethanolamide	C18 H37 N O2	544-31-0	299.2830	299.2824	1.89	98.05	FBF
45.591	N-stearoyl serine	C21 H41 N O4		371.3040	371.3036	1.22	98.55	FBF
45.591	MG(18:2(9Z,12Z)/0:0/0:0)[rac]	C21 H38 O4	26545-74-4	354.2774	354.2770	1.08	98.02	FBF
45.608	MG(0:0/20:5(5Z,8Z,11Z,14Z,17Z)/0:0)	C23 H36 O4		376.2599	376.2614	-3.85	89.82	FBF
45.625	1α-hydroxy-22-[3-(1-hydroxy-1-methylethyl)phenyl]-23,24,25,26,27-pentanorvitamin D3 / 1α-hydroxy-22-[3-(1-hydroxy-1-methylethyl)phenyl]-23,24,25,26,27-pentanorcholecalciferol	C31 H44 O3		464.3272	464.3290	-3.89	88.48	FBF
45.625	<Ipecac (Emetine)>	C29 H40 N2 O4	483-18-1	480.3004	480.2988	3.30	91.45	FBF
45.748	Schleicherastatin 3	C29 H50 O3	256445-53-1	446.3762	446.3760	0.41	99.32	FBF
45.800	<Palmitic amide>	C16 H33 N O	629-54-9	255.2566	255.2562	1.41	98.25	FBF
46.236	N-palmitoyl threonine	C20 H39 N O4		357.2882	357.2879	0.70	98.28	FBF
46.323	Oleoyl Ethanolamide	C20 H39 N O2	111-58-0	325.2983	325.2981	0.72	98.72	FBF
46.341	<9-deoxy-9-methylene-16,16-dimethyl -PGE2>	C23 H38 O4	61263-35-2	378.2782	378.2770	3.05	93.63	FBF
46.568	<Dodemorph>	C18 H35 N O	1593-77-7	281.2725	281.2719	2.22	97.24	FBF
46.882	PS(12:0/15:0)	C33 H64 N O10 P		665.4273	665.4268	0.77	99.20	FBF
46.899	Voacamine	C43 H52 N4 O5	3371-85-5	704.3961	704.3938	3.36	90.07	FBF
46.951	R.g.-Keto III	C42 H60 O4		628.4490	628.4492	-0.23	81.35	FBF
46.986	Armillatin	C38 H58 O6	139051-17-5	610.4211	610.4233	-3.72	93.77	FBF
47.091	Kni 102	C31 H41 N5 O7	139694-65-8	595.3026	595.3006	3.38	89.55	FBF
47.161	Armillatin	C38 H58 O6	139051-17-5	610.4214	610.4233	-3.17	94.52	FBF
47.196	Cyclopassifloic acid B	C31 H52 O6	292167-35-2	520.3777	520.3764	2.52	82.37	FBF
47.300	Chondrillasterol 3-[glucosyl-(1->4)-glucoside]	C41 H68 O11		736.4743	736.4762	-2.53	91.86	FBF
47.318	Goyaglycoside a	C37 H60 O9	333332-41-5	648.4215	648.4237	-3.52	87.45	FBF
47.318	PC(P-16:0/13:0)	C37 H75 N O7 P		676.5291	676.5281	1.39	84.26	FBF
47.527	DG(13:0/18:3(9Z,12Z,15Z)/0:0)[iso2]	C34 H60 O5		548.4441	548.4441	0.11	99.58	FBF
47.719	PE(21:0/0:0)	C26 H54 N O7 P		523.3643	523.3638	1.03	99.20	FBF
47.754	Cucurbitacin P	C30 H48 O7		520.3379	520.3400	-4.02	91.07	FBF
47.824	3alpha-Acetomethoxy-11alpha-oxo-12-ursen-24-oic acid	C33 H50 O5	17019-95-3	526.3663	526.3658	0.97	99.40	FBF
47.824	PG(22:1(11Z)/0:0)	C28 H55 O9 P		566.3585	566.3584	0.19	95.07	FBF
48.103	Hovenidulcigenin B	C32 H50 O7	182173-50-8	546.3534	546.3557	-4.11	91.63	FBF
48.312	N-stearoyl valine	C23 H45 N O3		383.3402	383.3399	0.76	99.51	FBF
48.469	Ganoderiol I	C31 H50 O5	114567-49-6	502.3635	502.3658	-4.65	81.84	FBF
48.556	Vitamin D3 glucosiduronate	C33 H52 O7		560.3715	560.3713	0.29	95.49	FBF
48.556	Phorbol 12-tiglate 13-decanoate	C35 H52 O8	59086-92-9	600.3639	600.3662	-3.87	93.30	FBF
48.609	24,24-difluoro-1α,25-dihydroxy-26,27-dimethyl-24a-homovitamin D3 / 24,24-difluoro-1α,25-dihydroxy-26,27-dimethyl-24a-homocholecalciferol	C30 H48 F2 O3		494.3582	494.3572	2.12	96.88	FBF
48.731	<Stearamide>	C18 H37 N O	124-26-5	283.2877	283.2875	0.62	99.50	FBF
48.783	28-Homobrassinolide	C29 H50 O6	80483-89-2	494.3583	494.3607	-4.97	87.30	FBF
49.028	PG(20:1(11Z)/0:0)	C26 H51 O9 P		538.3271	538.3271	-0.02	90.74	FBF
49.062	MG(22:4(7Z,10Z,13Z,16Z)/0:0/0:0)	C25 H42 O4		406.3096	406.3083	3.18	93.48	FBF
49.062	Spirolide B	C42 H63 N O7	170713-72-1	693.4584	693.4605	-2.94	87.75	FBF
49.272	Pheophorbide a	C35 H36 N4 O5	15664-29-6	592.2687	592.2686	0.22	99.83	FBF
49.307	GlcCer(d15:2(4E,6E)/18:0)	C39 H73 N O8		683.5339	683.5336	0.38	99.84	FBF
49.394	LysoPE(18:2(9Z,12Z)/0:0)	C23 H44 N O7 P		477.2857	477.2855	0.38	99.28	FBF
49.429	Boviquinone 4	C26 H36 O4	28129-52-4	412.2596	412.2614	-4.24	92.11	FBF
49.429	Aspidoalbine	C24 H32 N2 O5	2122-25-0	428.2330	428.2311	4.44	80.66	FBF
49.429	<Irbesartan>	C25 H28 N6 O	138402-11-6	428.2331	428.2325	1.50	87.27	FBF
49.656	Di(2-ethylhexyl) adipate	C22 H42 O4	103-23-1	370.3089	370.3083	1.59	97.85	FBF
49.708	Ecalcidene	C29 H45 N O3		455.3400	455.3399	0.23	99.48	FBF
50.092	adenosylhopane	C40 H63 N5 O3		661.4912	661.4931	-2.84	90.31	FBF
50.179	MGDG(18:3(9Z,12Z,15Z)/18:4(6Z,9Z,12Z,15Z))	C45 H72 O10		772.5127	772.5125	0.22	99.42	FBF
50.179	C18 Sulfatide	C42 H81 N O11 S		807.5500	807.5530	-3.81	90.22	FBF
50.633	2-methyl-1,3-Cyclohexanedione	C7 H10 O2	1193-55-1	126.0683	126.0681	1.51	98.69	FBF
50.633	Diisononyl phthalate	C26 H42 O4	28553-12-0	418.3064	418.3083	-4.56	90.69	FBF
50.633	(10S)-Juvenile hormone III diol	C16 H28 O4		284.1990	284.1988	0.83	99.34	FBF
50.633	2,6-Dihydroxy-4-methoxytoluene	C8 H10 O3	6307-89-7	154.0632	154.0630	1.66	98.21	FBF
51.017	DG(14:1(9Z)/17:2(9Z,12Z)/0:0)[iso2]	C34 H60 O5		548.4442	548.4441	0.31	99.71	FBF
51.174	DG(13:0/20:5(5Z,8Z,11Z,14Z,17Z)/0:0)[iso2]	C36 H60 O5		572.4443	572.4441	0.41	99.44	FBF
51.174	<U-74389G>	C37 H50 N6 O2	153190-29-5	610.3999	610.3995	0.60	93.77	FBF
51.174	bacteriohopane-32,33, 34-triol-35-carbamate	C36 H63 N O5		589.4710	589.4706	0.57	99.72	FBF
51.208	Elaidylphosphocholine	C23 H49 N O4 P	156161-89-6	434.3407	434.3399	1.77	97.58	FBF
51.296	PS(P-18:0/22:6(4Z,7Z,10Z,13Z,16Z,19Z))	C46 H78 N O9 P		819.5394	819.5414	-2.48	96.93	FBF
51.435	PS(O-16:0/20:3(8Z,11Z,14Z))	C42 H78 N O9 P		771.5414	771.5414	-0.02	94.89	FBF
51.645	Z-Arg-Arg-NHMec	C30 H39 N9 O6	88937-61-5	621.3032	621.3023	1.43	81.74	FBF
51.732	<Oligomycin A>	C45 H74 O11	579-13-5	790.5203	790.5231	-3.58	89.37	FBF
51.802	(3beta,22R,23R,24S)-3,22,23-Trihydroxystigmastan-6-one	C29 H50 O4	90524-90-6	462.3714	462.3709	1.16	99.00	FBF
51.941	Mayolene-16	C34 H60 O4		532.4495	532.4492	0.70	98.94	FBF
52.081	PS(P-20:0/16:1(9Z))	C42 H80 N O9 P		773.5579	773.5571	1.13	99.19	FBF

RT	Name	Formula	CAS	Mass	Mass (Tgt)	Diff (Tgt, ppm)	Score	Algorithm
52.081	Neoannonin B	C37 H66 O6	170312-94-4	606.4863	606.4859	0.63	99.28	FBF
52.168	Ergosterol acetate	C32 H50 O2	2418-45-3	466.3788	466.3811	-4.82	86.97	FBF
52.203	DG(13:0/18:2(9Z,12Z)/0:0)[iso2]	C34 H62 O5		550.4600	550.4597	0.42	98.34	FBF
52.552	7-methyl-cis-8,9-Epoxy-tricosane	C23 H46 O		338.3553	338.3549	1.34	98.65	FBF
52.552	9,10-Tricosadiene	C23 H44		320.3446	320.3443	1.01	99.32	FBF
52.988	DG(15:0/14:0/0:0)	C32 H62 O5		526.4600	526.4597	0.45	99.68	FBF
53.040	PG(P-16:0/12:0)	C34 H67 O9 P		650.4520	650.4523	-0.45	96.36	FBF
53.110	Caffeoylcycloartenol	C39 H56 O4	75884-34-3	588.4154	588.4179	-4.25	86.91	FBF
53.180	24-Norursa-3,12-diene	C29 H46		394.3602	394.3600	0.73	99.17	FBF
53.320	Caffeoylcycloartenol	C39 H56 O4	75884-34-3	588.4156	588.4179	-3.88	87.37	FBF
53.354	DG(14:0/15:0/0:0)	C32 H62 O5		526.4599	526.4597	0.26	98.29	FBF
53.424	Araliacerebroside	C40 H77 N O10		731.5550	731.5547	0.35	95.95	FBF
53.477	DG(15:1(9Z)/20:5(5Z,8Z,11Z,14Z,17Z)/0:0)[iso2]	C38 H62 O5		598.4582	598.4597	-2.47	81.30	FBF
53.477	SM(d18:0/12:0)	C35 H74 N2 O6 P		649.5282	649.5284	-0.43	97.63	FBF
53.599	DG(12:0/22:4(7Z,10Z,13Z,16Z)/0:0)[iso2]	C37 H64 O5		588.4751	588.4754	-0.51	95.40	FBF
53.983	Stigmastan-3,5-diene	C29 H48		396.3758	396.3756	0.49	99.62	FBF
53.983	Hericenone D	C37 H58 O6	137592-04-2	598.4211	598.4233	-3.73	93.60	FBF
54.262	Soyacerebroside II	C40 H75 N O9	115074-93-6	713.5449	713.5442	0.97	98.71	FBF
54.262	<Thioetheramide-PC>	C40 H84 N2 O5 P S	116457-99-9	735.5849	735.5839	1.35	83.83	FBF
54.279	Cer(d16:1(4E)/18:1(9Z)(2OH))	C34 H65 N O4		551.4913	551.4914	-0.05	97.11	FBF
54.384	PG(O-16:0/12:0)	C34 H69 O9 P		652.4683	652.4679	0.58	97.90	FBF
54.401	PE(17:0/22:6(4Z,7Z,10Z,13Z,16Z,19Z))	C44 H76 N O8 P		777.5313	777.5309	0.57	99.62	FBF
54.506	Megalomicin C1	C48 H84 N2 O17		960.5791	960.5770	2.18	96.27	FBF
54.576	Soyacerebroside II	C40 H75 N O9	115074-93-6	713.5443	713.5442	0.16	98.54	FBF
54.576	N-(3-(15-methyl-hexadecanoyloxy)-13-methyl-tetradecanoyl)-L-serine	C37 H71 N O6		625.5282	625.5281	0.04	99.62	FBF
54.628	SM(d18:1/12:0)	C35 H72 N2 O6 P		647.5125	647.5128	-0.53	98.35	FBF
54.785	PI(22:1(11Z)/21:0)	C52 H99 O13 P		962.6820	962.6823	-0.31	96.95	FBF
54.925	PI(17:0/14:0)	C40 H77 O13 P		796.5108	796.5102	0.81	98.21	FBF
54.925	MGDG(20:5(5Z,8Z,11Z,14Z,17Z)/18:4(6Z,9Z,12Z,15Z))	C47 H72 O10		796.5108	796.5125	-2.18	97.09	FBF
55.274	PA(P-16:0/12:0)	C31 H61 O7 P		576.4157	576.4155	0.31	98.91	FBF
55.343	DG(14:0/20:4(5Z,8Z,11Z,14Z)/0:0)	C37 H64 O5		588.4752	588.4754	-0.24	96.89	FBF
55.396	Aragusteroketal	C31 H54 O4		490.4024	490.4022	0.32	99.70	FBF
55.413	PC(O-20:0/22:6(4Z,7Z,10Z,13Z,16Z,19Z))	C50 H91 N O7 P		848.6532	848.6533	-0.18	98.22	FBF
55.902	Rollidecin D	C37 H66 O6	200703-17-9	606.4862	606.4859	0.47	98.77	FBF
55.919	Montecristin	C37 H66 O4	185336-15-6	574.4950	574.4961	-1.90	95.44	FBF
55.937	Cer(d14:1(4E)/20:1(11Z)(2OH))	C34 H65 N O4		551.4919	551.4914	0.92	99.39	FBF
55.937	Cer(d14:2(4E,6E)/20:1(11Z))	C34 H63 N O3		533.4812	533.4808	0.68	99.65	FBF
56.094	1,2-Di-(9Z,12Z,15Z-octadecatrienoyl)-3-(Galactosyl-alpha-1-6-Galactosyl-beta-1)-glycerol	C51 H84 O15		936.5791	936.5810	-2.10	97.69	FBF
56.181	PE(22:4(7Z,10Z,13Z,16Z)/17:1(9Z))	C44 H78 N O8 P		779.5480	779.5465	1.86	97.53	FBF
56.303	Cer(d14:2(4E,6E)/20:0(2OH))	C34 H65 N O4		551.4916	551.4914	0.43	97.71	FBF
56.477	7Z-Tricosen-11-one	C23 H44 O		336.3397	336.3392	1.32	97.69	FBF
56.896	MGDG(20:5(5Z,8Z,11Z,14Z,17Z)/18:3(9Z,12Z,15Z))	C47 H74 O10		798.5260	798.5282	-2.69	96.44	FBF
57.158	20:3-Glc-Campesterol	C54 H90 O7		850.6686	850.6687	-0.02	95.67	FBF
57.158	PC(P-20:0/22:4(7Z,10Z,13Z,16Z))	C50 H93 N O7 P		850.6687	850.6690	-0.36	95.66	FBF
57.385	PA(O-16:0/12:0)	C31 H63 O7 P		578.4311	578.4311	-0.09	94.43	FBF
57.385	2-Hexaprenyl-3-methyl-5-hydroxy-6-methoxy-1,4-benzoquinol	C38 H58 O4		578.4311	578.4335	-4.22	91.87	FBF
58.275	PE(22:2(13Z,16Z)/15:1(9Z))	C42 H78 N O8 P		755.5480	755.5465	1.93	97.40	FBF
58.507	PE(22:4(7Z,10Z,13Z,16Z)/17:0)	C44 H80 N O8 P		781.5639	781.5622	2.29	96.63	FBF
58.571	PE(19:1(9Z)/22:6(4Z,7Z,10Z,13Z,16Z,19Z))	C46 H78 N O8 P		803.5445	803.5465	-2.48	96.85	FBF
59.129	DG(18:4(6Z,9Z,12Z,15Z)/20:5(5Z,8Z,11Z,14Z,17Z)/0:0)	C41 H62 O5		634.4575	634.4597	-3.43	94.67	FBF
59.409	18:1-Glc-Campesterol	C52 H90 O7		826.6680	826.6687	-0.76	97.23	FBF
59.548	cholest-5,24-dien-3beta-ol 3-O-beta-D-glucopyranoside	C33 H54 O6		546.3921	546.3920	0.10	99.54	FBF
59.548	endo-1,4-beta-Xylanase	C34 H50 N4 O2	9025-57-4	546.3922	546.3934	-2.20	97.55	FBF
60.421	Cer(d14:2(4E,6E)/22:0(2OH))	C36 H69 N O4		579.5227	579.5227	0.09	99.48	FBF
61.246	1-Palmitoyl-2-linoleoyl PE	C39 H74 N O8 P	26662-95-3	715.5154	715.5152	0.24	99.89	FBF
61.293	C16-OH Sulfatide	C40 H77 N O12 S		795.5182	795.5166	1.93	97.35	FBF
61.328	PE(19:1(9Z)/20:4(5Z,8Z,11Z,14Z))	C44 H78 N O8 P		779.5445	779.5465	-2.53	96.20	FBF
62.218	PE(22:1(11Z)/17:2(9Z,12Z))	C44 H82 N O8 P		783.5783	783.5778	0.60	98.64	FBF
62.566	MGDG(18:2(9Z,12Z)/18:3(9Z,12Z,15Z))	C45 H76 O10		776.5417	776.5438	-2.77	94.65	FBF
62.601	PA(P-16:0/18:4(6Z,9Z,12Z,15Z))	C37 H65 O7 P		652.4467	652.4468	-0.20	94.83	FBF
62.619	DG(20:4(8Z,11Z,14Z,17Z)/18:4(6Z,9Z,12Z,15Z)/0:0)	C41 H64 O5		636.4733	636.4754	-3.26	94.71	FBF
62.671	alpha-Tochopheryl acetate	C31 H52 O3	52225-20-4	472.3917	472.3916	0.01	99.27	FBF
63.421	PC(P-18:0/17:2(9Z,12Z))	C43 H83 N O7 P		756.5878	756.5907	-3.90	91.32	FBF

Compound Details

Cpd 8: DL-pipecolic acid

Name	Formula	RT	RI	Mass	Diff (Tgt, ppm)	CAS	ID Source	Score	Algorithm
DL-pipecolic acid	C6 H11 N O2	1.035		129.0791	0.72	535-75-1	M-FBF-FragConfirm	99.59	FBF

Compound Screening Report

Species	m/z	Score (Tgt)	Score (Lib)	Score (DB)	Score (MFG)	Score (RT)
(M+H)+	130.0863	99.59				

Compound Chromatograms (overlaid)

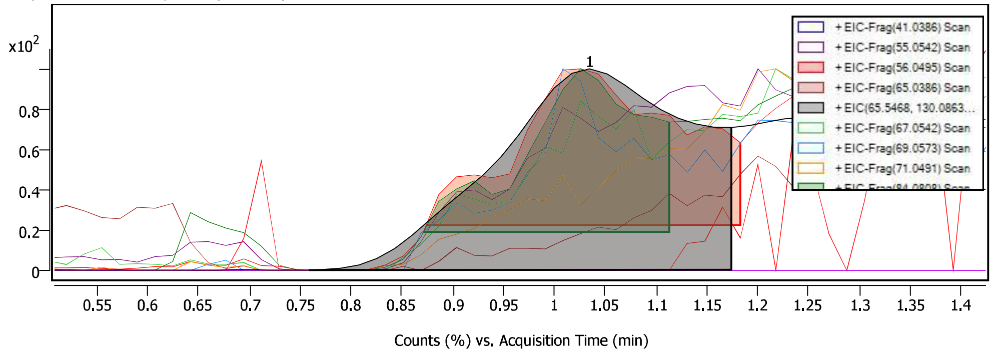

Structure

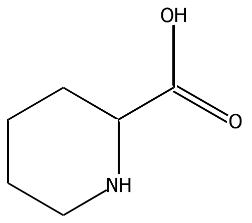

Coelution Plot

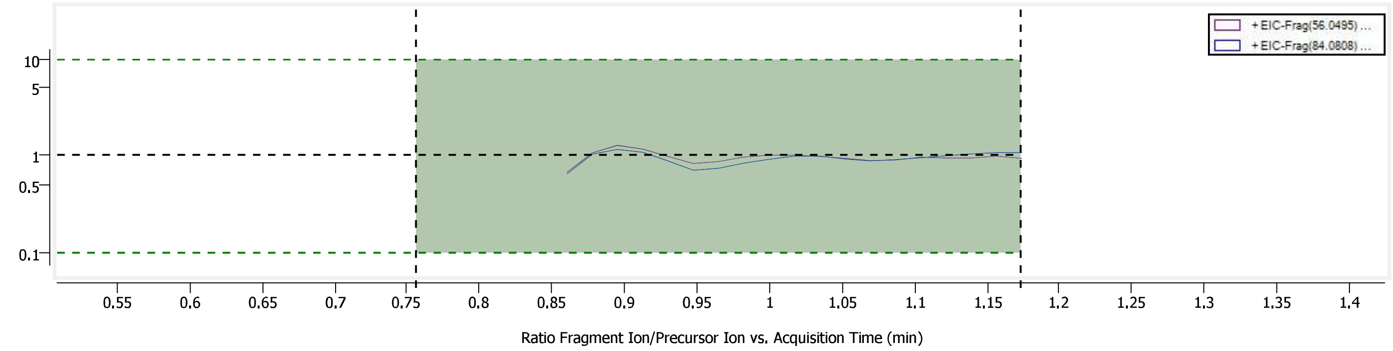

Compound Spectra (overlaid)

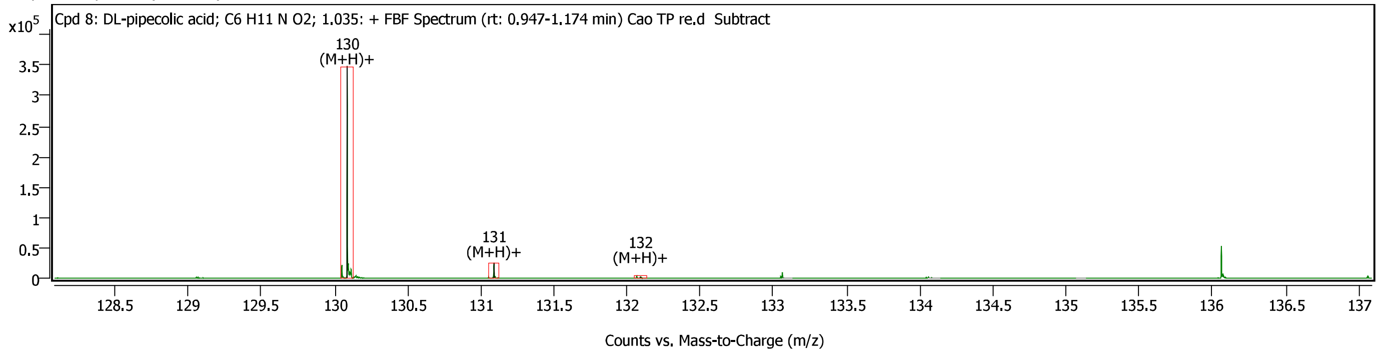

Fragment Spectrum (clean)

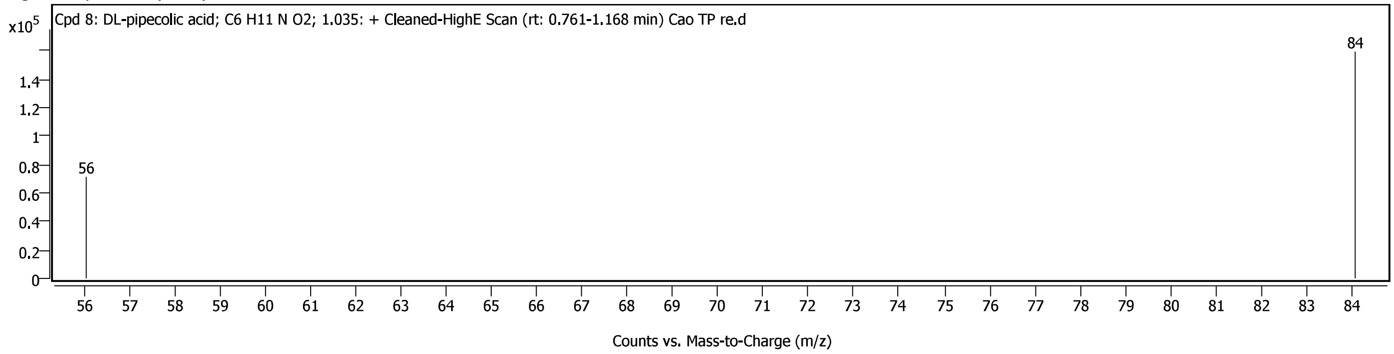

Compound Screening Report

Fragment Spectrum (raw)

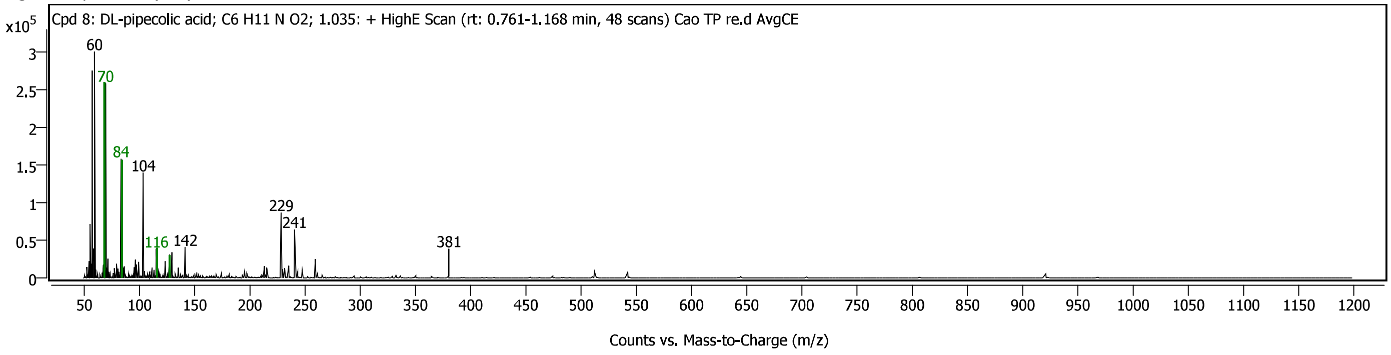

Compound ID Table

Name	Formula	Species	RT	RT Diff	Mass	CAS	ID Source	Score	Score (Lib)	Score (Tgt)
DL-pipecolic acid	C6 H11 N O2	(M+H)+	1.035		129.0791	535-75-1	FBF-FragConfirm	99.59		99.59
2,4-Dimethyl-2-oxazoline-4-methanol	C6 H11 N O2	(M+H)+	1.035		129.0791		FBF-FragConfirm	99.59		99.59
2-Pyrrolidineacetic acid	C6 H11 N O2	(M+H)+	1.035		129.0791		FBF-FragConfirm	99.59		99.59
3-acetamidobutanal	C6 H11 N O2	(M+H)+	1.035		129.0791		FBF-FragConfirm	99.59		99.59
Cycloleucine	C6 H11 N O2	(M+H)+	1.035		129.0791	52-52-8	FBF-FragConfirm	99.59		99.59
D-Pipecolic acid	C6 H11 N O2	(M+H)+	1.035		129.0791	1723-00-8	FBF-FragConfirm	99.59		99.59
L-Pipecolic acid	C6 H11 N O2	(M+H)+	1.035		129.0791	3105-95-1	FBF-FragConfirm	99.59		99.59
L-trans-4-Methyl-2-pyrrolidinecarboxylic acid	C6 H11 N O2	(M+H)+	1.035		129.0791	23009-50-9	FBF-FragConfirm	99.59		99.59
N4-Acetylaminobutanal	C6 H11 N O2	(M+H)+	1.035		129.0791		FBF-FragConfirm	99.59		99.59
Nipecotic acid	C6 H11 N O2	(M+H)+	1.035		129.0791	498-95-3	FBF-FragConfirm	99.59		99.59
trans-3-Aminocyclopentane-1-carboxylic acid	C6 H11 N O2	(M+H)+	1.035		129.0791	57376-72-4	FBF-FragConfirm	99.59		99.59
Vigabatrin	C6 H11 N O2	(M+H)+	1.035		129.0791	68506-86-5	FBF-FragConfirm	99.59		99.59

Cpd 1393: <Nicotinic acid>

Name	Formula	RT	RI	Mass	Diff (Tgt, ppm)	CAS	ID Source	Score	Algorithm
<Nicotinic acid>	C6 H5 N O2	1.174		123.0322	1.25	59-67-6	M-FBF	98.22	FBF

Species	m/z	Score (Tgt)	Score (Lib)	Score (DB)	Score (MFG)	Score (RT)
(M+H)+	124	98.22				

Compound Chromatograms (overlaid)

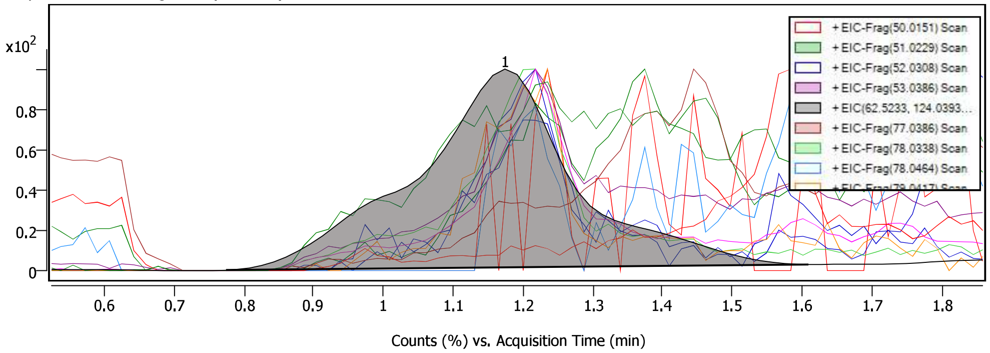

Structure

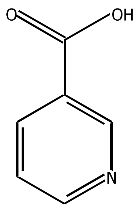

Coelution Plot

Compound Spectra (overlaid)

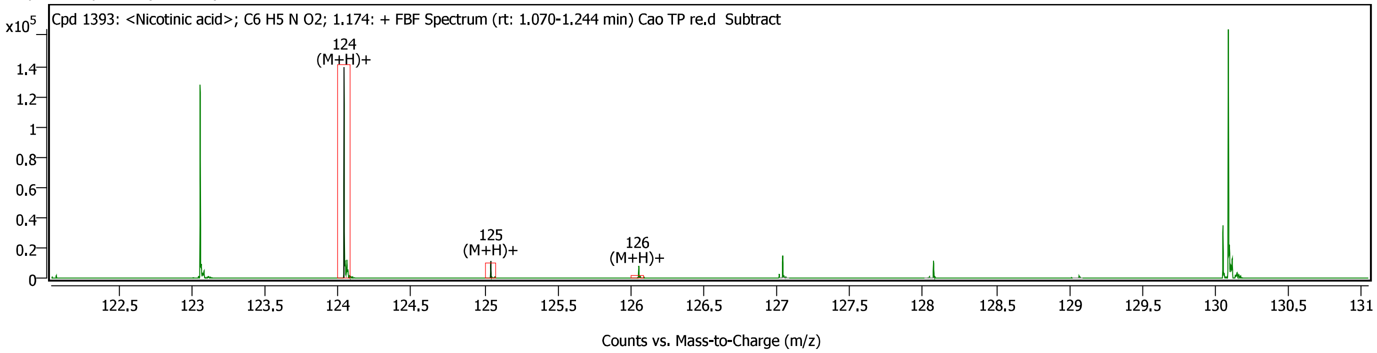

Compound Screening Report

Fragment Spectrum (raw)

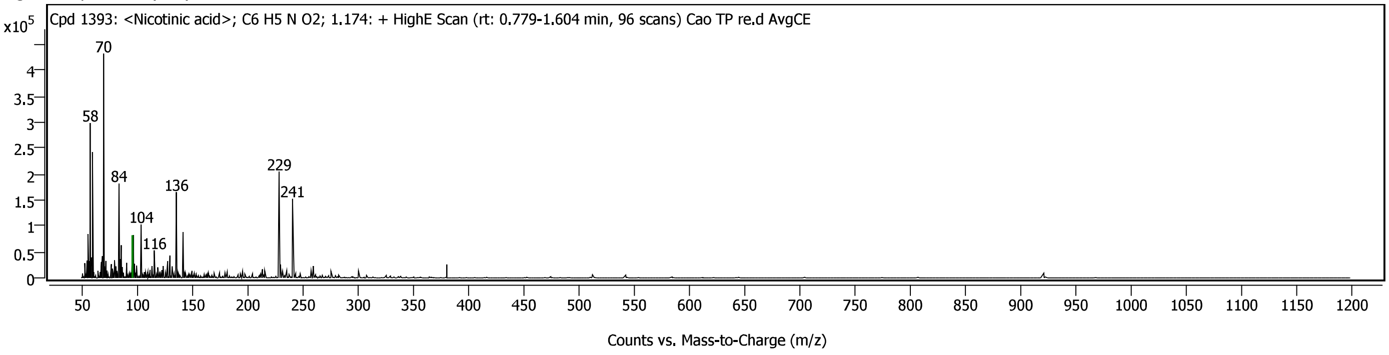

Compound ID Table

Name	Formula	Species	RT	RT Diff	Mass	CAS	ID Source	Score	Score (Lib)	Score (Tgt)
<Nicotinic acid>	C6 H5 N O2	(M+H)+	1.174		123.0322	59-67-6	FBF	98.22		98.22
<Picolinic acid>	C6 H5 N O2	(M+H)+	1.174		123.0322	14639-25-9	FBF	98.22		98.22
<Nitrobenzene>	C6 H5 N O2	(M+H)+	1.174		123.0322	98-95-3	FBF	98.22		98.22
<Isonicotinic acid>	C6 H5 N O2	(M+H)+	1.174		123.0322	55-22-1	FBF	98.22		98.22
<2-Hydroxy-4-imino-2,5-cyclohexadienone>	C6 H5 N O2	(M+H)+	1.174		123.0322	74331-93-4	FBF	98.22		98.22

Cpd 1584: <1-Aminocyclobutane carboxylic acid>

Name	Formula	RT	RI	Mass	Diff (Tgt, ppm)	CAS	ID Source	Score	Algorithm
<1-Aminocyclobutane carboxylic acid>	C5 H9 N O2	1.174		115.0635	1.06	22264-50-2	M-FBF	98.72	FBF

Species	m/z	Score (Tgt)	Score (Lib)	Score (DB)	Score (MFG)	Score (RT)
(M+H)+	116	98.72				

Compound Chromatograms (overlaid)

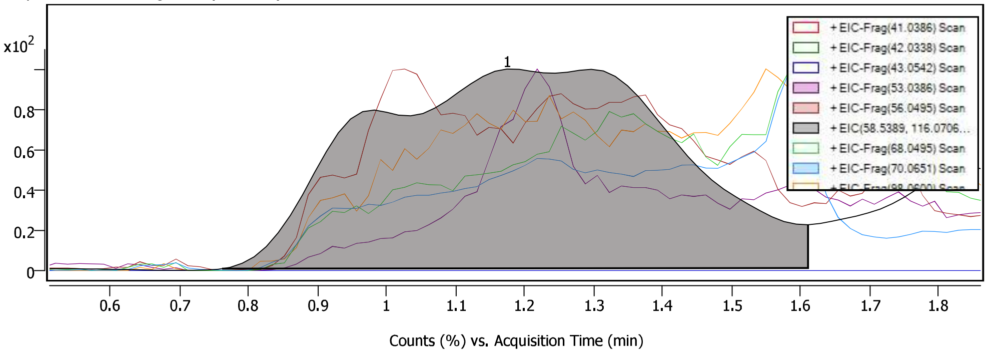

Structure

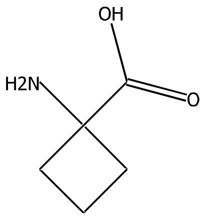

Coelution Plot

Compound Spectra (overlaid)

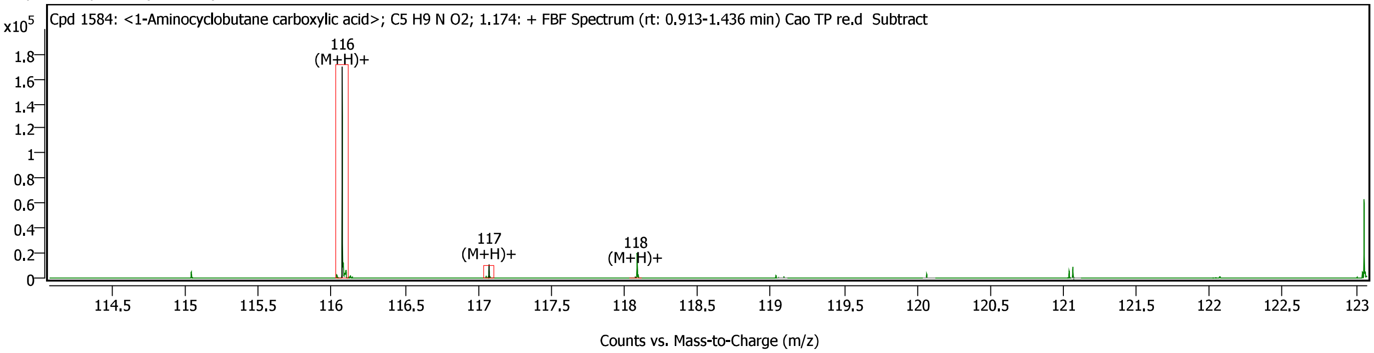

Fragment Spectrum (raw)

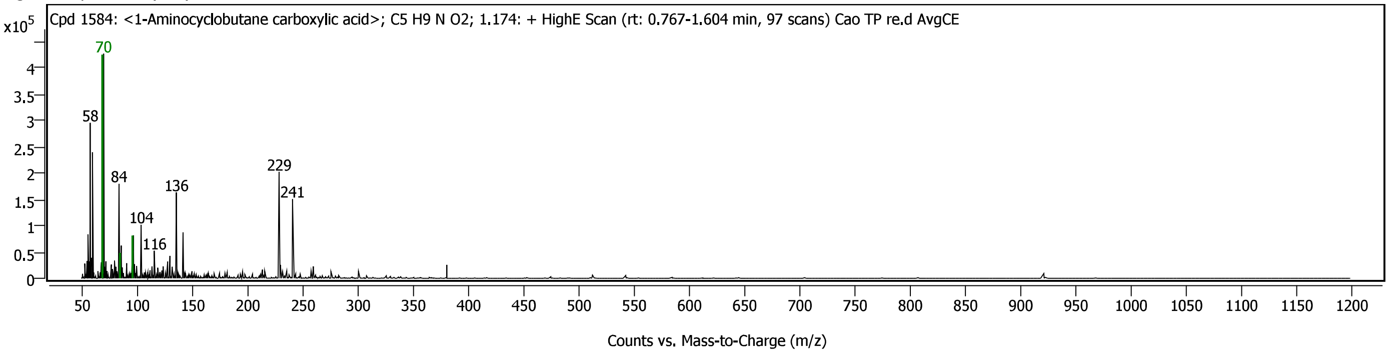

Compound ID Table

Name	Formula	Species	RT	RT Diff	Mass	CAS	ID Source	Score	Score (Lib)	Score (Tgt)
<1-Aminocyclobutane carboxylic acid>	C5 H9 N O2	(M+H)+	1.174		115.0635	22264-50-2	FBF	98.72		98.72
<Pterolactam>	C5 H9 N O2	(M+H)+	1.174		115.0635	38072-88-7	FBF	98.72		98.72
<L-Proline>	C5 H9 N O2	(M+H)+	1.174		115.0635	147-85-3	FBF	98.72		98.72
<D-Proline>	C5 H9 N O2	(M+H)+	1.174		115.0635	344-25-2	FBF	98.72		98.72
<4-Amino-2-methylenebutanoic acid>	C5 H9 N O2	(M+H)+	1.174		115.0635	65370-67-4	FBF	98.72		98.72
<3-Acetamidopropanal>	C5 H9 N O2	(M+H)+	1.174		115.0635		FBF	98.72		98.72

Cpd 1415: <Pirbuterol>

Name	Formula	RT	RI	Mass	Diff (Tgt, ppm)	CAS	ID Source	Score	Algorithm
<Pirbuterol>	C12 H20 N2 O3	1.244		240.1479	2.18	38677-81-5	FBF	97.67	FBF

Species	m/z	Score (Tgt)	Score (Lib)	Score (DB)	Score (MFG)	Score (RT)
(M+H)+	241	97.67				

Compound Chromatograms (overlaid)

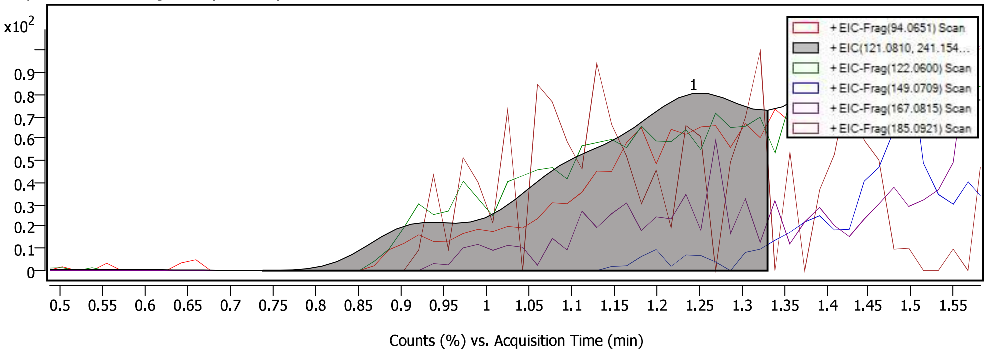

Structure

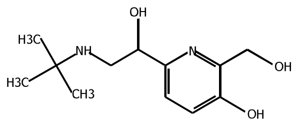

Coelution Plot

Compound Spectra (overlaid)

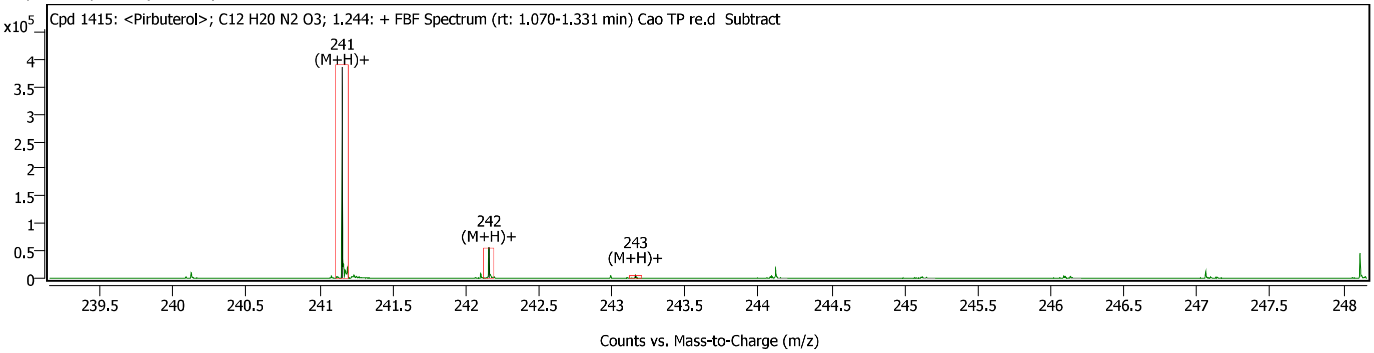

Compound Screening Report

Fragment Spectrum (raw)

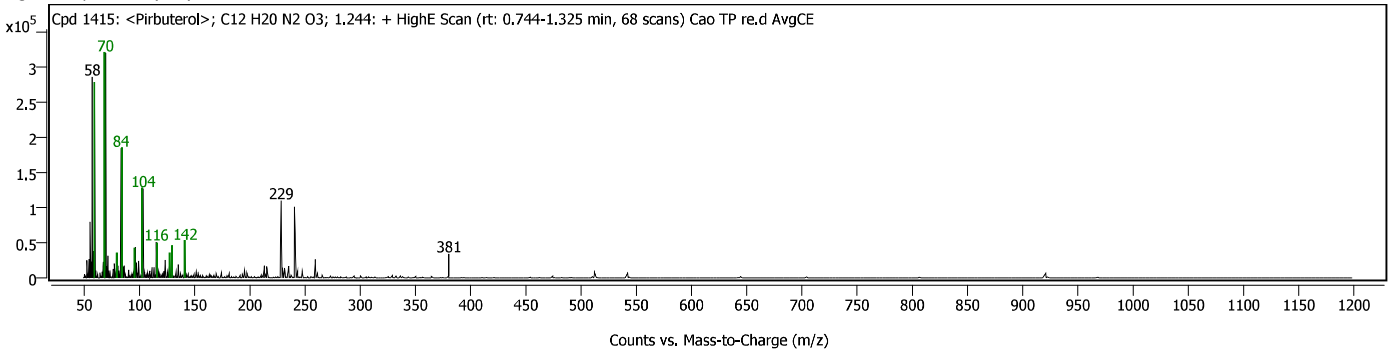

Compound ID Table

Name	Formula	Species	RT	RT Diff	Mass	CAS	ID Source	Score	Score (Lib)	Score (Tgt)
<Pirbuterol>	C12 H20 N2 O3	(M+H)+	1.244		240.1479	38677-81-5	FBF	97.67		97.67

Cpd 215: <Nitisinone>

Name	Formula	RT	RI	Mass	Diff (Tgt, ppm)	CAS	ID Source	Score	Algorithm
<Nitisinone>	C14 H10 F3 N O5	1.314		329.0518	2.20	104206-65-7	FBF	83.20	FBF

Species	m/z	Score (Tgt)	Score (Lib)	Score (DB)	Score (MFG)	Score (RT)
(M+H)+	330	83.20				

Compound Chromatograms (overlaid)

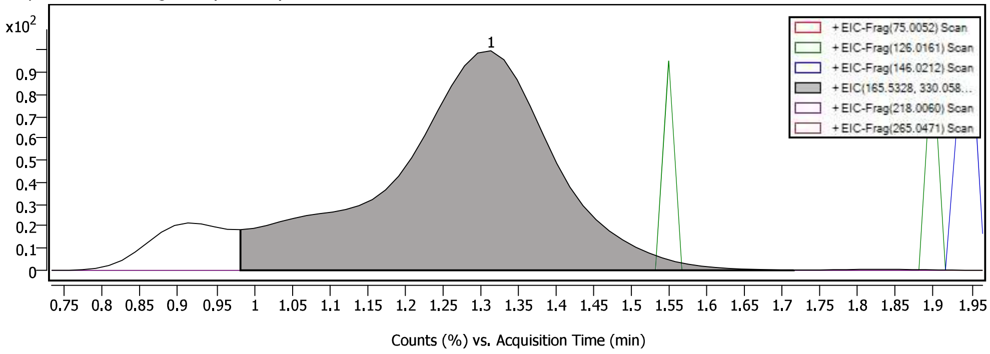

Structure

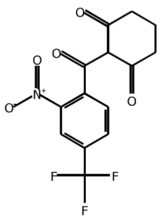

Coelution Plot

Compound Spectra (overlaid)

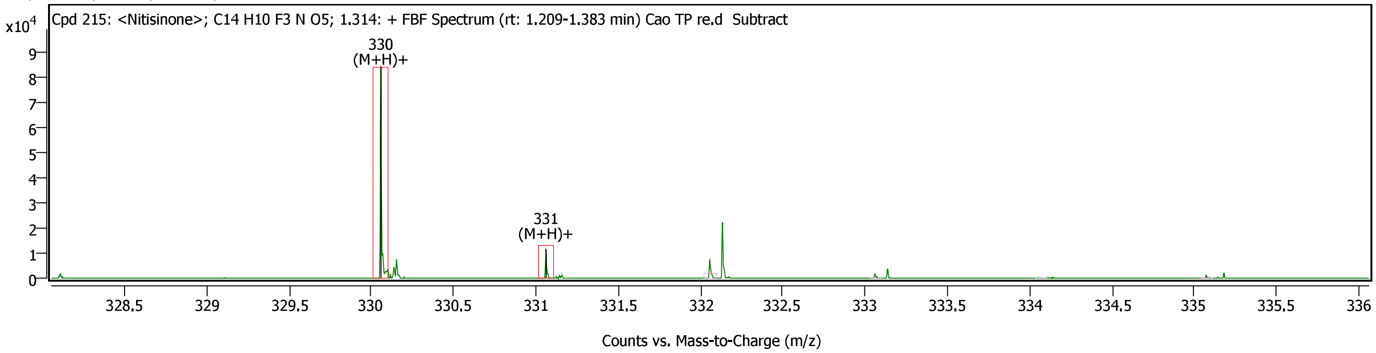

Fragment Spectrum (raw)

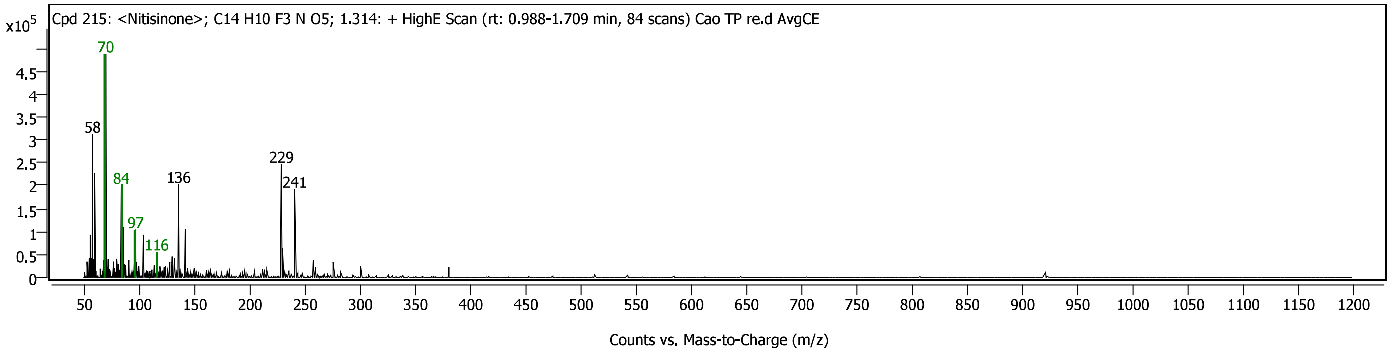

Compound Screening Report

Compound ID Table

Name	Formula	Species	RT	RT Diff	Mass	CAS	ID Source	Score	Score (Lib)	Score (Tgt)
<Nitisinone>	C14 H10 F3 N O5	(M+H)+	1.314		329.0518	104206-65-7	FBF	83.20		83.20

Cpd 467: <Mecarbam>

Name	Formula	RT	RI	Mass	Diff (Tgt, ppm)	CAS	ID Source	Score	Algorithm
<Mecarbam>	C10 H20 N O5 P S2	1.314		329.0520	-0.10	2595-54-2	FBF	96.47	FBF

Species	m/z	Score (Tgt)	Score (Lib)	Score (DB)	Score (MFG)	Score (RT)
(M+H)+	330	96.47				

Compound Chromatograms (overlaid)

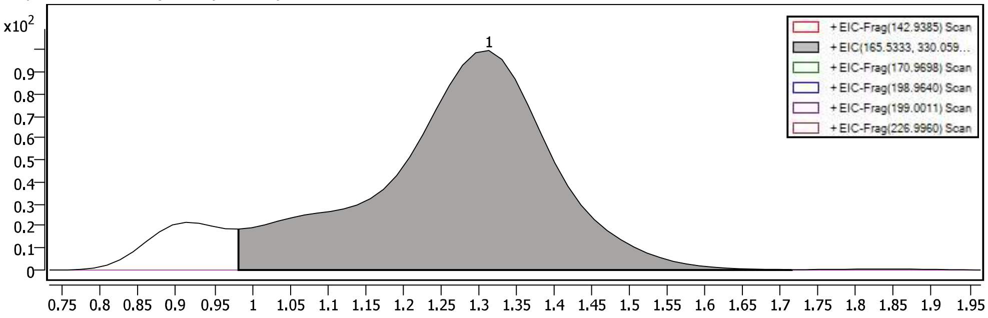

Structure

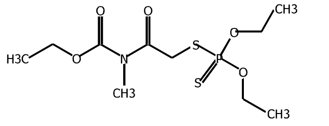

Coelution Plot

Compound Spectra (overlaid)

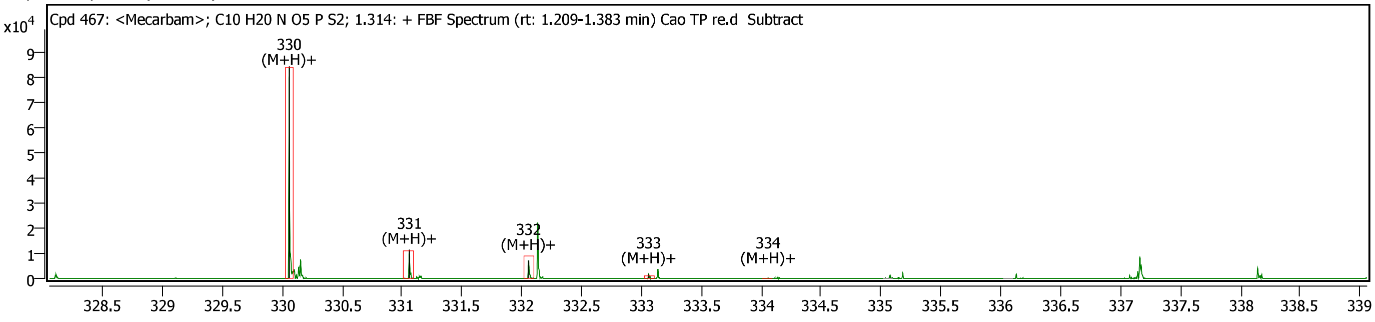

Fragment Spectrum (raw)

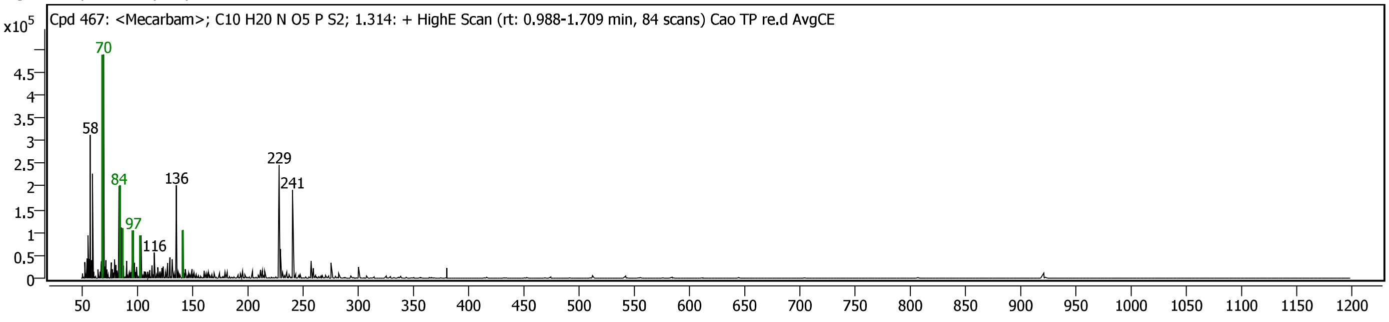

Compound ID Table

Name	Formula	Species	RT	RT Diff	Mass	CAS	ID Source	Score	Score (Lib)	Score (Tgt)
<Mecarbam>	C10 H20 N O5 P S2	(M+H)+	1.314		329.0520	2595-54-2	FBF	96.47		96.47

Cpd 218: <Ribothymidine>

Name	Formula	RT	RI	Mass	Diff (Tgt, ppm)	CAS	ID Source	Score	Algorithm
<Ribothymidine>	C10 H14 N2 O6	1.366		258.0857	1.89	1463-10-1	M-FBF	85.49	FBF

Species	m/z	Score (Tgt)	Score (Lib)	Score (DB)	Score (MFG)	Score (RT)
(M+2H)+2 (M+H)+	130 259	85.49				

Compound Screening Report

Compound Chromatograms (overlaid)

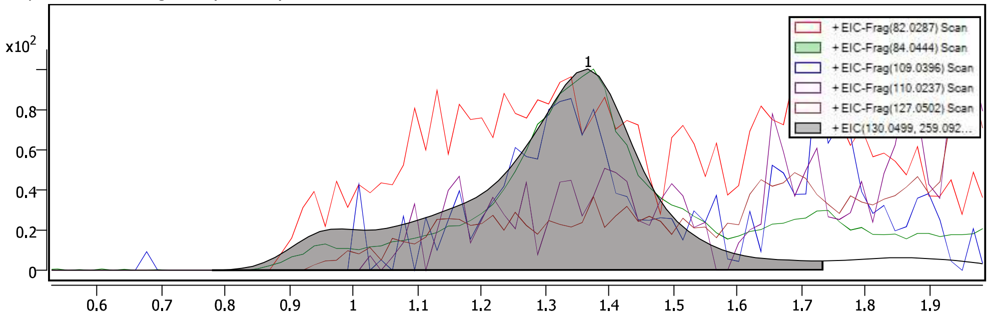

Structure

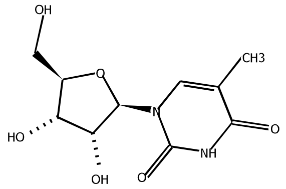

Coelution Plot

Compound Spectra (overlaid)

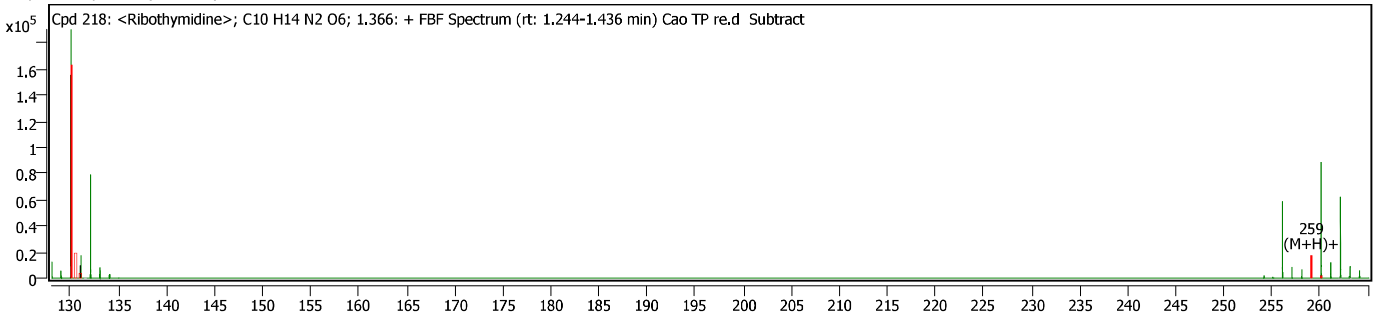

Fragment Spectrum (raw)

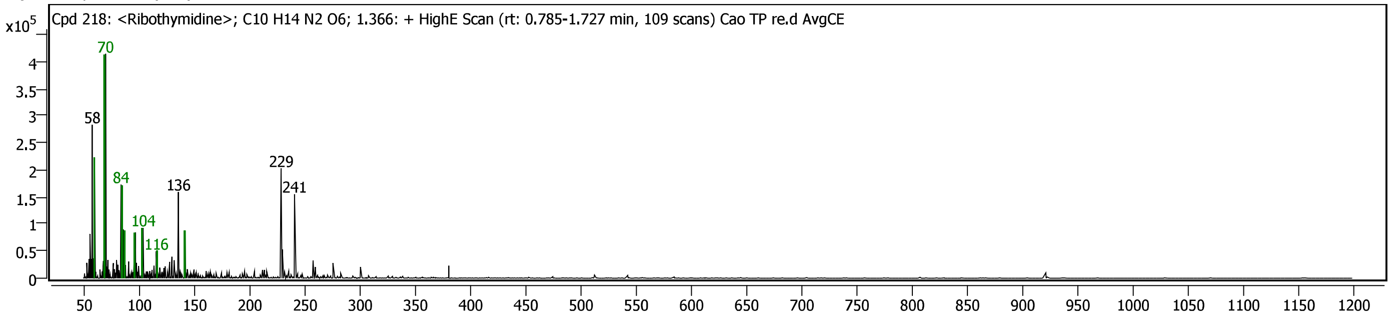

Compound ID Table

Name	Formula	Species	RT	RT Diff	Mass	CAS	ID Source	Score	Score (Lib)	Score (Tgt)
<Ribothymidine>	C10 H14 N2 O6	(M+2H)+2 (M+H)+	1.366		258.0857	1463-10-1	FBF	85.49		85.49
<Ribosylimidazoleacetic acid>	C10 H14 N2 O6	(M+2H)+2 (M+H)+	1.366		258.0857	29605-99-0	FBF	85.49		85.49
<3-Methyluridine>	C10 H14 N2 O6	(M+2H)+2 (M+H)+	1.366		258.0857	2140-69-4	FBF	85.49		85.49

Cpd 1454: Pyroglutamic acid

Name	Formula	RT	RI	Mass	Diff (Tgt, ppm)	CAS	ID Source	Score	Algorithm
Pyroglutamic acid	C5 H7 N O3	1.366		129.0428	1.22	98-79-3	FBF-FragConfirm	86.25	FBF

Species	m/z	Score (Tgt)	Score (Lib)	Score (DB)	Score (MFG)	Score (RT)
(M+H)+	130	86.25				

Compound Screening Report

Compound Chromatograms (overlaid)

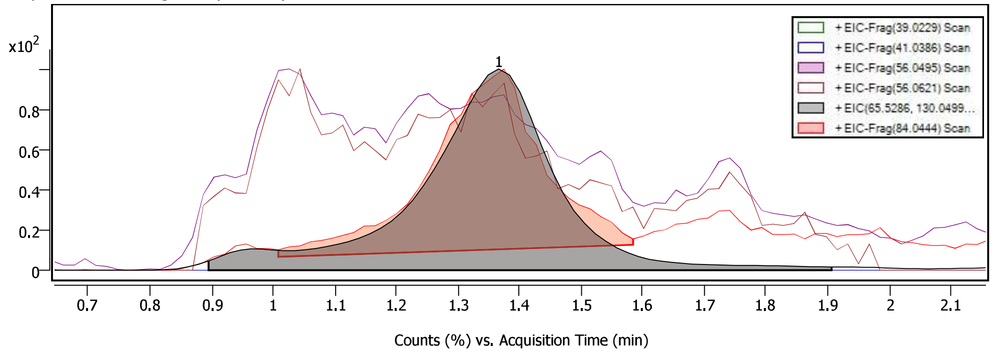

Structure

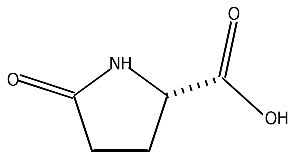

Coelution Plot

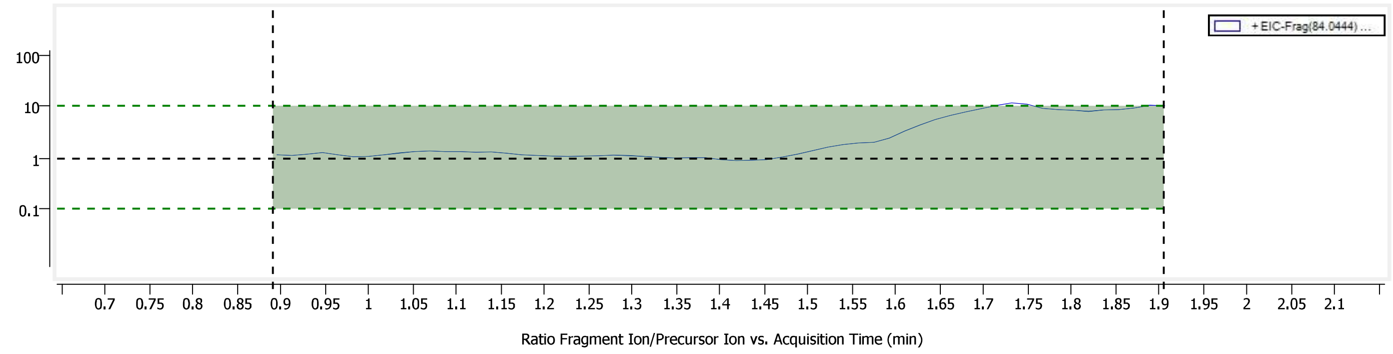

Compound Spectra (overlaid)

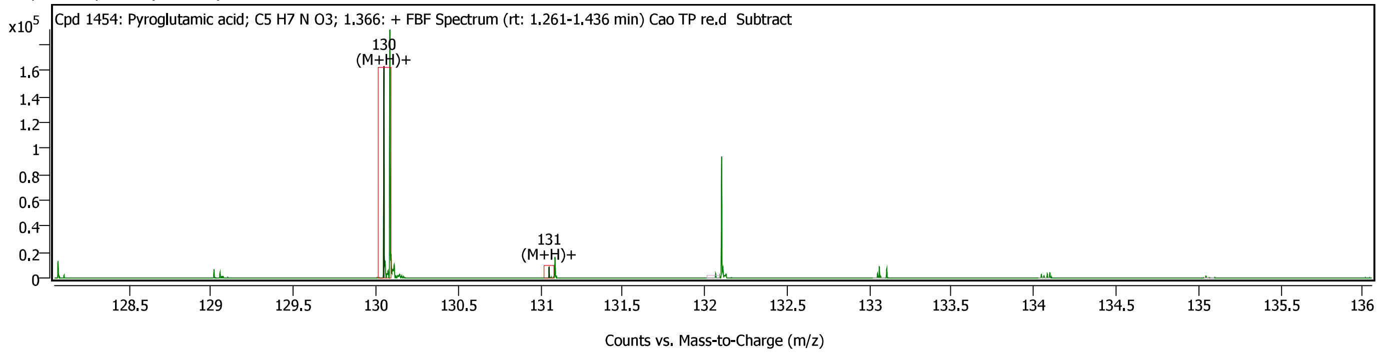

Fragment Spectrum (clean)

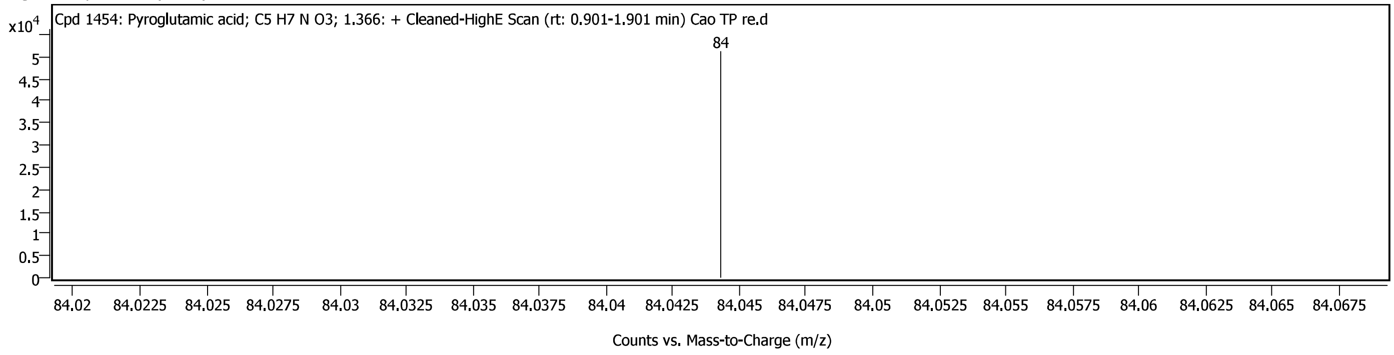

Compound Screening Report

Fragment Spectrum (raw)

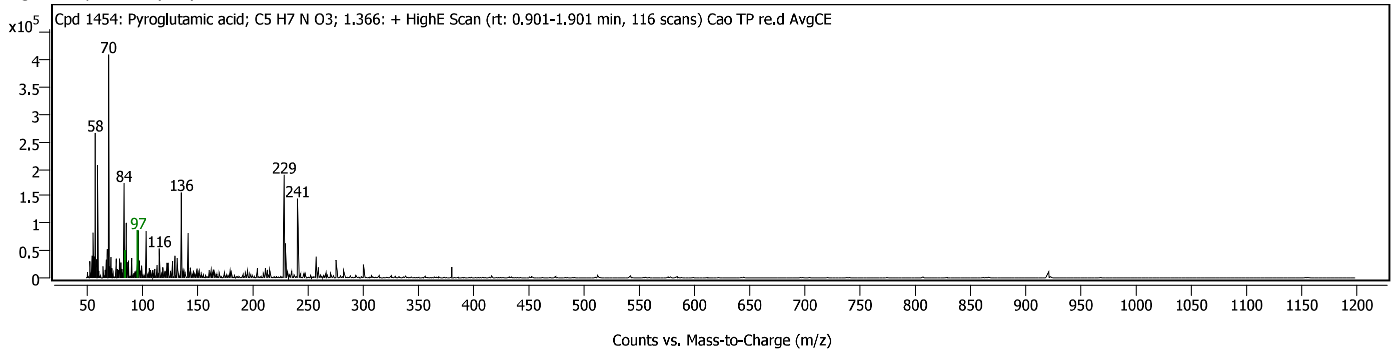

Compound ID Table

Name	Formula	Species	RT	RT Diff	Mass	CAS	ID Source	Score	Score (Lib)	Score (Tgt)
Pyroglutamic acid	C5 H7 N O3	(M+H)+	1.366		129.0428	98-79-3	FBF-FragConfirm	86.25		86.25
Pyrroline hydroxycarboxylic acid	C5 H7 N O3	(M+H)+	1.366		129.0428	22573-88-2	FBF-FragConfirm	86.25		86.25
N-Acryloylglycine	C5 H7 N O3	(M+H)+	1.366		129.0428	24599-25-5	FBF-FragConfirm	86.25		86.25
L-1-Pyrroline-3-hydroxy-5-carboxylate	C5 H7 N O3	(M+H)+	1.366		129.0428		FBF-FragConfirm	86.25		86.25
1-Pyrroline-4-hydroxy-2-carboxylate	C5 H7 N O3	(M+H)+	1.366		129.0428		FBF-FragConfirm	86.25		86.25
(R)-(+)-2-Pyrrolidone-5-carboxylic acid	C5 H7 N O3	(M+H)+	1.366		129.0428	4042-36-8	FBF-FragConfirm	86.25		86.25
4-Oxoproline	C5 H7 N O3	(M+H)+	1.366		129.0428		FBF-FragConfirm	86.25		86.25

Cpd 1414: <Pirbuterol>

Name	Formula	RT	RI	Mass	Diff (Tgt, ppm)	CAS	ID Source	Score	Algorithm
<Pirbuterol>	C12 H20 N2 O3	1.471		240.1480	2.68	38677-81-5	FBF	96.57	FBF

Species	m/z	Score (Tgt)	Score (Lib)	Score (DB)	Score (MFG)	Score (RT)
(M+H)+	241	96.57				

Compound Chromatograms (overlaid)

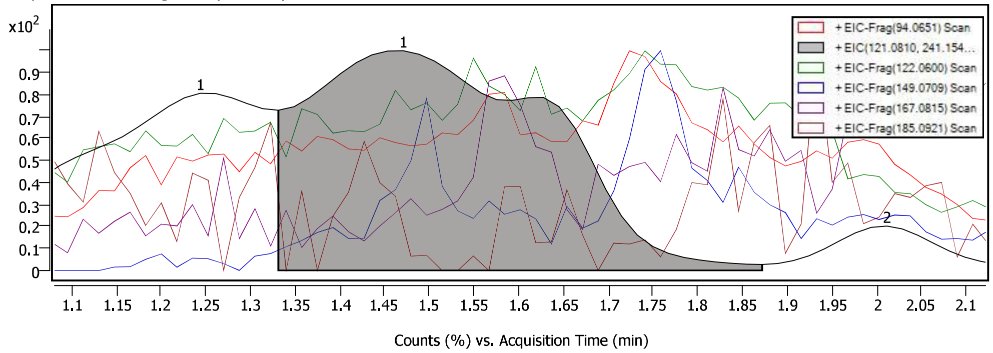

Structure

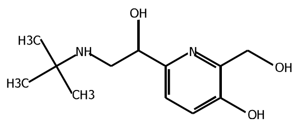

Coelution Plot

Compound Spectra (overlaid)

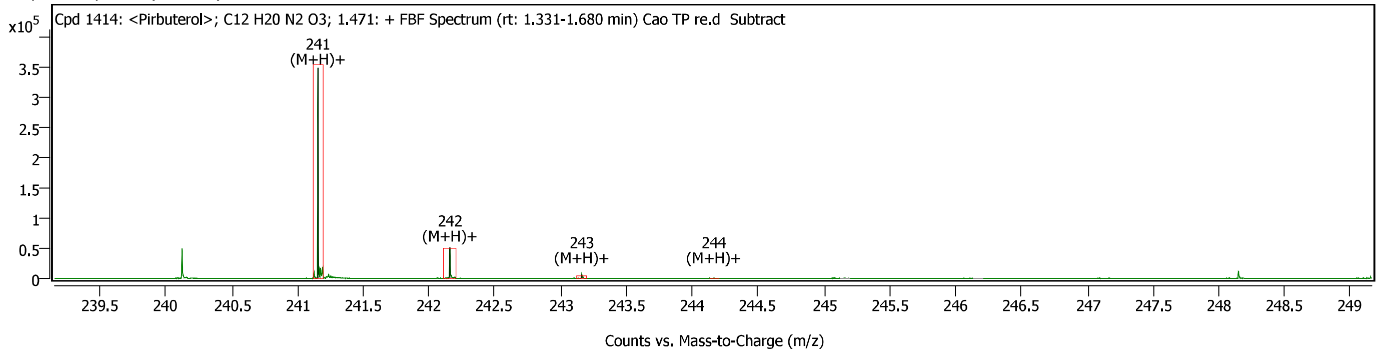

Fragment Spectrum (raw)

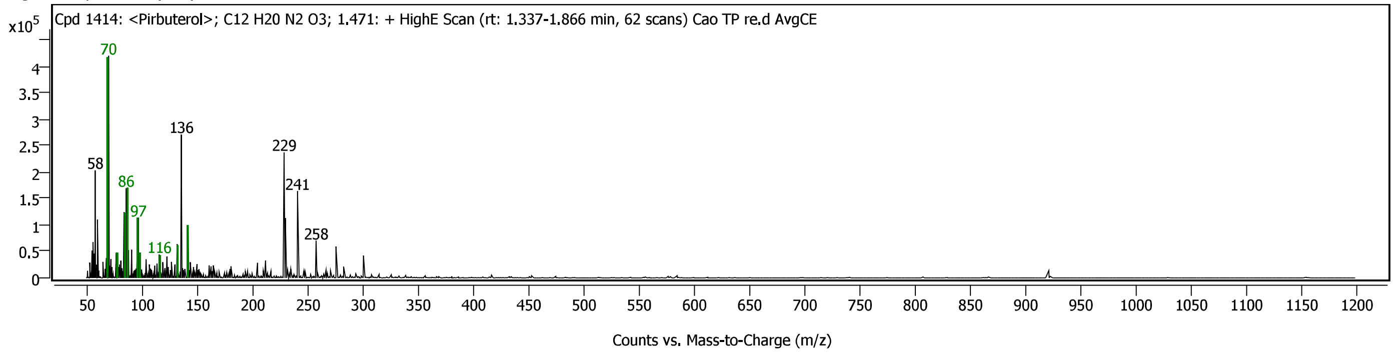

Compound ID Table

Name	Formula	Species	RT	RT Diff	Mass	CAS	ID Source	Score	Score (Lib)	Score (Tgt)
<Pirbuterol>	C12 H20 N2 O3	(M+H)+	1.471		240.1480	38677-81-5	FBF	96.57		96.57

Cpd 178: (S)-5'-Deoxy-5'-(methylsulfinyl)adenosine

Name	Formula	RT	RI	Mass	Diff (Tgt, ppm)	CAS	ID Source	Score	Algorithm
(S)-5'-Deoxy-5'-(methylsulfinyl)adenosine	C11 H15 N5 O4 S	1.488		313.0851	2.11	897-42-7	FBF	97.17	FBF

Species	m/z	Score (Tgt)	Score (Lib)	Score (DB)	Score (MFG)	Score (RT)
(M+H)+	314	97.17				

Compound Chromatograms (overlaid)

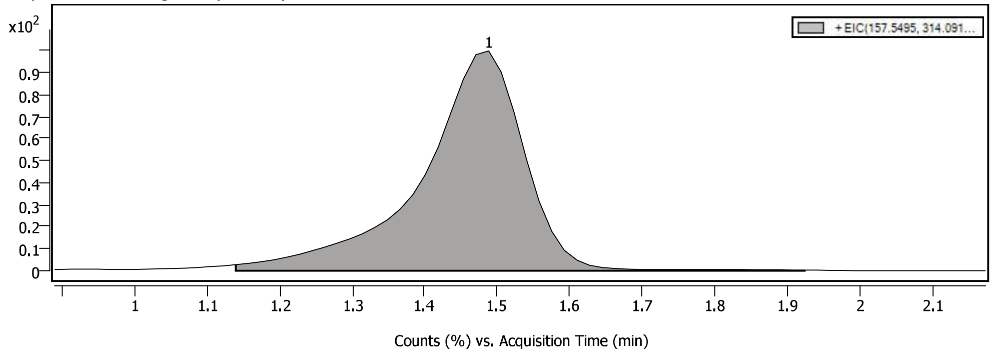

Structure

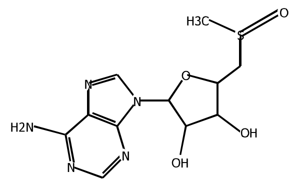

Compound Spectra (overlaid)

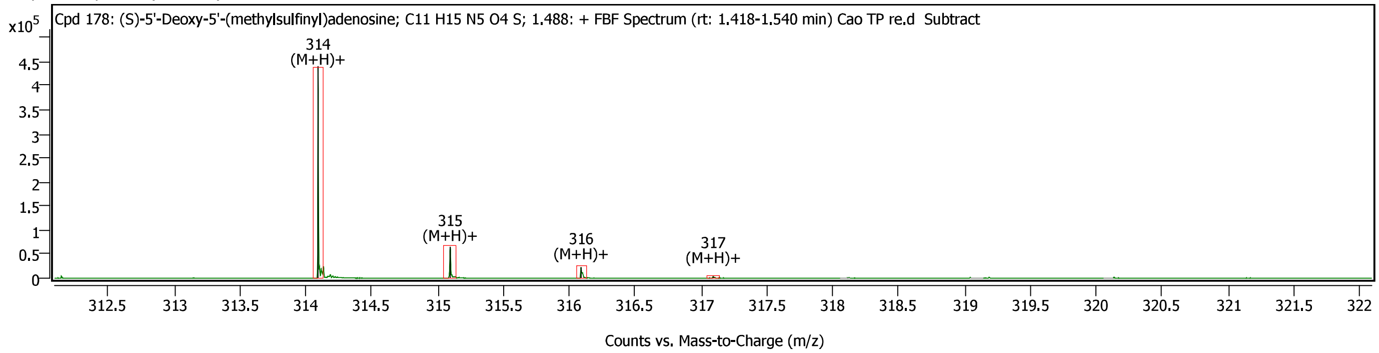

Compound ID Table

Name	Formula	Species	RT	RT Diff	Mass	CAS	ID Source	Score	Score (Lib)	Score (Tgt)
(S)-5'-Deoxy-5'-(methylsulfinyl)adenosine	C11 H15 N5 O4 S	(M+H)+	1.488		313.0851	897-42-7	FBF	97.17		97.17

Cpd 587: Piperidine

Name	Formula	RT	RI	Mass	Diff (Tgt, ppm)	CAS	ID Source	Score	Algorithm
Piperidine	C5 H11 N	1.540		85.0891	-0.52	110-89-4	FBF	95.99	FBF

Species	m/z	Score (Tgt)	Score (Lib)	Score (DB)	Score (MFG)	Score (RT)
(M+H)+	86	95.99				

Compound Chromatograms (overlaid)

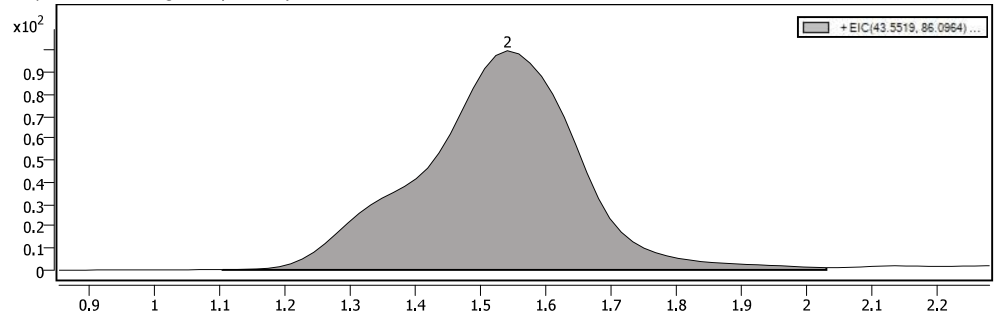

Structure

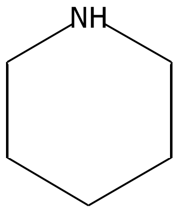

Compound Spectra (overlaid)

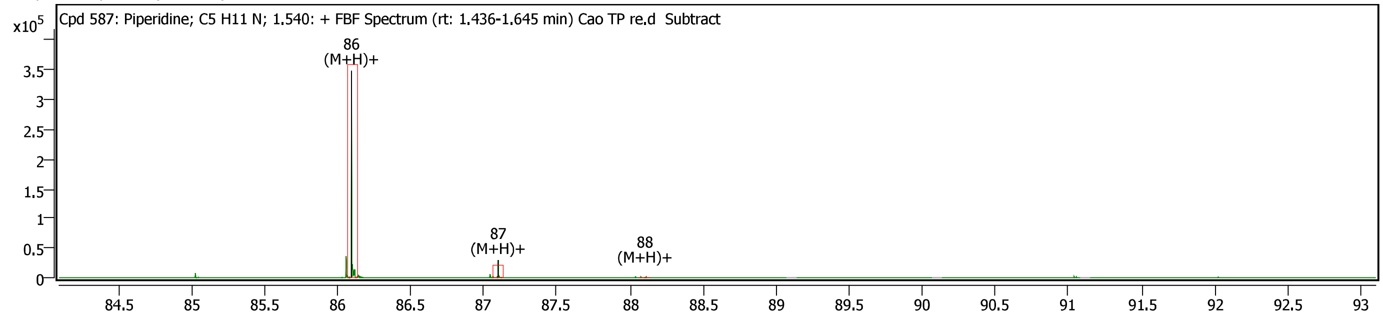

Compound ID Table

Name	Formula	Species	RT	RT Diff	Mass	CAS	ID Source	Score	Score (Lib)	Score (Tgt)
Piperidine	C5 H11 N	(M+H)+	1.540		85.0891	110-89-4	FBF	95.99		95.99

Cpd 96: <N-benzyl-1-methyl-1H-pyrazolo[3,4-d]pyrimidin-4-amine>

Name	Formula	RT	RI	Mass	Diff (Tgt, ppm)	CAS	ID Source	Score	Algorithm
<N-benzyl-1-methyl-1H-pyrazolo[3,4-d]pyrimidin-4-amine>	C13 H13 N5	1.575		239.1162	-3.90		FBF	92.19	FBF

Species	m/z	Score (Tgt)	Score (Lib)	Score (DB)	Score (MFG)	Score (RT)
(M+H)+	240	92.19				

Compound Chromatograms (overlaid)

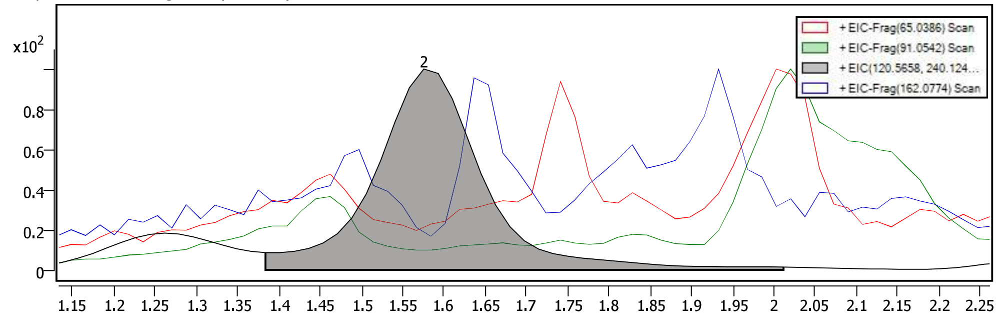

Structure

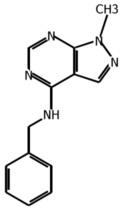

Coelution Plot

Compound Spectra (overlaid)

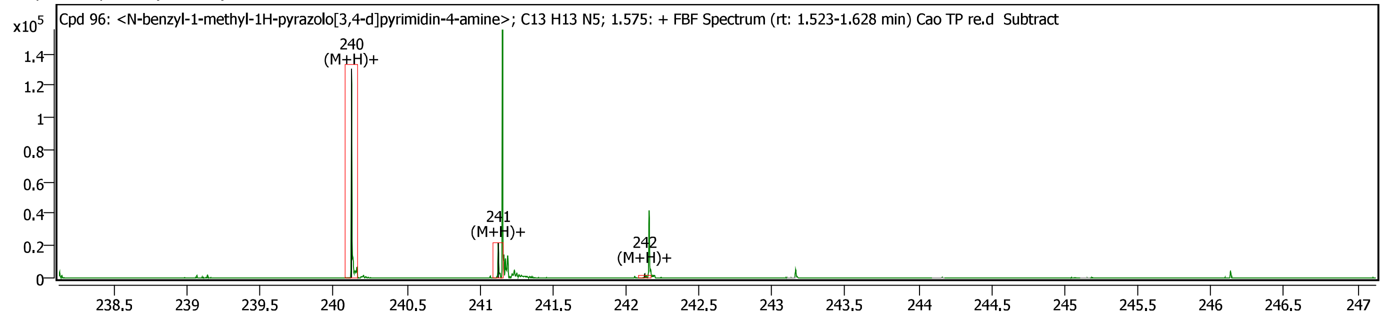

Fragment Spectrum (raw)

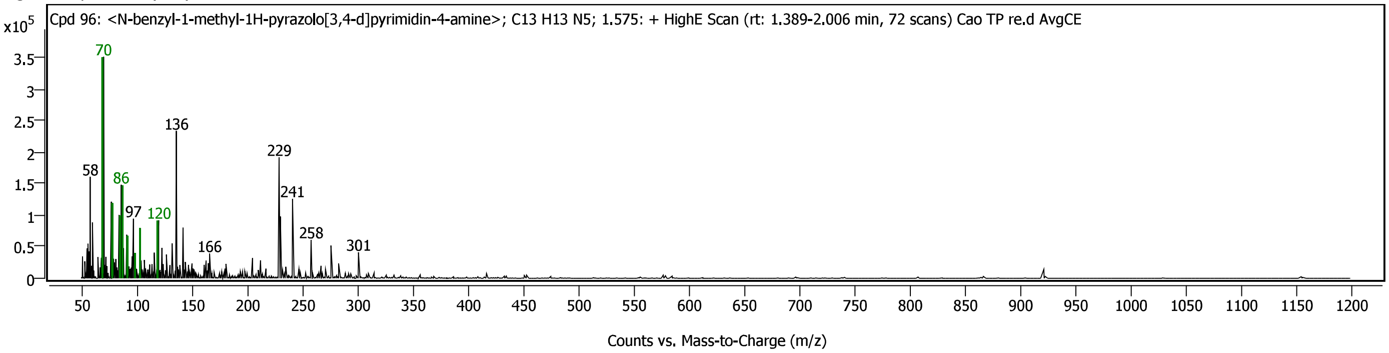

Compound ID Table

Name	Formula	Species	RT	RT Diff	Mass	CAS	ID Source	Score	Score (Lib)	Score (Tgt)
<N-benzyl-1-methyl-1H-pyrazolo[3,4-d]pyrimidin-4-amine>	C13 H13 N5	(M+H)+	1.575		239.1162		FBF	92.19		92.19

Cpd 941: Methyldopate

Name	Formula	RT	RI	Mass	Diff (Tgt, ppm)	CAS	ID Source	Score	Algorithm
Methyldopate	C12 H17 N O4	1.575		239.1161	1.36		FBF	96.53	FBF

Species	m/z	Score (Tgt)	Score (Lib)	Score (DB)	Score (MFG)	Score (RT)
(M+H)+	240	96.53				

Compound Chromatograms (overlaid)

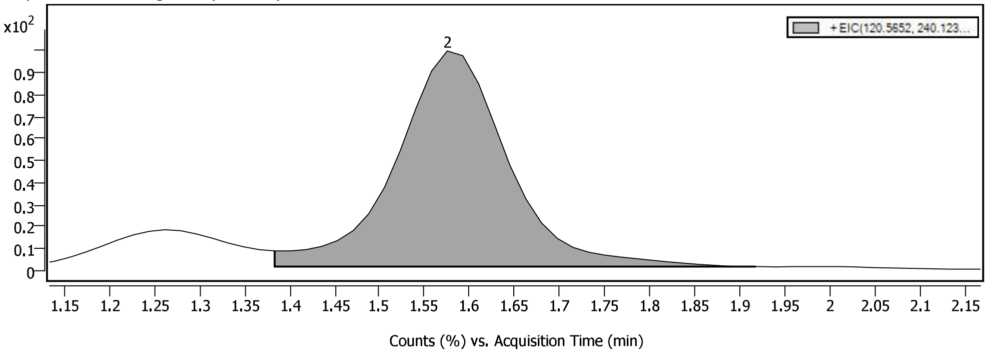

Structure

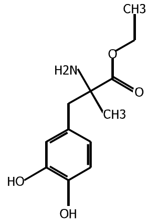

Compound Spectra (overlaid)

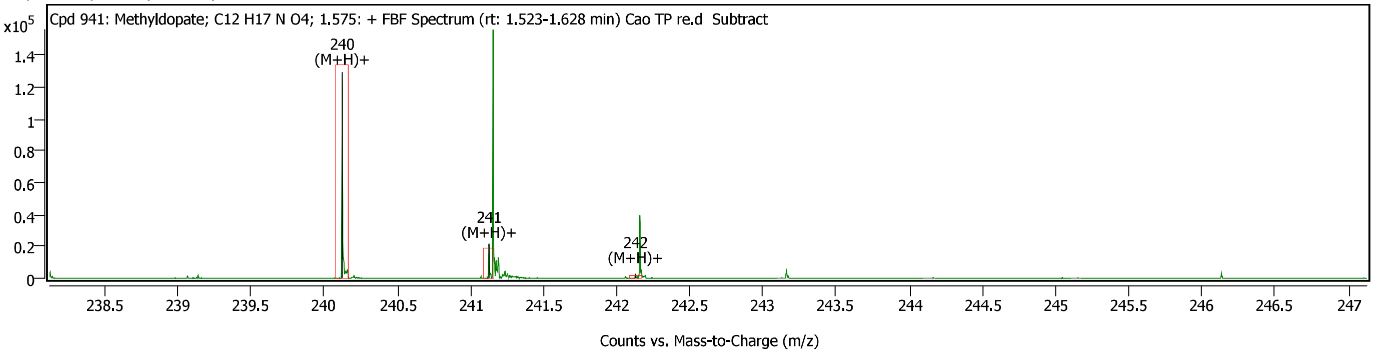

Compound ID Table

Name	Formula	Species	RT	RT Diff	Mass	CAS	ID Source	Score	Score (Lib)	Score (Tgt)
Methyldopate	C12 H17 N O4	(M+H)+	1.575		239.1161		FBF	96.53		96.53

Cpd 1151: <Pro Ile>

Name	Formula	RT	RI	Mass	Diff (Tgt, ppm)	CAS	ID Source	Score	Algorithm
<Pro Ile>	C11 H20 N2 O3	1.575		228.1484	4.34		M-FBF	90.72	FBF

Species	m/z	Score (Tgt)	Score (Lib)	Score (DB)	Score (MFG)	Score (RT)
(M+H)+	229	90.72				

Compound Screening Report

Compound Chromatograms (overlaid)

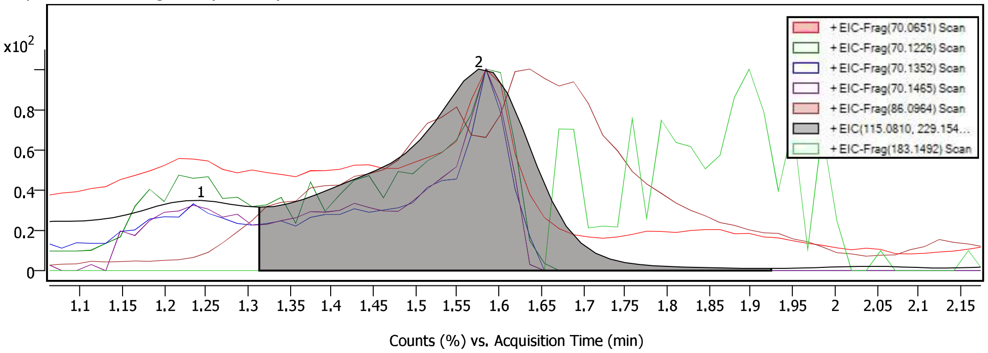

Structure

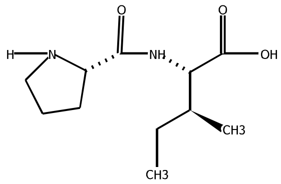

Coelution Plot

Compound Spectra (overlaid)

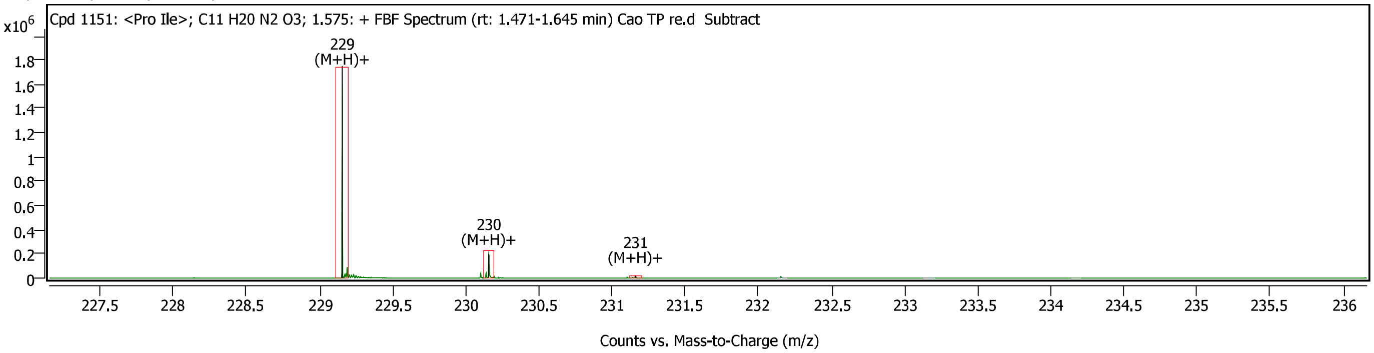

Fragment Spectrum (raw)

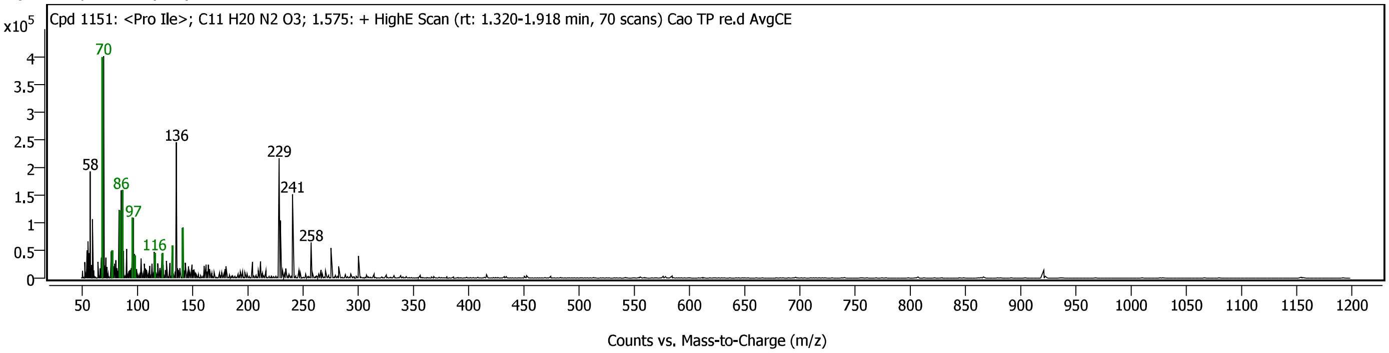

Compound ID Table

Name	Formula	Species	RT	RT Diff	Mass	CAS	ID Source	Score	Score (Lib)	Score (Tgt)
<Pro Ile>	C11 H20 N2 O3	(M+H)+	1.575		228.1484		FBF	90.72		90.72
<Leu Pro>	C11 H20 N2 O3	(M+H)+	1.575		228.1484		FBF	90.72		90.72
<Ile Pro>	C11 H20 N2 O3	(M+H)+	1.575		228.1484		FBF	90.72		90.72
<1-L-Leucyl-L-Proline>	C11 H20 N2 O3	(M+H)+	1.575		228.1484		FBF	90.72		90.72
<L-isoleucyl-L-proline>	C11 H20 N2 O3	(M+H)+	1.575		228.1484		FBF	90.72		90.72
<Pro Leu>	C11 H20 N2 O3	(M+H)+	1.575		228.1484		FBF	90.72		90.72

Cpd 1202: Adenine

Name	Formula	RT	RI	Mass	Diff (Tgt, ppm)	CAS	ID Source	Score	Algorithm
Adenine	C5 H5 N5	1.575		135.0548	2.07	73-24-5	FBF	80.64	FBF

Species	m/z	Score (Tgt)	Score (Lib)	Score (DB)	Score (MFG)	Score (RT)
(M+H)+	136	80.64				

Agilent

Compound Chromatograms (overlaid)

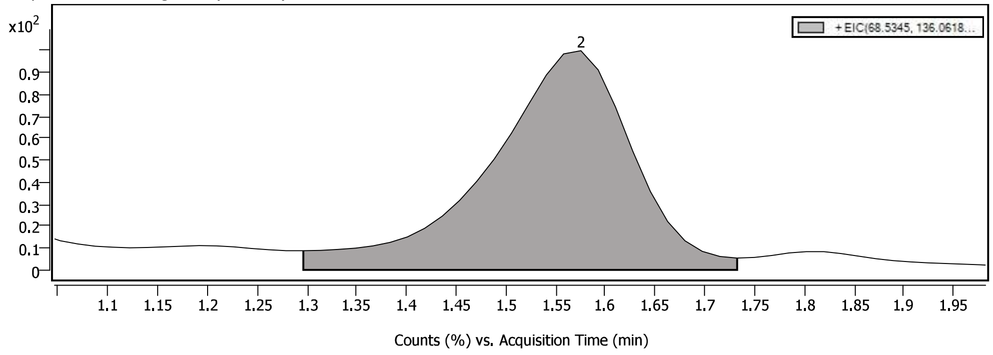

Structure

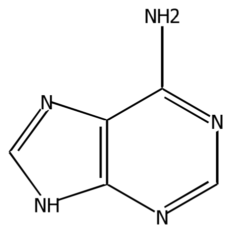

Compound Spectra (overlaid)

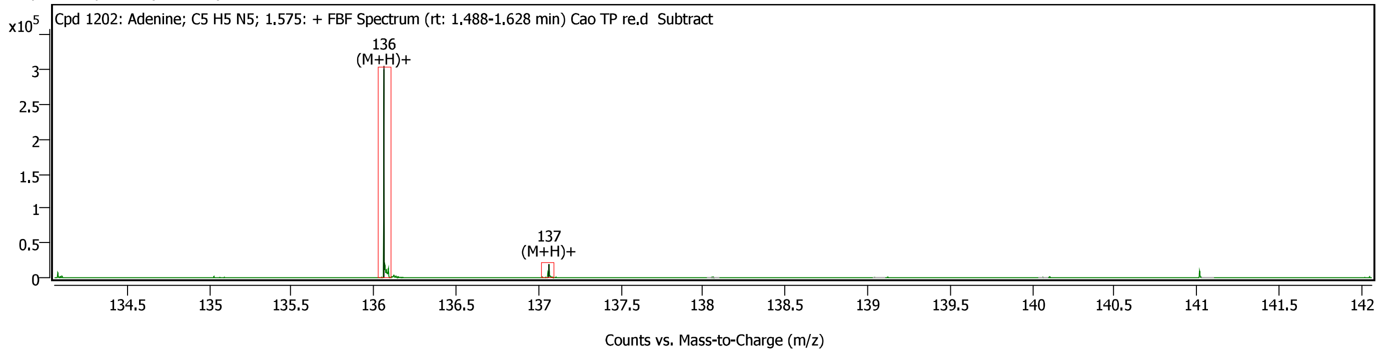

Compound ID Table

Name	Formula	Species	RT	RT Diff	Mass	CAS	ID Source	Score	Score (Lib)	Score (Tgt)
Adenine	C5 H5 N5	(M+H)+	1.575		135.0548	73-24-5	FBF	80.64		80.64

Cpd 1419: Adenosine

Name	Formula	RT	RI	Mass	Diff (Tgt, ppm)	CAS	ID Source	Score	Algorithm
Adenosine	C10 H13 N5 O4	1.575		267.0975	2.84	58-61-7	M-FBF-FragConfirm	95.94	FBF

Species	m/z	Score (Tgt)	Score (Lib)	Score (DB)	Score (MFG)	Score (RT)
(M+H)+	268	95.94				

Compound Chromatograms (overlaid)

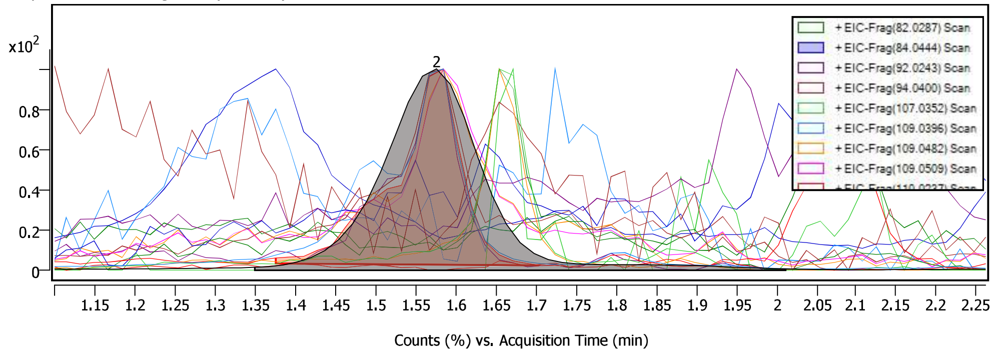

Structure

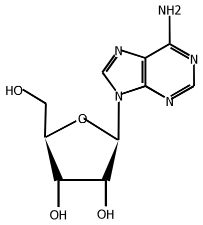

Coelution Plot

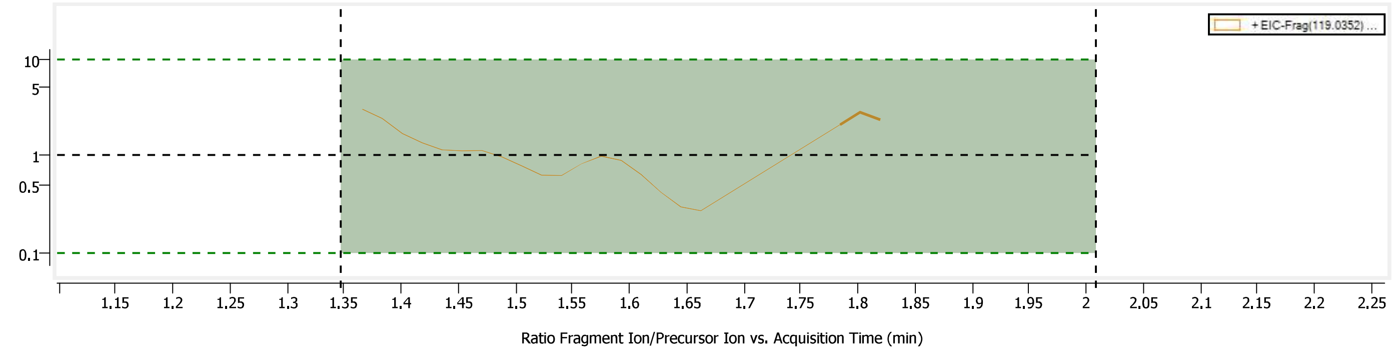

Compound Spectra (overlaid)

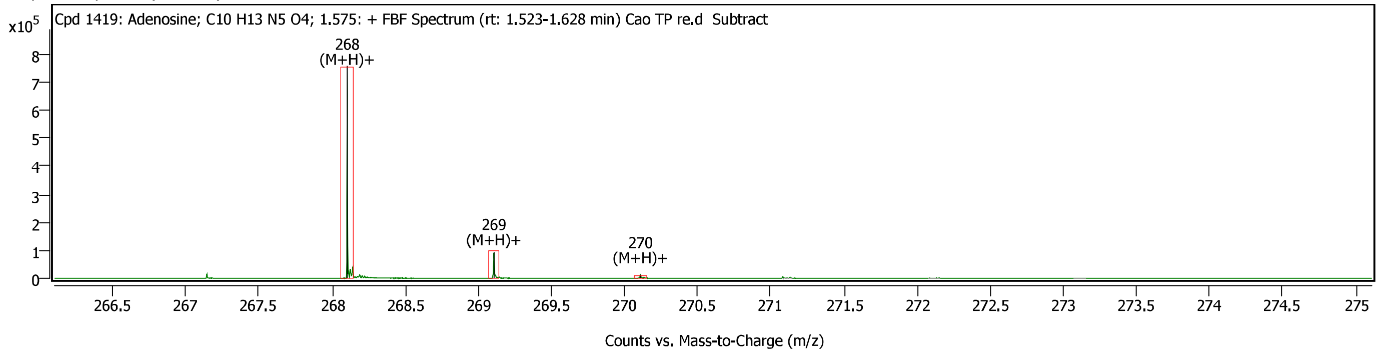

Fragment Spectrum (clean)

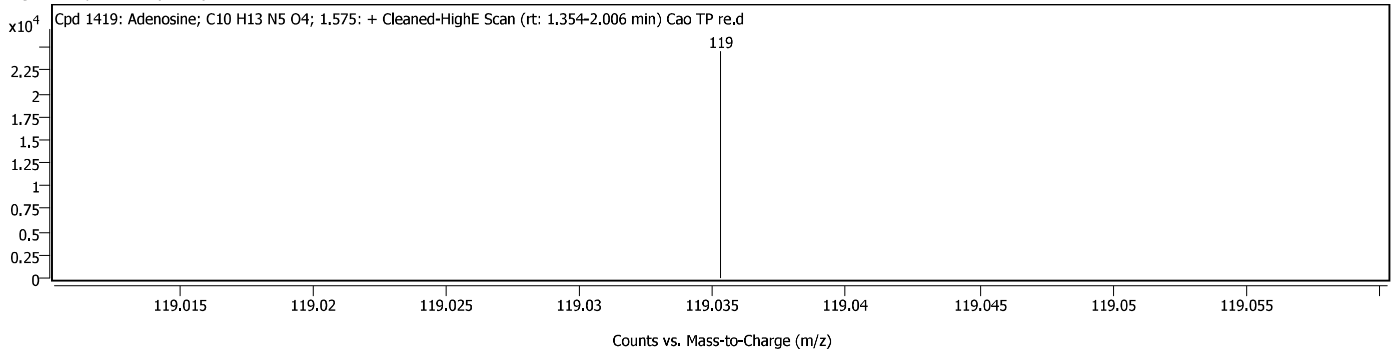

Fragment Spectrum (raw)

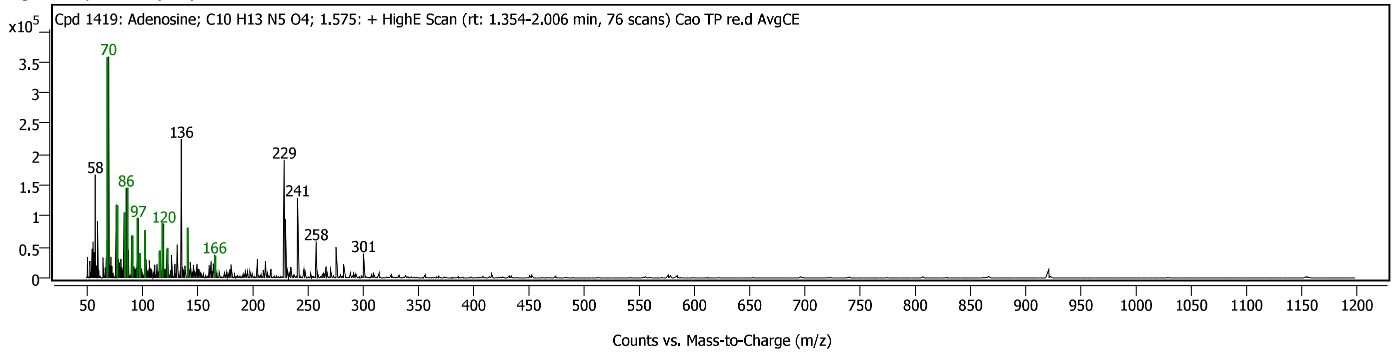

Compound ID Table

Name	Formula	Species	RT	RT Diff	Mass	CAS	ID Source	Score	Score (Lib)	Score (Tgt)
Adenosine	C10 H13 N5 O4	(M+H)+	1.575		267.0975	58-61-7	FBF-FragConfirm	95.94		95.94
Zidovudine	C10 H13 N5 O4	(M+H)+	1.575		267.0975	30516-87-1	FBF-FragConfirm	95.94		95.94
Vidarabine	C10 H13 N5 O4	(M+H)+	1.575		267.0975	5536-17-4	FBF-FragConfirm	95.94		95.94
3-Deoxyguanosine	C10 H13 N5 O4	(M+H)+	1.575		267.0975	3608-58-0	FBF-FragConfirm	95.94		95.94
2'-Deoxyguanosine	C10 H13 N5 O4	(M+H)+	1.575		267.0975	961-07-9	FBF-FragConfirm	95.94		95.94

Cpd 1665: <D-Cathinone>

Name	Formula	RT	RI	Mass	Diff (Tgt, ppm)	CAS	ID Source	Score	Algorithm
<D-Cathinone>	C9 H11 N O	1.593		149.0841	0.51	71031-15-7	M-FBF	99.69	FBF

Species	m/z	Score (Tgt)	Score (Lib)	Score (DB)	Score (MFG)	Score (RT)
(M+H)+	150	99.69				

Compound Chromatograms (overlaid)

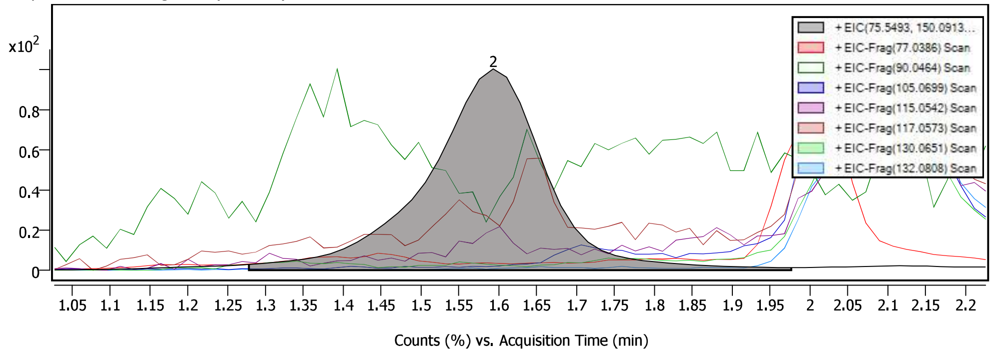

Structure

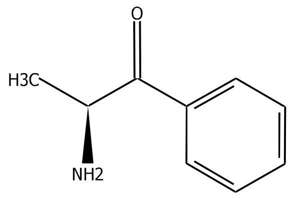

Coelution Plot

Compound Spectra (overlaid)

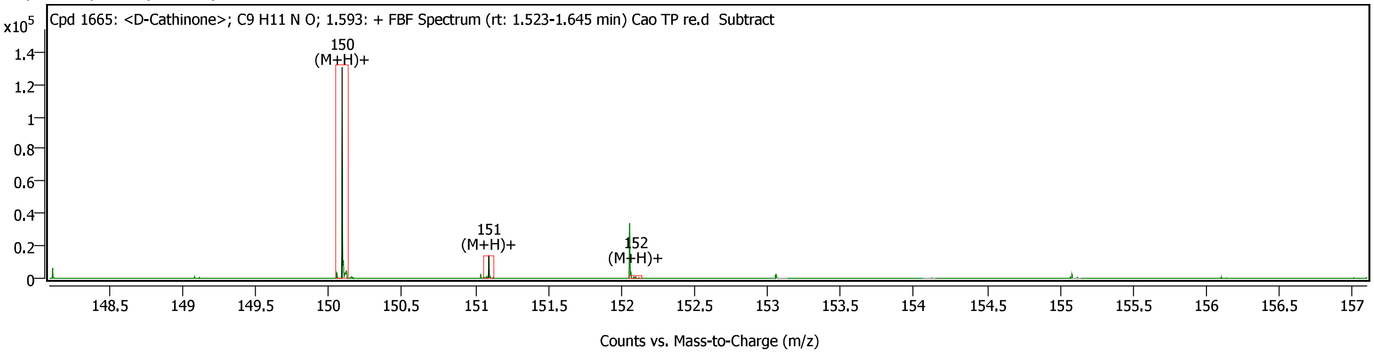

Fragment Spectrum (raw)

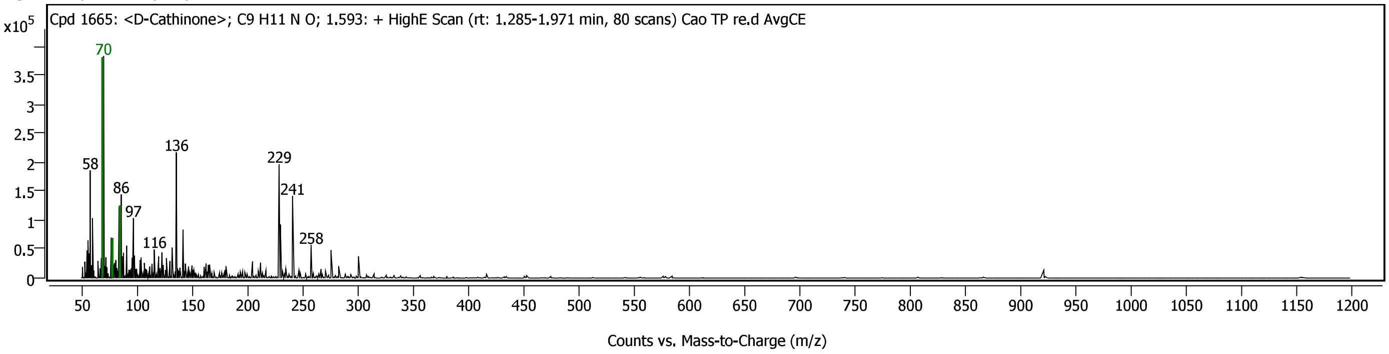

Compound ID Table

Name	Formula	Species	RT	RT Diff	Mass	CAS	ID Source	Score	Score (Lib)	Score (Tgt)
<D-Cathinone>	C9 H11 N O	(M+H)+	1.593		149.0841	71031-15-7	FBF	99.69		99.69
<3-(2-Furanylmethylene)pyrrolidine>	C9 H11 N O	(M+H)+	1.593		149.0841	118248-35-4	FBF	99.69		99.69
<2-(2-Furanyl)-3,4,5,6-tetrahydropyridine>	C9 H11 N O	(M+H)+	1.593		149.0841	104704-36-1	FBF	99.69		99.69
<2,3,6,7-Tetrahydrocyclopent[b]azepin-8(1H)-one>	C9 H11 N O	(M+H)+	1.593		149.0841	97826-65-8	FBF	99.69		99.69
<2,3-Dihydro-5-methyl-1H-pyrrolizine-7-carboxaldehyde>	C9 H11 N O	(M+H)+	1.593		149.0841	97073-07-9	FBF	99.69		99.69
<2,3-Dihydro-6-methyl-1H-pyrrolizine-5-carboxaldehyde>	C9 H11 N O	(M+H)+	1.593		149.0841	55041-87-7	FBF	99.69		99.69
<2-Aminopropiophenone>	C9 H11 N O	(M+H)+	1.593		149.0841	5265-18-9	FBF	99.69		99.69
<5,6,7,8-tetrahydro-2-Quinolone>	C9 H11 N O	(M+H)+	1.593		149.0841	54802-19-6	FBF	99.69		99.69
<3,4-Dihydro-5-(5-methyl-2-furanyl)-2H-pyrrole>	C9 H11 N O	(M+H)+	1.593		149.0841	118248-26-3	FBF	99.69		99.69
<3-Phenylpropionaldoxim>	C9 H11 N O	(M+H)+	1.593		149.0841		FBF	99.69		99.69
<5-Acetyl-2,3-dihydro-1H-pyrrolizine>	C9 H11 N O	(M+H)+	1.593		149.0841	55041-85-5	FBF	99.69		99.69
<Venoterpine>	C9 H11 N O	(M+H)+	1.593		149.0841	17948-42-4	FBF	99.69		99.69

Cpd 508: Tiapamil

Name	Formula	RT	RI	Mass	Diff (Tgt, ppm)	CAS	ID Source	Score	Algorithm
Tiapamil	C26 H37 N O8 S2	1.680		555.1955	-1.05	57010-31-8	FBF	83.82	FBF

Species	m/z	Score (Tgt)	Score (Lib)	Score (DB)	Score (MFG)	Score (RT)
(M+H)+	556	83.82				

Compound Chromatograms (overlaid)

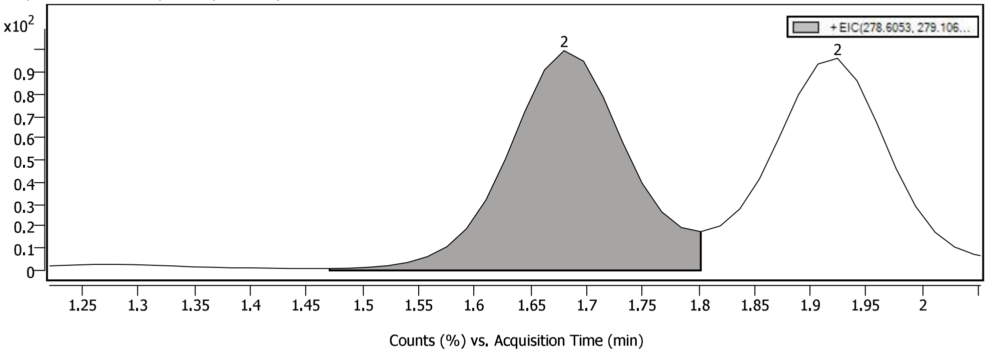

Structure

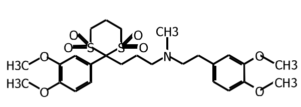

Compound Spectra (overlaid)

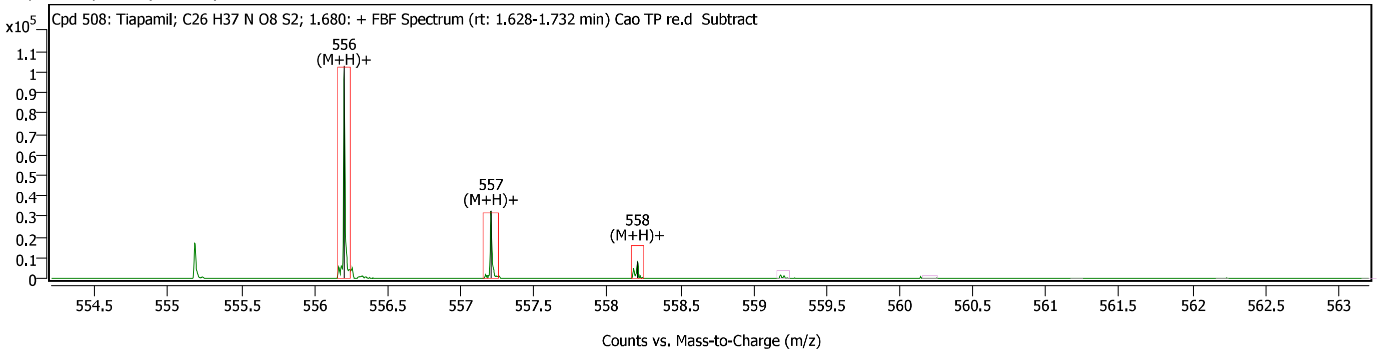

Compound ID Table

Name	Formula	Species	RT	RT Diff	Mass	CAS	ID Source	Score	Score (Lib)	Score (Tgt)
Tiapamil	C26 H37 N O8 S2	(M+H)+	1.680		555.1955	57010-31-8	FBF	83.82		83.82

Cpd 912: <2-Amino-3-methyl-1-butanol>

Name	Formula	RT	RI	Mass	Diff (Tgt, ppm)	CAS	ID Source	Score	Algorithm
<2-Amino-3-methyl-1-butanol>	C5 H13 N O	1.680		103.0998	0.63	473-75-6	FBF	99.19	FBF

Species	m/z	Score (Tgt)	Score (Lib)	Score (DB)	Score (MFG)	Score (RT)
(M+H)+	104	99.19				

Compound Chromatograms (overlaid)

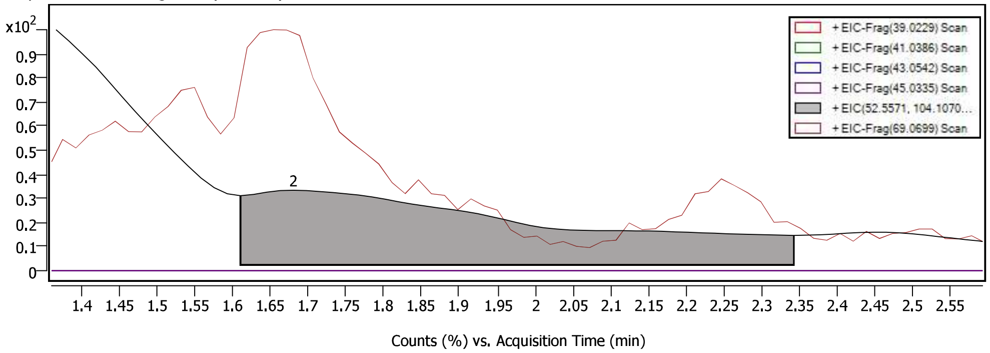

Structure

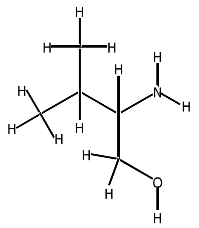

Coelution Plot

Compound Spectra (overlaid)

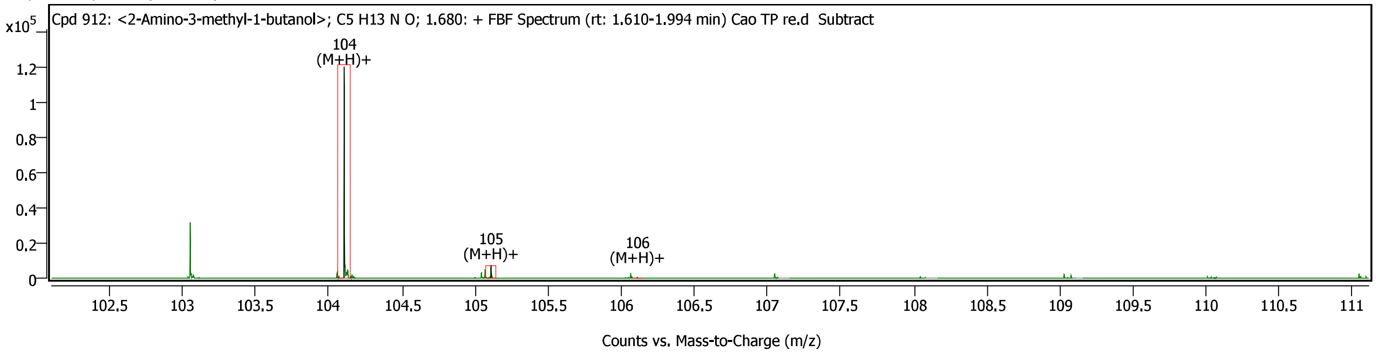

Fragment Spectrum (raw)

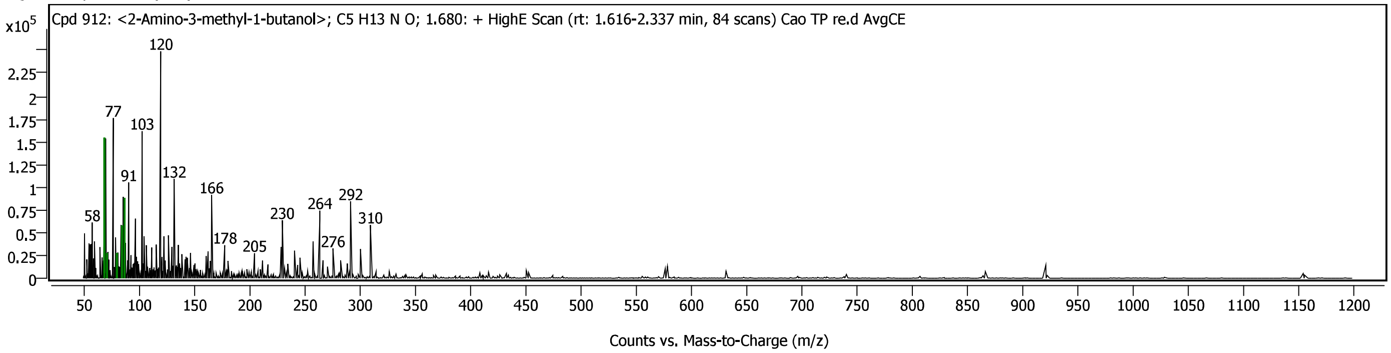

Compound Screening Report

Compound ID Table

Name	Formula	Species	RT	RT Diff	Mass	CAS	ID Source	Score	Score (Lib)	Score (Tgt)
<2-Amino-3-methyl-1-butanol>	C5 H13 N O	(M+H)+	1.680		103.0998	473-75-6	FBF	99.19		99.19

Cpd 1663: N-(1-Deoxy-1-fructosyl)isoleucine

Name	Formula	RT	RI	Mass	Diff (Tgt, ppm)	CAS	ID Source	Score	Algorithm
N-(1-Deoxy-1-fructosyl)isoleucine	C12 H23 N O7	1.680		293.1483	2.94	87304-79-8	M-FBF	95.59	FBF

Species	m/z	Score (Tgt)	Score (Lib)	Score (DB)	Score (MFG)	Score (RT)
(M+H)+	294	95.59				

Compound Chromatograms (overlaid)

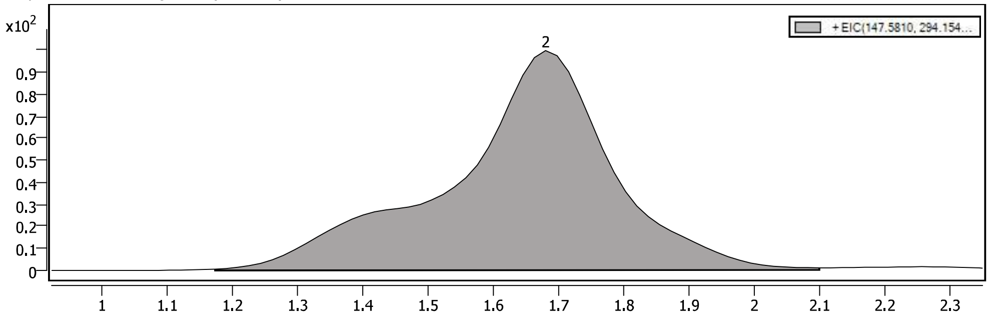

Structure

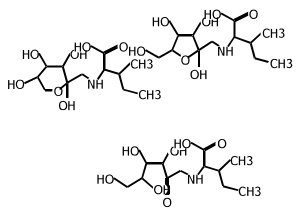

Compound Spectra (overlaid)

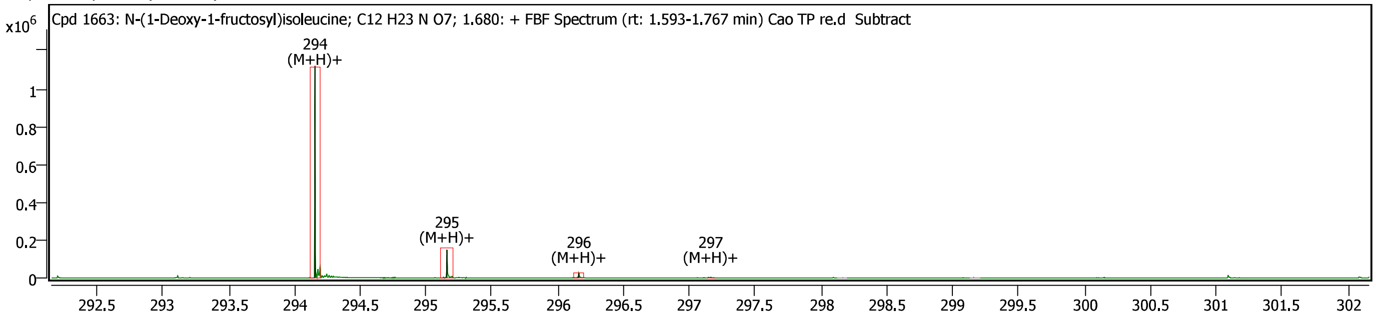

Compound ID Table

Name	Formula	Species	RT	RT Diff	Mass	CAS	ID Source	Score	Score (Lib)	Score (Tgt)
N-(1-Deoxy-1-fructosyl)isoleucine	C12 H23 N O7	(M+H)+	1.680		293.1483	87304-79-8	FBF	95.59		95.59
N-(1-Deoxy-1-fructosyl)leucine	C12 H23 N O7	(M+H)+	1.680		293.1483	34393-18-5	FBF	95.59		95.59

Cpd 564: L-Phenylalanine

Name	Formula	RT	RI	Mass	Diff (Tgt, ppm)	CAS	ID Source	Score	Algorithm
L-Phenylalanine	C9 H11 N O2	2.011		165.0794	2.75	63-91-2	M-FBF-FragConfirm	96.56	FBF

Species	m/z	Score (Tgt)	Score (Lib)	Score (DB)	Score (MFG)	Score (RT)
(M+H)+	166	96.56				

Compound Chromatograms (overlaid)

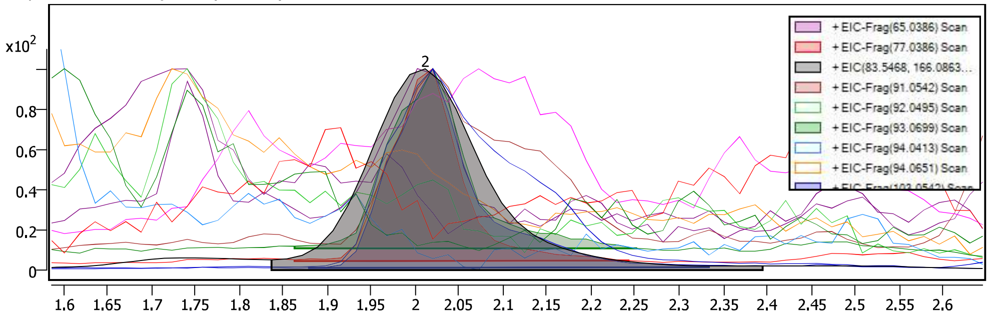

Structure

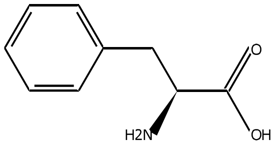

Coelution Plot

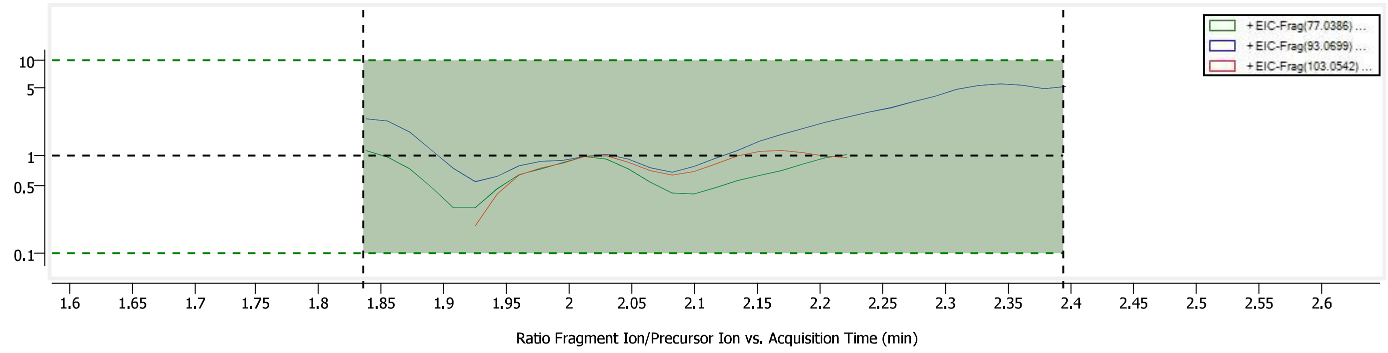

Compound Spectra (overlaid)

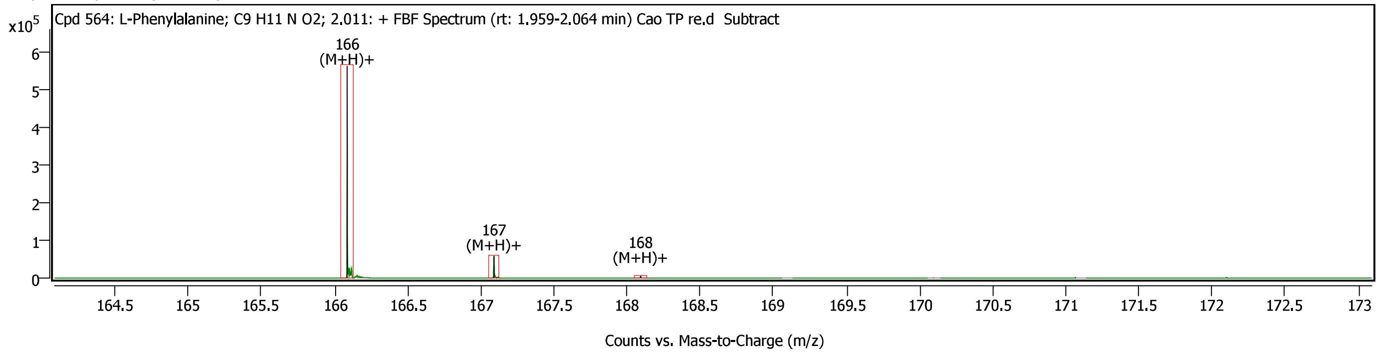

Fragment Spectrum (clean)

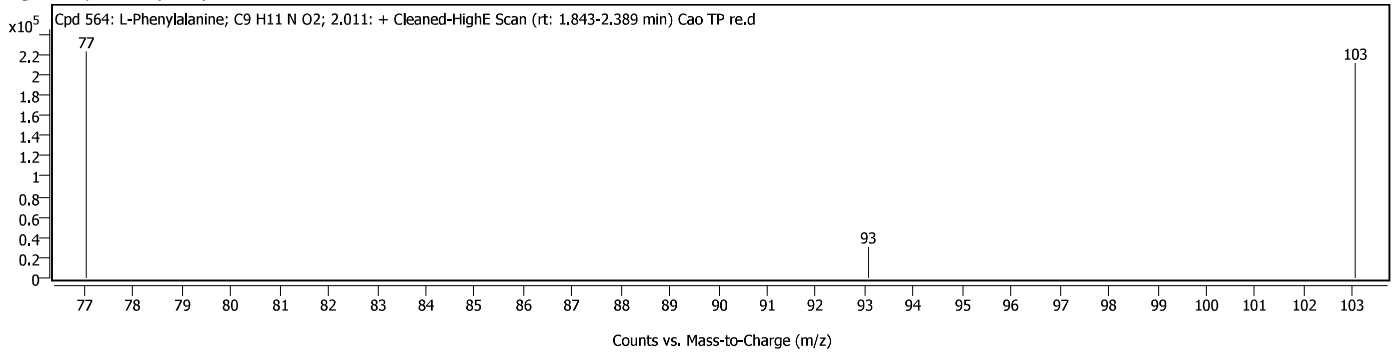

Fragment Spectrum (raw)

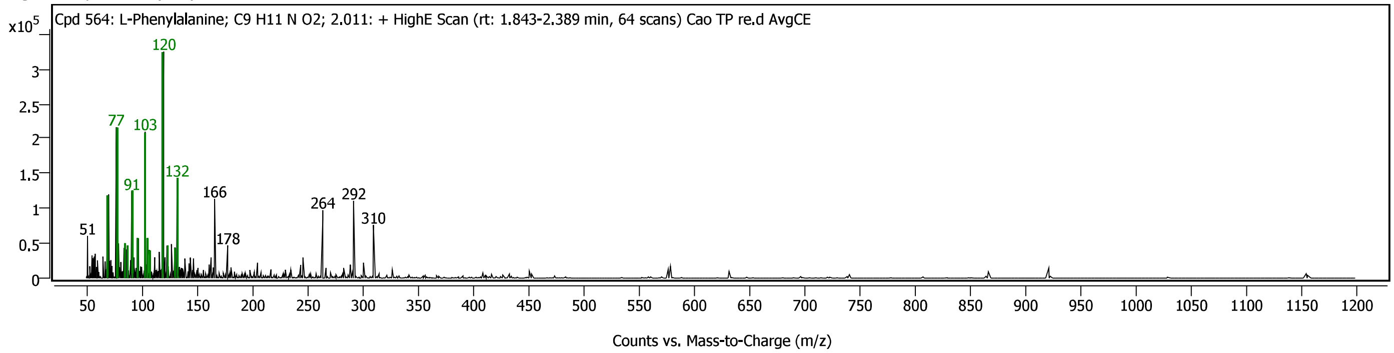

Compound ID Table

Name	Formula	Species	RT	RT Diff	Mass	CAS	ID Source	Score	Score (Lib)	Score (Tgt)
L-Phenylalanine	C9 H11 N O2	(M+H)+	2.011		165.0794	63-91-2	FBF-FragConfirm	96.56		96.56
DL-Phenylalanine	C9 H11 N O2	(M+H)+	2.011		165.0794	150-30-1	FBF-FragConfirm	96.56		96.56
2-amino-4'-hydroxy-Propiophenone	C9 H11 N O2	(M+H)+	2.011		165.0794	18259-42-2	FBF-FragConfirm	96.56		96.56
2-Propylisonicotinic acid	C9 H11 N O2	(M+H)+	2.011		165.0794	57663-82-8	FBF-FragConfirm	96.56		96.56
4-Hydroxy-1-(3-pyridinyl)-1-butanone	C9 H11 N O2	(M+H)+	2.011		165.0794	59578-62-0	FBF-FragConfirm	96.56		96.56
5-(3-Pyridyl)-2-hydroxytetrahydrofuran	C9 H11 N O2	(M+H)+	2.011		165.0794	53798-73-5	FBF-FragConfirm	96.56		96.56
Benzocaine	C9 H11 N O2	(M+H)+	2.011		165.0794	94-09-7	FBF-FragConfirm	96.56		96.56
Benzyl glycinate	C9 H11 N O2	(M+H)+	2.011		165.0794	1738-68-7	FBF-FragConfirm	96.56		96.56
4-(3-Pyridyl)-butanoic acid	C9 H11 N O2	(M+H)+	2.011		165.0794		FBF-FragConfirm	96.56		96.56
D-Phenylalanine	C9 H11 N O2	(M+H)+	2.011		165.0794	673-06-3	FBF-FragConfirm	96.56		96.56
Gentiatibetine	C9 H11 N O2	(M+H)+	2.011		165.0794	26005-36-7	FBF-FragConfirm	96.56		96.56
Methyl N-methylanthranilate	C9 H11 N O2	(M+H)+	2.011		165.0794	85-91-6	FBF-FragConfirm	96.56		96.56
Tricaine	C9 H11 N O2	(M+H)+	2.011		165.0794	582-33-2	FBF-FragConfirm	96.56		96.56
Metolcarb	C9 H11 N O2	(M+H)+	2.011		165.0794	1129-41-5	FBF-FragConfirm	96.56		96.56
N-(4-Hydroxyphenyl)propanamide	C9 H11 N O2	(M+H)+	2.011		165.0794	1693-37-4	FBF-FragConfirm	96.56		96.56
Ethyl 2-aminobenzoate	C9 H11 N O2	(M+H)+	2.011		165.0794	87-25-2	FBF-FragConfirm	96.56		96.56

Cpd 146: N-(1-Deoxy-1-fructosyl)phenylalanine

Name	Formula	RT	RI	Mass	Diff (Tgt, ppm)	CAS	ID Source	Score	Algorithm
N-(1-Deoxy-1-fructosyl)phenylalanine	C15 H21 N O7	2.099		327.1331	3.88	87251-83-0	FBF	91.24	FBF

Species	m/z	Score (Tgt)	Score (Lib)	Score (DB)	Score (MFG)	Score (RT)
(M+H)+	328	91.24				

Compound Chromatograms (overlaid)

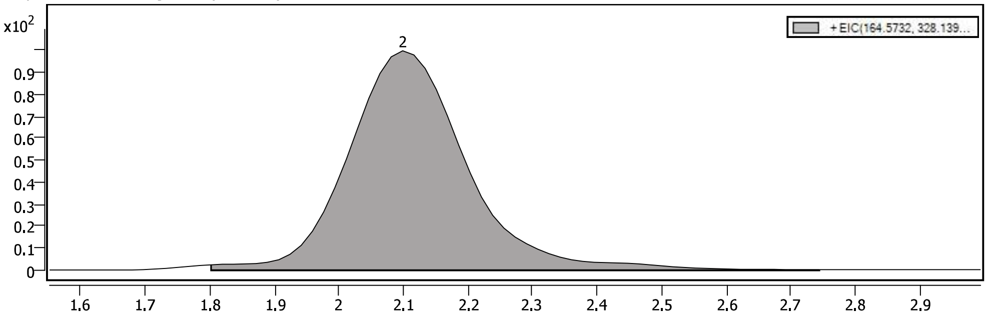

Structure

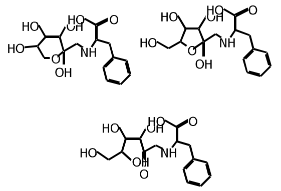

Compound Spectra (overlaid)

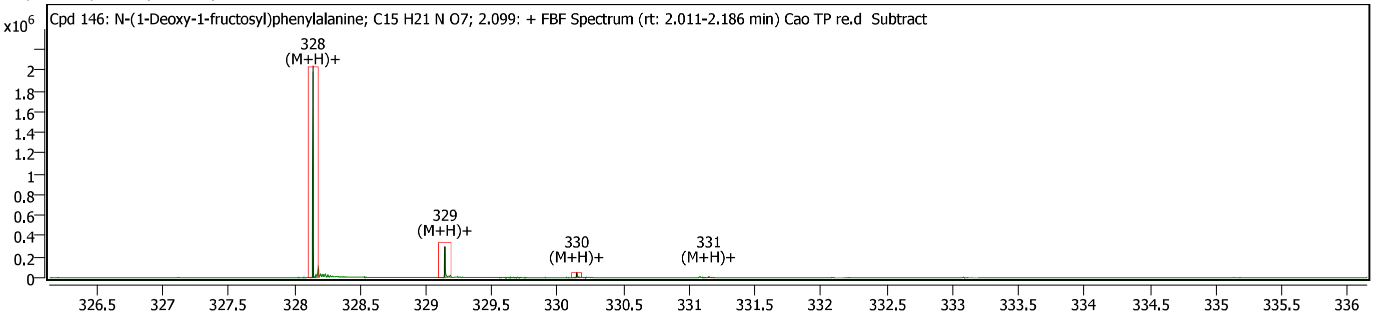

Compound ID Table

Name	Formula	Species	RT	RT Diff	Mass	CAS	ID Source	Score	Score (Lib)	Score (Tgt)
N-(1-Deoxy-1-fructosyl)phenylalanine	C15 H21 N O7	(M+H)+	2.099		327.1331	87251-83-0	FBF	91.24		91.24

Cpd 1233: Tranylcypromine glucuronide

Name	Formula	RT	RI	Mass	Diff (Tgt, ppm)	CAS	ID Source	Score	Algorithm
Tranylcypromine glucuronide	C15 H19 N O6	2.099		309.1223	3.46		FBF	93.03	FBF

Species	m/z	Score (Tgt)	Score (Lib)	Score (DB)	Score (MFG)	Score (RT)
(M+H)+	310	93.03				

Compound Chromatograms (overlaid)

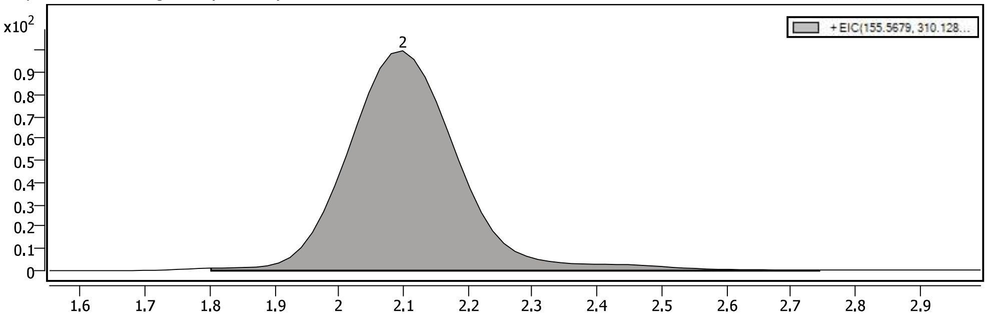

Structure

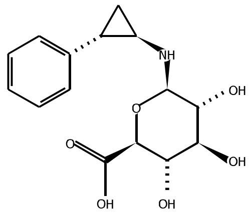

Compound Spectra (overlaid)

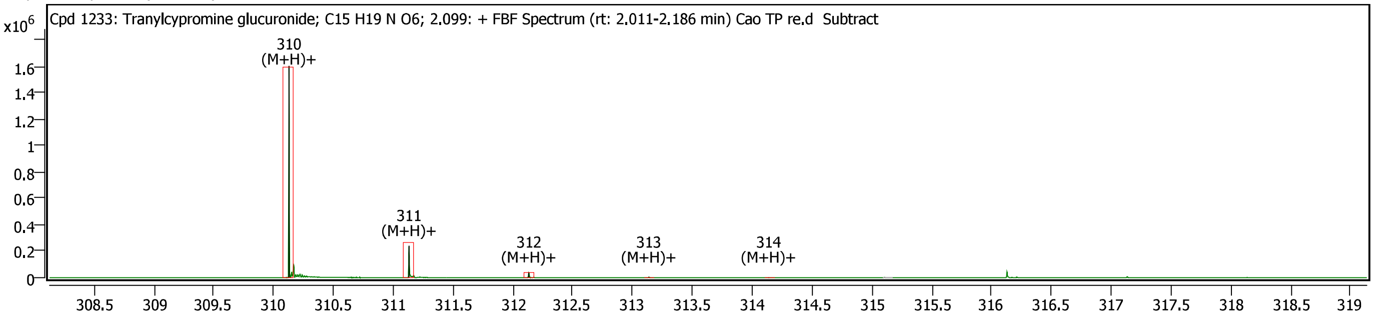

Compound ID Table

Name	Formula	Species	RT	RT Diff	Mass	CAS	ID Source	Score	Score (Lib)	Score (Tgt)
Tranylcypromine glucuronide	C15 H19 N O6	(M+H)+	2.099		309.1223		FBF	93.03		93.03

Cpd 1324: <trans-Zeatin>

Name	Formula	RT	RI	Mass	Diff (Tgt, ppm)	CAS	ID Source	Score	Algorithm
<trans-Zeatin>	C10 H13 N5 O	2.256		219.1112	-3.57	1637-39-4	M-FBF	91.90	FBF

Species	m/z	Score (Tgt)	Score (Lib)	Score (DB)	Score (MFG)	Score (RT)
(M+H)+	220	91.90				

Compound Chromatograms (overlaid)

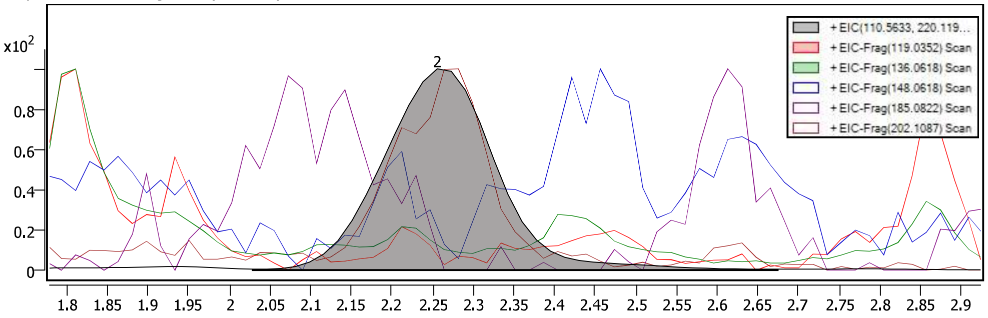

Structure

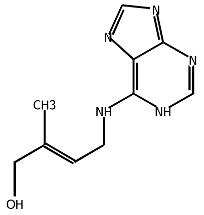

Coelution Plot

Compound Spectra (overlaid)

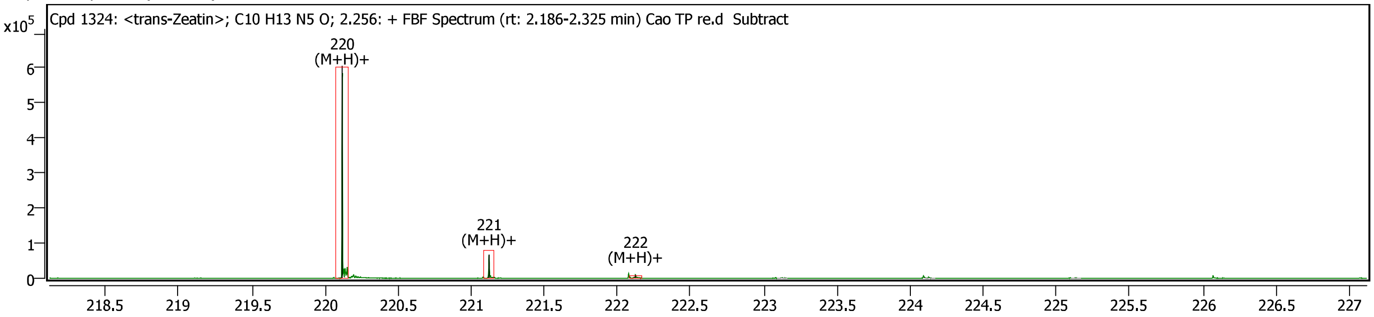

Fragment Spectrum (raw)

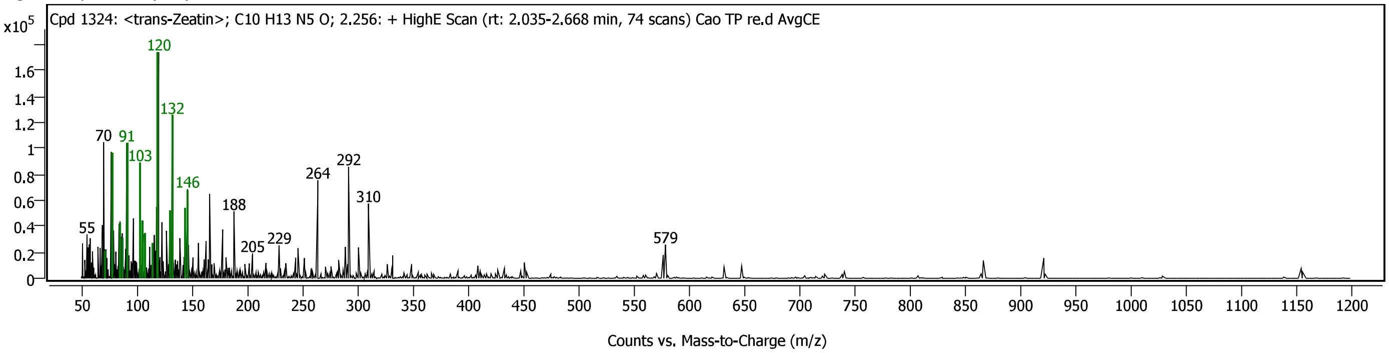

Compound ID Table

Name	Formula	Species	RT	RT Diff	Mass	CAS	ID Source	Score	Score (Lib)	Score (Tgt)
<trans-Zeatin>	C10 H13 N5 O	(M+H)+	2.256		219.1112	1637-39-4	FBF	91.90		91.90
<cis-Zeatin>	C10 H13 N5 O	(M+H)+	2.256		219.1112		FBF	91.90		91.90

Cpd 1447: D-Pantothenic acid

Name	Formula	RT	RI	Mass	Diff (Tgt, ppm)	CAS	ID Source	Score	Algorithm
D-Pantothenic acid	C9 H17 N O5	2.256		219.1112	2.20	79-83-4	FBF-FragConfirm	97.58	FBF

Species	m/z	Score (Tgt)	Score (Lib)	Score (DB)	Score (MFG)	Score (RT)
(M+H)+	220	97.58				

Compound Chromatograms (overlaid)

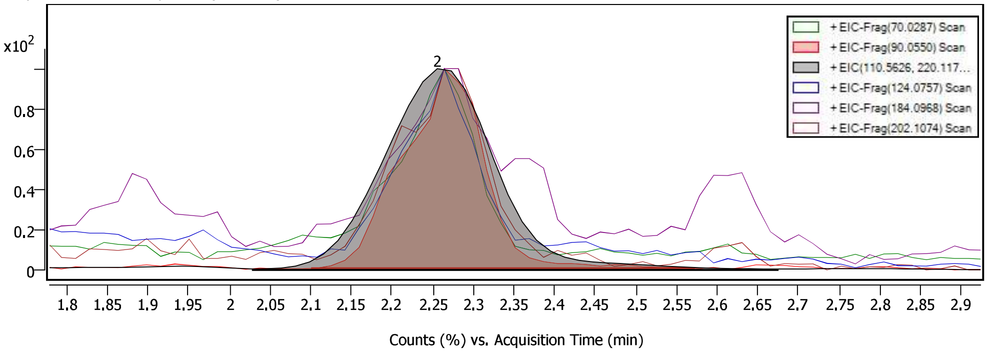

Structure

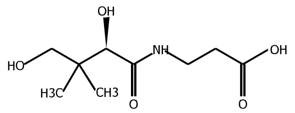

Coelution Plot

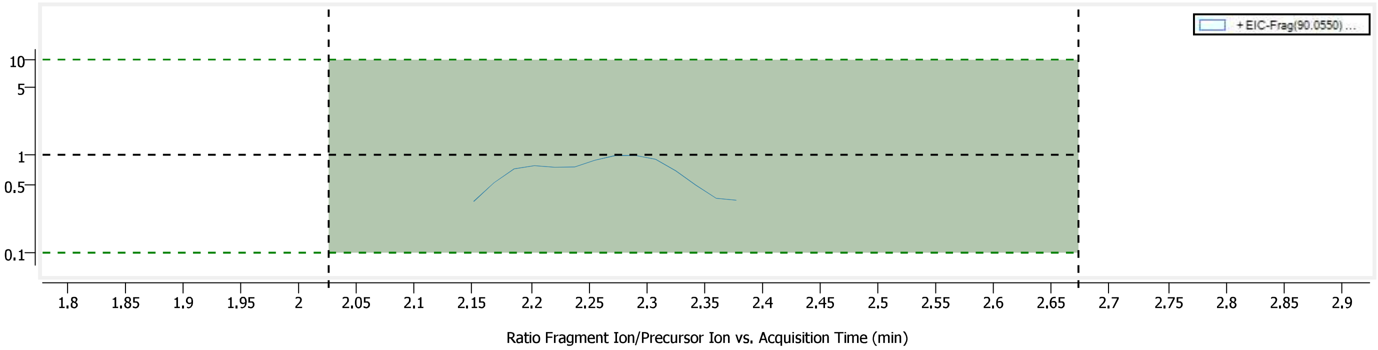

Compound Spectra (overlaid)

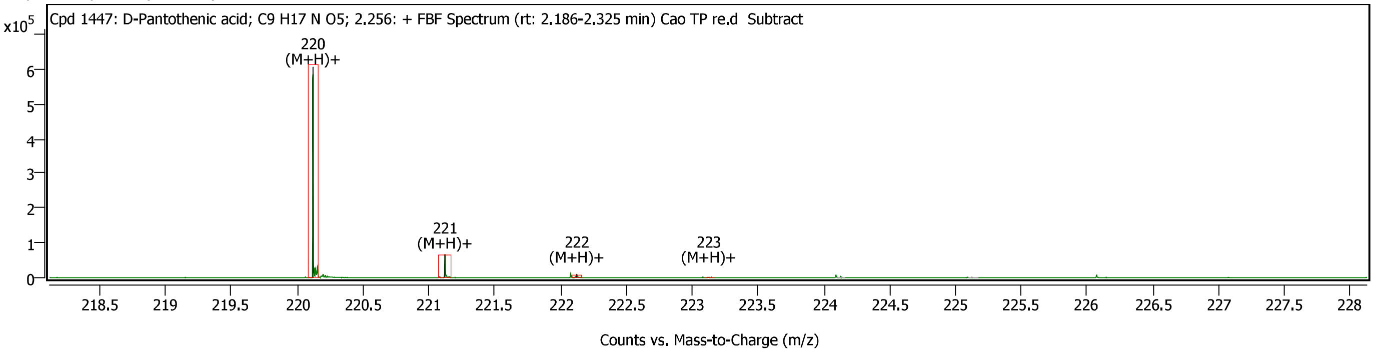

Fragment Spectrum (clean)

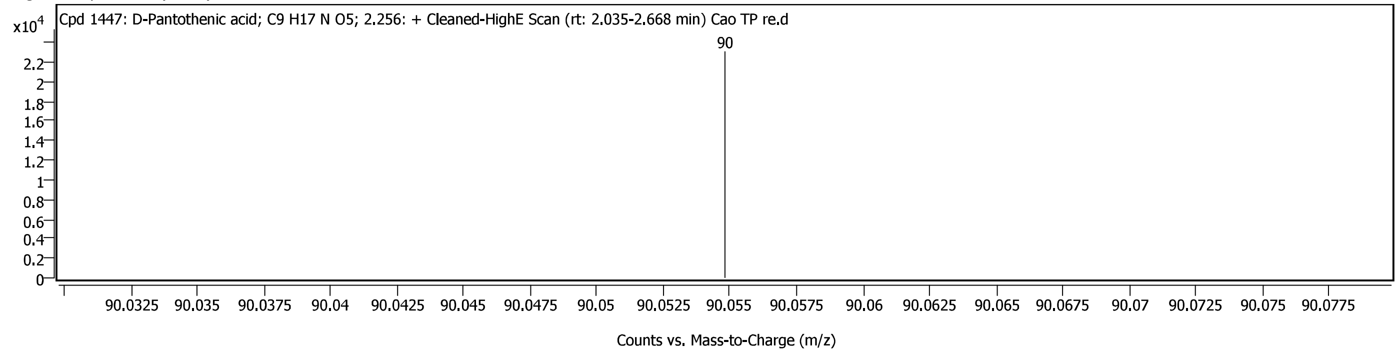

Fragment Spectrum (raw)

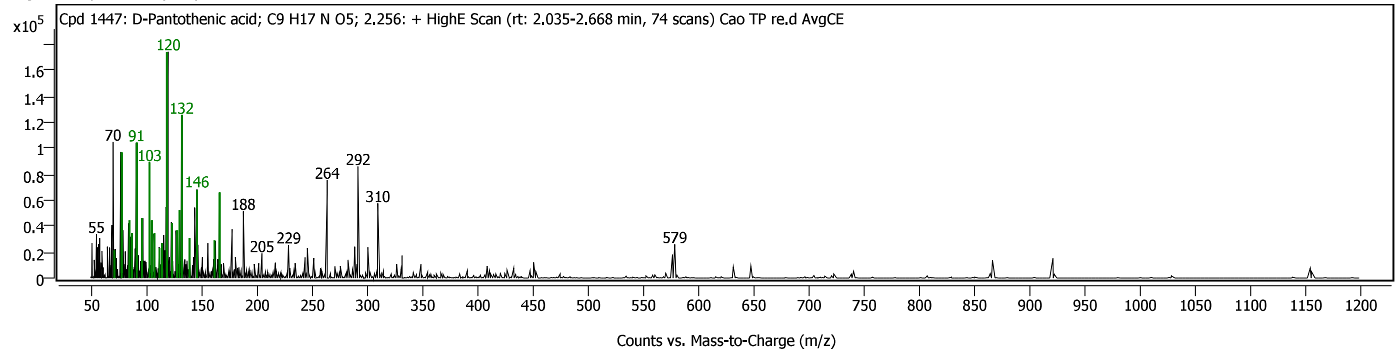

Compound ID Table

Name	Formula	Species	RT	RT Diff	Mass	CAS	ID Source	Score	Score (Lib)	Score (Tgt)
D-Pantothenic acid	C9 H17 N O5	(M+H)+	2.256		219.1112	79-83-4	FBF-FragConfirm	97.58		97.58

Cpd 507: Tiapamil

Name	Formula	RT	RI	Mass	Diff (Tgt, ppm)	CAS	ID Source	Score	Algorithm
Tiapamil	C26 H37 N O8 S2	2.325		555.1958	-0.41	57010-31-8	FBF	85.91	FBF

Species	m/z	Score (Tgt)	Score (Lib)	Score (DB)	Score (MFG)	Score (RT)
(M+H)+	556	85.91				

Compound Chromatograms (overlaid)

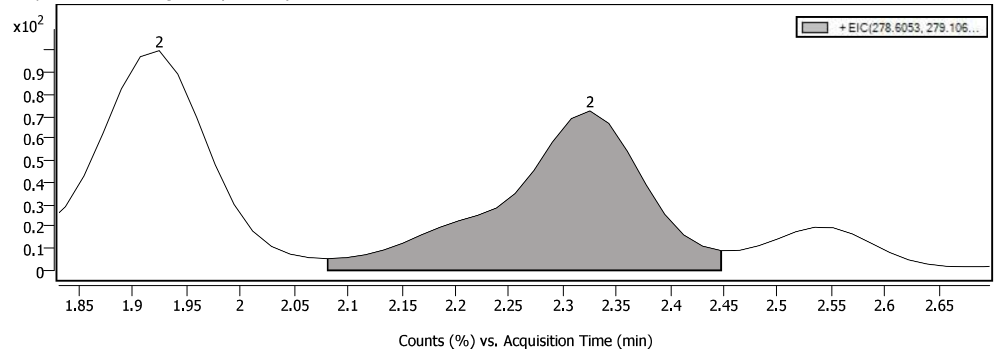

Structure

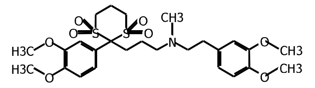

Compound Spectra (overlaid)

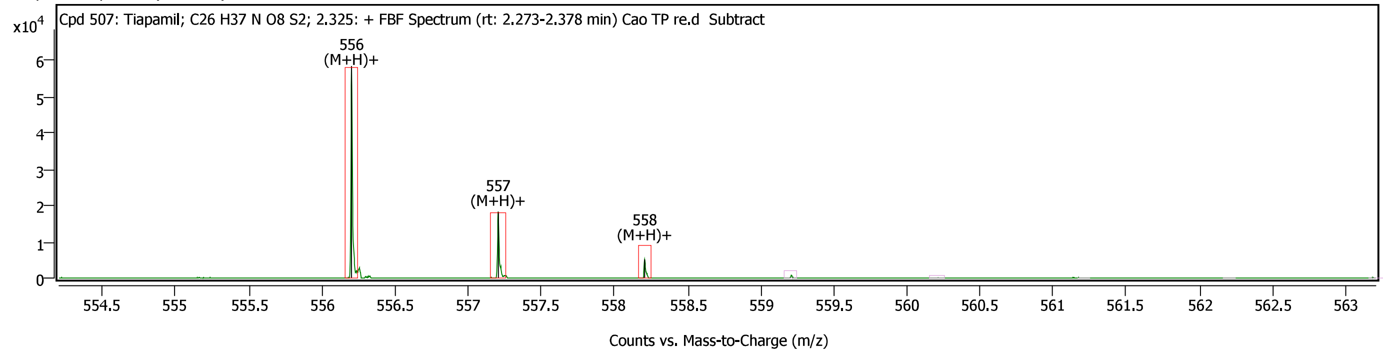

Compound ID Table

Name	Formula	Species	RT	RT Diff	Mass	CAS	ID Source	Score	Score (Lib)	Score (Tgt)
Tiapamil	C26 H37 N O8 S2	(M+H)+	2.325		555.1958	57010-31-8	FBF	85.91		85.91

Cpd 1453: Succinoadenosine

Compound Screening Report

Name	Formula	RT	RI	Mass	Diff (Tgt, ppm)	CAS	ID Source	Score	Algorithm
Succinoadenosine	C14 H17 N5 O8	2.430		383.1078	0.33	4542-23-8	FBF	98.83	FBF

Species	m/z	Score (Tgt)	Score (Lib)	Score (DB)	Score (MFG)	Score (RT)
(M+H)+	384	98.83				

Compound Chromatograms (overlaid)

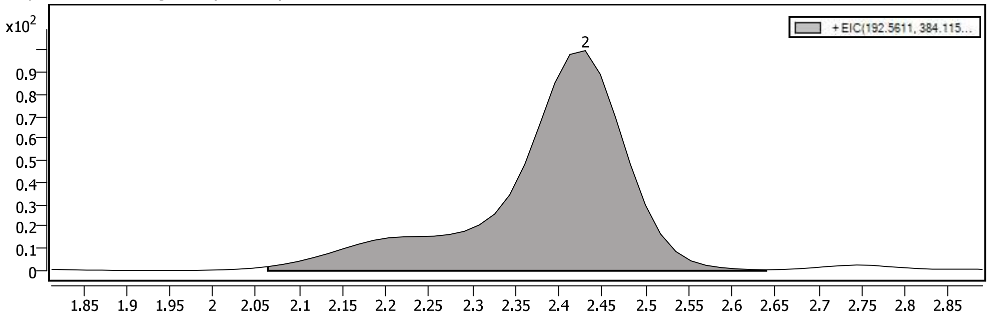

Structure

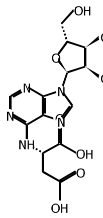

Compound Spectra (overlaid)

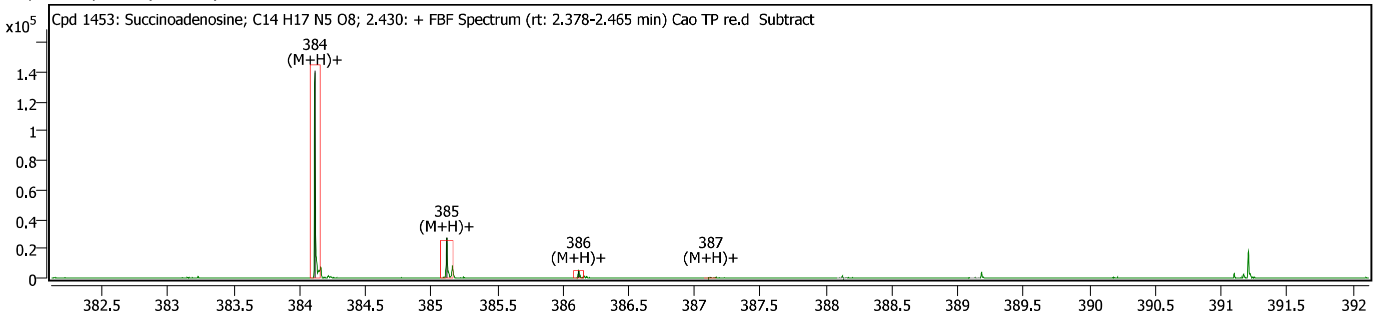

Compound ID Table

Name	Formula	Species	RT	RT Diff	Mass	CAS	ID Source	Score	Score (Lib)	Score (Tgt)
Succinoadenosine	C14 H17 N5 O8	(M+H)+	2.430		383.1078	4542-23-8	FBF	98.83		98.83

Cpd 1445: Octylamine

Name	Formula	RT	RI	Mass	Diff (Tgt, ppm)	CAS	ID Source	Score	Algorithm
Octylamine	C8 H19 N	2.604		129.1519	0.89	111-86-4	FBF	86.91	FBF

Species	m/z	Score (Tgt)	Score (Lib)	Score (DB)	Score (MFG)	Score (RT)
(M+H)+	130	86.91				

Compound Chromatograms (overlaid)

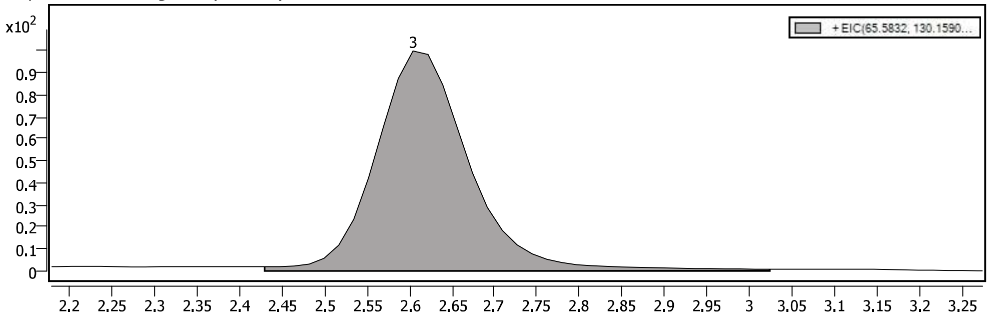

Structure

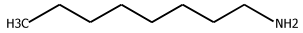

Compound Spectra (overlaid)

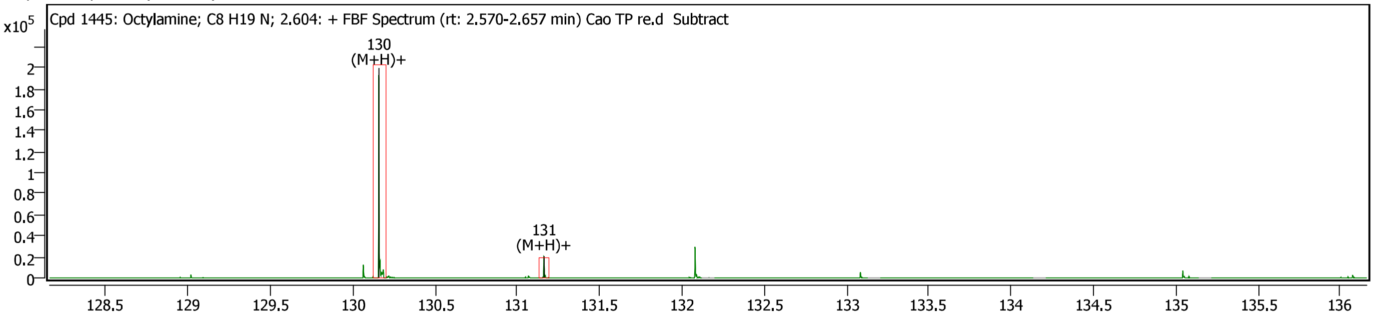

Compound ID Table

Name	Formula	Species	RT	RT Diff	Mass	CAS	ID Source	Score	Score (Lib)	Score (Tgt)
Octylamine	C8 H19 N	(M+H)+	2.604		129.1519	111-86-4	FBF	86.91		86.91

Cpd 1519: Semilepidinoside B

Name	Formula	RT	RI	Mass	Diff (Tgt, ppm)	CAS	ID Source	Score	Algorithm
Semilepidinoside B	C17 H22 N2 O7	2.622		366.1437	2.71		M-FBF	96.26	FBF

Species	m/z	Score (Tgt)	Score (Lib)	Score (DB)	Score (MFG)	Score (RT)
(M+H)+	367	96.26				

Compound Chromatograms (overlaid)

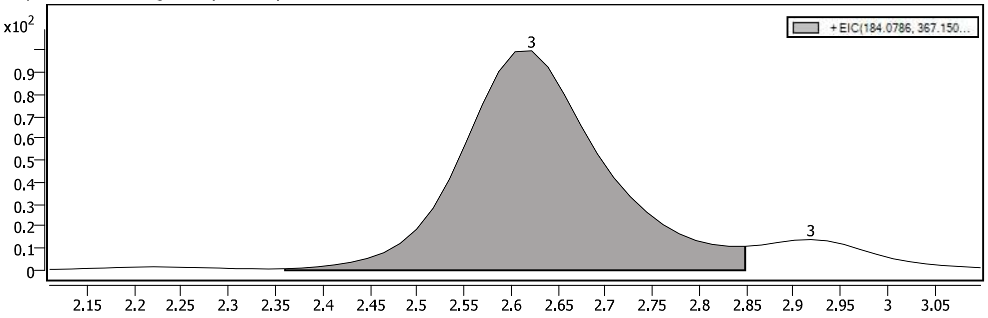

Structure

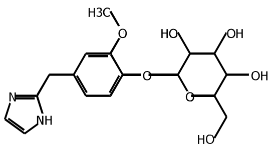

Compound Spectra (overlaid)

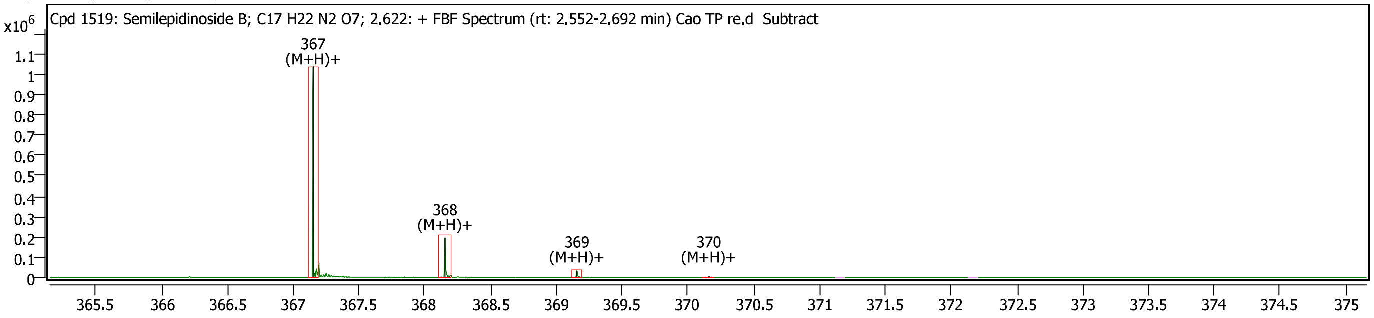

Compound ID Table

Name	Formula	Species	RT	RT Diff	Mass	CAS	ID Source	Score	Score (Lib)	Score (Tgt)
Semilepidinoside B	C17 H22 N2 O7	(M+H)+	2.622		366.1437		FBF	96.26		96.26
Tetrahydropentoxyline	C17 H22 N2 O7	(M+H)+	2.622		366.1437	154204-09-8	FBF	96.26		96.26
N-(1-Deoxy-1-fructosyl)tryptophan	C17 H22 N2 O7	(M+H)+	2.622		366.1437	25020-15-9	FBF	96.26		96.26

Cpd 216: DL-Tryptophan

Name	Formula	RT	RI	Mass	Diff (Tgt, ppm)	CAS	ID Source	Score	Algorithm
DL-Tryptophan	C11 H12 N2 O2	2.674		204.0901	1.26	54-12-6	M-FBF-FragConfirm	99.18	FBF

Species	m/z	Score (Tgt)	Score (Lib)	Score (DB)	Score (MFG)	Score (RT)
(M+H)+	205	99.18				

Compound Screening Report

Compound Chromatograms (overlaid)

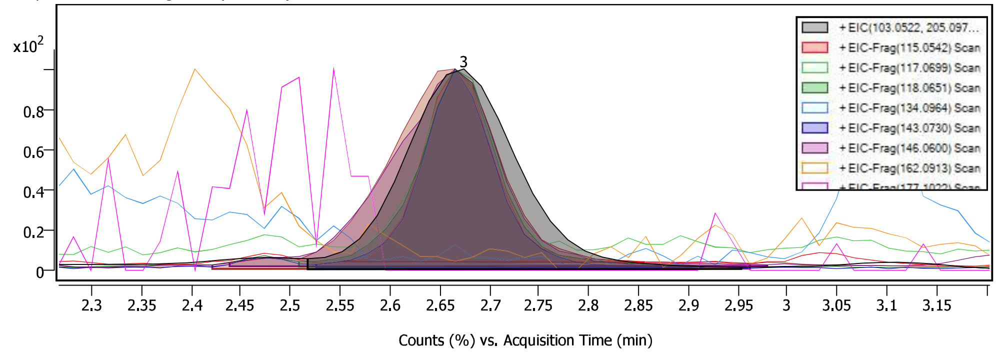

Structure

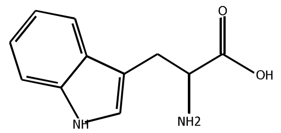

Coelution Plot

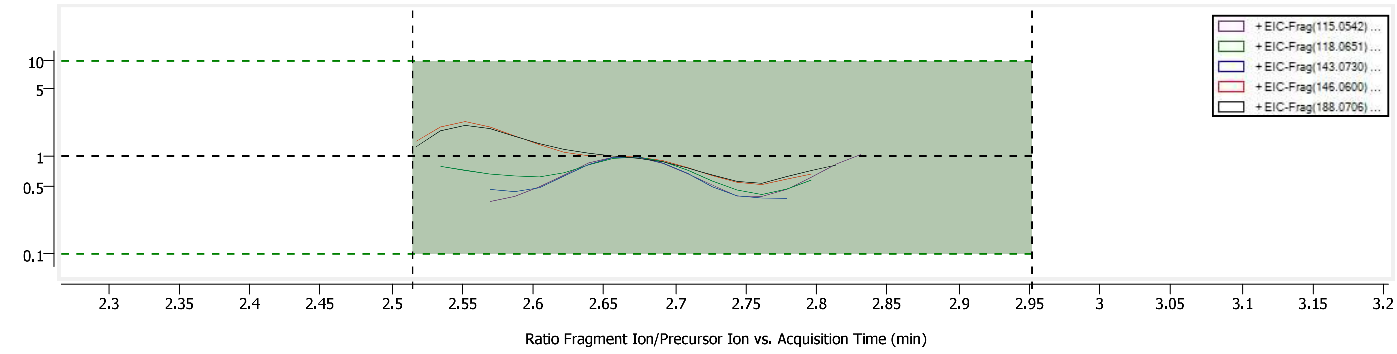

Compound Spectra (overlaid)

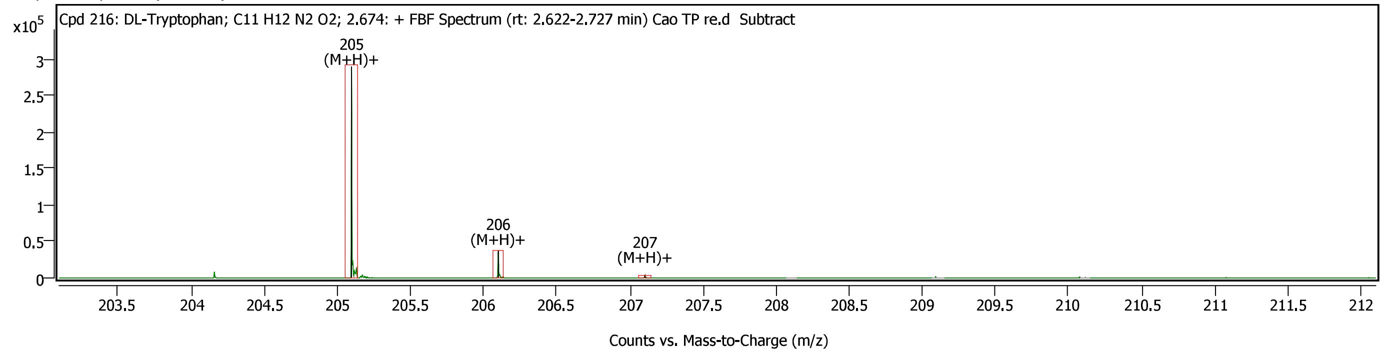

Fragment Spectrum (clean)

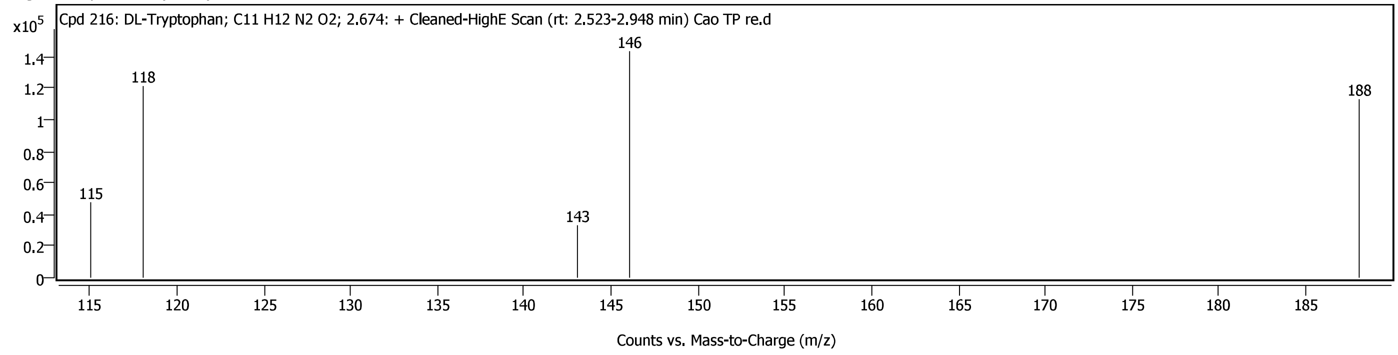

Fragment Spectrum (raw)

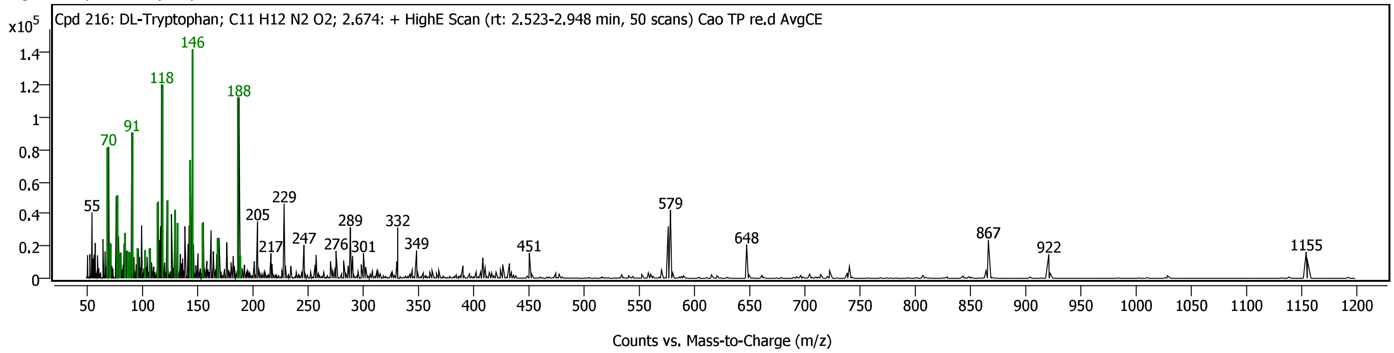

Compound ID Table

Name	Formula	Species	RT	RT Diff	Mass	CAS	ID Source	Score	Score (Lib)	Score (Tgt)
DL-Tryptophan	C11 H12 N2 O2	(M+H)+	2.674		204.0901	54-12-6	FBF-FragConfirm	99.18		99.18
3-Hydroxymethylantipyrine	C11 H12 N2 O2	(M+H)+	2.674		204.0901	18125-49-0	FBF-FragConfirm	99.18		99.18
Ethotoin	C11 H12 N2 O2	(M+H)+	2.674		204.0901	86-35-1	FBF-FragConfirm	99.18		99.18
D-Tryptophan	C11 H12 N2 O2	(M+H)+	2.674		204.0901	153-94-6	FBF-FragConfirm	99.18		99.18
L-Tryptophan	C11 H12 N2 O2	(M+H)+	2.674		204.0901	73-22-3	FBF-FragConfirm	99.18		99.18
Idazoxan	C11 H12 N2 O2	(M+H)+	2.674		204.0901	79944-58-4	FBF-FragConfirm	99.18		99.18
Nirvanol	C11 H12 N2 O2	(M+H)+	2.674		204.0901	631-07-2	FBF-FragConfirm	99.18		99.18
Vasicinol	C11 H12 N2 O2	(M+H)+	2.674		204.0901	5081-51-6	FBF-FragConfirm	99.18		99.18

Cpd 560: 4-Formyl Indole

Name	Formula	RT	RI	Mass	Diff (Tgt, ppm)	CAS	ID Source	Score	Algorithm
4-Formyl Indole	C9 H7 N O	2.674		145.0528	0.12	1074-86-8	M-FBF-FragConfirm	98.29	FBF

Species	m/z	Score (Tgt)	Score (Lib)	Score (DB)	Score (MFG)	Score (RT)
(M+H)+	146	98.29				

Compound Chromatograms (overlaid)

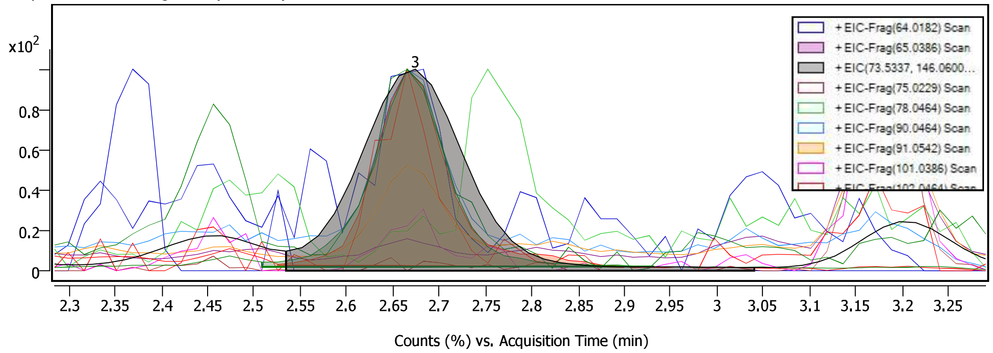

Structure

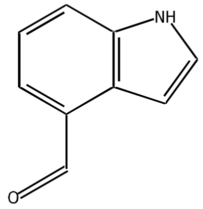

Coelution Plot

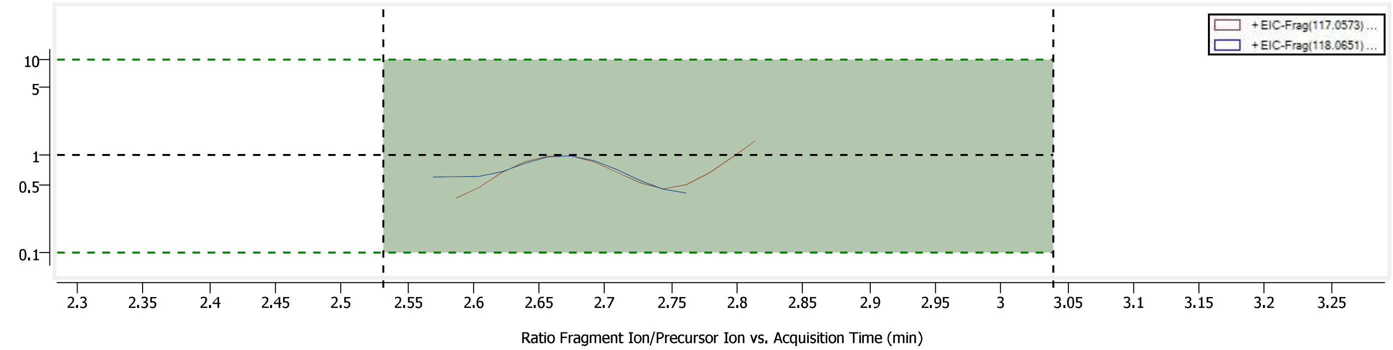

Compound Spectra (overlaid)

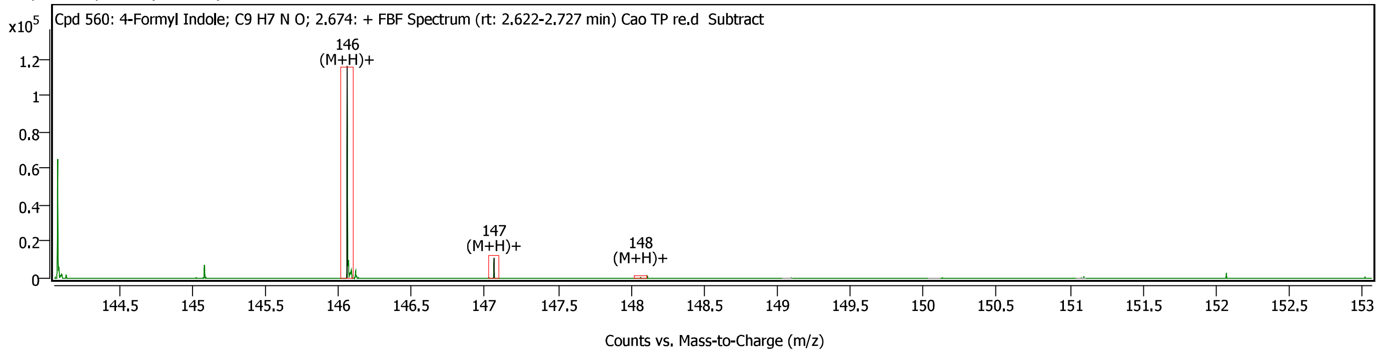

Fragment Spectrum (clean)

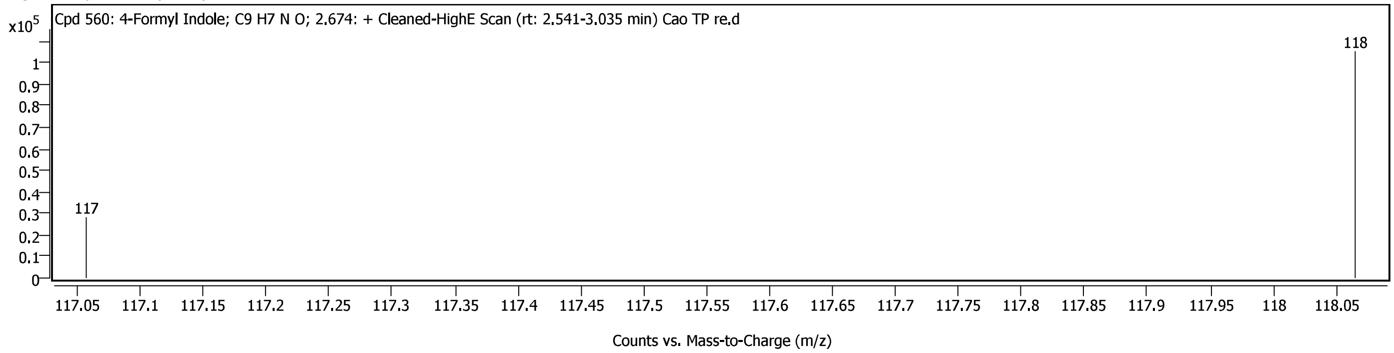

Fragment Spectrum (raw)

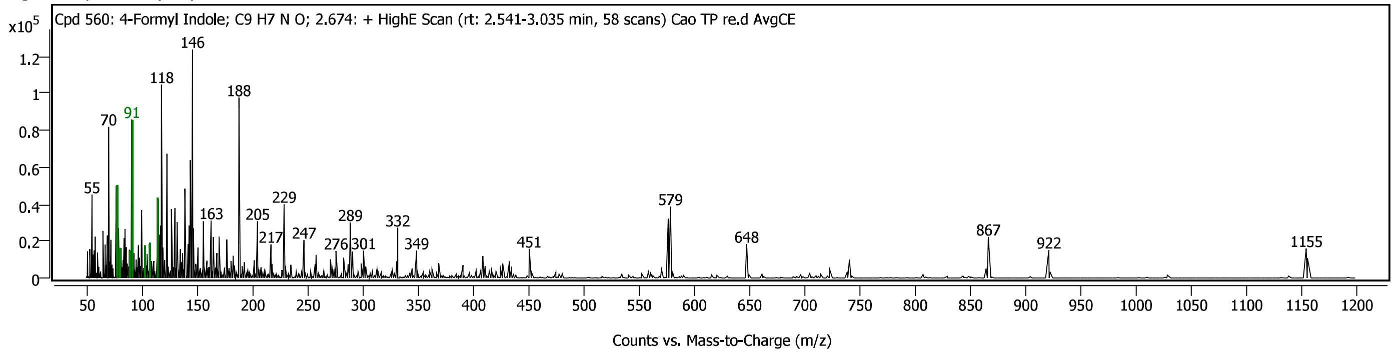

Compound ID Table

Name	Formula	Species	RT	RT Diff	Mass	CAS	ID Source	Score	Score (Lib)	Score (Tgt)
4-Formyl Indole	C9 H7 N O	(M+H)+	2.674		145.0528	1074-86-8	FBF-FragConfirm	98.29		98.29
Quinolin-2-ol	C9 H7 N O	(M+H)+	2.674		145.0528	70254-42-1	FBF-FragConfirm	98.29		98.29
Oxyquinoline	C9 H7 N O	(M+H)+	2.674		145.0528	148-24-3	FBF-FragConfirm	98.29		98.29
Isoquinoline N-oxide	C9 H7 N O	(M+H)+	2.674		145.0528	1532-72-5	FBF-FragConfirm	98.29		98.29
Isocarbostyril	C9 H7 N O	(M+H)+	2.674		145.0528	491-30-5	FBF-FragConfirm	98.29		98.29
4-Quinolinol	C9 H7 N O	(M+H)+	2.674		145.0528	611-36-9	FBF-FragConfirm	98.29		98.29
3-Methyleneoxindole	C9 H7 N O	(M+H)+	2.674		145.0528	1861-29-6	FBF-FragConfirm	98.29		98.29
2(1H)-Quinolinone	C9 H7 N O	(M+H)+	2.674		145.0528	59-31-4	FBF-FragConfirm	98.29		98.29
Indole-3-carboxaldehyde	C9 H7 N O	(M+H)+	2.674		145.0528	487-89-8	FBF-FragConfirm	98.29		98.29

Cpd 1452: 3-Amino-2-naphthoic acid

Name	Formula	RT	RI	Mass	Diff (Tgt, ppm)	CAS	ID Source	Score	Algorithm
3-Amino-2-naphthoic acid	C11 H9 N O2	2.674		187.0638	2.42	5959-52-4	M-FBF-FragConfirm	97.13	FBF

Species	m/z	Score (Tgt)	Score (Lib)	Score (DB)	Score (MFG)	Score (RT)
(M+H)+	188	97.13				

Compound Screening Report

Compound Chromatograms (overlaid)

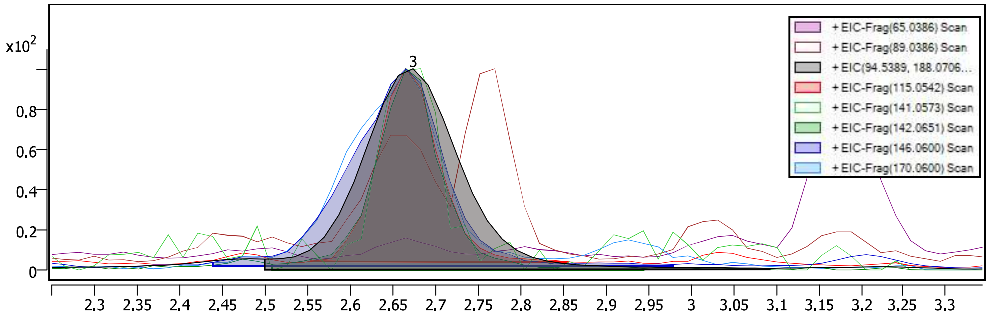

Structure

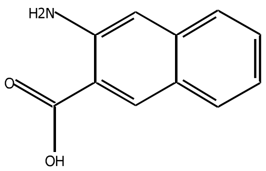

Coelution Plot

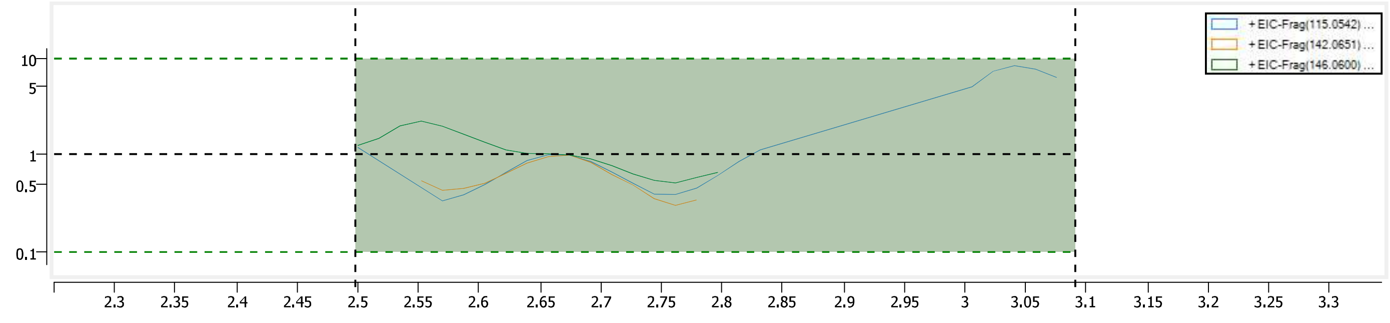

Compound Spectra (overlaid)

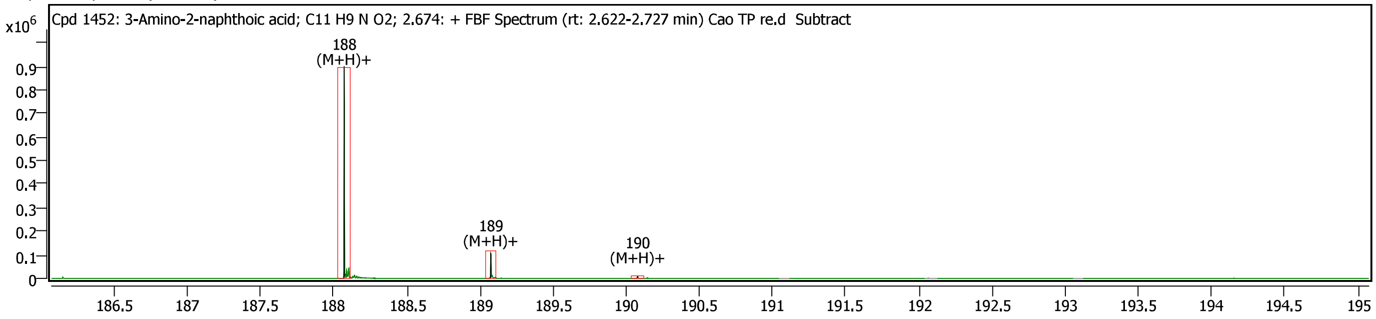

Fragment Spectrum (clean)

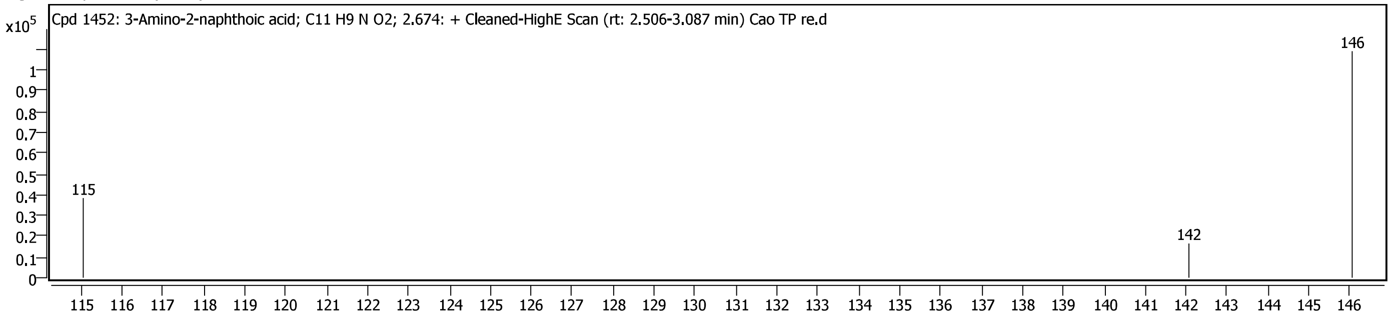

Fragment Spectrum (raw)

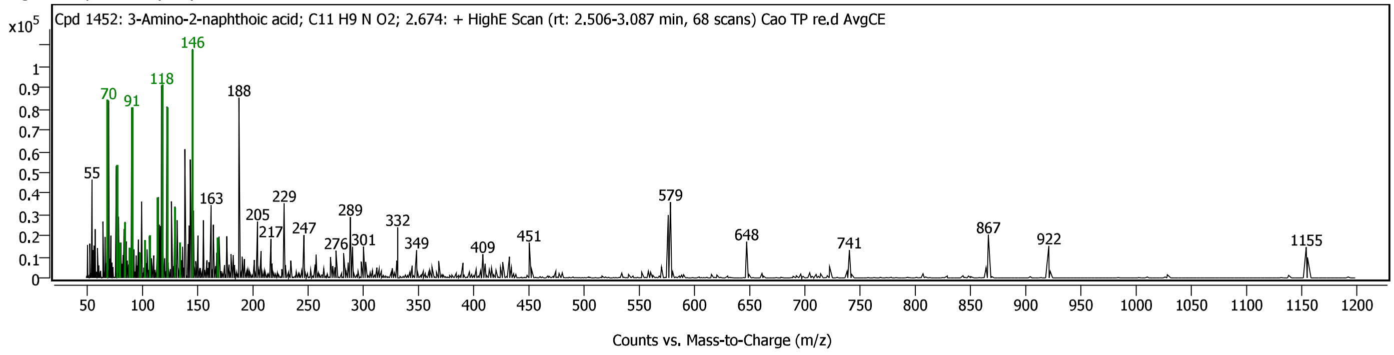

Compound ID Table

Name	Formula	Species	RT	RT Diff	Mass	CAS	ID Source	Score	Score (Lib)	Score (Tgt)
3-Amino-2-naphthoic acid	C11 H9 N O2	(M+H)+	2.674		187.0638	5959-52-4	FBF-FragConfirm	97.13		97.13
Indoleacrylic acid	C11 H9 N O2	(M+H)+	2.674		187.0638	1204-06-4	FBF-FragConfirm	97.13		97.13
Quinacetol	C11 H9 N O2	(M+H)+	2.674		187.0638	2598-31-4	FBF-FragConfirm	97.13		97.13

Cpd 473: Cinnamtannin A2

Name	Formula	RT	RI	Mass	Diff (Tgt, ppm)	CAS	ID Source	Score	Algorithm
Cinnamtannin A2	C60 H50 O24	2.901		1154.2684	-0.70		FBF	99.46	FBF

Species	m/z	Score (Tgt)	Score (Lib)	Score (DB)	Score (MFG)	Score (RT)
(M+2H)+2 (M+H)+	578 1155	99.46				

Compound Chromatograms (overlaid)

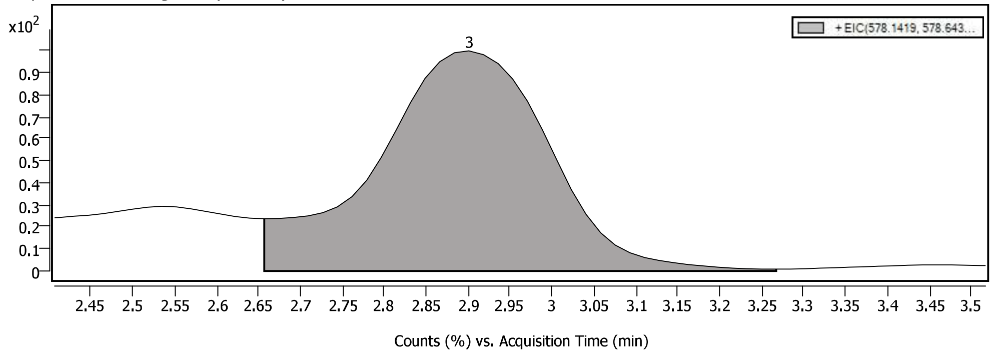

Structure

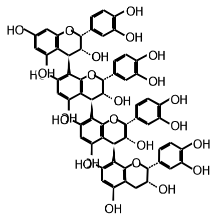

Compound Spectra (overlaid)

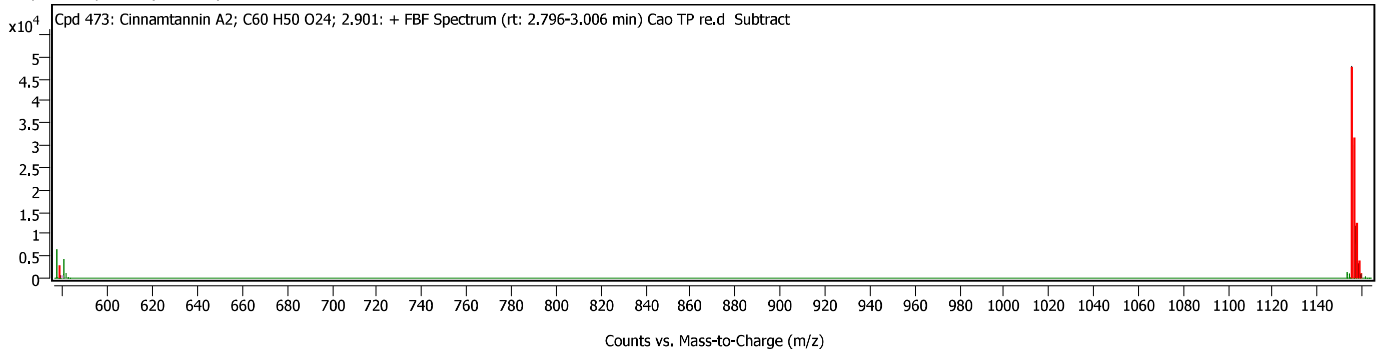

Compound ID Table

Name	Formula	Species	RT	RT Diff	Mass	CAS	ID Source	Score	Score (Lib)	Score (Tgt)
Cinnamtannin A2	C60 H50 O24	(M+2H)+2 (M+H)+	2.901		1154.2684		FBF	99.46		99.46

Cpd 563: 1,8-Diazacyclotetradecane-2,9-dione

Name	Formula	RT	RI	Mass	Diff (Tgt, ppm)	CAS	ID Source	Score	Algorithm
1,8-Diazacyclotetradecane-2,9-dione	C12 H22 N2 O2	2.971		226.1685	1.80		FBF	97.95	FBF

Species	m/z	Score (Tgt)	Score (Lib)	Score (DB)	Score (MFG)	Score (RT)
(M+2H)+2 (M+H)+	114 227	97.95				

Compound Chromatograms (overlaid)

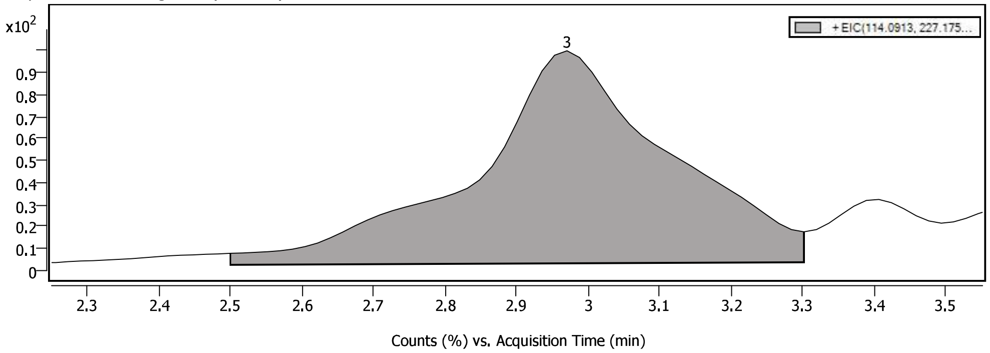

Structure

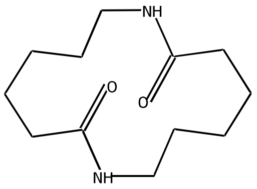

Compound Spectra (overlaid)

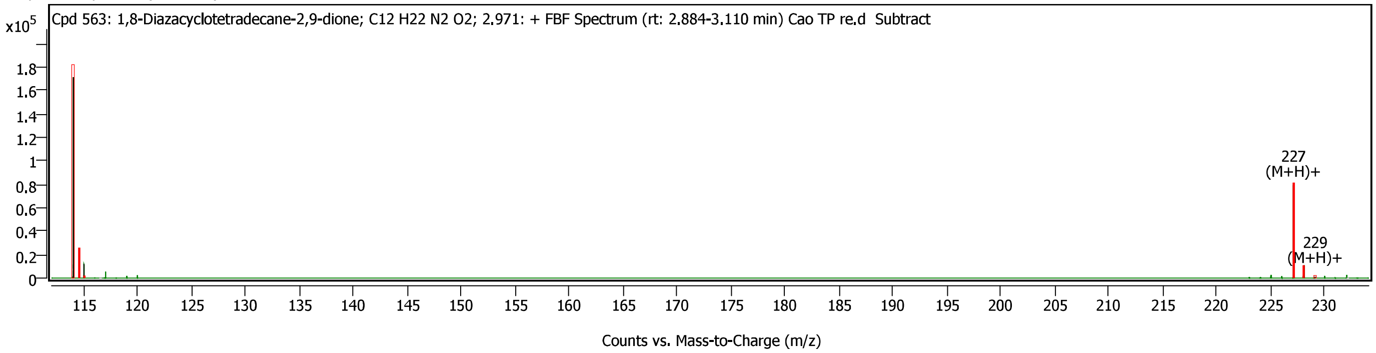

Compound ID Table

Name	Formula	Species	RT	RT Diff	Mass	CAS	ID Source	Score	Score (Lib)	Score (Tgt)
1,8-Diazacyclotetradecane-2,9-dione	C12 H22 N2 O2	(M+2H)+2 (M+H)+	2.971		226.1685		FBF	97.95		97.95

Cpd 1547: <ε-Caprolactam>

Name	Formula	RT	RI	Mass	Diff (Tgt, ppm)	CAS	ID Source	Score	Algorithm
<ε-Caprolactam>	C6 H11 N O	2.971		113.0842	1.36	105-60-2	M-FBF	98.45	FBF

Species	m/z	Score (Tgt)	Score (Lib)	Score (DB)	Score (MFG)	Score (RT)
(M+H)+	114	98.45				

Compound Chromatograms (overlaid)

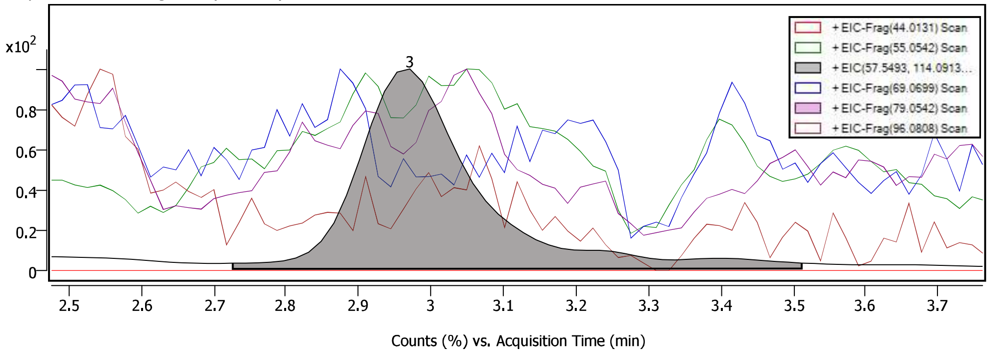

Structure

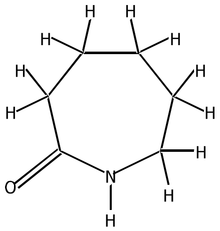

Coelution Plot

Compound Spectra (overlaid)

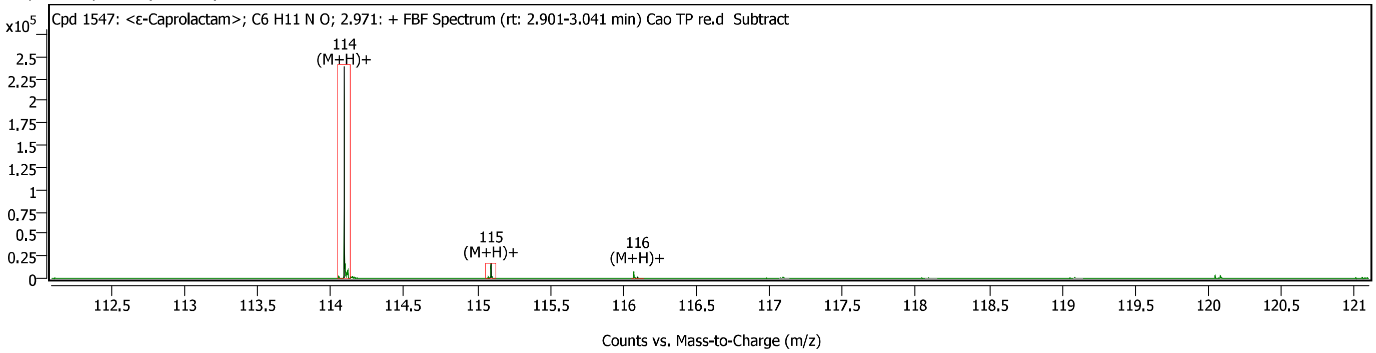

Compound Screening Report

Fragment Spectrum (raw)

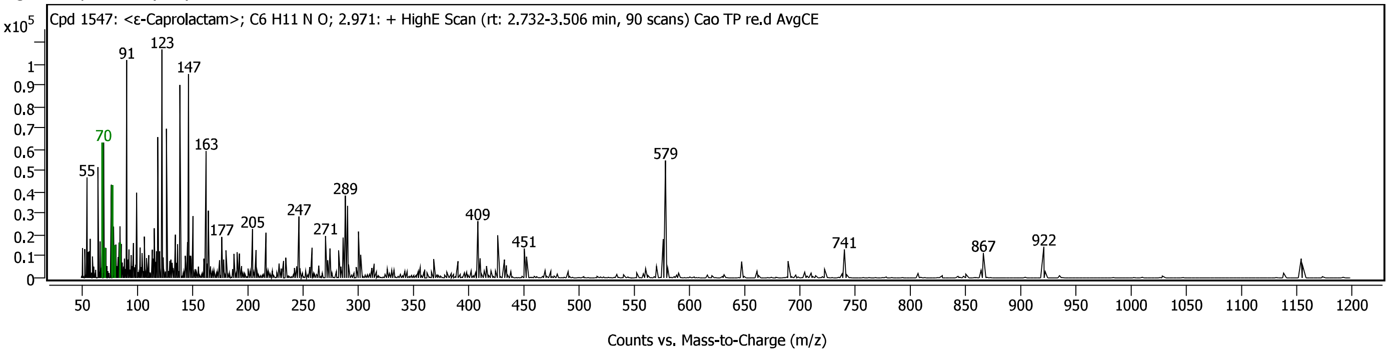

Compound ID Table

Name	Formula	Species	RT	RT Diff	Mass	CAS	ID Source	Score	Score (Lib)	Score (Tgt)
<ε-Caprolactam>	C6 H11 N O	(M+H)+	2.971		113.0842	105-60-2	FBF	98.45		98.45
<1-Piperidinecarboxaldehyde>	C6 H11 N O	(M+H)+	2.971		113.0842	2591-86-8	FBF	98.45		98.45
<2,5-Dihydro-2,4,5-trimethyloxazole>	C6 H11 N O	(M+H)+	2.971		113.0842	22694-96-8	FBF	98.45		98.45
<2-Acetylpyrrolidine>	C6 H11 N O	(M+H)+	2.971		113.0842	60026-20-2	FBF	98.45		98.45
<DMPO>	C6 H11 N O	(M+H)+	2.971		113.0842	3317-61-1	FBF	98.45		98.45
<N-Acetylpyrrolidine>	C6 H11 N O	(M+H)+	2.971		113.0842		FBF	98.45		98.45

Cpd 1642: <(-)-Catechin>

Name	Formula	RT	RI	Mass	Diff (Tgt, ppm)	CAS	ID Source	Score	Algorithm
<(-)-Catechin>	C15 H14 O6	3.041		290.0795	1.73	18829-70-4	M-FBF	96.73	FBF

Species	m/z	Score (Tgt)	Score (Lib)	Score (DB)	Score (MFG)	Score (RT)
(M+H)+	291	96.73				

Compound Chromatograms (overlaid)

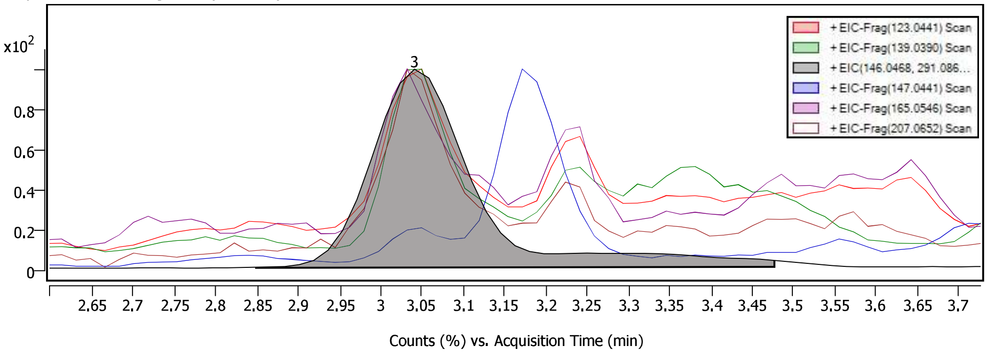

Structure

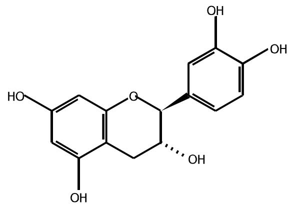

Coelution Plot

Compound Spectra (overlaid)

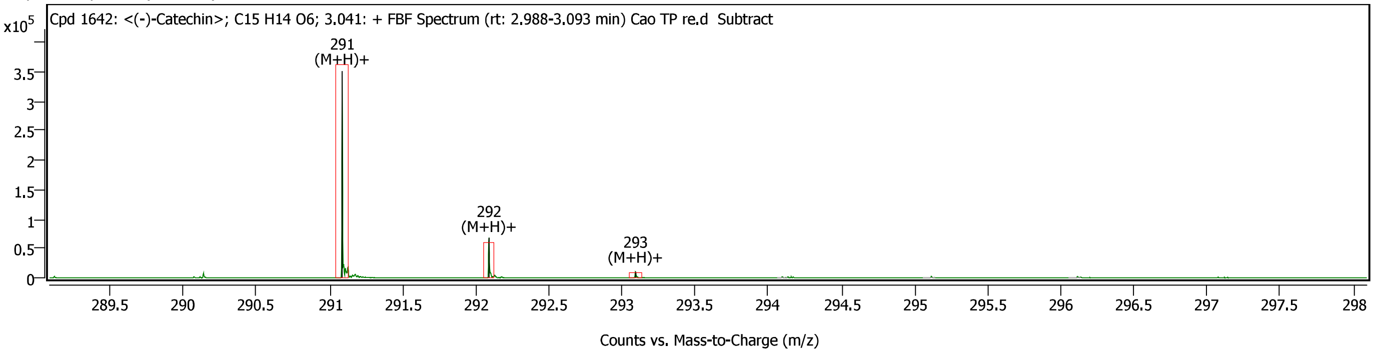

Compound Screening Report

Fragment Spectrum (raw)

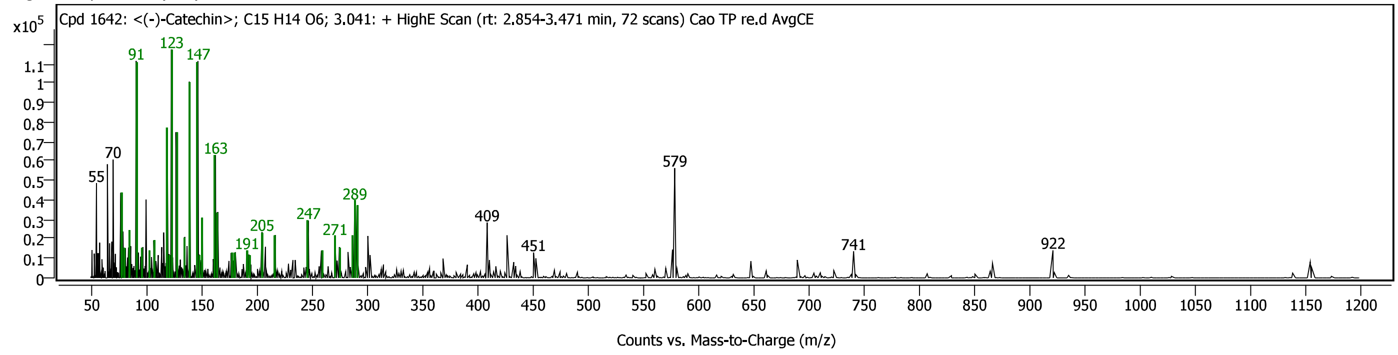

Compound ID Table

Name	Formula	Species	RT	RT Diff	Mass	CAS	ID Source	Score	Score (Lib)	Score (Tgt)
<(-)-Catechin>	C15 H14 O6	(M+H)+	3.041		290.0795	18829-70-4	FBF	96.73		96.73
<Fisetinidol-4beta-ol>	C15 H14 O6	(M+H)+	3.041		290.0795		FBF	96.73		96.73
<Fonsecin>	C15 H14 O6	(M+H)+	3.041		290.0795	3748-39-8	FBF	96.73		96.73
<ent-Fisetinidol-4beta-ol>	C15 H14 O6	(M+H)+	3.041		290.0795		FBF	96.73		96.73
<(-)-Epicatechin>	C15 H14 O6	(M+H)+	3.041		290.0795	490-46-0	FBF	96.73		96.73
<(+)-Epicatechin>	C15 H14 O6	(M+H)+	3.041		290.0795	35323-91-2	FBF	96.73		96.73
<ent-Robinetinidol>	C15 H14 O6	(M+H)+	3.041		290.0795		FBF	96.73		96.73
<(2R,3R)-3,5,7,3',5'-Pentahydroxyflavan>	C15 H14 O6	(M+H)+	3.041		290.0795		FBF	96.73		96.73
<(2R-cis)-2-(2,5-Dihydroxyphenyl)-3,4-dihydro-2H-1-benzopyran-3,5,7-triol>	C15 H14 O6	(M+H)+	3.041		290.0795		FBF	96.73		96.73
<2,8-Dihydroxy-3,4,7-trimethoxydibenzofuran>	C15 H14 O6	(M+H)+	3.041		290.0795	167278-42-4	FBF	96.73		96.73
<3,4,2',4',6'-Pentahydroxydihydrochalcone>	C15 H14 O6	(M+H)+	3.041		290.0795		FBF	96.73		96.73
<3,4,2',4',alpha-Pentahydroxydihydrochalcone>	C15 H14 O6	(M+H)+	3.041		290.0795		FBF	96.73		96.73
<6-Hydroxy-alpha-pyrufuran>	C15 H14 O6	(M+H)+	3.041		290.0795	167278-43-5	FBF	96.73		96.73
<alpha-Cotonefuran>	C15 H14 O6	(M+H)+	3.041		290.0795	93973-22-9	FBF	96.73		96.73
<Cartorimine>	C15 H14 O6	(M+H)+	3.041		290.0795		FBF	96.73		96.73
<Catechin>	C15 H14 O6	(M+H)+	3.041		290.0795	154-23-4	FBF	96.73		96.73
<cis-3,4-Leucopelargonidin>	C15 H14 O6	(M+H)+	3.041		290.0795		FBF	96.73		96.73
<ent-Epifisetinidol-4beta-ol>	C15 H14 O6	(M+H)+	3.041		290.0795		FBF	96.73		96.73
<Oritin-4beta-ol>	C15 H14 O6	(M+H)+	3.041		290.0795		FBF	96.73		96.73
<Fisetinidol-4alpha-ol>	C15 H14 O6	(M+H)+	3.041		290.0795		FBF	96.73		96.73
<Epifisetinidol-4alpha-ol>	C15 H14 O6	(M+H)+	3.041		290.0795		FBF	96.73		96.73
<Marshrin>	C15 H14 O6	(M+H)+	3.041		290.0795		FBF	96.73		96.73
<Epioritin-4alpha-ol>	C15 H14 O6	(M+H)+	3.041		290.0795		FBF	96.73		96.73
<Robinetinidol>	C15 H14 O6	(M+H)+	3.041		290.0795		FBF	96.73		96.73
<Ptaeroglycol>	C15 H14 O6	(M+H)+	3.041		290.0795	18836-12-9	FBF	96.73		96.73
<Plumericin>	C15 H14 O6	(M+H)+	3.041		290.0795	77-16-7	FBF	96.73		96.73
<Nubigenol>	C15 H14 O6	(M+H)+	3.041		290.0795		FBF	96.73		96.73
<Mesquitol>	C15 H14 O6	(M+H)+	3.041		290.0795		FBF	96.73		96.73
<Mikanolide>	C15 H14 O6	(M+H)+	3.041		290.0795	17928-61-9	FBF	96.73		96.73
<Luteoforol>	C15 H14 O6	(M+H)+	3.041		290.0795	24897-98-1	FBF	96.73		96.73
<Isoplumericin>	C15 H14 O6	(M+H)+	3.041		290.0795	31298-76-7	FBF	96.73		96.73
<gamma-Pyrufuran>	C15 H14 O6	(M+H)+	3.041		290.0795	93973-18-3	FBF	96.73		96.73
<Epioritin-4beta-ol>	C15 H14 O6	(M+H)+	3.041		290.0795		FBF	96.73		96.73

Cpd 106: (2S,4S)-Monatin

Name	Formula	RT	RI	Mass	Diff (Tgt, ppm)	CAS	ID Source	Score	Algorithm
(2S,4S)-Monatin	C14 H16 N2 O5	3.163		292.1060	0.12	146142-94-1	FBF	98.34	FBF

Species	m/z	Score (Tgt)	Score (Lib)	Score (DB)	Score (MFG)	Score (RT)
(M+H)+	293	98.34				

Compound Chromatograms (overlaid)

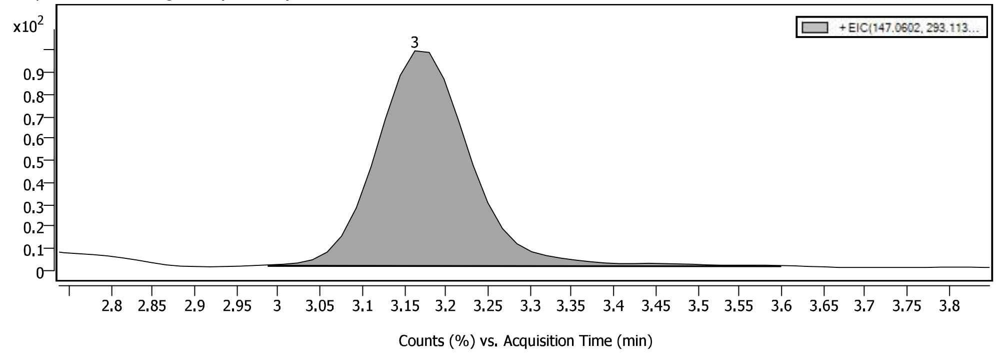

Structure

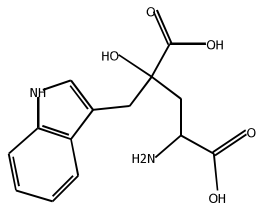

Compound Screening Report

Compound Spectra (overlaid)

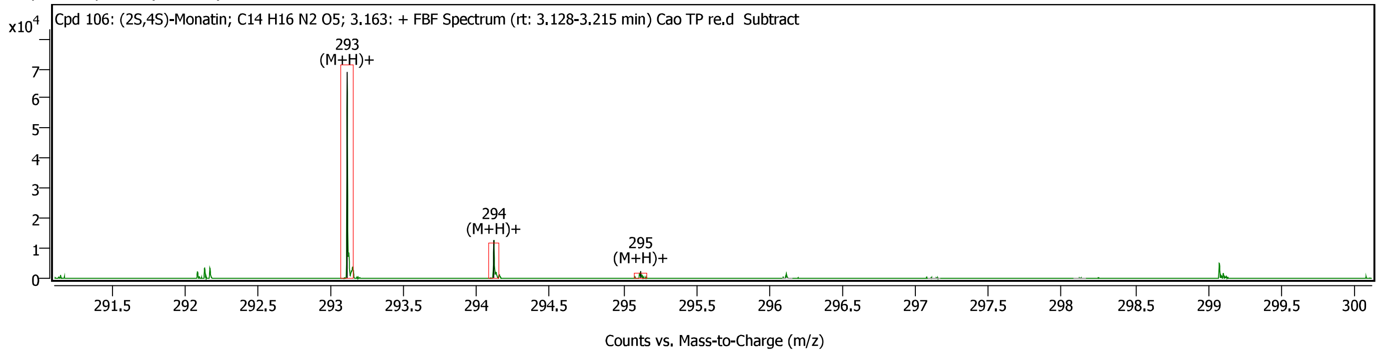

Compound ID Table

Name	Formula	Species	RT	RT Diff	Mass	CAS	ID Source	Score	Score (Lib)	Score (Tgt)
(2S,4S)-Monatin	C14 H16 N2 O5	(M+H)+	3.163		292.1060	146142-94-1	FBF	98.34		98.34

Cpd 1673: 2,4,6-Octatriyn-1-ol

Name	Formula	RT	RI	Mass	Diff (Tgt, ppm)	CAS	ID Source	Score	Algorithm
2,4,6-Octatriyn-1-ol	C8 H6 O	3.180		118.0416	-2.15	13033-21-1	M-FBF	97.04	FBF

Species	m/z	Score (Tgt)	Score (Lib)	Score (DB)	Score (MFG)	Score (RT)
(M+H)+	119	97.04				

Compound Chromatograms (overlaid)

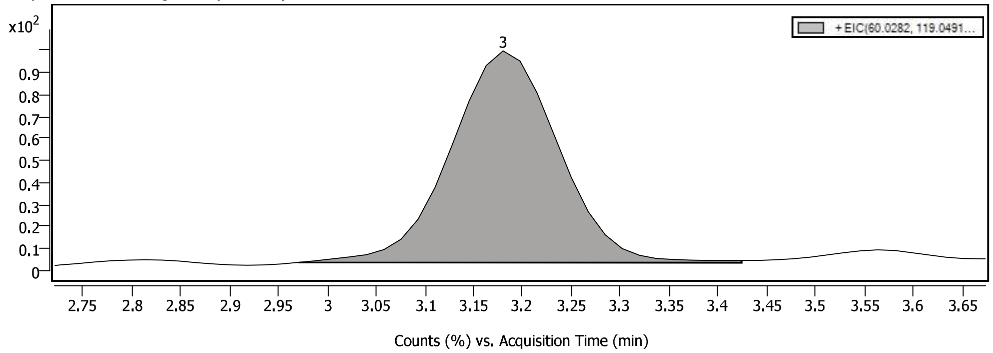

Structure

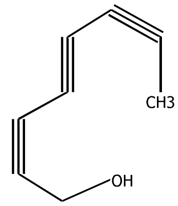

Compound Spectra (overlaid)

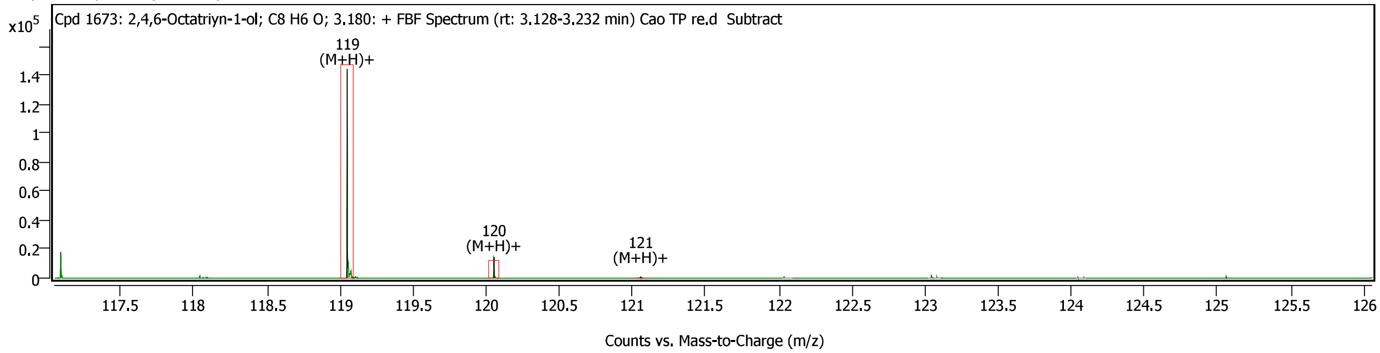

Compound ID Table

Name	Formula	Species	RT	RT Diff	Mass	CAS	ID Source	Score	Score (Lib)	Score (Tgt)
2,4,6-Octatriyn-1-ol	C8 H6 O	(M+H)+	3.180		118.0416	13033-21-1	FBF	97.04		97.04
xi-2,3-Octadiene-5,7-diyn-1-ol	C8 H6 O	(M+H)+	3.180		118.0416		FBF	97.04		97.04
Benzofuran	C8 H6 O	(M+H)+	3.180		118.0416	271-89-6	FBF	97.04		97.04
3,5,7-Octatriyn-1-ol	C8 H6 O	(M+H)+	3.180		118.0416	6071-20-1	FBF	97.04		97.04

Cpd 1432: Indol-3-ylacetyl-myo-inositol L-arabinoside

Name	Formula	RT	RI	Mass	Diff (Tgt, ppm)	CAS	ID Source	Score	Algorithm
Indol-3-ylacetyl-myo-inositol L-arabinoside	C21 H27 N O11	3.215		469.1586	0.45		FBF	99.74	FBF

Species	m/z	Score (Tgt)	Score (Lib)	Score (DB)	Score (MFG)	Score (RT)
(M+H)+	470	99.74				

Compound Chromatograms (overlaid)

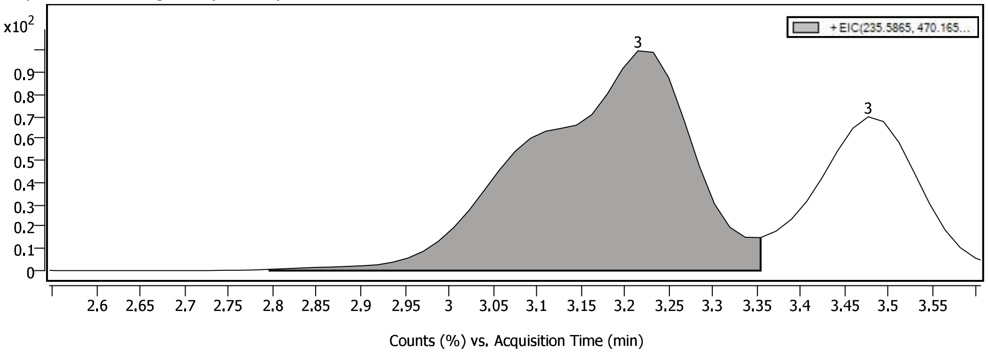

Structure

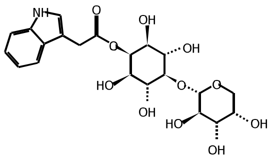

Compound Spectra (overlaid)

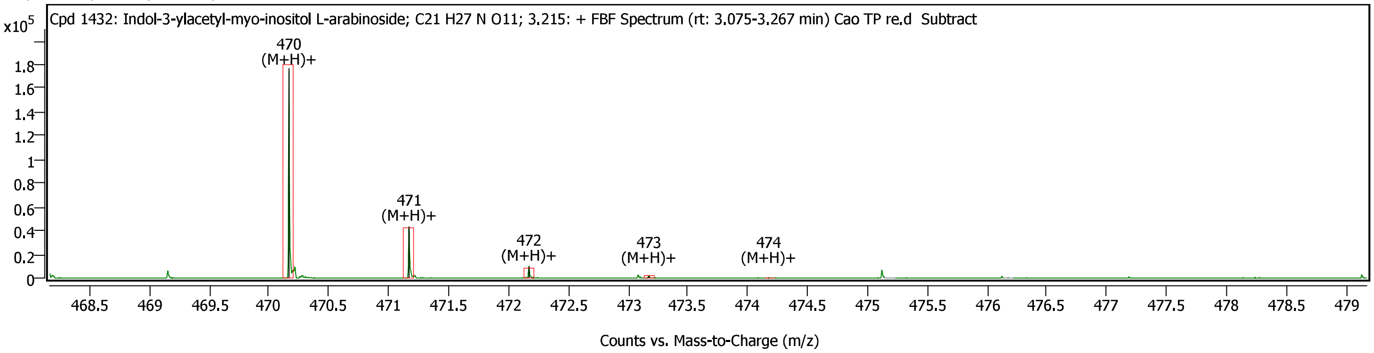

Compound ID Table

Name	Formula	Species	RT	RT Diff	Mass	CAS	ID Source	Score	Score (Lib)	Score (Tgt)
Indol-3-ylacetyl-myo-inositol L-arabinoside	C21 H27 N O11	(M+H)+	3.215		469.1586		FBF	99.74		99.74

Cpd 1709: Procyanidin B2

Name	Formula	RT	RI	Mass	Diff (Tgt, ppm)	CAS	ID Source	Score	Algorithm
Procyanidin B2	C30 H26 O12	3.320		578.1434	1.61		FBF-FragConfirm	98.59	FBF

Species	m/z	Score (Tgt)	Score (Lib)	Score (DB)	Score (MFG)	Score (RT)
(M+H)+	579	98.59				

Compound Chromatograms (overlaid)

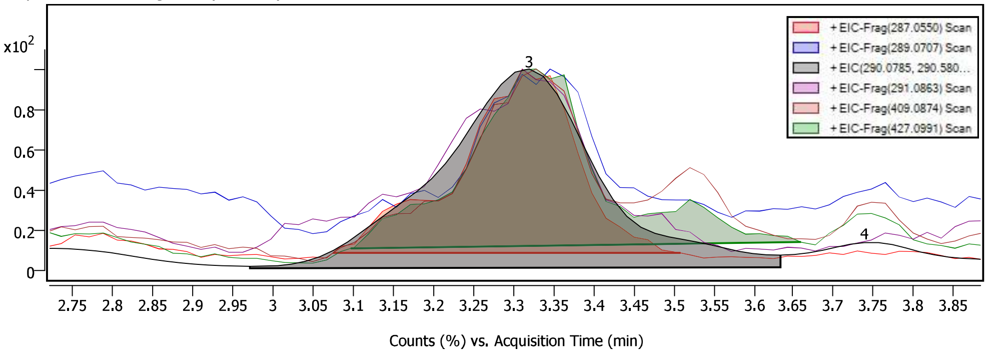

Structure

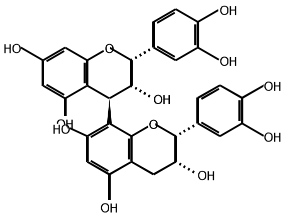

Coelution Plot

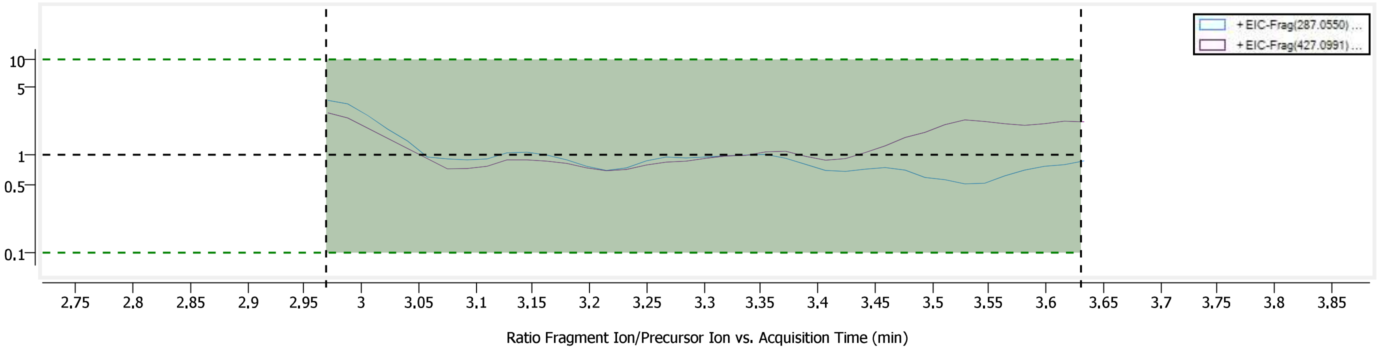

Compound Screening Report

Compound Spectra (overlaid)

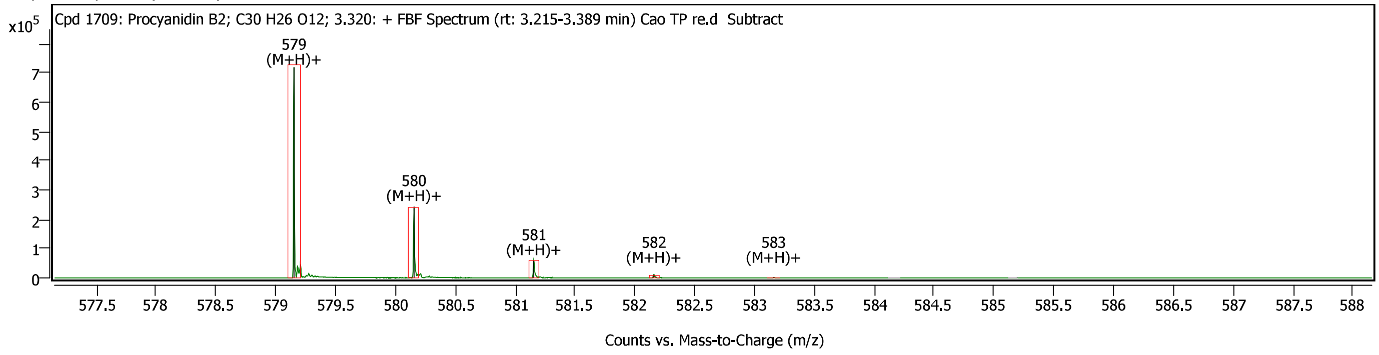

Fragment Spectrum (clean)

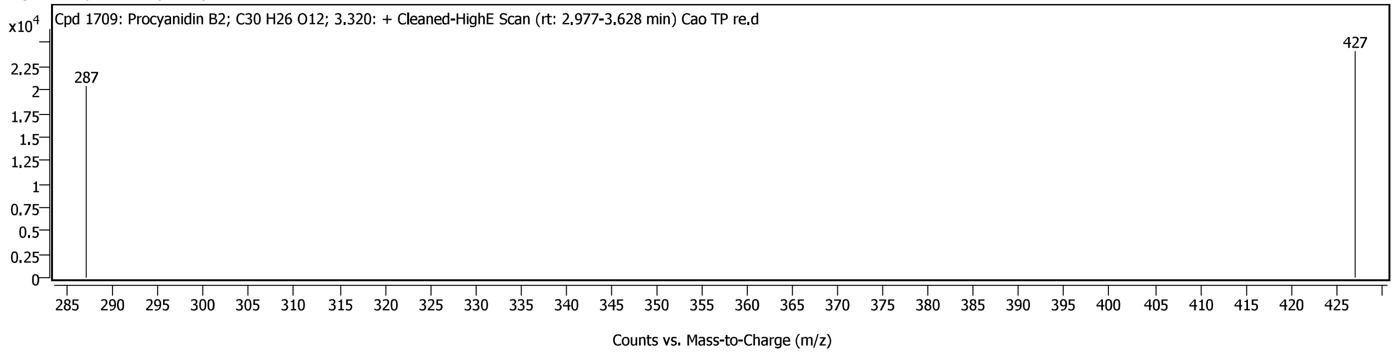

Fragment Spectrum (raw)

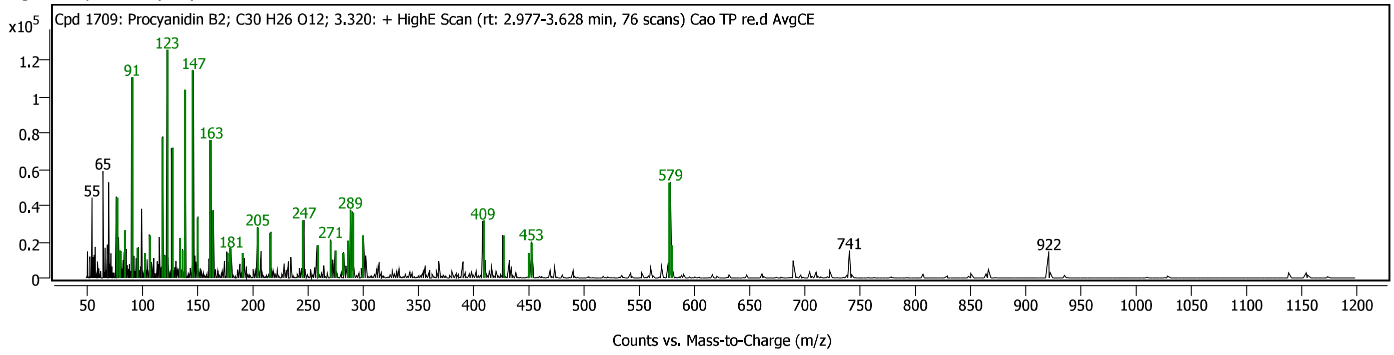

Compound Screening Report

Compound ID Table

Name	Formula	Species	RT	RT Diff	Mass	CAS	ID Source	Score	Score (Lib)	Score (Tgt)
Procyanidin B2	C30 H26 O12	(M+H)+	3.320		578.1434		FBF-FragConfirm	98.59		98.59
ent-Epicatechin-(4alpha->6)-ent-epicatechin	C30 H26 O12	(M+H)+	3.320		578.1434		FBF-FragConfirm	98.59		98.59
Luteolin 7-(6''-E-cinnamylglucoside)	C30 H26 O12	(M+H)+	3.320		578.1434		FBF-FragConfirm	98.59		98.59
Genistein 7-O-(2-p-coumaroylglucoside)	C30 H26 O12	(M+H)+	3.320		578.1434	106915-84-8	FBF-FragConfirm	98.59		98.59
Apigenin 7-glucoside-4'-p-coumarate	C30 H26 O12	(M+H)+	3.320		578.1434		FBF-FragConfirm	98.59		98.59
Genistein 7-O-(2''-p-coumaroylglucoside)	C30 H26 O12	(M+H)+	3.320		578.1434		FBF-FragConfirm	98.59		98.59
ent-Epicatechin(4alpha->8)catechin	C30 H26 O12	(M+H)+	3.320		578.1434	121842-80-6	FBF-FragConfirm	98.59		98.59
Epicatechin-(6'->8)-epicatechin	C30 H26 O12	(M+H)+	3.320		578.1434		FBF-FragConfirm	98.59		98.59
Apigenin 7-(6''-p-coumarylglucoside)	C30 H26 O12	(M+H)+	3.320		578.1434		FBF-FragConfirm	98.59		98.59
4-O-8',5'-5''-Dehydrotriferulic acid	C30 H26 O12	(M+H)+	3.320		578.1434		FBF-FragConfirm	98.59		98.59
Apigenin 7-(4''-Z-p-coumarylglucoside)	C30 H26 O12	(M+H)+	3.320		578.1434		FBF-FragConfirm	98.59		98.59
Apigenin 7-(4''-E-p-coumarylglucoside)	C30 H26 O12	(M+H)+	3.320		578.1434		FBF-FragConfirm	98.59		98.59
Apigenin 7-(3''-p-coumaroylglucoside)	C30 H26 O12	(M+H)+	3.320		578.1434		FBF-FragConfirm	98.59		98.59
Apigenin 7-(2''-E-p-coumaroylglucoside	C30 H26 O12	(M+H)+	3.320		578.1434		FBF-FragConfirm	98.59		98.59
2''-O-p-Coumaroylvitexin	C30 H26 O12	(M+H)+	3.320		578.1434	59282-55-2	FBF-FragConfirm	98.59		98.59
Kaempferol 3-(2-(E)-p-coumarylrhamnoside)	C30 H26 O12	(M+H)+	3.320		578.1434		FBF-FragConfirm	98.59		98.59
Apigenin 7-(6''-E-p-coumaroylgalactoside)	C30 H26 O12	(M+H)+	3.320		578.1434		FBF-FragConfirm	98.59		98.59
Kaempferol 3-(6''-(Z)-cinnamylglucoside)	C30 H26 O12	(M+H)+	3.320		578.1434		FBF-FragConfirm	98.59		98.59
Kaempferol 3-(2''-(Z)-p-coumaroylrhamnoside)	C30 H26 O12	(M+H)+	3.320		578.1434		FBF-FragConfirm	98.59		98.59
Procyanidin B5	C30 H26 O12	(M+H)+	3.320		578.1434	12798-57-1	FBF-FragConfirm	98.59		98.59
Vitexin 2''-O-p-coumarate	C30 H26 O12	(M+H)+	3.320		578.1434		FBF-FragConfirm	98.59		98.59
Terniflorin	C30 H26 O12	(M+H)+	3.320		578.1434	61237-19-2	FBF-FragConfirm	98.59		98.59
Procyanidin B8	C30 H26 O12	(M+H)+	3.320		578.1434	12798-60-6	FBF-FragConfirm	98.59		98.59
Procyanidin B7	C30 H26 O12	(M+H)+	3.320		578.1434	12798-59-3	FBF-FragConfirm	98.59		98.59
Procyanidin B6	C30 H26 O12	(M+H)+	3.320		578.1434	12798-58-2	FBF-FragConfirm	98.59		98.59
Kaempferol 3-(4''-p-coumarylrhamnoside)	C30 H26 O12	(M+H)+	3.320		578.1434		FBF-FragConfirm	98.59		98.59
Procyanidin B4	C30 H26 O12	(M+H)+	3.320		578.1434		FBF-FragConfirm	98.59		98.59
Procyanidin B1	C30 H26 O12	(M+H)+	3.320		578.1434		FBF-FragConfirm	98.59		98.59
Procyanidin B3	C30 H26 O12	(M+H)+	3.320		578.1434		FBF-FragConfirm	98.59		98.59

Cpd 1431: Indol-3-ylacetyl-myo-inositol L-arabinoside

Name	Formula	RT	RI	Mass	Diff (Tgt, ppm)	CAS	ID Source	Score	Algorithm
Indol-3-ylacetyl-myo-inositol L-arabinoside	C21 H27 N O11	3.477		469.1586	0.42		FBF	98.09	FBF

Species	m/z	Score (Tgt)	Score (Lib)	Score (DB)	Score (MFG)	Score (RT)
(M+H)+	470	98.09				

Compound Chromatograms (overlaid)

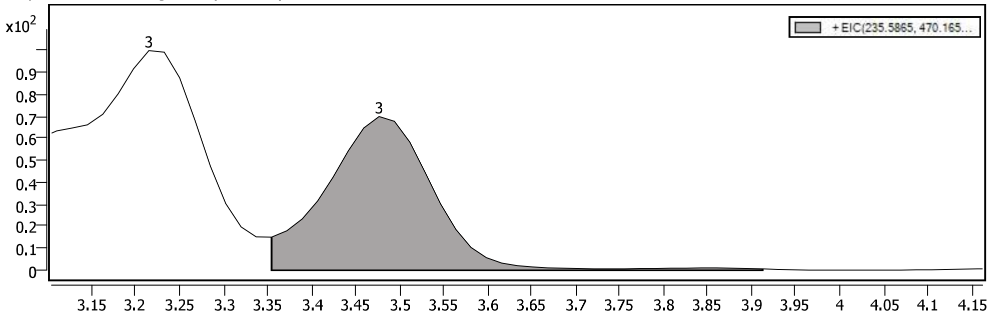

Structure

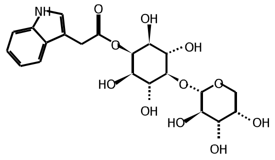

Compound Spectra (overlaid)

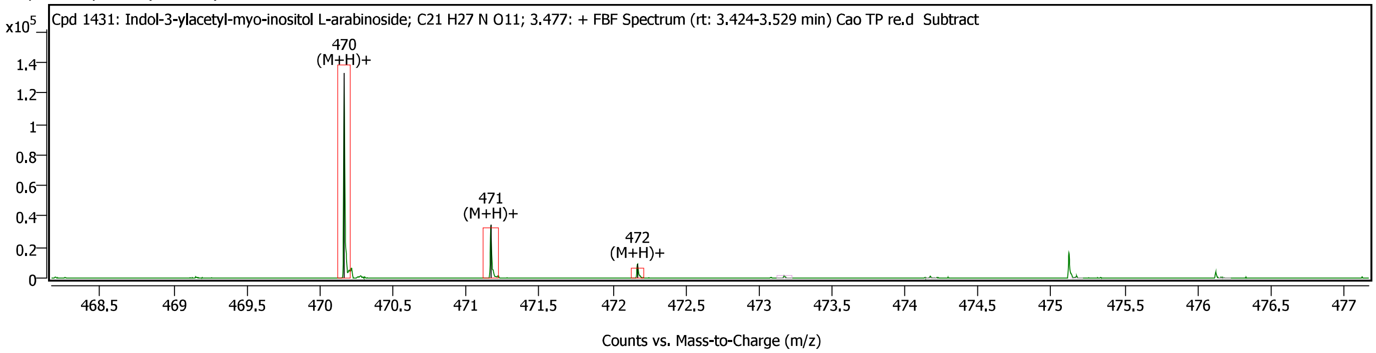

Compound Screening Report

Compound ID Table

Name	Formula	Species	RT	RT Diff	Mass	CAS	ID Source	Score	Score (Lib)	Score (Tgt)
Indol-3-ylacetyl-myo-inositol L-arabinoside	C21 H27 N O11	(M+H)+	3.477		469.1586		FBF	98.09		98.09

Cpd 109: De-O-methylsimmondsin

Name	Formula	RT	RI	Mass	Diff (Tgt, ppm)	CAS	ID Source	Score	Algorithm
De-O-methylsimmondsin	C15 H23 N O9	3.634		361.1376	0.87	135105-75-8	FBF	98.26	FBF

Species	m/z	Score (Tgt)	Score (Lib)	Score (DB)	Score (MFG)	Score (RT)
(M+H)+	362	98.26				

Compound Chromatograms (overlaid)

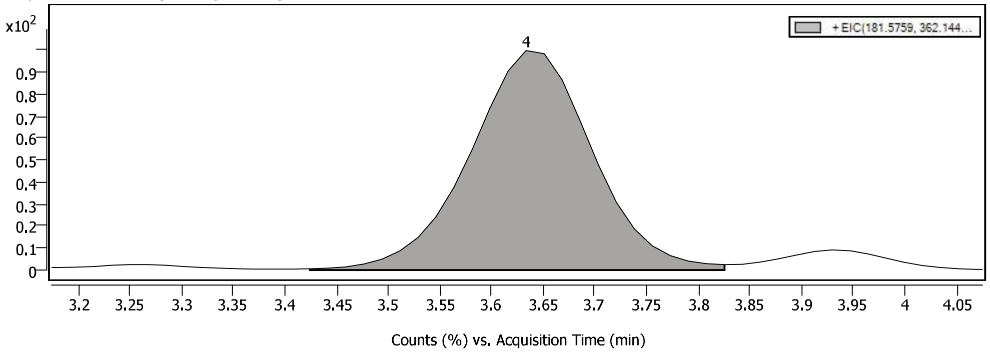

Structure

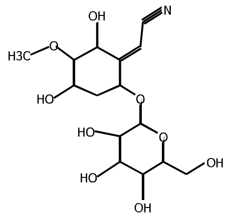

Compound Spectra (overlaid)

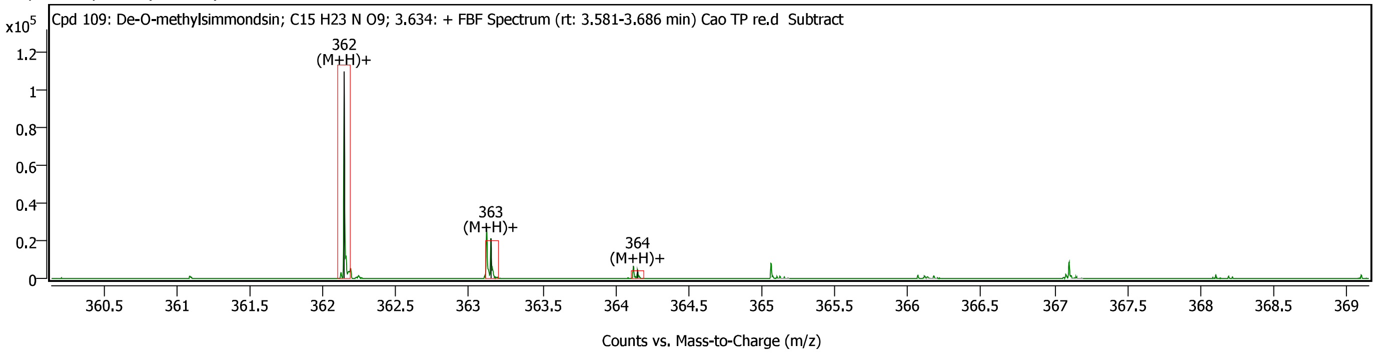

Compound ID Table

Name	Formula	Species	RT	RT Diff	Mass	CAS	ID Source	Score	Score (Lib)	Score (Tgt)
De-O-methylsimmondsin	C15 H23 N O9	(M+H)+	3.634		361.1376	135105-75-8	FBF	98.26		98.26

Cpd 1617: <Catechin 7-β-D-xylopyranoside>

Name	Formula	RT	RI	Mass	Diff (Tgt, ppm)	CAS	ID Source	Score	Algorithm
<Catechin 7-β-D-xylopyranoside>	C20 H22 O10	3.982		422.1217	0.85	42830-48-8	M-FBF	98.25	FBF

Species	m/z	Score (Tgt)	Score (Lib)	Score (DB)	Score (MFG)	Score (RT)
(M+H)+	423	98.25				

Compound Chromatograms (overlaid)

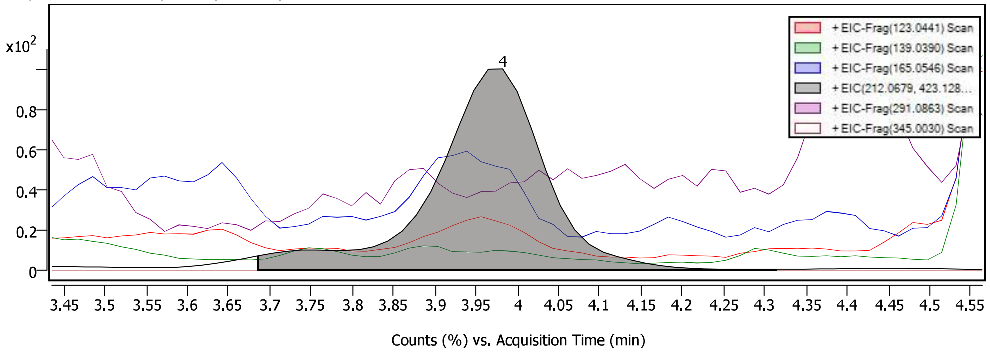

Structure

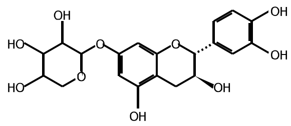

Coelution Plot

Compound Spectra (overlaid)

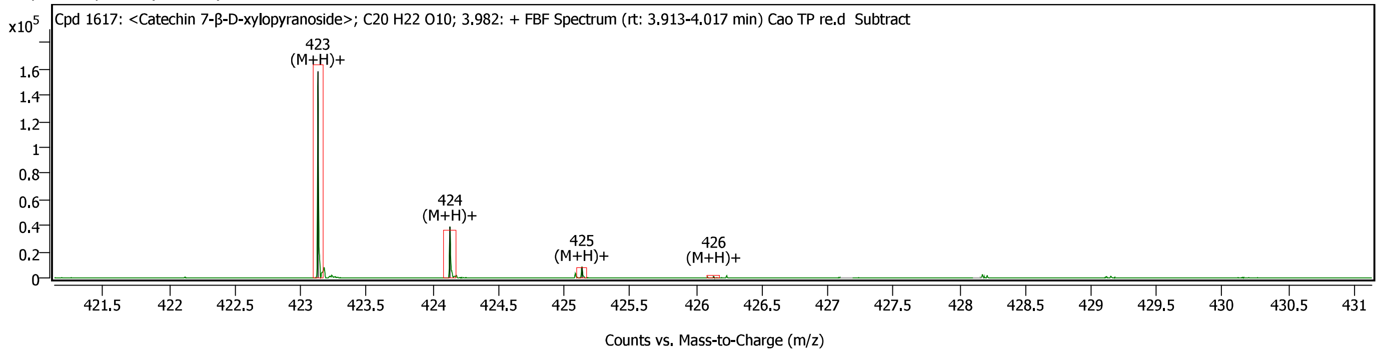

Fragment Spectrum (raw)

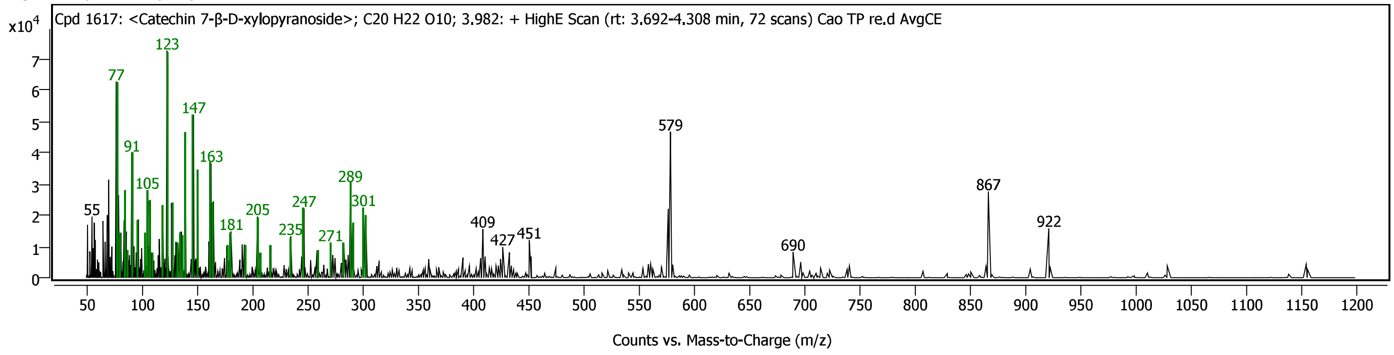

Compound ID Table

Name	Formula	Species	RT	RT Diff	Mass	CAS	ID Source	Score	Score (Lib)	Score (Tgt)
<Catechin 7-β-D-xylopyranoside>	C20 H22 O10	(M+H)+	3.982		422.1217	42830-48-8	FBF	98.25		98.25
<Rumexoside>	C20 H22 O10	(M+H)+	3.982		422.1217		FBF	98.25		98.25
<Polydine>	C20 H22 O10	(M+H)+	3.982		422.1217		FBF	98.25		98.25
<Plicatic acid>	C20 H22 O10	(M+H)+	3.982		422.1217	16462-65-0	FBF	98.25		98.25
<Catechin 7-O-apiofuranoside>	C20 H22 O10	(M+H)+	3.982		422.1217		FBF	98.25		98.25
<Catechin 5-O-beta-D-apiofuranoside>	C20 H22 O10	(M+H)+	3.982		422.1217		FBF	98.25		98.25
<2',4',3,4,alpha-Pentahydroxydihydrochalcone 3'-C-xyloside>	C20 H22 O10	(M+H)+	3.982		422.1217		FBF	98.25		98.25

Cpd 208: <Val Val>

Name	Formula	RT	RI	Mass	Diff (Tgt, ppm)	CAS	ID Source	Score	Algorithm
<Val Val>	C10 H20 N2 O3	4.052		216.1476	0.98		M-FBF	99.20	FBF

Species	m/z	Score (Tgt)	Score (Lib)	Score (DB)	Score (MFG)	Score (RT)
(M+H)+	217	99.20				

Compound Chromatograms (overlaid)

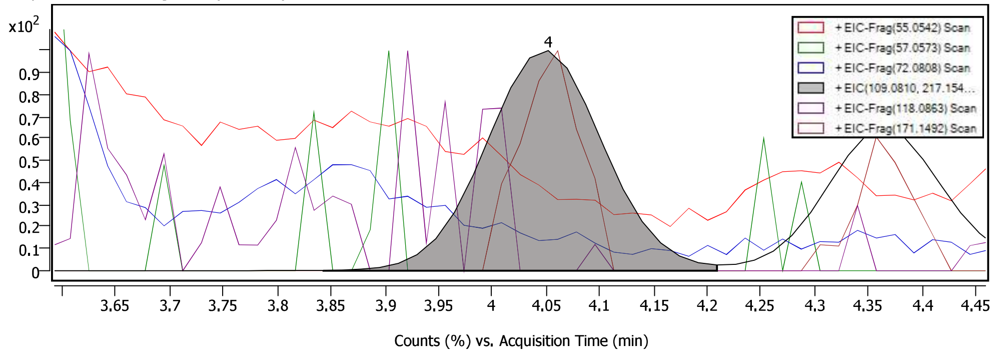

Structure

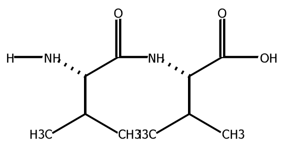

Coelution Plot

Compound Spectra (overlaid)

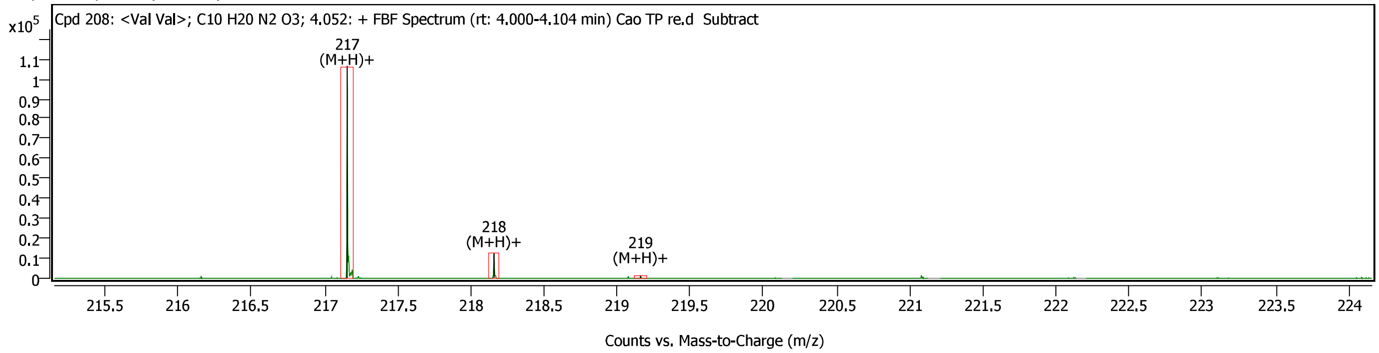

Fragment Spectrum (raw)

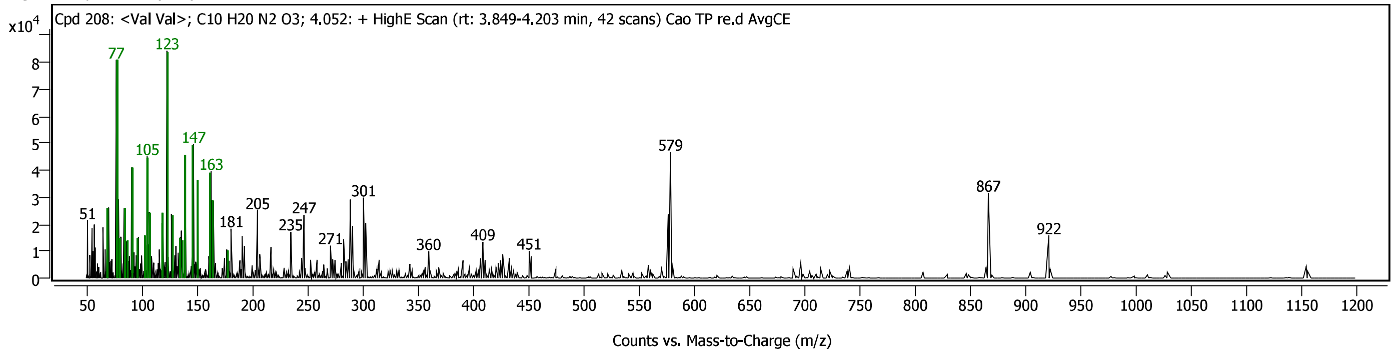

Compound ID Table

Name	Formula	Species	RT	RT Diff	Mass	CAS	ID Source	Score	Score (Lib)	Score (Tgt)
<Val Val>	C10 H20 N2 O3	(M+H)+	4.052		216.1476		FBF	99.20		99.20
<Valyl-Valine>	C10 H20 N2 O3	(M+H)+	4.052		216.1476		FBF	99.20		99.20

Cpd 1372: 1,2-O-Diacetylzephyranthine

Name	Formula	RT	RI	Mass	Diff (Tgt, ppm)	CAS	ID Source	Score	Algorithm
1,2-O-Diacetylzephyranthine	C20 H23 N O6	4.523		373.1529	0.91		FBF	99.44	FBF

Species	m/z	Score (Tgt)	Score (Lib)	Score (DB)	Score (MFG)	Score (RT)
(M+H)+	374	99.44				

Compound Chromatograms (overlaid)

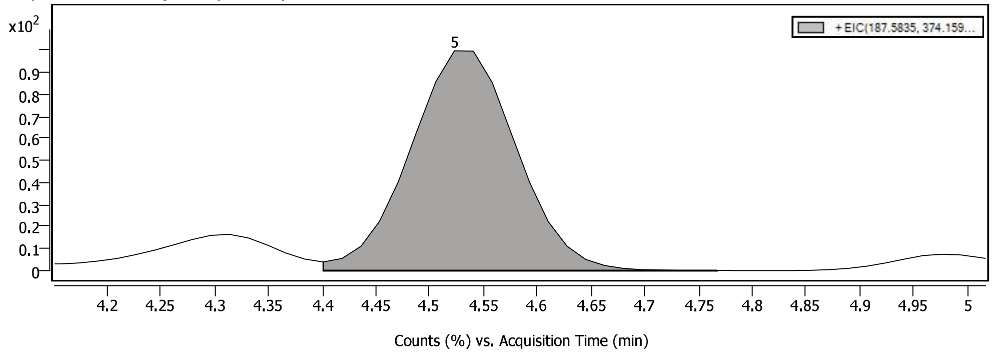

Structure

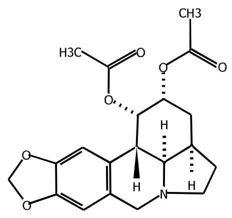

Compound Spectra (overlaid)

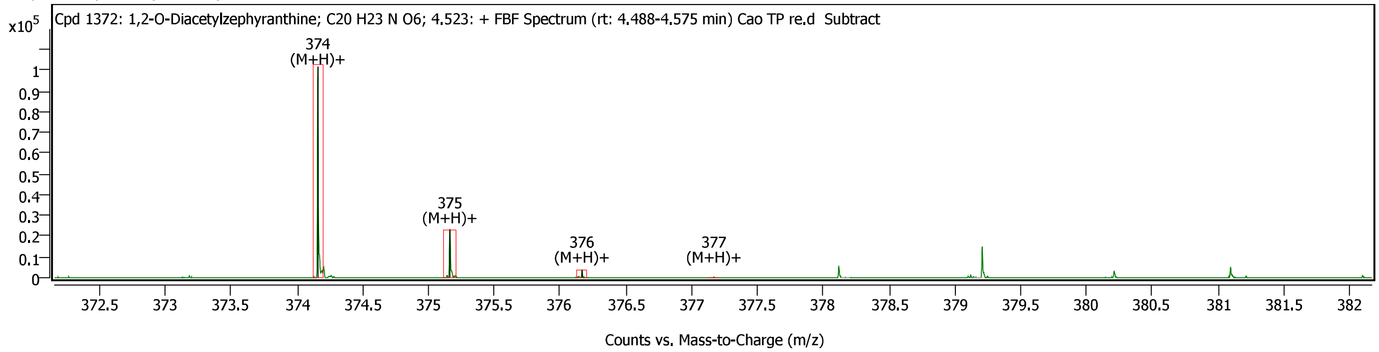

Compound ID Table

Name	Formula	Species	RT	RT Diff	Mass	CAS	ID Source	Score	Score (Lib)	Score (Tgt)
1,2-O-Diacetylzephyranthine	C20 H23 N O6	(M+H)+	4.523		373.1529		FBF	99.44		99.44

Cpd 746: Gambiriin A2

Name	Formula	RT	RI	Mass	Diff (Tgt, ppm)	CAS	ID Source	Score	Algorithm
Gambiriin A2	C30 H28 O12	4.715		580.1606	4.27		M-FBF	90.36	FBF

Species	m/z	Score (Tgt)	Score (Lib)	Score (DB)	Score (MFG)	Score (RT)
(M+2H)+2 (M+H)+	291 581	90.36				

Compound Chromatograms (overlaid)

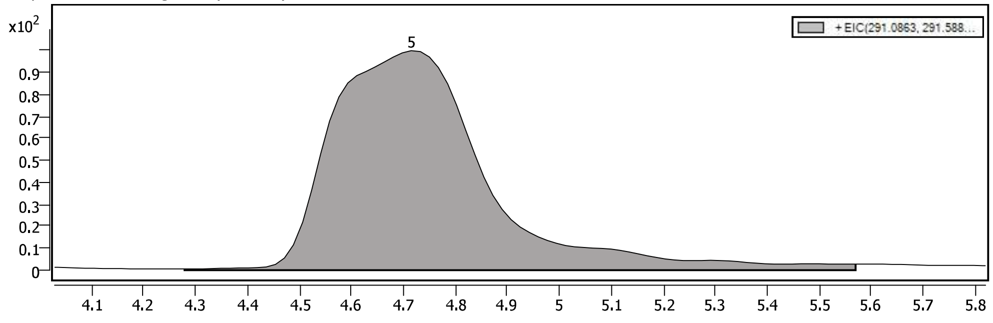

Structure

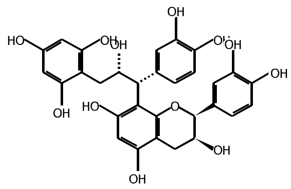

Compound Spectra (overlaid)

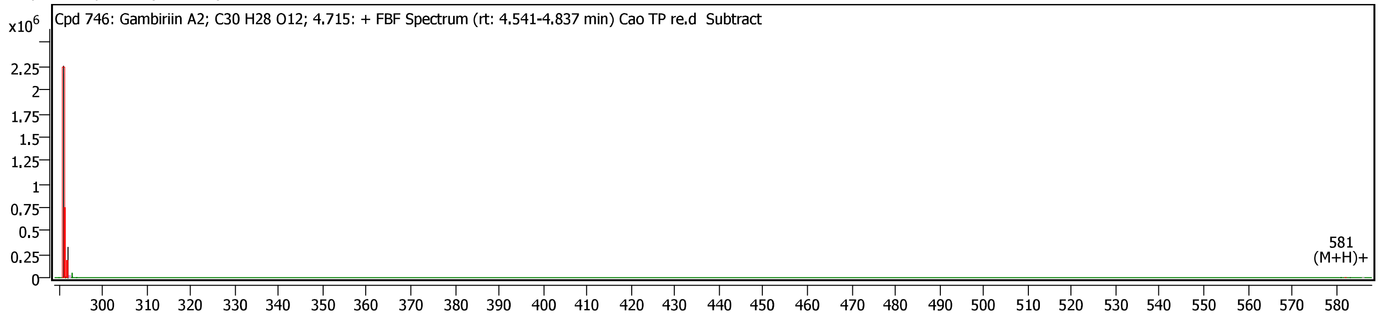

Compound ID Table

Name	Formula	Species	RT	RT Diff	Mass	CAS	ID Source	Score	Score (Lib)	Score (Tgt)
Gambiriin A2	C30 H28 O12	(M+2H)+2 (M+H)+	4.715		580.1606		FBF	90.36		90.36
4,2',3',4'-Tetrahydroxychalcone 4'-O-(2''-O-p-coumaroyl)glucoside	C30 H28 O12	(M+2H)+2 (M+H)+	4.715		580.1606		FBF	90.36		90.36
Gambiriin A1	C30 H28 O12	(M+2H)+2 (M+H)+	4.715		580.1606		FBF	90.36		90.36
4,2',3',4'-Tetrahydroxychalcone 4'-O-(6''-O-p-coumaroyl)glucoside	C30 H28 O12	(M+2H)+2 (M+H)+	4.715		580.1606		FBF	90.36		90.36
6''-p-Coumaroylprunin	C30 H28 O12	(M+2H)+2 (M+H)+	4.715		580.1606	96686-70-3	FBF	90.36		90.36
Chalconaringenin 2'-(6''-p-coumarylglucoside)	C30 H28 O12	(M+2H)+2 (M+H)+	4.715		580.1606		FBF	90.36		90.36
Prunin 3''-p-coumarate	C30 H28 O12	(M+2H)+2 (M+H)+	4.715		580.1606		FBF	90.36		90.36
Gambiriin A3	C30 H28 O12	(M+2H)+2 (M+H)+	4.715		580.1606		FBF	90.36		90.36
Naringenin 7-(2-p-Coumaroylglucoside)	C30 H28 O12	(M+2H)+2 (M+H)+	4.715		580.1606		FBF	90.36		90.36
Prunin 6''-p-coumarate	C30 H28 O12	(M+2H)+2 (M+H)+	4.715		580.1606		FBF	90.36		90.36

Cpd 1384: 2,5-Dihydroxybenzaldehyde

Name	Formula	RT	RI	Mass	Diff (Tgt, ppm)	CAS	ID Source	Score	Algorithm
2,5-Dihydroxybenzaldehyde	C7 H6 O3	4.715		138.0319	1.52	1194-98-5	M-FBF-FragConfirm	98.77	FBF

Species	m/z	Score (Tgt)	Score (Lib)	Score (DB)	Score (MFG)	Score (RT)
(M+H)+	139	98.77				

Compound Chromatograms (overlaid)

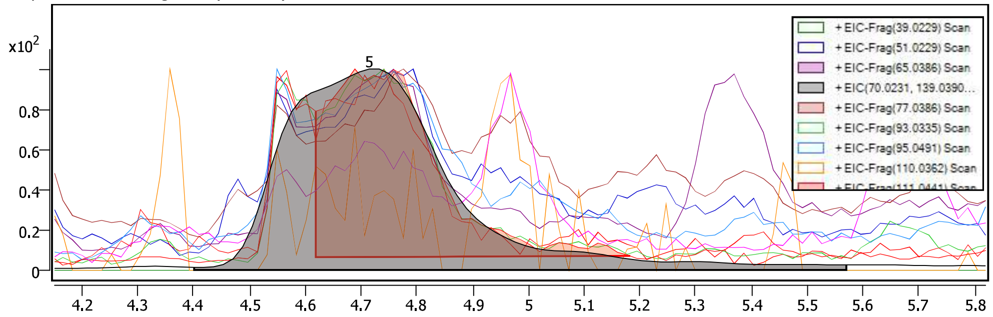

Structure

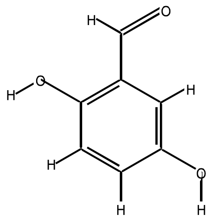

Coelution Plot

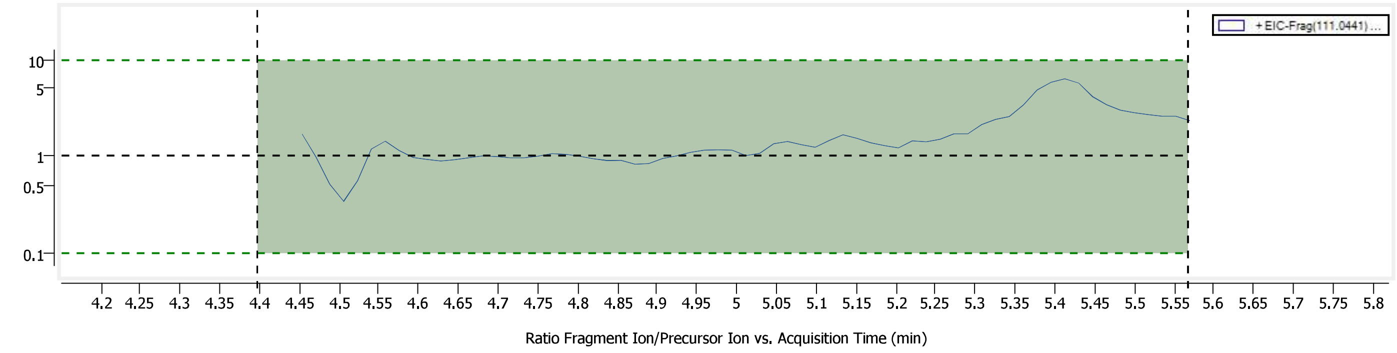

Compound Spectra (overlaid)

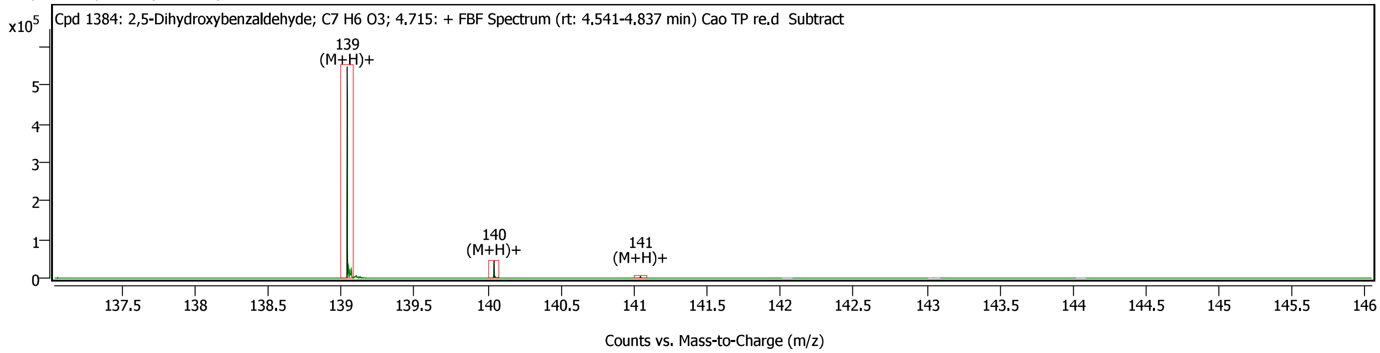

Fragment Spectrum (clean)

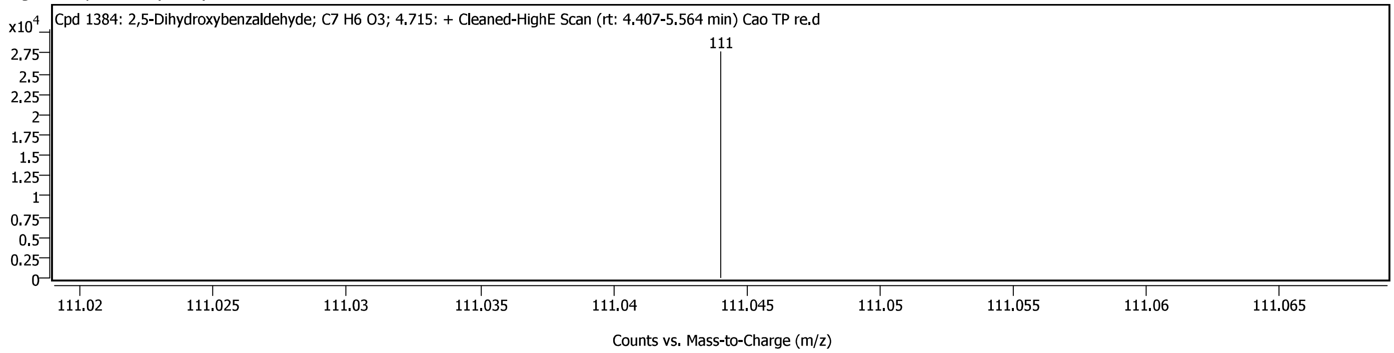

Fragment Spectrum (raw)

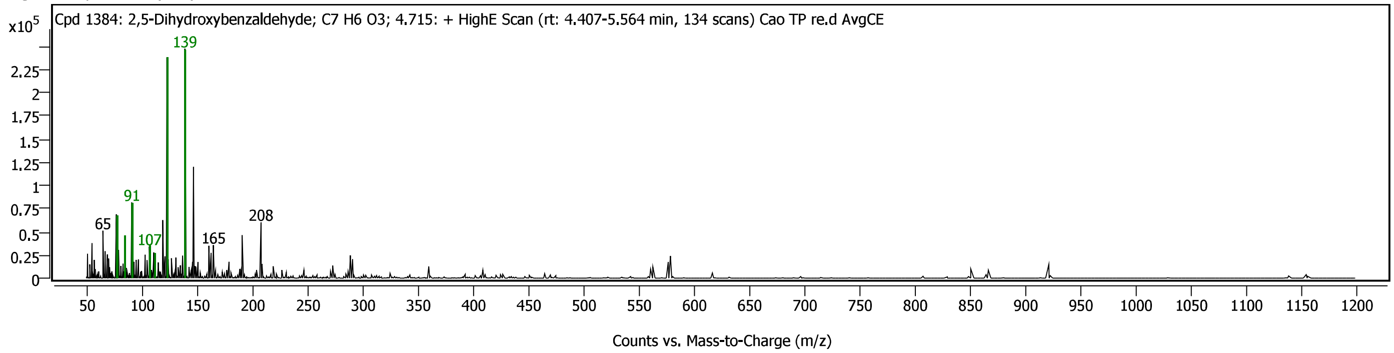

Compound ID Table

Name	Formula	Species	RT	RT Diff	Mass	CAS	ID Source	Score	Score (Lib)	Score (Tgt)
2,5-Dihydroxybenzaldehyde	C7 H6 O3	(M+H)+	4.715		138.0319	1194-98-5	FBF-FragConfirm	98.77		98.77
Sesamol	C7 H6 O3	(M+H)+	4.715		138.0319	533-31-3	FBF-FragConfirm	98.77		98.77
m-Hydroxybenzoic acid	C7 H6 O3	(M+H)+	4.715		138.0319	99-06-9	FBF-FragConfirm	98.77		98.77
alpha-Furyl methyl diketone	C7 H6 O3	(M+H)+	4.715		138.0319	1438-92-2	FBF-FragConfirm	98.77		98.77
4-Hydroxybenzoic acid	C7 H6 O3	(M+H)+	4.715		138.0319	99-96-7	FBF-FragConfirm	98.77		98.77
3,4-Dihydroxybenzaldehyde	C7 H6 O3	(M+H)+	4.715		138.0319	139-85-5	FBF-FragConfirm	98.77		98.77
2-Methoxy-1,4-benzoquinone	C7 H6 O3	(M+H)+	4.715		138.0319	2880-58-2	FBF-FragConfirm	98.77		98.77
2-Hydroxy-5-methylquinone	C7 H6 O3	(M+H)+	4.715		138.0319	615-91-8	FBF-FragConfirm	98.77		98.77
Salicylic acid	C7 H6 O3	(M+H)+	4.715		138.0319	69-72-7	FBF-FragConfirm	98.77		98.77

Cpd 1641: (-)-Catechin

Compound Screening Report

Name	Formula	RT	RI	Mass	Diff (Tgt, ppm)	CAS	ID Source	Score	Algorithm
(-)-Catechin	C15 H14 O6	4.715		290.0802	4.04	18829-70-4	M-FBF-FragConfirm	90.41	FBF

Species	m/z	Score (Tgt)	Score (Lib)	Score (DB)	Score (MFG)	Score (RT)
(M+H)+	291	90.41				

Compound Chromatograms (overlaid)

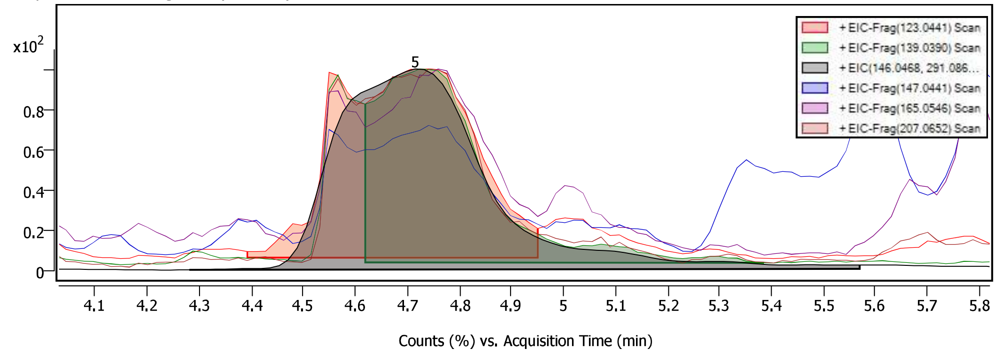

Structure

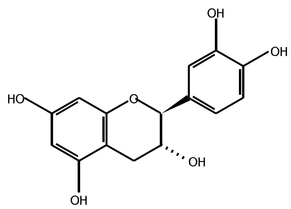

Coelution Plot

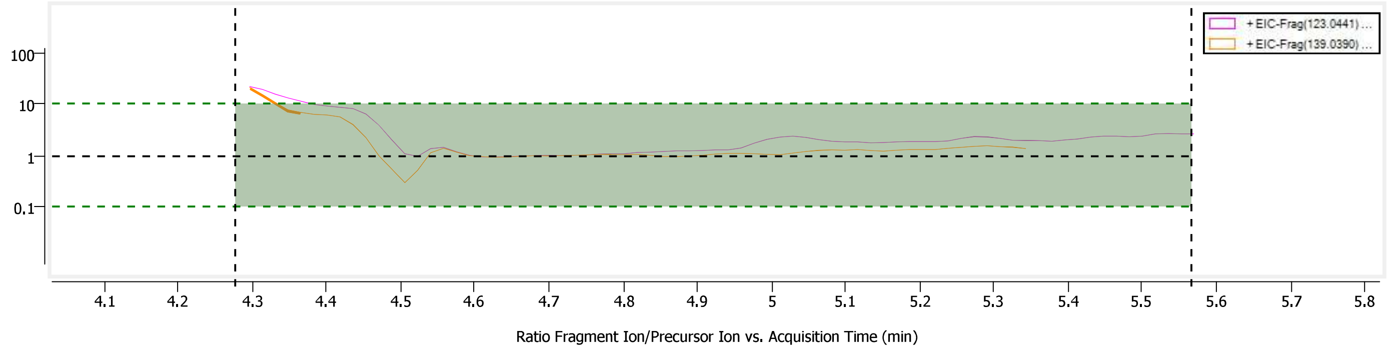

Compound Spectra (overlaid)

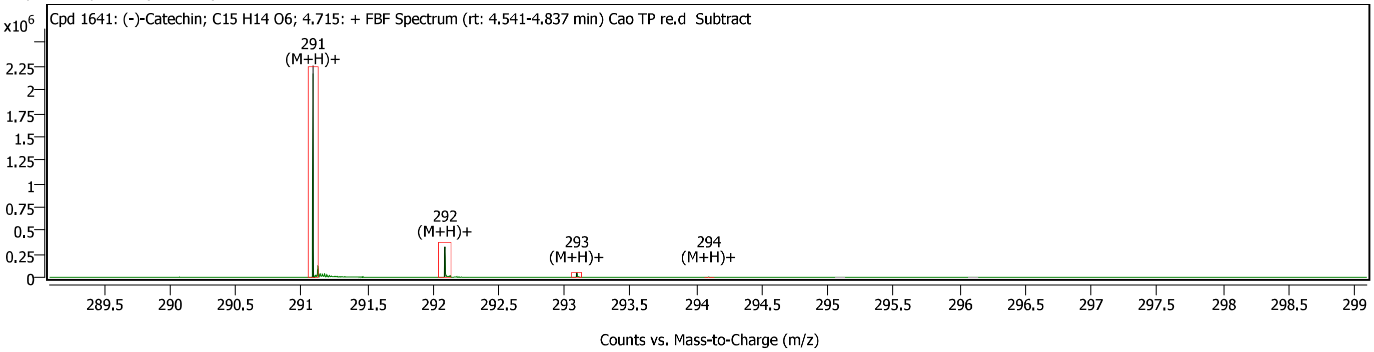

Fragment Spectrum (clean)

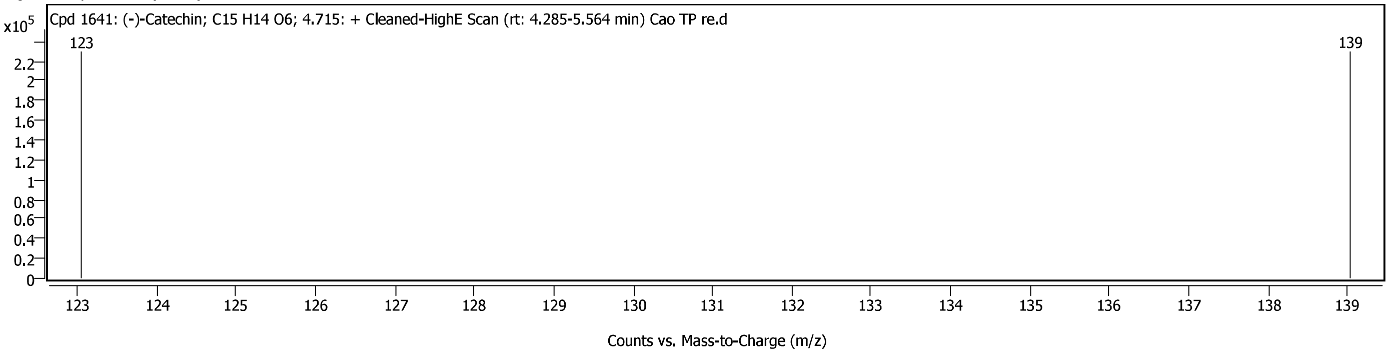

Fragment Spectrum (raw)

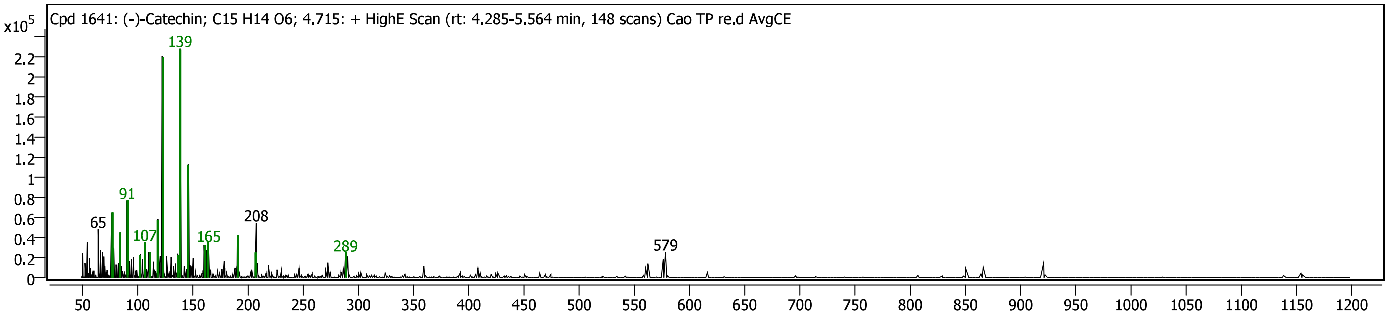

Compound ID Table

Name	Formula	Species	RT	RT Diff	Mass	CAS	ID Source	Score	Score (Lib)	Score (Tgt)
(-)-Catechin	C15 H14 O6	(M+H)+	4.715		290.0802	18829-70-4	FBF-FragConfirm	90.41		90.41
Fisetinidol-4beta-ol	C15 H14 O6	(M+H)+	4.715		290.0802		FBF-FragConfirm	90.41		90.41
Fonsecin	C15 H14 O6	(M+H)+	4.715		290.0802	3748-39-8	FBF-FragConfirm	90.41		90.41
ent-Fisetinidol-4beta-ol	C15 H14 O6	(M+H)+	4.715		290.0802		FBF-FragConfirm	90.41		90.41
(-)-Epicatechin	C15 H14 O6	(M+H)+	4.715		290.0802	490-46-0	FBF-FragConfirm	90.41		90.41
(+)-Epicatechin	C15 H14 O6	(M+H)+	4.715		290.0802	35323-91-2	FBF-FragConfirm	90.41		90.41
ent-Robinetinidol	C15 H14 O6	(M+H)+	4.715		290.0802		FBF-FragConfirm	90.41		90.41
(2R,3R)-3,5,7,3',5'-Pentahydroxyflavan	C15 H14 O6	(M+H)+	4.715		290.0802		FBF-FragConfirm	90.41		90.41
(2R-cis)-2-(2,5-Dihydroxyphenyl)-3,4-dihydro-2H-1-benzopyran-3,5,7-triol	C15 H14 O6	(M+H)+	4.715		290.0802		FBF-FragConfirm	90.41		90.41
2,8-Dihydroxy-3,4,7-trimethoxydibenzofuran	C15 H14 O6	(M+H)+	4.715		290.0802	167278-42-4	FBF-FragConfirm	90.41		90.41
3,4,2',4',6'-Pentahydroxydihydrochalcone	C15 H14 O6	(M+H)+	4.715		290.0802		FBF-FragConfirm	90.41		90.41
3,4,2',4',alpha-Pentahydroxydihydrochalcone	C15 H14 O6	(M+H)+	4.715		290.0802		FBF-FragConfirm	90.41		90.41
6-Hydroxy-alpha-pyrufuran	C15 H14 O6	(M+H)+	4.715		290.0802	167278-43-5	FBF-FragConfirm	90.41		90.41
alpha-Cotonefuran	C15 H14 O6	(M+H)+	4.715		290.0802	93973-22-9	FBF-FragConfirm	90.41		90.41
Cartorimine	C15 H14 O6	(M+H)+	4.715		290.0802		FBF-FragConfirm	90.41		90.41
Catechin	C15 H14 O6	(M+H)+	4.715		290.0802	154-23-4	FBF-FragConfirm	90.41		90.41
cis-3,4-Leucopelargonidin	C15 H14 O6	(M+H)+	4.715		290.0802		FBF-FragConfirm	90.41		90.41
ent-Epifisetinidol-4beta-ol	C15 H14 O6	(M+H)+	4.715		290.0802		FBF-FragConfirm	90.41		90.41
Oritin-4beta-ol	C15 H14 O6	(M+H)+	4.715		290.0802		FBF-FragConfirm	90.41		90.41
Fisetinidol-4alpha-ol	C15 H14 O6	(M+H)+	4.715		290.0802		FBF-FragConfirm	90.41		90.41
Epifisetinidol-4alpha-ol	C15 H14 O6	(M+H)+	4.715		290.0802		FBF-FragConfirm	90.41		90.41
Marshrin	C15 H14 O6	(M+H)+	4.715		290.0802		FBF-FragConfirm	90.41		90.41
Epioritin-4alpha-ol	C15 H14 O6	(M+H)+	4.715		290.0802		FBF-FragConfirm	90.41		90.41
Robinetinidol	C15 H14 O6	(M+H)+	4.715		290.0802		FBF-FragConfirm	90.41		90.41
Ptaeroglycol	C15 H14 O6	(M+H)+	4.715		290.0802	18836-12-9	FBF-FragConfirm	90.41		90.41
Plumericin	C15 H14 O6	(M+H)+	4.715		290.0802	77-16-7	FBF-FragConfirm	90.41		90.41
Nubigenol	C15 H14 O6	(M+H)+	4.715		290.0802		FBF-FragConfirm	90.41		90.41
Mesquitol	C15 H14 O6	(M+H)+	4.715		290.0802		FBF-FragConfirm	90.41		90.41
Mikanolide	C15 H14 O6	(M+H)+	4.715		290.0802	17928-61-9	FBF-FragConfirm	90.41		90.41
Luteoforol	C15 H14 O6	(M+H)+	4.715		290.0802	24897-98-1	FBF-FragConfirm	90.41		90.41
Isoplumericin	C15 H14 O6	(M+H)+	4.715		290.0802	31298-76-7	FBF-FragConfirm	90.41		90.41
gamma-Pyrufuran	C15 H14 O6	(M+H)+	4.715		290.0802	93973-18-3	FBF-FragConfirm	90.41		90.41
Epioritin-4beta-ol	C15 H14 O6	(M+H)+	4.715		290.0802		FBF-FragConfirm	90.41		90.41

Cpd 1546: <a-Asarone>

Name	Formula	RT	RI	Mass	Diff (Tgt, ppm)	CAS	ID Source	Score	Algorithm
<a-Asarone>	C12 H16 O3	4.855		208.1102	1.09	2883-98-9	M-FBF	98.91	FBF

Species	m/z	Score (Tgt)	Score (Lib)	Score (DB)	Score (MFG)	Score (RT)
(M+H)+	209	98.91				

Compound Chromatograms (overlaid)

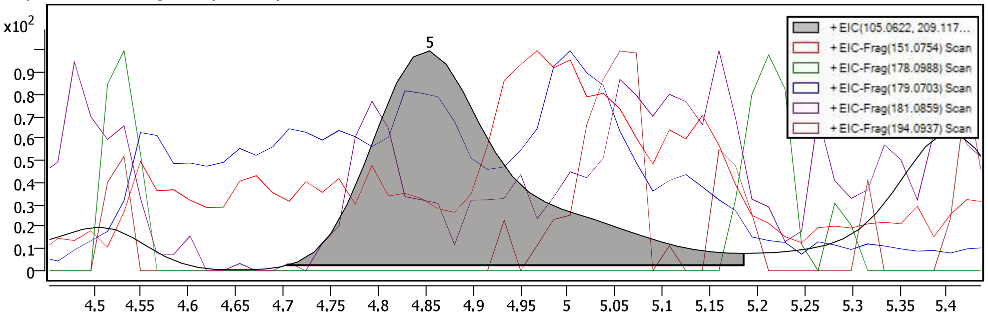

Structure

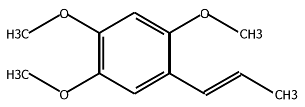

Coelution Plot

Compound Screening Report

Compound Spectra (overlaid)

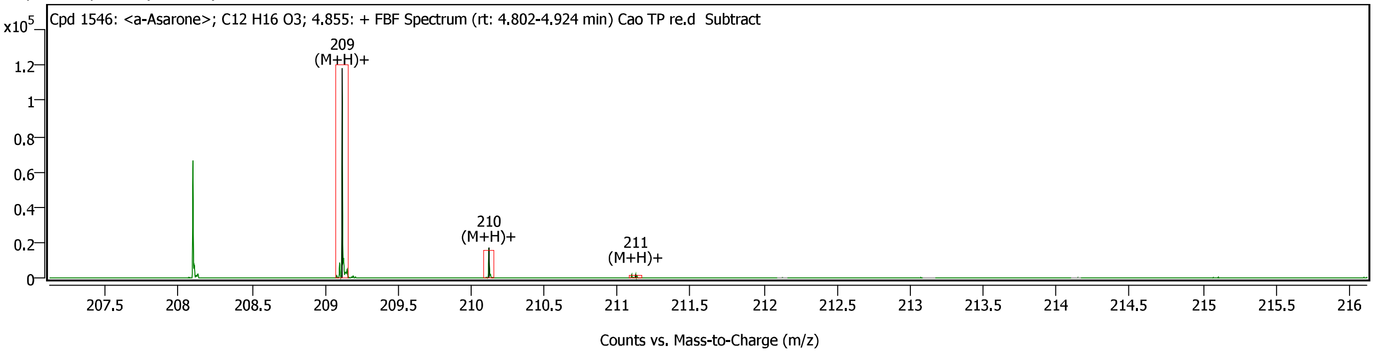

Fragment Spectrum (raw)

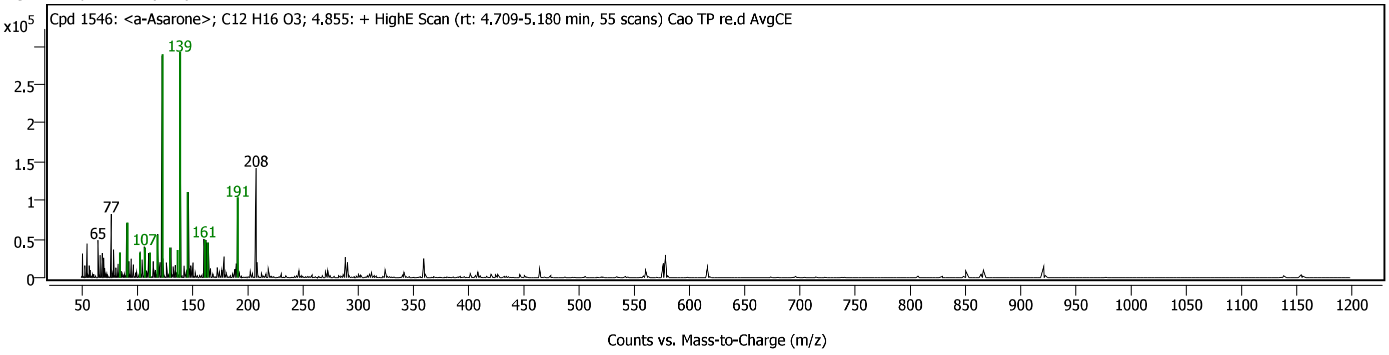

Compound ID Table

Name	Formula	Species	RT	RT Diff	Mass	CAS	ID Source	Score	Score (Lib)	Score (Tgt)
<a-Asarone>	C12 H16 O3	(M+H)+	4.855		208.1102	2883-98-9	FBF	98.91		98.91
<Jasmine ketolactone>	C12 H16 O3	(M+H)+	4.855		208.1102	70981-24-7	FBF	98.91		98.91
<Isoelemicin>	C12 H16 O3	(M+H)+	4.855		208.1102	5273-85-8	FBF	98.91		98.91
<5-Deoxydiplosporin>	C12 H16 O3	(M+H)+	4.855		208.1102		FBF	98.91		98.91
<(4-tert-Butyl-phenoxy)-acetic acid>	C12 H16 O3	(M+H)+	4.855		208.1102		FBF	98.91		98.91
<(Z)-3-Oxo-2-(2-pentenyl)-1-cyclopenteneacetic acid>	C12 H16 O3	(M+H)+	4.855		208.1102	120282-76-0	FBF	98.91		98.91
<12-oxo-5E,8E,10Z-dodecatrienoic acid>	C12 H16 O3	(M+H)+	4.855		208.1102		FBF	98.91		98.91
<1-Cyclohexene-1-acrylic acid, 2,6,6-trimethyl-3-oxo->	C12 H16 O3	(M+H)+	4.855		208.1102	28043-09-6	FBF	98.91		98.91
<2,4,5-Trimethoxy-1-allylbenzene>	C12 H16 O3	(M+H)+	4.855		208.1102	5353-15-1	FBF	98.91		98.91
<3,4-Dihydro-6-methoxy-2,2-dimethyl-2H-1-benzopyran-4-ol>	C12 H16 O3	(M+H)+	4.855		208.1102	65383-71-3	FBF	98.91		98.91
<3,4-Dihydro-6-methoxy-3,7-dimethyl-1H-2-benzopyran-8-ol>	C12 H16 O3	(M+H)+	4.855		208.1102	70080-82-9	FBF	98.91		98.91
<4-Methoxybenzyl butanoate>	C12 H16 O3	(M+H)+	4.855		208.1102	6963-56-0	FBF	98.91		98.91
<2-Phenoxyethyl isobutyrate>	C12 H16 O3	(M+H)+	4.855		208.1102	103-60-6	FBF	98.91		98.91
<Primin>	C12 H16 O3	(M+H)+	4.855		208.1102	15121-94-5	FBF	98.91		98.91
<Amyl salicylate>	C12 H16 O3	(M+H)+	4.855		208.1102	2050-08-0	FBF	98.91		98.91
<Benzyl (2S,3S)-2-methyl-3-hydroxybutanoate>	C12 H16 O3	(M+H)+	4.855		208.1102		FBF	98.91		98.91
<beta-Asarone>	C12 H16 O3	(M+H)+	4.855		208.1102	5273-86-9	FBF	98.91		98.91
<Coronafacic acid>	C12 H16 O3	(M+H)+	4.855		208.1102	62251-98-3	FBF	98.91		98.91
<Elemicin>	C12 H16 O3	(M+H)+	4.855		208.1102	487-11-6	FBF	98.91		98.91
<Isoamyl salicylate>	C12 H16 O3	(M+H)+	4.855		208.1102	87-20-7	FBF	98.91		98.91
<Benzyl (2R,3S)-2-methyl-3-hydroxybutanoate>	C12 H16 O3	(M+H)+	4.855		208.1102		FBF	98.91		98.91

Cpd 1392: ent-Fisetinidol-(4beta->8)-catechin-(6->4beta)-ent-fisetinidol

Name	Formula	RT	RI	Mass	Diff (Tgt, ppm)	CAS	ID Source	Score	Algorithm
ent-Fisetinidol-(4beta->8)-catechin-(6->4beta)-ent-fisetinidol	C45 H38 O17	4.907		850.2112	0.30		FBF	99.28	FBF

Species	m/z	Score (Tgt)	Score (Lib)	Score (DB)	Score (MFG)	Score (RT)
(M+H)+	851	99.28				

Compound Screening Report

Compound Chromatograms (overlaid)

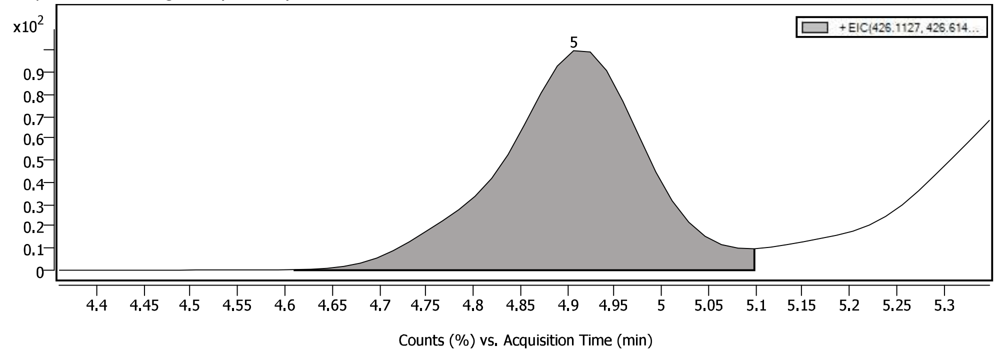

Structure

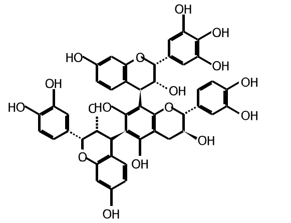

Compound Spectra (overlaid)

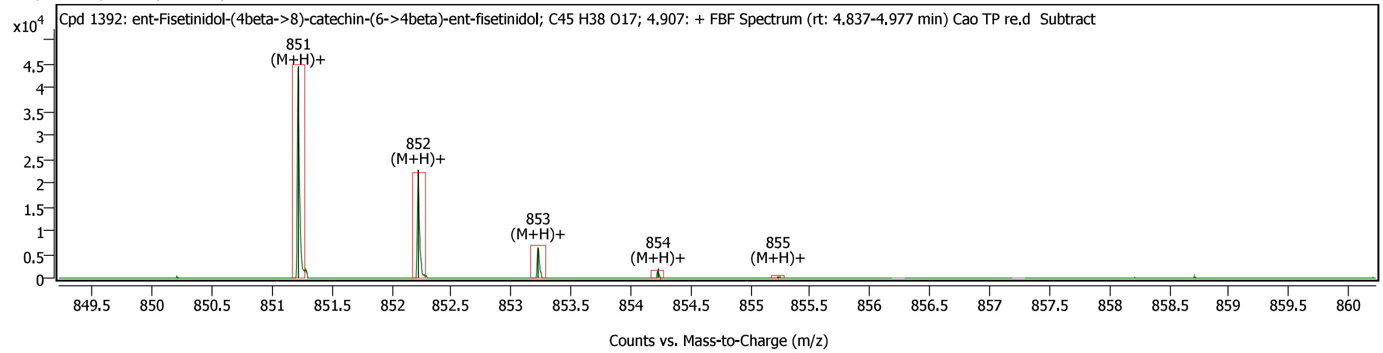

Compound ID Table

Name	Formula	Species	RT	RT Diff	Mass	CAS	ID Source	Score	Score (Lib)	Score (Tgt)
ent-Fisetinidol-(4beta->8)-catechin-(6->4beta)-ent-fisetinidol	C45 H38 O17	(M+H)+	4.907		850.2112		FBF	99.28		99.28

Cpd 1161: <Asn Asn Asn>

Name	Formula	RT	RI	Mass	Diff (Tgt, ppm)	CAS	ID Source	Score	Algorithm
<Asn Asn Asn>	C12 H20 N6 O7	5.011		360.1406	3.46		FBF	94.48	FBF

Species	m/z	Score (Tgt)	Score (Lib)	Score (DB)	Score (MFG)	Score (RT)
(M+H)+	361	94.48				

Compound Chromatograms (overlaid)

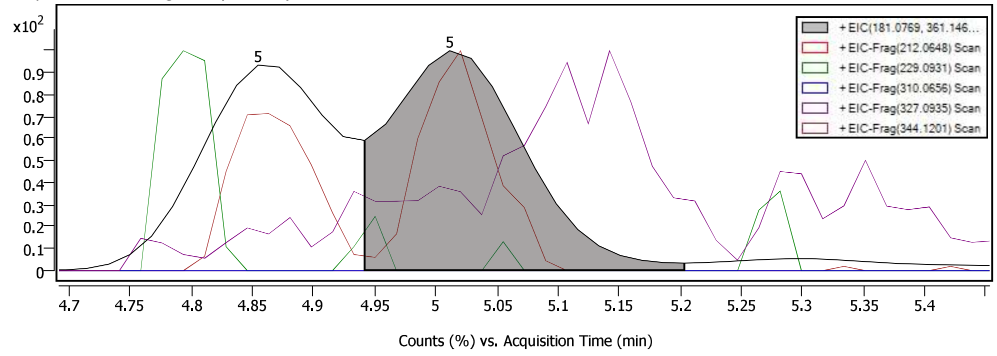

Structure

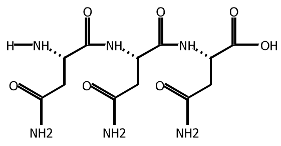

Coelution Plot

Compound Spectra (overlaid)

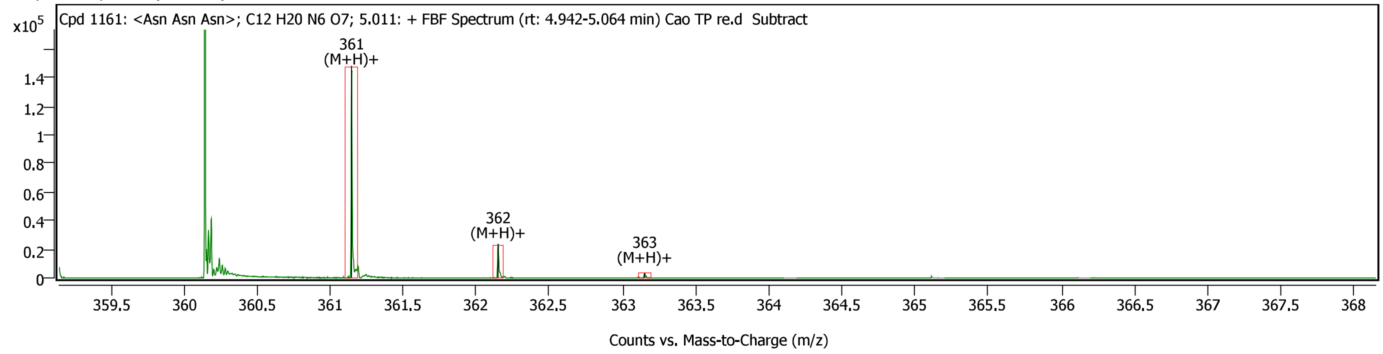

Compound Screening Report

Fragment Spectrum (raw)

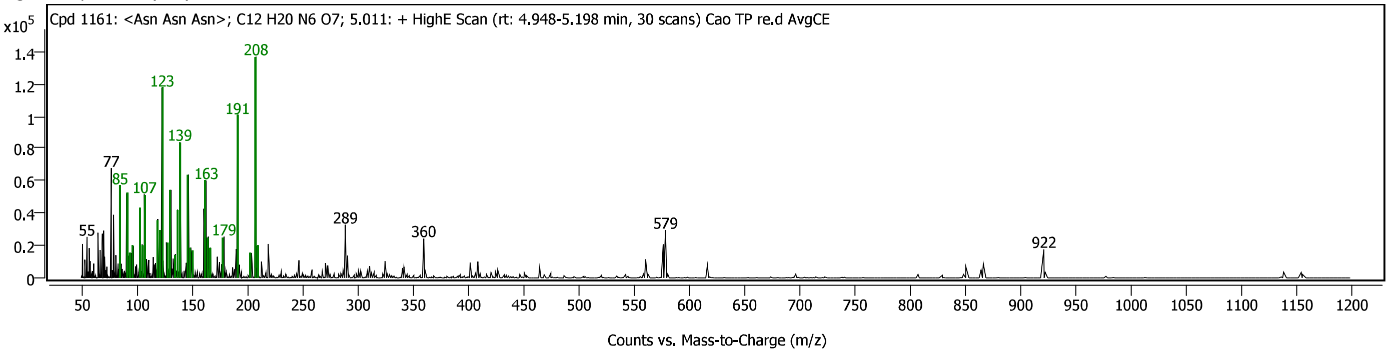

Compound ID Table

Name	Formula	Species	RT	RT Diff	Mass	CAS	ID Source	Score	Score (Lib)	Score (Tgt)
<Asn Asn Asn>	C12 H20 N6 O7	(M+H)+	5.011		360.1406		FBF	94.48		94.48

Cpd 94: <Pyridine-2-azo-p-dimethylaniline>

Name	Formula	RT	RI	Mass	Diff (Tgt, ppm)	CAS	ID Source	Score	Algorithm
<Pyridine-2-azo-p-dimethylaniline>	C13 H14 N4	5.413		226.1210	-3.54		FBF	93.61	FBF

Species	m/z	Score (Tgt)	Score (Lib)	Score (DB)	Score (MFG)	Score (RT)
(M+H)+	227	93.61				

Compound Chromatograms (overlaid)

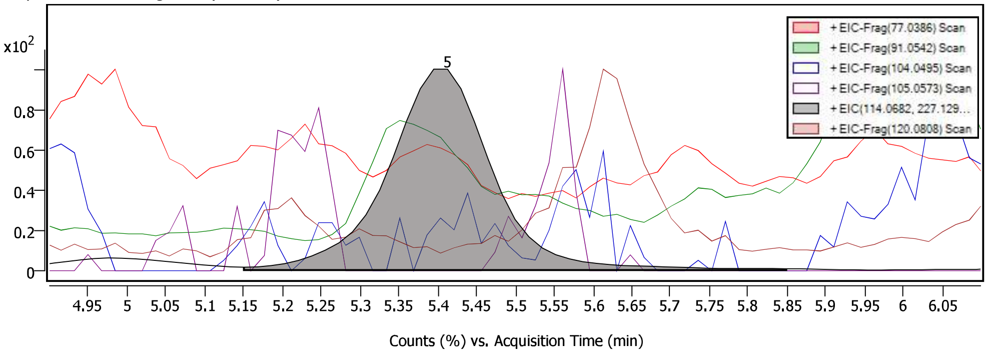

Structure

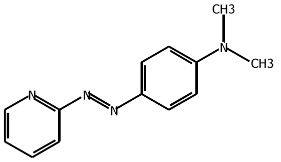

Coelution Plot

Compound Spectra (overlaid)

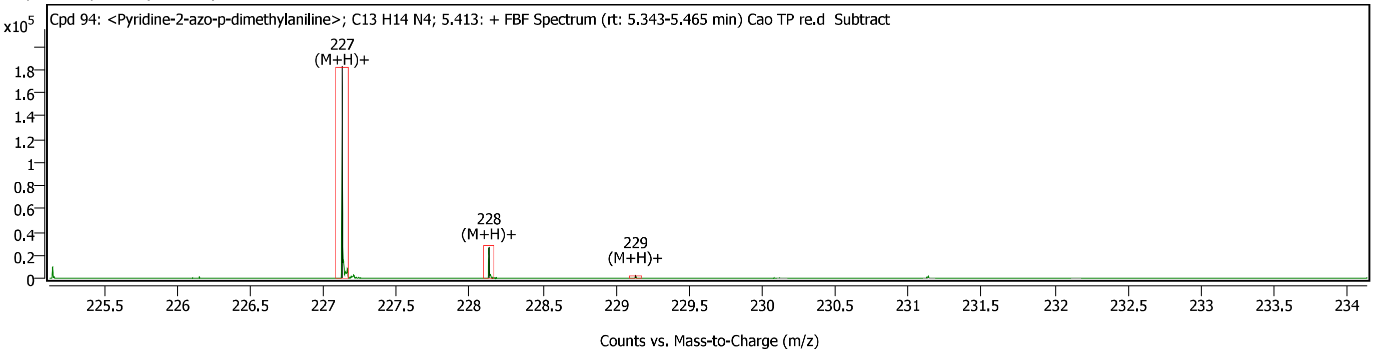

Fragment Spectrum (raw)

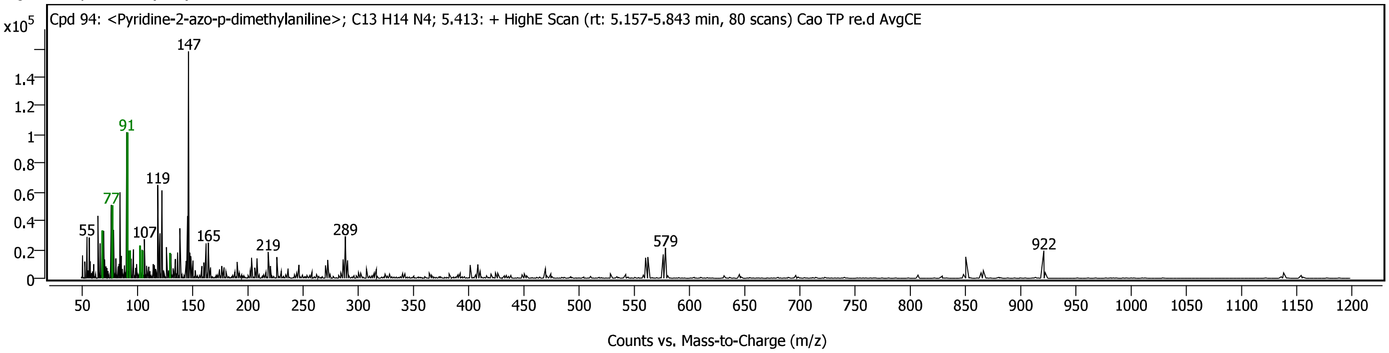

Compound Screening Report

Compound ID Table

Name	Formula	Species	RT	RT Diff	Mass	CAS	ID Source	Score	Score (Lib)	Score (Tgt)
<Pyridine-2-azo-p-dimethylaniline>	C13 H14 N4	(M+H)+	5.413		226.1210		FBF	93.61		93.61

Cpd 1687: Allixin

Name	Formula	RT	RI	Mass	Diff (Tgt, ppm)	CAS	ID Source	Score	Algorithm
Allixin	C12 H18 O4	5.413		226.1210	2.03	125263-70-9	M-FBF	97.26	FBF

Species	m/z	Score (Tgt)	Score (Lib)	Score (DB)	Score (MFG)	Score (RT)
(M+H)+	227	97.26				

Compound Chromatograms (overlaid)

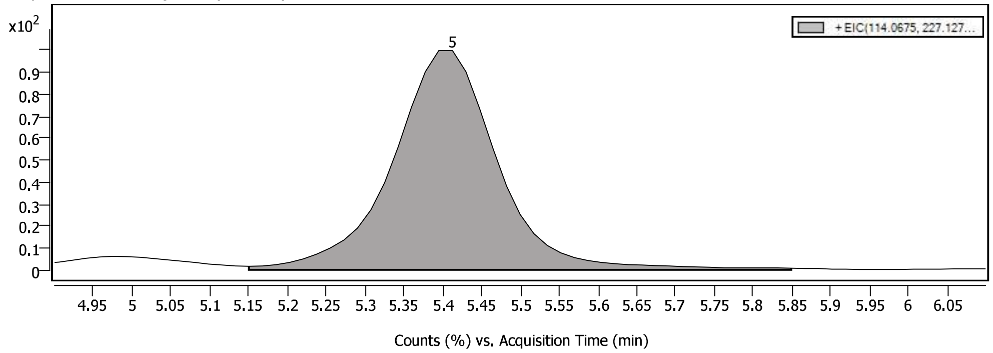

Structure

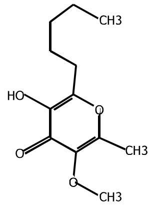

Compound Spectra (overlaid)

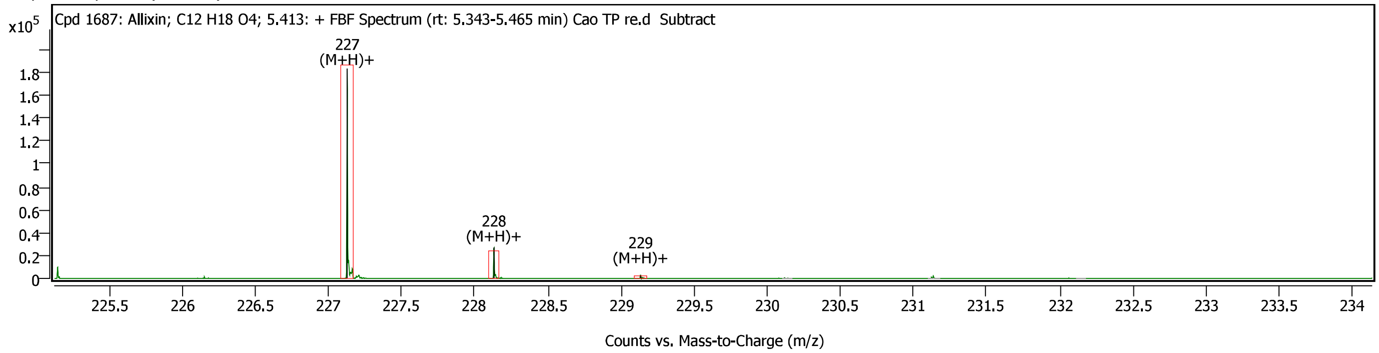

Compound ID Table

Name	Formula	Species	RT	RT Diff	Mass	CAS	ID Source	Score	Score (Lib)	Score (Tgt)
Allixin	C12 H18 O4	(M+H)+	5.413		226.1210	125263-70-9	FBF	97.26		97.26
12-hydroxyjasmonic acid	C12 H18 O4	(M+H)+	5.413		226.1210		FBF	97.26		97.26
3,7-Dimethyl-2E,6E-decadien-1,10-dioic acid	C12 H18 O4	(M+H)+	5.413		226.1210		FBF	97.26		97.26
Butopyronoxyl	C12 H18 O4	(M+H)+	5.413		226.1210	532-34-3	FBF	97.26		97.26
epi-4'-hydroxyjasmonic acid	C12 H18 O4	(M+H)+	5.413		226.1210		FBF	97.26		97.26
Tuberonic acid	C12 H18 O4	(M+H)+	5.413		226.1210		FBF	97.26		97.26

Cpd 1391: ent-Fisetinidol-(4beta->8)-catechin-(6->4beta)-ent-fisetinidol

Name	Formula	RT	RI	Mass	Diff (Tgt, ppm)	CAS	ID Source	Score	Algorithm
ent-Fisetinidol-(4beta->8)-catechin-(6->4beta)-ent-fisetinidol	C45 H38 O17	5.517		850.2112	0.31		FBF	99.46	FBF

Species	m/z	Score (Tgt)	Score (Lib)	Score (DB)	Score (MFG)	Score (RT)
(M+H)+	851	99.46				

Compound Chromatograms (overlaid)

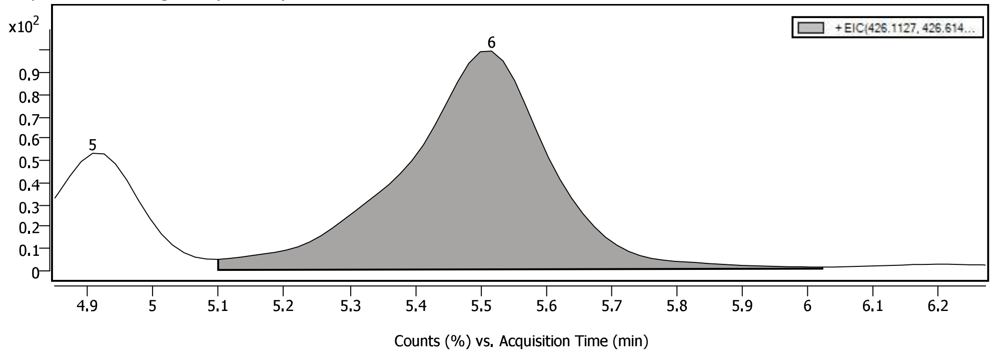

Structure

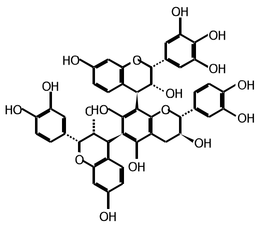

Compound Spectra (overlaid)

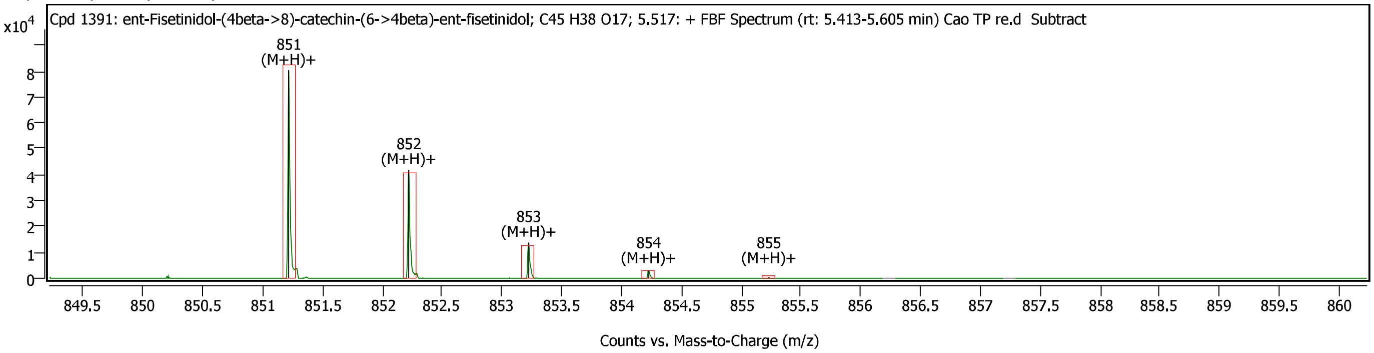

Compound ID Table

Name	Formula	Species	RT	RT Diff	Mass	CAS	ID Source	Score	Score (Lib)	Score (Tgt)
ent-Fisetinidol-(4beta->8)-catechin-(6->4beta)-ent-fisetinidol	C45 H38 O17	(M+H)+	5.517		850.2112		FBF	99.46		99.46

Cpd 1021: 4-Dedimethyl-6-dehydro-anhydrotetracycline

Name	Formula	RT	RI	Mass	Diff (Tgt, ppm)	CAS	ID Source	Score	Algorithm
4-Dedimethyl-6-dehydro-anhydrotetracycline	C20 H18 N2 O7	5.796		398.1116	0.62		FBF	99.27	FBF

Species	m/z	Score (Tgt)	Score (Lib)	Score (DB)	Score (MFG)	Score (RT)
(M+H)+	399	99.27				

Compound Chromatograms (overlaid)

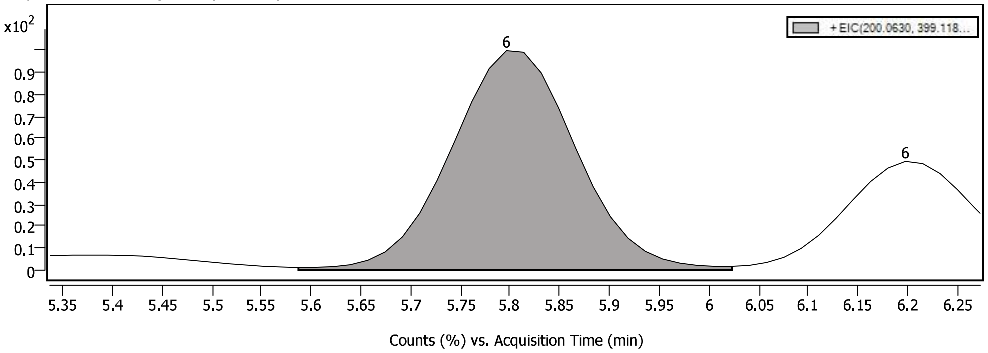

Structure

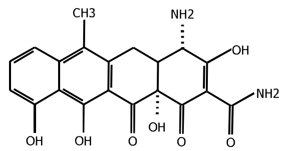

Compound Spectra (overlaid)

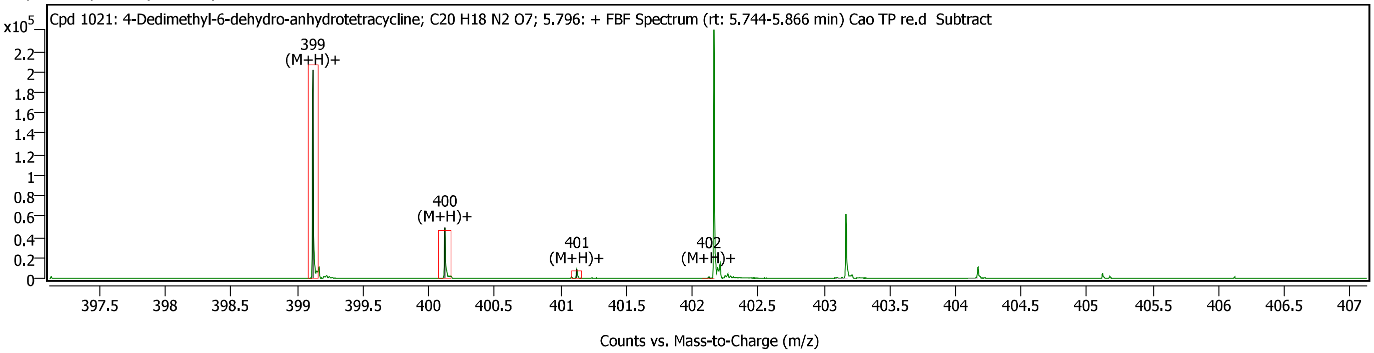

Compound ID Table

Name	Formula	Species	RT	RT Diff	Mass	CAS	ID Source	Score	Score (Lib)	Score (Tgt)
4-Dedimethyl-6-dehydro-anhydrotetracycline	C20 H18 N2 O7	(M+H)+	5.796		398.1116		FBF	99.27		99.27

Cpd 1222: S-Adenosyl-4-methylthio-2-oxobutanoate

Name	Formula	RT	RI	Mass	Diff (Tgt, ppm)	CAS	ID Source	Score	Algorithm
S-Adenosyl-4-methylthio-2-oxobutanoate	C15 H20 N5 O6 S	5.796		398.1120	-3.67	22365-11-3	FBF	81.56	FBF

Species	m/z	Score (Tgt)	Score (Lib)	Score (DB)	Score (MFG)	Score (RT)
(M+H)+	399	81.56				

Compound Screening Report

Compound Chromatograms (overlaid)

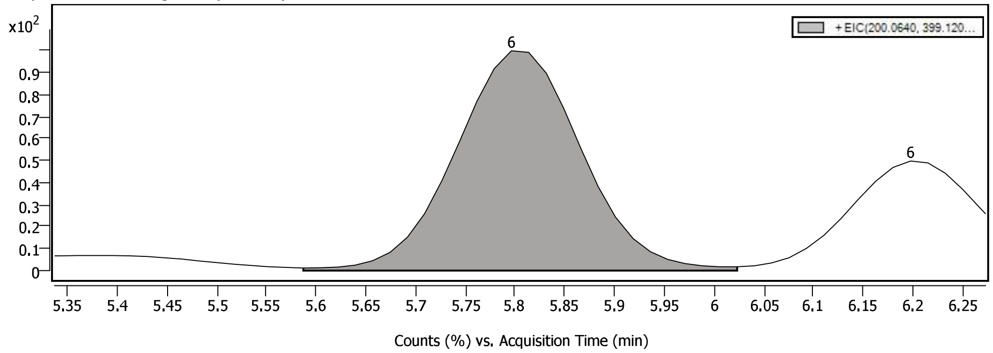

Structure

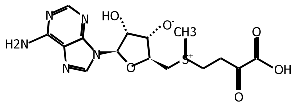

Compound Spectra (overlaid)

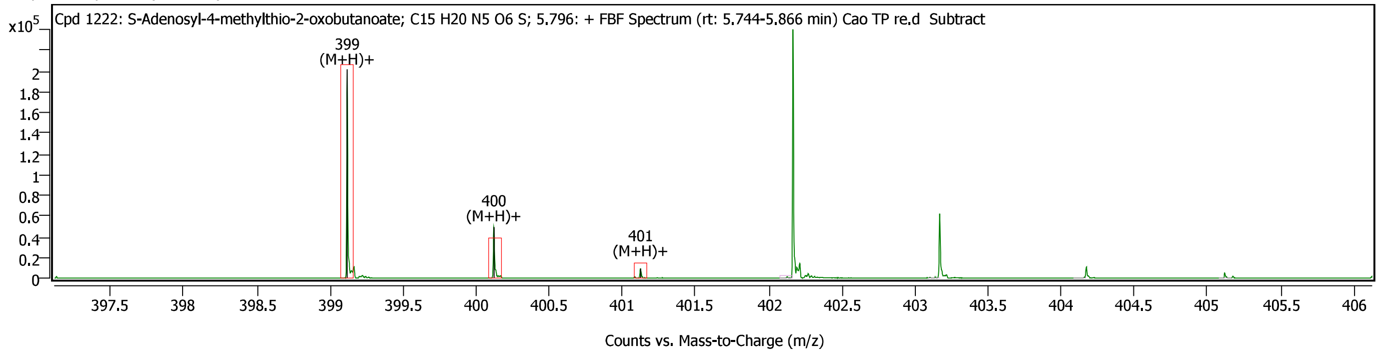

Compound ID Table

Name	Formula	Species	RT	RT Diff	Mass	CAS	ID Source	Score	Score (Lib)	Score (Tgt)
S-Adenosyl-4-methylthio-2-oxobutanoate	C15 H20 N5 O6 S	(M+H)+	5.796		398.1120	22365-11-3	FBF	81.56		81.56

Cpd 1331: Stilbamidine

Name	Formula	RT	RI	Mass	Diff (Tgt, ppm)	CAS	ID Source	Score	Algorithm
Stilbamidine	C16 H16 N4	5.866		264.1365	-3.88	122-06-5	FBF	92.54	FBF

Species	m/z	Score (Tgt)	Score (Lib)	Score (DB)	Score (MFG)	Score (RT)
(M+H)+	265	92.54				

Compound Chromatograms (overlaid)

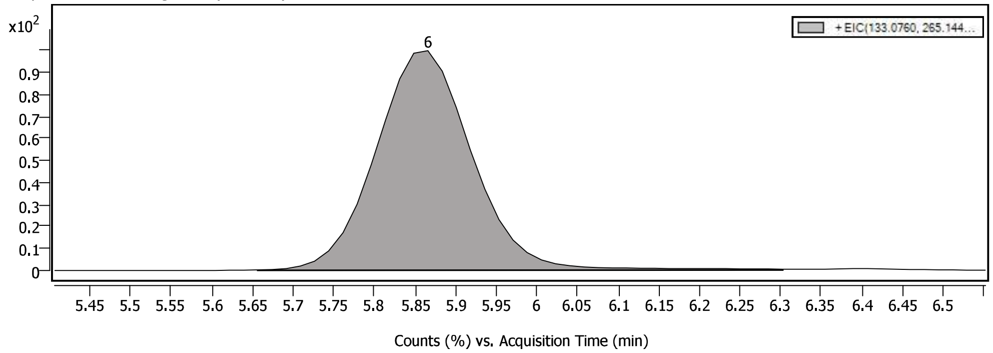

Structure

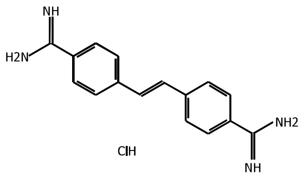

Compound Spectra (overlaid)

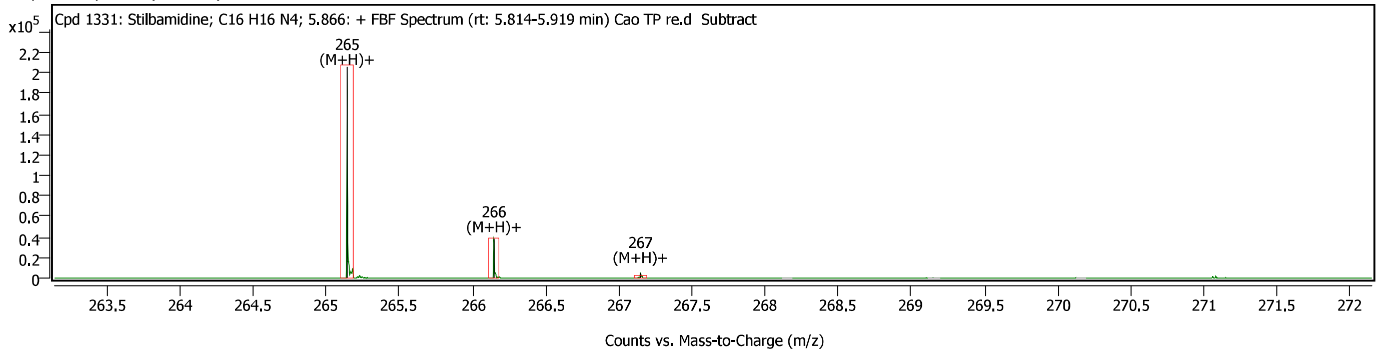

Compound ID Table

Name	Formula	Species	RT	RT Diff	Mass	CAS	ID Source	Score	Score (Lib)	Score (Tgt)
Stilbamidine	C16 H16 N4	(M+H)+	5.866		264.1365	122-06-5	FBF	92.54		92.54

Cpd 1386: <γ-CEHC>

Name	Formula	RT	RI	Mass	Diff (Tgt, ppm)	CAS	ID Source	Score	Algorithm
<γ-CEHC>	C15 H20 O4	5.866		264.1364	0.89	178167-75-4	M-FBF	98.61	FBF

Species	m/z	Score (Tgt)	Score (Lib)	Score (DB)	Score (MFG)	Score (RT)
(M+H)+	265	98.61				

Compound Chromatograms (overlaid)

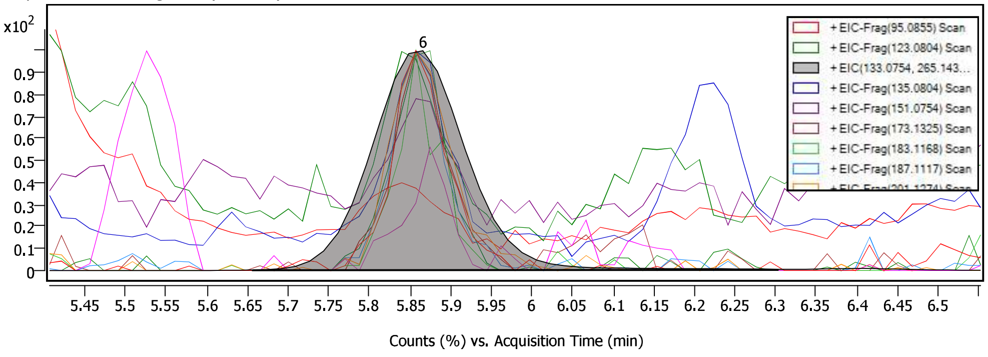

Structure

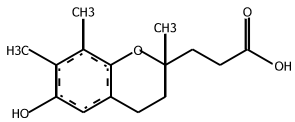

Coelution Plot

Compound Spectra (overlaid)

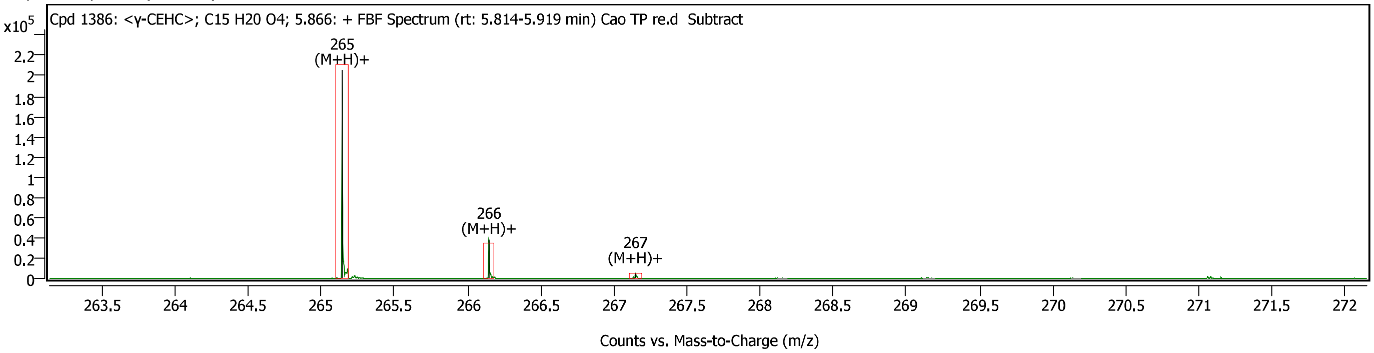

Fragment Spectrum (raw)

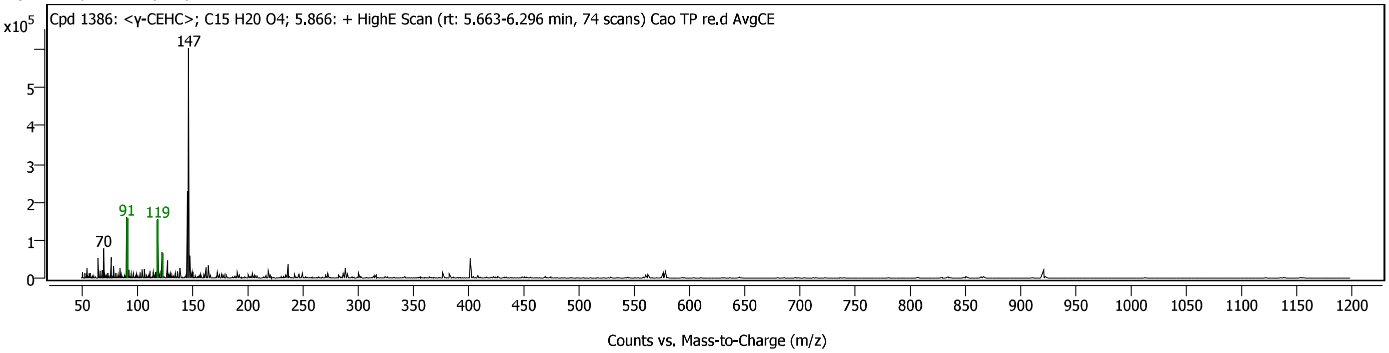

Compound ID Table

Name	Formula	Species	RT	RT Diff	Mass	CAS	ID Source	Score	Score (Lib)	Score (Tgt)
<γ-CEHC>	C15 H20 O4	(M+H)+	5.866		264.1364	178167-75-4	FBF	98.61		98.61
<Ascosalitoxin>	C15 H20 O4	(M+H)+	5.866		264.1364	152982-97-3	FBF	98.61		98.61
<2-Methyl-1-[2,4,6-trihydroxy-3-(3-methyl-2-butenyl)phenyl]-1-propanone>	C15 H20 O4	(M+H)+	5.866		264.1364	35932-36-6	FBF	98.61		98.61
<Alliacol A>	C15 H20 O4	(M+H)+	5.866		264.1364		FBF	98.61		98.61
<Artemisiifolin>	C15 H20 O4	(M+H)+	5.866		264.1364	26931-87-3	FBF	98.61		98.61
<Coronopilin>	C15 H20 O4	(M+H)+	5.866		264.1364	2571-81-5	FBF	98.61		98.61
<Baileyin>	C15 H20 O4	(M+H)+	5.866		264.1364	27875-37-2	FBF	98.61		98.61
<Blennin B>	C15 H20 O4	(M+H)+	5.866		264.1364	62824-37-7	FBF	98.61		98.61
<Chamissonin>	C15 H20 O4	(M+H)+	5.866		264.1364	24112-94-5	FBF	98.61		98.61
<Abscisic acid>	C15 H20 O4	(M+H)+	5.866		264.1364	7773-56-0	FBF	98.61		98.61
<Curcolonol>	C15 H20 O4	(M+H)+	5.866		264.1364	217817-09-9	FBF	98.61		98.61
<Abscisic acid (cis,trans)>	C15 H20 O4	(M+H)+	5.866		264.1364	14375-45-2	FBF	98.61		98.61
<Enokipodin C>	C15 H20 O4	(M+H)+	5.866		264.1364	359701-26-1	FBF	98.61		98.61
<4-Hydroxydehydromyoporone>	C15 H20 O4	(M+H)+	5.866		264.1364	72896-64-1	FBF	98.61		98.61
<Plenolin>	C15 H20 O4	(M+H)+	5.866		264.1364	34257-95-9	FBF	98.61		98.61
<3-Epiarmefolin>	C15 H20 O4	(M+H)+	5.866		264.1364	293313-26-5	FBF	98.61		98.61
<Florilenalin>	C15 H20 O4	(M+H)+	5.866		264.1364	54964-49-7	FBF	98.61		98.61
<15-Hydroxymarasmen-3-one>	C15 H20 O4	(M+H)+	5.866		264.1364	124894-86-6	FBF	98.61		98.61
<(S)-(+)-Abscisic acid>	C15 H20 O4	(M+H)+	5.866		264.1364		FBF	98.61		98.61
<(8betaOH,10beta)-8-Hydroxy-3-oxo-7(11)-eremophilen-12,8-olide>	C15 H20 O4	(M+H)+	5.866		264.1364	171422-92-7	FBF	98.61		98.61
<(1beta,8beta)-1,8-Dihydroxy-3,7(11)-eudesmadien-12,8-olide>	C15 H20 O4	(M+H)+	5.866		264.1364	366494-92-0	FBF	98.61		98.61
<(10R,11R)-Pterosin L>	C15 H20 O4	(M+H)+	5.866		264.1364	41411-04-5	FBF	98.61		98.61
<(+)-Abscisic Acid>	C15 H20 O4	(M+H)+	5.866		264.1364	21293-29-8	FBF	98.61		98.61
<(-)-Illudin S>	C15 H20 O4	(M+H)+	5.866		264.1364		FBF	98.61		98.61
<7alpha,11alpha-Dihydroxy-4(13),8-coloratadien-12,11-olide>	C15 H20 O4	(M+H)+	5.866		264.1364		FBF	98.61		98.61
<Alkhanin>	C15 H20 O4	(M+H)+	5.866		264.1364	71327-31-6	FBF	98.61		98.61
<(-)-Abscisic acid>	C15 H20 O4	(M+H)+	5.866		264.1364	14398-53-9	FBF	98.61		98.61
<amaralin>	C15 H20 O4	(M+H)+	5.866		264.1364		FBF	98.61		98.61
<Michelenolide>	C15 H20 O4	(M+H)+	5.866		264.1364	66392-96-9	FBF	98.61		98.61
<Granilin>	C15 H20 O4	(M+H)+	5.866		264.1364	40737-97-1	FBF	98.61		98.61
<Vulgarin>	C15 H20 O4	(M+H)+	5.866		264.1364	3162-56-9	FBF	98.61		98.61
<Vermeerin>	C15 H20 O4	(M+H)+	5.866		264.1364	16983-23-6	FBF	98.61		98.61
<Umbellifolide>	C15 H20 O4	(M+H)+	5.866		264.1364	89026-40-4	FBF	98.61		98.61
<Thellungianin G>	C15 H20 O4	(M+H)+	5.866		264.1364	97180-28-4	FBF	98.61		98.61
<Tavulin>	C15 H20 O4	(M+H)+	5.866		264.1364	71030-09-6	FBF	98.61		98.61
<Tanacetin>	C15 H20 O4	(M+H)+	5.866		264.1364	1401-54-3	FBF	98.61		98.61
<Sequiterpene Lactone 326>	C15 H20 O4	(M+H)+	5.866		264.1364		FBF	98.61		98.61
<Geigerin>	C15 H20 O4	(M+H)+	5.866		264.1364	436-45-3	FBF	98.61		98.61
<Salonitenolide>	C15 H20 O4	(M+H)+	5.866		264.1364	26931-94-2	FBF	98.61		98.61
<Tatridin B>	C15 H20 O4	(M+H)+	5.866		264.1364	41653-76-3	FBF	98.61		98.61
<Pleniradin>	C15 H20 O4	(M+H)+	5.866		264.1364	25941-24-6	FBF	98.61		98.61
<Isoamberboin>	C15 H20 O4	(M+H)+	5.866		264.1364	30825-69-5	FBF	98.61		98.61
<Phomenone>	C15 H20 O4	(M+H)+	5.866		264.1364	55785-58-5	FBF	98.61		98.61
<Hirsutic acid C>	C15 H20 O4	(M+H)+	5.866		264.1364	3650-17-7	FBF	98.61		98.61
<Hulupinic acid>	C15 H20 O4	(M+H)+	5.866		264.1364		FBF	98.61		98.61
<Hymenolin>	C15 H20 O4	(M+H)+	5.866		264.1364	20555-05-9	FBF	98.61		98.61
<Heliespirone A>	C15 H20 O4	(M+H)+	5.866		264.1364	202533-71-9	FBF	98.61		98.61
<Istanbulin A>	C15 H20 O4	(M+H)+	5.866		264.1364	35481-83-5	FBF	98.61		98.61
<Ludovicin A>	C15 H20 O4	(M+H)+	5.866		264.1364	22740-13-2	FBF	98.61		98.61
<Ridentin>	C15 H20 O4	(M+H)+	5.866		264.1364	28148-84-7	FBF	98.61		98.61
<O-Formyloreadone>	C15 H20 O4	(M+H)+	5.866		264.1364		FBF	98.61		98.61

Cpd 1664: 1-(alpha-Methyl-4-(2-methylpropyl)benzeneacetate)-beta-D-Glucopyranuronic acid

Name	Formula	RT	RI	Mass	Diff (Tgt, ppm)	CAS	ID Source	Score	Algorithm
1-(alpha-Methyl-4-(2-methylpropyl)benzeneacetate)-beta-D-Glucopyranuronic acid	C19 H26 O8	5.971		382.1613	-3.86	115075-59-7	M-FBF	92.80	FBF

Species	m/z	Score (Tgt)	Score (Lib)	Score (DB)	Score (MFG)	Score (RT)
(M+H)+	383	92.80				

Compound Chromatograms (overlaid)

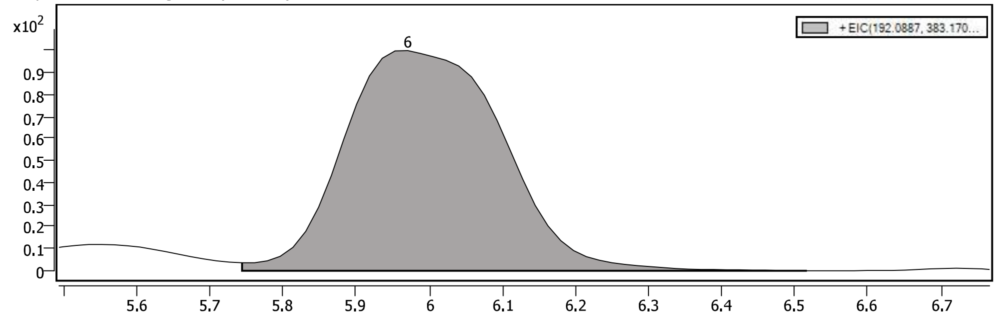

Structure

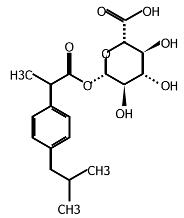

Compound Screening Report

Compound Spectra (overlaid)

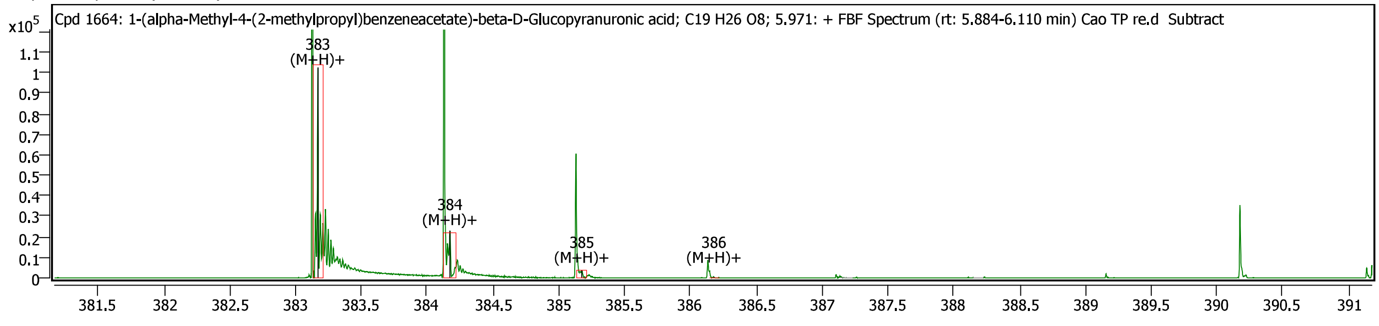

Compound ID Table

Name	Formula	Species	RT	RT Diff	Mass	CAS	ID Source	Score	Score (Lib)	Score (Tgt)
1-(alpha-Methyl-4-(2-methylpropyl)benzeneacetate)-beta-D-Glucopyranuronic acid	C19 H26 O8	(M+H)+	5.971		382.1613	115075-59-7	FBF	92.80		92.80
Cyclocalopin B	C19 H26 O8	(M+H)+	5.971		382.1613		FBF	92.80		92.80
7alpha,8alpha-Dihydroxycalonectrin	C19 H26 O8	(M+H)+	5.971		382.1613	95673-99-7	FBF	92.80		92.80
4,8-Diacetyl-T2-tetrol	C19 H26 O8	(M+H)+	5.971		382.1613	65180-29-2	FBF	92.80		92.80

Cpd 576: Riboflavin

Name	Formula	RT	RI	Mass	Diff (Tgt, ppm)	CAS	ID Source	Score	Algorithm
Riboflavin	C17 H20 N4 O6	6.145		376.1389	1.53	83-88-5	FBF-FragConfirm	98.79	FBF

Species	m/z	Score (Tgt)	Score (Lib)	Score (DB)	Score (MFG)	Score (RT)
(M+H)+	377	98.79				

Compound Chromatograms (overlaid)

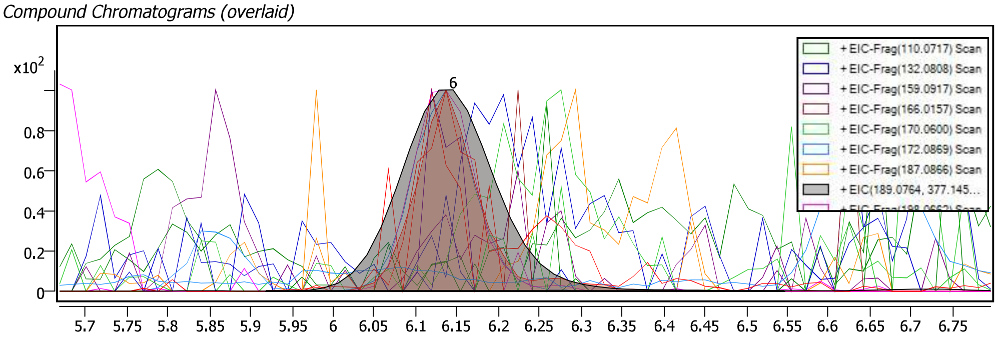

Structure

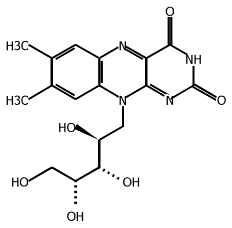

Coelution Plot

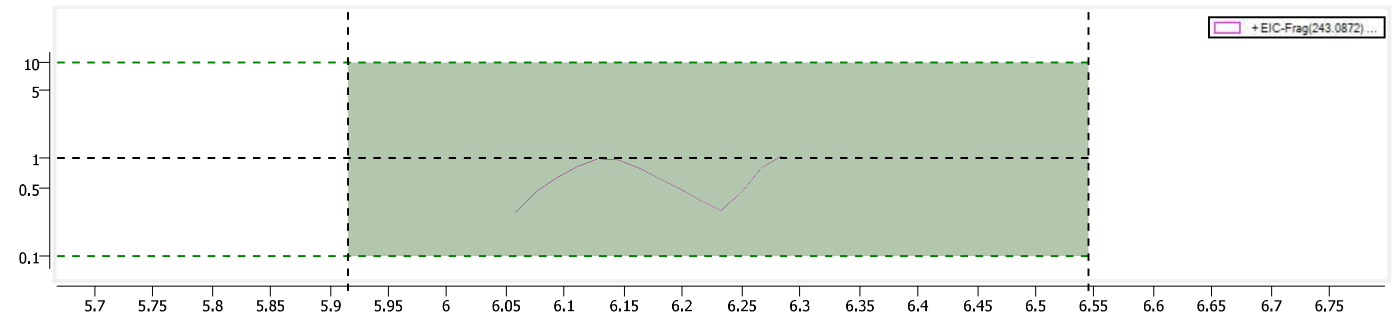

Compound Spectra (overlaid)

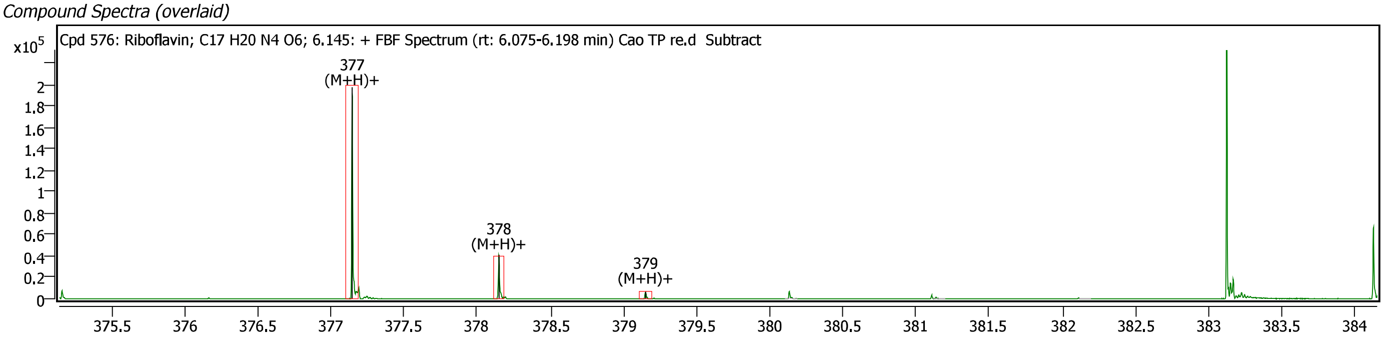

Fragment Spectrum (clean)

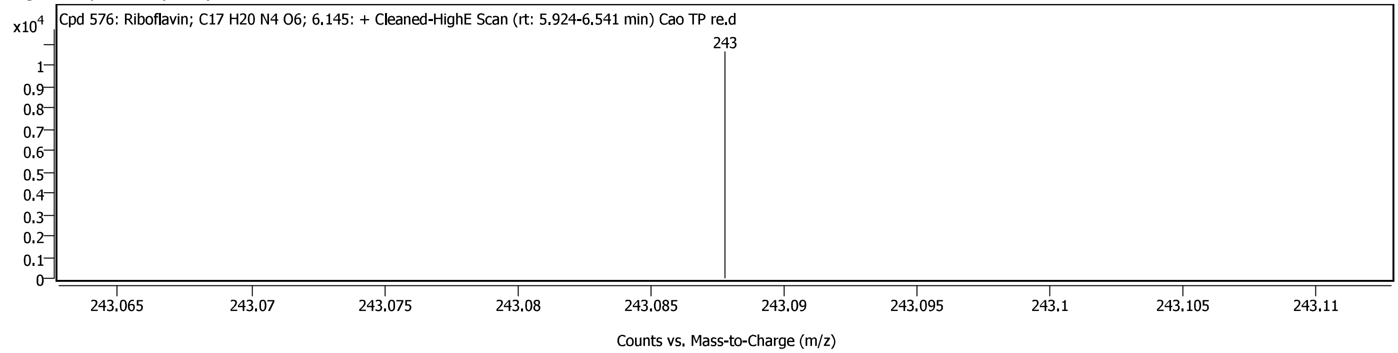

Fragment Spectrum (raw)

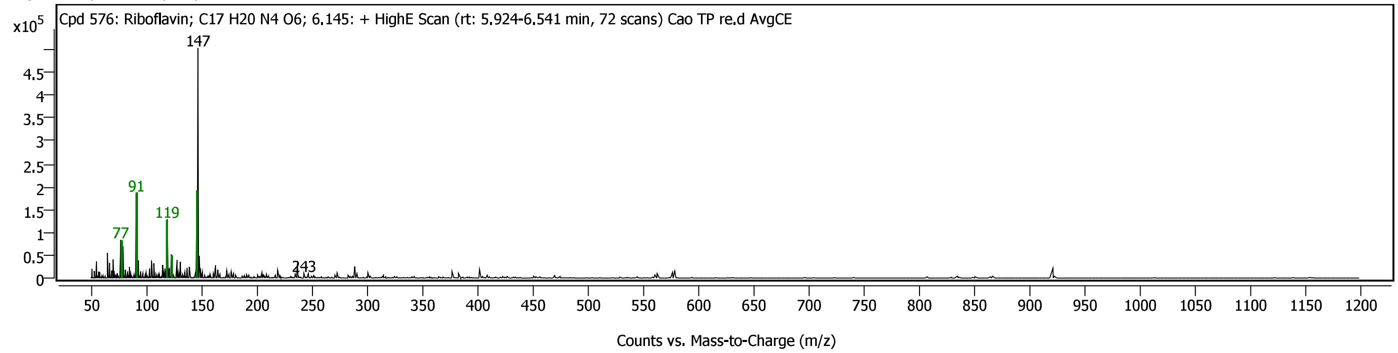

Compound ID Table

Name	Formula	Species	RT	RT Diff	Mass	CAS	ID Source	Score	Score (Lib)	Score (Tgt)
Riboflavin	C17 H20 N4 O6	(M+H)+	6.145		376.1389	83-88-5	FBF-FragConfirm	98.79		98.79
His-Val-OH	C17 H20 N4 O6	(M+H)+	6.145		376.1389		FBF-FragConfirm	98.79		98.79
Asp Gly Trp	C17 H20 N4 O6	(M+H)+	6.145		376.1389		FBF-FragConfirm	98.79		98.79
Asp Trp Gly	C17 H20 N4 O6	(M+H)+	6.145		376.1389		FBF-FragConfirm	98.79		98.79
Trp Gly Asp	C17 H20 N4 O6	(M+H)+	6.145		376.1389		FBF-FragConfirm	98.79		98.79
Gly Trp Asp	C17 H20 N4 O6	(M+H)+	6.145		376.1389		FBF-FragConfirm	98.79		98.79
Gly Asp Trp	C17 H20 N4 O6	(M+H)+	6.145		376.1389		FBF-FragConfirm	98.79		98.79
Ile-His-OH	C17 H20 N4 O6	(M+H)+	6.145		376.1389		FBF-FragConfirm	98.79		98.79
Leu-His-OH	C17 H20 N4 O6	(M+H)+	6.145		376.1389		FBF-FragConfirm	98.79		98.79
Trp Asp Gly	C17 H20 N4 O6	(M+H)+	6.145		376.1389		FBF-FragConfirm	98.79		98.79

Cpd 1020: 4-Dedimethyl-6-dehydro-anhydrotetracycline

Name	Formula	RT	RI	Mass	Diff (Tgt, ppm)	CAS	ID Source	Score	Algorithm
4-Dedimethyl-6-dehydro-anhydrotetracycline	C20 H18 N2 O7	6.198		398.1117	0.80		FBF	99.14	FBF

Species	m/z	Score (Tgt)	Score (Lib)	Score (DB)	Score (MFG)	Score (RT)
(M+H)+	399	99.14				

Compound Chromatograms (overlaid)

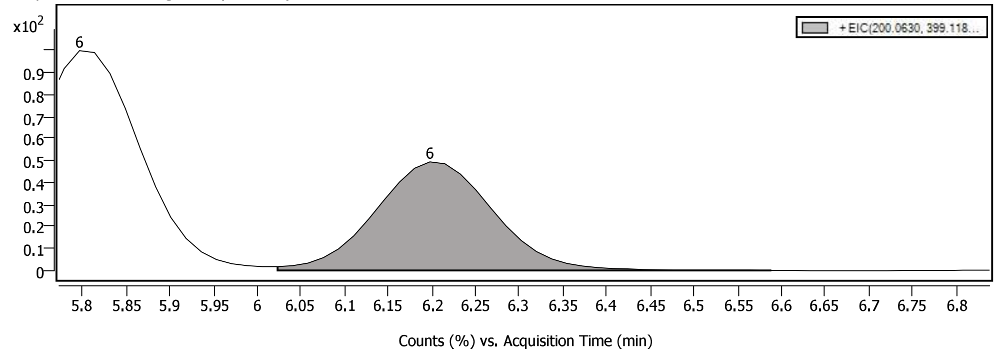

Structure

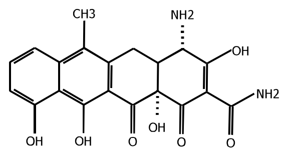

Compound Screening Report

Compound Spectra (overlaid)

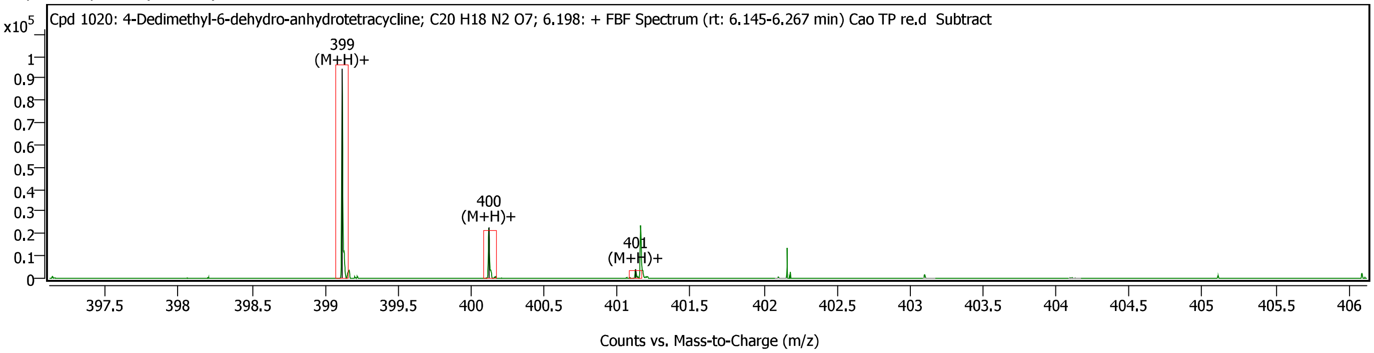

Compound ID Table

Name	Formula	Species	RT	RT Diff	Mass	CAS	ID Source	Score	Score (Lib)	Score (Tgt)
4-Dedimethyl-6-dehydro-anhydrotetracycline	C20 H18 N2 O7	(M+H)+	6.198		398.1117		FBF	99.14		99.14

Cpd 1221: S-Adenosyl-4-methylthio-2-oxobutanoate

Name	Formula	RT	RI	Mass	Diff (Tgt, ppm)	CAS	ID Source	Score	Algorithm
S-Adenosyl-4-methylthio-2-oxobutanoate	C15 H20 N5 O6 S	6.198		398.1120	-3.52	22365-11-3	FBF	82.16	FBF

Species	m/z	Score (Tgt)	Score (Lib)	Score (DB)	Score (MFG)	Score (RT)
(M+H)+	399	82.16				

Compound Chromatograms (overlaid)

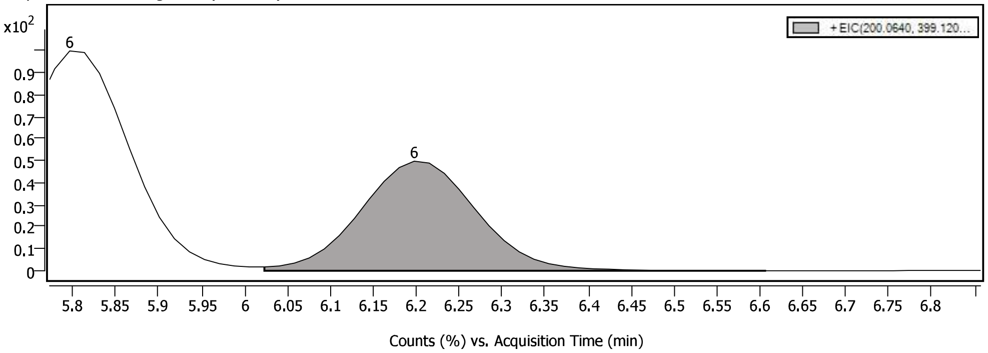

Structure

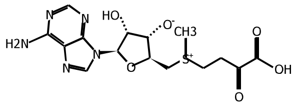

Compound Spectra (overlaid)

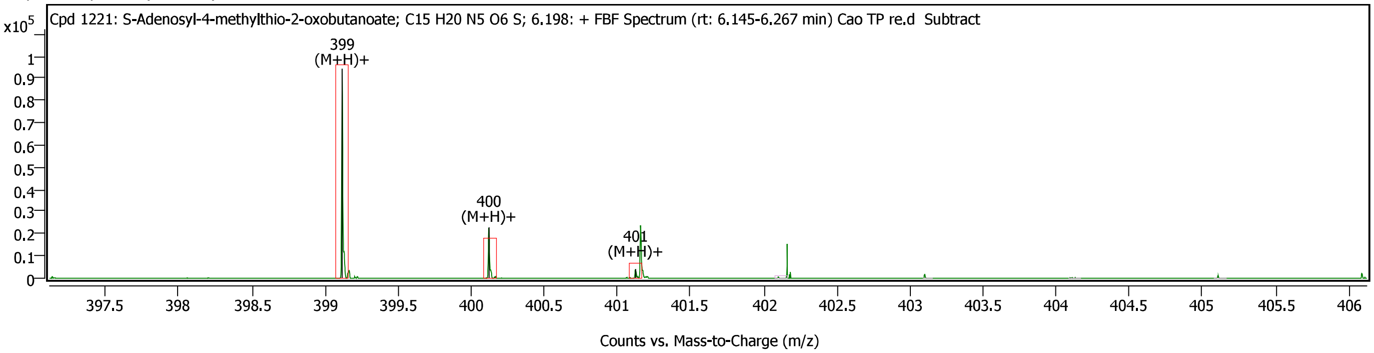

Compound ID Table

Name	Formula	Species	RT	RT Diff	Mass	CAS	ID Source	Score	Score (Lib)	Score (Tgt)
S-Adenosyl-4-methylthio-2-oxobutanoate	C15 H20 N5 O6 S	(M+H)+	6.198		398.1120	22365-11-3	FBF	82.16		82.16

Cpd 1569: <Cuminaldehyde>

Name	Formula	RT	RI	Mass	Diff (Tgt, ppm)	CAS	ID Source	Score	Algorithm
<Cuminaldehyde>	C10 H12 O	6.355		148.0888	0.07	122-03-2	M-FBF	99.65	FBF

Species	m/z	Score (Tgt)	Score (Lib)	Score (DB)	Score (MFG)	Score (RT)
(M+H)+	149	99.65				

Compound Screening Report

Compound Chromatograms (overlaid)

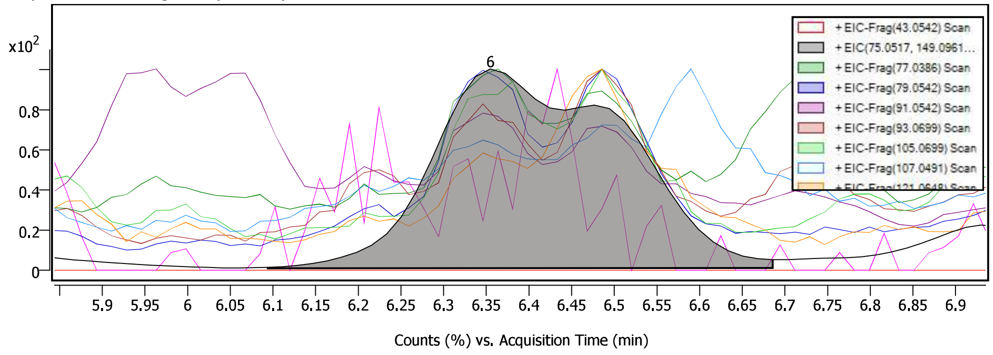

Structure

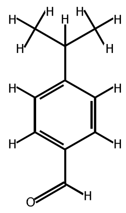

Coelution Plot

Compound Spectra (overlaid)

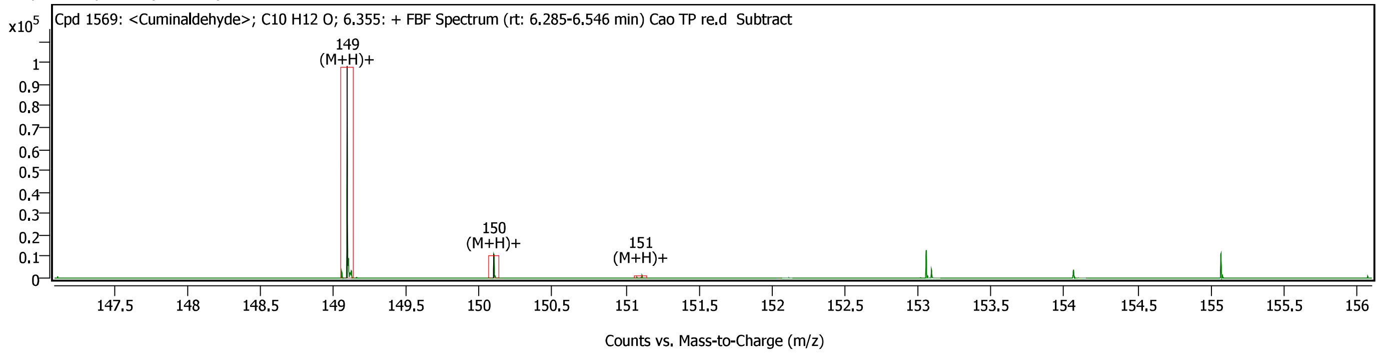

Fragment Spectrum (raw)

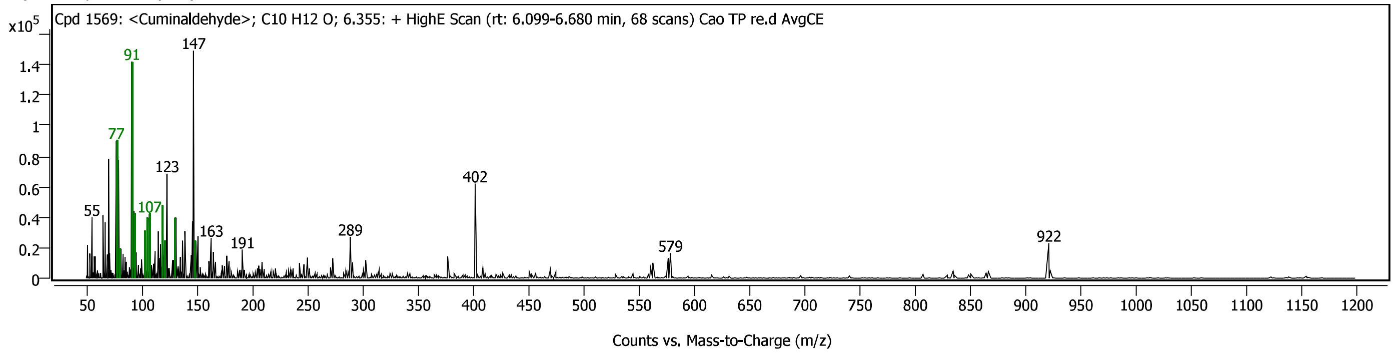

Compound ID Table

Name	Formula	Species	RT	RT Diff	Mass	CAS	ID Source	Score	Score (Lib)	Score (Tgt)
<Cuminaldehyde>	C10 H12 O	(M+H)+	6.355		148.0888	122-03-2	FBF	99.65		99.65
<2-(4-Methylphenyl)propanal>	C10 H12 O	(M+H)+	6.355		148.0888	99-72-9	FBF	99.65		99.65
<2,3,6-Trimethylbenzaldehyde>	C10 H12 O	(M+H)+	6.355		148.0888	34341-29-2	FBF	99.65		99.65
<2,4,6,8-decatetraenal>	C10 H12 O	(M+H)+	6.355		148.0888		FBF	99.65		99.65
<2,4,6-Trimethylbenzaldehyde>	C10 H12 O	(M+H)+	6.355		148.0888	487-68-3	FBF	99.65		99.65
<2',4'-Dimethylacetophenone>	C10 H12 O	(M+H)+	6.355		148.0888	89-74-7	FBF	99.65		99.65
<3,4,5-Trimethylbenzaldehyde>	C10 H12 O	(M+H)+	6.355		148.0888	5779-74-8	FBF	99.65		99.65
<4-Methylphenyl acetone>	C10 H12 O	(M+H)+	6.355		148.0888	2096-86-8	FBF	99.65		99.65
<4-Phenyl-3-buten-2-ol>	C10 H12 O	(M+H)+	6.355		148.0888	17488-65-2	FBF	99.65		99.65
<5,6,7,8-Tetrahydro-2-naphthol>	C10 H12 O	(M+H)+	6.355		148.0888	1125-78-6	FBF	99.65		99.65
<Anethole>	C10 H12 O	(M+H)+	6.355		148.0888	4180-23-8	FBF	99.65		99.65
<Duryl aldehyde>	C10 H12 O	(M+H)+	6.355		148.0888	5779-72-6	FBF	99.65		99.65
<Estragole>	C10 H12 O	(M+H)+	6.355		148.0888	140-67-0	FBF	99.65		99.65
<trans-Lachnophyllol>	C10 H12 O	(M+H)+	6.355		148.0888	23180-62-3	FBF	99.65		99.65

Cpd 1686: Butopyronoxyl

Name	Formula	RT	RI	Mass	Diff (Tgt, ppm)	CAS	ID Source	Score	Algorithm
Butopyronoxyl	C12 H18 O4	6.494		226.1206	0.52	532-34-3	M-FBF	99.02	FBF

Species	m/z	Score (Tgt)	Score (Lib)	Score (DB)	Score (MFG)	Score (RT)
(M+H)+	227	99.02				

Compound Chromatograms (overlaid)

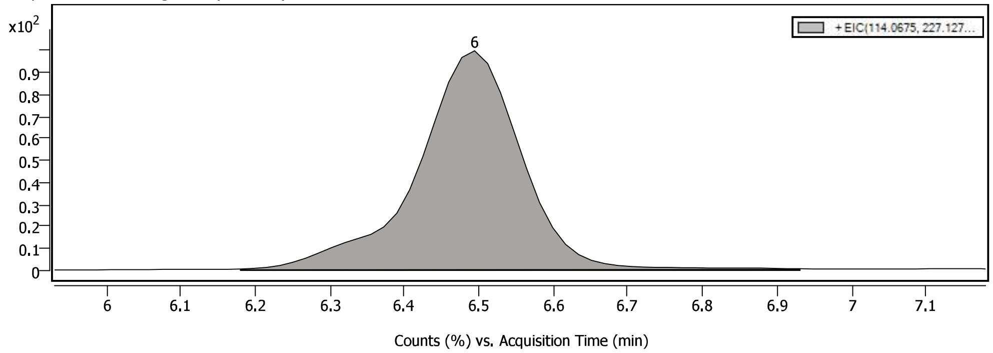

Structure

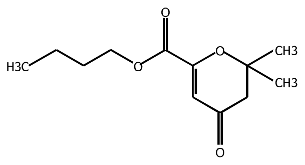

Compound Spectra (overlaid)

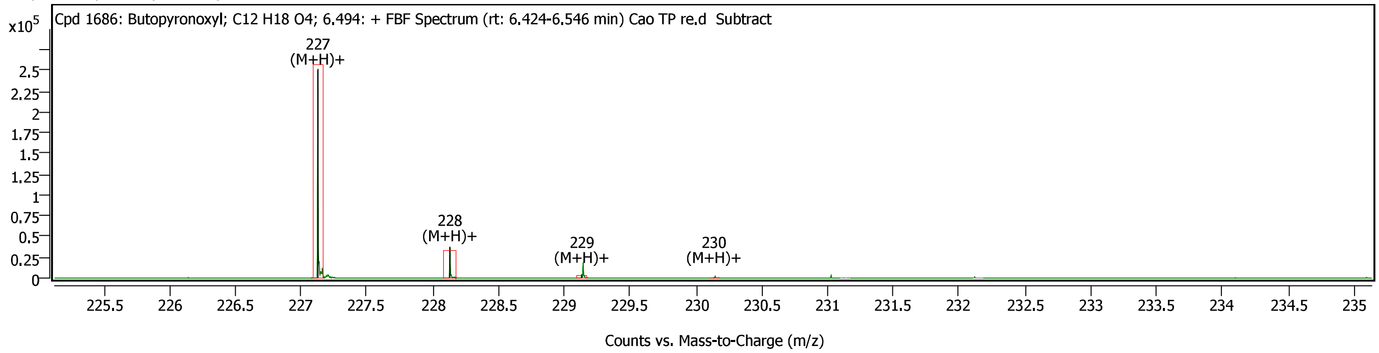

Compound ID Table

Name	Formula	Species	RT	RT Diff	Mass	CAS	ID Source	Score	Score (Lib)	Score (Tgt)
Butopyronoxyl	C12 H18 O4	(M+H)+	6.494		226.1206	532-34-3	FBF	99.02		99.02
Allixin	C12 H18 O4	(M+H)+	6.494		226.1206	125263-70-9	FBF	99.02		99.02
12-hydroxyjasmonic acid	C12 H18 O4	(M+H)+	6.494		226.1206		FBF	99.02		99.02
3,7-Dimethyl-2E,6E-decadien-1,10-dioic acid	C12 H18 O4	(M+H)+	6.494		226.1206		FBF	99.02		99.02
epi-4'-hydroxyjasmonic acid	C12 H18 O4	(M+H)+	6.494		226.1206		FBF	99.02		99.02
Tuberonic acid	C12 H18 O4	(M+H)+	6.494		226.1206		FBF	99.02		99.02

Cpd 1449: Cinnamoylglycine

Name	Formula	RT	RI	Mass	Diff (Tgt, ppm)	CAS	ID Source	Score	Algorithm
Cinnamoylglycine	C11 H11 N O3	6.721		205.0743	2.15	16534-24-0	M-FBF-FragConfirm	97.57	FBF

Species	m/z	Score (Tgt)	Score (Lib)	Score (DB)	Score (MFG)	Score (RT)
(M+H)+	206	97.57				

Compound Chromatograms (overlaid)

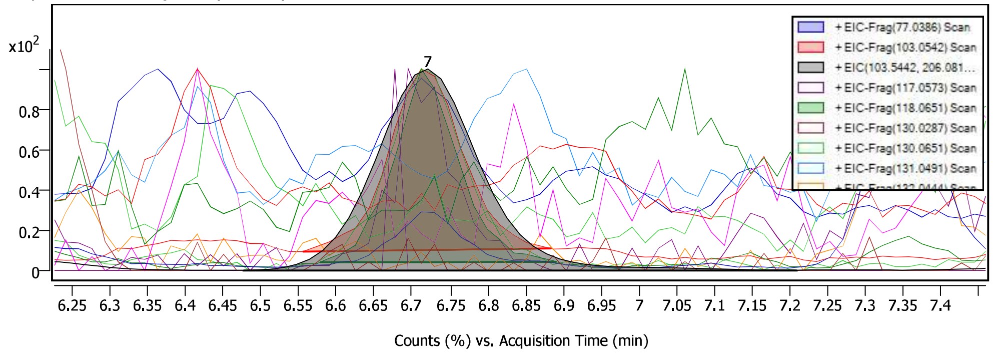

Structure

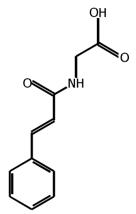

Compound Screening Report

Coelution Plot

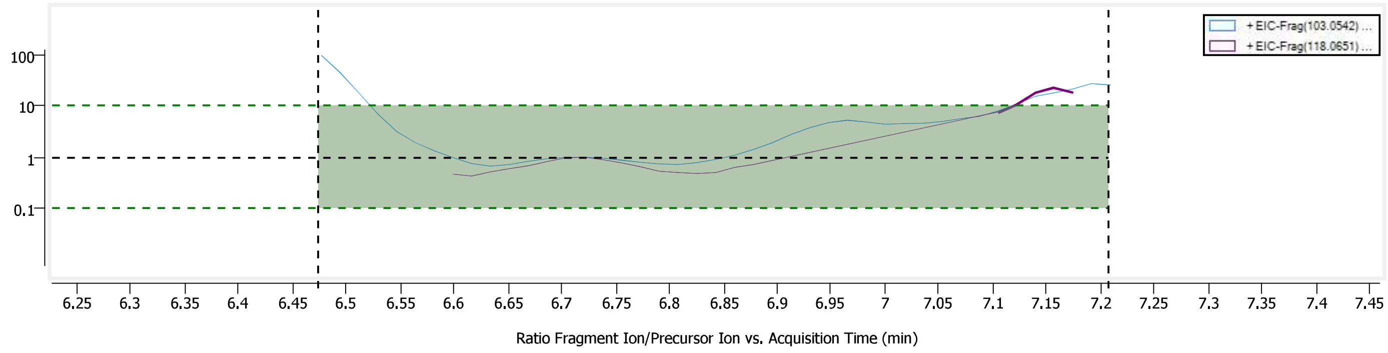

Compound Spectra (overlaid)

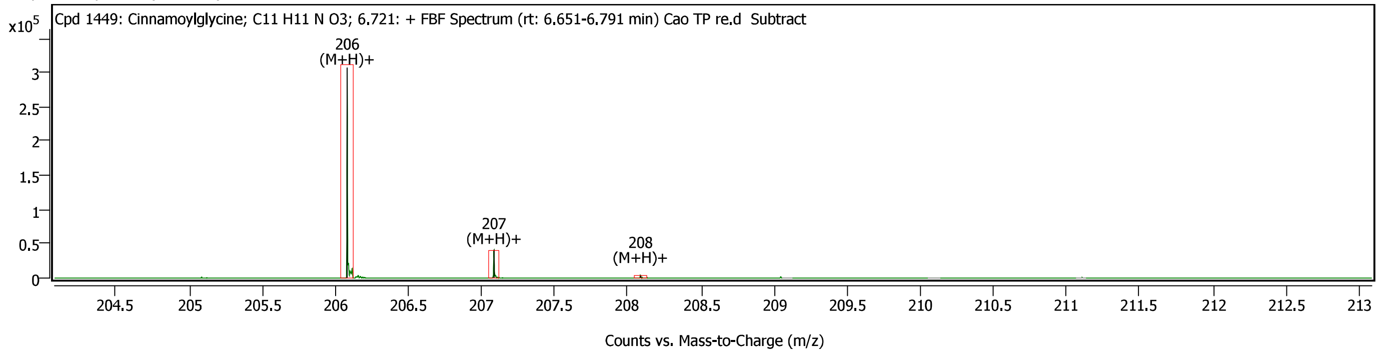

Fragment Spectrum (clean)

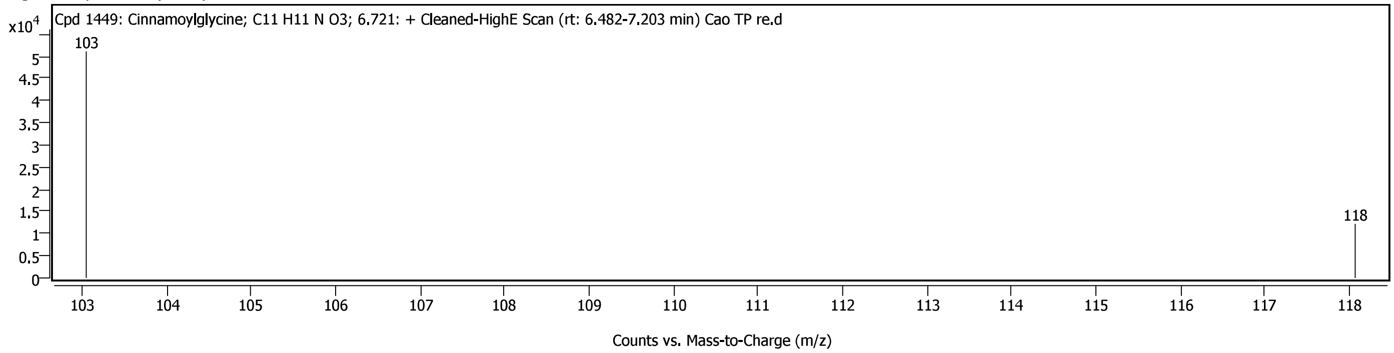

Fragment Spectrum (raw)

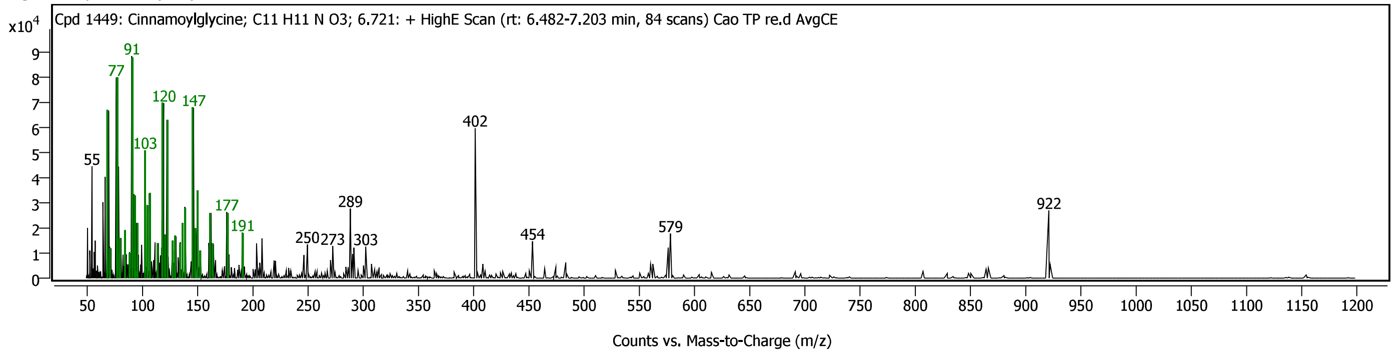

Compound ID Table

Name	Formula	Species	RT	RT Diff	Mass	CAS	ID Source	Score	Score (Lib)	Score (Tgt)
Cinnamoylglycine	C11 H11 N O3	(M+H)+	6.721		205.0743	16534-24-0	FBF-FragConfirm	97.57		97.57
Indolelactic acid	C11 H11 N O3	(M+H)+	6.721		205.0743	1821-52-9	FBF-FragConfirm	97.57		97.57
5-Methoxyindoleacetate	C11 H11 N O3	(M+H)+	6.721		205.0743	3471-31-6	FBF-FragConfirm	97.57		97.57
Gentianamine	C11 H11 N O3	(M+H)+	6.721		205.0743	22952-54-1	FBF-FragConfirm	97.57		97.57
Methyl 1-methoxy-1H-indole-3-carboxylate	C11 H11 N O3	(M+H)+	6.721		205.0743	18377-50-9	FBF-FragConfirm	97.57		97.57
N-(3-Hydroxypropyl)phthalimide	C11 H11 N O3	(M+H)+	6.721		205.0743	883-44-3	FBF-FragConfirm	97.57		97.57
Swietenidin B	C11 H11 N O3	(M+H)+	6.721		205.0743	2721-56-4	FBF-FragConfirm	97.57		97.57

Cpd 1283: Istamycin C1

Name	Formula	RT	RI	Mass	Diff (Tgt, ppm)	CAS	ID Source	Score	Algorithm
Istamycin C1	C19 H37 N5 O6	6.808		431.2736	-1.83		FBF	97.20	FBF

Species	m/z	Score (Tgt)	Score (Lib)	Score (DB)	Score (MFG)	Score (RT)
(M+H)+	432	97.20				

Compound Chromatograms (overlaid)

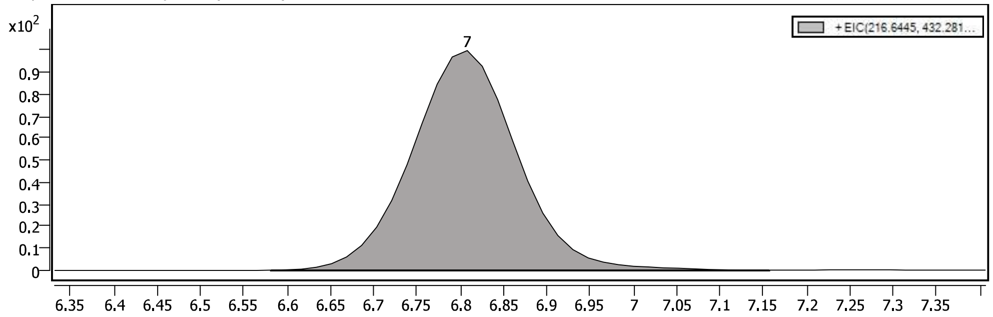

Structure

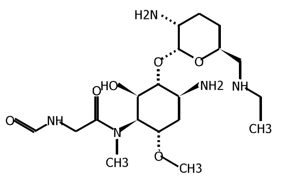

Compound Spectra (overlaid)

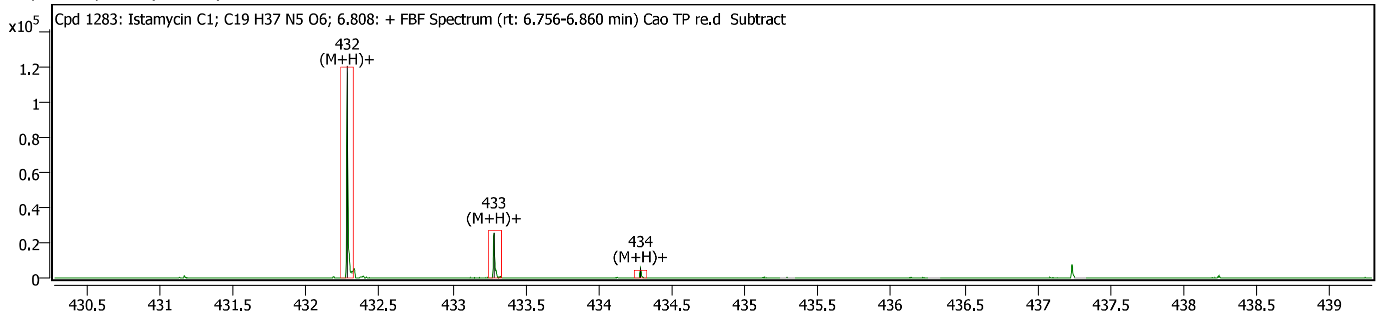

Compound ID Table

Name	Formula	Species	RT	RT Diff	Mass	CAS	ID Source	Score	Score (Lib)	Score (Tgt)
Istamycin C1	C19 H37 N5 O6	(M+H)+	6.808		431.2736		FBF	97.20		97.20

Cpd 132: N1-Caffeoyl-N10-feruloylspermidine

Name	Formula	RT	RI	Mass	Diff (Tgt, ppm)	CAS	ID Source	Score	Algorithm
N1-Caffeoyl-N10-feruloylspermidine	C26 H33 N3 O6	7.087		483.2374	0.88	114916-07-3	FBF	99.49	FBF

Species	m/z	Score (Tgt)	Score (Lib)	Score (DB)	Score (MFG)	Score (RT)
(M+H)+	484	99.49				

Compound Chromatograms (overlaid)

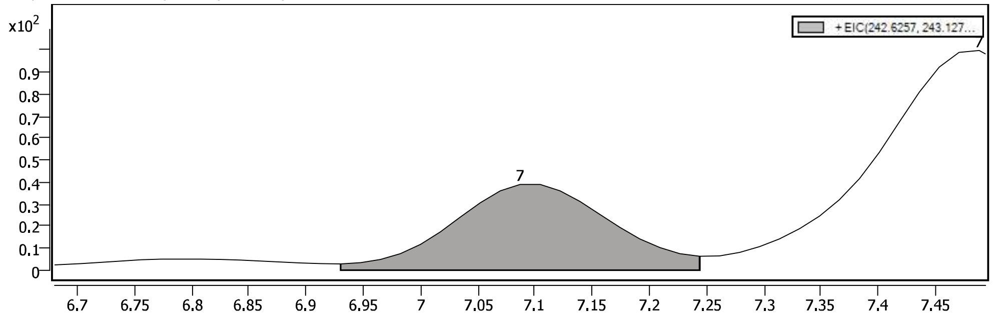

Structure

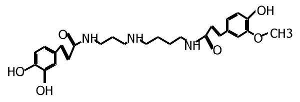

Compound Spectra (overlaid)

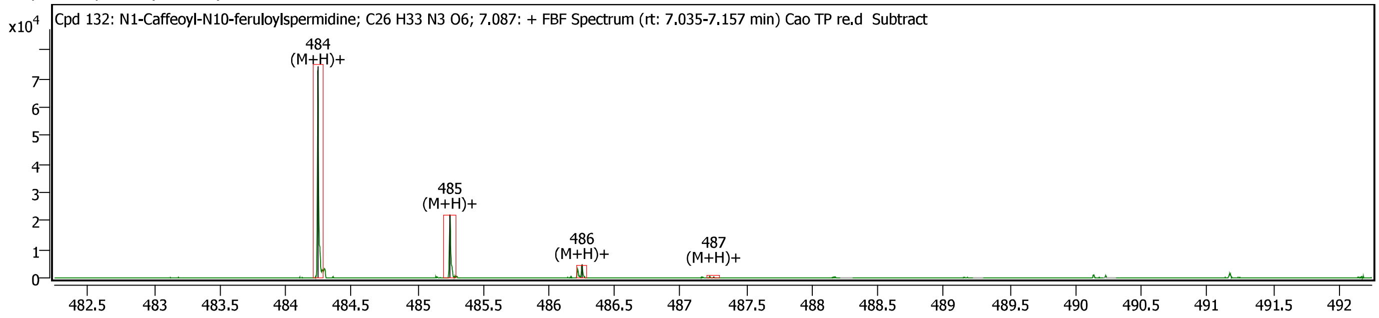

Compound Screening Report

Compound ID Table

Name	Formula	Species	RT	RT Diff	Mass	CAS	ID Source	Score	Score (Lib)	Score (Tgt)
N1-Caffeoyl-N10-feruloylspermidine	C26 H33 N3 O6	(M+H)+	7.087		483.2374	114916-07-3	FBF	99.49		99.49

Cpd 715: 5,7,3'-Trihydroxy-6,4',5'-trimethoxyflavanone

Name	Formula	RT	RI	Mass	Diff (Tgt, ppm)	CAS	ID Source	Score	Algorithm
5,7,3'-Trihydroxy-6,4',5'-trimethoxyflavanone	C18 H18 O8	7.436		362.1004	0.73		FBF	81.27	FBF

Species	m/z	Score (Tgt)	Score (Lib)	Score (DB)	Score (MFG)	Score (RT)
(M+H)+	363	81.27				

Compound Chromatograms (overlaid)

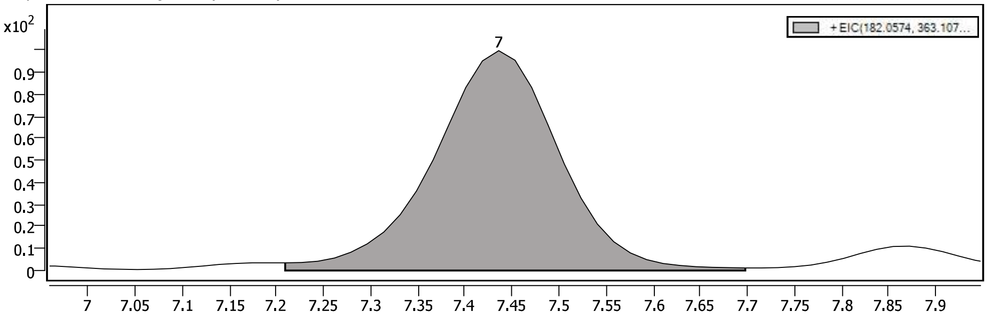

Structure

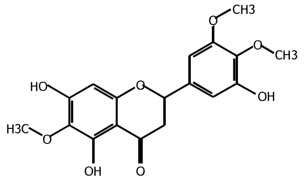

Compound Spectra (overlaid)

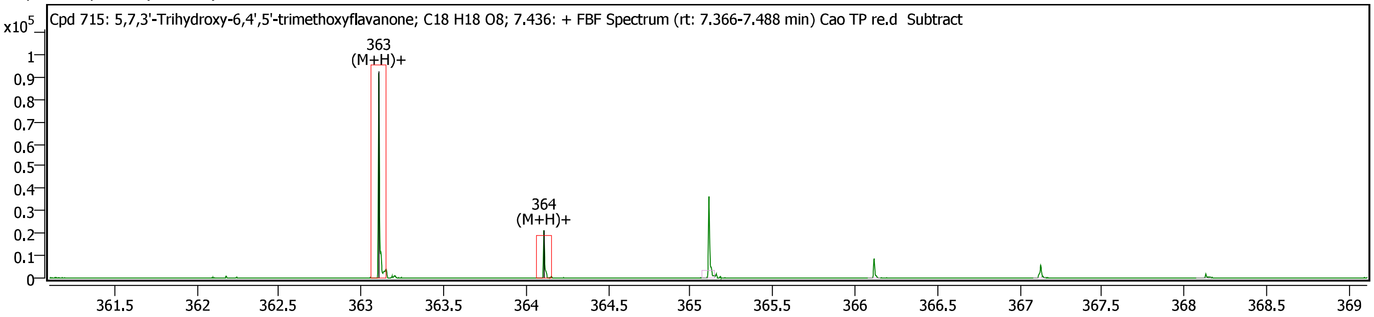

Compound ID Table

Name	Formula	Species	RT	RT Diff	Mass	CAS	ID Source	Score	Score (Lib)	Score (Tgt)
5,7,3'-Trihydroxy-6,4',5'-trimethoxyflavanone	C18 H18 O8	(M+H)+	7.436		362.1004		FBF	81.27		81.27

Cpd 753: Myricetin 3-galactoside

Name	Formula	RT	RI	Mass	Diff (Tgt, ppm)	CAS	ID Source	Score	Algorithm
Myricetin 3-galactoside	C21 H20 O13	7.471		480.0906	0.48	15648-86-9	FBF-FragConfirm	99.50	FBF

Species	m/z	Score (Tgt)	Score (Lib)	Score (DB)	Score (MFG)	Score (RT)
(M+H)+	481	99.50				

Compound Chromatograms (overlaid)

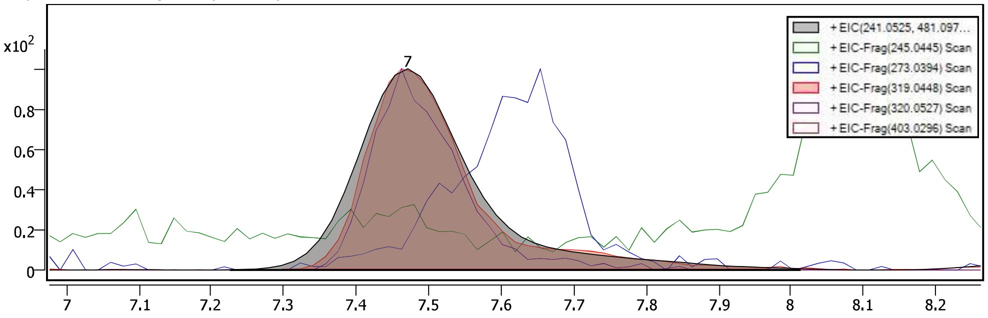

Structure

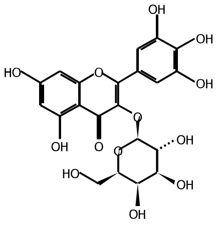

Compound Screening Report

Coelution Plot

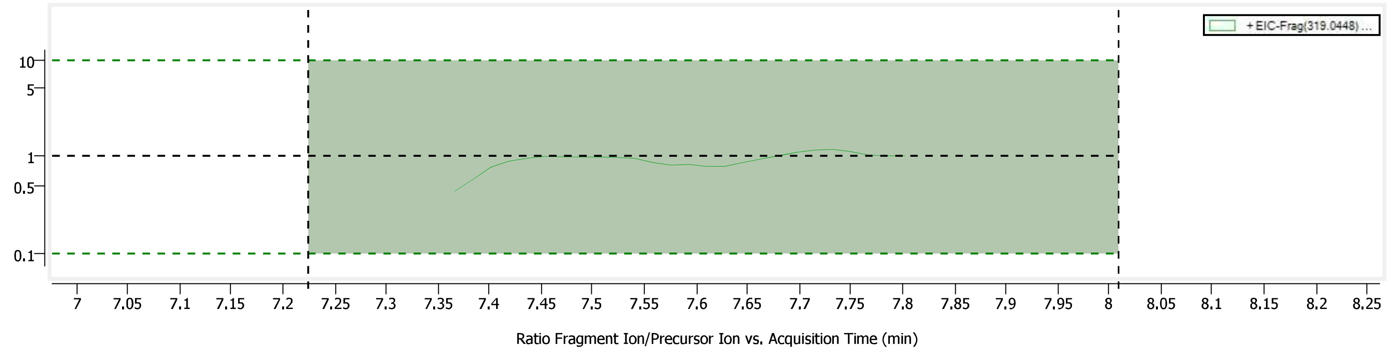

Compound Spectra (overlaid)

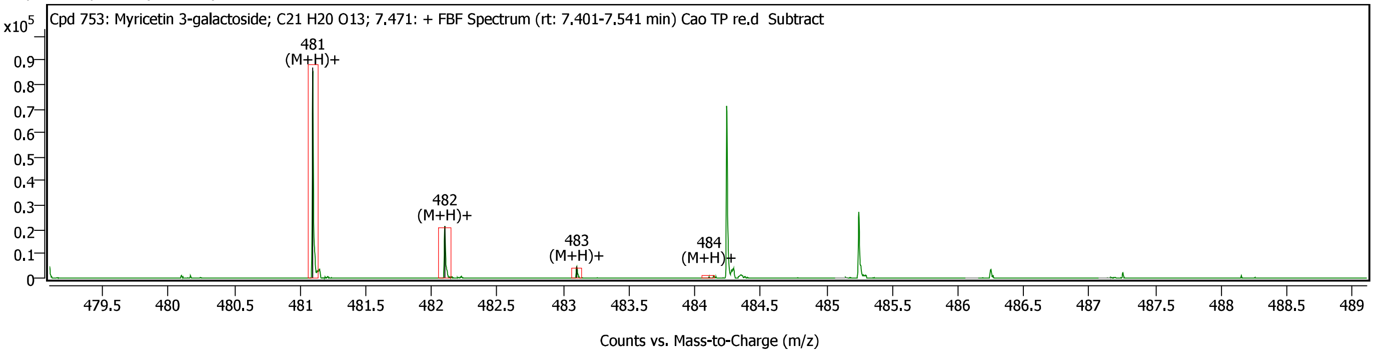

Fragment Spectrum (clean)

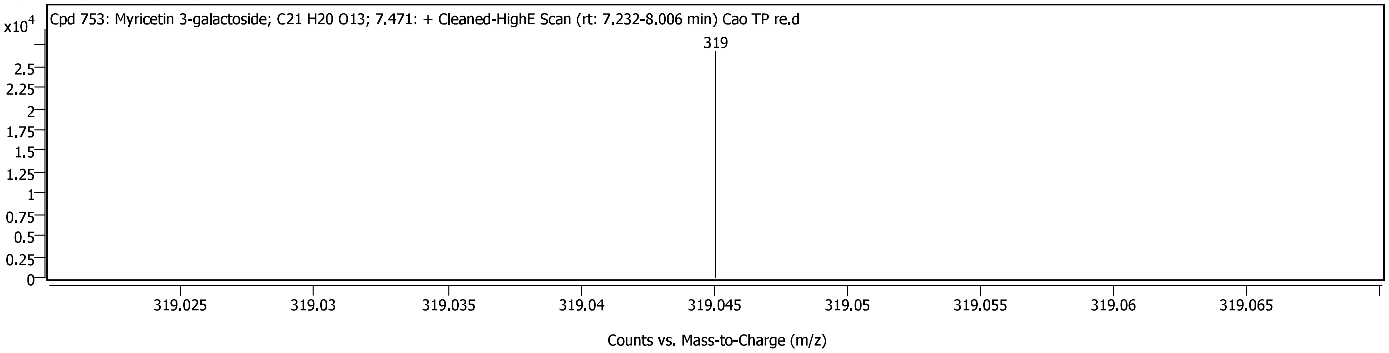

Fragment Spectrum (raw)

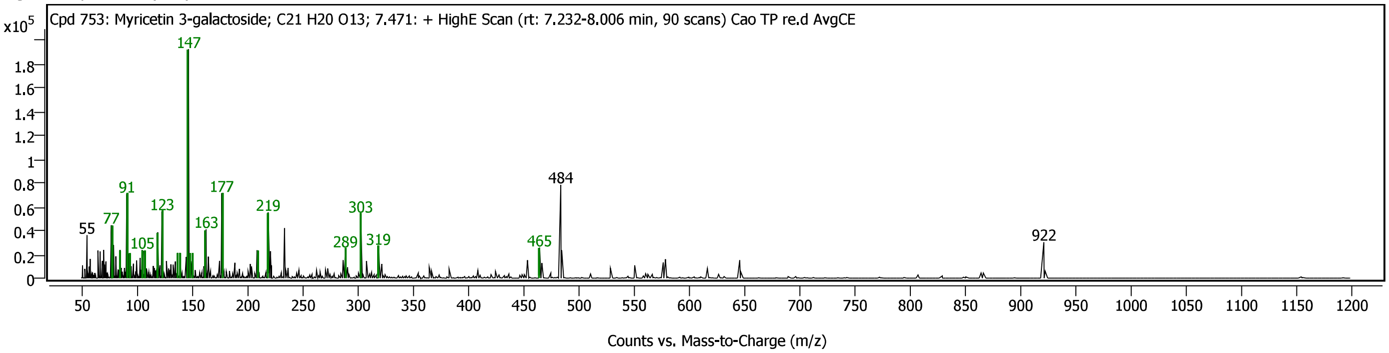

Compound Screening Report

Compound ID Table

Name	Formula	Species	RT	RT Diff	Mass	CAS	ID Source	Score	Score (Lib)	Score (Tgt)
Myricetin 3-galactoside	C21 H20 O13	(M+H)+	7.471		480.0906	15648-86-9	FBF-FragConfirm	99.50		99.50
Gossypetin 3-galactoside	C21 H20 O13	(M+H)+	7.471		480.0906		FBF-FragConfirm	99.50		99.50
Telephioidin	C21 H20 O13	(M+H)+	7.471		480.0906		FBF-FragConfirm	99.50		99.50
11-o-Galloylbergenin	C21 H20 O13	(M+H)+	7.471		480.0906		FBF-FragConfirm	99.50		99.50
Gossypetin 8-glucoside	C21 H20 O13	(M+H)+	7.471		480.0906		FBF-FragConfirm	99.50		99.50
Gossypetin 7-glucoside	C21 H20 O13	(M+H)+	7.471		480.0906		FBF-FragConfirm	99.50		99.50
Gossypetin 3-glucoside	C21 H20 O13	(M+H)+	7.471		480.0906		FBF-FragConfirm	99.50		99.50
6-Hydroxytricetin 5-glucoside	C21 H20 O13	(M+H)+	7.471		480.0906		FBF-FragConfirm	99.50		99.50
4-o-Galloylbergenin	C21 H20 O13	(M+H)+	7.471		480.0906		FBF-FragConfirm	99.50		99.50
3,5,7,2',3',4'-Hexahydroxyflavone 3-glucoside	C21 H20 O13	(M+H)+	7.471		480.0906		FBF-FragConfirm	99.50		99.50
Myricetin 3-glucoside	C21 H20 O13	(M+H)+	7.471		480.0906		FBF-FragConfirm	99.50		99.50
Zeravschanoside	C21 H20 O13	(M+H)+	7.471		480.0906		FBF-FragConfirm	99.50		99.50
Myricetin 3'-glucoside	C21 H20 O13	(M+H)+	7.471		480.0906		FBF-FragConfirm	99.50		99.50
Quercetagetin 6-glucoside	C21 H20 O13	(M+H)+	7.471		480.0906		FBF-FragConfirm	99.50		99.50
Quercetagetin 7-glucoside	C21 H20 O13	(M+H)+	7.471		480.0906		FBF-FragConfirm	99.50		99.50
Quercetagitrin	C21 H20 O13	(M+H)+	7.471		480.0906	548-75-4	FBF-FragConfirm	99.50		99.50
Quercetin-3'-glucuronide	C21 H20 O13	(M+H)+	7.471		480.0906		FBF-FragConfirm	99.50		99.50
Tagetiin	C21 H20 O13	(M+H)+	7.471		480.0906		FBF-FragConfirm	99.50		99.50
Myricetin 7-glucoside	C21 H20 O13	(M+H)+	7.471		480.0906		FBF-FragConfirm	99.50		99.50

Cpd 1371: 1,2-O-Diacetylzephyranthine

Name	Formula	RT	RI	Mass	Diff (Tgt, ppm)	CAS	ID Source	Score	Algorithm
1,2-O-Diacetylzephyranthine	C20 H23 N O6	7.698		373.1529	0.94		FBF	99.29	FBF

Species	m/z	Score (Tgt)	Score (Lib)	Score (DB)	Score (MFG)	Score (RT)
(M+H)+	374	99.29				

Compound Chromatograms (overlaid)

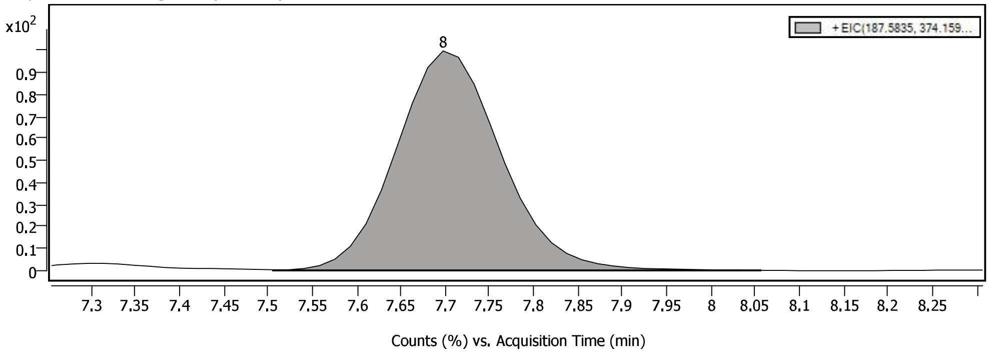

Structure

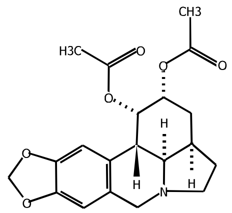

Compound Spectra (overlaid)

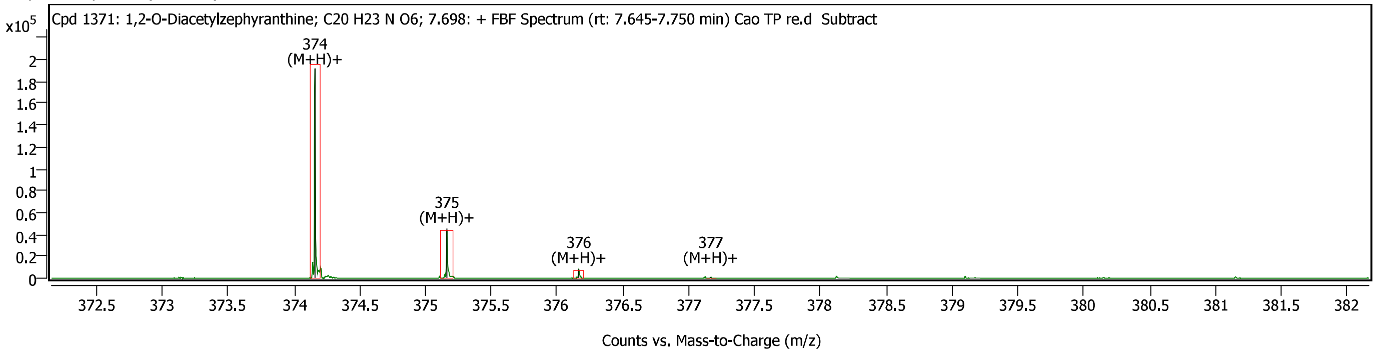

Compound ID Table

Name	Formula	Species	RT	RT Diff	Mass	CAS	ID Source	Score	Score (Lib)	Score (Tgt)
1,2-O-Diacetylzephyranthine	C20 H23 N O6	(M+H)+	7.698		373.1529		FBF	99.29		99.29

Cpd 1313: Isoquercitrin

Name	Formula	RT	RI	Mass	Diff (Tgt, ppm)	CAS	ID Source	Score	Algorithm
Isoquercitrin	C21 H20 O12	7.942		464.0958	0.73		M-FBF-FragConfirm	98.80	FBF

Species	m/z	Score (Tgt)	Score (Lib)	Score (DB)	Score (MFG)	Score (RT)
(M+H)+	465	98.80				

Compound Screening Report

Compound Chromatograms (overlaid)

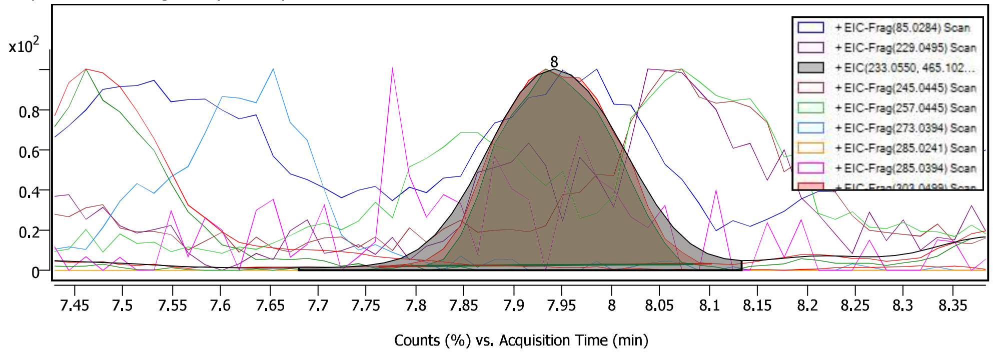

Structure

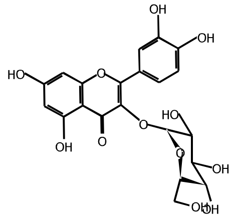

Coelution Plot

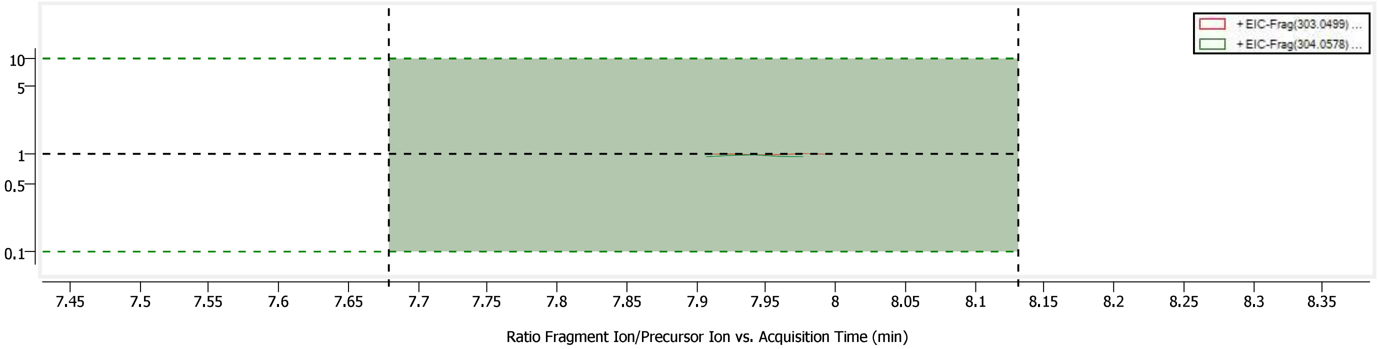

Compound Spectra (overlaid)

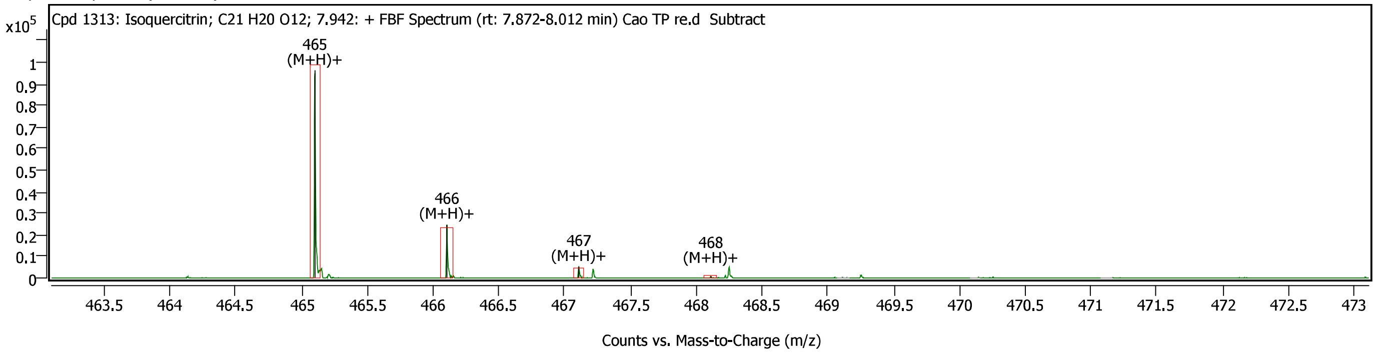

Fragment Spectrum (clean)

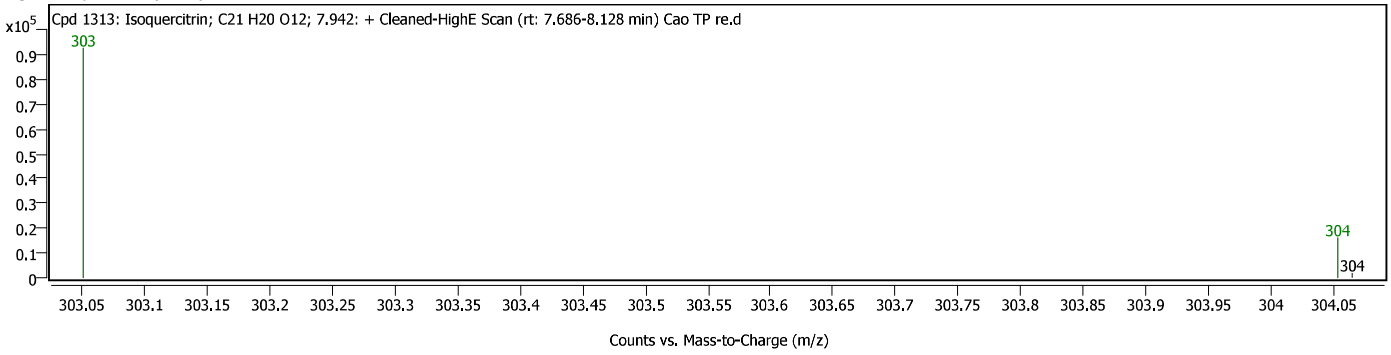

Compound Screening Report

Fragment Spectrum (raw)

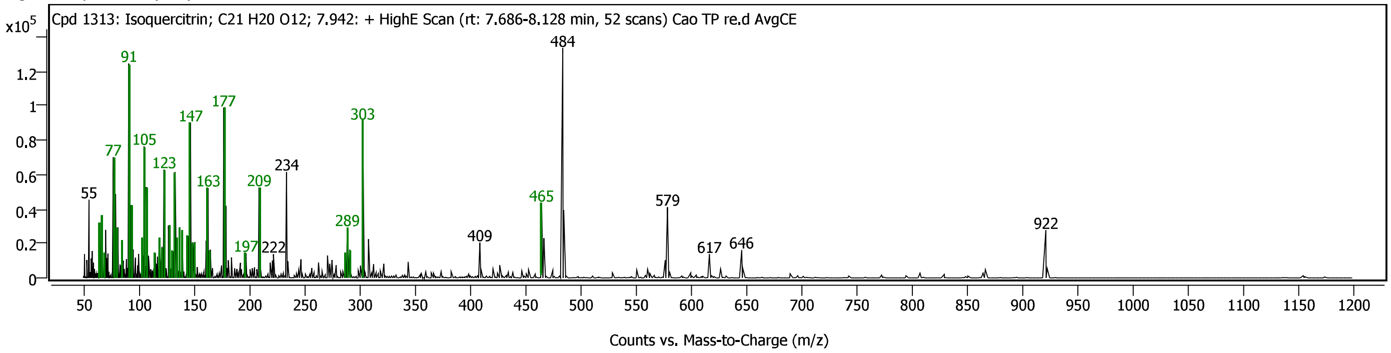

Compound ID Table

Name	Formula	Species	RT	RT Diff	Mass	CAS	ID Source	Score	Score (Lib)	Score (Tgt)
Isoquercitrin	C21 H20 O12	(M+H)+	7.942		464.0958		FBF-FragConfirm	98.80		98.80
Bracteatin 6-O-glucoside	C21 H20 O12	(M+H)+	7.942		464.0958		FBF-FragConfirm	98.80		98.80
Quercetagetin 3-rhamnoside	C21 H20 O12	(M+H)+	7.942		464.0958		FBF-FragConfirm	98.80		98.80
8-Hydroxyluteolin 8-glucoside	C21 H20 O12	(M+H)+	7.942		464.0958		FBF-FragConfirm	98.80		98.80
Annulatin 3'-xyloside	C21 H20 O12	(M+H)+	7.942		464.0958		FBF-FragConfirm	98.80		98.80
Bracteatin 6-glucoside	C21 H20 O12	(M+H)+	7.942		464.0958		FBF-FragConfirm	98.80		98.80
Gossypetin 7-rhamnoside	C21 H20 O12	(M+H)+	7.942		464.0958		FBF-FragConfirm	98.80		98.80
Bractein	C21 H20 O12	(M+H)+	7.942		464.0958		FBF-FragConfirm	98.80		98.80
Corniculatusin 3-alpha-L-arabinofuranoside	C21 H20 O12	(M+H)+	7.942		464.0958		FBF-FragConfirm	98.80		98.80
Eriodictyol 7-glucuronide	C21 H20 O12	(M+H)+	7.942		464.0958		FBF-FragConfirm	98.80		98.80
6-Hydroxyluteolin 7-glucoside	C21 H20 O12	(M+H)+	7.942		464.0958		FBF-FragConfirm	98.80		98.80
Gossypetin 8-rhamnoside	C21 H20 O12	(M+H)+	7.942		464.0958		FBF-FragConfirm	98.80		98.80
6-Hydroxytricetin 5-rhamnoside	C21 H20 O12	(M+H)+	7.942		464.0958		FBF-FragConfirm	98.80		98.80
6-Hydroxykaempferol 3-glucoside	C21 H20 O12	(M+H)+	7.942		464.0958		FBF-FragConfirm	98.80		98.80
6-Hydroxyluteolin 7-galactoside	C21 H20 O12	(M+H)+	7.942		464.0958		FBF-FragConfirm	98.80		98.80
6-Hydroxyluteolin 6-glucoside	C21 H20 O12	(M+H)+	7.942		464.0958		FBF-FragConfirm	98.80		98.80
6-Hydroxyluteolin 5-glucoside	C21 H20 O12	(M+H)+	7.942		464.0958		FBF-FragConfirm	98.80		98.80
6-Hydroxykaempferol 7-glucoside	C21 H20 O12	(M+H)+	7.942		464.0958		FBF-FragConfirm	98.80		98.80
6-C-Glucosylquercetin	C21 H20 O12	(M+H)+	7.942		464.0958		FBF-FragConfirm	98.80		98.80
6-C-beta-D-Glucopyranosyl-5,7,2',4',5'-pentahydroxyflavone	C21 H20 O12	(M+H)+	7.942		464.0958		FBF-FragConfirm	98.80		98.80
5,7,3',4',5'-Pentahydroxyflavone 8-C-glucopyranoside	C21 H20 O12	(M+H)+	7.942		464.0958		FBF-FragConfirm	98.80		98.80
5,6,7,3',4'-Pentahydroxy-8-methoxyflavone 7-apioside	C21 H20 O12	(M+H)+	7.942		464.0958		FBF-FragConfirm	98.80		98.80
3,5,7,2',6'-Pentahydroxyflavone 2'-glucoside	C21 H20 O12	(M+H)+	7.942		464.0958		FBF-FragConfirm	98.80		98.80
2'-Hydroxyisoorientin	C21 H20 O12	(M+H)+	7.942		464.0958		FBF-FragConfirm	98.80		98.80
(2S)-5,7,3',4'-Tetrahydroxyflavanone 7-glucuronide	C21 H20 O12	(M+H)+	7.942		464.0958		FBF-FragConfirm	98.80		98.80
Herbacetin 3-beta-D-glucofuranoside	C21 H20 O12	(M+H)+	7.942		464.0958		FBF-FragConfirm	98.80		98.80
8-Hydroxyluteolin 7-glucoside	C21 H20 O12	(M+H)+	7.942		464.0958		FBF-FragConfirm	98.80		98.80
Herbacetin 3-glucoside	C21 H20 O12	(M+H)+	7.942		464.0958		FBF-FragConfirm	98.80		98.80
Tricetin 7-glucoside	C21 H20 O12	(M+H)+	7.942		464.0958		FBF-FragConfirm	98.80		98.80
Quercetin 3-alloside	C21 H20 O12	(M+H)+	7.942		464.0958		FBF-FragConfirm	98.80		98.80
Herbacetin 7-glucoside	C21 H20 O12	(M+H)+	7.942		464.0958		FBF-FragConfirm	98.80		98.80
Tricetin 3'-glucoside	C21 H20 O12	(M+H)+	7.942		464.0958		FBF-FragConfirm	98.80		98.80
Spiraeoside	C21 H20 O12	(M+H)+	7.942		464.0958		FBF-FragConfirm	98.80		98.80
Robinetin 7-glucoside	C21 H20 O12	(M+H)+	7.942		464.0958		FBF-FragConfirm	98.80		98.80
Quercimeritrin	C21 H20 O12	(M+H)+	7.942		464.0958		FBF-FragConfirm	98.80		98.80
Quercetin 5-glucoside	C21 H20 O12	(M+H)+	7.942		464.0958		FBF-FragConfirm	98.80		98.80
Quercetin 4'-glucoside	C21 H20 O12	(M+H)+	7.942		464.0958	20229-56-5	FBF-FragConfirm	98.80		98.80
Quercetin 3-β-D-glucoside	C21 H20 O12	(M+H)+	7.942		464.0958	482-35-9	FBF-FragConfirm	98.80		98.80
Quercetin 3-O-glucoside	C21 H20 O12	(M+H)+	7.942		464.0958	21637-25-2	FBF-FragConfirm	98.80		98.80
Quercetin 3'-glucoside	C21 H20 O12	(M+H)+	7.942		464.0958		FBF-FragConfirm	98.80		98.80
Quercetin 7-galactoside	C21 H20 O12	(M+H)+	7.942		464.0958		FBF-FragConfirm	98.80		98.80
Myricetin 3'-rhamnoside	C21 H20 O12	(M+H)+	7.942		464.0958		FBF-FragConfirm	98.80		98.80
Patuletin 3-xyloside	C21 H20 O12	(M+H)+	7.942		464.0958		FBF-FragConfirm	98.80		98.80
Isoetin 5'-glucoside	C21 H20 O12	(M+H)+	7.942		464.0958		FBF-FragConfirm	98.80		98.80
Myricitrin	C21 H20 O12	(M+H)+	7.942		464.0958	17912-87-7	FBF-FragConfirm	98.80		98.80
Herbacetin 8-glucoside	C21 H20 O12	(M+H)+	7.942		464.0958		FBF-FragConfirm	98.80		98.80
Hyperin	C21 H20 O12	(M+H)+	7.942		464.0958		FBF-FragConfirm	98.80		98.80
Hyperoside	C21 H20 O12	(M+H)+	7.942		464.0958	482-36-0	FBF-FragConfirm	98.80		98.80
Isoaffinetin	C21 H20 O12	(M+H)+	7.942		464.0958		FBF-FragConfirm	98.80		98.80
Isoetin 7-glucoside	C21 H20 O12	(M+H)+	7.942		464.0958		FBF-FragConfirm	98.80		98.80
Larycitrin 3-alpha-L-arabinofuranoside	C21 H20 O12	(M+H)+	7.942		464.0958		FBF-FragConfirm	98.80		98.80
Herbacetin 4'-glucoside	C21 H20 O12	(M+H)+	7.942		464.0958		FBF-FragConfirm	98.80		98.80
Myricetin 7-rhamnoside	C21 H20 O12	(M+H)+	7.942		464.0958	184533-14-0	FBF-FragConfirm	98.80		98.80

Cpd 1416: Netilmicin

Name	Formula	RT	RI	Mass	Diff (Tgt, ppm)	CAS	ID Source	Score	Algorithm
Netilmicin	C21 H41 N5 O7	8.081		475.2996	-2.19		FBF	97.46	FBF

Species	m/z	Score (Tgt)	Score (Lib)	Score (DB)	Score (MFG)	Score (RT)
(M+H)+	476	97.46				

Compound Chromatograms (overlaid)

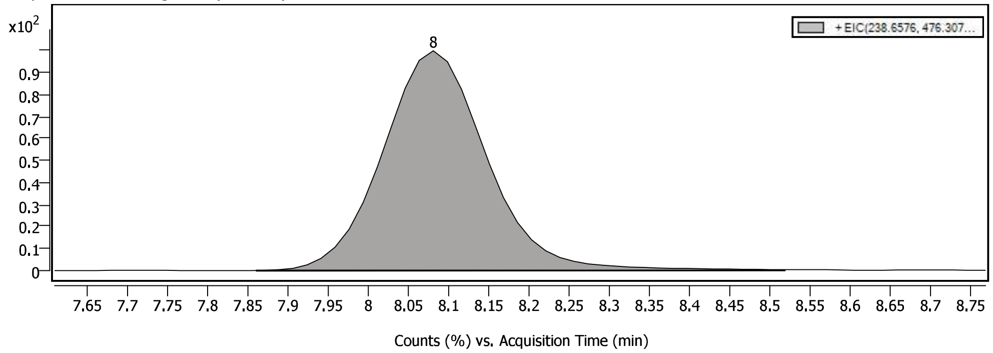

Structure

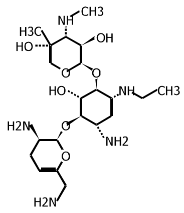

Compound Spectra (overlaid)

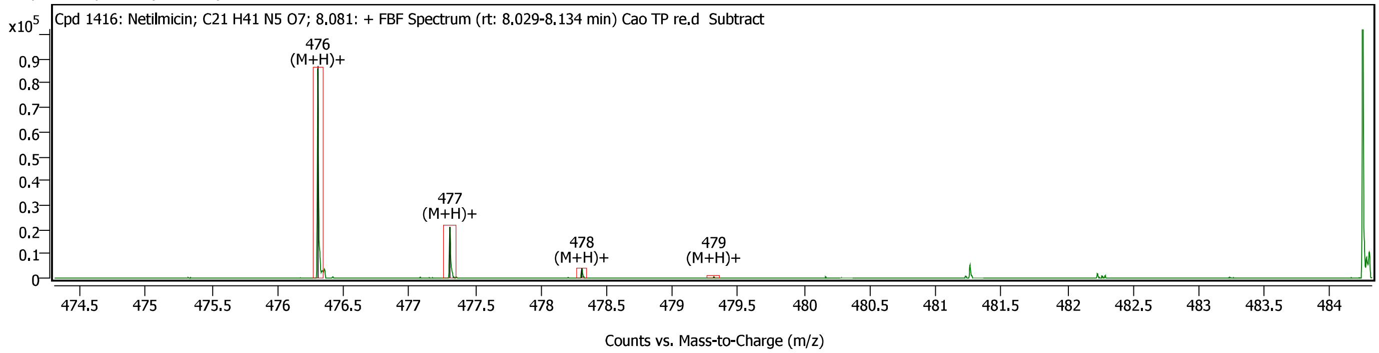

Compound ID Table

Name	Formula	Species	RT	RT Diff	Mass	CAS	ID Source	Score	Score (Lib)	Score (Tgt)
Netilmicin	C21 H41 N5 O7	(M+H)+	8.081		475.2996		FBF	97.46		97.46

Cpd 585: PAC-1

Name	Formula	RT	RI	Mass	Diff (Tgt, ppm)	CAS	ID Source	Score	Algorithm
PAC-1	C23 H28 N4 O2	8.116		392.2218	1.35	315183-21-2	FBF	96.79	FBF

Species	m/z	Score (Tgt)	Score (Lib)	Score (DB)	Score (MFG)	Score (RT)
(M+2H)+2 (M+H)+	197 393	96.79				

Compound Chromatograms (overlaid)

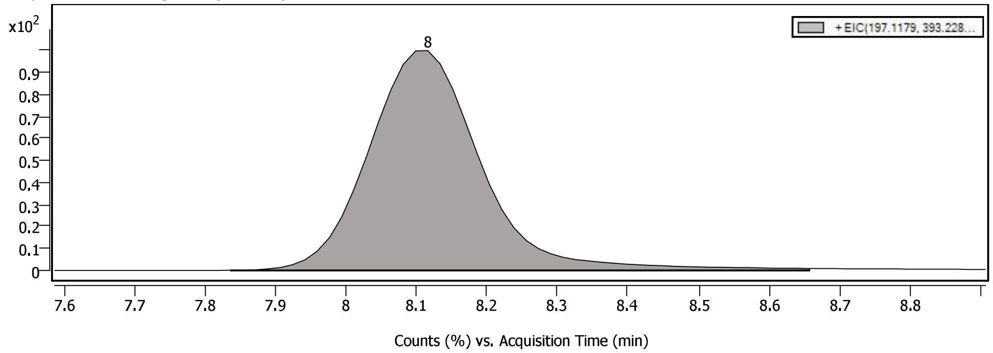

Structure

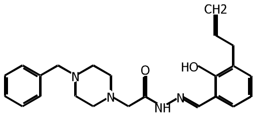

Compound Spectra (overlaid)

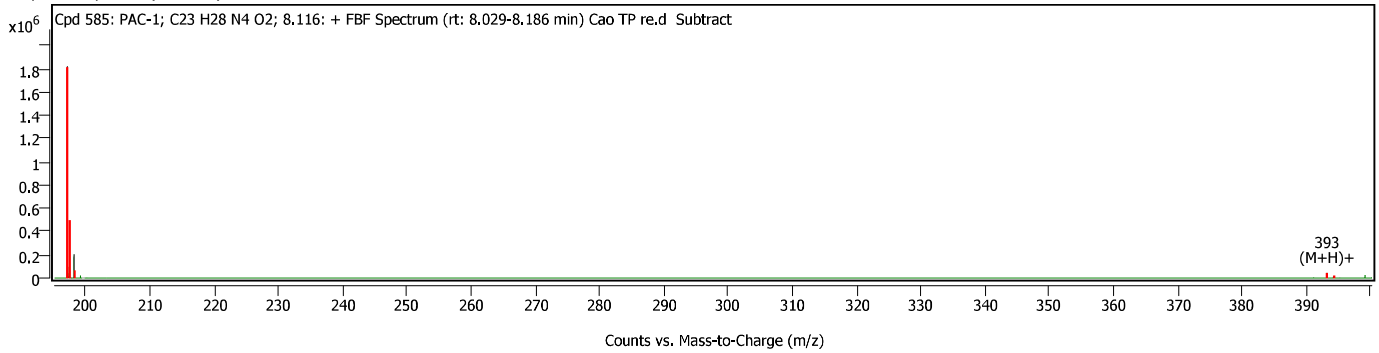

Compound ID Table

Name	Formula	Species	RT	RT Diff	Mass	CAS	ID Source	Score	Score (Lib)	Score (Tgt)
PAC-1	C23 H28 N4 O2	(M+2H)+2 (M+H)+	8.116		392.2218	315183-21-2	FBF	96.79		96.79

Compound Screening Report

Cpd 1295: Dihydrofukinolide

Name	Formula	RT	RI	Mass	Diff (Tgt, ppm)	CAS	ID Source	Score	Algorithm
Dihydrofukinolide	C22 H32 O6	8.116		392.2217	4.72	41059-95-4	M-FBF	98.62	FBF

Species	m/z	Score (Tgt)	Score (Lib)	Score (DB)	Score (MFG)	Score (RT)
(M+2H)+2 (M+H)+	197 393	98.62				

Compound Chromatograms (overlaid)

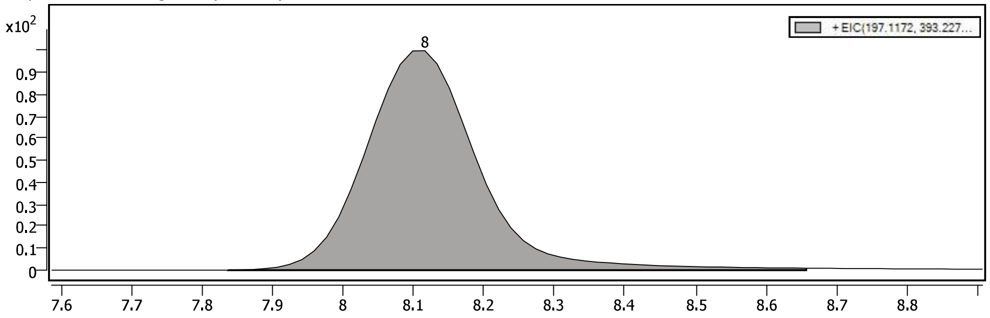

Counts (%) vs. Acquisition Time (min)

Structure

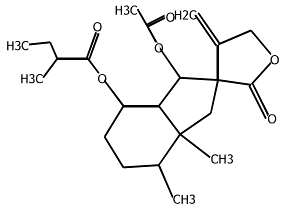

Compound Spectra (overlaid)

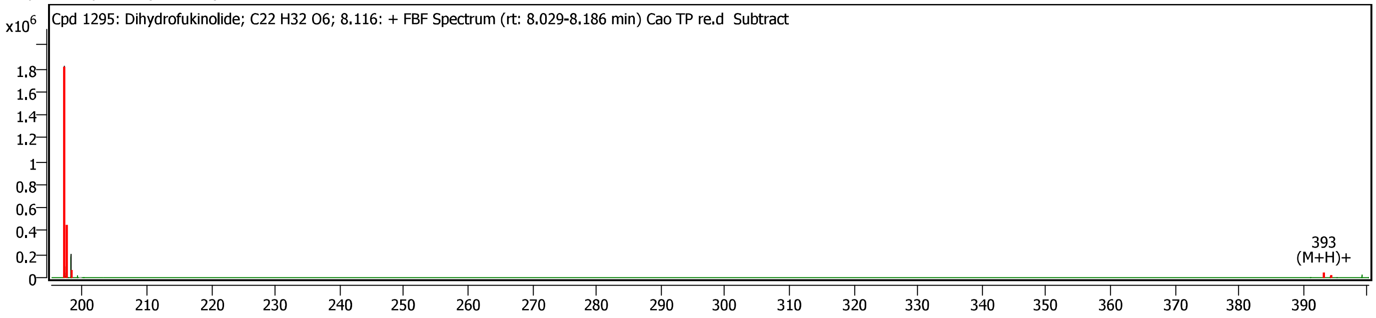

Counts vs. Mass-to-Charge (m/z)

Compound ID Table

Name	Formula	Species	RT	RT Diff	Mass	CAS	ID Source	Score	Score (Lib)	Score (Tgt)
Dihydrofukinolide	C22 H32 O6	(M+2H)+2 (M+H)+	8.116		392.2217	41059-95-4	FBF	98.62		98.62
Nigakilactone B	C22 H32 O6	(M+2H)+2 (M+H)+	8.116		392.2217	24148-77-4	FBF	98.62		98.62
Isodomedin	C22 H32 O6	(M+2H)+2 (M+H)+	8.116		392.2217	39388-61-9	FBF	98.62		98.62

Cpd 1493: <Trospium>

Name	Formula	RT	RI	Mass	Diff (Tgt, ppm)	CAS	ID Source	Score	Algorithm
<Trospium>	C25 H30 N O3	8.116		392.2217	-2.17	10405-02-4	FBF	84.73	FBF

Species	m/z	Score (Tgt)	Score (Lib)	Score (DB)	Score (MFG)	Score (RT)
(M+2H)+2 (M+H)+	197 393	84.73				

Compound Chromatograms (overlaid)

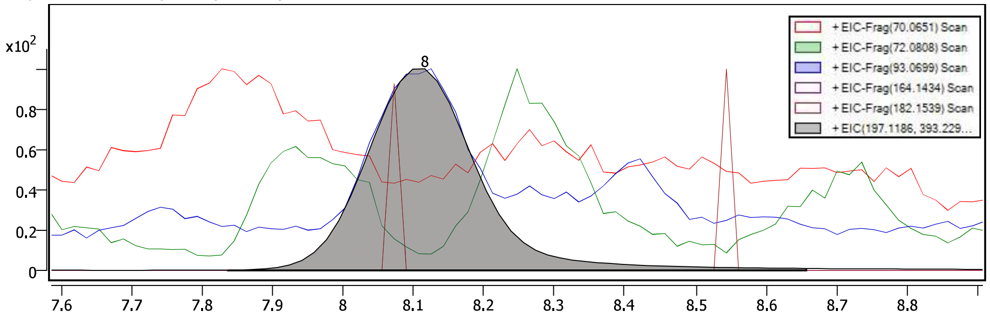

Counts (%) vs. Acquisition Time (min)

Structure

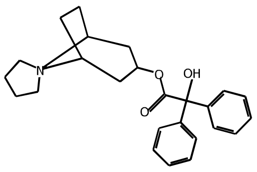

Coelution Plot

Compound Spectra (overlaid)

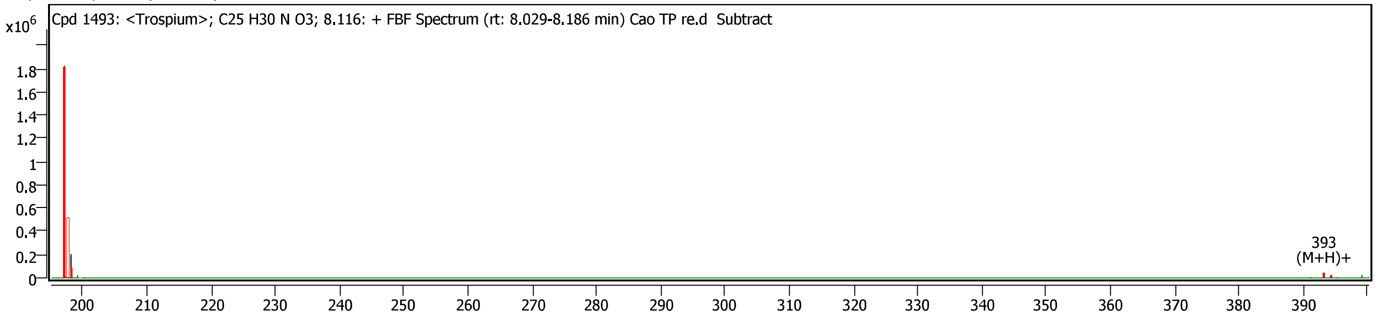

Fragment Spectrum (raw)

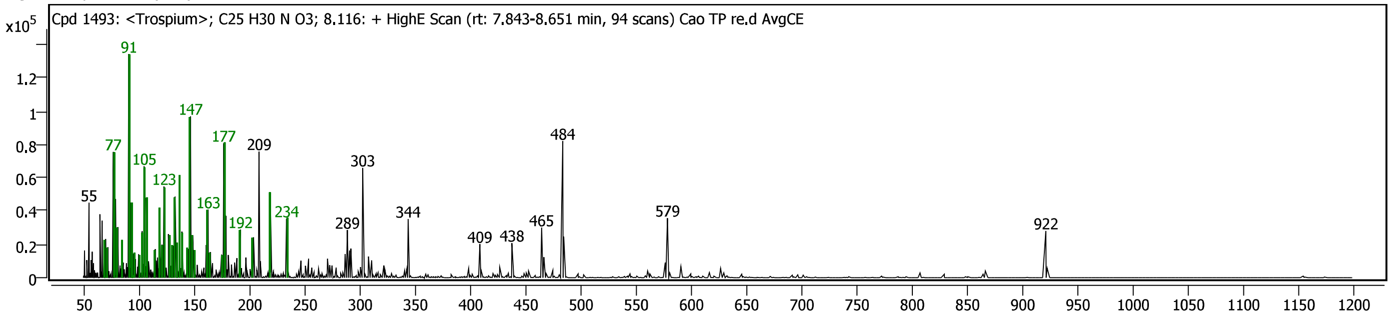

Compound ID Table

Name	Formula	Species	RT	RT Diff	Mass	CAS	ID Source	Score	Score (Lib)	Score (Tgt)
<Trospium>	C25 H30 N O3	(M+2H)+2 (M+H)+	8.116		392.2217	10405-02-4	FBF	84.73		84.73

Cpd 1330: <Sophoraflavanone B>

Name	Formula	RT	RI	Mass	Diff (Tgt, ppm)	CAS	ID Source	Score	Algorithm
<Sophoraflavanone B>	C20 H20 O5	8.134		340.1315	1.14		M-FBF	98.33	FBF

Species	m/z	Score (Tgt)	Score (Lib)	Score (DB)	Score (MFG)	Score (RT)
(M+H)+	341	98.33				

Compound Chromatograms (overlaid)

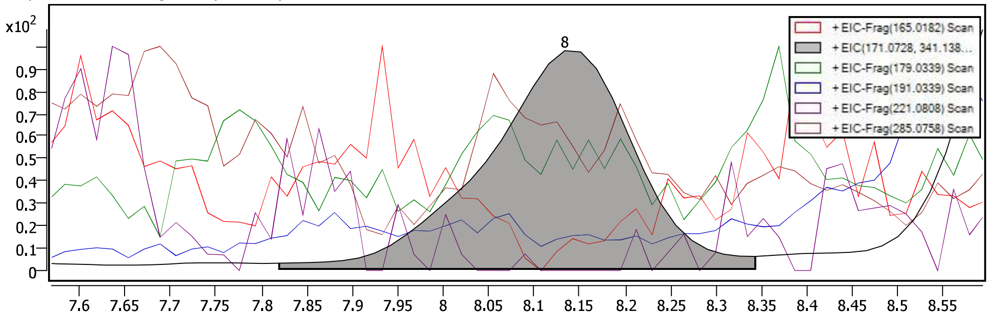

Structure

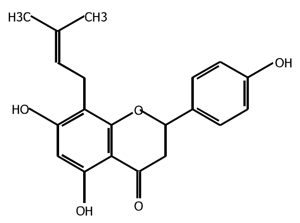

Coelution Plot

Compound Spectra (overlaid)

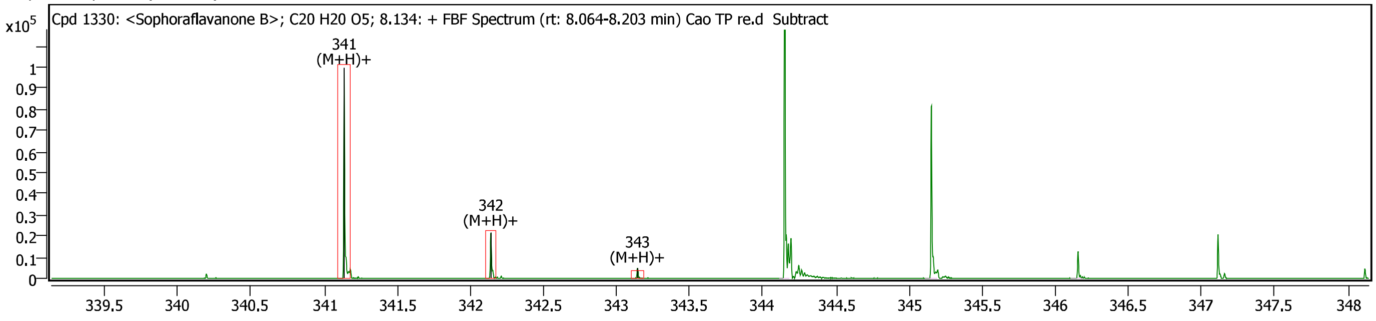

Compound Screening Report

Fragment Spectrum (raw)

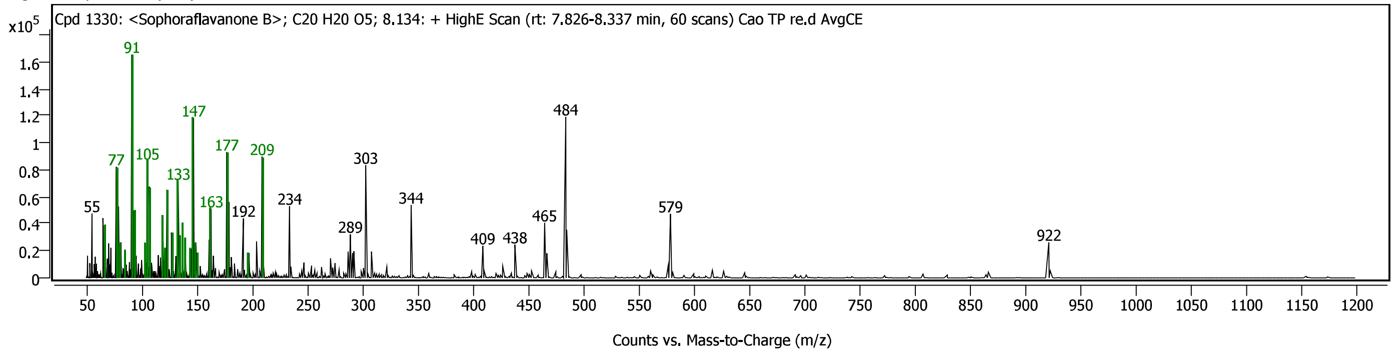

Compound ID Table

Name	Formula	Species	RT	RT Diff	Mass	CAS	ID Source	Score	Score (Lib)	Score (Tgt)
<Sophoraflavanone B>	C20 H20 O5	(M+H)+	8.134		340.1315		FBF	98.33		98.33
<4-Dimethylallyl-(6aS,11aS)-3,6a,9-trihydroxypterocarpan>	C20 H20 O5	(M+H)+	8.134		340.1315		FBF	98.33		98.33
<5,4,-Dihydroxy-4'',4''-Dimethyl-5''-[2'',3'':7,6]methyldihydrofuranoflavanone>	C20 H20 O5	(M+H)+	8.134		340.1315		FBF	98.33		98.33
<6-Prenylnaringenin>	C20 H20 O5	(M+H)+	8.134		340.1315		FBF	98.33		98.33
<5'-Prenyllicodione>	C20 H20 O5	(M+H)+	8.134		340.1315		FBF	98.33		98.33
<5-Prenyl-6'',6''-Dimethyl-5''-hydroxy-4'',5''-dihydropyrano[2'',3'':4',3']-4,2'-dihydroxychalcone>	C20 H20 O5	(M+H)+	8.134		340.1315		FBF	98.33		98.33
<5-Hydroxy-7-(3-methyl-2,3-epoxybutoxy)flavanone>	C20 H20 O5	(M+H)+	8.134		340.1315		FBF	98.33		98.33
<5,7-Dihydroxy-8-C-(4-hydroxy-3-methyl-2-butenyl)flavanone>	C20 H20 O5	(M+H)+	8.134		340.1315		FBF	98.33		98.33
<5,4'-Dihydroxy-7-O-prenylflavanone>	C20 H20 O5	(M+H)+	8.134		340.1315		FBF	98.33		98.33
<Bavachromanol>	C20 H20 O5	(M+H)+	8.134		340.1315		FBF	98.33		98.33
<4,2',6'-Trihydroxy-4'-prenyloxychalcone>	C20 H20 O5	(M+H)+	8.134		340.1315		FBF	98.33		98.33
<Helikrausichalcone>	C20 H20 O5	(M+H)+	8.134		340.1315		FBF	98.33		98.33
<3'-(2-Hydroxy-3-methylbut-3-enyl)-4,2',4'-trihydroxychalcone>	C20 H20 O5	(M+H)+	8.134		340.1315		FBF	98.33		98.33
<1-Hydroxy-3,5-dimethoxy-2-prenylxanthone>	C20 H20 O5	(M+H)+	8.134		340.1315	35323-89-8	FBF	98.33		98.33
<Brosimacutin D>	C20 H20 O5	(M+H)+	8.134		340.1315		FBF	98.33		98.33
<(6aS,11aS)-2-dimethylallyl-3,6a,9-trihydroxypterocarpan>	C20 H20 O5	(M+H)+	8.134		340.1315		FBF	98.33		98.33
<(2S)-5,7,4'-Trihydroxy-6-(1,1-dimethylallyl)flavanone>	C20 H20 O5	(M+H)+	8.134		340.1315		FBF	98.33		98.33
<(+)-Dihydrowighteone>	C20 H20 O5	(M+H)+	8.134		340.1315		FBF	98.33		98.33
<(+)-Galbacin>	C20 H20 O5	(M+H)+	8.134		340.1315	61891-31-4	FBF	98.33		98.33
<Bakuchalcone>	C20 H20 O5	(M+H)+	8.134		340.1315		FBF	98.33		98.33
<(+-)-5-Deoxykievitone>	C20 H20 O5	(M+H)+	8.134		340.1315		FBF	98.33		98.33
<Licoflavanone>	C20 H20 O5	(M+H)+	8.134		340.1315		FBF	98.33		98.33
<Desmethylxanthohumol>	C20 H20 O5	(M+H)+	8.134		340.1315	115063-39-3	FBF	98.33		98.33
<Ugonin E>	C20 H20 O5	(M+H)+	8.134		340.1315		FBF	98.33		98.33
<Selinone>	C20 H20 O5	(M+H)+	8.134		340.1315		FBF	98.33		98.33
<Sandwicarpin>	C20 H20 O5	(M+H)+	8.134		340.1315		FBF	98.33		98.33
<Morachalcone A>	C20 H20 O5	(M+H)+	8.134		340.1315		FBF	98.33		98.33
<Licocoumarone>	C20 H20 O5	(M+H)+	8.134		340.1315		FBF	98.33		98.33
<Brosimacutin E>	C20 H20 O5	(M+H)+	8.134		340.1315		FBF	98.33		98.33
<Kushenol S>	C20 H20 O5	(M+H)+	8.134		340.1315		FBF	98.33		98.33
<Broussochalcone A>	C20 H20 O5	(M+H)+	8.134		340.1315		FBF	98.33		98.33
<Glyceollidin II>	C20 H20 O5	(M+H)+	8.134		340.1315		FBF	98.33		98.33
<Glyceollidin I>	C20 H20 O5	(M+H)+	8.134		340.1315		FBF	98.33		98.33
<Glepidotin B>	C20 H20 O5	(M+H)+	8.134		340.1315	87440-56-0	FBF	98.33		98.33
<Euchrenone a7>	C20 H20 O5	(M+H)+	8.134		340.1315		FBF	98.33		98.33
<Dolichin B>	C20 H20 O5	(M+H)+	8.134		340.1315		FBF	98.33		98.33
<Dolichin A>	C20 H20 O5	(M+H)+	8.134		340.1315		FBF	98.33		98.33
<Kanzonol A>	C20 H20 O5	(M+H)+	8.134		340.1315		FBF	98.33		98.33
<Crotin (chalcone)>	C20 H20 O5	(M+H)+	8.134		340.1315		FBF	98.33		98.33

Cpd 121: N-trans-Feruloyl-4-O-methyldopamine

Name	Formula	RT	RI	Mass	Diff (Tgt, ppm)	CAS	ID Source	Score	Algorithm
N-trans-Feruloyl-4-O-methyldopamine	C19 H21 N O5	8.169		343.1426	1.87	78510-20-0	FBF	97.51	FBF

Species	m/z	Score (Tgt)	Score (Lib)	Score (DB)	Score (MFG)	Score (RT)
(M+H)+	344	97.51				

Compound Screening Report

Compound Chromatograms (overlaid)

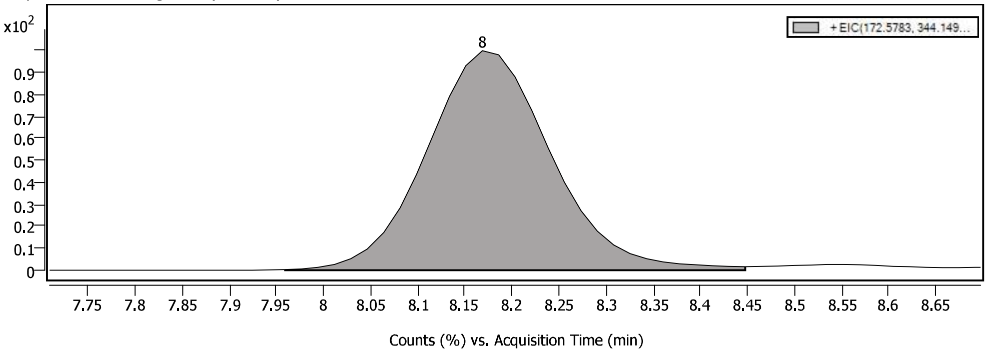

Structure

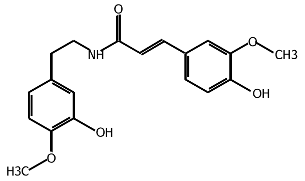

Compound Spectra (overlaid)

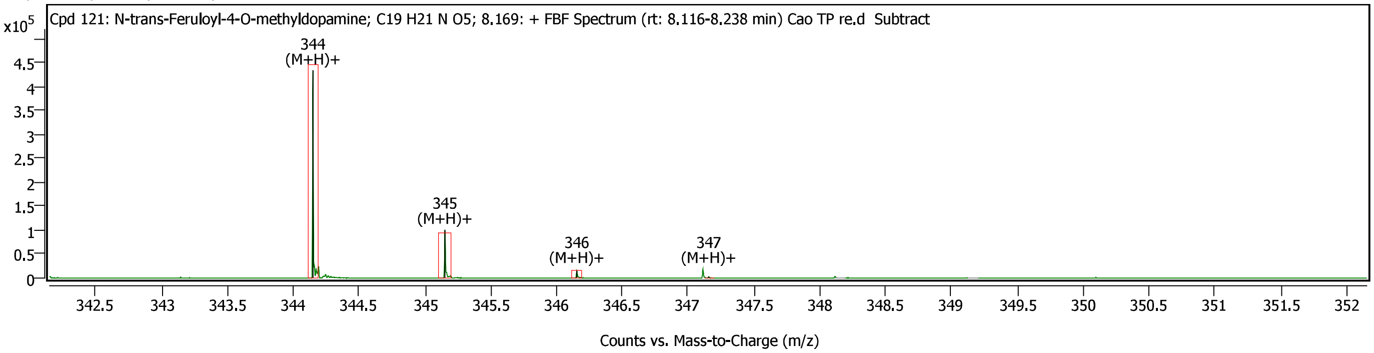

Compound ID Table

Name	Formula	Species	RT	RT Diff	Mass	CAS	ID Source	Score	Score (Lib)	Score (Tgt)
N-trans-Feruloyl-4-O-methyldopamine	C19 H21 N O5	(M+H)+	8.169		343.1426	78510-20-0	FBF	97.51		97.51

Cpd 1578: <Lunarine>

Name	Formula	RT	RI	Mass	Diff (Tgt, ppm)	CAS	ID Source	Score	Algorithm
<Lunarine>	C25 H31 N3 O4	8.273		437.2319	1.04	24185-51-1	M-FBF	98.10	FBF

Species	m/z	Score (Tgt)	Score (Lib)	Score (DB)	Score (MFG)	Score (RT)
(M+H)+	438	98.10				

Compound Chromatograms (overlaid)

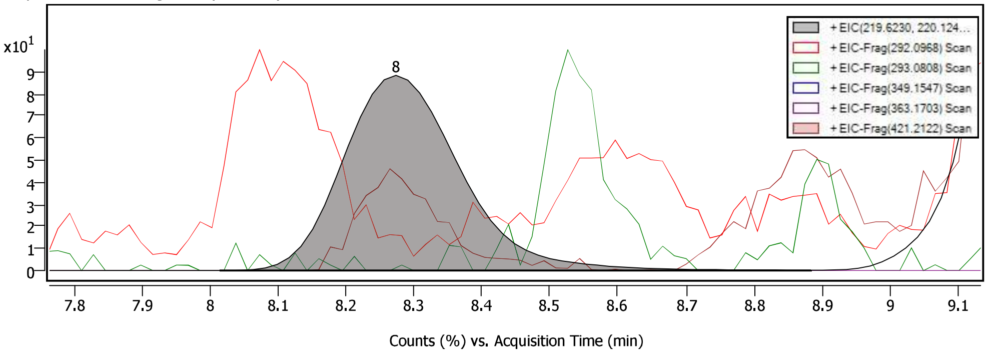

Structure

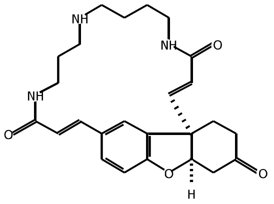

Coelution Plot

Compound Spectra (overlaid)

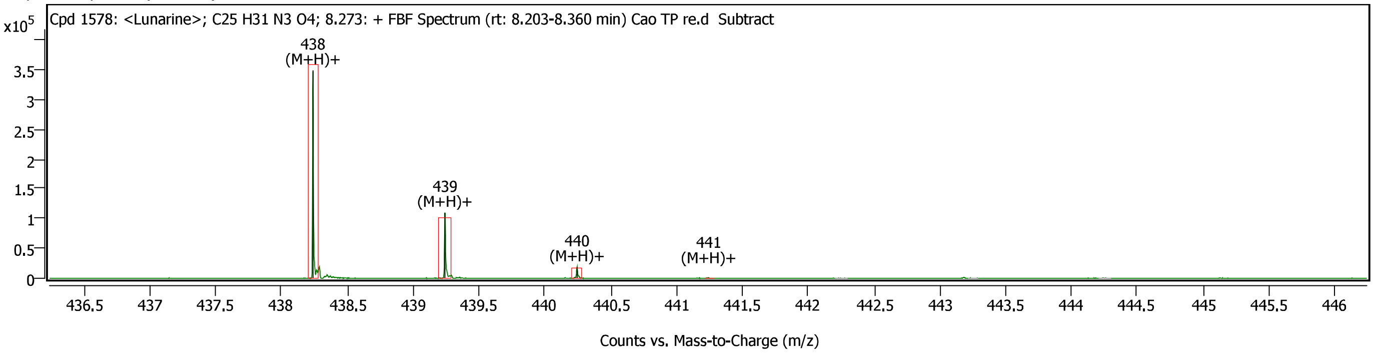

Fragment Spectrum (raw)

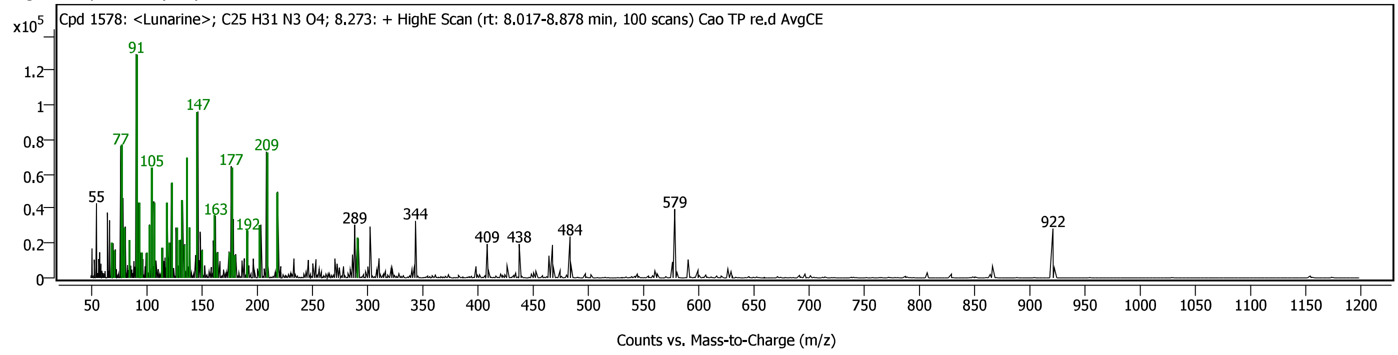

Compound ID Table

Name	Formula	Species	RT	RT Diff	Mass	CAS	ID Source	Score	Score (Lib)	Score (Tgt)
<Lunarine>	C25 H31 N3 O4	(M+H)+	8.273		437.2319	24185-51-1	FBF	98.10		98.10
<N1,N10-Dicoumaroylspermidine>	C25 H31 N3 O4	(M+H)+	8.273		437.2319	65715-79-9	FBF	98.10		98.10

Cpd 553: Dianthramine

Name	Formula	RT	RI	Mass	Diff (Tgt, ppm)	CAS	ID Source	Score	Algorithm
Dianthramine	C14 H11 N O6	8.343		289.0589	0.82	136945-65-8	FBF	98.88	FBF

Species	m/z	Score (Tgt)	Score (Lib)	Score (DB)	Score (MFG)	Score (RT)
(M+H)+	290	98.88				

Compound Chromatograms (overlaid)

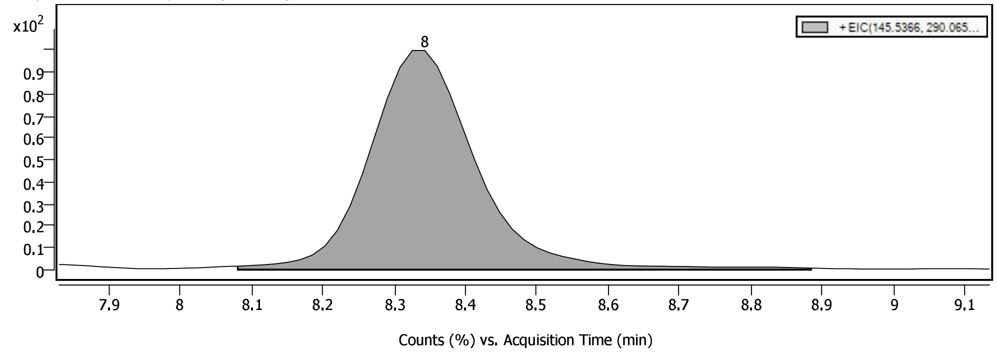

Structure

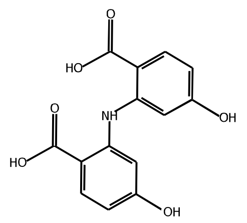

Compound Spectra (overlaid)

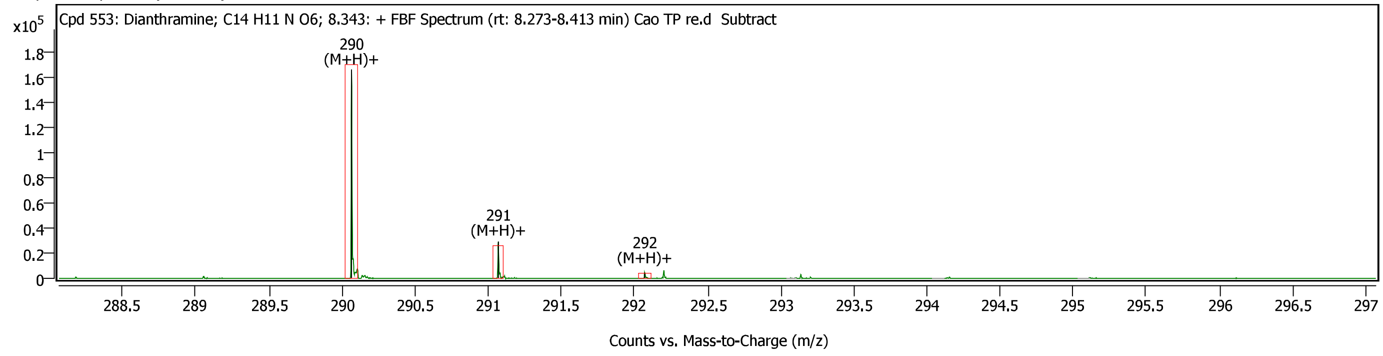

Compound ID Table

Name	Formula	Species	RT	RT Diff	Mass	CAS	ID Source	Score	Score (Lib)	Score (Tgt)
Dianthramine	C14 H11 N O6	(M+H)+	8.343		289.0589	136945-65-8	FBF	98.88		98.88

Cpd 131: N1-Caffeoyl-N10-feruloylspermidine

Name	Formula	RT	RI	Mass	Diff (Tgt, ppm)	CAS	ID Source	Score	Algorithm
N1-Caffeoyl-N10-feruloylspermidine	C26 H33 N3 O6	8.430		483.2368	-0.27	114916-07-3	FBF	99.12	FBF

Species	m/z	Score (Tgt)	Score (Lib)	Score (DB)	Score (MFG)	Score (RT)
(M+H)+	484	99.12				

Compound Chromatograms (overlaid)

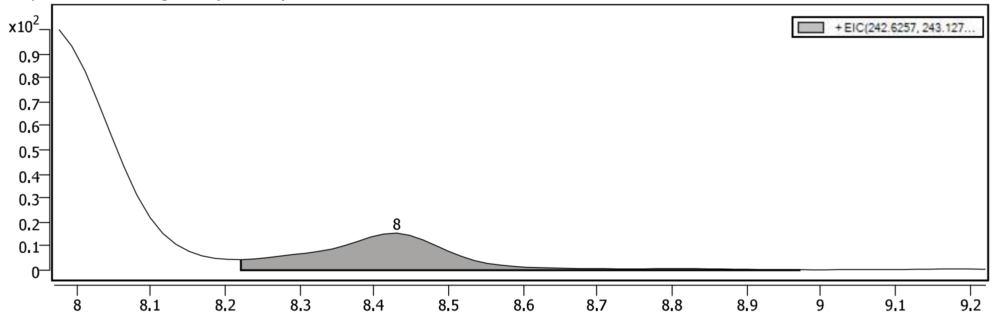

Structure

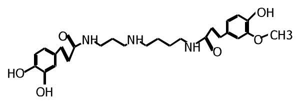

Compound Spectra (overlaid)

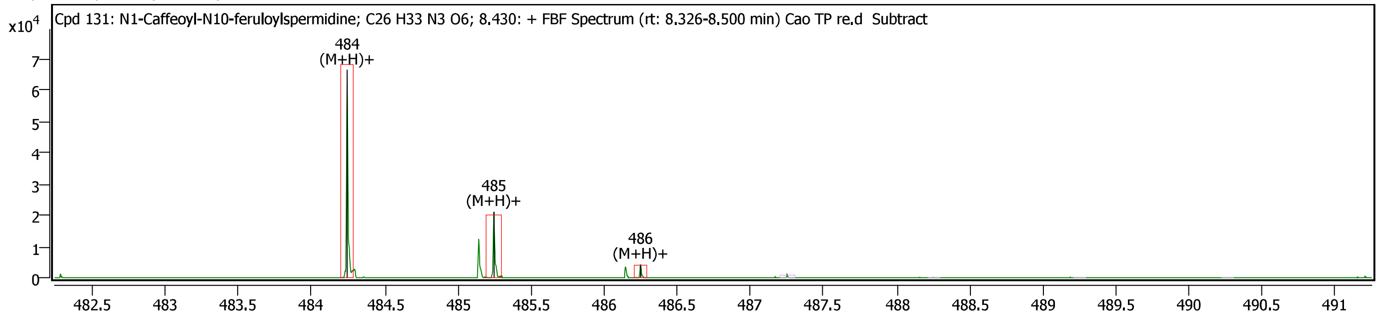

Compound ID Table

Name	Formula	Species	RT	RT Diff	Mass	CAS	ID Source	Score	Score (Lib)	Score (Tgt)
N1-Caffeoyl-N10-feruloylspermidine	C26 H33 N3 O6	(M+H)+	8.430		483.2368	114916-07-3	FBF	99.12		99.12

Cpd 1608: Pyranodelphinin A

Name	Formula	RT	RI	Mass	Diff (Tgt, ppm)	CAS	ID Source	Score	Algorithm
Pyranodelphinin A	C30 H33 O16	8.552		649.1793	3.81		FBF	88.35	FBF

Species	m/z	Score (Tgt)	Score (Lib)	Score (DB)	Score (MFG)	Score (RT)
(M+H)+	650	88.35				

Compound Chromatograms (overlaid)

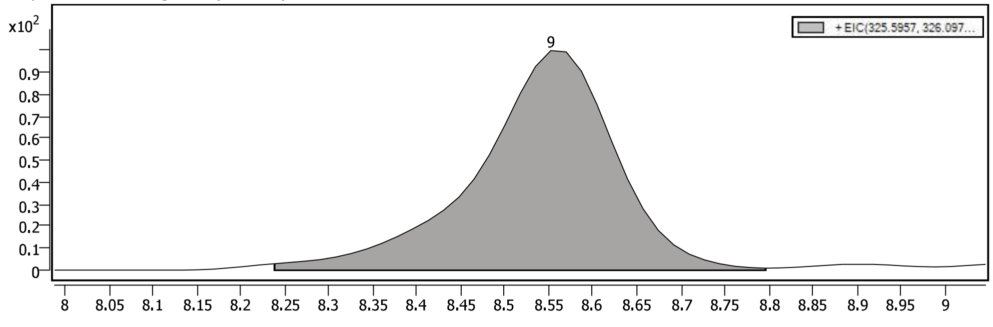

Structure

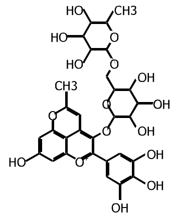

Compound Spectra (overlaid)

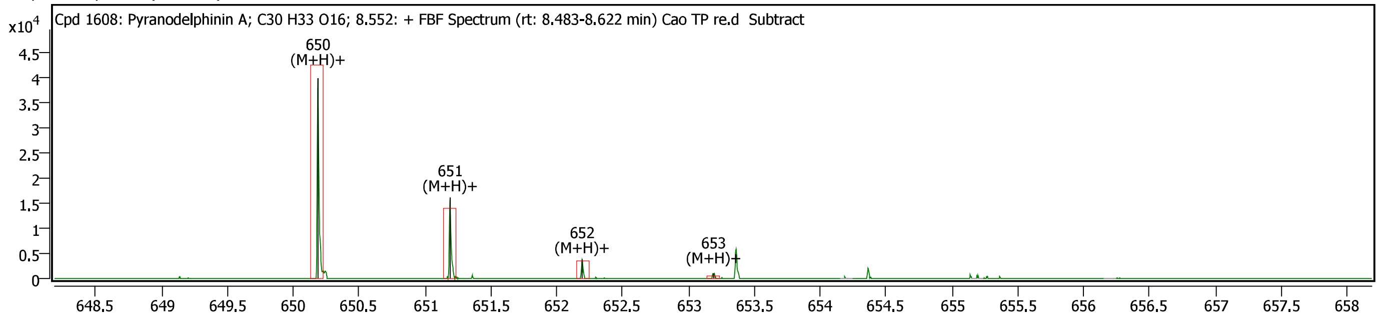

Compound ID Table

Name	Formula	Species	RT	RT Diff	Mass	CAS	ID Source	Score	Score (Lib)	Score (Tgt)
Pyranodelphinin A	C30 H33 O16	(M+H)+	8.552		649.1793		FBF	88.35		88.35

Cpd 851: Matairesinol

Name	Formula	RT	RI	Mass	Diff (Tgt, ppm)	CAS	ID Source	Score	Algorithm
Matairesinol	C20 H22 O6	8.639		358.1419	0.80	580-72-3	FBF-FragConfirm	98.06	FBF

Species	m/z	Score (Tgt)	Score (Lib)	Score (DB)	Score (MFG)	Score (RT)
(M+H)+	359	98.06				

Compound Chromatograms (overlaid)

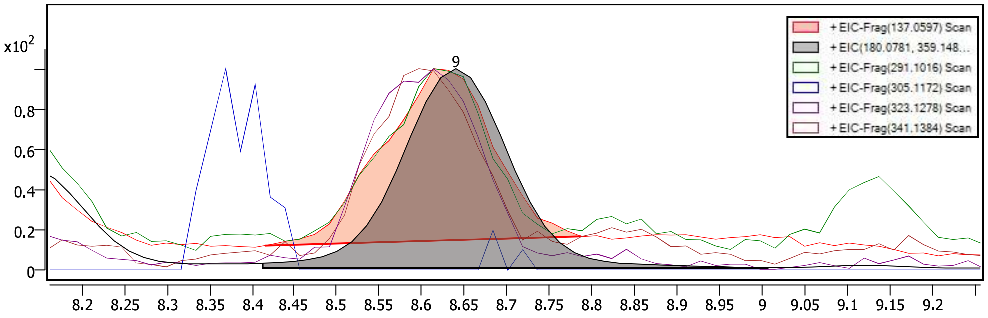

Structure

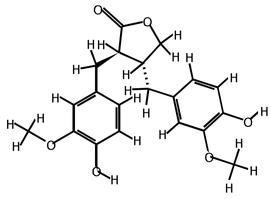

Coelution Plot

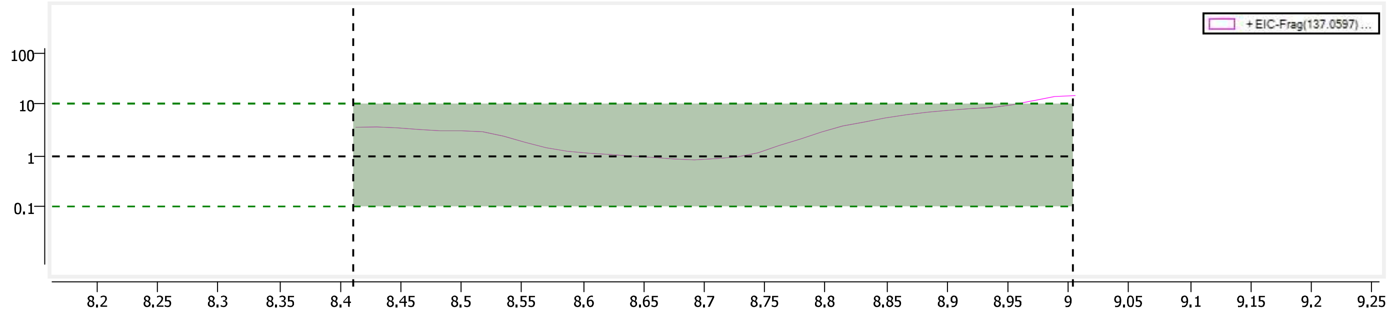

Compound Spectra (overlaid)

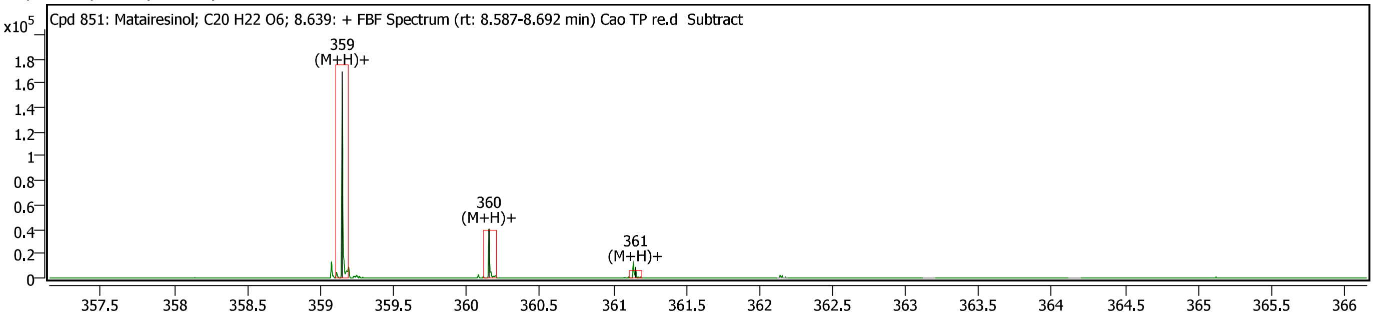

Fragment Spectrum (clean)

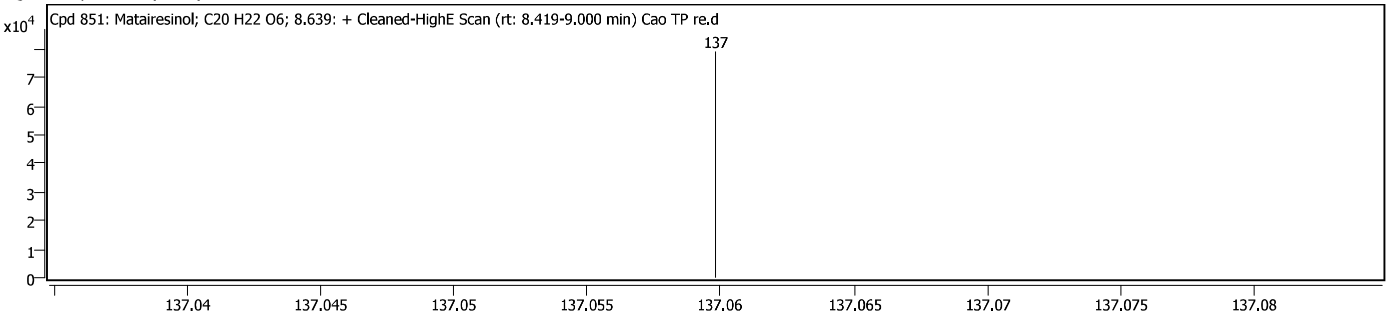

Fragment Spectrum (raw)

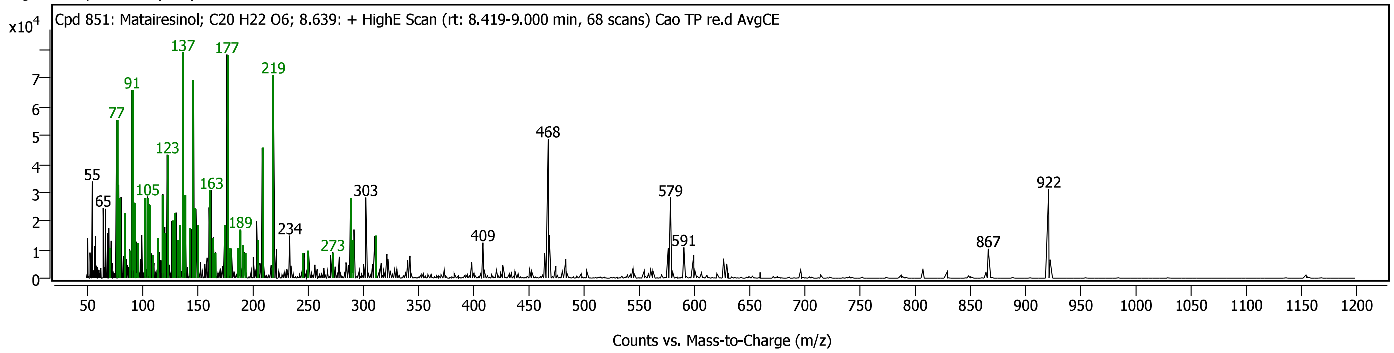

Compound ID Table

Name	Formula	Species	RT	RT Diff	Mass	CAS	ID Source	Score	Score (Lib)	Score (Tgt)
Matairesinol	C20 H22 O6	(M+H)+	8.639		358.1419	580-72-3	FBF-FragConfirm	98.06		98.06
6-Prenylcatechin	C20 H22 O6	(M+H)+	8.639		358.1419		FBF-FragConfirm	98.06		98.06
Columbin	C20 H22 O6	(M+H)+	8.639		358.1419	546-97-4	FBF-FragConfirm	98.06		98.06
Sanshodiol	C20 H22 O6	(M+H)+	8.639		358.1419	54854-91-0	FBF-FragConfirm	98.06		98.06
Clemaphenol A	C20 H22 O6	(M+H)+	8.639		358.1419		FBF-FragConfirm	98.06		98.06
Brosimacutin A	C20 H22 O6	(M+H)+	8.639		358.1419		FBF-FragConfirm	98.06		98.06
8-Prenylcatechin	C20 H22 O6	(M+H)+	8.639		358.1419		FBF-FragConfirm	98.06		98.06
(+)-Pinoresinol	C20 H22 O6	(M+H)+	8.639		358.1419	487-36-5	FBF-FragConfirm	98.06		98.06
5-Deoxykievitone hydrate	C20 H22 O6	(M+H)+	8.639		358.1419		FBF-FragConfirm	98.06		98.06
2',4',5,7-Tetramethoxy-8-methylflavanone	C20 H22 O6	(M+H)+	8.639		358.1419		FBF-FragConfirm	98.06		98.06
(7'x,8'x)-4,7'-Epoxy-3,8'-bilign-7-ene-3,5'-dimethoxy-4',9,9'-triol	C20 H22 O6	(M+H)+	8.639		358.1419		FBF-FragConfirm	98.06		98.06
Coleone A	C20 H22 O6	(M+H)+	8.639		358.1419	1984-44-7	FBF-FragConfirm	98.06		98.06
Pedicellin	C20 H22 O6	(M+H)+	8.639		358.1419		FBF-FragConfirm	98.06		98.06
Brosimacutin B	C20 H22 O6	(M+H)+	8.639		358.1419		FBF-FragConfirm	98.06		98.06
Dihydrocubebin	C20 H22 O6	(M+H)+	8.639		358.1419	24563-03-9	FBF-FragConfirm	98.06		98.06
Isobutylshikonin	C20 H22 O6	(M+H)+	8.639		358.1419		FBF-FragConfirm	98.06		98.06
Triptonide	C20 H22 O6	(M+H)+	8.639		358.1419	38647-11-9	FBF-FragConfirm	98.06		98.06
Isocolumbin	C20 H22 O6	(M+H)+	8.639		358.1419		FBF-FragConfirm	98.06		98.06
Miroestrol	C20 H22 O6	(M+H)+	8.639		358.1419	2618-41-9	FBF-FragConfirm	98.06		98.06
Multiradiatin	C20 H22 O6	(M+H)+	8.639		358.1419	58262-52-5	FBF-FragConfirm	98.06		98.06
Multistatin	C20 H22 O6	(M+H)+	8.639		358.1419	64937-26-4	FBF-FragConfirm	98.06		98.06
Glicophenone	C20 H22 O6	(M+H)+	8.639		358.1419		FBF-FragConfirm	98.06		98.06

Cpd 1644: Dide-O-methyl-4-O-alpha-D-glucopyranosylsimmondsin

Name	Formula	RT	RI	Mass	Diff (Tgt, ppm)	CAS	ID Source	Score	Algorithm
Dide-O-methyl-4-O-alpha-D-glucopyranosylsimmondsin	C20 H31 N O14	8.639		509.1720	-4.92	370068-00-1	FBF	82.62	FBF

Species	m/z	Score (Tgt)	Score (Lib)	Score (DB)	Score (MFG)	Score (RT)
(M+H)+	510	82.62				

Compound Chromatograms (overlaid)

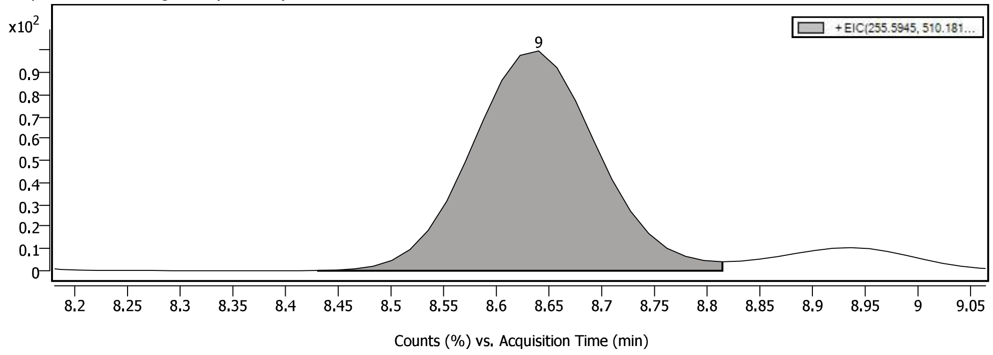

Structure

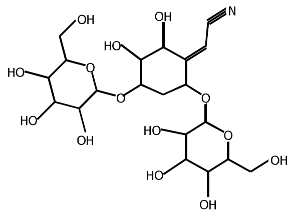

Compound Spectra (overlaid)

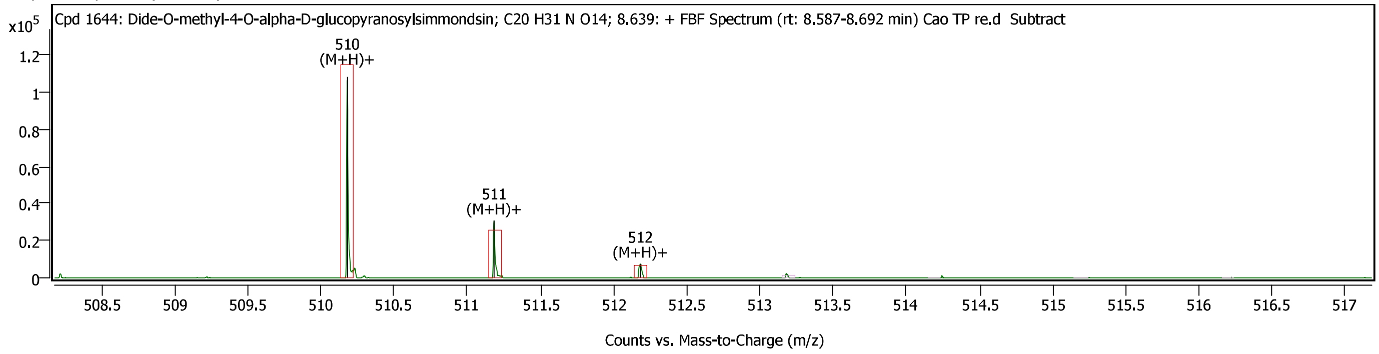

Compound ID Table

Name	Formula	Species	RT	RT Diff	Mass	CAS	ID Source	Score	Score (Lib)	Score (Tgt)
Dide-O-methyl-4-O-alpha-D-glucopyranosylsimmondsin	C20 H31 N O14	(M+H)+	8.639		509.1720	370068-00-1	FBF	82.62		82.62

Cpd 120: 8,8'-Methylenebiscatechin

Name	Formula	RT	RI	Mass	Diff (Tgt, ppm)	CAS	ID Source	Score	Algorithm
8,8'-Methylenebiscatechin	C31 H28 O12	9.128		592.1581	0.08	81555-08-0	M-FBF	99.24	FBF

Species	m/z	Score (Tgt)	Score (Lib)	Score (DB)	Score (MFG)	Score (RT)
(M+H)+	593	99.24				

Compound Chromatograms (overlaid)

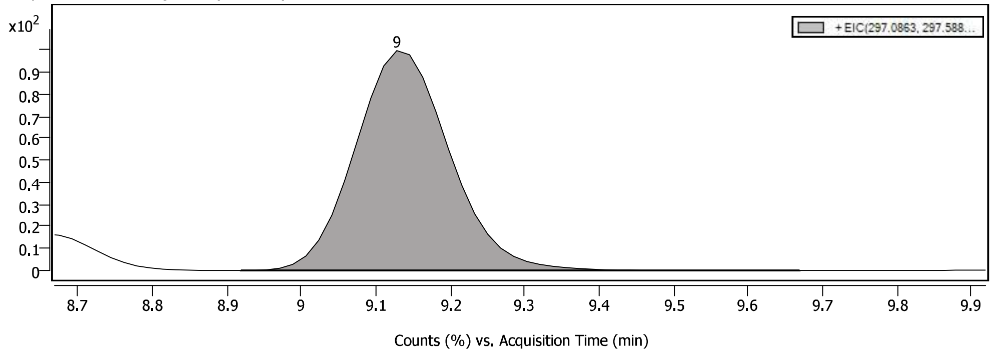

Structure

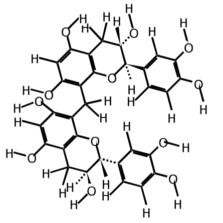

Compound Spectra (overlaid)

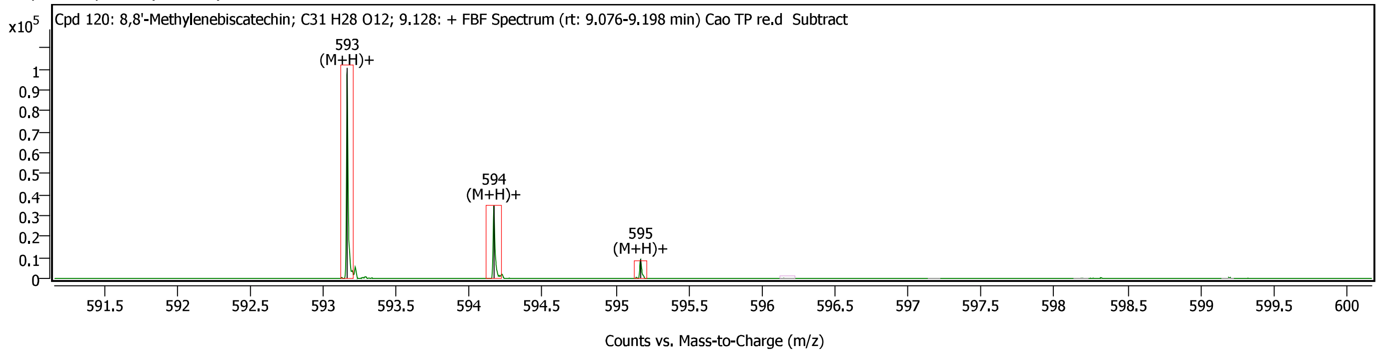

Compound ID Table

Name	Formula	Species	RT	RT Diff	Mass	CAS	ID Source	Score	Score (Lib)	Score (Tgt)
8,8'-Methylenebiscatechin	C31 H28 O12	(M+H)+	9.128		592.1581	81555-08-0	FBF	99.24		99.24
Aurasperone C	C31 H28 O12	(M+H)+	9.128		592.1581	41689-66-1	FBF	99.24		99.24

Cpd 1577: Lunarine

Name	Formula	RT	RI	Mass	Diff (Tgt, ppm)	CAS	ID Source	Score	Algorithm
Lunarine	C25 H31 N3 O4	9.180		437.2322	1.72	24185-51-1	FBF-FragConfirm	98.40	FBF

Species	m/z	Score (Tgt)	Score (Lib)	Score (DB)	Score (MFG)	Score (RT)
(M+H)+	438	98.40				

Compound Chromatograms (overlaid)

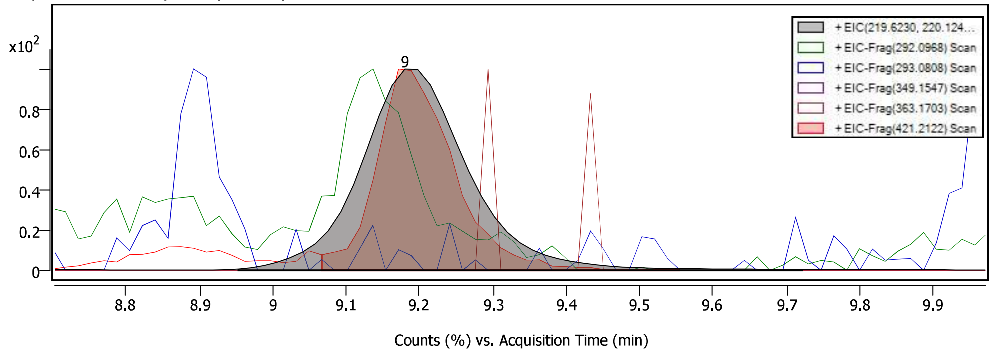

Structure

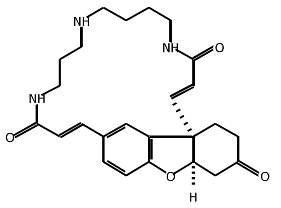

Coelution Plot

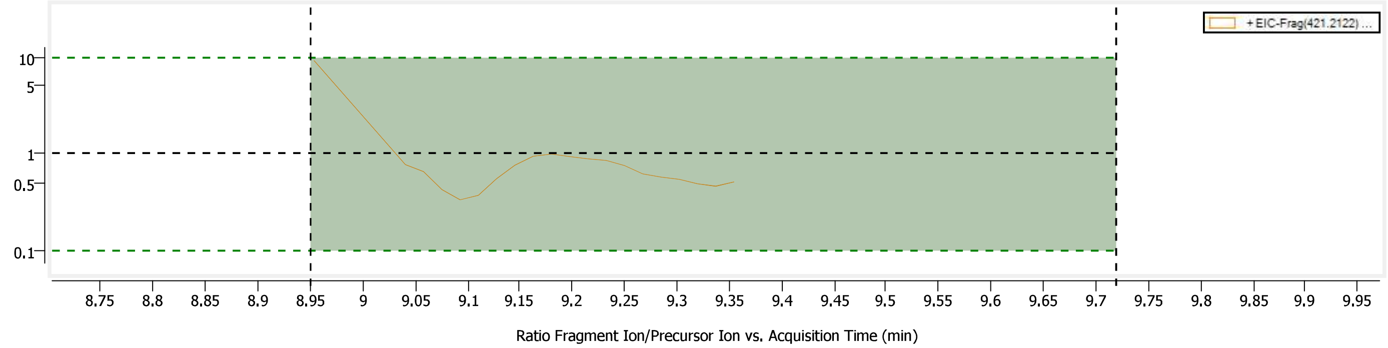

Compound Spectra (overlaid)

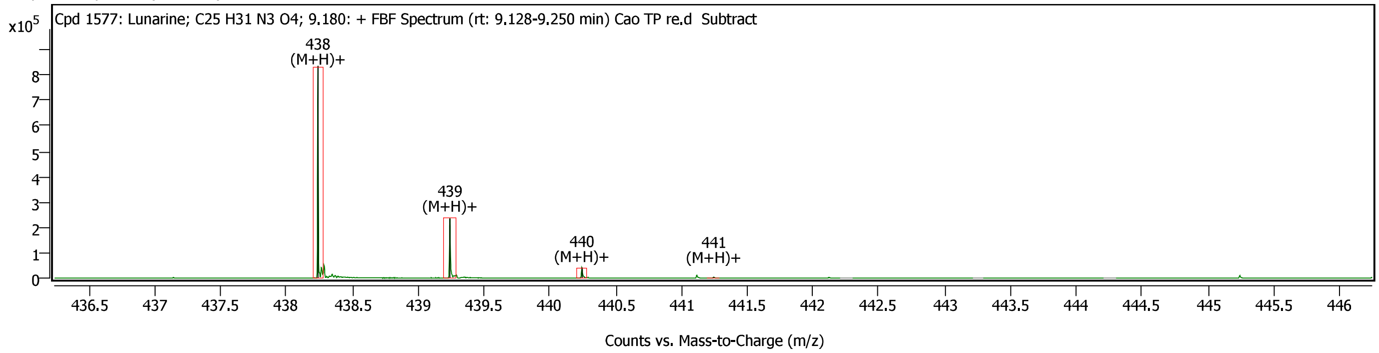

Fragment Spectrum (clean)

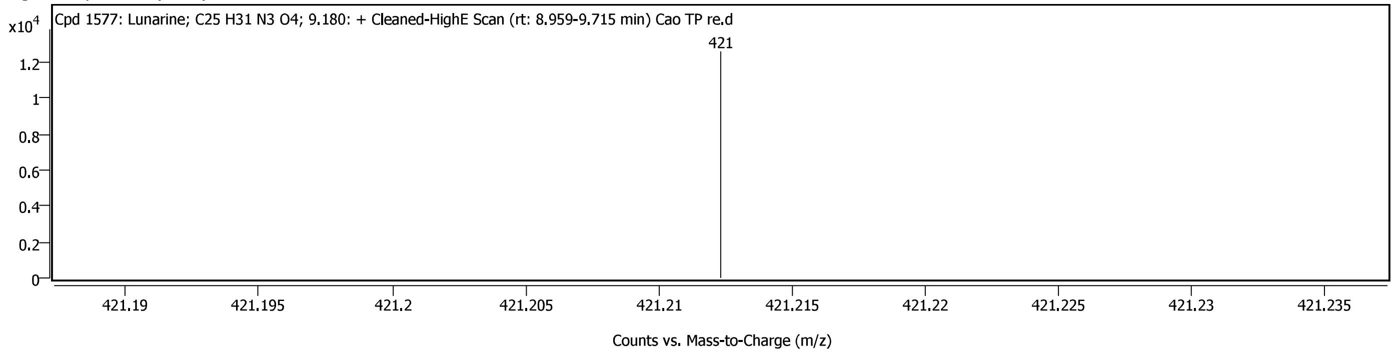

Fragment Spectrum (raw)

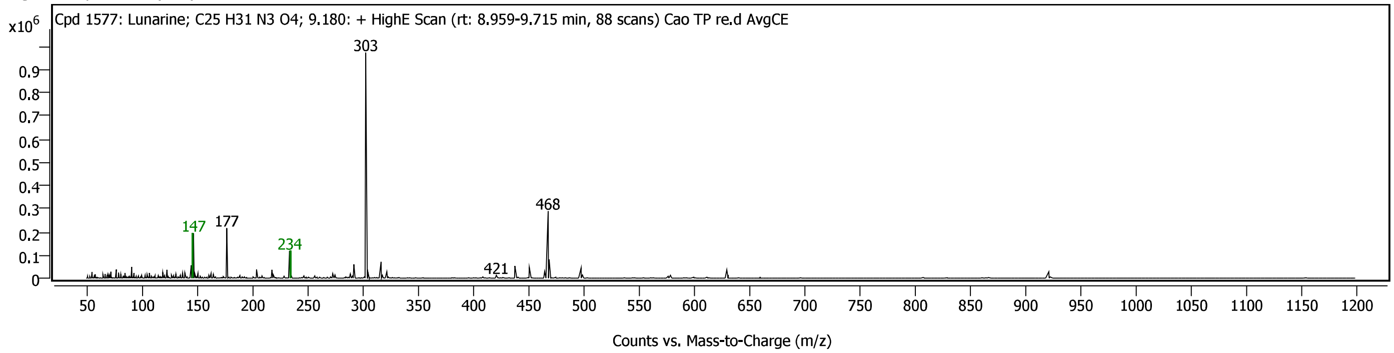

Compound ID Table

Name	Formula	Species	RT	RT Diff	Mass	CAS	ID Source	Score	Score (Lib)	Score (Tgt)
Lunarine	C25 H31 N3 O4	(M+H)+	9.180		437.2322	24185-51-1	FBF-FragConfirm	98.40		98.40
N1,N10-Dicoumaroylspermidine	C25 H31 N3 O4	(M+H)+	9.180		437.2322	65715-79-9	FBF-FragConfirm	98.40		98.40

Cpd 1580: N1,N10-Diferuloylspermidine

Name	Formula	RT	RI	Mass	Diff (Tgt, ppm)	CAS	ID Source	Score	Algorithm
N1,N10-Diferuloylspermidine	C27 H35 N3 O6	9.267		497.2528	0.49	70185-61-4	FBF	99.54	FBF

Species	m/z	Score (Tgt)	Score (Lib)	Score (DB)	Score (MFG)	Score (RT)
(M+H)+	498	99.54				

Compound Chromatograms (overlaid)

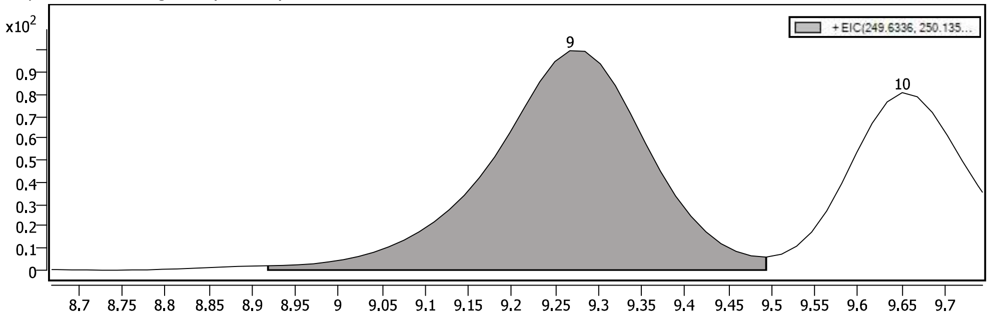

Structure

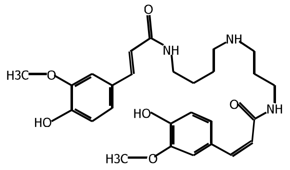

Compound Spectra (overlaid)

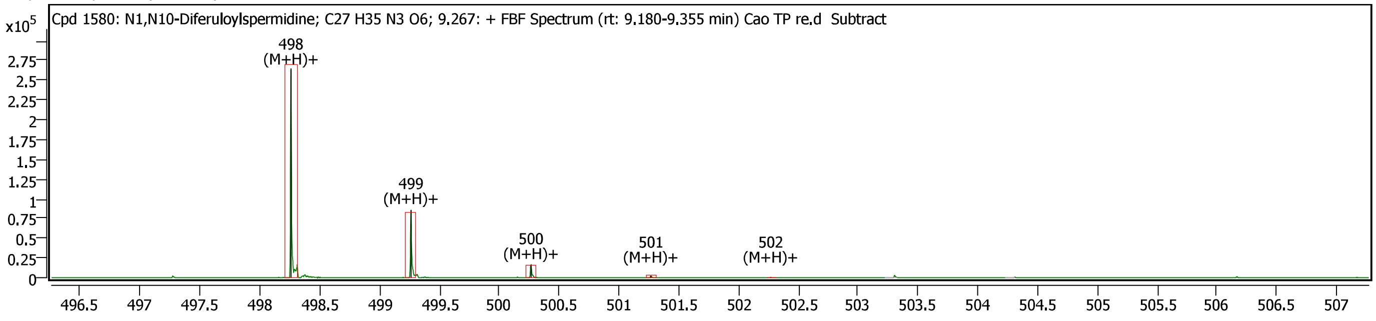

Compound ID Table

Name	Formula	Species	RT	RT Diff	Mass	CAS	ID Source	Score	Score (Lib)	Score (Tgt)
N1,N10-Diferuloylspermidine	C27 H35 N3 O6	(M+H)+	9.267		497.2528	70185-61-4	FBF	99.54		99.54

Cpd 758: Rhamnetin

Name	Formula	RT	RI	Mass	Diff (Tgt, ppm)	CAS	ID Source	Score	Algorithm
Rhamnetin	C16 H12 O7	9.390		316.0586	0.95	90-19-7	M-FBF-FragConfirm	98.42	FBF

Species	m/z	Score (Tgt)	Score (Lib)	Score (DB)	Score (MFG)	Score (RT)
(M+H)+	317	98.42				

Compound Chromatograms (overlaid)

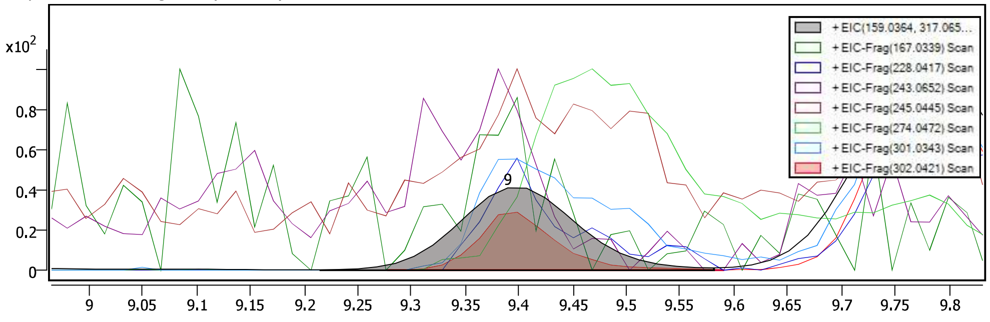

Structure

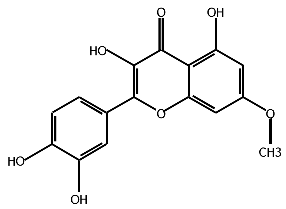

Coelution Plot

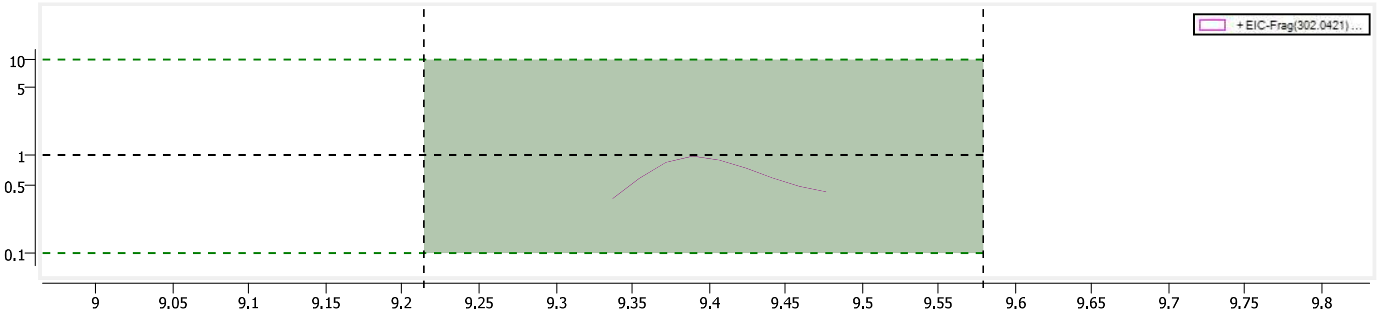

Compound Screening Report

Compound Spectra (overlaid)

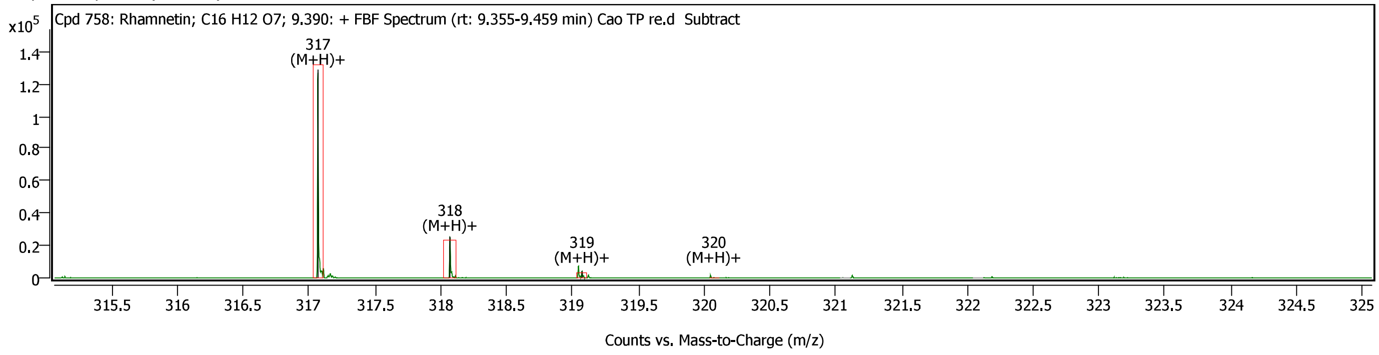

Fragment Spectrum (clean)

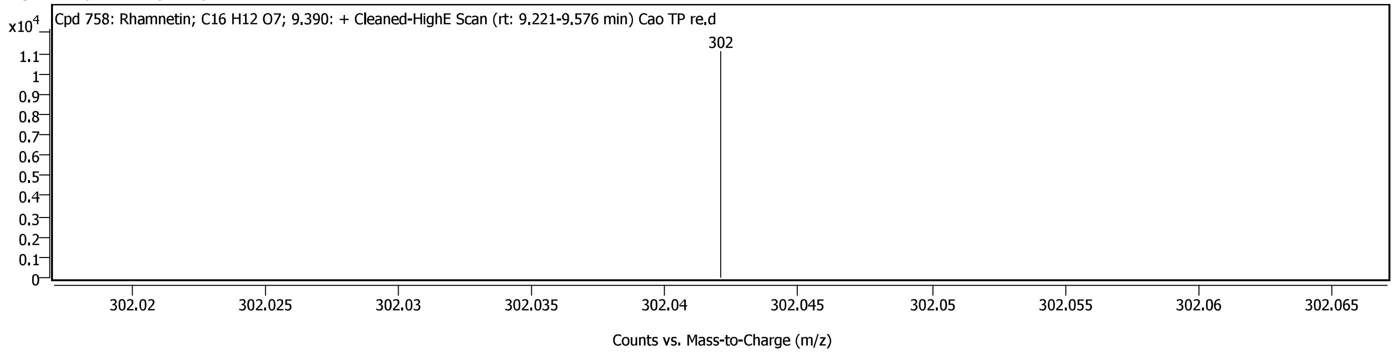

Fragment Spectrum (raw)

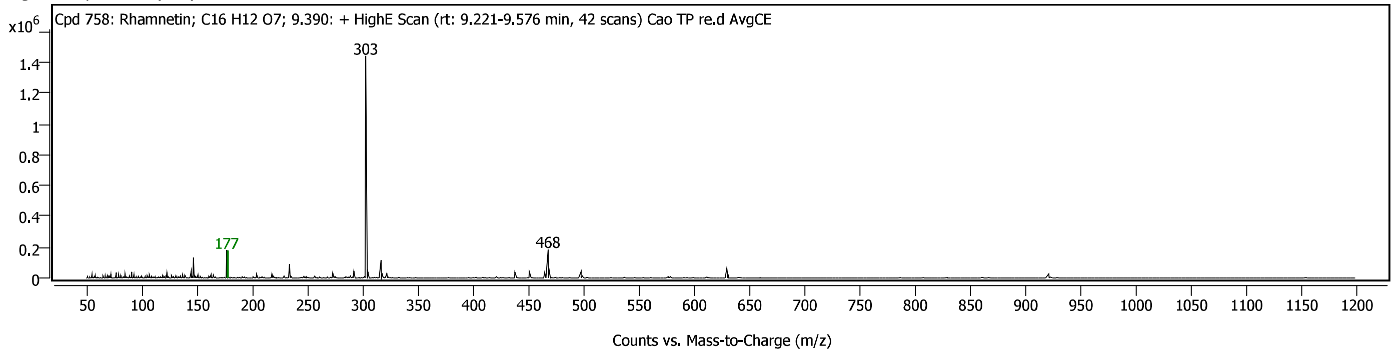

Compound Screening Report

Compound ID Table

Name	Formula	Species	RT	RT Diff	Mass	CAS	ID Source	Score	Score (Lib)	Score (Tgt)
Rhamnetin	C16 H12 O7	(M+H)+	9.390		316.0586	90-19-7	FBF-FragConfirm	98.42		98.42
3,5,6,7-Tetrahydroxy-4'-methoxyflavone	C16 H12 O7	(M+H)+	9.390		316.0586		FBF-FragConfirm	98.42		98.42
3'-O-Methyltricetin	C16 H12 O7	(M+H)+	9.390		316.0586		FBF-FragConfirm	98.42		98.42
6-Methoxykaempferol	C16 H12 O7	(M+H)+	9.390		316.0586		FBF-FragConfirm	98.42		98.42
6-Hydroxyluteolin 4'-methyl ether	C16 H12 O7	(M+H)+	9.390		316.0586		FBF-FragConfirm	98.42		98.42
6a,7-Dihydroxymaackiain	C16 H12 O7	(M+H)+	9.390		316.0586		FBF-FragConfirm	98.42		98.42
5,6,7,4'-Tetrahydroxy-8-methoxyisoflavone	C16 H12 O7	(M+H)+	9.390		316.0586		FBF-FragConfirm	98.42		98.42
5,6,7,4'-Tetrahydroxy-3'-methoxyisoflavone	C16 H12 O7	(M+H)+	9.390		316.0586		FBF-FragConfirm	98.42		98.42
5,2',3'-Trihydroxy-6,7-methylenedioxyflavanone	C16 H12 O7	(M+H)+	9.390		316.0586		FBF-FragConfirm	98.42		98.42
7-Methoxy-3,6,3',4'-tetrahydroxyflavone	C16 H12 O7	(M+H)+	9.390		316.0586		FBF-FragConfirm	98.42		98.42
3'-O-Methylmelanoxetin	C16 H12 O7	(M+H)+	9.390		316.0586		FBF-FragConfirm	98.42		98.42
Pedalitin	C16 H12 O7	(M+H)+	9.390		316.0586		FBF-FragConfirm	98.42		98.42
3-Methylherbacetin	C16 H12 O7	(M+H)+	9.390		316.0586		FBF-FragConfirm	98.42		98.42
Azaleatin	C16 H12 O7	(M+H)+	9.390		316.0586		FBF-FragConfirm	98.42		98.42
3,7,3',4'-Tetrahydroxy-8-methoxyflavone	C16 H12 O7	(M+H)+	9.390		316.0586		FBF-FragConfirm	98.42		98.42
3,5,8,2'-Tetrahydroxy-7-methoxyflavone	C16 H12 O7	(M+H)+	9.390		316.0586		FBF-FragConfirm	98.42		98.42
3,5,6,4'-Tetrahydroxy-7-methoxyflavone	C16 H12 O7	(M+H)+	9.390		316.0586		FBF-FragConfirm	98.42		98.42
1,3,5,8-Tetrahydroxy-6-methoxy-2-methylanthraquinone	C16 H12 O7	(M+H)+	9.390		316.0586	101508-13-8	FBF-FragConfirm	98.42		98.42
3-Methoxy-6,7,3',4'-tetrahydroxyflavone	C16 H12 O7	(M+H)+	9.390		316.0586		FBF-FragConfirm	98.42		98.42
3-O-Methylquercetin	C16 H12 O7	(M+H)+	9.390		316.0586	1486-70-0	FBF-FragConfirm	98.42		98.42
(-)-4,9,11,12a-Tetrahydroxyrotenone	C16 H12 O7	(M+H)+	9.390		316.0586		FBF-FragConfirm	98.42		98.42
5,6,7,4'-Tetrahydroxy-3-methoxyflavone	C16 H12 O7	(M+H)+	9.390		316.0586		FBF-FragConfirm	98.42		98.42
Herbacetin 4'-methyl ether	C16 H12 O7	(M+H)+	9.390		316.0586		FBF-FragConfirm	98.42		98.42
Vogeletin	C16 H12 O7	(M+H)+	9.390		316.0586		FBF-FragConfirm	98.42		98.42
Tricetin 4'-methyl ether	C16 H12 O7	(M+H)+	9.390		316.0586		FBF-FragConfirm	98.42		98.42
Carajuflavone	C16 H12 O7	(M+H)+	9.390		316.0586		FBF-FragConfirm	98.42		98.42
Tamarixetin	C16 H12 O7	(M+H)+	9.390		316.0586	603-61-2	FBF-FragConfirm	98.42		98.42
Sexangularetin	C16 H12 O7	(M+H)+	9.390		316.0586		FBF-FragConfirm	98.42		98.42
Capillarisin	C16 H12 O7	(M+H)+	9.390		316.0586	56365-38-9	FBF-FragConfirm	98.42		98.42
Pollenitin	C16 H12 O7	(M+H)+	9.390		316.0586		FBF-FragConfirm	98.42		98.42
Transilitin	C16 H12 O7	(M+H)+	9.390		316.0586		FBF-FragConfirm	98.42		98.42
Onopordin	C16 H12 O7	(M+H)+	9.390		316.0586		FBF-FragConfirm	98.42		98.42
Nepetin	C16 H12 O7	(M+H)+	9.390		316.0586		FBF-FragConfirm	98.42		98.42
Milimorin	C16 H12 O7	(M+H)+	9.390		316.0586		FBF-FragConfirm	98.42		98.42
Junipegenin A	C16 H12 O7	(M+H)+	9.390		316.0586		FBF-FragConfirm	98.42		98.42
Isorhamnetin	C16 H12 O7	(M+H)+	9.390		316.0586	480-19-3	FBF-FragConfirm	98.42		98.42
Hypolaetin 3'-methyl ether	C16 H12 O7	(M+H)+	9.390		316.0586		FBF-FragConfirm	98.42		98.42
Pinoquercetin	C16 H12 O7	(M+H)+	9.390		316.0586	491-49-6	FBF-FragConfirm	98.42		98.42
Nodifloretin	C16 H12 O7	(M+H)+	9.390		316.0586		FBF-FragConfirm	98.42		98.42

Cpd 1438: N-Adenylylanthranilate

Name	Formula	RT	RI	Mass	Diff (Tgt, ppm)	CAS	ID Source	Score	Algorithm
N-Adenylylanthranilate	C17 H19 N6 O8 P	9.494		466.1013	2.41		FBF	93.16	FBF

Species	m/z	Score (Tgt)	Score (Lib)	Score (DB)	Score (MFG)	Score (RT)
(M+H)+	467	93.16				

Compound Chromatograms (overlaid)

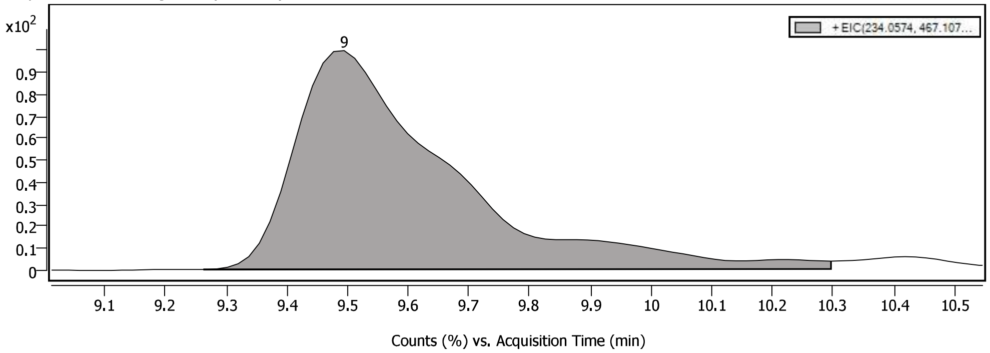

Structure

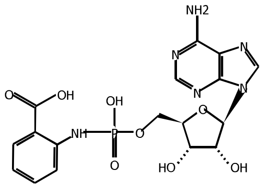

Compound Spectra (overlaid)

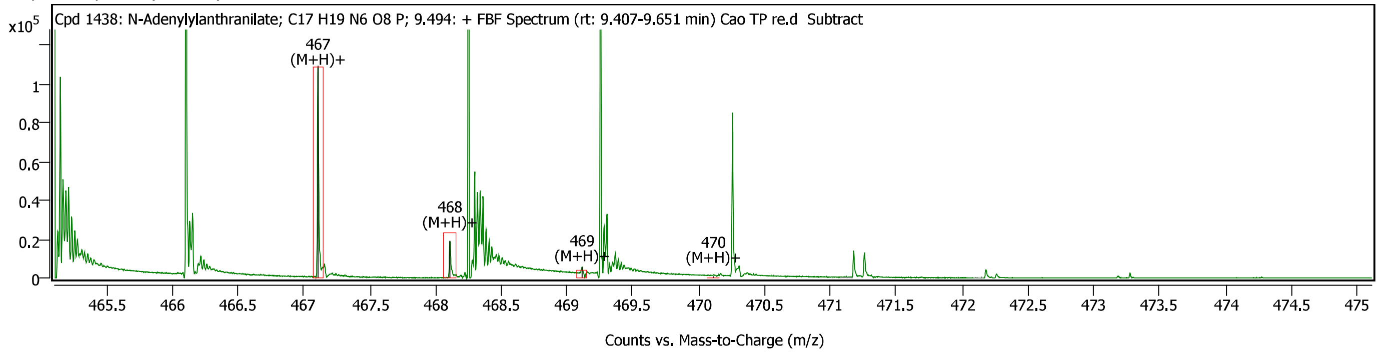

Compound ID Table

Name	Formula	Species	RT	RT Diff	Mass	CAS	ID Source	Score	Score (Lib)	Score (Tgt)
N-Adenylylanthranilate	C17 H19 N6 O8 P	(M+H)+	9.494		466.1013		FBF	93.16		93.16

Cpd 1312: Quercetin 3-O-glucoside

Name	Formula	RT	RI	Mass	Diff (Tgt, ppm)	CAS	ID Source	Score	Algorithm
Quercetin 3-O-glucoside	C21 H20 O12	9.500		464.0968	2.90	21637-25-2	M-FBF-FragConfirm	94.76	FBF

Species	m/z	Score (Tgt)	Score (Lib)	Score (DB)	Score (MFG)	Score (RT)
(M+H)+	465	94.76				

Compound Chromatograms (overlaid)

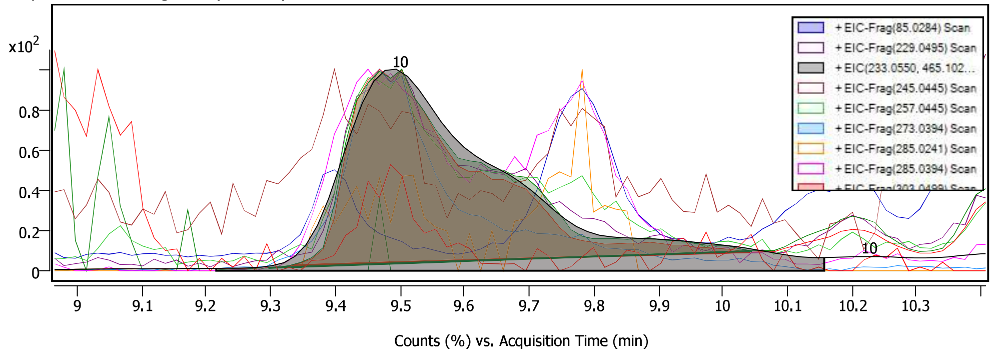

Structure

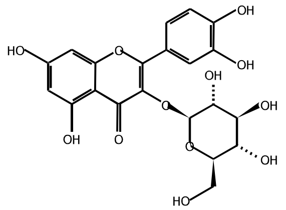

Coelution Plot

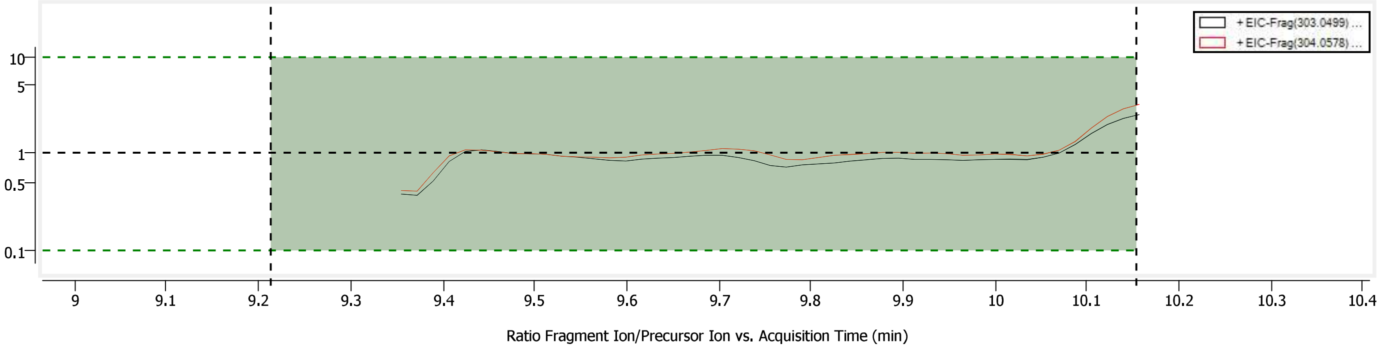

Compound Spectra (overlaid)

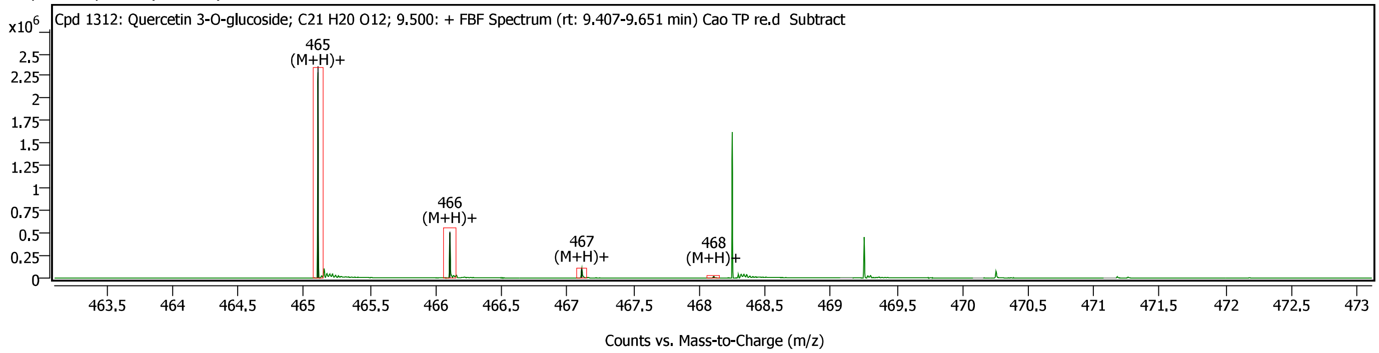

Fragment Spectrum (clean)

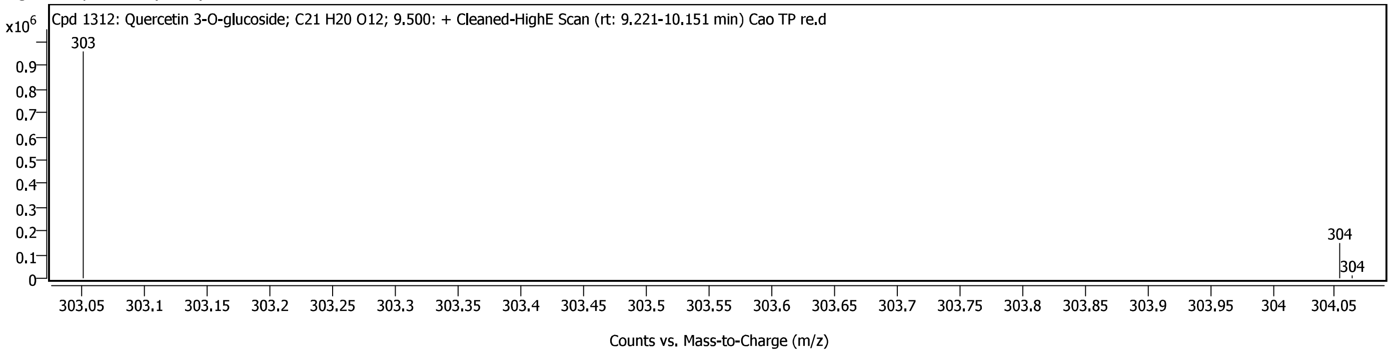

Fragment Spectrum (raw)

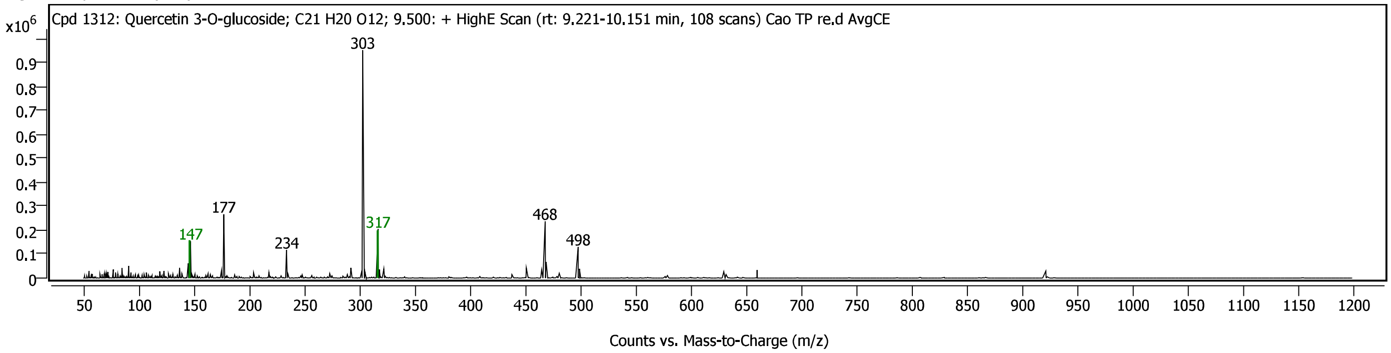

Compound Screening Report

Compound ID Table

Name	Formula	Species	RT	RT Diff	Mass	CAS	ID Source	Score	Score (Lib)	Score (Tgt)
Quercetin 3-O-glucoside	C21 H20 O12	(M+H)+	9.500		464.0968	21637-25-2	FBF-FragConfirm	94.76		94.76
Bracteatin 6-O-glucoside	C21 H20 O12	(M+H)+	9.500		464.0968		FBF-FragConfirm	94.76		94.76
Quercetagetin 3-rhamnoside	C21 H20 O12	(M+H)+	9.500		464.0968		FBF-FragConfirm	94.76		94.76
8-Hydroxyluteolin 8-glucoside	C21 H20 O12	(M+H)+	9.500		464.0968		FBF-FragConfirm	94.76		94.76
Annulatin 3'-xyloside	C21 H20 O12	(M+H)+	9.500		464.0968		FBF-FragConfirm	94.76		94.76
Bracteatin 6-glucoside	C21 H20 O12	(M+H)+	9.500		464.0968		FBF-FragConfirm	94.76		94.76
Gossypetin 7-rhamnoside	C21 H20 O12	(M+H)+	9.500		464.0968		FBF-FragConfirm	94.76		94.76
Bractein	C21 H20 O12	(M+H)+	9.500		464.0968		FBF-FragConfirm	94.76		94.76
Corniculatusin 3-alpha-L-arabinofuranoside	C21 H20 O12	(M+H)+	9.500		464.0968		FBF-FragConfirm	94.76		94.76
Eriodictyol 7-glucuronide	C21 H20 O12	(M+H)+	9.500		464.0968		FBF-FragConfirm	94.76		94.76
6-Hydroxyluteolin 7-glucoside	C21 H20 O12	(M+H)+	9.500		464.0968		FBF-FragConfirm	94.76		94.76
Gossypetin 8-rhamnoside	C21 H20 O12	(M+H)+	9.500		464.0968		FBF-FragConfirm	94.76		94.76
6-Hydroxytricetin 5-rhamnoside	C21 H20 O12	(M+H)+	9.500		464.0968		FBF-FragConfirm	94.76		94.76
6-Hydroxykaempferol 3-glucoside	C21 H20 O12	(M+H)+	9.500		464.0968		FBF-FragConfirm	94.76		94.76
6-Hydroxyluteolin 7-galactoside	C21 H20 O12	(M+H)+	9.500		464.0968		FBF-FragConfirm	94.76		94.76
6-Hydroxyluteolin 6-glucoside	C21 H20 O12	(M+H)+	9.500		464.0968		FBF-FragConfirm	94.76		94.76
6-Hydroxyluteolin 5-glucoside	C21 H20 O12	(M+H)+	9.500		464.0968		FBF-FragConfirm	94.76		94.76
6-Hydroxykaempferol 7-glucoside	C21 H20 O12	(M+H)+	9.500		464.0968		FBF-FragConfirm	94.76		94.76
6-C-Glucosylquercetin	C21 H20 O12	(M+H)+	9.500		464.0968		FBF-FragConfirm	94.76		94.76
6-C-beta-D-Glucopyranosyl-5,7,2',4',5'-pentahydroxyflavone	C21 H20 O12	(M+H)+	9.500		464.0968		FBF-FragConfirm	94.76		94.76
5,7,3',4',5'-Pentahydroxyflavone 8-C-glucopyranoside	C21 H20 O12	(M+H)+	9.500		464.0968		FBF-FragConfirm	94.76		94.76
5,6,7,3',4'-Pentahydroxy-8-methoxyflavone 7-apioside	C21 H20 O12	(M+H)+	9.500		464.0968		FBF-FragConfirm	94.76		94.76
3,5,7,2',6'-Pentahydroxyflavone 2'-glucoside	C21 H20 O12	(M+H)+	9.500		464.0968		FBF-FragConfirm	94.76		94.76
2'-Hydroxyisoorientin	C21 H20 O12	(M+H)+	9.500		464.0968		FBF-FragConfirm	94.76		94.76
(2S)-5,7,3',4'-Tetrahydroxyflavanone 7-glucuronide	C21 H20 O12	(M+H)+	9.500		464.0968		FBF-FragConfirm	94.76		94.76
Herbacetin 3-beta-D-glucofuranoside	C21 H20 O12	(M+H)+	9.500		464.0968		FBF-FragConfirm	94.76		94.76
8-Hydroxyluteolin 7-glucoside	C21 H20 O12	(M+H)+	9.500		464.0968		FBF-FragConfirm	94.76		94.76
Herbacetin 3-glucoside	C21 H20 O12	(M+H)+	9.500		464.0968		FBF-FragConfirm	94.76		94.76
Tricetin 7-glucoside	C21 H20 O12	(M+H)+	9.500		464.0968		FBF-FragConfirm	94.76		94.76
Quercetin 7-galactoside	C21 H20 O12	(M+H)+	9.500		464.0968		FBF-FragConfirm	94.76		94.76
Herbacetin 7-glucoside	C21 H20 O12	(M+H)+	9.500		464.0968		FBF-FragConfirm	94.76		94.76
Tricetin 3'-glucoside	C21 H20 O12	(M+H)+	9.500		464.0968		FBF-FragConfirm	94.76		94.76
Spiraeoside	C21 H20 O12	(M+H)+	9.500		464.0968		FBF-FragConfirm	94.76		94.76
Robinetin 7-glucoside	C21 H20 O12	(M+H)+	9.500		464.0968		FBF-FragConfirm	94.76		94.76
Quercimeritrin	C21 H20 O12	(M+H)+	9.500		464.0968		FBF-FragConfirm	94.76		94.76
Quercetin 5-glucoside	C21 H20 O12	(M+H)+	9.500		464.0968		FBF-FragConfirm	94.76		94.76
Quercetin 4'-glucoside	C21 H20 O12	(M+H)+	9.500		464.0968	20229-56-5	FBF-FragConfirm	94.76		94.76
Quercetin 3-β-D-glucoside	C21 H20 O12	(M+H)+	9.500		464.0968	482-35-9	FBF-FragConfirm	94.76		94.76
Quercetin 3'-glucoside	C21 H20 O12	(M+H)+	9.500		464.0968		FBF-FragConfirm	94.76		94.76
Quercetin 3-alloside	C21 H20 O12	(M+H)+	9.500		464.0968		FBF-FragConfirm	94.76		94.76
Patuletin 3-xyloside	C21 H20 O12	(M+H)+	9.500		464.0968		FBF-FragConfirm	94.76		94.76
Myricetin 3'-rhamnoside	C21 H20 O12	(M+H)+	9.500		464.0968		FBF-FragConfirm	94.76		94.76
Isoetin 5'-glucoside	C21 H20 O12	(M+H)+	9.500		464.0968		FBF-FragConfirm	94.76		94.76
Myricitrin	C21 H20 O12	(M+H)+	9.500		464.0968	17912-87-7	FBF-FragConfirm	94.76		94.76
Herbacetin 8-glucoside	C21 H20 O12	(M+H)+	9.500		464.0968		FBF-FragConfirm	94.76		94.76
Hyperin	C21 H20 O12	(M+H)+	9.500		464.0968		FBF-FragConfirm	94.76		94.76
Hyperoside	C21 H20 O12	(M+H)+	9.500		464.0968	482-36-0	FBF-FragConfirm	94.76		94.76
Isoaffinetin	C21 H20 O12	(M+H)+	9.500		464.0968		FBF-FragConfirm	94.76		94.76
Isoetin 7-glucoside	C21 H20 O12	(M+H)+	9.500		464.0968		FBF-FragConfirm	94.76		94.76
Isoquercitrin	C21 H20 O12	(M+H)+	9.500		464.0968		FBF-FragConfirm	94.76		94.76
Larycitrin 3-alpha-L-arabinofuranoside	C21 H20 O12	(M+H)+	9.500		464.0968		FBF-FragConfirm	94.76		94.76
Herbacetin 4'-glucoside	C21 H20 O12	(M+H)+	9.500		464.0968		FBF-FragConfirm	94.76		94.76
Myricetin 7-rhamnoside	C21 H20 O12	(M+H)+	9.500		464.0968	184533-14-0	FBF-FragConfirm	94.76		94.76

Cpd 1403: <Herbacetin>

Name	Formula	RT	RI	Mass	Diff (Tgt, ppm)	CAS	ID Source	Score	Algorithm
<Herbacetin>	C15 H10 O7	9.512		302.0429	0.95		M-FBF	99.23	FBF

Species	m/z	Score (Tgt)	Score (Lib)	Score (DB)	Score (MFG)	Score (RT)
(M+H)+	303	99.23				

Compound Chromatograms (overlaid)

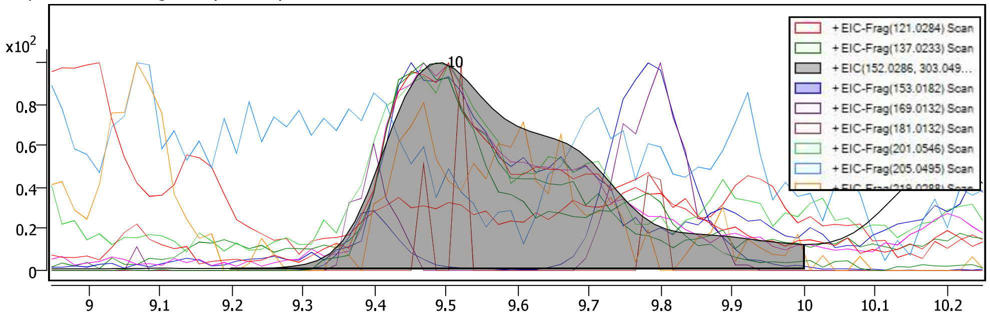

Counts (%) vs. Acquisition Time (min)

Structure

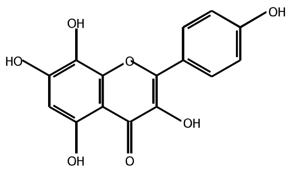

Coelution Plot

Compound Screening Report

Compound Spectra (overlaid)

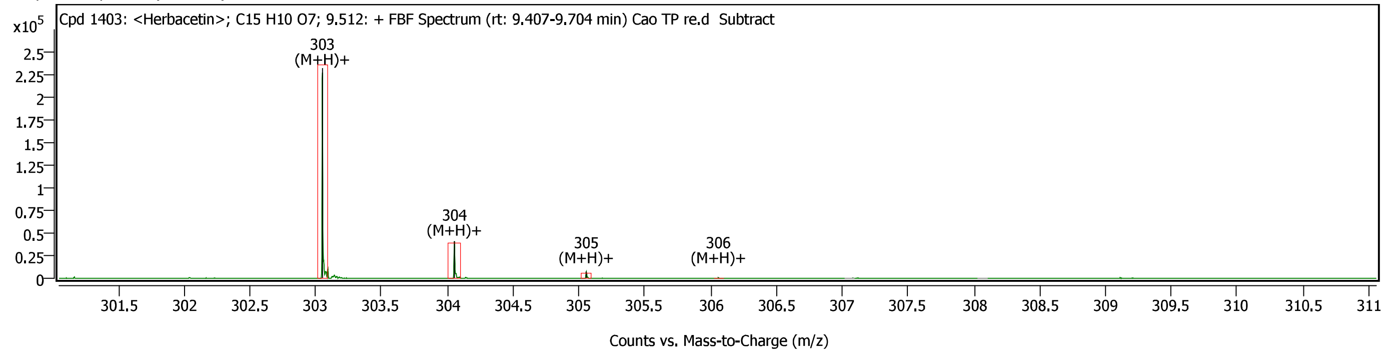

Fragment Spectrum (raw)

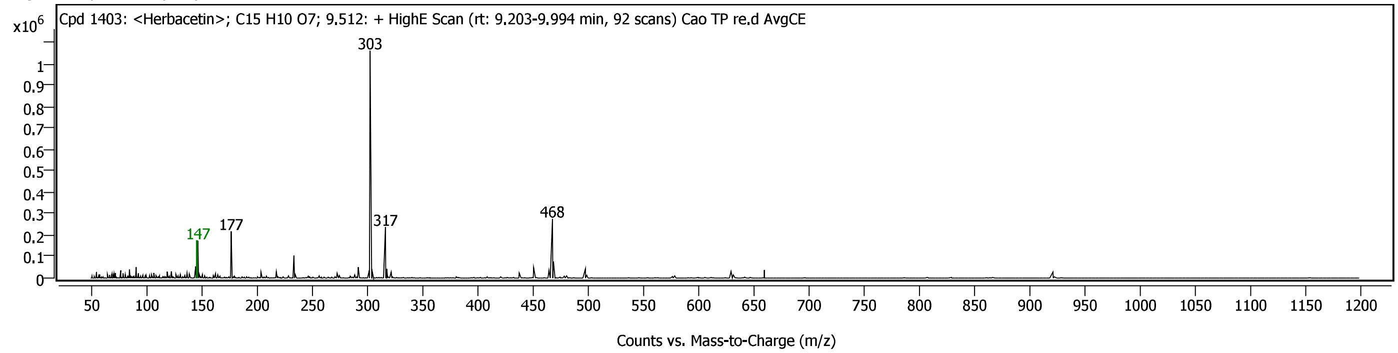

Compound ID Table

Name	Formula	Species	RT	RT Diff	Mass	CAS	ID Source	Score	Score (Lib)	Score (Tgt)
<Herbacetin>	C15 H10 O7	(M+H)+	9.512		302.0429		FBF	99.23		99.23
<6-Hydroxyluteolin>	C15 H10 O7	(M+H)+	9.512		302.0429		FBF	99.23		99.23
<6-Hydroxykaempferol>	C15 H10 O7	(M+H)+	9.512		302.0429		FBF	99.23		99.23
<2-(4-Hydroxyphenyl)-5,6,7,8-tetrahydroxy-4H-1-benzopyran-4-one>	C15 H10 O7	(M+H)+	9.512		302.0429	577-26-4	FBF	99.23		99.23
<2',3',4',5,7-Pentahydroxyflavone>	C15 H10 O7	(M+H)+	9.512		302.0429	144707-17-5	FBF	99.23		99.23
<2'-Hydroxypseudobaptigenin>	C15 H10 O7	(M+H)+	9.512		302.0429		FBF	99.23		99.23
<3,5,7,2',5'-Pentahydroxyflavone>	C15 H10 O7	(M+H)+	9.512		302.0429		FBF	99.23		99.23
<5,6,7,3',4'-Pentahydroxyisoflavone>	C15 H10 O7	(M+H)+	9.512		302.0429		FBF	99.23		99.23
<5,7,8,2',4'-Pentahydroxyisoflavone>	C15 H10 O7	(M+H)+	9.512		302.0429		FBF	99.23		99.23
<5,7,8,3',4'-Pentahydroxyisoflavone>	C15 H10 O7	(M+H)+	9.512		302.0429		FBF	99.23		99.23
<Bracteatin>	C15 H10 O7	(M+H)+	9.512		302.0429		FBF	99.23		99.23
<Tricetin>	C15 H10 O7	(M+H)+	9.512		302.0429	520-31-0	FBF	99.23		99.23
<Hypolaetin>	C15 H10 O7	(M+H)+	9.512		302.0429		FBF	99.23		99.23
<Isoetin>	C15 H10 O7	(M+H)+	9.512		302.0429		FBF	99.23		99.23
<Melanoxetin>	C15 H10 O7	(M+H)+	9.512		302.0429		FBF	99.23		99.23
<Morin>	C15 H10 O7	(M+H)+	9.512		302.0429	480-16-0	FBF	99.23		99.23
<Quercetin>	C15 H10 O7	(M+H)+	9.512		302.0429	117-39-5	FBF	99.23		99.23
<Rhynchosin>	C15 H10 O7	(M+H)+	9.512		302.0429		FBF	99.23		99.23
<Robinetin>	C15 H10 O7	(M+H)+	9.512		302.0429		FBF	99.23		99.23
<Viscidulin I>	C15 H10 O7	(M+H)+	9.512		302.0429	92519-95-4	FBF	99.23		99.23

Cpd 1579: N1,N10-Diferuloylspermidine

Name	Formula	RT	RI	Mass	Diff (Tgt, ppm)	CAS	ID Source	Score	Algorithm
N1,N10-Diferuloylspermidine	C27 H35 N3 O6	9.651		497.2529	0.61	70185-61-4	FBF	99.33	FBF

Species	m/z	Score (Tgt)	Score (Lib)	Score (DB)	Score (MFG)	Score (RT)
(M+H)+	498	99.33				

Compound Chromatograms (overlaid)

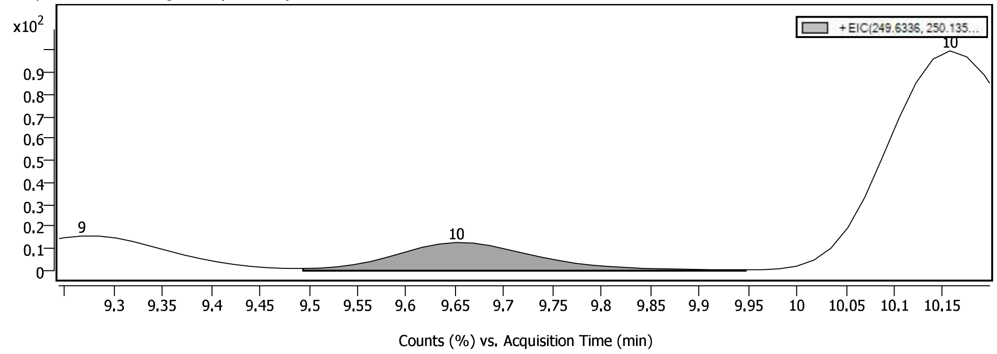

Structure

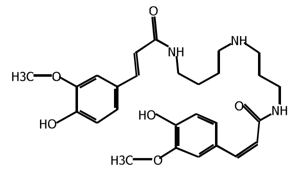

Compound Spectra (overlaid)

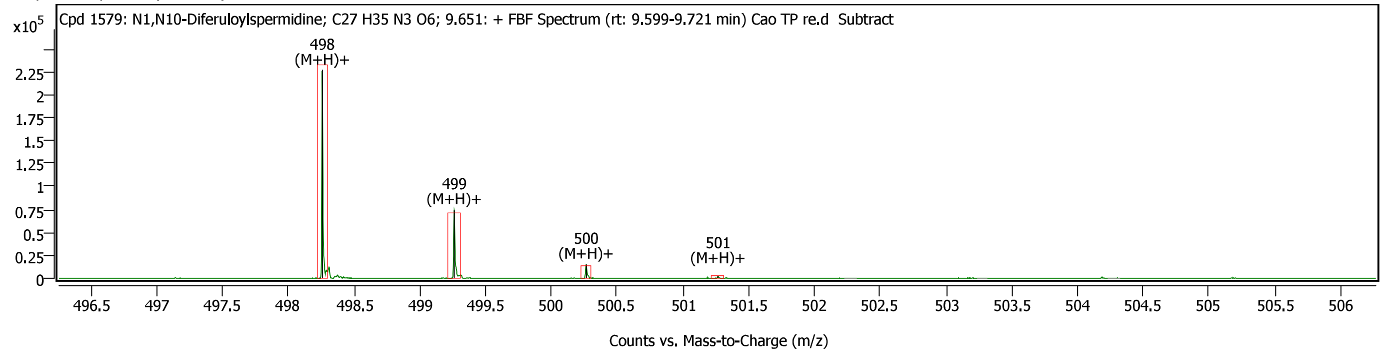

Compound ID Table

Name	Formula	Species	RT	RT Diff	Mass	CAS	ID Source	Score	Score (Lib)	Score (Tgt)
N1,N10-Diferuloylspermidine	C27 H35 N3 O6	(M+H)+	9.651		497.2529	70185-61-4	FBF	99.33		99.33

Cpd 757: 3-O-Methylquercetin

Name	Formula	RT	RI	Mass	Diff (Tgt, ppm)	CAS	ID Source	Score	Algorithm
3-O-Methylquercetin	C16 H12 O7	9.791		316.0586	1.09	1486-70-0	M-FBF-FragConfirm	98.34	FBF

Species	m/z	Score (Tgt)	Score (Lib)	Score (DB)	Score (MFG)	Score (RT)
(M+H)+	317	98.34				

Compound Chromatograms (overlaid)

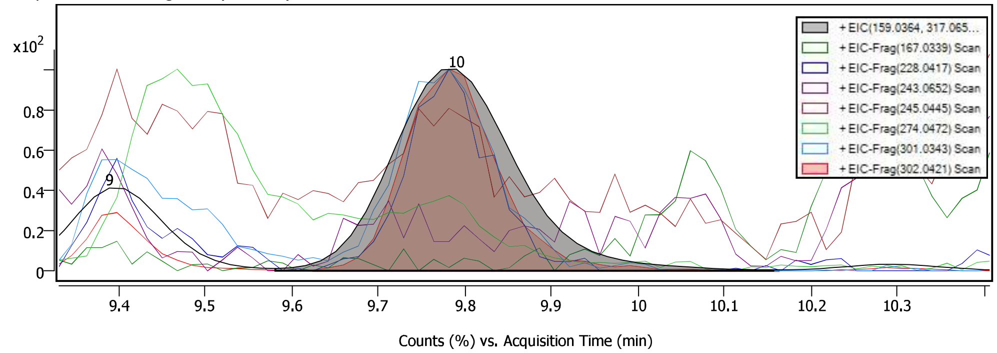

Structure

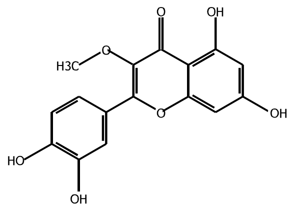

Coelution Plot

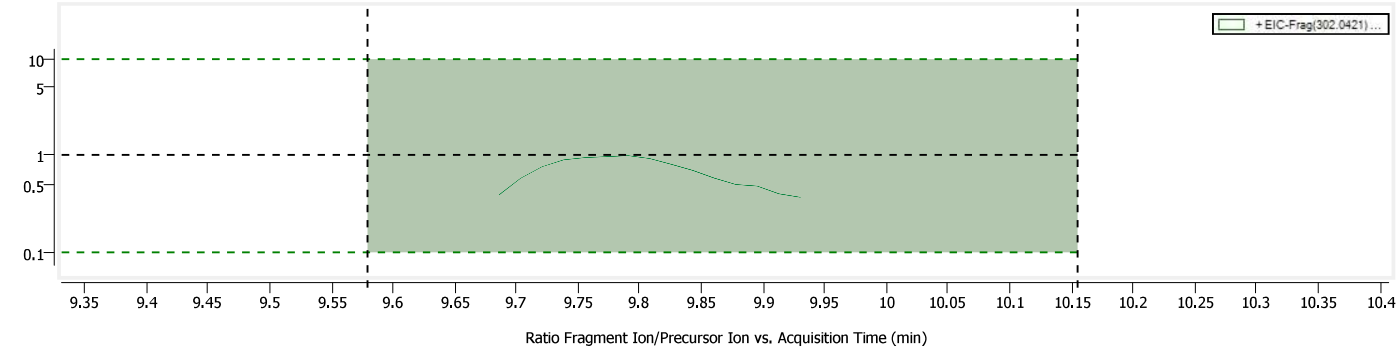

Compound Spectra (overlaid)

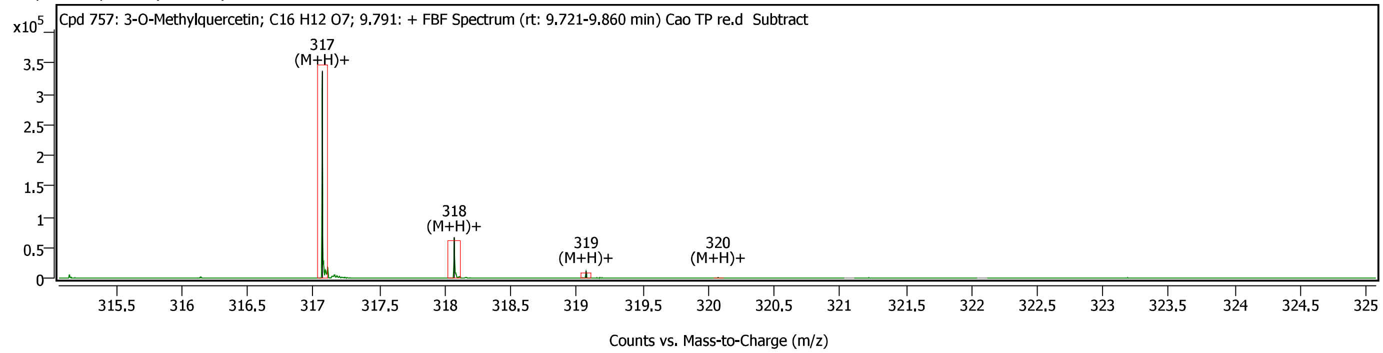

Fragment Spectrum (clean)

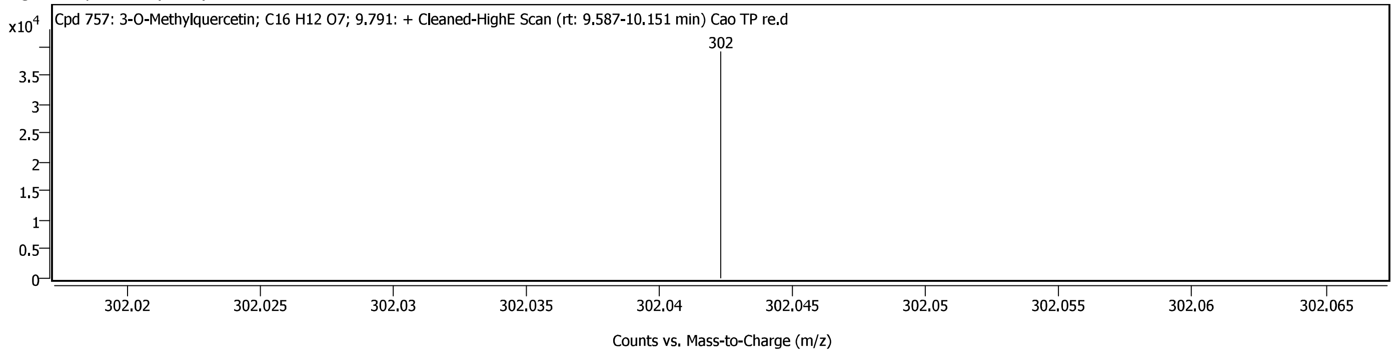

Fragment Spectrum (raw)

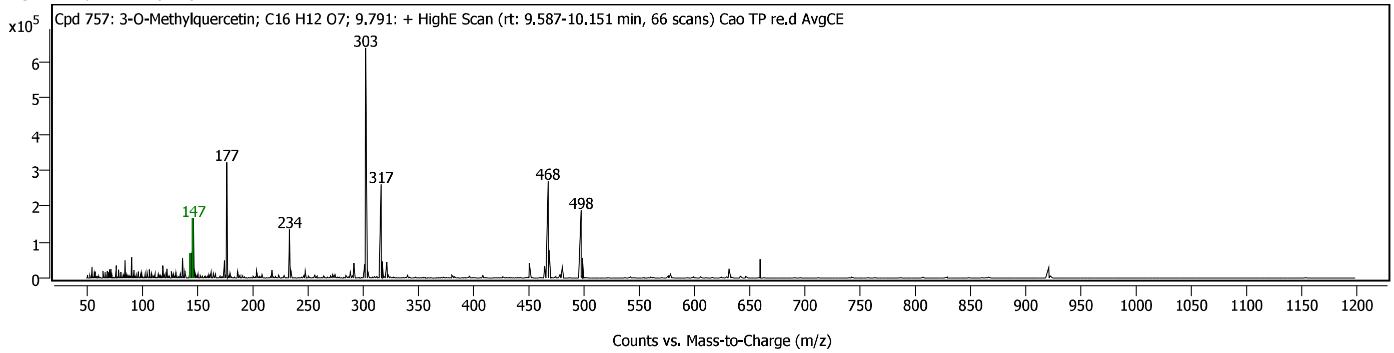

Compound ID Table

Name	Formula	Species	RT	RT Diff	Mass	CAS	ID Source	Score	Score (Lib)	Score (Tgt)
3-O-Methylquercetin	C16 H12 O7	(M+H)+	9.791		316.0586	1486-70-0	FBF-FragConfirm	98.34		98.34
3,5,6,7-Tetrahydroxy-4'-methoxyflavone	C16 H12 O7	(M+H)+	9.791		316.0586		FBF-FragConfirm	98.34		98.34
3'-O-Methyltricetin	C16 H12 O7	(M+H)+	9.791		316.0586		FBF-FragConfirm	98.34		98.34
6-Methoxykaempferol	C16 H12 O7	(M+H)+	9.791		316.0586		FBF-FragConfirm	98.34		98.34
6-Hydroxyluteolin 4'-methyl ether	C16 H12 O7	(M+H)+	9.791		316.0586		FBF-FragConfirm	98.34		98.34
6a,7-Dihydroxymaackiain	C16 H12 O7	(M+H)+	9.791		316.0586		FBF-FragConfirm	98.34		98.34
5,6,7,4'-Tetrahydroxy-8-methoxyisoflavone	C16 H12 O7	(M+H)+	9.791		316.0586		FBF-FragConfirm	98.34		98.34
5,6,7,4'-Tetrahydroxy-3'-methoxyisoflavone	C16 H12 O7	(M+H)+	9.791		316.0586		FBF-FragConfirm	98.34		98.34
5,2',3'-Trihydroxy-6,7-methylenedioxyflavanone	C16 H12 O7	(M+H)+	9.791		316.0586		FBF-FragConfirm	98.34		98.34
7-Methoxy-3,6,3',4'-tetrahydroxyflavone	C16 H12 O7	(M+H)+	9.791		316.0586		FBF-FragConfirm	98.34		98.34
3'-O-Methylmelanoxetin	C16 H12 O7	(M+H)+	9.791		316.0586		FBF-FragConfirm	98.34		98.34
Pedalitin	C16 H12 O7	(M+H)+	9.791		316.0586		FBF-FragConfirm	98.34		98.34
3-Methylherbacetin	C16 H12 O7	(M+H)+	9.791		316.0586		FBF-FragConfirm	98.34		98.34
Azaleatin	C16 H12 O7	(M+H)+	9.791		316.0586		FBF-FragConfirm	98.34		98.34
3,7,3',4'-Tetrahydroxy-8-methoxyflavone	C16 H12 O7	(M+H)+	9.791		316.0586		FBF-FragConfirm	98.34		98.34
3,5,8,2'-Tetrahydroxy-7-methoxyflavone	C16 H12 O7	(M+H)+	9.791		316.0586		FBF-FragConfirm	98.34		98.34
3,5,6,4'-Tetrahydroxy-7-methoxyflavone	C16 H12 O7	(M+H)+	9.791		316.0586		FBF-FragConfirm	98.34		98.34
1,3,5,8-Tetrahydroxy-6-methoxy-2-methylanthraquinone	C16 H12 O7	(M+H)+	9.791		316.0586	101508-13-8	FBF-FragConfirm	98.34		98.34
3-Methoxy-6,7,3',4'-tetrahydroxyflavone	C16 H12 O7	(M+H)+	9.791		316.0586		FBF-FragConfirm	98.34		98.34
(-)-4,9,11,12a-Tetrahydroxyrotenone	C16 H12 O7	(M+H)+	9.791		316.0586		FBF-FragConfirm	98.34		98.34
5,6,7,4'-Tetrahydroxy-3-methoxyflavone	C16 H12 O7	(M+H)+	9.791		316.0586		FBF-FragConfirm	98.34		98.34
Herbacetin 4'-methyl ether	C16 H12 O7	(M+H)+	9.791		316.0586		FBF-FragConfirm	98.34		98.34
Pinoquercetin	C16 H12 O7	(M+H)+	9.791		316.0586	491-49-6	FBF-FragConfirm	98.34		98.34
Vogeletin	C16 H12 O7	(M+H)+	9.791		316.0586		FBF-FragConfirm	98.34		98.34
Tricetin 4'-methyl ether	C16 H12 O7	(M+H)+	9.791		316.0586		FBF-FragConfirm	98.34		98.34
Carajuflavone	C16 H12 O7	(M+H)+	9.791		316.0586		FBF-FragConfirm	98.34		98.34
Tamarixetin	C16 H12 O7	(M+H)+	9.791		316.0586	603-61-2	FBF-FragConfirm	98.34		98.34
Sexangularetin	C16 H12 O7	(M+H)+	9.791		316.0586		FBF-FragConfirm	98.34		98.34
Rhamnetin	C16 H12 O7	(M+H)+	9.791		316.0586	90-19-7	FBF-FragConfirm	98.34		98.34
Capillarisin	C16 H12 O7	(M+H)+	9.791		316.0586	56365-38-9	FBF-FragConfirm	98.34		98.34
Pollenitin	C16 H12 O7	(M+H)+	9.791		316.0586		FBF-FragConfirm	98.34		98.34
Transilitin	C16 H12 O7	(M+H)+	9.791		316.0586		FBF-FragConfirm	98.34		98.34
Onopordin	C16 H12 O7	(M+H)+	9.791		316.0586		FBF-FragConfirm	98.34		98.34
Nepetin	C16 H12 O7	(M+H)+	9.791		316.0586		FBF-FragConfirm	98.34		98.34
Milimorin	C16 H12 O7	(M+H)+	9.791		316.0586		FBF-FragConfirm	98.34		98.34
Junipegenin A	C16 H12 O7	(M+H)+	9.791		316.0586		FBF-FragConfirm	98.34		98.34
Isorhamnetin	C16 H12 O7	(M+H)+	9.791		316.0586	480-19-3	FBF-FragConfirm	98.34		98.34
Hypolaetin 3'-methyl ether	C16 H12 O7	(M+H)+	9.791		316.0586		FBF-FragConfirm	98.34		98.34
Nodifloretin	C16 H12 O7	(M+H)+	9.791		316.0586		FBF-FragConfirm	98.34		98.34

Cpd 763: 7-O-Methylgossypetin 3-rhamnoside

Name	Formula	RT	RI	Mass	Diff (Tgt, ppm)	CAS	ID Source	Score	Algorithm
7-O-Methylgossypetin 3-rhamnoside	C22 H22 O12	9.791		478.1114	0.67	56768-33-3	M-FBF-FragConfirm	98.78	FBF

Compound Screening Report

Species	m/z	Score (Tgt)	Score (Lib)	Score (DB)	Score (MFG)	Score (RT)
(M+H)+	479	98.78				

Compound Chromatograms (overlaid)

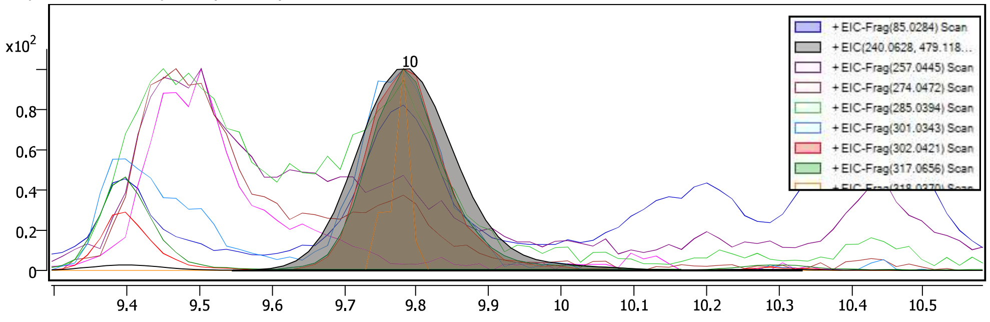

Structure

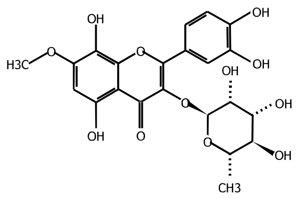

Coelution Plot

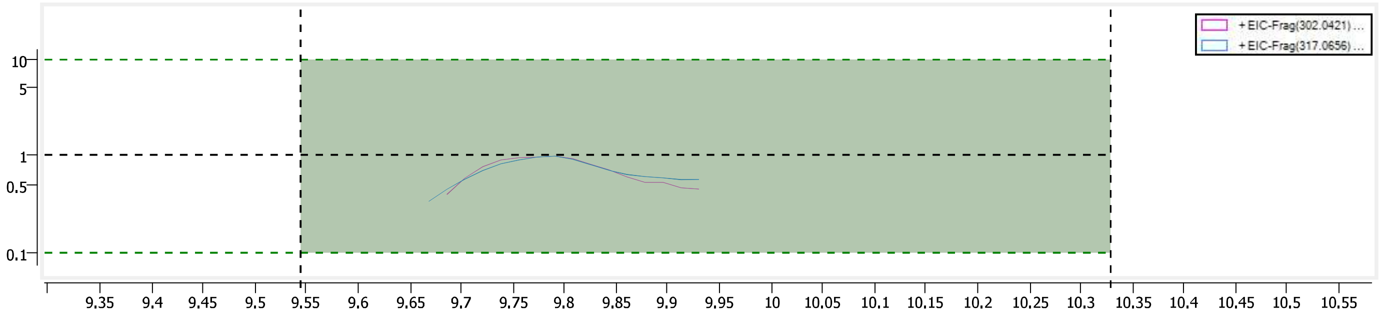

Compound Spectra (overlaid)

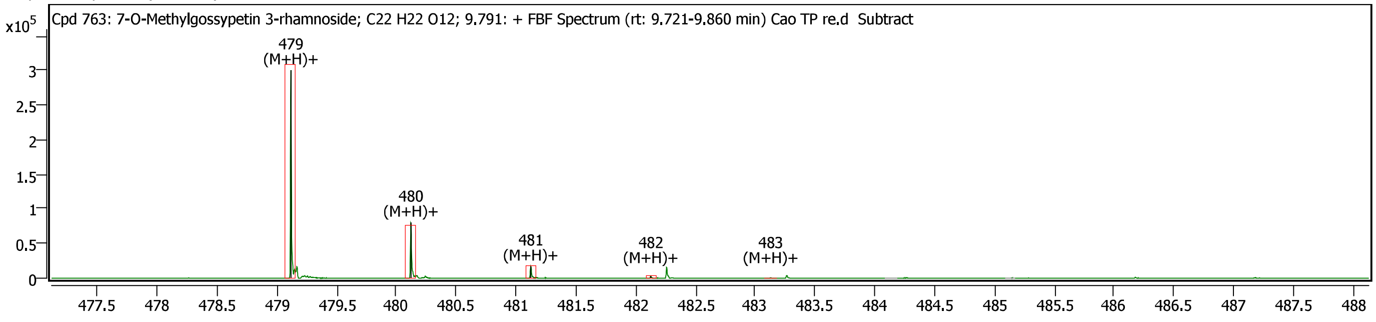

Fragment Spectrum (clean)

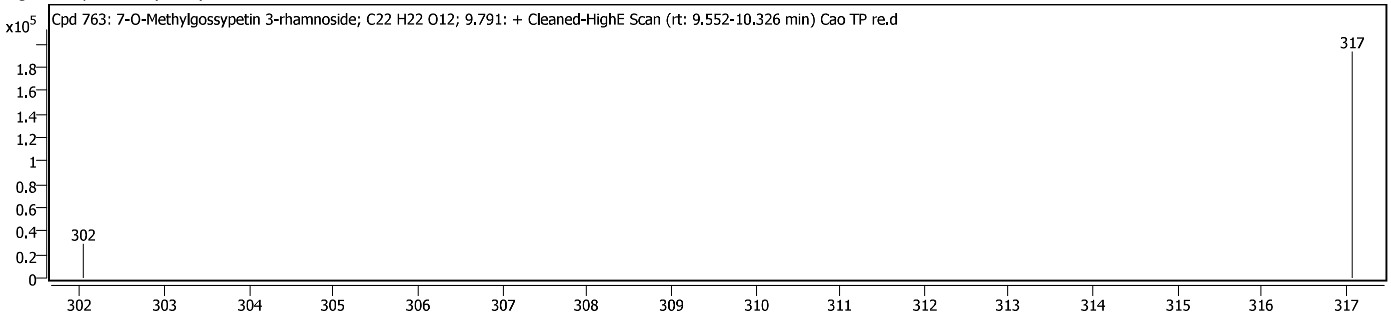

Fragment Spectrum (raw)

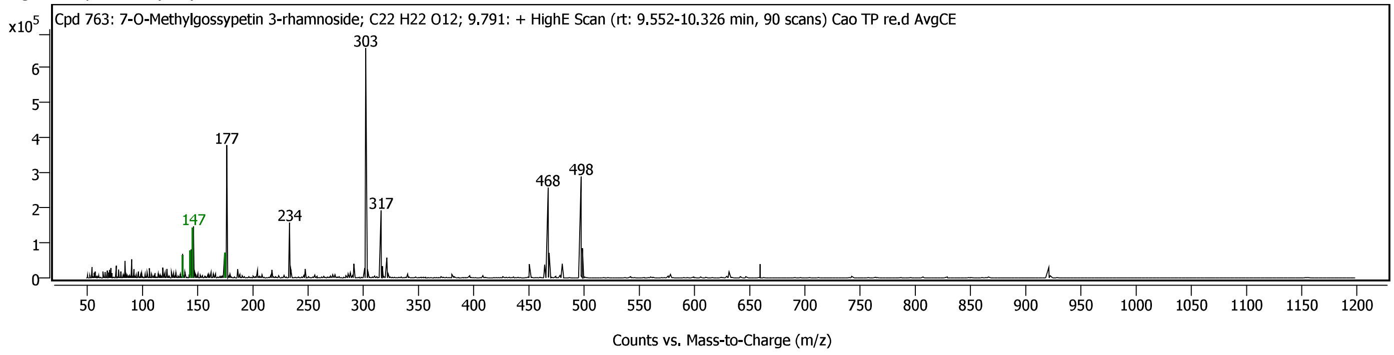

Compound ID Table

Name	Formula	Species	RT	RT Diff	Mass	CAS	ID Source	Score	Score (Lib)	Score (Tgt)
7-O-Methylgossypetin 3-rhamnoside	C22 H22 O12	(M+H)+	9.791		478.1114	56768-33-3	FBF-FragConfirm	98.78		98.78
Azaleatin 3-glucoside	C22 H22 O12	(M+H)+	9.791		478.1114		FBF-FragConfirm	98.78		98.78
6-Methoxyluteolin 3'-glucoside	C22 H22 O12	(M+H)+	9.791		478.1114	112208-84-1	FBF-FragConfirm	98.78		98.78
8-Hydroxyluteolin 3'-methyl ether 7-glucoside	C22 H22 O12	(M+H)+	9.791		478.1114		FBF-FragConfirm	98.78		98.78
8-Hydroxyluteolin 4'-methyl ether 8-glucoside	C22 H22 O12	(M+H)+	9.791		478.1114		FBF-FragConfirm	98.78		98.78
Alliumoside A	C22 H22 O12	(M+H)+	9.791		478.1114	58902-89-9	FBF-FragConfirm	98.78		98.78
Azaleatin 3-galactoside	C22 H22 O12	(M+H)+	9.791		478.1114		FBF-FragConfirm	98.78		98.78
Eupatolitin 3-apioside	C22 H22 O12	(M+H)+	9.791		478.1114		FBF-FragConfirm	98.78		98.78
Estragonoside	C22 H22 O12	(M+H)+	9.791		478.1114	181020-33-7	FBF-FragConfirm	98.78		98.78
Eupafolin 4'-glucoside	C22 H22 O12	(M+H)+	9.791		478.1114	112208-83-0	FBF-FragConfirm	98.78		98.78
Europetin 3-rhamnoside	C22 H22 O12	(M+H)+	9.791		478.1114		FBF-FragConfirm	98.78		98.78
Herbacetin 7-methyl ether 3-glucoside	C22 H22 O12	(M+H)+	9.791		478.1114		FBF-FragConfirm	98.78		98.78
6-Methoxyluteolin 7-glucoside	C22 H22 O12	(M+H)+	9.791		478.1114		FBF-FragConfirm	98.78		98.78
6-Hydroxykaempferol 3-methyl ether 7-glucoside	C22 H22 O12	(M+H)+	9.791		478.1114		FBF-FragConfirm	98.78		98.78
6-Methoxykaempferol 7-glucoside	C22 H22 O12	(M+H)+	9.791		478.1114		FBF-FragConfirm	98.78		98.78
Isorhamnetin 3-galactoside	C22 H22 O12	(M+H)+	9.791		478.1114	5041-82-7	FBF-FragConfirm	98.78		98.78
6-Methoxykaempferol 3-glucoside	C22 H22 O12	(M+H)+	9.791		478.1114		FBF-FragConfirm	98.78		98.78
6-Methoxykaempferol 3-galactoside	C22 H22 O12	(M+H)+	9.791		478.1114		FBF-FragConfirm	98.78		98.78
6-Hydroxyluteolin 7-methyl ether 6-glucoside	C22 H22 O12	(M+H)+	9.791		478.1114		FBF-FragConfirm	98.78		98.78
6-Hydroxyluteolin 7-methyl ether 6-galactoside	C22 H22 O12	(M+H)+	9.791		478.1114		FBF-FragConfirm	98.78		98.78
6-Hydroxykaempferol 4'-methyl ether 7-glucoside	C22 H22 O12	(M+H)+	9.791		478.1114		FBF-FragConfirm	98.78		98.78
Isorhamnetin 3-glucoside	C22 H22 O12	(M+H)+	9.791		478.1114		FBF-FragConfirm	98.78		98.78
6-Hydroxykaempferol 3-methyl ether 6-glucoside	C22 H22 O12	(M+H)+	9.791		478.1114		FBF-FragConfirm	98.78		98.78
5,2',4',5'-Tetrahydroxy-7-methoxy-4-phenylcoumarin 5-O-glucoside	C22 H22 O12	(M+H)+	9.791		478.1114		FBF-FragConfirm	98.78		98.78
2-O-(4-Hydroxycinnamoyl)-1-O-galloyl-beta-D-glucopyranoside	C22 H22 O12	(M+H)+	9.791		478.1114	94356-18-0	FBF-FragConfirm	98.78		98.78
1,3,5,8-Tetrahydroxy-6-methoxy-2-methylanthraquinone 8-O-b-D-glucoside	C22 H22 O12	(M+H)+	9.791		478.1114	101508-15-0	FBF-FragConfirm	98.78		98.78
Pedaliin	C22 H22 O12	(M+H)+	9.791		478.1114	22860-72-6	FBF-FragConfirm	98.78		98.78
8-C-Rhamnosyleuropetin	C22 H22 O12	(M+H)+	9.791		478.1114		FBF-FragConfirm	98.78		98.78
Annulatin 7-rhamnoside	C22 H22 O12	(M+H)+	9.791		478.1114		FBF-FragConfirm	98.78		98.78
Tricetin 3'-methyl ether 7-glucoside	C22 H22 O12	(M+H)+	9.791		478.1114		FBF-FragConfirm	98.78		98.78
Quercetin 3-methyl ether 7-glucoside	C22 H22 O12	(M+H)+	9.791		478.1114		FBF-FragConfirm	98.78		98.78
Isorhamnetin 7-glucoside	C22 H22 O12	(M+H)+	9.791		478.1114		FBF-FragConfirm	98.78		98.78
Tamarixin	C22 H22 O12	(M+H)+	9.791		478.1114		FBF-FragConfirm	98.78		98.78
Tamarixetin 7-glucoside	C22 H22 O12	(M+H)+	9.791		478.1114		FBF-FragConfirm	98.78		98.78
Tamarixetin 3-galactoside	C22 H22 O12	(M+H)+	9.791		478.1114		FBF-FragConfirm	98.78		98.78
Syringetin 3-xyloside	C22 H22 O12	(M+H)+	9.791		478.1114		FBF-FragConfirm	98.78		98.78
Sexangularetin 3-glucoside	C22 H22 O12	(M+H)+	9.791		478.1114		FBF-FragConfirm	98.78		98.78
Rhamnetin 5-glucoside	C22 H22 O12	(M+H)+	9.791		478.1114		FBF-FragConfirm	98.78		98.78
Rhamnetin 3-glucoside	C22 H22 O12	(M+H)+	9.791		478.1114		FBF-FragConfirm	98.78		98.78
Rhamnetin 3-galactoside	C22 H22 O12	(M+H)+	9.791		478.1114		FBF-FragConfirm	98.78		98.78
Ranupenin 3-rhamnoside	C22 H22 O12	(M+H)+	9.791		478.1114		FBF-FragConfirm	98.78		98.78
Quercetin 3-methyl ether 5-glucoside	C22 H22 O12	(M+H)+	9.791		478.1114		FBF-FragConfirm	98.78		98.78
Sexangularetin 3-galactoside	C22 H22 O12	(M+H)+	9.791		478.1114		FBF-FragConfirm	98.78		98.78
Isorhamnetin 4'-glucoside	C22 H22 O12	(M+H)+	9.791		478.1114		FBF-FragConfirm	98.78		98.78
Quercetin 3-methyl ether 4'-glucoside	C22 H22 O12	(M+H)+	9.791		478.1114		FBF-FragConfirm	98.78		98.78
Nepetin 4'-glucoside	C22 H22 O12	(M+H)+	9.791		478.1114		FBF-FragConfirm	98.78		98.78
Laricitrin 3-rhamnoside	C22 H22 O12	(M+H)+	9.791		478.1114		FBF-FragConfirm	98.78		98.78
Quercetin 3-methyl ether 3'-glucoside	C22 H22 O12	(M+H)+	9.791		478.1114		FBF-FragConfirm	98.78		98.78
Keyakinin B	C22 H22 O12	(M+H)+	9.791		478.1114		FBF-FragConfirm	98.78		98.78
Mearnsitrin	C22 H22 O12	(M+H)+	9.791		478.1114	30484-88-9	FBF-FragConfirm	98.78		98.78
Myricetin 3,4'-dimethyl ether 3'-xyloside	C22 H22 O12	(M+H)+	9.791		478.1114		FBF-FragConfirm	98.78		98.78
Myricetin 5-methyl ether 3-rhamnoside	C22 H22 O12	(M+H)+	9.791		478.1114		FBF-FragConfirm	98.78		98.78
Nepitrin	C22 H22 O12	(M+H)+	9.791		478.1114	569-90-4	FBF-FragConfirm	98.78		98.78
Patuletin 3-rhamnoside	C22 H22 O12	(M+H)+	9.791		478.1114		FBF-FragConfirm	98.78		98.78
Quercetin 3-methyl ether 7-galactoside	C22 H22 O12	(M+H)+	9.791		478.1114		FBF-FragConfirm	98.78		98.78
Pollenin B	C22 H22 O12	(M+H)+	9.791		478.1114	30484-94-7	FBF-FragConfirm	98.78		98.78

Compound Screening Report

Cpd 1636: 5,7-Dihydroxy-2-(4-hydroxy-3-methoxyphenyl)-3-[3,4,5-trihydroxy-6-[[(2R,3R,4R,5R,6S)-3,4,5-trihydroxy-6-methyloxan-2-yl]oxymethyl]oxan-2-yl]oxychromen-4-one

Name	Formula	RT	RI	Mass	Diff (Tgt, ppm)	CAS	ID Source	Score	Algorithm
5,7-Dihydroxy-2-(4-hydroxy-3-methoxyphenyl)-3-[3,4,5-trihydroxy-6-[[(2R,3R,4R,5R,6S)-3,4,5-trihydroxy-6-methyloxan-2-yl]oxymethyl]oxan-2-yl]oxychromen-4-one	C28 H32 O16	9.791		624.1694	0.65	1393485-18-1	M-FBF-FragConfirm	99.65	FBF

Species	m/z	Score (Tgt)	Score (Lib)	Score (DB)	Score (MFG)	Score (RT)
(M+H)+	625	99.65				

Compound Chromatograms (overlaid)

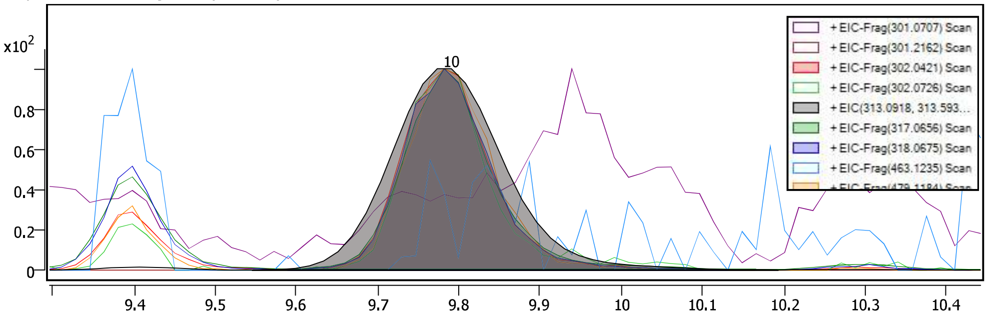

Structure

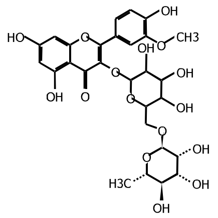

Coelution Plot

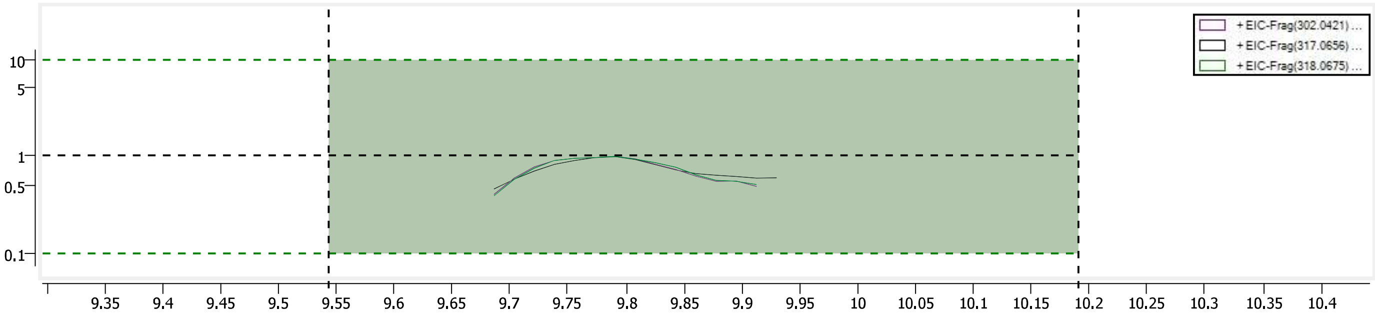

Compound Spectra (overlaid)

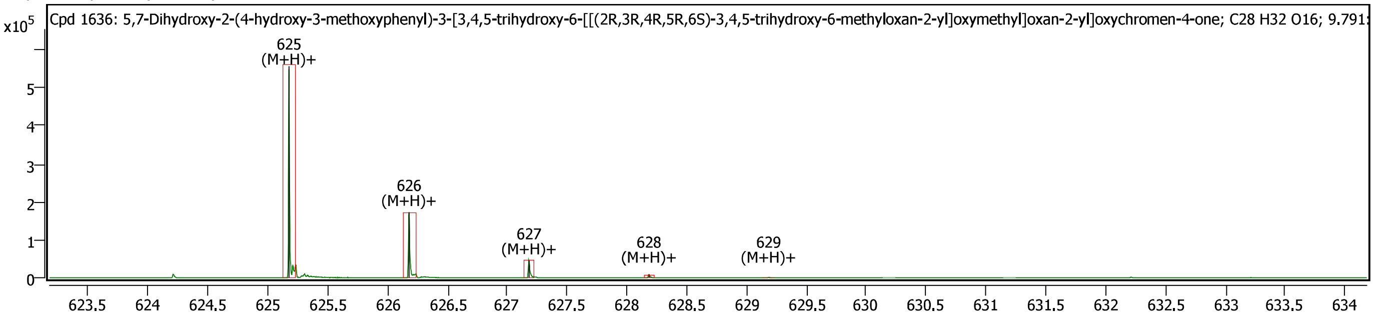

Fragment Spectrum (clean)

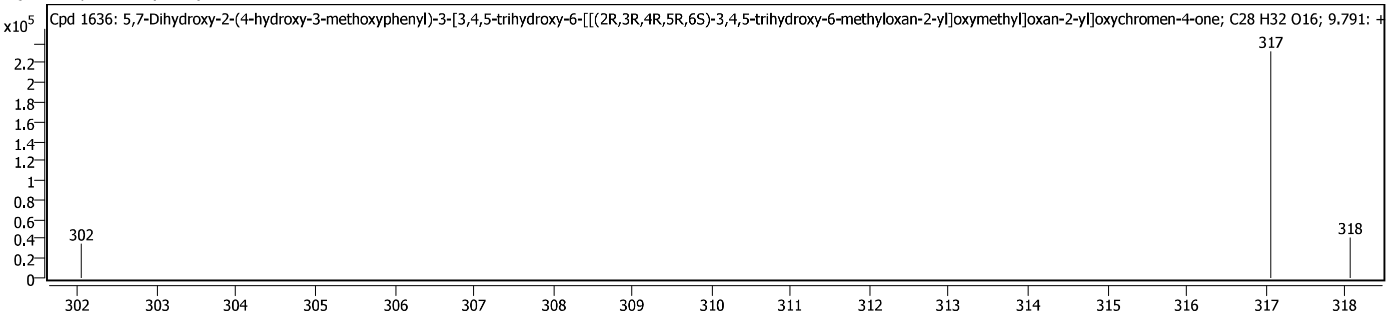

Fragment Spectrum (raw)

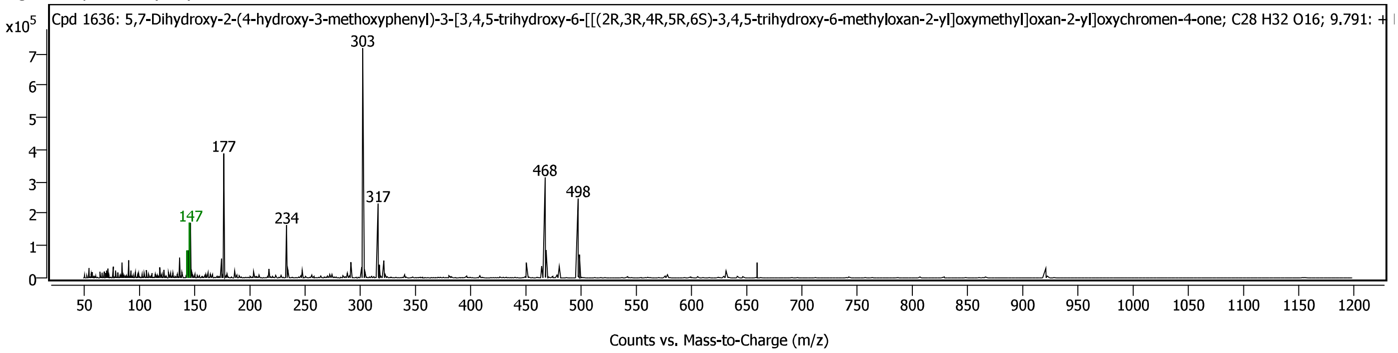

Compound Screening Report

Compound ID Table

Name	Formula	Species	RT	RT Diff	Mass	CAS	ID Source	Score	Score (Lib)	Score (Tgt)
5,7-Dihydroxy-2-(4-hydroxy-3-methoxyphenyl)-3-[3,4,5-trihydroxy-6-[[(2R,3R,4R,5R,6S)-3,4,5-trihydroxy-6-methyloxan-2-yl]oxymethyl]oxan-2-yl]oxychromen-4-one	C28 H32 O16	(M+H)+	9.791		624.1694	1393485-18-1	FBF-FragConfirm	99.65		99.65
Isorhamnetin 3-O-[b-L-rhamnofuranosyl-(1->6)-D-glucopyranoside]	C28 H32 O16	(M+H)+	9.791		624.1694	37138-79-7	FBF-FragConfirm	99.65		99.65
Isoscoparin 2''-O-glucoside	C28 H32 O16	(M+H)+	9.791		624.1694		FBF-FragConfirm	99.65		99.65
Isoscoparin 4'-O-glucoside	C28 H32 O16	(M+H)+	9.791		624.1694		FBF-FragConfirm	99.65		99.65
Isoscoparin 7-O-glucoside	C28 H32 O16	(M+H)+	9.791		624.1694		FBF-FragConfirm	99.65		99.65
Isorhamnetin 3-rhamnoside-7-glucoside	C28 H32 O16	(M+H)+	9.791		624.1694		FBF-FragConfirm	99.65		99.65
Isorhamnetin 3-rhamnosyl-(1->2)-galactoside	C28 H32 O16	(M+H)+	9.791		624.1694		FBF-FragConfirm	99.65		99.65
Isorhamnetin 3-robinobioside	C28 H32 O16	(M+H)+	9.791		624.1694		FBF-FragConfirm	99.65		99.65
Isorhamnetin 4'-neohesperidoside	C28 H32 O16	(M+H)+	9.791		624.1694		FBF-FragConfirm	99.65		99.65
Kaempferol 5-methyl ether 3-galactoside-4'-glucoside	C28 H32 O16	(M+H)+	9.791		624.1694		FBF-FragConfirm	99.65		99.65
6-C-Glucopyranosyl-8-C-arabinopyranosyltricin	C28 H32 O16	(M+H)+	9.791		624.1694		FBF-FragConfirm	99.65		99.65
Isorhamnetin 3-neohesperidoside	C28 H32 O16	(M+H)+	9.791		624.1694		FBF-FragConfirm	99.65		99.65
Isoscutellarein 4'-methyl ether 7-allosyl-(1->2)-glucoside	C28 H32 O16	(M+H)+	9.791		624.1694		FBF-FragConfirm	99.65		99.65
Kaempferide 3,7-diglucoside	C28 H32 O16	(M+H)+	9.791		624.1694		FBF-FragConfirm	99.65		99.65
Luteolin 3'-methyl ether 5,4'-diglucoside	C28 H32 O16	(M+H)+	9.791		624.1694		FBF-FragConfirm	99.65		99.65
Keioside	C28 H32 O16	(M+H)+	9.791		624.1694	107740-46-5	FBF-FragConfirm	99.65		99.65
Knoutinoside	C28 H32 O16	(M+H)+	9.791		624.1694		FBF-FragConfirm	99.65		99.65
Isorhamnetin 3-O-[b-D-glucopyranosyl-(1->2)-a-L-rhamnopyranoside]	C28 H32 O16	(M+H)+	9.791		624.1694		FBF-FragConfirm	99.65		99.65
Isorhamnetin 3-glucoside-7-rhamnoside	C28 H32 O16	(M+H)+	9.791		624.1694		FBF-FragConfirm	99.65		99.65
Isorhamnetin 3-glucoside 4'-rhamnoside	C28 H32 O16	(M+H)+	9.791		624.1694	28289-13-6	FBF-FragConfirm	99.65		99.65
6,8-Diglucosyldiosmetin	C28 H32 O16	(M+H)+	9.791		624.1694	98813-28-6	FBF-FragConfirm	99.65		99.65
Rhamnocitrin 3,4'-diglucoside	C28 H32 O16	(M+H)+	9.791		624.1694		FBF-FragConfirm	99.65		99.65
Isorhamnetin 3-glucoside-4'-rhamnoside	C28 H32 O16	(M+H)+	9.791		624.1694		FBF-FragConfirm	99.65		99.65
Luteolin 3'-methyl ether 7-allosyl-(1->2)-glucoside	C28 H32 O16	(M+H)+	9.791		624.1694		FBF-FragConfirm	99.65		99.65
3,8-Di-C-glucopyranosyldiosmetin	C28 H32 O16	(M+H)+	9.791		624.1694		FBF-FragConfirm	99.65		99.65
3,8-Diglucosyldiosmetin	C28 H32 O16	(M+H)+	9.791		624.1694	97218-32-1	FBF-FragConfirm	99.65		99.65
6,8-Di-C-glucopyranosyldiosmetin	C28 H32 O16	(M+H)+	9.791		624.1694		FBF-FragConfirm	99.65		99.65
6-C-Arabinopyranosyl-8-C-glucopyranosyltricin	C28 H32 O16	(M+H)+	9.791		624.1694		FBF-FragConfirm	99.65		99.65
Isorhamnetin 3-galactoside-7-rhamnoside	C28 H32 O16	(M+H)+	9.791		624.1694		FBF-FragConfirm	99.65		99.65
6-C-Rhamnopyranosylrhamnetin 3-O-glucopyranoside	C28 H32 O16	(M+H)+	9.791		624.1694		FBF-FragConfirm	99.65		99.65
6-Methoxykaempferol 3-robinobioside	C28 H32 O16	(M+H)+	9.791		624.1694		FBF-FragConfirm	99.65		99.65
Azaleatin 3-rutinoside	C28 H32 O16	(M+H)+	9.791		624.1694		FBF-FragConfirm	99.65		99.65
Calendoflavoside	C28 H32 O16	(M+H)+	9.791		624.1694	55033-90-4	FBF-FragConfirm	99.65		99.65
Chrysoeriol 7-O-8-C-bisglucoside	C28 H32 O16	(M+H)+	9.791		624.1694		FBF-FragConfirm	99.65		99.65
Crosatoside A	C28 H32 O16	(M+H)+	9.791		624.1694	139742-27-1	FBF-FragConfirm	99.65		99.65
Luteoayamenin	C28 H32 O16	(M+H)+	9.791		624.1694		FBF-FragConfirm	99.65		99.65
Luteolin 3'-methyl ether 7,4'-diglucoside	C28 H32 O16	(M+H)+	9.791		624.1694		FBF-FragConfirm	99.65		99.65
Tamarixetin 7-rutinoside	C28 H32 O16	(M+H)+	9.791		624.1694		FBF-FragConfirm	99.65		99.65
Sexangularetin 3-rutinoside	C28 H32 O16	(M+H)+	9.791		624.1694		FBF-FragConfirm	99.65		99.65
Stellarin 2	C28 H32 O16	(M+H)+	9.791		624.1694		FBF-FragConfirm	99.65		99.65
Scoparin 2''-O-glucoside	C28 H32 O16	(M+H)+	9.791		624.1694		FBF-FragConfirm	99.65		99.65
Sexangularetin 3-glucoside 7-rhamnoside	C28 H32 O16	(M+H)+	9.791		624.1694	47850-51-1	FBF-FragConfirm	99.65		99.65
Sexangularetin 3-glucoside-7-rhamnoside	C28 H32 O16	(M+H)+	9.791		624.1694		FBF-FragConfirm	99.65		99.65
Sexangularetin 3-neohesperidoside	C28 H32 O16	(M+H)+	9.791		624.1694		FBF-FragConfirm	99.65		99.65
Sexangularetin 3-rhamnoside-7-glucoside	C28 H32 O16	(M+H)+	9.791		624.1694		FBF-FragConfirm	99.65		99.65
Subulin	C28 H32 O16	(M+H)+	9.791		624.1694		FBF-FragConfirm	99.65		99.65
Tricetin 7-methyl ether 3'-glucoside-5'-rhamnoside	C28 H32 O16	(M+H)+	9.791		624.1694		FBF-FragConfirm	99.65		99.65
Tectorigenin 7-O-gentiobioside	C28 H32 O16	(M+H)+	9.791		624.1694		FBF-FragConfirm	99.65		99.65
Swertiajaponin 3'-O-glucoside	C28 H32 O16	(M+H)+	9.791		624.1694		FBF-FragConfirm	99.65		99.65
Syringetin 3-rhamnosyl-(1->5)-alpha-L-arabinofuranoside	C28 H32 O16	(M+H)+	9.791		624.1694		FBF-FragConfirm	99.65		99.65
Tamarixetin 3-neohesperidoside	C28 H32 O16	(M+H)+	9.791		624.1694		FBF-FragConfirm	99.65		99.65
Luteolin 4'-methyl ether 7-sophoroside	C28 H32 O16	(M+H)+	9.791		624.1694		FBF-FragConfirm	99.65		99.65
Tamarixetin 3-rutinoside	C28 H32 O16	(M+H)+	9.791		624.1694		FBF-FragConfirm	99.65		99.65
Luteolin 3'-methyl ether 7-mannosyl-(1->2)-alloside	C28 H32 O16	(M+H)+	9.791		624.1694		FBF-FragConfirm	99.65		99.65
Tamarixetin 3-robinobioside	C28 H32 O16	(M+H)+	9.791		624.1694		FBF-FragConfirm	99.65		99.65
Rhamnocitrin 3-galactoside-4'-glucoside	C28 H32 O16	(M+H)+	9.791		624.1694		FBF-FragConfirm	99.65		99.65
Narcissin	C28 H32 O16	(M+H)+	9.791		624.1694		FBF-FragConfirm	99.65		99.65
Pasternoside	C28 H32 O16	(M+H)+	9.791		624.1694	10576-85-9	FBF-FragConfirm	99.65		99.65
Scoparin 2''-glucoside	C28 H32 O16	(M+H)+	9.791		624.1694	124902-15-4	FBF-FragConfirm	99.65		99.65
Rhamnazin 3-glucosyl-(1->5)-alpha-L-arabinofuranoside	C28 H32 O16	(M+H)+	9.791		624.1694		FBF-FragConfirm	99.65		99.65
Rhamnocitrin 3-glucosyl-(1->2)-galactoside	C28 H32 O16	(M+H)+	9.791		624.1694		FBF-FragConfirm	99.65		99.65
Mearnsetin 3,7-dirhamnoside	C28 H32 O16	(M+H)+	9.791		624.1694		FBF-FragConfirm	99.65		99.65
Myricetin 3,4'-dimethyl ether 7-rhamnoside-3'-xyloside	C28 H32 O16	(M+H)+	9.791		624.1694		FBF-FragConfirm	99.65		99.65
Patuletin 3,7-dirhamnoside	C28 H32 O16	(M+H)+	9.791		624.1694		FBF-FragConfirm	99.65		99.65
Rhamnetin 3-rutinoside	C28 H32 O16	(M+H)+	9.791		624.1694		FBF-FragConfirm	99.65		99.65
Scoparin 6''-O-glucoside	C28 H32 O16	(M+H)+	9.791		624.1694		FBF-FragConfirm	99.65		99.65

Compound Screening Report

Compound ID Table

Name	Formula	Species	RT	RT Diff	Mass	CAS	ID Source	Score	Score (Lib)	Score (Tgt)
Quercetin 3,4'-dimethyl ether 7-alpha-L-Arabinofuranosyl-(1->6)-glucoside	C28 H32 O16	(M+H)+	9.791		624.1694		FBF-FragConfirm	99.65		99.65
Myricetin 7-methyl ether 3,4'-di-O-alpha-L-rhamnopyranoside	C28 H32 O16	(M+H)+	9.791		624.1694		FBF-FragConfirm	99.65		99.65
Rhamnazin 3-xylosyl-(1->2)-glucoside	C28 H32 O16	(M+H)+	9.791		624.1694		FBF-FragConfirm	99.65		99.65
Rhamnetin 3-neohesperidoside	C28 H32 O16	(M+H)+	9.791		624.1694		FBF-FragConfirm	99.65		99.65
Rhamnetin 3-robinobioside	C28 H32 O16	(M+H)+	9.791		624.1694		FBF-FragConfirm	99.65		99.65
Rhamnetin 3-galactoside-3'-rhamnoside	C28 H32 O16	(M+H)+	9.791		624.1694		FBF-FragConfirm	99.65		99.65

Cpd 788: Ikarisoside F

Name	Formula	RT	RI	Mass	Diff (Tgt, ppm)	CAS	ID Source	Score	Algorithm
Ikarisoside F	C31 H36 O14	9.948		632.2085	-3.20		FBF	81.89	FBF

Species	m/z	Score (Tgt)	Score (Lib)	Score (DB)	Score (MFG)	Score (RT)
(M+H)+	633	81.89				

Compound Chromatograms (overlaid)

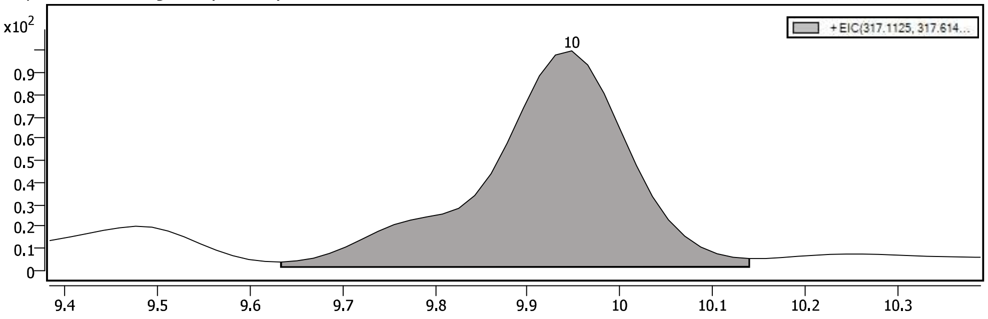

Structure

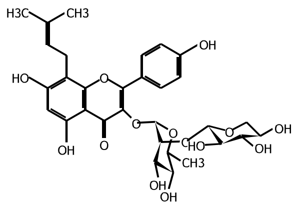

Compound Spectra (overlaid)

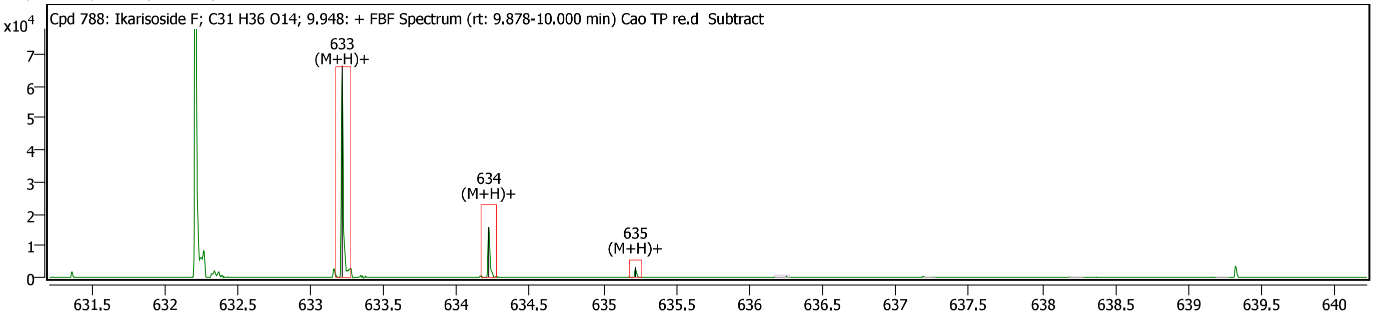

Compound ID Table

Name	Formula	Species	RT	RT Diff	Mass	CAS	ID Source	Score	Score (Lib)	Score (Tgt)
Ikarisoside F	C31 H36 O14	(M+H)+	9.948		632.2085		FBF	81.89		81.89

Cpd 1310: Myricitrin

Name	Formula	RT	RI	Mass	Diff (Tgt, ppm)	CAS	ID Source	Score	Algorithm
Myricitrin	C21 H20 O12	10.419		464.0958	0.68	17912-87-7	M-FBF-FragConfirm	98.92	FBF

Species	m/z	Score (Tgt)	Score (Lib)	Score (DB)	Score (MFG)	Score (RT)
(M+H)+	465	98.92				

Compound Chromatograms (overlaid)

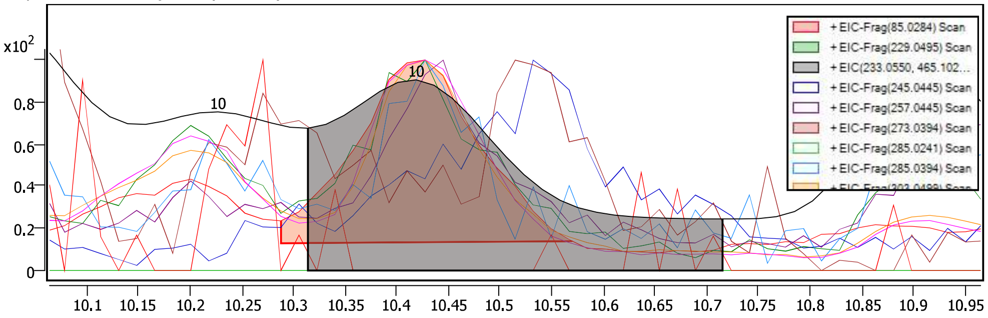

Structure

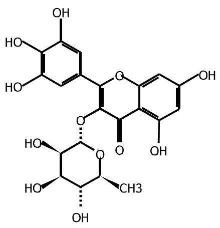

Compound Screening Report

Coelution Plot

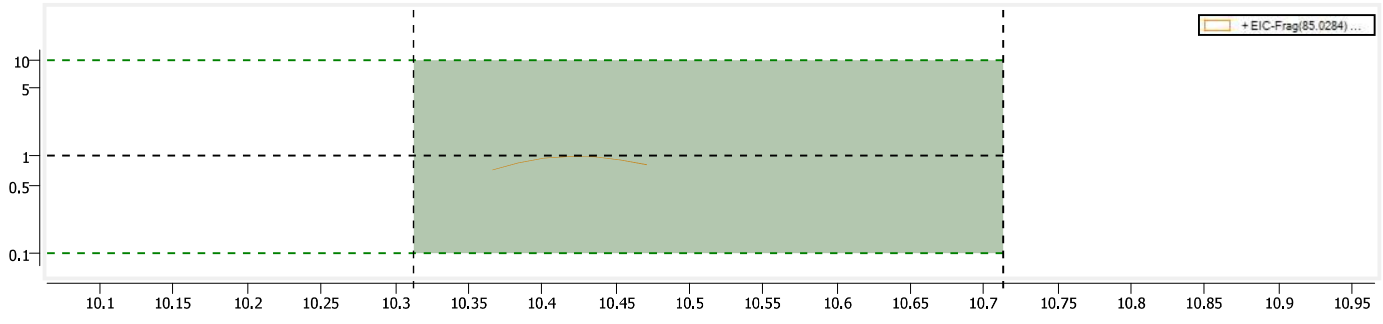

Compound Spectra (overlaid)

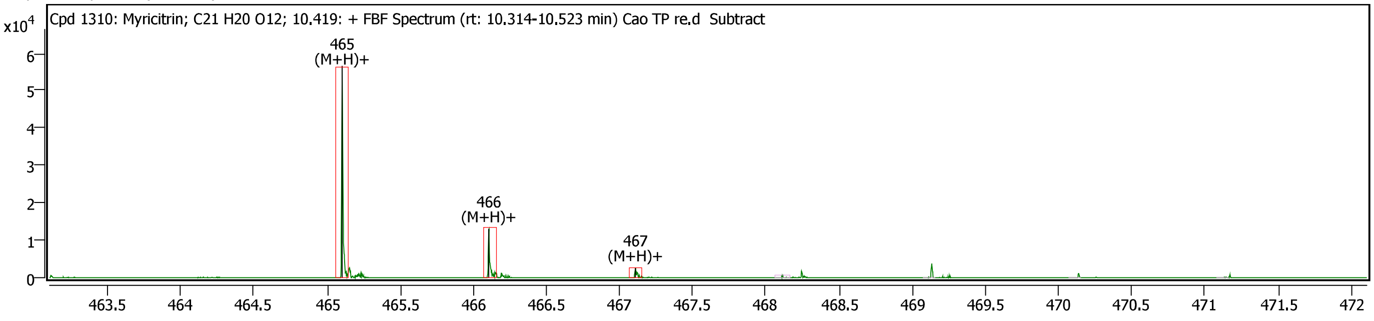

Fragment Spectrum (clean)

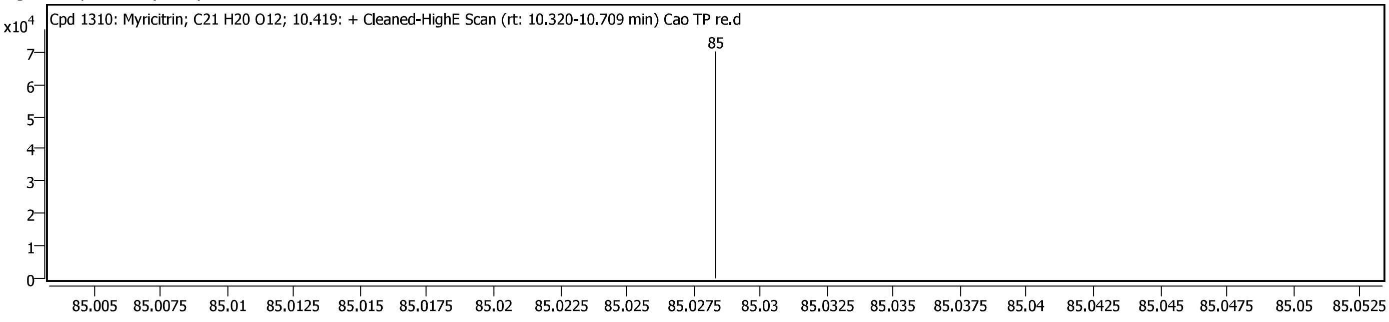

Fragment Spectrum (raw)

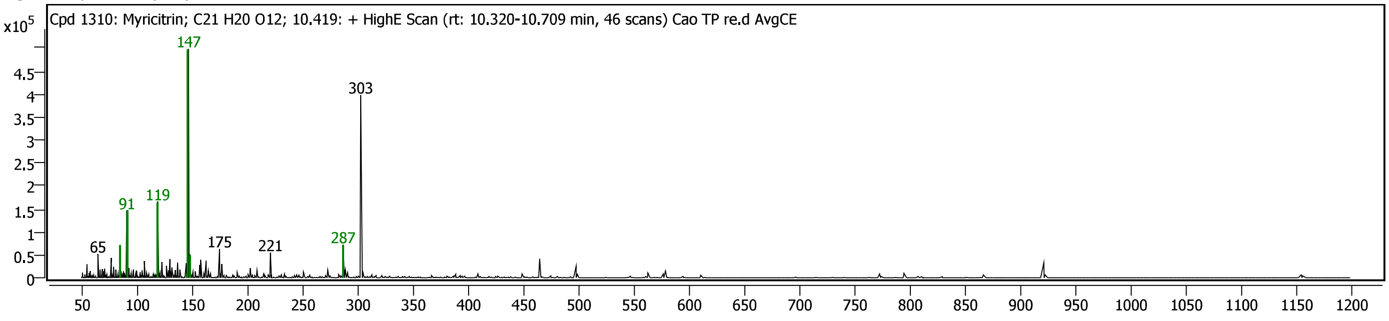

Compound Screening Report

Compound ID Table

Name	Formula	Species	RT	RT Diff	Mass	CAS	ID Source	Score	Score (Lib)	Score (Tgt)
Myricitrin	C21 H20 O12	(M+H)+	10.419		464.0958	17912-87-7	FBF-FragConfirm	98.92		98.92
Bracteatin 6-O-glucoside	C21 H20 O12	(M+H)+	10.419		464.0958		FBF-FragConfirm	98.92		98.92
Quercetagetin 3-rhamnoside	C21 H20 O12	(M+H)+	10.419		464.0958		FBF-FragConfirm	98.92		98.92
8-Hydroxyluteolin 8-glucoside	C21 H20 O12	(M+H)+	10.419		464.0958		FBF-FragConfirm	98.92		98.92
Annulatin 3'-xyloside	C21 H20 O12	(M+H)+	10.419		464.0958		FBF-FragConfirm	98.92		98.92
Bracteatin 6-glucoside	C21 H20 O12	(M+H)+	10.419		464.0958		FBF-FragConfirm	98.92		98.92
Gossypetin 7-rhamnoside	C21 H20 O12	(M+H)+	10.419		464.0958		FBF-FragConfirm	98.92		98.92
Bractein	C21 H20 O12	(M+H)+	10.419		464.0958		FBF-FragConfirm	98.92		98.92
Corniculatusin 3-alpha-L-arabinofuranoside	C21 H20 O12	(M+H)+	10.419		464.0958		FBF-FragConfirm	98.92		98.92
Eriodictyol 7-glucuronide	C21 H20 O12	(M+H)+	10.419		464.0958		FBF-FragConfirm	98.92		98.92
6-Hydroxyluteolin 7-glucoside	C21 H20 O12	(M+H)+	10.419		464.0958		FBF-FragConfirm	98.92		98.92
Gossypetin 8-rhamnoside	C21 H20 O12	(M+H)+	10.419		464.0958		FBF-FragConfirm	98.92		98.92
6-Hydroxytricetin 5-rhamnoside	C21 H20 O12	(M+H)+	10.419		464.0958		FBF-FragConfirm	98.92		98.92
6-Hydroxykaempferol 3-glucoside	C21 H20 O12	(M+H)+	10.419		464.0958		FBF-FragConfirm	98.92		98.92
6-Hydroxyluteolin 7-galactoside	C21 H20 O12	(M+H)+	10.419		464.0958		FBF-FragConfirm	98.92		98.92
6-Hydroxyluteolin 6-glucoside	C21 H20 O12	(M+H)+	10.419		464.0958		FBF-FragConfirm	98.92		98.92
6-Hydroxyluteolin 5-glucoside	C21 H20 O12	(M+H)+	10.419		464.0958		FBF-FragConfirm	98.92		98.92
6-Hydroxykaempferol 7-glucoside	C21 H20 O12	(M+H)+	10.419		464.0958		FBF-FragConfirm	98.92		98.92
6-C-Glucosylquercetin	C21 H20 O12	(M+H)+	10.419		464.0958		FBF-FragConfirm	98.92		98.92
6-C-beta-D-Glucopyranosyl-5,7,2',4',5'-pentahydroxyflavone	C21 H20 O12	(M+H)+	10.419		464.0958		FBF-FragConfirm	98.92		98.92
5,7,3',4',5'-Pentahydroxyflavone 8-C-glucopyranoside	C21 H20 O12	(M+H)+	10.419		464.0958		FBF-FragConfirm	98.92		98.92
5,6,7,3',4'-Pentahydroxy-8-methoxyflavone 7-apioside	C21 H20 O12	(M+H)+	10.419		464.0958		FBF-FragConfirm	98.92		98.92
3,5,7,2',6'-Pentahydroxyflavone 2'-glucoside	C21 H20 O12	(M+H)+	10.419		464.0958		FBF-FragConfirm	98.92		98.92
2'-Hydroxyisoorientin	C21 H20 O12	(M+H)+	10.419		464.0958		FBF-FragConfirm	98.92		98.92
(2S)-5,7,3',4'-Tetrahydroxyflavanone 7-glucuronide	C21 H20 O12	(M+H)+	10.419		464.0958		FBF-FragConfirm	98.92		98.92
Herbacetin 3-beta-D-glucofuranoside	C21 H20 O12	(M+H)+	10.419		464.0958		FBF-FragConfirm	98.92		98.92
8-Hydroxyluteolin 7-glucoside	C21 H20 O12	(M+H)+	10.419		464.0958		FBF-FragConfirm	98.92		98.92
Herbacetin 3-glucoside	C21 H20 O12	(M+H)+	10.419		464.0958		FBF-FragConfirm	98.92		98.92
Tricetin 7-glucoside	C21 H20 O12	(M+H)+	10.419		464.0958		FBF-FragConfirm	98.92		98.92
Quercetin 3-alloside	C21 H20 O12	(M+H)+	10.419		464.0958		FBF-FragConfirm	98.92		98.92
Herbacetin 7-glucoside	C21 H20 O12	(M+H)+	10.419		464.0958		FBF-FragConfirm	98.92		98.92
Tricetin 3'-glucoside	C21 H20 O12	(M+H)+	10.419		464.0958		FBF-FragConfirm	98.92		98.92
Spiraeoside	C21 H20 O12	(M+H)+	10.419		464.0958		FBF-FragConfirm	98.92		98.92
Robinetin 7-glucoside	C21 H20 O12	(M+H)+	10.419		464.0958		FBF-FragConfirm	98.92		98.92
Quercimeritrin	C21 H20 O12	(M+H)+	10.419		464.0958		FBF-FragConfirm	98.92		98.92
Quercetin 5-glucoside	C21 H20 O12	(M+H)+	10.419		464.0958		FBF-FragConfirm	98.92		98.92
Quercetin 4'-glucoside	C21 H20 O12	(M+H)+	10.419		464.0958	20229-56-5	FBF-FragConfirm	98.92		98.92
Quercetin 3-β-D-glucoside	C21 H20 O12	(M+H)+	10.419		464.0958	482-35-9	FBF-FragConfirm	98.92		98.92
Quercetin 3-O-glucoside	C21 H20 O12	(M+H)+	10.419		464.0958	21637-25-2	FBF-FragConfirm	98.92		98.92
Quercetin 3'-glucoside	C21 H20 O12	(M+H)+	10.419		464.0958		FBF-FragConfirm	98.92		98.92
Quercetin 7-galactoside	C21 H20 O12	(M+H)+	10.419		464.0958		FBF-FragConfirm	98.92		98.92
Myricetin 3'-rhamnoside	C21 H20 O12	(M+H)+	10.419		464.0958		FBF-FragConfirm	98.92		98.92
Patuletin 3-xyloside	C21 H20 O12	(M+H)+	10.419		464.0958		FBF-FragConfirm	98.92		98.92
Isoetin 5'-glucoside	C21 H20 O12	(M+H)+	10.419		464.0958		FBF-FragConfirm	98.92		98.92
Herbacetin 8-glucoside	C21 H20 O12	(M+H)+	10.419		464.0958		FBF-FragConfirm	98.92		98.92
Hyperin	C21 H20 O12	(M+H)+	10.419		464.0958		FBF-FragConfirm	98.92		98.92
Hyperoside	C21 H20 O12	(M+H)+	10.419		464.0958	482-36-0	FBF-FragConfirm	98.92		98.92
Isoaffinetin	C21 H20 O12	(M+H)+	10.419		464.0958		FBF-FragConfirm	98.92		98.92
Isoetin 7-glucoside	C21 H20 O12	(M+H)+	10.419		464.0958		FBF-FragConfirm	98.92		98.92
Isoquercitrin	C21 H20 O12	(M+H)+	10.419		464.0958		FBF-FragConfirm	98.92		98.92
Larycitrin 3-alpha-L-arabinofuranoside	C21 H20 O12	(M+H)+	10.419		464.0958		FBF-FragConfirm	98.92		98.92
Herbacetin 4'-glucoside	C21 H20 O12	(M+H)+	10.419		464.0958		FBF-FragConfirm	98.92		98.92
Myricetin 7-rhamnoside	C21 H20 O12	(M+H)+	10.419		464.0958	184533-14-0	FBF-FragConfirm	98.92		98.92

Cpd 768: Rutin

Name	Formula	RT	RI	Mass	Diff (Tgt, ppm)	CAS	ID Source	Score	Algorithm
Rutin	C27 H30 O16	10.436		610.1534	0.07	153-18-4	M-FBF-FragConfirm	98.52	FBF

Species	m/z	Score (Tgt)	Score (Lib)	Score (DB)	Score (MFG)	Score (RT)
(M+H)+	611	98.52				

Compound Chromatograms (overlaid)

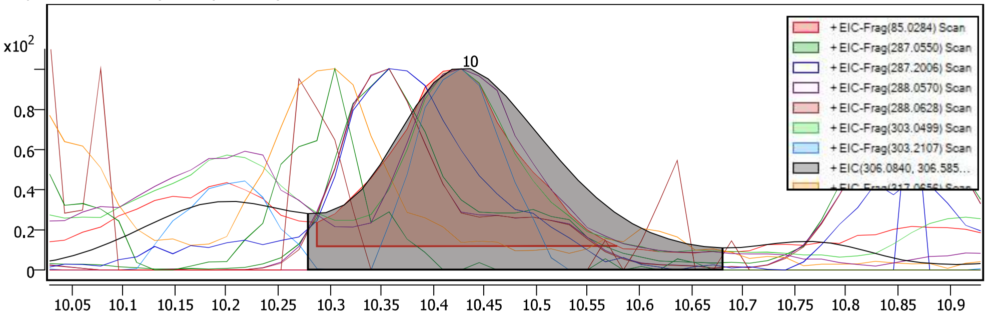

Counts (%) vs. Acquisition Time (min)

Structure

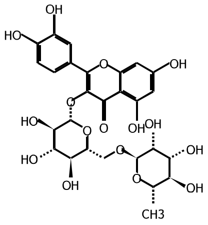

Compound Screening Report

Coelution Plot

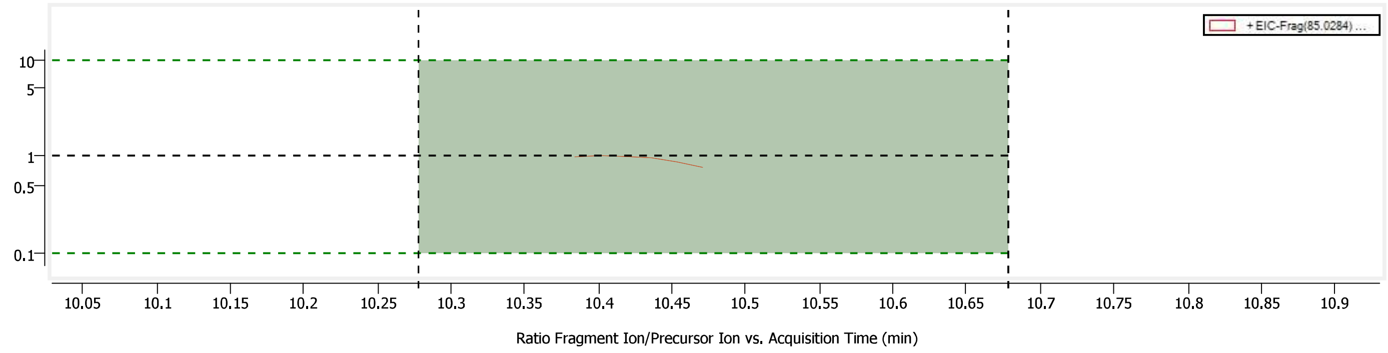

Compound Spectra (overlaid)

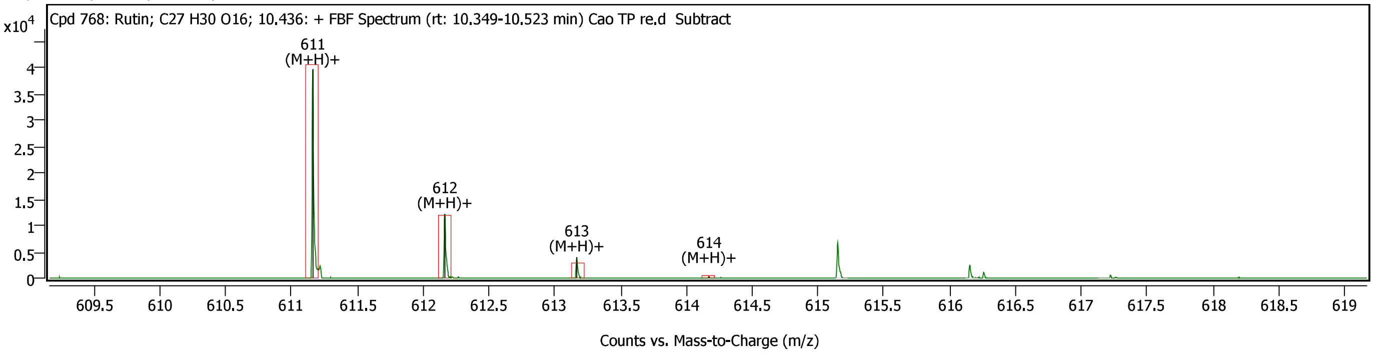

Fragment Spectrum (clean)

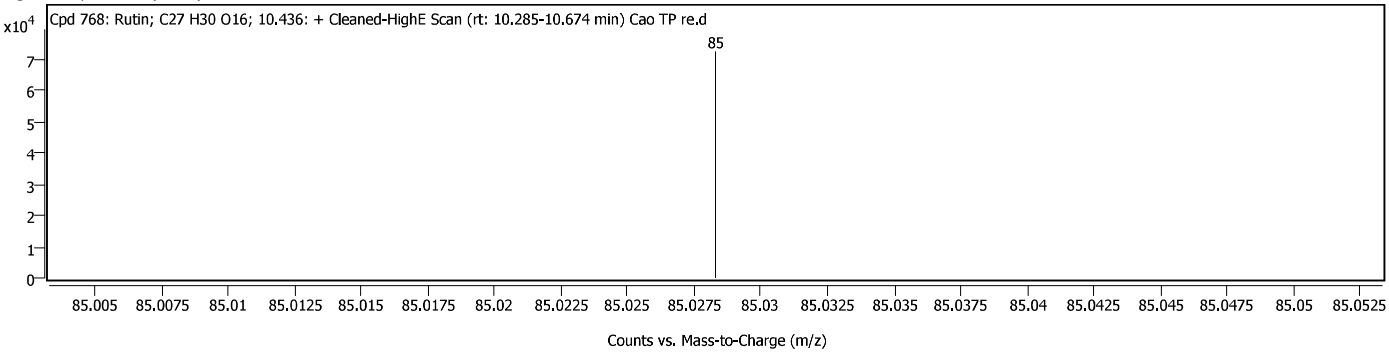

Fragment Spectrum (raw)

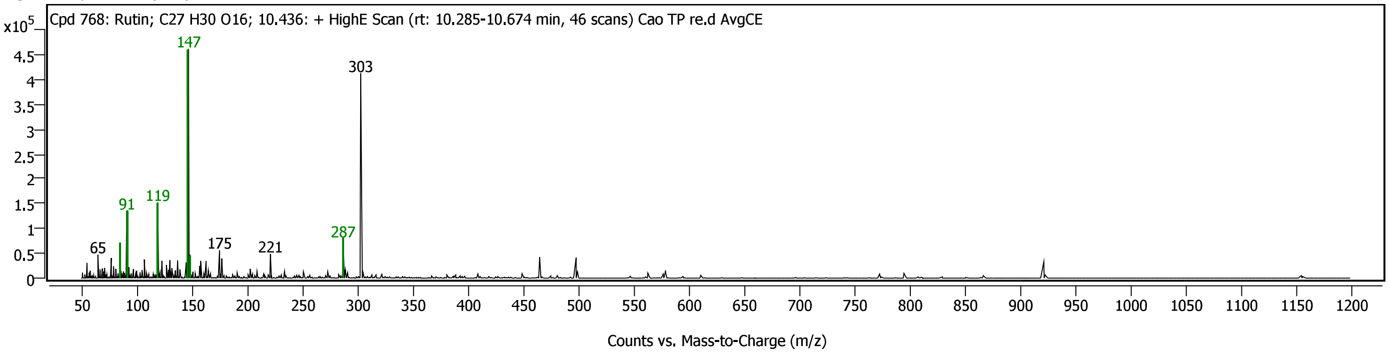

Compound ID Table

Name	Formula	Species	RT	RT Diff	Mass	CAS	ID Source	Score	Score (Lib)	Score (Tgt)
Rutin	C27 H30 O16	(M+H)+	10.436		610.1534	153-18-4	FBF-FragConfirm	98.52		98.52
Isorhamnetin 3-apiosyl-(1->2)-galactoside	C27 H30 O16	(M+H)+	10.436		610.1534		FBF-FragConfirm	98.52		98.52
Isorhamnetin 3-sambubioside	C27 H30 O16	(M+H)+	10.436		610.1534		FBF-FragConfirm	98.52		98.52
Isorhamnetin 3-O-[b-D-xylopyranosyl-(1->6)-b-D-glucopyranoside]	C27 H30 O16	(M+H)+	10.436		610.1534	142905-19-9	FBF-FragConfirm	98.52		98.52
Isorhamnetin 3-L-arabinopyranosyl-(1->6)-galactoside	C27 H30 O16	(M+H)+	10.436		610.1534		FBF-FragConfirm	98.52		98.52
Isorhamnetin 3-glucoside-7-xyloside	C27 H30 O16	(M+H)+	10.436		610.1534		FBF-FragConfirm	98.52		98.52
Isorhamnetin 3-glucoside-7-alpha-L-arabinopyranoside	C27 H30 O16	(M+H)+	10.436		610.1534		FBF-FragConfirm	98.52		98.52
Isorhamnetin 3-apiosyl-(1->2)-glucoside	C27 H30 O16	(M+H)+	10.436		610.1534		FBF-FragConfirm	98.52		98.52
Isoorientin 4'-O-glucoside	C27 H30 O16	(M+H)+	10.436		610.1534		FBF-FragConfirm	98.52		98.52
Isoquercitrin 4''-rhamnoside	C27 H30 O16	(M+H)+	10.436		610.1534	123160-33-8	FBF-FragConfirm	98.52		98.52
Isoorientin 7-O-galactoside	C27 H30 O16	(M+H)+	10.436		610.1534		FBF-FragConfirm	98.52		98.52
Isoorientin 7-glucoside	C27 H30 O16	(M+H)+	10.436		610.1534	35450-86-3	FBF-FragConfirm	98.52		98.52
Kaempferol 3-galactoside-4'-glucoside	C27 H30 O16	(M+H)+	10.436		610.1534		FBF-FragConfirm	98.52		98.52
Isoorientin 3'-O-glucoside	C27 H30 O16	(M+H)+	10.436		610.1534		FBF-FragConfirm	98.52		98.52
Isoorientin 2''-O-alpha-D-mannoside	C27 H30 O16	(M+H)+	10.436		610.1534		FBF-FragConfirm	98.52		98.52
Isorhamnetin 3-vicianoside	C27 H30 O16	(M+H)+	10.436		610.1534		FBF-FragConfirm	98.52		98.52
Isorhamnetin 3-xylosyl-(1->2)-galactoside	C27 H30 O16	(M+H)+	10.436		610.1534		FBF-FragConfirm	98.52		98.52
Isorhamnetin 3-xylosyl-(1->6)-glucoside	C27 H30 O16	(M+H)+	10.436		610.1534		FBF-FragConfirm	98.52		98.52
Isoscutellarein 7-allosyl-(1->2)-glucoside	C27 H30 O16	(M+H)+	10.436		610.1534		FBF-FragConfirm	98.52		98.52
Kaempferol 3,4'-diglucoside	C27 H30 O16	(M+H)+	10.436		610.1534		FBF-FragConfirm	98.52		98.52
Kaempferol 3,5-digalactoside	C27 H30 O16	(M+H)+	10.436		610.1534		FBF-FragConfirm	98.52		98.52
Kaempferol 3,5-diglucoside	C27 H30 O16	(M+H)+	10.436		610.1534		FBF-FragConfirm	98.52		98.52
Kaempferol 3,7-digalactoside	C27 H30 O16	(M+H)+	10.436		610.1534		FBF-FragConfirm	98.52		98.52
Kaempferol 3-gentiobioside	C27 H30 O16	(M+H)+	10.436		610.1534		FBF-FragConfirm	98.52		98.52
Herbacetin 8-rutinoside	C27 H30 O16	(M+H)+	10.436		610.1534		FBF-FragConfirm	98.52		98.52
Rheinoside A	C27 H30 O16	(M+H)+	10.436		610.1534	111545-28-9	FBF-FragConfirm	98.52		98.52
6-Hydroxyluteolin 7-rutinoside	C27 H30 O16	(M+H)+	10.436		610.1534		FBF-FragConfirm	98.52		98.52
8-C-Glucosylquercetin 2''-O-rhamnoside	C27 H30 O16	(M+H)+	10.436		610.1534		FBF-FragConfirm	98.52		98.52
8-Hydroxyapigenin 8-sophoroside	C27 H30 O16	(M+H)+	10.436		610.1534		FBF-FragConfirm	98.52		98.52
2''-O-beta-L-galactopyranosylorientin	C27 H30 O16	(M+H)+	10.436		610.1534		FBF-FragConfirm	98.52		98.52
3,7,2',3',4'-Pentahydroxyflavone 3-neohesperidoside	C27 H30 O16	(M+H)+	10.436		610.1534		FBF-FragConfirm	98.52		98.52
3-[(2S,3R,4S,5S,6R)-4,5-Dihydroxy-6-(hydroxymethyl)-3-[(2S,3R,4S,5R)-3,4,5-trihydroxyoxan-2-yl]oxyoxan-2-yl]oxy-2-(3,4-dihydroxyphenyl)-5-hydroxy-7-methoxychromen-4-one	C27 H30 O16	(M+H)+	10.436		610.1534		FBF-FragConfirm	98.52		98.52
6-C-Glucosylkaempferol 3-O-glucoside	C27 H30 O16	(M+H)+	10.436		610.1534		FBF-FragConfirm	98.52		98.52
6-Hydroxykaempferol 3-rutinoside	C27 H30 O16	(M+H)+	10.436		610.1534		FBF-FragConfirm	98.52		98.52
6-Hydroxykaempferol 7-rutinoside	C27 H30 O16	(M+H)+	10.436		610.1534		FBF-FragConfirm	98.52		98.52
6-Hydroxyluteolin 6-glucoside-3'-rhamnoside	C27 H30 O16	(M+H)+	10.436		610.1534		FBF-FragConfirm	98.52		98.52
Allivicin	C27 H30 O16	(M+H)+	10.436		610.1534	71939-16-7	FBF-FragConfirm	98.52		98.52
Herbacetin 3-rhamnoside-8-glucoside	C27 H30 O16	(M+H)+	10.436		610.1534		FBF-FragConfirm	98.52		98.52
Aureusidin 4,6-diglucoside	C27 H30 O16	(M+H)+	10.436		610.1534		FBF-FragConfirm	98.52		98.52
Herbacetin 7-rhamnoside-8-glucoside	C27 H30 O16	(M+H)+	10.436		610.1534		FBF-FragConfirm	98.52		98.52
Annulatin 7-rhamnoside-3'-xyloside	C27 H30 O16	(M+H)+	10.436		610.1534		FBF-FragConfirm	98.52		98.52
Kaempferol 3-glucoside-7-galactoside	C27 H30 O16	(M+H)+	10.436		610.1534		FBF-FragConfirm	98.52		98.52
Calendoflavobioside	C27 H30 O16	(M+H)+	10.436		610.1534	32453-36-4	FBF-FragConfirm	98.52		98.52
Camelliaside C	C27 H30 O16	(M+H)+	10.436		610.1534		FBF-FragConfirm	98.52		98.52
Fisetin 3,7-diglucoside	C27 H30 O16	(M+H)+	10.436		610.1534		FBF-FragConfirm	98.52		98.52
Flavocannabiside	C27 H30 O16	(M+H)+	10.436		610.1534		FBF-FragConfirm	98.52		98.52
Gossypetin 8-methyl ether 3-xylosyl-(1->2)-rhamnoside	C27 H30 O16	(M+H)+	10.436		610.1534		FBF-FragConfirm	98.52		98.52
Kaempferol 3,7-diglucoside	C27 H30 O16	(M+H)+	10.436		610.1534		FBF-FragConfirm	98.52		98.52
Herbacetin 7-glucosyl-(1->3)-rhamnoside	C27 H30 O16	(M+H)+	10.436		610.1534		FBF-FragConfirm	98.52		98.52
Isoorientin 2''-O-glucopyranoside	C27 H30 O16	(M+H)+	10.436		610.1534		FBF-FragConfirm	98.52		98.52
Quercetin 3-glucosyl-(1->2)-rhamnoside	C27 H30 O16	(M+H)+	10.436		610.1534		FBF-FragConfirm	98.52		98.52
Quercetin 3-glucosyl-(1->4)-rhamnoside	C27 H30 O16	(M+H)+	10.436		610.1534		FBF-FragConfirm	98.52		98.52
Orobol 6,8-di-C-glucoside	C27 H30 O16	(M+H)+	10.436		610.1534		FBF-FragConfirm	98.52		98.52
Panasenoside	C27 H30 O16	(M+H)+	10.436		610.1534		FBF-FragConfirm	98.52		98.52
Quercetin 3-(2-glucosylrhamnoside)	C27 H30 O16	(M+H)+	10.436		610.1534	143016-74-4	FBF-FragConfirm	98.52		98.52
Quercetin 3-galactoside 7-rhamnoside	C27 H30 O16	(M+H)+	10.436		610.1534	38784-81-5	FBF-FragConfirm	98.52		98.52
Quercetin 3-galactoside-7-rhamnoside	C27 H30 O16	(M+H)+	10.436		610.1534		FBF-FragConfirm	98.52		98.52
Quercetin 3-galactosyl-(1->2)-rhamnoside	C27 H30 O16	(M+H)+	10.436		610.1534		FBF-FragConfirm	98.52		98.52
Quercetin 3-galactosyl-(1->4)-rhamnoside	C27 H30 O16	(M+H)+	10.436		610.1534		FBF-FragConfirm	98.52		98.52
Quercetin 3-glucoside-7-rhamnoside	C27 H30 O16	(M+H)+	10.436		610.1534		FBF-FragConfirm	98.52		98.52
Quercetin 3-methyl ether 7-alpha-L-arabinofuranosyl-(1->6)-glucoside	C27 H30 O16	(M+H)+	10.436		610.1534		FBF-FragConfirm	98.52		98.52
Luteolin 7-gentiobioside	C27 H30 O16	(M+H)+	10.436		610.1534		FBF-FragConfirm	98.52		98.52
Robinetin 3-rutinoside	C27 H30 O16	(M+H)+	10.436		610.1534		FBF-FragConfirm	98.52		98.52

Compound Screening Report

Compound ID Table

Name	Formula	Species	RT	RT Diff	Mass	CAS	ID Source	Score	Score (Lib)	Score (Tgt)
Quercetin 3-rhamnoside-3'-glucoside	C27 H30 O16	(M+H)+	10.436		610.1534		FBF-FragConfirm	98.52		98.52
Quercetin 3-rhamnoside-7-glucoside	C27 H30 O16	(M+H)+	10.436		610.1534		FBF-FragConfirm	98.52		98.52
Quercetin 3-rhamnosyl-(1->2)-galactoside	C27 H30 O16	(M+H)+	10.436		610.1534		FBF-FragConfirm	98.52		98.52
Quercetin 3-robinobioside	C27 H30 O16	(M+H)+	10.436		610.1534		FBF-FragConfirm	98.52		98.52
Quercetin 7-(rhamnosylglucoside)	C27 H30 O16	(M+H)+	10.436		610.1534	73432-00-5	FBF-FragConfirm	98.52		98.52
Quercetin 7-methyl ether 3-alpha-L-arabinopyranosyl-(1->3)-galactoside	C27 H30 O16	(M+H)+	10.436		610.1534		FBF-FragConfirm	98.52		98.52
Quercetin 7-rutinoside	C27 H30 O16	(M+H)+	10.436		610.1534		FBF-FragConfirm	98.52		98.52
Quercetin 8-C-(2''-rhamnosylglucoside)	C27 H30 O16	(M+H)+	10.436		610.1534	182062-20-0	FBF-FragConfirm	98.52		98.52
Kaempferol 3-glucosyl-(1->6)-galactoside	C27 H30 O16	(M+H)+	10.436		610.1534		FBF-FragConfirm	98.52		98.52
Kaempferol 3-O-β-D-glucosyl-(1->2)-β-D-glucoside	C27 H30 O16	(M+H)+	10.436		610.1534	19895-95-5	FBF-FragConfirm	98.52		98.52
Quercetin 3-neohesperidoside	C27 H30 O16	(M+H)+	10.436		610.1534		FBF-FragConfirm	98.52		98.52
Myricetin 3-rhamnosyl-(1->2)-rhamnoside	C27 H30 O16	(M+H)+	10.436		610.1534		FBF-FragConfirm	98.52		98.52
Luteolin 7,4'-diglucoside	C27 H30 O16	(M+H)+	10.436		610.1534		FBF-FragConfirm	98.52		98.52
Luteolin 7-galactosyl-(1->6)-galactoside	C27 H30 O16	(M+H)+	10.436		610.1534		FBF-FragConfirm	98.52		98.52
Kaempferol 3-O-β-D-glucosylgalactoside	C27 H30 O16	(M+H)+	10.436		610.1534		FBF-FragConfirm	98.52		98.52
Orientin 7-glucoside	C27 H30 O16	(M+H)+	10.436		610.1534		FBF-FragConfirm	98.52		98.52
Kaempferol 7,4'-diglucoside	C27 H30 O16	(M+H)+	10.436		610.1534		FBF-FragConfirm	98.52		98.52
Kaempferol 7-sophoroside	C27 H30 O16	(M+H)+	10.436		610.1534		FBF-FragConfirm	98.52		98.52
Lucenin 2	C27 H30 O16	(M+H)+	10.436		610.1534		FBF-FragConfirm	98.52		98.52
Luteolin 3',4'-diglucoside	C27 H30 O16	(M+H)+	10.436		610.1534		FBF-FragConfirm	98.52		98.52
Luteolin 7,3'-diglucoside	C27 H30 O16	(M+H)+	10.436		610.1534		FBF-FragConfirm	98.52		98.52
Luteolin 7-allosyl-(1->2)-glucoside	C27 H30 O16	(M+H)+	10.436		610.1534		FBF-FragConfirm	98.52		98.52
Orobol 7-O-sophoroside	C27 H30 O16	(M+H)+	10.436		610.1534		FBF-FragConfirm	98.52		98.52
Luteolin 7-galactoside-4'-glucoside	C27 H30 O16	(M+H)+	10.436		610.1534		FBF-FragConfirm	98.52		98.52
Luteolin 6-C-glucoside 8-C-arabinoside	C27 H30 O16	(M+H)+	10.436		610.1534		FBF-FragConfirm	98.52		98.52
Orientin 3'-O-glucoside	C27 H30 O16	(M+H)+	10.436		610.1534		FBF-FragConfirm	98.52		98.52
Luteolin 7-laminaribioside	C27 H30 O16	(M+H)+	10.436		610.1534		FBF-FragConfirm	98.52		98.52
Lutonarin	C27 H30 O16	(M+H)+	10.436		610.1534		FBF-FragConfirm	98.52		98.52
Meloside L	C27 H30 O16	(M+H)+	10.436		610.1534	55196-48-0	FBF-FragConfirm	98.52		98.52
Multinoside A	C27 H30 O16	(M+H)+	10.436		610.1534	59262-54-3	FBF-FragConfirm	98.52		98.52
Myricetin 3,4'-di-O-alpha-L-rhamnopyranoside	C27 H30 O16	(M+H)+	10.436		610.1534		FBF-FragConfirm	98.52		98.52
Orientin 4'-glucoside	C27 H30 O16	(M+H)+	10.436		610.1534		FBF-FragConfirm	98.52		98.52

Cpd 472: Cinnamtannin A2

Name	Formula	RT	RI	Mass	Diff (Tgt, ppm)	CAS	ID Source	Score	Algorithm
Cinnamtannin A2	C60 H50 O24	10.663		1154.2695	0.29		FBF	99.15	FBF

Species	m/z	Score (Tgt)	Score (Lib)	Score (DB)	Score (MFG)	Score (RT)
(M+H)+	1155	99.15				

Compound Chromatograms (overlaid)

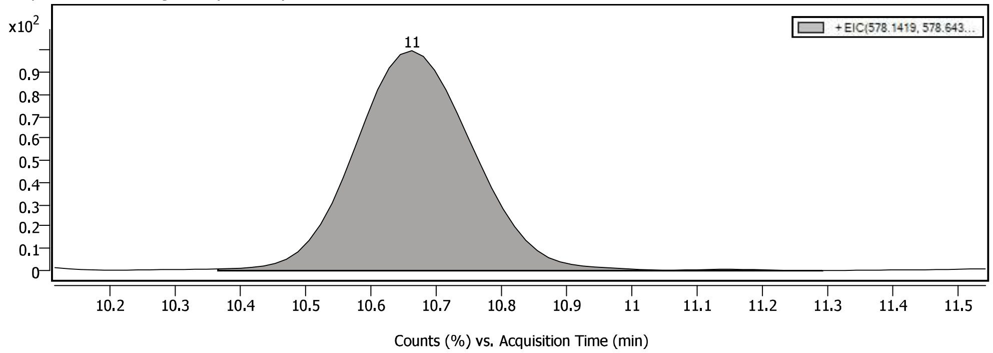

Structure

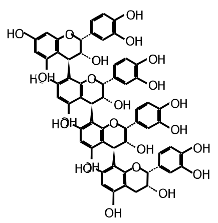

Compound Spectra (overlaid)

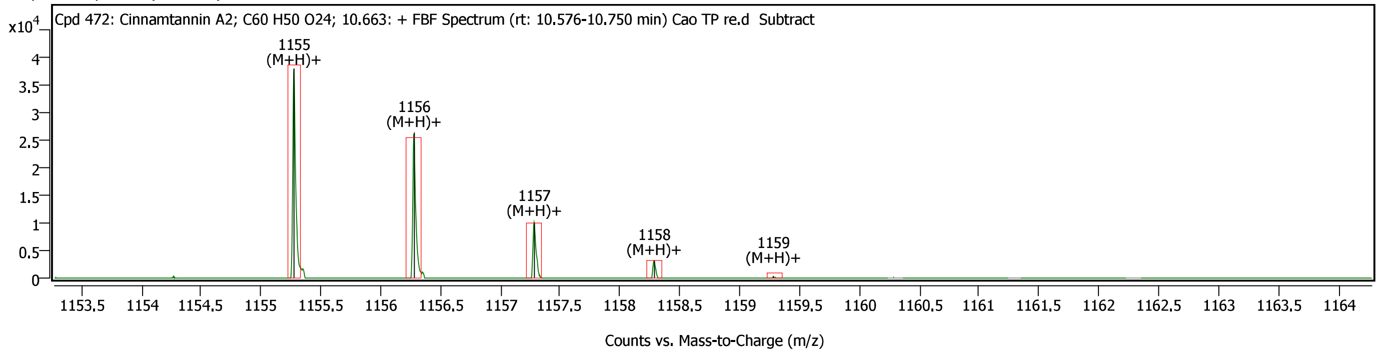

Compound ID Table

Name	Formula	Species	RT	RT Diff	Mass	CAS	ID Source	Score	Score (Lib)	Score (Tgt)
Cinnamtannin A2	C60 H50 O24	(M+H)+	10.663		1154.2695		FBF	99.15		99.15

Cpd 99: (±)-Naringenin

Name	Formula	RT	RI	Mass	Diff (Tgt, ppm)	CAS	ID Source	Score	Algorithm
(±)-Naringenin	C15 H12 O5	10.750		272.0688	1.31	67604-48-2	FBF-FragConfirm	97.45	FBF

Species	m/z	Score (Tgt)	Score (Lib)	Score (DB)	Score (MFG)	Score (RT)
(M+H)+	273	97.45				

Compound Chromatograms (overlaid)

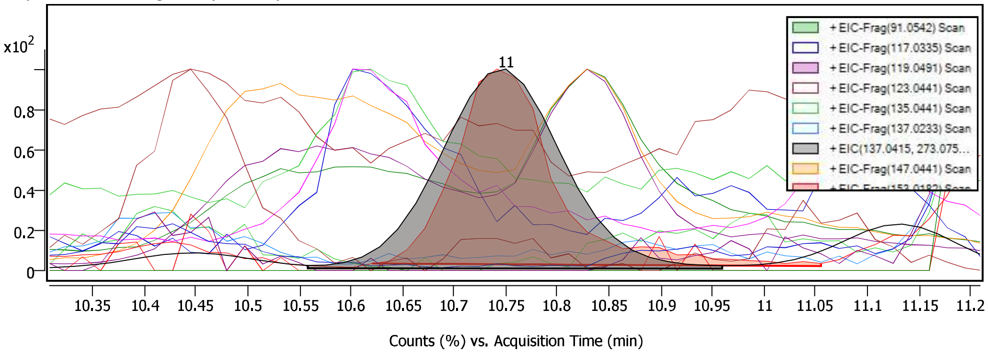

Structure

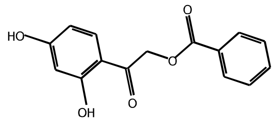

Coelution Plot

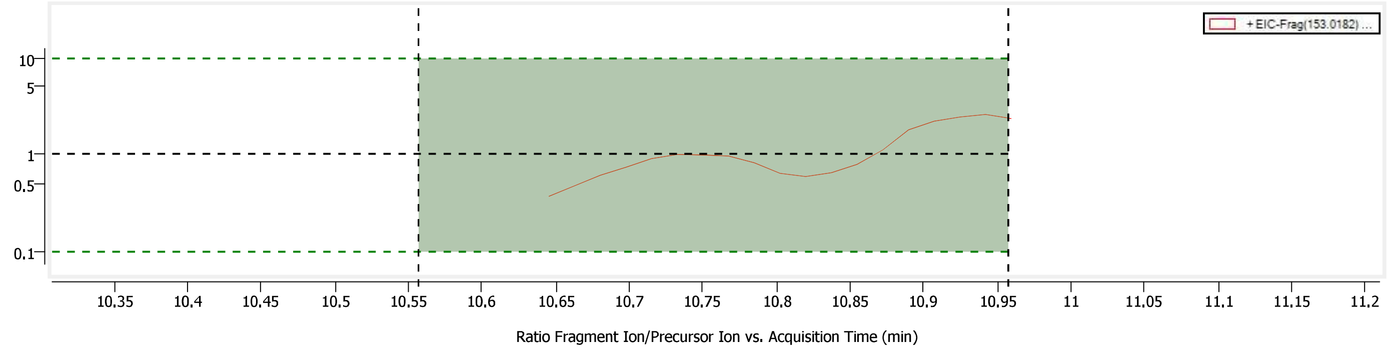

Compound Spectra (overlaid)

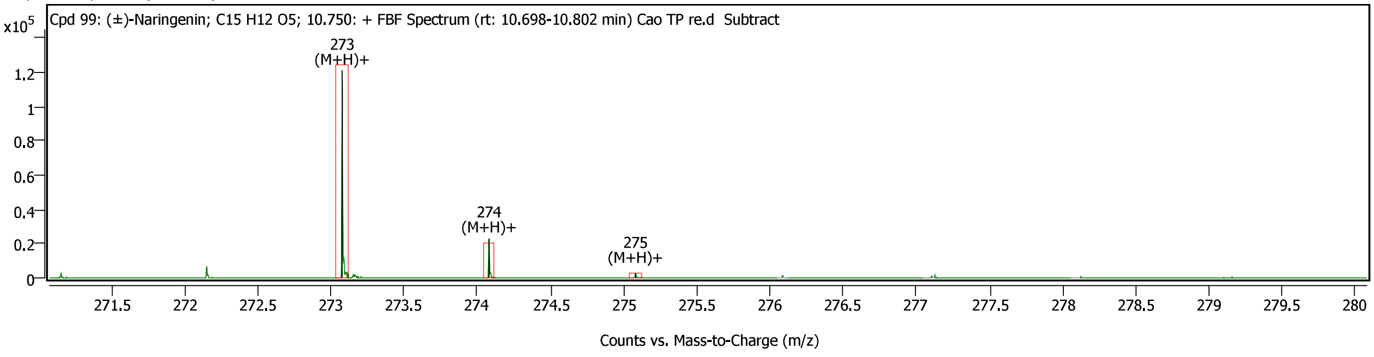

Fragment Spectrum (clean)

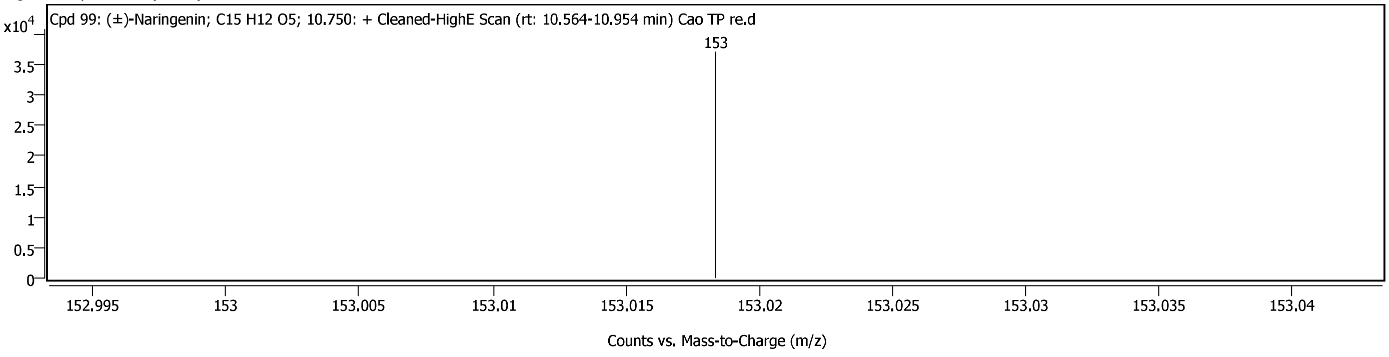

Fragment Spectrum (raw)

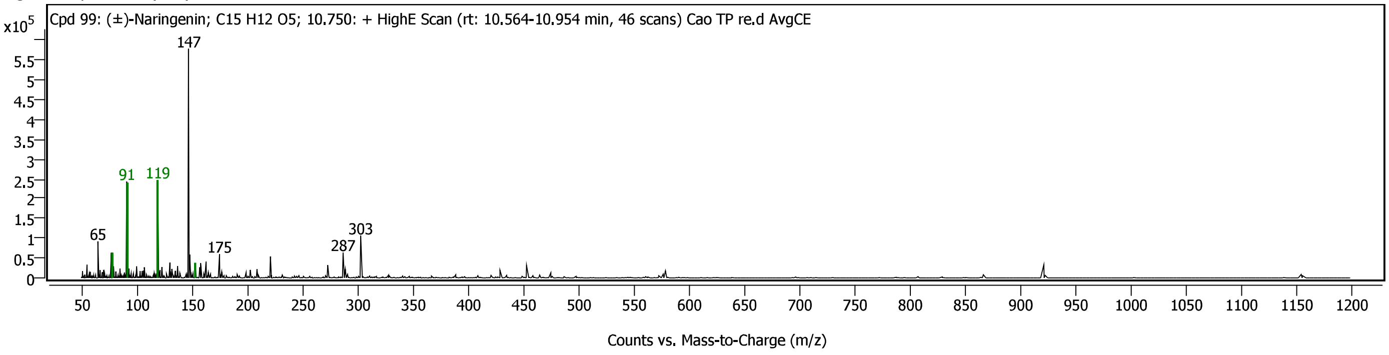

Compound ID Table

Name	Formula	Species	RT	RT Diff	Mass	CAS	ID Source	Score	Score (Lib)	Score (Tgt)
(±)-Naringenin	C15 H12 O5	(M+H)+	10.750		272.0688	67604-48-2	FBF-FragConfirm	97.45		97.45
3'-Hydroxydihydrodaidzein	C15 H12 O5	(M+H)+	10.750		272.0688		FBF-FragConfirm	97.45		97.45
Dihydrogenistein	C15 H12 O5	(M+H)+	10.750		272.0688	21554-71-2	FBF-FragConfirm	97.45		97.45
6-Hydroxydihydrodaidzein	C15 H12 O5	(M+H)+	10.750		272.0688		FBF-FragConfirm	97.45		97.45
6,7,4'-Trihydroxyflavanone	C15 H12 O5	(M+H)+	10.750		272.0688		FBF-FragConfirm	97.45		97.45
5,8,4'-Trihydroxyflavanone	C15 H12 O5	(M+H)+	10.750		272.0688		FBF-FragConfirm	97.45		97.45
5,7,3'-Trihydroxyflavanone	C15 H12 O5	(M+H)+	10.750		272.0688		FBF-FragConfirm	97.45		97.45
5,7,2'-Trihydroxyflavanone	C15 H12 O5	(M+H)+	10.750		272.0688		FBF-FragConfirm	97.45		97.45
4-Hydroxydemethylmedicarpin	C15 H12 O5	(M+H)+	10.750		272.0688		FBF-FragConfirm	97.45		97.45
4,2',3',4'-Tetrahydroxychalcone	C15 H12 O5	(M+H)+	10.750		272.0688		FBF-FragConfirm	97.45		97.45
2,7,4'-Trihydroxyisoflavanone	C15 H12 O5	(M+H)+	10.750		272.0688		FBF-FragConfirm	97.45		97.45
2'-Hydroxydihydrodaidzein	C15 H12 O5	(M+H)+	10.750		272.0688		FBF-FragConfirm	97.45		97.45
2beta-5,7-Trihydroxyflavanone	C15 H12 O5	(M+H)+	10.750		272.0688		FBF-FragConfirm	97.45		97.45
2,4,2',4'-Tetrahydroxychalcone	C15 H12 O5	(M+H)+	10.750		272.0688		FBF-FragConfirm	97.45		97.45
1-Hydroxy-3,7-dimethoxyxanthone	C15 H12 O5	(M+H)+	10.750		272.0688	13379-35-6	FBF-FragConfirm	97.45		97.45
(-)-Glycinol	C15 H12 O5	(M+H)+	10.750		272.0688		FBF-FragConfirm	97.45		97.45
7,8,4'-Trihydroxyflavanone	C15 H12 O5	(M+H)+	10.750		272.0688		FBF-FragConfirm	97.45		97.45
p-Coumaroyltriacetic acid lactone	C15 H12 O5	(M+H)+	10.750		272.0688		FBF-FragConfirm	97.45		97.45
Dihydrobaicalein	C15 H12 O5	(M+H)+	10.750		272.0688		FBF-FragConfirm	97.45		97.45
8-Hydroxydihydrodaidzein	C15 H12 O5	(M+H)+	10.750		272.0688		FBF-FragConfirm	97.45		97.45
Moracin J	C15 H12 O5	(M+H)+	10.750		272.0688	73338-89-3	FBF-FragConfirm	97.45		97.45
Toralactone	C15 H12 O5	(M+H)+	10.750		272.0688	41743-74-2	FBF-FragConfirm	97.45		97.45
Rubrofusarin	C15 H12 O5	(M+H)+	10.750		272.0688	3567-00-8	FBF-FragConfirm	97.45		97.45
Pseudosindorin	C15 H12 O5	(M+H)+	10.750		272.0688		FBF-FragConfirm	97.45		97.45
Butein	C15 H12 O5	(M+H)+	10.750		272.0688	487-52-5	FBF-FragConfirm	97.45		97.45
Naringenin chalcone	C15 H12 O5	(M+H)+	10.750		272.0688	25515-46-2	FBF-FragConfirm	97.45		97.45
Naringenin	C15 H12 O5	(M+H)+	10.750		272.0688	480-41-1	FBF-FragConfirm	97.45		97.45
Pinobanksin	C15 H12 O5	(M+H)+	10.750		272.0688	548-82-3	FBF-FragConfirm	97.45		97.45
Methylnorlichexanthone	C15 H12 O5	(M+H)+	10.750		272.0688	22938-77-8	FBF-FragConfirm	97.45		97.45
Garbanzol	C15 H12 O5	(M+H)+	10.750		272.0688	1226-22-8	FBF-FragConfirm	97.45		97.45
Butin	C15 H12 O5	(M+H)+	10.750		272.0688	492-14-8	FBF-FragConfirm	97.45		97.45
Dihydronorwogonin	C15 H12 O5	(M+H)+	10.750		272.0688		FBF-FragConfirm	97.45		97.45
Licodione	C15 H12 O5	(M+H)+	10.750		272.0688		FBF-FragConfirm	97.45		97.45

Cpd 731: Helichrysin A

Name	Formula	RT	RI	Mass	Diff (Tgt, ppm)	CAS	ID Source	Score	Algorithm
Helichrysin A	C21 H22 O10	10.750		434.1215	0.38	529-41-9	M-FBF-FragConfirm	98.55	FBF

Species	m/z	Score (Tgt)	Score (Lib)	Score (DB)	Score (MFG)	Score (RT)
(M+H)+	435	98.55				

Compound Chromatograms (overlaid)

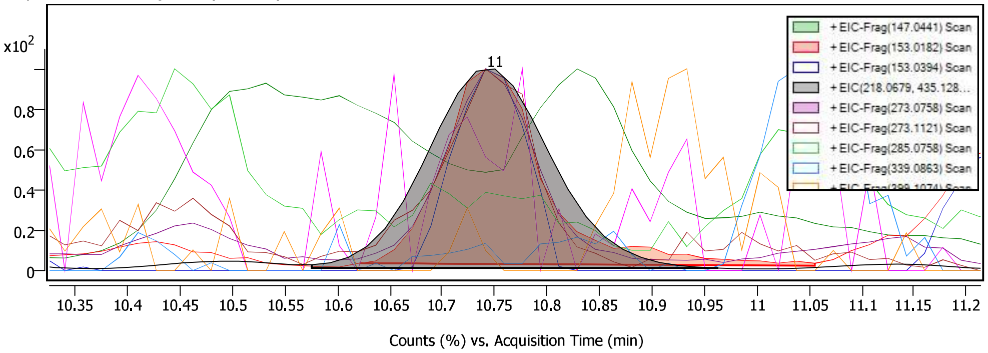

Structure

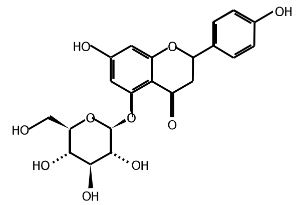

Compound Screening Report

Coelution Plot

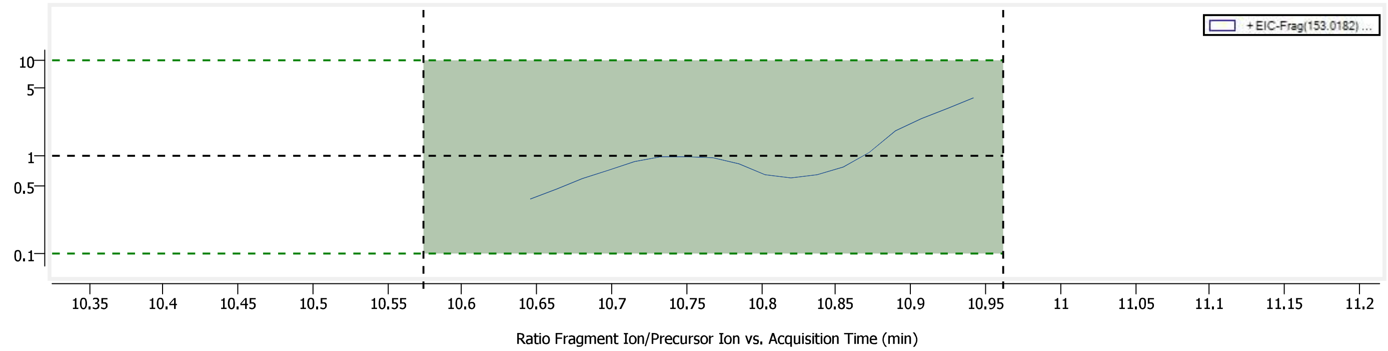

Compound Spectra (overlaid)

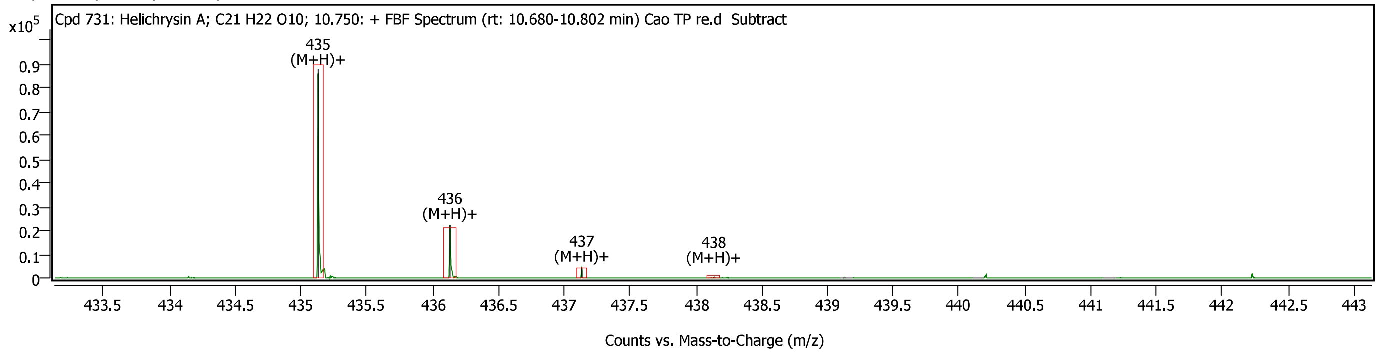

Fragment Spectrum (clean)

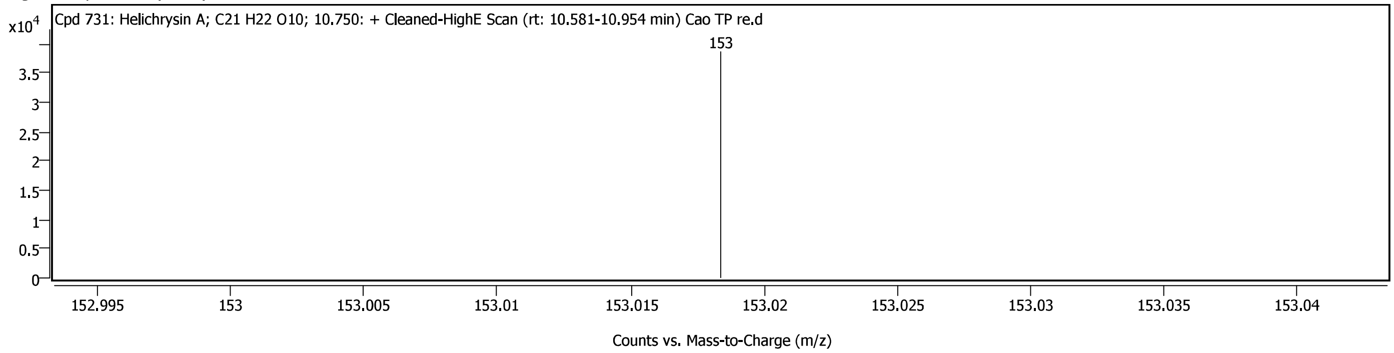

Fragment Spectrum (raw)

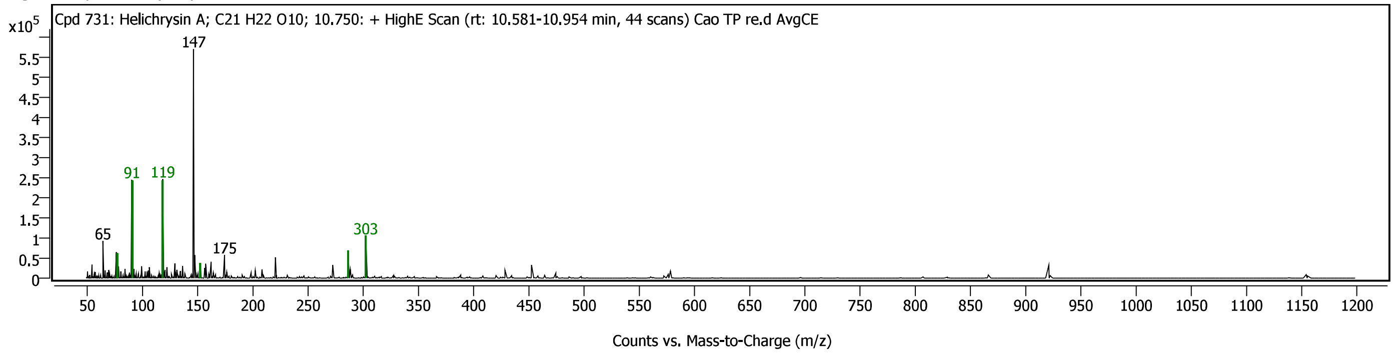

Compound ID Table

Name	Formula	Species	RT	RT Diff	Mass	CAS	ID Source	Score	Score (Lib)	Score (Tgt)
Helichrysin A	C21 H22 O10	(M+H)+	10.750		434.1215	529-41-9	FBF-FragConfirm	98.55		98.55
5,7,2'-Trihydroxyflavanone 7-glucoside	C21 H22 O10	(M+H)+	10.750		434.1215		FBF-FragConfirm	98.55		98.55
5,2'-Dihydroxy-3,6,7,8,4',5'-hexamethoxyflavone	C21 H22 O10	(M+H)+	10.750		434.1215		FBF-FragConfirm	98.55		98.55
8-C-Glucosylnaringenin	C21 H22 O10	(M+H)+	10.750		434.1215		FBF-FragConfirm	98.55		98.55
7-Hydroxyaloin B	C21 H22 O10	(M+H)+	10.750		434.1215	82461-12-9	FBF-FragConfirm	98.55		98.55
5-Hydroxyaloin A	C21 H22 O10	(M+H)+	10.750		434.1215	138373-23-6	FBF-FragConfirm	98.55		98.55
5,7-Dihydroxy-3,6,8,3',4',5'-hexamethoxyflavone	C21 H22 O10	(M+H)+	10.750		434.1215		FBF-FragConfirm	98.55		98.55
5,7-Dihydroxy-3,6,8,2',4',5'-hexamethoxyflavone	C21 H22 O10	(M+H)+	10.750		434.1215		FBF-FragConfirm	98.55		98.55
5,7,8-Trihydroxyflavanone 7-glucoside	C21 H22 O10	(M+H)+	10.750		434.1215		FBF-FragConfirm	98.55		98.55
2',4',6',beta-Tetrahydroxychalcone 2'-glucoside	C21 H22 O10	(M+H)+	10.750		434.1215		FBF-FragConfirm	98.55		98.55
4'-Hydroxyfenoprofen glucuronide	C21 H22 O10	(M+H)+	10.750		434.1215	35440-37-0	FBF-FragConfirm	98.55		98.55
Aloin	C21 H22 O10	(M+H)+	10.750		434.1215	61502-40-7	FBF-FragConfirm	98.55		98.55
3',5'-Dihydroxy-3,5,6,7,8,4'-hexamethoxyflavone	C21 H22 O10	(M+H)+	10.750		434.1215		FBF-FragConfirm	98.55		98.55
2-O-Caffeoylarbutin	C21 H22 O10	(M+H)+	10.750		434.1215	230955-36-9	FBF-FragConfirm	98.55		98.55
2',4',6',beta-Tetrahydroxychalcone 4'-glucoside	C21 H22 O10	(M+H)+	10.750		434.1215		FBF-FragConfirm	98.55		98.55
Chalconaringenin 4-glucoside	C21 H22 O10	(M+H)+	10.750		434.1215		FBF-FragConfirm	98.55		98.55
2',4,4',6'-Tetrahydroxychalcone 4'-O-glucoside	C21 H22 O10	(M+H)+	10.750		434.1215		FBF-FragConfirm	98.55		98.55
(2S)-5,6,7-Trihydroxyflavanone 7-glucoside	C21 H22 O10	(M+H)+	10.750		434.1215		FBF-FragConfirm	98.55		98.55
Eriodictin	C21 H22 O10	(M+H)+	10.750		434.1215		FBF-FragConfirm	98.55		98.55
Agehoustin D	C21 H22 O10	(M+H)+	10.750		434.1215		FBF-FragConfirm	98.55		98.55
(2R,3R)-3,3',4',7-Tetrahydroxyflavanone 7-O-alpha-L-Rhamnopyranoside	C21 H22 O10	(M+H)+	10.750		434.1215	114728-43-7	FBF-FragConfirm	98.55		98.55
Isomonospermoside	C21 H22 O10	(M+H)+	10.750		434.1215		FBF-FragConfirm	98.55		98.55
Isosalipurposide	C21 H22 O10	(M+H)+	10.750		434.1215		FBF-FragConfirm	98.55		98.55
Phlorizin chalcone	C21 H22 O10	(M+H)+	10.750		434.1215	4547-85-7	FBF-FragConfirm	98.55		98.55
Palodulcin B	C21 H22 O10	(M+H)+	10.750		434.1215		FBF-FragConfirm	98.55		98.55
Naringenin 7-O-glucoside	C21 H22 O10	(M+H)+	10.750		434.1215	529-55-5	FBF-FragConfirm	98.55		98.55
Naringenin 4'-O-glucoside	C21 H22 O10	(M+H)+	10.750		434.1215		FBF-FragConfirm	98.55		98.55
Naringenin 4'-O-galactoside	C21 H22 O10	(M+H)+	10.750		434.1215		FBF-FragConfirm	98.55		98.55
Monospermoside	C21 H22 O10	(M+H)+	10.750		434.1215		FBF-FragConfirm	98.55		98.55
Coreopsin	C21 H22 O10	(M+H)+	10.750		434.1215		FBF-FragConfirm	98.55		98.55
Isookaninrhamnoside	C21 H22 O10	(M+H)+	10.750		434.1215		FBF-FragConfirm	98.55		98.55
Chalconaringenin 4'-glucoside	C21 H22 O10	(M+H)+	10.750		434.1215		FBF-FragConfirm	98.55		98.55
Isohemiphloin	C21 H22 O10	(M+H)+	10.750		434.1215		FBF-FragConfirm	98.55		98.55
Isocoreopsin	C21 H22 O10	(M+H)+	10.750		434.1215		FBF-FragConfirm	98.55		98.55
Isocarthamidin 7-O-rhamnoside	C21 H22 O10	(M+H)+	10.750		434.1215		FBF-FragConfirm	98.55		98.55
Hemiphloin	C21 H22 O10	(M+H)+	10.750		434.1215		FBF-FragConfirm	98.55		98.55
Eriodictyol 5-O-rhamnoside	C21 H22 O10	(M+H)+	10.750		434.1215		FBF-FragConfirm	98.55		98.55
Digicitrin	C21 H22 O10	(M+H)+	10.750		434.1215		FBF-FragConfirm	98.55		98.55
Floribundoside	C21 H22 O10	(M+H)+	10.750		434.1215		FBF-FragConfirm	98.55		98.55
Dihydrogenistin	C21 H22 O10	(M+H)+	10.750		434.1215		FBF-FragConfirm	98.55		98.55

Cpd 1589: Citbismine F

Name	Formula	RT	RI	Mass	Diff (Tgt, ppm)	CAS	ID Source	Score	Algorithm
Citbismine F	C36 H34 N2 O10	10.837		654.2225	1.69		FBF	97.54	FBF

Species	m/z	Score (Tgt)	Score (Lib)	Score (DB)	Score (MFG)	Score (RT)
(M+2H)+2 (M+H)+	328.655	97.54				

Compound Chromatograms (overlaid)

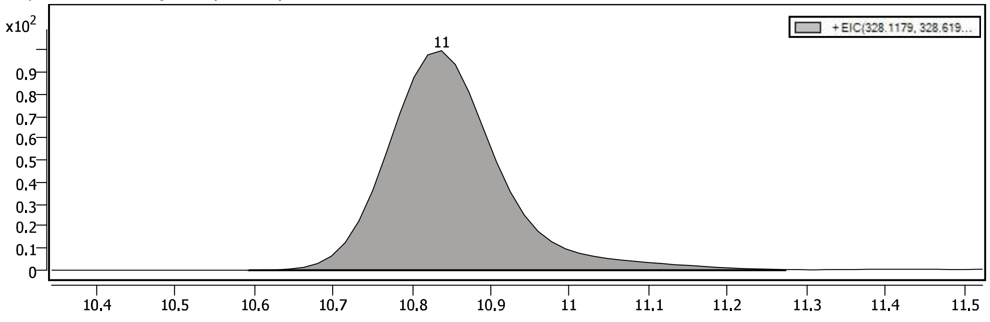

Structure

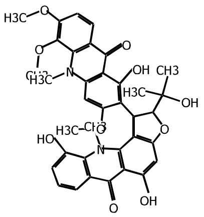

Compound Spectra (overlaid)

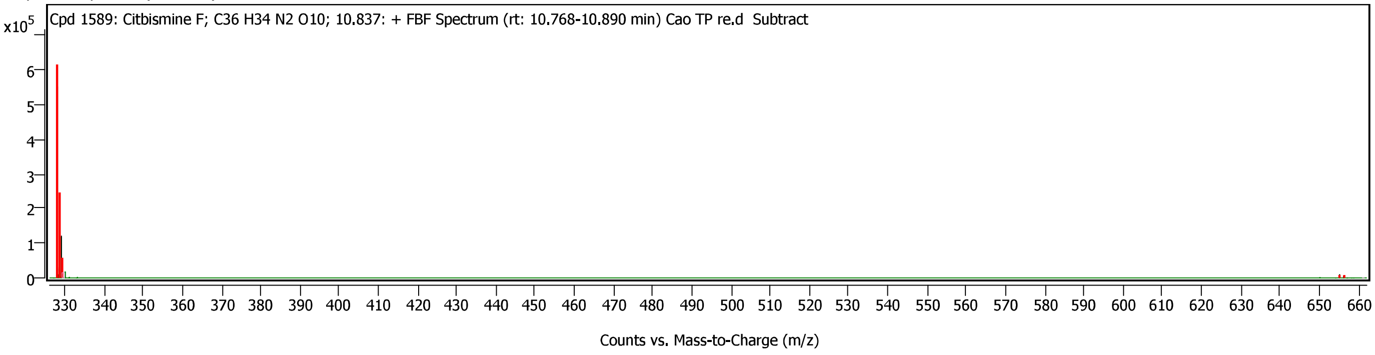

Compound ID Table

Name	Formula	Species	RT	RT Diff	Mass	CAS	ID Source	Score	Score (Lib)	Score (Tgt)
Citbismine F	C36 H34 N2 O10	(M+2H)+2<br>(M+H)+	10.837		654.2225		FBF	97.54		97.54

Cpd 745: Kaempferol 7-galactoside

Name	Formula	RT	RI	Mass	Diff (Tgt, ppm)	CAS	ID Source	Score	Algorithm
Kaempferol 7-galactoside	C21 H20 O11	10.872		448.1008	0.44		M-FBF-FragConfirm	98.28	FBF

Species	m/z	Score (Tgt)	Score (Lib)	Score (DB)	Score (MFG)	Score (RT)
(M+H)+	449	98.28				

Compound Chromatograms (overlaid)

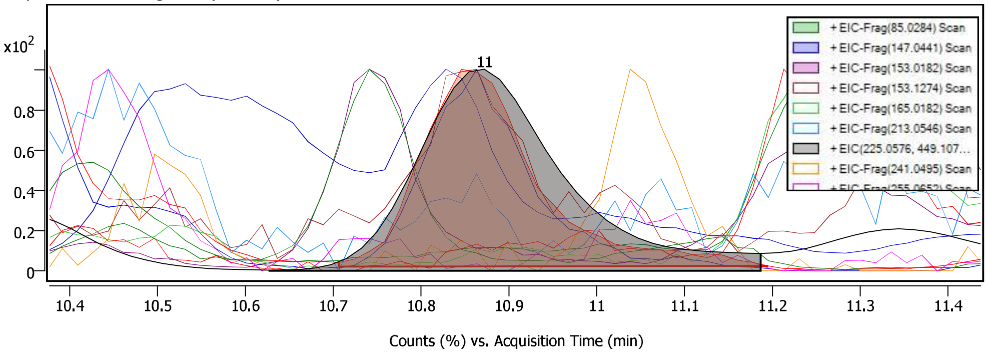

Structure

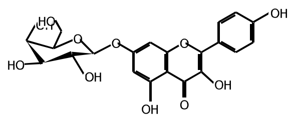

Coelution Plot

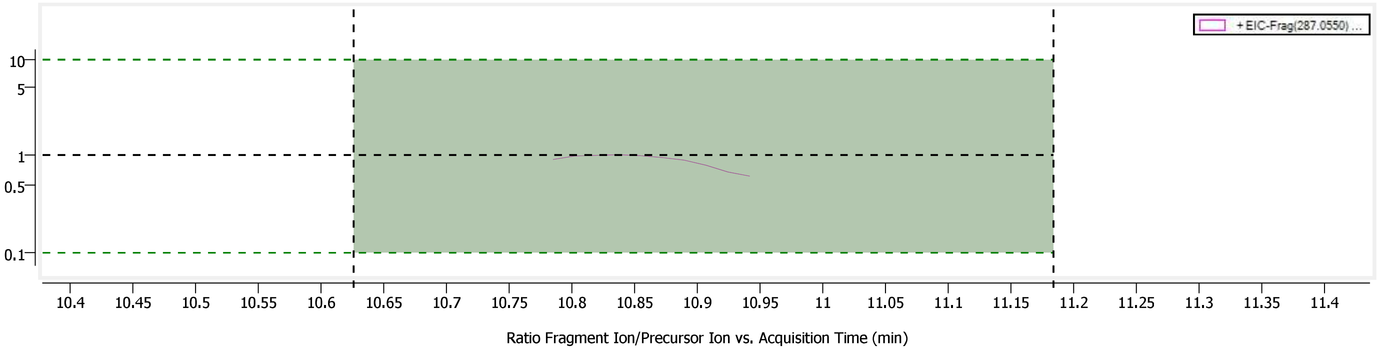

Compound Spectra (overlaid)

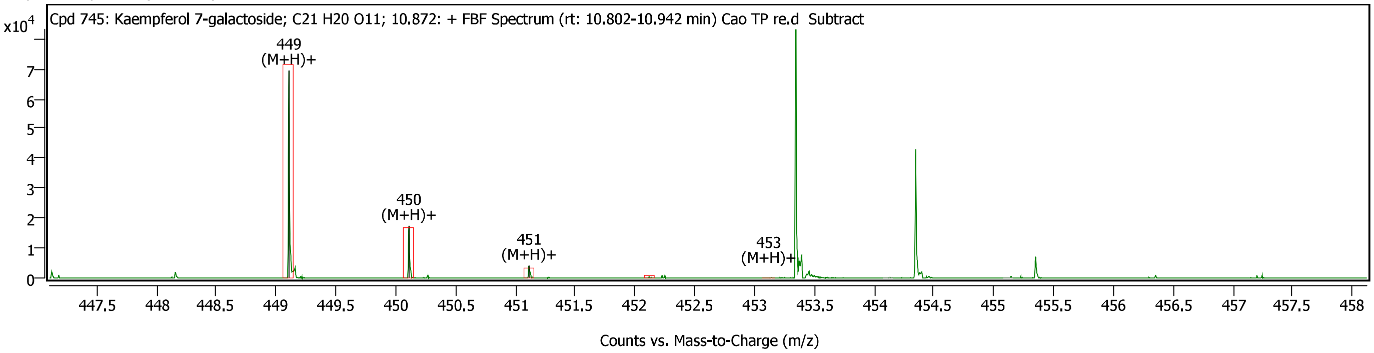

Fragment Spectrum (clean)

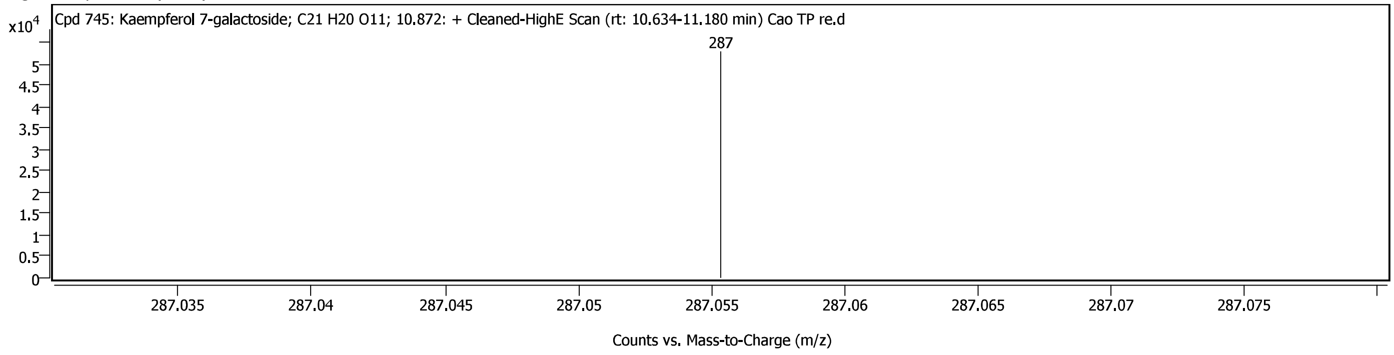

Fragment Spectrum (raw)

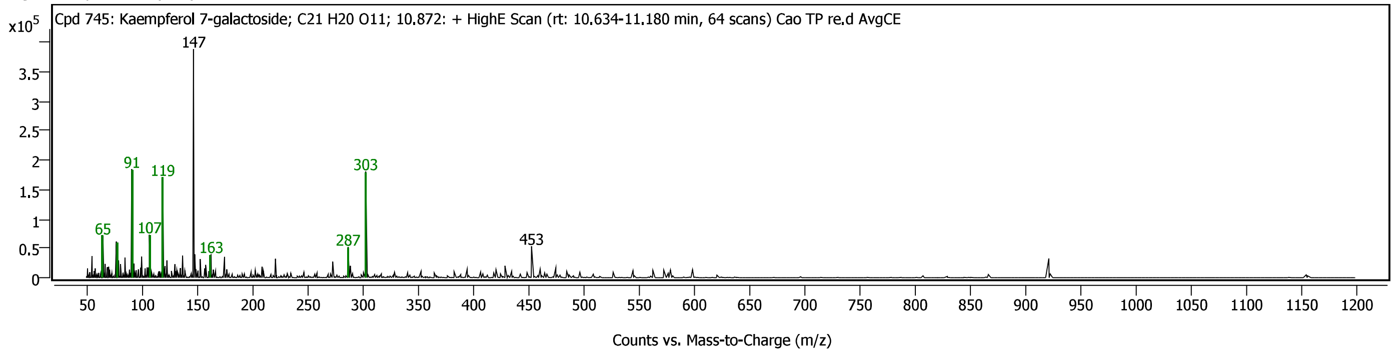

Compound ID Table

Name	Formula	Species	RT	RT Diff	Mass	CAS	ID Source	Score	Score (Lib)	Score (Tgt)
Kaempferol 7-galactoside	C21 H20 O11	(M+H)+	10.872		448.1008		FBF-FragConfirm	98.28		98.28
Carthamone	C21 H20 O11	(M+H)+	10.872		448.1008		FBF-FragConfirm	98.28		98.28
Fisetin 3-glucoside	C21 H20 O11	(M+H)+	10.872		448.1008		FBF-FragConfirm	98.28		98.28
Azaleatin 3-arabinoside	C21 H20 O11	(M+H)+	10.872		448.1008	63742-73-4	FBF-FragConfirm	98.28		98.28
Aureusin	C21 H20 O11	(M+H)+	10.872		448.1008		FBF-FragConfirm	98.28		98.28
Herbacetin 8-rhamnoside	C21 H20 O11	(M+H)+	10.872		448.1008		FBF-FragConfirm	98.28		98.28
Fisetin 8-C-glucoside	C21 H20 O11	(M+H)+	10.872		448.1008		FBF-FragConfirm	98.28		98.28
Fisetin 7-glucoside	C21 H20 O11	(M+H)+	10.872		448.1008		FBF-FragConfirm	98.28		98.28
Fisetin 4'-glucoside	C21 H20 O11	(M+H)+	10.872		448.1008		FBF-FragConfirm	98.28		98.28
evolvuside B	C21 H20 O11	(M+H)+	10.872		448.1008		FBF-FragConfirm	98.28		98.28
Petunidin-3-O-arabinoside	C21 H20 O11	(M+H)+	10.872		448.1008		FBF-FragConfirm	98.28		98.28
Herbacetin 7-rhamnoside	C21 H20 O11	(M+H)+	10.872		448.1008		FBF-FragConfirm	98.28		98.28
Dihydronorwogonin 7-O-glucuronide	C21 H20 O11	(M+H)+	10.872		448.1008		FBF-FragConfirm	98.28		98.28
Dihydrobaicalein 7-O-glucuronide	C21 H20 O11	(M+H)+	10.872		448.1008		FBF-FragConfirm	98.28		98.28
Datiscanin	C21 H20 O11	(M+H)+	10.872		448.1008		FBF-FragConfirm	98.28		98.28
Cynaroside	C21 H20 O11	(M+H)+	10.872		448.1008	5373-11-5	FBF-FragConfirm	98.28		98.28
Cernuoside	C21 H20 O11	(M+H)+	10.872		448.1008		FBF-FragConfirm	98.28		98.28
6-C-Galactosylisoscutellarein	C21 H20 O11	(M+H)+	10.872		448.1008		FBF-FragConfirm	98.28		98.28
Aureusidin 6-O-glucoside	C21 H20 O11	(M+H)+	10.872		448.1008	633-15-8	FBF-FragConfirm	98.28		98.28
8-C-Glucosylorobol	C21 H20 O11	(M+H)+	10.872		448.1008		FBF-FragConfirm	98.28		98.28
8-C-Galactosylluteolin	C21 H20 O11	(M+H)+	10.872		448.1008		FBF-FragConfirm	98.28		98.28
8-C-beta-D-Glucopyranosylkaempferol	C21 H20 O11	(M+H)+	10.872		448.1008		FBF-FragConfirm	98.28		98.28
6-Hydroxyluteolin 7-rhamnoside	C21 H20 O11	(M+H)+	10.872		448.1008		FBF-FragConfirm	98.28		98.28
6-Hydroxyluteolin 6-rhamnoside	C21 H20 O11	(M+H)+	10.872		448.1008		FBF-FragConfirm	98.28		98.28
Astragalin	C21 H20 O11	(M+H)+	10.872		448.1008	480-10-4	FBF-FragConfirm	98.28		98.28
Isoorientin	C21 H20 O11	(M+H)+	10.872		448.1008		FBF-FragConfirm	98.28		98.28
6-Hydroxyluteolin 5-rhamnoside	C21 H20 O11	(M+H)+	10.872		448.1008		FBF-FragConfirm	98.28		98.28
6-C-Galactosylluteolin	C21 H20 O11	(M+H)+	10.872		448.1008		FBF-FragConfirm	98.28		98.28
Isorhamnetin 3-alpha-L-arabinofuranoside	C21 H20 O11	(M+H)+	10.872		448.1008		FBF-FragConfirm	98.28		98.28
5,7,3',4'-Tetrahydroxy-4-phenylcoumarin 5-O-glucoside	C21 H20 O11	(M+H)+	10.872		448.1008		FBF-FragConfirm	98.28		98.28
5,7,2',6'-Tetrahydroxyflavone 2'-O-glucoside	C21 H20 O11	(M+H)+	10.872		448.1008		FBF-FragConfirm	98.28		98.28
1,2,6,8-Tetrahydroxy-3-methylanthraquinone 2-O-b-D-glucoside	C21 H20 O11	(M+H)+	10.872		448.1008		FBF-FragConfirm	98.28		98.28
6-C-Glucosylorobol	C21 H20 O11	(M+H)+	10.872		448.1008		FBF-FragConfirm	98.28		98.28
6-C-Glucopyranosylkaempferol	C21 H20 O11	(M+H)+	10.872		448.1008		FBF-FragConfirm	98.28		98.28
Distichin	C21 H20 O11	(M+H)+	10.872		448.1008		FBF-FragConfirm	98.28		98.28
8-C-Methylquercetin 3-xyloside	C21 H20 O11	(M+H)+	10.872		448.1008		FBF-FragConfirm	98.28		98.28
Hypolaetin 8-rhamnoside	C21 H20 O11	(M+H)+	10.872		448.1008		FBF-FragConfirm	98.28		98.28
Naringenin 5-O-glucuronide	C21 H20 O11	(M+H)+	10.872		448.1008		FBF-FragConfirm	98.28		98.28
Naringenin-4'-O-glucuronide	C21 H20 O11	(M+H)+	10.872		448.1008		FBF-FragConfirm	98.28		98.28
Naringenin-4'-O-β-D-Glucuronide	C21 H20 O11	(M+H)+	10.872		448.1008	158196-35-1	FBF-FragConfirm	98.28		98.28
Naringenin-7-O-glucuronide	C21 H20 O11	(M+H)+	10.872		448.1008		FBF-FragConfirm	98.28		98.28
Naringenin-7-O-β-D-Glucuronide	C21 H20 O11	(M+H)+	10.872		448.1008		FBF-FragConfirm	98.28		98.28
Orientin	C21 H20 O11	(M+H)+	10.872		448.1008		FBF-FragConfirm	98.28		98.28
Orobol 7-O-glucoside	C21 H20 O11	(M+H)+	10.872		448.1008		FBF-FragConfirm	98.28		98.28
Quercetin 3-methyl ether 3'-xyloside	C21 H20 O11	(M+H)+	10.872		448.1008		FBF-FragConfirm	98.28		98.28
Naringenin 4'-O-glucuronide	C21 H20 O11	(M+H)+	10.872		448.1008		FBF-FragConfirm	98.28		98.28
Quercetin 7-rhamnoside	C21 H20 O11	(M+H)+	10.872		448.1008		FBF-FragConfirm	98.28		98.28
Quercitrin	C21 H20 O11	(M+H)+	10.872		448.1008	522-12-3	FBF-FragConfirm	98.28		98.28
Rhamnetin 3-alpha-L-arabinofuranoside	C21 H20 O11	(M+H)+	10.872		448.1008		FBF-FragConfirm	98.28		98.28
Scutellarein 6-glucoside	C21 H20 O11	(M+H)+	10.872		448.1008		FBF-FragConfirm	98.28		98.28
Scutellarein 7-glucoside	C21 H20 O11	(M+H)+	10.872		448.1008		FBF-FragConfirm	98.28		98.28
Trifolin	C21 H20 O11	(M+H)+	10.872		448.1008		FBF-FragConfirm	98.28		98.28
Rhamnetin 3-alpha-L-arabinopyranoside	C21 H20 O11	(M+H)+	10.872		448.1008		FBF-FragConfirm	98.28		98.28
Luteolin 7-galactoside	C21 H20 O11	(M+H)+	10.872		448.1008		FBF-FragConfirm	98.28		98.28
Luteolin 5-glucoside	C21 H20 O11	(M+H)+	10.872		448.1008		FBF-FragConfirm	98.28		98.28
Luteolin 4'-O-glucoside	C21 H20 O11	(M+H)+	10.872		448.1008	6920-38-3	FBF-FragConfirm	98.28		98.28
Luteolin 3'-glucoside	C21 H20 O11	(M+H)+	10.872		448.1008		FBF-FragConfirm	98.28		98.28
Luteolin 7-glucoside	C21 H20 O11	(M+H)+	10.872		448.1008		FBF-FragConfirm	98.28		98.28
Kaempferol 3-alpha-D-galactoside	C21 H20 O11	(M+H)+	10.872		448.1008		FBF-FragConfirm	98.28		98.28
Isorhamnetin 3-xyloside	C21 H20 O11	(M+H)+	10.872		448.1008		FBF-FragConfirm	98.28		98.28
Kaempferol 5-glucoside	C21 H20 O11	(M+H)+	10.872		448.1008		FBF-FragConfirm	98.28		98.28
Kaempferol 3-O-β-D-galactoside	C21 H20 O11	(M+H)+	10.872		448.1008		FBF-FragConfirm	98.28		98.28
Kaempferol 3-alpha-D-glucoside	C21 H20 O11	(M+H)+	10.872		448.1008		FBF-FragConfirm	98.28		98.28
Isoscutellarein 7-glucoside	C21 H20 O11	(M+H)+	10.872		448.1008		FBF-FragConfirm	98.28		98.28
Maritimein	C21 H20 O11	(M+H)+	10.872		448.1008		FBF-FragConfirm	98.28		98.28
Kaempferol 7-alloside	C21 H20 O11	(M+H)+	10.872		448.1008	765949-92-6	FBF-FragConfirm	98.28		98.28
Maritimetin 7-glucoside	C21 H20 O11	(M+H)+	10.872		448.1008		FBF-FragConfirm	98.28		98.28
Kaempferol 4'-glucoside	C21 H20 O11	(M+H)+	10.872		448.1008		FBF-FragConfirm	98.28		98.28
Kaempferol 7-O-glucoside	C21 H20 O11	(M+H)+	10.872		448.1008	16290-07-6	FBF-FragConfirm	98.28		98.28

Cpd 516: SB 243213

Name	Formula	RT	RI	Mass	Diff (Tgt, ppm)	CAS	ID Source	Score	Algorithm
SB 243213	C22 H19 F3 N4 O2	10.942		428.1451	-2.23		FBF	97.21	FBF

Species	m/z	Score (Tgt)	Score (Lib)	Score (DB)	Score (MFG)	Score (RT)
(M+H)+	429	97.21				

Compound Chromatograms (overlaid)

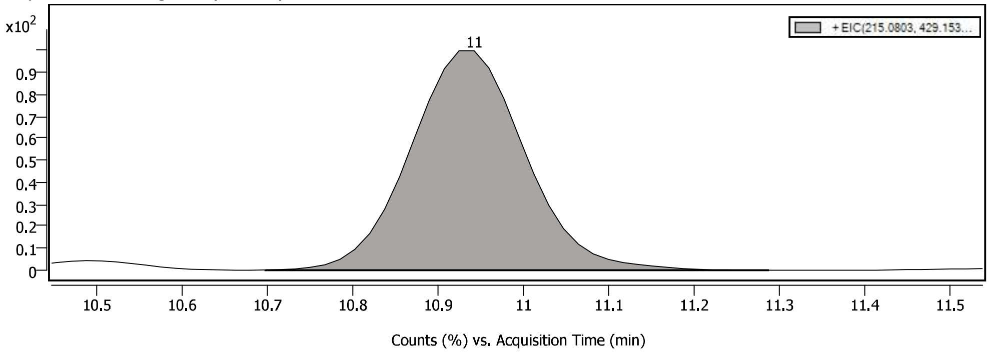

Structure

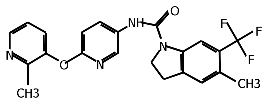

Compound Spectra (overlaid)

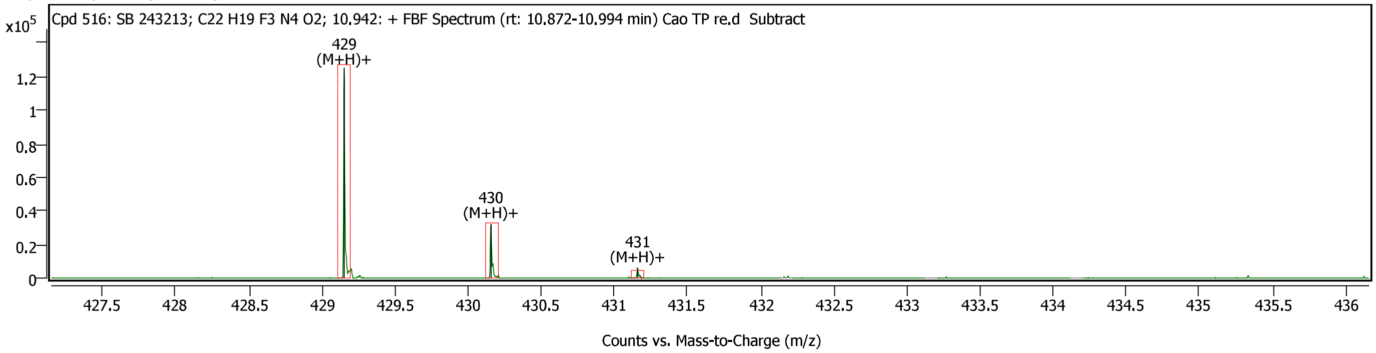

Compound ID Table

Name	Formula	Species	RT	RT Diff	Mass	CAS	ID Source	Score	Score (Lib)	Score (Tgt)
SB 243213	C22 H19 F3 N4 O2	(M+H)+	10.942		428.1451		FBF	97.21		97.21

Cpd 736: 10-Acetoxyoleuropein

Name	Formula	RT	RI	Mass	Diff (Tgt, ppm)	CAS	ID Source	Score	Algorithm
10-Acetoxyoleuropein	C27 H34 O15	11.047		598.1906	1.34		M-FBF	98.52	FBF

Species	m/z	Score (Tgt)	Score (Lib)	Score (DB)	Score (MFG)	Score (RT)
(M+H)+	599	98.52				

Compound Chromatograms (overlaid)

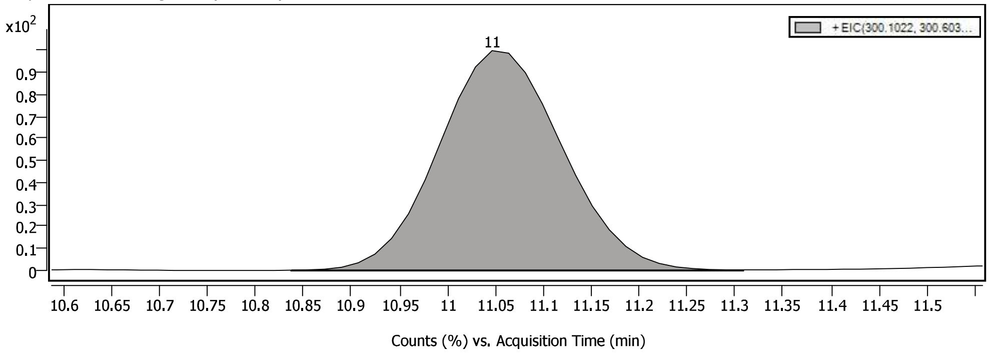

Structure

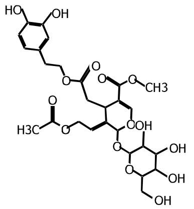

Compound Spectra (overlaid)

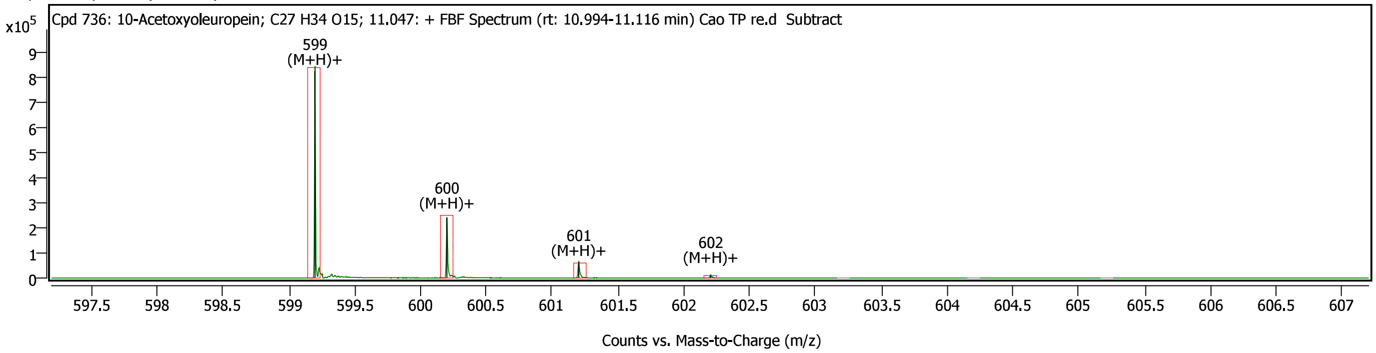

Compound ID Table

Name	Formula	Species	RT	RT Diff	Mass	CAS	ID Source	Score	Score (Lib)	Score (Tgt)
10-Acetoxyoleuropein	C27 H34 O15	(M+H)+	11.047		598.1906		FBF	98.52		98.52
Phloretin 3',5'-Di-C-glucoside	C27 H34 O15	(M+H)+	11.047		598.1906		FBF	98.52		98.52
Geniposide pentaacetate	C27 H34 O15	(M+H)+	11.047		598.1906	49776-64-9	FBF	98.52		98.52
Catechin 3-O-rutinoside	C27 H34 O15	(M+H)+	11.047		598.1906		FBF	98.52		98.52

Cpd 1232: 7-Hydroxytrifluoperazine glucuronide

Name	Formula	RT	RI	Mass	Diff (Tgt, ppm)	CAS	ID Source	Score	Algorithm
7-Hydroxytrifluoperazine glucuronide	C27 H32 F3 N3 O7 S	11.047		599.1935	3.71		FBF	85.28	FBF

Species	m/z	Score (Tgt)	Score (Lib)	Score (DB)	Score (MFG)	Score (RT)
(M+H)+	600	85.28				

Compound Chromatograms (overlaid)

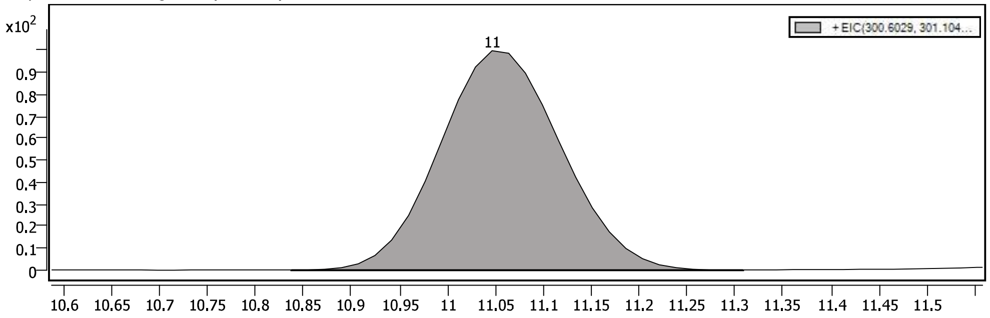

Counts (%) vs. Acquisition Time (min)

Structure

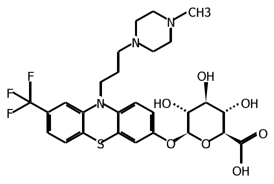

Compound Spectra (overlaid)

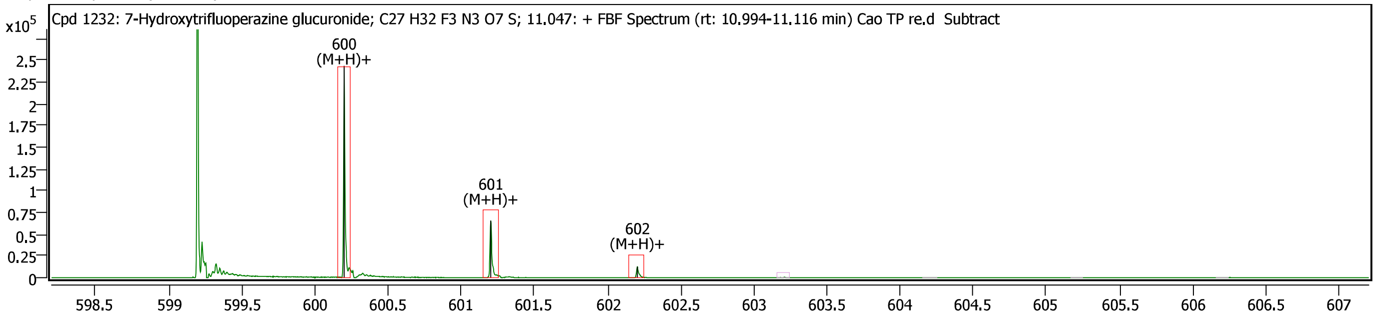

Counts vs. Mass-to-Charge (m/z)

Compound ID Table

Name	Formula	Species	RT	RT Diff	Mass	CAS	ID Source	Score	Score (Lib)	Score (Tgt)
7-Hydroxytrifluoperazine glucuronide	C27 H32 F3 N3 O7 S	(M+H)+	11.047		599.1935		FBF	85.28		85.28

Cpd 1661: Naringin

Name	Formula	RT	RI	Mass	Diff (Tgt, ppm)	CAS	ID Source	Score	Algorithm
Naringin	C27 H32 O14	11.047		580.1795	0.50	10236-47-2	FBF-FragConfirm	98.83	FBF

Species	m/z	Score (Tgt)	Score (Lib)	Score (DB)	Score (MFG)	Score (RT)
(M+H)+	581	98.83				

Compound Chromatograms (overlaid)

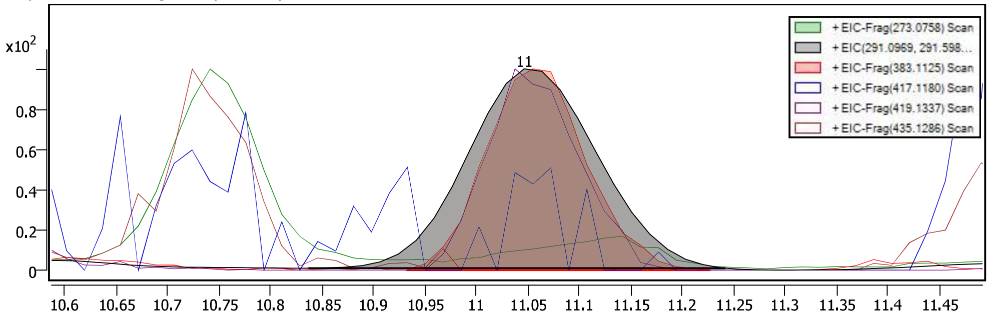

Counts (%) vs. Acquisition Time (min)

Structure

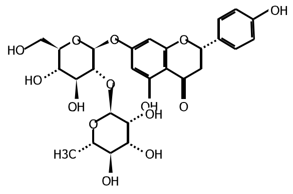

Compound Screening Report

Coelution Plot

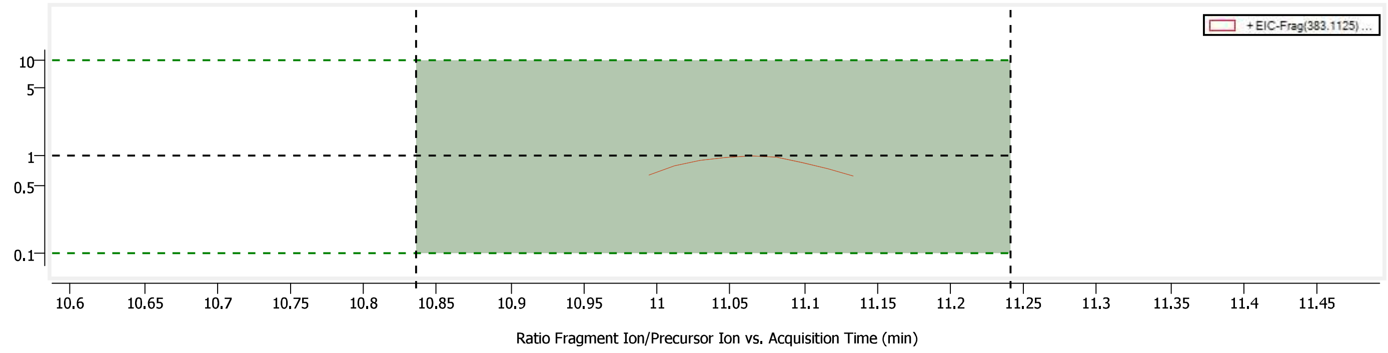

Compound Spectra (overlaid)

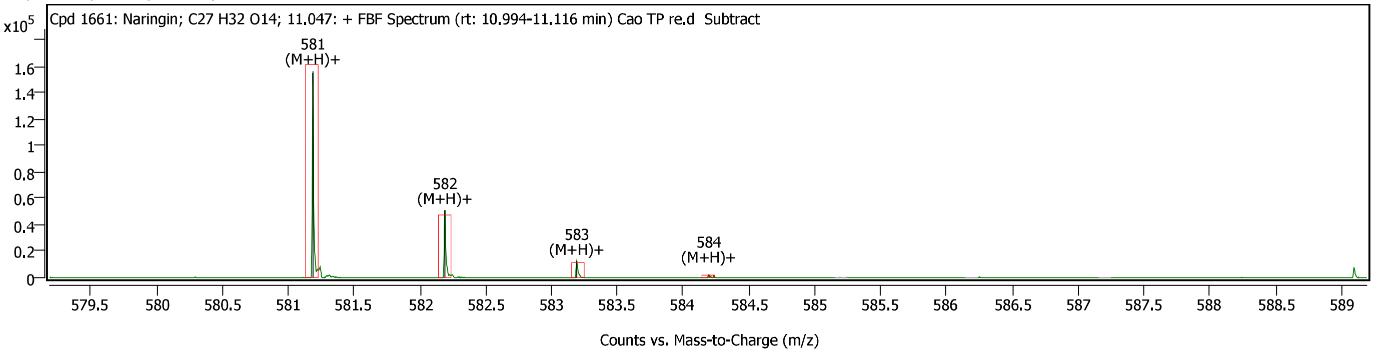

Fragment Spectrum (clean)

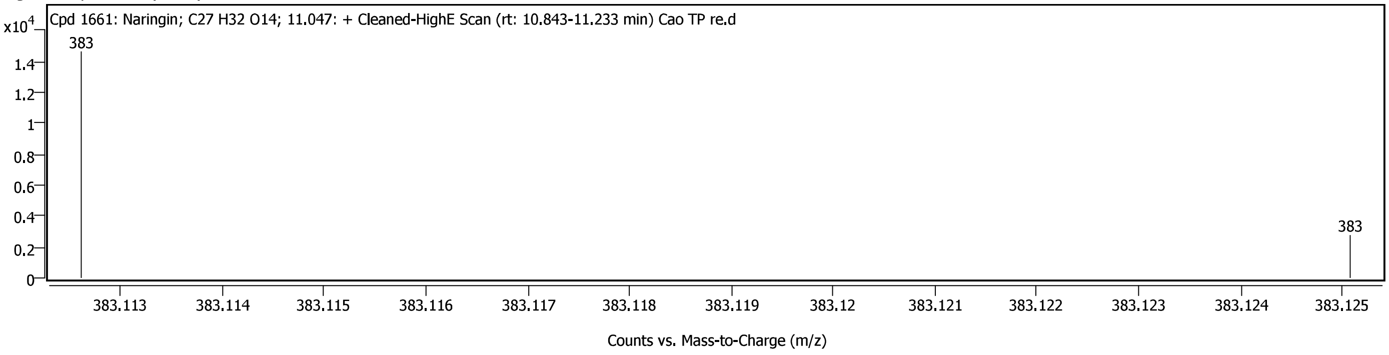

Fragment Spectrum (raw)

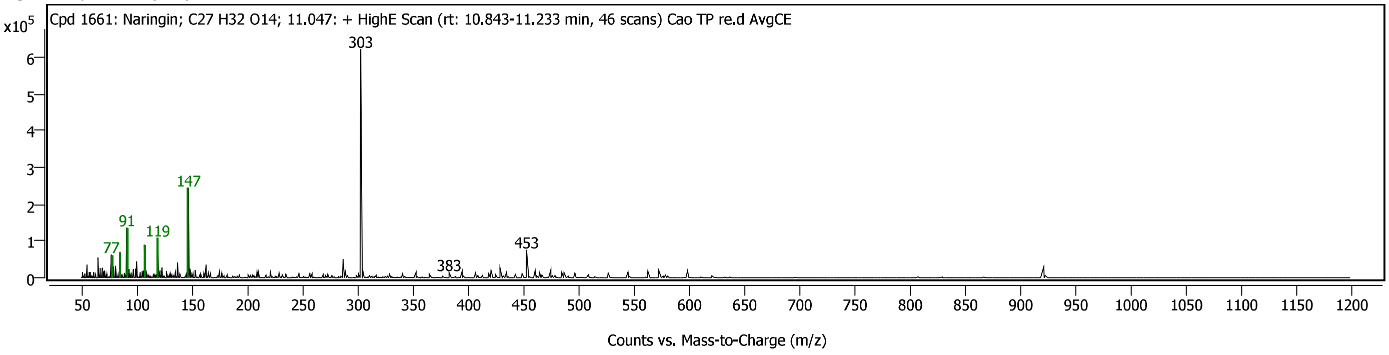

Compound ID Table

Name	Formula	Species	RT	RT Diff	Mass	CAS	ID Source	Score	Score (Lib)	Score (Tgt)
Naringin	C27 H32 O14	(M+H)+	11.047		580.1795	10236-47-2	FBF-FragConfirm	98.83		98.83
Glucoliquiritin	C27 H32 O14	(M+H)+	11.047		580.1795	93446-18-5	FBF-FragConfirm	98.83		98.83
(S)-Naringenin 8-C-(2''-rhamnosylglucoside)	C27 H32 O14	(M+H)+	11.047		580.1795		FBF-FragConfirm	98.83		98.83
5,7,4'-Trihydroxyflavanone 4'-O-rutinoside	C27 H32 O14	(M+H)+	11.047		580.1795		FBF-FragConfirm	98.83		98.83
5'-Methoxycastavinol	C27 H32 O14	(M+H)+	11.047		580.1795	183607-16-1	FBF-FragConfirm	98.83		98.83
Cascaroside F	C27 H32 O14	(M+H)+	11.047		580.1795	164322-83-2	FBF-FragConfirm	98.83		98.83
Chalconaringenin 2'-rhamnosyl-(1->4)-glucoside	C27 H32 O14	(M+H)+	11.047		580.1795		FBF-FragConfirm	98.83		98.83
Cascaroside A	C27 H32 O14	(M+H)+	11.047		580.1795		FBF-FragConfirm	98.83		98.83
Isoliquiritigenin 4,4'-diglucoside	C27 H32 O14	(M+H)+	11.047		580.1795		FBF-FragConfirm	98.83		98.83
Isosakuranetin 7-alpha-L-arabinofuranosyl-(1->6)-glucoside	C27 H32 O14	(M+H)+	11.047		580.1795		FBF-FragConfirm	98.83		98.83
Naringenin 5-O-neohesperidoside	C27 H32 O14	(M+H)+	11.047		580.1795		FBF-FragConfirm	98.83		98.83
Naringin chalcone	C27 H32 O14	(M+H)+	11.047		580.1795		FBF-FragConfirm	98.83		98.83
Narirutin	C27 H32 O14	(M+H)+	11.047		580.1795		FBF-FragConfirm	98.83		98.83
Natsudaidain 3-glucoside	C27 H32 O14	(M+H)+	11.047		580.1795	98891-93-1	FBF-FragConfirm	98.83		98.83

Cpd 1437: N-Adenylylanthranilate

Name	Formula	RT	RI	Mass	Diff (Tgt, ppm)	CAS	ID Source	Score	Algorithm
N-Adenylylanthranilate	C17 H19 N6 O8 P	11.239		466.1014	2.50		FBF	92.39	FBF

Species	m/z	Score (Tgt)	Score (Lib)	Score (DB)	Score (MFG)	Score (RT)
(M+H)+	467	92.39				

Compound Chromatograms (overlaid)

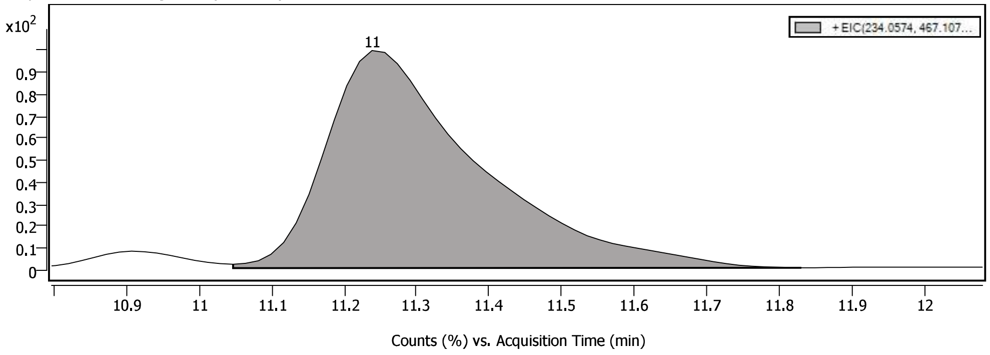

Structure

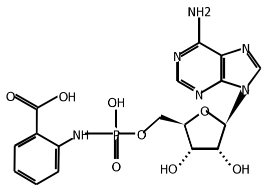

Compound Spectra (overlaid)

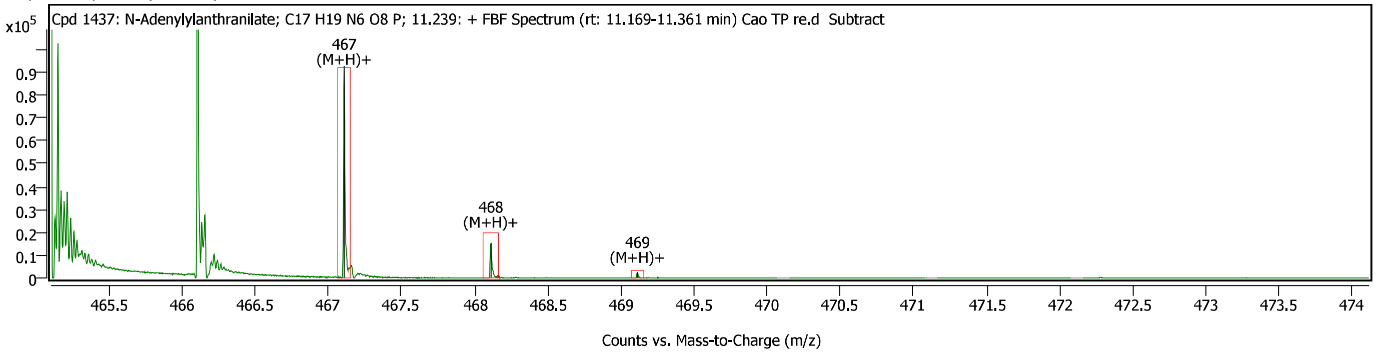

Compound ID Table

Name	Formula	Species	RT	RT Diff	Mass	CAS	ID Source	Score	Score (Lib)	Score (Tgt)
N-Adenylylanthranilate	C17 H19 N6 O8 P	(M+H)+	11.239		466.1014		FBF	92.39		92.39

Cpd 1399: Quercetin

Name	Formula	RT	RI	Mass	Diff (Tgt, ppm)	CAS	ID Source	Score	Algorithm
Quercetin	C15 H10 O7	11.256		302.0435	2.93	117-39-5	M-FBF-FragConfirm	95.08	FBF

Species	m/z	Score (Tgt)	Score (Lib)	Score (DB)	Score (MFG)	Score (RT)
(M+H)+	303	95.08				

Compound Screening Report

Agilent

Compound Chromatograms (overlaid)

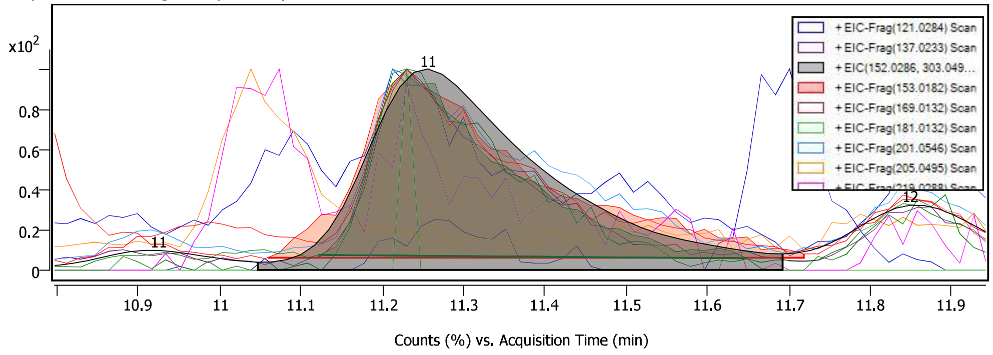

Structure

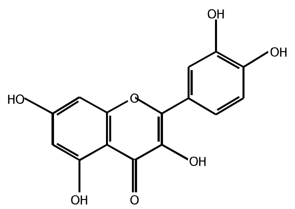

Coelution Plot

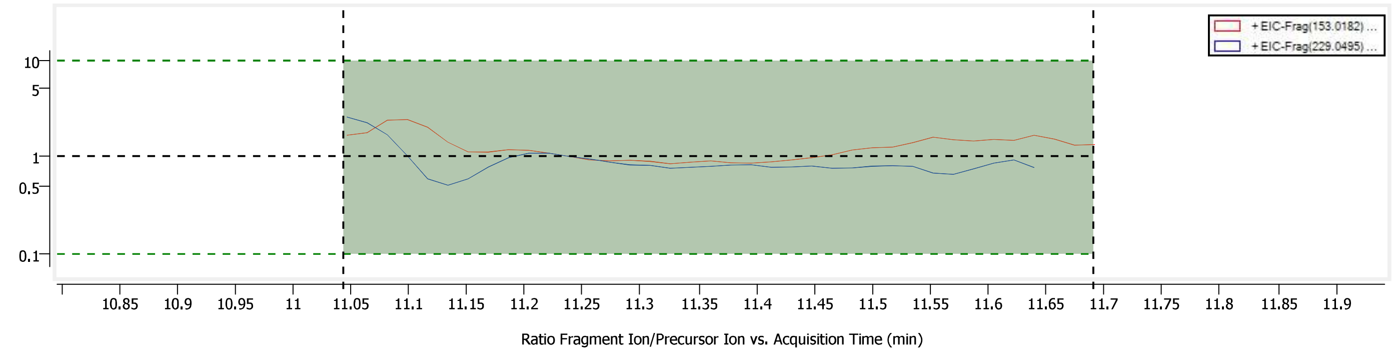

Compound Spectra (overlaid)

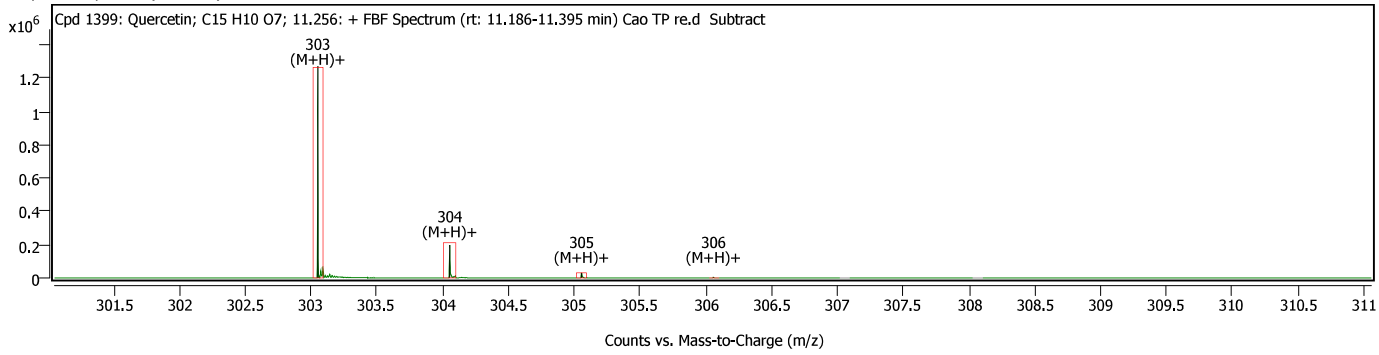

Fragment Spectrum (clean)

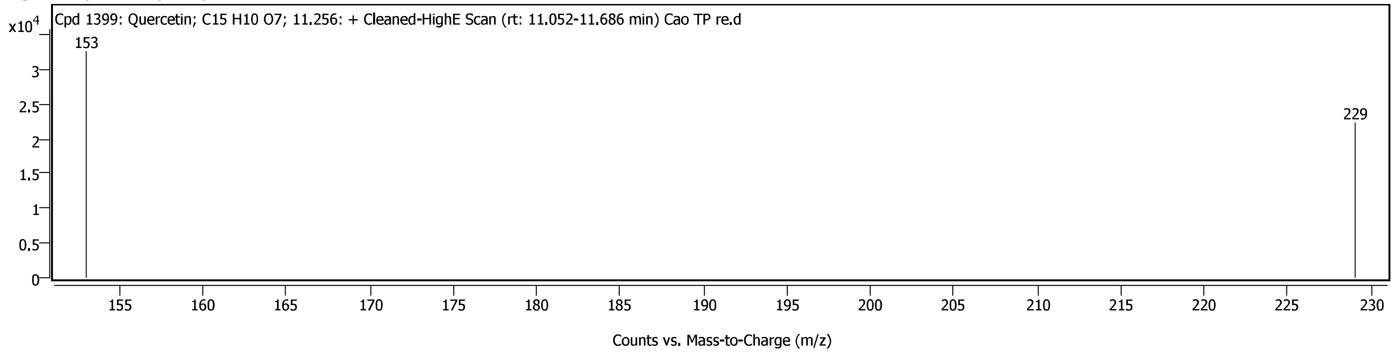

Compound Screening Report

Fragment Spectrum (raw)

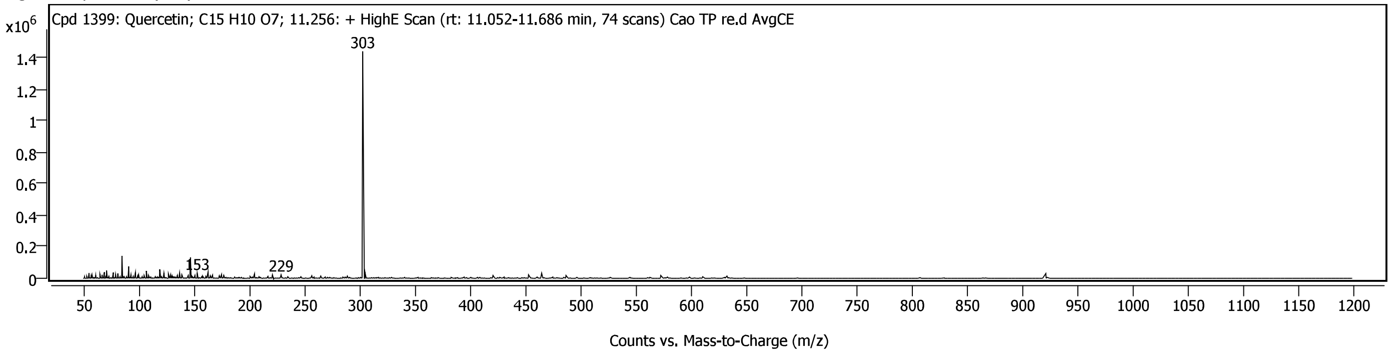

Compound ID Table

Name	Formula	Species	RT	RT Diff	Mass	CAS	ID Source	Score	Score (Lib)	Score (Tgt)
Quercetin	C15 H10 O7	(M+H)+	11.256		302.0435	117-39-5	FBF-FragConfirm	95.08		95.08
6-Hydroxyluteolin	C15 H10 O7	(M+H)+	11.256		302.0435		FBF-FragConfirm	95.08		95.08
5,7,8,3',4'-Pentahydroxyisoflavone	C15 H10 O7	(M+H)+	11.256		302.0435		FBF-FragConfirm	95.08		95.08
Herbacetin	C15 H10 O7	(M+H)+	11.256		302.0435		FBF-FragConfirm	95.08		95.08
2-(4-Hydroxyphenyl)-5,6,7,8-tetrahydroxy-4H-1-benzopyran-4-one	C15 H10 O7	(M+H)+	11.256		302.0435	577-26-4	FBF-FragConfirm	95.08		95.08
2',3',4',5,7-Pentahydroxyflavone	C15 H10 O7	(M+H)+	11.256		302.0435	144707-17-5	FBF-FragConfirm	95.08		95.08
2'-Hydroxypseudobaptigenin	C15 H10 O7	(M+H)+	11.256		302.0435		FBF-FragConfirm	95.08		95.08
3,5,7,2',5'-Pentahydroxyflavone	C15 H10 O7	(M+H)+	11.256		302.0435		FBF-FragConfirm	95.08		95.08
5,6,7,3',4'-Pentahydroxyisoflavone	C15 H10 O7	(M+H)+	11.256		302.0435		FBF-FragConfirm	95.08		95.08
5,7,8,2',4'-Pentahydroxyisoflavone	C15 H10 O7	(M+H)+	11.256		302.0435		FBF-FragConfirm	95.08		95.08
6-Hydroxykaempferol	C15 H10 O7	(M+H)+	11.256		302.0435		FBF-FragConfirm	95.08		95.08
Tricetin	C15 H10 O7	(M+H)+	11.256		302.0435	520-31-0	FBF-FragConfirm	95.08		95.08
Bracteatin	C15 H10 O7	(M+H)+	11.256		302.0435		FBF-FragConfirm	95.08		95.08
Hypolaetin	C15 H10 O7	(M+H)+	11.256		302.0435		FBF-FragConfirm	95.08		95.08
Isoetin	C15 H10 O7	(M+H)+	11.256		302.0435		FBF-FragConfirm	95.08		95.08
Melanoxetin	C15 H10 O7	(M+H)+	11.256		302.0435		FBF-FragConfirm	95.08		95.08
Morin	C15 H10 O7	(M+H)+	11.256		302.0435	480-16-0	FBF-FragConfirm	95.08		95.08
Rhynchosin	C15 H10 O7	(M+H)+	11.256		302.0435		FBF-FragConfirm	95.08		95.08
Robinetin	C15 H10 O7	(M+H)+	11.256		302.0435		FBF-FragConfirm	95.08		95.08
Viscidulin I	C15 H10 O7	(M+H)+	11.256		302.0435	92519-95-4	FBF-FragConfirm	95.08		95.08

Cpd 767: Rutin

Name	Formula	RT	RI	Mass	Diff (Tgt, ppm)	CAS	ID Source	Score	Algorithm
Rutin	C27 H30 O16	11.343		610.1543	1.55	153-18-4	FBF-FragConfirm	98.26	FBF

Species	m/z	Score (Tgt)	Score (Lib)	Score (DB)	Score (MFG)	Score (RT)
(M+H)+	611	98.26				

Compound Chromatograms (overlaid)

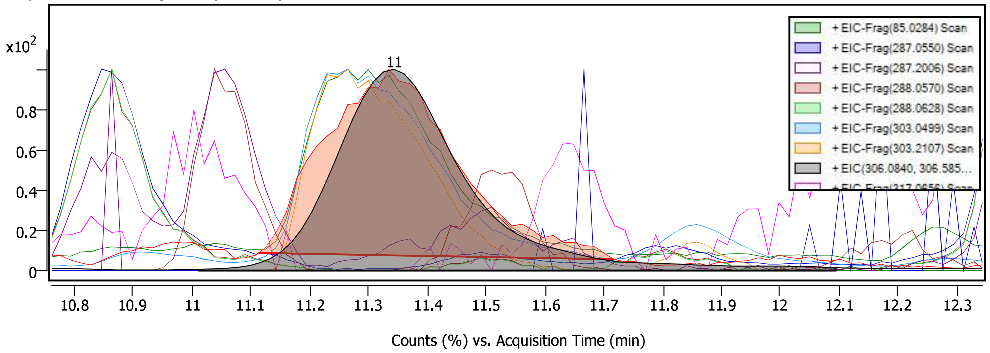

Structure

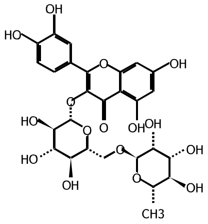

Coelution Plot

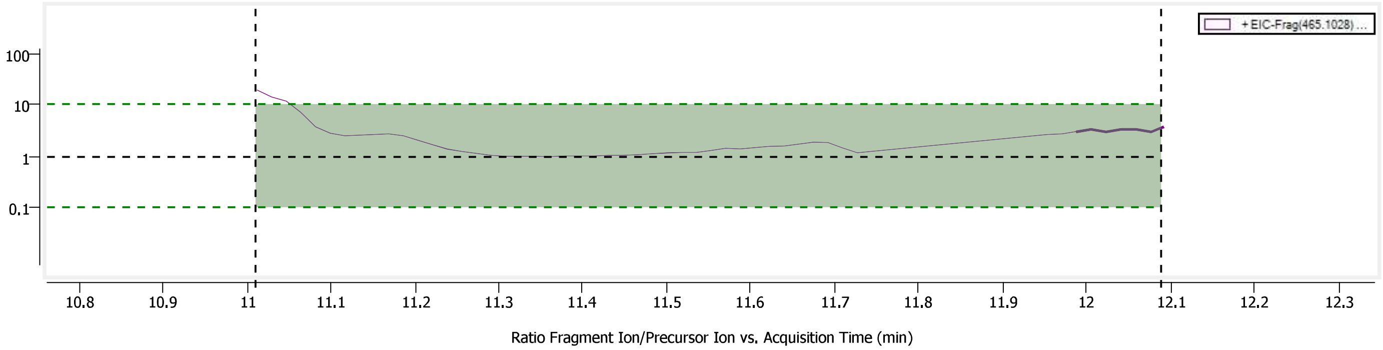

Compound Spectra (overlaid)

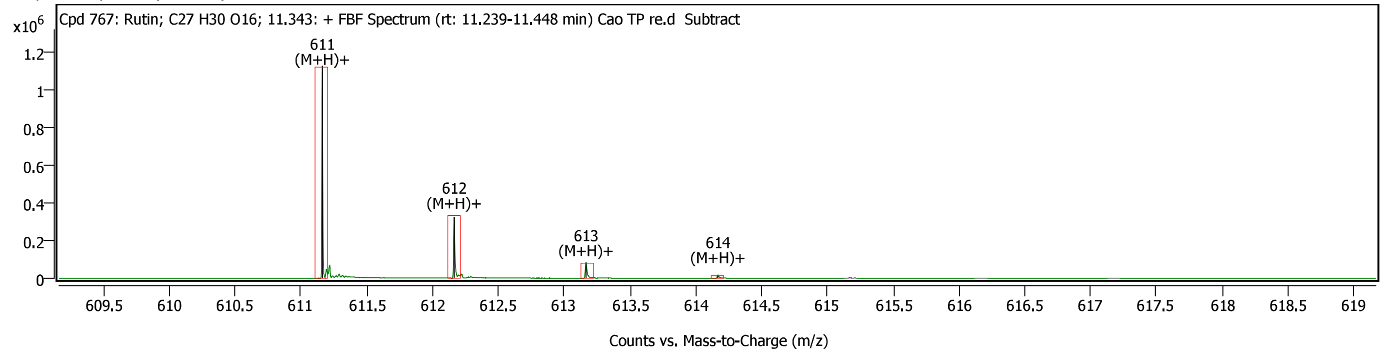

Fragment Spectrum (clean)

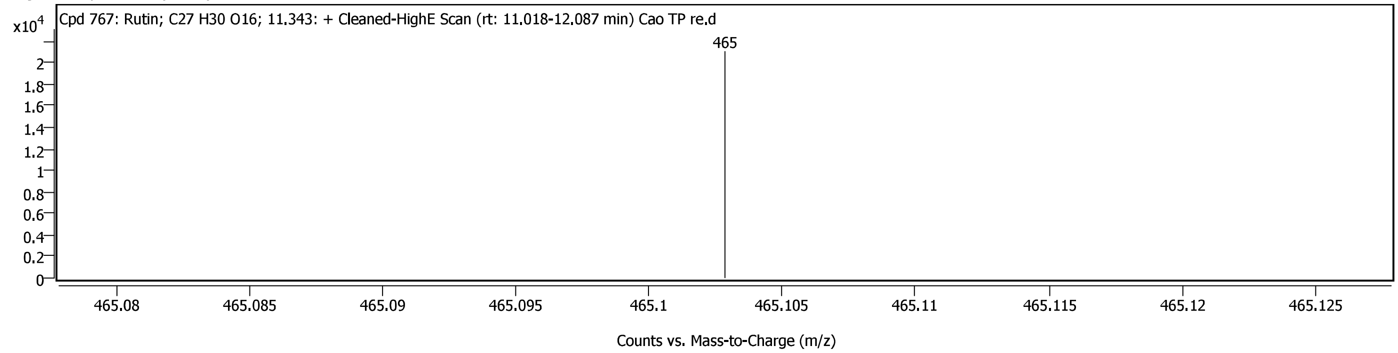

Fragment Spectrum (raw)

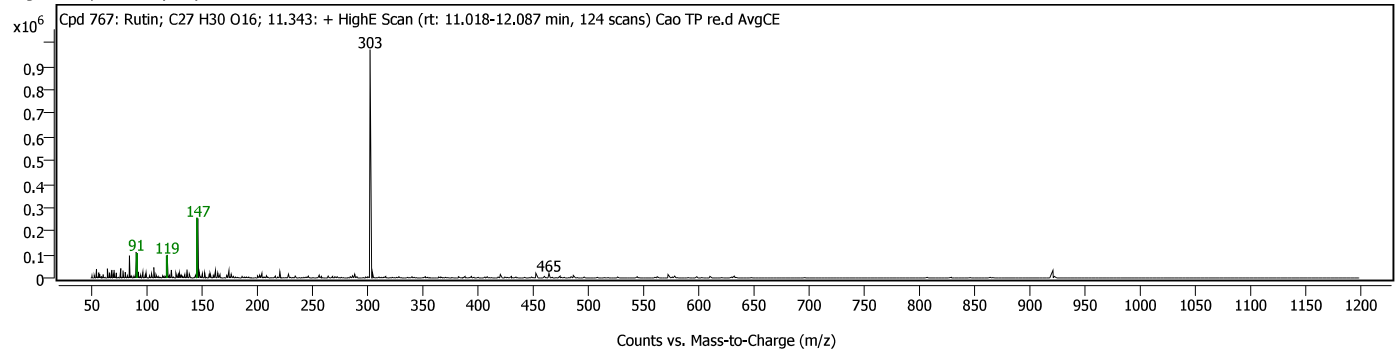

Compound ID Table

Name	Formula	Species	RT	RT Diff	Mass	CAS	ID Source	Score	Score (Lib)	Score (Tgt)
Rutin	C27 H30 O16	(M+H)+	11.343		610.1543	153-18-4	FBF-FragConfirm	98.26		98.26
Isorhamnetin 3-apiosyl-(1->2)-galactoside	C27 H30 O16	(M+H)+	11.343		610.1543		FBF-FragConfirm	98.26		98.26
Isorhamnetin 3-sambubioside	C27 H30 O16	(M+H)+	11.343		610.1543		FBF-FragConfirm	98.26		98.26
Isorhamnetin 3-O-[b-D-xylopyranosyl-(1->6)-b-D-glucopyranoside]	C27 H30 O16	(M+H)+	11.343		610.1543	142905-19-9	FBF-FragConfirm	98.26		98.26
Isorhamnetin 3-L-arabinopyranosyl-(1->6)-galactoside	C27 H30 O16	(M+H)+	11.343		610.1543		FBF-FragConfirm	98.26		98.26
Isorhamnetin 3-glucoside-7-xyloside	C27 H30 O16	(M+H)+	11.343		610.1543		FBF-FragConfirm	98.26		98.26
Isorhamnetin 3-glucoside-7-alpha-L-arabinopyranoside	C27 H30 O16	(M+H)+	11.343		610.1543		FBF-FragConfirm	98.26		98.26
Isorhamnetin 3-apiosyl-(1->2)-glucoside	C27 H30 O16	(M+H)+	11.343		610.1543		FBF-FragConfirm	98.26		98.26
Isoorientin 4'-O-glucoside	C27 H30 O16	(M+H)+	11.343		610.1543		FBF-FragConfirm	98.26		98.26
Isoquercitrin 4''-rhamnoside	C27 H30 O16	(M+H)+	11.343		610.1543	123160-33-8	FBF-FragConfirm	98.26		98.26
Isoorientin 7-O-galactoside	C27 H30 O16	(M+H)+	11.343		610.1543		FBF-FragConfirm	98.26		98.26
Isoorientin 7-glucoside	C27 H30 O16	(M+H)+	11.343		610.1543	35450-86-3	FBF-FragConfirm	98.26		98.26
Kaempferol 3-galactoside-4'-glucoside	C27 H30 O16	(M+H)+	11.343		610.1543		FBF-FragConfirm	98.26		98.26
Isoorientin 3'-O-glucoside	C27 H30 O16	(M+H)+	11.343		610.1543		FBF-FragConfirm	98.26		98.26
Isoorientin 2''-O-alpha-D-mannoside	C27 H30 O16	(M+H)+	11.343		610.1543		FBF-FragConfirm	98.26		98.26
Isorhamnetin 3-vicianoside	C27 H30 O16	(M+H)+	11.343		610.1543		FBF-FragConfirm	98.26		98.26
Isorhamnetin 3-xylosyl-(1->2)-galactoside	C27 H30 O16	(M+H)+	11.343		610.1543		FBF-FragConfirm	98.26		98.26
Isorhamnetin 3-xylosyl-(1->6)-glucoside	C27 H30 O16	(M+H)+	11.343		610.1543		FBF-FragConfirm	98.26		98.26
Isoscutellarein 7-allosyl-(1->2)-glucoside	C27 H30 O16	(M+H)+	11.343		610.1543		FBF-FragConfirm	98.26		98.26
Kaempferol 3,4'-diglucoside	C27 H30 O16	(M+H)+	11.343		610.1543		FBF-FragConfirm	98.26		98.26
Kaempferol 3,5-digalactoside	C27 H30 O16	(M+H)+	11.343		610.1543		FBF-FragConfirm	98.26		98.26
Kaempferol 3,5-diglucoside	C27 H30 O16	(M+H)+	11.343		610.1543		FBF-FragConfirm	98.26		98.26
Kaempferol 3,7-digalactoside	C27 H30 O16	(M+H)+	11.343		610.1543		FBF-FragConfirm	98.26		98.26
Kaempferol 3-gentiobioside	C27 H30 O16	(M+H)+	11.343		610.1543		FBF-FragConfirm	98.26		98.26
Herbacetin 8-rutinoside	C27 H30 O16	(M+H)+	11.343		610.1543		FBF-FragConfirm	98.26		98.26
Rheinoside A	C27 H30 O16	(M+H)+	11.343		610.1543	111545-28-9	FBF-FragConfirm	98.26		98.26
6-Hydroxyluteolin 7-rutinoside	C27 H30 O16	(M+H)+	11.343		610.1543		FBF-FragConfirm	98.26		98.26
8-C-Glucosylquercetin 2''-O-rhamnoside	C27 H30 O16	(M+H)+	11.343		610.1543		FBF-FragConfirm	98.26		98.26
8-Hydroxyapigenin 8-sophoroside	C27 H30 O16	(M+H)+	11.343		610.1543		FBF-FragConfirm	98.26		98.26
2''-O-beta-L-galactopyranosylorientin	C27 H30 O16	(M+H)+	11.343		610.1543		FBF-FragConfirm	98.26		98.26
3,7,2',3',4'-Pentahydroxyflavone 3-neohesperidoside	C27 H30 O16	(M+H)+	11.343		610.1543		FBF-FragConfirm	98.26		98.26
3-[(2S,3R,4S,5S,6R)-4,5-Dihydroxy-6-(hydroxymethyl)-3-[(2S,3R,4S,5R)-3,4,5-trihydroxyoxan-2-yl]oxyoxan-2-yl]oxy-2-(3,4-dihydroxyphenyl)-5-hydroxy-7-methoxychromen-4-one	C27 H30 O16	(M+H)+	11.343		610.1543		FBF-FragConfirm	98.26		98.26
6-C-Glucosylkaempferol 3-O-glucoside	C27 H30 O16	(M+H)+	11.343		610.1543		FBF-FragConfirm	98.26		98.26
6-Hydroxykaempferol 3-rutinoside	C27 H30 O16	(M+H)+	11.343		610.1543		FBF-FragConfirm	98.26		98.26
6-Hydroxykaempferol 7-rutinoside	C27 H30 O16	(M+H)+	11.343		610.1543		FBF-FragConfirm	98.26		98.26
6-Hydroxyluteolin 6-glucoside-3'-rhamnoside	C27 H30 O16	(M+H)+	11.343		610.1543		FBF-FragConfirm	98.26		98.26
Allivicin	C27 H30 O16	(M+H)+	11.343		610.1543	71939-16-7	FBF-FragConfirm	98.26		98.26
Herbacetin 3-rhamnoside-8-glucoside	C27 H30 O16	(M+H)+	11.343		610.1543		FBF-FragConfirm	98.26		98.26
Aureusidin 4,6-diglucoside	C27 H30 O16	(M+H)+	11.343		610.1543		FBF-FragConfirm	98.26		98.26
Herbacetin 7-rhamnoside-8-glucoside	C27 H30 O16	(M+H)+	11.343		610.1543		FBF-FragConfirm	98.26		98.26
Annulatin 7-rhamnoside-3'-xyloside	C27 H30 O16	(M+H)+	11.343		610.1543		FBF-FragConfirm	98.26		98.26
Kaempferol 3-glucoside-7-galactoside	C27 H30 O16	(M+H)+	11.343		610.1543		FBF-FragConfirm	98.26		98.26
Calendoflavobioside	C27 H30 O16	(M+H)+	11.343		610.1543	32453-36-4	FBF-FragConfirm	98.26		98.26
Camelliaside C	C27 H30 O16	(M+H)+	11.343		610.1543		FBF-FragConfirm	98.26		98.26
Fisetin 3,7-diglucoside	C27 H30 O16	(M+H)+	11.343		610.1543		FBF-FragConfirm	98.26		98.26
Flavocannabiside	C27 H30 O16	(M+H)+	11.343		610.1543		FBF-FragConfirm	98.26		98.26
Gossypetin 8-methyl ether 3-xylosyl-(1->2)-rhamnoside	C27 H30 O16	(M+H)+	11.343		610.1543		FBF-FragConfirm	98.26		98.26
Kaempferol 3,7-diglucoside	C27 H30 O16	(M+H)+	11.343		610.1543		FBF-FragConfirm	98.26		98.26
Herbacetin 7-glucosyl-(1->3)-rhamnoside	C27 H30 O16	(M+H)+	11.343		610.1543		FBF-FragConfirm	98.26		98.26
Isoorientin 2''-O-glucopyranoside	C27 H30 O16	(M+H)+	11.343		610.1543		FBF-FragConfirm	98.26		98.26
Quercetin 3-glucosyl-(1->2)-rhamnoside	C27 H30 O16	(M+H)+	11.343		610.1543		FBF-FragConfirm	98.26		98.26
Quercetin 3-glucosyl-(1->4)-rhamnoside	C27 H30 O16	(M+H)+	11.343		610.1543		FBF-FragConfirm	98.26		98.26
Orobol 6,8-di-C-glucoside	C27 H30 O16	(M+H)+	11.343		610.1543		FBF-FragConfirm	98.26		98.26
Panasenoside	C27 H30 O16	(M+H)+	11.343		610.1543		FBF-FragConfirm	98.26		98.26
Quercetin 3-(2-glucosylrhamnoside)	C27 H30 O16	(M+H)+	11.343		610.1543	143016-74-4	FBF-FragConfirm	98.26		98.26
Quercetin 3-galactoside 7-rhamnoside	C27 H30 O16	(M+H)+	11.343		610.1543	38784-81-5	FBF-FragConfirm	98.26		98.26
Quercetin 3-galactoside-7-rhamnoside	C27 H30 O16	(M+H)+	11.343		610.1543		FBF-FragConfirm	98.26		98.26
Quercetin 3-galactosyl-(1->2)-rhamnoside	C27 H30 O16	(M+H)+	11.343		610.1543		FBF-FragConfirm	98.26		98.26
Quercetin 3-galactosyl-(1->4)-rhamnoside	C27 H30 O16	(M+H)+	11.343		610.1543		FBF-FragConfirm	98.26		98.26
Quercetin 3-glucoside-7-rhamnoside	C27 H30 O16	(M+H)+	11.343		610.1543		FBF-FragConfirm	98.26		98.26
Quercetin 3-methyl ether 7-alpha-L-arabinofuranosyl-(1->6)-glucoside	C27 H30 O16	(M+H)+	11.343		610.1543		FBF-FragConfirm	98.26		98.26
Luteolin 7-gentiobioside	C27 H30 O16	(M+H)+	11.343		610.1543		FBF-FragConfirm	98.26		98.26
Robinetin 3-rutinoside	C27 H30 O16	(M+H)+	11.343		610.1543		FBF-FragConfirm	98.26		98.26

Compound ID Table

Name	Formula	Species	RT	RT Diff	Mass	CAS	ID Source	Score	Score (Lib)	Score (Tgt)
Quercetin 3-rhamnoside-3'-glucoside	C27 H30 O16	(M+H)+	11.343		610.1543		FBF-FragConfirm	98.26		98.26
Quercetin 3-rhamnoside-7-glucoside	C27 H30 O16	(M+H)+	11.343		610.1543		FBF-FragConfirm	98.26		98.26
Quercetin 3-rhamnosyl-(1->2)-galactoside	C27 H30 O16	(M+H)+	11.343		610.1543		FBF-FragConfirm	98.26		98.26
Quercetin 3-robinobioside	C27 H30 O16	(M+H)+	11.343		610.1543		FBF-FragConfirm	98.26		98.26
Quercetin 7-(rhamnosylglucoside)	C27 H30 O16	(M+H)+	11.343		610.1543	73432-00-5	FBF-FragConfirm	98.26		98.26
Quercetin 7-methyl ether 3-alpha-L-arabinopyranosyl-(1->3)-galactoside	C27 H30 O16	(M+H)+	11.343		610.1543		FBF-FragConfirm	98.26		98.26
Quercetin 7-rutinoside	C27 H30 O16	(M+H)+	11.343		610.1543		FBF-FragConfirm	98.26		98.26
Quercetin 8-C-(2''-rhamnosylglucoside)	C27 H30 O16	(M+H)+	11.343		610.1543	182062-20-0	FBF-FragConfirm	98.26		98.26
Kaempferol 3-glucosyl-(1->6)-galactoside	C27 H30 O16	(M+H)+	11.343		610.1543		FBF-FragConfirm	98.26		98.26
Kaempferol 3-O-β-D-glucosyl-(1->2)-β-D-glucoside	C27 H30 O16	(M+H)+	11.343		610.1543	19895-95-5	FBF-FragConfirm	98.26		98.26
Quercetin 3-neohesperidoside	C27 H30 O16	(M+H)+	11.343		610.1543		FBF-FragConfirm	98.26		98.26
Myricetin 3-rhamnosyl-(1->2)-rhamnoside	C27 H30 O16	(M+H)+	11.343		610.1543		FBF-FragConfirm	98.26		98.26
Luteolin 7,4'-diglucoside	C27 H30 O16	(M+H)+	11.343		610.1543		FBF-FragConfirm	98.26		98.26
Luteolin 7-galactosyl-(1->6)-galactoside	C27 H30 O16	(M+H)+	11.343		610.1543		FBF-FragConfirm	98.26		98.26
Kaempferol 3-O-β-D-glucosylgalactoside	C27 H30 O16	(M+H)+	11.343		610.1543		FBF-FragConfirm	98.26		98.26
Orientin 7-glucoside	C27 H30 O16	(M+H)+	11.343		610.1543		FBF-FragConfirm	98.26		98.26
Kaempferol 7,4'-diglucoside	C27 H30 O16	(M+H)+	11.343		610.1543		FBF-FragConfirm	98.26		98.26
Kaempferol 7-sophoroside	C27 H30 O16	(M+H)+	11.343		610.1543		FBF-FragConfirm	98.26		98.26
Lucenin 2	C27 H30 O16	(M+H)+	11.343		610.1543		FBF-FragConfirm	98.26		98.26
Luteolin 3',4'-diglucoside	C27 H30 O16	(M+H)+	11.343		610.1543		FBF-FragConfirm	98.26		98.26
Luteolin 7,3'-diglucoside	C27 H30 O16	(M+H)+	11.343		610.1543		FBF-FragConfirm	98.26		98.26
Luteolin 7-allosyl-(1->2)-glucoside	C27 H30 O16	(M+H)+	11.343		610.1543		FBF-FragConfirm	98.26		98.26
Orobol 7-O-sophoroside	C27 H30 O16	(M+H)+	11.343		610.1543		FBF-FragConfirm	98.26		98.26
Luteolin 7-galactoside-4'-glucoside	C27 H30 O16	(M+H)+	11.343		610.1543		FBF-FragConfirm	98.26		98.26
Luteolin 6-C-glucoside 8-C-arabinoside	C27 H30 O16	(M+H)+	11.343		610.1543		FBF-FragConfirm	98.26		98.26
Orientin 3'-O-glucoside	C27 H30 O16	(M+H)+	11.343		610.1543		FBF-FragConfirm	98.26		98.26
Luteolin 7-laminaribioside	C27 H30 O16	(M+H)+	11.343		610.1543		FBF-FragConfirm	98.26		98.26
Lutonarin	C27 H30 O16	(M+H)+	11.343		610.1543		FBF-FragConfirm	98.26		98.26
Meloside L	C27 H30 O16	(M+H)+	11.343		610.1543	55196-48-0	FBF-FragConfirm	98.26		98.26
Multinoside A	C27 H30 O16	(M+H)+	11.343		610.1543	59262-54-3	FBF-FragConfirm	98.26		98.26
Myricetin 3,4'-di-O-alpha-L-rhamnopyranoside	C27 H30 O16	(M+H)+	11.343		610.1543		FBF-FragConfirm	98.26		98.26
Orientin 4'-glucoside	C27 H30 O16	(M+H)+	11.343		610.1543		FBF-FragConfirm	98.26		98.26

Cpd 1515: N-Caffeoyltryptophan

Name	Formula	RT	RI	Mass	Diff (Tgt, ppm)	CAS	ID Source	Score	Algorithm
N-Caffeoyltryptophan	C20 H18 N2 O5	11.762		366.1223	2.06	109163-69-1	FBF	97.65	FBF

Species	m/z	Score (Tgt)	Score (Lib)	Score (DB)	Score (MFG)	Score (RT)
(M+H)+	367	97.65				

Compound Chromatograms (overlaid)

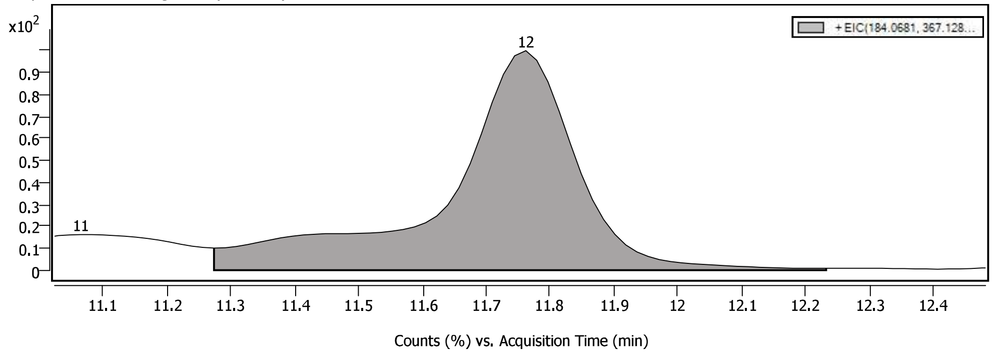

Structure

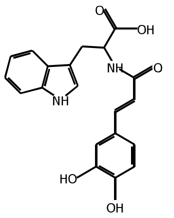

Compound Spectra (overlaid)

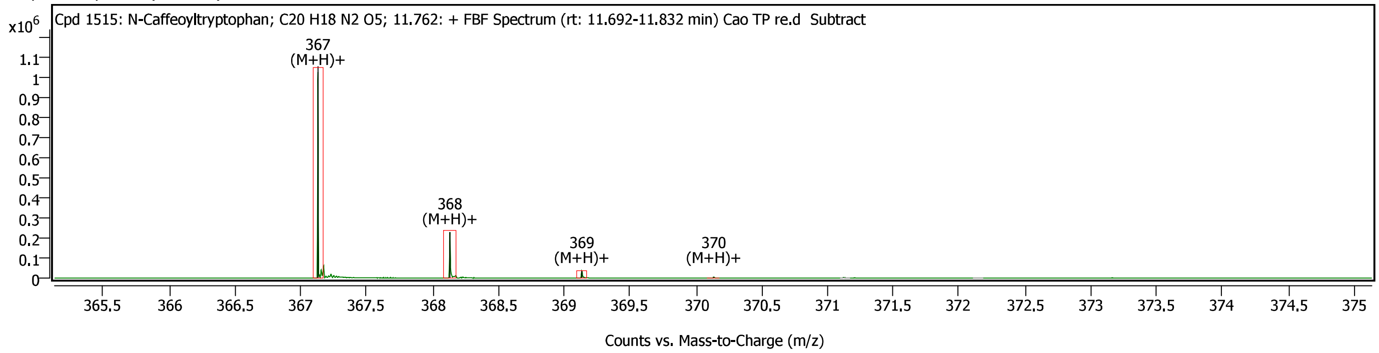

Compound ID Table

Name	Formula	Species	RT	RT Diff	Mass	CAS	ID Source	Score	Score (Lib)	Score (Tgt)
N-Caffeoyltryptophan	C20 H18 N2 O5	(M+H)+	11.762		366.1223	109163-69-1	FBF	97.65		97.65

Cpd 1685: epi-4'-hydroxyjasmonic acid

Name	Formula	RT	RI	Mass	Diff (Tgt, ppm)	CAS	ID Source	Score	Algorithm
epi-4'-hydroxyjasmonic acid	C12 H18 O4	11.989		226.1208	1.35		M-FBF	97.71	FBF

Species	m/z	Score (Tgt)	Score (Lib)	Score (DB)	Score (MFG)	Score (RT)
(M+H)+	227	97.71				

Compound Chromatograms (overlaid)

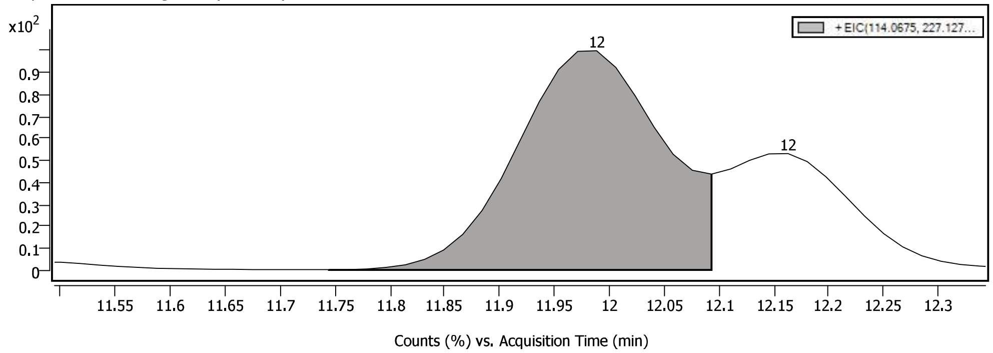

Structure

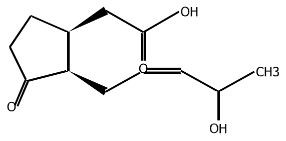

Compound Spectra (overlaid)

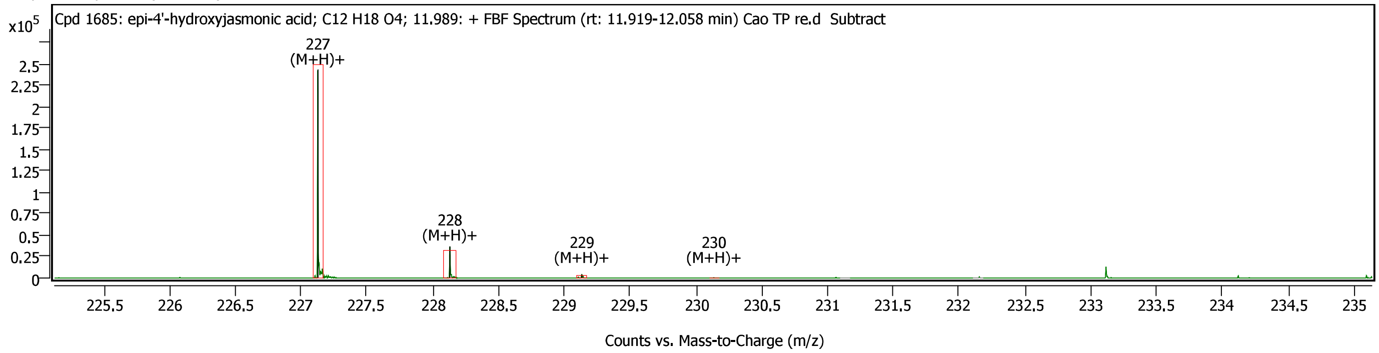

Compound ID Table

Name	Formula	Species	RT	RT Diff	Mass	CAS	ID Source	Score	Score (Lib)	Score (Tgt)
epi-4'-hydroxyjasmonic acid	C12 H18 O4	(M+H)+	11.989		226.1208		FBF	97.71		97.71
Allixin	C12 H18 O4	(M+H)+	11.989		226.1208	125263-70-9	FBF	97.71		97.71
12-hydroxyjasmonic acid	C12 H18 O4	(M+H)+	11.989		226.1208		FBF	97.71		97.71
3,7-Dimethyl-2E,6E-decadien-1,10-dioic acid	C12 H18 O4	(M+H)+	11.989		226.1208		FBF	97.71		97.71
Butopyronoxyl	C12 H18 O4	(M+H)+	11.989		226.1208	532-34-3	FBF	97.71		97.71
Tuberonic acid	C12 H18 O4	(M+H)+	11.989		226.1208		FBF	97.71		97.71

Cpd 1557: Polyethylene, oxidized

Name	Formula	RT	RI	Mass	Diff (Tgt, ppm)	CAS	ID Source	Score	Algorithm
Polyethylene, oxidized	C12 H20 O5	12.163		244.1315	1.83	68441-17-8	FBF	97.59	FBF

Species	m/z	Score (Tgt)	Score (Lib)	Score (DB)	Score (MFG)	Score (RT)
(M+H)+	245	97.59				

Compound Chromatograms (overlaid)

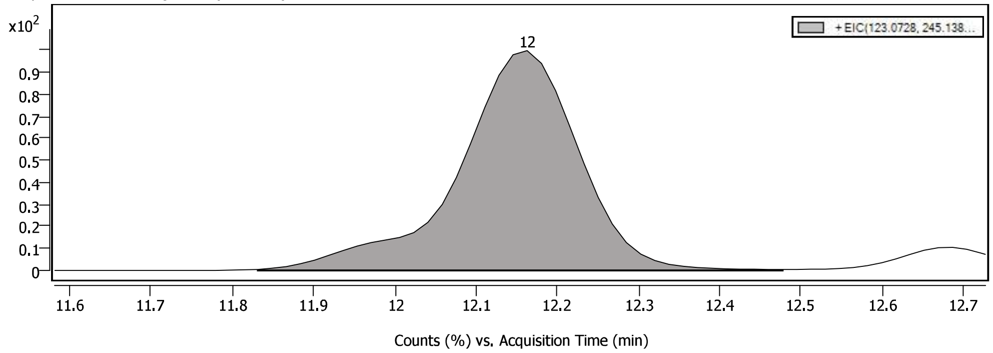

Structure

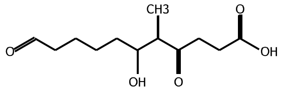

Compound Screening Report

Compound Spectra (overlaid)

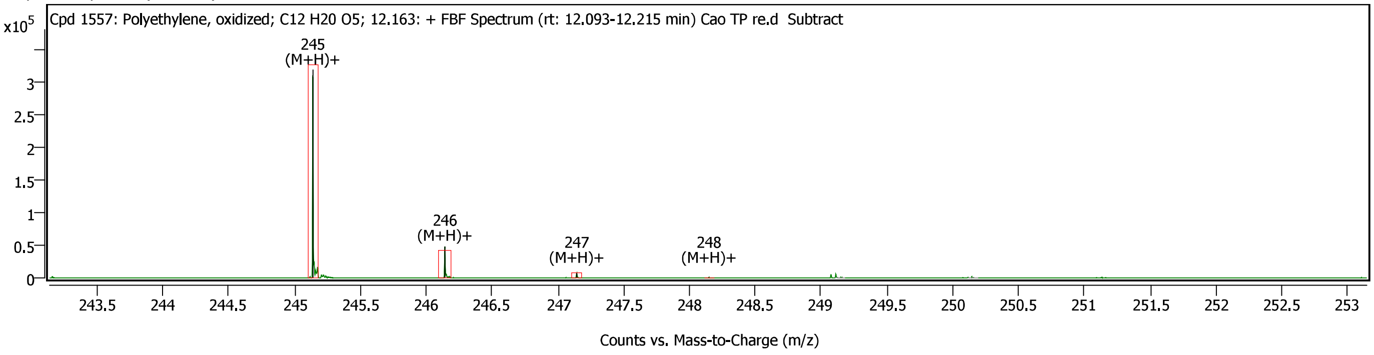

Compound ID Table

Name	Formula	Species	RT	RT Diff	Mass	CAS	ID Source	Score	Score (Lib)	Score (Tgt)
Polyethylene, oxidized	C12 H20 O5	(M+H)+	12.163		244.1315	68441-17-8	FBF	97.59		97.59

Cpd 1466: <Troxerutin>

Name	Formula	RT	RI	Mass	Diff (Tgt, ppm)	CAS	ID Source	Score	Algorithm
<Troxerutin>	C33 H42 O19	12.268		742.2323	0.43	7085-55-4	M-FBF	98.72	FBF

Species	m/z	Score (Tgt)	Score (Lib)	Score (DB)	Score (MFG)	Score (RT)
(M+H)+	743	98.72				

Compound Chromatograms (overlaid)

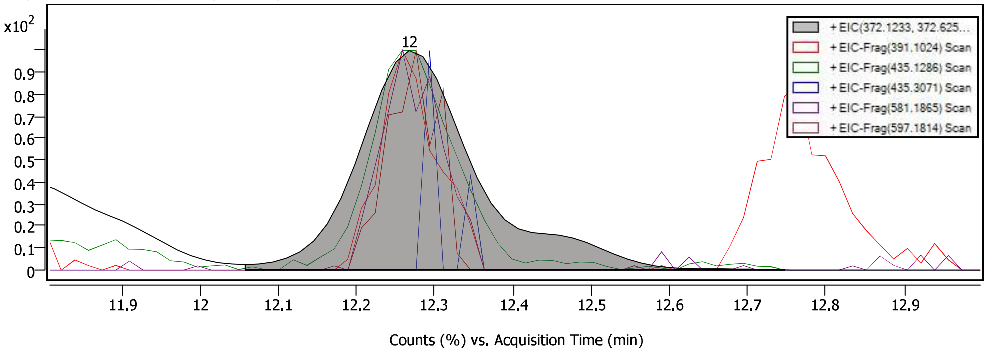

Structure

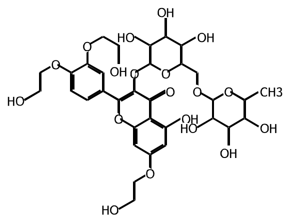

Coelution Plot

Compound Spectra (overlaid)

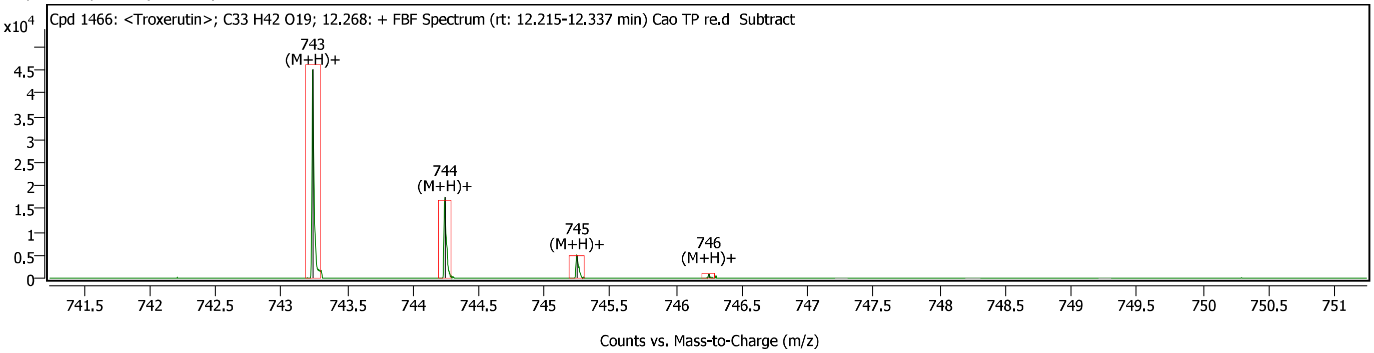

Fragment Spectrum (raw)

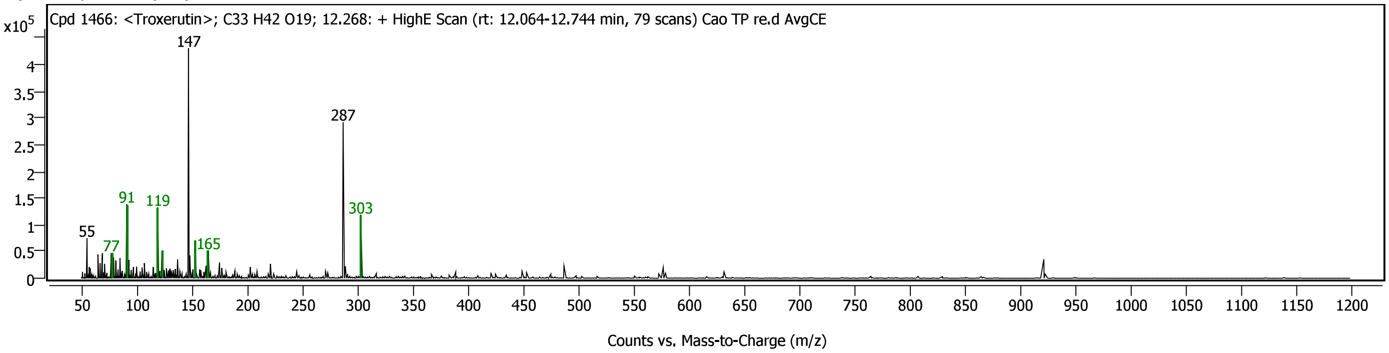

Compound Screening Report

Compound ID Table

Name	Formula	Species	RT	RT Diff	Mass	CAS	ID Source	Score	Score (Lib)	Score (Tgt)
<Troxerutin>	C33 H42 O19	(M+H)+	12.268		742.2323	7085-55-4	FBF	98.72		98.72
<Narirutin 4'-glucoside>	C33 H42 O19	(M+H)+	12.268		742.2323		FBF	98.72		98.72
<Naringin 4'-glucoside>	C33 H42 O19	(M+H)+	12.268		742.2323		FBF	98.72		98.72

Cpd 725: Prunin 6''-O-gallate

Name	Formula	RT	RI	Mass	Diff (Tgt, ppm)	CAS	ID Source	Score	Algorithm
Prunin 6''-O-gallate	C28 H26 O14	12.285		586.1323	0.13		FBF	99.31	FBF

Species	m/z	Score (Tgt)	Score (Lib)	Score (DB)	Score (MFG)	Score (RT)
(M+H)+	587	99.31				

Compound Chromatograms (overlaid)

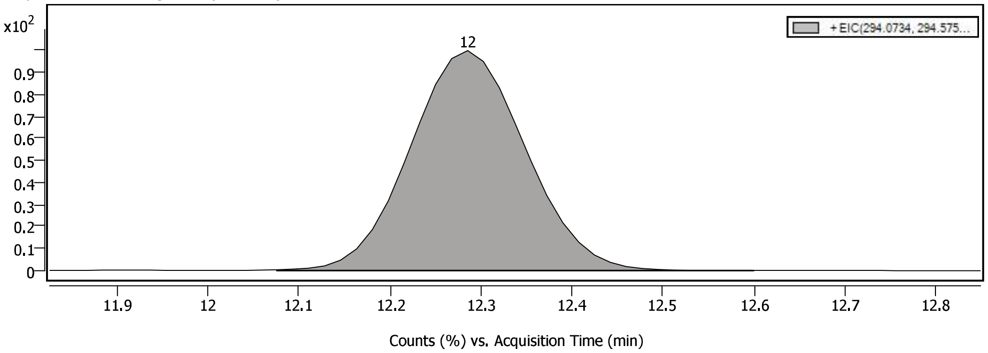

Structure

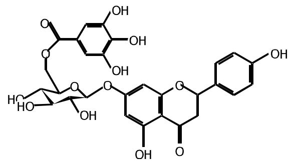

Compound Spectra (overlaid)

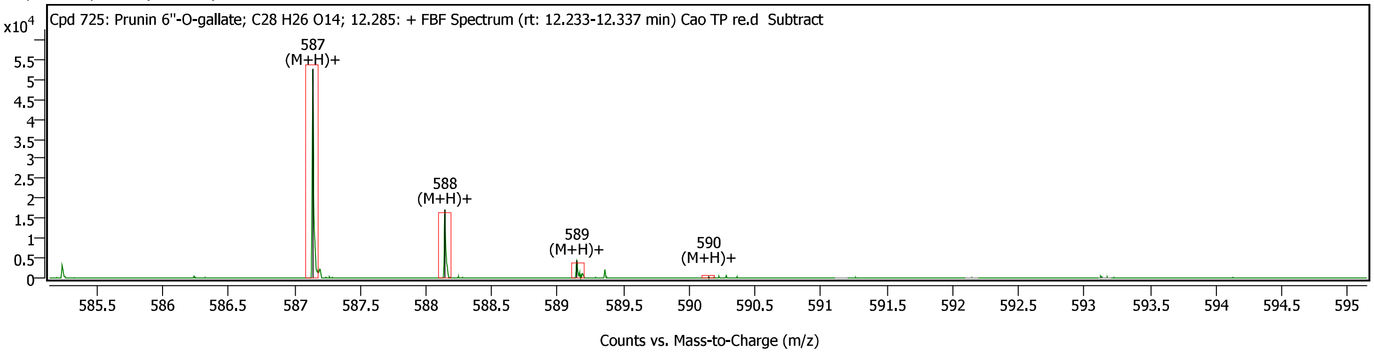

Compound ID Table

Name	Formula	Species	RT	RT Diff	Mass	CAS	ID Source	Score	Score (Lib)	Score (Tgt)
Prunin 6''-O-gallate	C28 H26 O14	(M+H)+	12.285		586.1323		FBF	99.31		99.31

Cpd 165: 15-Acetoxyscirpene-3,4-diol 4-O-a-D-glucopyranoside

Name	Formula	RT	RI	Mass	Diff (Tgt, ppm)	CAS	ID Source	Score	Algorithm
15-Acetoxyscirpene-3,4-diol 4-O-a-D-glucopyranoside	C23 H34 O11	12.442		486.2079	-4.55	99124-45-5	FBF	90.63	FBF

Species	m/z	Score (Tgt)	Score (Lib)	Score (DB)	Score (MFG)	Score (RT)
(M+H)+	487	90.63				

Compound Chromatograms (overlaid)

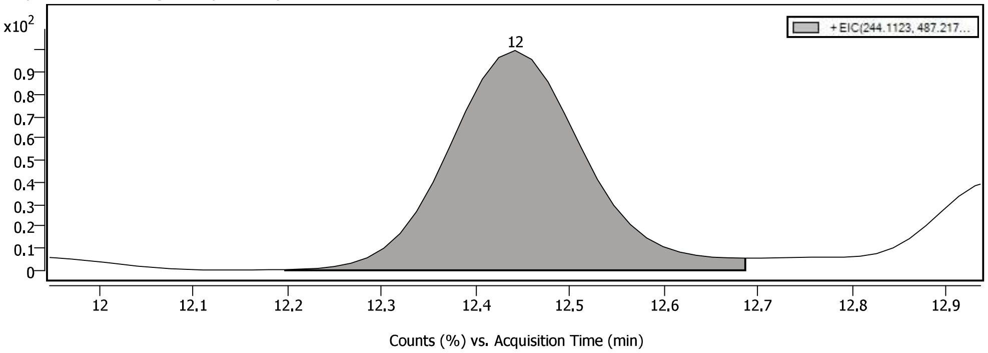

Structure

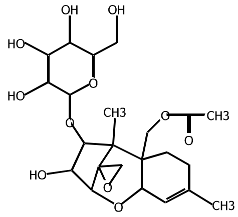

Compound Screening Report

Compound Spectra (overlaid)

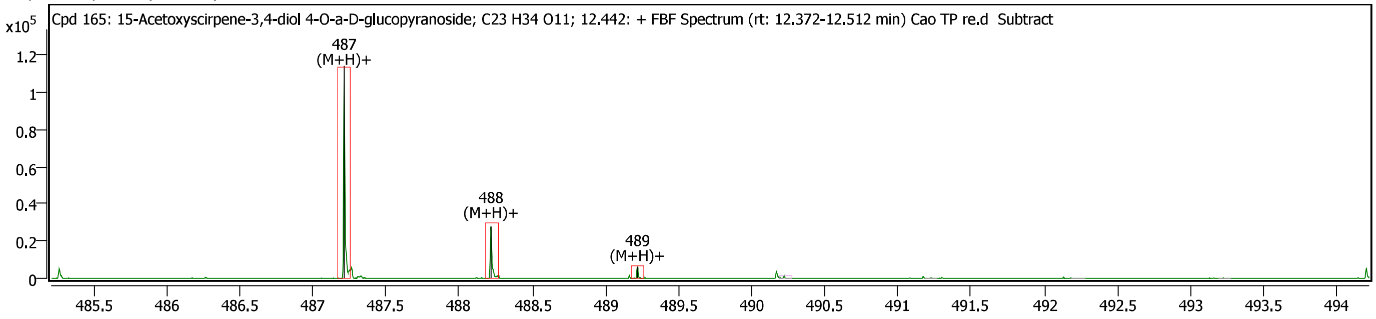

Compound ID Table

Name	Formula	Species	RT	RT Diff	Mass	CAS	ID Source	Score	Score (Lib)	Score (Tgt)
15-Acetoxyscirpene-3,4-diol 4-O-a-D-glucopyranoside	C23 H34 O11	(M+H)+	12.442		486.2079	99124-45-5	FBF	90.63		90.63

Cpd 1448: Glucosylgalactosyl hydroxylysine

Name	Formula	RT	RI	Mass	Diff (Tgt, ppm)	CAS	ID Source	Score	Algorithm
Glucosylgalactosyl hydroxylysine	C18 H34 N2 O13	12.442		486.2080	3.85	32448-35-4	FBF	90.91	FBF

Species	m/z	Score (Tgt)	Score (Lib)	Score (DB)	Score (MFG)	Score (RT)
(M+H)+	487	90.91				

Compound Chromatograms (overlaid)

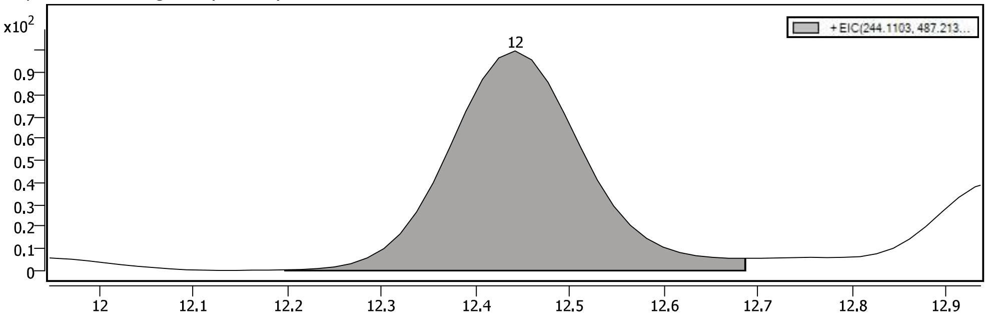

Structure

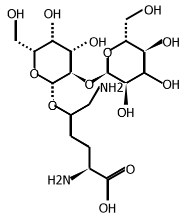

Compound Spectra (overlaid)

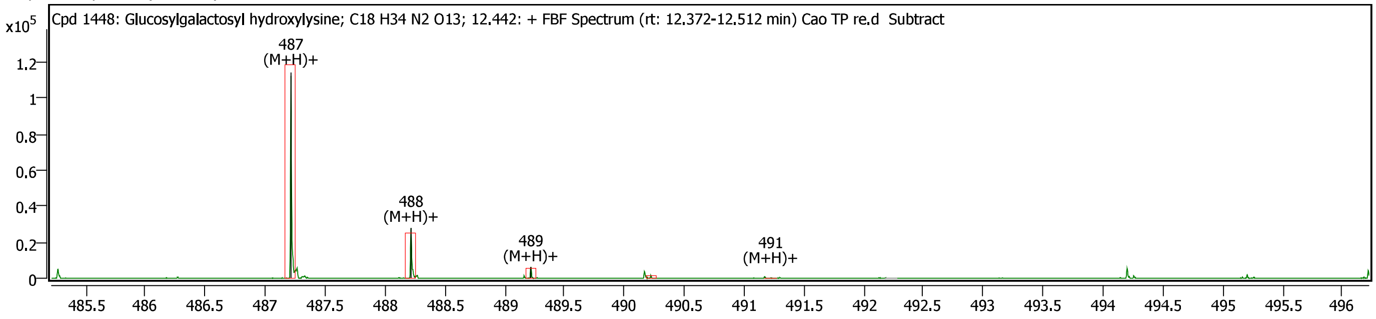

Compound ID Table

Name	Formula	Species	RT	RT Diff	Mass	CAS	ID Source	Score	Score (Lib)	Score (Tgt)
Glucosylgalactosyl hydroxylysine	C18 H34 N2 O13	(M+H)+	12.442		486.2080	32448-35-4	FBF	90.91		90.91

Cpd 95: N,N'-(((4-methyl-1,3-phenylene)bis(azanediyl))bis(carbonothioyl))dibenzamide

Name	Formula	RT	RI	Mass	Diff (Tgt, ppm)	CAS	ID Source	Score	Algorithm
N,N'-(((4-methyl-1,3-phenylene)bis(azanediyl))bis(carbonothioyl))dibenzamide	C23 H20 N4 O2 S2	12.460		448.1016	-2.59		FBF	80.73	FBF

Species	m/z	Score (Tgt)	Score (Lib)	Score (DB)	Score (MFG)	Score (RT)
(M+H)+	449	80.73				

Compound Screening Report

Compound Chromatograms (overlaid)

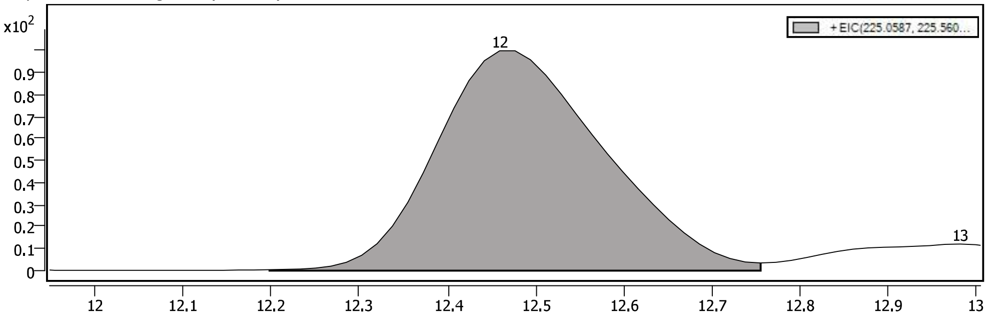

Counts (%) vs. Acquisition Time (min)

Structure

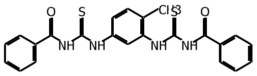

Compound Spectra (overlaid)

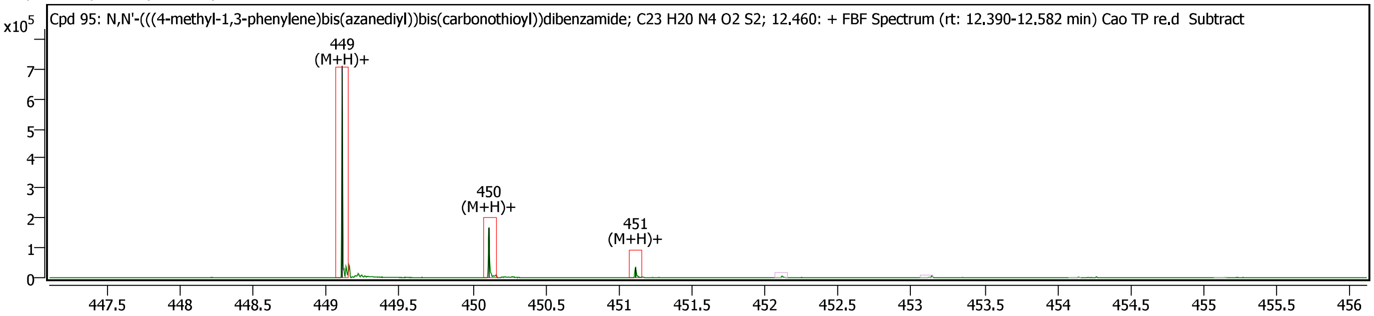

Counts vs. Mass-to-Charge (m/z)

Compound ID Table

Name	Formula	Species	RT	RT Diff	Mass	CAS	ID Source	Score	Score (Lib)	Score (Tgt)
N,N'-(((4-methyl-1,3-phenylene)bis(azanediyl))bis(carbonothioyl))dibenzamide	C23 H20 N4 O2 S2	(M+H)+	12.460		448.1016		FBF	80.73		80.73

Cpd 744: Luteolin 7-galactoside

Name	Formula	RT	RI	Mass	Diff (Tgt, ppm)	CAS	ID Source	Score	Algorithm
Luteolin 7-galactoside	C21 H20 O11	12.460		448.1013	1.57		M-FBF-FragConfirm	98.59	FBF

Species	m/z	Score (Tgt)	Score (Lib)	Score (DB)	Score (MFG)	Score (RT)
(M+H)+	449	98.59				

Compound Chromatograms (overlaid)

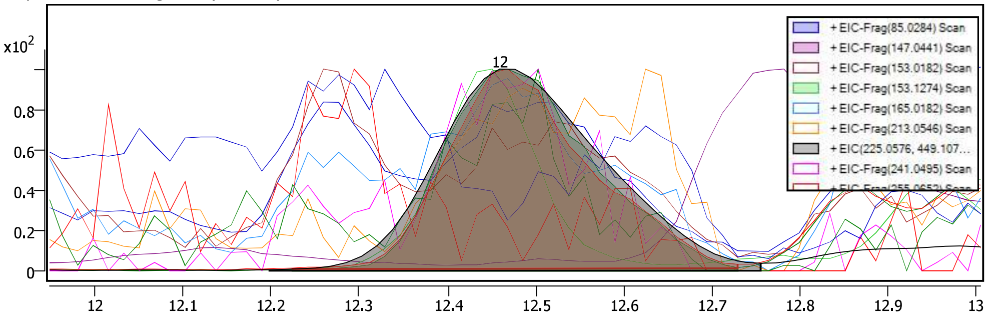

Counts (%) vs. Acquisition Time (min)

Structure

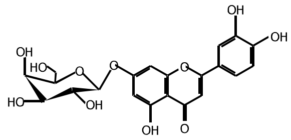

Coelution Plot

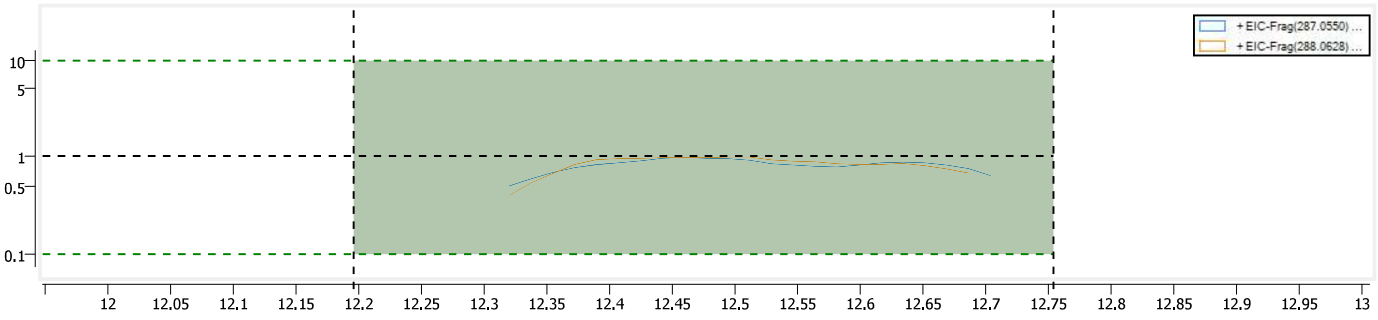

Ratio Fragment Ion/Precursor Ion vs. Acquisition Time (min)

Compound Spectra (overlaid)

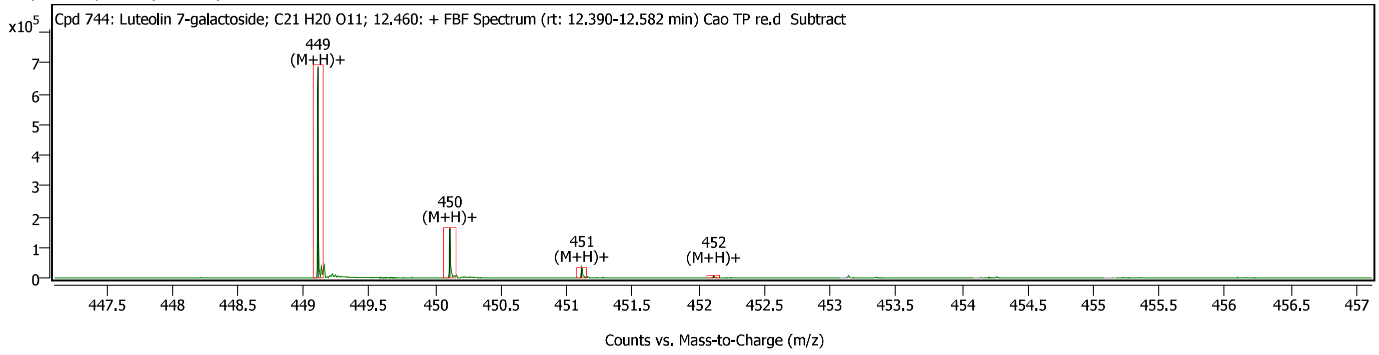

Fragment Spectrum (clean)

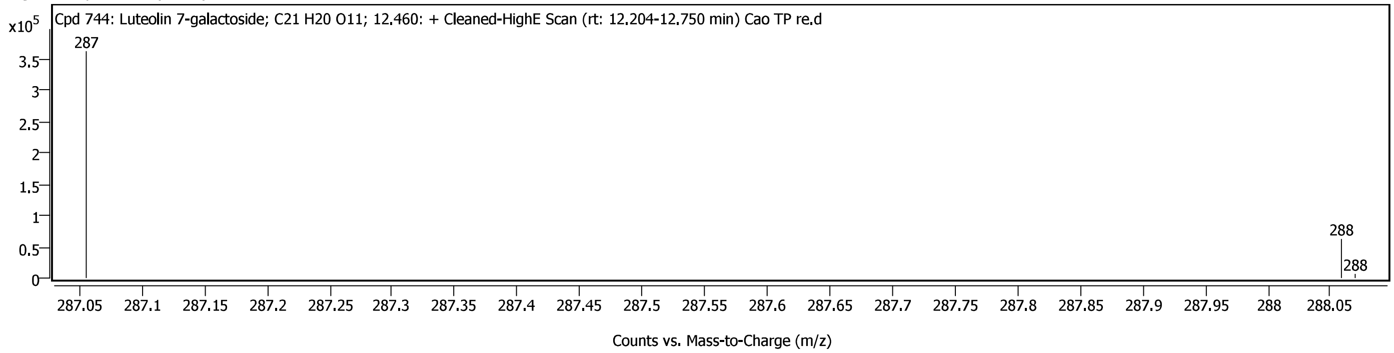

Fragment Spectrum (raw)

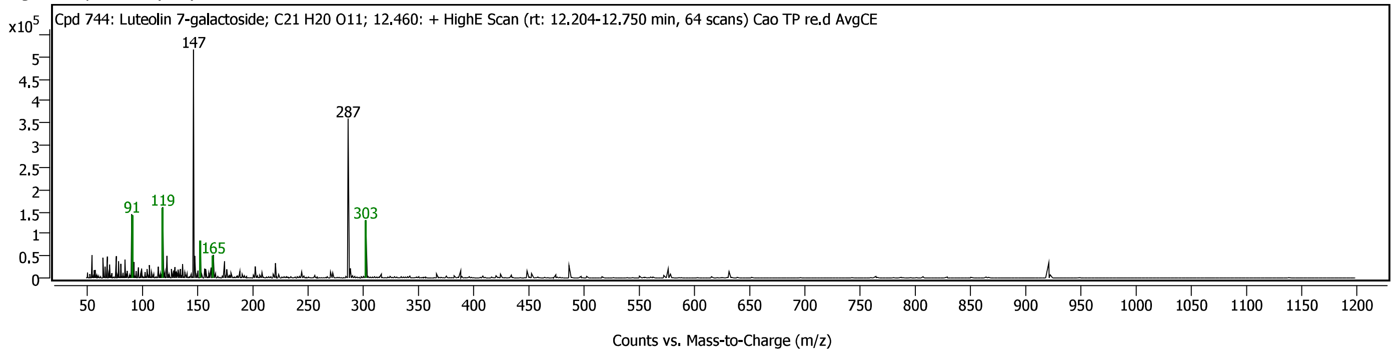

Compound Screening Report

Compound ID Table

Name	Formula	Species	RT	RT Diff	Mass	CAS	ID Source	Score	Score (Lib)	Score (Tgt)
Luteolin 7-galactoside	C21 H20 O11	(M+H)+	12.460		448.1013		FBF-FragConfirm	98.59		98.59
Carthamone	C21 H20 O11	(M+H)+	12.460		448.1013		FBF-FragConfirm	98.59		98.59
Fisetin 3-glucoside	C21 H20 O11	(M+H)+	12.460		448.1013		FBF-FragConfirm	98.59		98.59
Azaleatin 3-arabinoside	C21 H20 O11	(M+H)+	12.460		448.1013	63742-73-4	FBF-FragConfirm	98.59		98.59
Aureusin	C21 H20 O11	(M+H)+	12.460		448.1013		FBF-FragConfirm	98.59		98.59
Herbacetin 8-rhamnoside	C21 H20 O11	(M+H)+	12.460		448.1013		FBF-FragConfirm	98.59		98.59
Fisetin 8-C-glucoside	C21 H20 O11	(M+H)+	12.460		448.1013		FBF-FragConfirm	98.59		98.59
Fisetin 7-glucoside	C21 H20 O11	(M+H)+	12.460		448.1013		FBF-FragConfirm	98.59		98.59
Fisetin 4'-glucoside	C21 H20 O11	(M+H)+	12.460		448.1013		FBF-FragConfirm	98.59		98.59
evolvuside B	C21 H20 O11	(M+H)+	12.460		448.1013		FBF-FragConfirm	98.59		98.59
Petunidin-3-O-arabinoside	C21 H20 O11	(M+H)+	12.460		448.1013		FBF-FragConfirm	98.59		98.59
Herbacetin 7-rhamnoside	C21 H20 O11	(M+H)+	12.460		448.1013		FBF-FragConfirm	98.59		98.59
Dihydronorwogonin 7-O-glucuronide	C21 H20 O11	(M+H)+	12.460		448.1013		FBF-FragConfirm	98.59		98.59
Dihydrobaicalein 7-O-glucuronide	C21 H20 O11	(M+H)+	12.460		448.1013		FBF-FragConfirm	98.59		98.59
Datiscanin	C21 H20 O11	(M+H)+	12.460		448.1013		FBF-FragConfirm	98.59		98.59
Cynaroside	C21 H20 O11	(M+H)+	12.460		448.1013	5373-11-5	FBF-FragConfirm	98.59		98.59
Cernuoside	C21 H20 O11	(M+H)+	12.460		448.1013		FBF-FragConfirm	98.59		98.59
6-C-Galactosylisoscutellarein	C21 H20 O11	(M+H)+	12.460		448.1013		FBF-FragConfirm	98.59		98.59
Aureusidin 6-O-glucoside	C21 H20 O11	(M+H)+	12.460		448.1013	633-15-8	FBF-FragConfirm	98.59		98.59
8-C-Glucosylorobol	C21 H20 O11	(M+H)+	12.460		448.1013		FBF-FragConfirm	98.59		98.59
8-C-Galactosylluteolin	C21 H20 O11	(M+H)+	12.460		448.1013		FBF-FragConfirm	98.59		98.59
8-C-beta-D-Glucopyranosylkaempferol	C21 H20 O11	(M+H)+	12.460		448.1013		FBF-FragConfirm	98.59		98.59
6-Hydroxyluteolin 7-rhamnoside	C21 H20 O11	(M+H)+	12.460		448.1013		FBF-FragConfirm	98.59		98.59
6-Hydroxyluteolin 6-rhamnoside	C21 H20 O11	(M+H)+	12.460		448.1013		FBF-FragConfirm	98.59		98.59
Astragalin	C21 H20 O11	(M+H)+	12.460		448.1013	480-10-4	FBF-FragConfirm	98.59		98.59
Isoorientin	C21 H20 O11	(M+H)+	12.460		448.1013		FBF-FragConfirm	98.59		98.59
6-Hydroxyluteolin 5-rhamnoside	C21 H20 O11	(M+H)+	12.460		448.1013		FBF-FragConfirm	98.59		98.59
6-C-Galactosylluteolin	C21 H20 O11	(M+H)+	12.460		448.1013		FBF-FragConfirm	98.59		98.59
Isorhamnetin 3-alpha-L-arabinofuranoside	C21 H20 O11	(M+H)+	12.460		448.1013		FBF-FragConfirm	98.59		98.59
5,7,3',4'-Tetrahydroxy-4-phenylcoumarin 5-O-glucoside	C21 H20 O11	(M+H)+	12.460		448.1013		FBF-FragConfirm	98.59		98.59
5,7,2',6'-Tetrahydroxyflavone 2'-O-glucoside	C21 H20 O11	(M+H)+	12.460		448.1013		FBF-FragConfirm	98.59		98.59
1,2,6,8-Tetrahydroxy-3-methylanthraquinone 2-O-b-D-glucoside	C21 H20 O11	(M+H)+	12.460		448.1013		FBF-FragConfirm	98.59		98.59
6-C-Glucosylorobol	C21 H20 O11	(M+H)+	12.460		448.1013		FBF-FragConfirm	98.59		98.59
6-C-Glucopyranosylkaempferol	C21 H20 O11	(M+H)+	12.460		448.1013		FBF-FragConfirm	98.59		98.59
Distichin	C21 H20 O11	(M+H)+	12.460		448.1013		FBF-FragConfirm	98.59		98.59
8-C-Methylquercetin 3-xyloside	C21 H20 O11	(M+H)+	12.460		448.1013		FBF-FragConfirm	98.59		98.59
Hypolaetin 8-rhamnoside	C21 H20 O11	(M+H)+	12.460		448.1013		FBF-FragConfirm	98.59		98.59
Naringenin 5-O-glucuronide	C21 H20 O11	(M+H)+	12.460		448.1013		FBF-FragConfirm	98.59		98.59
Naringenin-4'-O-glucuronide	C21 H20 O11	(M+H)+	12.460		448.1013		FBF-FragConfirm	98.59		98.59
Naringenin-4'-O-β-D-Glucuronide	C21 H20 O11	(M+H)+	12.460		448.1013	158196-35-1	FBF-FragConfirm	98.59		98.59
Naringenin-7-O-glucuronide	C21 H20 O11	(M+H)+	12.460		448.1013		FBF-FragConfirm	98.59		98.59
Naringenin-7-O-β-D-Glucuronide	C21 H20 O11	(M+H)+	12.460		448.1013		FBF-FragConfirm	98.59		98.59
Orientin	C21 H20 O11	(M+H)+	12.460		448.1013		FBF-FragConfirm	98.59		98.59
Orobol 7-O-glucoside	C21 H20 O11	(M+H)+	12.460		448.1013		FBF-FragConfirm	98.59		98.59
Quercetin 3-methyl ether 3'-xyloside	C21 H20 O11	(M+H)+	12.460		448.1013		FBF-FragConfirm	98.59		98.59
Naringenin 4'-O-glucuronide	C21 H20 O11	(M+H)+	12.460		448.1013		FBF-FragConfirm	98.59		98.59
Quercetin 7-rhamnoside	C21 H20 O11	(M+H)+	12.460		448.1013		FBF-FragConfirm	98.59		98.59
Quercitrin	C21 H20 O11	(M+H)+	12.460		448.1013	522-12-3	FBF-FragConfirm	98.59		98.59
Rhamnetin 3-alpha-L-arabinofuranoside	C21 H20 O11	(M+H)+	12.460		448.1013		FBF-FragConfirm	98.59		98.59
Scutellarein 6-glucoside	C21 H20 O11	(M+H)+	12.460		448.1013		FBF-FragConfirm	98.59		98.59
Scutellarein 7-glucoside	C21 H20 O11	(M+H)+	12.460		448.1013		FBF-FragConfirm	98.59		98.59
Trifolin	C21 H20 O11	(M+H)+	12.460		448.1013		FBF-FragConfirm	98.59		98.59
Rhamnetin 3-alpha-L-arabinopyranoside	C21 H20 O11	(M+H)+	12.460		448.1013		FBF-FragConfirm	98.59		98.59
Kaempferol 4'-glucoside	C21 H20 O11	(M+H)+	12.460		448.1013		FBF-FragConfirm	98.59		98.59
Luteolin 5-glucoside	C21 H20 O11	(M+H)+	12.460		448.1013		FBF-FragConfirm	98.59		98.59
Luteolin 4'-O-glucoside	C21 H20 O11	(M+H)+	12.460		448.1013	6920-38-3	FBF-FragConfirm	98.59		98.59
Luteolin 3'-glucoside	C21 H20 O11	(M+H)+	12.460		448.1013		FBF-FragConfirm	98.59		98.59
Luteolin 7-glucoside	C21 H20 O11	(M+H)+	12.460		448.1013		FBF-FragConfirm	98.59		98.59
Kaempferol 7-galactoside	C21 H20 O11	(M+H)+	12.460		448.1013		FBF-FragConfirm	98.59		98.59
Kaempferol 3-alpha-D-galactoside	C21 H20 O11	(M+H)+	12.460		448.1013		FBF-FragConfirm	98.59		98.59
Isorhamnetin 3-xyloside	C21 H20 O11	(M+H)+	12.460		448.1013		FBF-FragConfirm	98.59		98.59
Kaempferol 5-glucoside	C21 H20 O11	(M+H)+	12.460		448.1013		FBF-FragConfirm	98.59		98.59
Kaempferol 3-O-β-D-galactoside	C21 H20 O11	(M+H)+	12.460		448.1013		FBF-FragConfirm	98.59		98.59
Kaempferol 3-alpha-D-glucoside	C21 H20 O11	(M+H)+	12.460		448.1013		FBF-FragConfirm	98.59		98.59
Isoscutellarein 7-glucoside	C21 H20 O11	(M+H)+	12.460		448.1013		FBF-FragConfirm	98.59		98.59
Maritimein	C21 H20 O11	(M+H)+	12.460		448.1013		FBF-FragConfirm	98.59		98.59
Kaempferol 7-alloside	C21 H20 O11	(M+H)+	12.460		448.1013	765949-92-6	FBF-FragConfirm	98.59		98.59
Maritimetin 7-glucoside	C21 H20 O11	(M+H)+	12.460		448.1013		FBF-FragConfirm	98.59		98.59
Kaempferol 7-O-glucoside	C21 H20 O11	(M+H)+	12.460		448.1013	16290-07-6	FBF-FragConfirm	98.59		98.59

Cpd 1194: Coumarin

Name	Formula	RT	RI	Mass	Diff (Tgt, ppm)	CAS	ID Source	Score	Algorithm
Coumarin	C9 H6 O2	12.786		146.0368	0.14	91-64-5	FBF-FragConfirm	99.65	FBF

Species	m/z	Score (Tgt)	Score (Lib)	Score (DB)	Score (MFG)	Score (RT)
(M+H)+	147	99.65				

Compound Screening Report

Agilent

Compound Chromatograms (overlaid)

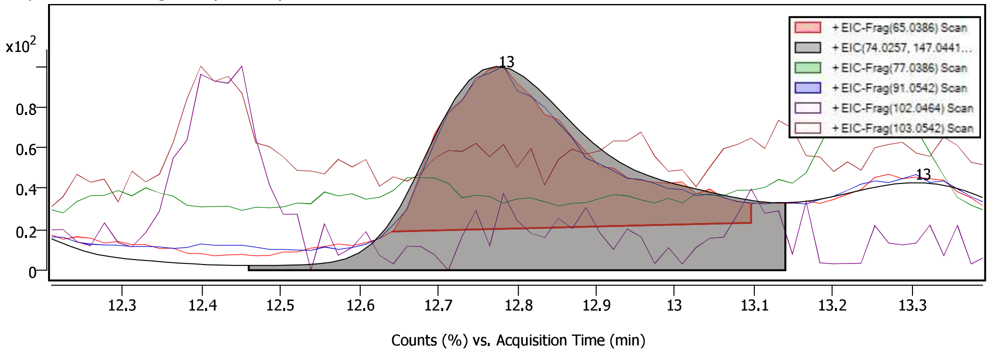

Structure

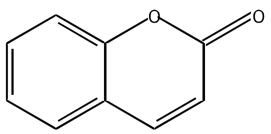

Coelution Plot

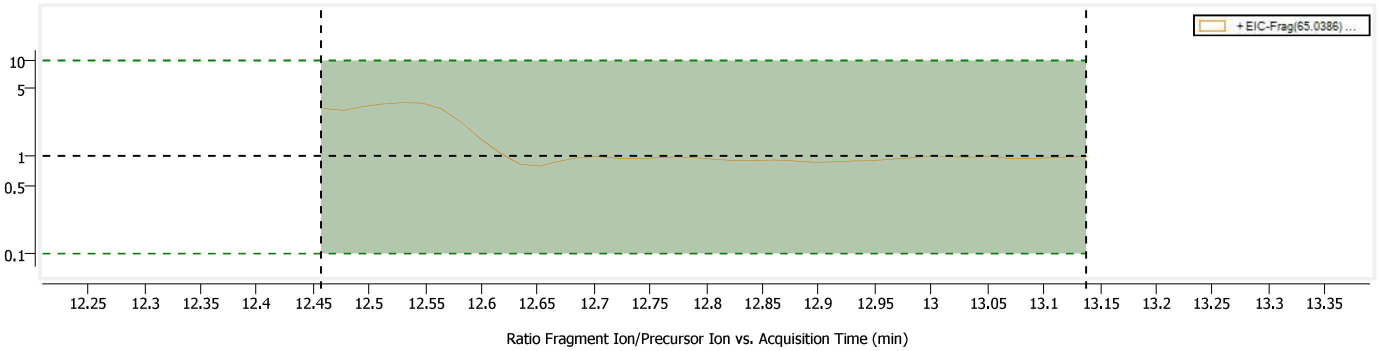

Compound Spectra (overlaid)

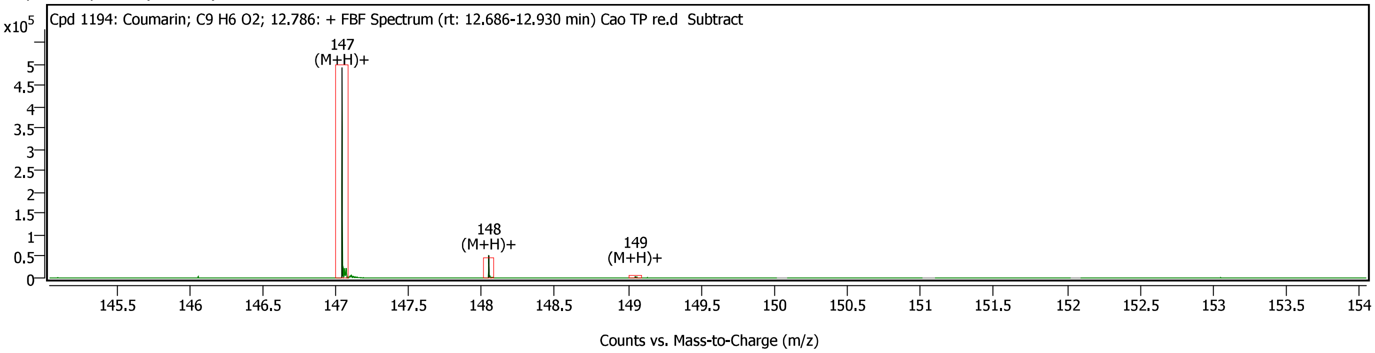

Fragment Spectrum (clean)

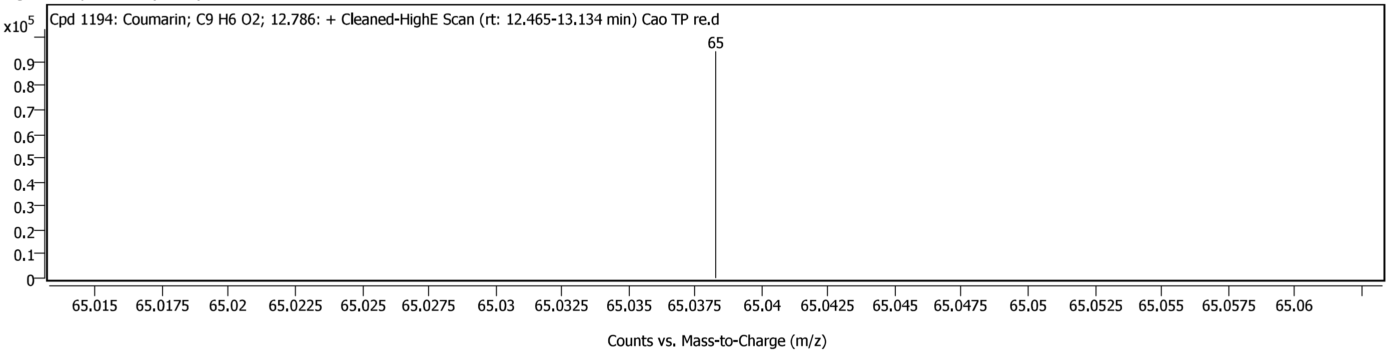

Compound Screening Report

Fragment Spectrum (raw)

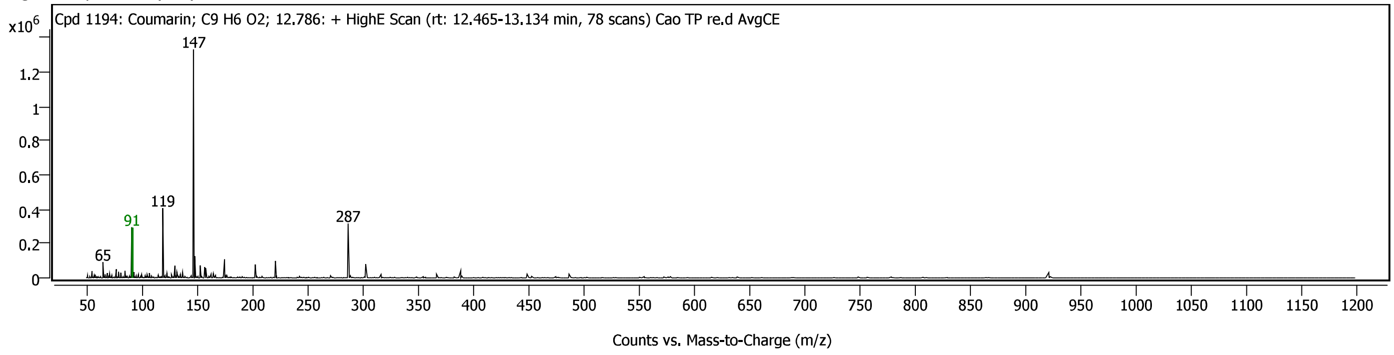

Compound ID Table

Name	Formula	Species	RT	RT Diff	Mass	CAS	ID Source	Score	Score (Lib)	Score (Tgt)
Coumarin	C9 H6 O2	(M+H)+	12.786		146.0368	91-64-5	FBF-FragConfirm	99.65		99.65
Phenylpropiolic acid	C9 H6 O2	(M+H)+	12.786		146.0368	637-44-5	FBF-FragConfirm	99.65		99.65
Chromone	C9 H6 O2	(M+H)+	12.786		146.0368	491-38-3	FBF-FragConfirm	99.65		99.65
2-Benzofurancarboxaldehyde	C9 H6 O2	(M+H)+	12.786		146.0368	4265-16-1	FBF-FragConfirm	99.65		99.65

Cpd 762: Isorhamnetin 3-glucoside

Name	Formula	RT	RI	Mass	Diff (Tgt, ppm)	CAS	ID Source	Score	Algorithm
Isorhamnetin 3-glucoside	C22 H22 O12	13.157		478.1115	0.69		M-FBF-FragConfirm	98.11	FBF

Species	m/z	Score (Tgt)	Score (Lib)	Score (DB)	Score (MFG)	Score (RT)
(M+H)+	479	98.11				

Compound Chromatograms (overlaid)

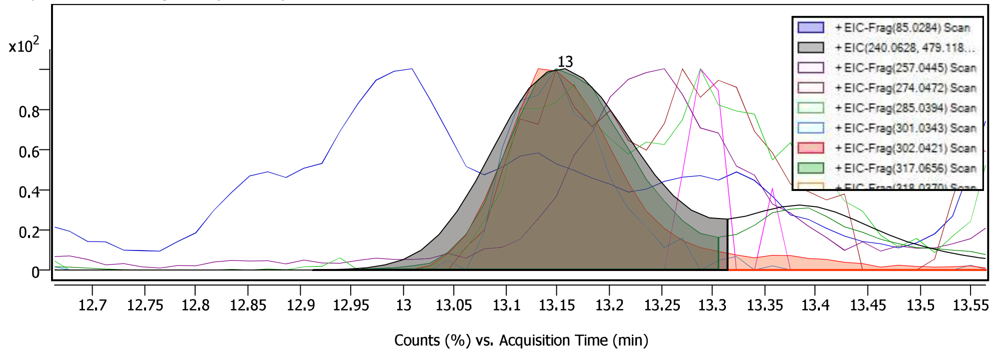

Structure

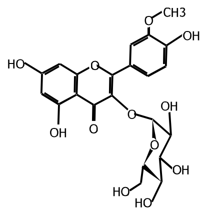

Coelution Plot

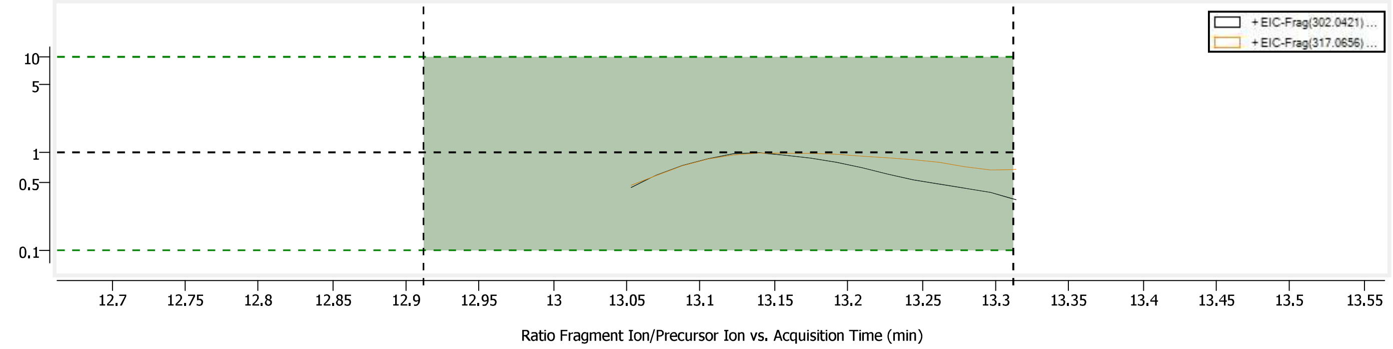

Compound Spectra (overlaid)

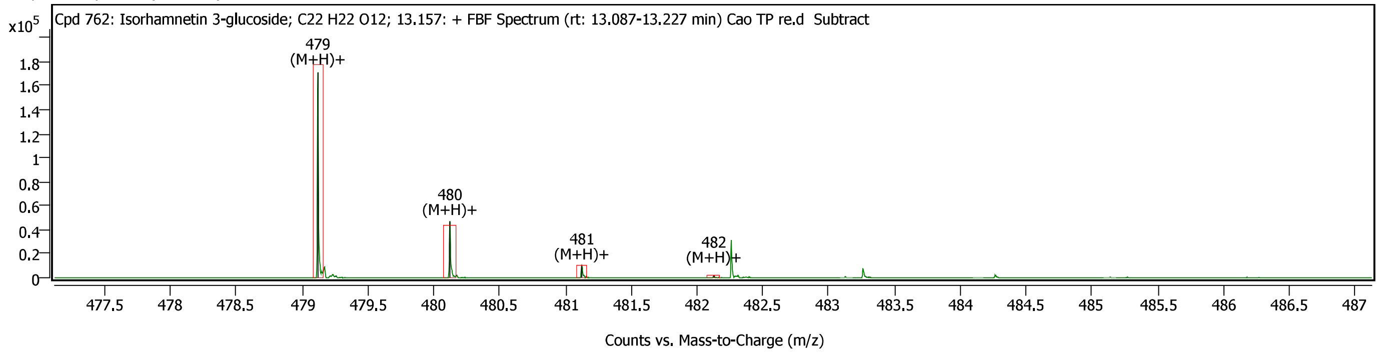

Fragment Spectrum (clean)

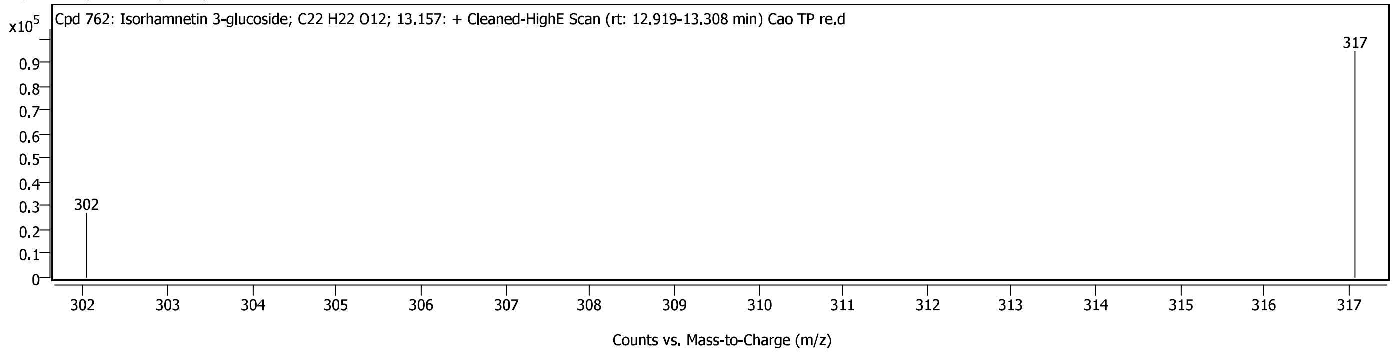

Fragment Spectrum (raw)

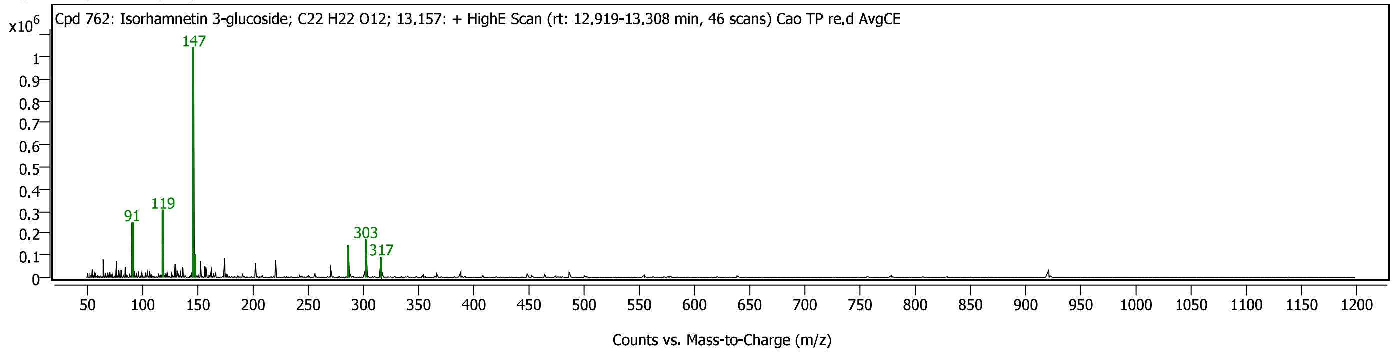

Compound ID Table

Name	Formula	Species	RT	RT Diff	Mass	CAS	ID Source	Score	Score (Lib)	Score (Tgt)
Isorhamnetin 3-glucoside	C22 H22 O12	(M+H)+	13.157		478.1115		FBF-FragConfirm	98.11		98.11
Azaleatin 3-glucoside	C22 H22 O12	(M+H)+	13.157		478.1115		FBF-FragConfirm	98.11		98.11
6-Hydroxykaempferol 3-methyl ether 7-glucoside	C22 H22 O12	(M+H)+	13.157		478.1115		FBF-FragConfirm	98.11		98.11
8-Hydroxyluteolin 3'-methyl ether 7-glucoside	C22 H22 O12	(M+H)+	13.157		478.1115		FBF-FragConfirm	98.11		98.11
8-Hydroxyluteolin 4'-methyl ether 8-glucoside	C22 H22 O12	(M+H)+	13.157		478.1115		FBF-FragConfirm	98.11		98.11
Alliumoside A	C22 H22 O12	(M+H)+	13.157		478.1115	58902-89-9	FBF-FragConfirm	98.11		98.11
Azaleatin 3-galactoside	C22 H22 O12	(M+H)+	13.157		478.1115		FBF-FragConfirm	98.11		98.11
Eupatolitin 3-apioside	C22 H22 O12	(M+H)+	13.157		478.1115		FBF-FragConfirm	98.11		98.11
Estragonoside	C22 H22 O12	(M+H)+	13.157		478.1115	181020-33-7	FBF-FragConfirm	98.11		98.11
Eupafolin 4'-glucoside	C22 H22 O12	(M+H)+	13.157		478.1115	112208-83-0	FBF-FragConfirm	98.11		98.11
Europetin 3-rhamnoside	C22 H22 O12	(M+H)+	13.157		478.1115		FBF-FragConfirm	98.11		98.11
Herbacetin 7-methyl ether 3-glucoside	C22 H22 O12	(M+H)+	13.157		478.1115		FBF-FragConfirm	98.11		98.11
6-Methoxyluteolin 7-glucoside	C22 H22 O12	(M+H)+	13.157		478.1115		FBF-FragConfirm	98.11		98.11
7-O-Methylgossypetin 3-rhamnoside	C22 H22 O12	(M+H)+	13.157		478.1115	56768-33-3	FBF-FragConfirm	98.11		98.11
6-Methoxyluteolin 3'-glucoside	C22 H22 O12	(M+H)+	13.157		478.1115	112208-84-1	FBF-FragConfirm	98.11		98.11
Isorhamnetin 3-galactoside	C22 H22 O12	(M+H)+	13.157		478.1115	5041-82-7	FBF-FragConfirm	98.11		98.11
6-Methoxykaempferol 7-glucoside	C22 H22 O12	(M+H)+	13.157		478.1115		FBF-FragConfirm	98.11		98.11
6-Methoxykaempferol 3-glucoside	C22 H22 O12	(M+H)+	13.157		478.1115		FBF-FragConfirm	98.11		98.11
6-Methoxykaempferol 3-galactoside	C22 H22 O12	(M+H)+	13.157		478.1115		FBF-FragConfirm	98.11		98.11
6-Hydroxyluteolin 7-methyl ether 6-glucoside	C22 H22 O12	(M+H)+	13.157		478.1115		FBF-FragConfirm	98.11		98.11
6-Hydroxyluteolin 7-methyl ether 6-galactoside	C22 H22 O12	(M+H)+	13.157		478.1115		FBF-FragConfirm	98.11		98.11
6-Hydroxykaempferol 4'-methyl ether 7-glucoside	C22 H22 O12	(M+H)+	13.157		478.1115		FBF-FragConfirm	98.11		98.11
6-Hydroxykaempferol 3-methyl ether 6-glucoside	C22 H22 O12	(M+H)+	13.157		478.1115		FBF-FragConfirm	98.11		98.11
5,2',4',5'-Tetrahydroxy-7-methoxy-4-phenylcoumarin 5-O-glucoside	C22 H22 O12	(M+H)+	13.157		478.1115		FBF-FragConfirm	98.11		98.11
2-O-(4-Hydroxycinnamoyl)-1-O-galloyl-beta-D-glucopyranoside	C22 H22 O12	(M+H)+	13.157		478.1115	94356-18-0	FBF-FragConfirm	98.11		98.11
1,3,5,8-Tetrahydroxy-6-methoxy-2-methylanthraquinone 8-O-b-D-glucoside	C22 H22 O12	(M+H)+	13.157		478.1115	101508-15-0	FBF-FragConfirm	98.11		98.11
Pedaliin	C22 H22 O12	(M+H)+	13.157		478.1115	22860-72-6	FBF-FragConfirm	98.11		98.11
8-C-Rhamnosyleuropetin	C22 H22 O12	(M+H)+	13.157		478.1115		FBF-FragConfirm	98.11		98.11
Annulatin 7-rhamnoside	C22 H22 O12	(M+H)+	13.157		478.1115		FBF-FragConfirm	98.11		98.11
Tricetin 3'-methyl ether 7-glucoside	C22 H22 O12	(M+H)+	13.157		478.1115		FBF-FragConfirm	98.11		98.11
Quercetin 3-methyl ether 7-glucoside	C22 H22 O12	(M+H)+	13.157		478.1115		FBF-FragConfirm	98.11		98.11
Isorhamnetin 7-glucoside	C22 H22 O12	(M+H)+	13.157		478.1115		FBF-FragConfirm	98.11		98.11
Tamarixin	C22 H22 O12	(M+H)+	13.157		478.1115		FBF-FragConfirm	98.11		98.11
Tamarixetin 7-glucoside	C22 H22 O12	(M+H)+	13.157		478.1115		FBF-FragConfirm	98.11		98.11
Tamarixetin 3-galactoside	C22 H22 O12	(M+H)+	13.157		478.1115		FBF-FragConfirm	98.11		98.11
Syringetin 3-xyloside	C22 H22 O12	(M+H)+	13.157		478.1115		FBF-FragConfirm	98.11		98.11
Sexangularetin 3-glucoside	C22 H22 O12	(M+H)+	13.157		478.1115		FBF-FragConfirm	98.11		98.11
Rhamnetin 5-glucoside	C22 H22 O12	(M+H)+	13.157		478.1115		FBF-FragConfirm	98.11		98.11
Rhamnetin 3-glucoside	C22 H22 O12	(M+H)+	13.157		478.1115		FBF-FragConfirm	98.11		98.11
Rhamnetin 3-galactoside	C22 H22 O12	(M+H)+	13.157		478.1115		FBF-FragConfirm	98.11		98.11
Ranupenin 3-rhamnoside	C22 H22 O12	(M+H)+	13.157		478.1115		FBF-FragConfirm	98.11		98.11
Quercetin 3-methyl ether 5-glucoside	C22 H22 O12	(M+H)+	13.157		478.1115		FBF-FragConfirm	98.11		98.11
Sexangularetin 3-galactoside	C22 H22 O12	(M+H)+	13.157		478.1115		FBF-FragConfirm	98.11		98.11
Isorhamnetin 4'-glucoside	C22 H22 O12	(M+H)+	13.157		478.1115		FBF-FragConfirm	98.11		98.11
Quercetin 3-methyl ether 4'-glucoside	C22 H22 O12	(M+H)+	13.157		478.1115		FBF-FragConfirm	98.11		98.11
Nepetin 4'-glucoside	C22 H22 O12	(M+H)+	13.157		478.1115		FBF-FragConfirm	98.11		98.11
Laricitrin 3-rhamnoside	C22 H22 O12	(M+H)+	13.157		478.1115		FBF-FragConfirm	98.11		98.11
Quercetin 3-methyl ether 3'-glucoside	C22 H22 O12	(M+H)+	13.157		478.1115		FBF-FragConfirm	98.11		98.11
Keyakinin B	C22 H22 O12	(M+H)+	13.157		478.1115		FBF-FragConfirm	98.11		98.11
Mearnsitrin	C22 H22 O12	(M+H)+	13.157		478.1115	30484-88-9	FBF-FragConfirm	98.11		98.11
Myricetin 3,4'-dimethyl ether 3'-xyloside	C22 H22 O12	(M+H)+	13.157		478.1115		FBF-FragConfirm	98.11		98.11
Myricetin 5-methyl ether 3-rhamnoside	C22 H22 O12	(M+H)+	13.157		478.1115		FBF-FragConfirm	98.11		98.11
Nepitrin	C22 H22 O12	(M+H)+	13.157		478.1115	569-90-4	FBF-FragConfirm	98.11		98.11
Patuletin 3-rhamnoside	C22 H22 O12	(M+H)+	13.157		478.1115		FBF-FragConfirm	98.11		98.11
Quercetin 3-methyl ether 7-galactoside	C22 H22 O12	(M+H)+	13.157		478.1115		FBF-FragConfirm	98.11		98.11
Pollenin B	C22 H22 O12	(M+H)+	13.157		478.1115	30484-94-7	FBF-FragConfirm	98.11		98.11

Cpd 810: Genistein 4'-O-glucoside

Name	Formula	RT	RI	Mass	Diff (Tgt, ppm)	CAS	ID Source	Score	Algorithm
Genistein 4'-O-glucoside	C21 H20 O10	13.210		432.1056	-0.15	152-95-4	FBF-FragConfirm	97.60	FBF

Species	m/z	Score (Tgt)	Score (Lib)	Score (DB)	Score (MFG)	Score (RT)
(M+H)+	433	97.60				

Compound Chromatograms (overlaid)

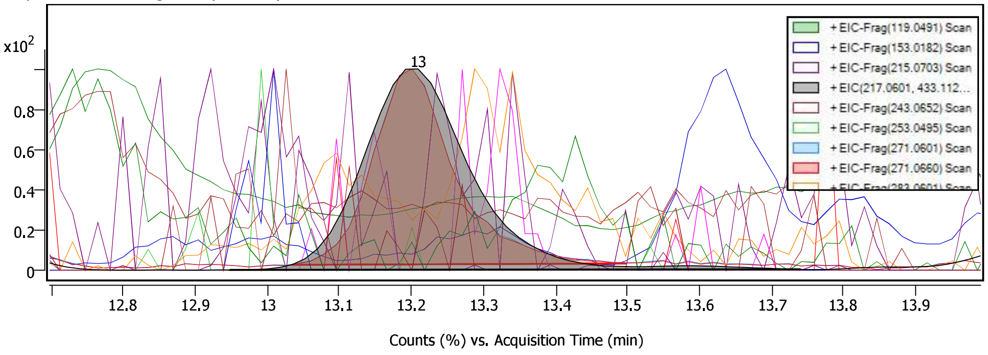

Structure

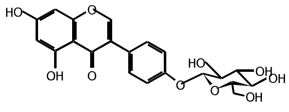

Coelution Plot

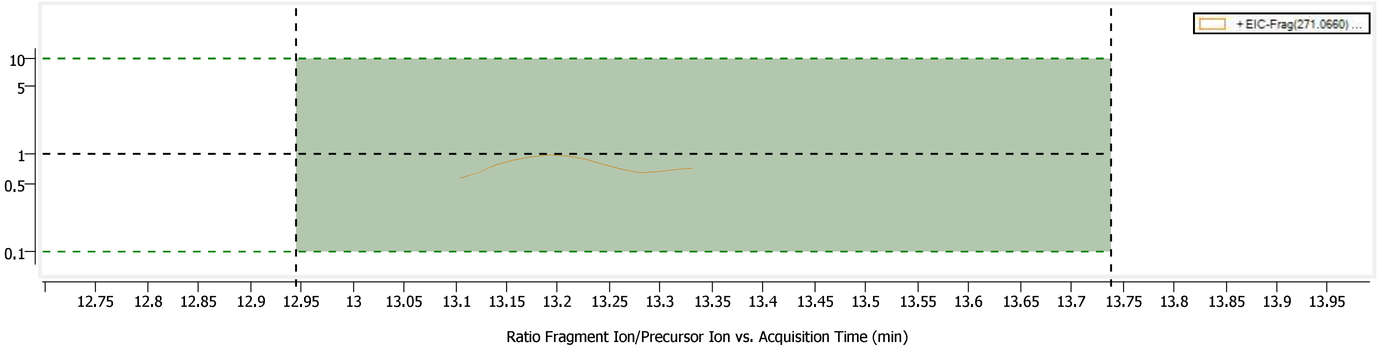

Compound Spectra (overlaid)

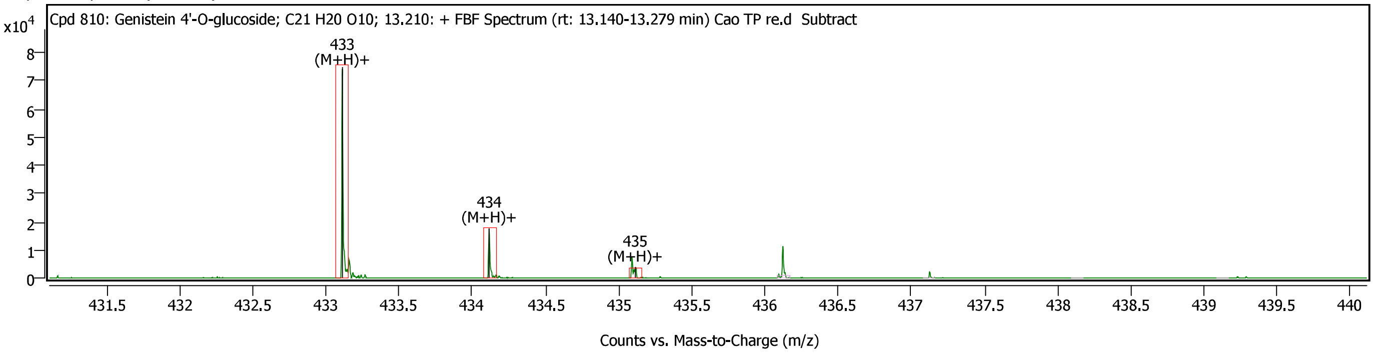

Fragment Spectrum (clean)

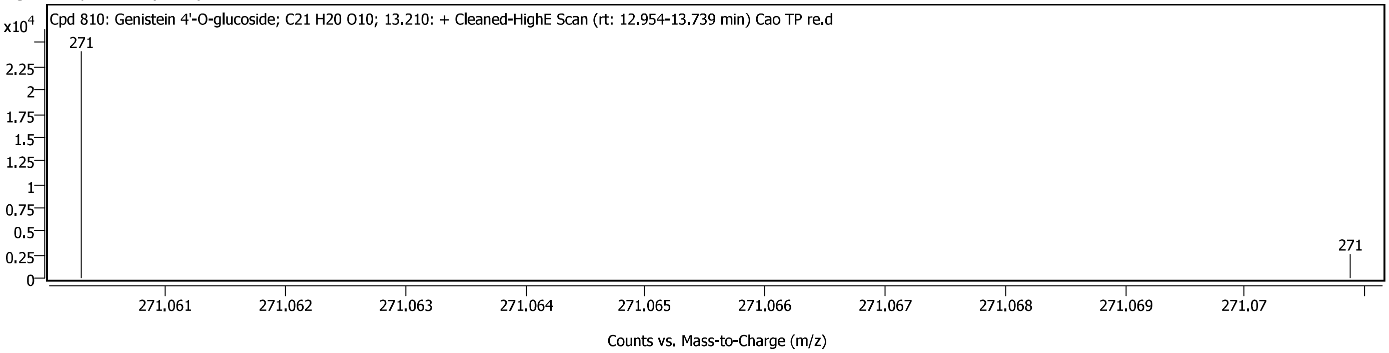

Fragment Spectrum (raw)

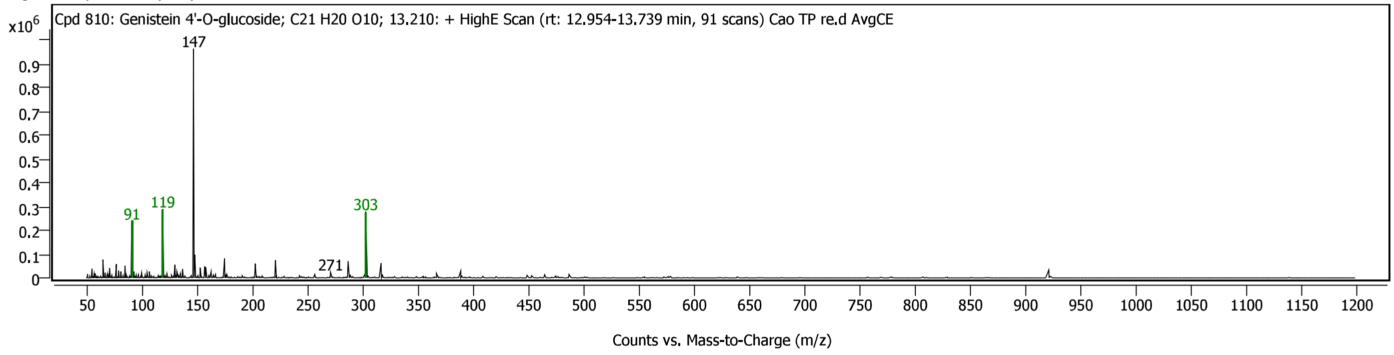

Compound ID Table

Name	Formula	Species	RT	RT Diff	Mass	CAS	ID Source	Score	Score (Lib)	Score (Tgt)
Genistein 4'-O-glucoside	C21 H20 O10	(M+H)+	13.210		432.1056	152-95-4	FBF-FragConfirm	97.60		97.60
8-C-Rhamnopyranosylluteolin	C21 H20 O10	(M+H)+	13.210		432.1056		FBF-FragConfirm	97.60		97.60
6-C-beta-D-Galactosylapigenin	C21 H20 O10	(M+H)+	13.210		432.1056		FBF-FragConfirm	97.60		97.60
alpha-Rhamnorobin	C21 H20 O10	(M+H)+	13.210		432.1056	5041-74-7	FBF-FragConfirm	97.60		97.60
Apigenin 4'-glucoside	C21 H20 O10	(M+H)+	13.210		432.1056		FBF-FragConfirm	97.60		97.60
Apigenin 4'-O-glucoside	C21 H20 O10	(M+H)+	13.210		432.1056	20486-34-4	FBF-FragConfirm	97.60		97.60
Apigenin 5-galactoside	C21 H20 O10	(M+H)+	13.210		432.1056		FBF-FragConfirm	97.60		97.60
Apigenin 7-glucoside	C21 H20 O10	(M+H)+	13.210		432.1056	578-74-5	FBF-FragConfirm	97.60		97.60
Apigenin 5-glucoside	C21 H20 O10	(M+H)+	13.210		432.1056		FBF-FragConfirm	97.60		97.60
Apigenin 7-galactoside	C21 H20 O10	(M+H)+	13.210		432.1056		FBF-FragConfirm	97.60		97.60
Aureusidin 6-rhamnoside	C21 H20 O10	(M+H)+	13.210		432.1056		FBF-FragConfirm	97.60		97.60
Baicalein 6-glucoside	C21 H20 O10	(M+H)+	13.210		432.1056		FBF-FragConfirm	97.60		97.60
8-C-beta-D-Galactopyranosylapigenin	C21 H20 O10	(M+H)+	13.210		432.1056		FBF-FragConfirm	97.60		97.60
Demethyltexasin 4'-O-glucoside	C21 H20 O10	(M+H)+	13.210		432.1056		FBF-FragConfirm	97.60		97.60
8-C-Glucosyl-5-deoxykaempferol	C21 H20 O10	(M+H)+	13.210		432.1056		FBF-FragConfirm	97.60		97.60
7,3',4'-Trihydroxyflavone 7-glucoside	C21 H20 O10	(M+H)+	13.210		432.1056		FBF-FragConfirm	97.60		97.60
Dihydrodaidzein 7-O-glucuronide	C21 H20 O10	(M+H)+	13.210		432.1056		FBF-FragConfirm	97.60		97.60
7,3',4'-Trihydroxyflavone 7-galactoside	C21 H20 O10	(M+H)+	13.210		432.1056		FBF-FragConfirm	97.60		97.60
7,3',4',5'-Tetrahydroxyflavone 7-rhamnoside	C21 H20 O10	(M+H)+	13.210		432.1056		FBF-FragConfirm	97.60		97.60
6-Hydroxydaidzein 4'-glucoside	C21 H20 O10	(M+H)+	13.210		432.1056		FBF-FragConfirm	97.60		97.60
6-C-Fucosylluteolin	C21 H20 O10	(M+H)+	13.210		432.1056		FBF-FragConfirm	97.60		97.60
6-C-Fucopyranosylluteolin	C21 H20 O10	(M+H)+	13.210		432.1056		FBF-FragConfirm	97.60		97.60
6-C-Chinovopyranosylluteolin	C21 H20 O10	(M+H)+	13.210		432.1056		FBF-FragConfirm	97.60		97.60
5,7,8-Trihydroxyflavone 7-galactoside	C21 H20 O10	(M+H)+	13.210		432.1056		FBF-FragConfirm	97.60		97.60
5,7,8-Trihydroxyflavone 5-glucoside	C21 H20 O10	(M+H)+	13.210		432.1056		FBF-FragConfirm	97.60		97.60
5,7,2'-Trihydroxy 7-glucoside	C21 H20 O10	(M+H)+	13.210		432.1056		FBF-FragConfirm	97.60		97.60
3'-Hydroxy-3,5,8,4',5'-pentamethoxy-6,7-methylenedioxyflavone	C21 H20 O10	(M+H)+	13.210		432.1056		FBF-FragConfirm	97.60		97.60
1-O-beta-D-Glucopyranosylaloeemodin	C21 H20 O10	(M+H)+	13.210		432.1056		FBF-FragConfirm	97.60		97.60
Emodin 8-glucoside	C21 H20 O10	(M+H)+	13.210		432.1056	23313-21-5	FBF-FragConfirm	97.60		97.60
Afzelin	C21 H20 O10	(M+H)+	13.210		432.1056	482-39-3	FBF-FragConfirm	97.60		97.60
1,8-Dihydroxy-3-hydroxymethylanthraquinone 8-O-b-D-glucoside	C21 H20 O10	(M+H)+	13.210		432.1056	33037-46-6	FBF-FragConfirm	97.60		97.60
w-O-beta-D-Glucopyranosylaloeemodin	C21 H20 O10	(M+H)+	13.210		432.1056	50488-89-6	FBF-FragConfirm	97.60		97.60
Luteolin 3'-methyl ether 7-xyloside	C21 H20 O10	(M+H)+	13.210		432.1056		FBF-FragConfirm	97.60		97.60
Vitexin	C21 H20 O10	(M+H)+	13.210		432.1056	3681-93-4	FBF-FragConfirm	97.60		97.60
Sulfurein	C21 H20 O10	(M+H)+	13.210		432.1056		FBF-FragConfirm	97.60		97.60
Scutellarein 7-rhamnoside	C21 H20 O10	(M+H)+	13.210		432.1056		FBF-FragConfirm	97.60		97.60
Resokaempferol 7-glucoside	C21 H20 O10	(M+H)+	13.210		432.1056		FBF-FragConfirm	97.60		97.60
Resokaempferol 4'-glucoside	C21 H20 O10	(M+H)+	13.210		432.1056		FBF-FragConfirm	97.60		97.60
Galanginin	C21 H20 O10	(M+H)+	13.210		432.1056		FBF-FragConfirm	97.60		97.60
Pueraria glycoside 1	C21 H20 O10	(M+H)+	13.210		432.1056		FBF-FragConfirm	97.60		97.60
Peonidin pentose	C21 H20 O10	(M+H)+	13.210		432.1056	741197-64-8	FBF-FragConfirm	97.60		97.60
Neovitexin	C21 H20 O10	(M+H)+	13.210		432.1056		FBF-FragConfirm	97.60		97.60
Luteolin 7-rhamnoside	C21 H20 O10	(M+H)+	13.210		432.1056		FBF-FragConfirm	97.60		97.60
Luteolin 3'-rhamnoside	C21 H20 O10	(M+H)+	13.210		432.1056		FBF-FragConfirm	97.60		97.60
Kaempferol 7-rhamnoside	C21 H20 O10	(M+H)+	13.210		432.1056		FBF-FragConfirm	97.60		97.60
Kaempferol 5-rhamnoside	C21 H20 O10	(M+H)+	13.210		432.1056		FBF-FragConfirm	97.60		97.60
Galangin 7-glucoside	C21 H20 O10	(M+H)+	13.210		432.1056		FBF-FragConfirm	97.60		97.60
Kaempferol 4'-rhamnoside	C21 H20 O10	(M+H)+	13.210		432.1056		FBF-FragConfirm	97.60		97.60
Kaempferol 3-O-alpha-L-rhamnofuranoside	C21 H20 O10	(M+H)+	13.210		432.1056	5041-73-6	FBF-FragConfirm	97.60		97.60
Kaempferide 3-alpha-L-arabinopyranoside	C21 H20 O10	(M+H)+	13.210		432.1056		FBF-FragConfirm	97.60		97.60
Isovitexin	C21 H20 O10	(M+H)+	13.210		432.1056	38953-85-4	FBF-FragConfirm	97.60		97.60
Isogenistein 7-O-glucoside	C21 H20 O10	(M+H)+	13.210		432.1056		FBF-FragConfirm	97.60		97.60
Isogenistein 7-glucoside	C21 H20 O10	(M+H)+	13.210		432.1056	70943-69-0	FBF-FragConfirm	97.60		97.60
Glucoemodin	C21 H20 O10	(M+H)+	13.210		432.1056	34298-85-6	FBF-FragConfirm	97.60		97.60
Genistin	C21 H20 O10	(M+H)+	13.210		432.1056	529-59-9	FBF-FragConfirm	97.60		97.60
Genistein 8-C-glucoside	C21 H20 O10	(M+H)+	13.210		432.1056		FBF-FragConfirm	97.60		97.60
Genistein 7-O-glucoside	C21 H20 O10	(M+H)+	13.210		432.1056		FBF-FragConfirm	97.60		97.60
Genistein 5-O-glucoside	C21 H20 O10	(M+H)+	13.210		432.1056		FBF-FragConfirm	97.60		97.60
Resokaempferol 3-glucoside	C21 H20 O10	(M+H)+	13.210		432.1056		FBF-FragConfirm	97.60		97.60
Genistein 5-glucoside	C21 H20 O10	(M+H)+	13.210		432.1056	128508-06-5	FBF-FragConfirm	97.60		97.60

Cpd 1265: 1-Methyl-4-nitro-5-(S-Gluctathionyl) Imidazole

Compound Screening Report

Name	Formula	RT	RI	Mass	Diff (Tgt, ppm)	CAS	ID Source	Score	Algorithm
1-Methyl-4-nitro-5-(S-Gluctathionyl) Imidazole	C14 H20 N6 O8 S	13.210		432.1060	-0.79	36892-55-4	FBF	90.30	FBF

Species	m/z	Score (Tgt)	Score (Lib)	Score (DB)	Score (MFG)	Score (RT)
(M+H)+	433	90.30				

Compound Chromatograms (overlaid)

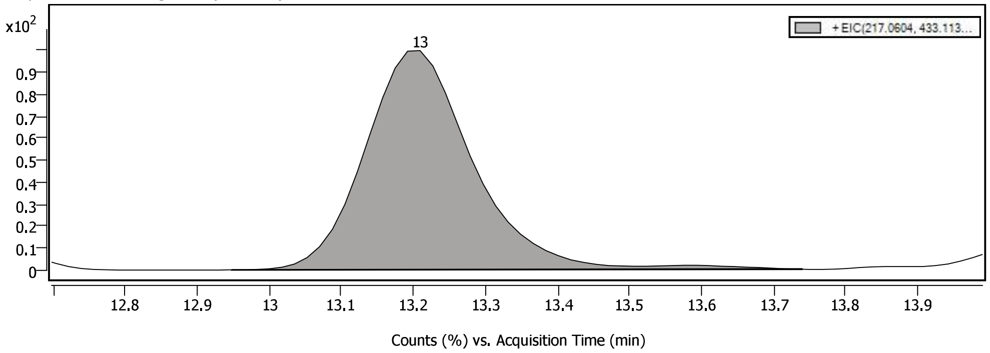

Structure

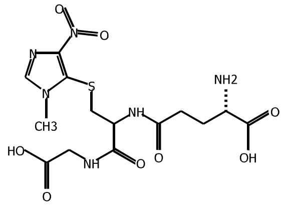

Compound Spectra (overlaid)

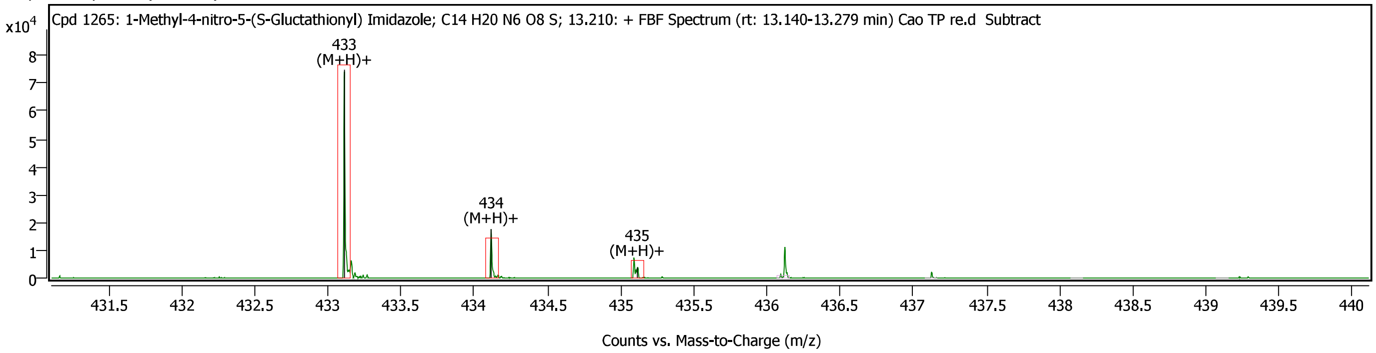

Compound ID Table

Name	Formula	Species	RT	RT Diff	Mass	CAS	ID Source	Score	Score (Lib)	Score (Tgt)
1-Methyl-4-nitro-5-(S-Gluctathionyl) Imidazole	C14 H20 N6 O8 S	(M+H)+	13.210		432.1060	36892-55-4	FBF	90.30		90.30

Cpd 1307: <Quercetin 3-O-glucoside>

Name	Formula	RT	RI	Mass	Diff (Tgt, ppm)	CAS	ID Source	Score	Algorithm
<Quercetin 3-O-glucoside>	C21 H20 O12	13.297		464.0960	1.20	21637-25-2	M-FBF	98.83	FBF

Species	m/z	Score (Tgt)	Score (Lib)	Score (DB)	Score (MFG)	Score (RT)
(M+H)+	465	98.83				

Compound Chromatograms (overlaid)

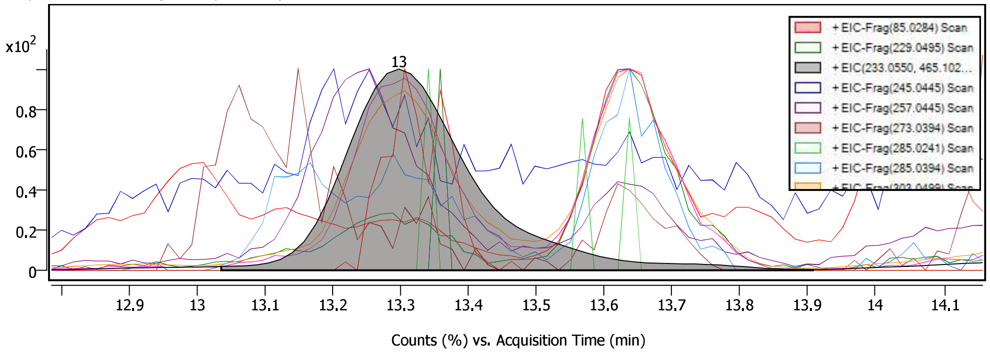

Structure

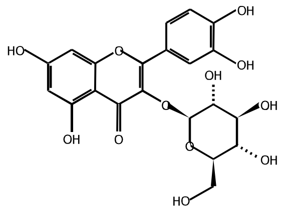

Coelution Plot

Compound Spectra (overlaid)

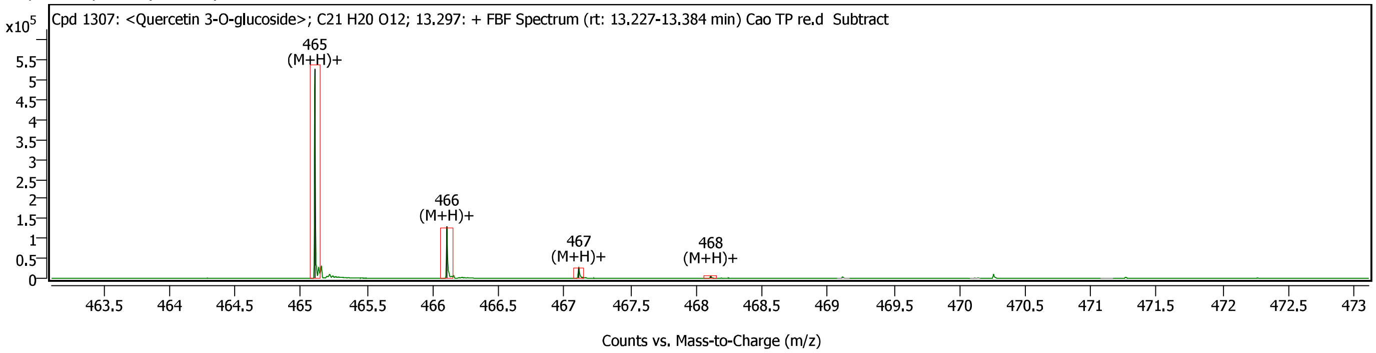

Fragment Spectrum (raw)

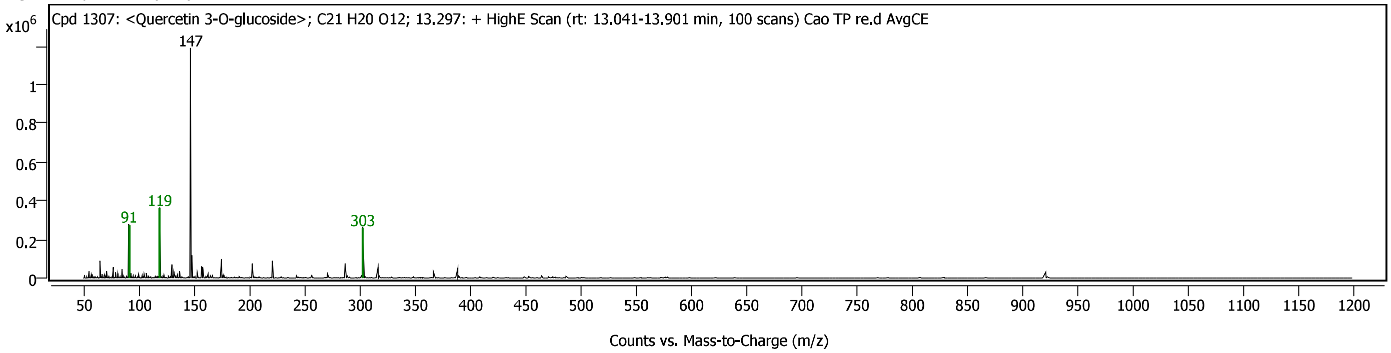

Compound Screening Report

Compound ID Table

Name	Formula	Species	RT	RT Diff	Mass	CAS	ID Source	Score	Score (Lib)	Score (Tgt)
<Quercetin 3-O-glucoside>	C21 H20 O12	(M+H)+	13.297		464.0960	21637-25-2	FBF	98.83		98.83
<Bracteatin 6-O-glucoside>	C21 H20 O12	(M+H)+	13.297		464.0960		FBF	98.83		98.83
<Quercetagetin 3-rhamnoside>	C21 H20 O12	(M+H)+	13.297		464.0960		FBF	98.83		98.83
<8-Hydroxyluteolin 8-glucoside>	C21 H20 O12	(M+H)+	13.297		464.0960		FBF	98.83		98.83
<Annulatin 3'-xyloside>	C21 H20 O12	(M+H)+	13.297		464.0960		FBF	98.83		98.83
<Bracteatin 6-glucoside>	C21 H20 O12	(M+H)+	13.297		464.0960		FBF	98.83		98.83
<Gossypetin 7-rhamnoside>	C21 H20 O12	(M+H)+	13.297		464.0960		FBF	98.83		98.83
<Bractein>	C21 H20 O12	(M+H)+	13.297		464.0960		FBF	98.83		98.83
<Corniculatusin 3-alpha-L-arabinofuranoside>	C21 H20 O12	(M+H)+	13.297		464.0960		FBF	98.83		98.83
<Eriodictyol 7-glucuronide>	C21 H20 O12	(M+H)+	13.297		464.0960		FBF	98.83		98.83
<6-Hydroxyluteolin 7-glucoside>	C21 H20 O12	(M+H)+	13.297		464.0960		FBF	98.83		98.83
<Gossypetin 8-rhamnoside>	C21 H20 O12	(M+H)+	13.297		464.0960		FBF	98.83		98.83
<6-Hydroxytricetin 5-rhamnoside>	C21 H20 O12	(M+H)+	13.297		464.0960		FBF	98.83		98.83
<6-Hydroxykaempferol 3-glucoside>	C21 H20 O12	(M+H)+	13.297		464.0960		FBF	98.83		98.83
<6-Hydroxyluteolin 7-galactoside>	C21 H20 O12	(M+H)+	13.297		464.0960		FBF	98.83		98.83
<6-Hydroxyluteolin 6-glucoside>	C21 H20 O12	(M+H)+	13.297		464.0960		FBF	98.83		98.83
<6-Hydroxyluteolin 5-glucoside>	C21 H20 O12	(M+H)+	13.297		464.0960		FBF	98.83		98.83
<6-Hydroxykaempferol 7-glucoside>	C21 H20 O12	(M+H)+	13.297		464.0960		FBF	98.83		98.83
<6-C-Glucosylquercetin>	C21 H20 O12	(M+H)+	13.297		464.0960		FBF	98.83		98.83
<6-C-beta-D-Glucopyranosyl-5,7,2',4',5'-pentahydroxyflavone>	C21 H20 O12	(M+H)+	13.297		464.0960		FBF	98.83		98.83
<5,7,3',4',5'-Pentahydroxyflavone 8-C-glucopyranoside>	C21 H20 O12	(M+H)+	13.297		464.0960		FBF	98.83		98.83
<5,6,7,3',4'-Pentahydroxy-8-methoxyflavone 7-apioside>	C21 H20 O12	(M+H)+	13.297		464.0960		FBF	98.83		98.83
<3,5,7,2',6'-Pentahydroxyflavone 2'-glucoside>	C21 H20 O12	(M+H)+	13.297		464.0960		FBF	98.83		98.83
<2'-Hydroxyisoorientin>	C21 H20 O12	(M+H)+	13.297		464.0960		FBF	98.83		98.83
<(2S)-5,7,3',4'-Tetrahydroxyflavanone 7-glucuronide>	C21 H20 O12	(M+H)+	13.297		464.0960		FBF	98.83		98.83
<Herbacetin 3-beta-D-glucofuranoside>	C21 H20 O12	(M+H)+	13.297		464.0960		FBF	98.83		98.83
<8-Hydroxyluteolin 7-glucoside>	C21 H20 O12	(M+H)+	13.297		464.0960		FBF	98.83		98.83
<Herbacetin 3-glucoside>	C21 H20 O12	(M+H)+	13.297		464.0960		FBF	98.83		98.83
<Tricetin 7-glucoside>	C21 H20 O12	(M+H)+	13.297		464.0960		FBF	98.83		98.83
<Quercetin 7-galactoside>	C21 H20 O12	(M+H)+	13.297		464.0960		FBF	98.83		98.83
<Herbacetin 7-glucoside>	C21 H20 O12	(M+H)+	13.297		464.0960		FBF	98.83		98.83
<Tricetin 3'-glucoside>	C21 H20 O12	(M+H)+	13.297		464.0960		FBF	98.83		98.83
<Spiraeoside>	C21 H20 O12	(M+H)+	13.297		464.0960		FBF	98.83		98.83
<Robinetin 7-glucoside>	C21 H20 O12	(M+H)+	13.297		464.0960		FBF	98.83		98.83
<Quercimeritrin>	C21 H20 O12	(M+H)+	13.297		464.0960		FBF	98.83		98.83
<Quercetin 5-glucoside>	C21 H20 O12	(M+H)+	13.297		464.0960		FBF	98.83		98.83
<Quercetin 4'-glucoside>	C21 H20 O12	(M+H)+	13.297		464.0960	20229-56-5	FBF	98.83		98.83
<Quercetin 3-β-D-glucoside>	C21 H20 O12	(M+H)+	13.297		464.0960	482-35-9	FBF	98.83		98.83
<Quercetin 3'-glucoside>	C21 H20 O12	(M+H)+	13.297		464.0960		FBF	98.83		98.83
<Quercetin 3-alloside>	C21 H20 O12	(M+H)+	13.297		464.0960		FBF	98.83		98.83
<Patuletin 3-xyloside>	C21 H20 O12	(M+H)+	13.297		464.0960		FBF	98.83		98.83
<Myricetin 3'-rhamnoside>	C21 H20 O12	(M+H)+	13.297		464.0960		FBF	98.83		98.83
<Isoetin 5'-glucoside>	C21 H20 O12	(M+H)+	13.297		464.0960		FBF	98.83		98.83
<Myricitrin>	C21 H20 O12	(M+H)+	13.297		464.0960	17912-87-7	FBF	98.83		98.83
<Herbacetin 8-glucoside>	C21 H20 O12	(M+H)+	13.297		464.0960		FBF	98.83		98.83
<Hyperin>	C21 H20 O12	(M+H)+	13.297		464.0960		FBF	98.83		98.83
<Hyperoside>	C21 H20 O12	(M+H)+	13.297		464.0960	482-36-0	FBF	98.83		98.83
<Isoaffinetin>	C21 H20 O12	(M+H)+	13.297		464.0960		FBF	98.83		98.83
<Isoetin 7-glucoside>	C21 H20 O12	(M+H)+	13.297		464.0960		FBF	98.83		98.83
<Isoquercitrin>	C21 H20 O12	(M+H)+	13.297		464.0960		FBF	98.83		98.83
<Larycitrin 3-alpha-L-arabinofuranoside>	C21 H20 O12	(M+H)+	13.297		464.0960		FBF	98.83		98.83
<Herbacetin 4'-glucoside>	C21 H20 O12	(M+H)+	13.297		464.0960		FBF	98.83		98.83
<Myricetin 7-rhamnoside>	C21 H20 O12	(M+H)+	13.297		464.0960	184533-14-0	FBF	98.83		98.83

Cpd 194: C.I. Pigment Red 149

Name	Formula	RT	RI	Mass	Diff (Tgt, ppm)	CAS	ID Source	Score	Algorithm
C.I. Pigment Red 149	C40 H26 N2 O4	13.401		598.1897	0.74	4948-15-6	FBF	81.58	FBF

Species	m/z	Score (Tgt)	Score (Lib)	Score (DB)	Score (MFG)	Score (RT)
(M+H)+	599	81.58				

Compound Chromatograms (overlaid)

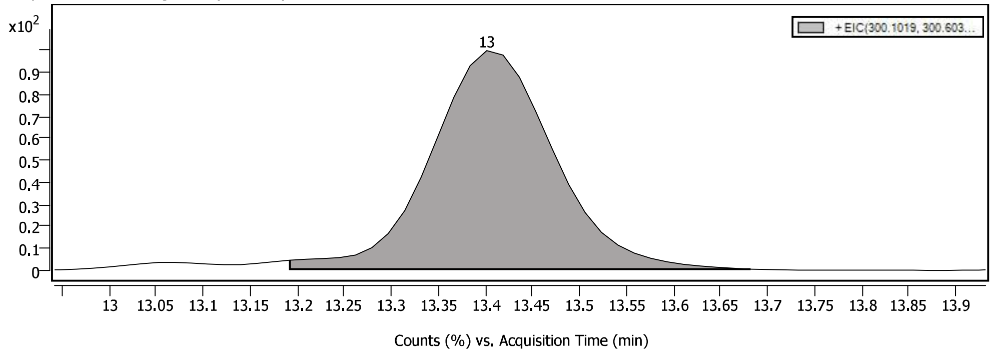

Structure

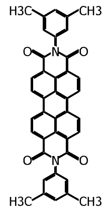

Compound Screening Report

Compound Spectra (overlaid)

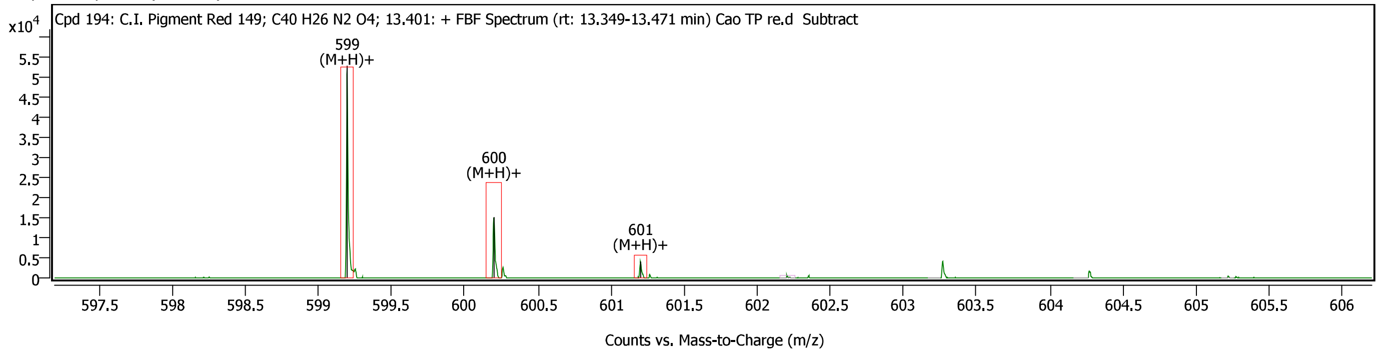

Compound ID Table

Name	Formula	Species	RT	RT Diff	Mass	CAS	ID Source	Score	Score (Lib)	Score (Tgt)
C.I. Pigment Red 149	C40 H26 N2 O4	(M+H)+	13.401		598.1897	4948-15-6	FBF	81.58		81.58

Cpd 143: Carnocin CP 5

Name	Formula	RT	RI	Mass	Diff (Tgt, ppm)	CAS	ID Source	Score	Algorithm
Carnocin CP 5	C23 H19 N3 O5 S	13.628		449.1040	-1.12	149983-81-3	FBF	89.07	FBF

Species	m/z	Score (Tgt)	Score (Lib)	Score (DB)	Score (MFG)	Score (RT)
(M+H)+	450	89.07				

Compound Chromatograms (overlaid)

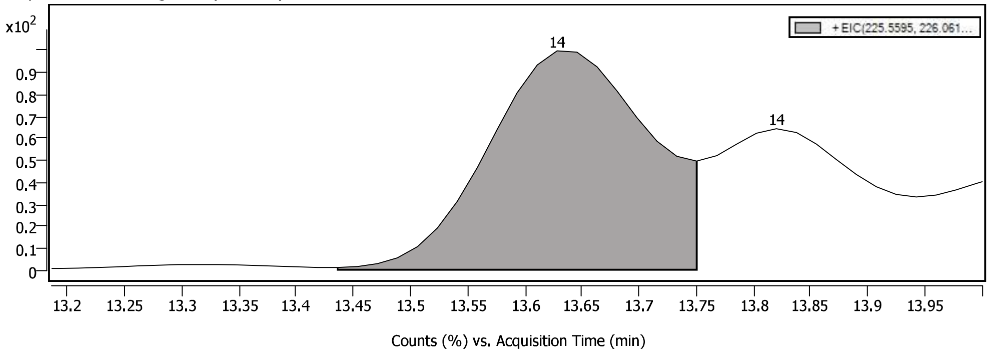

Structure

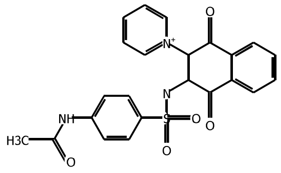

Compound Spectra (overlaid)

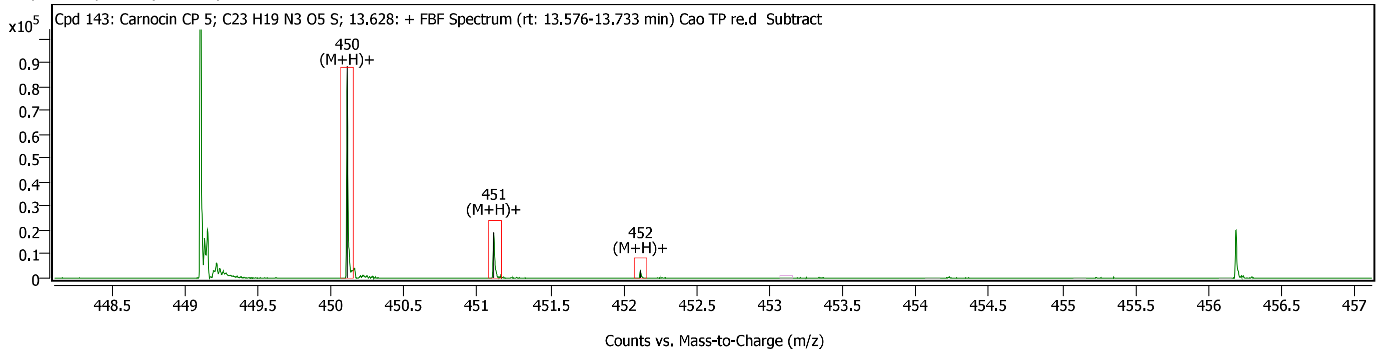

Compound ID Table

Name	Formula	Species	RT	RT Diff	Mass	CAS	ID Source	Score	Score (Lib)	Score (Tgt)
Carnocin CP 5	C23 H19 N3 O5 S	(M+H)+	13.628		449.1040	149983-81-3	FBF	89.07		89.07

Cpd 142: Carnocin CP 5

Name	Formula	RT	RI	Mass	Diff (Tgt, ppm)	CAS	ID Source	Score	Algorithm
Carnocin CP 5	C23 H19 N3 O5 S	13.820		449.1040	-1.29	149983-81-3	FBF	92.78	FBF

Species	m/z	Score (Tgt)	Score (Lib)	Score (DB)	Score (MFG)	Score (RT)
(M+H)+	450	92.78				

Compound Chromatograms (overlaid)

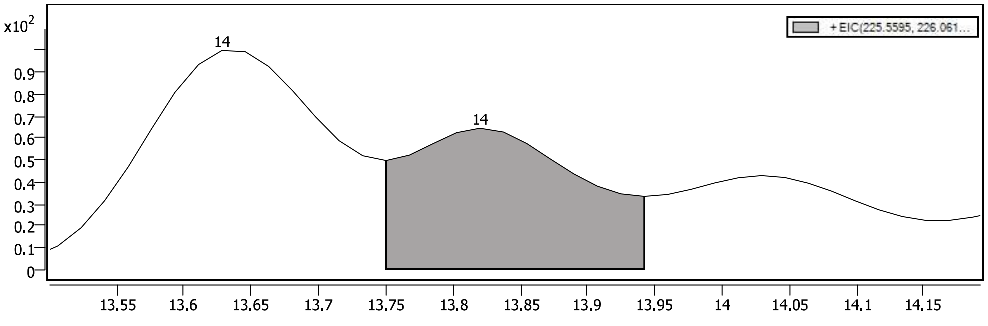

Structure

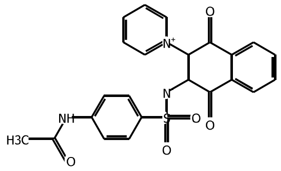

Compound Spectra (overlaid)

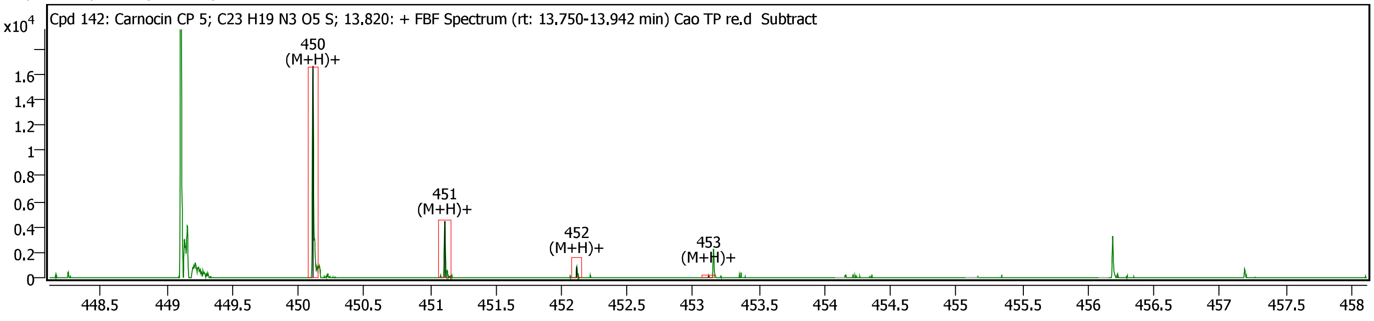

Compound ID Table

Name	Formula	Species	RT	RT Diff	Mass	CAS	ID Source	Score	Score (Lib)	Score (Tgt)
Carnocin CP 5	C23 H19 N3 O5 S	(M+H)+	13.820		449.1040	149983-81-3	FBF	92.78		92.78

Cpd 1193: Coumarin

Name	Formula	RT	RI	Mass	Diff (Tgt, ppm)	CAS	ID Source	Score	Algorithm
Coumarin	C9 H6 O2	13.890		146.0369	0.63	91-64-5	FBF-FragConfirm	99.38	FBF

Species	m/z	Score (Tgt)	Score (Lib)	Score (DB)	Score (MFG)	Score (RT)
(M+H)+	147	99.38				

Compound Chromatograms (overlaid)

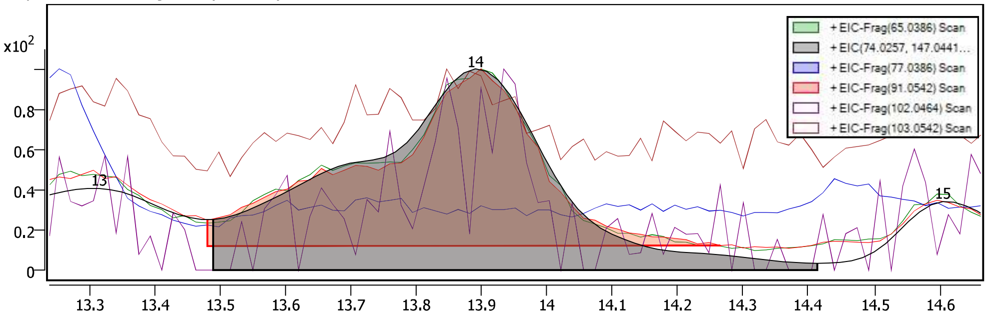

Structure

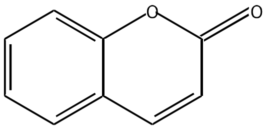

Coelution Plot

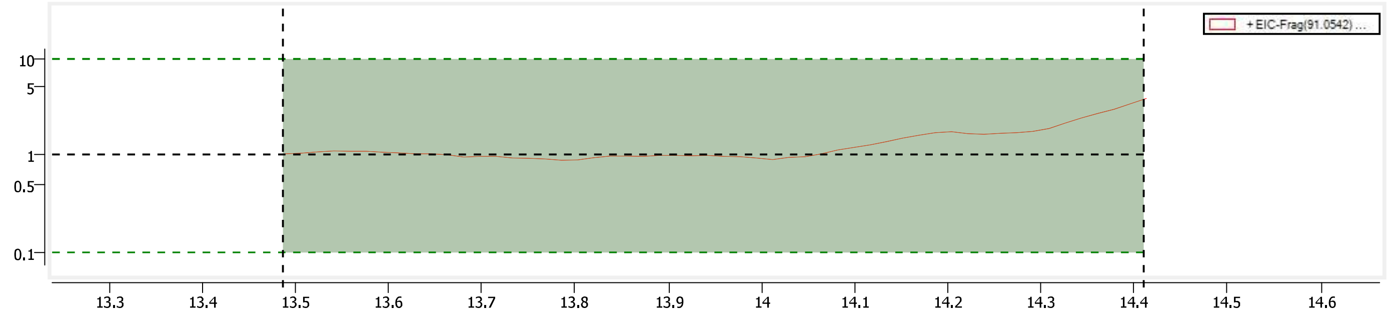

Compound Spectra (overlaid)

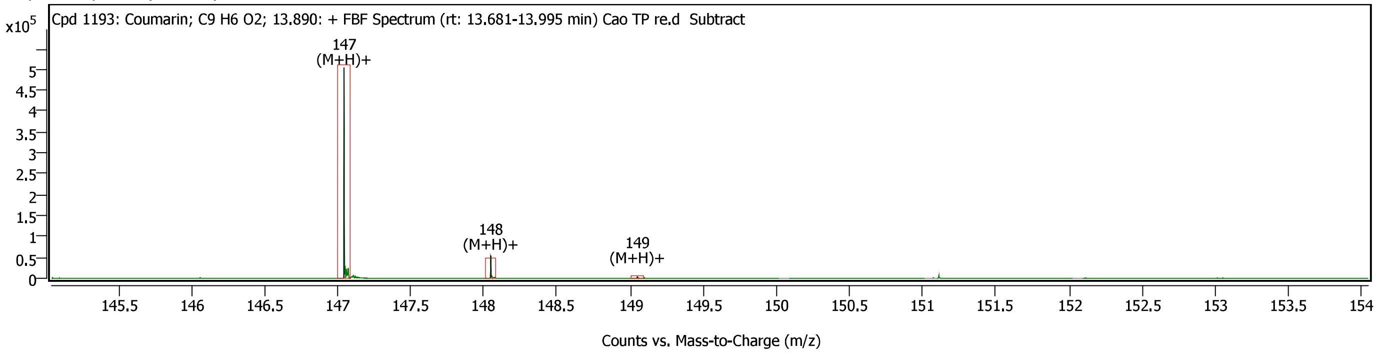

Fragment Spectrum (clean)

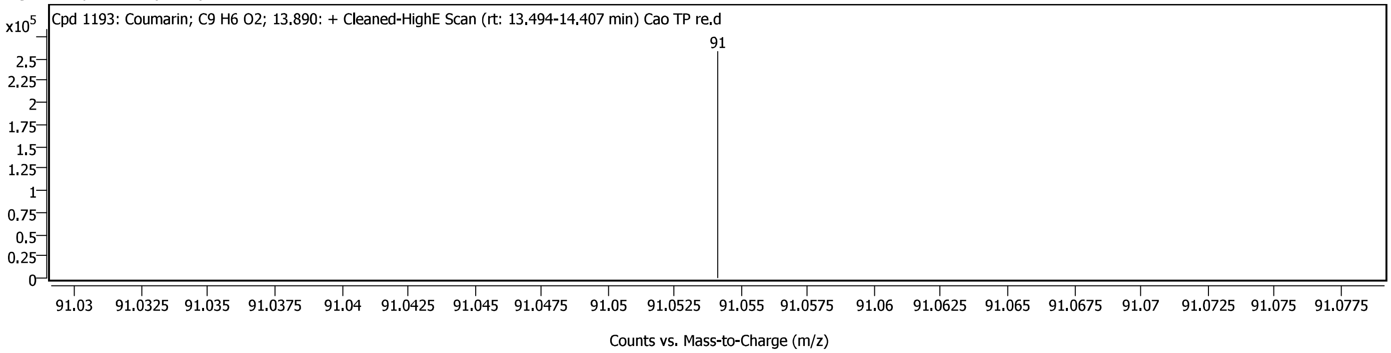

Fragment Spectrum (raw)

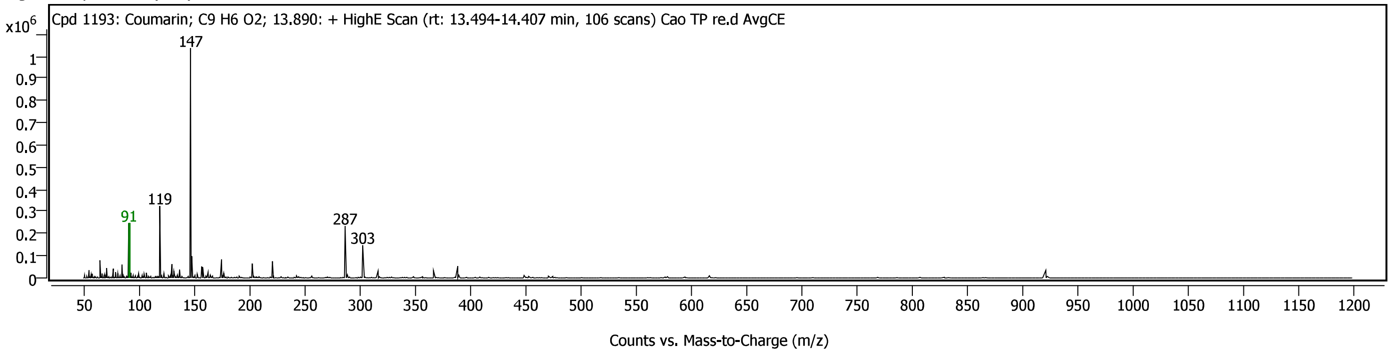

Compound ID Table

Name	Formula	Species	RT	RT Diff	Mass	CAS	ID Source	Score	Score (Lib)	Score (Tgt)
Coumarin	C9 H6 O2	(M+H)+	13.890		146.0369	91-64-5	FBF-FragConfirm	99.38		99.38
Phenylpropiolic acid	C9 H6 O2	(M+H)+	13.890		146.0369	637-44-5	FBF-FragConfirm	99.38		99.38
Chromone	C9 H6 O2	(M+H)+	13.890		146.0369	491-38-3	FBF-FragConfirm	99.38		99.38
2-Benzofurancarboxaldehyde	C9 H6 O2	(M+H)+	13.890		146.0369	4265-16-1	FBF-FragConfirm	99.38		99.38

Cpd 811: <7-[(2S,3R,4S,5S,6R)-6-[[(2S,3R,4R)-3,4-Dihydroxy-4-(hydroxymethyl)oxolan-2-yl]oxymethyl]-3,4,5-trihydroxyoxan-2-yl]oxy-5-hydroxy-3-(4-hydroxyphenyl)-6-methoxychromen-4-one>

Name	Formula	RT	RI	Mass	Diff (Tgt, ppm)	CAS	ID Source	Score	Algorithm
<7-[(2S,3R,4S,5S,6R)-6-[[(2S,3R,4R)-3,4-Dihydroxy-4-(hydroxymethyl)oxolan-2-yl]oxymethyl]-3,4,5-trihydroxyoxan-2-yl]oxy-5-hydroxy-3-(4-hydroxyphenyl)-6-methoxychromen-4-one>	C27 H30 O15	14.047		594.1590	0.88	365544-05-4	M-FBF	99.25	FBF

Species	m/z	Score (Tgt)	Score (Lib)	Score (DB)	Score (MFG)	Score (RT)
(M+H)+	595	99.25				

Compound Chromatograms (overlaid)

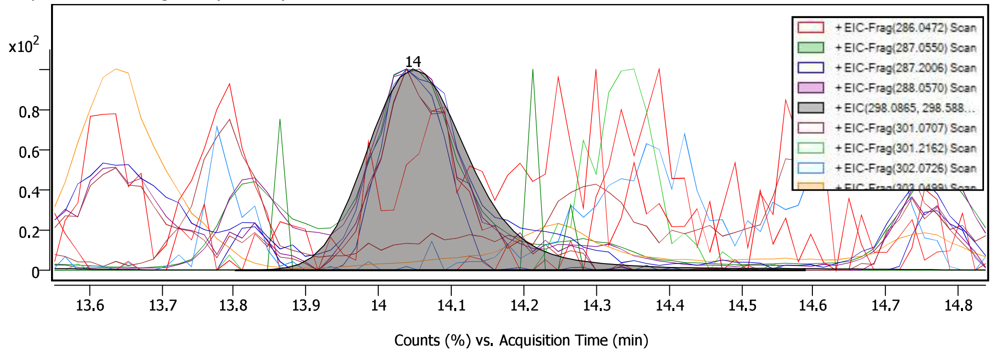

Structure

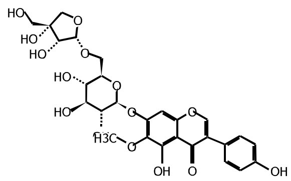

Coelution Plot

Compound Spectra (overlaid)

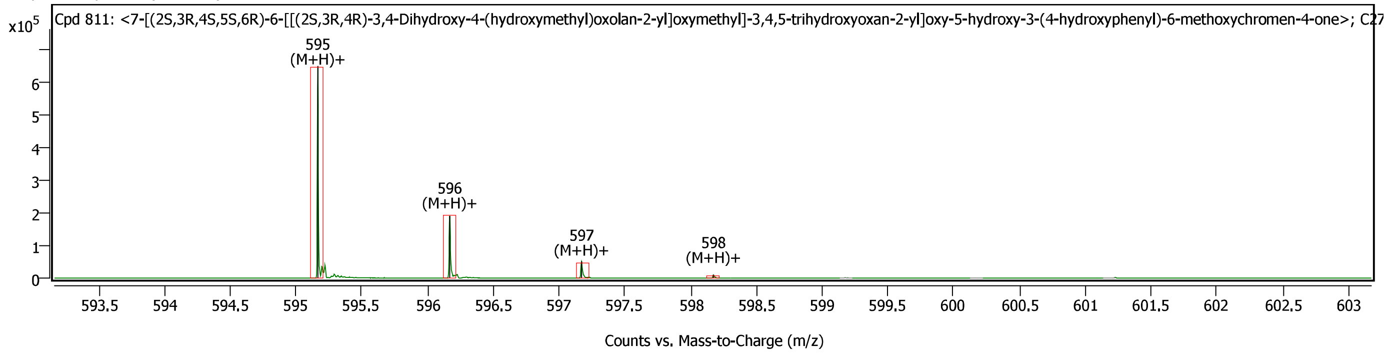

Fragment Spectrum (raw)

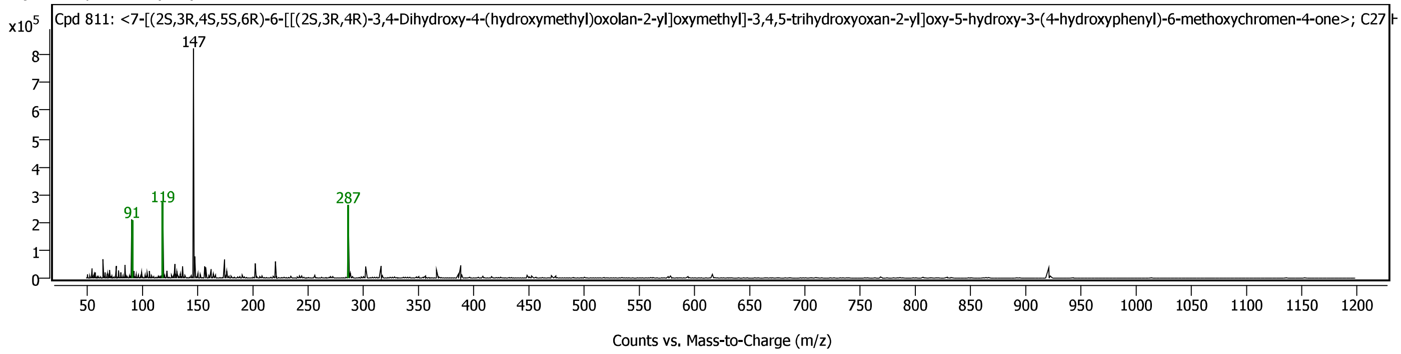

Compound Screening Report

Compound ID Table

Name	Formula	Species	RT	RT Diff	Mass	CAS	ID Source	Score	Score (Lib)	Score (Tgt)
<7-[(2S,3R,4S,5S,6R)-6-[[(2S,3R,4R)-3,4-Dihydroxy-4-(hydroxymethyl)oxolan-2-yl]oxymethyl]-3,4,5-trihydroxyoxan-2-yl]oxy-5-hydroxy-3-(4-hydroxyphenyl)-6-methoxychromen-4-one>	C27 H30 O15	(M+H)+	14.047		594.1590	365544-05-4	FBF	99.25		99.25
<Elatin(flavonoid)>	C27 H30 O15	(M+H)+	14.047		594.1590		FBF	99.25		99.25
<Vitexin 4'-O-glucoside>	C27 H30 O15	(M+H)+	14.047		594.1590		FBF	99.25		99.25
<Horridin (flavone)>	C27 H30 O15	(M+H)+	14.047		594.1590		FBF	99.25		99.25
<Graveobioside B>	C27 H30 O15	(M+H)+	14.047		594.1590	33579-63-4	FBF	99.25		99.25
<Genistein 7,4'-di-O-glucoside>	C27 H30 O15	(M+H)+	14.047		594.1590		FBF	99.25		99.25
<Flavosativaside>	C27 H30 O15	(M+H)+	14.047		594.1590		FBF	99.25		99.25
<Fisetin 7-rutinoside>	C27 H30 O15	(M+H)+	14.047		594.1590		FBF	99.25		99.25
<Chrysoeriol 6-C-glucoside-8-C-arabinopyranoside>	C27 H30 O15	(M+H)+	14.047		594.1590		FBF	99.25		99.25
<Datiscin>	C27 H30 O15	(M+H)+	14.047		594.1590		FBF	99.25		99.25
<Crenuloside>	C27 H30 O15	(M+H)+	14.047		594.1590		FBF	99.25		99.25
<Chrysoeriol 7-apiosyl-(1->6)-glucoside>	C27 H30 O15	(M+H)+	14.047		594.1590		FBF	99.25		99.25
<Chrysoeriol 7-alpha-L-arabinofuranosyl-(1->6)-glucoside>	C27 H30 O15	(M+H)+	14.047		594.1590		FBF	99.25		99.25
<Isoorientin 2''-O-rhamnoside>	C27 H30 O15	(M+H)+	14.047		594.1590		FBF	99.25		99.25
<Isovitexin 2''-O-glucoside>	C27 H30 O15	(M+H)+	14.047		594.1590	60767-80-8	FBF	99.25		99.25
<Biorobin>	C27 H30 O15	(M+H)+	14.047		594.1590	17297-56-2	FBF	99.25		99.25
<Isoorientin 6''-O-rhamnoside>	C27 H30 O15	(M+H)+	14.047		594.1590		FBF	99.25		99.25
<Isoorientin 6''-rhamnoside>	C27 H30 O15	(M+H)+	14.047		594.1590	108602-35-3	FBF	99.25		99.25
<Isoorientin 7-O-rhamnoside>	C27 H30 O15	(M+H)+	14.047		594.1590		FBF	99.25		99.25
<Isorhamnetin 3-alpha-L-arabinopyranoside-7-rhamnoside>	C27 H30 O15	(M+H)+	14.047		594.1590		FBF	99.25		99.25
<Isorhamnetin 3-alpha-L-arabinopyranosyl-(1->2)-rhamnoside>	C27 H30 O15	(M+H)+	14.047		594.1590		FBF	99.25		99.25
<Isorhamnetin 3-O-a-L-arabinopyranoside 7-O-a-L-rhamnopyranoside>	C27 H30 O15	(M+H)+	14.047		594.1590	69760-70-9	FBF	99.25		99.25
<Isosaponarin>	C27 H30 O15	(M+H)+	14.047		594.1590		FBF	99.25		99.25
<Isoscutellarein 7-neohesperidoside>	C27 H30 O15	(M+H)+	14.047		594.1590		FBF	99.25		99.25
<Isovitexin 2''-O-galactoside>	C27 H30 O15	(M+H)+	14.047		594.1590		FBF	99.25		99.25
<Isovitexin 6''-O-glucoside>	C27 H30 O15	(M+H)+	14.047		594.1590		FBF	99.25		99.25
<Chrysoeriol 6-C-arabinopyranoside-8-C-glucoside>	C27 H30 O15	(M+H)+	14.047		594.1590		FBF	99.25		99.25
<2'-Hydroxydaidzein 7,4'-di-O-glucoside>	C27 H30 O15	(M+H)+	14.047		594.1590		FBF	99.25		99.25
<Astragalin 7-rhamnoside>	C27 H30 O15	(M+H)+	14.047		594.1590	38784-79-1	FBF	99.25		99.25
<5,3',4'-Trihydroxy-7-methoxy-4-phenylcoumarin 5-O-xylosyl-(1->6)-glucoside>	C27 H30 O15	(M+H)+	14.047		594.1590		FBF	99.25		99.25
<Kaempferol 3-alpha-D-glucoside-7-rhamnoside>	C27 H30 O15	(M+H)+	14.047		594.1590		FBF	99.25		99.25
<Apigenin 7-cellobioside>	C27 H30 O15	(M+H)+	14.047		594.1590		FBF	99.25		99.25
<1,8-Dihydroxy-3-hydroxymethylanthraquinone 1,8-di-O-b-D-glucoside>	C27 H30 O15	(M+H)+	14.047		594.1590		FBF	99.25		99.25
<2'-Hydroxydaidzein 4',7-diglucoside>	C27 H30 O15	(M+H)+	14.047		594.1590	88048-22-0	FBF	99.25		99.25
<Kaempferol 3-galactoside-7-rhamnoside>	C27 H30 O15	(M+H)+	14.047		594.1590		FBF	99.25		99.25
<2''-O-beta-L-galactopyranosylvitexin>	C27 H30 O15	(M+H)+	14.047		594.1590		FBF	99.25		99.25
<3,6-Di-C-glucosylapigenin>	C27 H30 O15	(M+H)+	14.047		594.1590		FBF	99.25		99.25
<3,6-Diglucopyranosyl-4',5,7-trihydroxyflavone>	C27 H30 O15	(M+H)+	14.047		594.1590	90456-55-6	FBF	99.25		99.25
<3,8-Di-C-glucopyranosylapigenin>	C27 H30 O15	(M+H)+	14.047		594.1590		FBF	99.25		99.25
<4,6,4'-Trihydroxyaurone 4,6-di-O-glucoside>	C27 H30 O15	(M+H)+	14.047		594.1590		FBF	99.25		99.25
<6,8-Di-C-arabinopyranosylapometzgerin>	C27 H30 O15	(M+H)+	14.047		594.1590		FBF	99.25		99.25
<Apigenin 7-[galactosyl-(1->4)-mannoside]>	C27 H30 O15	(M+H)+	14.047		594.1590	84638-36-8	FBF	99.25		99.25
<6,8-Di-C-arabinopyranosyltricin>	C27 H30 O15	(M+H)+	14.047		594.1590		FBF	99.25		99.25
<6,8-Di-C-galactopyranosylapigenin>	C27 H30 O15	(M+H)+	14.047		594.1590		FBF	99.25		99.25
<6-C-Galactosylapigenin 6''-O-galactoside>	C27 H30 O15	(M+H)+	14.047		594.1590		FBF	99.25		99.25
<6-C-Glucopyranosyl-8-C-galactopyranosylapigenin>	C27 H30 O15	(M+H)+	14.047		594.1590		FBF	99.25		99.25
<6-C-Glucopyranosyl-8-C-xylopyranosylchrysoeriol>	C27 H30 O15	(M+H)+	14.047		594.1590		FBF	99.25		99.25
<6-C-Xylopyranosyl-8-C-glucopyranosylchrysoeriol>	C27 H30 O15	(M+H)+	14.047		594.1590		FBF	99.25		99.25
<8-C-Glucopyranosylgenistein 4'-O-glucoside>	C27 H30 O15	(M+H)+	14.047		594.1590		FBF	99.25		99.25
<8-C-xylopyranosylchrysoeriol 2''-O-glucoside>	C27 H30 O15	(M+H)+	14.047		594.1590		FBF	99.25		99.25
<Apigenin 7,4'-dialloside>	C27 H30 O15	(M+H)+	14.047		594.1590		FBF	99.25		99.25
<Apigenin 7,4'-diglucoside>	C27 H30 O15	(M+H)+	14.047		594.1590		FBF	99.25		99.25
<Apigenin 7-allosyl-(1->2)-glucoside>	C27 H30 O15	(M+H)+	14.047		594.1590		FBF	99.25		99.25
<Kaempferol 7-galactosyl-(1->4)-rhamnoside>	C27 H30 O15	(M+H)+	14.047		594.1590		FBF	99.25		99.25
<Luteolin 5-O-rutinoside>	C27 H30 O15	(M+H)+	14.047		594.1590		FBF	99.25		99.25
<Rheinoside C>	C27 H30 O15	(M+H)+	14.047		594.1590	111545-29-0	FBF	99.25		99.25
<Safflor Yellow A>	C27 H30 O15	(M+H)+	14.047		594.1590	85532-77-0	FBF	99.25		99.25
<Orientin 2''-rhamnoside>	C27 H30 O15	(M+H)+	14.047		594.1590	81398-30-3	FBF	99.25		99.25
<Orientin 7-rhamnoside>	C27 H30 O15	(M+H)+	14.047		594.1590	29432-29-9	FBF	99.25		99.25
<Palasitrin>	C27 H30 O15	(M+H)+	14.047		594.1590		FBF	99.25		99.25
<Paniculatin>	C27 H30 O15	(M+H)+	14.047		594.1590		FBF	99.25		99.25
<Peumoside>	C27 H30 O15	(M+H)+	14.047		594.1590		FBF	99.25		99.25

Compound ID Table

Name	Formula	Species	RT	RT Diff	Mass	CAS	ID Source	Score	Score (Lib)	Score (Tgt)
<Quercetin 3,7-dirhamnoside>	C27 H30 O15	(M+H)+	14.047		594.1590	28638-13-3	FBF	99.25		99.25
<Quercetin 3-methyl ether 7-rhamnoside-3'-xyloside>	C27 H30 O15	(M+H)+	14.047		594.1590		FBF	99.25		99.25
<Rhamnocitrin 3-apiosyl-(1->2)-glucoside>	C27 H30 O15	(M+H)+	14.047		594.1590		FBF	99.25		99.25
<Rhamnocitrin 3-lathyroside>	C27 H30 O15	(M+H)+	14.047		594.1590		FBF	99.25		99.25
<Saponarin>	C27 H30 O15	(M+H)+	14.047		594.1590		FBF	99.25		99.25
<Orientin 7-O-rhamnoside>	C27 H30 O15	(M+H)+	14.047		594.1590		FBF	99.25		99.25
<Vitexin 6''-O-glucoside>	C27 H30 O15	(M+H)+	14.047		594.1590		FBF	99.25		99.25
<Scoparin 2''-O-xyloside>	C27 H30 O15	(M+H)+	14.047		594.1590		FBF	99.25		99.25
<Kaempferol 3-glucosyl-(1->2)-rhamnoside>	C27 H30 O15	(M+H)+	14.047		594.1590		FBF	99.25		99.25
<Scutellarein 6-rhamnosyl-(1->2)-galactoside>	C27 H30 O15	(M+H)+	14.047		594.1590		FBF	99.25		99.25
<Scutellarein 7-glucosyl-(1->4)-rhamnoside>	C27 H30 O15	(M+H)+	14.047		594.1590		FBF	99.25		99.25
<Scutellarein 7-neohesperidoside>	C27 H30 O15	(M+H)+	14.047		594.1590		FBF	99.25		99.25
<Scutellarein 7-rutinoside>	C27 H30 O15	(M+H)+	14.047		594.1590		FBF	99.25		99.25
<Tricetin 3'-rhamnosyl-(1->4)-rhamnoside>	C27 H30 O15	(M+H)+	14.047		594.1590		FBF	99.25		99.25
<Vicenin 2>	C27 H30 O15	(M+H)+	14.047		594.1590		FBF	99.25		99.25
<Vitexin 4'-O-galactoside>	C27 H30 O15	(M+H)+	14.047		594.1590		FBF	99.25		99.25
<Vitexin 7-O-glucoside>	C27 H30 O15	(M+H)+	14.047		594.1590		FBF	99.25		99.25
<Scoparin 2''-xyloside>	C27 H30 O15	(M+H)+	14.047		594.1590	124934-30-1	FBF	99.25		99.25
<Kaempferol 3-glucoside-7-rhamnoside>	C27 H30 O15	(M+H)+	14.047		594.1590	2392-95-2	FBF	99.25		99.25
<Luteolin 3'-methyl ether 7-apiosyl-(1->2)-glucoside>	C27 H30 O15	(M+H)+	14.047		594.1590		FBF	99.25		99.25
<Orientin 2''-O-rhamnoside>	C27 H30 O15	(M+H)+	14.047		594.1590		FBF	99.25		99.25
<Luteolin 4'-methyl ether 7-xylosyl-(1->6)-glucoside>	C27 H30 O15	(M+H)+	14.047		594.1590		FBF	99.25		99.25
<Nicotiflorin>	C27 H30 O15	(M+H)+	14.047		594.1590	17650-84-9	FBF	99.25		99.25
<Kaempferol 3-neohesperidoside>	C27 H30 O15	(M+H)+	14.047		594.1590		FBF	99.25		99.25
<Kaempferol 3-O-glucosyl-(1->2)-rhamnoside>	C27 H30 O15	(M+H)+	14.047		594.1590	142451-65-8	FBF	99.25		99.25
<Kaempferol 3-rhamnoside-7-glucoside>	C27 H30 O15	(M+H)+	14.047		594.1590		FBF	99.25		99.25
<Kaempferol 3-rhamnosyl-(1->2)-galactoside>	C27 H30 O15	(M+H)+	14.047		594.1590		FBF	99.25		99.25
<Kaempferol 3-rungioside>	C27 H30 O15	(M+H)+	14.047		594.1590	28447-29-2	FBF	99.25		99.25
<Kaempferol 7-neohesperidoside>	C27 H30 O15	(M+H)+	14.047		594.1590	17353-03-6	FBF	99.25		99.25
<Kaempferol 7-rhamnoside-4'-glucoside>	C27 H30 O15	(M+H)+	14.047		594.1590		FBF	99.25		99.25
<Luteolin 3'-methyl ether 7-arabinosyl-(1->2)-galactoside>	C27 H30 O15	(M+H)+	14.047		594.1590		FBF	99.25		99.25
<Luteolin 7-neohesperidoside>	C27 H30 O15	(M+H)+	14.047		594.1590		FBF	99.25		99.25
<Luteolin 4'-methyl ether 7-alpha-L-arabinofuranosyl-(1->6)-beta-D-glucoside>	C27 H30 O15	(M+H)+	14.047		594.1590		FBF	99.25		99.25
<Kaempferol 4'-glucoside 7-rhamnoside>	C27 H30 O15	(M+H)+	14.047		594.1590	205876-37-5	FBF	99.25		99.25
<Luteolin 4'-neohesperidoside>	C27 H30 O15	(M+H)+	14.047		594.1590		FBF	99.25		99.25
<Luteolin 7-β-rutinoside>	C27 H30 O15	(M+H)+	14.047		594.1590	20633-84-5	FBF	99.25		99.25
<Nepetin 7-rhamnoside-3'-xyloside>	C27 H30 O15	(M+H)+	14.047		594.1590		FBF	99.25		99.25
<Luteolin 4'-rutinoside>	C27 H30 O15	(M+H)+	14.047		594.1590		FBF	99.25		99.25
<Neosaponarin>	C27 H30 O15	(M+H)+	14.047		594.1590		FBF	99.25		99.25
<Multiflorin B>	C27 H30 O15	(M+H)+	14.047		594.1590		FBF	99.25		99.25
<Meloside A>	C27 H30 O15	(M+H)+	14.047		594.1590		FBF	99.25		99.25
<Luteolin 7-rhamnosyl(1->6)galactoside>	C27 H30 O15	(M+H)+	14.047		594.1590		FBF	99.25		99.25
<Luteolin 5-methyl ether 7-xylosyl-(1->6)-glucoside>	C27 H30 O15	(M+H)+	14.047		594.1590		FBF	99.25		99.25

Cpd 1517: 3,5-Dicaffeoyl-4-succinoylquinic acid

Name	Formula	RT	RI	Mass	Diff (Tgt, ppm)	CAS	ID Source	Score	Algorithm
3,5-Dicaffeoyl-4-succinoylquinic acid	C29 H28 O15	14.064		616.1405	-3.78	179761-31-0	FBF	92.17	FBF

Species	m/z	Score (Tgt)	Score (Lib)	Score (DB)	Score (MFG)	Score (RT)
(M+H)+	617	92.17				

Compound Chromatograms (overlaid)

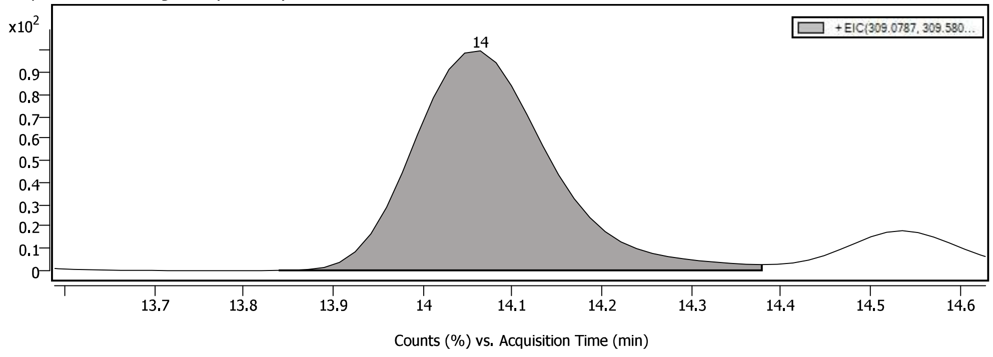

Structure

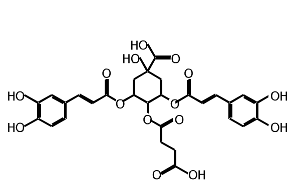

Compound Screening Report

Compound Spectra (overlaid)

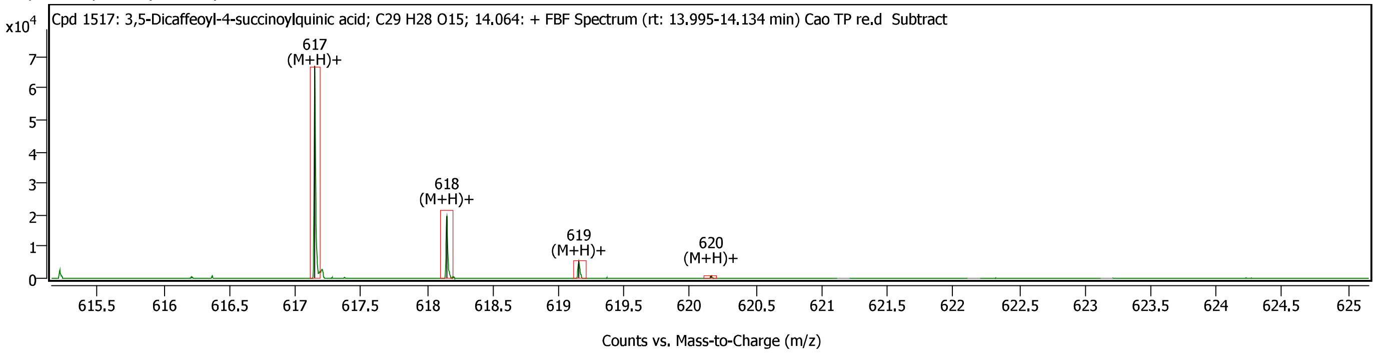

Compound ID Table

Name	Formula	Species	RT	RT Diff	Mass	CAS	ID Source	Score	Score (Lib)	Score (Tgt)
3,5-Dicaffeoyl-4-succinoylquinic acid	C29 H28 O15	(M+H)+	14.064		616.1405	179761-31-0	FBF	92.17		92.17

Cpd 11: <Foramsulfuron>

Name	Formula	RT	RI	Mass	Diff (Tgt, ppm)	CAS	ID Source	Score	Algorithm
<Foramsulfuron>	C17 H20 N6 O7 S	14.675		452.1112	-0.41	173159-57-4	FBF	86.40	FBF

Species	m/z	Score (Tgt)	Score (Lib)	Score (DB)	Score (MFG)	Score (RT)
(M+H)+	453	86.40				

Compound Chromatograms (overlaid)

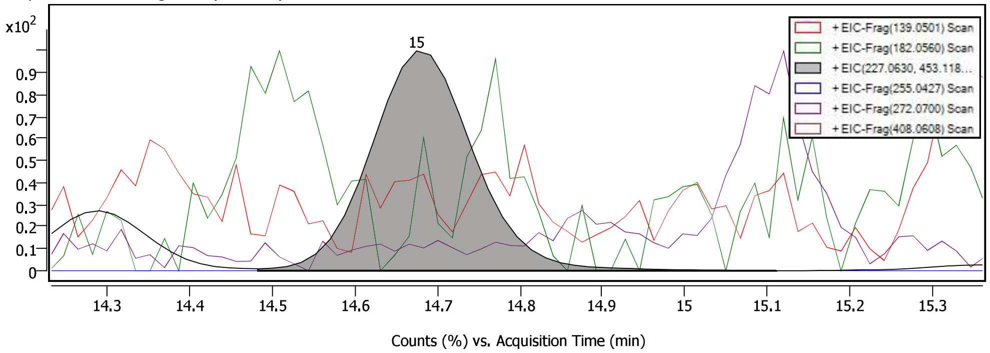

Structure

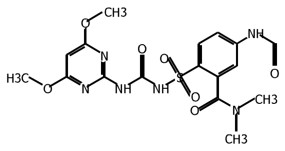

Coelution Plot

Compound Spectra (overlaid)

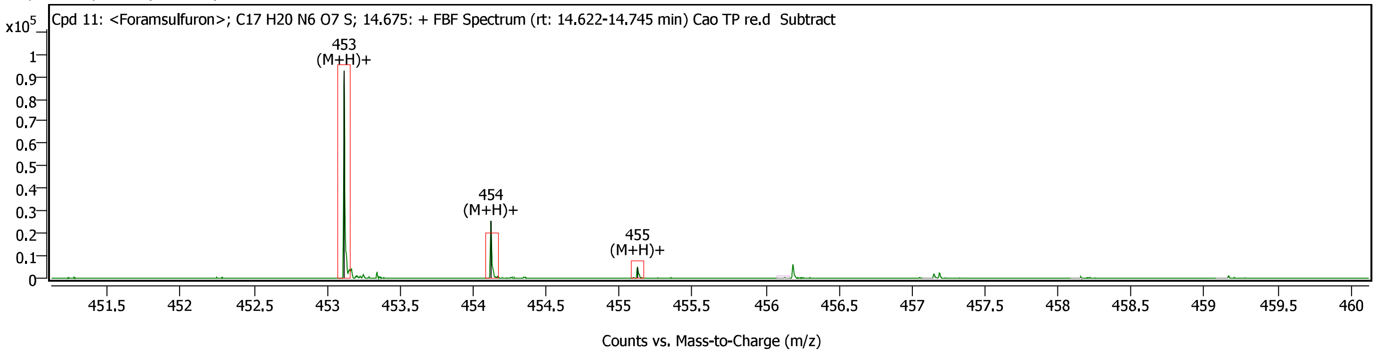

Fragment Spectrum (raw)

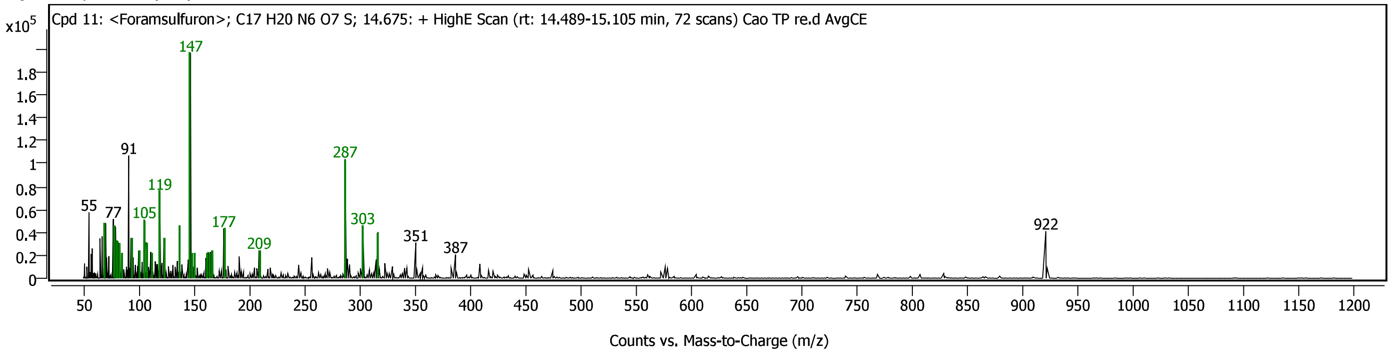

Compound Screening Report

Compound ID Table

Name	Formula	Species	RT	RT Diff	Mass	CAS	ID Source	Score	Score (Lib)	Score (Tgt)
<Foramsulfuron>	C17 H20 N6 O7 S	(M+H)+	14.675		452.1112	173159-57-4	FBF	86.40		86.40

Cpd 1635: <5,7-Dihydroxy-2-(4-hydroxy-3-methoxyphenyl)-3-[3,4,5-trihydroxy-6-[[(2R,3R,4R,5R,6S)-3,4,5-trihydroxy-6-methyloxan-2-yl]oxymethyl]oxan-2-yl]oxychromen-4-one>

Name	Formula	RT	RI	Mass	Diff (Tgt, ppm)	CAS	ID Source	Score	Algorithm
<5,7-Dihydroxy-2-(4-hydroxy-3-methoxyphenyl)-3-[3,4,5-trihydroxy-6-[[(2R,3R,4R,5R,6S)-3,4,5-trihydroxy-6-methyloxan-2-yl]oxymethyl]oxan-2-yl]oxychromen-4-one>	C28 H32 O16	14.675		624.1691	0.09	1393485-18-1	M-FBF	99.75	FBF

Species	m/z	Score (Tgt)	Score (Lib)	Score (DB)	Score (MFG)	Score (RT)
(M+H)+	625	99.75				

Compound Chromatograms (overlaid)

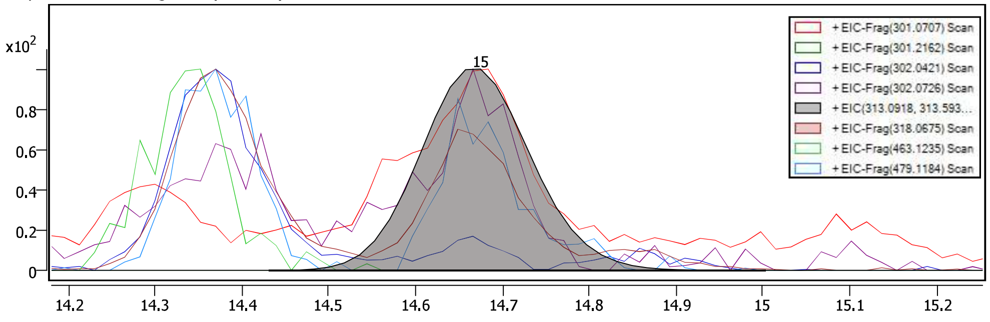

Structure

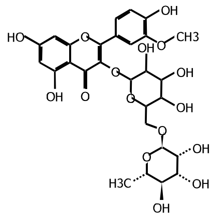

Coelution Plot

Compound Spectra (overlaid)

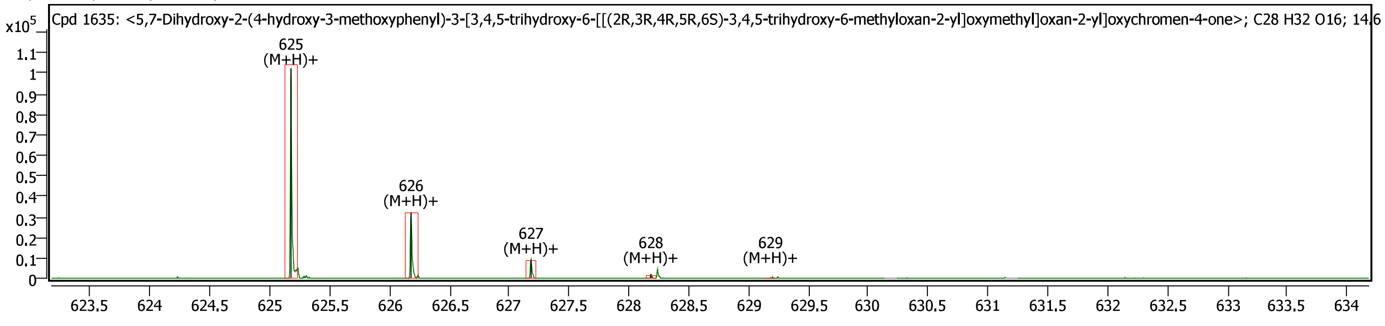

Fragment Spectrum (raw)

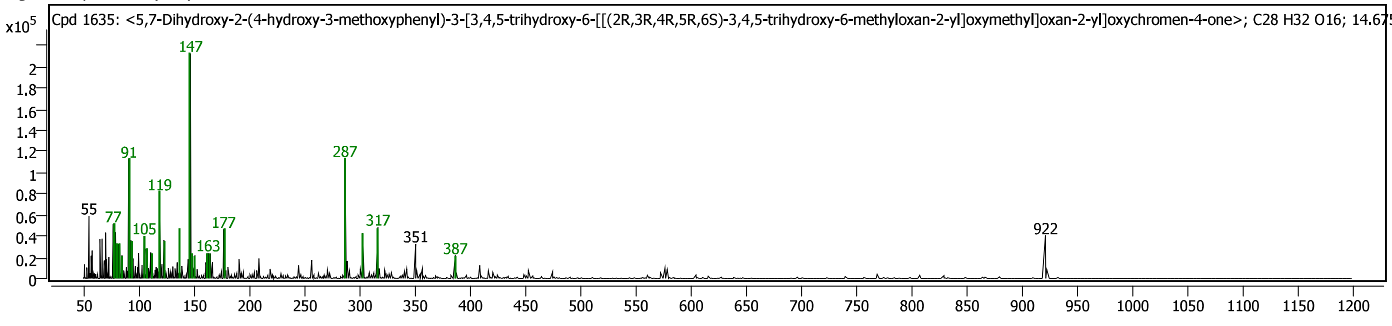

Compound ID Table

Name	Formula	Species	RT	RT Diff	Mass	CAS	ID Source	Score	Score (Lib)	Score (Tgt)
<5,7-Dihydroxy-2-(4-hydroxy-3-methoxyphenyl)-3-[3,4,5-trihydroxy-6-[[(2R,3R,4R,5R,6S)-3,4,5-trihydroxy-6-methyloxan-2-yl]oxymethyl]oxan-2-yl]oxychromen-4-one>	C28 H32 O16	(M+H)+	14.675		624.1691	1393485-18-1	FBF	99.75		99.75
<Isorhamnetin 3-O-[b-L-rhamnofuranosyl-(1->6)-D-glucopyranoside]>	C28 H32 O16	(M+H)+	14.675		624.1691	37138-79-7	FBF	99.75		99.75
<Isoscoparin 2''-O-glucoside>	C28 H32 O16	(M+H)+	14.675		624.1691		FBF	99.75		99.75
<Isoscoparin 4'-O-glucoside>	C28 H32 O16	(M+H)+	14.675		624.1691		FBF	99.75		99.75
<Isoscoparin 7-O-glucoside>	C28 H32 O16	(M+H)+	14.675		624.1691		FBF	99.75		99.75
<Isorhamnetin 3-rhamnoside-7-glucoside>	C28 H32 O16	(M+H)+	14.675		624.1691		FBF	99.75		99.75
<Isorhamnetin 3-rhamnosyl-(1->2)-galactoside>	C28 H32 O16	(M+H)+	14.675		624.1691		FBF	99.75		99.75
<Isorhamnetin 3-robinobioside>	C28 H32 O16	(M+H)+	14.675		624.1691		FBF	99.75		99.75
<Isorhamnetin 4'-neohesperidoside>	C28 H32 O16	(M+H)+	14.675		624.1691		FBF	99.75		99.75
<Kaempferol 5-methyl ether 3-galactoside-4'-glucoside>	C28 H32 O16	(M+H)+	14.675		624.1691		FBF	99.75		99.75
<6-C-Glucopyranosyl-8-C-arabinopyranosyltricin>	C28 H32 O16	(M+H)+	14.675		624.1691		FBF	99.75		99.75
<Isorhamnetin 3-neohesperidoside>	C28 H32 O16	(M+H)+	14.675		624.1691		FBF	99.75		99.75
<Isoscutellarein 4'-methyl ether 7-allosyl-(1->2)-glucoside>	C28 H32 O16	(M+H)+	14.675		624.1691		FBF	99.75		99.75
<Kaempferide 3,7-diglucoside>	C28 H32 O16	(M+H)+	14.675		624.1691		FBF	99.75		99.75
<Luteolin 3'-methyl ether 5,4'-diglucoside>	C28 H32 O16	(M+H)+	14.675		624.1691		FBF	99.75		99.75
<Keioside>	C28 H32 O16	(M+H)+	14.675		624.1691	107740-46-5	FBF	99.75		99.75
<Knoutinoside>	C28 H32 O16	(M+H)+	14.675		624.1691		FBF	99.75		99.75
<Isorhamnetin 3-O-[b-D-glucopyranosyl-(1->2)-a-L-rhamnopyranoside]>	C28 H32 O16	(M+H)+	14.675		624.1691		FBF	99.75		99.75
<Isorhamnetin 3-glucoside-7-rhamnoside>	C28 H32 O16	(M+H)+	14.675		624.1691		FBF	99.75		99.75
<Isorhamnetin 3-glucoside 4'-rhamnoside>	C28 H32 O16	(M+H)+	14.675		624.1691	28289-13-6	FBF	99.75		99.75
<6,8-Diglucosyldiosmetin>	C28 H32 O16	(M+H)+	14.675		624.1691	98813-28-6	FBF	99.75		99.75
<Rhamnocitrin 3,4'-diglucoside>	C28 H32 O16	(M+H)+	14.675		624.1691		FBF	99.75		99.75
<Isorhamnetin 3-glucoside-4'-rhamnoside>	C28 H32 O16	(M+H)+	14.675		624.1691		FBF	99.75		99.75
<Luteolin 3'-methyl ether 7-allosyl-(1->2)-glucoside>	C28 H32 O16	(M+H)+	14.675		624.1691		FBF	99.75		99.75
<3,8-Di-C-glucopyranosyldiosmetin>	C28 H32 O16	(M+H)+	14.675		624.1691		FBF	99.75		99.75
<3,8-Diglucosyldiosmetin>	C28 H32 O16	(M+H)+	14.675		624.1691	97218-32-1	FBF	99.75		99.75
<6,8-Di-C-glucopyranosyldiosmetin>	C28 H32 O16	(M+H)+	14.675		624.1691		FBF	99.75		99.75
<6-C-Arabinopyranosyl-8-C-glucopyranosyltricin>	C28 H32 O16	(M+H)+	14.675		624.1691		FBF	99.75		99.75
<Isorhamnetin 3-galactoside-7-rhamnoside>	C28 H32 O16	(M+H)+	14.675		624.1691		FBF	99.75		99.75
<6-C-Rhamnopyranosylrhamnetin 3-O-glucopyranoside>	C28 H32 O16	(M+H)+	14.675		624.1691		FBF	99.75		99.75
<6-Methoxykaempferol 3-robinobioside>	C28 H32 O16	(M+H)+	14.675		624.1691		FBF	99.75		99.75
<Azaleatin 3-rutinoside>	C28 H32 O16	(M+H)+	14.675		624.1691		FBF	99.75		99.75
<Calendoflavoside>	C28 H32 O16	(M+H)+	14.675		624.1691	55033-90-4	FBF	99.75		99.75
<Chrysoeriol 7-O-8-C-bisglucoside>	C28 H32 O16	(M+H)+	14.675		624.1691		FBF	99.75		99.75
<Crosatoside A>	C28 H32 O16	(M+H)+	14.675		624.1691	139742-27-1	FBF	99.75		99.75
<Luteoayamenin>	C28 H32 O16	(M+H)+	14.675		624.1691		FBF	99.75		99.75
<Luteolin 3'-methyl ether 7,4'-diglucoside>	C28 H32 O16	(M+H)+	14.675		624.1691		FBF	99.75		99.75
<Tamarixetin 7-rutinoside>	C28 H32 O16	(M+H)+	14.675		624.1691		FBF	99.75		99.75
<Sexangularetin 3-rutinoside>	C28 H32 O16	(M+H)+	14.675		624.1691		FBF	99.75		99.75
<Stellarin 2>	C28 H32 O16	(M+H)+	14.675		624.1691		FBF	99.75		99.75
<Scoparin 2''-O-glucoside>	C28 H32 O16	(M+H)+	14.675		624.1691		FBF	99.75		99.75
<Sexangularetin 3-glucoside 7-rhamnoside>	C28 H32 O16	(M+H)+	14.675		624.1691	47850-51-1	FBF	99.75		99.75
<Sexangularetin 3-glucoside-7-rhamnoside>	C28 H32 O16	(M+H)+	14.675		624.1691		FBF	99.75		99.75
<Sexangularetin 3-neohesperidoside>	C28 H32 O16	(M+H)+	14.675		624.1691		FBF	99.75		99.75
<Sexangularetin 3-rhamnoside-7-glucoside>	C28 H32 O16	(M+H)+	14.675		624.1691		FBF	99.75		99.75
<Subulin>	C28 H32 O16	(M+H)+	14.675		624.1691		FBF	99.75		99.75
<Tricetin 7-methyl ether 3'-glucoside-5'-rhamnoside>	C28 H32 O16	(M+H)+	14.675		624.1691		FBF	99.75		99.75
<Tectorigenin 7-O-gentiobioside>	C28 H32 O16	(M+H)+	14.675		624.1691		FBF	99.75		99.75
<Swertiajaponin 3'-O-glucoside>	C28 H32 O16	(M+H)+	14.675		624.1691		FBF	99.75		99.75
<Syringetin 3-rhamnosyl-(1->5)-alpha-L-arabinofuranoside>	C28 H32 O16	(M+H)+	14.675		624.1691		FBF	99.75		99.75
<Tamarixetin 3-neohesperidoside>	C28 H32 O16	(M+H)+	14.675		624.1691		FBF	99.75		99.75
<Luteolin 4'-methyl ether 7-sophoroside>	C28 H32 O16	(M+H)+	14.675		624.1691		FBF	99.75		99.75
<Tamarixetin 3-rutinoside>	C28 H32 O16	(M+H)+	14.675		624.1691		FBF	99.75		99.75
<Luteolin 3'-methyl ether 7-mannosyl-(1->2)-alloside>	C28 H32 O16	(M+H)+	14.675		624.1691		FBF	99.75		99.75
<Tamarixetin 3-robinobioside>	C28 H32 O16	(M+H)+	14.675		624.1691		FBF	99.75		99.75
<Rhamnocitrin 3-galactoside-4'-glucoside>	C28 H32 O16	(M+H)+	14.675		624.1691		FBF	99.75		99.75
<Narcissin>	C28 H32 O16	(M+H)+	14.675		624.1691		FBF	99.75		99.75
<Pasternoside>	C28 H32 O16	(M+H)+	14.675		624.1691	10576-85-9	FBF	99.75		99.75
<Scoparin 2''-glucoside>	C28 H32 O16	(M+H)+	14.675		624.1691	124902-15-4	FBF	99.75		99.75
<Rhamnazin 3-glucosyl-(1->5)-alpha-L-arabinofuranoside>	C28 H32 O16	(M+H)+	14.675		624.1691		FBF	99.75		99.75
<Rhamnocitrin 3-glucosyl-(1->2)-galactoside>	C28 H32 O16	(M+H)+	14.675		624.1691		FBF	99.75		99.75
<Mearnsetin 3,7-dirhamnoside>	C28 H32 O16	(M+H)+	14.675		624.1691		FBF	99.75		99.75

Compound ID Table

Name	Formula	Species	RT	RT Diff	Mass	CAS	ID Source	Score	Score (Lib)	Score (Tgt)
<Myricetin 3,4'-dimethyl ether 7-rhamnoside-3'-xyloside>	C28 H32 O16	(M+H)+	14.675		624.1691		FBF	99.75		99.75
<Patuletin 3,7-dirhamnoside>	C28 H32 O16	(M+H)+	14.675		624.1691		FBF	99.75		99.75
<Rhamnetin 3-rutinoside>	C28 H32 O16	(M+H)+	14.675		624.1691		FBF	99.75		99.75
<Scoparin 6''-O-glucoside>	C28 H32 O16	(M+H)+	14.675		624.1691		FBF	99.75		99.75
<Quercetin 3,4'-dimethyl ether 7-alpha-L-Arabinofuranosyl-(1->6)-glucoside>	C28 H32 O16	(M+H)+	14.675		624.1691		FBF	99.75		99.75
<Myricetin 7-methyl ether 3,4'-di-O-alpha-L-rhamnopyranoside>	C28 H32 O16	(M+H)+	14.675		624.1691		FBF	99.75		99.75
<Rhamnazin 3-xylosyl-(1->2)-glucoside>	C28 H32 O16	(M+H)+	14.675		624.1691		FBF	99.75		99.75
<Rhamnetin 3-neohesperidoside>	C28 H32 O16	(M+H)+	14.675		624.1691		FBF	99.75		99.75
<Rhamnetin 3-robinobioside>	C28 H32 O16	(M+H)+	14.675		624.1691		FBF	99.75		99.75
<Rhamnetin 3-galactoside-3'-rhamnoside>	C28 H32 O16	(M+H)+	14.675		624.1691		FBF	99.75		99.75

Cpd 1396: <Morin>

Name	Formula	RT	RI	Mass	Diff (Tgt, ppm)	CAS	ID Source	Score	Algorithm
<Morin>	C15 H10 O7	14.745		302.0430	0.99	480-16-0	M-FBF	99.10	FBF

Species	m/z	Score (Tgt)	Score (Lib)	Score (DB)	Score (MFG)	Score (RT)
(M+H)+	303	99.10				

Compound Chromatograms (overlaid)

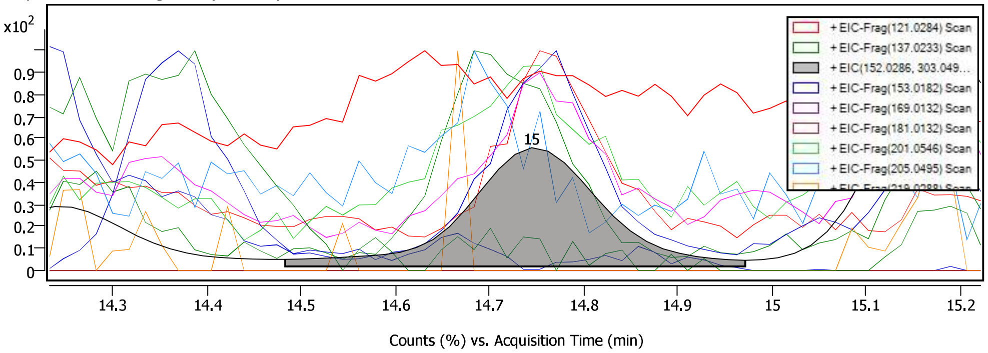

Structure

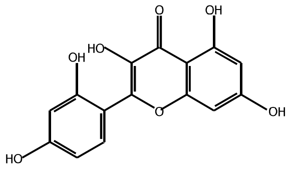

Coelution Plot

Compound Spectra (overlaid)

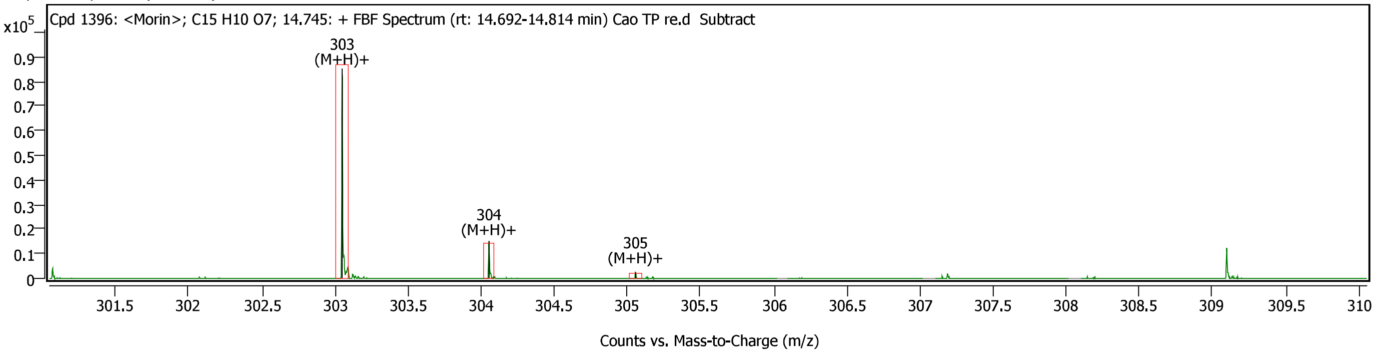

Fragment Spectrum (raw)

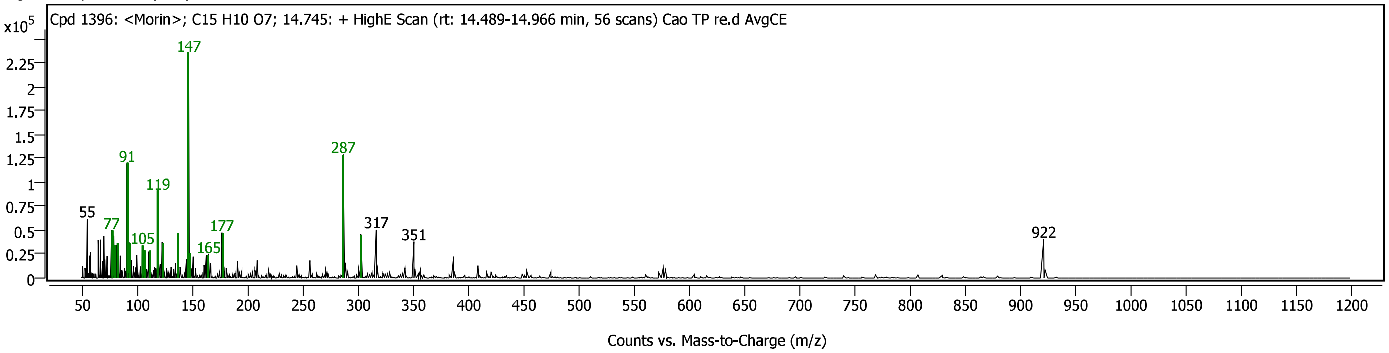

Compound ID Table

Name	Formula	Species	RT	RT Diff	Mass	CAS	ID Source	Score	Score (Lib)	Score (Tgt)
<Morin>	C15 H10 O7	(M+H)+	14.745		302.0430	480-16-0	FBF	99.10		99.10
<6-Hydroxyluteolin>	C15 H10 O7	(M+H)+	14.745		302.0430		FBF	99.10		99.10
<5,7,8,3',4'-Pentahydroxyisoflavone>	C15 H10 O7	(M+H)+	14.745		302.0430		FBF	99.10		99.10
<Herbacetin>	C15 H10 O7	(M+H)+	14.745		302.0430		FBF	99.10		99.10
<2-(4-Hydroxyphenyl)-5,6,7,8-tetrahydroxy-4H-1-benzopyran-4-one>	C15 H10 O7	(M+H)+	14.745		302.0430	577-26-4	FBF	99.10		99.10
<2',3',4',5,7-Pentahydroxyflavone>	C15 H10 O7	(M+H)+	14.745		302.0430	144707-17-5	FBF	99.10		99.10
<2'-Hydroxypseudobaptigenin>	C15 H10 O7	(M+H)+	14.745		302.0430		FBF	99.10		99.10
<3,5,7,2',5'-Pentahydroxyflavone>	C15 H10 O7	(M+H)+	14.745		302.0430		FBF	99.10		99.10
<5,6,7,3',4'-Pentahydroxyisoflavone>	C15 H10 O7	(M+H)+	14.745		302.0430		FBF	99.10		99.10
<5,7,8,2',4'-Pentahydroxyisoflavone>	C15 H10 O7	(M+H)+	14.745		302.0430		FBF	99.10		99.10
<6-Hydroxykaempferol>	C15 H10 O7	(M+H)+	14.745		302.0430		FBF	99.10		99.10
<Tricetin>	C15 H10 O7	(M+H)+	14.745		302.0430	520-31-0	FBF	99.10		99.10
<Bracteatin>	C15 H10 O7	(M+H)+	14.745		302.0430		FBF	99.10		99.10
<Hypolaetin>	C15 H10 O7	(M+H)+	14.745		302.0430		FBF	99.10		99.10
<Isoetin>	C15 H10 O7	(M+H)+	14.745		302.0430		FBF	99.10		99.10
<Melanoxetin>	C15 H10 O7	(M+H)+	14.745		302.0430		FBF	99.10		99.10
<Quercetin>	C15 H10 O7	(M+H)+	14.745		302.0430	117-39-5	FBF	99.10		99.10
<Rhynchosin>	C15 H10 O7	(M+H)+	14.745		302.0430		FBF	99.10		99.10
<Robinetin>	C15 H10 O7	(M+H)+	14.745		302.0430		FBF	99.10		99.10
<Viscidulin I>	C15 H10 O7	(M+H)+	14.745		302.0430	92519-95-4	FBF	99.10		99.10

Cpd 1424: 5'-Butyrylphosphoinosine

Name	Formula	RT	RI	Mass	Diff (Tgt, ppm)	CAS	ID Source	Score	Algorithm
5'-Butyrylphosphoinosine	C14 H19 N4 O9 P	14.762		418.0905	3.56		FBF	84.77	FBF

Species	m/z	Score (Tgt)	Score (Lib)	Score (DB)	Score (MFG)	Score (RT)
(M+H)+	419	84.77				

Compound Chromatograms (overlaid)

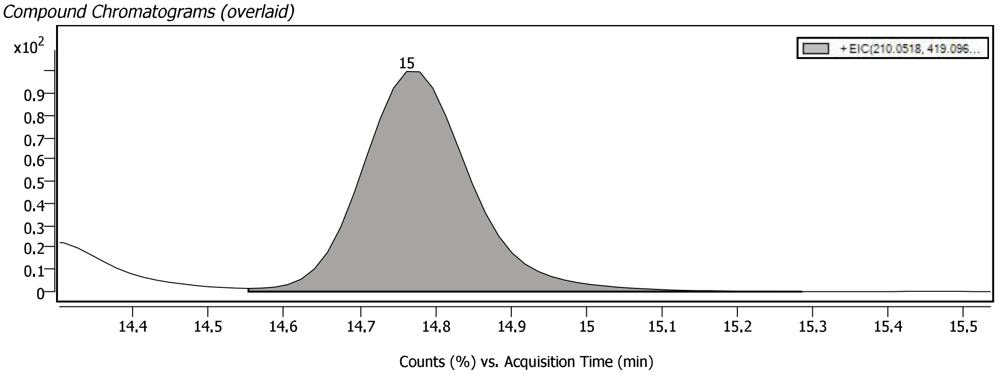

Structure

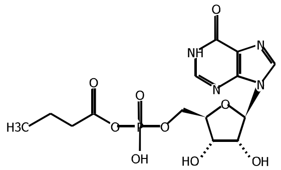

Compound Spectra (overlaid)

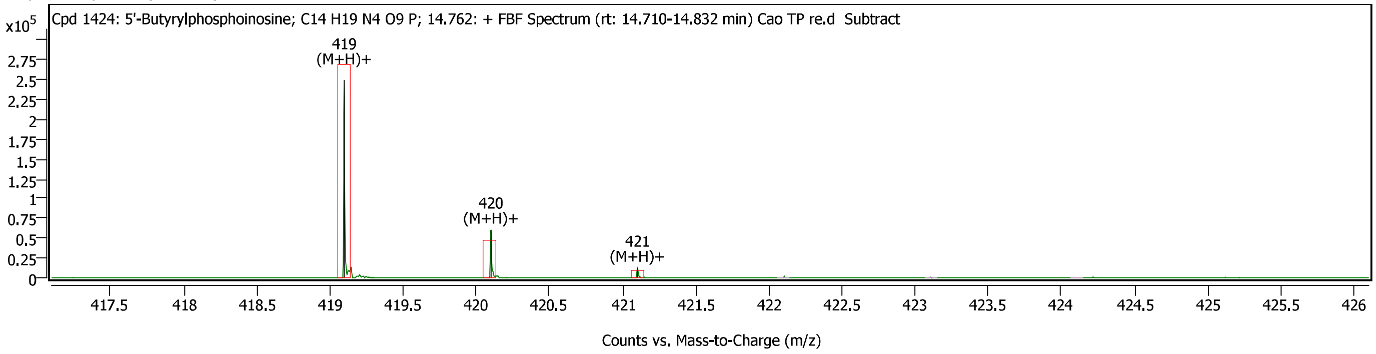

Compound ID Table

Name	Formula	Species	RT	RT Diff	Mass	CAS	ID Source	Score	Score (Lib)	Score (Tgt)
5'-Butyrylphosphoinosine	C14 H19 N4 O9 P	(M+H)+	14.762		418.0905		FBF	84.77		84.77

Cpd 149: <n-Propyl cinnamate>

Name	Formula	RT	RI	Mass	Diff (Tgt, ppm)	CAS	ID Source	Score	Algorithm
<n-Propyl cinnamate>	C12 H14 O2	14.867		190.0996	1.02	7778-83-8	M-FBF	99.04	FBF

Species	m/z	Score (Tgt)	Score (Lib)	Score (DB)	Score (MFG)	Score (RT)
(M+H)+	191	99.04				

Compound Chromatograms (overlaid)

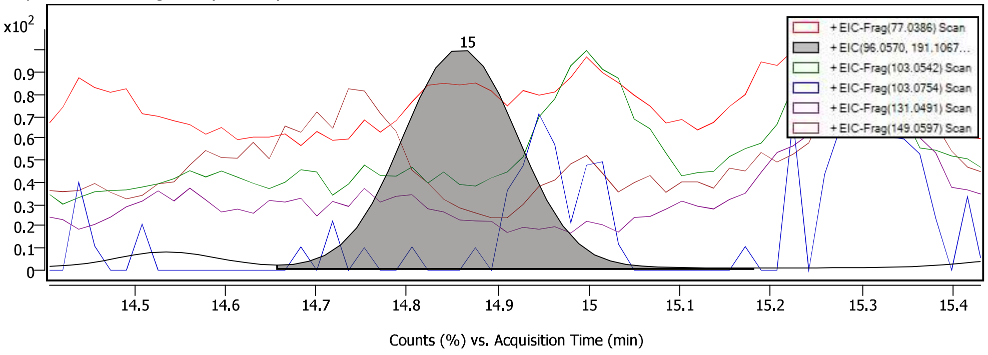

Structure

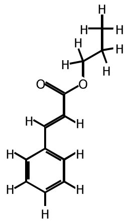

Coelution Plot

Compound Spectra (overlaid)

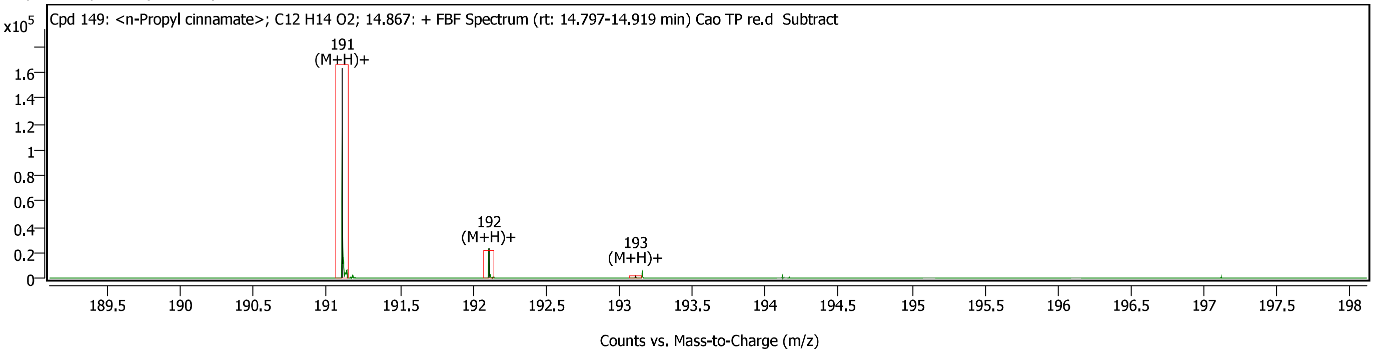

Fragment Spectrum (raw)

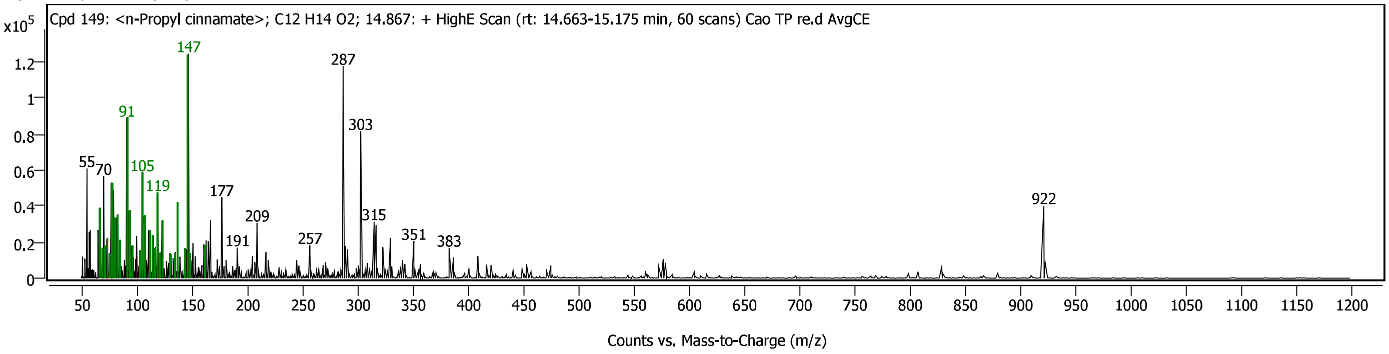

Compound ID Table

Name	Formula	Species	RT	RT Diff	Mass	CAS	ID Source	Score	Score (Lib)	Score (Tgt)
<n-Propyl cinnamate>	C12 H14 O2	(M+H)+	14.867		190.0996	7778-83-8	FBF	99.04		99.04
<Benzyl tiglate>	C12 H14 O2	(M+H)+	14.867		190.0996	37576-88-8	FBF	99.04		99.04
<(R)-3,7-Dimethyl-5-indanecarboxylic acid>	C12 H14 O2	(M+H)+	14.867		190.0996	217802-61-4	FBF	99.04		99.04
<(S)-3-Butyl-1(3H)-isobenzofuranone>	C12 H14 O2	(M+H)+	14.867		190.0996	3413-15-8	FBF	99.04		99.04
<1-(4-Methoxyphenyl)-1-penten-3-one>	C12 H14 O2	(M+H)+	14.867		190.0996	104-27-8	FBF	99.04		99.04
<3-(2-Methylpropylidene)-3alpha,4-dihydro-1(3H)-isobenzofuranone>	C12 H14 O2	(M+H)+	14.867		190.0996	66983-88-8	FBF	99.04		99.04
<3,5,7,9,11-Dodecapentaenoic acid>	C12 H14 O2	(M+H)+	14.867		190.0996	30409-26-8	FBF	99.04		99.04
<3-Dimethylallyl-4-hydroxybenzaldehyde>	C12 H14 O2	(M+H)+	14.867		190.0996		FBF	99.04		99.04
<Benzyl trans-2-methyl-2-butenoate>	C12 H14 O2	(M+H)+	14.867		190.0996	37526-88-8	FBF	99.04		99.04
<C12:5n-1,3,5,7,9>	C12 H14 O2	(M+H)+	14.867		190.0996		FBF	99.04		99.04
<Cinnamyl propionate>	C12 H14 O2	(M+H)+	14.867		190.0996	103-56-0	FBF	99.04		99.04
<Isopropyl cinnamate>	C12 H14 O2	(M+H)+	14.867		190.0996	7780-06-5	FBF	99.04		99.04
<Ligustilide>	C12 H14 O2	(M+H)+	14.867		190.0996	4431-01-0	FBF	99.04		99.04
<Precocene I>	C12 H14 O2	(M+H)+	14.867		190.0996	17598-02-6	FBF	99.04		99.04

Cpd 1234: <Tolmetin>

Name	Formula	RT	RI	Mass	Diff (Tgt, ppm)	CAS	ID Source	Score	Algorithm
<Tolmetin>	C15 H15 N O3	15.006		257.1055	1.11	26171-23-3	M-FBF	98.90	FBF

Species	m/z	Score (Tgt)	Score (Lib)	Score (DB)	Score (MFG)	Score (RT)
(M+H)+	258	98.90				

Compound Screening Report

Compound Chromatograms (overlaid)

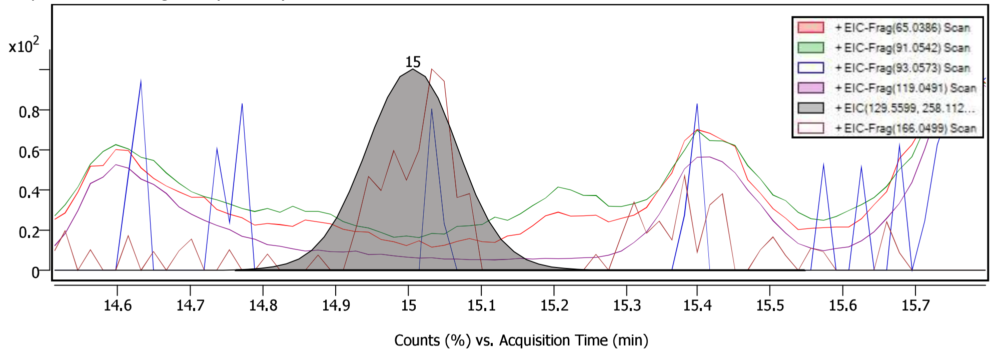

Structure

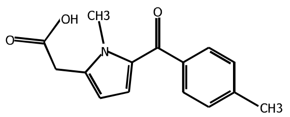

Coelution Plot

Compound Spectra (overlaid)

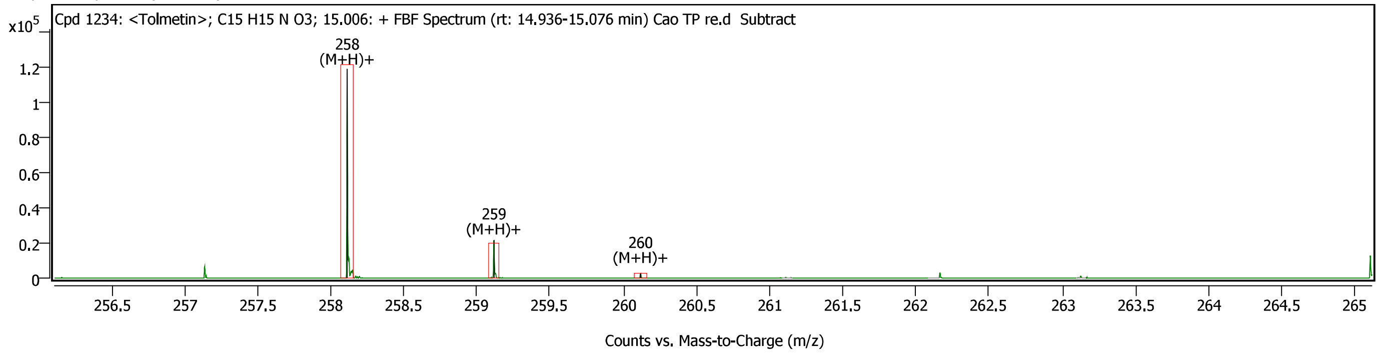

Fragment Spectrum (raw)

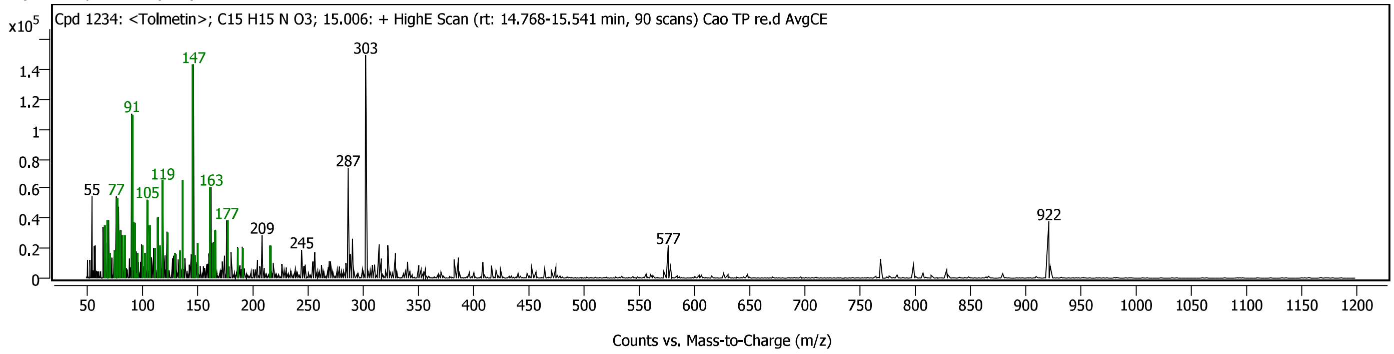

Compound ID Table

Name	Formula	Species	RT	RT Diff	Mass	CAS	ID Source	Score	Score (Lib)	Score (Tgt)
<Tolmetin>	C15 H15 N O3	(M+H)+	15.006		257.1055	26171-23-3	FBF	98.90		98.90
<Mefenamic acid metabolite>	C15 H15 N O3	(M+H)+	15.006		257.1055	5129-20-4	FBF	98.90		98.90
<Ismine>	C15 H15 N O3	(M+H)+	15.006		257.1055	1805-78-3	FBF	98.90		98.90

Cpd 126: Liatrin

Name	Formula	RT	RI	Mass	Diff (Tgt, ppm)	CAS	ID Source	Score	Algorithm
Liatrin	C22 H26 O8	15.111		418.1629	0.28	34175-79-6	M-FBF	99.75	FBF

Species	m/z	Score (Tgt)	Score (Lib)	Score (DB)	Score (MFG)	Score (RT)
(M+H)+	419	99.75				

Compound Chromatograms (overlaid)

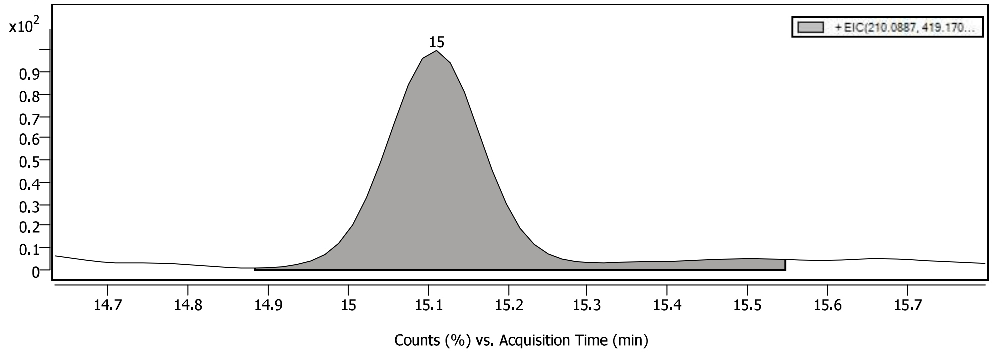

Structure

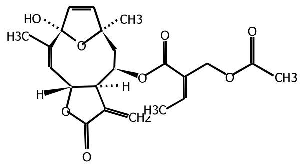

Compound Spectra (overlaid)

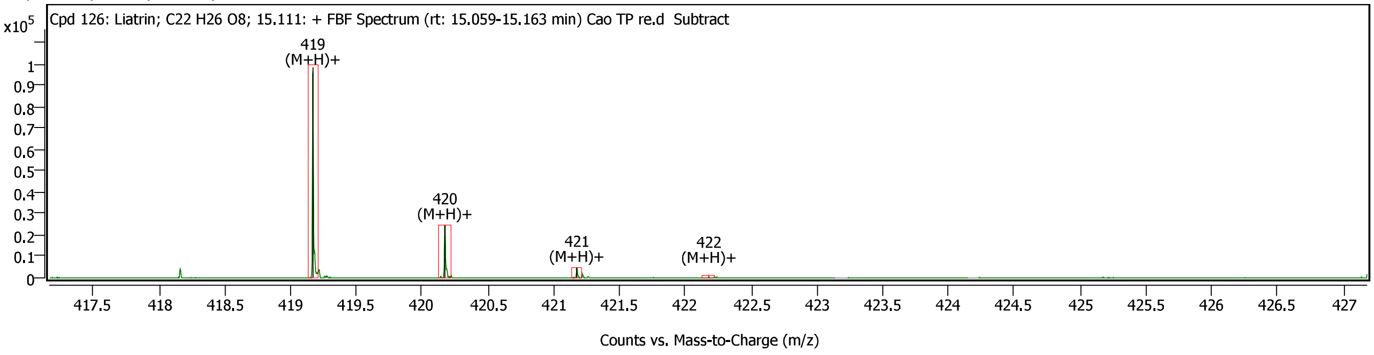

Compound ID Table

Name	Formula	Species	RT	RT Diff	Mass	CAS	ID Source	Score	Score (Lib)	Score (Tgt)
Liatrin	C22 H26 O8	(M+H)+	15.111		418.1629	34175-79-6	FBF	99.75		99.75
(R)-Byakangelicinn 2'-(3-methylbutanoate)	C22 H26 O8	(M+H)+	15.111		418.1629		FBF	99.75		99.75
Euparotin acetate	C22 H26 O8	(M+H)+	15.111		418.1629	10215-89-1	FBF	99.75		99.75
Lirioresinol A	C22 H26 O8	(M+H)+	15.111		418.1629	21453-71-4	FBF	99.75		99.75
Syringaresinol	C22 H26 O8	(M+H)+	15.111		418.1629	21453-69-0	FBF	99.75		99.75

Cpd 1276: <Fluridone>

Name	Formula	RT	RI	Mass	Diff (Tgt, ppm)	CAS	ID Source	Score	Algorithm
<Fluridone>	C19 H14 F3 N O	15.111		329.1028	0.29	59756-60-4	FBF	98.35	FBF

Species	m/z	Score (Tgt)	Score (Lib)	Score (DB)	Score (MFG)	Score (RT)
(M+H)+	330	98.35				

Compound Chromatograms (overlaid)

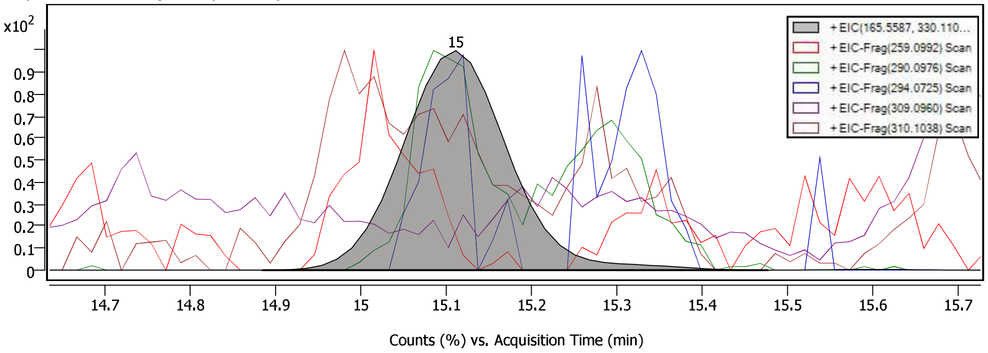

Structure

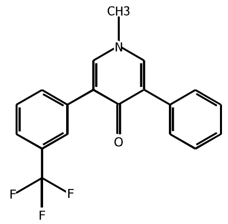

Coelution Plot

Compound Spectra (overlaid)

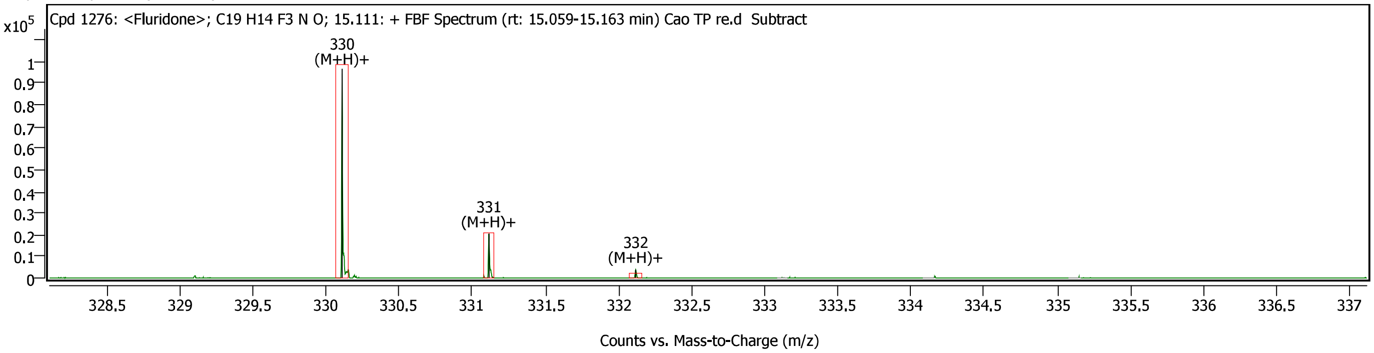

Compound Screening Report

Agilent

Fragment Spectrum (raw)

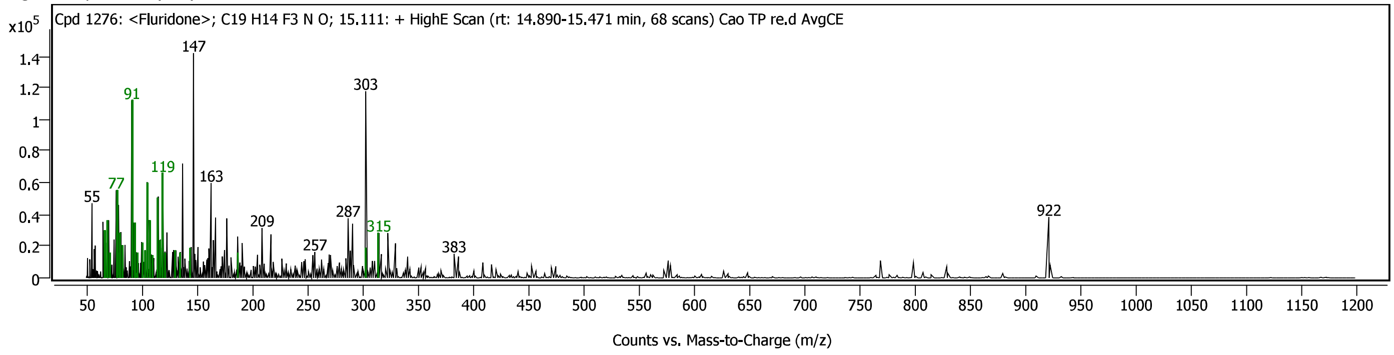

Compound ID Table

Name	Formula	Species	RT	RT Diff	Mass	CAS	ID Source	Score	Score (Lib)	Score (Tgt)
<Fluridone>	C19 H14 F3 N O	(M+H)+	15.111		329.1028	59756-60-4	FBF	98.35		98.35

Cpd 1368: <12-Hydroxy-4,4-bisnor-4,8,11,13-podocarpatetraen-3-one>

Name	Formula	RT	RI	Mass	Diff (Tgt, ppm)	CAS	ID Source	Score	Algorithm
<12-Hydroxy-4,4-bisnor-4,8,11,13-podocarpatetraen-3-one>	C15 H16 O2	15.216		228.1154	1.74	108123-97-3	M-FBF	98.18	FBF

Species	m/z	Score (Tgt)	Score (Lib)	Score (DB)	Score (MFG)	Score (RT)
(M+H)+	229	98.18				

Compound Chromatograms (overlaid)

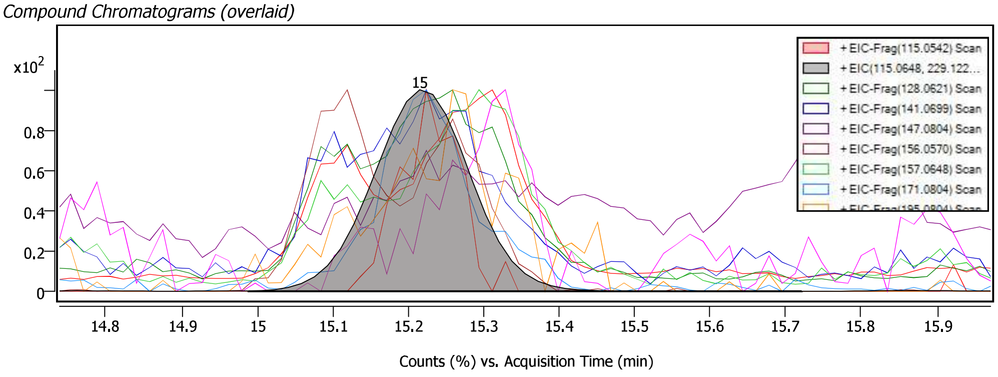

Structure

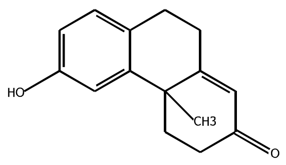

Coelution Plot

Compound Spectra (overlaid)

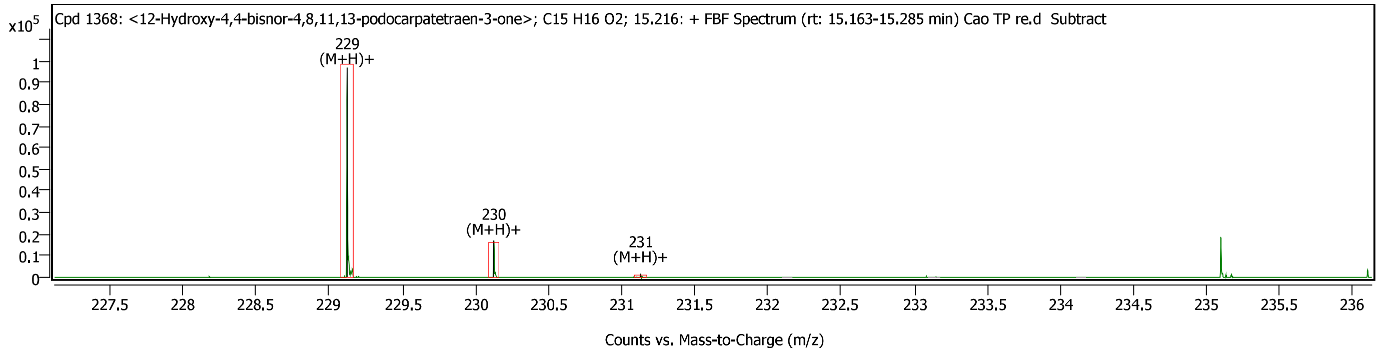

Fragment Spectrum (raw)

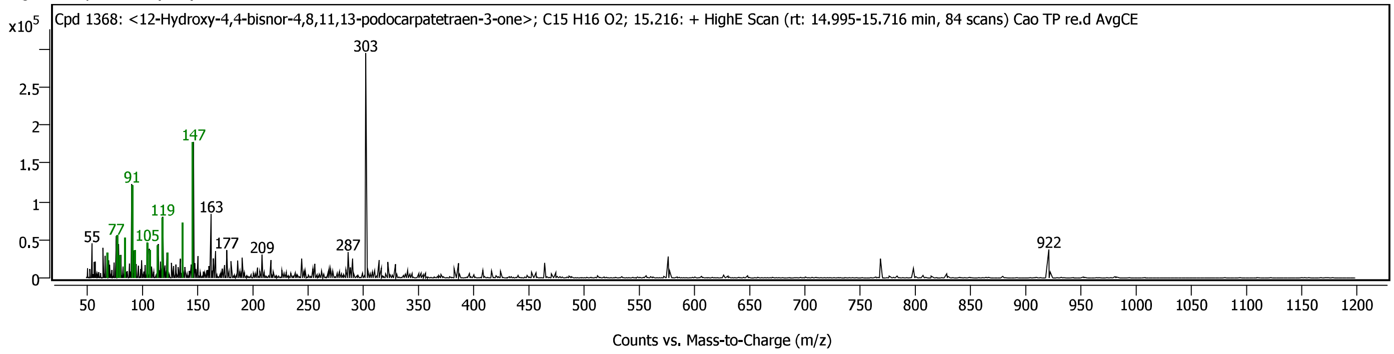

Compound Screening Report

Compound ID Table

Name	Formula	Species	RT	RT Diff	Mass	CAS	ID Source	Score	Score (Lib)	Score (Tgt)
<12-Hydroxy-4,4-bisnor-4,8,11,13-podocarpatetraen-3-one>	C15 H16 O2	(M+H)+	15.216		228.1154	108123-97-3	FBF	98.18		98.18
<Bisphenol A>	C15 H16 O2	(M+H)+	15.216		228.1154	80-05-7	FBF	98.18		98.18
<(S)-Curzeone>	C15 H16 O2	(M+H)+	15.216		228.1154		FBF	98.18		98.18
<1,1-Bis(4-hydroxyphenyl)propane>	C15 H16 O2	(M+H)+	15.216		228.1154		FBF	98.18		98.18
<Cacalol>	C15 H16 O2	(M+H)+	15.216		228.1154		FBF	98.18		98.18
<Lindenenone>	C15 H16 O2	(M+H)+	15.216		228.1154		FBF	98.18		98.18
<Mansonone C>	C15 H16 O2	(M+H)+	15.216		228.1154	5574-34-5	FBF	98.18		98.18
<Nabumetone>	C15 H16 O2	(M+H)+	15.216		228.1154	42924-53-8	FBF	98.18		98.18

Cpd 819: Neobavaisoflavone

Name	Formula	RT	RI	Mass	Diff (Tgt, ppm)	CAS	ID Source	Score	Algorithm
Neobavaisoflavone	C20 H18 O4	15.320		322.1208	0.81	41060-15-5	FBF-FragConfirm	99.40	FBF

Species	m/z	Score (Tgt)	Score (Lib)	Score (DB)	Score (MFG)	Score (RT)
(M+H)+	323	99.40				

Compound Chromatograms (overlaid)

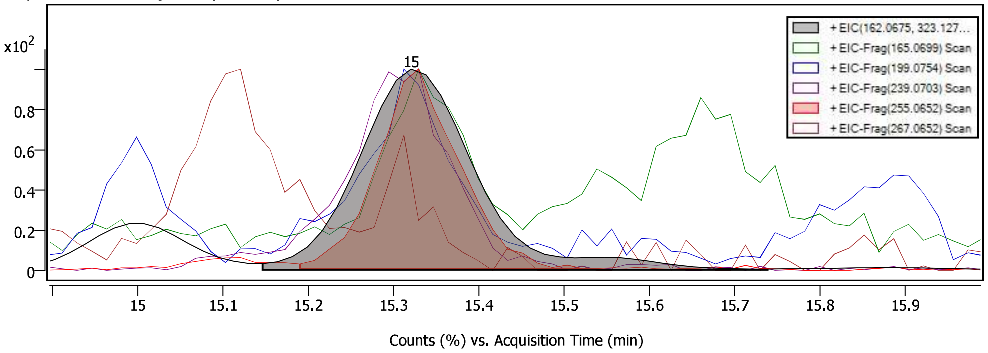

Structure

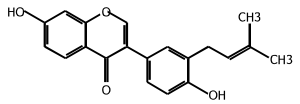

Coelution Plot

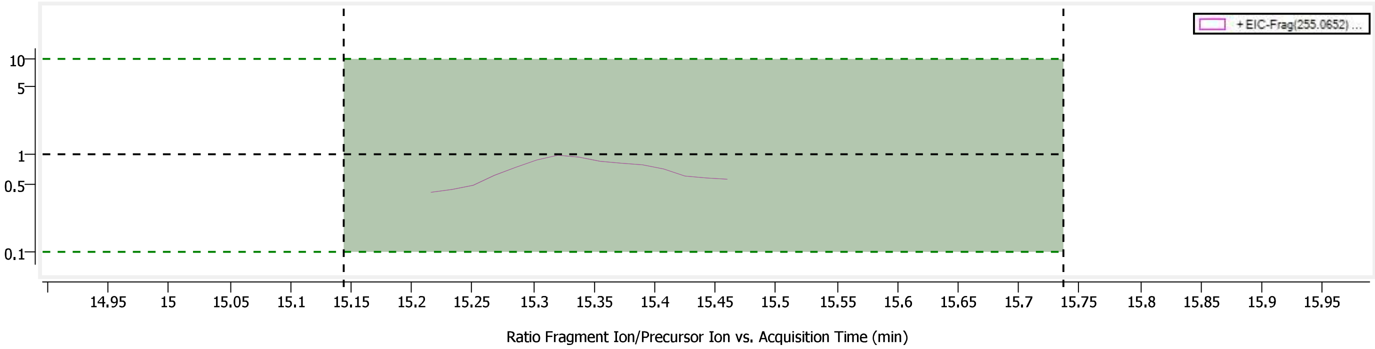

Compound Spectra (overlaid)

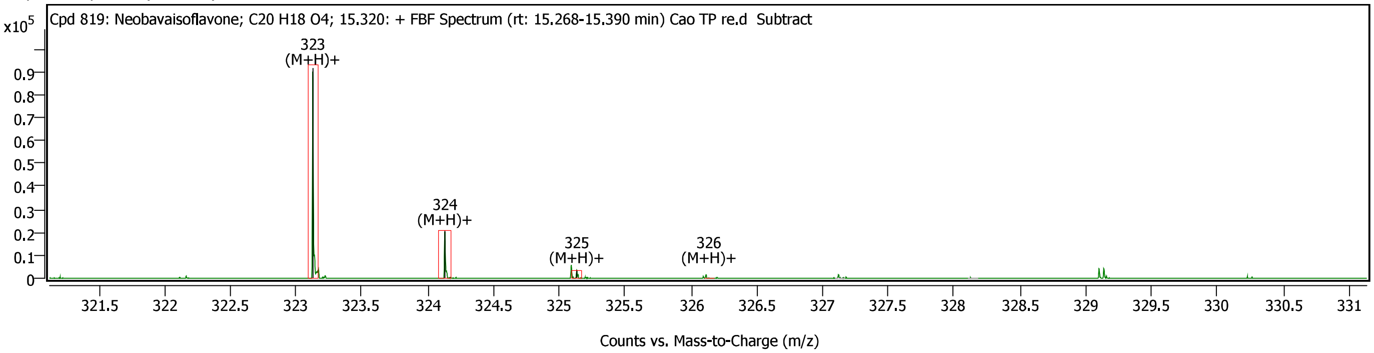

Fragment Spectrum (clean)

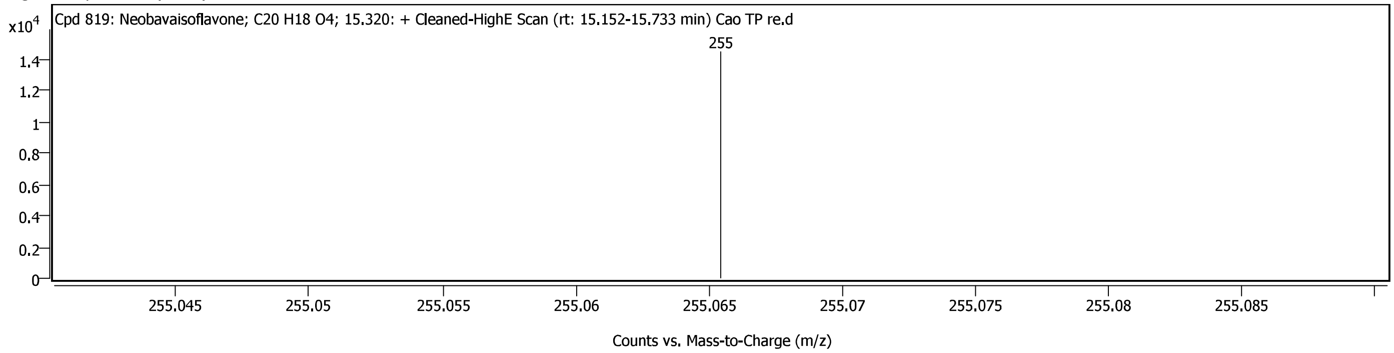

Fragment Spectrum (raw)

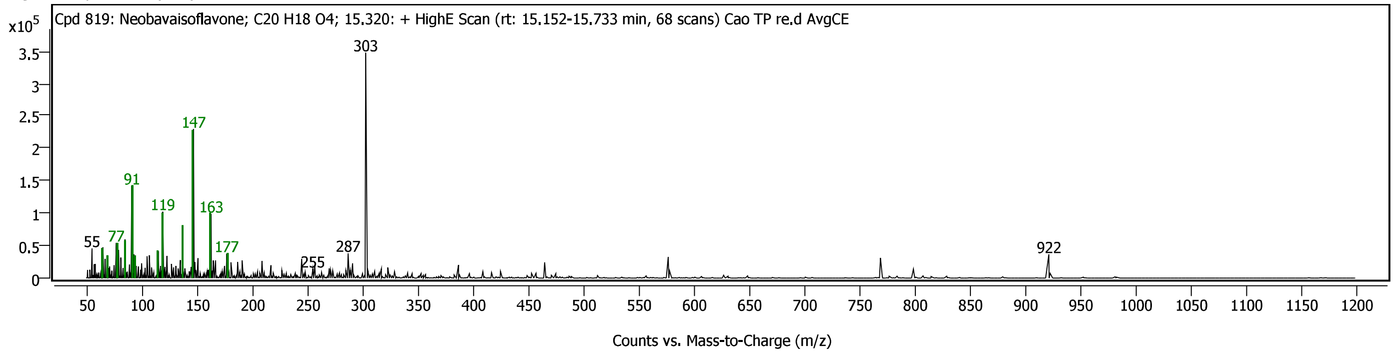

Compound ID Table

Name	Formula	Species	RT	RT Diff	Mass	CAS	ID Source	Score	Score (Lib)	Score (Tgt)
Neobavaisoflavone	C20 H18 O4	(M+H)+	15.320		322.1208	41060-15-5	FBF-FragConfirm	99.40		99.40
5,7-Dihydroxy-8-prenylflavone	C20 H18 O4	(M+H)+	15.320		322.1208		FBF-FragConfirm	99.40		99.40
Licoflavone A	C20 H18 O4	(M+H)+	15.320		322.1208		FBF-FragConfirm	99.40		99.40
Flemichapparin A	C20 H18 O4	(M+H)+	15.320		322.1208		FBF-FragConfirm	99.40		99.40
Bavachromene	C20 H18 O4	(M+H)+	15.320		322.1208		FBF-FragConfirm	99.40		99.40
Abyssinone I	C20 H18 O4	(M+H)+	15.320		322.1208		FBF-FragConfirm	99.40		99.40
6-(3,3-Dimethylallyl)chrysin	C20 H18 O4	(M+H)+	15.320		322.1208		FBF-FragConfirm	99.40		99.40
(-)-Shinpterocarpin	C20 H18 O4	(M+H)+	15.320		322.1208	157414-04-5	FBF-FragConfirm	99.40		99.40
4'-Hydroxyisolonchocarpin	C20 H18 O4	(M+H)+	15.320		322.1208		FBF-FragConfirm	99.40		99.40
2,3-Dihydro-4-(4-methoxyphenyl)-1H-phenalene-1,2,3-triol	C20 H18 O4	(M+H)+	15.320		322.1208	163811-78-7	FBF-FragConfirm	99.40		99.40
(-)-Phaseollin	C20 H18 O4	(M+H)+	15.320		322.1208		FBF-FragConfirm	99.40		99.40
Glabrene	C20 H18 O4	(M+H)+	15.320		322.1208		FBF-FragConfirm	99.40		99.40
7,4'-Dihydroxy-8-prenylflavone	C20 H18 O4	(M+H)+	15.320		322.1208		FBF-FragConfirm	99.40		99.40
Kanzonol D	C20 H18 O4	(M+H)+	15.320		322.1208		FBF-FragConfirm	99.40		99.40
Isobavachromene	C20 H18 O4	(M+H)+	15.320		322.1208		FBF-FragConfirm	99.40		99.40
Obovatin	C20 H18 O4	(M+H)+	15.320		322.1208		FBF-FragConfirm	99.40		99.40
Isoneorautenol	C20 H18 O4	(M+H)+	15.320		322.1208		FBF-FragConfirm	99.40		99.40
Neorautenol	C20 H18 O4	(M+H)+	15.320		322.1208		FBF-FragConfirm	99.40		99.40
Nordurlettone	C20 H18 O4	(M+H)+	15.320		322.1208		FBF-FragConfirm	99.40		99.40
Munsericin	C20 H18 O4	(M+H)+	15.320		322.1208		FBF-FragConfirm	99.40		99.40
Kanzonol B	C20 H18 O4	(M+H)+	15.320		322.1208		FBF-FragConfirm	99.40		99.40

Cpd 600: Rhizocticin A

Name	Formula	RT	RI	Mass	Diff (Tgt, ppm)	CAS	ID Source	Score	Algorithm
Rhizocticin A	C11 H22 N5 O6 P	15.407		351.1303	-1.45	114301-25-6	FBF	98.44	FBF

Species	m/z	Score (Tgt)	Score (Lib)	Score (DB)	Score (MFG)	Score (RT)
(M+H)+	352	98.44				

Compound Chromatograms (overlaid)

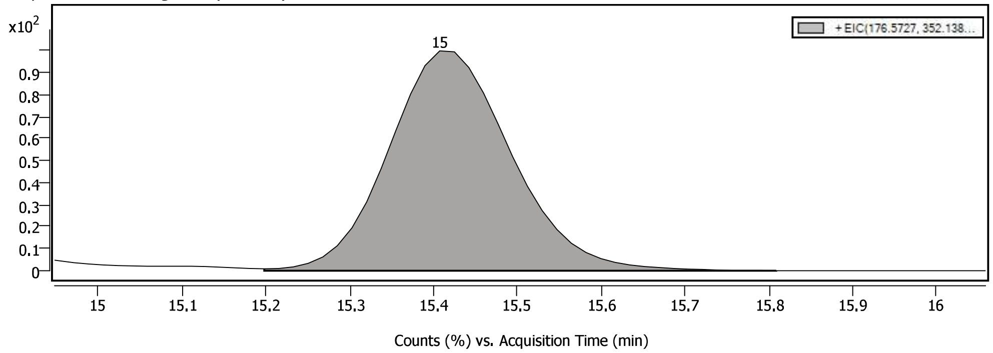

Structure

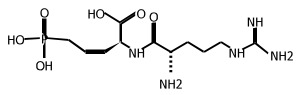

Compound Spectra (overlaid)

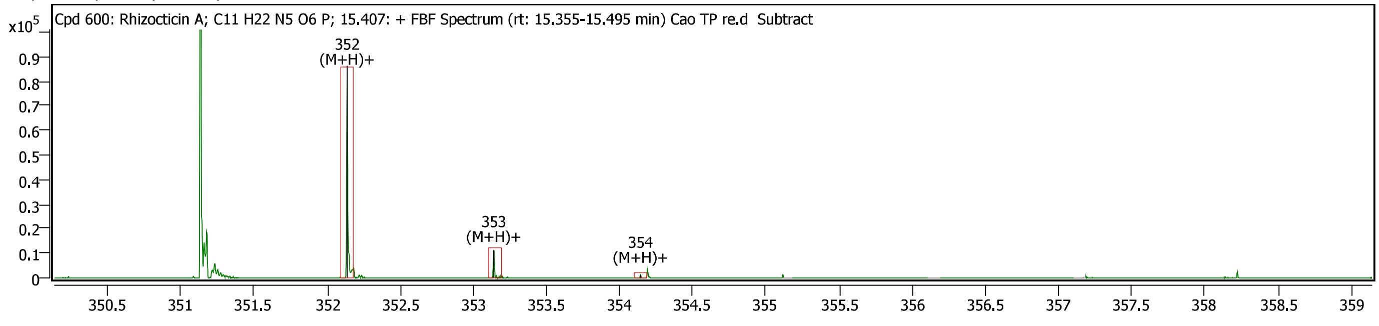

Compound ID Table

Name	Formula	Species	RT	RT Diff	Mass	CAS	ID Source	Score	Score (Lib)	Score (Tgt)
Rhizocticin A	C11 H22 N5 O6 P	(M+H)+	15.407		351.1303	114301-25-6	FBF	98.44		98.44

Cpd 183: Na-p-Hydroxycoumaroyltryptophan

Name	Formula	RT	RI	Mass	Diff (Tgt, ppm)	CAS	ID Source	Score	Algorithm
Na-p-Hydroxycoumaroyltryptophan	C20 H18 N2 O4	15.425		350.1272	1.42		M-FBF	98.31	FBF

Species	m/z	Score (Tgt)	Score (Lib)	Score (DB)	Score (MFG)	Score (RT)
(M+H)+	351	98.31				

Compound Chromatograms (overlaid)

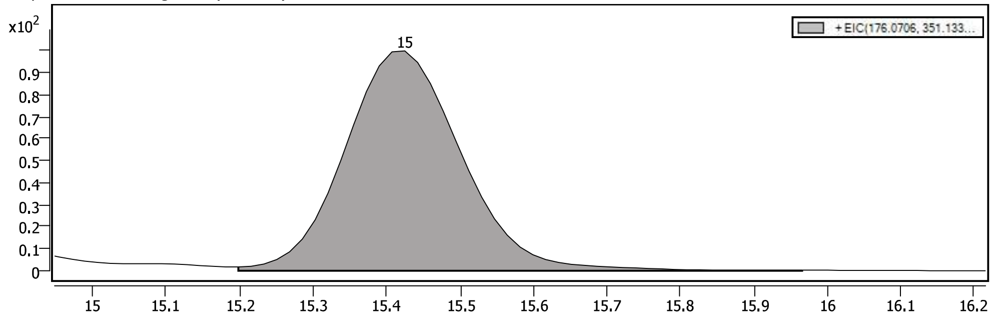

Structure

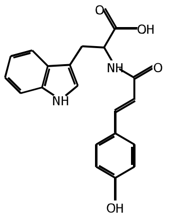

Compound Spectra (overlaid)

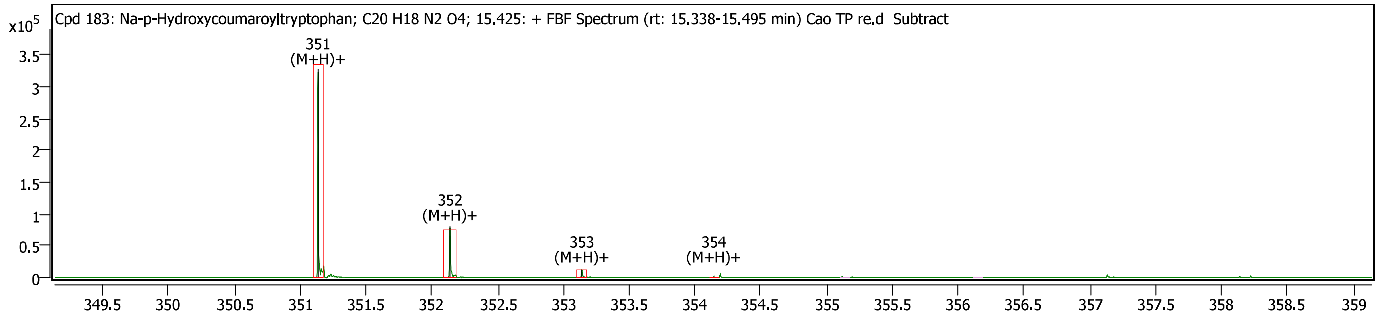

Compound ID Table

Name	Formula	Species	RT	RT Diff	Mass	CAS	ID Source	Score	Score (Lib)	Score (Tgt)
Na-p-Hydroxycoumaroyltryptophan	C20 H18 N2 O4	(M+H)+	15.425		350.1272		FBF	98.31		98.31
Moschamindole	C20 H18 N2 O4	(M+H)+	15.425		350.1272	99615-94-8	FBF	98.31		98.31

Cpd 1514: N-Caffeoyltryptophan

Name	Formula	RT	RI	Mass	Diff (Tgt, ppm)	CAS	ID Source	Score	Algorithm
N-Caffeoyltryptophan	C20 H18 N2 O5	15.564		366.1220	1.15	109163-69-1	FBF	98.94	FBF

Species	m/z	Score (Tgt)	Score (Lib)	Score (DB)	Score (MFG)	Score (RT)
(M+H)+	367	98.94				

Compound Chromatograms (overlaid)

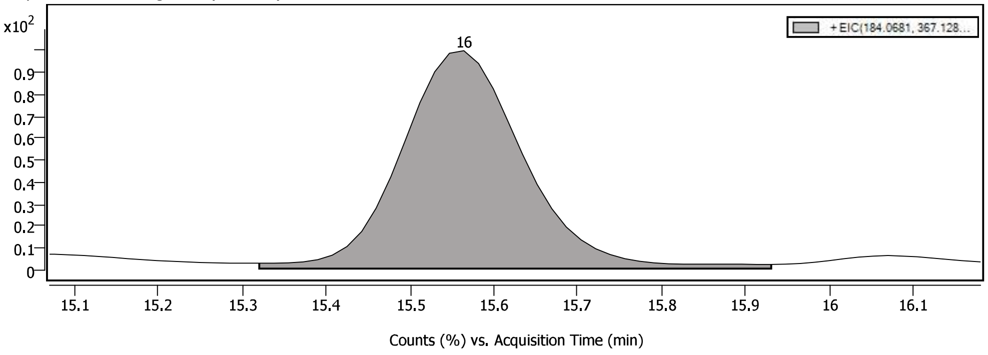

Structure

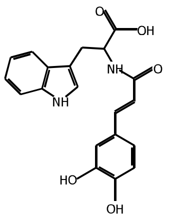

Compound Spectra (overlaid)

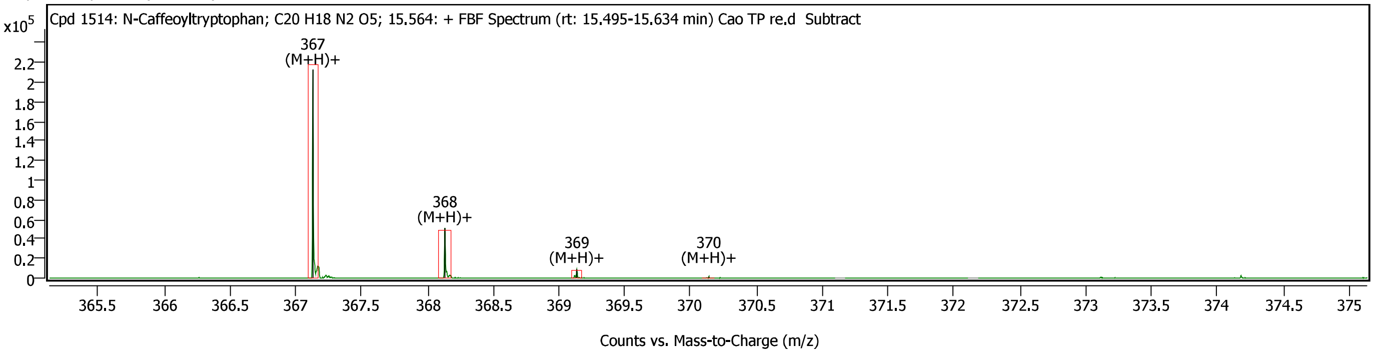

Compound ID Table

Name	Formula	Species	RT	RT Diff	Mass	CAS	ID Source	Score	Score (Lib)	Score (Tgt)
N-Caffeoyltryptophan	C20 H18 N2 O5	(M+H)+	15.564		366.1220	109163-69-1	FBF	98.94		98.94

Cpd 1306: Hyperoside

Name	Formula	RT	RI	Mass	Diff (Tgt, ppm)	CAS	ID Source	Score	Algorithm
Hyperoside	C21 H20 O12	15.582		464.0963	1.86	482-36-0	M-FBF-FragConfirm	97.50	FBF

Species	m/z	Score (Tgt)	Score (Lib)	Score (DB)	Score (MFG)	Score (RT)
(M+H)+	465	97.50				

Compound Chromatograms (overlaid)

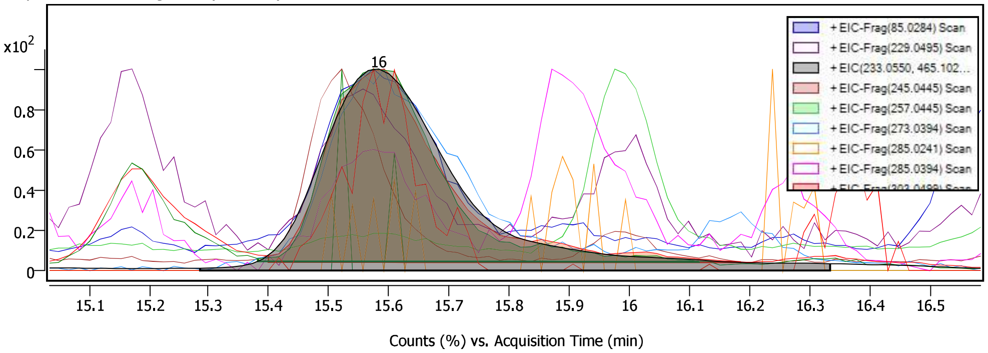

Structure

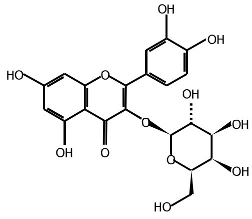

Coelution Plot

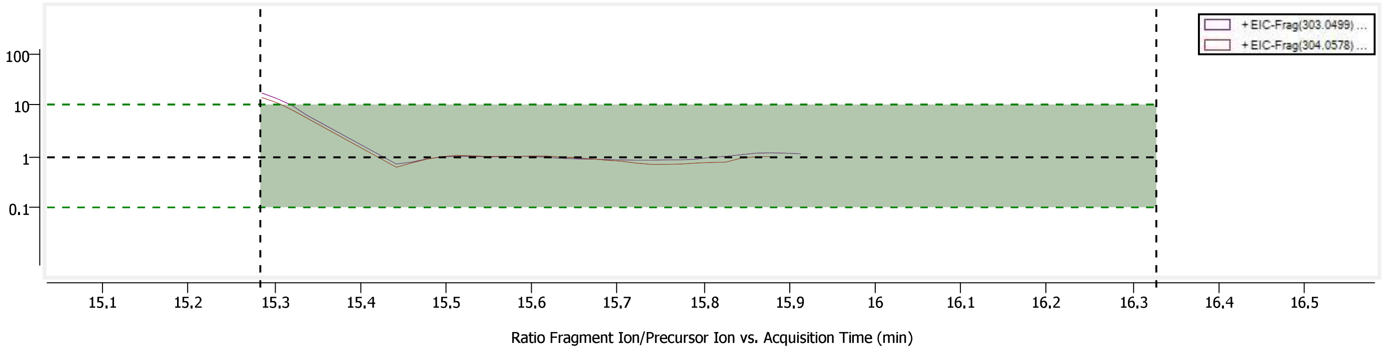

Compound Screening Report

Compound Spectra (overlaid)

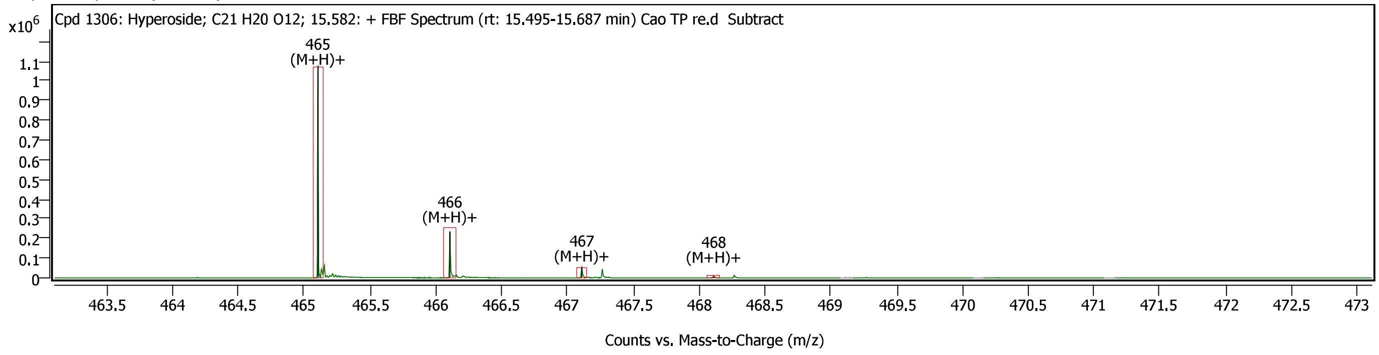

Fragment Spectrum (clean)

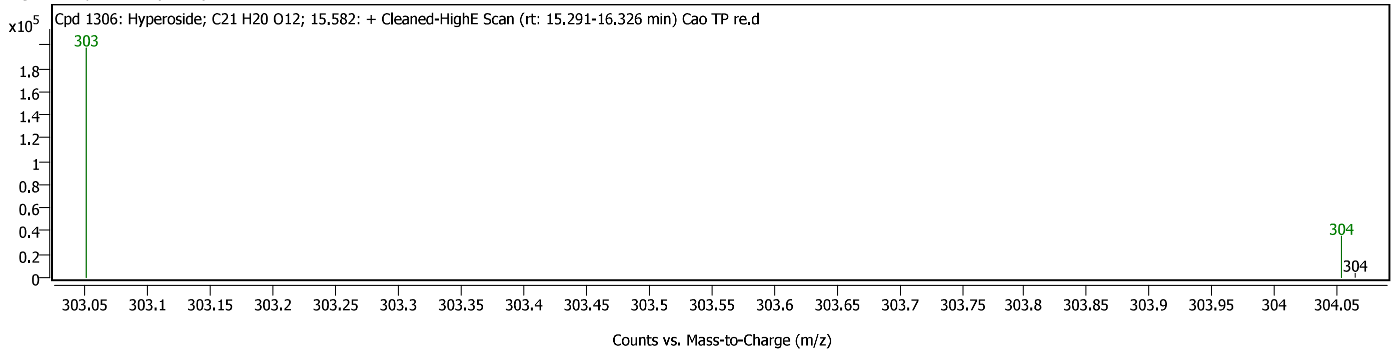

Fragment Spectrum (raw)

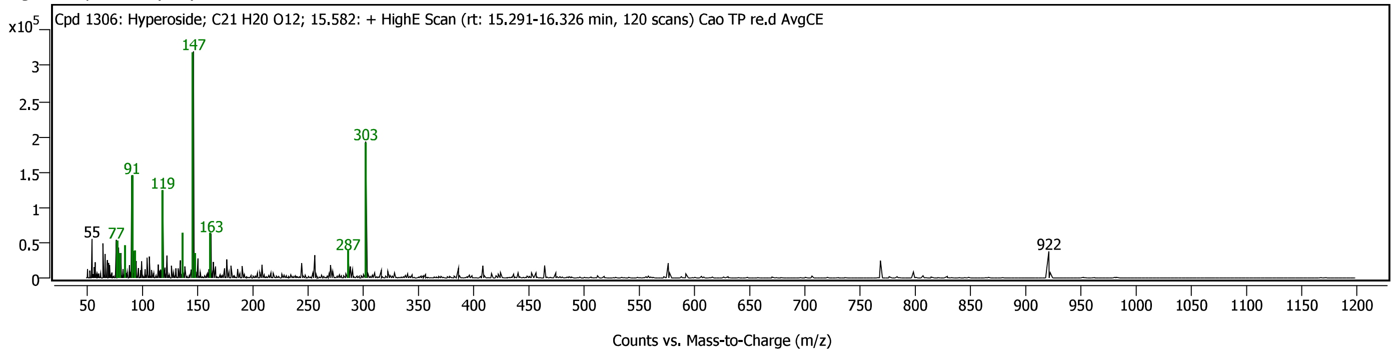

Compound ID Table

Name	Formula	Species	RT	RT Diff	Mass	CAS	ID Source	Score	Score (Lib)	Score (Tgt)
Hyperoside	C21 H20 O12	(M+H)+	15.582		464.0963	482-36-0	FBF-FragConfirm	97.50		97.50
Bracteatin 6-O-glucoside	C21 H20 O12	(M+H)+	15.582		464.0963		FBF-FragConfirm	97.50		97.50
Quercetagetin 3-rhamnoside	C21 H20 O12	(M+H)+	15.582		464.0963		FBF-FragConfirm	97.50		97.50
8-Hydroxyluteolin 8-glucoside	C21 H20 O12	(M+H)+	15.582		464.0963		FBF-FragConfirm	97.50		97.50
Annulatin 3'-xyloside	C21 H20 O12	(M+H)+	15.582		464.0963		FBF-FragConfirm	97.50		97.50
Bracteatin 6-glucoside	C21 H20 O12	(M+H)+	15.582		464.0963		FBF-FragConfirm	97.50		97.50
Gossypetin 7-rhamnoside	C21 H20 O12	(M+H)+	15.582		464.0963		FBF-FragConfirm	97.50		97.50
Bractein	C21 H20 O12	(M+H)+	15.582		464.0963		FBF-FragConfirm	97.50		97.50
Corniculatusin 3-alpha-L-arabinofuranoside	C21 H20 O12	(M+H)+	15.582		464.0963		FBF-FragConfirm	97.50		97.50
Eriodictyol 7-glucuronide	C21 H20 O12	(M+H)+	15.582		464.0963		FBF-FragConfirm	97.50		97.50
6-Hydroxyluteolin 7-glucoside	C21 H20 O12	(M+H)+	15.582		464.0963		FBF-FragConfirm	97.50		97.50
Gossypetin 8-rhamnoside	C21 H20 O12	(M+H)+	15.582		464.0963		FBF-FragConfirm	97.50		97.50
6-Hydroxytricetin 5-rhamnoside	C21 H20 O12	(M+H)+	15.582		464.0963		FBF-FragConfirm	97.50		97.50
6-Hydroxykaempferol 3-glucoside	C21 H20 O12	(M+H)+	15.582		464.0963		FBF-FragConfirm	97.50		97.50
6-Hydroxyluteolin 7-galactoside	C21 H20 O12	(M+H)+	15.582		464.0963		FBF-FragConfirm	97.50		97.50
6-Hydroxyluteolin 6-glucoside	C21 H20 O12	(M+H)+	15.582		464.0963		FBF-FragConfirm	97.50		97.50
6-Hydroxyluteolin 5-glucoside	C21 H20 O12	(M+H)+	15.582		464.0963		FBF-FragConfirm	97.50		97.50
6-Hydroxykaempferol 7-glucoside	C21 H20 O12	(M+H)+	15.582		464.0963		FBF-FragConfirm	97.50		97.50
6-C-Glucosylquercetin	C21 H20 O12	(M+H)+	15.582		464.0963		FBF-FragConfirm	97.50		97.50
6-C-beta-D-Glucopyranosyl-5,7,2',4',5'-pentahydroxyflavone	C21 H20 O12	(M+H)+	15.582		464.0963		FBF-FragConfirm	97.50		97.50
5,7,3',4',5'-Pentahydroxyflavone 8-C-glucopyranoside	C21 H20 O12	(M+H)+	15.582		464.0963		FBF-FragConfirm	97.50		97.50
5,6,7,3',4'-Pentahydroxy-8-methoxyflavone 7-apioside	C21 H20 O12	(M+H)+	15.582		464.0963		FBF-FragConfirm	97.50		97.50
3,5,7,2',6'-Pentahydroxyflavone 2'-glucoside	C21 H20 O12	(M+H)+	15.582		464.0963		FBF-FragConfirm	97.50		97.50
2'-Hydroxyisoorientin	C21 H20 O12	(M+H)+	15.582		464.0963		FBF-FragConfirm	97.50		97.50
(2S)-5,7,3',4'-Tetrahydroxyflavanone 7-glucuronide	C21 H20 O12	(M+H)+	15.582		464.0963		FBF-FragConfirm	97.50		97.50
Herbacetin 3-beta-D-glucofuranoside	C21 H20 O12	(M+H)+	15.582		464.0963		FBF-FragConfirm	97.50		97.50
8-Hydroxyluteolin 7-glucoside	C21 H20 O12	(M+H)+	15.582		464.0963		FBF-FragConfirm	97.50		97.50
Herbacetin 3-glucoside	C21 H20 O12	(M+H)+	15.582		464.0963		FBF-FragConfirm	97.50		97.50
Tricetin 7-glucoside	C21 H20 O12	(M+H)+	15.582		464.0963		FBF-FragConfirm	97.50		97.50
Quercetin 3-alloside	C21 H20 O12	(M+H)+	15.582		464.0963		FBF-FragConfirm	97.50		97.50
Herbacetin 7-glucoside	C21 H20 O12	(M+H)+	15.582		464.0963		FBF-FragConfirm	97.50		97.50
Tricetin 3'-glucoside	C21 H20 O12	(M+H)+	15.582		464.0963		FBF-FragConfirm	97.50		97.50
Spiraeoside	C21 H20 O12	(M+H)+	15.582		464.0963		FBF-FragConfirm	97.50		97.50
Robinetin 7-glucoside	C21 H20 O12	(M+H)+	15.582		464.0963		FBF-FragConfirm	97.50		97.50
Quercimeritrin	C21 H20 O12	(M+H)+	15.582		464.0963		FBF-FragConfirm	97.50		97.50
Quercetin 5-glucoside	C21 H20 O12	(M+H)+	15.582		464.0963		FBF-FragConfirm	97.50		97.50
Quercetin 4'-glucoside	C21 H20 O12	(M+H)+	15.582		464.0963	20229-56-5	FBF-FragConfirm	97.50		97.50
Quercetin 3-β-D-glucoside	C21 H20 O12	(M+H)+	15.582		464.0963	482-35-9	FBF-FragConfirm	97.50		97.50
Quercetin 3-O-glucoside	C21 H20 O12	(M+H)+	15.582		464.0963	21637-25-2	FBF-FragConfirm	97.50		97.50
Quercetin 3'-glucoside	C21 H20 O12	(M+H)+	15.582		464.0963		FBF-FragConfirm	97.50		97.50
Quercetin 7-galactoside	C21 H20 O12	(M+H)+	15.582		464.0963		FBF-FragConfirm	97.50		97.50
Myricetin 3'-rhamnoside	C21 H20 O12	(M+H)+	15.582		464.0963		FBF-FragConfirm	97.50		97.50
Patuletin 3-xyloside	C21 H20 O12	(M+H)+	15.582		464.0963		FBF-FragConfirm	97.50		97.50
Isoetin 5'-glucoside	C21 H20 O12	(M+H)+	15.582		464.0963		FBF-FragConfirm	97.50		97.50
Myricitrin	C21 H20 O12	(M+H)+	15.582		464.0963	17912-87-7	FBF-FragConfirm	97.50		97.50
Herbacetin 8-glucoside	C21 H20 O12	(M+H)+	15.582		464.0963		FBF-FragConfirm	97.50		97.50
Hyperin	C21 H20 O12	(M+H)+	15.582		464.0963		FBF-FragConfirm	97.50		97.50
Isoaffinetin	C21 H20 O12	(M+H)+	15.582		464.0963		FBF-FragConfirm	97.50		97.50
Isoetin 7-glucoside	C21 H20 O12	(M+H)+	15.582		464.0963		FBF-FragConfirm	97.50		97.50
Isoquercitrin	C21 H20 O12	(M+H)+	15.582		464.0963		FBF-FragConfirm	97.50		97.50
Larycitrin 3-alpha-L-arabinofuranoside	C21 H20 O12	(M+H)+	15.582		464.0963		FBF-FragConfirm	97.50		97.50
Herbacetin 4'-glucoside	C21 H20 O12	(M+H)+	15.582		464.0963		FBF-FragConfirm	97.50		97.50
Myricetin 7-rhamnoside	C21 H20 O12	(M+H)+	15.582		464.0963	184533-14-0	FBF-FragConfirm	97.50		97.50

Cpd 1380: Morfamquat

Name	Formula	RT	RI	Mass	Diff (Tgt, ppm)	CAS	ID Source	Score	Algorithm
Morfamquat	C26 H34 N4 O4	15.687		466.2568	-2.48	4636-83-3	FBF	96.15	FBF

Species	m/z	Score (Tgt)	Score (Lib)	Score (DB)	Score (MFG)	Score (RT)
(M+H)+	467	96.15				

Compound Chromatograms (overlaid)

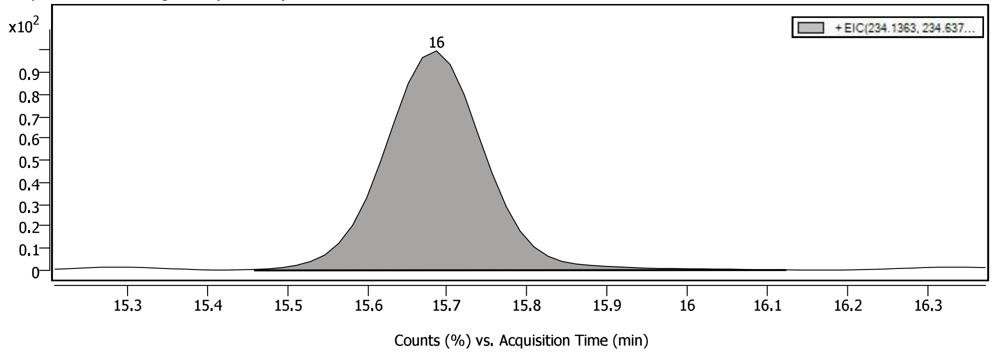

Structure

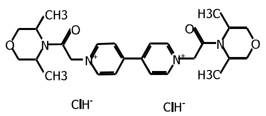

Compound Spectra (overlaid)

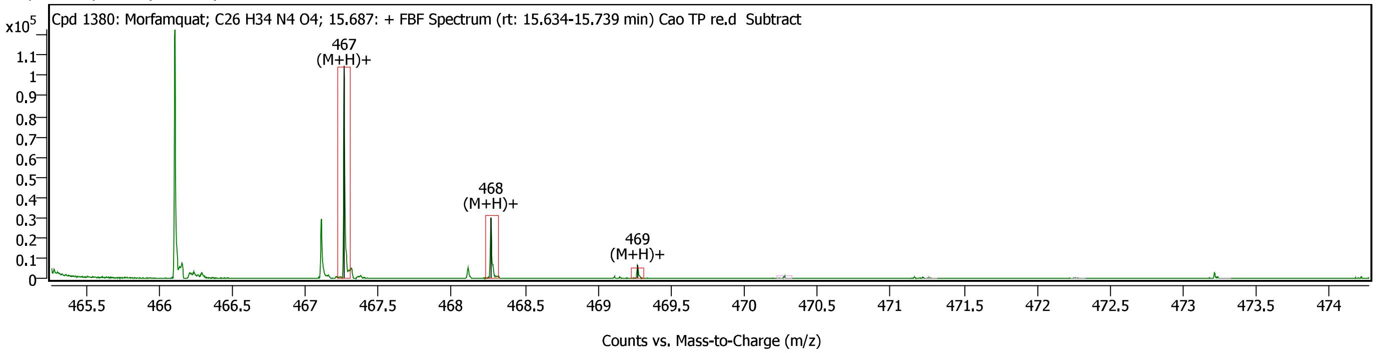

Compound ID Table

Name	Formula	Species	RT	RT Diff	Mass	CAS	ID Source	Score	Score (Lib)	Score (Tgt)
Morfamquat	C26 H34 N4 O4	(M+H)+	15.687		466.2568	4636-83-3	FBF	96.15		96.15

Cpd 119: Aurasperone C

Name	Formula	RT	RI	Mass	Diff (Tgt, ppm)	CAS	ID Source	Score	Algorithm
Aurasperone C	C31 H28 O12	16.001		592.1577	-0.66	41689-66-1	M-FBF	80.23	FBF

Species	m/z	Score (Tgt)	Score (Lib)	Score (DB)	Score (MFG)	Score (RT)
(M+H)+	593	80.23				

Compound Chromatograms (overlaid)

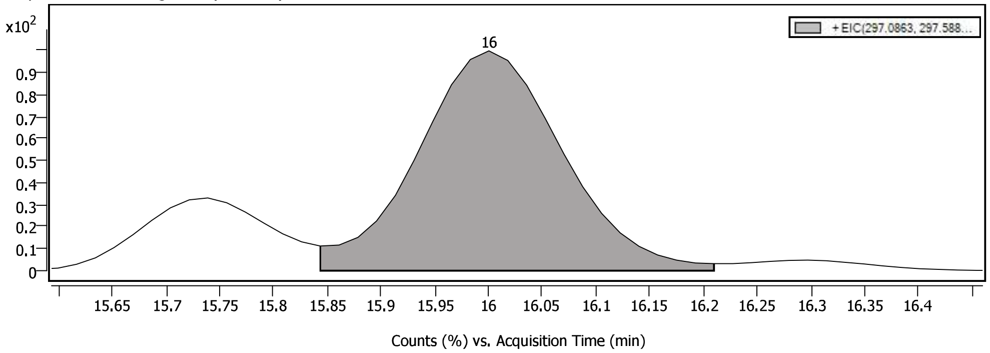

Structure

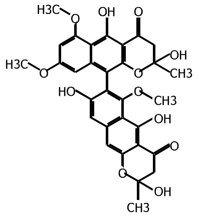

Compound Spectra (overlaid)

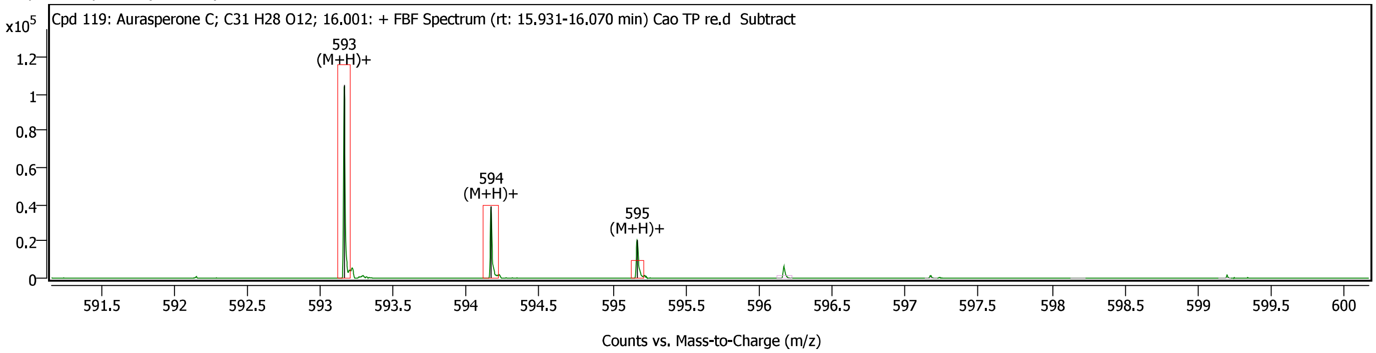

Compound ID Table

Name	Formula	Species	RT	RT Diff	Mass	CAS	ID Source	Score	Score (Lib)	Score (Tgt)
Aurasperone C	C31 H28 O12	(M+H)+	16.001		592.1577	41689-66-1	FBF	80.23		80.23
8,8'-Methylenebiscatechin	C31 H28 O12	(M+H)+	16.001		592.1577	81555-08-0	FBF	80.23		80.23

Cpd 1285: <10-Hydroxycamtothecin>

Name	Formula	RT	RI	Mass	Diff (Tgt, ppm)	CAS	ID Source	Score	Algorithm
<10-Hydroxycamtothecin>	C20 H16 N2 O5	16.088		364.1061	0.61	19685-09-7	FBF	99.34	FBF

Species	m/z	Score (Tgt)	Score (Lib)	Score (DB)	Score (MFG)	Score (RT)
(M+H)+	365	99.34				

Compound Chromatograms (overlaid)

Structure

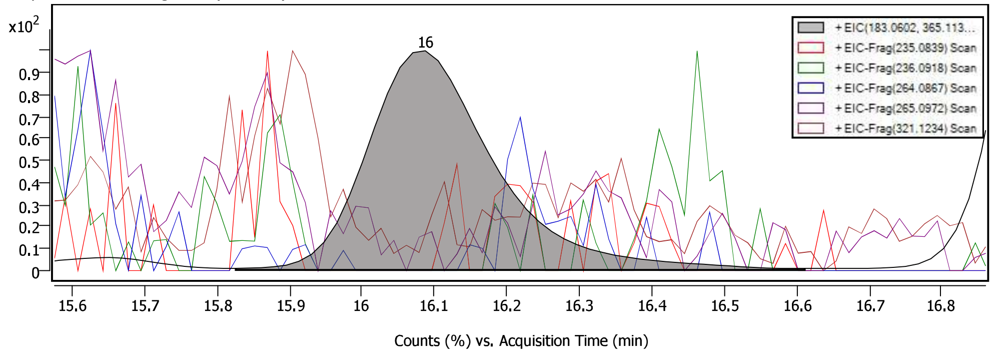

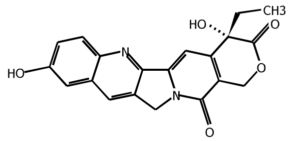

Coelution Plot

Compound Spectra (overlaid)

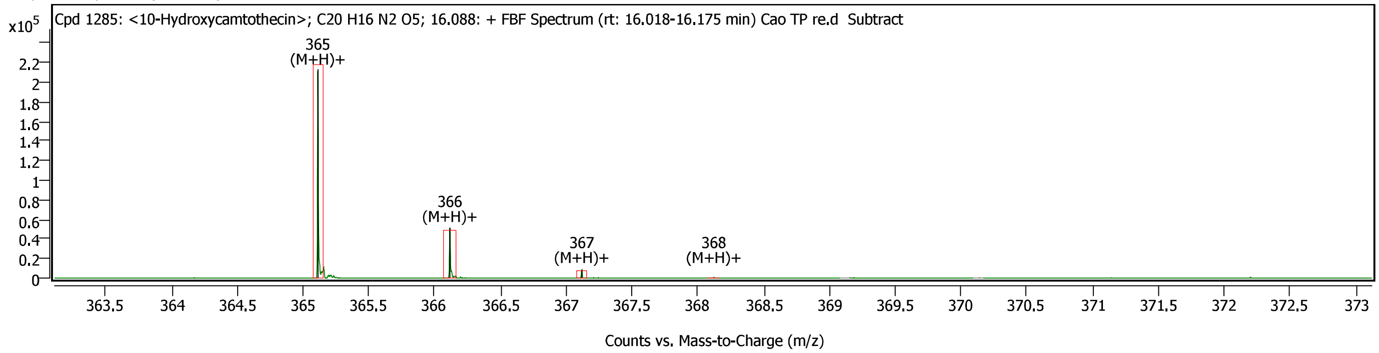

Fragment Spectrum (raw)

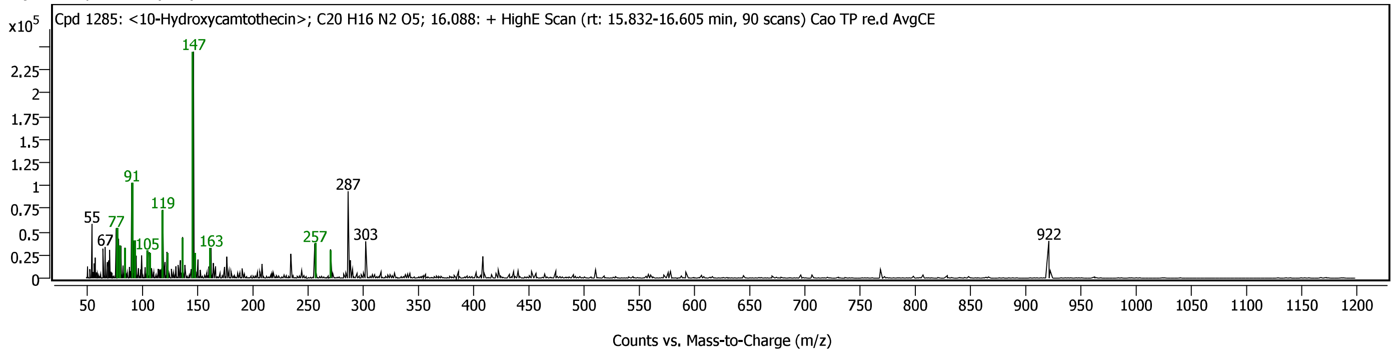

Compound ID Table

Name	Formula	Species	RT	RT Diff	Mass	CAS	ID Source	Score	Score (Lib)	Score (Tgt)
<10-Hydroxycamtothecin>	C20 H16 N2 O5	(M+H)+	16.088		364.1061	19685-09-7	FBF	99.34		99.34

Cpd 102: Piperenol A triacetate

Name	Formula	RT	RI	Mass	Diff (Tgt, ppm)	CAS	ID Source	Score	Algorithm
Piperenol A triacetate	C27 H26 O10	16.384		510.1503	-4.56	134476-92-9	FBF	90.55	FBF

Species	m/z	Score (Tgt)	Score (Lib)	Score (DB)	Score (MFG)	Score (RT)
(M+H)+	511	90.55				

Compound Chromatograms (overlaid)

Structure

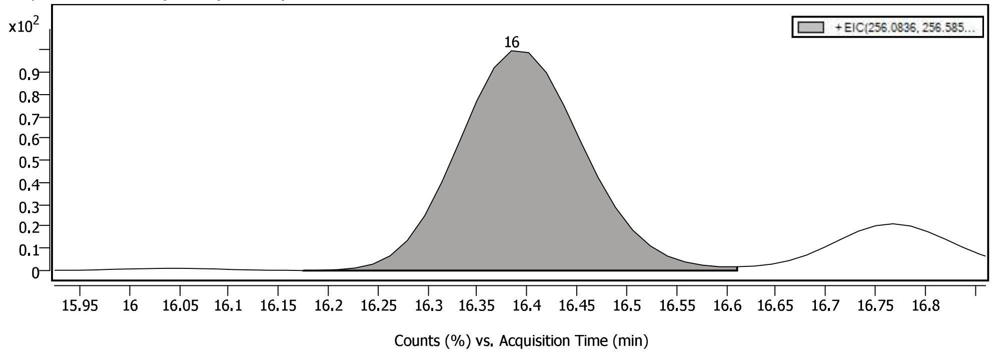

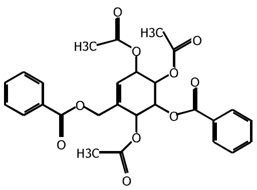

Compound Spectra (overlaid)

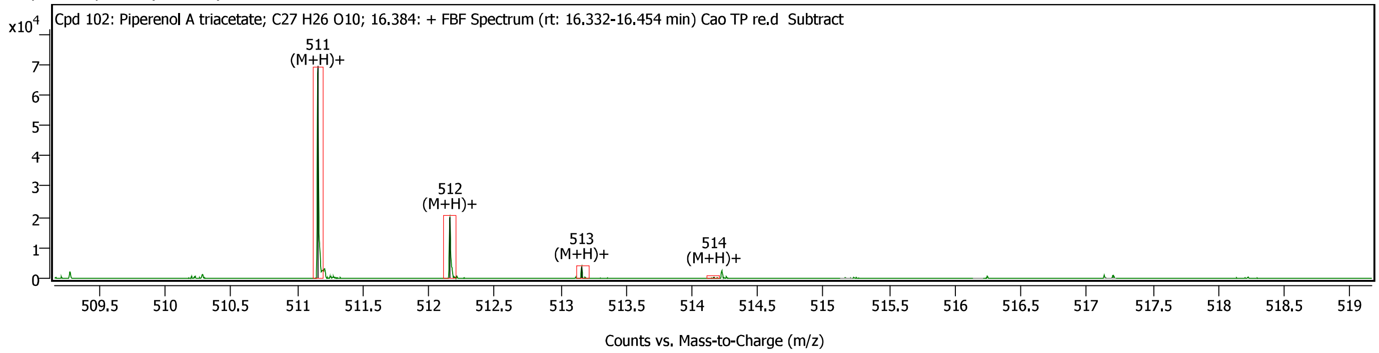

Compound ID Table

Name	Formula	Species	RT	RT Diff	Mass	CAS	ID Source	Score	Score (Lib)	Score (Tgt)
Piperenol A triacetate	C27 H26 O10	(M+H)+	16.384		510.1503	134476-92-9	FBF	90.55		90.55

Cpd 528: Petasitenine

Name	Formula	RT	RI	Mass	Diff (Tgt, ppm)	CAS	ID Source	Score	Algorithm
Petasitenine	C19 H27 N O7	16.576		381.1772	-4.20	60102-37-6	FBF	85.95	FBF

Species	m/z	Score (Tgt)	Score (Lib)	Score (DB)	Score (MFG)	Score (RT)
(M+H)+	382	85.95				

Compound Chromatograms (overlaid)

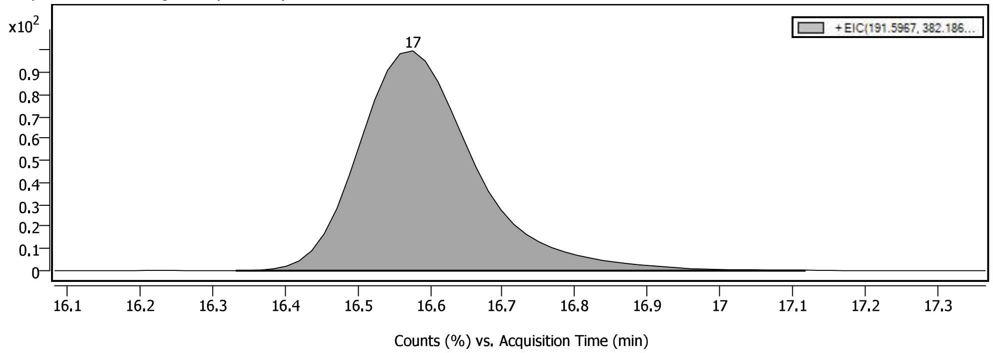

Structure

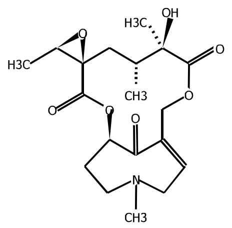

Compound Spectra (overlaid)

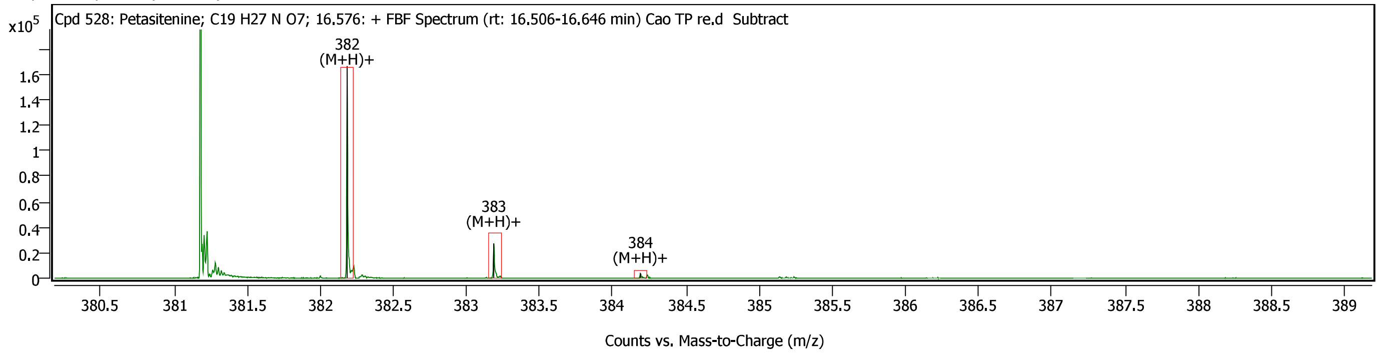

Compound ID Table

Name	Formula	Species	RT	RT Diff	Mass	CAS	ID Source	Score	Score (Lib)	Score (Tgt)
Petasitenine	C19 H27 N O7	(M+H)+	16.576		381.1772	60102-37-6	FBF	85.95		85.95

Cpd 536: Vomicine

Name	Formula	RT	RI	Mass	Diff (Tgt, ppm)	CAS	ID Source	Score	Algorithm
Vomicine	C22 H24 N2 O4	16.576		380.1742	1.68	125-15-5	M-FBF	98.56	FBF

Species	m/z	Score (Tgt)	Score (Lib)	Score (DB)	Score (MFG)	Score (RT)
(M+H)+	381	98.56				

Compound Chromatograms (overlaid)

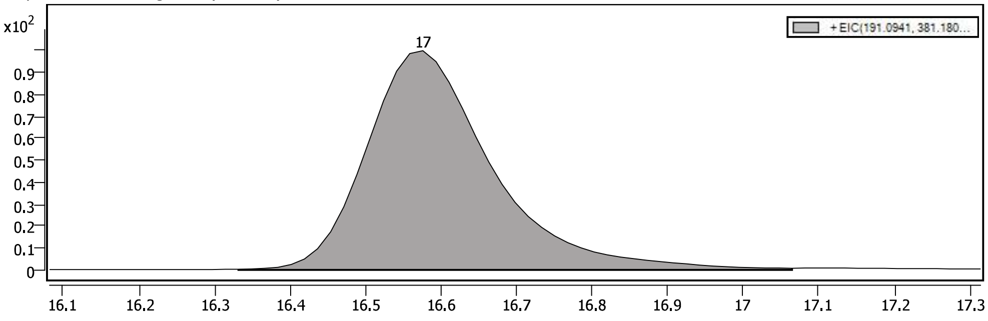

Structure

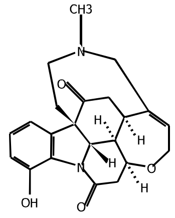

Compound Spectra (overlaid)

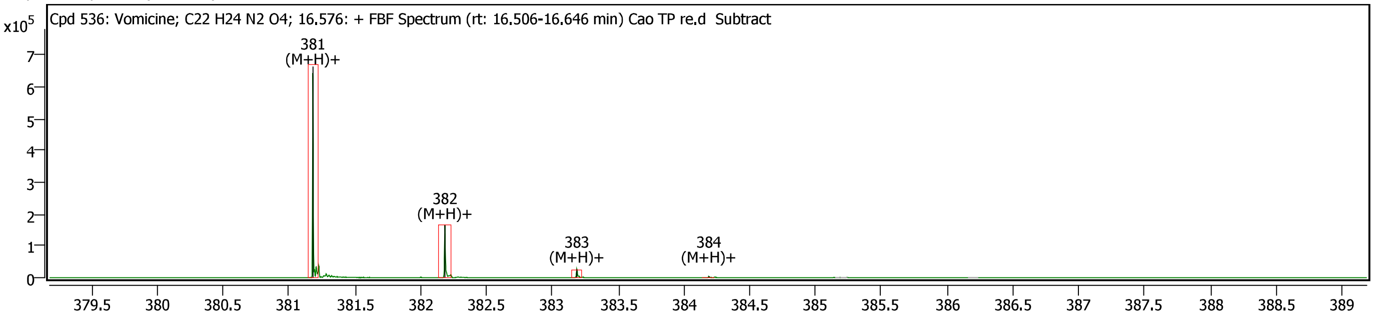

Compound ID Table

Name	Formula	Species	RT	RT Diff	Mass	CAS	ID Source	Score	Score (Lib)	Score (Tgt)
Vomicine	C22 H24 N2 O4	(M+H)+	16.576		380.1742	125-15-5	FBF	98.56		98.56
Di-4-coumaroylputrescine	C22 H24 N2 O4	(M+H)+	16.576		380.1742	37946-59-1	FBF	98.56		98.56

Cpd 91: 10-Fluoro-capric acid

Name	Formula	RT	RI	Mass	Diff (Tgt, ppm)	CAS	ID Source	Score	Algorithm
10-Fluoro-capric acid	C10 H19 F O2	16.925		190.1360	-4.85		FBF	84.22	FBF

Species	m/z	Score (Tgt)	Score (Lib)	Score (DB)	Score (MFG)	Score (RT)
(M+H)+	191	84.22				

Compound Chromatograms (overlaid)

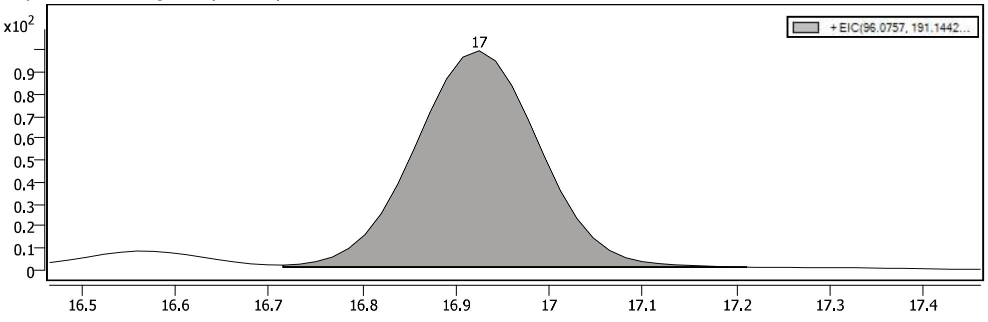

Structure

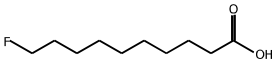

Compound Spectra (overlaid)

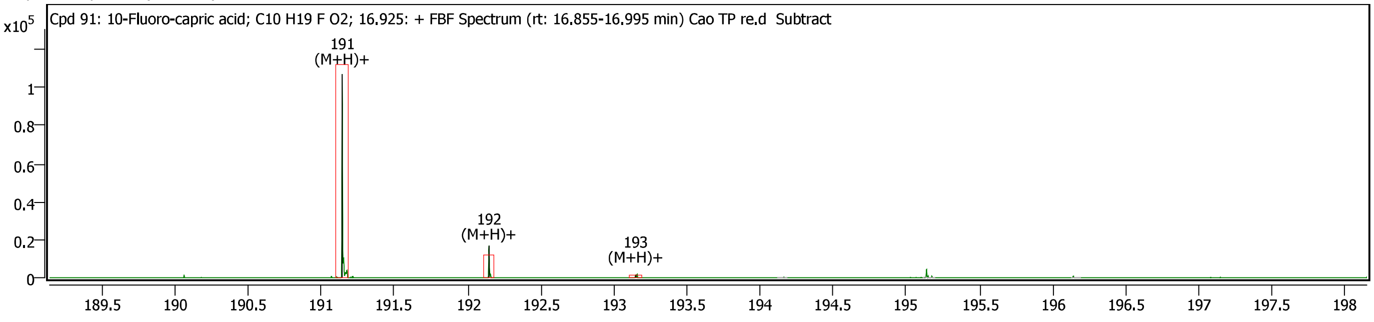

Compound ID Table

Name	Formula	Species	RT	RT Diff	Mass	CAS	ID Source	Score	Score (Lib)	Score (Tgt)
10-Fluoro-capric acid	C10 H19 F O2	(M+H)+	16.925		190.1360		FBF	84.22		84.22

Cpd 1284: <10-Hydroxycamtothecin>

Name	Formula	RT	RI	Mass	Diff (Tgt, ppm)	CAS	ID Source	Score	Algorithm
<10-Hydroxycamtothecin>	C20 H16 N2 O5	16.995		364.1066	1.90	19685-09-7	FBF	98.16	FBF

Species	m/z	Score (Tgt)	Score (Lib)	Score (DB)	Score (MFG)	Score (RT)
(M+H)+	365	98.16				

Compound Chromatograms (overlaid)

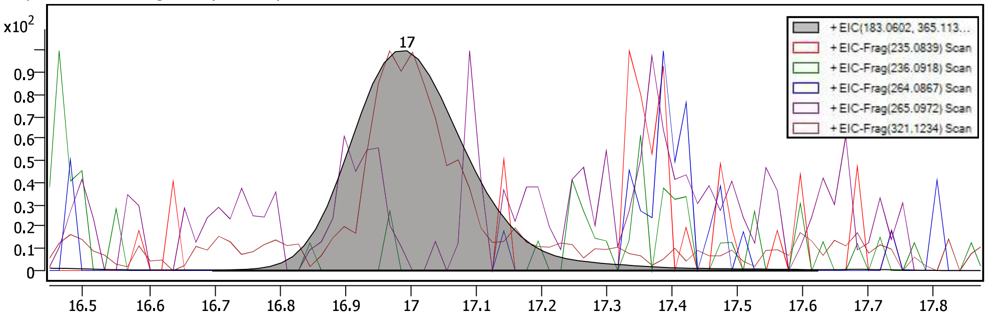

Structure

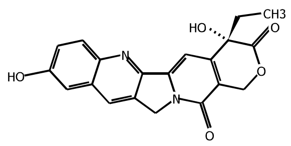

Coelution Plot

Compound Spectra (overlaid)

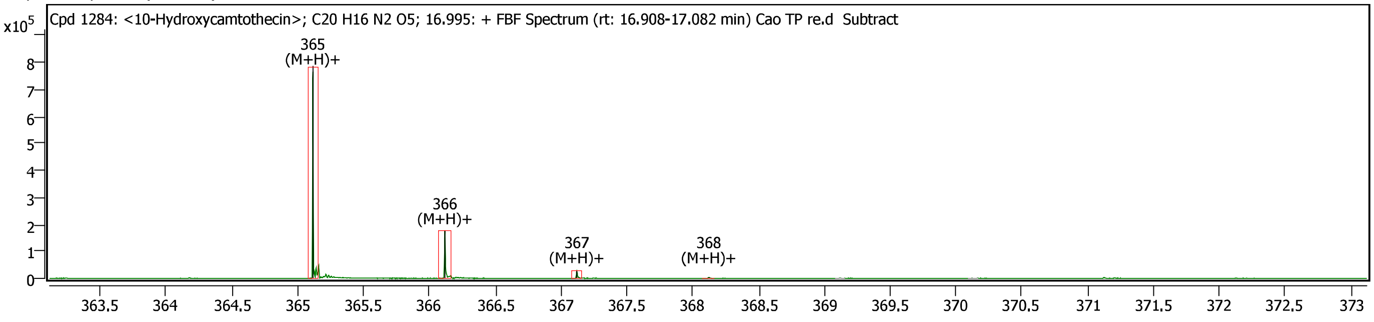

Fragment Spectrum (raw)

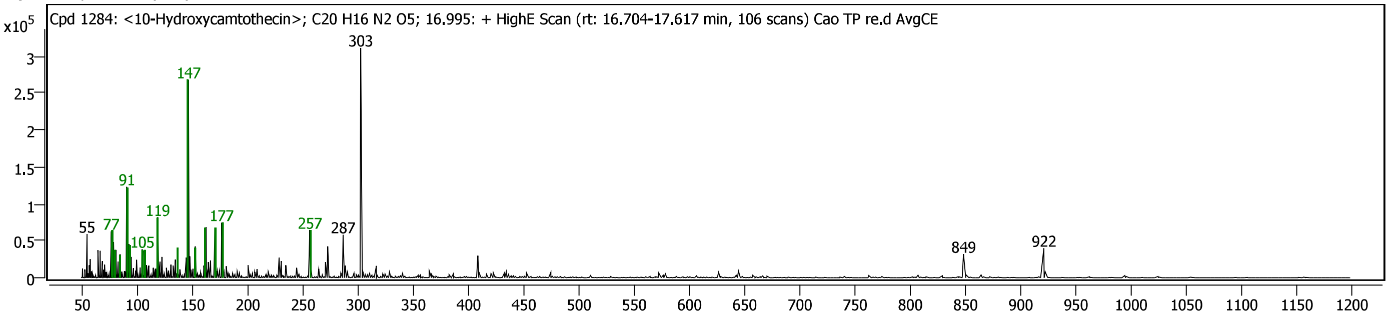

Compound ID Table

Name	Formula	Species	RT	RT Diff	Mass	CAS	ID Source	Score	Score (Lib)	Score (Tgt)
<10-Hydroxycamtothecin>	C20 H16 N2 O5	(M+H)+	16.995		364.1066	19685-09-7	FBF	98.16		98.16

Cpd 1715: <Quinaprilat>

Name	Formula	RT	RI	Mass	Diff (Tgt, ppm)	CAS	ID Source	Score	Algorithm
<Quinaprilat>	C23 H26 N2 O5	17.222		410.1848	1.45	82768-85-2	FBF	98.55	FBF

Species	m/z	Score (Tgt)	Score (Lib)	Score (DB)	Score (MFG)	Score (RT)
(M+H)+	411	98.55				

Compound Screening Report

Compound Chromatograms (overlaid)

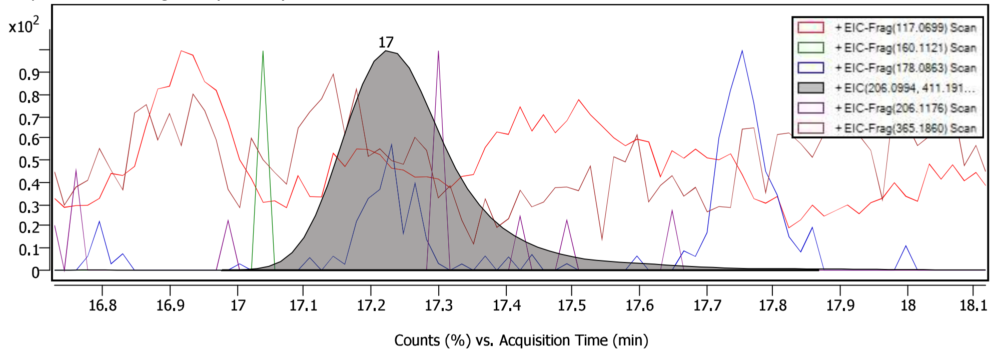

Structure

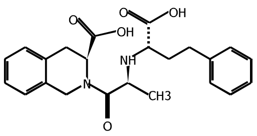

Coelution Plot

Compound Spectra (overlaid)

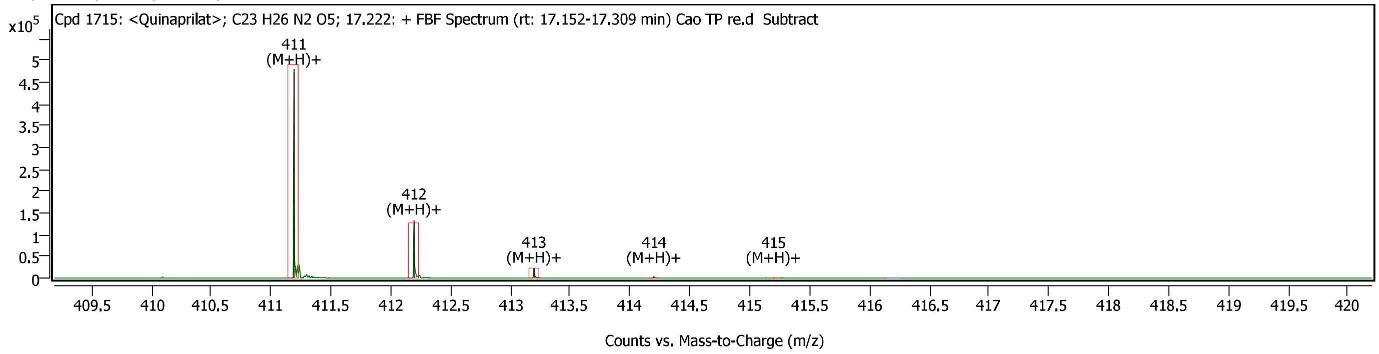

Fragment Spectrum (raw)

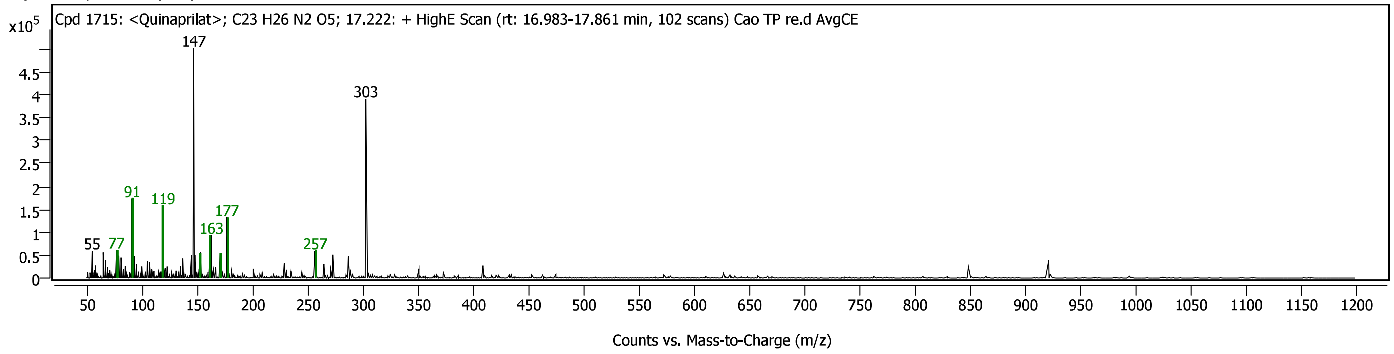

Compound ID Table

Name	Formula	Species	RT	RT Diff	Mass	CAS	ID Source	Score	Score (Lib)	Score (Tgt)
<Quinaprilat>	C23 H26 N2 O5	(M+H)+	17.222		410.1848	82768-85-2	FBF	98.55		98.55

Cpd 1674: Kaempferol 7-methyl ether 3-xylosyl-(1->2)-[rhamnosyl-(1->6)-glucoside]

Name	Formula	RT	RI	Mass	Diff (Tgt, ppm)	CAS	ID Source	Score	Algorithm
Kaempferol 7-methyl ether 3-xylosyl-(1->2)-[rhamnosyl-(1->6)-glucoside]	C33 H40 O19	17.396		740.2163	-0.14		M-FBF-FragConfirm	99.93	FBF

Species	m/z	Score (Tgt)	Score (Lib)	Score (DB)	Score (MFG)	Score (RT)
(M+2H)+2 (M+H)+	371 741	99.93				

Compound Chromatograms (overlaid)

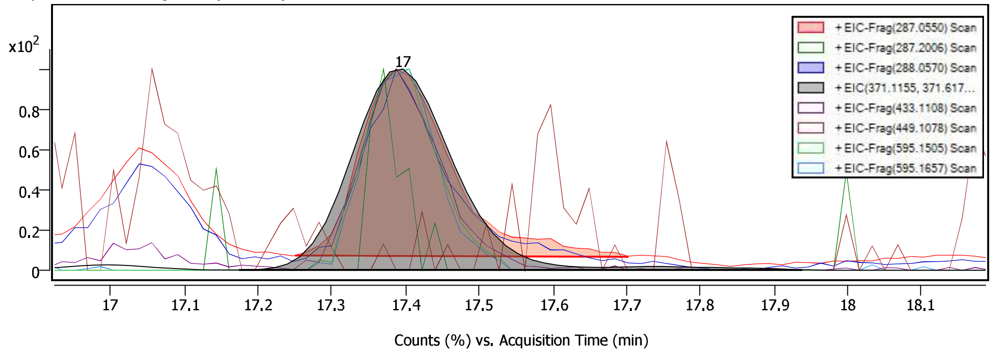

Structure

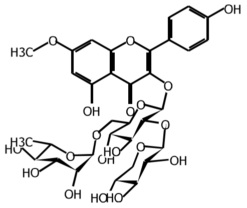

Compound Screening Report

Agilent

Coelution Plot

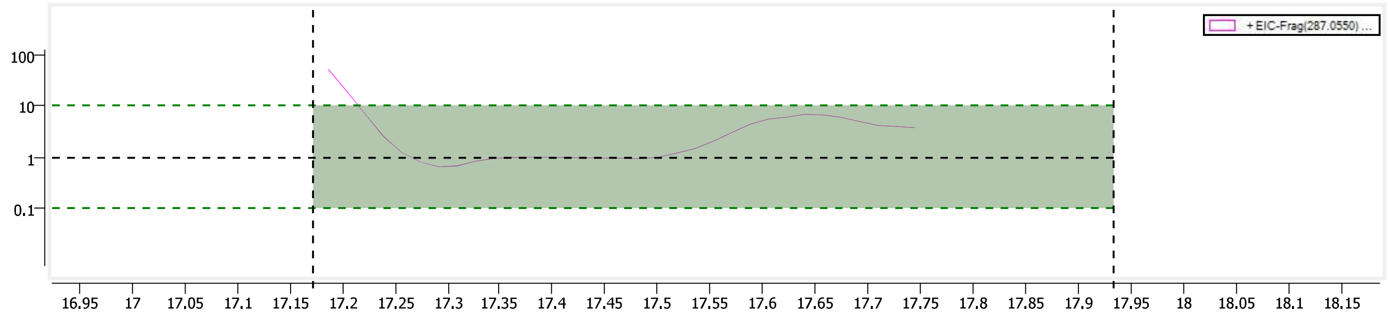

Compound Spectra (overlaid)

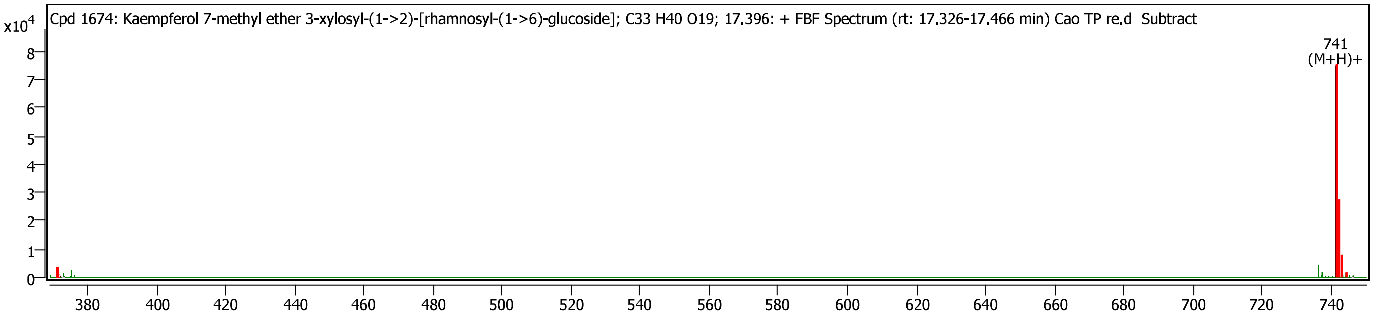

Fragment Spectrum (clean)

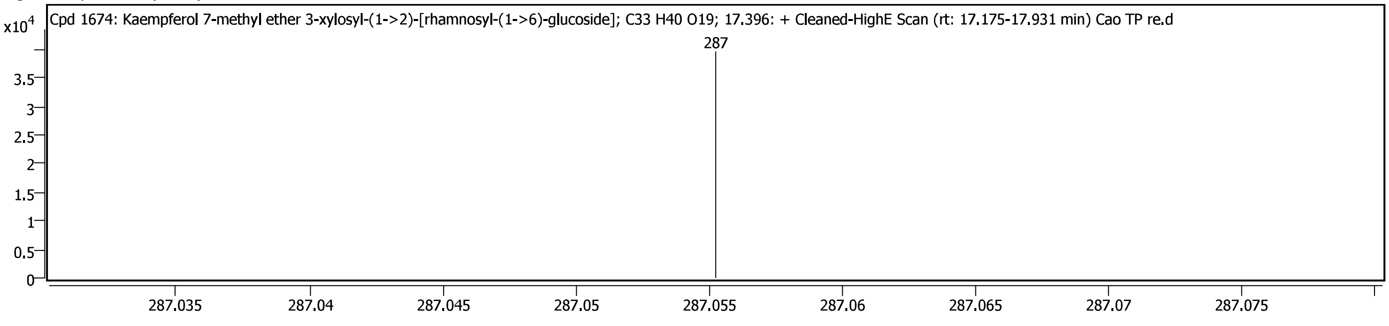

Fragment Spectrum (raw)

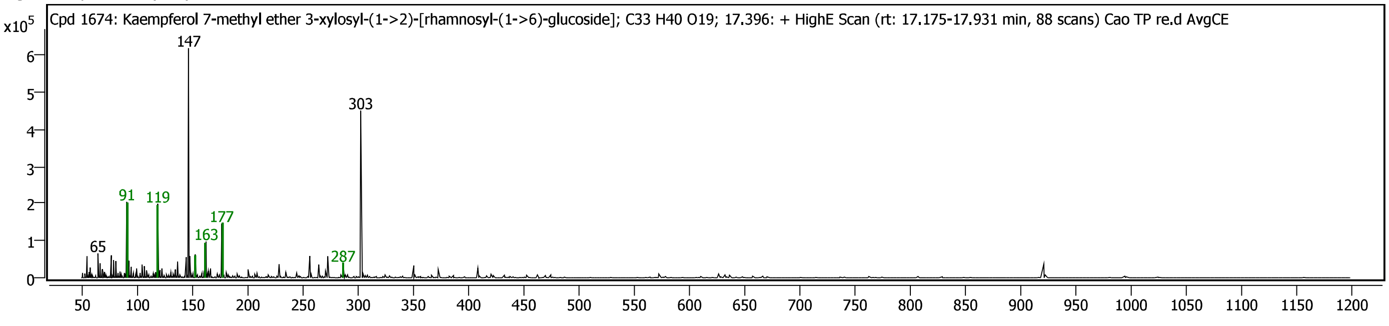

Compound Screening Report

Compound ID Table

Name	Formula	Species	RT	RT Diff	Mass	CAS	ID Source	Score	Score (Lib)	Score (Tgt)
Kaempferol 7-methyl ether 3-xylosyl-(1->2)-[rhamnosyl-(1->6)-glucoside]	C33 H40 O19	(M+2H)+2 (M+H)+	17.396		740.2163		FBF-FragConfirm	99.93		99.93
Clitorin	C33 H40 O19	(M+2H)+2 (M+H)+	17.396		740.2163	55804-74-5	FBF-FragConfirm	99.93		99.93
Kaempferol 3-isorhamninoside	C33 H40 O19	(M+2H)+2 (M+H)+	17.396		740.2163		FBF-FragConfirm	99.93		99.93
Kaempferol 3-rhamnosyl-(1->2)-galactoside-7-rhamnoside	C33 H40 O19	(M+2H)+2 (M+H)+	17.396		740.2163		FBF-FragConfirm	99.93		99.93
Kaempferol 3-glucoside-7,4'-dirhamnoside	C33 H40 O19	(M+2H)+2 (M+H)+	17.396		740.2163		FBF-FragConfirm	99.93		99.93
Kaempferol 3-(2''-rhamnosylrutinoside)	C33 H40 O19	(M+2H)+2 (M+H)+	17.396		740.2163	32602-83-8	FBF-FragConfirm	99.93		99.93
Kaempferol 3-(2''-rhamnosylgalactoside) 7-rhamnoside	C33 H40 O19	(M+2H)+2 (M+H)+	17.396		740.2163	124027-49-2	FBF-FragConfirm	99.93		99.93
Isovitexin 7-O-galactoside-2''-O-rhamnoside	C33 H40 O19	(M+2H)+2 (M+H)+	17.396		740.2163		FBF-FragConfirm	99.93		99.93
Apigenin 7-O-(2G-rhamnosyl)gentiobioside	C33 H40 O19	(M+2H)+2 (M+H)+	17.396		740.2163		FBF-FragConfirm	99.93		99.93
Chrysophanol 1-triglucoside	C33 H40 O19	(M+2H)+2 (M+H)+	17.396		740.2163	120181-07-9	FBF-FragConfirm	99.93		99.93
Apigenin 7-rutinoside-4'-glucoside	C33 H40 O19	(M+2H)+2 (M+H)+	17.396		740.2163		FBF-FragConfirm	99.93		99.93
Apigenin 7-neohesperidoside-4'-glucoside	C33 H40 O19	(M+2H)+2 (M+H)+	17.396		740.2163		FBF-FragConfirm	99.93		99.93
7-[6-[[3,4-Dihydroxy-6-methyl-5-[3,4,5-trihydroxy-6-(hydroxymethyl)oxan-2-yl]oxyoxan-2-yl]oxymethyl]-3,4,5-trihydroxyoxan-2-yl]oxy-5-hydroxy-3-(4-hydroxyphenyl)chromen-4-one	C33 H40 O19	(M+2H)+2 (M+H)+	17.396		740.2163		FBF-FragConfirm	99.93		99.93
6,8-Di-C-glucosylgenkwanin 2'''-O-xyloside	C33 H40 O19	(M+2H)+2 (M+H)+	17.396		740.2163		FBF-FragConfirm	99.93		99.93
Kaempferol 3-glucosyl-(1->3)-rhamnoside-7-rhamnoside	C33 H40 O19	(M+2H)+2 (M+H)+	17.396		740.2163		FBF-FragConfirm	99.93		99.93
Kaempferol 3-rhamnosyl-(1->2)-rhamnosyl-(1->6)-glucoside	C33 H40 O19	(M+2H)+2 (M+H)+	17.396		740.2163		FBF-FragConfirm	99.93		99.93
Isovitexin 7-O-glucoside-2''-O-rhamnoside	C33 H40 O19	(M+2H)+2 (M+H)+	17.396		740.2163		FBF-FragConfirm	99.93		99.93
Kaempferol 3-neohesperidoside-7-rhamnoside	C33 H40 O19	(M+2H)+2 (M+H)+	17.396		740.2163		FBF-FragConfirm	99.93		99.93
Kaempferol 7-methyl ether 3-rhamnosyl-(1->3)-[apiosyl-(1->6)-glucoside]	C33 H40 O19	(M+2H)+2 (M+H)+	17.396		740.2163		FBF-FragConfirm	99.93		99.93
Kaempferol 3-rhamninoside	C33 H40 O19	(M+2H)+2 (M+H)+	17.396		740.2163		FBF-FragConfirm	99.93		99.93
Vitexin 7-O-rutinoside	C33 H40 O19	(M+2H)+2 (M+H)+	17.396		740.2163		FBF-FragConfirm	99.93		99.93
Vitexin 4'-O-glucoside-2''-O-rhamnoside	C33 H40 O19	(M+2H)+2 (M+H)+	17.396		740.2163		FBF-FragConfirm	99.93		99.93
robinin	C33 H40 O19	(M+2H)+2 (M+H)+	17.396		740.2163		FBF-FragConfirm	99.93		99.93
Kaempferol 4'-rhamninoside	C33 H40 O19	(M+2H)+2 (M+H)+	17.396		740.2163		FBF-FragConfirm	99.93		99.93
Kaempferol 3-rutinoside-7-rhamnoside	C33 H40 O19	(M+2H)+2 (M+H)+	17.396		740.2163		FBF-FragConfirm	99.93		99.93
Kaempferol 3-rhamnosyl-(1->4)-rhamnosyl-(1->6)-glucoside	C33 H40 O19	(M+2H)+2 (M+H)+	17.396		740.2163		FBF-FragConfirm	99.93		99.93
Kaempferol 3-rhamnosyl-(1->3)-rhamnosyl-(1->6)-glucoside	C33 H40 O19	(M+2H)+2 (M+H)+	17.396		740.2163	83170-31-4	FBF-FragConfirm	99.93		99.93
Kaempferol 3-rhamnoside-7-glucosyl-(1->2)-rhamnoside	C33 H40 O19	(M+2H)+2 (M+H)+	17.396		740.2163		FBF-FragConfirm	99.93		99.93

Cpd 935: Quinalizarin

Name	Formula	RT	RI	Mass	Diff (Tgt, ppm)	CAS	ID Source	Score	Algorithm
Quinalizarin	C14 H8 O6	17.448		272.0324	1.00	81-61-8	FBF	98.27	FBF

Species	m/z	Score (Tgt)	Score (Lib)	Score (DB)	Score (MFG)	Score (RT)
(M+2H)+2 (M+H)+	137 273	98.27				

Compound Chromatograms (overlaid)

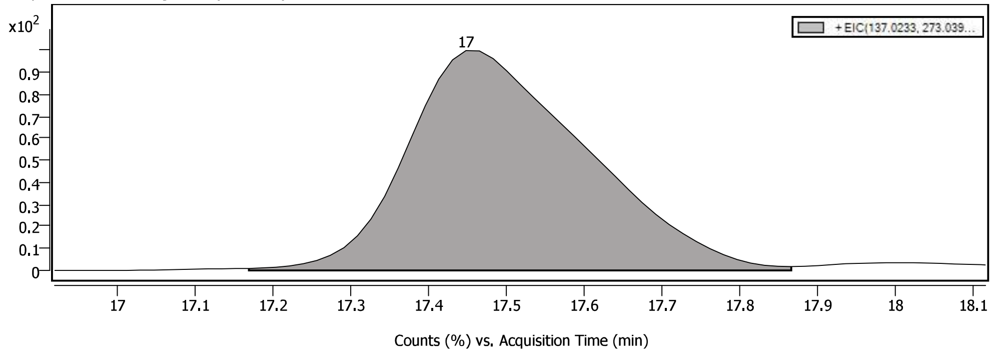

Structure

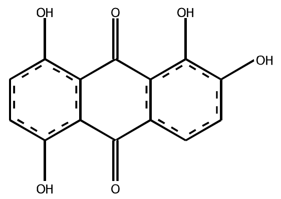

Compound Screening Report

Compound Spectra (overlaid)

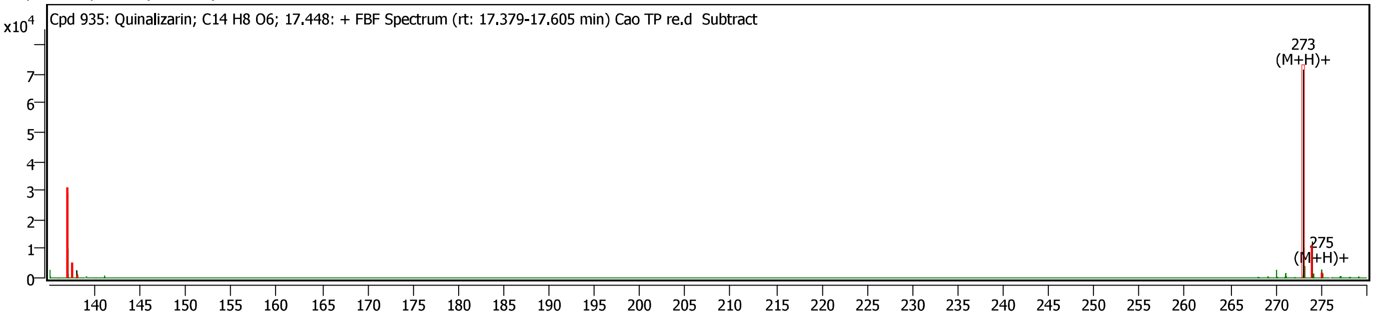

Compound ID Table

Name	Formula	Species	RT	RT Diff	Mass	CAS	ID Source	Score	Score (Lib)	Score (Tgt)
Quinalizarin	C14 H8 O6	(M+2H)+2 (M+H)+	17.448		272.0324	81-61-8	FBF	98.27		98.27

Cpd 1394: Morin

Name	Formula	RT	RI	Mass	Diff (Tgt, ppm)	CAS	ID Source	Score	Algorithm
Morin	C15 H10 O7	17.489		302.0437	3.46	480-16-0	M-FBF-FragConfirm	92.87	FBF

Species	m/z	Score (Tgt)	Score (Lib)	Score (DB)	Score (MFG)	Score (RT)
(M+H)+	303	92.87				

Compound Chromatograms (overlaid)

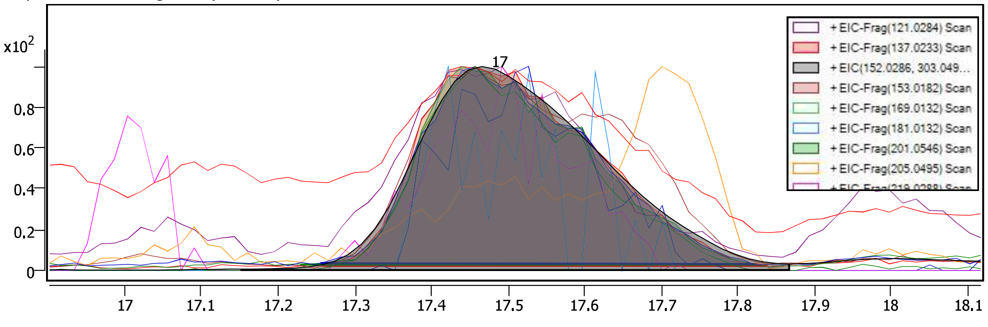

Structure

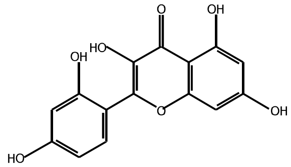

Coelution Plot

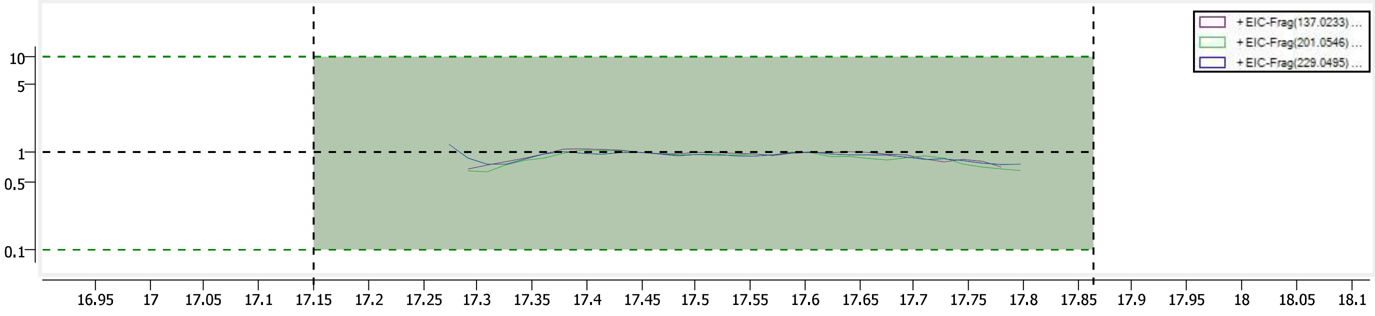

Compound Spectra (overlaid)

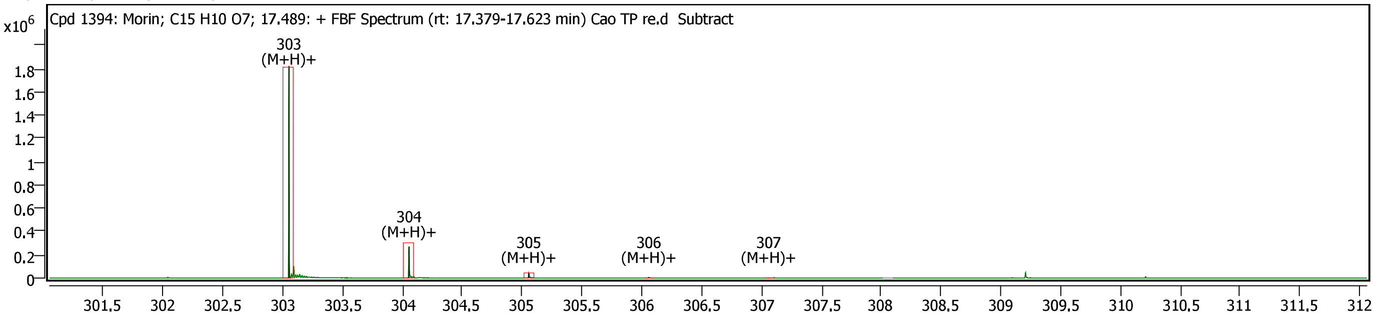

Fragment Spectrum (clean)

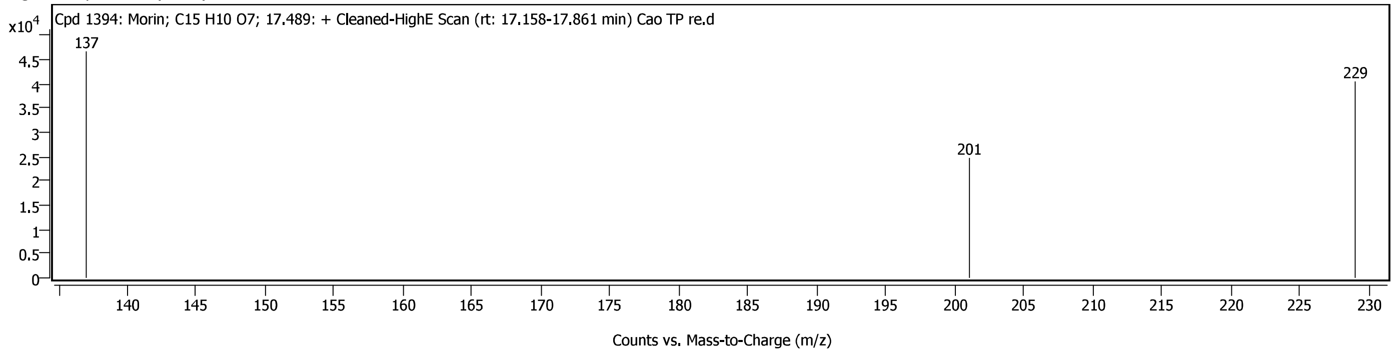

Fragment Spectrum (raw)

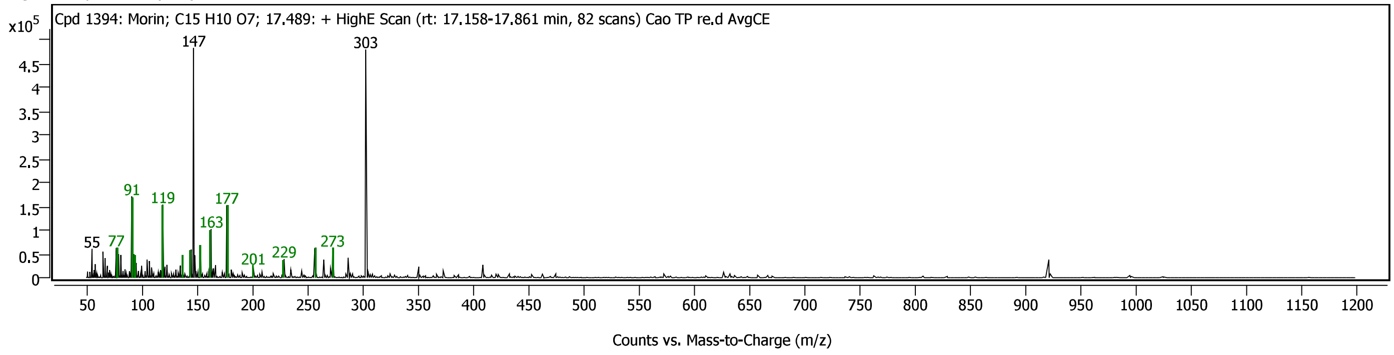

Compound ID Table

Name	Formula	Species	RT	RT Diff	Mass	CAS	ID Source	Score	Score (Lib)	Score (Tgt)
Morin	C15 H10 O7	(M+H)+	17.489		302.0437	480-16-0	FBF-FragConfirm	92.87		92.87
6-Hydroxyluteolin	C15 H10 O7	(M+H)+	17.489		302.0437		FBF-FragConfirm	92.87		92.87
5,7,8,3',4'-Pentahydroxyisoflavone	C15 H10 O7	(M+H)+	17.489		302.0437		FBF-FragConfirm	92.87		92.87
Herbacetin	C15 H10 O7	(M+H)+	17.489		302.0437		FBF-FragConfirm	92.87		92.87
2-(4-Hydroxyphenyl)-5,6,7,8-tetrahydroxy-4H-1-benzopyran-4-one	C15 H10 O7	(M+H)+	17.489		302.0437	577-26-4	FBF-FragConfirm	92.87		92.87
2',3',4',5,7-Pentahydroxyflavone	C15 H10 O7	(M+H)+	17.489		302.0437	144707-17-5	FBF-FragConfirm	92.87		92.87
2'-Hydroxypseudobaptigenin	C15 H10 O7	(M+H)+	17.489		302.0437		FBF-FragConfirm	92.87		92.87
3,5,7,2',5'-Pentahydroxyflavone	C15 H10 O7	(M+H)+	17.489		302.0437		FBF-FragConfirm	92.87		92.87
5,6,7,3',4'-Pentahydroxyisoflavone	C15 H10 O7	(M+H)+	17.489		302.0437		FBF-FragConfirm	92.87		92.87
5,7,8,2',4'-Pentahydroxyisoflavone	C15 H10 O7	(M+H)+	17.489		302.0437		FBF-FragConfirm	92.87		92.87
6-Hydroxykaempferol	C15 H10 O7	(M+H)+	17.489		302.0437		FBF-FragConfirm	92.87		92.87
Tricetin	C15 H10 O7	(M+H)+	17.489		302.0437	520-31-0	FBF-FragConfirm	92.87		92.87
Bracteatin	C15 H10 O7	(M+H)+	17.489		302.0437		FBF-FragConfirm	92.87		92.87
Hypolaetin	C15 H10 O7	(M+H)+	17.489		302.0437		FBF-FragConfirm	92.87		92.87
Isoetin	C15 H10 O7	(M+H)+	17.489		302.0437		FBF-FragConfirm	92.87		92.87
Melanoxetin	C15 H10 O7	(M+H)+	17.489		302.0437		FBF-FragConfirm	92.87		92.87
Quercetin	C15 H10 O7	(M+H)+	17.489		302.0437	117-39-5	FBF-FragConfirm	92.87		92.87
Rhynchosin	C15 H10 O7	(M+H)+	17.489		302.0437		FBF-FragConfirm	92.87		92.87
Robinetin	C15 H10 O7	(M+H)+	17.489		302.0437		FBF-FragConfirm	92.87		92.87
Viscidulin I	C15 H10 O7	(M+H)+	17.489		302.0437	92519-95-4	FBF-FragConfirm	92.87		92.87

Cpd 765: Nympholide A

Name	Formula	RT	RI	Mass	Diff (Tgt, ppm)	CAS	ID Source	Score	Algorithm
Nympholide A	C30 H26 O15	17.571		626.1275	0.51		M-FBF	98.10	FBF

Species	m/z	Score (Tgt)	Score (Lib)	Score (DB)	Score (MFG)	Score (RT)
(M+H)+	627	98.10				

Compound Chromatograms (overlaid)

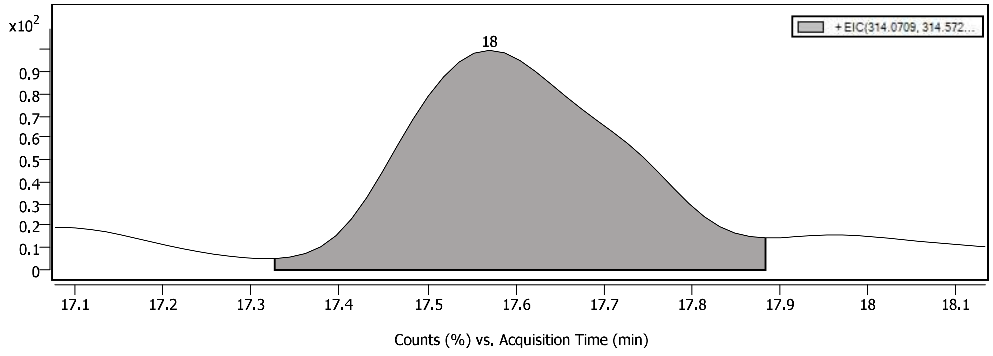

Structure

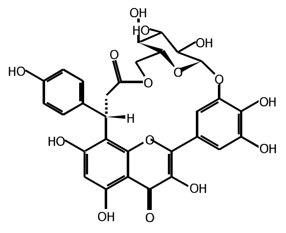

Compound Screening Report

Compound Spectra (overlaid)

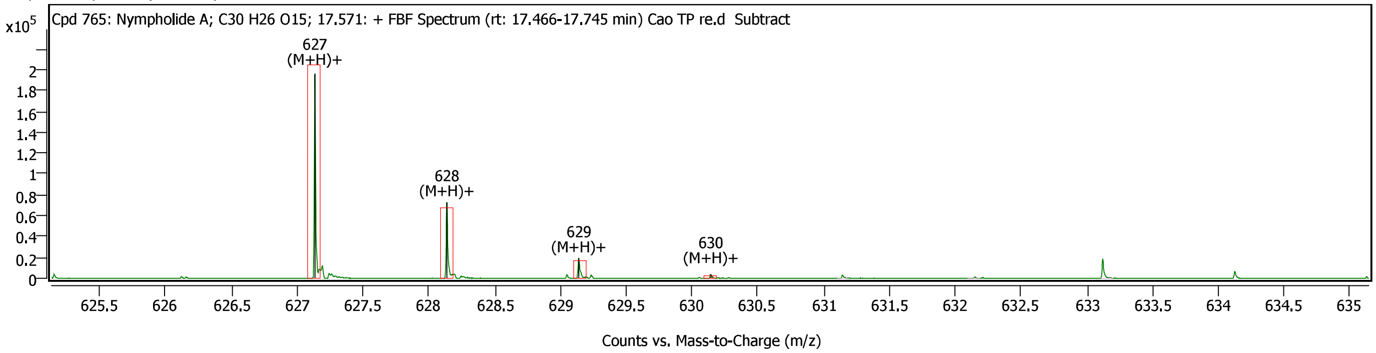

Compound ID Table

Name	Formula	Species	RT	RT Diff	Mass	CAS	ID Source	Score	Score (Lib)	Score (Tgt)
Nympholide A	C30 H26 O15	(M+H)+	17.571		626.1275		FBF	98.10		98.10
Myricetin 3'-O-(6"-p-coumaroyl)glucoside	C30 H26 O15	(M+H)+	17.571		626.1275		FBF	98.10		98.10
6"-Caffeoylhyperin	C30 H26 O15	(M+H)+	17.571		626.1275	84575-22-4	FBF	98.10		98.10
6-Hydroxykaempferol 7-(6"-(E)-caffeylglucoside)	C30 H26 O15	(M+H)+	17.571		626.1275		FBF	98.10		98.10
Myricetin 3-(6"-p-coumaroylglucoside)	C30 H26 O15	(M+H)+	17.571		626.1275		FBF	98.10		98.10
Nympholide B	C30 H26 O15	(M+H)+	17.571		626.1275		FBF	98.10		98.10
Quercetin 3-(6"-caffeylgalactoside)	C30 H26 O15	(M+H)+	17.571		626.1275		FBF	98.10		98.10
Spicoside A	C30 H26 O15	(M+H)+	17.571		626.1275		FBF	98.10		98.10

Cpd 100: <(±)-Jasmonic Acid>

Name	Formula	RT	RI	Mass	Diff (Tgt, ppm)	CAS	ID Source	Score	Algorithm
<(±)-Jasmonic Acid>	C12 H18 O3	17.728		210.1259	1.33	77026-92-7	M-FBF	98.20	FBF

Species	m/z	Score (Tgt)	Score (Lib)	Score (DB)	Score (MFG)	Score (RT)
(M+H)+	211	98.20				

Compound Chromatograms (overlaid)

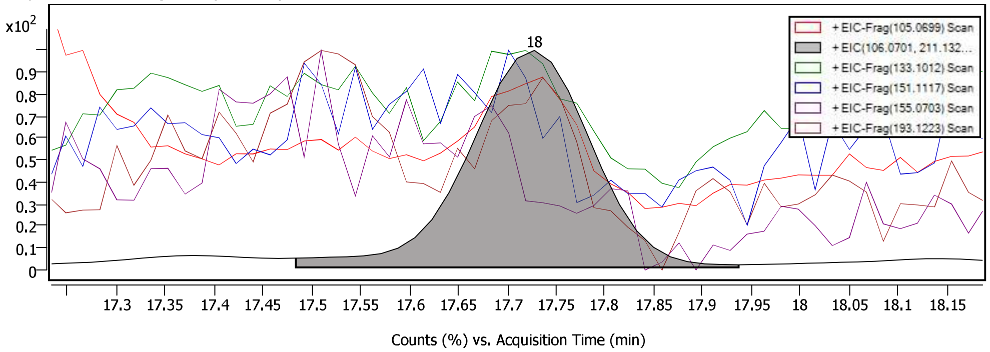

Structure

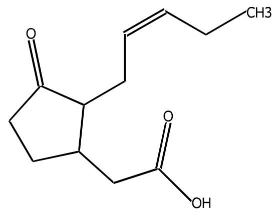

Coelution Plot

Compound Spectra (overlaid)

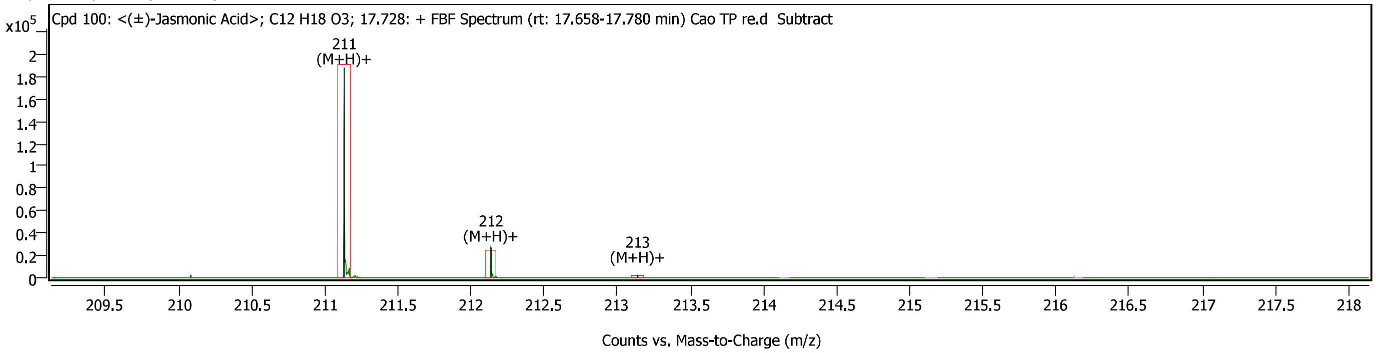

Fragment Spectrum (raw)

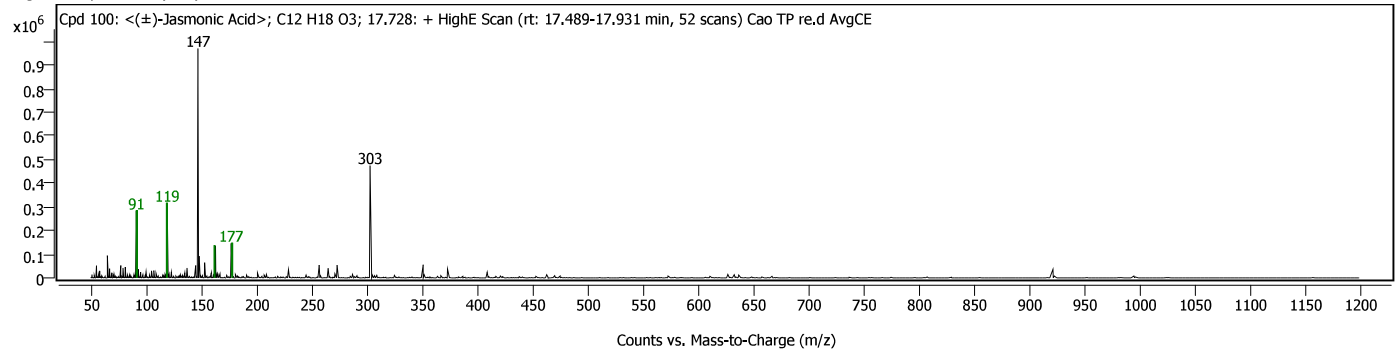

Compound ID Table

Name	Formula	Species	RT	RT Diff	Mass	CAS	ID Source	Score	Score (Lib)	Score (Tgt)
<(±)-Jasmonic Acid>	C12 H18 O3	(M+H)+	17.728		210.1259	77026-92-7	FBF	98.20		98.20
<3-Ethenyl-2,5-dimethyl-4-oxohex-5-en-2-yl acetate>	C12 H18 O3	(M+H)+	17.728		210.1259	79507-89-4	FBF	98.20		98.20
<(±)7-epi Jasmonic Acid>	C12 H18 O3	(M+H)+	17.728		210.1259	62653-85-4	FBF	98.20		98.20
<(3S,7R)-iso-jasmonic acid>	C12 H18 O3	(M+H)+	17.728		210.1259		FBF	98.20		98.20
<(3S,7S)-Jasmonic acid>	C12 H18 O3	(M+H)+	17.728		210.1259		FBF	98.20		98.20
<(R)-8-Acetoxycarvotanacetone>	C12 H18 O3	(M+H)+	17.728		210.1259	56691-69-1	FBF	98.20		98.20
<1-Benzyloxy-1-(2-methoxyethoxy)ethane>	C12 H18 O3	(M+H)+	17.728		210.1259	7492-39-9	FBF	98.20		98.20
<Cymather aldehyde methyl ester>	C12 H18 O3	(M+H)+	17.728		210.1259		FBF	98.20		98.20
<4-(Butoxymethyl)-2-methoxyphenol>	C12 H18 O3	(M+H)+	17.728		210.1259	82654-98-6	FBF	98.20		98.20
<Dihydro-3-(2-octenyl)-2,5-furandione>	C12 H18 O3	(M+H)+	17.728		210.1259	42482-06-4	FBF	98.20		98.20
<Isoamyl 2-furonpropionate>	C12 H18 O3	(M+H)+	17.728		210.1259	7779-67-1	FBF	98.20		98.20
<Jasmonic acid>	C12 H18 O3	(M+H)+	17.728		210.1259	6894-38-8	FBF	98.20		98.20
<Sedanonic acid>	C12 H18 O3	(M+H)+	17.728		210.1259	6697-07-0	FBF	98.20		98.20
<Dihydro-3-(1-octenyl)-2,5-furandione>	C12 H18 O3	(M+H)+	17.728		210.1259	7757-96-2	FBF	98.20		98.20

Cpd 1252: Oxprenolol glucuronide

Name	Formula	RT	RI	Mass	Diff (Tgt, ppm)	CAS	ID Source	Score	Algorithm
Oxprenolol glucuronide	C21 H31 N O9	17.762		441.1982	-3.76	53564-66-2	FBF	85.97	FBF

Species	m/z	Score (Tgt)	Score (Lib)	Score (DB)	Score (MFG)	Score (RT)
(M+H)+	442	85.97				

Compound Chromatograms (overlaid)

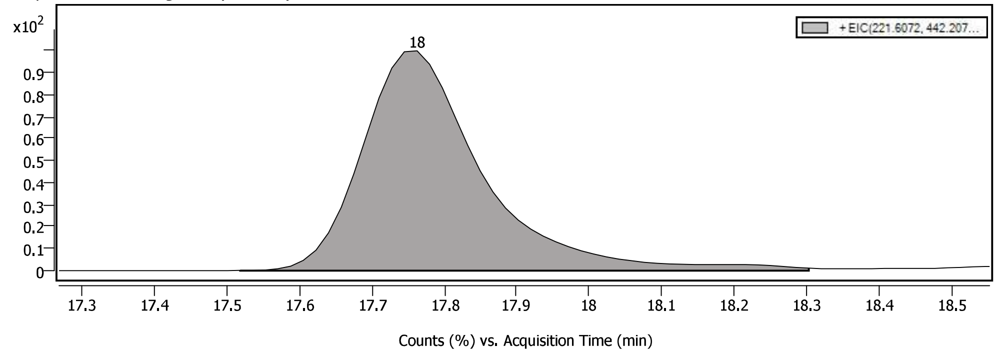

Structure

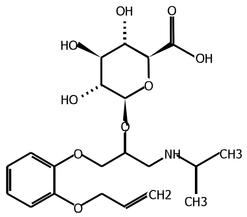

Compound Spectra (overlaid)

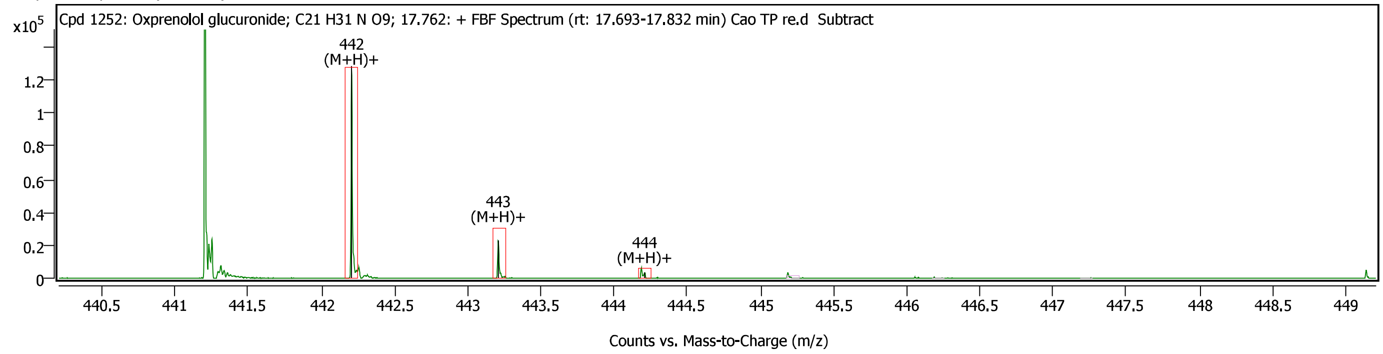

Compound ID Table

Name	Formula	Species	RT	RT Diff	Mass	CAS	ID Source	Score	Score (Lib)	Score (Tgt)
Oxprenolol glucuronide	C21 H31 N O9	(M+H)+	17.762		441.1982	53564-66-2	FBF	85.97		85.97

Cpd 1576: Diferuloylputrescine

Compound Screening Report

Name	Formula	RT	RI	Mass	Diff (Tgt, ppm)	CAS	ID Source	Score	Algorithm
Diferuloylputrescine	C24 H28 N2 O6	17.762		440.1953	1.18	42369-86-8	FBF	99.04	FBF

Species	m/z	Score (Tgt)	Score (Lib)	Score (DB)	Score (MFG)	Score (RT)
(M+H)+	441	99.04				

Compound Chromatograms (overlaid)

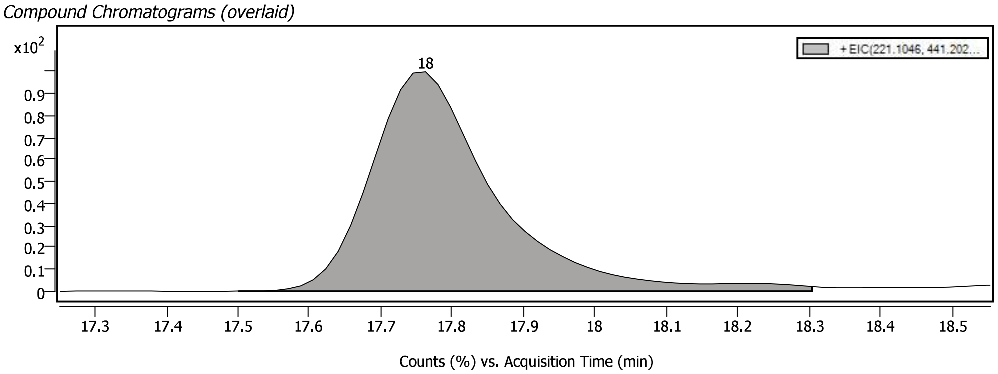

Structure

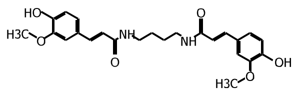

Compound Spectra (overlaid)

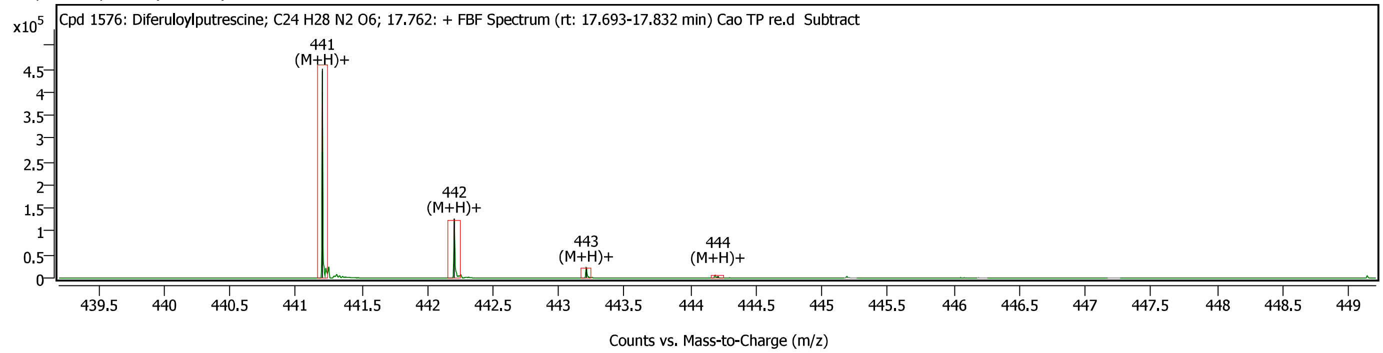

Compound ID Table

Name	Formula	Species	RT	RT Diff	Mass	CAS	ID Source	Score	Score (Lib)	Score (Tgt)
Diferuloylputrescine	C24 H28 N2 O6	(M+H)+	17.762		440.1953	42369-86-8	FBF	99.04		99.04

Cpd 579: N-dodecanoyl-L-Homoserine lactone-3-hydrazone-fluorescein

Name	Formula	RT	RI	Mass	Diff (Tgt, ppm)	CAS	ID Source	Score	Algorithm
N-dodecanoyl-L-Homoserine lactone-3-hydrazone-fluorescein	C37 H40 N4 O8 S	17.850		700.2558	-1.27		FBF	87.20	FBF

Species	m/z	Score (Tgt)	Score (Lib)	Score (DB)	Score (MFG)	Score (RT)
(M+2H)+2 (M+H)+	351 701	87.20				

Compound Chromatograms (overlaid)

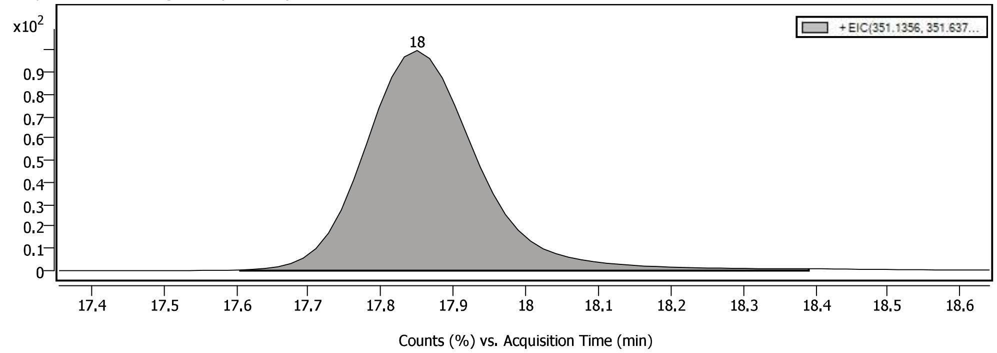

Structure

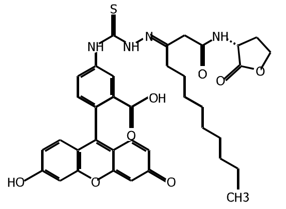

Compound Screening Report

Compound Spectra (overlaid)

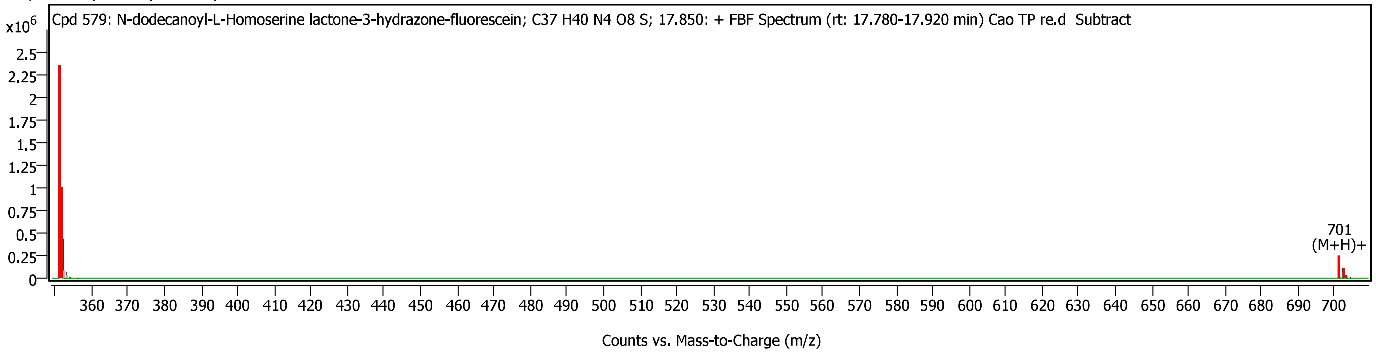

Compound ID Table

Name	Formula	Species	RT	RT Diff	Mass	CAS	ID Source	Score	Score (Lib)	Score (Tgt)
N-dodecanoyl-L-Homoserine lactone-3-hydrazone-fluorescein	C37 H40 N4 O8 S	(M+2H)+2 (M+H)+	17.850		700.2558		FBF	87.20		87.20

Cpd 599: Rhizocticin A

Name	Formula	RT	RI	Mass	Diff (Tgt, ppm)	CAS	ID Source	Score	Algorithm
Rhizocticin A	C11 H22 N5 O6 P	17.850		351.1304	-0.94	114301-25-6	FBF	99.18	FBF

Species	m/z	Score (Tgt)	Score (Lib)	Score (DB)	Score (MFG)	Score (RT)
(M+H)+	352	99.18				

Compound Chromatograms (overlaid)

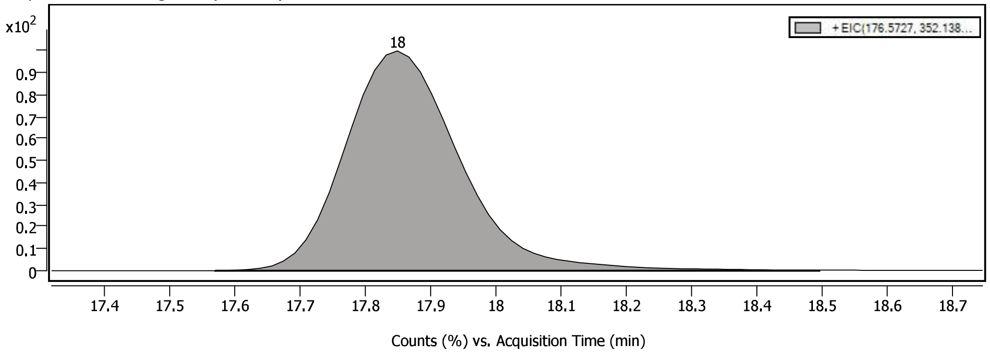

Structure

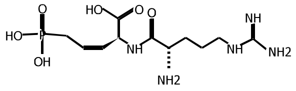

Compound Spectra (overlaid)

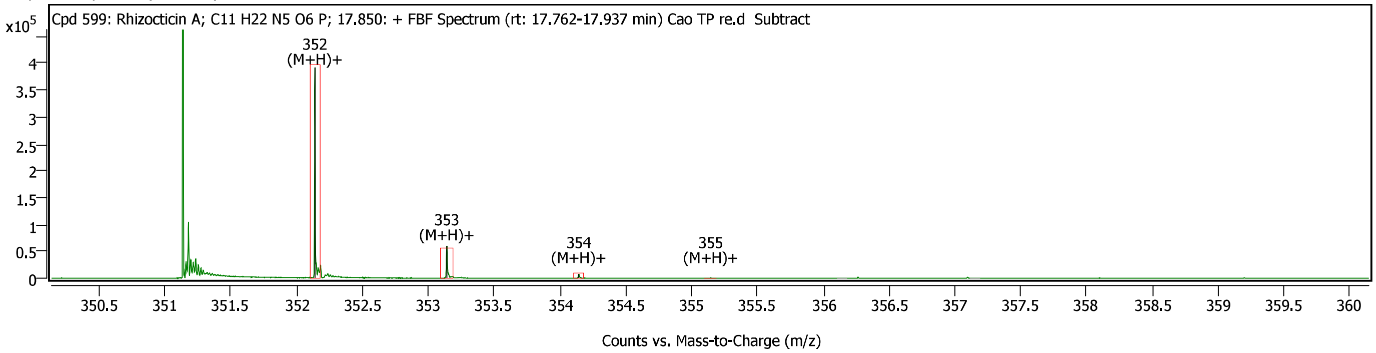

Compound ID Table

Name	Formula	Species	RT	RT Diff	Mass	CAS	ID Source	Score	Score (Lib)	Score (Tgt)
Rhizocticin A	C11 H22 N5 O6 P	(M+H)+	17.850		351.1304	114301-25-6	FBF	99.18		99.18

Cpd 1279: N1,N5,N10-Tricaffeoyl spermidine

Name	Formula	RT	RI	Mass	Diff (Tgt, ppm)	CAS	ID Source	Score	Algorithm
N1,N5,N10-Tricaffeoyl spermidine	C34 H37 N3 O9	18.006		631.2532	0.38		FBF	99.16	FBF

Species	m/z	Score (Tgt)	Score (Lib)	Score (DB)	Score (MFG)	Score (RT)
(M+H)+	632	99.16				

Compound Screening Report

Compound Chromatograms (overlaid)

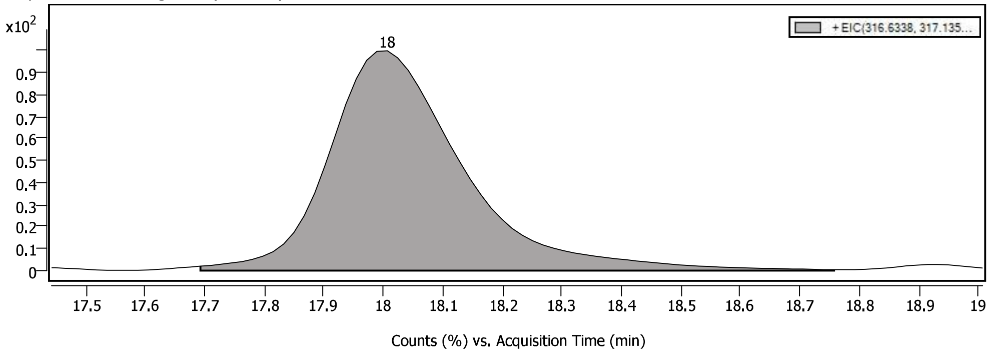

Structure

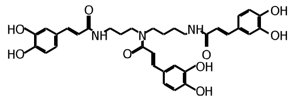

Compound Spectra (overlaid)

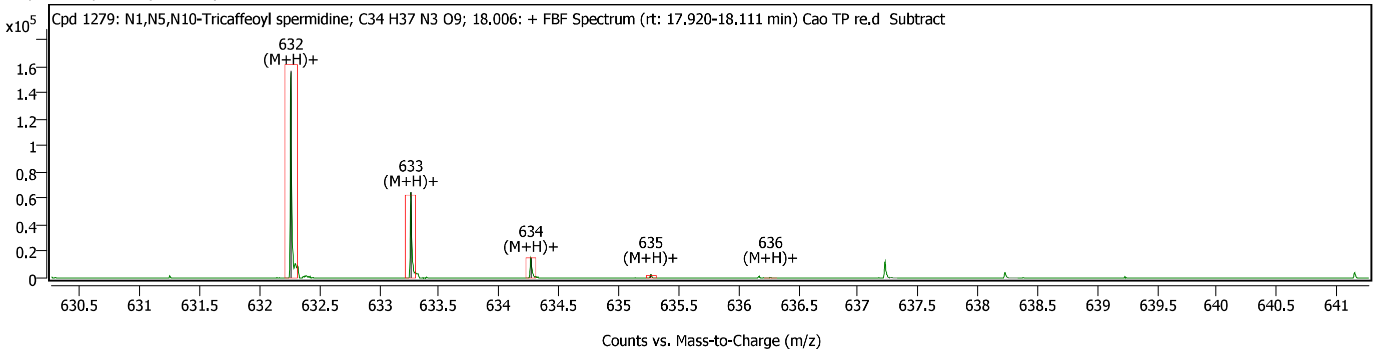

Compound ID Table

Name	Formula	Species	RT	RT Diff	Mass	CAS	ID Source	Score	Score (Lib)	Score (Tgt)
N1,N5,N10-Tricaffeoyl spermidine	C34 H37 N3 O9	(M+H)+	18.006		631.2532		FBF	99.16		99.16

Cpd 535: Isopimpinellin

Name	Formula	RT	RI	Mass	Diff (Tgt, ppm)	CAS	ID Source	Score	Algorithm
Isopimpinellin	C13 H10 O5	18.303		246.0531	1.14	482-27-9	FBF-FragConfirm	97.88	FBF

Species	m/z	Score (Tgt)	Score (Lib)	Score (DB)	Score (MFG)	Score (RT)
(M+H)+	247	97.88				

Compound Chromatograms (overlaid)

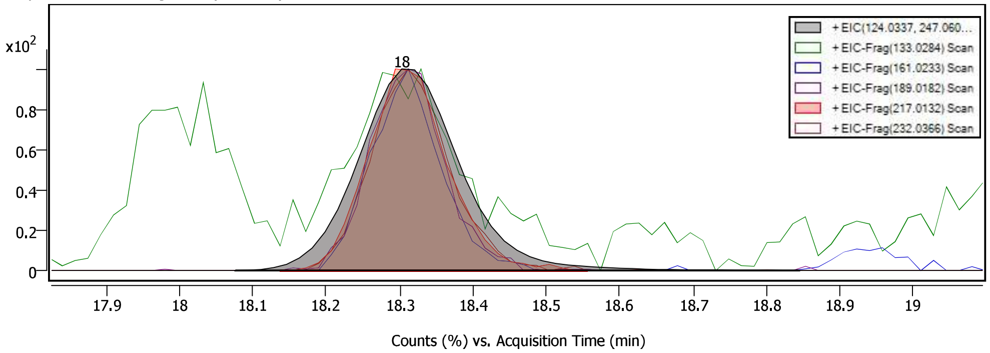

Structure

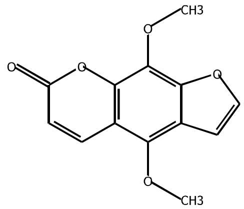

Coelution Plot

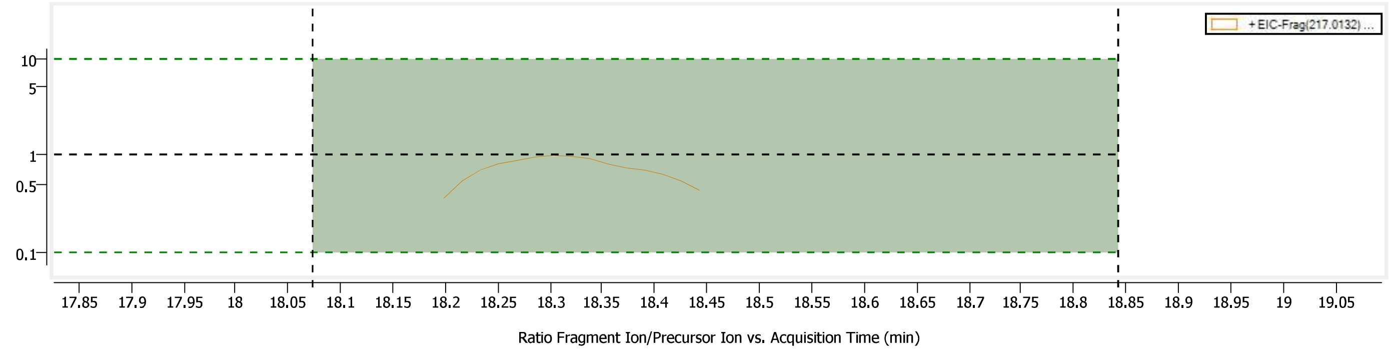

Compound Spectra (overlaid)

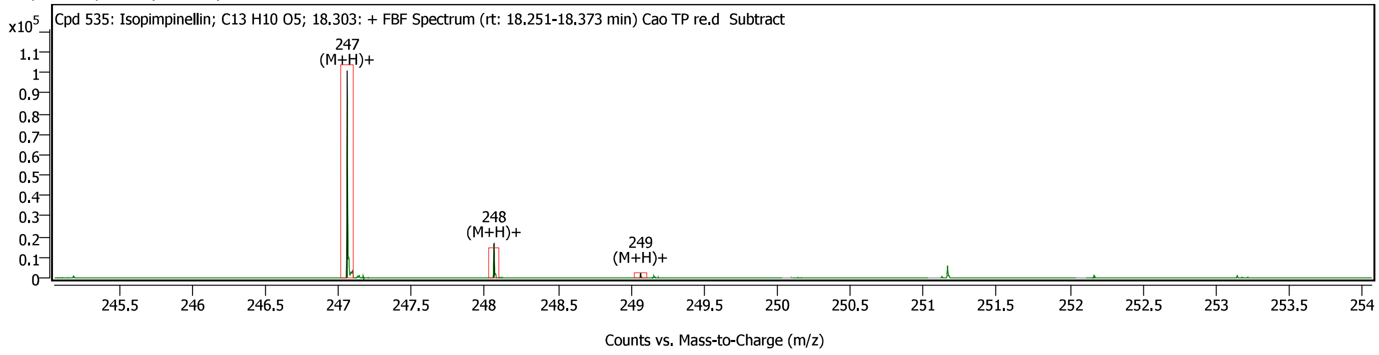

Fragment Spectrum (clean)

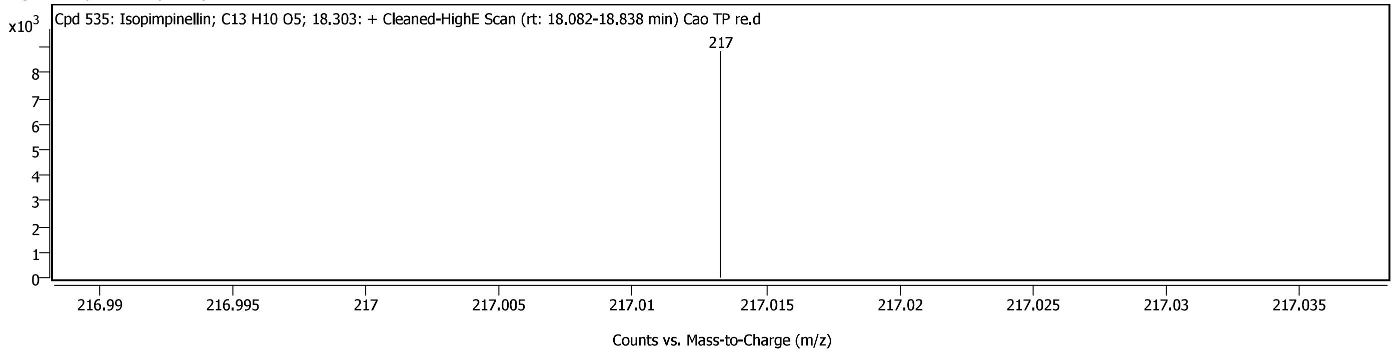

Fragment Spectrum (raw)

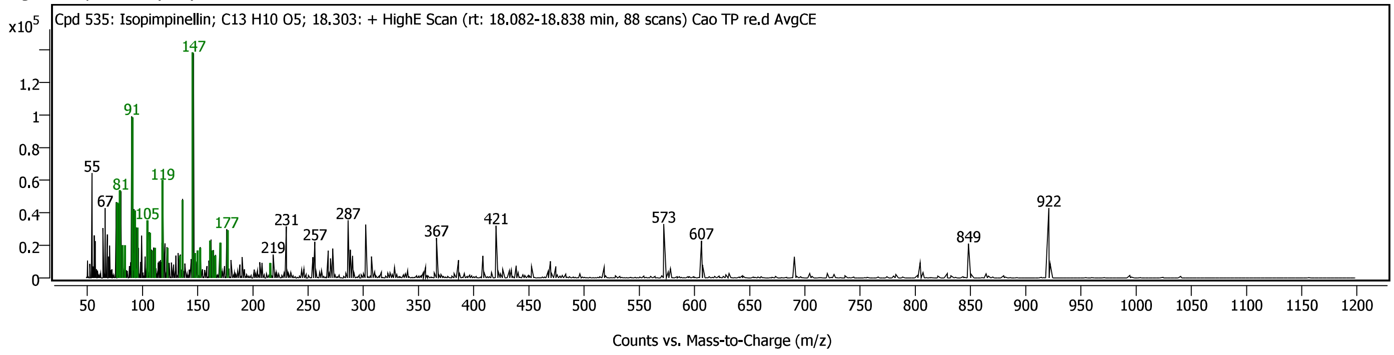

Compound ID Table

Name	Formula	Species	RT	RT Diff	Mass	CAS	ID Source	Score	Score (Lib)	Score (Tgt)
Isopimpinellin	C13 H10 O5	(M+H)+	18.303		246.0531	482-27-9	FBF-FragConfirm	97.88		97.88
Pimpinellin	C13 H10 O5	(M+H)+	18.303		246.0531	131-12-4	FBF-FragConfirm	97.88		97.88
Coriandrone C	C13 H10 O5	(M+H)+	18.303		246.0531	177795-32-3	FBF-FragConfirm	97.88		97.88
2,3',4,6-Tetrahydroxybenzophenone	C13 H10 O5	(M+H)+	18.303		246.0531		FBF-FragConfirm	97.88		97.88

Cpd 1333: 6alpha,9-Difluoro-11beta-hydroxypregn-4-ene-3,20-dione

Name	Formula	RT	RI	Mass	Diff (Tgt, ppm)	CAS	ID Source	Score	Algorithm
6alpha,9-Difluoro-11beta-hydroxypregn-4-ene-3,20-dione	C21 H28 F2 O3	18.774		366.2022	4.27		FBF	90.51	FBF

Species	m/z	Score (Tgt)	Score (Lib)	Score (DB)	Score (MFG)	Score (RT)
(M+H)+	367	90.51				

Compound Chromatograms (overlaid)

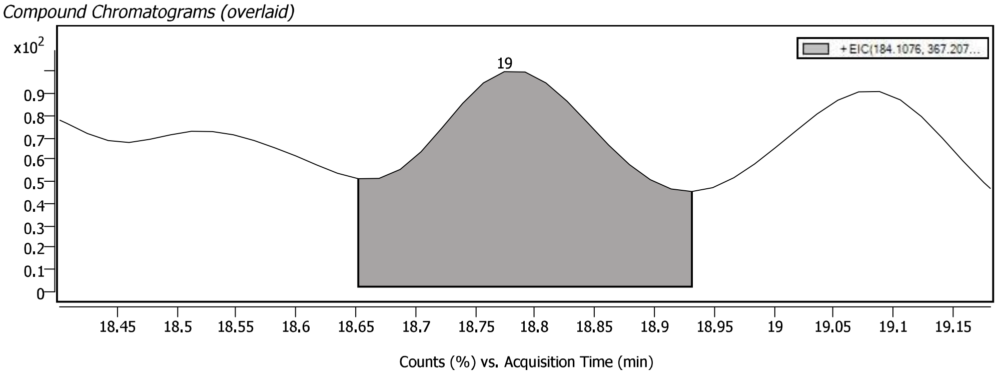

Structure

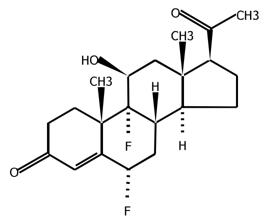

Compound Spectra (overlaid)

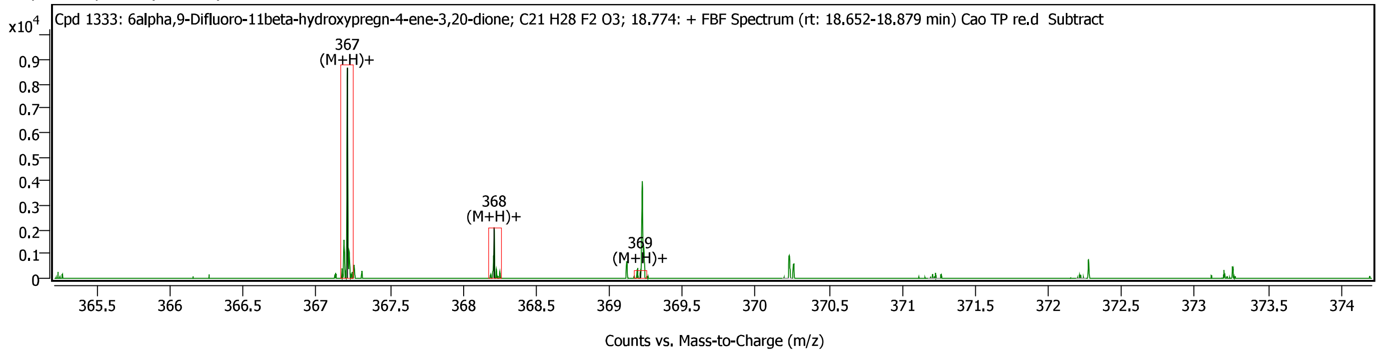

Compound ID Table

Name	Formula	Species	RT	RT Diff	Mass	CAS	ID Source	Score	Score (Lib)	Score (Tgt)
6alpha,9-Difluoro-11beta-hydroxypregn-4-ene-3,20-dione	C21 H28 F2 O3	(M+H)+	18.774		366.2022		FBF	90.51		90.51

Cpd 844: Kolaflavanone

Name	Formula	RT	RI	Mass	Diff (Tgt, ppm)	CAS	ID Source	Score	Algorithm
Kolaflavanone	C31 H24 O12	20.240		588.1266	-0.22		FBF	99.52	FBF

Species	m/z	Score (Tgt)	Score (Lib)	Score (DB)	Score (MFG)	Score (RT)
(M+H)+	589	99.52				

Compound Chromatograms (overlaid)

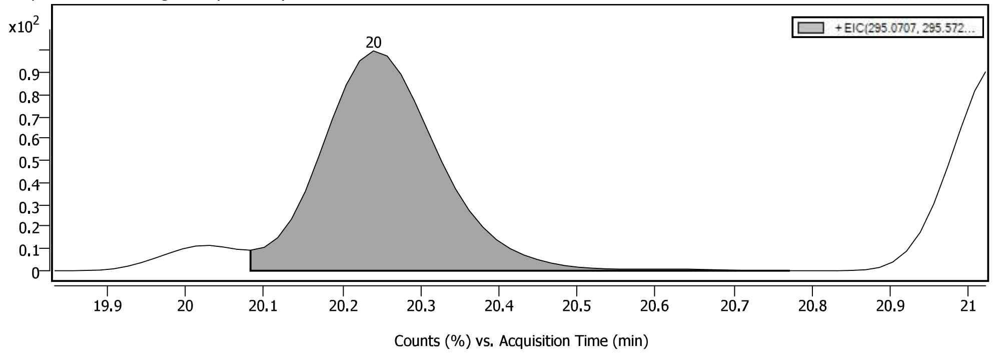

Structure

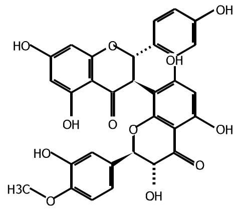

Compound Spectra (overlaid)

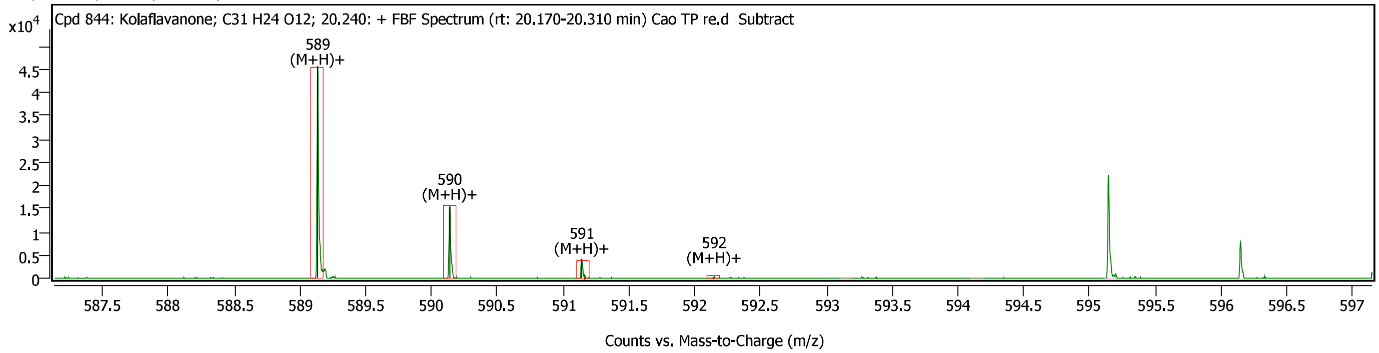

Compound ID Table

Name	Formula	Species	RT	RT Diff	Mass	CAS	ID Source	Score	Score (Lib)	Score (Tgt)
Kolaflavanone	C31 H24 O12	(M+H)+	20.240		588.1266		FBF	99.52		99.52

Cpd 1281: N1,N5,N10-Tricoumaroyl spermidine

Name	Formula	RT	RI	Mass	Diff (Tgt, ppm)	CAS	ID Source	Score	Algorithm
N1,N5,N10-Tricoumaroyl spermidine	C34 H37 N3 O6	20.711		583.2685	0.53		FBF	98.49	FBF

Species	m/z	Score (Tgt)	Score (Lib)	Score (DB)	Score (MFG)	Score (RT)
(M+H)+	584	98.49				

Compound Chromatograms (overlaid)

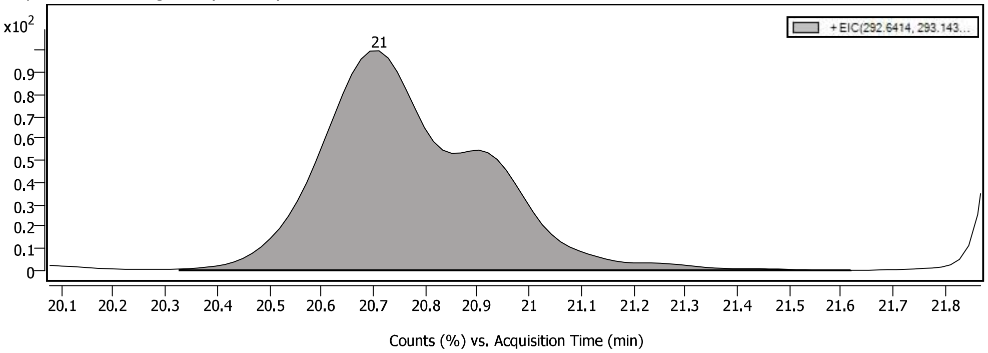

Structure

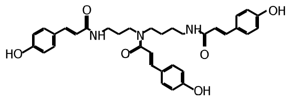

Compound Spectra (overlaid)

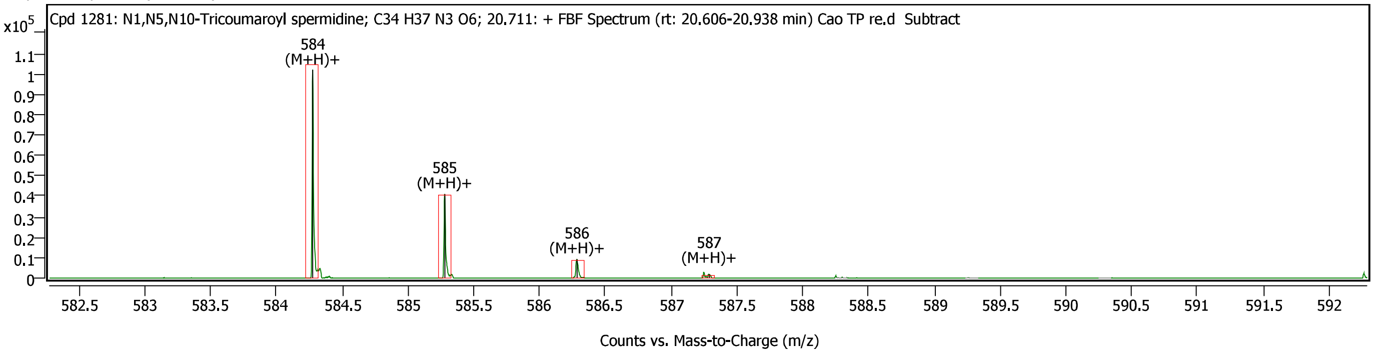

Compound ID Table

Name	Formula	Species	RT	RT Diff	Mass	CAS	ID Source	Score	Score (Lib)	Score (Tgt)
N1,N5,N10-Tricoumaroyl spermidine	C34 H37 N3 O6	(M+H)+	20.711		583.2685		FBF	98.49		98.49

Cpd 807: Luteolin 7-(6''-ferulylglucoside)

Name	Formula	RT	RI	Mass	Diff (Tgt, ppm)	CAS	ID Source	Score	Algorithm
Luteolin 7-(6''-ferulylglucoside)	C31 H28 O14	20.798		624.1485	1.00		M-FBF	99.02	FBF

Species	m/z	Score (Tgt)	Score (Lib)	Score (DB)	Score (MFG)	Score (RT)
(M+H)+	625	99.02				

Compound Chromatograms (overlaid)

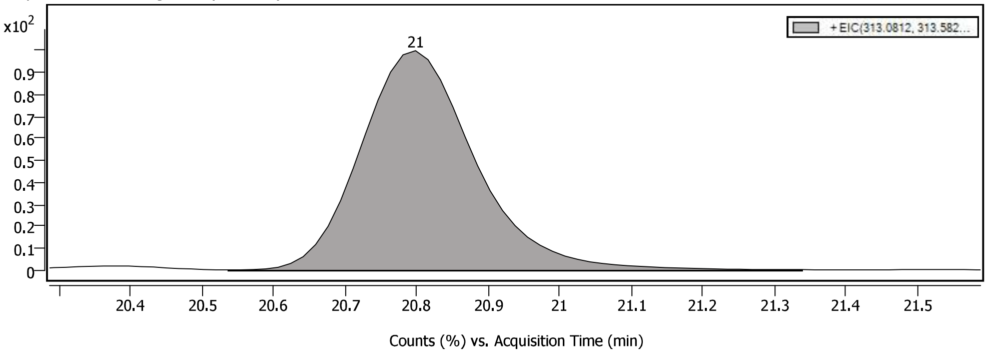

Structure

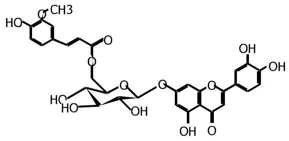

Compound Spectra (overlaid)

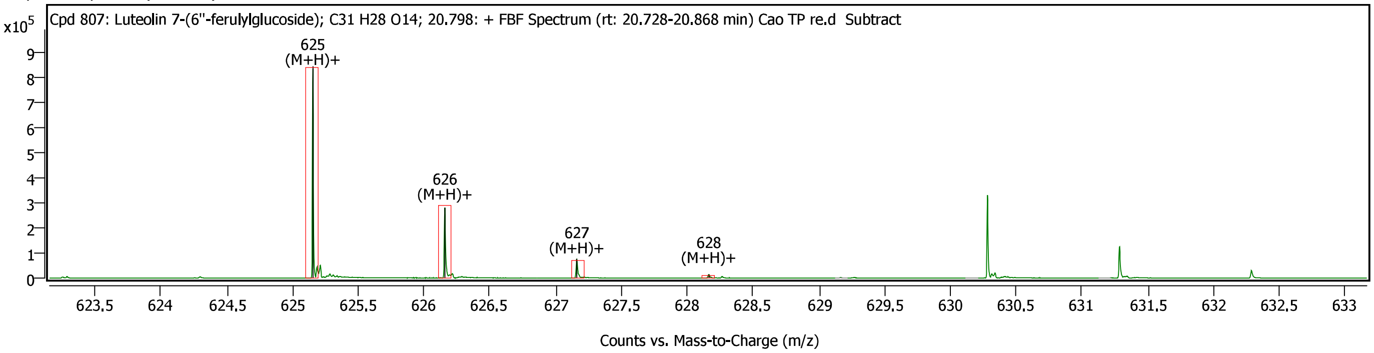

Compound Screening Report

Compound ID Table

Name	Formula	Species	RT	RT Diff	Mass	CAS	ID Source	Score	Score (Lib)	Score (Tgt)
Luteolin 7-(6''-ferulylglucoside)	C31 H28 O14	(M+H)+	20.798		624.1485		FBF	99.02		99.02
2''-O-Feruloylorientin	C31 H28 O14	(M+H)+	20.798		624.1485		FBF	99.02		99.02
4''-Methyl-6''-(3,4-dihydroxy-E-cinnamoyl)isoorientin	C31 H28 O14	(M+H)+	20.798		624.1485		FBF	99.02		99.02
Isoorientin 2''-O-(E)-ferulate	C31 H28 O14	(M+H)+	20.798		624.1485		FBF	99.02		99.02
6-Hydroxykaempferol 6-methyl ether 3-(6''-p-coumaroylglucoside)	C31 H28 O14	(M+H)+	20.798		624.1485		FBF	99.02		99.02
Isorhamnetin 3-(6''-p-coumarylglucoside)	C31 H28 O14	(M+H)+	20.798		624.1485		FBF	99.02		99.02
Isorhamnetin 7-(6''-p-coumarylglucoside)	C31 H28 O14	(M+H)+	20.798		624.1485		FBF	99.02		99.02
Kaempferol 3-(6''-ferulylglucoside)	C31 H28 O14	(M+H)+	20.798		624.1485		FBF	99.02		99.02

Cpd 1682: Allamandin

Name	Formula	RT	RI	Mass	Diff (Tgt, ppm)	CAS	ID Source	Score	Algorithm
Allamandin	C15 H16 O7	20.798		308.0900	1.29	51820-82-7	M-FBF	98.47	FBF

Species	m/z	Score (Tgt)	Score (Lib)	Score (DB)	Score (MFG)	Score (RT)
(M+H)+	309	98.47				

Compound Chromatograms (overlaid)

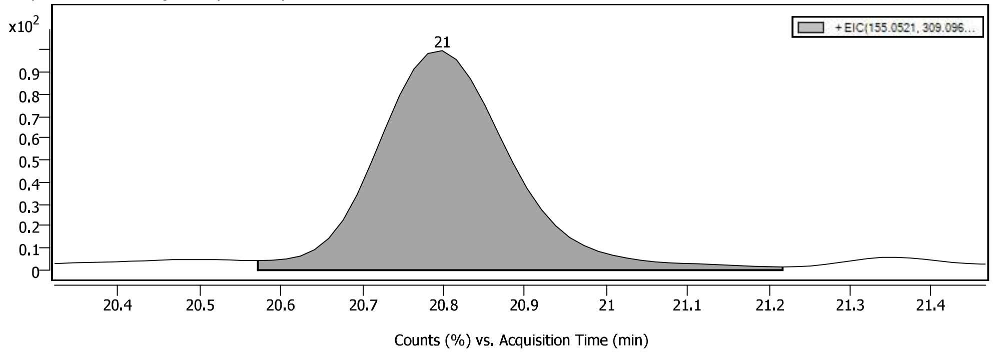

Structure

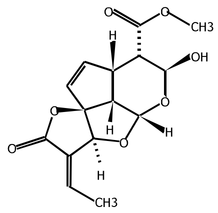

Compound Spectra (overlaid)

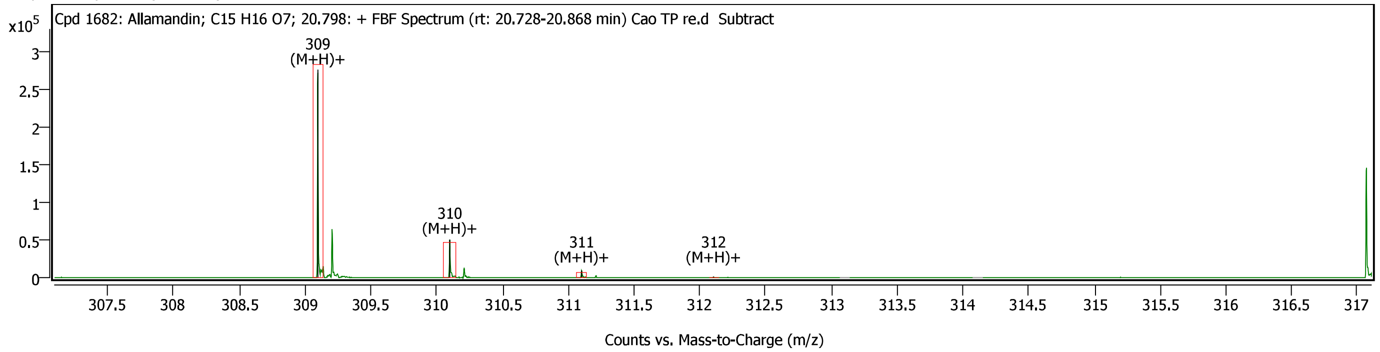

Compound ID Table

Name	Formula	Species	RT	RT Diff	Mass	CAS	ID Source	Score	Score (Lib)	Score (Tgt)
Allamandin	C15 H16 O7	(M+H)+	20.798		308.0900	51820-82-7	FBF	98.47		98.47
4R,5R,6S-Trihydroxy-2-hydroxymethyl-2-cyclohexen-1-one 6-(2-hydroxy-6-methylbenzoate)	C15 H16 O7	(M+H)+	20.798		308.0900	146475-71-0	FBF	98.47		98.47

Cpd 515: Loxtidine

Name	Formula	RT	RI	Mass	Diff (Tgt, ppm)	CAS	ID Source	Score	Algorithm
Loxtidine	C19 H29 N5 O2	21.042		359.2312	-2.64	76956-02-0	FBF	96.51	FBF

Species	m/z	Score (Tgt)	Score (Lib)	Score (DB)	Score (MFG)	Score (RT)
(M+H)+	360	96.51				

Compound Chromatograms (overlaid)

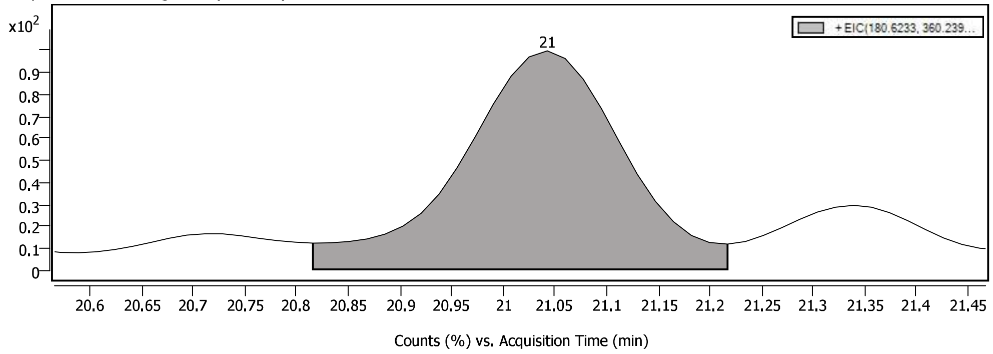

Structure

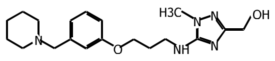

Compound Spectra (overlaid)

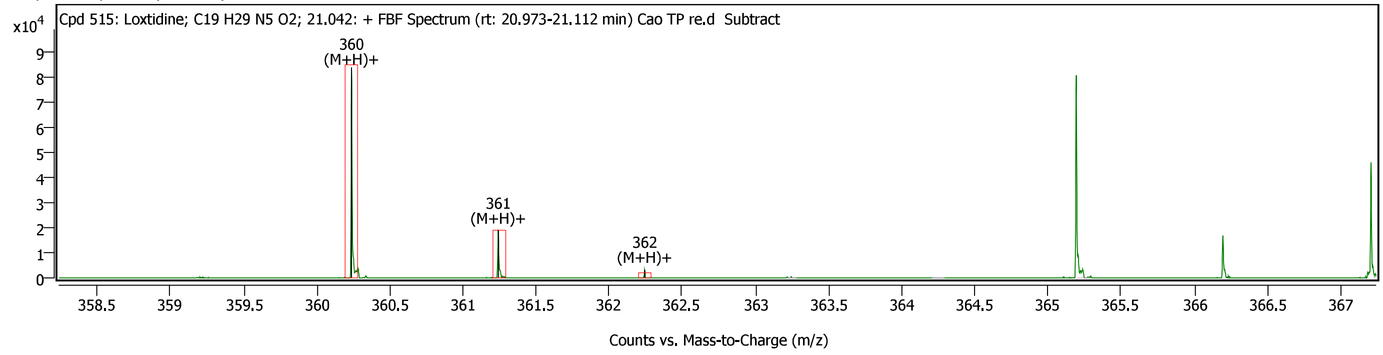

Compound ID Table

Name	Formula	Species	RT	RT Diff	Mass	CAS	ID Source	Score	Score (Lib)	Score (Tgt)
Loxtidine	C19 H29 N5 O2	(M+H)+	21.042		359.2312	76956-02-0	FBF	96.51		96.51

Cpd 843: Kolaflavanone

Name	Formula	RT	RI	Mass	Diff (Tgt, ppm)	CAS	ID Source	Score	Algorithm
Kolaflavanone	C31 H24 O12	21.042		588.1269	0.20		FBF	99.38	FBF

Species	m/z	Score (Tgt)	Score (Lib)	Score (DB)	Score (MFG)	Score (RT)
(M+H)+	589	99.38				

Compound Chromatograms (overlaid)

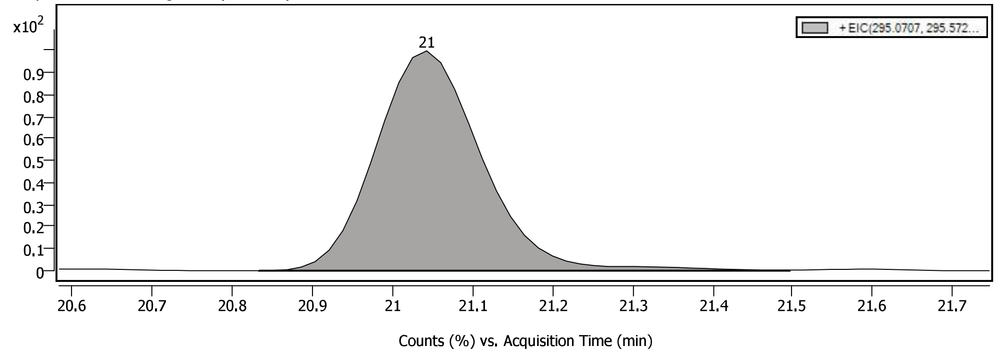

Structure

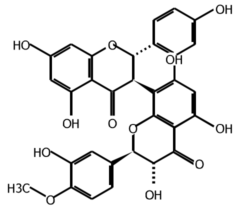

Compound Spectra (overlaid)

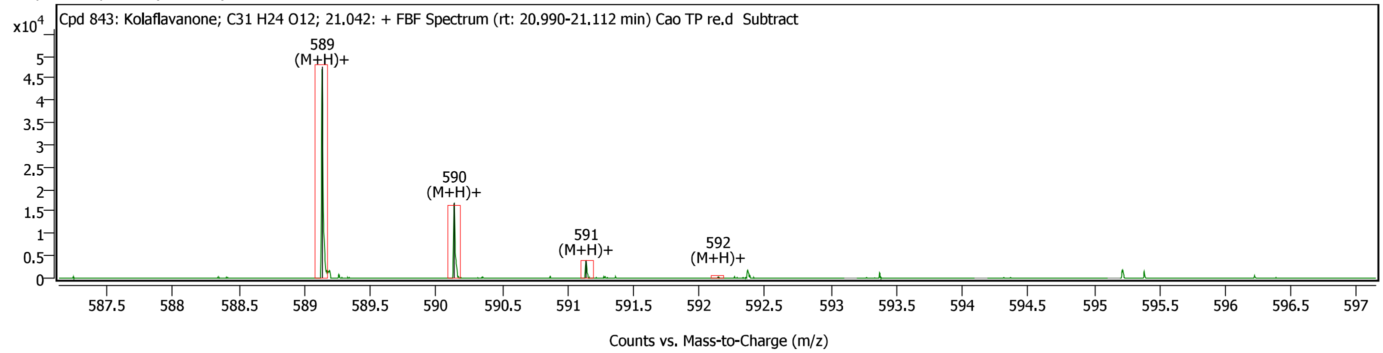

Compound ID Table

Name	Formula	Species	RT	RT Diff	Mass	CAS	ID Source	Score	Score (Lib)	Score (Tgt)
Kolaflavanone	C31 H24 O12	(M+H)+	21.042		588.1269		FBF	99.38		99.38

Cpd 1332: 6alpha,9-Difluoro-11beta-hydroxypregn-4-ene-3,20-dione

Name	Formula	RT	RI	Mass	Diff (Tgt, ppm)	CAS	ID Source	Score	Algorithm
6alpha,9-Difluoro-11beta-hydroxypregn-4-ene-3,20-dione	C21 H28 F2 O3	21.042		366.2020	3.71		FBF	90.43	FBF

Species	m/z	Score (Tgt)	Score (Lib)	Score (DB)	Score (MFG)	Score (RT)
(M+H)+	367	90.43				

Compound Chromatograms (overlaid)

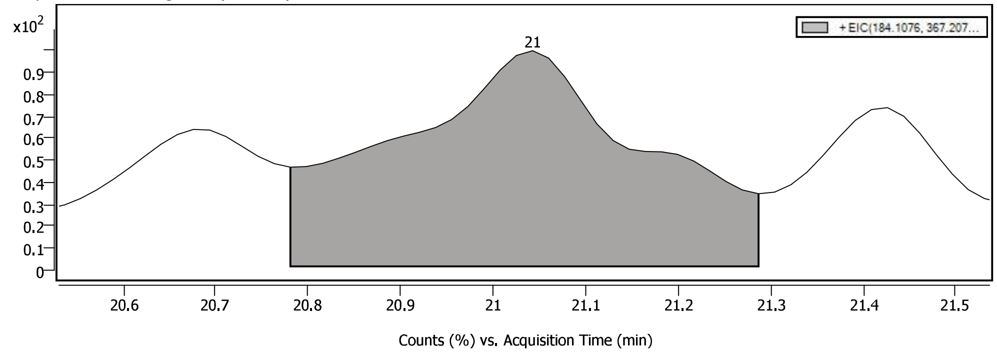

Structure

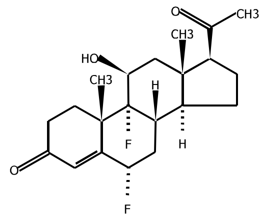

Compound Spectra (overlaid)

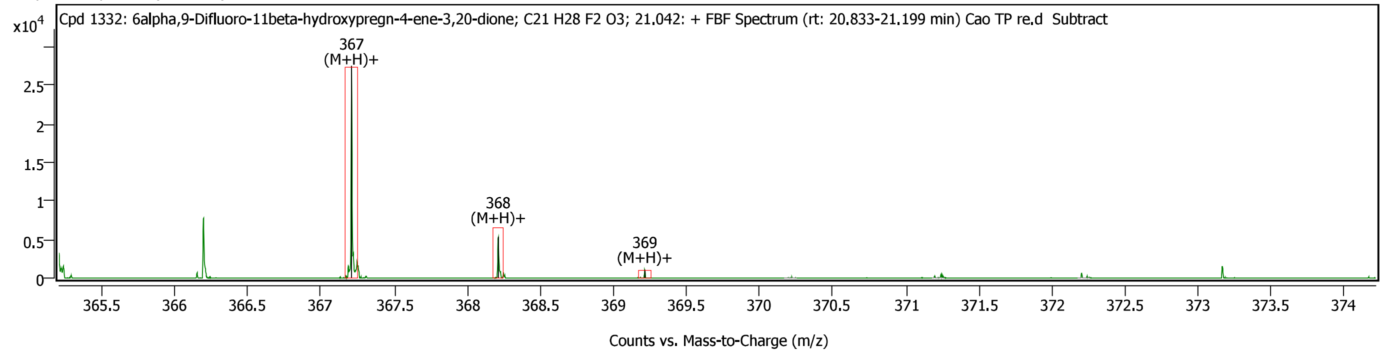

Compound ID Table

Name	Formula	Species	RT	RT Diff	Mass	CAS	ID Source	Score	Score (Lib)	Score (Tgt)
6alpha,9-Difluoro-11beta-hydroxypregn-4-ene-3,20-dione	C21 H28 F2 O3	(M+H)+	21.042		366.2020		FBF	90.43		90.43

Cpd 198: Dihydromorelloflavone

Name	Formula	RT	RI	Mass	Diff (Tgt, ppm)	CAS	ID Source	Score	Algorithm
Dihydromorelloflavone	C30 H22 O11	21.182		558.1162	-0.06		M-FBF	99.29	FBF

Species	m/z	Score (Tgt)	Score (Lib)	Score (DB)	Score (MFG)	Score (RT)
(M+H)+	559	99.29				

Compound Chromatograms (overlaid)

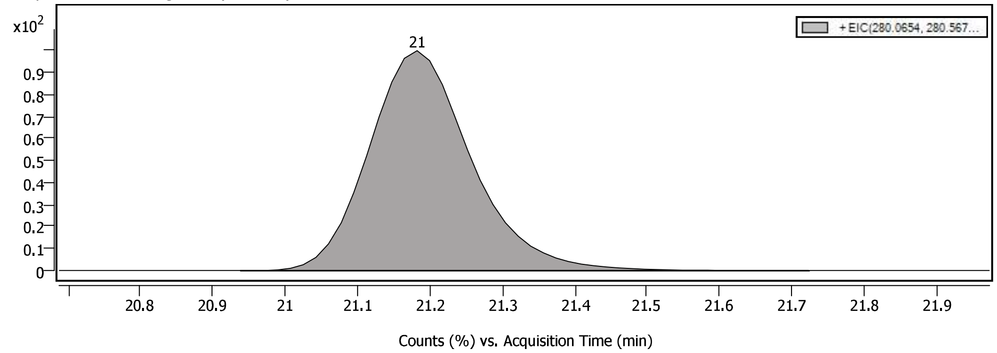

Structure

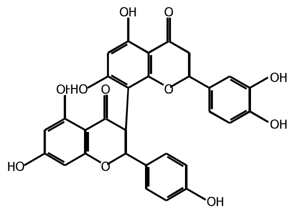

Compound Spectra (overlaid)

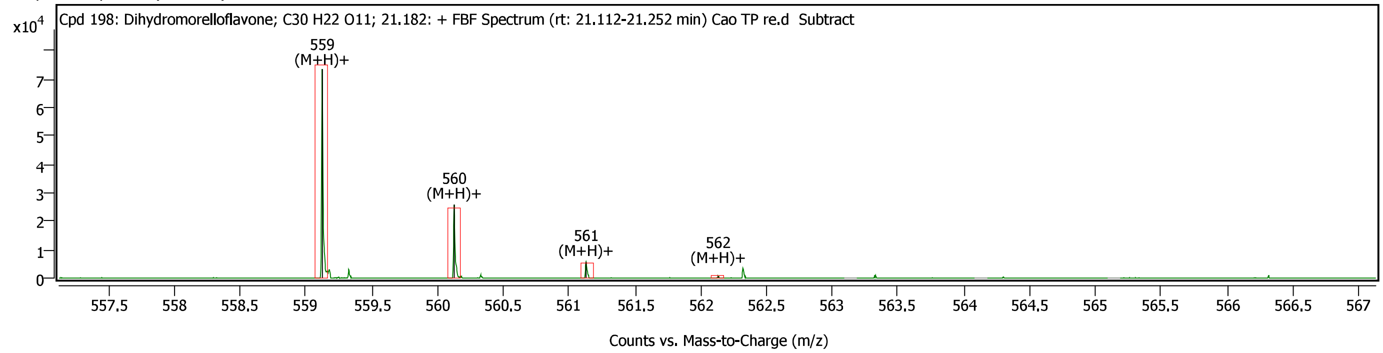

Compound Screening Report

Compound ID Table

Name	Formula	Species	RT	RT Diff	Mass	CAS	ID Source	Score	Score (Lib)	Score (Tgt)
Dihydromorelloflavone	C30 H22 O11	(M+H)+	21.182		558.1162		FBF	99.29		99.29
Deoxyrubroskyrin	C30 H22 O11	(M+H)+	21.182		558.1162	52680-04-3	FBF	99.29		99.29

Cpd 590: N1,N5,N10-Triferuloyl spermidine

Name	Formula	RT	RI	Mass	Diff (Tgt, ppm)	CAS	ID Source	Score	Algorithm
N1,N5,N10-Triferuloyl spermidine	C37 H43 N3 O9	21.484		673.3000	0.09		FBF	99.15	FBF

Species	m/z	Score (Tgt)	Score (Lib)	Score (DB)	Score (MFG)	Score (RT)
(M+H)+	674	99.15				

Compound Chromatograms (overlaid)

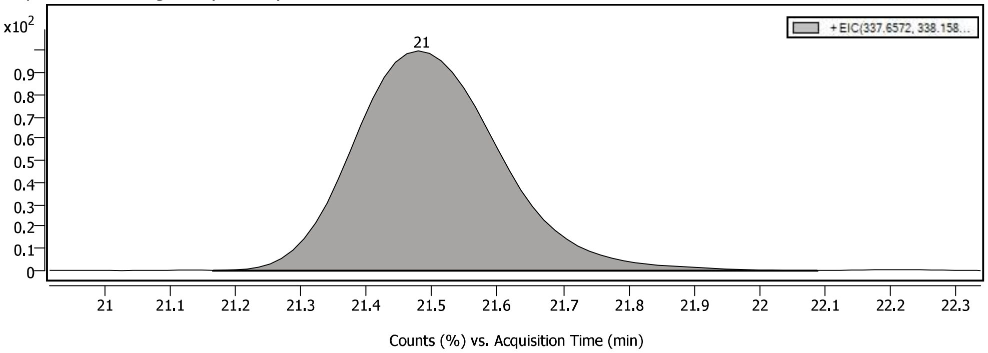

Structure

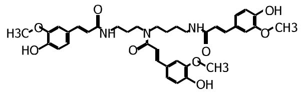

Compound Spectra (overlaid)

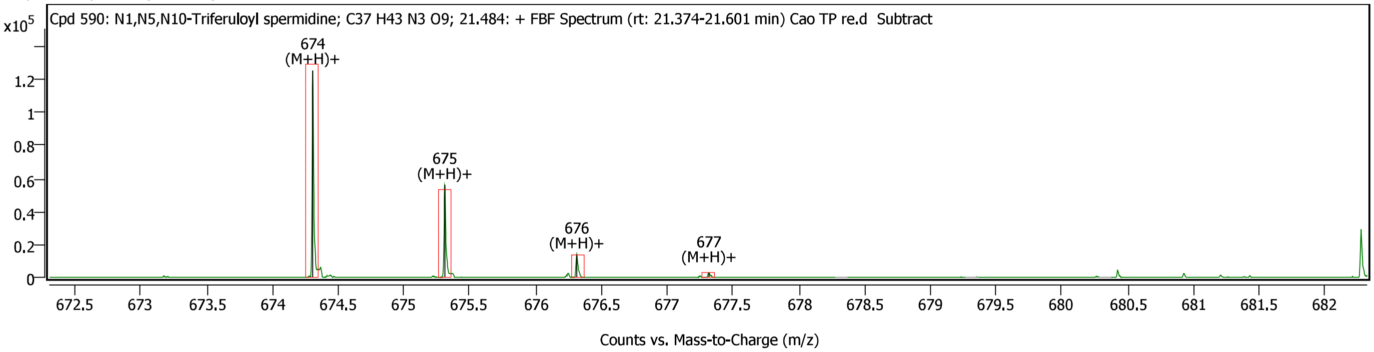

Compound ID Table

Name	Formula	Species	RT	RT Diff	Mass	CAS	ID Source	Score	Score (Lib)	Score (Tgt)
N1,N5,N10-Triferuloyl spermidine	C37 H43 N3 O9	(M+H)+	21.484		673.3000		FBF	99.15		99.15

Cpd 911: <KOdiA-PC>

Name	Formula	RT	RI	Mass	Diff (Tgt, ppm)	CAS	ID Source	Score	Algorithm
<KOdiA-PC>	C32 H59 N O11 P	21.828		664.3821	-0.66	439904-33-3	FBF	97.25	FBF

Species	m/z	Score (Tgt)	Score (Lib)	Score (DB)	Score (MFG)	Score (RT)
(M+H)+	665	97.25				

Compound Chromatograms (overlaid)

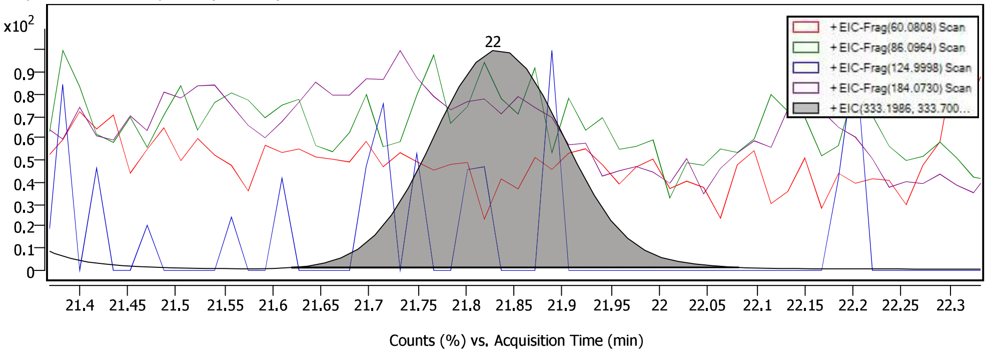

Structure

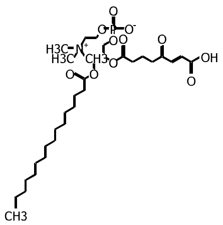

Coelution Plot

Compound Screening Report

Compound Spectra (overlaid)

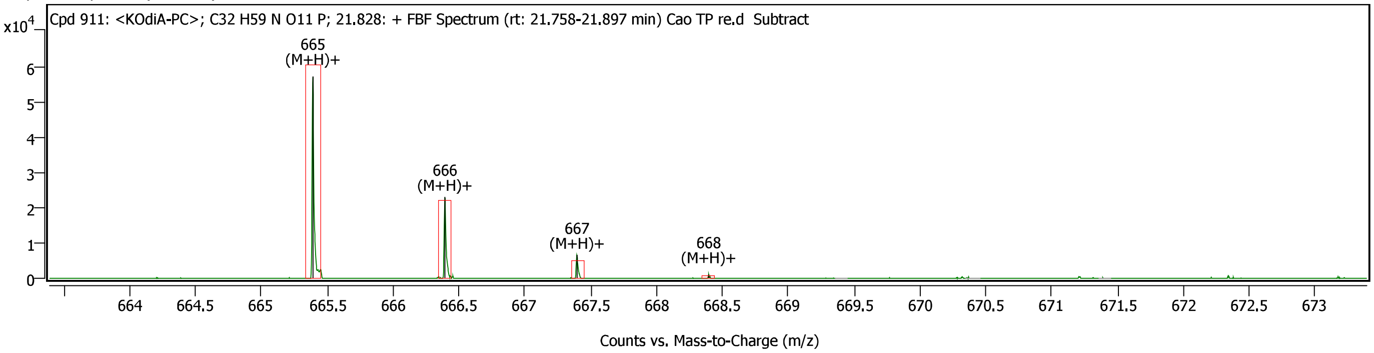

Fragment Spectrum (raw)

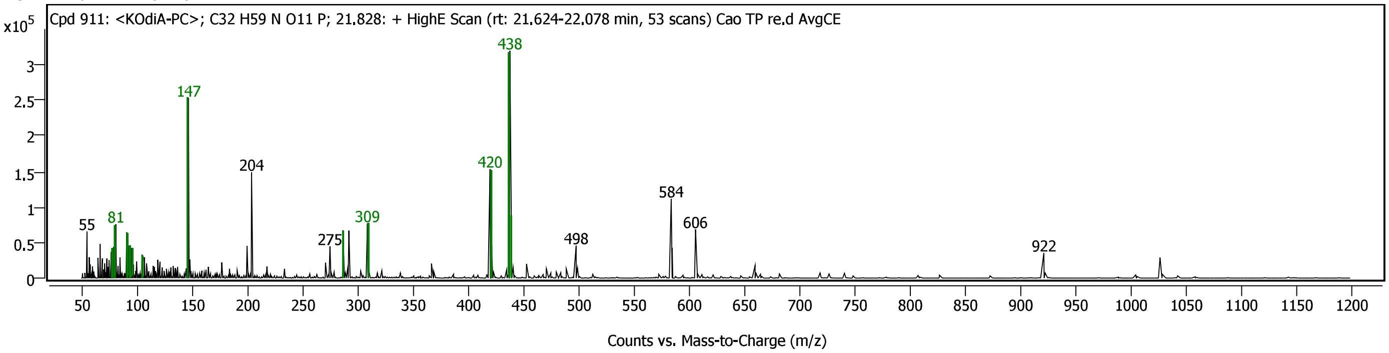

Compound ID Table

Name	Formula	Species	RT	RT Diff	Mass	CAS	ID Source	Score	Score (Lib)	Score (Tgt)
<KOdiA-PC>	C32 H59 N O11 P	(M+H)+	21.828		664.3821	439904-33-3	FBF	97.25		97.25

Cpd 1012: Caloxanthin sulfate

Name	Formula	RT	RI	Mass	Diff (Tgt, ppm)	CAS	ID Source	Score	Algorithm
Caloxanthin sulfate	C40 H56 O6 S	21.828		664.3824	3.96		FBF	88.69	FBF

Species	m/z	Score (Tgt)	Score (Lib)	Score (DB)	Score (MFG)	Score (RT)
(M+H)+	665	88.69				

Compound Chromatograms (overlaid)

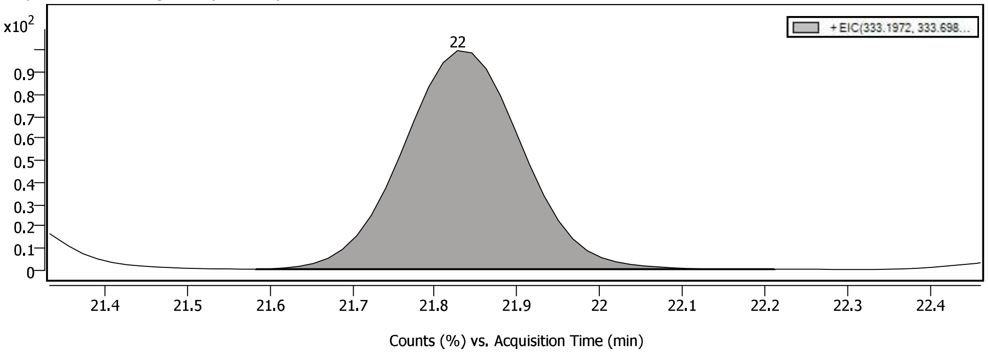

Structure

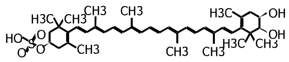

Compound Spectra (overlaid)

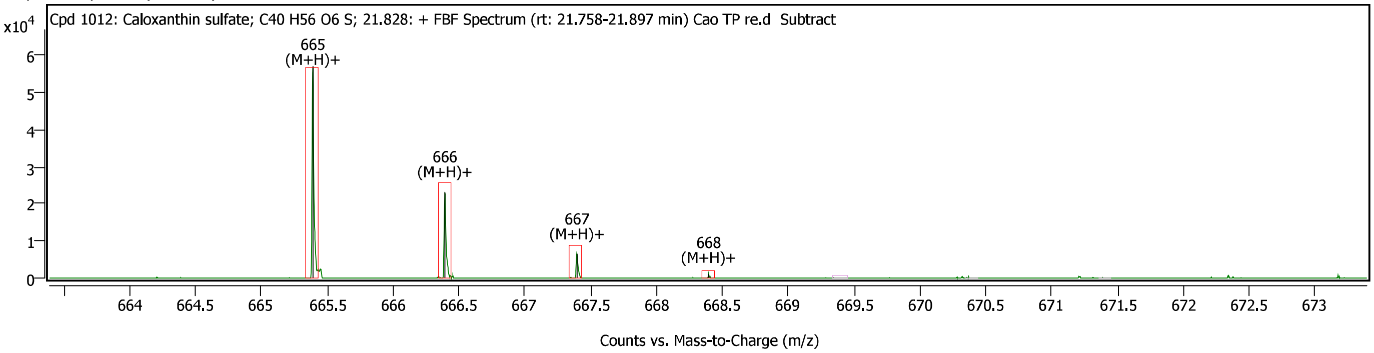

Compound ID Table

Name	Formula	Species	RT	RT Diff	Mass	CAS	ID Source	Score	Score (Lib)	Score (Tgt)
Caloxanthin sulfate	C40 H56 O6 S	(M+H)+	21.828		664.3824		FBF	88.69		88.69

Cpd 1623: <18α-Glycyrrhetinic acid>

Compound Screening Report

Name	Formula	RT	RI	Mass	Diff (Tgt, ppm)	CAS	ID Source	Score	Algorithm
<18α-Glycyrrhetinic acid>	C30 H46 O4	21.828		470.3395	-0.14	1449-05-4	M-FBF	98.89	FBF

Species	m/z	Score (Tgt)	Score (Lib)	Score (DB)	Score (MFG)	Score (RT)
(M+H)+	471	98.89				

Compound Chromatograms (overlaid)

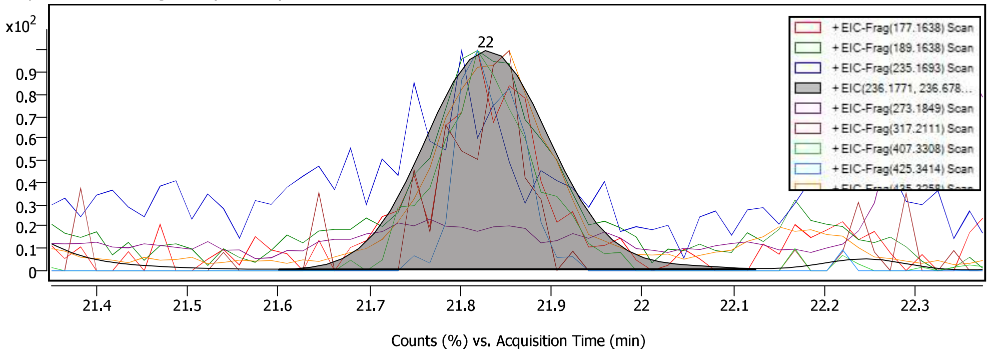

Structure

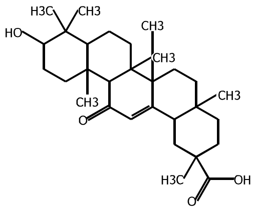

Coelution Plot

Compound Spectra (overlaid)

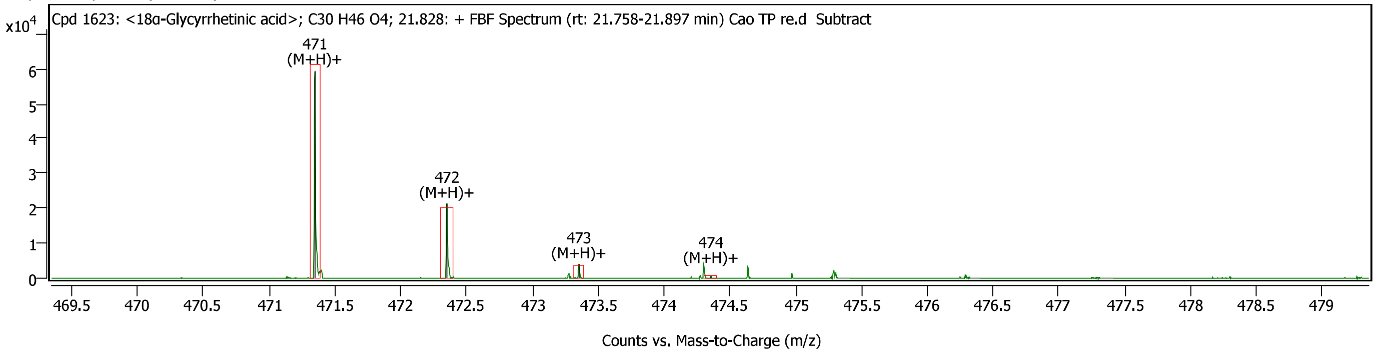

Fragment Spectrum (raw)

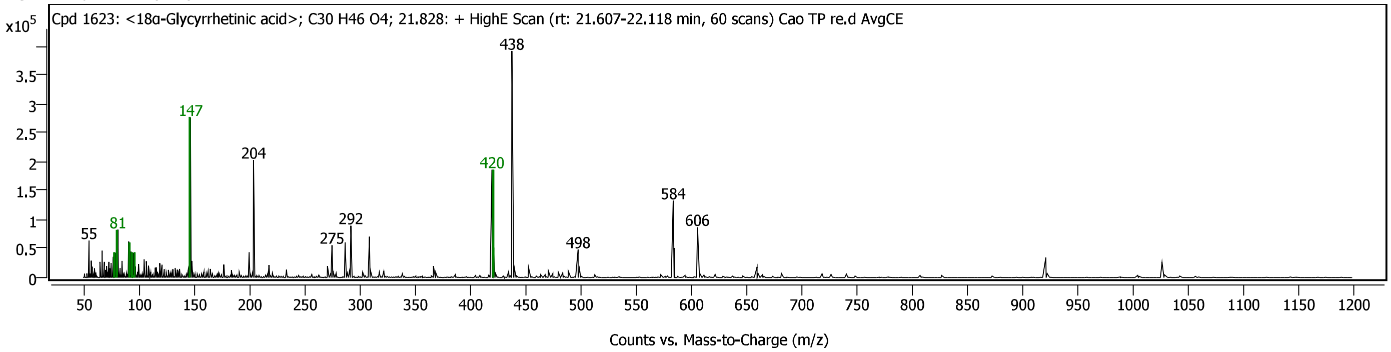

Compound ID Table

Name	Formula	Species	RT	RT Diff	Mass	CAS	ID Source	Score	Score (Lib)	Score (Tgt)
<18α-Glycyrrhetinic acid>	C30 H46 O4	(M+H)+	21.828		470.3395	1449-05-4	FBF	98.89		98.89
<11-Keto-beta-boswellic acid>	C30 H46 O4	(M+H)+	21.828		470.3395	17019-92-0	FBF	98.89		98.89
<3alpha-hydroxyglycyrrhetinic acid>	C30 H46 O4	(M+H)+	21.828		470.3395		FBF	98.89		98.89
<Ganoderiol B>	C30 H46 O4	(M+H)+	21.828		470.3395	106518-62-1	FBF	98.89		98.89
<3beta-3,24-Dihydroxy-9(11),12-oleanadien-30-oic acid>	C30 H46 O4	(M+H)+	21.828		470.3395	56262-33-0	FBF	98.89		98.89
<28-Hydroxymangiferonic acid>	C30 H46 O4	(M+H)+	21.828		470.3395	155511-27-6	FBF	98.89		98.89
<23-Hydroxy-3-oxocycloart-24-en-26-oic acid>	C30 H46 O4	(M+H)+	21.828		470.3395		FBF	98.89		98.89
<1α-hydroxy-18-(4-hydroxy-4-ethyl-2-hexynyloxy)-23,24,25,26,27-pentanorvitamin D3 / 1α-hydroxy-18-(4-hydroxy-4-ethyl-2-hexynyloxy)-23,24,25,26,27-pentanorcholecalciferol>	C30 H46 O4	(M+H)+	21.828		470.3395		FBF	98.89		98.89
<16-Hydroxy-3-oxo-12-oleanen-28-oic acid>	C30 H46 O4	(M+H)+	21.828		470.3395	77625-71-9	FBF	98.89		98.89
<(22R)-1α,22,25-trihydroxy-26,27-dimethyl-23,24-tetradehydro-24a-homo-20-epivitamin D3 / (22R)-1α,22,25-trihydroxy-26,27-dimethyl-23,24-tetradehydro-24a-homo-20-epicholecalciferol>	C30 H46 O4	(M+H)+	21.828		470.3395		FBF	98.89		98.89
<(E,6S)-7-Hydroxy-2-methyl-6-[(10S,13S,14S,17S)-4,4,10,13,14-pentamethyl-3-oxo-1,2,5,6,7,11,12,15,16,17-decahydrocyclopenta[a]phenanthren-17-yl]hept-2-enoic acid>	C30 H46 O4	(M+H)+	21.828		470.3395	1273558-72-7	FBF	98.89		98.89
<(3alpha,20R,24Z)-3-Hydroxy-21-oxoeupha-8,24-dien-26-oic acid>	C30 H46 O4	(M+H)+	21.828		470.3395	195204-05-8	FBF	98.89		98.89
<(22S)-1α,25-dihydroxy-22-methoxy-26,27-dimethyl-23,24-tetradehydro-20-epivitamin D3 / (22S)-1α,25-dihydroxy-22-methoxy-26,27-dimethyl-23,24-tetradehydro-20-epicholecalciferol>	C30 H46 O4	(M+H)+	21.828		470.3395		FBF	98.89		98.89
<(22S)-1α,22,25-trihydroxy-26,27-dimethyl-23,24-tetradehydro-24a-homo-20-epivitamin D3 / (22S)-1α,22,25-trihydroxy-26,27-dimethyl-23,24-tetradehydro-24a-homo-20-epicholecalciferol>	C30 H46 O4	(M+H)+	21.828		470.3395		FBF	98.89		98.89
<(22S)-1α,22,25-trihydroxy-26,27-dimethyl-23,23,24,24-tetradehydro-24a-homovitamin D3 / (22S)-1α,22,25-trihydroxy-26,27-dimethyl-23,23,24,24-tetradehydro-24a-homocholecalciferol>	C30 H46 O4	(M+H)+	21.828		470.3395		FBF	98.89		98.89
<(22R)-1α,25-dihydroxy-22-methoxy-26,27-dimethyl-23,23,24,24-tetradehydrovitamin D3 / (22R)-1α,25-dihydroxy-22-methoxy-26,27-dimethyl-23,23,24,24-tetradehydrocholecalciferol>	C30 H46 O4	(M+H)+	21.828		470.3395		FBF	98.89		98.89
<2-Hydroxy-3-oxo-12-oleanen-28-oic acid>	C30 H46 O4	(M+H)+	21.828		470.3395	73584-62-0	FBF	98.89		98.89
<Ganodermic acid Jb>	C30 H46 O4	(M+H)+	21.828		470.3395	112430-68-9	FBF	98.89		98.89
<6beta-Hydroxy-3-oxo-12-oleanen-28-oic acid>	C30 H46 O4	(M+H)+	21.828		470.3395	32337-18-1	FBF	98.89		98.89
<Liquiritic acid>	C30 H46 O4	(M+H)+	21.828		470.3395	10379-72-3	FBF	98.89		98.89
<Colubrinic acid>	C30 H46 O4	(M+H)+	21.828		470.3395	67594-73-4	FBF	98.89		98.89
<Secoisobryononic acid>	C30 H46 O4	(M+H)+	21.828		470.3395	162898-23-9	FBF	98.89		98.89
<Rubinic acid>	C30 H46 O4	(M+H)+	21.828		470.3395	94662-96-1	FBF	98.89		98.89
<Pomonic acid>	C30 H46 O4	(M+H)+	21.828		470.3395	13849-90-6	FBF	98.89		98.89
<Nebrosteroid L>	C30 H46 O4	(M+H)+	21.828		470.3395		FBF	98.89		98.89
<Murrayenol>	C30 H46 O4	(M+H)+	21.828		470.3395		FBF	98.89		98.89
<Secobryononic acid>	C30 H46 O4	(M+H)+	21.828		470.3395	162898-22-8	FBF	98.89		98.89
<Lansic acid>	C30 H46 O4	(M+H)+	21.828		470.3395	19954-99-5	FBF	98.89		98.89
<gypsogenin>	C30 H46 O4	(M+H)+	21.828		470.3395		FBF	98.89		98.89
<Glycyrrhetinic acid>	C30 H46 O4	(M+H)+	21.828		470.3395		FBF	98.89		98.89
<Glycyrrhetic acid>	C30 H46 O4	(M+H)+	21.828		470.3395	471-53-4	FBF	98.89		98.89
<Koetjapic acid>	C30 H46 O4	(M+H)+	21.828		470.3395	142905-27-9	FBF	98.89		98.89

Cpd 35: Cer(t18:0/18:0)

Name	Formula	RT	RI	Mass	Diff (Tgt, ppm)	CAS	ID Source	Score	Algorithm
Cer(t18:0/18:0)	C36 H73 N O4	22.089		583.5561	3.64		M-FBF	84.13	FBF

Species	m/z	Score (Tgt)	Score (Lib)	Score (DB)	Score (MFG)	Score (RT)
(M+H)+	585	84.13				

Compound Chromatograms (overlaid)

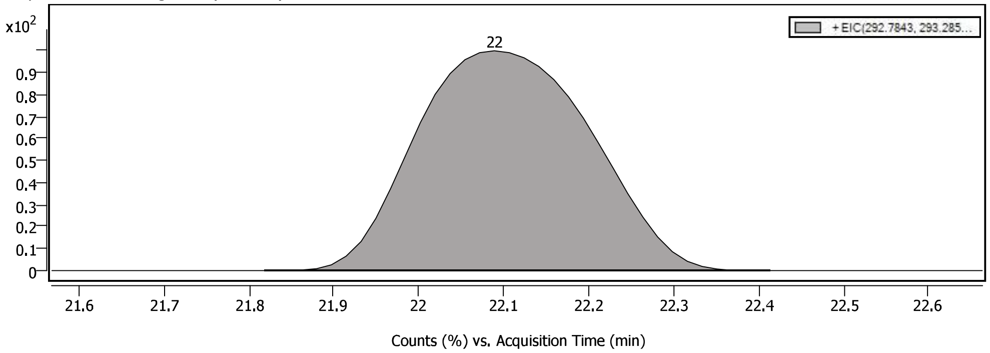

Structure

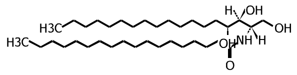

Compound Spectra (overlaid)

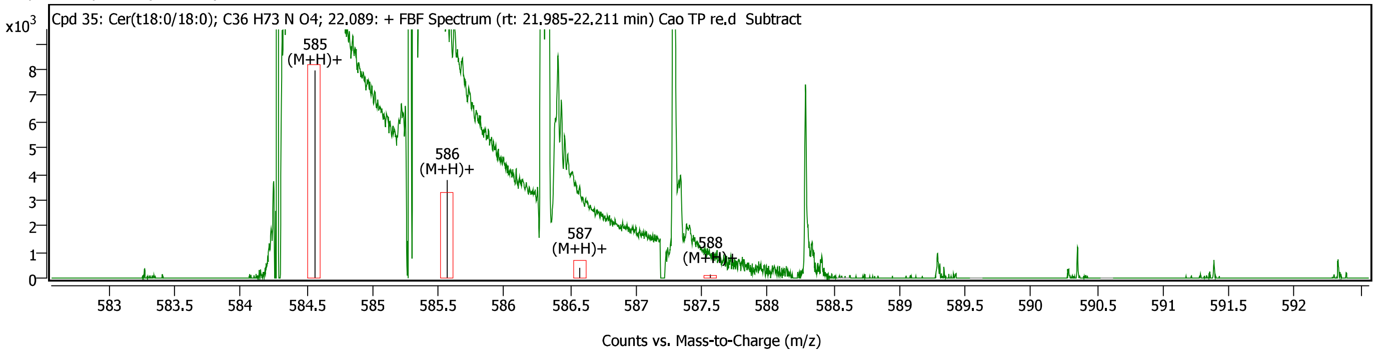

Compound ID Table

Name	Formula	Species	RT	RT Diff	Mass	CAS	ID Source	Score	Score (Lib)	Score (Tgt)
Cer(t18:0/18:0)	C36 H73 N O4	(M+H)+	22.089		583.5561		FBF	84.13		84.13
Cer(t20:0/16:0)	C36 H73 N O4	(M+H)+	22.089		583.5561		FBF	84.13		84.13
Cer(d20:0/16:0(2OH))	C36 H73 N O4	(M+H)+	22.089		583.5561		FBF	84.13		84.13
Cer(d18:0/18:0(2OH))	C36 H73 N O4	(M+H)+	22.089		583.5561		FBF	84.13		84.13

Cpd 1280: N1,N5,N10-Tricoumaroyl spermidine

Name	Formula	RT	RI	Mass	Diff (Tgt, ppm)	CAS	ID Source	Score	Algorithm
N1,N5,N10-Tricoumaroyl spermidine	C34 H37 N3 O6	22.089		583.2699	2.89		FBF	95.49	FBF

Species	m/z	Score (Tgt)	Score (Lib)	Score (DB)	Score (MFG)	Score (RT)
(M+H)+	584	95.49				

Compound Chromatograms (overlaid)

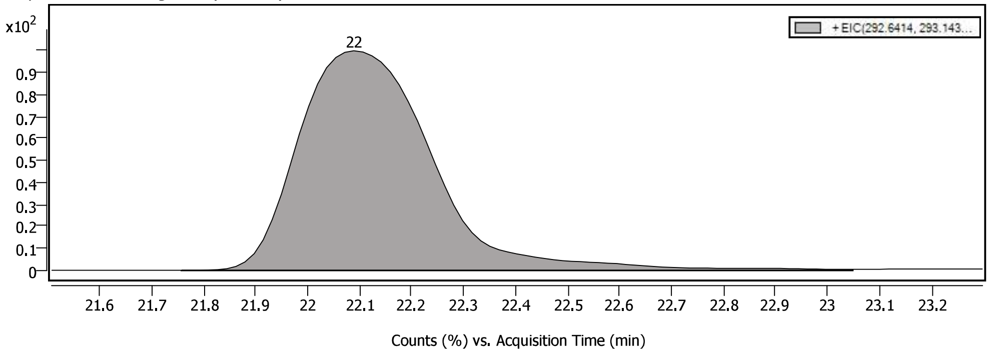

Structure

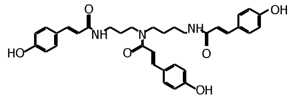

Compound Spectra (overlaid)

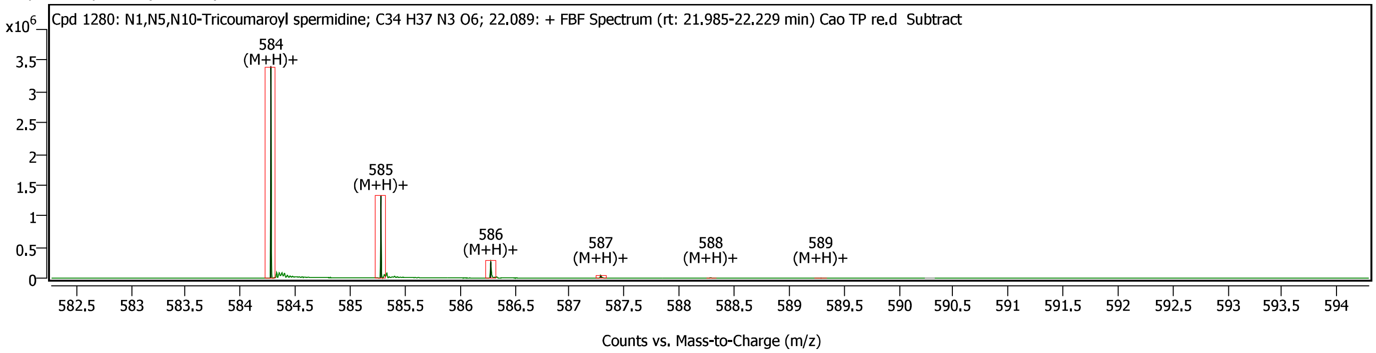

Compound Screening Report

Compound ID Table

Name	Formula	Species	RT	RT Diff	Mass	CAS	ID Source	Score	Score (Lib)	Score (Tgt)
N1,N5,N10-Tricoumaroyl spermidine	C34 H37 N3 O6	(M+H)+	22.089		583.2699		FBF	95.49		95.49

Cpd 730: Helichrysin A

Name	Formula	RT	RI	Mass	Diff (Tgt, ppm)	CAS	ID Source	Score	Algorithm
Helichrysin A	C21 H22 O10	22.334		434.1215	0.51	529-41-9	FBF-FragConfirm	99.38	FBF

Species	m/z	Score (Tgt)	Score (Lib)	Score (DB)	Score (MFG)	Score (RT)
(M+H)+	435	99.38				

Compound Chromatograms (overlaid)

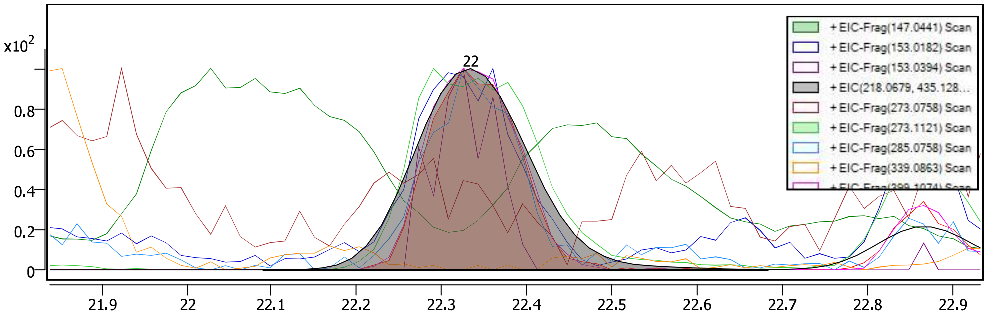

Structure

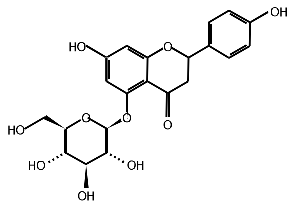

Coelution Plot

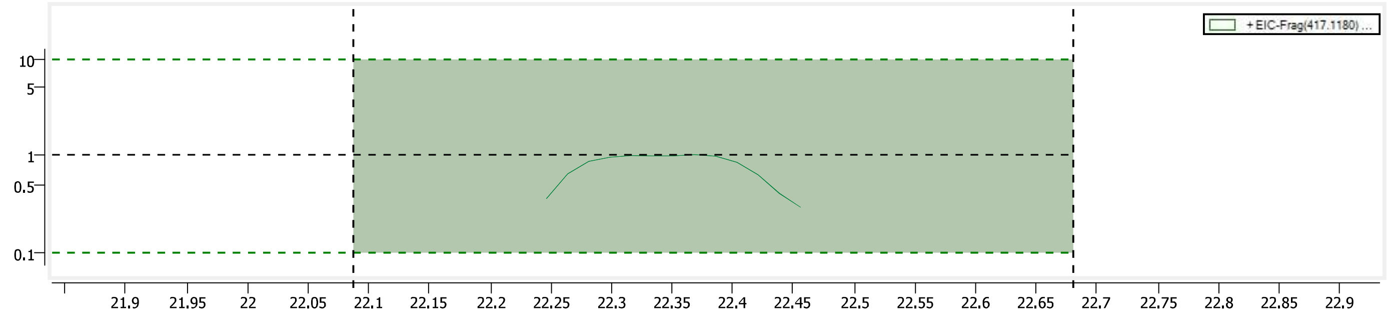

Compound Spectra (overlaid)

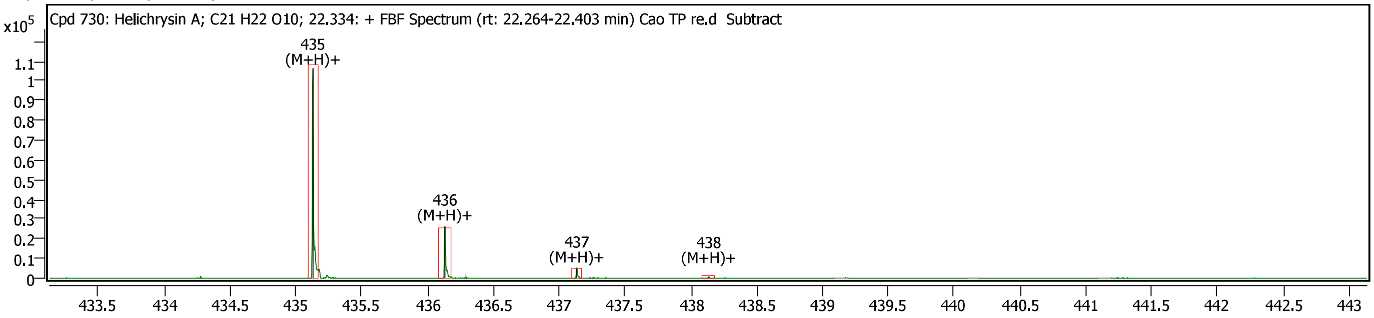

Fragment Spectrum (clean)

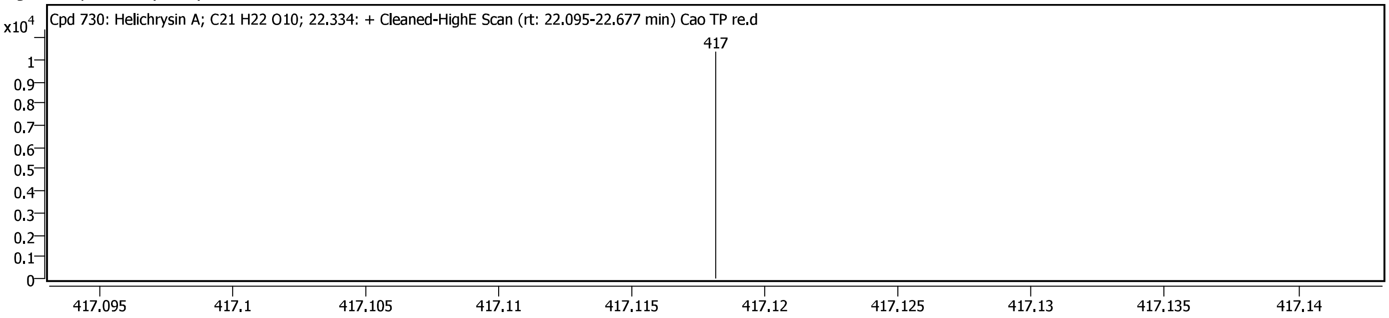

Compound Screening Report

Fragment Spectrum (raw)

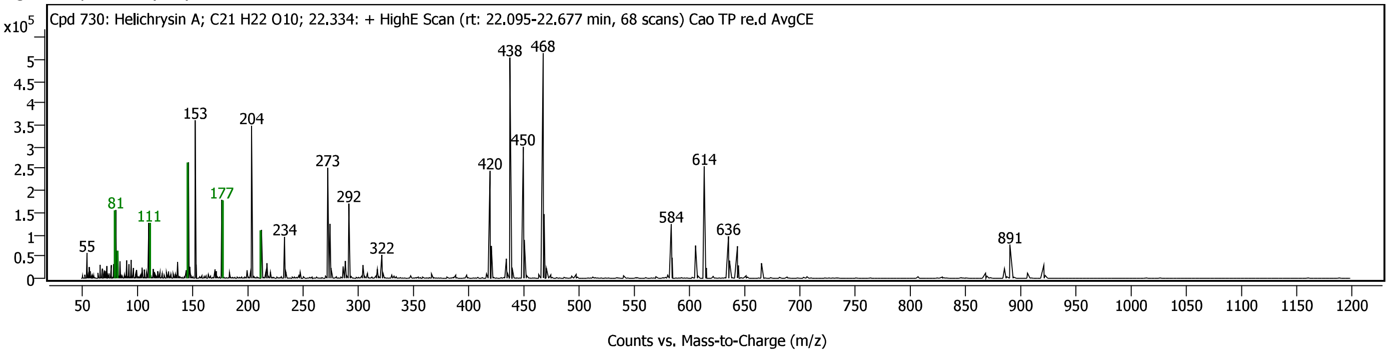

Compound ID Table

Name	Formula	Species	RT	RT Diff	Mass	CAS	ID Source	Score	Score (Lib)	Score (Tgt)
Helichrysin A	C21 H22 O10	(M+H)+	22.334		434.1215	529-41-9	FBF-FragConfirm	99.38		99.38
5,7,2'-Trihydroxyflavanone 7-glucoside	C21 H22 O10	(M+H)+	22.334		434.1215		FBF-FragConfirm	99.38		99.38
5,2'-Dihydroxy-3,6,7,8,4',5'-hexamethoxyflavone	C21 H22 O10	(M+H)+	22.334		434.1215		FBF-FragConfirm	99.38		99.38
8-C-Glucosylnaringenin	C21 H22 O10	(M+H)+	22.334		434.1215		FBF-FragConfirm	99.38		99.38
7-Hydroxyaloin B	C21 H22 O10	(M+H)+	22.334		434.1215	82461-12-9	FBF-FragConfirm	99.38		99.38
5-Hydroxyaloin A	C21 H22 O10	(M+H)+	22.334		434.1215	138373-23-6	FBF-FragConfirm	99.38		99.38
5,7-Dihydroxy-3,6,8,3',4',5'-hexamethoxyflavone	C21 H22 O10	(M+H)+	22.334		434.1215		FBF-FragConfirm	99.38		99.38
5,7-Dihydroxy-3,6,8,2',4',5'-hexamethoxyflavone	C21 H22 O10	(M+H)+	22.334		434.1215		FBF-FragConfirm	99.38		99.38
5,7,8-Trihydroxyflavanone 7-glucoside	C21 H22 O10	(M+H)+	22.334		434.1215		FBF-FragConfirm	99.38		99.38
2',4',6',beta-Tetrahydroxychalcone 2'-glucoside	C21 H22 O10	(M+H)+	22.334		434.1215		FBF-FragConfirm	99.38		99.38
4'-Hydroxyfenoprofen glucuronide	C21 H22 O10	(M+H)+	22.334		434.1215	35440-37-0	FBF-FragConfirm	99.38		99.38
Aloin	C21 H22 O10	(M+H)+	22.334		434.1215	61502-40-7	FBF-FragConfirm	99.38		99.38
3',5'-Dihydroxy-3,5,6,7,8,4'-hexamethoxyflavone	C21 H22 O10	(M+H)+	22.334		434.1215		FBF-FragConfirm	99.38		99.38
2-O-Caffeoylarbutin	C21 H22 O10	(M+H)+	22.334		434.1215	230955-36-9	FBF-FragConfirm	99.38		99.38
2',4',6',beta-Tetrahydroxychalcone 4'-glucoside	C21 H22 O10	(M+H)+	22.334		434.1215		FBF-FragConfirm	99.38		99.38
Chalconaringenin 4-glucoside	C21 H22 O10	(M+H)+	22.334		434.1215		FBF-FragConfirm	99.38		99.38
2',4,4',6'-Tetrahydroxychalcone 4'-O-glucoside	C21 H22 O10	(M+H)+	22.334		434.1215		FBF-FragConfirm	99.38		99.38
(2S)-5,6,7-Trihydroxyflavanone 7-glucoside	C21 H22 O10	(M+H)+	22.334		434.1215		FBF-FragConfirm	99.38		99.38
Eriodictin	C21 H22 O10	(M+H)+	22.334		434.1215		FBF-FragConfirm	99.38		99.38
Agehoustin D	C21 H22 O10	(M+H)+	22.334		434.1215		FBF-FragConfirm	99.38		99.38
(2R,3R)-3,3',4',7-Tetrahydroxyflavanone 7-O-alpha-L-Rhamnopyranoside	C21 H22 O10	(M+H)+	22.334		434.1215	114728-43-7	FBF-FragConfirm	99.38		99.38
Isomonospermoside	C21 H22 O10	(M+H)+	22.334		434.1215		FBF-FragConfirm	99.38		99.38
Isosalipurposide	C21 H22 O10	(M+H)+	22.334		434.1215		FBF-FragConfirm	99.38		99.38
Phlorizin chalcone	C21 H22 O10	(M+H)+	22.334		434.1215	4547-85-7	FBF-FragConfirm	99.38		99.38
Palodulcin B	C21 H22 O10	(M+H)+	22.334		434.1215		FBF-FragConfirm	99.38		99.38
Naringenin 7-O-glucoside	C21 H22 O10	(M+H)+	22.334		434.1215	529-55-5	FBF-FragConfirm	99.38		99.38
Naringenin 4'-O-glucoside	C21 H22 O10	(M+H)+	22.334		434.1215		FBF-FragConfirm	99.38		99.38
Naringenin 4'-O-galactoside	C21 H22 O10	(M+H)+	22.334		434.1215		FBF-FragConfirm	99.38		99.38
Monospermoside	C21 H22 O10	(M+H)+	22.334		434.1215		FBF-FragConfirm	99.38		99.38
Coreopsin	C21 H22 O10	(M+H)+	22.334		434.1215		FBF-FragConfirm	99.38		99.38
Isookaninrhamnoside	C21 H22 O10	(M+H)+	22.334		434.1215		FBF-FragConfirm	99.38		99.38
Chalconaringenin 4'-glucoside	C21 H22 O10	(M+H)+	22.334		434.1215		FBF-FragConfirm	99.38		99.38
Isohemiphloin	C21 H22 O10	(M+H)+	22.334		434.1215		FBF-FragConfirm	99.38		99.38
Isocoreopsin	C21 H22 O10	(M+H)+	22.334		434.1215		FBF-FragConfirm	99.38		99.38
Isocarthamidin 7-O-rhamnoside	C21 H22 O10	(M+H)+	22.334		434.1215		FBF-FragConfirm	99.38		99.38
Hemiphloin	C21 H22 O10	(M+H)+	22.334		434.1215		FBF-FragConfirm	99.38		99.38
Eriodictyol 5-O-rhamnoside	C21 H22 O10	(M+H)+	22.334		434.1215		FBF-FragConfirm	99.38		99.38
Digicitrin	C21 H22 O10	(M+H)+	22.334		434.1215		FBF-FragConfirm	99.38		99.38
Floribundoside	C21 H22 O10	(M+H)+	22.334		434.1215		FBF-FragConfirm	99.38		99.38
Dihydrogenistin	C21 H22 O10	(M+H)+	22.334		434.1215		FBF-FragConfirm	99.38		99.38

Cpd 1290: 6'''-Deamino-6'''-dehydro-6'''-oxoneomycin C

Name	Formula	RT	RI	Mass	Diff (Tgt, ppm)	CAS	ID Source	Score	Algorithm
6'''-Deamino-6'''-dehydro-6'''-oxoneomycin C	C23 H43 N5 O14	22.477		613.2805	-0.24		FBF	84.50	FBF

Species	m/z	Score (Tgt)	Score (Lib)	Score (DB)	Score (MFG)	Score (RT)
(M+H)+	614	84.50				

Compound Chromatograms (overlaid)

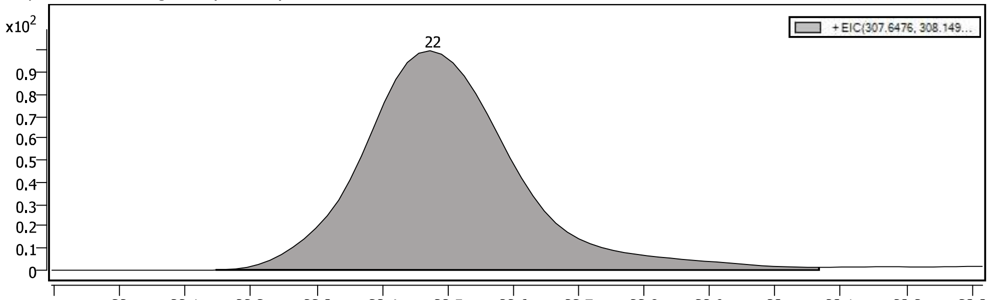

Structure

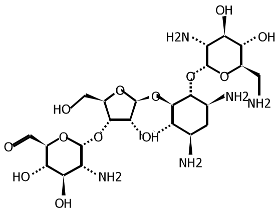

Compound Spectra (overlaid)

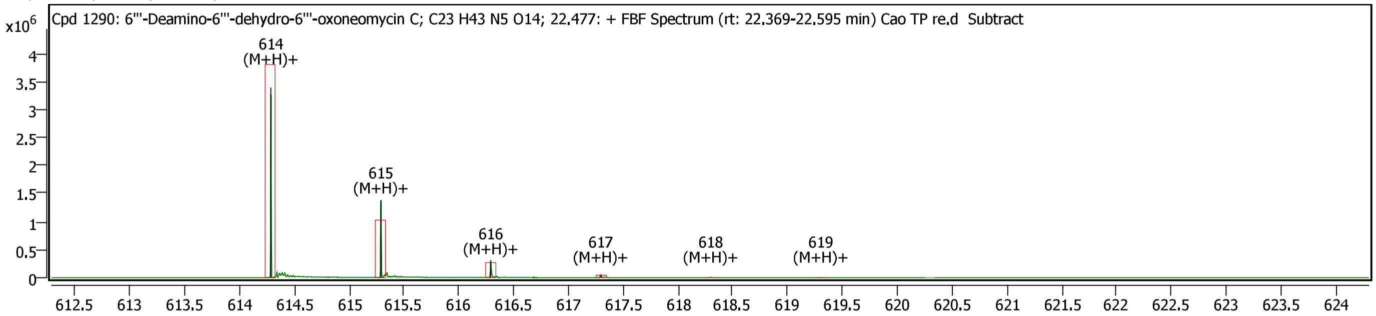

Compound ID Table

Name	Formula	Species	RT	RT Diff	Mass	CAS	ID Source	Score	Score (Lib)	Score (Tgt)
6'''-Deamino-6'''-dehydro-6'''-oxoneomycin C	C23 H43 N5 O14	(M+H)+	22.477		613.2805		FBF	84.50		84.50

Cpd 1323: GW 6471

Name	Formula	RT	RI	Mass	Diff (Tgt, ppm)	CAS	ID Source	Score	Algorithm
GW 6471	C35 H36 F3 N3 O5	22.506		635.2609	0.28	436159-64-7	FBF	99.38	FBF

Species	m/z	Score (Tgt)	Score (Lib)	Score (DB)	Score (MFG)	Score (RT)
(M+H)+	636	99.38				

Compound Chromatograms (overlaid)

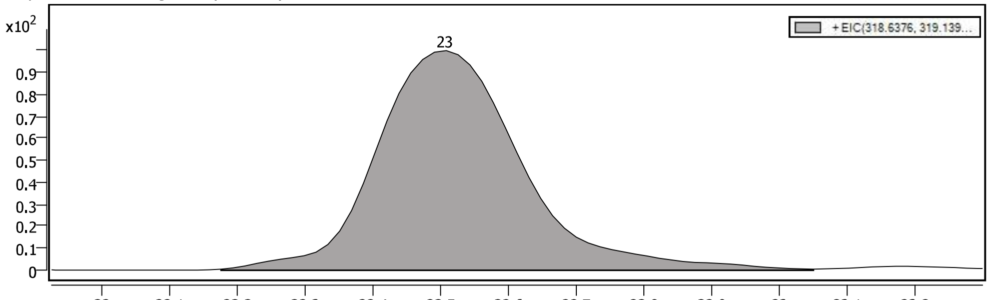

Structure

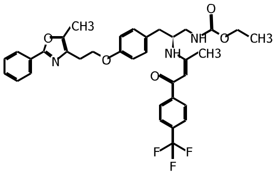

Compound Spectra (overlaid)

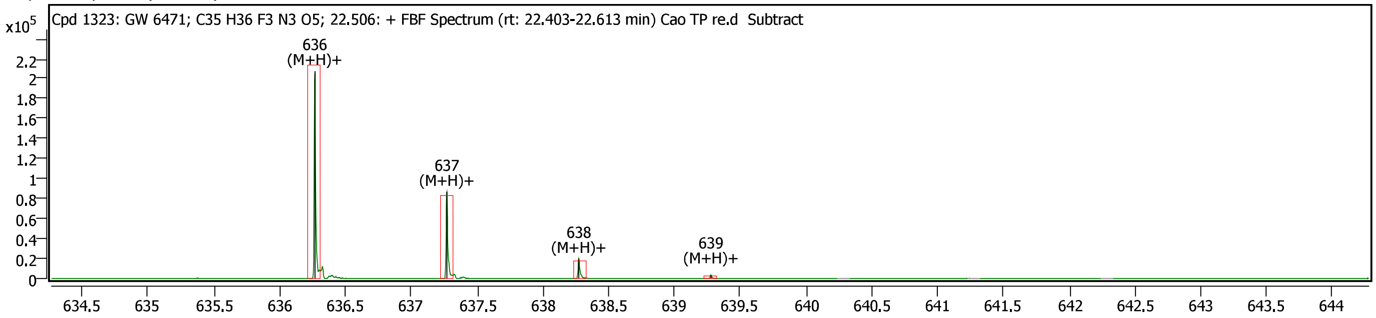

Compound ID Table

Name	Formula	Species	RT	RT Diff	Mass	CAS	ID Source	Score	Score (Lib)	Score (Tgt)
GW 6471	C35 H36 F3 N3 O5	(M+H)+	22.506		635.2609	436159-64-7	FBF	99.38		99.38

Cpd 589: N1,N5,N10-Triferuloyl spermidine

Name	Formula	RT	RI	Mass	Diff (Tgt, ppm)	CAS	ID Source	Score	Algorithm
N1,N5,N10-Triferuloyl spermidine	C37 H43 N3 O9	23.032		673.3016	2.53		FBF	96.32	FBF

Species	m/z	Score (Tgt)	Score (Lib)	Score (DB)	Score (MFG)	Score (RT)
(M+H)+	674	96.32				

Compound Chromatograms (overlaid)

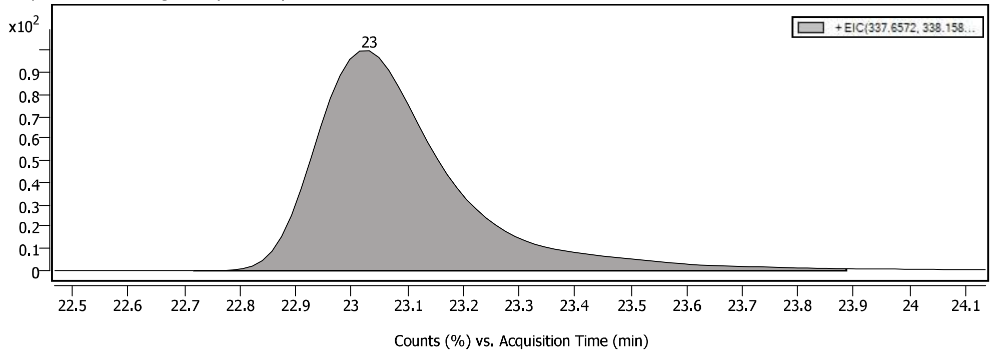

Structure

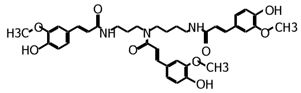

Compound Spectra (overlaid)

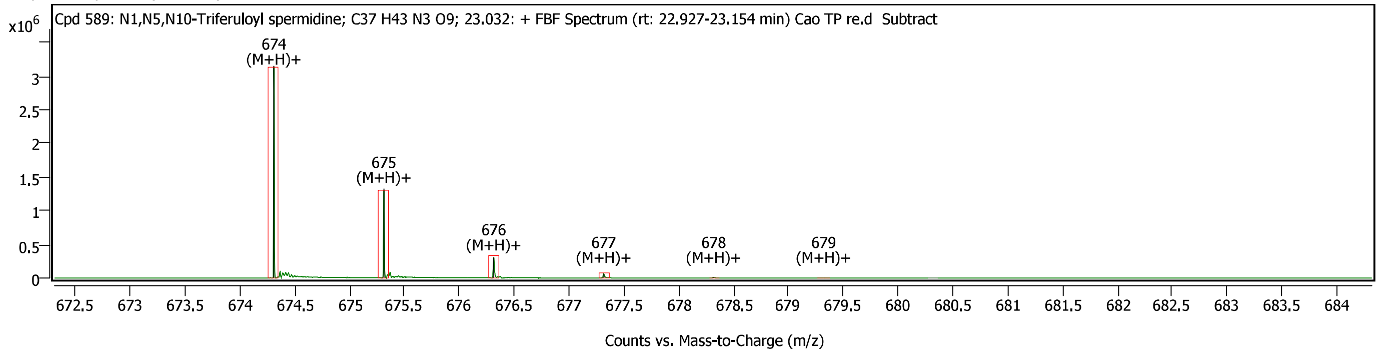

Compound ID Table

Name	Formula	Species	RT	RT Diff	Mass	CAS	ID Source	Score	Score (Lib)	Score (Tgt)
N1,N5,N10-Triferuloyl spermidine	C37 H43 N3 O9	(M+H)+	23.032		673.3016		FBF	96.32		96.32

Cpd 127: Louisfieserone A

Name	Formula	RT	RI	Mass	Diff (Tgt, ppm)	CAS	ID Source	Score	Algorithm
Louisfieserone A	C22 H24 O5	23.642		368.1637	3.66		M-FBF	92.03	FBF

Species	m/z	Score (Tgt)	Score (Lib)	Score (DB)	Score (MFG)	Score (RT)
(M+H)+	369	92.03				

Compound Chromatograms (overlaid)

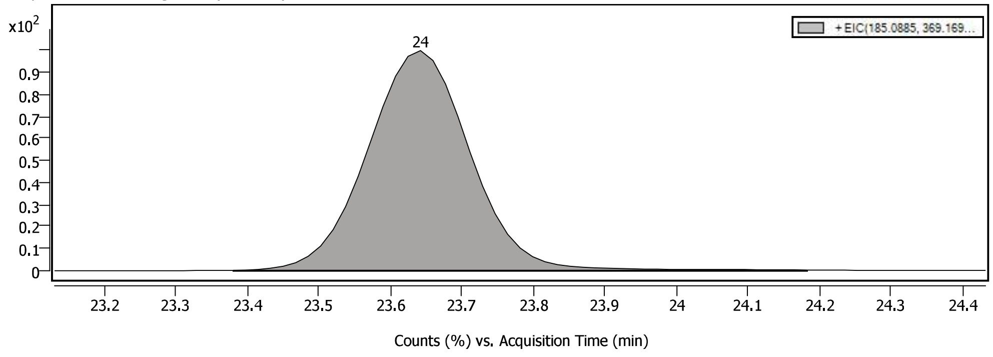

Structure

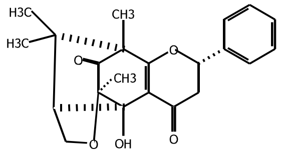

Compound Spectra (overlaid)

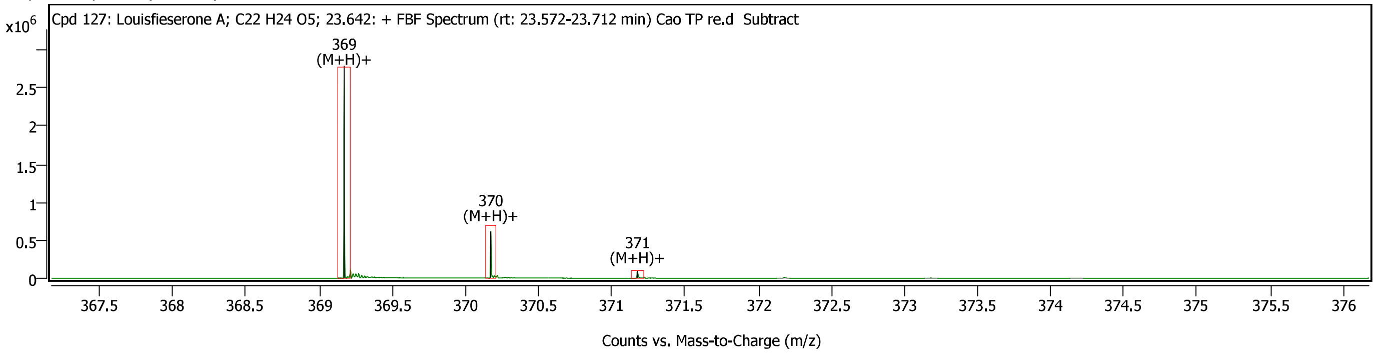

Compound ID Table

Name	Formula	Species	RT	RT Diff	Mass	CAS	ID Source	Score	Score (Lib)	Score (Tgt)
Louisfieserone A	C22 H24 O5	(M+H)+	23.642		368.1637		FBF	92.03		92.03
9-Hydroxy-4-(3,7-dimethyl-2,6-octadienyloxy)-psoralen	C22 H24 O5	(M+H)+	23.642		368.1637	69239-53-8	FBF	92.03		92.03
Edulane	C22 H24 O5	(M+H)+	23.642		368.1637		FBF	92.03		92.03
Epoxycandidone	C22 H24 O5	(M+H)+	23.642		368.1637		FBF	92.03		92.03
4,4-Bis[4-(acetyloxy)phenyl]3-hexanone	C22 H24 O5	(M+H)+	23.642		368.1637		FBF	92.03		92.03
Edulenol	C22 H24 O5	(M+H)+	23.642		368.1637		FBF	92.03		92.03
4'-O-Methylxanthohumol	C22 H24 O5	(M+H)+	23.642		368.1637		FBF	92.03		92.03
Flowerine	C22 H24 O5	(M+H)+	23.642		368.1637		FBF	92.03		92.03
Quercetol C	C22 H24 O5	(M+H)+	23.642		368.1637		FBF	92.03		92.03
Sphaerosinin	C22 H24 O5	(M+H)+	23.642		368.1637		FBF	92.03		92.03
Kanzonol P	C22 H24 O5	(M+H)+	23.642		368.1637	156250-72-5	FBF	92.03		92.03
Louisfieserone B	C22 H24 O5	(M+H)+	23.642		368.1637		FBF	92.03		92.03

Cpd 1162: Asp His Val

Name	Formula	RT	RI	Mass	Diff (Tgt, ppm)	CAS	ID Source	Score	Algorithm
Asp His Val	C15 H23 N5 O6	23.642		369.1664	4.26		M-FBF-FragConfirm	90.63	FBF

Species	m/z	Score (Tgt)	Score (Lib)	Score (DB)	Score (MFG)	Score (RT)
(M+H)+	370	90.63				

Compound Chromatograms (overlaid)

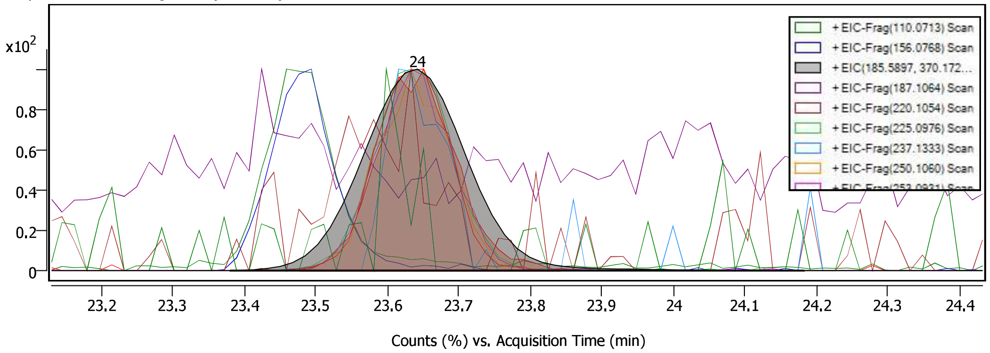

Structure

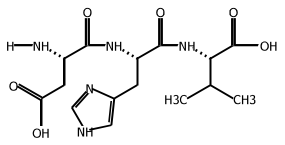

Coelution Plot

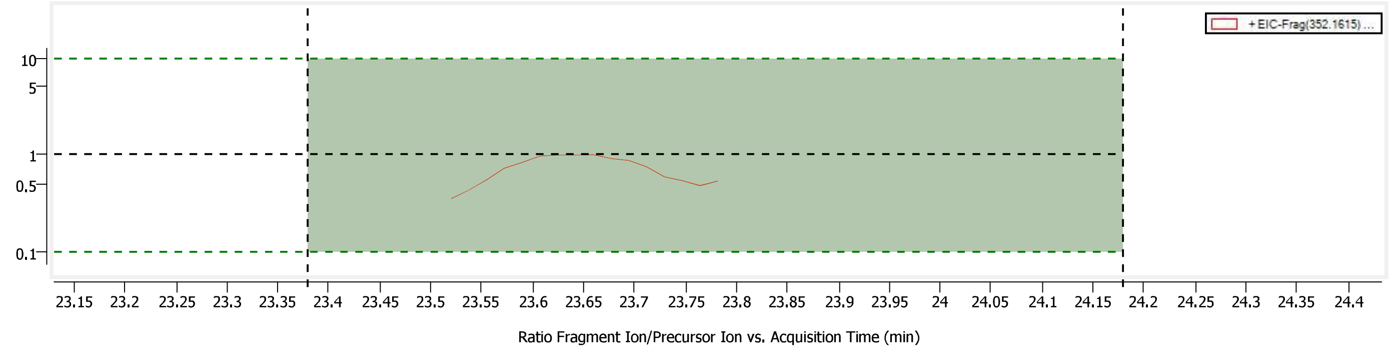

Compound Screening Report

Compound Spectra (overlaid)

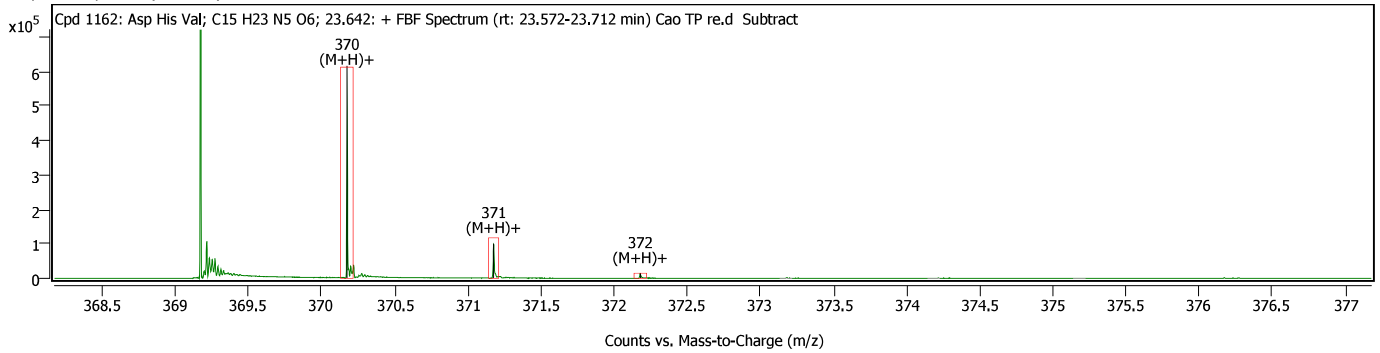

Fragment Spectrum (clean)

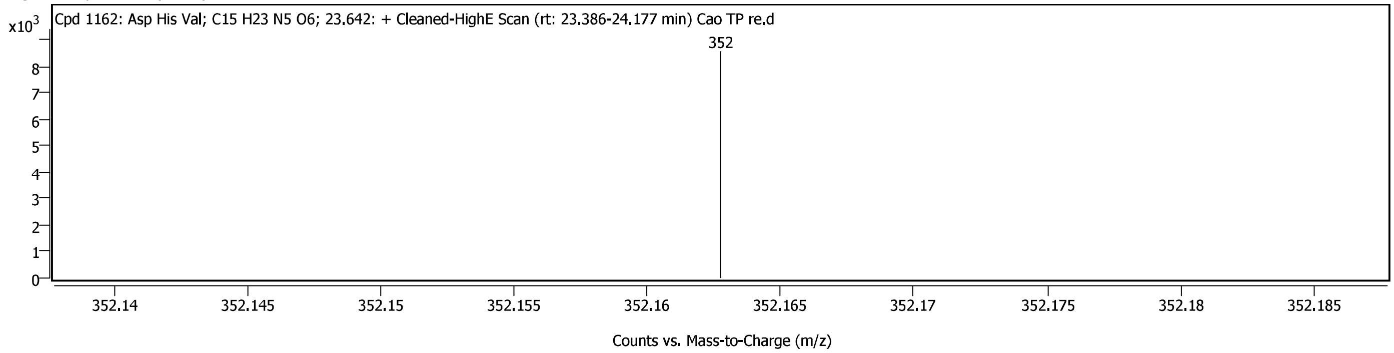

Fragment Spectrum (raw)

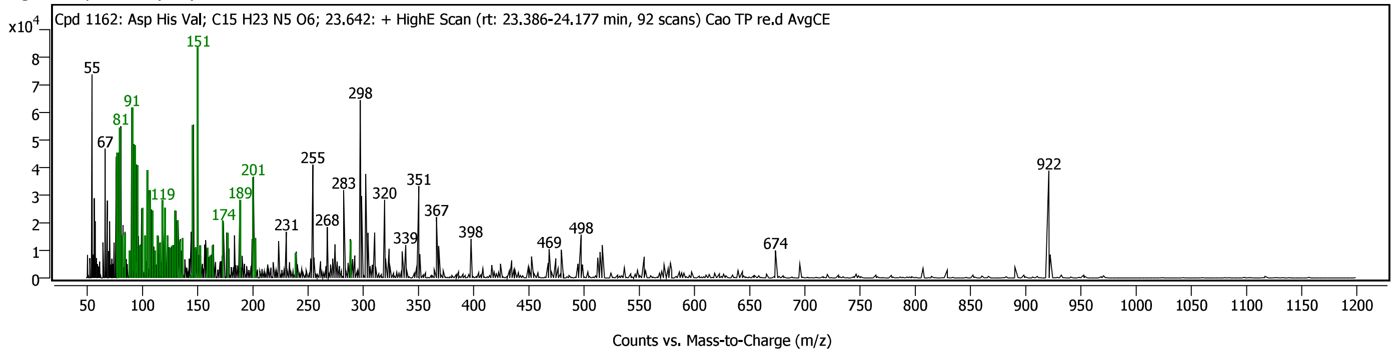

Compound ID Table

Name	Formula	Species	RT	RT Diff	Mass	CAS	ID Source	Score	Score (Lib)	Score (Tgt)
Asp His Val	C15 H23 N5 O6	(M+H)+	23.642		369.1664		FBF-FragConfirm	90.63		90.63
Val His Asp	C15 H23 N5 O6	(M+H)+	23.642		369.1664		FBF-FragConfirm	90.63		90.63
Val Asp His	C15 H23 N5 O6	(M+H)+	23.642		369.1664		FBF-FragConfirm	90.63		90.63
His Val Asp	C15 H23 N5 O6	(M+H)+	23.642		369.1664		FBF-FragConfirm	90.63		90.63
His Asp Val	C15 H23 N5 O6	(M+H)+	23.642		369.1664		FBF-FragConfirm	90.63		90.63
Asp Val His	C15 H23 N5 O6	(M+H)+	23.642		369.1664		FBF-FragConfirm	90.63		90.63

Cpd 1220: Prasterone sulfate

Name	Formula	RT	RI	Mass	Diff (Tgt, ppm)	CAS	ID Source	Score	Algorithm
Prasterone sulfate	C19 H28 O5 S	23.642		368.1639	-4.96	651-48-9	M-FBF	82.01	FBF

Species	m/z	Score (Tgt)	Score (Lib)	Score (DB)	Score (MFG)	Score (RT)
(M+H)+	369	82.01				

Compound Chromatograms (overlaid)

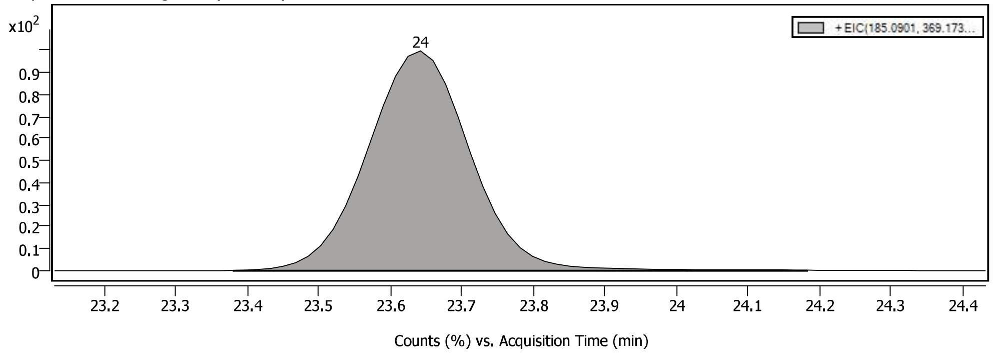

Structure

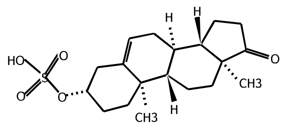

Compound Spectra (overlaid)

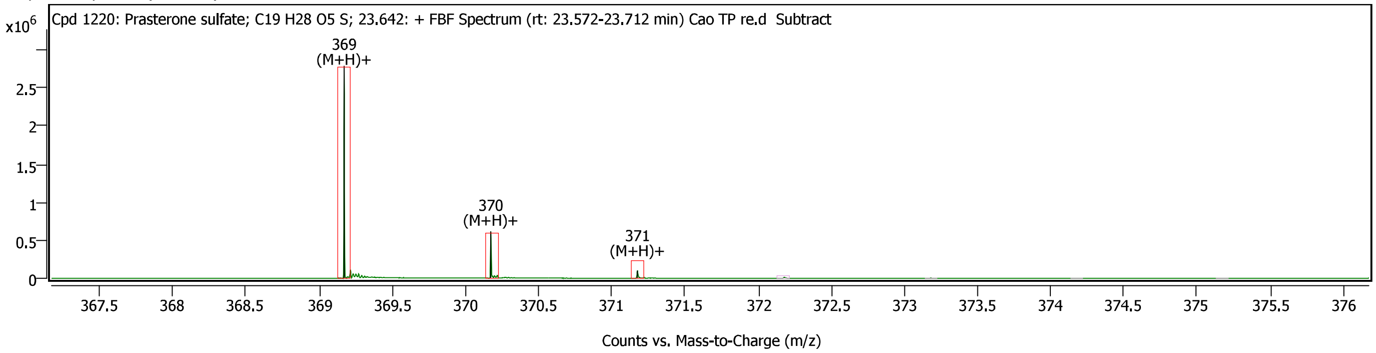

Compound ID Table

Name	Formula	Species	RT	RT Diff	Mass	CAS	ID Source	Score	Score (Lib)	Score (Tgt)
Prasterone sulfate	C19 H28 O5 S	(M+H)+	23.642		368.1639	651-48-9	FBF	82.01		82.01
Testosterone sulfate	C19 H28 O5 S	(M+H)+	23.642		368.1639	651-45-6	FBF	82.01		82.01
3-β-Hydroxyandrost-5-en-17-one sulfate	C19 H28 O5 S	(M+H)+	23.642		368.1639		FBF	82.01		82.01

Cpd 1706: (E)-3-(2-Methylpropylidene)-1(3H)-isobenzofuranone

Name	Formula	RT	RI	Mass	Diff (Tgt, ppm)	CAS	ID Source	Score	Algorithm
(E)-3-(2-Methylpropylidene)-1(3H)-isobenzofuranone	C12 H12 O2	23.642		188.0839	1.16	56014-69-8	M-FBF	98.80	FBF

Species	m/z	Score (Tgt)	Score (Lib)	Score (DB)	Score (MFG)	Score (RT)
(M+H)+	189	98.80				

Compound Chromatograms (overlaid)

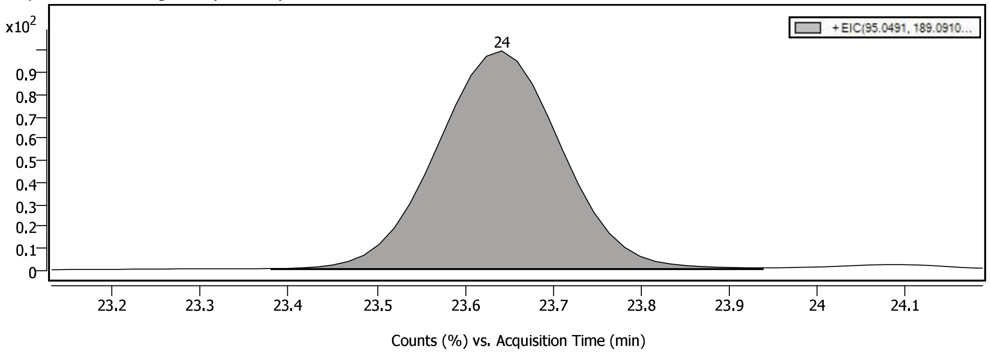

Structure

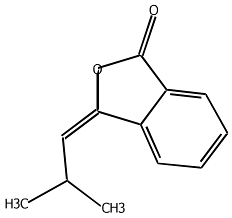

Compound Spectra (overlaid)

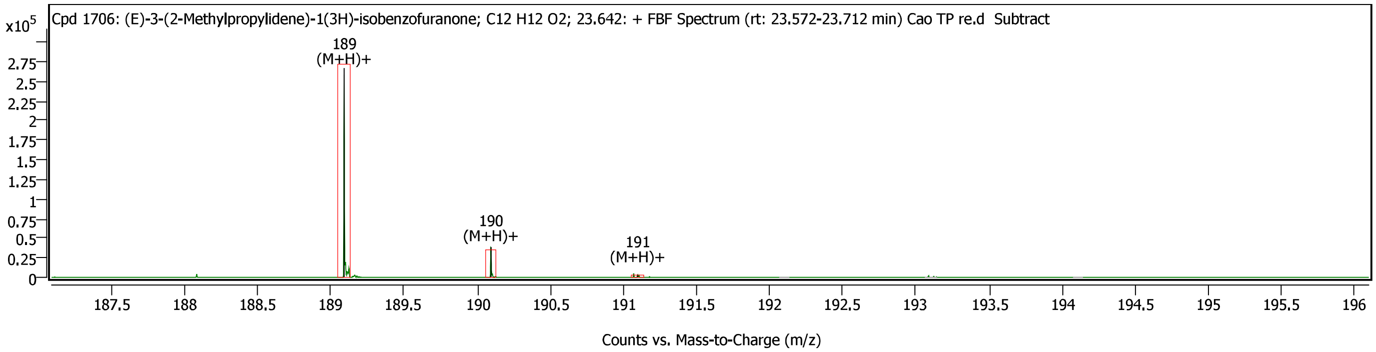

Compound ID Table

Name	Formula	Species	RT	RT Diff	Mass	CAS	ID Source	Score	Score (Lib)	Score (Tgt)
(E)-3-(2-Methylpropylidene)-1(3H)-isobenzofuranone	C12 H12 O2	(M+H)+	23.642		188.0839	56014-69-8	FBF	98.80		98.80
Allyl cinnamate	C12 H12 O2	(M+H)+	23.642		188.0839	56289-56-6	FBF	98.80		98.80
5,8,11-Dodecatriynoic acid	C12 H12 O2	(M+H)+	23.642		188.0839		FBF	98.80		98.80
Trigoforin	C12 H12 O2	(M+H)+	23.642		188.0839	14002-93-8	FBF	98.80		98.80
Butylidenephthalide	C12 H12 O2	(M+H)+	23.642		188.0839	551-08-6	FBF	98.80		98.80
cis-2,3-Dihydro-2,3-dihydroxybiphenyl	C12 H12 O2	(M+H)+	23.642		188.0839		FBF	98.80		98.80

Cpd 1353: <13-epi-12-oxo Phytodienoic Acid>

Name	Formula	RT	RI	Mass	Diff (Tgt, ppm)	CAS	ID Source	Score	Algorithm
<13-epi-12-oxo Phytodienoic Acid>	C18 H28 O3	24.619		292.2042	1.28	71606-07-0	M-FBF	98.96	FBF

Species	m/z	Score (Tgt)	Score (Lib)	Score (DB)	Score (MFG)	Score (RT)
(M+H)+	293	98.96				

Compound Screening Report

Compound Chromatograms (overlaid)

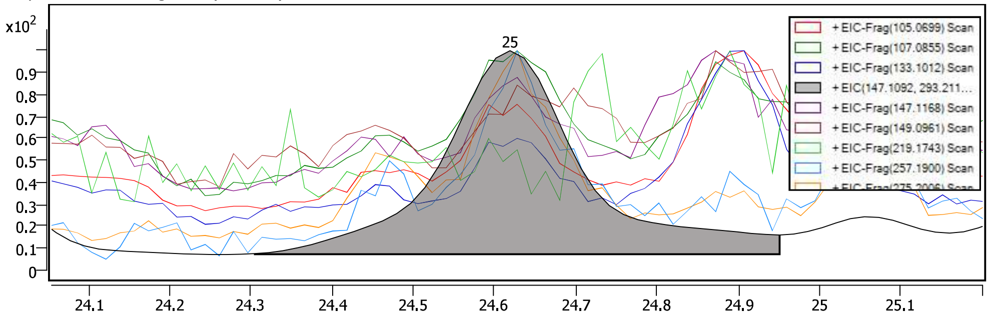

Structure

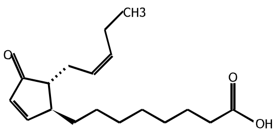

Coelution Plot

Compound Spectra (overlaid)

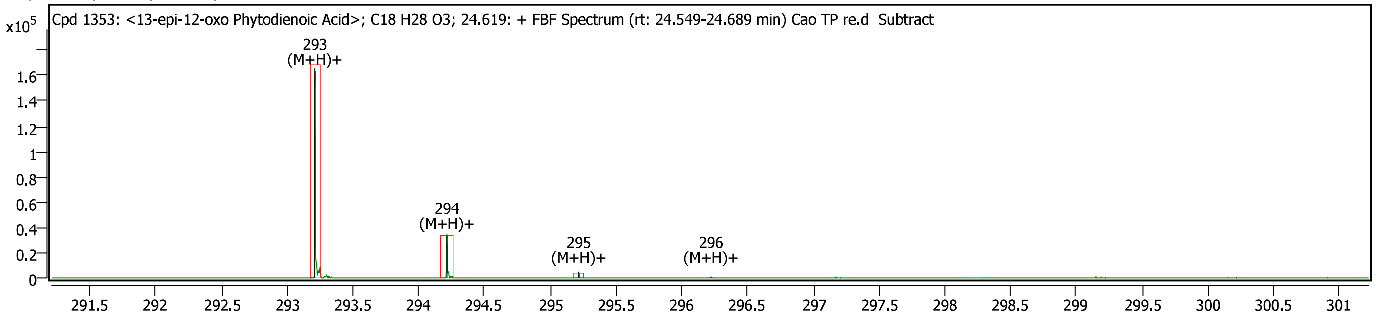

Fragment Spectrum (raw)

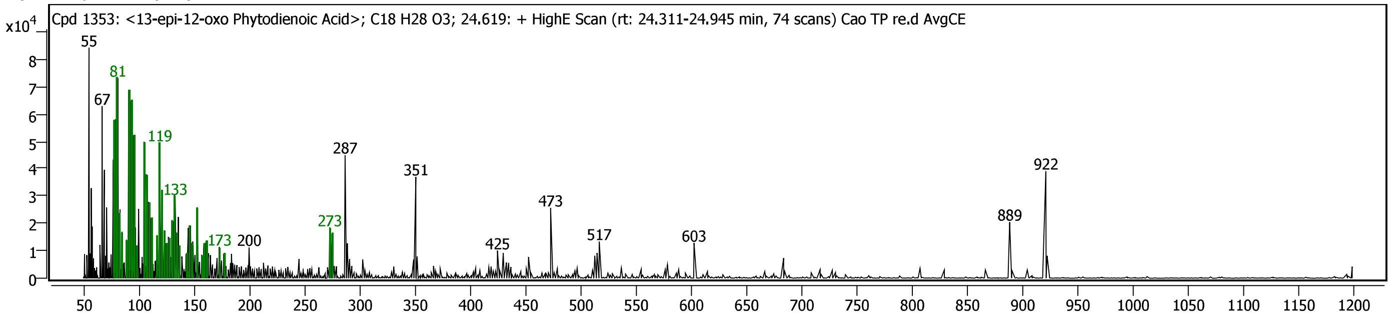

Compound ID Table

Name	Formula	Species	RT	RT Diff	Mass	CAS	ID Source	Score	Score (Lib)	Score (Tgt)
<13-epi-12-oxo Phytodienoic Acid>	C18 H28 O3	(M+H)+	24.619		292.2042	71606-07-0	FBF	98.96		98.96
<17beta-Hydroxy-2-oxa-5alpha-androstan-3-one>	C18 H28 O3	(M+H)+	24.619		292.2042		FBF	98.96		98.96
<Etherolenic acid>	C18 H28 O3	(M+H)+	24.619		292.2042		FBF	98.96		98.96
<13-keto-9Z,11E,15Z-octadecatrienoic acid>	C18 H28 O3	(M+H)+	24.619		292.2042		FBF	98.96		98.96
<(-)-8-hydroxy-11E,17-octadecadien-9-ynoic acid>	C18 H28 O3	(M+H)+	24.619		292.2042		FBF	98.96		98.96
<(9R,13R)-12-oxo-phytodienoic acid>	C18 H28 O3	(M+H)+	24.619		292.2042		FBF	98.96		98.96
<[7]-Paradol>	C18 H28 O3	(M+H)+	24.619		292.2042	53172-04-6	FBF	98.96		98.96
<12,13S-epoxy-9Z,11,15Z-octadecatrienoic acid>	C18 H28 O3	(M+H)+	24.619		292.2042		FBF	98.96		98.96
<12-OPDA>	C18 H28 O3	(M+H)+	24.619		292.2042	85551-10-6	FBF	98.96		98.96
<12-oxo-PDA>	C18 H28 O3	(M+H)+	24.619		292.2042		FBF	98.96		98.96
<10-OPDA>	C18 H28 O3	(M+H)+	24.619		292.2042		FBF	98.96		98.96
<Panaquinquecol 1>	C18 H28 O3	(M+H)+	24.619		292.2042	133921-57-0	FBF	98.96		98.96
<8-hydroxy-10,12-octadecadiynoic acid>	C18 H28 O3	(M+H)+	24.619		292.2042		FBF	98.96		98.96
<9,10-EOT>	C18 H28 O3	(M+H)+	24.619		292.2042		FBF	98.96		98.96
<9-hydroxy-10E,14Z-octadecadien-12-ynoic acid>	C18 H28 O3	(M+H)+	24.619		292.2042		FBF	98.96		98.96
<9-OxoOTrE>	C18 H28 O3	(M+H)+	24.619		292.2042	125559-74-2	FBF	98.96		98.96
<alpha-licanic acid>	C18 H28 O3	(M+H)+	24.619		292.2042		FBF	98.96		98.96
<Colnelenic acid>	C18 H28 O3	(M+H)+	24.619		292.2042		FBF	98.96		98.96
<8-hydroxy-9,11-octadecadiynoic acid>	C18 H28 O3	(M+H)+	24.619		292.2042		FBF	98.96		98.96

Cpd 776: Kaempferol 3-apioside-7-rhamnosyl-(1->6)-(2''-(E)-caffeoylglactoside)

Compound Screening Report

Name	Formula	RT	RI	Mass	Diff (Tgt, ppm)	CAS	ID Source	Score	Algorithm
Kaempferol 3-apioside-7-rhamnosyl-(1->6)-(2''-(E)-caffeoylglactoside)	C41 H44 O22	24.706		888.2300	-2.69		FBF	96.36	FBF

Species	m/z	Score (Tgt)	Score (Lib)	Score (DB)	Score (MFG)	Score (RT)
(M+H)+	889	96.36				

Compound Chromatograms (overlaid)

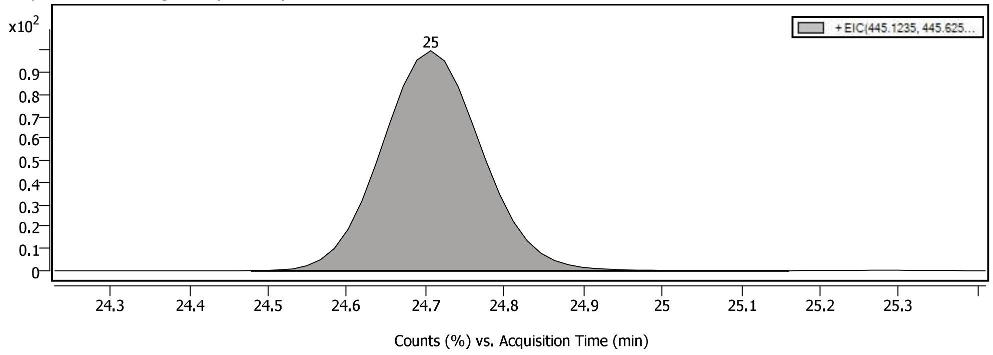

Structure

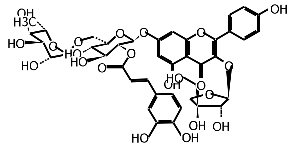

Compound Spectra (overlaid)

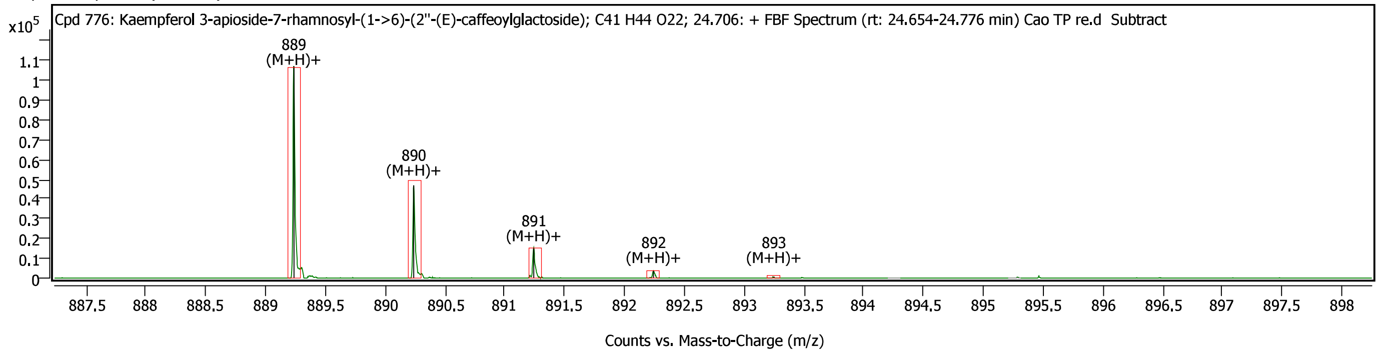

Compound ID Table

Name	Formula	Species	RT	RT Diff	Mass	CAS	ID Source	Score	Score (Lib)	Score (Tgt)
Kaempferol 3-apioside-7-rhamnosyl-(1->6)-(2''-(E)-caffeoylglactoside)	C41 H44 O22	(M+H)+	24.706		888.2300		FBF	96.36		96.36

Cpd 1373: L-Olivosyl-oleandolide

Name	Formula	RT	RI	Mass	Diff (Tgt, ppm)	CAS	ID Source	Score	Algorithm
L-Olivosyl-oleandolide	C26 H44 O10	25.055		516.2913	-4.11		FBF	92.20	FBF

Species	m/z	Score (Tgt)	Score (Lib)	Score (DB)	Score (MFG)	Score (RT)
(M+H)+	517	92.20				

Compound Chromatograms (overlaid)

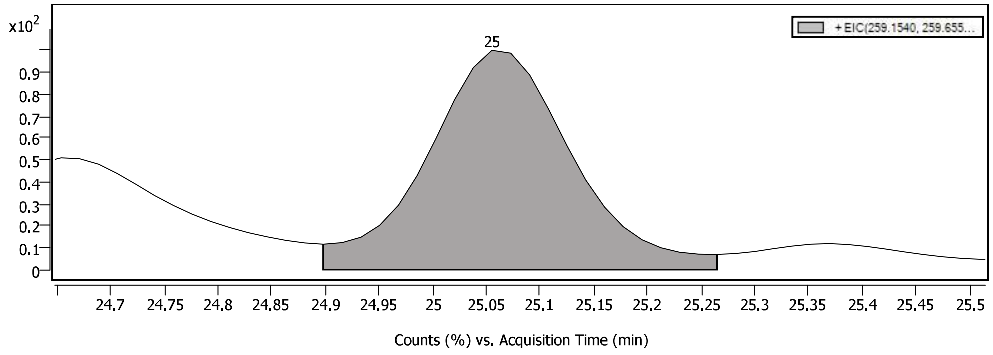

Structure

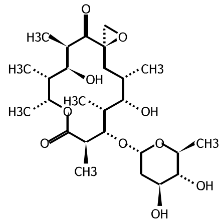

Compound Spectra (overlaid)

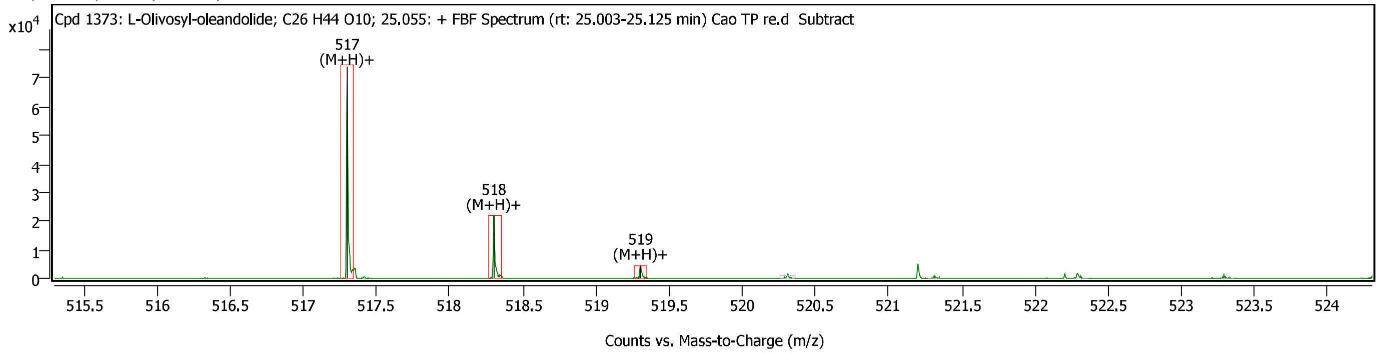

Compound ID Table

Name	Formula	Species	RT	RT Diff	Mass	CAS	ID Source	Score	Score (Lib)	Score (Tgt)
L-Olivosyl-oleandolide	C26 H44 O10	(M+H)+	25.055		516.2913		FBF	92.20		92.20

Cpd 5: (8S,Z)-6-((S)-3-Hydroxy-2-methylpropylidene)-8-methyloctahydroindolizin-8-ol

Name	Formula	RT	RI	Mass	Diff (Tgt, ppm)	CAS	ID Source	Score	Algorithm
(8S,Z)-6-((S)-3-Hydroxy-2-methylpropylidene)-8-methyloctahydroindolizin-8-ol	C13 H23 N O2	25.160		225.1731	0.83		M-FBF	98.86	FBF

Species	m/z	Score (Tgt)	Score (Lib)	Score (DB)	Score (MFG)	Score (RT)
(M+H)+	226	98.86				

Compound Chromatograms (overlaid)

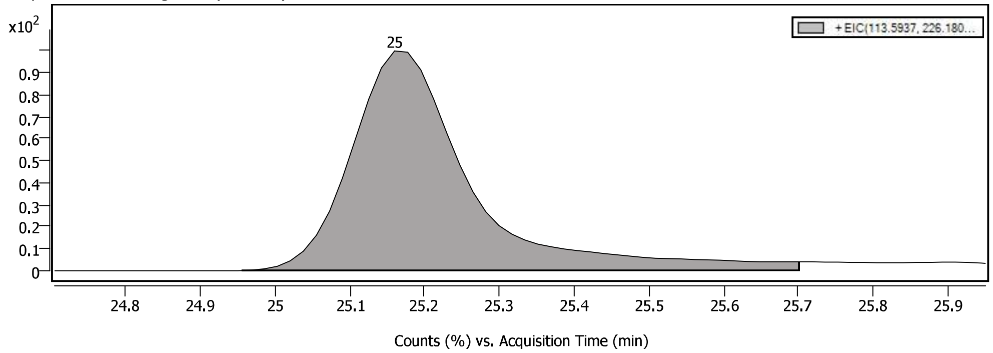

Structure

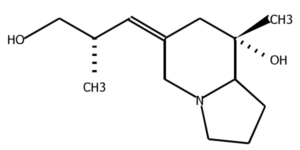

Compound Spectra (overlaid)

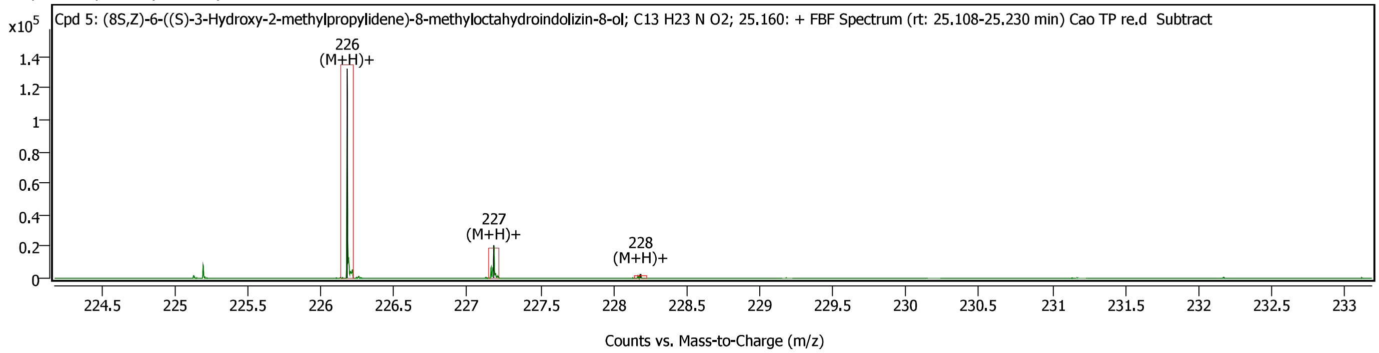

Compound ID Table

Name	Formula	Species	RT	RT Diff	Mass	CAS	ID Source	Score	Score (Lib)	Score (Tgt)
(8S,Z)-6-((S)-3-Hydroxy-2-methylpropylidene)-8-methyloctahydroindolizin-8-ol	C13 H23 N O2	(M+H)+	25.160		225.1731		FBF	98.86		98.86
(7R,8R,E)-8-Methyl-6-(2-methylpropylidene)octahydroindolizine-7,8-diol	C13 H23 N O2	(M+H)+	25.160		225.1731		FBF	98.86		98.86

Cpd 1352: <9-OxoOTrE>

Name	Formula	RT	RI	Mass	Diff (Tgt, ppm)	CAS	ID Source	Score	Algorithm
<9-OxoOTrE>	C18 H28 O3	25.404		292.2043	1.45	125559-74-2	M-FBF	98.79	FBF

Species	m/z	Score (Tgt)	Score (Lib)	Score (DB)	Score (MFG)	Score (RT)
(M+H)+	293	98.79				

Compound Screening Report

Compound Chromatograms (overlaid)

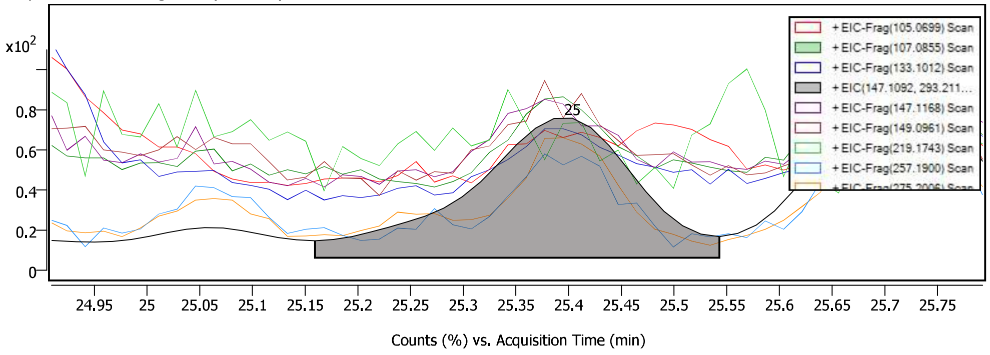

Structure

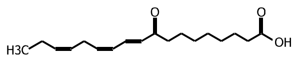

Coelution Plot

Compound Spectra (overlaid)

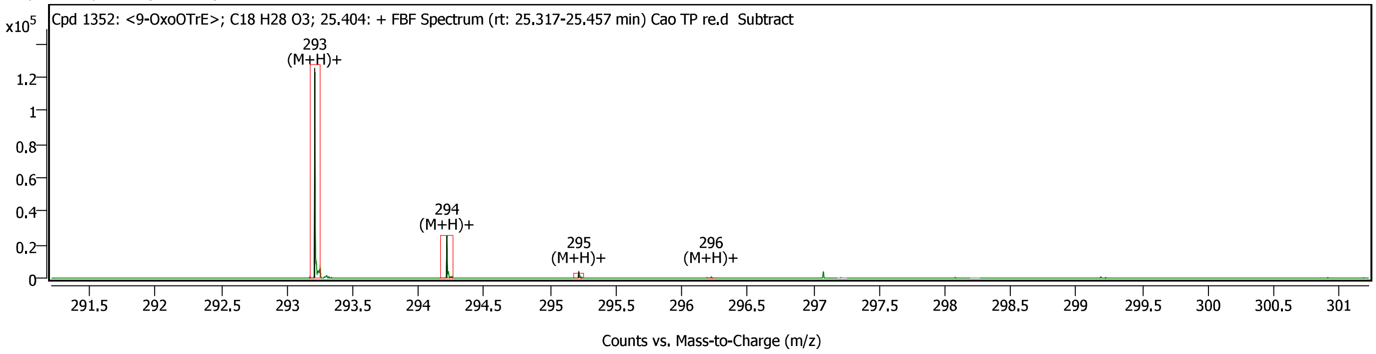

Fragment Spectrum (raw)

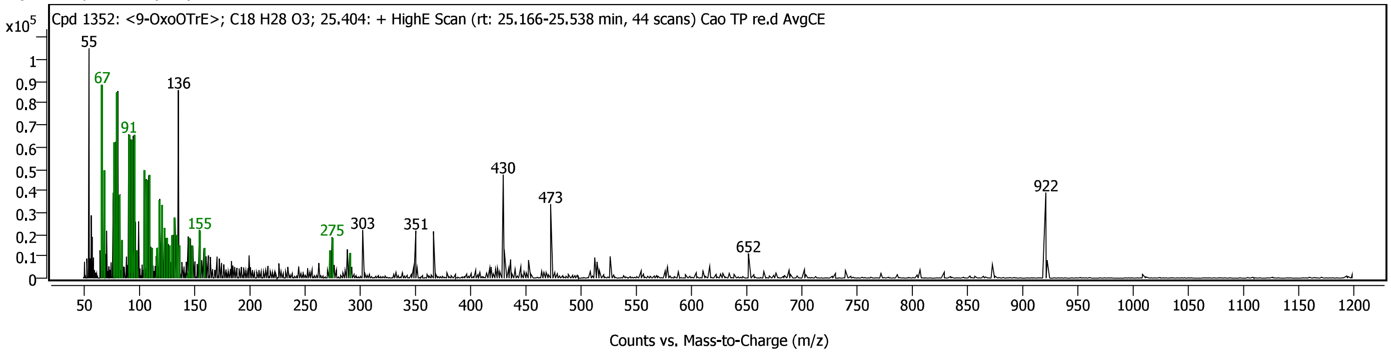

Compound ID Table

Name	Formula	Species	RT	RT Diff	Mass	CAS	ID Source	Score	Score (Lib)	Score (Tgt)
<9-OxoOTrE>	C18 H28 O3	(M+H)+	25.404		292.2043	125559-74-2	FBF	98.79		98.79
<17beta-Hydroxy-2-oxa-5alpha-androstan-3-one>	C18 H28 O3	(M+H)+	25.404		292.2043		FBF	98.79		98.79
<Etherolenic acid>	C18 H28 O3	(M+H)+	25.404		292.2043		FBF	98.79		98.79
<13-keto-9Z,11E,15Z-octadecatrienoic acid>	C18 H28 O3	(M+H)+	25.404		292.2043		FBF	98.79		98.79
<(-)-8-hydroxy-11E,17-octadecadien-9-ynoic acid>	C18 H28 O3	(M+H)+	25.404		292.2043		FBF	98.79		98.79
<(9R,13R)-12-oxo-phytodienoic acid>	C18 H28 O3	(M+H)+	25.404		292.2043		FBF	98.79		98.79
<[7]-Paradol>	C18 H28 O3	(M+H)+	25.404		292.2043	53172-04-6	FBF	98.79		98.79
<12,13S-epoxy-9Z,11,15Z-octadecatrienoic acid>	C18 H28 O3	(M+H)+	25.404		292.2043		FBF	98.79		98.79
<12-OPDA>	C18 H28 O3	(M+H)+	25.404		292.2043	85551-10-6	FBF	98.79		98.79
<12-oxo-PDA>	C18 H28 O3	(M+H)+	25.404		292.2043		FBF	98.79		98.79
<13-epi-12-oxo Phytodienoic Acid>	C18 H28 O3	(M+H)+	25.404		292.2043	71606-07-0	FBF	98.79		98.79
<10-OPDA>	C18 H28 O3	(M+H)+	25.404		292.2043		FBF	98.79		98.79
<Panaquinquecol 1>	C18 H28 O3	(M+H)+	25.404		292.2043	133921-57-0	FBF	98.79		98.79
<8-hydroxy-10,12-octadecadiynoic acid>	C18 H28 O3	(M+H)+	25.404		292.2043		FBF	98.79		98.79
<9,10-EOT>	C18 H28 O3	(M+H)+	25.404		292.2043		FBF	98.79		98.79
<9-hydroxy-10E,14Z-octadecadien-12-ynoic acid>	C18 H28 O3	(M+H)+	25.404		292.2043		FBF	98.79		98.79
<alpha-licanic acid>	C18 H28 O3	(M+H)+	25.404		292.2043		FBF	98.79		98.79
<Colnelenic acid>	C18 H28 O3	(M+H)+	25.404		292.2043		FBF	98.79		98.79
<8-hydroxy-9,11-octadecadiynoic acid>	C18 H28 O3	(M+H)+	25.404		292.2043		FBF	98.79		98.79

Cpd 501: 4,4-Difluoropregn-5-ene-3,20-dione

Name	Formula	RT	RI	Mass	Diff (Tgt, ppm)	CAS	ID Source	Score	Algorithm
4,4-Difluoropregn-5-ene-3,20-dione	C21 H28 F2 O2	25.701		350.2070	3.72		FBF	92.77	FBF

Species	m/z	Score (Tgt)	Score (Lib)	Score (DB)	Score (MFG)	Score (RT)
(M+H)+	351	92.77				

Compound Chromatograms (overlaid)

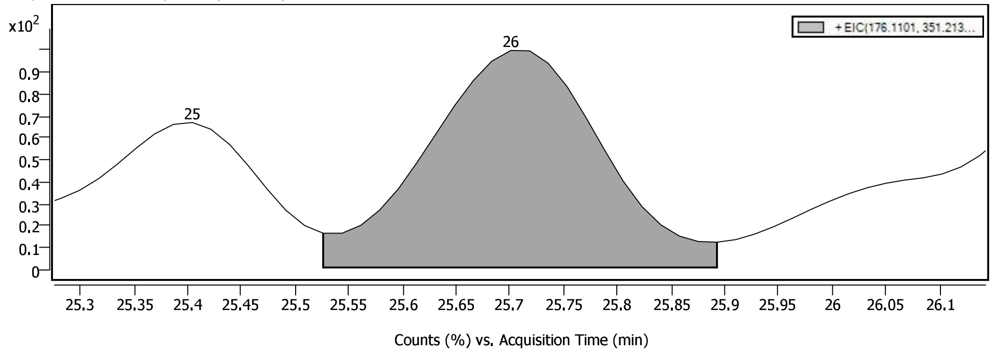

Structure

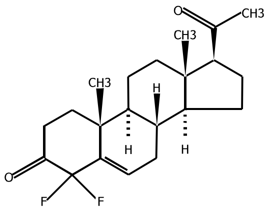

Compound Spectra (overlaid)

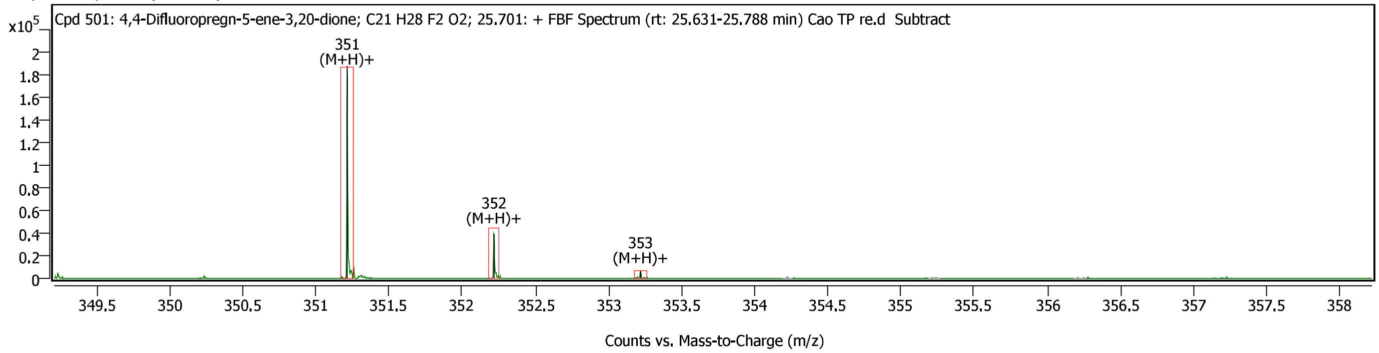

Compound ID Table

Name	Formula	Species	RT	RT Diff	Mass	CAS	ID Source	Score	Score (Lib)	Score (Tgt)
4,4-Difluoropregn-5-ene-3,20-dione	C21 H28 F2 O2	(M+H)+	25.701		350.2070		FBF	92.77		92.77

Cpd 1594: <Confertifoline>

Name	Formula	RT	RI	Mass	Diff (Tgt, ppm)	CAS	ID Source	Score	Algorithm
<Confertifoline>	C15 H22 O2	26.660		234.1624	1.85	1811-23-0	M-FBF	97.65	FBF

Species	m/z	Score (Tgt)	Score (Lib)	Score (DB)	Score (MFG)	Score (RT)
(M+H)+	235	97.65				

Compound Chromatograms (overlaid)

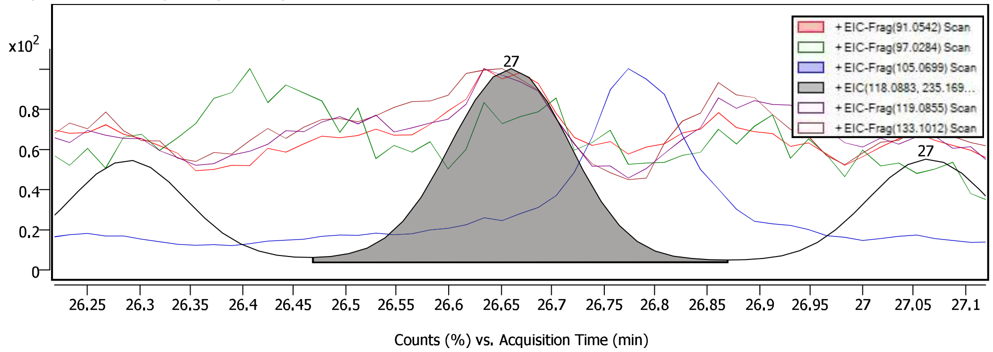

Structure

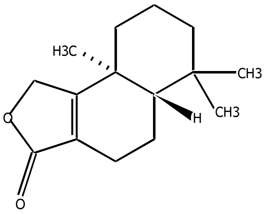

Coelution Plot

Compound Spectra (overlaid)

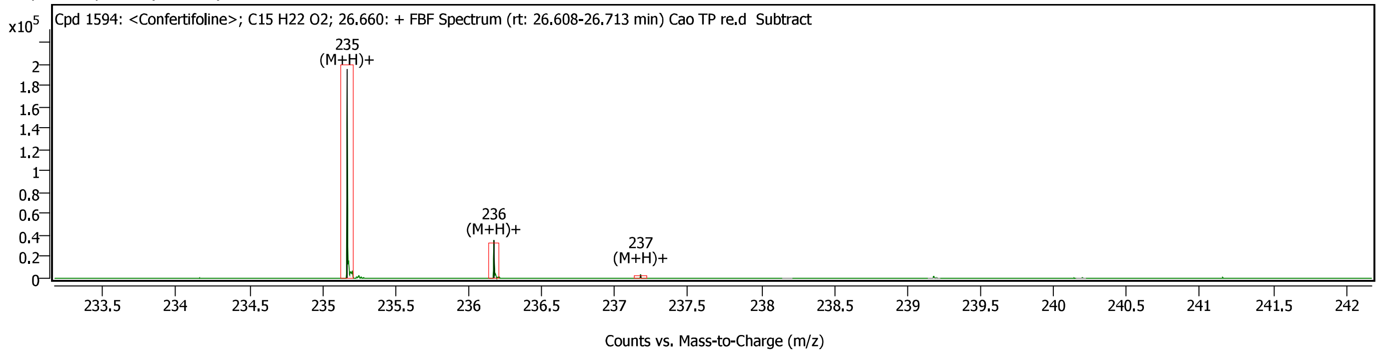

Compound Screening Report

Fragment Spectrum (raw)

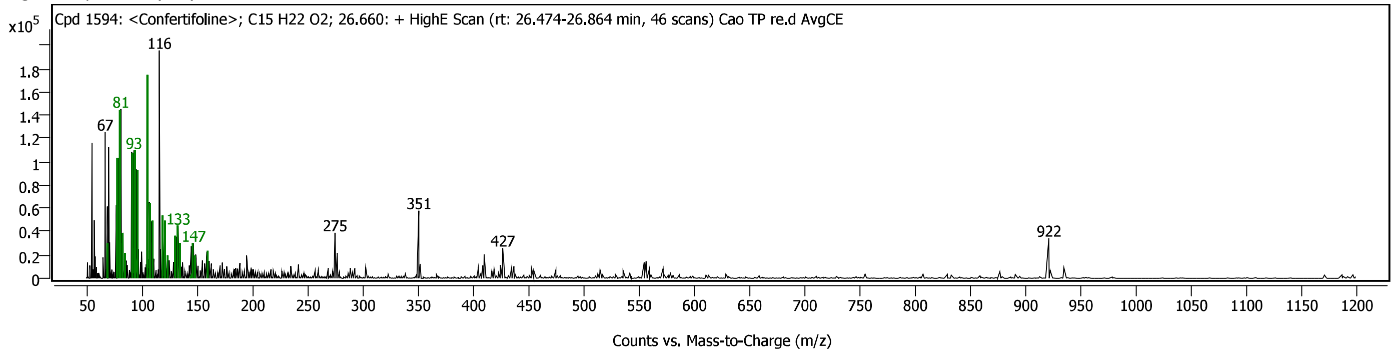

Compound ID Table

Name	Formula	Species	RT	RT Diff	Mass	CAS	ID Source	Score	Score (Lib)	Score (Tgt)
<Confertifoline>	C15 H22 O2	(M+H)+	26.660		234.1624	1811-23-0	FBF	97.65		97.65
<9-Pentadecene-12,14-diyne-1,11-diol>	C15 H22 O2	(M+H)+	26.660		234.1624	108112-83-0	FBF	97.65		97.65
<7-(1,3-Cyclohexadienyl)-5-hydroxy-2,6-dimethyl-2-hepten-4-one>	C15 H22 O2	(M+H)+	26.660		234.1624		FBF	97.65		97.65
<7-Hydroxycostal>	C15 H22 O2	(M+H)+	26.660		234.1624	86703-04-0	FBF	97.65		97.65
<8beta-Hydroxy-3-longipinen-5-one>	C15 H22 O2	(M+H)+	26.660		234.1624		FBF	97.65		97.65
<alpha-Rotunol>	C15 H22 O2	(M+H)+	26.660		234.1624	24405-56-9	FBF	97.65		97.65
<Alcyopterosins O>	C15 H22 O2	(M+H)+	26.660		234.1624		FBF	97.65		97.65
<alpha-Cyperone>	C15 H22 O2	(M+H)+	26.660		234.1624		FBF	97.65		97.65
<Bakkenolide A>	C15 H22 O2	(M+H)+	26.660		234.1624	19906-72-0	FBF	97.65		97.65
<beta-Costic acid>	C15 H22 O2	(M+H)+	26.660		234.1624	3650-43-9	FBF	97.65		97.65
<beta-Santalic acid>	C15 H22 O2	(M+H)+	26.660		234.1624	73590-17-7	FBF	97.65		97.65
<Chrysantherol>	C15 H22 O2	(M+H)+	26.660		234.1624		FBF	97.65		97.65
<4-Methylphenyl octanoate>	C15 H22 O2	(M+H)+	26.660		234.1624	59558-23-5	FBF	97.65		97.65
<Hydroxyisopatchoulenone>	C15 H22 O2	(M+H)+	26.660		234.1624	63512-80-1	FBF	97.65		97.65
<13-Hydroxygermacrone>	C15 H22 O2	(M+H)+	26.660		234.1624		FBF	97.65		97.65
<4,10-Longipinanedione>	C15 H22 O2	(M+H)+	26.660		234.1624	88198-34-9	FBF	97.65		97.65
<3-Phenylpropyl hexanoate>	C15 H22 O2	(M+H)+	26.660		234.1624	6281-40-9	FBF	97.65		97.65
<3,7-Bisaboladiene-2,8-dione>	C15 H22 O2	(M+H)+	26.660		234.1624	57095-92-8	FBF	97.65		97.65
<2-Methyl-4-phenyl-2-butyl 2-methylpropanoate>	C15 H22 O2	(M+H)+	26.660		234.1624	10031-71-7	FBF	97.65		97.65
<2,7(14)-Illudadiene-10,15-diol>	C15 H22 O2	(M+H)+	26.660		234.1624	141940-52-5	FBF	97.65		97.65
<12-Hydroxy-chiloscyphone>	C15 H22 O2	(M+H)+	26.660		234.1624		FBF	97.65		97.65
<1,13-Dihydroxy-herbertene>	C15 H22 O2	(M+H)+	26.660		234.1624		FBF	97.65		97.65
<1,10-Epoxygermacrone>	C15 H22 O2	(M+H)+	26.660		234.1624	52061-45-7	FBF	97.65		97.65
<(Z)-alpha-Bergamotenoic acid>	C15 H22 O2	(M+H)+	26.660		234.1624	124439-27-6	FBF	97.65		97.65
<(4S,5S)-(+)-Germacrone 4,5-epoxide>	C15 H22 O2	(M+H)+	26.660		234.1624		FBF	97.65		97.65
<(4alpha,5alpha)-11-Eremophilene-2,9-dione>	C15 H22 O2	(M+H)+	26.660		234.1624		FBF	97.65		97.65
<(1alpha,4beta,5beta)-4-Hydroxy-7(11),10(14)-guaiadien-8-one>	C15 H22 O2	(M+H)+	26.660		234.1624		FBF	97.65		97.65
<4,6-Decadiyn-1-ol isovalerate>	C15 H22 O2	(M+H)+	26.660		234.1624	29314-16-7	FBF	97.65		97.65
<7,9-Illudadiene-3,14-diol>	C15 H22 O2	(M+H)+	26.660		234.1624	141940-51-4	FBF	97.65		97.65
<Curcumanolide A>	C15 H22 O2	(M+H)+	26.660		234.1624	97550-04-4	FBF	97.65		97.65
<Oxysolavetivone>	C15 H22 O2	(M+H)+	26.660		234.1624	103573-06-4	FBF	97.65		97.65
<Marasmene>	C15 H22 O2	(M+H)+	26.660		234.1624	124869-12-1	FBF	97.65		97.65
<Curcumenone>	C15 H22 O2	(M+H)+	26.660		234.1624		FBF	97.65		97.65
<Zerumbone oxide>	C15 H22 O2	(M+H)+	26.660		234.1624	22471-70-1	FBF	97.65		97.65
<Valerenic acid>	C15 H22 O2	(M+H)+	26.660		234.1624		FBF	97.65		97.65
<Tetradymol>	C15 H22 O2	(M+H)+	26.660		234.1624	52279-13-7	FBF	97.65		97.65
<Sugeonol>	C15 H22 O2	(M+H)+	26.660		234.1624		FBF	97.65		97.65
<Sclerosporin>	C15 H22 O2	(M+H)+	26.660		234.1624	66419-03-2	FBF	97.65		97.65
<Saussurea lactone>	C15 H22 O2	(M+H)+	26.660		234.1624	23527-07-3	FBF	97.65		97.65
<Rishitinol>	C15 H22 O2	(M+H)+	26.660		234.1624	31316-42-4	FBF	97.65		97.65
<Procurcumenol>	C15 H22 O2	(M+H)+	26.660		234.1624	21698-40-8	FBF	97.65		97.65
<Polygodial>	C15 H22 O2	(M+H)+	26.660		234.1624	6754-20-7	FBF	97.65		97.65
<p-Hydroxynonanophenone>	C15 H22 O2	(M+H)+	26.660		234.1624	14392-69-9	FBF	97.65		97.65
<Petasalbin>	C15 H22 O2	(M+H)+	26.660		234.1624	4176-11-8	FBF	97.65		97.65
<xi-2-Hydroxy-1,3,5-bisabolatrien-9-one>	C15 H22 O2	(M+H)+	26.660		234.1624		FBF	97.65		97.65
<Onitin>	C15 H22 O2	(M+H)+	26.660		234.1624		FBF	97.65		97.65
<Epiacoronene>	C15 H22 O2	(M+H)+	26.660		234.1624		FBF	97.65		97.65
<Macrophyllic acid A>	C15 H22 O2	(M+H)+	26.660		234.1624		FBF	97.65		97.65
<Dehydrocurdione>	C15 H22 O2	(M+H)+	26.660		234.1624	38230-32-9	FBF	97.65		97.65
<Dihydroisoalantolactone>	C15 H22 O2	(M+H)+	26.660		234.1624		FBF	97.65		97.65
<Drimenin>	C15 H22 O2	(M+H)+	26.660		234.1624	2326-89-8	FBF	97.65		97.65
<Cyclodehydroisolubimin>	C15 H22 O2	(M+H)+	26.660		234.1624	72055-93-7	FBF	97.65		97.65
<Eremophilenolide>	C15 H22 O2	(M+H)+	26.660		234.1624	4871-90-3	FBF	97.65		97.65
<Germacrene A acid>	C15 H22 O2	(M+H)+	26.660		234.1624		FBF	97.65		97.65
<Isocurcumenol>	C15 H22 O2	(M+H)+	26.660		234.1624	24063-71-6	FBF	97.65		97.65
<Curmadione>	C15 H22 O2	(M+H)+	26.660		234.1624		FBF	97.65		97.65
<Curcumenol>	C15 H22 O2	(M+H)+	26.660		234.1624		FBF	97.65		97.65
<Helminthosporal>	C15 H22 O2	(M+H)+	26.660		234.1624	723-61-5	FBF	97.65		97.65

Cpd 1639: Tragopogonsaponin K

Name	Formula	RT	RI	Mass	Diff (Tgt, ppm)	CAS	ID Source	Score	Algorithm
Tragopogonsaponin K	C50 H72 O15	26.748		912.4871	-0.01		FBF	99.81	FBF

Species	m/z	Score (Tgt)	Score (Lib)	Score (DB)	Score (MFG)	Score (RT)
(M+H)+	913	99.81				

Compound Chromatograms (overlaid)

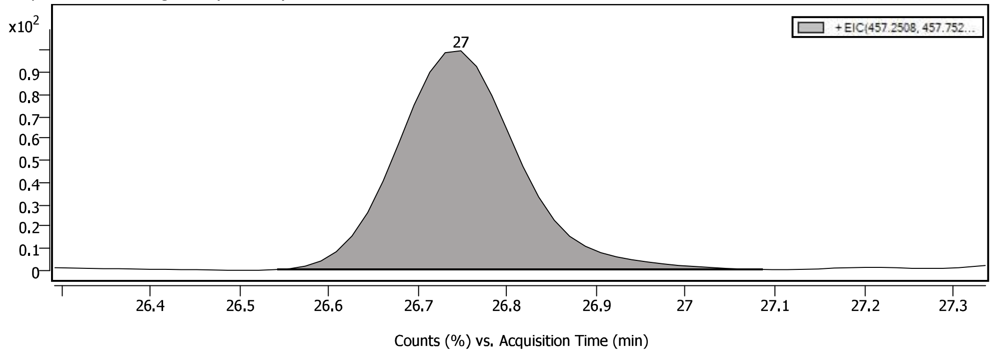

Structure

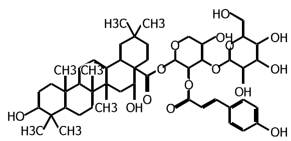

Compound Spectra (overlaid)

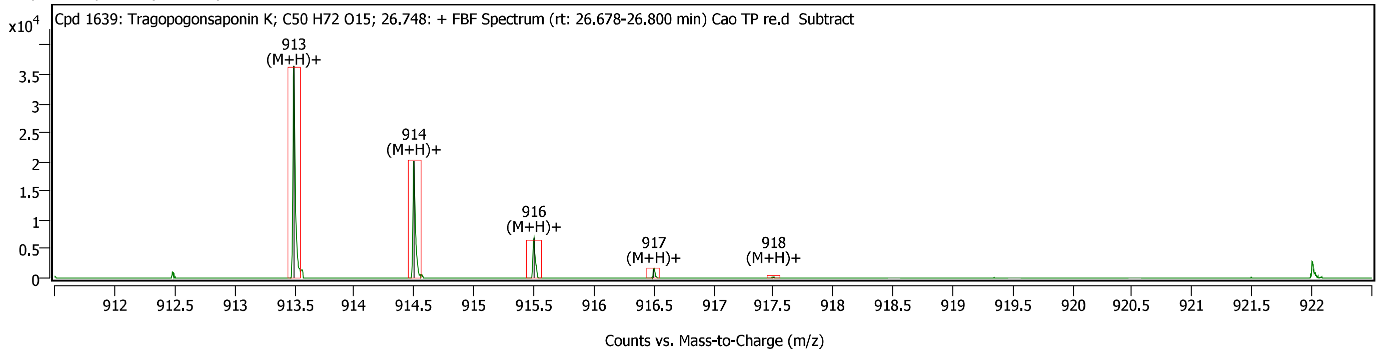

Compound ID Table

Name	Formula	Species	RT	RT Diff	Mass	CAS	ID Source	Score	Score (Lib)	Score (Tgt)
Tragopogonsaponin K	C50 H72 O15	(M+H)+	26.748		912.4871		FBF	99.81		99.81

Cpd 148: Ephedrannin A

Name	Formula	RT	RI	Mass	Diff (Tgt, ppm)	CAS	ID Source	Score	Algorithm
Ephedrannin A	C30 H20 O11	26.765		556.1005	-0.13	82001-39-6	FBF	99.44	FBF

Species	m/z	Score (Tgt)	Score (Lib)	Score (DB)	Score (MFG)	Score (RT)
(M+H)+	557	99.44				

Compound Chromatograms (overlaid)

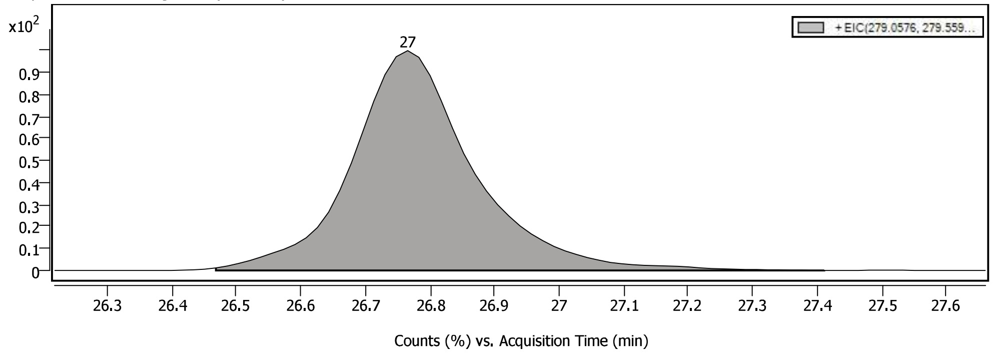

Structure

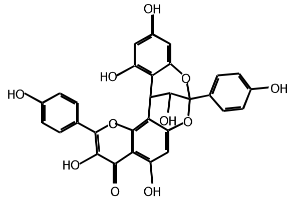

Compound Spectra (overlaid)

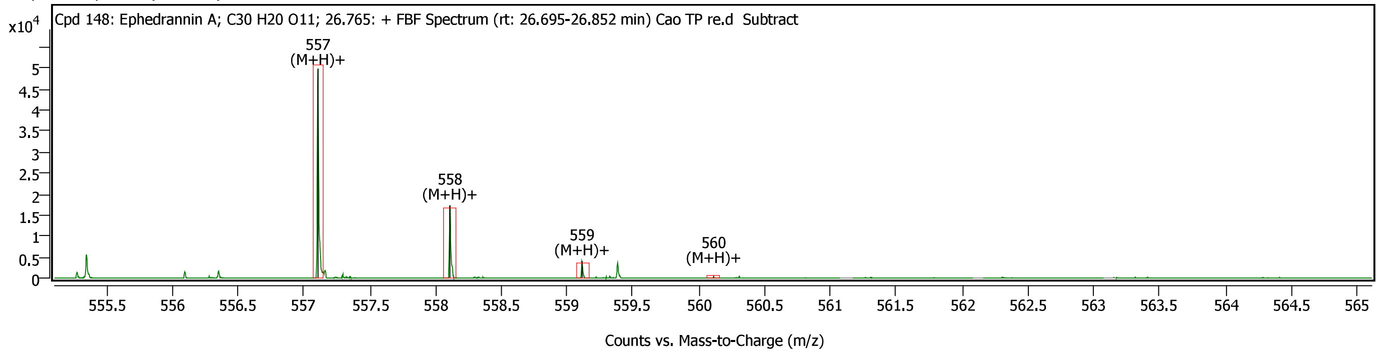

Compound ID Table

Name	Formula	Species	RT	RT Diff	Mass	CAS	ID Source	Score	Score (Lib)	Score (Tgt)
Ephedrannin A	C30 H20 O11	(M+H)+	26.765		556.1005	82001-39-6	FBF	99.44		99.44

Cpd 1366: C16 Sphinganine

Name	Formula	RT	RI	Mass	Diff (Tgt, ppm)	CAS	ID Source	Score	Algorithm
C16 Sphinganine	C16 H35 N O2	27.254		273.2674	2.26		FBF	96.92	FBF

Species	m/z	Score (Tgt)	Score (Lib)	Score (DB)	Score (MFG)	Score (RT)
(M+H)+	274	96.92				

Compound Chromatograms (overlaid)

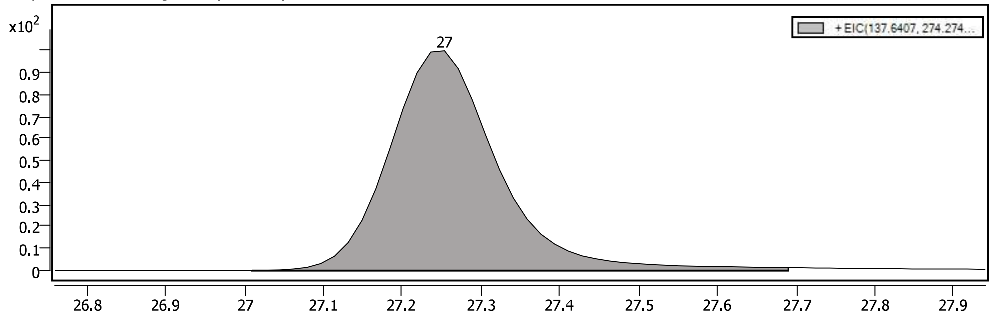

Structure

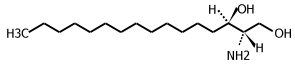

Compound Spectra (overlaid)

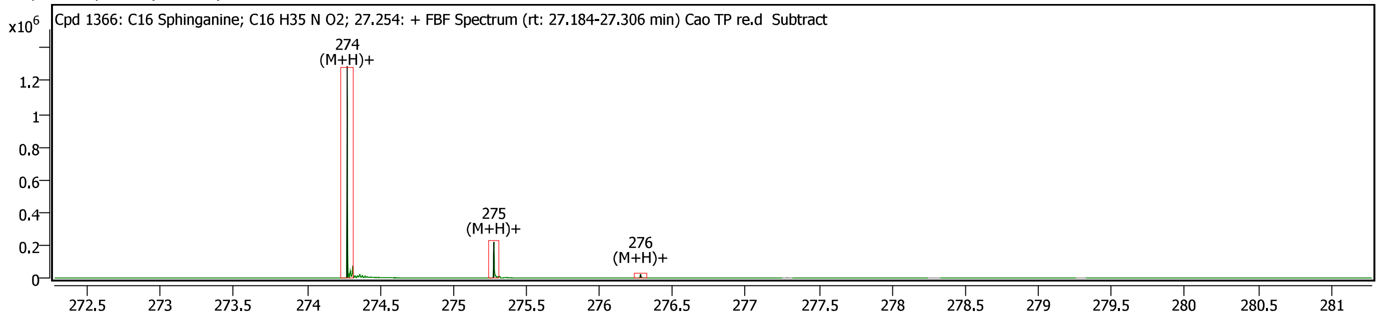

Compound ID Table

Name	Formula	Species	RT	RT Diff	Mass	CAS	ID Source	Score	Score (Lib)	Score (Tgt)
C16 Sphinganine	C16 H35 N O2	(M+H)+	27.254		273.2674		FBF	96.92		96.92

Cpd 220: Kurilensoside G

Name	Formula	RT	RI	Mass	Diff (Tgt, ppm)	CAS	ID Source	Score	Algorithm
Kurilensoside G	C32 H54 O10	27.585		598.3718	0.21		FBF	98.99	FBF

Species	m/z	Score (Tgt)	Score (Lib)	Score (DB)	Score (MFG)	Score (RT)
(M+H)+	599	98.99				

Compound Chromatograms (overlaid)

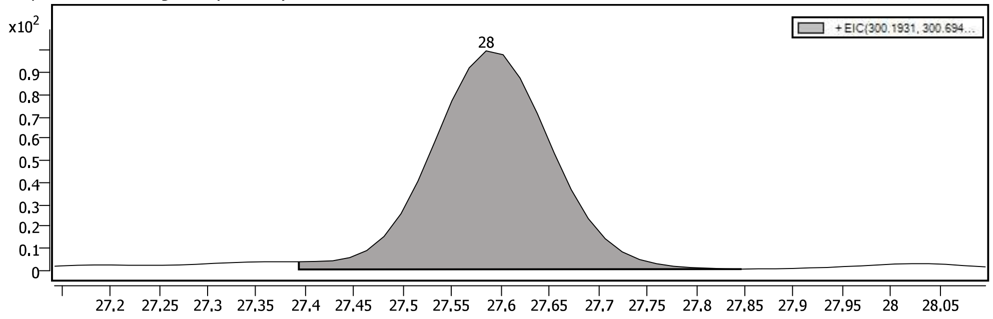

Structure

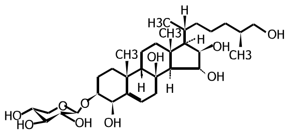

Compound Screening Report

Compound Spectra (overlaid)

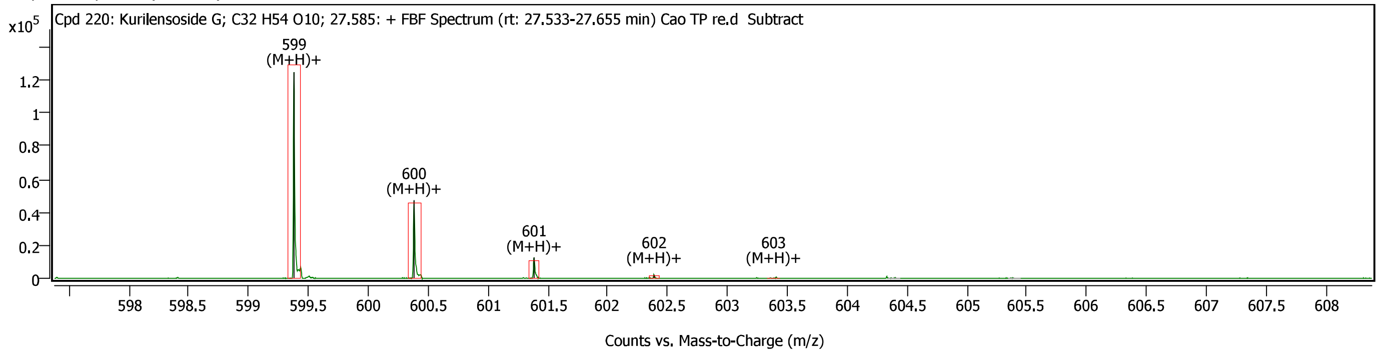

Compound ID Table

Name	Formula	Species	RT	RT Diff	Mass	CAS	ID Source	Score	Score (Lib)	Score (Tgt)
Kurilensoside G	C32 H54 O10	(M+H)+	27.585		598.3718		FBF	98.99		98.99

Cpd 475: Chikusetsusaponin Ia

Name	Formula	RT	RI	Mass	Diff (Tgt, ppm)	CAS	ID Source	Score	Algorithm
Chikusetsusaponin Ia	C41 H70 O12	27.672		754.4845	-2.92		FBF	95.15	FBF

Species	m/z	Score (Tgt)	Score (Lib)	Score (DB)	Score (MFG)	Score (RT)
(M+H)+	755	95.15				

Compound Chromatograms (overlaid)

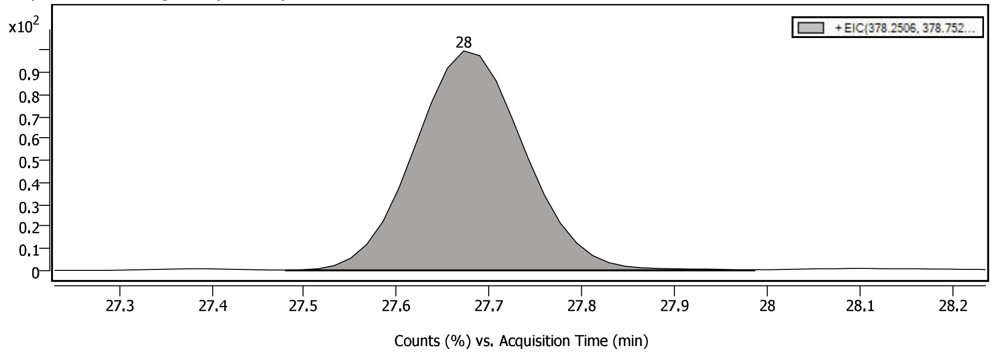

Structure

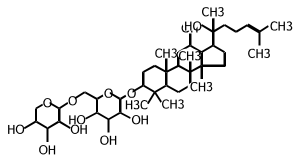

Compound Spectra (overlaid)

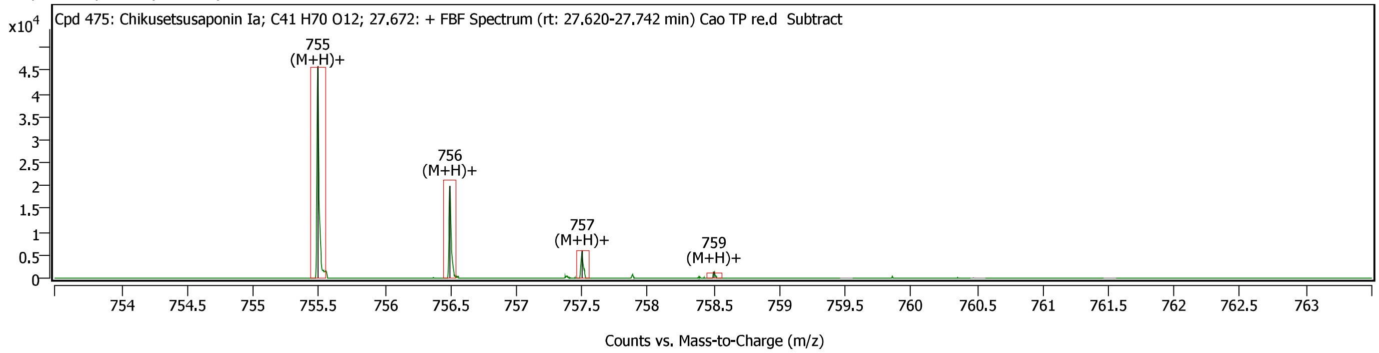

Compound ID Table

Name	Formula	Species	RT	RT Diff	Mass	CAS	ID Source	Score	Score (Lib)	Score (Tgt)
Chikusetsusaponin Ia	C41 H70 O12	(M+H)+	27.672		754.4845		FBF	95.15		95.15

Cpd 890: <thio-Miltefosine>

Name	Formula	RT	RI	Mass	Diff (Tgt, ppm)	CAS	ID Source	Score	Algorithm
<thio-Miltefosine>	C21 H47 N O3 P S	27.777		424.3021	1.56	943022-11-5	FBF	86.61	FBF

Species	m/z	Score (Tgt)	Score (Lib)	Score (DB)	Score (MFG)	Score (RT)
(M+2H)+2 (M+H)+	213 425	86.61				

Compound Chromatograms (overlaid)

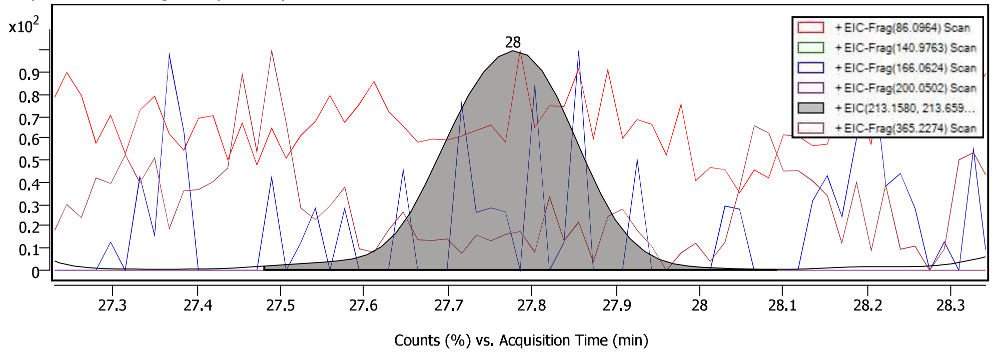

Structure

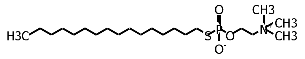

Coelution Plot

Compound Spectra (overlaid)

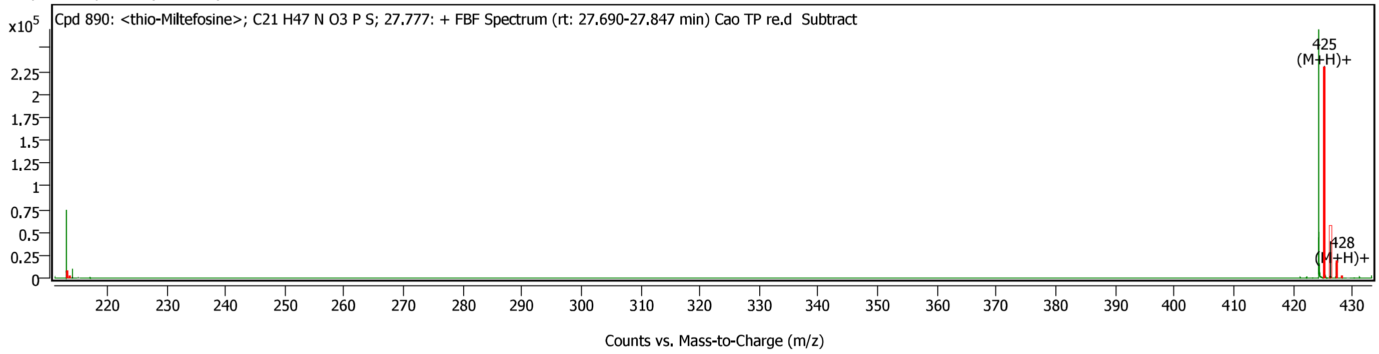

Fragment Spectrum (raw)

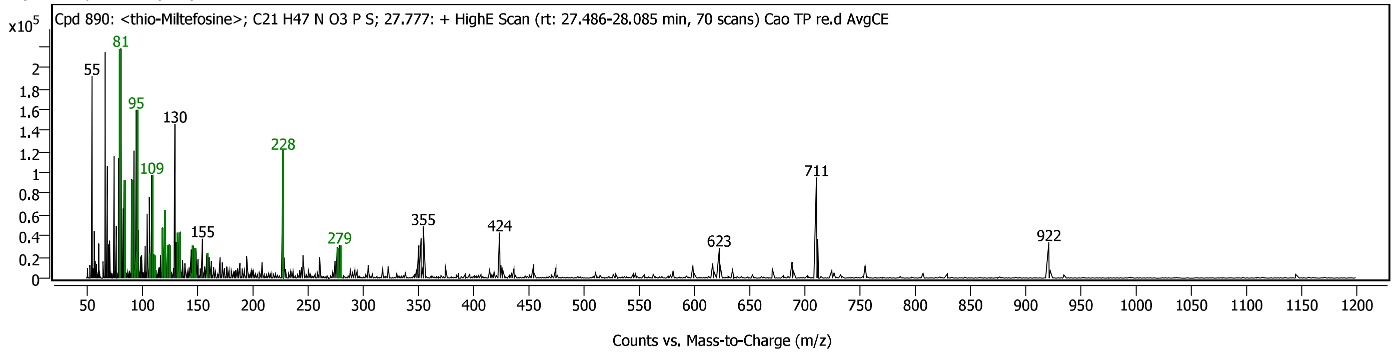

Compound ID Table

Name	Formula	Species	RT	RT Diff	Mass	CAS	ID Source	Score	Score (Lib)	Score (Tgt)
<thio-Miltefosine>	C21 H47 N O3 P S	(M+2H)+2<br>(M+H)+	27.777		424.3021	943022-11-5	FBF	86.61		86.61

Cpd 500: 4,4-Difluoropregn-5-ene-3,20-dione

Name	Formula	RT	RI	Mass	Diff (Tgt, ppm)	CAS	ID Source	Score	Algorithm
4,4-Difluoropregn-5-ene-3,20-dione	C21 H28 F2 O2	27.864		350.2070	3.70		FBF	93.59	FBF

Species	m/z	Score (Tgt)	Score (Lib)	Score (DB)	Score (MFG)	Score (RT)
(M+H)+	351	93.59				

Compound Chromatograms (overlaid)

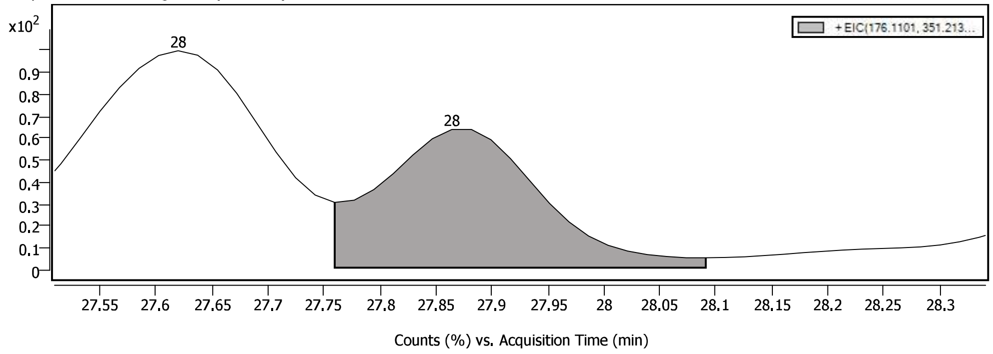

Structure

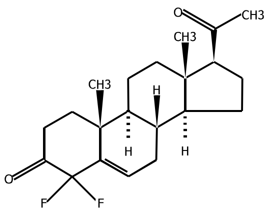

Compound Screening Report

Compound Spectra (overlaid)

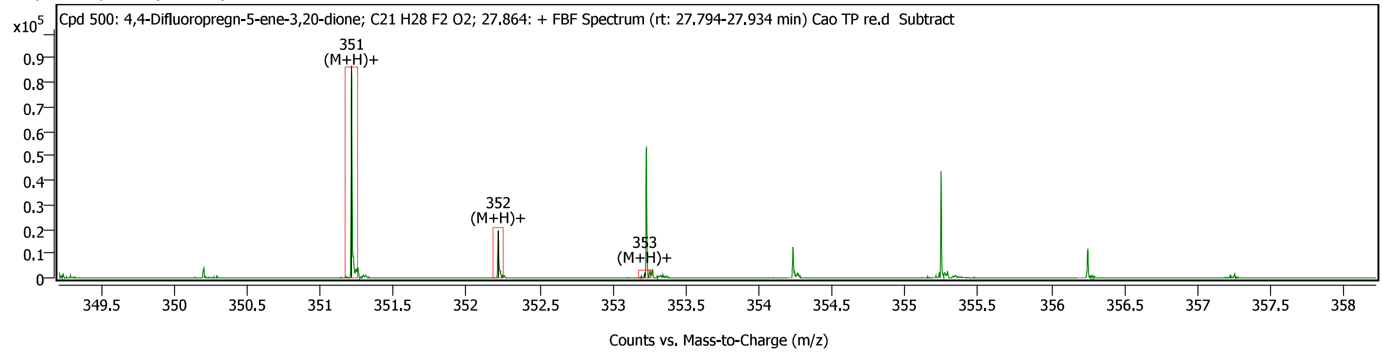

Compound ID Table

Name	Formula	Species	RT	RT Diff	Mass	CAS	ID Source	Score	Score (Lib)	Score (Tgt)
4,4-Difluoropregn-5-ene-3,20-dione	C21 H28 F2 O2	(M+H)+	27.864		350.2070		FBF	93.59		93.59

Cpd 1694: Botrydial

Name	Formula	RT	RI	Mass	Diff (Tgt, ppm)	CAS	ID Source	Score	Algorithm
Botrydial	C17 H26 O5	27.917		310.1782	0.43		M-FBF	98.68	FBF

Species	m/z	Score (Tgt)	Score (Lib)	Score (DB)	Score (MFG)	Score (RT)
(M+H)+	311	98.68				

Compound Chromatograms (overlaid)

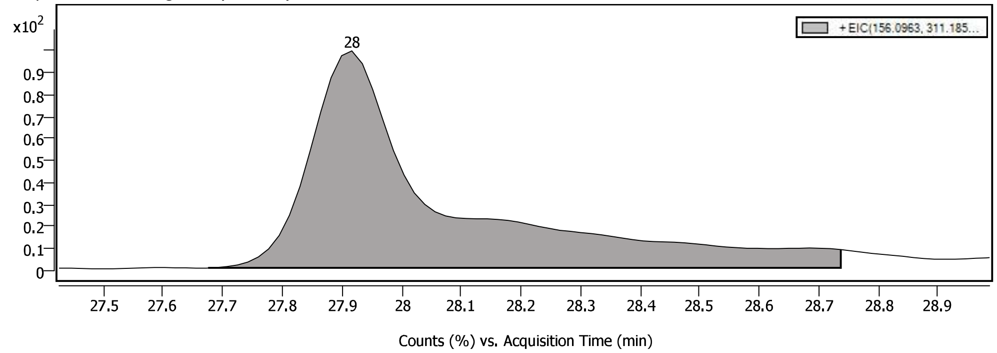

Structure

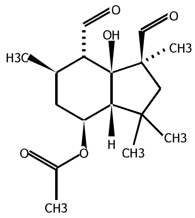

Compound Spectra (overlaid)

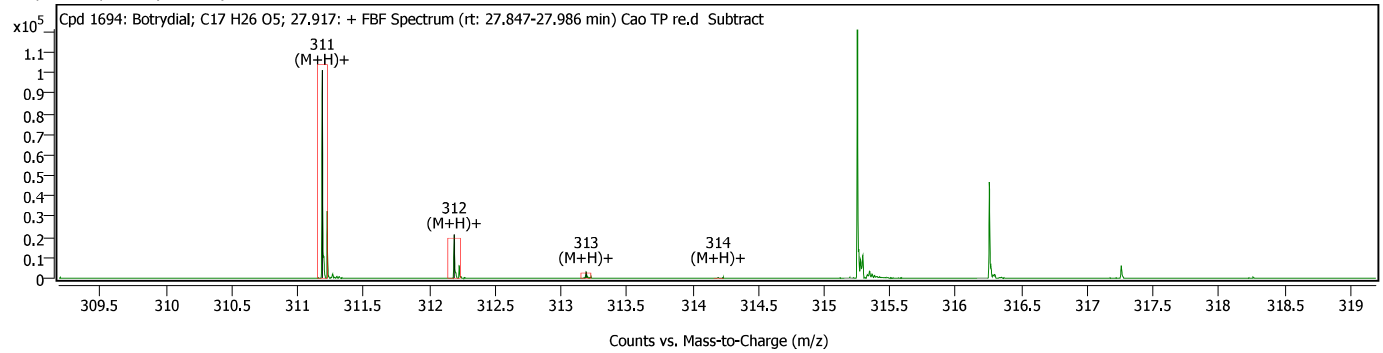

Compound ID Table

Name	Formula	Species	RT	RT Diff	Mass	CAS	ID Source	Score	Score (Lib)	Score (Tgt)
Botrydial	C17 H26 O5	(M+H)+	27.917		310.1782		FBF	98.68		98.68
Valdiate	C17 H26 O5	(M+H)+	27.917		310.1782	162232-32-8	FBF	98.68		98.68
methyl 8-[2-(2-formyl-vinyl)-3-hydroxy-5-oxo-cyclopentyl]-octanoate	C17 H26 O5	(M+H)+	27.917		310.1782		FBF	98.68		98.68
3-Hydroxy-6,8-dimethoxy-7(11)-eremophilen-12,8-olide	C17 H26 O5	(M+H)+	27.917		310.1782	162613-65-2	FBF	98.68		98.68

Cpd 1357: 3,3-Difluoro-5alpha-androstan-17beta-yl acetate

Name	Formula	RT	RI	Mass	Diff (Tgt, ppm)	CAS	ID Source	Score	Algorithm
3,3-Difluoro-5alpha-androstan-17beta-yl acetate	C21 H32 F2 O2	27.986		354.2387	4.63	1827-75-4	FBF	90.42	FBF

Species	m/z	Score (Tgt)	Score (Lib)	Score (DB)	Score (MFG)	Score (RT)
(M+H)+	355	90.42				

Compound Chromatograms (overlaid)

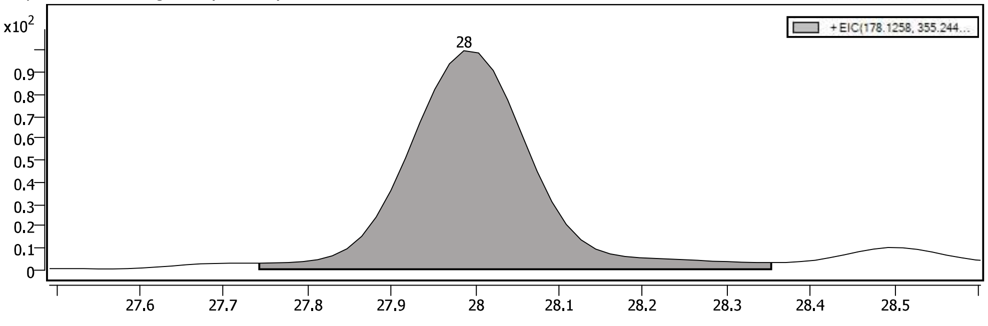

Structure

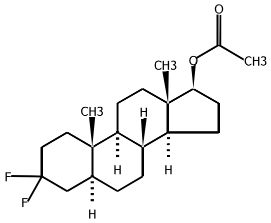

Compound Spectra (overlaid)

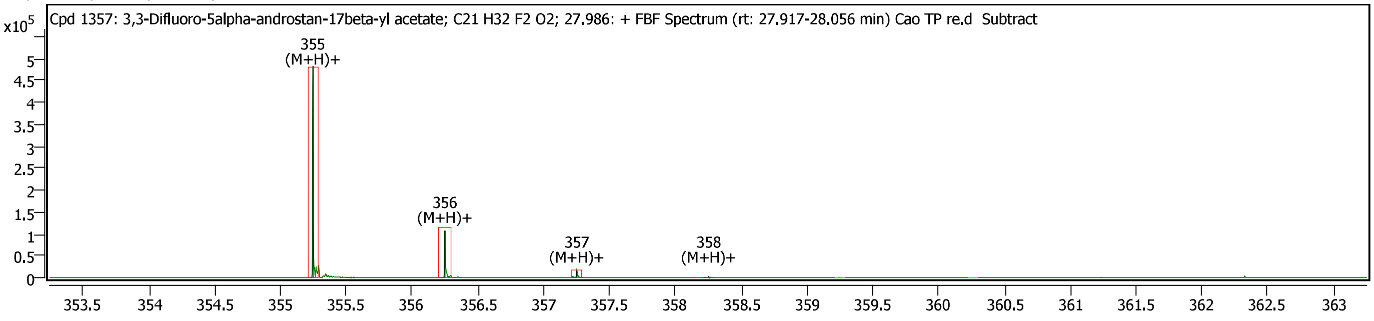

Compound ID Table

Name	Formula	Species	RT	RT Diff	Mass	CAS	ID Source	Score	Score (Lib)	Score (Tgt)
3,3-Difluoro-5alpha-androstan-17beta-yl acetate	C21 H32 F2 O2	(M+H)+	27.986		354.2387	1827-75-4	FBF	90.42		90.42

Cpd 86: 12,13-DiHOME

Name	Formula	RT	RI	Mass	Diff (Tgt, ppm)	CAS	ID Source	Score	Algorithm
12,13-DiHOME	C18 H34 O4	28.004		314.2461	1.39		M-FBF-FragConfirm	98.09	FBF

Species	m/z	Score (Tgt)	Score (Lib)	Score (DB)	Score (MFG)	Score (RT)
(M+H)+	315	98.09				

Compound Chromatograms (overlaid)

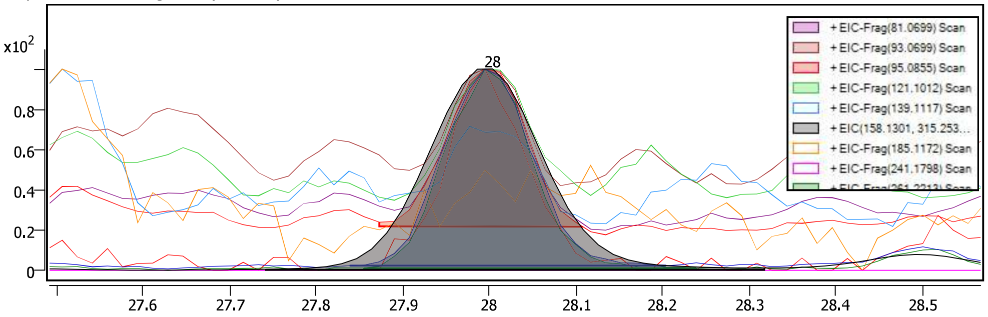

Structure

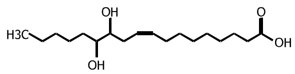

Coelution Plot

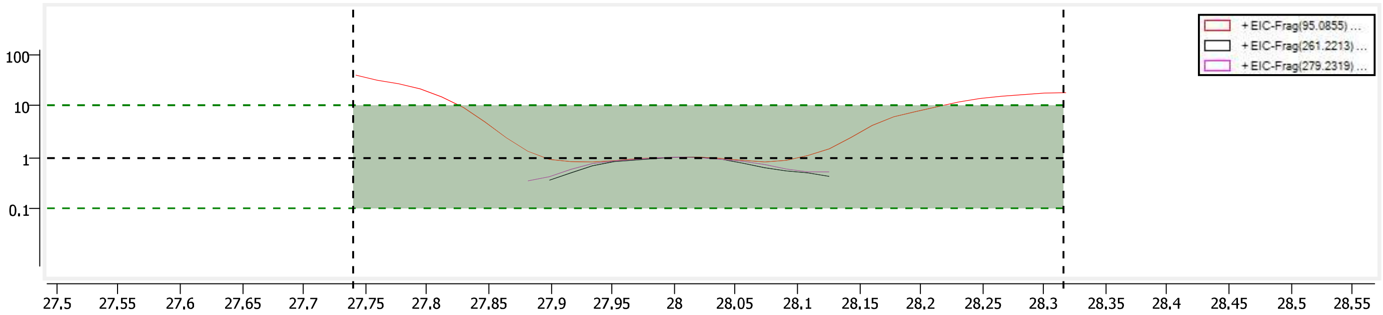

Compound Screening Report

Compound Spectra (overlaid)

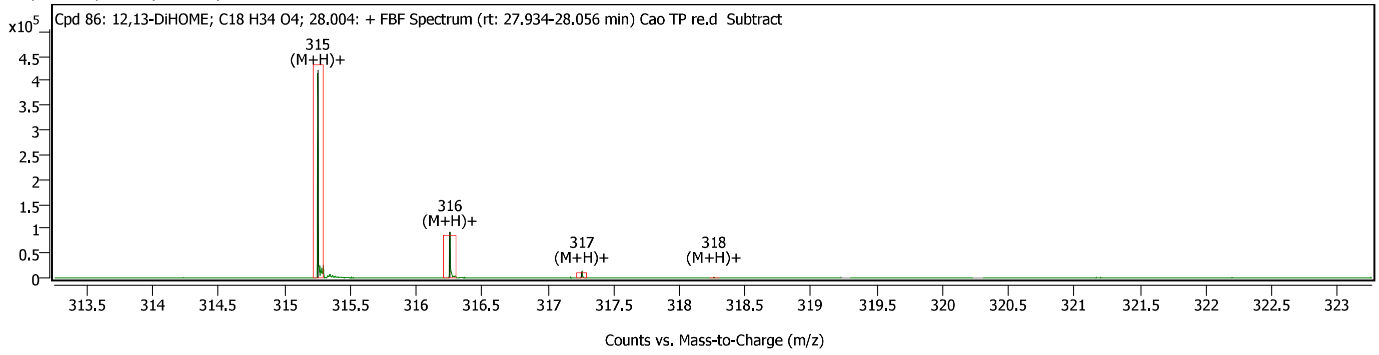

Fragment Spectrum (clean)

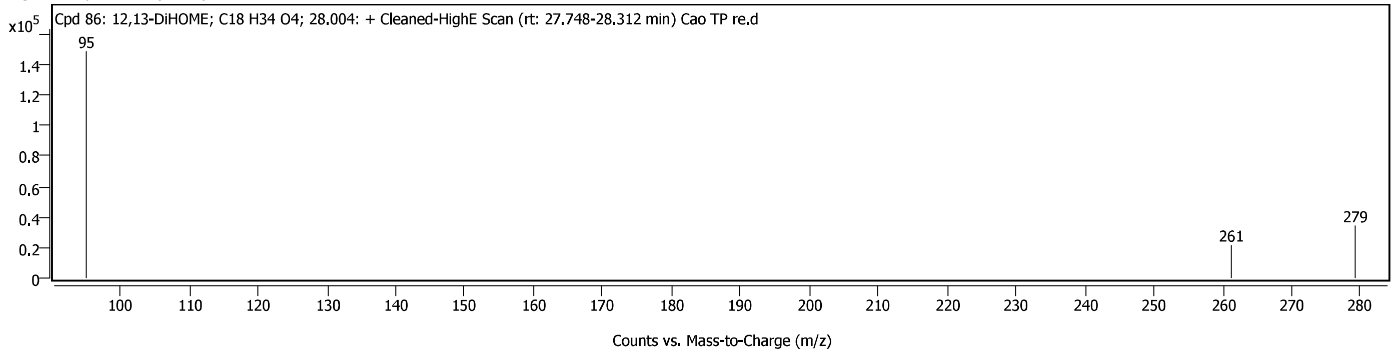

Fragment Spectrum (raw)

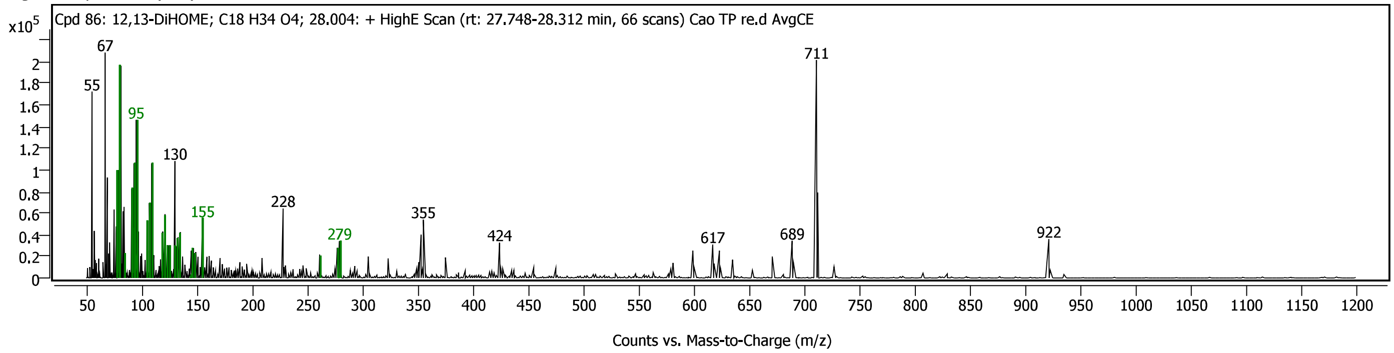

Compound Screening Report

Compound ID Table

Name	Formula	Species	RT	RT Diff	Mass	CAS	ID Source	Score	Score (Lib)	Score (Tgt)
12,13-DiHOME	C18 H34 O4	(M+H)+	28.004		314.2461		FBF-FragConfirm	98.09		98.09
12S-HpOME(13Z)	C18 H34 O4	(M+H)+	28.004		314.2461		FBF-FragConfirm	98.09		98.09
(±)12,13-DiHOME	C18 H34 O4	(M+H)+	28.004		314.2461	263399-35-5	FBF-FragConfirm	98.09		98.09
12R-HpOME(13Z)	C18 H34 O4	(M+H)+	28.004		314.2461		FBF-FragConfirm	98.09		98.09
12S-HpOME(13E)	C18 H34 O4	(M+H)+	28.004		314.2461		FBF-FragConfirm	98.09		98.09
14S-HpOME(12E)	C18 H34 O4	(M+H)+	28.004		314.2461		FBF-FragConfirm	98.09		98.09
13R-HpOME(11E)	C18 H34 O4	(M+H)+	28.004		314.2461		FBF-FragConfirm	98.09		98.09
13S-HpOME(11E)	C18 H34 O4	(M+H)+	28.004		314.2461		FBF-FragConfirm	98.09		98.09
14R-HpOME(12E)	C18 H34 O4	(M+H)+	28.004		314.2461		FBF-FragConfirm	98.09		98.09
12,13-dihydroxy-9-octadecenoic acid	C18 H34 O4	(M+H)+	28.004		314.2461		FBF-FragConfirm	98.09		98.09
8R-HpOME	C18 H34 O4	(M+H)+	28.004		314.2461		FBF-FragConfirm	98.09		98.09
12-HpOME(10E)	C18 H34 O4	(M+H)+	28.004		314.2461		FBF-FragConfirm	98.09		98.09
12,13-DiHOME(9)	C18 H34 O4	(M+H)+	28.004		314.2461		FBF-FragConfirm	98.09		98.09
11R-HpOME	C18 H34 O4	(M+H)+	28.004		314.2461		FBF-FragConfirm	98.09		98.09
11-HpOME(9Z)	C18 H34 O4	(M+H)+	28.004		314.2461		FBF-FragConfirm	98.09		98.09
11-HpOME	C18 H34 O4	(M+H)+	28.004		314.2461		FBF-FragConfirm	98.09		98.09
10S-HpOME	C18 H34 O4	(M+H)+	28.004		314.2461		FBF-FragConfirm	98.09		98.09
10R-HpOME(11Z)	C18 H34 O4	(M+H)+	28.004		314.2461		FBF-FragConfirm	98.09		98.09
10-HpOME	C18 H34 O4	(M+H)+	28.004		314.2461		FBF-FragConfirm	98.09		98.09
18-hydroxy-9S,10R-epoxy-stearic acid	C18 H34 O4	(M+H)+	28.004		314.2461		FBF-FragConfirm	98.09		98.09
12R-HpOME(13E)	C18 H34 O4	(M+H)+	28.004		314.2461		FBF-FragConfirm	98.09		98.09
18-hydroxy-9R,10S-epoxy-stearic acid	C18 H34 O4	(M+H)+	28.004		314.2461		FBF-FragConfirm	98.09		98.09
9,10-dihydroxy-12-octadecenoic acid	C18 H34 O4	(M+H)+	28.004		314.2461		FBF-FragConfirm	98.09		98.09
Octadecanedioic acid	C18 H34 O4	(M+H)+	28.004		314.2461	871-70-5	FBF-FragConfirm	98.09		98.09
6-HpOME(7E)	C18 H34 O4	(M+H)+	28.004		314.2461		FBF-FragConfirm	98.09		98.09
9-HpOME(7E)	C18 H34 O4	(M+H)+	28.004		314.2461		FBF-FragConfirm	98.09		98.09
9-HpOME	C18 H34 O4	(M+H)+	28.004		314.2461		FBF-FragConfirm	98.09		98.09
9,13-dihydroxy-11-octadecenoic acid	C18 H34 O4	(M+H)+	28.004		314.2461		FBF-FragConfirm	98.09		98.09
9,13-dihydroxy-10-octadecenoic acid	C18 H34 O4	(M+H)+	28.004		314.2461		FBF-FragConfirm	98.09		98.09
9,13-DiHOME(11)	C18 H34 O4	(M+H)+	28.004		314.2461		FBF-FragConfirm	98.09		98.09
9,13-DiHOME(10)	C18 H34 O4	(M+H)+	28.004		314.2461		FBF-FragConfirm	98.09		98.09
9,10-Epoxy-18-hydroxystearate	C18 H34 O4	(M+H)+	28.004		314.2461		FBF-FragConfirm	98.09		98.09
Dibutyl decanedioate	C18 H34 O4	(M+H)+	28.004		314.2461	109-43-3	FBF-FragConfirm	98.09		98.09
9,10-DiHOME(12)	C18 H34 O4	(M+H)+	28.004		314.2461		FBF-FragConfirm	98.09		98.09
8-HpOME	C18 H34 O4	(M+H)+	28.004		314.2461		FBF-FragConfirm	98.09		98.09
9,10-DiHOME	C18 H34 O4	(M+H)+	28.004		314.2461	263399-34-4	FBF-FragConfirm	98.09		98.09
7S,10S-diHOME	C18 H34 O4	(M+H)+	28.004		314.2461		FBF-FragConfirm	98.09		98.09
7S,8S-DiHOME(9Z)	C18 H34 O4	(M+H)+	28.004		314.2461		FBF-FragConfirm	98.09		98.09
7-HpOME(5E)	C18 H34 O4	(M+H)+	28.004		314.2461		FBF-FragConfirm	98.09		98.09
8-HpOME(6E)	C18 H34 O4	(M+H)+	28.004		314.2461		FBF-FragConfirm	98.09		98.09
5S,8R-DiHOME	C18 H34 O4	(M+H)+	28.004		314.2461		FBF-FragConfirm	98.09		98.09
8R,11S-DiHOME	C18 H34 O4	(M+H)+	28.004		314.2461		FBF-FragConfirm	98.09		98.09

Cpd 279: PI(10:0/16:0)

Name	Formula	RT	RI	Mass	Diff (Tgt, ppm)	CAS	ID Source	Score	Algorithm
PI(10:0/16:0)	C35 H67 O13 P	28.091		726.4317	-0.29		M-FBF	91.29	FBF

Species	m/z	Score (Tgt)	Score (Lib)	Score (DB)	Score (MFG)	Score (RT)
(M+H)+	727	91.29				

Compound Chromatograms (overlaid)

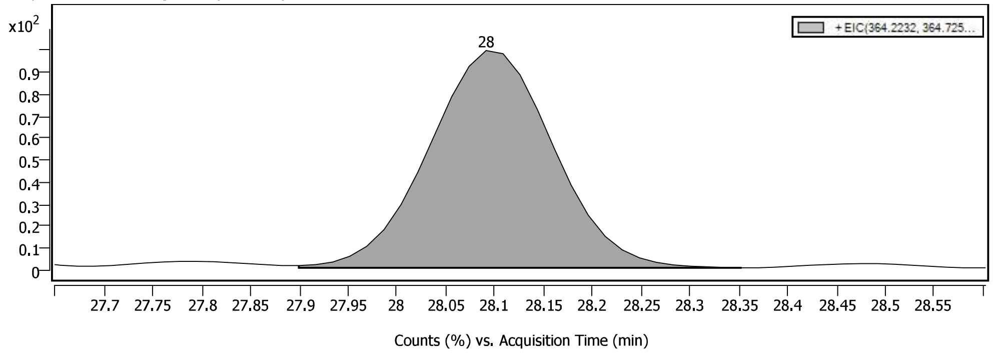

Structure

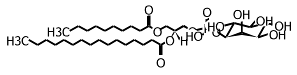

Compound Screening Report

Compound Spectra (overlaid)

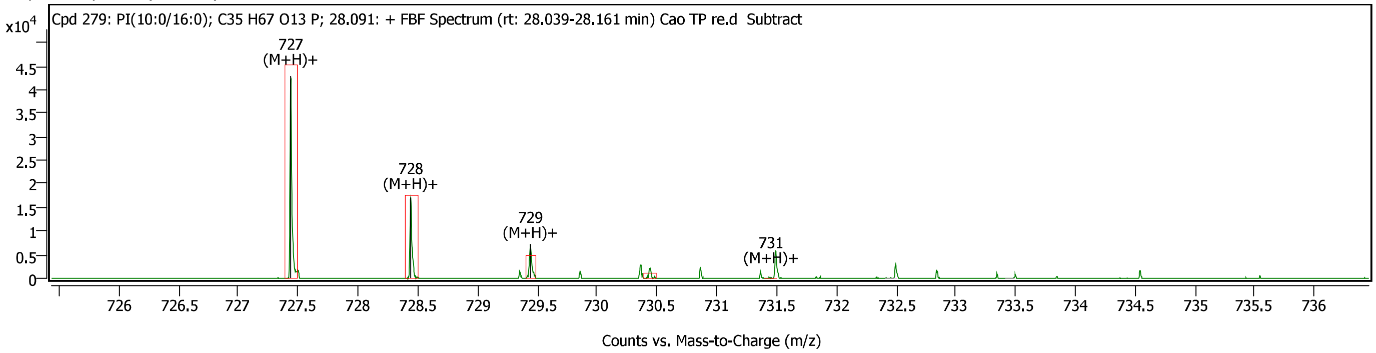

Compound ID Table

Name	Formula	Species	RT	RT Diff	Mass	CAS	ID Source	Score	Score (Lib)	Score (Tgt)
PI(10:0/16:0)	C35 H67 O13 P	(M+H)+	28.091		726.4317		FBF	91.29		91.29
PI(14:0/12:0)	C35 H67 O13 P	(M+H)+	28.091		726.4317		FBF	91.29		91.29
PI(13:0/13:0)	C35 H67 O13 P	(M+H)+	28.091		726.4317		FBF	91.29		91.29
PI(12:0/14:0)	C35 H67 O13 P	(M+H)+	28.091		726.4317		FBF	91.29		91.29

Cpd 1655: Notoginsenoside R10

Name	Formula	RT	RI	Mass	Diff (Tgt, ppm)	CAS	ID Source	Score	Algorithm
Notoginsenoside R10	C30 H50 O9	28.283		554.3455	0.08	335157-20-5	FBF	99.73	FBF

Species	m/z	Score (Tgt)	Score (Lib)	Score (DB)	Score (MFG)	Score (RT)
(M+H)+	555	99.73				

Compound Chromatograms (overlaid)

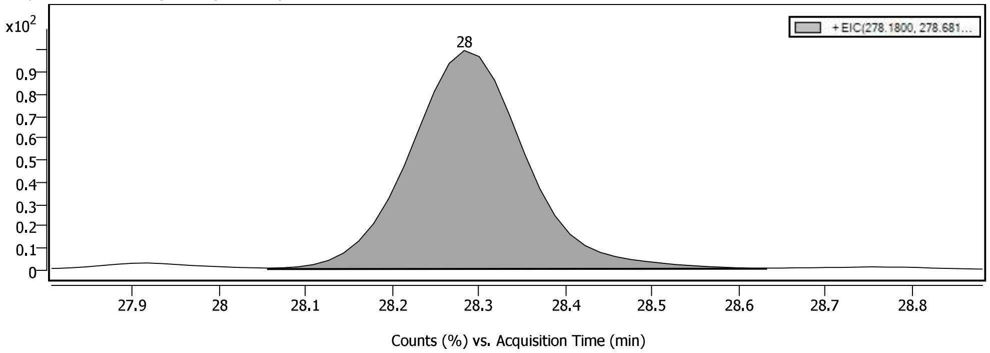

Structure

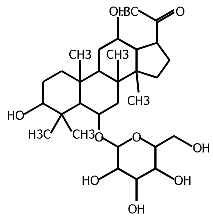

Compound Spectra (overlaid)

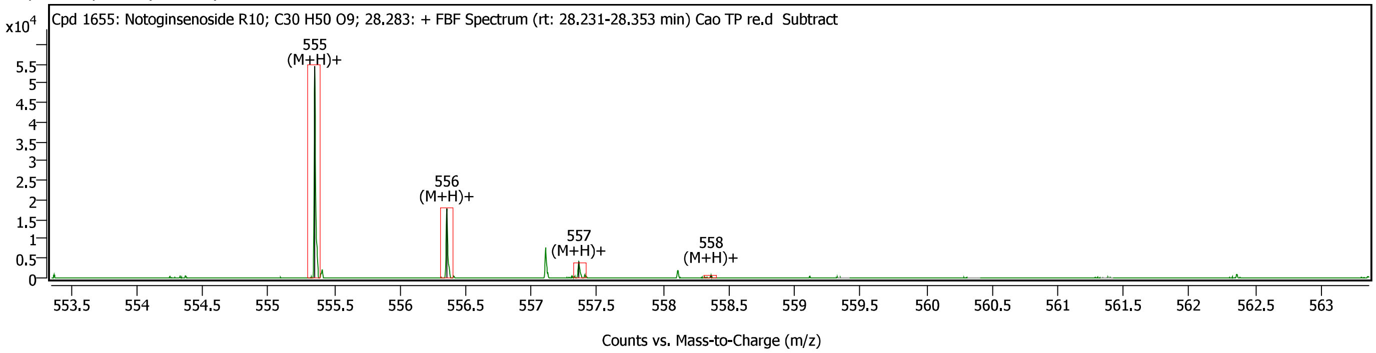

Compound ID Table

Name	Formula	Species	RT	RT Diff	Mass	CAS	ID Source	Score	Score (Lib)	Score (Tgt)
Notoginsenoside R10	C30 H50 O9	(M+H)+	28.283		554.3455	335157-20-5	FBF	99.73		99.73

Cpd 186: Evasterioside D

Name	Formula	RT	RI	Mass	Diff (Tgt, ppm)	CAS	ID Source	Score	Algorithm
Evasterioside D	C33 H58 O10	28.423		614.4025	-0.74		M-FBF	99.61	FBF

Species	m/z	Score (Tgt)	Score (Lib)	Score (DB)	Score (MFG)	Score (RT)
(M+H)+	615	99.61				

Compound Screening Report

Compound Chromatograms (overlaid)

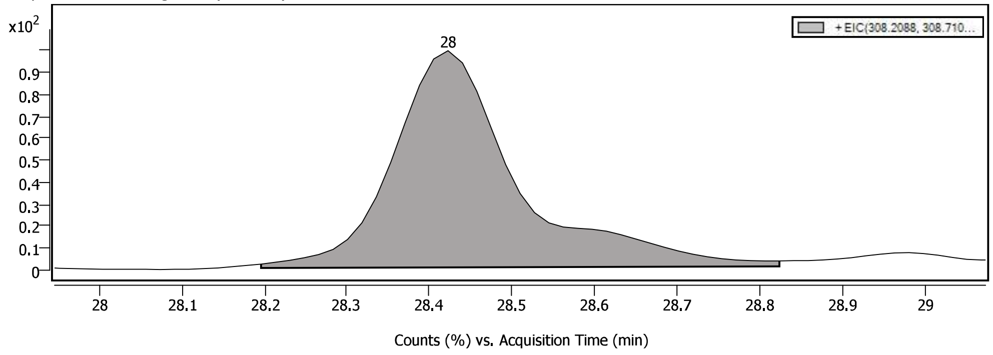

Structure

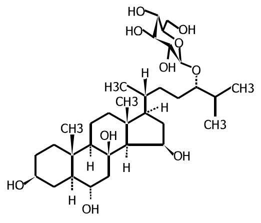

Compound Spectra (overlaid)

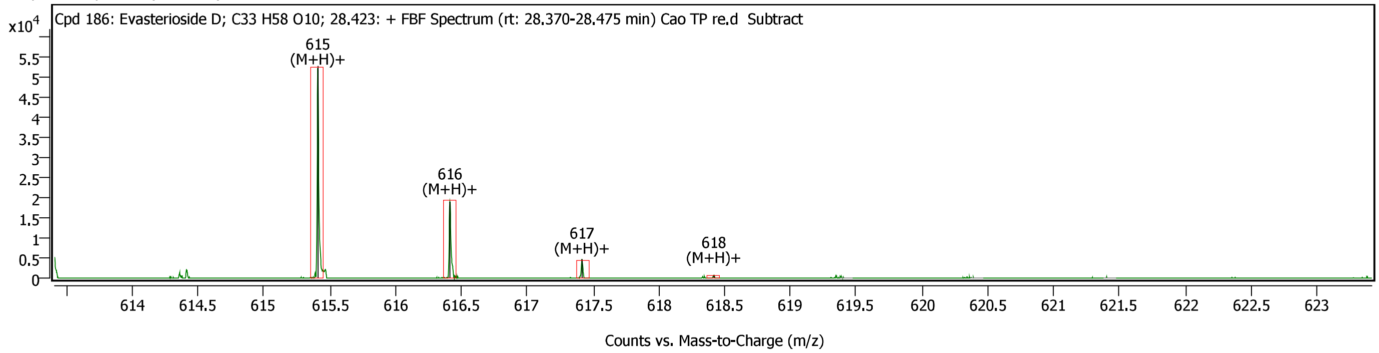

Compound ID Table

Name	Formula	Species	RT	RT Diff	Mass	CAS	ID Source	Score	Score (Lib)	Score (Tgt)
Evasterioside D	C33 H58 O10	(M+H)+	28.423		614.4025		FBF	99.61		99.61
Antibiotic X 14889C	C33 H58 O10	(M+H)+	28.423		614.4025	97671-95-9	FBF	99.61		99.61
Kurilensoside E	C33 H58 O10	(M+H)+	28.423		614.4025		FBF	99.61		99.61

Cpd 1462: Corrinoid

Name	Formula	RT	RI	Mass	Diff (Tgt, ppm)	CAS	ID Source	Score	Algorithm
Corrinoid	C19 H22 N4	28.440		306.1834	-3.28	262-76-0	FBF	94.39	FBF

Species	m/z	Score (Tgt)	Score (Lib)	Score (DB)	Score (MFG)	Score (RT)
(M+H)+	307	94.39				

Compound Chromatograms (overlaid)

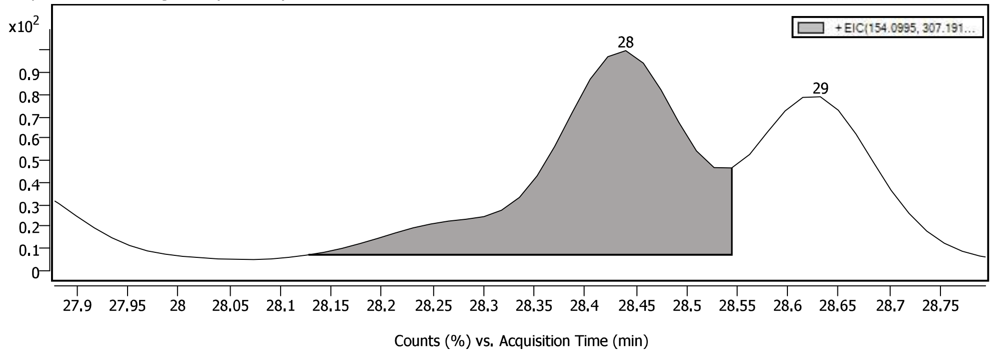

Structure

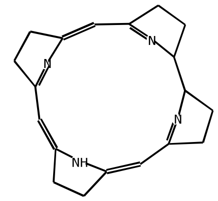

Compound Spectra (overlaid)

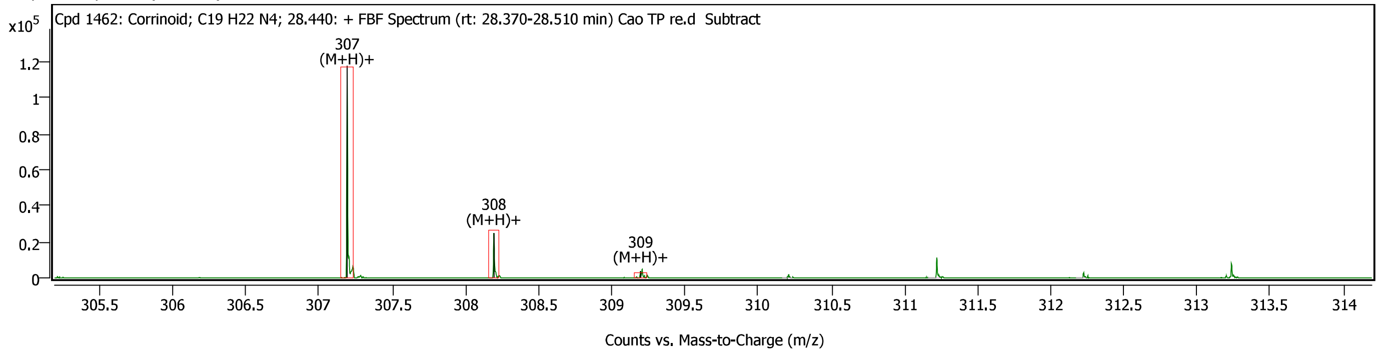

Compound ID Table

Name	Formula	Species	RT	RT Diff	Mass	CAS	ID Source	Score	Score (Lib)	Score (Tgt)
Corrinoid	C19 H22 N4	(M+H)+	28.440		306.1834	262-76-0	FBF	94.39		94.39

Cpd 128: Notoginsenoside T2

Name	Formula	RT	RI	Mass	Diff (Tgt, ppm)	CAS	ID Source	Score	Algorithm
Notoginsenoside T2	C37 H62 O10	28.510		666.4320	-3.47	343962-54-9	FBF	92.63	FBF

Species	m/z	Score (Tgt)	Score (Lib)	Score (DB)	Score (MFG)	Score (RT)
(M+H)+	667	92.63				

Compound Chromatograms (overlaid)

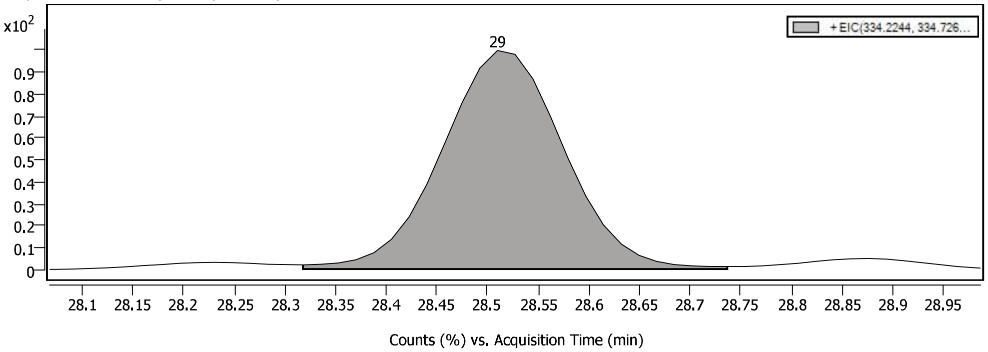

Structure

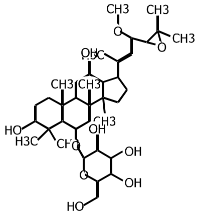

Compound Spectra (overlaid)

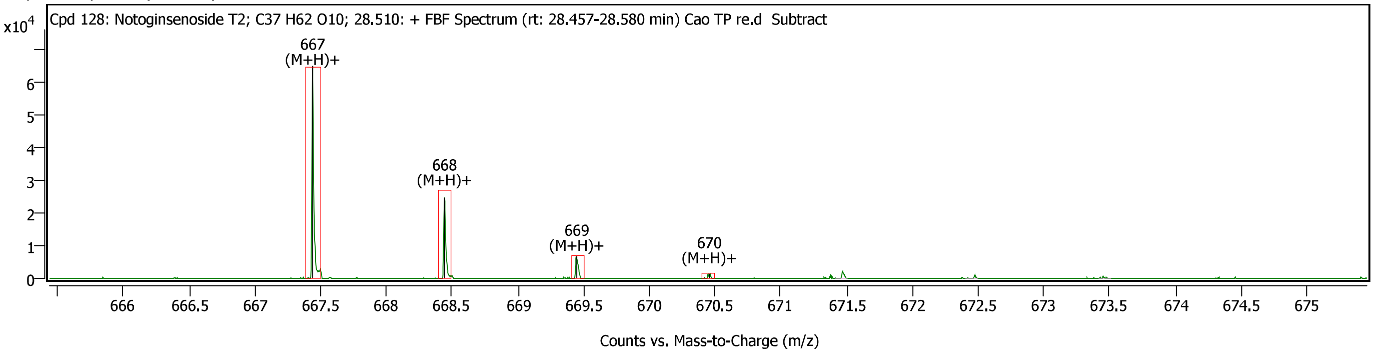

Compound ID Table

Name	Formula	Species	RT	RT Diff	Mass	CAS	ID Source	Score	Score (Lib)	Score (Tgt)
Notoginsenoside T2	C37 H62 O10	(M+H)+	28.510		666.4320	343962-54-9	FBF	92.63		92.63

Cpd 604: <PAz-PC>

Name	Formula	RT	RI	Mass	Diff (Tgt, ppm)	CAS	ID Source	Score	Algorithm
<PAz-PC>	C33 H65 N O10 P	28.510		666.4320	-3.90	117746-89-1	FBF	91.88	FBF

Species	m/z	Score (Tgt)	Score (Lib)	Score (DB)	Score (MFG)	Score (RT)
(M+H)+	667	91.88				

Compound Chromatograms (overlaid)

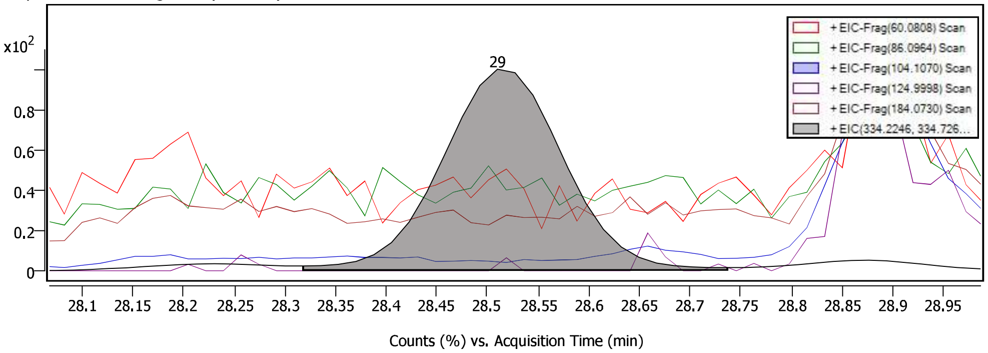

Structure

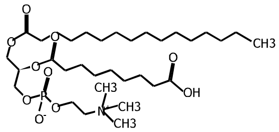

Coelution Plot

Compound Spectra (overlaid)

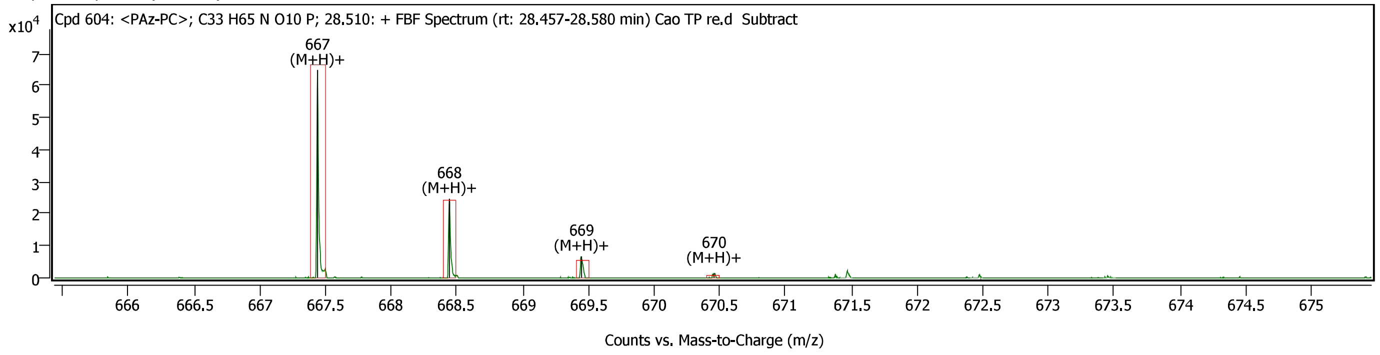

Fragment Spectrum (raw)

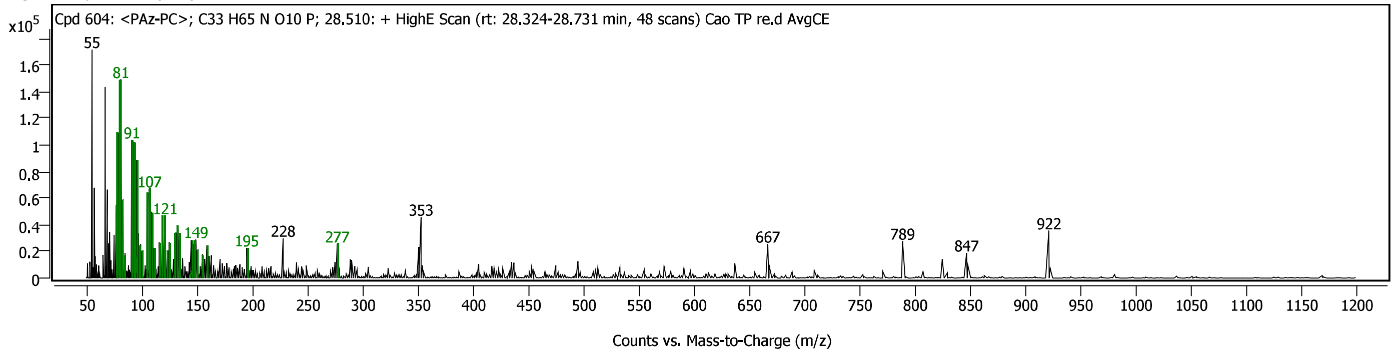

Compound ID Table

Name	Formula	Species	RT	RT Diff	Mass	CAS	ID Source	Score	Score (Lib)	Score (Tgt)
<PAz-PC>	C33 H65 N O10 P	(M+H)+	28.510		666.4320	117746-89-1	FBF	91.88		91.88

Cpd 1461: Corrinoid

Name	Formula	RT	RI	Mass	Diff (Tgt, ppm)	CAS	ID Source	Score	Algorithm
Corrinoid	C19 H22 N4	28.632		306.1833	-3.70	262-76-0	FBF	93.49	FBF

Species	m/z	Score (Tgt)	Score (Lib)	Score (DB)	Score (MFG)	Score (RT)
(M+H)+	307	93.49				

Compound Chromatograms (overlaid)

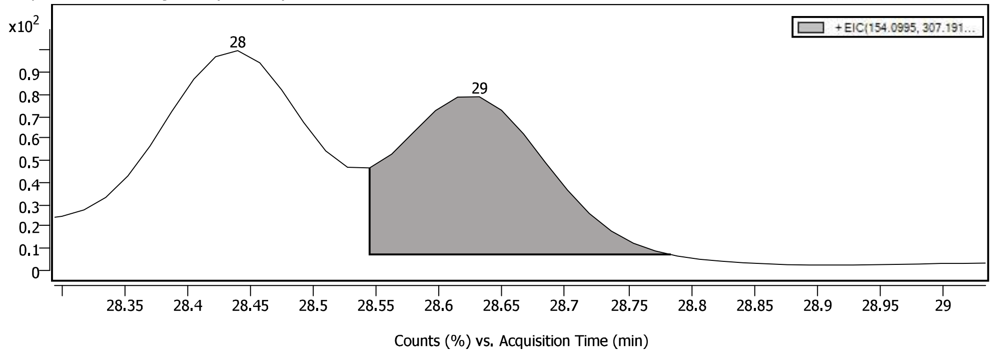

Structure

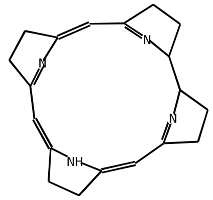

Compound Spectra (overlaid)

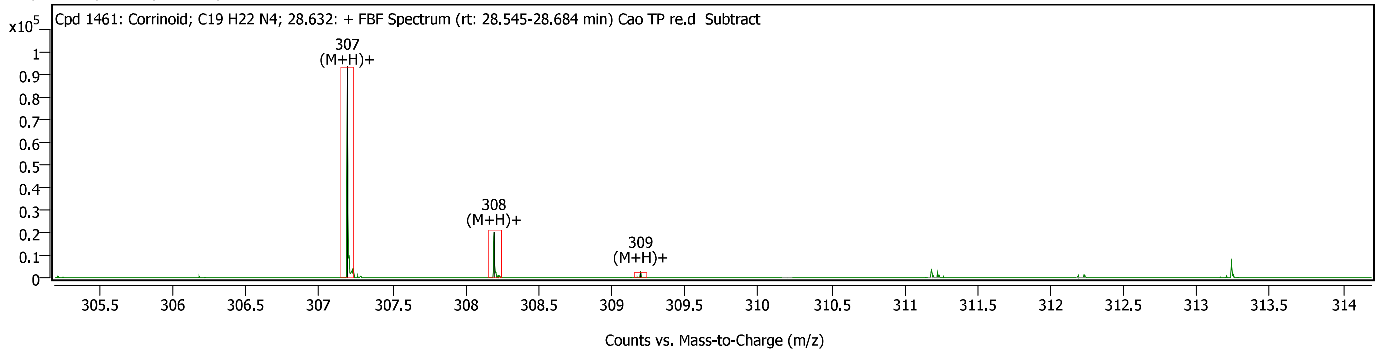

Compound ID Table

Name	Formula	Species	RT	RT Diff	Mass	CAS	ID Source	Score	Score (Lib)	Score (Tgt)
Corrinoid	C19 H22 N4	(M+H)+	28.632		306.1833	262-76-0	FBF	93.49		93.49

Cpd 712: (+)-Vulgraon B

Name	Formula	RT	RI	Mass	Diff (Tgt, ppm)	CAS	ID Source	Score	Algorithm
(+)-Vulgraon B	C16 H24	28.894		216.1881	1.27		FBF	99.01	FBF

Species	m/z	Score (Tgt)	Score (Lib)	Score (DB)	Score (MFG)	Score (RT)
(M+2H)+2 (M+H)+	109 217	99.01				

Compound Chromatograms (overlaid)

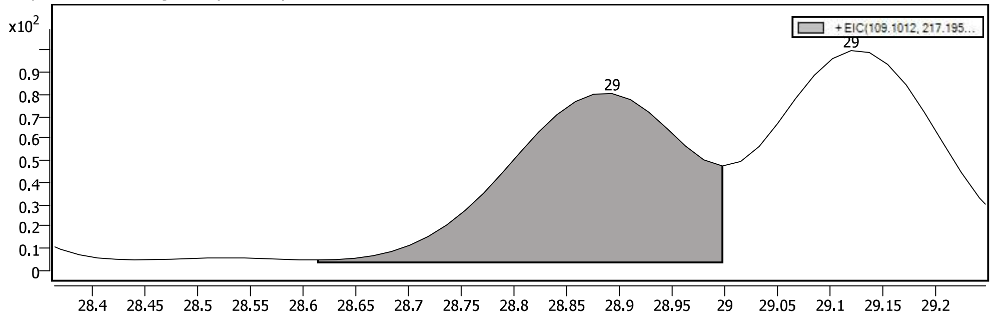

Structure

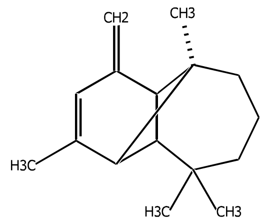

Compound Spectra (overlaid)

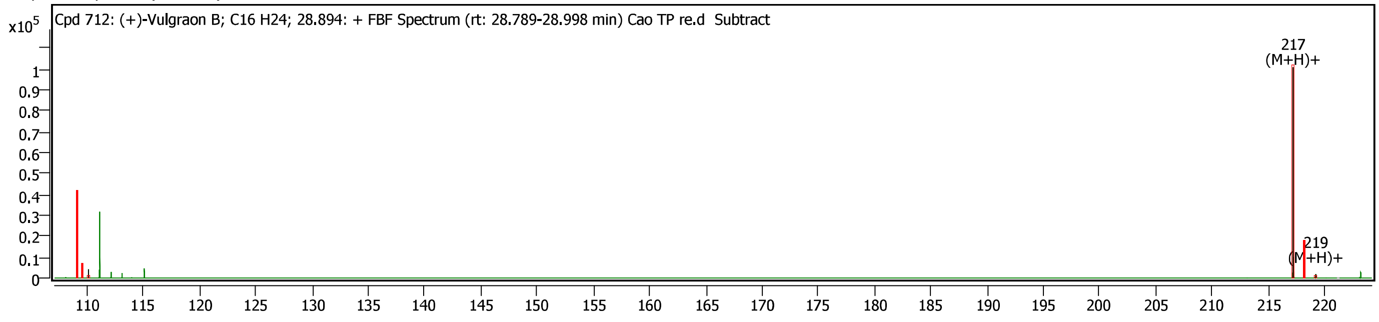

Compound ID Table

Name	Formula	Species	RT	RT Diff	Mass	CAS	ID Source	Score	Score (Lib)	Score (Tgt)
(+)-Vulgraon B	C16 H24	(M+2H)+2 (M+H)+	28.894		216.1881		FBF	99.01		99.01

Cpd 1667: endo-1,4-beta-Xylanase

Name	Formula	RT	RI	Mass	Diff (Tgt, ppm)	CAS	ID Source	Score	Algorithm
endo-1,4-beta-Xylanase	C34 H50 N4 O2	29.016		546.3922	-2.17	9025-57-4	FBF	97.57	FBF

Species	m/z	Score (Tgt)	Score (Lib)	Score (DB)	Score (MFG)	Score (RT)
(M+H)+	547	97.57				

Compound Chromatograms (overlaid)

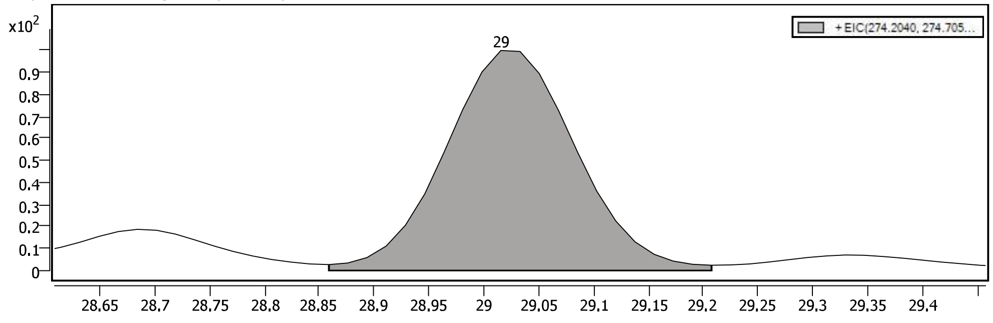

Structure

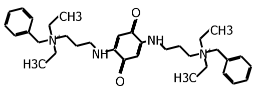

Compound Spectra (overlaid)

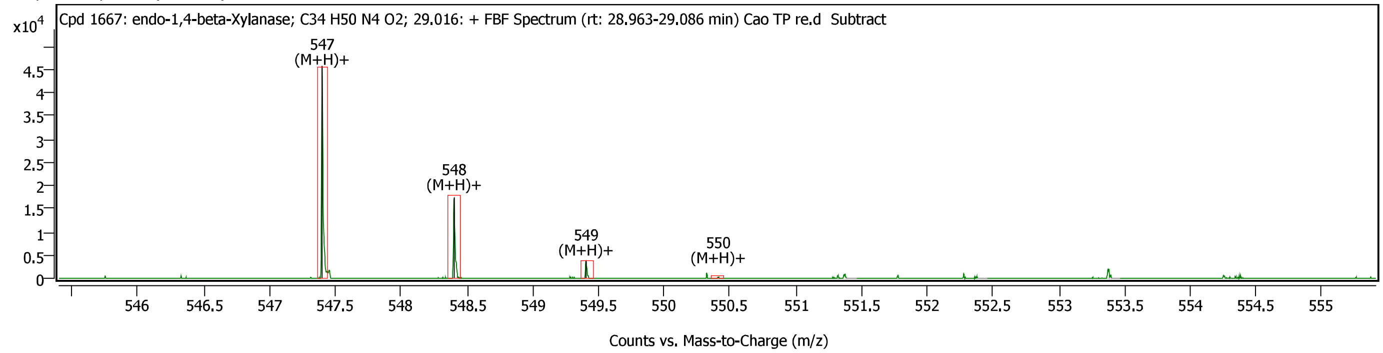

Compound ID Table

Name	Formula	Species	RT	RT Diff	Mass	CAS	ID Source	Score	Score (Lib)	Score (Tgt)
endo-1,4-beta-Xylanase	C34 H50 N4 O2	(M+H)+	29.016		546.3922	9025-57-4	FBF	97.57		97.57

Cpd 79: 5-Ethyl-7-methyl-3E,5E,7E-undecatriene

Name	Formula	RT	RI	Mass	Diff (Tgt, ppm)	CAS	ID Source	Score	Algorithm
5-Ethyl-7-methyl-3E,5E,7E-undecatriene	C14 H24	29.120		192.1878	0.03		FBF	86.72	FBF

Species	m/z	Score (Tgt)	Score (Lib)	Score (DB)	Score (MFG)	Score (RT)
(M+2H)+2 (M+H)+	97 193	86.72				

Compound Chromatograms (overlaid)

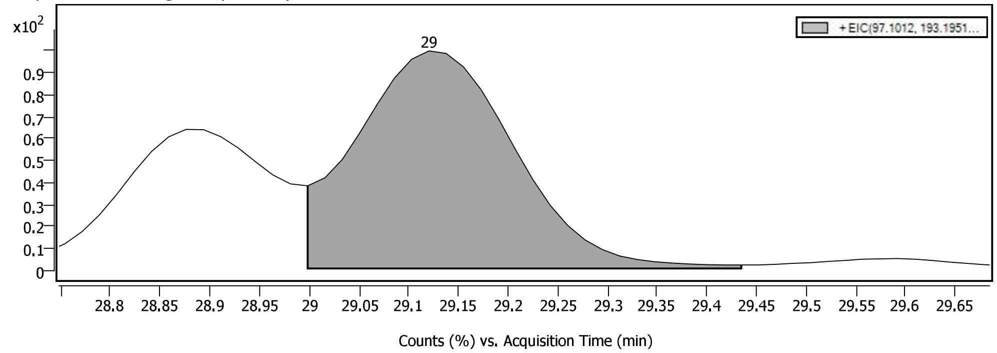

Structure

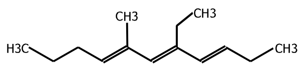

Compound Spectra (overlaid)

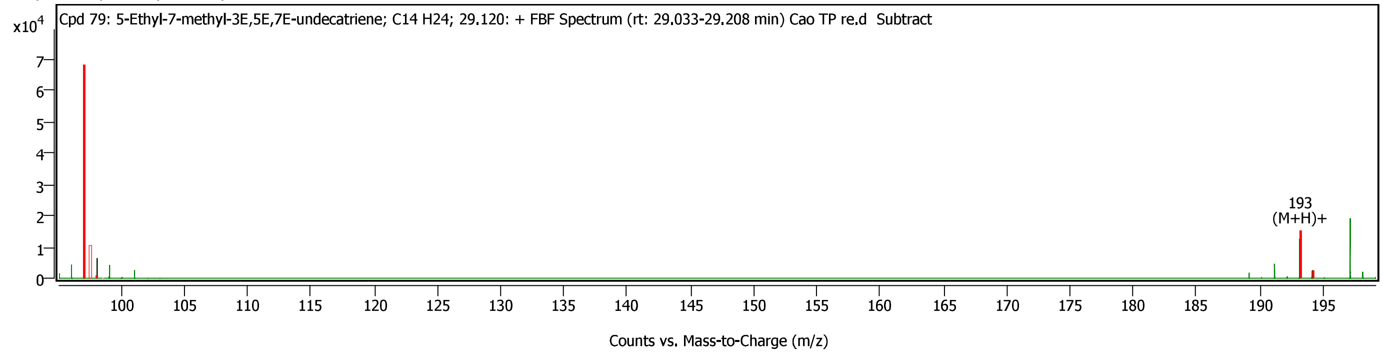

Compound ID Table

Name	Formula	Species	RT	RT Diff	Mass	CAS	ID Source	Score	Score (Lib)	Score (Tgt)
5-Ethyl-7-methyl-3E,5E,7E-undecatriene	C14 H24	(M+2H)+2 (M+H)+	29.120		192.1878		FBF	86.72		86.72

Cpd 201: 28-Homobrassinolide

Name	Formula	RT	RI	Mass	Diff (Tgt, ppm)	CAS	ID Source	Score	Algorithm
28-Homobrassinolide	C29 H50 O6	29.435		494.3609	0.40	80483-89-2	FBF	99.75	FBF

Species	m/z	Score (Tgt)	Score (Lib)	Score (DB)	Score (MFG)	Score (RT)
(M+H)+	495	99.75				

Compound Chromatograms (overlaid)

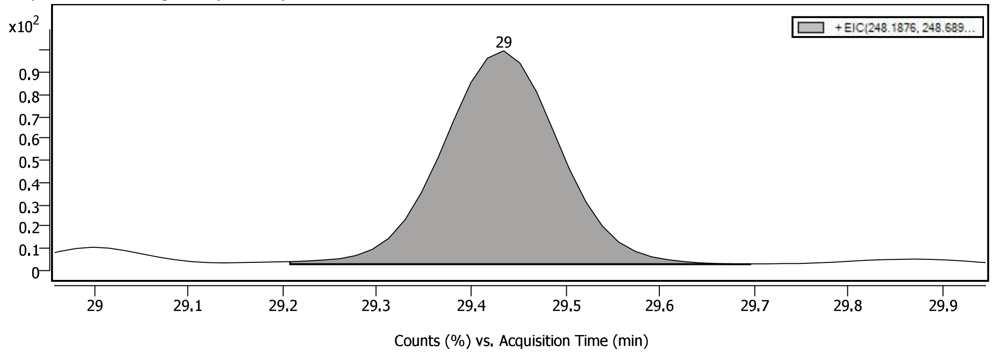

Structure

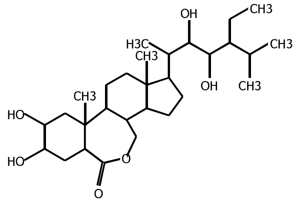

Compound Spectra (overlaid)

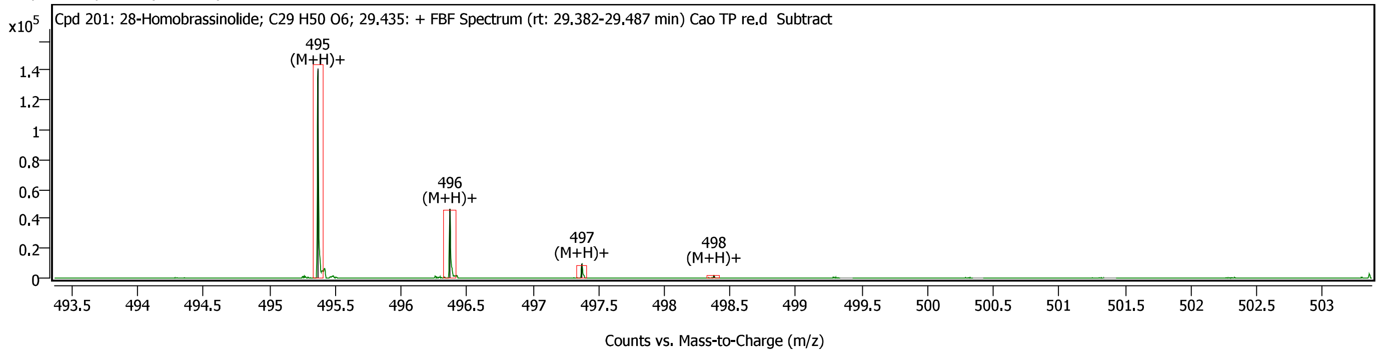

Compound ID Table

Name	Formula	Species	RT	RT Diff	Mass	CAS	ID Source	Score	Score (Lib)	Score (Tgt)
28-Homobrassinolide	C29 H50 O6	(M+H)+	29.435		494.3609	80483-89-2	FBF	99.75		99.75

Cpd 1029: PC(P-17:0/0:0)

Name	Formula	RT	RI	Mass	Diff (Tgt, ppm)	CAS	ID Source	Score	Algorithm
PC(P-17:0/0:0)	C25 H53 N O6 P	29.435		494.3610	-0.17		FBF	96.20	FBF

Species	m/z	Score (Tgt)	Score (Lib)	Score (DB)	Score (MFG)	Score (RT)
(M+H)+	495	96.20				

Compound Chromatograms (overlaid)

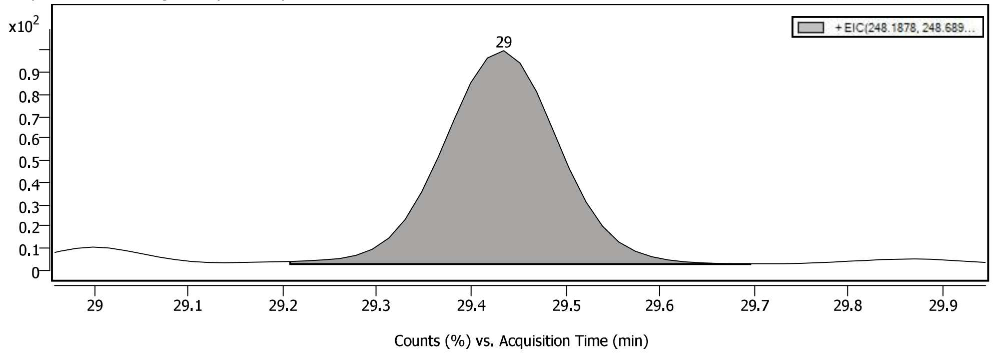

Structure

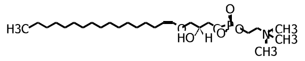

Compound Spectra (overlaid)

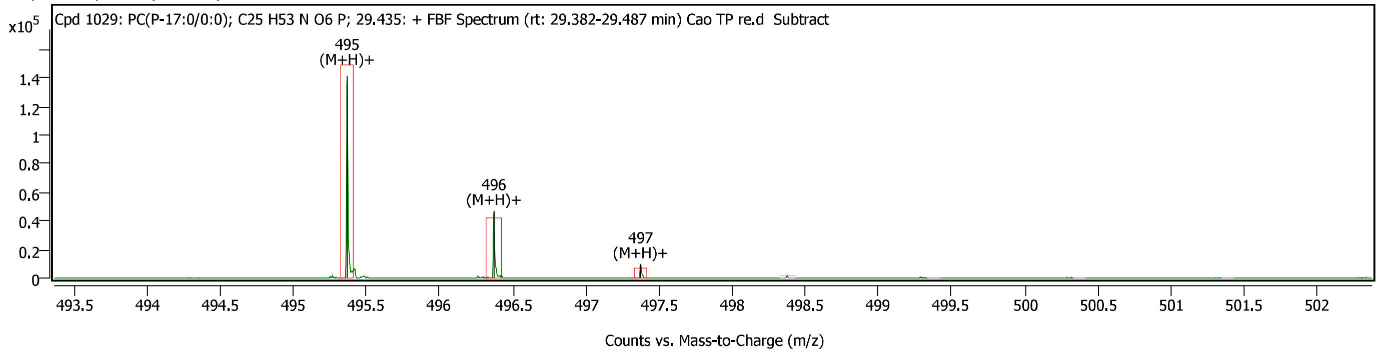

Compound ID Table

Name	Formula	Species	RT	RT Diff	Mass	CAS	ID Source	Score	Score (Lib)	Score (Tgt)
PC(P-17:0/0:0)	C25 H53 N O6 P	(M+H)+	29.435		494.3610		FBF	96.20		96.20

Cpd 860: PC(P-19:1(12Z)/0:0)

Name	Formula	RT	RI	Mass	Diff (Tgt, ppm)	CAS	ID Source	Score	Algorithm
PC(P-19:1(12Z)/0:0)	C27 H55 N O6 P	29.609		520.3765	-0.34		FBF	97.90	FBF

Species	m/z	Score (Tgt)	Score (Lib)	Score (DB)	Score (MFG)	Score (RT)
(M+H)+	521	97.90				

Compound Chromatograms (overlaid)

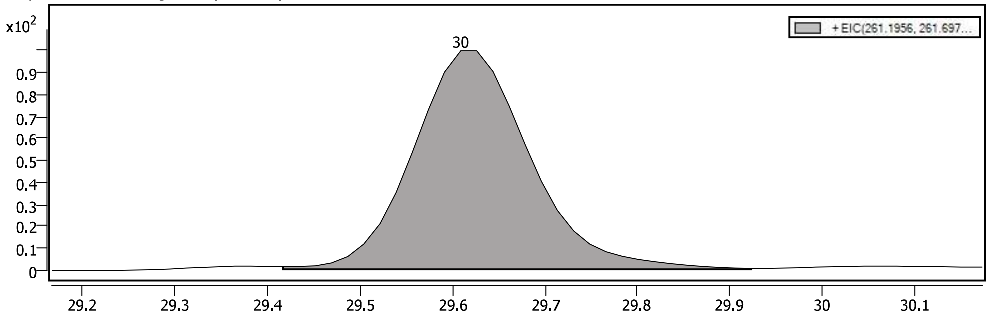

Structure

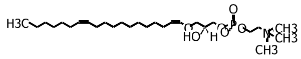

Compound Spectra (overlaid)

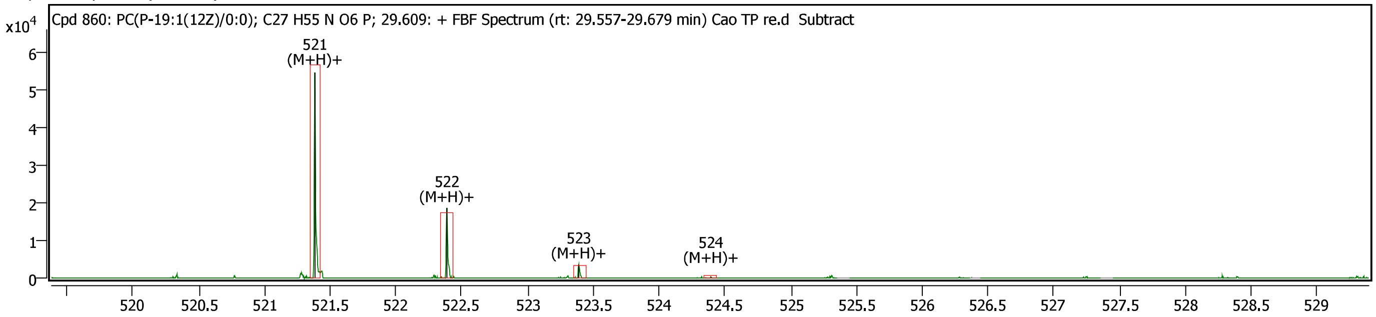

Compound ID Table

Name	Formula	Species	RT	RT Diff	Mass	CAS	ID Source	Score	Score (Lib)	Score (Tgt)
PC(P-19:1(12Z)/0:0)	C27 H55 N O6 P	(M+H)+	29.609		520.3765		FBF	97.90		97.90

Cpd 1646: Cyclopassifloic acid B

Name	Formula	RT	RI	Mass	Diff (Tgt, ppm)	CAS	ID Source	Score	Algorithm
Cyclopassifloic acid B	C31 H52 O6	29.609		520.3765	0.20	292167-35-2	FBF	99.83	FBF

Species	m/z	Score (Tgt)	Score (Lib)	Score (DB)	Score (MFG)	Score (RT)
(M+H)+	521	99.83				

Compound Chromatograms (overlaid)

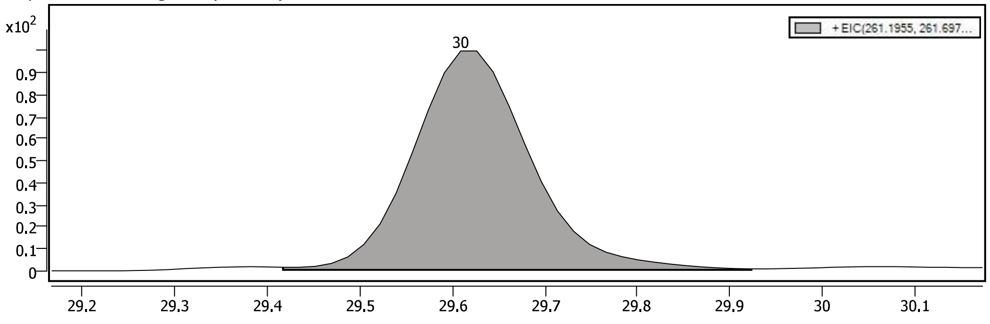

Structure

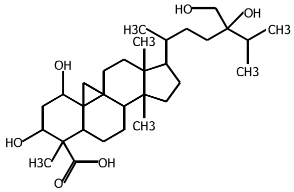

Compound Screening Report

Compound Spectra (overlaid)

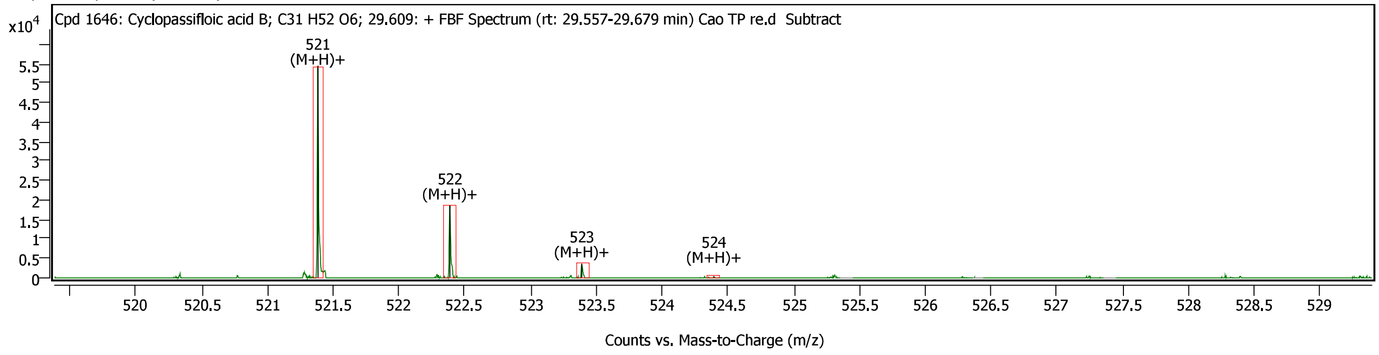

Compound ID Table

Name	Formula	Species	RT	RT Diff	Mass	CAS	ID Source	Score	Score (Lib)	Score (Tgt)
Cyclopassifloic acid B	C31 H52 O6	(M+H)+	29.609		520.3765	292167-35-2	FBF	99.83		99.83

Cpd 161: Cyclopassifloic acid E

Name	Formula	RT	RI	Mass	Diff (Tgt, ppm)	CAS	ID Source	Score	Algorithm
Cyclopassifloic acid E	C31 H52 O8	29.801		552.3665	0.57	301540-74-9	FBF	82.72	FBF

Species	m/z	Score (Tgt)	Score (Lib)	Score (DB)	Score (MFG)	Score (RT)
(M+2H)+2 (M+H)+	277 553	82.72				

Compound Chromatograms (overlaid)

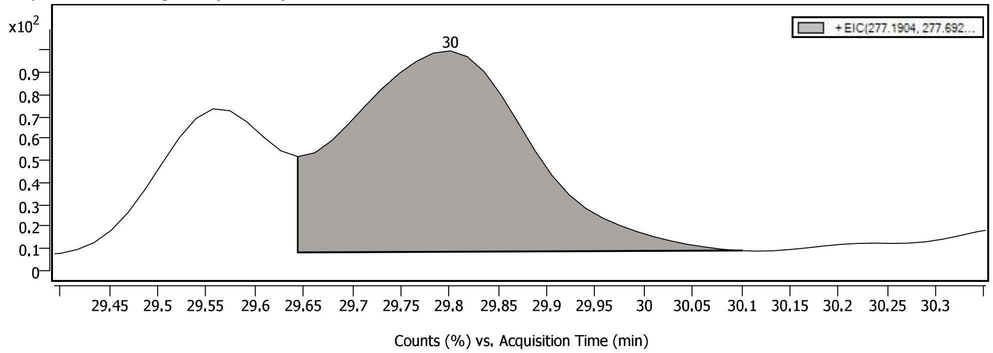

Structure

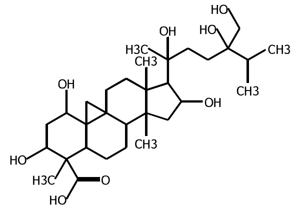

Compound Spectra (overlaid)

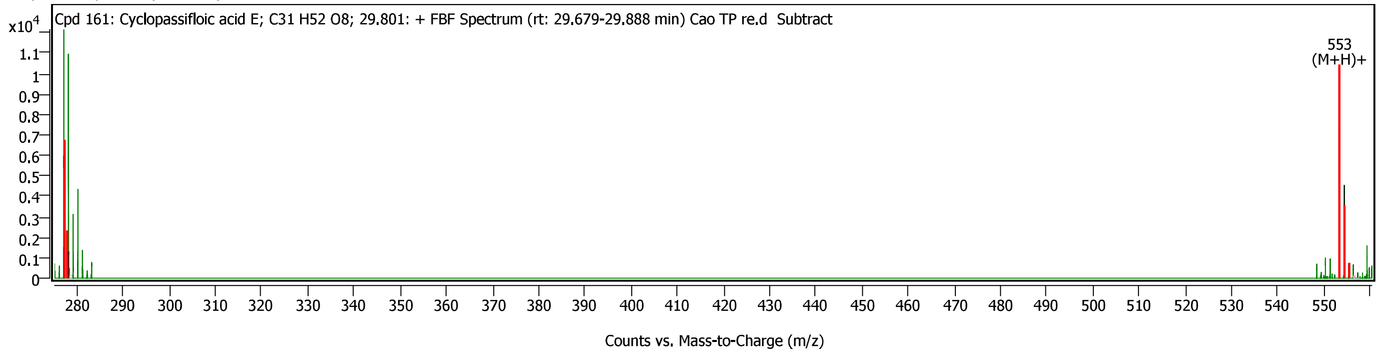

Compound ID Table

Name	Formula	Species	RT	RT Diff	Mass	CAS	ID Source	Score	Score (Lib)	Score (Tgt)
Cyclopassifloic acid E	C31 H52 O8	(M+2H)+2 (M+H)+	29.801		552.3665	301540-74-9	FBF	82.72		82.72

Cpd 3: <Linoleoyl glycine>

Name	Formula	RT	RI	Mass	Diff (Tgt, ppm)	CAS	ID Source	Score	Algorithm
<Linoleoyl glycine>	C20 H35 N O3	29.818		337.2621	1.17	2764-03-6	M-FBF	98.62	FBF

Species	m/z	Score (Tgt)	Score (Lib)	Score (DB)	Score (MFG)	Score (RT)
(M+H)+	338	98.62				

Compound Screening Report

Compound Chromatograms (overlaid)

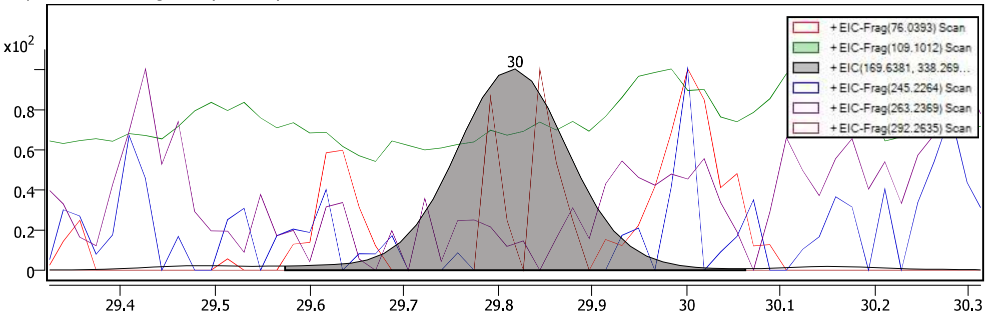

Structure

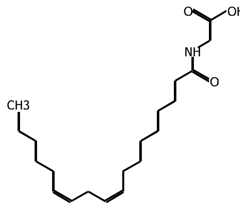

Coelution Plot

Compound Spectra (overlaid)

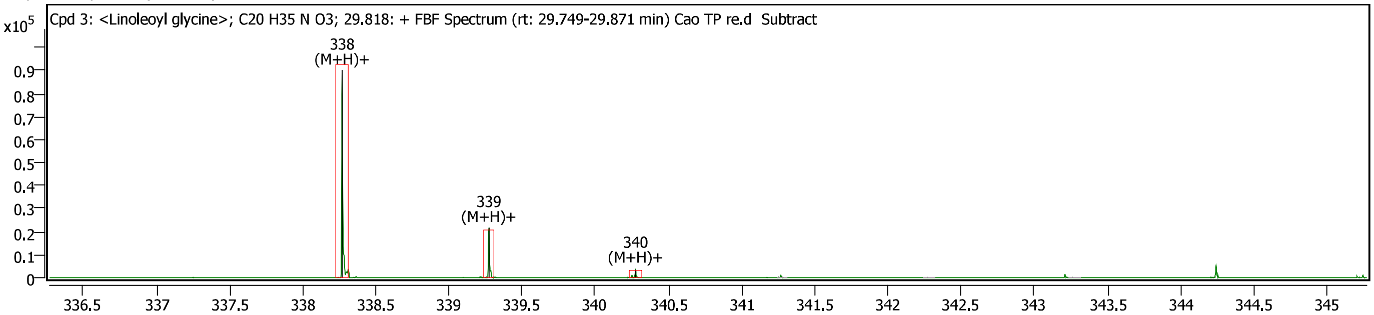

Fragment Spectrum (raw)

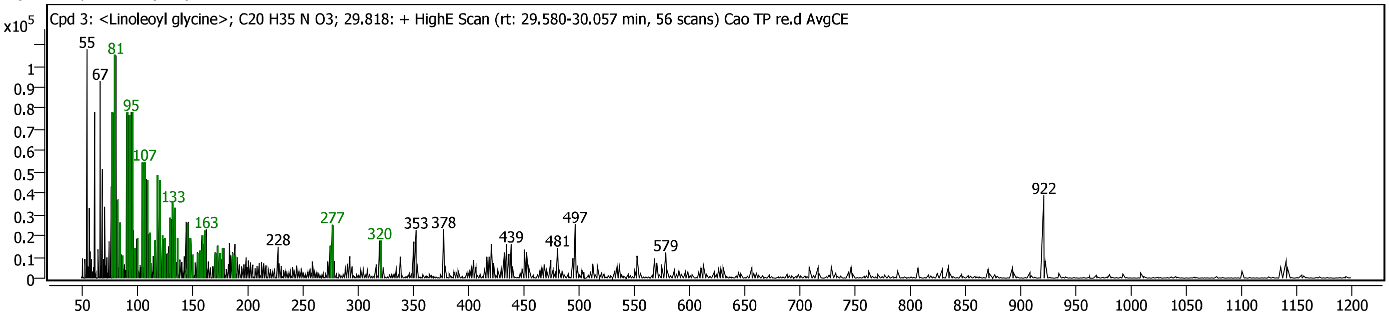

Compound ID Table

Name	Formula	Species	RT	RT Diff	Mass	CAS	ID Source	Score	Score (Lib)	Score (Tgt)
<Linoleoyl glycine>	C20 H35 N O3	(M+H)+	29.818		337.2621	2764-03-6	FBF	98.62		98.62
<(7R,E)-8-((1S,Z)-1-Hydroxy-1-methylhexahydro-2H-quinolizin-3(4H)-ylidene)-4,7-dimethyloct-4-ene-2,3-diol>	C20 H35 N O3	(M+H)+	29.818		337.2621		FBF	98.62		98.62
<Palmyrolide A>	C20 H35 N O3	(M+H)+	29.818		337.2621		FBF	98.62		98.62
<N-cis-hexadec-9Z-enoyl-L-Homoserine lactone>	C20 H35 N O3	(M+H)+	29.818		337.2621	479050-94-7	FBF	98.62		98.62

Cpd 479: trans-EKODE-(E)-Ib

Name	Formula	RT	RI	Mass	Diff (Tgt, ppm)	CAS	ID Source	Score	Algorithm
trans-EKODE-(E)-Ib	C18 H30 O4	30.115		310.2148	1.33	478931-82-7	FBF-FragConfirm	98.01	FBF

Species	m/z	Score (Tgt)	Score (Lib)	Score (DB)	Score (MFG)	Score (RT)
(M+H)+	311	98.01				

Compound Screening Report

Compound Chromatograms (overlaid)

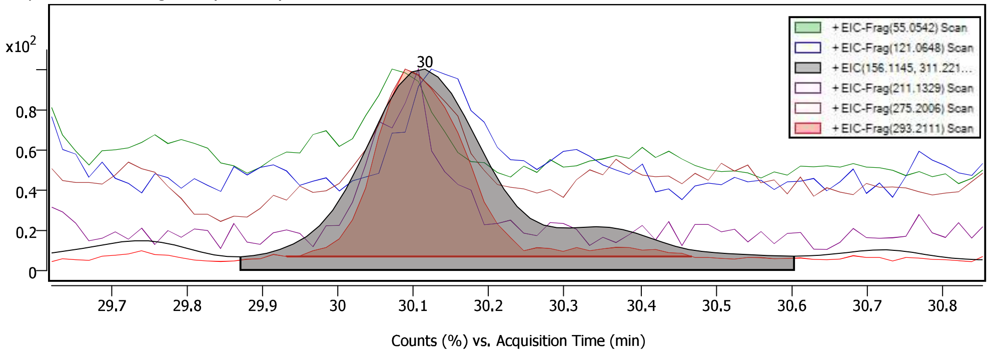

Structure

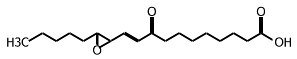

Coelution Plot

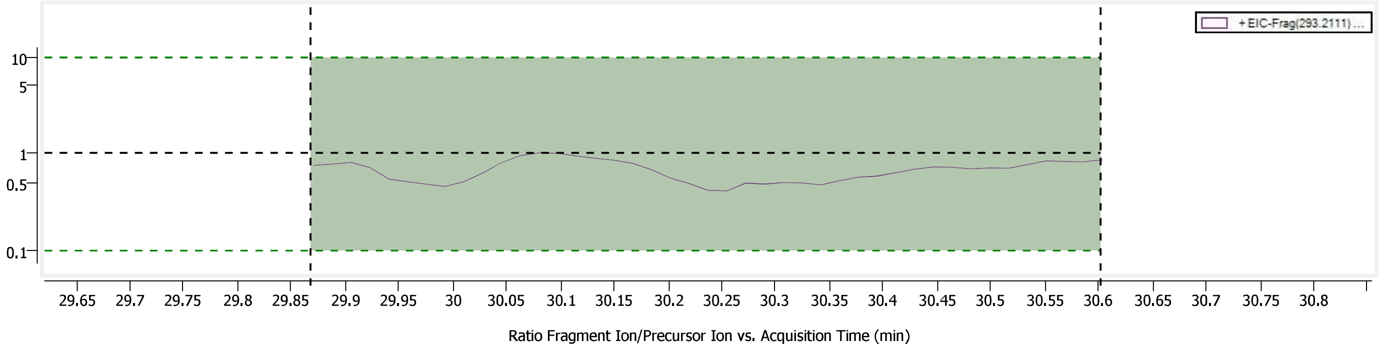

Compound Spectra (overlaid)

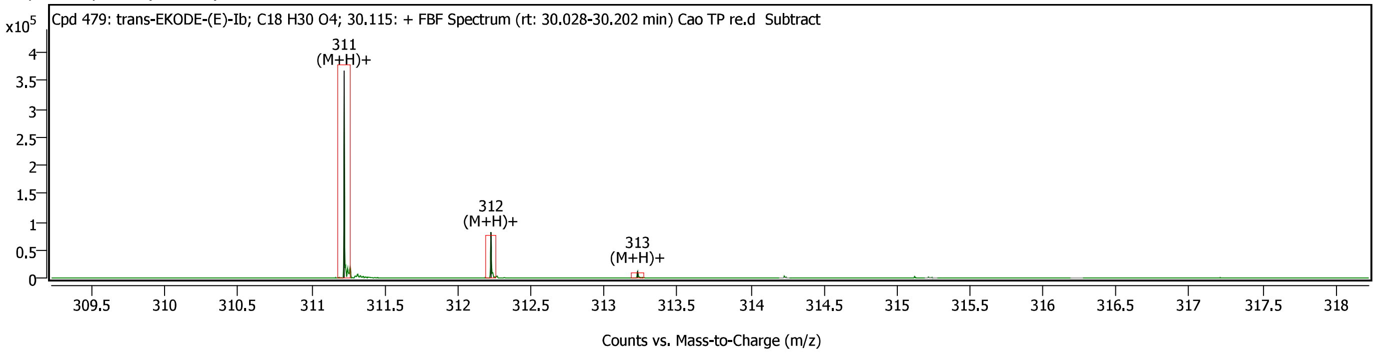

Fragment Spectrum (clean)

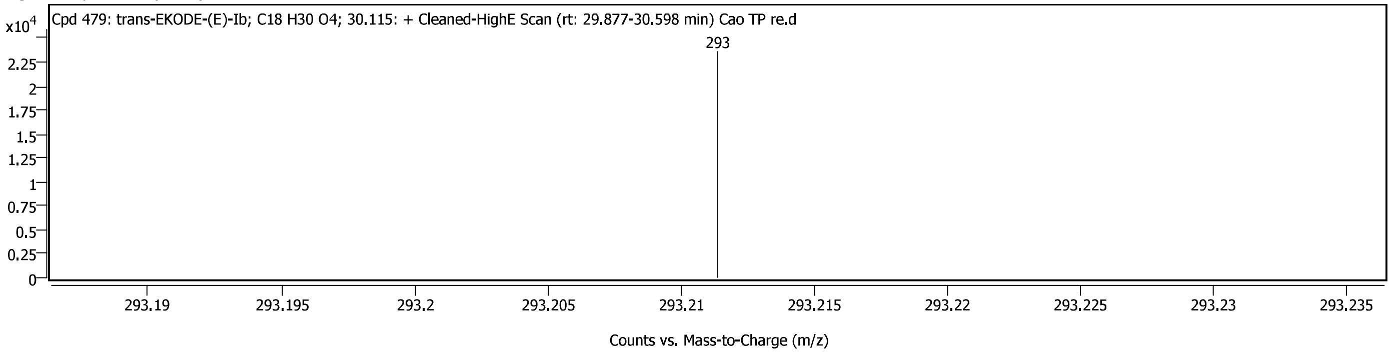

Compound Screening Report

Fragment Spectrum (raw)

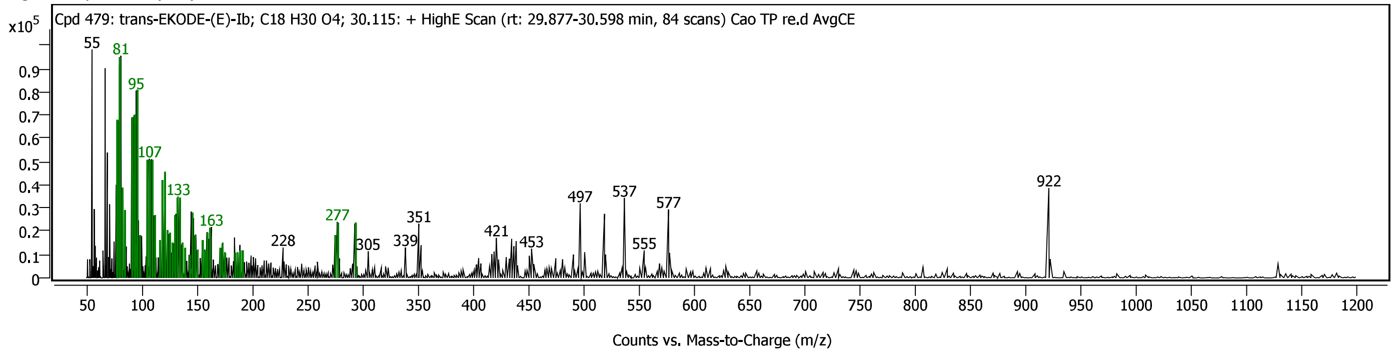

Compound ID Table

Name	Formula	Species	RT	RT Diff	Mass	CAS	ID Source	Score	Score (Lib)	Score (Tgt)
trans-EKODE-(E)-Ib	C18 H30 O4	(M+H)+	30.115		310.2148	478931-82-7	FBF-FragConfirm	98.01		98.01
2(R)-HPOT	C18 H30 O4	(M+H)+	30.115		310.2148		FBF-FragConfirm	98.01		98.01
Dihydroalbocycline	C18 H30 O4	(M+H)+	30.115		310.2148	67003-69-4	FBF-FragConfirm	98.01		98.01
(9Z,11R,12S,13S,15Z)-12,13-Epoxy-11-hydroxy-9,15-octadecadienoic acid	C18 H30 O4	(M+H)+	30.115		310.2148	106034-49-5	FBF-FragConfirm	98.01		98.01
12-HpOTrE	C18 H30 O4	(M+H)+	30.115		310.2148		FBF-FragConfirm	98.01		98.01
12-hydroperoxy-9Z,13E,15-octadecatrienoic acid	C18 H30 O4	(M+H)+	30.115		310.2148		FBF-FragConfirm	98.01		98.01
13(S)-HpOTrE	C18 H30 O4	(M+H)+	30.115		310.2148	67597-26-6	FBF-FragConfirm	98.01		98.01
13-HpOTrE	C18 H30 O4	(M+H)+	30.115		310.2148		FBF-FragConfirm	98.01		98.01
13-hydroperoxy-9,11E,15Z-octadecatrienoic acid	C18 H30 O4	(M+H)+	30.115		310.2148		FBF-FragConfirm	98.01		98.01
13S-HpOTrE	C18 H30 O4	(M+H)+	30.115		310.2148		FBF-FragConfirm	98.01		98.01
13S-HpOTrE(gamma)	C18 H30 O4	(M+H)+	30.115		310.2148	121107-97-9	FBF-FragConfirm	98.01		98.01
15,16-epoxy-13-OH-9Z,11E-octadecadienoic acid	C18 H30 O4	(M+H)+	30.115		310.2148		FBF-FragConfirm	98.01		98.01
16-HpOTrE	C18 H30 O4	(M+H)+	30.115		310.2148		FBF-FragConfirm	98.01		98.01
9-oxo-12,13-epoxy-10-octadecenoic acid	C18 H30 O4	(M+H)+	30.115		310.2148		FBF-FragConfirm	98.01		98.01
Sterebin A	C18 H30 O4	(M+H)+	30.115		310.2148	107647-14-3	FBF-FragConfirm	98.01		98.01
7S,8S-DiHOTrE	C18 H30 O4	(M+H)+	30.115		310.2148		FBF-FragConfirm	98.01		98.01
9(S)-HpOTrE	C18 H30 O4	(M+H)+	30.115		310.2148	111004-08-1	FBF-FragConfirm	98.01		98.01
9,10-epoxy-13-oxo-11-octadecenoic acid	C18 H30 O4	(M+H)+	30.115		310.2148		FBF-FragConfirm	98.01		98.01
9H-12(13)-EpODE	C18 H30 O4	(M+H)+	30.115		310.2148		FBF-FragConfirm	98.01		98.01
9-HpOTrE	C18 H30 O4	(M+H)+	30.115		310.2148		FBF-FragConfirm	98.01		98.01
9-hydroperoxy-10E,12,15Z-octadecatrienoic acid	C18 H30 O4	(M+H)+	30.115		310.2148		FBF-FragConfirm	98.01		98.01
9S-hydroxy-10S,11S-epoxy-12Z,15Z-octadecadienoic acid	C18 H30 O4	(M+H)+	30.115		310.2148		FBF-FragConfirm	98.01		98.01
9S-hydroxy-12R,13S-epoxy-10E,15Z-octadecadienoic acid	C18 H30 O4	(M+H)+	30.115		310.2148		FBF-FragConfirm	98.01		98.01
Auxin b	C18 H30 O4	(M+H)+	30.115		310.2148		FBF-FragConfirm	98.01		98.01
16-hydroperoxy-9Z,12,14E-octadecatrienoic acid	C18 H30 O4	(M+H)+	30.115		310.2148		FBF-FragConfirm	98.01		98.01

Cpd 164: Cyclopassifloic acid A

Name	Formula	RT	RI	Mass	Diff (Tgt, ppm)	CAS	ID Source	Score	Algorithm
Cyclopassifloic acid A	C31 H52 O7	30.150		536.3712	-0.17	292167-34-1	M-FBF	99.64	FBF

Species	m/z	Score (Tgt)	Score (Lib)	Score (DB)	Score (MFG)	Score (RT)
(M+H)+	537	99.64				

Compound Chromatograms (overlaid)

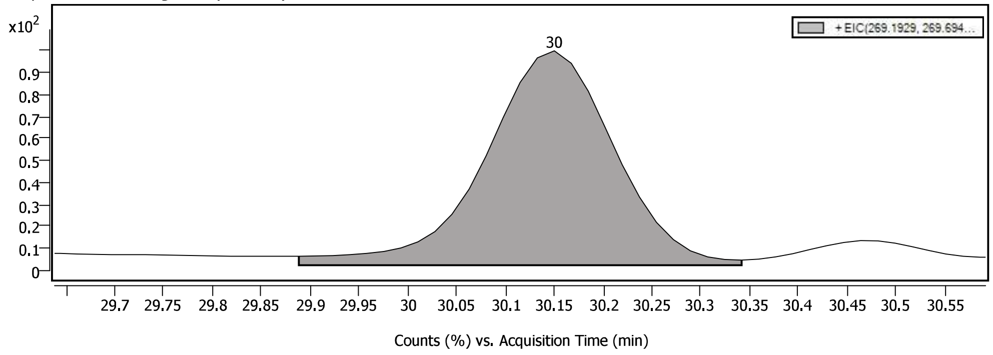

Structure

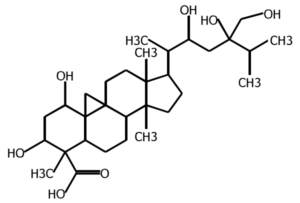

Compound Spectra (overlaid)

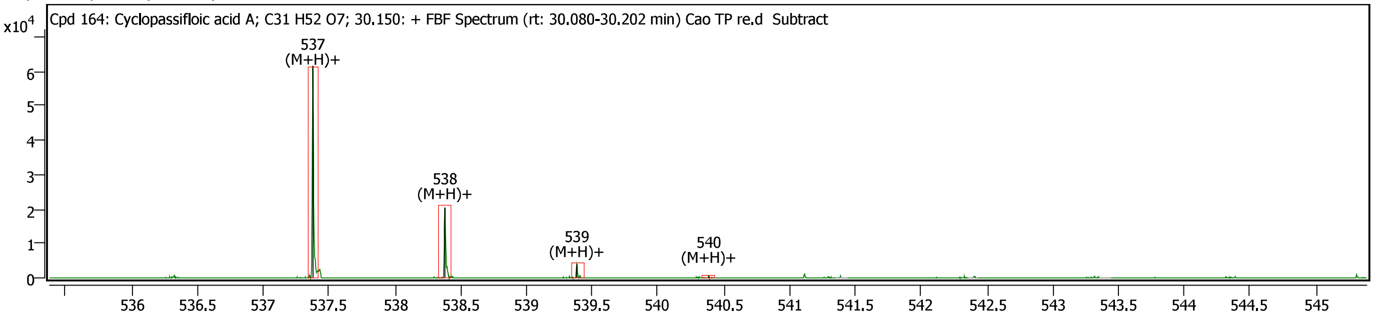

Compound ID Table

Name	Formula	Species	RT	RT Diff	Mass	CAS	ID Source	Score	Score (Lib)	Score (Tgt)
Cyclopassifloic acid A	C31 H52 O7	(M+H)+	30.150		536.3712	292167-34-1	FBF	99.64		99.64
Cyclopassifloic acid C	C31 H52 O7	(M+H)+	30.150		536.3712	292167-36-3	FBF	99.64		99.64
Cyclotricuspidogenin C	C31 H52 O7	(M+H)+	30.150		536.3712	239794-30-0	FBF	99.64		99.64

Cpd 239: 1-Hydroxyvitamin D3 3-D-glucopyranoside

Name	Formula	RT	RI	Mass	Diff (Tgt, ppm)	CAS	ID Source	Score	Algorithm
1-Hydroxyvitamin D3 3-D-glucopyranoside	C33 H52 O8	30.150		576.3639	-4.00		M-FBF	92.76	FBF

Species	m/z	Score (Tgt)	Score (Lib)	Score (DB)	Score (MFG)	Score (RT)
(M+H)+	577	92.76				

Compound Chromatograms (overlaid)

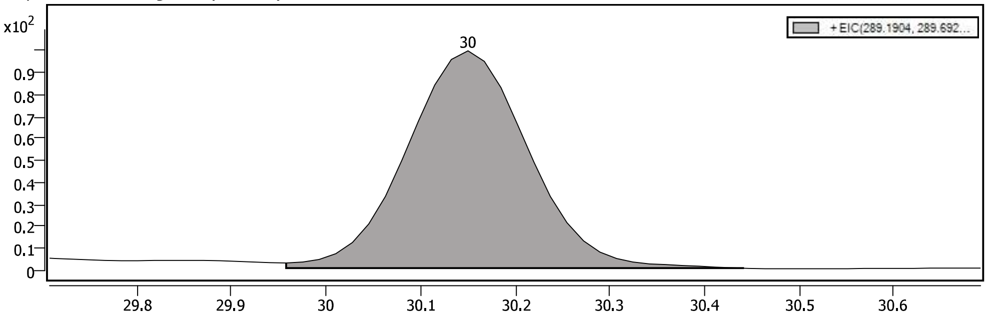

Structure

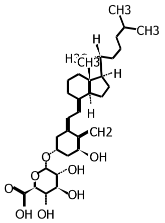

Compound Spectra (overlaid)

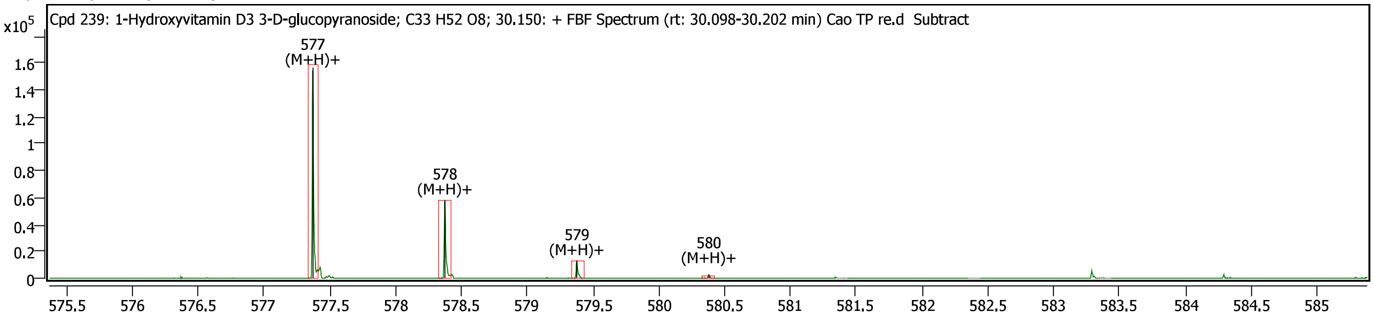

Compound ID Table

Name	Formula	Species	RT	RT Diff	Mass	CAS	ID Source	Score	Score (Lib)	Score (Tgt)
1-Hydroxyvitamin D3 3-D-glucopyranoside	C33 H52 O8	(M+H)+	30.150		576.3639		FBF	92.76		92.76
Trillin	C33 H52 O8	(M+H)+	30.150		576.3639		FBF	92.76		92.76

Cpd 1305: <ponasterone A>

Name	Formula	RT	RI	Mass	Diff (Tgt, ppm)	CAS	ID Source	Score	Algorithm
<ponasterone A>	C27 H44 O6	30.150		464.3130	-1.67		M-FBF	97.74	FBF

Species	m/z	Score (Tgt)	Score (Lib)	Score (DB)	Score (MFG)	Score (RT)
(M+H)+	465	97.74				

Compound Screening Report

Compound Chromatograms (overlaid)

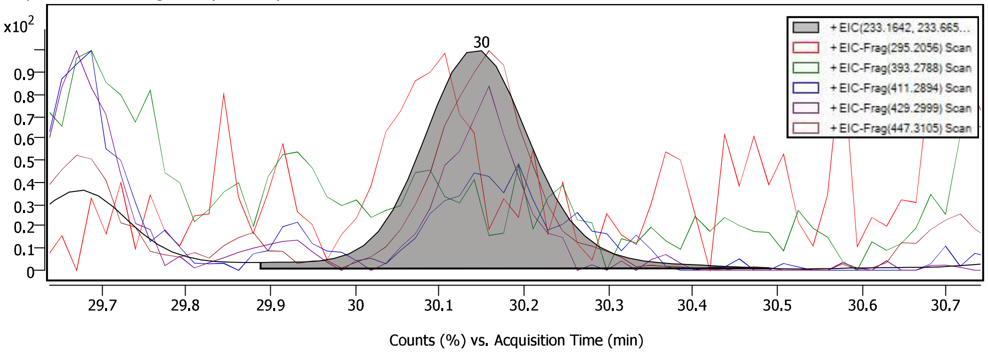

Structure

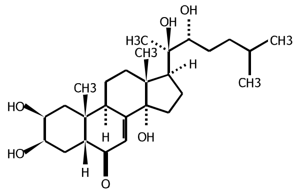

Coelution Plot

Compound Spectra (overlaid)

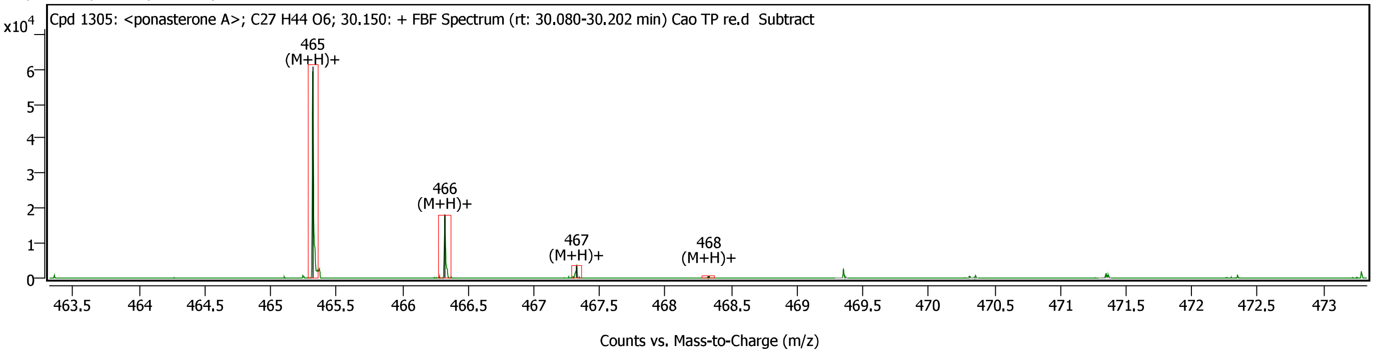

Fragment Spectrum (raw)

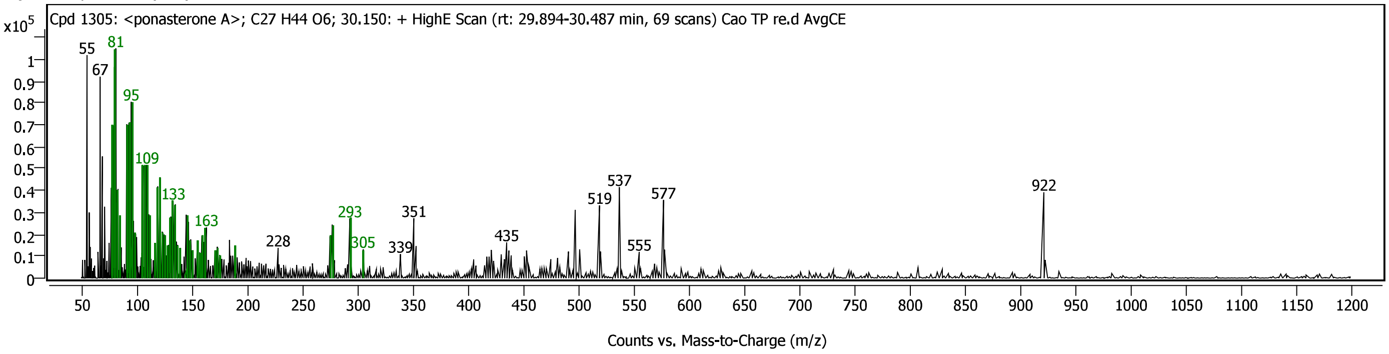

Compound ID Table

Name	Formula	Species	RT	RT Diff	Mass	CAS	ID Source	Score	Score (Lib)	Score (Tgt)
<ponasterone A>	C27 H44 O6	(M+H)+	30.150		464.3130		FBF	97.74		97.74
<3-Epiecdysone>	C27 H44 O6	(M+H)+	30.150		464.3130		FBF	97.74		97.74
<3α,6α,7α,12α-Tetrahydroxy-5β-cholest-24-en-26-oic acid>	C27 H44 O6	(M+H)+	30.150		464.3130		FBF	97.74		97.74
<22-iso-ecdysone>	C27 H44 O6	(M+H)+	30.150		464.3130		FBF	97.74		97.74
<3alpha,6alpha,7alpha,12alpha-Tetrahydroxy-5beta-cholest-24-en-26-oic acid>	C27 H44 O6	(M+H)+	30.150		464.3130		FBF	97.74		97.74
<3alpha,7alpha,12alpha,26-Tetrahydroxy-5beta-23E-cholestan-27-oic acid>	C27 H44 O6	(M+H)+	30.150		464.3130		FBF	97.74		97.74
<Neoallilogenin>	C27 H44 O6	(M+H)+	30.150		464.3130		FBF	97.74		97.74
<Alliogenin>	C27 H44 O6	(M+H)+	30.150		464.3130		FBF	97.74		97.74
<Convallagenin B>	C27 H44 O6	(M+H)+	30.150		464.3130		FBF	97.74		97.74
<ecdysone>	C27 H44 O6	(M+H)+	30.150		464.3130		FBF	97.74		97.74

Cpd 249: 9,11alpha-epoxy-6alpha-acetoxy-cholest-7-en-3beta,5alpha,19-triol

Name	Formula	RT	RI	Mass	Diff (Tgt, ppm)	CAS	ID Source	Score	Algorithm
9,11alpha-epoxy-6alpha-acetoxy-cholest-7-en-3beta,5alpha,19-triol	C29 H46 O6	30.307		490.3272	-4.48		FBF	89.28	FBF

Species	m/z	Score (Tgt)	Score (Lib)	Score (DB)	Score (MFG)	Score (RT)
(M+H)+	491	89.28				

Compound Chromatograms (overlaid)

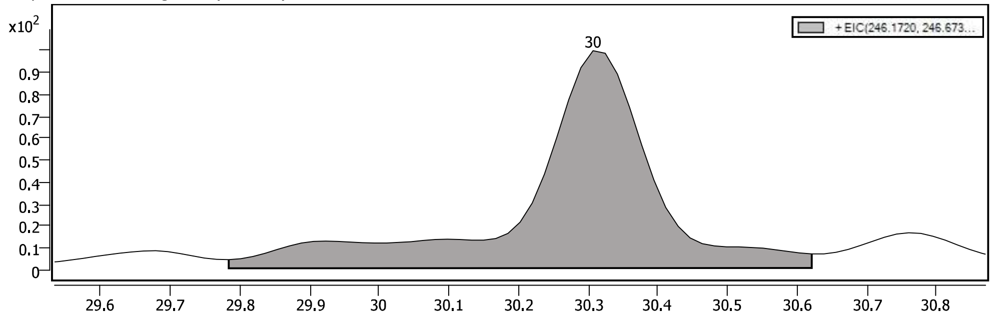

Structure

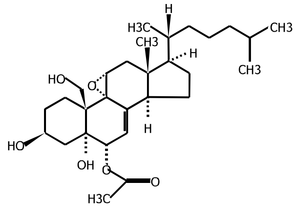

Compound Spectra (overlaid)

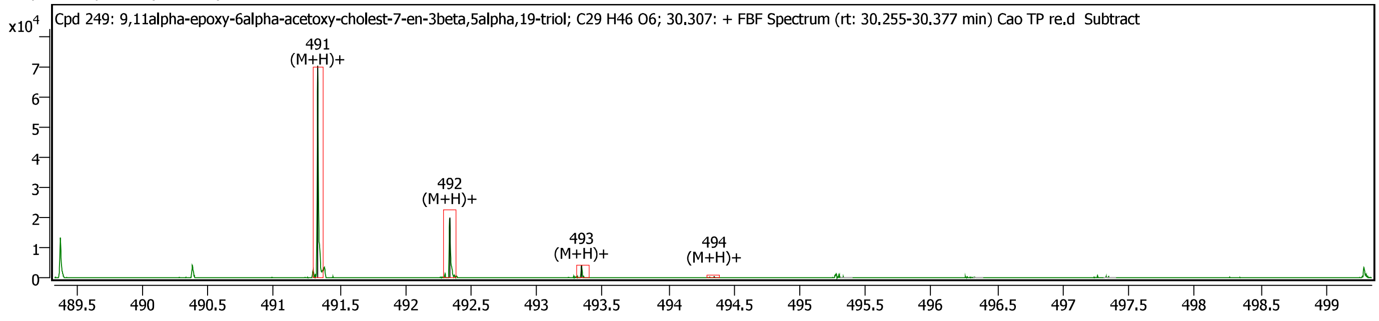

Compound ID Table

Name	Formula	Species	RT	RT Diff	Mass	CAS	ID Source	Score	Score (Lib)	Score (Tgt)
9,11alpha-epoxy-6alpha-acetoxy-cholest-7-en-3beta,5alpha,19-triol	C29 H46 O6	(M+H)+	30.307		490.3272		FBF	89.28		89.28

Cpd 241: (25R)-5alpha-spirostan-3beta,6alpha,23S-triol 6-O-beta-D-glucopyranoside

Name	Formula	RT	RI	Mass	Diff (Tgt, ppm)	CAS	ID Source	Score	Algorithm
(25R)-5alpha-spirostan-3beta,6alpha,23S-triol 6-O-beta-D-glucopyranoside	C34 H58 O10	31.040		626.4029	-0.17		FBF	99.84	FBF

Species	m/z	Score (Tgt)	Score (Lib)	Score (DB)	Score (MFG)	Score (RT)
(M+H)+	627	99.84				

Compound Chromatograms (overlaid)

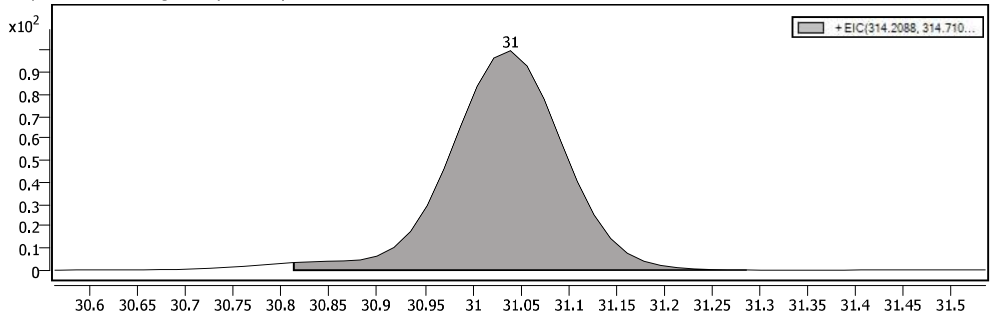

Structure

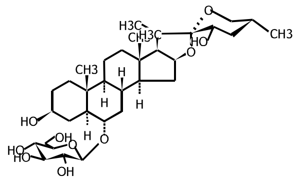

Compound Spectra (overlaid)

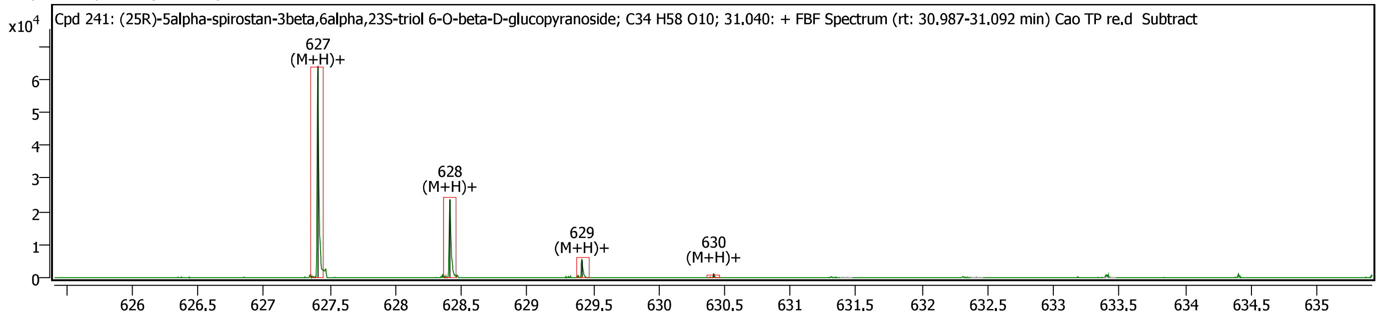

Compound Screening Report

Compound ID Table

Name	Formula	Species	RT	RT Diff	Mass	CAS	ID Source	Score	Score (Lib)	Score (Tgt)
(25R)-5alpha-spirostan-3beta,6alpha,23S-triol 6-O-beta-D-glucopyranoside	C34 H58 O10	(M+H)+	31.040		626.4029		FBF	99.84		99.84

Cpd 1229: <15-methyl-15(S)-PGE1>

Name	Formula	RT	RI	Mass	Diff (Tgt, ppm)	CAS	ID Source	Score	Algorithm
<15-methyl-15(S)-PGE1>	C21 H36 O5	31.528		368.2566	0.85	35700-26-6	M-FBF	99.38	FBF

Species	m/z	Score (Tgt)	Score (Lib)	Score (DB)	Score (MFG)	Score (RT)
(M+H)+	369	99.38				

Compound Chromatograms (overlaid)

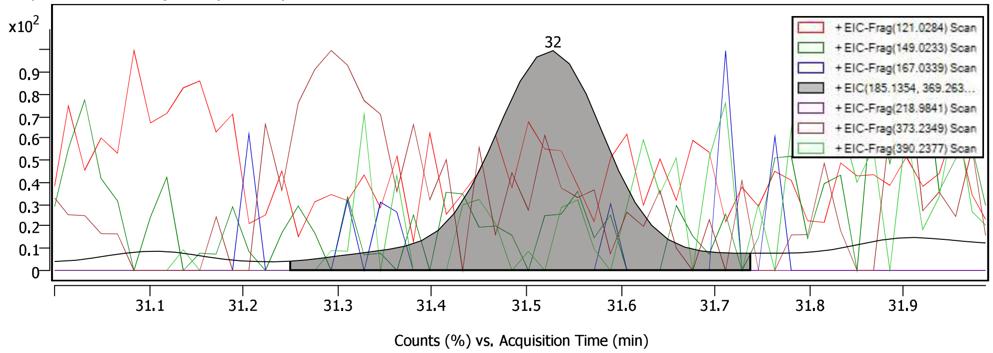

Structure

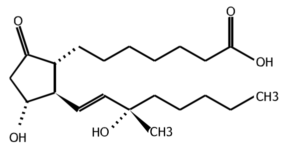

Coelution Plot

Compound Spectra (overlaid)

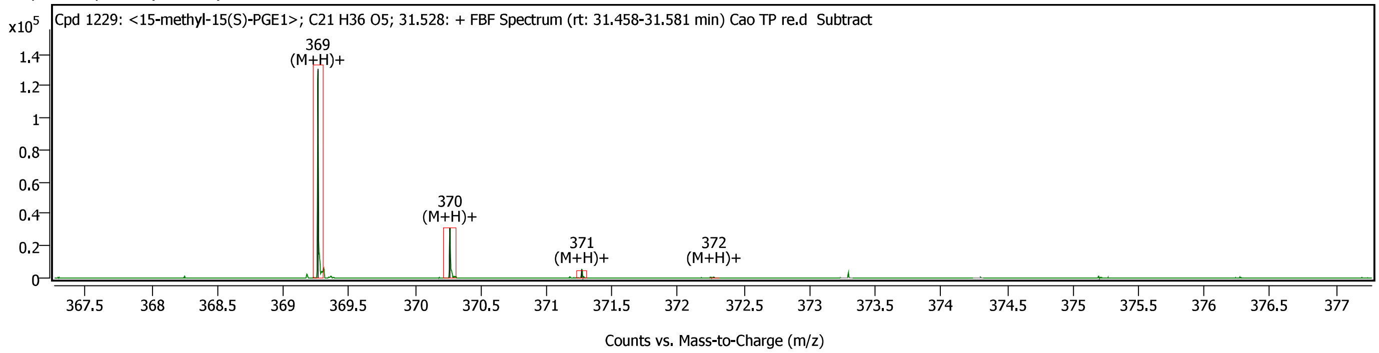

Fragment Spectrum (raw)

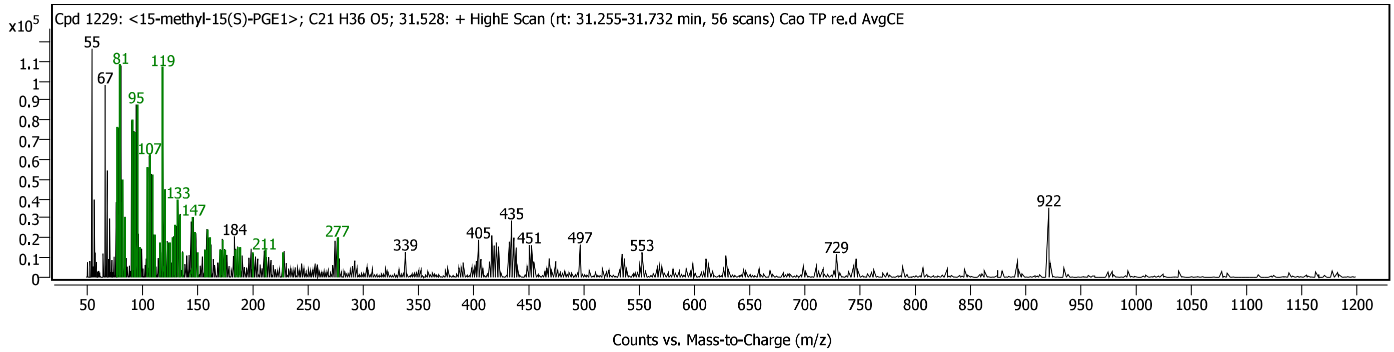

Compound ID Table

Name	Formula	Species	RT	RT Diff	Mass	CAS	ID Source	Score	Score (Lib)	Score (Tgt)
<15-methyl-15(S)-PGE1>	C21 H36 O5	(M+H)+	31.528		368.2566	35700-26-6	FBF	99.38		99.38
<Allocortol>	C21 H36 O5	(M+H)+	31.528		368.2566	1169-36-4	FBF	99.38		99.38
<15(R)-15-methyl PGF2α>	C21 H36 O5	(M+H)+	31.528		368.2566	35864-81-4	FBF	99.38		99.38
<15(S)-15-methyl PGF2α>	C21 H36 O5	(M+H)+	31.528		368.2566	35700-23-3	FBF	99.38		99.38
<Misoprostol (free acid)>	C21 H36 O5	(M+H)+	31.528		368.2566	112137-89-0	FBF	99.38		99.38
<Beta-Cortol>	C21 H36 O5	(M+H)+	31.528		368.2566	667-65-2	FBF	99.38		99.38
<Cortol>	C21 H36 O5	(M+H)+	31.528		368.2566	516-38-1	FBF	99.38		99.38
<gamma-Eudesmol rhamnoside>	C21 H36 O5	(M+H)+	31.528		368.2566	349112-31-8	FBF	99.38		99.38
<PGF2α methyl ester>	C21 H36 O5	(M+H)+	31.528		368.2566	33854-16-9	FBF	99.38		99.38

Cpd 1499: <Eplerenone>

Name	Formula	RT	RI	Mass	Diff (Tgt, ppm)	CAS	ID Source	Score	Algorithm
<Eplerenone>	C24 H30 O6	31.546		414.2046	0.77	107724-20-9	M-FBF	98.21	FBF

Species	m/z	Score (Tgt)	Score (Lib)	Score (DB)	Score (MFG)	Score (RT)
(M+H)+	415	98.21				

Compound Chromatograms (overlaid)

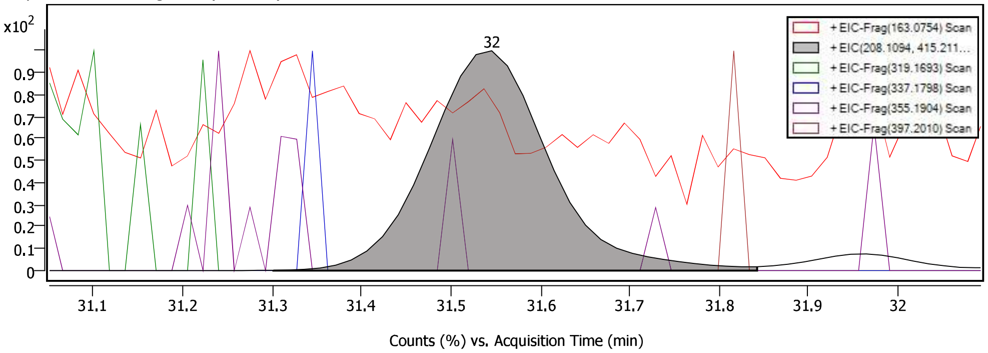

Structure

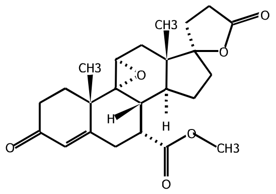

Coelution Plot

Compound Spectra (overlaid)

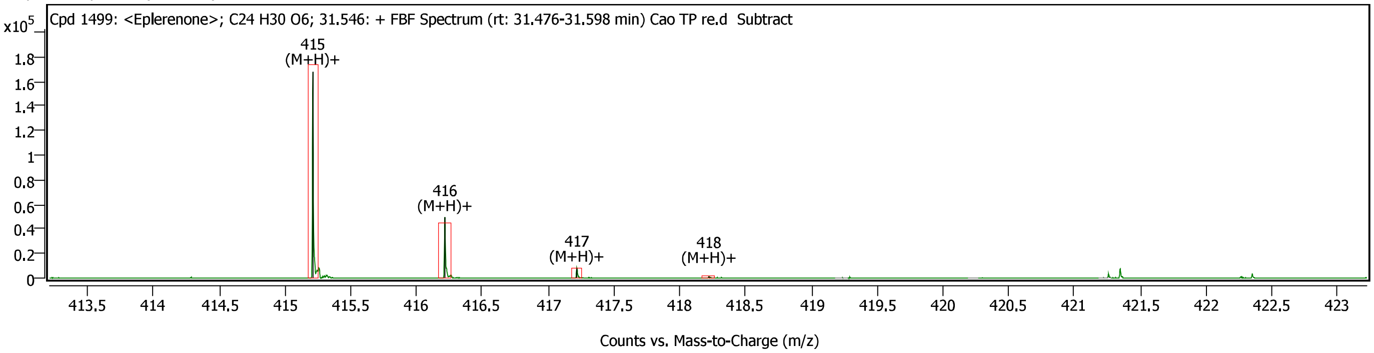

Fragment Spectrum (raw)

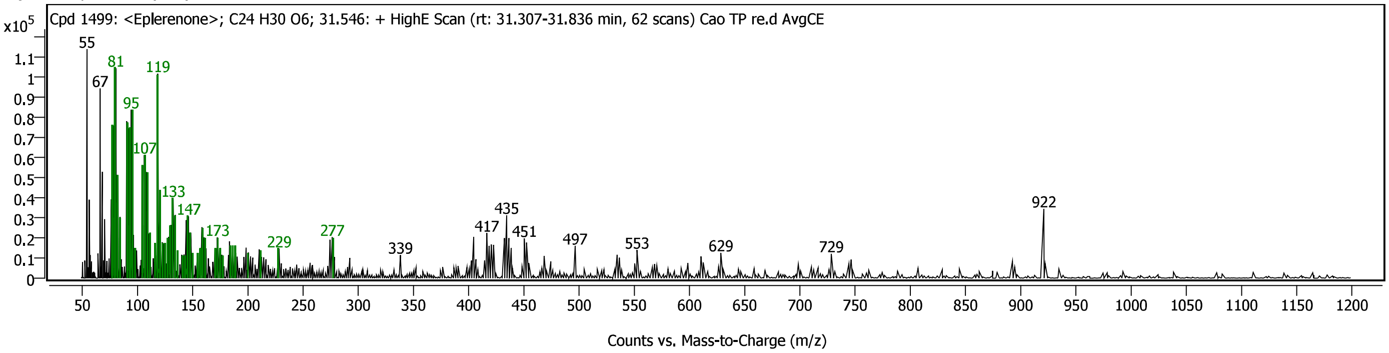

Compound ID Table

Name	Formula	Species	RT	RT Diff	Mass	CAS	ID Source	Score	Score (Lib)	Score (Tgt)
<Eplerenone>	C24 H30 O6	(M+H)+	31.546		414.2046	107724-20-9	FBF	98.21		98.21
<Armillaripin>	C24 H30 O6	(M+H)+	31.546		414.2046	129741-56-6	FBF	98.21		98.21
<Clausarinol>	C24 H30 O6	(M+H)+	31.546		414.2046		FBF	98.21		98.21
<4-O-Methylmelleolide>	C24 H30 O6	(M+H)+	31.546		414.2046	96627-13-3	FBF	98.21		98.21
<Estra-1,3,5(10)-triene-3,6alpha,17beta-triol triacetate>	C24 H30 O6	(M+H)+	31.546		414.2046		FBF	98.21		98.21
<Magnoshinin>	C24 H30 O6	(M+H)+	31.546		414.2046	86702-02-5	FBF	98.21		98.21
<Estra-1,3,5(10)-triene-3,6beta,17beta-triol triacetate>	C24 H30 O6	(M+H)+	31.546		414.2046		FBF	98.21		98.21
<Armillarin>	C24 H30 O6	(M+H)+	31.546		414.2046	83329-14-0	FBF	98.21		98.21

Cpd 10: Asebotoxin II

Name	Formula	RT	RI	Mass	Diff (Tgt, ppm)	CAS	ID Source	Score	Algorithm
Asebotoxin II	C23 H36 O6	32.331		408.2492	-4.95	23984-18-1	M-FBF	88.50	FBF

Species	m/z	Score (Tgt)	Score (Lib)	Score (DB)	Score (MFG)	Score (RT)
(M+H)+	409	88.50				

Compound Chromatograms (overlaid)

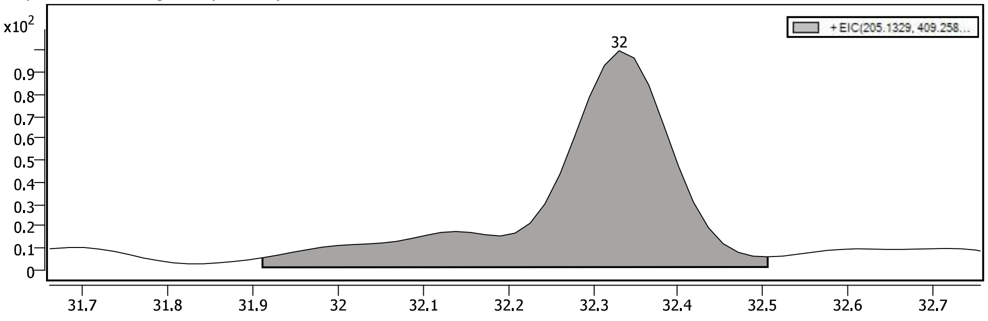

Counts (%) vs. Acquisition Time (min)

Structure

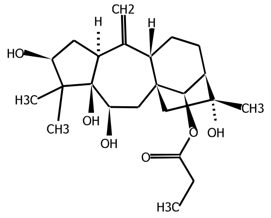

Compound Spectra (overlaid)

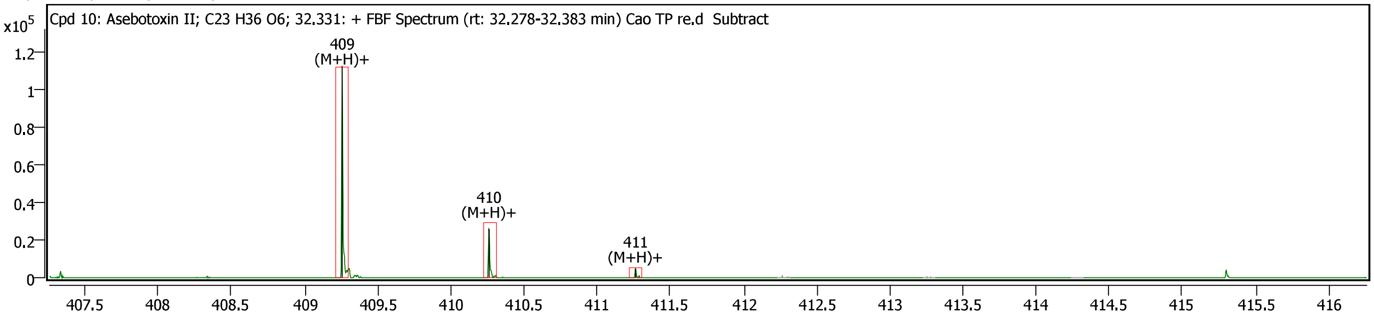

Counts vs. Mass-to-Charge (m/z)

Compound ID Table

Name	Formula	Species	RT	RT Diff	Mass	CAS	ID Source	Score	Score (Lib)	Score (Tgt)
Asebotoxin II	C23 H36 O6	(M+H)+	32.331		408.2492	23984-18-1	FBF	88.50		88.50
Diacetyl-8-gingerdiol	C23 H36 O6	(M+H)+	32.331		408.2492		FBF	88.50		88.50
15R-PGE2 methyl ester, 15-acetate	C23 H36 O6	(M+H)+	32.331		408.2492		FBF	88.50		88.50

Cpd 145: <3-methoxy Prostaglandin F1α>

Name	Formula	RT	RI	Mass	Diff (Tgt, ppm)	CAS	ID Source	Score	Algorithm
<3-methoxy Prostaglandin F1α>	C21 H38 O6	32.331		386.2672	0.89	54432-43-8	M-FBF	99.05	FBF

Species	m/z	Score (Tgt)	Score (Lib)	Score (DB)	Score (MFG)	Score (RT)
(M+H)+	387	99.05				

Compound Chromatograms (overlaid)

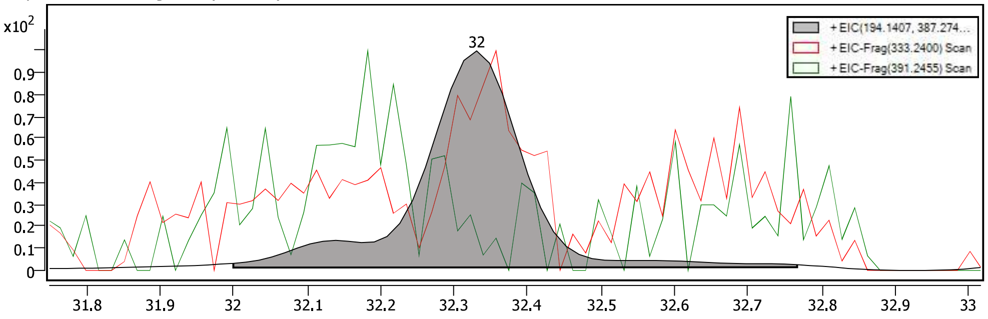

Counts (%) vs. Acquisition Time (min)

Structure

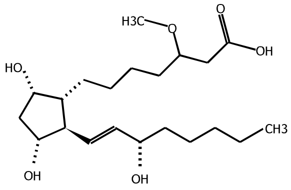

Coelution Plot

Compound Spectra (overlaid)

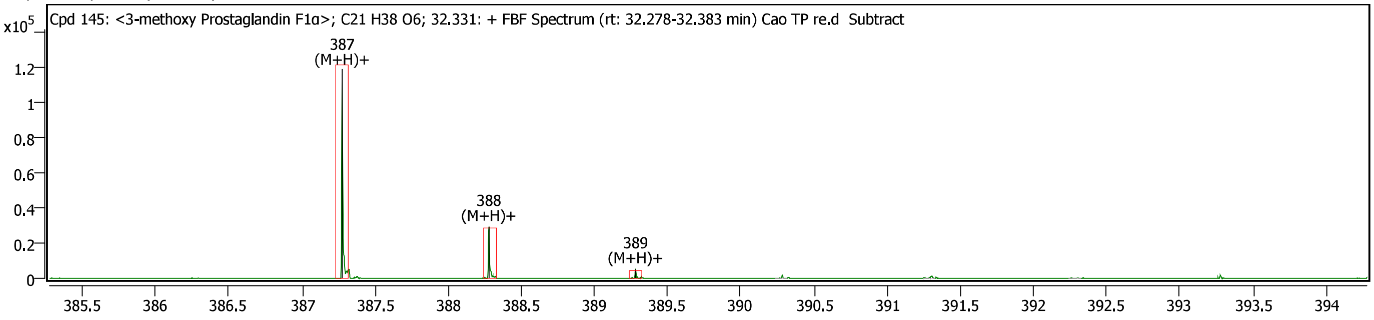

Counts vs. Mass-to-Charge (m/z)

Fragment Spectrum (raw)

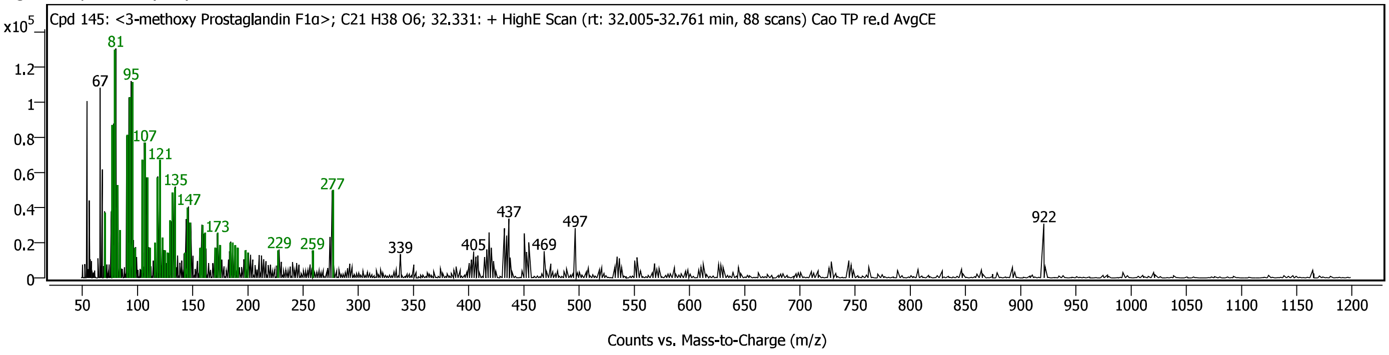

Compound ID Table

Name	Formula	Species	RT	RT Diff	Mass	CAS	ID Source	Score	Score (Lib)	Score (Tgt)
<3-methoxy Prostaglandin F1α>	C21 H38 O6	(M+H)+	32.331		386.2672	54432-43-8	FBF	99.05		99.05
<Cryptomeridiol 11-rhamnoside>	C21 H38 O6	(M+H)+	32.331		386.2672	349112-30-7	FBF	99.05		99.05
<6-Deoxyerythronolide B>	C21 H38 O6	(M+H)+	32.331		386.2672	15797-36-1	FBF	99.05		99.05
<Glycerol trihexanoate>	C21 H38 O6	(M+H)+	32.331		386.2672	621-70-5	FBF	99.05		99.05
<Mangalkanyl glucoside>	C21 H38 O6	(M+H)+	32.331		386.2672	259144-63-3	FBF	99.05		99.05

Cpd 1598: <Dehydro (11,12)ursolic acid lactone>

Name	Formula	RT	RI	Mass	Diff (Tgt, ppm)	CAS	ID Source	Score	Algorithm
<Dehydro (11,12)ursolic acid lactone>	C30 H46 O3	32.401		454.3449	0.51	35959-05-8	M-FBF	99.14	FBF

Species	m/z	Score (Tgt)	Score (Lib)	Score (DB)	Score (MFG)	Score (RT)
(M+H)+	455	99.14				

Compound Chromatograms (overlaid)

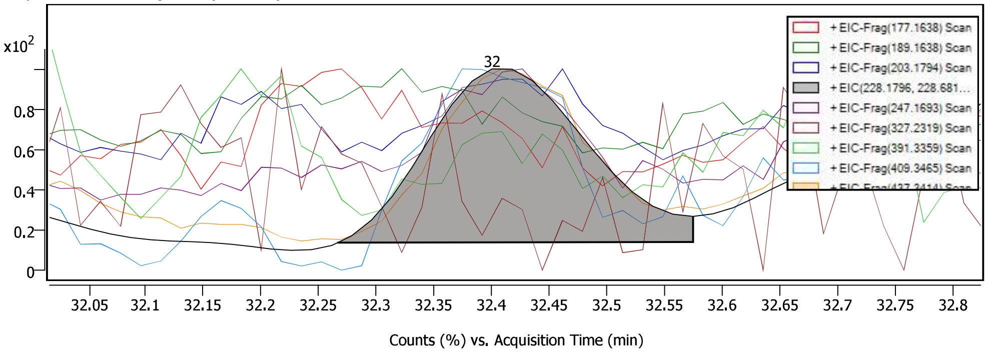

Structure

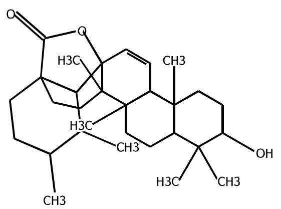

Coelution Plot

Compound Spectra (overlaid)

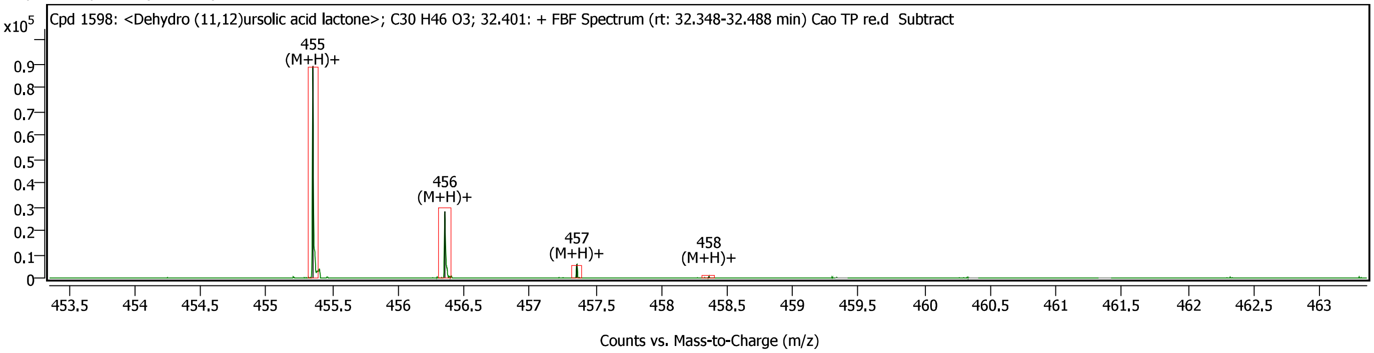

Compound Screening Report

Fragment Spectrum (raw)

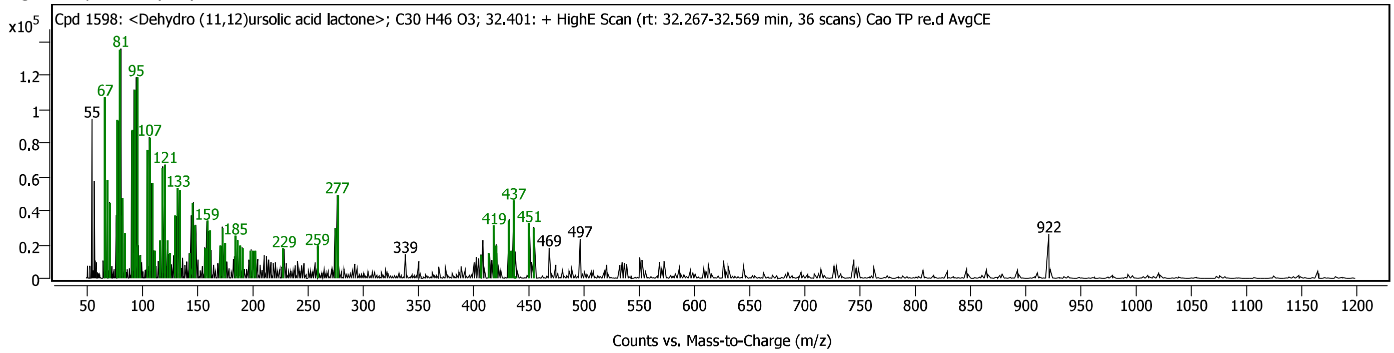

Compound ID Table

Name	Formula	Species	RT	RT Diff	Mass	CAS	ID Source	Score	Score (Lib)	Score (Tgt)
<Dehydro (11,12)ursolic acid lactone>	C30 H46 O3	(M+H)+	32.401		454.3449	35959-05-8	FBF	99.14		99.14
<beta-Elemonic acid>	C30 H46 O3	(M+H)+	32.401		454.3449	28282-25-9	FBF	99.14		99.14
<EB 1129>	C30 H46 O3	(M+H)+	32.401		454.3449		FBF	99.14		99.14
<Glypallidifloric acid>	C30 H46 O3	(M+H)+	32.401		454.3449	17991-81-0	FBF	99.14		99.14
<Desoxoglabrolide>	C30 H46 O3	(M+H)+	32.401		454.3449	10379-62-1	FBF	99.14		99.14
<Bryononic acid>	C30 H46 O3	(M+H)+	32.401		454.3449	24480-44-2	FBF	99.14		99.14
<Betulonic acid>	C30 H46 O3	(M+H)+	32.401		454.3449	4481-62-3	FBF	99.14		99.14
<1α,24-Dihydroxy-22-ene-24-cyclopropylvitamin D3>	C30 H46 O3	(M+H)+	32.401		454.3449		FBF	99.14		99.14
<9(11)-Dehydroglycyrrhetic acid>	C30 H46 O3	(M+H)+	32.401		454.3449	14884-88-9	FBF	99.14		99.14
<3beta-3-Hydroxy-11-oxolanosta-8,24-dien-26-al>	C30 H46 O3	(M+H)+	32.401		454.3449	126313-85-7	FBF	99.14		99.14
<1α,25-dihydroxy-26,27-dimethyl-22,22,23,23-tetradehydro-24a-homovitamin D3 / 1α,25-dihydroxy-26,27-dimethyl-22,22,23,23-tetradehydro-24a-homocholecalciferol>	C30 H46 O3	(M+H)+	32.401		454.3449		FBF	99.14		99.14
<(22E,24E)-1α,25-dihydroxy-26,27-dimethyl-22,23,24,24a-tetradehydro-24a-homovitamin D3 / (22E,24E)-1α,25-dihydroxy-26,27-dimethyl-22,23,24,24a-tetradehydro-24a-homocholecalciferol>	C30 H46 O3	(M+H)+	32.401		454.3449		FBF	99.14		99.14
<Epoxyganoderiol B>	C30 H46 O3	(M+H)+	32.401		454.3449	114020-57-4	FBF	99.14		99.14
<1α,25-dihydroxy-26,27-dimethyl-22,22,23,23-tetradehydro-24a-homo-20-epivitamin D3 / 1α,25-dihydroxy-26,27-dimethyl-22,22,23,23-tetradehydro-24a-homo-20-epicholecalciferol>	C30 H46 O3	(M+H)+	32.401		454.3449		FBF	99.14		99.14
<Isomasticadienonic acid>	C30 H46 O3	(M+H)+	32.401		454.3449	5956-26-3	FBF	99.14		99.14
<Ganoderal B>	C30 H46 O3	(M+H)+	32.401		454.3449	114020-55-2	FBF	99.14		99.14
<Mangiferonic acid>	C30 H46 O3	(M+H)+	32.401		454.3449	13878-90-5	FBF	99.14		99.14
<Ganoderic acid Y>	C30 H46 O3	(M+H)+	32.401		454.3449	86377-52-8	FBF	99.14		99.14
<Moronic acid>	C30 H46 O3	(M+H)+	32.401		454.3449		FBF	99.14		99.14
<Ursonic acid>	C30 H46 O3	(M+H)+	32.401		454.3449	6246-46-4	FBF	99.14		99.14
<Micromeric acid>	C30 H46 O3	(M+H)+	32.401		454.3449	22477-85-6	FBF	99.14		99.14
<Masticadienonic acid>	C30 H46 O3	(M+H)+	32.401		454.3449	514-49-8	FBF	99.14		99.14
<Tomentosolic acid>	C30 H46 O3	(M+H)+	32.401		454.3449	6812-98-2	FBF	99.14		99.14
<Lucidal>	C30 H46 O3	(M+H)+	32.401		454.3449	252351-96-5	FBF	99.14		99.14
<Katononic acid>	C30 H46 O3	(M+H)+	32.401		454.3449	76094-29-6	FBF	99.14		99.14
<Ganoderiol F>	C30 H46 O3	(M+H)+	32.401		454.3449	114567-47-4	FBF	99.14		99.14

Cpd 188: delta5-Demissine

Name	Formula	RT	RI	Mass	Diff (Tgt, ppm)	CAS	ID Source	Score	Algorithm
delta5-Demissine	C50 H81 N O20	32.575		1015.5355	0.32	195433-57-9	FBF	99.58	FBF

Species	m/z	Score (Tgt)	Score (Lib)	Score (DB)	Score (MFG)	Score (RT)
(M+H)+	1017	99.58				

Compound Chromatograms (overlaid)

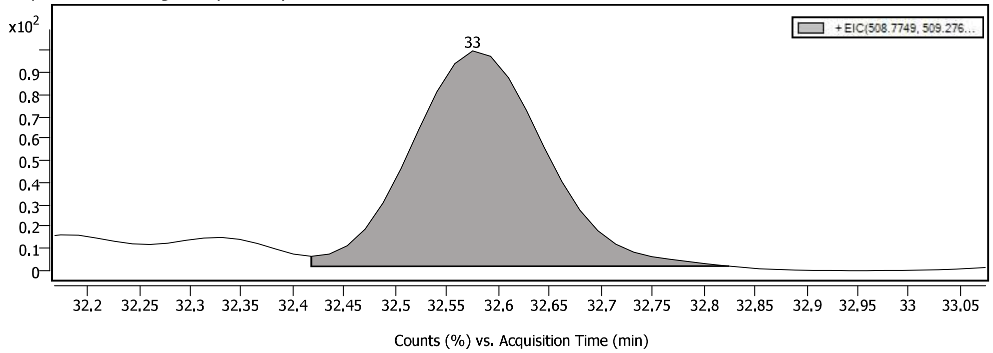

Structure

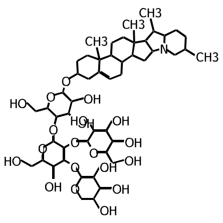

Compound Spectra (overlaid)

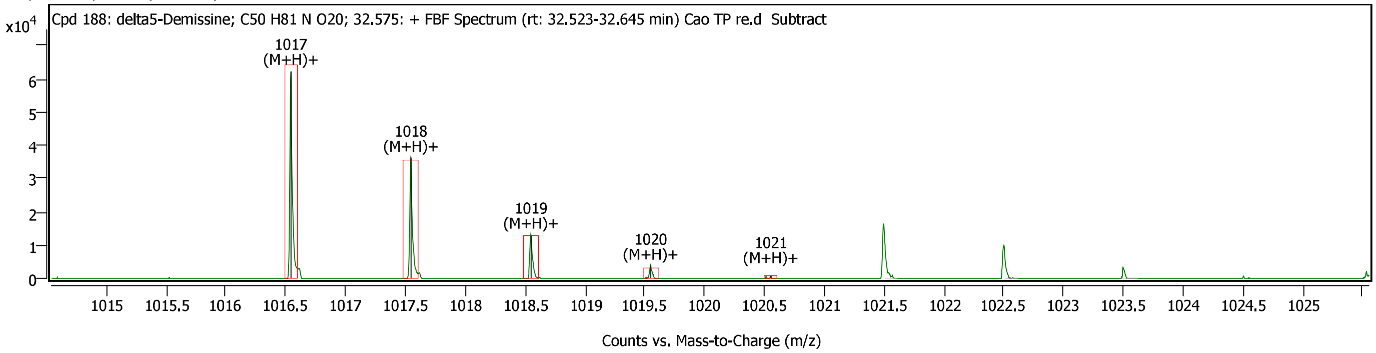

Compound ID Table

Name	Formula	Species	RT	RT Diff	Mass	CAS	ID Source	Score	Score (Lib)	Score (Tgt)
delta5-Demissine	C50 H81 N O20	(M+H)+	32.575		1015.5355	195433-57-9	FBF	99.58		99.58

Cpd 187: delta5-Demissine

Name	Formula	RT	RI	Mass	Diff (Tgt, ppm)	CAS	ID Source	Score	Algorithm
delta5-Demissine	C50 H81 N O20	33.290		1015.5354	0.16	195433-57-9	FBF	99.64	FBF

Species	m/z	Score (Tgt)	Score (Lib)	Score (DB)	Score (MFG)	Score (RT)
(M+H)+	1017	99.64				

Compound Chromatograms (overlaid)

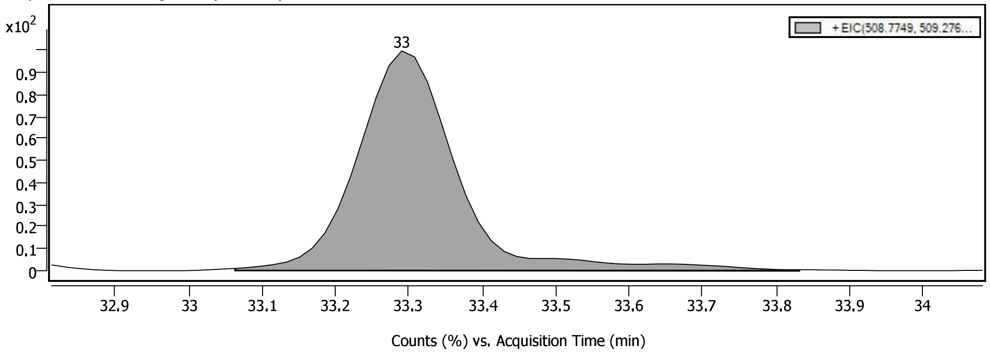

Structure

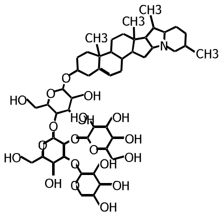

Compound Spectra (overlaid)

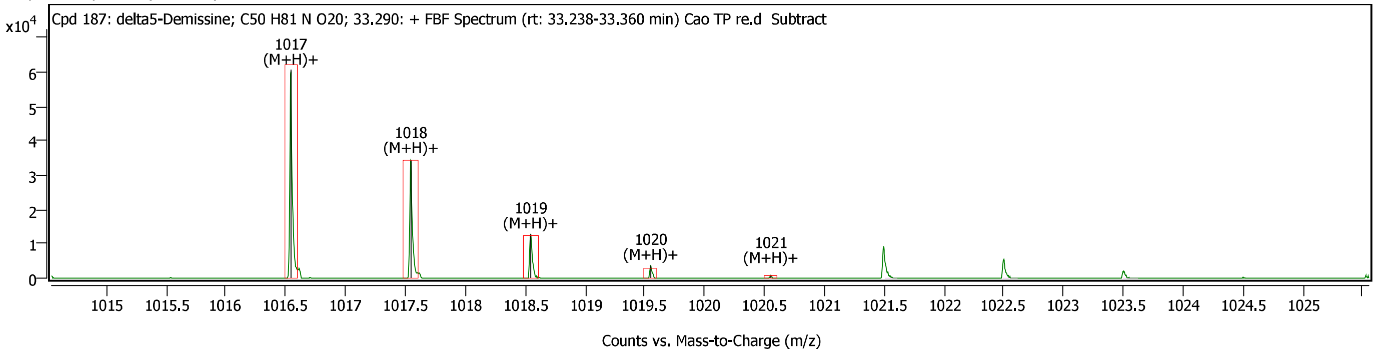

Compound ID Table

Name	Formula	Species	RT	RT Diff	Mass	CAS	ID Source	Score	Score (Lib)	Score (Tgt)
delta5-Demissine	C50 H81 N O20	(M+H)+	33.290		1015.5354	195433-57-9	FBF	99.64		99.64

Cpd 1140: 9,11-Octadecadiynoic acid

Name	Formula	RT	RI	Mass	Diff (Tgt, ppm)	CAS	ID Source	Score	Algorithm
9,11-Octadecadiynoic acid	C18 H28 O2	34.006		276.2096	2.36		M-FBF-FragConfirm	96.95	FBF

Species	m/z	Score (Tgt)	Score (Lib)	Score (DB)	Score (MFG)	Score (RT)
(M+2H)+2 (M+H)+	139 277	96.95				

Compound Screening Report

Compound Chromatograms (overlaid)

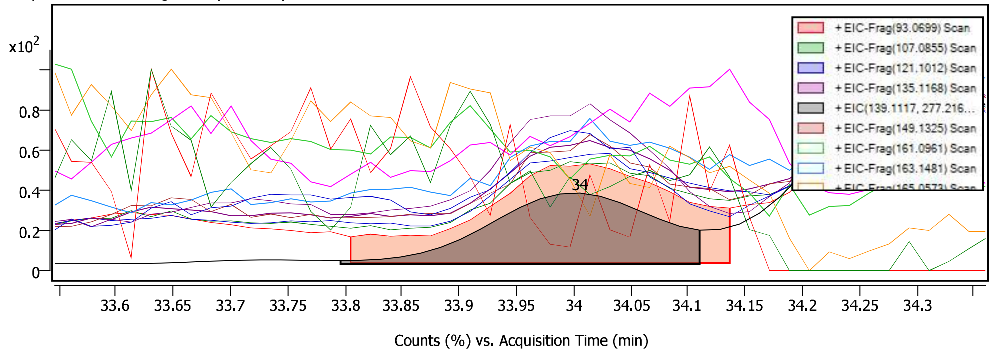

Structure

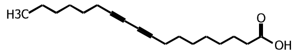

Coelution Plot

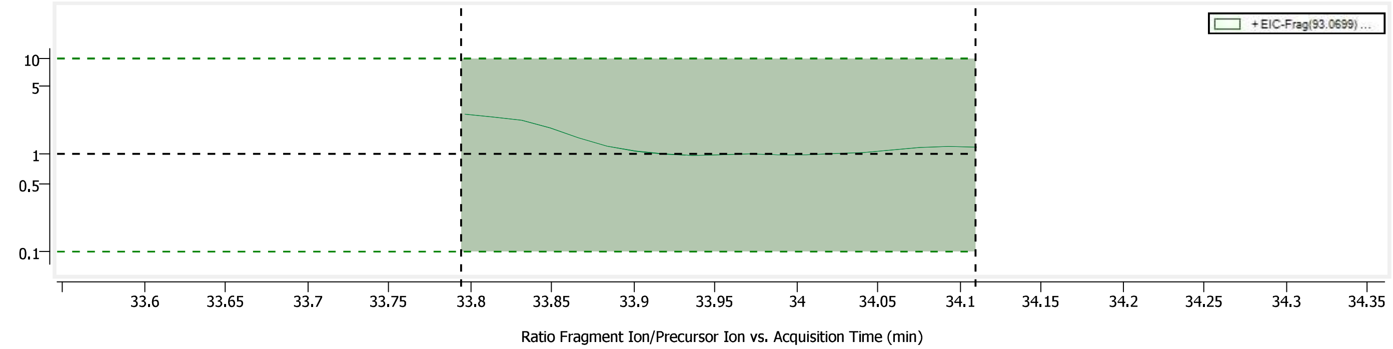

Compound Spectra (overlaid)

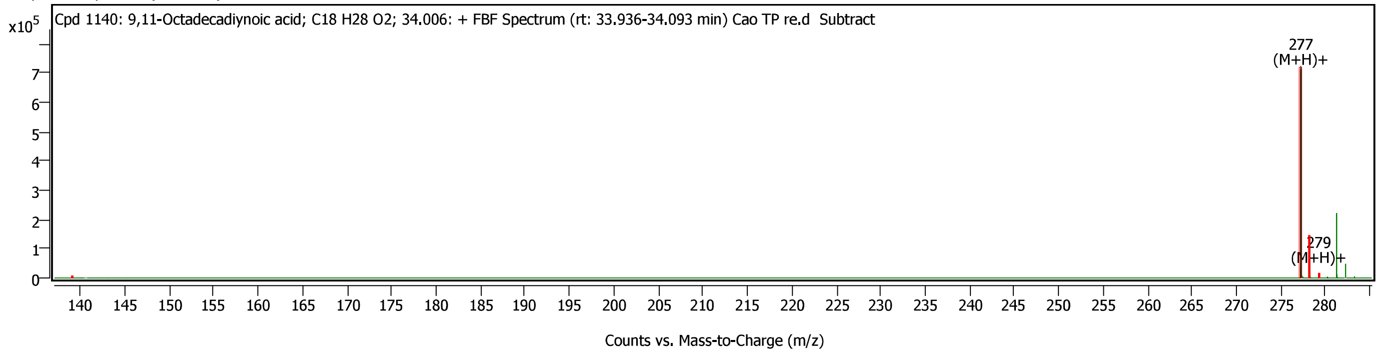

Fragment Spectrum (clean)

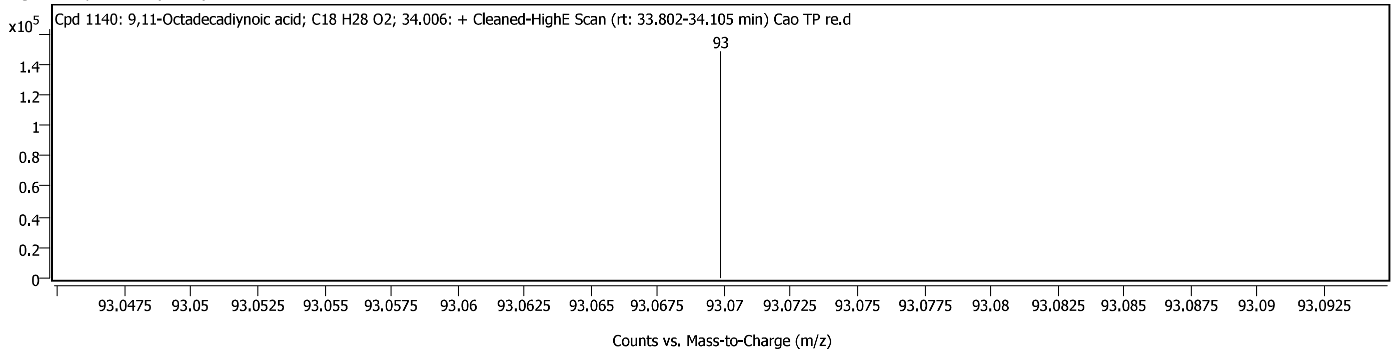

Fragment Spectrum (raw)

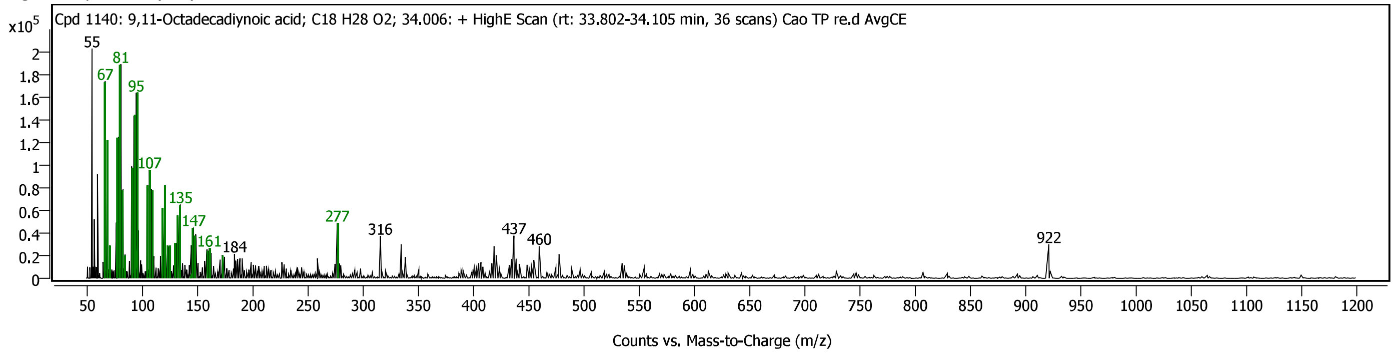

Compound Screening Report

Compound ID Table

Name	Formula	Species	RT	RT Diff	Mass	CAS	ID Source	Score	Score (Lib)	Score (Tgt)
9,11-Octadecadiynoic acid	C18 H28 O2	(M+2H)+2 (M+H)+	34.006		276.2096		FBF-FragConfirm	96.95		96.95
3E,9Z,12Z,15Z-Octadecatetraenoic acid	C18 H28 O2	(M+2H)+2 (M+H)+	34.006		276.2096	693-78-7	FBF-FragConfirm	96.95		96.95
3,8-Octadecadiynoic acid	C18 H28 O2	(M+2H)+2 (M+H)+	34.006		276.2096		FBF-FragConfirm	96.95		96.95
4,8-Octadecadiynoic acid	C18 H28 O2	(M+2H)+2 (M+H)+	34.006		276.2096		FBF-FragConfirm	96.95		96.95
2,5-Octadecadiynoic acid	C18 H28 O2	(M+2H)+2 (M+H)+	34.006		276.2096		FBF-FragConfirm	96.95		96.95
2,6-Octadecadiynoic acid	C18 H28 O2	(M+2H)+2 (M+H)+	34.006		276.2096		FBF-FragConfirm	96.95		96.95
2,7-Octadecadiynoic acid	C18 H28 O2	(M+2H)+2 (M+H)+	34.006		276.2096		FBF-FragConfirm	96.95		96.95
3,6-Octadecadiynoic acid	C18 H28 O2	(M+2H)+2 (M+H)+	34.006		276.2096		FBF-FragConfirm	96.95		96.95
3,7-Octadecadiynoic acid	C18 H28 O2	(M+2H)+2 (M+H)+	34.006		276.2096		FBF-FragConfirm	96.95		96.95
4,6-Octadecadiynoic acid	C18 H28 O2	(M+2H)+2 (M+H)+	34.006		276.2096		FBF-FragConfirm	96.95		96.95
14,17-Octadecadiynoic acid	C18 H28 O2	(M+2H)+2 (M+H)+	34.006		276.2096		FBF-FragConfirm	96.95		96.95
5,7-Octadecadiynoic acid	C18 H28 O2	(M+2H)+2 (M+H)+	34.006		276.2096		FBF-FragConfirm	96.95		96.95
4,7-Octadecadiynoic acid	C18 H28 O2	(M+2H)+2 (M+H)+	34.006		276.2096		FBF-FragConfirm	96.95		96.95
5,8-Octadecadiynoic acid	C18 H28 O2	(M+2H)+2 (M+H)+	34.006		276.2096		FBF-FragConfirm	96.95		96.95
19-Noretiocholan-3b-ol-17-one	C18 H28 O2	(M+2H)+2 (M+H)+	34.006		276.2096	64044-10-6	FBF-FragConfirm	96.95		96.95
4,9-Octadecadiynoic acid	C18 H28 O2	(M+2H)+2 (M+H)+	34.006		276.2096		FBF-FragConfirm	96.95		96.95
5,10-Octadecadiynoic acid	C18 H28 O2	(M+2H)+2 (M+H)+	34.006		276.2096		FBF-FragConfirm	96.95		96.95
19-Noretiocholanolone	C18 H28 O2	(M+2H)+2 (M+H)+	34.006		276.2096	33036-33-8	FBF-FragConfirm	96.95		96.95
19-Norandrosterone	C18 H28 O2	(M+2H)+2 (M+H)+	34.006		276.2096	1225-01-0	FBF-FragConfirm	96.95		96.95
5,8,11,14-Octadecatetraenoic acid	C18 H28 O2	(M+2H)+2 (M+H)+	34.006		276.2096	75236-11-2	FBF-FragConfirm	96.95		96.95
11,14-Octadecadiynoic acid	C18 H28 O2	(M+2H)+2 (M+H)+	34.006		276.2096		FBF-FragConfirm	96.95		96.95
11,15-Octadecadiynoic acid	C18 H28 O2	(M+2H)+2 (M+H)+	34.006		276.2096		FBF-FragConfirm	96.95		96.95
(9Z,12Z)-octadeca-9,12-dien-6-ynoic acid	C18 H28 O2	(M+2H)+2 (M+H)+	34.006		276.2096		FBF-FragConfirm	96.95		96.95
(9Z,14Z)-octadeca-9,14-dien-6-ynoic acid	C18 H28 O2	(M+2H)+2 (M+H)+	34.006		276.2096		FBF-FragConfirm	96.95		96.95
10,12-Octadecadiynoic acid	C18 H28 O2	(M+2H)+2 (M+H)+	34.006		276.2096		FBF-FragConfirm	96.95		96.95
10,13-Octadecadiynoic acid	C18 H28 O2	(M+2H)+2 (M+H)+	34.006		276.2096		FBF-FragConfirm	96.95		96.95
10,14-Octadecadiynoic acid	C18 H28 O2	(M+2H)+2 (M+H)+	34.006		276.2096		FBF-FragConfirm	96.95		96.95
11E,13E-octadecadien-9-ynoic acid	C18 H28 O2	(M+2H)+2 (M+H)+	34.006		276.2096		FBF-FragConfirm	96.95		96.95
18:4(5Z,9Z,12Z,15Z)	C18 H28 O2	(M+2H)+2 (M+H)+	34.006		276.2096		FBF-FragConfirm	96.95		96.95
19-Nor-5-androstenediol	C18 H28 O2	(M+2H)+2 (M+H)+	34.006		276.2096		FBF-FragConfirm	96.95		96.95
12,14-Octadecadiynoic acid	C18 H28 O2	(M+2H)+2 (M+H)+	34.006		276.2096		FBF-FragConfirm	96.95		96.95
12,15-Octadecadiynoic acid	C18 H28 O2	(M+2H)+2 (M+H)+	34.006		276.2096		FBF-FragConfirm	96.95		96.95
12,16-Octadecadiynoic acid	C18 H28 O2	(M+2H)+2 (M+H)+	34.006		276.2096		FBF-FragConfirm	96.95		96.95
13,16-Octadecadiynoic acid	C18 H28 O2	(M+2H)+2 (M+H)+	34.006		276.2096		FBF-FragConfirm	96.95		96.95
13,17-Octadecadiynoic acid	C18 H28 O2	(M+2H)+2 (M+H)+	34.006		276.2096		FBF-FragConfirm	96.95		96.95
5,12-Octadecadiynoic acid	C18 H28 O2	(M+2H)+2 (M+H)+	34.006		276.2096		FBF-FragConfirm	96.95		96.95
4,8,12,15-Octadecatetraenoic acid	C18 H28 O2	(M+2H)+2 (M+H)+	34.006		276.2096	67329-10-6	FBF-FragConfirm	96.95		96.95
trans-3, cis-9, cis-12, cis-15-octadecatetraenoic acid; C18:4n-3,6,9,15	C18 H28 O2	(M+2H)+2 (M+H)+	34.006		276.2096		FBF-FragConfirm	96.95		96.95
C18:4n-1,3,6,9	C18 H28 O2	(M+2H)+2 (M+H)+	34.006		276.2096		FBF-FragConfirm	96.95		96.95
C18:4n-3,5,7,9	C18 H28 O2	(M+2H)+2 (M+H)+	34.006		276.2096		FBF-FragConfirm	96.95		96.95
9Z,11Z,13E,15E-octadecatetraenoic acid	C18 H28 O2	(M+2H)+2 (M+H)+	34.006		276.2096		FBF-FragConfirm	96.95		96.95
9Z,12Z-Octadecadien-6-ynoic acid	C18 H28 O2	(M+2H)+2 (M+H)+	34.006		276.2096		FBF-FragConfirm	96.95		96.95
9Z,14Z-Octadecadien-12-ynoic acid	C18 H28 O2	(M+2H)+2 (M+H)+	34.006		276.2096		FBF-FragConfirm	96.95		96.95
Acetylenic acids; 11,13-Octadecadien-9-ynoic acid, (E,E)-	C18 H28 O2	(M+2H)+2 (M+H)+	34.006		276.2096		FBF-FragConfirm	96.95		96.95
beta-parinaric acid	C18 H28 O2	(M+2H)+2 (M+H)+	34.006		276.2096		FBF-FragConfirm	96.95		96.95
cis-Parinaric Acid	C18 H28 O2	(M+2H)+2 (M+H)+	34.006		276.2096	18427-44-6	FBF-FragConfirm	96.95		96.95
9,13-Octadecadiynoic acid	C18 H28 O2	(M+2H)+2 (M+H)+	34.006		276.2096		FBF-FragConfirm	96.95		96.95
α-Parinaric acid	C18 H28 O2	(M+2H)+2 (M+H)+	34.006		276.2096	593-38-4	FBF-FragConfirm	96.95		96.95
Kinoprene	C18 H28 O2	(M+2H)+2 (M+H)+	34.006		276.2096	42588-37-4	FBF-FragConfirm	96.95		96.95
Moroctic acid	C18 H28 O2	(M+2H)+2 (M+H)+	34.006		276.2096		FBF-FragConfirm	96.95		96.95
Phenethyl decanoate	C18 H28 O2	(M+2H)+2 (M+H)+	34.006		276.2096	61810-55-7	FBF-FragConfirm	96.95		96.95
Stearidonic Acid	C18 H28 O2	(M+2H)+2 (M+H)+	34.006		276.2096	20290-75-9	FBF-FragConfirm	96.95		96.95

Compound ID Table

Name	Formula	Species	RT	RT Diff	Mass	CAS	ID Source	Score	Score (Lib)	Score (Tgt)
trans-2,trans-4,trans-6,cis-11-octadecatetraenoic acid	C18 H28 O2	(M+2H)+2 (M+H)+	34.006		276.2096		FBF-FragConfirm	96.95		96.95
9,12-Octadecadiynoic Acid	C18 H28 O2	(M+2H)+2 (M+H)+	34.006		276.2096	2012-14-8	FBF-FragConfirm	96.95		96.95
9,12,15,17-Octadecatetraenoic acid	C18 H28 O2	(M+2H)+2 (M+H)+	34.006		276.2096	958762-34-0	FBF-FragConfirm	96.95		96.95
5,9,12,15-octadecatetraenoic acid	C18 H28 O2	(M+2H)+2 (M+H)+	34.006		276.2096		FBF-FragConfirm	96.95		96.95
6,8-Octadecadiynoic acid	C18 H28 O2	(M+2H)+2 (M+H)+	34.006		276.2096		FBF-FragConfirm	96.95		96.95
6,9-Octadecadiynoic acid	C18 H28 O2	(M+2H)+2 (M+H)+	34.006		276.2096		FBF-FragConfirm	96.95		96.95
6-[3]-ladderane-hexanoic acid	C18 H28 O2	(M+2H)+2 (M+H)+	34.006		276.2096		FBF-FragConfirm	96.95		96.95
5Z,8Z,11Z,14Z-Octadecatetraenoic acid	C18 H28 O2	(M+2H)+2 (M+H)+	34.006		276.2096		FBF-FragConfirm	96.95		96.95
6,10-Octadecadiynoic acid	C18 H28 O2	(M+2H)+2 (M+H)+	34.006		276.2096		FBF-FragConfirm	96.95		96.95
6,11-Octadecadiynoic acid	C18 H28 O2	(M+2H)+2 (M+H)+	34.006		276.2096		FBF-FragConfirm	96.95		96.95
6,12-Octadecadiynoic acid	C18 H28 O2	(M+2H)+2 (M+H)+	34.006		276.2096		FBF-FragConfirm	96.95		96.95
6,9,12,15-Octadecatetraenoic acid	C18 H28 O2	(M+2H)+2 (M+H)+	34.006		276.2096	2091-28-3	FBF-FragConfirm	96.95		96.95
8,11-Octadecadiynoic acid	C18 H28 O2	(M+2H)+2 (M+H)+	34.006		276.2096		FBF-FragConfirm	96.95		96.95
5,9-Octadecadiynoic acid	C18 H28 O2	(M+2H)+2 (M+H)+	34.006		276.2096		FBF-FragConfirm	96.95		96.95
7,10-Octadecadiynoic acid	C18 H28 O2	(M+2H)+2 (M+H)+	34.006		276.2096		FBF-FragConfirm	96.95		96.95
7,11-Octadecadiynoic acid	C18 H28 O2	(M+2H)+2 (M+H)+	34.006		276.2096		FBF-FragConfirm	96.95		96.95
7,12-Octadecadiynoic acid	C18 H28 O2	(M+2H)+2 (M+H)+	34.006		276.2096		FBF-FragConfirm	96.95		96.95
7,9-Octadecadiynoic acid	C18 H28 O2	(M+2H)+2 (M+H)+	34.006		276.2096		FBF-FragConfirm	96.95		96.95
8,10-Octadecadiynoic acid	C18 H28 O2	(M+2H)+2 (M+H)+	34.006		276.2096		FBF-FragConfirm	96.95		96.95
8,12-Octadecadiynoic acid	C18 H28 O2	(M+2H)+2 (M+H)+	34.006		276.2096		FBF-FragConfirm	96.95		96.95

Cpd 551: Delcorine

Name	Formula	RT	RI	Mass	Diff (Tgt, ppm)	CAS	ID Source	Score	Algorithm
Delcorine	C26 H41 N O7	34.058		479.2883	0.03	52358-55-1	FBF	99.77	FBF

Species	m/z	Score (Tgt)	Score (Lib)	Score (DB)	Score (MFG)	Score (RT)
(M+H)+	480	99.77				

Compound Chromatograms (overlaid)

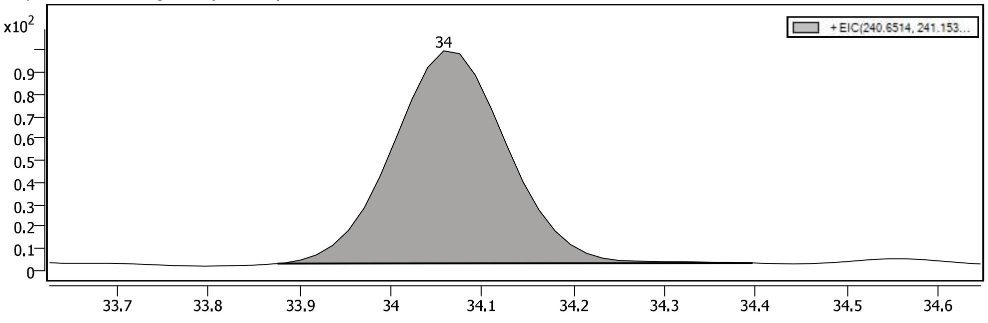

Structure

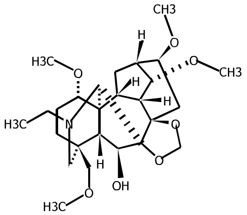

Compound Spectra (overlaid)

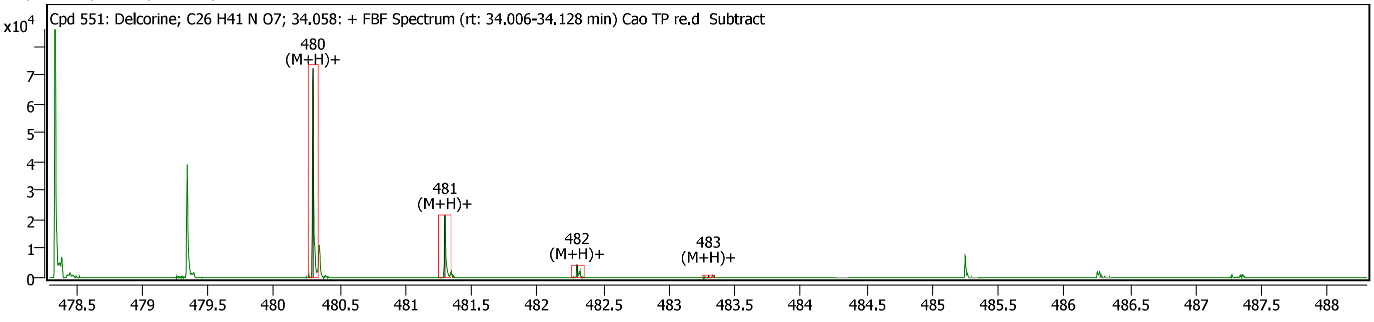

Compound ID Table

Name	Formula	Species	RT	RT Diff	Mass	CAS	ID Source	Score	Score (Lib)	Score (Tgt)
Delcorine	C26 H41 N O7	(M+H)+	34.058		479.2883	52358-55-1	FBF	99.77		99.77

Cpd 278: PI(12:0/12:0)

Name	Formula	RT	RI	Mass	Diff (Tgt, ppm)	CAS	ID Source	Score	Algorithm
PI(12:0/12:0)	C33 H63 O13 P	34.180		698.4031	3.51		FBF	89.87	FBF

Species	m/z	Score (Tgt)	Score (Lib)	Score (DB)	Score (MFG)	Score (RT)
(M+H)+	699	89.87				

Compound Chromatograms (overlaid)

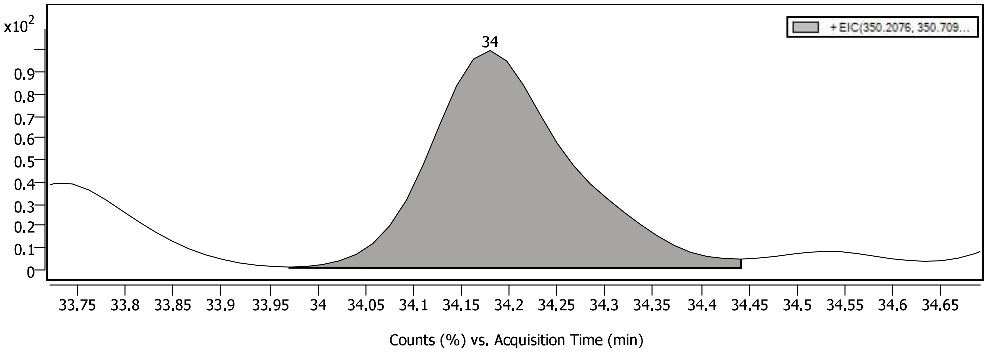

Structure

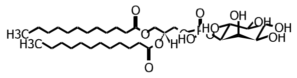

Compound Spectra (overlaid)

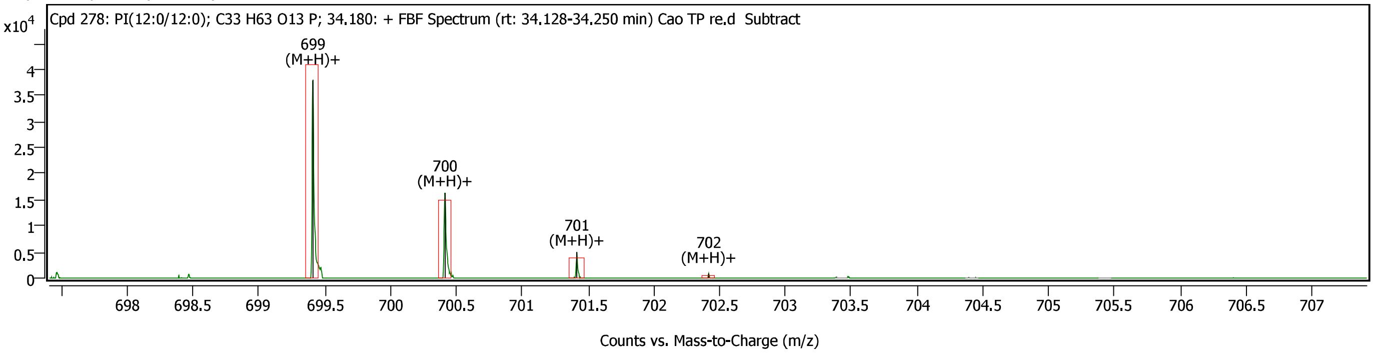

Compound ID Table

Name	Formula	Species	RT	RT Diff	Mass	CAS	ID Source	Score	Score (Lib)	Score (Tgt)
PI(12:0/12:0)	C33 H63 O13 P	(M+H)+	34.180		698.4031		FBF	89.87		89.87

Cpd 1139: Stearidonic Acid

Name	Formula	RT	RI	Mass	Diff (Tgt, ppm)	CAS	ID Source	Score	Algorithm
Stearidonic Acid	C18 H28 O2	34.302		276.2102	4.63	20290-75-9	M-FBF-FragConfirm	86.97	FBF

Species	m/z	Score (Tgt)	Score (Lib)	Score (DB)	Score (MFG)	Score (RT)
(M+2H)+2 (M+H)+	139 277	86.97				

Compound Chromatograms (overlaid)

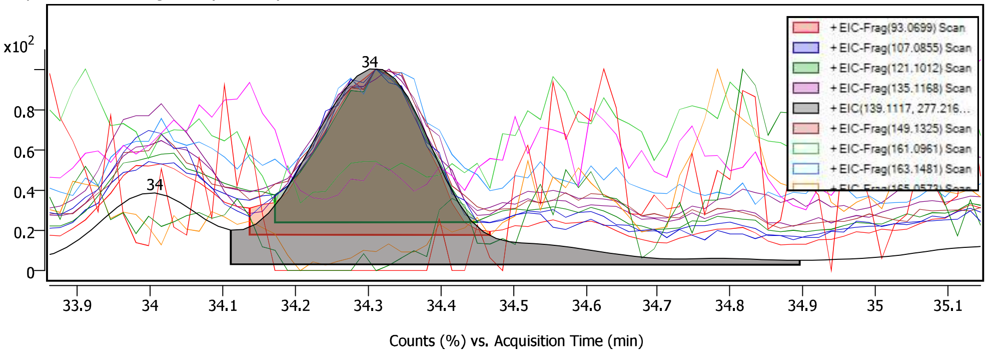

Structure

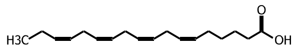

Coelution Plot

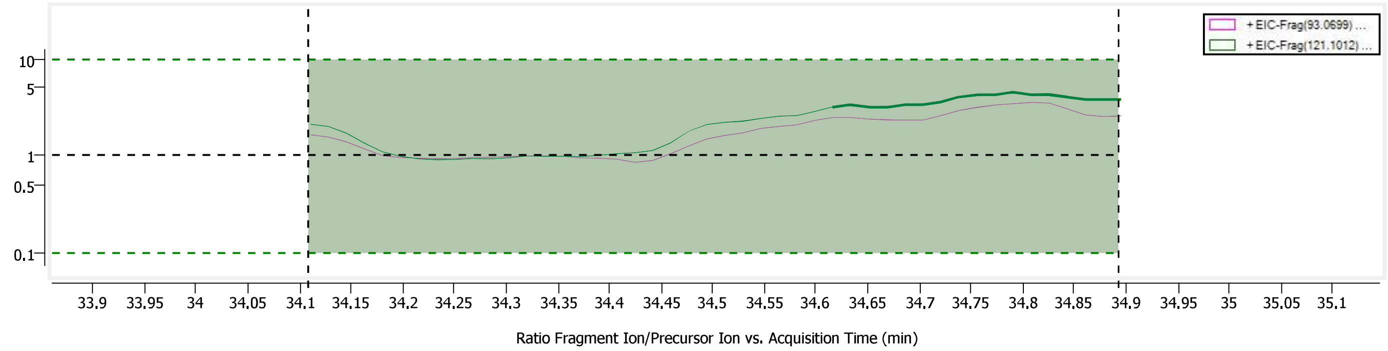

Compound Spectra (overlaid)

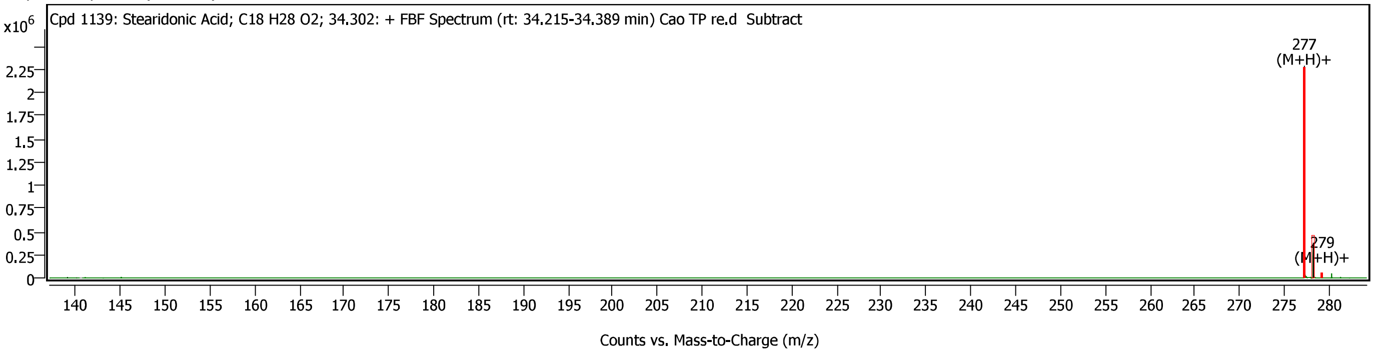

Fragment Spectrum (clean)

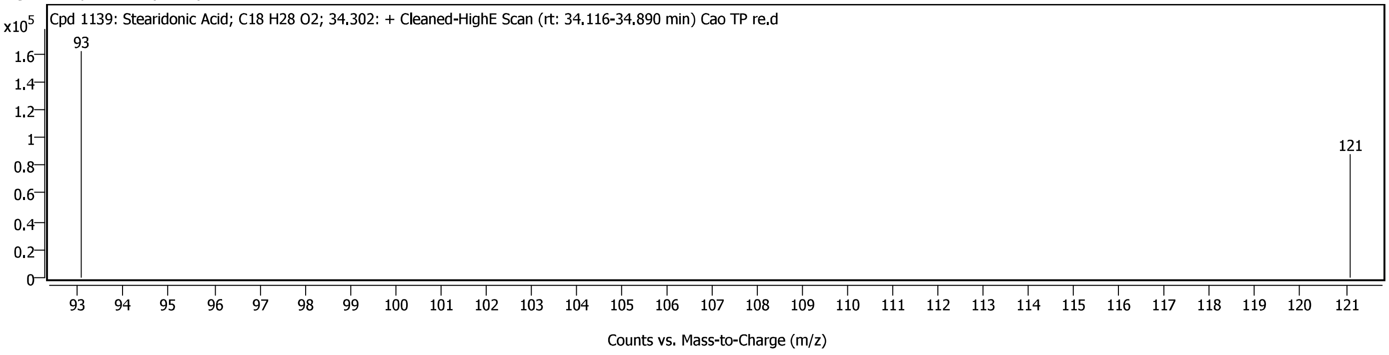

Fragment Spectrum (raw)

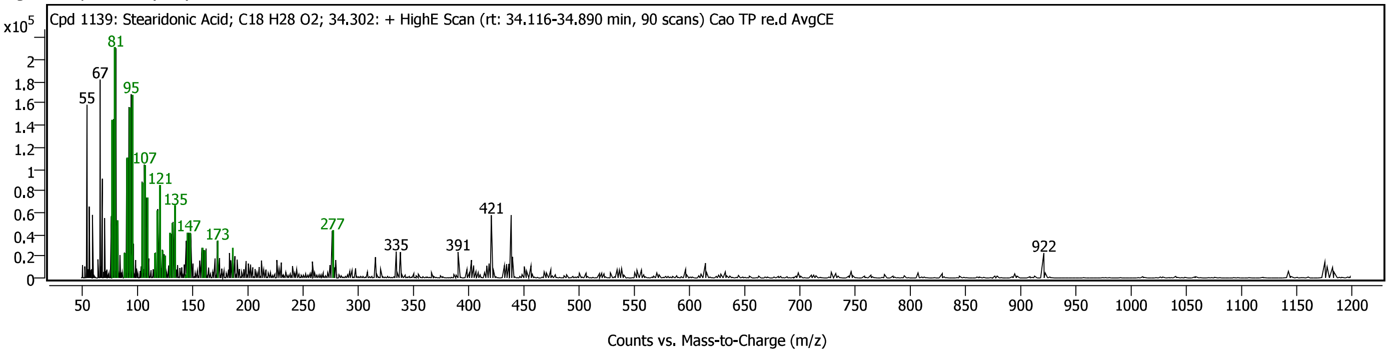

Compound ID Table

Name	Formula	Species	RT	RT Diff	Mass	CAS	ID Source	Score	Score (Lib)	Score (Tgt)
Stearidonic Acid	C18 H28 O2	(M+2H)+2 (M+H)+	34.302		276.2102	20290-75-9	FBF-FragConfirm	86.97		86.97
3E,9Z,12Z,15Z-Octadecatetraenoic acid	C18 H28 O2	(M+2H)+2 (M+H)+	34.302		276.2102	693-78-7	FBF-FragConfirm	86.97		86.97
3,7-Octadecadiynoic acid	C18 H28 O2	(M+2H)+2 (M+H)+	34.302		276.2102		FBF-FragConfirm	86.97		86.97
19-Noretiocholanolone	C18 H28 O2	(M+2H)+2 (M+H)+	34.302		276.2102	33036-33-8	FBF-FragConfirm	86.97		86.97
4,8-Octadecadiynoic acid	C18 H28 O2	(M+2H)+2 (M+H)+	34.302		276.2102		FBF-FragConfirm	86.97		86.97
2,5-Octadecadiynoic acid	C18 H28 O2	(M+2H)+2 (M+H)+	34.302		276.2102		FBF-FragConfirm	86.97		86.97
2,6-Octadecadiynoic acid	C18 H28 O2	(M+2H)+2 (M+H)+	34.302		276.2102		FBF-FragConfirm	86.97		86.97
2,7-Octadecadiynoic acid	C18 H28 O2	(M+2H)+2 (M+H)+	34.302		276.2102		FBF-FragConfirm	86.97		86.97
3,6-Octadecadiynoic acid	C18 H28 O2	(M+2H)+2 (M+H)+	34.302		276.2102		FBF-FragConfirm	86.97		86.97
3,8-Octadecadiynoic acid	C18 H28 O2	(M+2H)+2 (M+H)+	34.302		276.2102		FBF-FragConfirm	86.97		86.97
19-Norandrosterone	C18 H28 O2	(M+2H)+2 (M+H)+	34.302		276.2102	1225-01-0	FBF-FragConfirm	86.97		86.97
4,6-Octadecadiynoic acid	C18 H28 O2	(M+2H)+2 (M+H)+	34.302		276.2102		FBF-FragConfirm	86.97		86.97
5,7-Octadecadiynoic acid	C18 H28 O2	(M+2H)+2 (M+H)+	34.302		276.2102		FBF-FragConfirm	86.97		86.97
4,7-Octadecadiynoic acid	C18 H28 O2	(M+2H)+2 (M+H)+	34.302		276.2102		FBF-FragConfirm	86.97		86.97
5,8-Octadecadiynoic acid	C18 H28 O2	(M+2H)+2 (M+H)+	34.302		276.2102		FBF-FragConfirm	86.97		86.97
19-Noretiocholan-3b-ol-17-one	C18 H28 O2	(M+2H)+2 (M+H)+	34.302		276.2102	64044-10-6	FBF-FragConfirm	86.97		86.97
4,9-Octadecadiynoic acid	C18 H28 O2	(M+2H)+2 (M+H)+	34.302		276.2102		FBF-FragConfirm	86.97		86.97
14,17-Octadecadiynoic acid	C18 H28 O2	(M+2H)+2 (M+H)+	34.302		276.2102		FBF-FragConfirm	86.97		86.97
18:4(5Z,9Z,12Z,15Z)	C18 H28 O2	(M+2H)+2 (M+H)+	34.302		276.2102		FBF-FragConfirm	86.97		86.97
5,12-Octadecadiynoic acid	C18 H28 O2	(M+2H)+2 (M+H)+	34.302		276.2102		FBF-FragConfirm	86.97		86.97
10,14-Octadecadiynoic acid	C18 H28 O2	(M+2H)+2 (M+H)+	34.302		276.2102		FBF-FragConfirm	86.97		86.97
8,12-Octadecadiynoic acid	C18 H28 O2	(M+2H)+2 (M+H)+	34.302		276.2102		FBF-FragConfirm	86.97		86.97
11,15-Octadecadiynoic acid	C18 H28 O2	(M+2H)+2 (M+H)+	34.302		276.2102		FBF-FragConfirm	86.97		86.97
(9Z,12Z)-octadeca-9,12-dien-6-ynoic acid	C18 H28 O2	(M+2H)+2 (M+H)+	34.302		276.2102		FBF-FragConfirm	86.97		86.97
(9Z,14Z)-octadeca-9,14-dien-6-ynoic acid	C18 H28 O2	(M+2H)+2 (M+H)+	34.302		276.2102		FBF-FragConfirm	86.97		86.97
10,12-Octadecadiynoic acid	C18 H28 O2	(M+2H)+2 (M+H)+	34.302		276.2102		FBF-FragConfirm	86.97		86.97
10,13-Octadecadiynoic acid	C18 H28 O2	(M+2H)+2 (M+H)+	34.302		276.2102		FBF-FragConfirm	86.97		86.97
11,14-Octadecadiynoic acid	C18 H28 O2	(M+2H)+2 (M+H)+	34.302		276.2102		FBF-FragConfirm	86.97		86.97
13,17-Octadecadiynoic acid	C18 H28 O2	(M+2H)+2 (M+H)+	34.302		276.2102		FBF-FragConfirm	86.97		86.97
11E,13E-octadecadien-9-ynoic acid	C18 H28 O2	(M+2H)+2 (M+H)+	34.302		276.2102		FBF-FragConfirm	86.97		86.97
19-Nor-5-androstenediol	C18 H28 O2	(M+2H)+2 (M+H)+	34.302		276.2102		FBF-FragConfirm	86.97		86.97
12,14-Octadecadiynoic acid	C18 H28 O2	(M+2H)+2 (M+H)+	34.302		276.2102		FBF-FragConfirm	86.97		86.97
12,15-Octadecadiynoic acid	C18 H28 O2	(M+2H)+2 (M+H)+	34.302		276.2102		FBF-FragConfirm	86.97		86.97
12,16-Octadecadiynoic acid	C18 H28 O2	(M+2H)+2 (M+H)+	34.302		276.2102		FBF-FragConfirm	86.97		86.97
13,16-Octadecadiynoic acid	C18 H28 O2	(M+2H)+2 (M+H)+	34.302		276.2102		FBF-FragConfirm	86.97		86.97
5,10-Octadecadiynoic acid	C18 H28 O2	(M+2H)+2 (M+H)+	34.302		276.2102		FBF-FragConfirm	86.97		86.97
5,8,11,14-Octadecatetraenoic acid	C18 H28 O2	(M+2H)+2 (M+H)+	34.302		276.2102	75236-11-2	FBF-FragConfirm	86.97		86.97
trans-3, cis-9, cis-12, cis-15-octadecatetraenoic acid; C18:4n-3,6,9,15	C18 H28 O2	(M+2H)+2 (M+H)+	34.302		276.2102		FBF-FragConfirm	86.97		86.97
beta-parinaric acid	C18 H28 O2	(M+2H)+2 (M+H)+	34.302		276.2102		FBF-FragConfirm	86.97		86.97
9,12-Octadecadiynoic Acid	C18 H28 O2	(M+2H)+2 (M+H)+	34.302		276.2102	2012-14-8	FBF-FragConfirm	86.97		86.97
C18:4n-3,5,7,9	C18 H28 O2	(M+2H)+2 (M+H)+	34.302		276.2102		FBF-FragConfirm	86.97		86.97
9Z,11Z,13E,15E-octadecatetraenoic acid	C18 H28 O2	(M+2H)+2 (M+H)+	34.302		276.2102		FBF-FragConfirm	86.97		86.97
9Z,12Z-Octadecadien-6-ynoic acid	C18 H28 O2	(M+2H)+2 (M+H)+	34.302		276.2102		FBF-FragConfirm	86.97		86.97
9Z,14Z-Octadecadien-12-ynoic acid	C18 H28 O2	(M+2H)+2 (M+H)+	34.302		276.2102		FBF-FragConfirm	86.97		86.97
Acetylenic acids; 11,13-Octadecadien-9-ynoic acid, (E,E)-	C18 H28 O2	(M+2H)+2 (M+H)+	34.302		276.2102		FBF-FragConfirm	86.97		86.97
C18:4n-1,3,6,9	C18 H28 O2	(M+2H)+2 (M+H)+	34.302		276.2102		FBF-FragConfirm	86.97		86.97
9,12,15,17-Octadecatetraenoic acid	C18 H28 O2	(M+2H)+2 (M+H)+	34.302		276.2102	958762-34-0	FBF-FragConfirm	86.97		86.97
cis-Parinaric Acid	C18 H28 O2	(M+2H)+2 (M+H)+	34.302		276.2102	18427-44-6	FBF-FragConfirm	86.97		86.97
α-Parinaric acid	C18 H28 O2	(M+2H)+2 (M+H)+	34.302		276.2102	593-38-4	FBF-FragConfirm	86.97		86.97
Kinoprene	C18 H28 O2	(M+2H)+2 (M+H)+	34.302		276.2102	42588-37-4	FBF-FragConfirm	86.97		86.97
Moroctic acid	C18 H28 O2	(M+2H)+2 (M+H)+	34.302		276.2102		FBF-FragConfirm	86.97		86.97
Phenethyl decanoate	C18 H28 O2	(M+2H)+2 (M+H)+	34.302		276.2102	61810-55-7	FBF-FragConfirm	86.97		86.97

Compound Screening Report

Compound ID Table

Name	Formula	Species	RT	RT Diff	Mass	CAS	ID Source	Score	Score (Lib)	Score (Tgt)
trans-2,trans-4,trans-6,cis-11-octadecatetraenoic acid	C18 H28 O2	(M+2H)+2 (M+H)+	34.302		276.2102		FBF-FragConfirm	86.97		86.97
9,13-Octadecadiynoic acid	C18 H28 O2	(M+2H)+2 (M+H)+	34.302		276.2102		FBF-FragConfirm	86.97		86.97
8,11-Octadecadiynoic acid	C18 H28 O2	(M+2H)+2 (M+H)+	34.302		276.2102		FBF-FragConfirm	86.97		86.97
4,8,12,15-Octadecatetraenoic acid	C18 H28 O2	(M+2H)+2 (M+H)+	34.302		276.2102	67329-10-6	FBF-FragConfirm	86.97		86.97
6,12-Octadecadiynoic acid	C18 H28 O2	(M+2H)+2 (M+H)+	34.302		276.2102		FBF-FragConfirm	86.97		86.97
5,9,12,15-octadecatetraenoic acid	C18 H28 O2	(M+2H)+2 (M+H)+	34.302		276.2102		FBF-FragConfirm	86.97		86.97
6,9-Octadecadiynoic acid	C18 H28 O2	(M+2H)+2 (M+H)+	34.302		276.2102		FBF-FragConfirm	86.97		86.97
6-[3]-ladderane-hexanoic acid	C18 H28 O2	(M+2H)+2 (M+H)+	34.302		276.2102		FBF-FragConfirm	86.97		86.97
5Z,8Z,11Z,14Z-Octadecatetraenoic acid	C18 H28 O2	(M+2H)+2 (M+H)+	34.302		276.2102		FBF-FragConfirm	86.97		86.97
6,10-Octadecadiynoic acid	C18 H28 O2	(M+2H)+2 (M+H)+	34.302		276.2102		FBF-FragConfirm	86.97		86.97
6,11-Octadecadiynoic acid	C18 H28 O2	(M+2H)+2 (M+H)+	34.302		276.2102		FBF-FragConfirm	86.97		86.97
6,8-Octadecadiynoic acid	C18 H28 O2	(M+2H)+2 (M+H)+	34.302		276.2102		FBF-FragConfirm	86.97		86.97
8,10-Octadecadiynoic acid	C18 H28 O2	(M+2H)+2 (M+H)+	34.302		276.2102		FBF-FragConfirm	86.97		86.97
6,9,12,15-Octadecatetraenoic acid	C18 H28 O2	(M+2H)+2 (M+H)+	34.302		276.2102	2091-28-3	FBF-FragConfirm	86.97		86.97
5,9-Octadecadiynoic acid	C18 H28 O2	(M+2H)+2 (M+H)+	34.302		276.2102		FBF-FragConfirm	86.97		86.97
7,10-Octadecadiynoic acid	C18 H28 O2	(M+2H)+2 (M+H)+	34.302		276.2102		FBF-FragConfirm	86.97		86.97
7,11-Octadecadiynoic acid	C18 H28 O2	(M+2H)+2 (M+H)+	34.302		276.2102		FBF-FragConfirm	86.97		86.97
7,12-Octadecadiynoic acid	C18 H28 O2	(M+2H)+2 (M+H)+	34.302		276.2102		FBF-FragConfirm	86.97		86.97
7,9-Octadecadiynoic acid	C18 H28 O2	(M+2H)+2 (M+H)+	34.302		276.2102		FBF-FragConfirm	86.97		86.97
9,11-Octadecadiynoic acid	C18 H28 O2	(M+2H)+2 (M+H)+	34.302		276.2102		FBF-FragConfirm	86.97		86.97

Cpd 273: PI(O-20:0/0:0)

Name	Formula	RT	RI	Mass	Diff (Tgt, ppm)	CAS	ID Source	Score	Algorithm
PI(O-20:0/0:0)	C29 H59 O11 P	34.459		614.3821	4.31		FBF	85.38	FBF

Species	m/z	Score (Tgt)	Score (Lib)	Score (DB)	Score (MFG)	Score (RT)
(M+H)+	615	85.38				

Compound Chromatograms (overlaid)

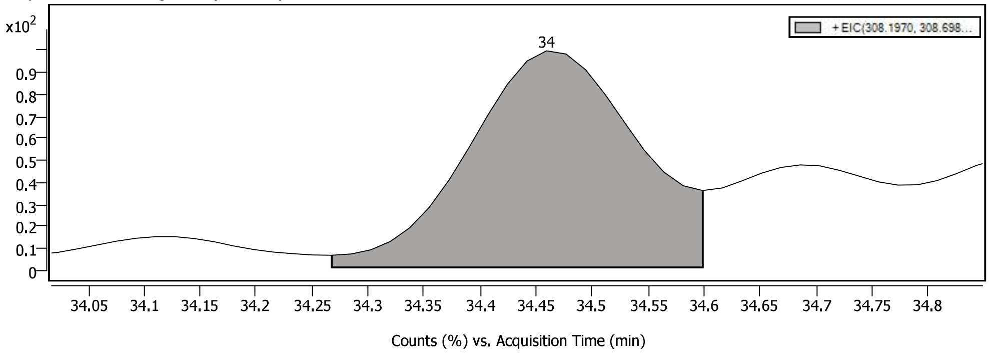

Structure

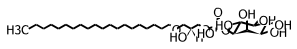

Compound Spectra (overlaid)

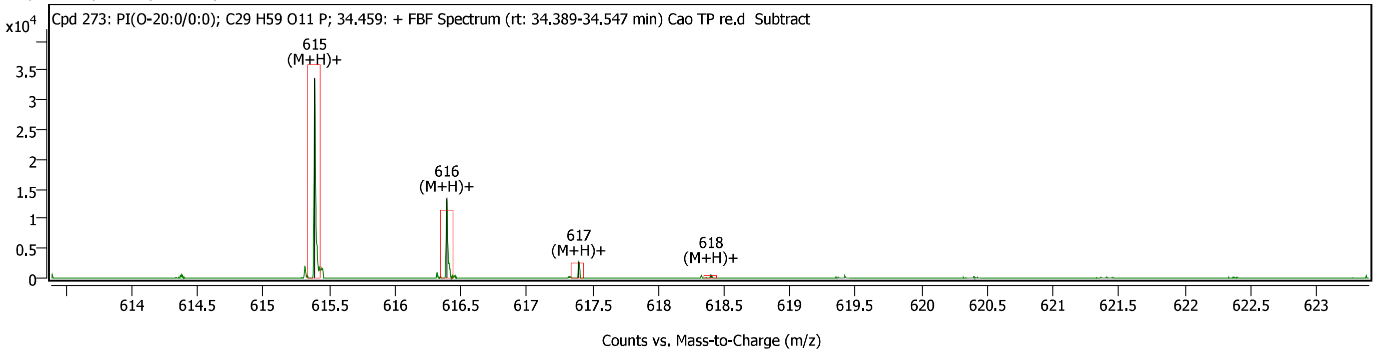

Compound ID Table

Name	Formula	Species	RT	RT Diff	Mass	CAS	ID Source	Score	Score (Lib)	Score (Tgt)
PI(O-20:0/0:0)	C29 H59 O11 P	(M+H)+	34.459		614.3821		FBF	85.38		85.38

Cpd 1409: Synaptolepis factor K1

Name	Formula	RT	RI	Mass	Diff (Tgt, ppm)	CAS	ID Source	Score	Algorithm
Synaptolepis factor K1	C36 H54 O8	34.459		614.3821	0.43	66268-94-8	FBF	99.47	FBF

Species	m/z	Score (Tgt)	Score (Lib)	Score (DB)	Score (MFG)	Score (RT)
(M+H)+	615	99.47				

Compound Chromatograms (overlaid)

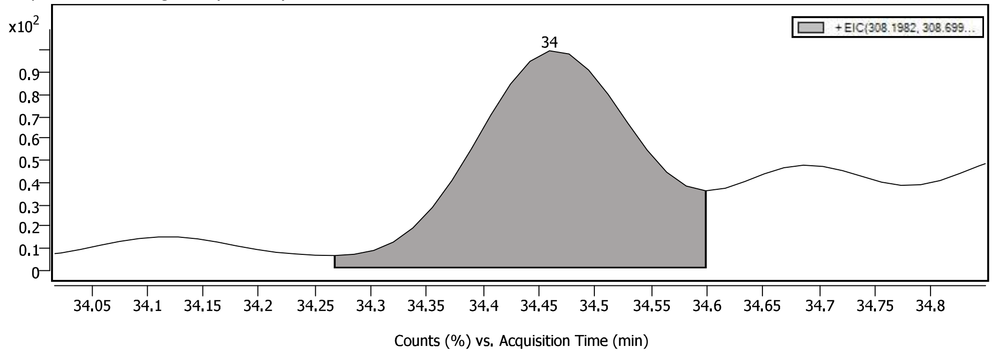

Structure

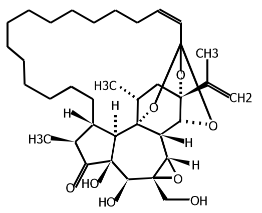

Compound Spectra (overlaid)

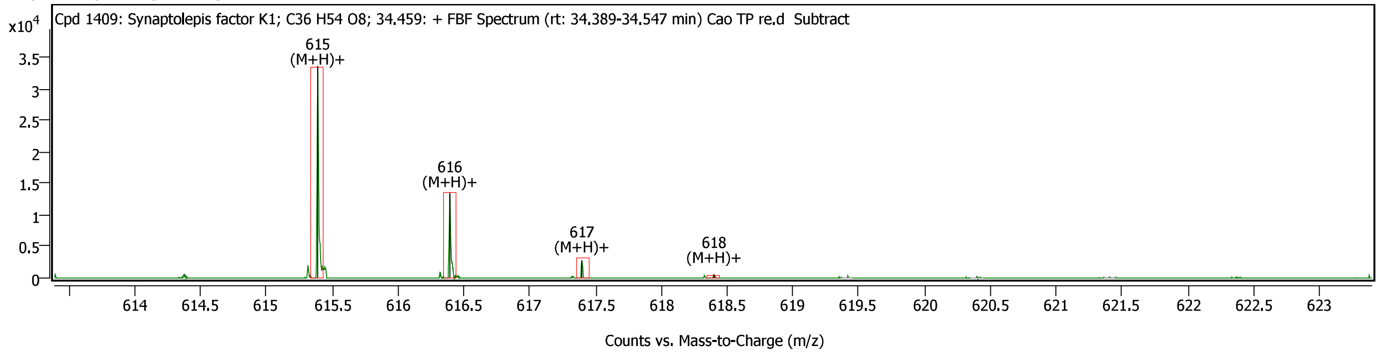

Compound ID Table

Name	Formula	Species	RT	RT Diff	Mass	CAS	ID Source	Score	Score (Lib)	Score (Tgt)
Synaptolepis factor K1	C36 H54 O8	(M+H)+	34.459		614.3821	66268-94-8	FBF	99.47		99.47

Cpd 1302: Brevetoxin B

Name	Formula	RT	RI	Mass	Diff (Tgt, ppm)	CAS	ID Source	Score	Algorithm
Brevetoxin B	C50 H70 O14	34.669		894.4769	0.35	79580-28-2	FBF	90.78	FBF

Species	m/z	Score (Tgt)	Score (Lib)	Score (DB)	Score (MFG)	Score (RT)
(M+H)+	895	90.78				

Compound Chromatograms (overlaid)

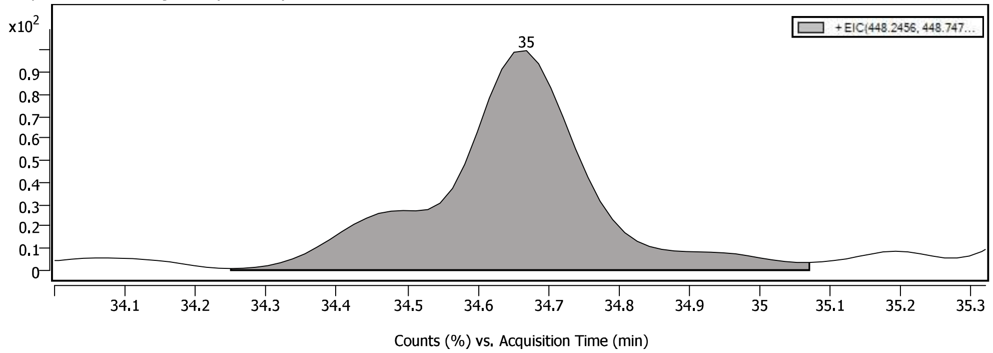

Structure

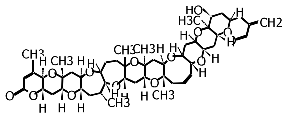

Compound Spectra (overlaid)

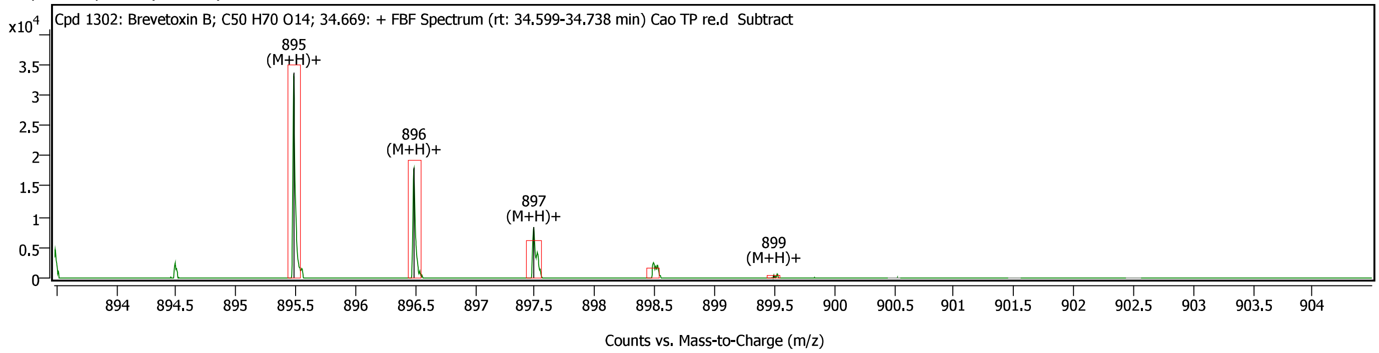

Compound ID Table

Name	Formula	Species	RT	RT Diff	Mass	CAS	ID Source	Score	Score (Lib)	Score (Tgt)
Brevetoxin B	C50 H70 O14	(M+H)+	34.669		894.4769	79580-28-2	FBF	90.78		90.78

Cpd 1272: Dequalinium

Name	Formula	RT	RI	Mass	Diff (Tgt, ppm)	CAS	ID Source	Score	Algorithm
Dequalinium	C30 H40 N4	34.721		456.3242	-2.35	522-51-0	FBF	97.18	FBF

Species	m/z	Score (Tgt)	Score (Lib)	Score (DB)	Score (MFG)	Score (RT)
(M+H)+	457	97.18				

Compound Chromatograms (overlaid)

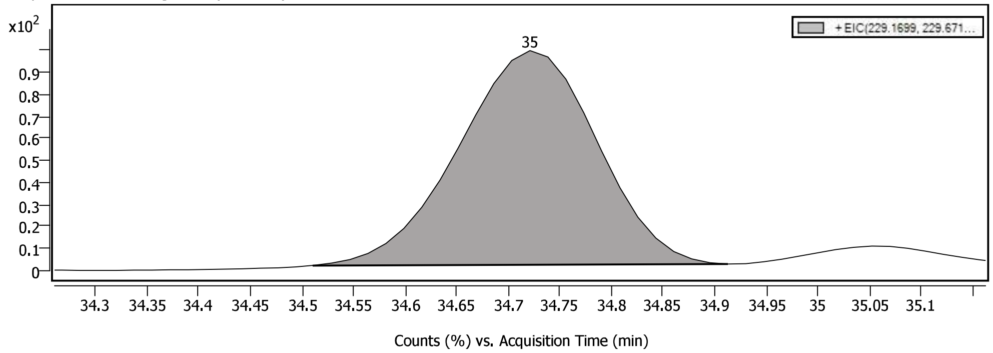

Structure

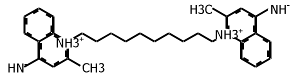

Compound Spectra (overlaid)

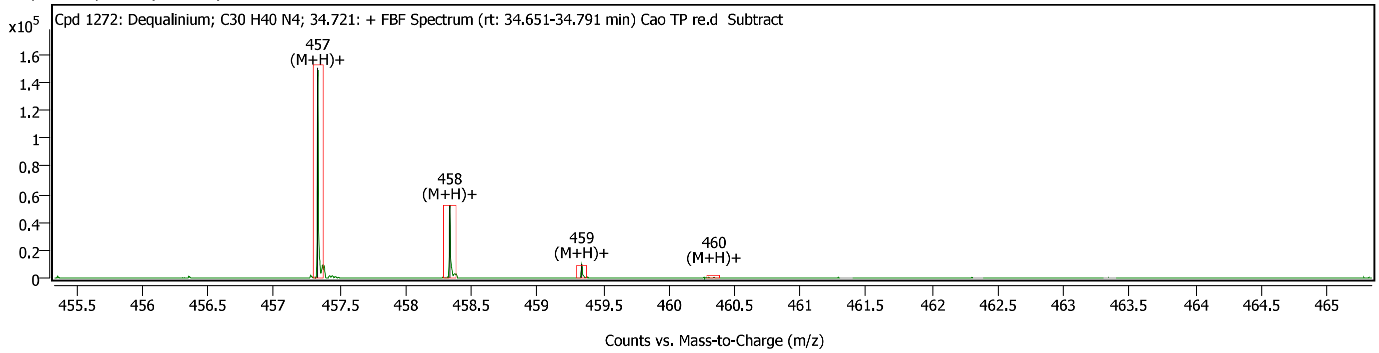

Compound ID Table

Name	Formula	Species	RT	RT Diff	Mass	CAS	ID Source	Score	Score (Lib)	Score (Tgt)
Dequalinium	C30 H40 N4	(M+H)+	34.721		456.3242	522-51-0	FBF	97.18		97.18

Cpd 1404: Zizyphine A

Name	Formula	RT	RI	Mass	Diff (Tgt, ppm)	CAS	ID Source	Score	Algorithm
Zizyphine A	C33 H49 N5 O6	34.948		611.3674	-1.49	51059-42-8	FBF	97.67	FBF

Species	m/z	Score (Tgt)	Score (Lib)	Score (DB)	Score (MFG)	Score (RT)
(M+H)+	612	97.67				

Compound Chromatograms (overlaid)

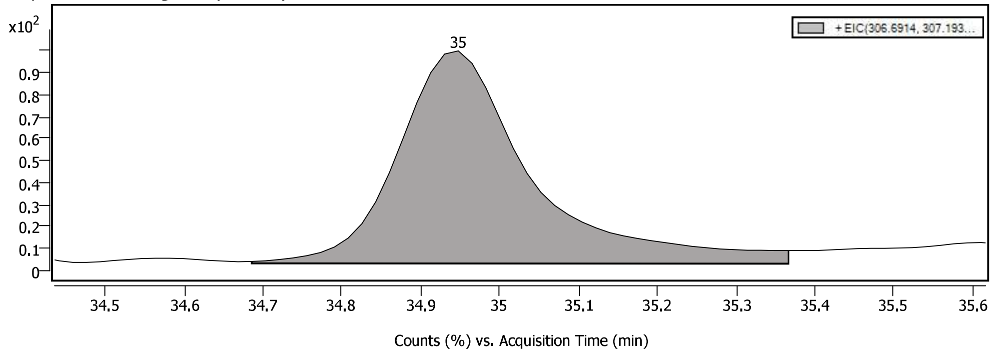

Structure

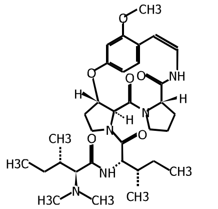

Compound Screening Report

Compound Spectra (overlaid)

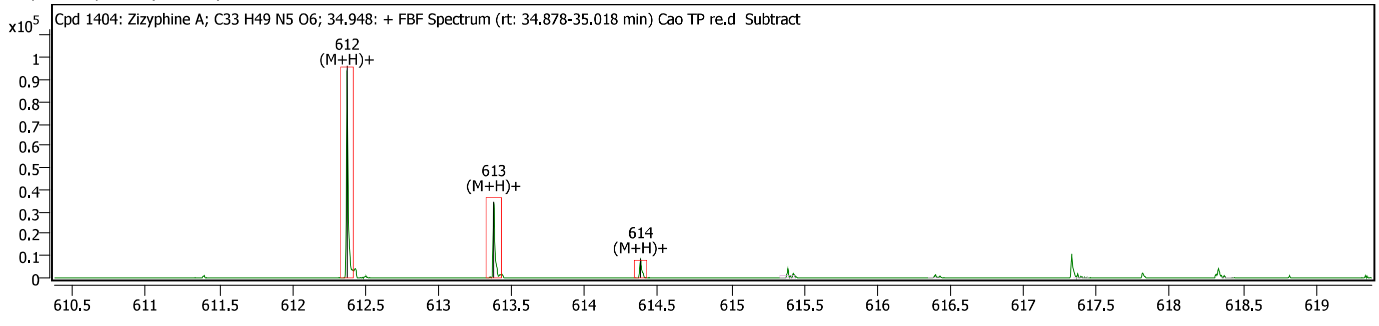

Compound ID Table

Name	Formula	Species	RT	RT Diff	Mass	CAS	ID Source	Score	Score (Lib)	Score (Tgt)
Zizyphine A	C33 H49 N5 O6	(M+H)+	34.948		611.3674	51059-42-8	FBF	97.67		97.67

Cpd 1385: Terminaline

Name	Formula	RT	RI	Mass	Diff (Tgt, ppm)	CAS	ID Source	Score	Algorithm
Terminaline	C23 H41 N O2	35.715		363.3140	0.68	15112-49-9	FBF	99.06	FBF

Species	m/z	Score (Tgt)	Score (Lib)	Score (DB)	Score (MFG)	Score (RT)
(M+H)+	364	99.06				

Compound Chromatograms (overlaid)

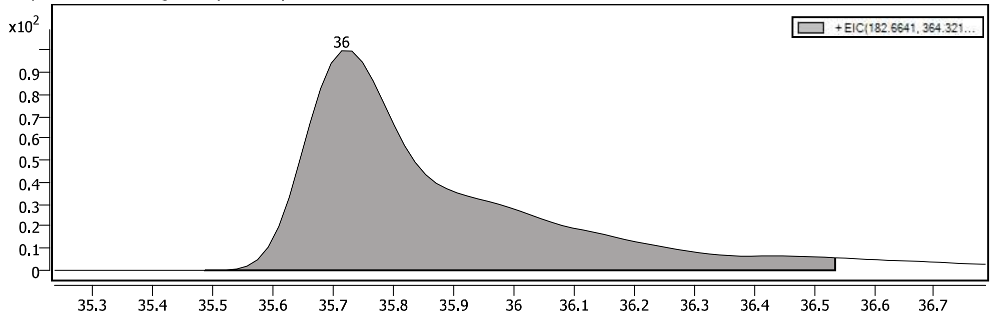

Structure

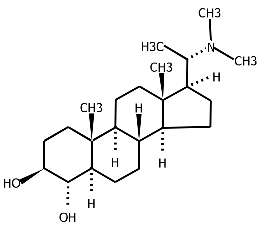

Compound Spectra (overlaid)

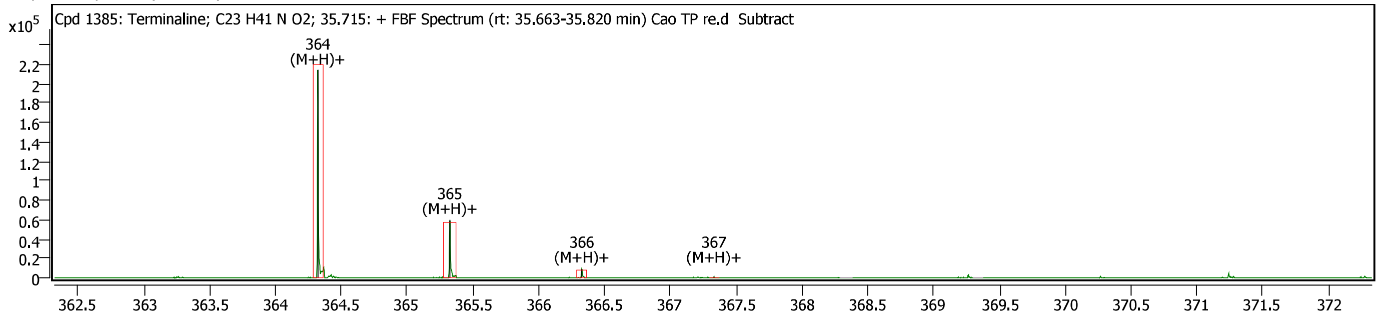

Compound ID Table

Name	Formula	Species	RT	RT Diff	Mass	CAS	ID Source	Score	Score (Lib)	Score (Tgt)
Terminaline	C23 H41 N O2	(M+H)+	35.715		363.3140	15112-49-9	FBF	99.06		99.06

Cpd 938: 12(S),20-DiHETE

Name	Formula	RT	RI	Mass	Diff (Tgt, ppm)	CAS	ID Source	Score	Algorithm
12(S),20-DiHETE	C20 H32 O4	35.977		336.2286	-4.32	89614-44-8	M-FBF-FragConfirm	91.57	FBF

Species	m/z	Score (Tgt)	Score (Lib)	Score (DB)	Score (MFG)	Score (RT)
(M+H)+	337	91.57				

Compound Screening Report

Compound Chromatograms (overlaid)

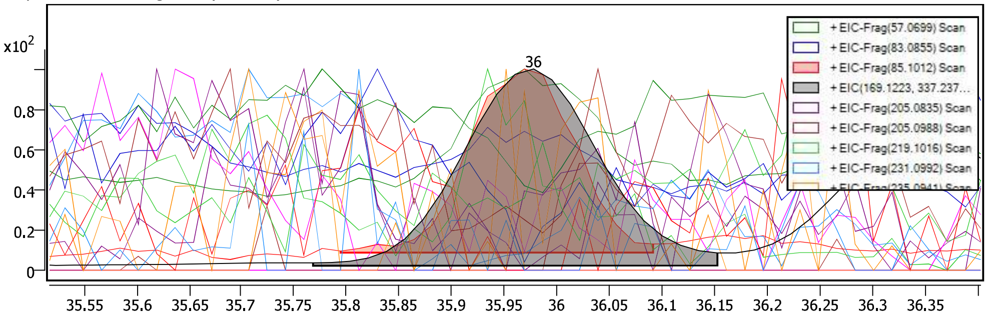

Structure

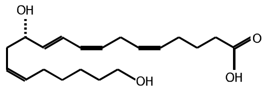

Coelution Plot

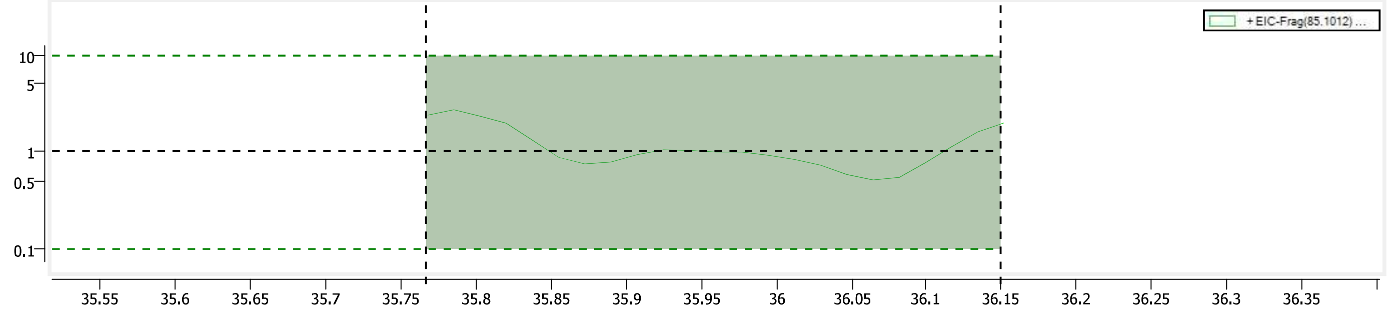

Compound Spectra (overlaid)

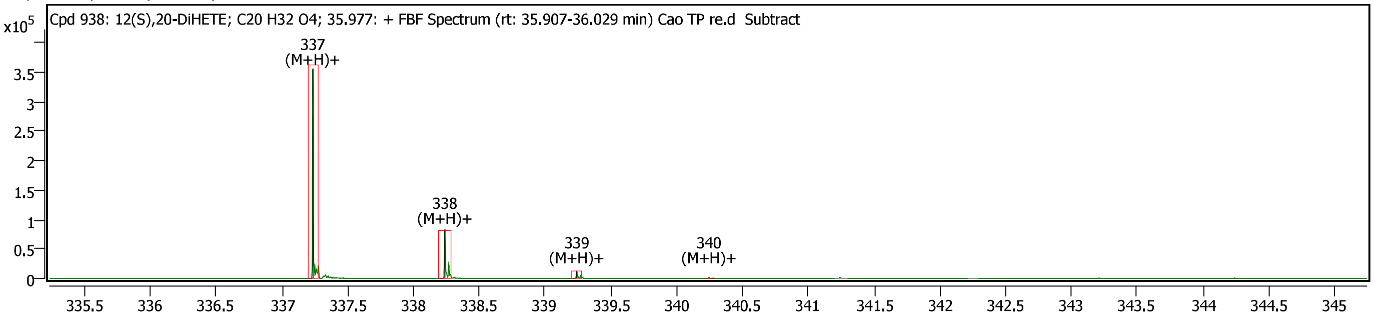

Fragment Spectrum (clean)

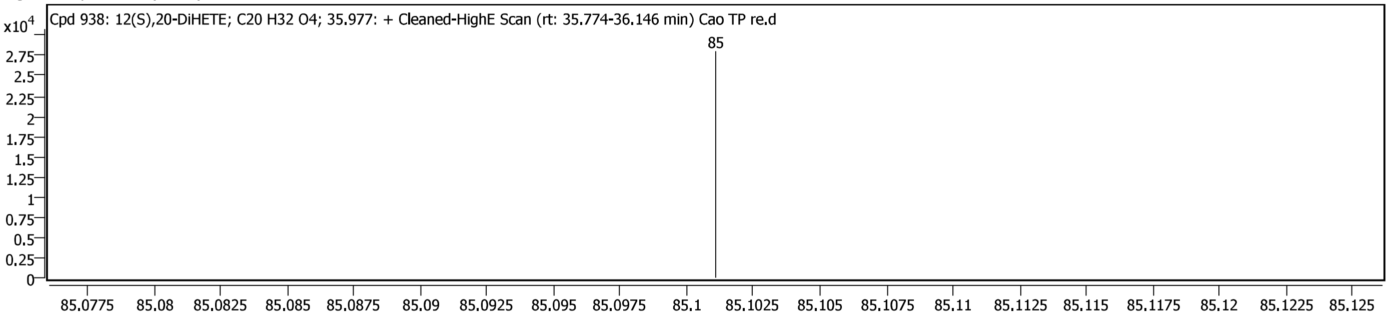

Fragment Spectrum (raw)

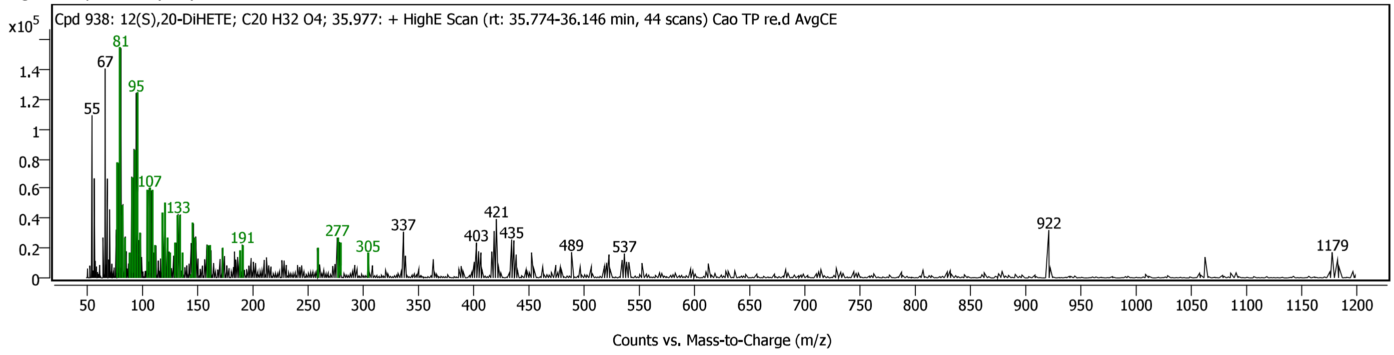

Compound ID Table

Name	Formula	Species	RT	RT Diff	Mass	CAS	ID Source	Score	Score (Lib)	Score (Tgt)
12(S),20-DiHETE	C20 H32 O4	(M+H)+	35.977		336.2286	89614-44-8	FBF-FragConfirm	91.57		91.57
5(S),12(S)-DiHETE	C20 H32 O4	(M+H)+	35.977		336.2286	73151-67-4	FBF-FragConfirm	91.57		91.57
5(S),6(R)-11-trans DiHETE	C20 H32 O4	(M+H)+	35.977		336.2286		FBF-FragConfirm	91.57		91.57
14,15-HxB3 (13R)	C20 H32 O4	(M+H)+	35.977		336.2286		FBF-FragConfirm	91.57		91.57
14-hydroperoxy-5Z,8Z,11Z,15E-eicosatetraenoic acid	C20 H32 O4	(M+H)+	35.977		336.2286		FBF-FragConfirm	91.57		91.57
15(S)-HpETE	C20 H32 O4	(M+H)+	35.977		336.2286	70981-96-3	FBF-FragConfirm	91.57		91.57
15-epi-PGA1	C20 H32 O4	(M+H)+	35.977		336.2286	20897-92-1	FBF-FragConfirm	91.57		91.57
17,18-DiHETE	C20 H32 O4	(M+H)+	35.977		336.2286		FBF-FragConfirm	91.57		91.57
5(S),12(R)-DiHETE	C20 H32 O4	(M+H)+	35.977		336.2286	71652-82-9	FBF-FragConfirm	91.57		91.57
5,6-DiHETE	C20 H32 O4	(M+H)+	35.977		336.2286	845673-97-4	FBF-FragConfirm	91.57		91.57
5-Hydroperoxy-6-trans-8,11,14-cis-eicosatetraenoate (5-HPETE)	C20 H32 O4	(M+H)+	35.977		336.2286	70968-82-0	FBF-FragConfirm	91.57		91.57
5(S),6(R)-DiHETE	C20 H32 O4	(M+H)+	35.977		336.2286	82948-88-7	FBF-FragConfirm	91.57		91.57
14,15-Ep-11-HETrE	C20 H32 O4	(M+H)+	35.977		336.2286		FBF-FragConfirm	91.57		91.57
5(S)-HpETE	C20 H32 O4	(M+H)+	35.977		336.2286	71774-08-8	FBF-FragConfirm	91.57		91.57
5,12-DiHETE	C20 H32 O4	(M+H)+	35.977		336.2286		FBF-FragConfirm	91.57		91.57
5,12-dihydroxy-6,8,10,14-eicosatetraenoic acid	C20 H32 O4	(M+H)+	35.977		336.2286		FBF-FragConfirm	91.57		91.57
5,9,11,13-Eicosatetraenoic acid, 8,15-dihydroxy-	C20 H32 O4	(M+H)+	35.977		336.2286		FBF-FragConfirm	91.57		91.57
5(S),6(S)-DiHETE	C20 H32 O4	(M+H)+	35.977		336.2286	82948-87-6	FBF-FragConfirm	91.57		91.57
14,15-HxA3 (11S)	C20 H32 O4	(M+H)+	35.977		336.2286		FBF-FragConfirm	91.57		91.57
PGF2α 1,15-lactone	C20 H32 O4	(M+H)+	35.977		336.2286	55314-49-3	FBF-FragConfirm	91.57		91.57
(±)-17,18-DiHETE	C20 H32 O4	(M+H)+	35.977		336.2286		FBF-FragConfirm	91.57		91.57
(14S)-14,15-Dihydroxy-8(17),13(16)-labdadien-19-oic acid	C20 H32 O4	(M+H)+	35.977		336.2286		FBF-FragConfirm	91.57		91.57
11,12-DiHETE	C20 H32 O4	(M+H)+	35.977		336.2286		FBF-FragConfirm	91.57		91.57
5S,11R-DiHETE	C20 H32 O4	(M+H)+	35.977		336.2286		FBF-FragConfirm	91.57		91.57
(-)-Stolondiol	C20 H32 O4	(M+H)+	35.977		336.2286		FBF-FragConfirm	91.57		91.57
(±)12-HpETE	C20 H32 O4	(M+H)+	35.977		336.2286	71030-35-8	FBF-FragConfirm	91.57		91.57
(±)-14,15-DiHETE	C20 H32 O4	(M+H)+	35.977		336.2286		FBF-FragConfirm	91.57		91.57
11-deoxy-PGE2	C20 H32 O4	(M+H)+	35.977		336.2286	35535-53-9	FBF-FragConfirm	91.57		91.57
14,15-dihydroxy-5,8,10,12-eicosatetraenoic acid	C20 H32 O4	(M+H)+	35.977		336.2286		FBF-FragConfirm	91.57		91.57
(±)11,12-Ep-15(S)-HETrE	C20 H32 O4	(M+H)+	35.977		336.2286		FBF-FragConfirm	91.57		91.57
14,15-DiHETE	C20 H32 O4	(M+H)+	35.977		336.2286		FBF-FragConfirm	91.57		91.57
11-HpETE	C20 H32 O4	(M+H)+	35.977		336.2286		FBF-FragConfirm	91.57		91.57
11R-HpETE	C20 H32 O4	(M+H)+	35.977		336.2286		FBF-FragConfirm	91.57		91.57
12(S)-HpETE	C20 H32 O4	(M+H)+	35.977		336.2286	71774-10-2	FBF-FragConfirm	91.57		91.57
12-epi-LTB4	C20 H32 O4	(M+H)+	35.977		336.2286	83709-73-3	FBF-FragConfirm	91.57		91.57
12R-HpETE	C20 H32 O4	(M+H)+	35.977		336.2286		FBF-FragConfirm	91.57		91.57
ent-1(10)-Halimene-15,19-dioic acid	C20 H32 O4	(M+H)+	35.977		336.2286		FBF-FragConfirm	91.57		91.57
8,15-DiHETE	C20 H32 O4	(M+H)+	35.977		336.2286	77667-08-4	FBF-FragConfirm	91.57		91.57
Hepoxilin B3	C20 H32 O4	(M+H)+	35.977		336.2286	71799-95-6	FBF-FragConfirm	91.57		91.57
LTB4	C20 H32 O4	(M+H)+	35.977		336.2286	71160-24-2	FBF-FragConfirm	91.57		91.57
9alpha-(3-Methylbutanoyloxy)-4S-hydroxy-10(14)-oplopen-3-one	C20 H32 O4	(M+H)+	35.977		336.2286	348113-04-2	FBF-FragConfirm	91.57		91.57
9S-HpETE	C20 H32 O4	(M+H)+	35.977		336.2286		FBF-FragConfirm	91.57		91.57
Bicyclo Prostaglandin E1	C20 H32 O4	(M+H)+	35.977		336.2286		FBF-FragConfirm	91.57		91.57
Diterpenoid SP-II	C20 H32 O4	(M+H)+	35.977		336.2286	3301-61-9	FBF-FragConfirm	91.57		91.57
Hepoxilin A3	C20 H32 O4	(M+H)+	35.977		336.2286	85589-24-8	FBF-FragConfirm	91.57		91.57
Methyl 8-gingerol	C20 H32 O4	(M+H)+	35.977		336.2286		FBF-FragConfirm	91.57		91.57
6-trans-LTB4	C20 H32 O4	(M+H)+	35.977		336.2286		FBF-FragConfirm	91.57		91.57
PGF2α 1,9-lactone	C20 H32 O4	(M+H)+	35.977		336.2286	55314-48-2	FBF-FragConfirm	91.57		91.57
PGA1	C20 H32 O4	(M+H)+	35.977		336.2286	14152-28-4	FBF-FragConfirm	91.57		91.57
5S,15R-diHETE	C20 H32 O4	(M+H)+	35.977		336.2286		FBF-FragConfirm	91.57		91.57
PGC1	C20 H32 O4	(M+H)+	35.977		336.2286		FBF-FragConfirm	91.57		91.57
PGF2alpha-1,9-lactone	C20 H32 O4	(M+H)+	35.977		336.2286		FBF-FragConfirm	91.57		91.57
PGF2α 1,11-lactone	C20 H32 O4	(M+H)+	35.977		336.2286	62410-84-8	FBF-FragConfirm	91.57		91.57
5S,12S-DiHETE	C20 H32 O4	(M+H)+	35.977		336.2286		FBF-FragConfirm	91.57		91.57
Portulal	C20 H32 O4	(M+H)+	35.977		336.2286	22571-65-9	FBF-FragConfirm	91.57		91.57
PGB1	C20 H32 O4	(M+H)+	35.977		336.2286	13345-51-2	FBF-FragConfirm	91.57		91.57
8R-HpETE	C20 H32 O4	(M+H)+	35.977		336.2286		FBF-FragConfirm	91.57		91.57
8-iso-PGA1	C20 H32 O4	(M+H)+	35.977		336.2286	211186-29-7	FBF-FragConfirm	91.57		91.57
8,9-DiHETE	C20 H32 O4	(M+H)+	35.977		336.2286		FBF-FragConfirm	91.57		91.57
8,15-dihydroxy-5,9,11,13-eicosatetraenoic acid	C20 H32 O4	(M+H)+	35.977		336.2286		FBF-FragConfirm	91.57		91.57
8,12-iso-iPF2α-VI 1,5- lactone	C20 H32 O4	(M+H)+	35.977		336.2286		FBF-FragConfirm	91.57		91.57
6-hydroperoxy-4E,8Z,11Z,14Z-eicosatetraenoic acid	C20 H32 O4	(M+H)+	35.977		336.2286		FBF-FragConfirm	91.57		91.57
9-HpETE	C20 H32 O4	(M+H)+	35.977		336.2286		FBF-FragConfirm	91.57		91.57
8(R),15(S)-DiHETE	C20 H32 O4	(M+H)+	35.977		336.2286	80234-67-9	FBF-FragConfirm	91.57		91.57
6-trans-12-epi-LTB4	C20 H32 O4	(M+H)+	35.977		336.2286		FBF-FragConfirm	91.57		91.57
5Z,8Z,11Z-Eicosatriene-1,20-dioic acid	C20 H32 O4	(M+H)+	35.977		336.2286		FBF-FragConfirm	91.57		91.57
5S,15S-DiHETE	C20 H32 O4	(M+H)+	35.977		336.2286		FBF-FragConfirm	91.57		91.57
8S-HpETE	C20 H32 O4	(M+H)+	35.977		336.2286		FBF-FragConfirm	91.57		91.57
8(S),15(S)-DiHETE(Z,E,Z,E)	C20 H32 O4	(M+H)+	35.977		336.2286	80234-65-7	FBF-FragConfirm	91.57		91.57
8S-hydroxy-11R,12S-epoxy-5Z,9E,14Z-eicosatrienoic acid	C20 H32 O4	(M+H)+	35.977		336.2286		FBF-FragConfirm	91.57		91.57
8S,15S-DiHETE(Z,E,E,E)	C20 H32 O4	(M+H)+	35.977		336.2286	80234-66-8	FBF-FragConfirm	91.57		91.57

Cpd 190: Lauroyl diethanolamide

Name	Formula	RT	RI	Mass	Diff (Tgt, ppm)	CAS	ID Source	Score	Algorithm
Lauroyl diethanolamide	C16 H33 N O3	36.117		287.2464	1.14	120-40-1	M-FBF	98.39	FBF

Species	m/z	Score (Tgt)	Score (Lib)	Score (DB)	Score (MFG)	Score (RT)
(M+H)+	288	98.39				

Compound Screening Report

Compound Chromatograms (overlaid)

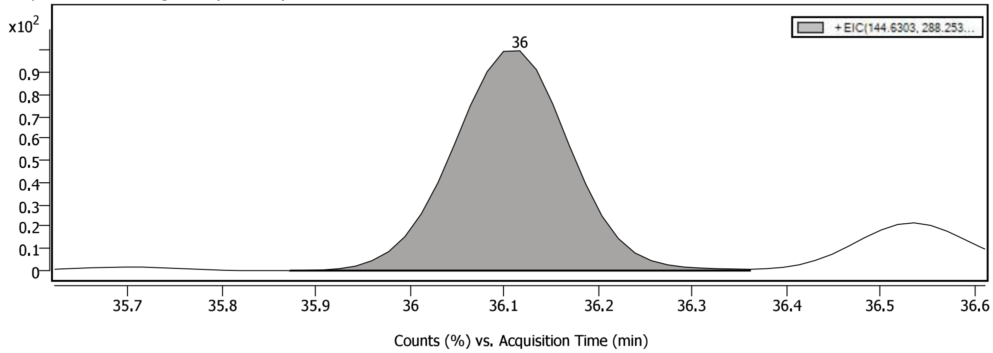

Structure

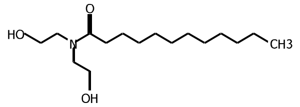

Compound Spectra (overlaid)

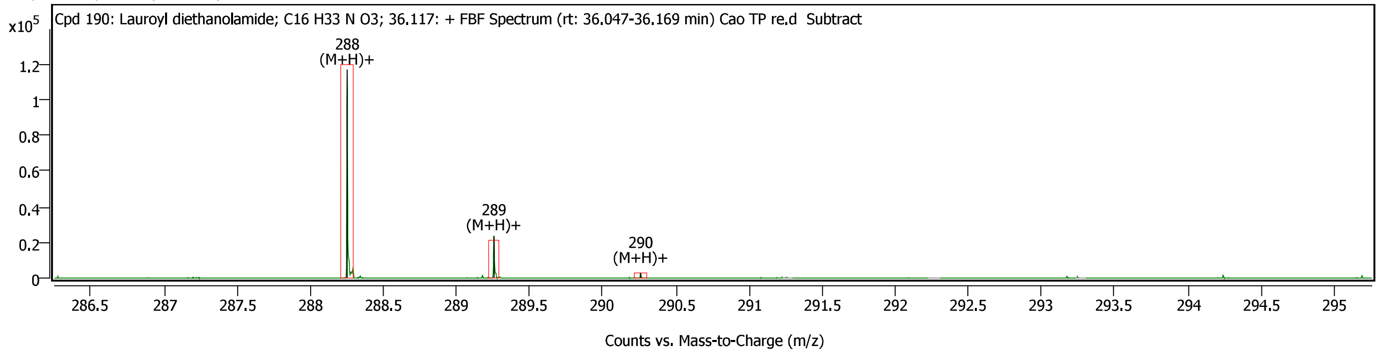

Compound ID Table

Name	Formula	Species	RT	RT Diff	Mass	CAS	ID Source	Score	Score (Lib)	Score (Tgt)
Lauroyl diethanolamide	C16 H33 N O3	(M+H)+	36.117		287.2464	120-40-1	FBF	98.39		98.39
Prosopinine	C16 H33 N O3	(M+H)+	36.117		287.2464		FBF	98.39		98.39

Cpd 1463: Phytosphingosine

Name	Formula	RT	RI	Mass	Diff (Tgt, ppm)	CAS	ID Source	Score	Algorithm
Phytosphingosine	C18 H39 N O3	36.256		317.2933	0.94	554-62-1	FBF-FragConfirm	98.42	FBF

Species	m/z	Score (Tgt)	Score (Lib)	Score (DB)	Score (MFG)	Score (RT)
(M+H)+	318	98.42				

Compound Chromatograms (overlaid)

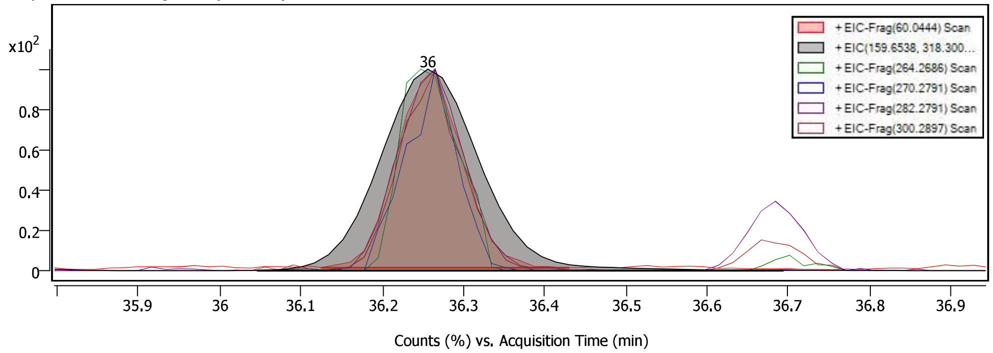

Structure

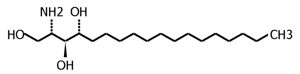

Coelution Plot

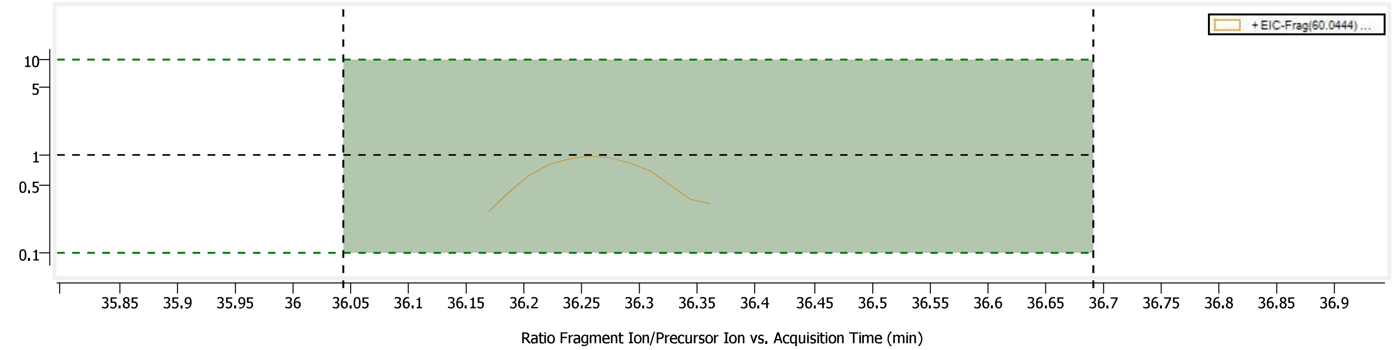

Compound Spectra (overlaid)

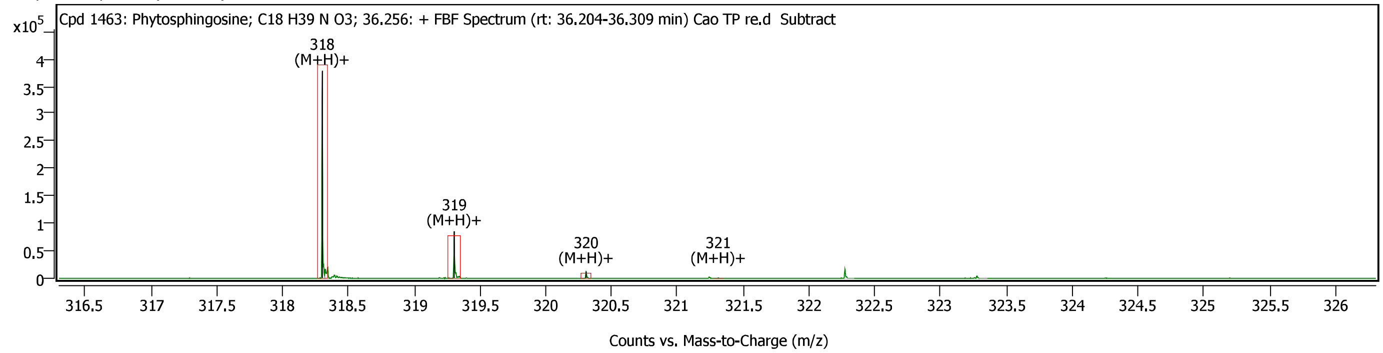

Fragment Spectrum (clean)

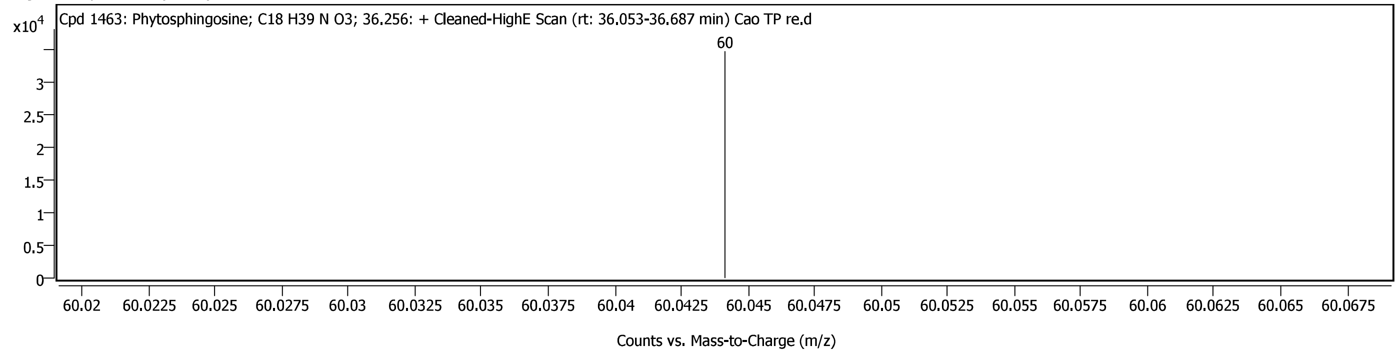

Fragment Spectrum (raw)

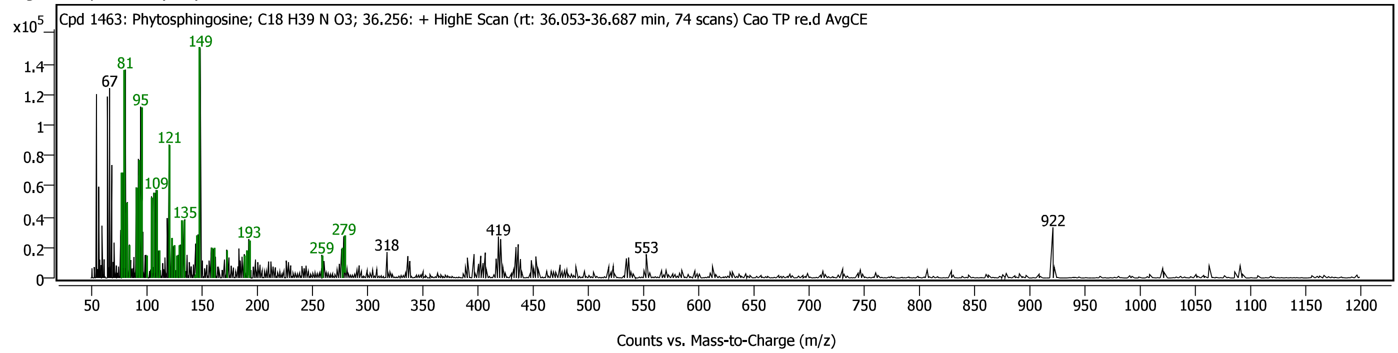

Compound ID Table

Name	Formula	Species	RT	RT Diff	Mass	CAS	ID Source	Score	Score (Lib)	Score (Tgt)
Phytosphingosine	C18 H39 N O3	(M+H)+	36.256		317.2933	554-62-1	FBF-FragConfirm	98.42		98.42

Cpd 939: Hypromellose

Name	Formula	RT	RI	Mass	Diff (Tgt, ppm)	CAS	ID Source	Score	Algorithm
Hypromellose	C32 H60 O19	36.623		748.3723	-0.84		FBF	96.10	FBF

Species	m/z	Score (Tgt)	Score (Lib)	Score (DB)	Score (MFG)	Score (RT)
(M+2H)+2 (M+H)+	375 749	96.10				

Compound Chromatograms (overlaid)

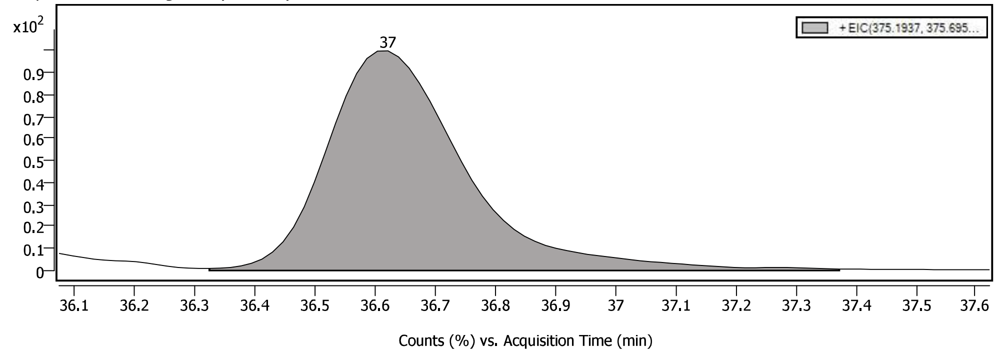

Structure

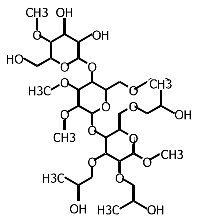

Compound Spectra (overlaid)

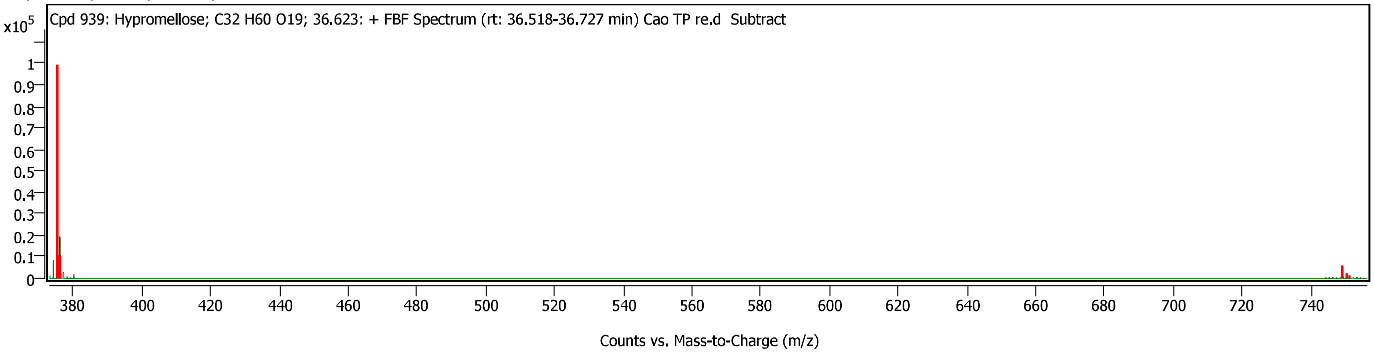

Compound ID Table

Name	Formula	Species	RT	RT Diff	Mass	CAS	ID Source	Score	Score (Lib)	Score (Tgt)
Hypromellose	C32 H60 O19	(M+2H)+2 (M+H)+	36.623		748.3723		FBF	96.10		96.10

Cpd 136: Soyasaponin bg

Name	Formula	RT	RI	Mass	Diff (Tgt, ppm)	CAS	ID Source	Score	Algorithm
Soyasaponin bg	C54 H84 O21	37.164		1068.5506	0.11	143519-54-4	FBF	94.23	FBF

Species	m/z	Score (Tgt)	Score (Lib)	Score (DB)	Score (MFG)	Score (RT)
(M+H)+	1070	94.23				

Compound Chromatograms (overlaid)

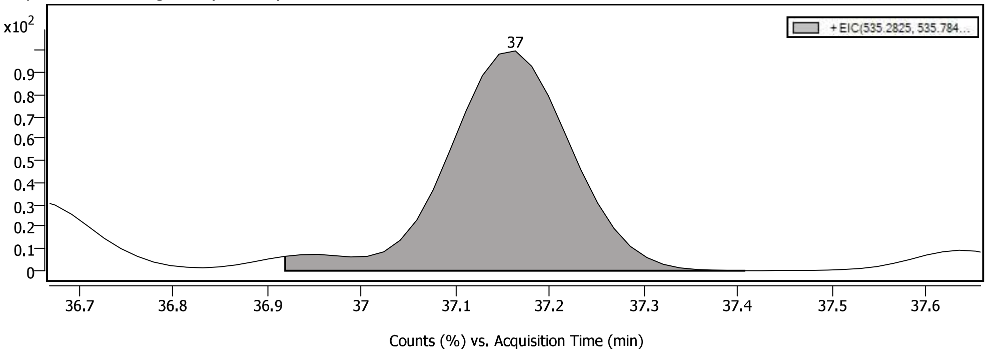

Structure

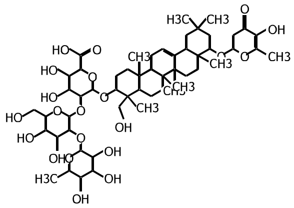

Compound Spectra (overlaid)

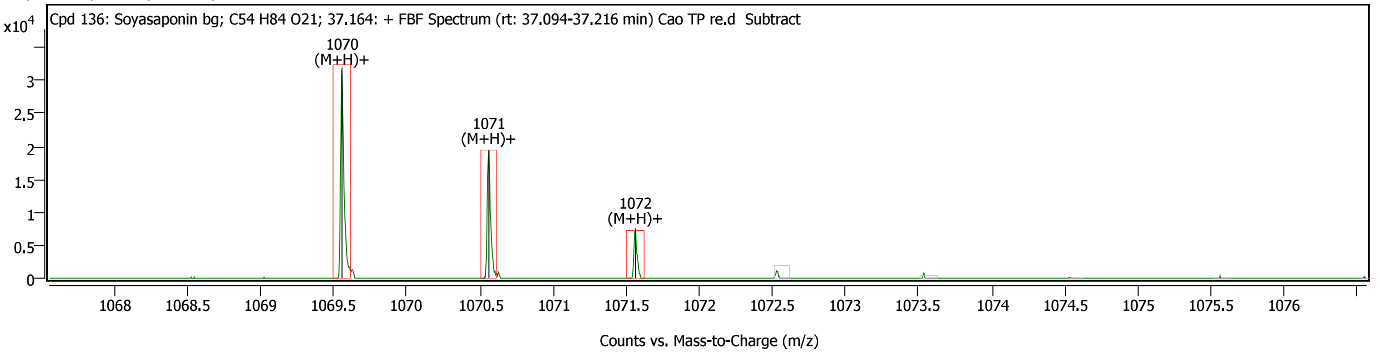

Compound ID Table

Name	Formula	Species	RT	RT Diff	Mass	CAS	ID Source	Score	Score (Lib)	Score (Tgt)
Soyasaponin bg	C54 H84 O21	(M+H)+	37.164		1068.5506	143519-54-4	FBF	94.23		94.23

Cpd 1147: <Dodecylphosphocholine>

Name	Formula	RT	RI	Mass	Diff (Tgt, ppm)	CAS	ID Source	Score	Algorithm
<Dodecylphosphocholine>	C17 H39 N O4 P	37.443		352.2615	-0.47	29557-51-5	FBF	97.35	FBF

Species	m/z	Score (Tgt)	Score (Lib)	Score (DB)	Score (MFG)	Score (RT)
(M+H)+	353	97.35				

Compound Chromatograms (overlaid)

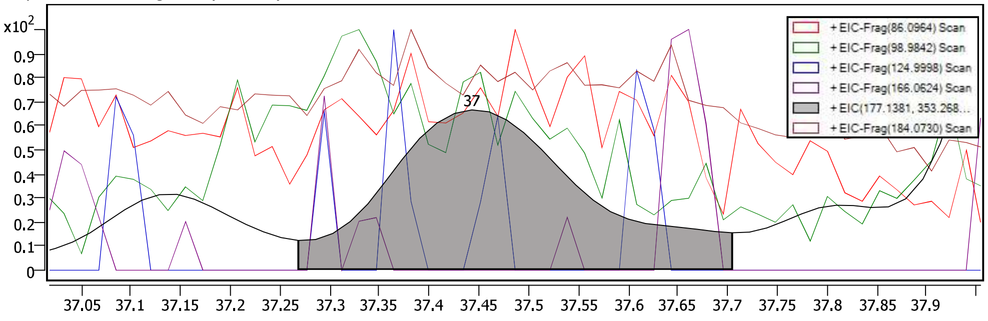

Counts (%) vs. Acquisition Time (min)

Structure

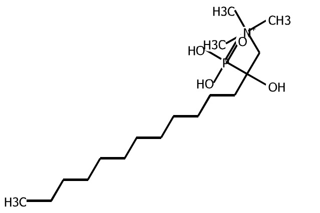

Coelution Plot

Compound Spectra (overlaid)

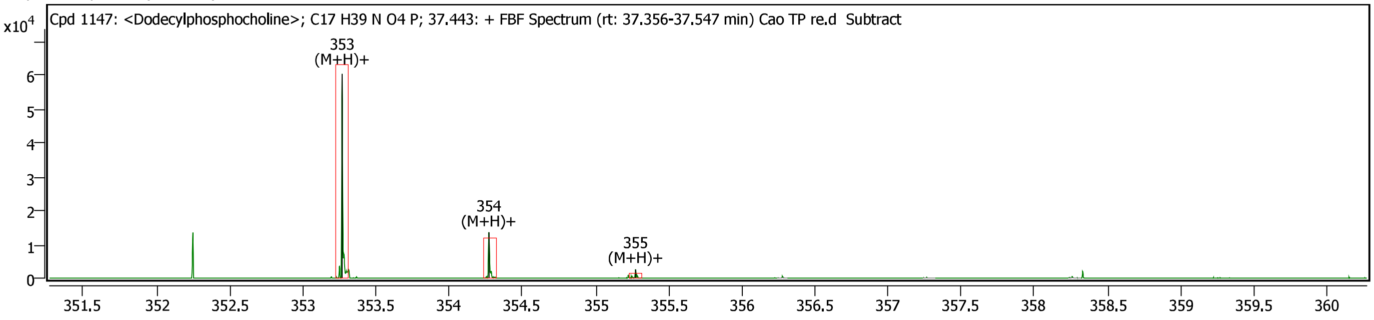

Counts vs. Mass-to-Charge (m/z)

Fragment Spectrum (raw)

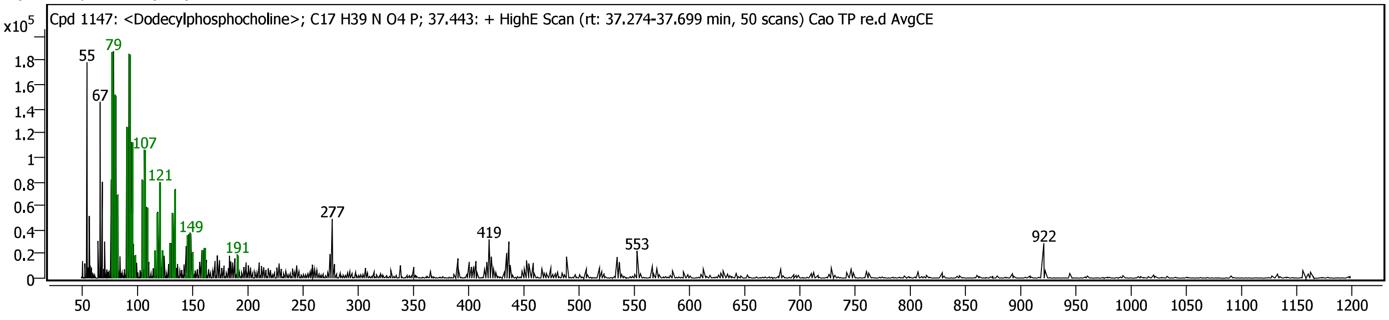

Counts vs. Mass-to-Charge (m/z)

Compound ID Table

Name	Formula	Species	RT	RT Diff	Mass	CAS	ID Source	Score	Score (Lib)	Score (Tgt)
<Dodecylphosphocholine>	C17 H39 N O4 P	(M+H)+	37.443		352.2615	29557-51-5	FBF	97.35		97.35

Cpd 1634: <Etiocholanolone>

Name	Formula	RT	RI	Mass	Diff (Tgt, ppm)	CAS	ID Source	Score	Algorithm
<Etiocholanolone>	C19 H30 O2	37.879		290.2248	0.83	53-42-9	M-FBF	99.35	FBF

Species	m/z	Score (Tgt)	Score (Lib)	Score (DB)	Score (MFG)	Score (RT)
(M+H)+	291	99.35				

Compound Chromatograms (overlaid)

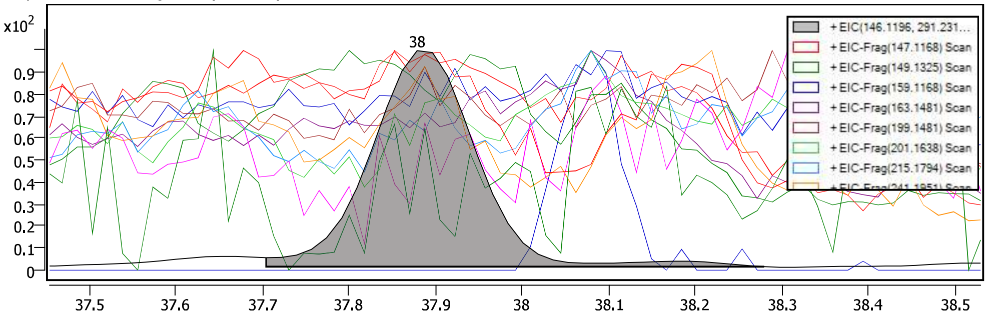

Counts (%) vs. Acquisition Time (min)

Structure

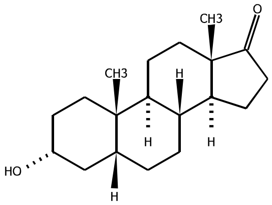

Coelution Plot

Compound Spectra (overlaid)

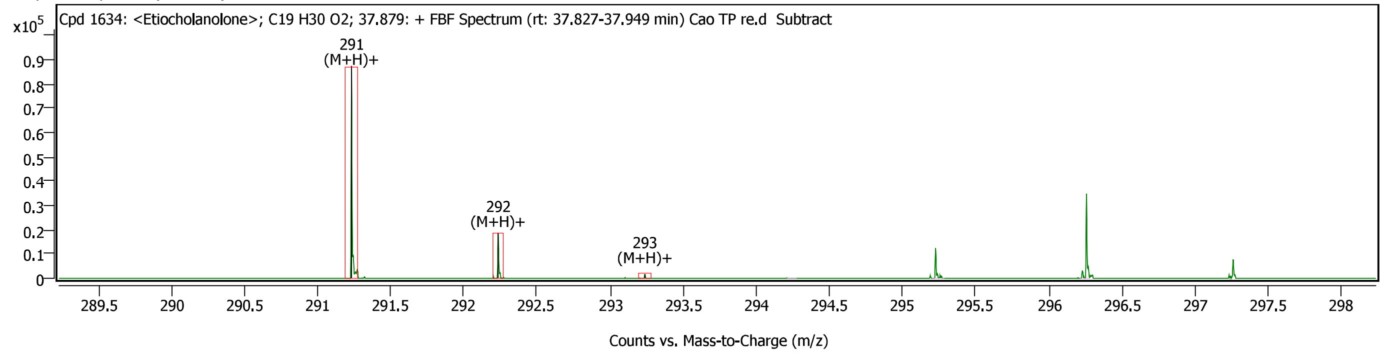

Fragment Spectrum (raw)

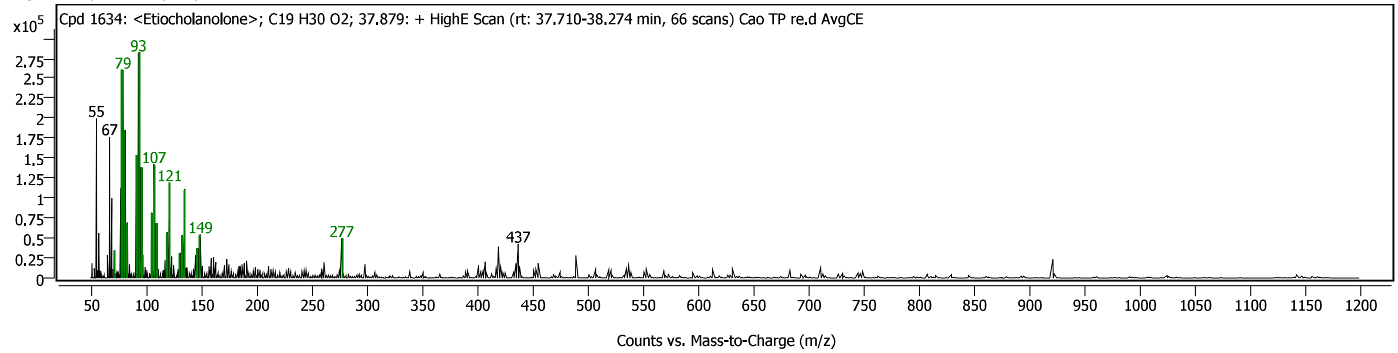

Compound ID Table

Name	Formula	Species	RT	RT Diff	Mass	CAS	ID Source	Score	Score (Lib)	Score (Tgt)
<Etiocholanolone>	C19 H30 O2	(M+H)+	37.879		290.2248	53-42-9	FBF	99.35		99.35
<4-Methylphenyl dodecanoate>	C19 H30 O2	(M+H)+	37.879		290.2248	10024-57-4	FBF	99.35		99.35
<11beta-Hydroxy-5alpha-androstan-17-one>	C19 H30 O2	(M+H)+	37.879		290.2248		FBF	99.35		99.35
<Sterculynic acid>	C19 H30 O2	(M+H)+	37.879		290.2248		FBF	99.35		99.35
<4-Androstenediol>	C19 H30 O2	(M+H)+	37.879		290.2248		FBF	99.35		99.35
<16-Oxosteroid>	C19 H30 O2	(M+H)+	37.879		290.2248		FBF	99.35		99.35
<17beta-Hydroxy-2alpha-methyl-5alpha-estran-3-one>	C19 H30 O2	(M+H)+	37.879		290.2248		FBF	99.35		99.35
<5β-Dihydrotestosterone>	C19 H30 O2	(M+H)+	37.879		290.2248	571-22-2	FBF	99.35		99.35
<16:4(2E,4E,8E,10E)(7Me[R],9Me,14Me[R])>	C19 H30 O2	(M+H)+	37.879		290.2248		FBF	99.35		99.35
<13-Phenyltridecanoic acid>	C19 H30 O2	(M+H)+	37.879		290.2248		FBF	99.35		99.35
<10,13-Nonadecadiynoic acid>	C19 H30 O2	(M+H)+	37.879		290.2248		FBF	99.35		99.35
<Stearidonic Acid methyl ester>	C19 H30 O2	(M+H)+	37.879		290.2248		FBF	99.35		99.35
<5α-Dihydrotestosterone>	C19 H30 O2	(M+H)+	37.879		290.2248	521-18-6	FBF	99.35		99.35
<Androsterone>	C19 H30 O2	(M+H)+	37.879		290.2248	53-41-8	FBF	99.35		99.35
<androst-5-en-3beta,16alpha-diol>	C19 H30 O2	(M+H)+	37.879		290.2248		FBF	99.35		99.35
<Androstenediol>	C19 H30 O2	(M+H)+	37.879		290.2248		FBF	99.35		99.35
<cis-Parinaric Acid methyl ester>	C19 H30 O2	(M+H)+	37.879		290.2248	26474-40-8	FBF	99.35		99.35
<Epiandrosterone>	C19 H30 O2	(M+H)+	37.879		290.2248	481-29-8	FBF	99.35		99.35
<Epietiocholanolone>	C19 H30 O2	(M+H)+	37.879		290.2248		FBF	99.35		99.35
<androst-5-ene-3β,16α-diol>	C19 H30 O2	(M+H)+	37.879		290.2248		FBF	99.35		99.35

Cpd 167: TR-Saponin B

Name	Formula	RT	RI	Mass	Diff (Tgt, ppm)	CAS	ID Source	Score	Algorithm
TR-Saponin B	C52 H80 O20	38.647		1024.5244	0.12	288153-02-6	FBF	98.77	FBF

Species	m/z	Score (Tgt)	Score (Lib)	Score (DB)	Score (MFG)	Score (RT)
(M+H)+	1026	98.77				

Compound Chromatograms (overlaid)

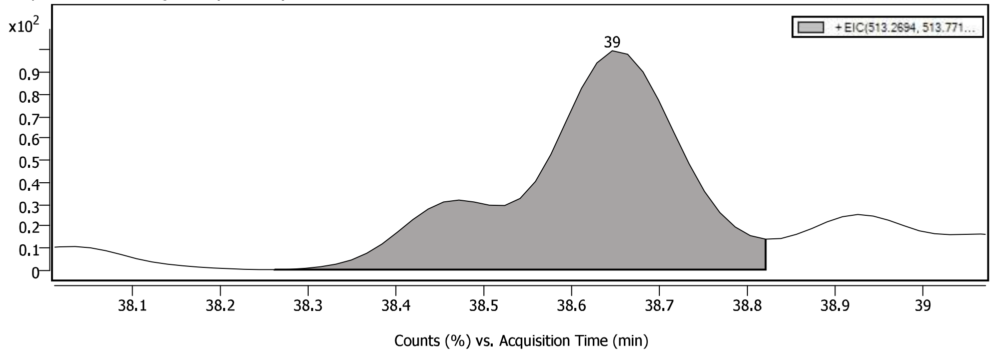

Structure

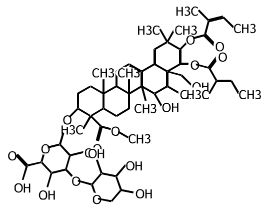

Compound Screening Report

Compound Spectra (overlaid)

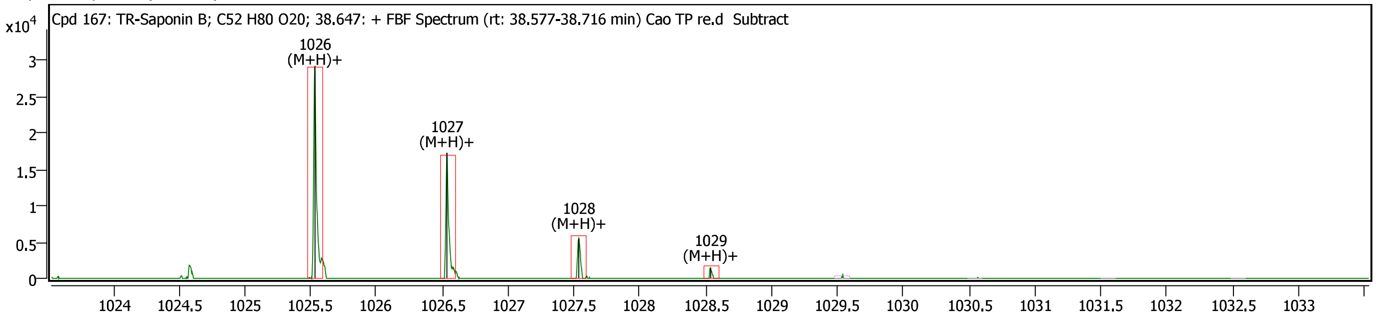

Counts vs. Mass-to-Charge (m/z)

Compound ID Table

Name	Formula	Species	RT	RT Diff	Mass	CAS	ID Source	Score	Score (Lib)	Score (Tgt)
TR-Saponin B	C52 H80 O20	(M+H)+	38.647		1024.5244	288153-02-6	FBF	98.77		98.77

Cpd 598: 5-Oxoavermectin "1b" aglycone

Name	Formula	RT	RI	Mass	Diff (Tgt, ppm)	CAS	ID Source	Score	Algorithm
5-Oxoavermectin "1b" aglycone	C33 H44 O8	38.839		568.3037	0.19		FBF	99.04	FBF

Species	m/z	Score (Tgt)	Score (Lib)	Score (DB)	Score (MFG)	Score (RT)
(M+H)+	569	99.04				

Compound Chromatograms (overlaid)

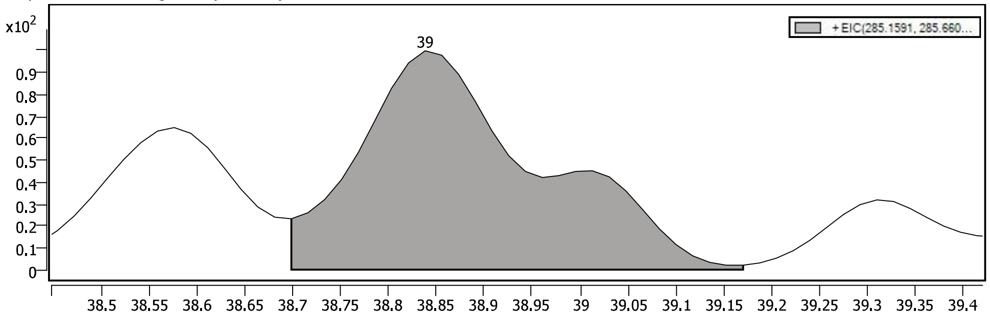

Counts (%) vs. Acquisition Time (min)

Structure

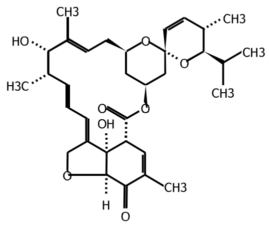

Compound Spectra (overlaid)

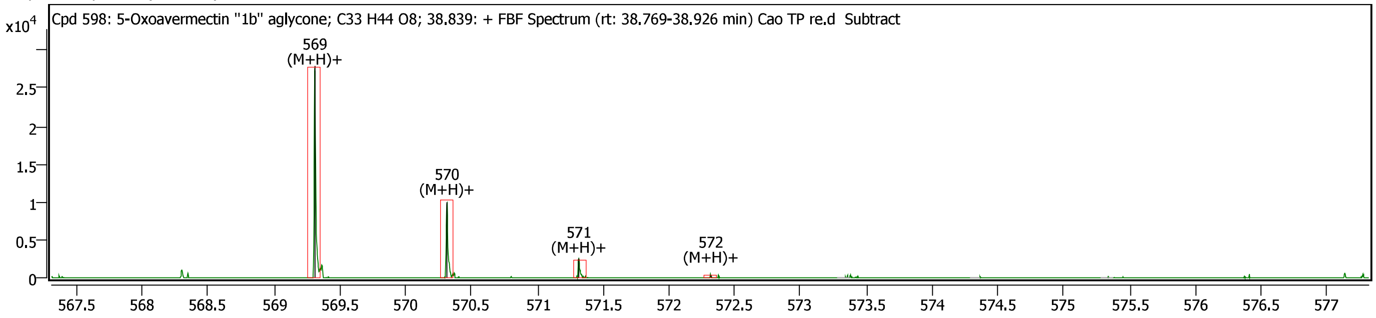

Counts vs. Mass-to-Charge (m/z)

Compound ID Table

Name	Formula	Species	RT	RT Diff	Mass	CAS	ID Source	Score	Score (Lib)	Score (Tgt)
5-Oxoavermectin "1b" aglycone	C33 H44 O8	(M+H)+	38.839		568.3037		FBF	99.04		99.04

Cpd 1132: 19-Norandrosterone

Name	Formula	RT	RI	Mass	Diff (Tgt, ppm)	CAS	ID Source	Score	Algorithm
19-Norandrosterone	C18 H28 O2	39.118		276.2097	2.71	1225-01-0	M-FBF-FragConfirm	94.81	FBF

Species	m/z	Score (Tgt)	Score (Lib)	Score (DB)	Score (MFG)	Score (RT)
(M+H)+	277	94.81				

Compound Screening Report

Compound Chromatograms (overlaid)

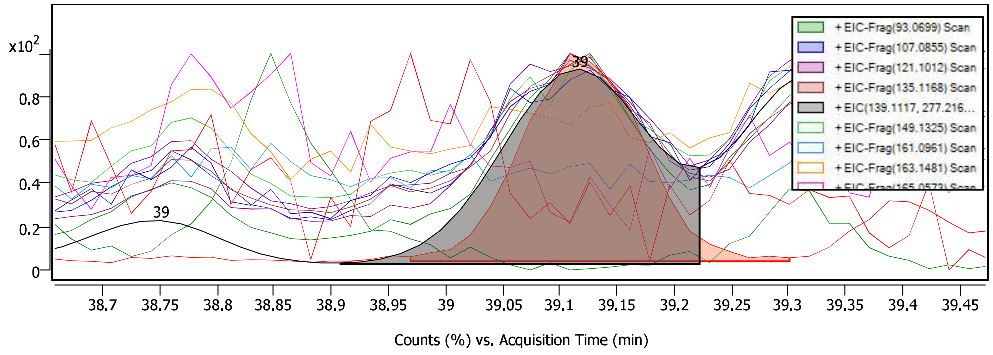

Structure

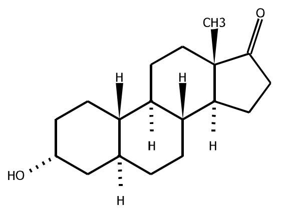

Coelution Plot

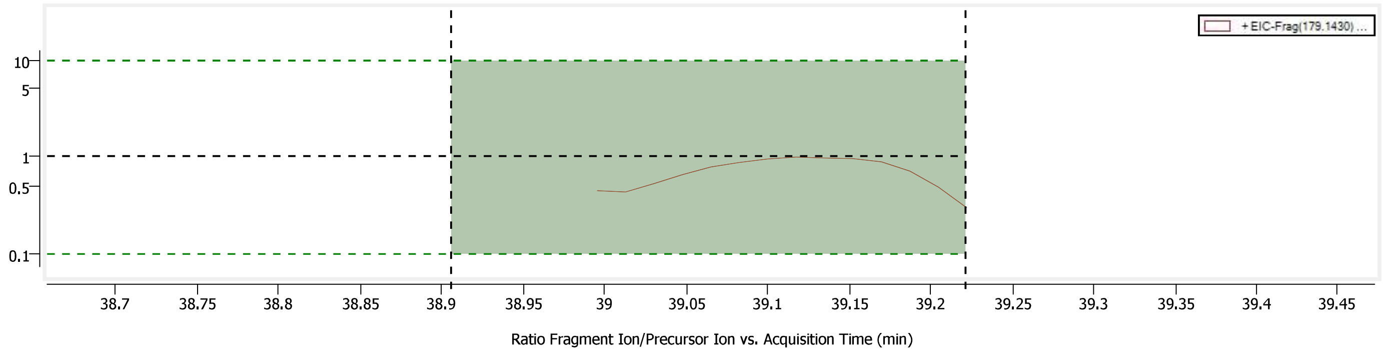

Compound Spectra (overlaid)

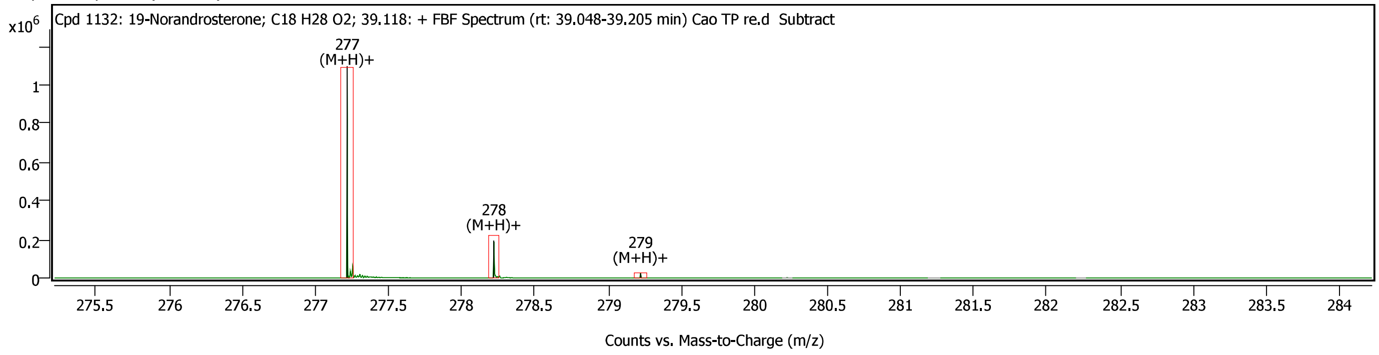

Fragment Spectrum (clean)

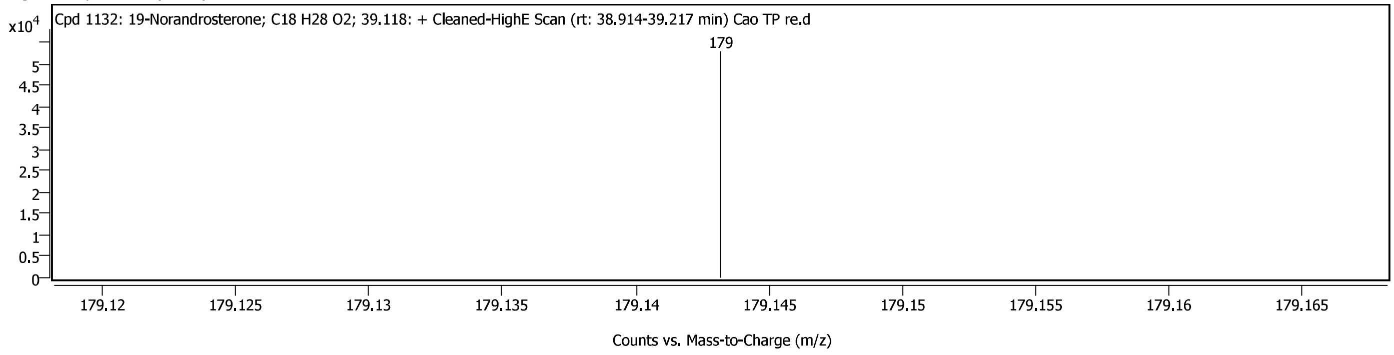

Fragment Spectrum (raw)

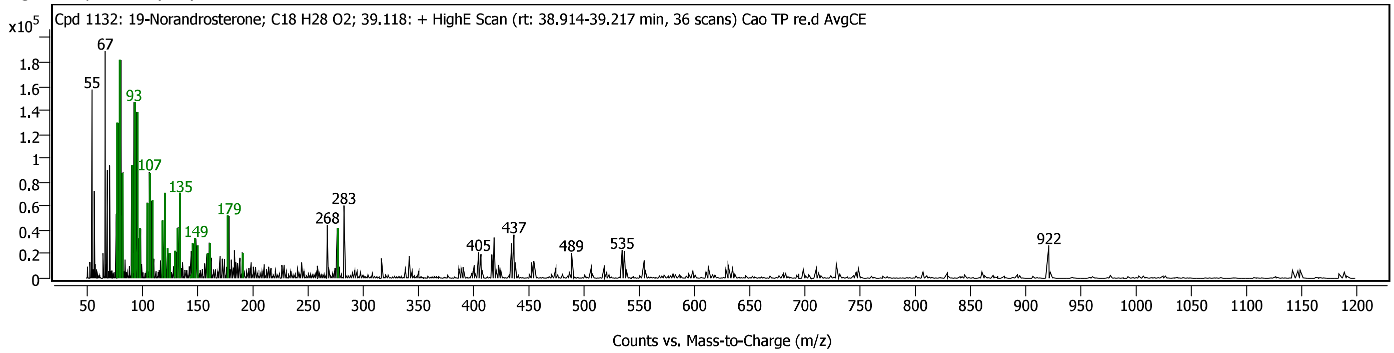

Compound ID Table

Name	Formula	Species	RT	RT Diff	Mass	CAS	ID Source	Score	Score (Lib)	Score (Tgt)
19-Norandrosterone	C18 H28 O2	(M+H)+	39.118		276.2097	1225-01-0	FBF-FragConfirm	94.81		94.81
3E,9Z,12Z,15Z-Octadecatetraenoic acid	C18 H28 O2	(M+H)+	39.118		276.2097	693-78-7	FBF-FragConfirm	94.81		94.81
3,8-Octadecadiynoic acid	C18 H28 O2	(M+H)+	39.118		276.2097		FBF-FragConfirm	94.81		94.81
4,8-Octadecadiynoic acid	C18 H28 O2	(M+H)+	39.118		276.2097		FBF-FragConfirm	94.81		94.81
2,5-Octadecadiynoic acid	C18 H28 O2	(M+H)+	39.118		276.2097		FBF-FragConfirm	94.81		94.81
2,6-Octadecadiynoic acid	C18 H28 O2	(M+H)+	39.118		276.2097		FBF-FragConfirm	94.81		94.81
2,7-Octadecadiynoic acid	C18 H28 O2	(M+H)+	39.118		276.2097		FBF-FragConfirm	94.81		94.81
3,6-Octadecadiynoic acid	C18 H28 O2	(M+H)+	39.118		276.2097		FBF-FragConfirm	94.81		94.81
3,7-Octadecadiynoic acid	C18 H28 O2	(M+H)+	39.118		276.2097		FBF-FragConfirm	94.81		94.81
4,6-Octadecadiynoic acid	C18 H28 O2	(M+H)+	39.118		276.2097		FBF-FragConfirm	94.81		94.81
14,17-Octadecadiynoic acid	C18 H28 O2	(M+H)+	39.118		276.2097		FBF-FragConfirm	94.81		94.81
5,7-Octadecadiynoic acid	C18 H28 O2	(M+H)+	39.118		276.2097		FBF-FragConfirm	94.81		94.81
4,7-Octadecadiynoic acid	C18 H28 O2	(M+H)+	39.118		276.2097		FBF-FragConfirm	94.81		94.81
5,8-Octadecadiynoic acid	C18 H28 O2	(M+H)+	39.118		276.2097		FBF-FragConfirm	94.81		94.81
19-Noretiocholan-3b-ol-17-one	C18 H28 O2	(M+H)+	39.118		276.2097	64044-10-6	FBF-FragConfirm	94.81		94.81
4,9-Octadecadiynoic acid	C18 H28 O2	(M+H)+	39.118		276.2097		FBF-FragConfirm	94.81		94.81
5,10-Octadecadiynoic acid	C18 H28 O2	(M+H)+	39.118		276.2097		FBF-FragConfirm	94.81		94.81
19-Noretiocholanolone	C18 H28 O2	(M+H)+	39.118		276.2097	33036-33-8	FBF-FragConfirm	94.81		94.81
18:4(5Z,9Z,12Z,15Z)	C18 H28 O2	(M+H)+	39.118		276.2097		FBF-FragConfirm	94.81		94.81
5,8,11,14-Octadecatetraenoic acid	C18 H28 O2	(M+H)+	39.118		276.2097	75236-11-2	FBF-FragConfirm	94.81		94.81
10,14-Octadecadiynoic acid	C18 H28 O2	(M+H)+	39.118		276.2097		FBF-FragConfirm	94.81		94.81
8,12-Octadecadiynoic acid	C18 H28 O2	(M+H)+	39.118		276.2097		FBF-FragConfirm	94.81		94.81
11,15-Octadecadiynoic acid	C18 H28 O2	(M+H)+	39.118		276.2097		FBF-FragConfirm	94.81		94.81
(9Z,12Z)-octadeca-9,12-dien-6-ynoic acid	C18 H28 O2	(M+H)+	39.118		276.2097		FBF-FragConfirm	94.81		94.81
(9Z,14Z)-octadeca-9,14-dien-6-ynoic acid	C18 H28 O2	(M+H)+	39.118		276.2097		FBF-FragConfirm	94.81		94.81
10,12-Octadecadiynoic acid	C18 H28 O2	(M+H)+	39.118		276.2097		FBF-FragConfirm	94.81		94.81
10,13-Octadecadiynoic acid	C18 H28 O2	(M+H)+	39.118		276.2097		FBF-FragConfirm	94.81		94.81
11,14-Octadecadiynoic acid	C18 H28 O2	(M+H)+	39.118		276.2097		FBF-FragConfirm	94.81		94.81
13,17-Octadecadiynoic acid	C18 H28 O2	(M+H)+	39.118		276.2097		FBF-FragConfirm	94.81		94.81
11E,13E-octadecadien-9-ynoic acid	C18 H28 O2	(M+H)+	39.118		276.2097		FBF-FragConfirm	94.81		94.81
19-Nor-5-androstenediol	C18 H28 O2	(M+H)+	39.118		276.2097		FBF-FragConfirm	94.81		94.81
12,14-Octadecadiynoic acid	C18 H28 O2	(M+H)+	39.118		276.2097		FBF-FragConfirm	94.81		94.81
12,15-Octadecadiynoic acid	C18 H28 O2	(M+H)+	39.118		276.2097		FBF-FragConfirm	94.81		94.81
12,16-Octadecadiynoic acid	C18 H28 O2	(M+H)+	39.118		276.2097		FBF-FragConfirm	94.81		94.81
13,16-Octadecadiynoic acid	C18 H28 O2	(M+H)+	39.118		276.2097		FBF-FragConfirm	94.81		94.81
5,12-Octadecadiynoic acid	C18 H28 O2	(M+H)+	39.118		276.2097		FBF-FragConfirm	94.81		94.81
4,8,12,15-Octadecatetraenoic acid	C18 H28 O2	(M+H)+	39.118		276.2097	67329-10-6	FBF-FragConfirm	94.81		94.81
trans-3, cis-9, cis-12, cis-15-octadecatetraenoic acid; C18:4n-3,6,9,15	C18 H28 O2	(M+H)+	39.118		276.2097		FBF-FragConfirm	94.81		94.81
C18:4n-1,3,6,9	C18 H28 O2	(M+H)+	39.118		276.2097		FBF-FragConfirm	94.81		94.81
C18:4n-3,5,7,9	C18 H28 O2	(M+H)+	39.118		276.2097		FBF-FragConfirm	94.81		94.81
9Z,11Z,13E,15E-octadecatetraenoic acid	C18 H28 O2	(M+H)+	39.118		276.2097		FBF-FragConfirm	94.81		94.81
9Z,12Z-Octadecadien-6-ynoic acid	C18 H28 O2	(M+H)+	39.118		276.2097		FBF-FragConfirm	94.81		94.81
9Z,14Z-Octadecadien-12-ynoic acid	C18 H28 O2	(M+H)+	39.118		276.2097		FBF-FragConfirm	94.81		94.81
Acetylenic acids; 11,13-Octadecadien-9-ynoic acid, (E,E)-	C18 H28 O2	(M+H)+	39.118		276.2097		FBF-FragConfirm	94.81		94.81
beta-parinaric acid	C18 H28 O2	(M+H)+	39.118		276.2097		FBF-FragConfirm	94.81		94.81
cis-Parinaric Acid	C18 H28 O2	(M+H)+	39.118		276.2097	18427-44-6	FBF-FragConfirm	94.81		94.81
9,13-Octadecadiynoic acid	C18 H28 O2	(M+H)+	39.118		276.2097		FBF-FragConfirm	94.81		94.81
α-Parinaric acid	C18 H28 O2	(M+H)+	39.118		276.2097	593-38-4	FBF-FragConfirm	94.81		94.81
Kinoprene	C18 H28 O2	(M+H)+	39.118		276.2097	42588-37-4	FBF-FragConfirm	94.81		94.81
Moroctic acid	C18 H28 O2	(M+H)+	39.118		276.2097		FBF-FragConfirm	94.81		94.81
Phenethyl decanoate	C18 H28 O2	(M+H)+	39.118		276.2097	61810-55-7	FBF-FragConfirm	94.81		94.81
Stearidonic Acid	C18 H28 O2	(M+H)+	39.118		276.2097	20290-75-9	FBF-FragConfirm	94.81		94.81
trans-2,trans-4,trans-6,cis-11-octadecatetraenoic acid	C18 H28 O2	(M+H)+	39.118		276.2097		FBF-FragConfirm	94.81		94.81
9,12-Octadecadiynoic Acid	C18 H28 O2	(M+H)+	39.118		276.2097	2012-14-8	FBF-FragConfirm	94.81		94.81
9,12,15,17-Octadecatetraenoic acid	C18 H28 O2	(M+H)+	39.118		276.2097	958762-34-0	FBF-FragConfirm	94.81		94.81
5,9,12,15-octadecatetraenoic acid	C18 H28 O2	(M+H)+	39.118		276.2097		FBF-FragConfirm	94.81		94.81
6,8-Octadecadiynoic acid	C18 H28 O2	(M+H)+	39.118		276.2097		FBF-FragConfirm	94.81		94.81
6,9-Octadecadiynoic acid	C18 H28 O2	(M+H)+	39.118		276.2097		FBF-FragConfirm	94.81		94.81
6-[3]-ladderane-hexanoic acid	C18 H28 O2	(M+H)+	39.118		276.2097		FBF-FragConfirm	94.81		94.81
5Z,8Z,11Z,14Z-Octadecatetraenoic acid	C18 H28 O2	(M+H)+	39.118		276.2097		FBF-FragConfirm	94.81		94.81
6,10-Octadecadiynoic acid	C18 H28 O2	(M+H)+	39.118		276.2097		FBF-FragConfirm	94.81		94.81
6,11-Octadecadiynoic acid	C18 H28 O2	(M+H)+	39.118		276.2097		FBF-FragConfirm	94.81		94.81
6,12-Octadecadiynoic acid	C18 H28 O2	(M+H)+	39.118		276.2097		FBF-FragConfirm	94.81		94.81
6,9,12,15-Octadecatetraenoic acid	C18 H28 O2	(M+H)+	39.118		276.2097	2091-28-3	FBF-FragConfirm	94.81		94.81
8,11-Octadecadiynoic acid	C18 H28 O2	(M+H)+	39.118		276.2097		FBF-FragConfirm	94.81		94.81
5,9-Octadecadiynoic acid	C18 H28 O2	(M+H)+	39.118		276.2097		FBF-FragConfirm	94.81		94.81
7,10-Octadecadiynoic acid	C18 H28 O2	(M+H)+	39.118		276.2097		FBF-FragConfirm	94.81		94.81
7,11-Octadecadiynoic acid	C18 H28 O2	(M+H)+	39.118		276.2097		FBF-FragConfirm	94.81		94.81
7,12-Octadecadiynoic acid	C18 H28 O2	(M+H)+	39.118		276.2097		FBF-FragConfirm	94.81		94.81
7,9-Octadecadiynoic acid	C18 H28 O2	(M+H)+	39.118		276.2097		FBF-FragConfirm	94.81		94.81
8,10-Octadecadiynoic acid	C18 H28 O2	(M+H)+	39.118		276.2097		FBF-FragConfirm	94.81		94.81
9,11-Octadecadiynoic acid	C18 H28 O2	(M+H)+	39.118		276.2097		FBF-FragConfirm	94.81		94.81

Cpd 1130: <19-Norandrosterone>

Name	Formula	RT	RI	Mass	Diff (Tgt, ppm)	CAS	ID Source	Score	Algorithm
<19-Norandrosterone>	C18 H28 O2	39.484		276.2098	3.22	1225-01-0	M-FBF	92.00	FBF

Species	m/z	Score (Tgt)	Score (Lib)	Score (DB)	Score (MFG)	Score (RT)
(M+H)+	277	92.00				

Compound Chromatograms (overlaid)

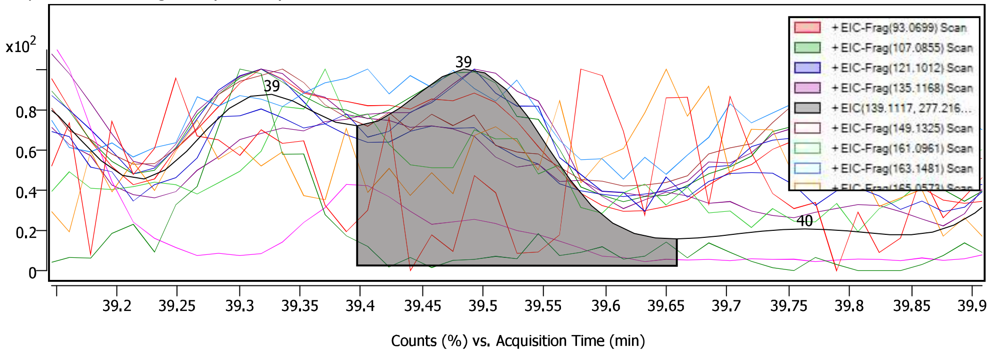

Structure

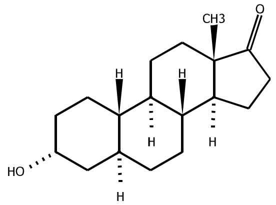

Coelution Plot

Compound Spectra (overlaid)

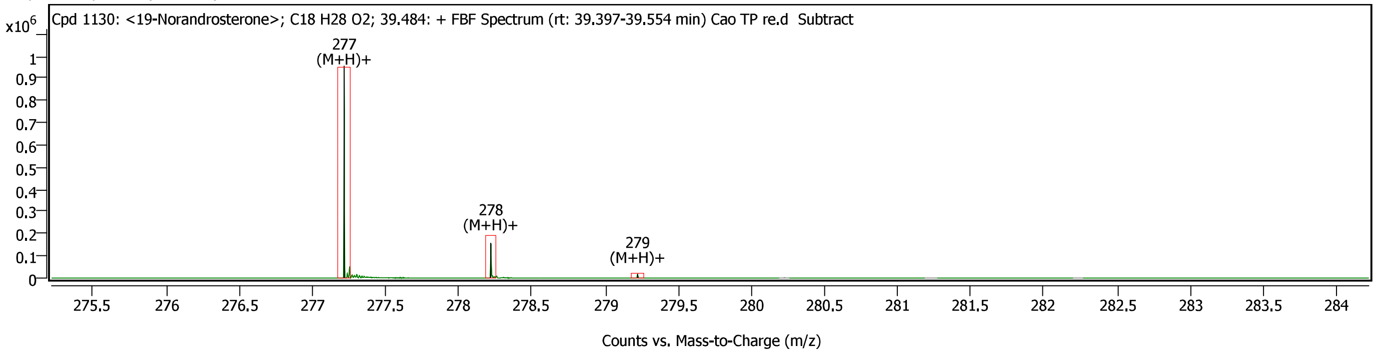

Fragment Spectrum (raw)

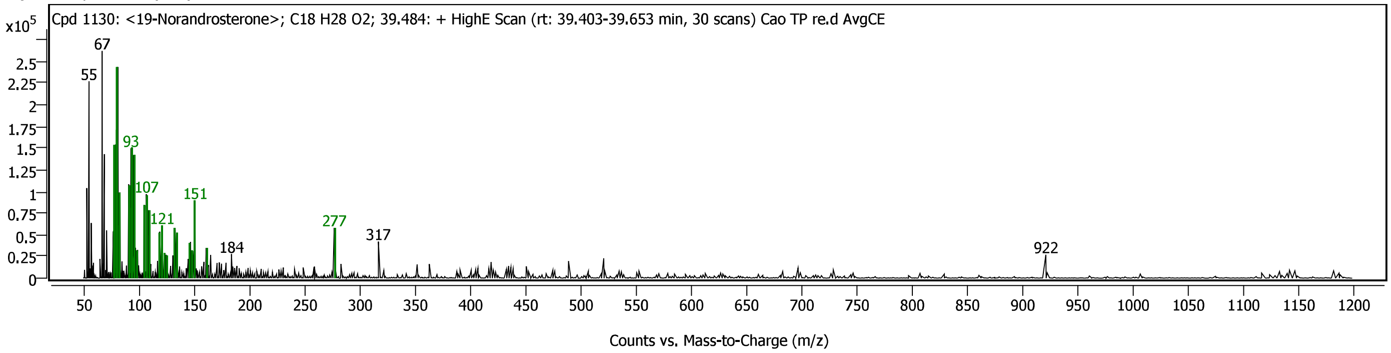

Compound ID Table

Name	Formula	Species	RT	RT Diff	Mass	CAS	ID Source	Score	Score (Lib)	Score (Tgt)
<19-Norandrosterone>	C18 H28 O2	(M+H)+	39.484		276.2098	1225-01-0	FBF	92.00		92.00
<3E,9Z,12Z,15Z-Octadecatetraenoic acid>	C18 H28 O2	(M+H)+	39.484		276.2098	693-78-7	FBF	92.00		92.00
<3,8-Octadecadiynoic acid>	C18 H28 O2	(M+H)+	39.484		276.2098		FBF	92.00		92.00
<4,8-Octadecadiynoic acid>	C18 H28 O2	(M+H)+	39.484		276.2098		FBF	92.00		92.00
<2,5-Octadecadiynoic acid>	C18 H28 O2	(M+H)+	39.484		276.2098		FBF	92.00		92.00
<2,6-Octadecadiynoic acid>	C18 H28 O2	(M+H)+	39.484		276.2098		FBF	92.00		92.00
<2,7-Octadecadiynoic acid>	C18 H28 O2	(M+H)+	39.484		276.2098		FBF	92.00		92.00
<3,6-Octadecadiynoic acid>	C18 H28 O2	(M+H)+	39.484		276.2098		FBF	92.00		92.00
<3,7-Octadecadiynoic acid>	C18 H28 O2	(M+H)+	39.484		276.2098		FBF	92.00		92.00
<4,6-Octadecadiynoic acid>	C18 H28 O2	(M+H)+	39.484		276.2098		FBF	92.00		92.00
<14,17-Octadecadiynoic acid>	C18 H28 O2	(M+H)+	39.484		276.2098		FBF	92.00		92.00
<5,7-Octadecadiynoic acid>	C18 H28 O2	(M+H)+	39.484		276.2098		FBF	92.00		92.00
<4,7-Octadecadiynoic acid>	C18 H28 O2	(M+H)+	39.484		276.2098		FBF	92.00		92.00
<5,8-Octadecadiynoic acid>	C18 H28 O2	(M+H)+	39.484		276.2098		FBF	92.00		92.00
<19-Noretiocholan-3b-ol-17-one>	C18 H28 O2	(M+H)+	39.484		276.2098	64044-10-6	FBF	92.00		92.00
<4,9-Octadecadiynoic acid>	C18 H28 O2	(M+H)+	39.484		276.2098		FBF	92.00		92.00
<5,10-Octadecadiynoic acid>	C18 H28 O2	(M+H)+	39.484		276.2098		FBF	92.00		92.00
<19-Noretiocholanolone>	C18 H28 O2	(M+H)+	39.484		276.2098	33036-33-8	FBF	92.00		92.00
<18:4(5Z,9Z,12Z,15Z)>	C18 H28 O2	(M+H)+	39.484		276.2098		FBF	92.00		92.00
<5,8,11,14-Octadecatetraenoic acid>	C18 H28 O2	(M+H)+	39.484		276.2098	75236-11-2	FBF	92.00		92.00
<10,14-Octadecadiynoic acid>	C18 H28 O2	(M+H)+	39.484		276.2098		FBF	92.00		92.00
<8,12-Octadecadiynoic acid>	C18 H28 O2	(M+H)+	39.484		276.2098		FBF	92.00		92.00
<11,15-Octadecadiynoic acid>	C18 H28 O2	(M+H)+	39.484		276.2098		FBF	92.00		92.00
<(9Z,12Z)-octadeca-9,12-dien-6-ynoic acid>	C18 H28 O2	(M+H)+	39.484		276.2098		FBF	92.00		92.00
<(9Z,14Z)-octadeca-9,14-dien-6-ynoic acid>	C18 H28 O2	(M+H)+	39.484		276.2098		FBF	92.00		92.00
<10,12-Octadecadiynoic acid>	C18 H28 O2	(M+H)+	39.484		276.2098		FBF	92.00		92.00
<10,13-Octadecadiynoic acid>	C18 H28 O2	(M+H)+	39.484		276.2098		FBF	92.00		92.00
<11,14-Octadecadiynoic acid>	C18 H28 O2	(M+H)+	39.484		276.2098		FBF	92.00		92.00
<13,17-Octadecadiynoic acid>	C18 H28 O2	(M+H)+	39.484		276.2098		FBF	92.00		92.00
<11E,13E-octadecadien-9-ynoic acid>	C18 H28 O2	(M+H)+	39.484		276.2098		FBF	92.00		92.00
<19-Nor-5-androstenediol>	C18 H28 O2	(M+H)+	39.484		276.2098		FBF	92.00		92.00
<12,14-Octadecadiynoic acid>	C18 H28 O2	(M+H)+	39.484		276.2098		FBF	92.00		92.00
<12,15-Octadecadiynoic acid>	C18 H28 O2	(M+H)+	39.484		276.2098		FBF	92.00		92.00
<12,16-Octadecadiynoic acid>	C18 H28 O2	(M+H)+	39.484		276.2098		FBF	92.00		92.00
<13,16-Octadecadiynoic acid>	C18 H28 O2	(M+H)+	39.484		276.2098		FBF	92.00		92.00
<5,12-Octadecadiynoic acid>	C18 H28 O2	(M+H)+	39.484		276.2098		FBF	92.00		92.00
<4,8,12,15-Octadecatetraenoic acid>	C18 H28 O2	(M+H)+	39.484		276.2098	67329-10-6	FBF	92.00		92.00
<trans-3, cis-9, cis-12, cis-15-octadecatetraenoic acid; C18:4n-3,6,9,15>	C18 H28 O2	(M+H)+	39.484		276.2098		FBF	92.00		92.00
<C18:4n-1,3,6,9>	C18 H28 O2	(M+H)+	39.484		276.2098		FBF	92.00		92.00
<C18:4n-3,5,7,9>	C18 H28 O2	(M+H)+	39.484		276.2098		FBF	92.00		92.00
<9Z,11Z,13E,15E-octadecatetraenoic acid>	C18 H28 O2	(M+H)+	39.484		276.2098		FBF	92.00		92.00
<9Z,12Z-Octadecadien-6-ynoic acid>	C18 H28 O2	(M+H)+	39.484		276.2098		FBF	92.00		92.00
<9Z,14Z-Octadecadien-12-ynoic acid>	C18 H28 O2	(M+H)+	39.484		276.2098		FBF	92.00		92.00
<Acetylenic acids; 11,13-Octadecadien-9-ynoic acid, (E,E)->	C18 H28 O2	(M+H)+	39.484		276.2098		FBF	92.00		92.00
<beta-parinaric acid>	C18 H28 O2	(M+H)+	39.484		276.2098		FBF	92.00		92.00
<cis-Parinaric Acid>	C18 H28 O2	(M+H)+	39.484		276.2098	18427-44-6	FBF	92.00		92.00
<9,13-Octadecadiynoic acid>	C18 H28 O2	(M+H)+	39.484		276.2098		FBF	92.00		92.00
<α-Parinaric acid>	C18 H28 O2	(M+H)+	39.484		276.2098	593-38-4	FBF	92.00		92.00
<Kinoprene>	C18 H28 O2	(M+H)+	39.484		276.2098	42588-37-4	FBF	92.00		92.00
<Moroctic acid>	C18 H28 O2	(M+H)+	39.484		276.2098		FBF	92.00		92.00
<Phenethyl decanoate>	C18 H28 O2	(M+H)+	39.484		276.2098	61810-55-7	FBF	92.00		92.00
<Stearidonic Acid>	C18 H28 O2	(M+H)+	39.484		276.2098	20290-75-9	FBF	92.00		92.00
<trans-2,trans-4,trans-6,cis-11-octadecatetraenoic acid>	C18 H28 O2	(M+H)+	39.484		276.2098		FBF	92.00		92.00
<9,12-Octadecadiynoic Acid>	C18 H28 O2	(M+H)+	39.484		276.2098	2012-14-8	FBF	92.00		92.00
<9,12,15,17-Octadecatetraenoic acid>	C18 H28 O2	(M+H)+	39.484		276.2098	958762-34-0	FBF	92.00		92.00
<5,9,12,15-octadecatetraenoic acid>	C18 H28 O2	(M+H)+	39.484		276.2098		FBF	92.00		92.00
<6,8-Octadecadiynoic acid>	C18 H28 O2	(M+H)+	39.484		276.2098		FBF	92.00		92.00
<6,9-Octadecadiynoic acid>	C18 H28 O2	(M+H)+	39.484		276.2098		FBF	92.00		92.00
<6-[3]-ladderane-hexanoic acid>	C18 H28 O2	(M+H)+	39.484		276.2098		FBF	92.00		92.00
<5Z,8Z,11Z,14Z-Octadecatetraenoic acid>	C18 H28 O2	(M+H)+	39.484		276.2098		FBF	92.00		92.00
<6,10-Octadecadiynoic acid>	C18 H28 O2	(M+H)+	39.484		276.2098		FBF	92.00		92.00
<6,11-Octadecadiynoic acid>	C18 H28 O2	(M+H)+	39.484		276.2098		FBF	92.00		92.00
<6,12-Octadecadiynoic acid>	C18 H28 O2	(M+H)+	39.484		276.2098		FBF	92.00		92.00
<6,9,12,15-Octadecatetraenoic acid>	C18 H28 O2	(M+H)+	39.484		276.2098	2091-28-3	FBF	92.00		92.00
<8,11-Octadecadiynoic acid>	C18 H28 O2	(M+H)+	39.484		276.2098		FBF	92.00		92.00
<5,9-Octadecadiynoic acid>	C18 H28 O2	(M+H)+	39.484		276.2098		FBF	92.00		92.00
<7,10-Octadecadiynoic acid>	C18 H28 O2	(M+H)+	39.484		276.2098		FBF	92.00		92.00
<7,11-Octadecadiynoic acid>	C18 H28 O2	(M+H)+	39.484		276.2098		FBF	92.00		92.00
<7,12-Octadecadiynoic acid>	C18 H28 O2	(M+H)+	39.484		276.2098		FBF	92.00		92.00
<7,9-Octadecadiynoic acid>	C18 H28 O2	(M+H)+	39.484		276.2098		FBF	92.00		92.00
<8,10-Octadecadiynoic acid>	C18 H28 O2	(M+H)+	39.484		276.2098		FBF	92.00		92.00
<9,11-Octadecadiynoic acid>	C18 H28 O2	(M+H)+	39.484		276.2098		FBF	92.00		92.00

Cpd 888: N-Oleoyl-L-Serine

Name	Formula	RT	RI	Mass	Diff (Tgt, ppm)	CAS	ID Source	Score	Algorithm
N-Oleoyl-L-Serine	C21 H39 N O4	39.519		369.2881	0.56		FBF	99.62	FBF

Species	m/z	Score (Tgt)	Score (Lib)	Score (DB)	Score (MFG)	Score (RT)
(M+H)+	370	99.62				

Compound Chromatograms (overlaid)

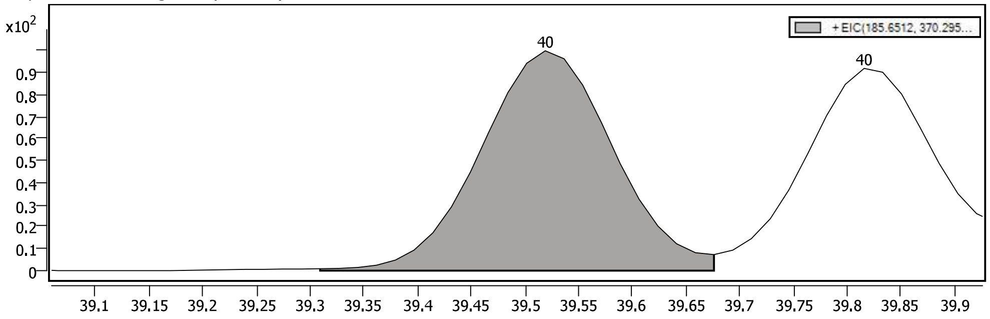

Counts (%) vs. Acquisition Time (min)

Structure

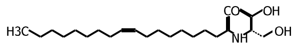

Compound Spectra (overlaid)

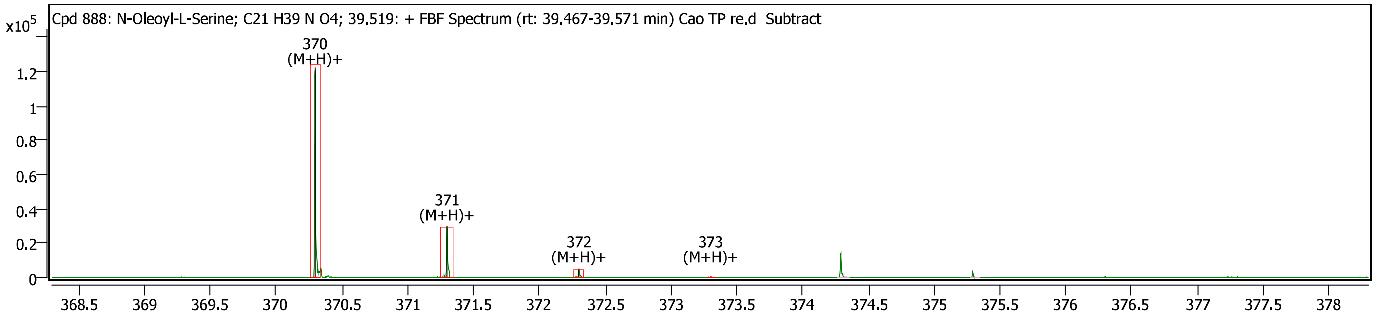

Counts vs. Mass-to-Charge (m/z)

Compound ID Table

Name	Formula	Species	RT	RT Diff	Mass	CAS	ID Source	Score	Score (Lib)	Score (Tgt)
N-Oleoyl-L-Serine	C21 H39 N O4	(M+H)+	39.519		369.2881		FBF	99.62		99.62

Cpd 447: <γ-Linolenic Acid>

Name	Formula	RT	RI	Mass	Diff (Tgt, ppm)	CAS	ID Source	Score	Algorithm
<γ-Linolenic Acid>	C18 H30 O2	39.763		278.2256	3.63	506-26-3	M-FBF	91.02	FBF

Species	m/z	Score (Tgt)	Score (Lib)	Score (DB)	Score (MFG)	Score (RT)
(M+H)+	279	91.02				

Compound Chromatograms (overlaid)

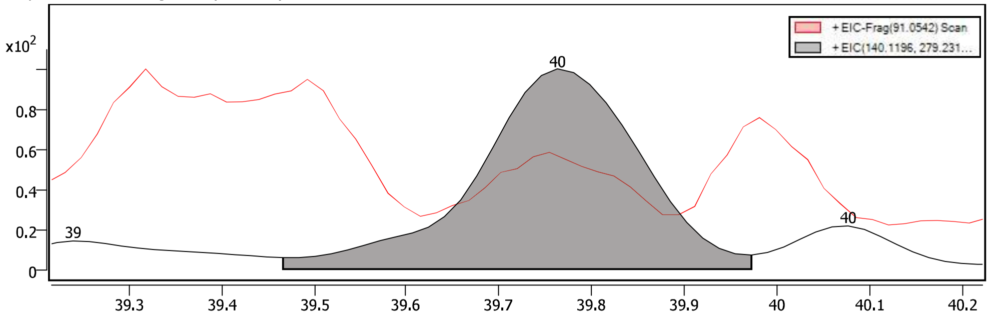

Counts (%) vs. Acquisition Time (min)

Structure

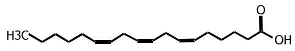

Coelution Plot

Compound Spectra (overlaid)

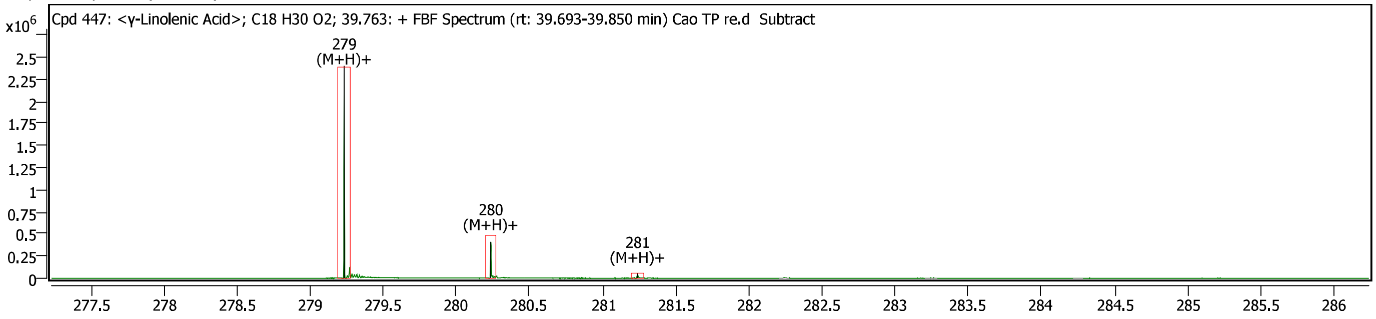

Counts vs. Mass-to-Charge (m/z)

Fragment Spectrum (raw)

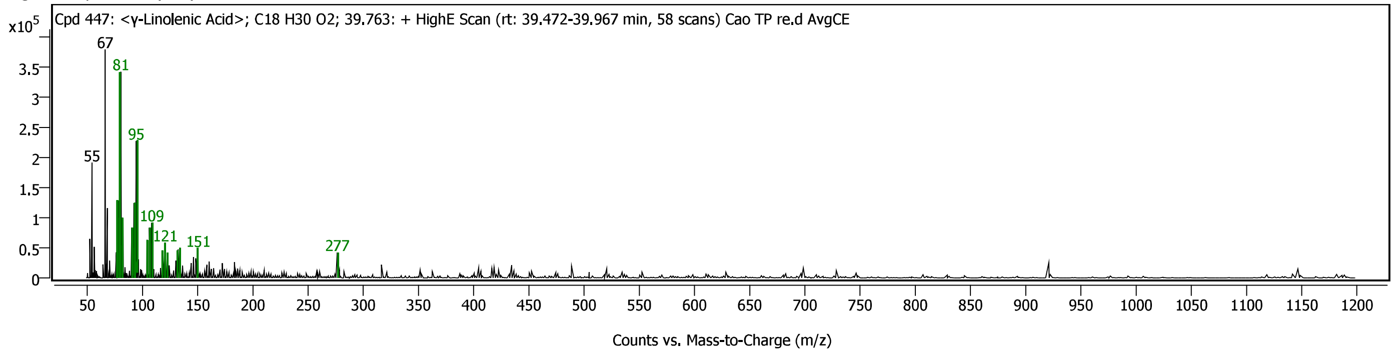

Compound ID Table

Name	Formula	Species	RT	RT Diff	Mass	CAS	ID Source	Score	Score (Lib)	Score (Tgt)
<γ-Linolenic Acid>	C18 H30 O2	(M+H)+	39.763		278.2256	506-26-3	FBF	91.02		91.02
<7Z,9Z,12Z-Octadecatrienoic acid>	C18 H30 O2	(M+H)+	39.763		278.2256		FBF	91.02		91.02
<3E,9Z,12Z-Octadecatrienoic acid>	C18 H30 O2	(M+H)+	39.763		278.2256	2277-04-5	FBF	91.02		91.02
<5,9,12-Octadecatrienoic acid>	C18 H30 O2	(M+H)+	39.763		278.2256		FBF	91.02		91.02
<5Z,9Z,12E-Octadecatrienoic acid>	C18 H30 O2	(M+H)+	39.763		278.2256		FBF	91.02		91.02
<6,10,14-Octadecatrienoic acid>	C18 H30 O2	(M+H)+	39.763		278.2256	873392-34-8	FBF	91.02		91.02
<7E,9Z,12Z-Octadecatrienoic acid>	C18 H30 O2	(M+H)+	39.763		278.2256		FBF	91.02		91.02
<9Z,12E,15E-Octadecatrienoic acid>	C18 H30 O2	(M+H)+	39.763		278.2256		FBF	91.02		91.02
<8-Hydroxy-15,16-bisnor-11-labden-13-one>	C18 H30 O2	(M+H)+	39.763		278.2256	16736-49-5	FBF	91.02		91.02
<8Z,10E,12Z-Octadecatrienoic acid>	C18 H30 O2	(M+H)+	39.763		278.2256	28872-28-8	FBF	91.02		91.02
<9,12,14-Octadecatrienoic acid>	C18 H30 O2	(M+H)+	39.763		278.2256	374621-93-9	FBF	91.02		91.02
<9E,12E,15Z-Octadecatrienoic acid>	C18 H30 O2	(M+H)+	39.763		278.2256	21661-13-2	FBF	91.02		91.02
<9E,12Z,15E-Octadecatrienoic acid>	C18 H30 O2	(M+H)+	39.763		278.2256		FBF	91.02		91.02
<9E,12Z,15Z-Octadecatrienoic acid>	C18 H30 O2	(M+H)+	39.763		278.2256	21661-10-9	FBF	91.02		91.02
<4E,6E,10Z-Hexadecatrienyl acetate>	C18 H30 O2	(M+H)+	39.763		278.2256		FBF	91.02		91.02
<4E,6E,11Z-Hexadecatrienyl acetate>	C18 H30 O2	(M+H)+	39.763		278.2256		FBF	91.02		91.02
<10E,12E,14E-Hexadecatrienyl acetate>	C18 H30 O2	(M+H)+	39.763		278.2256		FBF	91.02		91.02
<(E)-octadec-9-en-12-ynoic acid>	C18 H30 O2	(M+H)+	39.763		278.2256		FBF	91.02		91.02
<4E,6Z,10Z-Hexadecatrienyl acetate>	C18 H30 O2	(M+H)+	39.763		278.2256		FBF	91.02		91.02
<(E,E)-3,7,11-Trimethyl-2,6,10-dodecatrienyl propionate>	C18 H30 O2	(M+H)+	39.763		278.2256		FBF	91.02		91.02
<(R)-lamenallenic acid>	C18 H30 O2	(M+H)+	39.763		278.2256		FBF	91.02		91.02
<(S)-lamenallenic acid>	C18 H30 O2	(M+H)+	39.763		278.2256		FBF	91.02		91.02
<10,12,14-octadecatrienoic acid>	C18 H30 O2	(M+H)+	39.763		278.2256		FBF	91.02		91.02
<10,12,15-Octadecatrienoic acid>	C18 H30 O2	(M+H)+	39.763		278.2256	374621-94-0	FBF	91.02		91.02
<Columbinic acid>	C18 H30 O2	(M+H)+	39.763		278.2256		FBF	91.02		91.02
<10E,12E,14Z-Hexadecatrienyl acetate>	C18 H30 O2	(M+H)+	39.763		278.2256		FBF	91.02		91.02
<11Z,13E,15-Hexadecatrienyl acetate>	C18 H30 O2	(M+H)+	39.763		278.2256		FBF	91.02		91.02
<11Z-octadecen-9-ynoic acid>	C18 H30 O2	(M+H)+	39.763		278.2256		FBF	91.02		91.02
<13Z-Hexadecen-11-ynyl acetate>	C18 H30 O2	(M+H)+	39.763		278.2256		FBF	91.02		91.02
<16-methyl-6Z,9Z,12Z-heptadecatrienoic acid>	C18 H30 O2	(M+H)+	39.763		278.2256		FBF	91.02		91.02
<17-octadecen-9-ynoic acid>	C18 H30 O2	(M+H)+	39.763		278.2256		FBF	91.02		91.02
<2E,9Z,12Z-octadecatrienoic acid>	C18 H30 O2	(M+H)+	39.763		278.2256		FBF	91.02		91.02
<11E-octadecen-9-ynoic acid>	C18 H30 O2	(M+H)+	39.763		278.2256		FBF	91.02		91.02
<5,8,11-octadecatrienoic acid>	C18 H30 O2	(M+H)+	39.763		278.2256		FBF	91.02		91.02
<9E-Octadecen-12-ynoic acid>	C18 H30 O2	(M+H)+	39.763		278.2256		FBF	91.02		91.02
<Gorlic acid>	C18 H30 O2	(M+H)+	39.763		278.2256		FBF	91.02		91.02
<Elaidolinoleic acid>	C18 H30 O2	(M+H)+	39.763		278.2256		FBF	91.02		91.02
<octadeca-5S,6,16E-trienoic acid>	C18 H30 O2	(M+H)+	39.763		278.2256		FBF	91.02		91.02
<octadeca-9Z,11E,14Z-trienoic acid>	C18 H30 O2	(M+H)+	39.763		278.2256		FBF	91.02		91.02
<octadeca-9Z,11E,15Z-trienoic acid>	C18 H30 O2	(M+H)+	39.763		278.2256		FBF	91.02		91.02
<Pinolenic Acid>	C18 H30 O2	(M+H)+	39.763		278.2256	16833-54-8	FBF	91.02		91.02
<Pseudoeleostearic acid>	C18 H30 O2	(M+H)+	39.763		278.2256		FBF	91.02		91.02
<trans-3, cis-9, cis-12-octadecatrienoic acid; C18:3n-6,9,15>	C18 H30 O2	(M+H)+	39.763		278.2256		FBF	91.02		91.02
<estrane-3alpha,17alpha-diol>	C18 H30 O2	(M+H)+	39.763		278.2256		FBF	91.02		91.02
<α-Calendic acid>	C18 H30 O2	(M+H)+	39.763		278.2256	5204-87-5	FBF	91.02		91.02
<α-ESA>	C18 H30 O2	(M+H)+	39.763		278.2256	506-23-0	FBF	91.02		91.02
<α-Linolenic Acid>	C18 H30 O2	(M+H)+	39.763		278.2256	463-40-1	FBF	91.02		91.02
<β-Calendic acid>	C18 H30 O2	(M+H)+	39.763		278.2256	822-19-5	FBF	91.02		91.02
<β-Eleostearic acid>	C18 H30 O2	(M+H)+	39.763		278.2256	544-73-0	FBF	91.02		91.02
<Punicic acid>	C18 H30 O2	(M+H)+	39.763		278.2256		FBF	91.02		91.02
<estrane-3α,17α-diol>	C18 H30 O2	(M+H)+	39.763		278.2256		FBF	91.02		91.02
<C18:3n-4,8,12>	C18 H30 O2	(M+H)+	39.763		278.2256		FBF	91.02		91.02
<9E,11Z,13Z-octadecatrienoic acid>	C18 H30 O2	(M+H)+	39.763		278.2256		FBF	91.02		91.02
<9Z-Octadecen-12-ynoic acid>	C18 H30 O2	(M+H)+	39.763		278.2256		FBF	91.02		91.02
<Acetylenic acids; 11-Octadecen-9-ynoic acid, (E)-; Ximenynic acid; Santalbic acid; trans-11-Octadecen-9-ynoic acid; Ximeninic acid>	C18 H30 O2	(M+H)+	39.763		278.2256		FBF	91.02		91.02
<Acetylenic acids; 11-Octadecen-9-ynoic acid, (Z)-; cis-11-Octadecen-9-ynoic acid>	C18 H30 O2	(M+H)+	39.763		278.2256		FBF	91.02		91.02
<Acetylenic acids; 17-Octadecen-9-ynoic acid>	C18 H30 O2	(M+H)+	39.763		278.2256		FBF	91.02		91.02
<Crepenynic acid>	C18 H30 O2	(M+H)+	39.763		278.2256		FBF	91.02		91.02
<C18:3n-4,6,9>	C18 H30 O2	(M+H)+	39.763		278.2256		FBF	91.02		91.02
<C18:3n-3,6,8>	C18 H30 O2	(M+H)+	39.763		278.2256		FBF	91.02		91.02
<C18:3n-5,7,9>	C18 H30 O2	(M+H)+	39.763		278.2256		FBF	91.02		91.02
<C18:3n-6,9,16>	C18 H30 O2	(M+H)+	39.763		278.2256		FBF	91.02		91.02
<C18:3n-7,10,13>	C18 H30 O2	(M+H)+	39.763		278.2256		FBF	91.02		91.02
<Catalpic acid>	C18 H30 O2	(M+H)+	39.763		278.2256		FBF	91.02		91.02
<cis-8, trans-10, cis-12-octadecatrienoic acid; C18:3n-6,8,10>	C18 H30 O2	(M+H)+	39.763		278.2256		FBF	91.02		91.02
<octadeca-11E,13E,15Z-trienoic acid>	C18 H30 O2	(M+H)+	39.763		278.2256		FBF	91.02		91.02

Compound Screening Report

Compound ID Table

Name	Formula	Species	RT	RT Diff	Mass	CAS	ID Source	Score	Score (Lib)	Score (Tgt)
<9Z,12E,15Z-Octadecatrienoic acid>	C18 H30 O2	(M+H)+	39.763		278.2256		FBF	91.02		91.02
<9Z,12Z,15E-Octadecatrienoic acid>	C18 H30 O2	(M+H)+	39.763		278.2256	21661-08-5	FBF	91.02		91.02

Cpd 1583: Acetyl tributyl citrate

Name	Formula	RT	RI	Mass	Diff (Tgt, ppm)	CAS	ID Source	Score	Algorithm
Acetyl tributyl citrate	C20 H34 O8	40.252		402.2258	0.96	77-90-7	FBF-FragConfirm	98.17	FBF

Species	m/z	Score (Tgt)	Score (Lib)	Score (DB)	Score (MFG)	Score (RT)
(M+H)+	403	98.17				

Compound Chromatograms (overlaid)

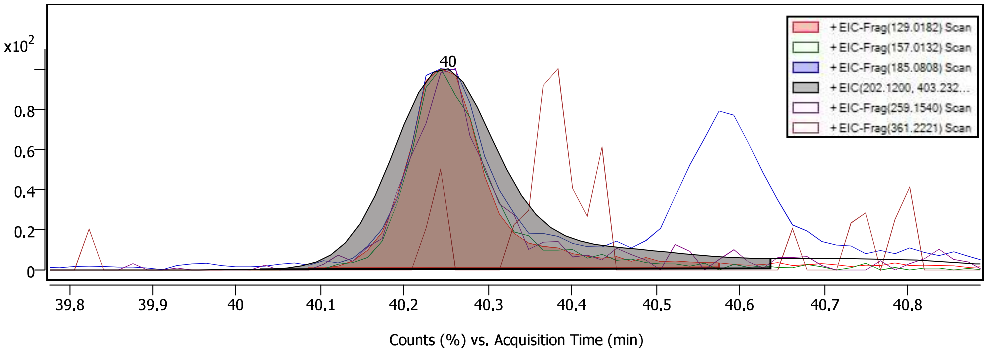

Structure

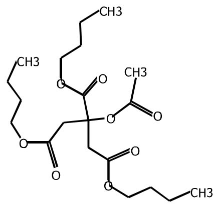

Coelution Plot

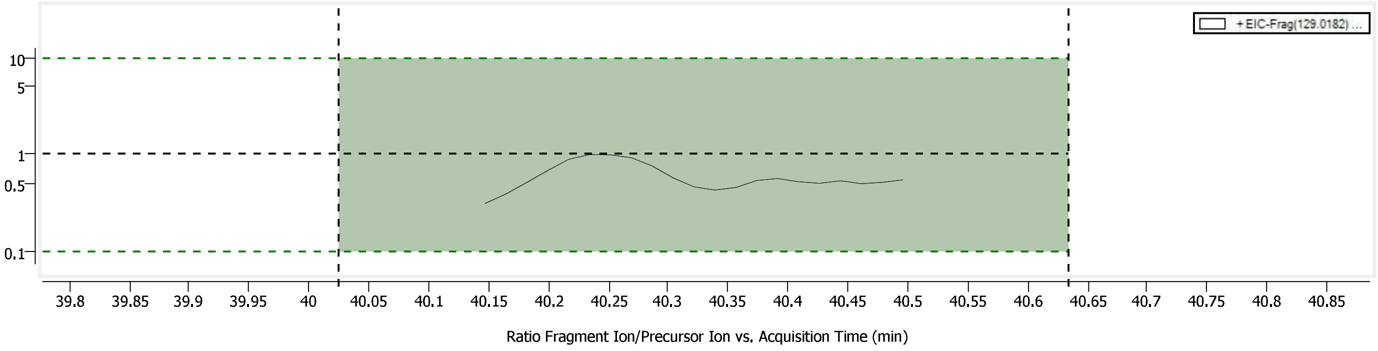

Compound Spectra (overlaid)

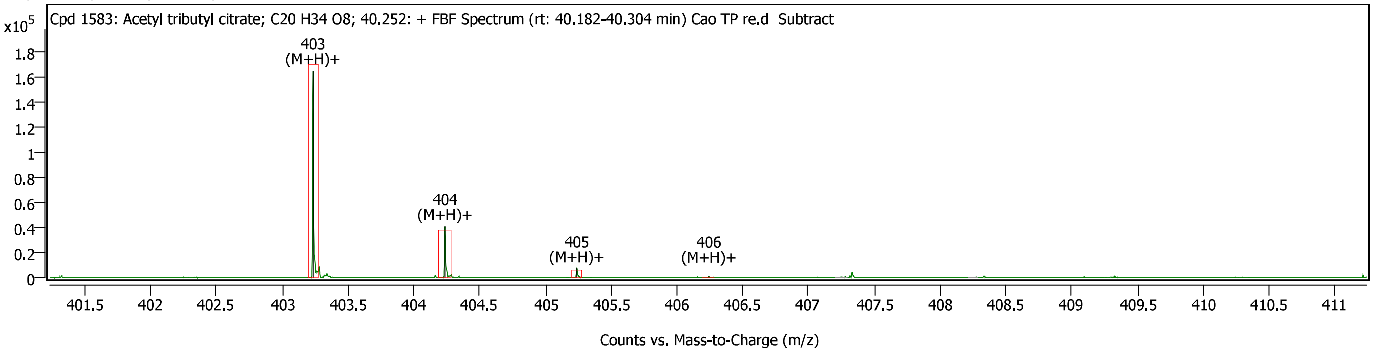

Fragment Spectrum (clean)

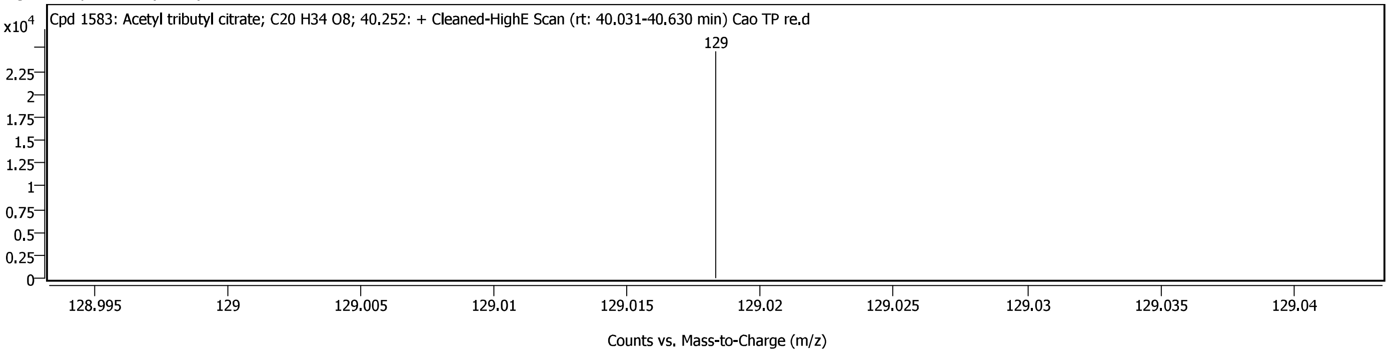

Compound Screening Report

Fragment Spectrum (raw)

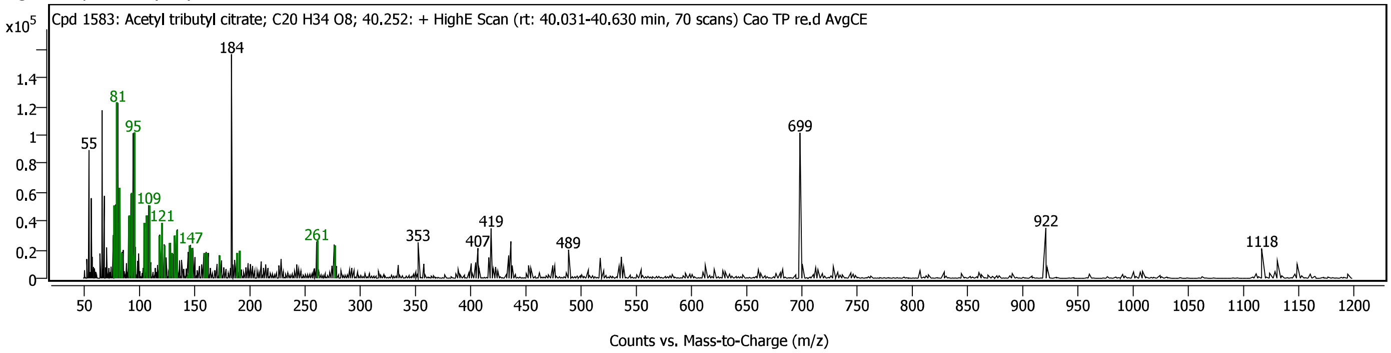

Compound ID Table

Name	Formula	Species	RT	RT Diff	Mass	CAS	ID Source	Score	Score (Lib)	Score (Tgt)
Acetyl tributyl citrate	C20 H34 O8	(M+H)+	40.252		402.2258	77-90-7	FBF-FragConfirm	98.17		98.17
5S-HETE di-endoperoxide	C20 H34 O8	(M+H)+	40.252		402.2258		FBF-FragConfirm	98.17		98.17

Cpd 1559: <Oxybutynin>

Name	Formula	RT	RI	Mass	Diff (Tgt, ppm)	CAS	ID Source	Score	Algorithm
<Oxybutynin>	C22 H31 N O3	40.496		357.2309	1.54	5633-20-5	M-FBF	97.50	FBF

Species	m/z	Score (Tgt)	Score (Lib)	Score (DB)	Score (MFG)	Score (RT)
(M+H)+	358	97.50				

Compound Chromatograms (overlaid)

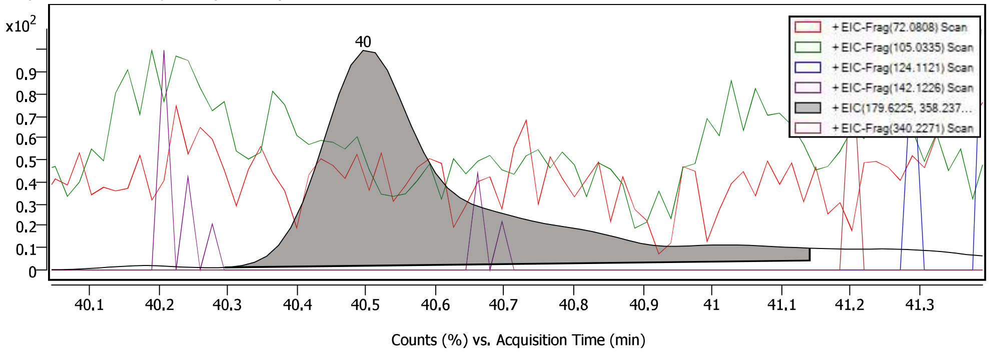

Structure

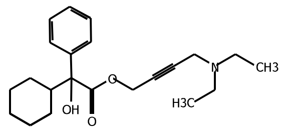

Coelution Plot

Compound Spectra (overlaid)

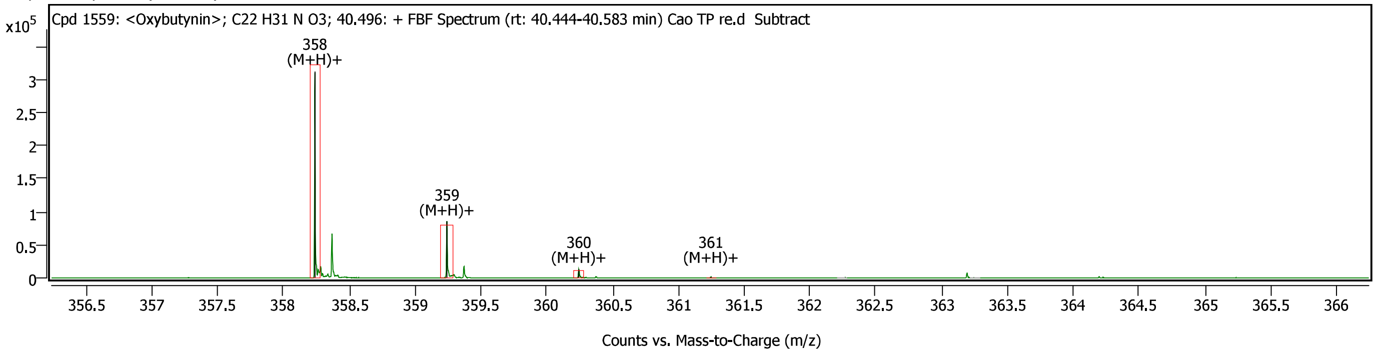

Fragment Spectrum (raw)

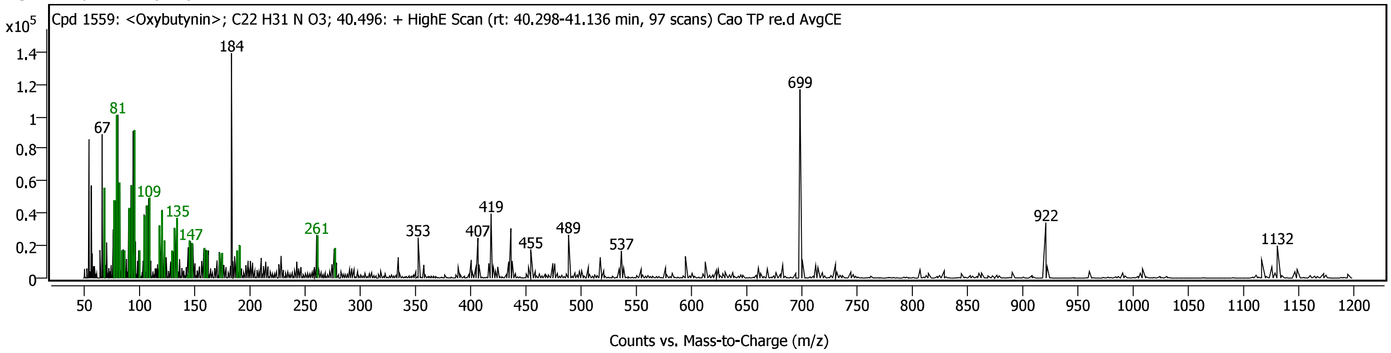

Compound Screening Report

Compound ID Table

Name	Formula	Species	RT	RT Diff	Mass	CAS	ID Source	Score	Score (Lib)	Score (Tgt)
<Oxybutynin>	C22 H31 N O3	(M+H)+	40.496		357.2309	5633-20-5	FBF	97.50		97.50
<Dihydroretrofractamide B>	C22 H31 N O3	(M+H)+	40.496		357.2309	75022-26-3	FBF	97.50		97.50
<Songorine>	C22 H31 N O3	(M+H)+	40.496		357.2309	509-24-0	FBF	97.50		97.50

Cpd 1244: Corchorusoside A

Name	Formula	RT	RI	Mass	Diff (Tgt, ppm)	CAS	ID Source	Score	Algorithm
Corchorusoside A	C35 H54 O14	40.618		698.3495	-2.66	210637-15-3	M-FBF	95.45	FBF

Species	m/z	Score (Tgt)	Score (Lib)	Score (DB)	Score (MFG)	Score (RT)
(M+H)+	699	95.45				

Compound Chromatograms (overlaid)

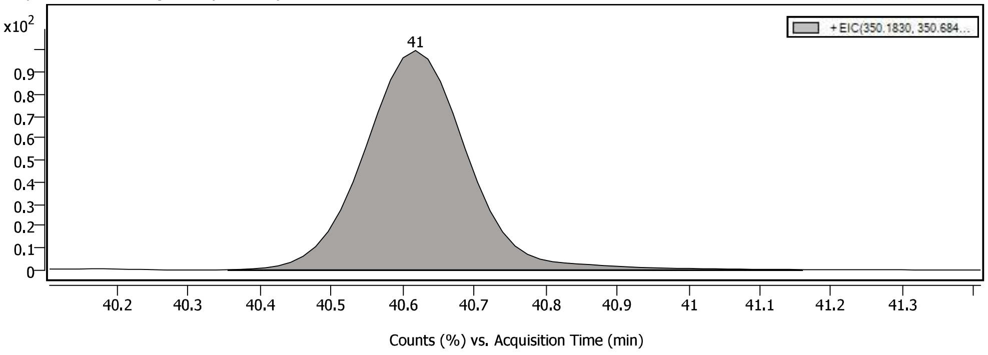

Structure

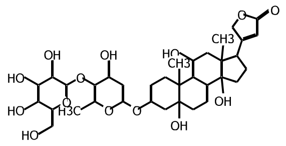

Compound Spectra (overlaid)

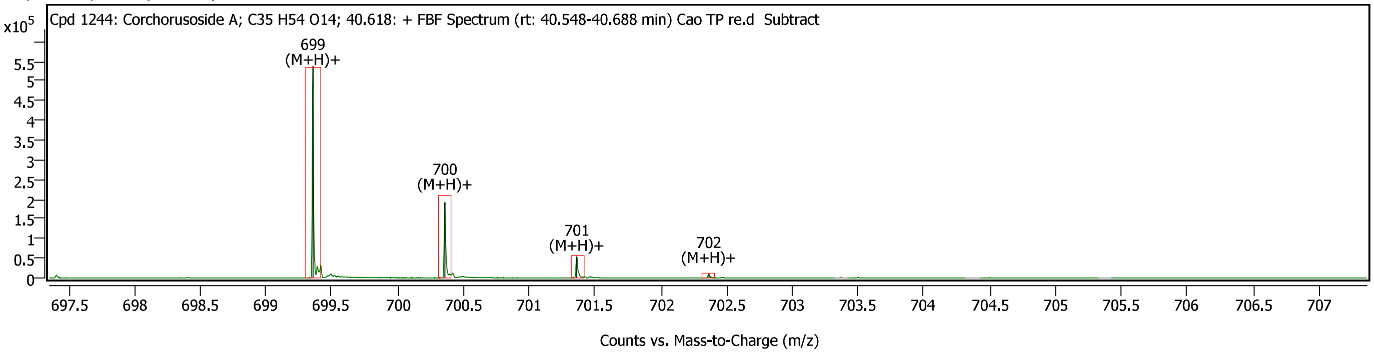

Compound ID Table

Name	Formula	Species	RT	RT Diff	Mass	CAS	ID Source	Score	Score (Lib)	Score (Tgt)
Corchorusoside A	C35 H54 O14	(M+H)+	40.618		698.3495	210637-15-3	FBF	95.45		95.45
Uzarin	C35 H54 O14	(M+H)+	40.618		698.3495	20231-81-6	FBF	95.45		95.45
Corchorusoside D	C35 H54 O14	(M+H)+	40.618		698.3495	210637-18-6	FBF	95.45		95.45
(15a,20R)-Dihydroxypregn-4-en-3-one 20-[glucosyl-(1->4)-6-acetyl-glucoside]	C35 H54 O14	(M+H)+	40.618		698.3495		FBF	95.45		95.45

Cpd 122: Hericenone E

Name	Formula	RT	RI	Mass	Diff (Tgt, ppm)	CAS	ID Source	Score	Algorithm
Hericenone E	C37 H54 O6	41.072		594.3896	-4.12	137592-05-3	M-FBF	92.43	FBF

Species	m/z	Score (Tgt)	Score (Lib)	Score (DB)	Score (MFG)	Score (RT)
(M+H)+	595	92.43				

Compound Chromatograms (overlaid)

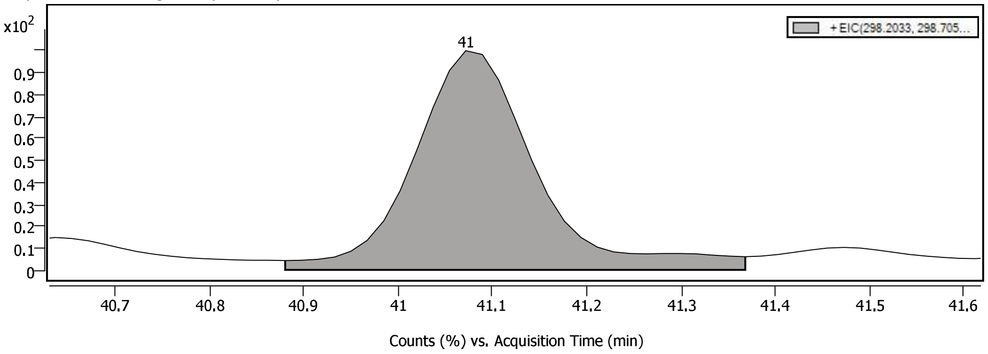

Structure

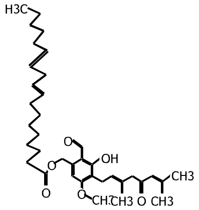

Compound Spectra (overlaid)

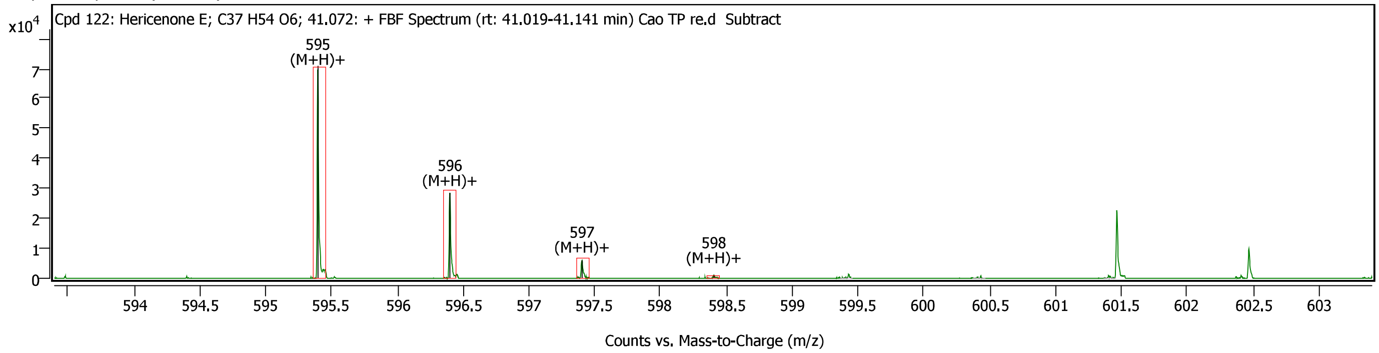

Compound ID Table

Name	Formula	Species	RT	RT Diff	Mass	CAS	ID Source	Score	Score (Lib)	Score (Tgt)
Hericenone E	C37 H54 O6	(M+H)+	41.072		594.3896	137592-05-3	FBF	92.43		92.43
Hericenone H	C37 H54 O6	(M+H)+	41.072		594.3896	141973-37-7	FBF	92.43		92.43
21-Benzoyl-Barringtogenol C	C37 H54 O6	(M+H)+	41.072		594.3896		FBF	92.43		92.43

Cpd 34: PE-Cer(d15:1(4E)/18:0)

Name	Formula	RT	RI	Mass	Diff (Tgt, ppm)	CAS	ID Source	Score	Algorithm
PE-Cer(d15:1(4E)/18:0)	C35 H71 N2 O6 P	41.124		646.5018	-4.90		M-FBF	89.80	FBF

Species	m/z	Score (Tgt)	Score (Lib)	Score (DB)	Score (MFG)	Score (RT)
(M+H)+	648	89.80				

Compound Chromatograms (overlaid)

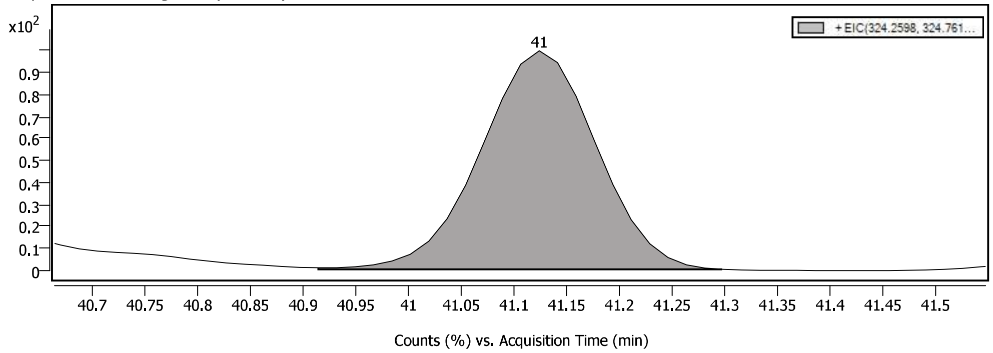

Structure

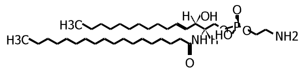

Compound Spectra (overlaid)

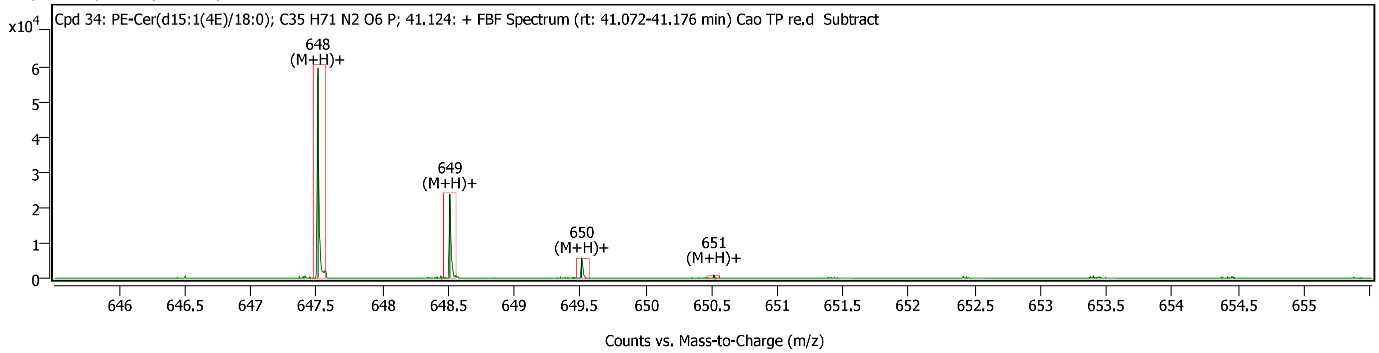

Compound ID Table

Name	Formula	Species	RT	RT Diff	Mass	CAS	ID Source	Score	Score (Lib)	Score (Tgt)
PE-Cer(d15:1(4E)/18:0)	C35 H71 N2 O6 P	(M+H)+	41.124		646.5018		FBF	89.80		89.80
PE-Cer(d14:1(4E)/19:0)	C35 H71 N2 O6 P	(M+H)+	41.124		646.5018		FBF	89.80		89.80

Cpd 1506: Pipecuronium

Name	Formula	RT	RI	Mass	Diff (Tgt, ppm)	CAS	ID Source	Score	Algorithm
Pipecuronium	C35 H62 N4 O4	41.211		602.4758	-2.18	68399-58-6	FBF	97.59	FBF

Species	m/z	Score (Tgt)	Score (Lib)	Score (DB)	Score (MFG)	Score (RT)
(M+H)+	603	97.59				

Compound Screening Report

Compound Chromatograms (overlaid)

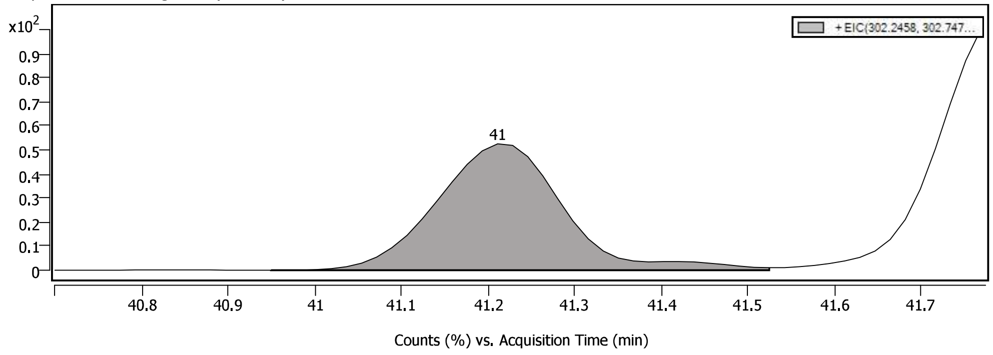

Structure

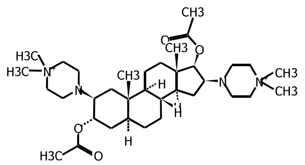

Compound Spectra (overlaid)

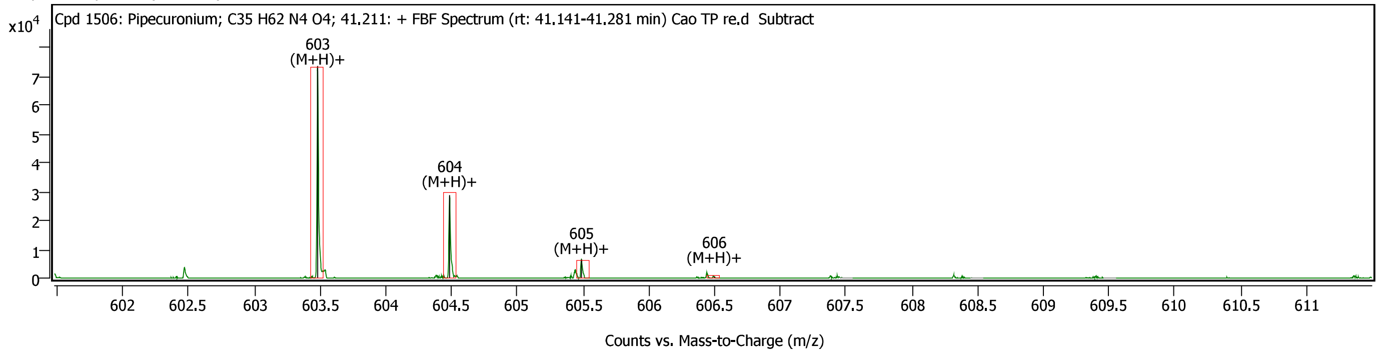

Compound ID Table

Name	Formula	Species	RT	RT Diff	Mass	CAS	ID Source	Score	Score (Lib)	Score (Tgt)
Pipecuronium	C35 H62 N4 O4	(M+H)+	41.211		602.4758	68399-58-6	FBF	97.59		97.59

Cpd 1704: Corchorosol A

Name	Formula	RT	RI	Mass	Diff (Tgt, ppm)	CAS	ID Source	Score	Algorithm
Corchorosol A	C29 H44 O9	41.333		536.2963	-4.17	23838-13-3	M-FBF	91.92	FBF

Species	m/z	Score (Tgt)	Score (Lib)	Score (DB)	Score (MFG)	Score (RT)
(M+H)+	537	91.92				

Compound Chromatograms (overlaid)

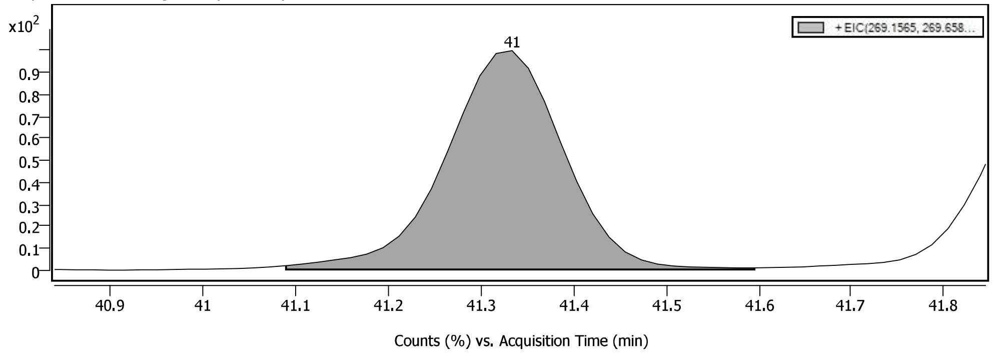

Structure

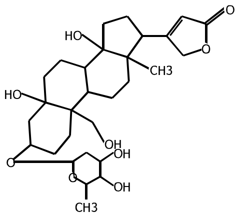

Compound Spectra (overlaid)

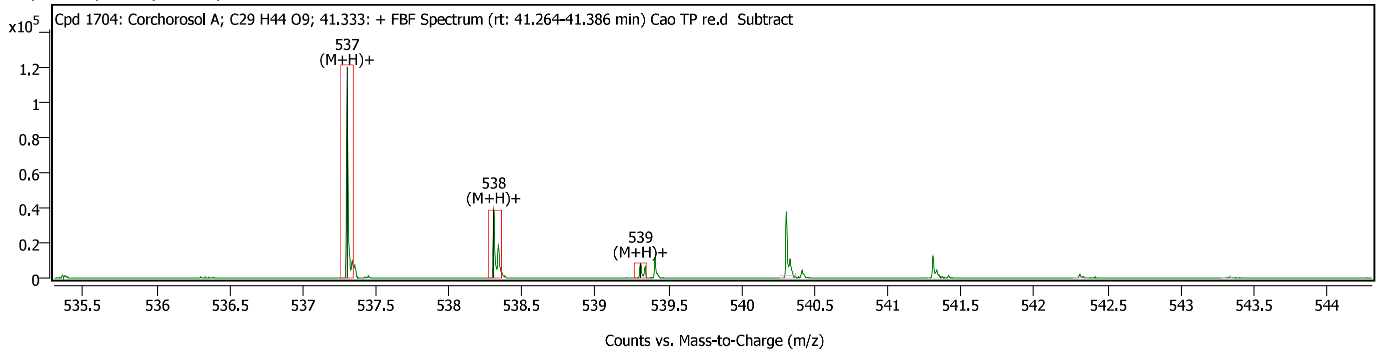

Compound ID Table

Name	Formula	Species	RT	RT Diff	Mass	CAS	ID Source	Score	Score (Lib)	Score (Tgt)
Corchorosol A	C29 H44 O9	(M+H)+	41.333		536.2963	23838-13-3	FBF	91.92		91.92
rhodexin A	C29 H44 O9	(M+H)+	41.333		536.2963		FBF	91.92		91.92
Coroglaucigenin-3-o-alpha-L-rhamnopyranoside	C29 H44 O9	(M+H)+	41.333		536.2963		FBF	91.92		91.92

Cpd 210: Drotaverine

Name	Formula	RT	RI	Mass	Diff (Tgt, ppm)	CAS	ID Source	Score	Algorithm
Drotaverine	C24 H31 N O4	41.473		397.2255	0.58	985-12-6	FBF	98.72	FBF

Species	m/z	Score (Tgt)	Score (Lib)	Score (DB)	Score (MFG)	Score (RT)
(M+H)+	398	98.72				

Compound Chromatograms (overlaid)

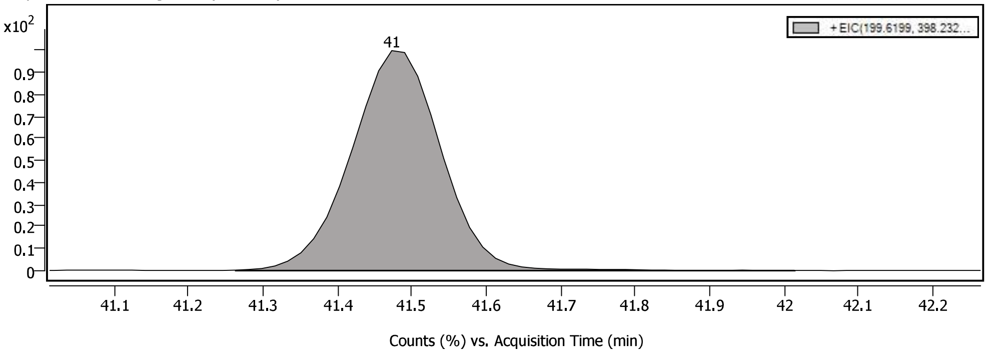

Structure

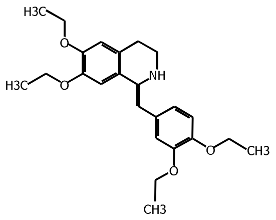

Compound Spectra (overlaid)

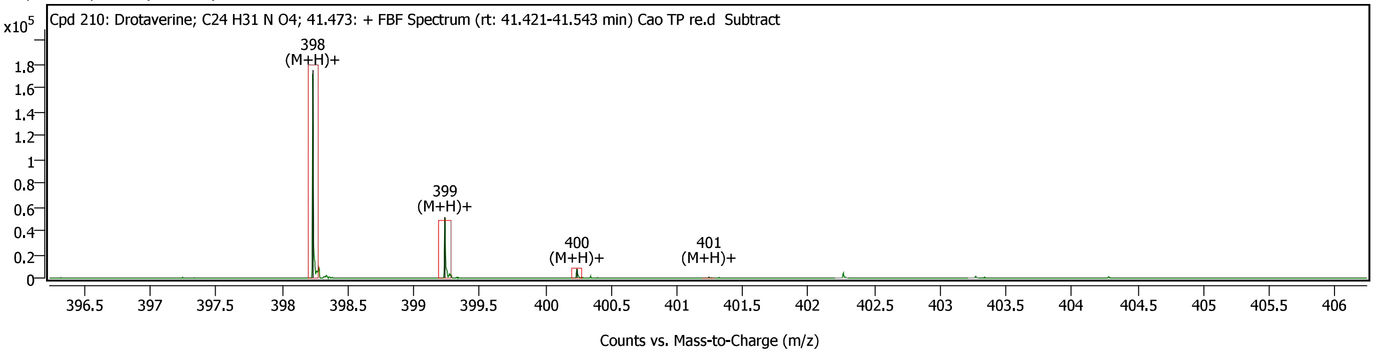

Compound ID Table

Name	Formula	Species	RT	RT Diff	Mass	CAS	ID Source	Score	Score (Lib)	Score (Tgt)
Drotaverine	C24 H31 N O4	(M+H)+	41.473		397.2255	985-12-6	FBF	98.72		98.72

Cpd 887: <AT-56>

Name	Formula	RT	RI	Mass	Diff (Tgt, ppm)	CAS	ID Source	Score	Algorithm
<AT-56>	C25 H27 N5	41.473		397.2256	-2.59	162640-98-4	FBF	96.77	FBF

Species	m/z	Score (Tgt)	Score (Lib)	Score (DB)	Score (MFG)	Score (RT)
(M+H)+	398	96.77				

Compound Chromatograms (overlaid)

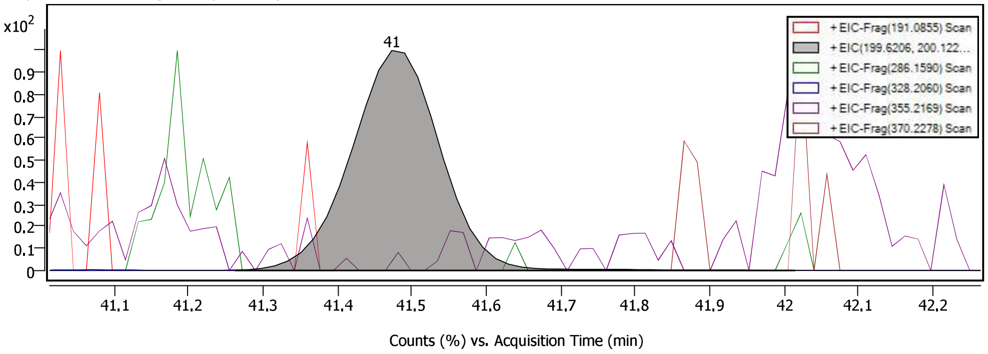

Structure

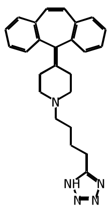

Coelution Plot

Compound Screening Report

Compound Spectra (overlaid)

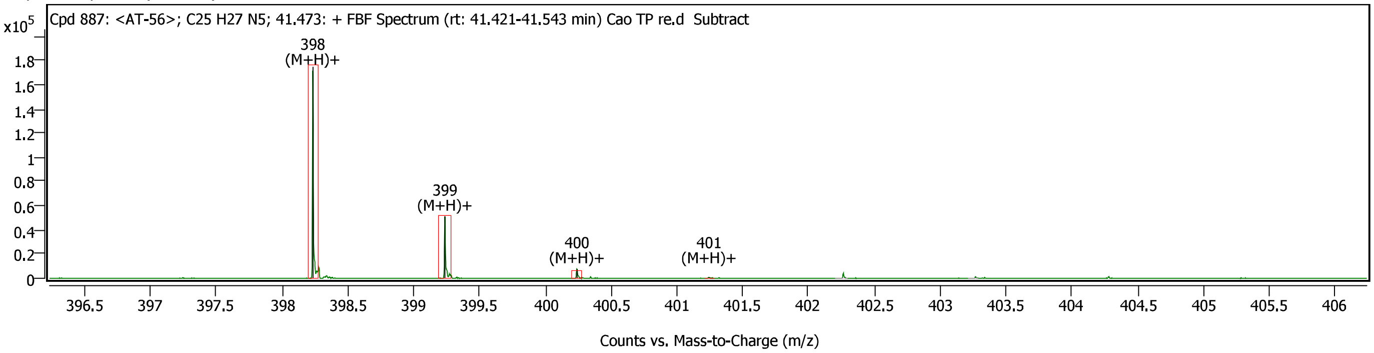

Fragment Spectrum (raw)

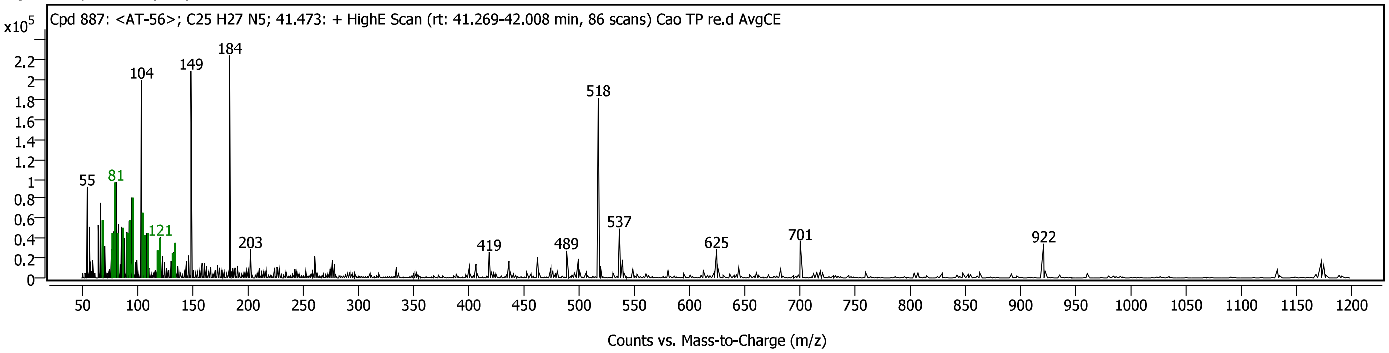

Compound ID Table

Name	Formula	Species	RT	RT Diff	Mass	CAS	ID Source	Score	Score (Lib)	Score (Tgt)
<AT-56>	C25 H27 N5	(M+H)+	41.473		397.2256	162640-98-4	FBF	96.77		96.77

Cpd 505: Dicyclohexyl phthalate

Name	Formula	RT	RI	Mass	Diff (Tgt, ppm)	CAS	ID Source	Score	Algorithm
Dicyclohexyl phthalate	C20 H26 O4	41.578		330.1834	0.95	84-61-7	FBF-FragConfirm	98.01	FBF

Species	m/z	Score (Tgt)	Score (Lib)	Score (DB)	Score (MFG)	Score (RT)
(M+H)+	331	98.01				

Compound Chromatograms (overlaid)

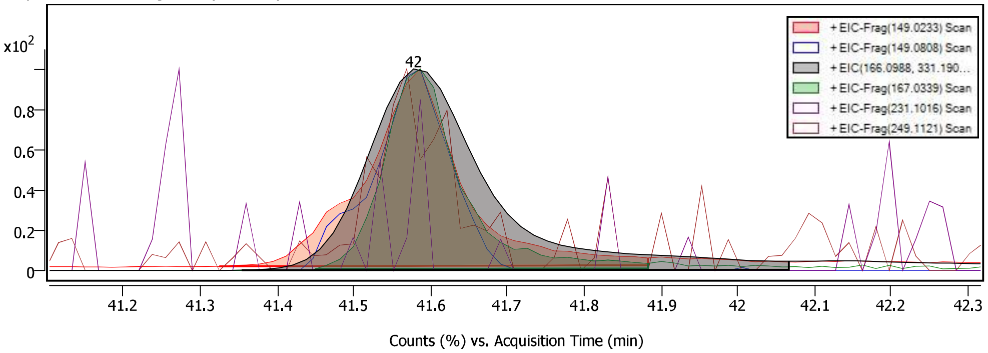

Structure

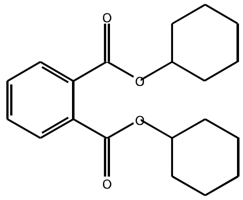

Coelution Plot

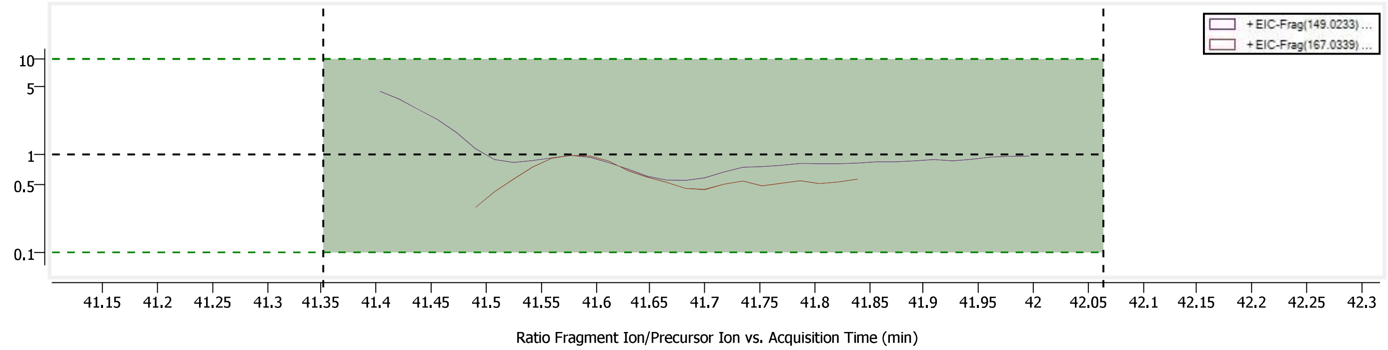

Compound Screening Report

Compound Spectra (overlaid)

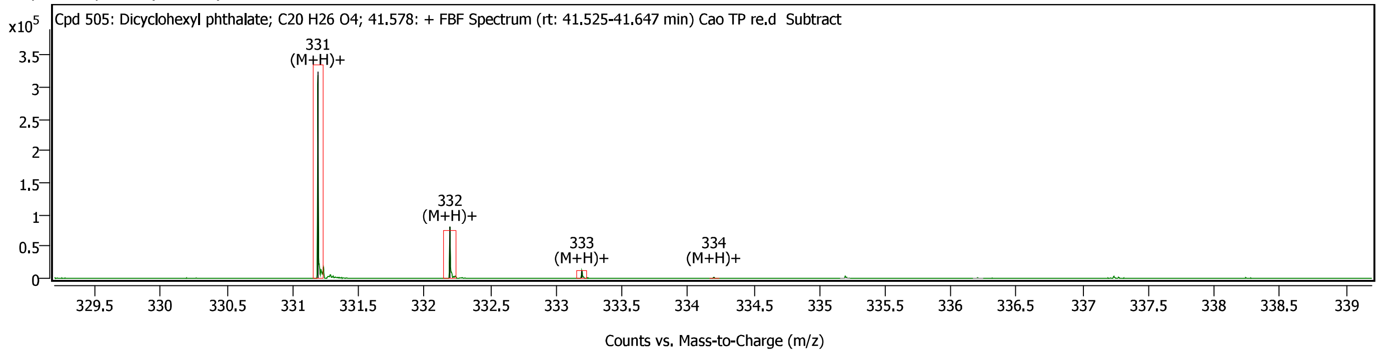

Fragment Spectrum (clean)

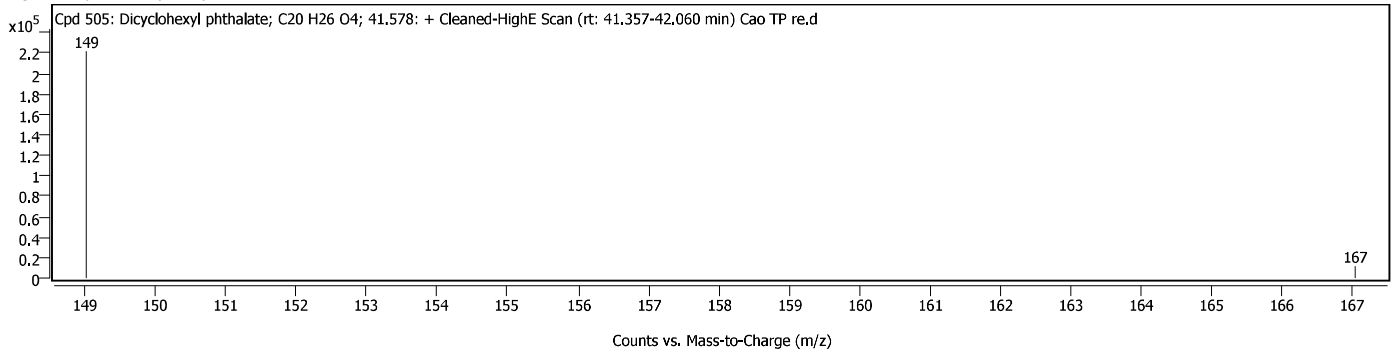

Fragment Spectrum (raw)

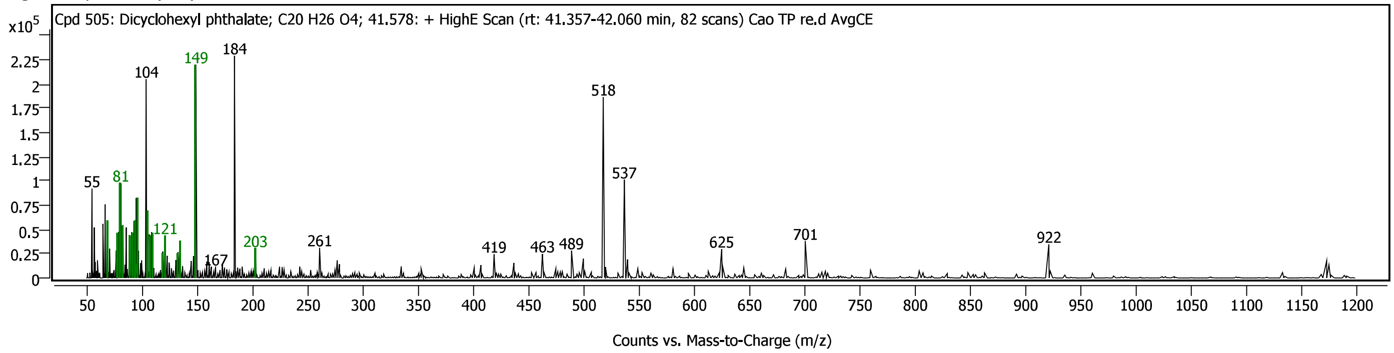

Compound ID Table

Name	Formula	Species	RT	RT Diff	Mass	CAS	ID Source	Score	Score (Lib)	Score (Tgt)
Dicyclohexyl phthalate	C20 H26 O4	(M+H)+	41.578		330.1834	84-61-7	FBF-FragConfirm	98.01		98.01
16-Hydroxy-4-oxoretinoic acid	C20 H26 O4	(M+H)+	41.578		330.1834	107257-23-8	FBF-FragConfirm	98.01		98.01
3,3',4,4'-Tetrahydroxy-5,5'-diisopropyl-2,2'-dimethylbiphenyl	C20 H26 O4	(M+H)+	41.578		330.1834	120901-51-1	FBF-FragConfirm	98.01		98.01
Carnosol	C20 H26 O4	(M+H)+	41.578		330.1834	5957-80-2	FBF-FragConfirm	98.01		98.01
4,4'-(2,3-Dimethyl-1,4-butanediyl)bis(2-methoxyphenol)	C20 H26 O4	(M+H)+	41.578		330.1834		FBF-FragConfirm	98.01		98.01
Gibberellin A15	C20 H26 O4	(M+H)+	41.578		330.1834		FBF-FragConfirm	98.01		98.01
Momilactone B	C20 H26 O4	(M+H)+	41.578		330.1834	51415-08-8	FBF-FragConfirm	98.01		98.01
Yucalexin P15	C20 H26 O4	(M+H)+	41.578		330.1834	119626-52-7	FBF-FragConfirm	98.01		98.01
Yucalexin P8	C20 H26 O4	(M+H)+	41.578		330.1834	119626-47-0	FBF-FragConfirm	98.01		98.01

Cpd 1239: Tacrolimus

Name	Formula	RT	RI	Mass	Diff (Tgt, ppm)	CAS	ID Source	Score	Algorithm
Tacrolimus	C44 H69 N O12	41.735		803.4818	-0.26	104987-11-3	FBF	98.80	FBF

Species	m/z	Score (Tgt)	Score (Lib)	Score (DB)	Score (MFG)	Score (RT)
(M+H)+	804	98.80				

Compound Chromatograms (overlaid)

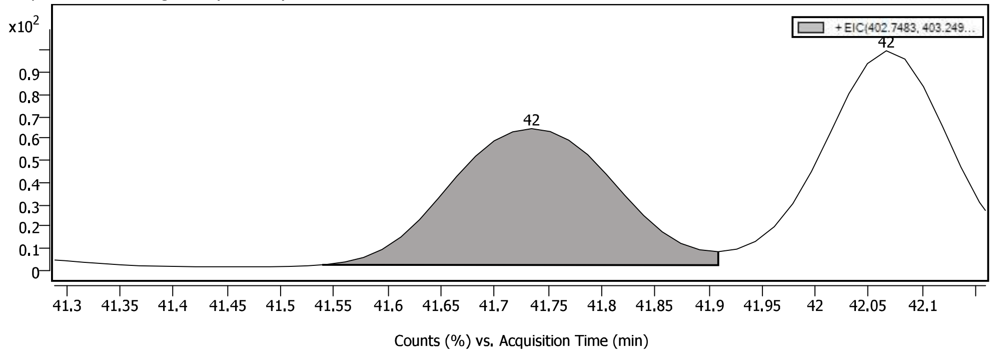

Structure

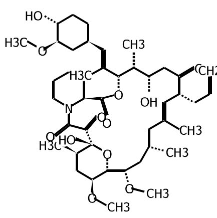

Compound Spectra (overlaid)

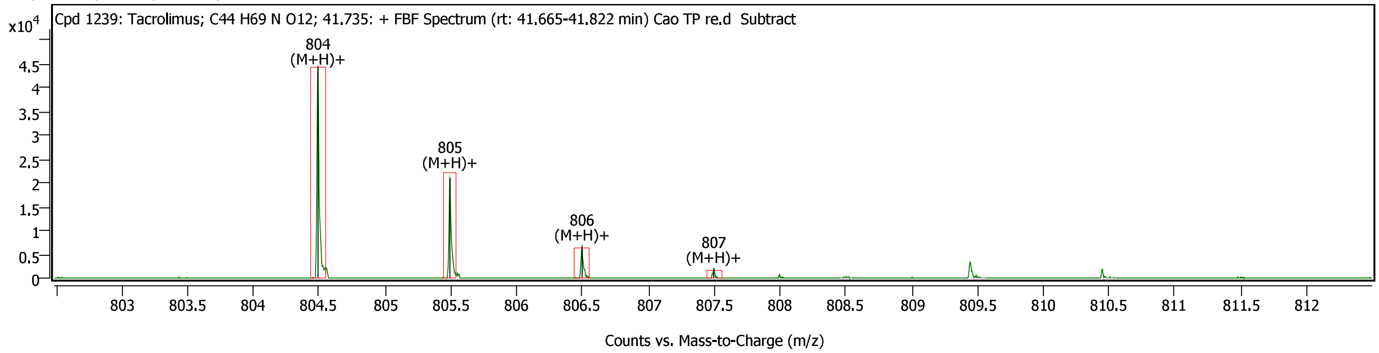

Compound ID Table

Name	Formula	Species	RT	RT Diff	Mass	CAS	ID Source	Score	Score (Lib)	Score (Tgt)
Tacrolimus	C44 H69 N O12	(M+H)+	41.735		803.4818	104987-11-3	FBF	98.80		98.80

Cpd 1505: Pipecuronium

Name	Formula	RT	RI	Mass	Diff (Tgt, ppm)	CAS	ID Source	Score	Algorithm
Pipecuronium	C35 H62 N4 O4	41.787		602.4760	-1.84	68399-58-6	FBF	98.07	FBF

Species	m/z	Score (Tgt)	Score (Lib)	Score (DB)	Score (MFG)	Score (RT)
(M+H)+	603	98.07				

Compound Chromatograms (overlaid)

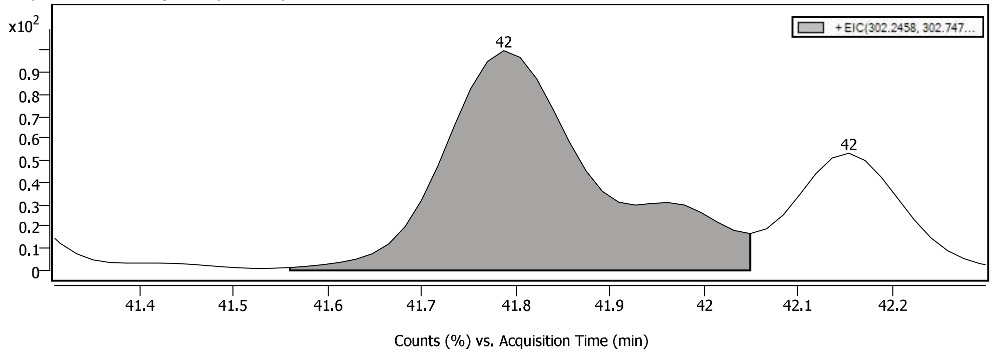

Structure

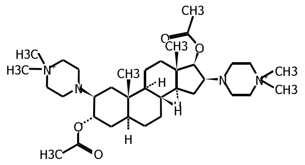

Compound Spectra (overlaid)

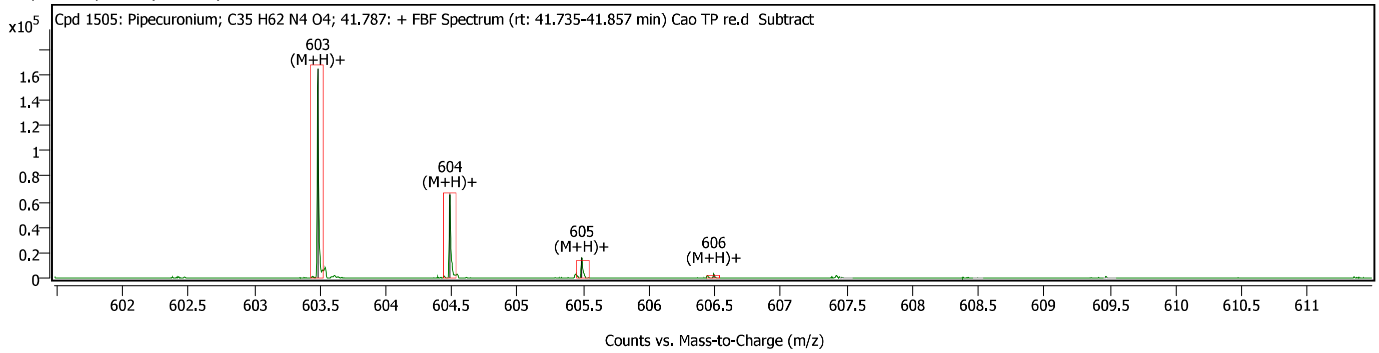

Compound ID Table

Name	Formula	Species	RT	RT Diff	Mass	CAS	ID Source	Score	Score (Lib)	Score (Tgt)
Pipecuronium	C35 H62 N4 O4	(M+H)+	41.787		602.4760	68399-58-6	FBF	98.07		98.07

Cpd 1320: Chalcomycin

Compound Screening Report

Name	Formula	RT	RI	Mass	Diff (Tgt, ppm)	CAS	ID Source	Score	Algorithm
Chalcomycin	C35 H56 O14	41.874		700.3648	-3.19	20283-48-1	FBF	95.25	FBF

Species	m/z	Score (Tgt)	Score (Lib)	Score (DB)	Score (MFG)	Score (RT)
(M+H)+	701	95.25				

Compound Chromatograms (overlaid)

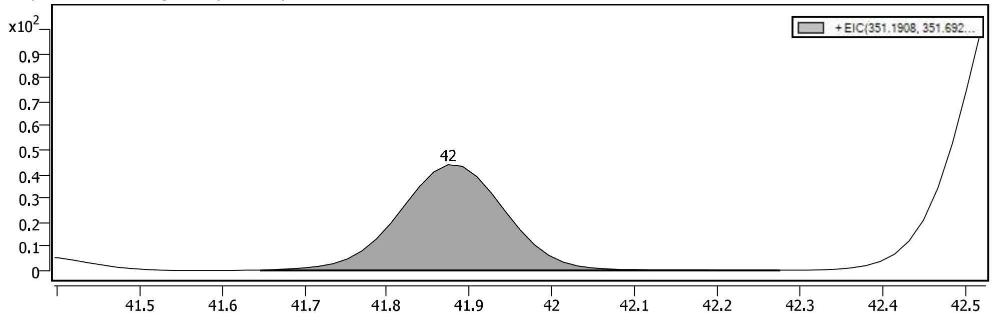

Structure

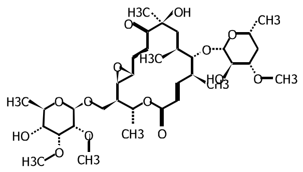

Compound Spectra (overlaid)

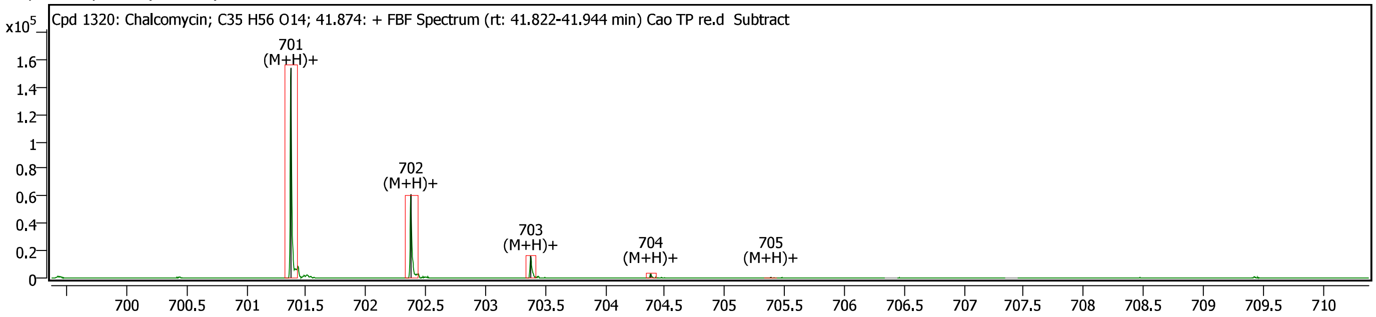

Compound ID Table

Name	Formula	Species	RT	RT Diff	Mass	CAS	ID Source	Score	Score (Lib)	Score (Tgt)
Chalcomycin	C35 H56 O14	(M+H)+	41.874		700.3648	20283-48-1	FBF	95.25		95.25

Cpd 1388: Bergamottin

Name	Formula	RT	RI	Mass	Diff (Tgt, ppm)	CAS	ID Source	Score	Algorithm
Bergamottin	C21 H22 O4	41.874		338.1522	1.14	7380-40-7	M-FBF-FragConfirm	98.95	FBF

Species	m/z	Score (Tgt)	Score (Lib)	Score (DB)	Score (MFG)	Score (RT)
(M+H)+	339	98.95				

Compound Chromatograms (overlaid)

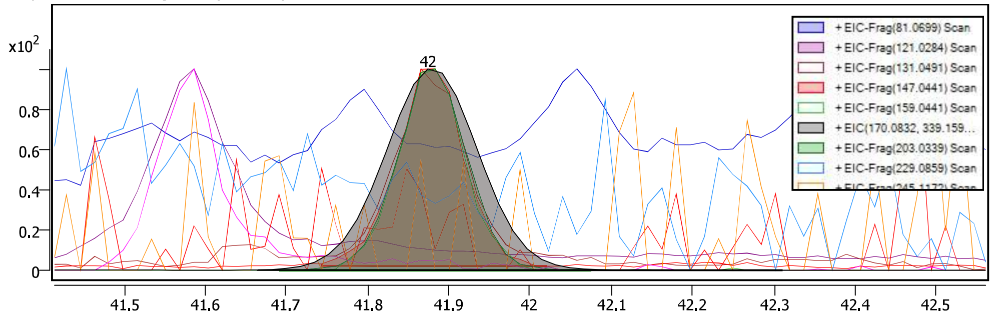

Structure

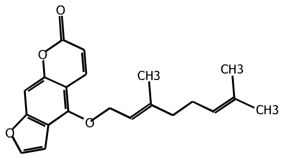

Coelution Plot

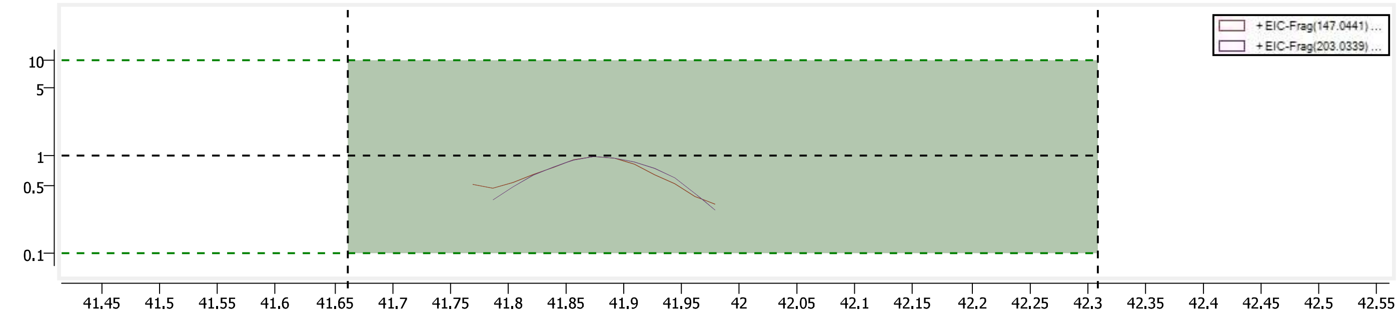

Compound Spectra (overlaid)

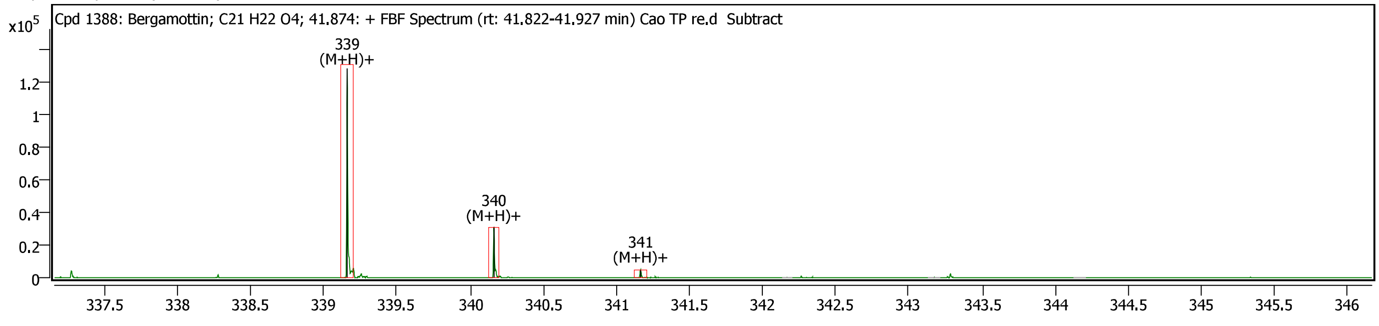

Fragment Spectrum (clean)

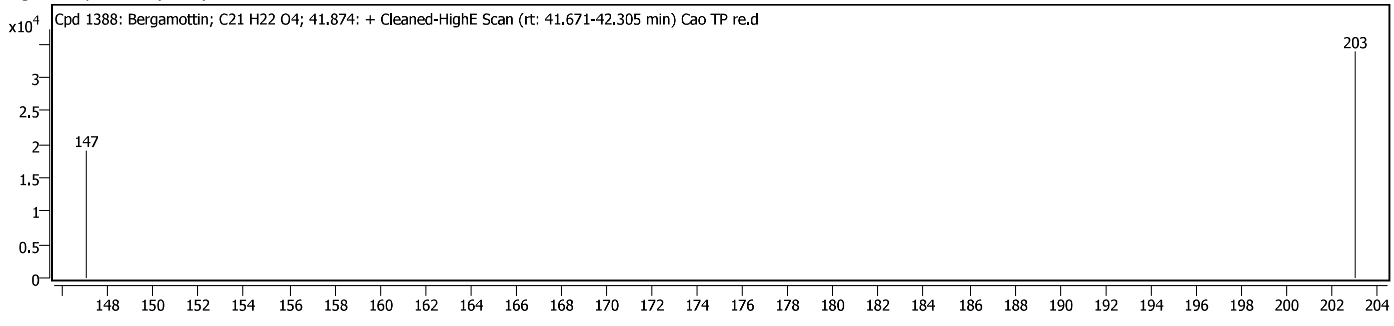

Fragment Spectrum (raw)

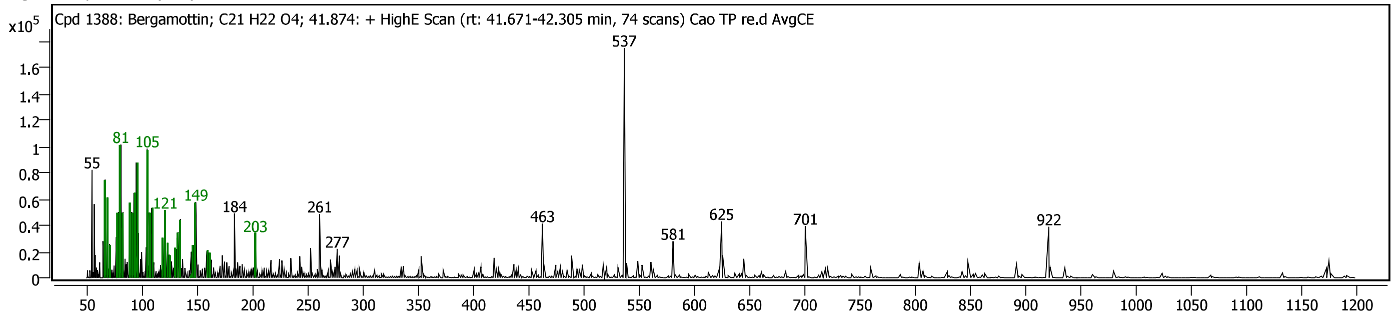

Compound Screening Report

Compound ID Table

Name	Formula	Species	RT	RT Diff	Mass	CAS	ID Source	Score	Score (Lib)	Score (Tgt)
Bergamottin	C21 H22 O4	(M+H)+	41.874		338.1522	7380-40-7	FBF-FragConfirm	98.95		98.95
3-hydroxy-5-methoxy-6-prenylstilbene-2-carboxylic acid	C21 H22 O4	(M+H)+	41.874		338.1522		FBF-FragConfirm	98.95		98.95
Crotaramin	C21 H22 O4	(M+H)+	41.874		338.1522		FBF-FragConfirm	98.95		98.95
7-O-Prenylcryptostrobin	C21 H22 O4	(M+H)+	41.874		338.1522		FBF-FragConfirm	98.95		98.95
6-C-Prenyl-8-C-methylpinocembrin	C21 H22 O4	(M+H)+	41.874		338.1522		FBF-FragConfirm	98.95		98.95
5-Methoxy-7-prenyloxyflavanone	C21 H22 O4	(M+H)+	41.874		338.1522		FBF-FragConfirm	98.95		98.95
5-Hydroxy-7-methoxy-6-C-prenylflavanone	C21 H22 O4	(M+H)+	41.874		338.1522		FBF-FragConfirm	98.95		98.95
5,7-Dihydroxy-6-methyl-8-prenylflavanone	C21 H22 O4	(M+H)+	41.874		338.1522		FBF-FragConfirm	98.95		98.95
4'-Hydroxyisoderricin	C21 H22 O4	(M+H)+	41.874		338.1522		FBF-FragConfirm	98.95		98.95
4-Hydroxyderricin	C21 H22 O4	(M+H)+	41.874		338.1522		FBF-FragConfirm	98.95		98.95
2-Hydroxy-4-methoxy-3-(3-methyl-2-butenyl)-6-(2-phenylethenyl)benzoic acid	C21 H22 O4	(M+H)+	41.874		338.1522	87402-84-4	FBF-FragConfirm	98.95		98.95
2'-O-Methylphaseollinisoflavan	C21 H22 O4	(M+H)+	41.874		338.1522		FBF-FragConfirm	98.95		98.95
2'-O-Methylglabridin	C21 H22 O4	(M+H)+	41.874		338.1522		FBF-FragConfirm	98.95		98.95
2'-Hydroxy-6'-methoxy-4'-prenyloxychalcone	C21 H22 O4	(M+H)+	41.874		338.1522		FBF-FragConfirm	98.95		98.95
2',4'-Dihydroxy-6'-methoxy-3'-prenylchalcone	C21 H22 O4	(M+H)+	41.874		338.1522		FBF-FragConfirm	98.95		98.95
2,2,4-Trimethyl-3-(4-methoxyphenyl)-2H-1-benzopyran-7-ol acetate	C21 H22 O4	(M+H)+	41.874		338.1522		FBF-FragConfirm	98.95		98.95
9-[(3,7-Dimethyl-2,6-octadienyl)oxy]-7H-furo[3,2-g][1]benzopyran-7-one	C21 H22 O4	(M+H)+	41.874		338.1522	7437-55-0	FBF-FragConfirm	98.95		98.95
4'-O-Methylglabridin	C21 H22 O4	(M+H)+	41.874		338.1522		FBF-FragConfirm	98.95		98.95
Archangelin	C21 H22 O4	(M+H)+	41.874		338.1522	21174-75-4	FBF-FragConfirm	98.95		98.95
Hildgardtol A	C21 H22 O4	(M+H)+	41.874		338.1522		FBF-FragConfirm	98.95		98.95
Bavachalcone	C21 H22 O4	(M+H)+	41.874		338.1522		FBF-FragConfirm	98.95		98.95
Sandwicensin	C21 H22 O4	(M+H)+	41.874		338.1522		FBF-FragConfirm	98.95		98.95
Licochalcone C	C21 H22 O4	(M+H)+	41.874		338.1522		FBF-FragConfirm	98.95		98.95
Licochalcone A	C21 H22 O4	(M+H)+	41.874		338.1522	58749-22-7	FBF-FragConfirm	98.95		98.95
Licoagrocarpin	C21 H22 O4	(M+H)+	41.874		338.1522	202815-29-0	FBF-FragConfirm	98.95		98.95
Hildgardtol B	C21 H22 O4	(M+H)+	41.874		338.1522		FBF-FragConfirm	98.95		98.95
Isoxanthohumol(Helichrysum)	C21 H22 O4	(M+H)+	41.874		338.1522		FBF-FragConfirm	98.95		98.95
Gancaonin X	C21 H22 O4	(M+H)+	41.874		338.1522	160825-65-0	FBF-FragConfirm	98.95		98.95
Falciformin	C21 H22 O4	(M+H)+	41.874		338.1522		FBF-FragConfirm	98.95		98.95
Tephrinone	C21 H22 O4	(M+H)+	41.874		338.1522		FBF-FragConfirm	98.95		98.95
Bavachinin	C21 H22 O4	(M+H)+	41.874		338.1522		FBF-FragConfirm	98.95		98.95

Cpd 1271: Dequalinium

Name	Formula	RT	RI	Mass	Diff (Tgt, ppm)	CAS	ID Source	Score	Algorithm
Dequalinium	C30 H40 N4	42.031		456.3235	-3.85	522-51-0	FBF	86.99	FBF

Species	m/z	Score (Tgt)	Score (Lib)	Score (DB)	Score (MFG)	Score (RT)
(M+H)+	457	86.99				

Compound Chromatograms (overlaid)

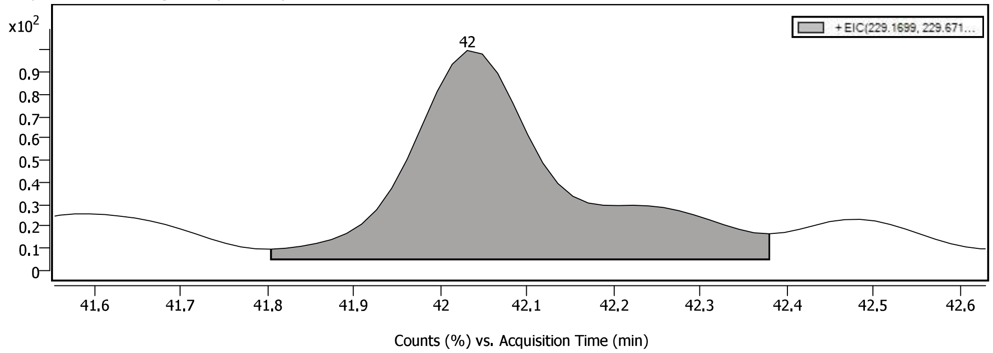

Structure

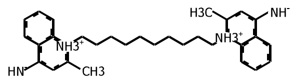

Compound Spectra (overlaid)

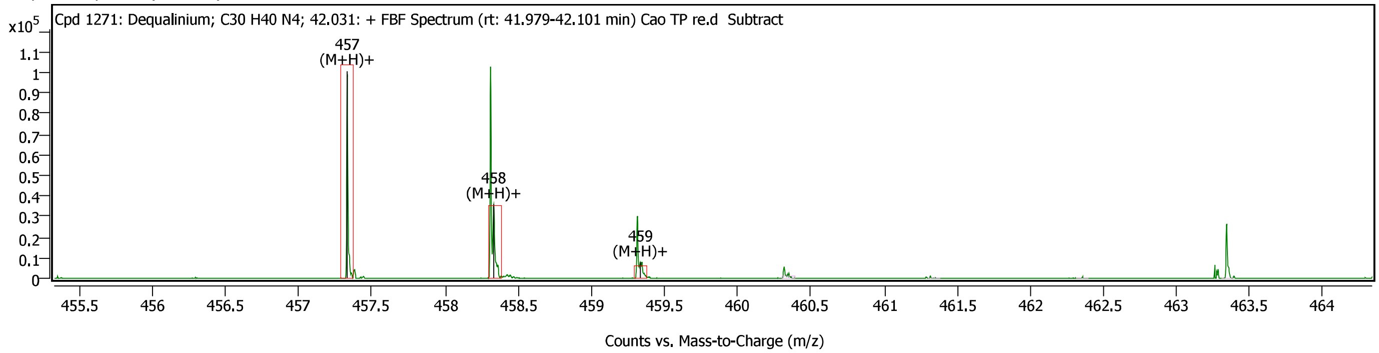

Compound ID Table

Name	Formula	Species	RT	RT Diff	Mass	CAS	ID Source	Score	Score (Lib)	Score (Tgt)
Dequalinium	C30 H40 N4	(M+H)+	42.031		456.3235	522-51-0	FBF	86.99		86.99

Cpd 1238: Tacrolimus

Compound Screening Report

Name	Formula	RT	RI	Mass	Diff (Tgt, ppm)	CAS	ID Source	Score	Algorithm
Tacrolimus	C44 H69 N O12	42.066		803.4819	-0.10	104987-11-3	FBF	99.75	FBF

Species	m/z	Score (Tgt)	Score (Lib)	Score (DB)	Score (MFG)	Score (RT)
(M+H)+	804	99.75				

Compound Chromatograms (overlaid)

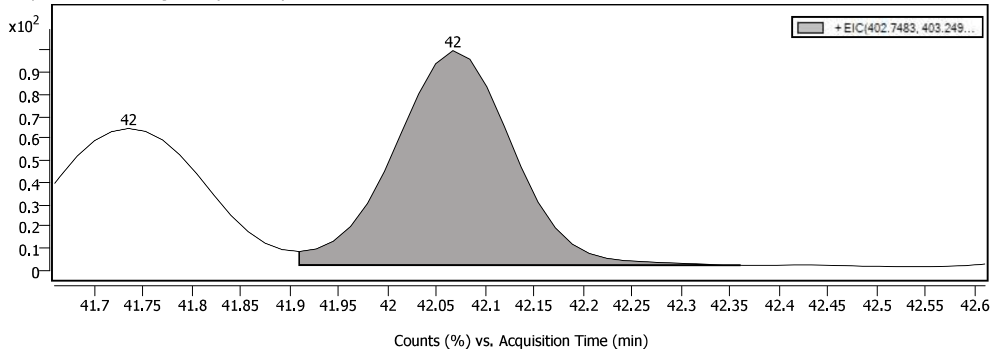

Structure

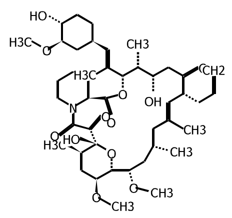

Compound Spectra (overlaid)

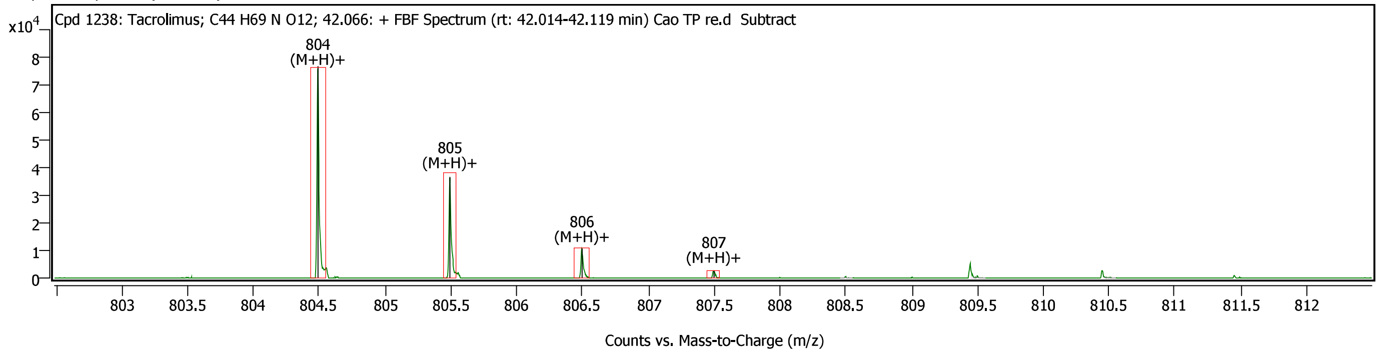

Compound ID Table

Name	Formula	Species	RT	RT Diff	Mass	CAS	ID Source	Score	Score (Lib)	Score (Tgt)
Tacrolimus	C44 H69 N O12	(M+H)+	42.066		803.4819	104987-11-3	FBF	99.75		99.75

Cpd 1703: rhodexin A

Name	Formula	RT	RI	Mass	Diff (Tgt, ppm)	CAS	ID Source	Score	Algorithm
rhodexin A	C29 H44 O9	42.066		536.2968	-3.18		M-FBF	94.41	FBF

Species	m/z	Score (Tgt)	Score (Lib)	Score (DB)	Score (MFG)	Score (RT)
(M+H)+	537	94.41				

Compound Chromatograms (overlaid)

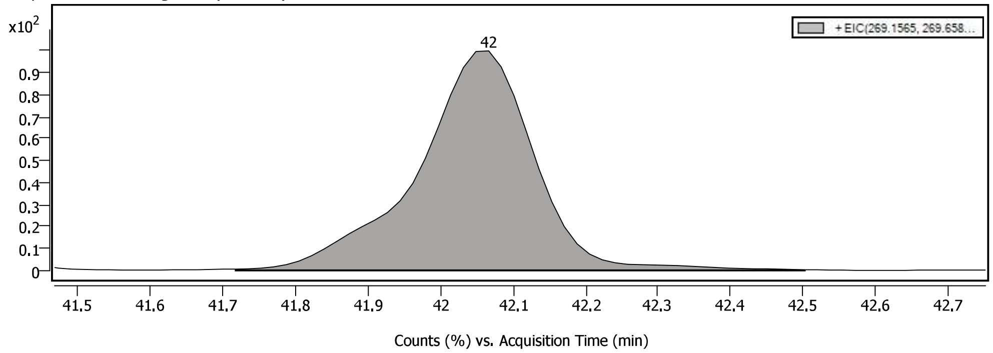

Structure

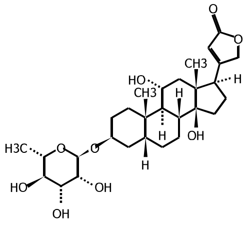

Compound Screening Report

Compound Spectra (overlaid)

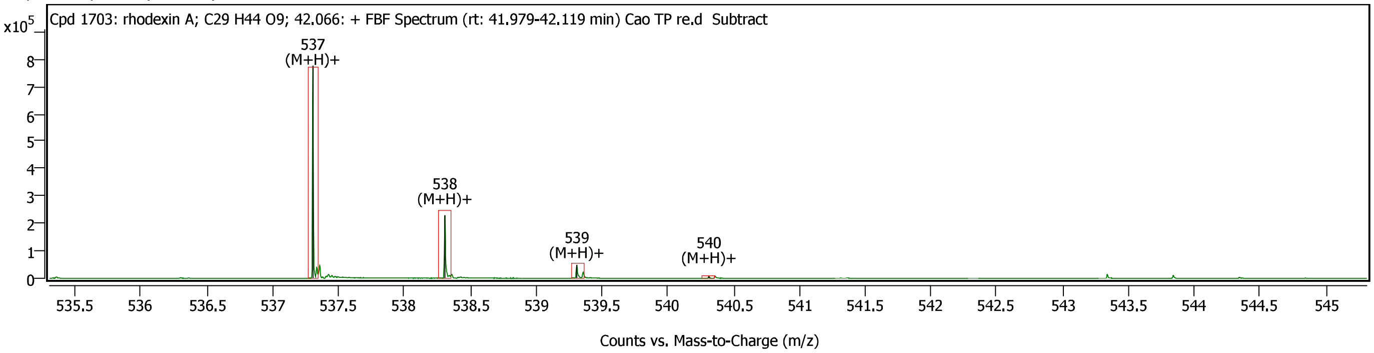

Compound ID Table

Name	Formula	Species	RT	RT Diff	Mass	CAS	ID Source	Score	Score (Lib)	Score (Tgt)
rhodexin A	C29 H44 O9	(M+H)+	42.066		536.2968		FBF	94.41		94.41
Corchorosol A	C29 H44 O9	(M+H)+	42.066		536.2968	23838-13-3	FBF	94.41		94.41
Coroglaucigenin-3-o-alpha-L-rhamnopyranoside	C29 H44 O9	(M+H)+	42.066		536.2968		FBF	94.41		94.41

Cpd 4: α-Linolenoyl Ethanolamide

Name	Formula	RT	RI	Mass	Diff (Tgt, ppm)	CAS	ID Source	Score	Algorithm
α-Linolenoyl Ethanolamide	C20 H35 N O2	42.363		321.2677	2.95	57086-93-8	FBF-FragConfirm	94.08	FBF

Species	m/z	Score (Tgt)	Score (Lib)	Score (DB)	Score (MFG)	Score (RT)
(M+H)+	322	94.08				

Compound Chromatograms (overlaid)

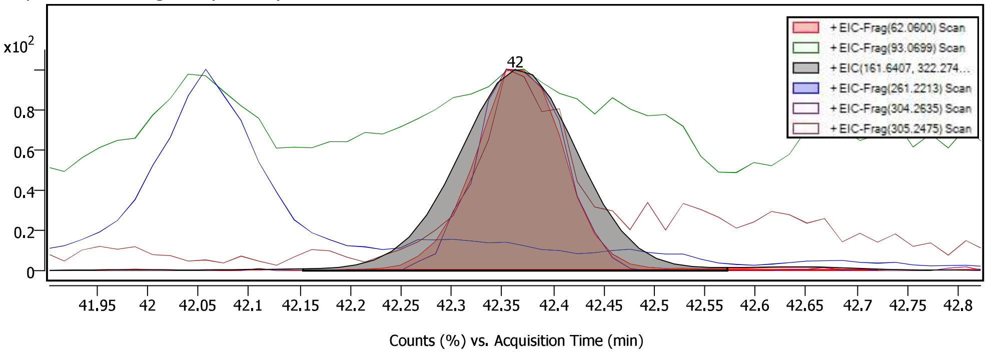

Structure

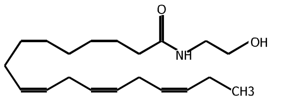

Coelution Plot

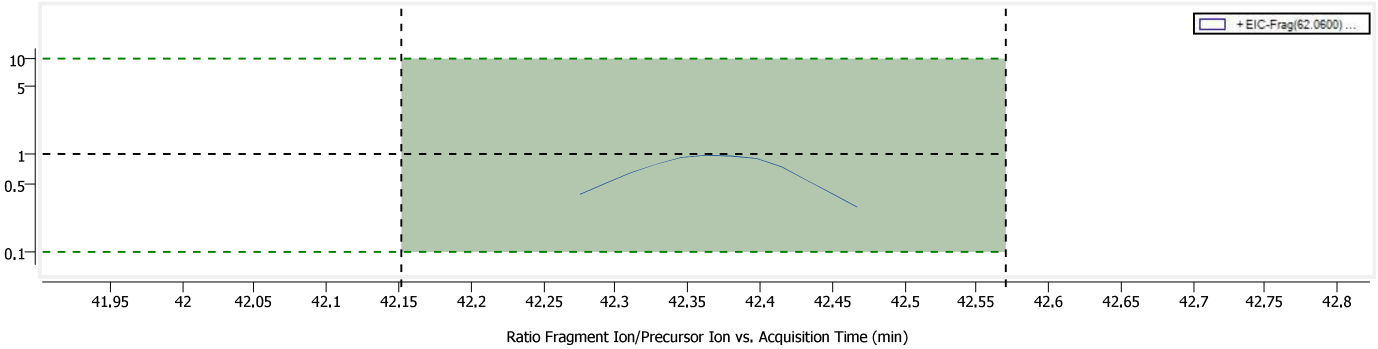

Compound Spectra (overlaid)

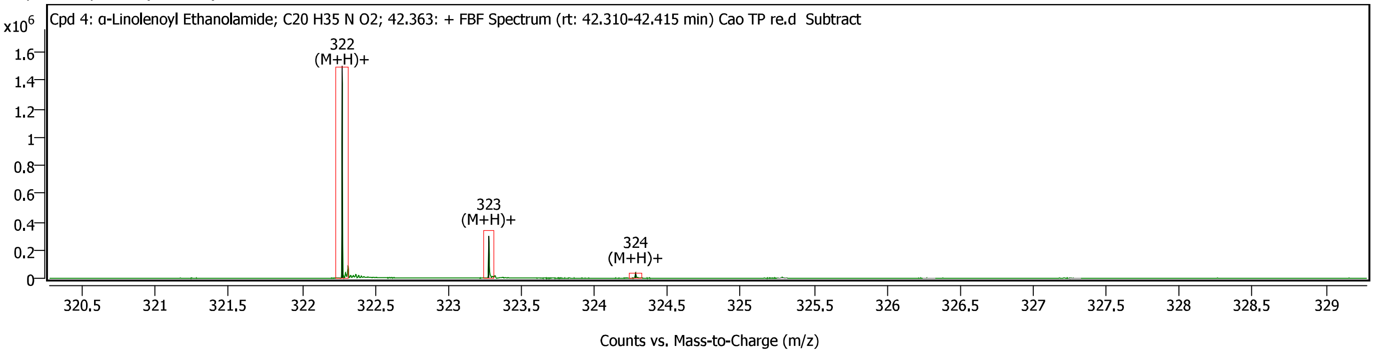

Fragment Spectrum (clean)

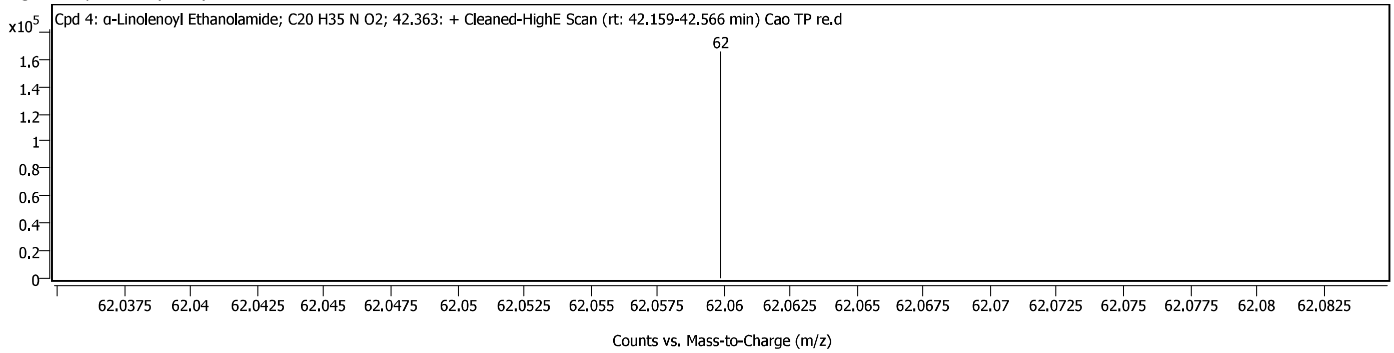

Fragment Spectrum (raw)

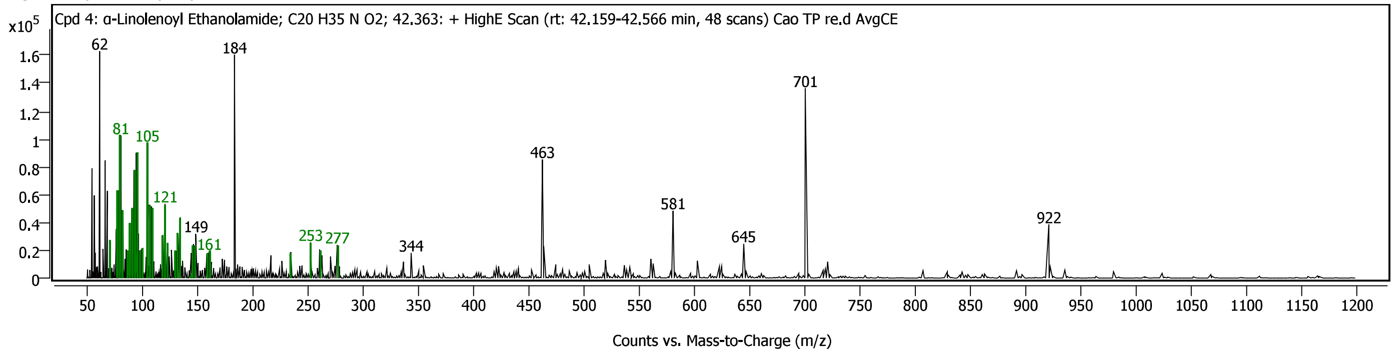

Compound ID Table

Name	Formula	Species	RT	RT Diff	Mass	CAS	ID Source	Score	Score (Lib)	Score (Tgt)
α-Linolenoyl Ethanolamide	C20 H35 N O2	(M+H)+	42.363		321.2677	57086-93-8	FBF-FragConfirm	94.08		94.08
(1S,Z)-3-((2R,E)-6-Hydroxy-2,5-dimethyloct-4-en-1-ylidene)-1-methyloctahydro-2H-quinolizin-1-ol	C20 H35 N O2	(M+H)+	42.363		321.2677		FBF-FragConfirm	94.08		94.08
(1S,Z)-3-((2R,E)-7-Hydroxy-2,5-dimethyloct-4-en-1-ylidene)-1-methyloctahydro-2H-quinolizin-1-ol	C20 H35 N O2	(M+H)+	42.363		321.2677		FBF-FragConfirm	94.08		94.08
(2R)-1-((1S,Z)-1-Hydroxy-1-methylhexahydro-2H-quinolizin-3(4H)-ylidene)-2,5-dimethyloctan-4-one	C20 H35 N O2	(M+H)+	42.363		321.2677		FBF-FragConfirm	94.08		94.08
Anandamide (18:3, n-6)	C20 H35 N O2	(M+H)+	42.363		321.2677		FBF-FragConfirm	94.08		94.08

Cpd 182: Ganoderic acid Mg

Name	Formula	RT	RI	Mass	Diff (Tgt, ppm)	CAS	ID Source	Score	Algorithm
Ganoderic acid Mg	C35 H54 O8	42.450		602.3820	0.26	110042-11-0	M-FBF	99.63	FBF

Species	m/z	Score (Tgt)	Score (Lib)	Score (DB)	Score (MFG)	Score (RT)
(M+H)+	603	99.63				

Compound Chromatograms (overlaid)

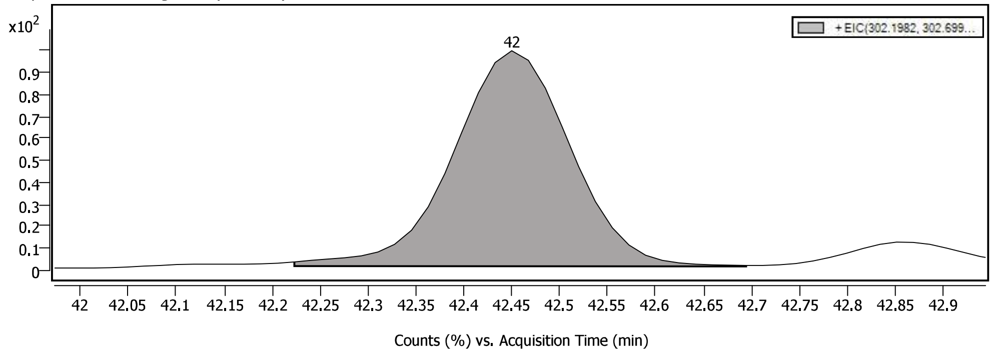

Structure

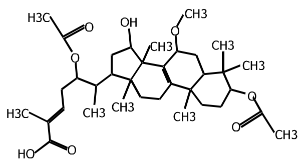

Compound Spectra (overlaid)

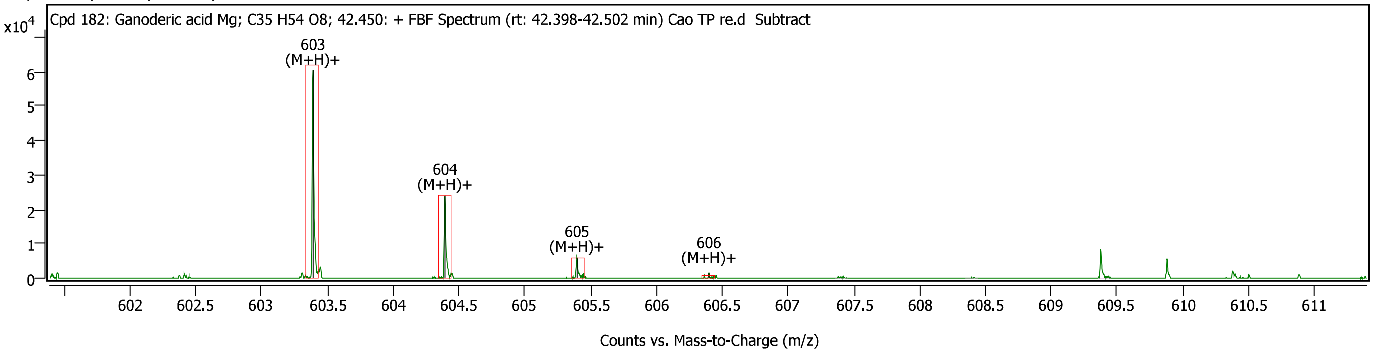

Compound ID Table

Name	Formula	Species	RT	RT Diff	Mass	CAS	ID Source	Score	Score (Lib)	Score (Tgt)
Ganoderic acid Mg	C35 H54 O8	(M+H)+	42.450		602.3820	110042-11-0	FBF	99.63		99.63
Tetronasin	C35 H54 O8	(M+H)+	42.450		602.3820	75139-06-9	FBF	99.63		99.63

Cpd 1011: 4-Ketonostoxanthin 3-sulfate

Name	Formula	RT	RI	Mass	Diff (Tgt, ppm)	CAS	ID Source	Score	Algorithm
4-Ketonostoxanthin 3-sulfate	C40 H53 Na O8 S	42.572		716.3384	3.51		FBF	89.78	FBF

Species	m/z	Score (Tgt)	Score (Lib)	Score (DB)	Score (MFG)	Score (RT)
(M+H)+	717	89.78				

Compound Chromatograms (overlaid)

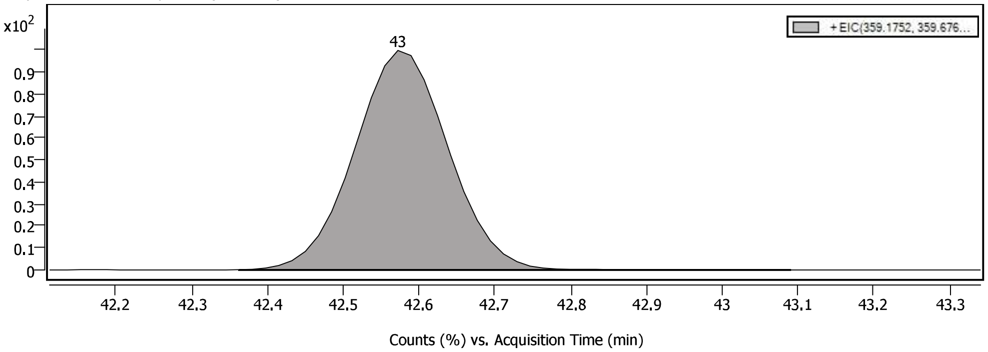

Structure

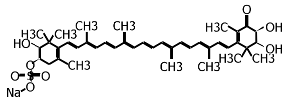

Compound Spectra (overlaid)

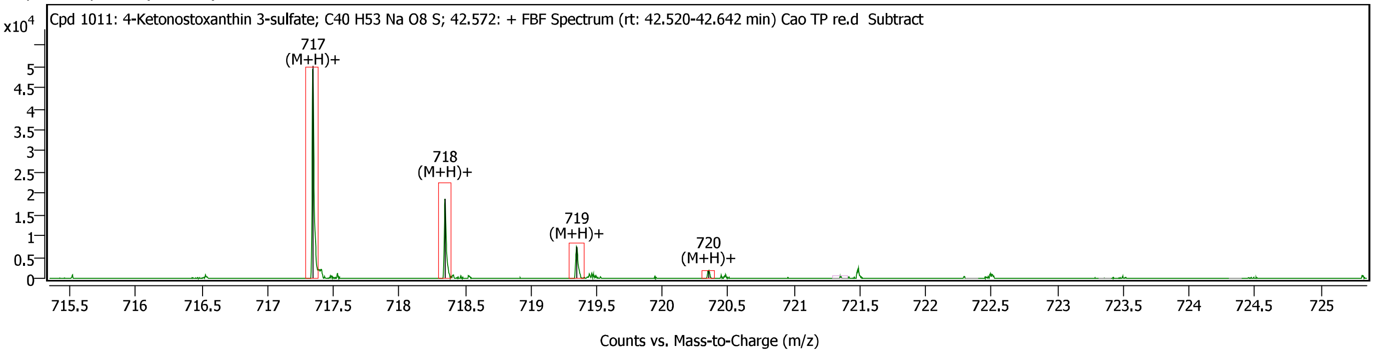

Compound ID Table

Name	Formula	Species	RT	RT Diff	Mass	CAS	ID Source	Score	Score (Lib)	Score (Tgt)
4-Ketonostoxanthin 3-sulfate	C40 H53 Na O8 S	(M+H)+	42.572		716.3384		FBF	89.78		89.78

Cpd 1319: Chalcomycin

Name	Formula	RT	RI	Mass	Diff (Tgt, ppm)	CAS	ID Source	Score	Algorithm
Chalcomycin	C35 H56 O14	42.572		700.3652	-2.62	20283-48-1	FBF	95.86	FBF

Species	m/z	Score (Tgt)	Score (Lib)	Score (DB)	Score (MFG)	Score (RT)
(M+H)+	701	95.86				

Compound Screening Report

Compound Chromatograms (overlaid)

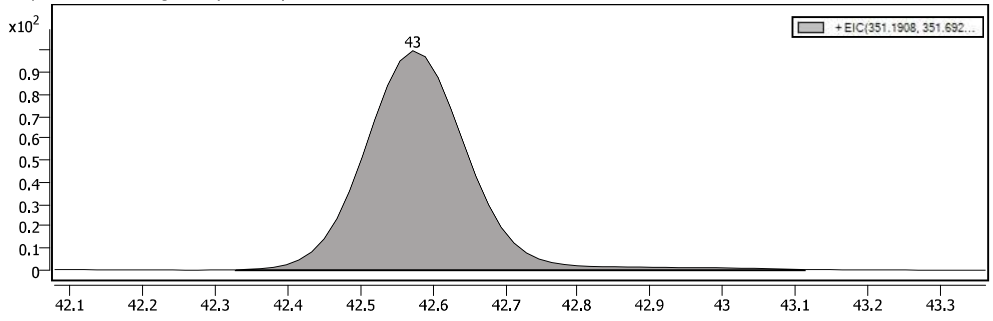

Structure

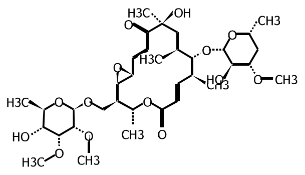

Compound Spectra (overlaid)

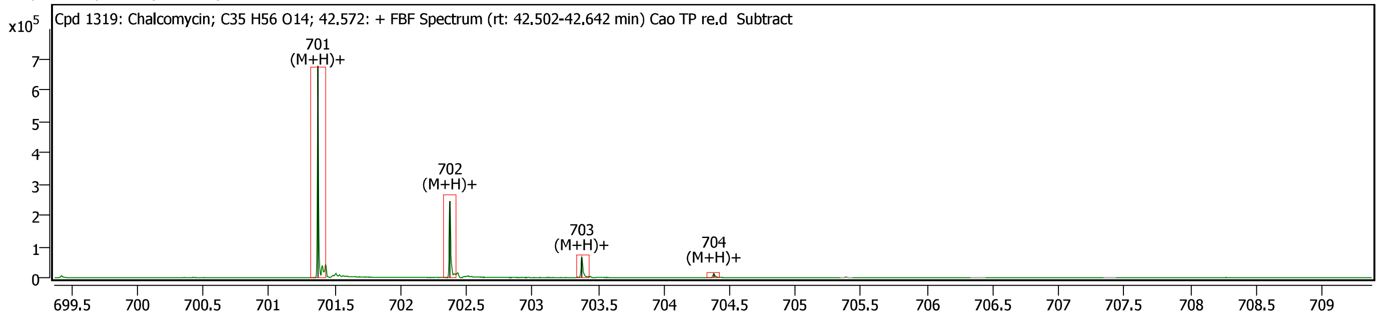

Compound ID Table

Name	Formula	Species	RT	RT Diff	Mass	CAS	ID Source	Score	Score (Lib)	Score (Tgt)
Chalcomycin	C35 H56 O14	(M+H)+	42.572		700.3652	20283-48-1	FBF	95.86		95.86

Cpd 1425: 5,7-Dihydroxy-4-methylcoumarin

Name	Formula	RT	RI	Mass	Diff (Tgt, ppm)	CAS	ID Source	Score	Algorithm
5,7-Dihydroxy-4-methylcoumarin	C10 H8 O4	42.642		192.0424	0.75	2107-76-8	M-FBF-FragConfirm	99.36	FBF

Species	m/z	Score (Tgt)	Score (Lib)	Score (DB)	Score (MFG)	Score (RT)
(M+H)+	193	99.36				

Compound Chromatograms (overlaid)

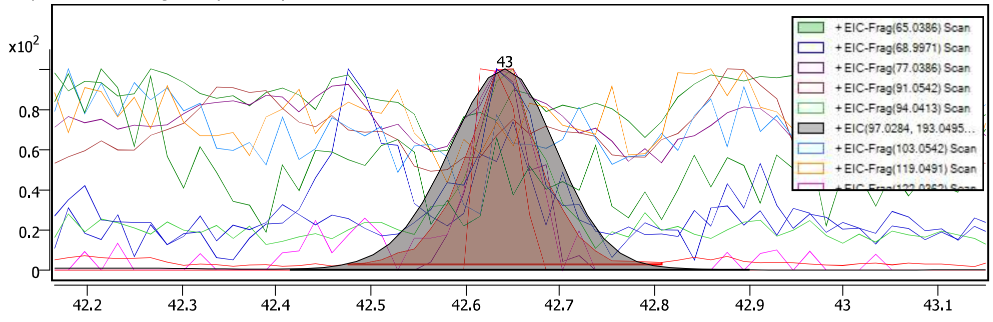

Structure

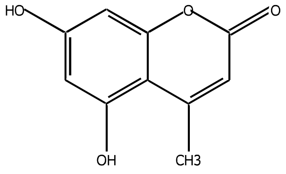

Coelution Plot

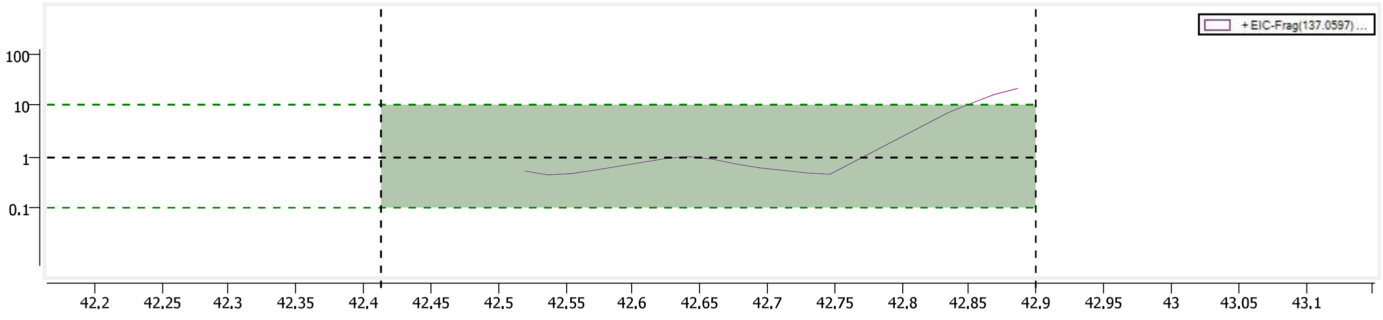

Compound Spectra (overlaid)

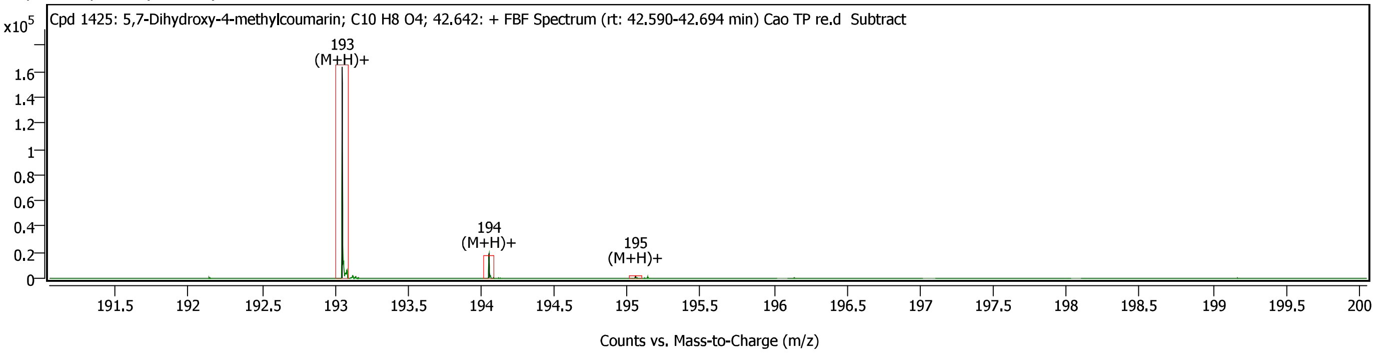

Fragment Spectrum (clean)

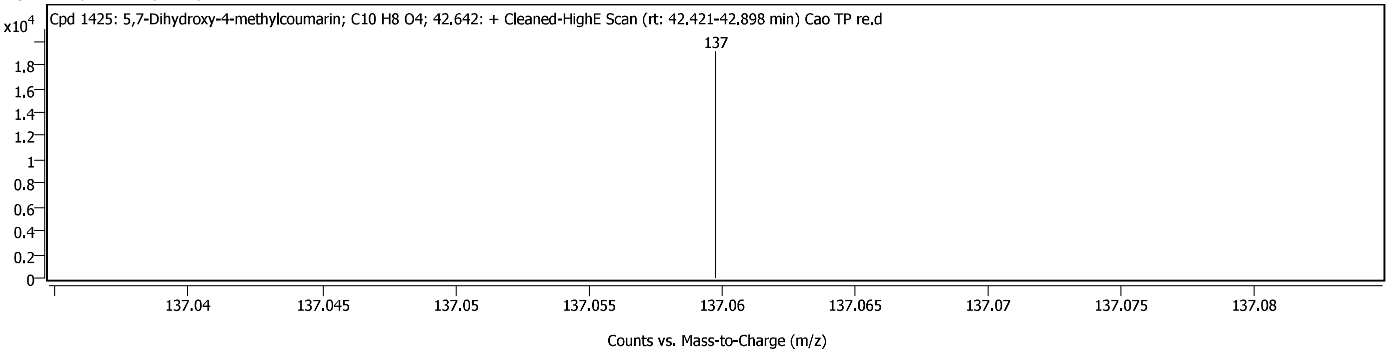

Fragment Spectrum (raw)

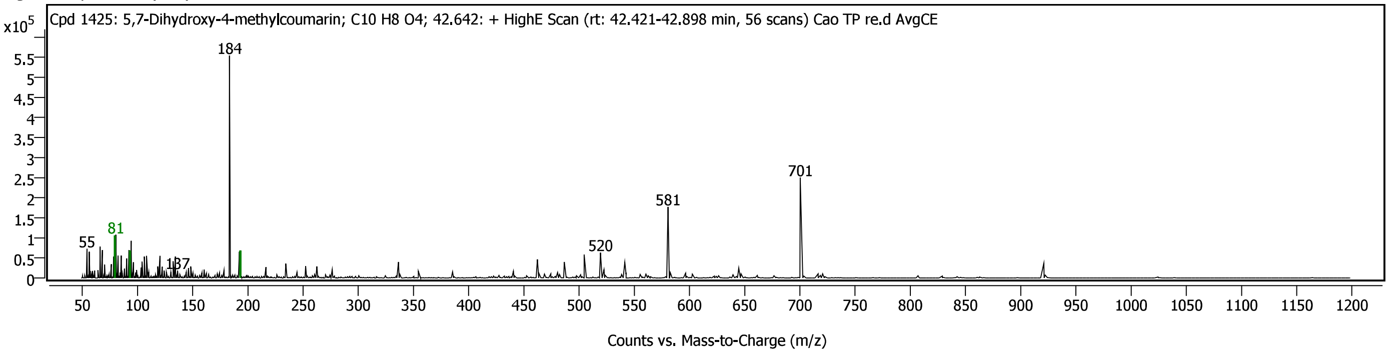

Compound ID Table

Name	Formula	Species	RT	RT Diff	Mass	CAS	ID Source	Score	Score (Lib)	Score (Tgt)
5,7-Dihydroxy-4-methylcoumarin	C10 H8 O4	(M+H)+	42.642		192.0424	2107-76-8	FBF-FragConfirm	99.36		99.36
trans-O-Hydroxybenzylidenepyruvate	C10 H8 O4	(M+H)+	42.642		192.0424		FBF-FragConfirm	99.36		99.36
4-Methylesculetin	C10 H8 O4	(M+H)+	42.642		192.0424	529-84-0	FBF-FragConfirm	99.36		99.36
1,3,6,8-Naphthalenetetrol	C10 H8 O4	(M+H)+	42.642		192.0424	18512-30-6	FBF-FragConfirm	99.36		99.36
2,10-dihydroxy-4,6,8-decatriynoic acid	C10 H8 O4	(M+H)+	42.642		192.0424		FBF-FragConfirm	99.36		99.36
2-Hydroxychromene-2-carboxylate	C10 H8 O4	(M+H)+	42.642		192.0424		FBF-FragConfirm	99.36		99.36
3,4-Dehydro-6-hydroxymellein	C10 H8 O4	(M+H)+	42.642		192.0424		FBF-FragConfirm	99.36		99.36
4,6,8-Decatriynoic acid, 2,10-dihydroxy-	C10 H8 O4	(M+H)+	42.642		192.0424		FBF-FragConfirm	99.36		99.36
4-Methyldaphnetin	C10 H8 O4	(M+H)+	42.642		192.0424	2107-77-9	FBF-FragConfirm	99.36		99.36
8-Hydroxy-7-methoxy-2H-1-benzopyran-2-one	C10 H8 O4	(M+H)+	42.642		192.0424	19492-03-6	FBF-FragConfirm	99.36		99.36
Acamelin	C10 H8 O4	(M+H)+	42.642		192.0424	74161-27-6	FBF-FragConfirm	99.36		99.36
Anemonin	C10 H8 O4	(M+H)+	42.642		192.0424	508-44-1	FBF-FragConfirm	99.36		99.36
Isoscopoletin	C10 H8 O4	(M+H)+	42.642		192.0424	776-86-3	FBF-FragConfirm	99.36		99.36
Methylenedioxycinnamic acid	C10 H8 O4	(M+H)+	42.642		192.0424		FBF-FragConfirm	99.36		99.36
Naphthazarin	C10 H8 O4	(M+H)+	42.642		192.0424	475-38-7	FBF-FragConfirm	99.36		99.36
Scopoletin	C10 H8 O4	(M+H)+	42.642		192.0424	92-61-5	FBF-FragConfirm	99.36		99.36

Cpd 925: Actinonin

Name	Formula	RT	RI	Mass	Diff (Tgt, ppm)	CAS	ID Source	Score	Algorithm
Actinonin	C19 H35 N3 O5	42.677		385.2594	4.55	13434-13-4	FBF	89.89	FBF

Species	m/z	Score (Tgt)	Score (Lib)	Score (DB)	Score (MFG)	Score (RT)
(M+H)+	386	89.89				

Compound Chromatograms (overlaid)

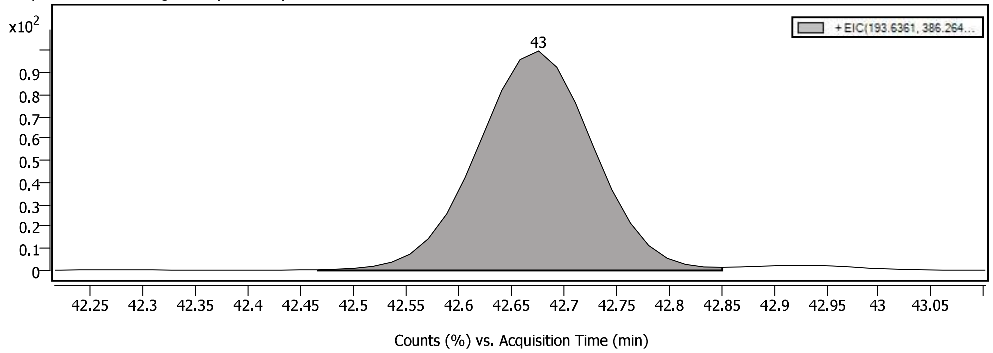

Structure

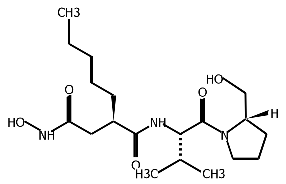

Compound Spectra (overlaid)

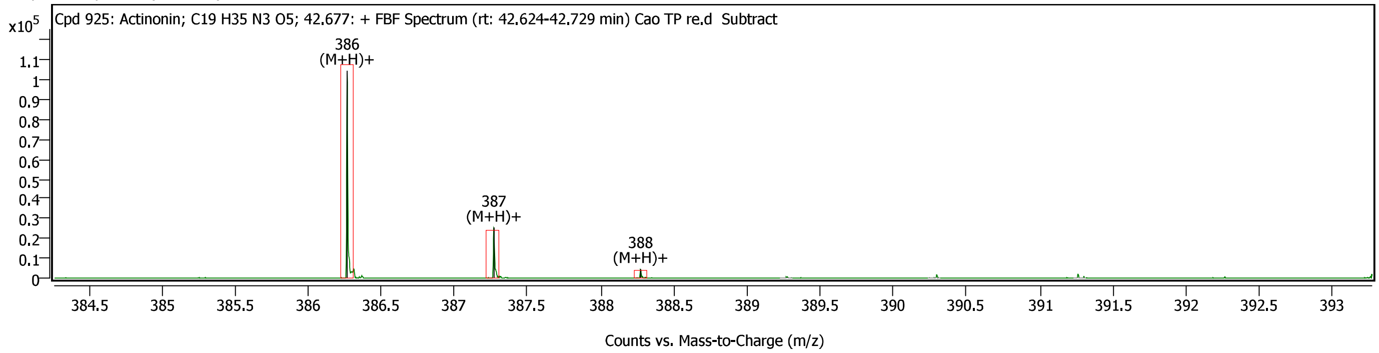

Compound ID Table

Name	Formula	Species	RT	RT Diff	Mass	CAS	ID Source	Score	Score (Lib)	Score (Tgt)
Actinonin	C19 H35 N3 O5	(M+H)+	42.677		385.2594	13434-13-4	FBF	89.89		89.89

Cpd 70: DG(12:0/17:2(9Z,12Z)/0:0)[iso2]

Name	Formula	RT	RI	Mass	Diff (Tgt, ppm)	CAS	ID Source	Score	Algorithm
DG(12:0/17:2(9Z,12Z)/0:0)[iso2]	C32 H58 O5	42.781		522.4285	0.18		FBF	99.89	FBF

Species	m/z	Score (Tgt)	Score (Lib)	Score (DB)	Score (MFG)	Score (RT)
(M+H)+	523	99.89				

Compound Chromatograms (overlaid)

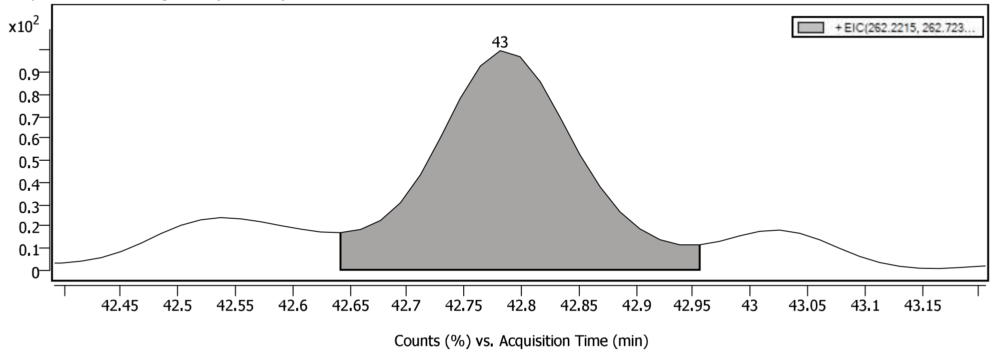

Structure

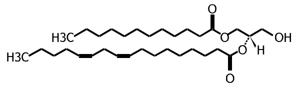

Compound Spectra (overlaid)

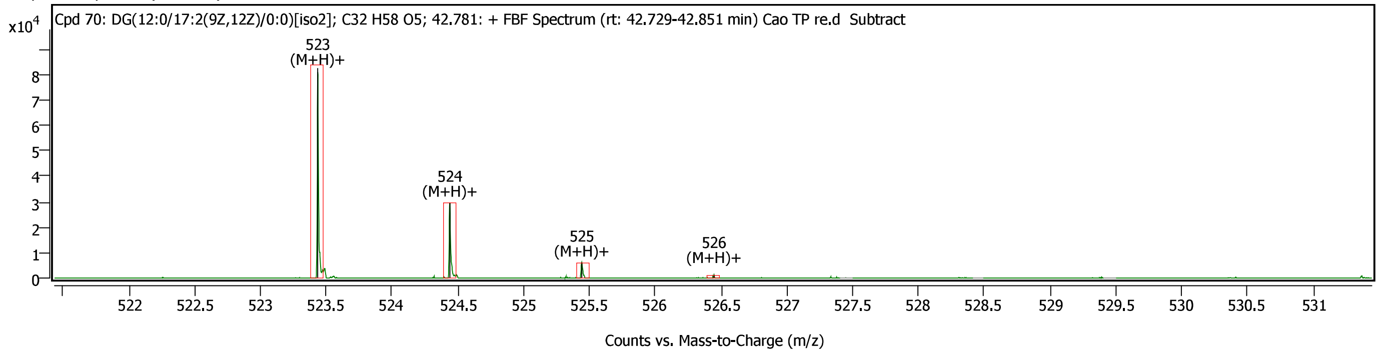

Compound ID Table

Name	Formula	Species	RT	RT Diff	Mass	CAS	ID Source	Score	Score (Lib)	Score (Tgt)
DG(12:0/17:2(9Z,12Z)/0:0)[iso2]	C32 H58 O5	(M+H)+	42.781		522.4285		FBF	99.89		99.89

Cpd 205: Epomusenin A

Compound Screening Report

Name	Formula	RT	RI	Mass	Diff (Tgt, ppm)	CAS	ID Source	Score	Algorithm
Epomusenin A	C37 H66 O3	42.781		558.5027	2.75	178330-55-7	M-FBF	94.71	FBF

Species	m/z	Score (Tgt)	Score (Lib)	Score (DB)	Score (MFG)	Score (RT)
(M+H)+	560	94.71				

Compound Chromatograms (overlaid)

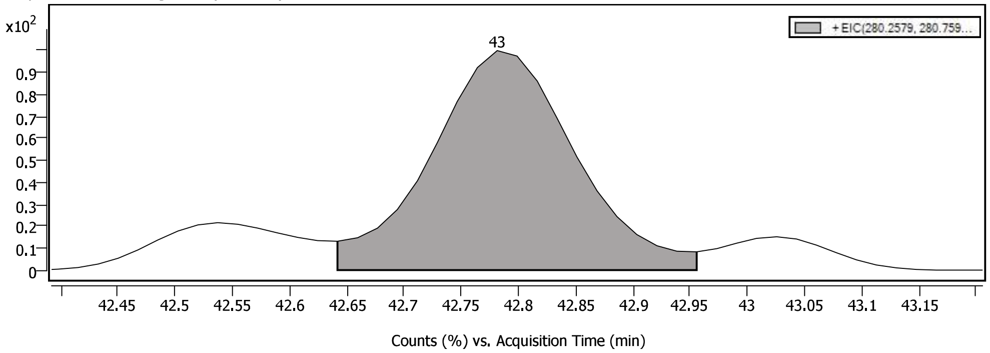

Structure

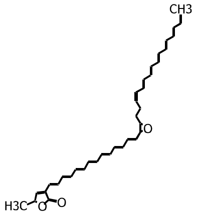

Compound Spectra (overlaid)

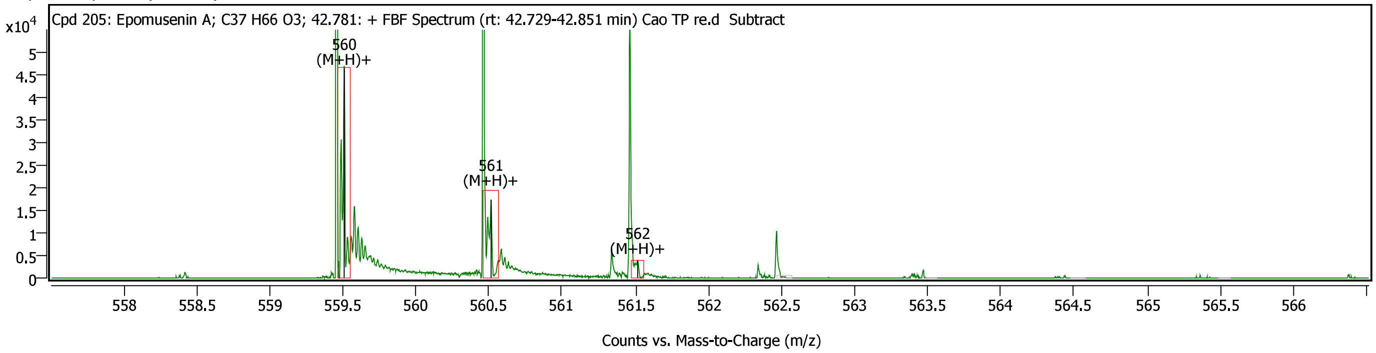

Compound ID Table

Name	Formula	Species	RT	RT Diff	Mass	CAS	ID Source	Score	Score (Lib)	Score (Tgt)
Epomusenin A	C37 H66 O3	(M+H)+	42.781		558.5027	178330-55-7	FBF	94.71		94.71
Epomusenin B	C37 H66 O3	(M+H)+	42.781		558.5027	178330-56-8	FBF	94.71		94.71

Cpd 607: PC(O-18:0/4:0)

Name	Formula	RT	RI	Mass	Diff (Tgt, ppm)	CAS	ID Source	Score	Algorithm
PC(O-18:0/4:0)	C30 H63 N O7 P	42.781		580.4320	-3.78		M-FBF	93.24	FBF

Species	m/z	Score (Tgt)	Score (Lib)	Score (DB)	Score (MFG)	Score (RT)
(M+H)+	581	93.24				

Compound Chromatograms (overlaid)

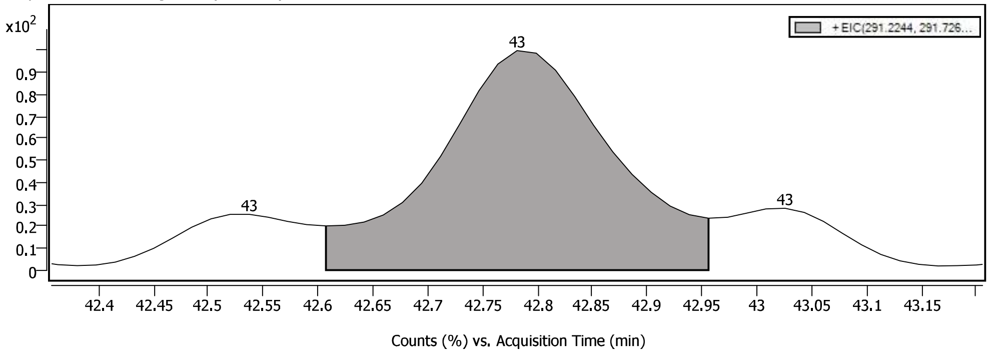

Structure

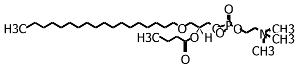

Compound Spectra (overlaid)

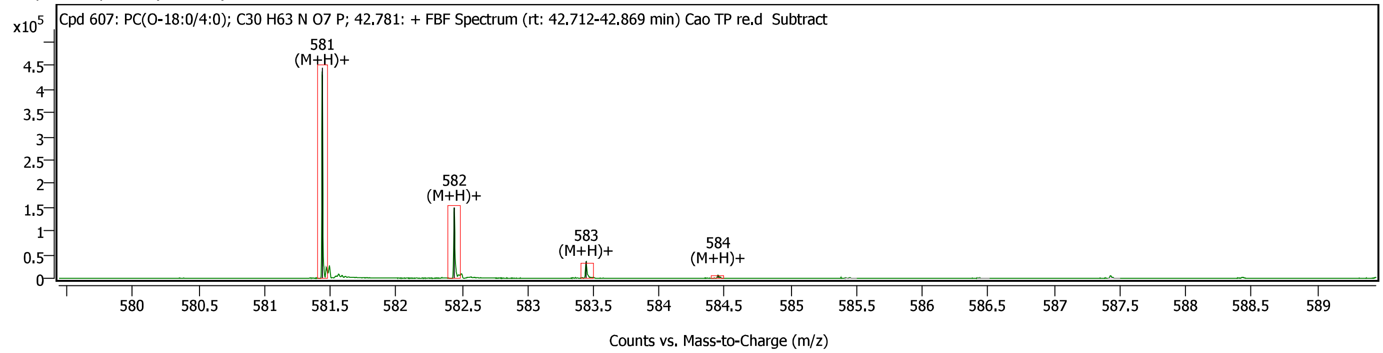

Compound ID Table

Name	Formula	Species	RT	RT Diff	Mass	CAS	ID Source	Score	Score (Lib)	Score (Tgt)
PC(O-18:0/4:0)	C30 H63 N O7 P	(M+H)+	42.781		580.4320		FBF	93.24		93.24
Hexanolamino PAF C-16	C30 H63 N O7 P	(M+H)+	42.781		580.4320	137566-83-7	FBF	93.24		93.24
LysoPC(22:0)	C30 H63 N O7 P	(M+H)+	42.781		580.4320		FBF	93.24		93.24
PC(O-12:0/10:0)[U]	C30 H63 N O7 P	(M+H)+	42.781		580.4320		FBF	93.24		93.24
PC(22:0/0:0)[U]	C30 H63 N O7 P	(M+H)+	42.781		580.4320		FBF	93.24		93.24
PC(6:0/O-16:0)[U]	C30 H63 N O7 P	(M+H)+	42.781		580.4320		FBF	93.24		93.24
PC(22:0/0:0)	C30 H63 N O7 P	(M+H)+	42.781		580.4320		FBF	93.24		93.24
PC(O-16:0/6:0)	C30 H63 N O7 P	(M+H)+	42.781		580.4320		FBF	93.24		93.24
PC(O-18:0/4:0)[U]	C30 H63 N O7 P	(M+H)+	42.781		580.4320		FBF	93.24		93.24
PC(O-20:0/2:0)	C30 H63 N O7 P	(M+H)+	42.781		580.4320		FBF	93.24		93.24
PC(O-20:0/2:0)[U]	C30 H63 N O7 P	(M+H)+	42.781		580.4320		FBF	93.24		93.24

Cpd 152: Ganoderol A

Name	Formula	RT	RI	Mass	Diff (Tgt, ppm)	CAS	ID Source	Score	Algorithm
Ganoderol A	C30 H46 O2	42.973		438.3500	0.40	104700-97-2	M-FBF	97.98	FBF

Species	m/z	Score (Tgt)	Score (Lib)	Score (DB)	Score (MFG)	Score (RT)
(M+H)+	439	97.98				

Compound Chromatograms (overlaid)

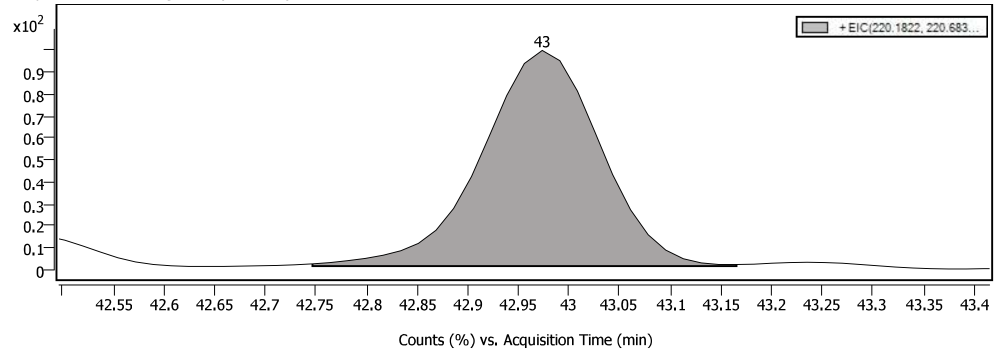

Structure

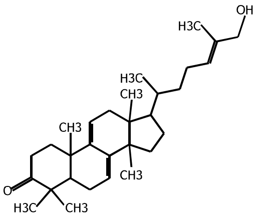

Compound Spectra (overlaid)

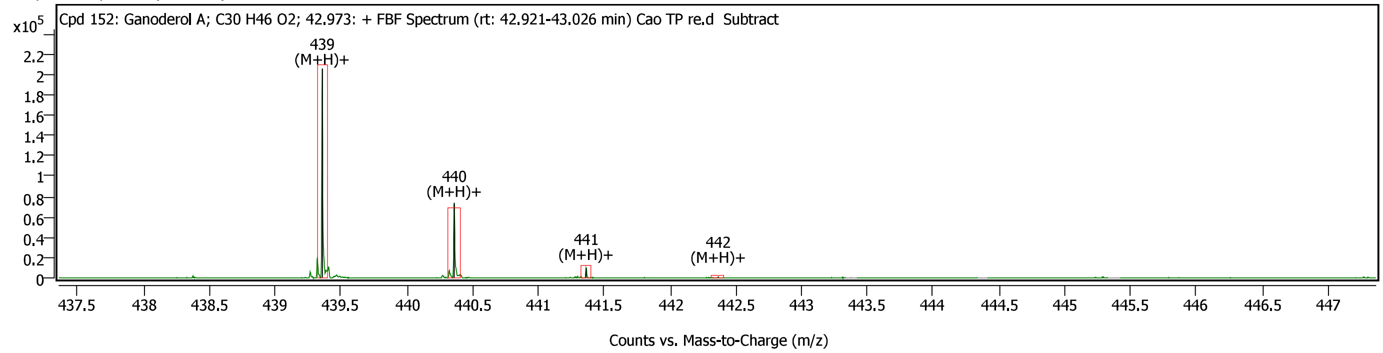

Compound ID Table

Name	Formula	Species	RT	RT Diff	Mass	CAS	ID Source	Score	Score (Lib)	Score (Tgt)
Ganoderol A	C30 H46 O2	(M+H)+	42.973		438.3500	104700-97-2	FBF	97.98		97.98
Thujyl 19-trachylobanoate	C30 H46 O2	(M+H)+	42.973		438.3500		FBF	97.98		97.98
Momordicinin	C30 H46 O2	(M+H)+	42.973		438.3500	128529-78-2	FBF	97.98		97.98
alpha,gamma-Onoceradienedione	C30 H46 O2	(M+H)+	42.973		438.3500	19940-78-4	FBF	97.98		97.98

Cpd 523: 8-Azaadenosine

Name	Formula	RT	RI	Mass	Diff (Tgt, ppm)	CAS	ID Source	Score	Algorithm
8-Azaadenosine	C9 H12 N6 O4	43.148		268.0932	4.29	10299-44-2	FBF	83.42	FBF

Species	m/z	Score (Tgt)	Score (Lib)	Score (DB)	Score (MFG)	Score (RT)
(M+H)+	269	83.42				

Compound Screening Report

Compound Chromatograms (overlaid)

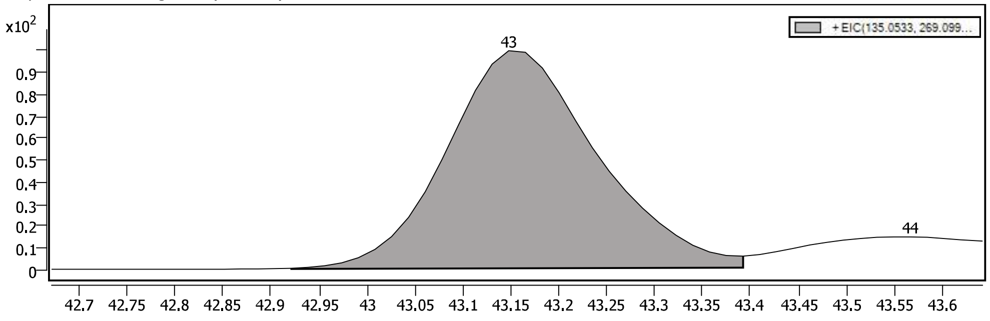

Structure

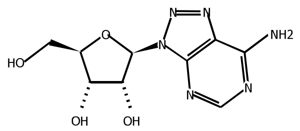

Compound Spectra (overlaid)

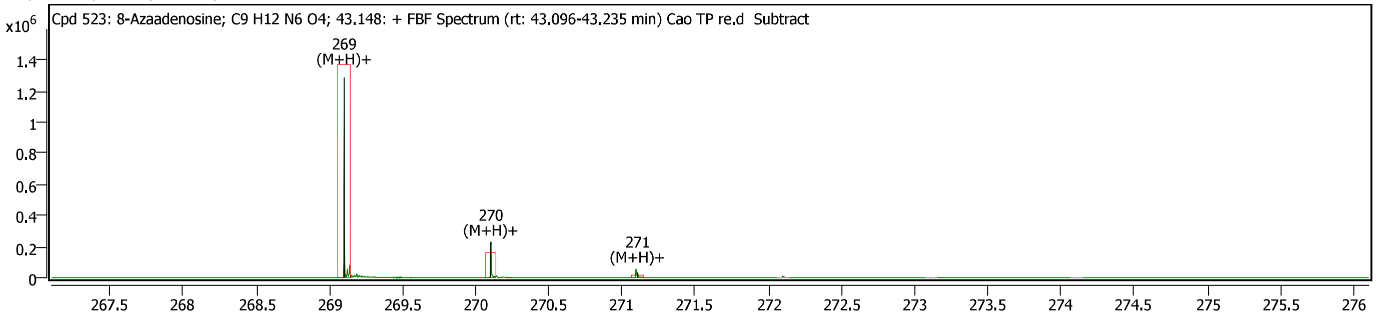

Compound ID Table

Name	Formula	Species	RT	RT Diff	Mass	CAS	ID Source	Score	Score (Lib)	Score (Tgt)
8-Azaadenosine	C9 H12 N6 O4	(M+H)+	43.148		268.0932	10299-44-2	FBF	83.42		83.42

Cpd 1693: (S)-Nerolidol 3-O-[a-L-Rhamnopyranosyl-(1->4)-a-L-rhamnopyranosyl-(1->2)-b-D-glucopyranoside]

Name	Formula	RT	RI	Mass	Diff (Tgt, ppm)	CAS	ID Source	Score	Algorithm
(S)-Nerolidol 3-O-[a-L-Rhamnopyranosyl-(1->4)-a-L-rhamnopyranosyl-(1->2)-b-D-glucopyranoside]	C33 H56 O14	43.165		676.3652	-2.69		M-FBF	94.87	FBF

Species	m/z	Score (Tgt)	Score (Lib)	Score (DB)	Score (MFG)	Score (RT)
(M+2H)+2 (M+H)+	339 677	94.87				

Compound Chromatograms (overlaid)

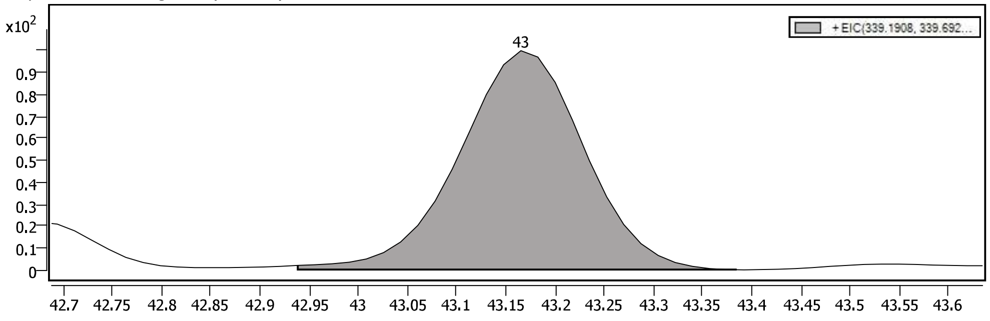

Structure

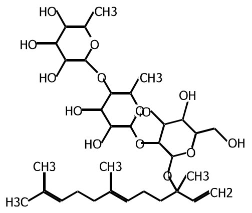

Compound Spectra (overlaid)

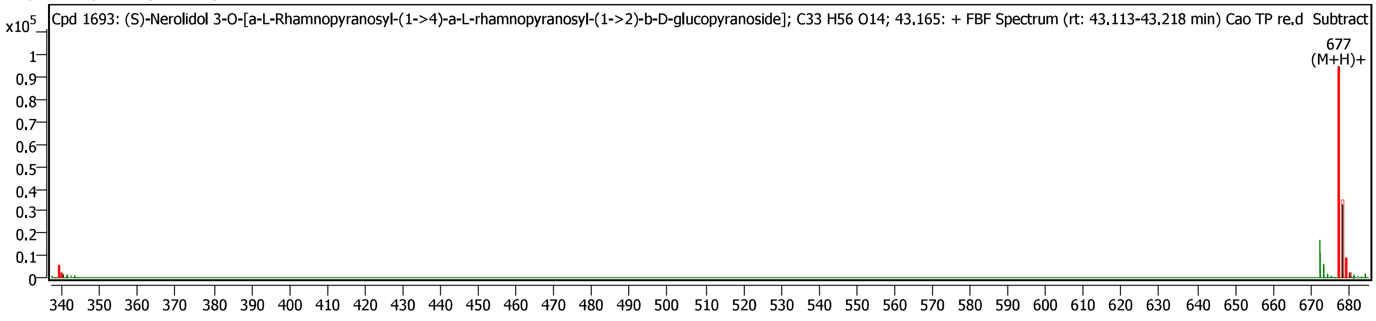

Compound Screening Report

Compound ID Table

Name	Formula	Species	RT	RT Diff	Mass	CAS	ID Source	Score	Score (Lib)	Score (Tgt)
(S)-Nerolidol 3-O-[a-L-Rhamnopyranosyl-(1->4)-a-L-rhamnopyranosyl-(1->2)-b-D-glucopyranoside]	C33 H56 O14	(M+2H)+2 (M+H)+	43.165		676.3652		FBF	94.87		94.87
(S)-Nerolidol 3-O-[a-L-rhamnopyranosyl-(1->4)-a-L-rhamnopyranosyl-(1->6)-b-D-glucopyranoside]	C33 H56 O14	(M+2H)+2 (M+H)+	43.165		676.3652	130466-33-0	FBF	94.87		94.87
Gingerglycolipid A	C33 H56 O14	(M+2H)+2 (M+H)+	43.165		676.3652	145937-22-0	FBF	94.87		94.87

Cpd 1495: <Doxapram>

Name	Formula	RT	RI	Mass	Diff (Tgt, ppm)	CAS	ID Source	Score	Algorithm
<Doxapram>	C24 H30 N2 O2	43.200		378.2312	1.33	309-29-5	FBF	98.00	FBF

Species	m/z	Score (Tgt)	Score (Lib)	Score (DB)	Score (MFG)	Score (RT)
(M+H)+	379	98.00				

Compound Chromatograms (overlaid)

Structure

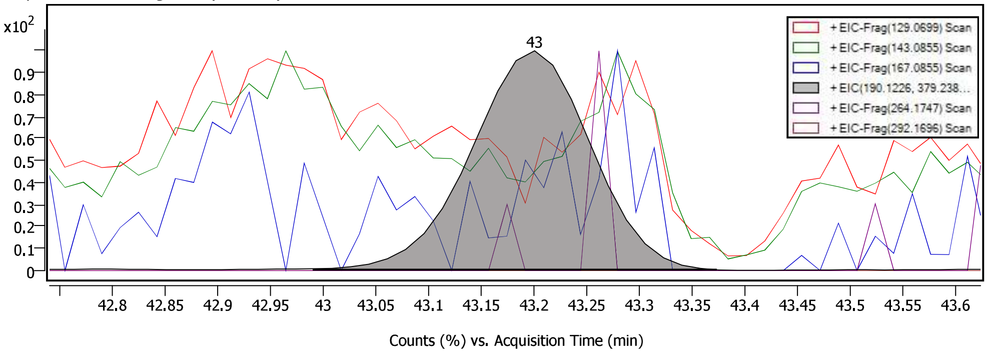

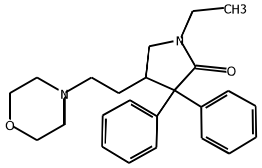

Coelution Plot

Compound Spectra (overlaid)

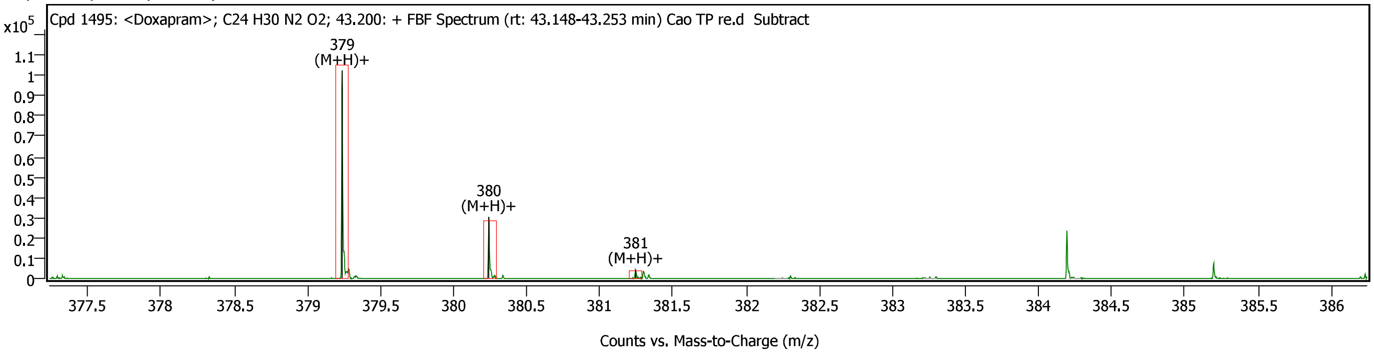

Fragment Spectrum (raw)

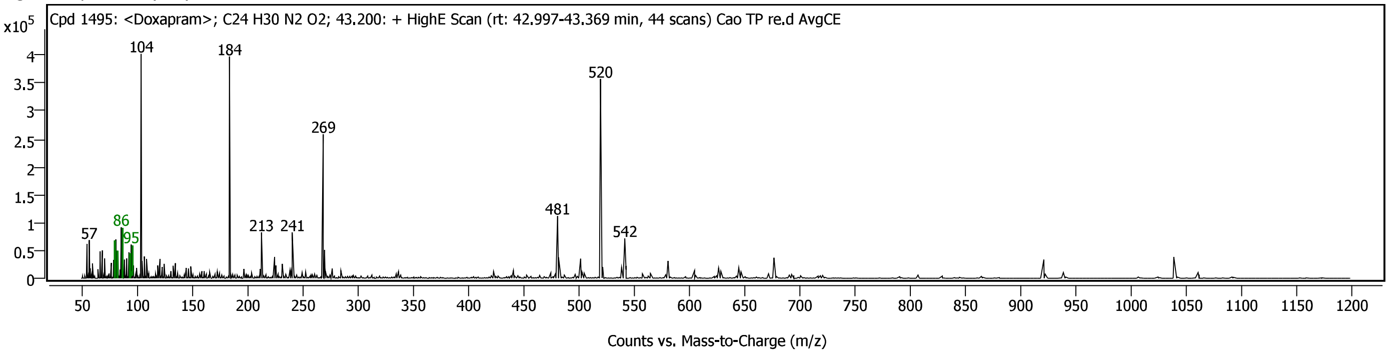

Compound ID Table

Name	Formula	Species	RT	RT Diff	Mass	CAS	ID Source	Score	Score (Lib)	Score (Tgt)
<Doxapram>	C24 H30 N2 O2	(M+H)+	43.200		378.2312	309-29-5	FBF	98.00		98.00

Cpd 259: PA(21:0/0:0)

Name	Formula	RT	RI	Mass	Diff (Tgt, ppm)	CAS	ID Source	Score	Algorithm
PA(21:0/0:0)	C24 H49 O7 P	43.270		480.3218	0.50		FBF	89.52	FBF

Species	m/z	Score (Tgt)	Score (Lib)	Score (DB)	Score (MFG)	Score (RT)
(M+H)+	481	89.52				

Compound Chromatograms (overlaid)

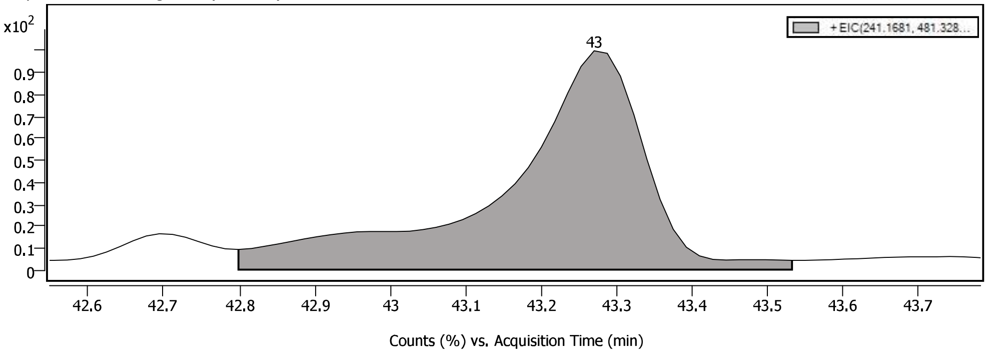

Structure

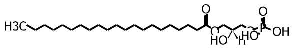

Compound Spectra (overlaid)

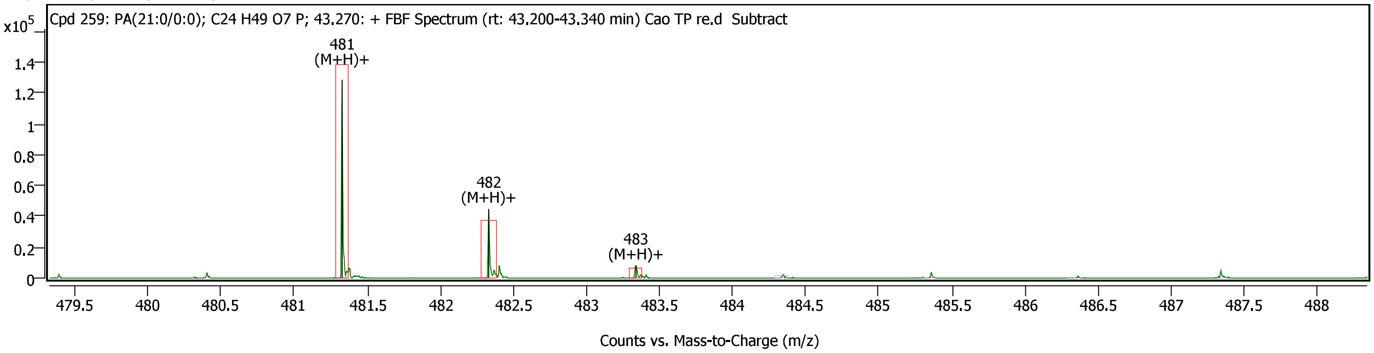

Compound ID Table

Name	Formula	Species	RT	RT Diff	Mass	CAS	ID Source	Score	Score (Lib)	Score (Tgt)
PA(21:0/0:0)	C24 H49 O7 P	(M+H)+	43.270		480.3218		FBF	89.52		89.52

Cpd 956: EB 1213

Name	Formula	RT	RI	Mass	Diff (Tgt, ppm)	CAS	ID Source	Score	Algorithm
EB 1213	C31 H44 O4	43.270		480.3219	-4.28		FBF	90.90	FBF

Species	m/z	Score (Tgt)	Score (Lib)	Score (DB)	Score (MFG)	Score (RT)
(M+H)+	481	90.90				

Compound Chromatograms (overlaid)

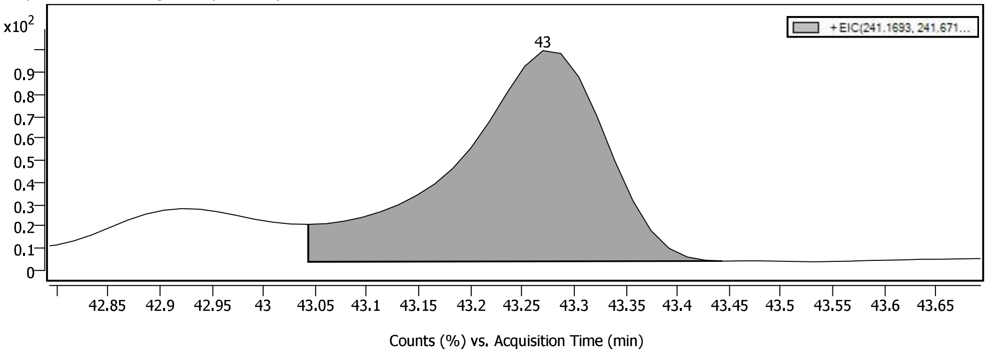

Structure

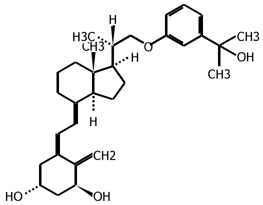

Compound Spectra (overlaid)

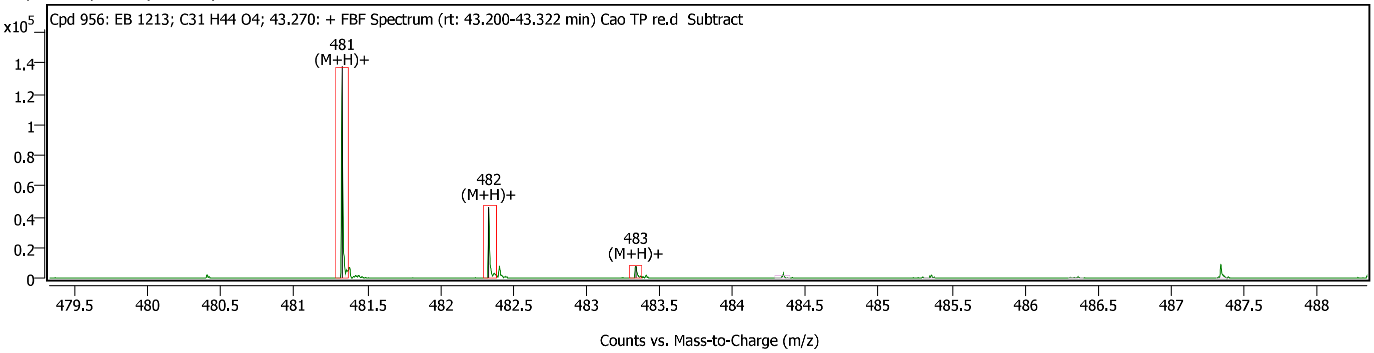

Compound ID Table

Name	Formula	Species	RT	RT Diff	Mass	CAS	ID Source	Score	Score (Lib)	Score (Tgt)
EB 1213	C31 H44 O4	(M+H)+	43.270		480.3219		FBF	90.90		90.90

Cpd 522: 8-Azaadenosine

Compound Screening Report

Name	Formula	RT	RI	Mass	Diff (Tgt, ppm)	CAS	ID Source	Score	Algorithm
8-Azaadenosine	C9 H12 N6 O4	43.567		268.0928	2.88	10299-44-2	FBF	82.46	FBF

Species	m/z	Score (Tgt)	Score (Lib)	Score (DB)	Score (MFG)	Score (RT)
(M+H)+	269	82.46				

Compound Chromatograms (overlaid)

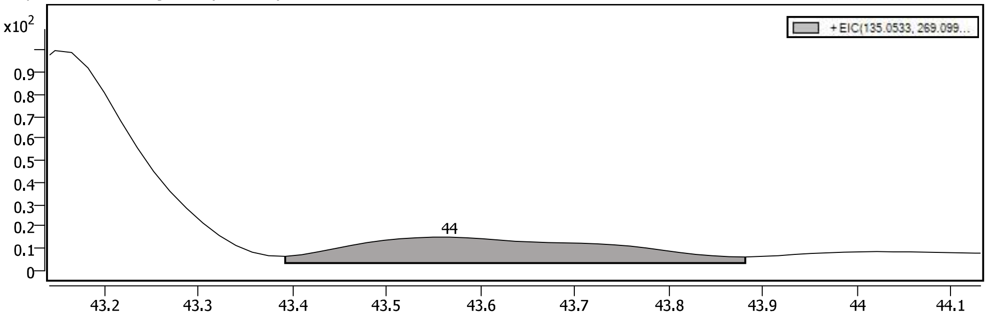

Structure

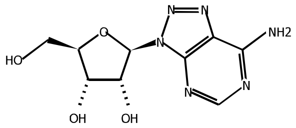

Compound Spectra (overlaid)

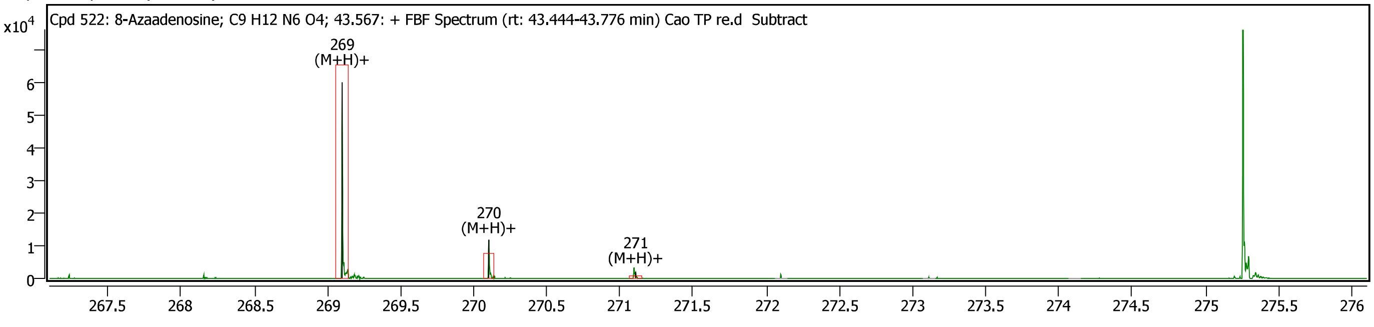

Compound ID Table

Name	Formula	Species	RT	RT Diff	Mass	CAS	ID Source	Score	Score (Lib)	Score (Tgt)
8-Azaadenosine	C9 H12 N6 O4	(M+H)+	43.567		268.0928	10299-44-2	FBF	82.46		82.46

Cpd 1692: Gingerglycolipid A

Name	Formula	RT	RI	Mass	Diff (Tgt, ppm)	CAS	ID Source	Score	Algorithm
Gingerglycolipid A	C33 H56 O14	43.881		676.3650	-2.91	145937-22-0	M-FBF	95.53	FBF

Species	m/z	Score (Tgt)	Score (Lib)	Score (DB)	Score (MFG)	Score (RT)
(M+H)+	677	95.53				

Compound Chromatograms (overlaid)

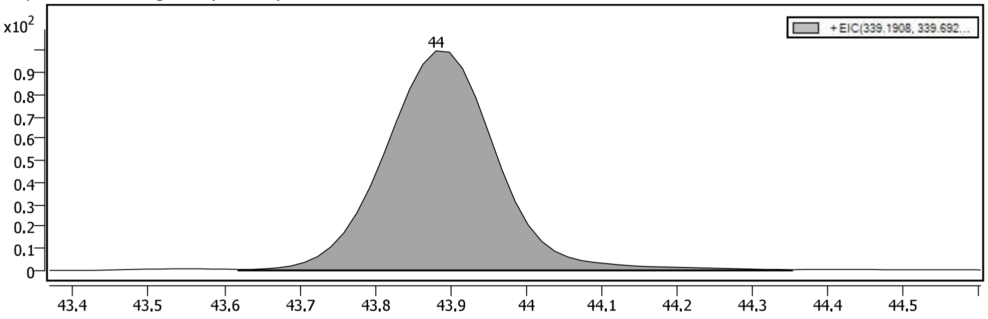

Structure

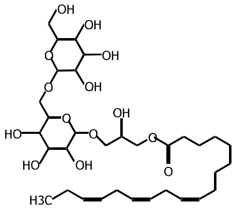

Compound Spectra (overlaid)

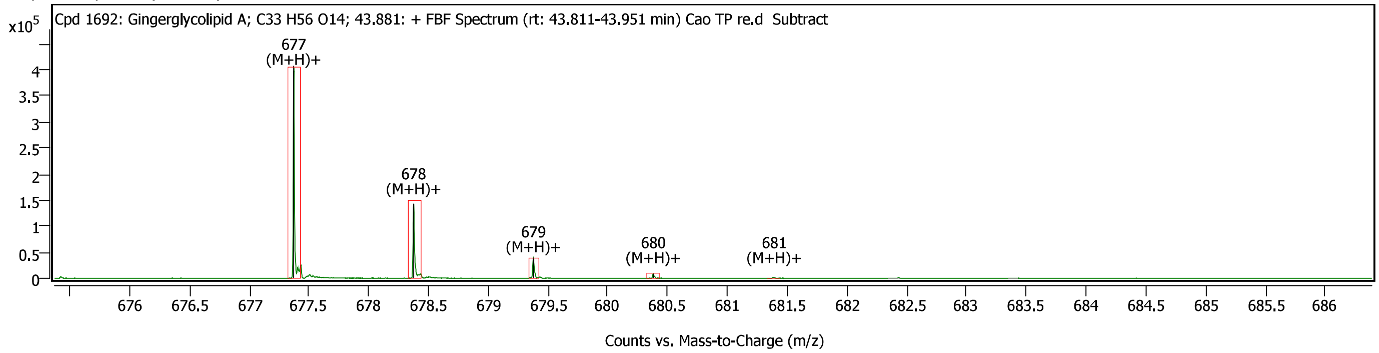

Compound ID Table

Name	Formula	Species	RT	RT Diff	Mass	CAS	ID Source	Score	Score (Lib)	Score (Tgt)
Gingerglycolipid A	C33 H56 O14	(M+H)+	43.881		676.3650	145937-22-0	FBF	95.53		95.53
(S)-Nerolidol 3-O-[a-L-rhamnopyranosyl-(1->4)-a-L-rhamnopyranosyl-(1->6)-b-D-glucopyranoside]	C33 H56 O14	(M+H)+	43.881		676.3650	130466-33-0	FBF	95.53		95.53
(S)-Nerolidol 3-O-[a-L-Rhamnopyranosyl-(1->4)-a-L-rhamnopyranosyl-(1->2)-b-D-glucopyranoside]	C33 H56 O14	(M+H)+	43.881		676.3650		FBF	95.53		95.53

Cpd 336: PE(19:0/0:0)

Name	Formula	RT	RI	Mass	Diff (Tgt, ppm)	CAS	ID Source	Score	Algorithm
PE(19:0/0:0)	C24 H50 N O7 P	43.916		495.3331	1.17		FBF	99.14	FBF

Species	m/z	Score (Tgt)	Score (Lib)	Score (DB)	Score (MFG)	Score (RT)
(M+H)+	496	99.14				

Compound Chromatograms (overlaid)

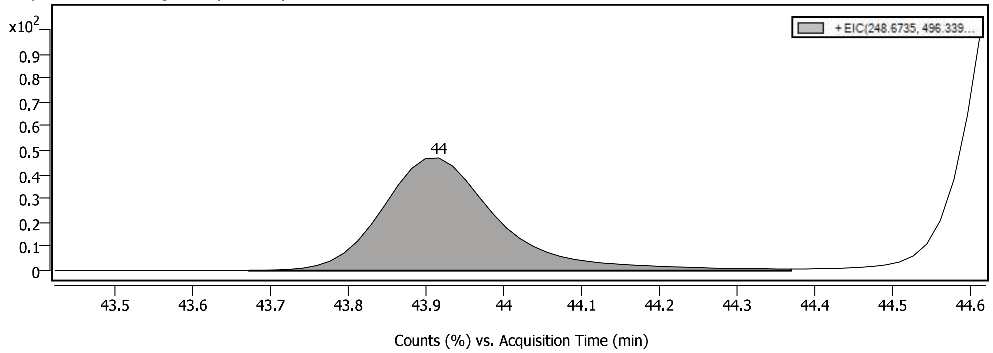

Structure

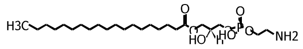

Compound Spectra (overlaid)

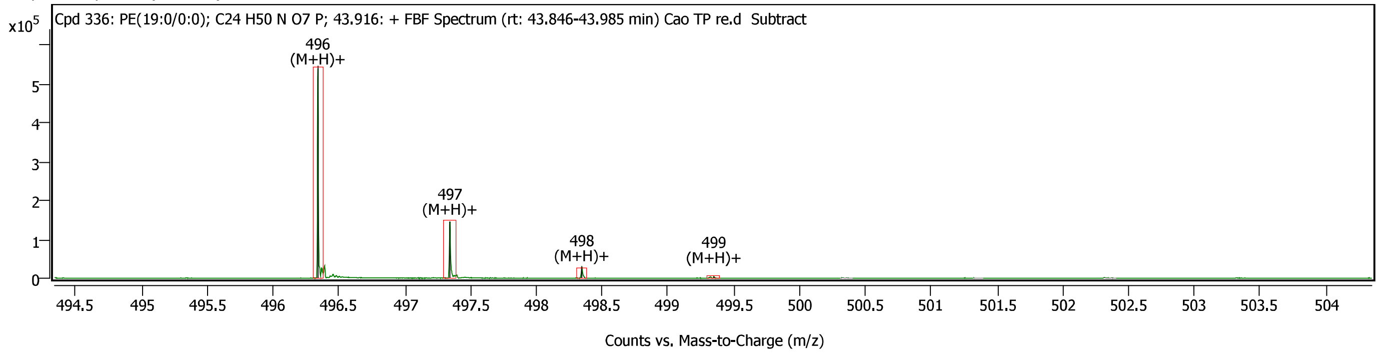

Compound ID Table

Name	Formula	Species	RT	RT Diff	Mass	CAS	ID Source	Score	Score (Lib)	Score (Tgt)
PE(19:0/0:0)	C24 H50 N O7 P	(M+H)+	43.916		495.3331		FBF	99.14		99.14

Cpd 30: PE-Cer(d15:2(4E,6E)/18:0)

Name	Formula	RT	RI	Mass	Diff (Tgt, ppm)	CAS	ID Source	Score	Algorithm
PE-Cer(d15:2(4E,6E)/18:0)	C35 H69 N2 O6 P	44.212		644.4905	1.75		M-FBF	80.92	FBF

Species	m/z	Score (Tgt)	Score (Lib)	Score (DB)	Score (MFG)	Score (RT)
(M+H)+	645	80.92				

Compound Chromatograms (overlaid)

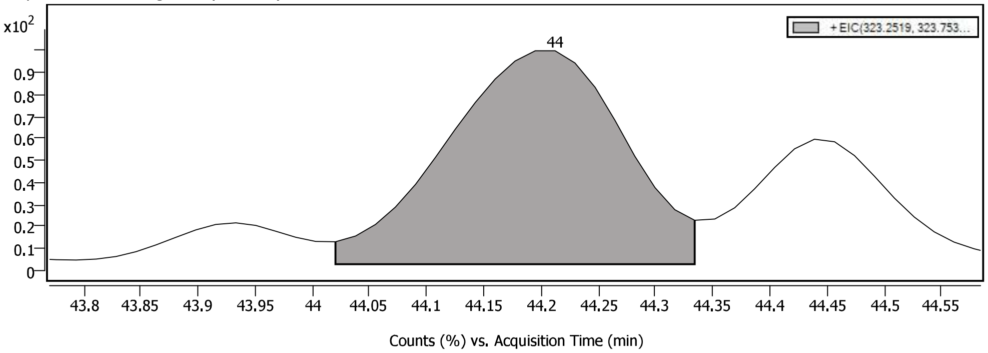

Structure

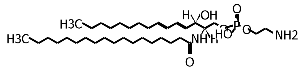

Compound Spectra (overlaid)

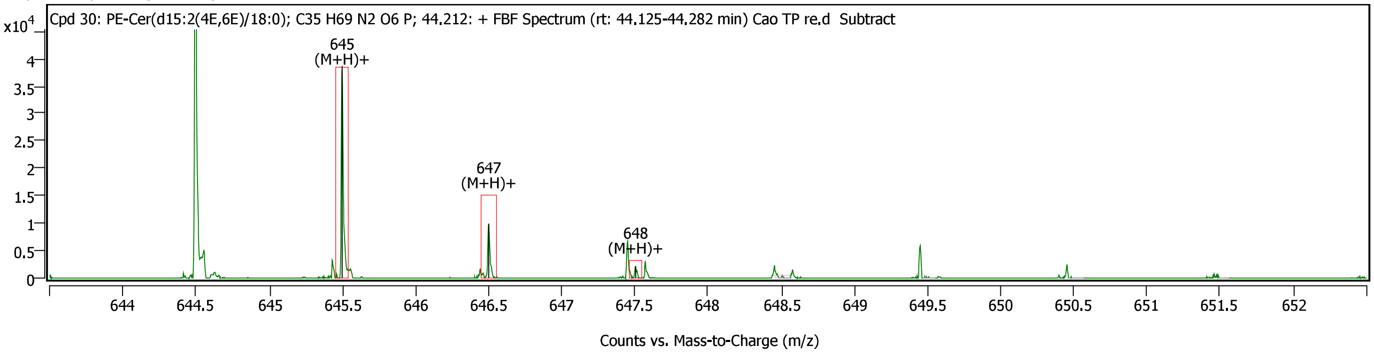

Compound ID Table

Name	Formula	Species	RT	RT Diff	Mass	CAS	ID Source	Score	Score (Lib)	Score (Tgt)
PE-Cer(d15:2(4E,6E)/18:0)	C35 H69 N2 O6 P	(M+H)+	44.212		644.4905		FBF	80.92		80.92
PE-Cer(d14:2(4E,6E)/19:0)	C35 H69 N2 O6 P	(M+H)+	44.212		644.4905		FBF	80.92		80.92

Cpd 1003: Cholesteryl linolenate

Name	Formula	RT	RI	Mass	Diff (Tgt, ppm)	CAS	ID Source	Score	Algorithm
Cholesteryl linolenate	C45 H74 O2	44.317		646.5670	-2.95		M-FBF	81.65	FBF

Species	m/z	Score (Tgt)	Score (Lib)	Score (DB)	Score (MFG)	Score (RT)
(M+2H)+2 (M+H)+	324 648	81.65				

Compound Chromatograms (overlaid)

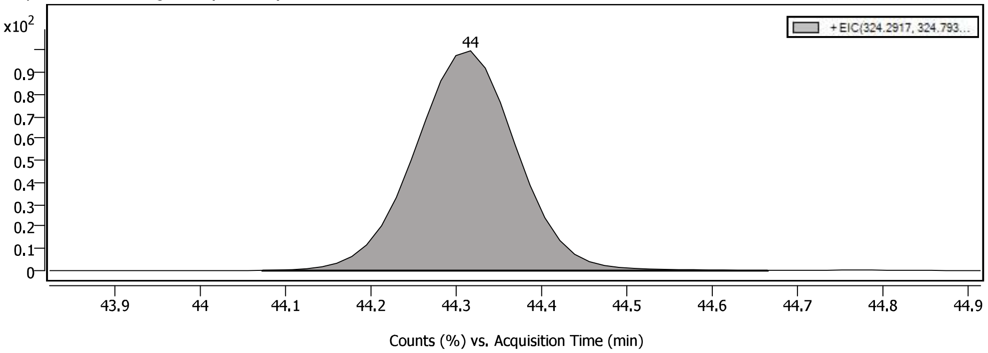

Structure

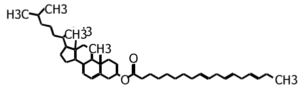

Compound Spectra (overlaid)

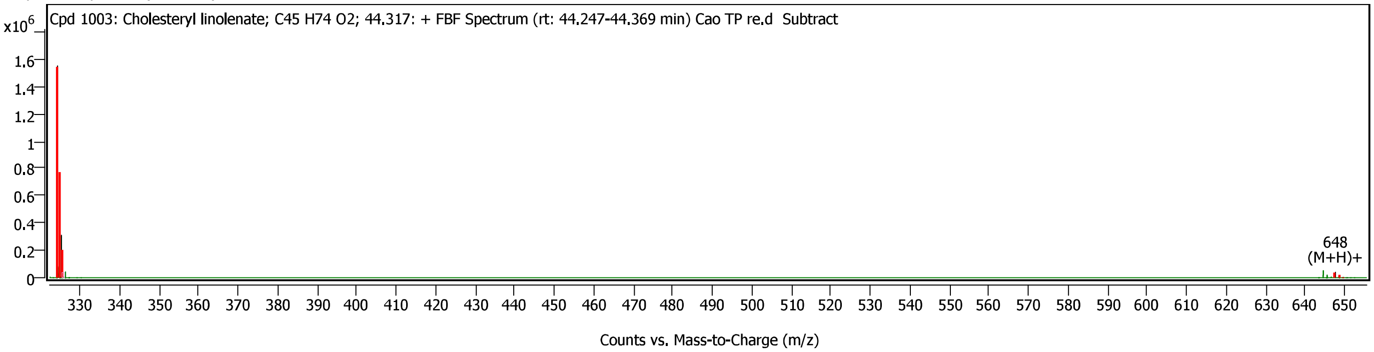

Compound Screening Report

Compound ID Table

Name	Formula	Species	RT	RT Diff	Mass	CAS	ID Source	Score	Score (Lib)	Score (Tgt)
Cholesteryl linolenate	C45 H74 O2	(M+2H)+2 (M+H)+	44.317		646.5670		FBF	81.65		81.65
18:3 Cholesteryl ester	C45 H74 O2	(M+2H)+2 (M+H)+	44.317		646.5670		FBF	81.65		81.65
16:2 Stigmasteryl ester	C45 H74 O2	(M+2H)+2 (M+H)+	44.317		646.5670		FBF	81.65		81.65
16:3 Sitosteryl ester	C45 H74 O2	(M+2H)+2 (M+H)+	44.317		646.5670		FBF	81.65		81.65
CE(18:3(9Z,12Z,15Z))	C45 H74 O2	(M+2H)+2 (M+H)+	44.317		646.5670		FBF	81.65		81.65
CE(18:3(6Z,9Z,12Z))	C45 H74 O2	(M+2H)+2 (M+H)+	44.317		646.5670		FBF	81.65		81.65

Cpd 1218: Linoleoyl Ethanolamide

Name	Formula	RT	RI	Mass	Diff (Tgt, ppm)	CAS	ID Source	Score	Algorithm
Linoleoyl Ethanolamide	C20 H37 N O2	44.317		323.2835	3.37	68171-52-8	FBF-FragConfirm	92.98	FBF

Species	m/z	Score (Tgt)	Score (Lib)	Score (DB)	Score (MFG)	Score (RT)
(M+H)+	324	92.98				

Compound Chromatograms (overlaid)

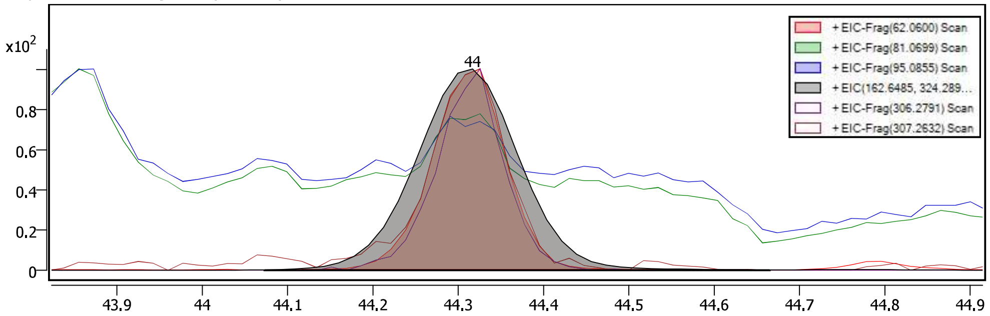

Counts (%) vs. Acquisition Time (min)

Structure

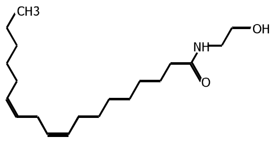

Coelution Plot

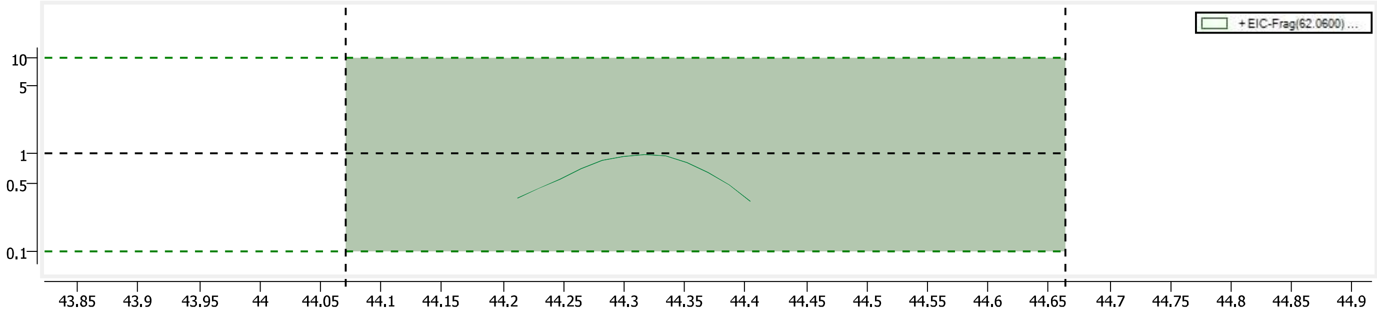

Ratio Fragment Ion/Precursor Ion vs. Acquisition Time (min)

Compound Spectra (overlaid)

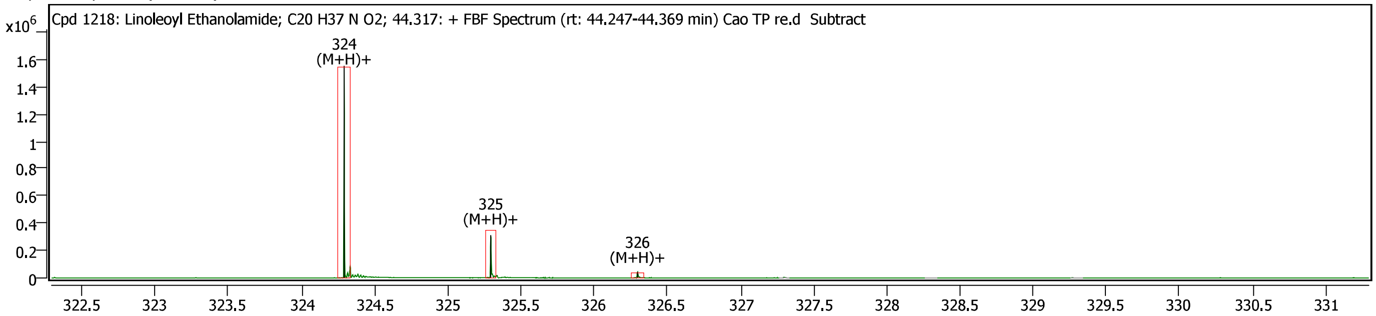

Counts vs. Mass-to-Charge (m/z)

Fragment Spectrum (clean)

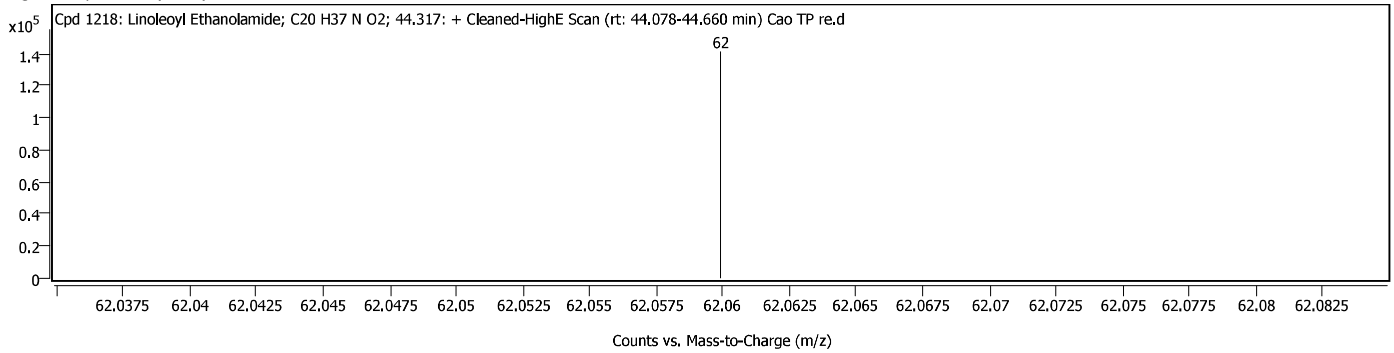

Fragment Spectrum (raw)

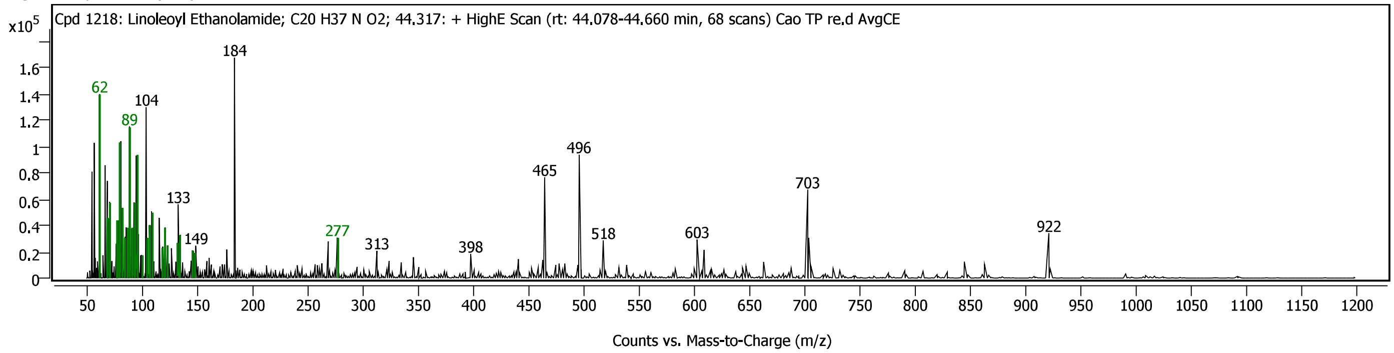

Compound ID Table

Name	Formula	Species	RT	RT Diff	Mass	CAS	ID Source	Score	Score (Lib)	Score (Tgt)
Linoleoyl Ethanolamide	C20 H37 N O2	(M+H)+	44.317		323.2835	68171-52-8	FBF-FragConfirm	92.98		92.98

Cpd 581: Progesterone 3-biotin

Name	Formula	RT	RI	Mass	Diff (Tgt, ppm)	CAS	ID Source	Score	Algorithm
Progesterone 3-biotin	C38 H59 N5 O5 S	44.614		697.4253	2.31		FBF	91.21	FBF

Species	m/z	Score (Tgt)	Score (Lib)	Score (DB)	Score (MFG)	Score (RT)
(M+H)+	698	91.21				

Compound Chromatograms (overlaid)

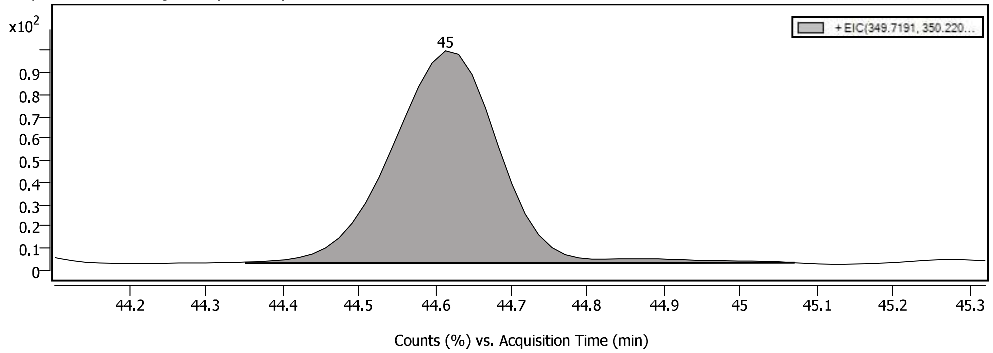

Structure

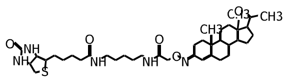

Compound Spectra (overlaid)

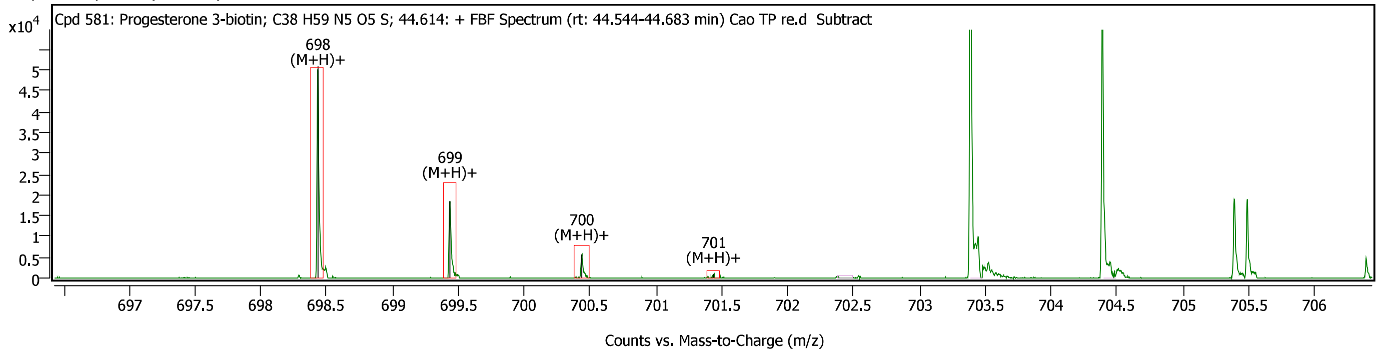

Compound ID Table

Name	Formula	Species	RT	RT Diff	Mass	CAS	ID Source	Score	Score (Lib)	Score (Tgt)
Progesterone 3-biotin	C38 H59 N5 O5 S	(M+H)+	44.614		697.4253		FBF	91.21		91.21

Cpd 111: Sambutoxin

Compound Screening Report

Name	Formula	RT	RI	Mass	Diff (Tgt, ppm)	CAS	ID Source	Score	Algorithm
Sambutoxin	C28 H39 N O4	44.788		453.2859	-4.54	160047-56-3	FBF	85.99	FBF

Species	m/z	Score (Tgt)	Score (Lib)	Score (DB)	Score (MFG)	Score (RT)
(M+H)+	454	85.99				

Compound Chromatograms (overlaid)

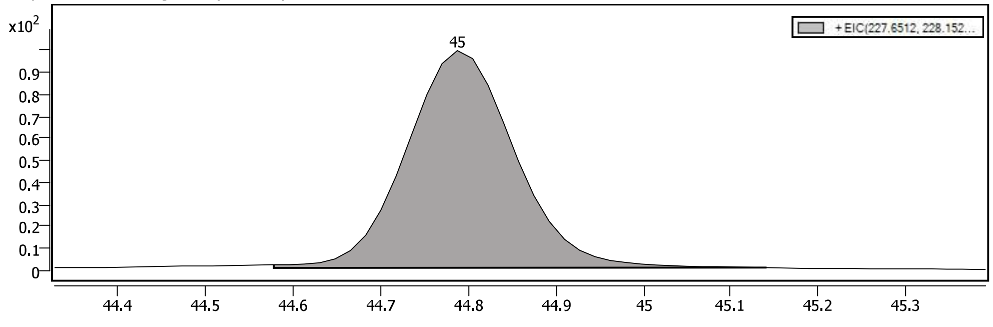

Structure

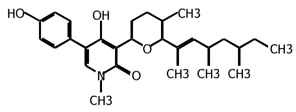

Compound Spectra (overlaid)

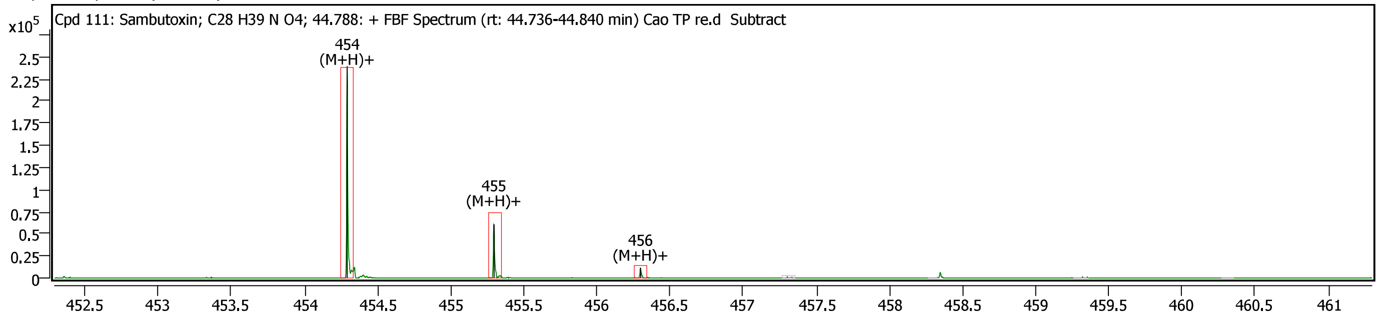

Compound ID Table

Name	Formula	Species	RT	RT Diff	Mass	CAS	ID Source	Score	Score (Lib)	Score (Tgt)
Sambutoxin	C28 H39 N O4	(M+H)+	44.788		453.2859	160047-56-3	FBF	85.99		85.99

Cpd 115: Hovenidulcioside A2

Name	Formula	RT	RI	Mass	Diff (Tgt, ppm)	CAS	ID Source	Score	Algorithm
Hovenidulcioside A2	C38 H58 O12	44.823		706.3904	-3.44	171499-80-2	FBF	93.76	FBF

Species	m/z	Score (Tgt)	Score (Lib)	Score (DB)	Score (MFG)	Score (RT)
(M+H)+	707	93.76				

Compound Chromatograms (overlaid)

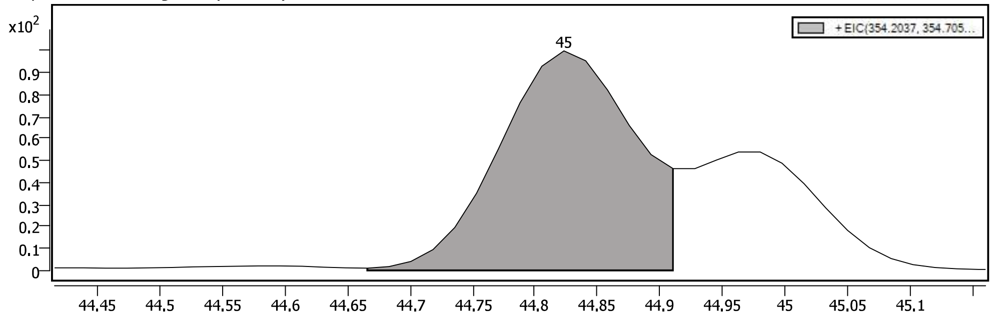

Structure

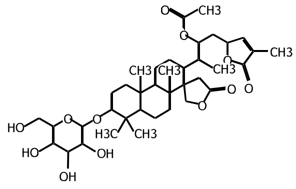

Compound Screening Report

Compound Spectra (overlaid)

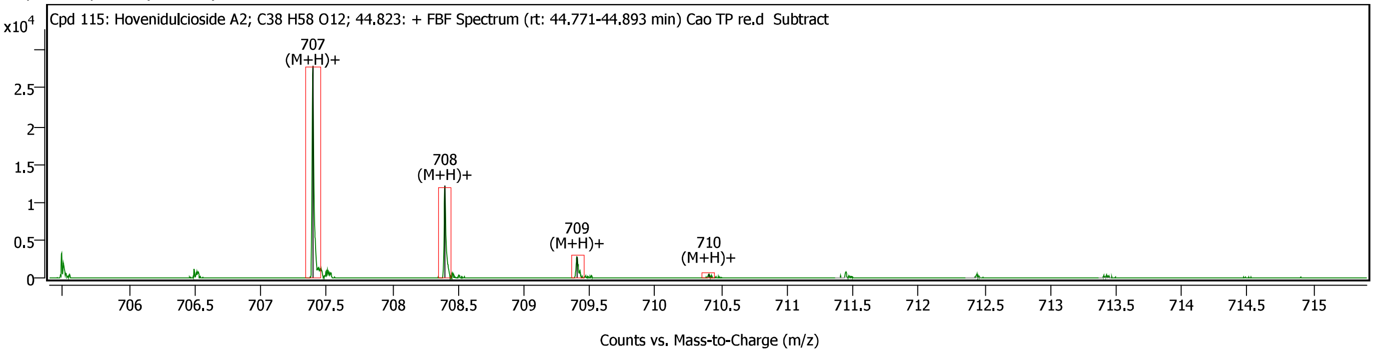

Compound ID Table

Name	Formula	Species	RT	RT Diff	Mass	CAS	ID Source	Score	Score (Lib)	Score (Tgt)
Hovenidulcioside A2	C38 H58 O12	(M+H)+	44.823		706.3904	171499-80-2	FBF	93.76		93.76

Cpd 1652: 4-Hydroxy-3-methoxycinnamaldehyde

Name	Formula	RT	RI	Mass	Diff (Tgt, ppm)	CAS	ID Source	Score	Algorithm
4-Hydroxy-3-methoxycinnamaldehyde	C10 H10 O3	44.823		178.0633	1.64	458-36-6	M-FBF-FragConfirm	98.11	FBF

Species	m/z	Score (Tgt)	Score (Lib)	Score (DB)	Score (MFG)	Score (RT)
(M+H)+	179	98.11				

Compound Chromatograms (overlaid)

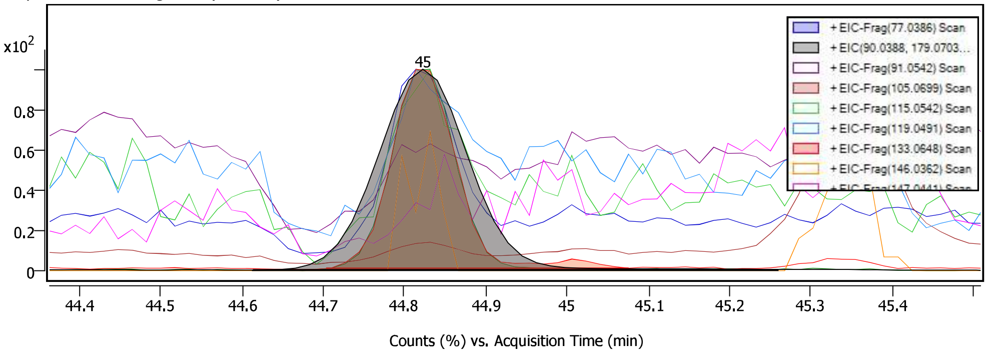

Structure

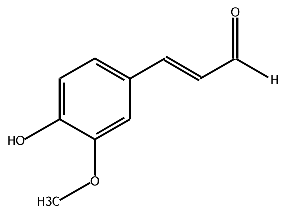

Coelution Plot

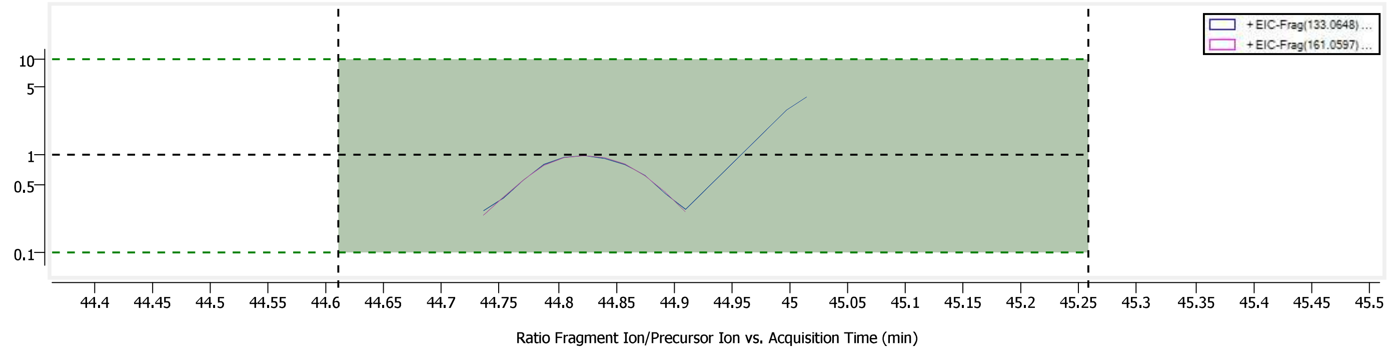

Compound Spectra (overlaid)

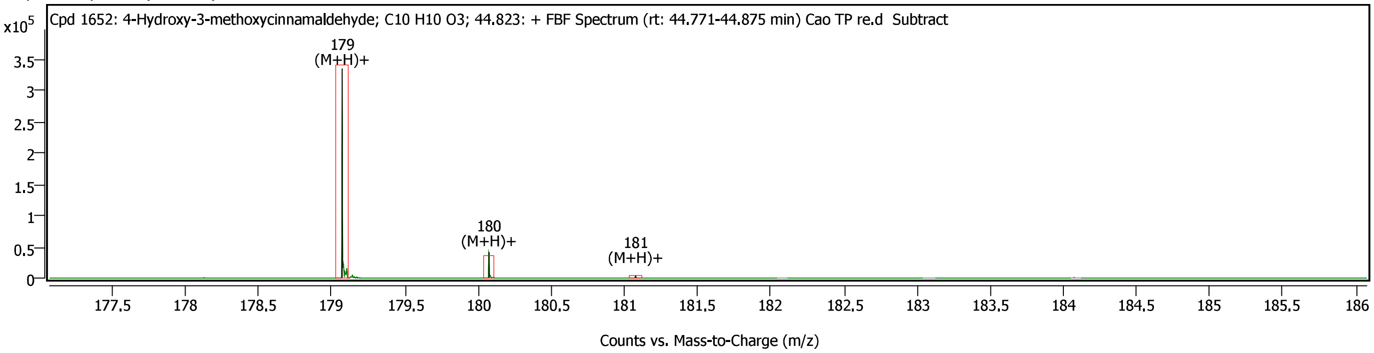

Fragment Spectrum (clean)

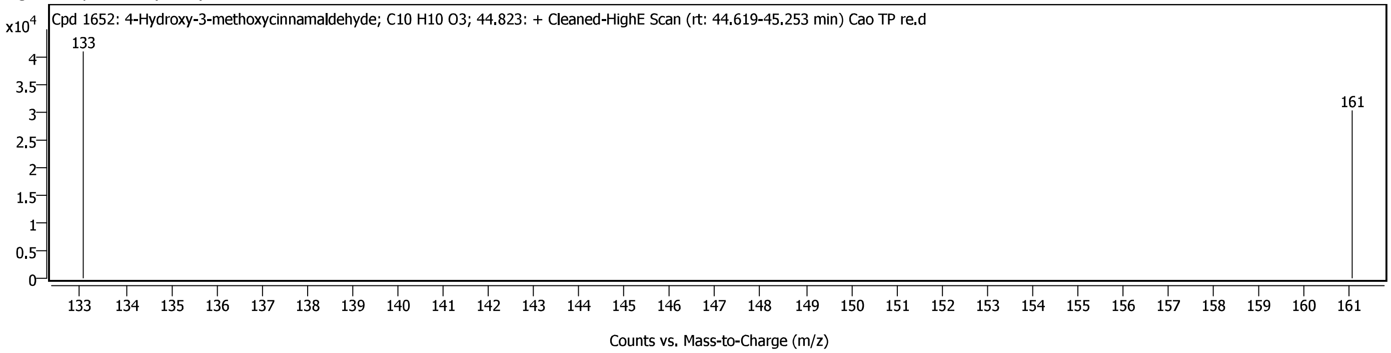

Fragment Spectrum (raw)

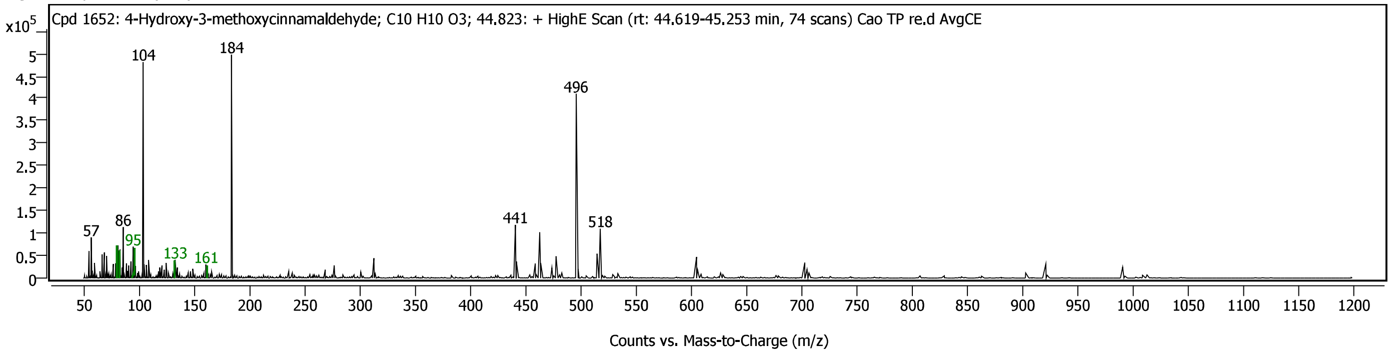

Compound ID Table

Name	Formula	Species	RT	RT Diff	Mass	CAS	ID Source	Score	Score (Lib)	Score (Tgt)
4-Hydroxy-3-methoxycinnamaldehyde	C10 H10 O3	(M+H)+	44.823		178.0633	458-36-6	FBF-FragConfirm	98.11		98.11
(E)-10-Hydroxy-2-decene-4,6-diynoic acid	C10 H10 O3	(M+H)+	44.823		178.0633		FBF-FragConfirm	98.11		98.11
3-(4-Hydroxy-2-methoxyphenyl)-2-propenal	C10 H10 O3	(M+H)+	44.823		178.0633	127321-19-1	FBF-FragConfirm	98.11		98.11
(R)-(-)-Mellein	C10 H10 O3	(M+H)+	44.823		178.0633	480-33-1	FBF-FragConfirm	98.11		98.11
(S)-Isosclerone	C10 H10 O3	(M+H)+	44.823		178.0633	54712-38-8	FBF-FragConfirm	98.11		98.11
3,4-Dihydro-6,7-dihydroxy-1(2H)-naphthalenone	C10 H10 O3	(M+H)+	44.823		178.0633		FBF-FragConfirm	98.11		98.11
1,2-Dihydroxy-3,4-epoxy-1,2,3,4-tetrahydronaphthalene	C10 H10 O3	(M+H)+	44.823		178.0633	75947-54-5	FBF-FragConfirm	98.11		98.11
10-hydroxy-8E-Decene-4,6-diynoic acid	C10 H10 O3	(M+H)+	44.823		178.0633		FBF-FragConfirm	98.11		98.11
10-hydroxy-8Z-Decene-4,6-diynoic acid	C10 H10 O3	(M+H)+	44.823		178.0633		FBF-FragConfirm	98.11		98.11
3-(4-Methylphenyl)oxiranecarboxylic acid	C10 H10 O3	(M+H)+	44.823		178.0633	81476-88-2	FBF-FragConfirm	98.11		98.11
Di-alpha-furfuryl ether	C10 H10 O3	(M+H)+	44.823		178.0633	4437-22-3	FBF-FragConfirm	98.11		98.11
9-oxo-2,4,5,7-decatetraenoic acid	C10 H10 O3	(M+H)+	44.823		178.0633		FBF-FragConfirm	98.11		98.11
3-Acetyl-6-methoxybenzaldehyde	C10 H10 O3	(M+H)+	44.823		178.0633	531-99-7	FBF-FragConfirm	98.11		98.11
4-Methoxycinnamic acid	C10 H10 O3	(M+H)+	44.823		178.0633	830-09-1	FBF-FragConfirm	98.11		98.11
8S-hydroxy-2-Decene-4,6-diynoic acid	C10 H10 O3	(M+H)+	44.823		178.0633		FBF-FragConfirm	98.11		98.11
8S-hydroxy-2E-Decene-4,6-diynoic acid	C10 H10 O3	(M+H)+	44.823		178.0633		FBF-FragConfirm	98.11		98.11
Coumarinic acid methyl ether	C10 H10 O3	(M+H)+	44.823		178.0633	6099-03-2	FBF-FragConfirm	98.11		98.11
Vermelone	C10 H10 O3	(M+H)+	44.823		178.0633	59796-04-2	FBF-FragConfirm	98.11		98.11

Cpd 124: Hydroxysintaxanthin 5,6-epoxide

Name	Formula	RT	RI	Mass	Diff (Tgt, ppm)	CAS	ID Source	Score	Algorithm
Hydroxysintaxanthin 5,6-epoxide	C31 H42 O3	44.945		462.3114	-4.42		FBF	91.23	FBF

Species	m/z	Score (Tgt)	Score (Lib)	Score (DB)	Score (MFG)	Score (RT)
(M+H)+	463	91.23				

Compound Chromatograms (overlaid)

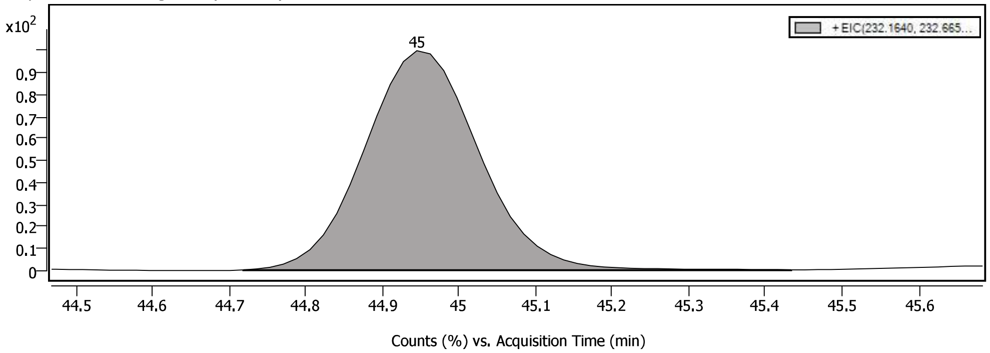

Structure

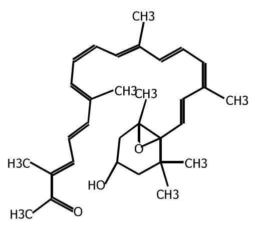

Compound Spectra (overlaid)

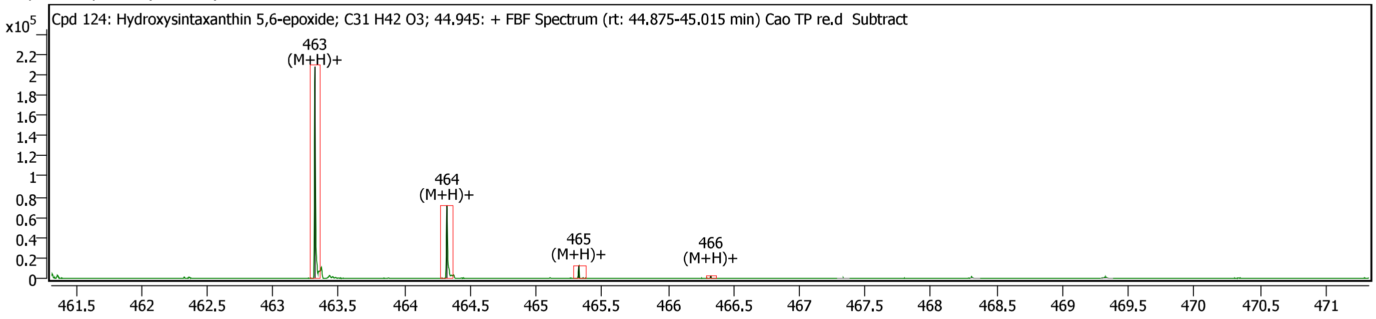

Compound ID Table

Name	Formula	Species	RT	RT Diff	Mass	CAS	ID Source	Score	Score (Lib)	Score (Tgt)
Hydroxysintaxanthin 5,6-epoxide	C31 H42 O3	(M+H)+	44.945		462.3114		FBF	91.23		91.23

Cpd 966: Pfaffic acid

Name	Formula	RT	RI	Mass	Diff (Tgt, ppm)	CAS	ID Source	Score	Algorithm
Pfaffic acid	C29 H44 O3	44.945		440.3299	2.03	86432-14-6	M-FBF	97.12	FBF

Species	m/z	Score (Tgt)	Score (Lib)	Score (DB)	Score (MFG)	Score (RT)
(M+H)+	441	97.12				

Compound Chromatograms (overlaid)

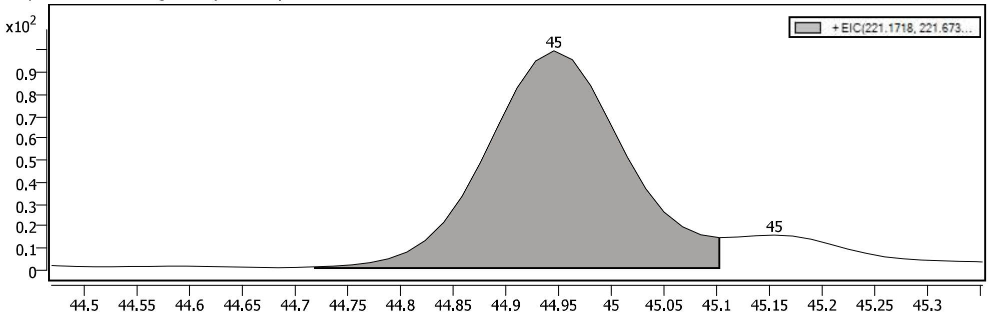

Structure

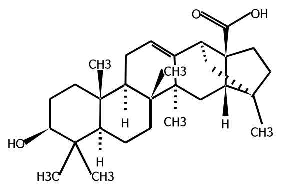

Compound Spectra (overlaid)

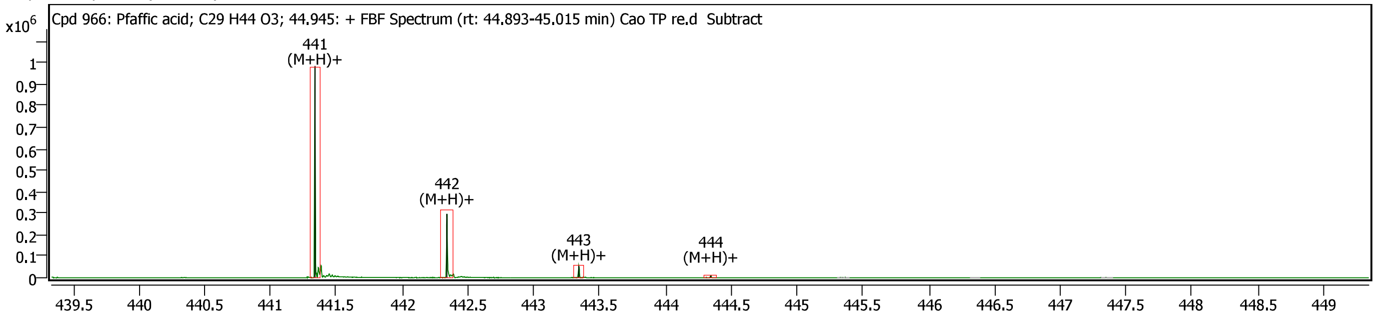

Compound Screening Report

Compound ID Table

Name	Formula	Species	RT	RT Diff	Mass	CAS	ID Source	Score	Score (Lib)	Score (Tgt)
Pfaffic acid	C29 H44 O3	(M+H)+	44.945		440.3299	86432-14-6	FBF	97.12		97.12
24a,24b-Dihomo-9,10-secocholesta-5,7,10(19),24a-tetraen-1α,3,25-triol	C29 H44 O3	(M+H)+	44.945		440.3299		FBF	97.12		97.12
(22E,24E)-1α,25-dihydroxy-22,23,24,24a-tetradehydro-24a,24b-dihomovitamin D3 / (22E,24E)-1α,25-dihydroxy-22,23,24,24a-tetradehydro-24a,24b-dihomocholecalciferol	C29 H44 O3	(M+H)+	44.945		440.3299		FBF	97.12		97.12
11α-ethynyl-1α,25-dihydroxyvitamin D3 / 11α-ethynyl-1α,25-dihydroxycholecalciferol	C29 H44 O3	(M+H)+	44.945		440.3299		FBF	97.12		97.12
1α,25-dihydroxy-26,27-dimethyl-22,22,23,23-tetradehydro-20-epivitamin D3 / 1α,25-dihydroxy-26,27-dimethyl-22,22,23,23-tetradehydro-20-epicholecalciferol	C29 H44 O3	(M+H)+	44.945		440.3299		FBF	97.12		97.12
1α,25-dihydroxy-26,27-dimethyl-22,22,23,23-tetradehydrovitamin D3 / 1α,25-dihydroxy-26,27-dimethyl-22,22,23,23-tetradehydro-cholecalciferol	C29 H44 O3	(M+H)+	44.945		440.3299		FBF	97.12		97.12
(20S)-20-cyclopropyl-1α,25-dihydroxy-16,17-didehydro-21-norvitamin D3 / (20S)-20-cyclopropyl-1α,25-dihydroxy-16,17-didehydro-21-norcholecalciferol	C29 H44 O3	(M+H)+	44.945		440.3299		FBF	97.12		97.12
5alpha,8alpha-epidioxy-stigmasta-6,9(11),22E-trien-3beta-ol	C29 H44 O3	(M+H)+	44.945		440.3299		FBF	97.12		97.12
Stoloniferone D	C29 H44 O3	(M+H)+	44.945		440.3299		FBF	97.12		97.12
Stoloniferone Q	C29 H44 O3	(M+H)+	44.945		440.3299		FBF	97.12		97.12
Camelledionol	C29 H44 O3	(M+H)+	44.945		440.3299	81426-90-6	FBF	97.12		97.12

Cpd 246: Homodolichosterone

Name	Formula	RT	RI	Mass	Diff (Tgt, ppm)	CAS	ID Source	Score	Algorithm
Homodolichosterone	C29 H48 O5	45.276		476.3503	0.34	85797-14-4	M-FBF	98.63	FBF

Species	m/z	Score (Tgt)	Score (Lib)	Score (DB)	Score (MFG)	Score (RT)
(M+H)+	477	98.63				

Compound Chromatograms (overlaid)

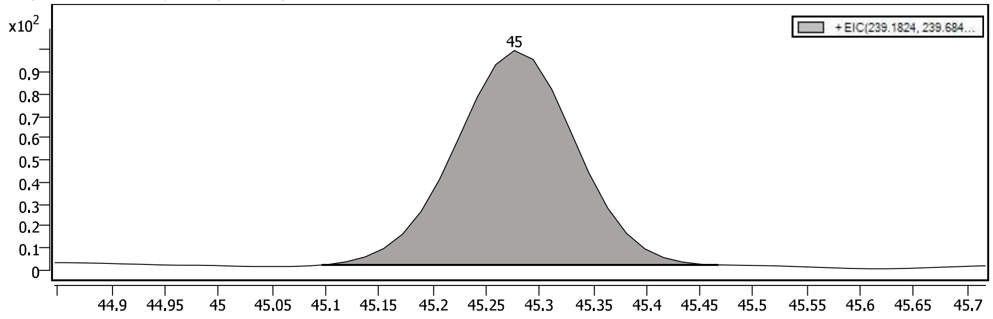

Counts (%) vs. Acquisition Time (min)

Structure

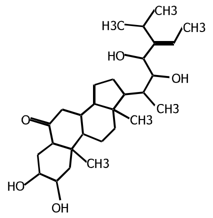

Compound Spectra (overlaid)

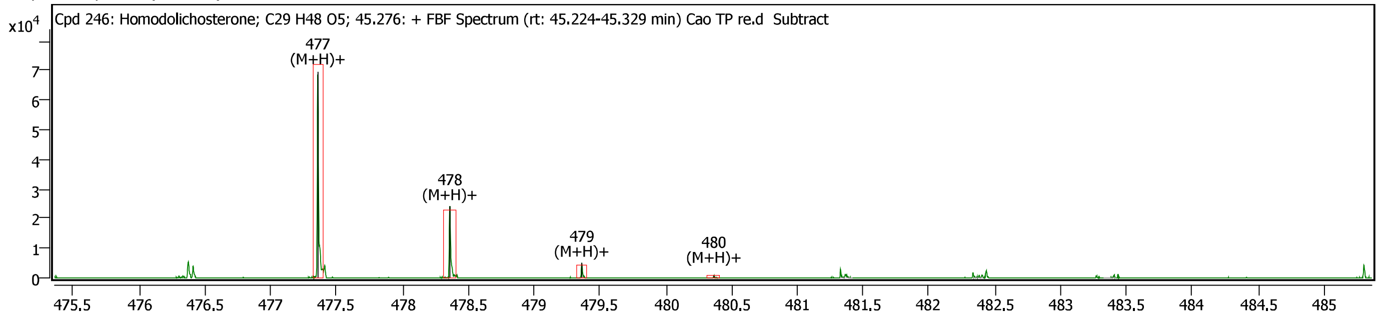

Counts vs. Mass-to-Charge (m/z)

Compound ID Table

Name	Formula	Species	RT	RT Diff	Mass	CAS	ID Source	Score	Score (Lib)	Score (Tgt)
Homodolichosterone	C29 H48 O5	(M+H)+	45.276		476.3503	85797-14-4	FBF	98.63		98.63
11-acetoxy-3beta,6alpha-dihydroxy-9,11-seco-5alpha-cholest-7-en-9-one.	C29 H48 O5	(M+H)+	45.276		476.3503		FBF	98.63		98.63
(2alpha,3alpha,5alpha,22R,23R)-2,3,22,23-Tetrahydroxy-25-methylergost-24(28)en-6-one	C29 H48 O5	(M+H)+	45.276		476.3503	111618-87-2	FBF	98.63		98.63
1α,25-dihydroxy-2β-(2-hydroxyethoxy)vitamin D3 / 1α,25-dihydroxy-2β-(2-hydroxyethoxy)cholecalciferol	C29 H48 O5	(M+H)+	45.276		476.3503		FBF	98.63		98.63

Cpd 1270: Palmitoyl Ethanolamide

Name	Formula	RT	RI	Mass	Diff (Tgt, ppm)	CAS	ID Source	Score	Algorithm
Palmitoyl Ethanolamide	C18 H37 N O2	45.521		299.2830	1.89	544-31-0	FBF-FragConfirm	98.05	FBF

Compound Screening Report

Species	m/z	Score (Tgt)	Score (Lib)	Score (DB)	Score (MFG)	Score (RT)
(M+H)+	300	98.05				

Compound Chromatograms (overlaid)

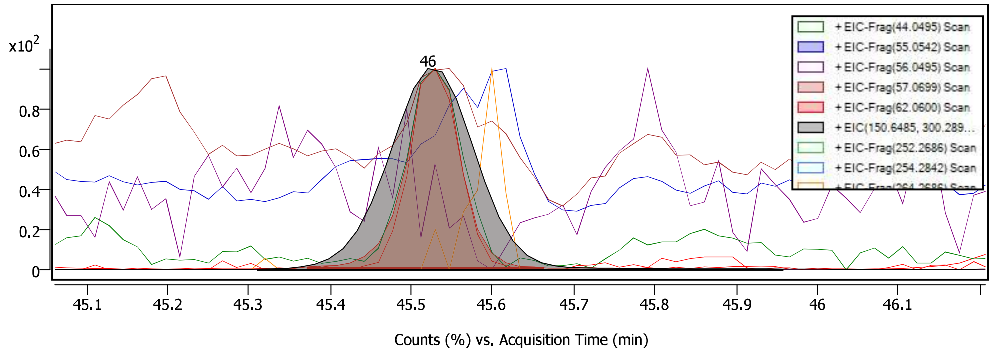

Structure

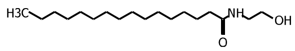

Coelution Plot

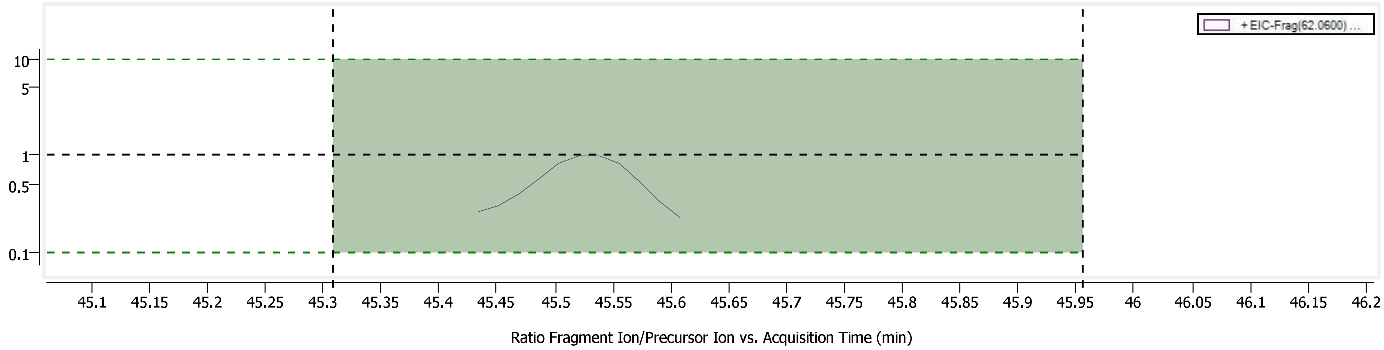

Compound Spectra (overlaid)

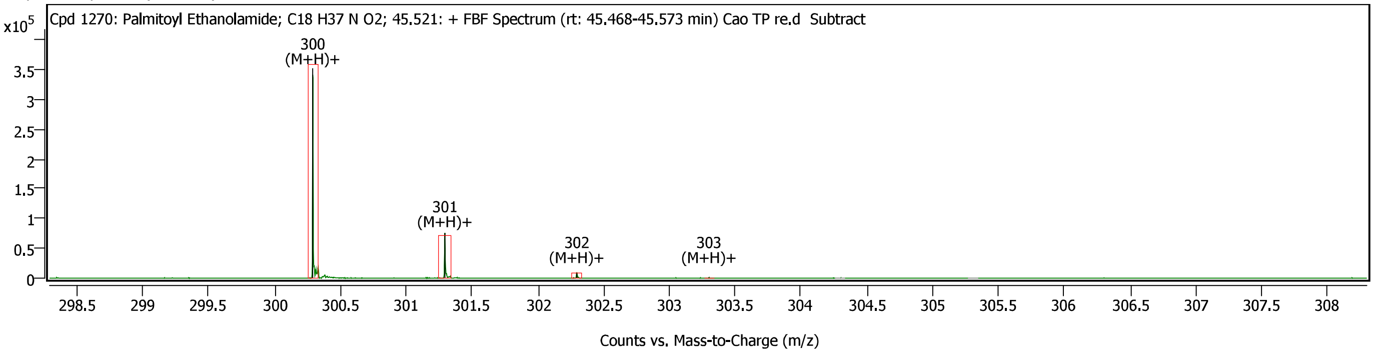

Fragment Spectrum (clean)

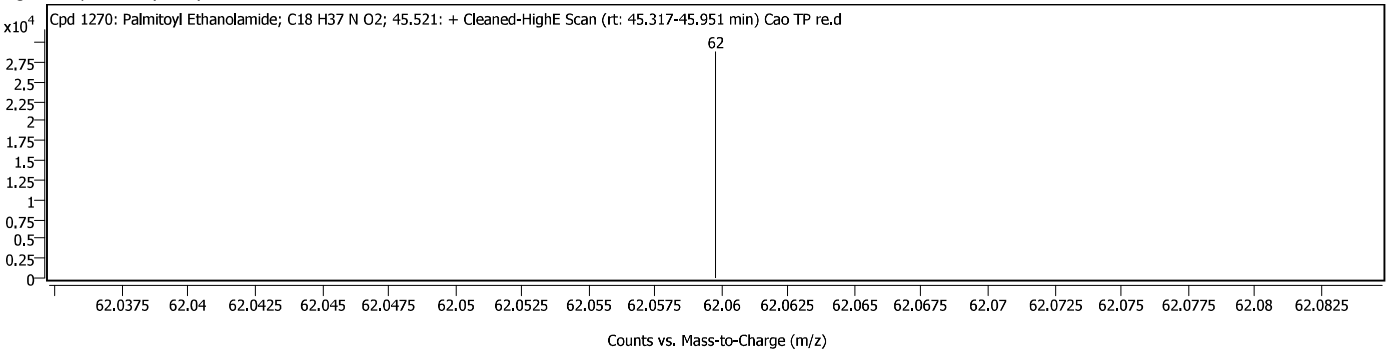

Fragment Spectrum (raw)

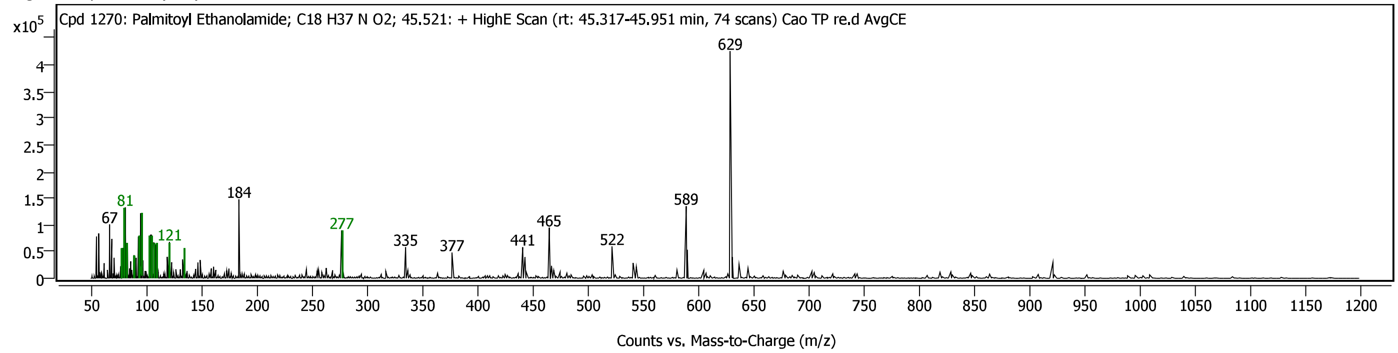

Compound ID Table

Name	Formula	Species	RT	RT Diff	Mass	CAS	ID Source	Score	Score (Lib)	Score (Tgt)
Palmitoyl Ethanolamide	C18 H37 N O2	(M+H)+	45.521		299.2830	544-31-0	FBF-FragConfirm	98.05		98.05
Sphingosine	C18 H37 N O2	(M+H)+	45.521		299.2830	123-78-4	FBF-FragConfirm	98.05		98.05
Palmitoyl-EA	C18 H37 N O2	(M+H)+	45.521		299.2830		FBF-FragConfirm	98.05		98.05
L-threo-Sphingosine C-18	C18 H37 N O2	(M+H)+	45.521		299.2830	25695-95-8	FBF-FragConfirm	98.05		98.05
5-hydroxy,3E-sphingosine	C18 H37 N O2	(M+H)+	45.521		299.2830		FBF-FragConfirm	98.05		98.05
3-Ketosphinganine	C18 H37 N O2	(M+H)+	45.521		299.2830	16105-69-4	FBF-FragConfirm	98.05		98.05
2R-aminooctadec-4Z-ene-1,3S-diol	C18 H37 N O2	(M+H)+	45.521		299.2830		FBF-FragConfirm	98.05		98.05
2-amino-octadecanoic acid	C18 H37 N O2	(M+H)+	45.521		299.2830		FBF-FragConfirm	98.05		98.05
12-amino-octadecanoic acid	C18 H37 N O2	(M+H)+	45.521		299.2830		FBF-FragConfirm	98.05		98.05
(8Z,d18:1) sphingosine	C18 H37 N O2	(M+H)+	45.521		299.2830		FBF-FragConfirm	98.05		98.05

Cpd 390: N-stearoyl serine

Name	Formula	RT	RI	Mass	Diff (Tgt, ppm)	CAS	ID Source	Score	Algorithm
N-stearoyl serine	C21 H41 N O4	45.591		371.3040	1.22		FBF	98.55	FBF

Species	m/z	Score (Tgt)	Score (Lib)	Score (DB)	Score (MFG)	Score (RT)
(M+H)+	372	98.55				

Compound Chromatograms (overlaid)

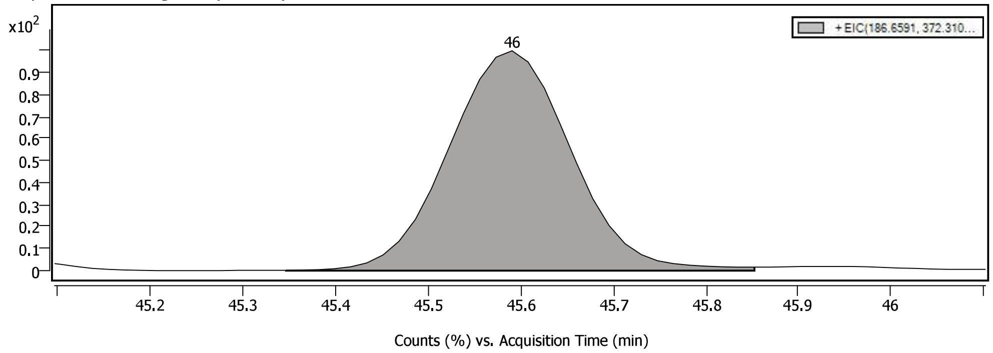

Structure

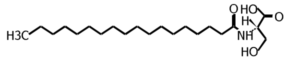

Compound Spectra (overlaid)

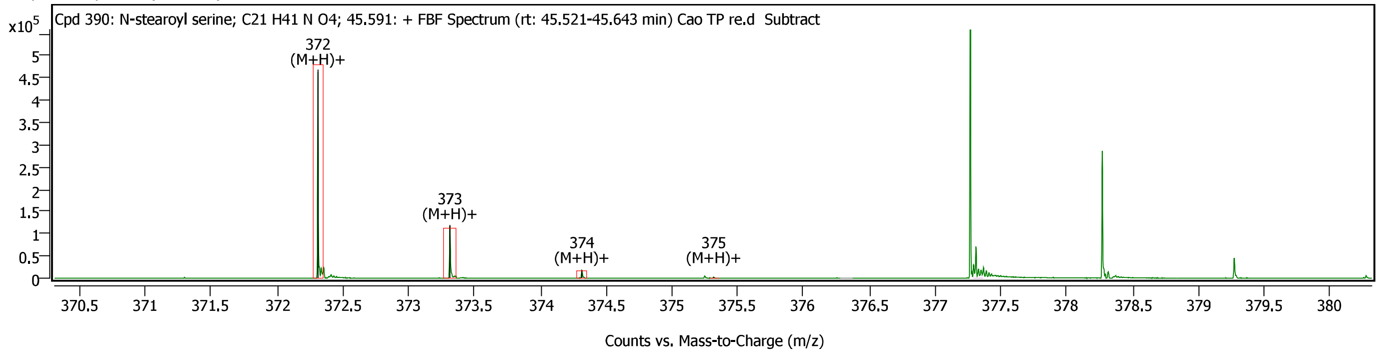

Compound ID Table

Name	Formula	Species	RT	RT Diff	Mass	CAS	ID Source	Score	Score (Lib)	Score (Tgt)
N-stearoyl serine	C21 H41 N O4	(M+H)+	45.591		371.3040		FBF	98.55		98.55

Cpd 513: MG(18:2(9Z,12Z)/0:0/0:0)[rac]

Name	Formula	RT	RI	Mass	Diff (Tgt, ppm)	CAS	ID Source	Score	Algorithm
MG(18:2(9Z,12Z)/0:0/0:0)[rac]	C21 H38 O4	45.591		354.2774	1.08	26545-74-4	M-FBF-FragConfirm	98.02	FBF

Species	m/z	Score (Tgt)	Score (Lib)	Score (DB)	Score (MFG)	Score (RT)
(M+H)+	355	98.02				

Compound Screening Report

Compound Chromatograms (overlaid)

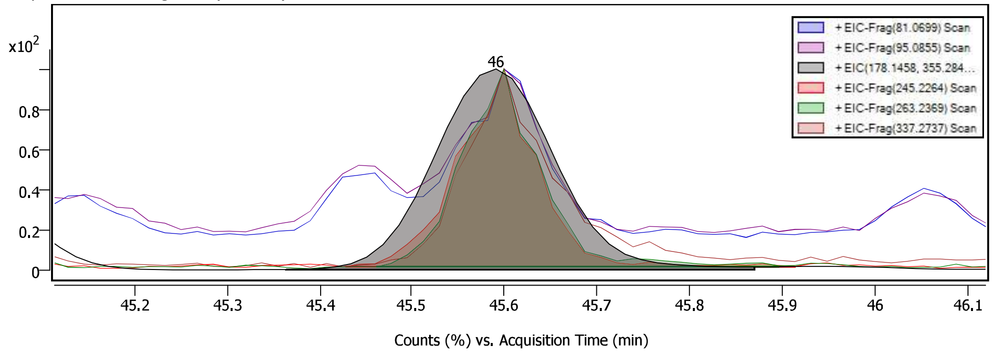

Structure

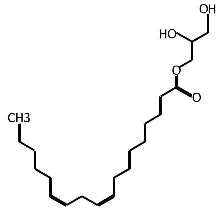

Coelution Plot

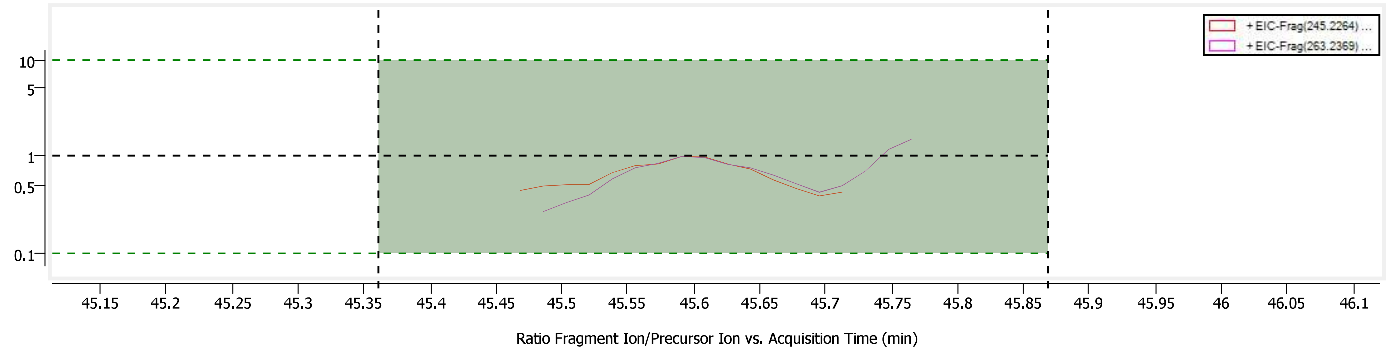

Compound Spectra (overlaid)

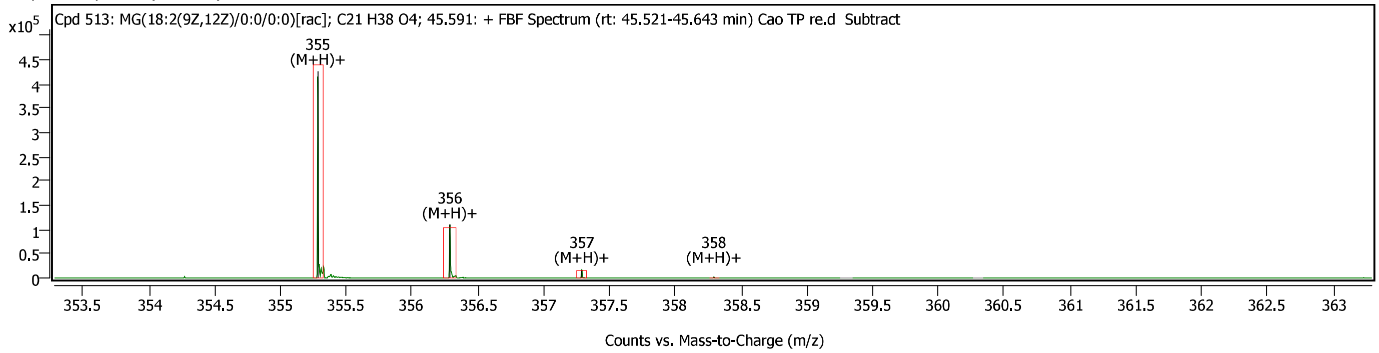

Fragment Spectrum (clean)

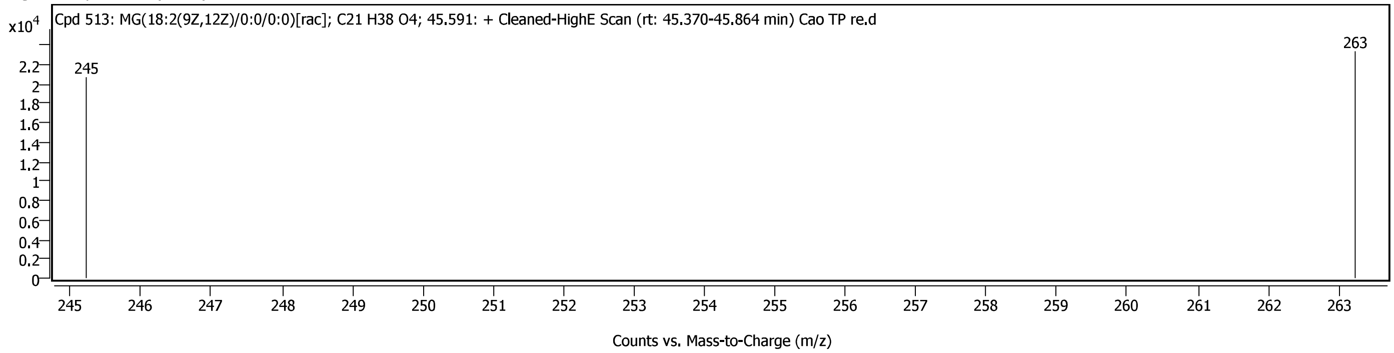

Fragment Spectrum (raw)

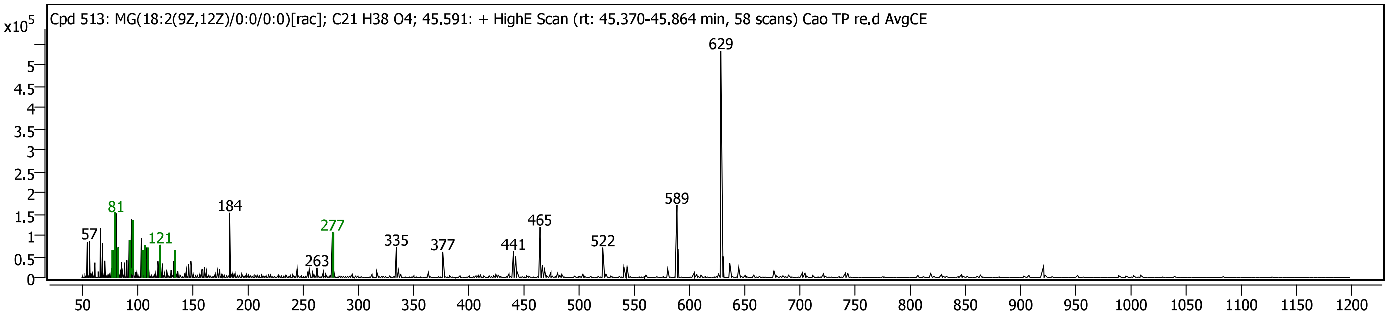

Compound ID Table

Name	Formula	Species	RT	RT Diff	Mass	CAS	ID Source	Score	Score (Lib)	Score (Tgt)
MG(18:2(9Z,12Z)/0:0/0:0)[rac]	C21 H38 O4	(M+H)+	45.591		354.2774	26545-74-4	FBF-FragConfirm	98.02		98.02
Methyl acetyl ricinoleate	C21 H38 O4	(M+H)+	45.591		354.2774	140-03-4	FBF-FragConfirm	98.02		98.02
1-Linoleoyl Glycerol	C21 H38 O4	(M+H)+	45.591		354.2774		FBF-FragConfirm	98.02		98.02
Ceriporic acid B	C21 H38 O4	(M+H)+	45.591		354.2774		FBF-FragConfirm	98.02		98.02
2-Linoleoyl Glycerol	C21 H38 O4	(M+H)+	45.591		354.2774	3443-82-1	FBF-FragConfirm	98.02		98.02
MG(0:0/18:2(9Z,12Z)/0:0)	C21 H38 O4	(M+H)+	45.591		354.2774		FBF-FragConfirm	98.02		98.02
MG(18:2(9Z,12Z)/0:0/0:0)	C21 H38 O4	(M+H)+	45.591		354.2774		FBF-FragConfirm	98.02		98.02
PGF2α Alcohol methyl ether	C21 H38 O4	(M+H)+	45.591		354.2774	143656-18-2	FBF-FragConfirm	98.02		98.02

Cpd 1487: MG(0:0/20:5(5Z,8Z,11Z,14Z,17Z)/0:0)

Name	Formula	RT	RI	Mass	Diff (Tgt, ppm)	CAS	ID Source	Score	Algorithm
MG(0:0/20:5(5Z,8Z,11Z,14Z,17Z)/0:0)	C23 H36 O4	45.608		376.2599	-3.85		M-FBF	89.82	FBF

Species	m/z	Score (Tgt)	Score (Lib)	Score (DB)	Score (MFG)	Score (RT)
(M+H)+	377	89.82				

Compound Chromatograms (overlaid)

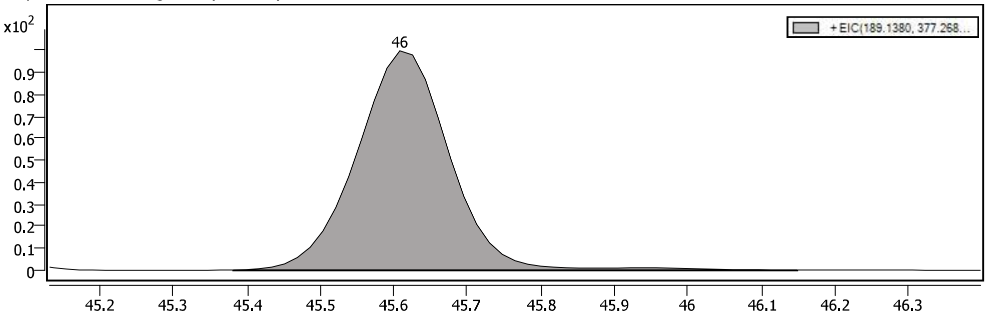

Structure

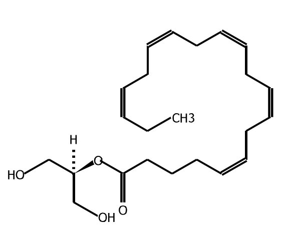

Compound Spectra (overlaid)

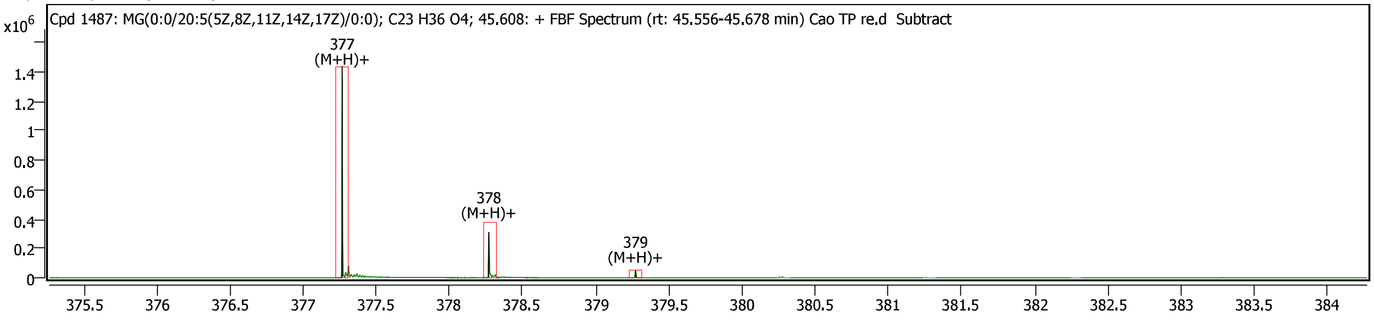

Compound ID Table

Name	Formula	Species	RT	RT Diff	Mass	CAS	ID Source	Score	Score (Lib)	Score (Tgt)
MG(0:0/20:5(5Z,8Z,11Z,14Z,17Z)/0:0)	C23 H36 O4	(M+H)+	45.608		376.2599		FBF	89.82		89.82
MG(20:5(5Z,8Z,11Z,14Z,17Z)/0:0/0:0)	C23 H36 O4	(M+H)+	45.608		376.2599		FBF	89.82		89.82
3-Acetyl-5alpha-androstane-3beta,17beta-diol 3-acetate	C23 H36 O4	(M+H)+	45.608		376.2599		FBF	89.82		89.82
[12]-Gingerdione	C23 H36 O4	(M+H)+	45.608		376.2599	91815-31-5	FBF	89.82		89.82
5alpha-Androstane-3beta,17beta-diol diacetate	C23 H36 O4	(M+H)+	45.608		376.2599		FBF	89.82		89.82

Cpd 982: 1α-hydroxy-22-[3-(1-hydroxy-1-methylethyl)phenyl]-23,24,25,26,27-pentanorvitamin D3 / 1α-hydroxy-22-[3-(1-hydroxy-1-methylethyl)phenyl]-23,24,25,26,27-pentanorcholecalciferol

Compound Screening Report

Name	Formula	RT	RI	Mass	Diff (Tgt, ppm)	CAS	ID Source	Score	Algorithm
1α-hydroxy-22-[3-(1-hydroxy-1-methylethyl)phenyl]-23,24,25,26,27-pentanorvitamin D3 / 1α-hydroxy-22-[3-(1-hydroxy-1-methylethyl)phenyl]-23,24,25,26,27-pentanorcholecalciferol	C31 H44 O3	45.625		464.3272	-3.89		FBF	88.48	FBF

Species	m/z	Score (Tgt)	Score (Lib)	Score (DB)	Score (MFG)	Score (RT)
(M+H)+	465	88.48				

Compound Chromatograms (overlaid)

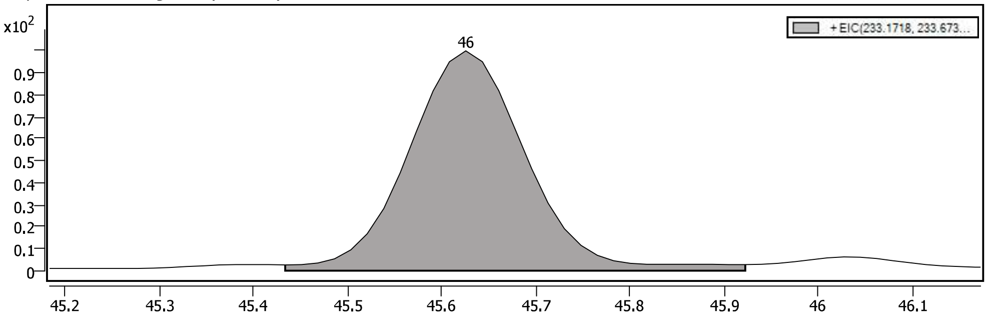

Structure

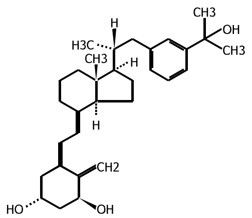

Compound Spectra (overlaid)

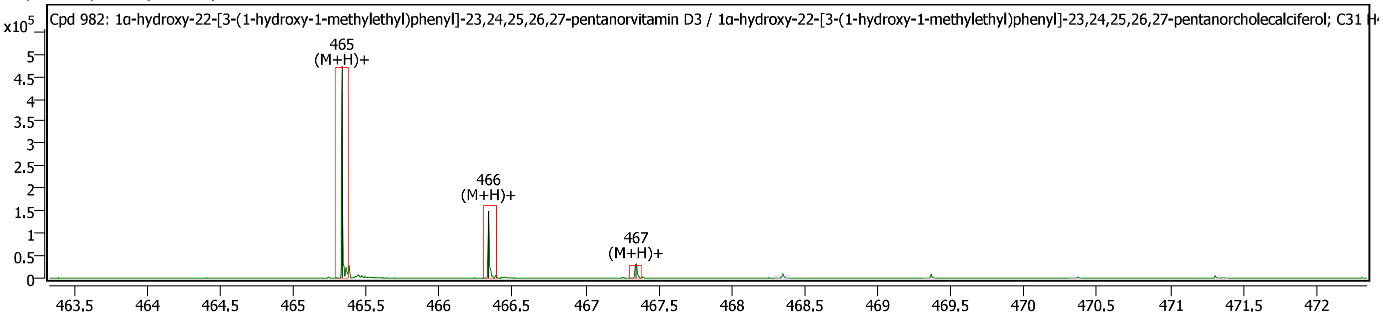

Compound ID Table

Name	Formula	Species	RT	RT Diff	Mass	CAS	ID Source	Score	Score (Lib)	Score (Tgt)
1α-hydroxy-22-[3-(1-hydroxy-1-methylethyl)phenyl]-23,24,25,26,27-pentanorvitamin D3 / 1α-hydroxy-22-[3-(1-hydroxy-1-methylethyl)phenyl]-23,24,25,26,27-pentanorcholecalciferol	C31 H44 O3	(M+H)+	45.625		464.3272		FBF	88.48		88.48

Cpd 1406: <Ipecac (Emetine)>

Name	Formula	RT	RI	Mass	Diff (Tgt, ppm)	CAS	ID Source	Score	Algorithm
<Ipecac (Emetine)>	C29 H40 N2 O4	45.625		480.3004	3.30	483-18-1	FBF	91.45	FBF

Species	m/z	Score (Tgt)	Score (Lib)	Score (DB)	Score (MFG)	Score (RT)
(M+H)+	481	91.45				

Compound Chromatograms (overlaid)

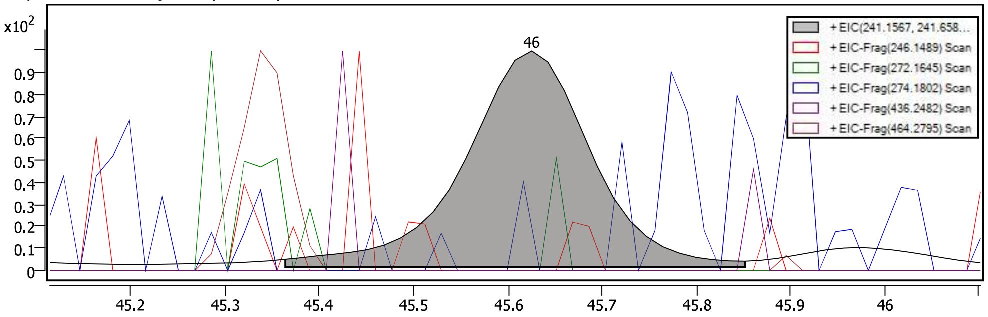

Structure

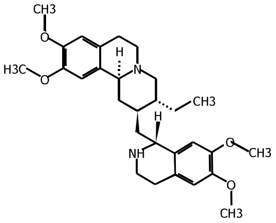

Coelution Plot

Compound Spectra (overlaid)

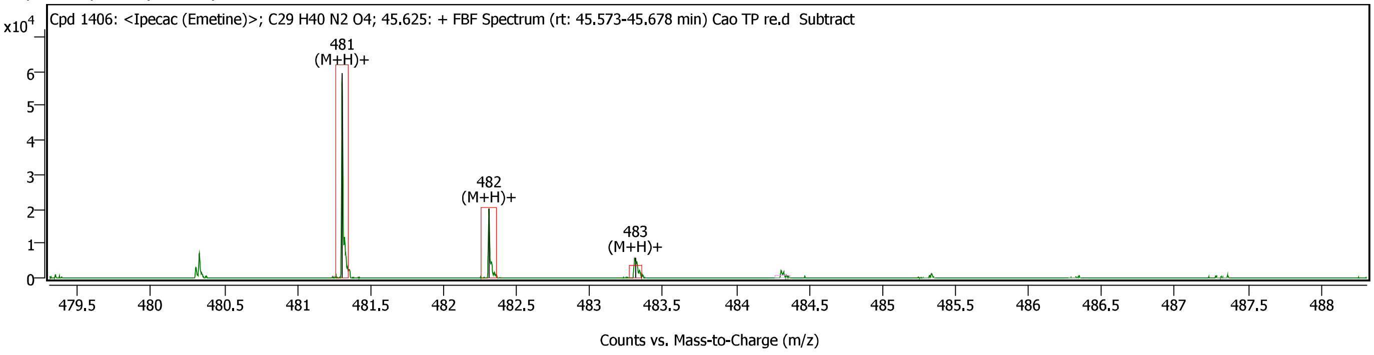

Fragment Spectrum (raw)

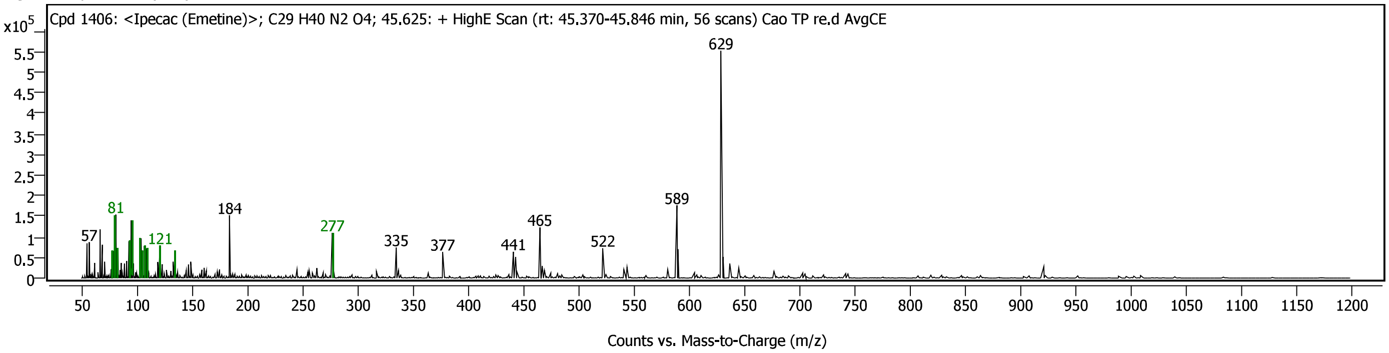

Compound ID Table

Name	Formula	Species	RT	RT Diff	Mass	CAS	ID Source	Score	Score (Lib)	Score (Tgt)
<Ipecac (Emetine)>	C29 H40 N2 O4	(M+H)+	45.625		480.3004	483-18-1	FBF	91.45		91.45

Cpd 242: Schleicherastatin 3

Name	Formula	RT	RI	Mass	Diff (Tgt, ppm)	CAS	ID Source	Score	Algorithm
Schleicherastatin 3	C29 H50 O3	45.748		446.3762	0.41	256445-53-1	M-FBF	99.32	FBF

Species	m/z	Score (Tgt)	Score (Lib)	Score (DB)	Score (MFG)	Score (RT)
(M+H)+	447	99.32				

Compound Chromatograms (overlaid)

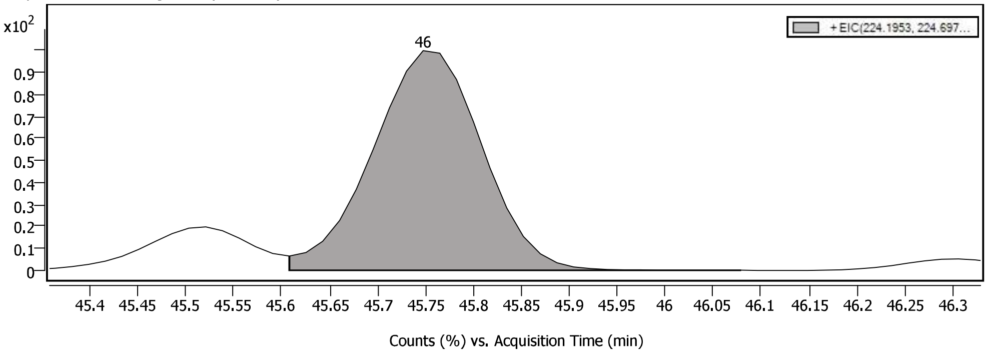

Structure

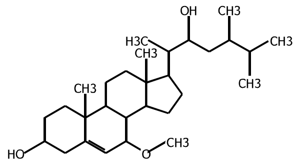

Compound Spectra (overlaid)

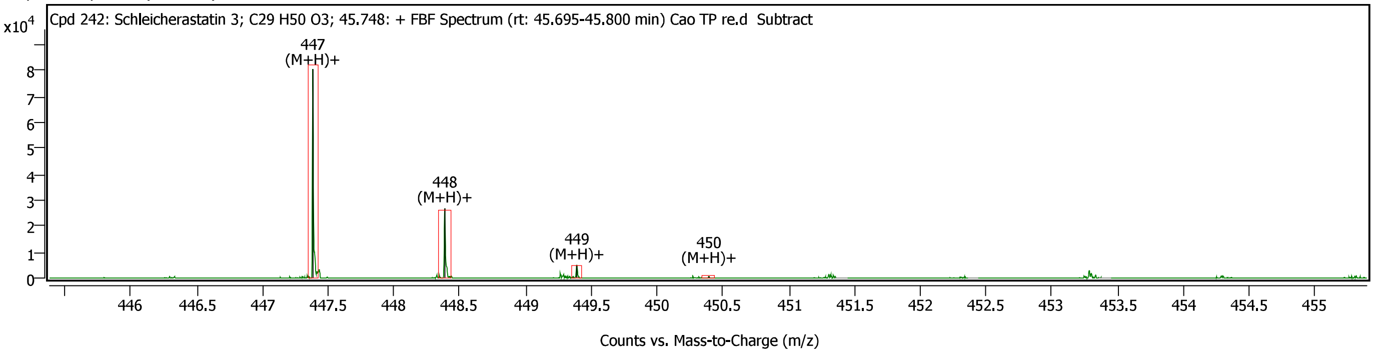

Compound Screening Report

Compound ID Table

Name	Formula	Species	RT	RT Diff	Mass	CAS	ID Source	Score	Score (Lib)	Score (Tgt)
Schleicherastatin 3	C29 H50 O3	(M+H)+	45.748		446.3762	256445-53-1	FBF	99.32		99.32
24-ethyl-5alpha-cholest-25-en-3alpha,12alpha,16alpha-triol	C29 H50 O3	(M+H)+	45.748		446.3762		FBF	99.32		99.32
4alpha-methyl-24-methylene-cholestan-3beta,8beta,11beta-triol	C29 H50 O3	(M+H)+	45.748		446.3762		FBF	99.32		99.32
24-hydroperoxy-24-vinylcholesterol	C29 H50 O3	(M+H)+	45.748		446.3762		FBF	99.32		99.32
alpha-Tocopherolquinone	C29 H50 O3	(M+H)+	45.748		446.3762	7559-04-8	FBF	99.32		99.32
Stigmastentriol	C29 H50 O3	(M+H)+	45.748		446.3762		FBF	99.32		99.32

Cpd 1492: <Palmitic amide>

Name	Formula	RT	RI	Mass	Diff (Tgt, ppm)	CAS	ID Source	Score	Algorithm
<Palmitic amide>	C16 H33 N O	45.800		255.2566	1.41	629-54-9	FBF	98.25	FBF

Species	m/z	Score (Tgt)	Score (Lib)	Score (DB)	Score (MFG)	Score (RT)
(M+H)+	256	98.25				

Compound Chromatograms (overlaid)

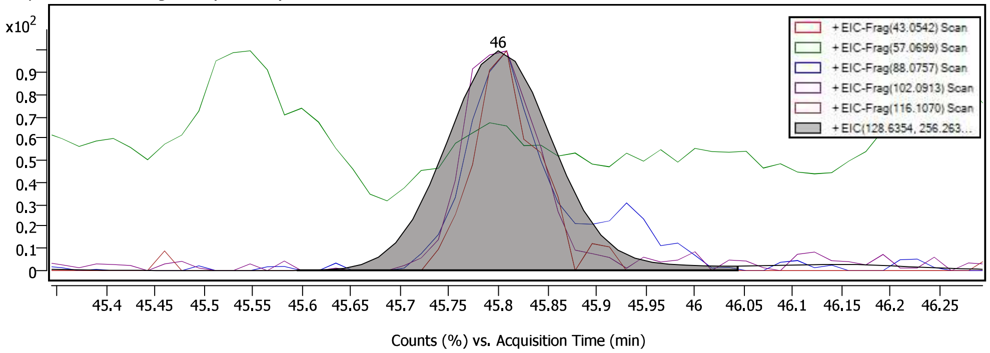

Structure

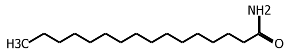

Coelution Plot

Compound Spectra (overlaid)

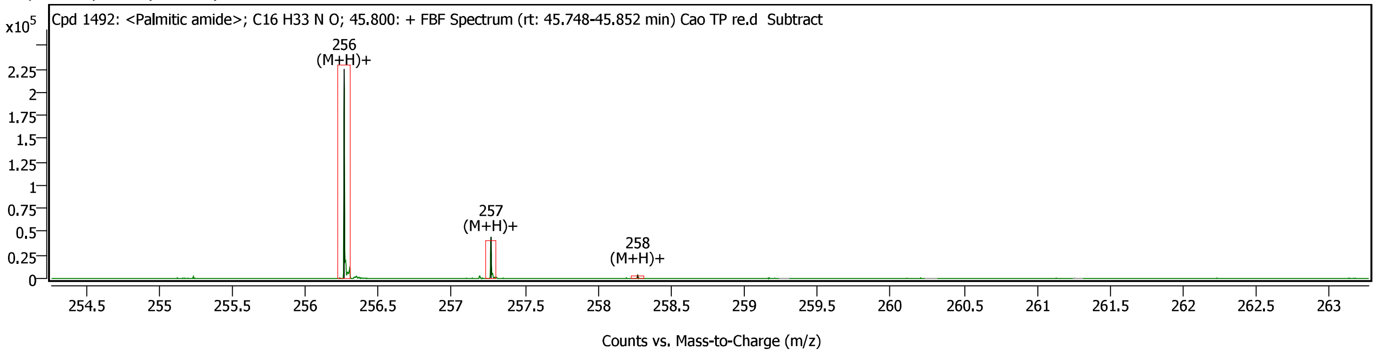

Fragment Spectrum (raw)

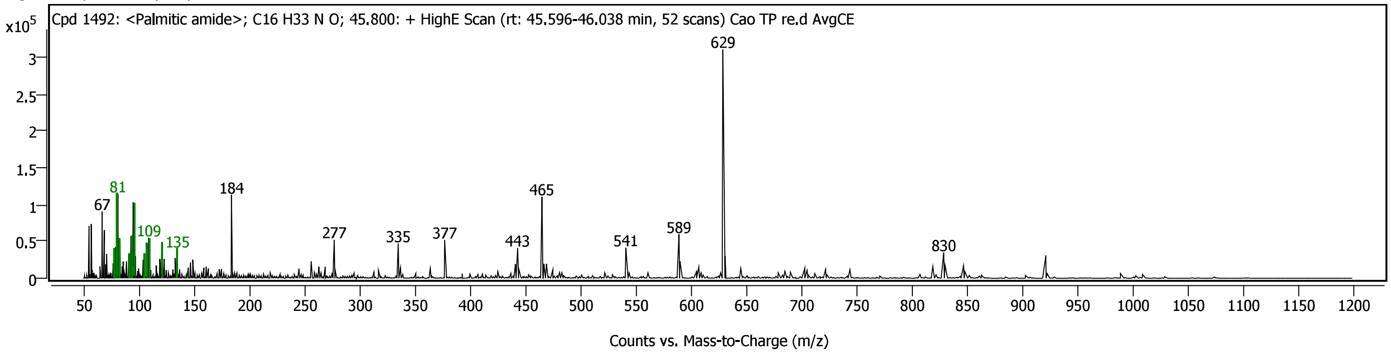

Compound ID Table

Name	Formula	Species	RT	RT Diff	Mass	CAS	ID Source	Score	Score (Lib)	Score (Tgt)
<Palmitic amide>	C16 H33 N O	(M+H)+	45.800		255.2566	629-54-9	FBF	98.25		98.25

Cpd 389: N-palmitoyl threonine

Name	Formula	RT	RI	Mass	Diff (Tgt, ppm)	CAS	ID Source	Score	Algorithm
N-palmitoyl threonine	C20 H39 N O4	46.236		357.2882	0.70		FBF	98.28	FBF

Species	m/z	Score (Tgt)	Score (Lib)	Score (DB)	Score (MFG)	Score (RT)
(M+H)+	358	98.28				

Compound Chromatograms (overlaid)

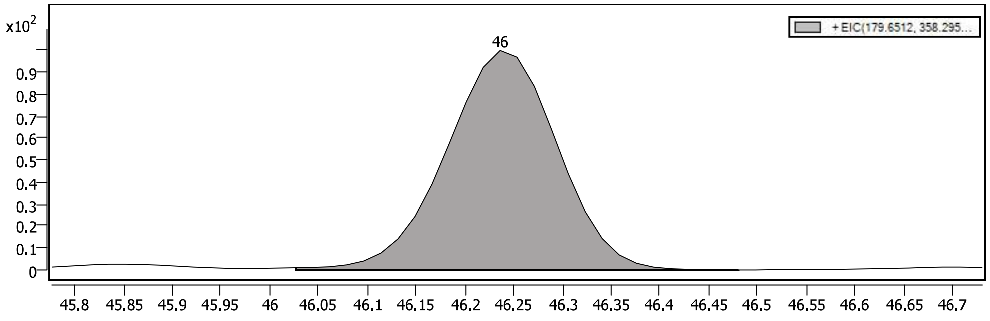

Structure

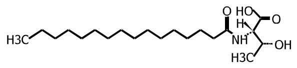

Compound Spectra (overlaid)

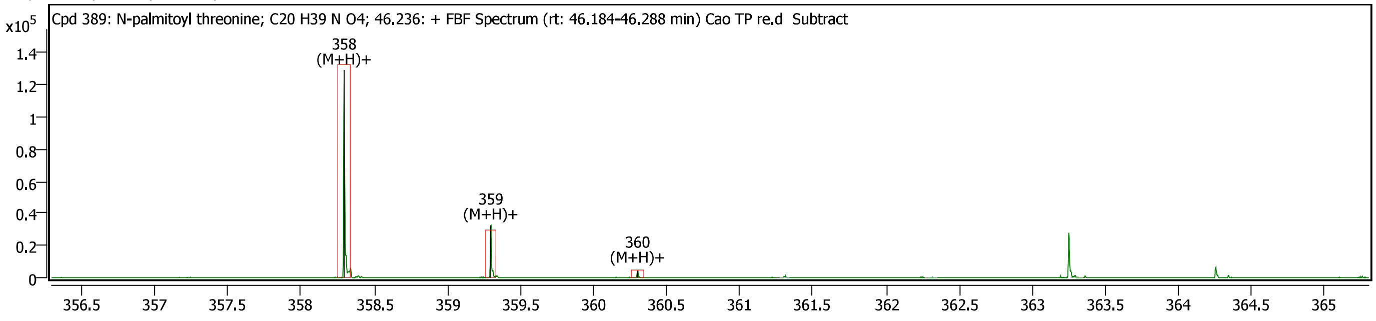

Compound ID Table

Name	Formula	Species	RT	RT Diff	Mass	CAS	ID Source	Score	Score (Lib)	Score (Tgt)
N-palmitoyl threonine	C20 H39 N O4	(M+H)+	46.236		357.2882		FBF	98.28		98.28

Cpd 1457: Oleoyl Ethanolamide

Name	Formula	RT	RI	Mass	Diff (Tgt, ppm)	CAS	ID Source	Score	Algorithm
Oleoyl Ethanolamide	C20 H39 N O2	46.323		325.2983	0.72	111-58-0	FBF-FragConfirm	98.72	FBF

Species	m/z	Score (Tgt)	Score (Lib)	Score (DB)	Score (MFG)	Score (RT)
(M+H)+	326	98.72				

Compound Chromatograms (overlaid)

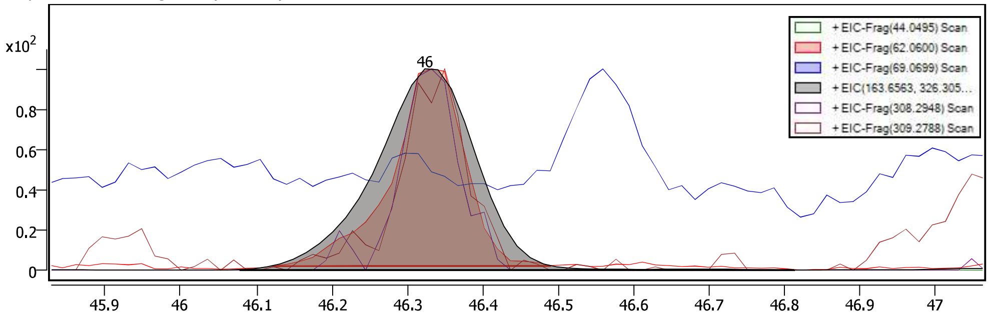

Structure

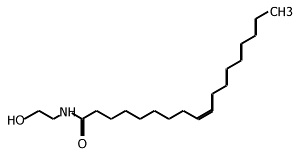

Coelution Plot

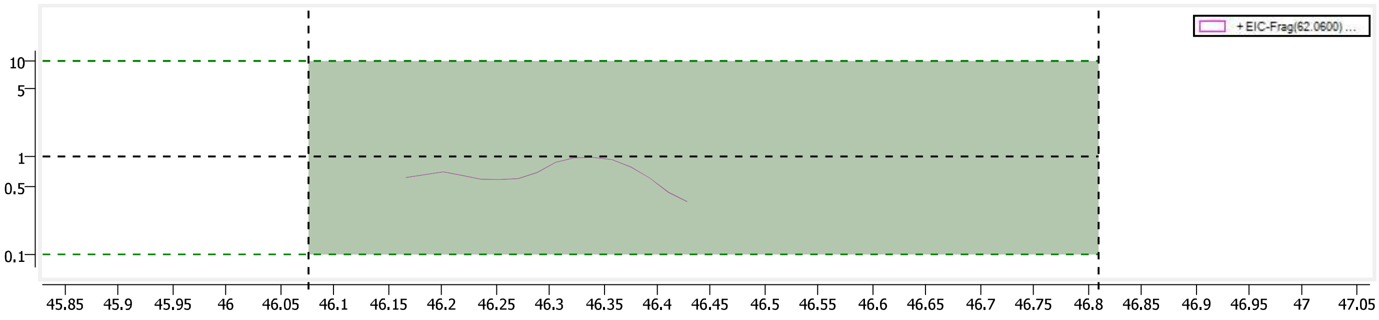

Compound Screening Report

Compound Spectra (overlaid)

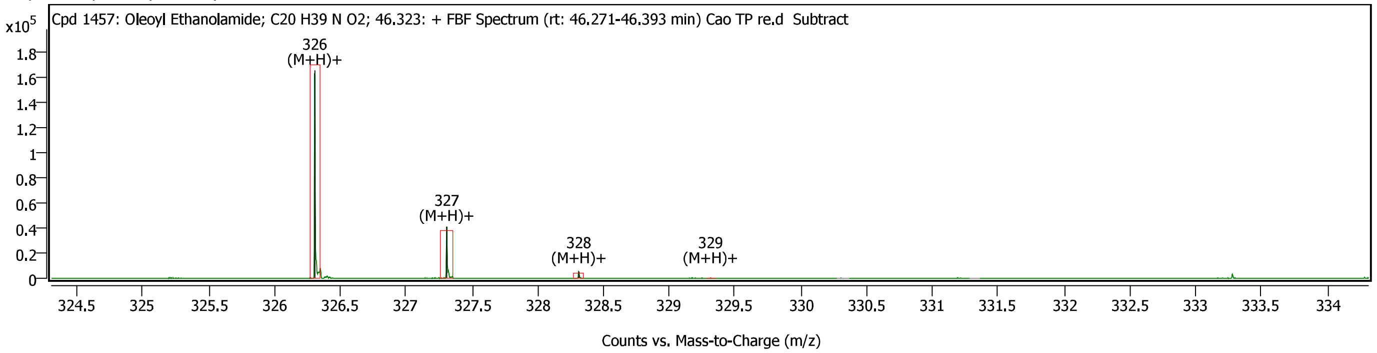

Fragment Spectrum (clean)

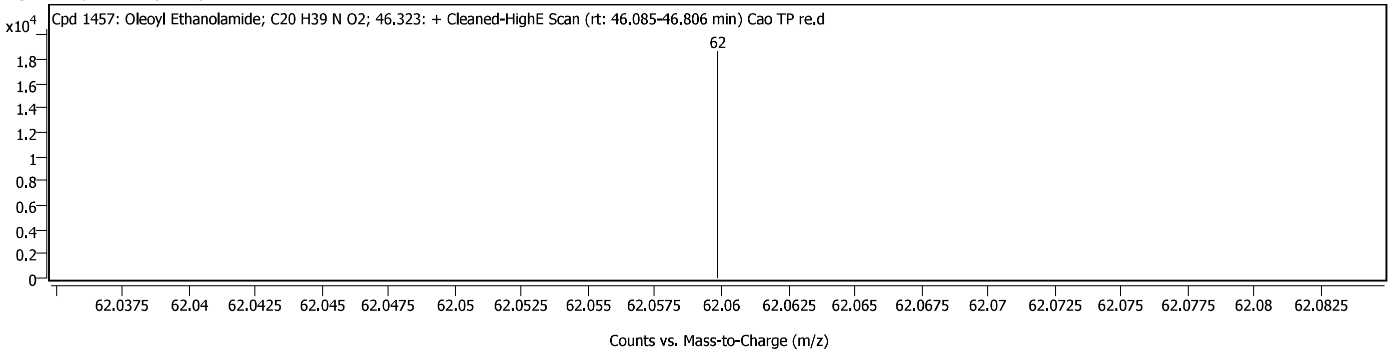

Fragment Spectrum (raw)

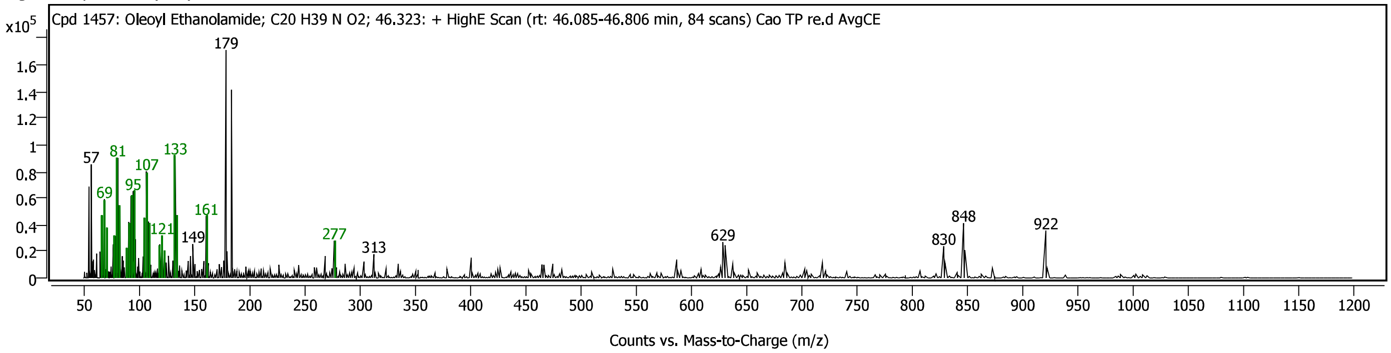

Compound ID Table

Name	Formula	Species	RT	RT Diff	Mass	CAS	ID Source	Score	Score (Lib)	Score (Tgt)
Oleoyl Ethanolamide	C20 H39 N O2	(M+H)+	46.323		325.2983	111-58-0	FBF-FragConfirm	98.72		98.72

Cpd 157: <9-deoxy-9-methylene-16,16-dimethyl -PGE2>

Name	Formula	RT	RI	Mass	Diff (Tgt, ppm)	CAS	ID Source	Score	Algorithm
<9-deoxy-9-methylene-16,16-dimethyl -PGE2>	C23 H38 O4	46.341		378.2782	3.05	61263-35-2	M-FBF	93.63	FBF

Species	m/z	Score (Tgt)	Score (Lib)	Score (DB)	Score (MFG)	Score (RT)
(M+H)+	379	93.63				

Compound Chromatograms (overlaid)

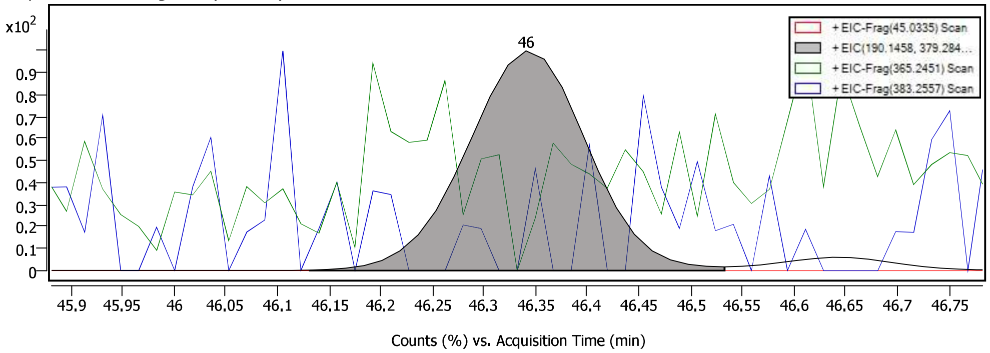

Structure

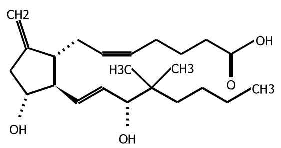

Coelution Plot

Compound Spectra (overlaid)

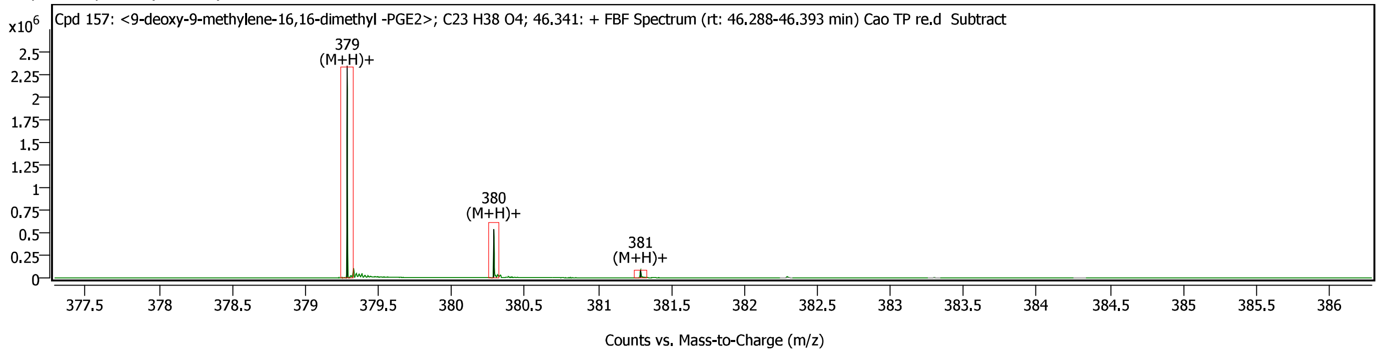

Fragment Spectrum (raw)

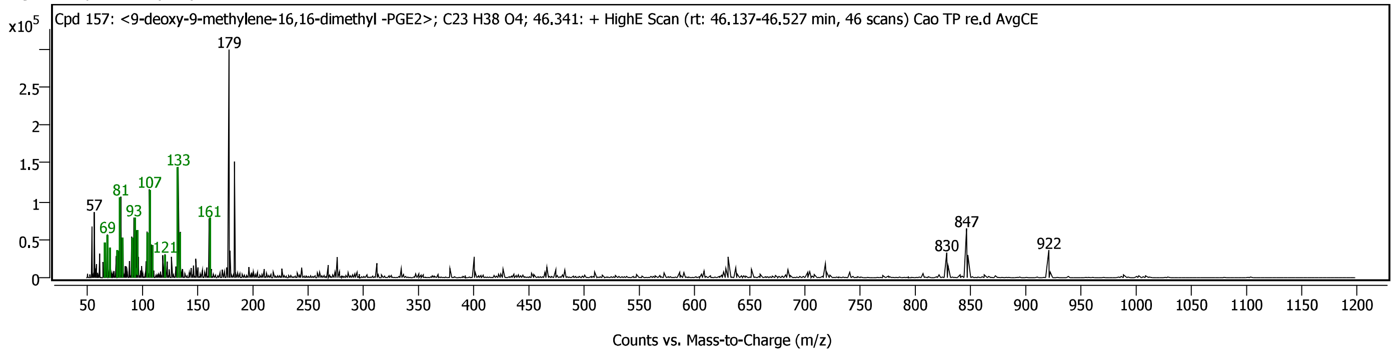

Compound ID Table

Name	Formula	Species	RT	RT Diff	Mass	CAS	ID Source	Score	Score (Lib)	Score (Tgt)
<9-deoxy-9-methylene-16,16-dimethyl -PGE2>	C23 H38 O4	(M+H)+	46.341		378.2782	61263-35-2	FBF	93.63		93.63
<Persenone A>	C23 H38 O4	(M+H)+	46.341		378.2782		FBF	93.63		93.63
<3α,7α,12α-Trihydroxy-24-nor-5β-cholan-23-al>	C23 H38 O4	(M+H)+	46.341		378.2782		FBF	93.63		93.63
<[12]-Gingerol>	C23 H38 O4	(M+H)+	46.341		378.2782	104264-55-3	FBF	93.63		93.63
<1-Acetoxy-2-hydroxy-5,12,15-heneicosatrien-4-one>	C23 H38 O4	(M+H)+	46.341		378.2782		FBF	93.63		93.63
<1-AG>	C23 H38 O4	(M+H)+	46.341		378.2782	35474-99-8	FBF	93.63		93.63
<3alpha,7alpha,12alpha-Trihydroxy-24-nor-5beta-cholan-23-al>	C23 H38 O4	(M+H)+	46.341		378.2782		FBF	93.63		93.63
<Norchenodeoxycholic acid>	C23 H38 O4	(M+H)+	46.341		378.2782		FBF	93.63		93.63
<MG(0:0/20:4(5Z,8Z,11Z,14Z)/0:0)>	C23 H38 O4	(M+H)+	46.341		378.2782	53847-30-6	FBF	93.63		93.63
<MG(0:0/20:4(8Z,11Z,14Z,17Z)/0:0)>	C23 H38 O4	(M+H)+	46.341		378.2782		FBF	93.63		93.63
<MG(20:4(8Z,11Z,14Z,17Z)/0:0/0:0)>	C23 H38 O4	(M+H)+	46.341		378.2782		FBF	93.63		93.63

Cpd 1458: <Dodemorph>

Name	Formula	RT	RI	Mass	Diff (Tgt, ppm)	CAS	ID Source	Score	Algorithm
<Dodemorph>	C18 H35 N O	46.568		281.2725	2.22	1593-77-7	M-FBF	97.24	FBF

Species	m/z	Score (Tgt)	Score (Lib)	Score (DB)	Score (MFG)	Score (RT)
(M+H)+	282	97.24				

Compound Chromatograms (overlaid)

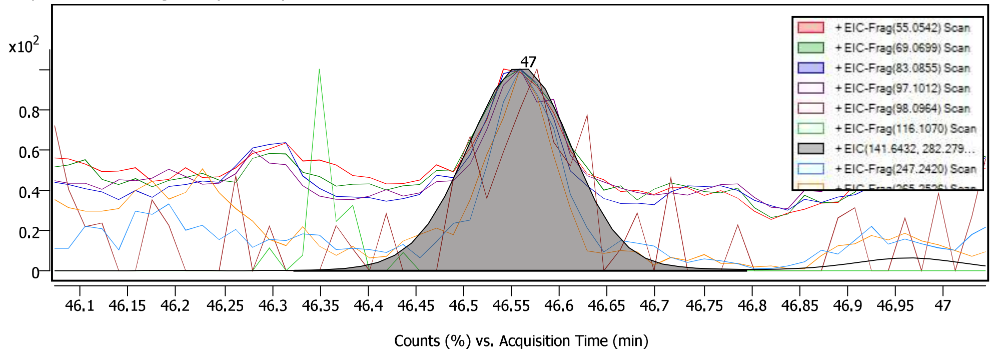

Structure

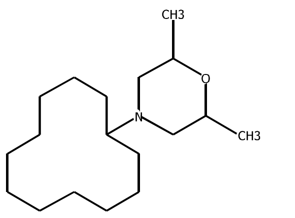

Coelution Plot

Compound Screening Report

Compound Spectra (overlaid)

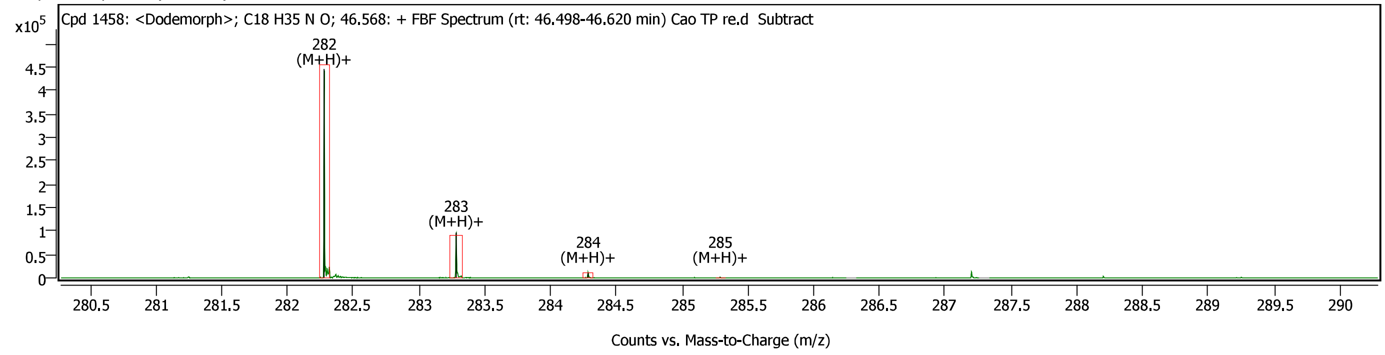

Fragment Spectrum (raw)

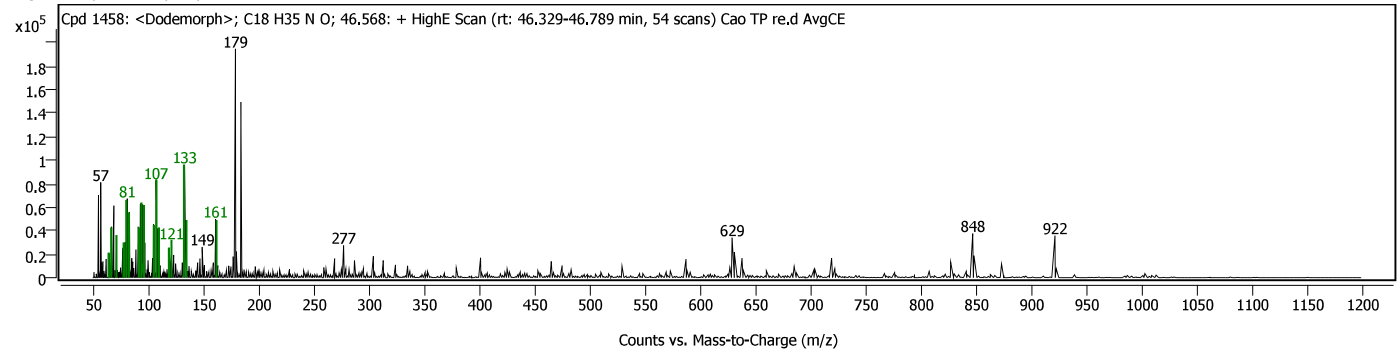

Compound ID Table

Name	Formula	Species	RT	RT Diff	Mass	CAS	ID Source	Score	Score (Lib)	Score (Tgt)
<Dodemorph>	C18 H35 N O	(M+H)+	46.568		281.2725	1593-77-7	FBF	97.24		97.24
<Oleamide>	C18 H35 N O	(M+H)+	46.568		281.2725	301-02-0	FBF	97.24		97.24
<Elaidamide>	C18 H35 N O	(M+H)+	46.568		281.2725	4303-70-2	FBF	97.24		97.24

Cpd 335: PS(12:0/15:0)

Name	Formula	RT	RI	Mass	Diff (Tgt, ppm)	CAS	ID Source	Score	Algorithm
PS(12:0/15:0)	C33 H64 N O10 P	46.882		665.4273	0.77		M-FBF	99.20	FBF

Species	m/z	Score (Tgt)	Score (Lib)	Score (DB)	Score (MFG)	Score (RT)
(M+H)+	666	99.20				

Compound Chromatograms (overlaid)

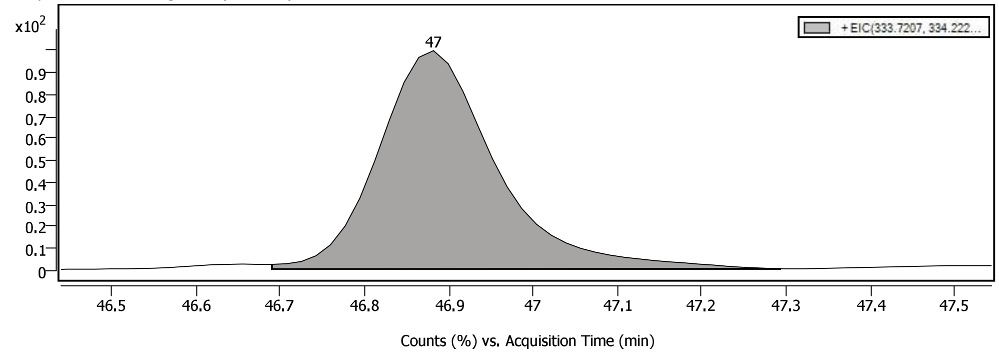

Structure

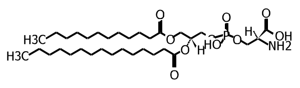

Compound Spectra (overlaid)

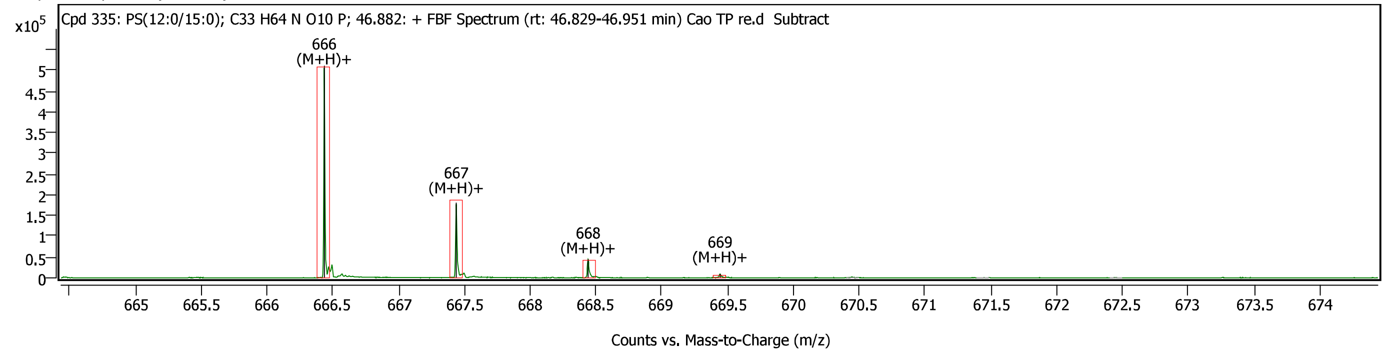

Compound Screening Report

Compound ID Table

Name	Formula	Species	RT	RT Diff	Mass	CAS	ID Source	Score	Score (Lib)	Score (Tgt)
PS(12:0/15:0)	C33 H64 N O10 P	(M+H)+	46.882		665.4273		FBF	99.20		99.20
PS(15:0/12:0)	C33 H64 N O10 P	(M+H)+	46.882		665.4273		FBF	99.20		99.20
PS(14:0/13:0)	C33 H64 N O10 P	(M+H)+	46.882		665.4273		FBF	99.20		99.20
PS(13:0/14:0)	C33 H64 N O10 P	(M+H)+	46.882		665.4273		FBF	99.20		99.20

Cpd 537: Voacamine

Name	Formula	RT	RI	Mass	Diff (Tgt, ppm)	CAS	ID Source	Score	Algorithm
Voacamine	C43 H52 N4 O5	46.899		704.3961	3.36	3371-85-5	FBF	90.07	FBF

Species	m/z	Score (Tgt)	Score (Lib)	Score (DB)	Score (MFG)	Score (RT)
(M+H)+	705	90.07				

Compound Chromatograms (overlaid)

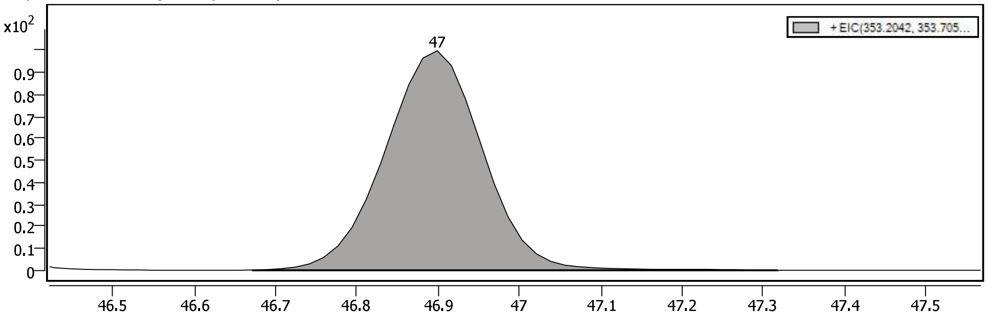

Counts (%) vs. Acquisition Time (min)

Structure

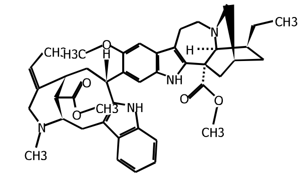

Compound Spectra (overlaid)

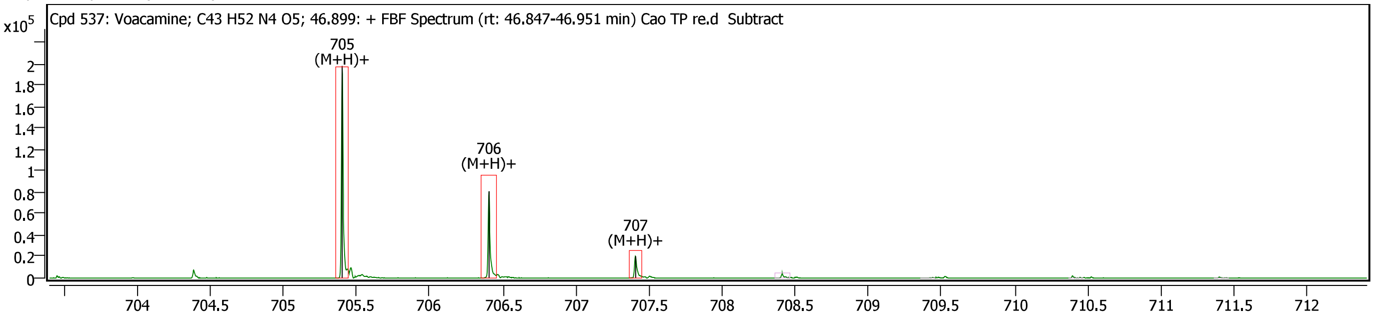

Counts vs. Mass-to-Charge (m/z)

Compound ID Table

Name	Formula	Species	RT	RT Diff	Mass	CAS	ID Source	Score	Score (Lib)	Score (Tgt)
Voacamine	C43 H52 N4 O5	(M+H)+	46.899		704.3961	3371-85-5	FBF	90.07		90.07

Cpd 591: R.g.-Keto III

Name	Formula	RT	RI	Mass	Diff (Tgt, ppm)	CAS	ID Source	Score	Algorithm
R.g.-Keto III	C42 H60 O4	46.951		628.4490	-0.23		FBF	81.35	FBF

Species	m/z	Score (Tgt)	Score (Lib)	Score (DB)	Score (MFG)	Score (RT)
(M+H)+	629	81.35				

Compound Chromatograms (overlaid)

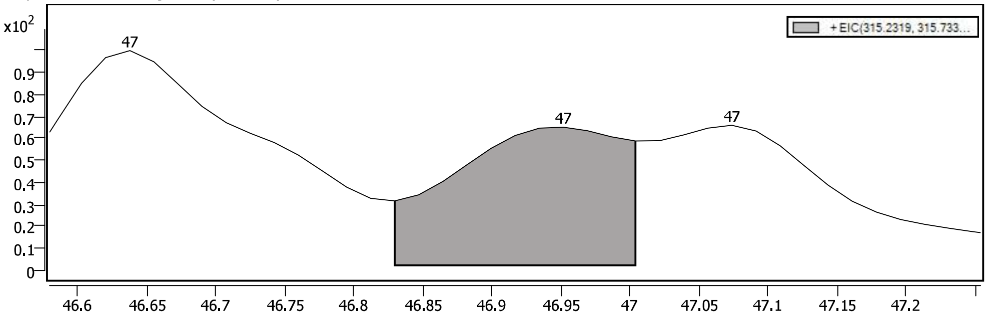

Counts (%) vs. Acquisition Time (min)

Structure

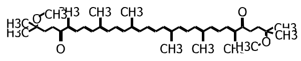

Compound Screening Report

Compound Spectra (overlaid)

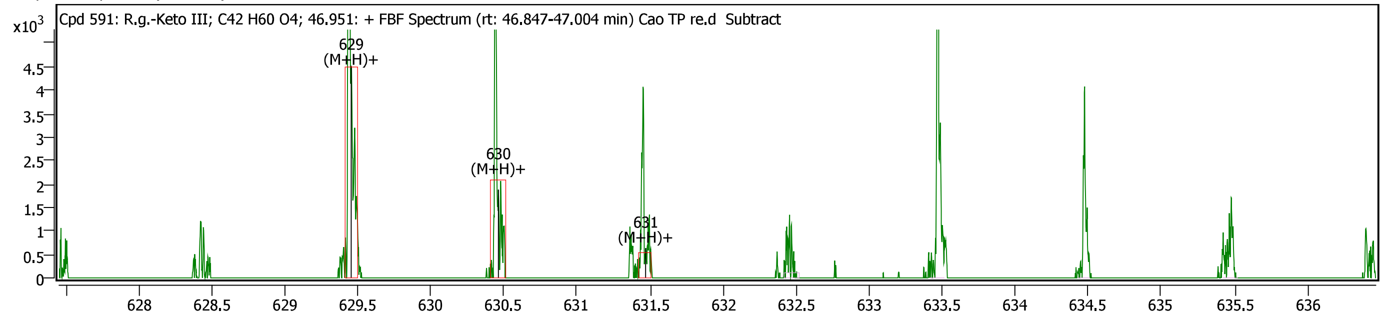

Compound ID Table

Name	Formula	Species	RT	RT Diff	Mass	CAS	ID Source	Score	Score (Lib)	Score (Tgt)
R.g.-Keto III	C42 H60 O4	(M+H)+	46.951		628.4490		FBF	81.35		81.35

Cpd 134: Armillatin

Name	Formula	RT	RI	Mass	Diff (Tgt, ppm)	CAS	ID Source	Score	Algorithm
Armillatin	C38 H58 O6	46.986		610.4211	-3.72	139051-17-5	FBF	93.77	FBF

Species	m/z	Score (Tgt)	Score (Lib)	Score (DB)	Score (MFG)	Score (RT)
(M+H)+	611	93.77				

Compound Chromatograms (overlaid)

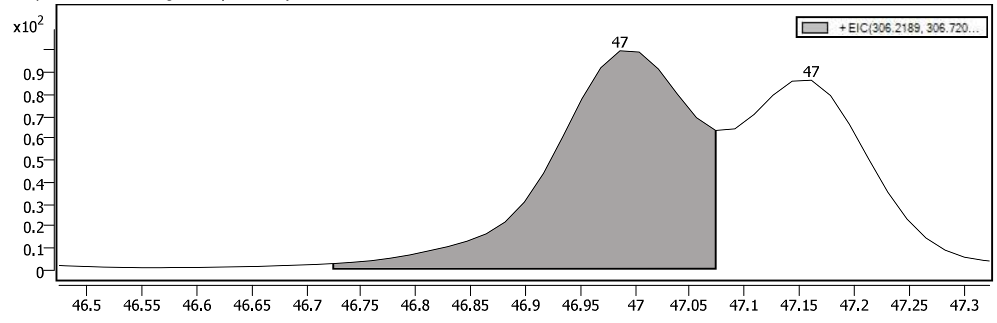

Structure

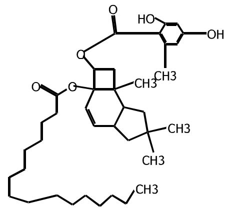

Compound Spectra (overlaid)

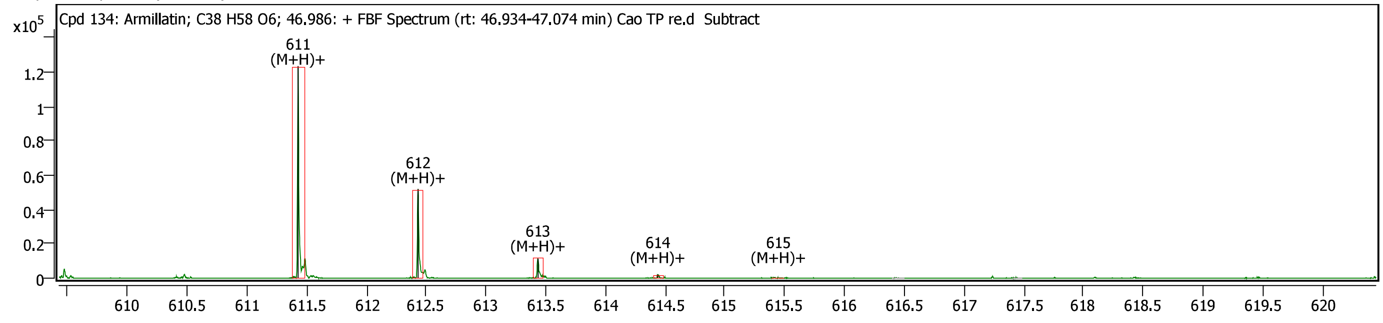

Compound ID Table

Name	Formula	Species	RT	RT Diff	Mass	CAS	ID Source	Score	Score (Lib)	Score (Tgt)
Armillatin	C38 H58 O6	(M+H)+	46.986		610.4211	139051-17-5	FBF	93.77		93.77

Cpd 1322: Kni 102

Name	Formula	RT	RI	Mass	Diff (Tgt, ppm)	CAS	ID Source	Score	Algorithm
Kni 102	C31 H41 N5 O7	47.091		595.3026	3.38	139694-65-8	FBF	89.55	FBF

Species	m/z	Score (Tgt)	Score (Lib)	Score (DB)	Score (MFG)	Score (RT)
(M+H)+	596	89.55				

Compound Chromatograms (overlaid)

Structure

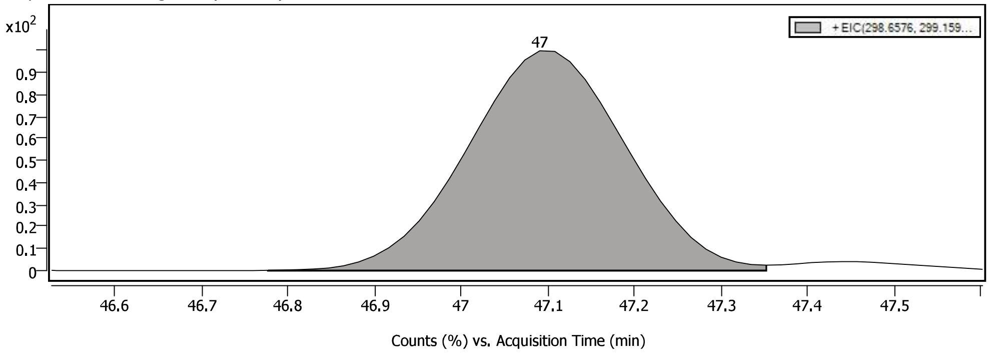

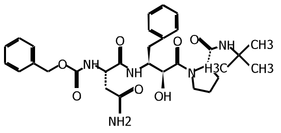

Compound Spectra (overlaid)

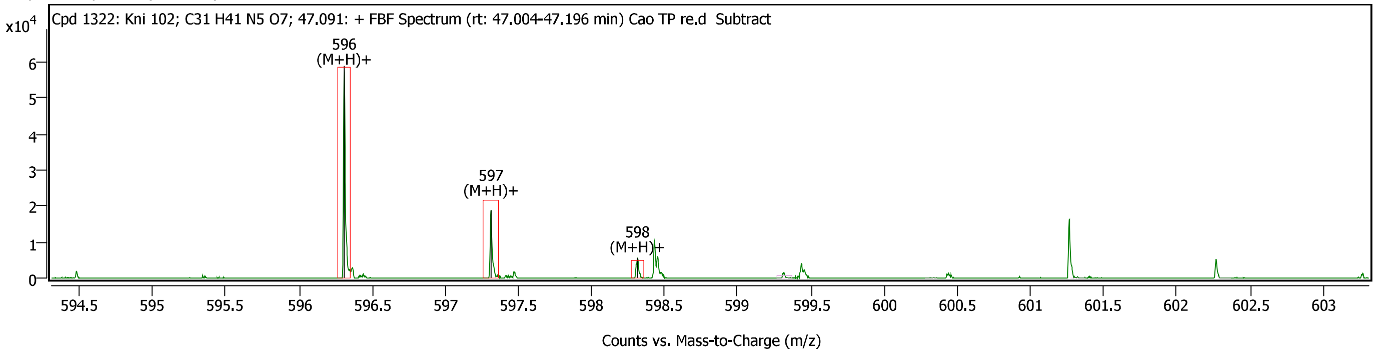

Compound ID Table

Name	Formula	Species	RT	RT Diff	Mass	CAS	ID Source	Score	Score (Lib)	Score (Tgt)
Kni 102	C31 H41 N5 O7	(M+H)+	47.091		595.3026	139694-65-8	FBF	89.55		89.55

Cpd 133: Armillatin

Name	Formula	RT	RI	Mass	Diff (Tgt, ppm)	CAS	ID Source	Score	Algorithm
Armillatin	C38 H58 O6	47.161		610.4214	-3.17	139051-17-5	FBF	94.52	FBF

Species	m/z	Score (Tgt)	Score (Lib)	Score (DB)	Score (MFG)	Score (RT)
(M+H)+	611	94.52				

Compound Chromatograms (overlaid)

Structure

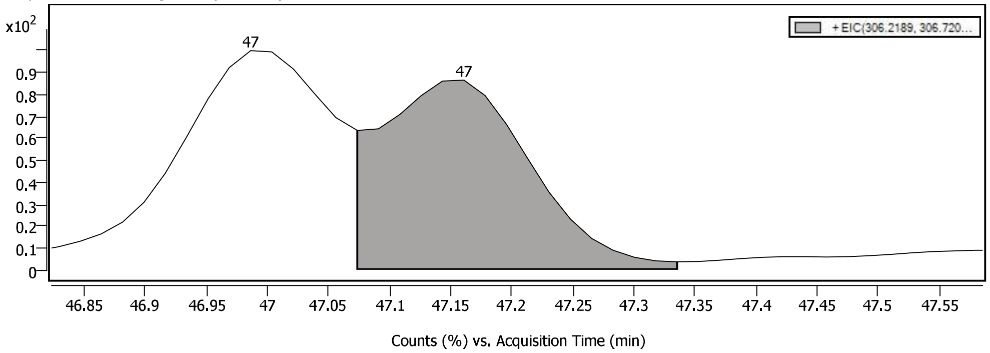

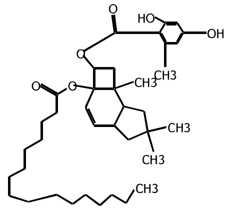

Compound Spectra (overlaid)

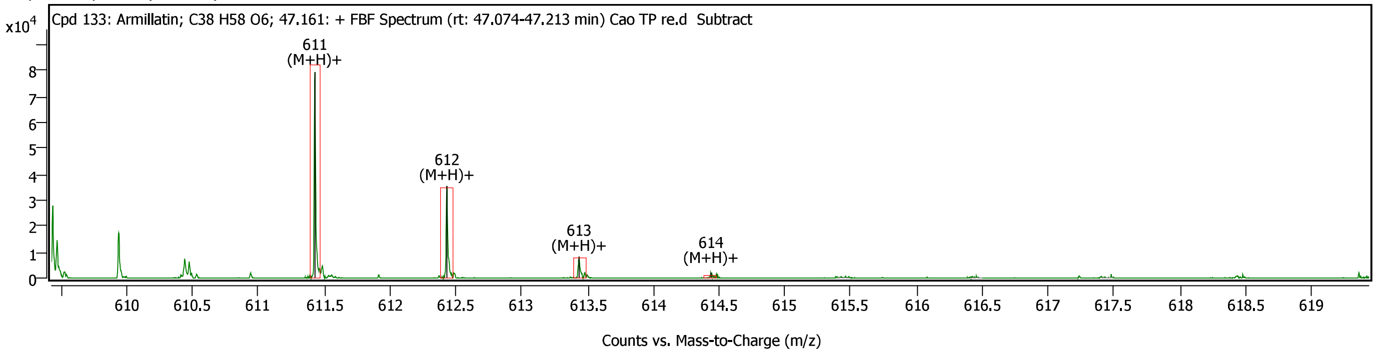

Compound ID Table

Name	Formula	Species	RT	RT Diff	Mass	CAS	ID Source	Score	Score (Lib)	Score (Tgt)
Armillatin	C38 H58 O6	(M+H)+	47.161		610.4214	139051-17-5	FBF	94.52		94.52

Cpd 1645: Cyclopassifloic acid B

Compound Screening Report

Name	Formula	RT	RI	Mass	Diff (Tgt, ppm)	CAS	ID Source	Score	Algorithm
Cyclopassifloic acid B	C31 H52 O6	47.196		520.3777	2.52	292167-35-2	FBF	82.37	FBF

Species	m/z	Score (Tgt)	Score (Lib)	Score (DB)	Score (MFG)	Score (RT)
(M+H)+	521	82.37				

Compound Chromatograms (overlaid)

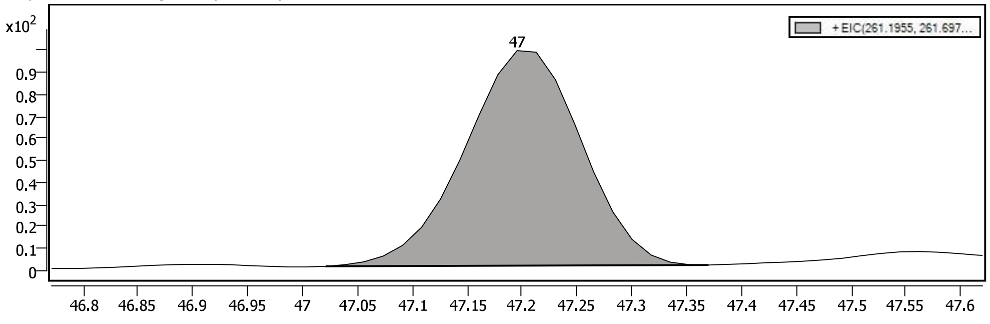

Structure

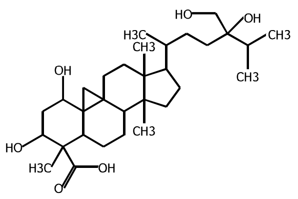

Compound Spectra (overlaid)

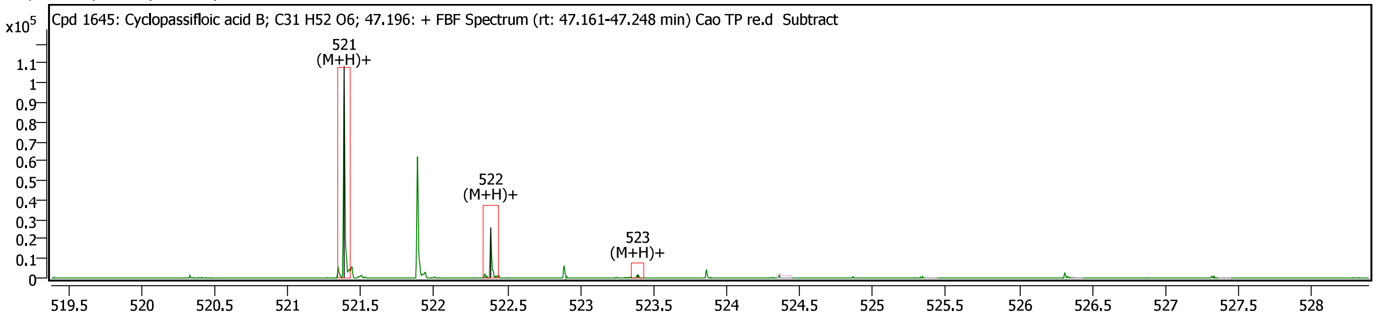

Compound ID Table

Name	Formula	Species	RT	RT Diff	Mass	CAS	ID Source	Score	Score (Lib)	Score (Tgt)
Cyclopassifloic acid B	C31 H52 O6	(M+H)+	47.196		520.3777	292167-35-2	FBF	82.37		82.37

Cpd 175: Chondrillasterol 3-[glucosyl-(1->4)-glucoside]

Name	Formula	RT	RI	Mass	Diff (Tgt, ppm)	CAS	ID Source	Score	Algorithm
Chondrillasterol 3-[glucosyl-(1->4)-glucoside]	C41 H68 O11	47.300		736.4743	-2.53		FBF	91.86	FBF

Species	m/z	Score (Tgt)	Score (Lib)	Score (DB)	Score (MFG)	Score (RT)
(M+H)+	737	91.86				

Compound Chromatograms (overlaid)

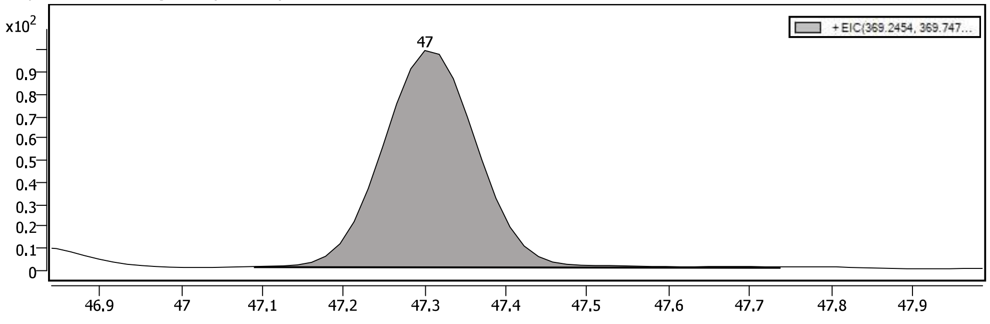

Structure

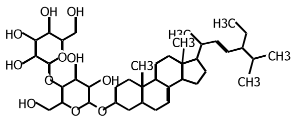

Compound Spectra (overlaid)

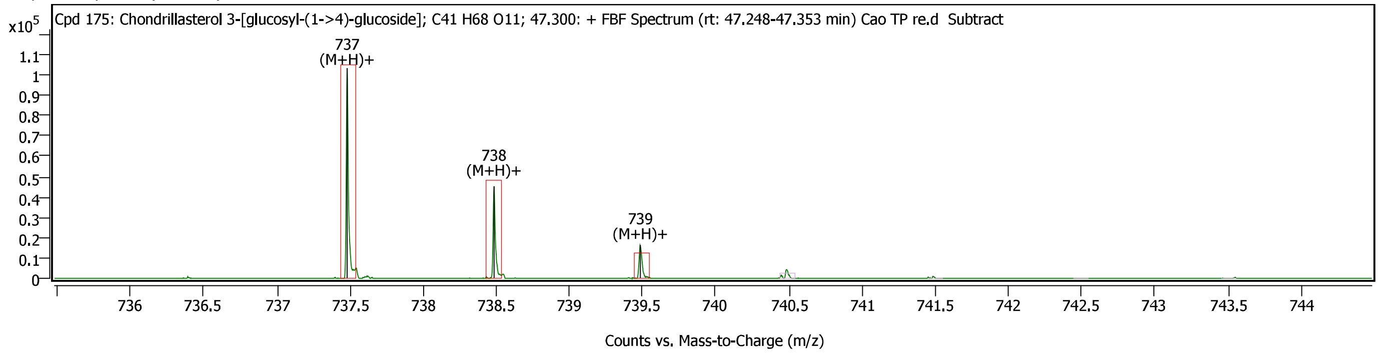

Compound ID Table

Name	Formula	Species	RT	RT Diff	Mass	CAS	ID Source	Score	Score (Lib)	Score (Tgt)
Chondrillasterol 3-[glucosyl-(1->4)-glucoside]	C41 H68 O11	(M+H)+	47.300		736.4743		FBF	91.86		91.86

Cpd 159: Goyaglycoside a

Name	Formula	RT	RI	Mass	Diff (Tgt, ppm)	CAS	ID Source	Score	Algorithm
Goyaglycoside a	C37 H60 O9	47.318		648.4215	-3.52	333332-41-5	M-FBF	87.45	FBF

Species	m/z	Score (Tgt)	Score (Lib)	Score (DB)	Score (MFG)	Score (RT)
(M+H)+	649	87.45				

Compound Chromatograms (overlaid)

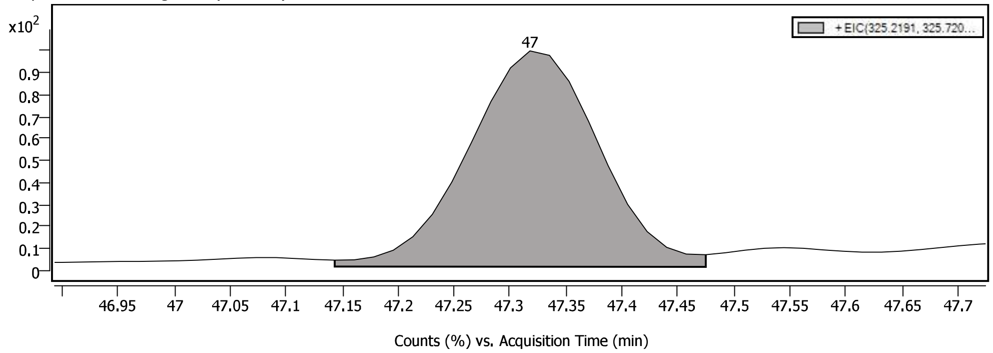

Structure

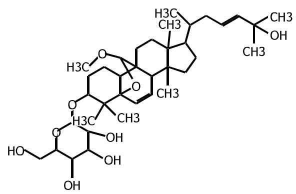

Compound Spectra (overlaid)

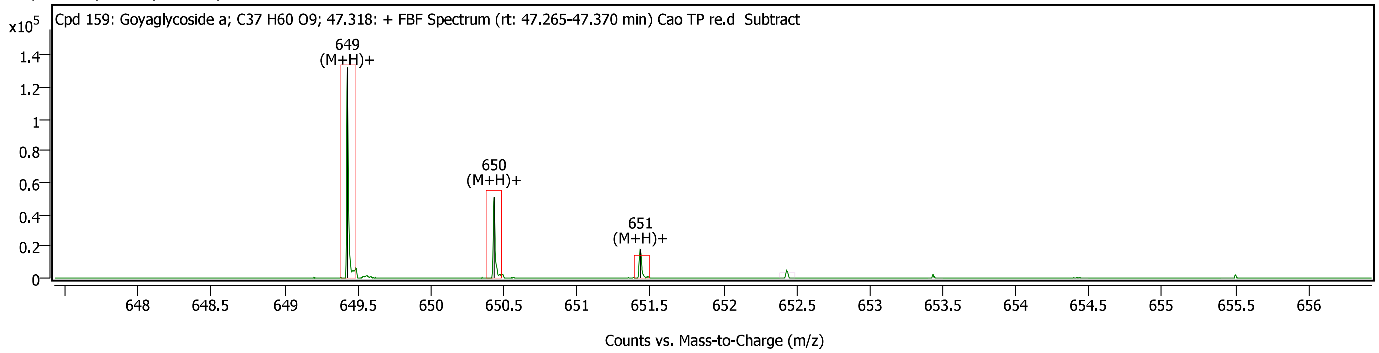

Compound ID Table

Name	Formula	Species	RT	RT Diff	Mass	CAS	ID Source	Score	Score (Lib)	Score (Tgt)
Goyaglycoside a	C37 H60 O9	(M+H)+	47.318		648.4215	333332-41-5	FBF	87.45		87.45
Momordicoside K	C37 H60 O9	(M+H)+	47.318		648.4215	81348-84-7	FBF	87.45		87.45

Cpd 367: PC(P-16:0/13:0)

Name	Formula	RT	RI	Mass	Diff (Tgt, ppm)	CAS	ID Source	Score	Algorithm
PC(P-16:0/13:0)	C37 H75 N O7 P	47.318		676.5291	1.39		FBF	84.26	FBF

Species	m/z	Score (Tgt)	Score (Lib)	Score (DB)	Score (MFG)	Score (RT)
(M+H)+	678	84.26				

Compound Screening Report

Compound Chromatograms (overlaid)

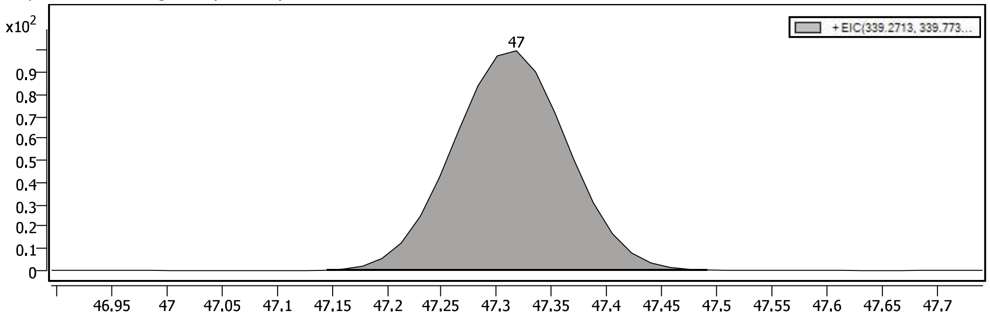

Counts (%) vs. Acquisition Time (min)

Structure

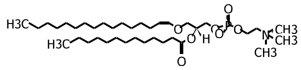

Compound Spectra (overlaid)

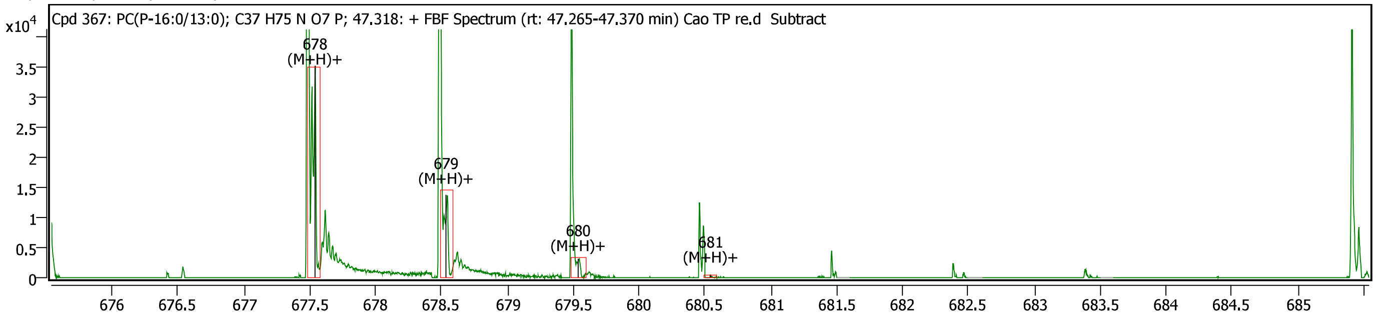

Counts vs. Mass-to-Charge (m/z)

Compound ID Table

Name	Formula	Species	RT	RT Diff	Mass	CAS	ID Source	Score	Score (Lib)	Score (Tgt)
PC(P-16:0/13:0)	C37 H75 N O7 P	(M+H)+	47.318		676.5291		FBF	84.26		84.26

Cpd 66: DG(13:0/18:3(9Z,12Z,15Z)/0:0)[iso2]

Name	Formula	RT	RI	Mass	Diff (Tgt, ppm)	CAS	ID Source	Score	Algorithm
DG(13:0/18:3(9Z,12Z,15Z)/0:0)[iso2]	C34 H60 O5	47.527		548.4441	0.11		M-FBF	99.58	FBF

Species	m/z	Score (Tgt)	Score (Lib)	Score (DB)	Score (MFG)	Score (RT)
(M+H)+	549	99.58				

Compound Chromatograms (overlaid)

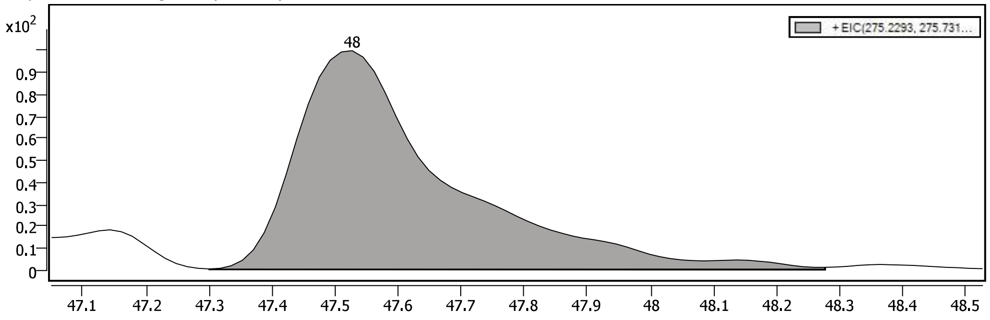

Counts (%) vs. Acquisition Time (min)

Structure

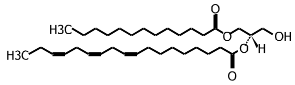

Compound Spectra (overlaid)

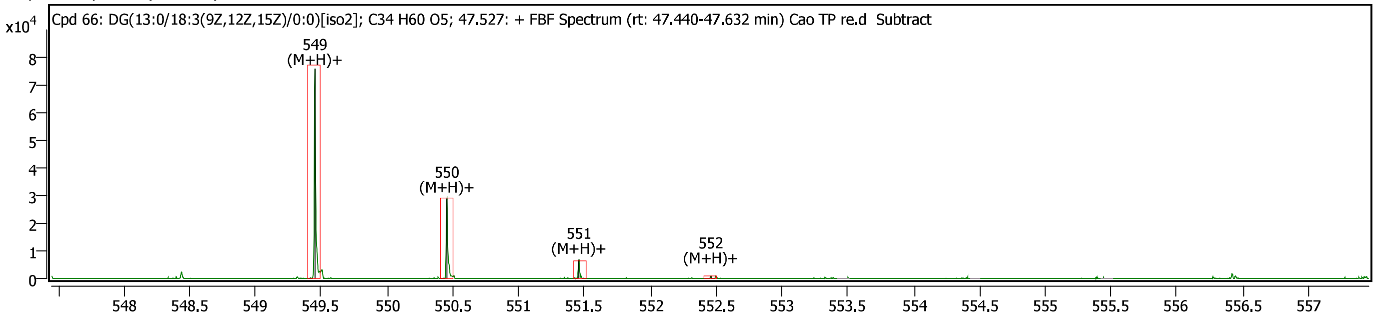

Counts vs. Mass-to-Charge (m/z)

Compound Screening Report

Compound ID Table

Name	Formula	Species	RT	RT Diff	Mass	CAS	ID Source	Score	Score (Lib)	Score (Tgt)
DG(13:0/18:3(9Z,12Z,15Z)/0:0)[iso2]	C34 H60 O5	(M+H)+	47.527		548.4441		FBF	99.58		99.58
DG(14:1(9Z)/17:2(9Z,12Z)/0:0)[iso2]	C34 H60 O5	(M+H)+	47.527		548.4441		FBF	99.58		99.58

Cpd 1040: PE(21:0/0:0)

Name	Formula	RT	RI	Mass	Diff (Tgt, ppm)	CAS	ID Source	Score	Algorithm
PE(21:0/0:0)	C26 H54 N O7 P	47.719		523.3643	1.03		M-FBF	99.20	FBF

Species	m/z	Score (Tgt)	Score (Lib)	Score (DB)	Score (MFG)	Score (RT)
(M+H)+	524	99.20				

Compound Chromatograms (overlaid)

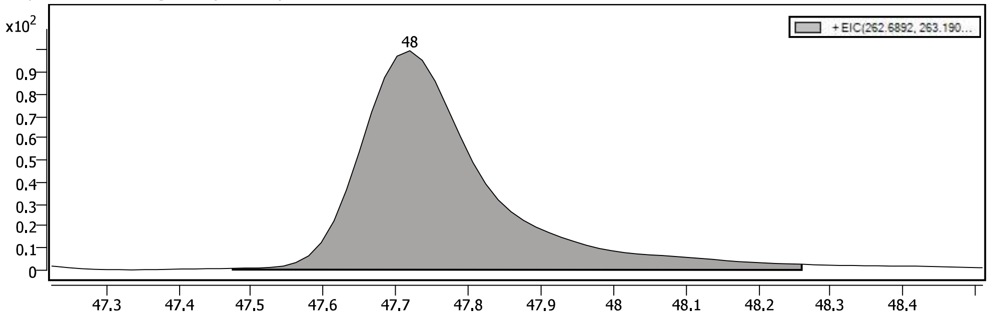

Structure

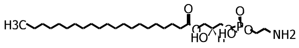

Compound Spectra (overlaid)

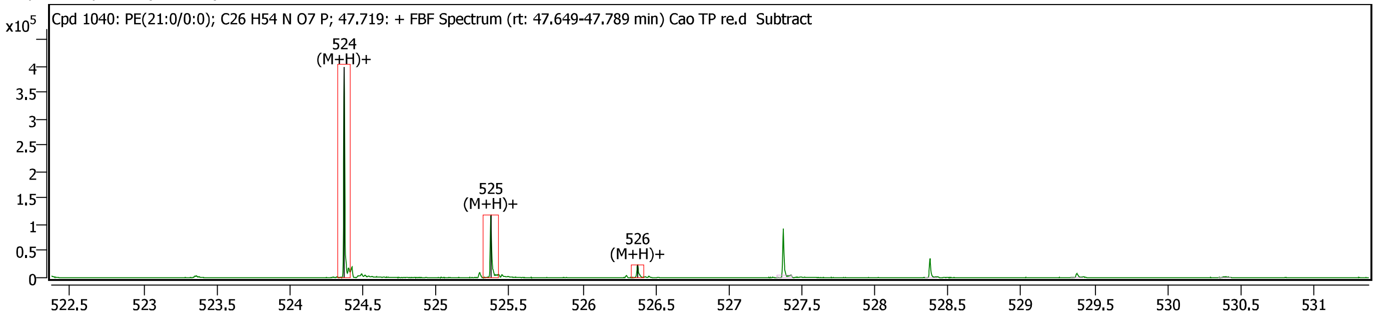

Compound ID Table

Name	Formula	Species	RT	RT Diff	Mass	CAS	ID Source	Score	Score (Lib)	Score (Tgt)
PE(21:0/0:0)	C26 H54 N O7 P	(M+H)+	47.719		523.3643		FBF	99.20		99.20
PC(2:0/O-16:0)[U]	C26 H54 N O7 P	(M+H)+	47.719		523.3643		FBF	99.20		99.20

Cpd 238: Cucurbitacin P

Name	Formula	RT	RI	Mass	Diff (Tgt, ppm)	CAS	ID Source	Score	Algorithm
Cucurbitacin P	C30 H48 O7	47.754		520.3379	-4.02		M-FBF	91.07	FBF

Species	m/z	Score (Tgt)	Score (Lib)	Score (DB)	Score (MFG)	Score (RT)
(M+H)+	521	91.07				

Compound Chromatograms (overlaid)

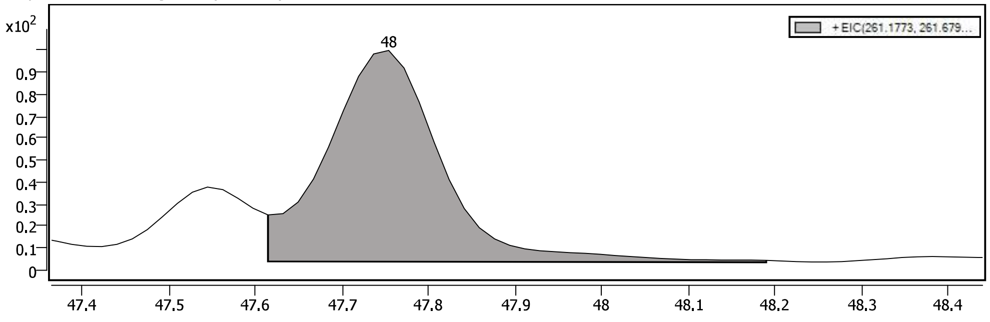

Structure

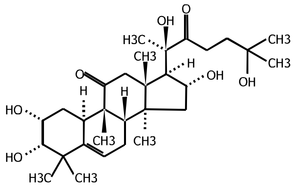

Compound Screening Report

Compound Spectra (overlaid)

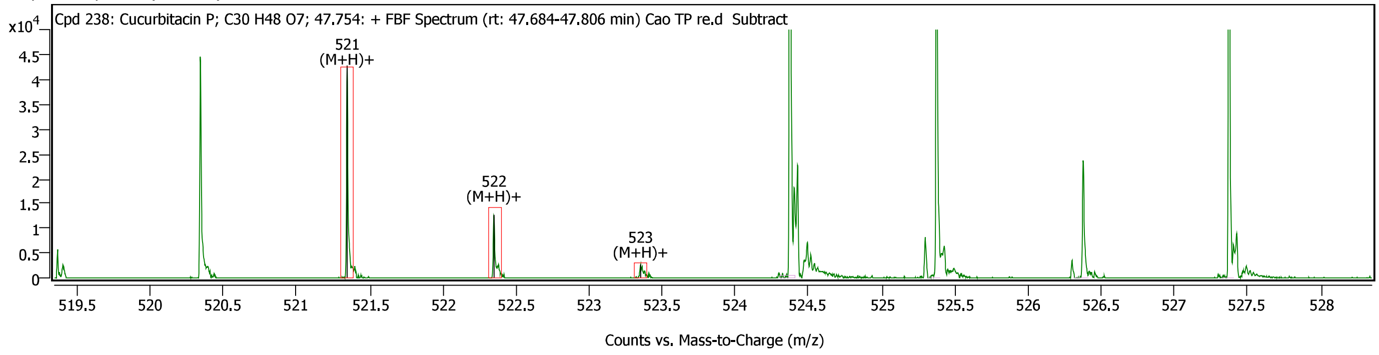

Compound ID Table

Name	Formula	Species	RT	RT Diff	Mass	CAS	ID Source	Score	Score (Lib)	Score (Tgt)
Cucurbitacin P	C30 H48 O7	(M+H)+	47.754		520.3379		FBF	91.07		91.07
Hippurin-1	C30 H48 O7	(M+H)+	47.754		520.3379		FBF	91.07		91.07
22-epi-Hippurin-1	C30 H48 O7	(M+H)+	47.754		520.3379		FBF	91.07		91.07

Cpd 156: 3alpha-Acetomethoxy-11alpha-oxo-12-ursen-24-oic acid

Name	Formula	RT	RI	Mass	Diff (Tgt, ppm)	CAS	ID Source	Score	Algorithm
3alpha-Acetomethoxy-11alpha-oxo-12-ursen-24-oic acid	C33 H50 O5	47.824		526.3663	0.97	17019-95-3	FBF	99.40	FBF

Species	m/z	Score (Tgt)	Score (Lib)	Score (DB)	Score (MFG)	Score (RT)
(M+H)+	527	99.40				

Compound Chromatograms (overlaid)

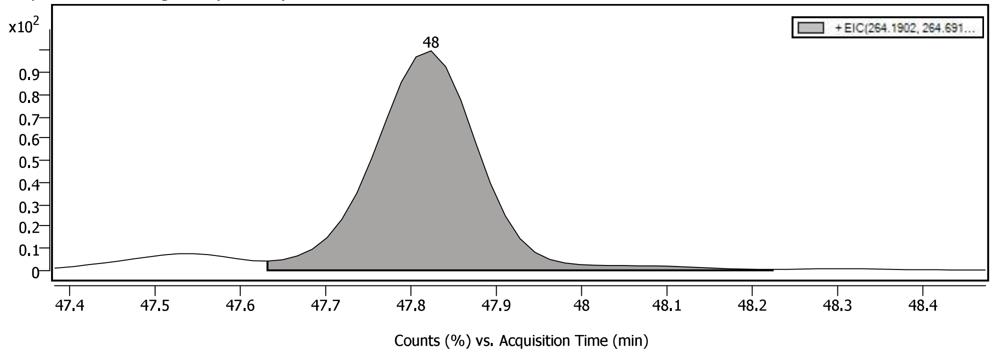

Structure

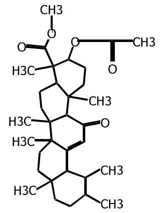

Compound Spectra (overlaid)

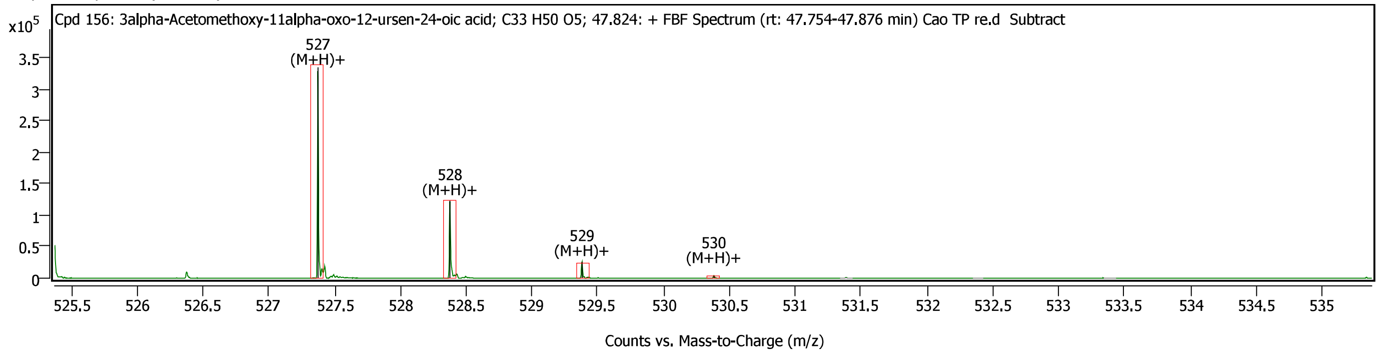

Compound ID Table

Name	Formula	Species	RT	RT Diff	Mass	CAS	ID Source	Score	Score (Lib)	Score (Tgt)
3alpha-Acetomethoxy-11alpha-oxo-12-ursen-24-oic acid	C33 H50 O5	(M+H)+	47.824		526.3663	17019-95-3	FBF	99.40		99.40

Cpd 291: PG(22:1(11Z)/0:0)

Name	Formula	RT	RI	Mass	Diff (Tgt, ppm)	CAS	ID Source	Score	Algorithm
PG(22:1(11Z)/0:0)	C28 H55 O9 P	47.824		566.3585	0.19		FBF	95.07	FBF

Species	m/z	Score (Tgt)	Score (Lib)	Score (DB)	Score (MFG)	Score (RT)
(M+H)+	567	95.07				

Compound Chromatograms (overlaid)

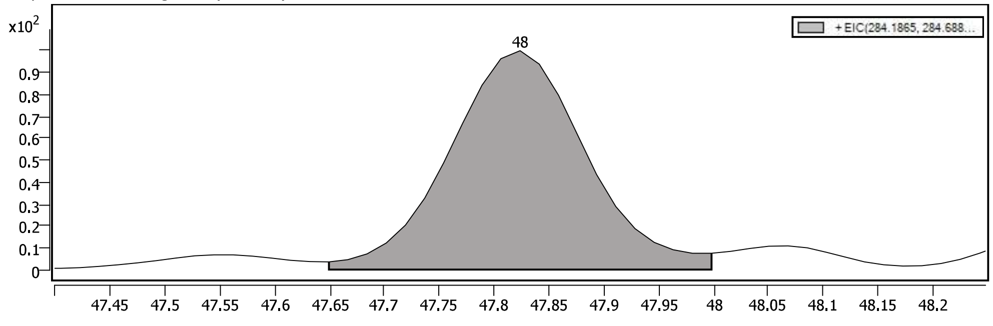

Structure

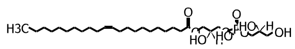

Compound Spectra (overlaid)

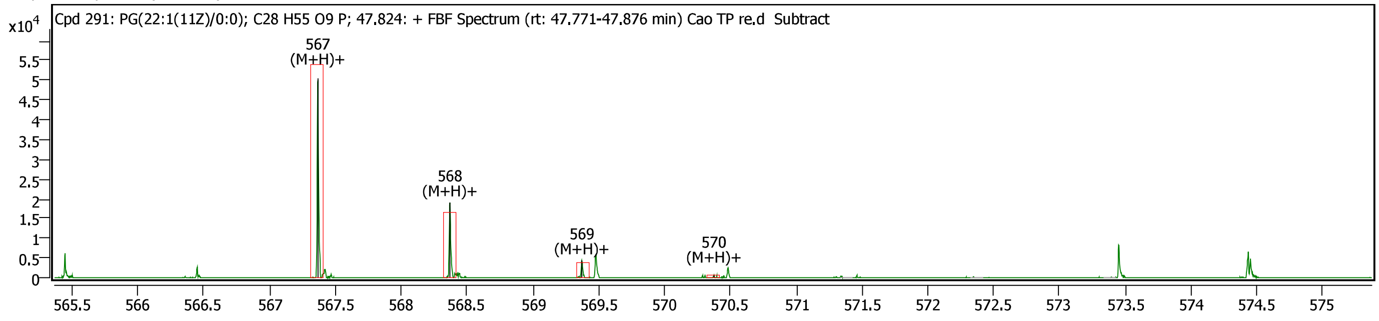

Compound ID Table

Name	Formula	Species	RT	RT Diff	Mass	CAS	ID Source	Score	Score (Lib)	Score (Tgt)
PG(22:1(11Z)/0:0)	C28 H55 O9 P	(M+H)+	47.824		566.3585		FBF	95.07		95.07

Cpd 1714: Hovenidulcigenin B

Name	Formula	RT	RI	Mass	Diff (Tgt, ppm)	CAS	ID Source	Score	Algorithm
Hovenidulcigenin B	C32 H50 O7	48.103		546.3534	-4.11	182173-50-8	M-FBF	91.63	FBF

Species	m/z	Score (Tgt)	Score (Lib)	Score (DB)	Score (MFG)	Score (RT)
(M+H)+	547	91.63				

Compound Chromatograms (overlaid)

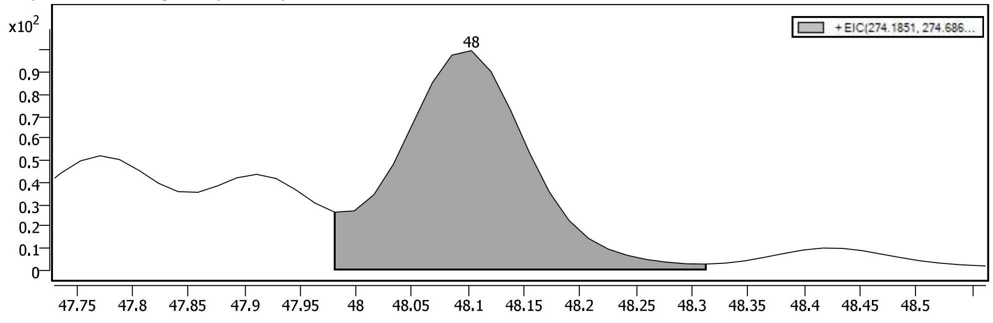

Structure

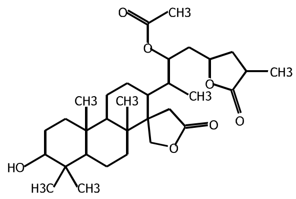

Compound Spectra (overlaid)

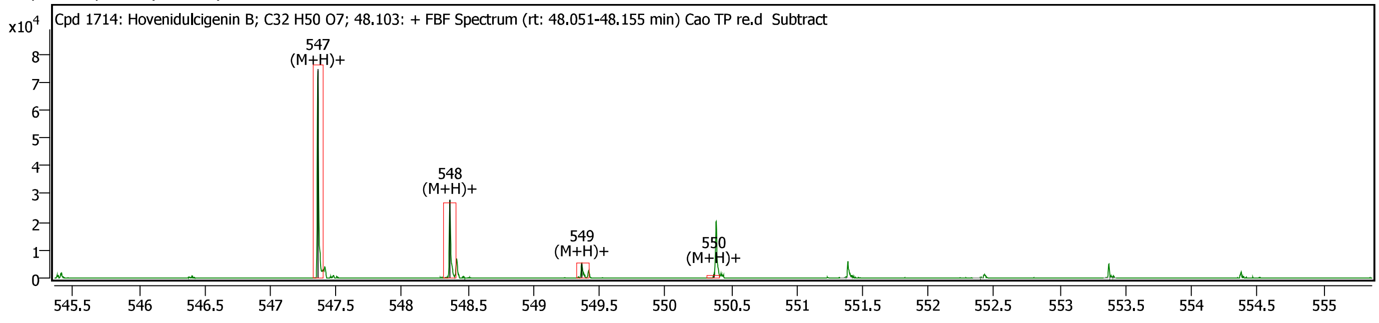

Compound ID Table

Name	Formula	Species	RT	RT Diff	Mass	CAS	ID Source	Score	Score (Lib)	Score (Tgt)
Hovenidulcigenin B	C32 H50 O7	(M+H)+	48.103		546.3534	182173-50-8	FBF	91.63		91.63
16-Glutaryloxy-1α,25-dihydroxyvitamin D3	C32 H50 O7	(M+H)+	48.103		546.3534		FBF	91.63		91.63
16-Glutaryloxy-1α,25-dihydroxy-20-epivitamin D3	C32 H50 O7	(M+H)+	48.103		546.3534		FBF	91.63		91.63

Cpd 388: N-stearoyl valine

Name	Formula	RT	RI	Mass	Diff (Tgt, ppm)	CAS	ID Source	Score	Algorithm
N-stearoyl valine	C23 H45 N O3	48.312		383.3402	0.76		FBF	99.51	FBF

Species	m/z	Score (Tgt)	Score (Lib)	Score (DB)	Score (MFG)	Score (RT)
(M+H)+	384	99.51				

Compound Chromatograms (overlaid)

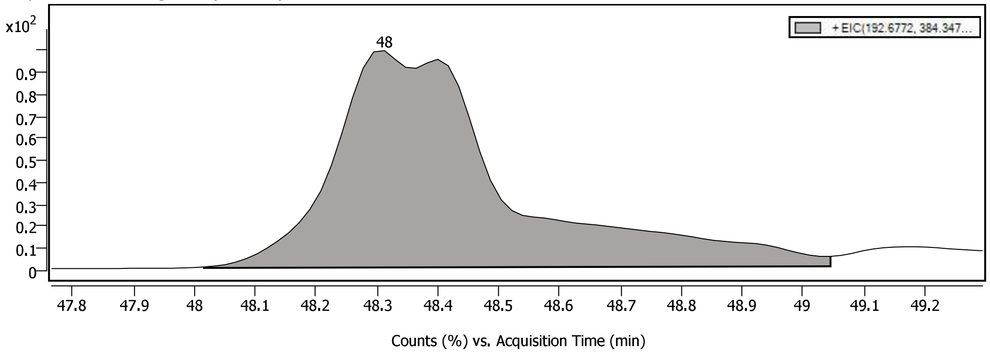

Structure

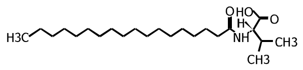

Compound Spectra (overlaid)

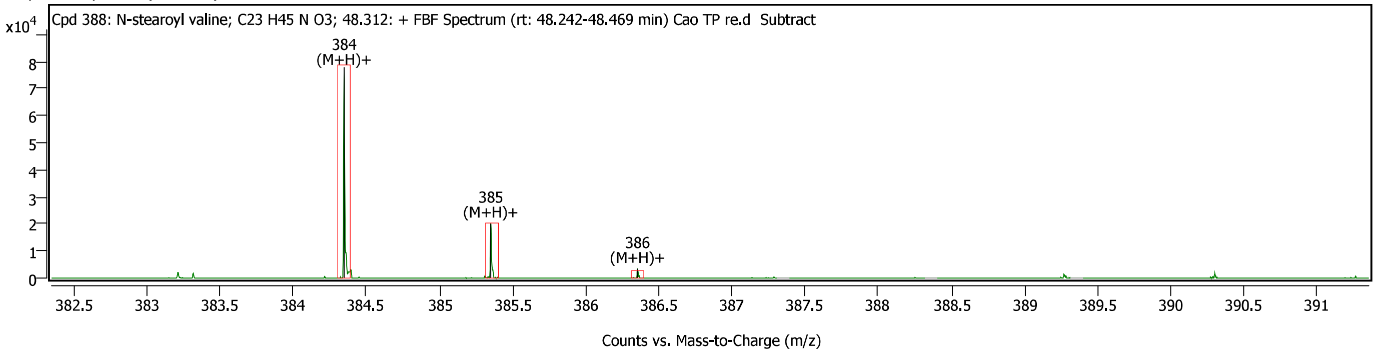

Compound ID Table

Name	Formula	Species	RT	RT Diff	Mass	CAS	ID Source	Score	Score (Lib)	Score (Tgt)
N-stearoyl valine	C23 H45 N O3	(M+H)+	48.312		383.3402		FBF	99.51		99.51

Cpd 147: Ganoderiol I

Name	Formula	RT	RI	Mass	Diff (Tgt, ppm)	CAS	ID Source	Score	Algorithm
Ganoderiol I	C31 H50 O5	48.469		502.3635	-4.65	114567-49-6	FBF	81.84	FBF

Species	m/z	Score (Tgt)	Score (Lib)	Score (DB)	Score (MFG)	Score (RT)
(M+H)+	503	81.84				

Compound Chromatograms (overlaid)

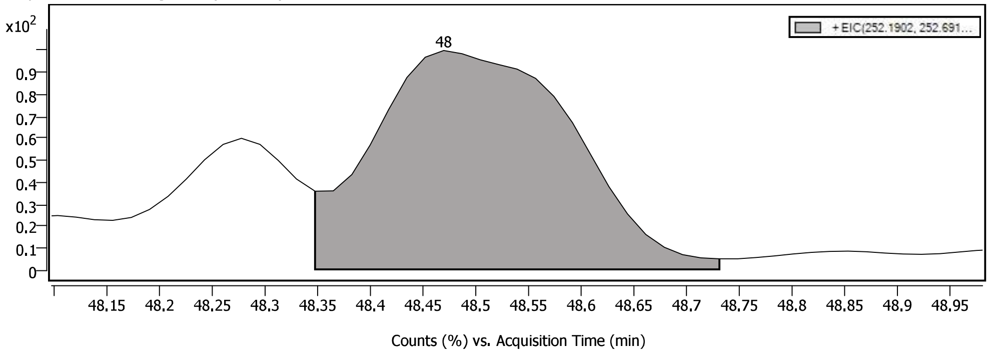

Structure

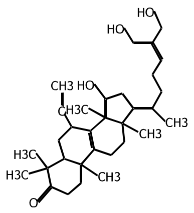

Compound Screening Report

Compound Spectra (overlaid)

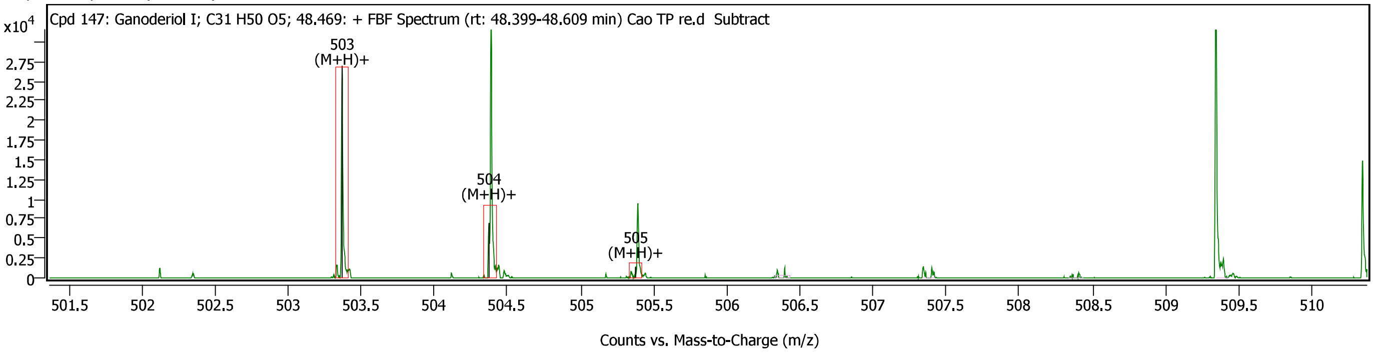

Compound ID Table

Name	Formula	Species	RT	RT Diff	Mass	CAS	ID Source	Score	Score (Lib)	Score (Tgt)
Ganoderiol I	C31 H50 O5	(M+H)+	48.469		502.3635	114567-49-6	FBF	81.84		81.84

Cpd 973: Vitamin D3 glucosiduronate

Name	Formula	RT	RI	Mass	Diff (Tgt, ppm)	CAS	ID Source	Score	Algorithm
Vitamin D3 glucosiduronate	C33 H52 O7	48.556		560.3715	0.29		FBF	95.49	FBF

Species	m/z	Score (Tgt)	Score (Lib)	Score (DB)	Score (MFG)	Score (RT)
(M+H)+	561	95.49				

Compound Chromatograms (overlaid)

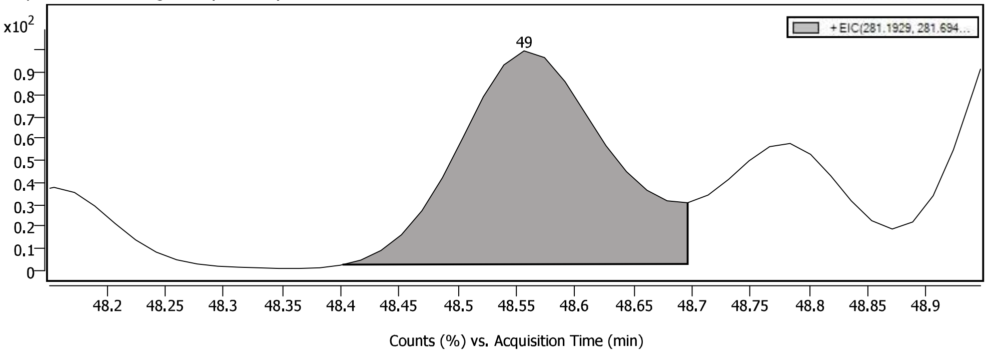

Structure

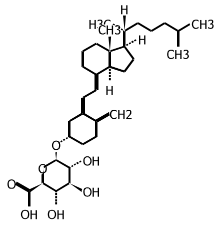

Compound Spectra (overlaid)

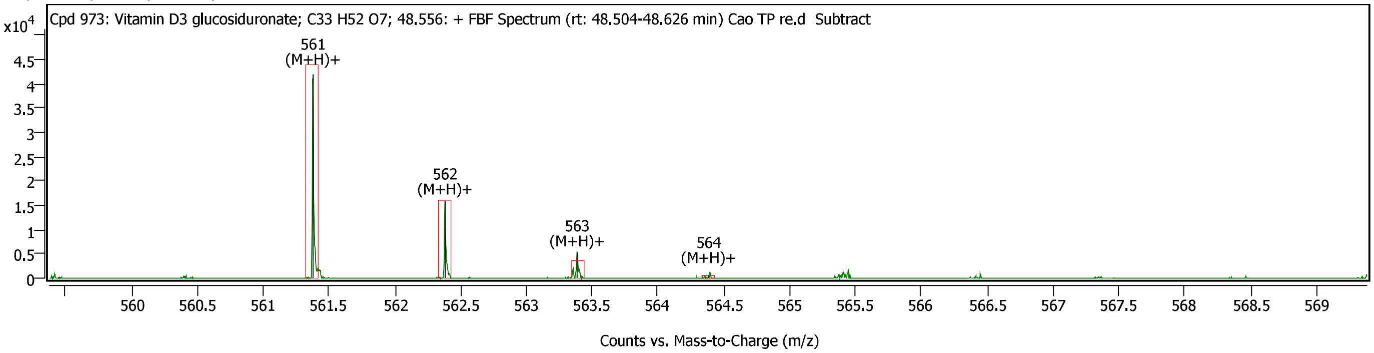

Compound ID Table

Name	Formula	Species	RT	RT Diff	Mass	CAS	ID Source	Score	Score (Lib)	Score (Tgt)
Vitamin D3 glucosiduronate	C33 H52 O7	(M+H)+	48.556		560.3715		FBF	95.49		95.49

Cpd 1410: Phorbol 12-tiglate 13-decanoate

Name	Formula	RT	RI	Mass	Diff (Tgt, ppm)	CAS	ID Source	Score	Algorithm
Phorbol 12-tiglate 13-decanoate	C35 H52 O8	48.556		600.3639	-3.87	59086-92-9	FBF	93.30	FBF

Species	m/z	Score (Tgt)	Score (Lib)	Score (DB)	Score (MFG)	Score (RT)
(M+H)+	601	93.30				

Compound Chromatograms (overlaid)

Structure

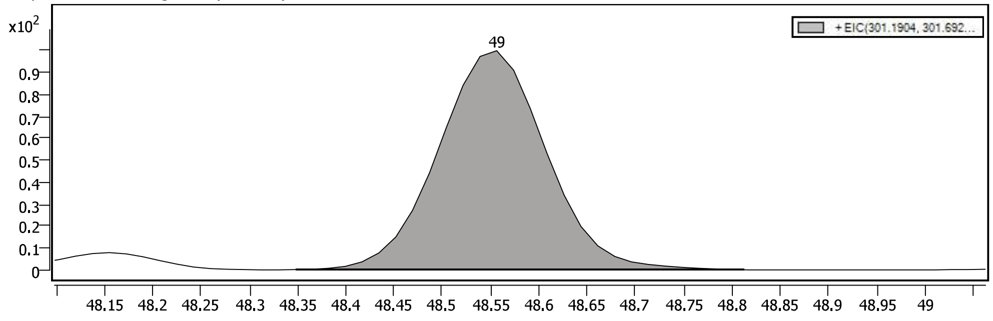

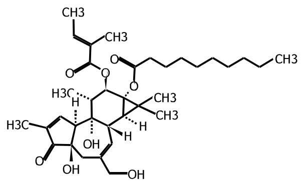

Compound Spectra (overlaid)

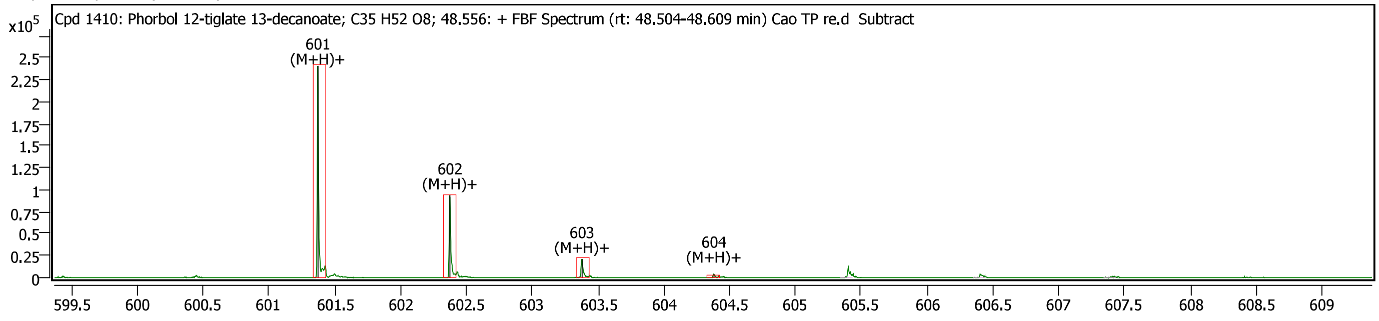

Compound ID Table

Name	Formula	Species	RT	RT Diff	Mass	CAS	ID Source	Score	Score (Lib)	Score (Tgt)
Phorbol 12-tiglate 13-decanoate	C35 H52 O8	(M+H)+	48.556		600.3639	59086-92-9	FBF	93.30		93.30

Cpd 983: 24,24-difluoro-1α,25-dihydroxy-26,27-dimethyl-24a-homovitamin D3 / 24,24-difluoro-1α,25-dihydroxy-26,27-dimethyl-24a-homocholecalciferol

Name	Formula	RT	RI	Mass	Diff (Tgt, ppm)	CAS	ID Source	Score	Algorithm
24,24-difluoro-1α,25-dihydroxy-26,27-dimethyl-24a-homovitamin D3 / 24,24-difluoro-1α,25-dihydroxy-26,27-dimethyl-24a-homocholecalciferol	C30 H48 F2 O3	48.609		494.3582	2.12		FBF	96.88	FBF

Species	m/z	Score (Tgt)	Score (Lib)	Score (DB)	Score (MFG)	Score (RT)
(M+H)+	495	96.88				

Compound Chromatograms (overlaid)

Structure

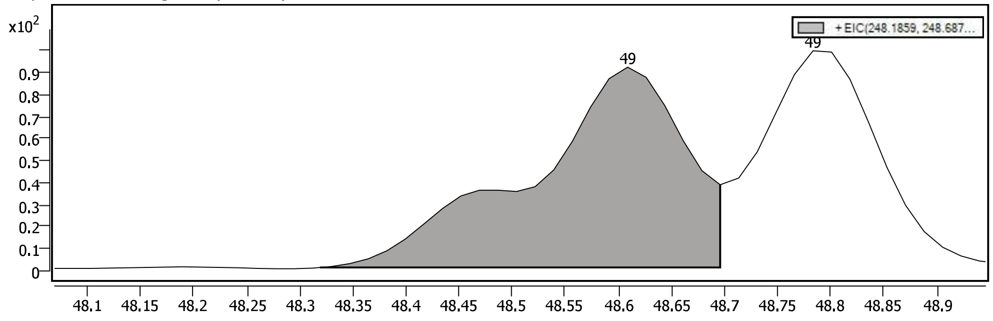

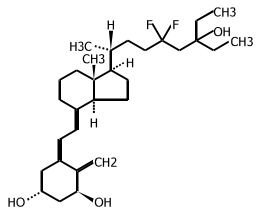

Compound Spectra (overlaid)

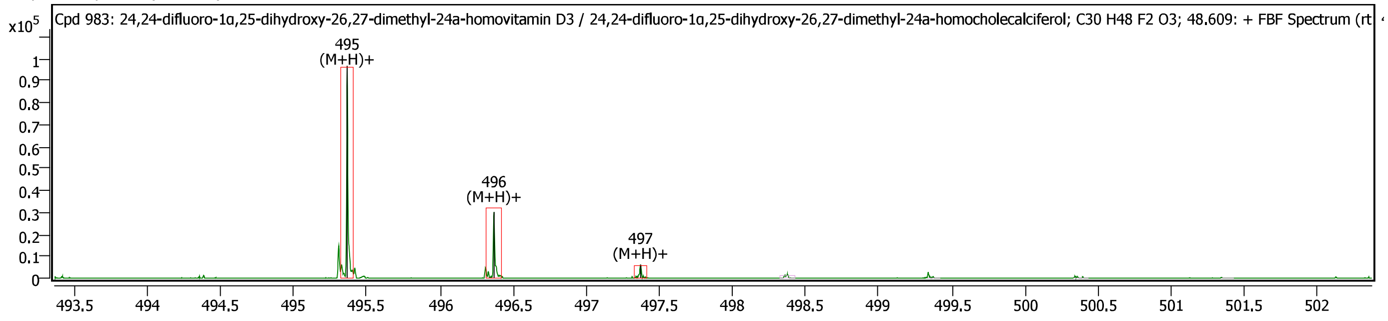

Compound Screening Report

Compound ID Table

Name	Formula	Species	RT	RT Diff	Mass	CAS	ID Source	Score	Score (Lib)	Score (Tgt)
24,24-difluoro-1α,25-dihydroxy-26,27-dimethyl-24a-homovitamin D3 / 24,24-difluoro-1α,25-dihydroxy-26,27-dimethyl-24a-homocholecalciferol	C30 H48 F2 O3	(M+H)+	48.609		494.3582		FBF	96.88		96.88

Cpd 1148: <Stearamide>

Name	Formula	RT	RI	Mass	Diff (Tgt, ppm)	CAS	ID Source	Score	Algorithm
<Stearamide>	C18 H37 N O	48.731		283.2877	0.62	124-26-5	FBF	99.50	FBF

Species	m/z	Score (Tgt)	Score (Lib)	Score (DB)	Score (MFG)	Score (RT)
(M+H)+	284	99.50				

Compound Chromatograms (overlaid)

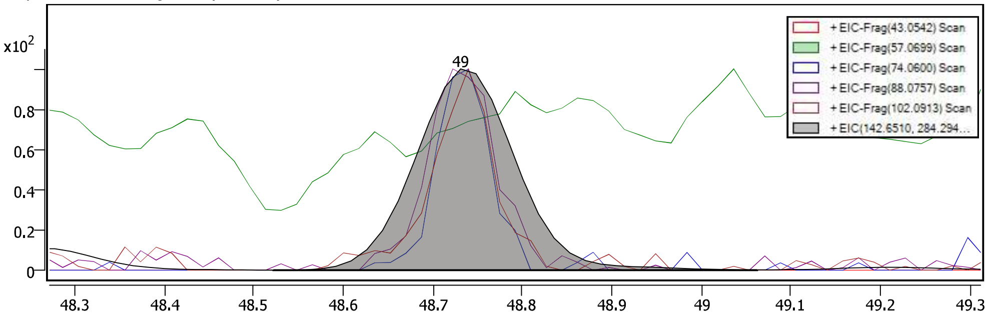

Structure

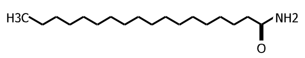

Coelution Plot

Compound Spectra (overlaid)

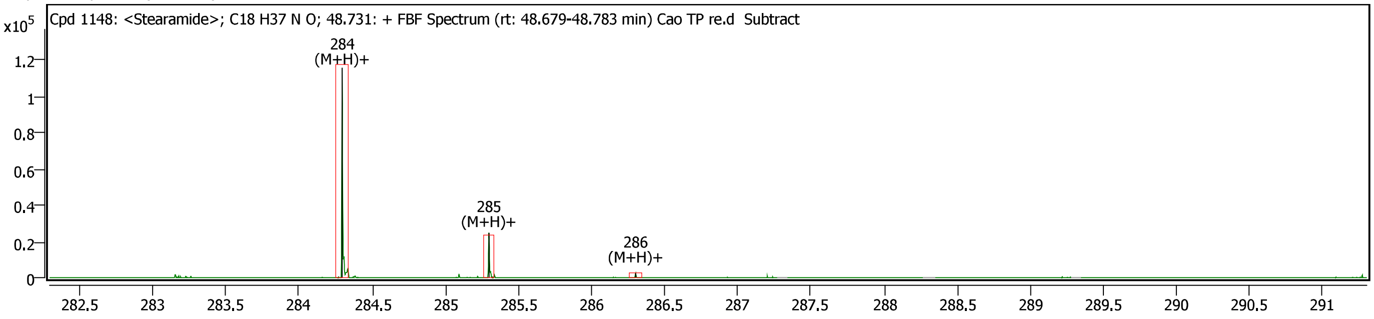

Fragment Spectrum (raw)

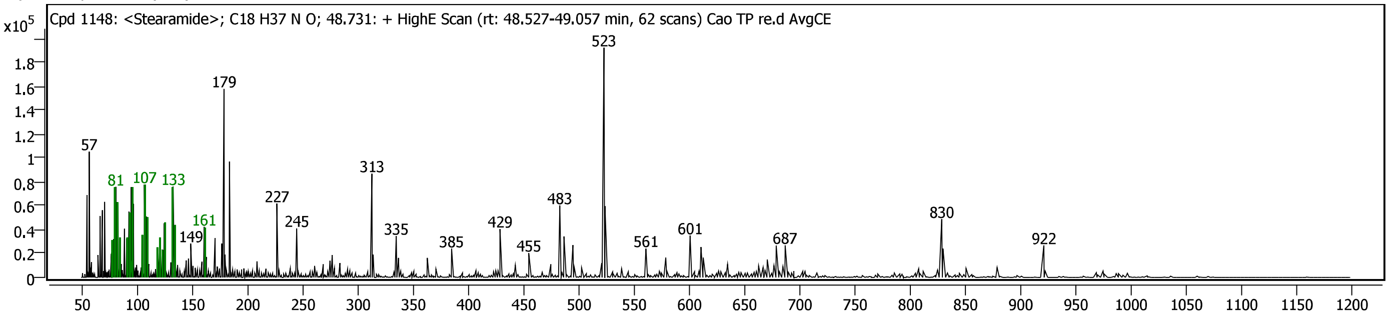

Compound ID Table

Name	Formula	Species	RT	RT Diff	Mass	CAS	ID Source	Score	Score (Lib)	Score (Tgt)
<Stearamide>	C18 H37 N O	(M+H)+	48.731		283.2877	124-26-5	FBF	99.50		99.50

Cpd 200: 28-Homobrassinolide

Name	Formula	RT	RI	Mass	Diff (Tgt, ppm)	CAS	ID Source	Score	Algorithm
28-Homobrassinolide	C29 H50 O6	48.783		494.3583	-4.97	80483-89-2	FBF	87.30	FBF

Species	m/z	Score (Tgt)	Score (Lib)	Score (DB)	Score (MFG)	Score (RT)
(M+H)+	495	87.30				

Compound Chromatograms (overlaid)

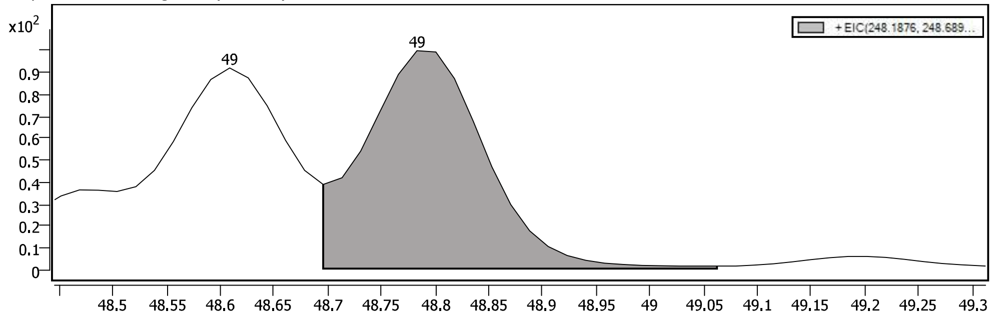

Structure

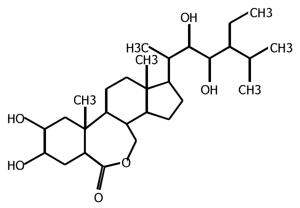

Compound Spectra (overlaid)

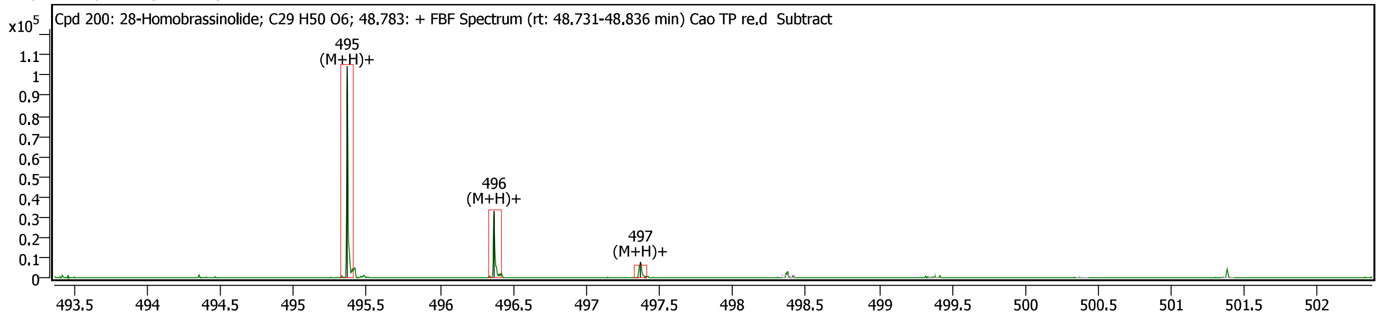

Compound ID Table

Name	Formula	Species	RT	RT Diff	Mass	CAS	ID Source	Score	Score (Lib)	Score (Tgt)
28-Homobrassinolide	C29 H50 O6	(M+H)+	48.783		494.3583	80483-89-2	FBF	87.30		87.30

Cpd 293: PG(20:1(11Z)/0:0)

Name	Formula	RT	RI	Mass	Diff (Tgt, ppm)	CAS	ID Source	Score	Algorithm
PG(20:1(11Z)/0:0)	C26 H51 O9 P	49.028		538.3271	-0.02		FBF	90.74	FBF

Species	m/z	Score (Tgt)	Score (Lib)	Score (DB)	Score (MFG)	Score (RT)
(M+H)+	539	90.74				

Compound Chromatograms (overlaid)

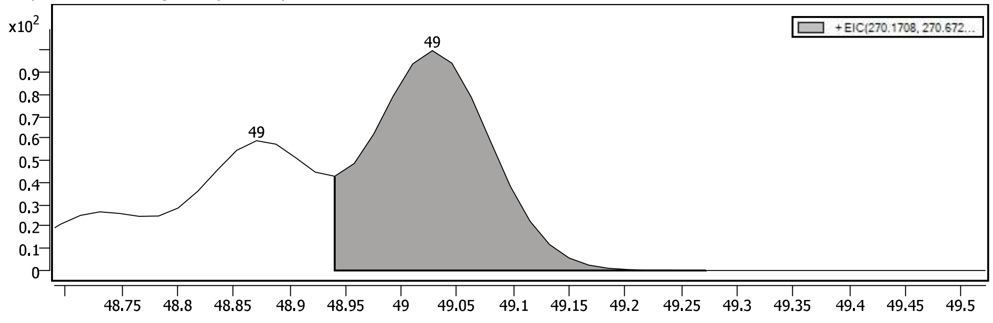

Structure

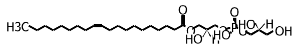

Compound Spectra (overlaid)

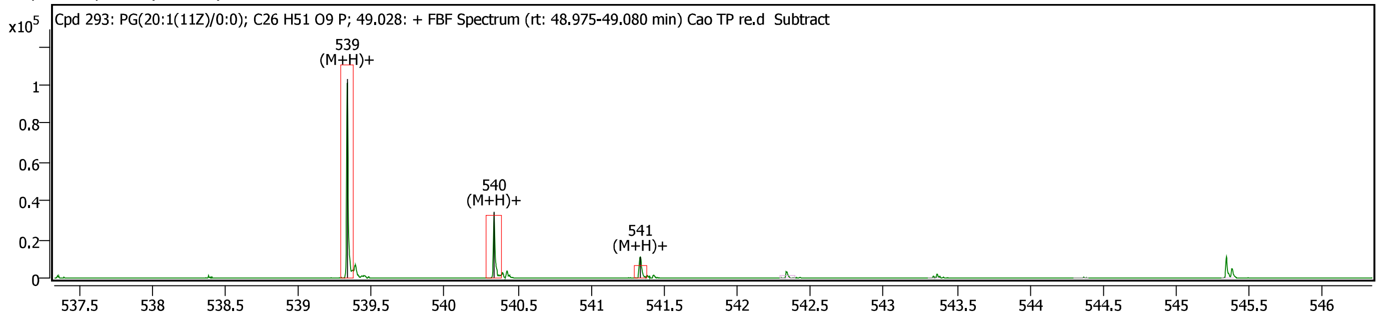

Compound ID Table

Name	Formula	Species	RT	RT Diff	Mass	CAS	ID Source	Score	Score (Lib)	Score (Tgt)
PG(20:1(11Z)/0:0)	C26 H51 O9 P	(M+H)+	49.028		538.3271		FBF	90.74		90.74

Cpd 949: MG(22:4(7Z,10Z,13Z,16Z)/0:0/0:0)

Compound Screening Report

Name	Formula	RT	RI	Mass	Diff (Tgt, ppm)	CAS	ID Source	Score	Algorithm
MG(22:4(7Z,10Z,13Z,16Z)/0:0/0:0)	C25 H42 O4	49.062		406.3096	3.18		M-FBF	93.48	FBF

Species	m/z	Score (Tgt)	Score (Lib)	Score (DB)	Score (MFG)	Score (RT)
(M+H)+	407	93.48				

Compound Chromatograms (overlaid)

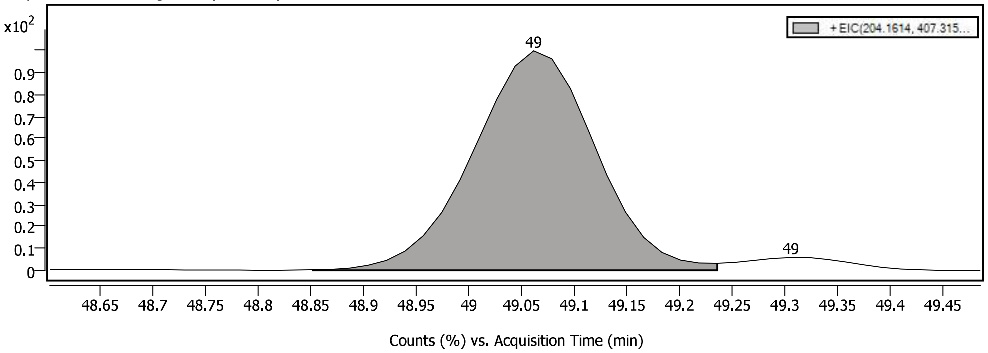

Structure

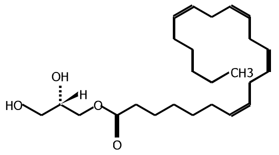

Compound Spectra (overlaid)

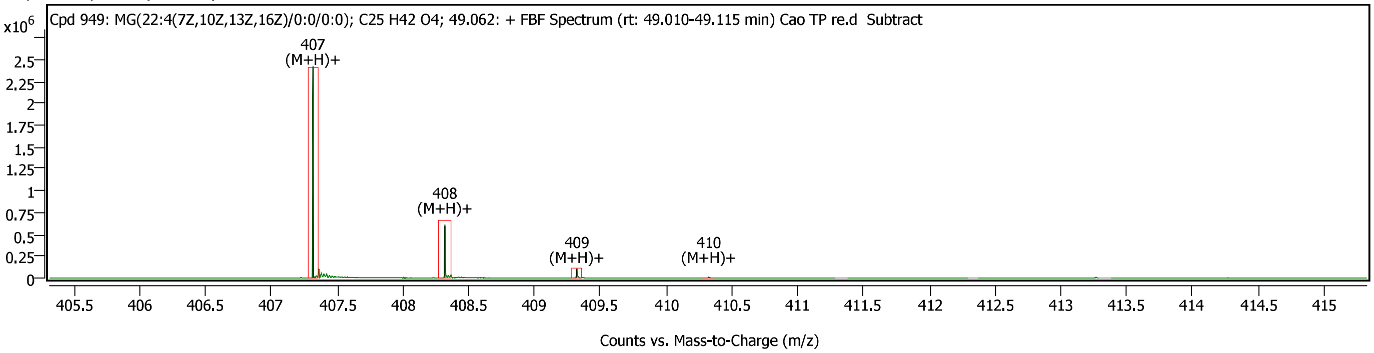

Compound ID Table

Name	Formula	Species	RT	RT Diff	Mass	CAS	ID Source	Score	Score (Lib)	Score (Tgt)
MG(22:4(7Z,10Z,13Z,16Z)/0:0/0:0)	C25 H42 O4	(M+H)+	49.062		406.3096		FBF	93.48		93.48
26,27-dinor-3α,6α,12α-trihydroxy-5β-cholestan-24-one	C25 H42 O4	(M+H)+	49.062		406.3096		FBF	93.48		93.48
1α,25-dihydroxy-19-nor-22-oxavitamin D3 / 1α,25-dihydroxy-19-nor-22-oxacholecalciferol	C25 H42 O4	(M+H)+	49.062		406.3096		FBF	93.48		93.48
MG(0:0/22:4(7Z,10Z,13Z,16Z)/0:0)	C25 H42 O4	(M+H)+	49.062		406.3096		FBF	93.48		93.48
26,27-dinor-3alpha,6alpha,12alpha-trihydroxy-5beta-cholestan-24-one	C25 H42 O4	(M+H)+	49.062		406.3096		FBF	93.48		93.48

Cpd 1524: Spirolide B

Name	Formula	RT	RI	Mass	Diff (Tgt, ppm)	CAS	ID Source	Score	Algorithm
Spirolide B	C42 H63 N O7	49.062		693.4584	-2.94	170713-72-1	FBF	87.75	FBF

Species	m/z	Score (Tgt)	Score (Lib)	Score (DB)	Score (MFG)	Score (RT)
(M+H)+	694	87.75				

Compound Chromatograms (overlaid)

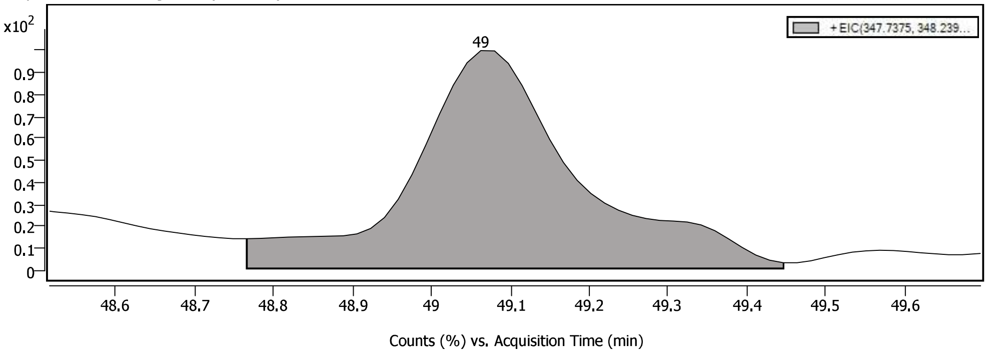

Structure

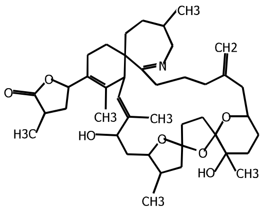

Compound Spectra (overlaid)

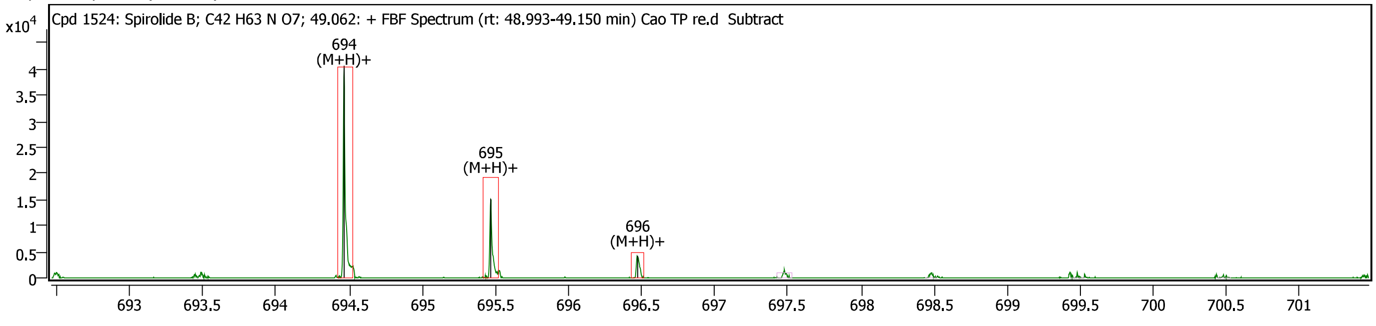

Compound ID Table

Name	Formula	Species	RT	RT Diff	Mass	CAS	ID Source	Score	Score (Lib)	Score (Tgt)
Spirolide B	C42 H63 N O7	(M+H)+	49.062		693.4584	170713-72-1	FBF	87.75		87.75

Cpd 594: Pheophorbide a

Name	Formula	RT	RI	Mass	Diff (Tgt, ppm)	CAS	ID Source	Score	Algorithm
Pheophorbide a	C35 H36 N4 O5	49.272		592.2687	0.22	15664-29-6	FBF	99.83	FBF

Species	m/z	Score (Tgt)	Score (Lib)	Score (DB)	Score (MFG)	Score (RT)
(M+H)+	593	99.83				

Compound Chromatograms (overlaid)

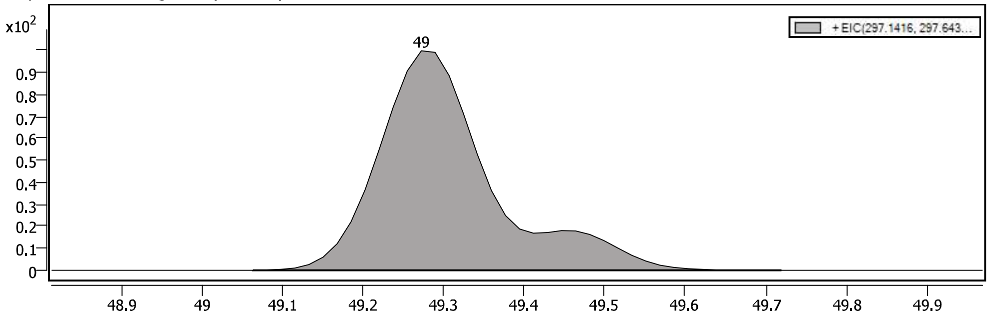

Structure

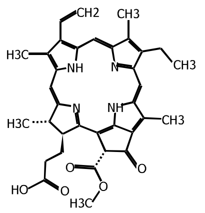

Compound Spectra (overlaid)

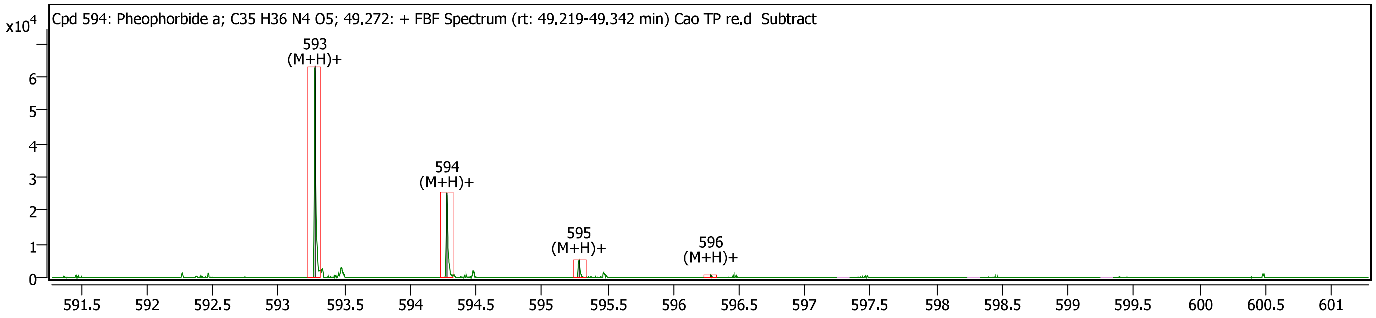

Compound ID Table

Name	Formula	Species	RT	RT Diff	Mass	CAS	ID Source	Score	Score (Lib)	Score (Tgt)
Pheophorbide a	C35 H36 N4 O5	(M+H)+	49.272		592.2687	15664-29-6	FBF	99.83		99.83

Cpd 20: GlcCer(d15:2(4E,6E)/18:0)

Name	Formula	RT	RI	Mass	Diff (Tgt, ppm)	CAS	ID Source	Score	Algorithm
GlcCer(d15:2(4E,6E)/18:0)	C39 H73 N O8	49.307		683.5339	0.38		FBF	99.84	FBF

Species	m/z	Score (Tgt)	Score (Lib)	Score (DB)	Score (MFG)	Score (RT)
(M+H)+	685	99.84				

Compound Chromatograms (overlaid)

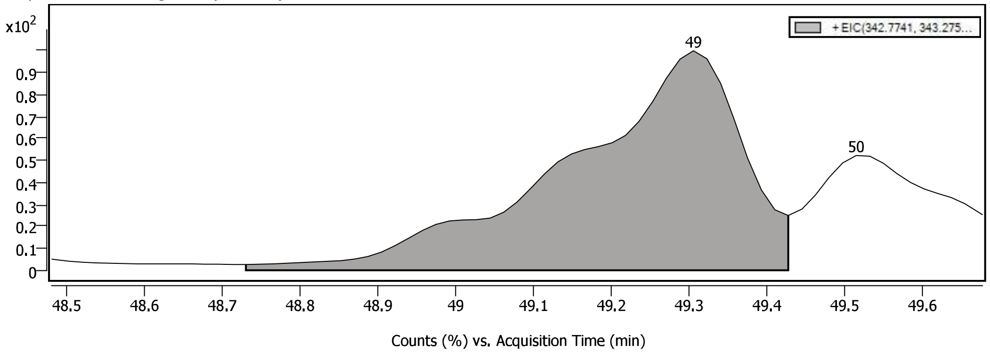

Structure

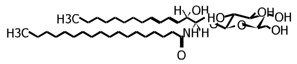

Compound Spectra (overlaid)

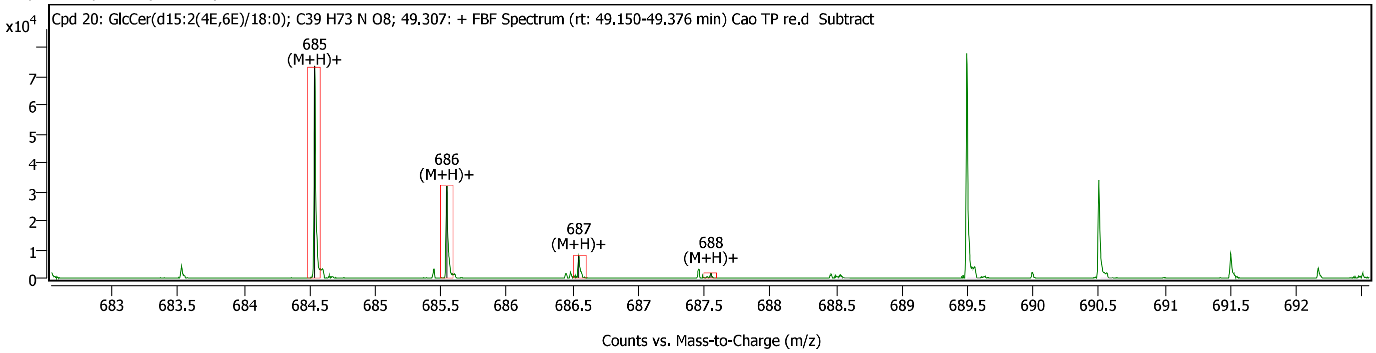

Compound ID Table

Name	Formula	Species	RT	RT Diff	Mass	CAS	ID Source	Score	Score (Lib)	Score (Tgt)
GlcCer(d15:2(4E,6E)/18:0)	C39 H73 N O8	(M+H)+	49.307		683.5339		FBF	99.84		99.84

Cpd 337: LysoPE(18:2(9Z,12Z)/0:0)

Name	Formula	RT	RI	Mass	Diff (Tgt, ppm)	CAS	ID Source	Score	Algorithm
LysoPE(18:2(9Z,12Z)/0:0)	C23 H44 N O7 P	49.394		477.2857	0.38		M-FBF	99.28	FBF

Species	m/z	Score (Tgt)	Score (Lib)	Score (DB)	Score (MFG)	Score (RT)
(M+H)+	478	99.28				

Compound Chromatograms (overlaid)

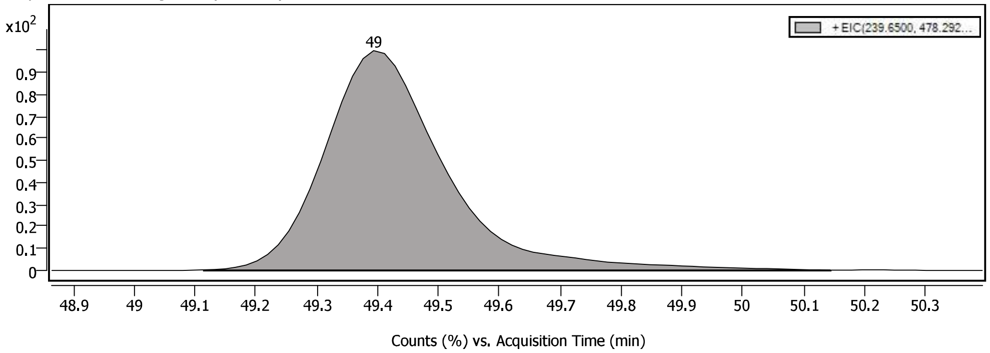

Structure

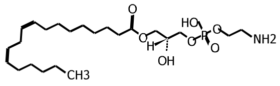

Compound Spectra (overlaid)

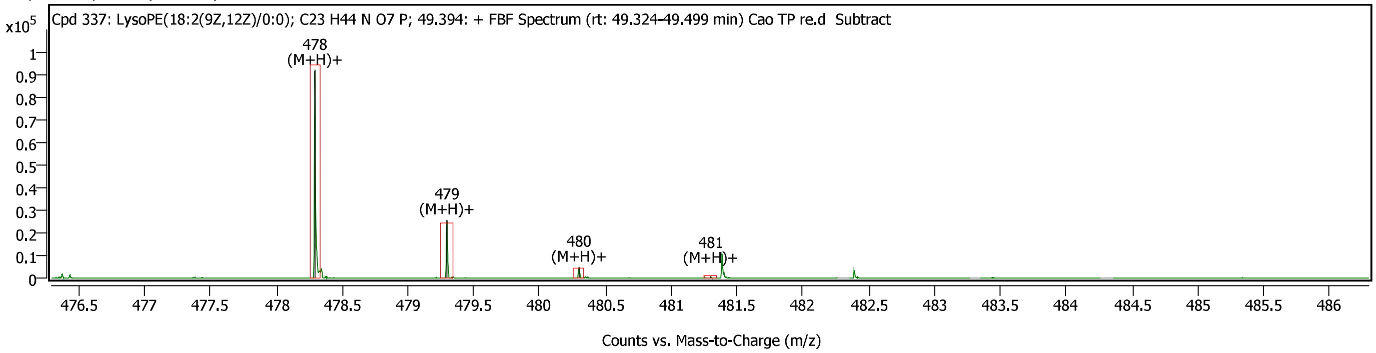

Compound Screening Report

Compound ID Table

Name	Formula	Species	RT	RT Diff	Mass	CAS	ID Source	Score	Score (Lib)	Score (Tgt)
LysoPE(18:2(9Z,12Z)/0:0)	C23 H44 N O7 P	(M+H)+	49.394		477.2857		FBF	99.28		99.28
PE(18:2(9Z,12Z)/0:0)	C23 H44 N O7 P	(M+H)+	49.394		477.2857		FBF	99.28		99.28
LysoPE(0:0/18:2(9Z,12Z))	C23 H44 N O7 P	(M+H)+	49.394		477.2857		FBF	99.28		99.28

Cpd 203: Boviquinone 4

Name	Formula	RT	RI	Mass	Diff (Tgt, ppm)	CAS	ID Source	Score	Algorithm
Boviquinone 4	C26 H36 O4	49.429		412.2596	-4.24	28129-52-4	FBF	92.11	FBF

Species	m/z	Score (Tgt)	Score (Lib)	Score (DB)	Score (MFG)	Score (RT)
(M+H)+	413	92.11				

Compound Chromatograms (overlaid)

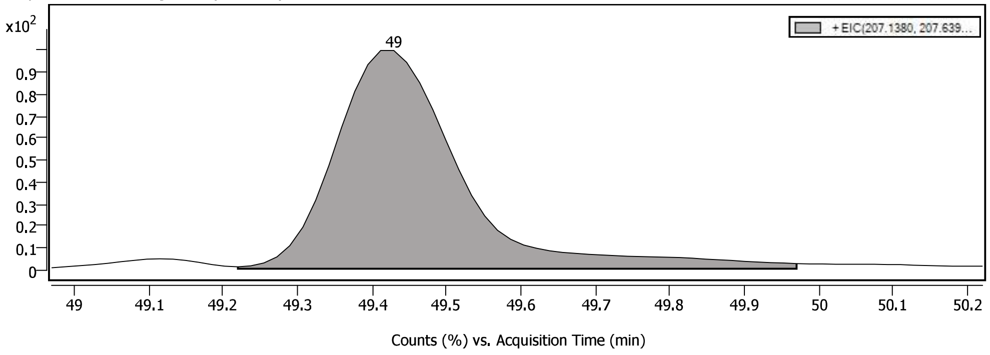

Structure

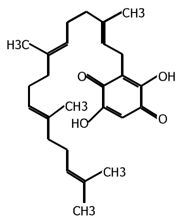

Compound Spectra (overlaid)

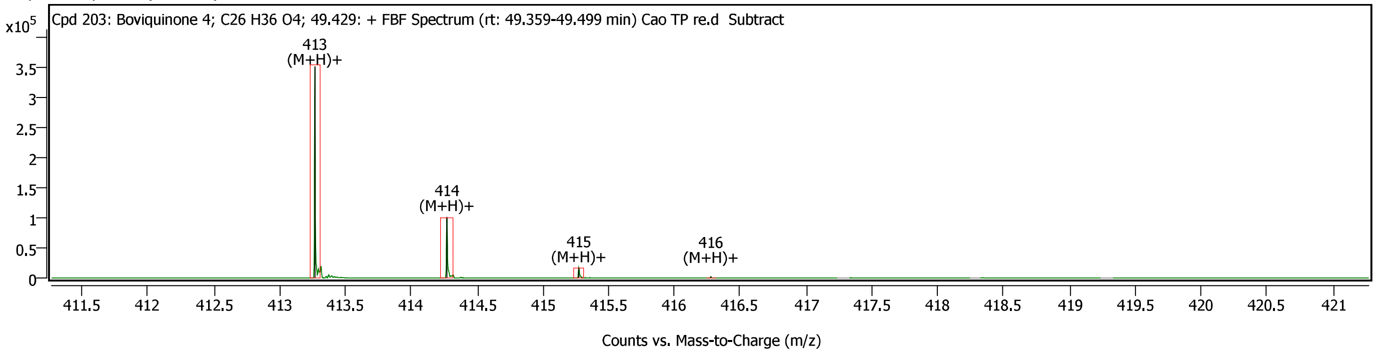

Compound ID Table

Name	Formula	Species	RT	RT Diff	Mass	CAS	ID Source	Score	Score (Lib)	Score (Tgt)
Boviquinone 4	C26 H36 O4	(M+H)+	49.429		412.2596	28129-52-4	FBF	92.11		92.11

Cpd 541: Aspidoalbine

Name	Formula	RT	RI	Mass	Diff (Tgt, ppm)	CAS	ID Source	Score	Algorithm
Aspidoalbine	C24 H32 N2 O5	49.429		428.2330	4.44	2122-25-0	FBF	80.66	FBF

Species	m/z	Score (Tgt)	Score (Lib)	Score (DB)	Score (MFG)	Score (RT)
(M+H)+	429	80.66				

Compound Chromatograms (overlaid)

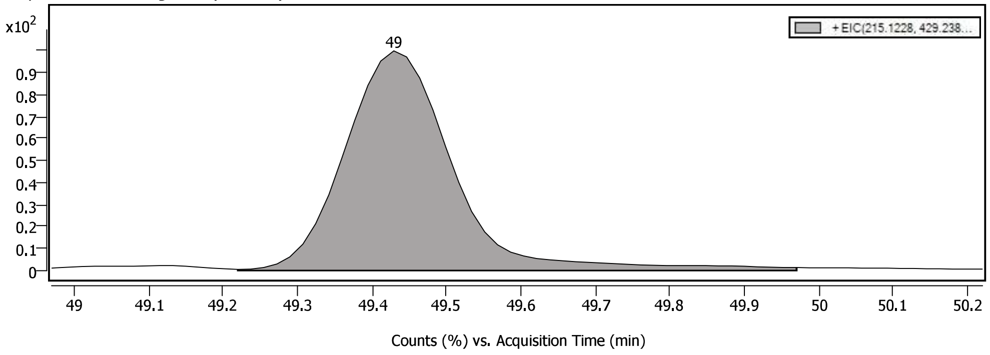

Structure

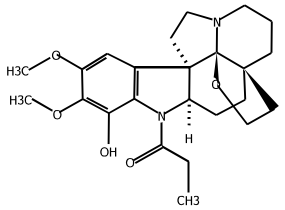

Compound Spectra (overlaid)

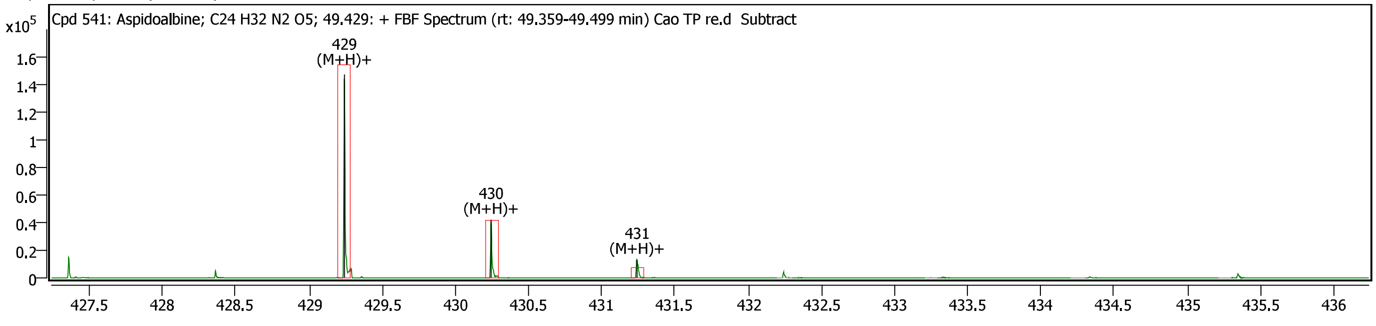

Compound ID Table

Name	Formula	Species	RT	RT Diff	Mass	CAS	ID Source	Score	Score (Lib)	Score (Tgt)
Aspidoalbine	C24 H32 N2 O5	(M+H)+	49.429		428.2330	2122-25-0	FBF	80.66		80.66

Cpd 942: <Irbesartan>

Name	Formula	RT	RI	Mass	Diff (Tgt, ppm)	CAS	ID Source	Score	Algorithm
<Irbesartan>	C25 H28 N6 O	49.429		428.2331	1.50	138402-11-6	FBF	87.27	FBF

Species	m/z	Score (Tgt)	Score (Lib)	Score (DB)	Score (MFG)	Score (RT)
(M+H)+	429	87.27				

Compound Chromatograms (overlaid)

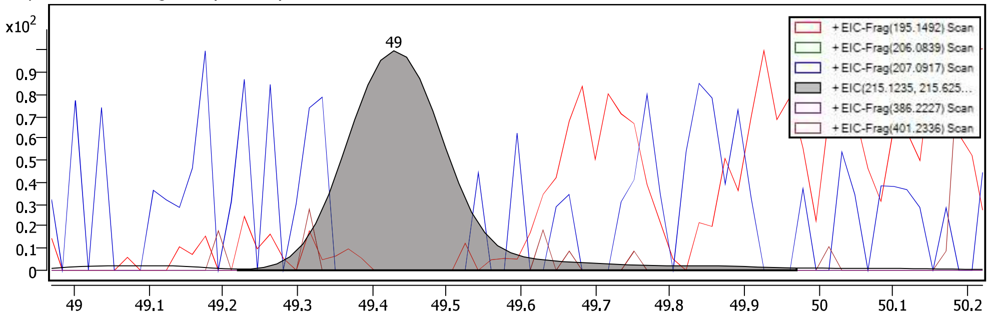

Structure

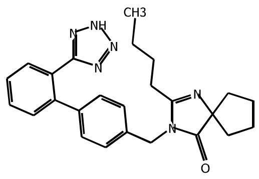

Coelution Plot

Compound Spectra (overlaid)

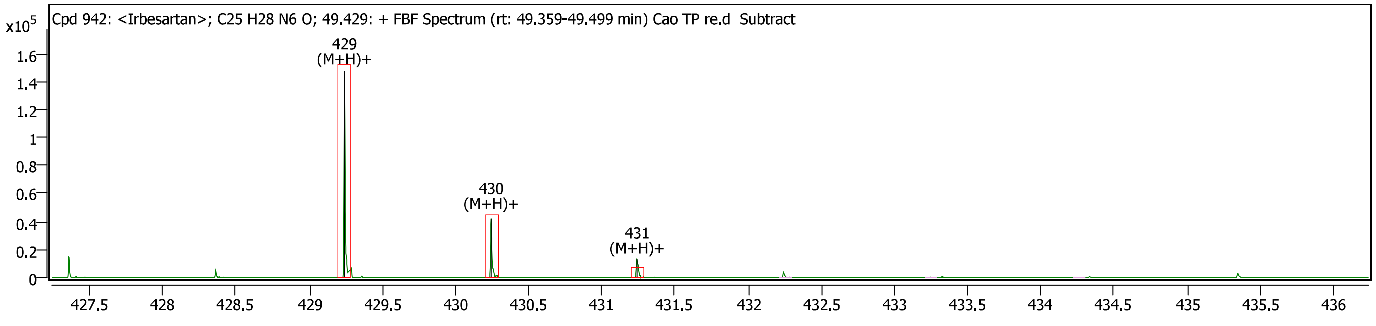

Fragment Spectrum (raw)

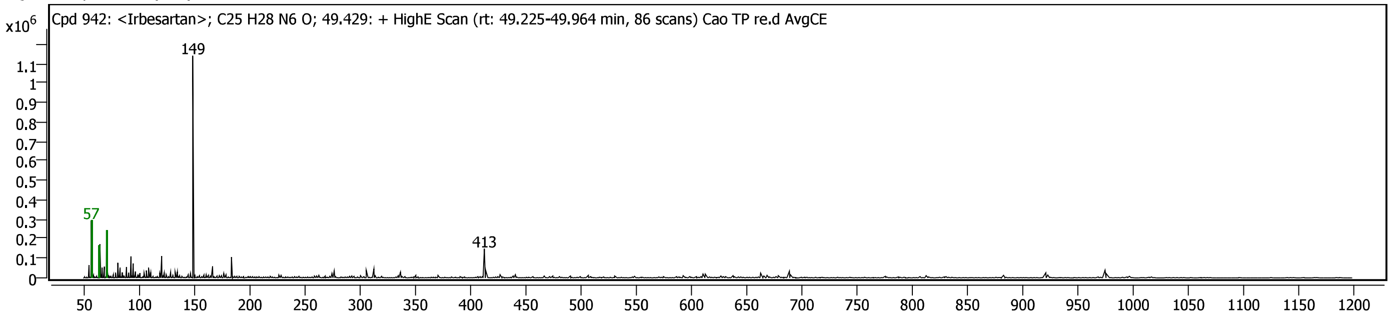

Compound Screening Report

Compound ID Table

Name	Formula	Species	RT	RT Diff	Mass	CAS	ID Source	Score	Score (Lib)	Score (Tgt)
<Irbesartan>	C25 H28 N6 O	(M+H)+	49.429		428.2331	138402-11-6	FBF	87.27		87.27

Cpd 1101: Di(2-ethylhexyl) adipate

Name	Formula	RT	RI	Mass	Diff (Tgt, ppm)	CAS	ID Source	Score	Algorithm
Di(2-ethylhexyl) adipate	C22 H42 O4	49.656		370.3089	1.59	103-23-1	FBF-FragConfirm	97.85	FBF

Species	m/z	Score (Tgt)	Score (Lib)	Score (DB)	Score (MFG)	Score (RT)
(M+H)+	371	97.85				

Compound Chromatograms (overlaid)

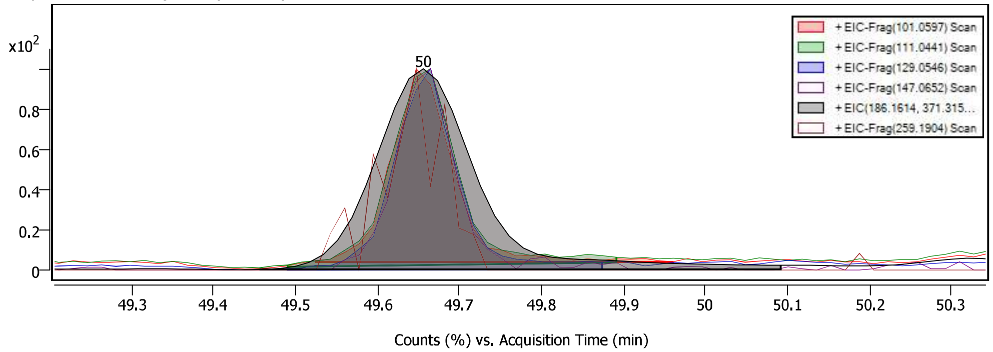

Structure

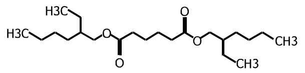

Coelution Plot

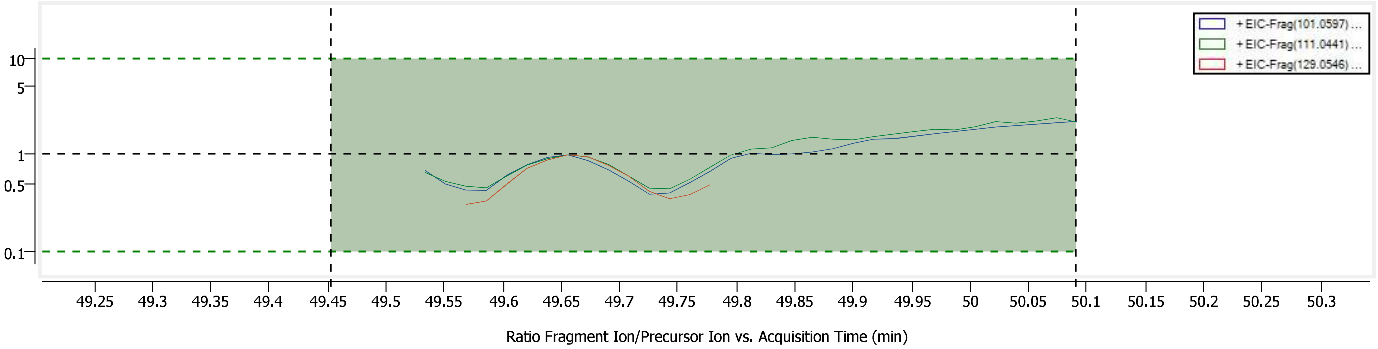

Compound Spectra (overlaid)

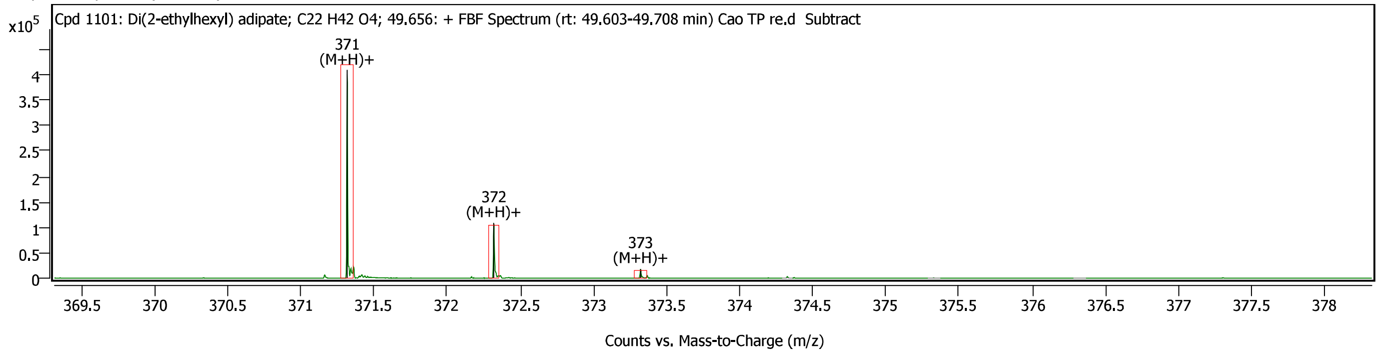

Fragment Spectrum (clean)

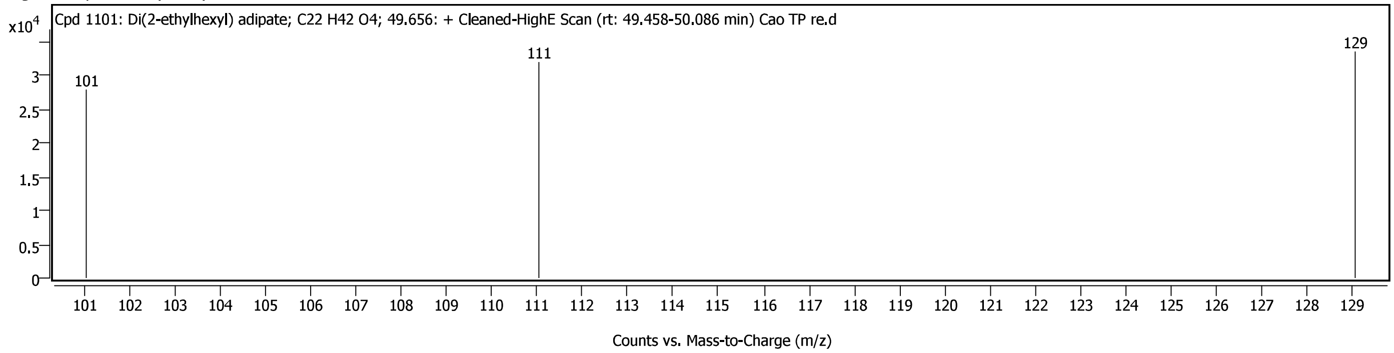

Fragment Spectrum (raw)

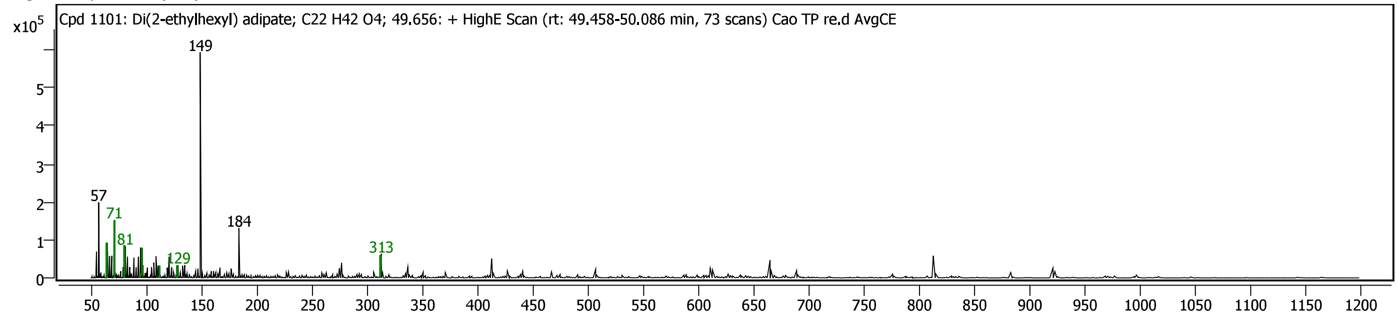

Compound ID Table

Name	Formula	Species	RT	RT Diff	Mass	CAS	ID Source	Score	Score (Lib)	Score (Tgt)
Di(2-ethylhexyl) adipate	C22 H42 O4	(M+H)+	49.656		370.3089	103-23-1	FBF-FragConfirm	97.85		97.85
Docosanedioic acid	C22 H42 O4	(M+H)+	49.656		370.3089		FBF-FragConfirm	97.85		97.85
Dioctyl hexanedioate	C22 H42 O4	(M+H)+	49.656		370.3089	123-79-5	FBF-FragConfirm	97.85		97.85

Cpd 957: Ecalcidene

Name	Formula	RT	RI	Mass	Diff (Tgt, ppm)	CAS	ID Source	Score	Algorithm
Ecalcidene	C29 H45 N O3	49.708		455.3400	0.23		FBF	99.48	FBF

Species	m/z	Score (Tgt)	Score (Lib)	Score (DB)	Score (MFG)	Score (RT)
(M+H)+	456	99.48				

Compound Chromatograms (overlaid)

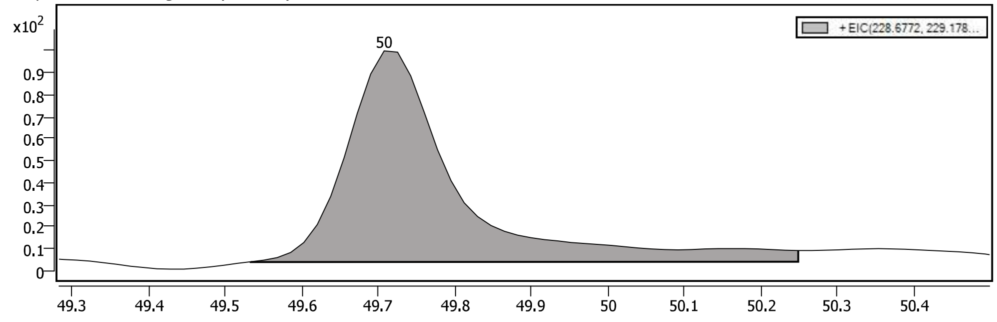

Structure

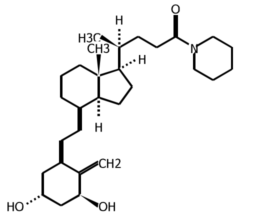

Compound Spectra (overlaid)

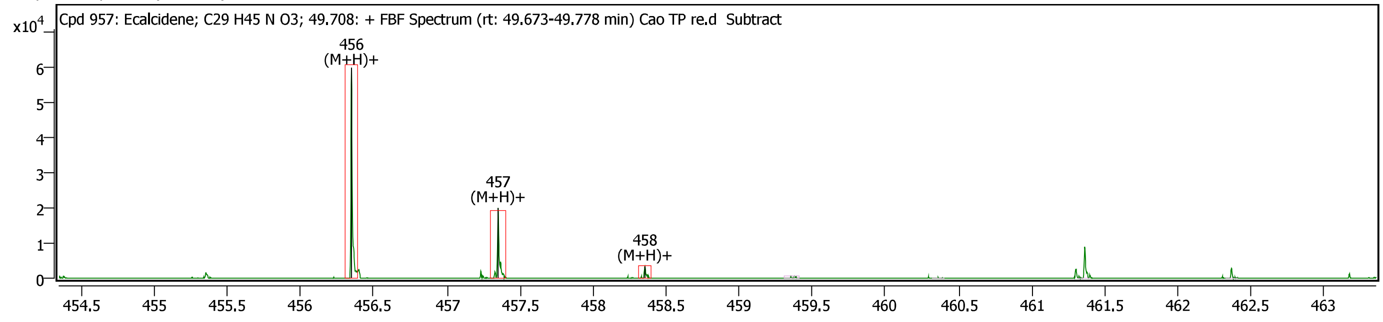

Compound ID Table

Name	Formula	Species	RT	RT Diff	Mass	CAS	ID Source	Score	Score (Lib)	Score (Tgt)
Ecalcidene	C29 H45 N O3	(M+H)+	49.708		455.3400		FBF	99.48		99.48

Cpd 701: adenosylhopane

Name	Formula	RT	RI	Mass	Diff (Tgt, ppm)	CAS	ID Source	Score	Algorithm
adenosylhopane	C40 H63 N5 O3	50.092		661.4912	-2.84		FBF	90.31	FBF

Species	m/z	Score (Tgt)	Score (Lib)	Score (DB)	Score (MFG)	Score (RT)
(M+H)+	662	90.31				

Compound Screening Report

Compound Chromatograms (overlaid)

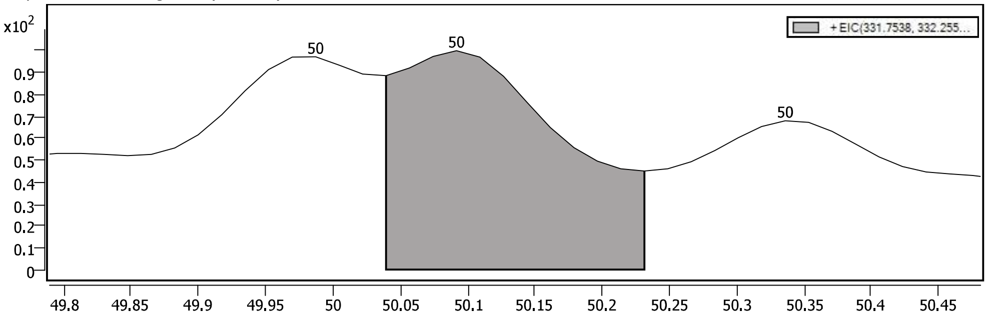

Structure

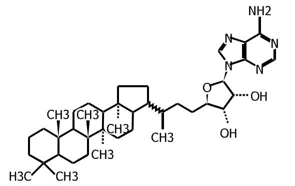

Compound Spectra (overlaid)

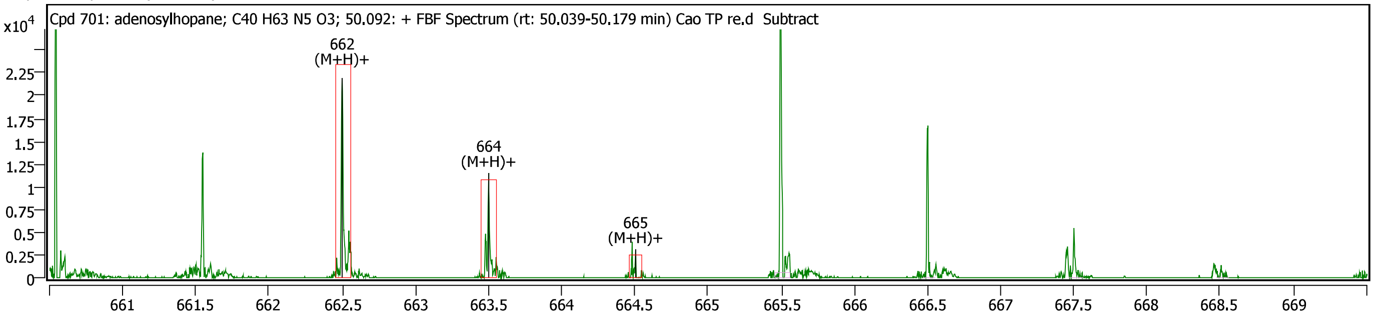

Compound ID Table

Name	Formula	Species	RT	RT Diff	Mass	CAS	ID Source	Score	Score (Lib)	Score (Tgt)
adenosylhopane	C40 H63 N5 O3	(M+H)+	50.092		661.4912		FBF	90.31		90.31

Cpd 863: MGDG(18:3(9Z,12Z,15Z)/18:4(6Z,9Z,12Z,15Z))

Name	Formula	RT	RI	Mass	Diff (Tgt, ppm)	CAS	ID Source	Score	Algorithm
MGDG(18:3(9Z,12Z,15Z)/18:4(6Z, 9Z,12Z,15Z))	C45 H72 O10	50.179		772.5127	0.22		FBF	99.42	FBF

Species	m/z	Score (Tgt)	Score (Lib)	Score (DB)	Score (MFG)	Score (RT)
(M+H)+	774	99.42				

Compound Chromatograms (overlaid)

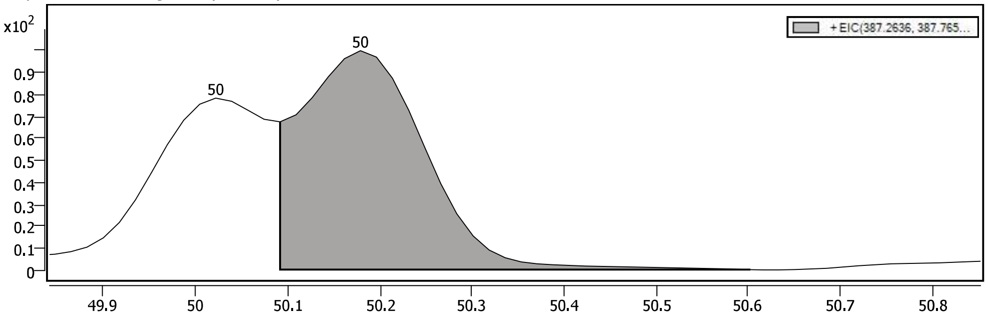

Structure

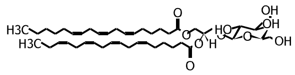

Compound Spectra (overlaid)

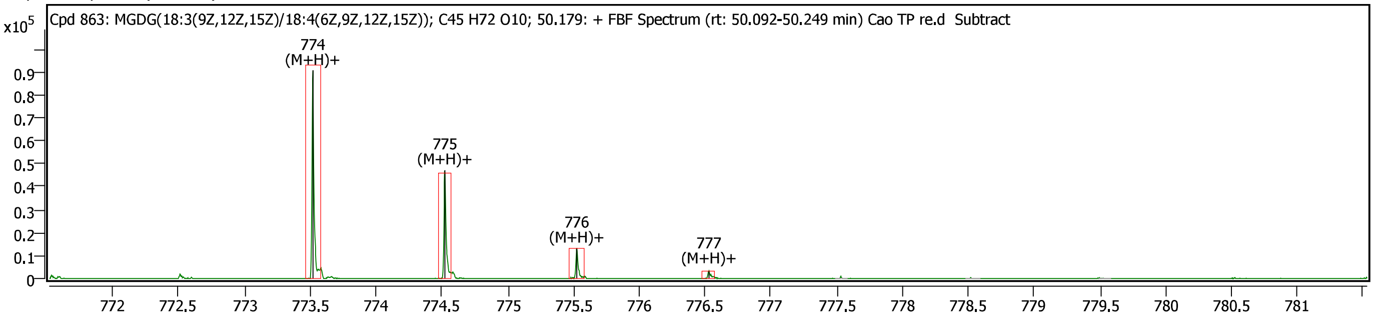

Compound ID Table

Name	Formula	Species	RT	RT Diff	Mass	CAS	ID Source	Score	Score (Lib)	Score (Tgt)
MGDG(18:3(9Z,12Z,15Z)/18:4(6Z, 9Z,12Z,15Z))	C45 H72 O10	(M+H)+	50.179		772.5127		FBF	99.42		99.42

Cpd 1006: C18 Sulfatide

Name	Formula	RT	RI	Mass	Diff (Tgt, ppm)	CAS	ID Source	Score	Algorithm
C18 Sulfatide	C42 H81 N O11 S	50.179		807.5500	-3.81		FBF	90.22	FBF

Species	m/z	Score (Tgt)	Score (Lib)	Score (DB)	Score (MFG)	Score (RT)
(M+H)+	809	90.22				

Compound Chromatograms (overlaid)

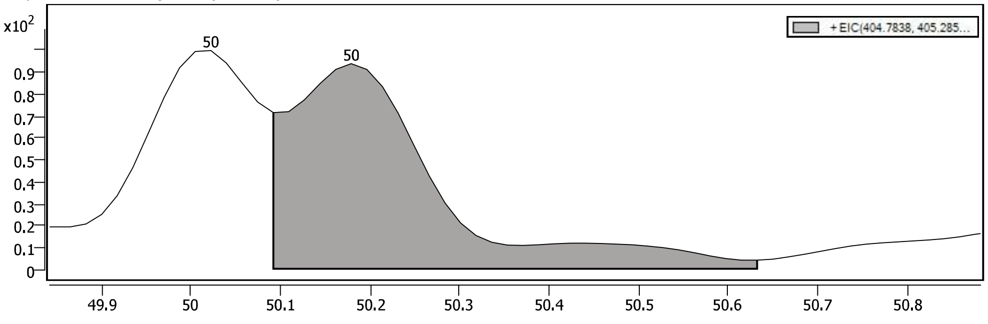

Structure

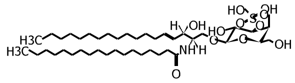

Compound Spectra (overlaid)

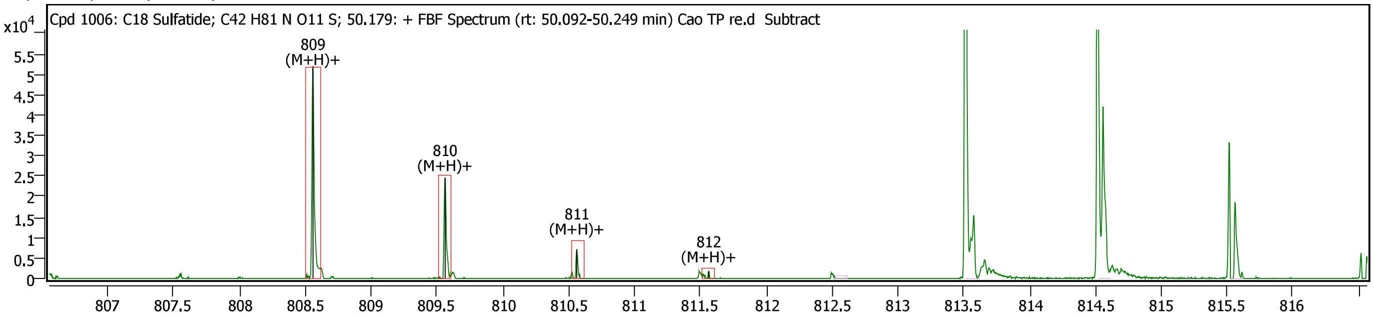

Compound ID Table

Name	Formula	Species	RT	RT Diff	Mass	CAS	ID Source	Score	Score (Lib)	Score (Tgt)
C18 Sulfatide	C42 H81 N O11 S	(M+H)+	50.179		807.5500		FBF	90.22		90.22

Cpd 75: 2-methyl-1,3-Cyclohexanedione

Name	Formula	RT	RI	Mass	Diff (Tgt, ppm)	CAS	ID Source	Score	Algorithm
2-methyl-1,3-Cyclohexanedione	C7 H10 O2	50.633		126.0683	1.51	1193-55-1	FBF-FragConfirm	98.69	FBF

Species	m/z	Score (Tgt)	Score (Lib)	Score (DB)	Score (MFG)	Score (RT)
(M+H)+	127	98.69				

Compound Chromatograms (overlaid)

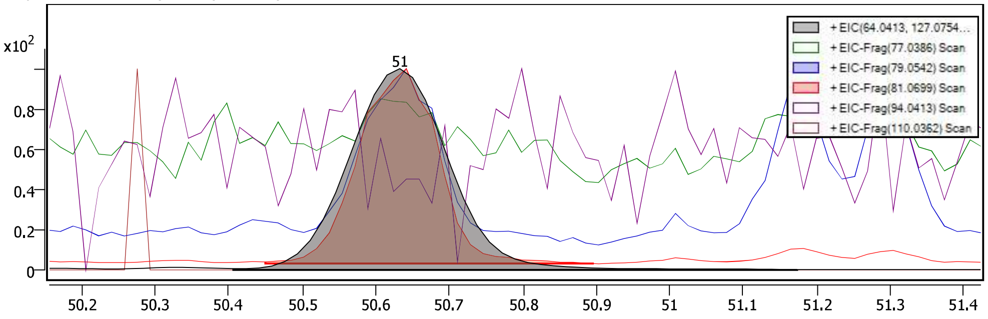

Structure

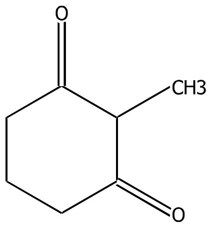

Compound Screening Report

Coelution Plot

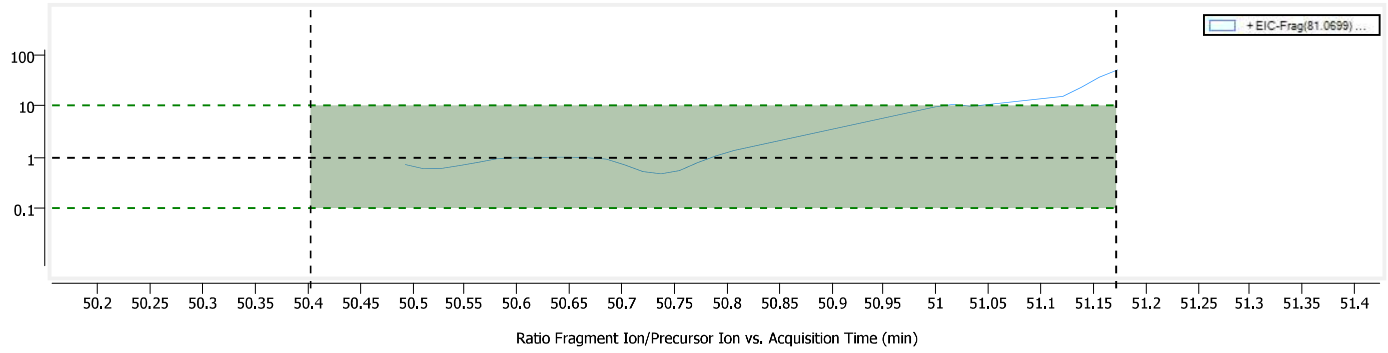

Compound Spectra (overlaid)

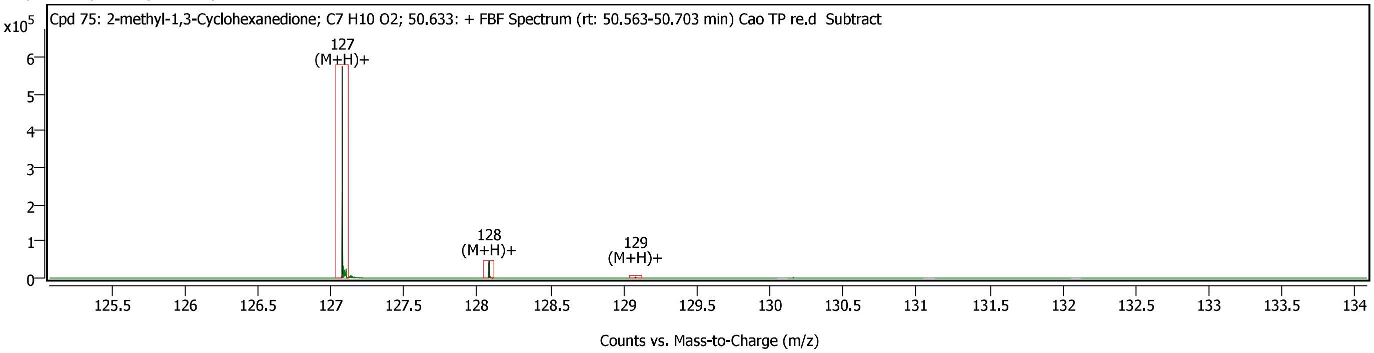

Fragment Spectrum (clean)

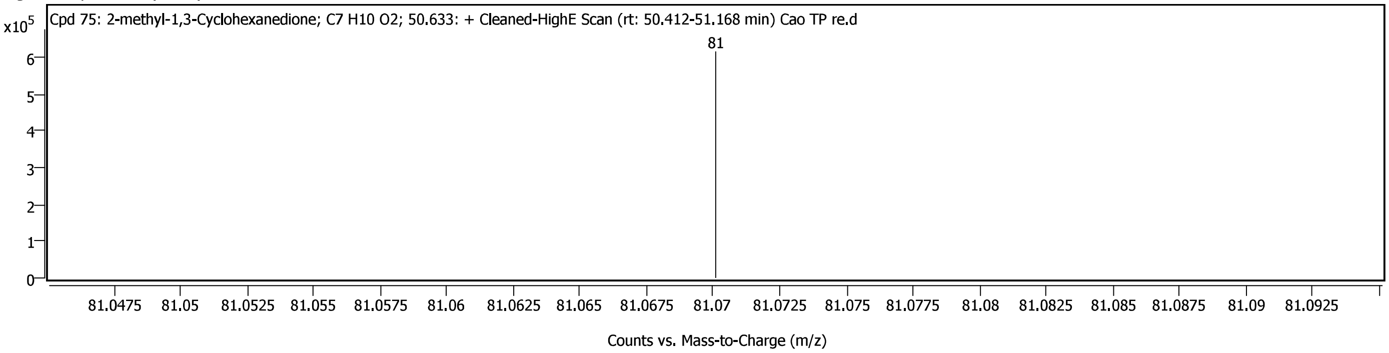

Fragment Spectrum (raw)

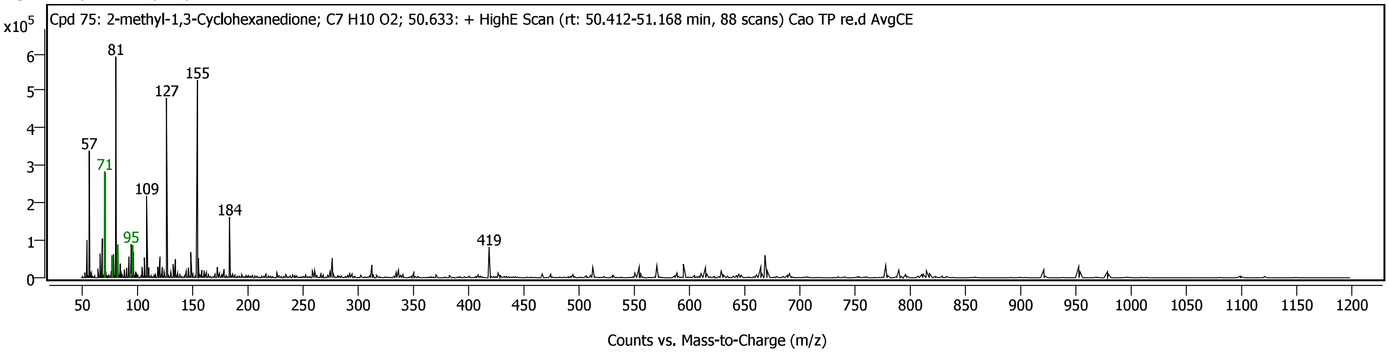

Compound Screening Report

Compound ID Table

Name	Formula	Species	RT	RT Diff	Mass	CAS	ID Source	Score	Score (Lib)	Score (Tgt)
2-methyl-1,3-Cyclohexanedione	C7 H10 O2	(M+H)+	50.633		126.0683	1193-55-1	FBF-FragConfirm	98.69		98.69
3Z-Hepten-2,5-dione	C7 H10 O2	(M+H)+	50.633		126.0683		FBF-FragConfirm	98.69		98.69
xi-4-Hydroxy-4-methyl-2-cyclohexen-1-one	C7 H10 O2	(M+H)+	50.633		126.0683		FBF-FragConfirm	98.69		98.69
3-Methyl-1,2-cyclohexanedione	C7 H10 O2	(M+H)+	50.633		126.0683	3008-43-3	FBF-FragConfirm	98.69		98.69
1-Cyclohexenecarboxylic acid	C7 H10 O2	(M+H)+	50.633		126.0683	636-82-8	FBF-FragConfirm	98.69		98.69
2-(Ethoxymethyl)furan	C7 H10 O2	(M+H)+	50.633		126.0683	6270-56-0	FBF-FragConfirm	98.69		98.69
2-heptenedial	C7 H10 O2	(M+H)+	50.633		126.0683		FBF-FragConfirm	98.69		98.69
3-(3-Ethyloxiranyl)-2-propenal	C7 H10 O2	(M+H)+	50.633		126.0683	78307-41-2	FBF-FragConfirm	98.69		98.69
3,4-Dimethyl-1,2-cyclopentanedione	C7 H10 O2	(M+H)+	50.633		126.0683	13494-06-9	FBF-FragConfirm	98.69		98.69
3,5-Dimethyl-1,2-cyclopentanedione	C7 H10 O2	(M+H)+	50.633		126.0683	13494-07-0	FBF-FragConfirm	98.69		98.69
3-Heptynoic acid	C7 H10 O2	(M+H)+	50.633		126.0683		FBF-FragConfirm	98.69		98.69
3E-Hepten-2,5-dione	C7 H10 O2	(M+H)+	50.633		126.0683		FBF-FragConfirm	98.69		98.69
xi-5-Isopropyl-2(5H)-furanone	C7 H10 O2	(M+H)+	50.633		126.0683	100019-33-8	FBF-FragConfirm	98.69		98.69
4-Heptynoic acid	C7 H10 O2	(M+H)+	50.633		126.0683		FBF-FragConfirm	98.69		98.69
4-Methyl-3Z,5-hexadienoic acid	C7 H10 O2	(M+H)+	50.633		126.0683		FBF-FragConfirm	98.69		98.69
6-Heptynoic acid	C7 H10 O2	(M+H)+	50.633		126.0683		FBF-FragConfirm	98.69		98.69
Allyl crotonate	C7 H10 O2	(M+H)+	50.633		126.0683	5453-44-1	FBF-FragConfirm	98.69		98.69
Enol-3-Ethyl-1,2-cyclopentanedione	C7 H10 O2	(M+H)+	50.633		126.0683	21835-01-8	FBF-FragConfirm	98.69		98.69
Methyl sorbate	C7 H10 O2	(M+H)+	50.633		126.0683	689-89-4	FBF-FragConfirm	98.69		98.69
Toluene-cis-dihydrodiol	C7 H10 O2	(M+H)+	50.633		126.0683		FBF-FragConfirm	98.69		98.69
4-Hydroxy-4-methyl-5-hexenoic acid gamma lactone	C7 H10 O2	(M+H)+	50.633		126.0683	1073-11-6	FBF-FragConfirm	98.69		98.69

Cpd 1000: Diisononyl phthalate

Name	Formula	RT	RI	Mass	Diff (Tgt, ppm)	CAS	ID Source	Score	Algorithm
Diisononyl phthalate	C26 H42 O4	50.633		418.3064	-4.56	28553-12-0	FBF-FragConfirm	90.69	FBF

Species	m/z	Score (Tgt)	Score (Lib)	Score (DB)	Score (MFG)	Score (RT)
(M+H)+	419	90.69				

Compound Chromatograms (overlaid)

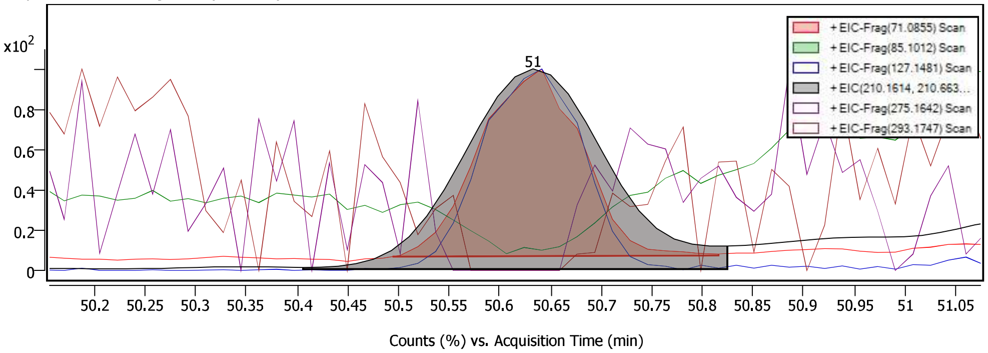

Structure

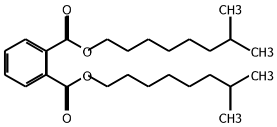

Coelution Plot

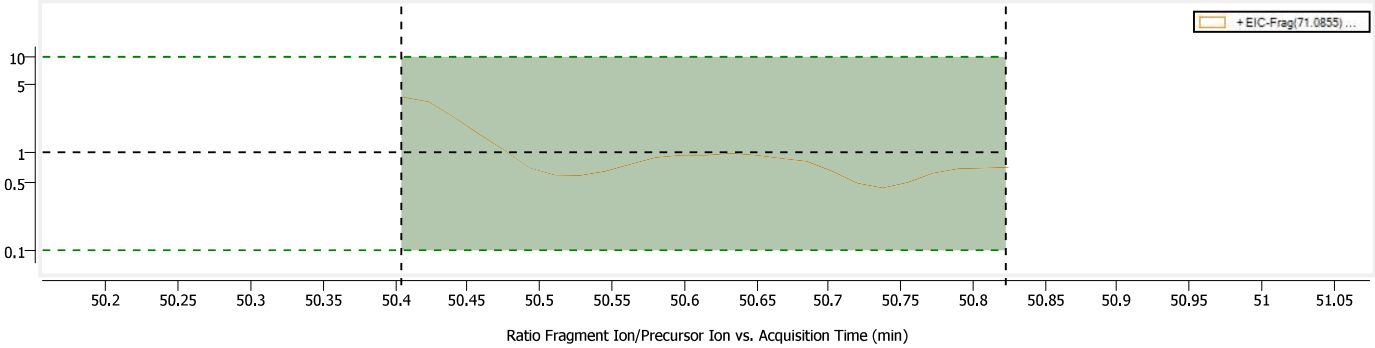

Compound Spectra (overlaid)

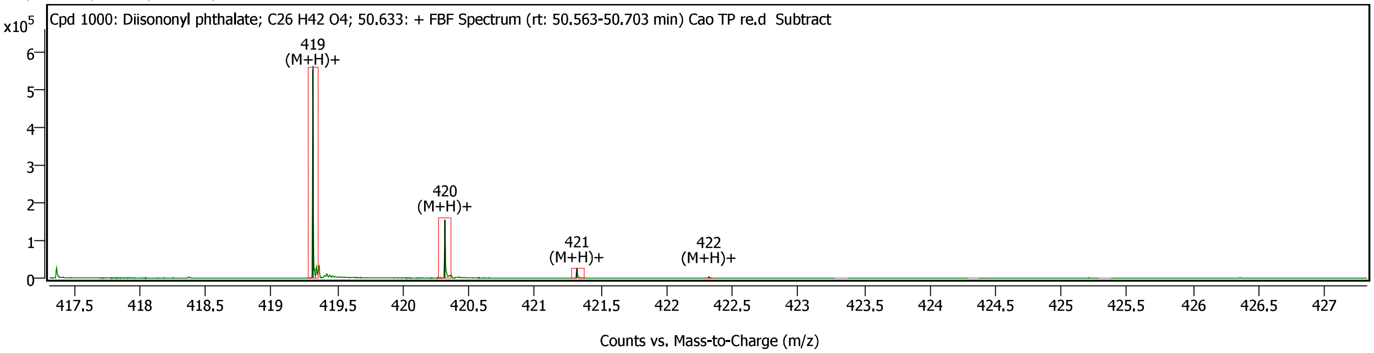

Fragment Spectrum (clean)

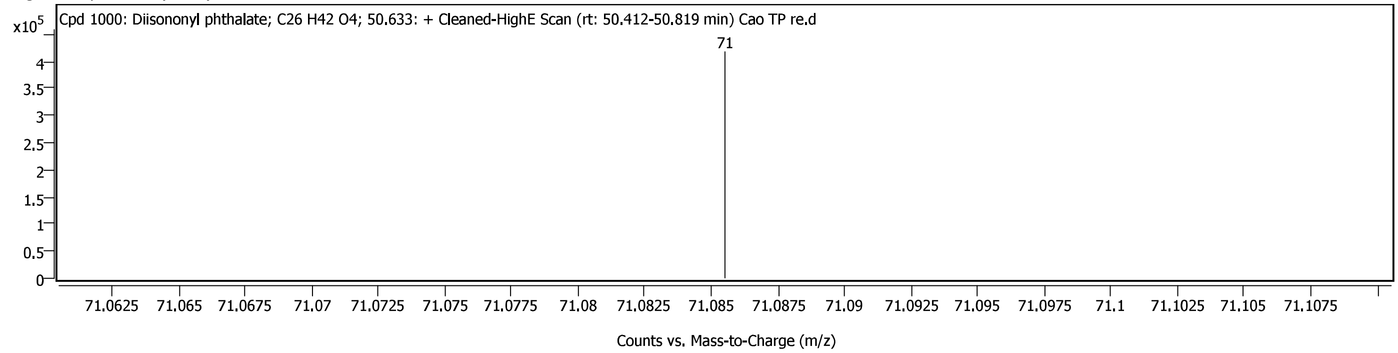

Fragment Spectrum (raw)

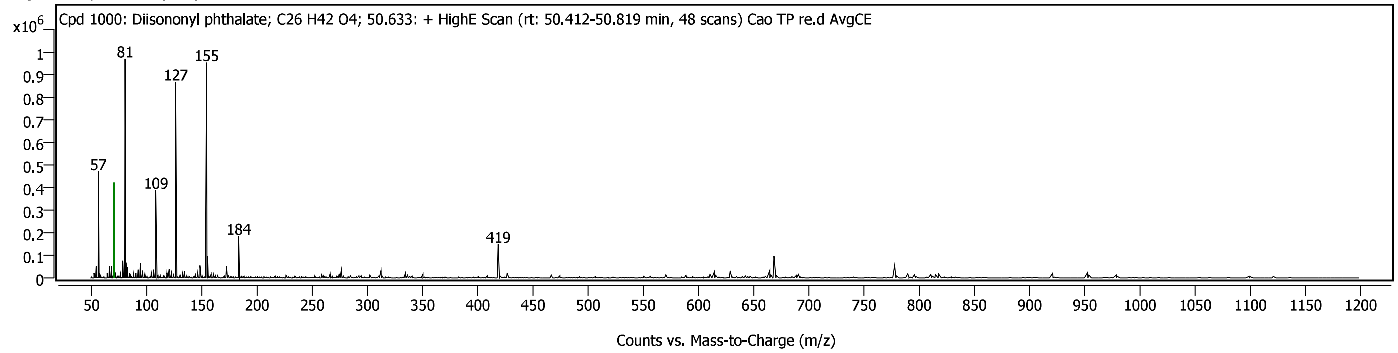

Compound ID Table

Name	Formula	Species	RT	RT Diff	Mass	CAS	ID Source	Score	Score (Lib)	Score (Tgt)
Diisononyl phthalate	C26 H42 O4	(M+H)+	50.633		418.3064	28553-12-0	FBF-FragConfirm	90.69		90.69
1α,25-dihydroxy-22-oxavitamin D3 / 1α,25-dihydroxy-22-oxacholecalciferol	C26 H42 O4	(M+H)+	50.633		418.3064		FBF-FragConfirm	90.69		90.69
(24R)-1α,24-dihydroxy-22-oxa-20-epivitamin D3 / (24R)-1α,24-dihydroxy-22-oxa-20-epicholecalciferol	C26 H42 O4	(M+H)+	50.633		418.3064		FBF-FragConfirm	90.69		90.69
(24R)-1α,24-dihydroxy-22-oxavitamin D3 / (24R)-1α,24-dihydroxy-22-oxacholecalciferol	C26 H42 O4	(M+H)+	50.633		418.3064		FBF-FragConfirm	90.69		90.69
(24S)-1α,24-dihydroxy-22-oxa-20-epivitamin D3 / (24S)-1α,24-dihydroxy-22-oxa-20-epicholecalciferol	C26 H42 O4	(M+H)+	50.633		418.3064		FBF-FragConfirm	90.69		90.69
(24S)-1α,24-dihydroxy-22-oxavitamin D3 / (24S)-1α,24-dihydroxy-22-oxacholecalciferol	C26 H42 O4	(M+H)+	50.633		418.3064		FBF-FragConfirm	90.69		90.69
1α,25-dihydroxy-22-oxa-20-epivitamin D3 / 1α,25-dihydroxy-22-oxa-20-epicholecalciferol	C26 H42 O4	(M+H)+	50.633		418.3064		FBF-FragConfirm	90.69		90.69
(20S)-1α,20,25-trihydroxy-24-norvitamin D3/(20S)-1α,20,25-trihydroxy-24-norcholecalciferol	C26 H42 O4	(M+H)+	50.633		418.3064		FBF-FragConfirm	90.69		90.69
1α,25-dihydroxy-23-oxavitamin D3 / 1α,25-dihydroxy-23-oxacholecalciferol	C26 H42 O4	(M+H)+	50.633		418.3064		FBF-FragConfirm	90.69		90.69
24-northornasterol A	C26 H42 O4	(M+H)+	50.633		418.3064		FBF-FragConfirm	90.69		90.69
Dichotellate A	C26 H42 O4	(M+H)+	50.633		418.3064		FBF-FragConfirm	90.69		90.69
Dichotellate B	C26 H42 O4	(M+H)+	50.633		418.3064		FBF-FragConfirm	90.69		90.69
Hexadecyl ferulate	C26 H42 O4	(M+H)+	50.633		418.3064	158306-36-6	FBF-FragConfirm	90.69		90.69
1β,25-dihydroxy-22-oxavitamin D3 / 1β,25-dihydroxy-22-oxacholecalciferol	C26 H42 O4	(M+H)+	50.633		418.3064		FBF-FragConfirm	90.69		90.69

Cpd 1304: (10S)-Juvenile hormone III diol

Name	Formula	RT	RI	Mass	Diff (Tgt, ppm)	CAS	ID Source	Score	Algorithm
(10S)-Juvenile hormone III diol	C16 H28 O4	50.633		284.1990	0.83		FBF	99.34	FBF

Species	m/z	Score (Tgt)	Score (Lib)	Score (DB)	Score (MFG)	Score (RT)
(M+H)+	285	99.34				

Compound Screening Report

Compound Chromatograms (overlaid)

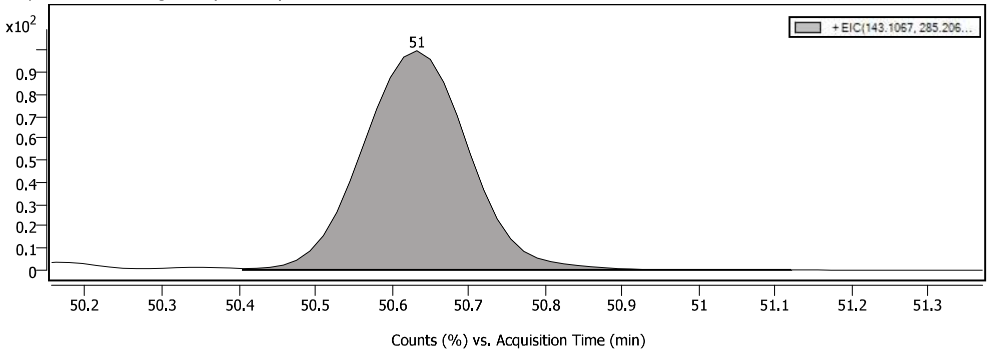

Structure

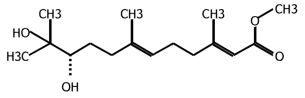

Compound Spectra (overlaid)

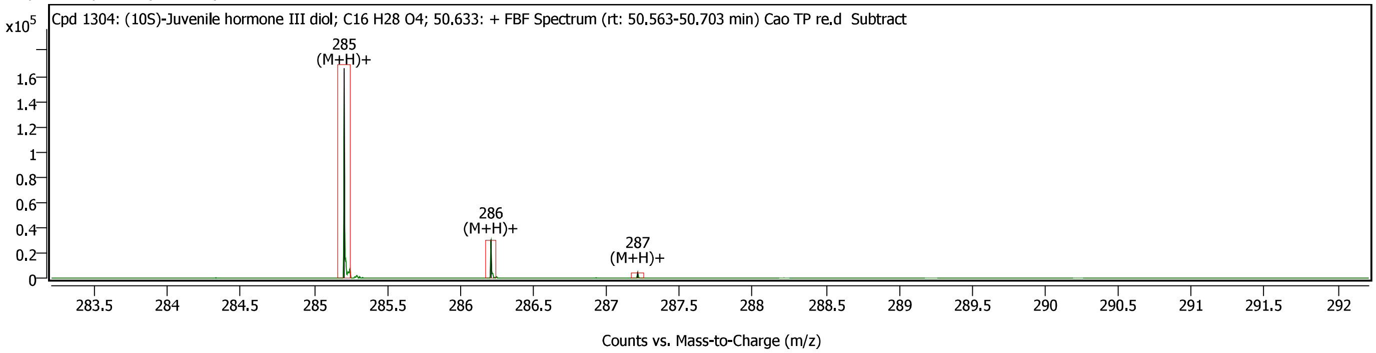

Compound ID Table

Name	Formula	Species	RT	RT Diff	Mass	CAS	ID Source	Score	Score (Lib)	Score (Tgt)
(10S)-Juvenile hormone III diol	C16 H28 O4	(M+H)+	50.633		284.1990		FBF	99.34		99.34

Cpd 1465: 2,6-Dihydroxy-4-methoxytoluene

Name	Formula	RT	RI	Mass	Diff (Tgt, ppm)	CAS	ID Source	Score	Algorithm
2,6-Dihydroxy-4-methoxytoluene	C8 H10 O3	50.633		154.0632	1.66	6307-89-7	M-FBF-FragConfirm	98.21	FBF

Species	m/z	Score (Tgt)	Score (Lib)	Score (DB)	Score (MFG)	Score (RT)
(M+H)+	155	98.21				

Compound Chromatograms (overlaid)

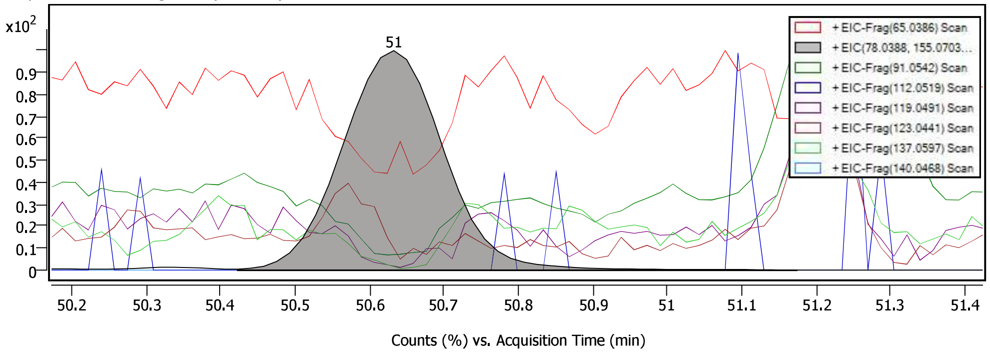

Structure

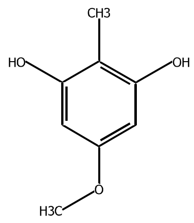

Coelution Plot

Compound Spectra (overlaid)

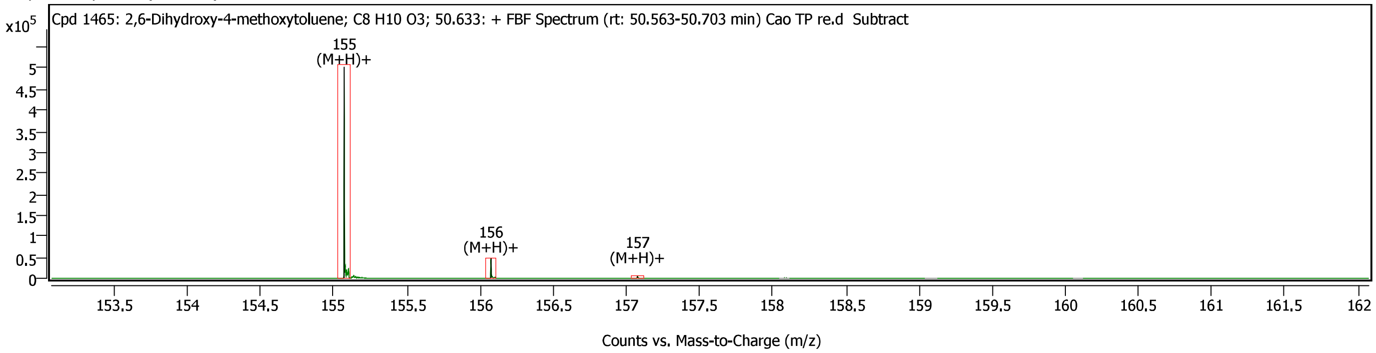

Fragment Spectrum (clean)

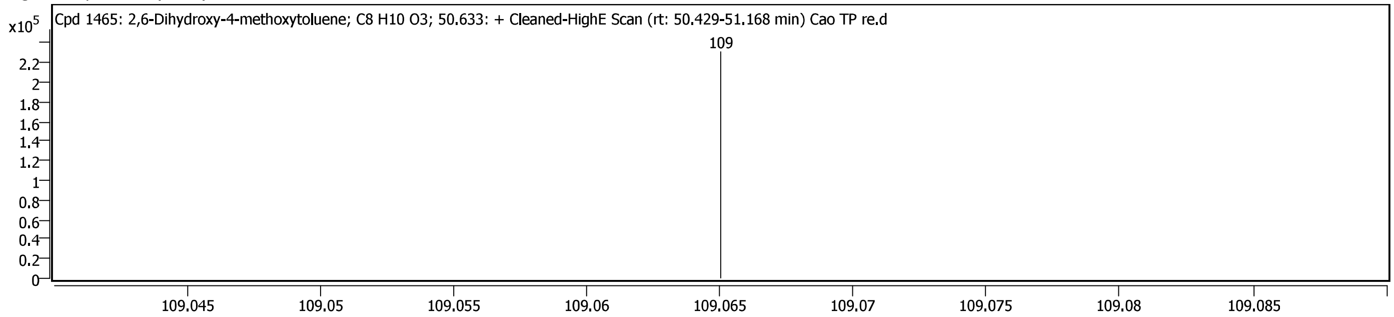

Fragment Spectrum (raw)

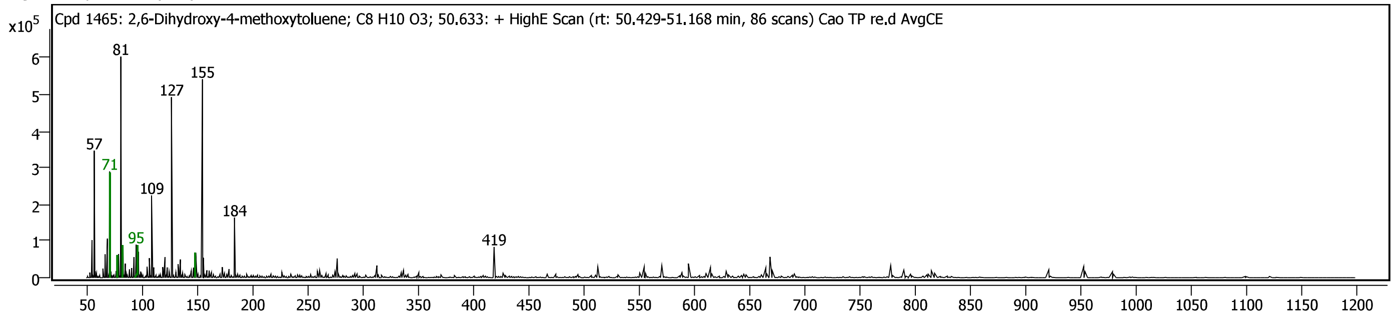

Compound ID Table

Name	Formula	Species	RT	RT Diff	Mass	CAS	ID Source	Score	Score (Lib)	Score (Tgt)
2,6-Dihydroxy-4-methoxytoluene	C8 H10 O3	(M+H)+	50.633		154.0632	6307-89-7	FBF-FragConfirm	98.21		98.21
3,4-Dihydroxyphenyl ethanol	C8 H10 O3	(M+H)+	50.633		154.0632	10597-60-1	FBF-FragConfirm	98.21		98.21
2-Furanylmethyl propanoate	C8 H10 O3	(M+H)+	50.633		154.0632	623-19-8	FBF-FragConfirm	98.21		98.21
2,6-Dimethoxyphenol	C8 H10 O3	(M+H)+	50.633		154.0632	91-10-1	FBF-FragConfirm	98.21		98.21
3-Hydroxy-phenylglycol	C8 H10 O3	(M+H)+	50.633		154.0632	20150-19-0	FBF-FragConfirm	98.21		98.21
Propyl 2-furoate	C8 H10 O3	(M+H)+	50.633		154.0632	615-10-1	FBF-FragConfirm	98.21		98.21
Sylvopinol	C8 H10 O3	(M+H)+	50.633		154.0632	30891-29-3	FBF-FragConfirm	98.21		98.21
Vanillyl alcohol	C8 H10 O3	(M+H)+	50.633		154.0632	498-00-0	FBF-FragConfirm	98.21		98.21

Cpd 65: DG(14:1(9Z)/17:2(9Z,12Z)/0:0)[iso2]

Name	Formula	RT	RI	Mass	Diff (Tgt, ppm)	CAS	ID Source	Score	Algorithm
DG(14:1(9Z)/17:2(9Z,12Z)/0:0)[iso2]	C34 H60 O5	51.017		548.4442	0.31		M-FBF	99.71	FBF

Species	m/z	Score (Tgt)	Score (Lib)	Score (DB)	Score (MFG)	Score (RT)
(M+H)+	549	99.71				

Compound Chromatograms (overlaid)

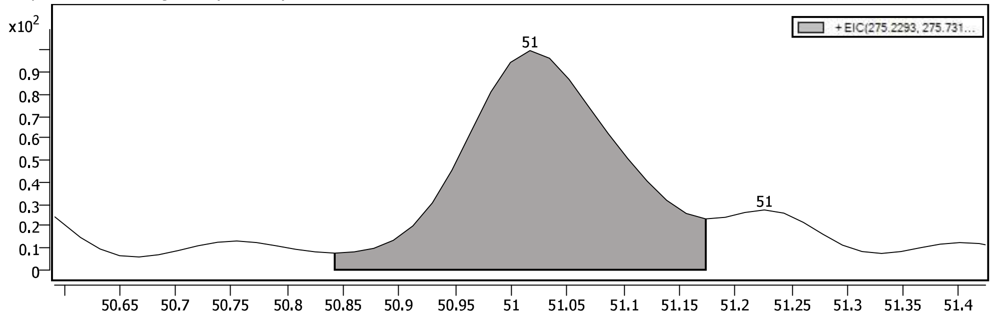

Structure

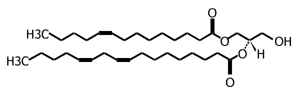

Compound Screening Report

Compound Spectra (overlaid)

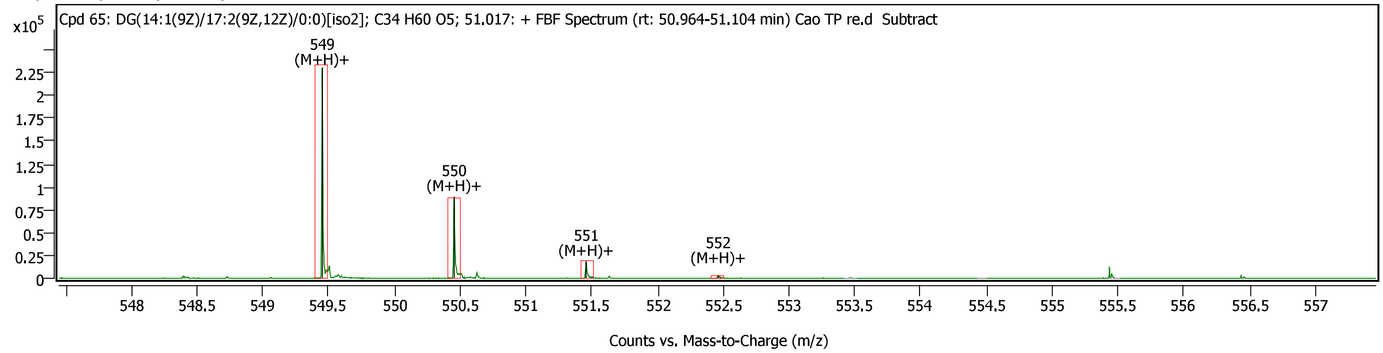

Compound ID Table

Name	Formula	Species	RT	RT Diff	Mass	CAS	ID Source	Score	Score (Lib)	Score (Tgt)
DG(14:1(9Z)/17:2(9Z,12Z)/0:0)[iso2]	C34 H60 O5	(M+H)+	51.017		548.4442		FBF	99.71		99.71
DG(13:0/18:3(9Z,12Z,15Z)/0:0)[iso2]	C34 H60 O5	(M+H)+	51.017		548.4442		FBF	99.71		99.71

Cpd 64: DG(13:0/20:5(5Z,8Z,11Z,14Z,17Z)/0:0)[iso2]

Name	Formula	RT	RI	Mass	Diff (Tgt, ppm)	CAS	ID Source	Score	Algorithm
DG(13:0/20:5(5Z,8Z,11Z,14Z,17Z)/0:0)[iso2]	C36 H60 O5	51.174		572.4443	0.41		FBF	99.44	FBF

Species	m/z	Score (Tgt)	Score (Lib)	Score (DB)	Score (MFG)	Score (RT)
(M+H)+	573	99.44				

Compound Chromatograms (overlaid)

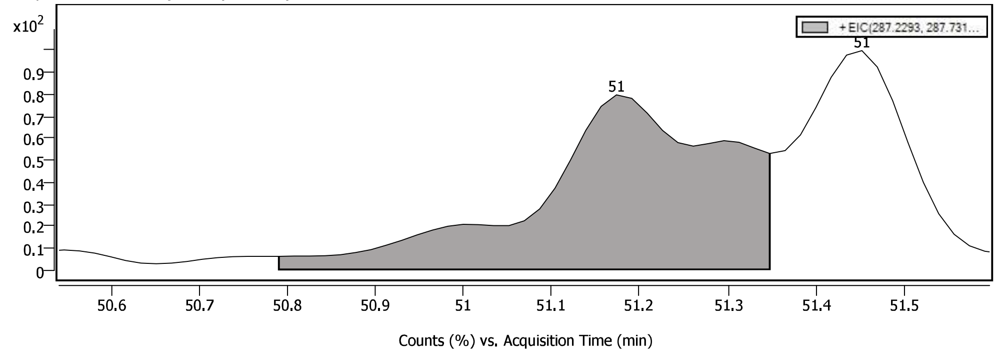

Structure

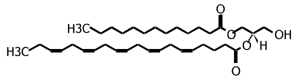

Compound Spectra (overlaid)

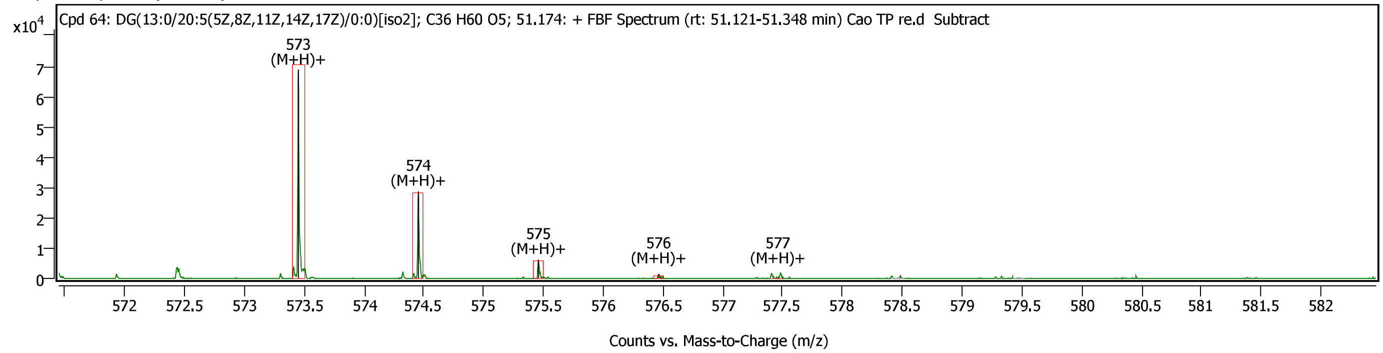

Compound ID Table

Name	Formula	Species	RT	RT Diff	Mass	CAS	ID Source	Score	Score (Lib)	Score (Tgt)
DG(13:0/20:5(5Z,8Z,11Z,14Z,17Z)/0:0)[iso2]	C36 H60 O5	(M+H)+	51.174		572.4443		FBF	99.44		99.44

Cpd 601: <U-74389G>

Name	Formula	RT	RI	Mass	Diff (Tgt, ppm)	CAS	ID Source	Score	Algorithm
<U-74389G>	C37 H50 N6 O2	51.174		610.3999	0.60	153190-29-5	FBF	93.77	FBF

Species	m/z	Score (Tgt)	Score (Lib)	Score (DB)	Score (MFG)	Score (RT)
(M+H)+	611	93.77				

Compound Screening Report

Compound Chromatograms (overlaid)

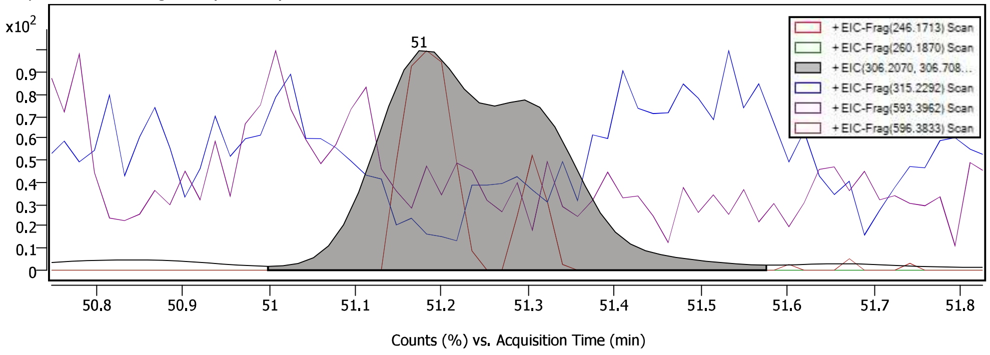

Structure

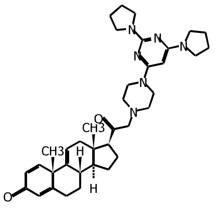

Coelution Plot

Compound Spectra (overlaid)

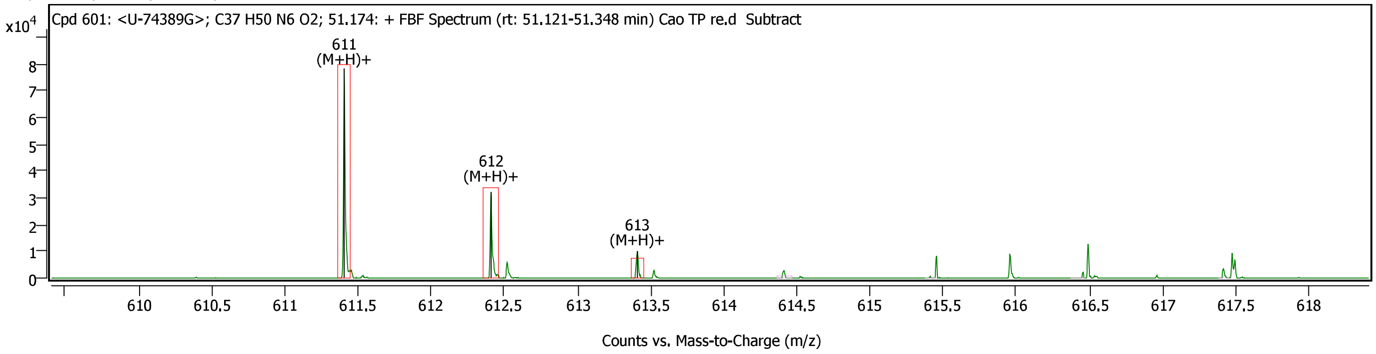

Fragment Spectrum (raw)

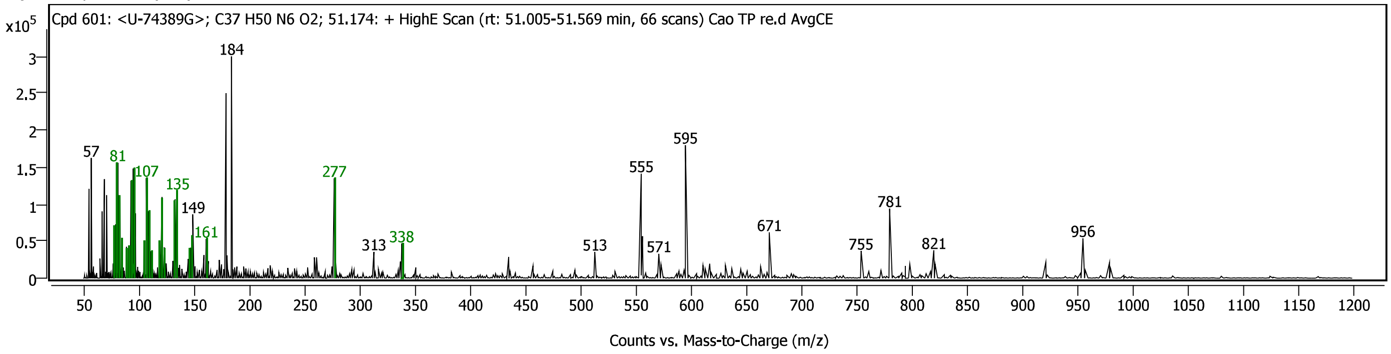

Compound ID Table

Name	Formula	Species	RT	RT Diff	Mass	CAS	ID Source	Score	Score (Lib)	Score (Tgt)
<U-74389G>	C37 H50 N6 O2	(M+H)+	51.174		610.3999	153190-29-5	FBF	93.77		93.77

Cpd 700: bacteriohopane-32,33, 34-triol-35-carbamate

Name	Formula	RT	RI	Mass	Diff (Tgt, ppm)	CAS	ID Source	Score	Algorithm
bacteriohopane-32,33, 34-triol-35-carbamate	C36 H63 N O5	51.174		589.4710	0.57		FBF	99.72	FBF

Species	m/z	Score (Tgt)	Score (Lib)	Score (DB)	Score (MFG)	Score (RT)
(M+H)+	590	99.72				

Compound Chromatograms (overlaid)

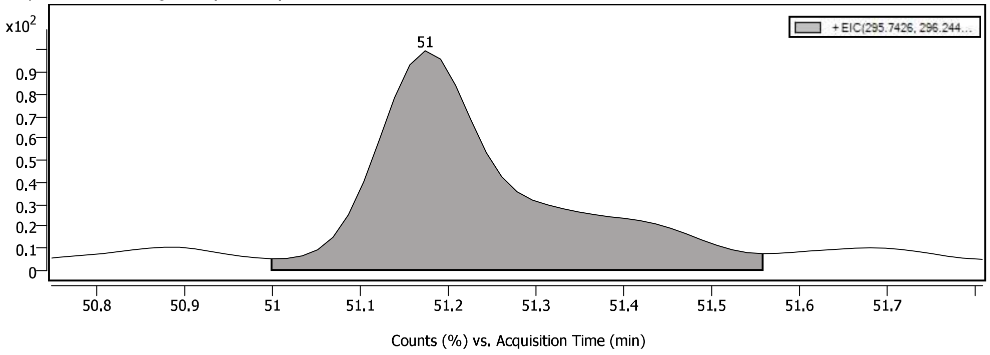

Structure

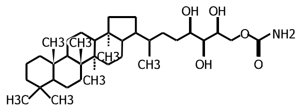

Compound Spectra (overlaid)

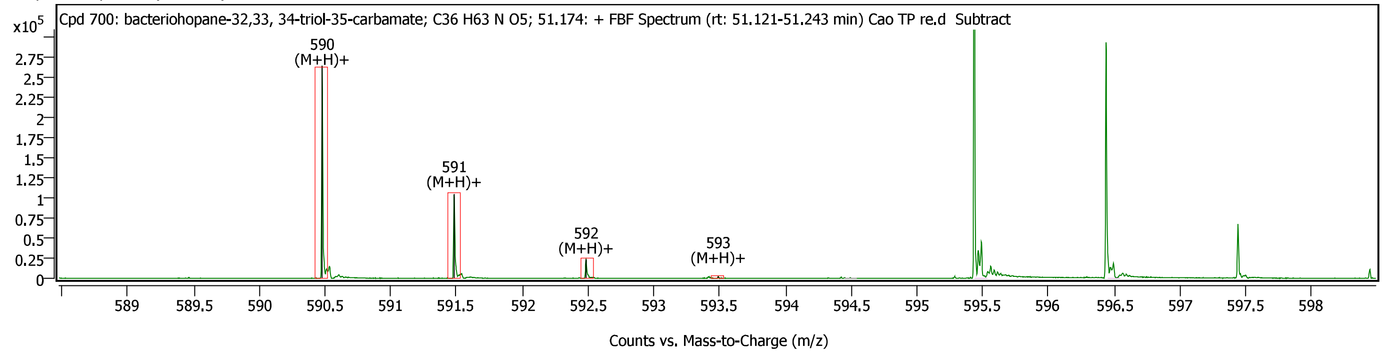

Compound ID Table

Name	Formula	Species	RT	RT Diff	Mass	CAS	ID Source	Score	Score (Lib)	Score (Tgt)
bacteriohopane-32,33, 34-triol-35-carbamate	C36 H63 N O5	(M+H)+	51.174		589.4710		FBF	99.72		99.72

Cpd 924: Elaidylphosphocholine

Name	Formula	RT	RI	Mass	Diff (Tgt, ppm)	CAS	ID Source	Score	Algorithm
Elaidylphosphocholine	C23 H49 N O4 P	51.208		434.3407	1.77	156161-89-6	FBF	97.58	FBF

Species	m/z	Score (Tgt)	Score (Lib)	Score (DB)	Score (MFG)	Score (RT)
(M+H)+	435	97.58				

Compound Chromatograms (overlaid)

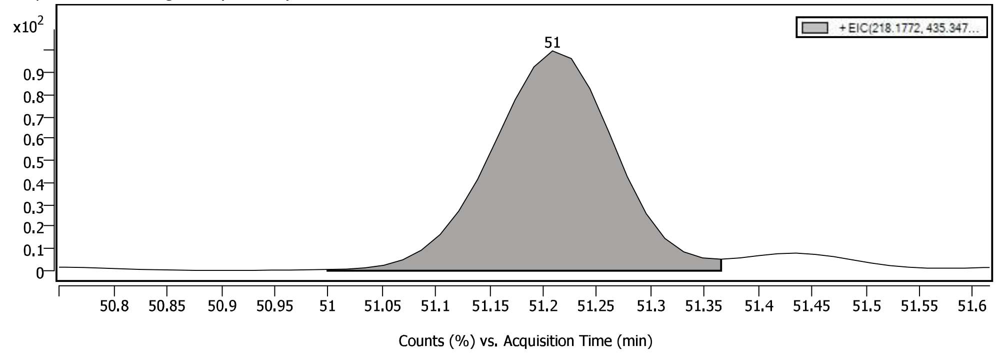

Structure

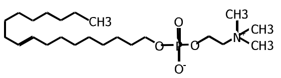

Compound Spectra (overlaid)

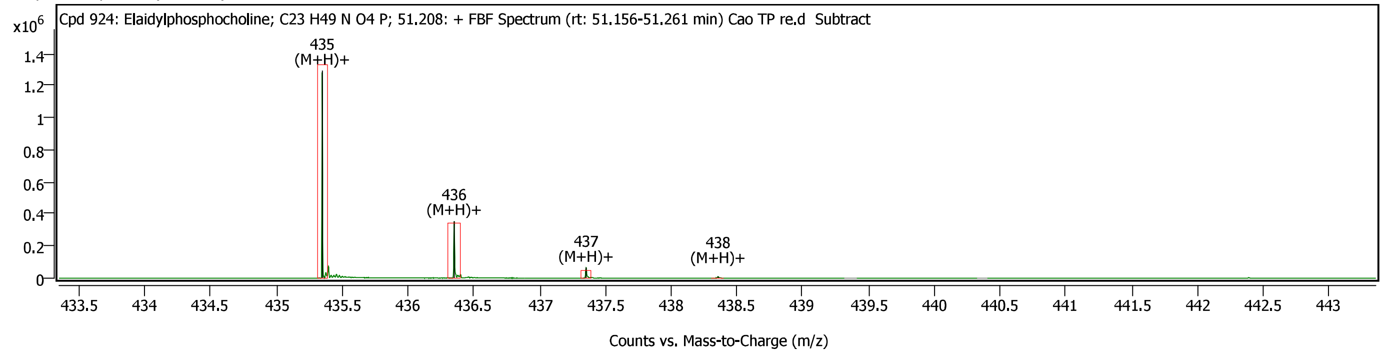

Compound ID Table

Name	Formula	Species	RT	RT Diff	Mass	CAS	ID Source	Score	Score (Lib)	Score (Tgt)
Elaidylphosphocholine	C23 H49 N O4 P	(M+H)+	51.208		434.3407	156161-89-6	FBF	97.58		97.58

Cpd 307: PS(P-18:0/22:6(4Z,7Z,10Z,13Z,16Z,19Z))

Name	Formula	RT	RI	Mass	Diff (Tgt, ppm)	CAS	ID Source	Score	Algorithm
PS(P-18:0/22:6(4Z,7Z,10Z,13Z,16Z,19Z))	C46 H78 N O9 P	51.296		819.5394	-2.48		FBF	96.93	FBF

Species	m/z	Score (Tgt)	Score (Lib)	Score (DB)	Score (MFG)	Score (RT)
(M+H)+	821	96.93				

Compound Screening Report

Compound Chromatograms (overlaid)

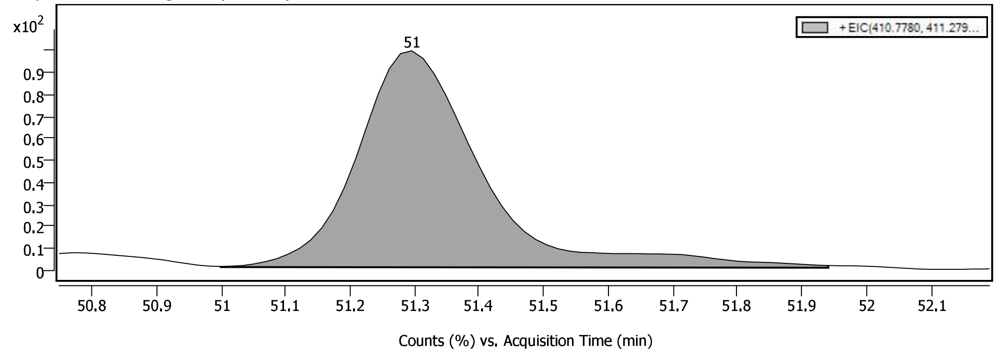

Structure

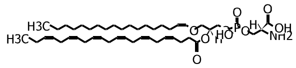

Compound Spectra (overlaid)

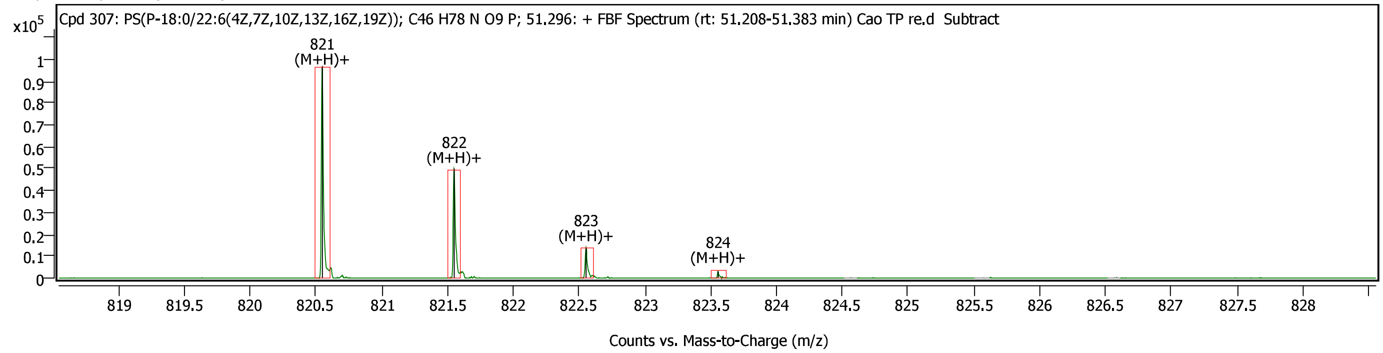

Compound ID Table

Name	Formula	Species	RT	RT Diff	Mass	CAS	ID Source	Score	Score (Lib)	Score (Tgt)
PS(P-18:0/22:6(4Z,7Z,10Z,13Z,16Z,19Z))	C46 H78 N O9 P	(M+H)+	51.296		819.5394		FBF	96.93		96.93

Cpd 317: PS(O-16:0/20:3(8Z,11Z,14Z))

Name	Formula	RT	RI	Mass	Diff (Tgt, ppm)	CAS	ID Source	Score	Algorithm
PS(O-16:0/20:3(8Z,11Z,14Z))	C42 H78 N O9 P	51.435		771.5414	-0.02		M-FBF	94.89	FBF

Species	m/z	Score (Tgt)	Score (Lib)	Score (DB)	Score (MFG)	Score (RT)
(M+H)+	773	94.89				

Compound Chromatograms (overlaid)

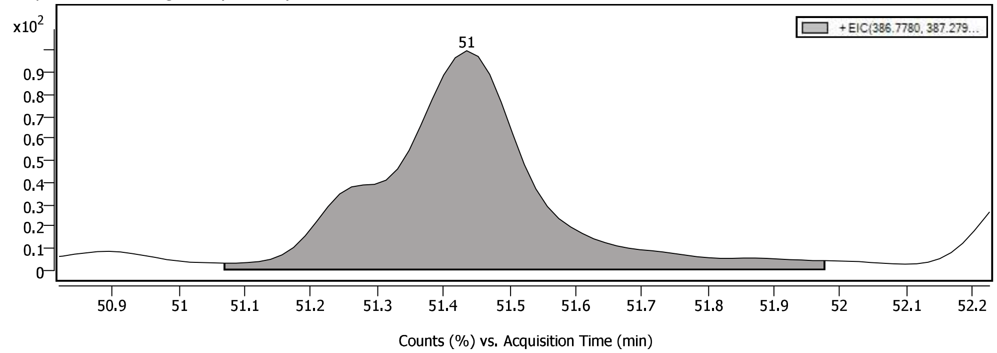

Structure

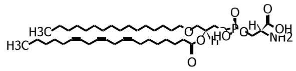

Compound Spectra (overlaid)

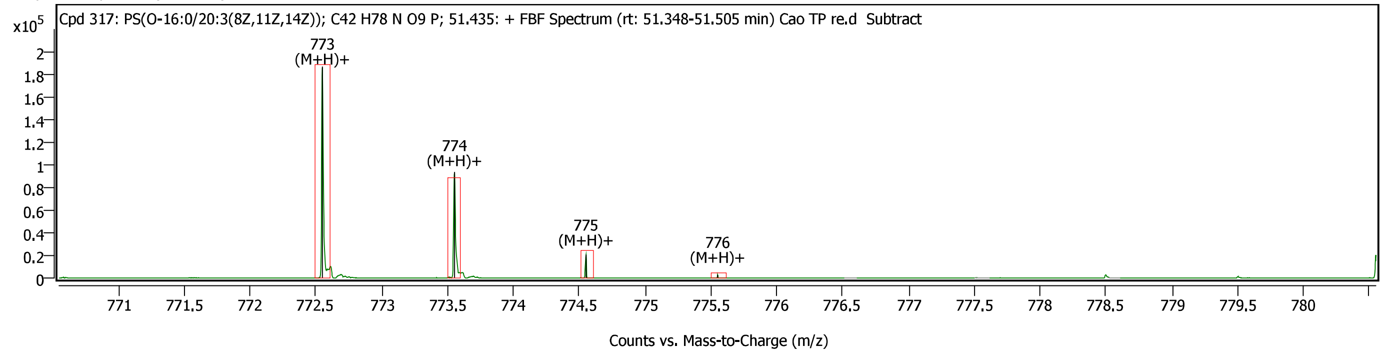

Compound Screening Report

Compound ID Table

Name	Formula	Species	RT	RT Diff	Mass	CAS	ID Source	Score	Score (Lib)	Score (Tgt)
PS(O-16:0/20:3(8Z,11Z,14Z))	C42 H78 N O9 P	(M+H)+	51.435		771.5414		FBF	94.89		94.89
PS(P-18:0/18:2(9Z,12Z))	C42 H78 N O9 P	(M+H)+	51.435		771.5414		FBF	94.89		94.89
PS(P-16:0/20:2(11Z,14Z))	C42 H78 N O9 P	(M+H)+	51.435		771.5414		FBF	94.89		94.89
PS(O-18:0/18:3(9Z,12Z,15Z))	C42 H78 N O9 P	(M+H)+	51.435		771.5414		FBF	94.89		94.89
PS(O-18:0/18:3(6Z,9Z,12Z))	C42 H78 N O9 P	(M+H)+	51.435		771.5414		FBF	94.89		94.89

Cpd 1443: Z-Arg-Arg-NHMec

Name	Formula	RT	RI	Mass	Diff (Tgt, ppm)	CAS	ID Source	Score	Algorithm
Z-Arg-Arg-NHMec	C30 H39 N9 O6	51.645		621.3032	1.43	88937-61-5	FBF	81.74	FBF

Species	m/z	Score (Tgt)	Score (Lib)	Score (DB)	Score (MFG)	Score (RT)
(M+H)+	622	81.74				

Compound Chromatograms (overlaid)

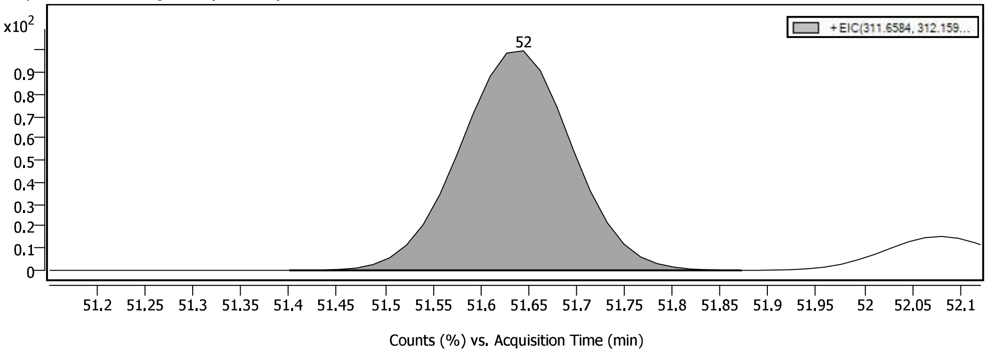

Structure

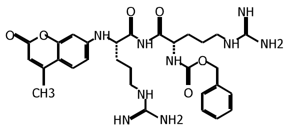

Compound Spectra (overlaid)

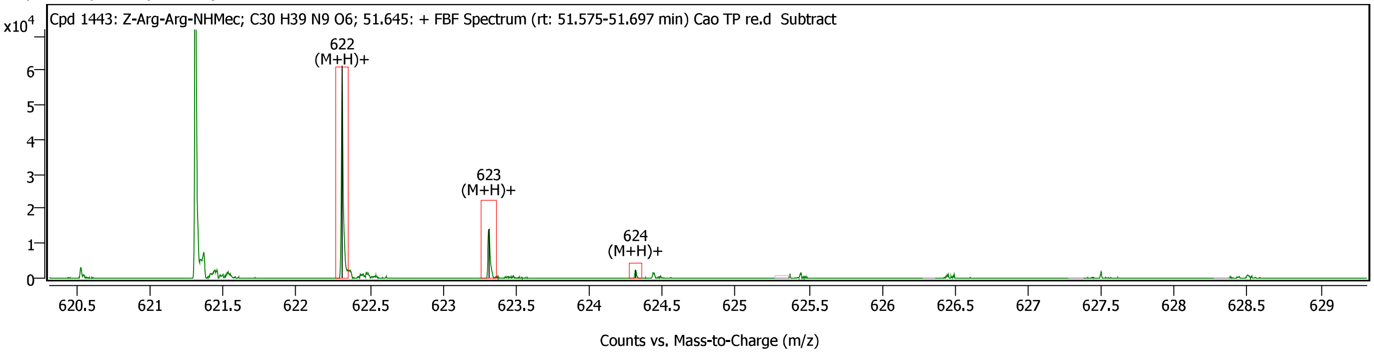

Compound ID Table

Name	Formula	Species	RT	RT Diff	Mass	CAS	ID Source	Score	Score (Lib)	Score (Tgt)
Z-Arg-Arg-NHMec	C30 H39 N9 O6	(M+H)+	51.645		621.3032	88937-61-5	FBF	81.74		81.74

Cpd 1379: <Oligomycin A>

Name	Formula	RT	RI	Mass	Diff (Tgt, ppm)	CAS	ID Source	Score	Algorithm
<Oligomycin A>	C45 H74 O11	51.732		790.5203	-3.58	579-13-5	FBF	89.37	FBF

Species	m/z	Score (Tgt)	Score (Lib)	Score (DB)	Score (MFG)	Score (RT)
(M+H)+	792	89.37				

Compound Chromatograms (overlaid)

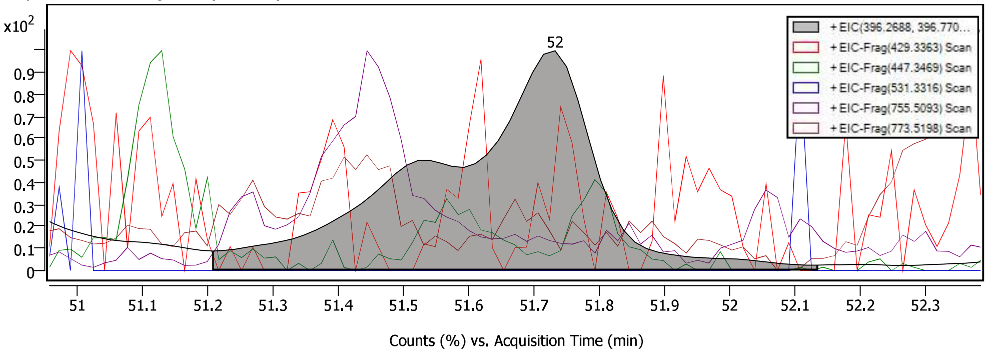

Structure

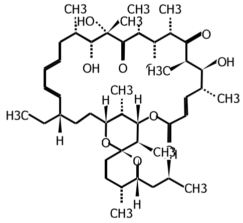

Coelution Plot

Compound Screening Report

Compound Spectra (overlaid)

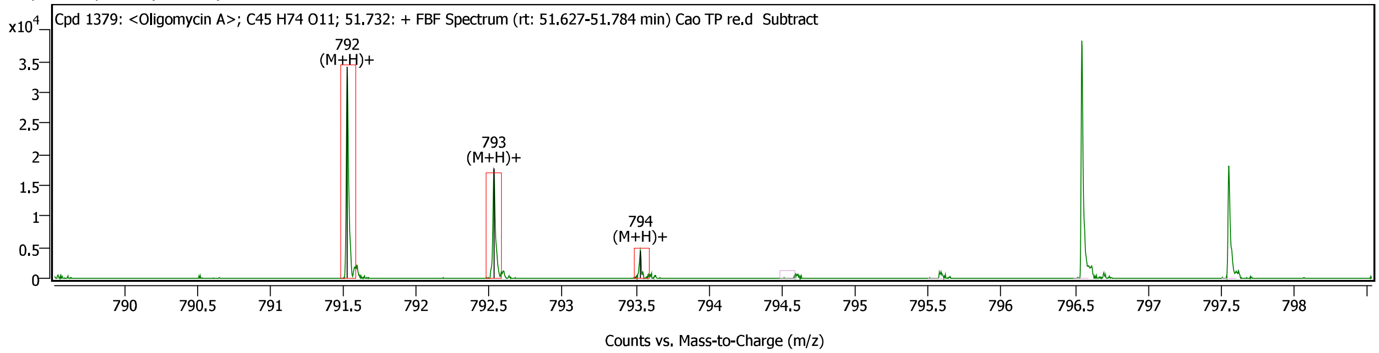

Fragment Spectrum (raw)

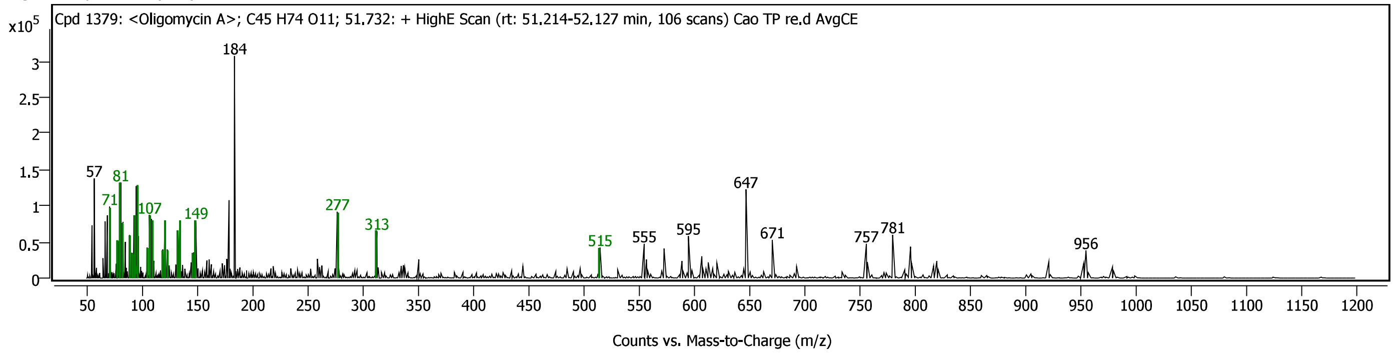

Compound ID Table

Name	Formula	Species	RT	RT Diff	Mass	CAS	ID Source	Score	Score (Lib)	Score (Tgt)
<Oligomycin A>	C45 H74 O11	(M+H)+	51.732		790.5203	579-13-5	FBF	89.37		89.37

Cpd 1588: (3beta,22R,23R,24S)-3,22,23-Trihydroxystigmastan-6-one

Name	Formula	RT	RI	Mass	Diff (Tgt, ppm)	CAS	ID Source	Score	Algorithm
(3beta,22R,23R,24S)-3,22,23-Trihydroxystigmastan-6-one	C29 H50 O4	51.802		462.3714	1.16	90524-90-6	M-FBF	99.00	FBF

Species	m/z	Score (Tgt)	Score (Lib)	Score (DB)	Score (MFG)	Score (RT)
(M+H)+	463	99.00				

Compound Chromatograms (overlaid)

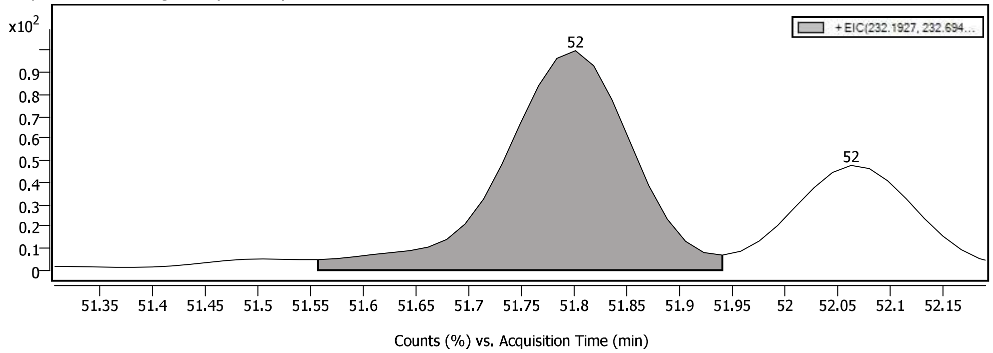

Structure

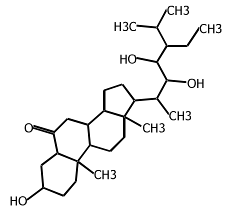

Compound Spectra (overlaid)

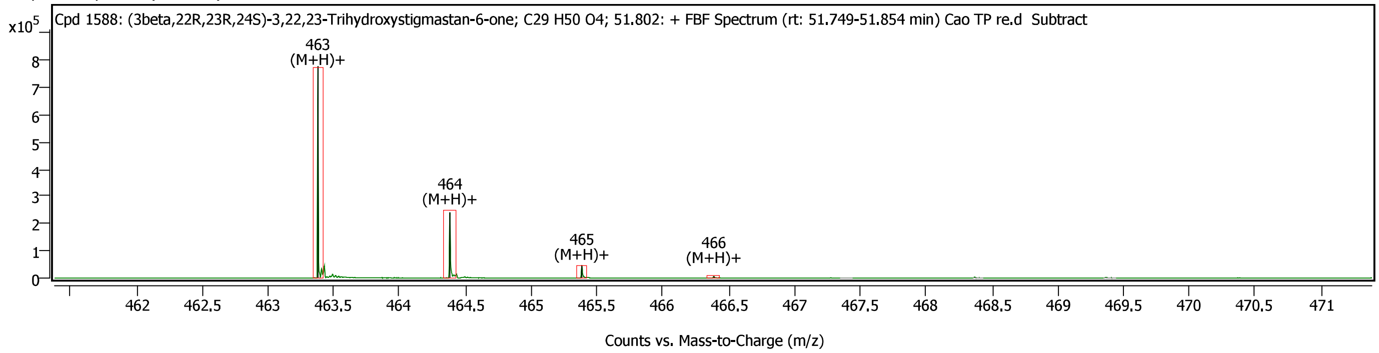

Compound ID Table

Name	Formula	Species	RT	RT Diff	Mass	CAS	ID Source	Score	Score (Lib)	Score (Tgt)
(3beta,22R,23R,24S)-3,22,23-Trihydroxystigmastan-6-one	C29 H50 O4	(M+H)+	51.802		462.3714	90524-90-6	FBF	99.00		99.00
6-Deoxohomodolichosterone	C29 H50 O4	(M+H)+	51.802		462.3714	110345-06-7	FBF	99.00		99.00
2α-(3-Hydroxypropyl)-1α,25-dihydroxy-19-norvitamin D3	C29 H50 O4	(M+H)+	51.802		462.3714		FBF	99.00		99.00

Cpd 873: Mayolene-16

Name	Formula	RT	RI	Mass	Diff (Tgt, ppm)	CAS	ID Source	Score	Algorithm
Mayolene-16	C34 H60 O4	51.941		532.4495	0.70		FBF	98.94	FBF

Species	m/z	Score (Tgt)	Score (Lib)	Score (DB)	Score (MFG)	Score (RT)
(M+H)+	533	98.94				

Compound Chromatograms (overlaid)

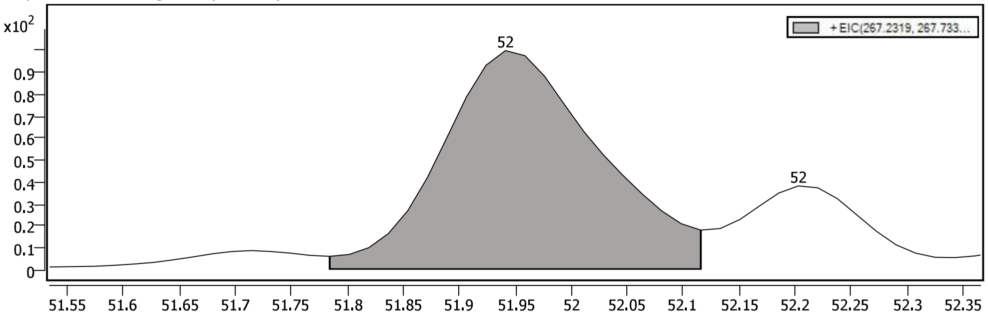

Structure

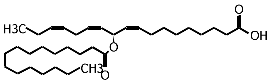

Compound Spectra (overlaid)

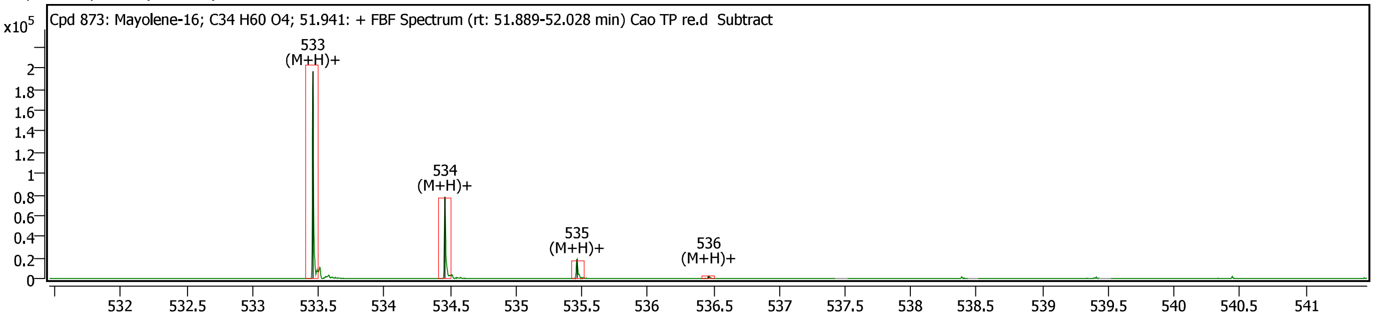

Compound ID Table

Name	Formula	Species	RT	RT Diff	Mass	CAS	ID Source	Score	Score (Lib)	Score (Tgt)
Mayolene-16	C34 H60 O4	(M+H)+	51.941		532.4495		FBF	98.94		98.94

Cpd 319: PS(P-20:0/16:1(9Z))

Name	Formula	RT	RI	Mass	Diff (Tgt, ppm)	CAS	ID Source	Score	Algorithm
PS(P-20:0/16:1(9Z))	C42 H80 N O9 P	52.081		773.5579	1.13		M-FBF	99.19	FBF

Species	m/z	Score (Tgt)	Score (Lib)	Score (DB)	Score (MFG)	Score (RT)
(M+H)+	775	99.19				

Compound Chromatograms (overlaid)

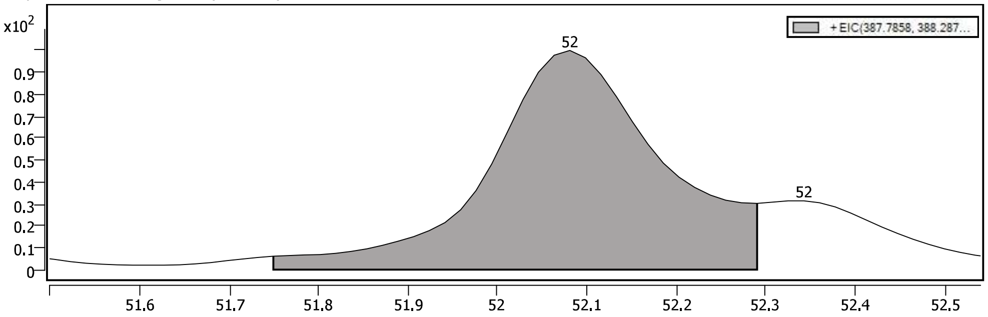

Structure

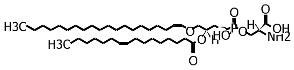

Compound Spectra (overlaid)

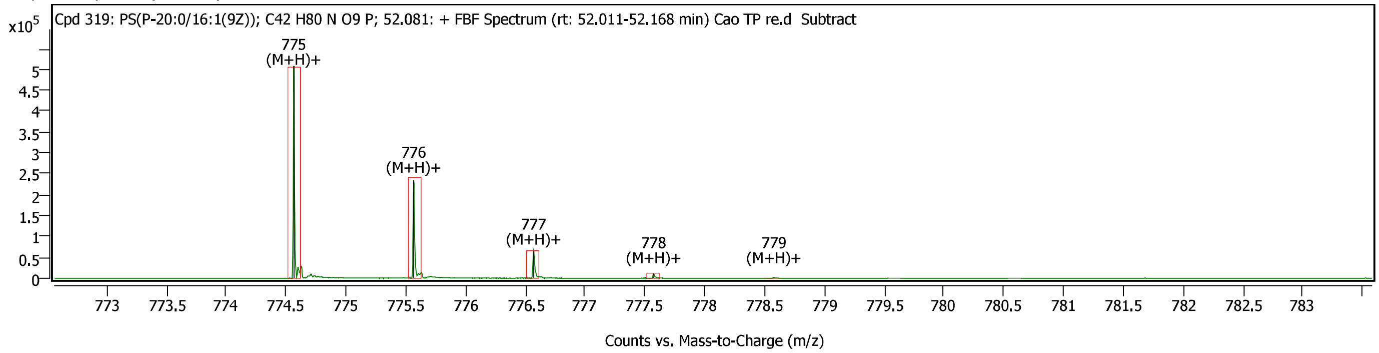

Compound ID Table

Name	Formula	Species	RT	RT Diff	Mass	CAS	ID Source	Score	Score (Lib)	Score (Tgt)
PS(P-20:0/16:1(9Z))	C42 H80 N O9 P	(M+H)+	52.081		773.5579		FBF	99.19		99.19
PS(P-16:0/20:1(11Z))	C42 H80 N O9 P	(M+H)+	52.081		773.5579		FBF	99.19		99.19
PS(O-16:0/20:2(11Z,14Z))	C42 H80 N O9 P	(M+H)+	52.081		773.5579		FBF	99.19		99.19
PS(O-18:0/18:2(9Z,12Z))	C42 H80 N O9 P	(M+H)+	52.081		773.5579		FBF	99.19		99.19
PS(P-18:0/18:1(9Z))	C42 H80 N O9 P	(M+H)+	52.081		773.5579		FBF	99.19		99.19

Cpd 1602: Neoannonin B

Name	Formula	RT	RI	Mass	Diff (Tgt, ppm)	CAS	ID Source	Score	Algorithm
Neoannonin B	C37 H66 O6	52.081		606.4863	0.63	170312-94-4	M-FBF	99.28	FBF

Species	m/z	Score (Tgt)	Score (Lib)	Score (DB)	Score (MFG)	Score (RT)
(M+H)+	607	99.28				

Compound Chromatograms (overlaid)

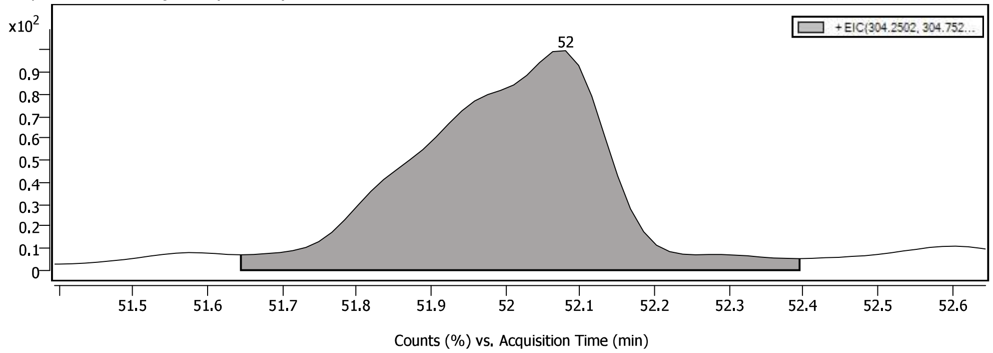

Structure

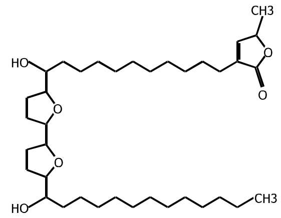

Compound Spectra (overlaid)

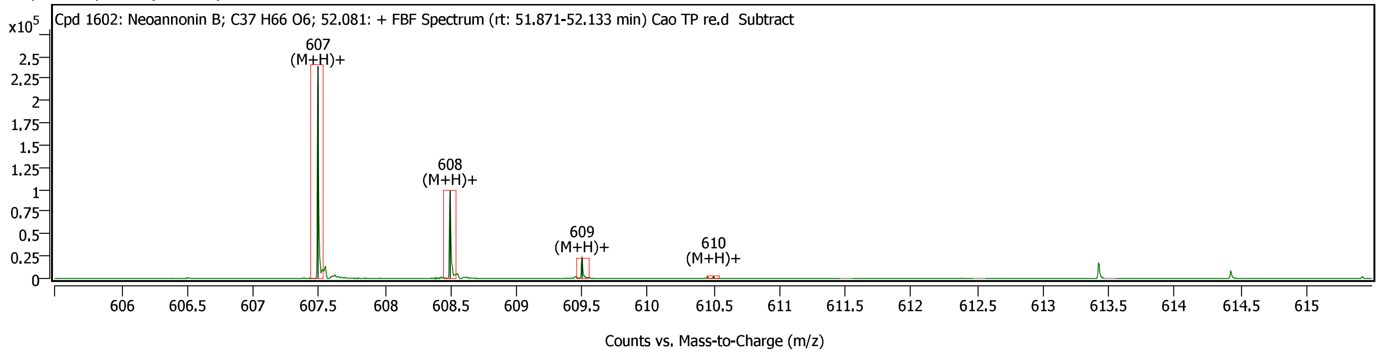

Compound ID Table

Name	Formula	Species	RT	RT Diff	Mass	CAS	ID Source	Score	Score (Lib)	Score (Tgt)
Neoannonin B	C37 H66 O6	(M+H)+	52.081		606.4863	170312-94-4	FBF	99.28		99.28
cis-Reticulatacin-10-one	C37 H66 O6	(M+H)+	52.081		606.4863	205439-33-4	FBF	99.28		99.28
Asiminenin A	C37 H66 O6	(M+H)+	52.081		606.4863	168075-11-4	FBF	99.28		99.28
Rollidecin D	C37 H66 O6	(M+H)+	52.081		606.4863	200703-17-9	FBF	99.28		99.28
Squamocin L	C37 H66 O6	(M+H)+	52.081		606.4863	98767-45-4	FBF	99.28		99.28

Cpd 913: Ergosterol acetate

Name	Formula	RT	RI	Mass	Diff (Tgt, ppm)	CAS	ID Source	Score	Algorithm
Ergosterol acetate	C32 H50 O2	52.168		466.3788	-4.82	2418-45-3	FBF	86.97	FBF

Species	m/z	Score (Tgt)	Score (Lib)	Score (DB)	Score (MFG)	Score (RT)
(M+H)+	467	86.97				

Compound Chromatograms (overlaid)

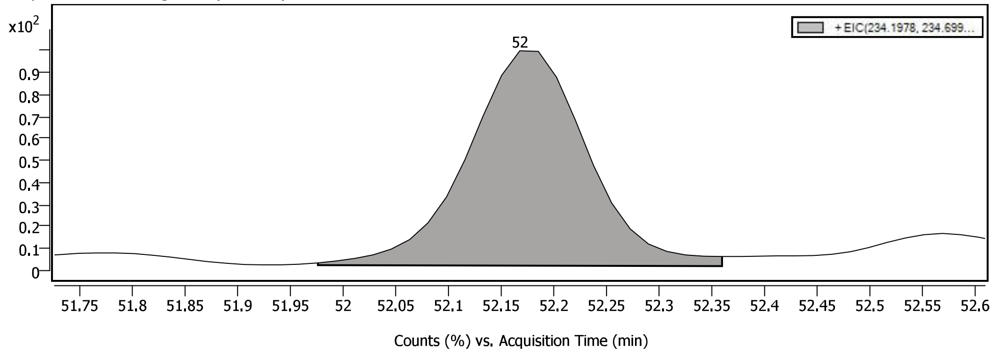

Structure

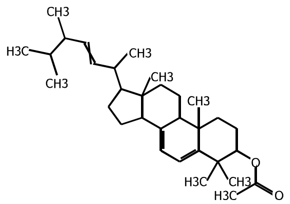

Compound Spectra (overlaid)

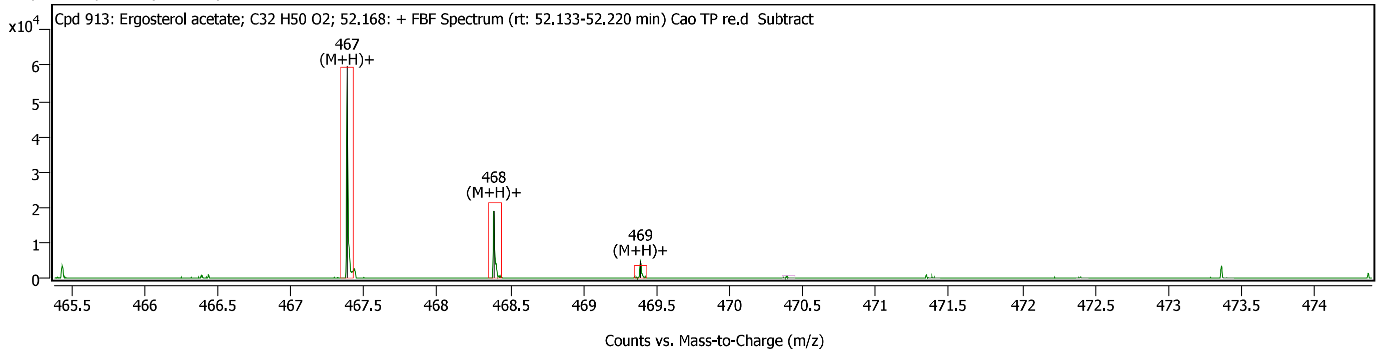

Compound ID Table

Name	Formula	Species	RT	RT Diff	Mass	CAS	ID Source	Score	Score (Lib)	Score (Tgt)
Ergosterol acetate	C32 H50 O2	(M+H)+	52.168		466.3788	2418-45-3	FBF	86.97		86.97

Cpd 59: DG(13:0/18:2(9Z,12Z)/0:0)[iso2]

Name	Formula	RT	RI	Mass	Diff (Tgt, ppm)	CAS	ID Source	Score	Algorithm
DG(13:0/18:2(9Z,12Z)/0:0)[iso2]	C34 H62 O5	52.203		550.4600	0.42		M-FBF	98.34	FBF

Species	m/z	Score (Tgt)	Score (Lib)	Score (DB)	Score (MFG)	Score (RT)
(M+H)+	551	98.34				

Compound Chromatograms (overlaid)

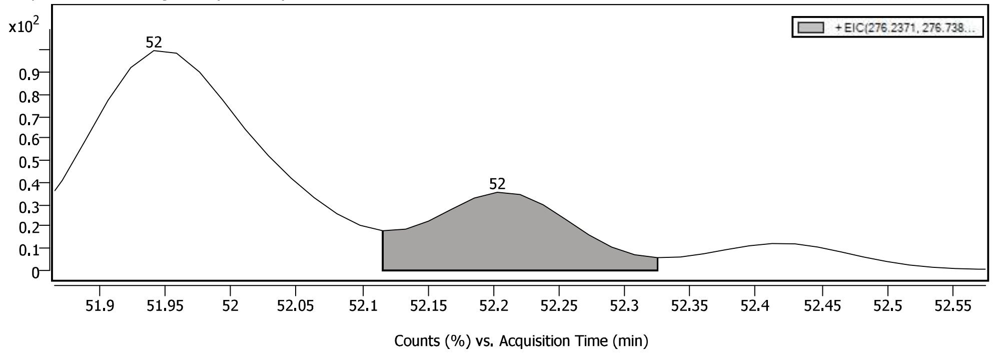

Structure

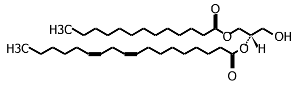

Compound Spectra (overlaid)

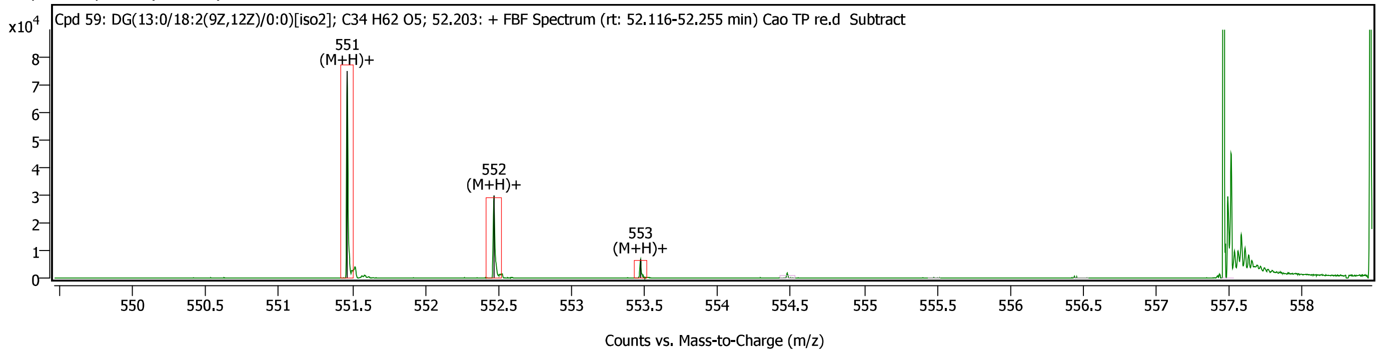

Compound ID Table

Name	Formula	Species	RT	RT Diff	Mass	CAS	ID Source	Score	Score (Lib)	Score (Tgt)
DG(13:0/18:2(9Z,12Z)/0:0)[iso2]	C34 H62 O5	(M+H)+	52.203		550.4600		FBF	98.34		98.34
DG(15:1(9Z)/16:1(9Z)/0:0)[iso2]	C34 H62 O5	(M+H)+	52.203		550.4600		FBF	98.34		98.34
DG(14:1(9Z)/17:1(9Z)/0:0)[iso2]	C34 H62 O5	(M+H)+	52.203		550.4600		FBF	98.34		98.34
DG(14:0/17:2(9Z,12Z)/0:0)[iso2]	C34 H62 O5	(M+H)+	52.203		550.4600		FBF	98.34		98.34

Cpd 72: 7-methyl-cis-8,9-Epoxy-tricosane

Name	Formula	RT	RI	Mass	Diff (Tgt, ppm)	CAS	ID Source	Score	Algorithm
7-methyl-cis-8,9-Epoxy-tricosane	C23 H46 O	52.552		338.3553	1.34		M-FBF	98.65	FBF

Species	m/z	Score (Tgt)	Score (Lib)	Score (DB)	Score (MFG)	Score (RT)
(M+H)+	339	98.65				

Compound Chromatograms (overlaid)

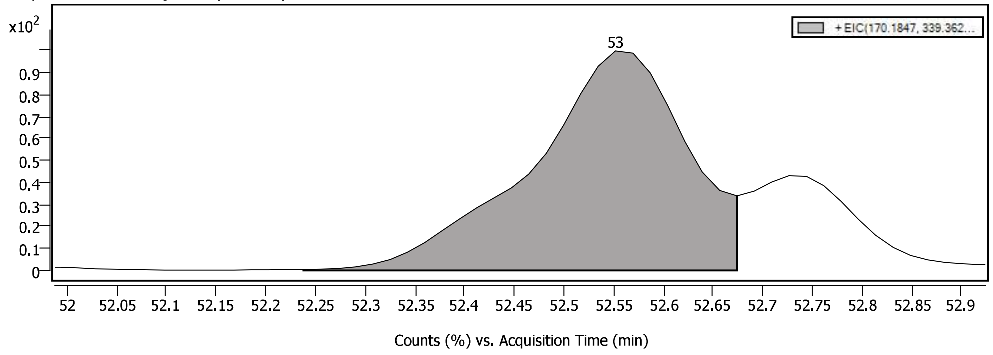

Structure

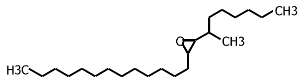

Compound Spectra (overlaid)

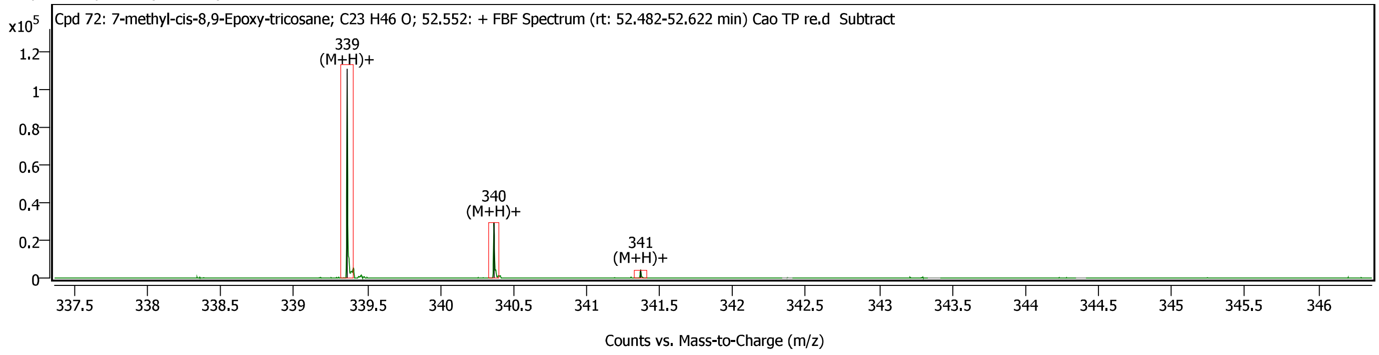

Compound ID Table

Name	Formula	Species	RT	RT Diff	Mass	CAS	ID Source	Score	Score (Lib)	Score (Tgt)
7-methyl-cis-8,9-Epoxy-tricosane	C23 H46 O	(M+H)+	52.552		338.3553		FBF	98.65		98.65
cis-9,10-Epoxytricosane	C23 H46 O	(M+H)+	52.552		338.3553		FBF	98.65		98.65

Cpd 77: 9,10-Tricosadiene

Name	Formula	RT	RI	Mass	Diff (Tgt, ppm)	CAS	ID Source	Score	Algorithm
9,10-Tricosadiene	C23 H44	52.552		320.3446	1.01		M-FBF	99.32	FBF

Species	m/z	Score (Tgt)	Score (Lib)	Score (DB)	Score (MFG)	Score (RT)
(M+H)+	321	99.32				

Compound Chromatograms (overlaid)

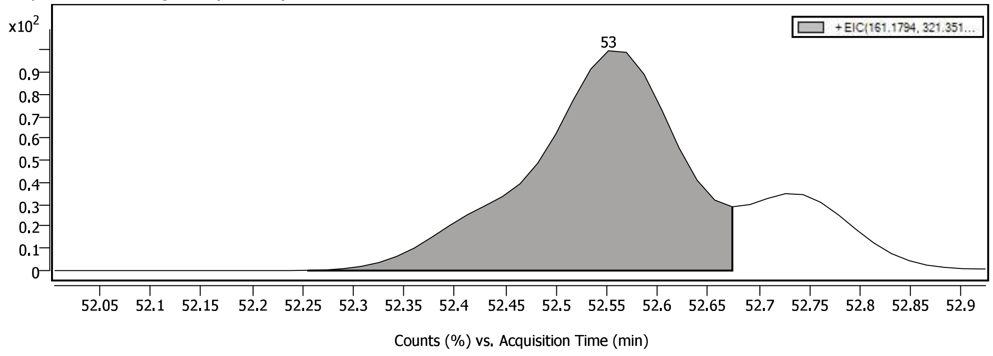

Structure

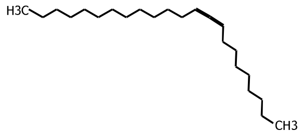

Compound Spectra (overlaid)

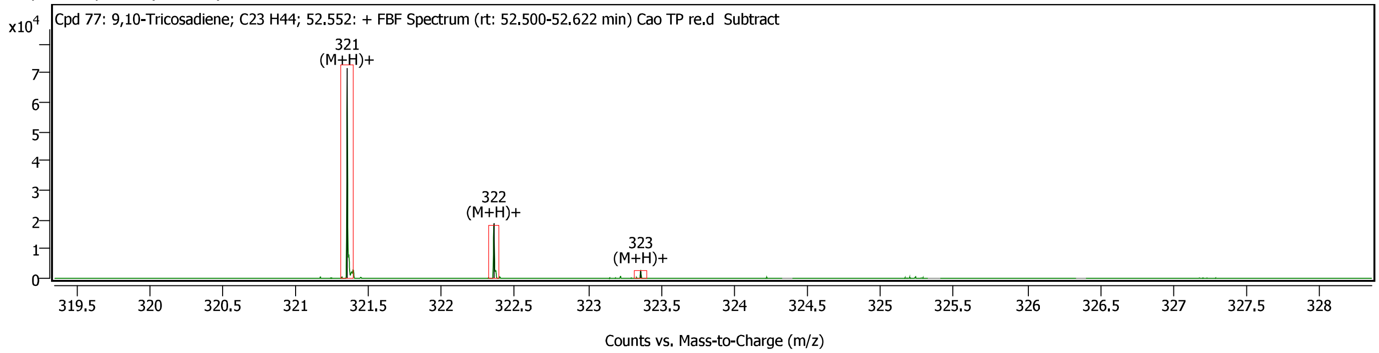

Compound ID Table

Name	Formula	Species	RT	RT Diff	Mass	CAS	ID Source	Score	Score (Lib)	Score (Tgt)
9,10-Tricosadiene	C23 H44	(M+H)+	52.552		320.3446		FBF	99.32		99.32
6Z,9Z-Tricosadiene	C23 H44	(M+H)+	52.552		320.3446		FBF	99.32		99.32
5,9-Tricosadiene	C23 H44	(M+H)+	52.552		320.3446		FBF	99.32		99.32

Cpd 68: DG(15:0/14:0/0:0)

Name	Formula	RT	RI	Mass	Diff (Tgt, ppm)	CAS	ID Source	Score	Algorithm
DG(15:0/14:0/0:0)	C32 H62 O5	52.988		526.4600	0.45		M-FBF	99.68	FBF

Species	m/z	Score (Tgt)	Score (Lib)	Score (DB)	Score (MFG)	Score (RT)
(M+H)+	527	99.68				

Compound Chromatograms (overlaid)

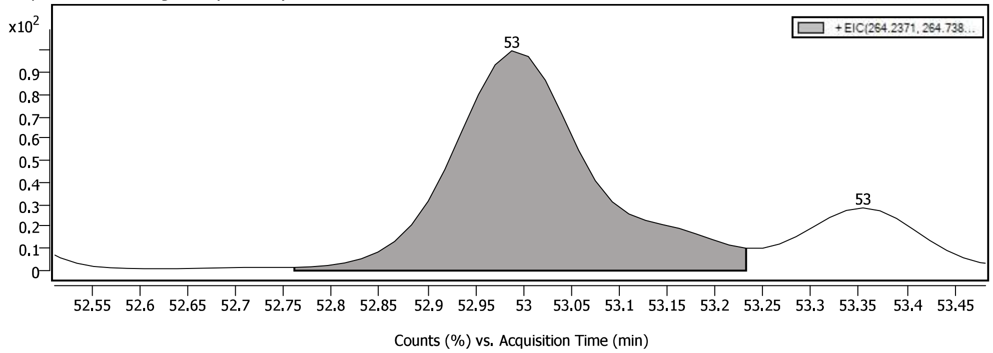

Structure

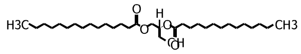

Compound Spectra (overlaid)

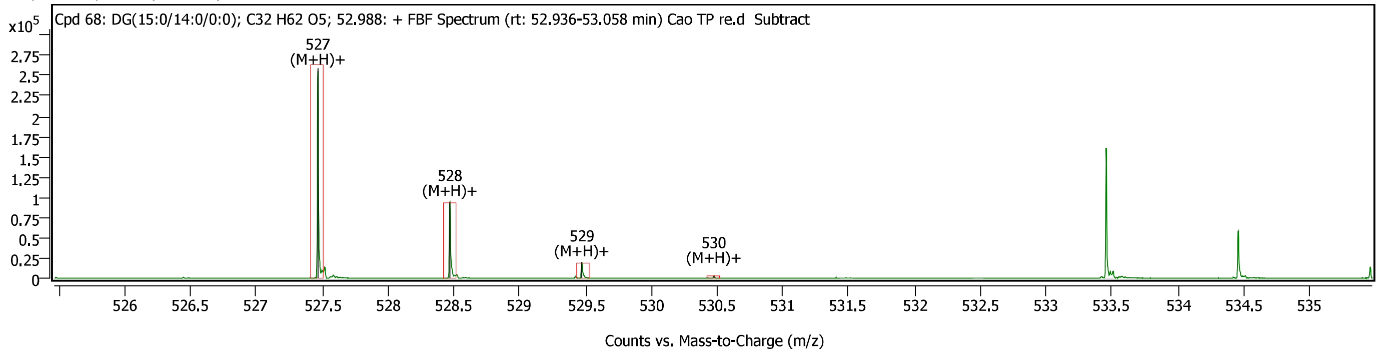

Compound ID Table

Name	Formula	Species	RT	RT Diff	Mass	CAS	ID Source	Score	Score (Lib)	Score (Tgt)
DG(15:0/14:0/0:0)	C32 H62 O5	(M+H)+	52.988		526.4600		FBF	99.68		99.68
DG(13:0/16:0/0:0)[iso2]	C32 H62 O5	(M+H)+	52.988		526.4600		FBF	99.68		99.68
DG(12:0/17:0/0:0)[iso2]	C32 H62 O5	(M+H)+	52.988		526.4600		FBF	99.68		99.68
DG(14:0/15:0/0:0)	C32 H62 O5	(M+H)+	52.988		526.4600		FBF	99.68		99.68

Cpd 296: PG(P-16:0/12:0)

Name	Formula	RT	RI	Mass	Diff (Tgt, ppm)	CAS	ID Source	Score	Algorithm
PG(P-16:0/12:0)	C34 H67 O9 P	53.040		650.4520	-0.45		FBF	96.36	FBF

Species	m/z	Score (Tgt)	Score (Lib)	Score (DB)	Score (MFG)	Score (RT)
(M+H)+	651	96.36				

Compound Chromatograms (overlaid)

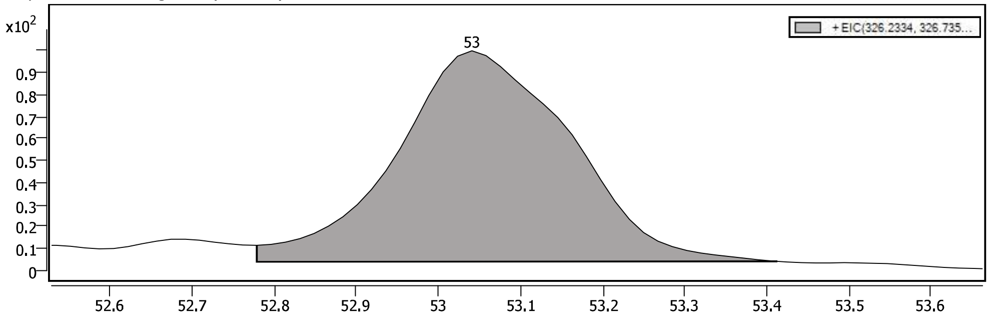

Counts (%) vs. Acquisition Time (min)

Structure

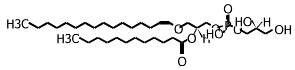

Compound Spectra (overlaid)

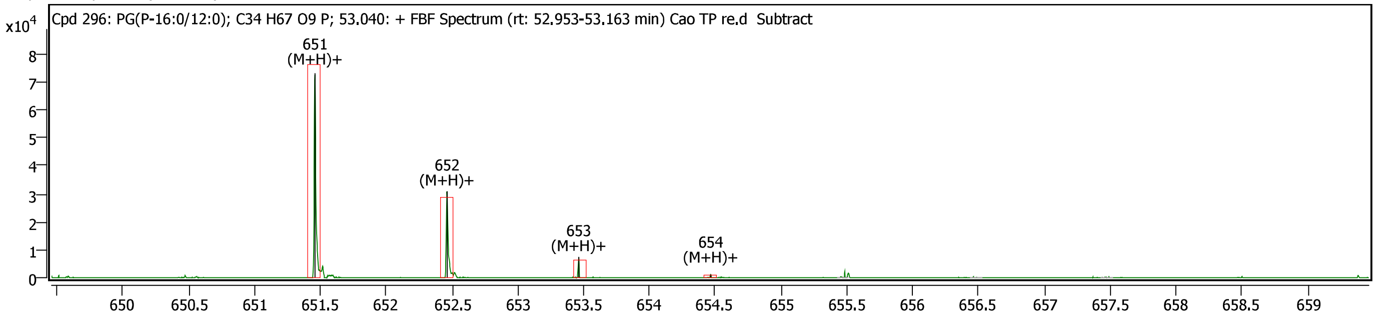

Counts vs. Mass-to-Charge (m/z)

Compound ID Table

Name	Formula	Species	RT	RT Diff	Mass	CAS	ID Source	Score	Score (Lib)	Score (Tgt)
PG(P-16:0/12:0)	C34 H67 O9 P	(M+H)+	53.040		650.4520		FBF	96.36		96.36

Cpd 1690: Caffeoylcycloartenol

Name	Formula	RT	RI	Mass	Diff (Tgt, ppm)	CAS	ID Source	Score	Algorithm
Caffeoylcycloartenol	C39 H56 O4	53.110		588.4154	-4.25	75884-34-3	FBF	86.91	FBF

Species	m/z	Score (Tgt)	Score (Lib)	Score (DB)	Score (MFG)	Score (RT)
(M+H)+	589	86.91				

Compound Chromatograms (overlaid)

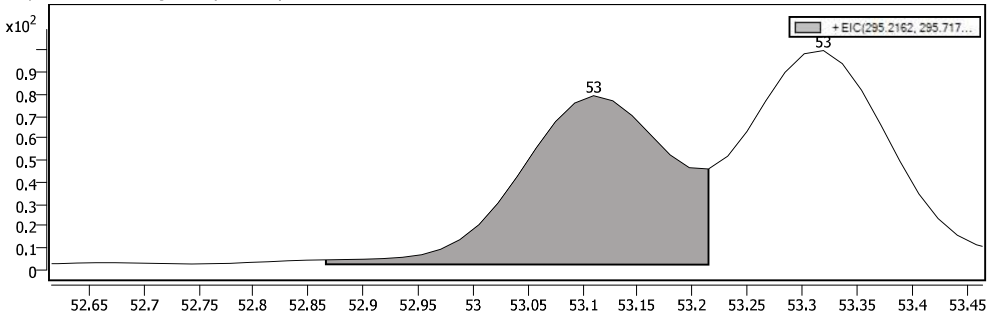

Counts (%) vs. Acquisition Time (min)

Structure

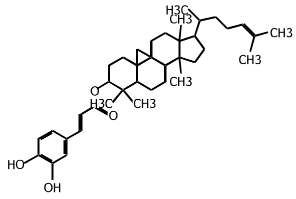

Compound Spectra (overlaid)

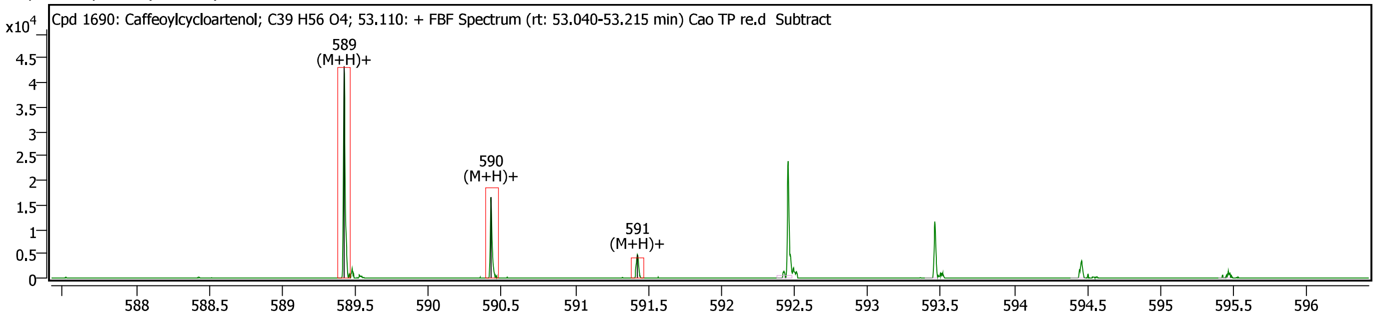

Counts vs. Mass-to-Charge (m/z)

Compound ID Table

Name	Formula	Species	RT	RT Diff	Mass	CAS	ID Source	Score	Score (Lib)	Score (Tgt)
Caffeoylcycloartenol	C39 H56 O4	(M+H)+	53.110		588.4154	75884-34-3	FBF	86.91		86.91

Cpd 1: 24-Norursa-3,12-diene

Name	Formula	RT	RI	Mass	Diff (Tgt, ppm)	CAS	ID Source	Score	Algorithm
24-Norursa-3,12-diene	C29 H46	53.180		394.3602	0.73		M-FBF	99.17	FBF

Species	m/z	Score (Tgt)	Score (Lib)	Score (DB)	Score (MFG)	Score (RT)
(M+H)+	395	99.17				

Compound Chromatograms (overlaid)

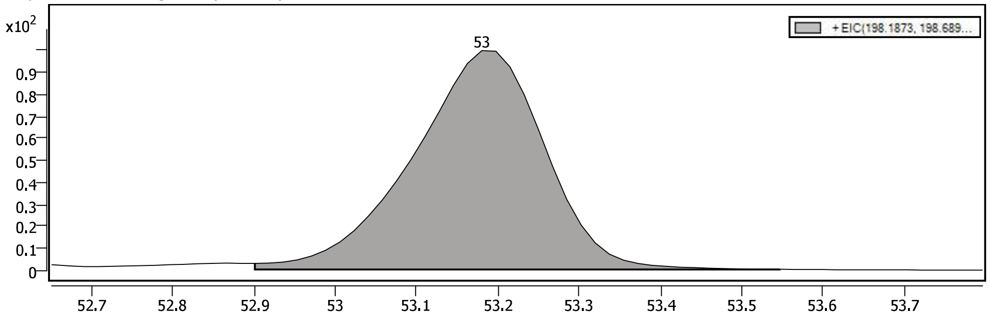

Structure

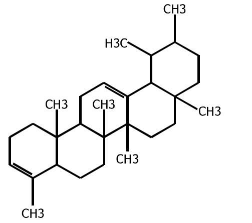

Compound Spectra (overlaid)

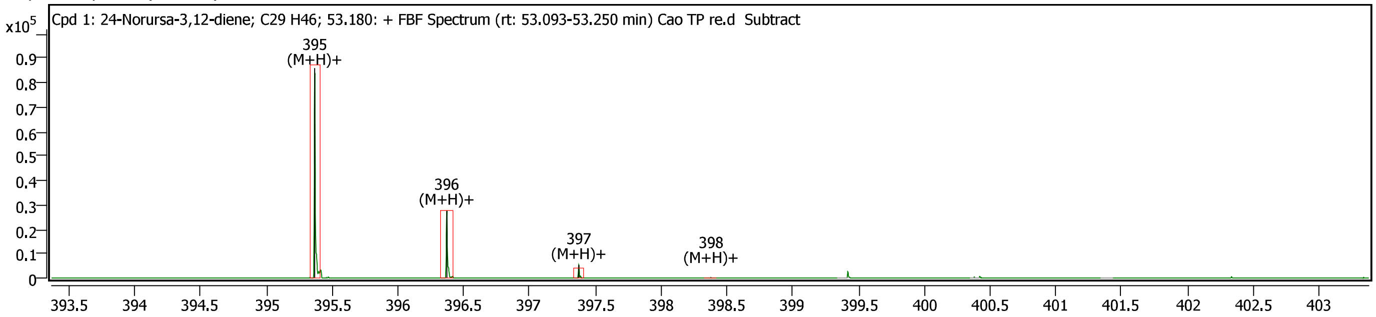

Compound ID Table

Name	Formula	Species	RT	RT Diff	Mass	CAS	ID Source	Score	Score (Lib)	Score (Tgt)
24-Norursa-3,12-diene	C29 H46	(M+H)+	53.180		394.3602		FBF	99.17		99.17
24-Noroleana-3,12-diene	C29 H46	(M+H)+	53.180		394.3602		FBF	99.17		99.17

Cpd 1689: Caffeoylcycloartenol

Name	Formula	RT	RI	Mass	Diff (Tgt, ppm)	CAS	ID Source	Score	Algorithm
Caffeoylcycloartenol	C39 H56 O4	53.320		588.4156	-3.88	75884-34-3	FBF	87.37	FBF

Species	m/z	Score (Tgt)	Score (Lib)	Score (DB)	Score (MFG)	Score (RT)
(M+H)+	589	87.37				

Compound Chromatograms (overlaid)

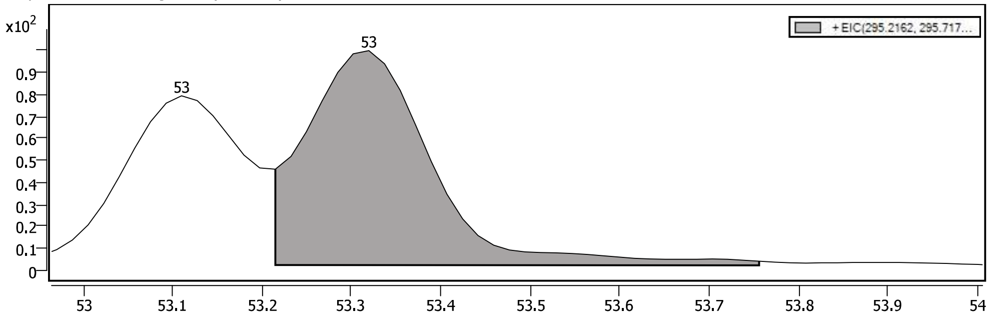

Structure

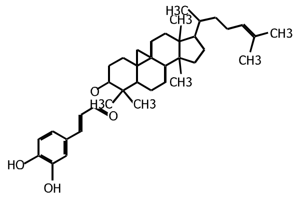

Compound Spectra (overlaid)

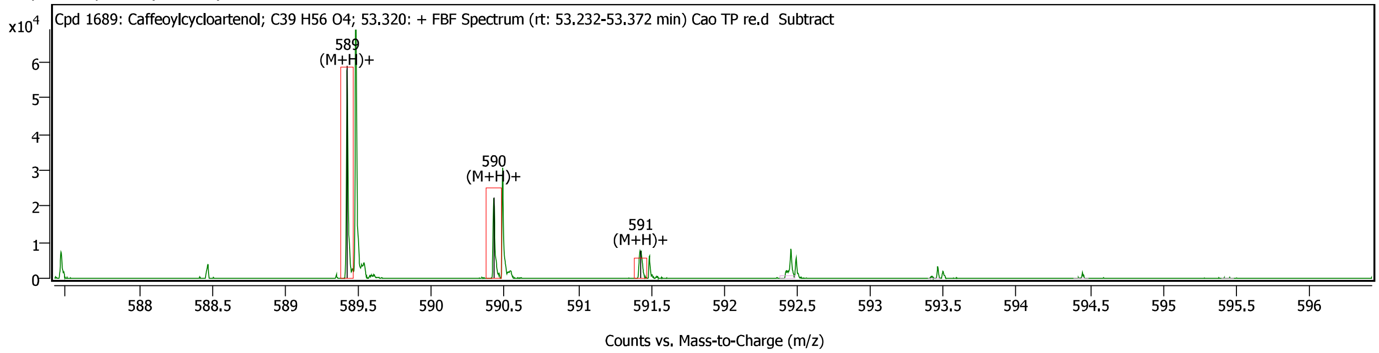

Compound ID Table

Name	Formula	Species	RT	RT Diff	Mass	CAS	ID Source	Score	Score (Lib)	Score (Tgt)
Caffeoylcycloartenol	C39 H56 O4	(M+H)+	53.320		588.4156	75884-34-3	FBF	87.37		87.37

Cpd 67: DG(14:0/15:0/0:0)

Name	Formula	RT	RI	Mass	Diff (Tgt, ppm)	CAS	ID Source	Score	Algorithm
DG(14:0/15:0/0:0)	C32 H62 O5	53.354		526.4599	0.26		M-FBF	98.29	FBF

Species	m/z	Score (Tgt)	Score (Lib)	Score (DB)	Score (MFG)	Score (RT)
(M+H)+	527	98.29				

Compound Chromatograms (overlaid)

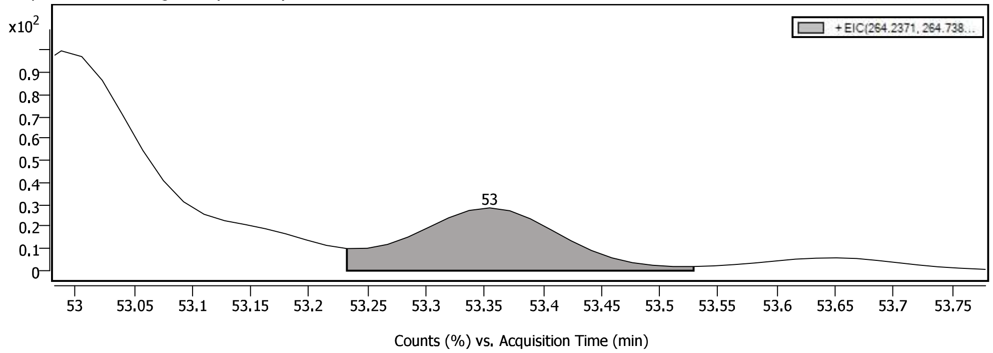

Structure

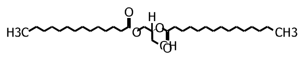

Compound Spectra (overlaid)

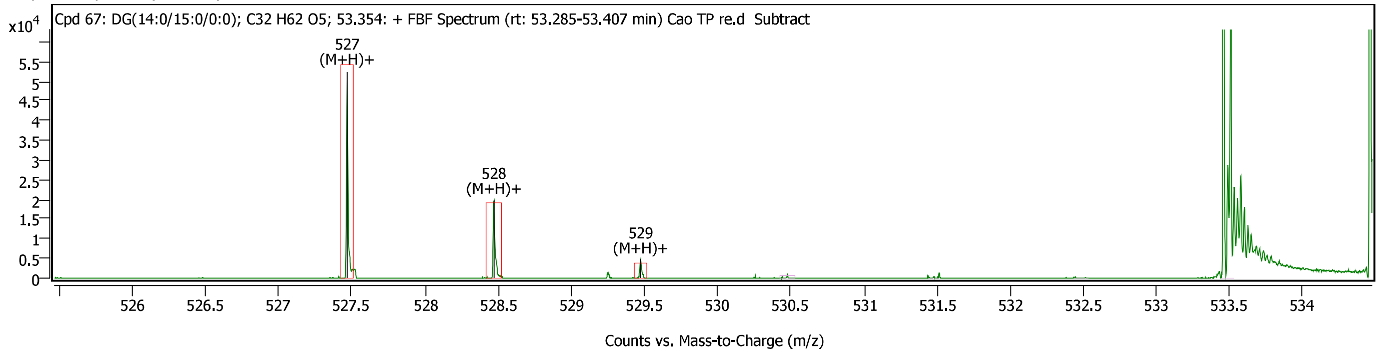

Compound ID Table

Name	Formula	Species	RT	RT Diff	Mass	CAS	ID Source	Score	Score (Lib)	Score (Tgt)
DG(14:0/15:0/0:0)	C32 H62 O5	(M+H)+	53.354		526.4599		FBF	98.29		98.29
DG(13:0/16:0/0:0)[iso2]	C32 H62 O5	(M+H)+	53.354		526.4599		FBF	98.29		98.29
DG(12:0/17:0/0:0)[iso2]	C32 H62 O5	(M+H)+	53.354		526.4599		FBF	98.29		98.29
DG(15:0/14:0/0:0)	C32 H62 O5	(M+H)+	53.354		526.4599		FBF	98.29		98.29

Cpd 181: Araliacerebroside

Name	Formula	RT	RI	Mass	Diff (Tgt, ppm)	CAS	ID Source	Score	Algorithm
Araliacerebroside	C40 H77 N O10	53.424		731.5550	0.35		FBF	95.95	FBF

Species	m/z	Score (Tgt)	Score (Lib)	Score (DB)	Score (MFG)	Score (RT)
(M+H)+	733	95.95				

Compound Chromatograms (overlaid)

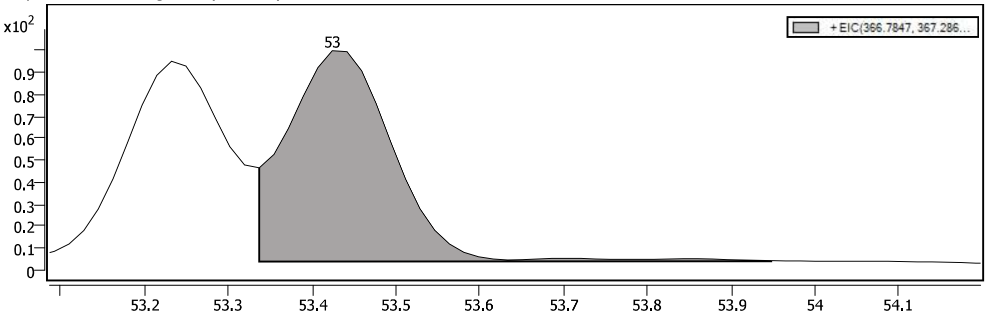

Structure

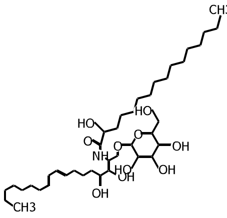

Compound Spectra (overlaid)

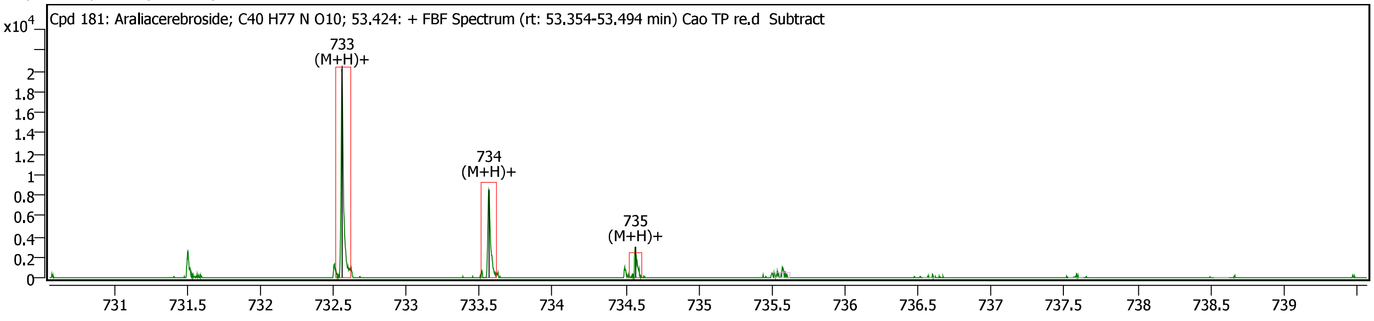

Compound ID Table

Name	Formula	Species	RT	RT Diff	Mass	CAS	ID Source	Score	Score (Lib)	Score (Tgt)
Araliacerebroside	C40 H77 N O10	(M+H)+	53.424		731.5550		FBF	95.95		95.95

Cpd 57: DG(15:1(9Z)/20:5(5Z,8Z,11Z,14Z,17Z)/0:0)[iso2]

Name	Formula	RT	RI	Mass	Diff (Tgt, ppm)	CAS	ID Source	Score	Algorithm
DG(15:1(9Z)/20:5(5Z,8Z,11Z,14Z, 17Z)/0:0)[iso2]	C38 H62 O5	53.477		598.4582	-2.47		M-FBF	81.30	FBF

Species	m/z	Score (Tgt)	Score (Lib)	Score (DB)	Score (MFG)	Score (RT)
(M+H)+	599	81.30				

Compound Chromatograms (overlaid)

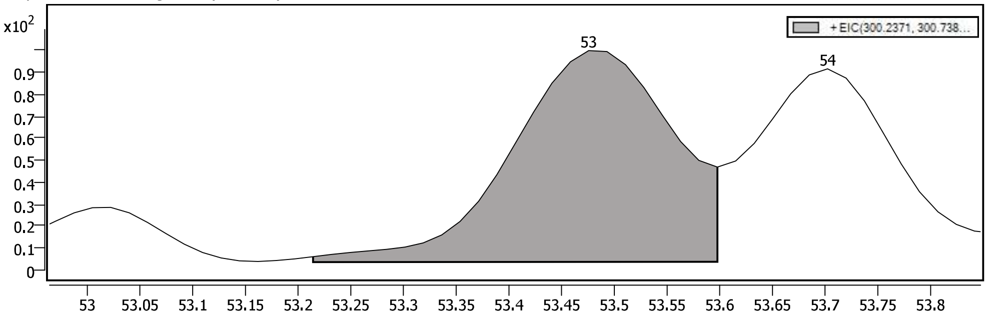

Structure

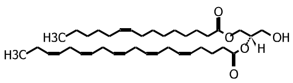

Compound Spectra (overlaid)

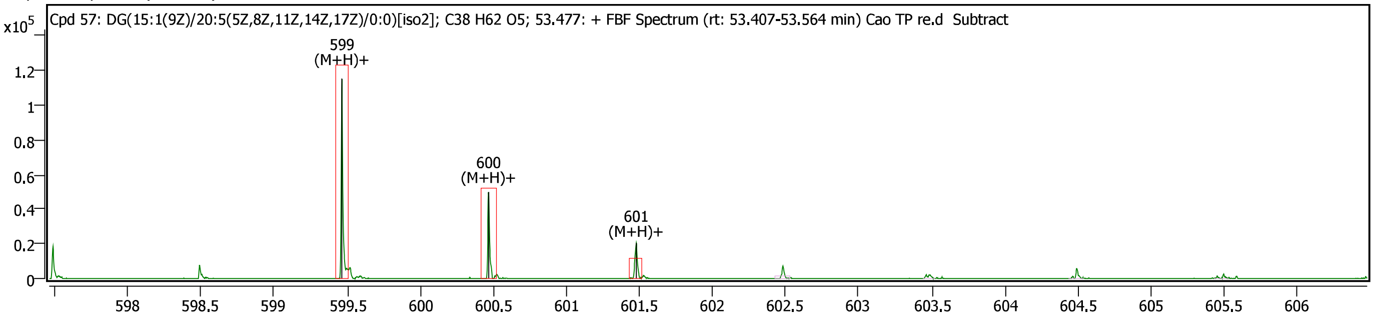

Compound ID Table

Name	Formula	Species	RT	RT Diff	Mass	CAS	ID Source	Score	Score (Lib)	Score (Tgt)
DG(15:1(9Z)/20:5(5Z,8Z,11Z,14Z, 17Z)/0:0)[iso2]	C38 H62 O5	(M+H)+	53.477		598.4582		FBF	81.30		81.30
DG(18:4(6Z,9Z,12Z,15Z)/17:2(9Z, 12Z)/0:0)[iso2]	C38 H62 O5	(M+H)+	53.477		598.4582		FBF	81.30		81.30
DG(13:0/22:6(4Z,7Z,10Z,13Z,16Z, 19Z)/0:0)[iso2]	C38 H62 O5	(M+H)+	53.477		598.4582		FBF	81.30		81.30

Cpd 251: SM(d18:0/12:0)

Name	Formula	RT	RI	Mass	Diff (Tgt, ppm)	CAS	ID Source	Score	Algorithm
SM(d18:0/12:0)	C35 H74 N2 O6 P	53.477		649.5282	-0.43		FBF	97.63	FBF

Species	m/z	Score (Tgt)	Score (Lib)	Score (DB)	Score (MFG)	Score (RT)
(M+H)+	651	97.63				

Compound Chromatograms (overlaid)

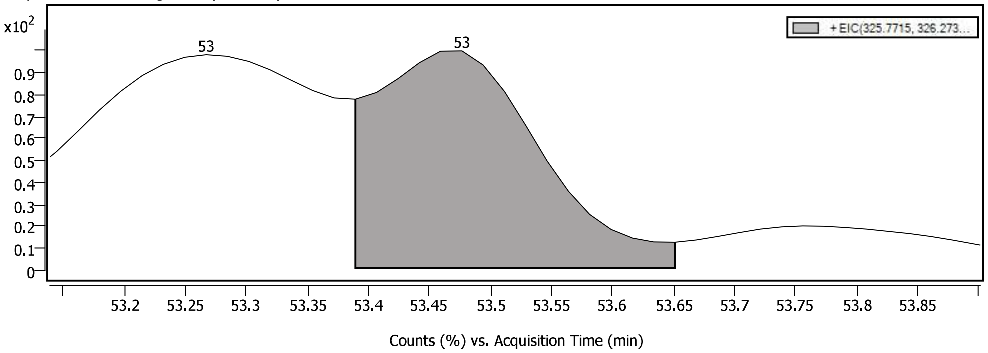

Structure

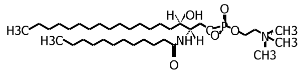

Compound Spectra (overlaid)

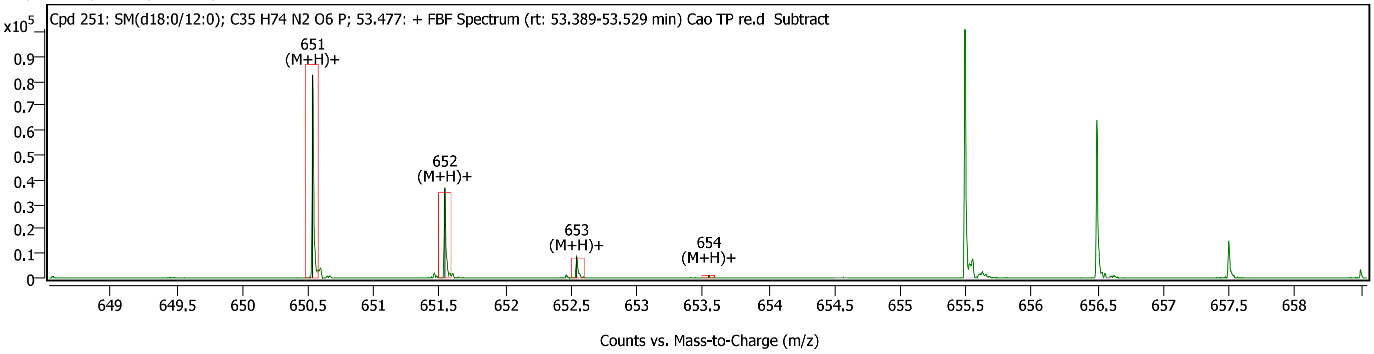

Compound ID Table

Name	Formula	Species	RT	RT Diff	Mass	CAS	ID Source	Score	Score (Lib)	Score (Tgt)
SM(d18:0/12:0)	C35 H74 N2 O6 P	(M+H)+	53.477		649.5282		FBF	97.63		97.63

Cpd 1699: DG(12:0/22:4(7Z,10Z,13Z,16Z)/0:0)[iso2]

Name	Formula	RT	RI	Mass	Diff (Tgt, ppm)	CAS	ID Source	Score	Algorithm
DG(12:0/22:4(7Z,10Z,13Z,16Z)/0:0)[iso2]	C37 H64 O5	53.599		588.4751	-0.51		M-FBF	95.40	FBF

Species	m/z	Score (Tgt)	Score (Lib)	Score (DB)	Score (MFG)	Score (RT)
(M+H)+	589	95.40				

Compound Chromatograms (overlaid)

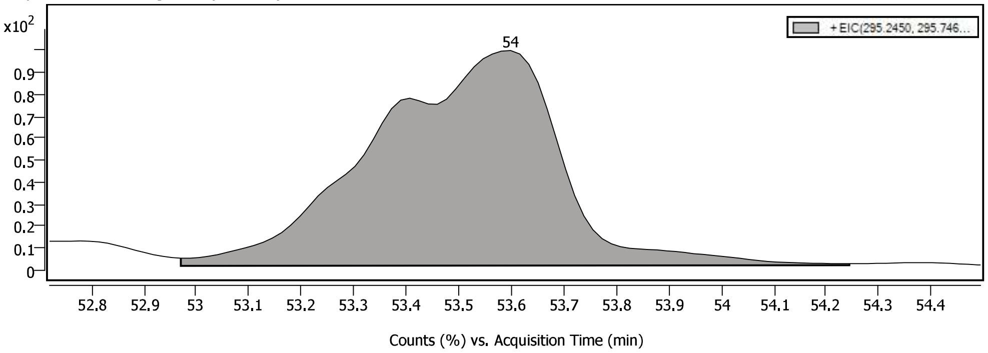

Structure

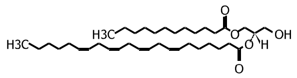

Compound Spectra (overlaid)

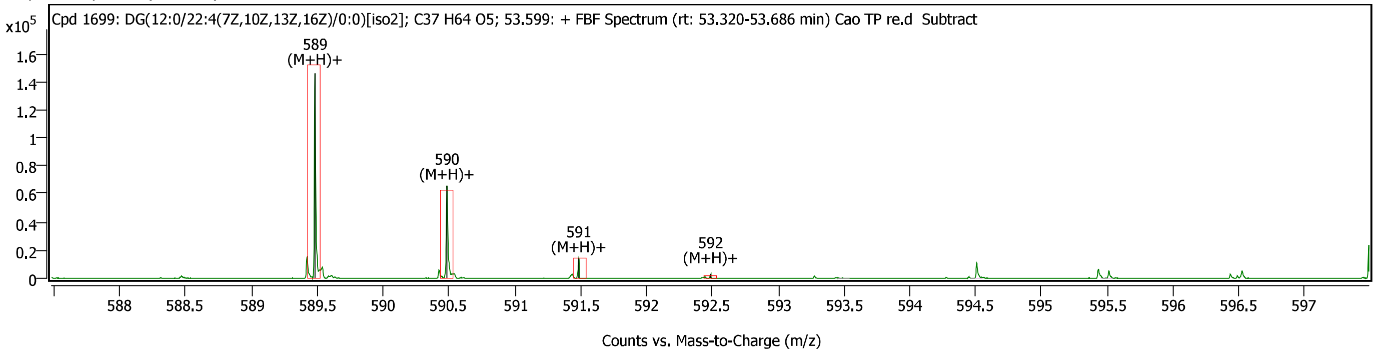

Compound ID Table

Name	Formula	Species	RT	RT Diff	Mass	CAS	ID Source	Score	Score (Lib)	Score (Tgt)
DG(12:0/22:4(7Z,10Z,13Z,16Z)/0:0)[iso2]	C37 H64 O5	(M+H)+	53.599		588.4751		FBF	95.40		95.40
Tripoxyrollin	C37 H64 O5	(M+H)+	53.599		588.4751	155606-59-0	FBF	95.40		95.40
DG(20:3(8Z,11Z,14Z)/14:1(9Z)/0:0)	C37 H64 O5	(M+H)+	53.599		588.4751		FBF	95.40		95.40
DG(20:3(5Z,8Z,11Z)/14:1(9Z)/0:0)	C37 H64 O5	(M+H)+	53.599		588.4751		FBF	95.40		95.40
DG(18:4(6Z,9Z,12Z,15Z)/16:0/0:0)	C37 H64 O5	(M+H)+	53.599		588.4751		FBF	95.40		95.40
DG(18:3(9Z,12Z,15Z)/16:1(9Z)/0:0)	C37 H64 O5	(M+H)+	53.599		588.4751		FBF	95.40		95.40
DG(18:3(6Z,9Z,12Z)/16:1(9Z)/0:0)	C37 H64 O5	(M+H)+	53.599		588.4751		FBF	95.40		95.40
DG(17:2(9Z,12Z)/17:2(9Z,12Z)/0:0)	C37 H64 O5	(M+H)+	53.599		588.4751		FBF	95.40		95.40
DG(20:4(5Z,8Z,11Z,14Z)/14:0/0:0)	C37 H64 O5	(M+H)+	53.599		588.4751		FBF	95.40		95.40
DG(16:1(9Z)/18:3(9Z,12Z,15Z)/0:0)[iso2]	C37 H64 O5	(M+H)+	53.599		588.4751		FBF	95.40		95.40
DG(14:1(9Z)/20:3(8Z,11Z,14Z)/0:0)	C37 H64 O5	(M+H)+	53.599		588.4751		FBF	95.40		95.40
DG(14:1(9Z)/20:3(5Z,8Z,11Z)/0:0)	C37 H64 O5	(M+H)+	53.599		588.4751		FBF	95.40		95.40
DG(14:0/20:4(8Z,11Z,14Z,17Z)/0:0)	C37 H64 O5	(M+H)+	53.599		588.4751		FBF	95.40		95.40
DG(14:0/20:4(5Z,8Z,11Z,14Z)/0:0)	C37 H64 O5	(M+H)+	53.599		588.4751		FBF	95.40		95.40
DG(16:1(9Z)/18:3(6Z,9Z,12Z)/0:0)	C37 H64 O5	(M+H)+	53.599		588.4751		FBF	95.40		95.40
DG(16:0/18:4(6Z,9Z,12Z,15Z)/0:0)	C37 H64 O5	(M+H)+	53.599		588.4751		FBF	95.40		95.40
DG(20:4(8Z,11Z,14Z,17Z)/14:0/0:0)	C37 H64 O5	(M+H)+	53.599		588.4751		FBF	95.40		95.40

Cpd 9: Stigmastan-3,5-diene

Name	Formula	RT	RI	Mass	Diff (Tgt, ppm)	CAS	ID Source	Score	Algorithm
Stigmastan-3,5-diene	C29 H48	53.983		396.3758	0.49		FBF	99.62	FBF

Species	m/z	Score (Tgt)	Score (Lib)	Score (DB)	Score (MFG)	Score (RT)
(M+H)+	397	99.62				

Compound Chromatograms (overlaid)

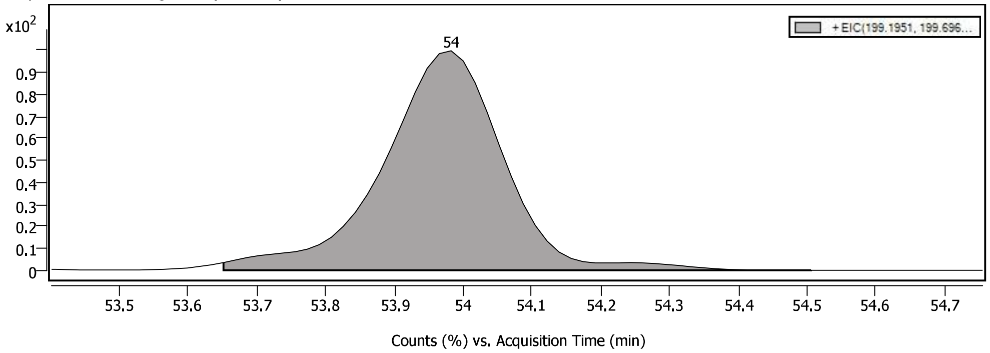

Structure

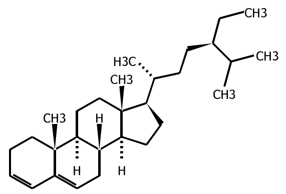

Compound Spectra (overlaid)

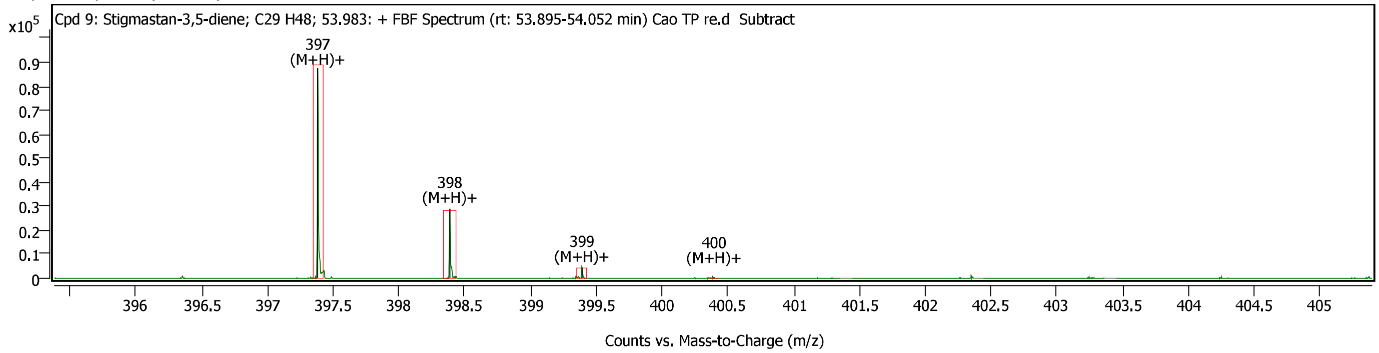

Compound ID Table

Name	Formula	Species	RT	RT Diff	Mass	CAS	ID Source	Score	Score (Lib)	Score (Tgt)
Stigmastan-3,5-diene	C29 H48	(M+H)+	53.983		396.3758		FBF	99.62		99.62

Cpd 1662: Hericenone D

Name	Formula	RT	RI	Mass	Diff (Tgt, ppm)	CAS	ID Source	Score	Algorithm
Hericenone D	C37 H58 O6	53.983		598.4211	-3.73	137592-04-2	M-FBF	93.60	FBF

Species	m/z	Score (Tgt)	Score (Lib)	Score (DB)	Score (MFG)	Score (RT)
(M+H)+	599	93.60				

Compound Chromatograms (overlaid)

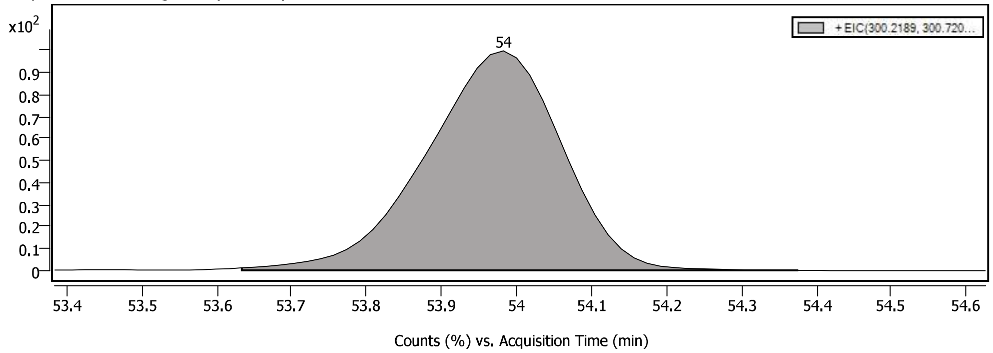

Structure

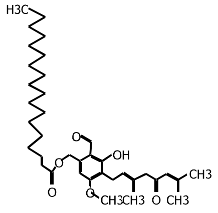

Compound Spectra (overlaid)

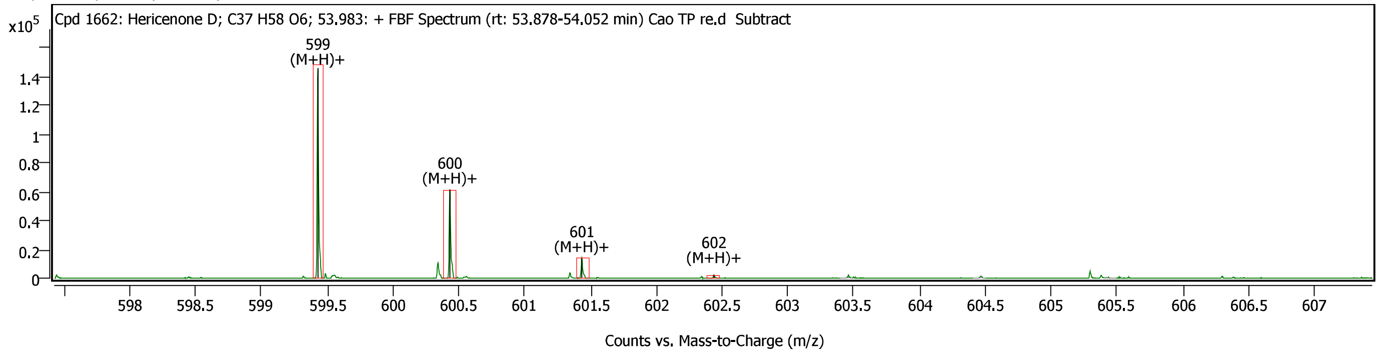

Compound ID Table

Name	Formula	Species	RT	RT Diff	Mass	CAS	ID Source	Score	Score (Lib)	Score (Tgt)
Hericenone D	C37 H58 O6	(M+H)+	53.983		598.4211	137592-04-2	FBF	93.60		93.60
Hericenone G	C37 H58 O6	(M+H)+	53.983		598.4211	141973-36-6	FBF	93.60		93.60

Cpd 19: Soyacerebroside II

Name	Formula	RT	RI	Mass	Diff (Tgt, ppm)	CAS	ID Source	Score	Algorithm
Soyacerebroside II	C40 H75 N O9	54.262		713.5449	0.97	115074-93-6	FBF-FragConfirm	98.71	FBF

Species	m/z	Score (Tgt)	Score (Lib)	Score (DB)	Score (MFG)	Score (RT)
(M+H)+	715	98.71				

Compound Screening Report

Compound Chromatograms (overlaid)

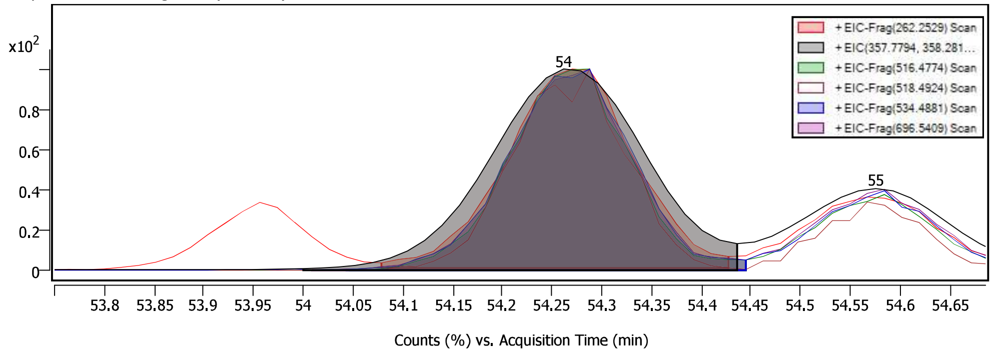

Structure

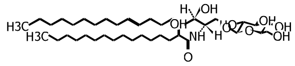

Coelution Plot

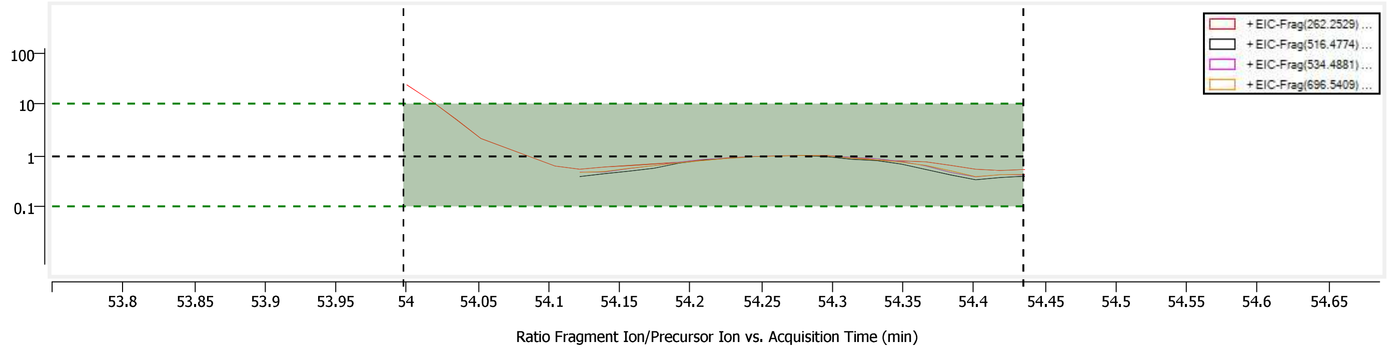

Compound Spectra (overlaid)

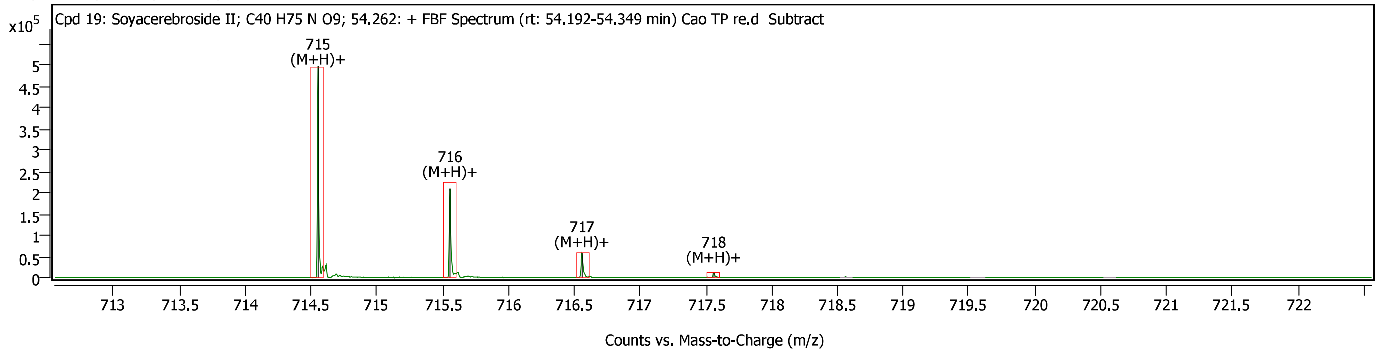

Fragment Spectrum (clean)

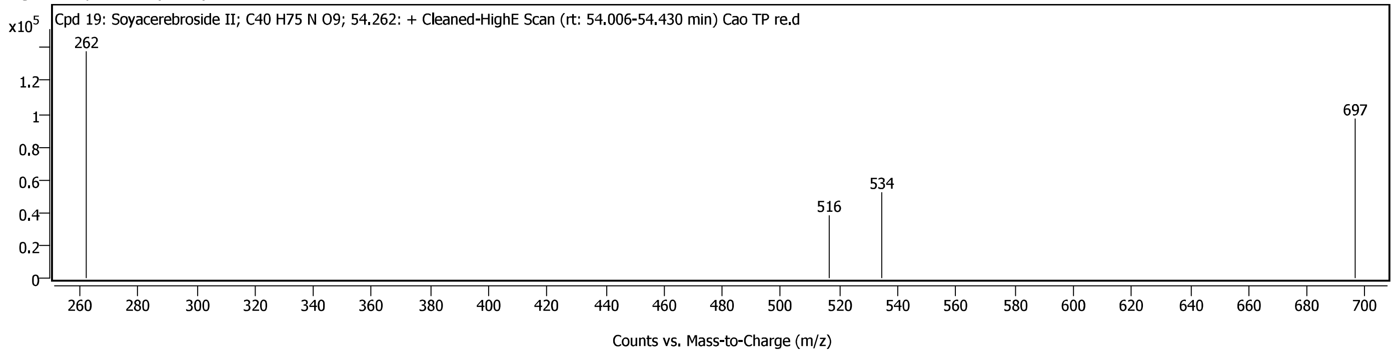

Fragment Spectrum (raw)

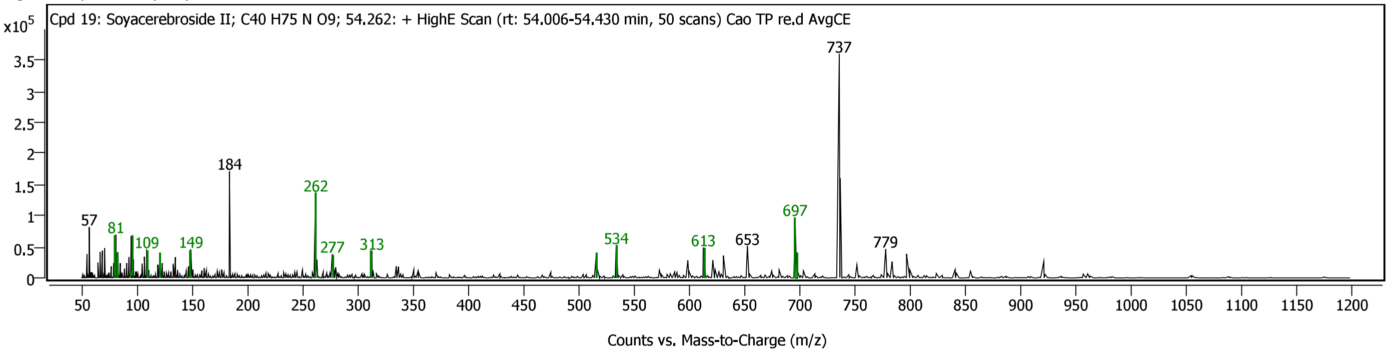

Compound ID Table

Name	Formula	Species	RT	RT Diff	Mass	CAS	ID Source	Score	Score (Lib)	Score (Tgt)
Soyacerebroside II	C40 H75 N O9	(M+H)+	54.262		713.5449	115074-93-6	FBF-FragConfirm	98.71		98.71
GlcCer(d16:2(4E,6E)/18:0(2OH))	C40 H75 N O9	(M+H)+	54.262		713.5449		FBF-FragConfirm	98.71		98.71
GlcCer(d14:2(4E,6E)/20:0(2OH))	C40 H75 N O9	(M+H)+	54.262		713.5449		FBF-FragConfirm	98.71		98.71
Soyacerebroside I	C40 H75 N O9	(M+H)+	54.262		713.5449	114297-20-0	FBF-FragConfirm	98.71		98.71

Cpd 1429: <Thioetheramide-PC>

Name	Formula	RT	RI	Mass	Diff (Tgt, ppm)	CAS	ID Source	Score	Algorithm
<Thioetheramide-PC>	C40 H84 N2 O5 P S	54.262		735.5849	1.35	116457-99-9	FBF	83.83	FBF

Species	m/z	Score (Tgt)	Score (Lib)	Score (DB)	Score (MFG)	Score (RT)
(M+H)+	737	83.83				

Compound Chromatograms (overlaid)

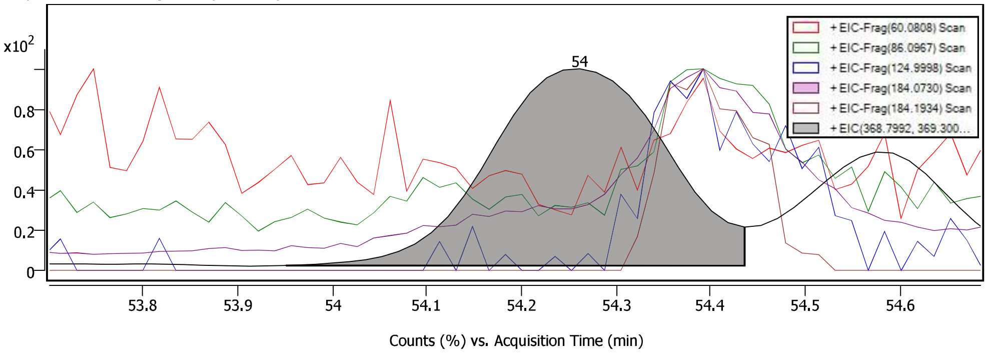

Structure

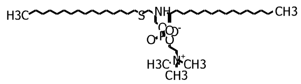

Coelution Plot

Compound Spectra (overlaid)

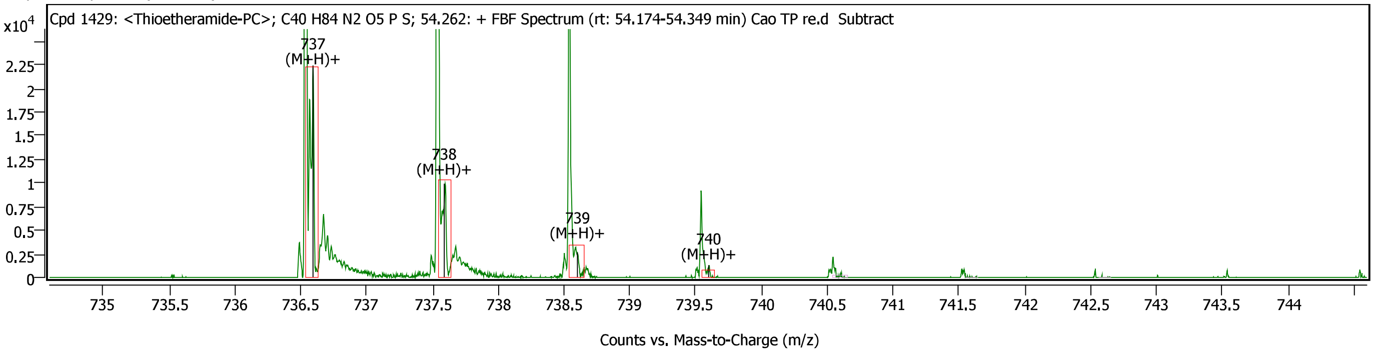

Fragment Spectrum (raw)

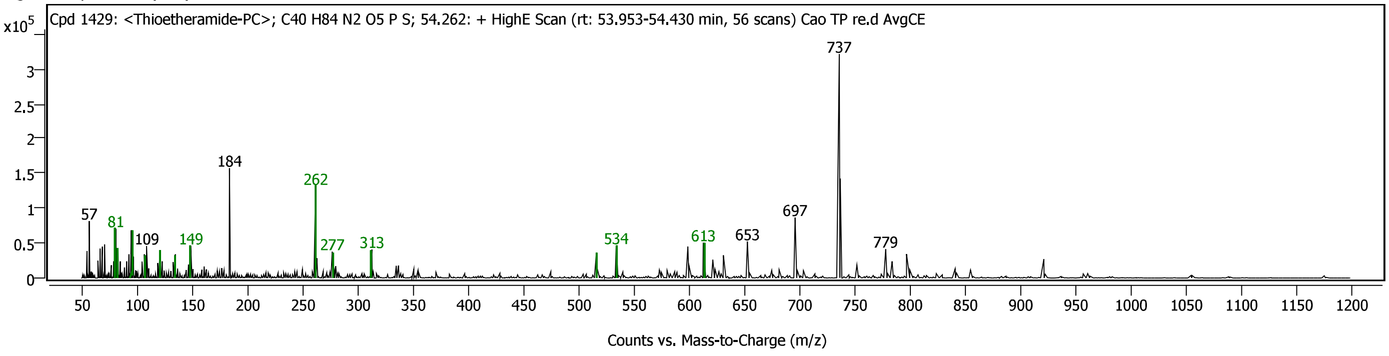

Compound Screening Report

Compound ID Table

Name	Formula	Species	RT	RT Diff	Mass	CAS	ID Source	Score	Score (Lib)	Score (Tgt)
<Thioetheramide-PC>	C40 H84 N2 O5 P S	(M+H)+	54.262		735.5849	116457-99-9	FBF	83.83		83.83

Cpd 39: Cer(d16:1(4E)/18:1(9Z)(2OH))

Name	Formula	RT	RI	Mass	Diff (Tgt, ppm)	CAS	ID Source	Score	Algorithm
Cer(d16:1(4E)/18:1(9Z)(2OH))	C34 H65 N O4	54.279		551.4913	-0.05		M-FBF	97.11	FBF

Species	m/z	Score (Tgt)	Score (Lib)	Score (DB)	Score (MFG)	Score (RT)
(M+H)+	552	97.11				

Compound Chromatograms (overlaid)

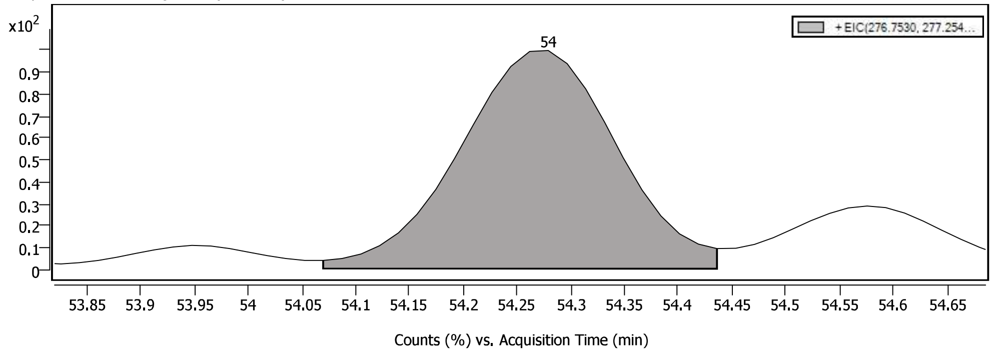

Structure

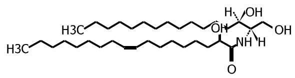

Compound Spectra (overlaid)

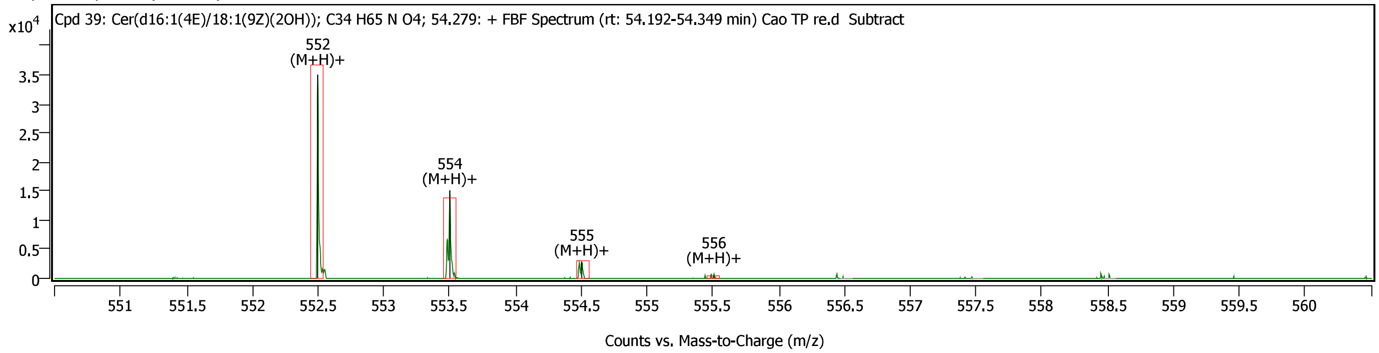

Compound ID Table

Name	Formula	Species	RT	RT Diff	Mass	CAS	ID Source	Score	Score (Lib)	Score (Tgt)
Cer(d16:1(4E)/18:1(9Z)(2OH))	C34 H65 N O4	(M+H)+	54.279		551.4913		FBF	97.11		97.11
Cer(d16:2(4E,6E)/18:0(2OH))	C34 H65 N O4	(M+H)+	54.279		551.4913		FBF	97.11		97.11
Cer(d14:2(4E,6E)/20:0(2OH))	C34 H65 N O4	(M+H)+	54.279		551.4913		FBF	97.11		97.11
Cer(d14:1(4E)/20:1(11Z)(2OH))	C34 H65 N O4	(M+H)+	54.279		551.4913		FBF	97.11		97.11

Cpd 298: PG(O-16:0/12:0)

Name	Formula	RT	RI	Mass	Diff (Tgt, ppm)	CAS	ID Source	Score	Algorithm
PG(O-16:0/12:0)	C34 H69 O9 P	54.384		652.4683	0.58		FBF	97.90	FBF

Species	m/z	Score (Tgt)	Score (Lib)	Score (DB)	Score (MFG)	Score (RT)
(M+H)+	653	97.90				

Compound Chromatograms (overlaid)

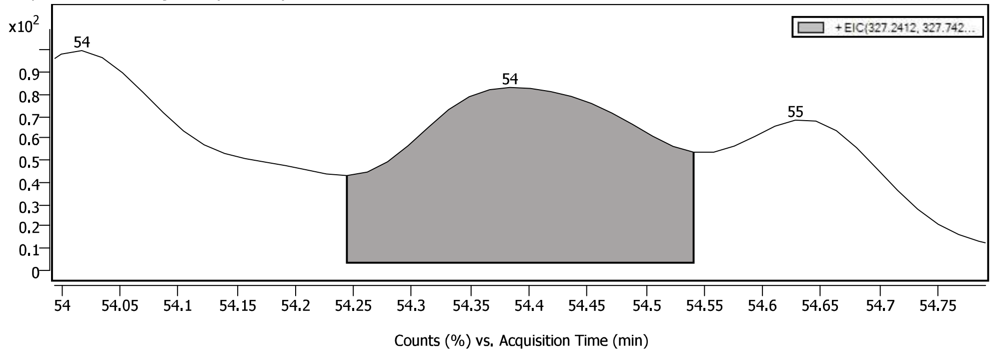

Structure

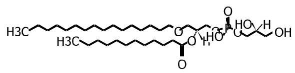

Compound Spectra (overlaid)

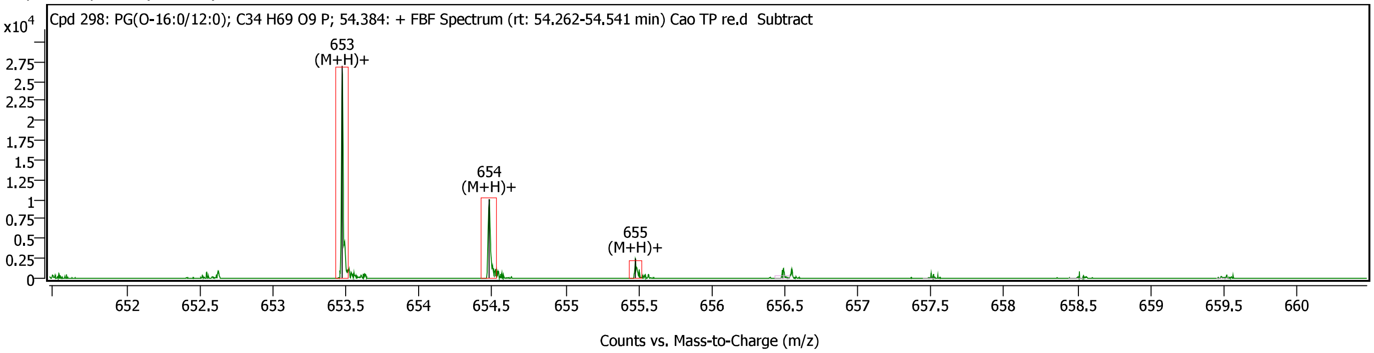

Compound ID Table

Name	Formula	Species	RT	RT Diff	Mass	CAS	ID Source	Score	Score (Lib)	Score (Tgt)
PG(O-16:0/12:0)	C34 H69 O9 P	(M+H)+	54.384		652.4683		FBF	97.90		97.90

Cpd 346: PE(17:0/22:6(4Z,7Z,10Z,13Z,16Z,19Z))

Name	Formula	RT	RI	Mass	Diff (Tgt, ppm)	CAS	ID Source	Score	Algorithm
PE(17:0/22:6(4Z,7Z,10Z,13Z,16Z, 19Z))	C44 H76 N O8 P	54.401		777.5313	0.57		M-FBF	99.62	FBF

Species	m/z	Score (Tgt)	Score (Lib)	Score (DB)	Score (MFG)	Score (RT)
(M+H)+	779	99.62				

Compound Chromatograms (overlaid)

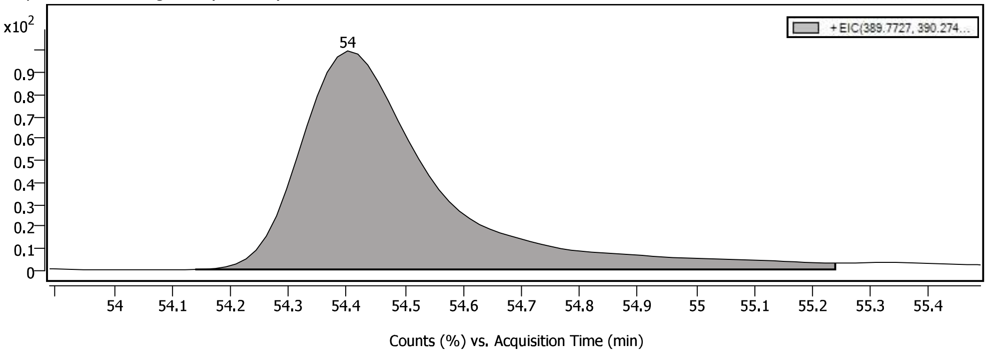

Structure

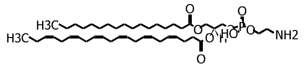

Compound Spectra (overlaid)

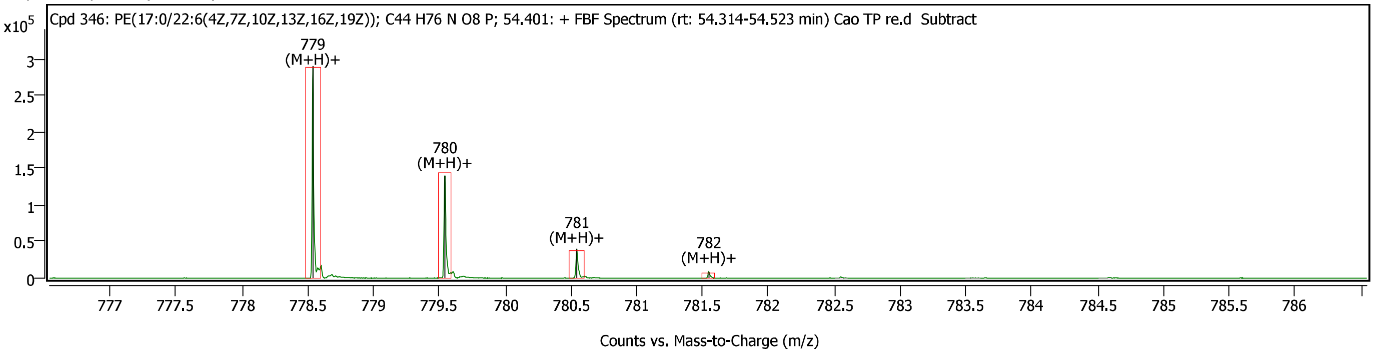

Compound ID Table

Name	Formula	Species	RT	RT Diff	Mass	CAS	ID Source	Score	Score (Lib)	Score (Tgt)
PE(17:0/22:6(4Z,7Z,10Z,13Z,16Z, 19Z))	C44 H76 N O8 P	(M+H)+	54.401		777.5313		FBF	99.62		99.62
PE(22:6(4Z,7Z,10Z,13Z,16Z,19Z)/ 17:0)	C44 H76 N O8 P	(M+H)+	54.401		777.5313		FBF	99.62		99.62
PE(22:4(7Z,10Z,13Z,16Z)/17:2(9Z ,12Z))	C44 H76 N O8 P	(M+H)+	54.401		777.5313		FBF	99.62		99.62
PE(20:5(5Z,8Z,11Z,14Z,17Z)/19:1 (9Z))	C44 H76 N O8 P	(M+H)+	54.401		777.5313		FBF	99.62		99.62
PE(19:1(9Z)/20:5(5Z,8Z,11Z,14Z, 17Z))	C44 H76 N O8 P	(M+H)+	54.401		777.5313		FBF	99.62		99.62
PE(17:2(9Z,12Z)/22:4(7Z,10Z,13Z ,16Z))	C44 H76 N O8 P	(M+H)+	54.401		777.5313		FBF	99.62		99.62

Cpd 1374: Megalomicin C1

Name	Formula	RT	RI	Mass	Diff (Tgt, ppm)	CAS	ID Source	Score	Algorithm
Megalomicin C1	C48 H84 N2 O17	54.506		960.5791	2.18		FBF	96.27	FBF

Species	m/z	Score (Tgt)	Score (Lib)	Score (DB)	Score (MFG)	Score (RT)
(M+H)+	962	96.27				

Compound Chromatograms (overlaid)

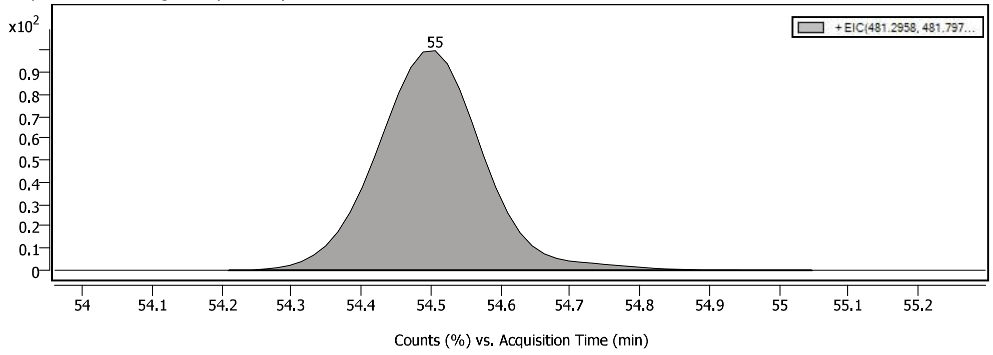

Structure

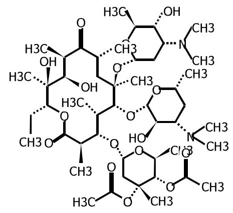

Compound Spectra (overlaid)

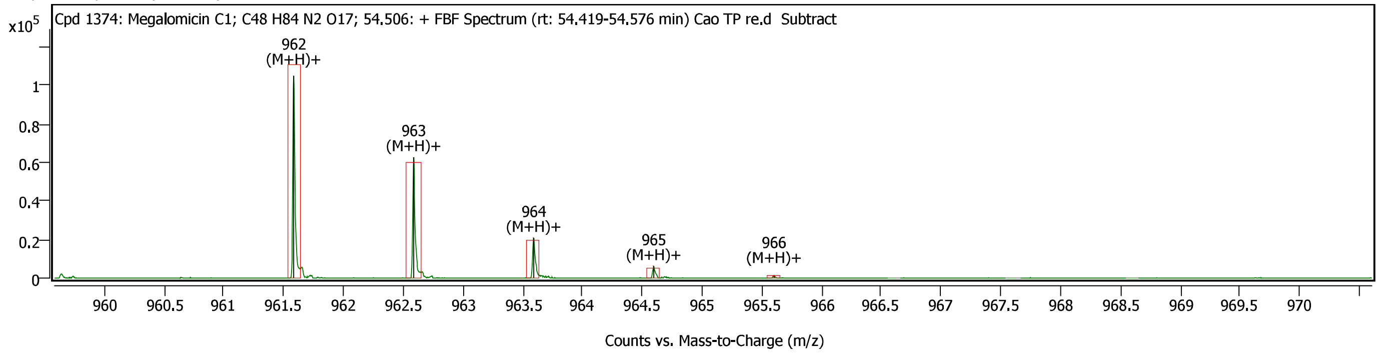

Compound ID Table

Name	Formula	Species	RT	RT Diff	Mass	CAS	ID Source	Score	Score (Lib)	Score (Tgt)
Megalomicin C1	C48 H84 N2 O17	(M+H)+	54.506		960.5791		FBF	96.27		96.27

Cpd 18: Soyacerebroside II

Name	Formula	RT	RI	Mass	Diff (Tgt, ppm)	CAS	ID Source	Score	Algorithm
Soyacerebroside II	C40 H75 N O9	54.576		713.5443	0.16	115074-93-6	FBF-FragConfirm	98.54	FBF

Species	m/z	Score (Tgt)	Score (Lib)	Score (DB)	Score (MFG)	Score (RT)
(M+H)+	715	98.54				

Compound Chromatograms (overlaid)

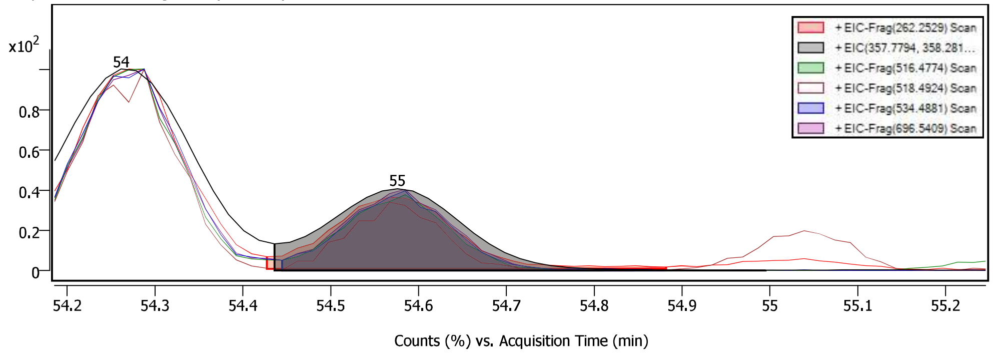

Structure

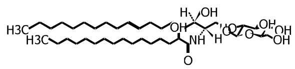

Coelution Plot

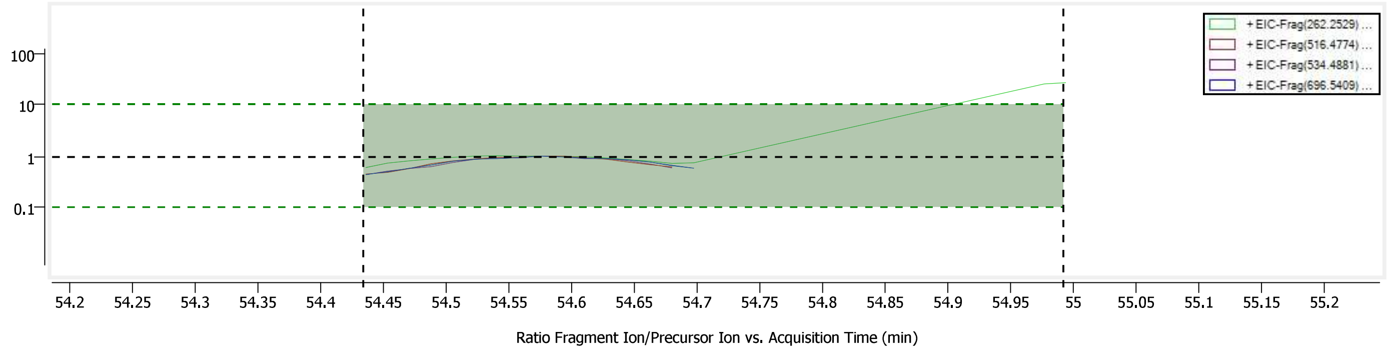

Compound Spectra (overlaid)

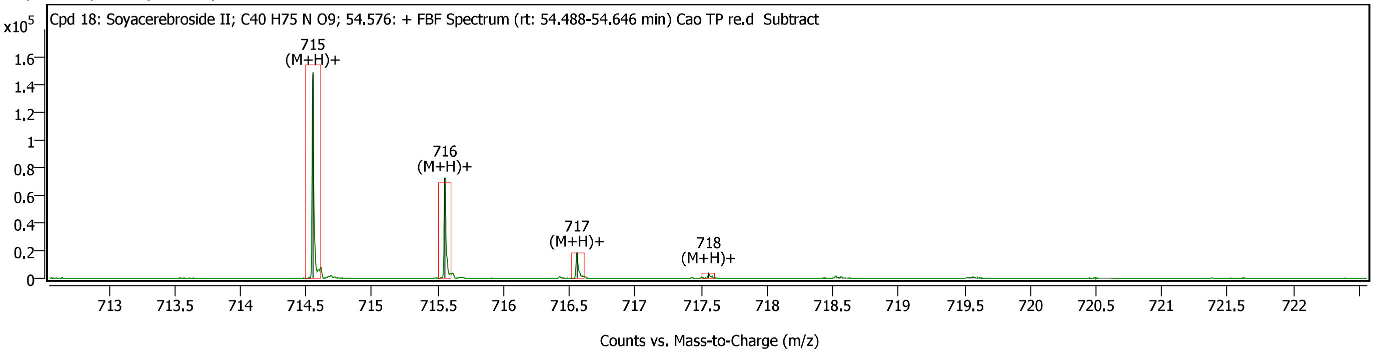

Fragment Spectrum (clean)

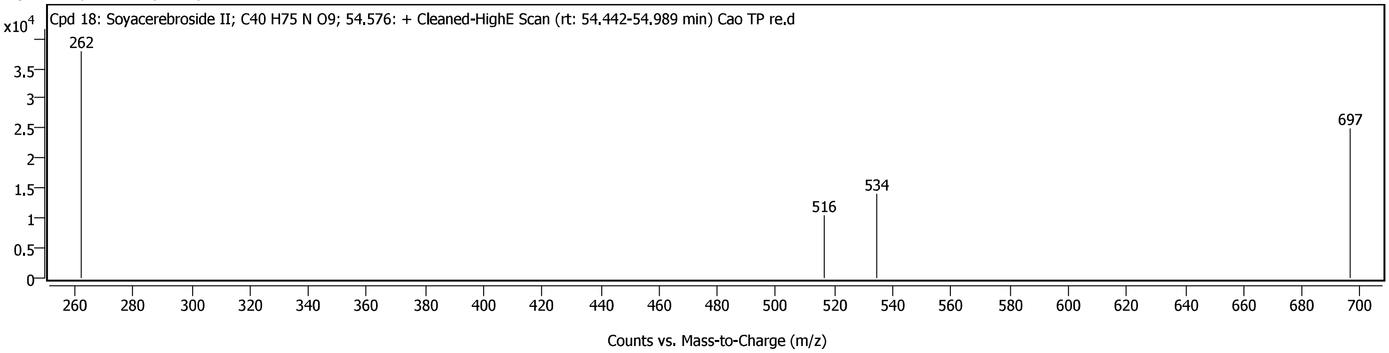

Fragment Spectrum (raw)

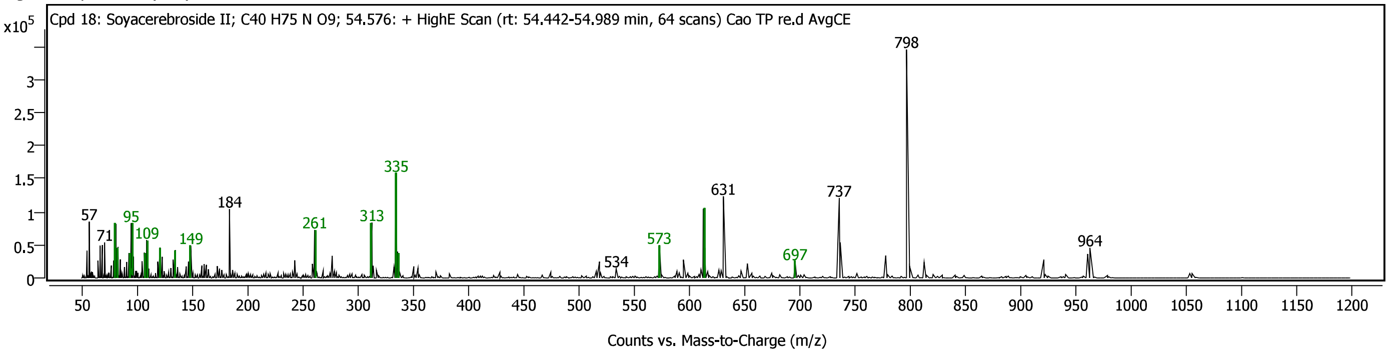

Compound ID Table

Name	Formula	Species	RT	RT Diff	Mass	CAS	ID Source	Score	Score (Lib)	Score (Tgt)
Soyacerebroside II	C40 H75 N O9	(M+H)+	54.576		713.5443	115074-93-6	FBF-FragConfirm	98.54		98.54
GlcCer(d16:2(4E,6E)/18:0(2OH))	C40 H75 N O9	(M+H)+	54.576		713.5443		FBF-FragConfirm	98.54		98.54
GlcCer(d14:2(4E,6E)/20:0(2OH))	C40 H75 N O9	(M+H)+	54.576		713.5443		FBF-FragConfirm	98.54		98.54
Soyacerebroside I	C40 H75 N O9	(M+H)+	54.576		713.5443	114297-20-0	FBF-FragConfirm	98.54		98.54

Cpd 883: N-(3-(15-methyl-hexadecanoyloxy)-13-methyl-tetradecanoyl)-L-serine

Name	Formula	RT	RI	Mass	Diff (Tgt, ppm)	CAS	ID Source	Score	Algorithm
N-(3-(15-methyl-hexadecanoyloxy)-13-methyl-tetradecanoyl)-L-serine	C37 H71 N O6	54.576		625.5282	0.04		FBF	99.62	FBF

Species	m/z	Score (Tgt)	Score (Lib)	Score (DB)	Score (MFG)	Score (RT)
(M+H)+	627	99.62				

Compound Chromatograms (overlaid)

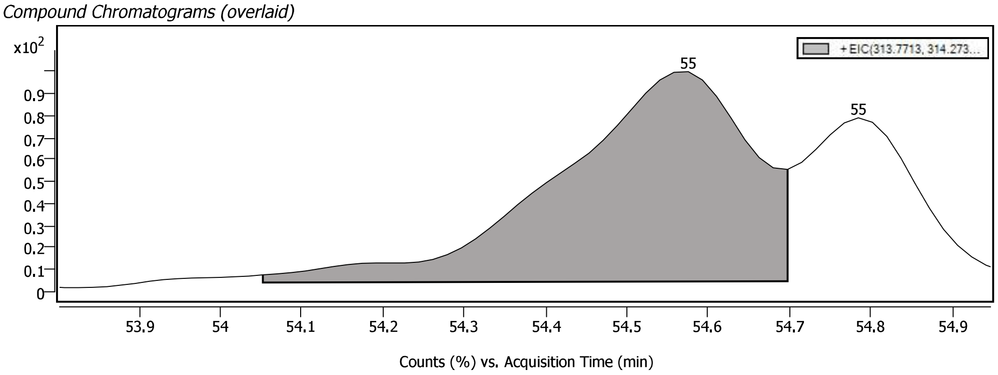

Structure

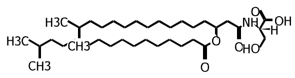

Compound Spectra (overlaid)

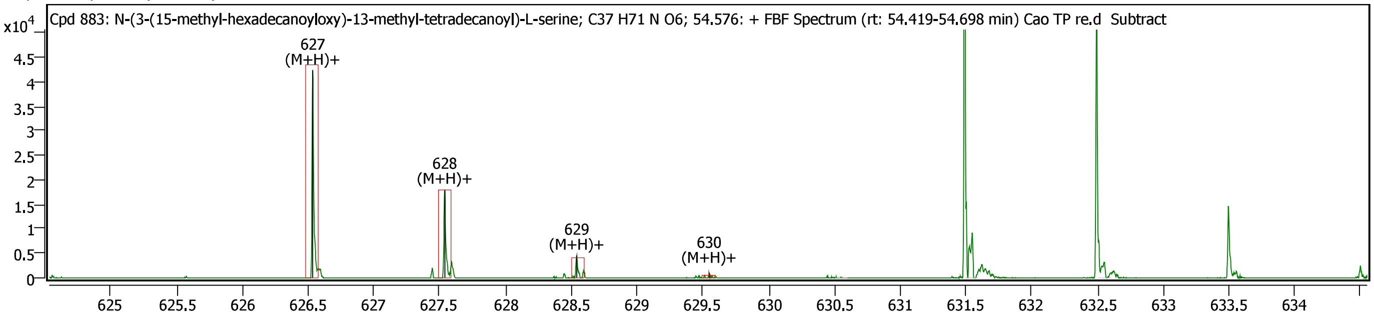

Compound ID Table

Name	Formula	Species	RT	RT Diff	Mass	CAS	ID Source	Score	Score (Lib)	Score (Tgt)
N-(3-(15-methyl-hexadecanoyloxy)-13-methyl-tetradecanoyl)-L-serine	C37 H71 N O6	(M+H)+	54.576		625.5282		FBF	99.62		99.62

Cpd 1007: SM(d18:1/12:0)

Name	Formula	RT	RI	Mass	Diff (Tgt, ppm)	CAS	ID Source	Score	Algorithm
SM(d18:1/12:0)	C35 H72 N2 O6 P	54.628		647.5125	-0.53		FBF	98.35	FBF

Species	m/z	Score (Tgt)	Score (Lib)	Score (DB)	Score (MFG)	Score (RT)
(M+H)+	649	98.35				

Compound Chromatograms (overlaid)

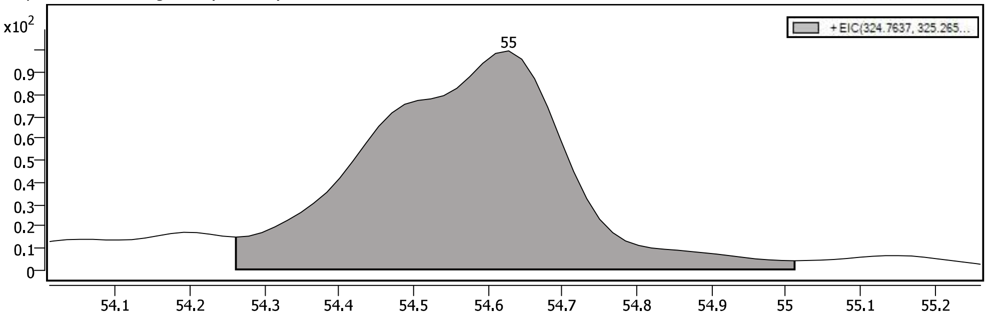

Structure

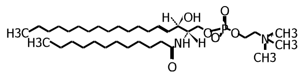

Compound Spectra (overlaid)

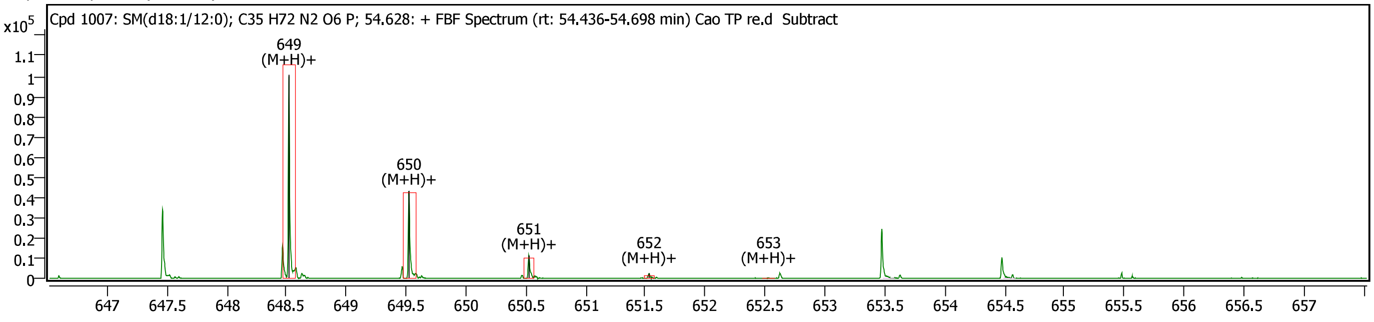

Compound ID Table

Name	Formula	Species	RT	RT Diff	Mass	CAS	ID Source	Score	Score (Lib)	Score (Tgt)
SM(d18:1/12:0)	C35 H72 N2 O6 P	(M+H)+	54.628		647.5125		FBF	98.35		98.35

Cpd 286: PI(22:1(11Z)/21:0)

Name	Formula	RT	RI	Mass	Diff (Tgt, ppm)	CAS	ID Source	Score	Algorithm
PI(22:1(11Z)/21:0)	C52 H99 O13 P	54.785		962.6820	-0.31		M-FBF	96.95	FBF

Species	m/z	Score (Tgt)	Score (Lib)	Score (DB)	Score (MFG)	Score (RT)
(M+H)+	964	96.95				

Compound Screening Report

Compound Chromatograms (overlaid)

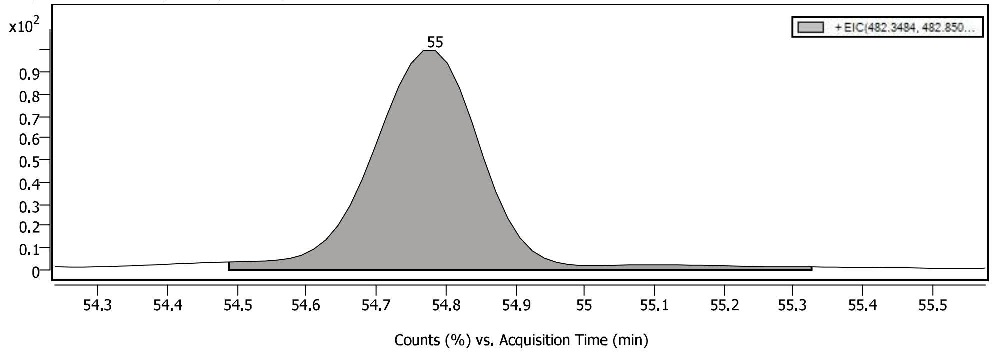

Structure

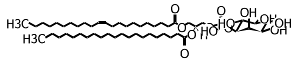

Compound Spectra (overlaid)

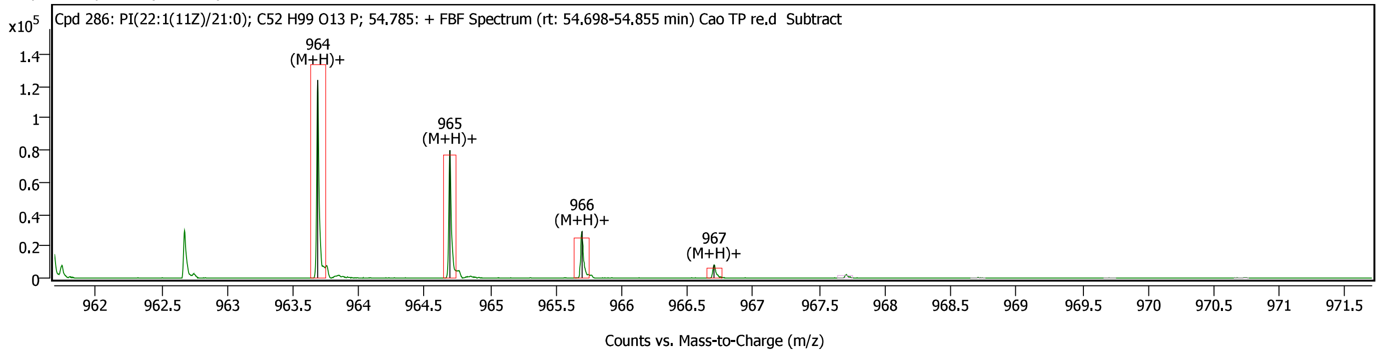

Compound ID Table

Name	Formula	Species	RT	RT Diff	Mass	CAS	ID Source	Score	Score (Lib)	Score (Tgt)
PI(22:1(11Z)/21:0)	C52 H99 O13 P	(M+H)+	54.785		962.6820		FBF	96.95		96.95
PI(21:0/22:1(11Z))	C52 H99 O13 P	(M+H)+	54.785		962.6820		FBF	96.95		96.95

Cpd 280: PI(17:0/14:0)

Name	Formula	RT	RI	Mass	Diff (Tgt, ppm)	CAS	ID Source	Score	Algorithm
PI(17:0/14:0)	C40 H77 O13 P	54.925		796.5108	0.81		M-FBF	98.21	FBF

Species	m/z	Score (Tgt)	Score (Lib)	Score (DB)	Score (MFG)	Score (RT)
(M+H)+	798	98.21				

Compound Chromatograms (overlaid)

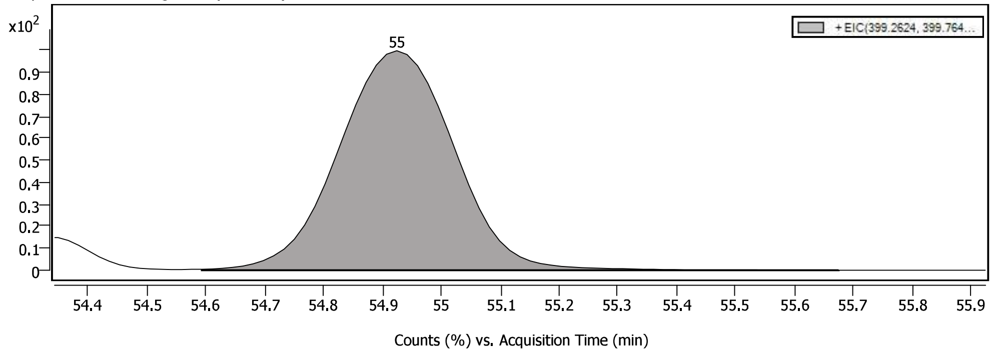

Structure

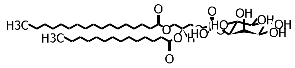

Compound Spectra (overlaid)

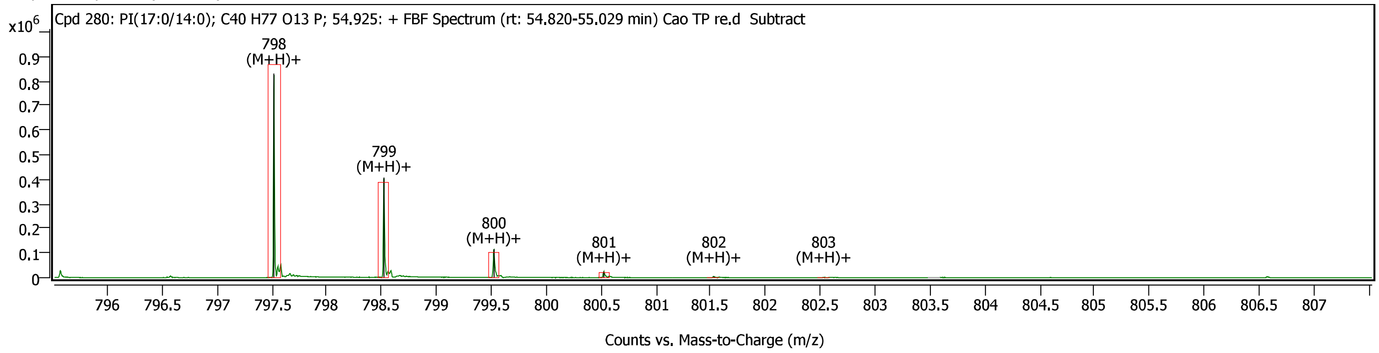

Compound ID Table

Name	Formula	Species	RT	RT Diff	Mass	CAS	ID Source	Score	Score (Lib)	Score (Tgt)
PI(17:0/14:0)	C40 H77 O13 P	(M+H)+	54.925		796.5108		FBF	98.21		98.21
PI(15:0/16:0)	C40 H77 O13 P	(M+H)+	54.925		796.5108		FBF	98.21		98.21
PI(12:0/19:0)	C40 H77 O13 P	(M+H)+	54.925		796.5108		FBF	98.21		98.21
PI(13:0/18:0)	C40 H77 O13 P	(M+H)+	54.925		796.5108		FBF	98.21		98.21
PI(14:0/17:0)	C40 H77 O13 P	(M+H)+	54.925		796.5108		FBF	98.21		98.21
PI(16:0/15:0)	C40 H77 O13 P	(M+H)+	54.925		796.5108		FBF	98.21		98.21
PI(19:0/12:0)	C40 H77 O13 P	(M+H)+	54.925		796.5108		FBF	98.21		98.21
PI(18:0/13:0)	C40 H77 O13 P	(M+H)+	54.925		796.5108		FBF	98.21		98.21

Cpd 862: MGDG(20:5(5Z,8Z,11Z,14Z,17Z)/18:4(6Z,9Z,12Z,15Z))

Name	Formula	RT	RI	Mass	Diff (Tgt, ppm)	CAS	ID Source	Score	Algorithm
MGDG(20:5(5Z,8Z,11Z,14Z,17Z)/18:4(6Z,9Z,12Z,15Z))	C47 H72 O10	54.925		796.5108	-2.18		FBF	97.09	FBF

Species	m/z	Score (Tgt)	Score (Lib)	Score (DB)	Score (MFG)	Score (RT)
(M+H)+	798	97.09				

Compound Chromatograms (overlaid)

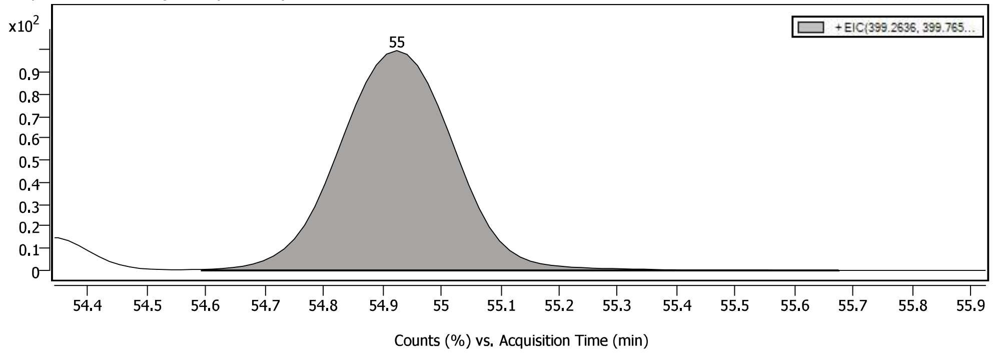

Structure

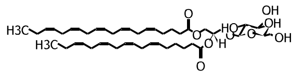

Compound Spectra (overlaid)

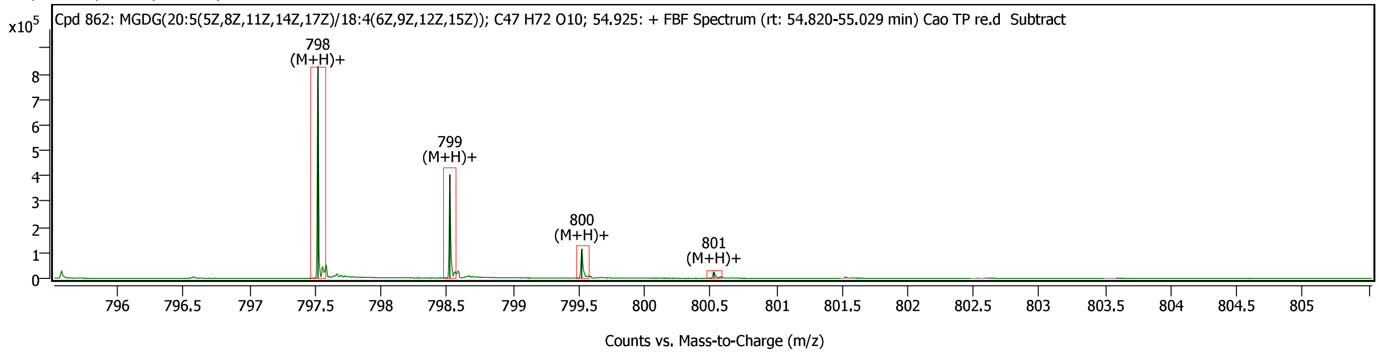

Compound ID Table

Name	Formula	Species	RT	RT Diff	Mass	CAS	ID Source	Score	Score (Lib)	Score (Tgt)
MGDG(20:5(5Z,8Z,11Z,14Z,17Z)/18:4(6Z,9Z,12Z,15Z))	C47 H72 O10	(M+H)+	54.925		796.5108		FBF	97.09		97.09

Cpd 261: PA(P-16:0/12:0)

Name	Formula	RT	RI	Mass	Diff (Tgt, ppm)	CAS	ID Source	Score	Algorithm
PA(P-16:0/12:0)	C31 H61 O7 P	55.274		576.4157	0.31		FBF	98.91	FBF

Species	m/z	Score (Tgt)	Score (Lib)	Score (DB)	Score (MFG)	Score (RT)
(M+H)+	577	98.91				

Compound Chromatograms (overlaid)

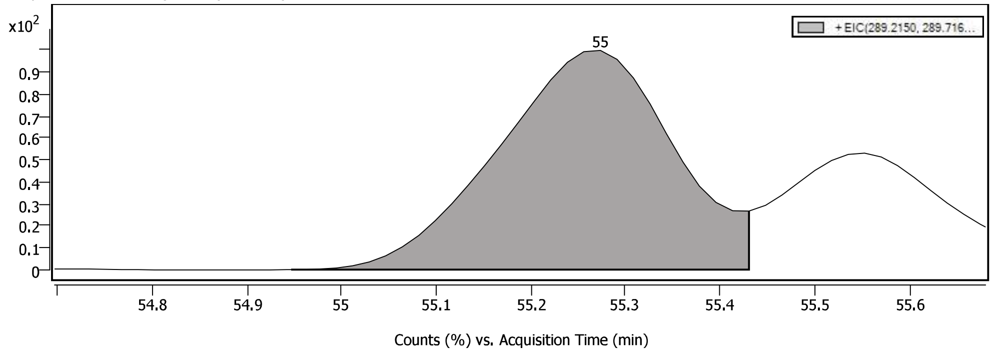

Structure

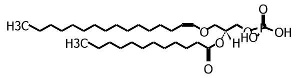

Compound Spectra (overlaid)

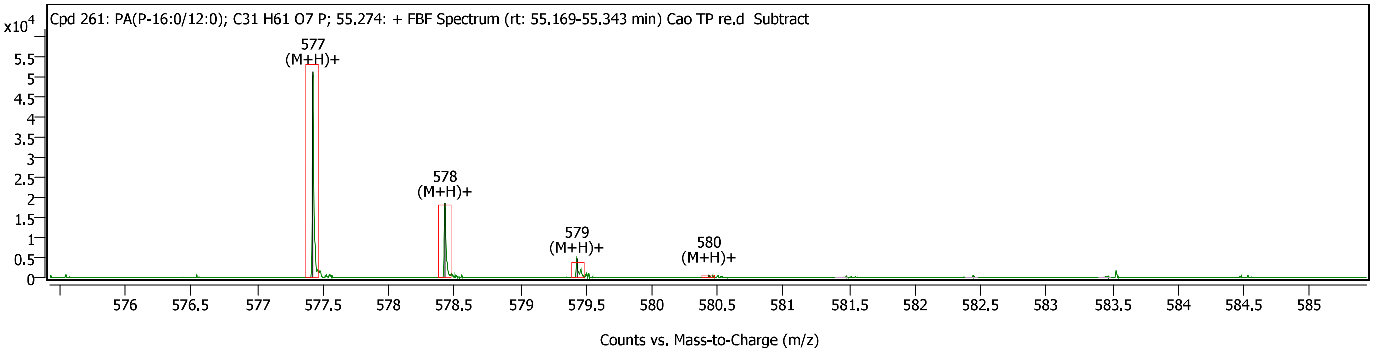

Compound ID Table

Name	Formula	Species	RT	RT Diff	Mass	CAS	ID Source	Score	Score (Lib)	Score (Tgt)
PA(P-16:0/12:0)	C31 H61 O7 P	(M+H)+	55.274		576.4157		FBF	98.91		98.91

Cpd 1698: DG(14:0/20:4(5Z,8Z,11Z,14Z)/0:0)

Name	Formula	RT	RI	Mass	Diff (Tgt, ppm)	CAS	ID Source	Score	Algorithm
DG(14:0/20:4(5Z,8Z,11Z,14Z)/0:0)	C37 H64 O5	55.343		588.4752	-0.24		M-FBF	96.89	FBF

Species	m/z	Score (Tgt)	Score (Lib)	Score (DB)	Score (MFG)	Score (RT)
(M+H)+	589	96.89				

Compound Chromatograms (overlaid)

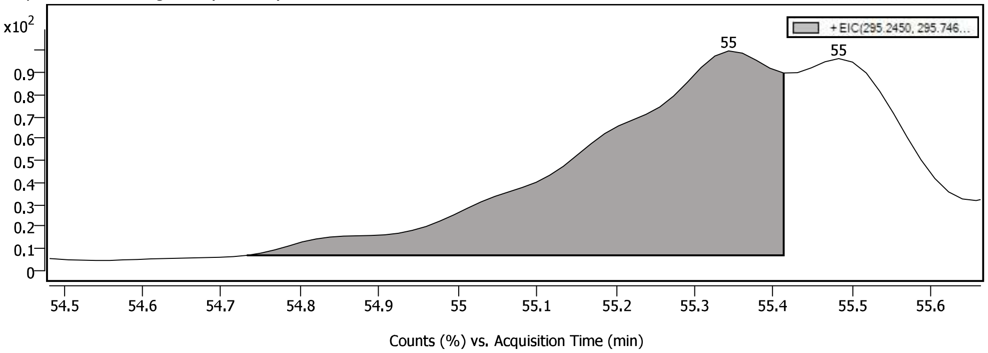

Structure

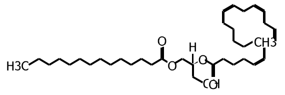

Compound Spectra (overlaid)

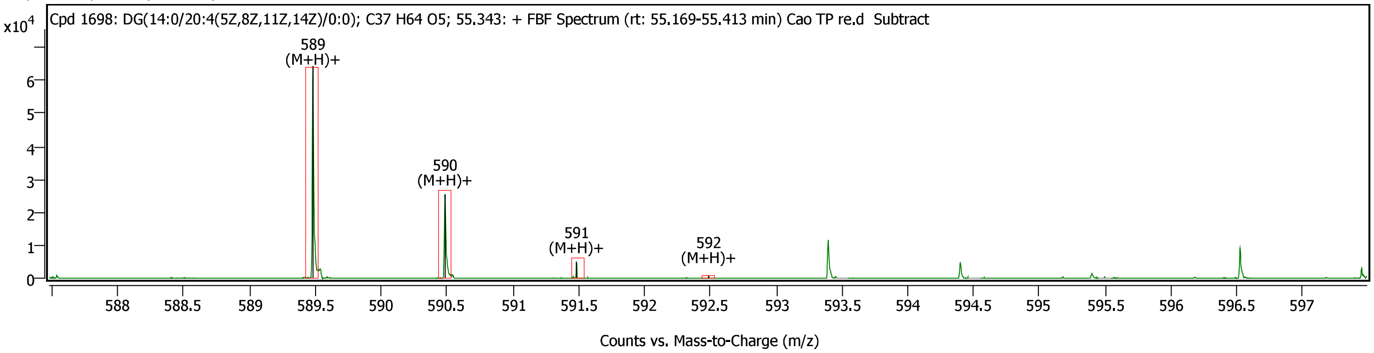

Compound Screening Report

Compound ID Table

Name	Formula	Species	RT	RT Diff	Mass	CAS	ID Source	Score	Score (Lib)	Score (Tgt)
DG(14:0/20:4(5Z,8Z,11Z,14Z)/0:0)	C37 H64 O5	(M+H)+	55.343		588.4752		FBF	96.89		96.89
Tripoxyrollin	C37 H64 O5	(M+H)+	55.343		588.4752	155606-59-0	FBF	96.89		96.89
DG(20:3(8Z,11Z,14Z)/14:1(9Z)/0:0)	C37 H64 O5	(M+H)+	55.343		588.4752		FBF	96.89		96.89
DG(20:3(5Z,8Z,11Z)/14:1(9Z)/0:0)	C37 H64 O5	(M+H)+	55.343		588.4752		FBF	96.89		96.89
DG(18:4(6Z,9Z,12Z,15Z)/16:0/0:0)	C37 H64 O5	(M+H)+	55.343		588.4752		FBF	96.89		96.89
DG(18:3(9Z,12Z,15Z)/16:1(9Z)/0:0)	C37 H64 O5	(M+H)+	55.343		588.4752		FBF	96.89		96.89
DG(18:3(6Z,9Z,12Z)/16:1(9Z)/0:0)	C37 H64 O5	(M+H)+	55.343		588.4752		FBF	96.89		96.89
DG(17:2(9Z,12Z)/17:2(9Z,12Z)/0:0)	C37 H64 O5	(M+H)+	55.343		588.4752		FBF	96.89		96.89
DG(20:4(5Z,8Z,11Z,14Z)/14:0/0:0)	C37 H64 O5	(M+H)+	55.343		588.4752		FBF	96.89		96.89
DG(16:1(9Z)/18:3(9Z,12Z,15Z)/0:0)[iso2]	C37 H64 O5	(M+H)+	55.343		588.4752		FBF	96.89		96.89
DG(14:1(9Z)/20:3(8Z,11Z,14Z)/0:0)	C37 H64 O5	(M+H)+	55.343		588.4752		FBF	96.89		96.89
DG(14:1(9Z)/20:3(5Z,8Z,11Z)/0:0)	C37 H64 O5	(M+H)+	55.343		588.4752		FBF	96.89		96.89
DG(14:0/20:4(8Z,11Z,14Z,17Z)/0:0)	C37 H64 O5	(M+H)+	55.343		588.4752		FBF	96.89		96.89
DG(12:0/22:4(7Z,10Z,13Z,16Z)/0:0)[iso2]	C37 H64 O5	(M+H)+	55.343		588.4752		FBF	96.89		96.89
DG(16:1(9Z)/18:3(6Z,9Z,12Z)/0:0)	C37 H64 O5	(M+H)+	55.343		588.4752		FBF	96.89		96.89
DG(16:0/18:4(6Z,9Z,12Z,15Z)/0:0)	C37 H64 O5	(M+H)+	55.343		588.4752		FBF	96.89		96.89
DG(20:4(8Z,11Z,14Z,17Z)/14:0/0:0)	C37 H64 O5	(M+H)+	55.343		588.4752		FBF	96.89		96.89

Cpd 234: Aragusteroketal

Name	Formula	RT	RI	Mass	Diff (Tgt, ppm)	CAS	ID Source	Score	Algorithm
Aragusteroketal	C31 H54 O4	55.396		490.4024	0.32		FBF	99.70	FBF

Species	m/z	Score (Tgt)	Score (Lib)	Score (DB)	Score (MFG)	Score (RT)
(M+H)+	491	99.70				

Compound Chromatograms (overlaid)

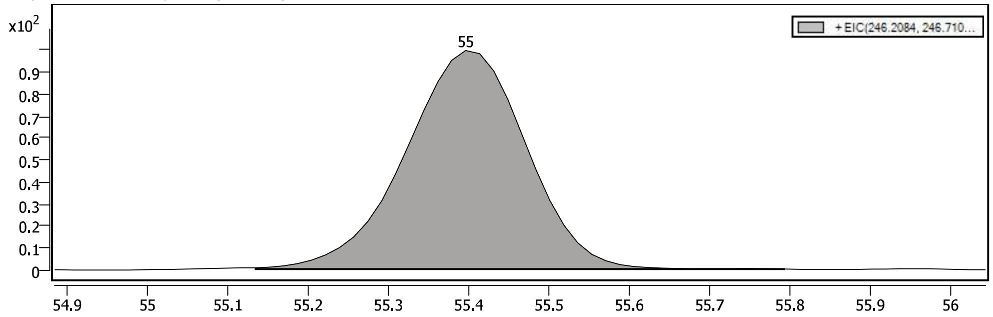

Structure

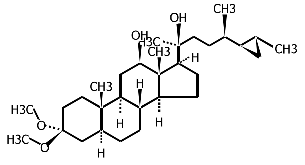

Compound Spectra (overlaid)

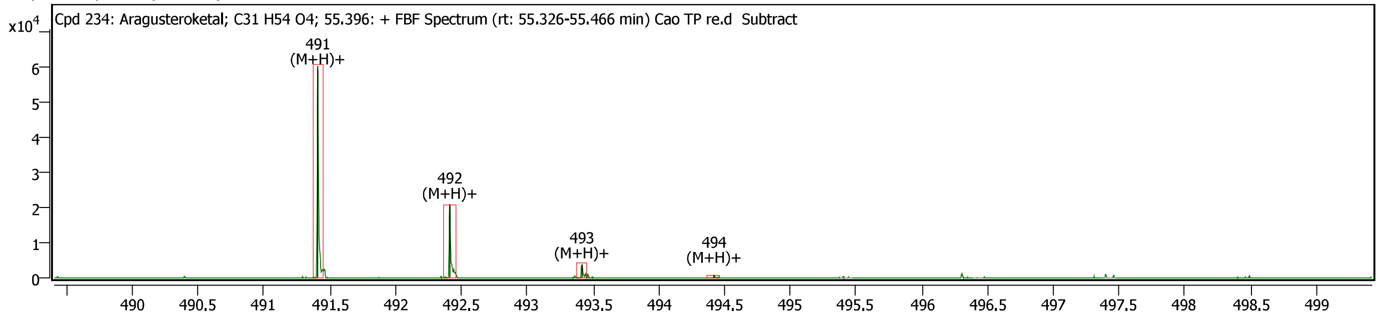

Compound ID Table

Name	Formula	Species	RT	RT Diff	Mass	CAS	ID Source	Score	Score (Lib)	Score (Tgt)
Aragusteroketal	C31 H54 O4	(M+H)+	55.396		490.4024		FBF	99.70		99.70

Cpd 368: PC(O-20:0/22:6(4Z,7Z,10Z,13Z,16Z,19Z))

Name	Formula	RT	RI	Mass	Diff (Tgt, ppm)	CAS	ID Source	Score	Algorithm
PC(O-20:0/22:6(4Z,7Z,10Z,13Z,16Z,19Z))	C50 H91 N O7 P	55.413		848.6532	-0.18		FBF	98.22	FBF

Species	m/z	Score (Tgt)	Score (Lib)	Score (DB)	Score (MFG)	Score (RT)
(M+H)+	850	98.22				

Compound Chromatograms (overlaid)

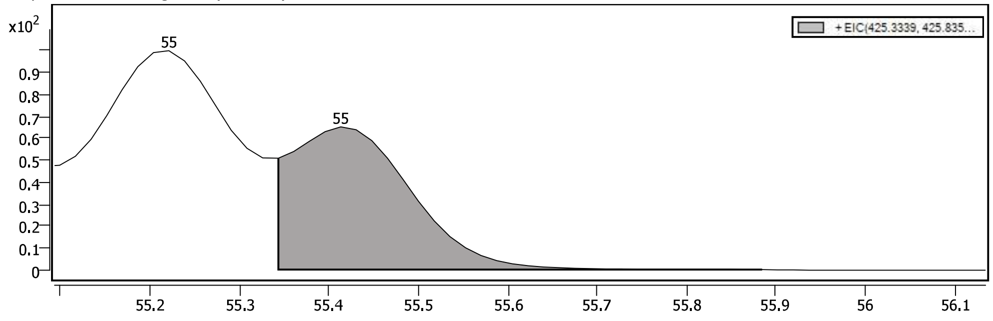

Structure

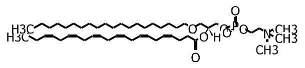

Compound Spectra (overlaid)

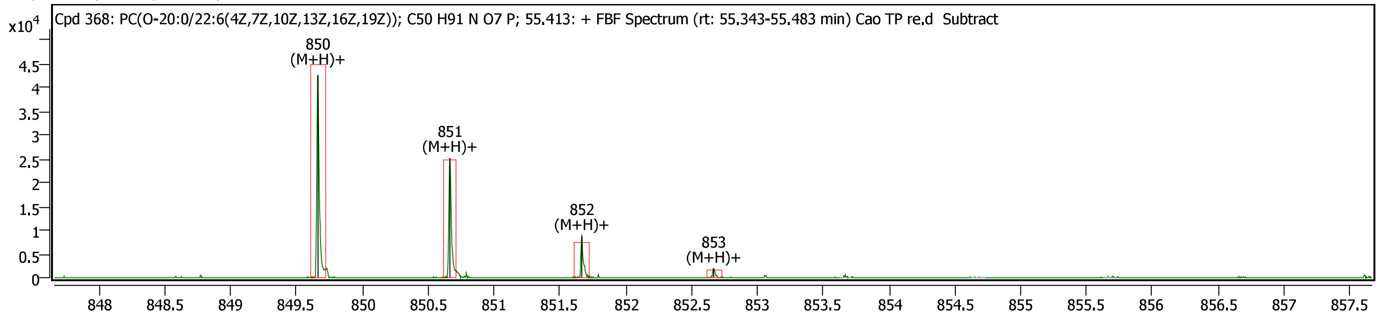

Compound ID Table

Name	Formula	Species	RT	RT Diff	Mass	CAS	ID Source	Score	Score (Lib)	Score (Tgt)
PC(O-20:0/22:6(4Z,7Z,10Z,13Z,16Z,19Z))	C50 H91 N O7 P	(M+H)+	55.413		848.6532		FBF	98.22		98.22

Cpd 1599: Rollidecin D

Name	Formula	RT	RI	Mass	Diff (Tgt, ppm)	CAS	ID Source	Score	Algorithm
Rollidecin D	C37 H66 O6	55.902		606.4862	0.47	200703-17-9	M-FBF	98.77	FBF

Species	m/z	Score (Tgt)	Score (Lib)	Score (DB)	Score (MFG)	Score (RT)
(M+H)+	607	98.77				

Compound Chromatograms (overlaid)

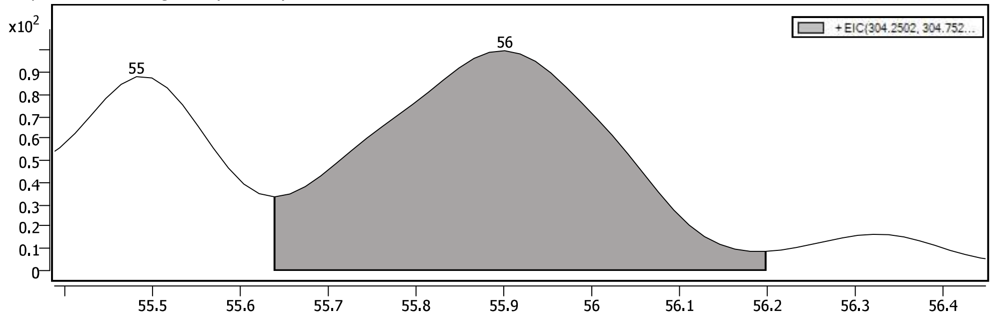

Structure

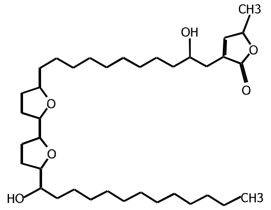

Compound Spectra (overlaid)

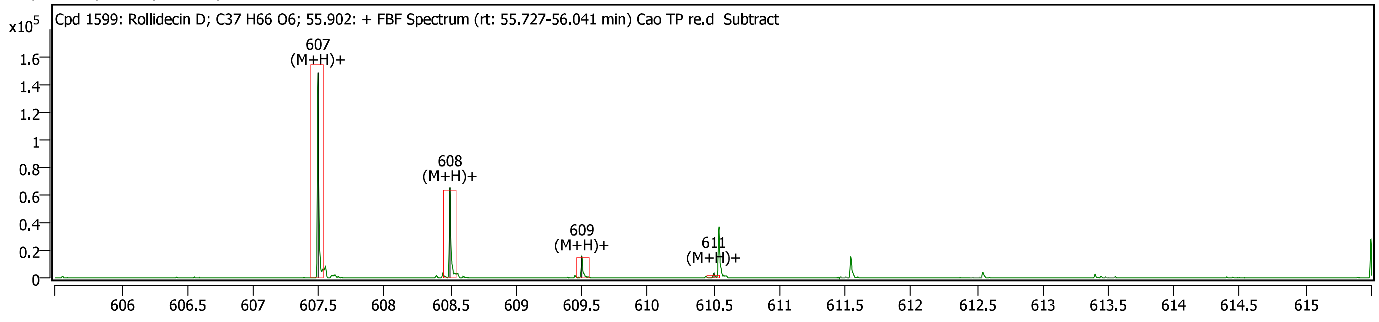

Compound Screening Report

Compound ID Table

Name	Formula	Species	RT	RT Diff	Mass	CAS	ID Source	Score	Score (Lib)	Score (Tgt)
Rollidecin D	C37 H66 O6	(M+H)+	55.902		606.4862	200703-17-9	FBF	98.77		98.77
cis-Reticulatacin-10-one	C37 H66 O6	(M+H)+	55.902		606.4862	205439-33-4	FBF	98.77		98.77
Asiminenin A	C37 H66 O6	(M+H)+	55.902		606.4862	168075-11-4	FBF	98.77		98.77
Neoannonin B	C37 H66 O6	(M+H)+	55.902		606.4862	170312-94-4	FBF	98.77		98.77
Squamocin L	C37 H66 O6	(M+H)+	55.902		606.4862	98767-45-4	FBF	98.77		98.77

Cpd 1513: Montecristin

Name	Formula	RT	RI	Mass	Diff (Tgt, ppm)	CAS	ID Source	Score	Algorithm
Montecristin	C37 H66 O4	55.919		574.4950	-1.90	185336-15-6	FBF	95.44	FBF

Species	m/z	Score (Tgt)	Score (Lib)	Score (DB)	Score (MFG)	Score (RT)
(M+H)+	576	95.44				

Compound Chromatograms (overlaid)

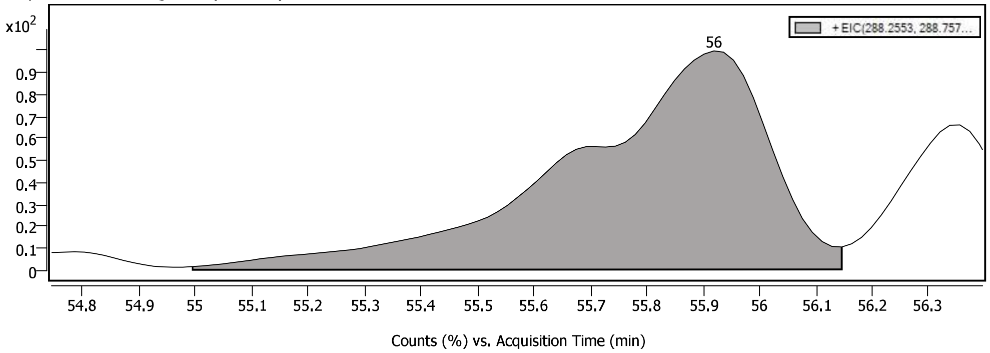

Structure

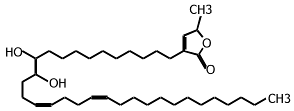

Compound Spectra (overlaid)

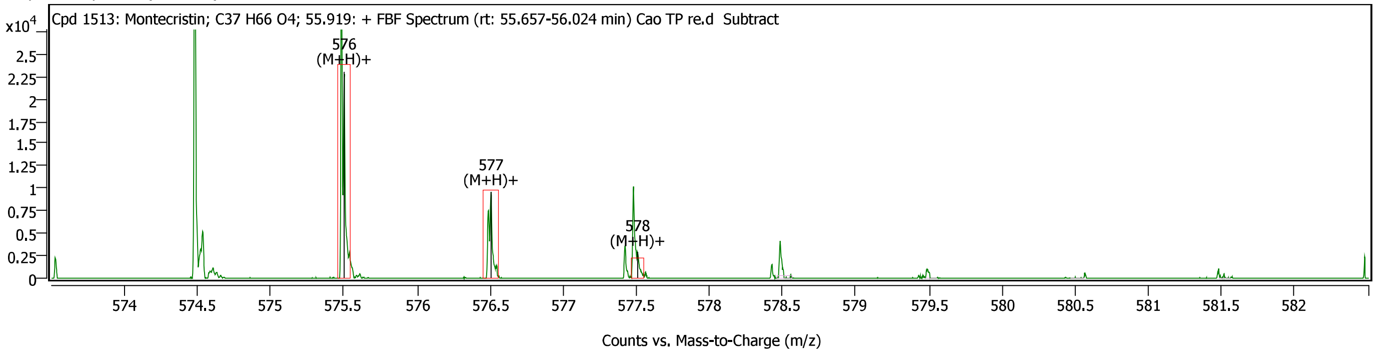

Compound ID Table

Name	Formula	Species	RT	RT Diff	Mass	CAS	ID Source	Score	Score (Lib)	Score (Tgt)
Montecristin	C37 H66 O4	(M+H)+	55.919		574.4950	185336-15-6	FBF	95.44		95.44

Cpd 38: Cer(d14:1(4E)/20:1(11Z)(2OH))

Name	Formula	RT	RI	Mass	Diff (Tgt, ppm)	CAS	ID Source	Score	Algorithm
Cer(d14:1(4E)/20:1(11Z)(2OH))	C34 H65 N O4	55.937		551.4919	0.92		M-FBF	99.39	FBF

Species	m/z	Score (Tgt)	Score (Lib)	Score (DB)	Score (MFG)	Score (RT)
(M+H)+	552	99.39				

Compound Chromatograms (overlaid)

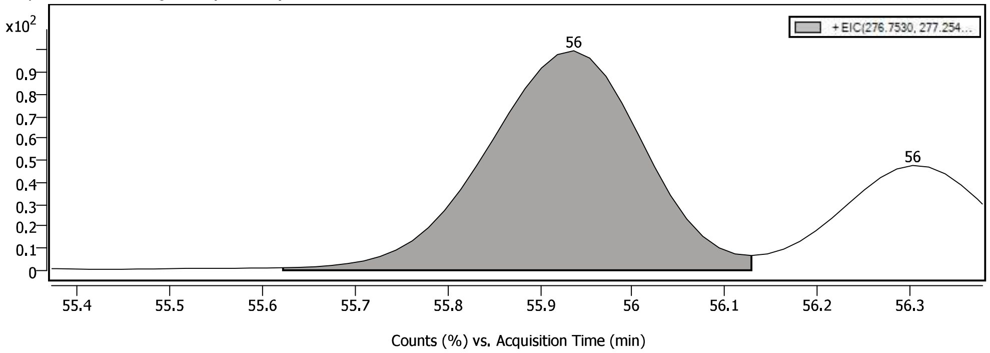

Structure

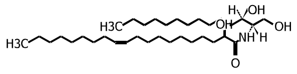

Compound Screening Report

Compound Spectra (overlaid)

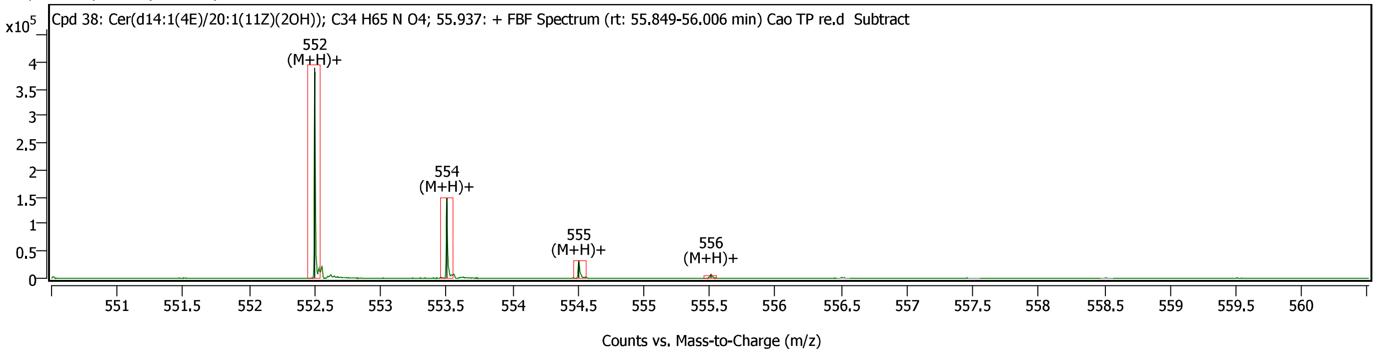

Compound ID Table

Name	Formula	Species	RT	RT Diff	Mass	CAS	ID Source	Score	Score (Lib)	Score (Tgt)
Cer(d14:1(4E)/20:1(11Z)(2OH))	C34 H65 N O4	(M+H)+	55.937		551.4919		FBF	99.39		99.39
Cer(d16:2(4E,6E)/18:0(2OH))	C34 H65 N O4	(M+H)+	55.937		551.4919		FBF	99.39		99.39
Cer(d16:1(4E)/18:1(9Z)(2OH))	C34 H65 N O4	(M+H)+	55.937		551.4919		FBF	99.39		99.39
Cer(d14:2(4E,6E)/20:0(2OH))	C34 H65 N O4	(M+H)+	55.937		551.4919		FBF	99.39		99.39

Cpd 40: Cer(d14:2(4E,6E)/20:1(11Z))

Name	Formula	RT	RI	Mass	Diff (Tgt, ppm)	CAS	ID Source	Score	Algorithm
Cer(d14:2(4E,6E)/20:1(11Z))	C34 H63 N O3	55.937		533.4812	0.68		FBF	99.65	FBF

Species	m/z	Score (Tgt)	Score (Lib)	Score (DB)	Score (MFG)	Score (RT)
(M+H)+	534	99.65				

Compound Chromatograms (overlaid)

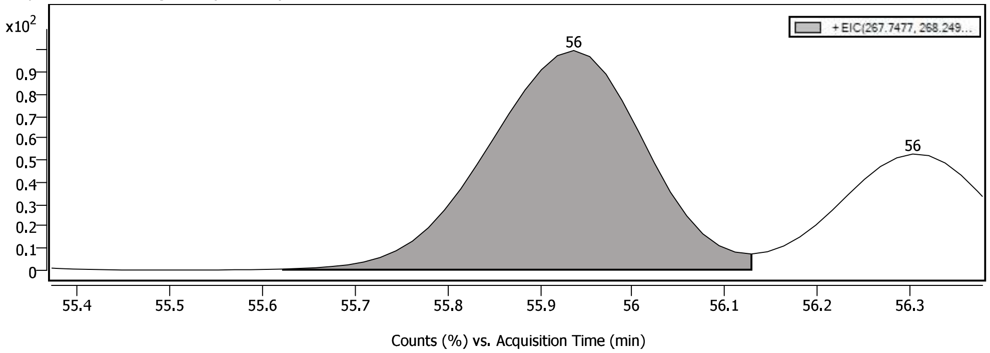

Structure

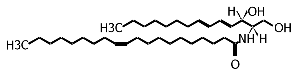

Compound Spectra (overlaid)

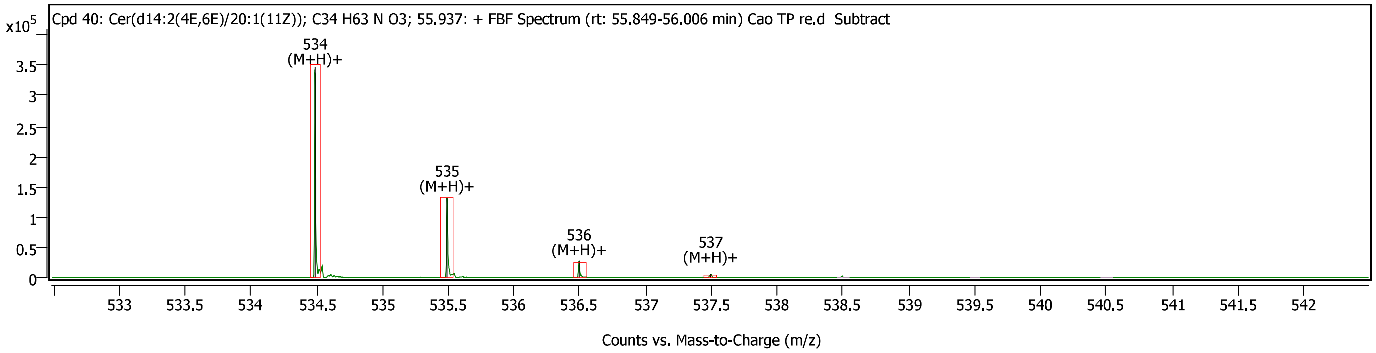

Compound ID Table

Name	Formula	Species	RT	RT Diff	Mass	CAS	ID Source	Score	Score (Lib)	Score (Tgt)
Cer(d14:2(4E,6E)/20:1(11Z))	C34 H63 N O3	(M+H)+	55.937		533.4812		FBF	99.65		99.65

Cpd 1484: 1,2-Di-(9Z,12Z,15Z-octadecatrienoyl)-3-(Galactosyl-alpha-1-6-Galactosyl-beta-1)-glycerol

Name	Formula	RT	RI	Mass	Diff (Tgt, ppm)	CAS	ID Source	Score	Algorithm
1,2-Di-(9Z,12Z,15Z-octadecatrienoyl)-3-(Galactosyl-alpha-1-6-Galactosyl-beta-1)-glycerol	C51 H84 O15	56.094		936.5791	-2.10		FBF	97.69	FBF

Species	m/z	Score (Tgt)	Score (Lib)	Score (DB)	Score (MFG)	Score (RT)
(M+H)+	938	97.69				

Compound Chromatograms (overlaid)

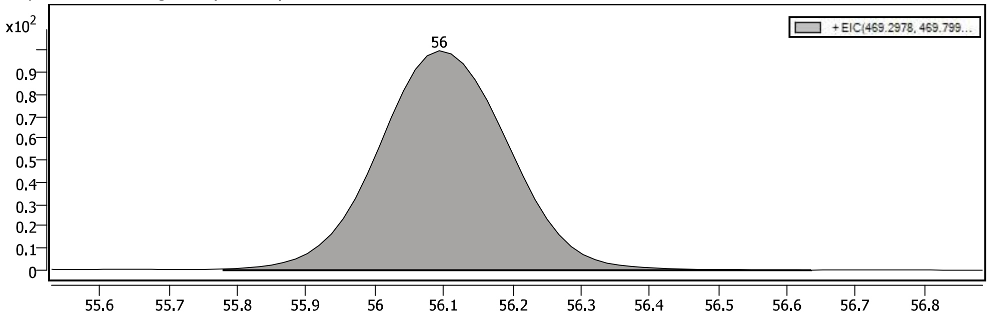

Structure

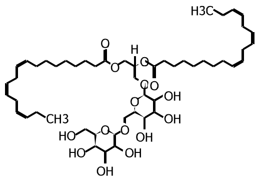

Compound Spectra (overlaid)

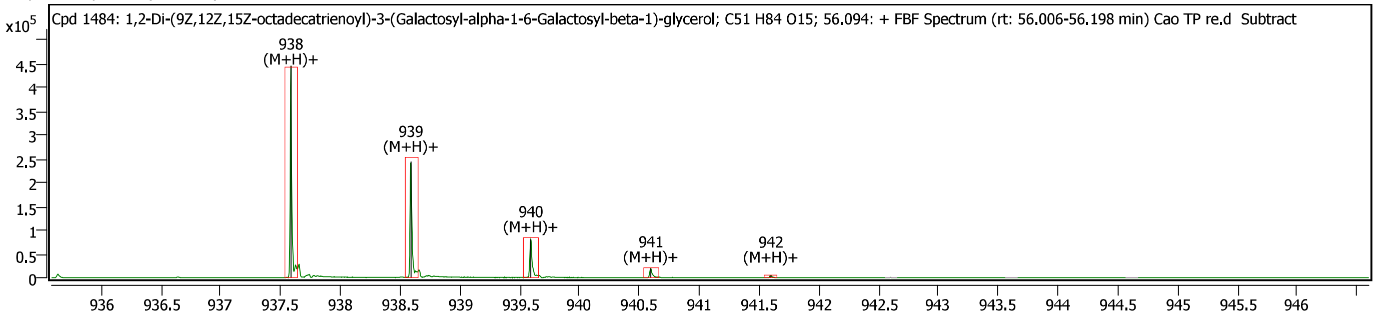

Compound ID Table

Name	Formula	Species	RT	RT Diff	Mass	CAS	ID Source	Score	Score (Lib)	Score (Tgt)
1,2-Di-(9Z,12Z,15Z-octadecatrienoyl)-3-(Galactosyl-alpha-1-6-Galactosyl-beta-1)-glycerol	C51 H84 O15	(M+H)+	56.094		936.5791		FBF	97.69		97.69

Cpd 349: PE(22:4(7Z,10Z,13Z,16Z)/17:1(9Z))

Name	Formula	RT	RI	Mass	Diff (Tgt, ppm)	CAS	ID Source	Score	Algorithm
PE(22:4(7Z,10Z,13Z,16Z)/17:1(9Z))	C44 H78 N O8 P	56.181		779.5480	1.86		M-FBF	97.53	FBF

Species	m/z	Score (Tgt)	Score (Lib)	Score (DB)	Score (MFG)	Score (RT)
(M+H)+	781	97.53				

Compound Chromatograms (overlaid)

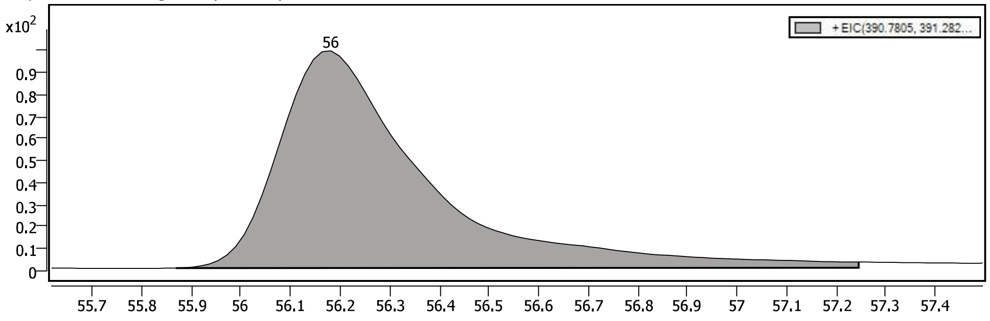

Structure

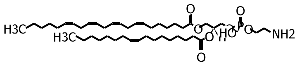

Compound Spectra (overlaid)

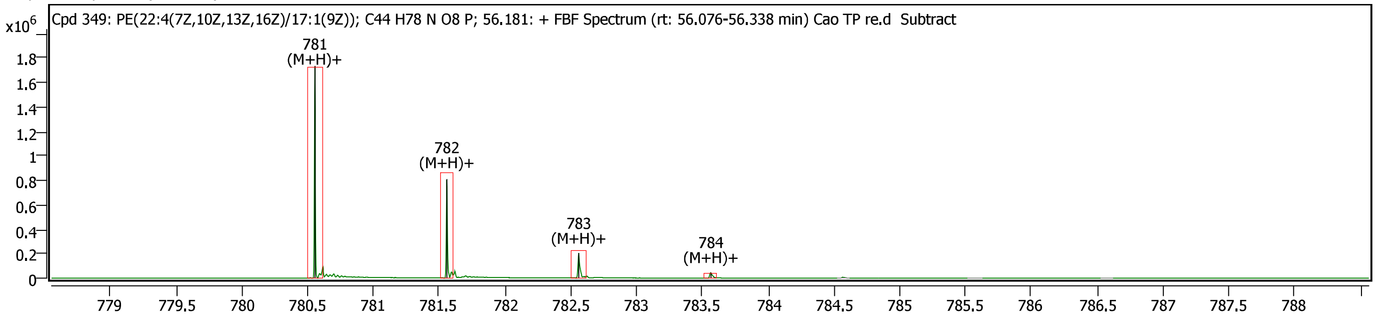

Compound ID Table

Name	Formula	Species	RT	RT Diff	Mass	CAS	ID Source	Score	Score (Lib)	Score (Tgt)
PE(22:4(7Z,10Z,13Z,16Z)/17:1(9Z))	C44 H78 N O8 P	(M+H)+	56.181		779.5480		FBF	97.53		97.53
PE(19:1(9Z)/20:4(5Z,8Z,11Z,14Z))	C44 H78 N O8 P	(M+H)+	56.181		779.5480		FBF	97.53		97.53
PE(17:1(9Z)/22:4(7Z,10Z,13Z,16Z))	C44 H78 N O8 P	(M+H)+	56.181		779.5480		FBF	97.53		97.53
PE(19:0/20:5(5Z,8Z,11Z,14Z,17Z))	C44 H78 N O8 P	(M+H)+	56.181		779.5480		FBF	97.53		97.53
PE(20:5(5Z,8Z,11Z,14Z,17Z)/19:0)	C44 H78 N O8 P	(M+H)+	56.181		779.5480		FBF	97.53		97.53
PE(20:4(5Z,8Z,11Z,14Z)/19:1(9Z))	C44 H78 N O8 P	(M+H)+	56.181		779.5480		FBF	97.53		97.53

Cpd 37: Cer(d14:2(4E,6E)/20:0(2OH))

Name	Formula	RT	RI	Mass	Diff (Tgt, ppm)	CAS	ID Source	Score	Algorithm
Cer(d14:2(4E,6E)/20:0(2OH))	C34 H65 N O4	56.303		551.4916	0.43		M-FBF	97.71	FBF

Species	m/z	Score (Tgt)	Score (Lib)	Score (DB)	Score (MFG)	Score (RT)
(M+H)+	552	97.71				

Compound Chromatograms (overlaid)

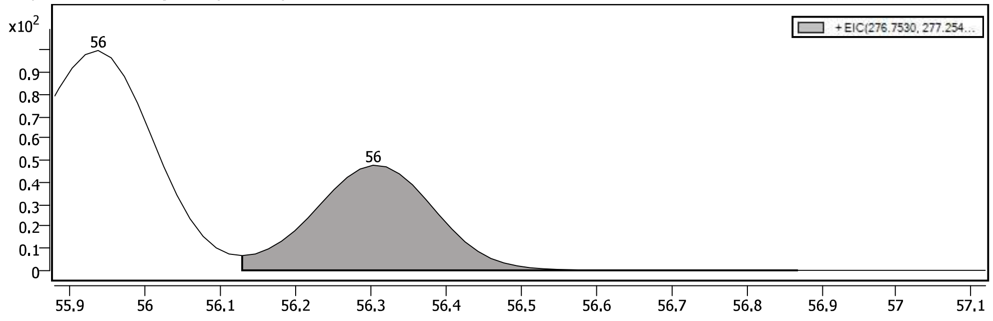

Counts (%) vs. Acquisition Time (min)

Structure

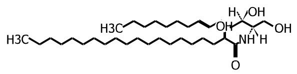

Compound Spectra (overlaid)

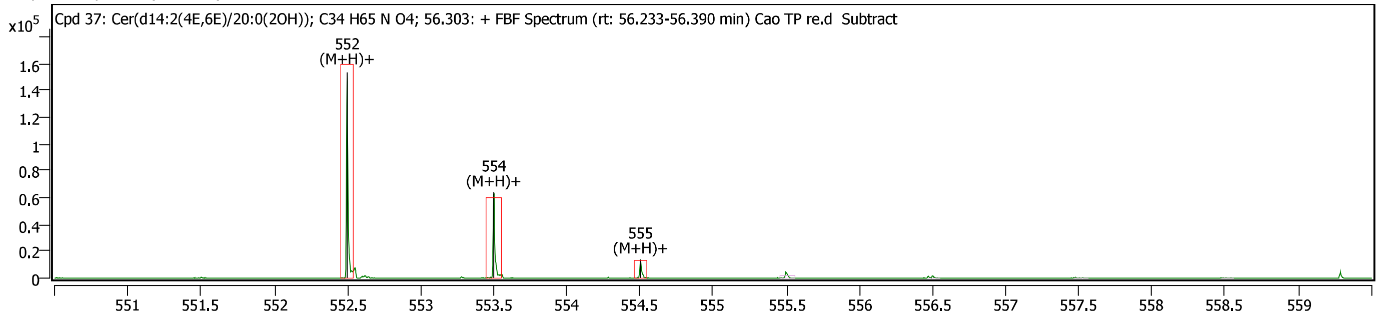

Counts vs. Mass-to-Charge (m/z)

Compound ID Table

Name	Formula	Species	RT	RT Diff	Mass	CAS	ID Source	Score	Score (Lib)	Score (Tgt)
Cer(d14:2(4E,6E)/20:0(2OH))	C34 H65 N O4	(M+H)+	56.303		551.4916		FBF	97.71		97.71
Cer(d16:2(4E,6E)/18:0(2OH))	C34 H65 N O4	(M+H)+	56.303		551.4916		FBF	97.71		97.71
Cer(d16:1(4E)/18:1(9Z)(2OH))	C34 H65 N O4	(M+H)+	56.303		551.4916		FBF	97.71		97.71
Cer(d14:1(4E)/20:1(11Z)(2OH))	C34 H65 N O4	(M+H)+	56.303		551.4916		FBF	97.71		97.71

Cpd 76: 7Z-Tricosen-11-one

Name	Formula	RT	RI	Mass	Diff (Tgt, ppm)	CAS	ID Source	Score	Algorithm
7Z-Tricosen-11-one	C23 H44 O	56.477		336.3397	1.32		M-FBF	97.69	FBF

Species	m/z	Score (Tgt)	Score (Lib)	Score (DB)	Score (MFG)	Score (RT)
(M+H)+	337	97.69				

Compound Chromatograms (overlaid)

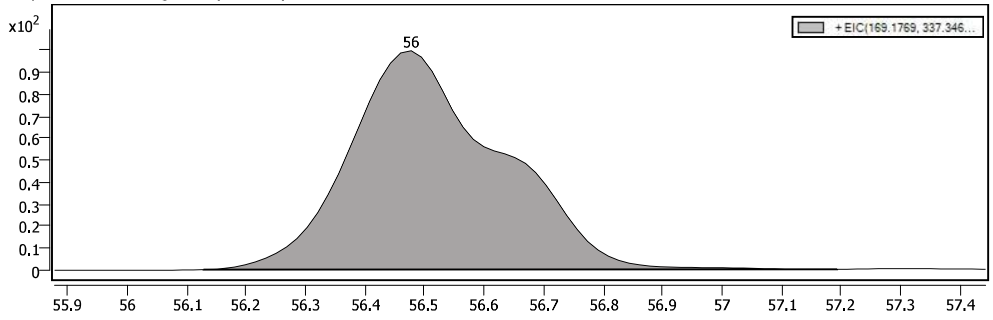

Counts (%) vs. Acquisition Time (min)

Structure

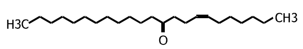

Compound Spectra (overlaid)

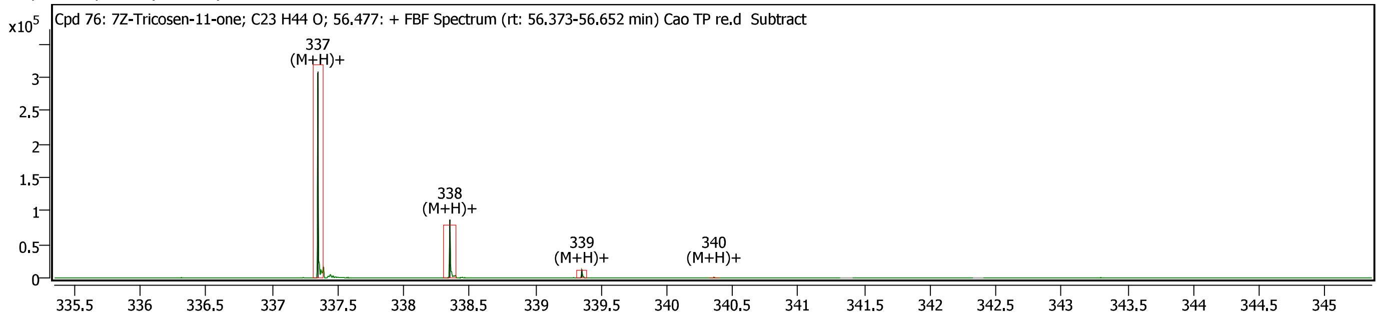

Compound ID Table

Name	Formula	Species	RT	RT Diff	Mass	CAS	ID Source	Score	Score (Lib)	Score (Tgt)
7Z-Tricosen-11-one	C23 H44 O	(M+H)+	56.477		336.3397		FBF	97.69		97.69
14Z-Tricosene-10-one	C23 H44 O	(M+H)+	56.477		336.3397		FBF	97.69		97.69

Cpd 864: MGDG(20:5(5Z,8Z,11Z,14Z,17Z)/18:3(9Z,12Z,15Z))

Name	Formula	RT	RI	Mass	Diff (Tgt, ppm)	CAS	ID Source	Score	Algorithm
MGDG(20:5(5Z,8Z,11Z,14Z,17Z)/ 18:3(9Z,12Z,15Z))	C47 H74 O10	56.896		798.5260	-2.69		FBF	96.44	FBF

Species	m/z	Score (Tgt)	Score (Lib)	Score (DB)	Score (MFG)	Score (RT)
(M+H)+	800	96.44				

Compound Chromatograms (overlaid)

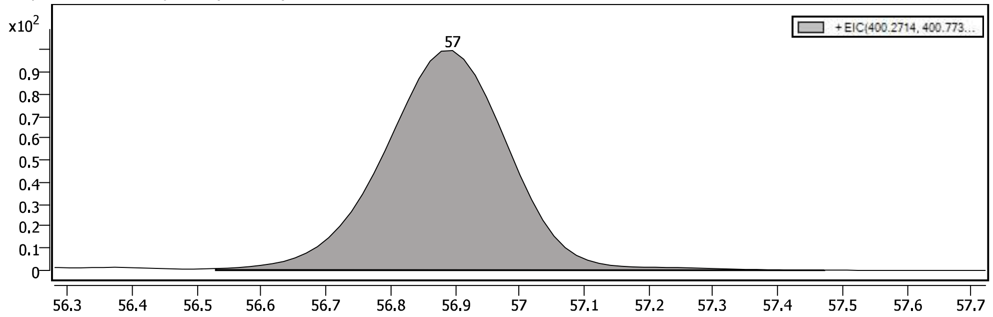

Structure

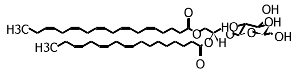

Compound Spectra (overlaid)

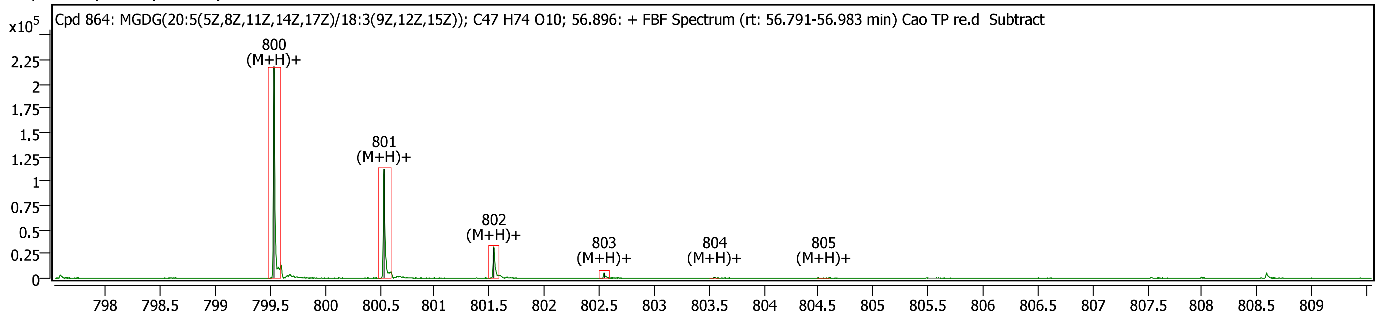

Compound ID Table

Name	Formula	Species	RT	RT Diff	Mass	CAS	ID Source	Score	Score (Lib)	Score (Tgt)
MGDG(20:5(5Z,8Z,11Z,14Z,17Z)/ 18:3(9Z,12Z,15Z))	C47 H74 O10	(M+H)+	56.896		798.5260		FBF	96.44		96.44

Cpd 12: 20:3-Glc-Campesterol

Name	Formula	RT	RI	Mass	Diff (Tgt, ppm)	CAS	ID Source	Score	Algorithm
20:3-Glc-Campesterol	C54 H90 O7	57.158		850.6686	-0.02		FBF	95.67	FBF

Species	m/z	Score (Tgt)	Score (Lib)	Score (DB)	Score (MFG)	Score (RT)
(M+H)+	852	95.67				

Compound Screening Report

Compound Chromatograms (overlaid)

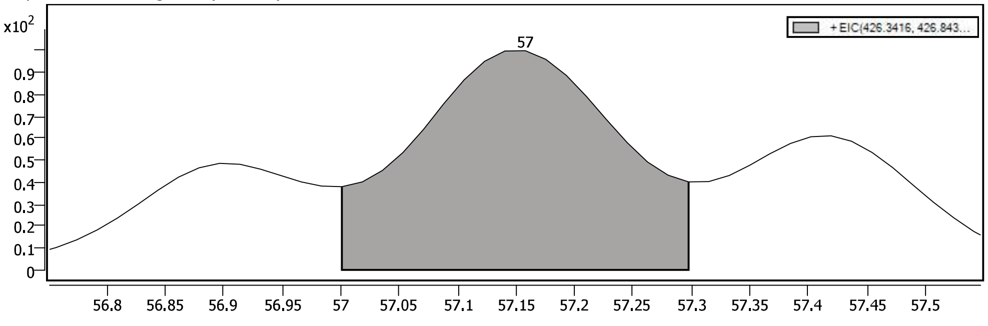

Structure

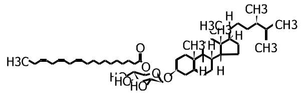

Compound Spectra (overlaid)

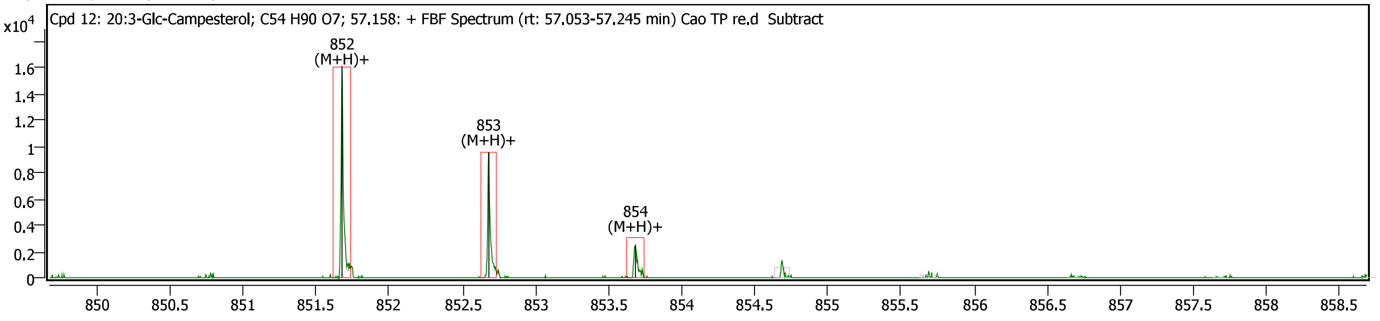

Compound ID Table

Name	Formula	Species	RT	RT Diff	Mass	CAS	ID Source	Score	Score (Lib)	Score (Tgt)
20:3-Glc-Campesterol	C54 H90 O7	(M+H)+	57.158		850.6686		FBF	95.67		95.67

Cpd 365: PC(P-20:0/22:4(7Z,10Z,13Z,16Z))

Name	Formula	RT	RI	Mass	Diff (Tgt, ppm)	CAS	ID Source	Score	Algorithm
PC(P-20:0/22:4(7Z,10Z,13Z,16Z))	C50 H93 N O7 P	57.158		850.6687	-0.36		FBF	95.66	FBF

Species	m/z	Score (Tgt)	Score (Lib)	Score (DB)	Score (MFG)	Score (RT)
(M+H)+	852	95.66				

Compound Chromatograms (overlaid)

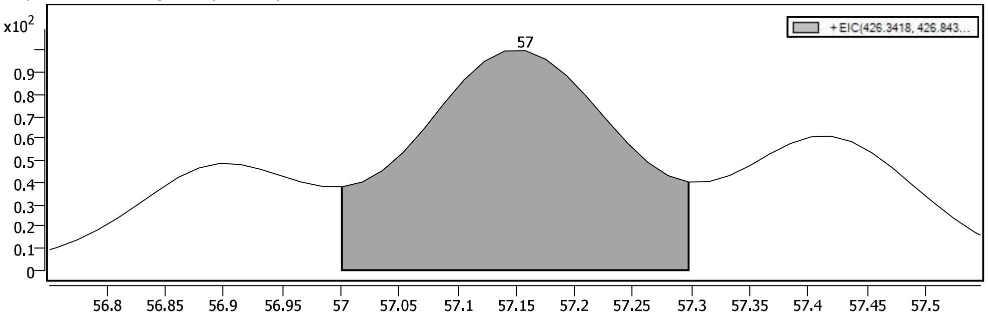

Structure

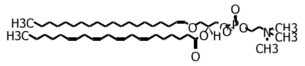

Compound Spectra (overlaid)

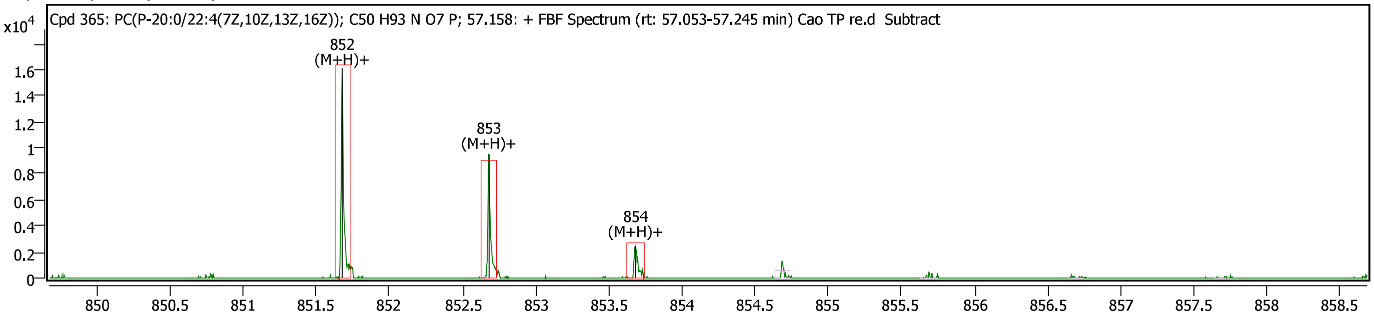

Compound ID Table

Name	Formula	Species	RT	RT Diff	Mass	CAS	ID Source	Score	Score (Lib)	Score (Tgt)
PC(P-20:0/22:4(7Z,10Z,13Z,16Z))	C50 H93 N O7 P	(M+H)+	57.158		850.6687		FBF	95.66		95.66

Cpd 263: PA(O-16:0/12:0)

Name	Formula	RT	RI	Mass	Diff (Tgt, ppm)	CAS	ID Source	Score	Algorithm
PA(O-16:0/12:0)	C31 H63 O7 P	57.385		578.4311	-0.09		FBF	94.43	FBF

Species	m/z	Score (Tgt)	Score (Lib)	Score (DB)	Score (MFG)	Score (RT)
(M+H)+	579	94.43				

Compound Chromatograms (overlaid)

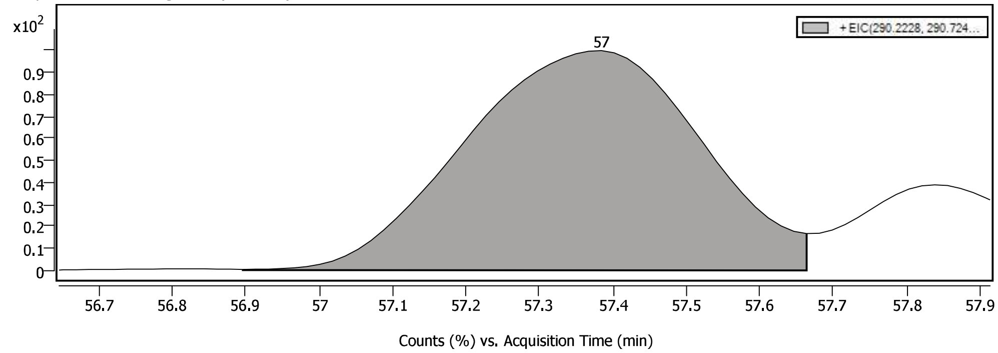

Structure

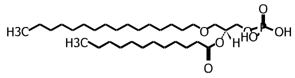

Compound Spectra (overlaid)

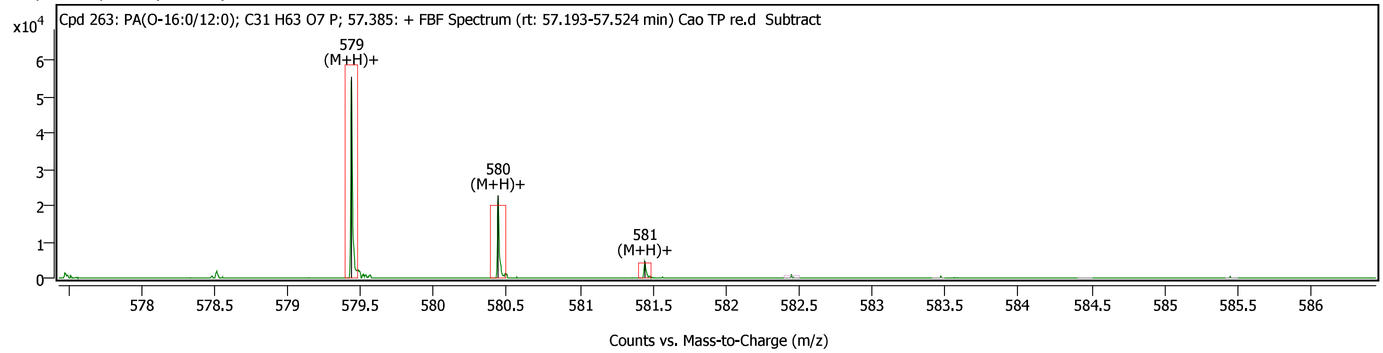

Compound ID Table

Name	Formula	Species	RT	RT Diff	Mass	CAS	ID Source	Score	Score (Lib)	Score (Tgt)
PA(O-16:0/12:0)	C31 H63 O7 P	(M+H)+	57.385		578.4311		FBF	94.43		94.43

Cpd 1491: 2-Hexaprenyl-3-methyl-5-hydroxy-6-methoxy-1,4-benzoquinol

Name	Formula	RT	RI	Mass	Diff (Tgt, ppm)	CAS	ID Source	Score	Algorithm
2-Hexaprenyl-3-methyl-5-hydroxy-6-methoxy-1,4-benzoquinol	C38 H58 O4	57.385		578.4311	-4.22		FBF	91.87	FBF

Species	m/z	Score (Tgt)	Score (Lib)	Score (DB)	Score (MFG)	Score (RT)
(M+H)+	579	91.87				

Compound Chromatograms (overlaid)

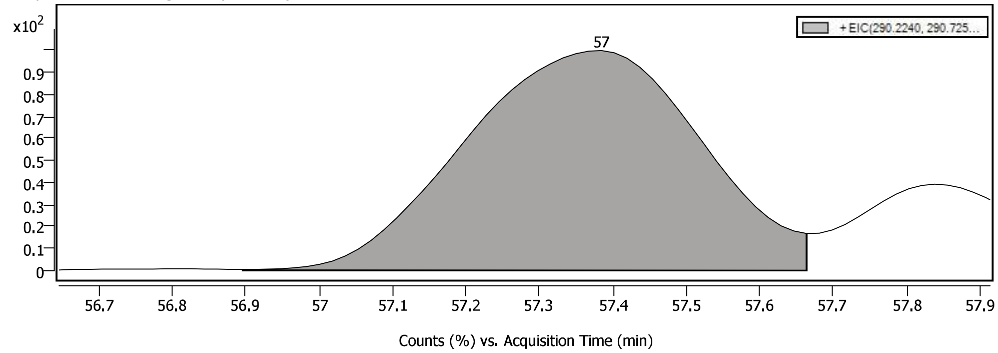

Structure

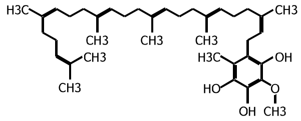

Compound Spectra (overlaid)

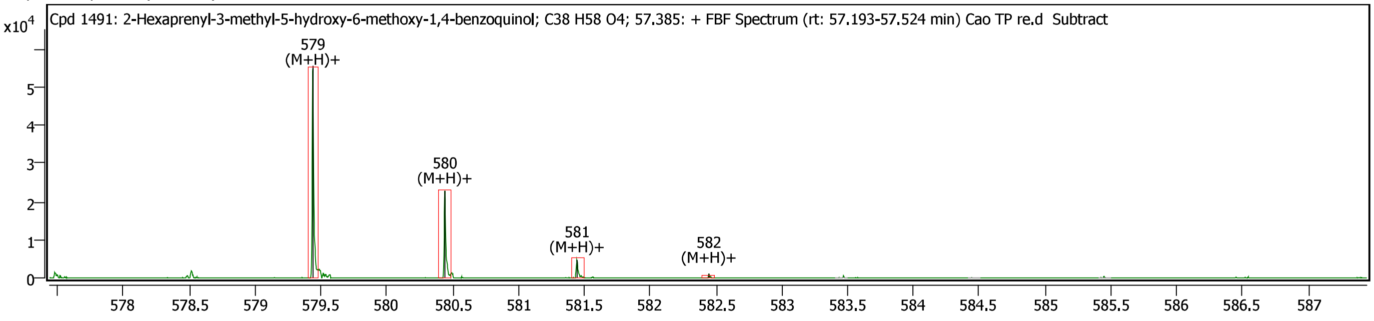

Compound ID Table

Name	Formula	Species	RT	RT Diff	Mass	CAS	ID Source	Score	Score (Lib)	Score (Tgt)
2-Hexaprenyl-3-methyl-5-hydroxy-6-methoxy-1,4-benzoquinol	C38 H58 O4	(M+H)+	57.385		578.4311		FBF	91.87		91.87

Cpd 355: PE(22:2(13Z,16Z)/15:1(9Z))

Name	Formula	RT	RI	Mass	Diff (Tgt, ppm)	CAS	ID Source	Score	Algorithm
PE(22:2(13Z,16Z)/15:1(9Z))	C42 H78 N O8 P	58.275		755.5480	1.93		M-FBF	97.40	FBF

Species	m/z	Score (Tgt)	Score (Lib)	Score (DB)	Score (MFG)	Score (RT)
(M+H)+	757	97.40				

Compound Chromatograms (overlaid)

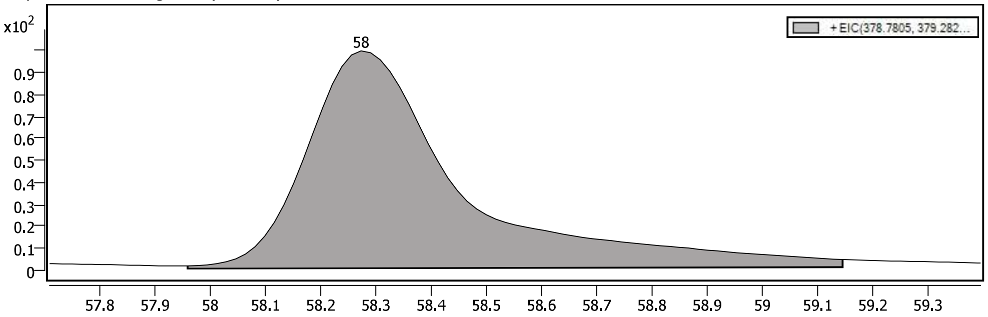

Structure

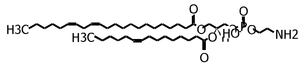

Compound Spectra (overlaid)

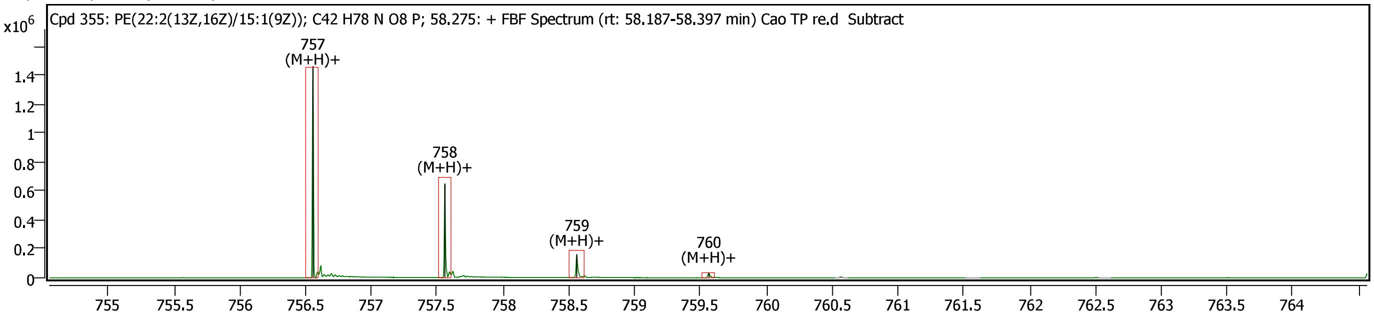

Compound ID Table

Name	Formula	Species	RT	RT Diff	Mass	CAS	ID Source	Score	Score (Lib)	Score (Tgt)
PE(22:2(13Z,16Z)/15:1(9Z))	C42 H78 N O8 P	(M+H)+	58.275		755.5480		FBF	97.40		97.40
PE(18:3(9Z,12Z,15Z)/19:0)	C42 H78 N O8 P	(M+H)+	58.275		755.5480		FBF	97.40		97.40
PE(15:1(9Z)/22:2(13Z,16Z))	C42 H78 N O8 P	(M+H)+	58.275		755.5480		FBF	97.40		97.40
PE(17:0/20:3(8Z,11Z,14Z))	C42 H78 N O8 P	(M+H)+	58.275		755.5480		FBF	97.40		97.40
PE(17:1(9Z)/20:2(11Z,14Z))	C42 H78 N O8 P	(M+H)+	58.275		755.5480		FBF	97.40		97.40
PE(17:2(9Z,12Z)/20:1(11Z))	C42 H78 N O8 P	(M+H)+	58.275		755.5480		FBF	97.40		97.40
PE(18:2(9Z,12Z)/19:1(9Z))	C42 H78 N O8 P	(M+H)+	58.275		755.5480		FBF	97.40		97.40
PE(18:3(6Z,9Z,12Z)/19:0)	C42 H78 N O8 P	(M+H)+	58.275		755.5480		FBF	97.40		97.40
PE(19:0/18:3(6Z,9Z,12Z))	C42 H78 N O8 P	(M+H)+	58.275		755.5480		FBF	97.40		97.40
PE(19:1(9Z)/18:2(9Z,12Z))	C42 H78 N O8 P	(M+H)+	58.275		755.5480		FBF	97.40		97.40
PE(20:1(11Z)/17:2(9Z,12Z))	C42 H78 N O8 P	(M+H)+	58.275		755.5480		FBF	97.40		97.40
PE(20:2(11Z,14Z)/17:1(9Z))	C42 H78 N O8 P	(M+H)+	58.275		755.5480		FBF	97.40		97.40
PE(20:3(8Z,11Z,14Z)/17:0)	C42 H78 N O8 P	(M+H)+	58.275		755.5480		FBF	97.40		97.40
PE(19:0/18:3(9Z,12Z,15Z))	C42 H78 N O8 P	(M+H)+	58.275		755.5480		FBF	97.40		97.40

Cpd 351: PE(22:4(7Z,10Z,13Z,16Z)/17:0)

Name	Formula	RT	RI	Mass	Diff (Tgt, ppm)	CAS	ID Source	Score	Algorithm
PE(22:4(7Z,10Z,13Z,16Z)/17:0)	C44 H80 N O8 P	58.507		781.5639	2.29		M-FBF	96.63	FBF

Species	m/z	Score (Tgt)	Score (Lib)	Score (DB)	Score (MFG)	Score (RT)
(M+H)+	783	96.63				

Compound Chromatograms (overlaid)

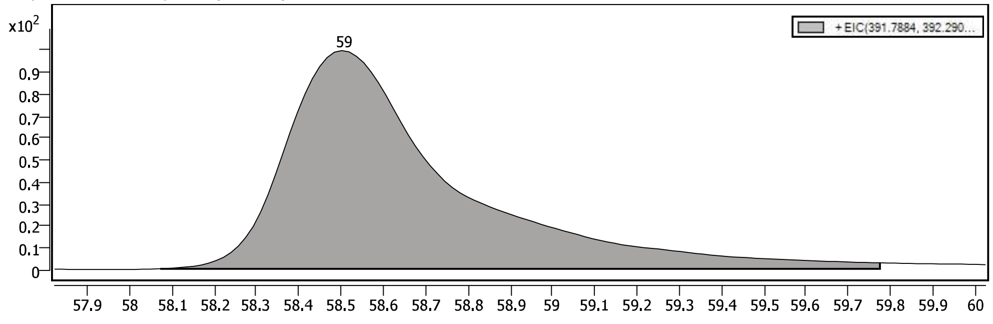

Structure

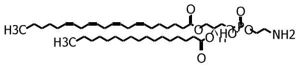

Compound Spectra (overlaid)

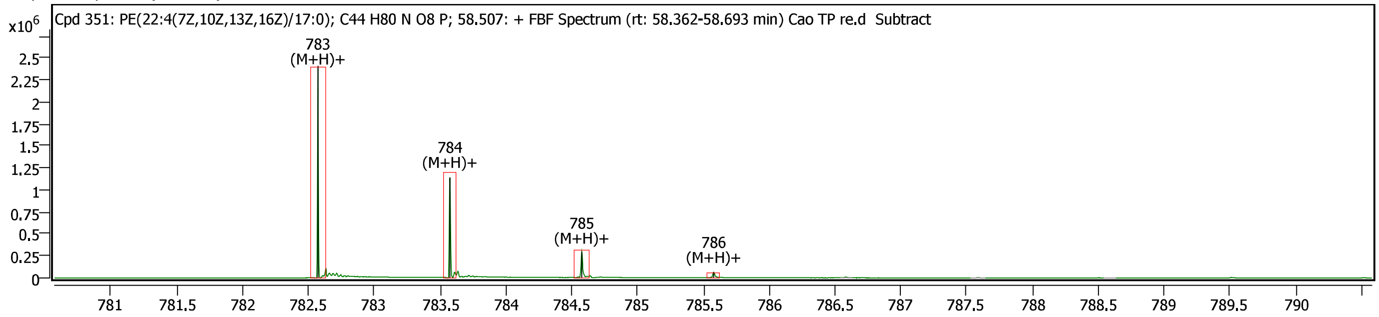

Compound ID Table

Name	Formula	Species	RT	RT Diff	Mass	CAS	ID Source	Score	Score (Lib)	Score (Tgt)
PE(22:4(7Z,10Z,13Z,16Z)/17:0)	C44 H80 N O8 P	(M+H)+	58.507		781.5639		FBF	96.63		96.63
PE(19:1(9Z)/20:3(8Z,11Z,14Z))	C44 H80 N O8 P	(M+H)+	58.507		781.5639		FBF	96.63		96.63
PE(17:0/22:4(7Z,10Z,13Z,16Z))	C44 H80 N O8 P	(M+H)+	58.507		781.5639		FBF	96.63		96.63
PE(17:2(9Z,12Z)/22:2(13Z,16Z))	C44 H80 N O8 P	(M+H)+	58.507		781.5639		FBF	96.63		96.63
PE(18:4(6Z,9Z,12Z,15Z)/21:0)	C44 H80 N O8 P	(M+H)+	58.507		781.5639		FBF	96.63		96.63
PE(19:0/20:4(5Z,8Z,11Z,14Z))	C44 H80 N O8 P	(M+H)+	58.507		781.5639		FBF	96.63		96.63
PE(20:3(8Z,11Z,14Z)/19:1(9Z))	C44 H80 N O8 P	(M+H)+	58.507		781.5639		FBF	96.63		96.63
PE(20:4(5Z,8Z,11Z,14Z)/19:0)	C44 H80 N O8 P	(M+H)+	58.507		781.5639		FBF	96.63		96.63
PE(21:0/18:4(6Z,9Z,12Z,15Z))	C44 H80 N O8 P	(M+H)+	58.507		781.5639		FBF	96.63		96.63
PE(22:2(13Z,16Z)/17:2(9Z,12Z))	C44 H80 N O8 P	(M+H)+	58.507		781.5639		FBF	96.63		96.63

Cpd 343: PE(19:1(9Z)/22:6(4Z,7Z,10Z,13Z,16Z,19Z))

Name	Formula	RT	RI	Mass	Diff (Tgt, ppm)	CAS	ID Source	Score	Algorithm
PE(19:1(9Z)/22:6(4Z,7Z,10Z,13Z, 16Z,19Z))	C46 H78 N O8 P	58.571		803.5445	-2.48		M-FBF	96.85	FBF

Species	m/z	Score (Tgt)	Score (Lib)	Score (DB)	Score (MFG)	Score (RT)
(M+H)+	805	96.85				

Compound Chromatograms (overlaid)

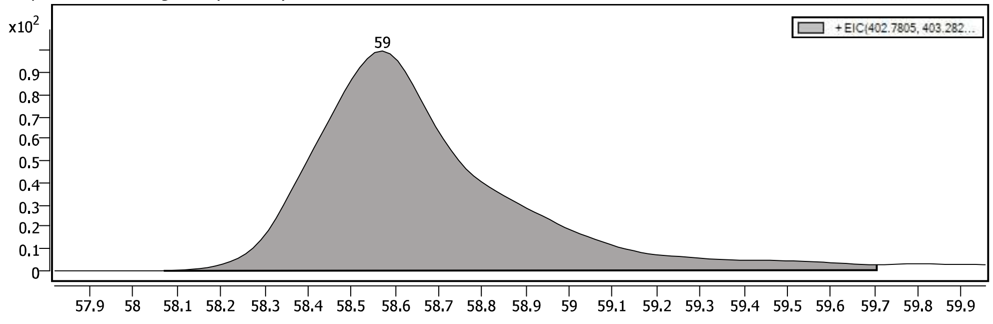

Structure

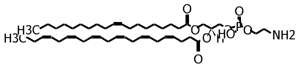

Compound Spectra (overlaid)

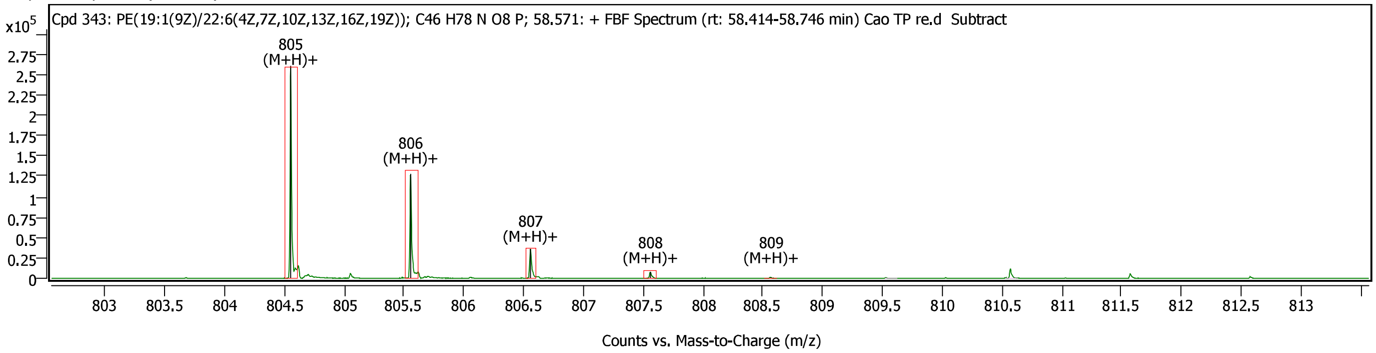

Compound ID Table

Name	Formula	Species	RT	RT Diff	Mass	CAS	ID Source	Score	Score (Lib)	Score (Tgt)
PE(19:1(9Z)/22:6(4Z,7Z,10Z,13Z, 16Z,19Z))	C46 H78 N O8 P	(M+H)+	58.571		803.5445		FBF	96.85		96.85
PE(22:6(4Z,7Z,10Z,13Z,16Z,19Z)/ 19:1(9Z))	C46 H78 N O8 P	(M+H)+	58.571		803.5445		FBF	96.85		96.85

Cpd 1468: DG(18:4(6Z,9Z,12Z,15Z)/20:5(5Z,8Z,11Z,14Z,17Z)/0:0)

Name	Formula	RT	RI	Mass	Diff (Tgt, ppm)	CAS	ID Source	Score	Algorithm
DG(18:4(6Z,9Z,12Z,15Z)/20:5(5Z, 8Z,11Z,14Z,17Z)/0:0)	C41 H62 O5	59.129		634.4575	-3.43		M-FBF	94.67	FBF

Species	m/z	Score (Tgt)	Score (Lib)	Score (DB)	Score (MFG)	Score (RT)
(M+H)+	635	94.67				

Compound Chromatograms (overlaid)

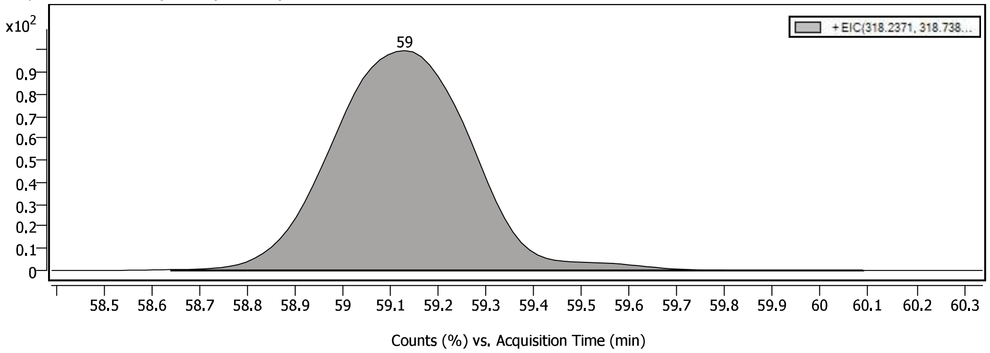

Structure

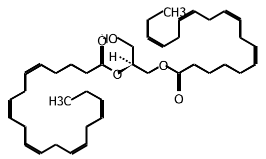

Compound Spectra (overlaid)

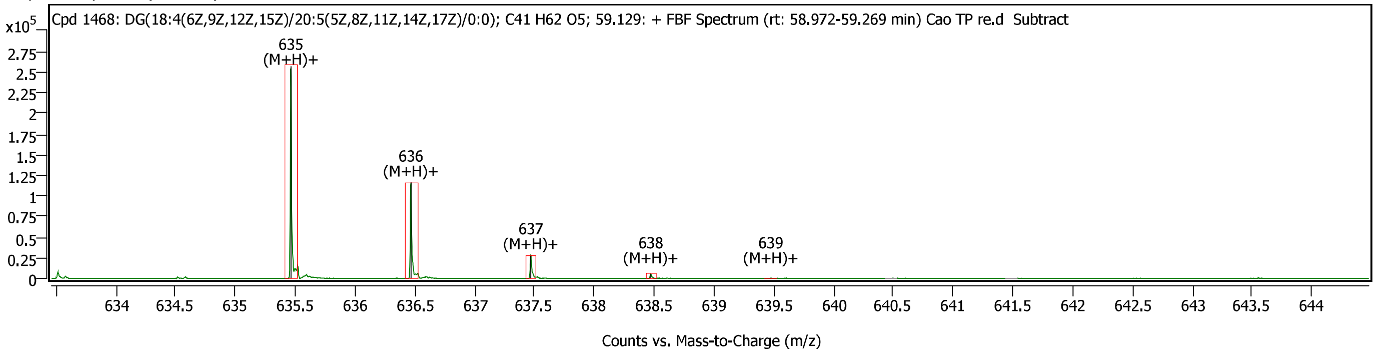

Compound ID Table

Name	Formula	Species	RT	RT Diff	Mass	CAS	ID Source	Score	Score (Lib)	Score (Tgt)
DG(18:4(6Z,9Z,12Z,15Z)/20:5(5Z, 8Z,11Z,14Z,17Z)/0:0)	C41 H62 O5	(M+H)+	59.129		634.4575		FBF	94.67		94.67
DG(20:5(5Z,8Z,11Z,14Z,17Z)/18: 4(6Z,9Z,12Z,15Z)/0:0)	C41 H62 O5	(M+H)+	59.129		634.4575		FBF	94.67		94.67

Cpd 13: 18:1-Glc-Campesterol

Name	Formula	RT	RI	Mass	Diff (Tgt, ppm)	CAS	ID Source	Score	Algorithm
18:1-Glc-Campesterol	C52 H90 O7	59.409		826.6680	-0.76		M-FBF	97.23	FBF

Species	m/z	Score (Tgt)	Score (Lib)	Score (DB)	Score (MFG)	Score (RT)
(M+H)+	828	97.23				

Compound Chromatograms (overlaid)

Structure

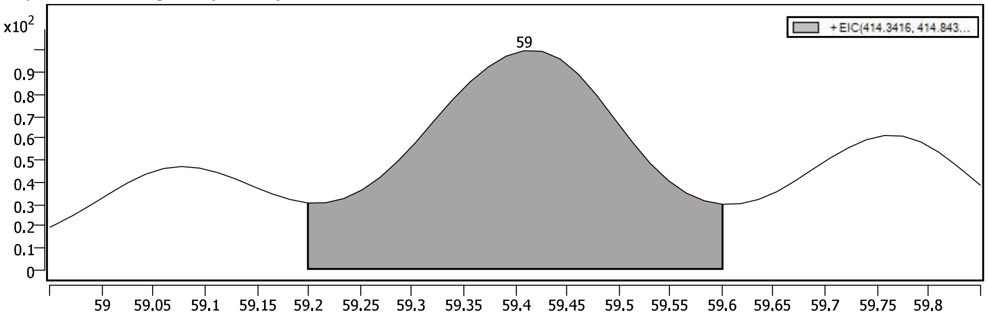

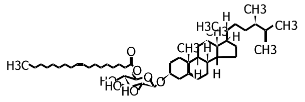

Compound Spectra (overlaid)

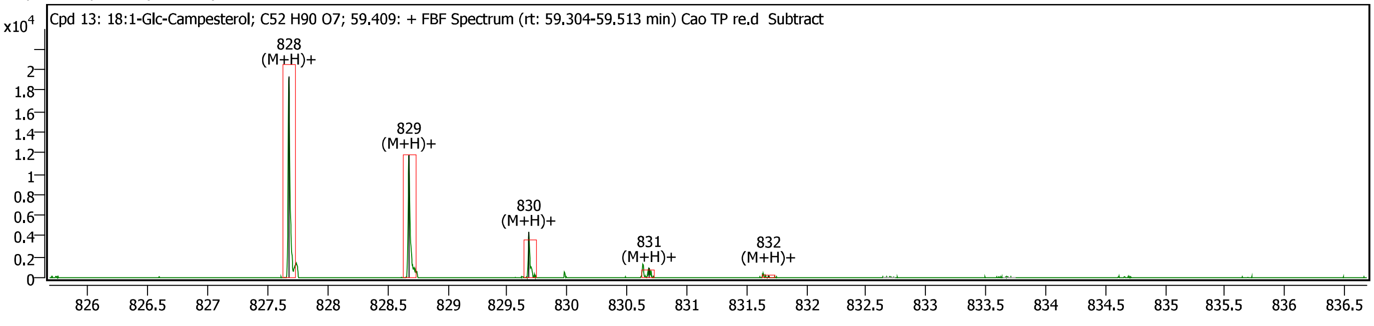

Compound ID Table

Name	Formula	Species	RT	RT Diff	Mass	CAS	ID Source	Score	Score (Lib)	Score (Tgt)
18:1-Glc-Campesterol	C52 H90 O7	(M+H)+	59.409		826.6680		FBF	97.23		97.23
Campesterol 6'-(9Z-octadecenoyl)-glucoside	C52 H90 O7	(M+H)+	59.409		826.6680	87168-08-9	FBF	97.23		97.23

Cpd 244: cholest-5,24-dien-3beta-ol 3-O-beta-D-glucopyranoside

Name	Formula	RT	RI	Mass	Diff (Tgt, ppm)	CAS	ID Source	Score	Algorithm
cholest-5,24-dien-3beta-ol 3-O-beta-D-glucopyranoside	C33 H54 O6	59.548		546.3921	0.10		FBF	99.54	FBF

Species	m/z	Score (Tgt)	Score (Lib)	Score (DB)	Score (MFG)	Score (RT)
(M+H)+	547	99.54				

Compound Chromatograms (overlaid)

Structure

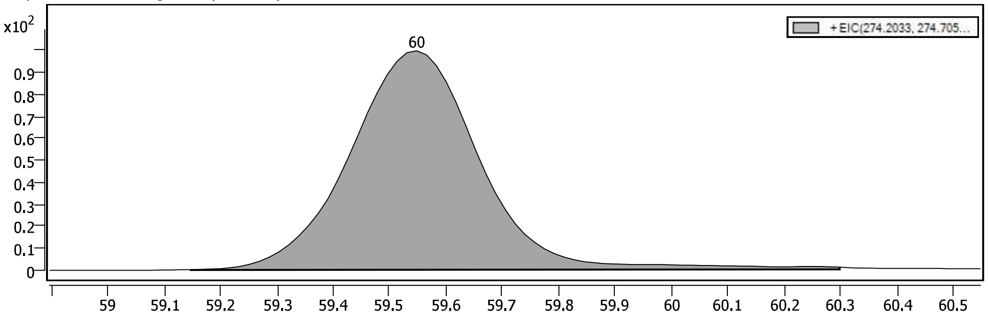

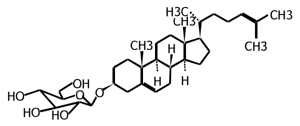

Compound Spectra (overlaid)

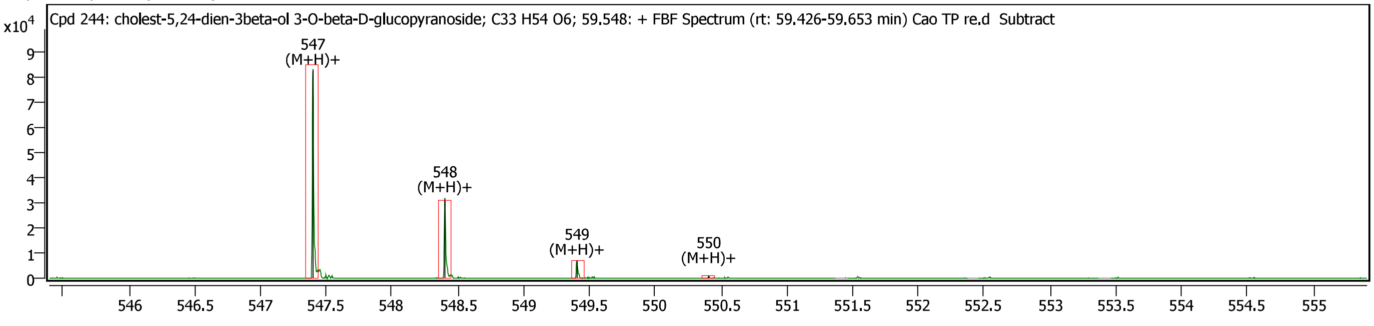

Compound Screening Report

Compound ID Table

Name	Formula	Species	RT	RT Diff	Mass	CAS	ID Source	Score	Score (Lib)	Score (Tgt)
cholest-5,24-dien-3beta-ol 3-O-beta-D-glucopyranoside	C33 H54 O6	(M+H)+	59.548		546.3921		FBF	99.54		99.54

Cpd 1666: endo-1,4-beta-Xylanase

Name	Formula	RT	RI	Mass	Diff (Tgt, ppm)	CAS	ID Source	Score	Algorithm
endo-1,4-beta-Xylanase	C34 H50 N4 O2	59.548		546.3922	-2.20	9025-57-4	FBF	97.55	FBF

Species	m/z	Score (Tgt)	Score (Lib)	Score (DB)	Score (MFG)	Score (RT)
(M+H)+	547	97.55				

Compound Chromatograms (overlaid)

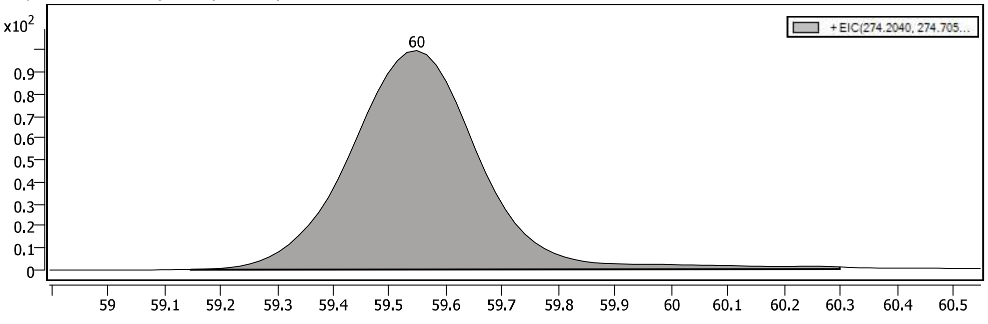

Structure

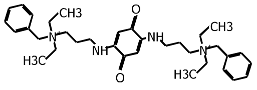

Compound Spectra (overlaid)

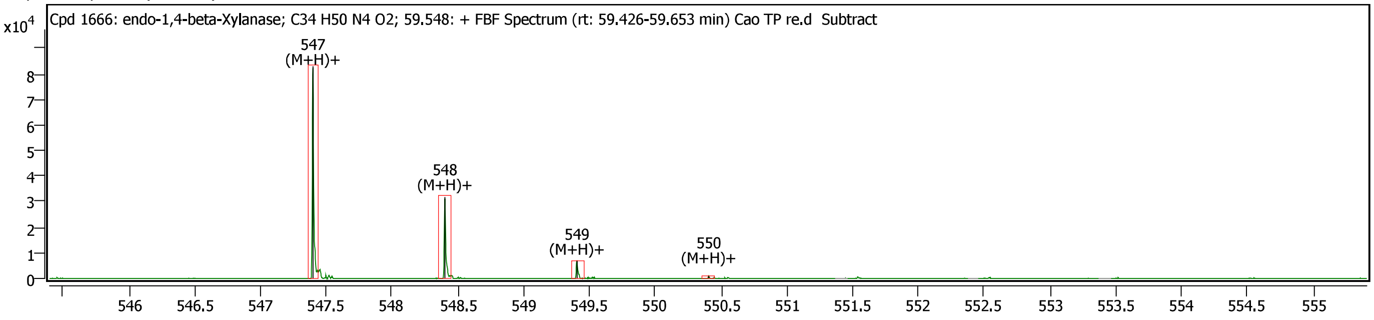

Compound ID Table

Name	Formula	Species	RT	RT Diff	Mass	CAS	ID Source	Score	Score (Lib)	Score (Tgt)
endo-1,4-beta-Xylanase	C34 H50 N4 O2	(M+H)+	59.548		546.3922	9025-57-4	FBF	97.55		97.55

Cpd 36: Cer(d14:2(4E,6E)/22:0(2OH))

Name	Formula	RT	RI	Mass	Diff (Tgt, ppm)	CAS	ID Source	Score	Algorithm
Cer(d14:2(4E,6E)/22:0(2OH))	C36 H69 N O4	60.421		579.5227	0.09		M-FBF	99.48	FBF

Species	m/z	Score (Tgt)	Score (Lib)	Score (DB)	Score (MFG)	Score (RT)
(M+H)+	581	99.48				

Compound Chromatograms (overlaid)

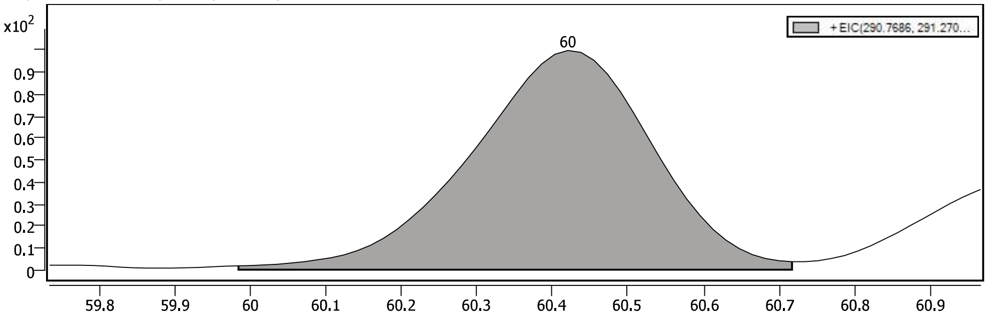

Structure

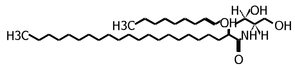

Compound Spectra (overlaid)

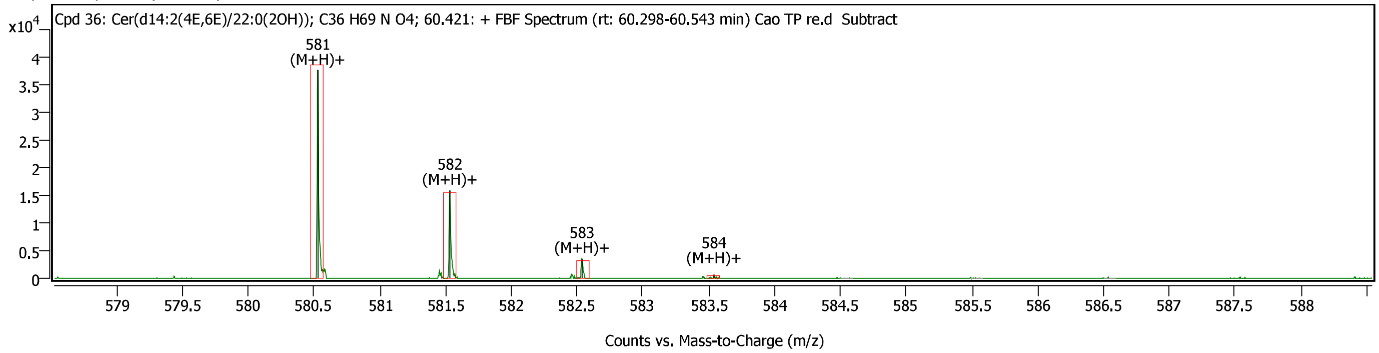

Compound ID Table

Name	Formula	Species	RT	RT Diff	Mass	CAS	ID Source	Score	Score (Lib)	Score (Tgt)
Cer(d14:2(4E,6E)/22:0(2OH))	C36 H69 N O4	(M+H)+	60.421		579.5227		FBF	99.48		99.48
Cer(d16:2(4E,6E)/20:0(2OH))	C36 H69 N O4	(M+H)+	60.421		579.5227		FBF	99.48		99.48
Cer(d14:1(4E)/22:1(13Z)(2OH))	C36 H69 N O4	(M+H)+	60.421		579.5227		FBF	99.48		99.48

Cpd 362: 1-Palmitoyl-2-linoleoyl PE

Name	Formula	RT	RI	Mass	Diff (Tgt, ppm)	CAS	ID Source	Score	Algorithm
1-Palmitoyl-2-linoleoyl PE	C39 H74 N O8 P	61.246		715.5154	0.24	26662-95-3	FBF-FragConfirm	99.89	FBF

Species	m/z	Score (Tgt)	Score (Lib)	Score (DB)	Score (MFG)	Score (RT)
(M+H)+	717	99.89				

Compound Chromatograms (overlaid)

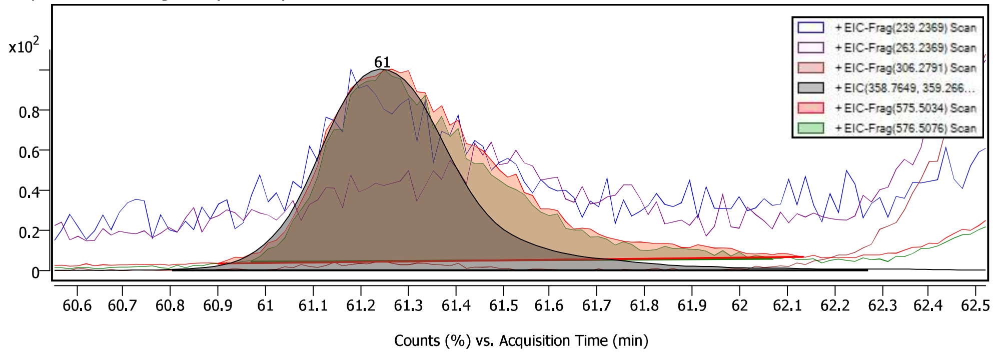

Structure

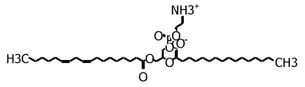

Coelution Plot

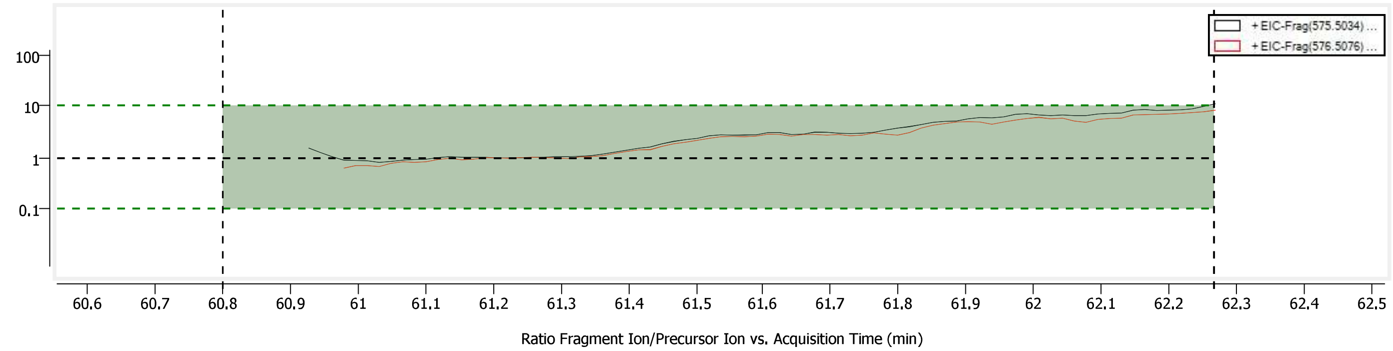

Compound Spectra (overlaid)

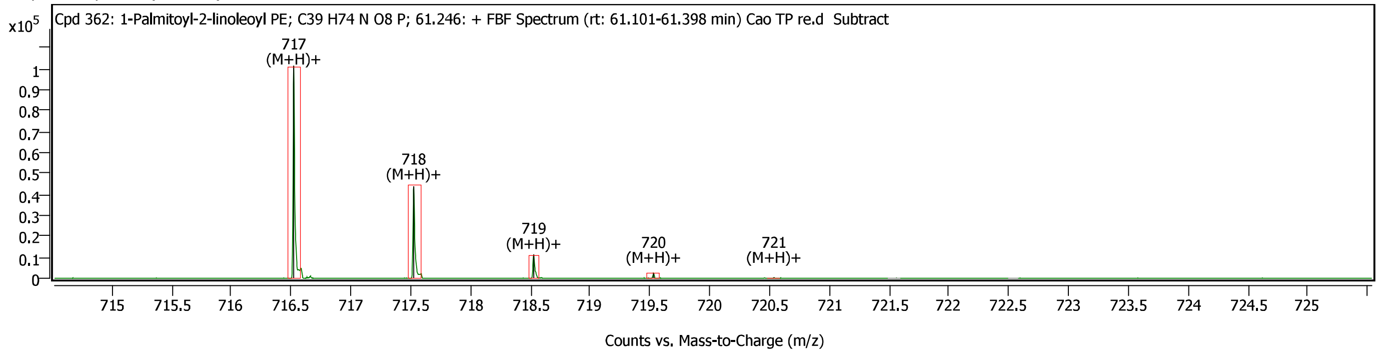

Compound Screening Report

Fragment Spectrum (clean)

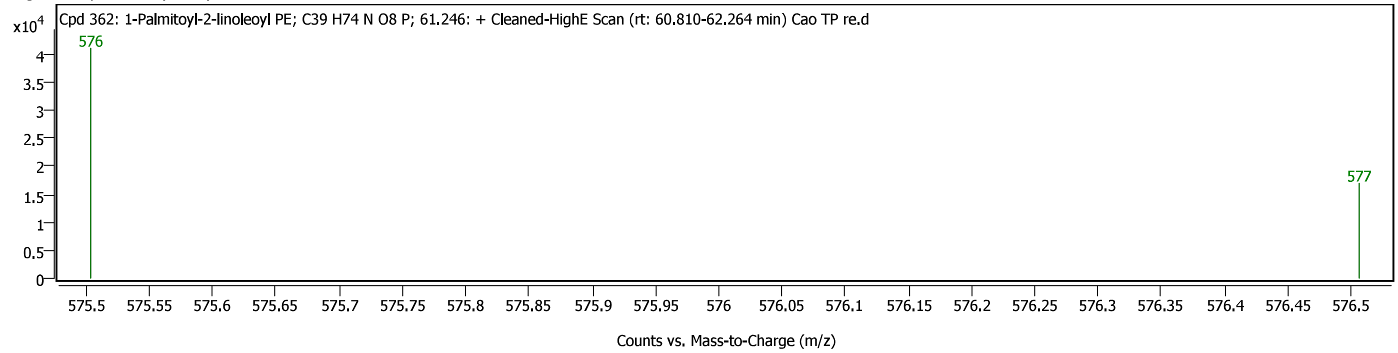

Fragment Spectrum (raw)

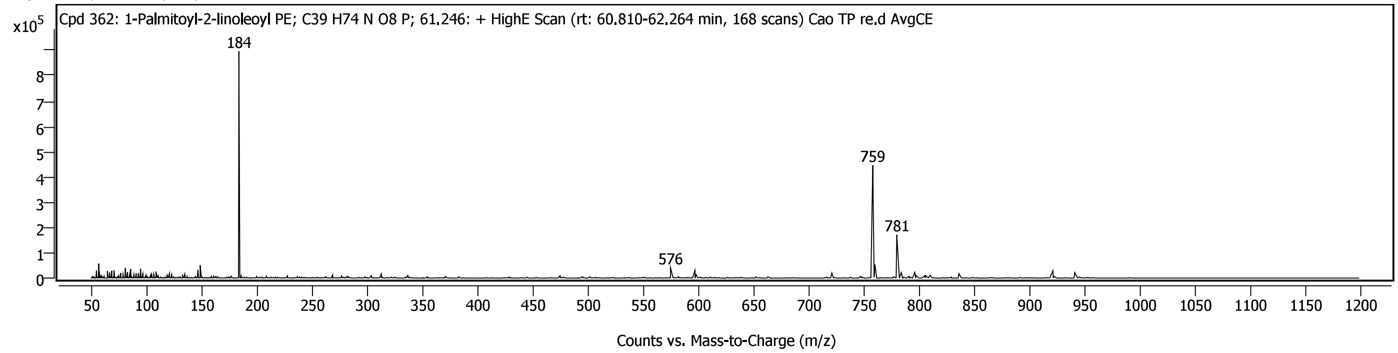

Compound ID Table

Name	Formula	Species	RT	RT Diff	Mass	CAS	ID Source	Score	Score (Lib)	Score (Tgt)
1-Palmitoyl-2-linoleoyl PE	C39 H74 N O8 P	(M+H)+	61.246		715.5154	26662-95-3	FBF-FragConfirm	99.89		99.89
PE(17:0/17:2(9Z,12Z))	C39 H74 N O8 P	(M+H)+	61.246		715.5154		FBF-FragConfirm	99.89		99.89
PE(16:1(9Z)/18:1(9Z))	C39 H74 N O8 P	(M+H)+	61.246		715.5154		FBF-FragConfirm	99.89		99.89
PE(20:2(11Z,14Z)/14:0)	C39 H74 N O8 P	(M+H)+	61.246		715.5154		FBF-FragConfirm	99.89		99.89
PE(12:0/22:2(13Z,16Z))	C39 H74 N O8 P	(M+H)+	61.246		715.5154		FBF-FragConfirm	99.89		99.89
PE(14:0/20:2(11Z,14Z))	C39 H74 N O8 P	(M+H)+	61.246		715.5154		FBF-FragConfirm	99.89		99.89
PE(14:1(9Z)/20:1(11Z))	C39 H74 N O8 P	(M+H)+	61.246		715.5154		FBF-FragConfirm	99.89		99.89
PE(16:0/18:2(9Z,12Z))	C39 H74 N O8 P	(M+H)+	61.246		715.5154		FBF-FragConfirm	99.89		99.89
PE(16:1(9Z)/18:1(11Z))	C39 H74 N O8 P	(M+H)+	61.246		715.5154		FBF-FragConfirm	99.89		99.89
PE(15:1(9Z)/19:1(9Z))	C39 H74 N O8 P	(M+H)+	61.246		715.5154		FBF-FragConfirm	99.89		99.89
PE(20:1(11Z)/14:1(9Z))	C39 H74 N O8 P	(M+H)+	61.246		715.5154		FBF-FragConfirm	99.89		99.89
PE(17:1(9Z)/17:1(9Z))	C39 H74 N O8 P	(M+H)+	61.246		715.5154		FBF-FragConfirm	99.89		99.89
PE(18:1(11Z)/16:1(9Z))	C39 H74 N O8 P	(M+H)+	61.246		715.5154		FBF-FragConfirm	99.89		99.89
PE(18:1(9Z)/16:1(9Z))	C39 H74 N O8 P	(M+H)+	61.246		715.5154		FBF-FragConfirm	99.89		99.89
PE(22:2(13Z,16Z)/12:0)	C39 H74 N O8 P	(M+H)+	61.246		715.5154		FBF-FragConfirm	99.89		99.89
PE(18:2(9Z,12Z)/16:0)	C39 H74 N O8 P	(M+H)+	61.246		715.5154		FBF-FragConfirm	99.89		99.89
PE(19:1(9Z)/15:1(9Z))	C39 H74 N O8 P	(M+H)+	61.246		715.5154		FBF-FragConfirm	99.89		99.89
PE(17:2(9Z,12Z)/17:0)	C39 H74 N O8 P	(M+H)+	61.246		715.5154		FBF-FragConfirm	99.89		99.89

Cpd 1005: C16-OH Sulfatide

Name	Formula	RT	RI	Mass	Diff (Tgt, ppm)	CAS	ID Source	Score	Algorithm
C16-OH Sulfatide	C40 H77 N O12 S	61.293		795.5182	1.93		FBF	97.35	FBF

Species	m/z	Score (Tgt)	Score (Lib)	Score (DB)	Score (MFG)	Score (RT)
(M+2H)+2 (M+H)+	399 797	97.35				

Compound Chromatograms (overlaid)

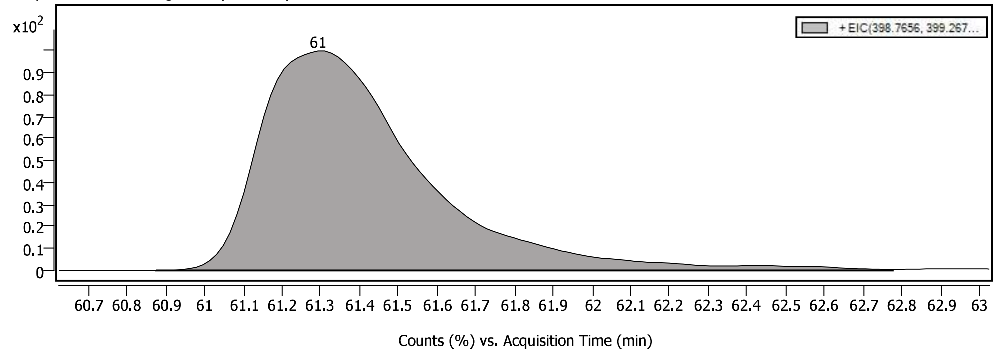

Structure

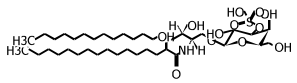

Compound Spectra (overlaid)

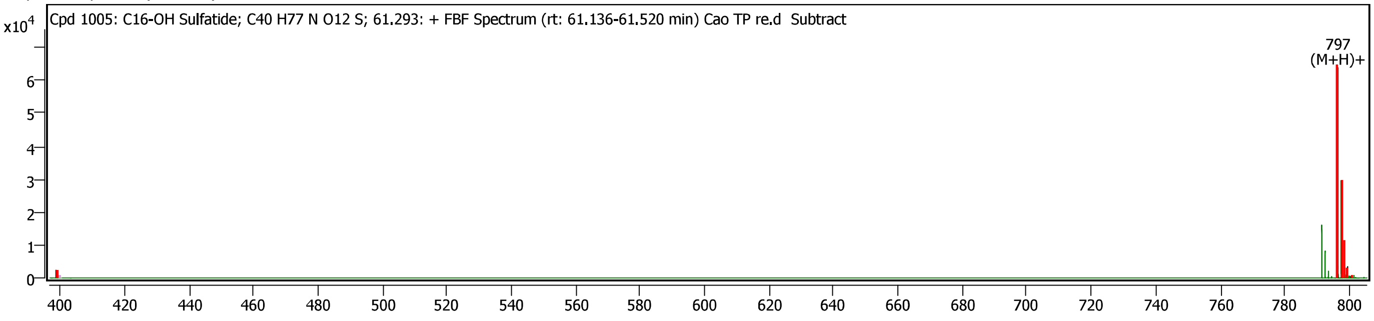

Compound ID Table

Name	Formula	Species	RT	RT Diff	Mass	CAS	ID Source	Score	Score (Lib)	Score (Tgt)
C16-OH Sulfatide	C40 H77 N O12 S	(M+2H)+2 (M+H)+	61.293		795.5182		FBF	97.35		97.35

Cpd 348: PE(19:1(9Z)/20:4(5Z,8Z,11Z,14Z))

Name	Formula	RT	RI	Mass	Diff (Tgt, ppm)	CAS	ID Source	Score	Algorithm
PE(19:1(9Z)/20:4(5Z,8Z,11Z,14Z))	C44 H78 N O8 P	61.328		779.5445	-2.53		M-FBF	96.20	FBF

Species	m/z	Score (Tgt)	Score (Lib)	Score (DB)	Score (MFG)	Score (RT)
(M+H)+	781	96.20				

Compound Chromatograms (overlaid)

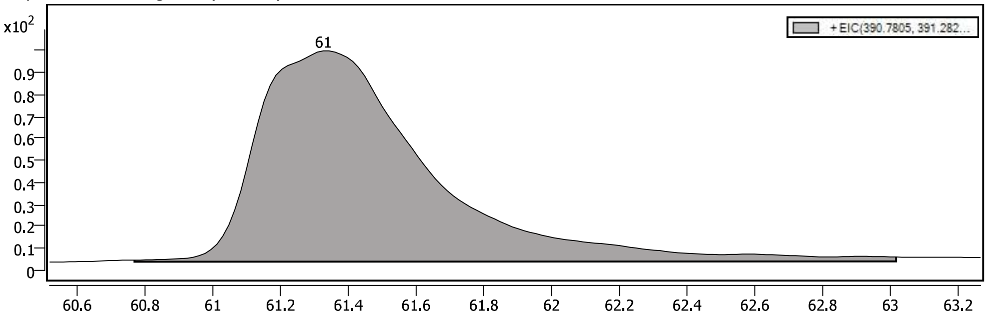

Structure

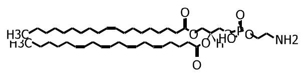

Compound Spectra (overlaid)

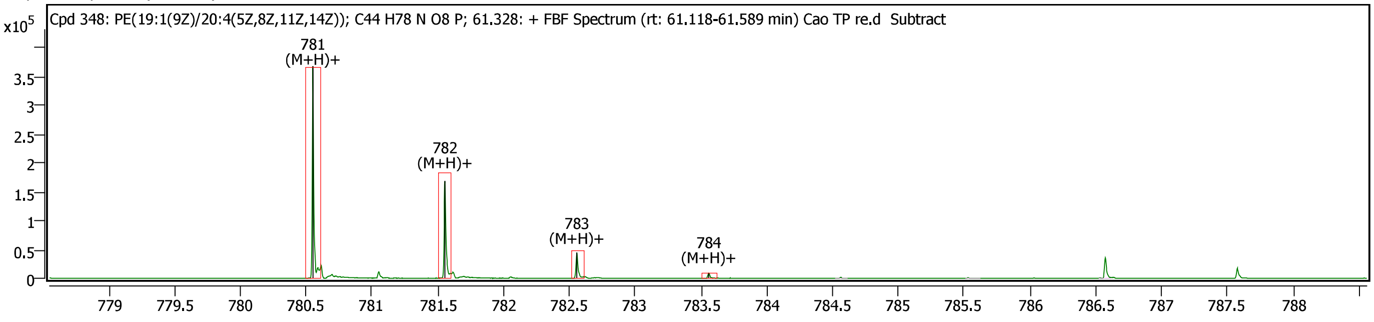

Compound ID Table

Name	Formula	Species	RT	RT Diff	Mass	CAS	ID Source	Score	Score (Lib)	Score (Tgt)
PE(19:1(9Z)/20:4(5Z,8Z,11Z,14Z))	C44 H78 N O8 P	(M+H)+	61.328		779.5445		FBF	96.20		96.20
PE(17:1(9Z)/22:4(7Z,10Z,13Z,16Z))	C44 H78 N O8 P	(M+H)+	61.328		779.5445		FBF	96.20		96.20
PE(19:0/20:5(5Z,8Z,11Z,14Z,17Z))	C44 H78 N O8 P	(M+H)+	61.328		779.5445		FBF	96.20		96.20
PE(20:5(5Z,8Z,11Z,14Z,17Z)/19:0)	C44 H78 N O8 P	(M+H)+	61.328		779.5445		FBF	96.20		96.20
PE(20:4(5Z,8Z,11Z,14Z)/19:1(9Z))	C44 H78 N O8 P	(M+H)+	61.328		779.5445		FBF	96.20		96.20
PE(22:4(7Z,10Z,13Z,16Z)/17:1(9Z))	C44 H78 N O8 P	(M+H)+	61.328		779.5445		FBF	96.20		96.20

Cpd 352: PE(22:1(11Z)/17:2(9Z,12Z))

Name	Formula	RT	RI	Mass	Diff (Tgt, ppm)	CAS	ID Source	Score	Algorithm
PE(22:1(11Z)/17:2(9Z,12Z))	C44 H82 N O8 P	62.218		783.5783	0.60		M-FBF	98.64	FBF

Species	m/z	Score (Tgt)	Score (Lib)	Score (DB)	Score (MFG)	Score (RT)
(M+H)+	785	98.64				

Compound Chromatograms (overlaid)

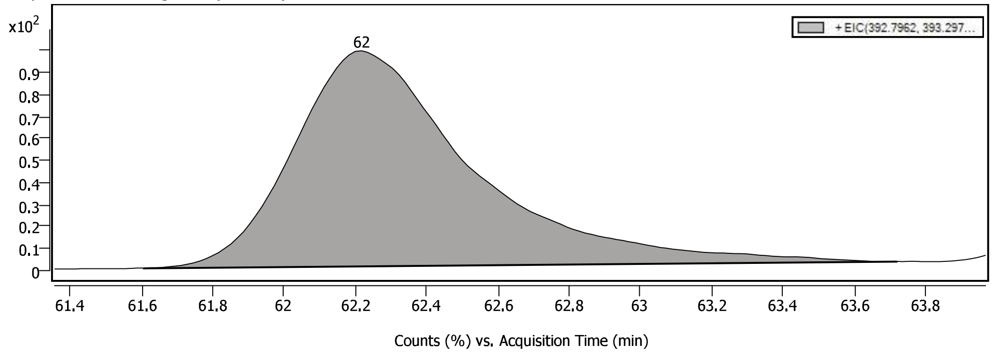

Structure

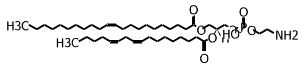

Compound Spectra (overlaid)

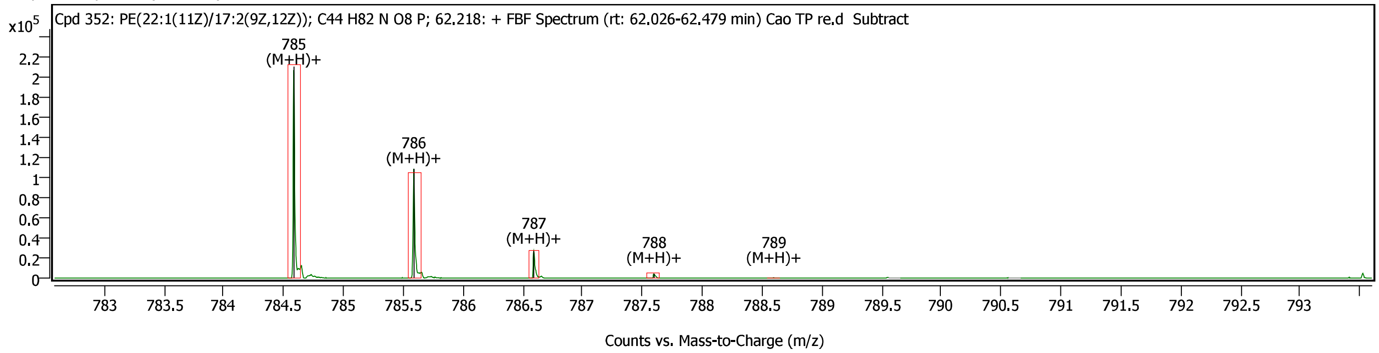

Compound ID Table

Name	Formula	Species	RT	RT Diff	Mass	CAS	ID Source	Score	Score (Lib)	Score (Tgt)
PE(22:1(11Z)/17:2(9Z,12Z))	C44 H82 N O8 P	(M+H)+	62.218		783.5783		FBF	98.64		98.64
PE(19:1(9Z)/20:2(11Z,14Z))	C44 H82 N O8 P	(M+H)+	62.218		783.5783		FBF	98.64		98.64
PE(17:1(9Z)/22:2(13Z,16Z))	C44 H82 N O8 P	(M+H)+	62.218		783.5783		FBF	98.64		98.64
PE(17:2(9Z,12Z)/22:1(11Z))	C44 H82 N O8 P	(M+H)+	62.218		783.5783		FBF	98.64		98.64
PE(18:3(6Z,9Z,12Z)/21:0)	C44 H82 N O8 P	(M+H)+	62.218		783.5783		FBF	98.64		98.64
PE(18:3(9Z,12Z,15Z)/21:0)	C44 H82 N O8 P	(M+H)+	62.218		783.5783		FBF	98.64		98.64
PE(19:0/20:3(8Z,11Z,14Z))	C44 H82 N O8 P	(M+H)+	62.218		783.5783		FBF	98.64		98.64
PE(21:0/18:3(6Z,9Z,12Z))	C44 H82 N O8 P	(M+H)+	62.218		783.5783		FBF	98.64		98.64
PE(20:2(11Z,14Z)/19:1(9Z))	C44 H82 N O8 P	(M+H)+	62.218		783.5783		FBF	98.64		98.64
PE(22:2(13Z,16Z)/17:1(9Z))	C44 H82 N O8 P	(M+H)+	62.218		783.5783		FBF	98.64		98.64
PE(21:0/18:3(9Z,12Z,15Z))	C44 H82 N O8 P	(M+H)+	62.218		783.5783		FBF	98.64		98.64
PE(20:3(8Z,11Z,14Z)/19:0)	C44 H82 N O8 P	(M+H)+	62.218		783.5783		FBF	98.64		98.64

Cpd 861: MGDG(18:2(9Z,12Z)/18:3(9Z,12Z,15Z))

Name	Formula	RT	RI	Mass	Diff (Tgt, ppm)	CAS	ID Source	Score	Algorithm
MGDG(18:2(9Z,12Z)/18:3(9Z,12Z, 15Z))	C45 H76 O10	62.566		776.5417	-2.77		FBF	94.65	FBF

Species	m/z	Score (Tgt)	Score (Lib)	Score (DB)	Score (MFG)	Score (RT)
(M+H)+	778	94.65				

Compound Chromatograms (overlaid)

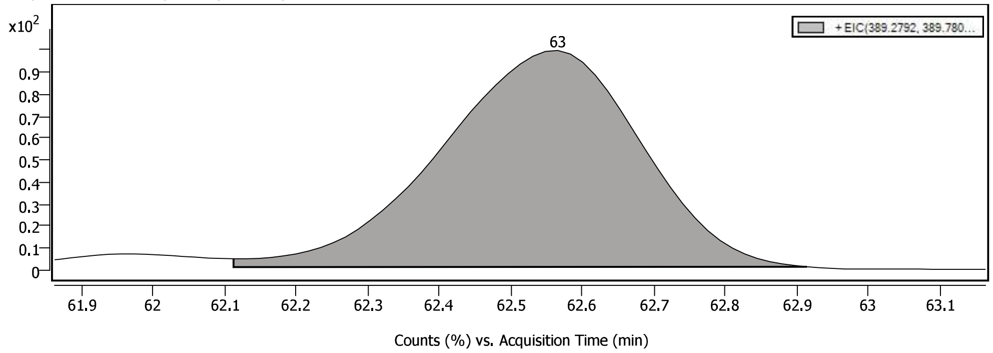

Structure

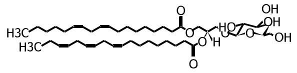

Compound Screening Report

Compound Spectra (overlaid)

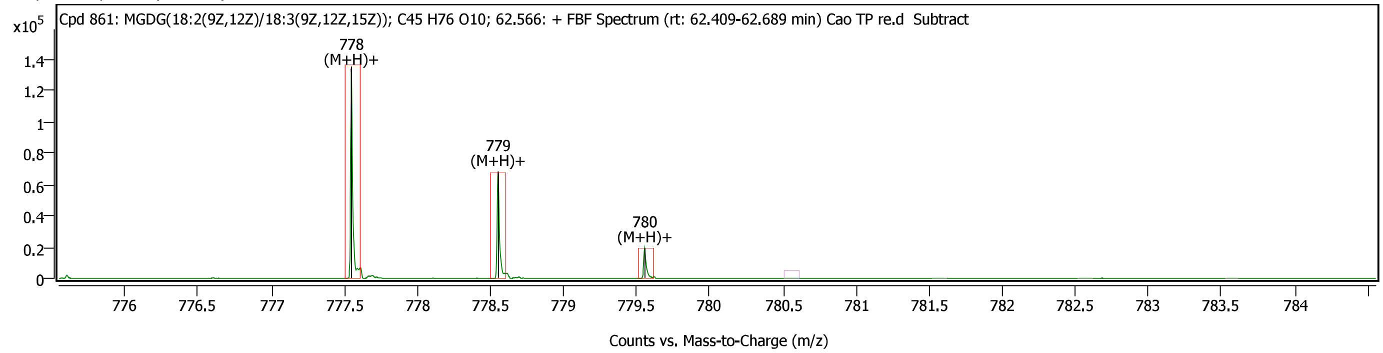

Compound ID Table

Name	Formula	Species	RT	RT Diff	Mass	CAS	ID Source	Score	Score (Lib)	Score (Tgt)
MGDG(18:2(9Z,12Z)/18:3(9Z,12Z, 15Z))	C45 H76 O10	(M+H)+	62.566		776.5417		FBF	94.65		94.65

Cpd 260: PA(P-16:0/18:4(6Z,9Z,12Z,15Z))

Name	Formula	RT	RI	Mass	Diff (Tgt, ppm)	CAS	ID Source	Score	Algorithm
PA(P-16:0/18:4(6Z,9Z,12Z,15Z))	C37 H65 O7 P	62.601		652.4467	-0.20		FBF	94.83	FBF

Species	m/z	Score (Tgt)	Score (Lib)	Score (DB)	Score (MFG)	Score (RT)
(M+H)+	653	94.83				

Compound Chromatograms (overlaid)

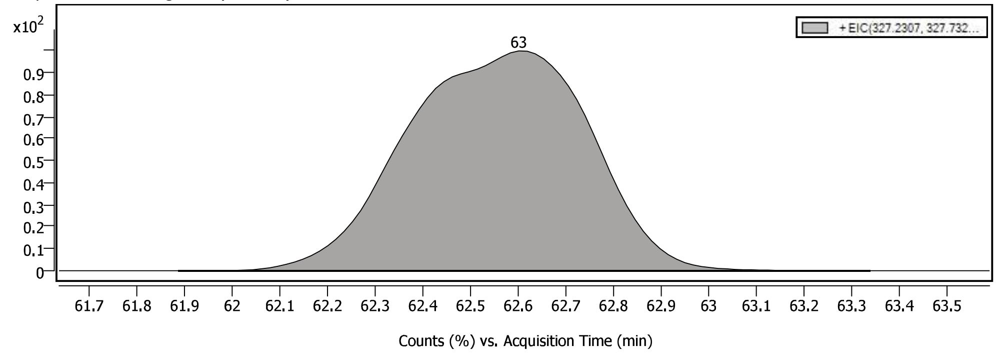

Structure

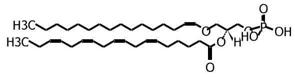

Compound Spectra (overlaid)

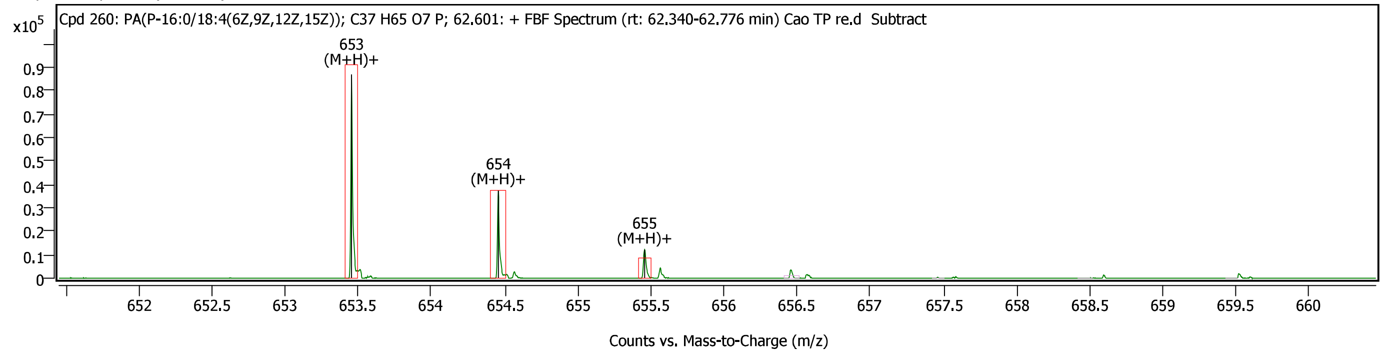

Compound ID Table

Name	Formula	Species	RT	RT Diff	Mass	CAS	ID Source	Score	Score (Lib)	Score (Tgt)
PA(P-16:0/18:4(6Z,9Z,12Z,15Z))	C37 H65 O7 P	(M+H)+	62.601		652.4467		FBF	94.83		94.83

Cpd 1204: DG(20:4(8Z,11Z,14Z,17Z)/18:4(6Z,9Z,12Z,15Z)/0:0)

Name	Formula	RT	RI	Mass	Diff (Tgt, ppm)	CAS	ID Source	Score	Algorithm
DG(20:4(8Z,11Z,14Z,17Z)/18:4(6 Z,9Z,12Z,15Z)/0:0)	C41 H64 O5	62.619		636.4733	-3.26		M-FBF	94.71	FBF

Species	m/z	Score (Tgt)	Score (Lib)	Score (DB)	Score (MFG)	Score (RT)
(M+H)+	637	94.71				

Compound Chromatograms (overlaid)

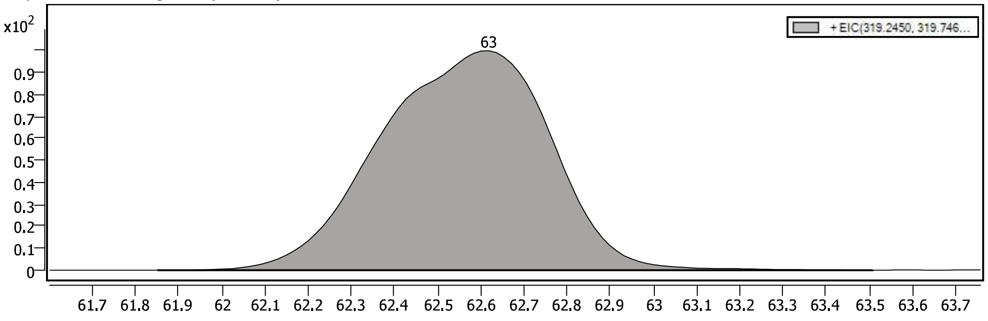

Structure

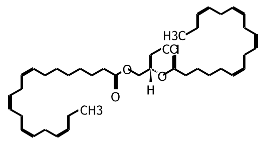

Compound Spectra (overlaid)

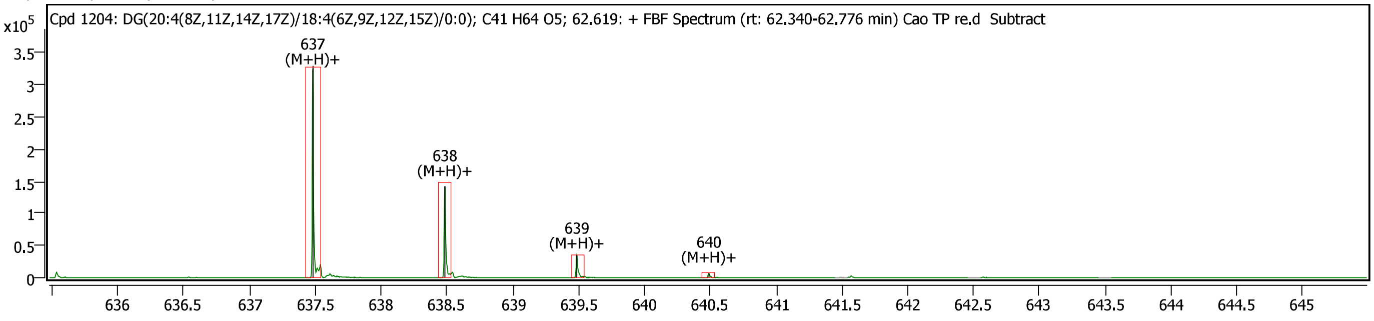

Compound ID Table

Name	Formula	Species	RT	RT Diff	Mass	CAS	ID Source	Score	Score (Lib)	Score (Tgt)
DG(20:4(8Z,11Z,14Z,17Z)/18:4(6Z,9Z,12Z,15Z)/0:0)	C41 H64 O5	(M+H)+	62.619		636.4733		FBF	94.71		94.71
DG(18:3(9Z,12Z,15Z)/20:5(5Z,8Z,11Z,14Z,17Z)/0:0)[iso2]	C41 H64 O5	(M+H)+	62.619		636.4733		FBF	94.71		94.71
DG(18:4(6Z,9Z,12Z,15Z)/20:4(8Z,11Z,14Z,17Z)/0:0)	C41 H64 O5	(M+H)+	62.619		636.4733		FBF	94.71		94.71
DG(18:4(6Z,9Z,12Z,15Z)/20:4(5Z,8Z,11Z,14Z)/0:0)	C41 H64 O5	(M+H)+	62.619		636.4733		FBF	94.71		94.71
DG(18:3(6Z,9Z,12Z)/20:5(5Z,8Z,11Z,14Z,17Z)/0:0)	C41 H64 O5	(M+H)+	62.619		636.4733		FBF	94.71		94.71
DG(20:4(5Z,8Z,11Z,14Z)/18:4(6Z,9Z,12Z,15Z)/0:0)	C41 H64 O5	(M+H)+	62.619		636.4733		FBF	94.71		94.71
DG(20:5(5Z,8Z,11Z,14Z,17Z)/18:3(6Z,9Z,12Z)/0:0)	C41 H64 O5	(M+H)+	62.619		636.4733		FBF	94.71		94.71
DG(20:5(5Z,8Z,11Z,14Z,17Z)/18:3(9Z,12Z,15Z)/0:0)	C41 H64 O5	(M+H)+	62.619		636.4733		FBF	94.71		94.71

Cpd 978: alpha-Tochopheryl acetate

Name	Formula	RT	RI	Mass	Diff (Tgt, ppm)	CAS	ID Source	Score	Algorithm
alpha-Tochopheryl acetate	C31 H52 O3	62.671		472.3917	0.01	52225-20-4	FBF-FragConfirm	99.27	FBF

Species	m/z	Score (Tgt)	Score (Lib)	Score (DB)	Score (MFG)	Score (RT)
(M+H)+	473	99.27				

Compound Chromatograms (overlaid)

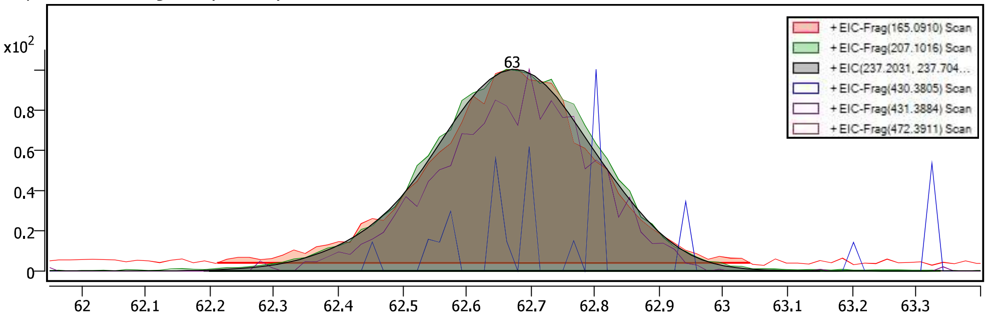

Structure

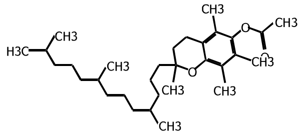

Compound Screening Report

Coelution Plot

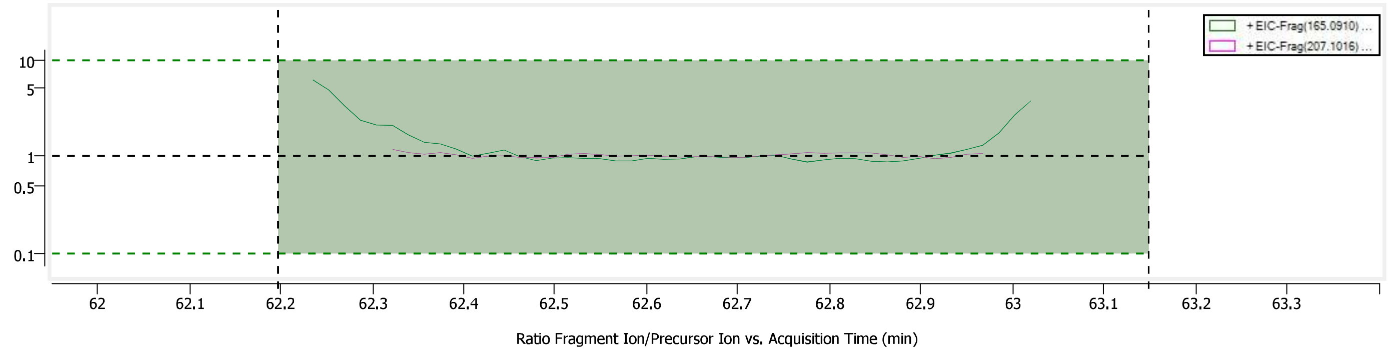

Compound Spectra (overlaid)

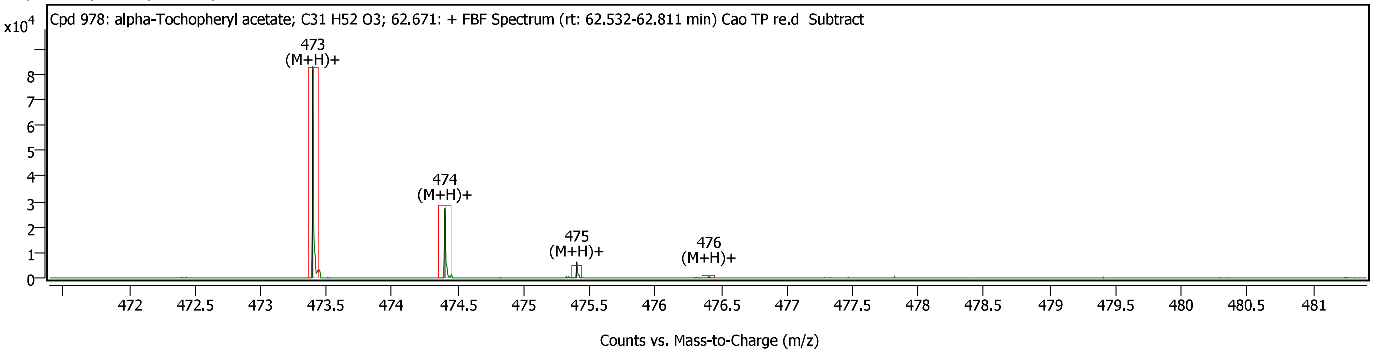

Fragment Spectrum (clean)

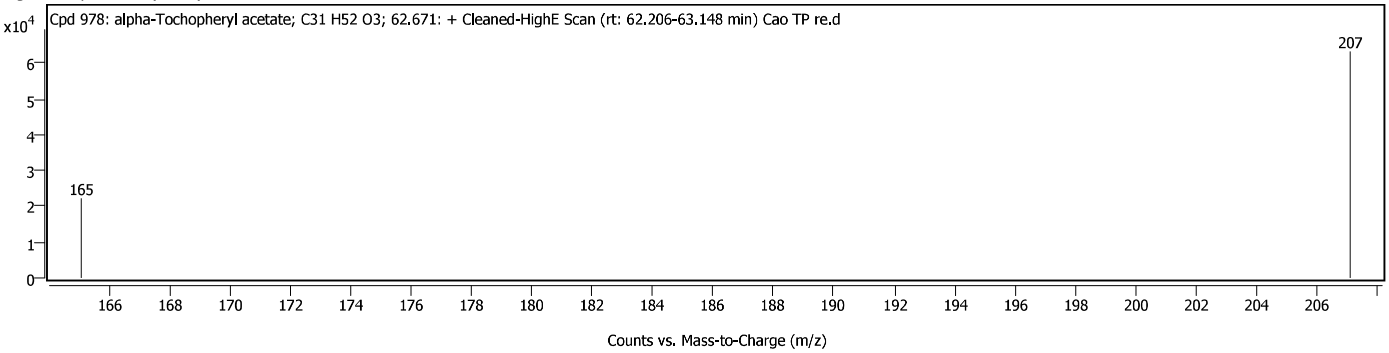

Fragment Spectrum (raw)

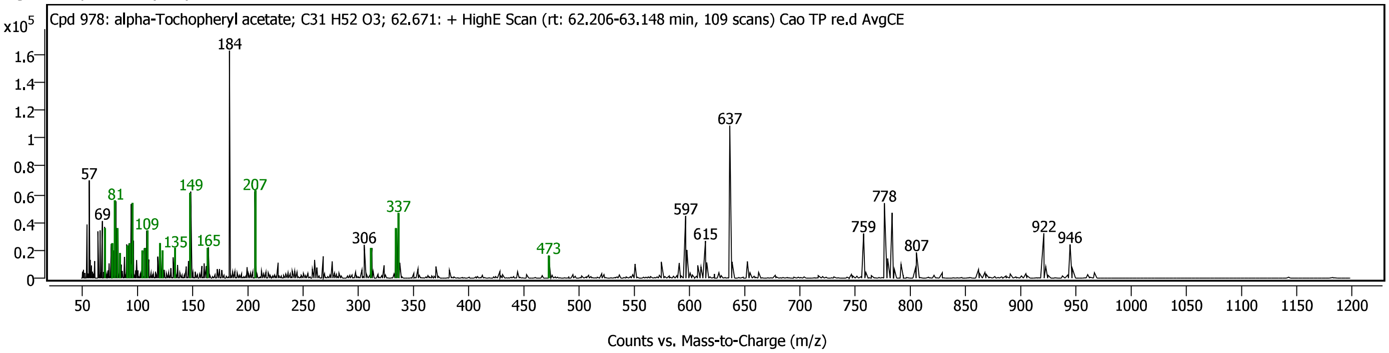

Compound Screening Report

Compound ID Table

Name	Formula	Species	RT	RT Diff	Mass	CAS	ID Source	Score	Score (Lib)	Score (Tgt)
alpha-Tochopheryl acetate	C31 H52 O3	(M+H)+	62.671		472.3917	52225-20-4	FBF-FragConfirm	99.27		99.27
1β-butyl-1α,25-dihydroxyvitamin D3 / 1β-butyl-1α,25-dihydroxycholecalciferol	C31 H52 O3	(M+H)+	62.671		472.3917		FBF-FragConfirm	99.27		99.27
1α,25-dihydroxy-26,27-dimethyl-24a,24b-dihomovitamin D3 / 1α,25-dihydroxy-26,27-dimethyl-24a,24b-dihomocholecalciferol	C31 H52 O3	(M+H)+	62.671		472.3917		FBF-FragConfirm	99.27		99.27
1α-butyl-1β,25-dihydroxyvitamin D3 / 1α-butyl-1β,25-dihydroxycholecalciferol	C31 H52 O3	(M+H)+	62.671		472.3917		FBF-FragConfirm	99.27		99.27
Theonellasterol D	C31 H52 O3	(M+H)+	62.671		472.3917		FBF-FragConfirm	99.27		99.27
26,27-diethyl-1α,25-dihydroxyvitamin D3 / 26,27-diethyl-1α,25-dihydroxycholecalciferol	C31 H52 O3	(M+H)+	62.671		472.3917		FBF-FragConfirm	99.27		99.27
Soyasapogenol D	C31 H52 O3	(M+H)+	62.671		472.3917	65892-76-4	FBF-FragConfirm	99.27		99.27

Cpd 366: PC(P-18:0/17:2(9Z,12Z))

Name	Formula	RT	RI	Mass	Diff (Tgt, ppm)	CAS	ID Source	Score	Algorithm
PC(P-18:0/17:2(9Z,12Z))	C43 H83 N O7 P	63.421		756.5878	-3.90		FBF	91.32	FBF

Species	m/z	Score (Tgt)	Score (Lib)	Score (DB)	Score (MFG)	Score (RT)
(M+H)+	758	91.32				

Compound Chromatograms (overlaid)

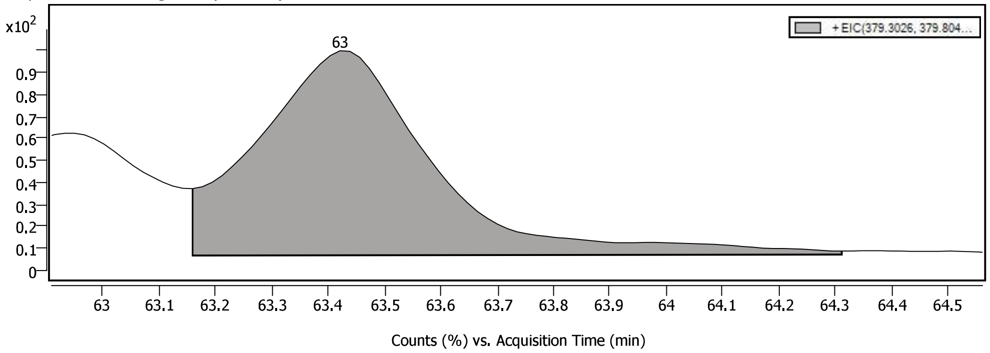

Structure

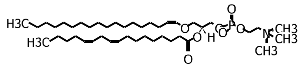

Compound Spectra (overlaid)

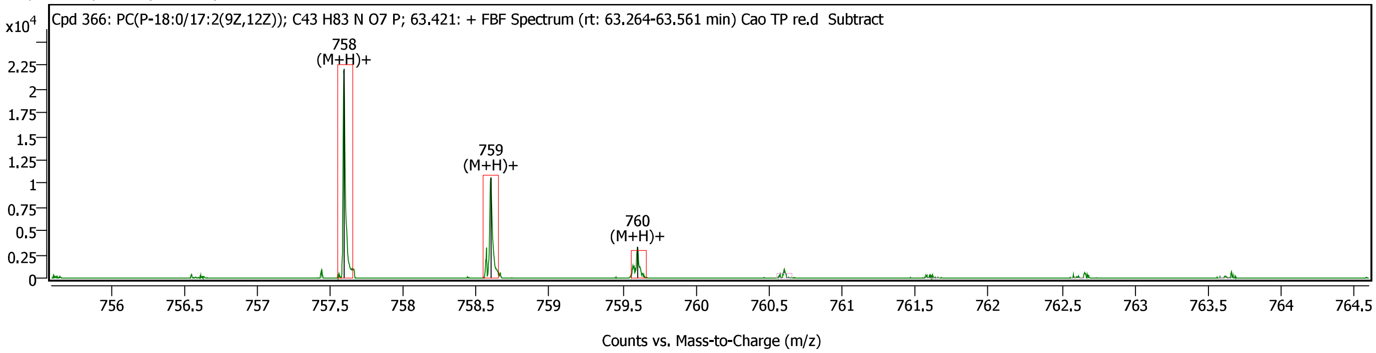

Compound ID Table

Name	Formula	Species	RT	RT Diff	Mass	CAS	ID Source	Score	Score (Lib)	Score (Tgt)
PC(P-18:0/17:2(9Z,12Z))	C43 H83 N O7 P	(M+H)+	63.421		756.5878		FBF	91.32		91.32

MassHunter Qual 12.0
(End of Report)
